# Supplementary material for: Optimized fragmentation schemes and data analysis strategies for proteome-wide cross-link identification
Source: Nat Commun. 2017 May 19;8:15473. doi: 10.1038/ncomms15473 (PMC5454533; doi:10.1038/ncomms15473)
Supplement: Supplementary Data 2 — Annotated mass spectra of all E.coli cross-links. [file ncomms15473-s3.pdf]

Supplementary Data 2. All annotated mass spectra of cross-linked *E. coli* peptides. Shown are the output files derived from XlinkX v2.0.

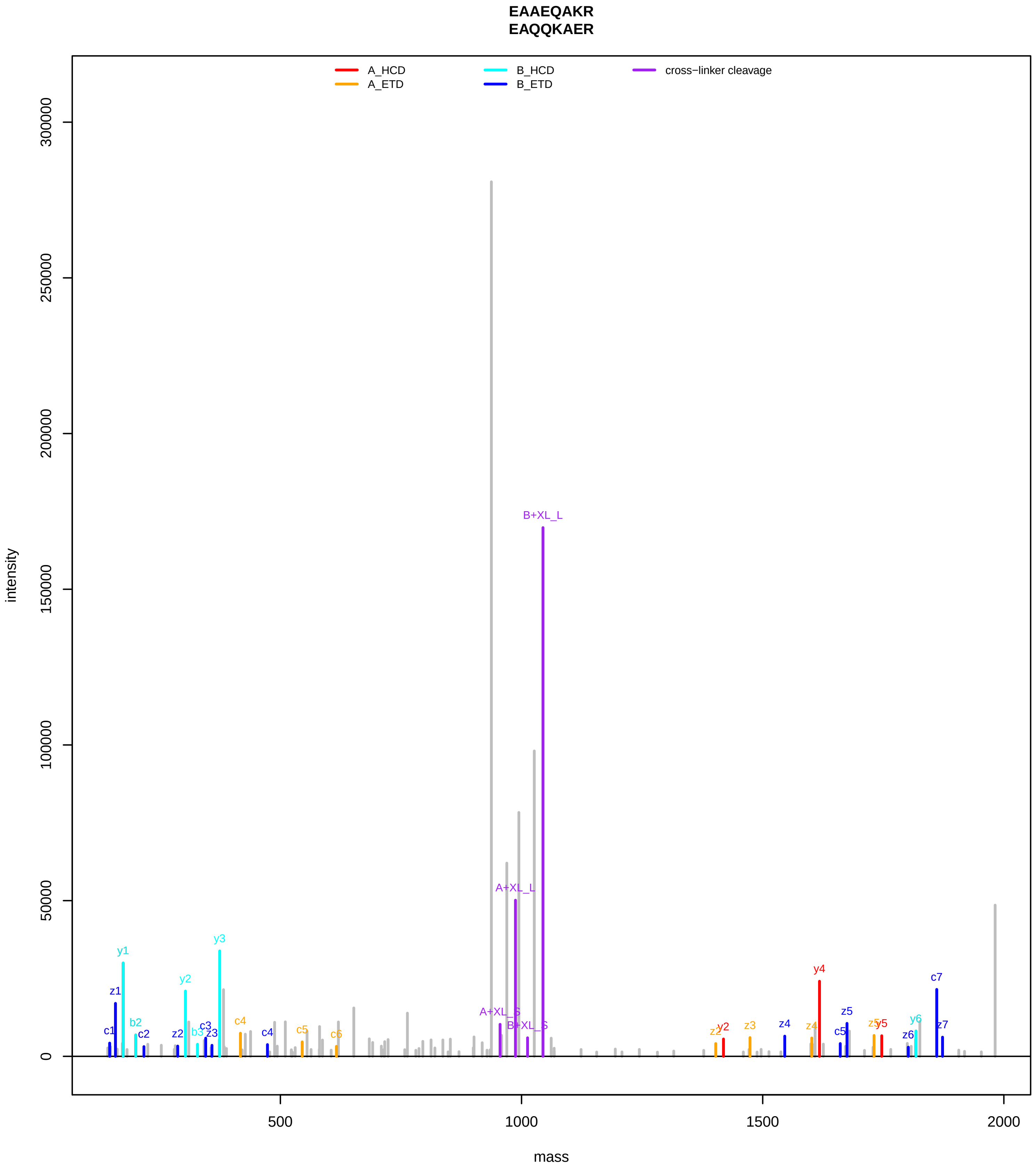

EAAEQAKR+XL_S

EAAEQAKR+XL_L

EAQQKAER+XL_S

EAQQKAER+XL_L

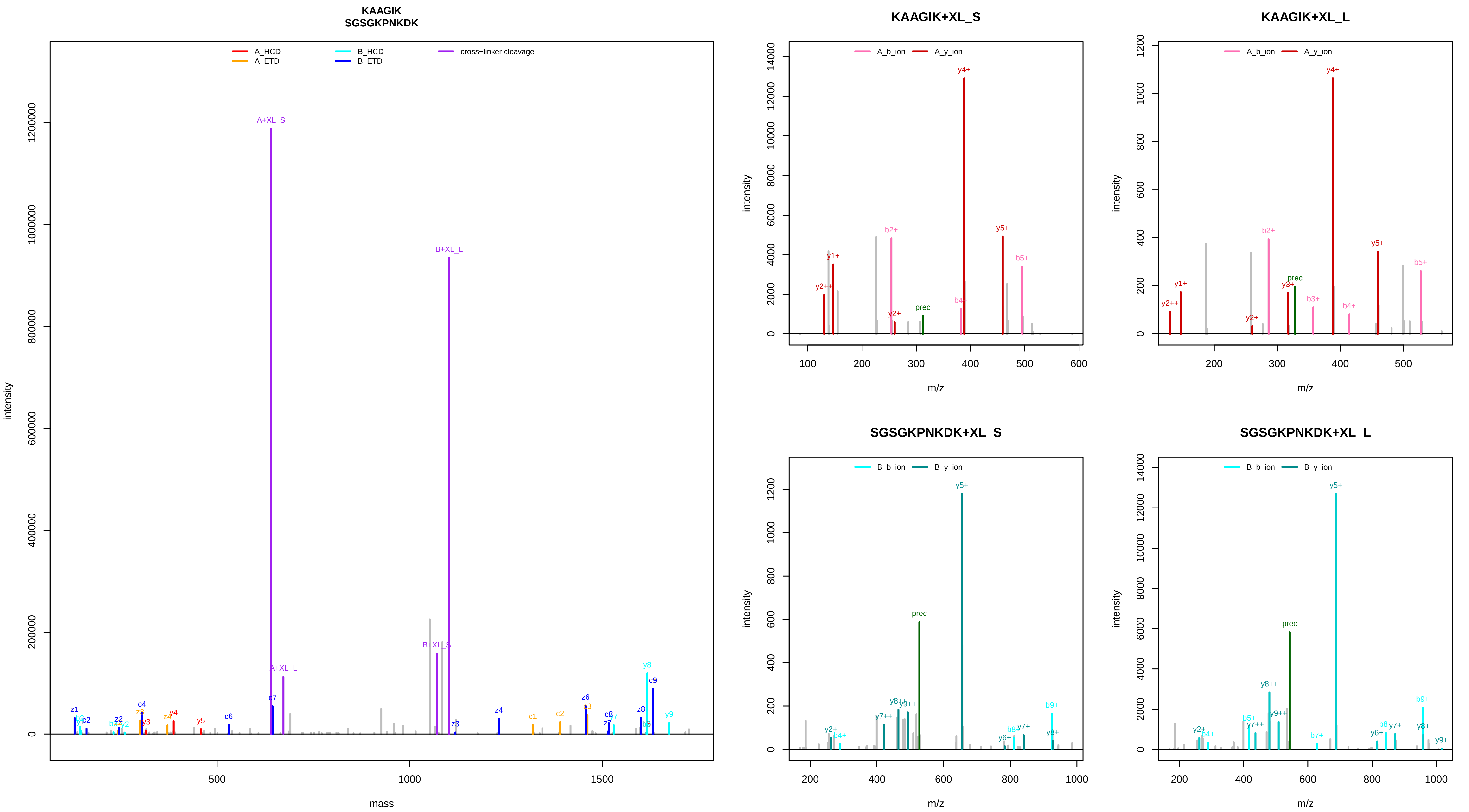
KAAGIK
SGSGKPNKDK
A_HCD
A_ETD
B_HCD
B_ETD
cross−linker cleavage
A+XL_S
B+XL_L
B+XL_S
A+XL_L
intensity
mass
KAAGIK+XL_S
KAAGIK+XL_L
A_b_ion
A_y_ion
SGSGKPNKDK+XL_S
SGSGKPNKDK+XL_L
B_b_ion
B_y_ion
prec
m/z

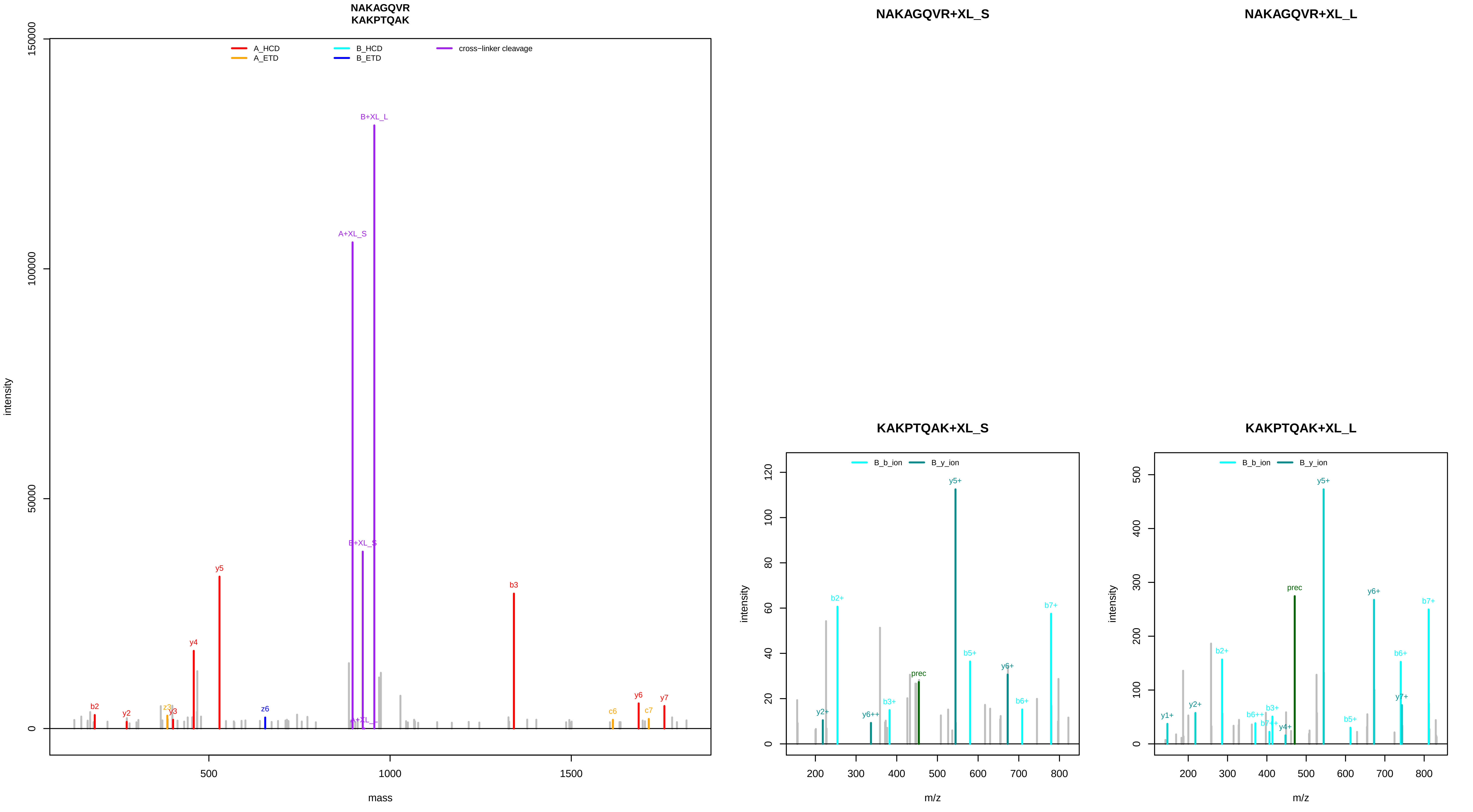

NAKAGQVR
KAKPTQAK
A_HCD
A_ETD
B_HCD
B_ETD
cross−linker cleavage
B+XL_L
A+XL_S
B+XL_S
y5
b3
y4
b2
y2
z3
y3
z6
c6
y6
c7
y7
intensity
mass
NAKAGQVR+XL_S
NAKAGQVR+XL_L
KAKPTQAK+XL_S
KAKPTQAK+XL_L
B_b_ion
B_y_ion
y5+
b2+
b7+
b5+
y6+
prec
b3+
b6+
y2+
y6++
y1+
b6++
y7+
y4+
intensity
m/z

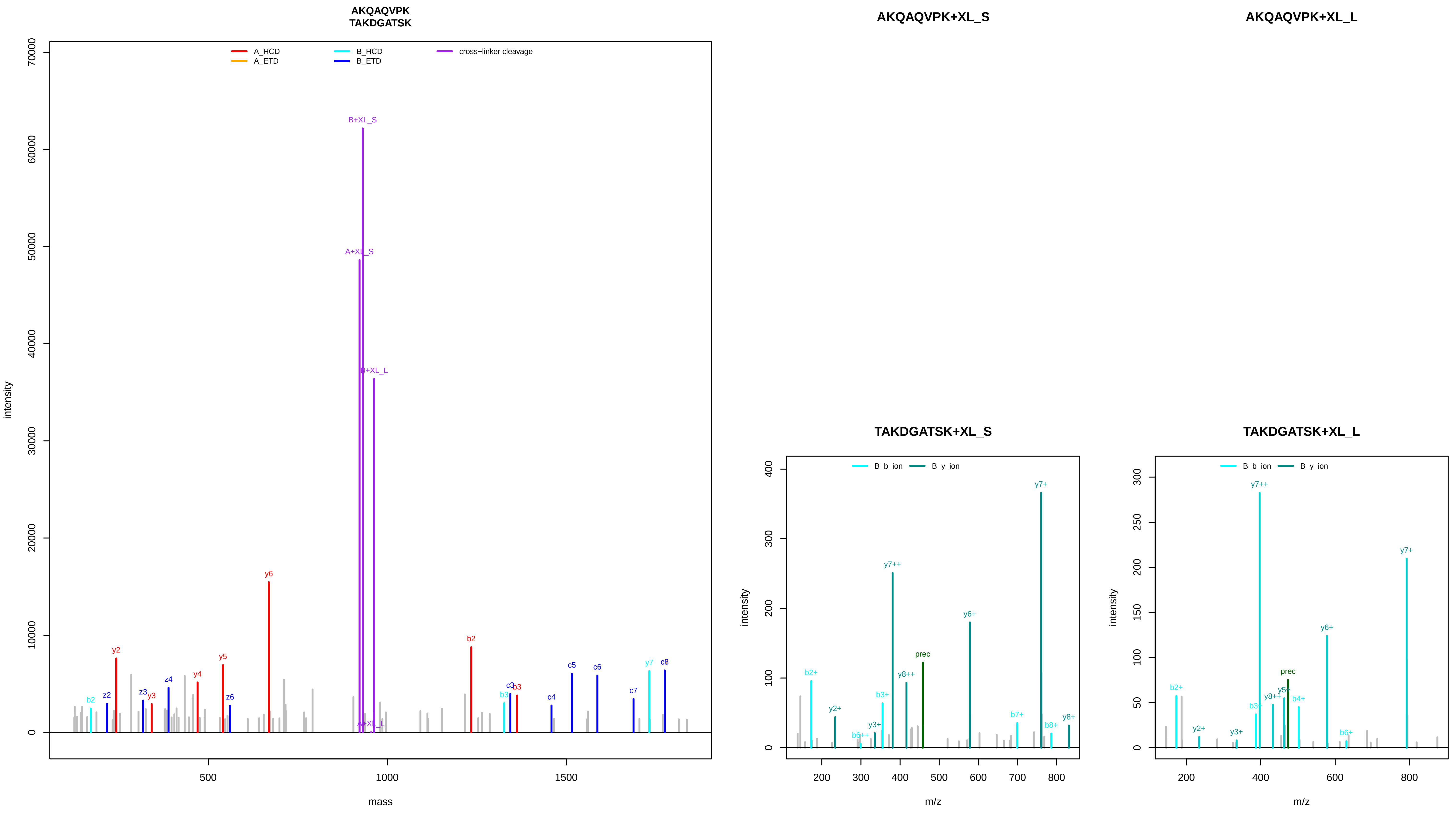
AKQAQVPK
TAKDGATSK
A_HCD
A_ETD
B_HCD
B_ETD
cross−linker cleavage
B+XL_S
A+XL_S
B+XL_L
A+XL_L
y2
y3
y4
y5
y6
b2
b3
b2
b3
y7
z2
z3
z4
z6
c3
c4
c5
c6
c7
c8
intensity
mass
AKQAQVPK+XL_S
AKQAQVPK+XL_L
TAKDGATSK+XL_S
TAKDGATSK+XL_L
B_b_ion
B_y_ion
b2+
y2+
b6++
y3+
b3+
y7++
y8++
prec
y6+
b7+
y7+
b8+
y8+
b3+
y5+
b4+
b6+
m/z

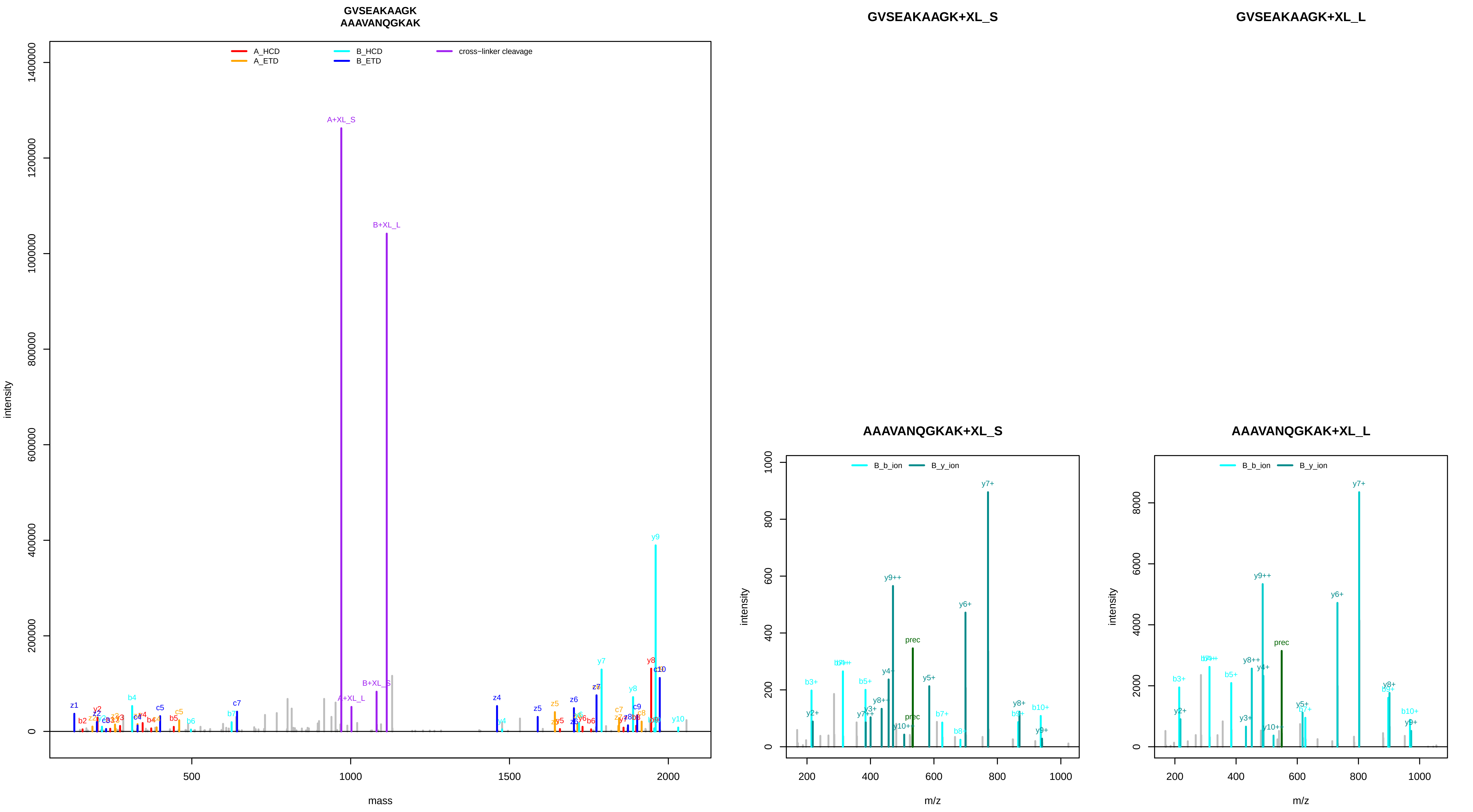
GVSEAKAAGK
AAAVANQGKAK
A_HCD
A_ETD
B_HCD
B_ETD
cross−linker cleavage
A+XL_S
B+XL_L
intensity
mass
GVSEAKAAGK+XL_S
GVSEAKAAGK+XL_L
AAAVANQGKAK+XL_S
AAAVANQGKAK+XL_L
B_b_ion
B_y_ion
prec
m/z

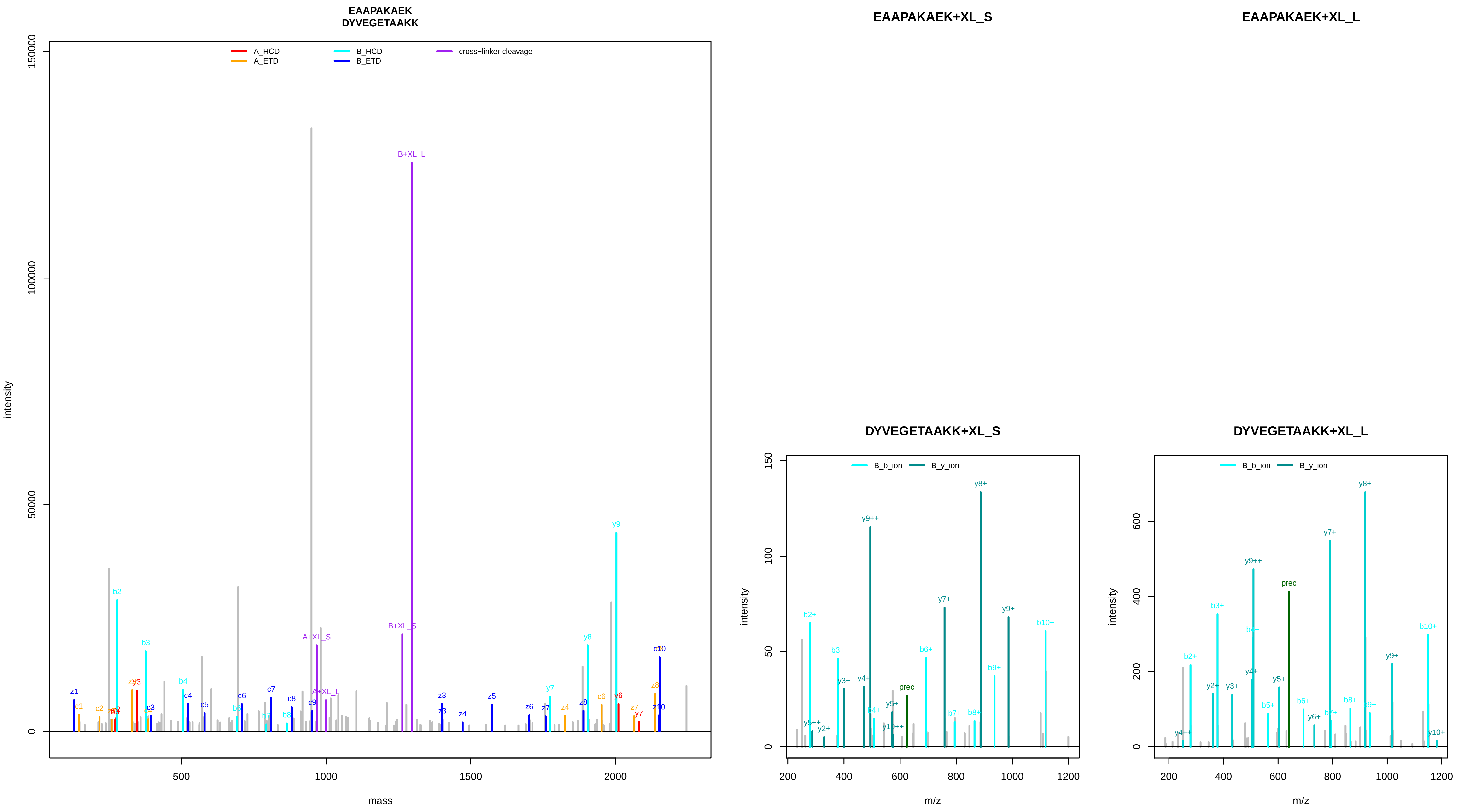
EAAPAKAEK
DYVEGETAAKK
A_HCD
A_ETD
B_HCD
B_ETD
cross−linker cleavage
B+XL_L
B+XL_S
A+XL_S
A+XL_L
intensity
mass
EAAPAKAEK+XL_S
EAAPAKAEK+XL_L
DYVEGETAAKK+XL_S
DYVEGETAAKK+XL_L
B_b_ion
B_y_ion
prec
m/z

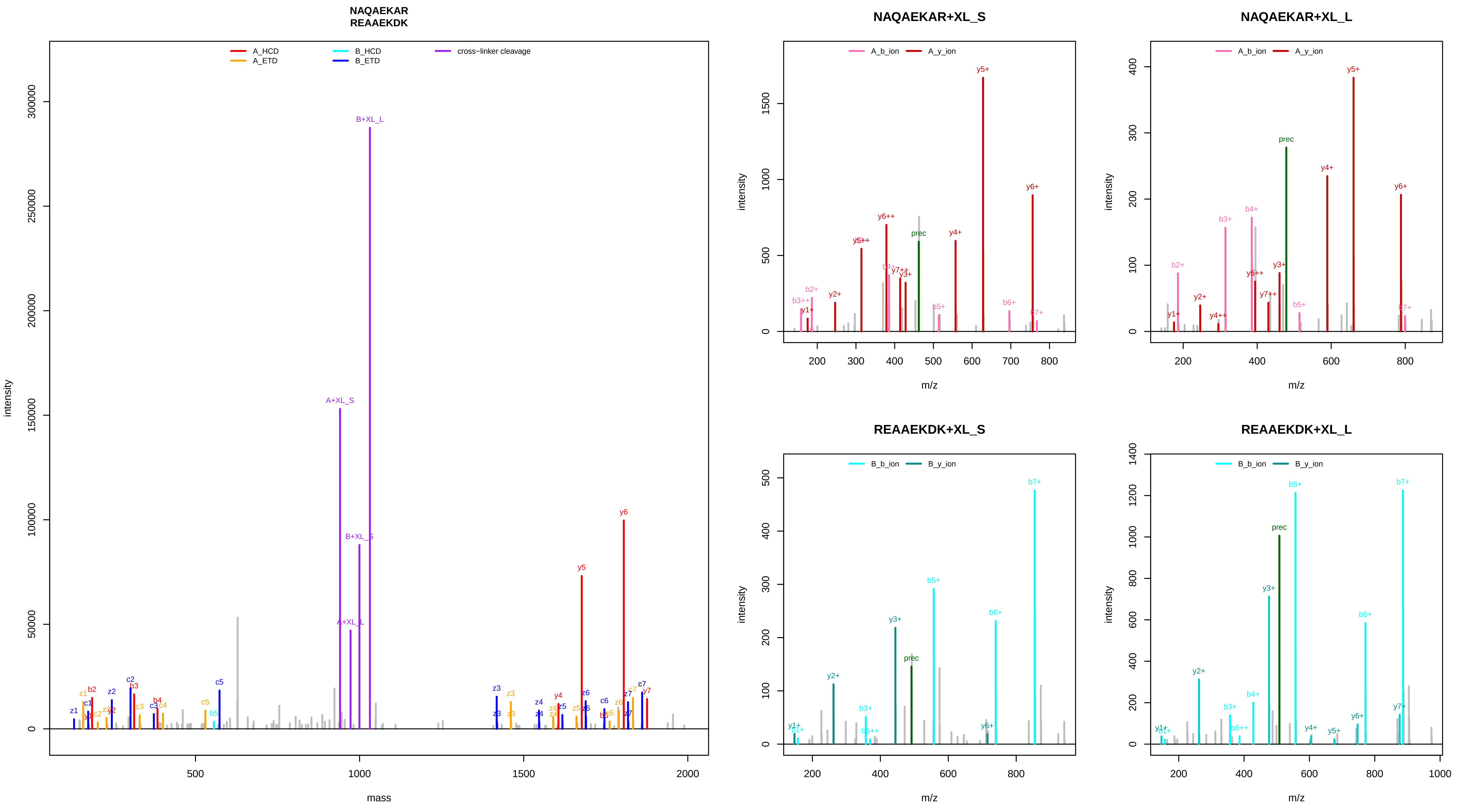
NAQAEKAR
REAAEKDK
A_HCD
A_ETD
B_HCD
B_ETD
cross−linker cleavage
B+XL_L
A+XL_S
B+XL_S
A+XL_L
intensity
mass
NAQAEKAR+XL_S
NAQAEKAR+XL_L
A_b_ion
A_y_ion
REAAEKDK+XL_S
REAAEKDK+XL_L
B_b_ion
B_y_ion
prec
intensity
m/z

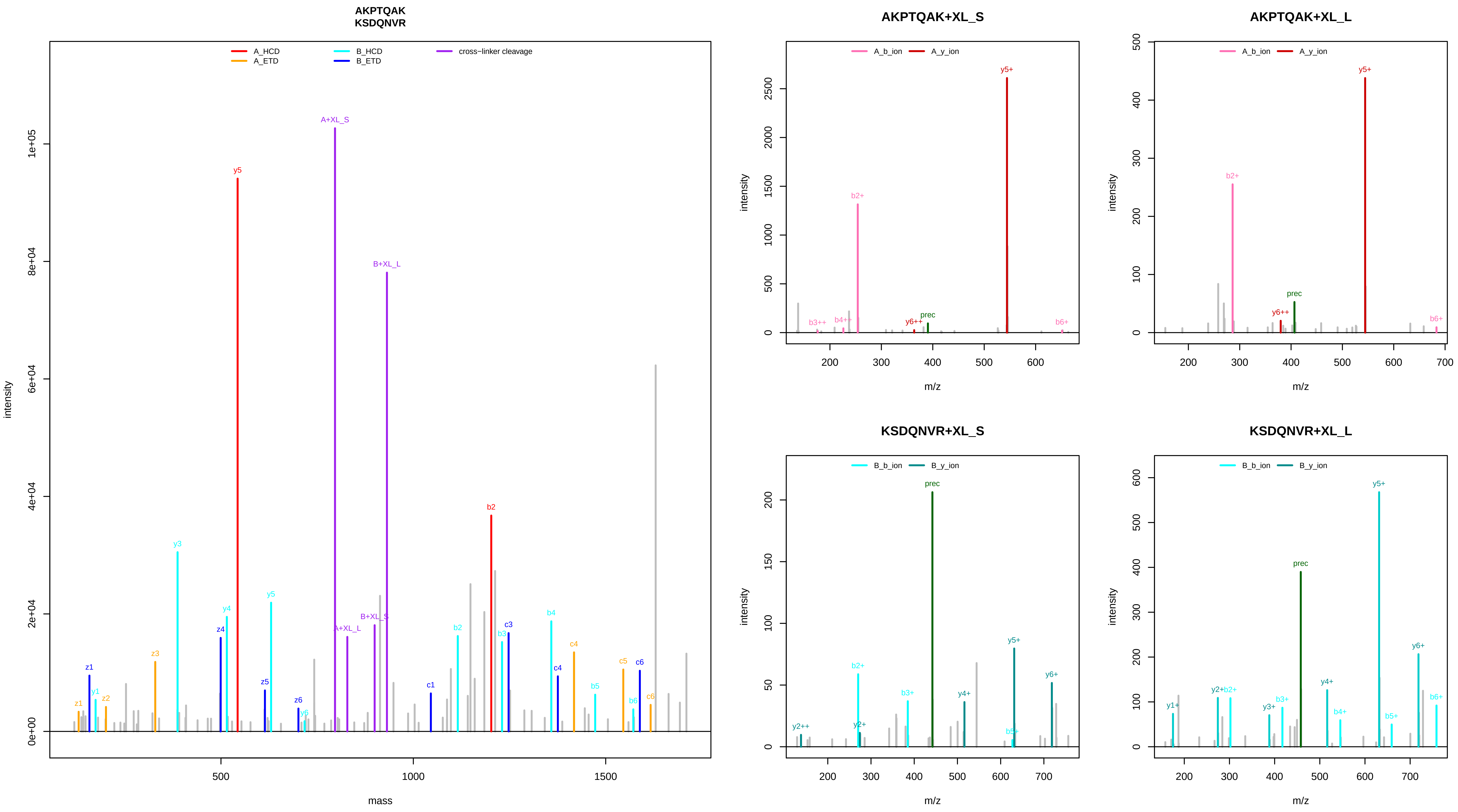
AKPTQAK
KSDQNVR
A_HCD
A_ETD
B_HCD
B_ETD
cross−linker cleavage
A+XL_S
B+XL_L
A+XL_L
B+XL_S
intensity
mass
AKPTQAK+XL_S
AKPTQAK+XL_L
KSDQNVR+XL_S
KSDQNVR+XL_L
A_b_ion
A_y_ion
B_b_ion
B_y_ion
prec
m/z
intensity

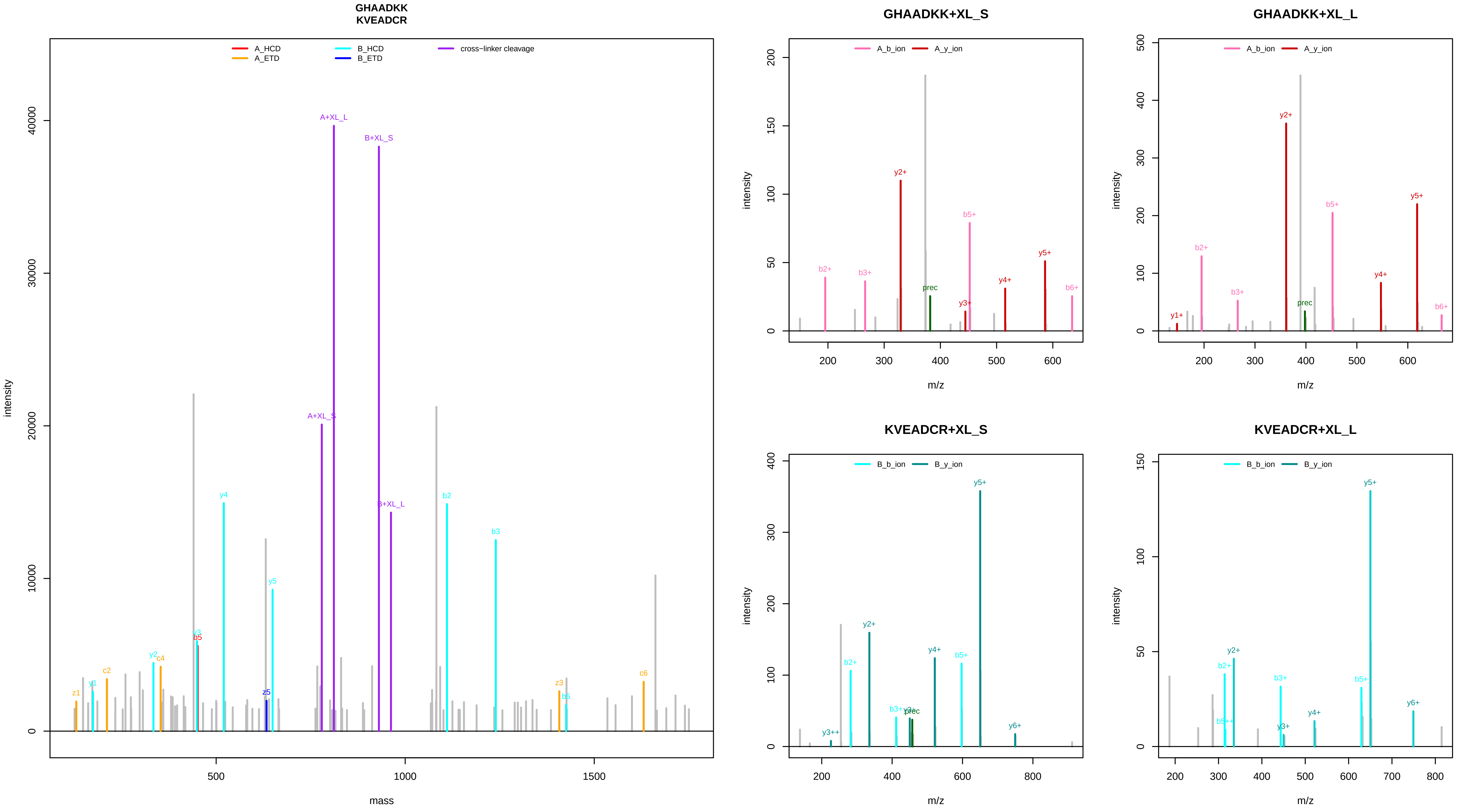
GHAADKK
KVEADCR
A_HCD
A_ETD
B_HCD
B_ETD
cross−linker cleavage
A+XL_L
B+XL_S
A+XL_S
B+XL_L
y4
b2
b3
y5
y3
b5
y2
c4
c2
y1
z1
z5
z3
b5
c6
intensity
mass
GHAADKK+XL_S
A_b_ion
A_y_ion
y2+
b5+
y5+
b2+
b3+
prec
y4+
y3+
b6+
m/z
GHAADKK+XL_L
A_b_ion
A_y_ion
y2+
b5+
y5+
b2+
b3+
y4+
prec
y1+
b6+
m/z
KVEADCR+XL_S
B_b_ion
B_y_ion
y5+
y2+
y4+
b5+
b2+
b3+
prec
y3++
y6+
m/z
KVEADCR+XL_L
B_b_ion
B_y_ion
y5+
y2+
b2+
b3+
b5+
y4+
y6+
y3+
m/z

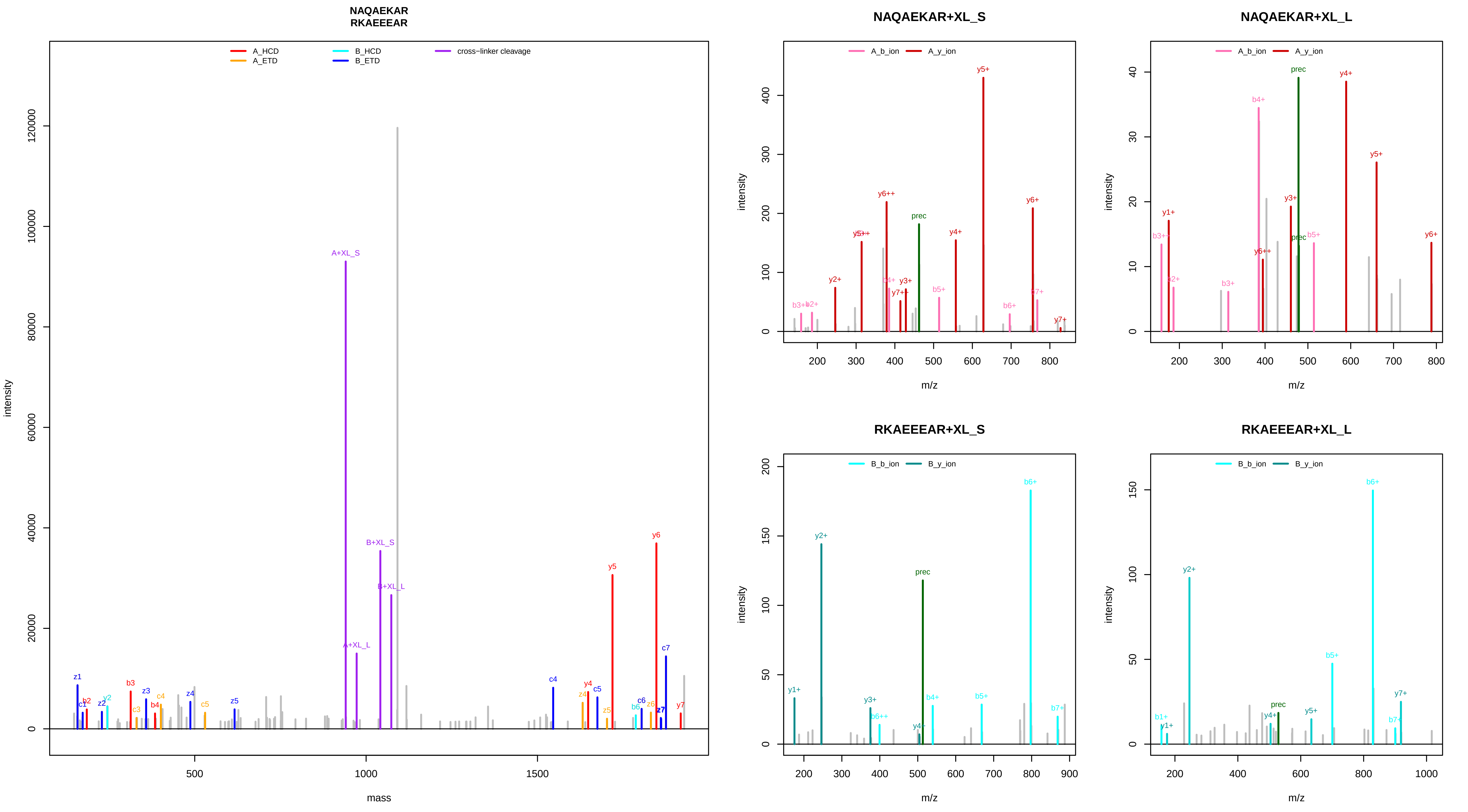
NAQAEKAR
RKAEEEAR
A_HCD
A_ETD
B_HCD
B_ETD
cross−linker cleavage
A+XL_S
B+XL_S
B+XL_L
A+XL_L
z1
c1
b2
z2
y2
b3
c3
z3
b4
c4
z4
c5
z5
c4
z4
y4
c5
z5
y5
b6
c6
y6
c7
z7
y7
intensity
mass
NAQAEKAR+XL_S
A_b_ion
A_y_ion
y5+
y6++
prec
y5++
y2+
y4+
y6+
b3++
b2+
b4+
y3+
y7++
b5+
b6+
b7+
y7+
m/z
NAQAEKAR+XL_L
prec
y4+
b4+
y5+
y1+
y3+
b3++
prec
b5+
y6++
y6+
b2+
b3+
RKAEEEAR+XL_S
B_b_ion
B_y_ion
b6+
y2+
prec
y1+
y3+
b6++
b4+
b5+
b7+
y4+
RKAEEEAR+XL_L
b6+
y2+
b5+
y7+
prec
b1+
y1+
y4+
y5+
b7+

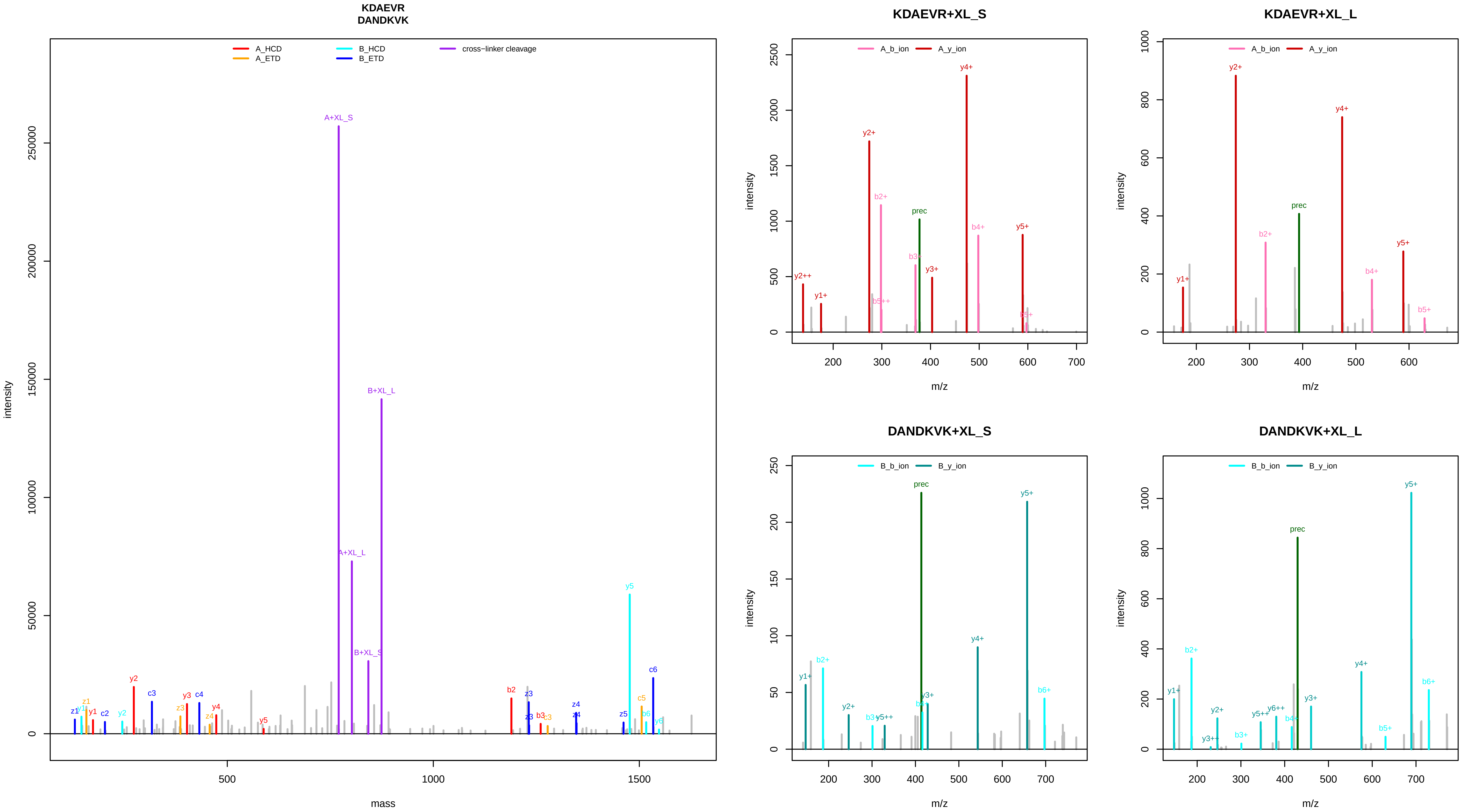
KDAEVR
DANDKVK
A_HCD
A_ETD
B_HCD
B_ETD
cross−linker cleavage
A+XL_S
B+XL_L
A+XL_L
B+XL_S
intensity
mass
KDAEVR+XL_S
KDAEVR+XL_L
DANDKVK+XL_S
DANDKVK+XL_L
A_b_ion
A_y_ion
B_b_ion
B_y_ion
prec
m/z
intensity

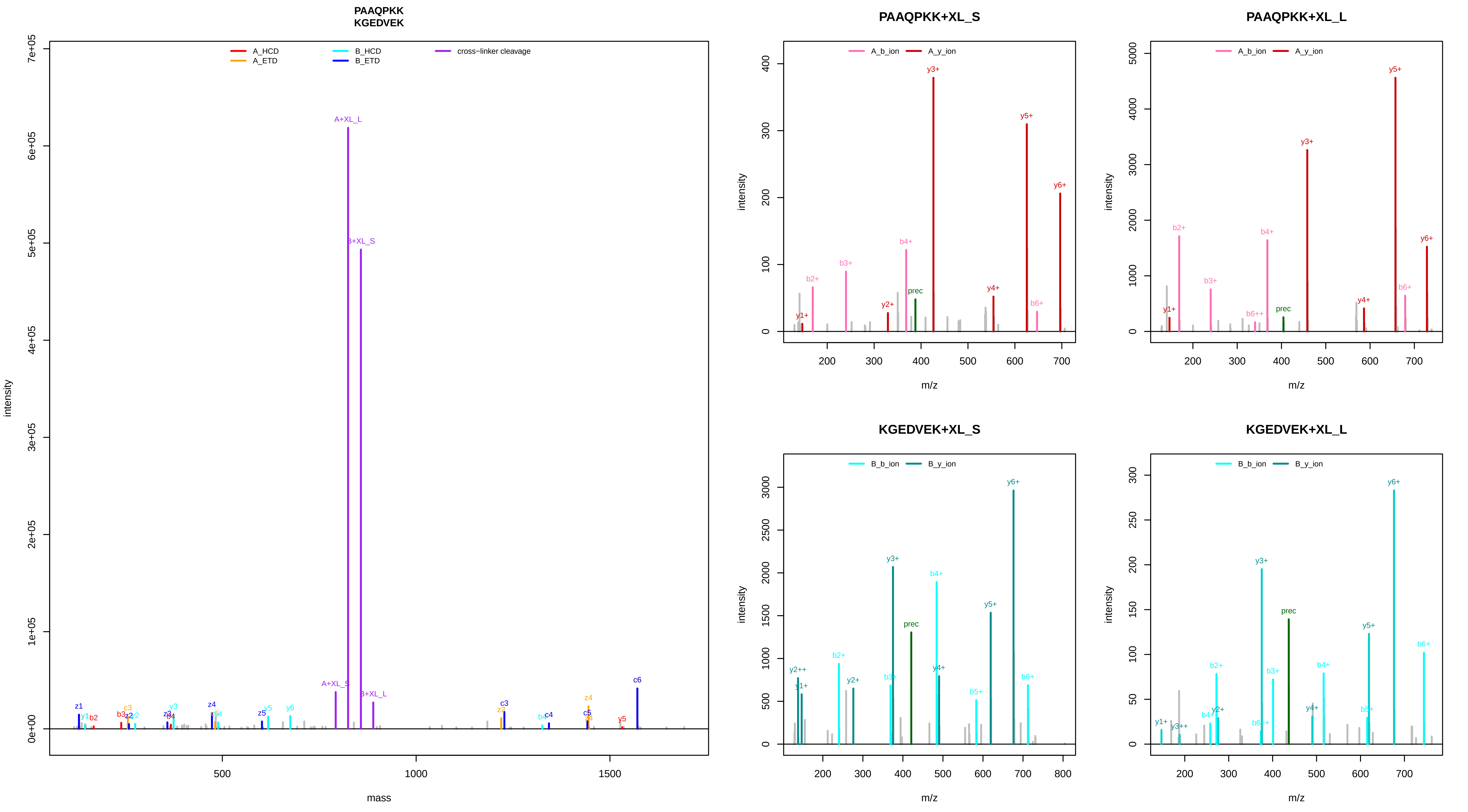

PAAQPKK
KGEDVEK
A_HCD
A_ETD
B_HCD
B_ETD
cross−linker cleavage
A+XL_L
B+XL_S
A+XL_S
B+XL_L
intensity
mass
PAAQPKK+XL_S
PAAQPKK+XL_L
A_b_ion
A_y_ion
KGEDVEK+XL_S
KGEDVEK+XL_L
B_b_ion
B_y_ion
prec
m/z

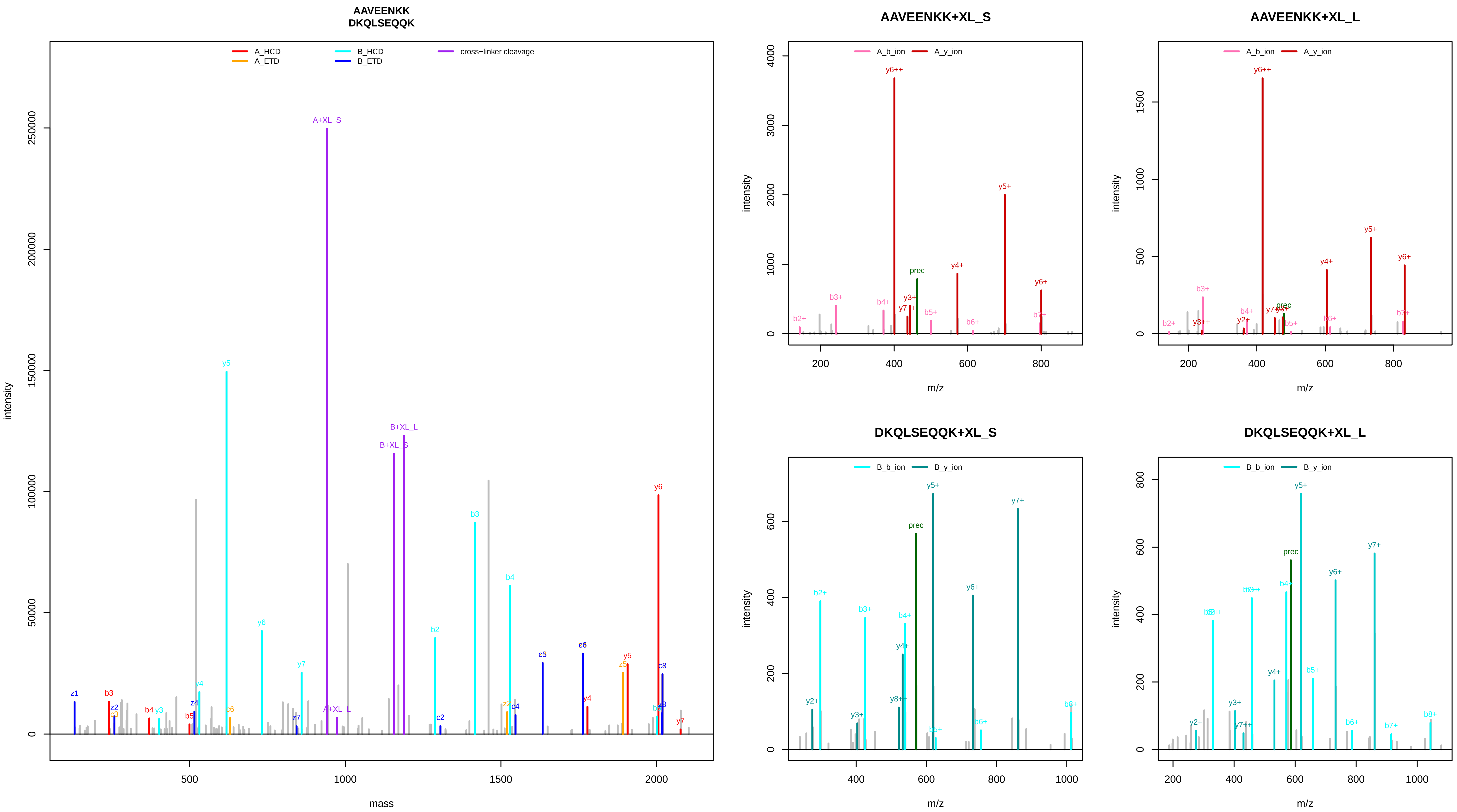
AAVEENKK
DKQLSEQQK
A_HCD
A_ETD
B_HCD
B_ETD
cross−linker cleavage
A+XL_S
B+XL_L
B+XL_S
A+XL_L
intensity
mass
AAVEENKK+XL_S
AAVEENKK+XL_L
DKQLSEQQK+XL_S
DKQLSEQQK+XL_L
A_b_ion
A_y_ion
B_b_ion
B_y_ion
prec
m/z

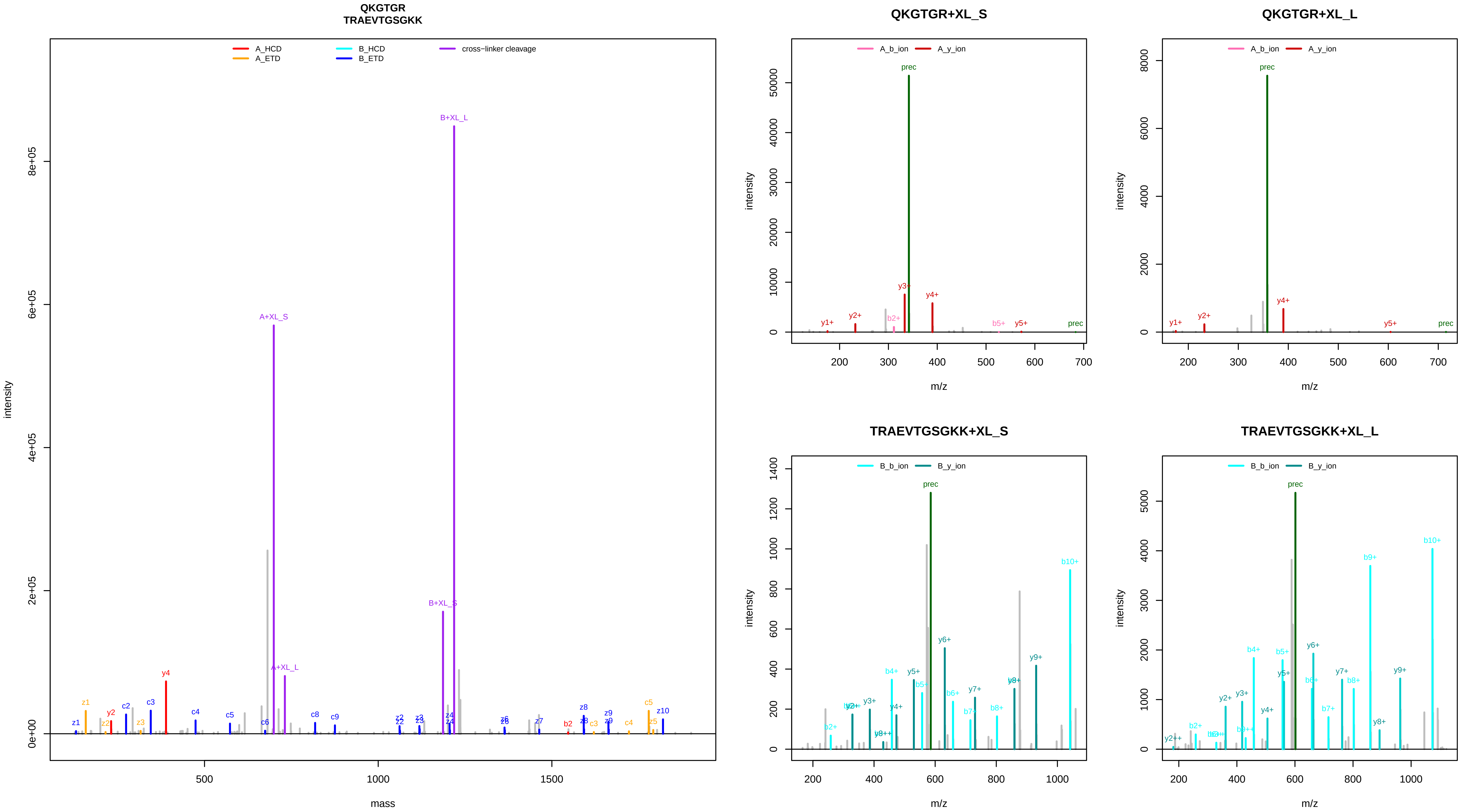

QKGTGR
TRAEVTGSGKK
A_HCD
A_ETD
B_HCD
B_ETD
cross−linker cleavage
intensity
mass
QKGTGR+XL_S
QKGTGR+XL_L
TRAEVTGSGKK+XL_S
TRAEVTGSGKK+XL_L
A_b_ion
A_y_ion
B_b_ion
B_y_ion
prec
m/z

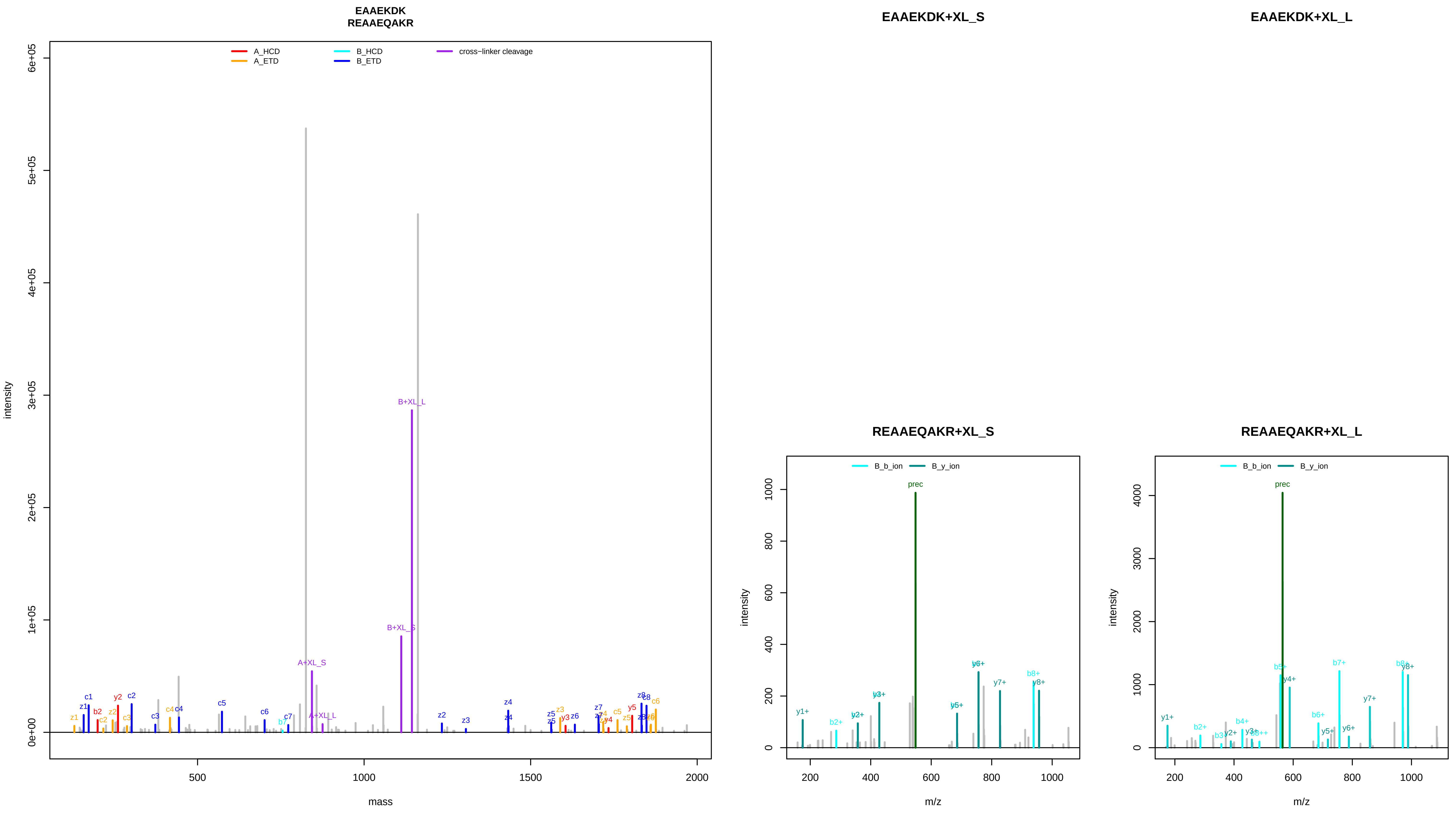
EAAEKDK
REAAEQAKR
A_HCD
A_ETD
B_HCD
B_ETD
cross−linker cleavage
B+XL_L
B+XL_S
A+XL_S
A+XL_L
intensity
mass
EAAEKDK+XL_S
EAAEKDK+XL_L
REAAEQAKR+XL_S
REAAEQAKR+XL_L
B_b_ion
B_y_ion
prec
m/z

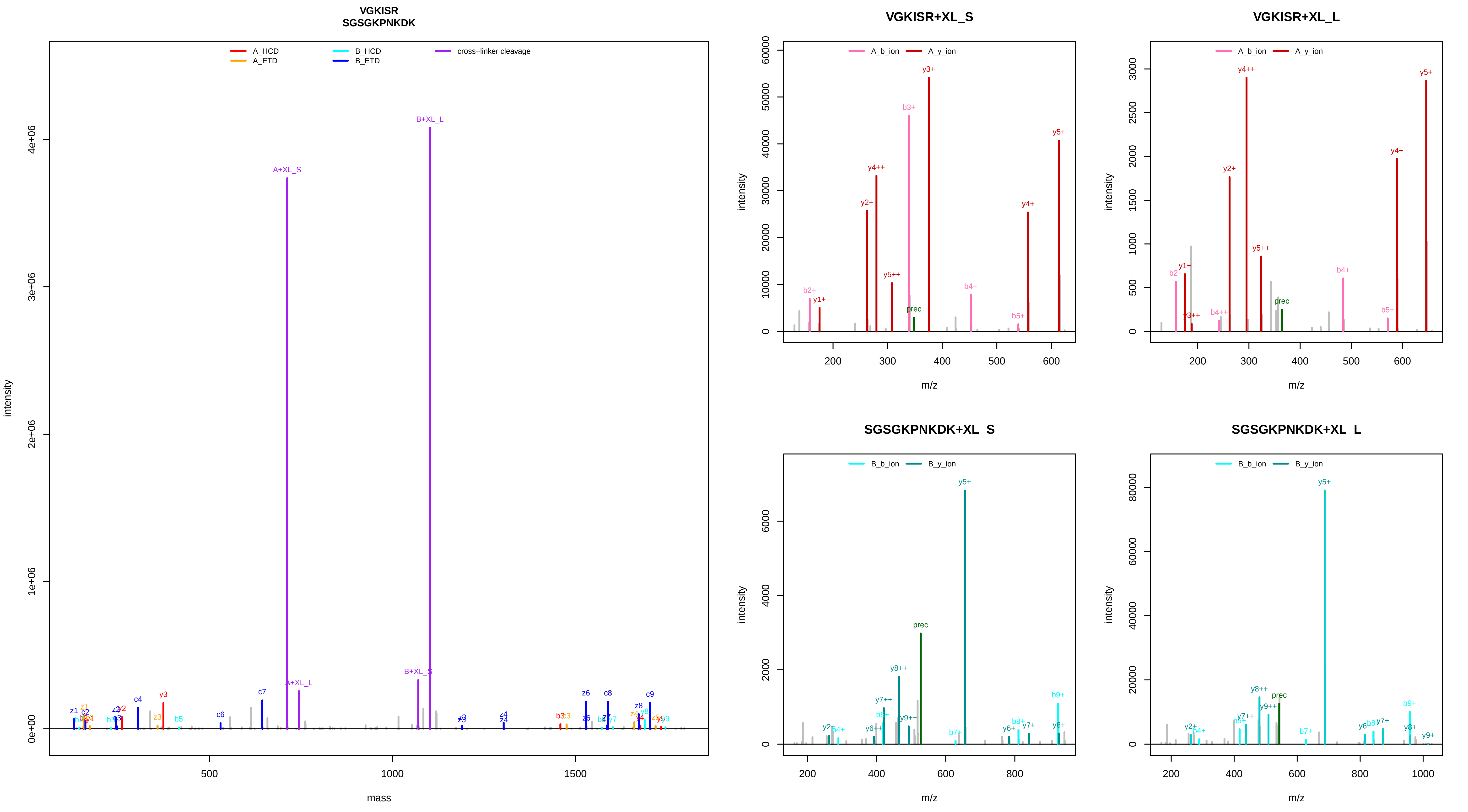
VGKISR
SGSGKPNKDK
A_HCD
A_ETD
B_HCD
B_ETD
cross−linker cleavage
A+XL_S
B+XL_L
A+XL_L
B+XL_S
intensity
mass
VGKISR+XL_S
VGKISR+XL_L
SGSGKPNKDK+XL_S
SGSGKPNKDK+XL_L
A_b_ion
A_y_ion
B_b_ion
B_y_ion
prec
m/z

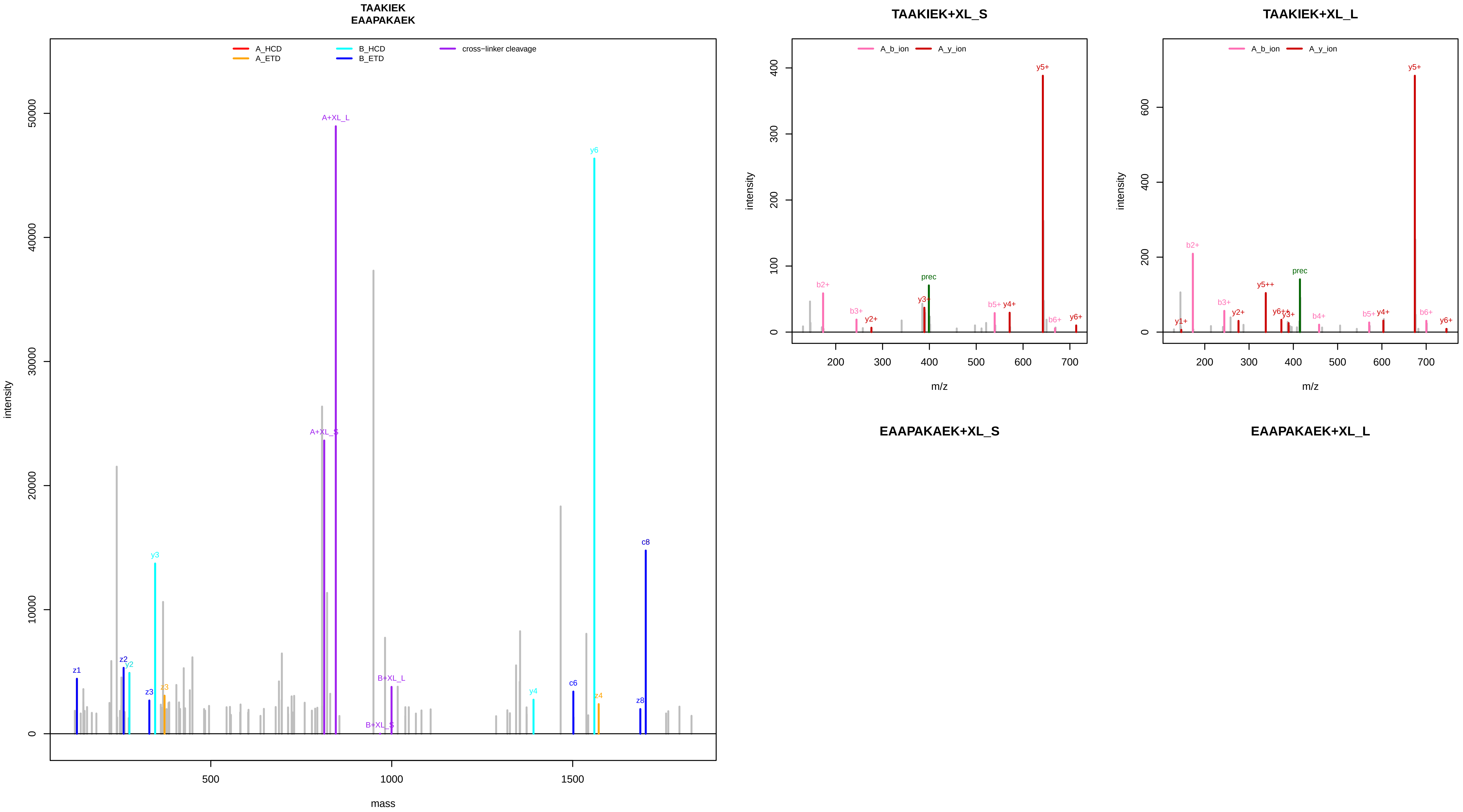
TAAKIEK
EAAPAKAEK
A_HCD
A_ETD
B_HCD
B_ETD
cross−linker cleavage
A+XL_L
A+XL_S
B+XL_L
B+XL_S
y6
y3
y2
y4
z1
z2
z3
z4
z8
c6
c8
intensity
mass
TAAKIEK+XL_S
TAAKIEK+XL_L
A_b_ion
A_y_ion
prec
y5+
y5++
b2+
b3+
b4+
b5+
b6+
y1+
y2+
y3+
y4+
y6+
y6++
m/z
EAAPAKAEK+XL_S
EAAPAKAEK+XL_L

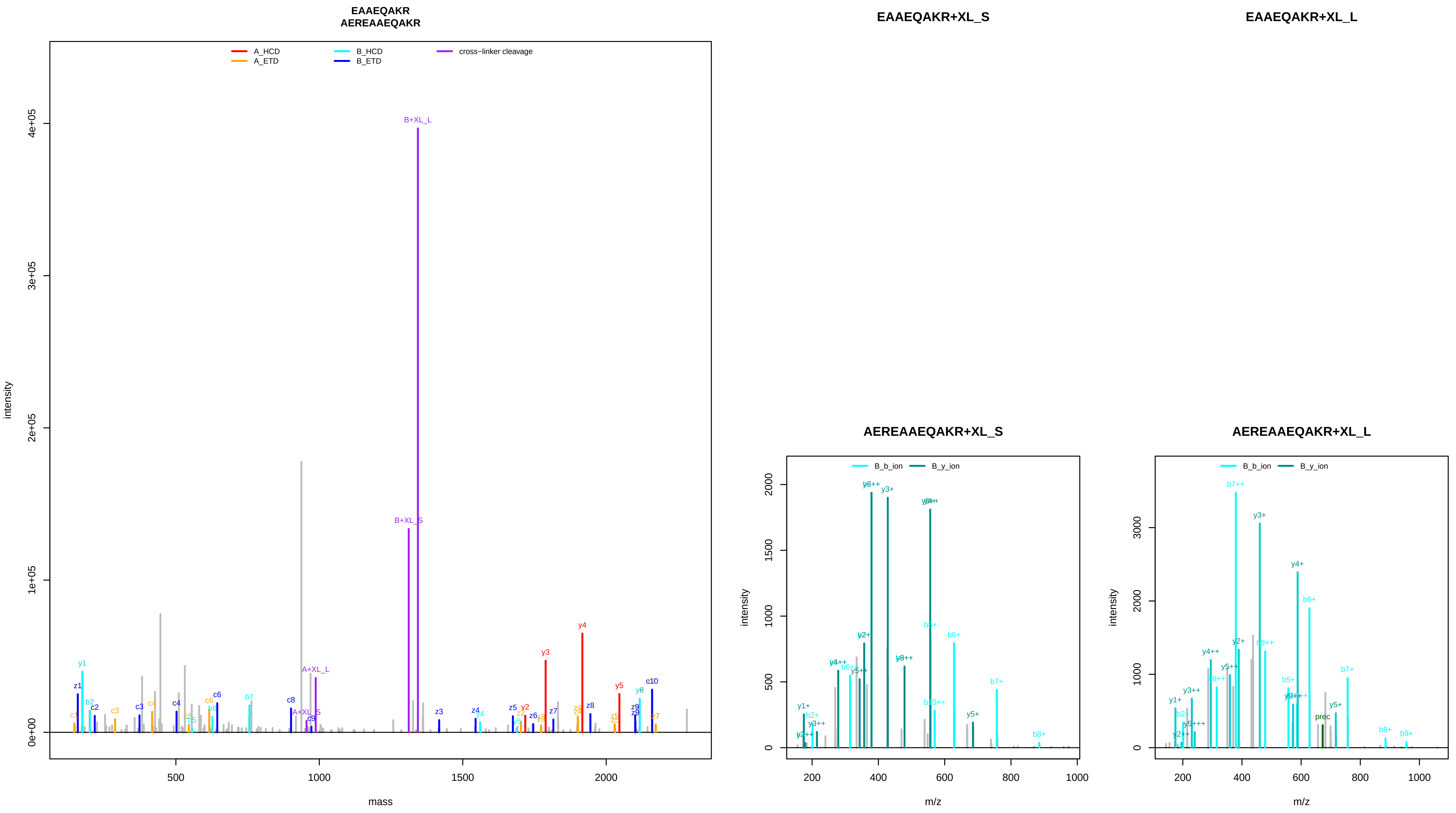
EAAEQAKR
AEREAAEQAKR
A_HCD
A_ETD
B_HCD
B_ETD
cross−linker cleavage
B+XL_L
B+XL_S
A+XL_L
A+XL_S
intensity
mass
EAAEQAKR+XL_S
EAAEQAKR+XL_L
AEREAAEQAKR+XL_S
AEREAAEQAKR+XL_L
B_b_ion
B_y_ion
intensity
m/z
prec

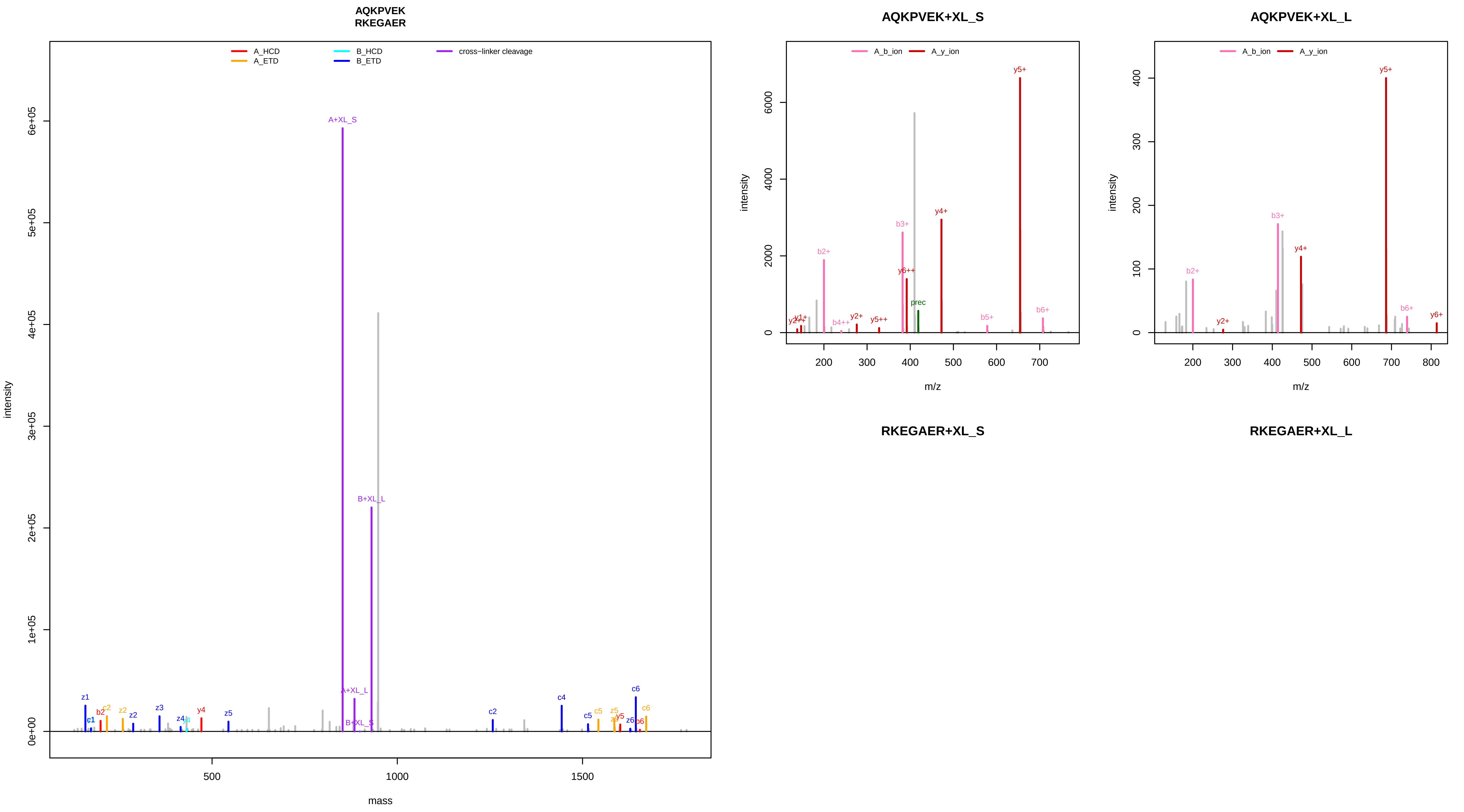
AQKPVEK
RKEGAER
A_HCD
A_ETD
B_HCD
B_ETD
cross−linker cleavage
A+XL_S
B+XL_L
A+XL_L
B+XL_S
intensity
mass
AQKPVEK+XL_S
AQKPVEK+XL_L
A_b_ion
A_y_ion
prec
m/z
RKEGAER+XL_S
RKEGAER+XL_L

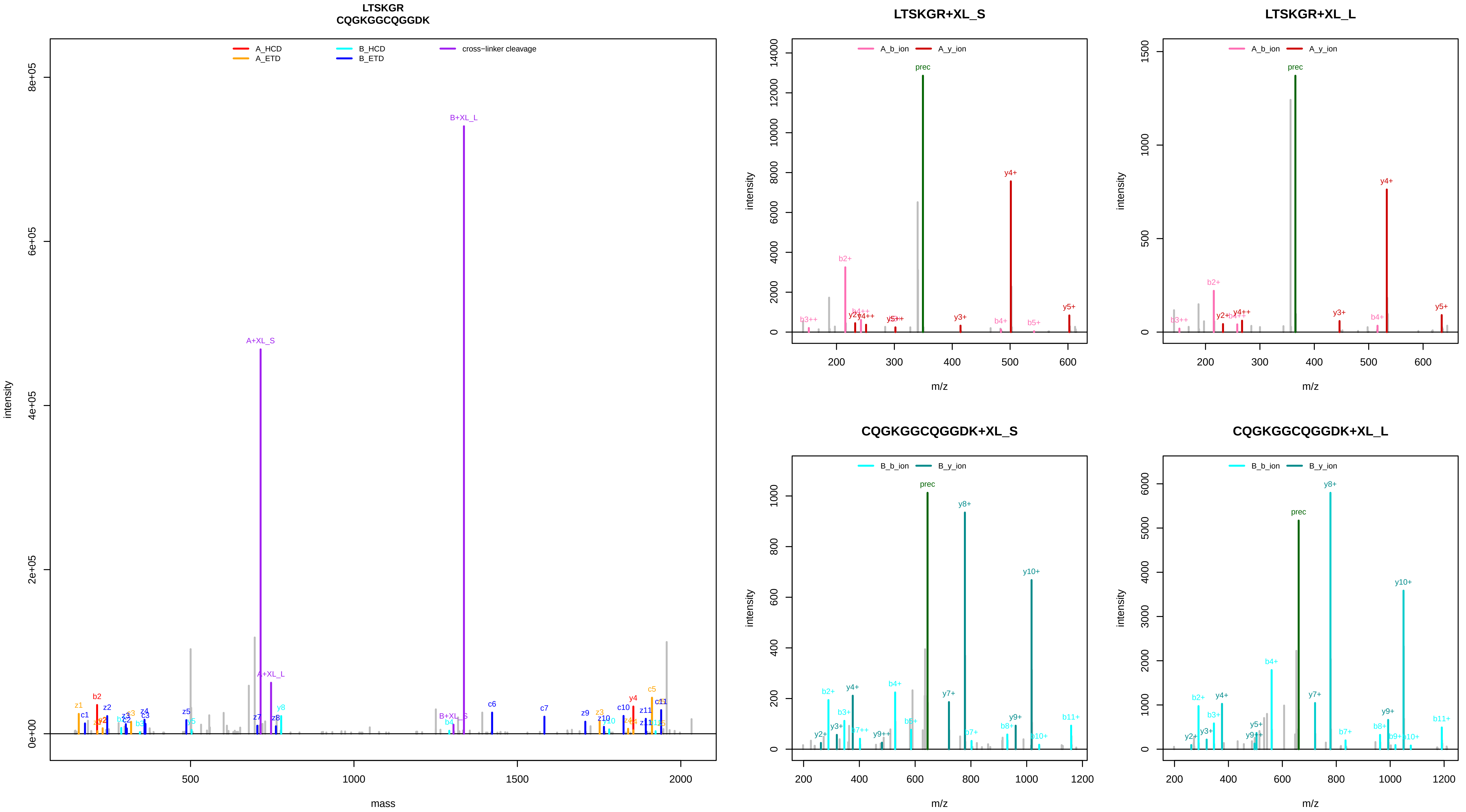
LTSKGR
CQGKGGCQGGDK
A_HCD
A_ETD
B_HCD
B_ETD
cross−linker cleavage
B+XL_L
A+XL_S
A+XL_L
B+XL_S
intensity
mass
LTSKGR+XL_S
LTSKGR+XL_L
CQGKGGCQGGDK+XL_S
CQGKGGCQGGDK+XL_L
A_b_ion
A_y_ion
B_b_ion
B_y_ion
prec
m/z

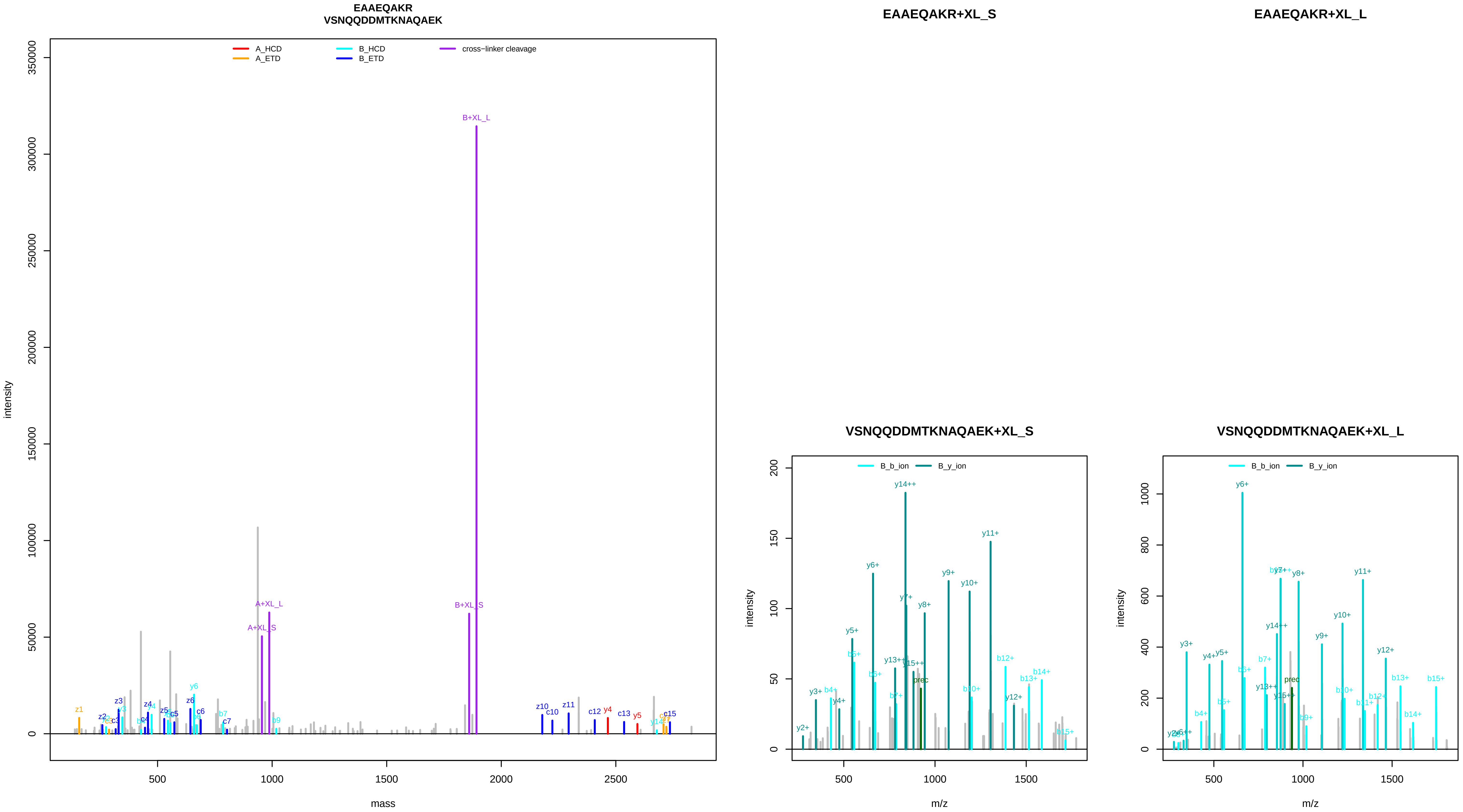
EAAEQAKR
VSNQQDDMTKNAQAEK
A_HCD
A_ETD
B_HCD
B_ETD
cross−linker cleavage
B+XL_L
B+XL_S
A+XL_L
A+XL_S
intensity
mass
EAAEQAKR+XL_S
EAAEQAKR+XL_L
VSNQQDDMTKNAQAEK+XL_S
VSNQQDDMTKNAQAEK+XL_L
B_b_ion
B_y_ion
prec
intensity
m/z

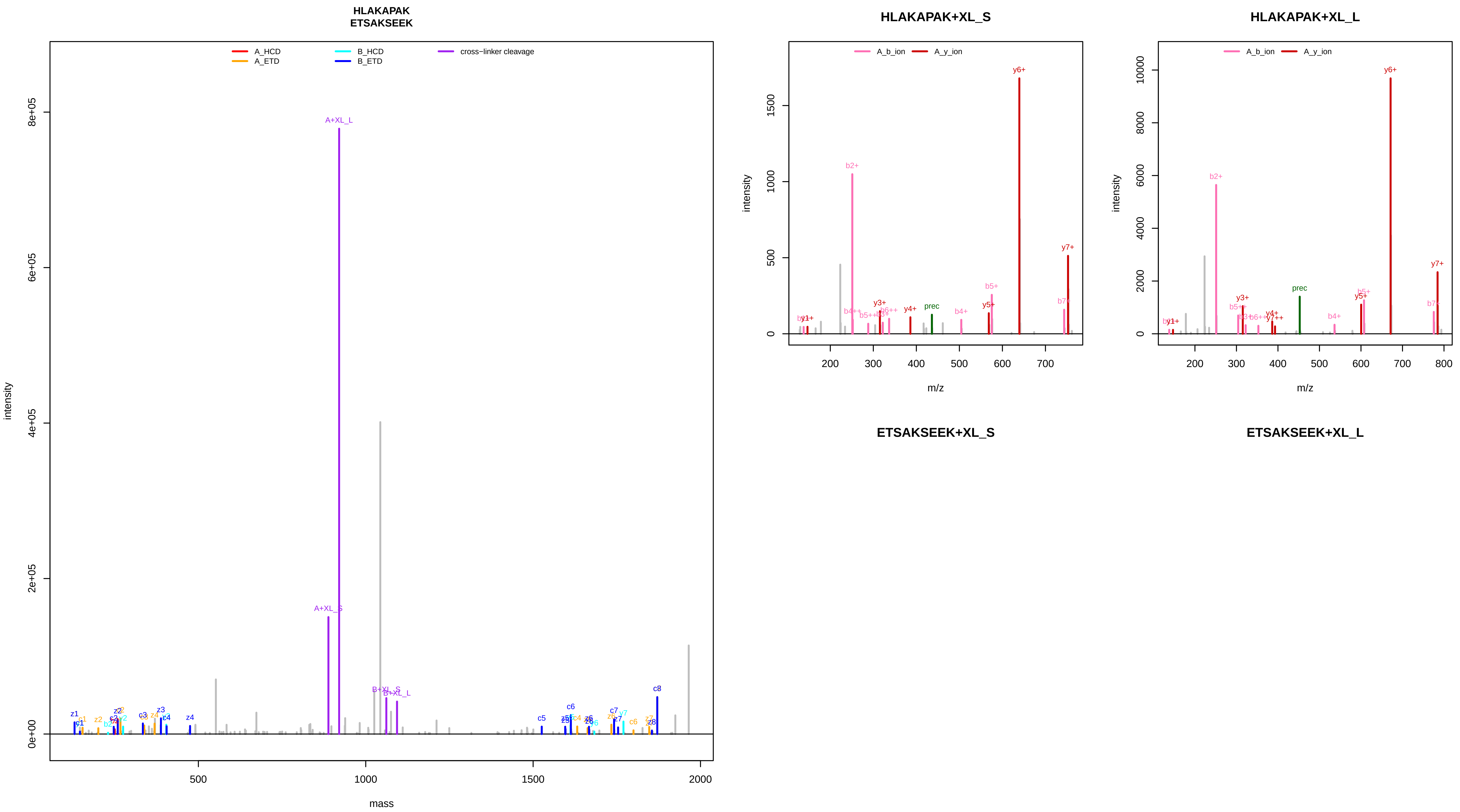
HLAKAPAK
ETSAKSEEK
A_HCD
A_ETD
B_HCD
B_ETD
cross−linker cleavage
A+XL_L
A+XL_S
B+XL_S
B+XL_L
intensity
mass
HLAKAPAK+XL_S
HLAKAPAK+XL_L
A_b_ion
A_y_ion
y6+
y7+
b2+
b5+
prec
m/z
ETSAKSEEK+XL_S
ETSAKSEEK+XL_L

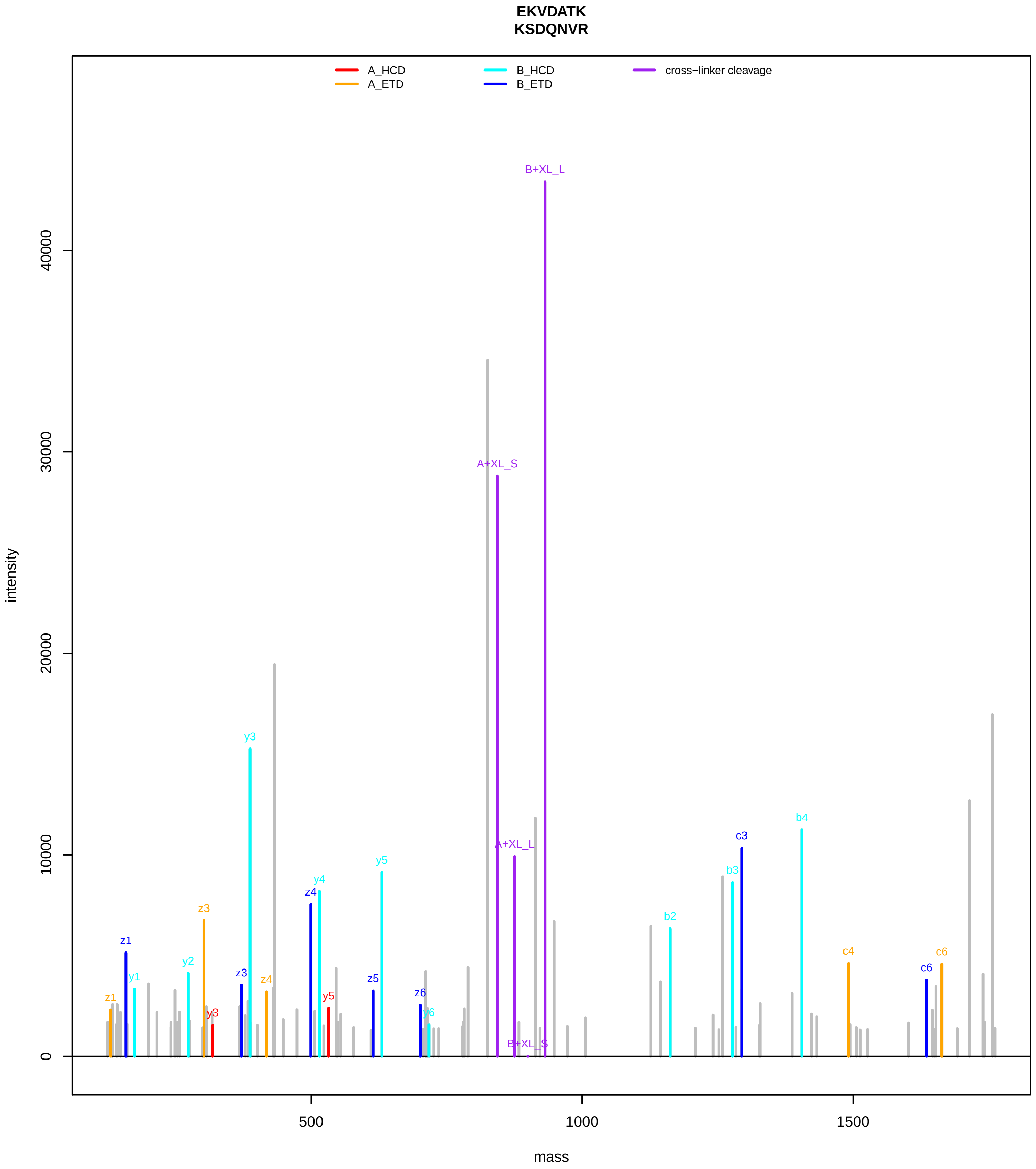

EKVDATK+XL_S

EKVDATK+XL_L

KSDQNVR+XL_S

KSDQNVR+XL_L

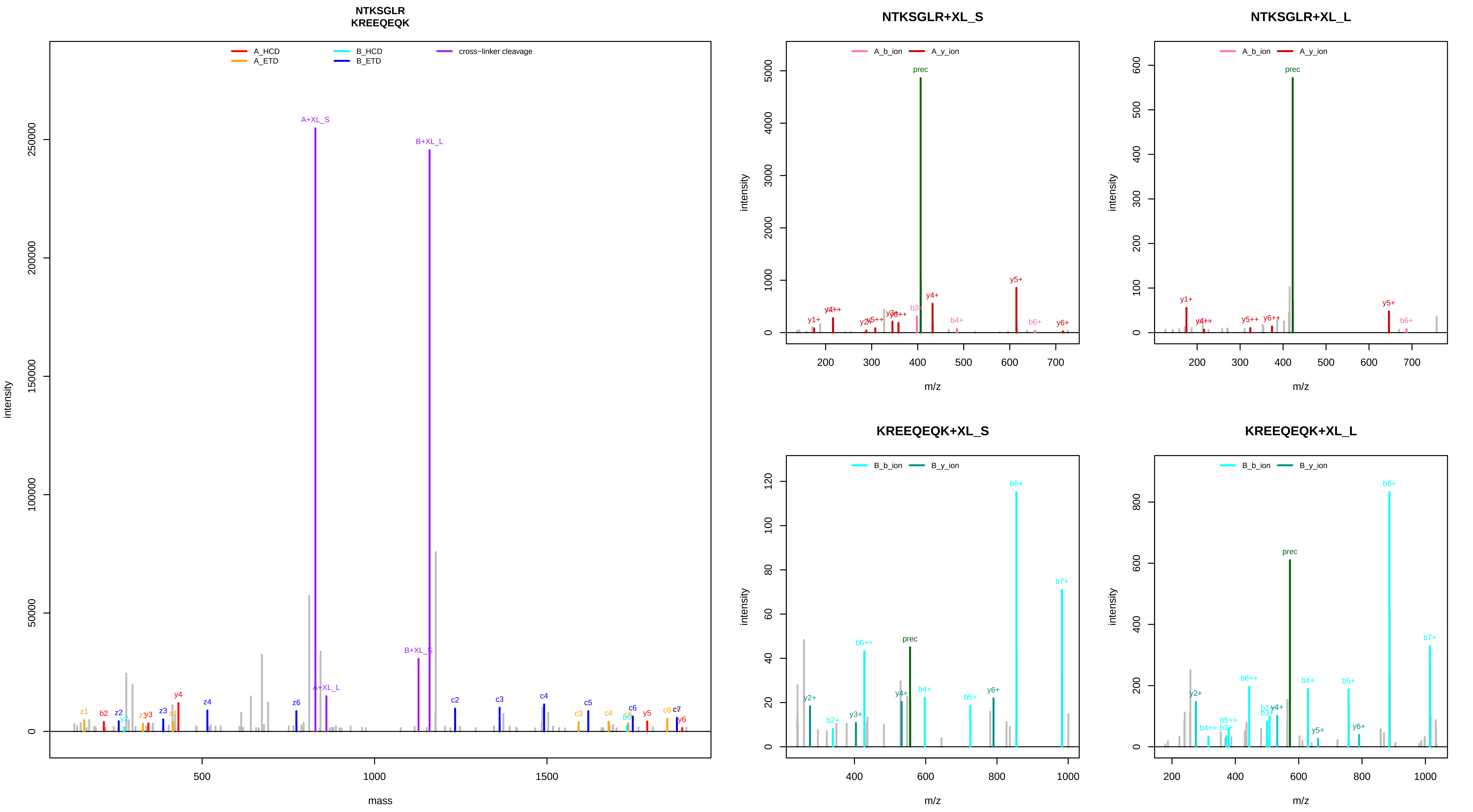
NTKSGLR
KREEQEQK
A_HCD
A_ETD
B_HCD
B_ETD
cross−linker cleavage
A+XL_S
B+XL_L
B+XL_S
A+XL_L
intensity
mass
NTKSGLR+XL_S
NTKSGLR+XL_L
KREEQEQK+XL_S
KREEQEQK+XL_L
A_b_ion
A_y_ion
B_b_ion
B_y_ion
prec
m/z

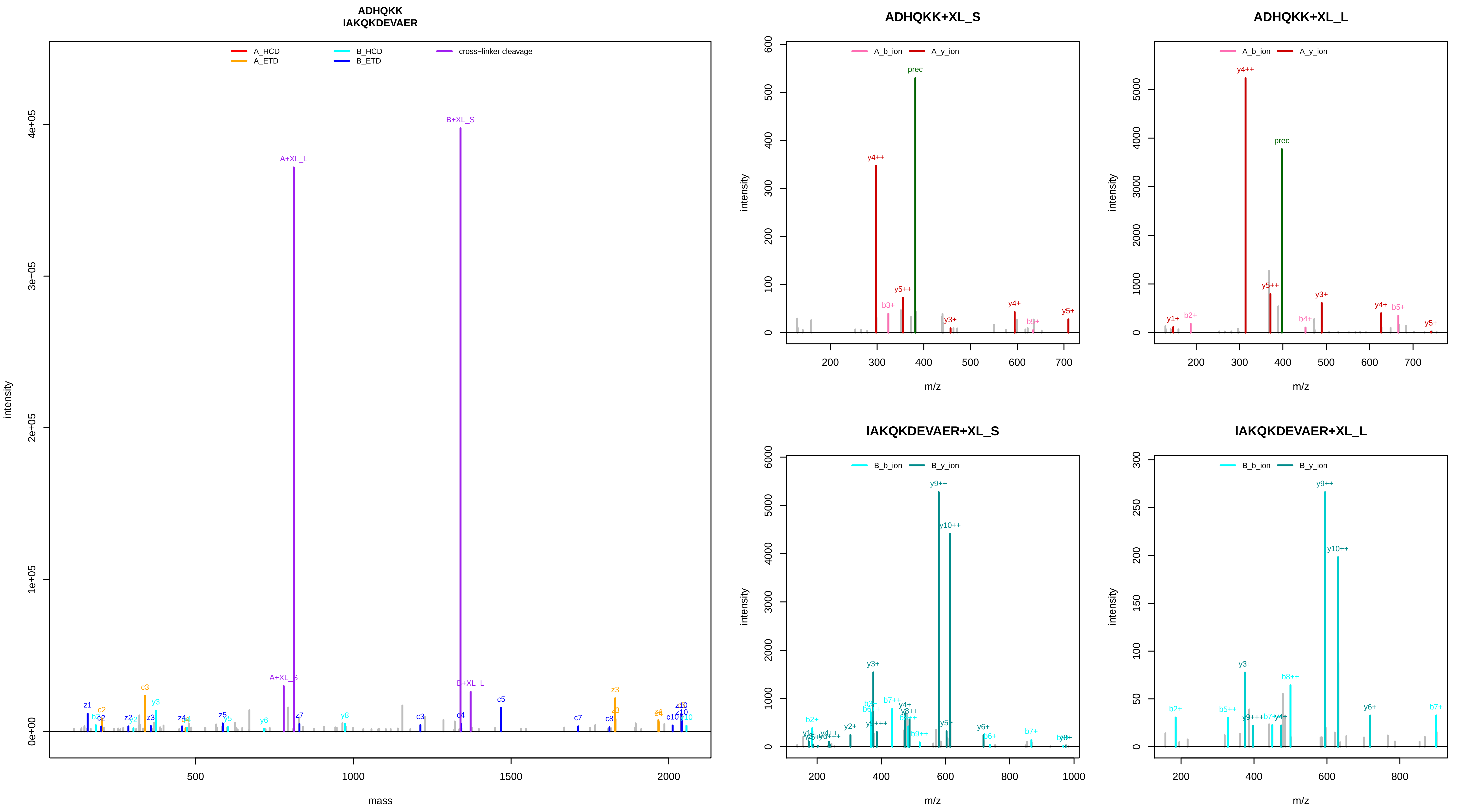
ADHQKK
IAKQKDEVAER
A_HCD
A_ETD
B_HCD
B_ETD
cross−linker cleavage
A+XL_L
B+XL_S
A+XL_S
B+XL_L
intensity
mass
ADHQKK+XL_S
ADHQKK+XL_L
IAKQKDEVAER+XL_S
IAKQKDEVAER+XL_L
A_b_ion
A_y_ion
B_b_ion
B_y_ion
prec
m/z

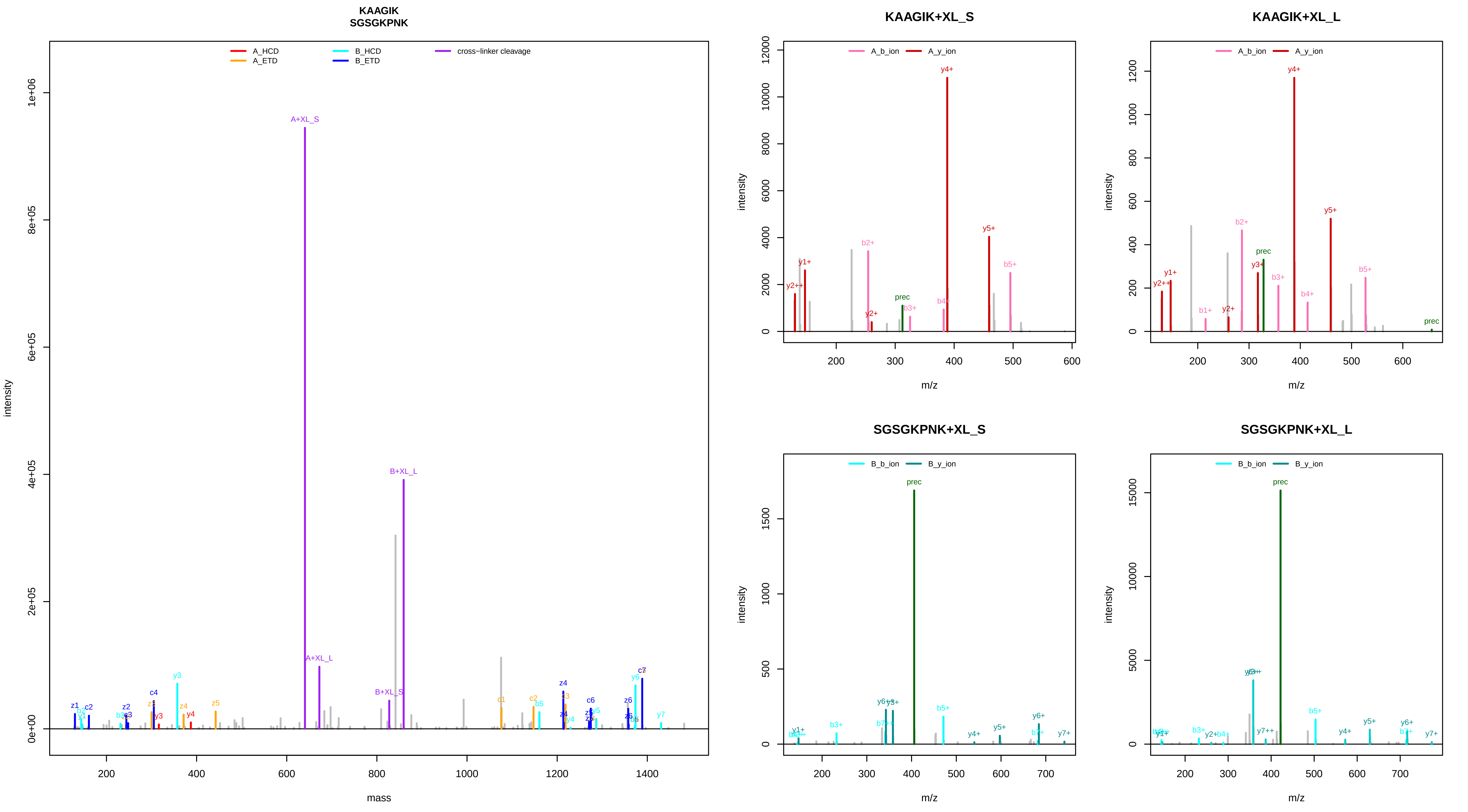
KAAGIK
SGSGKPNK
A_HCD
A_ETD
B_HCD
B_ETD
cross−linker cleavage
A+XL_S
B+XL_L
A+XL_L
B+XL_S
intensity
mass
KAAGIK+XL_S
KAAGIK+XL_L
SGSGKPNK+XL_S
SGSGKPNK+XL_L
A_b_ion
A_y_ion
B_b_ion
B_y_ion
prec
m/z

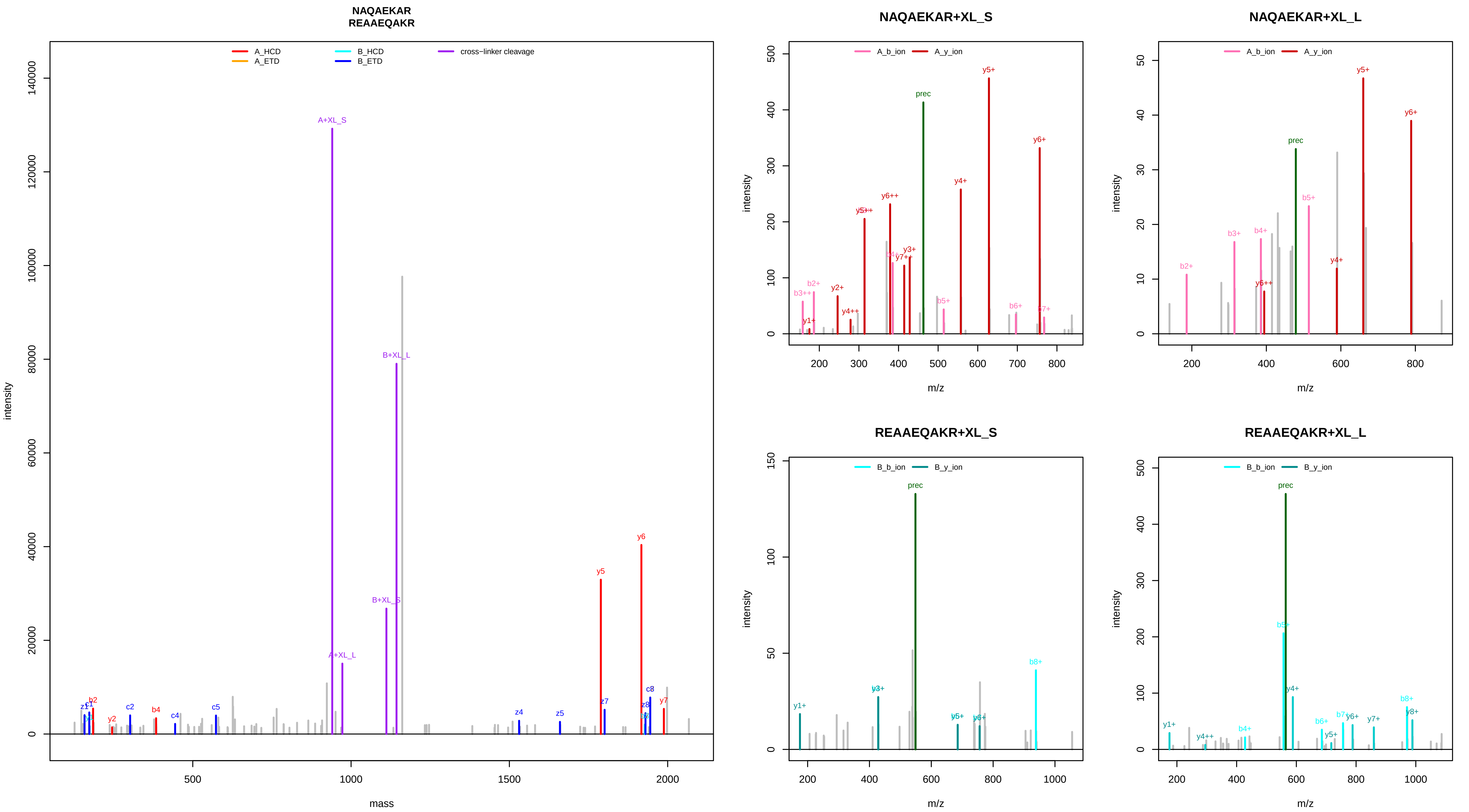
NAQAEKAR
REAAEQAKR
A_HCD
A_ETD
B_HCD
B_ETD
cross−linker cleavage
A+XL_S
B+XL_L
B+XL_S
A+XL_L
intensity
mass
NAQAEKAR+XL_S
NAQAEKAR+XL_L
A_b_ion
A_y_ion
REAAEQAKR+XL_S
REAAEQAKR+XL_L
B_b_ion
B_y_ion
prec
m/z

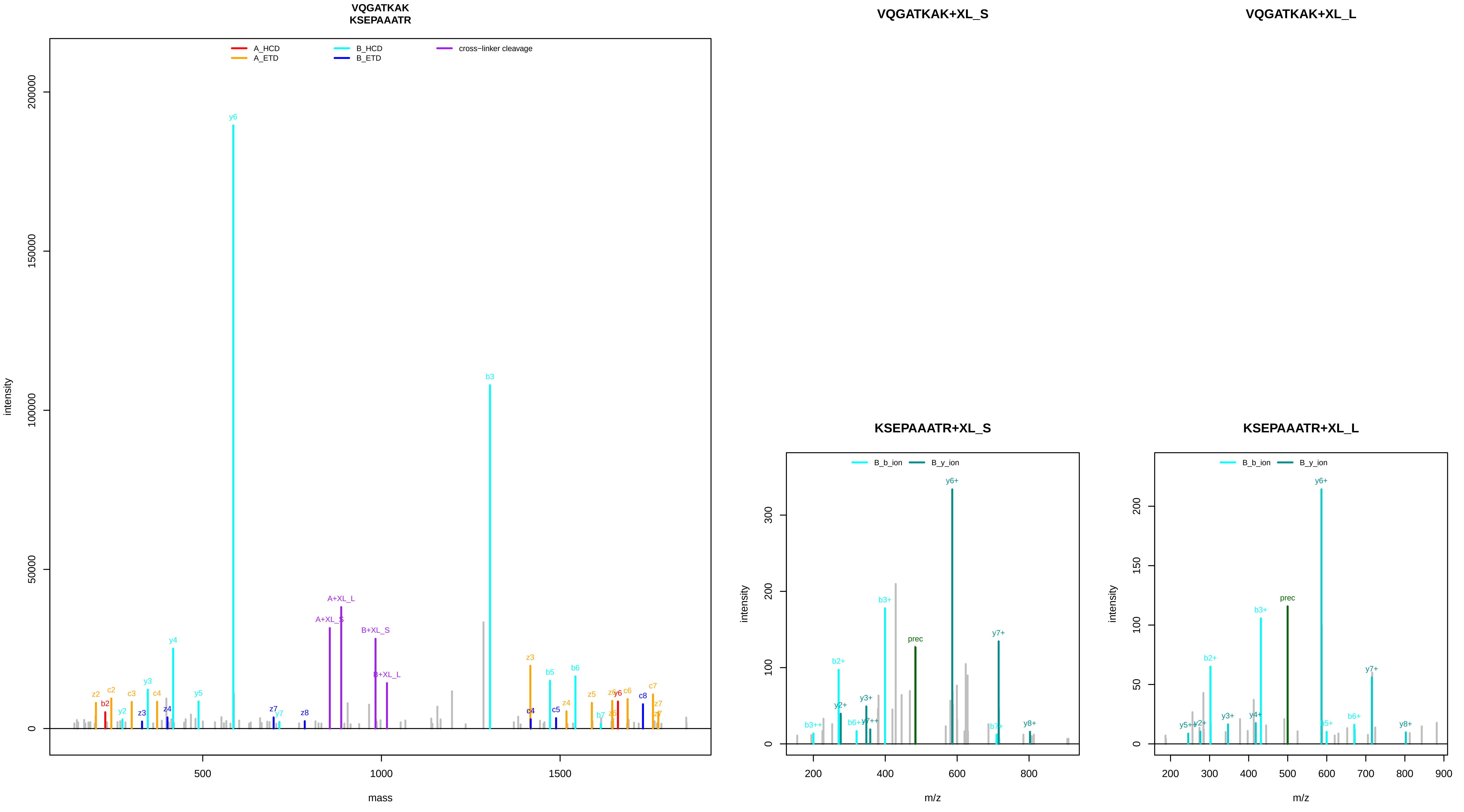
VQGATKAK
KSEPAAATR
A_HCD
A_ETD
B_HCD
B_ETD
cross−linker cleavage
intensity
mass
y6
b3
A+XL_L
A+XL_S
B+XL_S
B+XL_L
y4
y3
y5
y2
z2
c2
c3
c4
b2
z3
z4
z7
y7
z8
z3
c4
b5
b6
c5
z4
z5
b7
c6
c7
c8
z7
VQGATKAK+XL_S
VQGATKAK+XL_L
KSEPAAATR+XL_S
KSEPAAATR+XL_L
B_b_ion
B_y_ion
y6+
y7+
b3+
prec
b2+
y2+
y3+
y8+
b3++
b6+
y7++
y4+
b5+
b6+
m/z

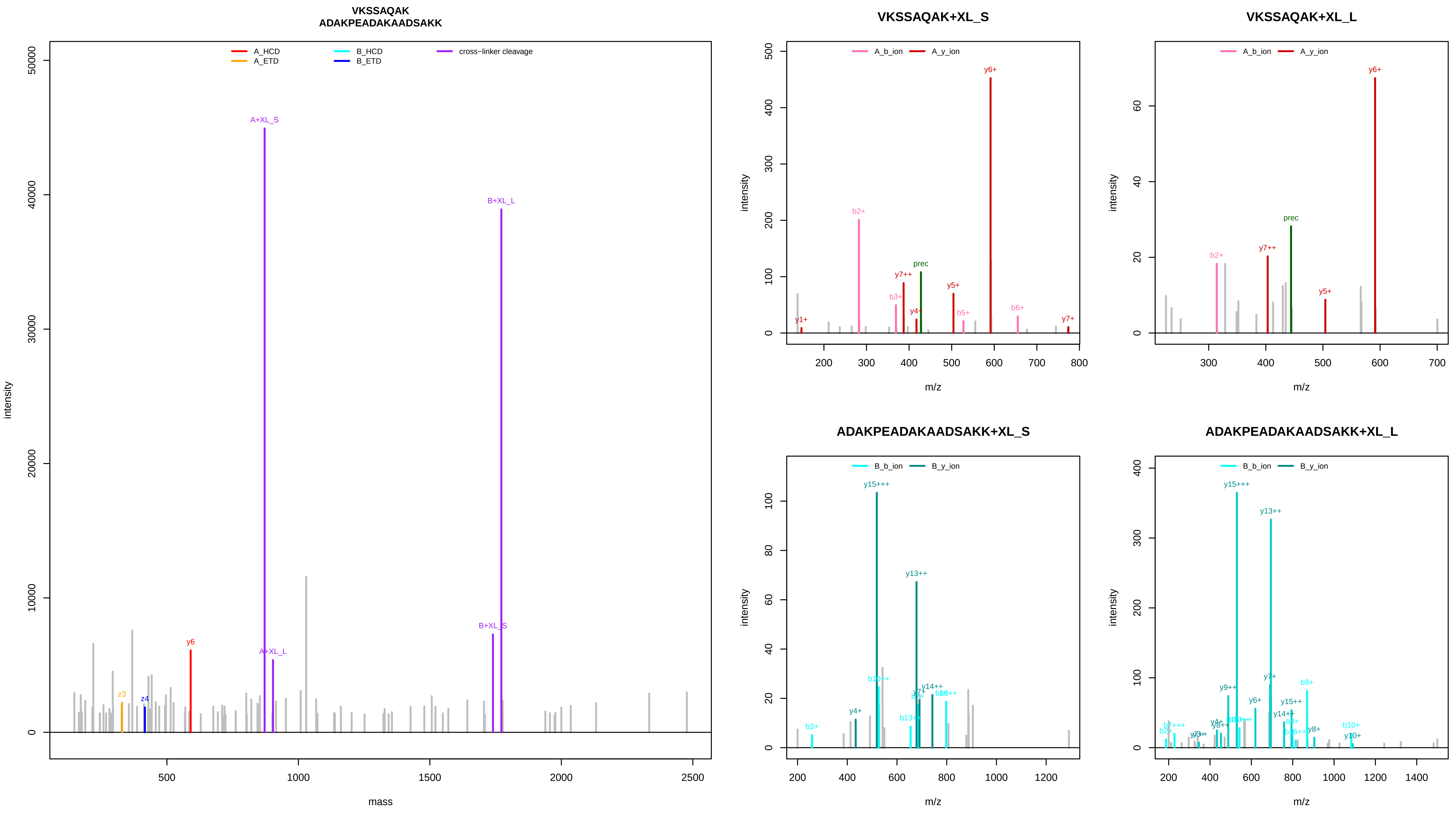

VKSSAQAK
ADAKPEADAKAADSAKK
A_HCD
A_ETD
B_HCD
B_ETD
cross−linker cleavage
A+XL_S
B+XL_L
B+XL_S
A+XL_L
y6
z3
z4
intensity
mass
VKSSAQAK+XL_S
VKSSAQAK+XL_L
ADAKPEADAKAADSAKK+XL_S
ADAKPEADAKAADSAKK+XL_L
A_b_ion
A_y_ion
B_b_ion
B_y_ion
prec
m/z

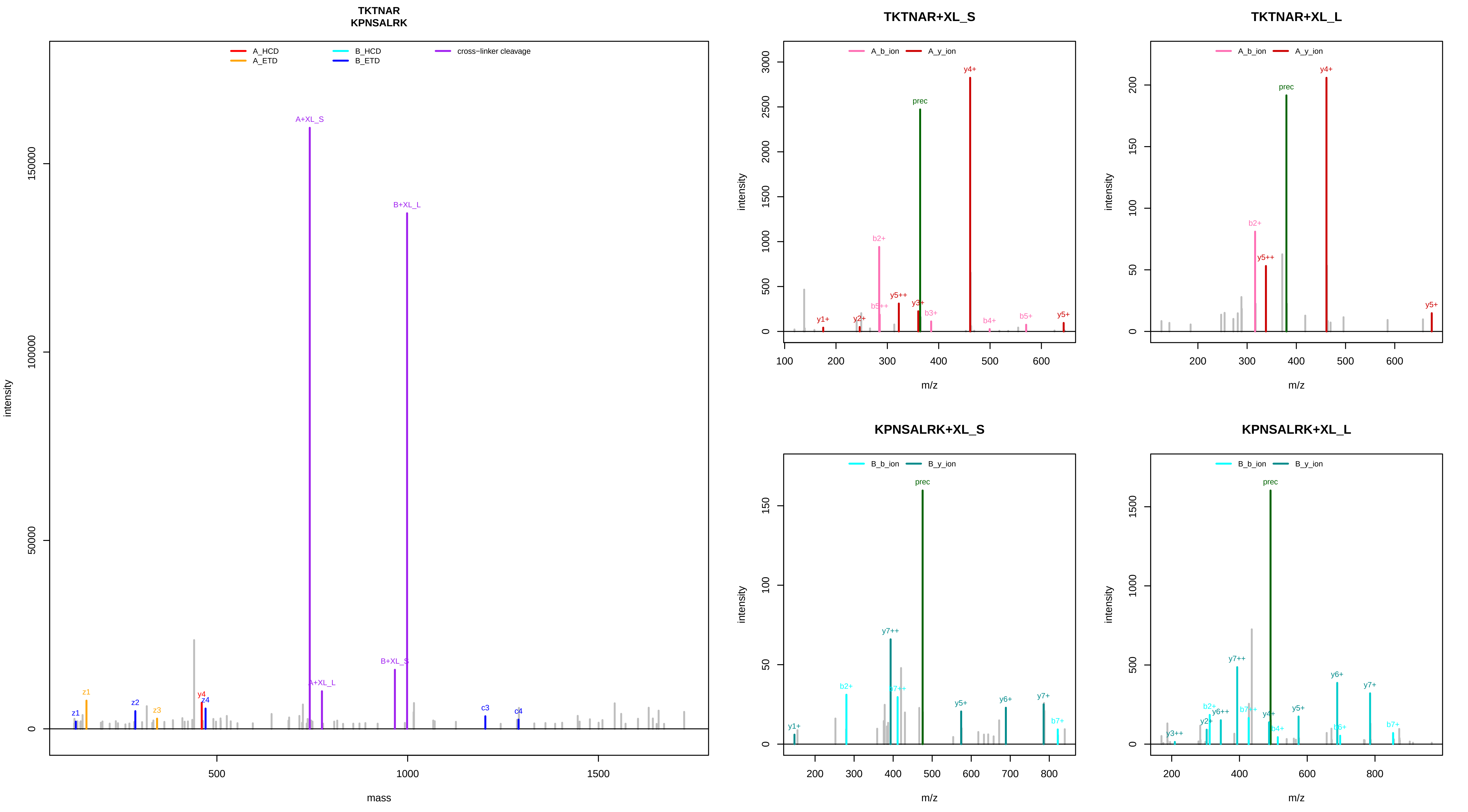
TKTNAR
KPNSALRK
A_HCD
A_ETD
B_HCD
B_ETD
cross−linker cleavage
A+XL_S
B+XL_L
B+XL_S
A+XL_L
z1
z2
z3
y4
z4
c3
c4
intensity
mass
TKTNAR+XL_S
TKTNAR+XL_L
KPNSALRK+XL_S
KPNSALRK+XL_L
A_b_ion
A_y_ion
B_b_ion
B_y_ion
prec
y1+
y2+
b5++
b2+
y5++
y3+
b3+
y4+
b4+
b5+
y5+
y7++
y7+
y6+
y6++
b7++
b7+
b6+
y3++
m/z

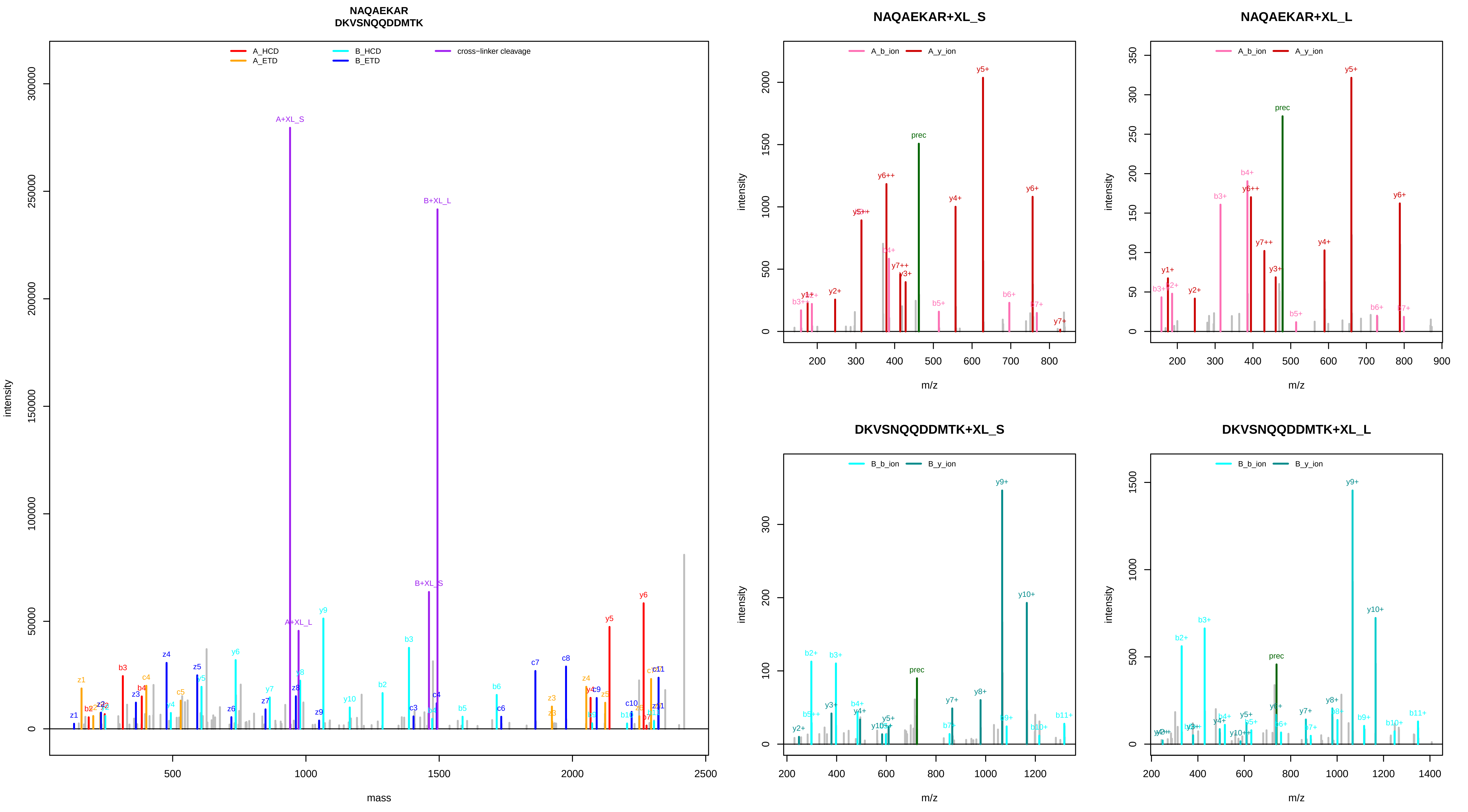

NAQAEKAR
DKVSNQQDDMTK
A_HCD
A_ETD
B_HCD
B_ETD
cross−linker cleavage
A+XL_S
B+XL_L
B+XL_S
A+XL_L
intensity
mass
NAQAEKAR+XL_S
NAQAEKAR+XL_L
DKVSNQQDDMTK+XL_S
DKVSNQQDDMTK+XL_L
A_b_ion
A_y_ion
B_b_ion
B_y_ion
prec
m/z

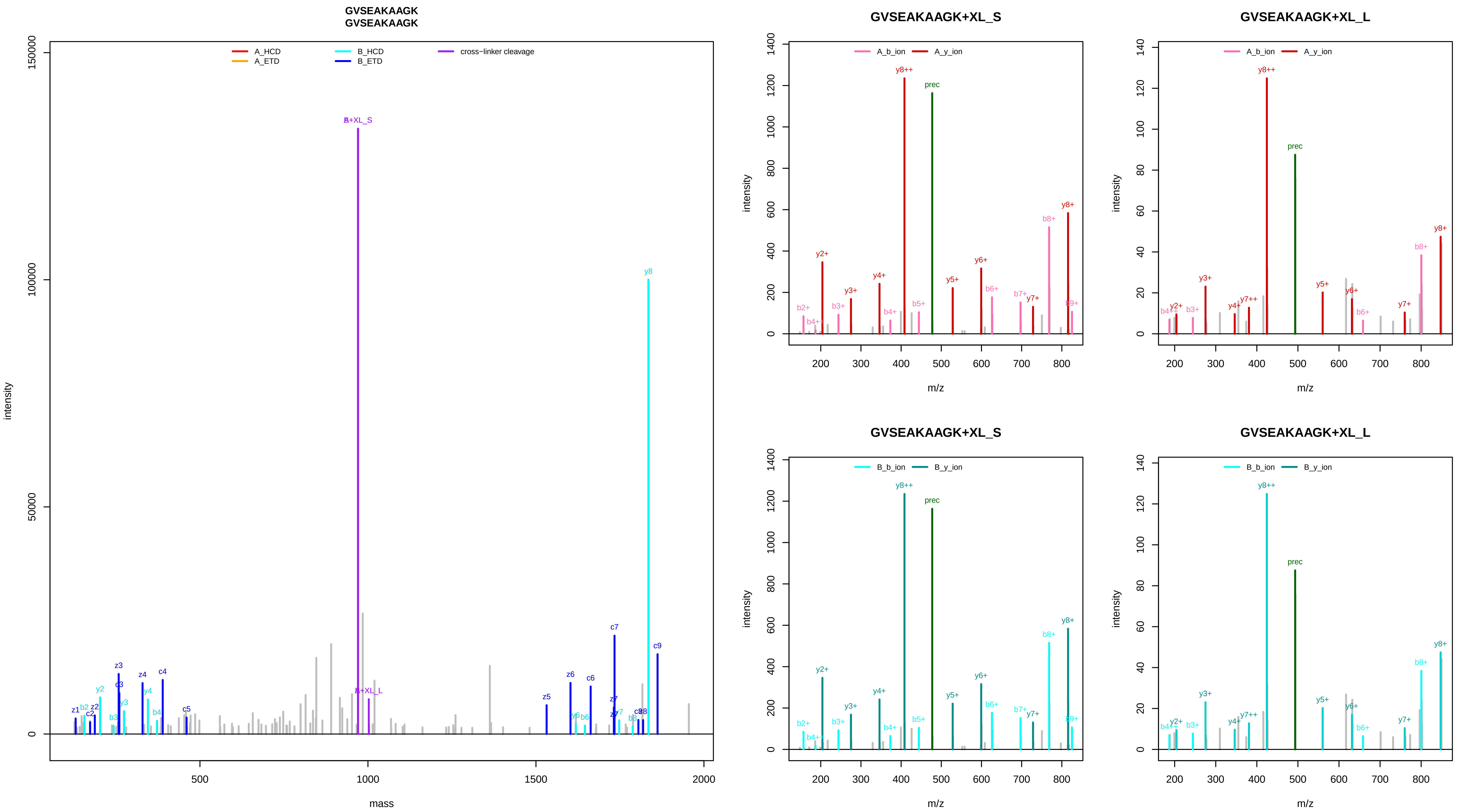
GVSEAKAAGK
GVSEAKAAGK
A_HCD
A_ETD
B_HCD
B_ETD
cross−linker cleavage
intensity
mass
GVSEAKAAGK+XL_S
GVSEAKAAGK+XL_L
A_b_ion
A_y_ion
B_b_ion
B_y_ion
prec
m/z

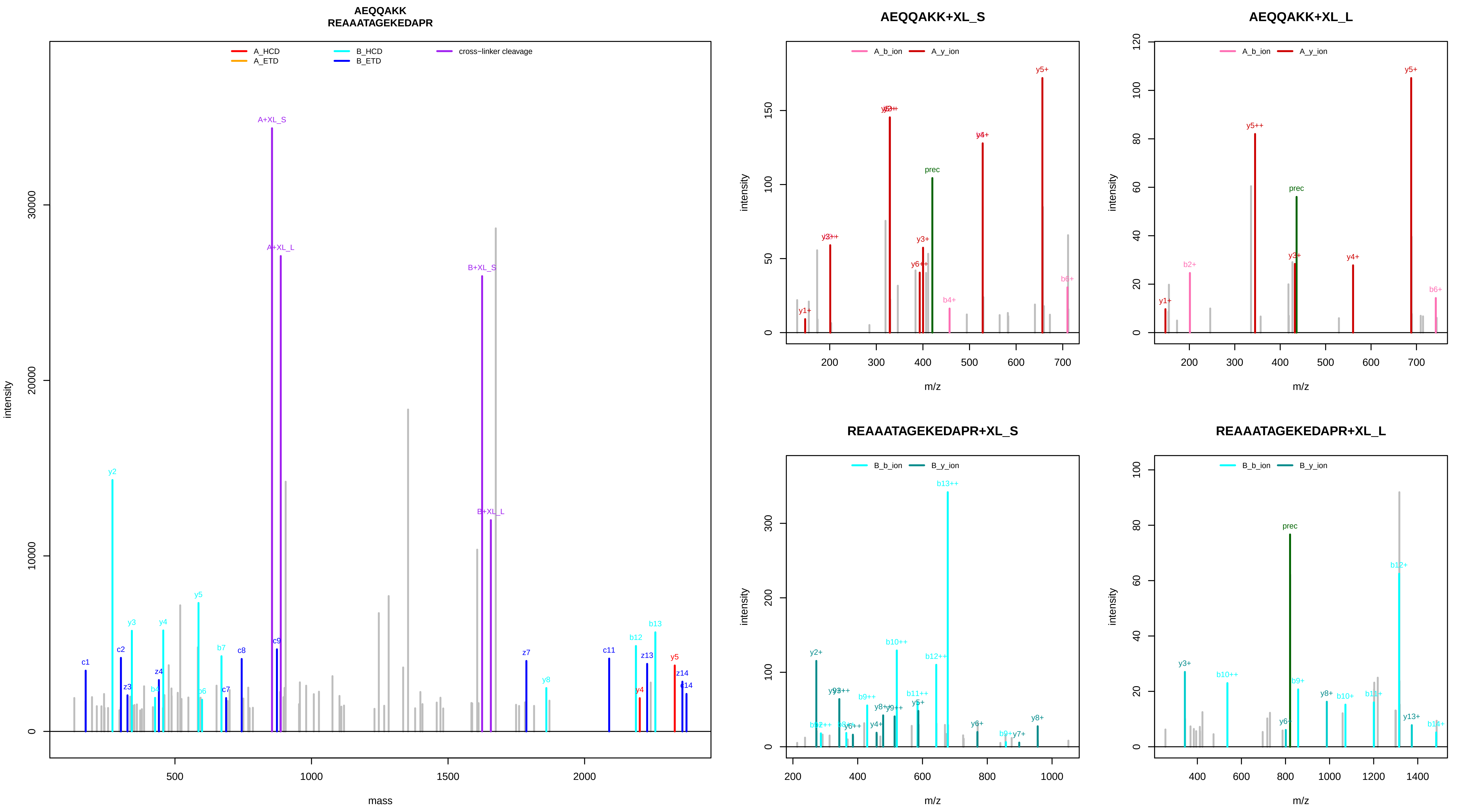
AEQQAKK
REAAATAGEKEDAPR
A_HCD
A_ETD
B_HCD
B_ETD
cross−linker cleavage
A+XL_S
A+XL_L
B+XL_S
B+XL_L
intensity
mass
AEQQAKK+XL_S
AEQQAKK+XL_L
A_b_ion
A_y_ion
prec
m/z
REAAATAGEKEDAPR+XL_S
REAAATAGEKEDAPR+XL_L
B_b_ion
B_y_ion
b13++
b10++
b12++
b12+

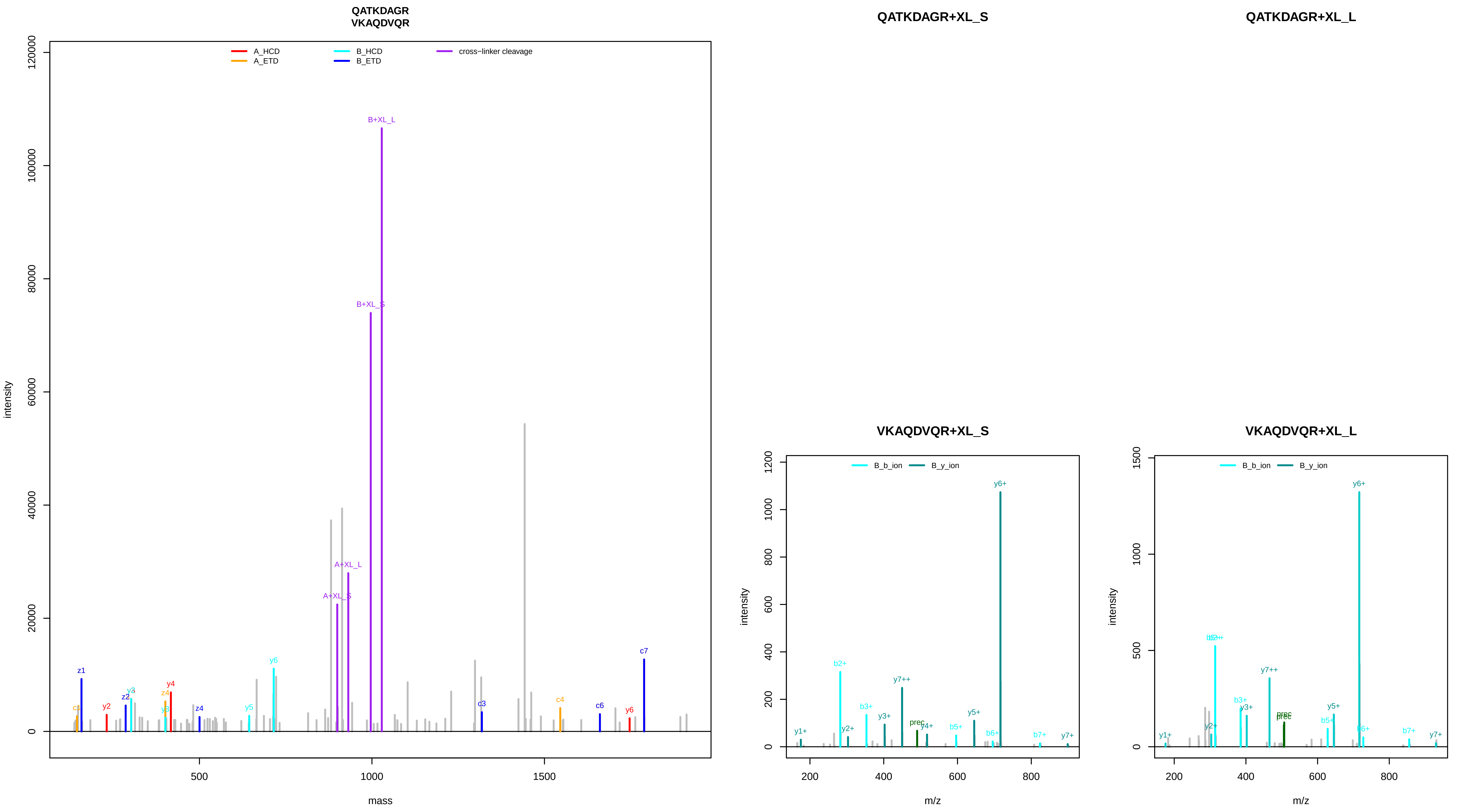
QATKDAGR
VKAQDVQR
A_HCD
A_ETD
B_HCD
B_ETD
cross−linker cleavage
B+XL_L
B+XL_S
A+XL_L
A+XL_S
intensity
mass
0
20000
40000
60000
80000
100000
120000
500
1000
1500
z1 c1 y2 z2 y3 z4 y4 z4 y5 y6 c3 c4 c6 y6 c7
QATKDAGR+XL_S
QATKDAGR+XL_L
VKAQDVQR+XL_S
VKAQDVQR+XL_L
B_b_ion
B_y_ion
intensity
m/z
0
200
400
600
800
1000
1200
1500
y1+ b2+ y2+ b3+ y3+ y7++ prec y4+ b5+ y5+ b6+ y6+ b7+ y7+

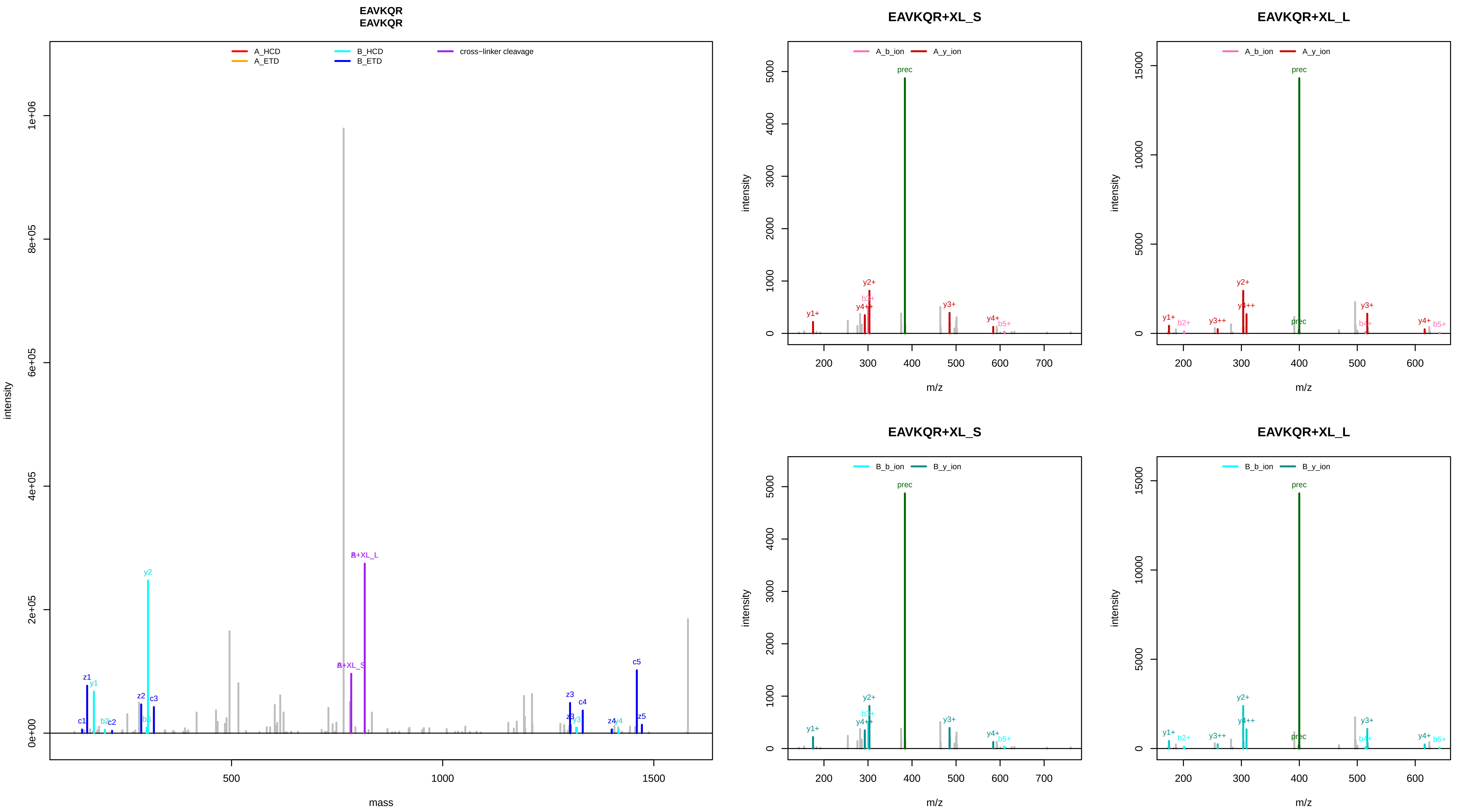
EAVKQR
EAVKQR
A_HCD
A_ETD
B_HCD
B_ETD
cross−linker cleavage
intensity
mass
EAVKQR+XL_S
EAVKQR+XL_L
A_b_ion
A_y_ion
B_b_ion
B_y_ion
prec
intensity
m/z

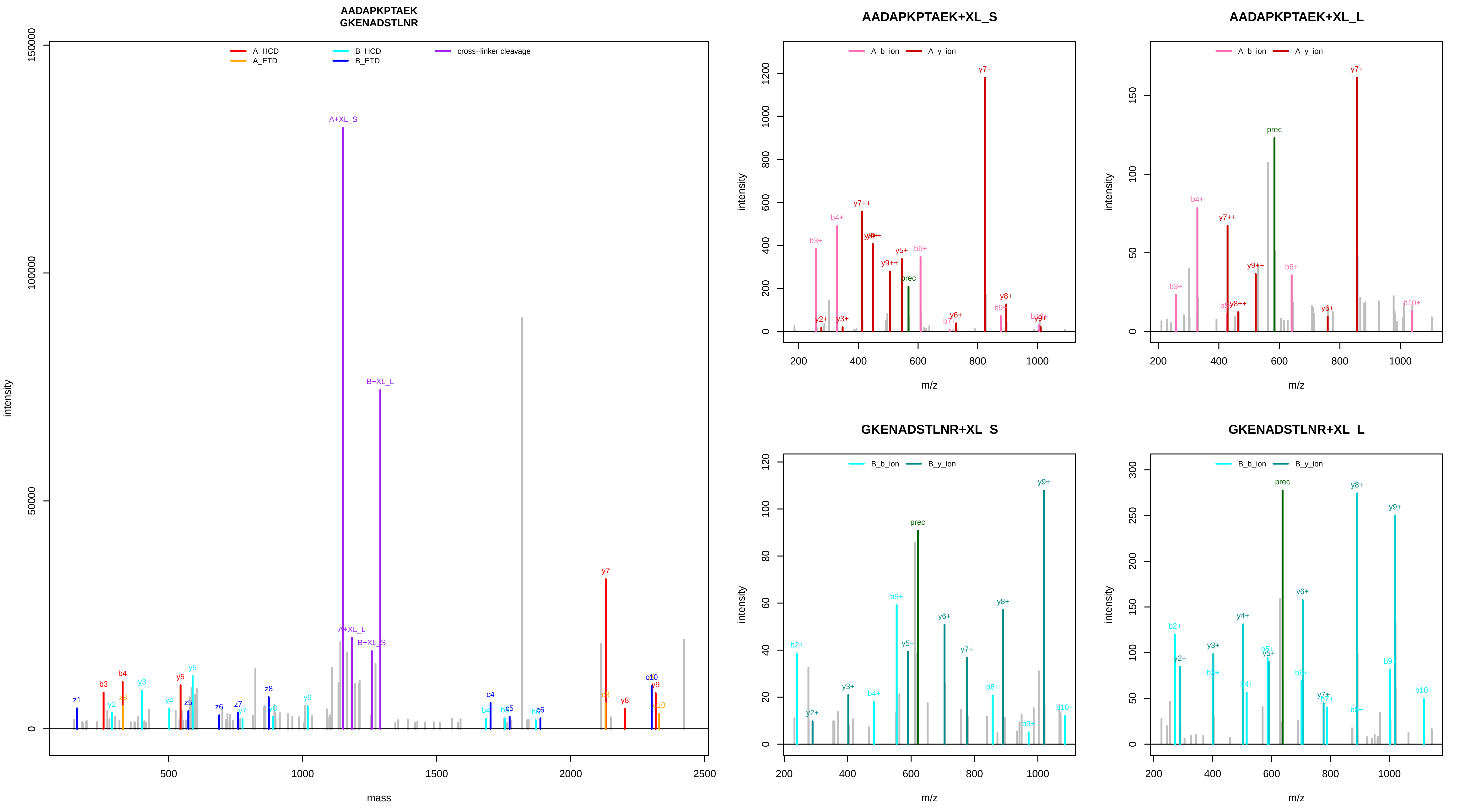
AADAPKPTAEK
GKENADSTLNR
A_HCD
A_ETD
B_HCD
B_ETD
cross−linker cleavage
A+XL_S
B+XL_L
A+XL_L
B+XL_S
intensity
mass
AADAPKPTAEK+XL_S
AADAPKPTAEK+XL_L
GKENADSTLNR+XL_S
GKENADSTLNR+XL_L
A_b_ion
A_y_ion
B_b_ion
B_y_ion
prec
m/z

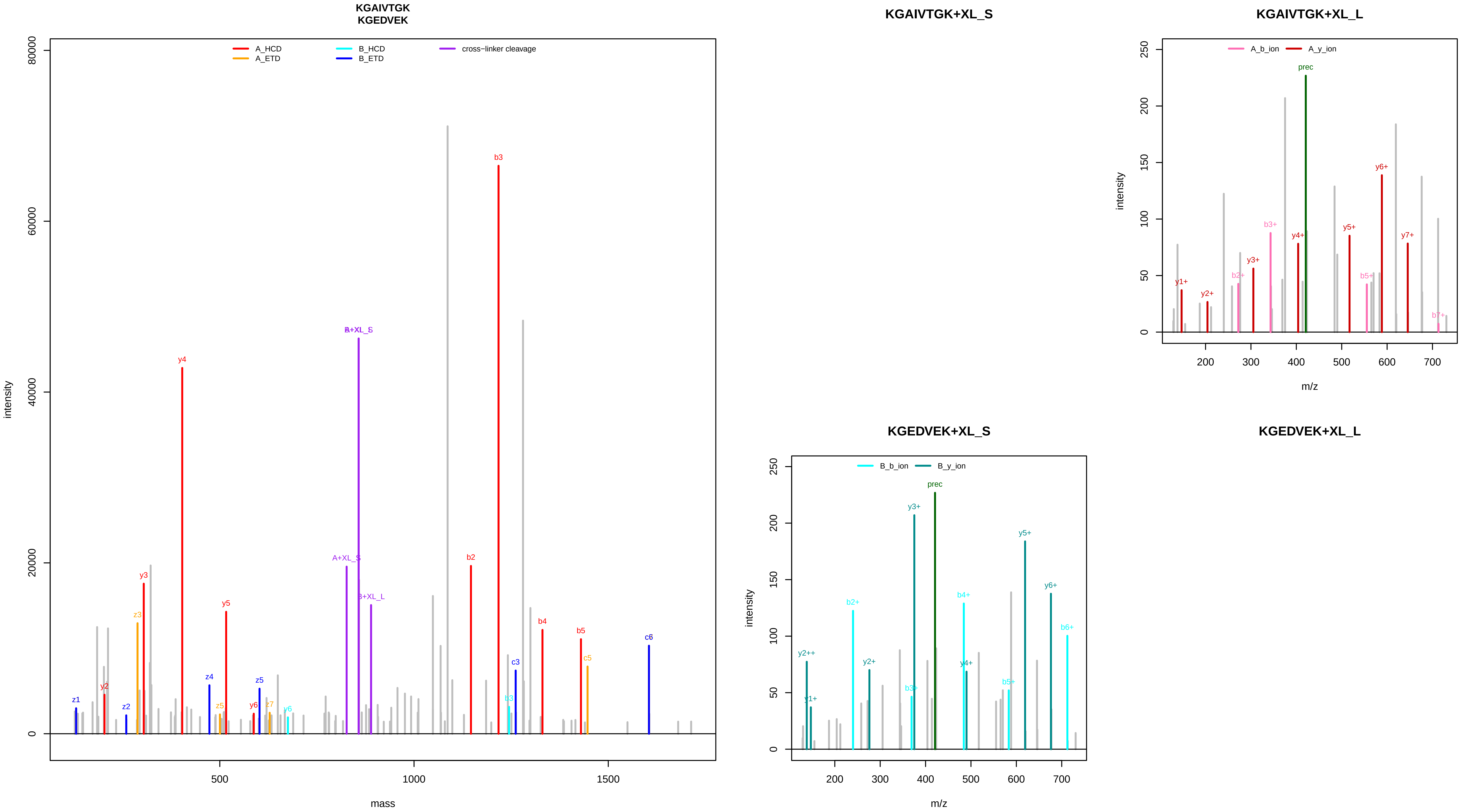
KGAIVTGK
KGEDVEK
A_HCD
A_ETD
B_HCD
B_ETD
cross−linker cleavage
intensity
mass
y4
b3
b2
y3
y5
z3
b4
b5
c5
c6
c3
z4
z5
z1
y2
z2
z5
y6
z7
y6
A+XL_S
B+XL_L
KGAIVTGK+XL_S
KGAIVTGK+XL_L
A_b_ion
A_y_ion
prec
y1+
y2+
b2+
y3+
b3+
y4+
y5+
b5+
y6+
y7+
b7+
m/z
KGEDVEK+XL_S
KGEDVEK+XL_L
B_b_ion
B_y_ion
y2++
y1+
b2+
y2+
y3+
b3+
b4+
y4+
b5+
y5+
y6+
b6+

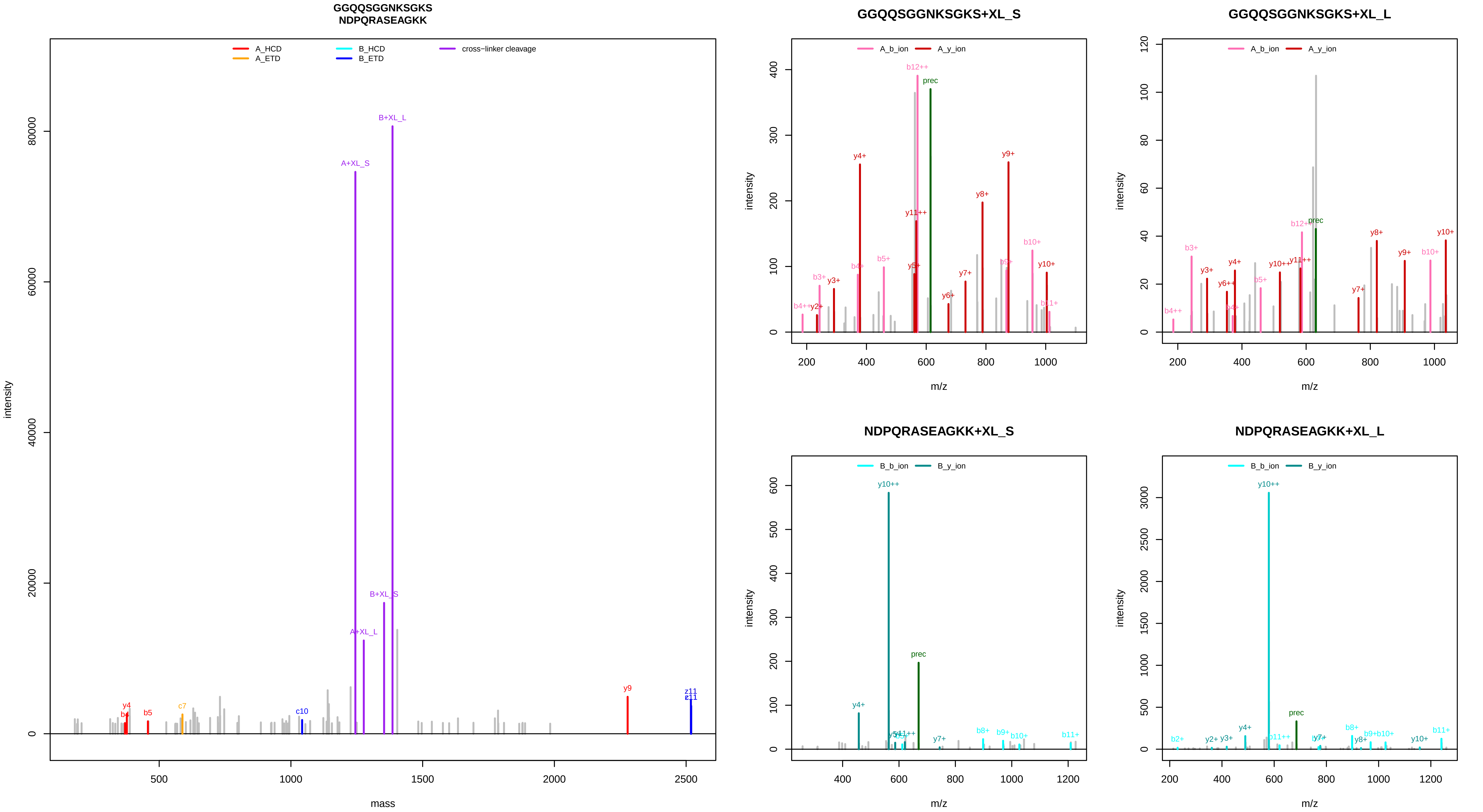

GGQQSGGNKSGKS
NDPQRASEAGKK
A_HCD
A_ETD
B_HCD
B_ETD
cross−linker cleavage
B+XL_L
A+XL_S
B+XL_S
A+XL_L
y4
b4
b5
c7
c10
y9
z11
z11
intensity
mass
0
20000
40000
60000
80000
500
1000
1500
2000
2500
GGQQSGGNKSGKS+XL_S
A_b_ion
A_y_ion
b12++
prec
y4+
y9+
y8+
y11++
b10+
b5+
y5+
y10+
b4+
b3+
y3+
y7+
b9+
b4++
y2+
y6+
b11+
intensity
m/z
GGQQSGGNKSGKS+XL_L
b12++
prec
y8+
y10+
b3+
y3+
y4+
y9+
b10+
y10++
y11++
y6++
b5+
y7+
b4++
b4+
intensity
m/z
NDPQRASEAGKK+XL_S
B_b_ion
B_y_ion
y10++
prec
y4+
y11++
y7+
b8+
b9+
b10+
b11+
intensity
m/z
NDPQRASEAGKK+XL_L
y10++
prec
y4+
b2+
y2+
y3+
b11++
y7+
b8+
y8+
b9+b10+
y10+
b11+
intensity
m/z

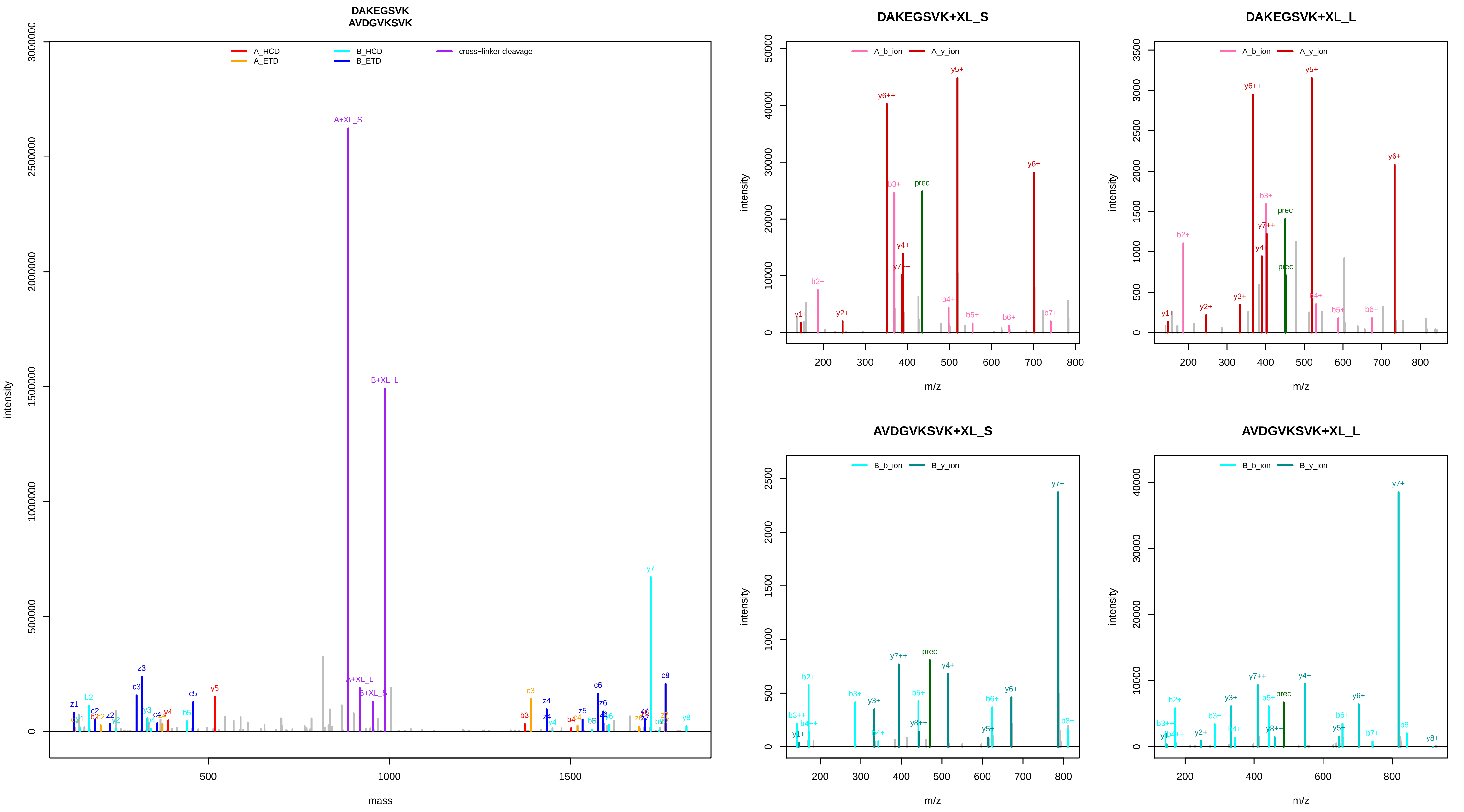
DAKEGSVK
AVDGVKSVK
A_HCD
A_ETD
B_HCD
B_ETD
cross−linker cleavage
A+XL_S
B+XL_L
A+XL_L
B+XL_S
intensity
mass
DAKEGSVK+XL_S
DAKEGSVK+XL_L
AVDGVKSVK+XL_S
AVDGVKSVK+XL_L
A_b_ion
A_y_ion
B_b_ion
B_y_ion
prec
m/z

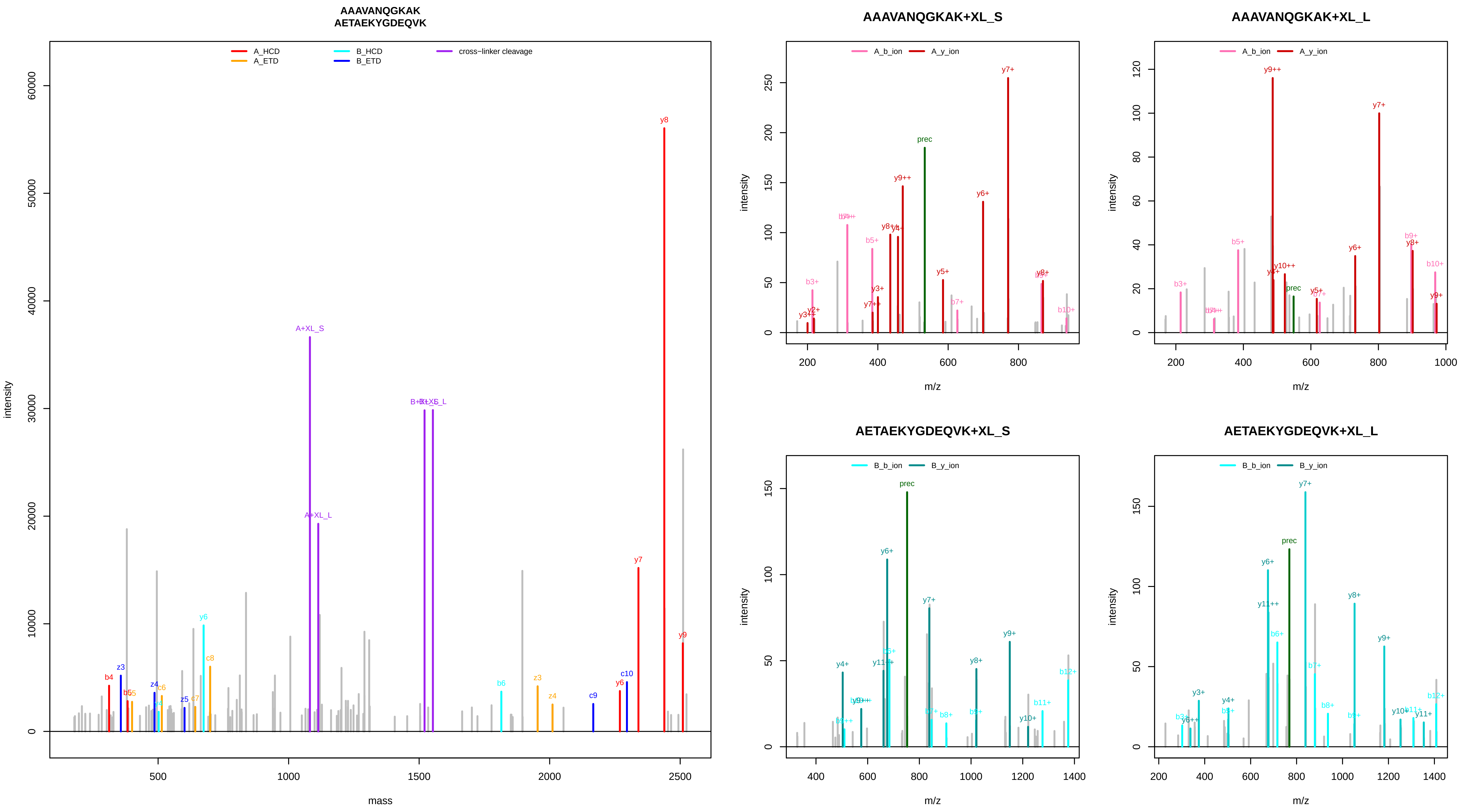
AAAVANQGKAK
AETAEKYGDEQVK
A_HCD
A_ETD
B_HCD
B_ETD
cross−linker cleavage
A+XL_S
A+XL_L
y8
y7
y9
y6
c10
c9
z4
z3
b6
c8
c7
c6
z5
z4
z3
b4
b5
y6
intensity
mass
AAAVANQGKAK+XL_S
A_b_ion
A_y_ion
y7+
prec
y9++
y6+
b5+
y5+
b3+
y3+
y2+
y7++
b7+
y8+
b10+
intensity
m/z
AAAVANQGKAK+XL_L
A_b_ion
A_y_ion
y9++
y7+
b5+
y6+
b9+
y8+
b10+
y10++
prec
y5+
b3+
y9+
intensity
m/z
AETAEKYGDEQVK+XL_S
B_b_ion
B_y_ion
prec
y6+
y7+
y9+
y8+
y4+
b12+
b11+
y10+
b8+
intensity
m/z
AETAEKYGDEQVK+XL_L
B_b_ion
B_y_ion
y7+
prec
y6+
y11++
b6+
y8+
y9+
b7+
b8+
y3+
y4+
b12+
y10+
y11+
intensity
m/z

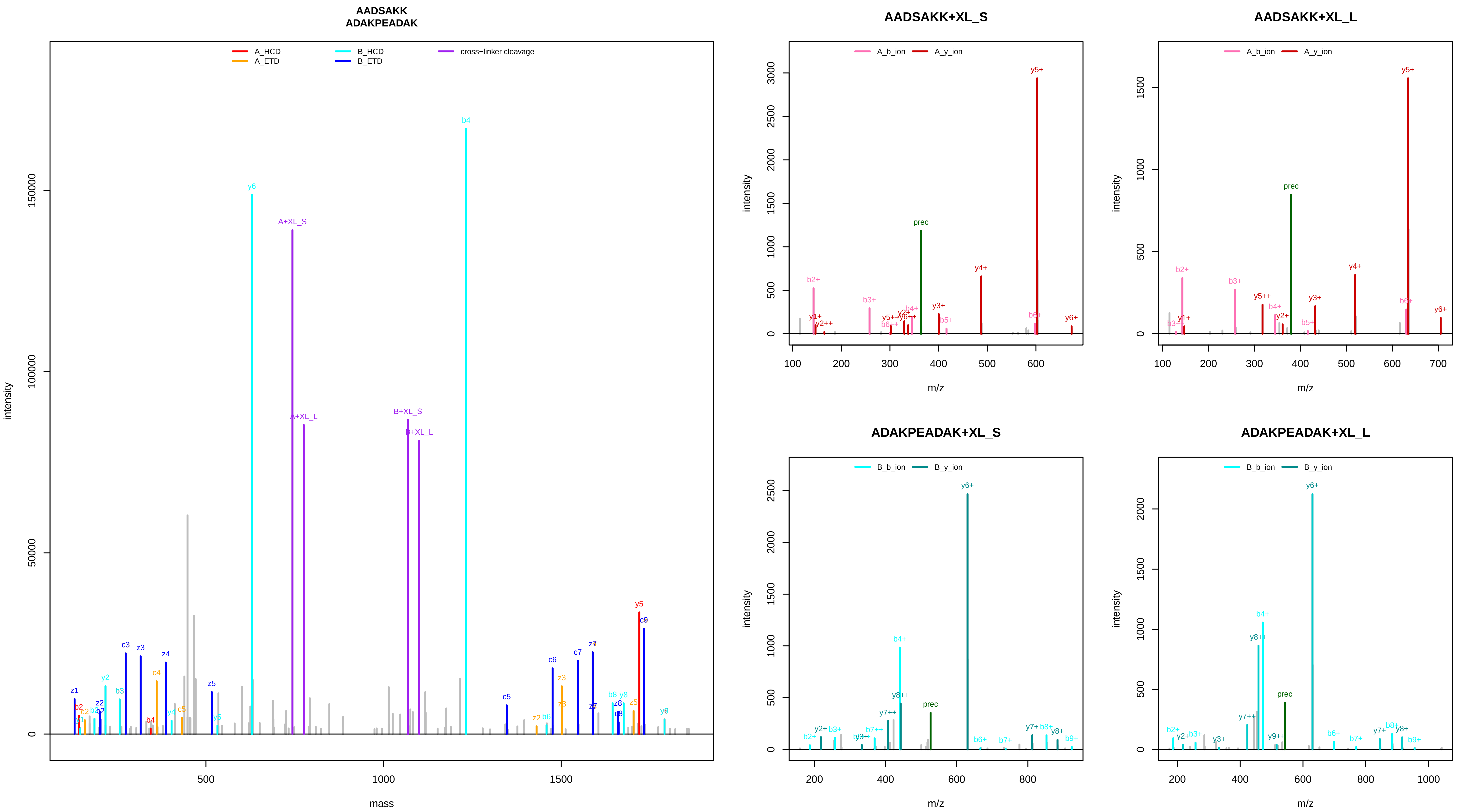

AADSAKK
ADAKPEADAK
A_HCD
A_ETD
B_HCD
B_ETD
cross−linker cleavage
A+XL_S
A+XL_L
B+XL_S
B+XL_L
intensity
mass
AADSAKK+XL_S
AADSAKK+XL_L
A_b_ion
A_y_ion
prec
m/z
ADAKPEADAK+XL_S
ADAKPEADAK+XL_L
B_b_ion
B_y_ion

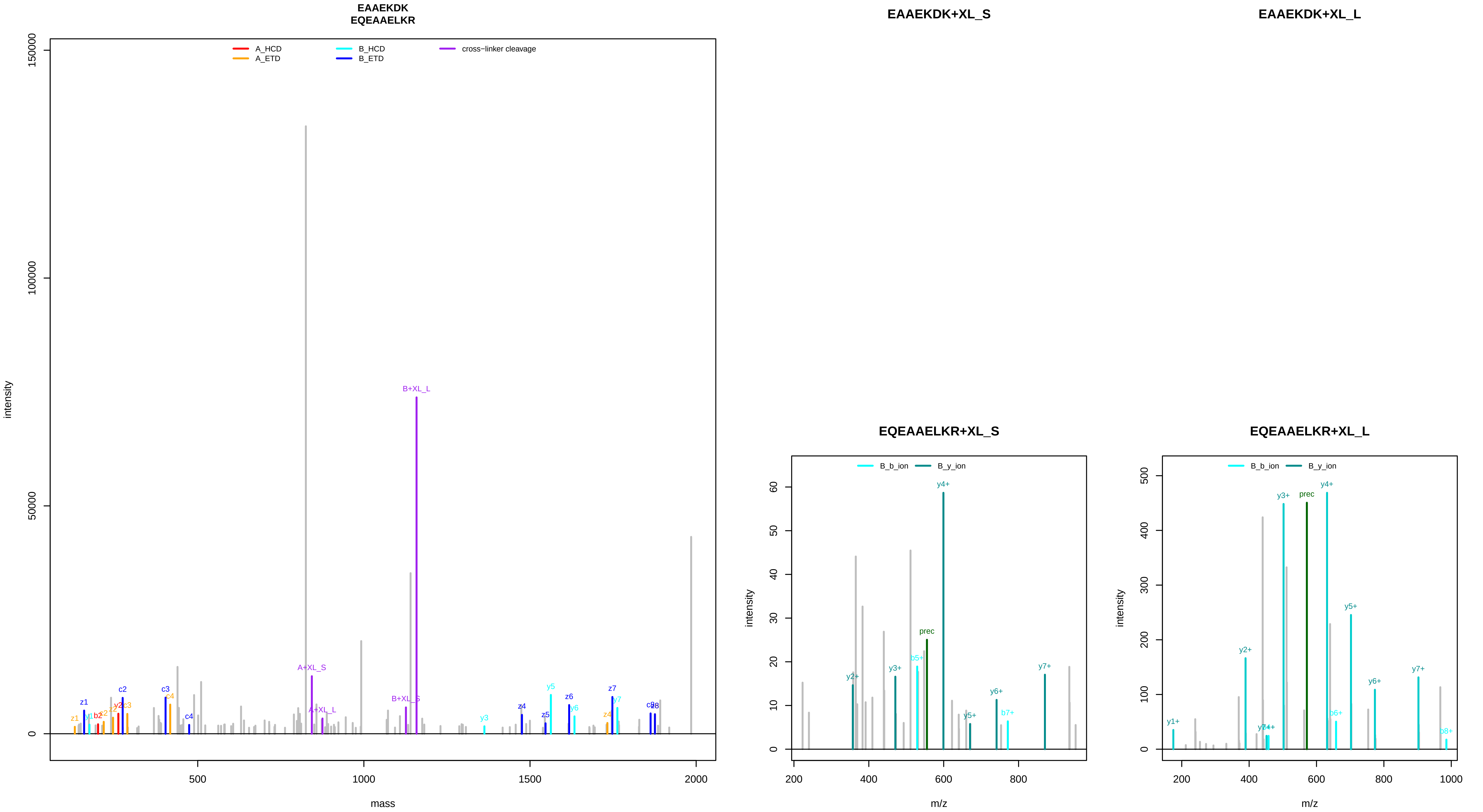

EAAEKDK
EQEAAELKR
A_HCD
A_ETD
B_HCD
B_ETD
cross−linker cleavage
B+XL_L
A+XL_S
A+XL_L
B+XL_S
intensity
mass
EAAEKDK+XL_S
EAAEKDK+XL_L
EQEAAELKR+XL_S
EQEAAELKR+XL_L
B_b_ion
B_y_ion
prec
m/z

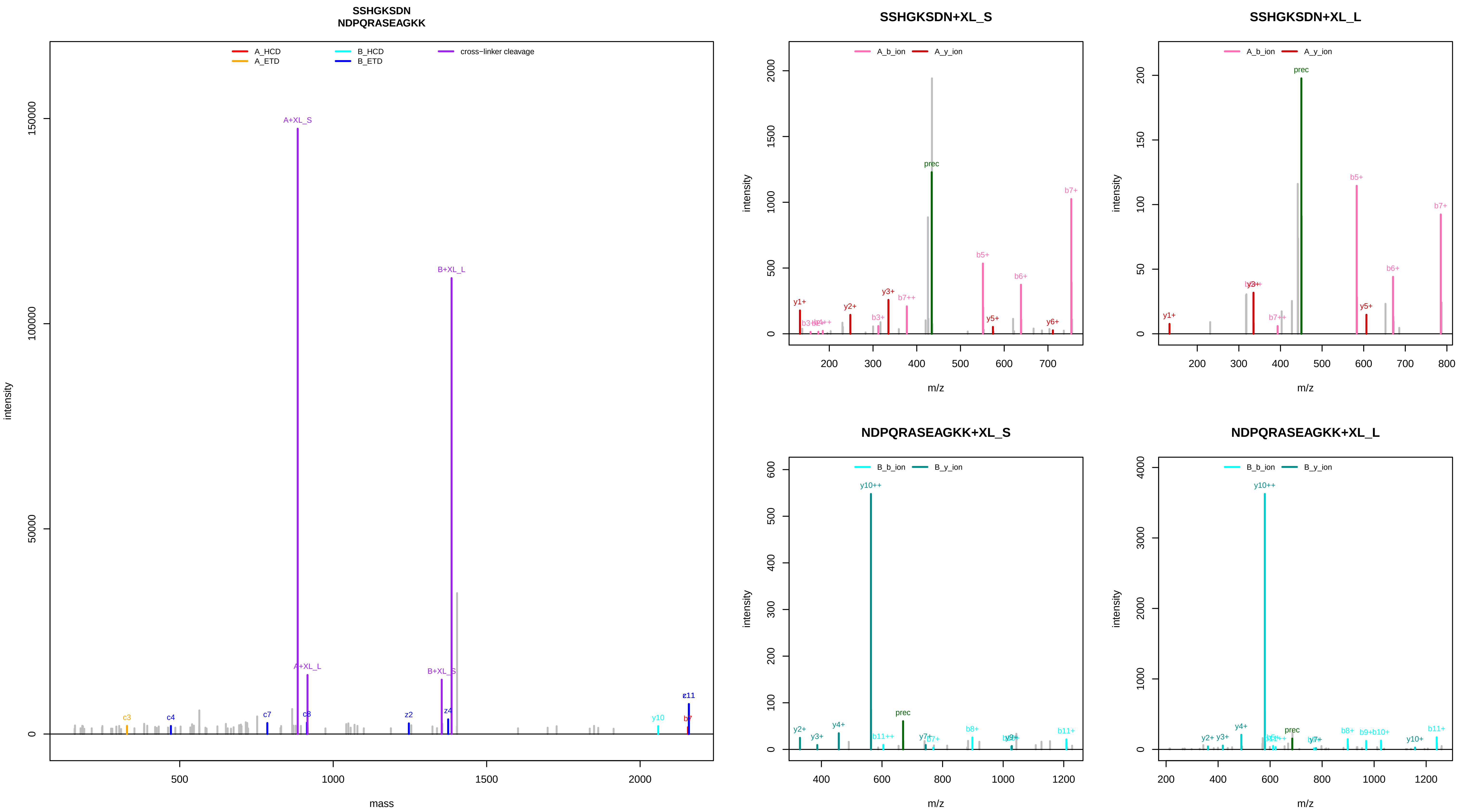
SSHGKSDN
NDPQRASEAGKK
A_HCD
A_ETD
B_HCD
B_ETD
cross−linker cleavage
A+XL_S
B+XL_L
A+XL_L
B+XL_S
c3
c4
c7
c8
z2
z4
y10
z11
intensity
mass
SSHGKSDN+XL_S
A_b_ion
A_y_ion
prec
b7+
b5+
b6+
y3+
b7++
y1+
y2+
b3+
y5+
y6+
intensity
m/z
SSHGKSDN+XL_L
A_b_ion
A_y_ion
prec
b5+
b7+
b6+
y3+
y1+
b7++
y5+
intensity
m/z
NDPQRASEAGKK+XL_S
B_b_ion
B_y_ion
y10++
prec
y4+
y2+
y3+
b11++
y7+
b7+
b8+
b11+
intensity
m/z
NDPQRASEAGKK+XL_L
B_b_ion
B_y_ion
y10++
prec
y4+
y2+ y3+
b8+
b9+b10+
b11+
y10+
intensity
m/z

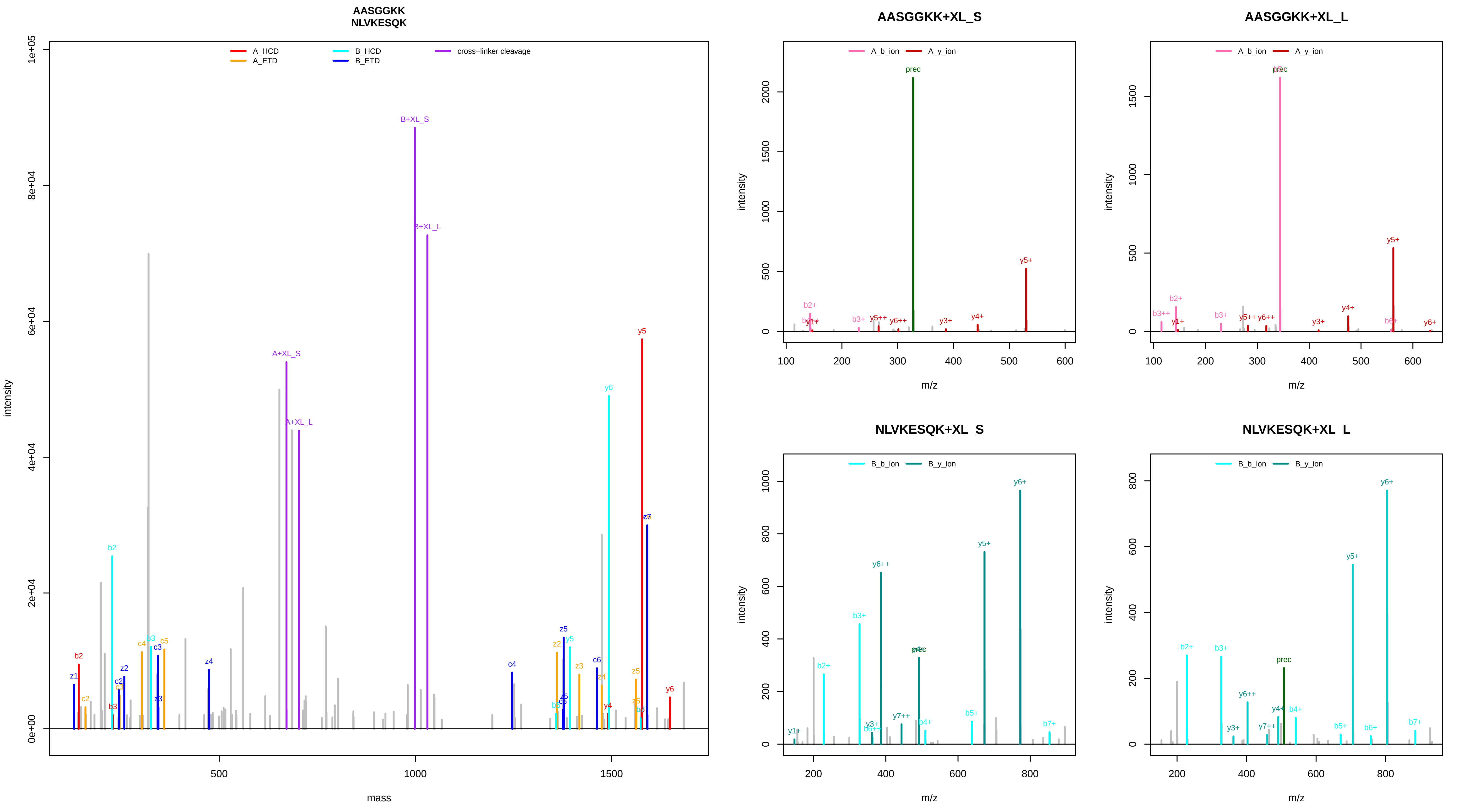
AASGGKK
NLVKESQK
A_HCD
A_ETD
B_HCD
B_ETD
cross−linker cleavage
B+XL_S
B+XL_L
A+XL_S
A+XL_L
intensity
mass
AASGGKK+XL_S
AASGGKK+XL_L
A_b_ion
A_y_ion
NLVKESQK+XL_S
NLVKESQK+XL_L
B_b_ion
B_y_ion
prec
m/z

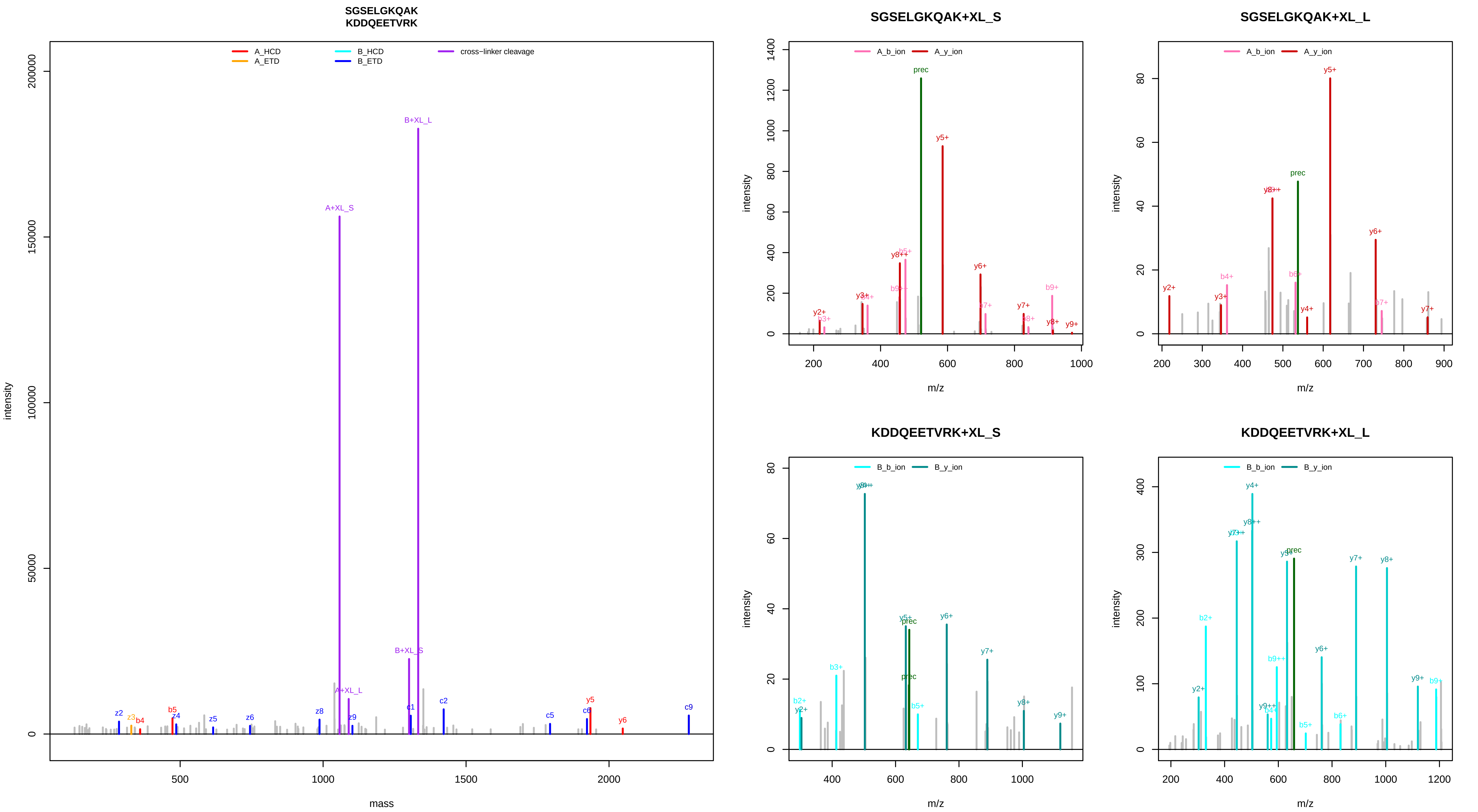
SGSELGKQAK
KDDQEETVRK
A_HCD
A_ETD
B_HCD
B_ETD
cross−linker cleavage
B+XL_L
A+XL_S
B+XL_S
A+XL_L
intensity
mass
SGSELGKQAK+XL_S
SGSELGKQAK+XL_L
KDDQEETVRK+XL_S
KDDQEETVRK+XL_L
A_b_ion
A_y_ion
B_b_ion
B_y_ion
prec
m/z

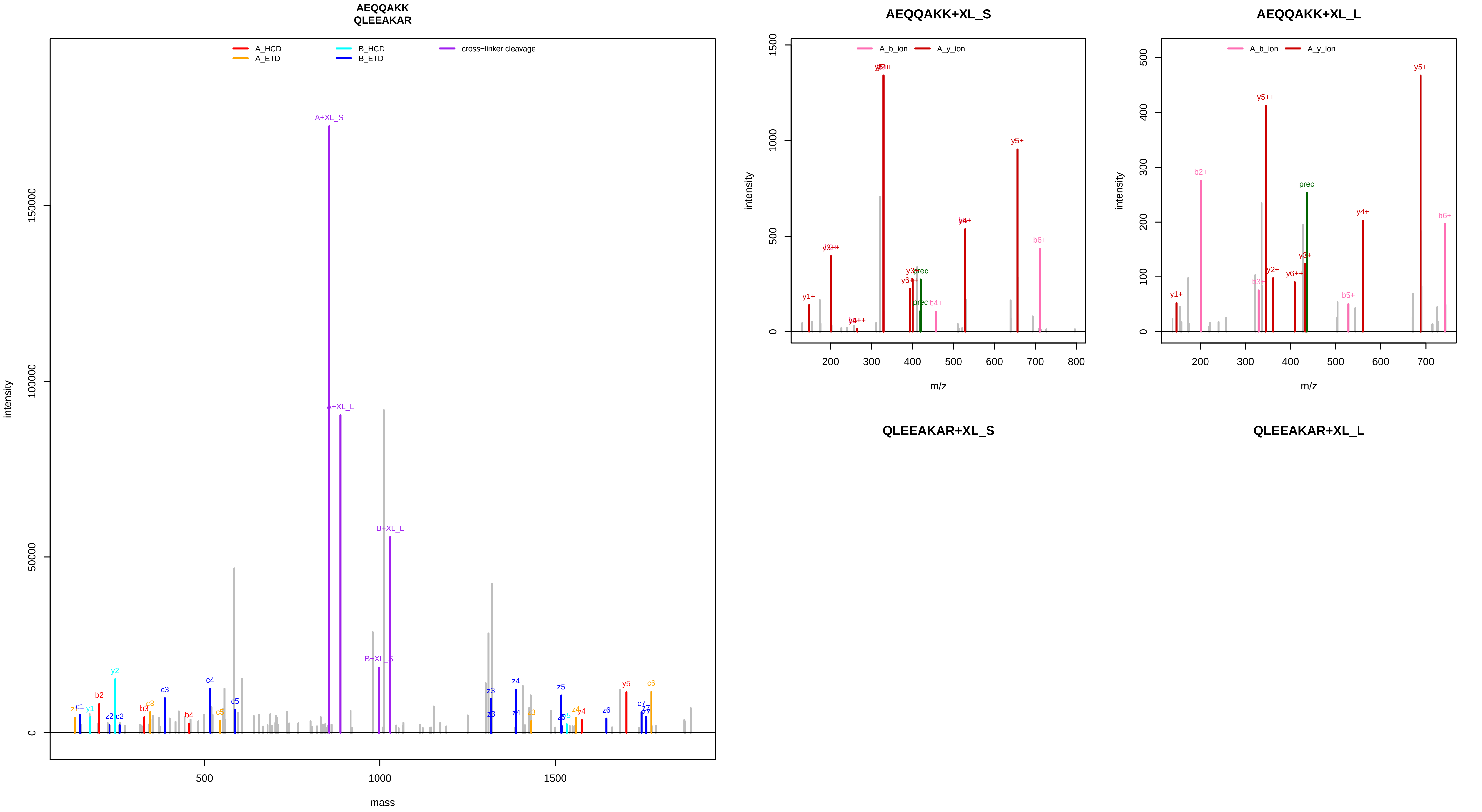
AEQQAKK
QLEEAKAR
A_HCD
A_ETD
B_HCD
B_ETD
cross−linker cleavage
A+XL_S
A+XL_L
B+XL_L
B+XL_S
intensity
mass
AEQQAKK+XL_S
AEQQAKK+XL_L
A_b_ion
A_y_ion
prec
intensity
m/z
QLEEAKAR+XL_S
QLEEAKAR+XL_L

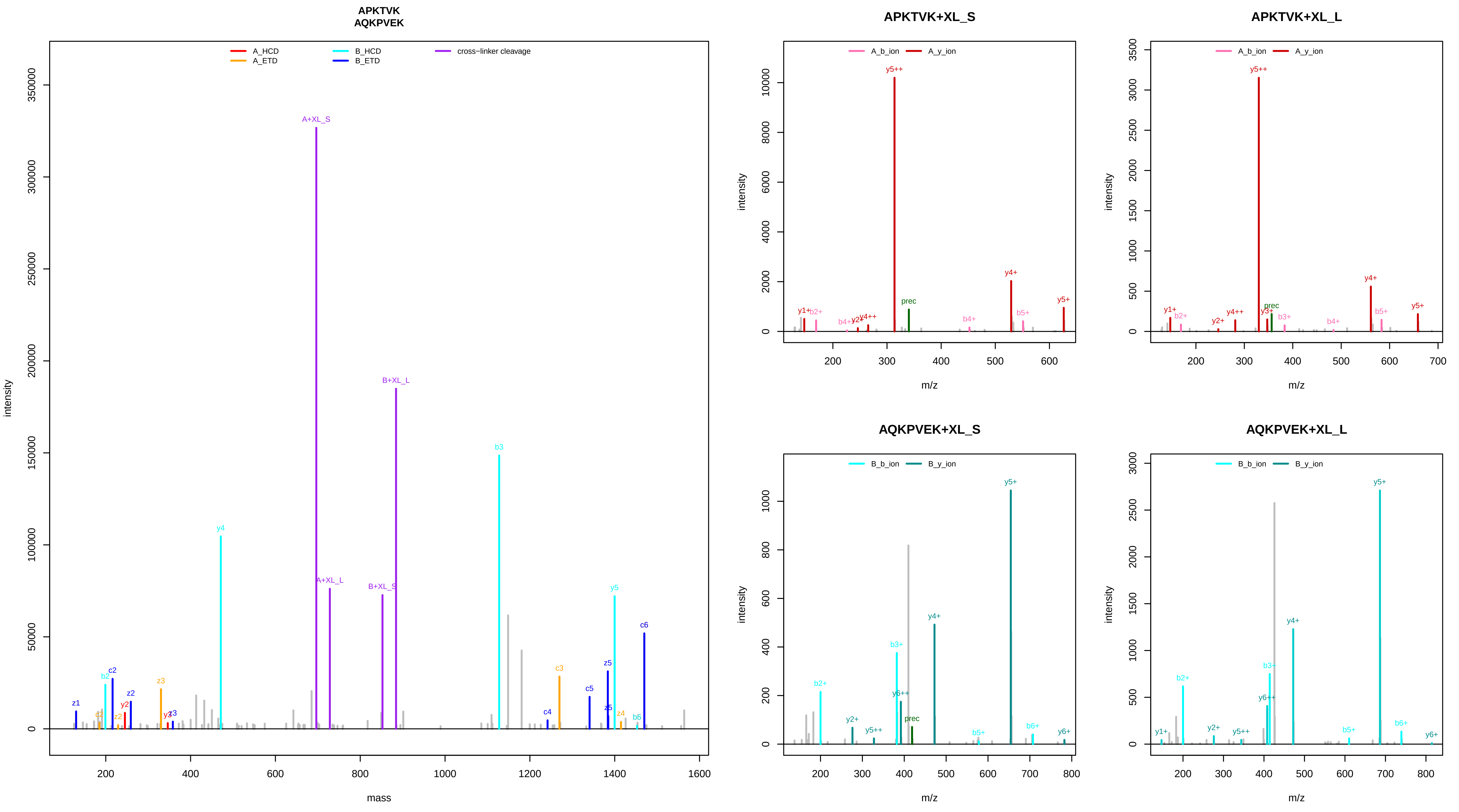
APKTVK
AQKPVEK
A_HCD
A_ETD
B_HCD
B_ETD
cross−linker cleavage
A+XL_S
B+XL_L
A+XL_L
B+XL_S
b3
y4
y5
c6
z5
c5
c3
c4
z3
c2
b2
z2
y2
z1
b5
z4
intensity
mass
APKTVK+XL_S
APKTVK+XL_L
AQKPVEK+XL_S
AQKPVEK+XL_L
A_b_ion
A_y_ion
B_b_ion
B_y_ion
y5++
y4+
y5+
prec
b5+
b4+
b3+
b2+
y1+
y2+
y3+
y4++
y6+
b6+
y6++
y5++
m/z

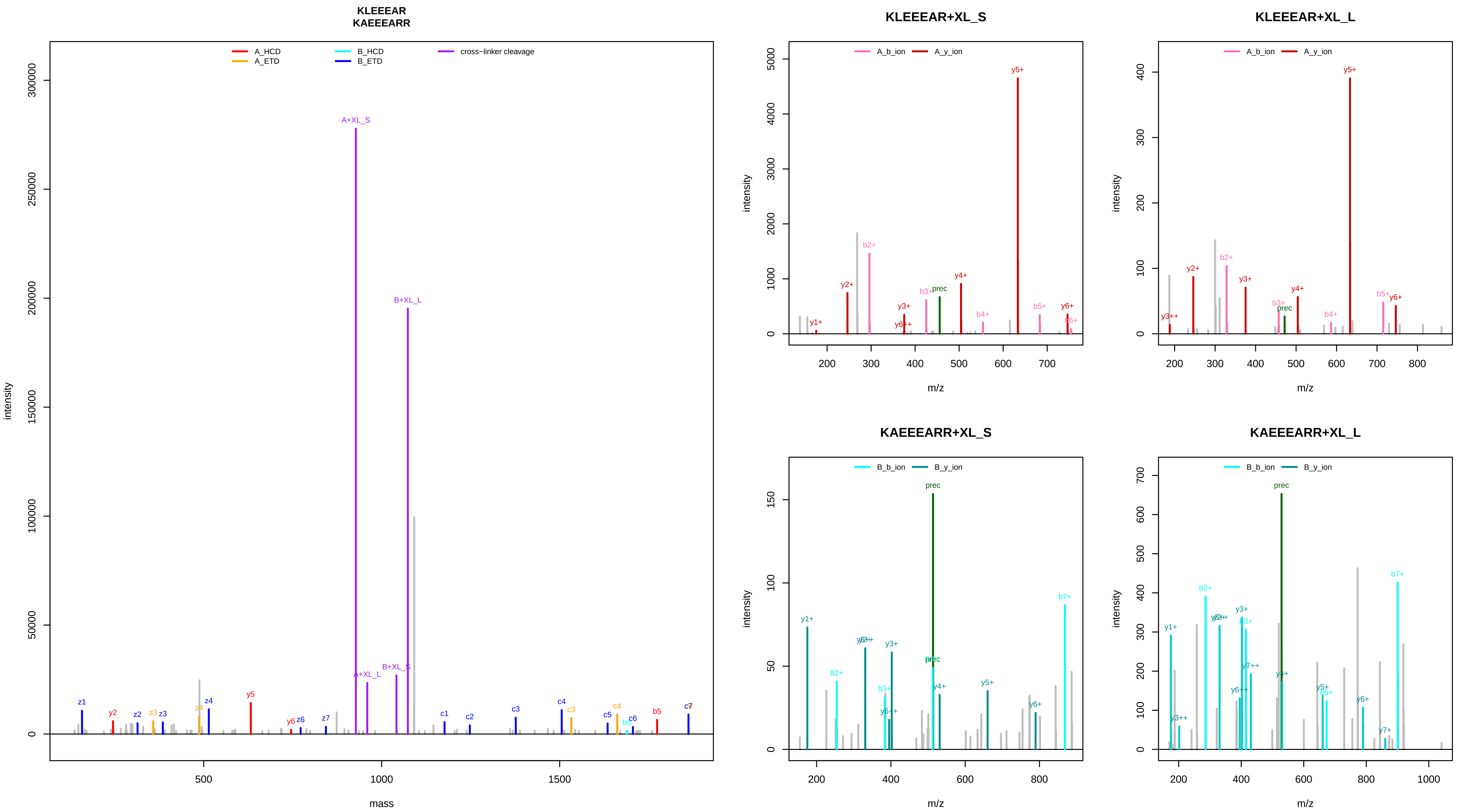
KLEEEAR
KAEEEARR
A_HCD
A_ETD
B_HCD
B_ETD
cross−linker cleavage
A+XL_S
B+XL_L
A+XL_L
B+XL_S
intensity
mass
KLEEEAR+XL_S
KLEEEAR+XL_L
KAEEEARR+XL_S
KAEEEARR+XL_L
A_b_ion
A_y_ion
B_b_ion
B_y_ion
prec
m/z

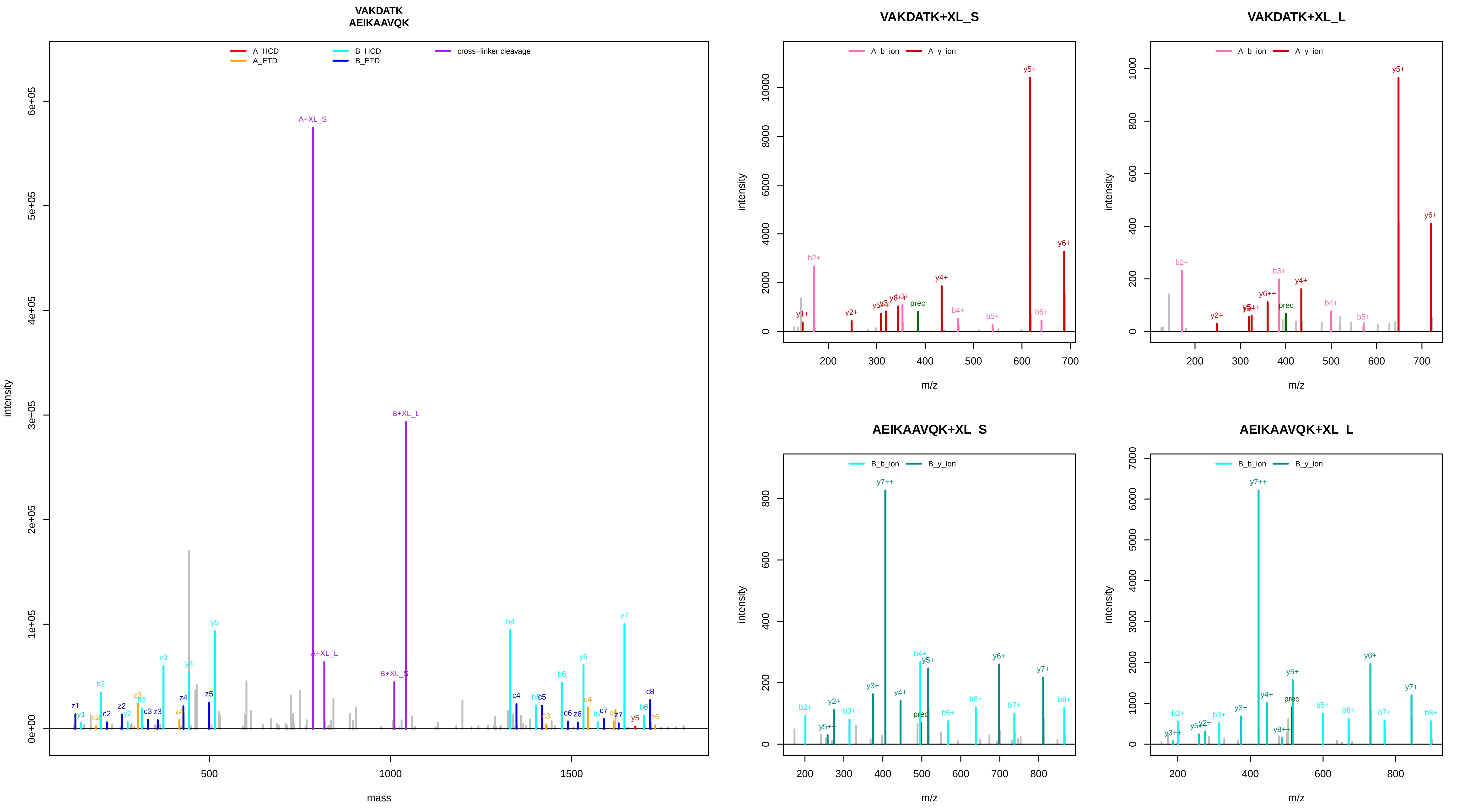
VAKDATK
AEIKAAVQK
A_HCD
A_ETD
B_HCD
B_ETD
cross−linker cleavage
A+XL_S
B+XL_L
A+XL_L
B+XL_S
intensity
mass
VAKDATK+XL_S
VAKDATK+XL_L
AEIKAAVQK+XL_S
AEIKAAVQK+XL_L
A_b_ion
A_y_ion
B_b_ion
B_y_ion
prec
m/z
intensity

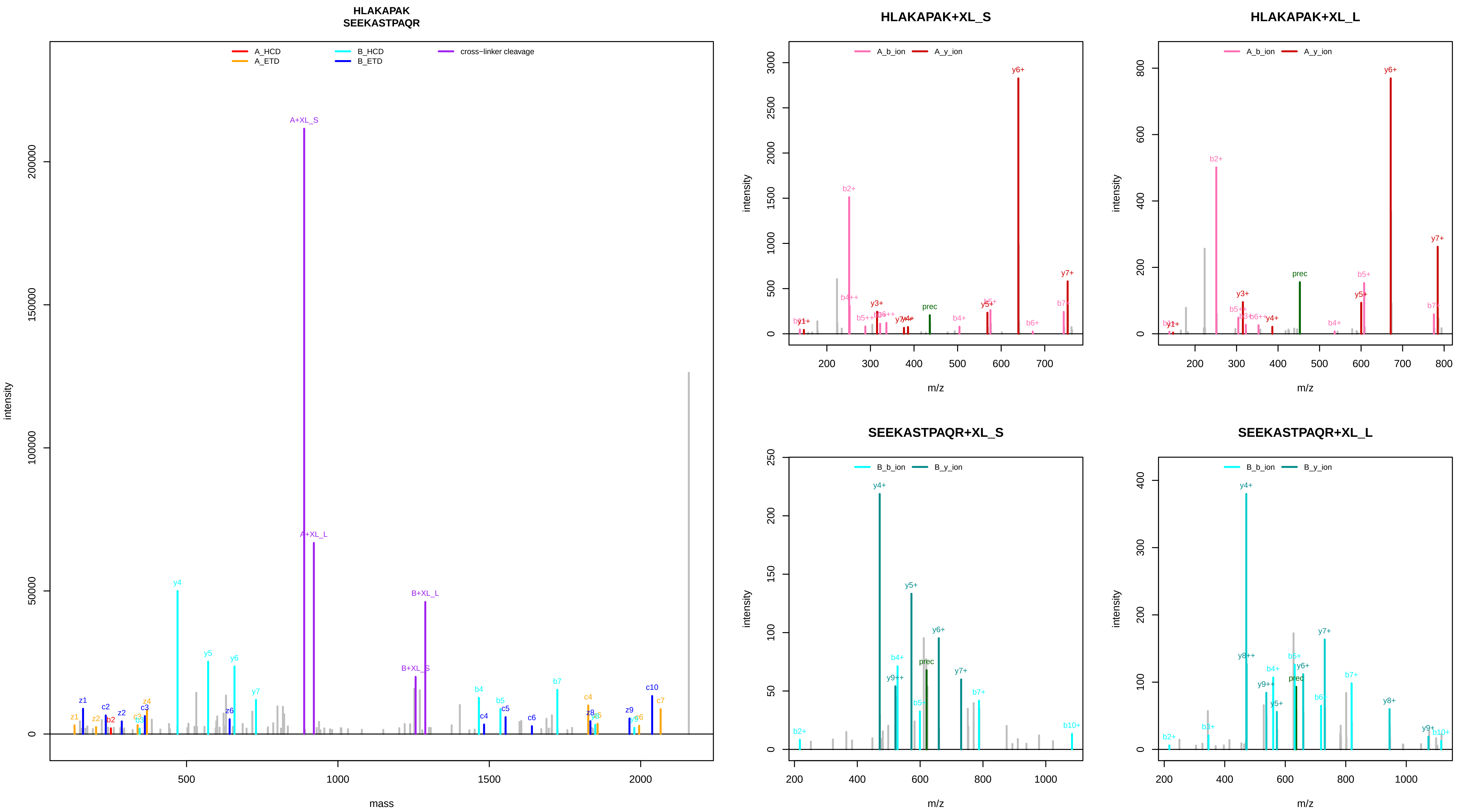
HLAKAPAK
SEEKASTPAQR
A_HCD
A_ETD
B_HCD
B_ETD
cross−linker cleavage
A+XL_S
A+XL_L
B+XL_L
B+XL_S
intensity
mass
HLAKAPAK+XL_S
HLAKAPAK+XL_L
SEEKASTPAQR+XL_S
SEEKASTPAQR+XL_L
A_b_ion
A_y_ion
B_b_ion
B_y_ion
prec
m/z

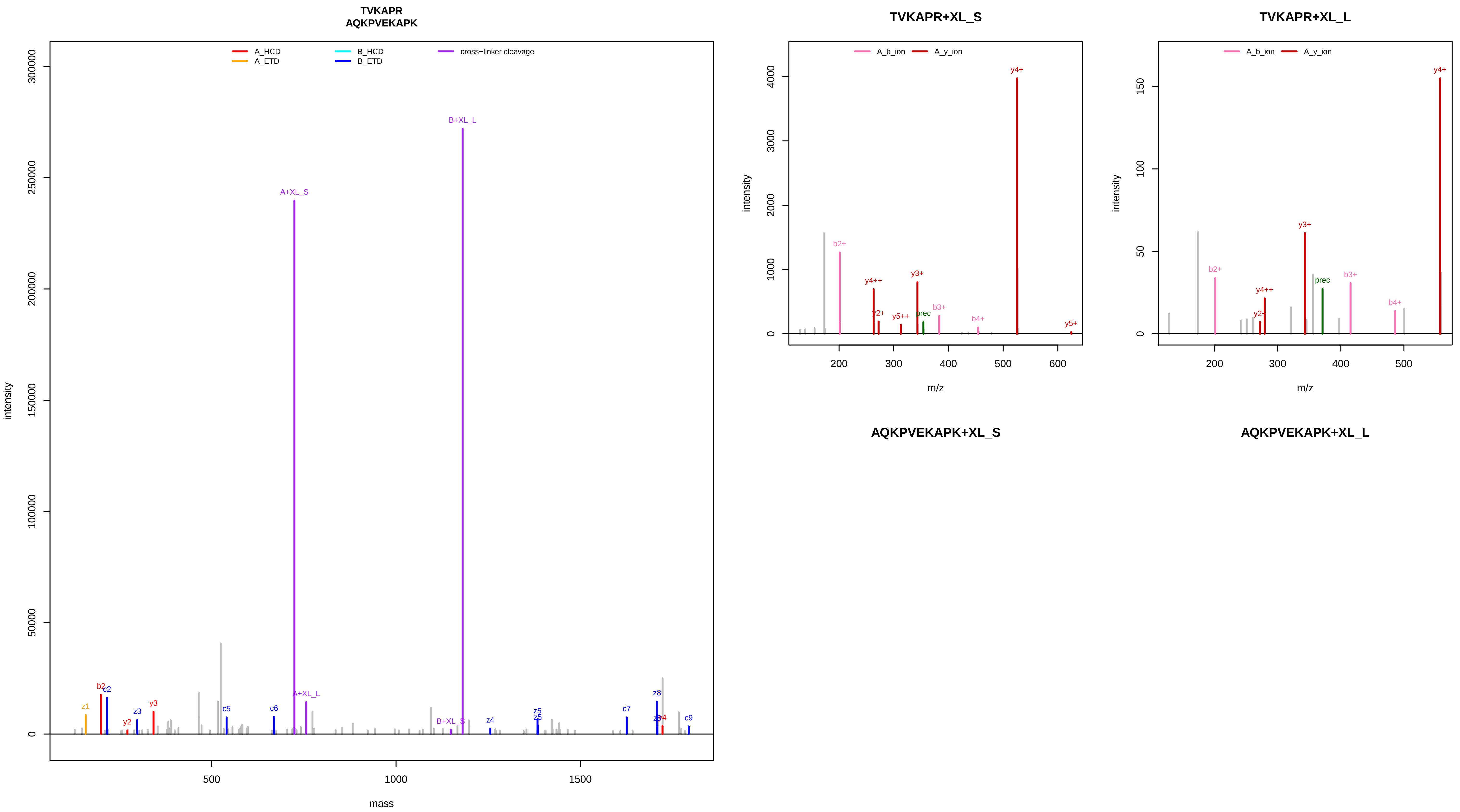
TVKAPR
AQKPVEKAPK
A_HCD
A_ETD
B_HCD
B_ETD
cross−linker cleavage
B+XL_L
A+XL_S
A+XL_L
B+XL_S
z1
b2
c2
y2
z3
y3
c5
c6
z4
z5
c7
z8
y4
c9
intensity
mass
TVKAPR+XL_S
A_b_ion
A_y_ion
y4+
b2+
y4++
y3+
y2+
y5++
prec
b3+
b4+
y5+
m/z
TVKAPR+XL_L
y3+
b2+
y4++
y2+
prec
b3+
b4+
y4+
AQKPVEKAPK+XL_S
AQKPVEKAPK+XL_L

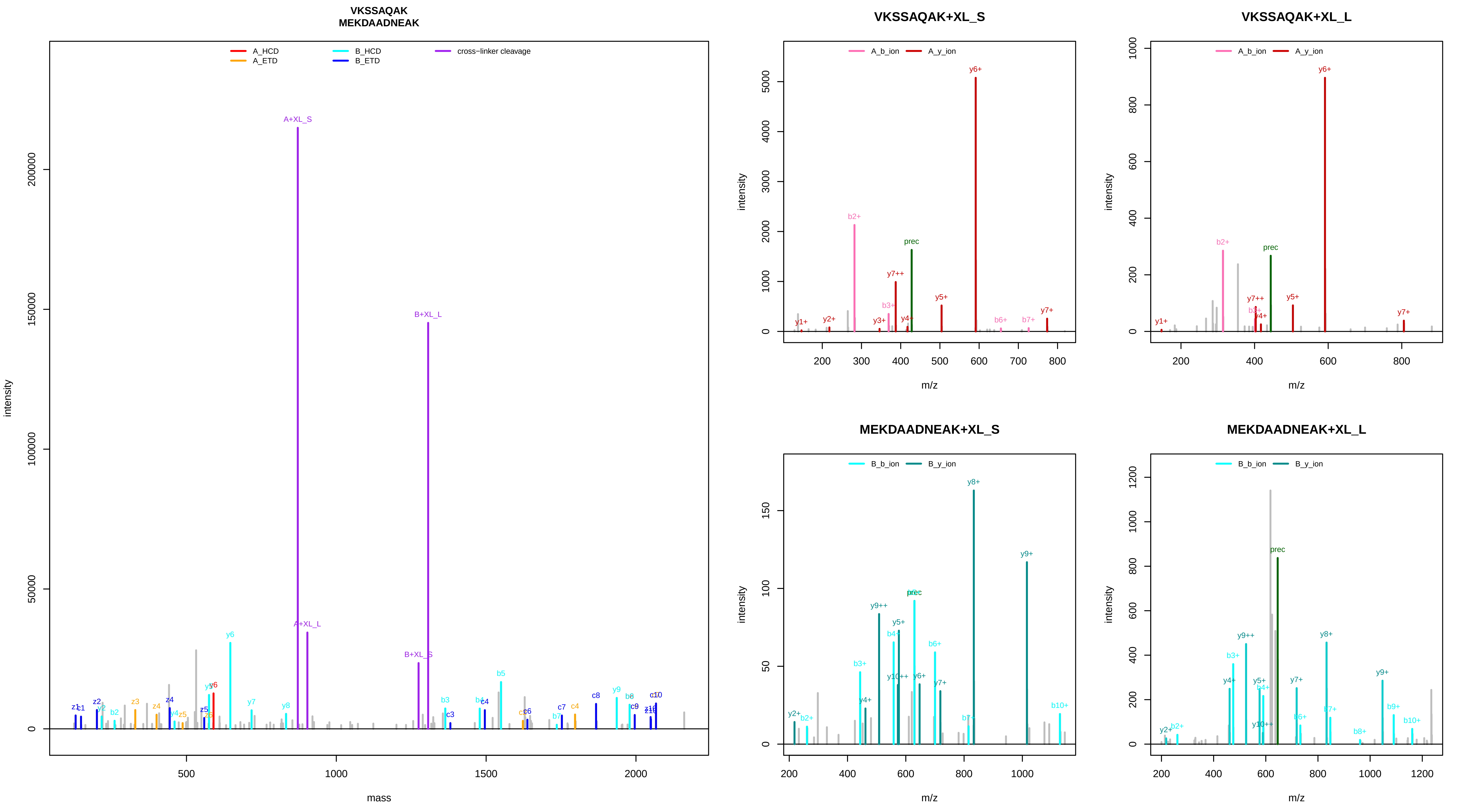
VKSSAQAK
MEKDAADNEAK
A_HCD
A_ETD
B_HCD
B_ETD
cross−linker cleavage
A+XL_S
B+XL_L
A+XL_L
B+XL_S
intensity
mass
0
50000
100000
150000
200000
500
1000
1500
2000
VKSSAQAK+XL_S
VKSSAQAK+XL_L
MEKDAADNEAK+XL_S
MEKDAADNEAK+XL_L
A_b_ion
A_y_ion
B_b_ion
B_y_ion
prec
y6+
b2+
y7++
y5+
y7+
y1+
y2+
y3+
y4+
b3+
b6+
b7+
y8+
y9+
y9++
b4+
b10+
m/z

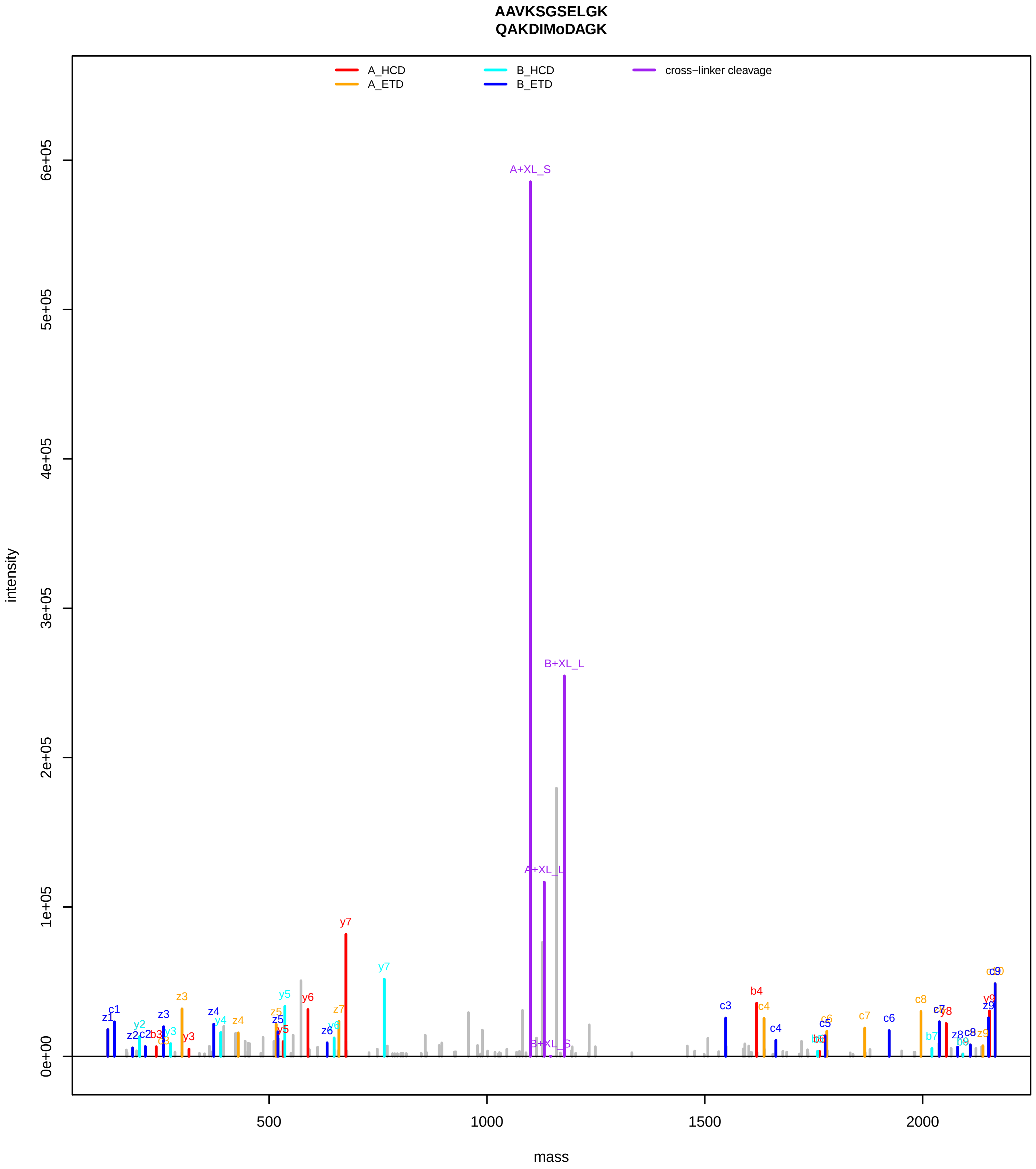
AAVKSGSELGK
QAKDIMoDAGK
A_HCD
A_ETD
B_HCD
B_ETD
cross−linker cleavage
A+XL_S
B+XL_L
A+XL_L
B+XL_S
intensity
mass
0e+00
1e+05
2e+05
3e+05
4e+05
5e+05
6e+05
500
1000
1500
2000

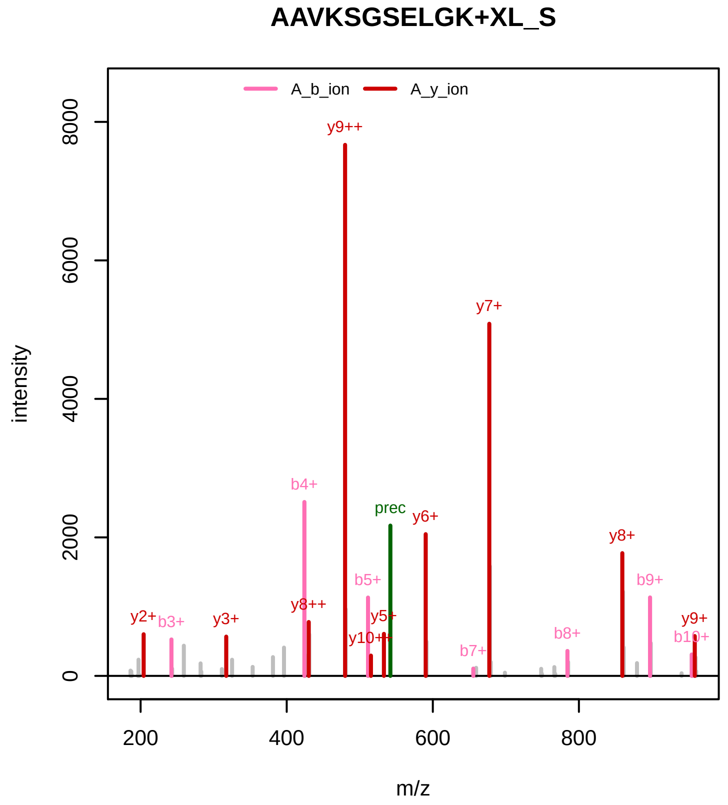
AAVKSGSELGK+XL_S
A_b_ion
A_y_ion
intensity
m/z
0
2000
4000
6000
8000
200
400
600
800
prec

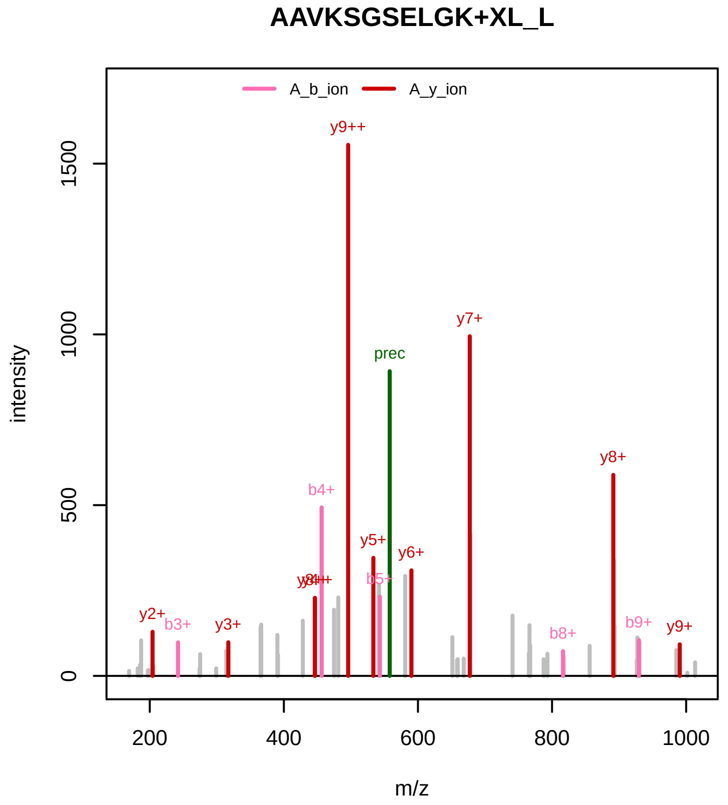
AAVKSGSELGK+XL_L
A_b_ion
A_y_ion
intensity
m/z
0
500
1000
1500
200
400
600
800
1000
prec

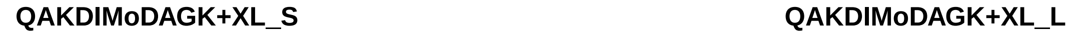
QAKDIMoDAGK+XL_S
QAKDIMoDAGK+XL_L

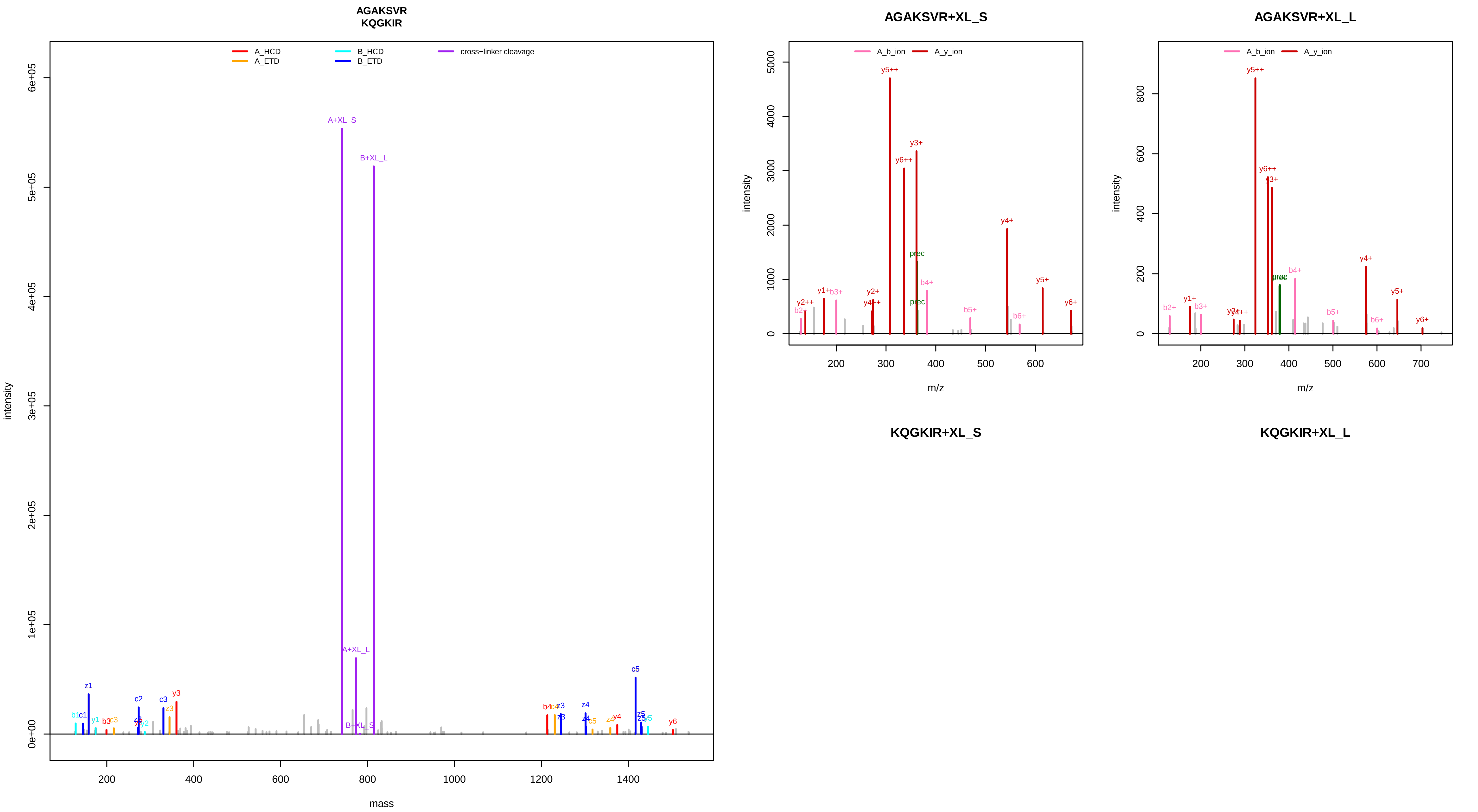
AGAKSVR
KQGKIR
A_HCD
A_ETD
B_HCD
B_ETD
cross−linker cleavage
A+XL_S
B+XL_L
A+XL_L
B+XL_S
intensity
mass
AGAKSVR+XL_S
AGAKSVR+XL_L
A_b_ion
A_y_ion
m/z
KQGKIR+XL_S
KQGKIR+XL_L

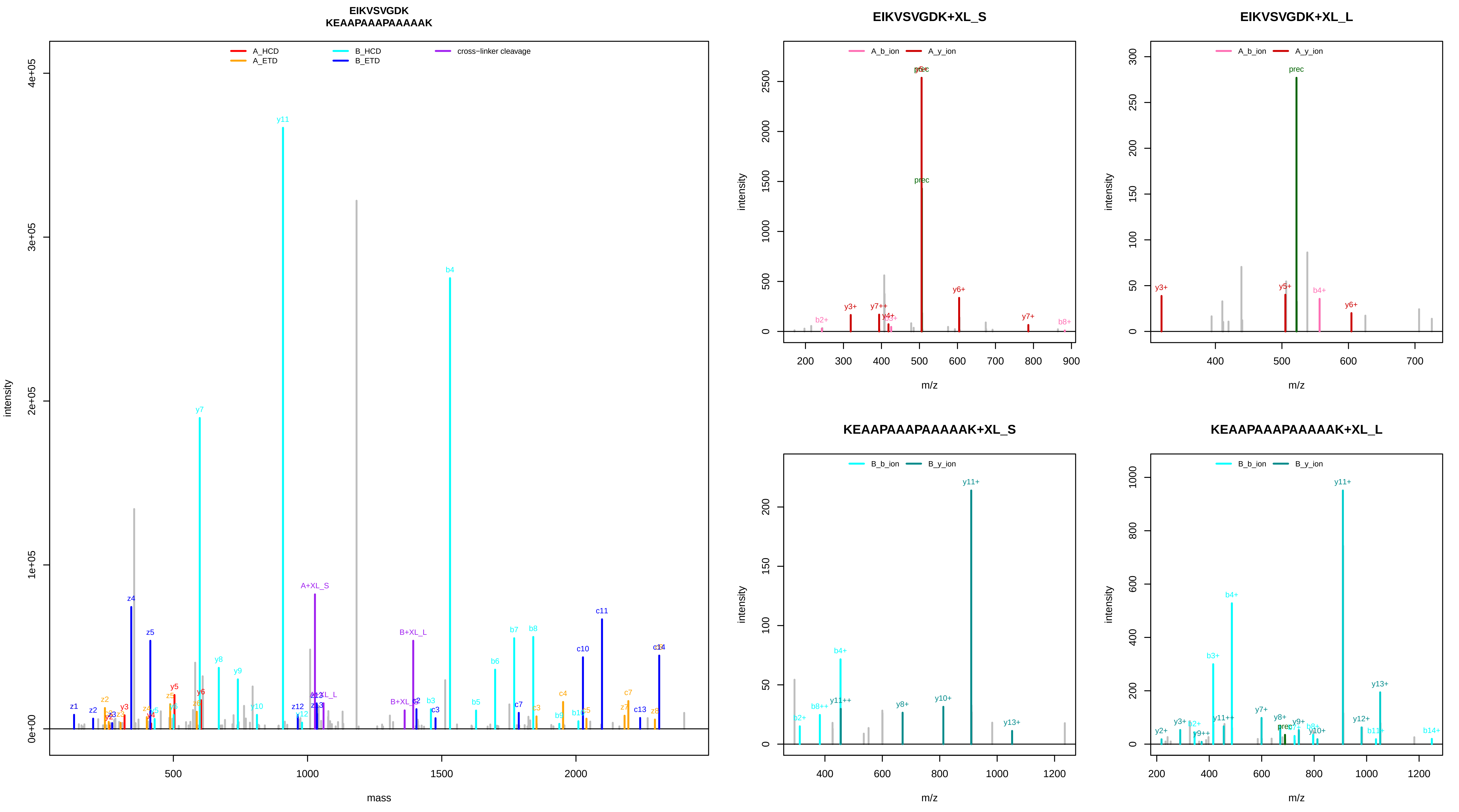
EIKVSVGDK
KEAAPAAAPAAAAAK
A_HCD
A_ETD
B_HCD
B_ETD
cross−linker cleavage
intensity
mass
A+XL_S
B+XL_L
EIKVSVGDK+XL_S
EIKVSVGDK+XL_L
KEAAPAAAPAAAAAK+XL_S
KEAAPAAAPAAAAAK+XL_L
A_b_ion
A_y_ion
B_b_ion
B_y_ion
prec
m/z

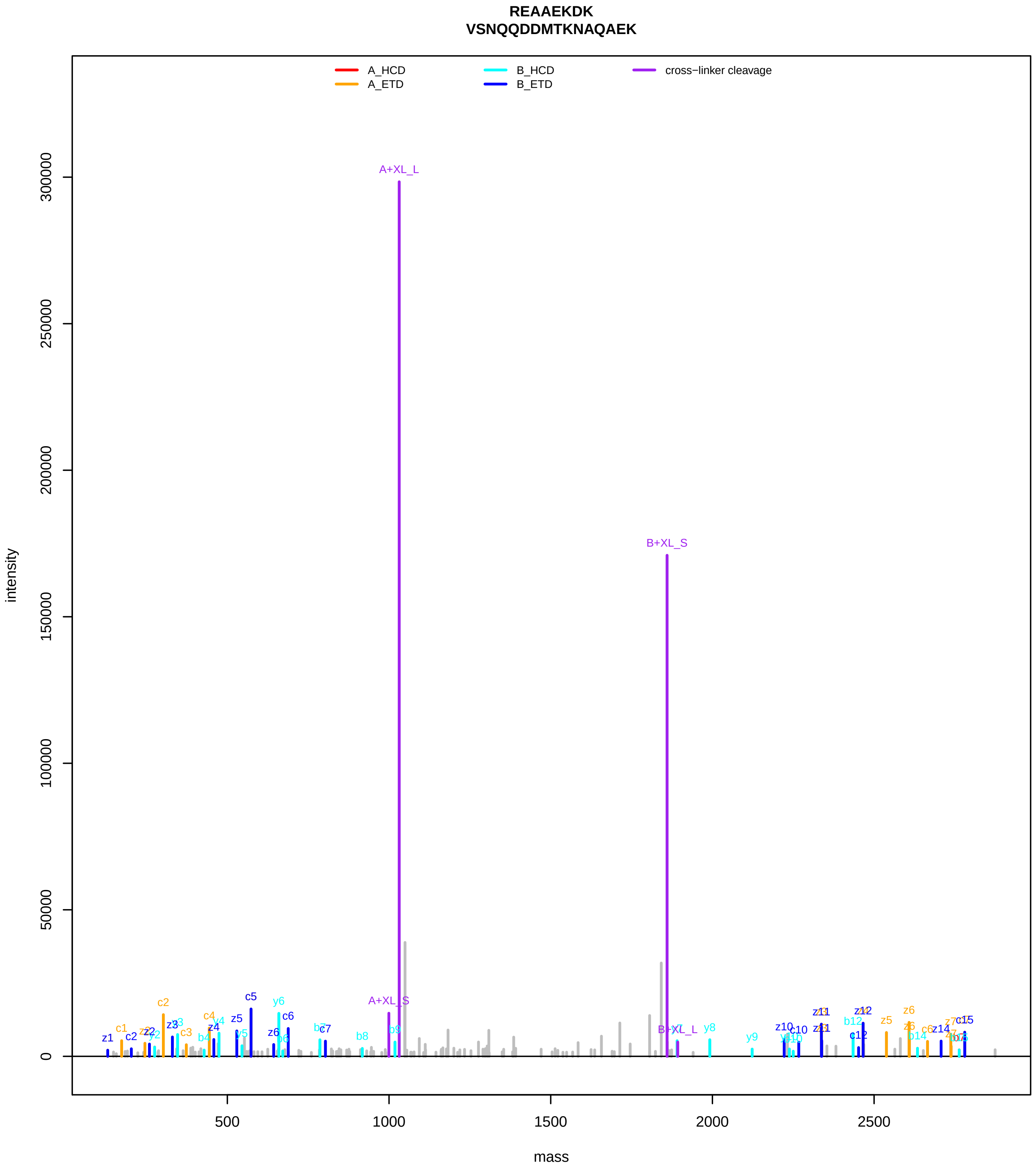

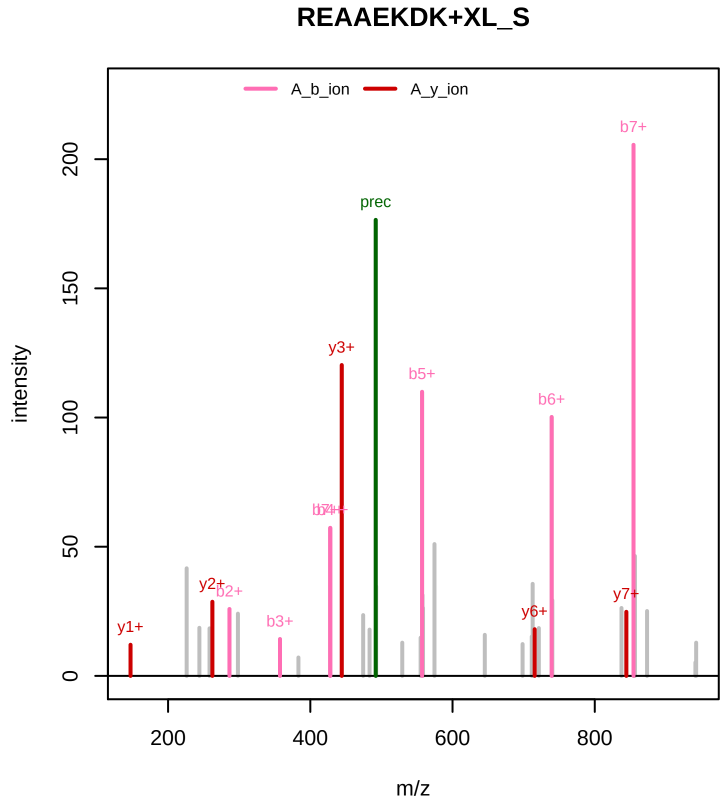

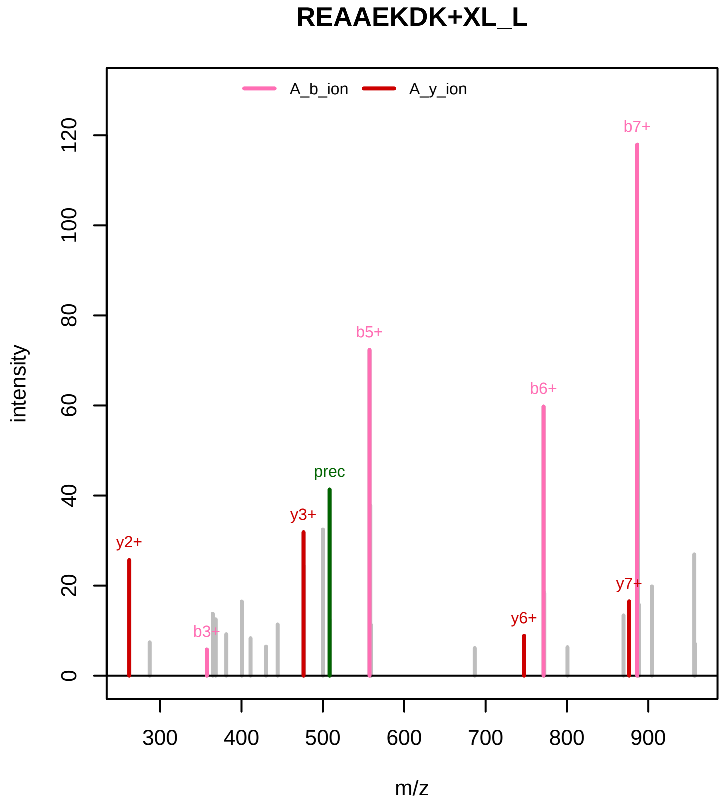

VSNQQDDMTKNAQAEK+XL_S

VSNQQDDMTKNAQAEK+XL_L

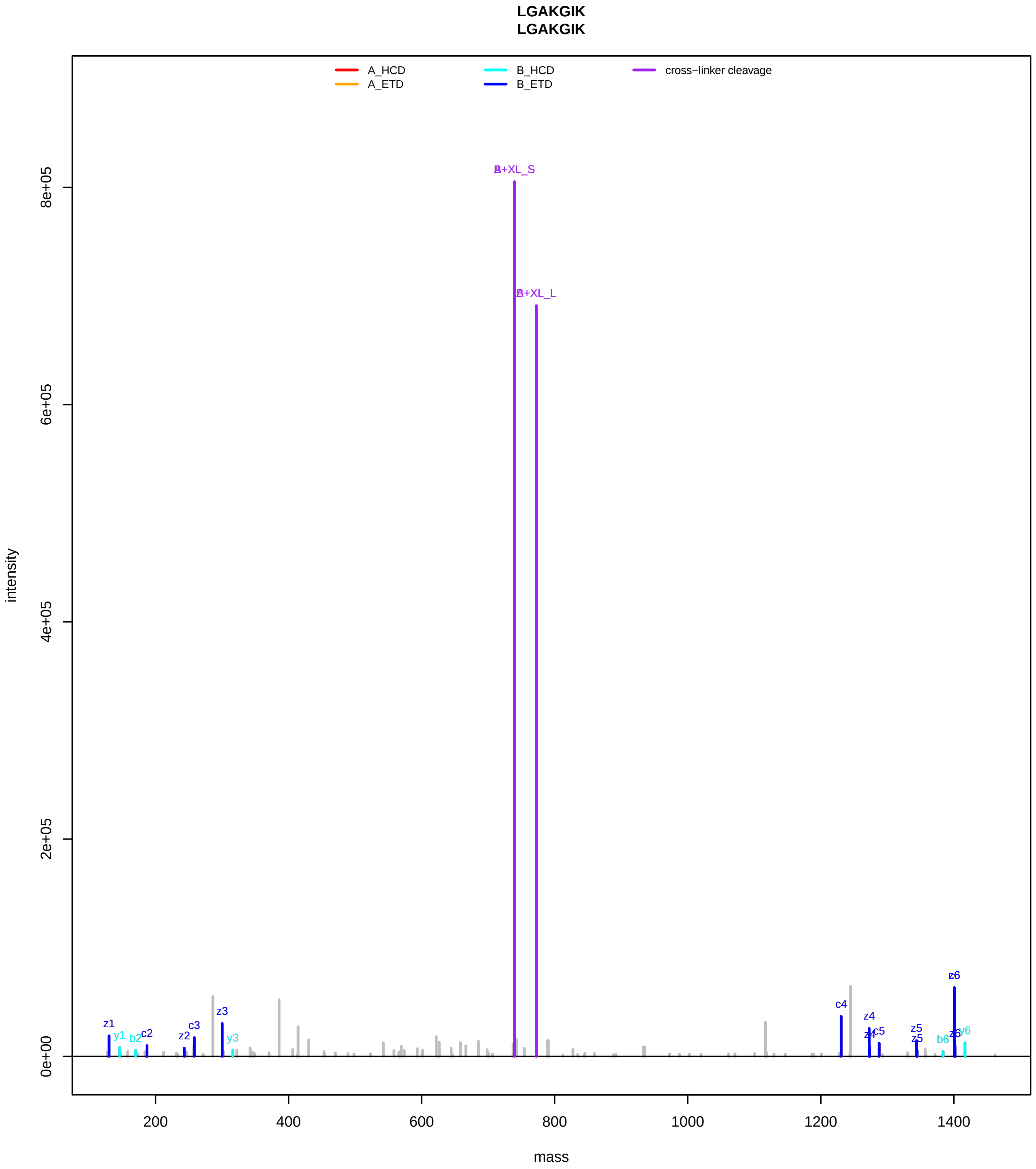

LGAKGIK+XL_S

LGAKGIK+XL_L

LGAKGIK+XL_S

LGAKGIK+XL_L

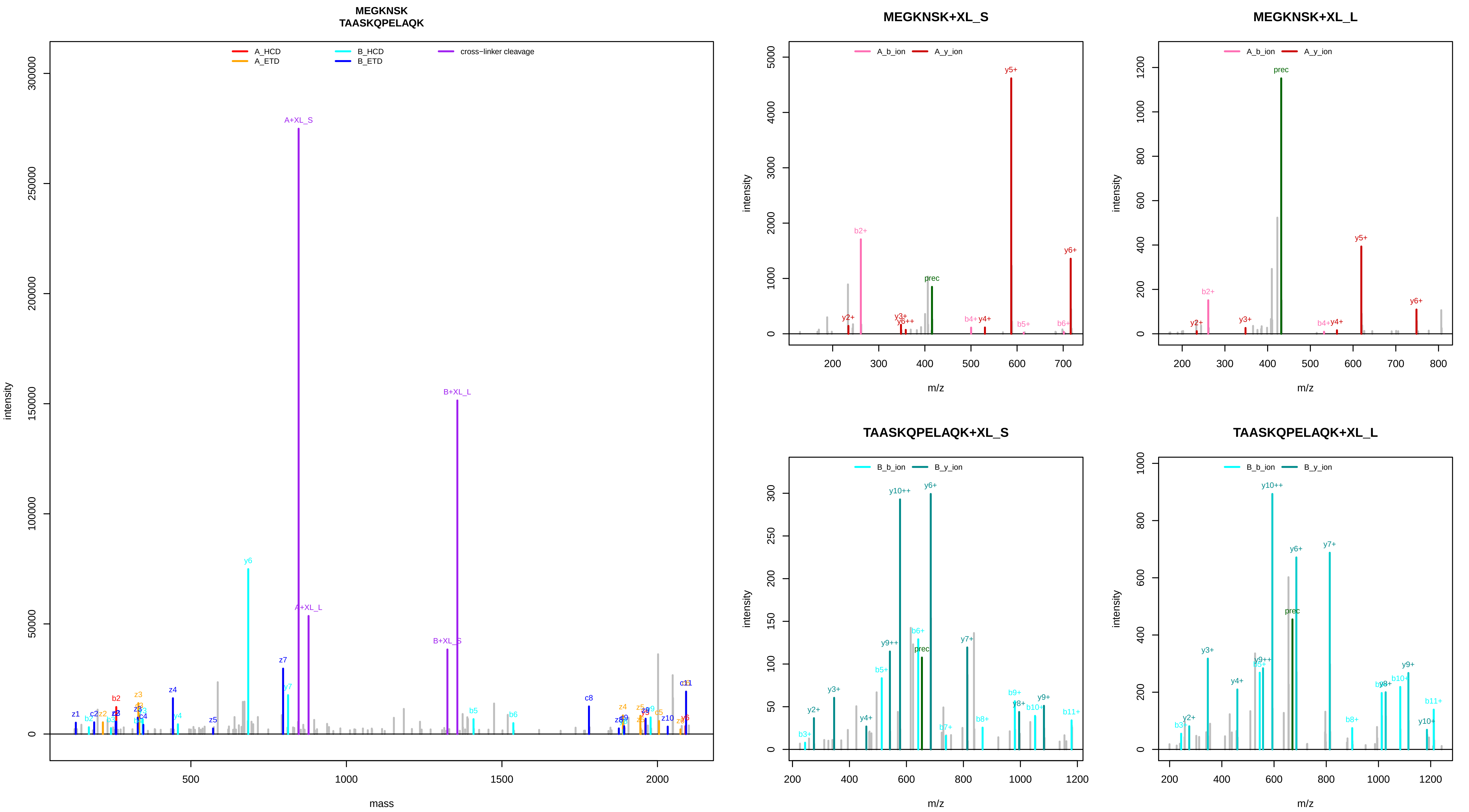
MEGKNSK
TAASKQPELAQK
A_HCD
A_ETD
B_HCD
B_ETD
cross−linker cleavage
A+XL_S
B+XL_L
A+XL_L
B+XL_S
intensity
mass
MEGKNSK+XL_S
MEGKNSK+XL_L
A_b_ion
A_y_ion
TAASKQPELAQK+XL_S
TAASKQPELAQK+XL_L
B_b_ion
B_y_ion
prec
m/z

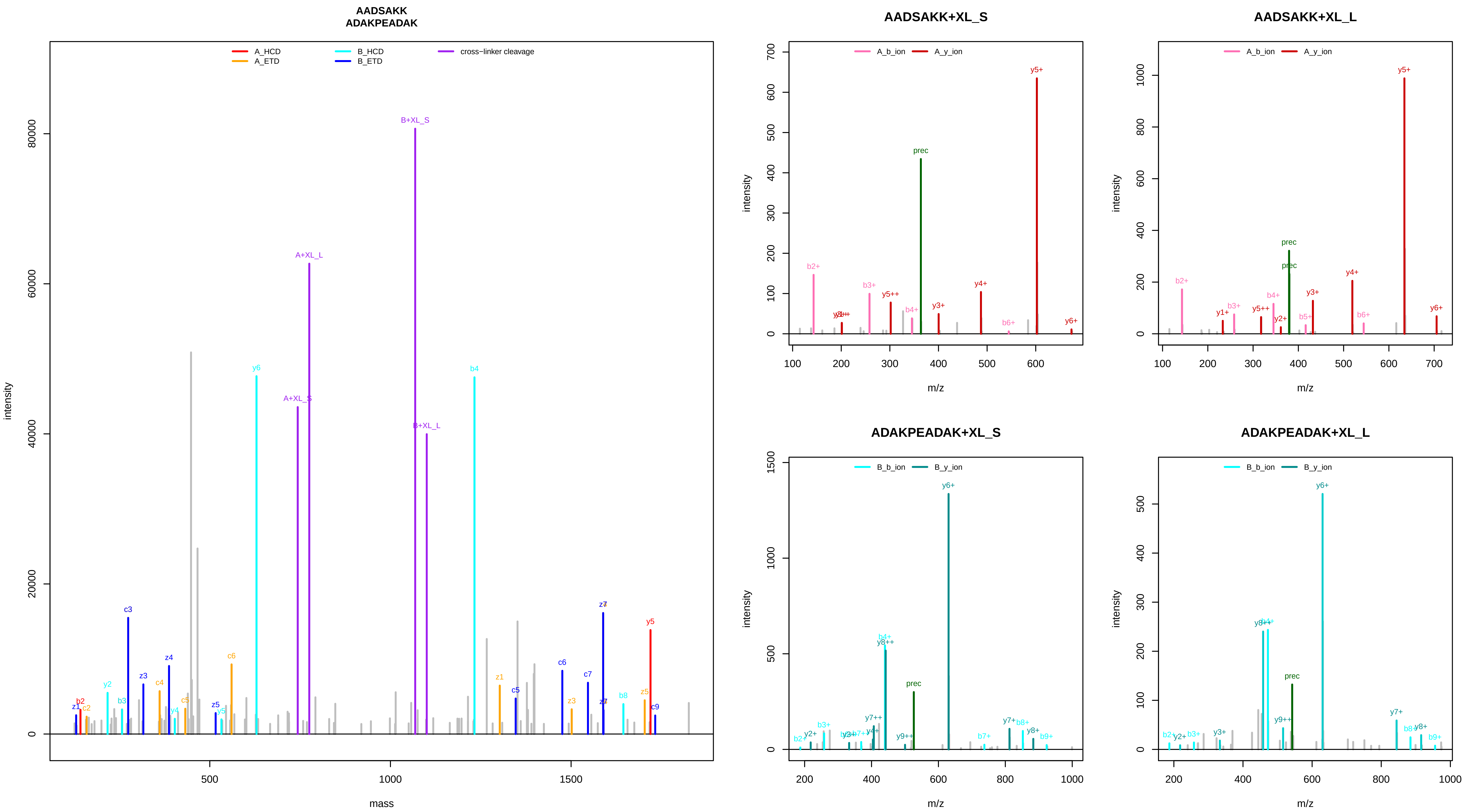

AADSAKK
ADAKPEADAK
A_HCD
A_ETD
B_HCD
B_ETD
cross−linker cleavage
B+XL_S
A+XL_L
A+XL_S
B+XL_L
y6
b4
intensity
mass
AADSAKK+XL_S
AADSAKK+XL_L
ADAKPEADAK+XL_S
ADAKPEADAK+XL_L
A_b_ion
A_y_ion
B_b_ion
B_y_ion
prec
m/z

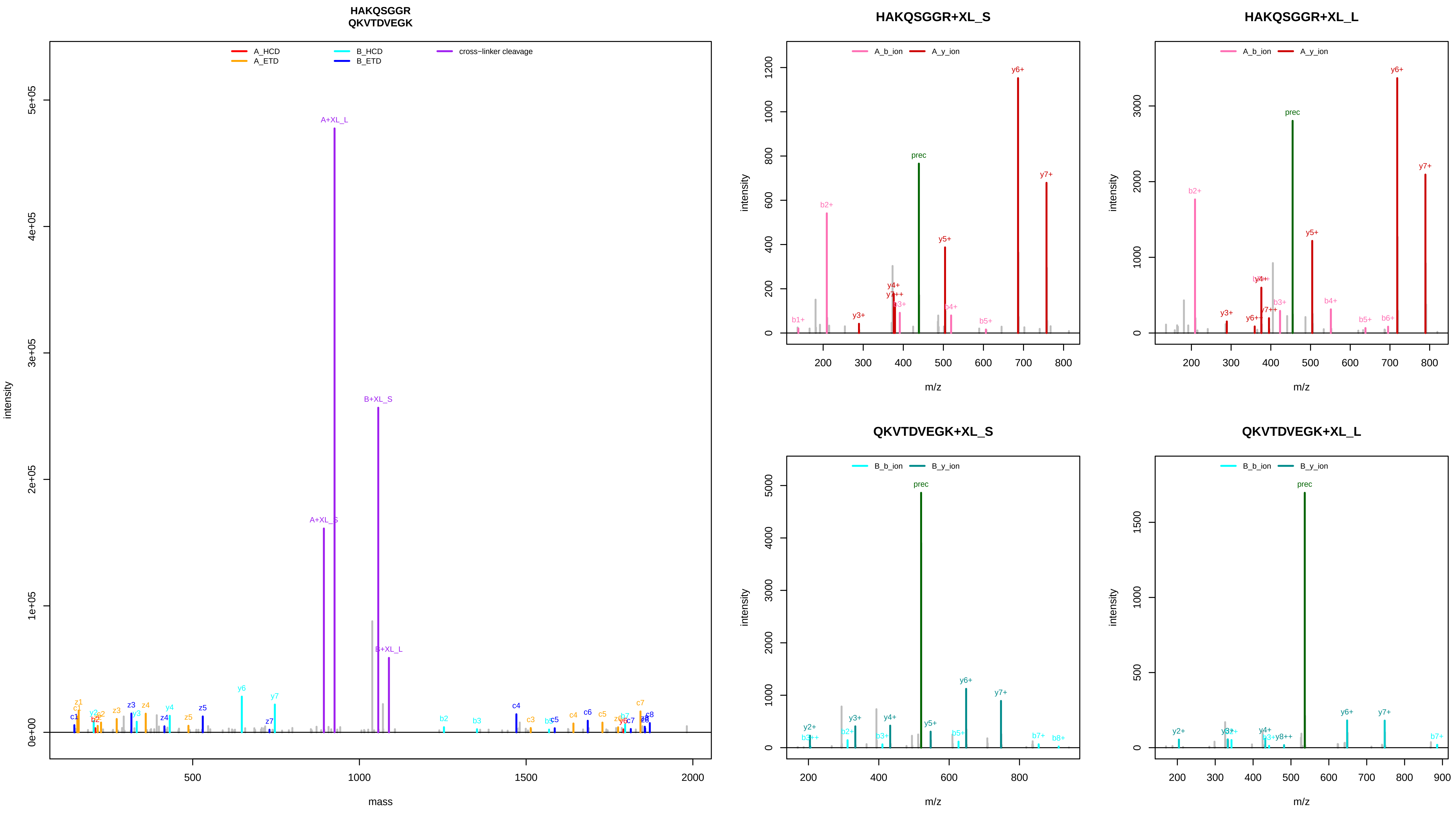
HAKQSGGR
QKVTDVEGK
HAKQSGGR+XL_S
HAKQSGGR+XL_L
QKVTDVEGK+XL_S
QKVTDVEGK+XL_L
A_HCD
A_ETD
B_HCD
B_ETD
cross−linker cleavage
A+XL_L
B+XL_S
A+XL_S
B+XL_L
A_b_ion
A_y_ion
B_b_ion
B_y_ion
prec
intensity
mass
m/z

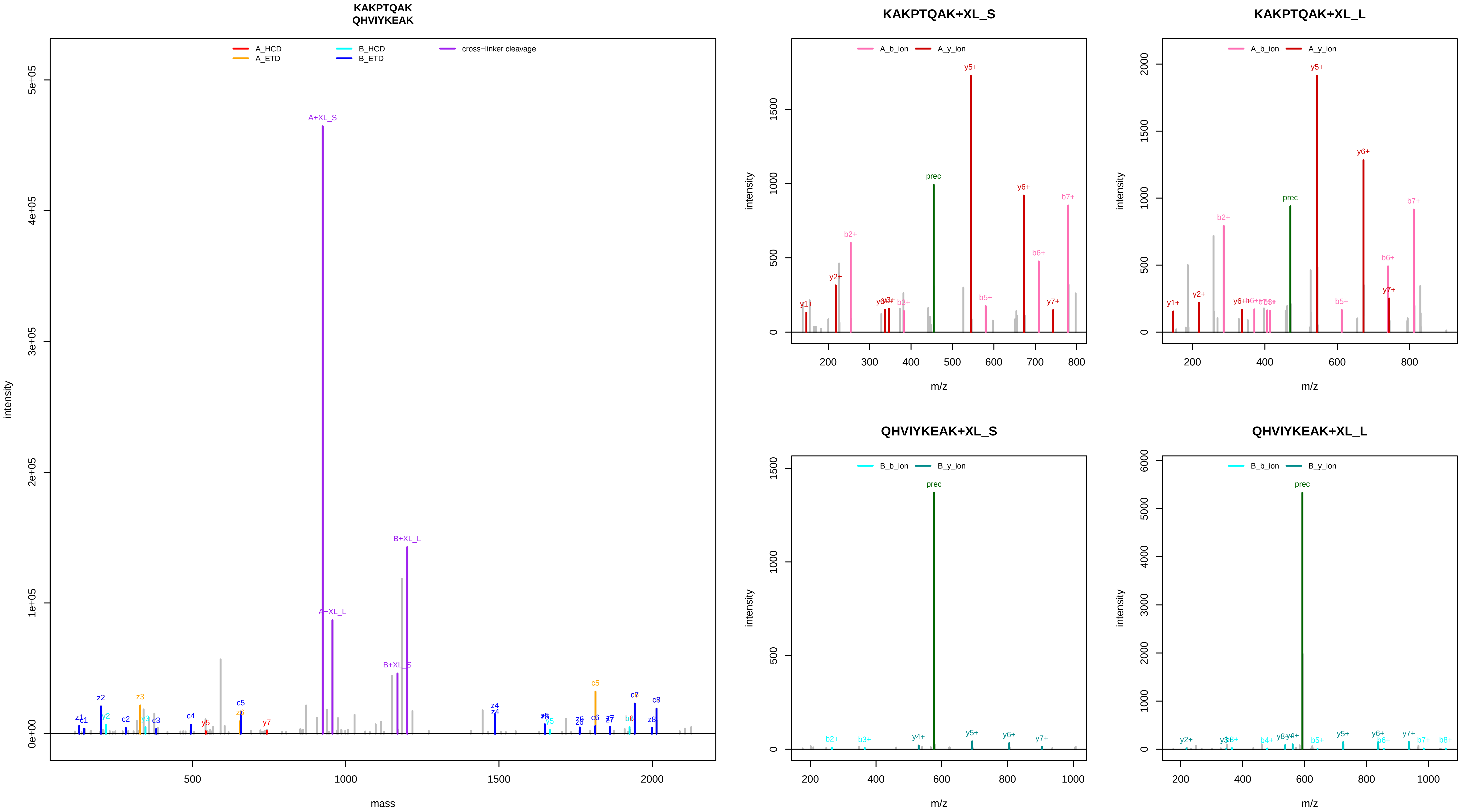
KAKPTQAK
QHVIYKEAK
A_HCD
A_ETD
B_HCD
B_ETD
cross−linker cleavage
A+XL_S
B+XL_L
A+XL_L
B+XL_S
intensity
mass
KAKPTQAK+XL_S
KAKPTQAK+XL_L
QHVIYKEAK+XL_S
QHVIYKEAK+XL_L
A_b_ion
A_y_ion
B_b_ion
B_y_ion
prec
m/z

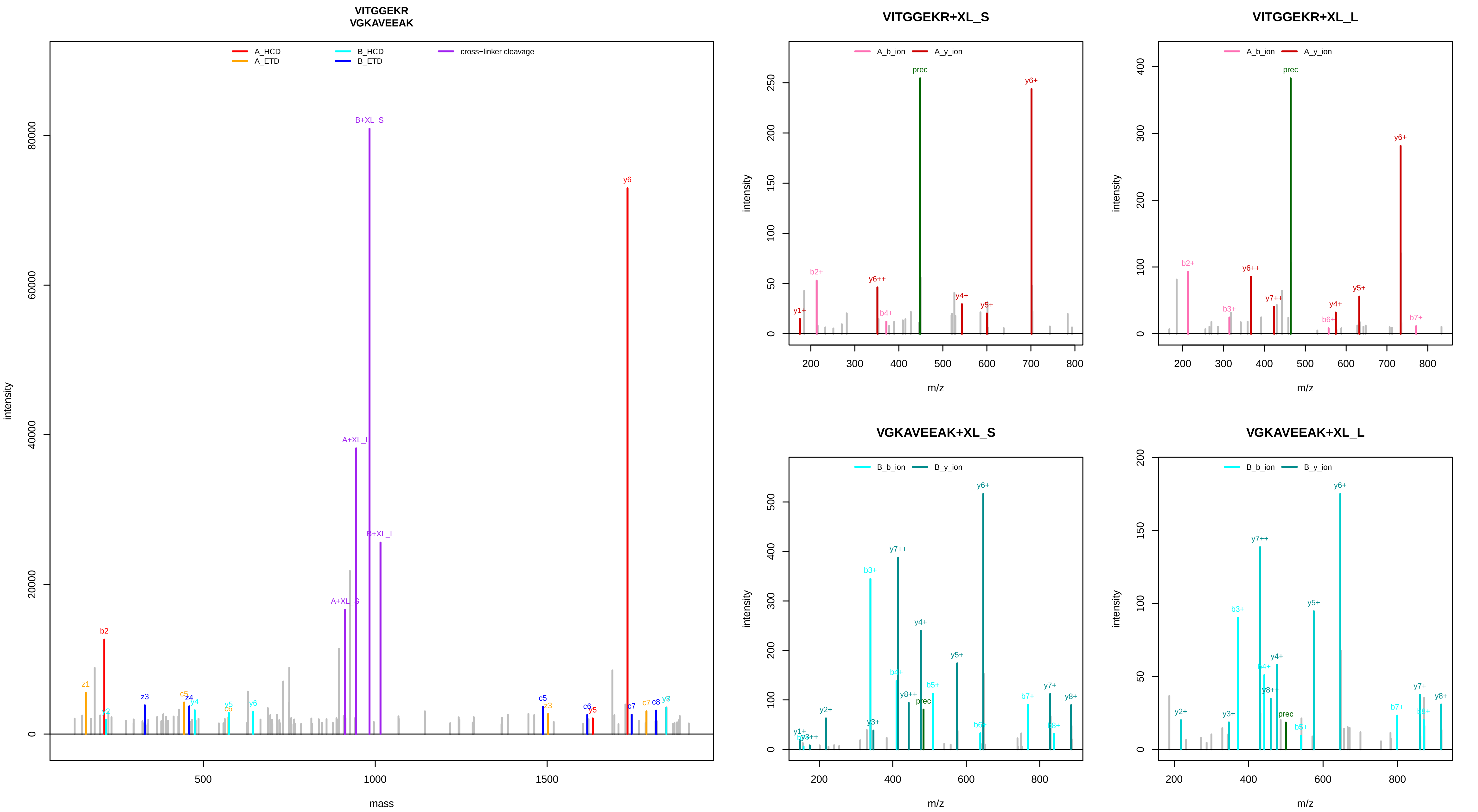
VITGGEKR
VGKAVEEAK
A_HCD
A_ETD
B_HCD
B_ETD
cross−linker cleavage
B+XL_S
A+XL_L
B+XL_L
A+XL_S
y6
b2
z1
z3
c5
z4
y4
y5
c6
c5
z3
c6
y5
c7
c8
intensity
mass
VITGGEKR+XL_S
A_b_ion
A_y_ion
prec
y6+
y6++
b2+
y1+
b4+
y4+
y5+
m/z
VITGGEKR+XL_L
A_b_ion
A_y_ion
prec
y6+
b2+
y6++
y7++
b3+
y4+
y5+
b6+
b7+
m/z
VGKAVEEAK+XL_S
B_b_ion
B_y_ion
y6+
y7++
b3+
y4+
y5+
b4+
y8++
prec
b5+
y7+
y8+
b7+
y2+
y3+
y1+
m/z
VGKAVEEAK+XL_L
B_b_ion
B_y_ion
y6+
y7++
b3+
y5+
y4+
y8++
prec
y2+
y3+
b5+
b7+
y7+
y8+
m/z

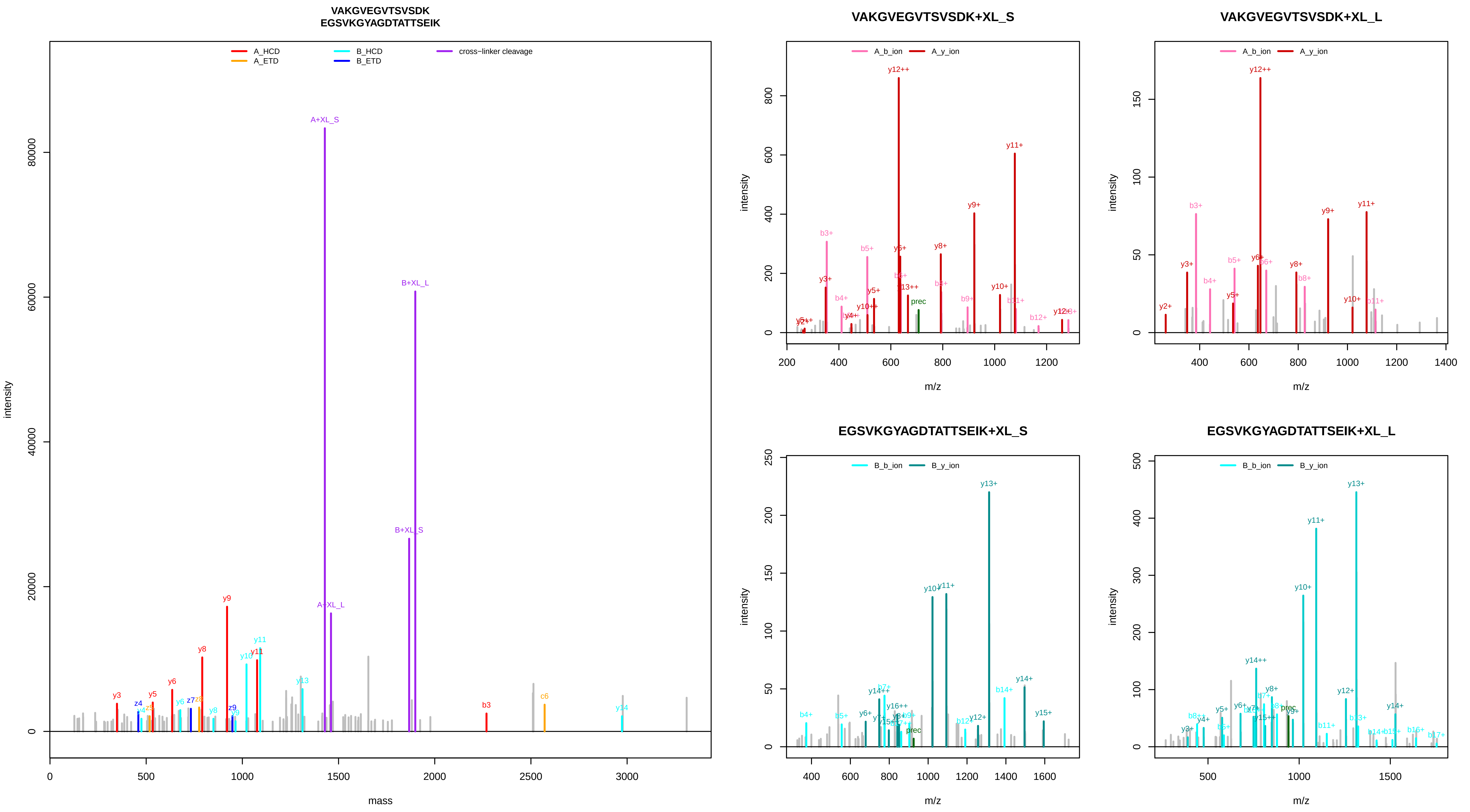
VAKGVEGVTSVSDK
EGSVKGYAGDTATTSEIK
A_HCD
A_ETD
B_HCD
B_ETD
cross−linker cleavage
A+XL_S
B+XL_L
B+XL_S
A+XL_L
intensity
mass
VAKGVEGVTSVSDK+XL_S
VAKGVEGVTSVSDK+XL_L
EGSVKGYAGDTATTSEIK+XL_S
EGSVKGYAGDTATTSEIK+XL_L
A_b_ion
A_y_ion
B_b_ion
B_y_ion
m/z
prec

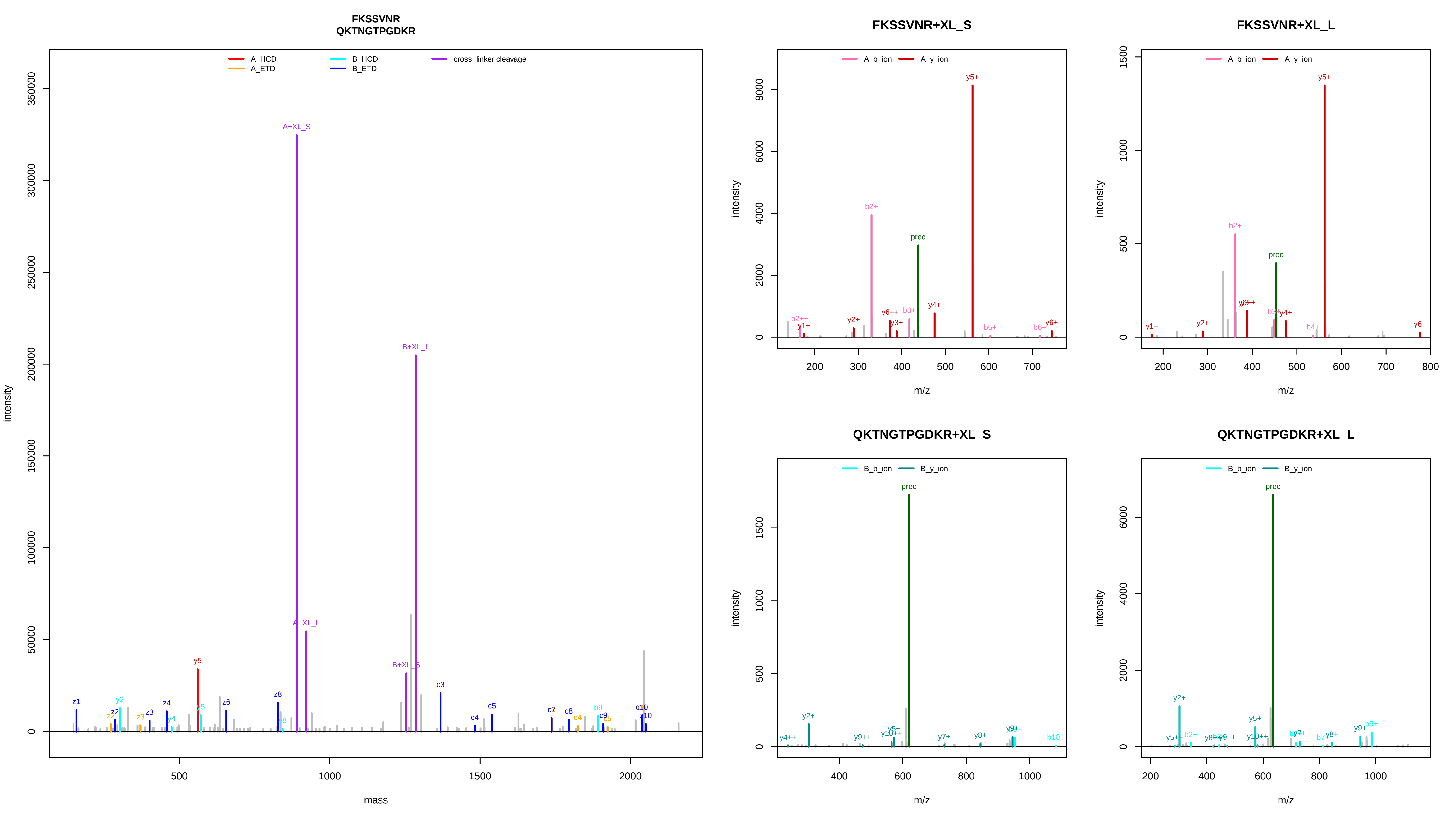

FKSSVNR
QKTNGTPGDKR
A_HCD
A_ETD
B_HCD
B_ETD
cross−linker cleavage
A+XL_S
B+XL_L
A+XL_L
B+XL_S
y5
z1
z2
z3
z4
z6
z8
c3
c4
c5
c7
c8
c9
c10
z10
y2
b9
intensity
mass
FKSSVNR+XL_S
A_b_ion
A_y_ion
y5+
b2+
prec
y4+
b3+
y6++
y3+
y2+
b2++
y1+
b5+
b6+
y6+
intensity
m/z
FKSSVNR+XL_L
A_b_ion
A_y_ion
y5+
b2+
prec
y4+
b3+
b4+
y1+
y2+
y6+
intensity
m/z
QKTNGTPGDKR+XL_S
B_b_ion
B_y_ion
prec
y2+
y4++
y9++
y5+
y10++
y7+
y8+
y9+
b10+
intensity
m/z
QKTNGTPGDKR+XL_L
B_b_ion
B_y_ion
prec
y2+
y5+
b2+
y5++
y8++
y9++
y10++
y7+
y8+
y9+
b9+
intensity
m/z

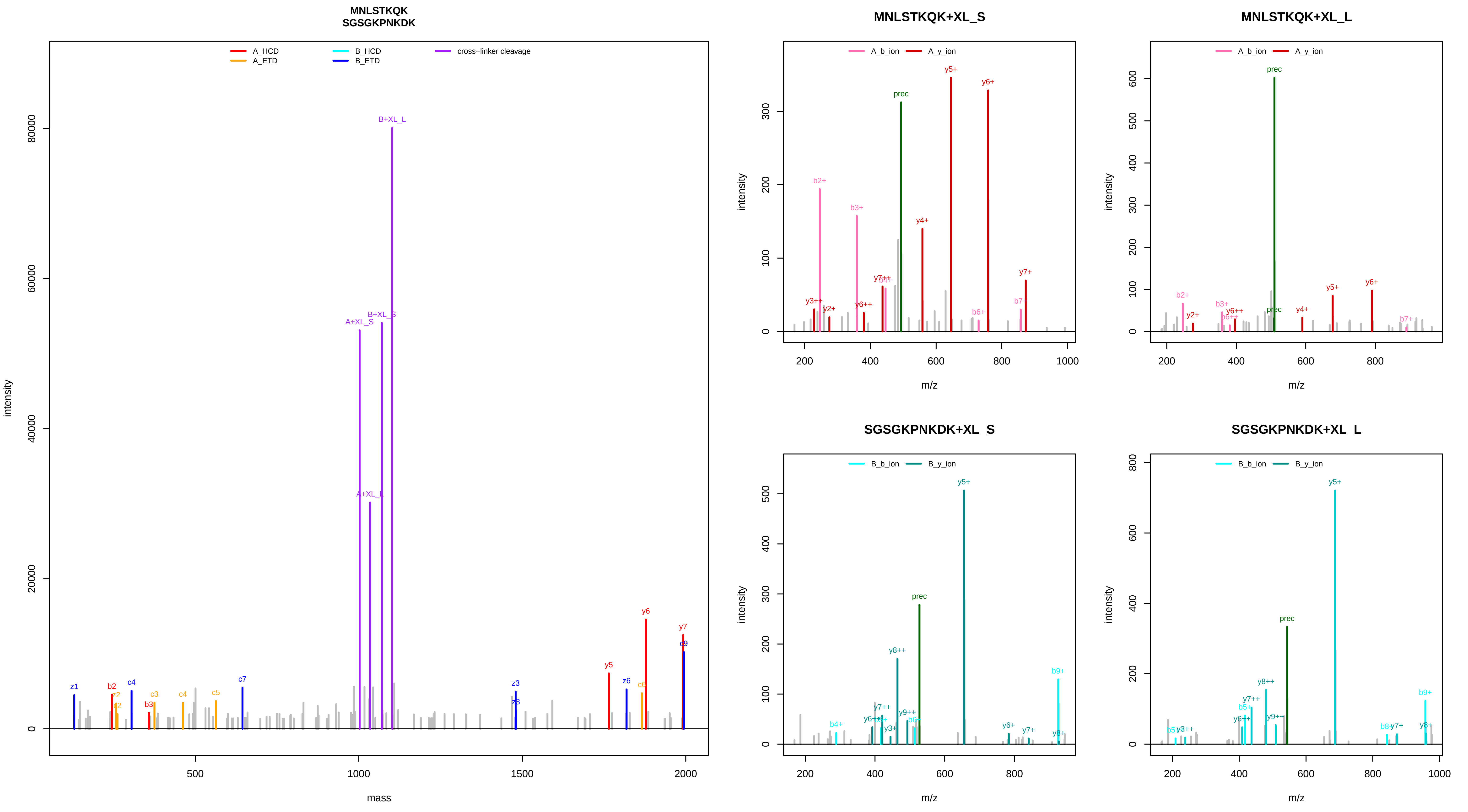
MNLSTKQK
SGSGKPNKDK
A_HCD
A_ETD
B_HCD
B_ETD
cross−linker cleavage
B+XL_L
A+XL_S
B+XL_S
A+XL_L
intensity
mass
MNLSTKQK+XL_S
MNLSTKQK+XL_L
A_b_ion
A_y_ion
prec
m/z
SGSGKPNKDK+XL_S
SGSGKPNKDK+XL_L
B_b_ion
B_y_ion

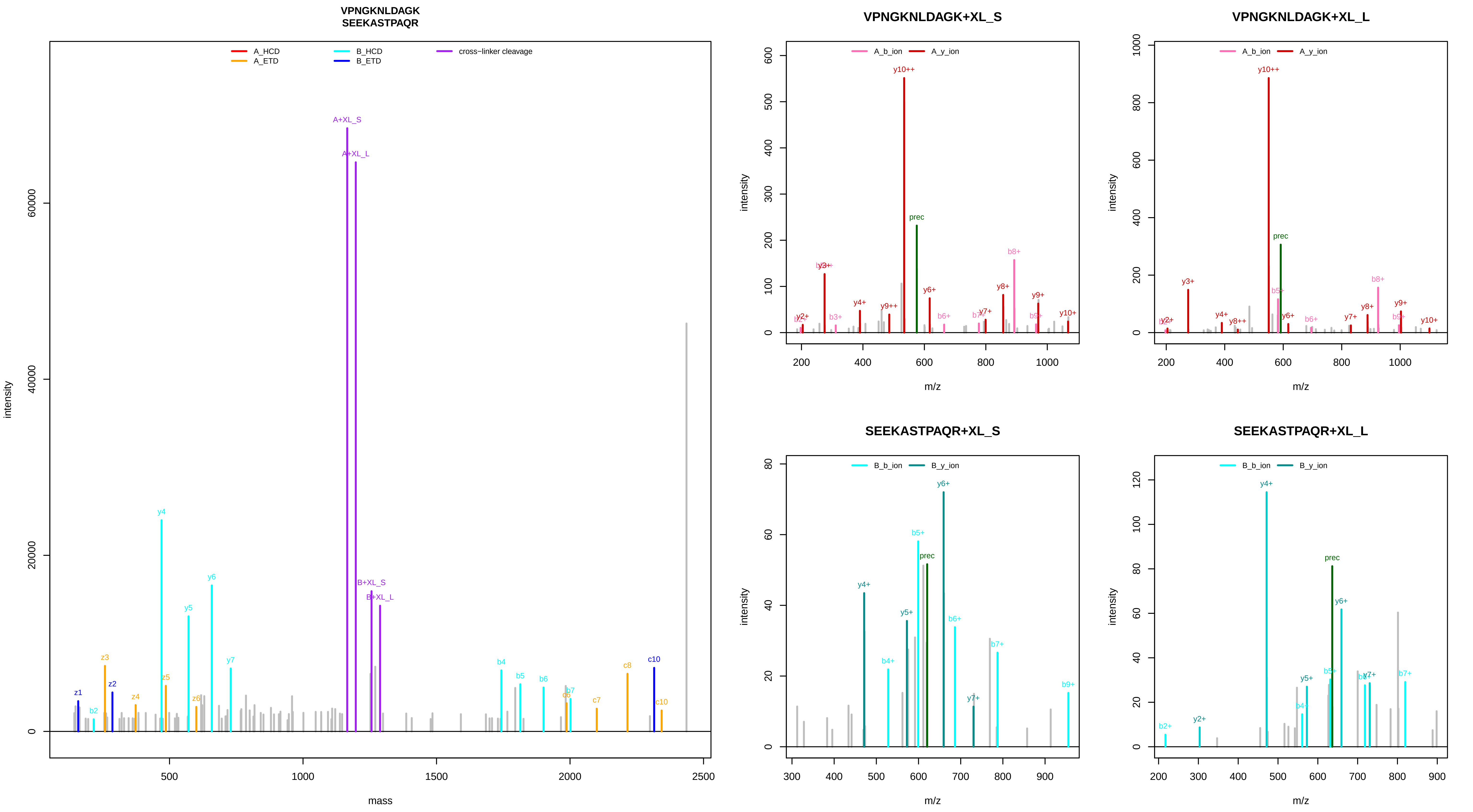
VPNGKNLDAGK
SEEKASTPAQR
A_HCD
A_ETD
B_HCD
B_ETD
cross−linker cleavage
A+XL_S
A+XL_L
B+XL_S
B+XL_L
intensity
mass
VPNGKNLDAGK+XL_S
VPNGKNLDAGK+XL_L
SEEKASTPAQR+XL_S
SEEKASTPAQR+XL_L
A_b_ion
A_y_ion
B_b_ion
B_y_ion
prec
m/z

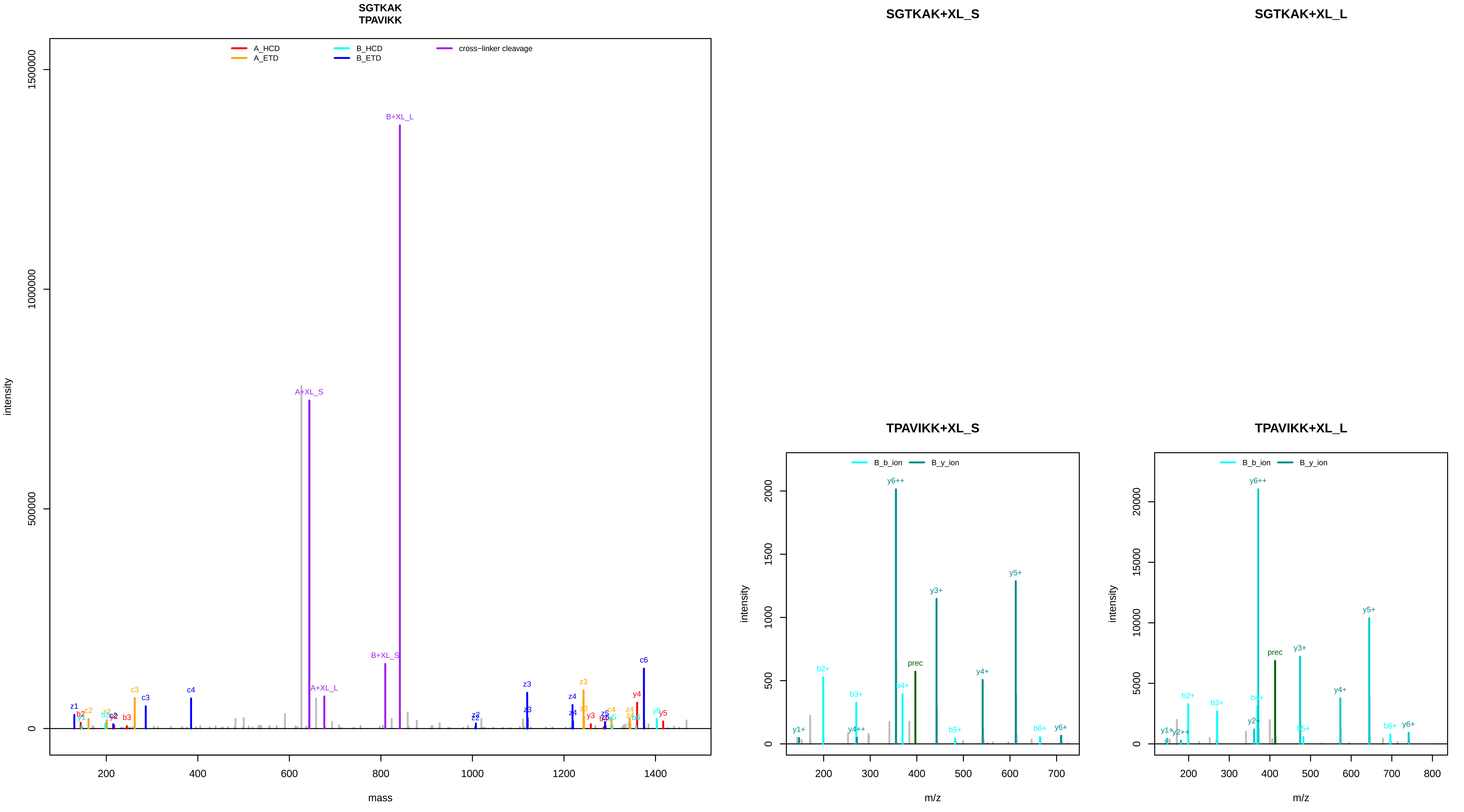
SGTKAK
TPAVIKK
A_HCD
A_ETD
B_HCD
B_ETD
cross−linker cleavage
B+XL_L
A+XL_S
B+XL_S
A+XL_L
intensity
mass
SGTKAK+XL_S
SGTKAK+XL_L
TPAVIKK+XL_S
TPAVIKK+XL_L
B_b_ion
B_y_ion
y6++
y5+
y3+
y4+
b2+
b3+
prec
m/z

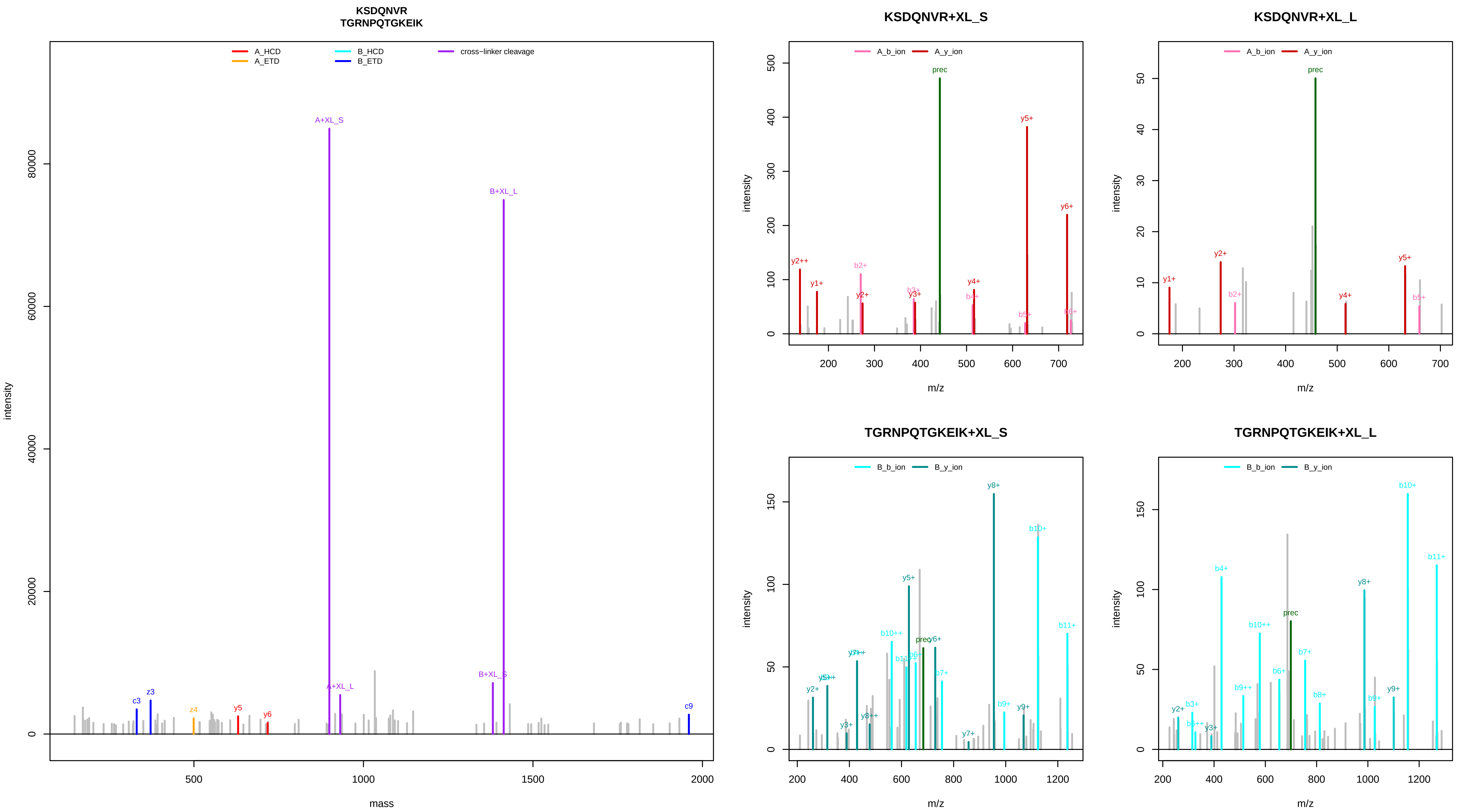
KSDQNVR
TGRNPQTGKEIK
A_HCD
A_ETD
B_HCD
B_ETD
cross−linker cleavage
A+XL_S
B+XL_L
B+XL_S
A+XL_L
c3
z3
z4
y5
y6
c9
intensity
mass
KSDQNVR+XL_S
KSDQNVR+XL_L
TGRNPQTGKEIK+XL_S
TGRNPQTGKEIK+XL_L
A_b_ion
A_y_ion
B_b_ion
B_y_ion
prec
m/z

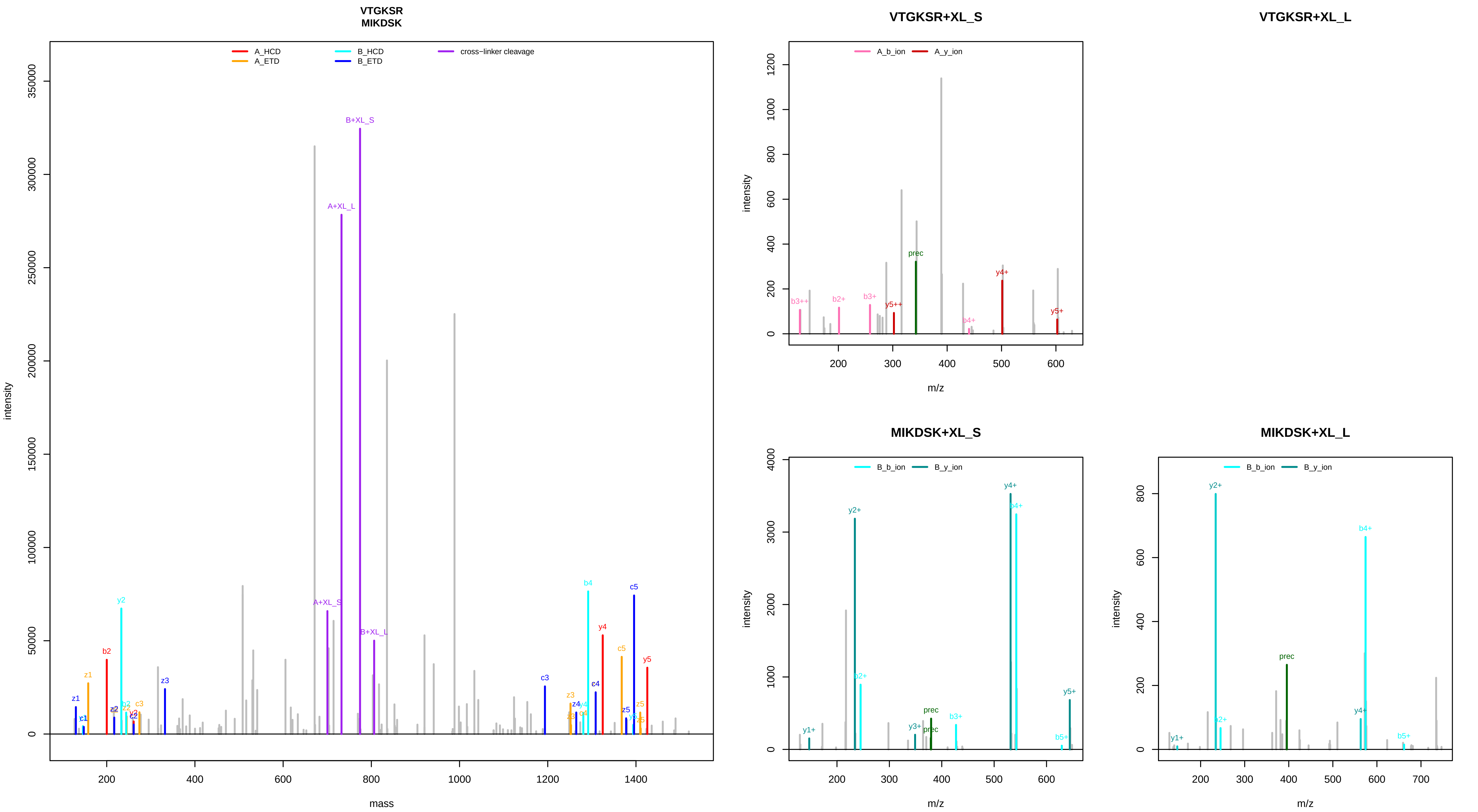
VTGKSR
MIKDSK
A_HCD
A_ETD
B_HCD
B_ETD
cross−linker cleavage
B+XL_S
A+XL_L
A+XL_S
B+XL_L
intensity
mass
VTGKSR+XL_S
A_b_ion
A_y_ion
prec
m/z
VTGKSR+XL_L
MIKDSK+XL_S
B_b_ion
B_y_ion
MIKDSK+XL_L

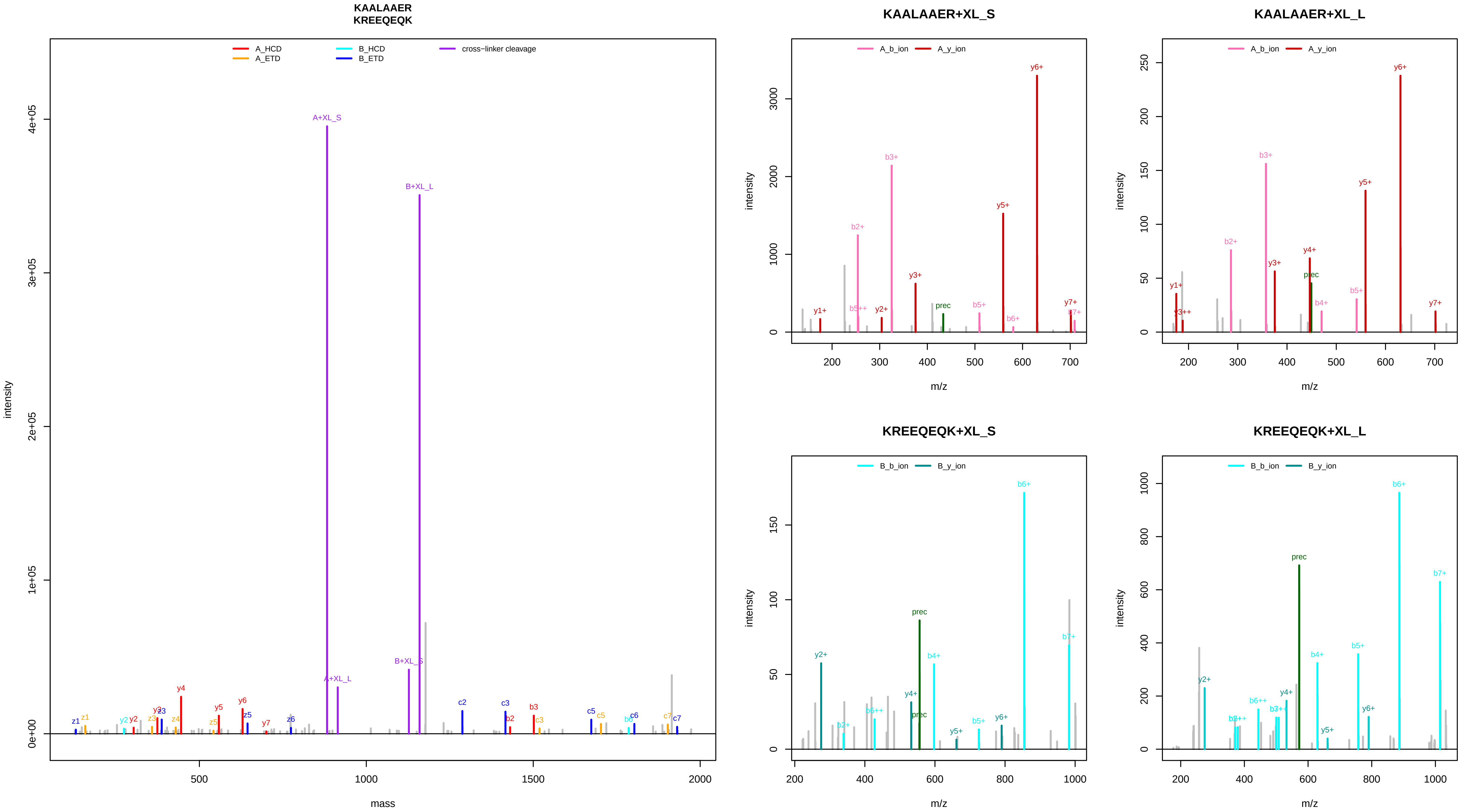
KAALAAER
KREEQEQK
A_HCD
A_ETD
B_HCD
B_ETD
cross−linker cleavage
A+XL_S
B+XL_L
B+XL_S
A+XL_L
intensity
mass
KAALAAER+XL_S
KAALAAER+XL_L
KREEQEQK+XL_S
KREEQEQK+XL_L
A_b_ion
A_y_ion
B_b_ion
B_y_ion
m/z
prec

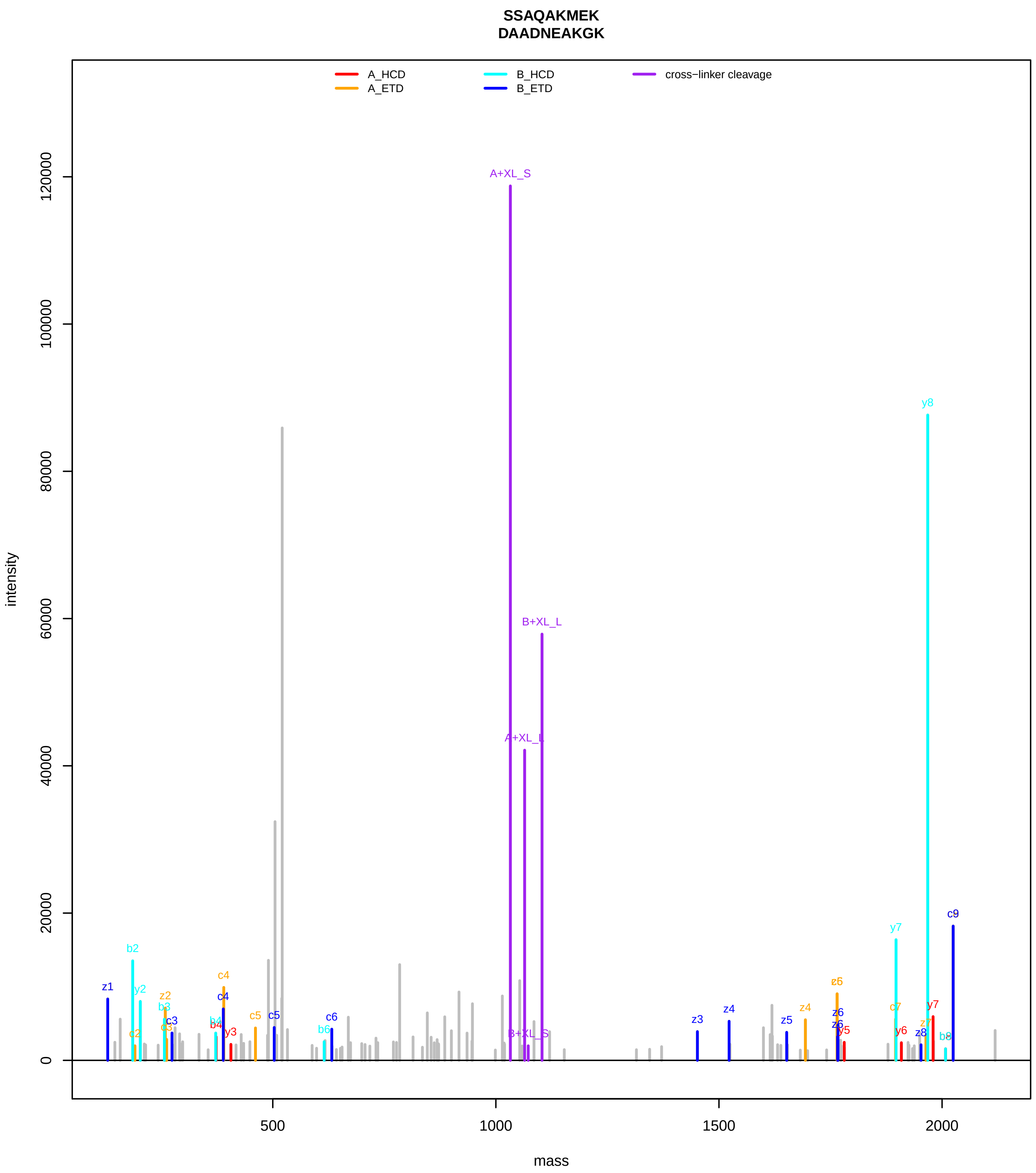

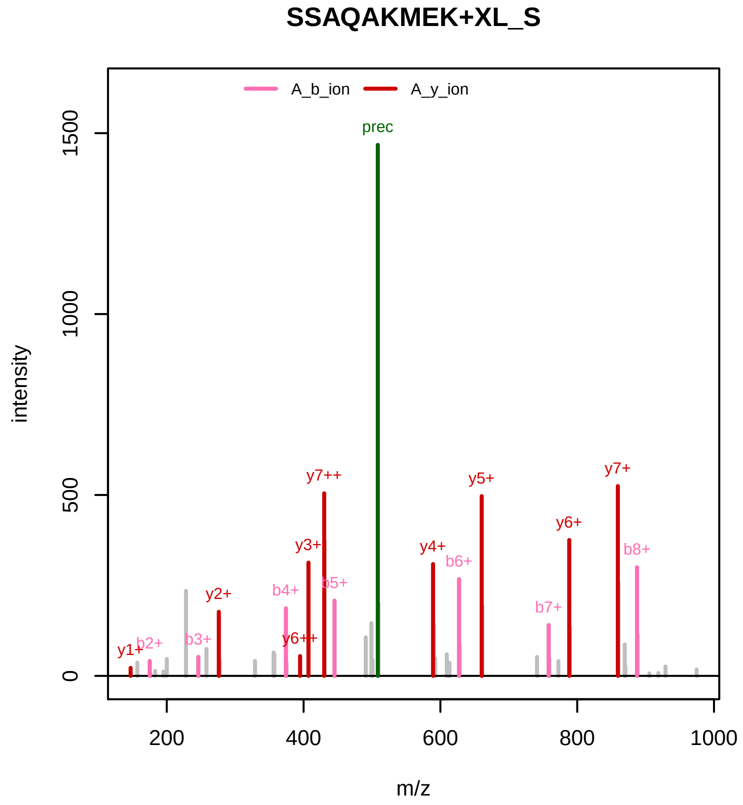

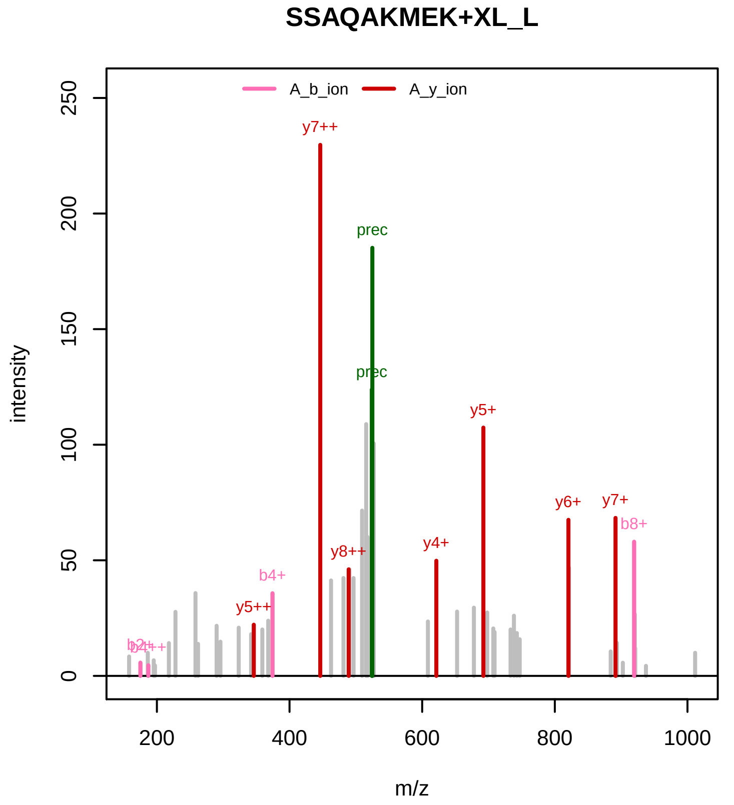

DAADNEAKGK+XL_S

DAADNEAKGK+XL_L

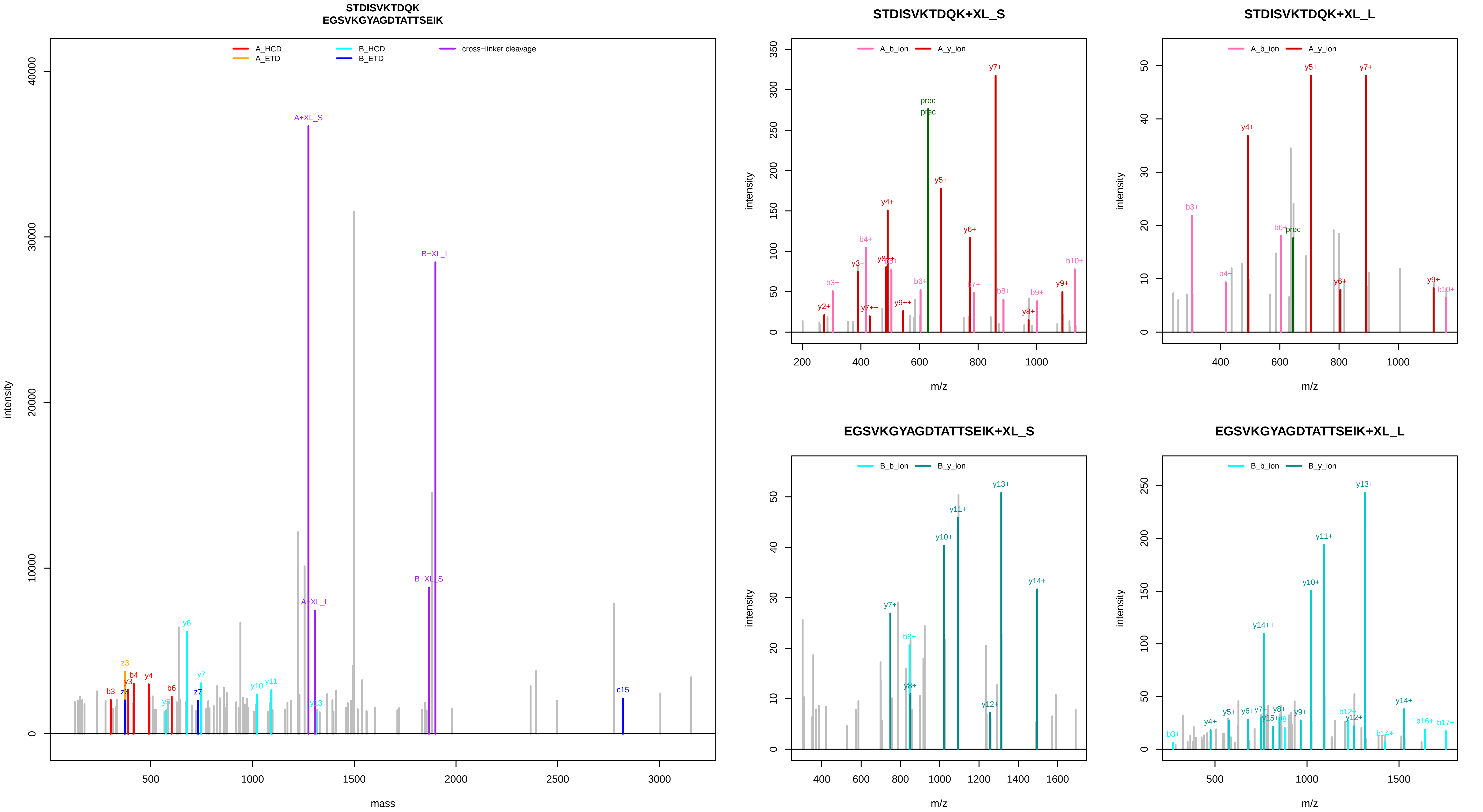
STDISVKTDQK
EGSVKGYAGDTATTSEIK
A_HCD
A_ETD
B_HCD
B_ETD
cross−linker cleavage
A+XL_S
B+XL_L
B+XL_S
A+XL_L
intensity
mass
STDISVKTDQK+XL_S
STDISVKTDQK+XL_L
EGSVKGYAGDTATTSEIK+XL_S
EGSVKGYAGDTATTSEIK+XL_L
A_b_ion
A_y_ion
B_b_ion
B_y_ion
m/z
prec

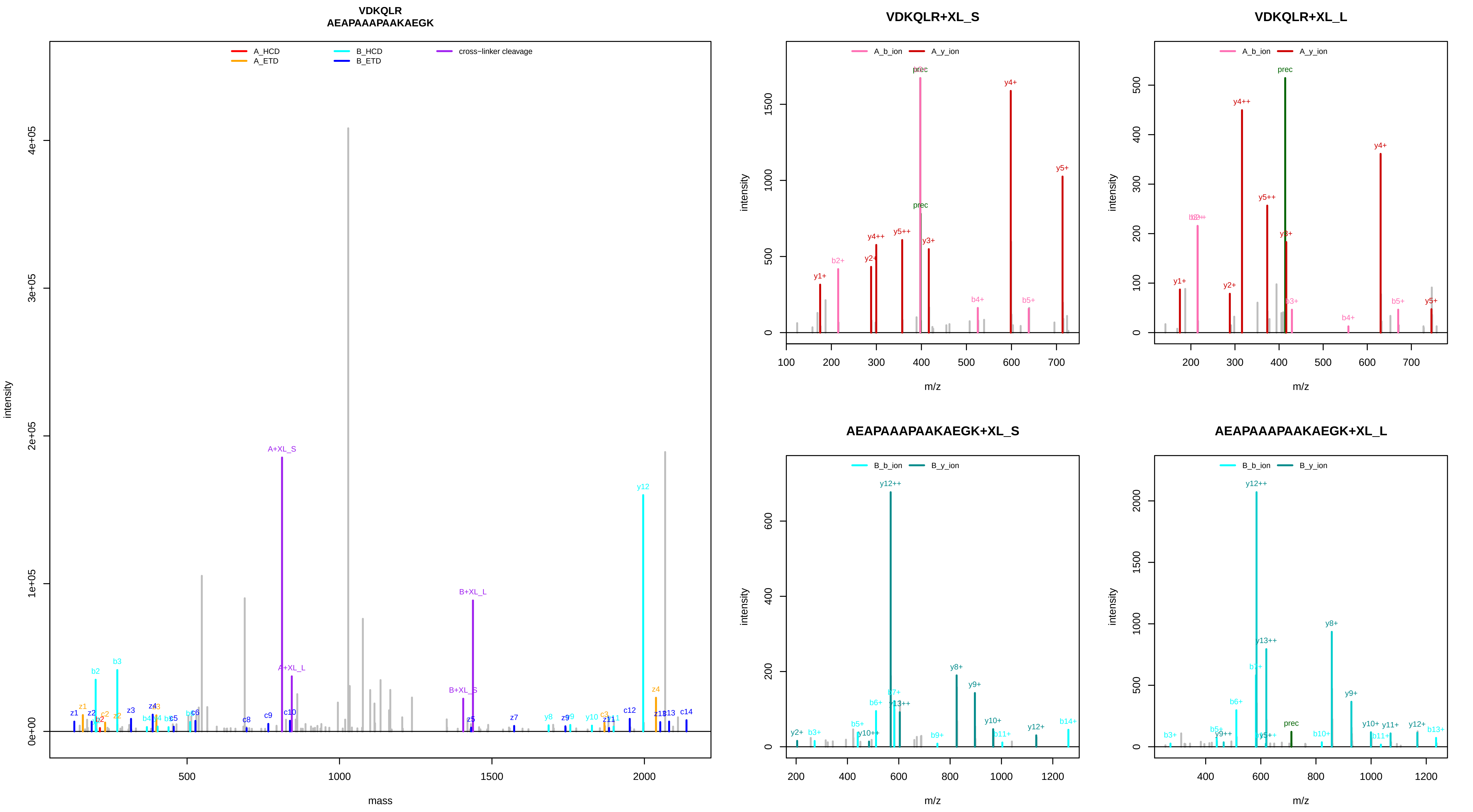
VDKQLR
AEAPAAAPAAKAEGK
A_HCD
A_ETD
B_HCD
B_ETD
cross−linker cleavage
intensity
mass
A+XL_S
A+XL_L
B+XL_L
B+XL_S
VDKQLR+XL_S
VDKQLR+XL_L
AEAPAAAPAAKAEGK+XL_S
AEAPAAAPAAKAEGK+XL_L
A_b_ion
A_y_ion
B_b_ion
B_y_ion
prec
m/z

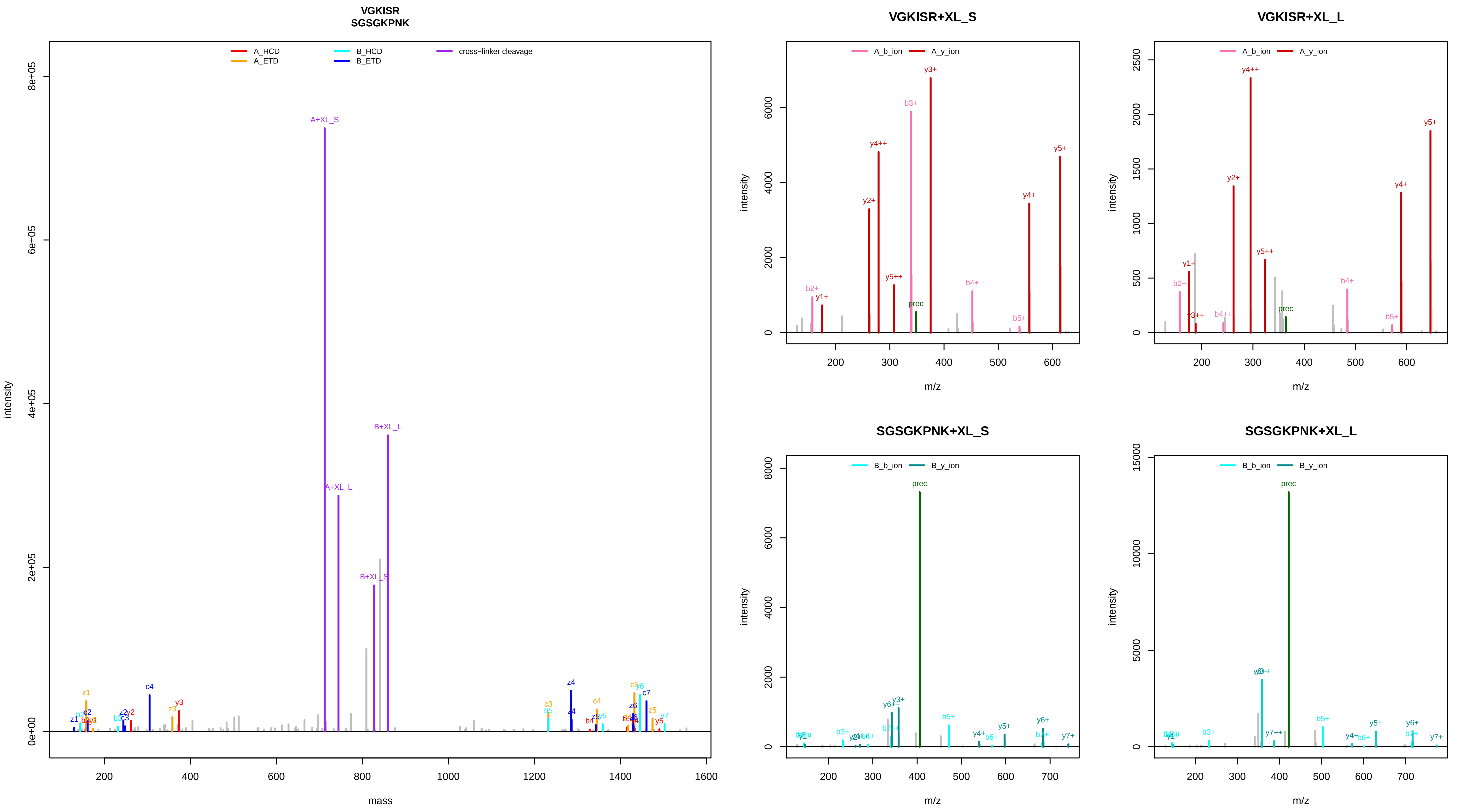
VGKISR
SGSGKPNK
A_HCD
A_ETD
B_HCD
B_ETD
cross−linker cleavage
A+XL_S
A+XL_L
B+XL_L
B+XL_S
intensity
mass
VGKISR+XL_S
VGKISR+XL_L
SGSGKPNK+XL_S
SGSGKPNK+XL_L
A_b_ion
A_y_ion
B_b_ion
B_y_ion
prec
m/z
intensity

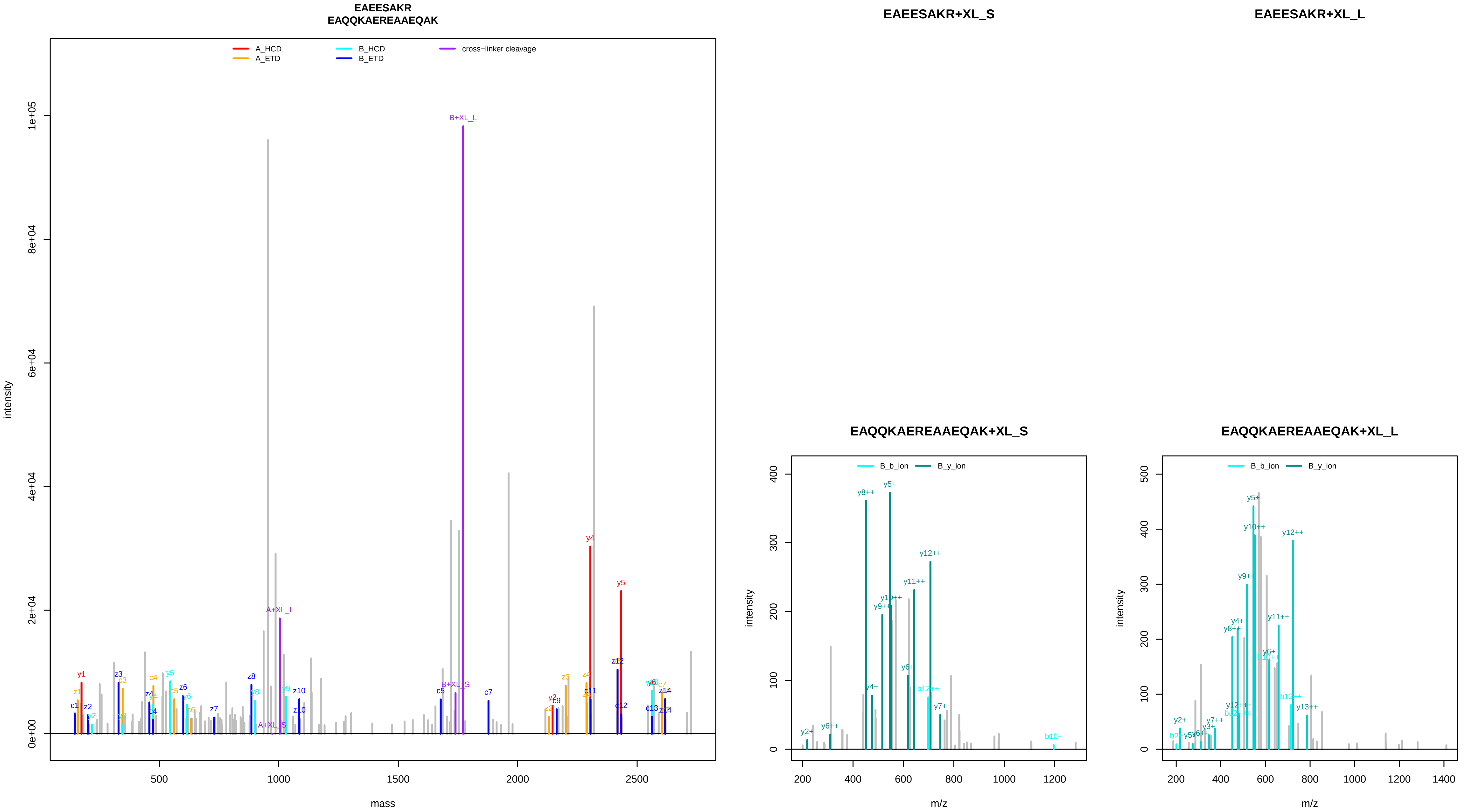
EAEESAKR
EAQQKAEREAAEQAK
A_HCD
A_ETD
B_HCD
B_ETD
cross−linker cleavage
B+XL_L
A+XL_L
B+XL_S
A+XL_S
intensity
mass
EAEESAKR+XL_S
EAEESAKR+XL_L
EAQQKAEREAAEQAK+XL_S
EAQQKAEREAAEQAK+XL_L
B_b_ion
B_y_ion
m/z

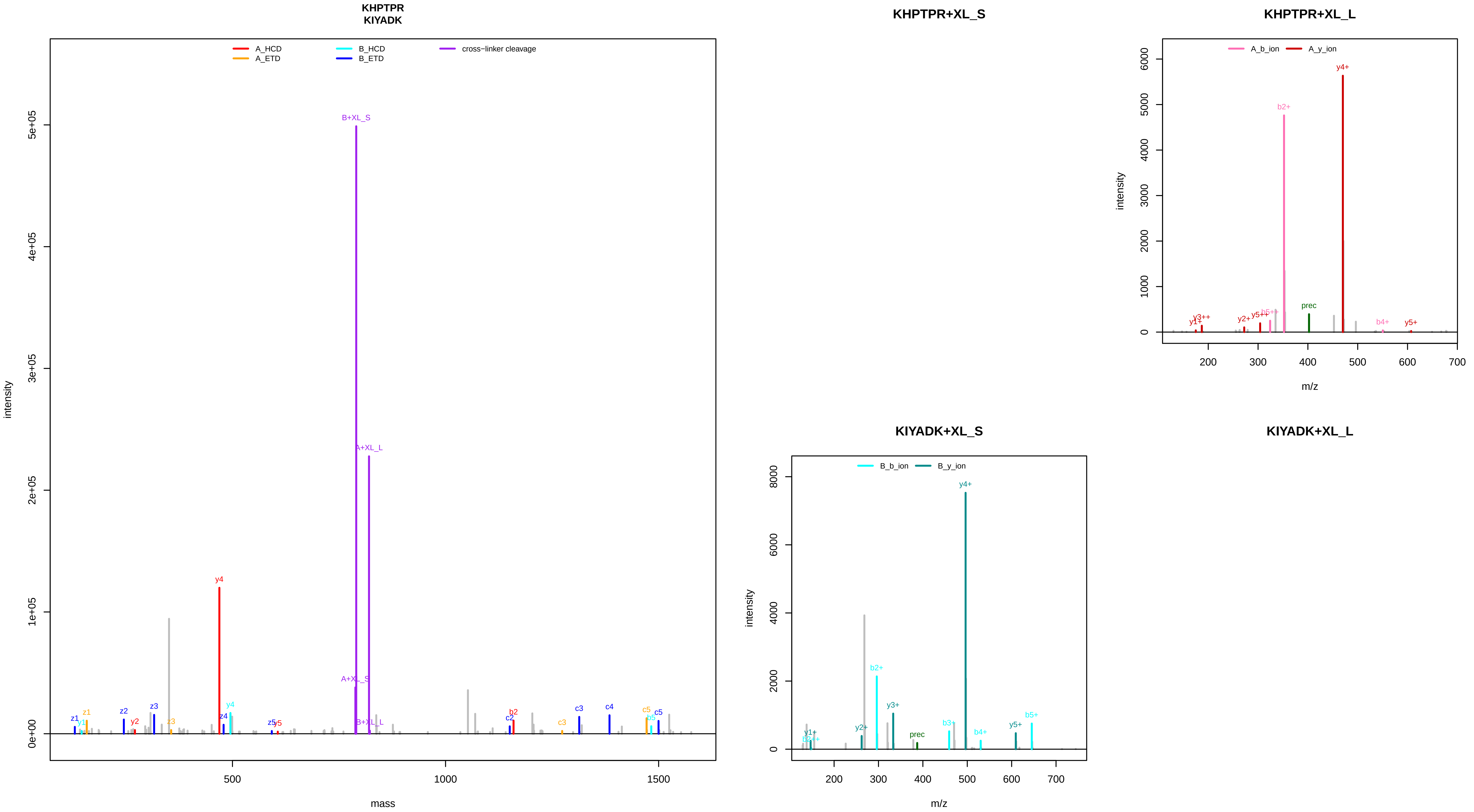

KHPTPR
KIYADK
A_HCD
A_ETD
B_HCD
B_ETD
cross−linker cleavage
B+XL_S
A+XL_L
A+XL_S
B+XL_L
y4
intensity
mass
KHPTPR+XL_S
KHPTPR+XL_L
A_b_ion
A_y_ion
y4+
b2+
prec
m/z
KIYADK+XL_S
KIYADK+XL_L
B_b_ion
B_y_ion

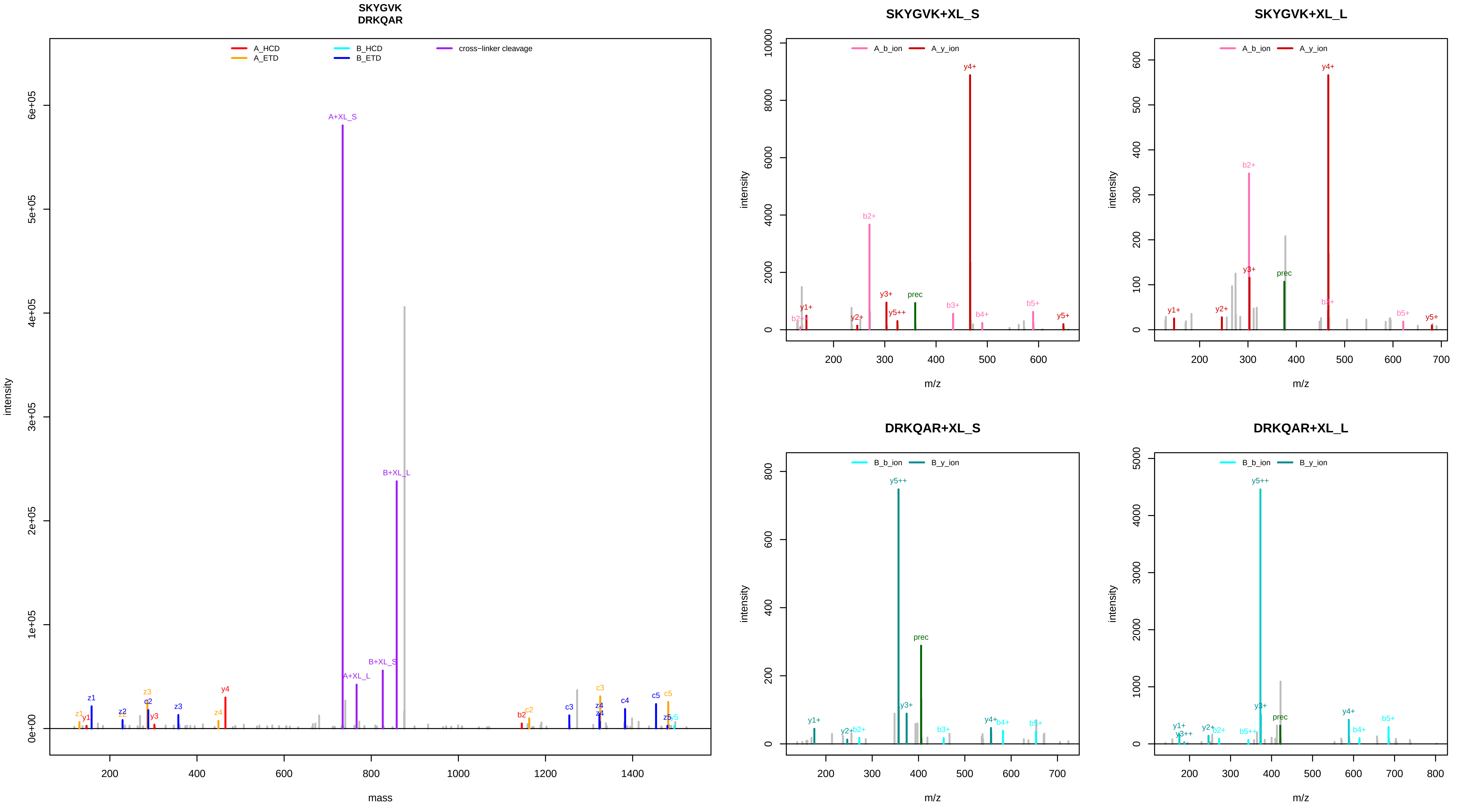

SKYGVK
DRKQAR
A_HCD
A_ETD
B_HCD
B_ETD
cross−linker cleavage
A+XL_S
B+XL_L
B+XL_S
A+XL_L
intensity
mass
SKYGVK+XL_S
SKYGVK+XL_L
DRKQAR+XL_S
DRKQAR+XL_L
A_b_ion
A_y_ion
B_b_ion
B_y_ion
prec
m/z

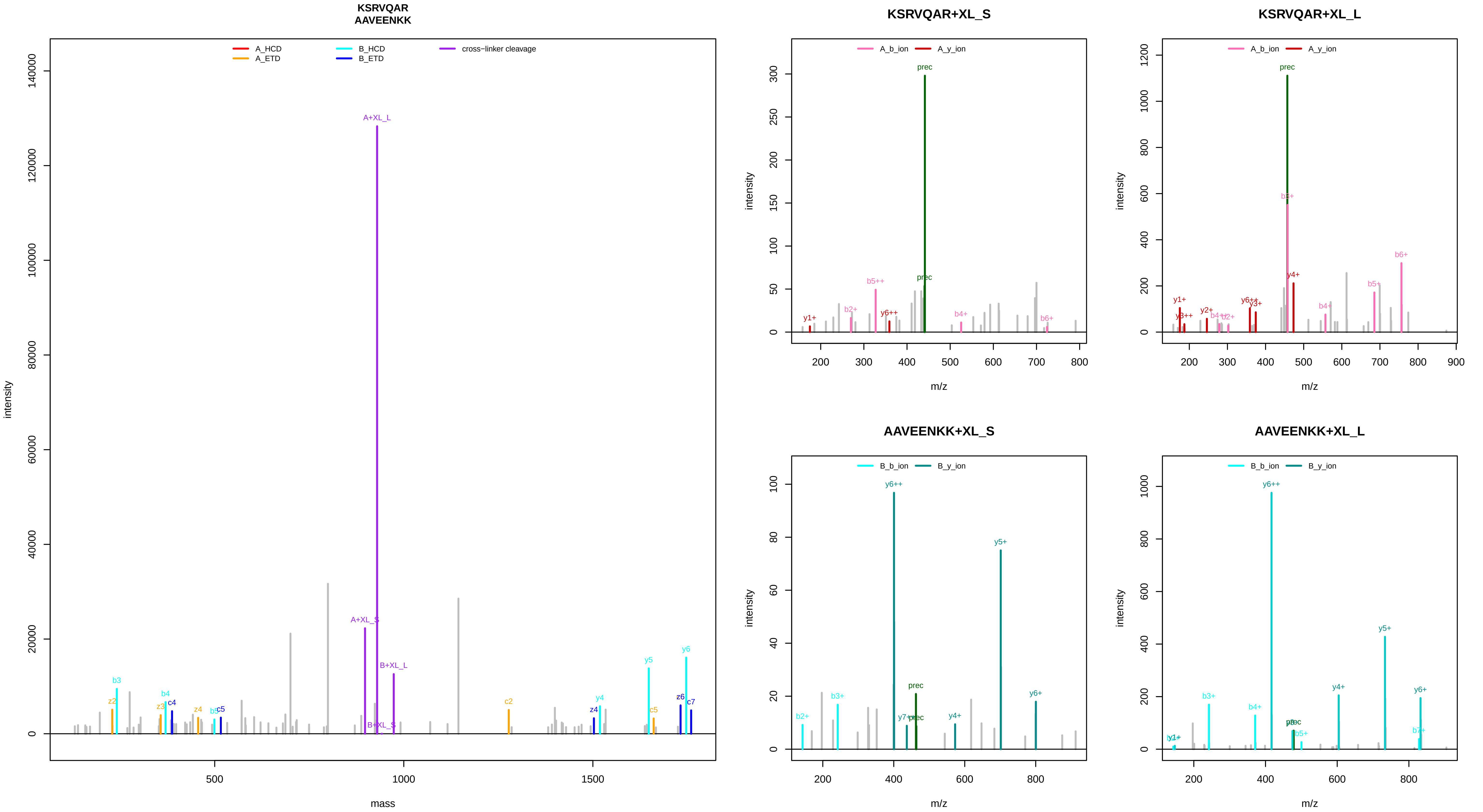
KSRVQAR
AAVEENKK
A_HCD
A_ETD
B_HCD
B_ETD
cross−linker cleavage
A+XL_L
A+XL_S
B+XL_L
B+XL_S
intensity
mass
KSRVQAR+XL_S
KSRVQAR+XL_L
AAVEENKK+XL_S
AAVEENKK+XL_L
A_b_ion
A_y_ion
B_b_ion
B_y_ion
prec
intensity
m/z

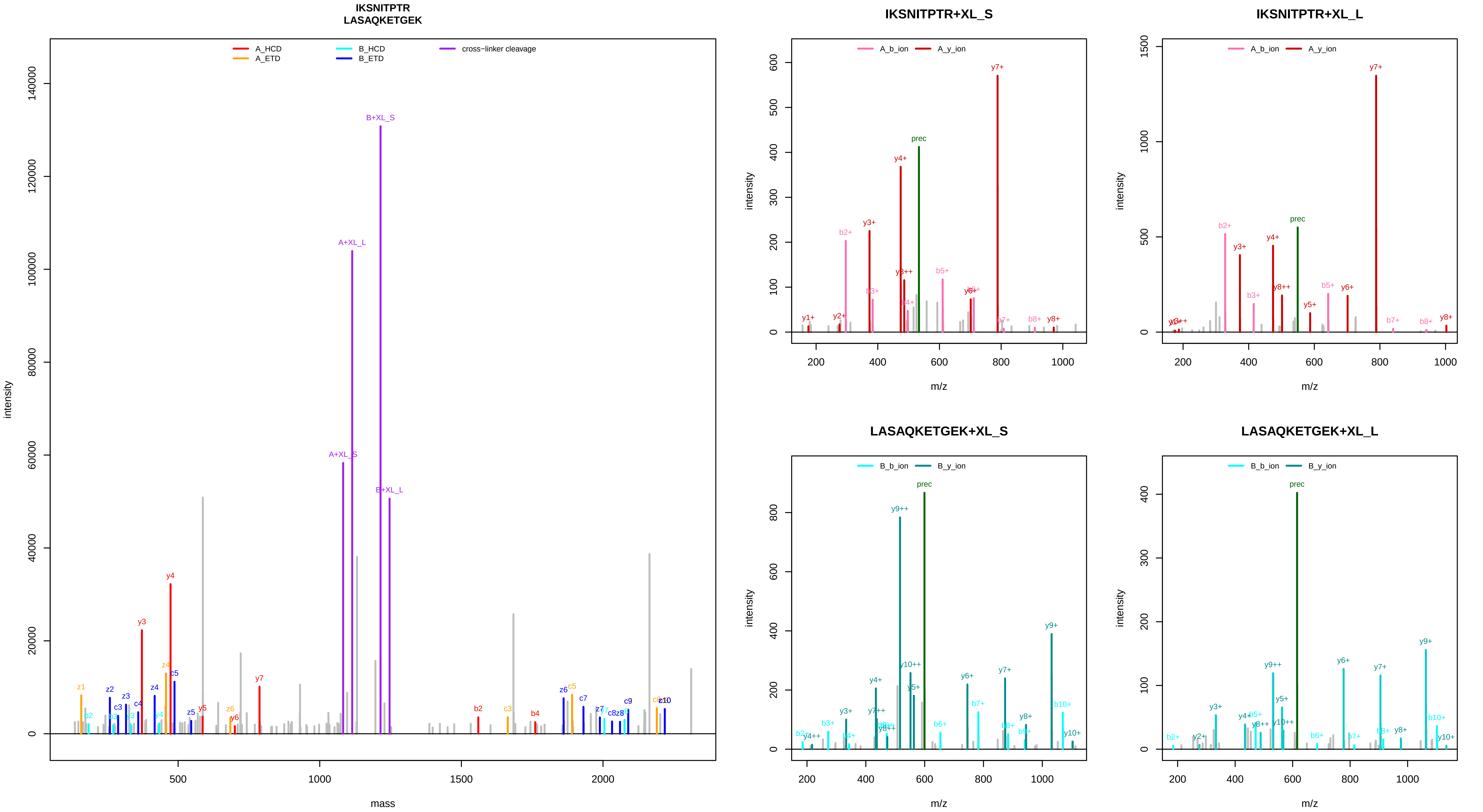
IKSNITPTR
LASAQKETGEK
A_HCD
A_ETD
B_HCD
B_ETD
cross−linker cleavage
B+XL_S
A+XL_L
A+XL_S
B+XL_L
intensity
mass
IKSNITPTR+XL_S
IKSNITPTR+XL_L
LASAQKETGEK+XL_S
LASAQKETGEK+XL_L
A_b_ion
A_y_ion
B_b_ion
B_y_ion
prec
m/z

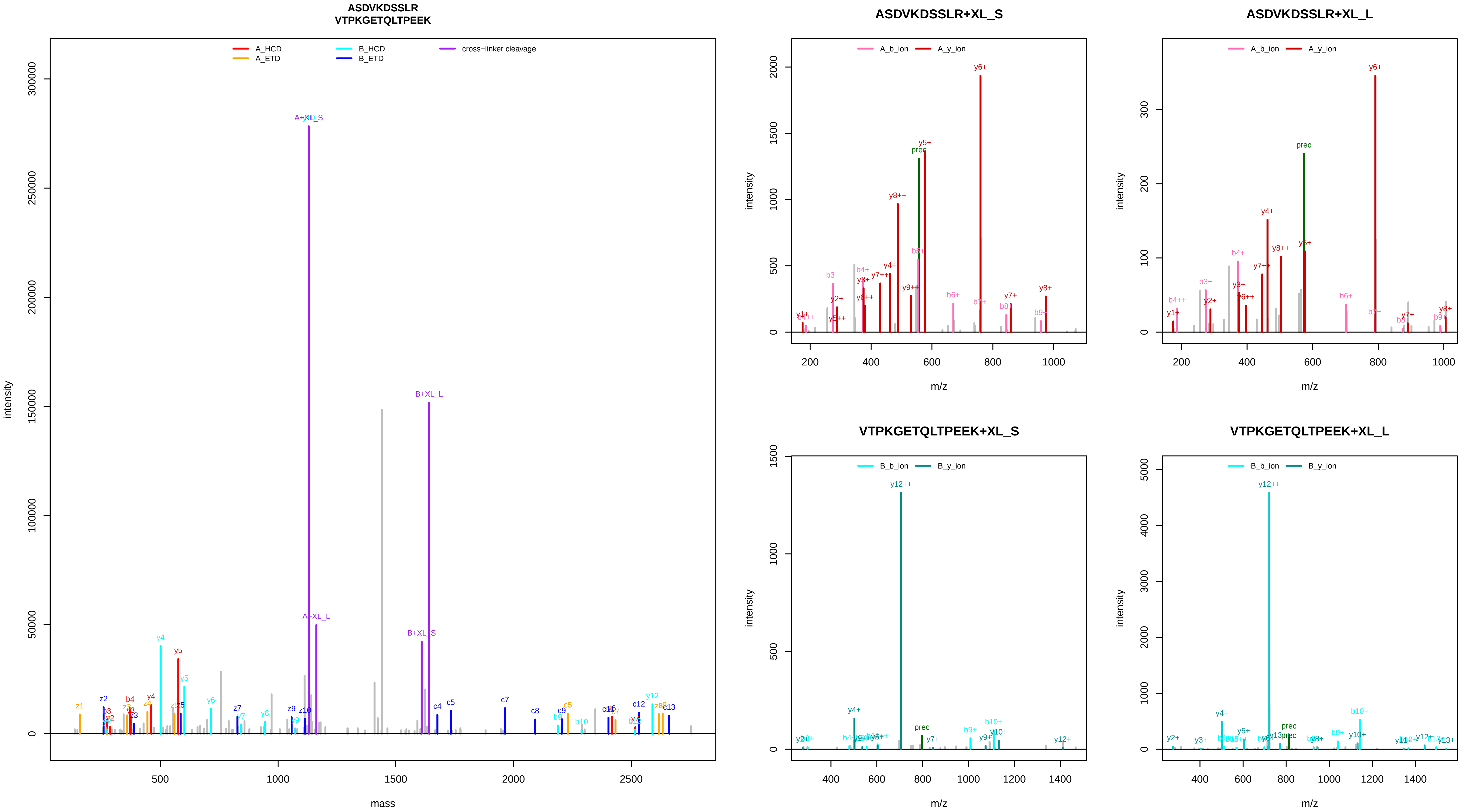
ASDVKDSSLR
VTPKGETQLTPEEK
A_HCD
A_ETD
B_HCD
B_ETD
cross−linker cleavage
A+XL_S
B+XL_L
A+XL_L
B+XL_S
intensity
mass
ASDVKDSSLR+XL_S
ASDVKDSSLR+XL_L
A_b_ion
A_y_ion
VTPKGETQLTPEEK+XL_S
VTPKGETQLTPEEK+XL_L
B_b_ion
B_y_ion
prec
y6+
y12++
m/z

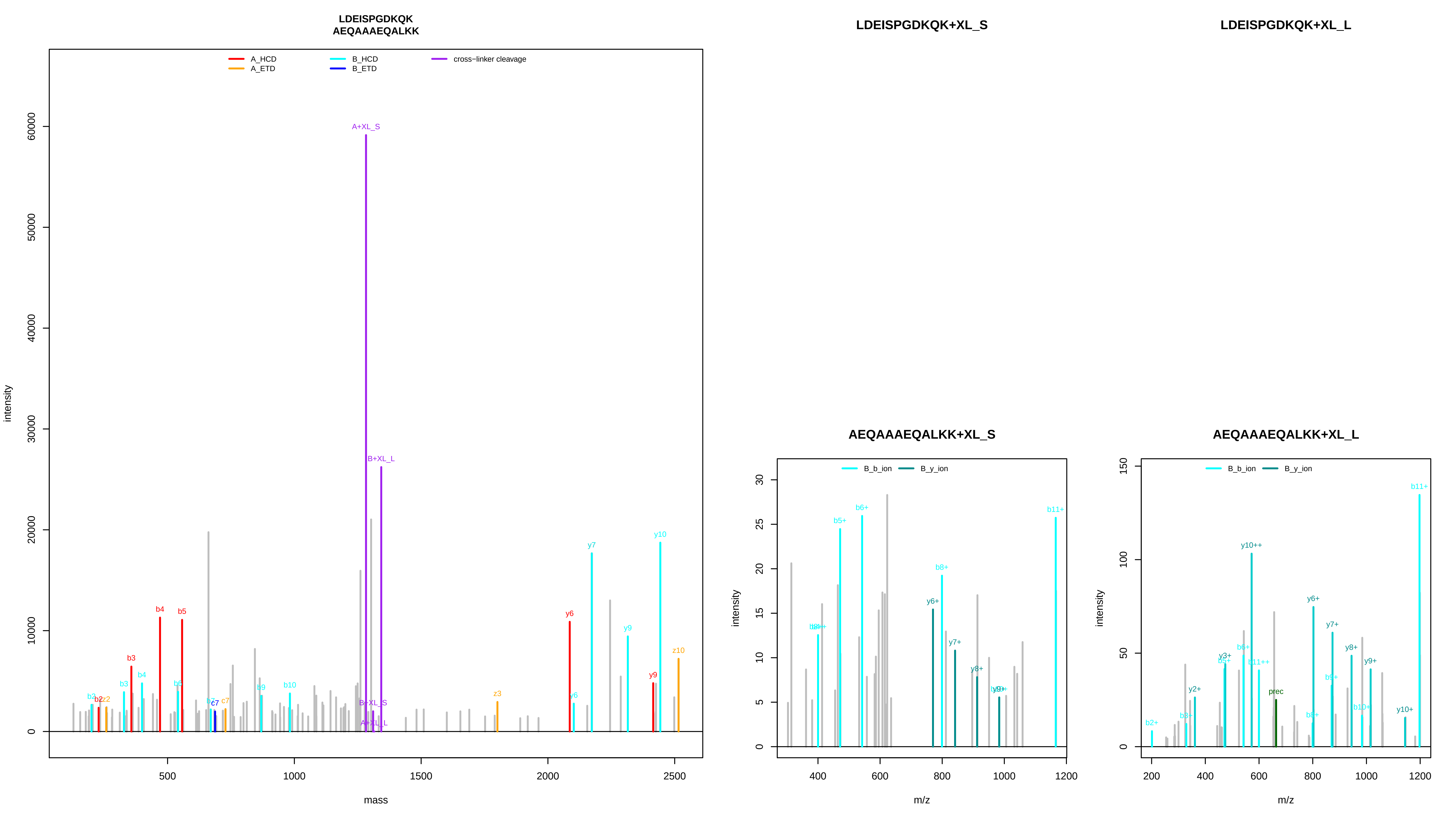
LDEISPGDKQK
AEQAAAEQALKK
A_HCD
A_ETD
B_HCD
B_ETD
cross−linker cleavage
A+XL_S
B+XL_L
B+XL_S
A+XL_L
b2
b3
b4
b5
b6
b9
b10
c7
z2
z3
z10
y6
y7
y9
y10
intensity
mass
LDEISPGDKQK+XL_S
LDEISPGDKQK+XL_L
AEQAAAEQALKK+XL_S
AEQAAAEQALKK+XL_L
B_b_ion
B_y_ion
b5+
b6+
b8+
b11+
y6+
y7+
y8+
b2+
y2+
y3+
b11++
y10++
prec
y9+
b10+
y10+
m/z

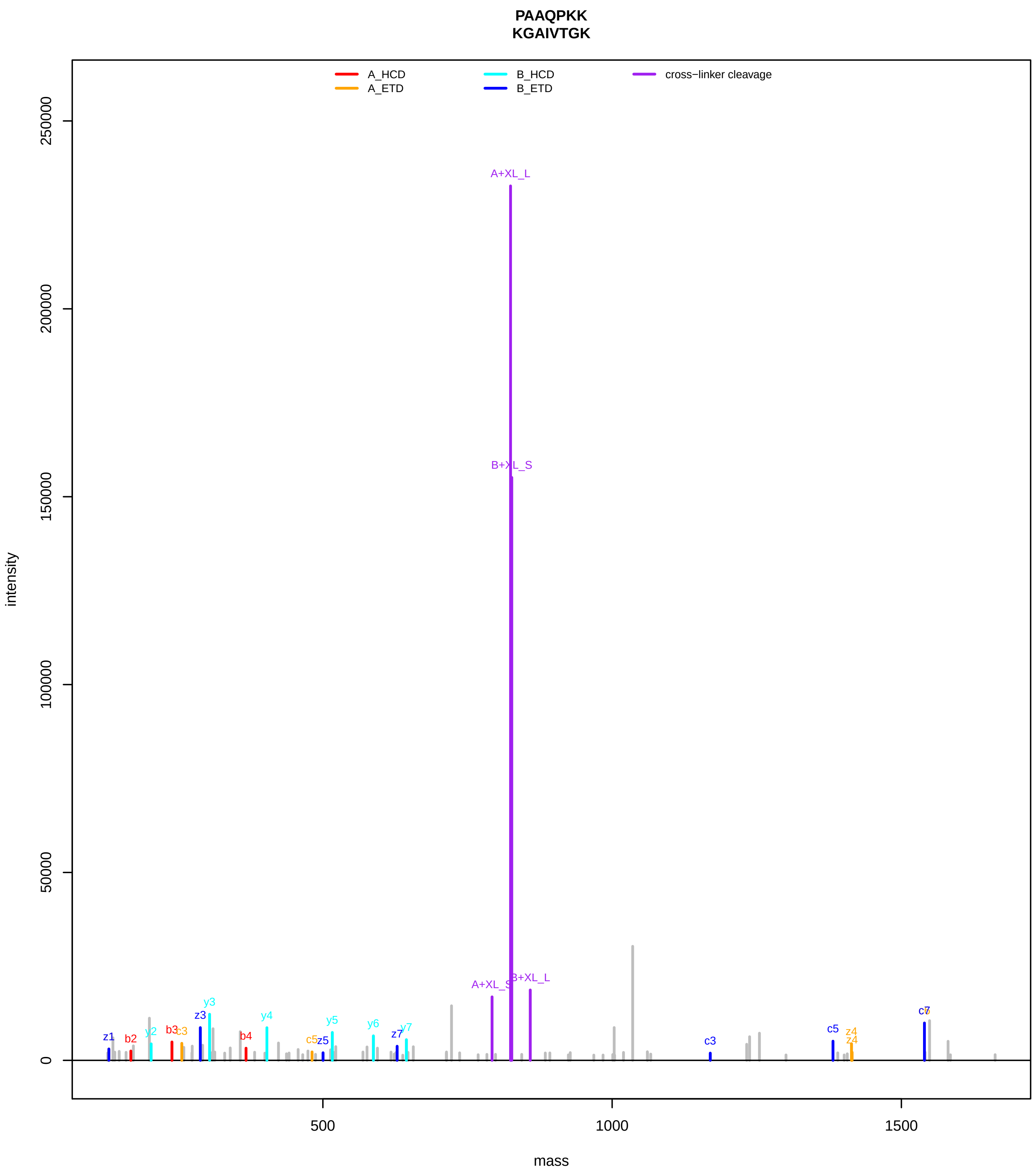

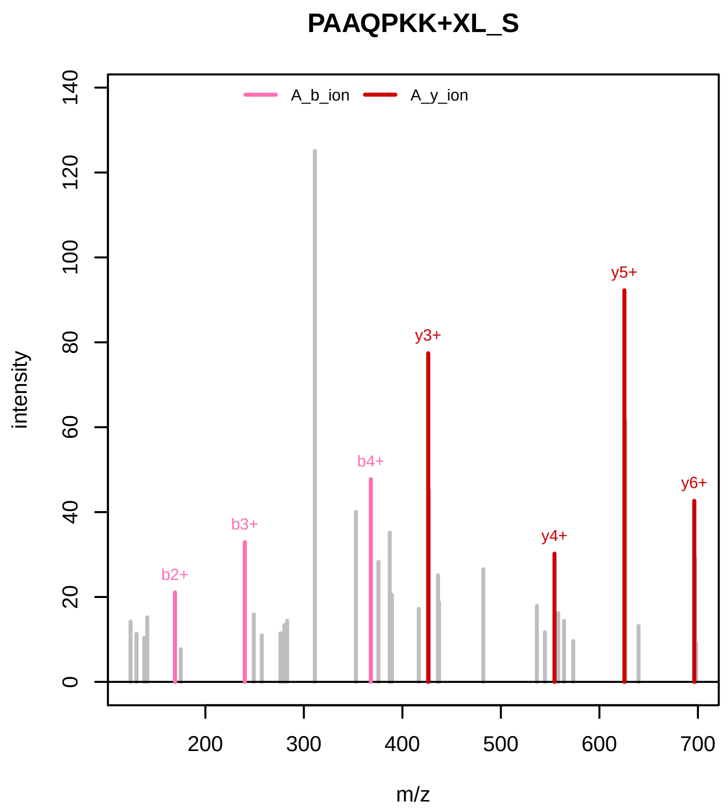

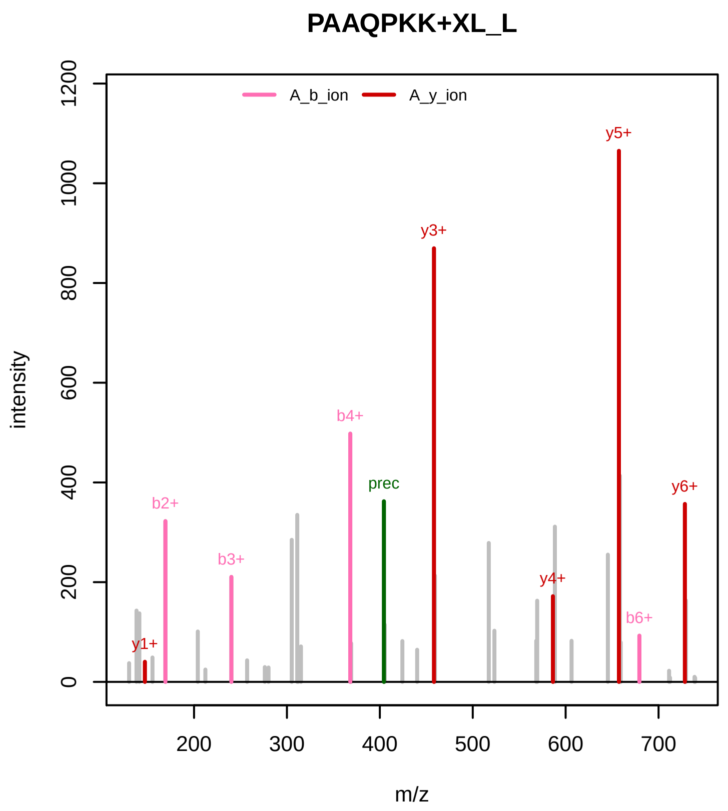

KGAIVTGK+XL_S

KGAIVTGK+XL_L

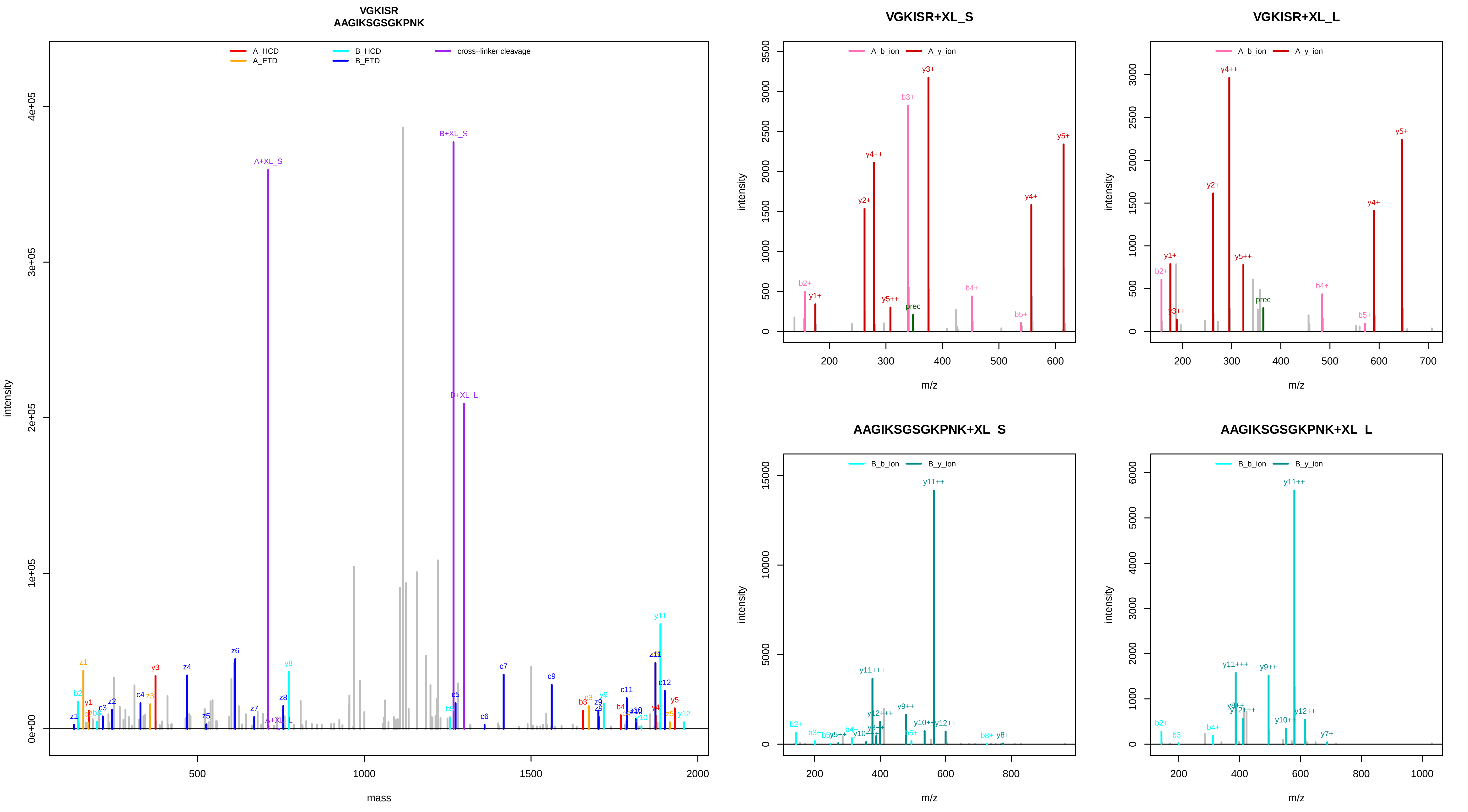
VGKISR
AAGIKSGSGKPNK
A_HCD
A_ETD
B_HCD
B_ETD
cross−linker cleavage
A+XL_S
B+XL_S
B+XL_L
A+XL_L
intensity
mass
VGKISR+XL_S
VGKISR+XL_L
A_b_ion
A_y_ion
AAGIKSGSGKPNK+XL_S
AAGIKSGSGKPNK+XL_L
B_b_ion
B_y_ion
m/z
prec

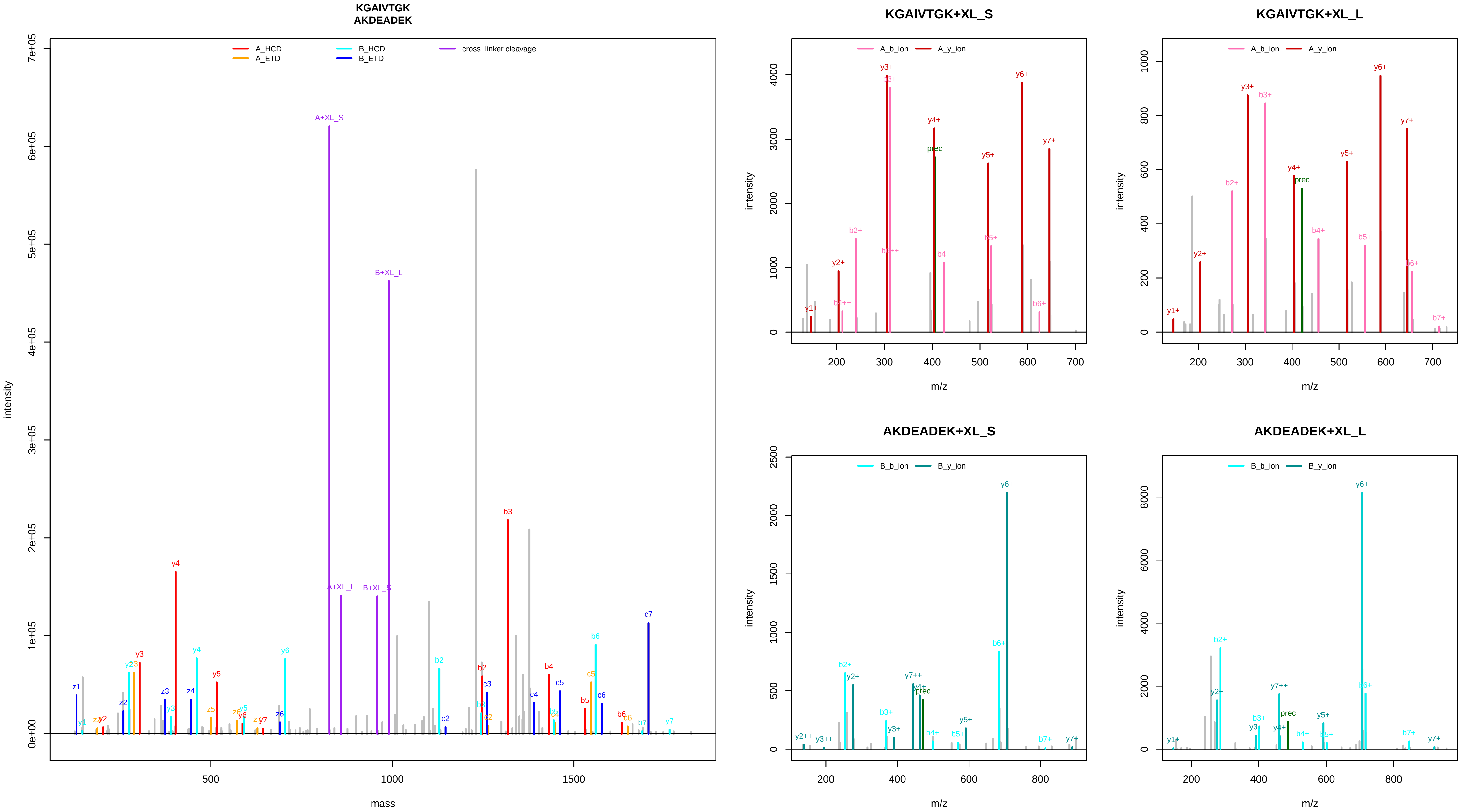
KGAIVTGK
AKDEADEK
A_HCD
A_ETD
B_HCD
B_ETD
cross−linker cleavage
A+XL_S
B+XL_L
A+XL_L
B+XL_S
intensity
mass
KGAIVTGK+XL_S
KGAIVTGK+XL_L
AKDEADEK+XL_S
AKDEADEK+XL_L
A_b_ion
A_y_ion
B_b_ion
B_y_ion
prec
m/z

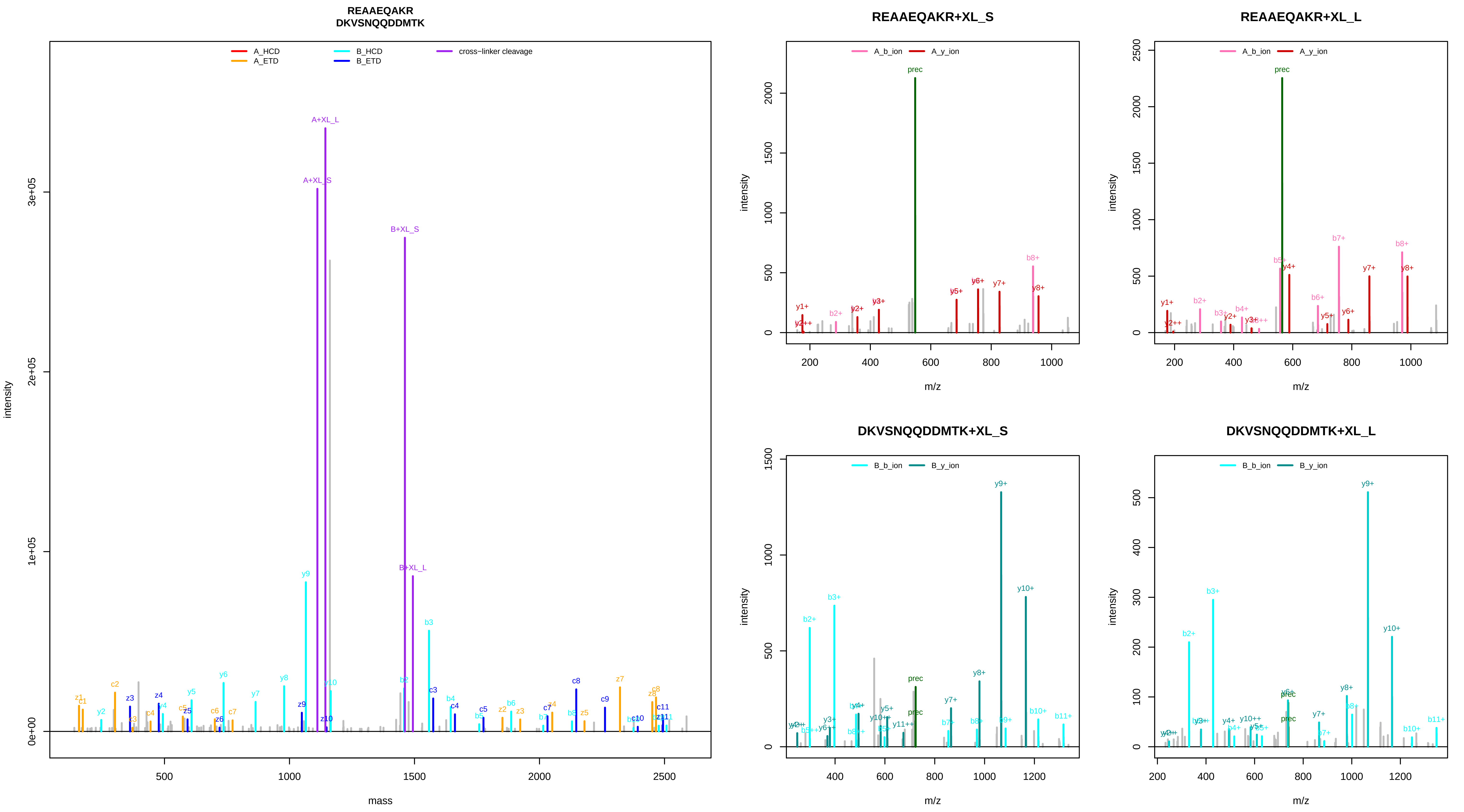

REAAEQAKR
DKVSNQQDDMTK
A_HCD
A_ETD
B_HCD
B_ETD
cross−linker cleavage
A+XL_L
A+XL_S
B+XL_S
B+XL_L
intensity
mass
REAAEQAKR+XL_S
REAAEQAKR+XL_L
DKVSNQQDDMTK+XL_S
DKVSNQQDDMTK+XL_L
A_b_ion
A_y_ion
B_b_ion
B_y_ion
prec
m/z

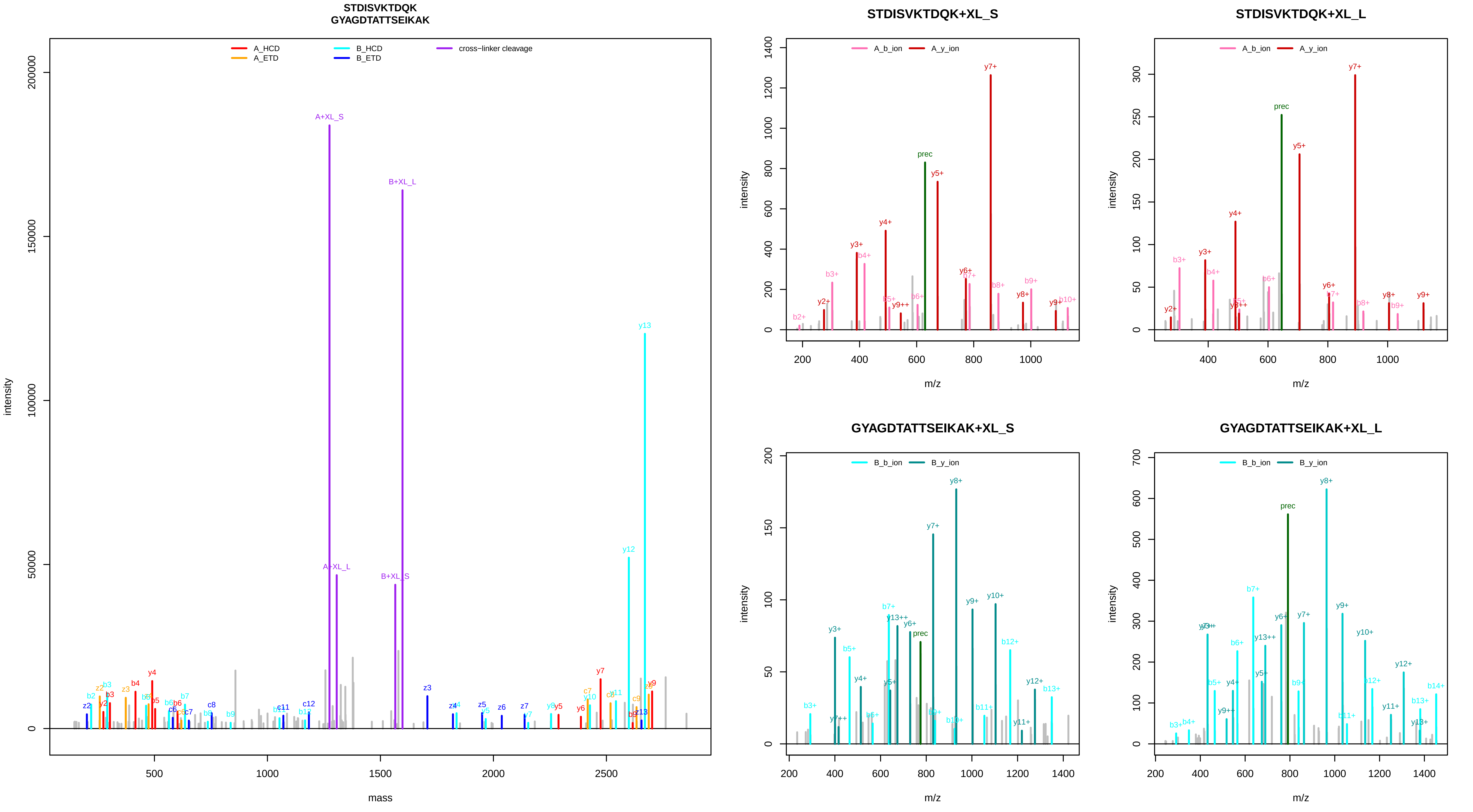
STDISVKTDQK
GYAGDTATTSEIKAK
A_HCD
A_ETD
B_HCD
B_ETD
cross−linker cleavage
A+XL_S
B+XL_L
A+XL_L
B+XL_S
intensity
mass
STDISVKTDQK+XL_S
STDISVKTDQK+XL_L
A_b_ion
A_y_ion
GYAGDTATTSEIKAK+XL_S
GYAGDTATTSEIKAK+XL_L
B_b_ion
B_y_ion
prec
m/z

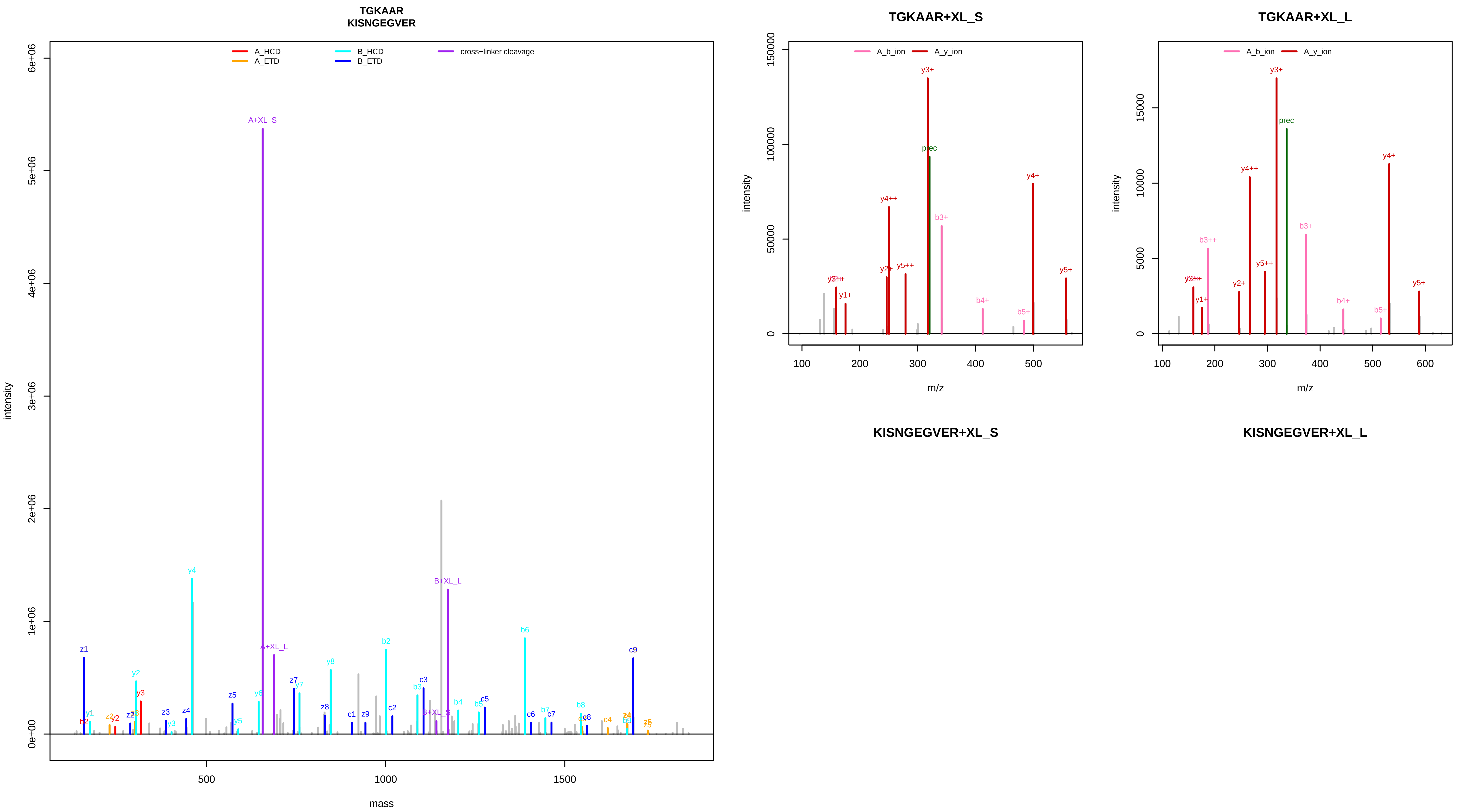

TGKAAR
KISNGEGVER
A_HCD
A_ETD
B_HCD
B_ETD
cross−linker cleavage
A+XL_S
A+XL_L
B+XL_L
B+XL_S
intensity
mass
TGKAAR+XL_S
TGKAAR+XL_L
A_b_ion
A_y_ion
prec
m/z
KISNGEGVER+XL_S
KISNGEGVER+XL_L

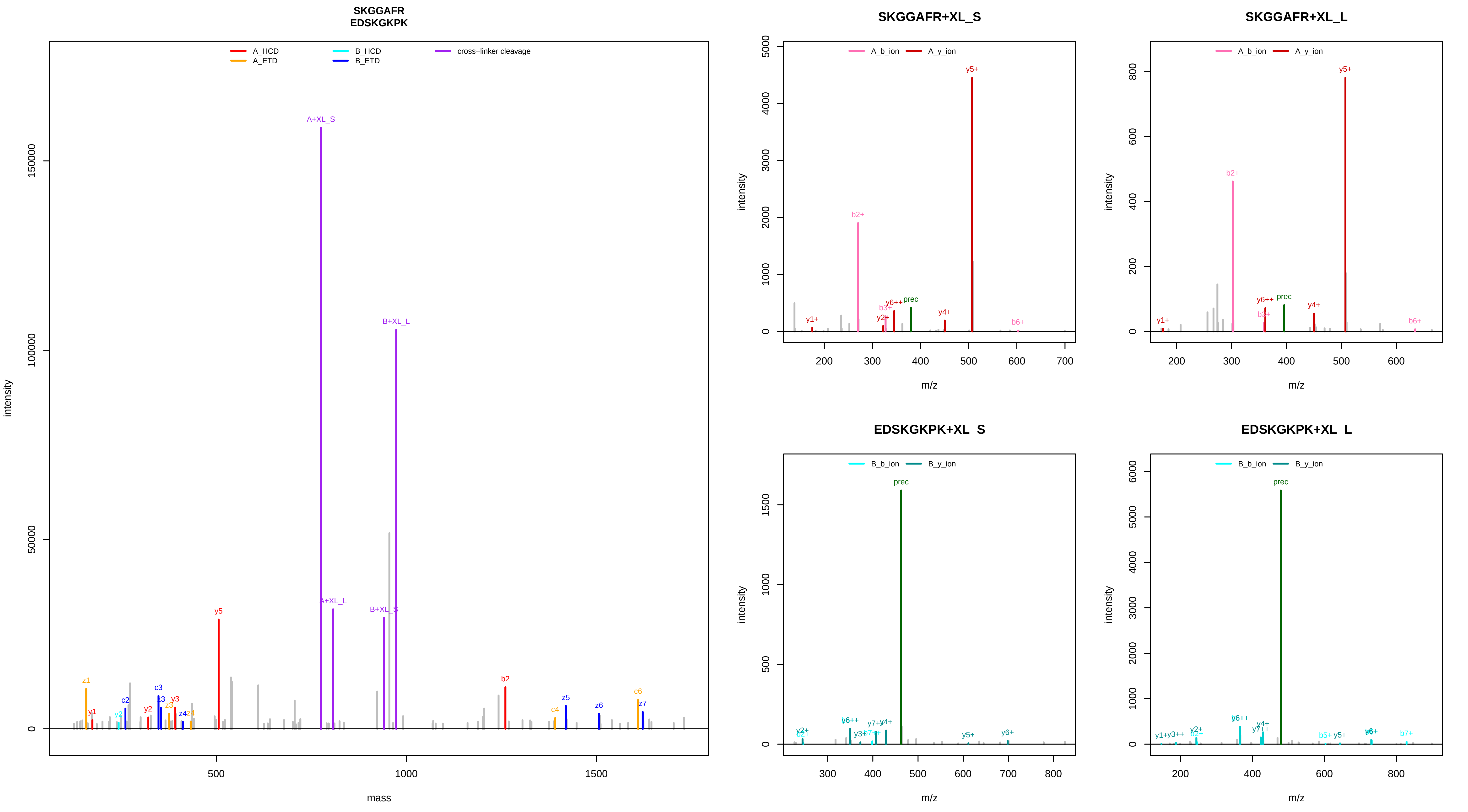
SKGGAFR
EDSKGKPK
A_HCD
A_ETD
B_HCD
B_ETD
cross−linker cleavage
A+XL_S
B+XL_L
A+XL_L
B+XL_S
intensity
mass
SKGGAFR+XL_S
SKGGAFR+XL_L
EDSKGKPK+XL_S
EDSKGKPK+XL_L
A_b_ion
A_y_ion
B_b_ion
B_y_ion
prec
m/z
intensity

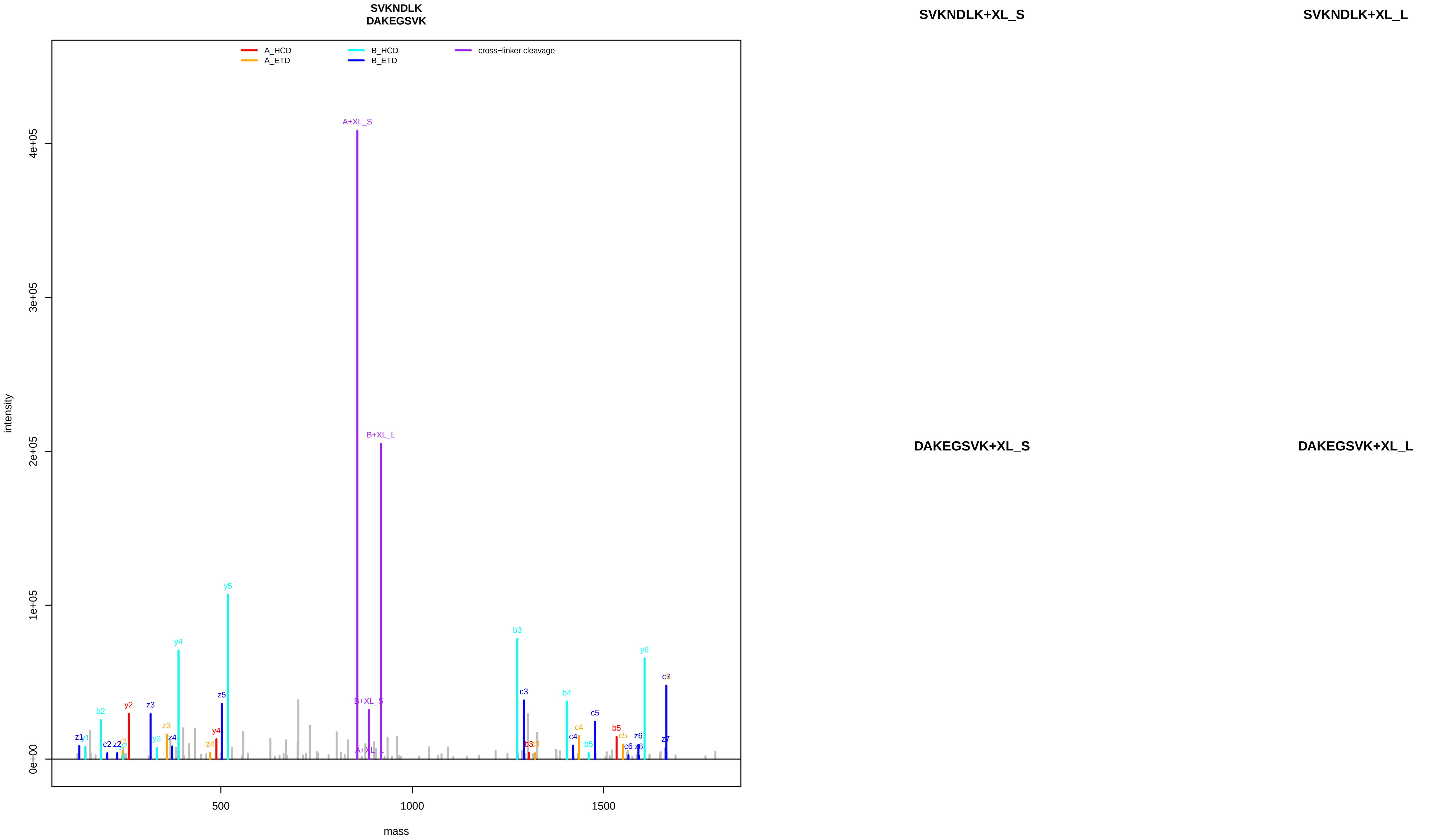
SVKNDLK
DAKEGSVK
A_HCD
A_ETD
B_HCD
B_ETD
cross−linker cleavage
A+XL_S
B+XL_L
B+XL_S
y5
y4
b3
y6
c7
c3
b4
c5
b5
c4
c5
c4
y2
z3
z5
b2
z3
y4
z4
z1
c2
z2
y3
z4
z6
c6
z7
intensity
0e+00
1e+05
2e+05
3e+05
4e+05
500
1000
1500
mass
SVKNDLK+XL_S
SVKNDLK+XL_L
DAKEGSVK+XL_S
DAKEGSVK+XL_L

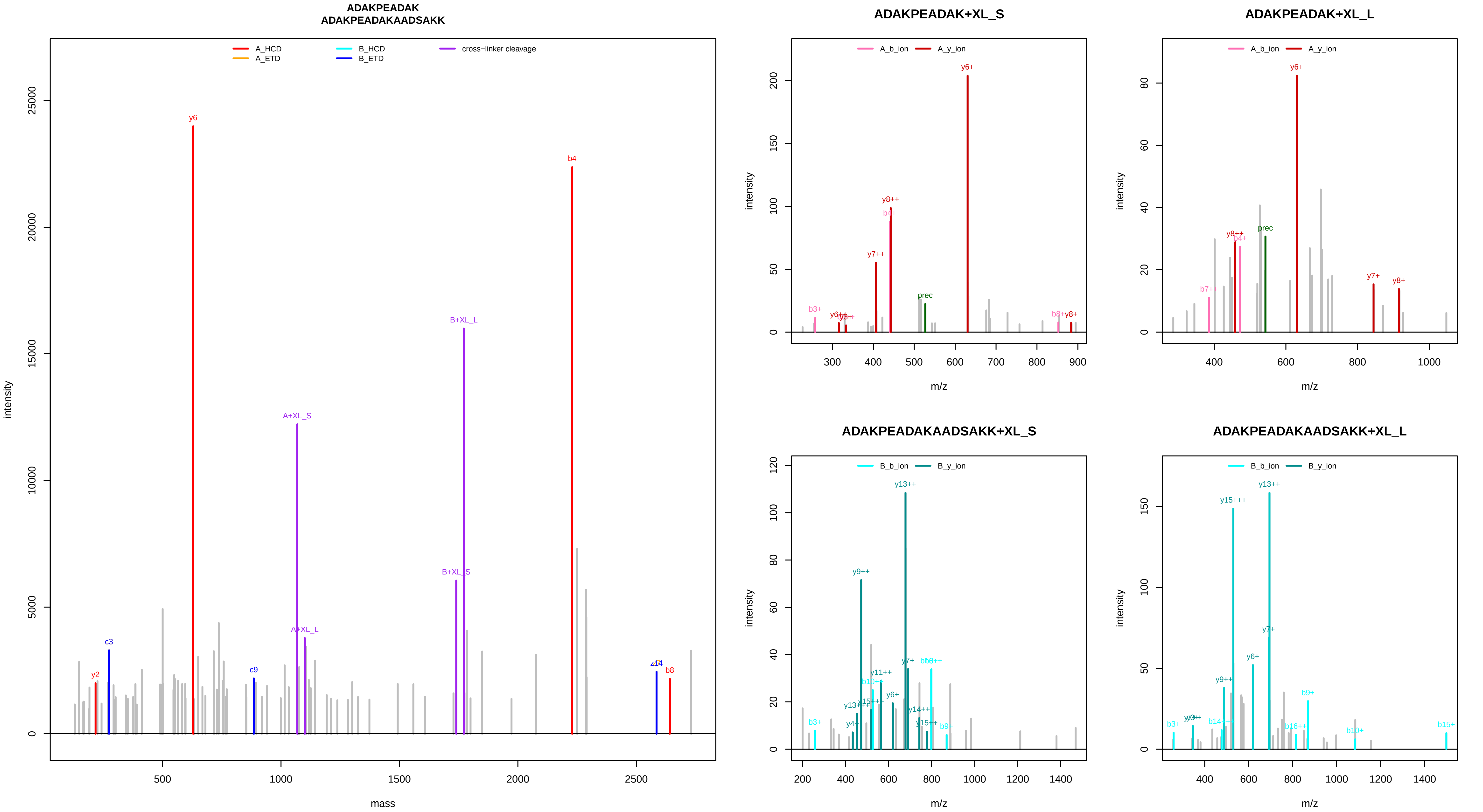
ADAKPEADAK
ADAKPEADAKAADSAKK
A_HCD
A_ETD
B_HCD
B_ETD
cross−linker cleavage
intensity
mass
y6
b4
B+XL_L
A+XL_S
B+XL_S
A+XL_L
c3
y2
c9
z14
b8
ADAKPEADAK+XL_S
A_b_ion
A_y_ion
y6+
y8++
y7++
prec
b3+
y8+
m/z
ADAKPEADAK+XL_L
y8++
b4+
b7++
y7+
y8+
ADAKPEADAKAADSAKK+XL_S
B_b_ion
B_y_ion
y13++
y9++
y7+
y11++
y6+
y14++
b3+
y4+
b9+
ADAKPEADAKAADSAKK+XL_L
y15+++
y6+
y9++
b9+
b3+
b14++
b16++
b10+
b15+

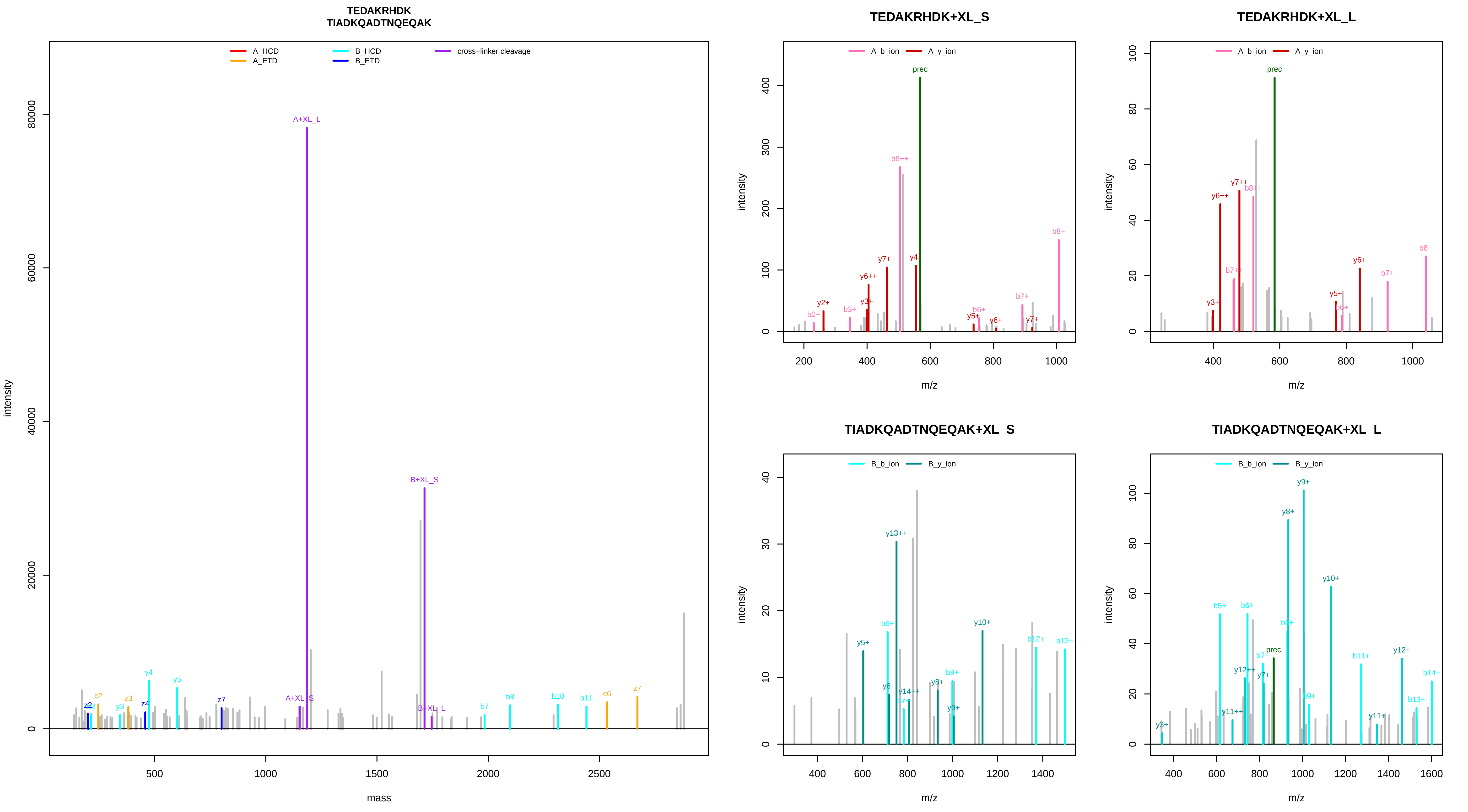
TEDAKRHDK
TIADKQADTNQEQAK
A_HCD
A_ETD
B_HCD
B_ETD
cross−linker cleavage
A+XL_L
B+XL_S
A+XL_S
B+XL_L
intensity
mass
TEDAKRHDK+XL_S
TEDAKRHDK+XL_L
A_b_ion
A_y_ion
prec
intensity
m/z
TIADKQADTNQEQAK+XL_S
TIADKQADTNQEQAK+XL_L
B_b_ion
B_y_ion
prec
intensity
m/z

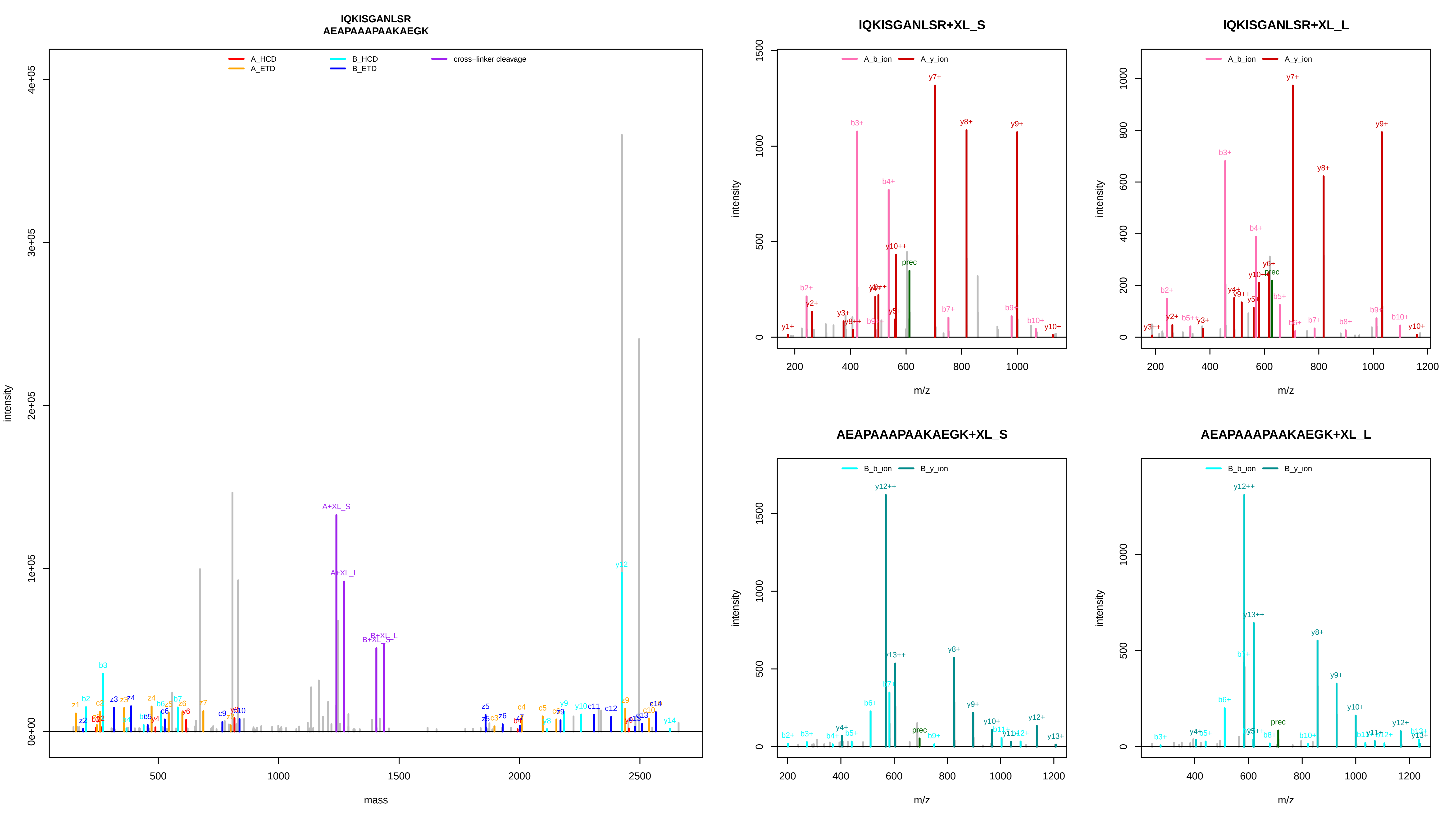
IQKISGANLSR
AEAPAAAPAAKAEGK
A_HCD
A_ETD
B_HCD
B_ETD
cross−linker cleavage
A+XL_S
A+XL_L
B+XL_S
B+XL_L
intensity
mass
IQKISGANLSR+XL_S
IQKISGANLSR+XL_L
A_b_ion
A_y_ion
AEAPAAAPAAKAEGK+XL_S
AEAPAAAPAAKAEGK+XL_L
B_b_ion
B_y_ion
prec
m/z

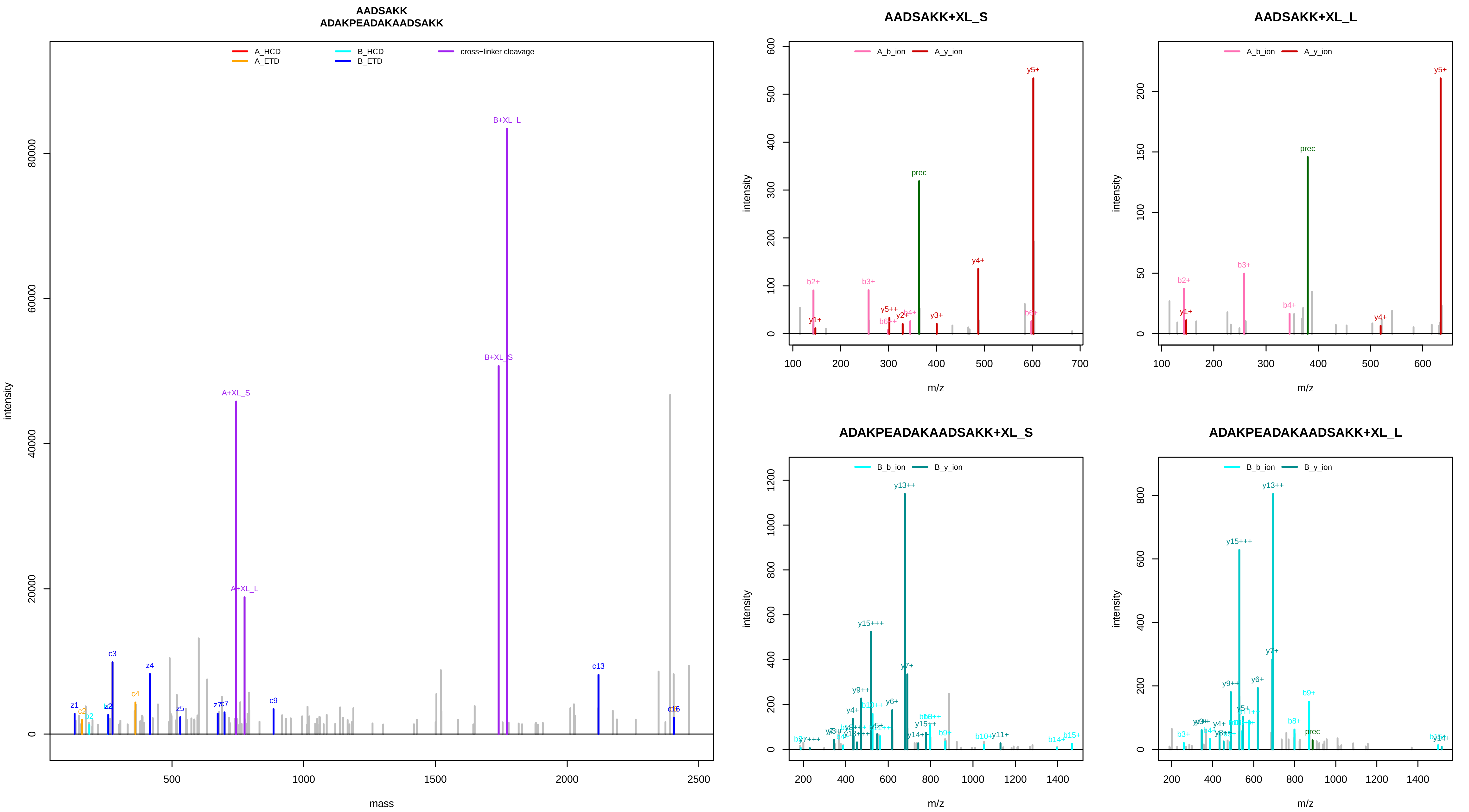

AADSAKK
ADAKPEADAKAADSAKK
A_HCD
A_ETD
B_HCD
B_ETD
cross−linker cleavage
B+XL_L
B+XL_S
A+XL_S
A+XL_L
c3
z4
c13
c9
c4
c16
z5
z1
c2
b2
z2
z7
c7
intensity
mass
AADSAKK+XL_S
AADSAKK+XL_L
ADAKPEADAKAADSAKK+XL_S
ADAKPEADAKAADSAKK+XL_L
A_b_ion
A_y_ion
B_b_ion
B_y_ion
y5+
y4+
prec
b2+
b3+
b4+
y1+
y3+
y2+
y5++
y13++
y15+++
y7+
y6+
y9++
y4+
b9+
b8+
y14+
y11+
b15+
m/z
intensity

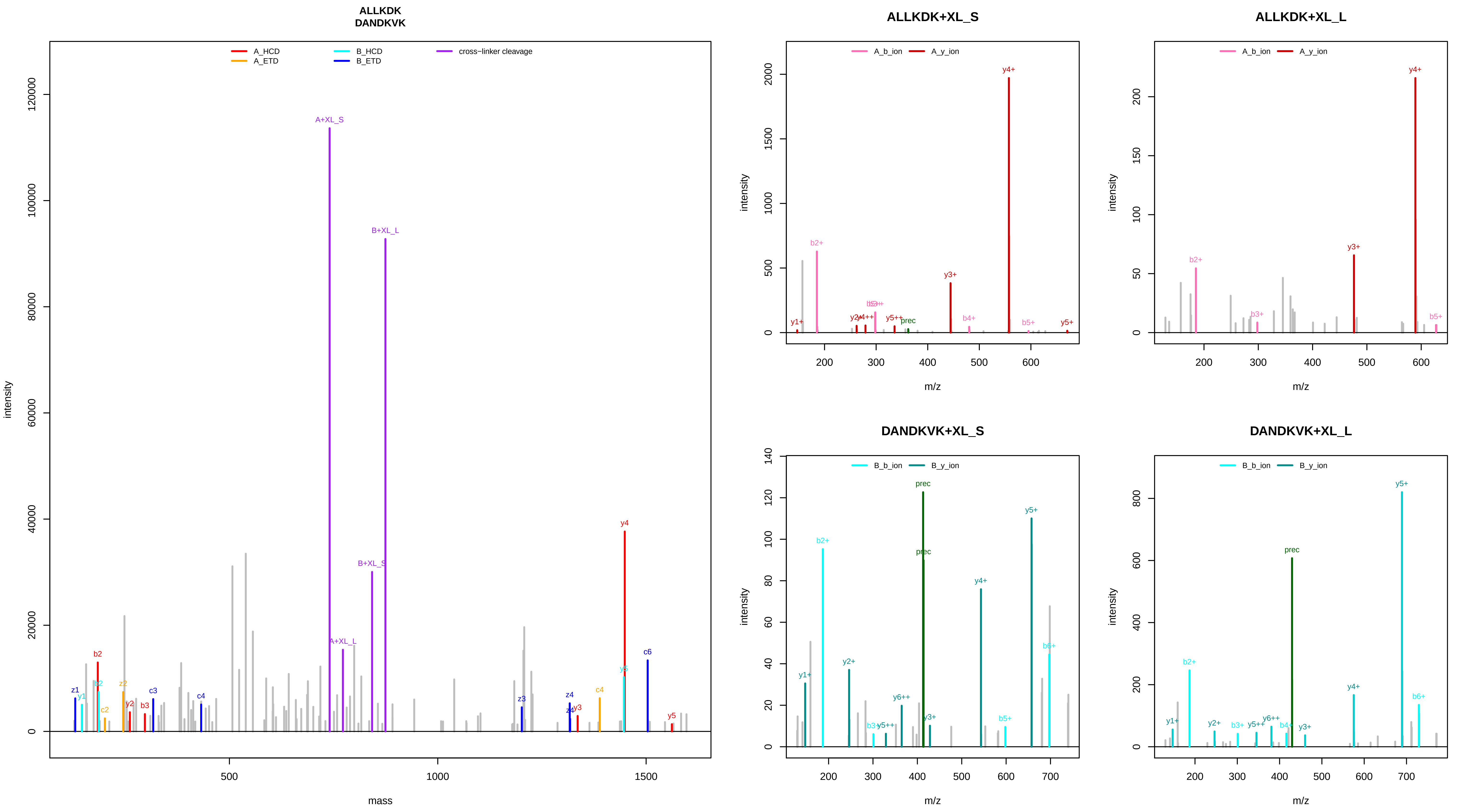
ALLKDK
DANDKVK
A_HCD
A_ETD
B_HCD
B_ETD
cross−linker cleavage
A+XL_S
B+XL_L
B+XL_S
A+XL_L
y4
c6
y5
mass
intensity
ALLKDK+XL_S
ALLKDK+XL_L
DANDKVK+XL_S
DANDKVK+XL_L
A_b_ion
A_y_ion
B_b_ion
B_y_ion
prec
m/z

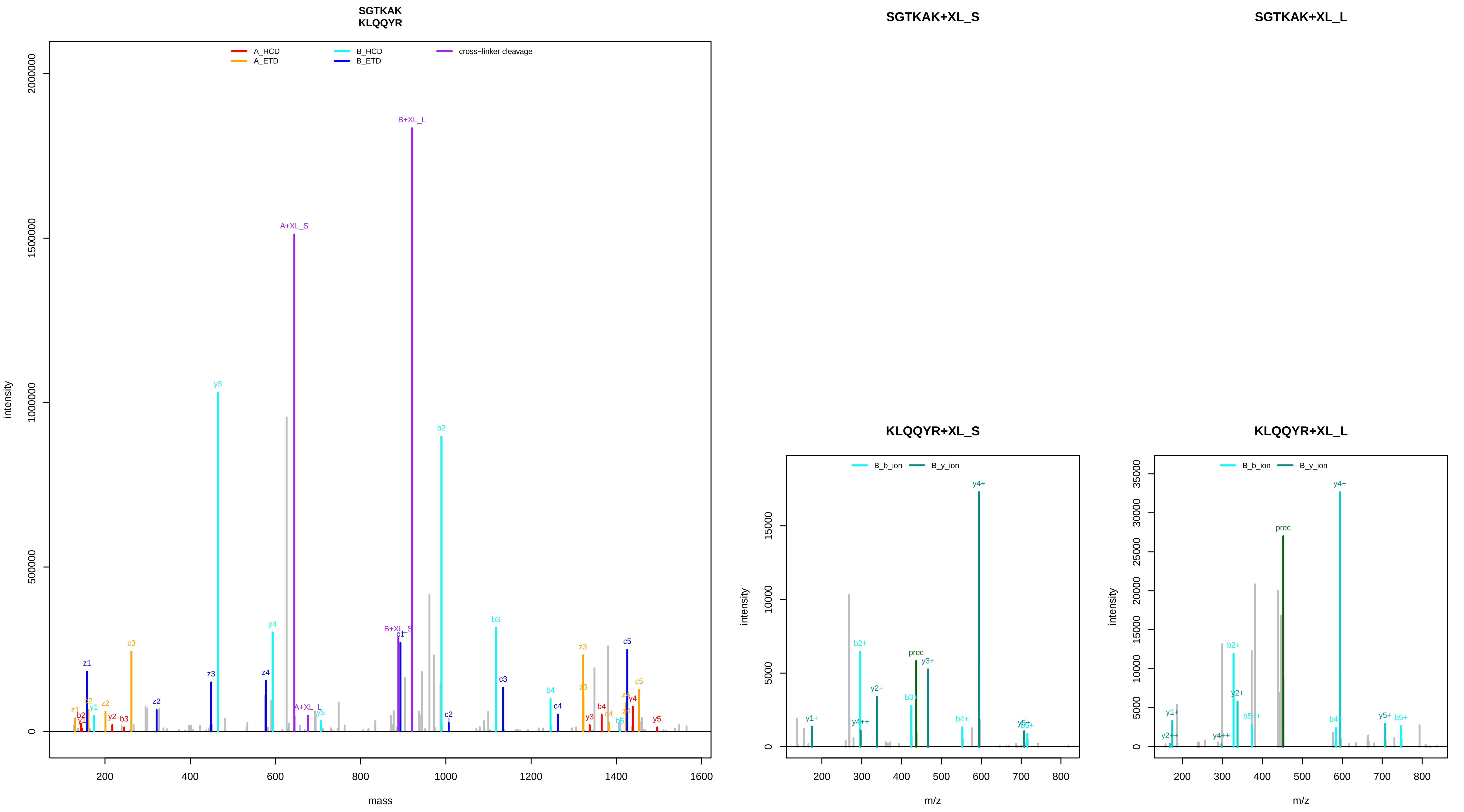
SGTKAK
KLQQYR
A_HCD
A_ETD
B_HCD
B_ETD
cross−linker cleavage
B+XL_L
A+XL_S
B+XL_S
A+XL_L
y3
b2
y4
b3
c1
c2
c3
c4
c5
z1
z2
z3
z4
b4
y5
intensity
mass
SGTKAK+XL_S
SGTKAK+XL_L
KLQQYR+XL_S
KLQQYR+XL_L
B_b_ion
B_y_ion
y4+
prec
y3+
y2+
b2+
y1+
y4++
b3+
b4+
y5+
b5+
y2++
intensity
m/z
m/z

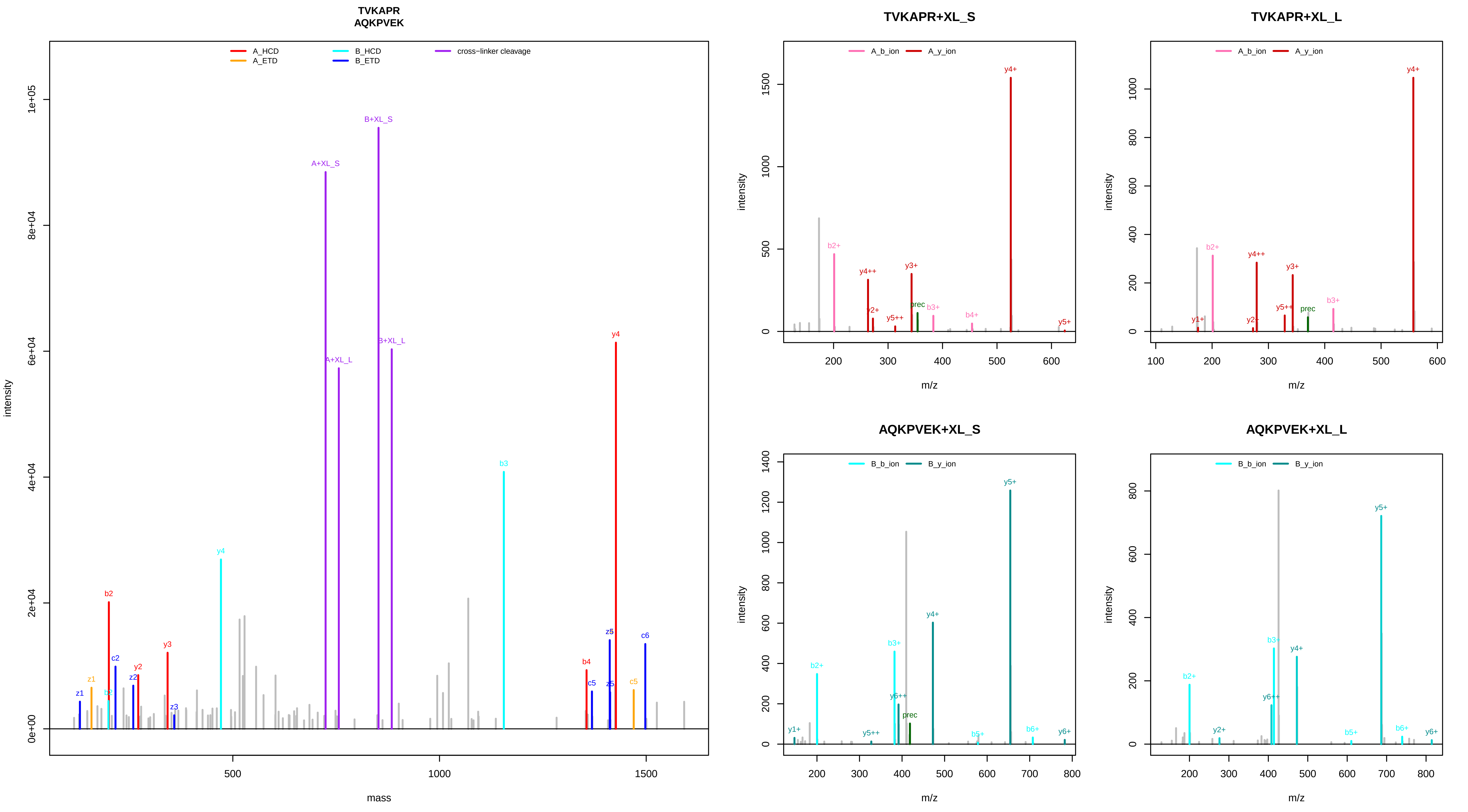

TVKAPR
AQKPVEK
A_HCD
A_ETD
B_HCD
B_ETD
cross−linker cleavage
B+XL_S
A+XL_S
B+XL_L
A+XL_L
y4
b3
b2
y3
y2
c2
z1
z1
z2
z3
b4
c5
z5
c6
c5
intensity
mass
TVKAPR+XL_S
TVKAPR+XL_L
A_b_ion
A_y_ion
b2+
y4++
y2+
y5++
y3+
prec
b3+
b4+
y4+
y5+
y1+
m/z
intensity
AQKPVEK+XL_S
AQKPVEK+XL_L
B_b_ion
B_y_ion
y1+
b2+
y5++
b3+
y6++
prec
y4+
b5+
y5+
b6+
y6+
y2+

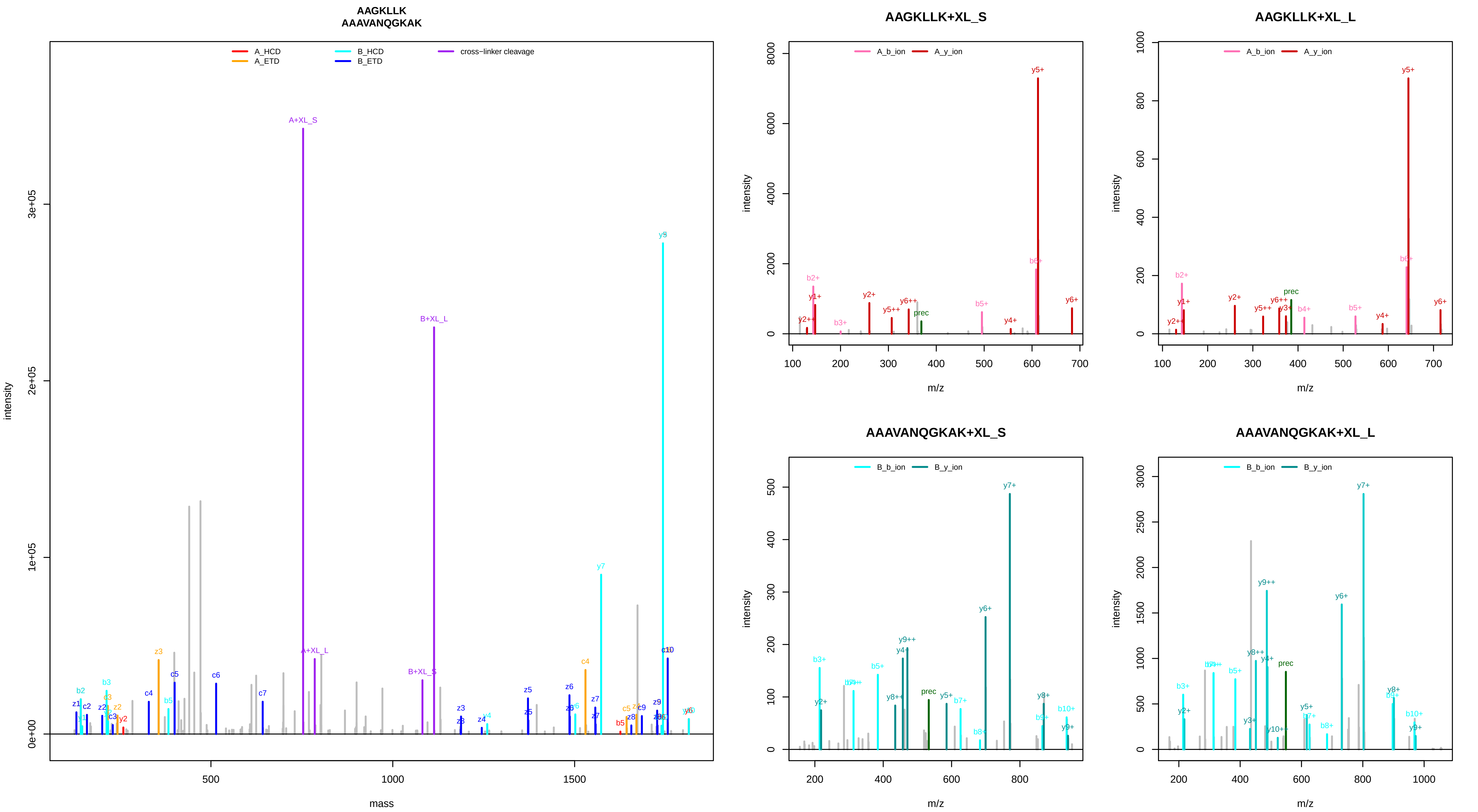
AAGKLLK
AAAVANQGKAK
A_HCD
A_ETD
B_HCD
B_ETD
cross−linker cleavage
A+XL_S
B+XL_L
intensity
mass
AAGKLLK+XL_S
AAGKLLK+XL_L
A_b_ion
A_y_ion
AAAVANQGKAK+XL_S
AAAVANQGKAK+XL_L
B_b_ion
B_y_ion
intensity
m/z

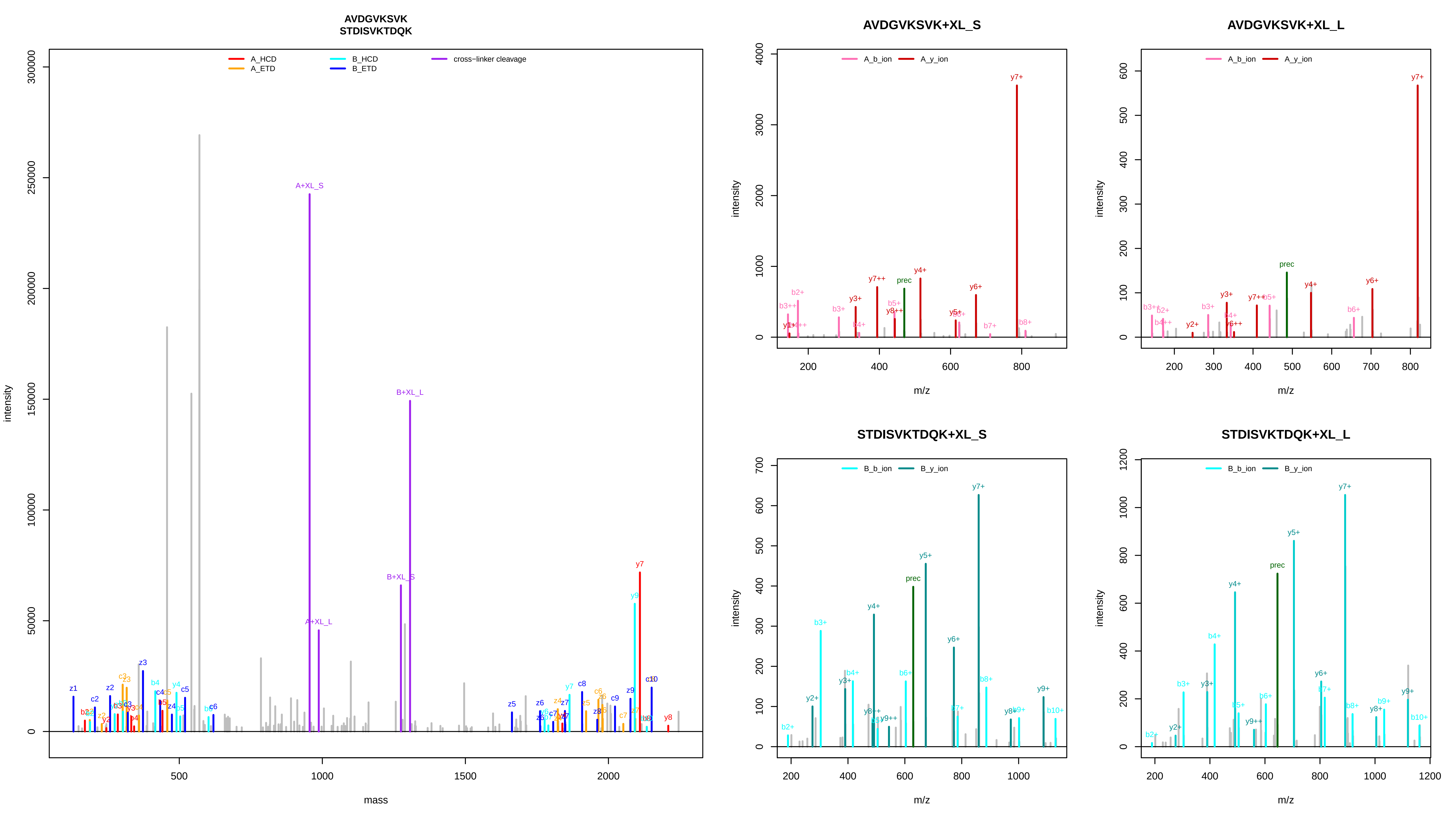
AVDGVKSVK
STDISVKTDQK
A_HCD
A_ETD
B_HCD
B_ETD
cross−linker cleavage
A+XL_S
A+XL_L
B+XL_L
B+XL_S
intensity
mass
AVDGVKSVK+XL_S
AVDGVKSVK+XL_L
STDISVKTDQK+XL_S
STDISVKTDQK+XL_L
A_b_ion
A_y_ion
B_b_ion
B_y_ion
prec
m/z

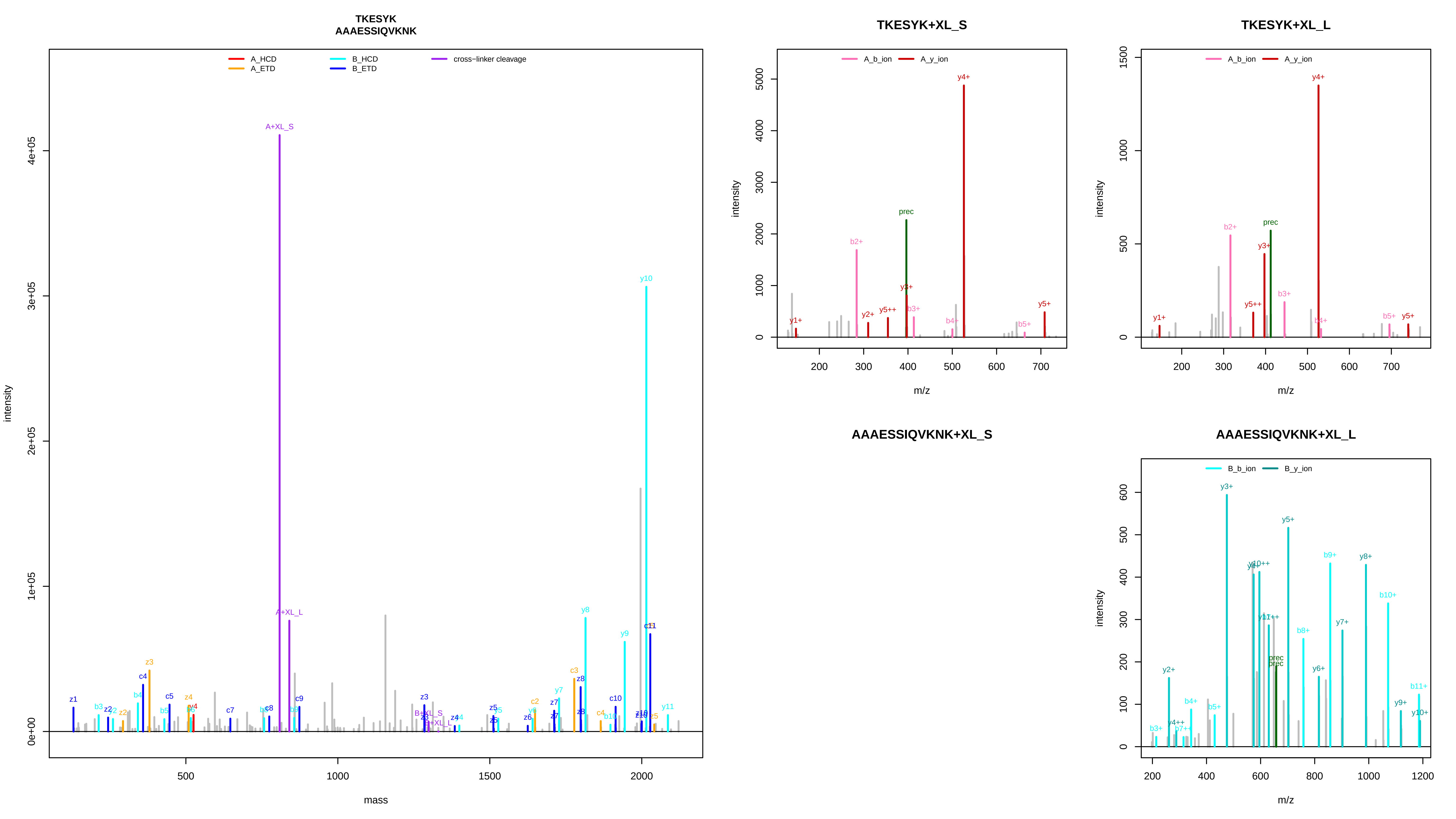

TKESYK
AAAESSIQVKNK
A_HCD
A_ETD
B_HCD
B_ETD
cross−linker cleavage
A+XL_S
A+XL_L
intensity
mass
TKESYK+XL_S
TKESYK+XL_L
A_b_ion
A_y_ion
prec
m/z
AAAESSIQVKNK+XL_S
AAAESSIQVKNK+XL_L
B_b_ion
B_y_ion

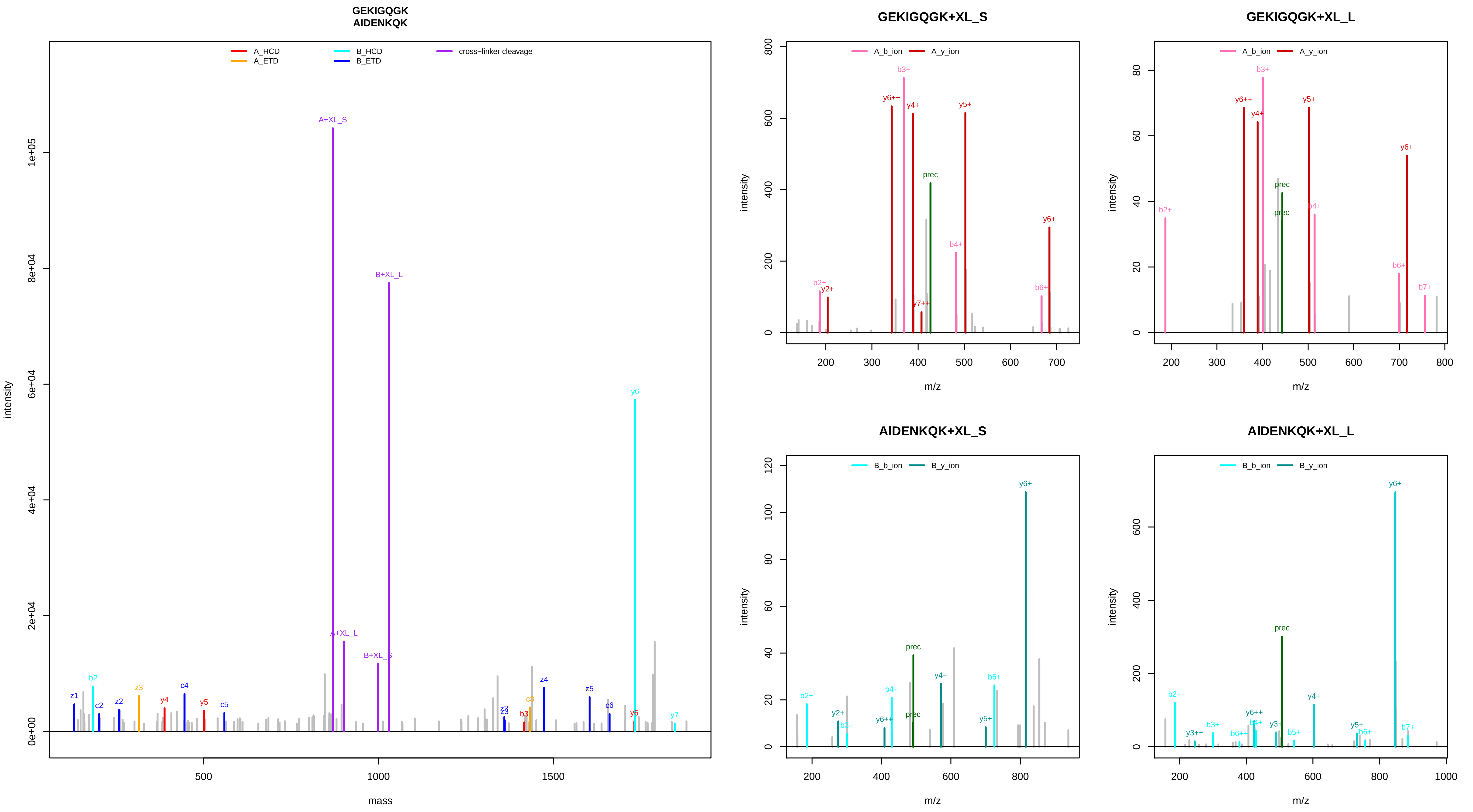
GEKIGQGK
AIDENKQK
A_HCD
A_ETD
B_HCD
B_ETD
cross−linker cleavage
A+XL_S
B+XL_L
A+XL_L
B+XL_S
intensity
mass
GEKIGQGK+XL_S
GEKIGQGK+XL_L
AIDENKQK+XL_S
AIDENKQK+XL_L
A_b_ion
A_y_ion
B_b_ion
B_y_ion
prec
m/z

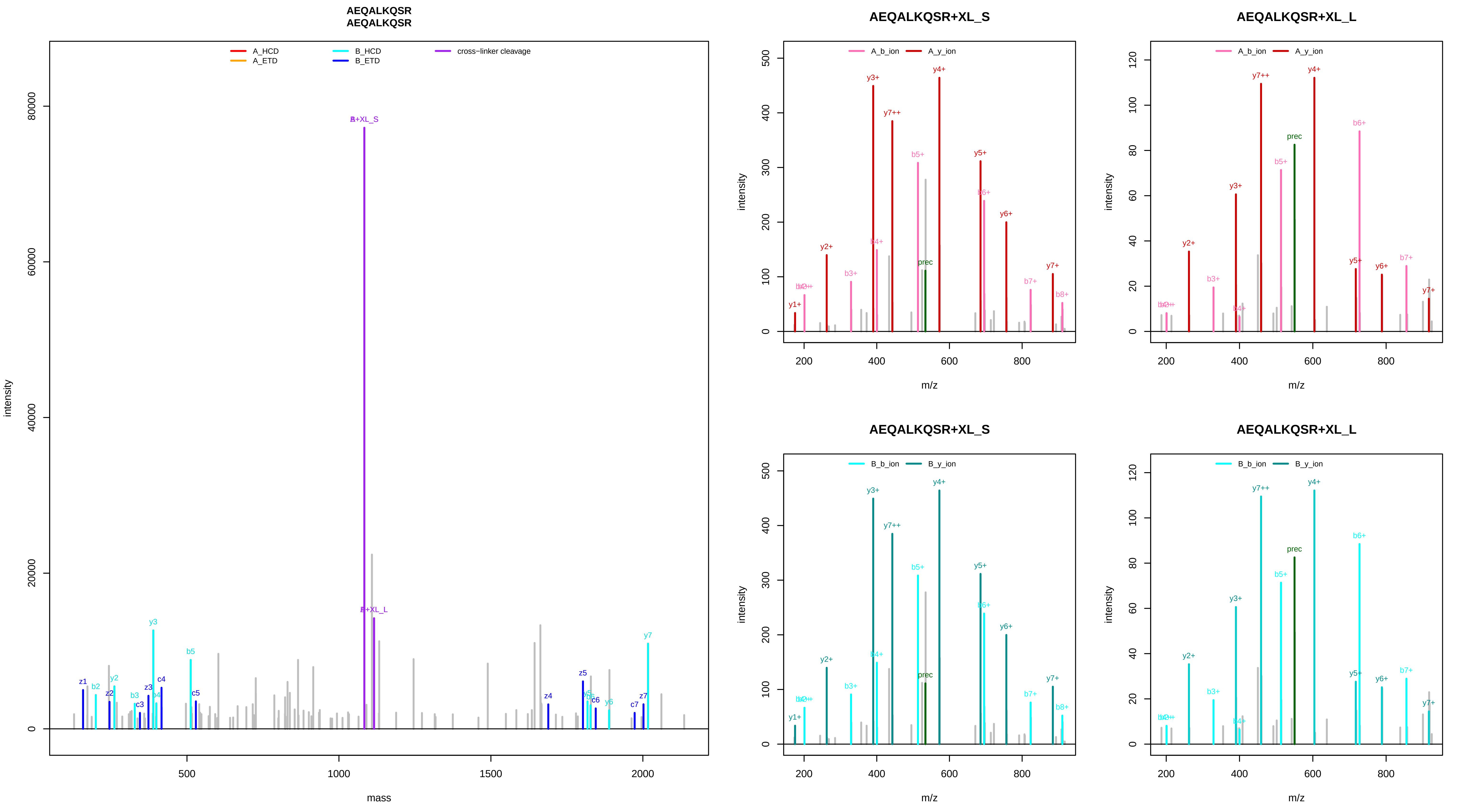
AEQALKQSR
AEQALKQSR
A_HCD
A_ETD
B_HCD
B_ETD
cross−linker cleavage
B+XL_S
B+XL_L
intensity
mass
AEQALKQSR+XL_S
AEQALKQSR+XL_L
A_b_ion
A_y_ion
B_b_ion
B_y_ion
prec
intensity
m/z

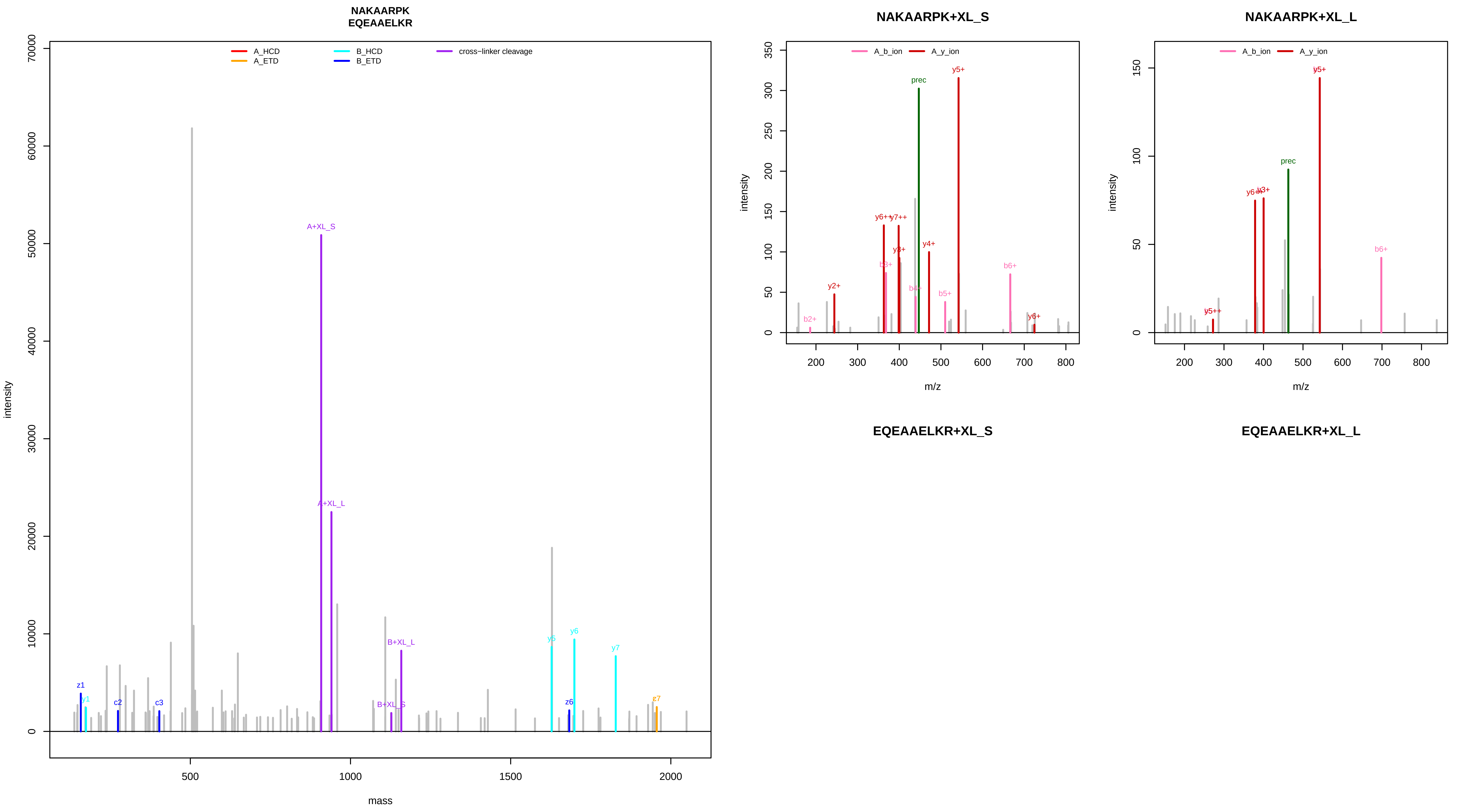
NAKAARPK
EQEAAELKR
A_HCD
A_ETD
B_HCD
B_ETD
cross−linker cleavage
A+XL_S
A+XL_L
B+XL_L
B+XL_S
z1
y1
c2
c3
y5
y6
y7
z6
z7
intensity
mass
NAKAARPK+XL_S
NAKAARPK+XL_L
A_b_ion
A_y_ion
prec
y5+
y6++
y7++
y3+
y4+
y2+
b3+
b4+
b5+
b6+
b2+
y6+
y5++
intensity
m/z
EQEAAELKR+XL_S
EQEAAELKR+XL_L

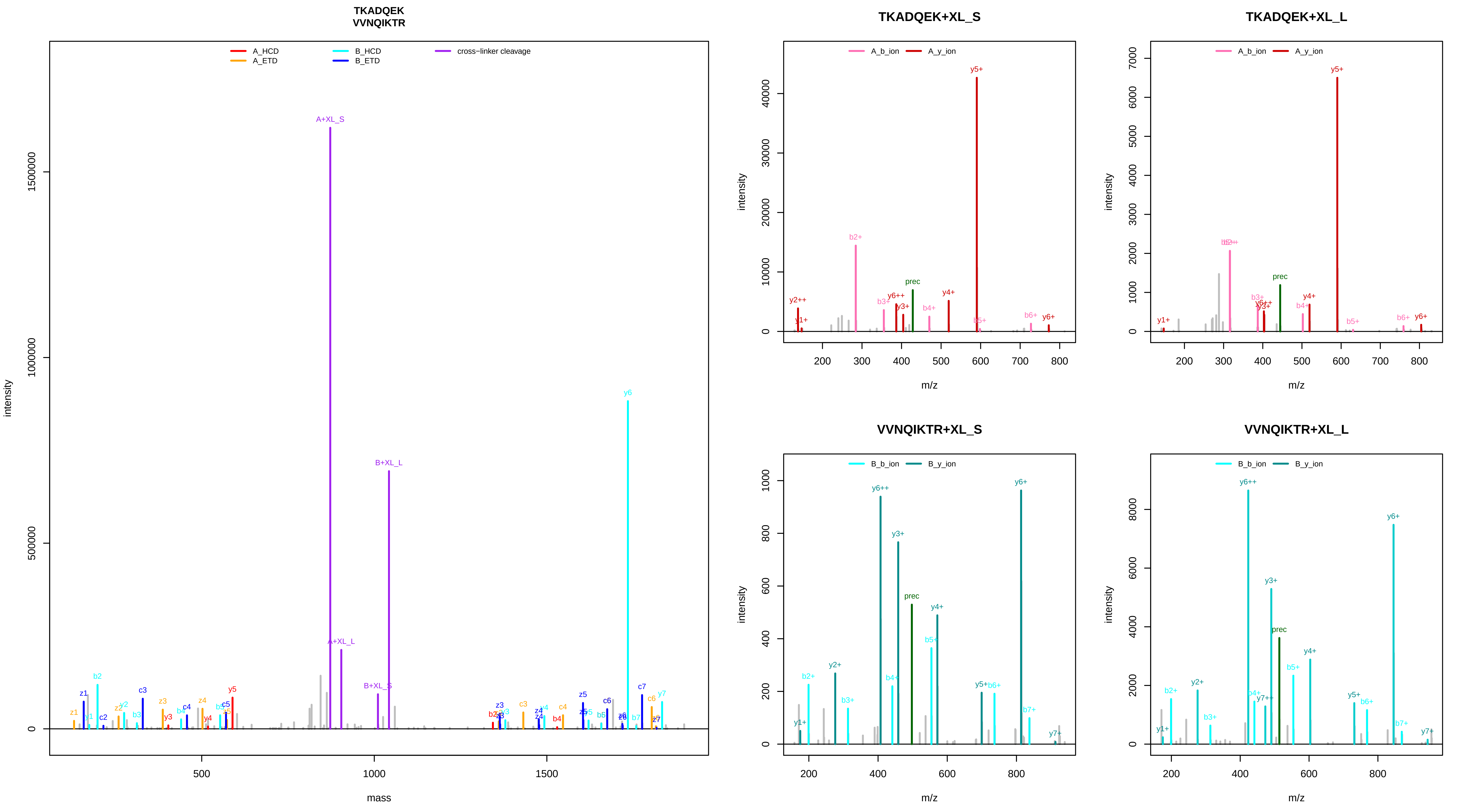
TKADQEK
VVNQIKTR
A_HCD
A_ETD
B_HCD
B_ETD
cross−linker cleavage
A+XL_S
B+XL_L
A+XL_L
B+XL_S
intensity
mass
TKADQEK+XL_S
TKADQEK+XL_L
A_b_ion
A_y_ion
VVNQIKTR+XL_S
VVNQIKTR+XL_L
B_b_ion
B_y_ion
prec
m/z
intensity

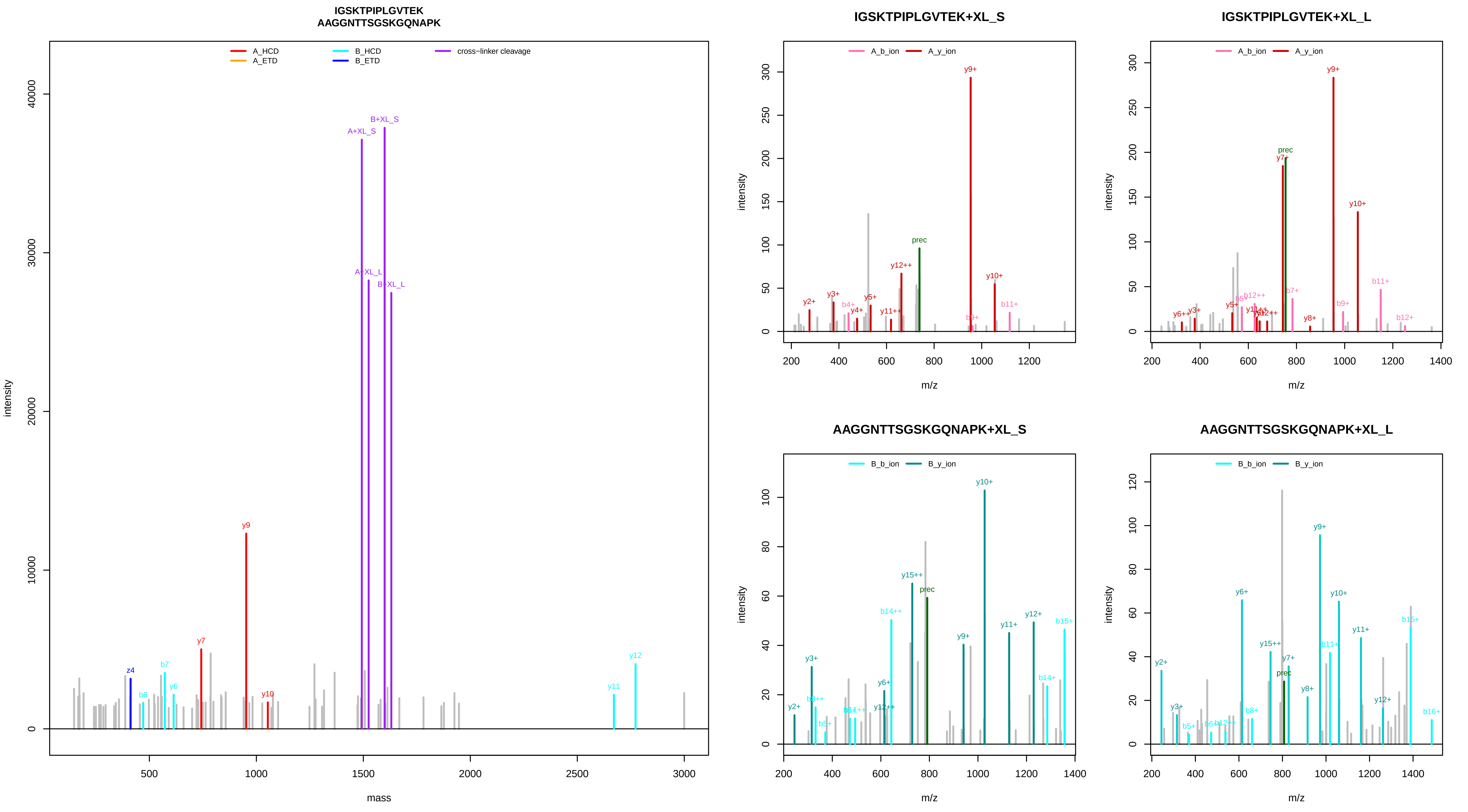

IGSKTPIPLGVTEK
AAGGNTTSGSKGQNAPK
A_HCD
A_ETD
B_HCD
B_ETD
cross−linker cleavage
B+XL_S
A+XL_S
A+XL_L
B+XL_L
y9
y7
y10
z4
b7
b6
y6
y11
y12
intensity
mass
IGSKTPIPLGVTEK+XL_S
IGSKTPIPLGVTEK+XL_L
AAGGNTTSGSKGQNAPK+XL_S
AAGGNTTSGSKGQNAPK+XL_L
A_b_ion
A_y_ion
B_b_ion
B_y_ion
prec
m/z

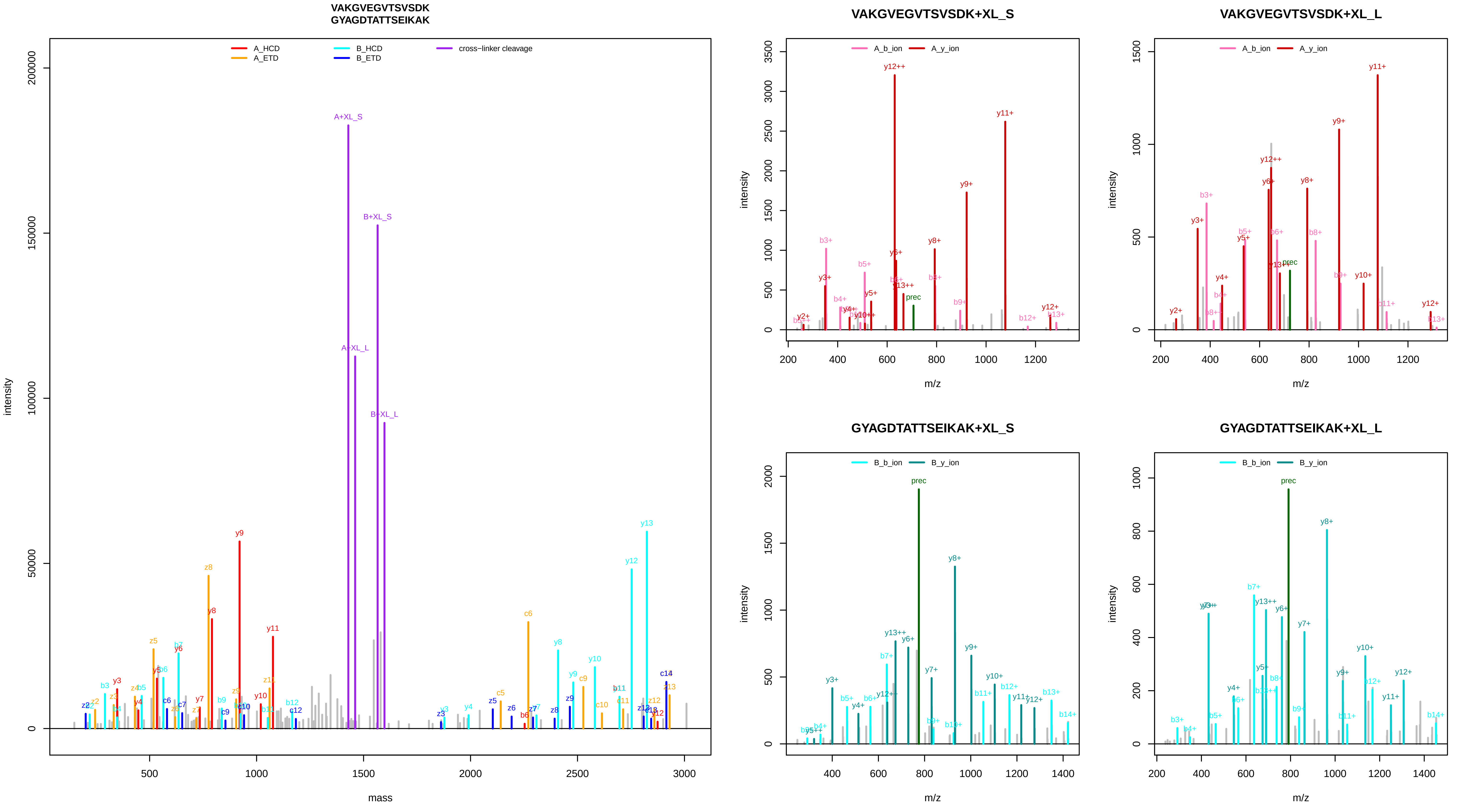
VAKGVEGVTSVSDK
GYAGDTATTSEIKAK
A_HCD
A_ETD
B_HCD
B_ETD
cross−linker cleavage
A+XL_S
B+XL_S
A+XL_L
B+XL_L
intensity
mass
VAKGVEGVTSVSDK+XL_S
VAKGVEGVTSVSDK+XL_L
GYAGDTATTSEIKAK+XL_S
GYAGDTATTSEIKAK+XL_L
A_b_ion
A_y_ion
B_b_ion
B_y_ion
prec
m/z

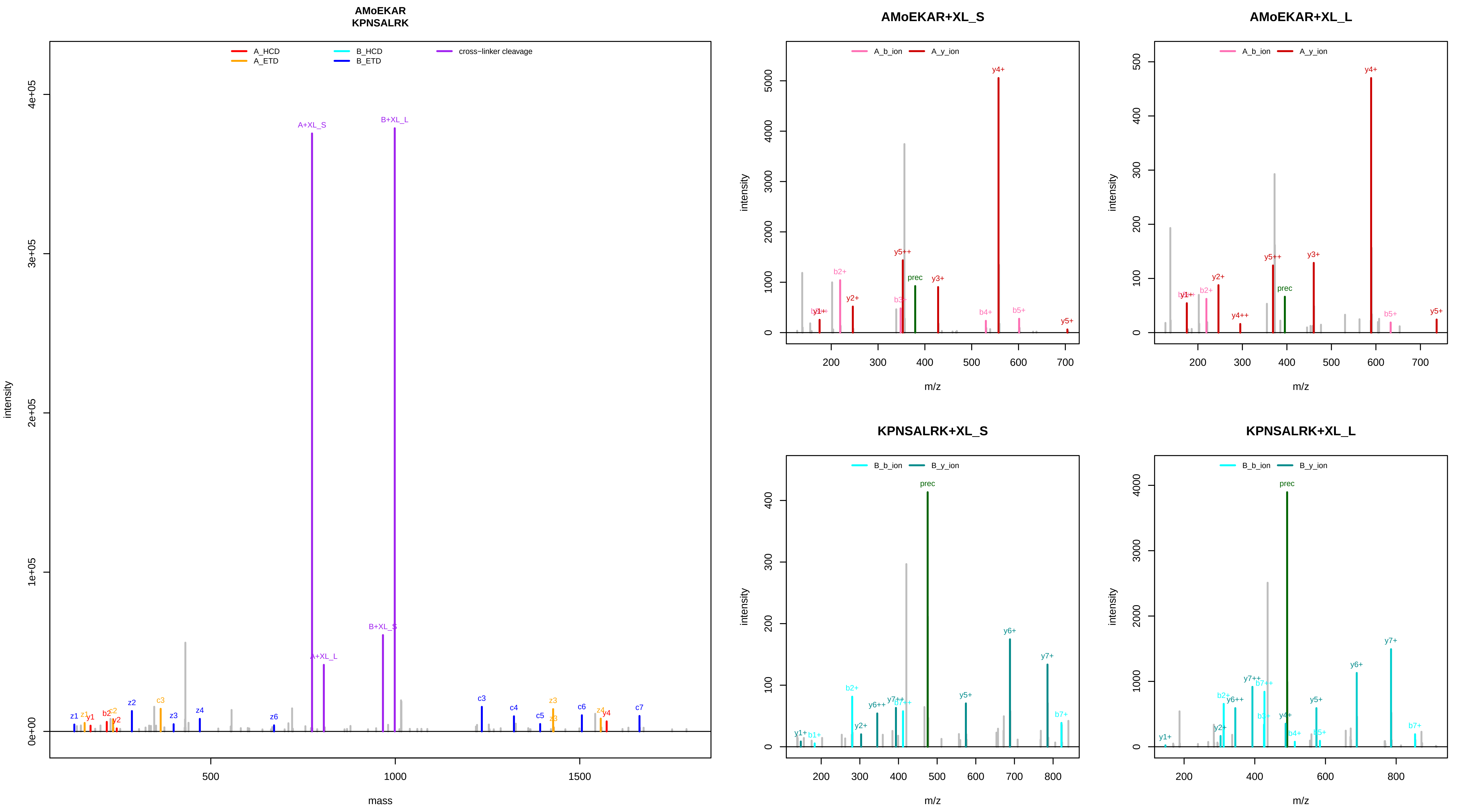
AMoEKAR
KPNSALRK
A_HCD
A_ETD
B_HCD
B_ETD
cross−linker cleavage
A+XL_S
B+XL_L
B+XL_S
A+XL_L
intensity
mass
AMoEKAR+XL_S
AMoEKAR+XL_L
KPNSALRK+XL_S
KPNSALRK+XL_L
A_b_ion
A_y_ion
B_b_ion
B_y_ion
prec
m/z

SDSNSR
LAAELKKK

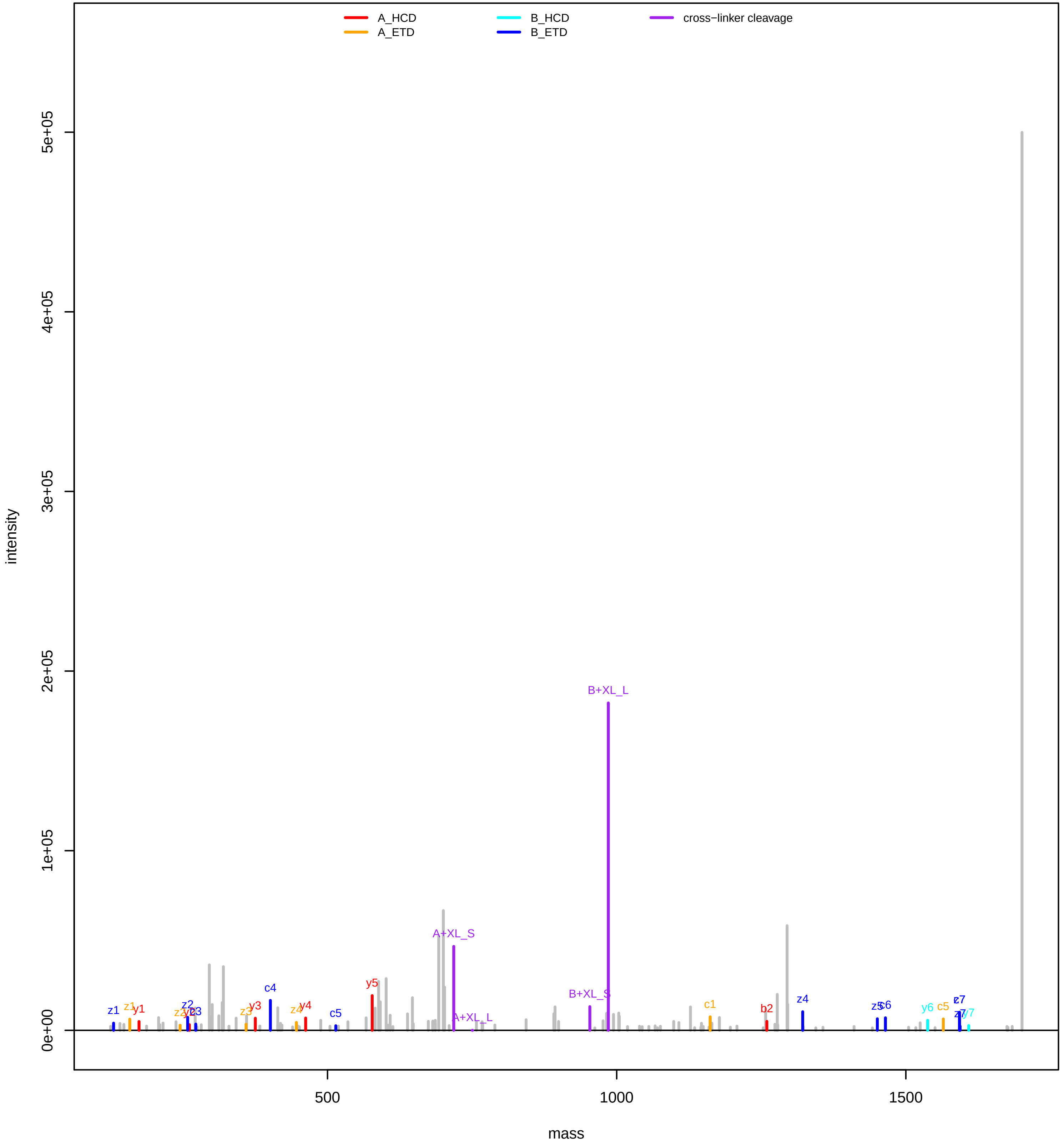

SDSNSR+XL_S

SDSNSR+XL_L

LAAELKKK+XL_S

LAAELKKK+XL_L

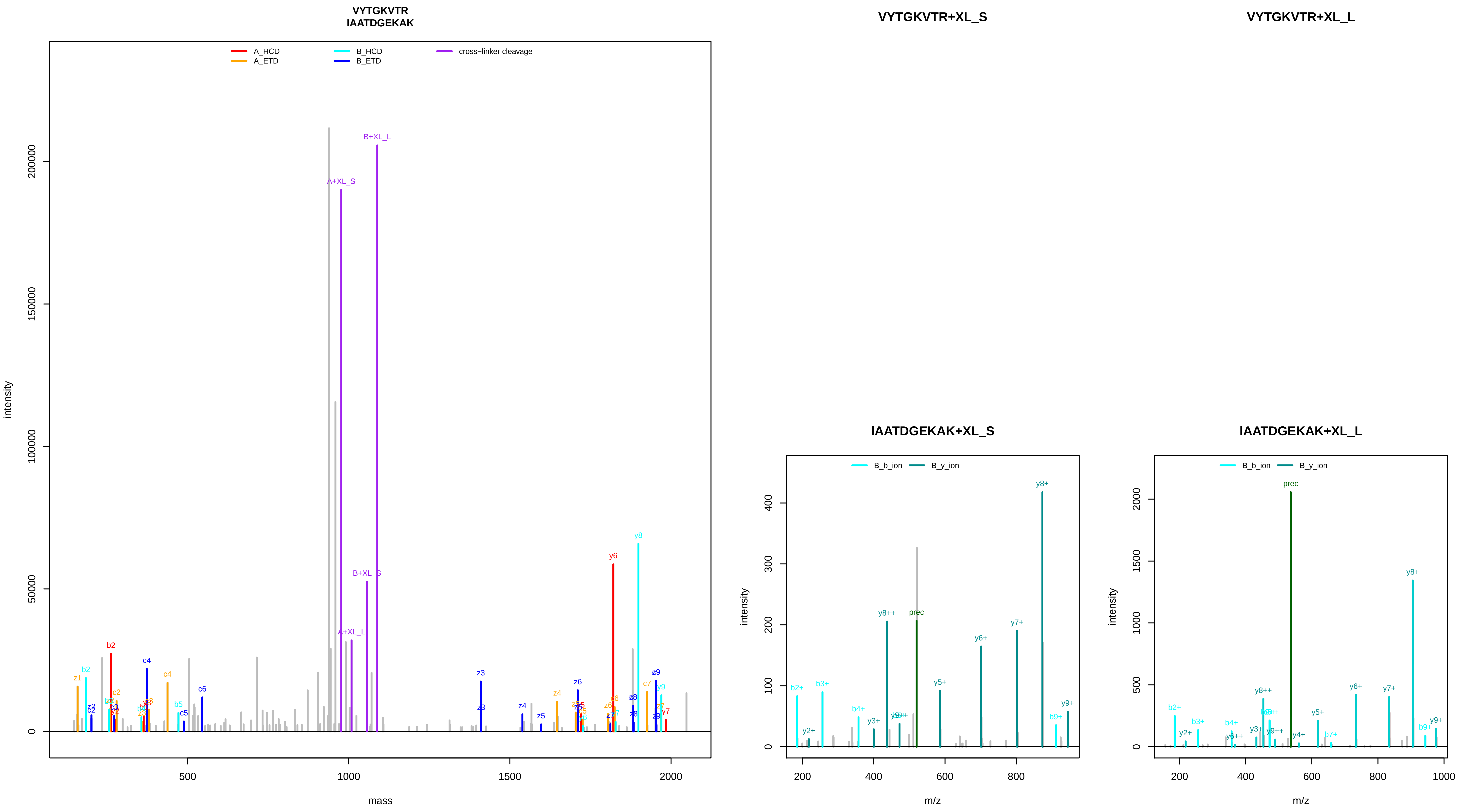

VYTGKVTR
IAATDGEKAK
A_HCD
A_ETD
B_HCD
B_ETD
cross−linker cleavage
A+XL_S
B+XL_L
B+XL_S
A+XL_L
intensity
mass
0
50000
100000
150000
200000
500
1000
1500
2000
VYTGKVTR+XL_S
VYTGKVTR+XL_L
IAATDGEKAK+XL_S
IAATDGEKAK+XL_L
B_b_ion
B_y_ion
prec
m/z

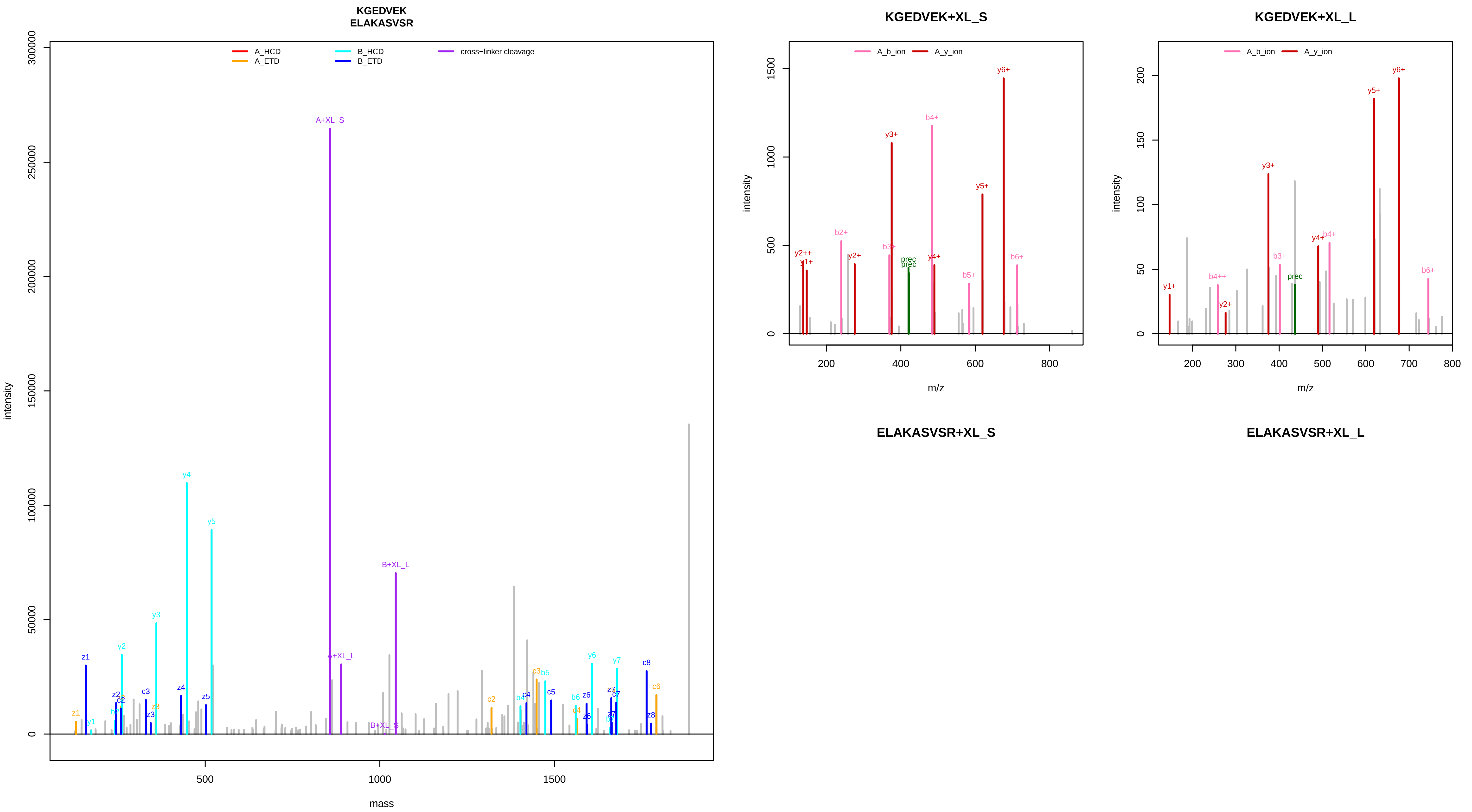
KGEDVEK
ELAKASVSR
A_HCD
A_ETD
B_HCD
B_ETD
cross−linker cleavage
A+XL_S
B+XL_L
A+XL_L
B+XL_S
intensity
mass
KGEDVEK+XL_S
KGEDVEK+XL_L
A_b_ion
A_y_ion
prec
m/z
ELAKASVSR+XL_S
ELAKASVSR+XL_L

NDLKTK
STDISVKTDQK

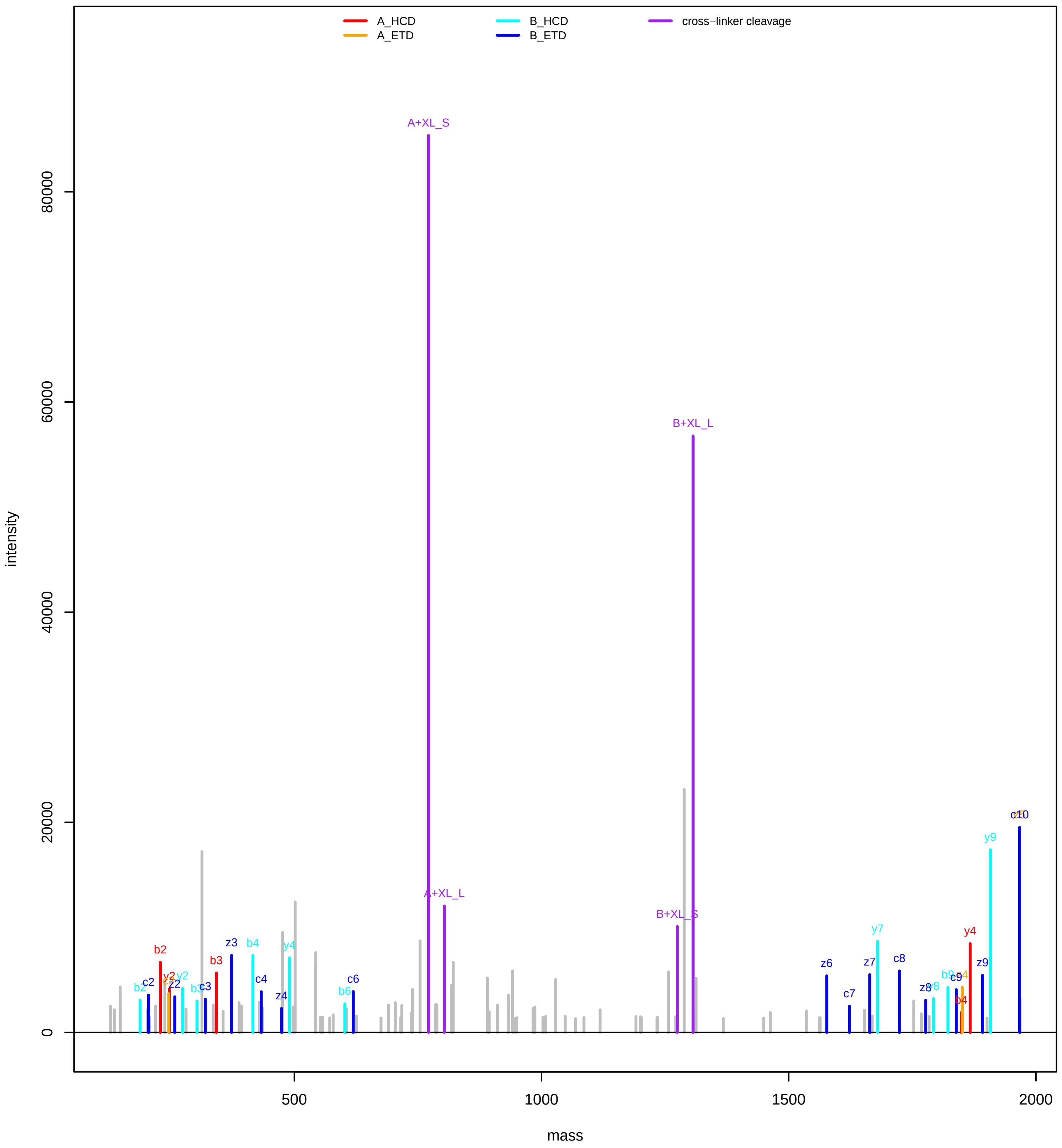

NDLKTK+XL_S

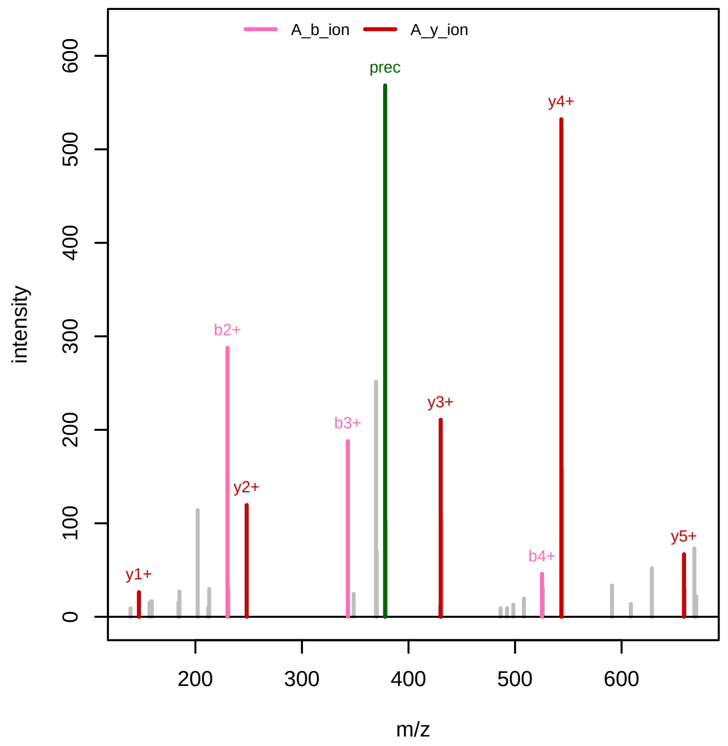

NDLKTK+XL_L

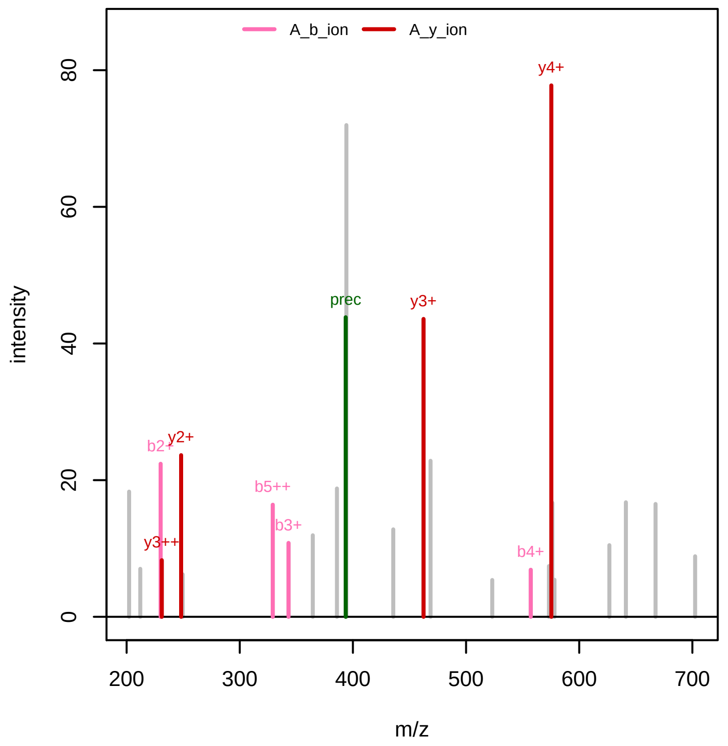

STDISVKTDQK+XL_S

STDISVKTDQK+XL_L

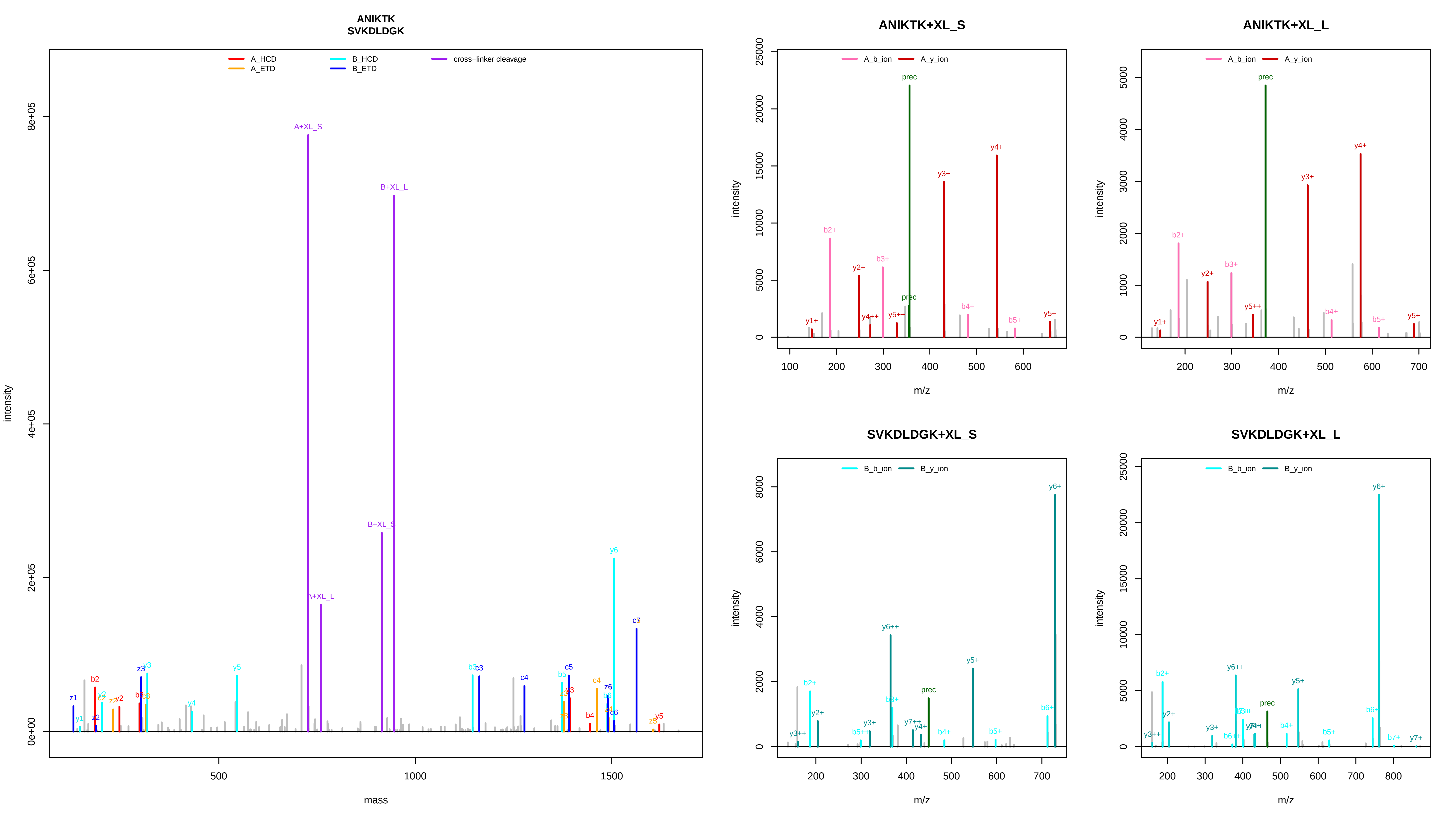

ANIKTK
SVKDLDGK
A_HCD
A_ETD
B_HCD
B_ETD
cross−linker cleavage
A+XL_S
B+XL_L
B+XL_S
A+XL_L
intensity
mass
0e+00
2e+05
4e+05
6e+05
8e+05
500
1000
1500
z1
y1
b2
z2
y2
c2
z3
y3
y4
y5
b3
c3
c4
b5
c5
z6
y6
c6
c7
b4
c4
y5
z5
ANIKTK+XL_S
A_b_ion
A_y_ion
prec
b2+
y2+
b3+
y3+
y4+
y1+
y4++
y5++
b4+
b5+
y5+
m/z
intensity
ANIKTK+XL_L
SVKDLDGK+XL_S
B_b_ion
B_y_ion
y6+
y6++
y5+
b2+
y2+
y3++
y3+
b5++
b3+
y7++
y4+
b4+
b5+
b6+
SVKDLDGK+XL_L
b6++
b7+
y7+

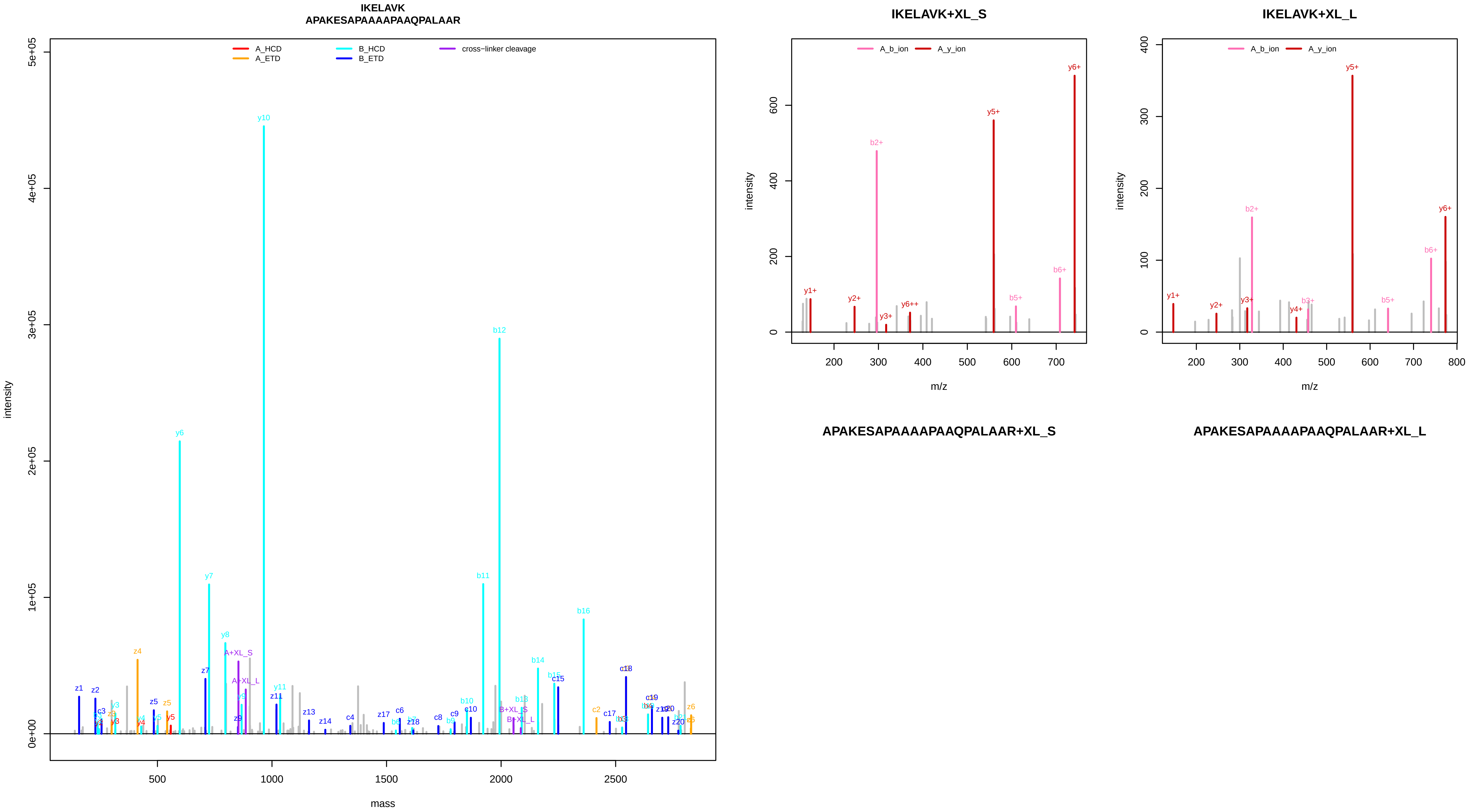
IKELAVK
APAKESAPAAAAPAAQPALAAR
A_HCD
A_ETD
B_HCD
B_ETD
cross−linker cleavage
intensity
mass
IKELAVK+XL_S
A_b_ion
A_y_ion
intensity
m/z
IKELAVK+XL_L
A_b_ion
A_y_ion
intensity
m/z
APAKESAPAAAAPAAQPALAAR+XL_S
APAKESAPAAAAPAAQPALAAR+XL_L

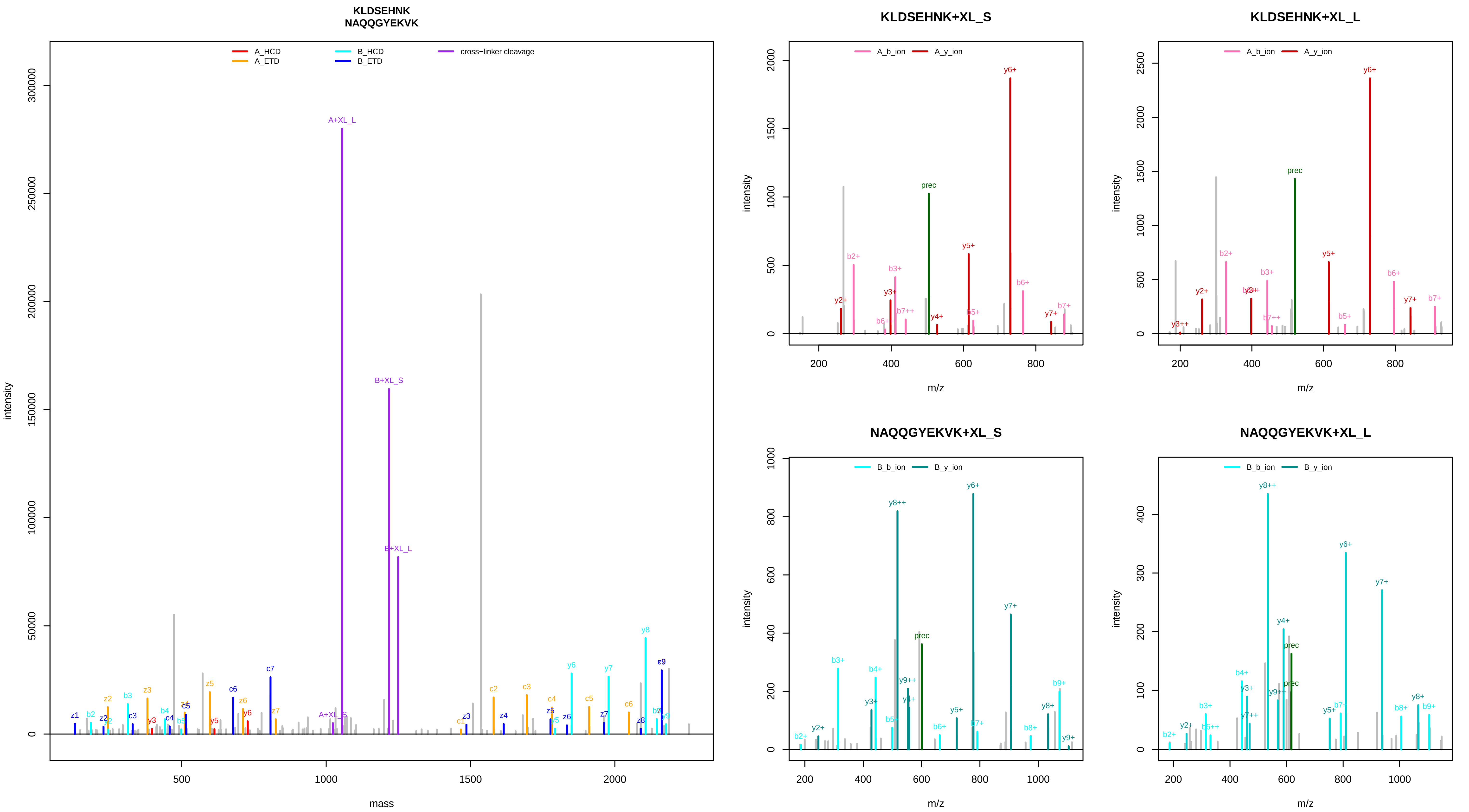

KLDSEHNK
NAQQGYEKVK
A_HCD
A_ETD
B_HCD
B_ETD
cross−linker cleavage
A+XL_L
B+XL_S
B+XL_L
A+XL_S
intensity
mass
KLDSEHNK+XL_S
KLDSEHNK+XL_L
NAQQGYEKVK+XL_S
NAQQGYEKVK+XL_L
A_b_ion
A_y_ion
B_b_ion
B_y_ion
prec
m/z

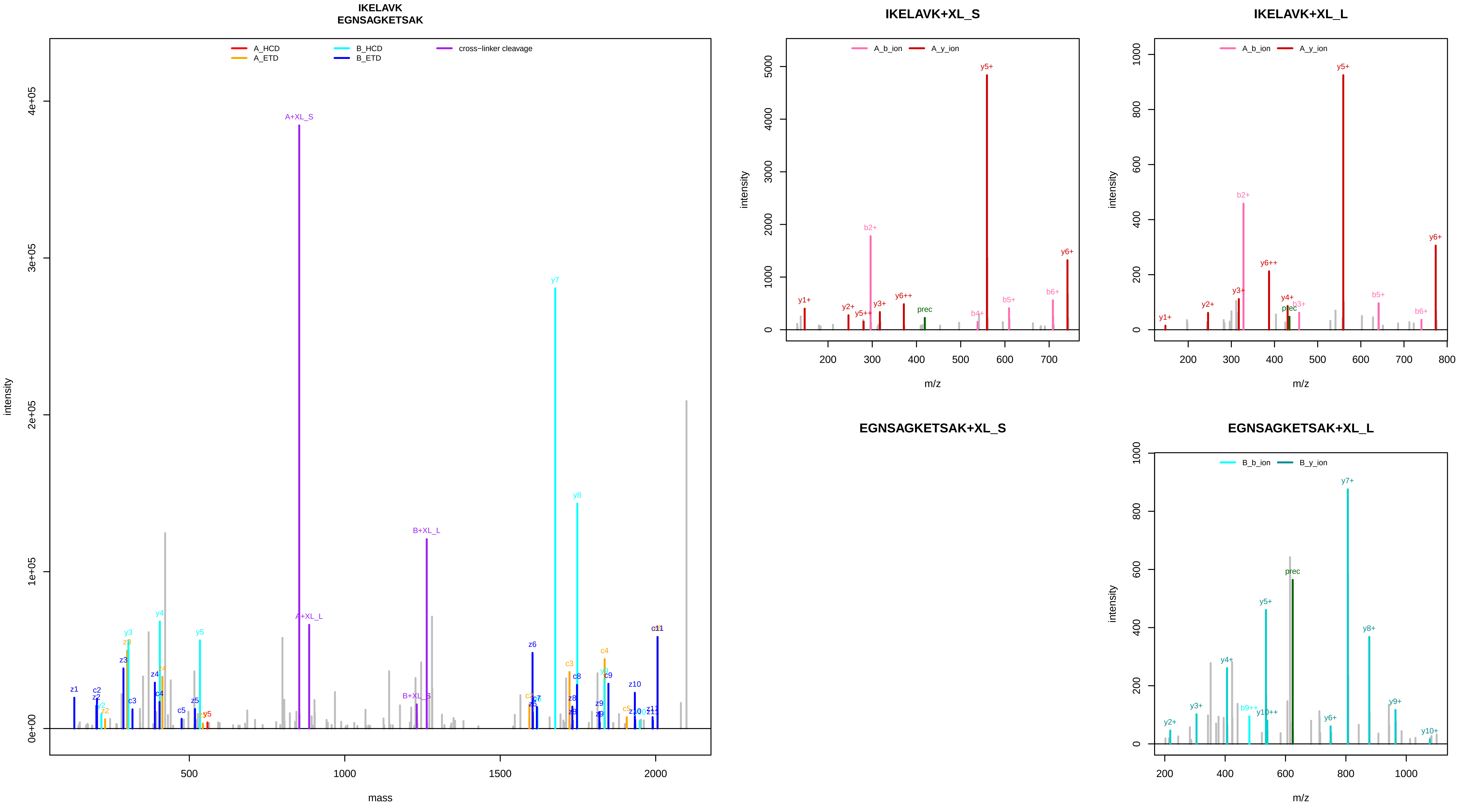
IKELAVK
EGNSAGKETSAK
A_HCD
A_ETD
B_HCD
B_ETD
cross−linker cleavage
A+XL_S
A+XL_L
B+XL_L
B+XL_S
y7
y8
y4
y5
y3
z6
c11
c4
c3
c8
c9
z10
z3
z4
c2
z1
c5
z5
intensity
mass
0e+00
1e+05
2e+05
3e+05
4e+05
500
1000
1500
2000
IKELAVK+XL_S
A_b_ion
A_y_ion
y5+
b2+
y6+
y1+
y2+
y5++
y3+
y6++
prec
b4+
b5+
b6+
m/z
IKELAVK+XL_L
y6++
y3+
y4+
b3+
EGNSAGKETSAK+XL_S
EGNSAGKETSAK+XL_L
B_b_ion
B_y_ion
y7+
y8+
y9+
y10+
y10++
y6+
b9++
y4+
y2+

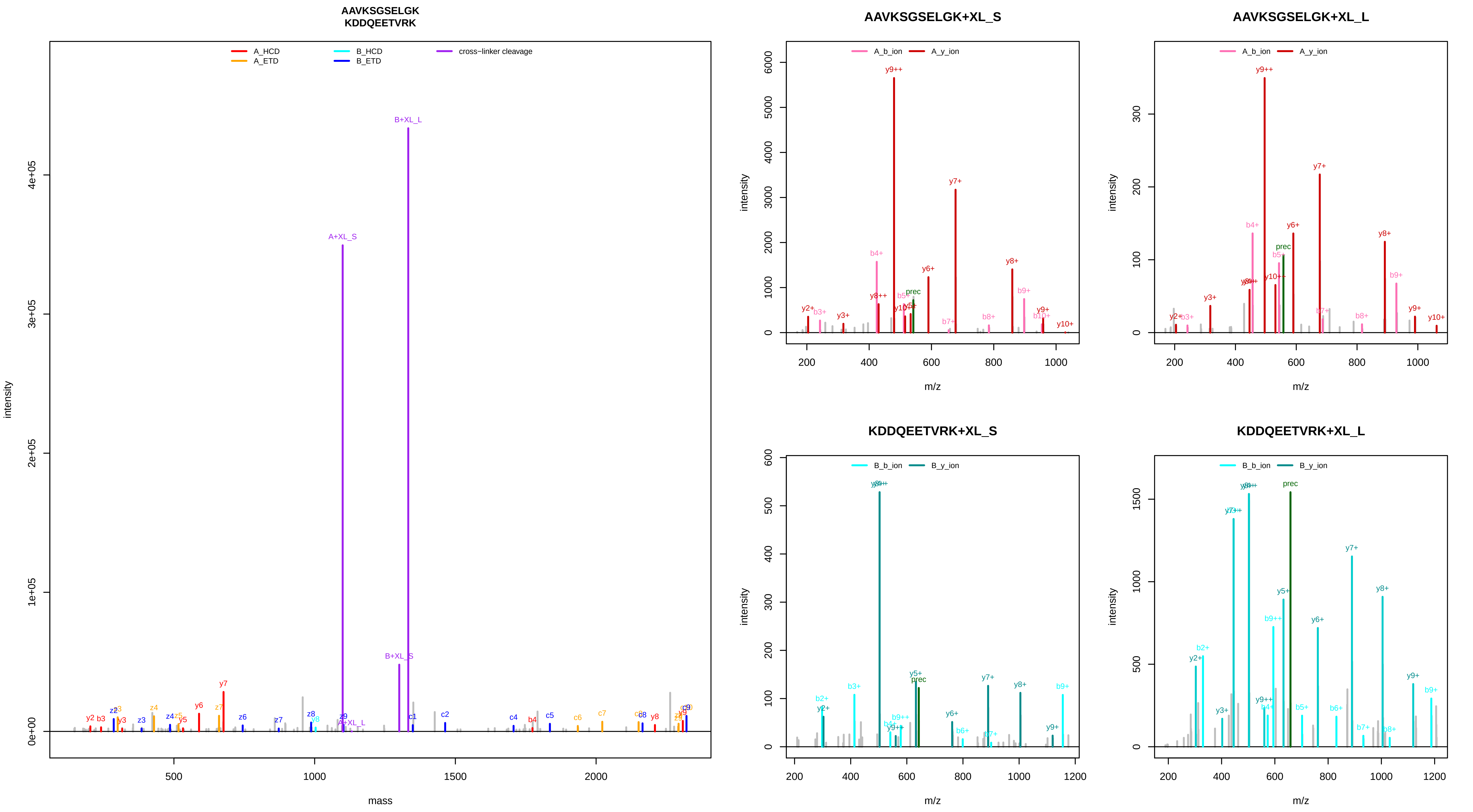
AAVKSGSELGK
KDDQEETVRK
A_HCD
A_ETD
B_HCD
B_ETD
cross−linker cleavage
B+XL_L
A+XL_S
B+XL_S
A+XL_L
intensity
mass
AAVKSGSELGK+XL_S
AAVKSGSELGK+XL_L
A_b_ion
A_y_ion
KDDQEETVRK+XL_S
KDDQEETVRK+XL_L
B_b_ion
B_y_ion
prec
m/z

AANDKGR
HSTKAFDASK

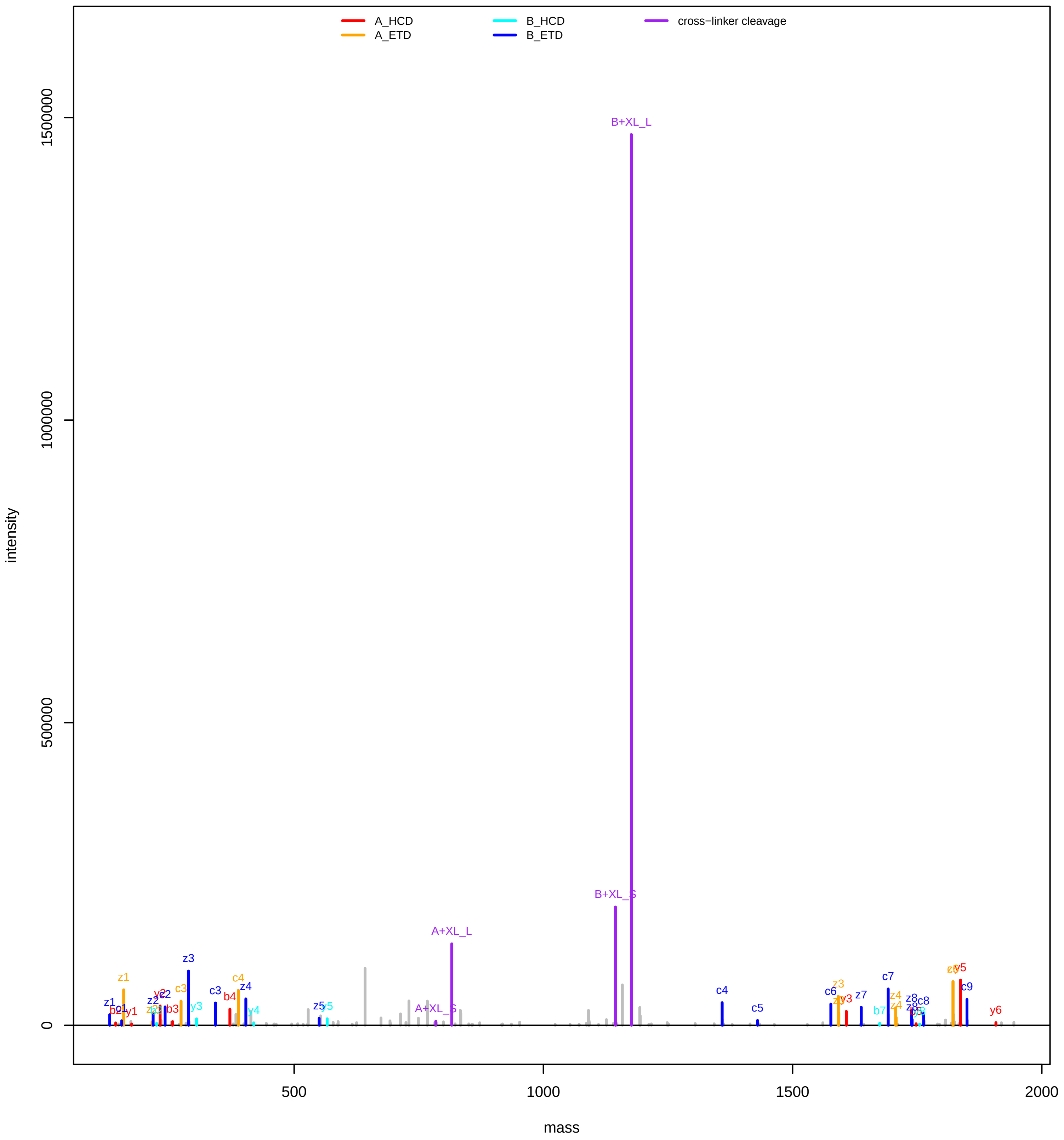

AANDKGR+XL_S

AANDKGR+XL_L

HSTKAFDASK+XL_S

HSTKAFDASK+XL_L

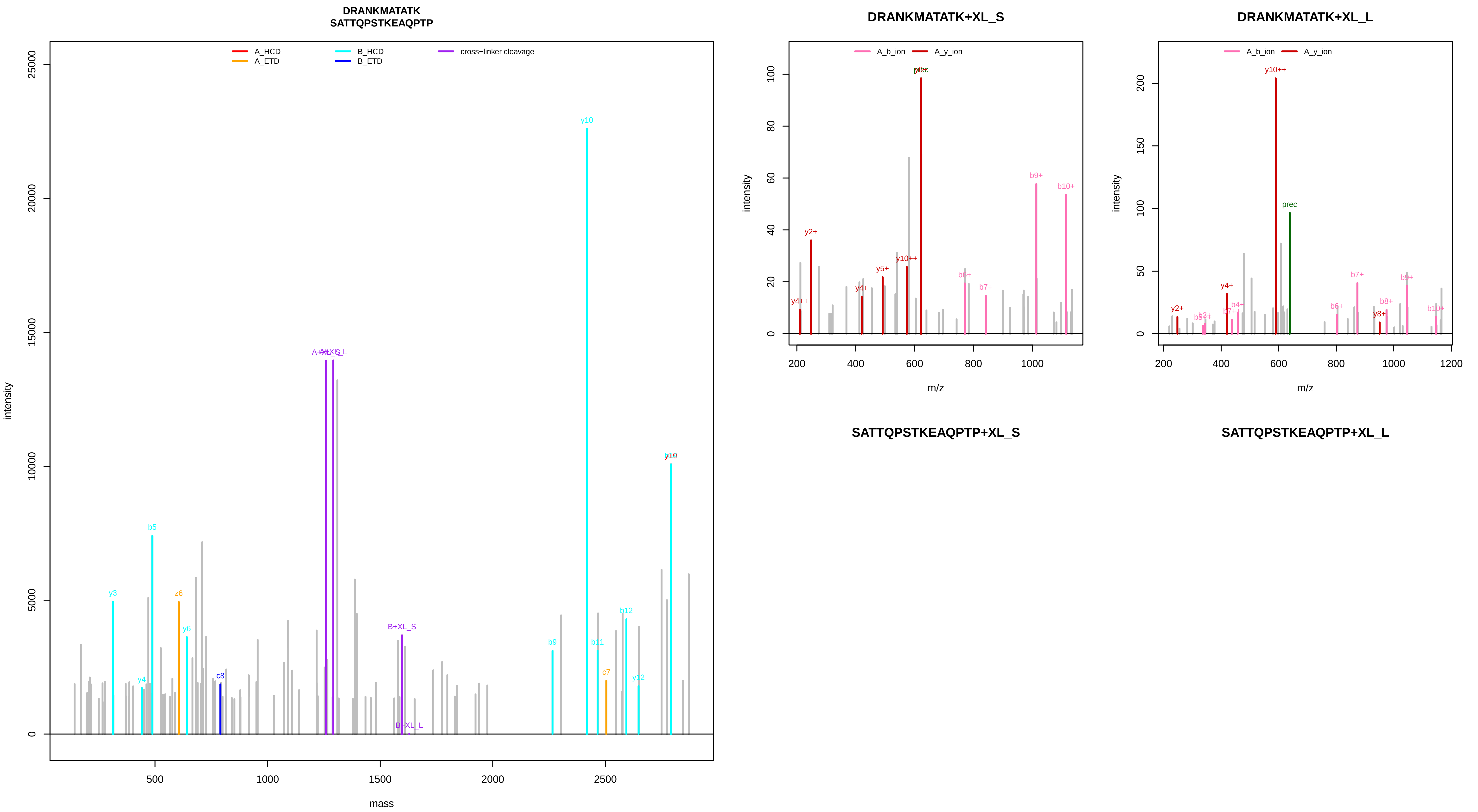
DRANKMATATK
SATTQPSTKEAQPTP
A_HCD
A_ETD
B_HCD
B_ETD
cross−linker cleavage
intensity
mass
y10
A+XL_S
A+XL_L
B+XL_S
B+XL_L
b5
y3
y4
z6
y6
c8
b9
b11
c7
b12
y12
DRANKMATATK+XL_S
A_b_ion
A_y_ion
prec
y2+
y4++
y4+
y5+
y10++
b6+
b7+
b9+
b10+
m/z
DRANKMATATK+XL_L
y8+
b8+
SATTQPSTKEAQPTP+XL_S
SATTQPSTKEAQPTP+XL_L

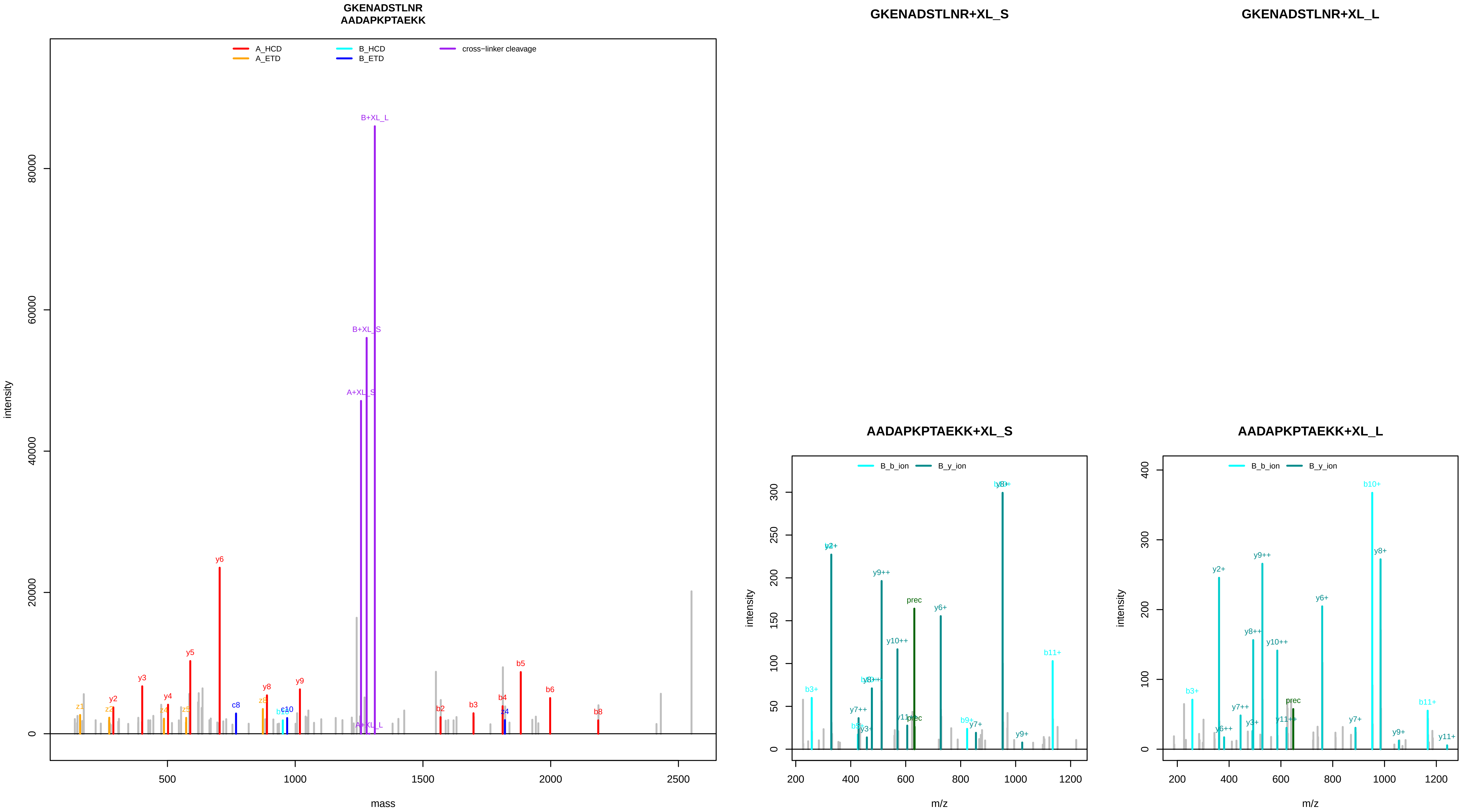

GKENADSTLNR
AADAPKPTAEKK
A_HCD
A_ETD
B_HCD
B_ETD
cross−linker cleavage
B+XL_L
B+XL_S
A+XL_S
A+XL_L
intensity
mass
GKENADSTLNR+XL_S
GKENADSTLNR+XL_L
AADAPKPTAEKK+XL_S
AADAPKPTAEKK+XL_L
B_b_ion
B_y_ion
intensity
m/z

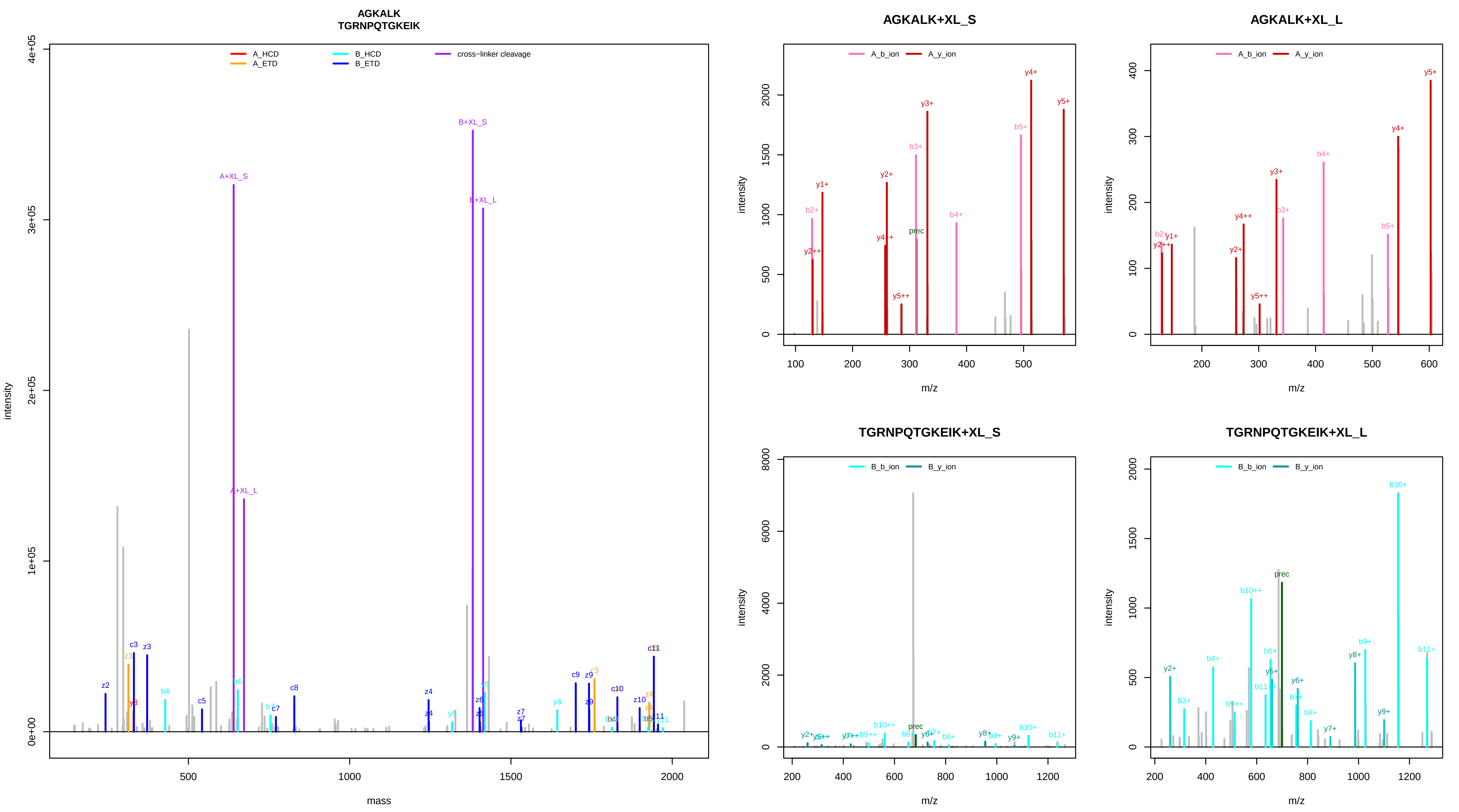
AGKALK
TGRNPQTGKEIK
A_HCD
A_ETD
B_HCD
B_ETD
cross−linker cleavage
B+XL_S
A+XL_S
B+XL_L
A+XL_L
intensity
mass
AGKALK+XL_S
AGKALK+XL_L
A_b_ion
A_y_ion
TGRNPQTGKEIK+XL_S
TGRNPQTGKEIK+XL_L
B_b_ion
B_y_ion
prec
m/z

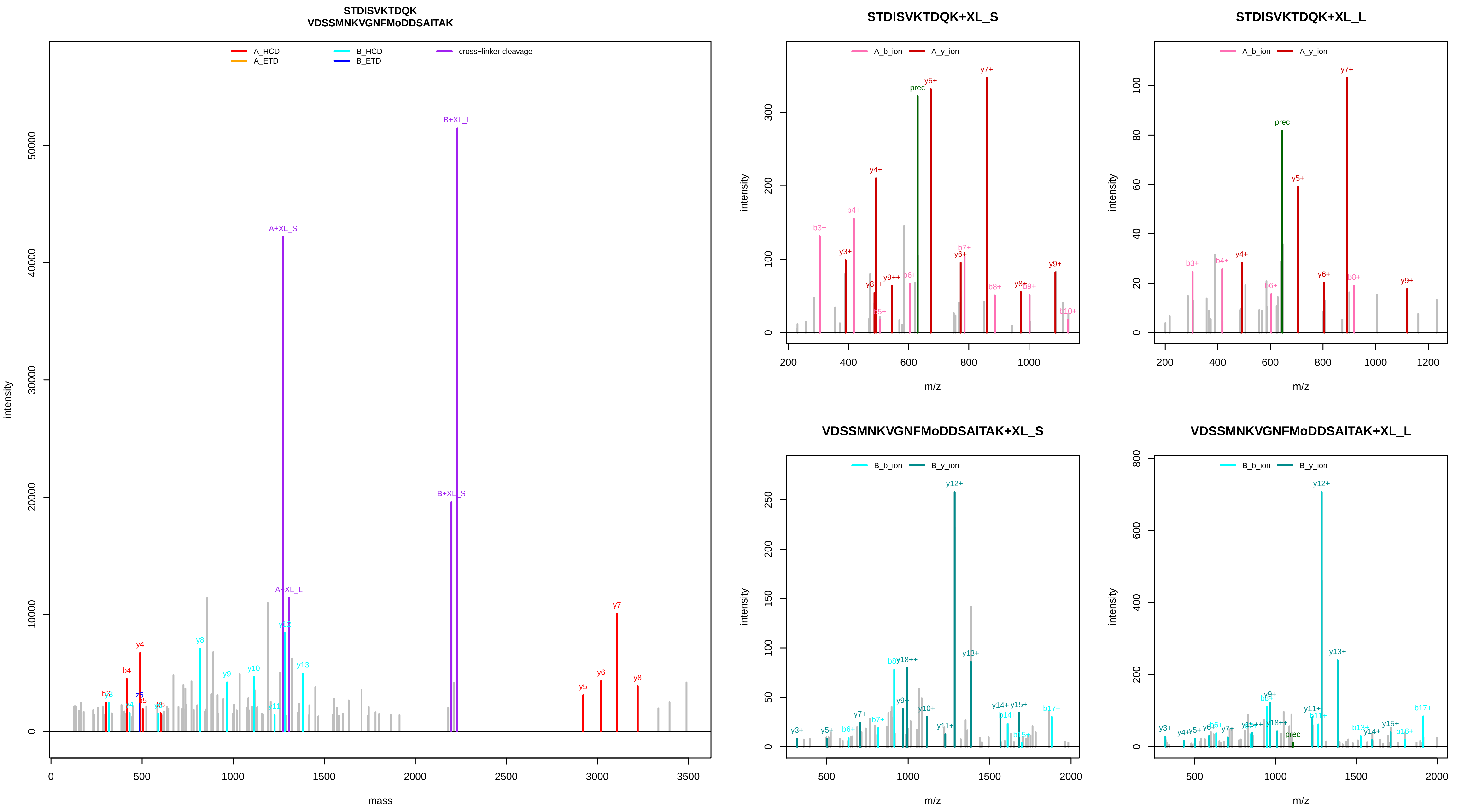
STDISVKTDQK
VDSSMNKVGNFMoDDSAITAK
A_HCD
A_ETD
B_HCD
B_ETD
cross−linker cleavage
B+XL_L
A+XL_S
B+XL_S
A+XL_L
y4
b4
b3
b5
b6
y8
y9
y10
y12
y11
y13
y5
y6
y7
y8
intensity
mass
STDISVKTDQK+XL_S
STDISVKTDQK+XL_L
A_b_ion
A_y_ion
prec
y3+
y4+
y5+
y6+
y7+
y8+
y9+
y8++
y9++
b3+
b4+
b5+
b6+
b7+
b8+
b9+
b10+
m/z
VDSSMNKVGNFMoDDSAITAK+XL_S
VDSSMNKVGNFMoDDSAITAK+XL_L
B_b_ion
B_y_ion
y12+
y13+
y18++
y7+
y9+
y10+
y11+
y14+
y15+
b17+
y3+
y5+
b6+
b7+
y4+
y6+
y15++
y11+
b13+

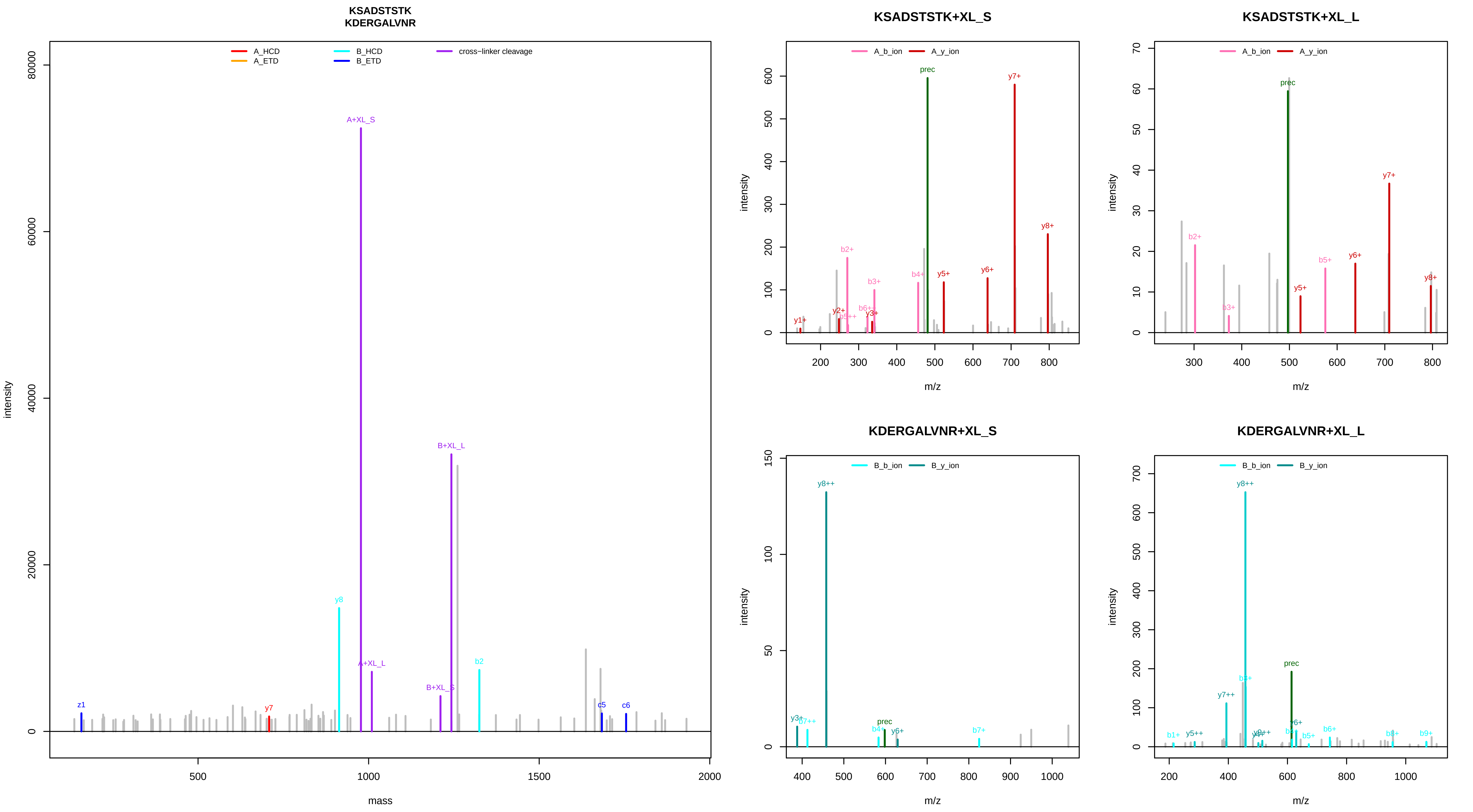

KSADSTSTK
KDERGALVNR
A_HCD
A_ETD
B_HCD
B_ETD
cross−linker cleavage
A+XL_S
B+XL_L
y8
A+XL_L
b2
B+XL_S
z1
y7
c5
c6
intensity
mass
KSADSTSTK+XL_S
KSADSTSTK+XL_L
KDERGALVNR+XL_S
KDERGALVNR+XL_L
A_b_ion
A_y_ion
B_b_ion
B_y_ion
prec
m/z

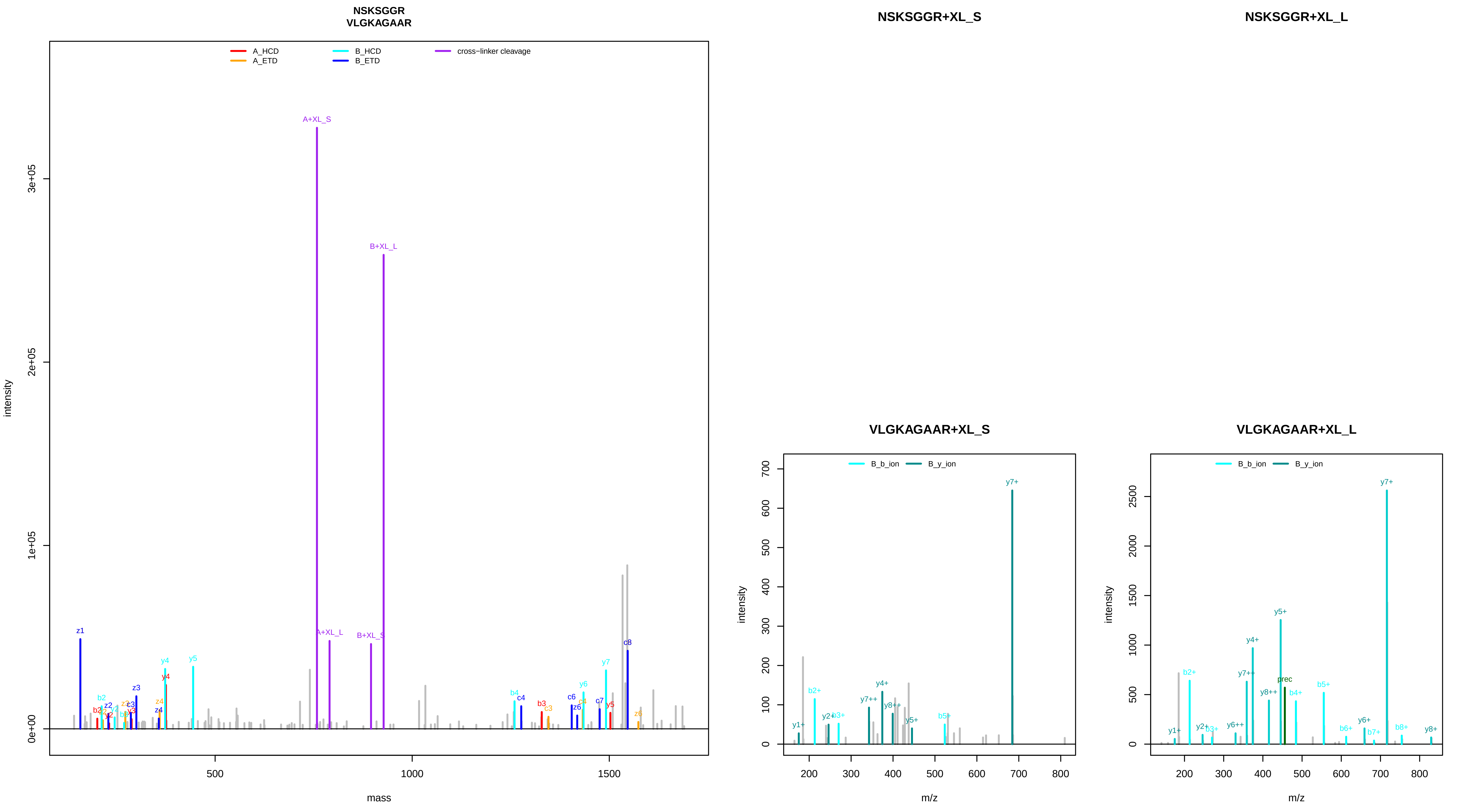

NSKSGGR
VLGKAGAAR
A_HCD
A_ETD
B_HCD
B_ETD
cross−linker cleavage
A+XL_S
B+XL_L
A+XL_L
B+XL_S
intensity
mass
NSKSGGR+XL_S
NSKSGGR+XL_L
VLGKAGAAR+XL_S
VLGKAGAAR+XL_L
B_b_ion
B_y_ion
m/z

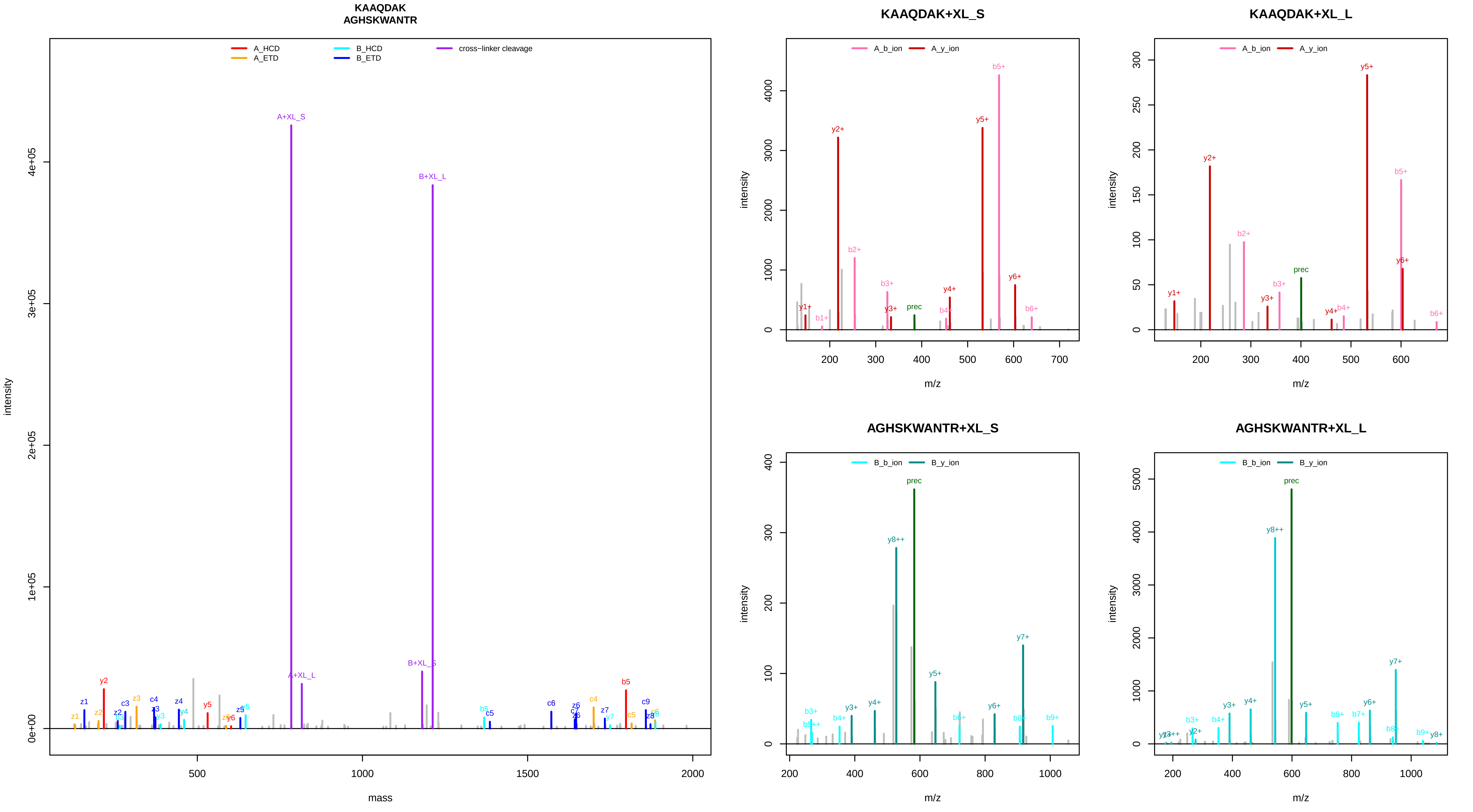
KAAQDAK
AGHSKWANTR
A_HCD
A_ETD
B_HCD
B_ETD
cross−linker cleavage
A+XL_S
B+XL_L
A+XL_L
B+XL_S
intensity
mass
KAAQDAK+XL_S
KAAQDAK+XL_L
A_b_ion
A_y_ion
AGHSKWANTR+XL_S
AGHSKWANTR+XL_L
B_b_ion
B_y_ion
prec
m/z

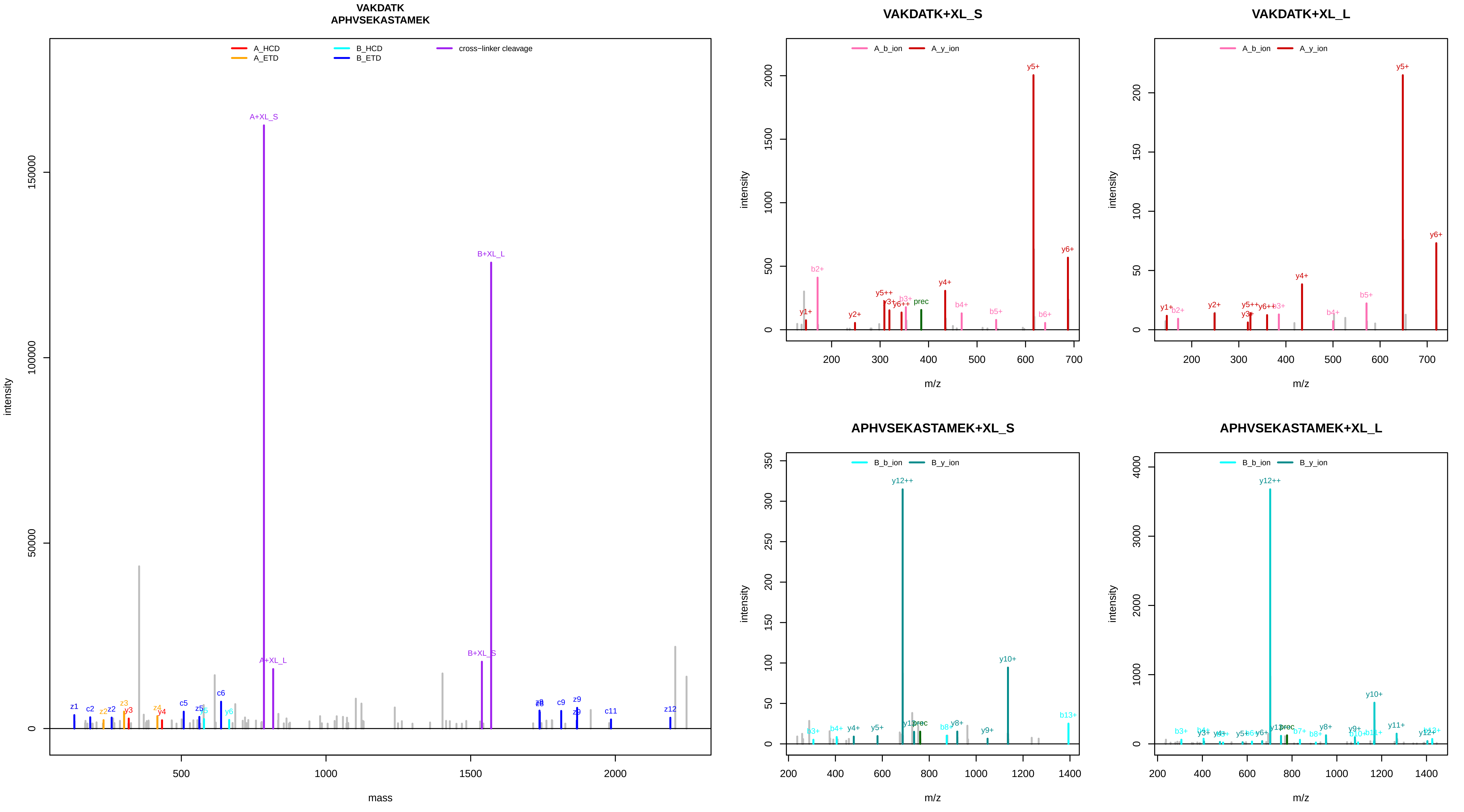

VAKDATK
APHVSEKASTAMEK
A_HCD
A_ETD
B_HCD
B_ETD
cross−linker cleavage
A+XL_S
B+XL_L
A+XL_L
B+XL_S
intensity
mass
VAKDATK+XL_S
VAKDATK+XL_L
APHVSEKASTAMEK+XL_S
APHVSEKASTAMEK+XL_L
A_b_ion
A_y_ion
B_b_ion
B_y_ion
m/z

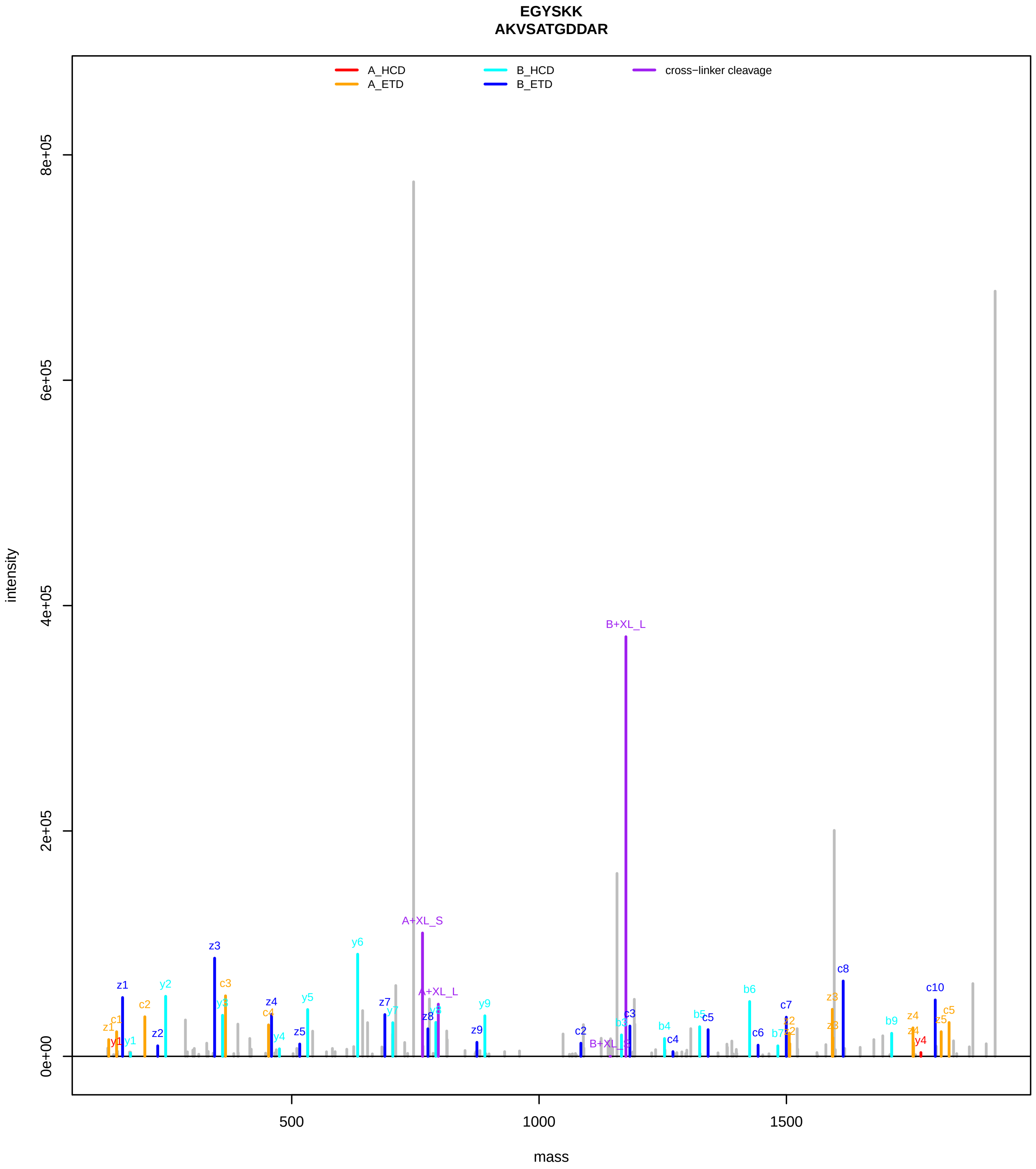

EGYSKK+XL_S

EGYSKK+XL_L

AKVSATGDDAR+XL_S

AKVSATGDDAR+XL_L

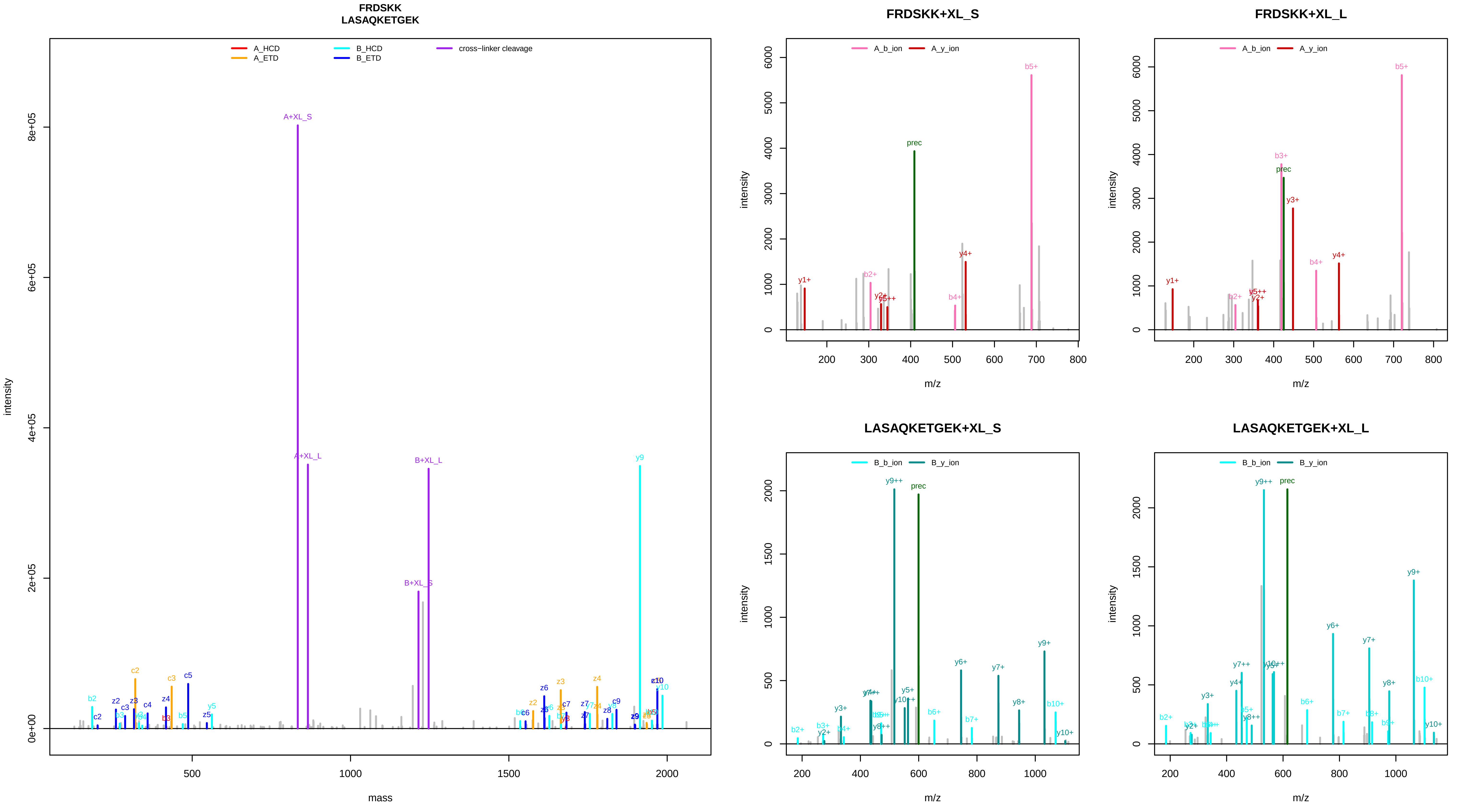
FRDSKK
LASAQKETGEK
A_HCD
A_ETD
B_HCD
B_ETD
cross−linker cleavage
A+XL_S
A+XL_L
B+XL_L
B+XL_S
y9
intensity
mass
FRDSKK+XL_S
FRDSKK+XL_L
LASAQKETGEK+XL_S
LASAQKETGEK+XL_L
A_b_ion
A_y_ion
B_b_ion
B_y_ion
prec
b5+
y4+
y9++
m/z
intensity

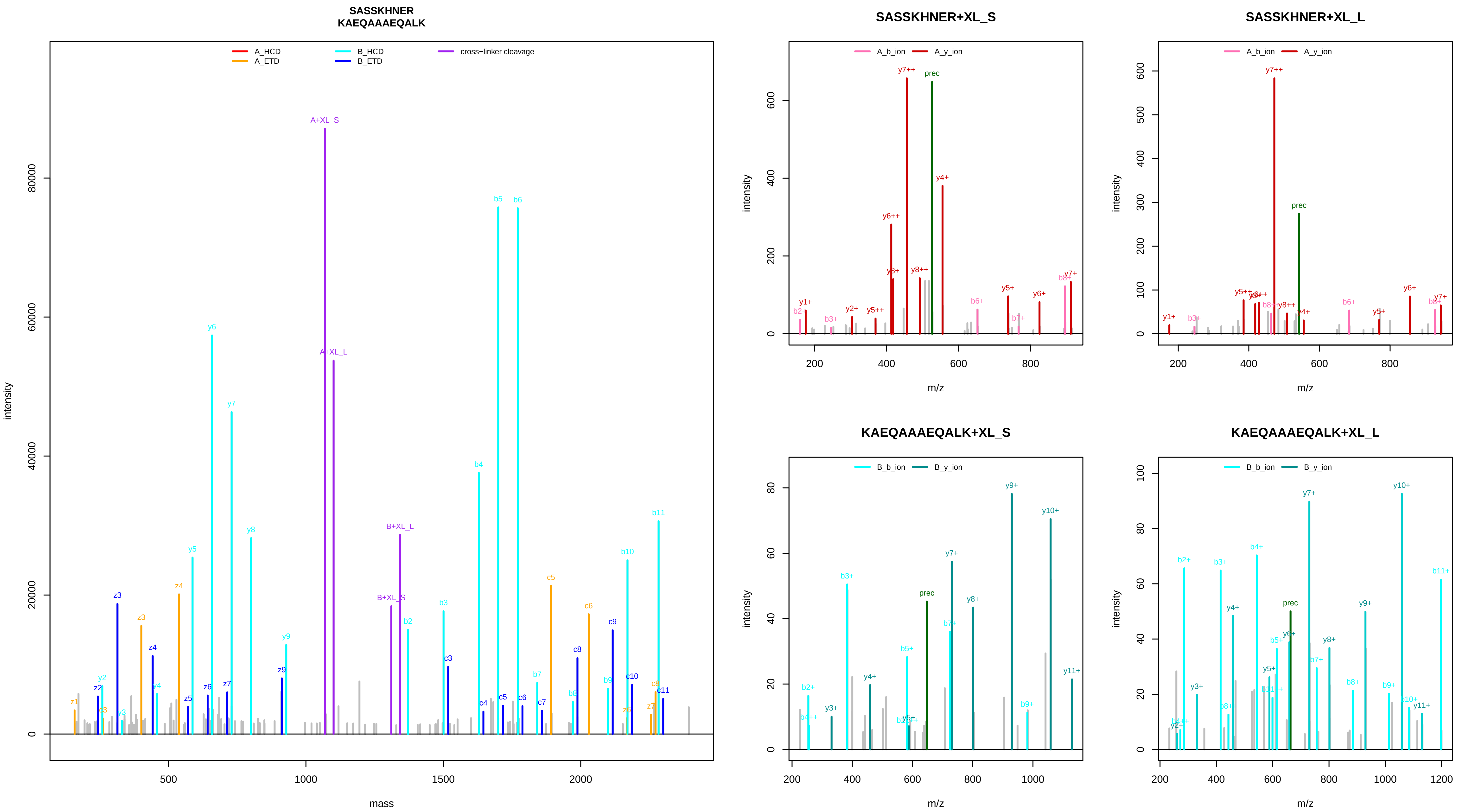
SASSKHNER
KAEQAAAEQALK
A_HCD
A_ETD
B_HCD
B_ETD
cross−linker cleavage
A+XL_S
A+XL_L
B+XL_L
B+XL_S
intensity
mass
SASSKHNER+XL_S
SASSKHNER+XL_L
A_b_ion
A_y_ion
prec
KAEQAAAEQALK+XL_S
KAEQAAAEQALK+XL_L
B_b_ion
B_y_ion
m/z

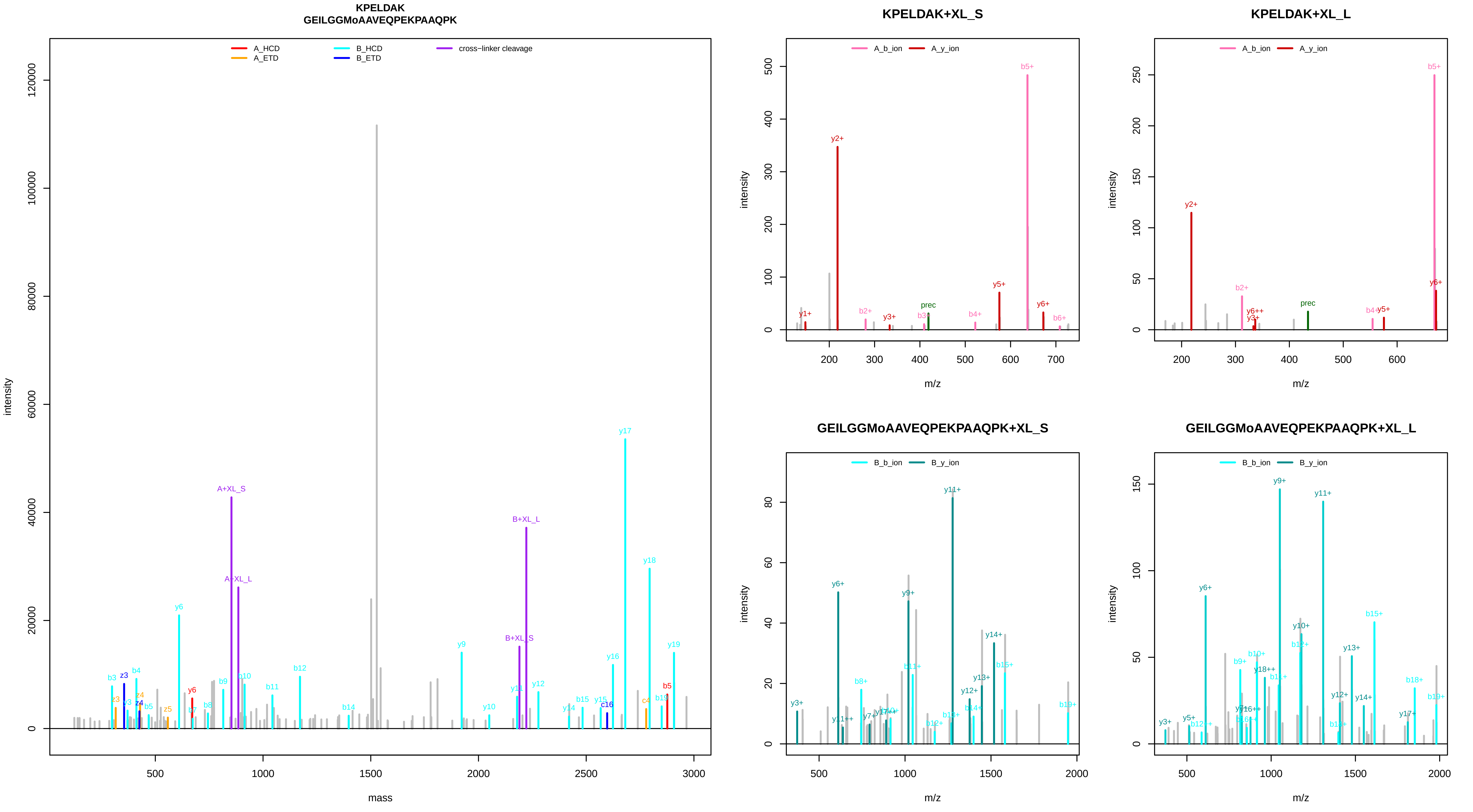
KPELDAK
GEILGGMoAAVEQPEKPAAQPK
A_HCD
A_ETD
B_HCD
B_ETD
cross−linker cleavage
A+XL_S
A+XL_L
B+XL_L
B+XL_S
intensity
mass
KPELDAK+XL_S
KPELDAK+XL_L
GEILGGMoAAVEQPEKPAAQPK+XL_S
GEILGGMoAAVEQPEKPAAQPK+XL_L
A_b_ion
A_y_ion
B_b_ion
B_y_ion
prec
m/z

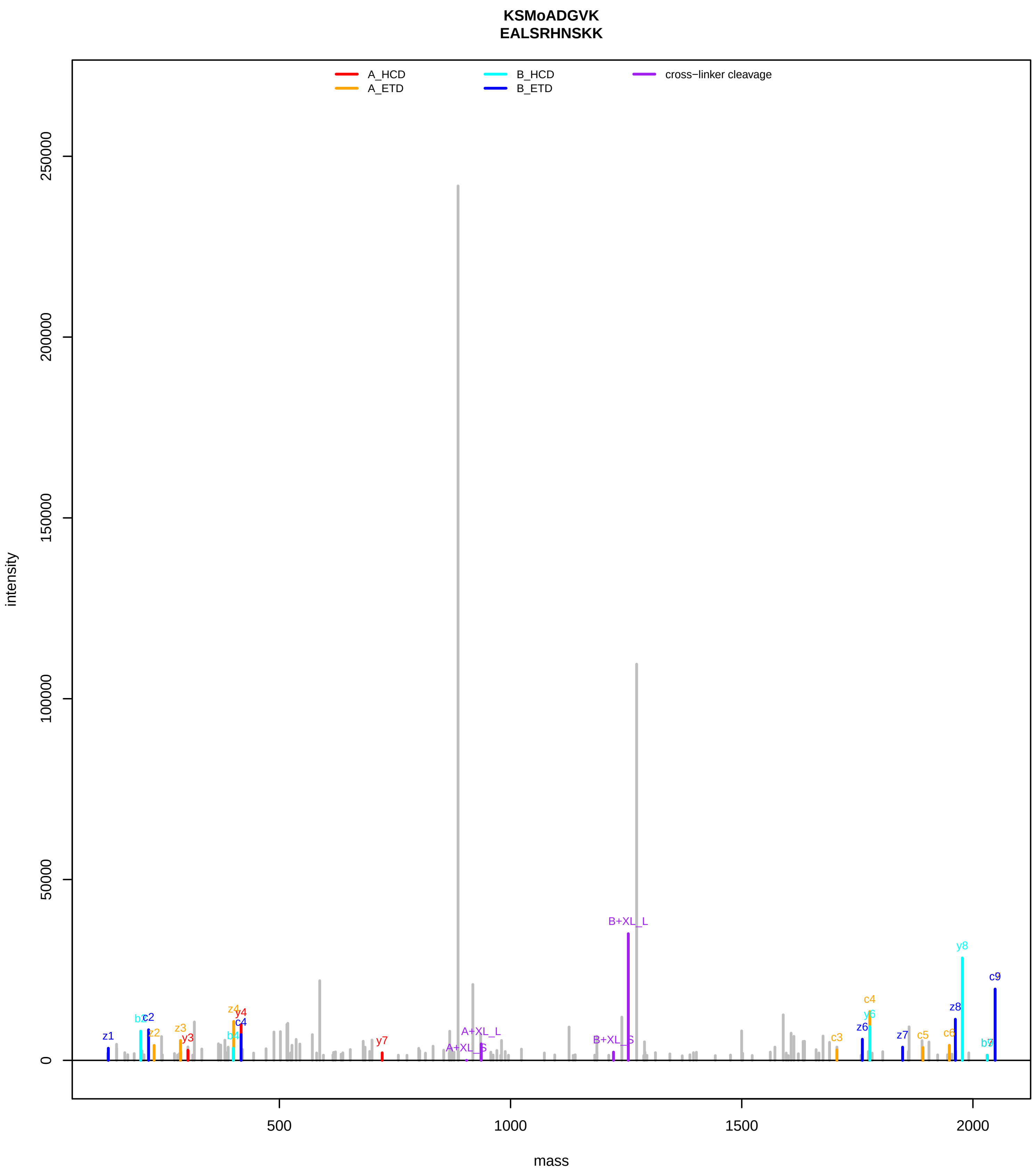

KSMoADGVK+XL_S

KSMoADGVK+XL_L

EALSRHNSKK+XL_S

EALSRHNSKK+XL_L

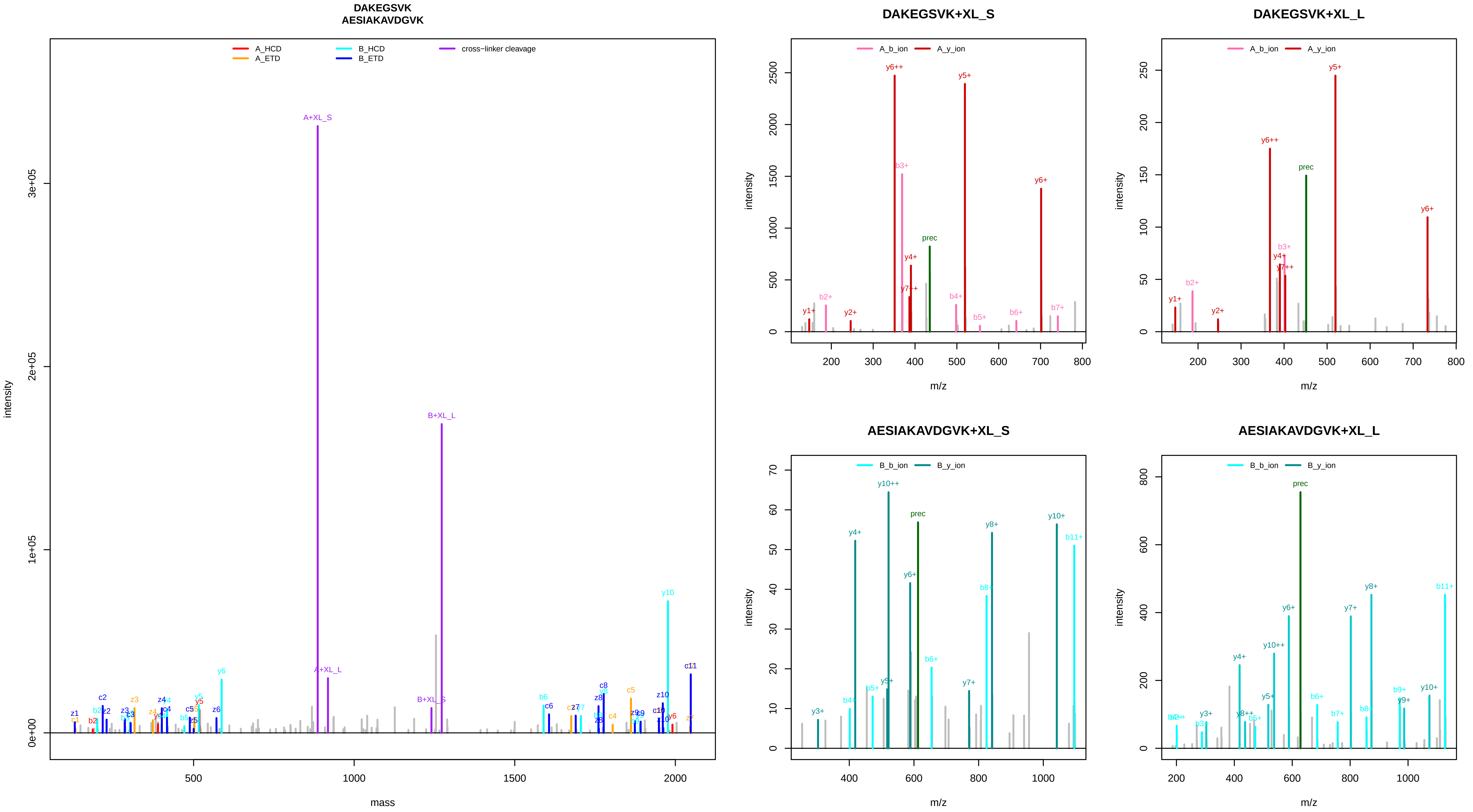

DAKEGSVK
AESIAKAVDGVK
A_HCD
A_ETD
B_HCD
B_ETD
cross−linker cleavage
A+XL_S
B+XL_L
A+XL_L
B+XL_S
intensity
mass
DAKEGSVK+XL_S
DAKEGSVK+XL_L
A_b_ion
A_y_ion
AESIAKAVDGVK+XL_S
AESIAKAVDGVK+XL_L
B_b_ion
B_y_ion
prec
m/z

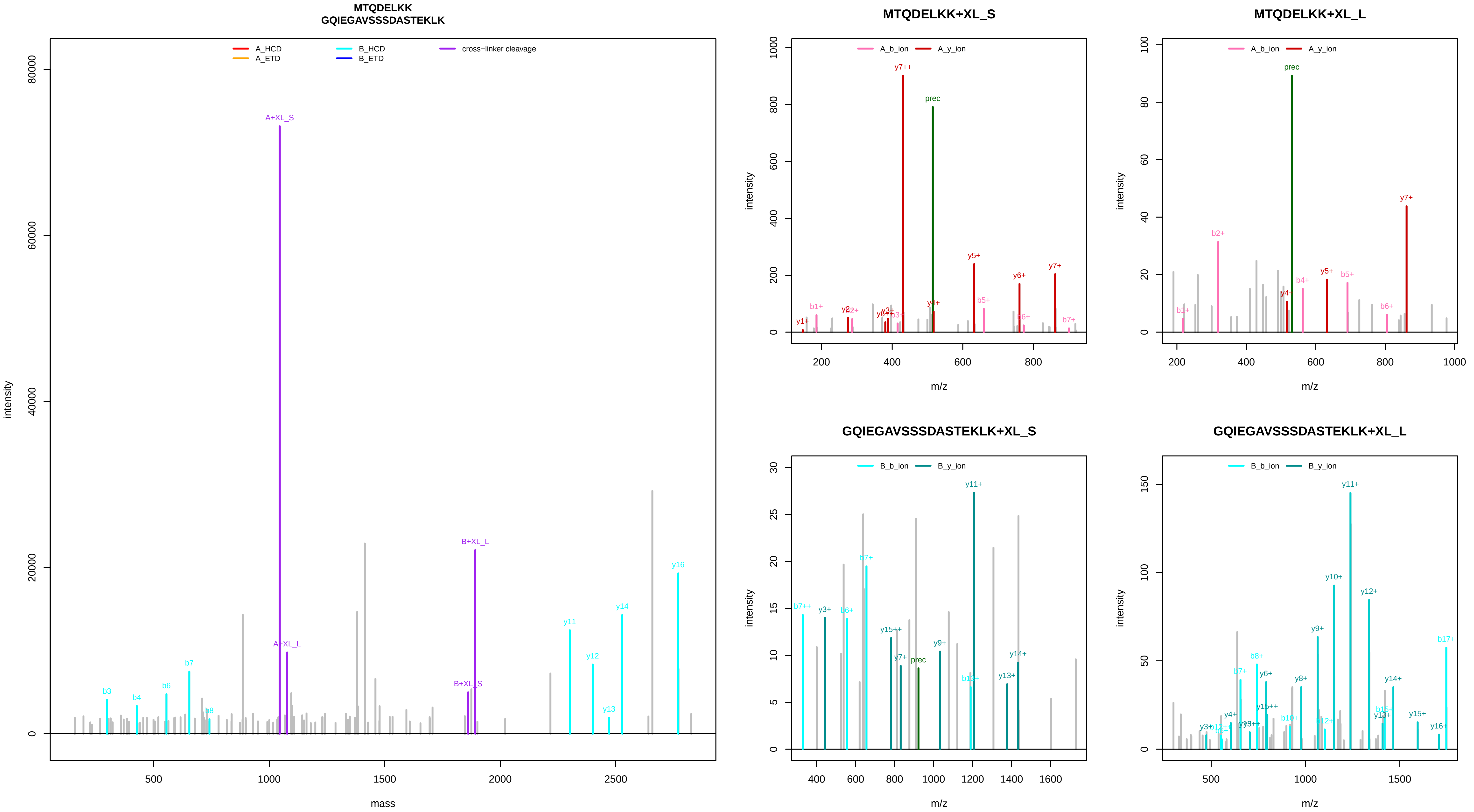

MTQDELKK
GQIEGAVSSSDASTEKLK
A_HCD
A_ETD
B_HCD
B_ETD
cross−linker cleavage
A+XL_S
A+XL_L
B+XL_L
B+XL_S
intensity
mass
MTQDELKK+XL_S
MTQDELKK+XL_L
GQIEGAVSSSDASTEKLK+XL_S
GQIEGAVSSSDASTEKLK+XL_L
A_b_ion
A_y_ion
B_b_ion
B_y_ion
prec
m/z

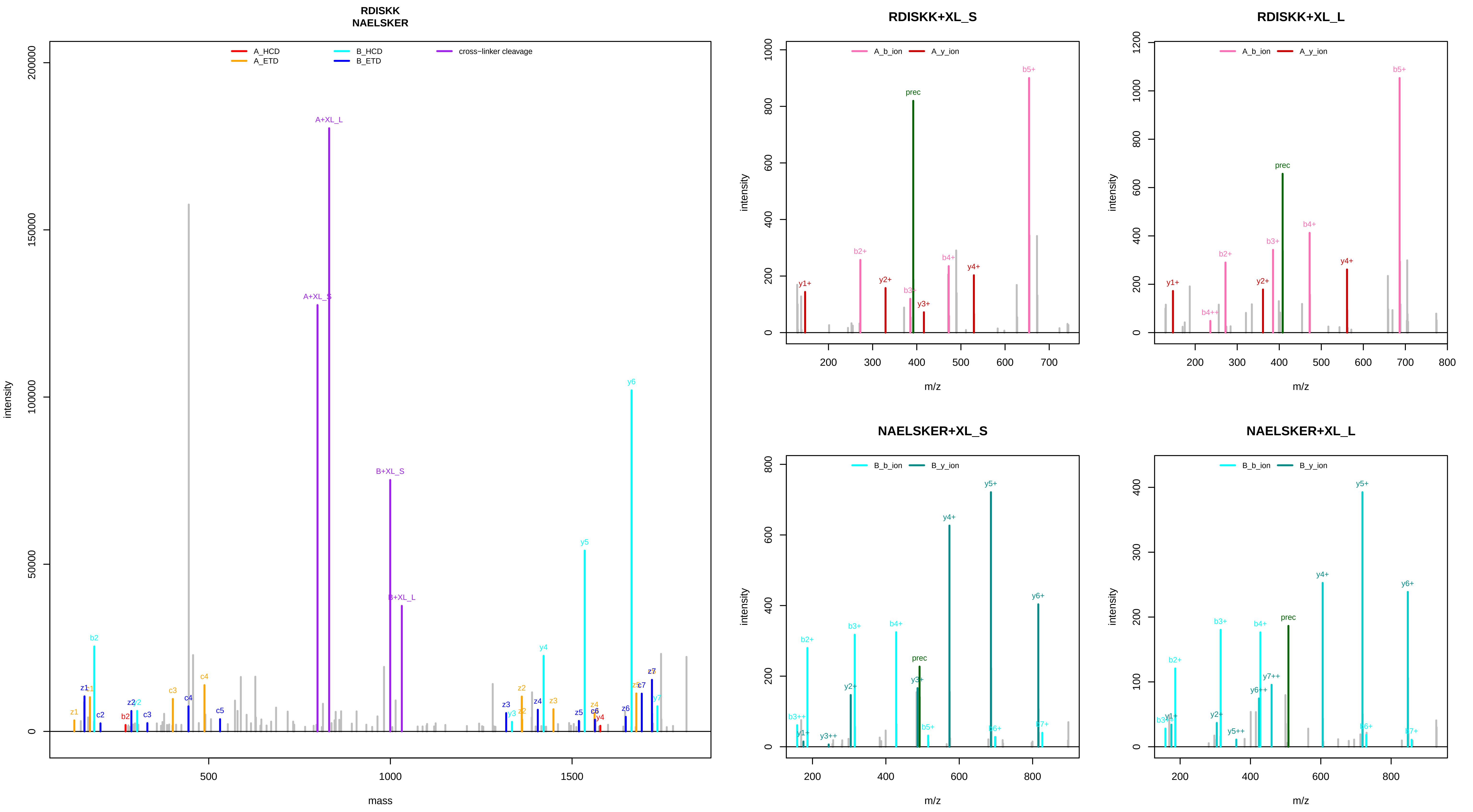
RDISKK
NAELSKER
A_HCD
A_ETD
B_HCD
B_ETD
cross−linker cleavage
A+XL_L
A+XL_S
B+XL_S
B+XL_L
intensity
mass
RDISKK+XL_S
RDISKK+XL_L
NAELSKER+XL_S
NAELSKER+XL_L
A_b_ion
A_y_ion
B_b_ion
B_y_ion
prec
m/z

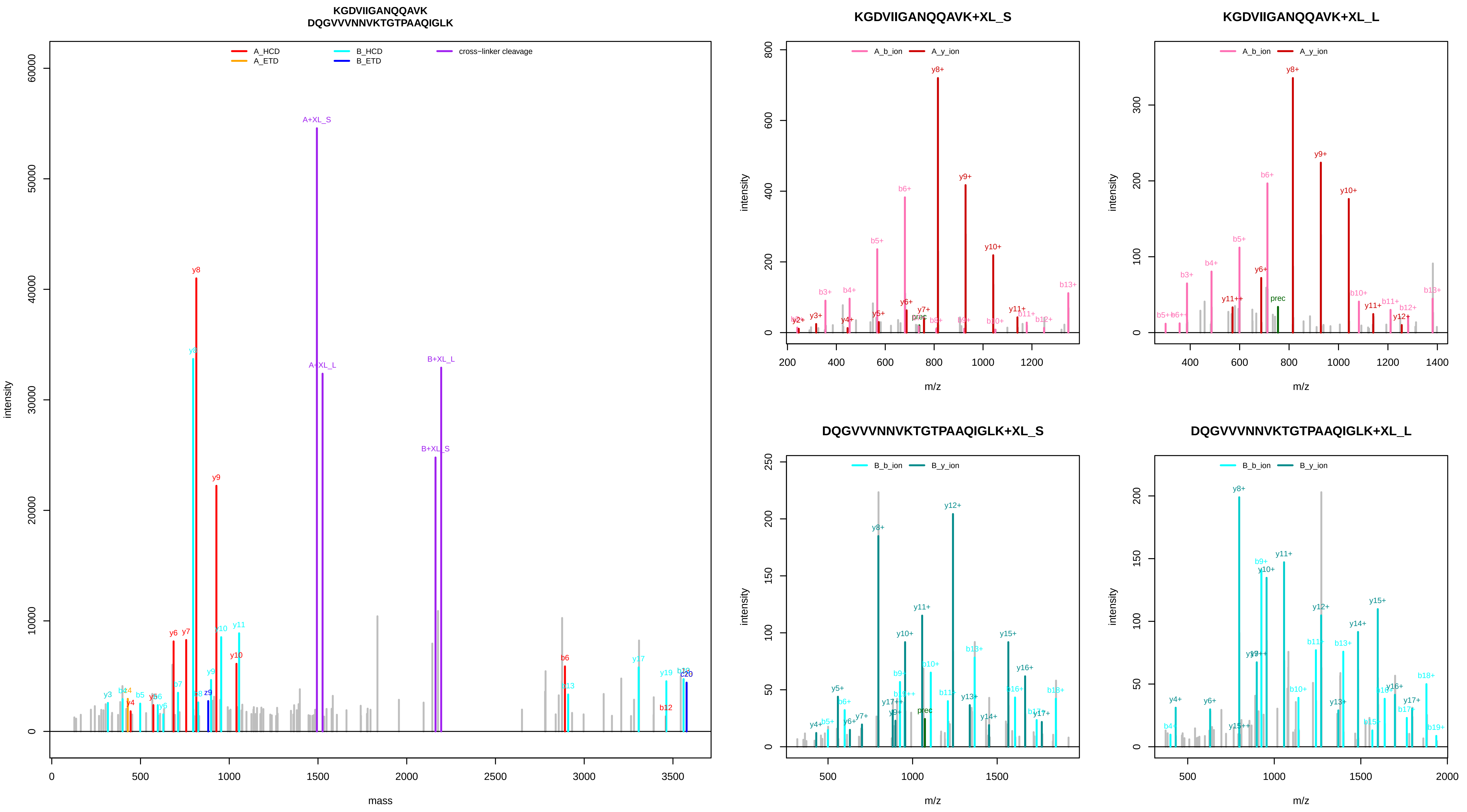
KGDVIIGANQQAVK
DQGVVVNNVKTGTPAAQIGLK
A_HCD
A_ETD
B_HCD
B_ETD
cross−linker cleavage
A+XL_S
A+XL_L
B+XL_L
B+XL_S
intensity
mass
KGDVIIGANQQAVK+XL_S
A_b_ion
A_y_ion
m/z
KGDVIIGANQQAVK+XL_L
DQGVVVNNVKTGTPAAQIGLK+XL_S
B_b_ion
B_y_ion
DQGVVVNNVKTGTPAAQIGLK+XL_L
prec

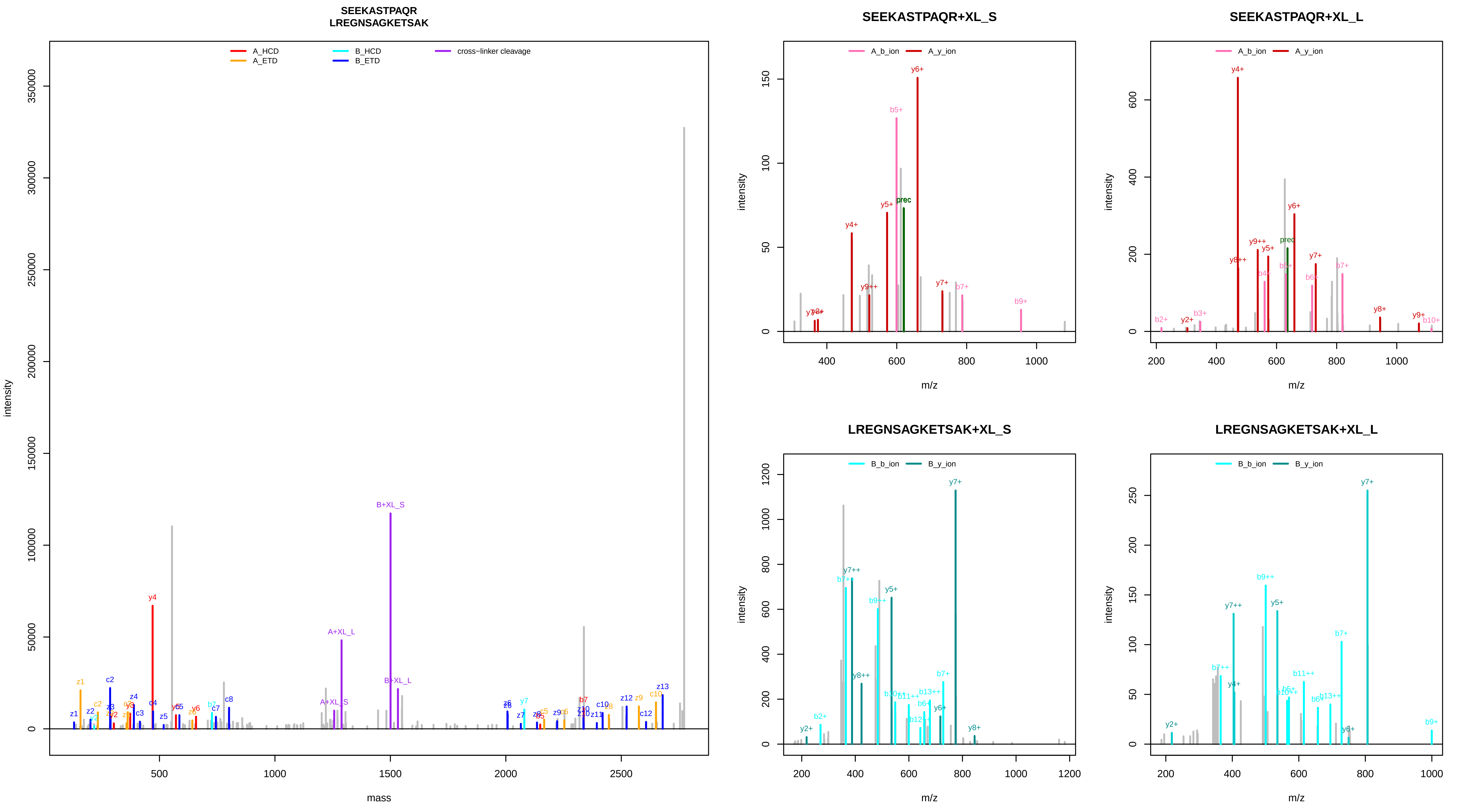
SEEKASTPAQR
LREGNSAGKETSAK
A_HCD
A_ETD
B_HCD
B_ETD
cross−linker cleavage
intensity
mass
B+XL_S
A+XL_L
B+XL_L
A+XL_S
SEEKASTPAQR+XL_S
SEEKASTPAQR+XL_L
LREGNSAGKETSAK+XL_S
LREGNSAGKETSAK+XL_L
A_b_ion
A_y_ion
B_b_ion
B_y_ion
prec
m/z

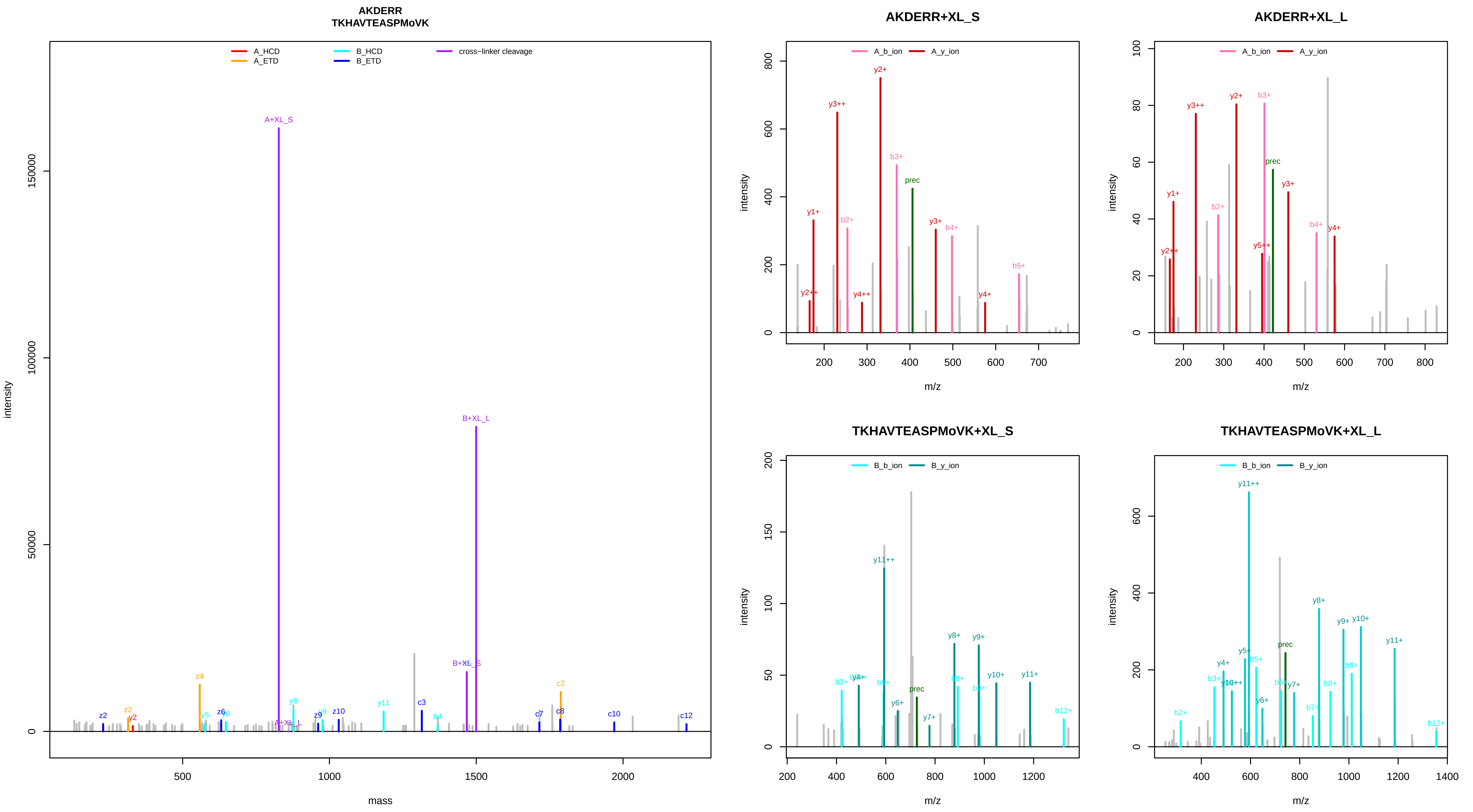
AKDERR
TKHAVTEASPMoVK
A_HCD
A_ETD
B_HCD
B_ETD
cross−linker cleavage
A+XL_S
B+XL_L
intensity
mass
AKDERR+XL_S
AKDERR+XL_L
TKHAVTEASPMoVK+XL_S
TKHAVTEASPMoVK+XL_L
A_b_ion
A_y_ion
B_b_ion
B_y_ion
prec
m/z

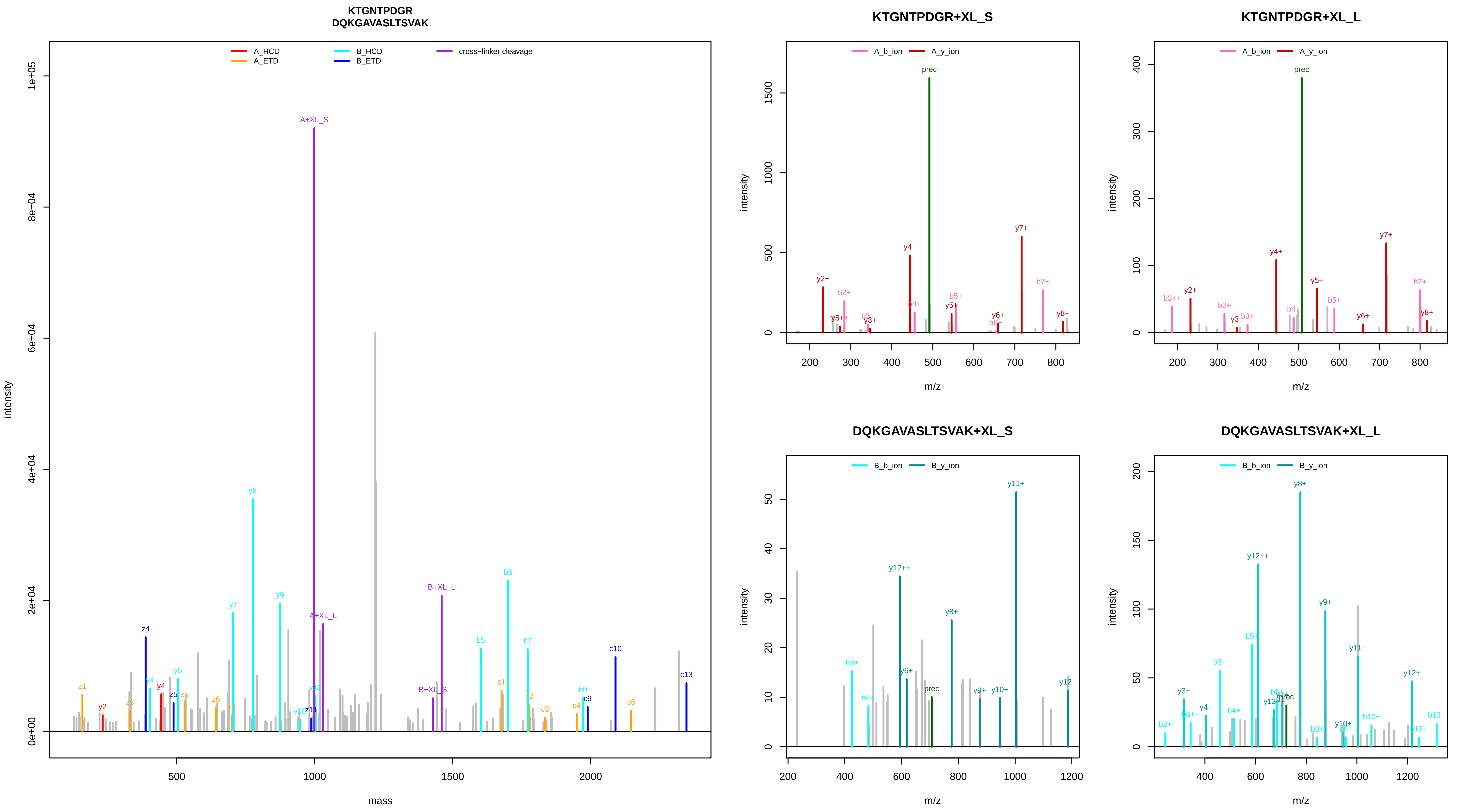
KTGNTPDGR
DQKGAVASLTSVAK
A_HCD
A_ETD
B_HCD
B_ETD
cross−linker cleavage
A+XL_S
A+XL_L
B+XL_L
B+XL_S
intensity
mass
KTGNTPDGR+XL_S
KTGNTPDGR+XL_L
DQKGAVASLTSVAK+XL_S
DQKGAVASLTSVAK+XL_L
A_b_ion
A_y_ion
B_b_ion
B_y_ion
prec
m/z

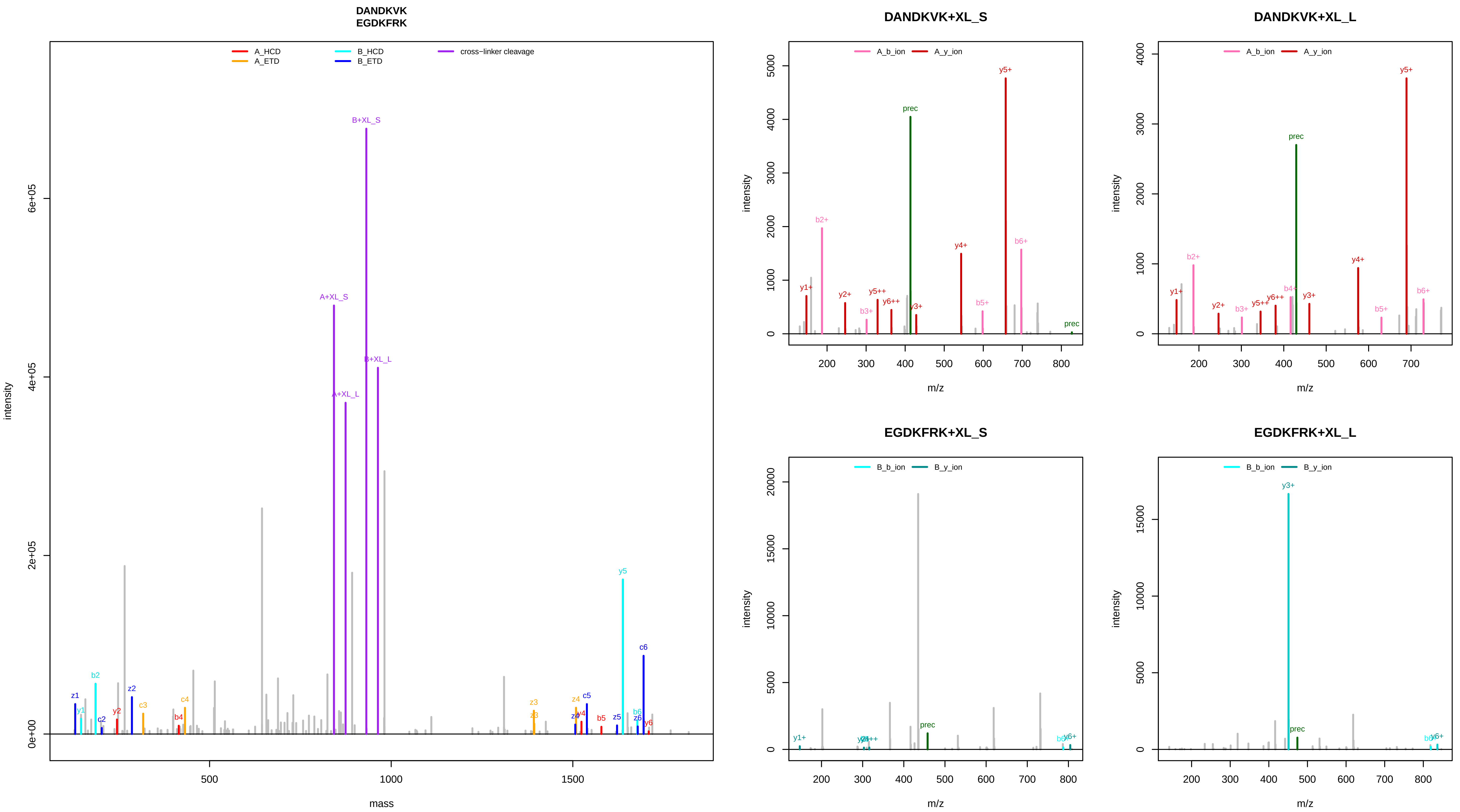
DANDKVK
EGDKFRK
A_HCD
A_ETD
B_HCD
B_ETD
cross−linker cleavage
B+XL_S
A+XL_S
B+XL_L
A+XL_L
intensity
mass
DANDKVK+XL_S
DANDKVK+XL_L
EGDKFRK+XL_S
EGDKFRK+XL_L
A_b_ion
A_y_ion
B_b_ion
B_y_ion
prec
m/z

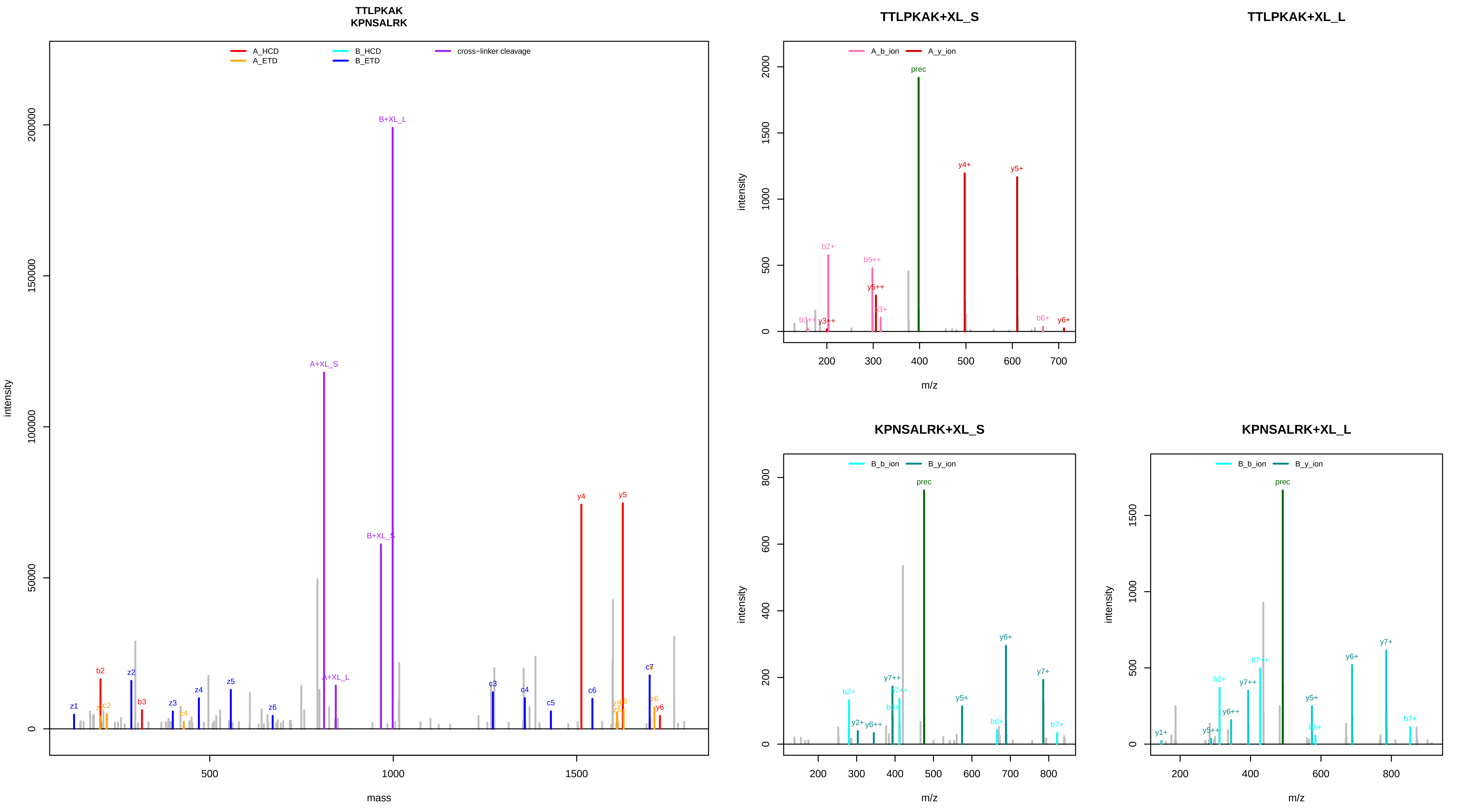

TTLPKAK
KPNSALRK
A_HCD
A_ETD
B_HCD
B_ETD
cross−linker cleavage
B+XL_L
A+XL_S
B+XL_S
A+XL_L
y4
y5
b2
b3
y6
z1
z2
z3
z4
z5
z6
c3
c4
c5
c6
c7
intensity
mass
TTLPKAK+XL_S
A_b_ion
A_y_ion
prec
y4+
y5+
b2+
b5++
y5++
b6+
y6+
m/z
TTLPKAK+XL_L
KPNSALRK+XL_S
B_b_ion
B_y_ion
y6+
y7+
y7++
b2+
y5+
b6+
b7+
KPNSALRK+XL_L
y1+
y5++
y6++
y7++
b7++

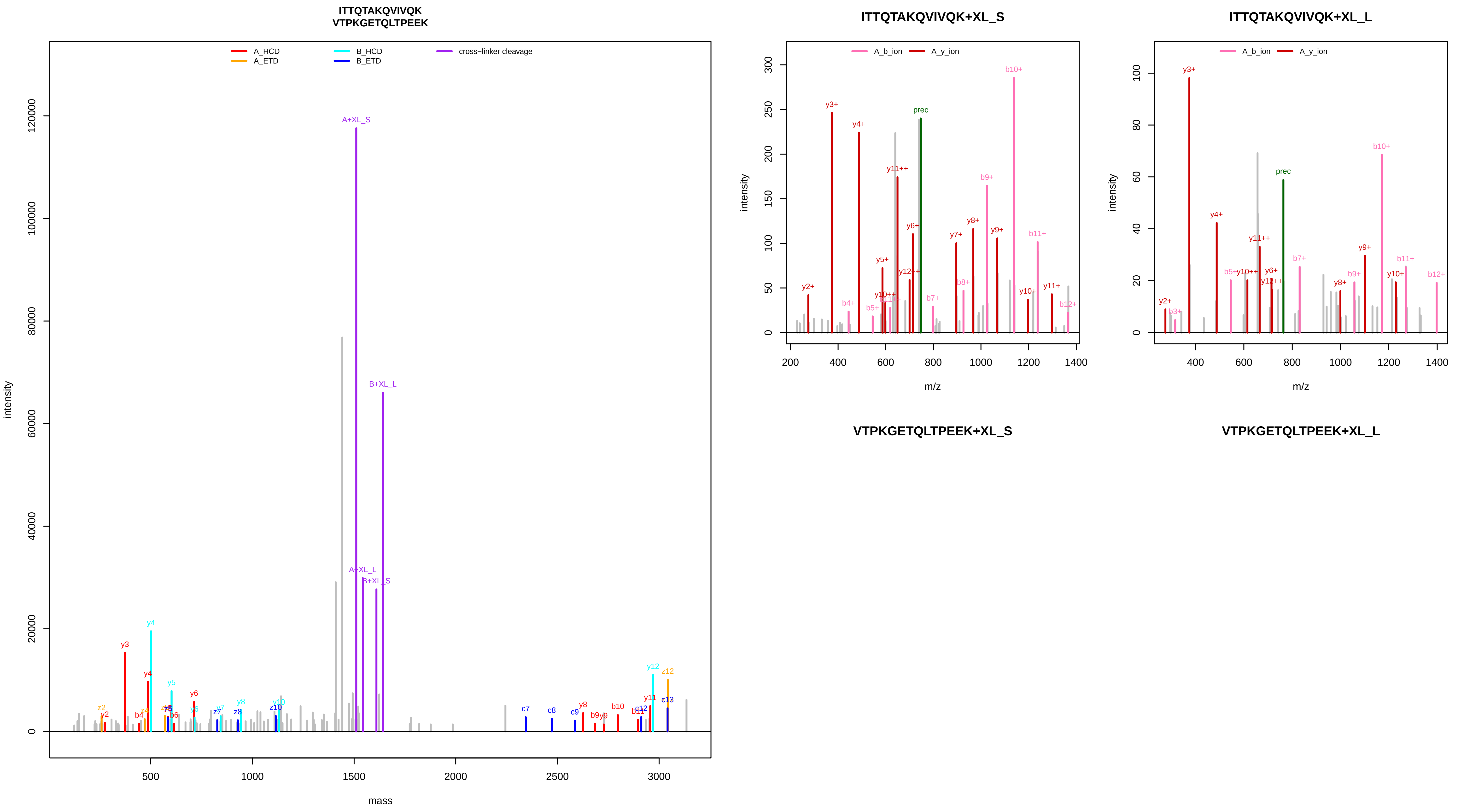
ITTQTAKQVIVQK
VTPKGETQLTPEEK
A_HCD
A_ETD
B_HCD
B_ETD
cross−linker cleavage
A+XL_S
B+XL_L
A+XL_L
B+XL_S
intensity
mass
0
20000
40000
60000
80000
100000
120000
500
1000
1500
2000
2500
3000
y2
y3
y4
y6
b4
b6
z2
z5
z7
z8
z10
y5
y8
y10
y12
z12
y8
y9
b9
b10
b11
y11
c7
c8
c9
c12
c13
ITTQTAKQVIVQK+XL_S
A_b_ion
A_y_ion
prec
intensity
m/z
y2+
y3+
y4+
y5+
y6+
y7+
y8+
y9+
y10+
y11+
y11++
b4+
b5+
b7+
b8+
b9+
b10+
b11+
b12+
ITTQTAKQVIVQK+XL_L
A_b_ion
A_y_ion
prec
intensity
m/z
y2+
y3+
y4+
y6+
y8+
y9+
y10+
y10++
y11++
b3+
b5+
b7+
b9+
b10+
b11+
b12+
VTPKGETQLTPEEK+XL_S
VTPKGETQLTPEEK+XL_L

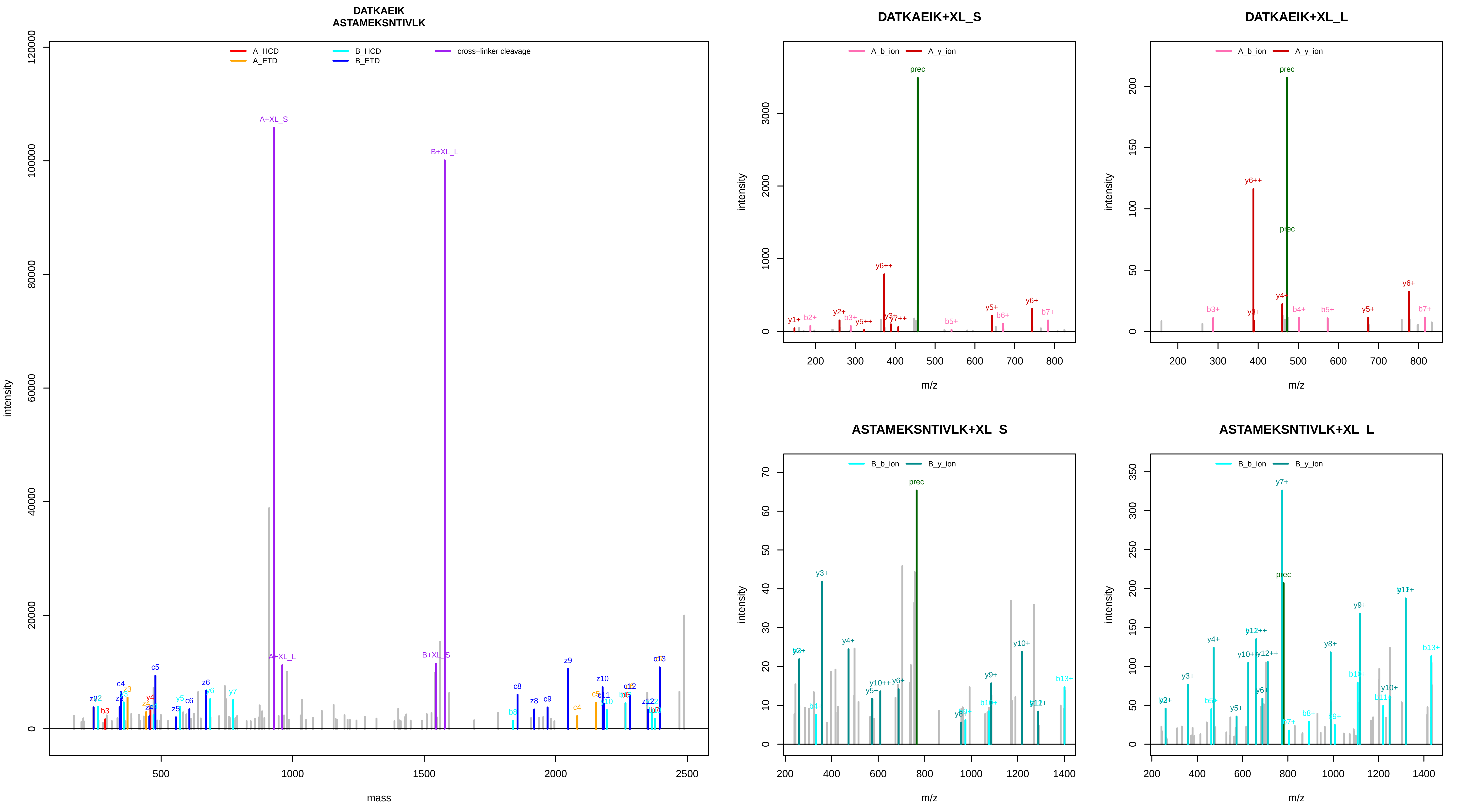
DATKAEIK
ASTAMEKSNTIVLK
A_HCD
A_ETD
B_HCD
B_ETD
cross−linker cleavage
A+XL_S
B+XL_L
A+XL_L
B+XL_S
intensity
mass
DATKAEIK+XL_S
DATKAEIK+XL_L
A_b_ion
A_y_ion
prec
ASTAMEKSNTIVLK+XL_S
ASTAMEKSNTIVLK+XL_L
B_b_ion
B_y_ion
m/z

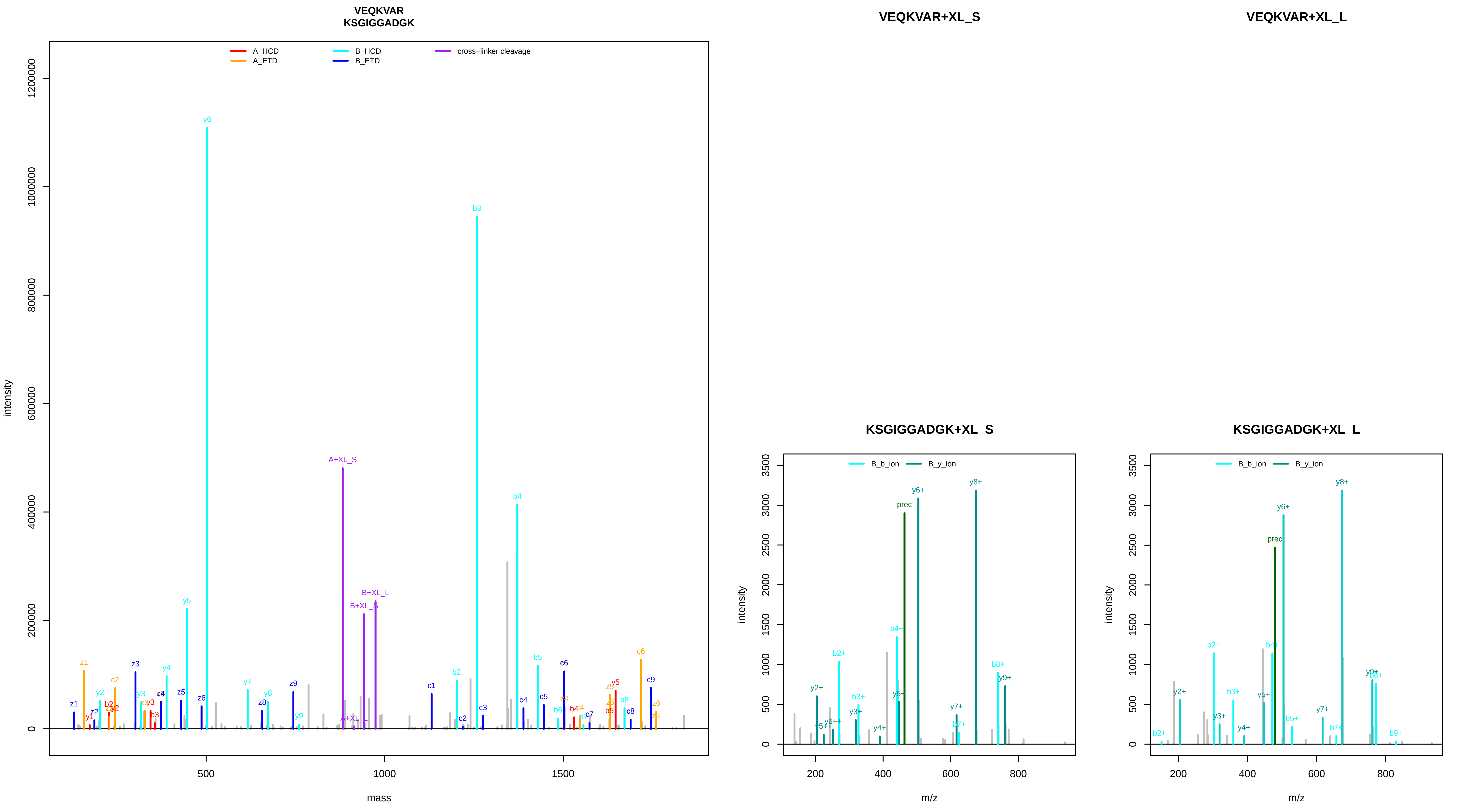
VEQKVAR
KSGIGGADGK
A_HCD
A_ETD
B_HCD
B_ETD
cross−linker cleavage
intensity
mass
A+XL_S
B+XL_L
B+XL_S
VEQKVAR+XL_S
VEQKVAR+XL_L
KSGIGGADGK+XL_S
KSGIGGADGK+XL_L
B_b_ion
B_y_ion
prec
intensity
m/z

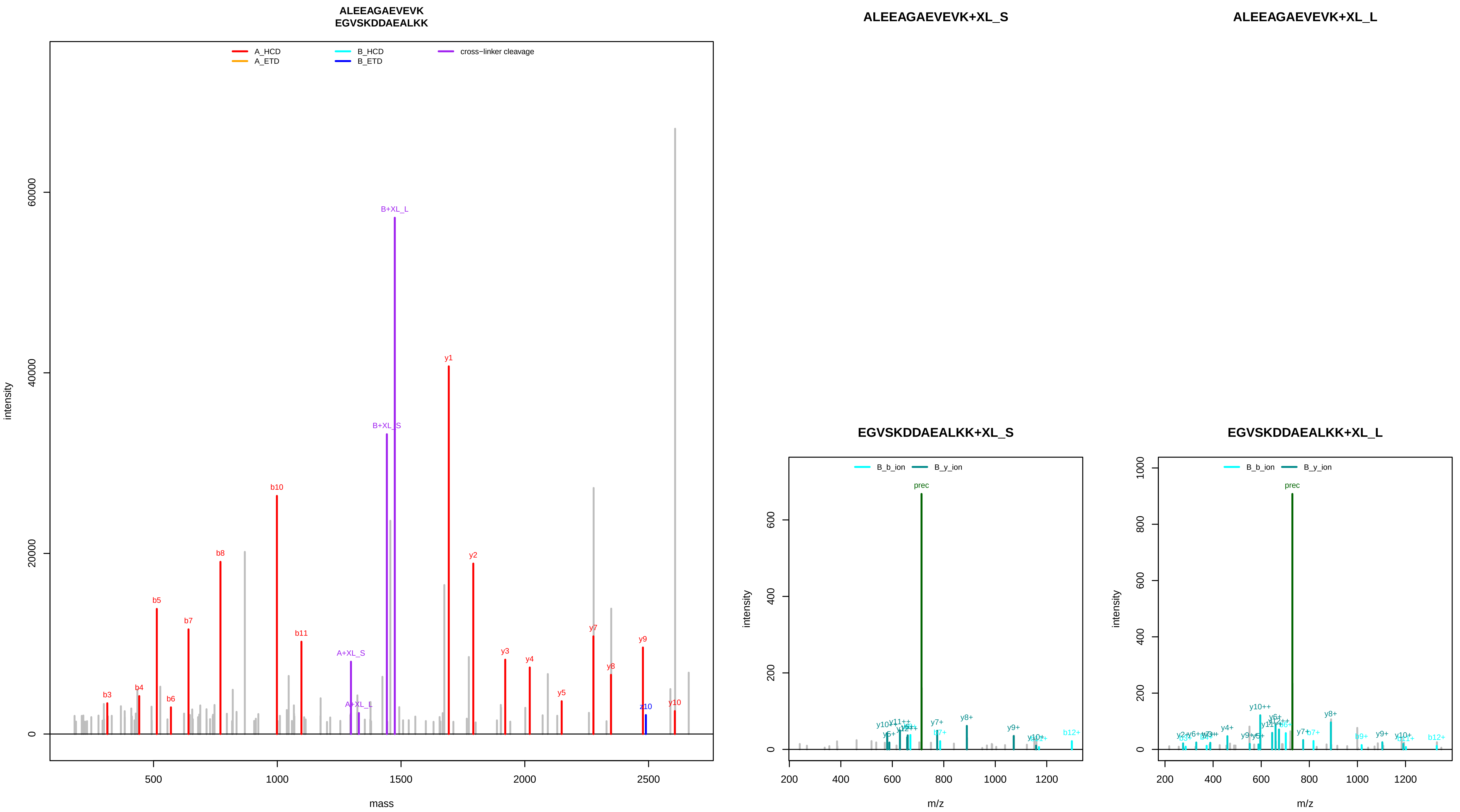
ALEEAGAEVEVK
EGVSKDDAEALKK
A_HCD
A_ETD
B_HCD
B_ETD
cross−linker cleavage
B+XL_L
B+XL_S
A+XL_S
A+XL_L
b3
b4
b5
b6
b7
b8
b10
b11
y1
y2
y3
y4
y5
y7
y8
y9
y10
z10
intensity
mass
ALEEAGAEVEVK+XL_S
ALEEAGAEVEVK+XL_L
EGVSKDDAEALKK+XL_S
EGVSKDDAEALKK+XL_L
B_b_ion
B_y_ion
prec
m/z

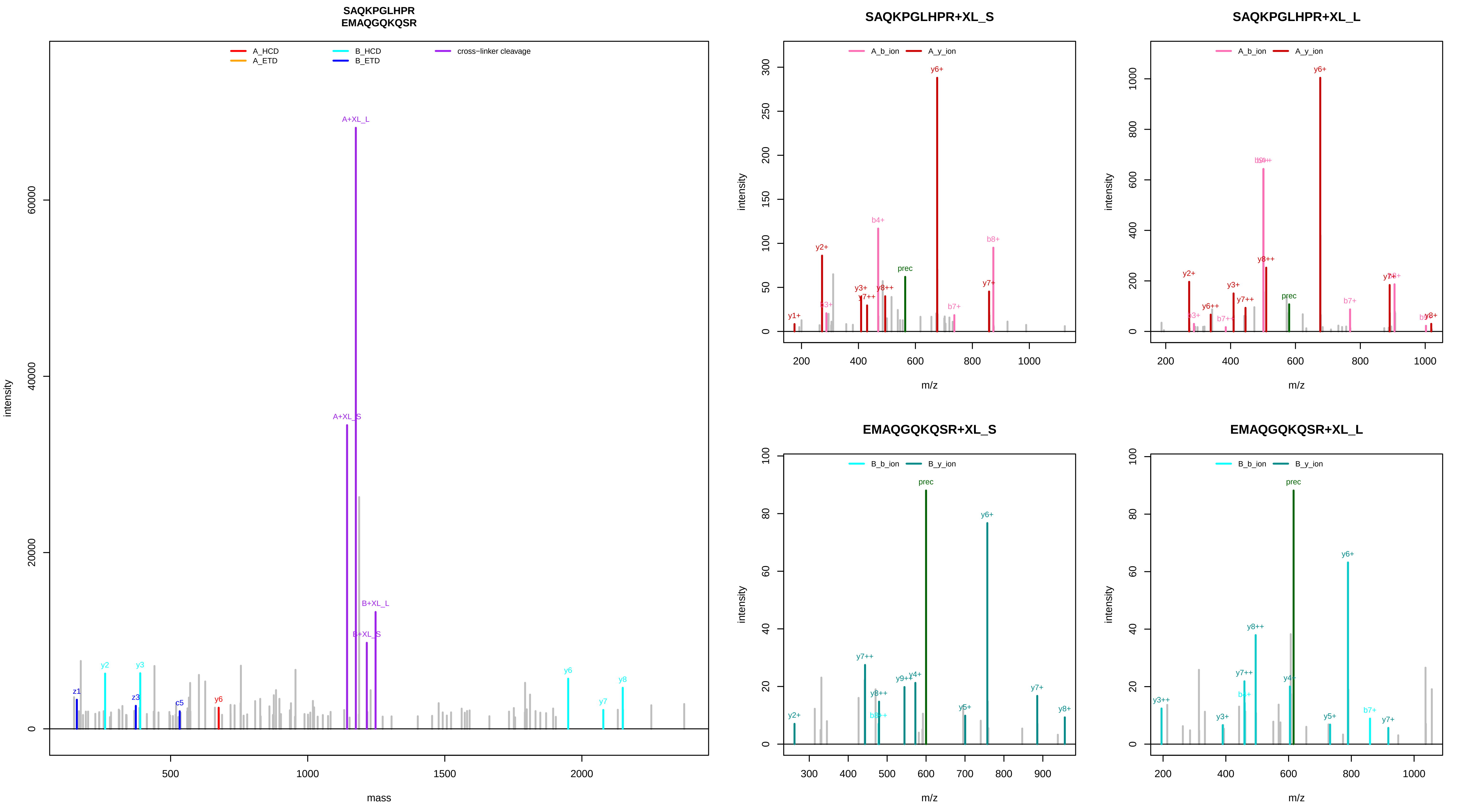

SAQKPGLHPR
EMAQGQKQSR
SAQKPGLHPR+XL_S
SAQKPGLHPR+XL_L
EMAQGQKQSR+XL_S
EMAQGQKQSR+XL_L
A_HCD
A_ETD
B_HCD
B_ETD
cross−linker cleavage
A_b_ion
A_y_ion
B_b_ion
B_y_ion
intensity
mass
m/z

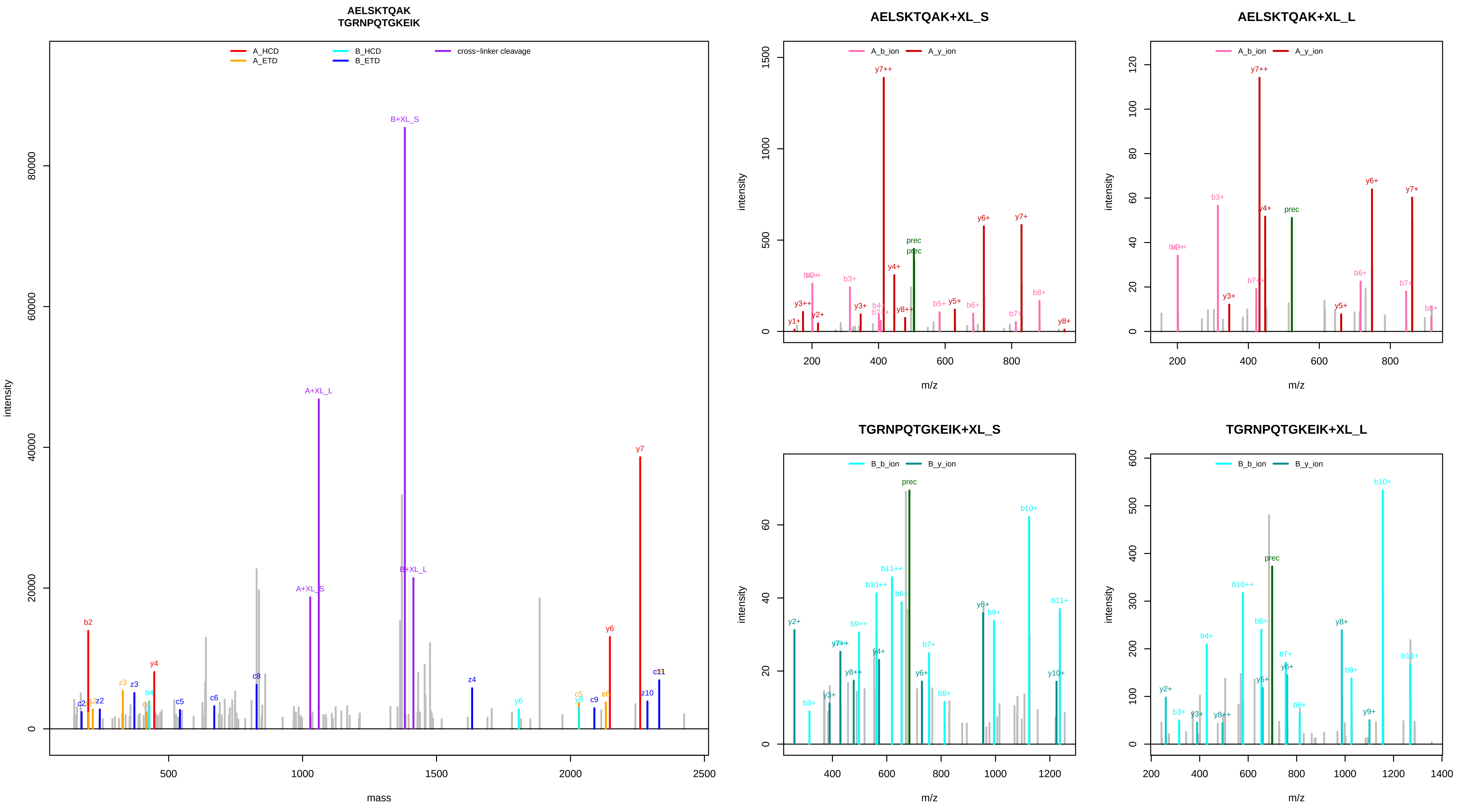
AELSKTQAK
TGRNPQTGKEIK
A_HCD
A_ETD
B_HCD
B_ETD
cross−linker cleavage
B+XL_S
A+XL_L
A+XL_S
B+XL_L
intensity
mass
b2
y4
y6
y7
z3
b4
c5
c6
c8
z4
y6
c9
z10
c11
AELSKTQAK+XL_S
AELSKTQAK+XL_L
TGRNPQTGKEIK+XL_S
TGRNPQTGKEIK+XL_L
A_b_ion
A_y_ion
B_b_ion
B_y_ion
y7++
y6+
y7+
y4+
y3+
b3+
y5+
b6+
b8+
prec
b10+
b11+
b10++
b11++
b9++
b9+
b7+
y8+
y2+
y10+
b4+
y9+
m/z
intensity

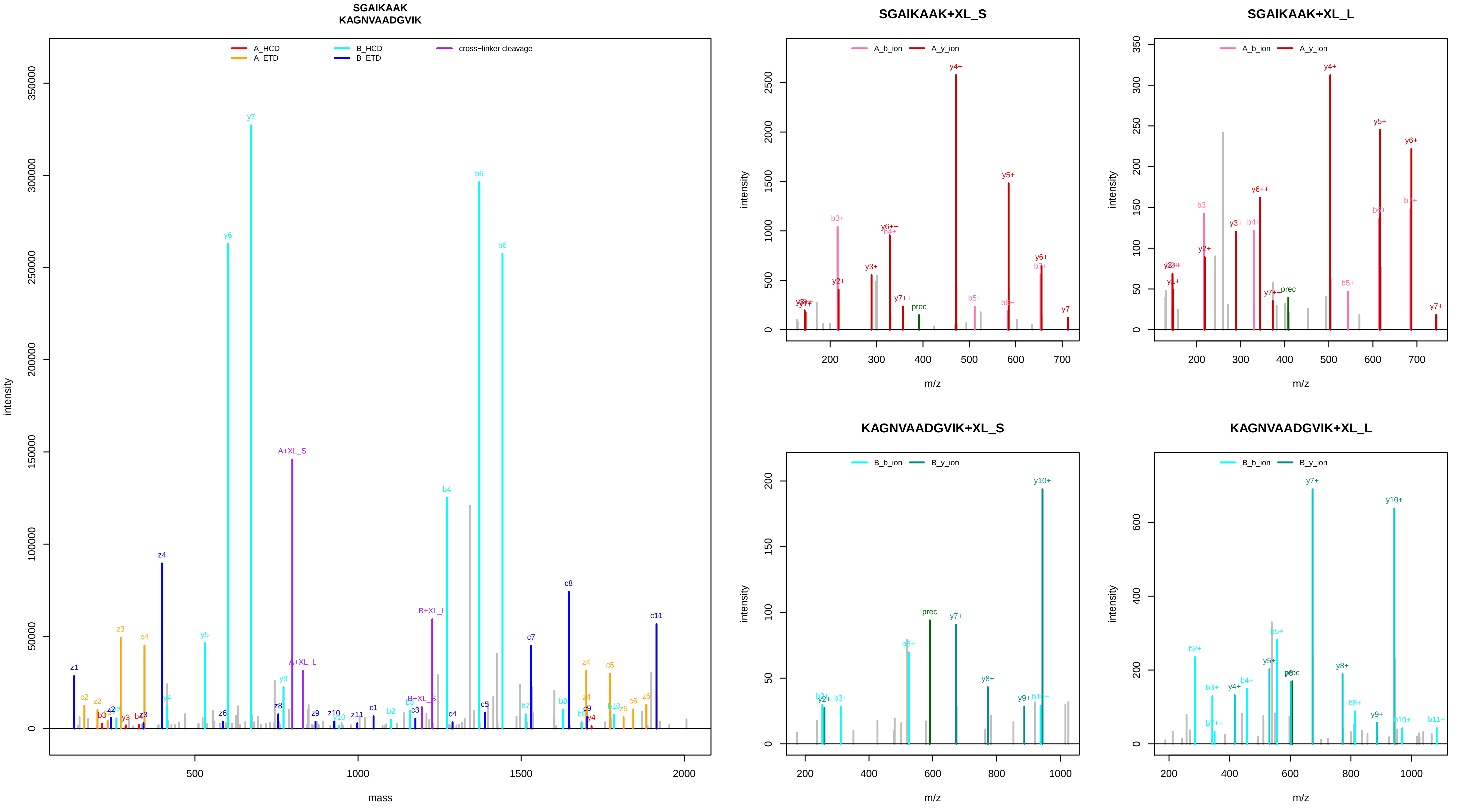

SGAIKAAK
KAGNVAADGVIK
A_HCD
A_ETD
B_HCD
B_ETD
cross−linker cleavage
intensity
mass
A+XL_S
A+XL_L
B+XL_L
B+XL_S
SGAIKAAK+XL_S
SGAIKAAK+XL_L
KAGNVAADGVIK+XL_S
KAGNVAADGVIK+XL_L
A_b_ion
A_y_ion
B_b_ion
B_y_ion
prec
m/z

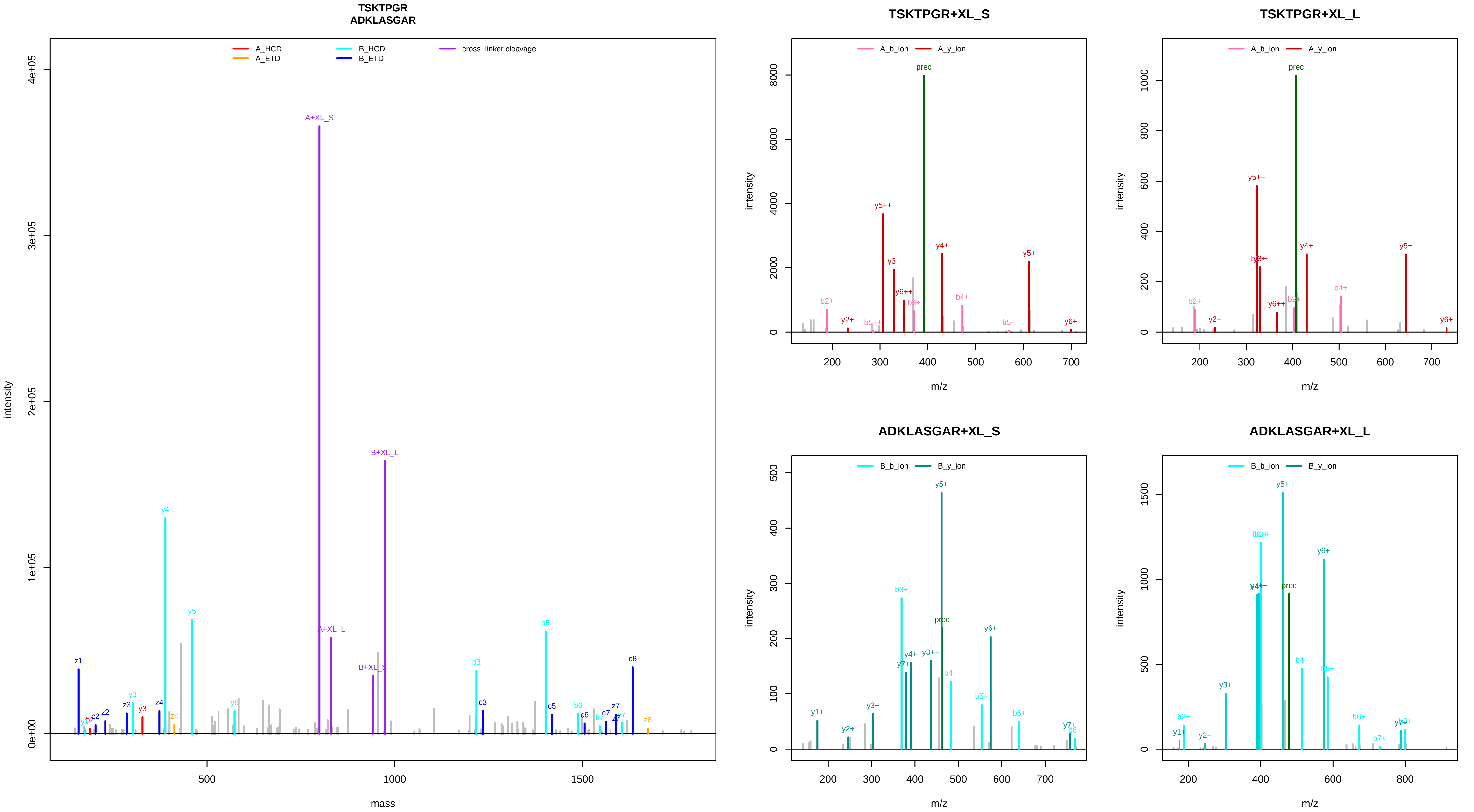

TSKTPGR
ADKLASGAR
A_HCD
A_ETD
B_HCD
B_ETD
cross−linker cleavage
A+XL_S
B+XL_L
A+XL_L
B+XL_S
intensity
mass
TSKTPGR+XL_S
TSKTPGR+XL_L
ADKLASGAR+XL_S
ADKLASGAR+XL_L
A_b_ion
A_y_ion
B_b_ion
B_y_ion
prec
m/z

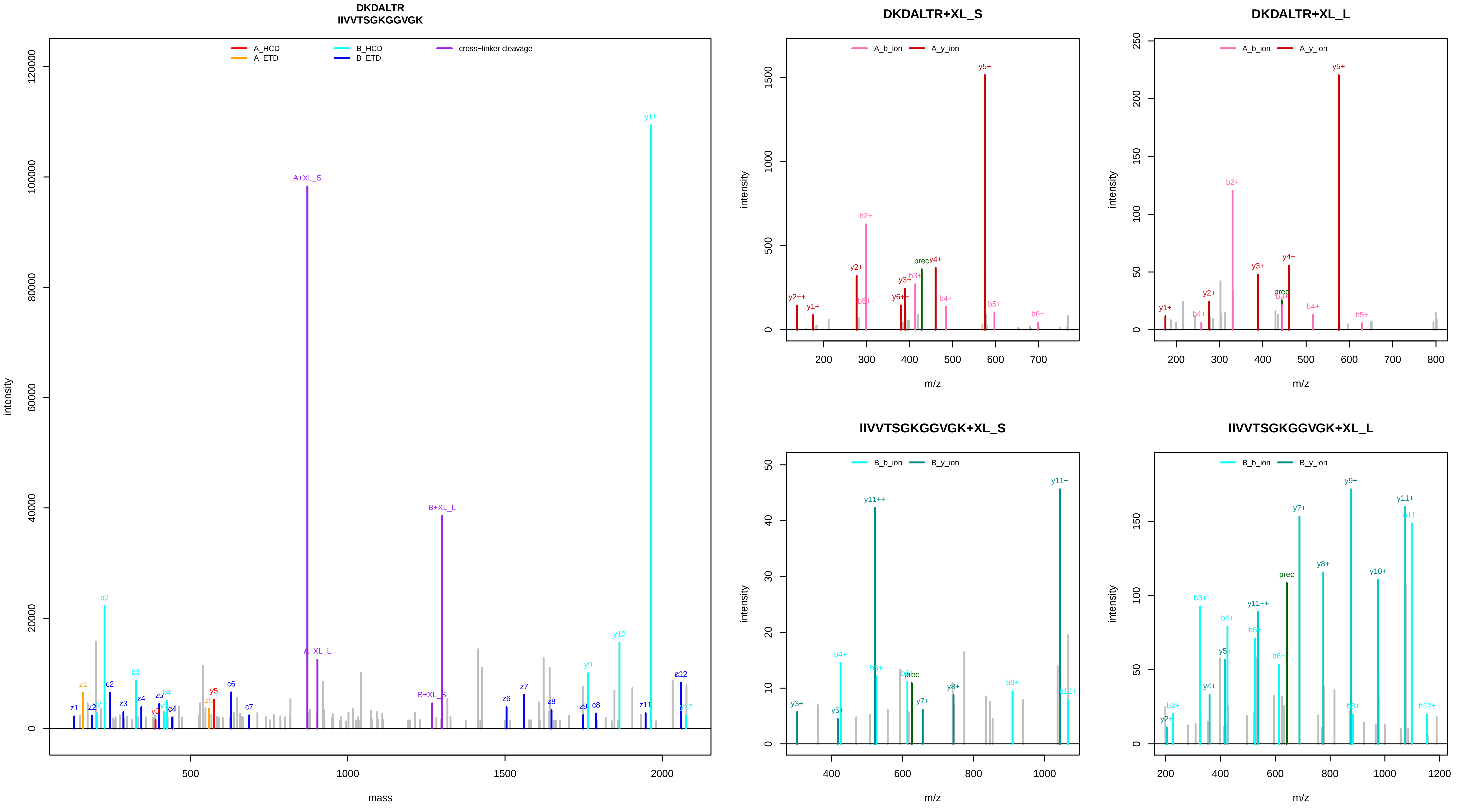

DKDALTR
IIVVTSGKGGVGK
A_HCD
A_ETD
B_HCD
B_ETD
cross−linker cleavage
intensity
mass
DKDALTR+XL_S
DKDALTR+XL_L
IIVVTSGKGGVGK+XL_S
IIVVTSGKGGVGK+XL_L
A_b_ion
A_y_ion
B_b_ion
B_y_ion
m/z

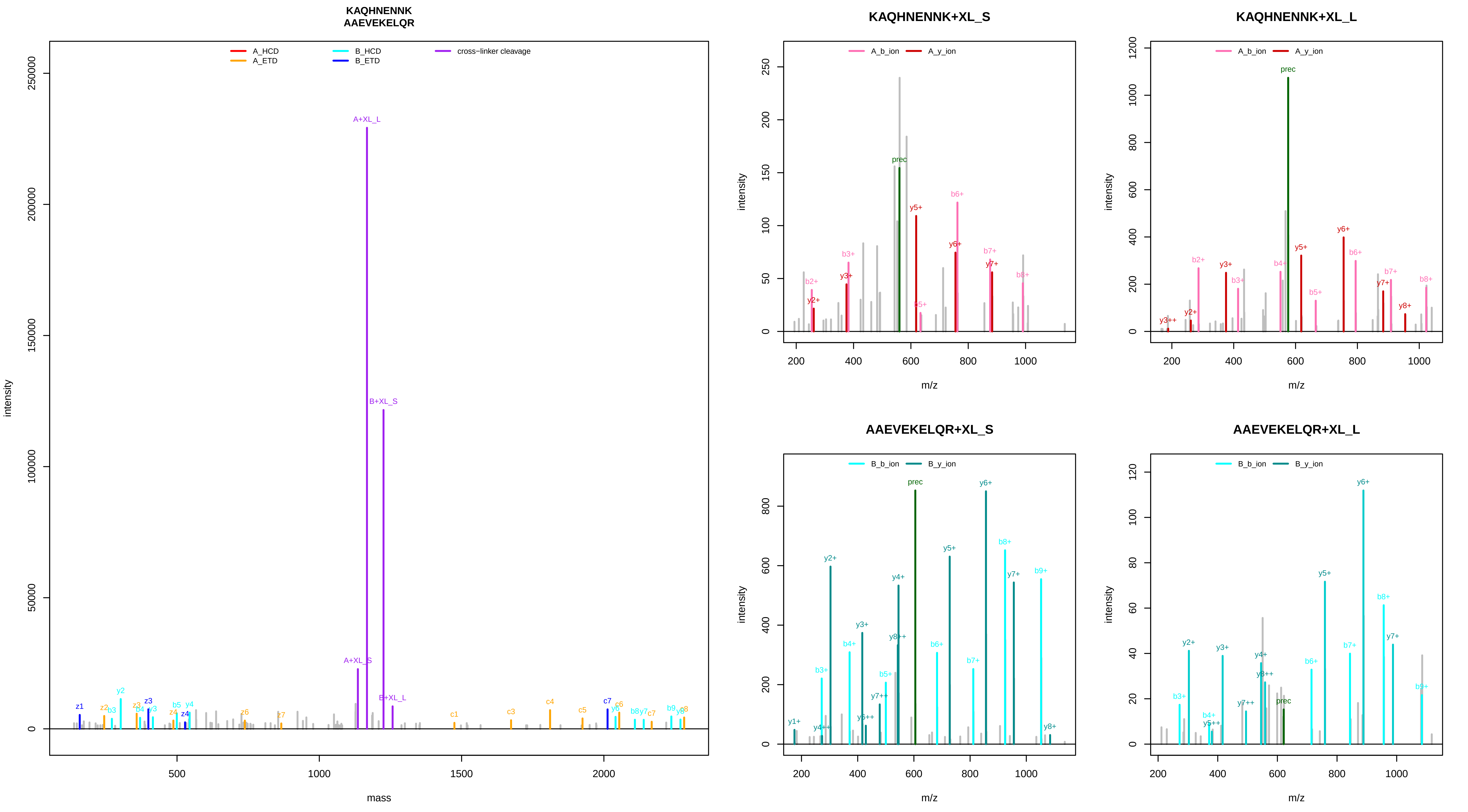
KAQHNENNK
AAEVEKELQR
A_HCD
A_ETD
B_HCD
B_ETD
cross−linker cleavage
A+XL_L
B+XL_S
A+XL_S
B+XL_L
intensity
mass
KAQHNENNK+XL_S
KAQHNENNK+XL_L
A_b_ion
A_y_ion
prec
m/z
AAEVEKELQR+XL_S
AAEVEKELQR+XL_L
B_b_ion
B_y_ion

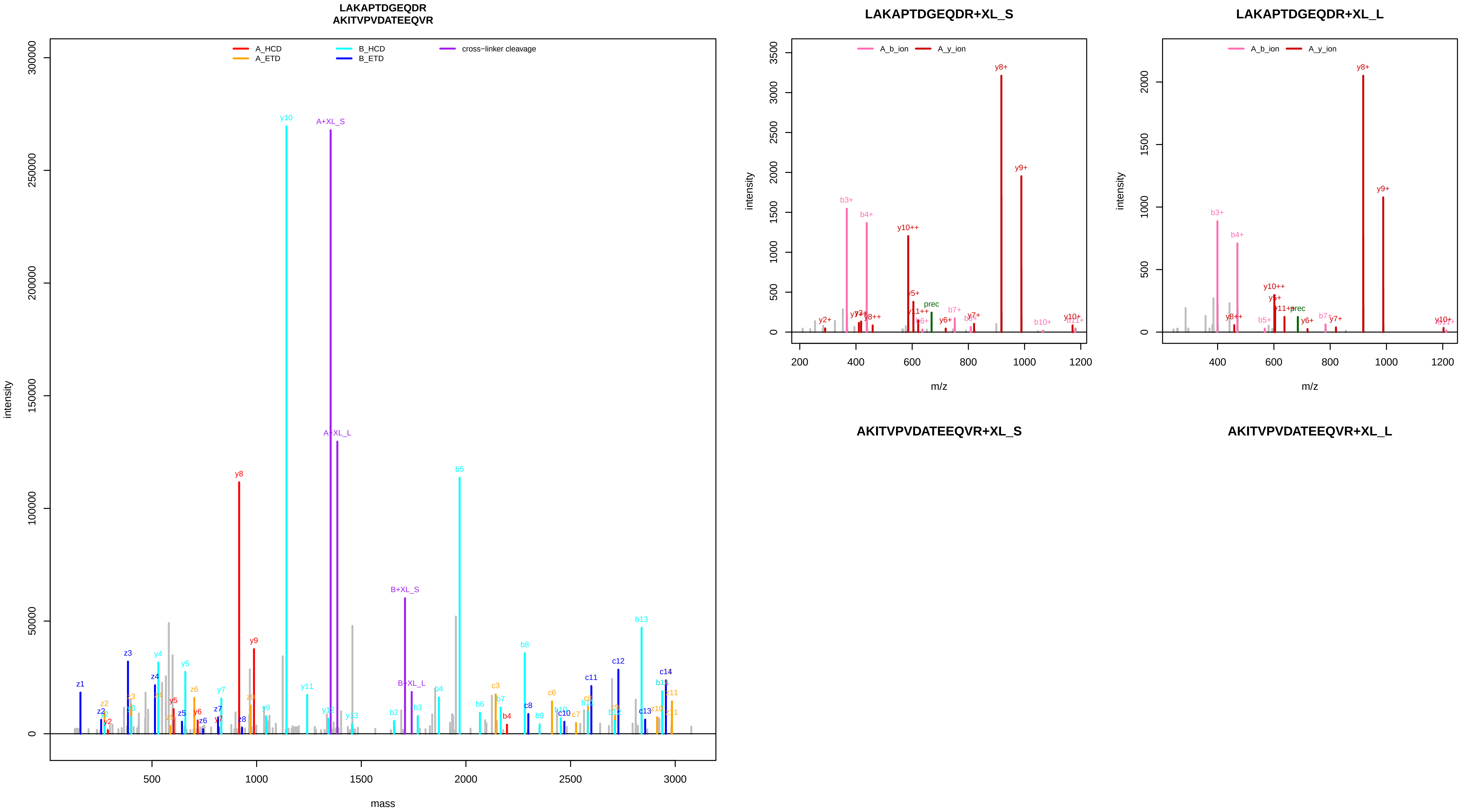
LAKAPTDGEQDR
AKITVPVDATEEQVR
A_HCD
A_ETD
B_HCD
B_ETD
cross−linker cleavage
intensity
mass
y10
A+XL_S
A+XL_L
y8
b5
B+XL_S
y9
b13
B+XL_L
LAKAPTDGEQDR+XL_S
LAKAPTDGEQDR+XL_L
A_b_ion
A_y_ion
y8+
y9+
b3+
b4+
y10++
prec
m/z
AKITVPVDATEEQVR+XL_S
AKITVPVDATEEQVR+XL_L

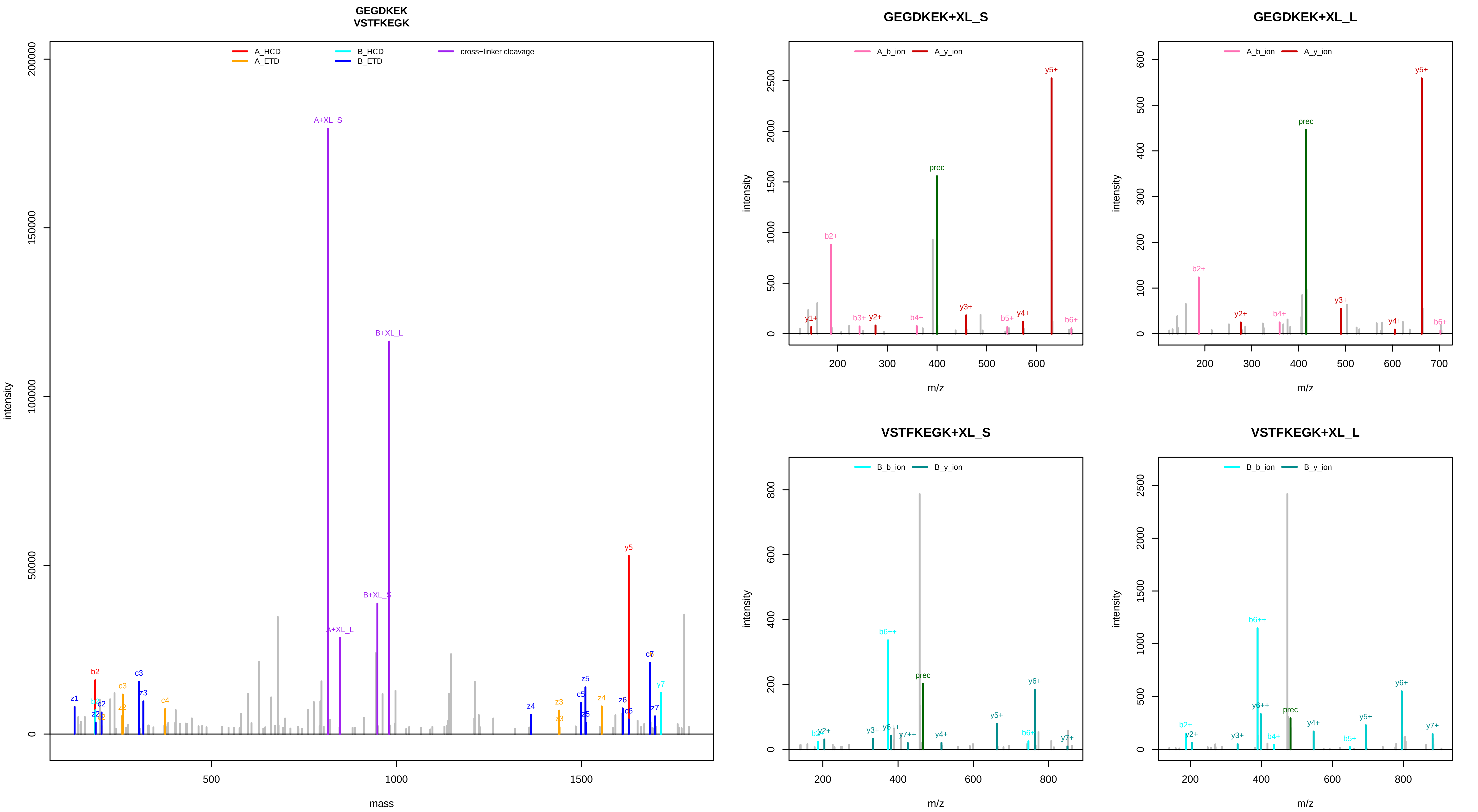
GEGDKEK
VSTFKEGK
A_HCD
A_ETD
B_HCD
B_ETD
cross−linker cleavage
A+XL_S
B+XL_L
B+XL_S
A+XL_L
y5
intensity
mass
GEGDKEK+XL_S
GEGDKEK+XL_L
VSTFKEGK+XL_S
VSTFKEGK+XL_L
A_b_ion
A_y_ion
B_b_ion
B_y_ion
prec
m/z

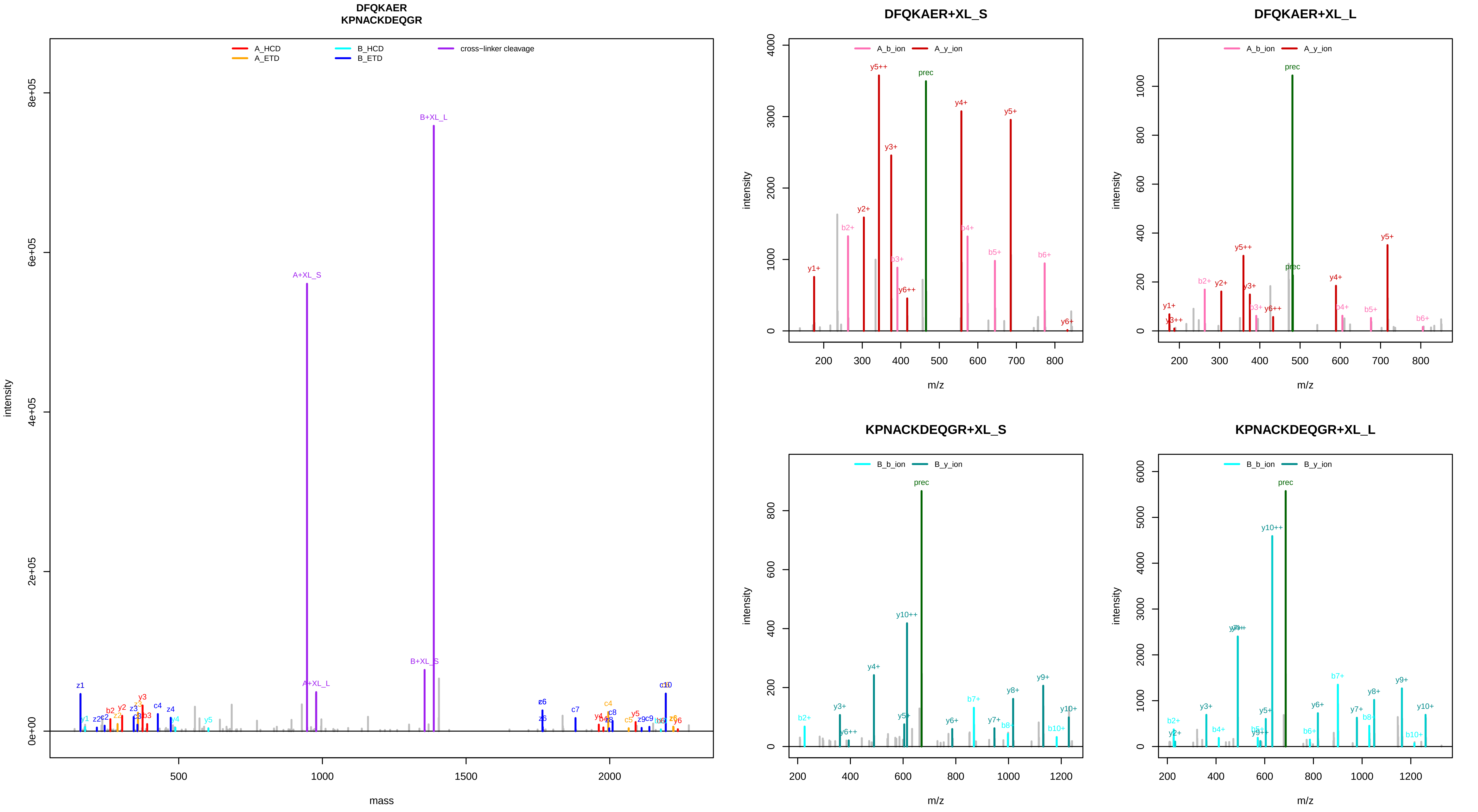
DFQKAER
KPNACKDEQGR
A_HCD
A_ETD
B_HCD
B_ETD
cross−linker cleavage
B+XL_L
A+XL_S
B+XL_S
A+XL_L
intensity
mass
DFQKAER+XL_S
DFQKAER+XL_L
A_b_ion
A_y_ion
prec
KPNACKDEQGR+XL_S
KPNACKDEQGR+XL_L
B_b_ion
B_y_ion
m/z

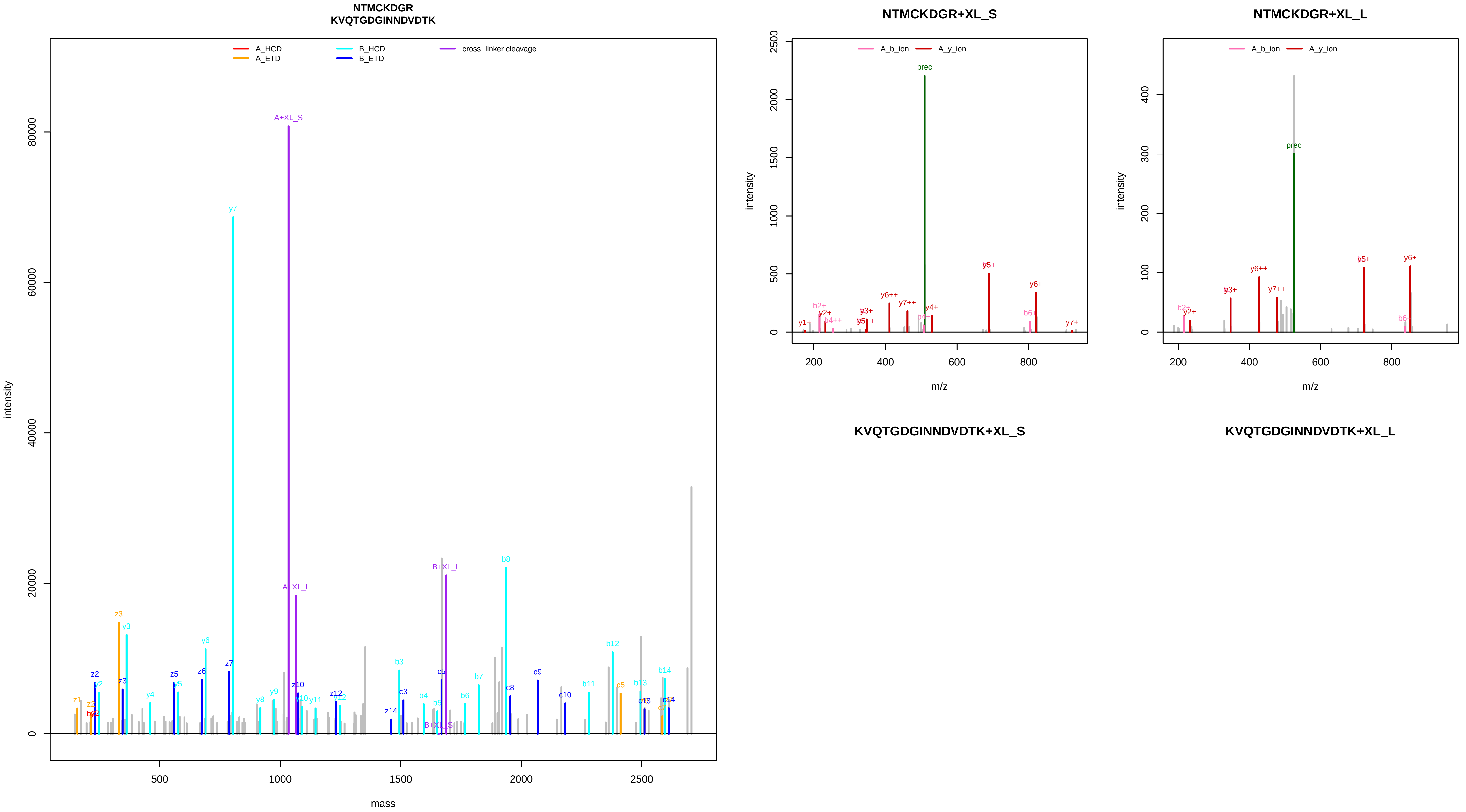
NTMCKDGR
KVQTGDGINNDVDTK
A_HCD
A_ETD
B_HCD
B_ETD
cross−linker cleavage
A+XL_S
A+XL_L
B+XL_L
B+XL_S
intensity
mass
NTMCKDGR+XL_S
NTMCKDGR+XL_L
A_b_ion
A_y_ion
prec
intensity
m/z
KVQTGDGINNDVDTK+XL_S
KVQTGDGINNDVDTK+XL_L

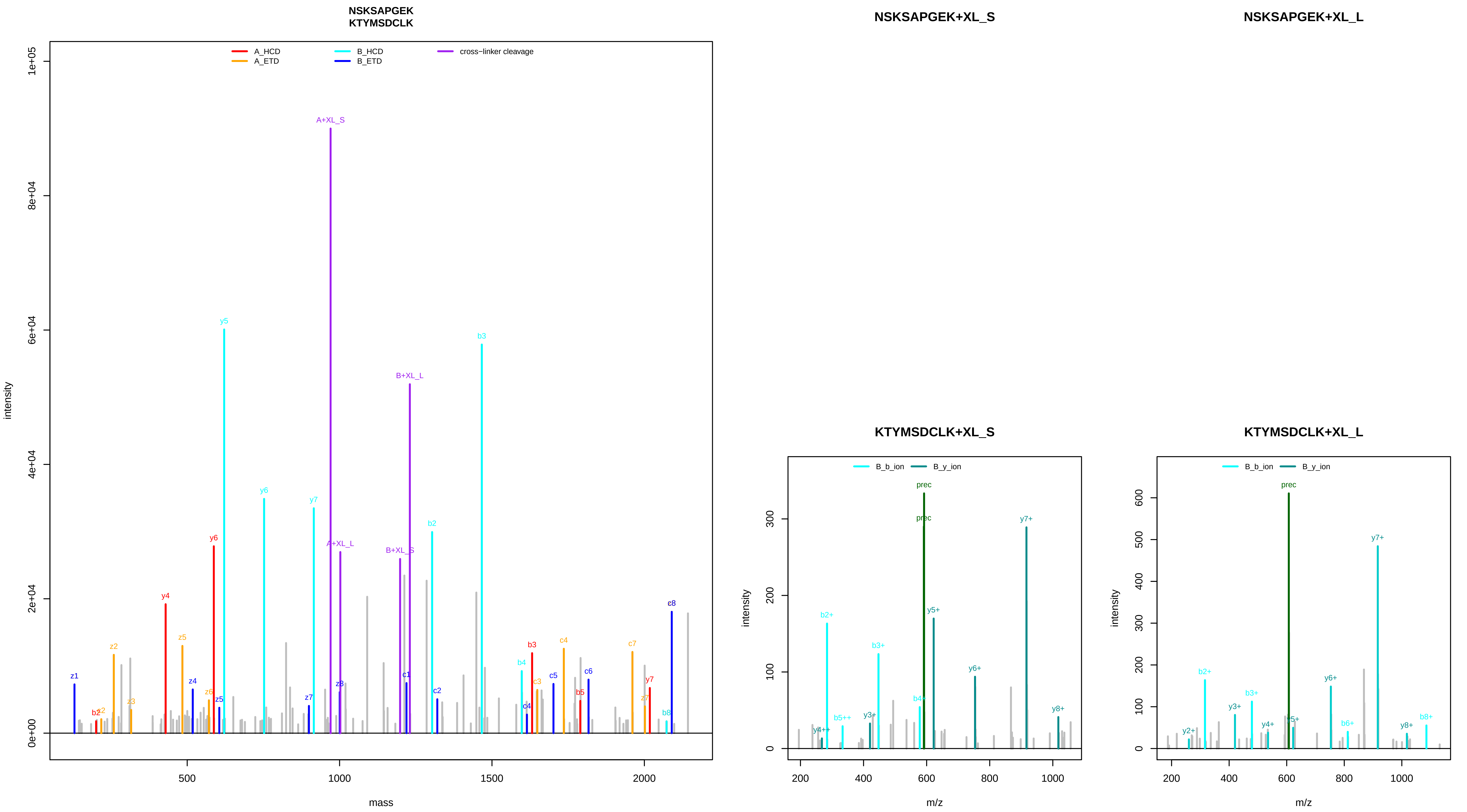
NSKSAPGEK
KTYMSDCLK
A_HCD
A_ETD
B_HCD
B_ETD
cross−linker cleavage
A+XL_S
B+XL_L
A+XL_L
B+XL_S
intensity
mass
NSKSAPGEK+XL_S
NSKSAPGEK+XL_L
KTYMSDCLK+XL_S
KTYMSDCLK+XL_L
B_b_ion
B_y_ion
prec
m/z

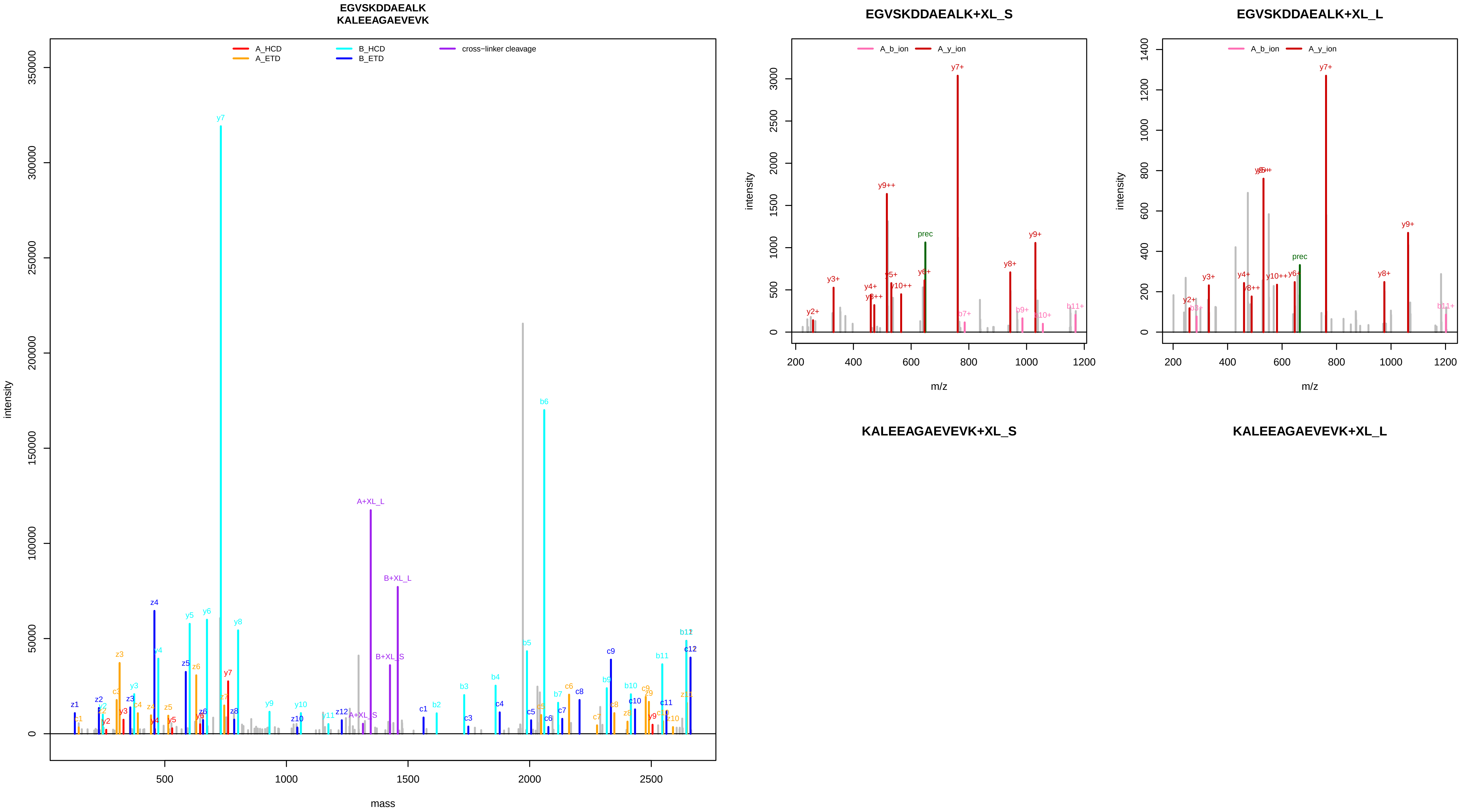

EGVSKDDAEALK
KALEEAGAEVEVK
A_HCD
A_ETD
B_HCD
B_ETD
cross−linker cleavage
A+XL_L
B+XL_L
B+XL_S
A+XL_S
intensity
mass
EGVSKDDAEALK+XL_S
EGVSKDDAEALK+XL_L
A_b_ion
A_y_ion
prec
intensity
m/z
KALEEAGAEVEVK+XL_S
KALEEAGAEVEVK+XL_L

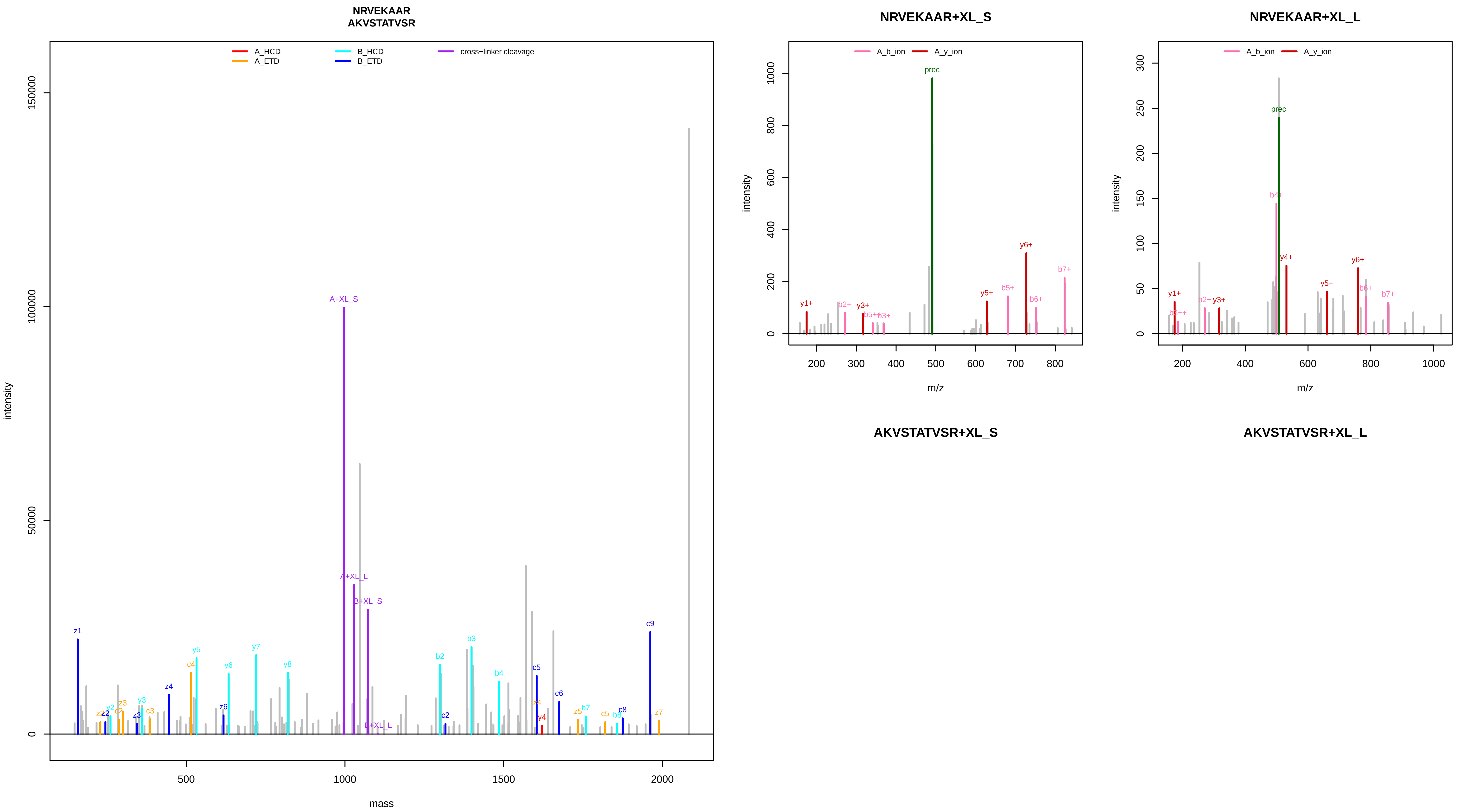
NRVEKAAR
AKVSTATVSR
A_HCD
A_ETD
B_HCD
B_ETD
cross−linker cleavage
intensity
mass
A+XL_S
A+XL_L
B+XL_S
B+XL_L
NRVEKAAR+XL_S
NRVEKAAR+XL_L
A_b_ion
A_y_ion
prec
m/z
AKVSTATVSR+XL_S
AKVSTATVSR+XL_L

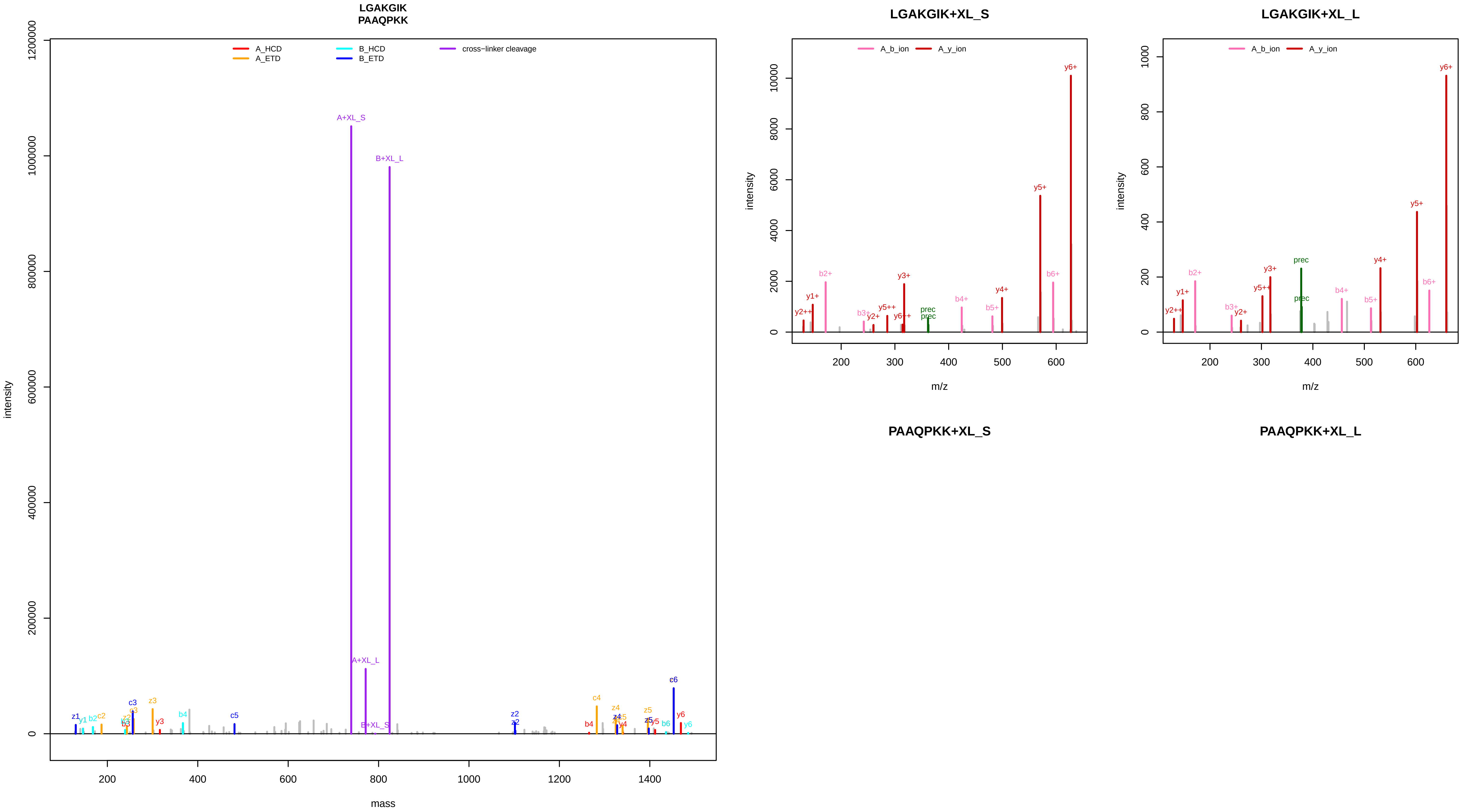
LGAKGIK
PAAQPKK
A_HCD
A_ETD
B_HCD
B_ETD
cross−linker cleavage
A+XL_S
B+XL_L
A+XL_L
B+XL_S
intensity
mass
LGAKGIK+XL_S
LGAKGIK+XL_L
A_b_ion
A_y_ion
m/z
PAAQPKK+XL_S
PAAQPKK+XL_L

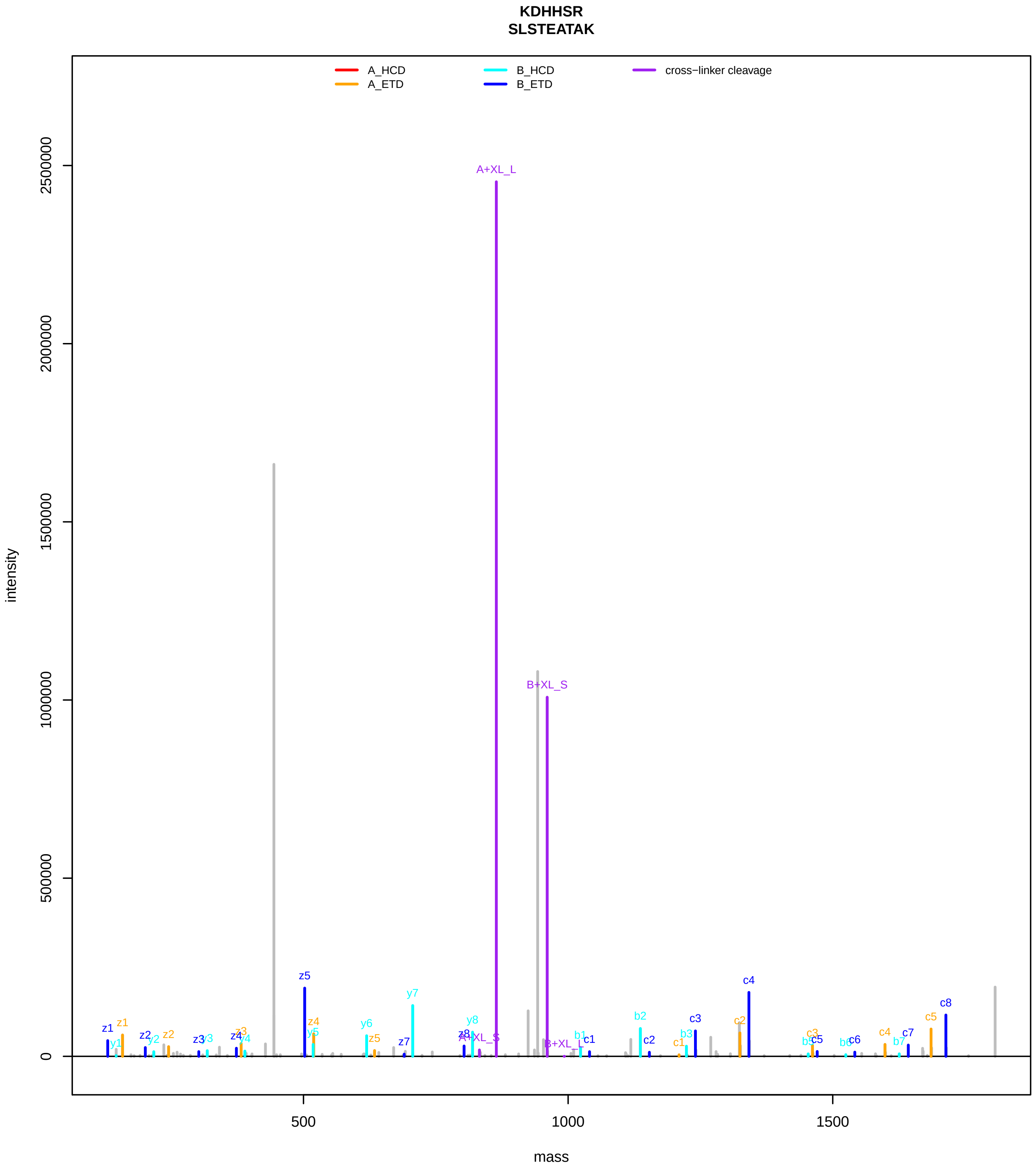

KDHHSR+XL_S

KDHHSR+XL_L

SLSTEATAK+XL_S

SLSTEATAK+XL_L

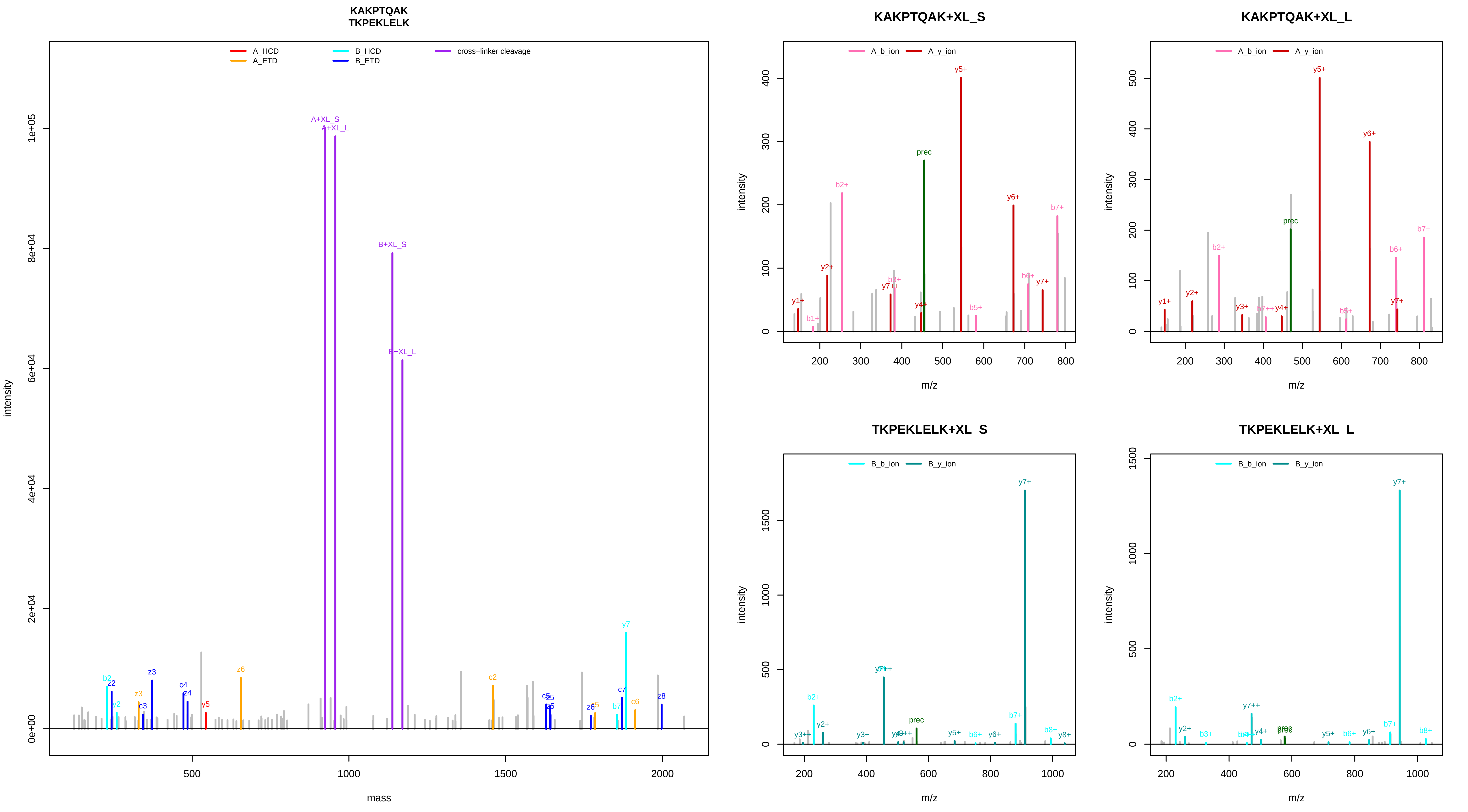

KAKPTQAK
TKPEKLELK
A_HCD
A_ETD
B_HCD
B_ETD
cross−linker cleavage
A+XL_S
A+XL_L
B+XL_S
B+XL_L
intensity
mass
KAKPTQAK+XL_S
KAKPTQAK+XL_L
TKPEKLELK+XL_S
TKPEKLELK+XL_L
A_b_ion
A_y_ion
B_b_ion
B_y_ion
prec
m/z

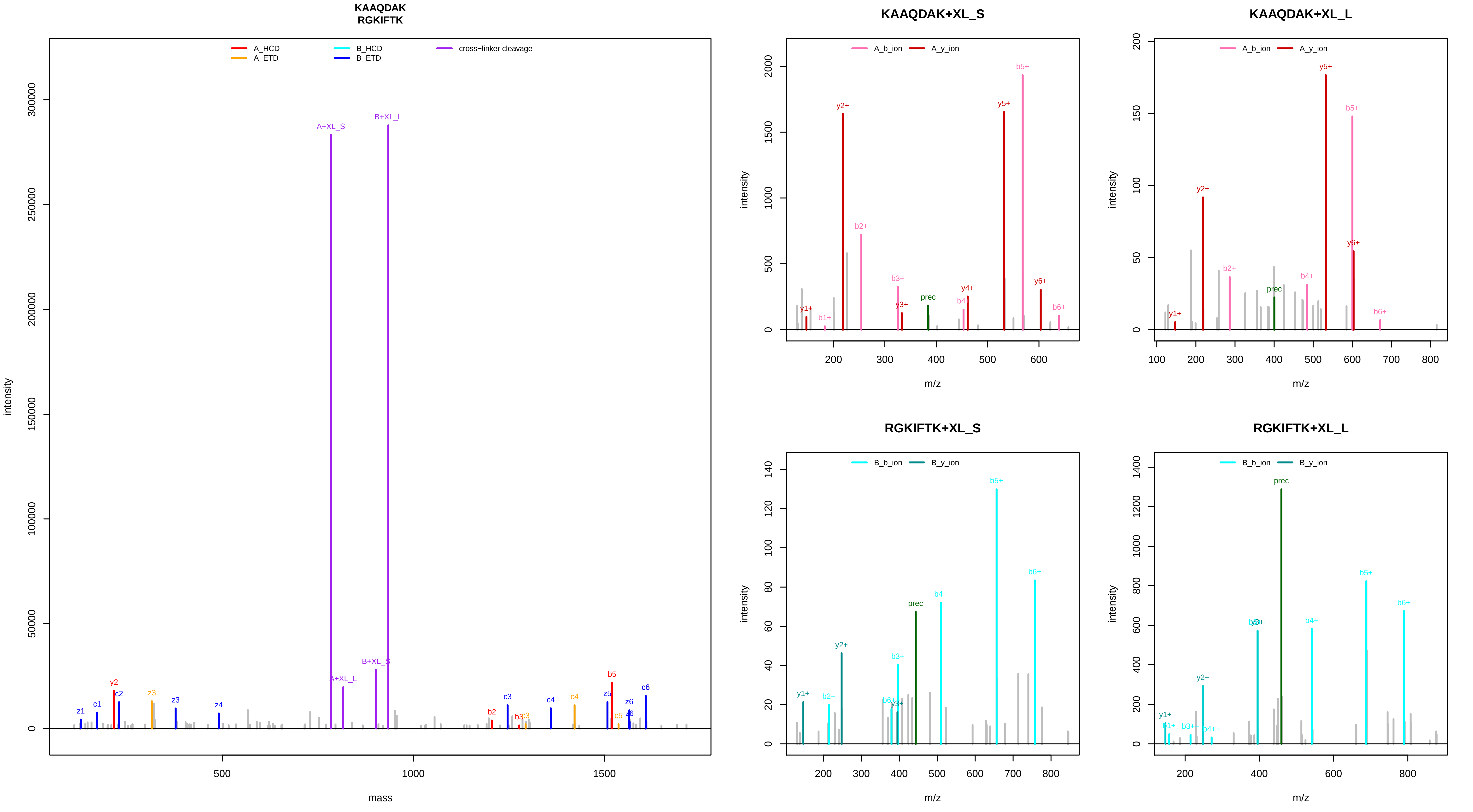

KAAQDAK
RGKIFTK
A_HCD
A_ETD
B_HCD
B_ETD
cross−linker cleavage
A+XL_S
B+XL_L
A+XL_L
B+XL_S
intensity
mass
KAAQDAK+XL_S
KAAQDAK+XL_L
RGKIFTK+XL_S
RGKIFTK+XL_L
A_b_ion
A_y_ion
B_b_ion
B_y_ion
prec
m/z

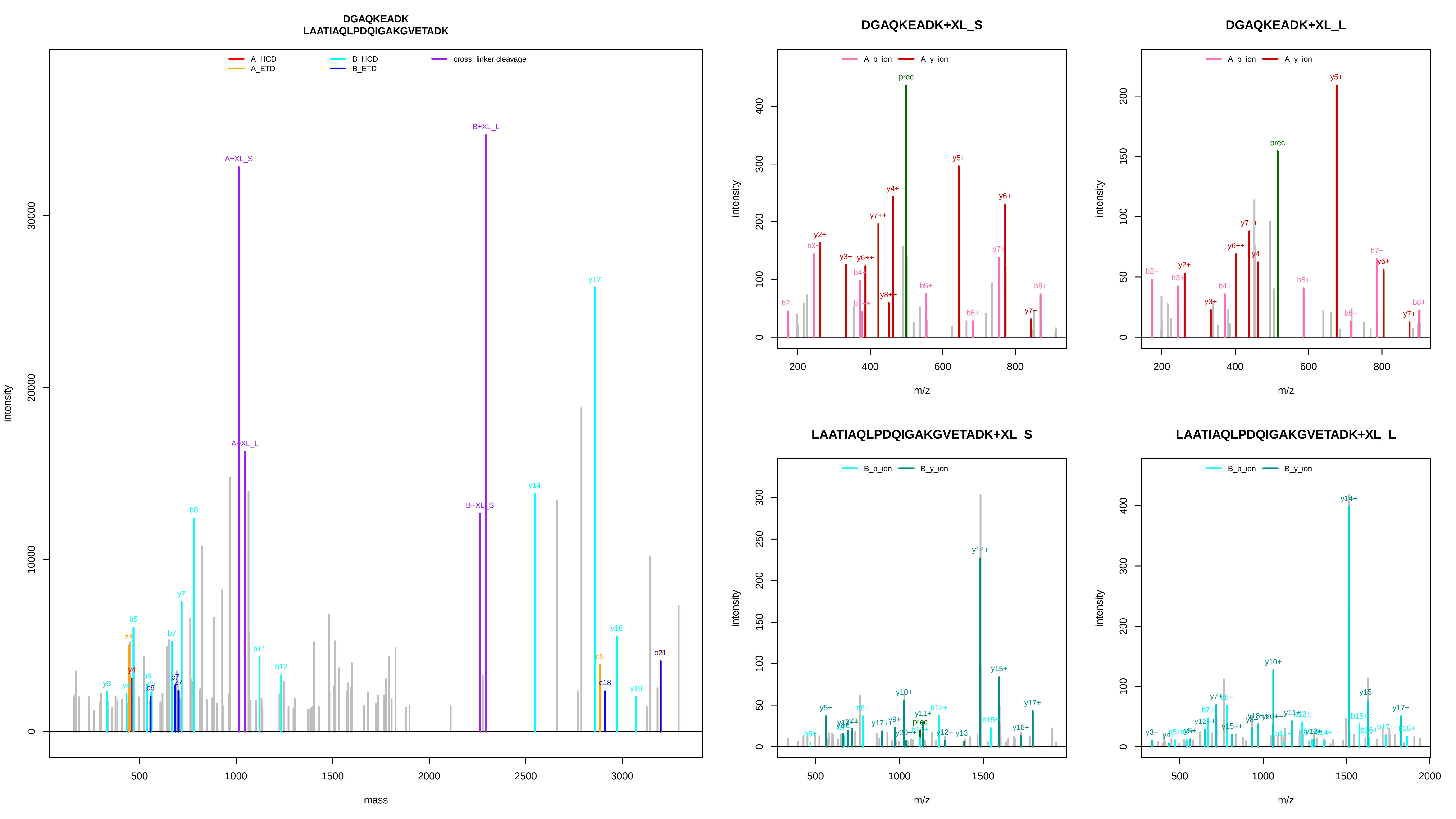
DGAQKEADK
LAATIAQLPDQIGAKGVETADK
A_HCD
A_ETD
B_HCD
B_ETD
cross−linker cleavage
B+XL_L
A+XL_S
A+XL_L
B+XL_S
intensity
mass
y17
y14
y18
y19
c21
c18
c5
b8
y7
b5
z4
y4
b7
b11
b12
y3
DGAQKEADK+XL_S
DGAQKEADK+XL_L
A_b_ion
A_y_ion
prec
y5+
y6+
y4+
y7++
y2+
b3+
y3+
y6++
b7+
b8+
b5+
b6+
y7+
b2+
m/z
LAATIAQLPDQIGAKGVETADK+XL_S
LAATIAQLPDQIGAKGVETADK+XL_L
B_b_ion
B_y_ion
y14+
y15+
y17+
y10+
y16+
y12+
y13+
b12+
y11+
y9+
y5+
b15+
b8+
b5+
y3+
y4+
b7+

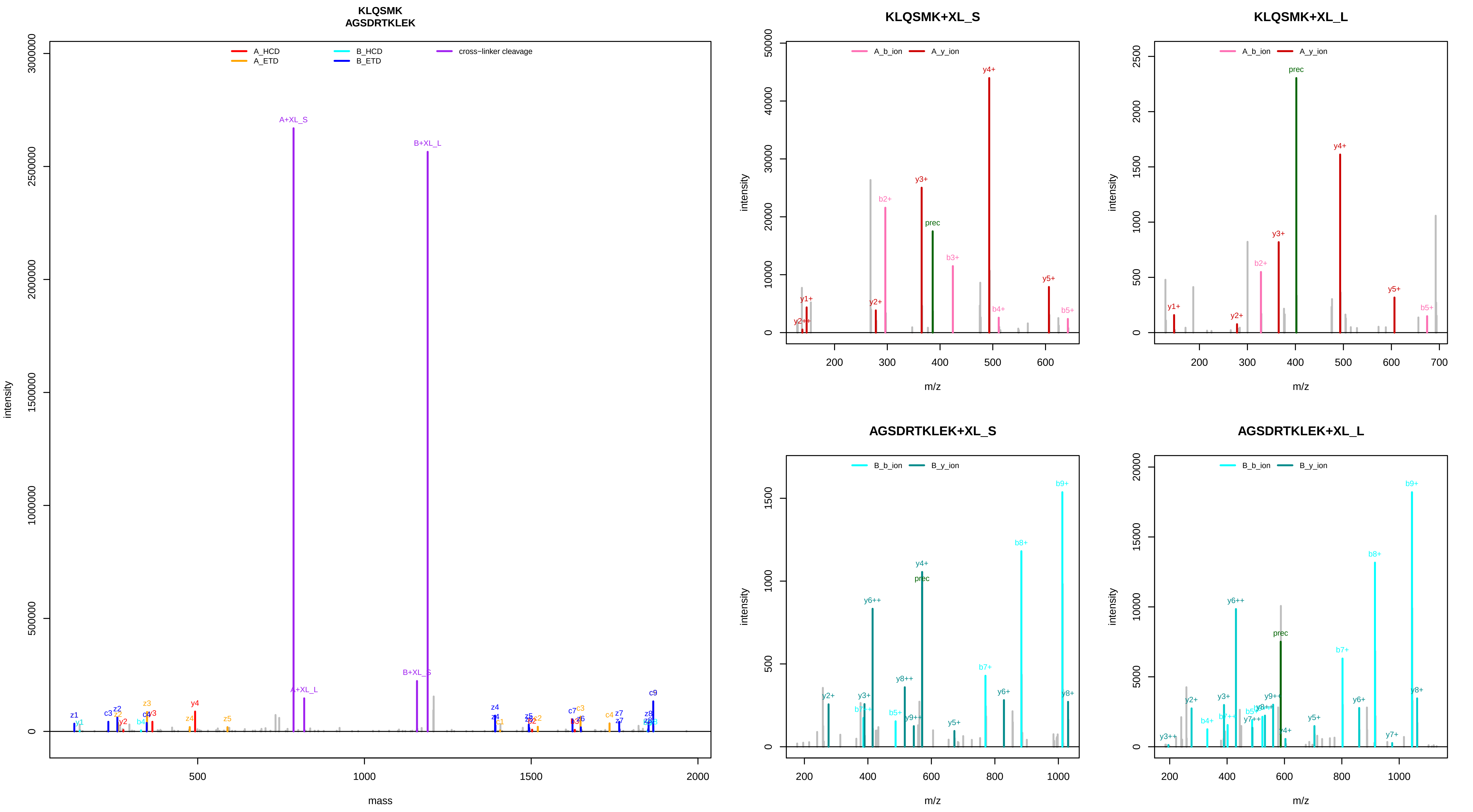

KLQSMK
AGSDRTKLEK
A_HCD
A_ETD
B_HCD
B_ETD
cross−linker cleavage
A+XL_S
B+XL_L
B+XL_S
A+XL_L
intensity
mass
KLQSMK+XL_S
KLQSMK+XL_L
A_b_ion
A_y_ion
AGSDRTKLEK+XL_S
AGSDRTKLEK+XL_L
B_b_ion
B_y_ion
prec
m/z

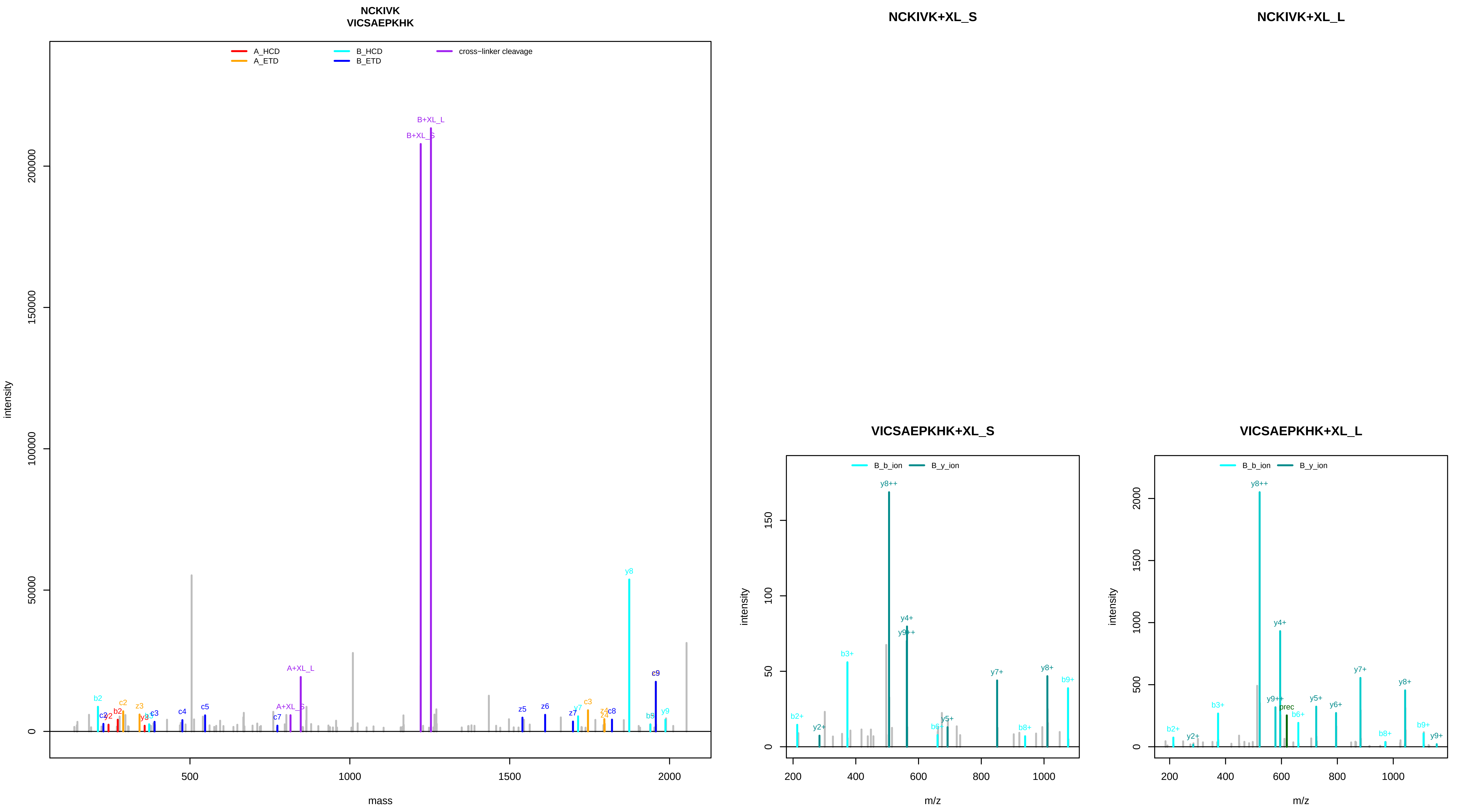
NCKIVK
VICSAEPKHK
A_HCD
A_ETD
B_HCD
B_ETD
cross−linker cleavage
B+XL_L
B+XL_S
A+XL_L
A+XL_S
intensity
mass
NCKIVK+XL_S
NCKIVK+XL_L
VICSAEPKHK+XL_S
VICSAEPKHK+XL_L
B_b_ion
B_y_ion
m/z
y8++
y4+
y9++
b3+
y7+
y8+
b9+
b2+
y2+
b6+
y5+
b8+
prec
y6+
y9+

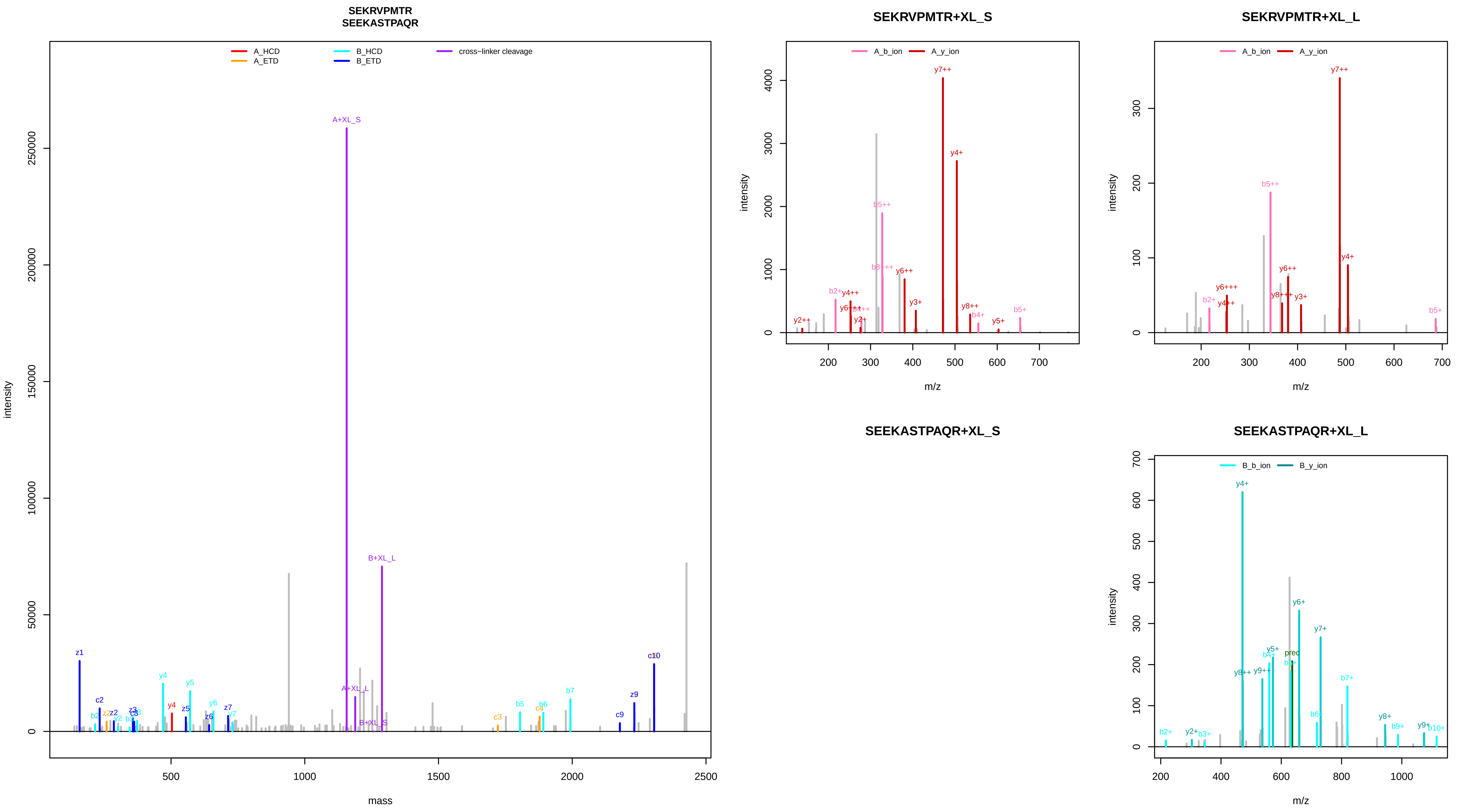
SEKRVPMTR
SEEKASTPAQR
A_HCD
A_ETD
B_HCD
B_ETD
cross−linker cleavage
A+XL_S
B+XL_L
A+XL_L
B+XL_S
intensity
mass
SEKRVPMTR+XL_S
SEKRVPMTR+XL_L
A_b_ion
A_y_ion
m/z
SEEKASTPAQR+XL_S
SEEKASTPAQR+XL_L
B_b_ion
B_y_ion
prec

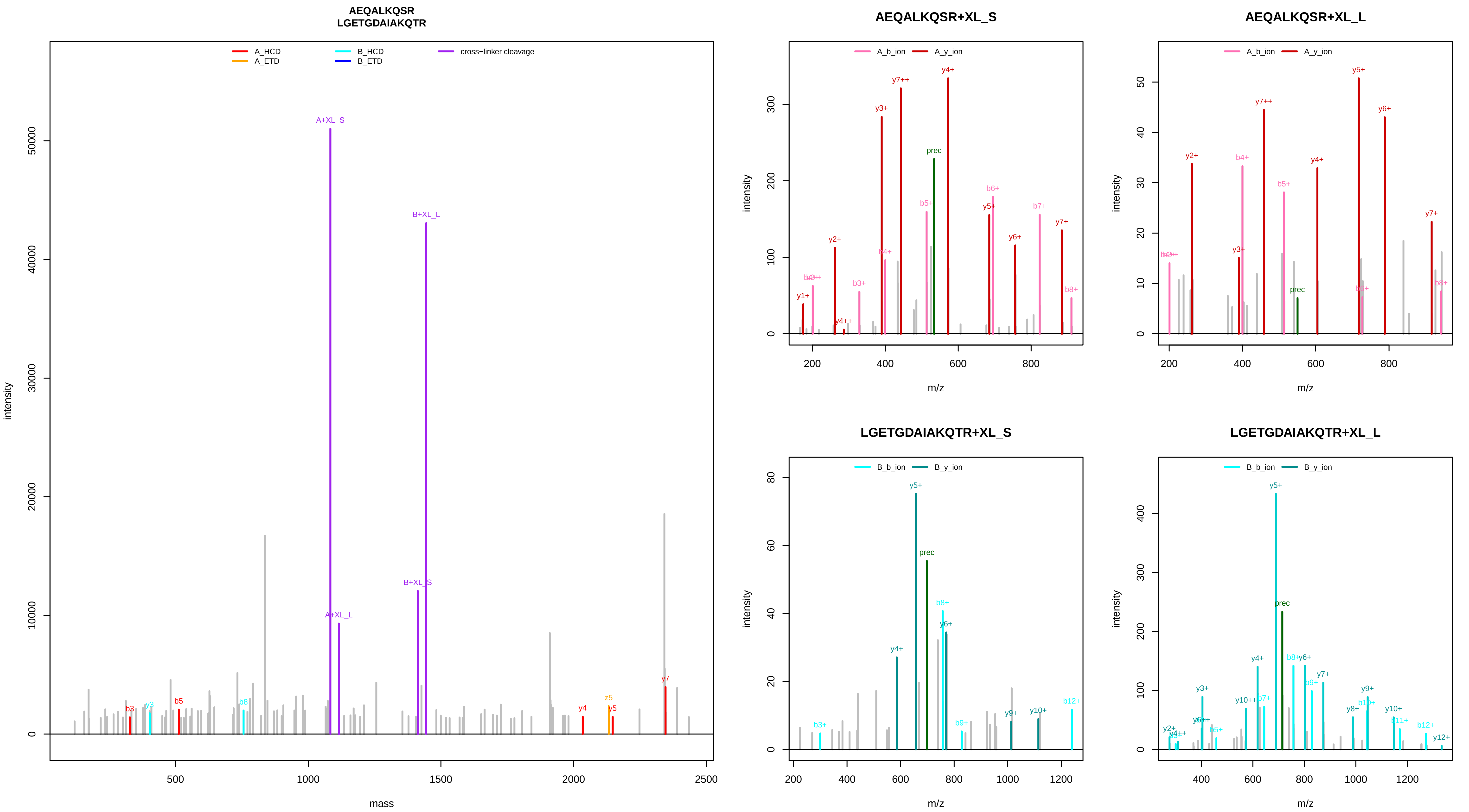
AEQALKQSR
LGETGDAIAKQTR
A_HCD
A_ETD
B_HCD
B_ETD
cross−linker cleavage
A+XL_S
B+XL_L
B+XL_S
A+XL_L
intensity
mass
AEQALKQSR+XL_S
AEQALKQSR+XL_L
LGETGDAIAKQTR+XL_S
LGETGDAIAKQTR+XL_L
A_b_ion
A_y_ion
B_b_ion
B_y_ion
prec
m/z

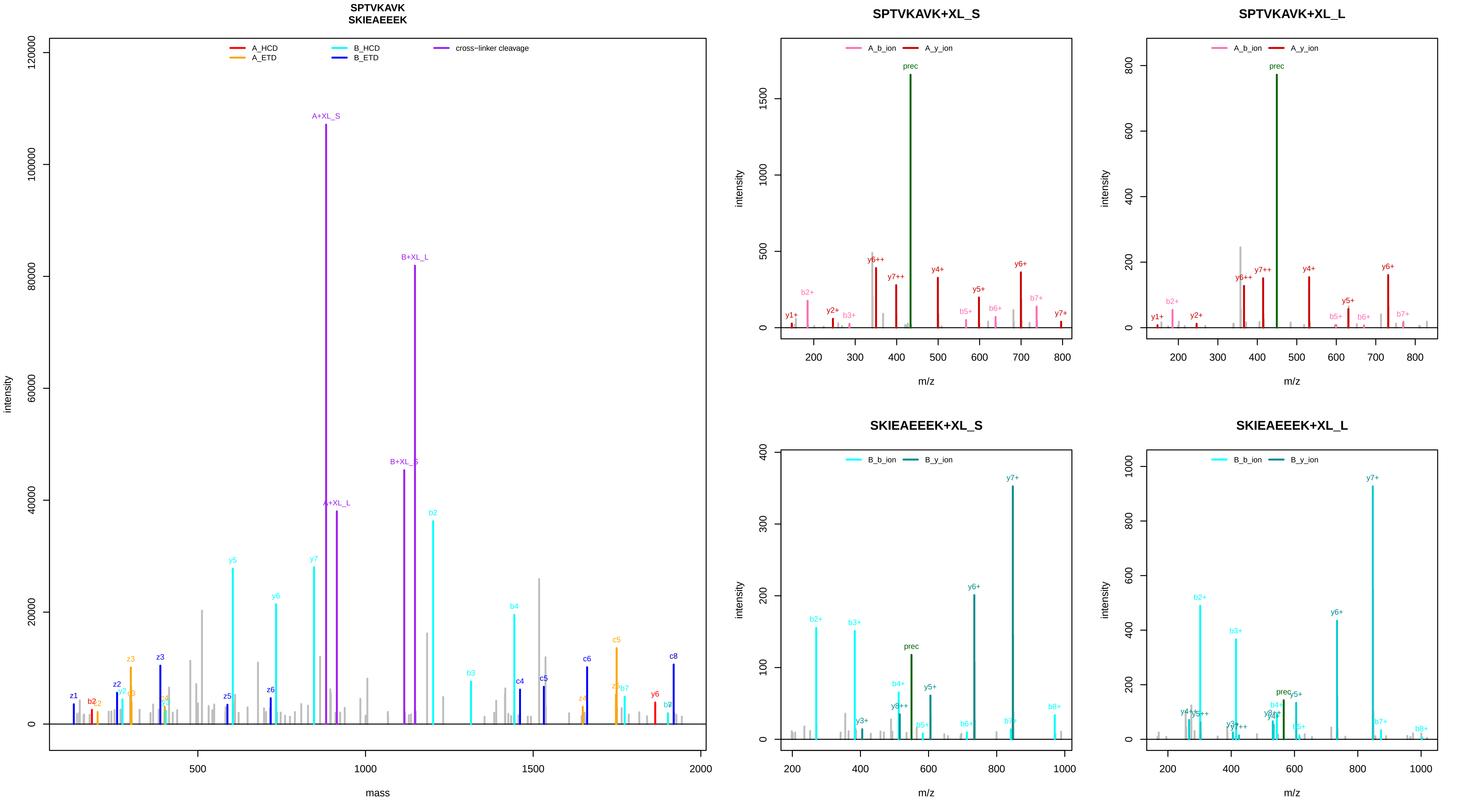

SPTVKAVK
SKIEAEEEK
A_HCD
A_ETD
B_HCD
B_ETD
cross−linker cleavage
intensity
mass
SPTVKAVK+XL_S
SPTVKAVK+XL_L
SKIEAEEEK+XL_S
SKIEAEEEK+XL_L
A_b_ion
A_y_ion
B_b_ion
B_y_ion
prec
m/z

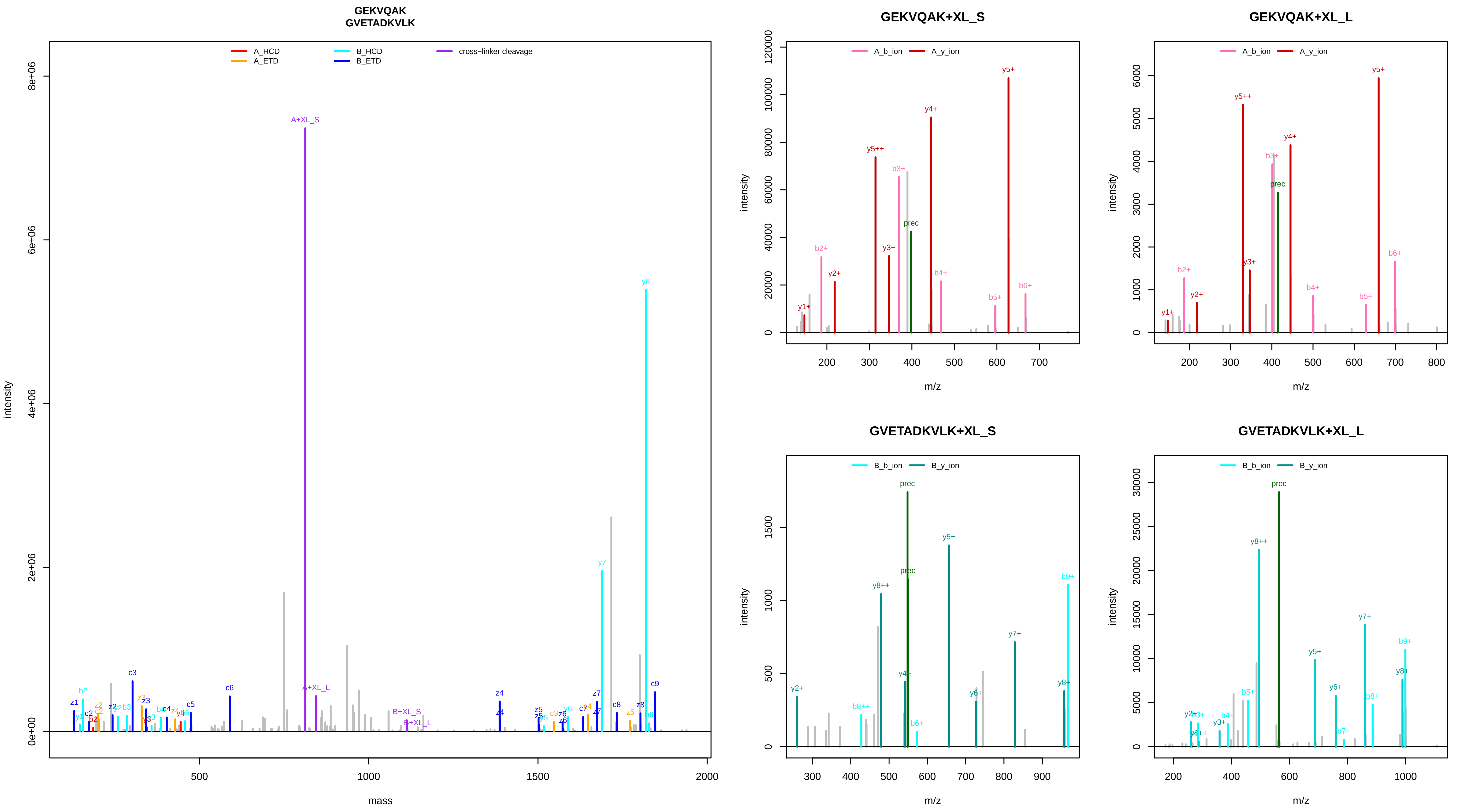
GEKVQAK
GVETADKVLK
A_HCD
A_ETD
B_HCD
B_ETD
cross−linker cleavage
A+XL_S
A+XL_L
B+XL_S
B+XL_L
y8
y7
c3
c6
c9
c8
z4
z7
z8
intensity
mass
GEKVQAK+XL_S
A_b_ion
A_y_ion
y5+
y4+
y5++
b3+
prec
y3+
b2+
y2+
y1+
b4+
b5+
b6+
intensity
m/z
GEKVQAK+XL_L
A_b_ion
A_y_ion
y5+
y5++
y4+
b3+
prec
y3+
b2+
y2+
y1+
b4+
b5+
b6+
intensity
m/z
GVETADKVLK+XL_S
B_b_ion
B_y_ion
prec
y5+
y8++
b9+
y7+
y2+
b8++
y4+
b6+
y6+
y8+
intensity
m/z
GVETADKVLK+XL_L
B_b_ion
B_y_ion
prec
y8++
y7+
y5+
y6+
b9+
y8+
b8+
b7+
b5+
b4+
y3+
y2+
b3+
y4++
intensity
m/z

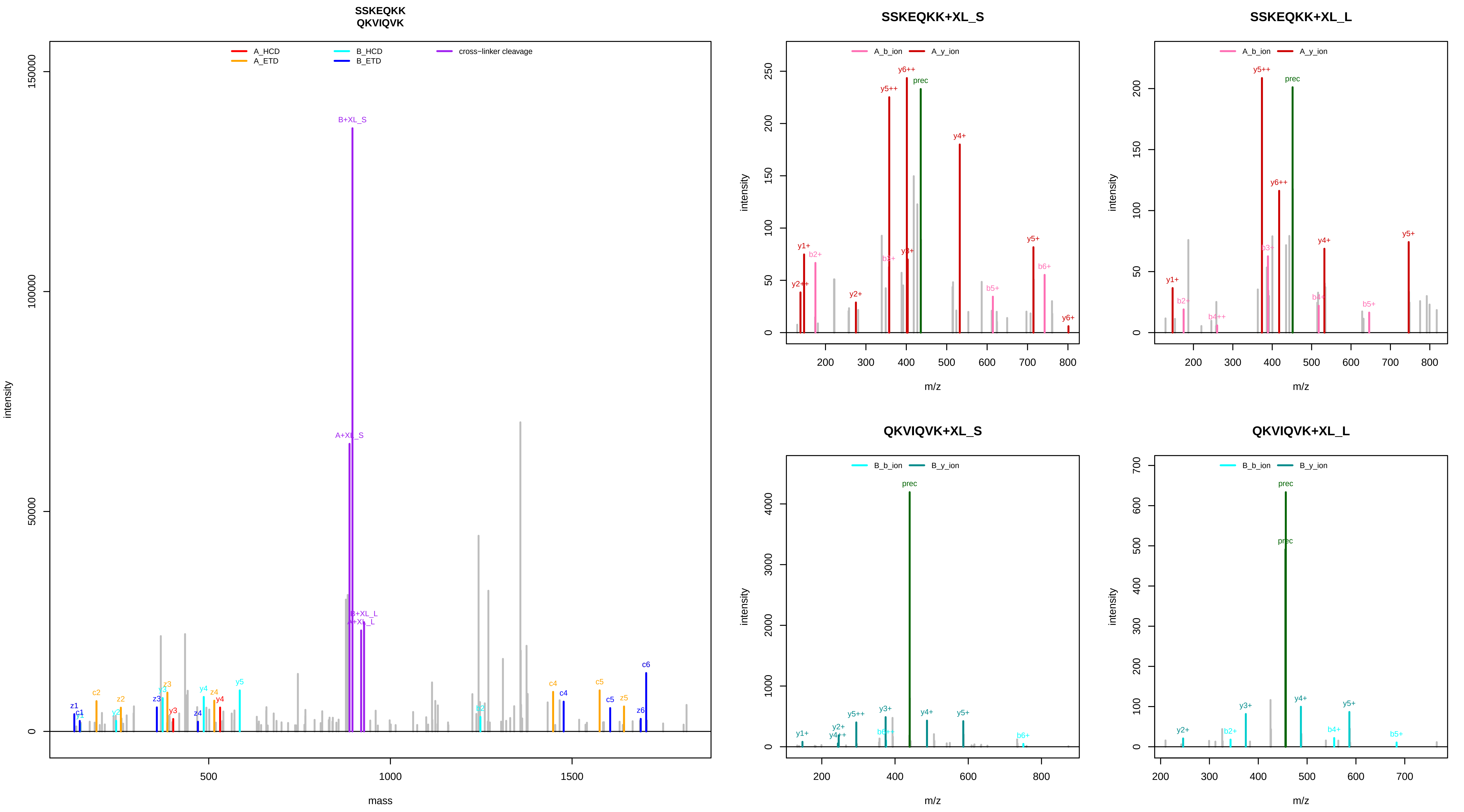

SSKEQKK
QKVIQVK
A_HCD
A_ETD
B_HCD
B_ETD
cross−linker cleavage
B+XL_S
A+XL_S
B+XL_L
A+XL_L
intensity
mass
SSKEQKK+XL_S
SSKEQKK+XL_L
QKVIQVK+XL_S
QKVIQVK+XL_L
A_b_ion
A_y_ion
B_b_ion
B_y_ion
prec
m/z

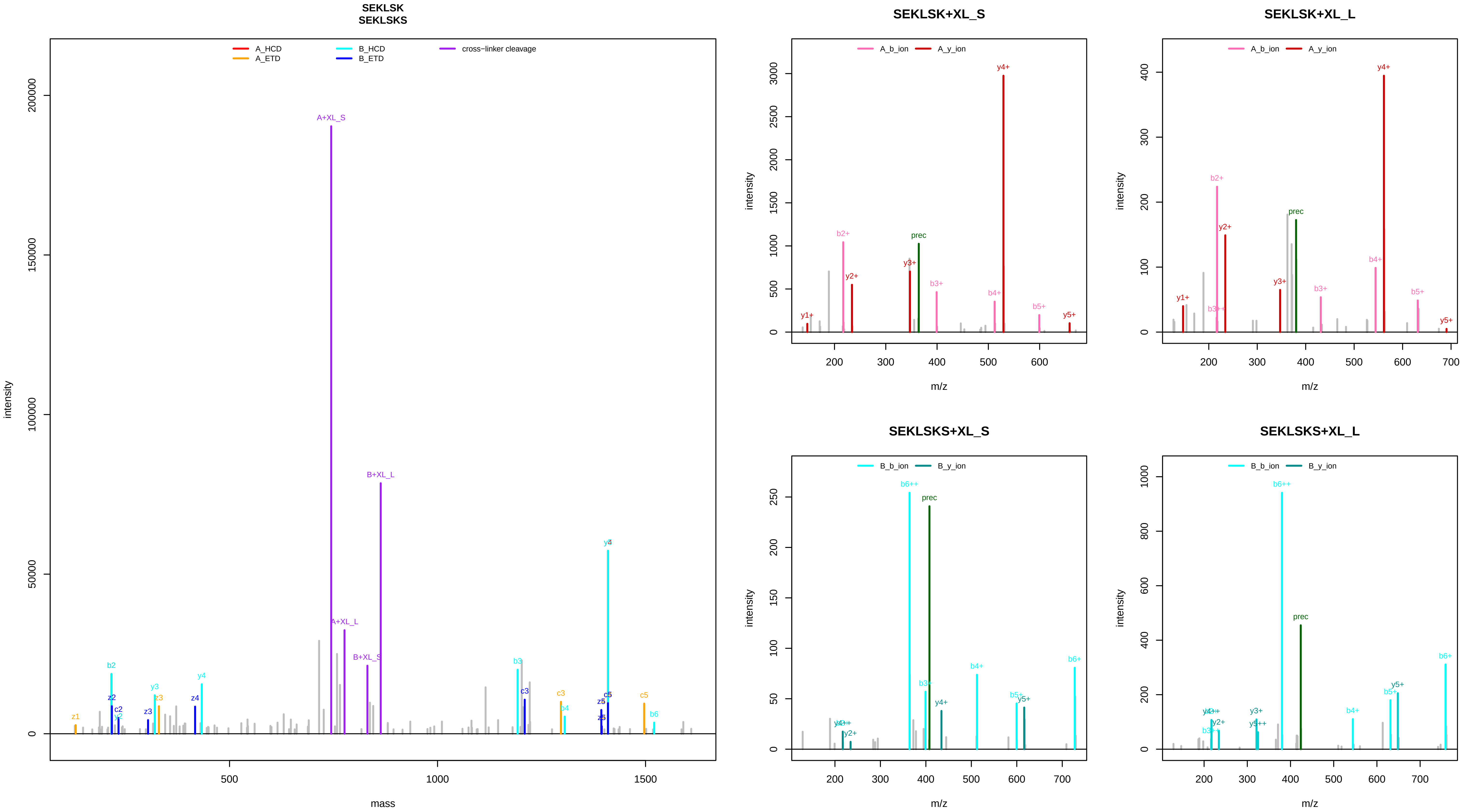

SEKLSK
SEKLSKS
A_HCD
A_ETD
B_HCD
B_ETD
cross−linker cleavage
A+XL_S
B+XL_L
A+XL_L
B+XL_S
intensity
mass
SEKLSK+XL_S
SEKLSK+XL_L
SEKLSKS+XL_S
SEKLSKS+XL_L
A_b_ion
A_y_ion
B_b_ion
B_y_ion
prec
m/z

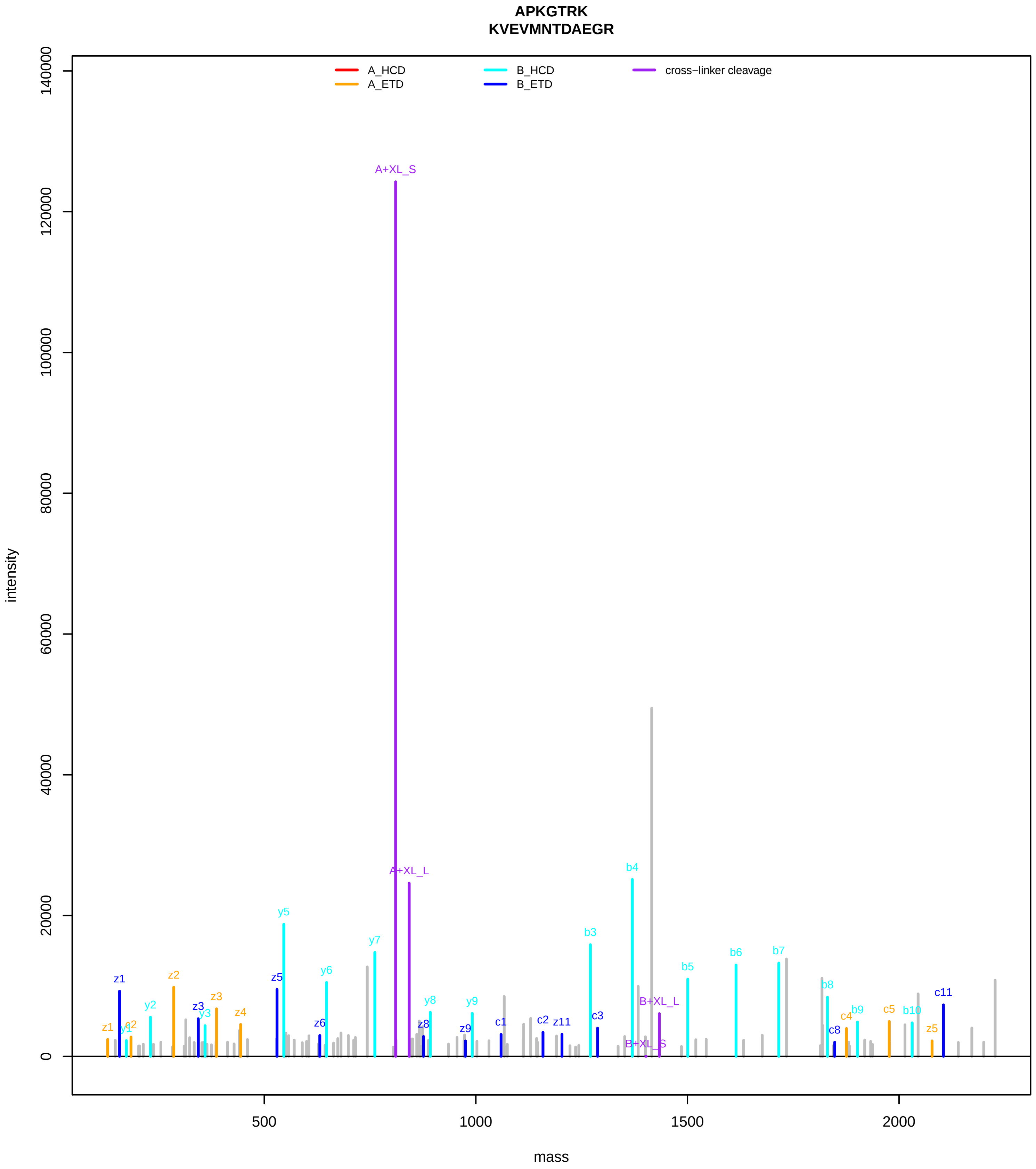

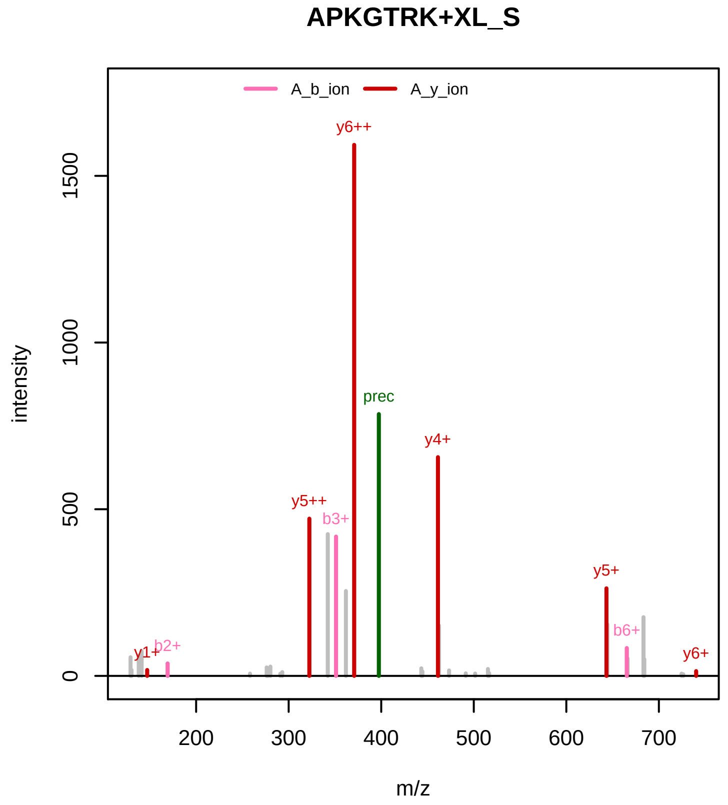

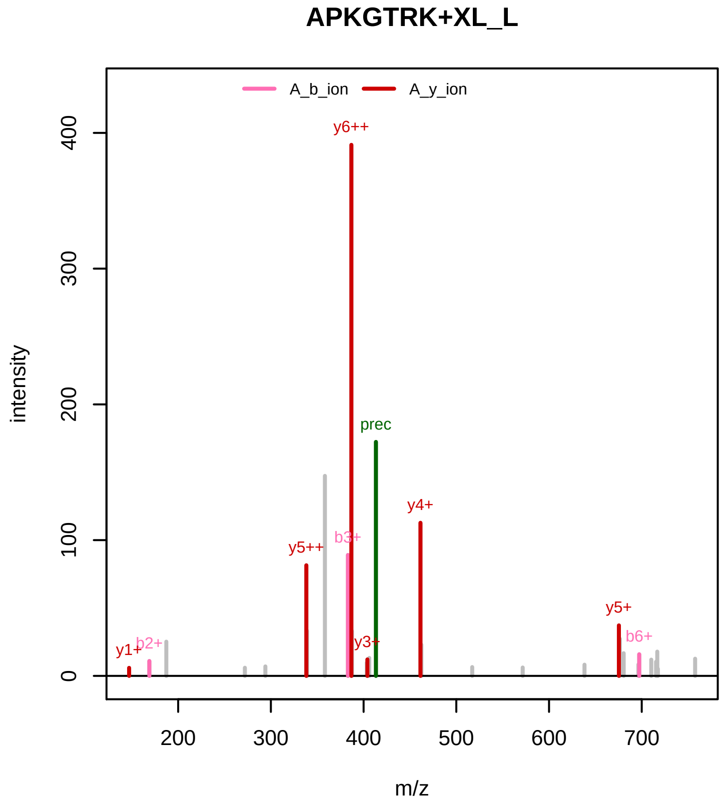

KVEVMNTDAEGR+XL_S

KVEVMNTDAEGR+XL_L

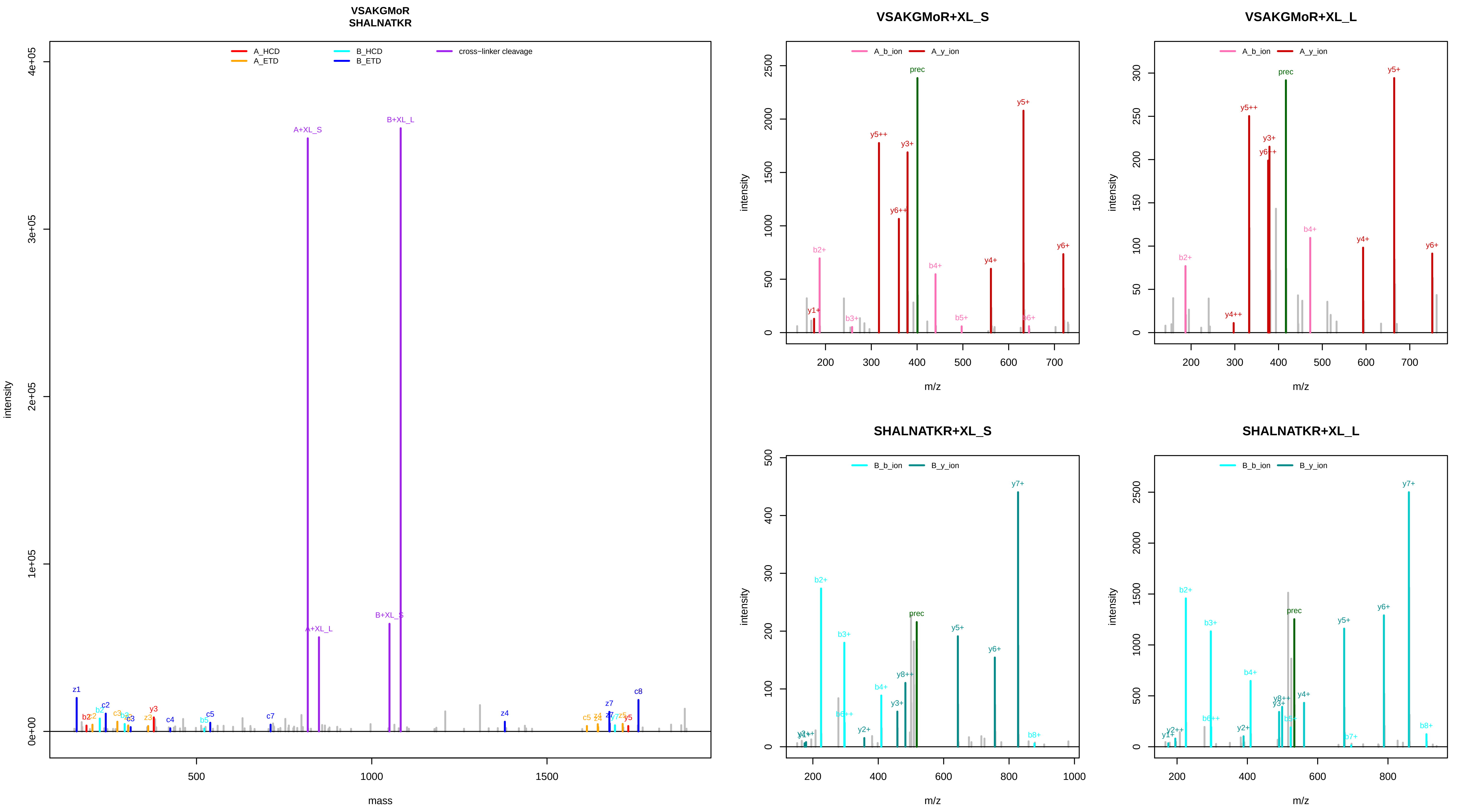
VSAKGMoR
SHALNATKR
A_HCD
A_ETD
B_HCD
B_ETD
cross−linker cleavage
A+XL_S
B+XL_L
A+XL_L
B+XL_S
intensity
mass
VSAKGMoR+XL_S
VSAKGMoR+XL_L
SHALNATKR+XL_S
SHALNATKR+XL_L
A_b_ion
A_y_ion
B_b_ion
B_y_ion
prec
intensity
m/z

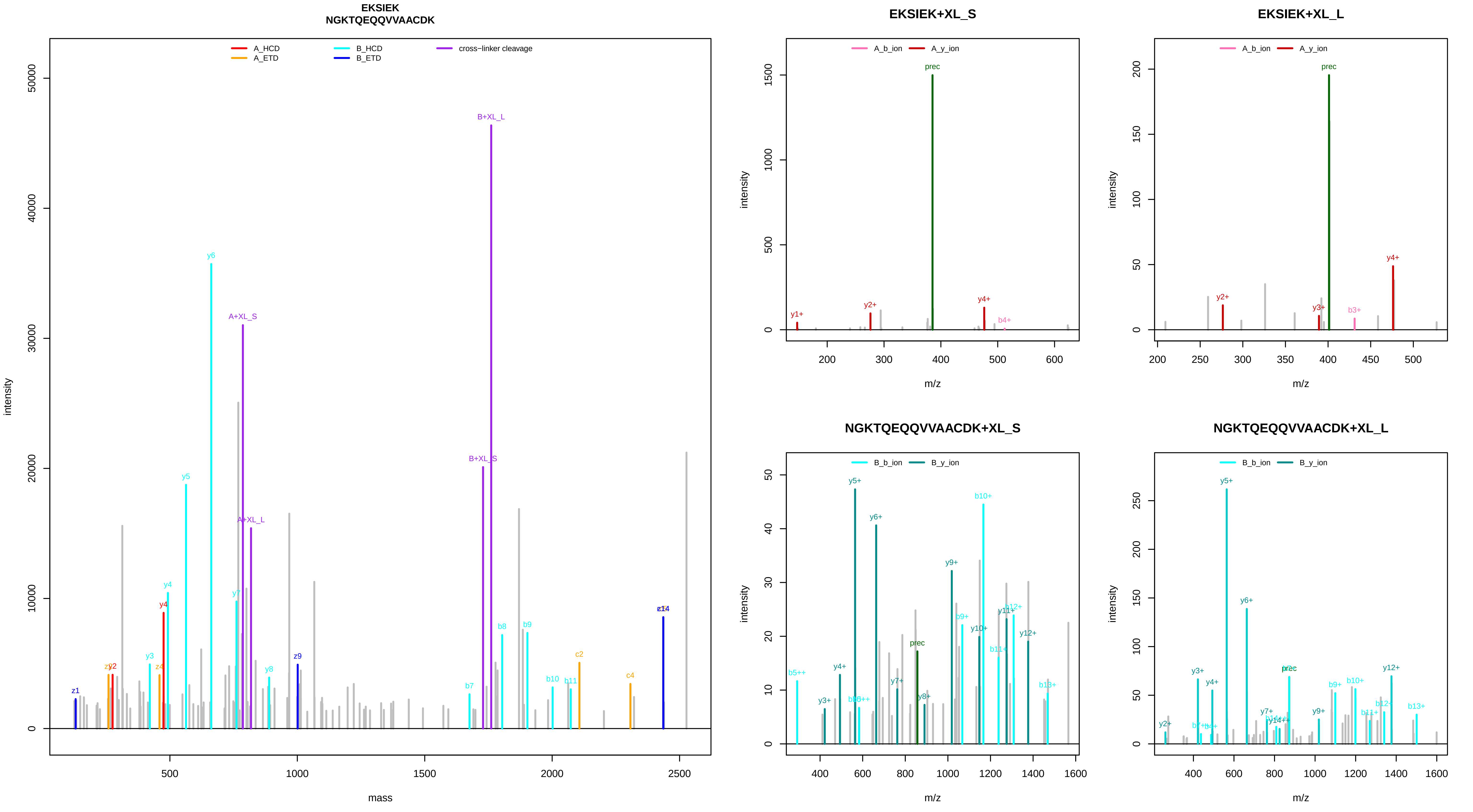
EKSIEK
NGKTQEQQVVAACDK
A_HCD
A_ETD
B_HCD
B_ETD
cross−linker cleavage
B+XL_L
A+XL_S
B+XL_S
A+XL_L
intensity
mass
EKSIEK+XL_S
EKSIEK+XL_L
A_b_ion
A_y_ion
prec
NGKTQEQQVVAACDK+XL_S
NGKTQEQQVVAACDK+XL_L
B_b_ion
B_y_ion
m/z

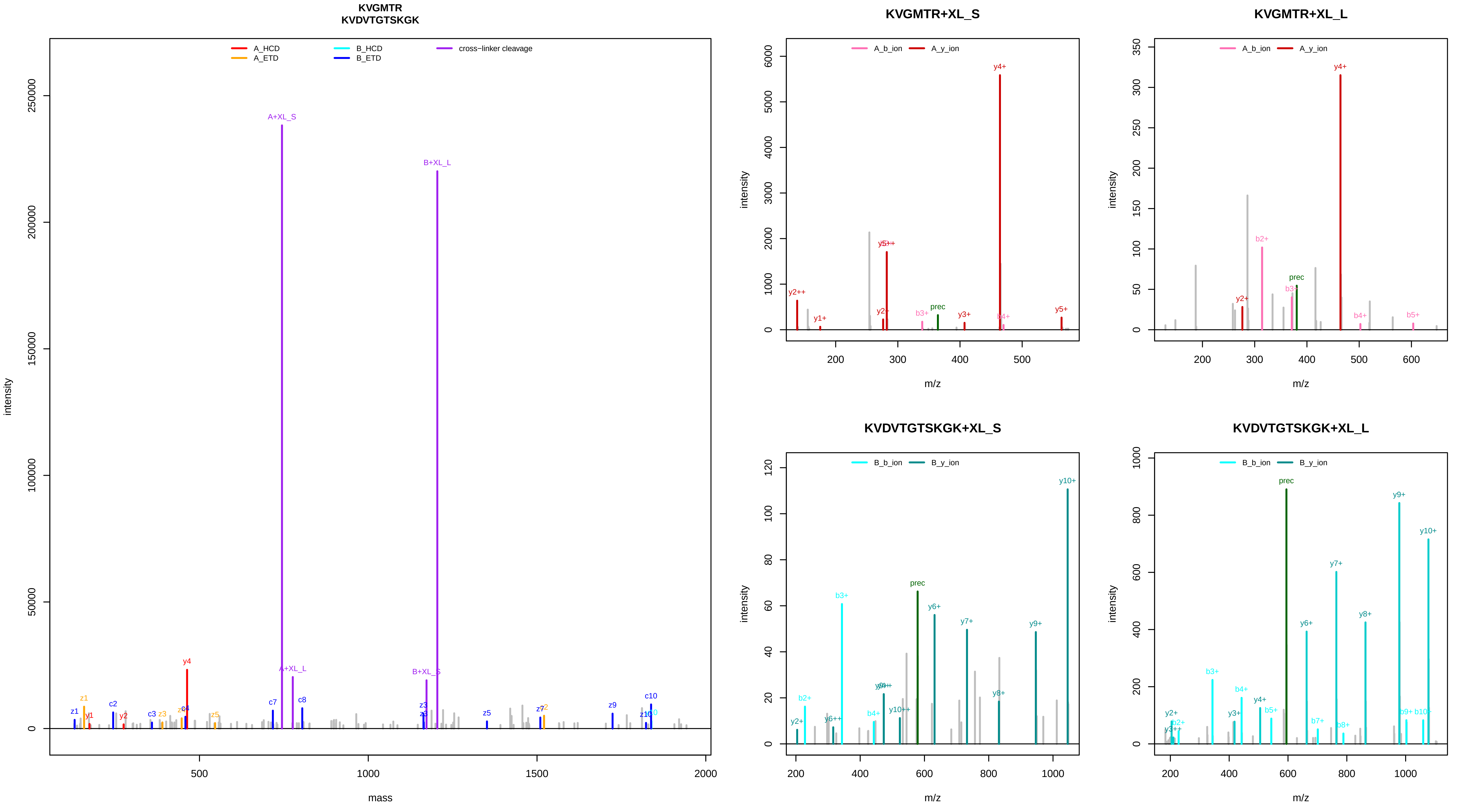
KVGMTR
KVDVTGTSKGK
A_HCD
A_ETD
B_HCD
B_ETD
cross−linker cleavage
A+XL_S
B+XL_L
A+XL_L
B+XL_S
intensity
mass
KVGMTR+XL_S
KVGMTR+XL_L
A_b_ion
A_y_ion
KVDVTGTSKGK+XL_S
KVDVTGTSKGK+XL_L
B_b_ion
B_y_ion
prec
m/z

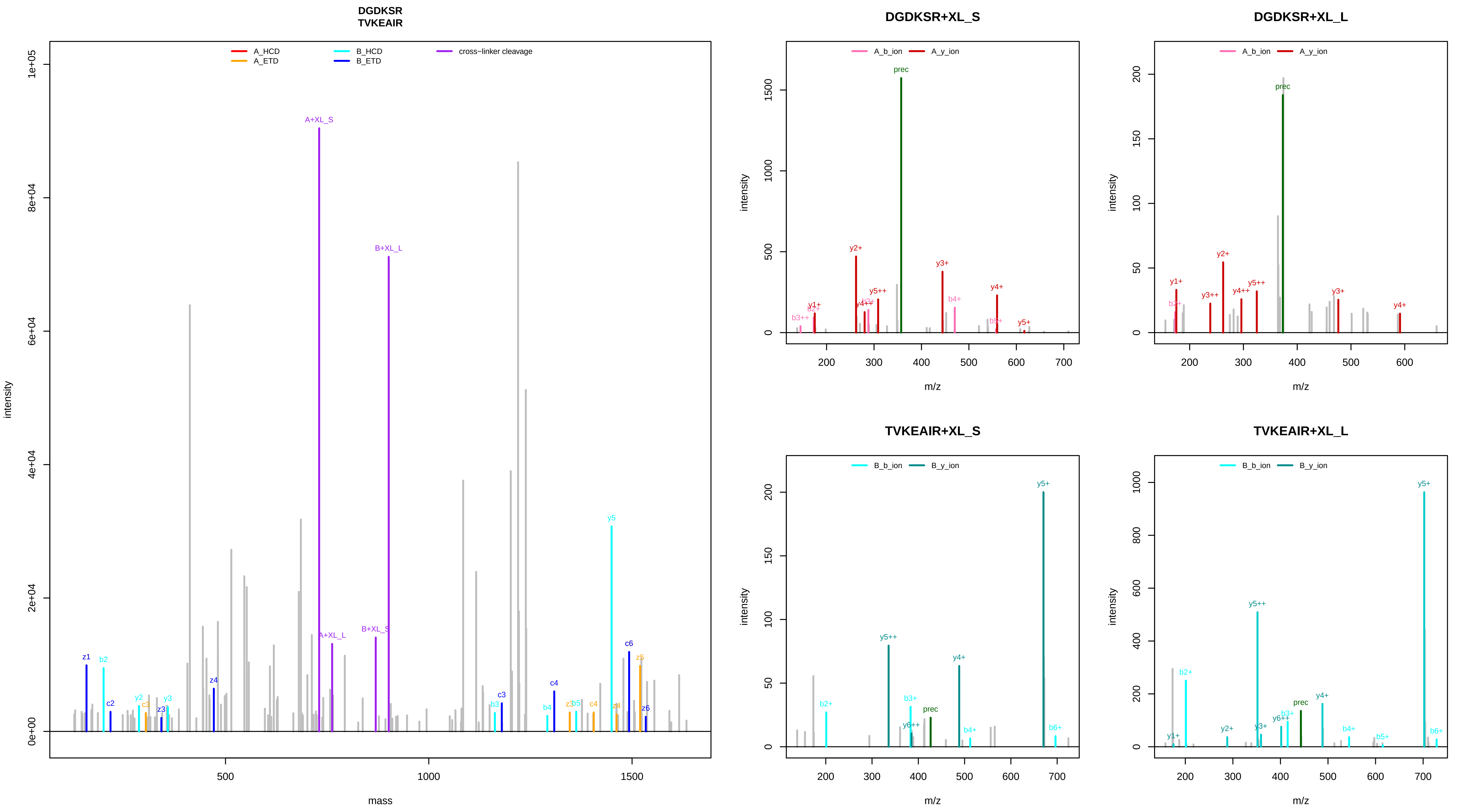

DGDKSR
TVKEAIR
A_HCD
A_ETD
B_HCD
B_ETD
cross−linker cleavage
A+XL_S
B+XL_L
A+XL_L
B+XL_S
intensity
mass
DGDKSR+XL_S
DGDKSR+XL_L
TVKEAIR+XL_S
TVKEAIR+XL_L
A_b_ion
A_y_ion
B_b_ion
B_y_ion
prec
m/z

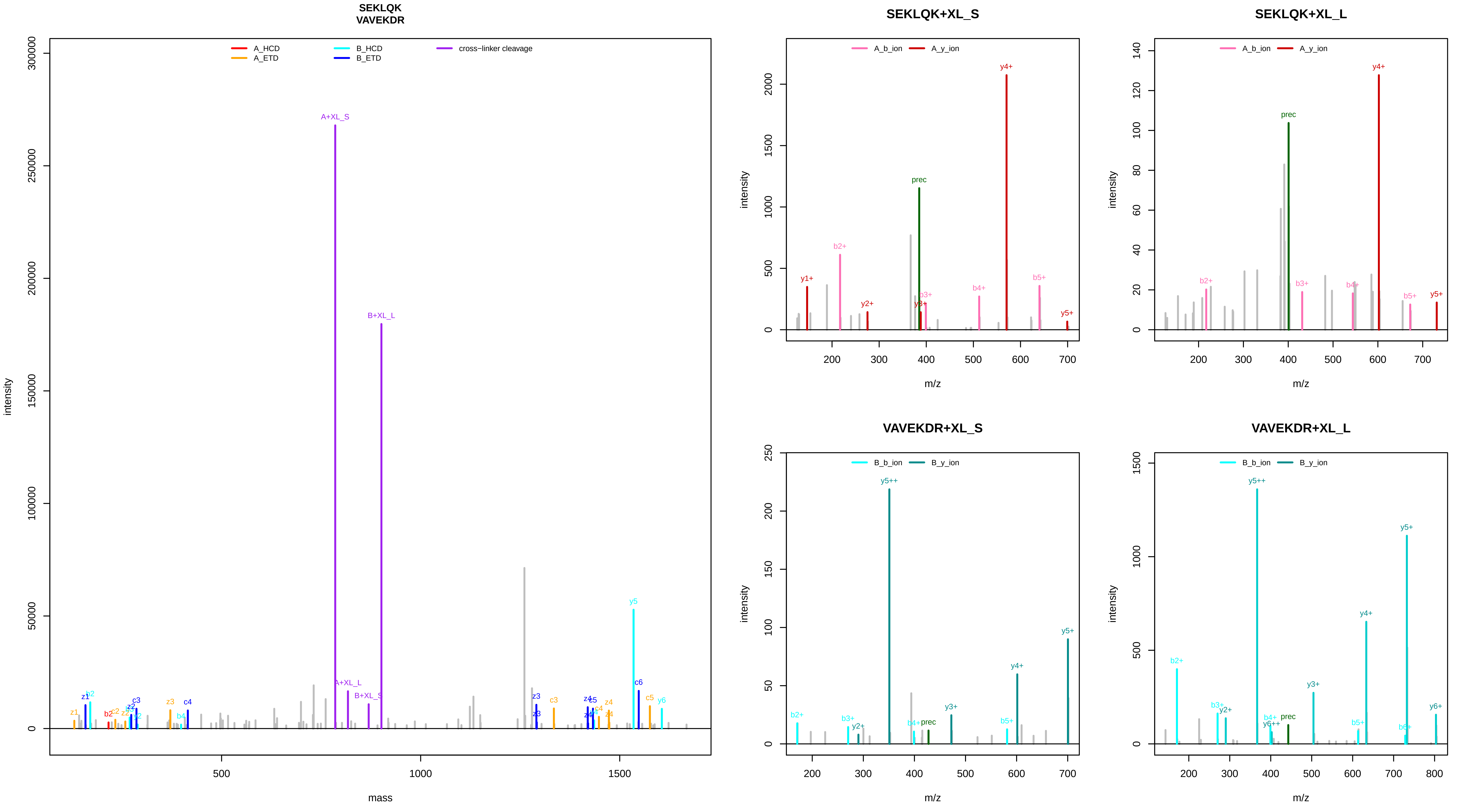

SEKLQK
VAVEKDR
A_HCD
A_ETD
B_HCD
B_ETD
cross−linker cleavage
A+XL_S
B+XL_L
A+XL_L
B+XL_S
y5
c6
c5
y6
intensity
mass
SEKLQK+XL_S
A_b_ion
A_y_ion
y4+
prec
b2+
y1+
y2+
b4+
b5+
y5+
m/z
SEKLQK+XL_L
b3+
VAVEKDR+XL_S
B_b_ion
B_y_ion
y5++
y3+
y4+
VAVEKDR+XL_L
b6+
y6+
y6++

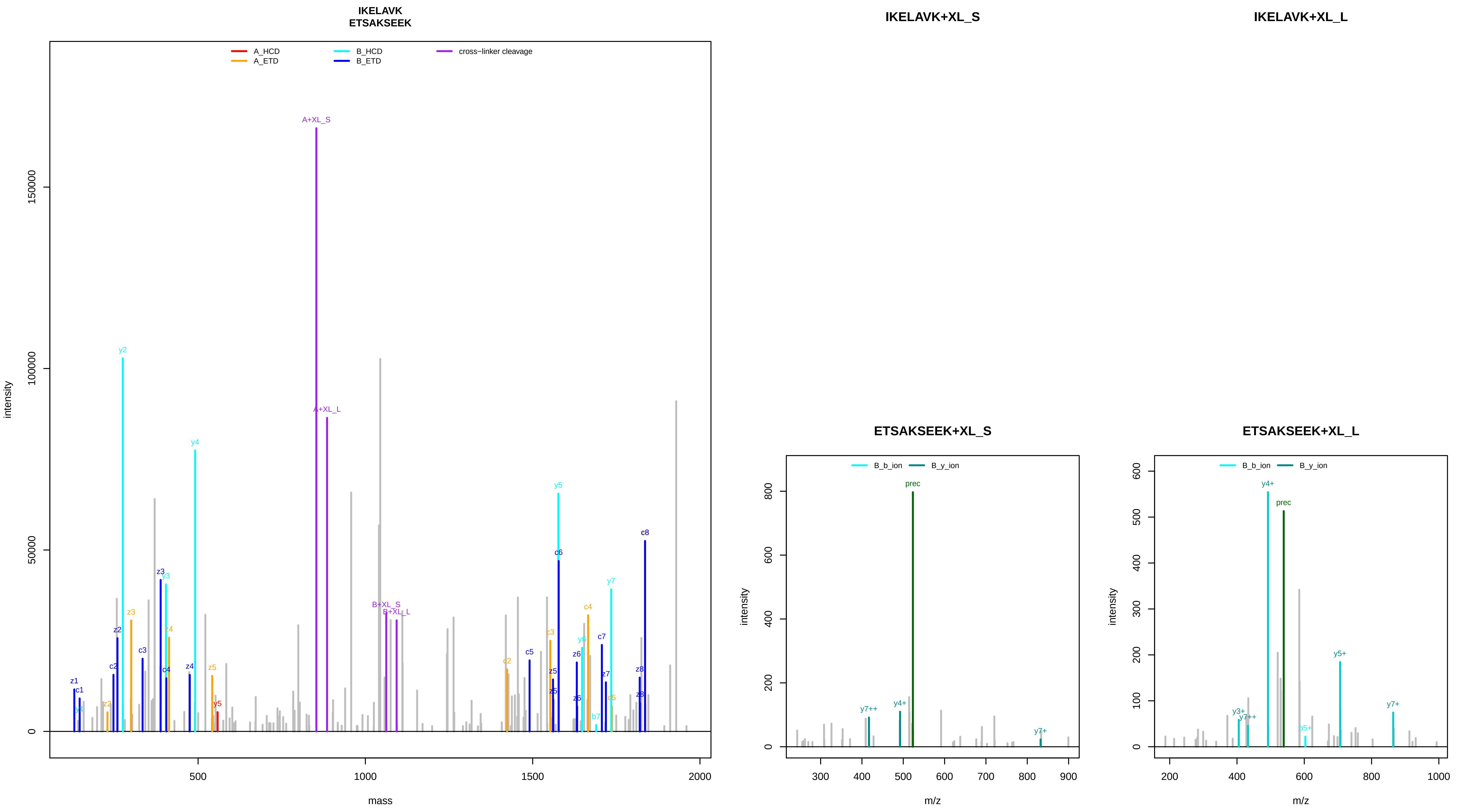
IKELAVK
ETSAKSEEK
A_HCD
A_ETD
B_HCD
B_ETD
cross−linker cleavage
A+XL_S
A+XL_L
B+XL_S
B+XL_L
intensity
mass
IKELAVK+XL_S
IKELAVK+XL_L
ETSAKSEEK+XL_S
ETSAKSEEK+XL_L
B_b_ion
B_y_ion
prec
m/z

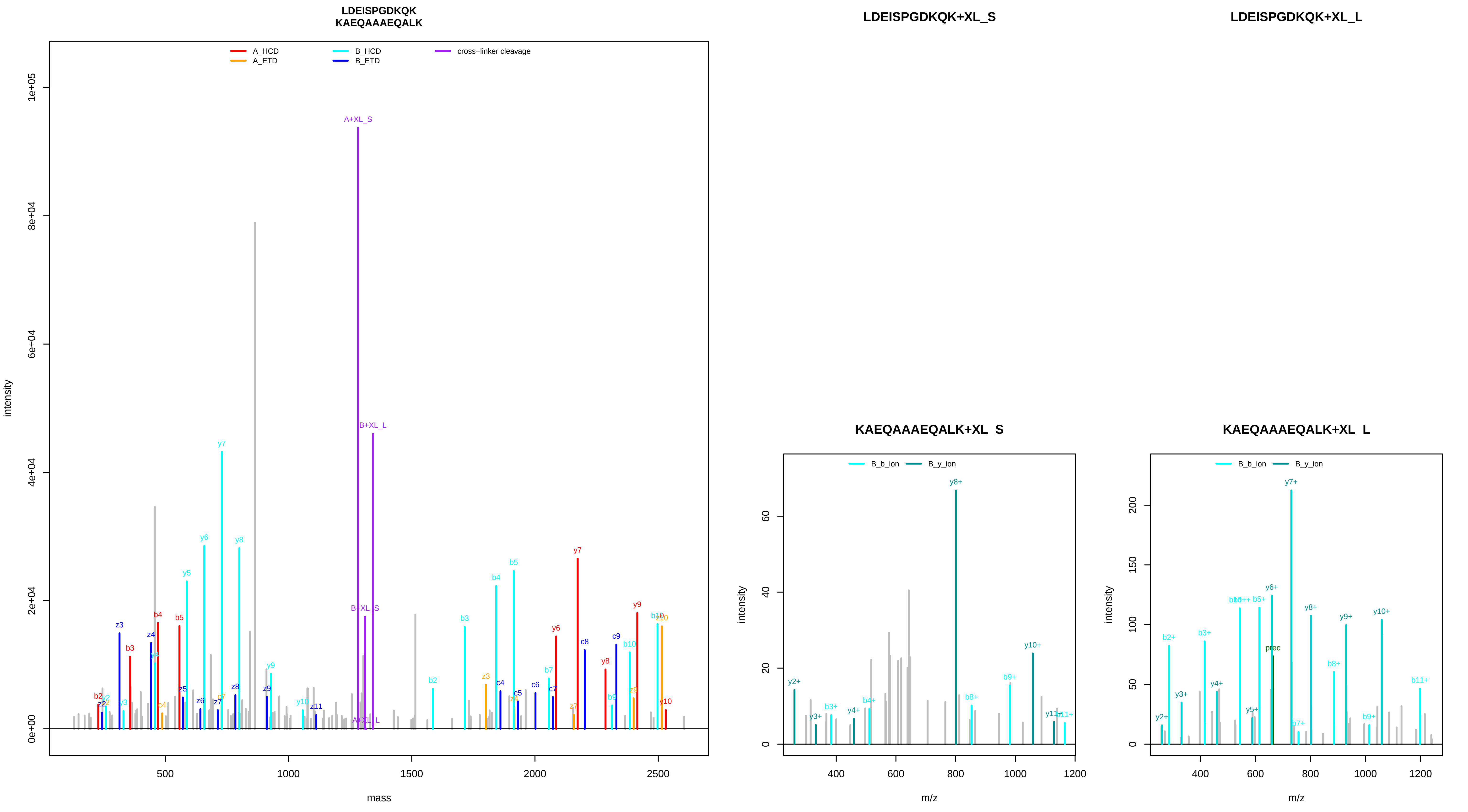

LDEISPGDKQK
KAEQAAAEQALK
A_HCD
A_ETD
B_HCD
B_ETD
cross−linker cleavage
A+XL_S
B+XL_L
B+XL_S
A+XL_L
intensity
mass
LDEISPGDKQK+XL_S
LDEISPGDKQK+XL_L
KAEQAAAEQALK+XL_S
KAEQAAAEQALK+XL_L
B_b_ion
B_y_ion
m/z

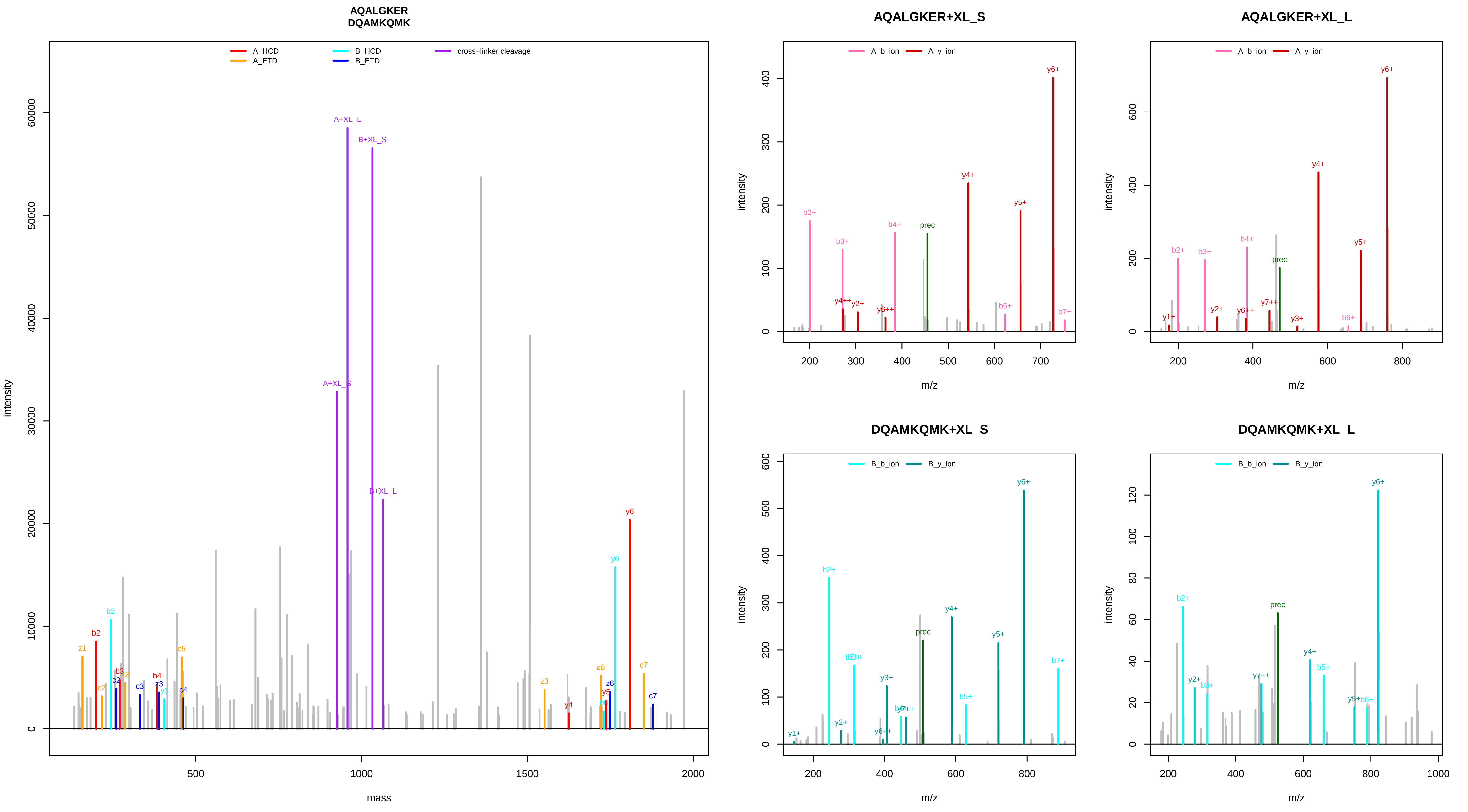
AQALGKER
DQAMKQMK
A_HCD
A_ETD
B_HCD
B_ETD
cross−linker cleavage
A+XL_L
B+XL_S
A+XL_S
B+XL_L
intensity
mass
AQALGKER+XL_S
AQALGKER+XL_L
DQAMKQMK+XL_S
DQAMKQMK+XL_L
A_b_ion
A_y_ion
B_b_ion
B_y_ion
prec
m/z

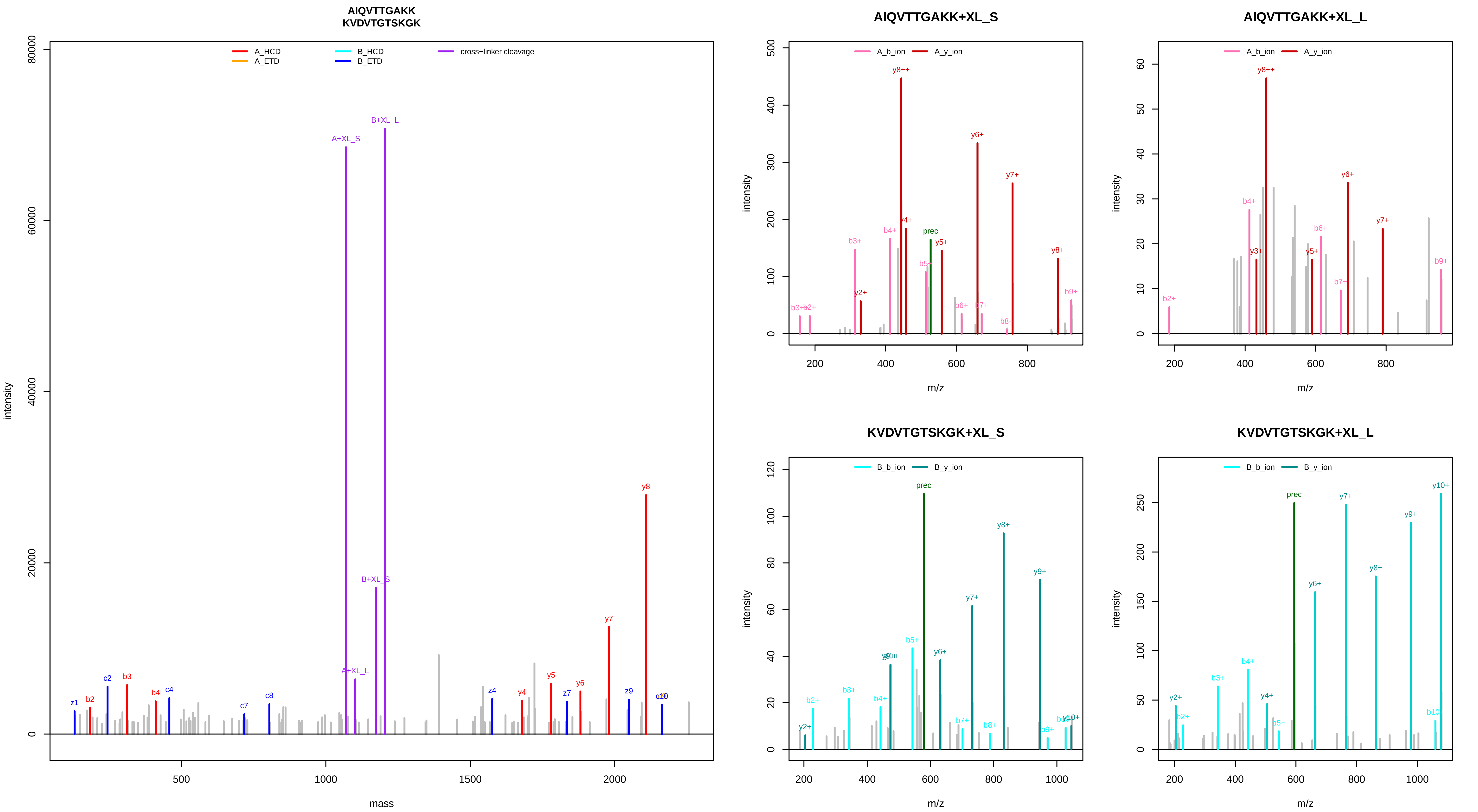

AIQVTTGAKK
KVDVTGTSKGK
A_HCD
A_ETD
B_HCD
B_ETD
cross−linker cleavage
A+XL_S
B+XL_L
B+XL_S
A+XL_L
z1
b2
c2
b3
b4
c4
c7
c8
z4
y4
y5
z7
y6
y7
z9
y8
c10
intensity
mass
AIQVTTGAKK+XL_S
AIQVTTGAKK+XL_L
KVDVTGTSKGK+XL_S
KVDVTGTSKGK+XL_L
A_b_ion
A_y_ion
B_b_ion
B_y_ion
prec
m/z

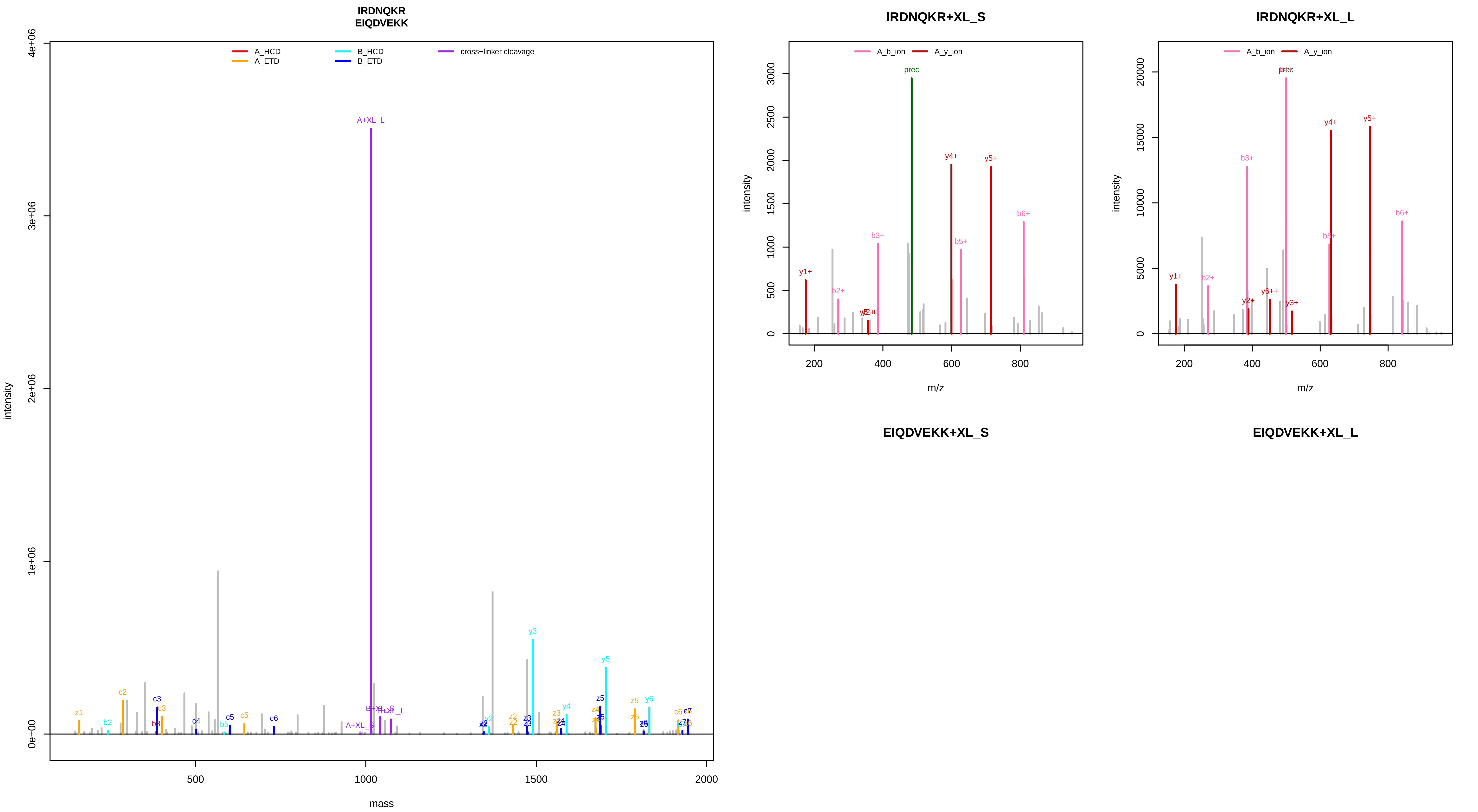
IRDNQKR
EIQDVEKK
A_HCD
A_ETD
B_HCD
B_ETD
cross−linker cleavage
A+XL_L
intensity
mass
IRDNQKR+XL_S
IRDNQKR+XL_L
A_b_ion
A_y_ion
prec
m/z
EIQDVEKK+XL_S
EIQDVEKK+XL_L

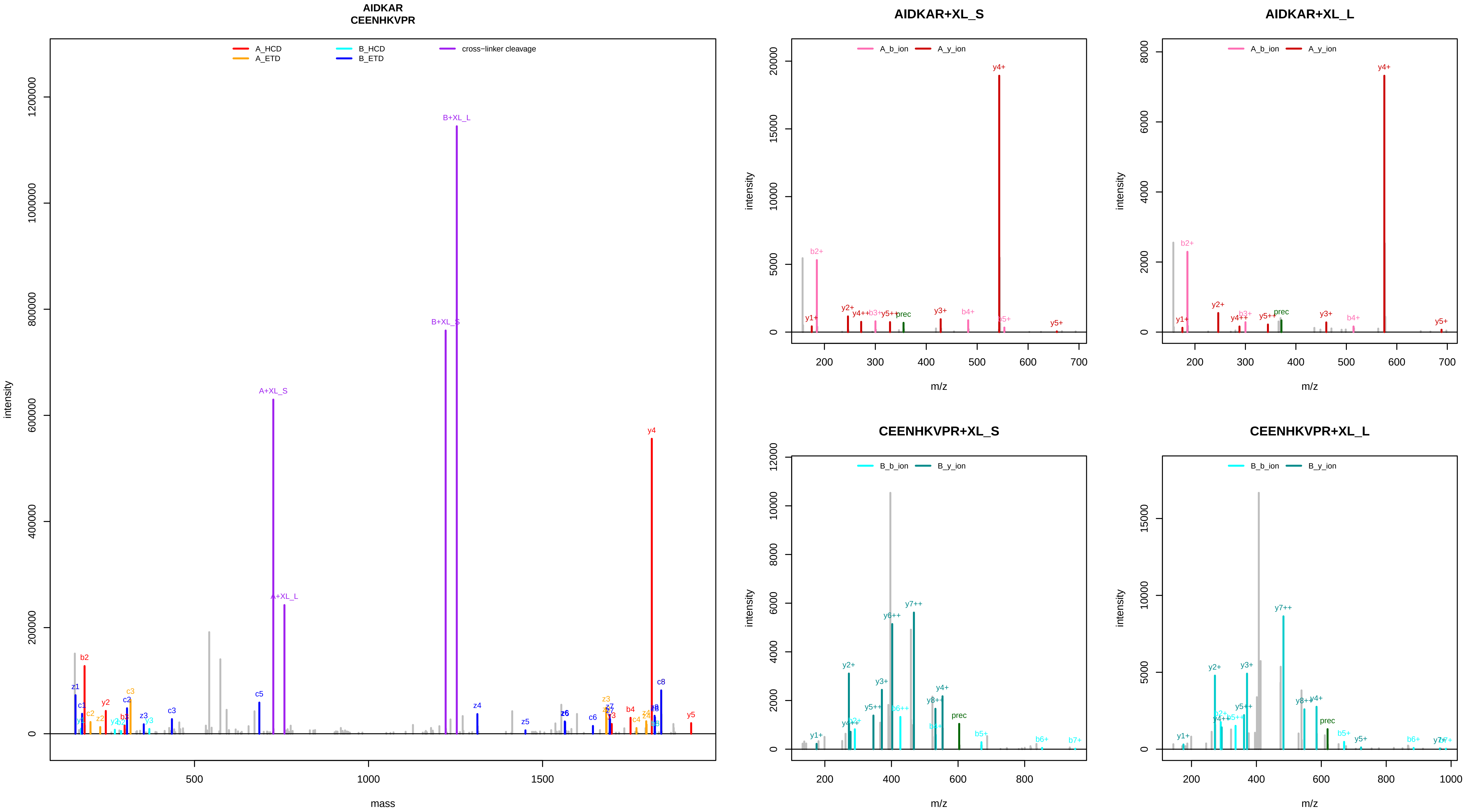
AIDKAR
CEENHKVPR
A_HCD
A_ETD
B_HCD
B_ETD
cross−linker cleavage
B+XL_L
B+XL_S
A+XL_S
A+XL_L
intensity
mass
AIDKAR+XL_S
AIDKAR+XL_L
A_b_ion
A_y_ion
CEENHKVPR+XL_S
CEENHKVPR+XL_L
B_b_ion
B_y_ion
prec
m/z

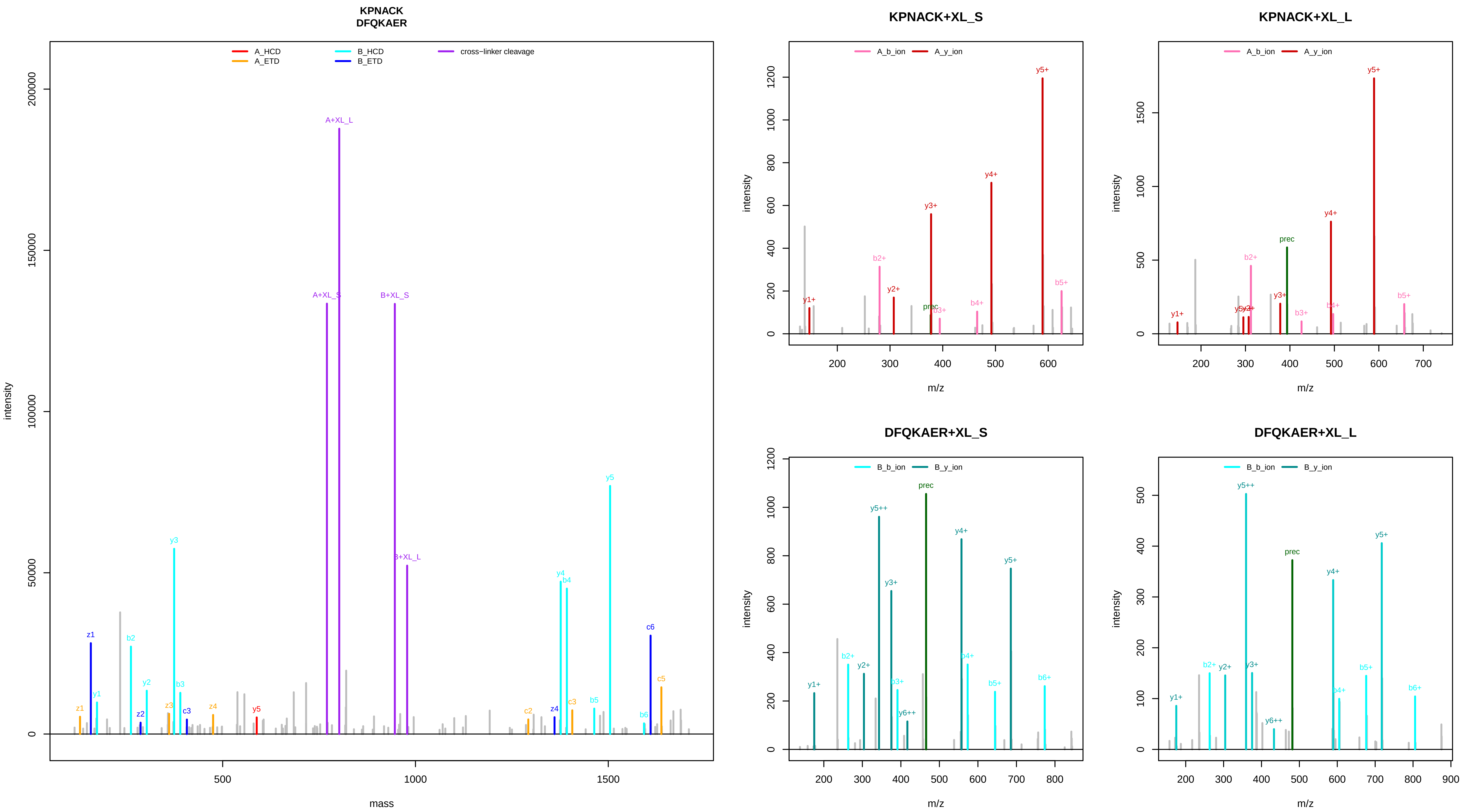
KPNACK
DFQKAER
A_HCD
A_ETD
B_HCD
B_ETD
cross−linker cleavage
A+XL_L
A+XL_S
B+XL_S
B+XL_L
z1
y1
b2
y2
z2
y3
b3
z3
c3
z4
y5
y4
b4
c2
c3
b5
y5
b6
c6
c5
intensity
mass
0
50000
100000
150000
200000
500
1000
1500
KPNACK+XL_S
A_b_ion
A_y_ion
y1+
b2+
y2+
y3+
prec
b3+
b4+
y4+
y5+
b5+
intensity
m/z
KPNACK+XL_L
A_b_ion
A_y_ion
y1+
y5y2+
b2+
y3+
b3+
prec
y4+
b4+
y5+
b5+
intensity
m/z
DFQKAER+XL_S
B_b_ion
B_y_ion
y1+
b2+
y2+
y5++
y3+
b3+
y6++
prec
y4+
b4+
b5+
y5+
b6+
intensity
m/z
DFQKAER+XL_L
B_b_ion
B_y_ion
y1+
b2+
y2+
y5++
y3+
y6++
prec
y4+
b4+
b5+
y5+
b6+
intensity
m/z

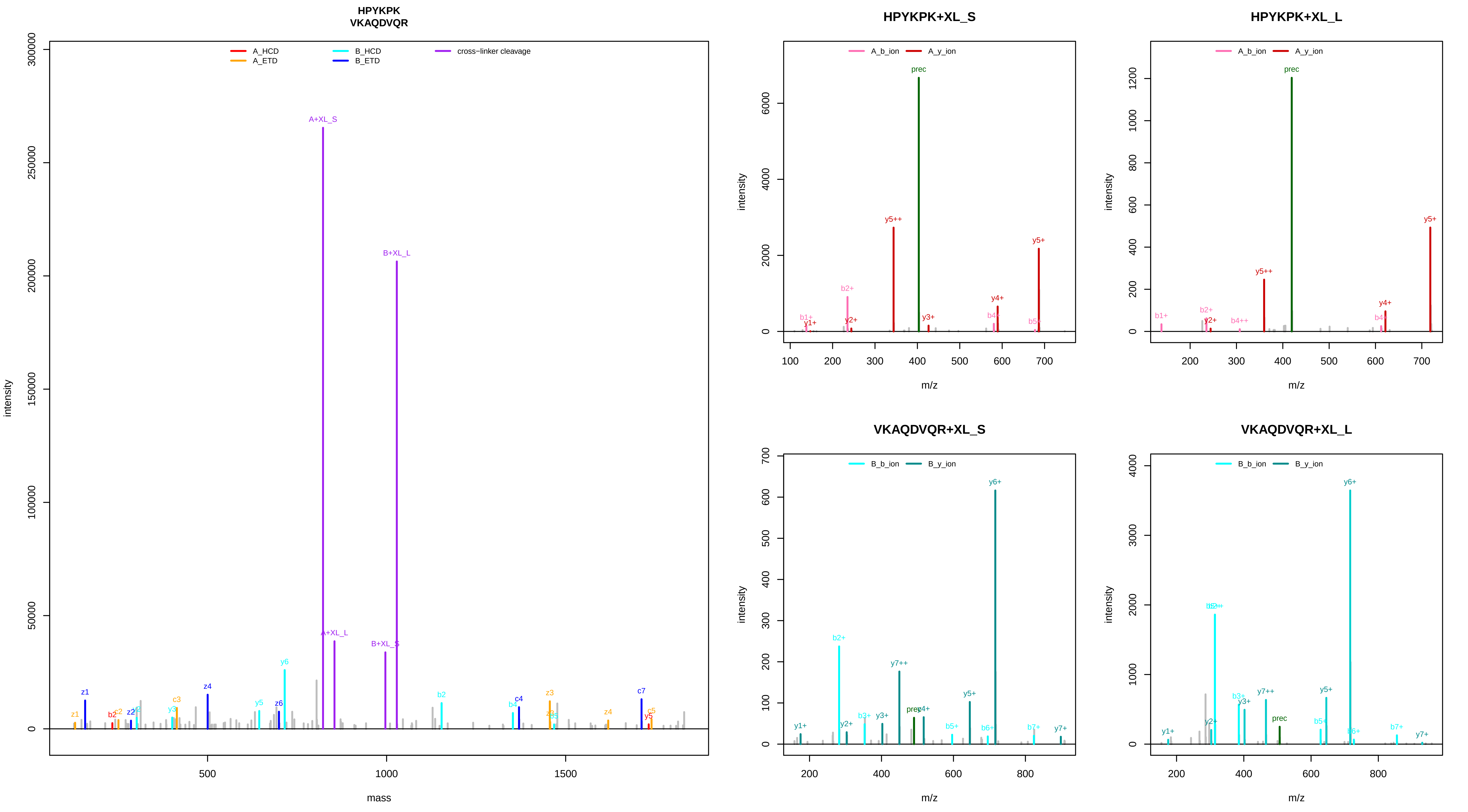
HPYKPK
VKAQDVQR
A_HCD
A_ETD
B_HCD
B_ETD
cross−linker cleavage
A+XL_S
B+XL_L
A+XL_L
B+XL_S
intensity
mass
HPYKPK+XL_S
HPYKPK+XL_L
VKAQDVQR+XL_S
VKAQDVQR+XL_L
A_b_ion
A_y_ion
B_b_ion
B_y_ion
prec
m/z

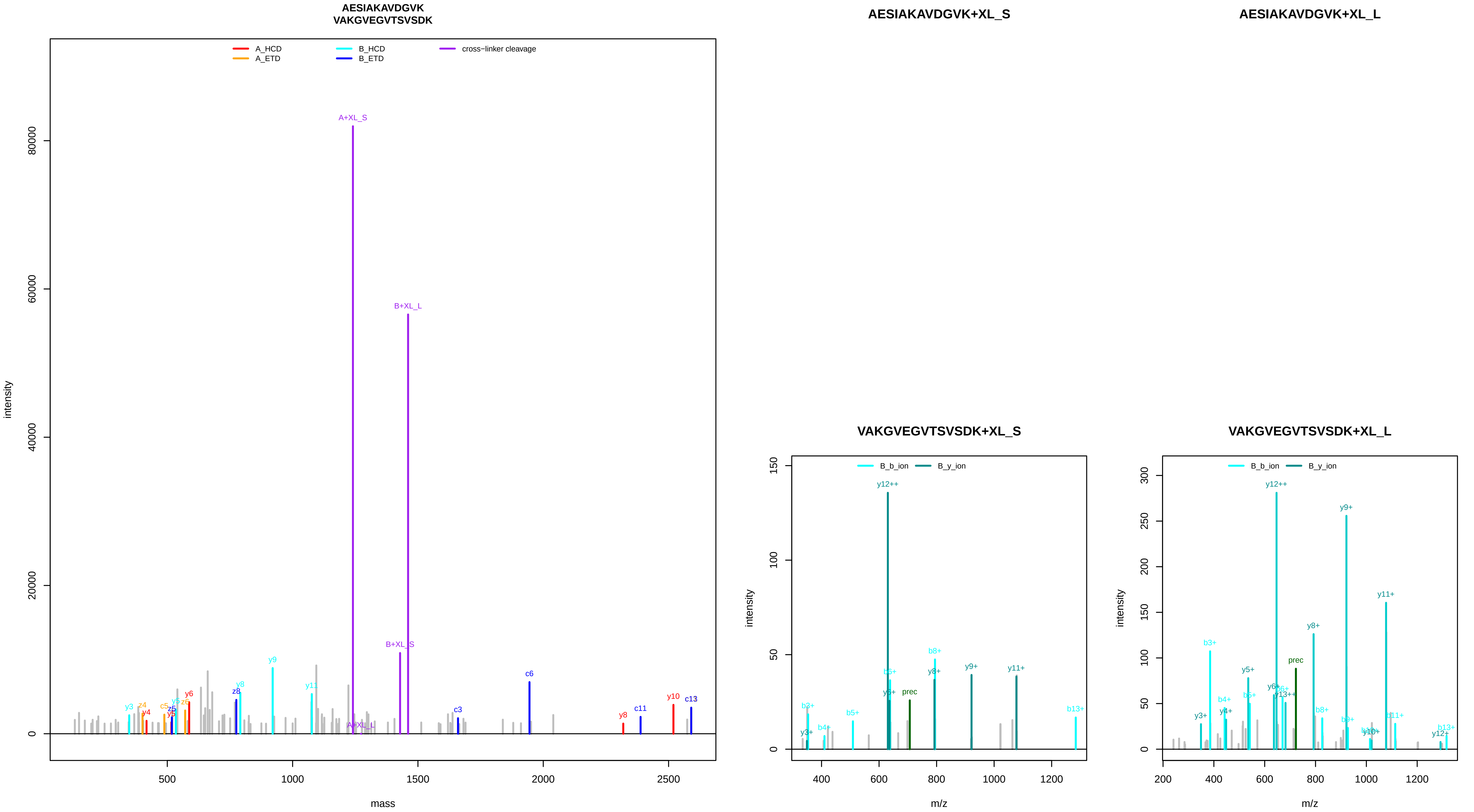
AESIAKAVDGVK
VAKGVEGVTSVSDK
A_HCD
A_ETD
B_HCD
B_ETD
cross−linker cleavage
A+XL_S
B+XL_L
B+XL_S
A+XL_L
intensity
mass
AESIAKAVDGVK+XL_S
AESIAKAVDGVK+XL_L
VAKGVEGVTSVSDK+XL_S
VAKGVEGVTSVSDK+XL_L
B_b_ion
B_y_ion
y12++
y9+
y11+
y8+
b8+
b3+
prec
b13+
m/z

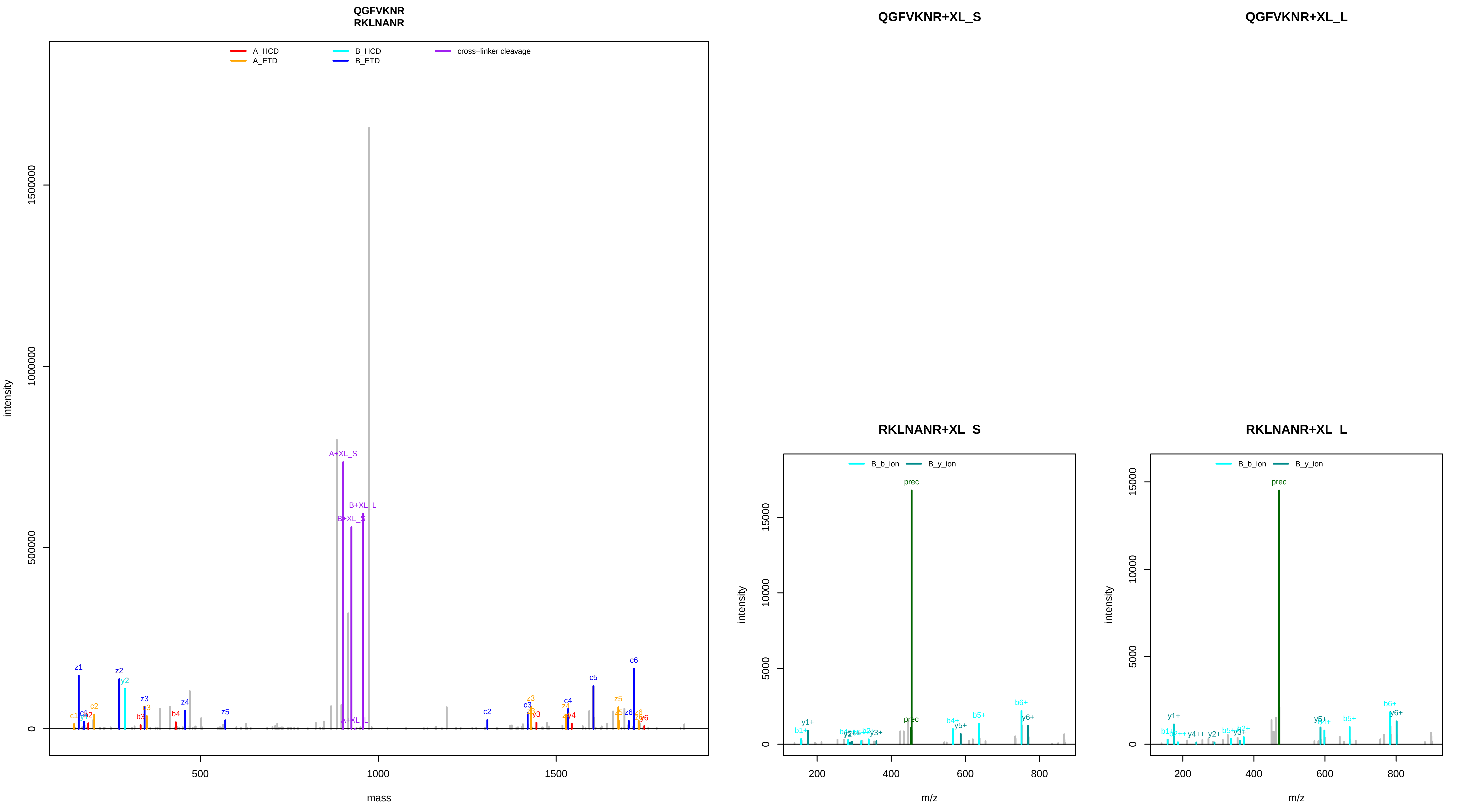

QGFVKNR
RKLNANR
A_HCD
A_ETD
B_HCD
B_ETD
cross−linker cleavage
A+XL_S
B+XL_L
B+XL_S
A+XL_L
z1
z2
y2
c2
z3
b3
b4
z4
z5
c2
c3
z3
y3
c4
z4
y4
c5
z5
z6
c6
y6
intensity
mass
0
500000
1000000
1500000
500
1000
1500
QGFVKNR+XL_S
QGFVKNR+XL_L
RKLNANR+XL_S
RKLNANR+XL_L
B_b_ion
B_y_ion
prec
y1+
b1+
y3+
b4+
y5+
b5+
b6+
y6+
b2++
y4++
y2+
b5+
y5+
intensity
m/z
0
5000
10000
15000
200
400
600
800

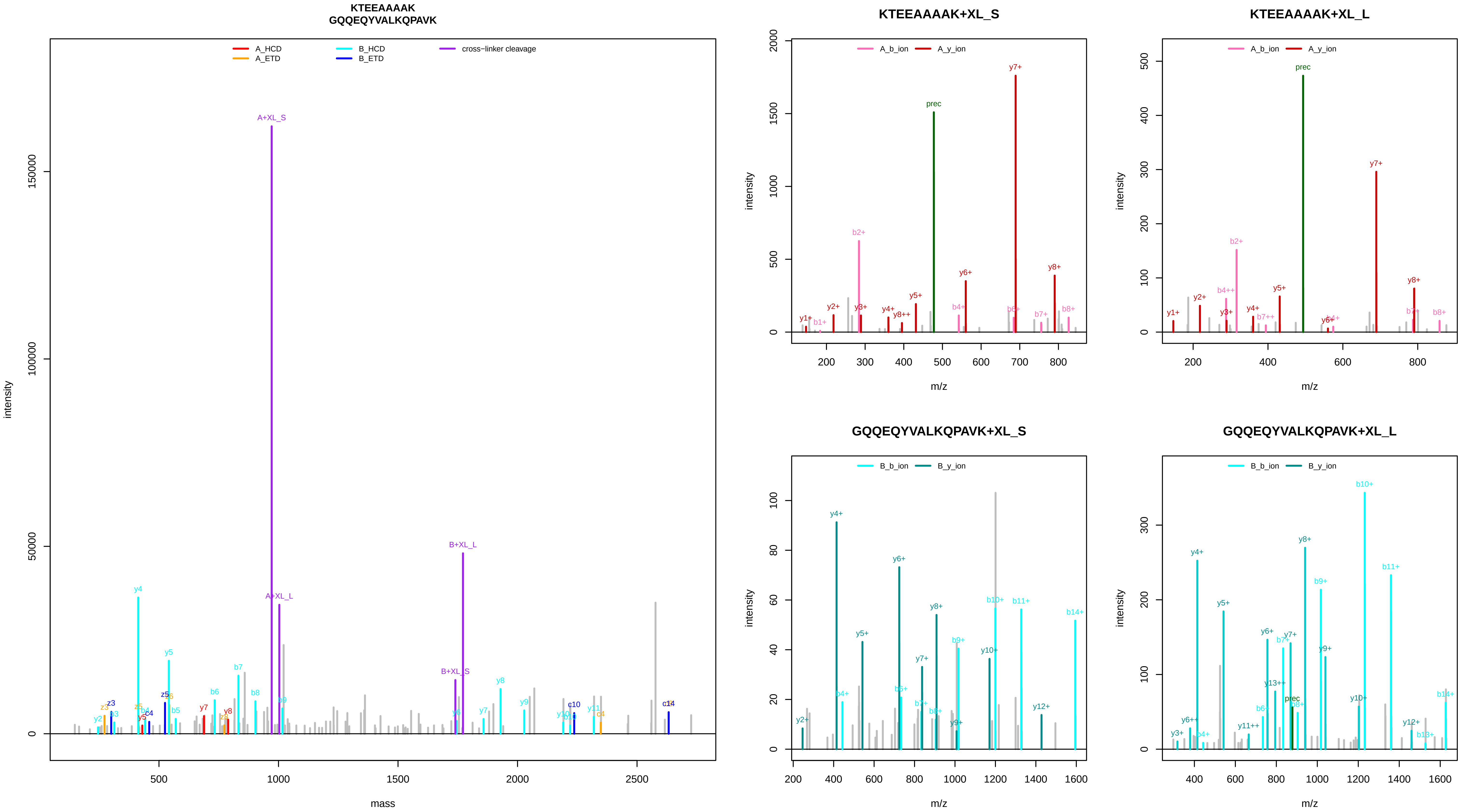
KTEEAAAAK
GQQEQYVALKQPAVK
A_HCD
A_ETD
B_HCD
B_ETD
cross−linker cleavage
A+XL_S
A+XL_L
B+XL_L
B+XL_S
intensity
mass
KTEEAAAAK+XL_S
KTEEAAAAK+XL_L
A_b_ion
A_y_ion
prec
GQQEQYVALKQPAVK+XL_S
GQQEQYVALKQPAVK+XL_L
B_b_ion
B_y_ion
m/z

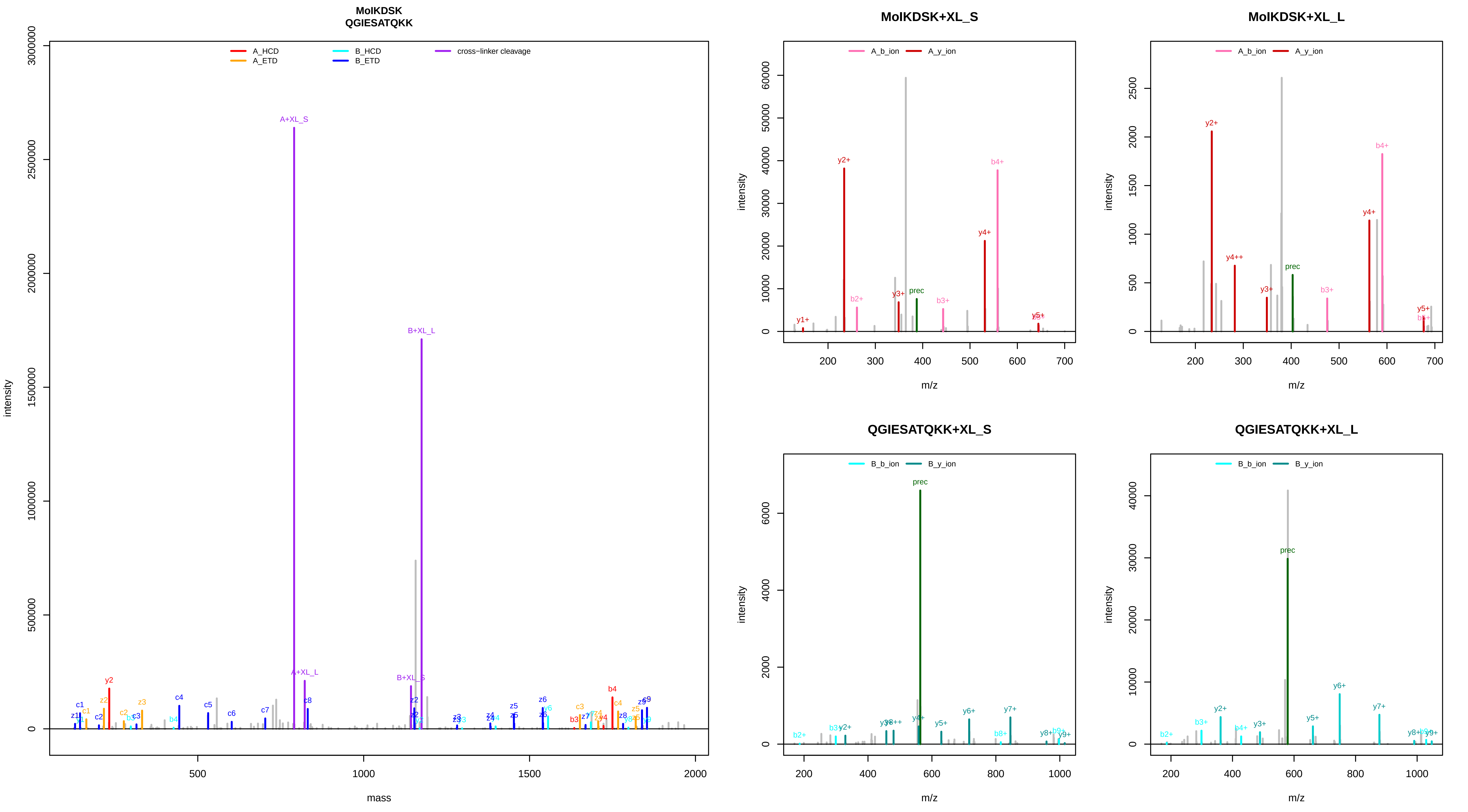

MoIKDSK
QGIESATQKK
A_HCD
A_ETD
B_HCD
B_ETD
cross−linker cleavage
A+XL_S
B+XL_L
A+XL_L
B+XL_S
intensity
mass
MoIKDSK+XL_S
MoIKDSK+XL_L
A_b_ion
A_y_ion
QGIESATQKK+XL_S
QGIESATQKK+XL_L
B_b_ion
B_y_ion
prec
intensity
m/z

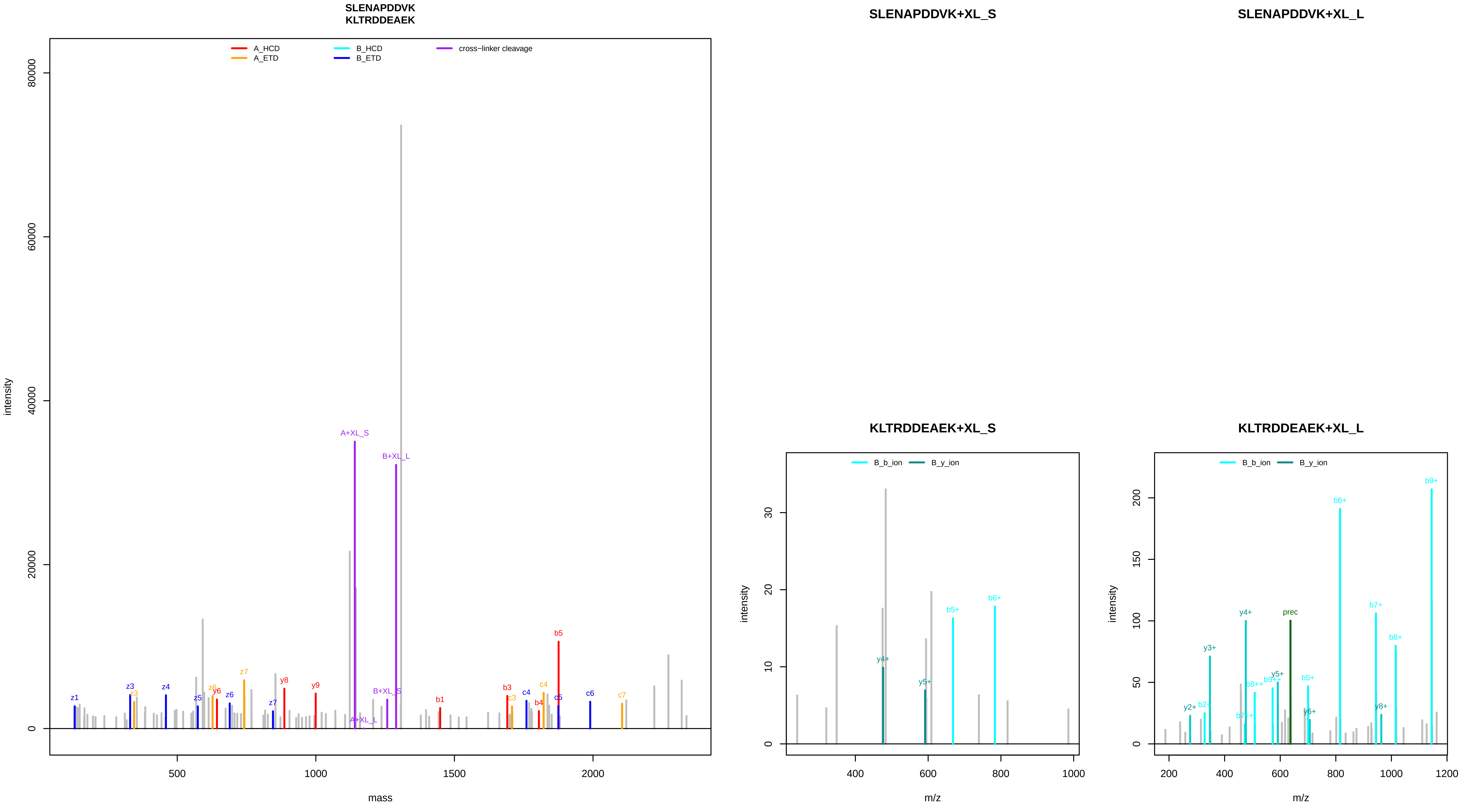
SLENAPDDVK
KLTRDDEAEK
A_HCD
A_ETD
B_HCD
B_ETD
cross−linker cleavage
intensity
mass
0
20000
40000
60000
80000
500
1000
1500
2000
A+XL_S
B+XL_L
B+XL_S
A+XL_L
z1
z3
z4
z5
z6
z7
y6
y8
y9
b1
b3
b4
b5
c3
c4
c5
c6
c7
SLENAPDDVK+XL_S
SLENAPDDVK+XL_L
KLTRDDEAEK+XL_S
B_b_ion
B_y_ion
intensity
m/z
0
10
20
30
400
600
800
1000
y4+
y5+
b5+
b6+
KLTRDDEAEK+XL_L
B_b_ion
B_y_ion
intensity
m/z
0
50
100
150
200
200
400
600
800
1000
1200
y2+
b2+
y3+
y4+
prec
b7++
b8++
b9++
y5+
b5+
y6+
b6+
b7+
b8+
y8+
b9+

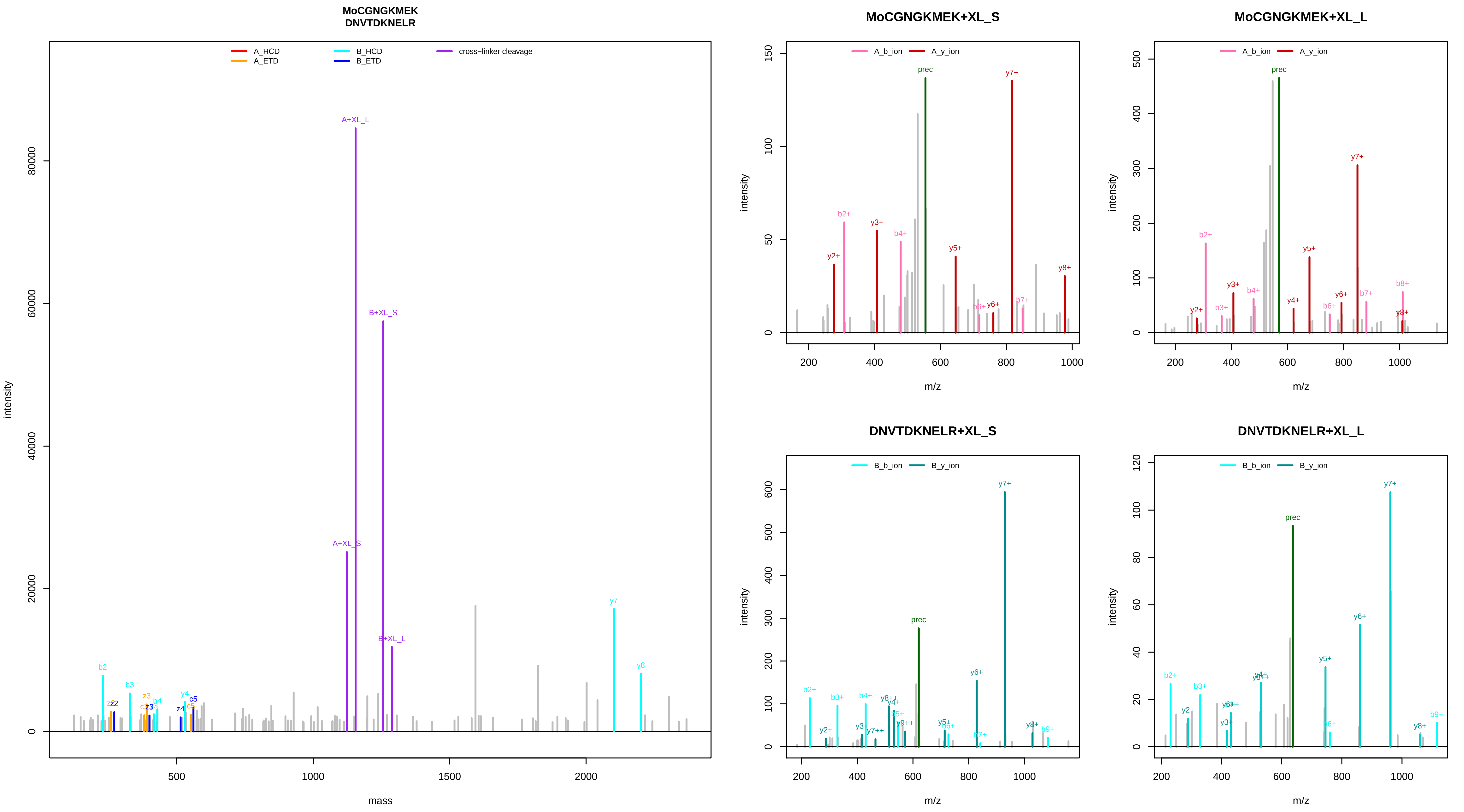

MoCGNGKMEK
DNVTDKNELR
A_HCD
A_ETD
B_HCD
B_ETD
cross−linker cleavage
A+XL_L
B+XL_S
A+XL_S
B+XL_L
b2
b3
b4
z3
z2
c3
z4
y4
c5
y7
y8
intensity
mass
0
20000
40000
60000
80000
500
1000
1500
2000
MoCGNGKMEK+XL_S
MoCGNGKMEK+XL_L
A_b_ion
A_y_ion
prec
b2+
y2+
y3+
b4+
y5+
y6+
y7+
y8+
b6+
b7+
b3+
y4+
b8+
DNVTDKNELR+XL_S
DNVTDKNELR+XL_L
B_b_ion
B_y_ion
b2+
b3+
b4+
y2+
y3+
y4+
y5+
y6+
y7+
y8+
b9+
y8++
y7++
y9++
y6++
m/z
intensity

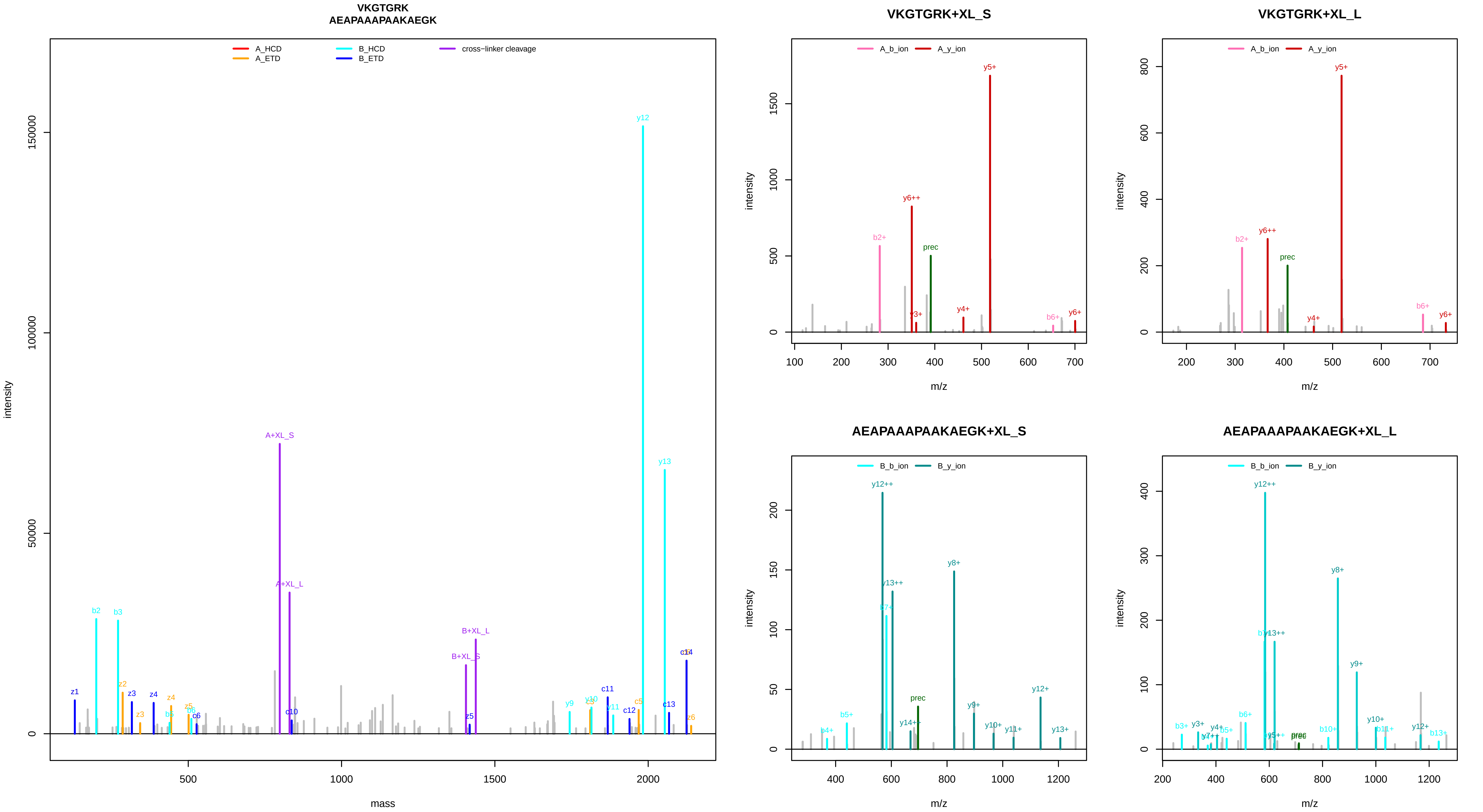

VKGTGRK
AEAPAAAPAAKAEGK
A_HCD
A_ETD
B_HCD
B_ETD
cross−linker cleavage
intensity
mass
A+XL_S
A+XL_L
B+XL_L
B+XL_S
y12
y13
VKGTGRK+XL_S
VKGTGRK+XL_L
A_b_ion
A_y_ion
AEAPAAAPAAKAEGK+XL_S
AEAPAAAPAAKAEGK+XL_L
B_b_ion
B_y_ion
m/z
prec

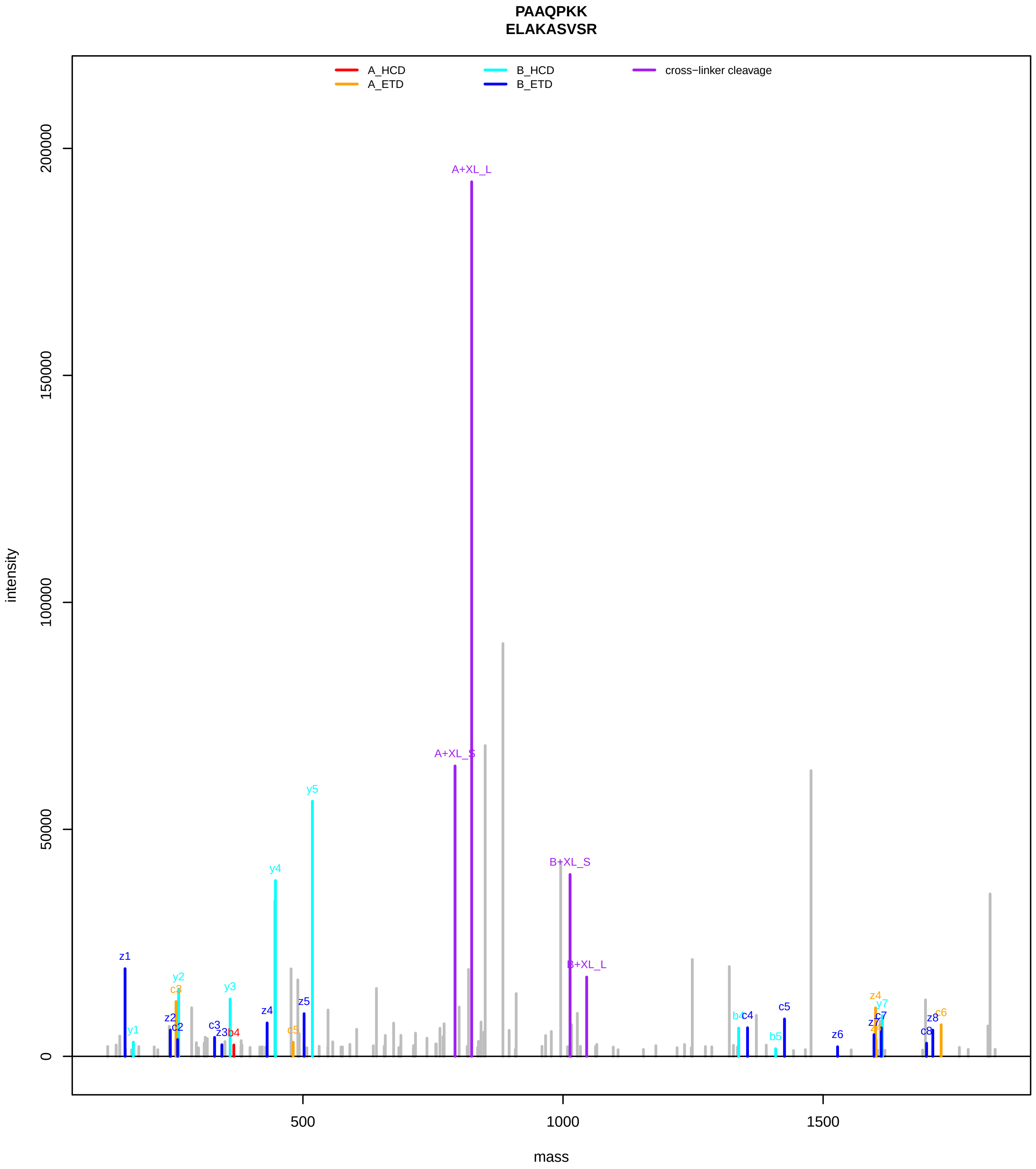

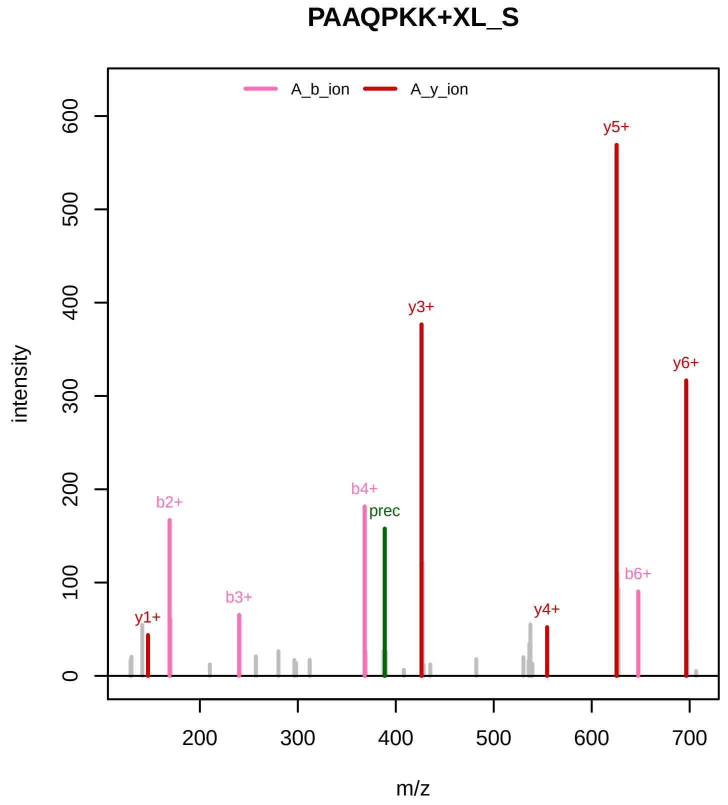

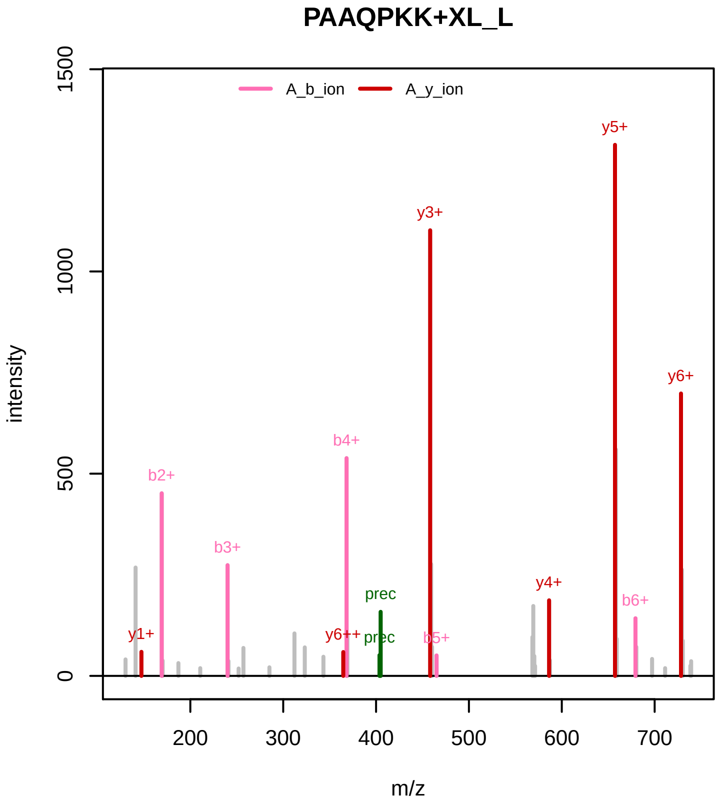

ELAKASVSR+XL_S

ELAKASVSR+XL_L

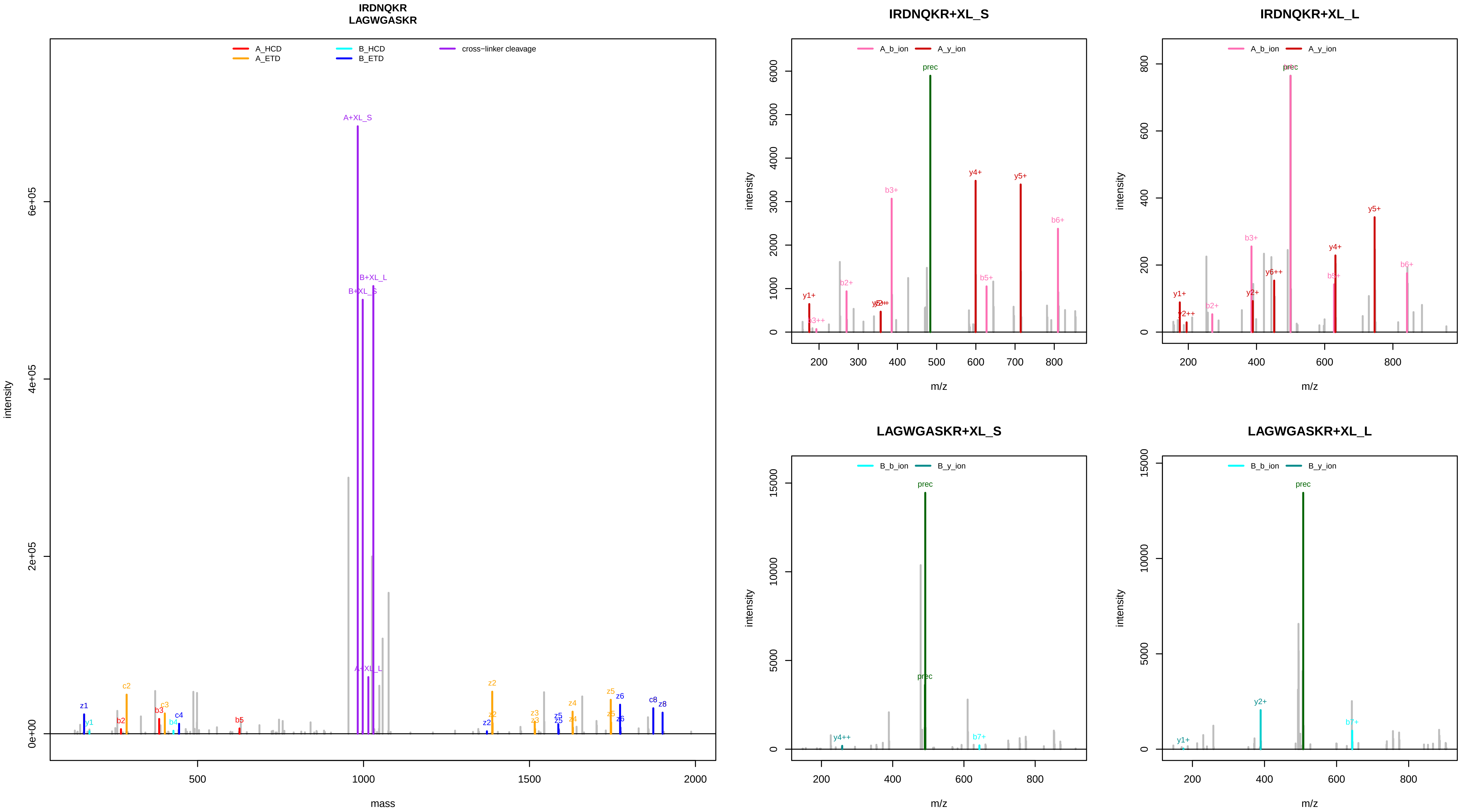
IRDNQKR
LAGWGASKR
A_HCD
A_ETD
B_HCD
B_ETD
cross−linker cleavage
A+XL_S
B+XL_L
B+XL_S
A+XL_L
intensity
mass
IRDNQKR+XL_S
IRDNQKR+XL_L
LAGWGASKR+XL_S
LAGWGASKR+XL_L
A_b_ion
A_y_ion
B_b_ion
B_y_ion
prec
m/z
intensity

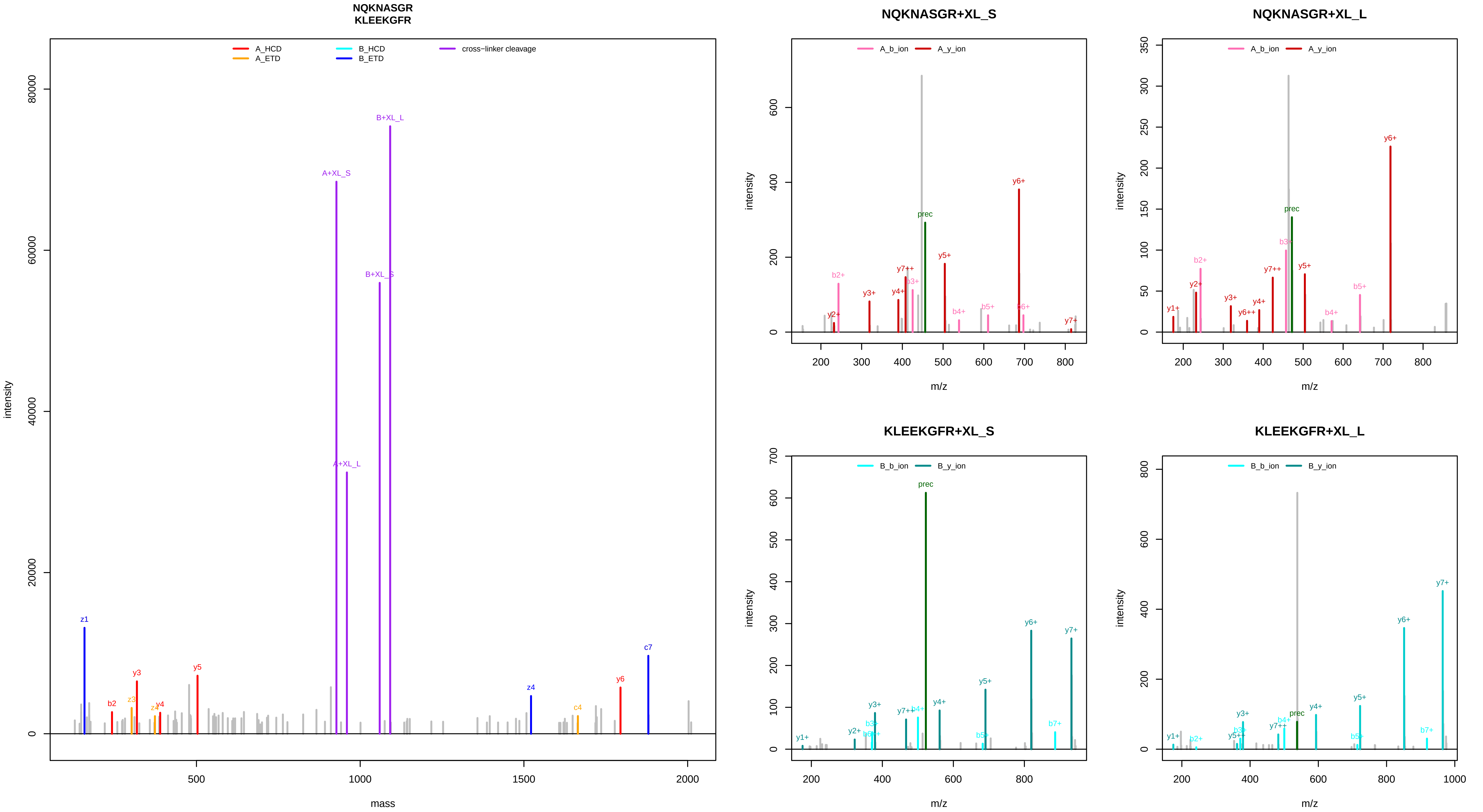
NQKNASGR
KLEEKGFR
A_HCD
A_ETD
B_HCD
B_ETD
cross−linker cleavage
B+XL_L
A+XL_S
B+XL_S
A+XL_L
z1
b2
y3
z3
z4
y4
y5
z4
c4
y6
c7
intensity
mass
NQKNASGR+XL_S
NQKNASGR+XL_L
KLEEKGFR+XL_S
KLEEKGFR+XL_L
A_b_ion
A_y_ion
B_b_ion
B_y_ion
prec
m/z

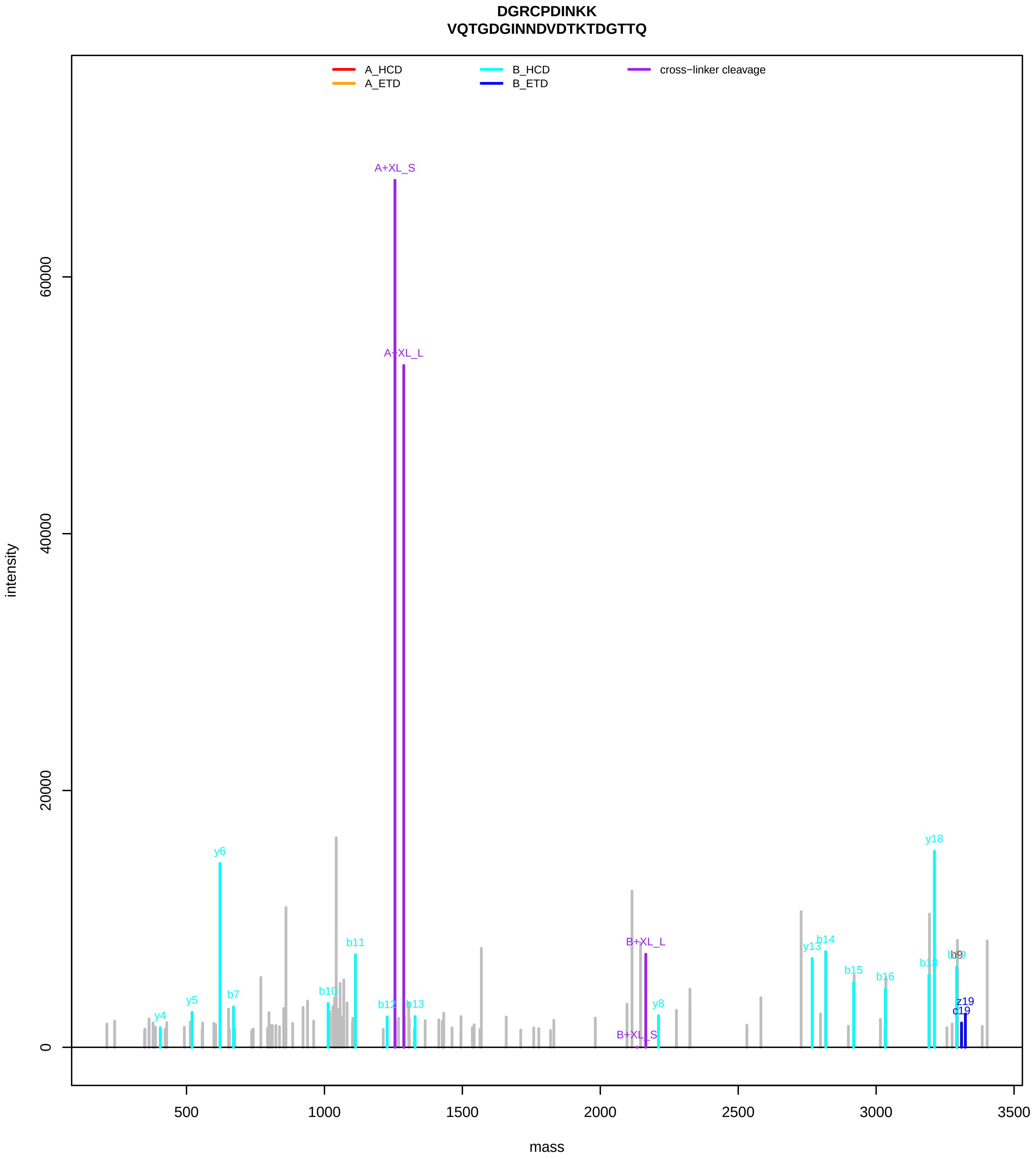

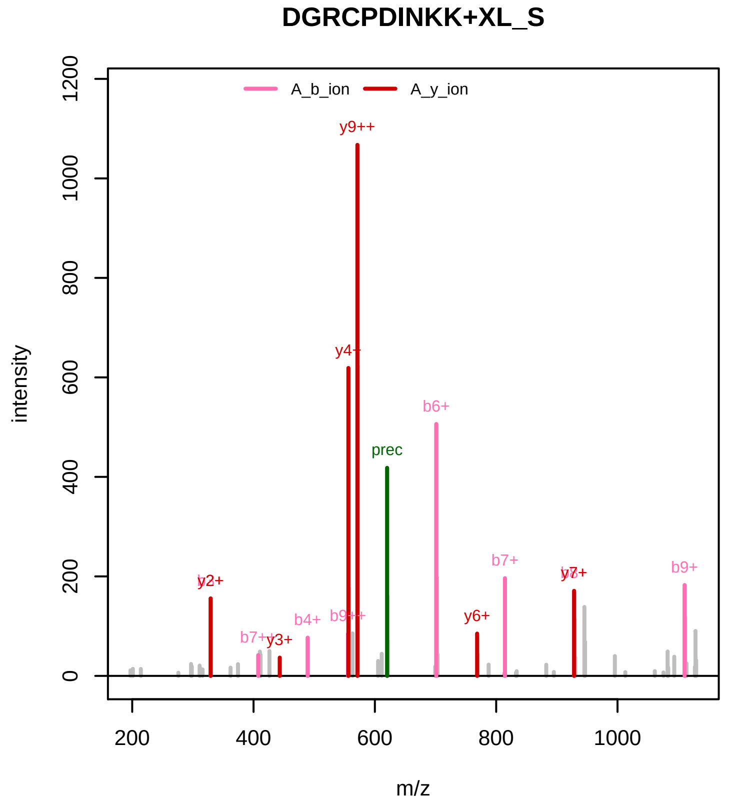

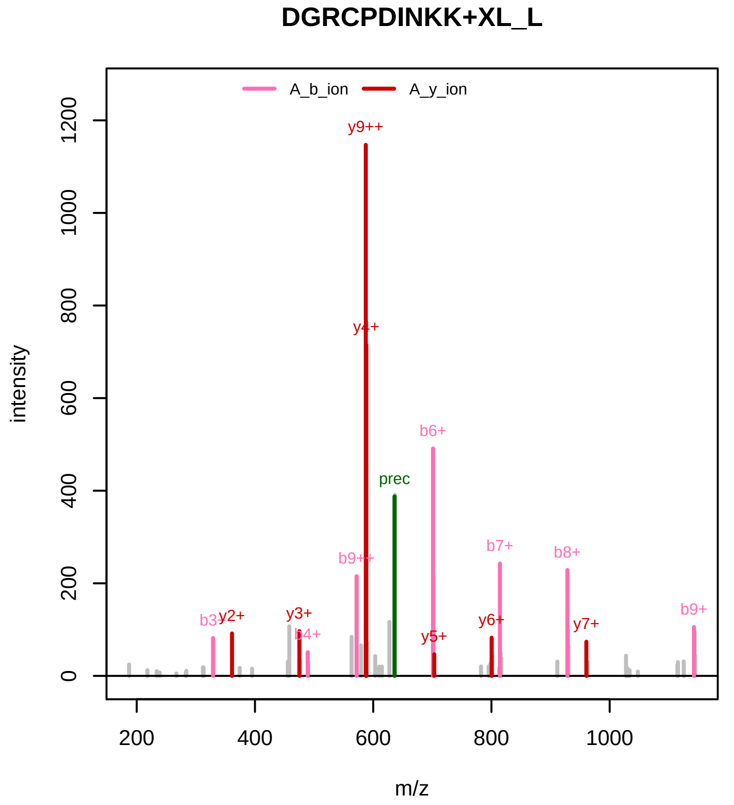

VQTGDGINNDVDTKTDGTTQ+XL_S

VQTGDGINNDVDTKTDGTTQ+XL_L

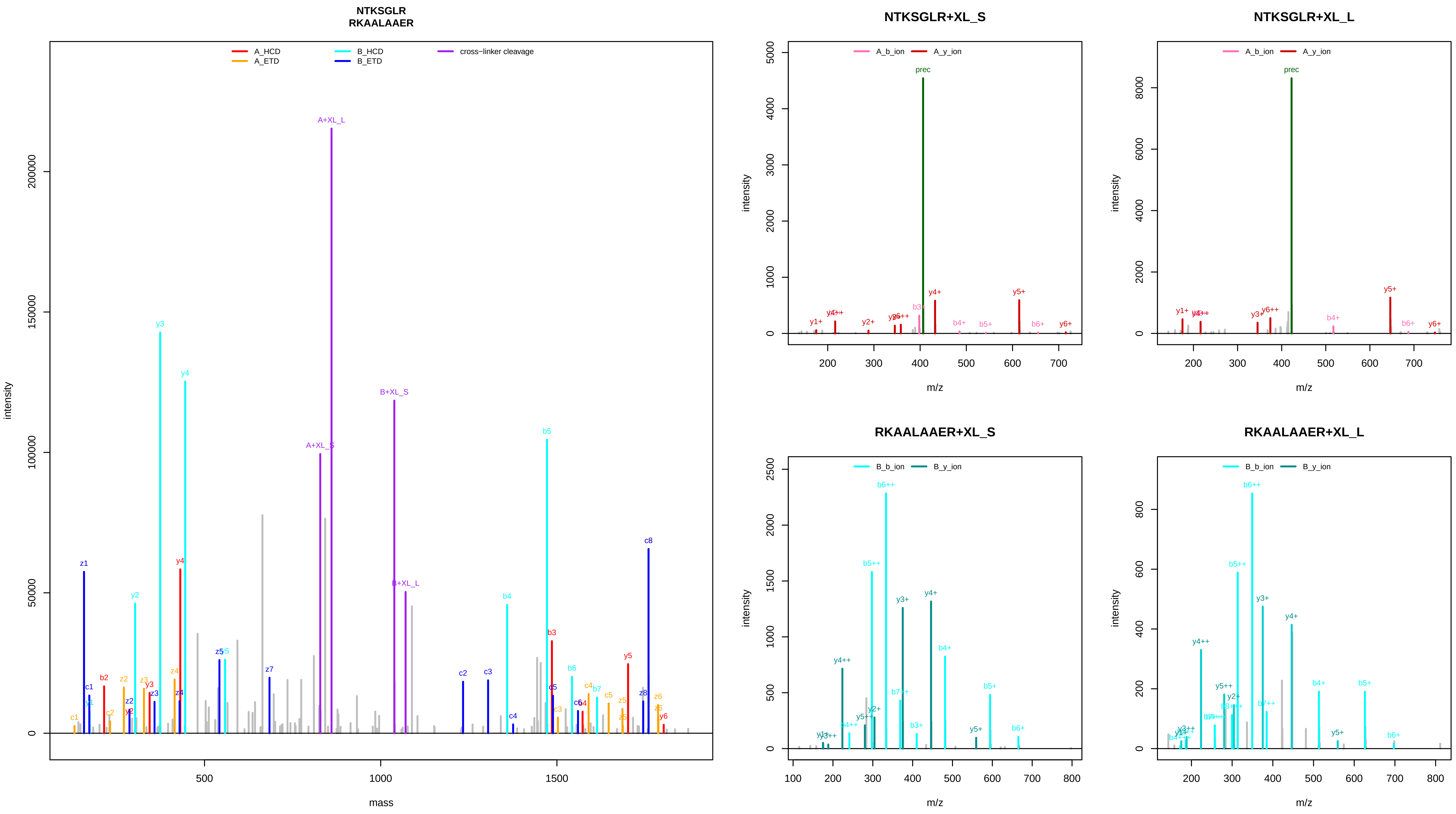

NTKSGLR
RKAALAAER
A_HCD
A_ETD
B_HCD
B_ETD
cross−linker cleavage
A+XL_L
B+XL_S
A+XL_S
B+XL_L
intensity
mass
NTKSGLR+XL_S
NTKSGLR+XL_L
RKAALAAER+XL_S
RKAALAAER+XL_L
A_b_ion
A_y_ion
B_b_ion
B_y_ion
prec
m/z
intensity

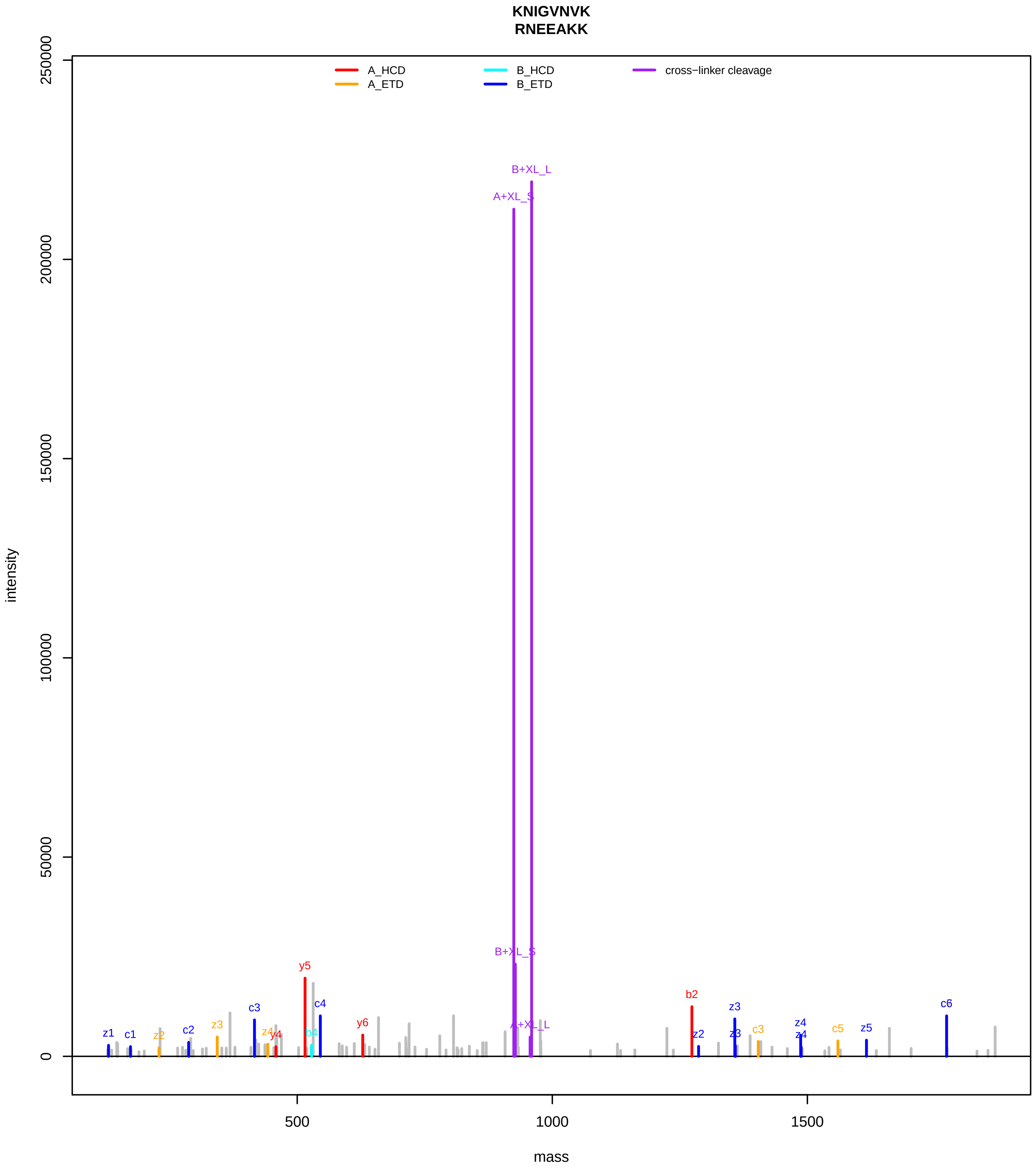

KNIGVNVK+XL_S

KNIGVNVK+XL_L

RNEEAKK+XL_S

RNEEAKK+XL_L

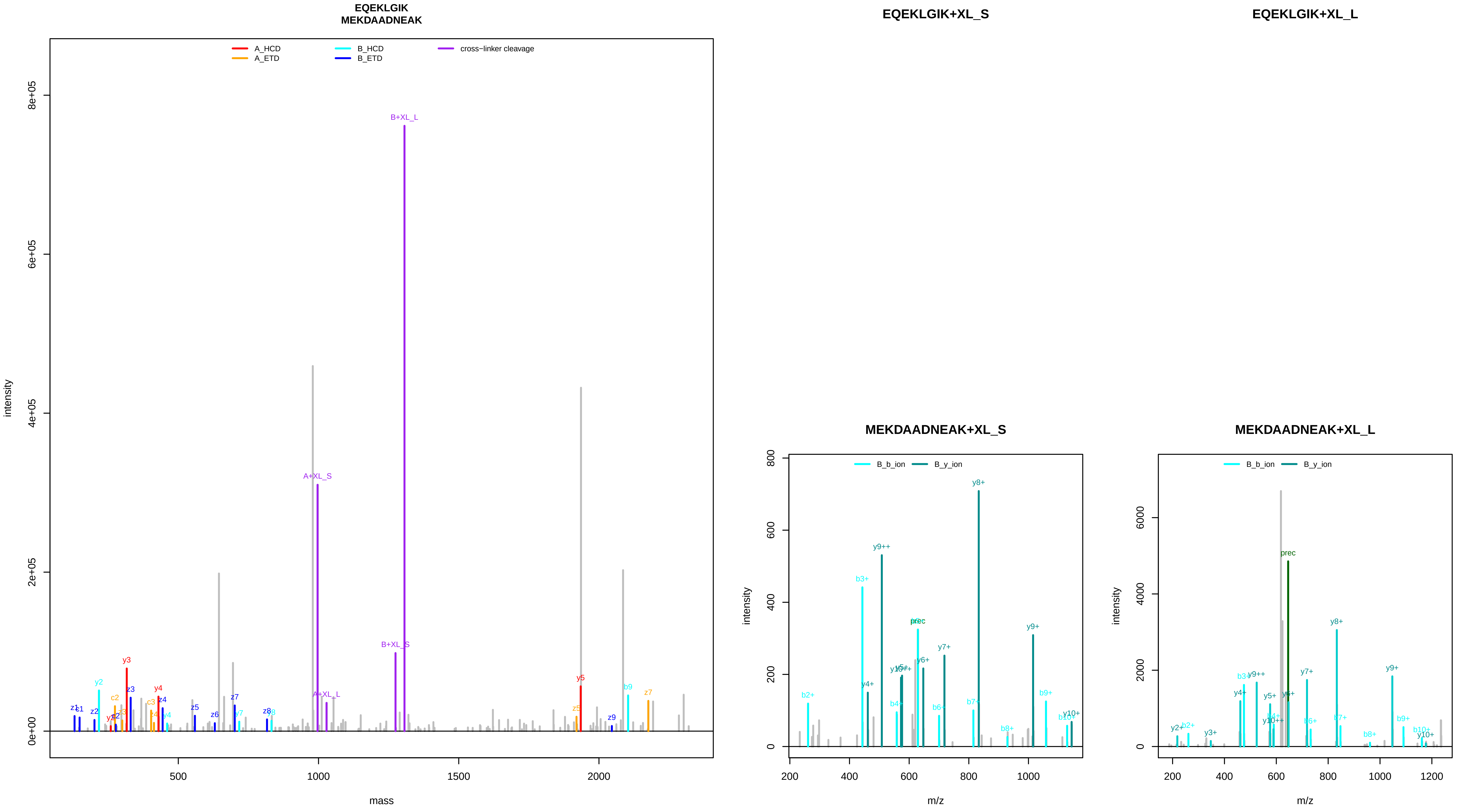
EQEKLGIK
MEKDAADNEAK
A_HCD
A_ETD
B_HCD
B_ETD
cross−linker cleavage
B+XL_L
A+XL_S
B+XL_S
A+XL_L
intensity
mass
0e+00
2e+05
4e+05
6e+05
8e+05
500
1000
1500
2000
EQEKLGIK+XL_S
EQEKLGIK+XL_L
MEKDAADNEAK+XL_S
MEKDAADNEAK+XL_L
B_b_ion
B_y_ion
prec
m/z

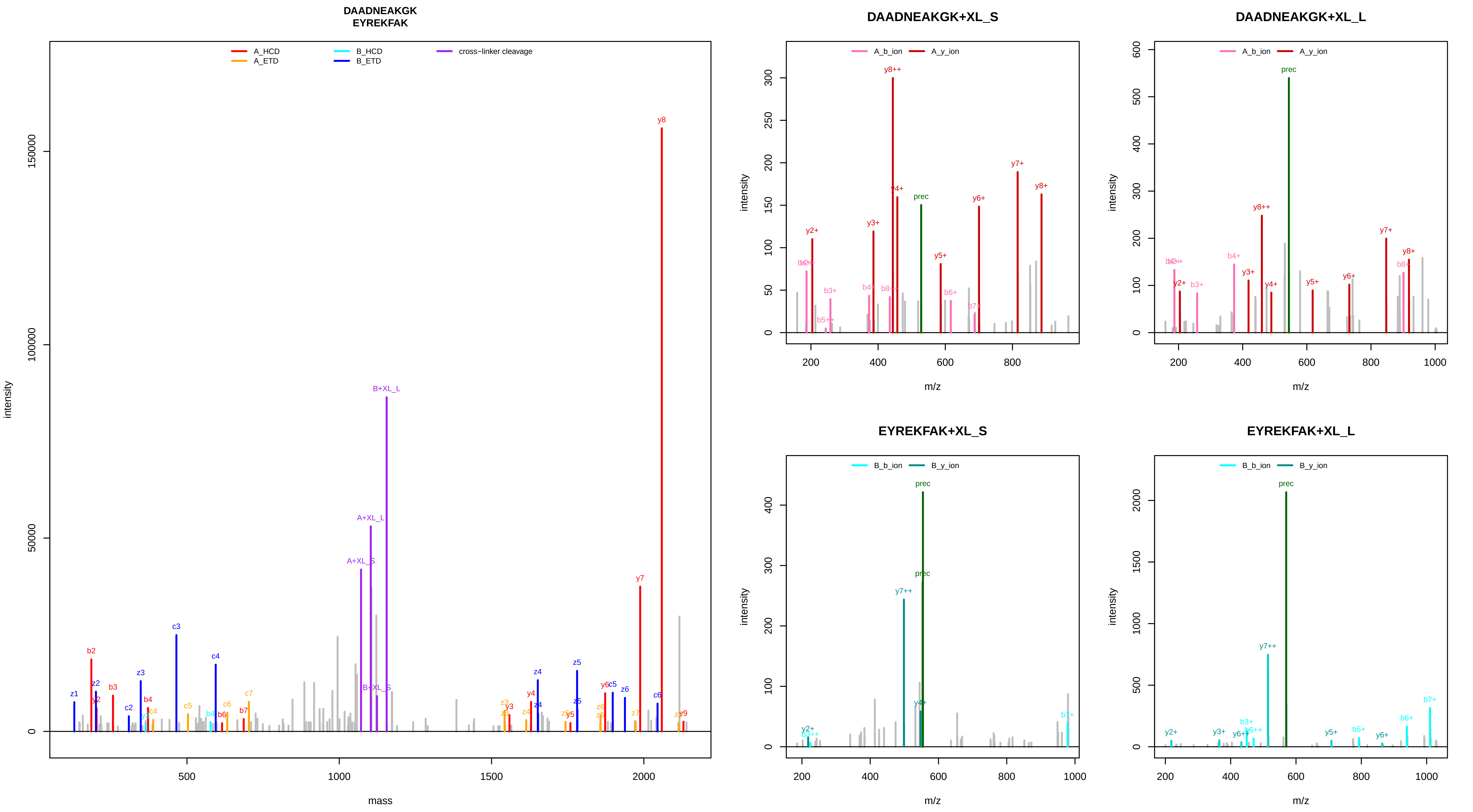
DAADNEAKGK
EYREKFAK
A_HCD
A_ETD
B_HCD
B_ETD
cross−linker cleavage
B+XL_L
A+XL_L
A+XL_S
B+XL_S
y8
y7
intensity
mass
DAADNEAKGK+XL_S
DAADNEAKGK+XL_L
EYREKFAK+XL_S
EYREKFAK+XL_L
A_b_ion
A_y_ion
B_b_ion
B_y_ion
prec
m/z
intensity

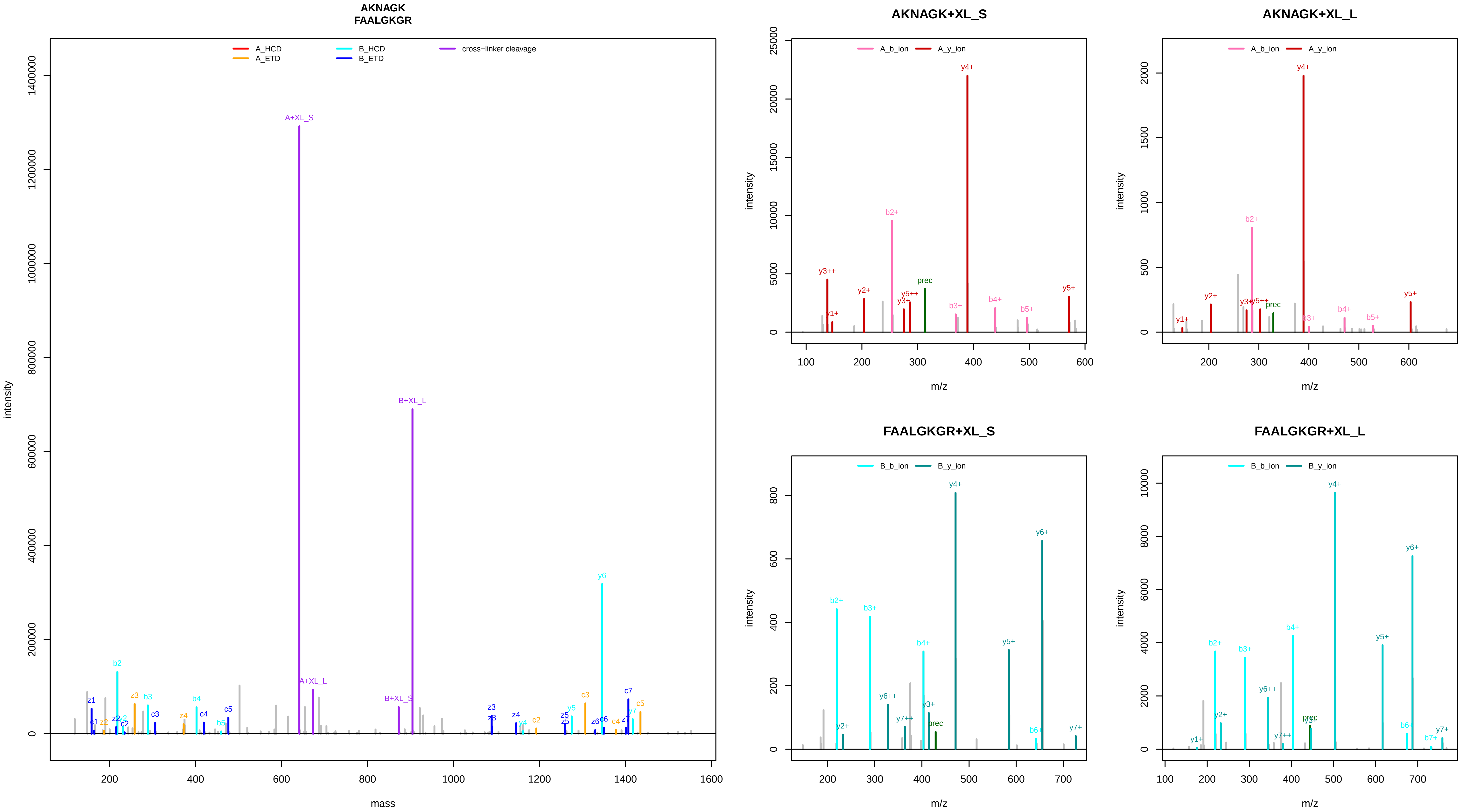
AKNAGK
FAALGKGR
A_HCD
A_ETD
B_HCD
B_ETD
cross−linker cleavage
A+XL_S
B+XL_L
A+XL_L
B+XL_S
intensity
mass
AKNAGK+XL_S
AKNAGK+XL_L
A_b_ion
A_y_ion
FAALGKGR+XL_S
FAALGKGR+XL_L
B_b_ion
B_y_ion
prec
m/z
intensity

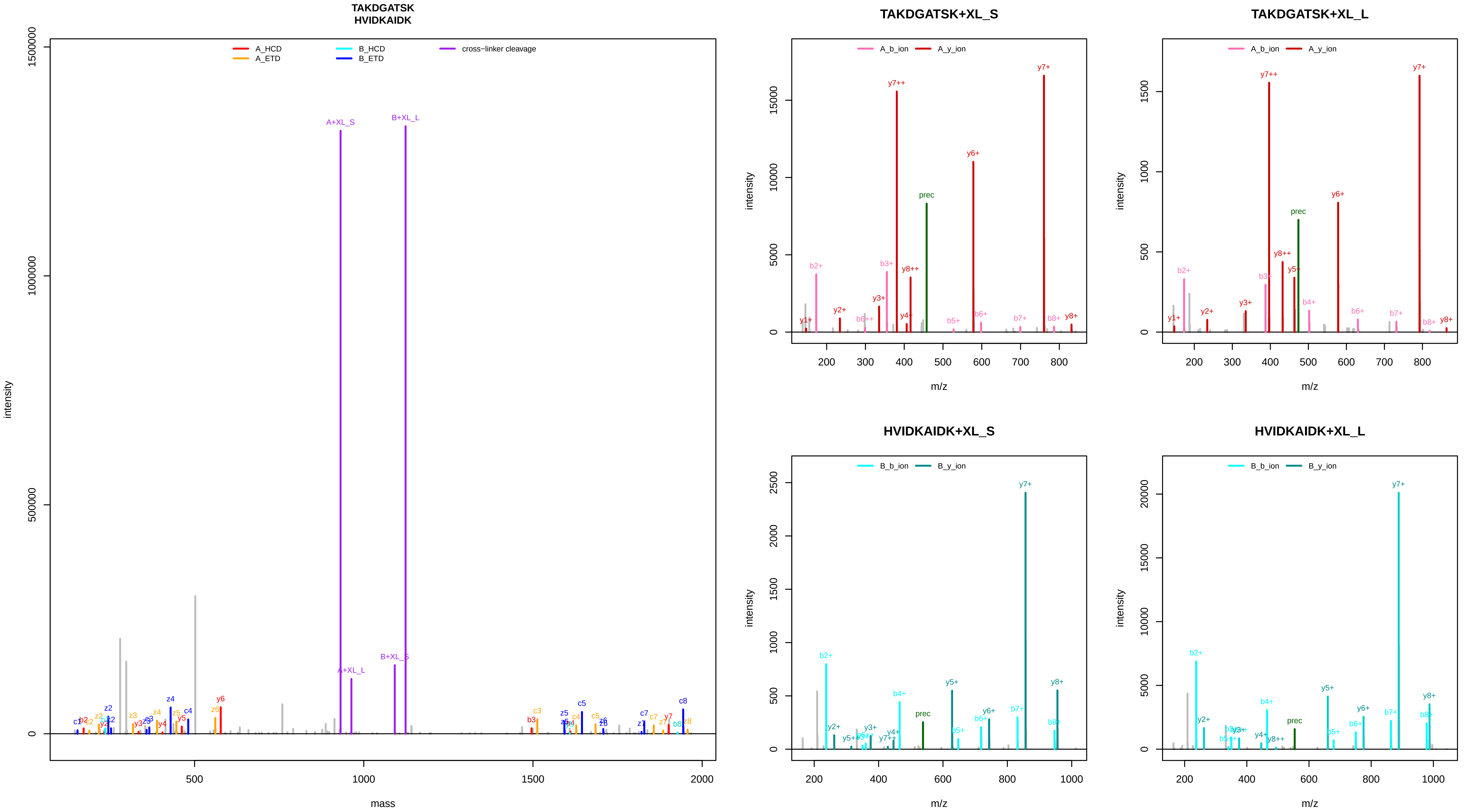
TAKDGATSK
HVIDKAIDK
A_HCD
A_ETD
B_HCD
B_ETD
cross−linker cleavage
A+XL_S
B+XL_L
A+XL_L
B+XL_S
intensity
mass
TAKDGATSK+XL_S
TAKDGATSK+XL_L
A_b_ion
A_y_ion
prec
HVIDKAIDK+XL_S
HVIDKAIDK+XL_L
B_b_ion
B_y_ion
m/z

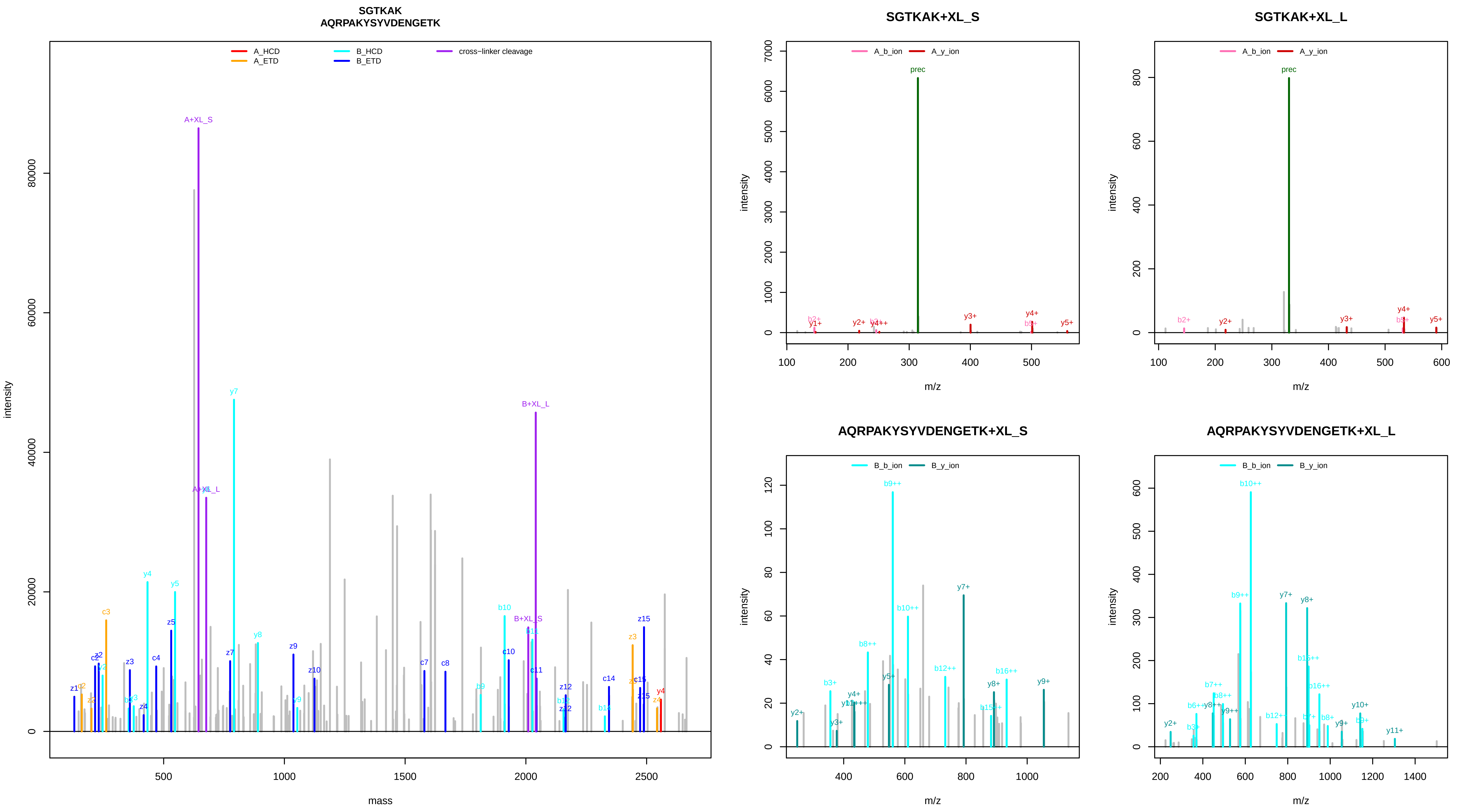
SGTKAK
AQRPAKYSYVDENGETK
A_HCD
A_ETD
B_HCD
B_ETD
cross−linker cleavage
A+XL_S
A+XL_L
B+XL_L
B+XL_S
intensity
mass
SGTKAK+XL_S
SGTKAK+XL_L
A_b_ion
A_y_ion
prec
intensity
m/z
AQRPAKYSYVDENGETK+XL_S
AQRPAKYSYVDENGETK+XL_L
B_b_ion
B_y_ion
intensity
m/z

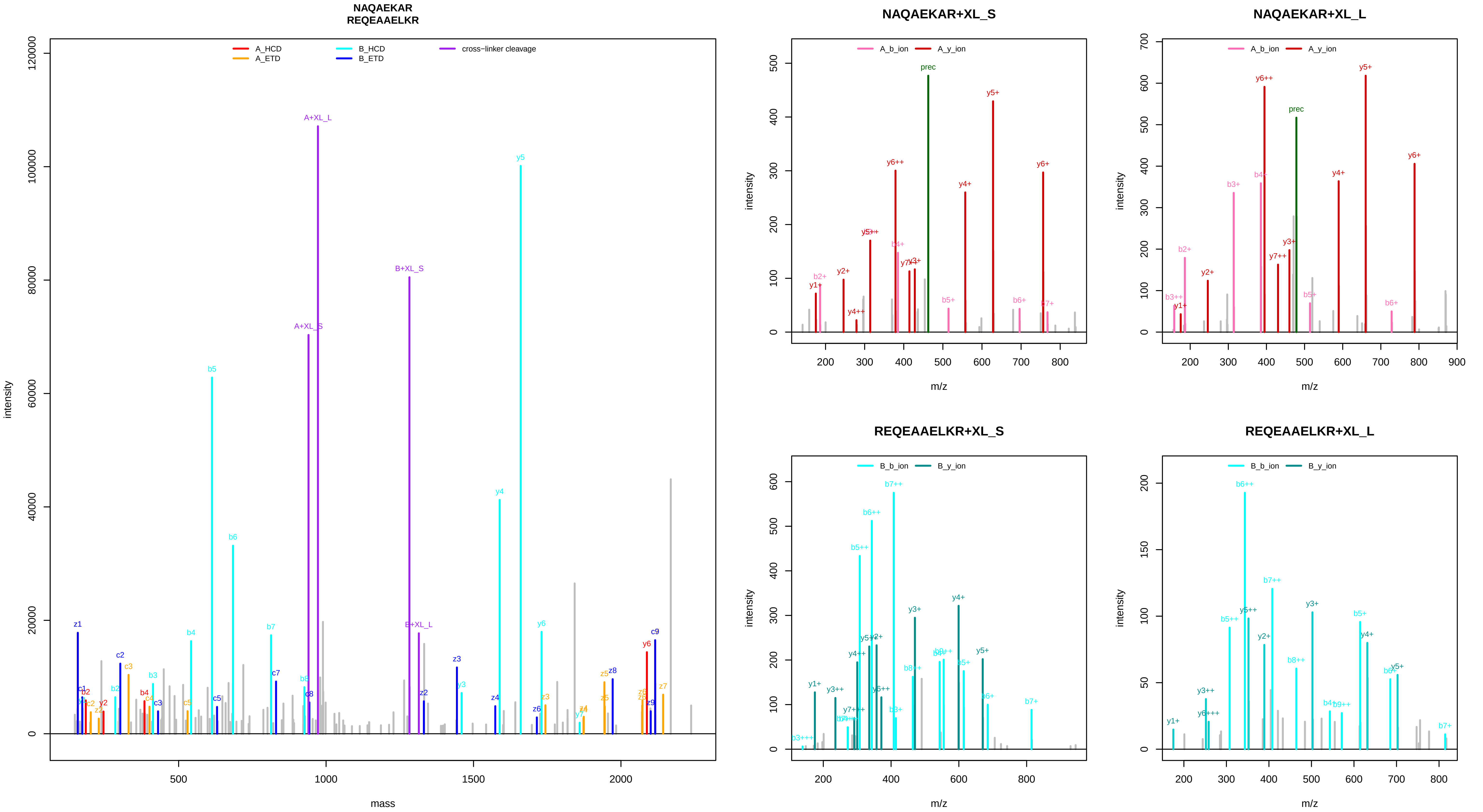
NAQAEKAR
REQEAAELKR
A_HCD
A_ETD
B_HCD
B_ETD
cross−linker cleavage
A+XL_L
A+XL_S
B+XL_S
B+XL_L
intensity
mass
NAQAEKAR+XL_S
NAQAEKAR+XL_L
A_b_ion
A_y_ion
prec
REQEAAELKR+XL_S
REQEAAELKR+XL_L
B_b_ion
B_y_ion
m/z

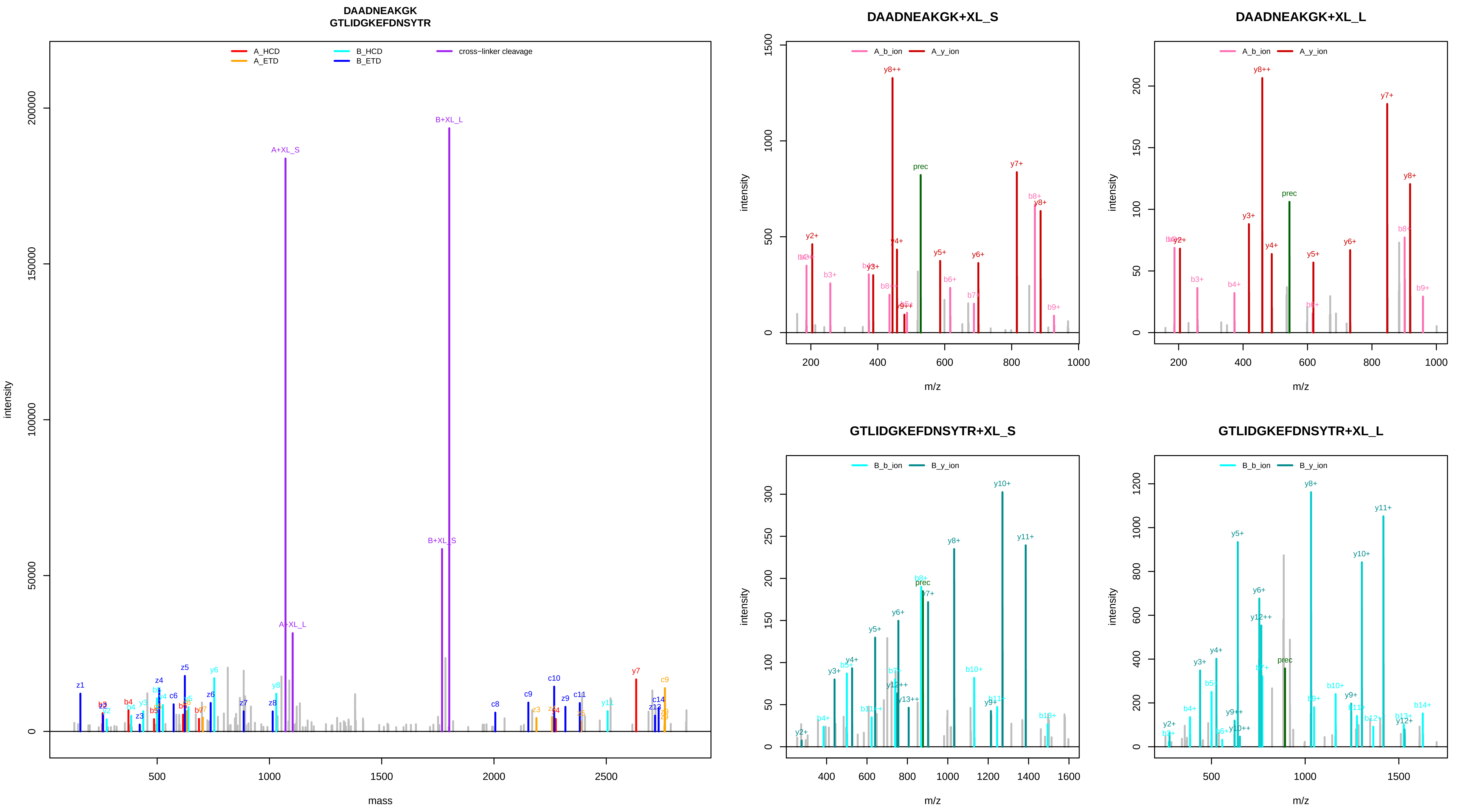
DAADNEAKGK
GTLIDGKEFDNSYTR
A_HCD
A_ETD
B_HCD
B_ETD
cross−linker cleavage
A+XL_S
B+XL_L
B+XL_S
A+XL_L
intensity
mass
DAADNEAKGK+XL_S
DAADNEAKGK+XL_L
GTLIDGKEFDNSYTR+XL_S
GTLIDGKEFDNSYTR+XL_L
A_b_ion
A_y_ion
B_b_ion
B_y_ion
prec
m/z

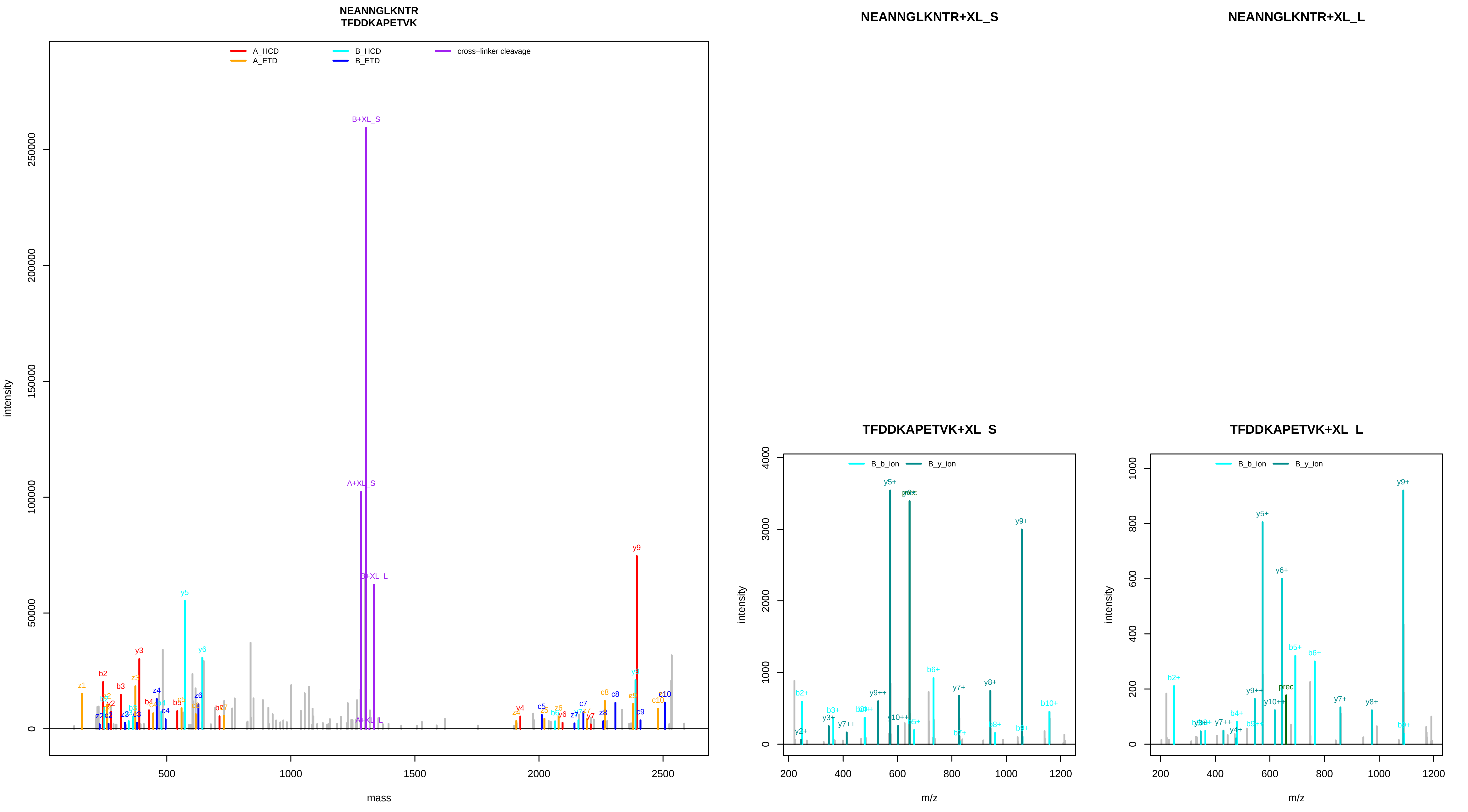
NEANNGLKNTR
TFDDKAPETVK
A_HCD
A_ETD
B_HCD
B_ETD
cross−linker cleavage
B+XL_S
A+XL_S
B+XL_L
A+XL_L
y9
y5
y6
y3
b2
b3
z1
intensity
mass
0
50000
100000
150000
200000
250000
500
1000
1500
2000
2500
NEANNGLKNTR+XL_S
NEANNGLKNTR+XL_L
TFDDKAPETVK+XL_S
TFDDKAPETVK+XL_L
B_b_ion
B_y_ion
y5+
prec
y9+
y6+
b5+
b6+
y7+
y8+
b2+
b10+
intensity
m/z
0
1000
2000
3000
4000
200
400
600
800
1000
1200

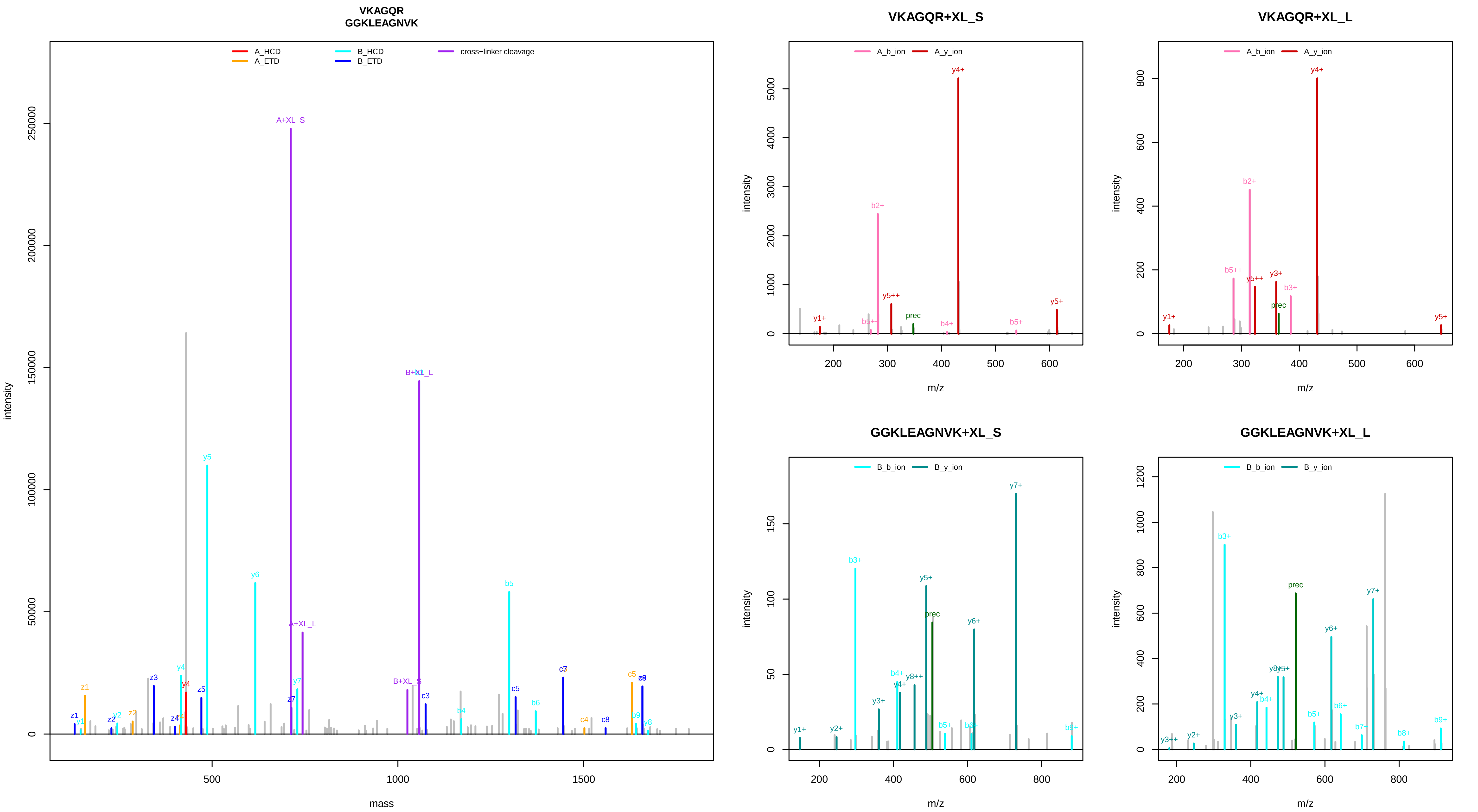
VKAGQR
GGKLEAGNVK
A_HCD
A_ETD
B_HCD
B_ETD
cross−linker cleavage
A+XL_S
B+XL_L
A+XL_L
B+XL_S
intensity
mass
VKAGQR+XL_S
VKAGQR+XL_L
GGKLEAGNVK+XL_S
GGKLEAGNVK+XL_L
A_b_ion
A_y_ion
B_b_ion
B_y_ion
prec
m/z

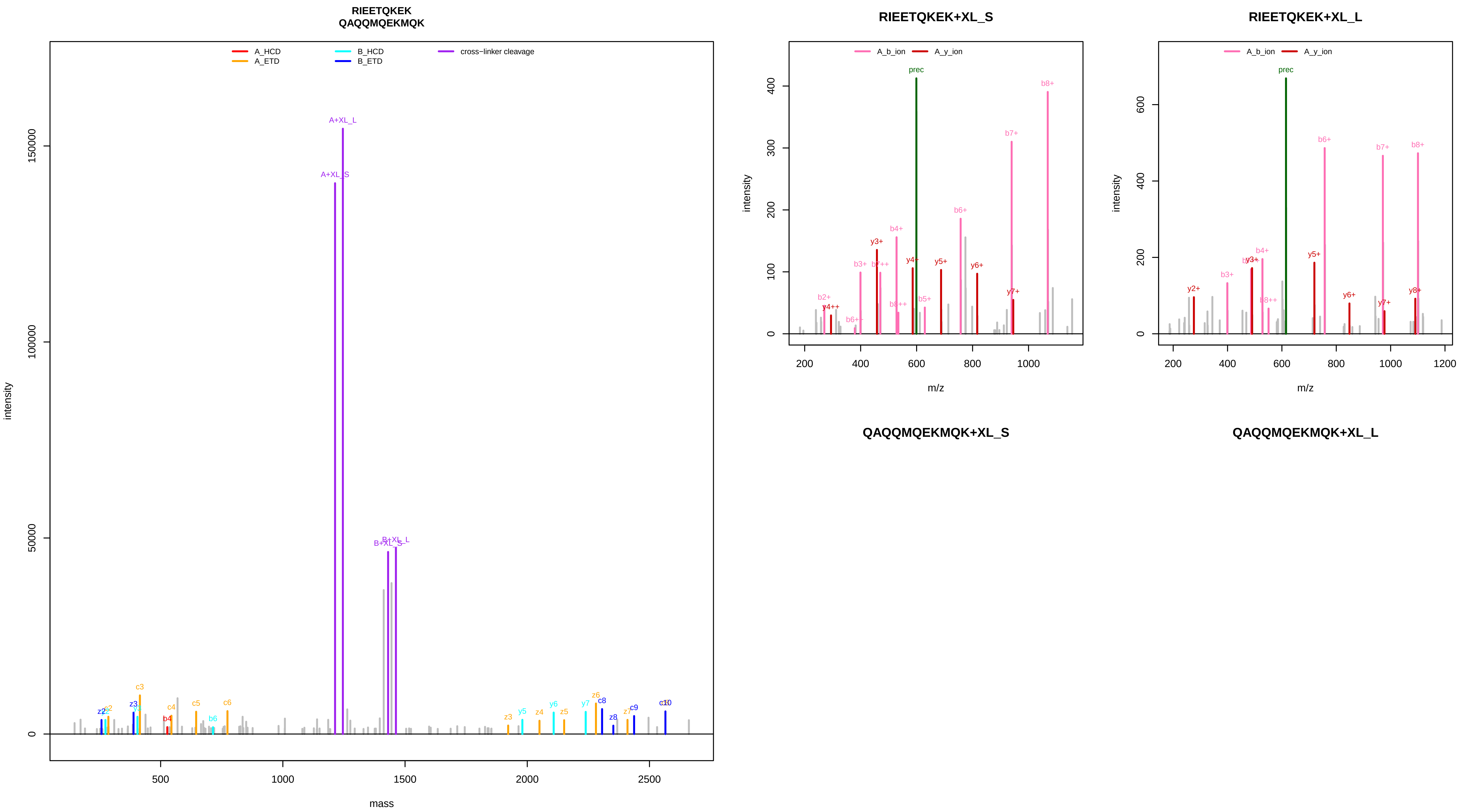
RIEETQKEK
QAQQMQEKMQK
A_HCD
A_ETD
B_HCD
B_ETD
cross−linker cleavage
A+XL_L
A+XL_S
B+XL_S
B+XL_L
intensity
mass
RIEETQKEK+XL_S
RIEETQKEK+XL_L
A_b_ion
A_y_ion
prec
m/z
QAQQMQEKMQK+XL_S
QAQQMQEKMQK+XL_L

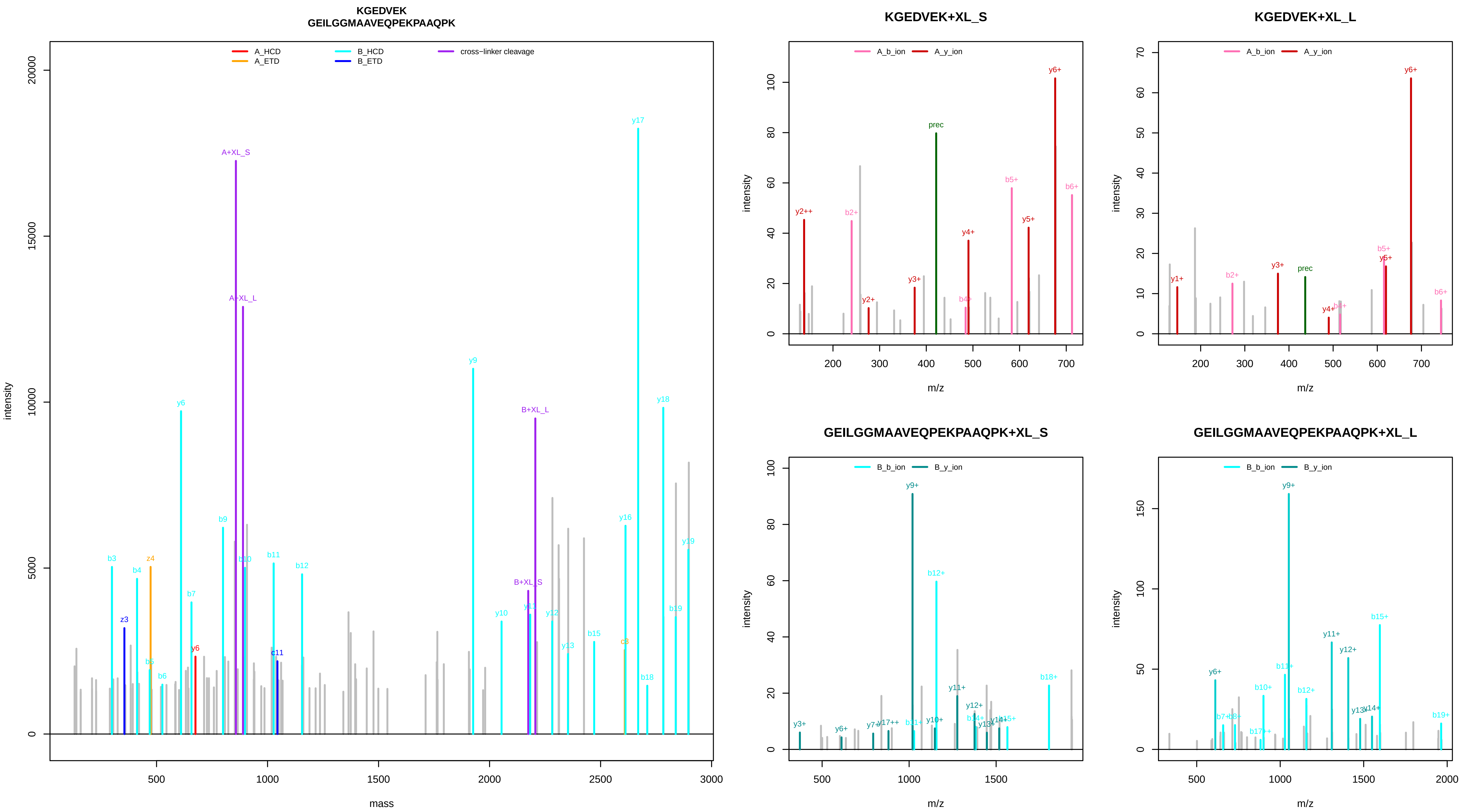
KGEDVEK
GEILGGMAAVEQPEKPAAQPK
A_HCD
A_ETD
B_HCD
B_ETD
cross−linker cleavage
intensity
mass
A+XL_S
A+XL_L
B+XL_L
B+XL_S
y17
y18
y19
y16
y9
y10
y12
y13
b15
b18
b19
c3
y6
b9
b10
b11
b12
b3
b4
z4
z3
b5
b6
b7
c11
KGEDVEK+XL_S
KGEDVEK+XL_L
A_b_ion
A_y_ion
prec
m/z
GEILGGMAAVEQPEKPAAQPK+XL_S
GEILGGMAAVEQPEKPAAQPK+XL_L
B_b_ion
B_y_ion
y9+
b12+
b18+
y11+
y12+
b15+

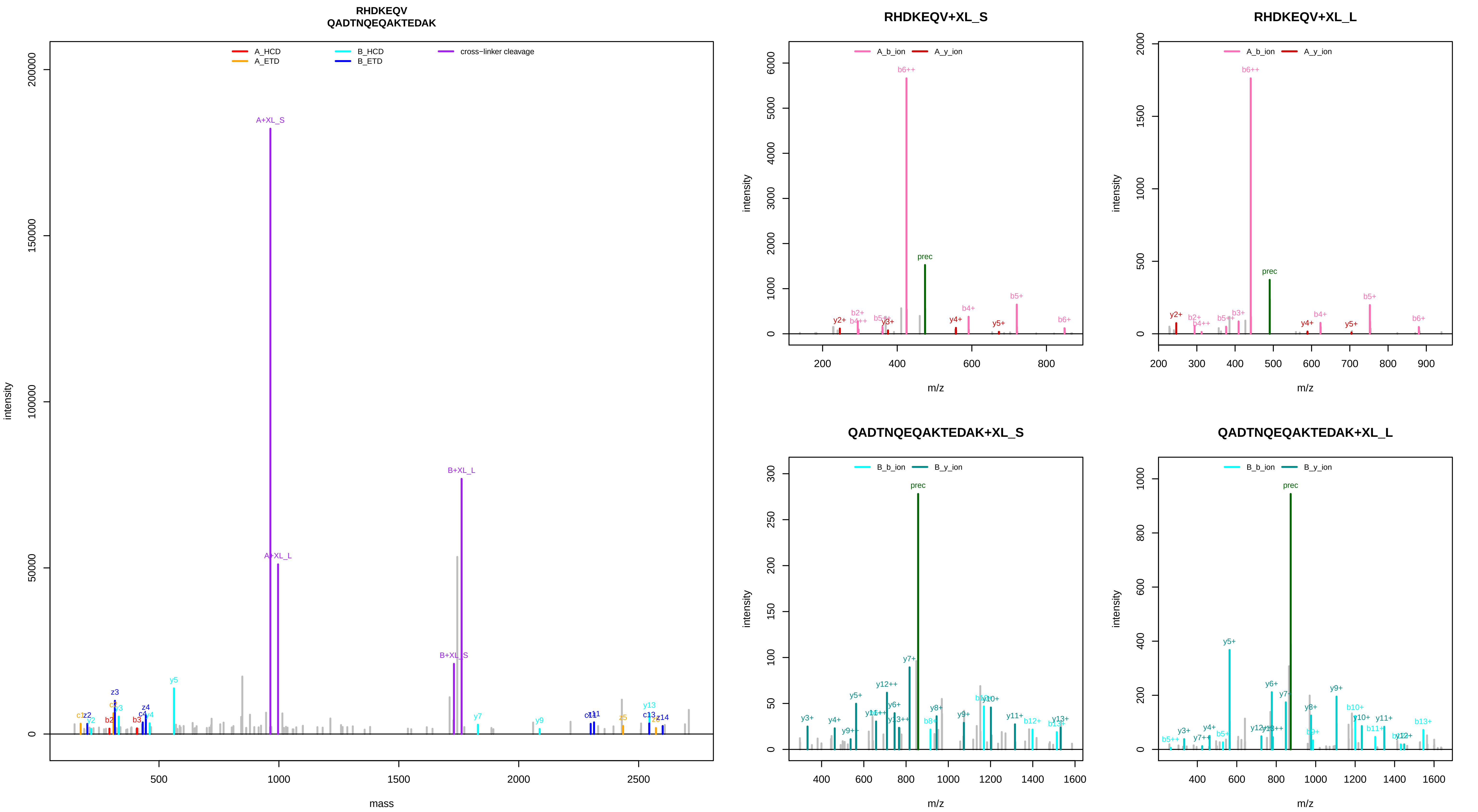
RHDKEQV
QADTNQEQAKTEDAK
A_HCD
A_ETD
B_HCD
B_ETD
cross−linker cleavage
A+XL_S
A+XL_L
B+XL_L
B+XL_S
intensity
mass
RHDKEQV+XL_S
RHDKEQV+XL_L
QADTNQEQAKTEDAK+XL_S
QADTNQEQAKTEDAK+XL_L
A_b_ion
A_y_ion
B_b_ion
B_y_ion
prec
b6++
m/z

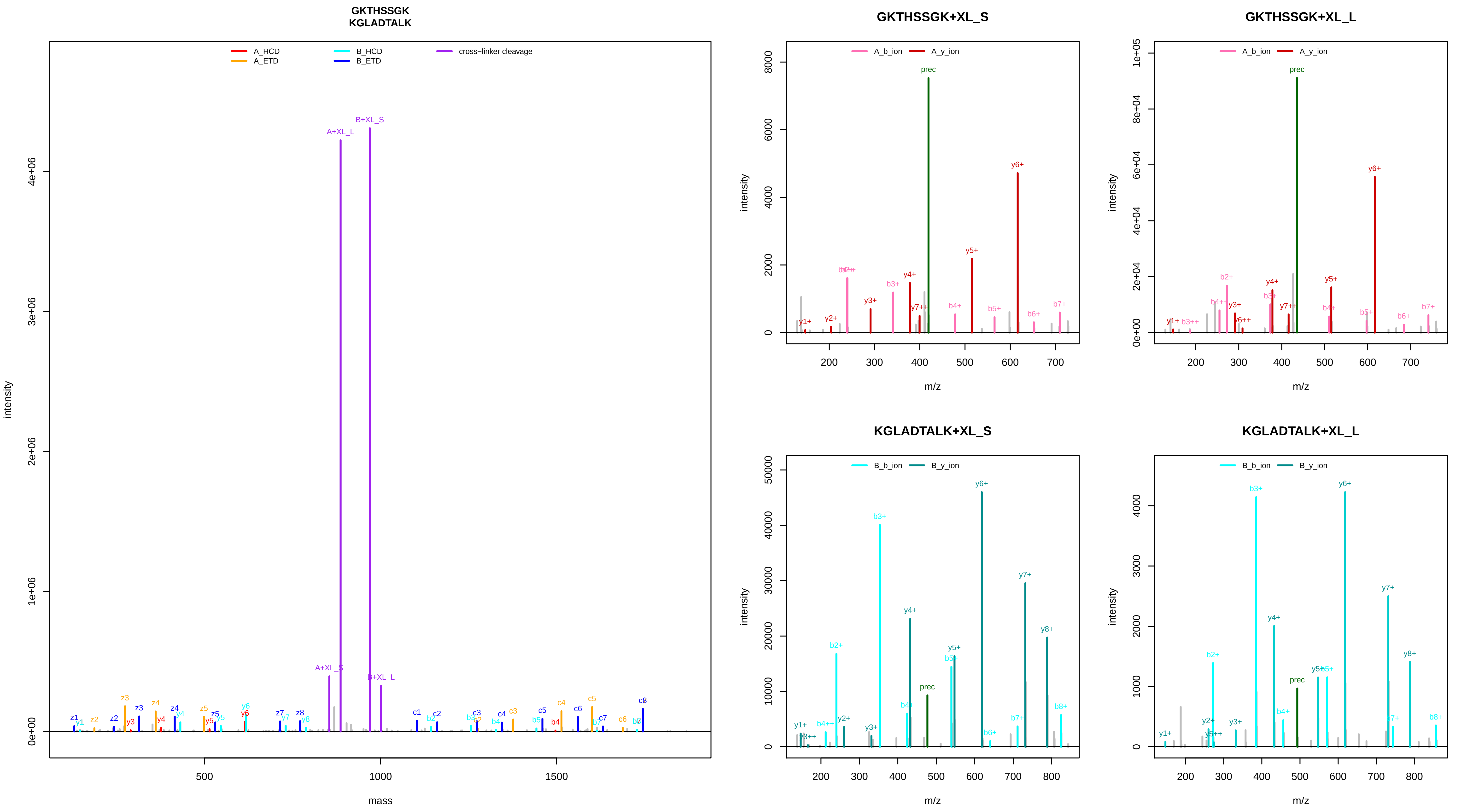
GKTHSSGK
KGLADTALK
A_HCD
A_ETD
B_HCD
B_ETD
cross−linker cleavage
A+XL_L
B+XL_S
A+XL_S
B+XL_L
intensity
mass
0e+00
1e+06
2e+06
3e+06
4e+06
500
1000
1500
GKTHSSGK+XL_S
GKTHSSGK+XL_L
KGLADTALK+XL_S
KGLADTALK+XL_L
A_b_ion
A_y_ion
B_b_ion
B_y_ion
prec
m/z

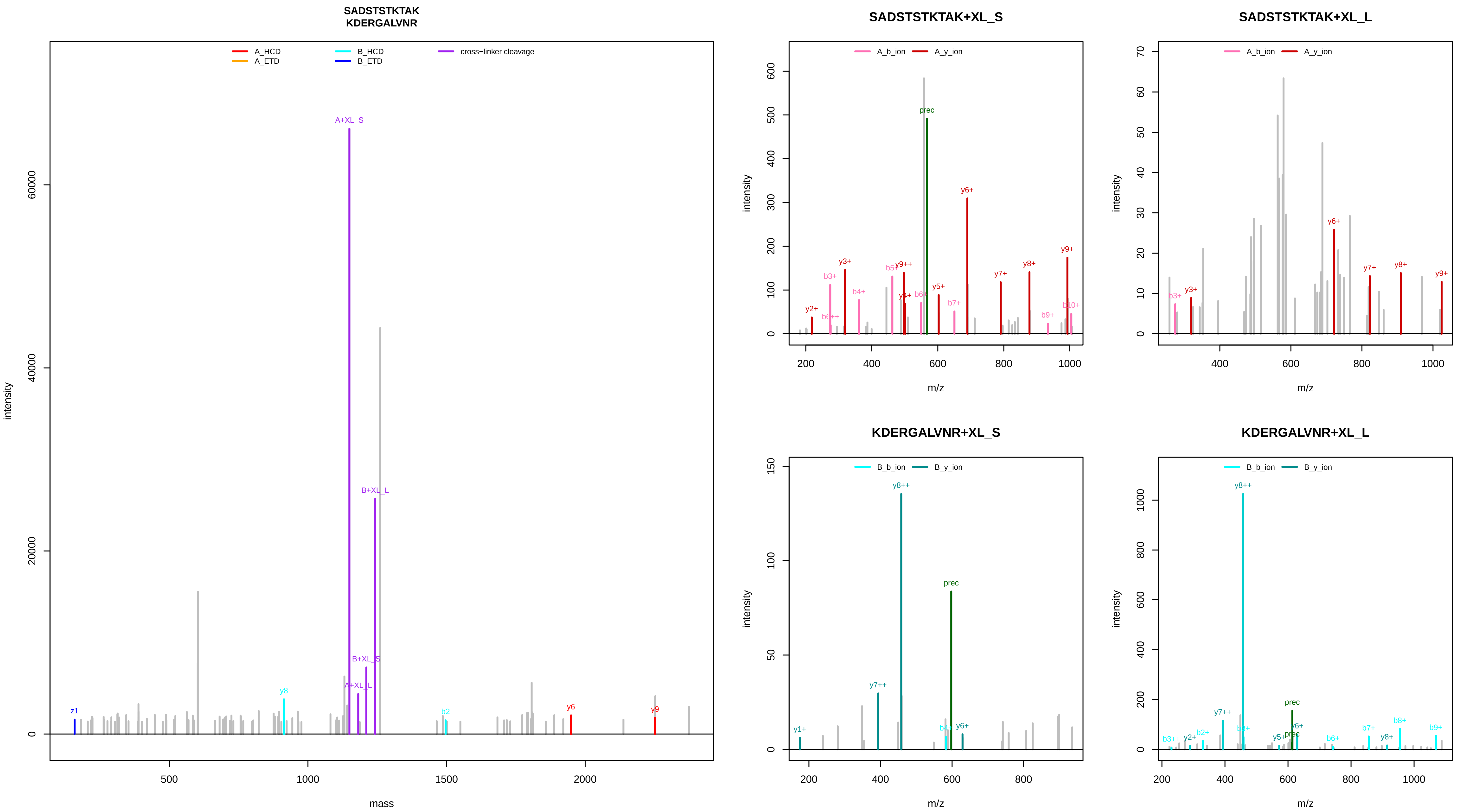

SADSTSTKTAK
KDERGALVNR
A_HCD
A_ETD
B_HCD
B_ETD
cross−linker cleavage
A+XL_S
B+XL_L
B+XL_S
A+XL_L
z1
y8
b2
y6
y9
intensity
mass
SADSTSTKTAK+XL_S
SADSTSTKTAK+XL_L
KDERGALVNR+XL_S
KDERGALVNR+XL_L
A_b_ion
A_y_ion
B_b_ion
B_y_ion
prec
m/z

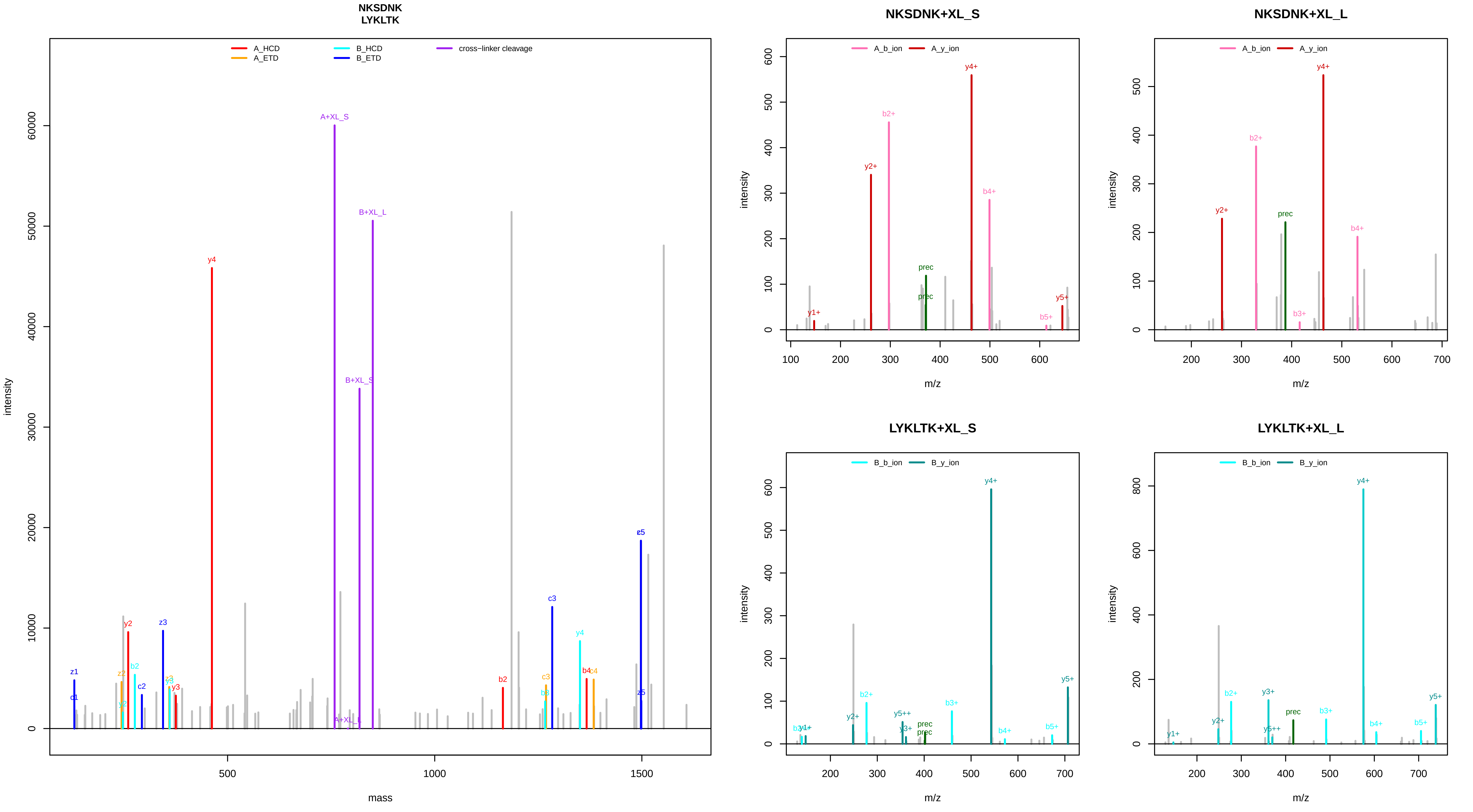

NKSDNK
LYKLTK
A_HCD
A_ETD
B_HCD
B_ETD
cross−linker cleavage
A+XL_S
B+XL_L
B+XL_S
A+XL_L
intensity
mass
NKSDNK+XL_S
NKSDNK+XL_L
LYKLTK+XL_S
LYKLTK+XL_L
A_b_ion
A_y_ion
B_b_ion
B_y_ion
prec
m/z

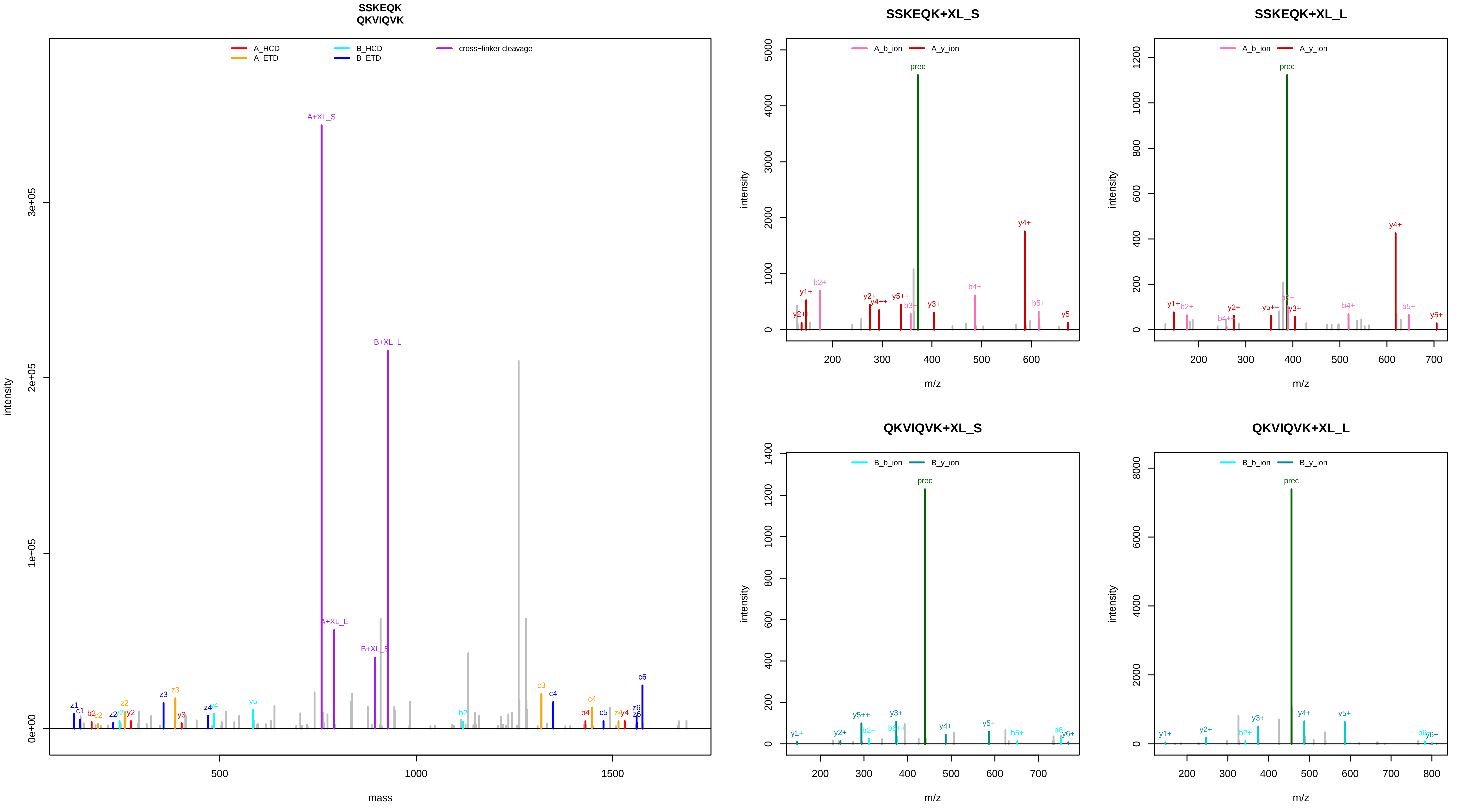
SSKEQK
QKVIQVK
A_HCD
A_ETD
B_HCD
B_ETD
cross−linker cleavage
A+XL_S
B+XL_L
A+XL_L
B+XL_S
intensity
mass
SSKEQK+XL_S
SSKEQK+XL_L
QKVIQVK+XL_S
QKVIQVK+XL_L
A_b_ion
A_y_ion
B_b_ion
B_y_ion
prec
m/z

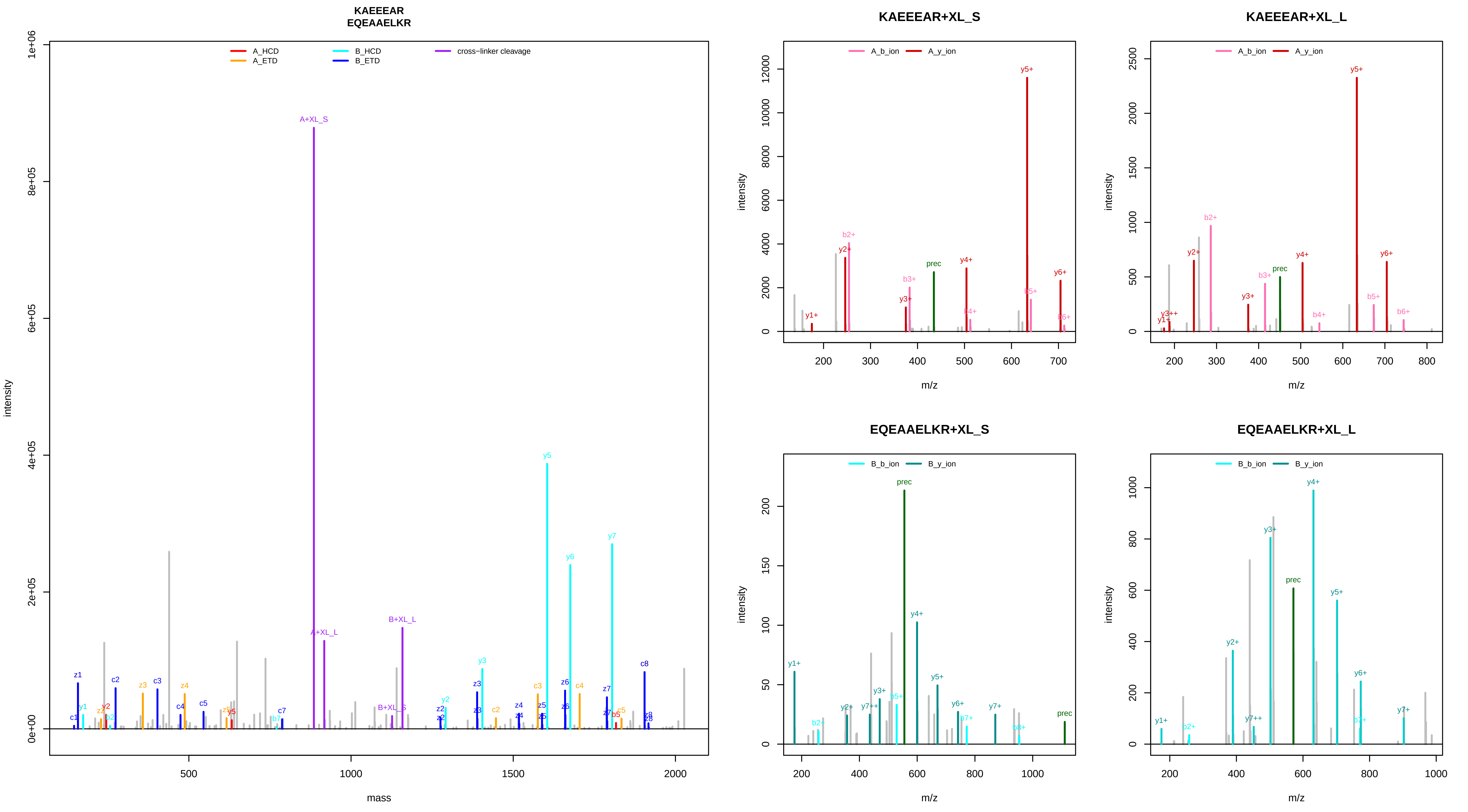
KAEEEAR
EQEAAELKR
A_HCD
A_ETD
B_HCD
B_ETD
cross−linker cleavage
A+XL_S
A+XL_L
B+XL_L
B+XL_S
intensity
mass
KAEEEAR+XL_S
KAEEEAR+XL_L
A_b_ion
A_y_ion
EQEAAELKR+XL_S
EQEAAELKR+XL_L
B_b_ion
B_y_ion
prec
m/z

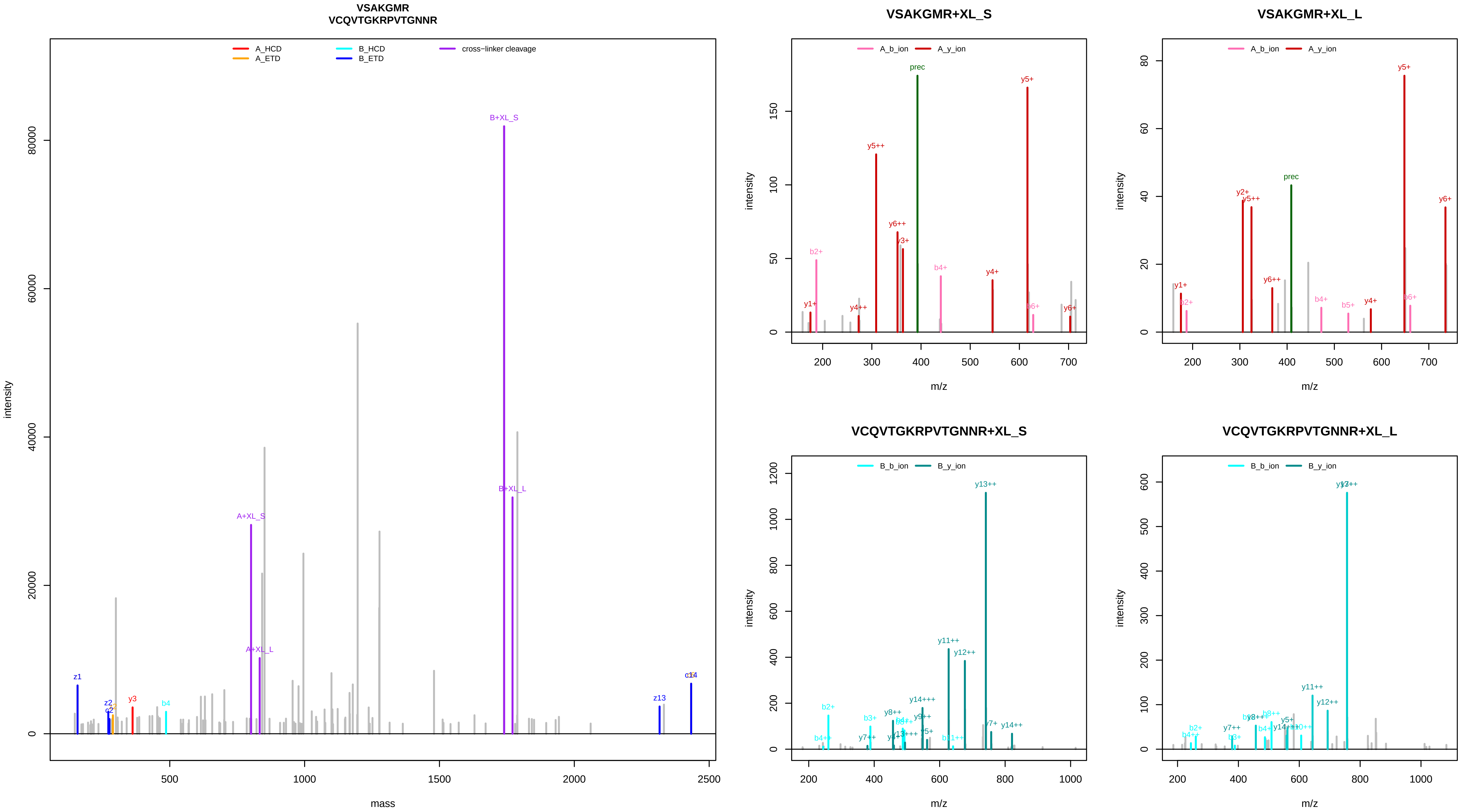
VSAKGMR
VCQVTGKRPVTGNNR
A_HCD
A_ETD
B_HCD
B_ETD
cross−linker cleavage
B+XL_S
B+XL_L
A+XL_S
A+XL_L
intensity
mass
VSAKGMR+XL_S
VSAKGMR+XL_L
VCQVTGKRPVTGNNR+XL_S
VCQVTGKRPVTGNNR+XL_L
A_b_ion
A_y_ion
B_b_ion
B_y_ion
prec
m/z

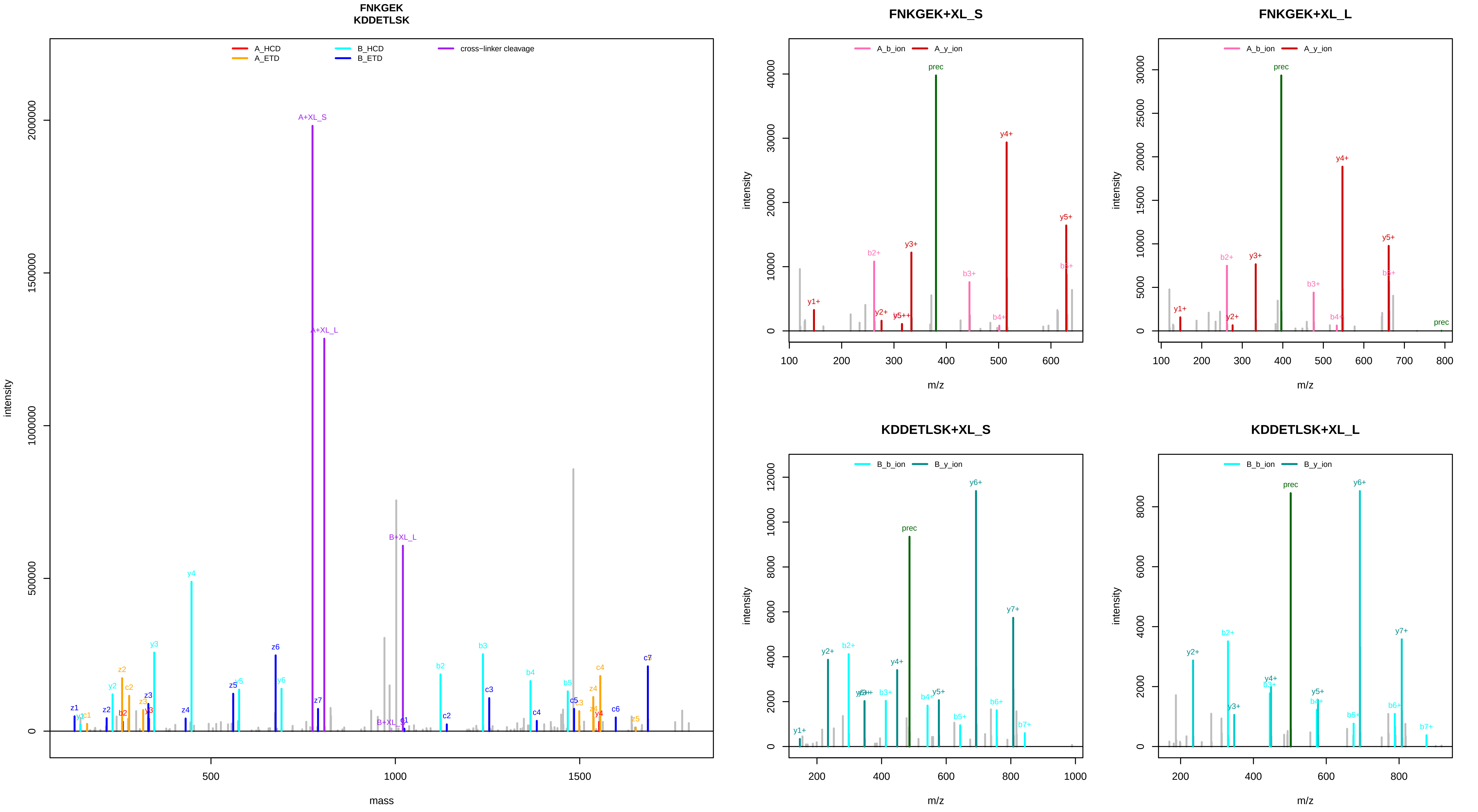

FNKGEK
KDDETLSK
A_HCD
A_ETD
B_HCD
B_ETD
cross−linker cleavage
A+XL_S
A+XL_L
B+XL_L
intensity
mass
FNKGEK+XL_S
FNKGEK+XL_L
A_b_ion
A_y_ion
KDDETLSK+XL_S
KDDETLSK+XL_L
B_b_ion
B_y_ion
prec
m/z

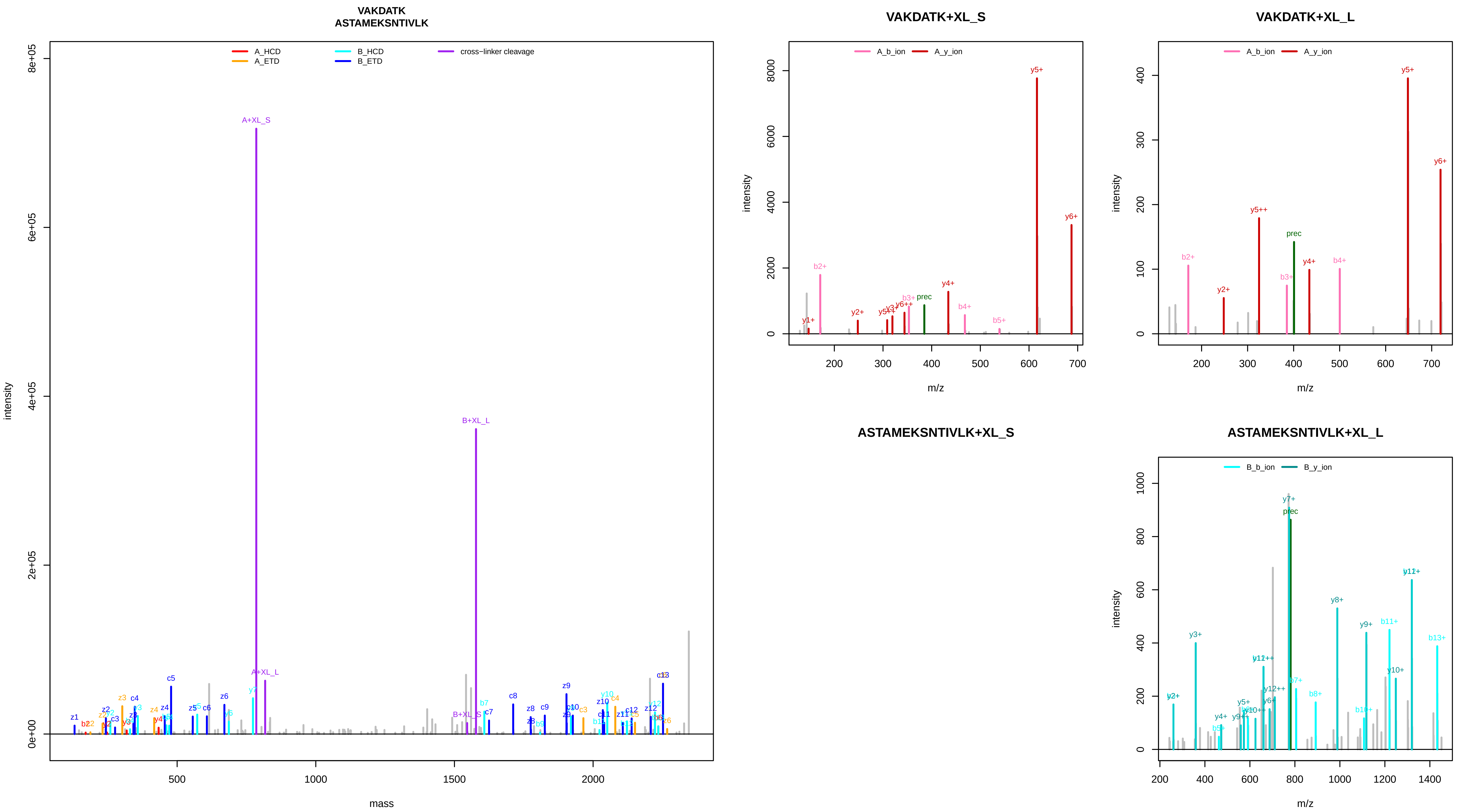
VAKDATK
ASTAMEKSNTIVLK
A_HCD
A_ETD
B_HCD
B_ETD
cross−linker cleavage
A+XL_S
B+XL_L
A+XL_L
B+XL_S
intensity
mass
VAKDATK+XL_S
VAKDATK+XL_L
ASTAMEKSNTIVLK+XL_S
ASTAMEKSNTIVLK+XL_L
A_b_ion
A_y_ion
B_b_ion
B_y_ion
prec
m/z

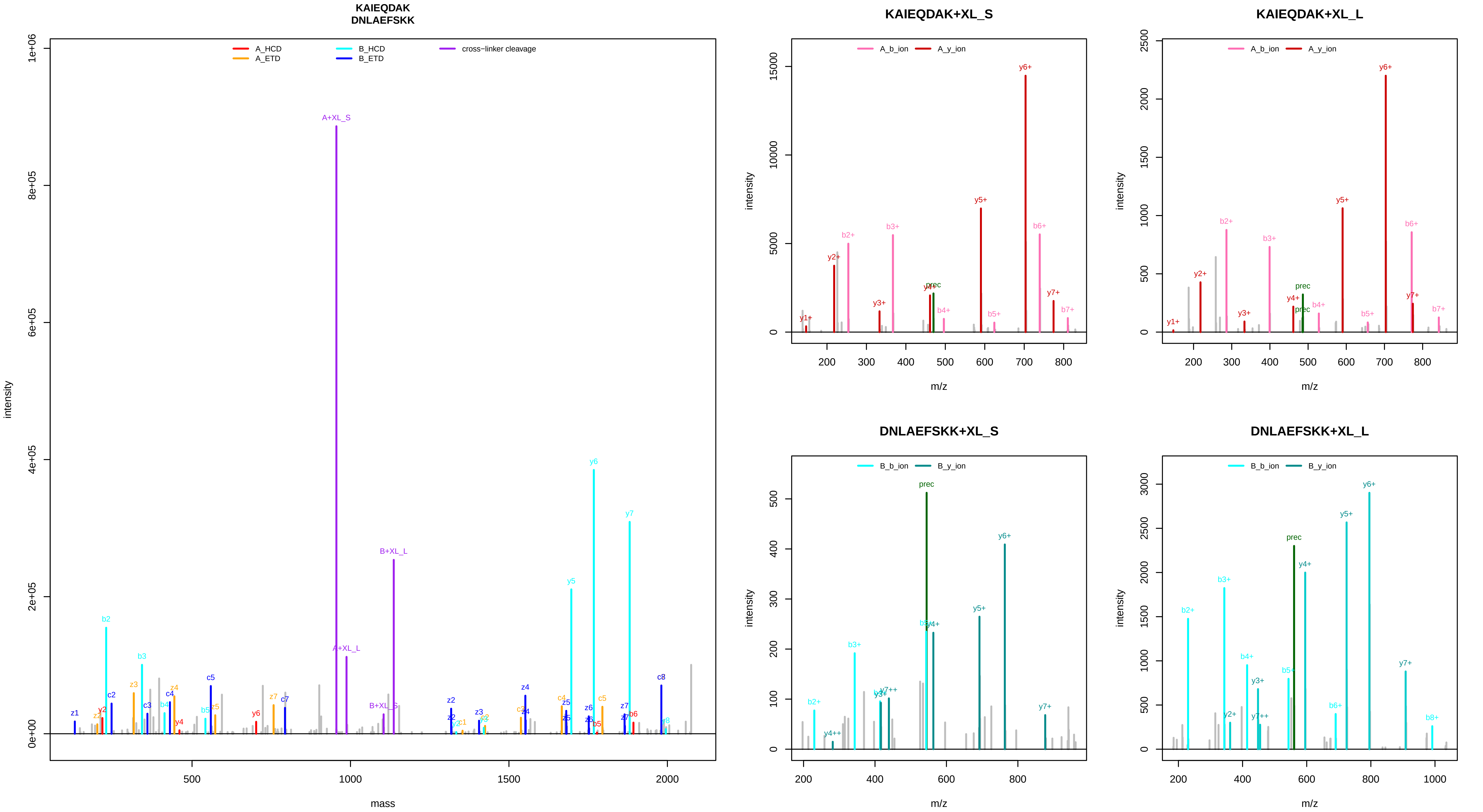
KAIEQDAK
DNLAEFSKK
A_HCD
A_ETD
B_HCD
B_ETD
cross−linker cleavage
A+XL_S
B+XL_L
A+XL_L
B+XL_S
intensity
mass
KAIEQDAK+XL_S
KAIEQDAK+XL_L
DNLAEFSKK+XL_S
DNLAEFSKK+XL_L
A_b_ion
A_y_ion
B_b_ion
B_y_ion
prec
m/z

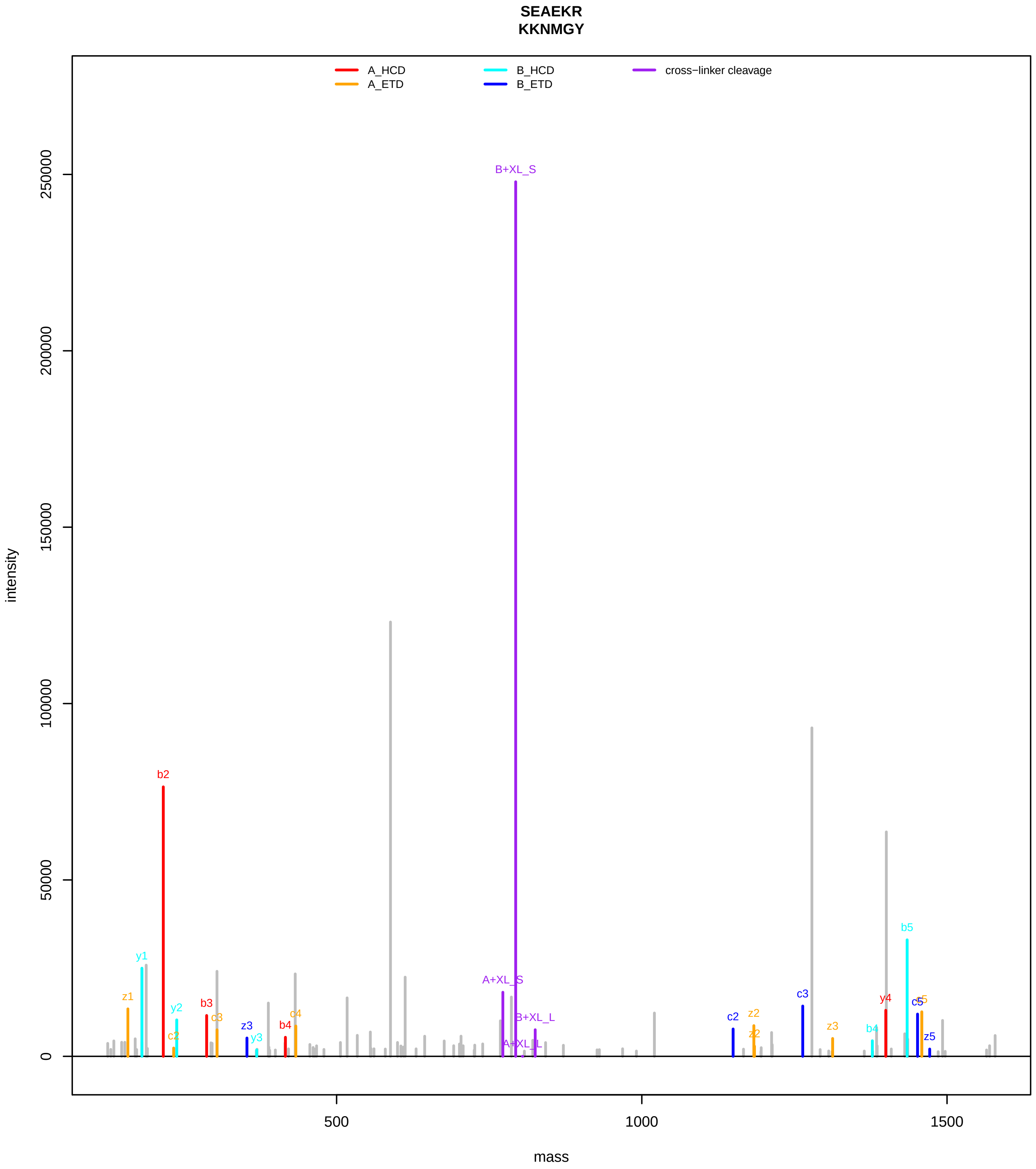

SEAEKR+XL_S

SEAEKR+XL_L

KKNMGY+XL_S

KKNMGY+XL_L

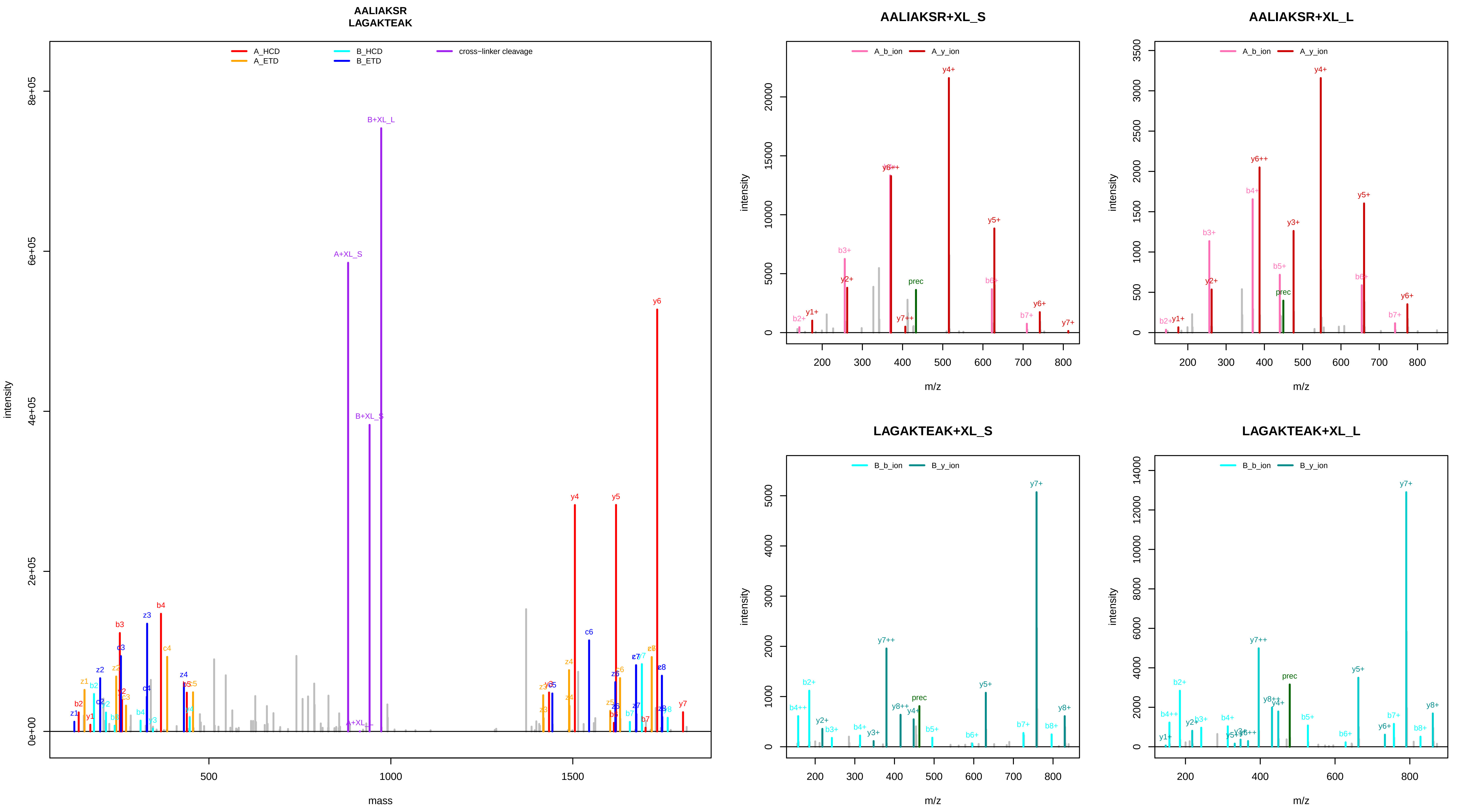
AALIAKSR
LAGAKTEAK
A_HCD
A_ETD
B_HCD
B_ETD
cross−linker cleavage
B+XL_L
A+XL_S
B+XL_S
intensity
mass
AALIAKSR+XL_S
AALIAKSR+XL_L
LAGAKTEAK+XL_S
LAGAKTEAK+XL_L
A_b_ion
A_y_ion
B_b_ion
B_y_ion
prec
m/z

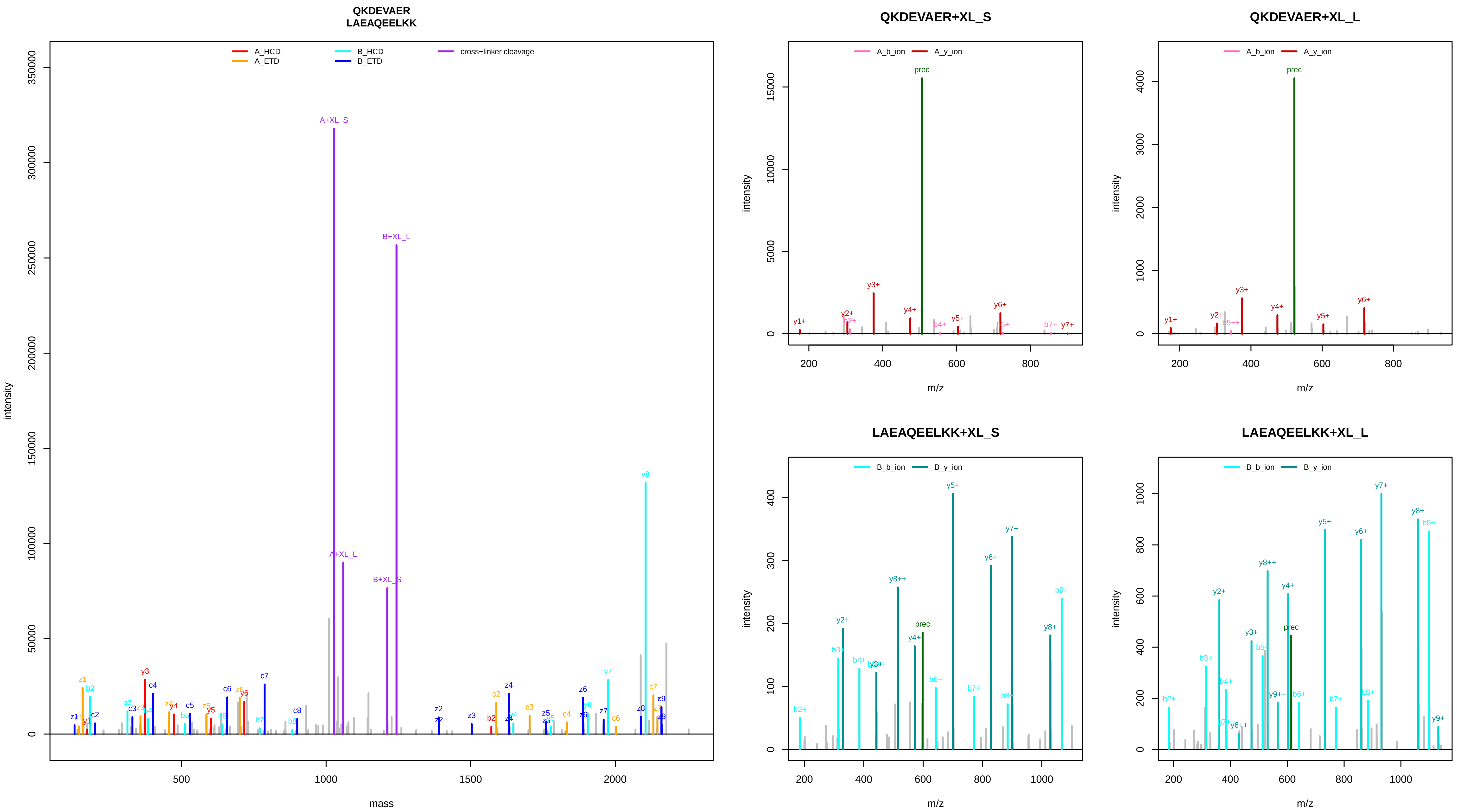
QKDEVAER
LAEAQEELKK
A_HCD
A_ETD
B_HCD
B_ETD
cross−linker cleavage
A+XL_S
B+XL_L
A+XL_L
B+XL_S
intensity
mass
QKDEVAER+XL_S
QKDEVAER+XL_L
LAEAQEELKK+XL_S
LAEAQEELKK+XL_L
A_b_ion
A_y_ion
B_b_ion
B_y_ion
prec
m/z

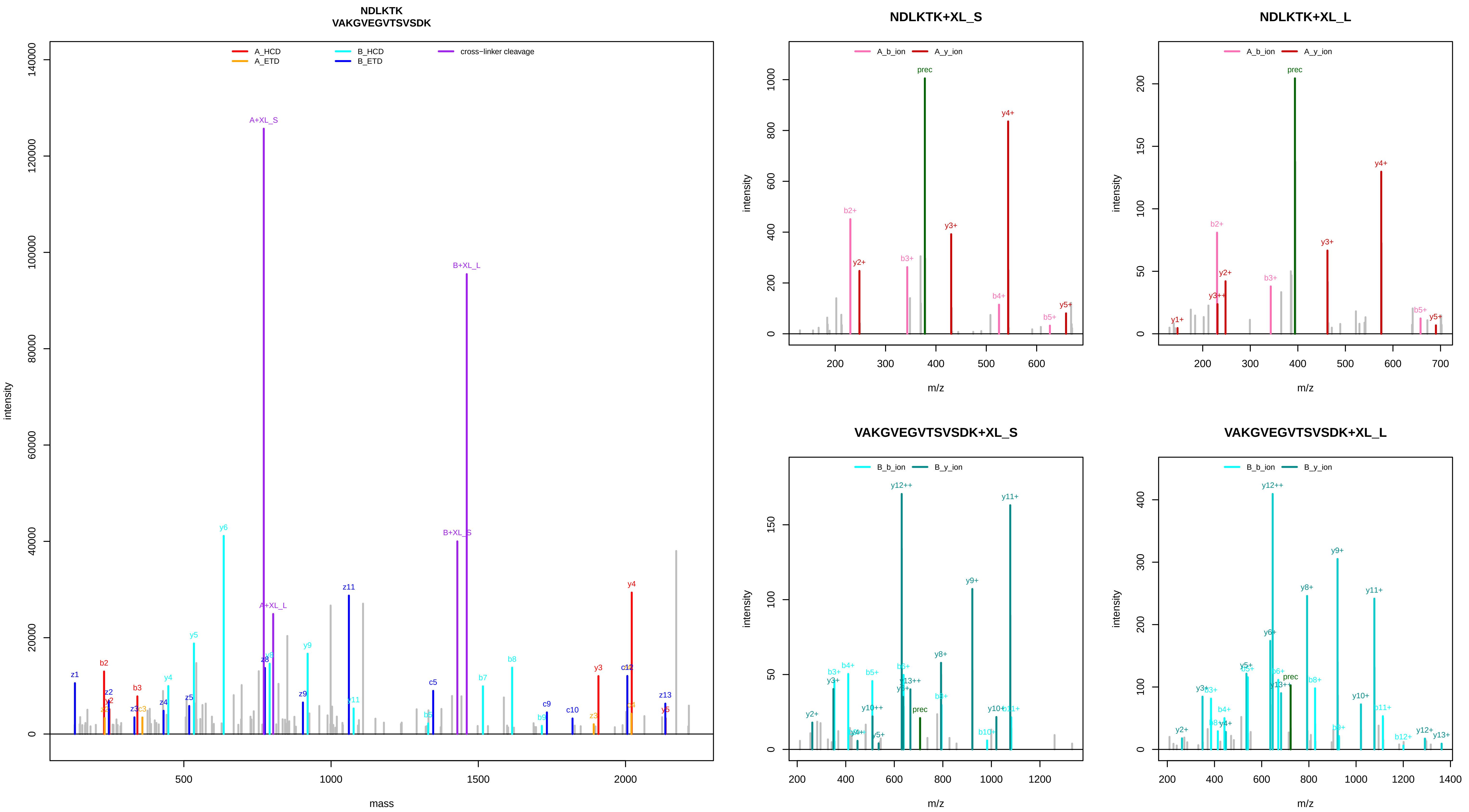
NDLKTK
VAKGVEGVTSVSDK
A_HCD
A_ETD
B_HCD
B_ETD
cross−linker cleavage
A+XL_S
B+XL_L
B+XL_S
A+XL_L
intensity
mass
NDLKTK+XL_S
NDLKTK+XL_L
A_b_ion
A_y_ion
prec
VAKGVEGVTSVSDK+XL_S
VAKGVEGVTSVSDK+XL_L
B_b_ion
B_y_ion
m/z

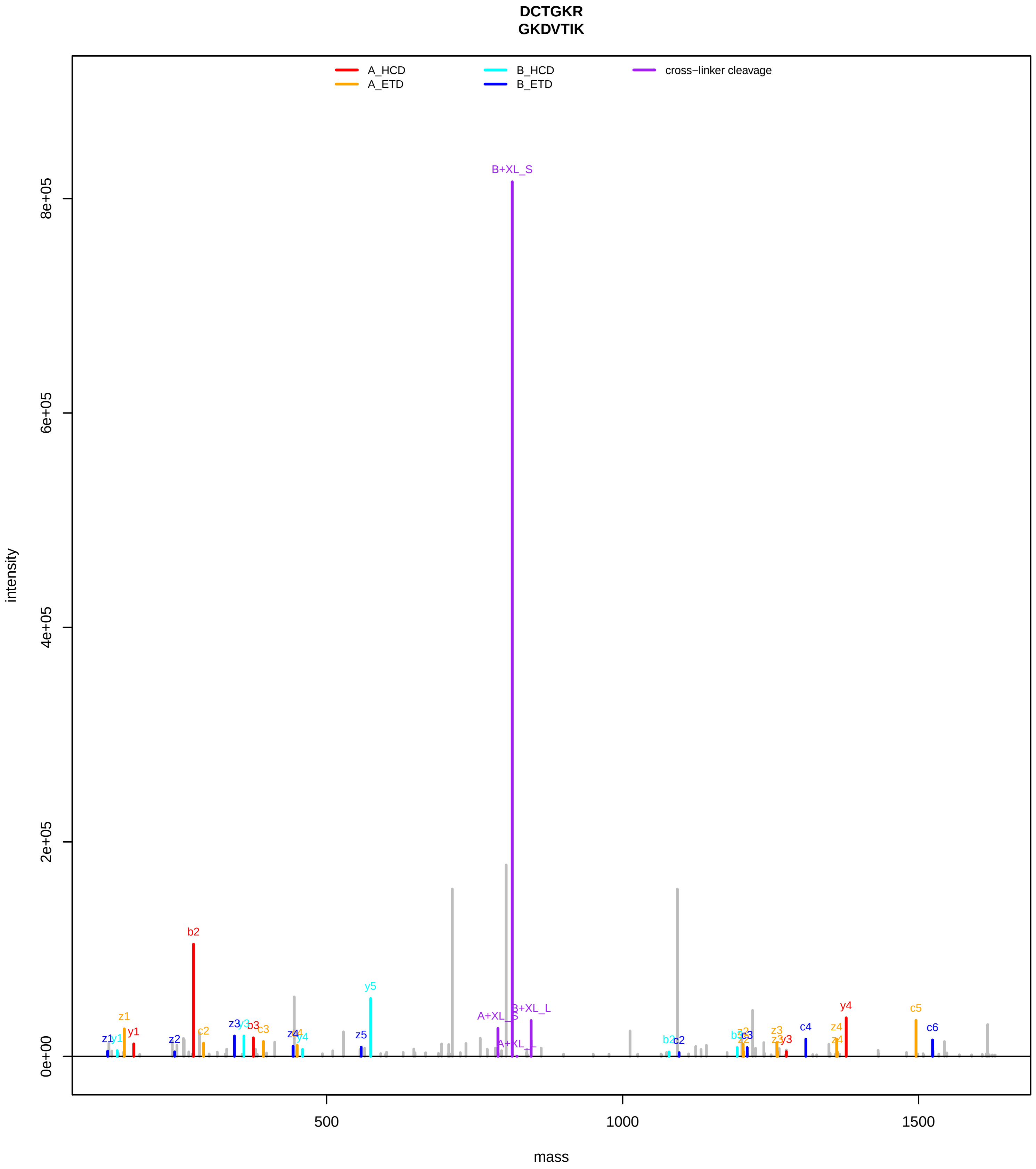

DCTGKR+XL_S

DCTGKR+XL_L

GKDVTIK+XL_S

GKDVTIK+XL_L

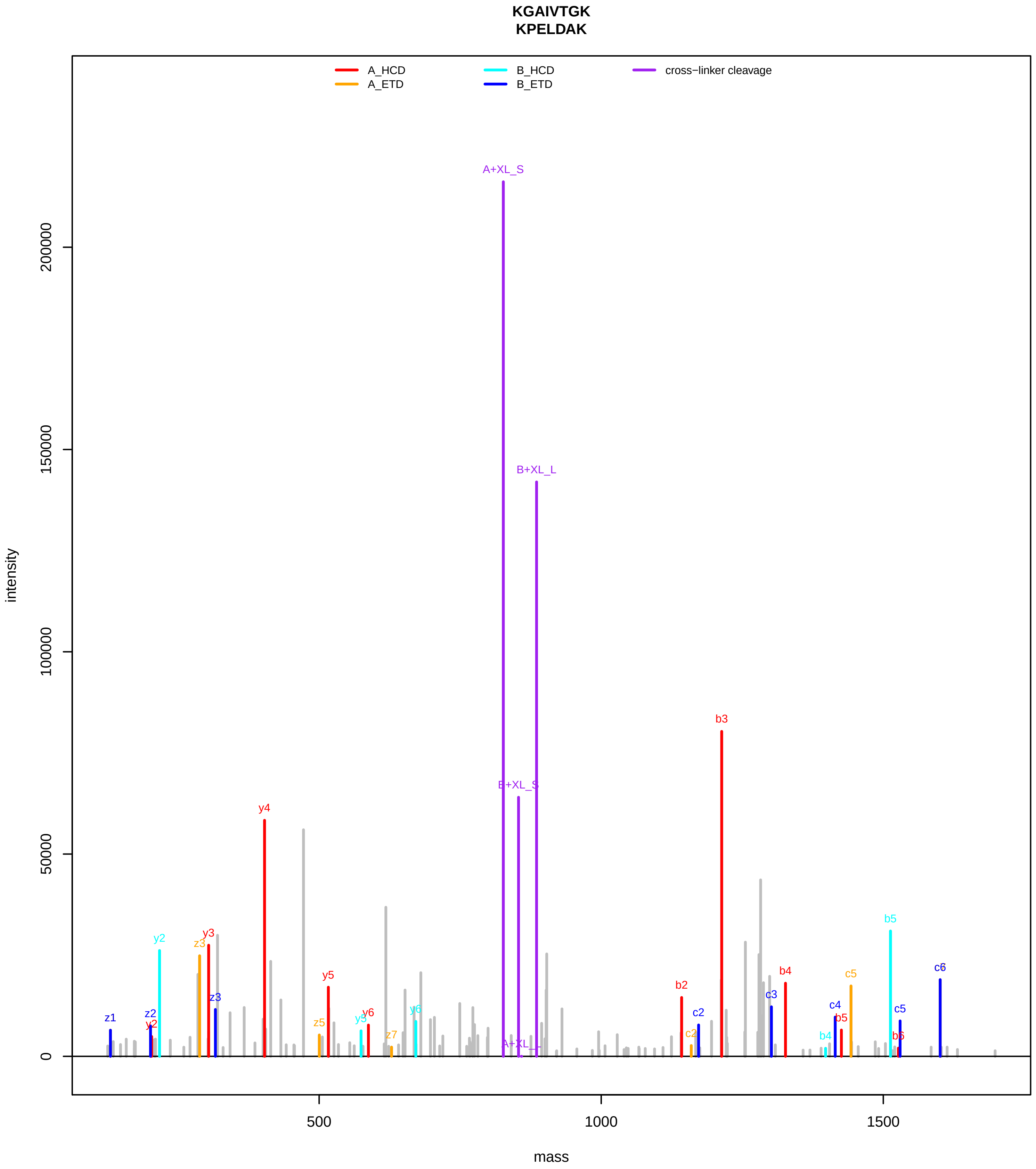

KGAIVTGK+XL_S

KGAIVTGK+XL_L

KPELDAK+XL_S

KPELDAK+XL_L

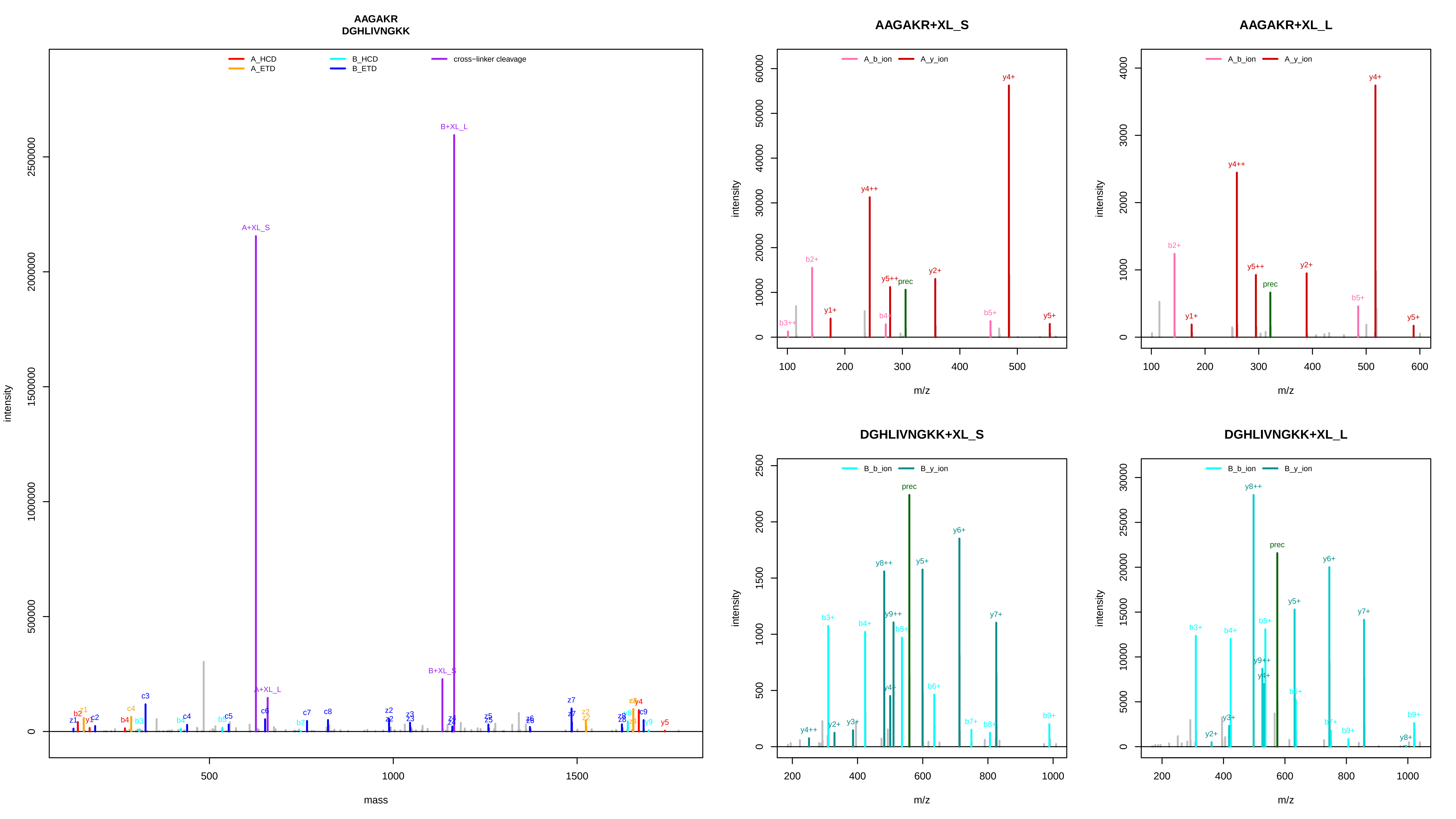
AAGAKR
DGHLIVNGKK
A_HCD
A_ETD
B_HCD
B_ETD
cross−linker cleavage
B+XL_L
A+XL_S
B+XL_S
A+XL_L
intensity
mass
AAGAKR+XL_S
AAGAKR+XL_L
A_b_ion
A_y_ion
y4+
y4++
y5++
prec
y2+
y1+
b2+
b4+
b5+
b3++
y5+
m/z
DGHLIVNGKK+XL_S
DGHLIVNGKK+XL_L
B_b_ion
B_y_ion
y8++
y9++
y6+
y5+
y7+
b3+
b4+
b5+
b6+
b7+
b8+
b9+
y4+
y3+
y2+
y4++
y8+

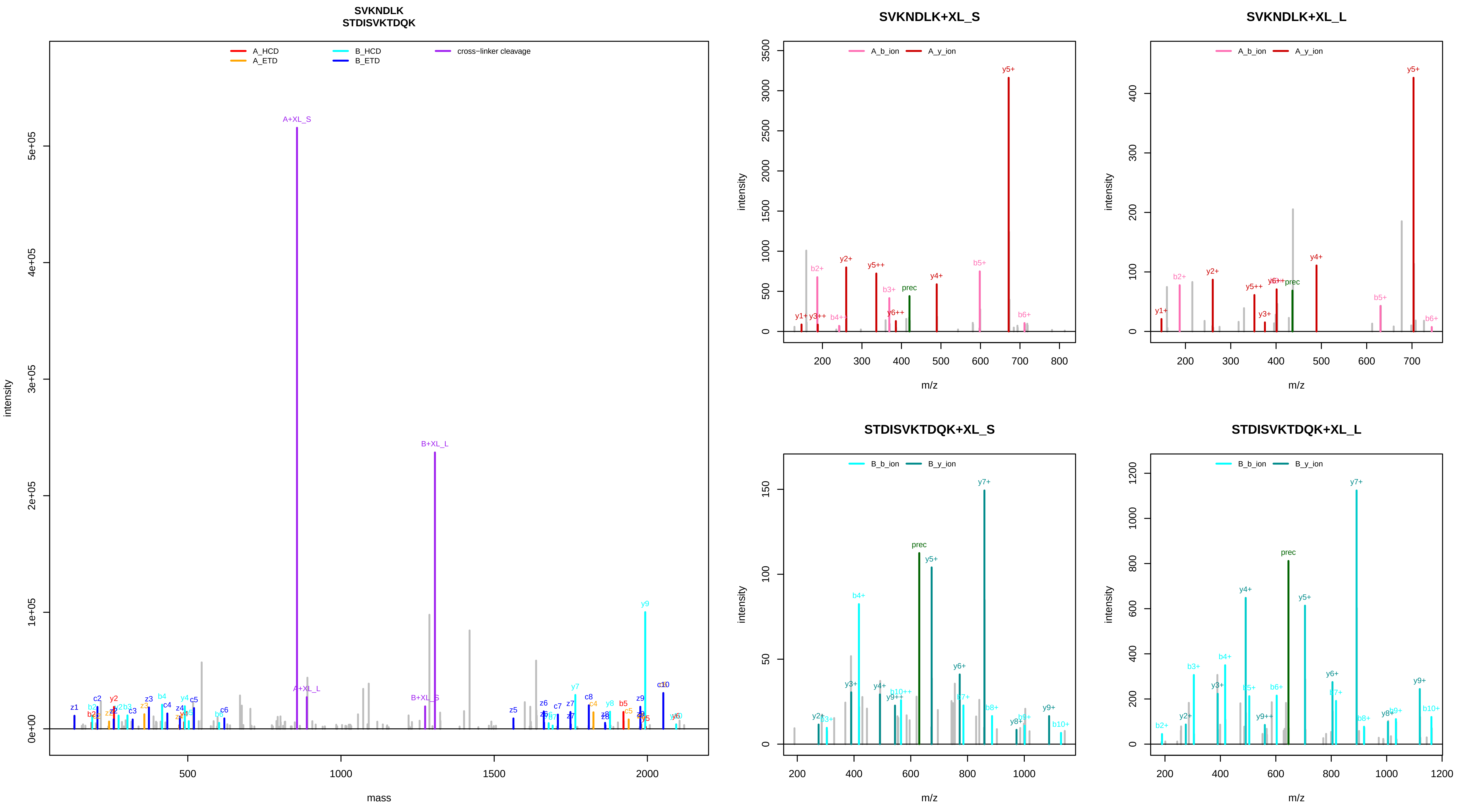

SVKNDLK
STDISVKTDQK
A_HCD
A_ETD
B_HCD
B_ETD
cross−linker cleavage
A+XL_S
B+XL_L
A+XL_L
B+XL_S
y9
c10
intensity
mass
0e+00
1e+05
2e+05
3e+05
4e+05
5e+05
500
1000
1500
2000
SVKNDLK+XL_S
A_b_ion
A_y_ion
y5+
y2+
y5++
y4+
b5+
b2+
b3+
prec
y6++
b6+
y1+
y3++
intensity
m/z
SVKNDLK+XL_L
A_b_ion
A_y_ion
y5+
y4+
y2+
b2+
y5++
prec
b5+
y1+
y3+
b6+
intensity
m/z
STDISVKTDQK+XL_S
B_b_ion
B_y_ion
y7+
prec
y5+
b4+
y6+
y3+
y4+
b10++
y9++
b7+
b8+
y8+
b9+
y9+
b10+
y2+
b3+
intensity
m/z
STDISVKTDQK+XL_L
B_b_ion
B_y_ion
y7+
prec
y4+
y5+
b4+
b3+
y3+
b5+
b6+
y6+
b7+
y9+
y9++
y2+
b2+
b8+
y8+
b9+
b10+
intensity
m/z

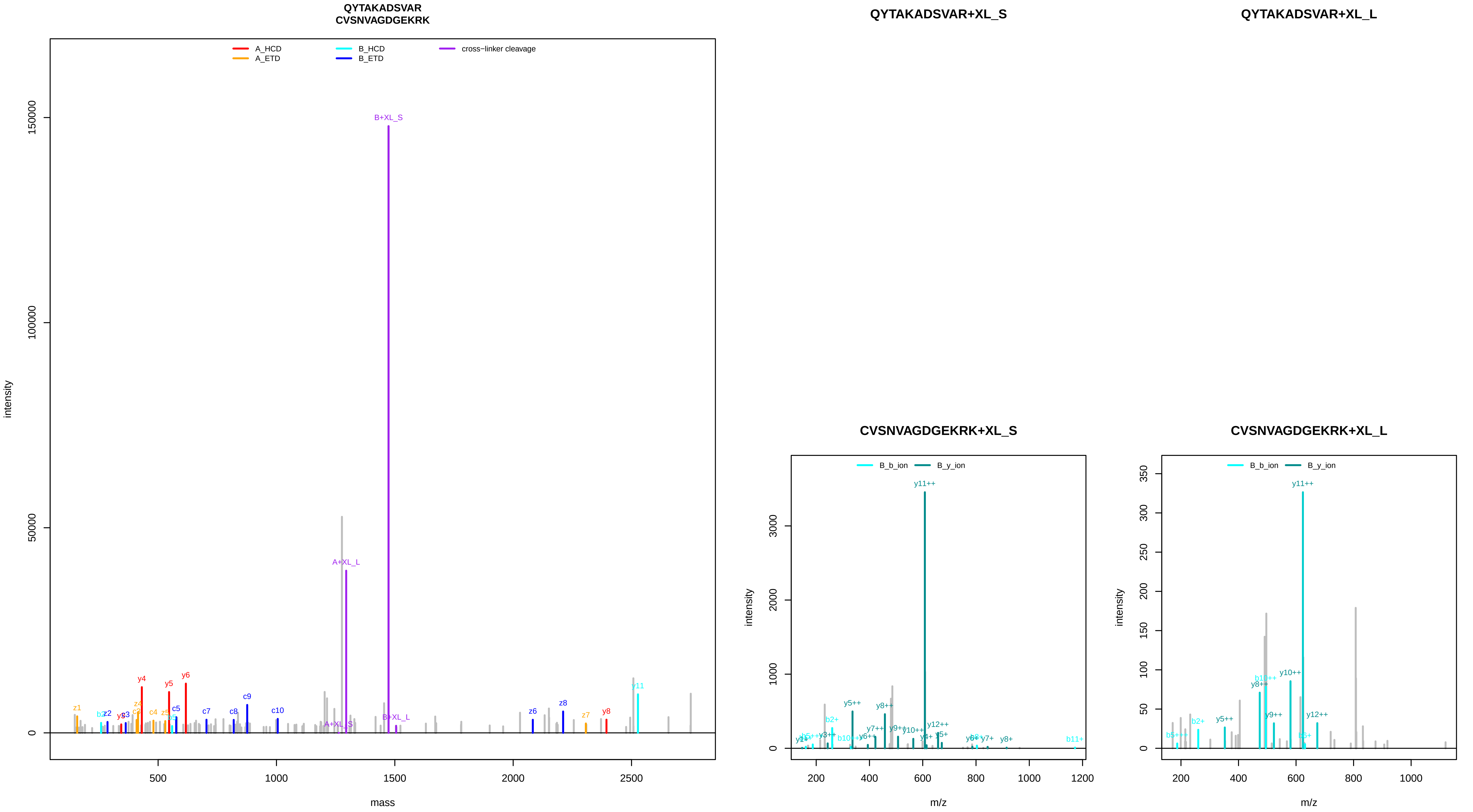
QYTAKADSVAR
CVSNVAGDGEKRK
A_HCD
A_ETD
B_HCD
B_ETD
cross−linker cleavage
B+XL_S
A+XL_L
A+XL_S
B+XL_L
intensity
mass
QYTAKADSVAR+XL_S
QYTAKADSVAR+XL_L
CVSNVAGDGEKRK+XL_S
CVSNVAGDGEKRK+XL_L
B_b_ion
B_y_ion
y11++
m/z

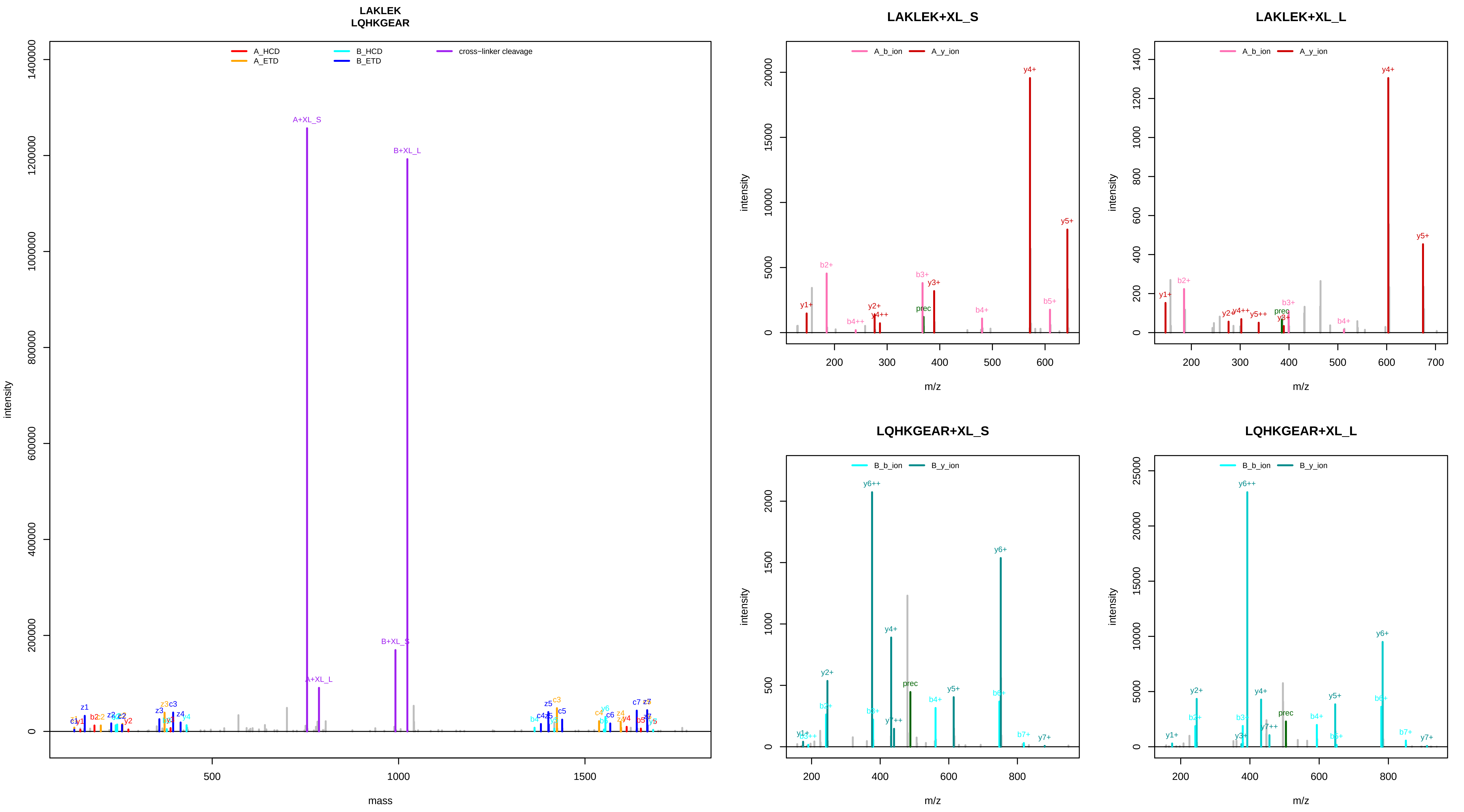
LAKLEK
LQHKGEAR
A_HCD
A_ETD
B_HCD
B_ETD
cross−linker cleavage
A+XL_S
B+XL_L
B+XL_S
A+XL_L
intensity
mass
LAKLEK+XL_S
LAKLEK+XL_L
LQHKGEAR+XL_S
LQHKGEAR+XL_L
A_b_ion
A_y_ion
B_b_ion
B_y_ion
y4+
y5+
b2+
b3+
y3+
prec
y6++
y6+
y4+
y2+
y5+
b4+
m/z

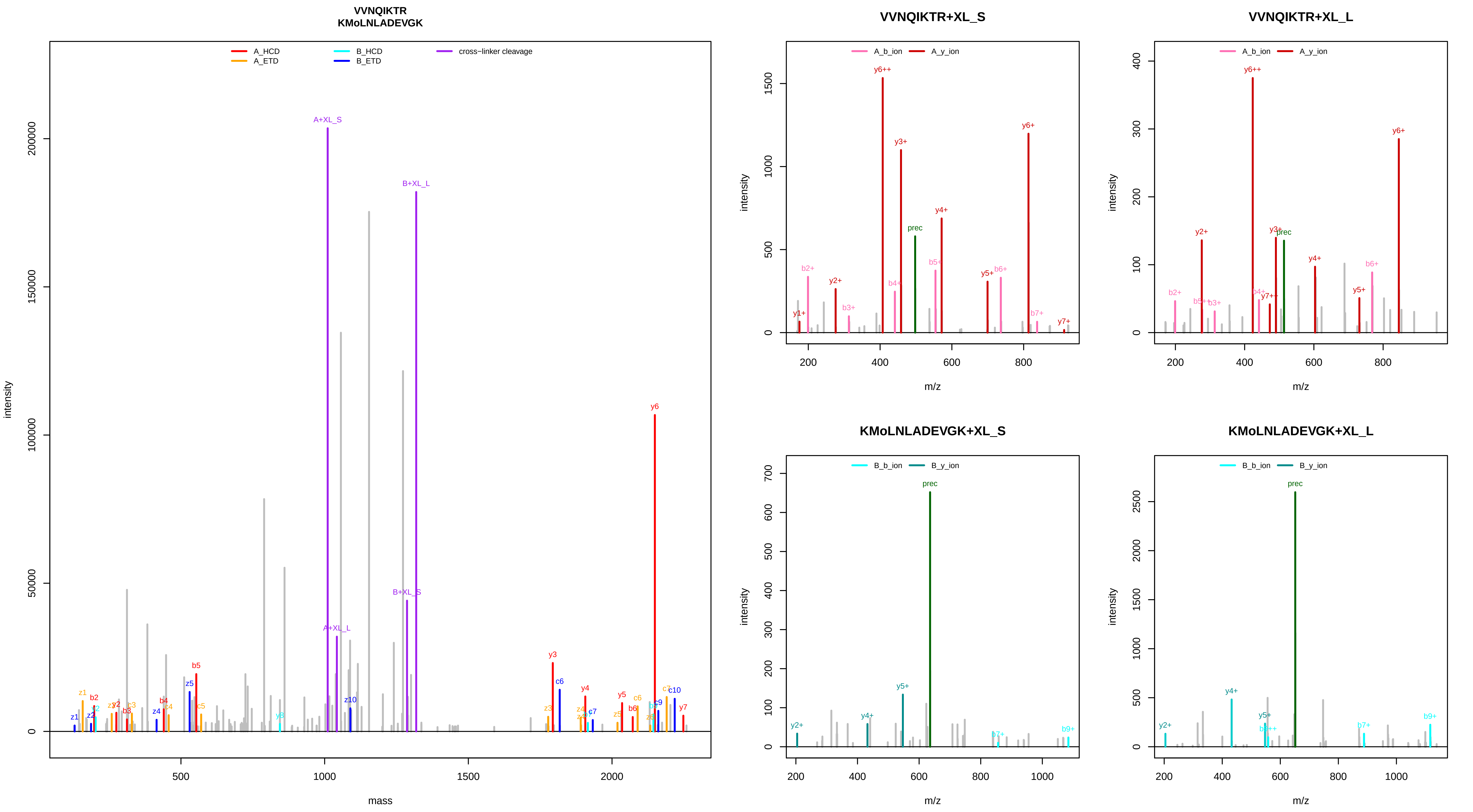

VVNQIKTR
KMoLNLADEVGK
A_HCD
A_ETD
B_HCD
B_ETD
cross−linker cleavage
A+XL_S
B+XL_L
B+XL_S
A+XL_L
intensity
mass
VVNQIKTR+XL_S
VVNQIKTR+XL_L
KMoLNLADEVGK+XL_S
KMoLNLADEVGK+XL_L
A_b_ion
A_y_ion
B_b_ion
B_y_ion
prec
m/z

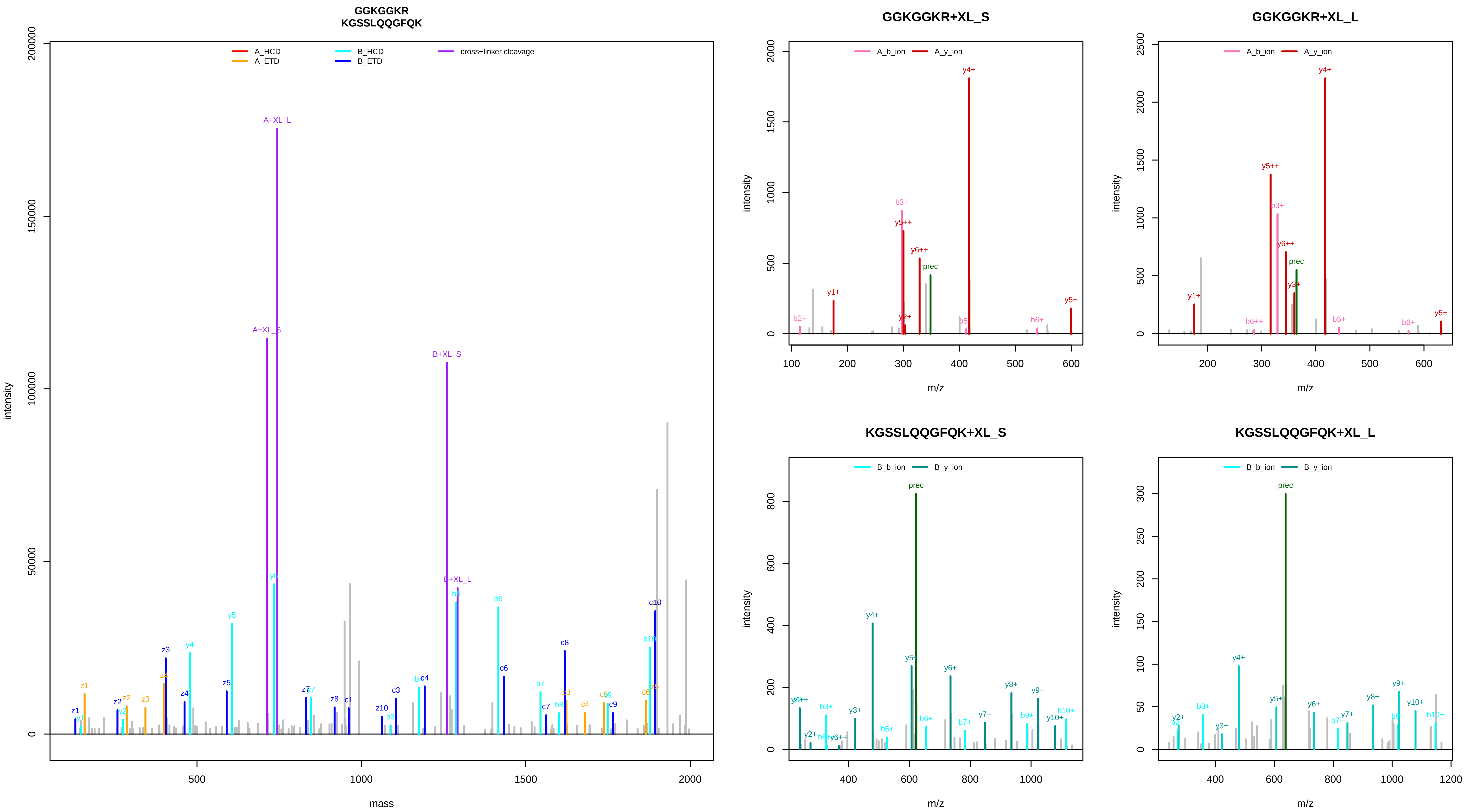
GGKGGKR
KGSSLQQGFQK
A_HCD
A_ETD
B_HCD
B_ETD
cross−linker cleavage
A+XL_L
A+XL_S
B+XL_S
B+XL_L
intensity
mass
GGKGGKR+XL_S
GGKGGKR+XL_L
KGSSLQQGFQK+XL_S
KGSSLQQGFQK+XL_L
A_b_ion
A_y_ion
B_b_ion
B_y_ion
prec
m/z

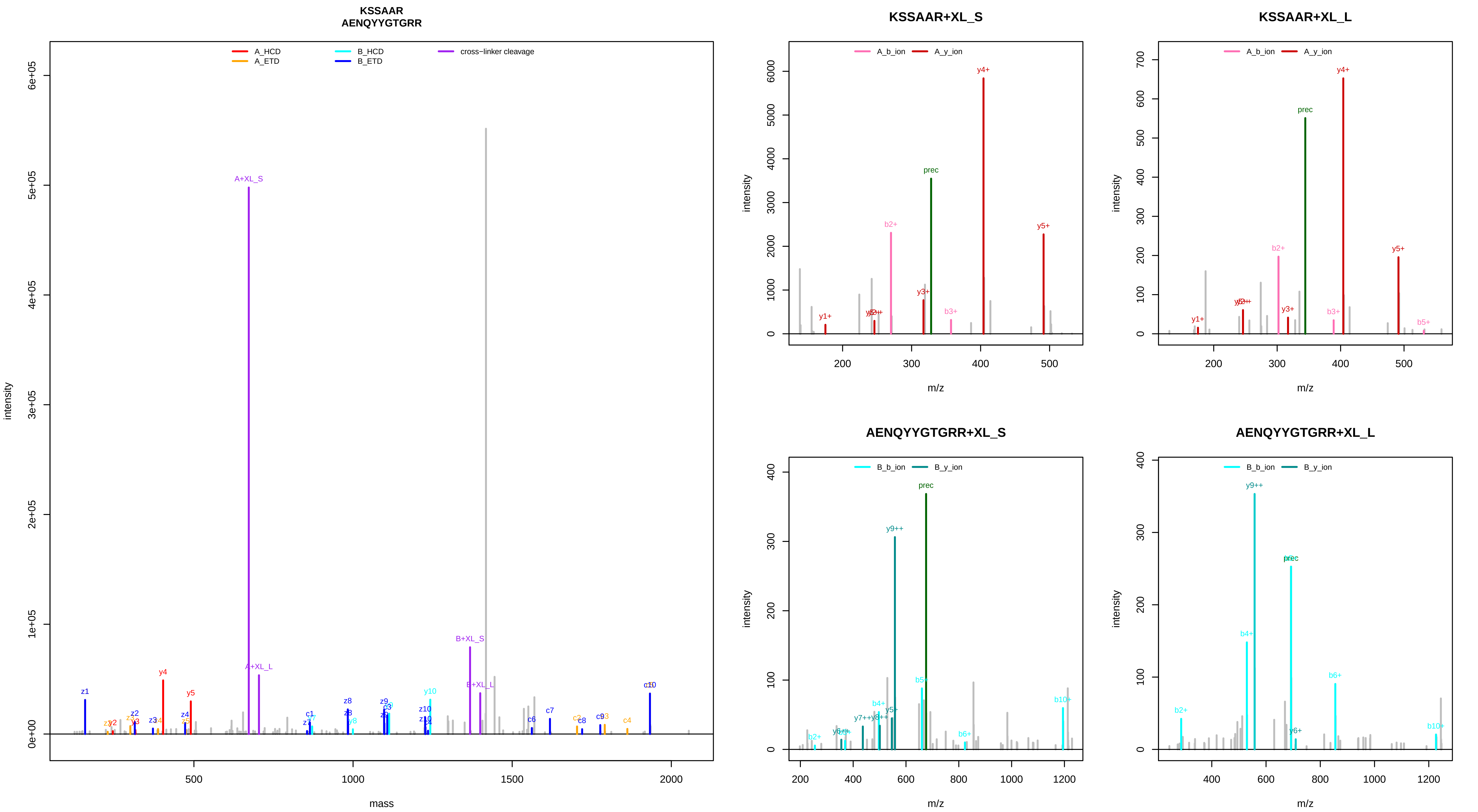
KSSAAR
AENQYYGTGRR
A_HCD
A_ETD
B_HCD
B_ETD
cross−linker cleavage
A+XL_S
A+XL_L
B+XL_S
B+XL_L
intensity
mass
KSSAAR+XL_S
KSSAAR+XL_L
AENQYYGTGRR+XL_S
AENQYYGTGRR+XL_L
A_b_ion
A_y_ion
B_b_ion
B_y_ion
prec
m/z
intensity

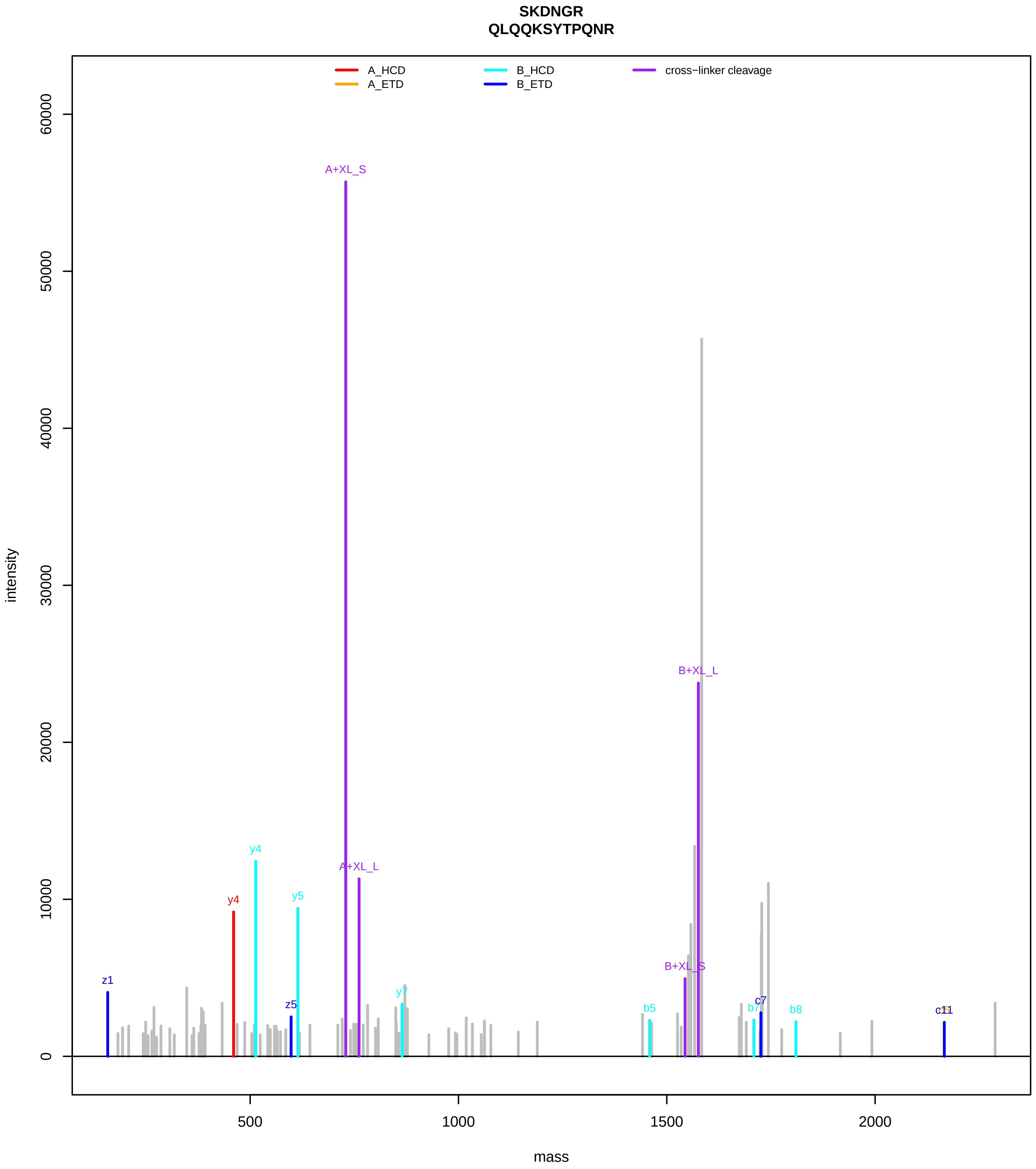

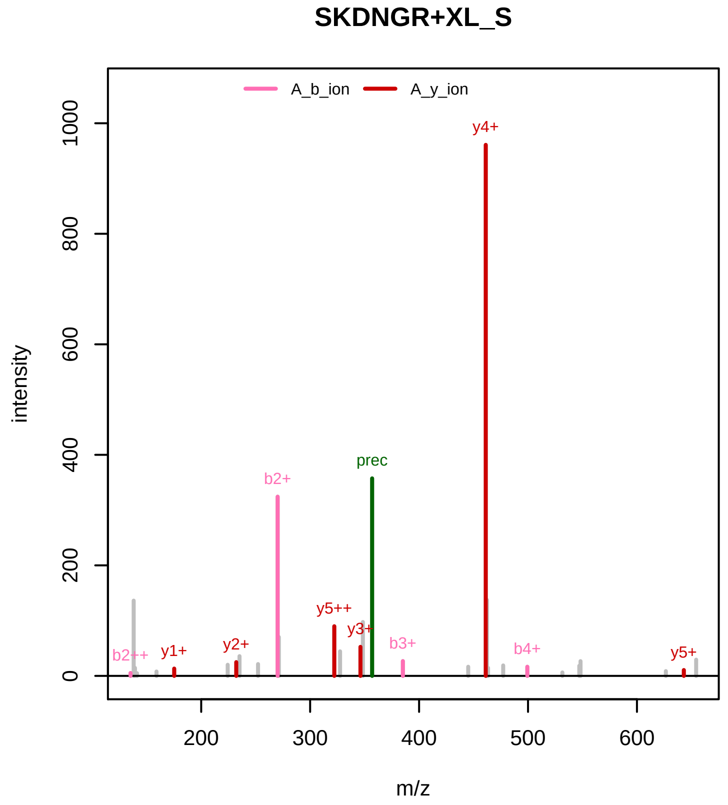

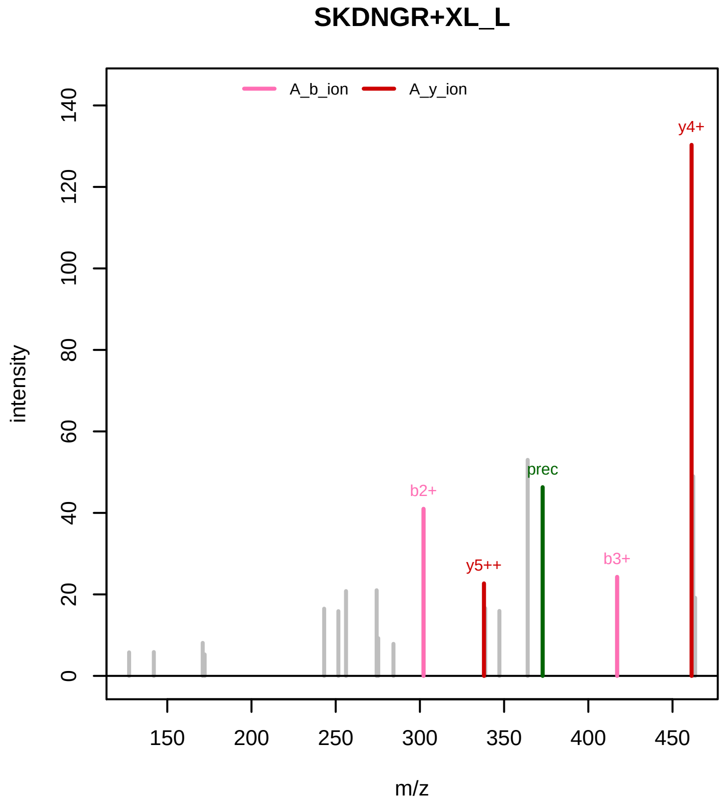

QLQQKSYTPQNR+XL_S

QLQQKSYTPQNR+XL_L

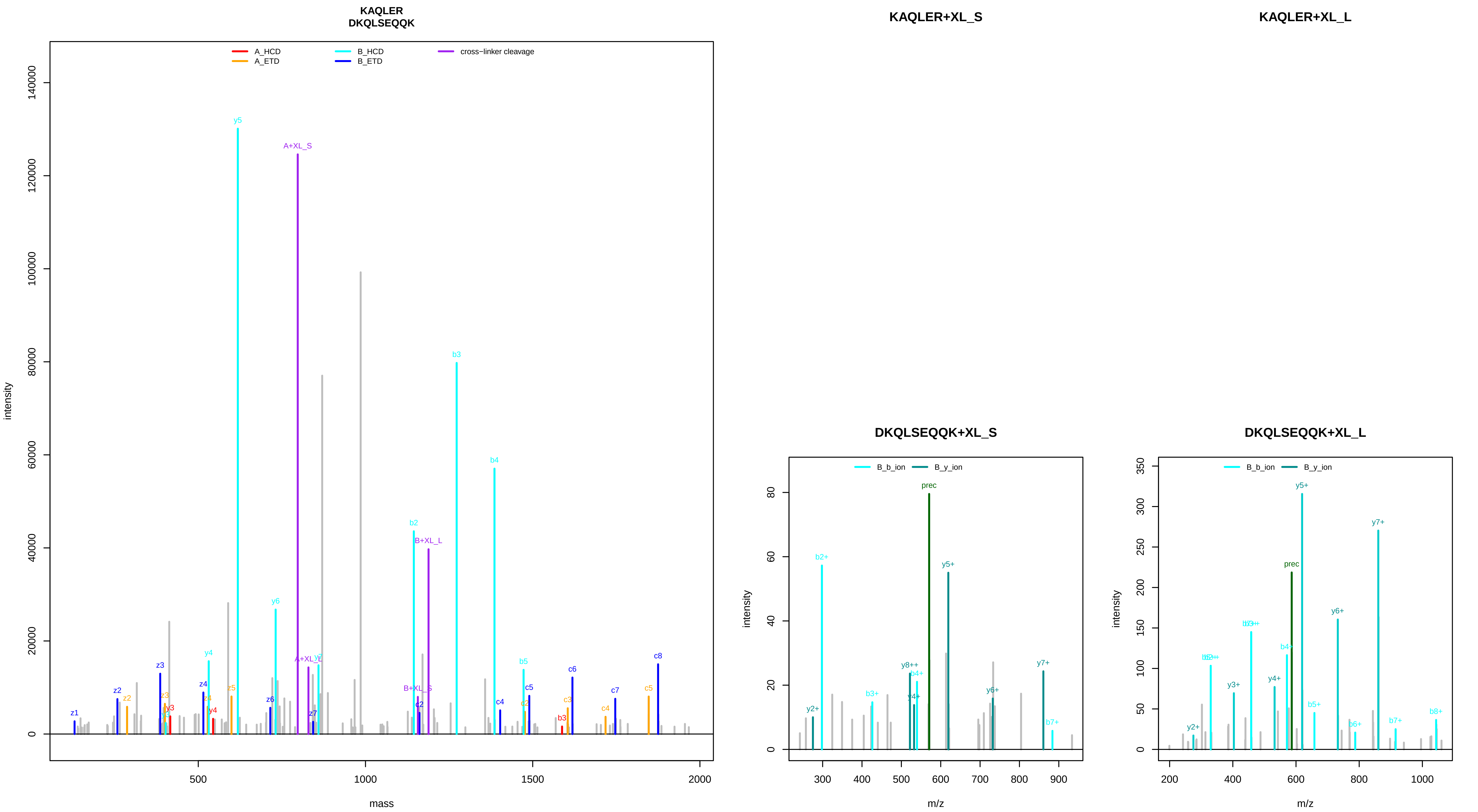
KAQLER
DKQLSEQQK
A_HCD
A_ETD
B_HCD
B_ETD
cross−linker cleavage
intensity
mass
KAQLER+XL_S
KAQLER+XL_L
DKQLSEQQK+XL_S
DKQLSEQQK+XL_L
B_b_ion
B_y_ion
m/z

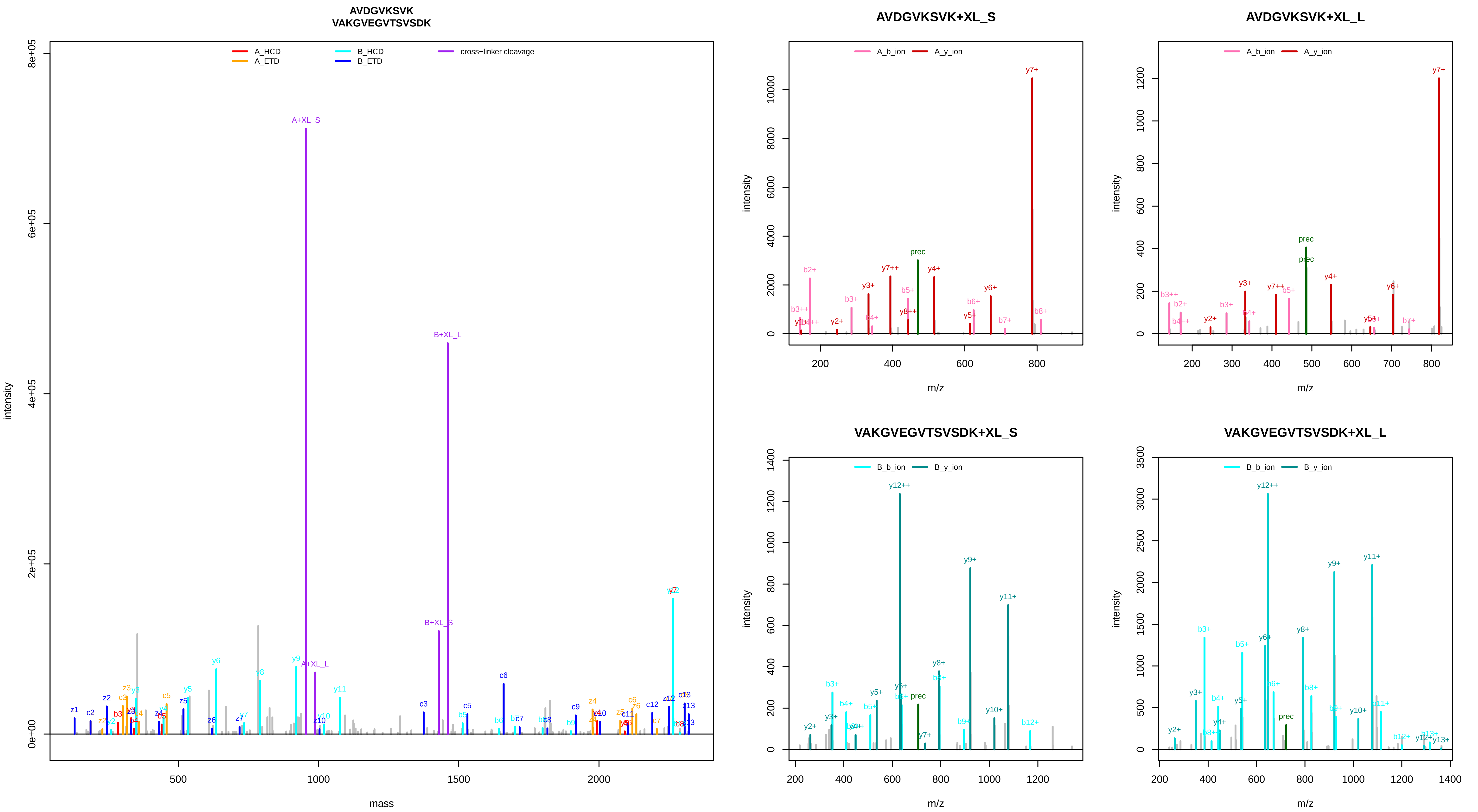
AVDGVKSVK
VAKGVEGVTSVSDK
A_HCD
A_ETD
B_HCD
B_ETD
cross−linker cleavage
A+XL_S
B+XL_L
B+XL_S
A+XL_L
intensity
mass
AVDGVKSVK+XL_S
AVDGVKSVK+XL_L
VAKGVEGVTSVSDK+XL_S
VAKGVEGVTSVSDK+XL_L
A_b_ion
A_y_ion
B_b_ion
B_y_ion
prec
m/z

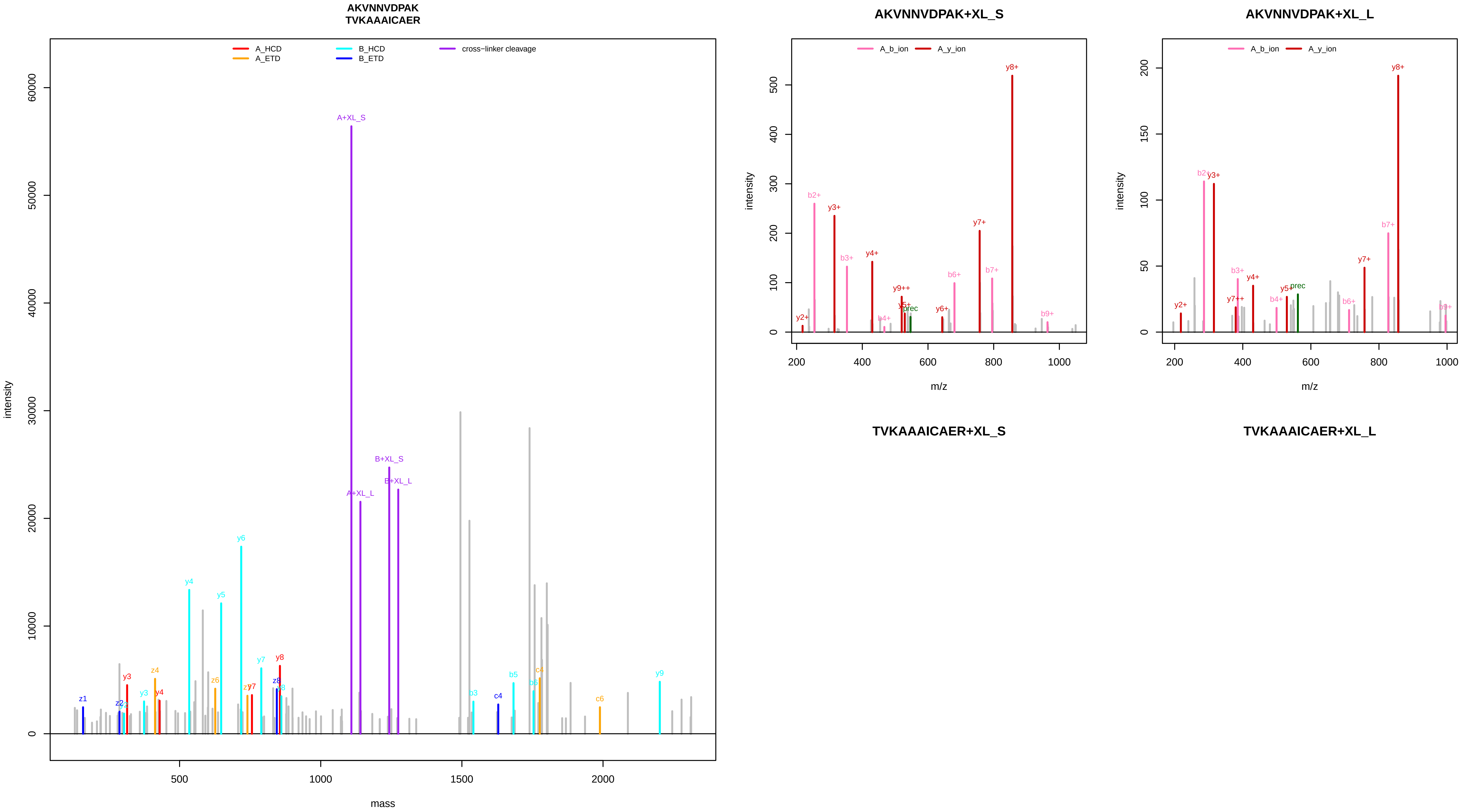

AKVNNVDPAK
TVKAAAICAER
A_HCD
A_ETD
B_HCD
B_ETD
cross−linker cleavage
A+XL_S
A+XL_L
B+XL_S
B+XL_L
intensity
mass
AKVNNVDPAK+XL_S
AKVNNVDPAK+XL_L
A_b_ion
A_y_ion
m/z
TVKAAAICAER+XL_S
TVKAAAICAER+XL_L

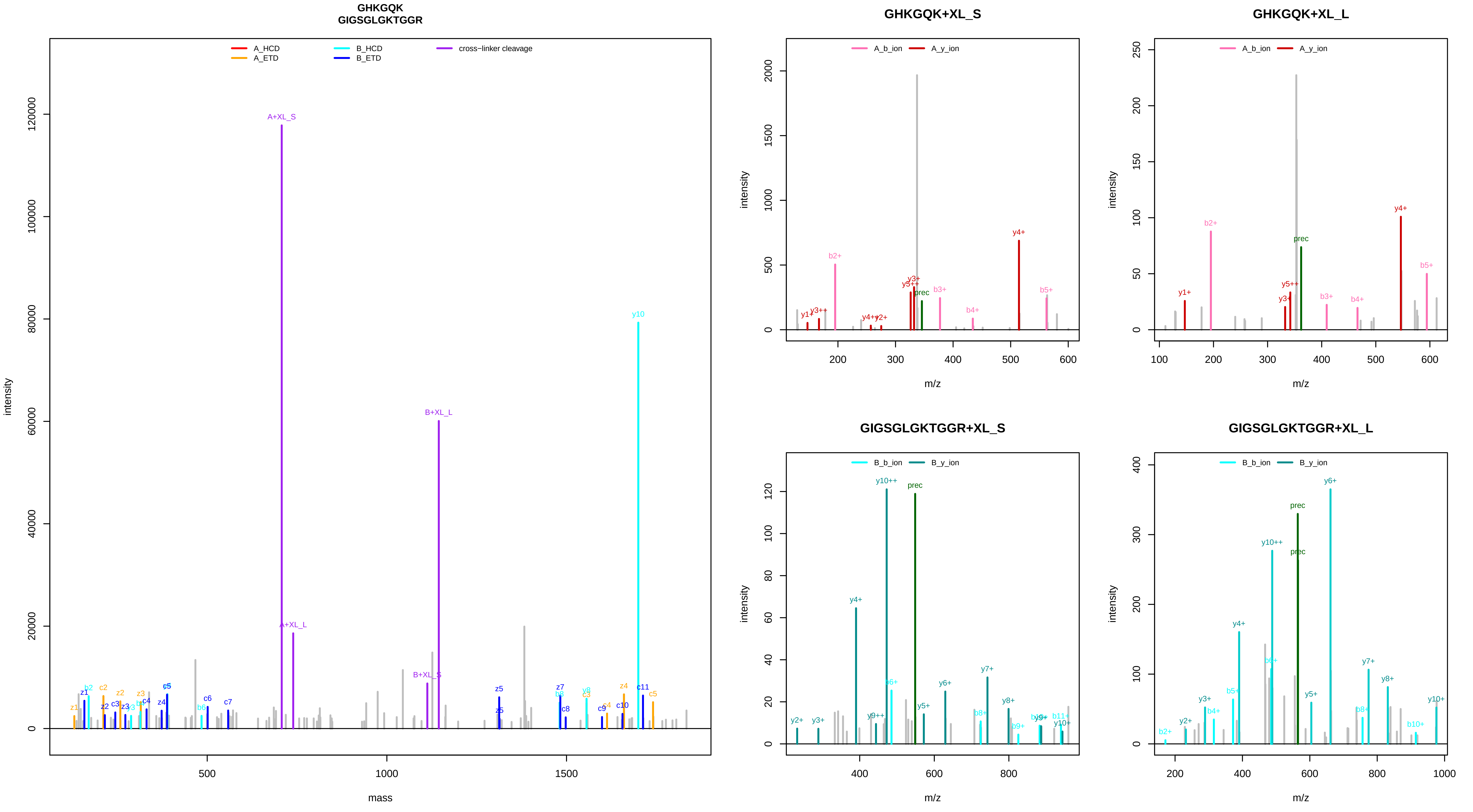
GHKGQK
GIGSGLGKTGGR
A_HCD
A_ETD
B_HCD
B_ETD
cross−linker cleavage
A+XL_S
A+XL_L
B+XL_L
B+XL_S
y10
intensity
mass
GHKGQK+XL_S
GHKGQK+XL_L
GIGSGLGKTGGR+XL_S
GIGSGLGKTGGR+XL_L
A_b_ion
A_y_ion
B_b_ion
B_y_ion
prec
m/z

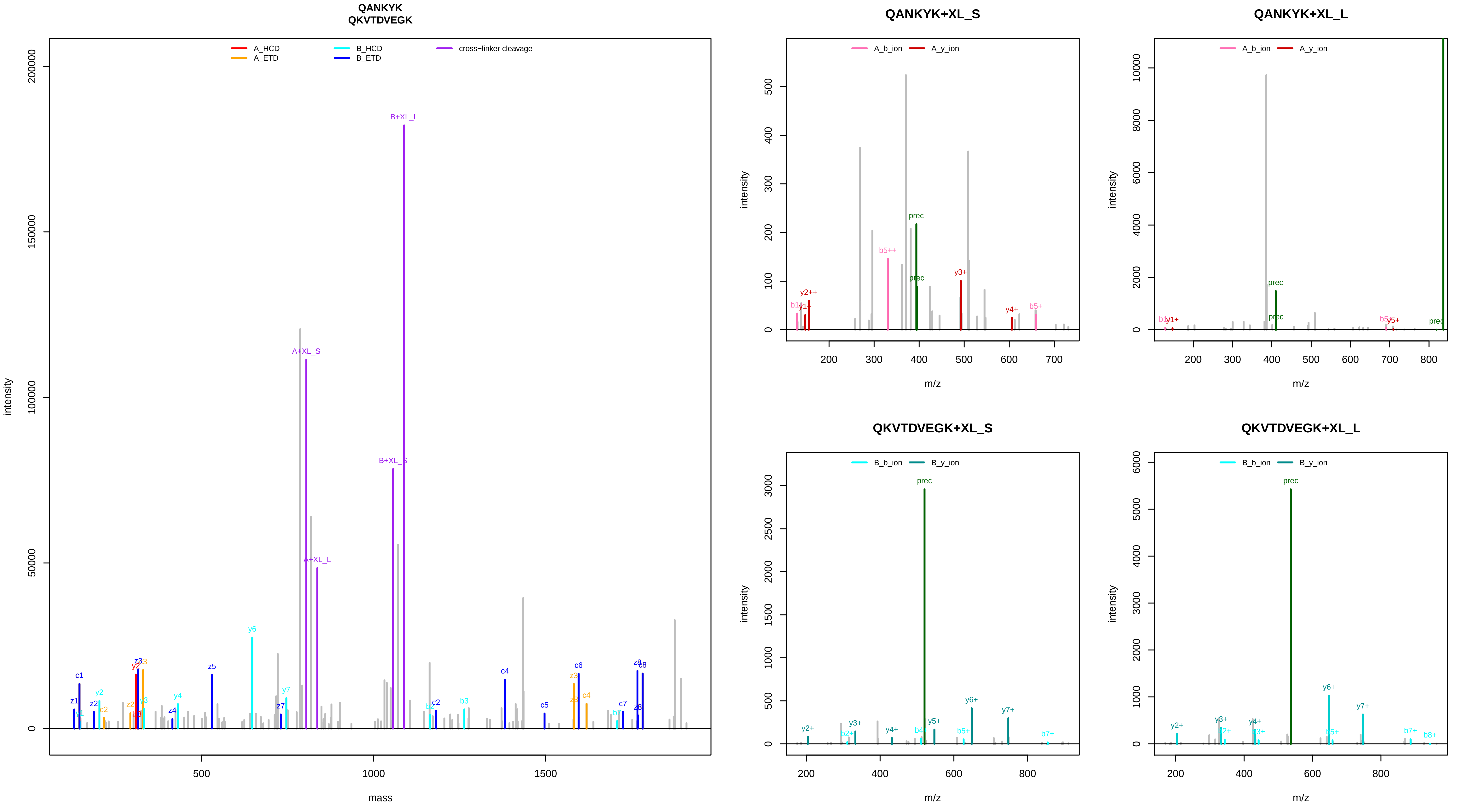
QANKYK
QKVTDVEGK
A_HCD
A_ETD
B_HCD
B_ETD
cross−linker cleavage
B+XL_L
A+XL_S
B+XL_S
A+XL_L
intensity
mass
0
50000
100000
150000
200000
500
1000
1500
QANKYK+XL_S
QANKYK+XL_L
A_b_ion
A_y_ion
prec
QKVTDVEGK+XL_S
QKVTDVEGK+XL_L
B_b_ion
B_y_ion
m/z

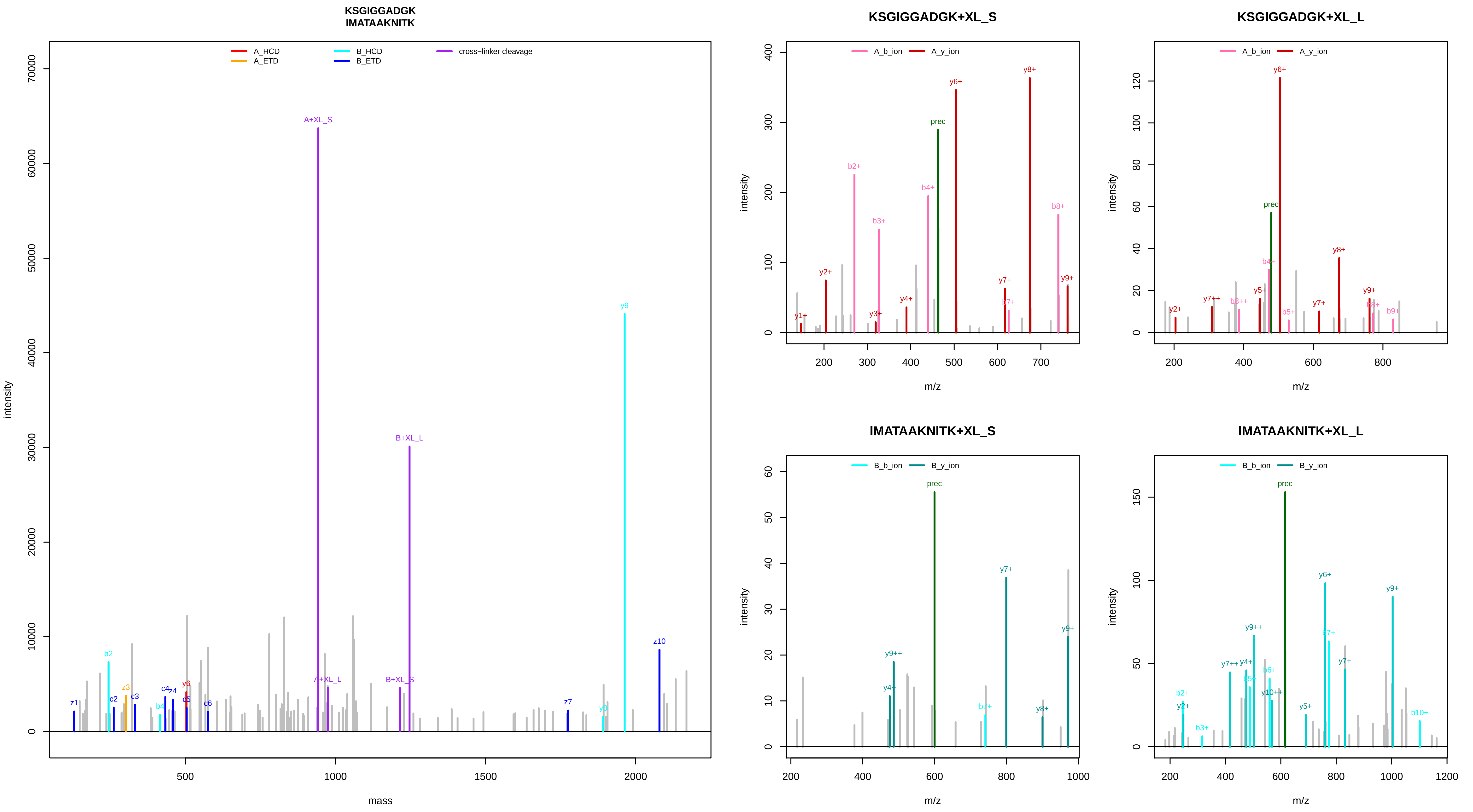

KSGIGGADGK
IMATAAKNITK
A_HCD
A_ETD
B_HCD
B_ETD
cross−linker cleavage
A+XL_S
B+XL_L
A+XL_L
B+XL_S
y9
z10
z7
y6
intensity
mass
KSGIGGADGK+XL_S
KSGIGGADGK+XL_L
IMATAAKNITK+XL_S
IMATAAKNITK+XL_L
A_b_ion
A_y_ion
B_b_ion
B_y_ion
prec
m/z

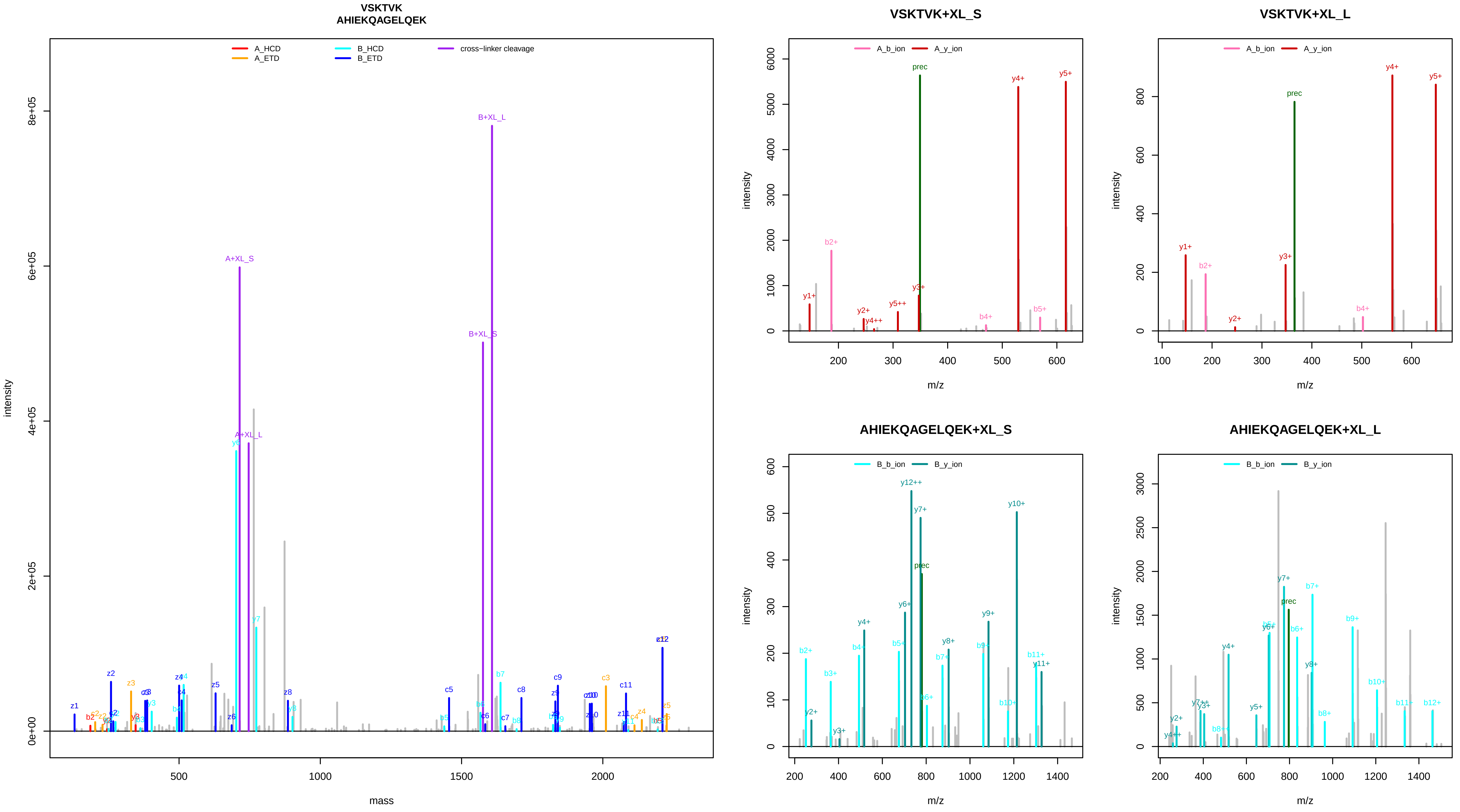

VSKTVK
AHIEKQAGELQEK
A_HCD
A_ETD
B_HCD
B_ETD
cross−linker cleavage
B+XL_L
A+XL_S
B+XL_S
A+XL_L
intensity
mass
VSKTVK+XL_S
VSKTVK+XL_L
A_b_ion
A_y_ion
prec
intensity
m/z
AHIEKQAGELQEK+XL_S
AHIEKQAGELQEK+XL_L
B_b_ion
B_y_ion
intensity
m/z

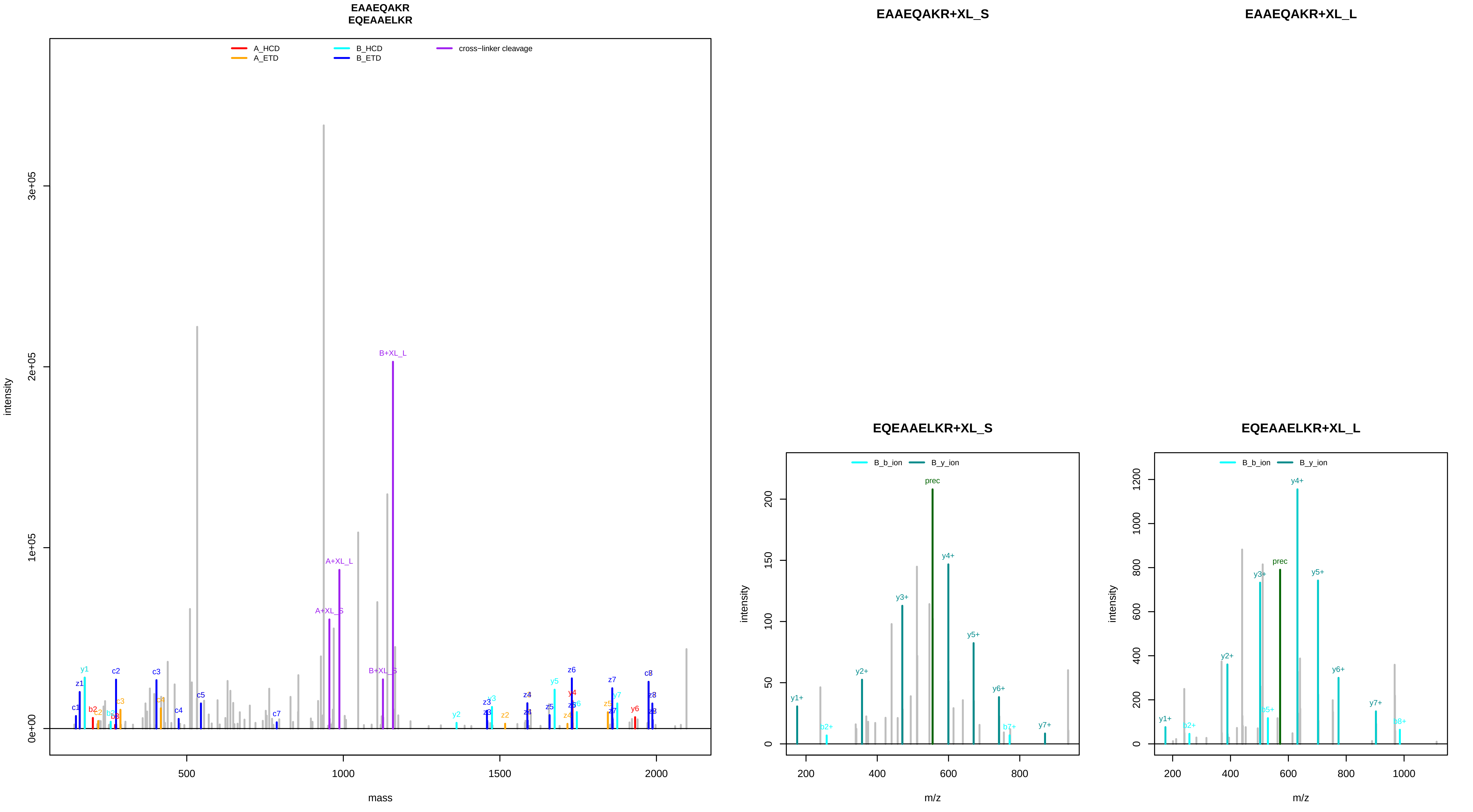
EAAEQAKR
EQEAAELKR
A_HCD
A_ETD
B_HCD
B_ETD
cross−linker cleavage
B+XL_L
A+XL_L
A+XL_S
B+XL_S
intensity
mass
0e+00
1e+05
2e+05
3e+05
500
1000
1500
2000
EAAEQAKR+XL_S
EAAEQAKR+XL_L
EQEAAELKR+XL_S
EQEAAELKR+XL_L
B_b_ion
B_y_ion
prec
m/z

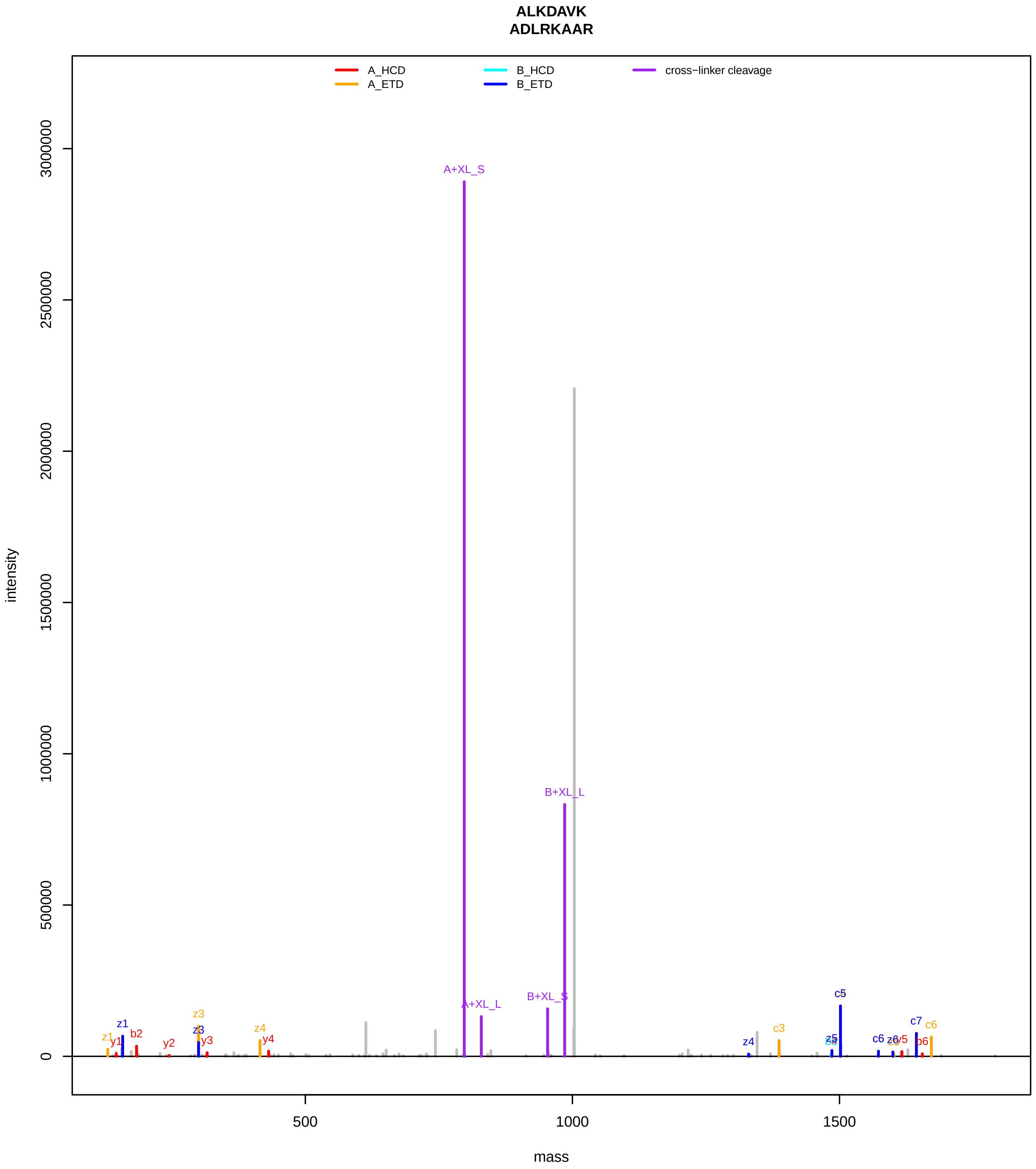
ALKDAVK
ADLRKAAR
A_HCD
A_ETD
B_HCD
B_ETD
cross−linker cleavage
A+XL_S
B+XL_L
A+XL_L
B+XL_S
intensity
mass

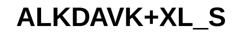
ALKDAVK+XL_S

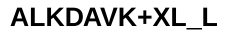
ALKDAVK+XL_L

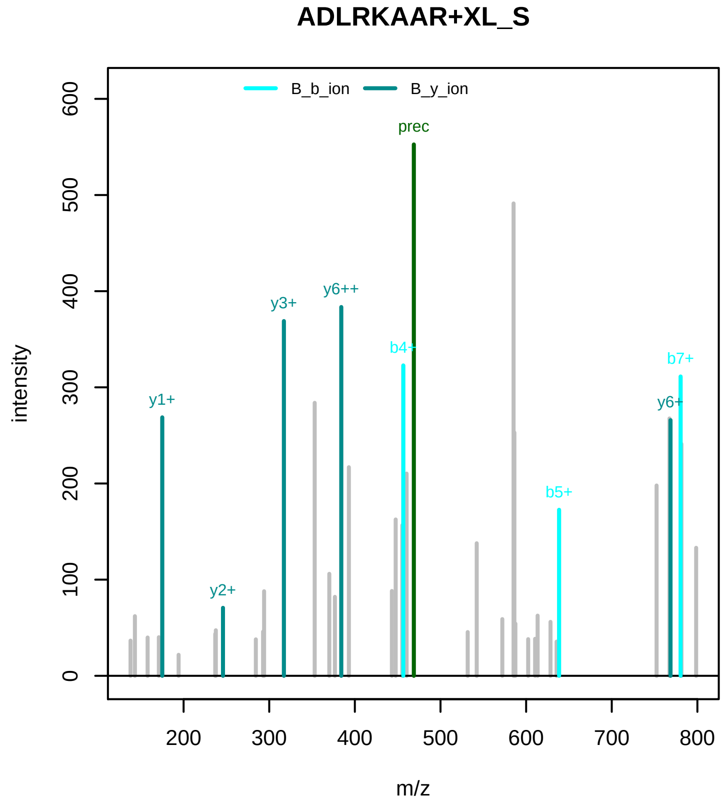
ADLRKAAR+XL_S
B_b_ion
B_y_ion
prec
intensity
m/z

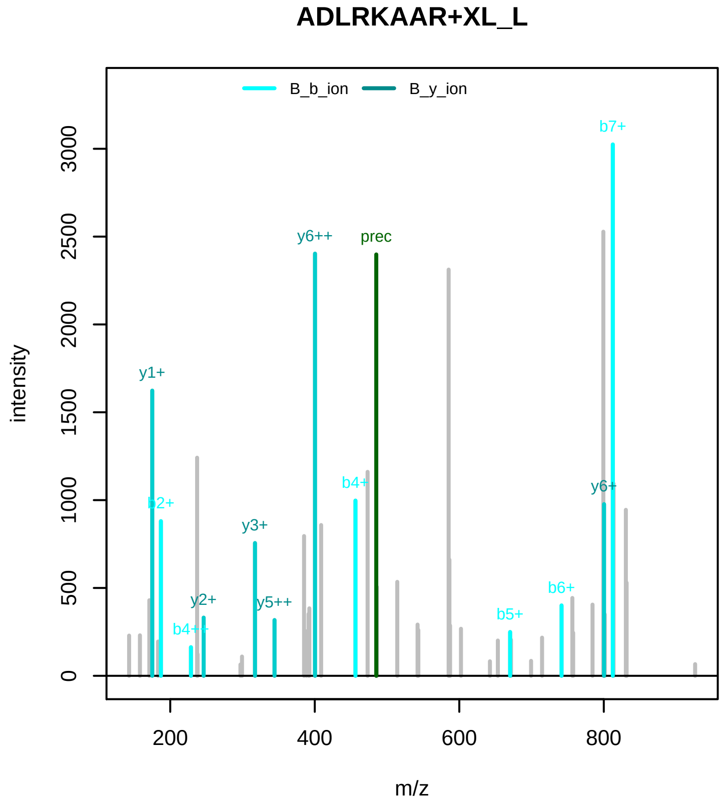
ADLRKAAR+XL_L
B_b_ion
B_y_ion
prec
intensity
m/z

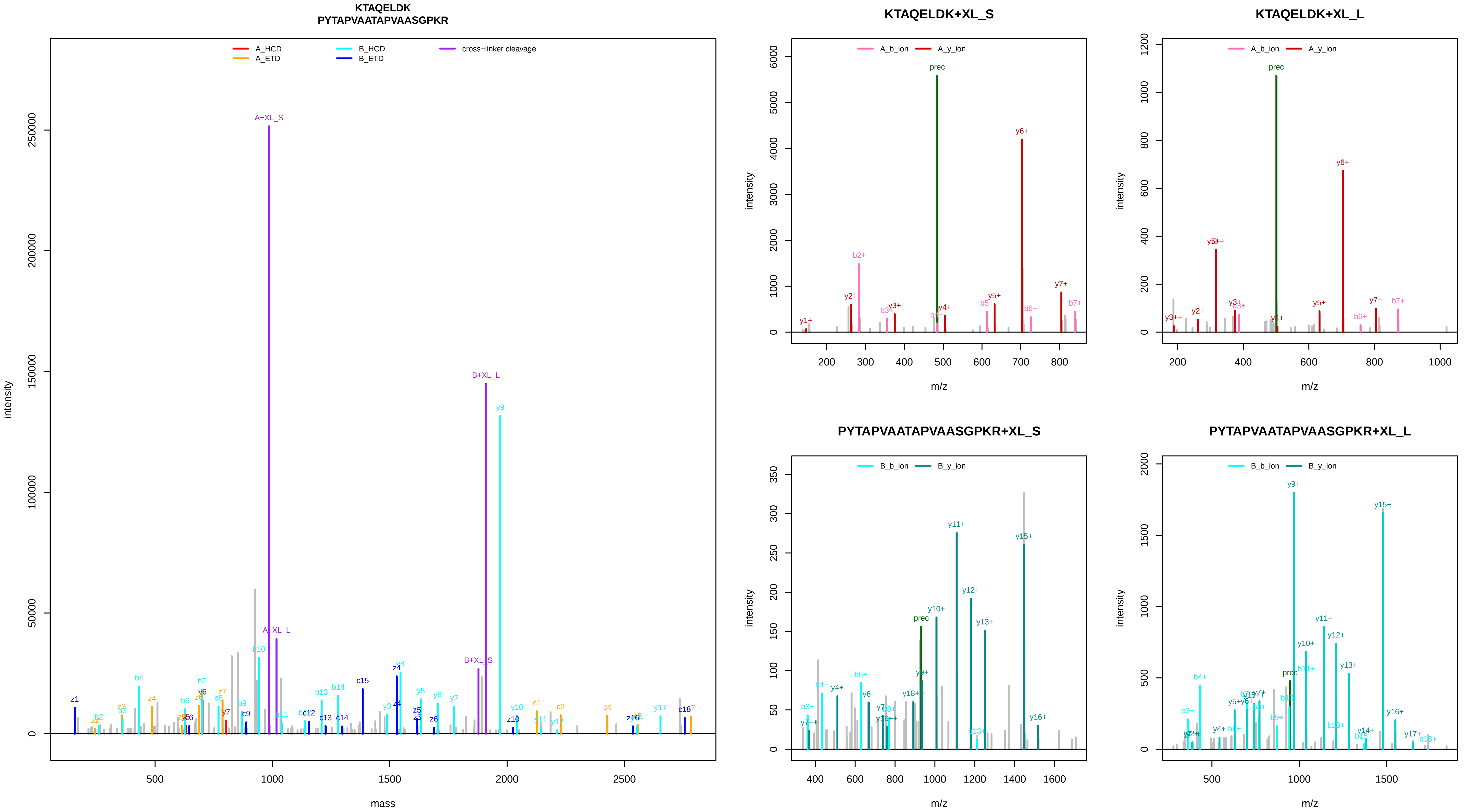
KTAQELDK
PYTAPVAATAPVAASGPKR
A_HCD
A_ETD
B_HCD
B_ETD
cross−linker cleavage
A+XL_S
B+XL_L
A+XL_L
B+XL_S
intensity
mass
KTAQELDK+XL_S
KTAQELDK+XL_L
PYTAPVAATAPVAASGPKR+XL_S
PYTAPVAATAPVAASGPKR+XL_L
A_b_ion
A_y_ion
B_b_ion
B_y_ion
prec
m/z

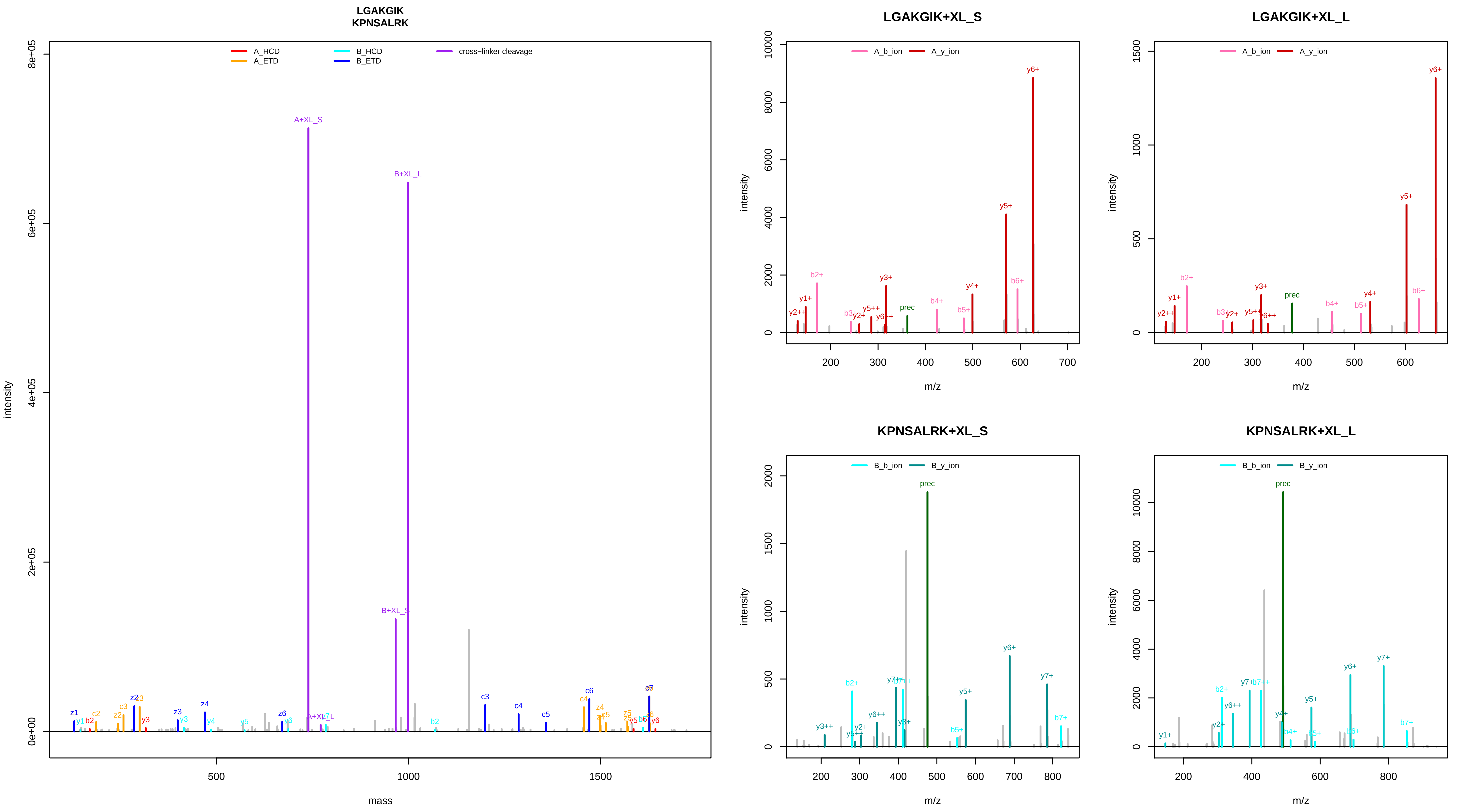
LGAKGIK
KPNSALRK
A_HCD
A_ETD
B_HCD
B_ETD
cross−linker cleavage
A+XL_S
B+XL_L
B+XL_S
intensity
mass
LGAKGIK+XL_S
LGAKGIK+XL_L
KPNSALRK+XL_S
KPNSALRK+XL_L
A_b_ion
A_y_ion
B_b_ion
B_y_ion
prec
intensity
m/z

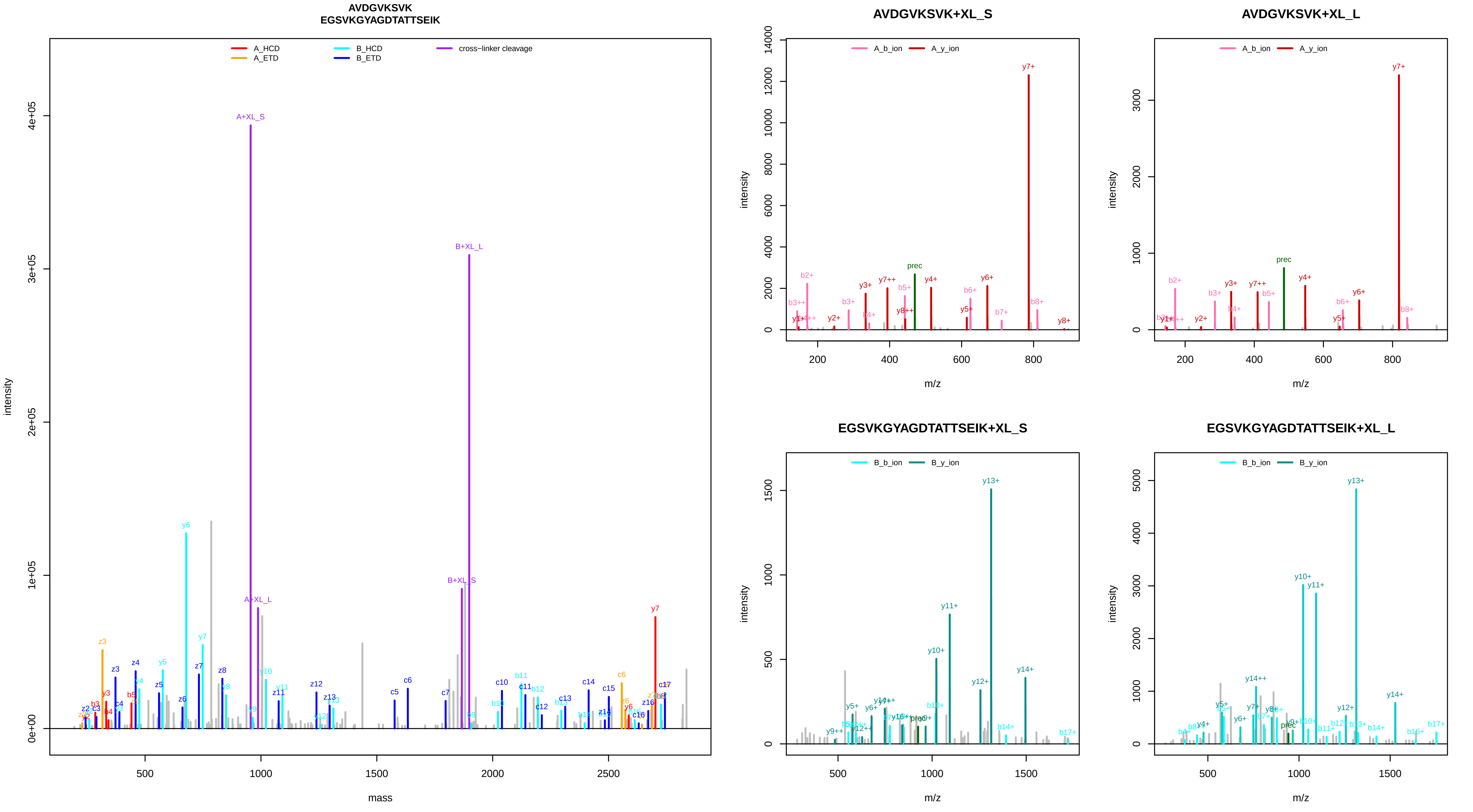
AVDGVKSVK
EGSVKGYAGDTATTSEIK
A_HCD
A_ETD
B_HCD
B_ETD
cross−linker cleavage
A+XL_S
B+XL_L
B+XL_S
A+XL_L
intensity
mass
AVDGVKSVK+XL_S
AVDGVKSVK+XL_L
A_b_ion
A_y_ion
prec
m/z
EGSVKGYAGDTATTSEIK+XL_S
EGSVKGYAGDTATTSEIK+XL_L
B_b_ion
B_y_ion

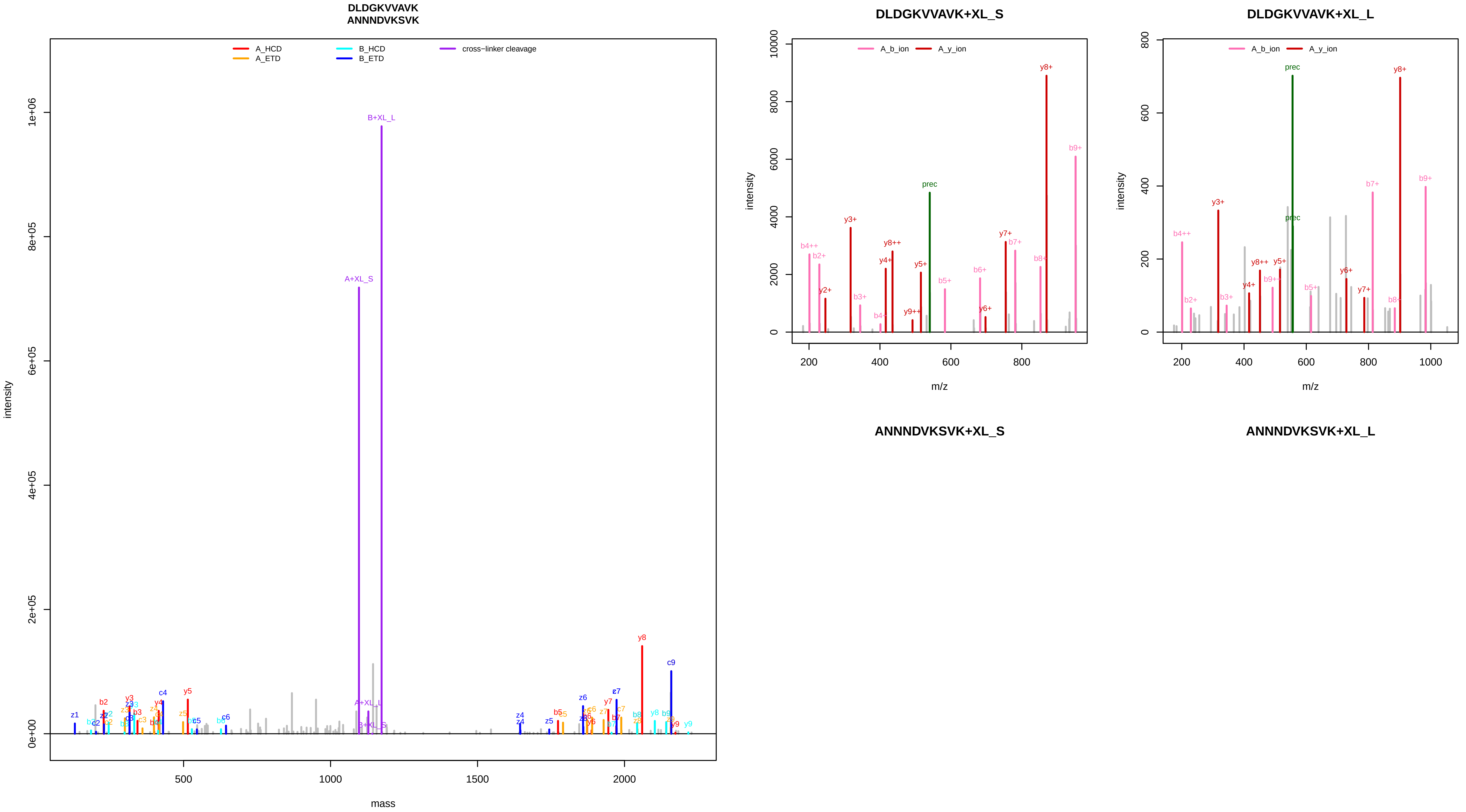
DLDGKVVAVK
ANNNDVKSVK
A_HCD
A_ETD
B_HCD
B_ETD
cross−linker cleavage
B+XL_L
A+XL_S
intensity
mass
0e+00
2e+05
4e+05
6e+05
8e+05
1e+06
500
1000
1500
2000
DLDGKVVAVK+XL_S
DLDGKVVAVK+XL_L
A_b_ion
A_y_ion
prec
m/z
intensity
ANNNDVKSVK+XL_S
ANNNDVKSVK+XL_L

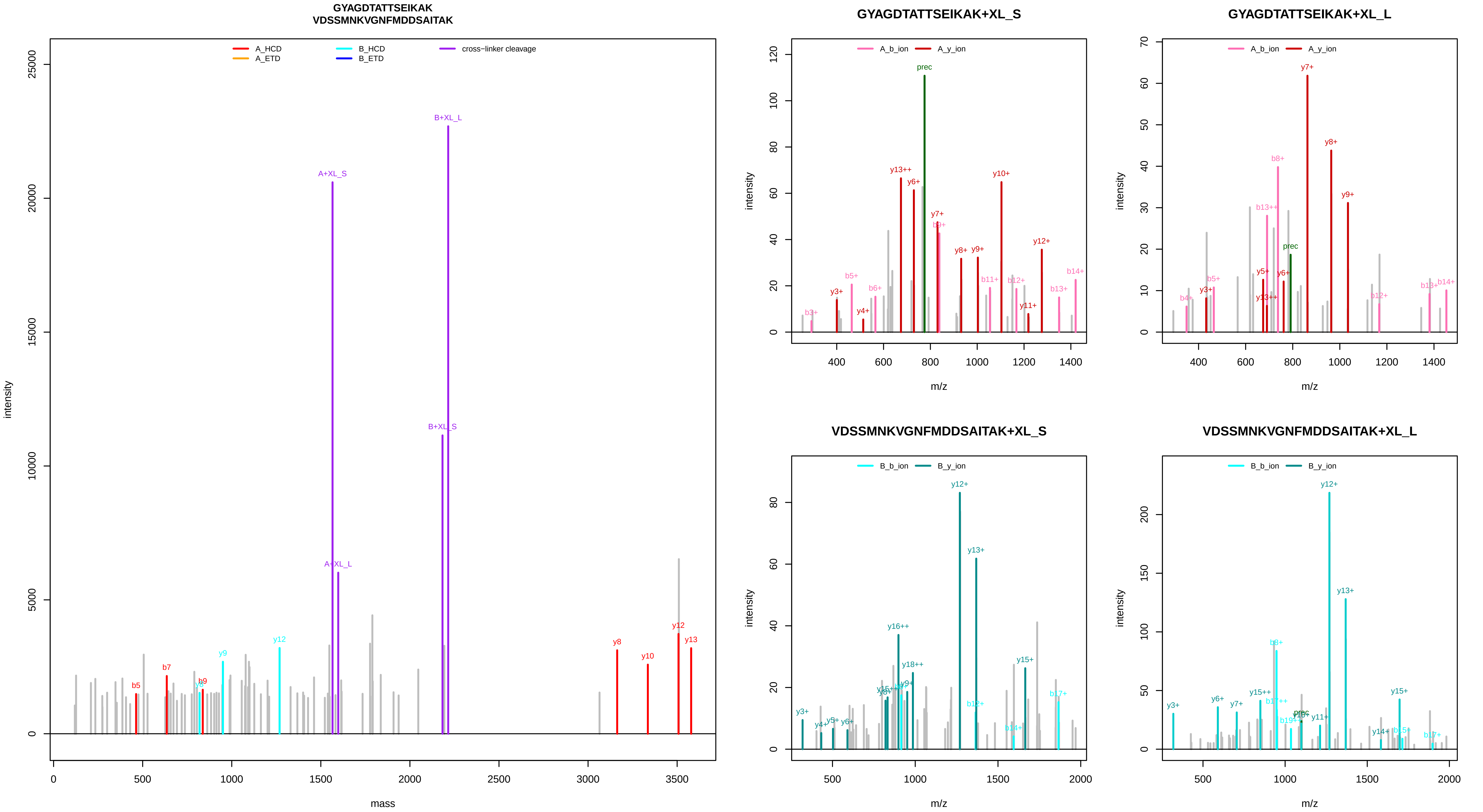

GYAGDTATTSEIKAK
VDSSMNKVGNFMDDSAITAK
A_HCD
A_ETD
B_HCD
B_ETD
cross−linker cleavage
B+XL_L
A+XL_S
B+XL_S
A+XL_L
intensity
mass
GYAGDTATTSEIKAK+XL_S
GYAGDTATTSEIKAK+XL_L
VDSSMNKVGNFMDDSAITAK+XL_S
VDSSMNKVGNFMDDSAITAK+XL_L
A_b_ion
A_y_ion
B_b_ion
B_y_ion
prec
m/z

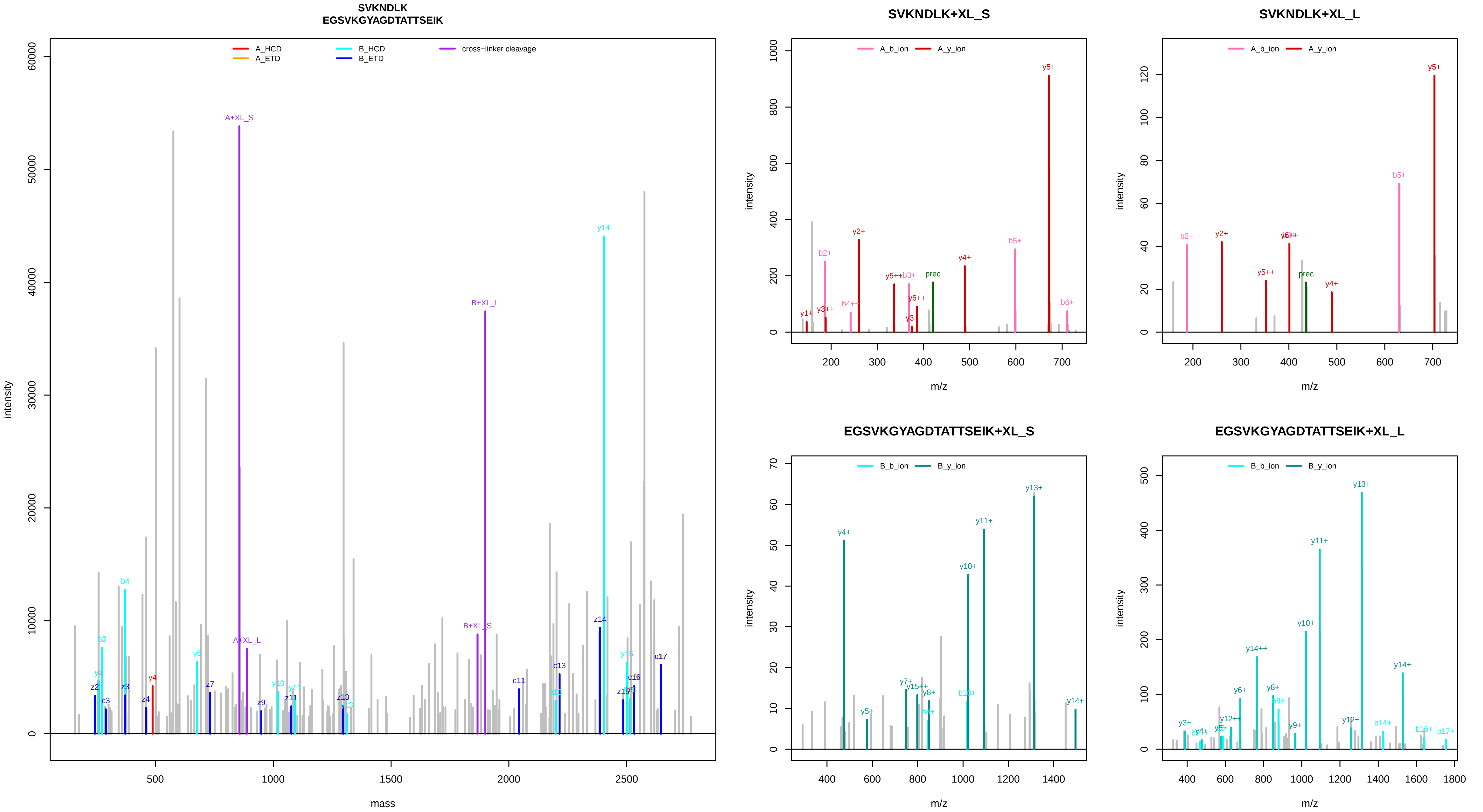
SVKNDLK
EGSVKGYAGDTATTSEIK
A_HCD
A_ETD
B_HCD
B_ETD
cross−linker cleavage
A+XL_S
B+XL_L
B+XL_S
A+XL_L
intensity
mass
SVKNDLK+XL_S
SVKNDLK+XL_L
EGSVKGYAGDTATTSEIK+XL_S
EGSVKGYAGDTATTSEIK+XL_L
A_b_ion
A_y_ion
B_b_ion
B_y_ion
prec
m/z

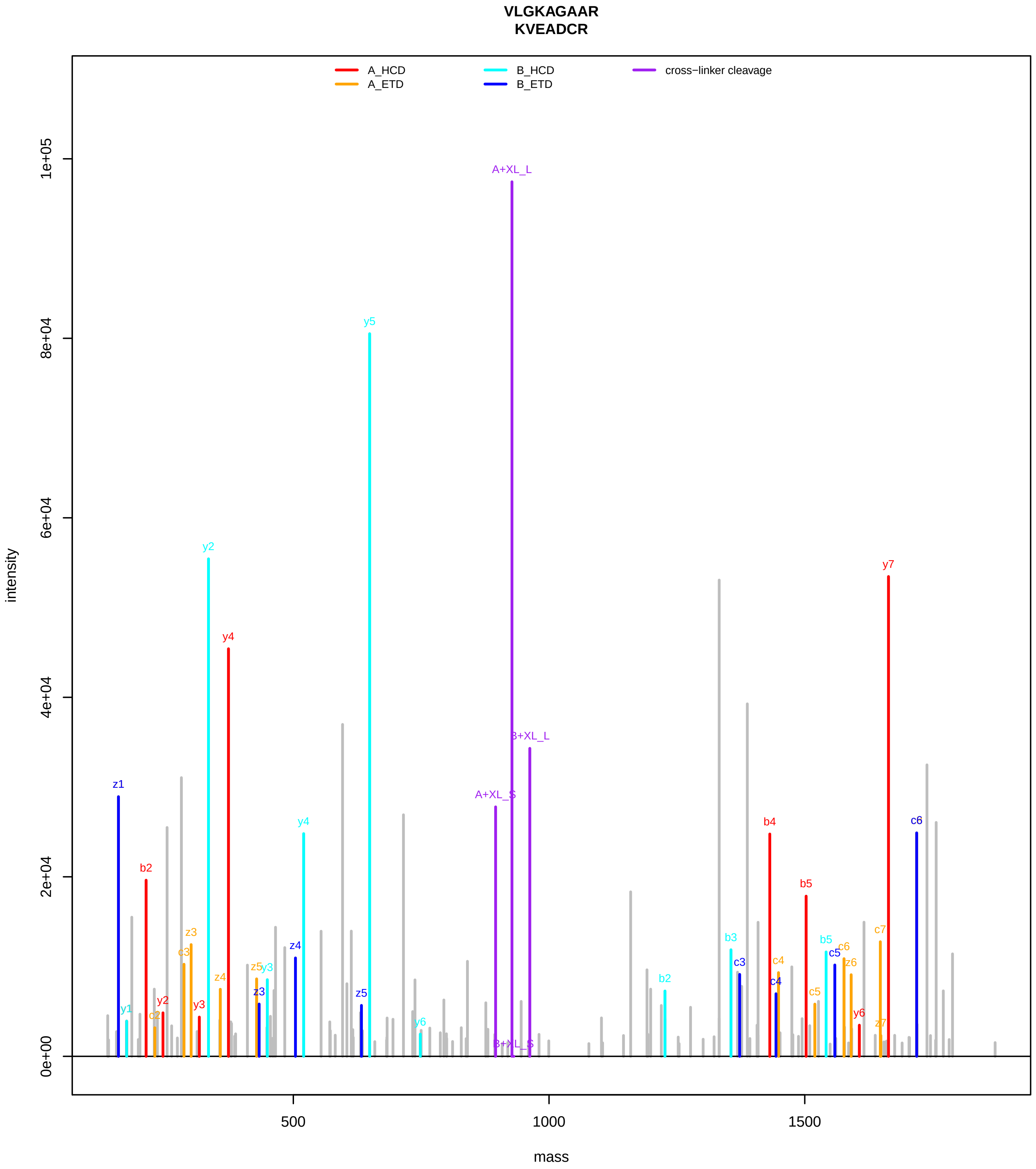

VLGKAGAAR+XL_S

VLGKAGAAR+XL_L

KVEADCR+XL_S

KVEADCR+XL_L

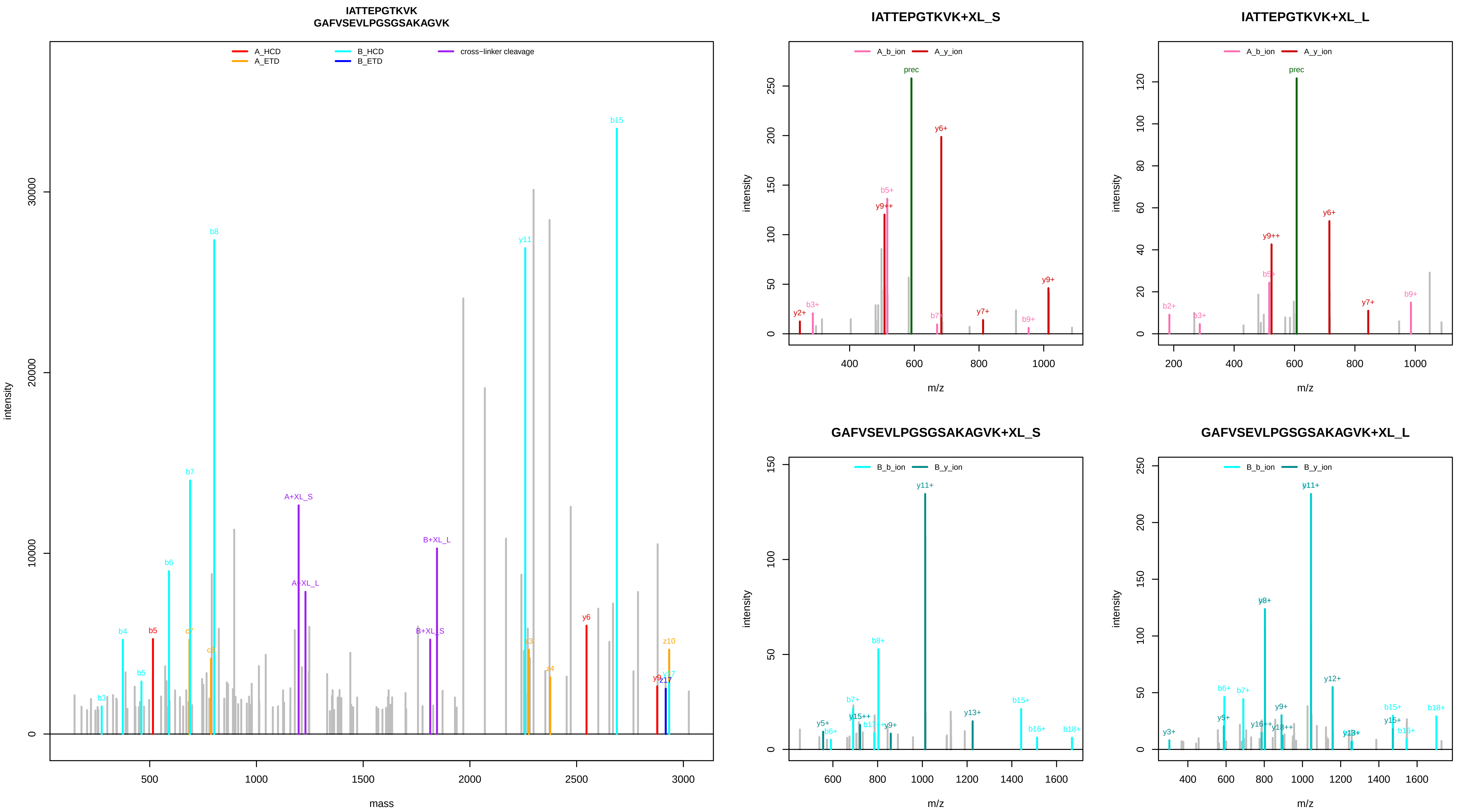
IATTEPGTKVK
GAFVSEVLPGSGSAKAGVK
A_HCD
A_ETD
B_HCD
B_ETD
cross−linker cleavage
intensity
mass
IATTEPGTKVK+XL_S
IATTEPGTKVK+XL_L
GAFVSEVLPGSGSAKAGVK+XL_S
GAFVSEVLPGSGSAKAGVK+XL_L
A_b_ion
A_y_ion
B_b_ion
B_y_ion
m/z
prec

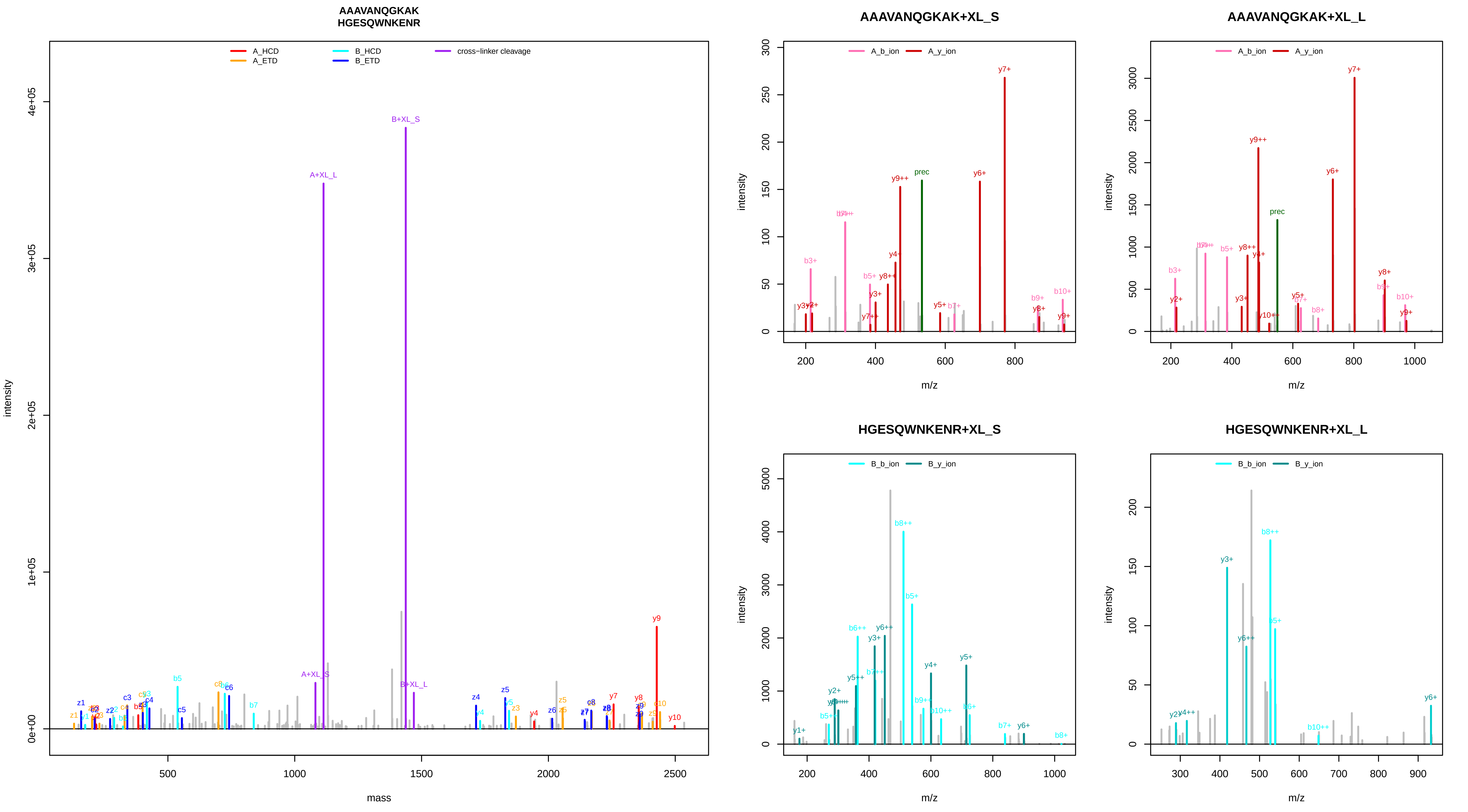
AAAVANQGKAK
HGESQWNKENR
A_HCD
A_ETD
B_HCD
B_ETD
cross−linker cleavage
B+XL_S
A+XL_L
intensity
mass
AAAVANQGKAK+XL_S
AAAVANQGKAK+XL_L
A_b_ion
A_y_ion
HGESQWNKENR+XL_S
HGESQWNKENR+XL_L
B_b_ion
B_y_ion
prec
m/z

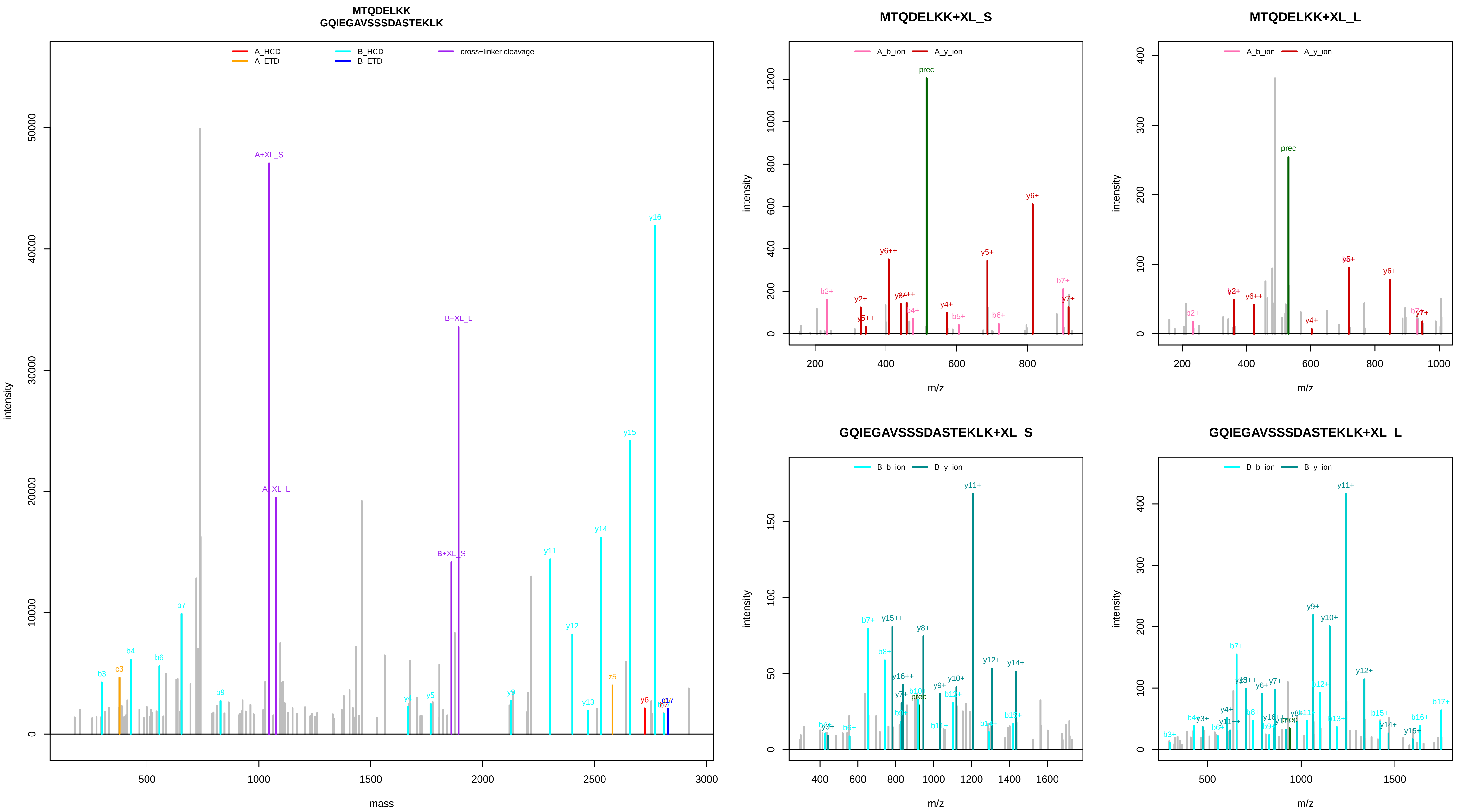
MTQDELKK
GQIEGAVSSSDASTEKLK
A_HCD
A_ETD
B_HCD
B_ETD
cross−linker cleavage
intensity
mass
MTQDELKK+XL_S
MTQDELKK+XL_L
GQIEGAVSSSDASTEKLK+XL_S
GQIEGAVSSSDASTEKLK+XL_L
A_b_ion
A_y_ion
B_b_ion
B_y_ion
m/z

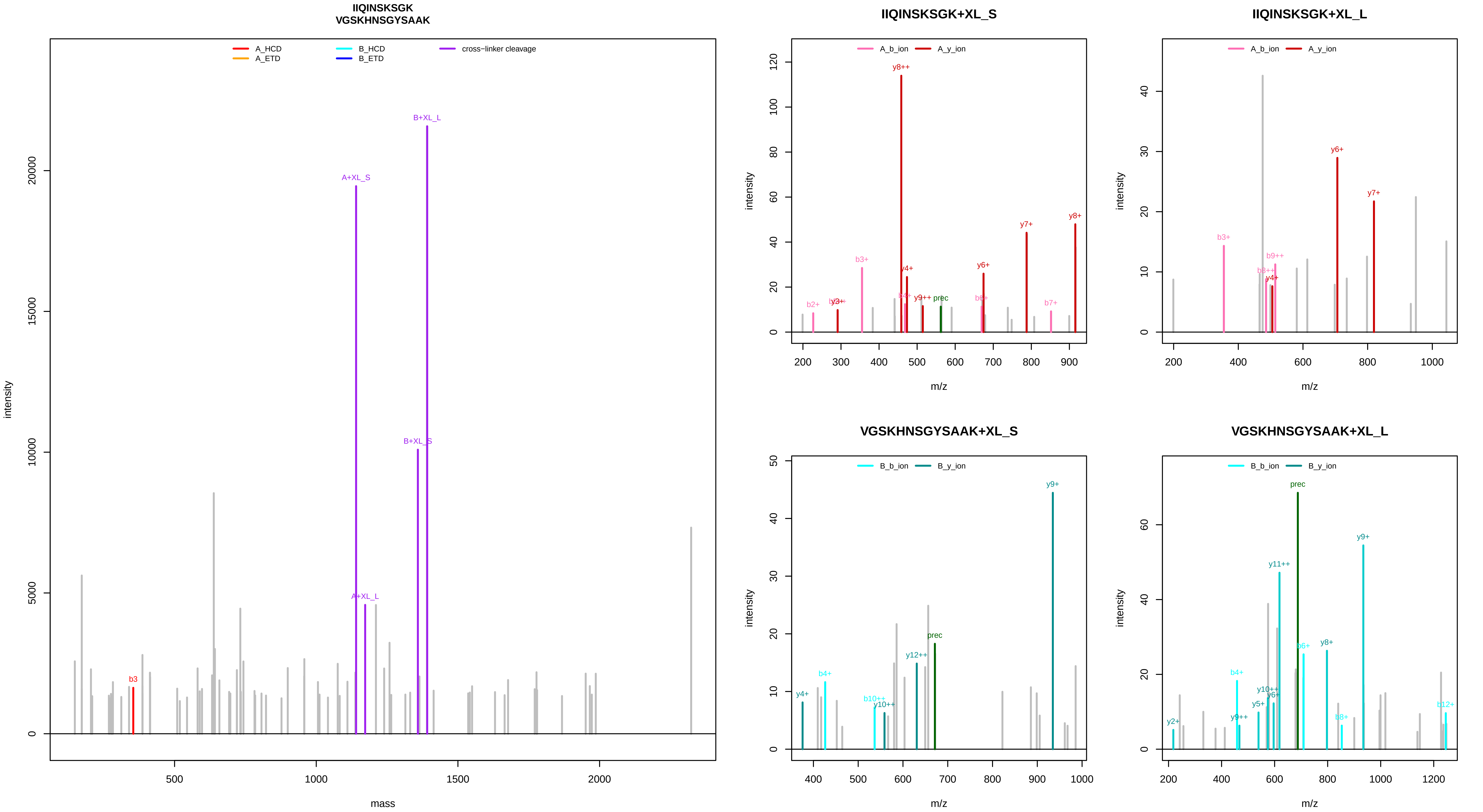
IIQINSKSGK
VGSKHNSGYSAAK
A_HCD
A_ETD
B_HCD
B_ETD
cross−linker cleavage
B+XL_L
A+XL_S
B+XL_S
A+XL_L
b3
intensity
mass
IIQINSKSGK+XL_S
IIQINSKSGK+XL_L
A_b_ion
A_y_ion
y8++
y7+
y8+
y6+
y4+
b3+
y9++
prec
b2+
b7+
b3+
b9++
y4+
m/z
VGSKHNSGYSAAK+XL_S
VGSKHNSGYSAAK+XL_L
B_b_ion
B_y_ion
y9+
prec
y12++
b4+
y4+
b10++
y10++
y11++
b6+
y8+
y9++
y5+
y10++
y6+
y2+
b8+
b12+

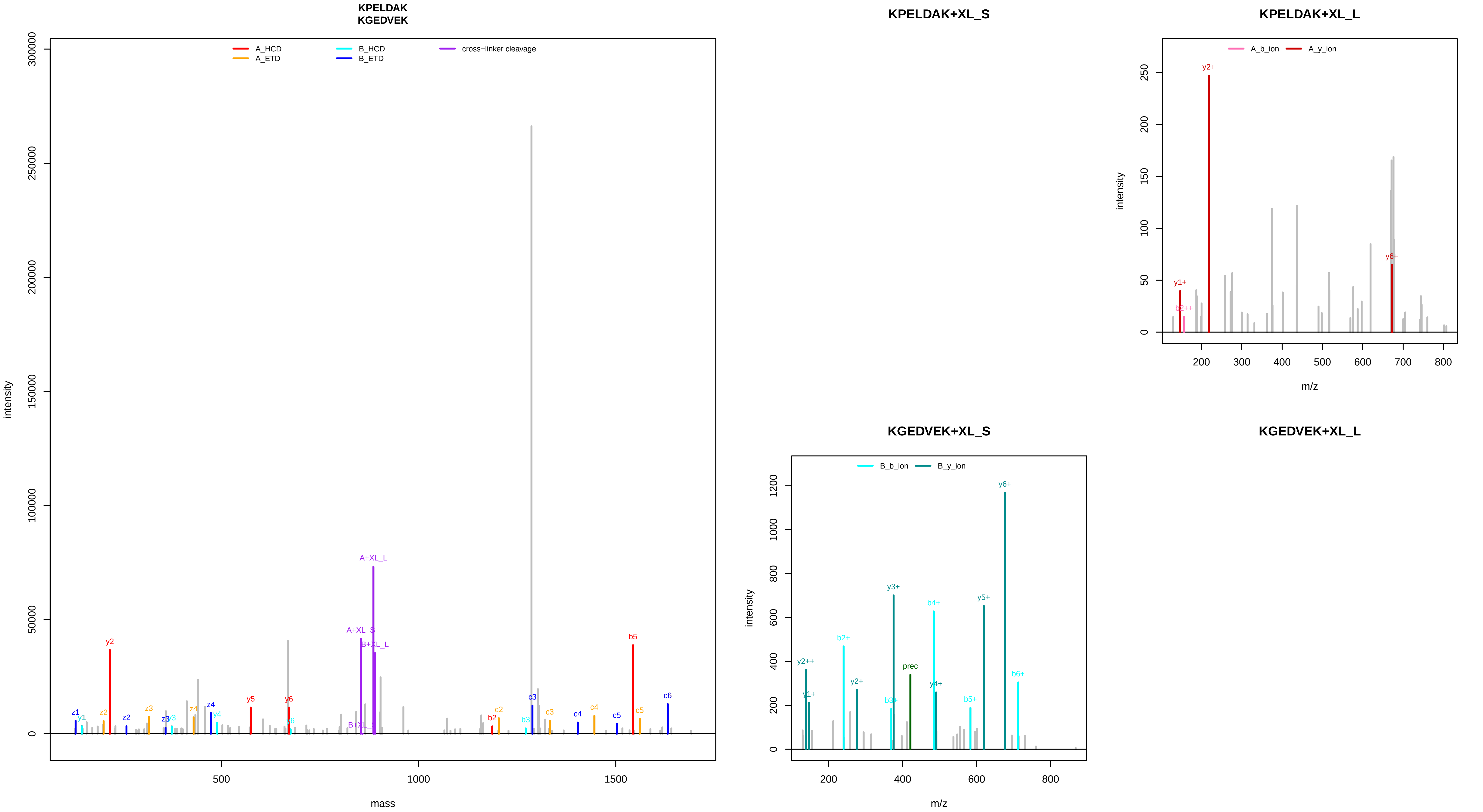
KPELDAK
KGEDVEK
A_HCD
A_ETD
B_HCD
B_ETD
cross−linker cleavage
intensity
mass
A+XL_L
A+XL_S
B+XL_L
B+XL_S
KPELDAK+XL_S
KPELDAK+XL_L
A_b_ion
A_y_ion
KGEDVEK+XL_S
B_b_ion
B_y_ion
prec
KGEDVEK+XL_L
m/z

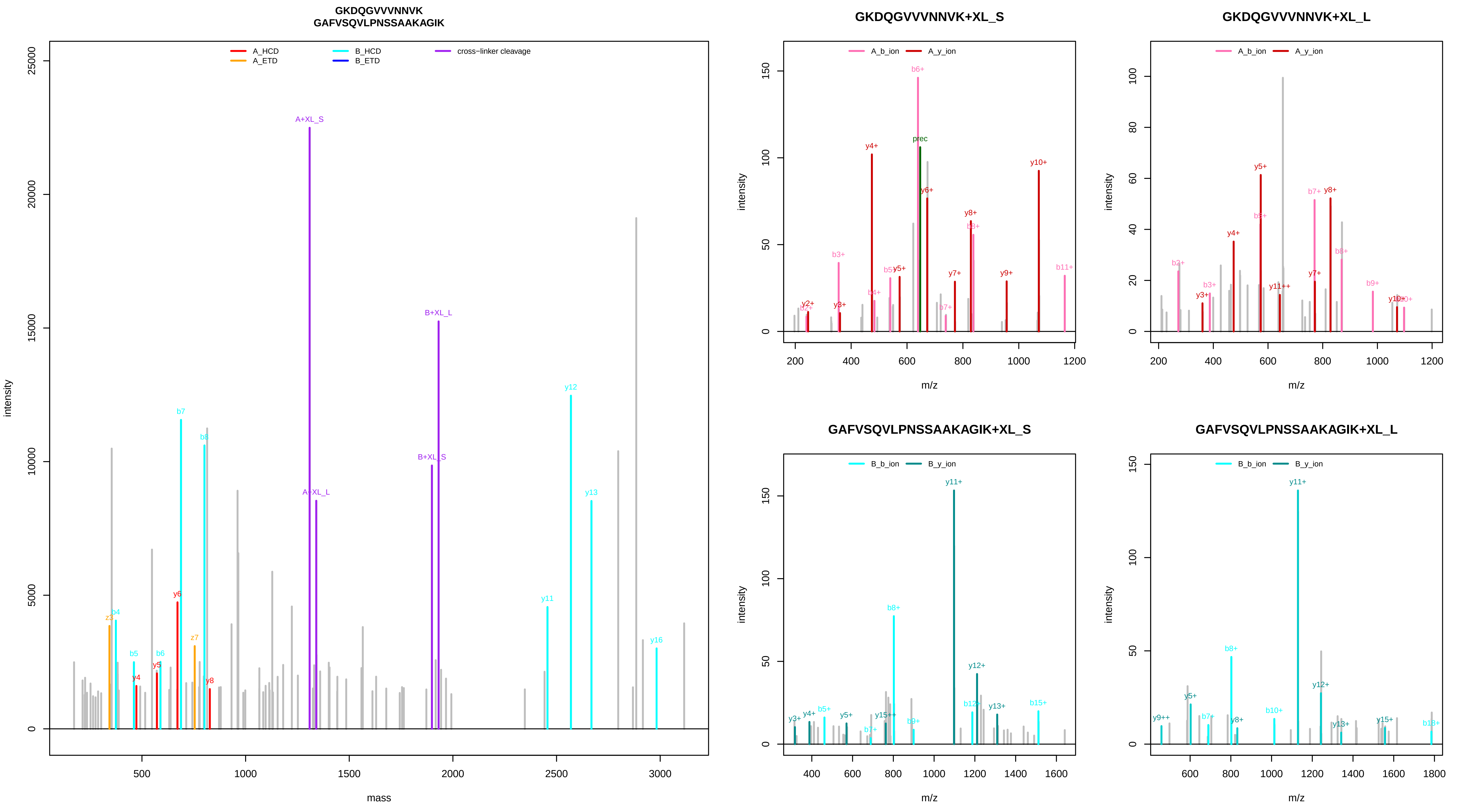
GKDQGVVVNNVK
GAFVSQVLPNSSAAKAGIK
A_HCD
A_ETD
B_HCD
B_ETD
cross−linker cleavage
A+XL_S
A+XL_L
B+XL_L
B+XL_S
intensity
mass
GKDQGVVVNNVK+XL_S
GKDQGVVVNNVK+XL_L
A_b_ion
A_y_ion
GAFVSQVLPNSSAAKAGIK+XL_S
GAFVSQVLPNSSAAKAGIK+XL_L
B_b_ion
B_y_ion
m/z
prec

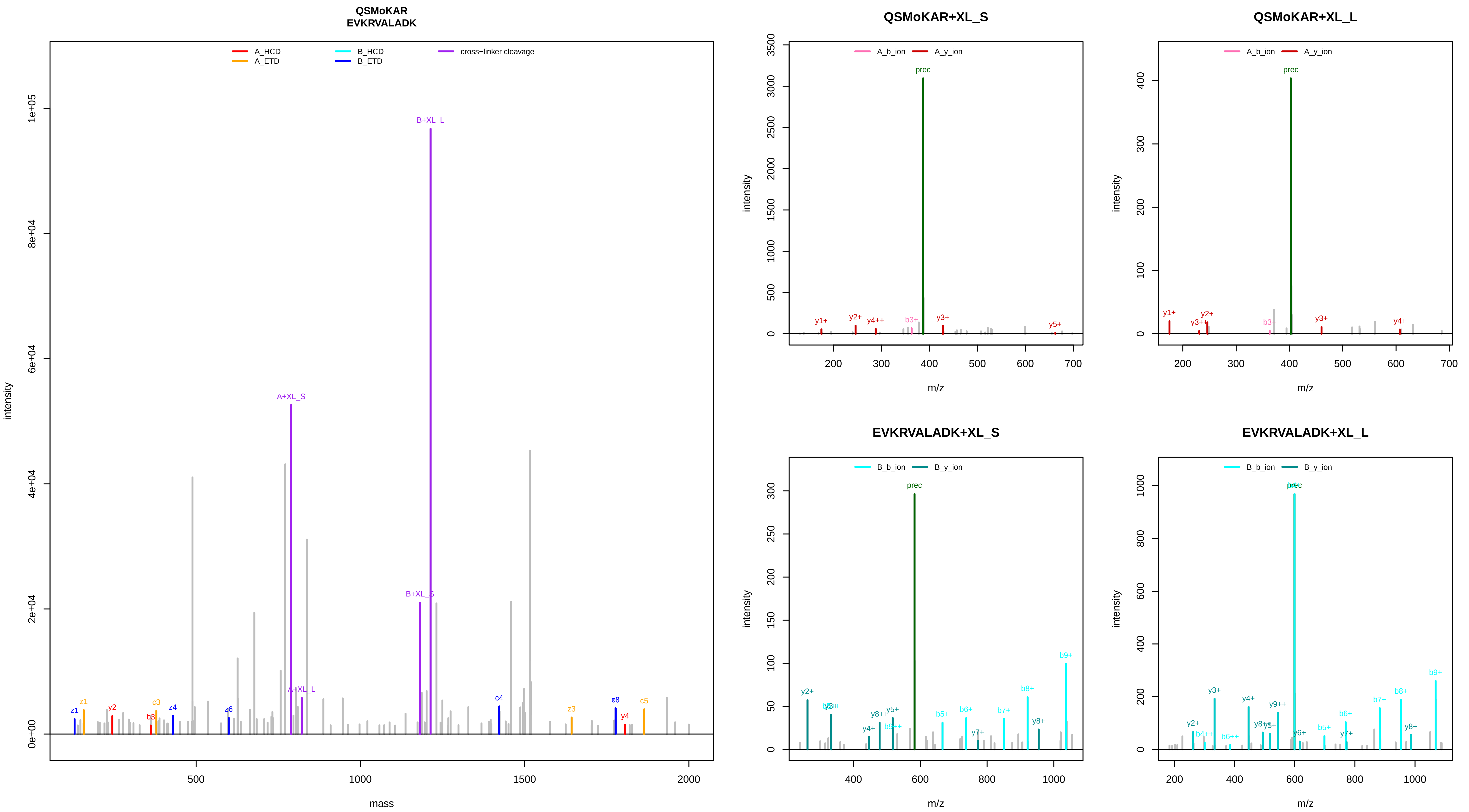
QSMoKAR
EVKRVALADK
A_HCD
A_ETD
B_HCD
B_ETD
cross−linker cleavage
B+XL_L
A+XL_S
B+XL_S
A+XL_L
intensity
mass
QSMoKAR+XL_S
QSMoKAR+XL_L
EVKRVALADK+XL_S
EVKRVALADK+XL_L
A_b_ion
A_y_ion
B_b_ion
B_y_ion
prec
m/z

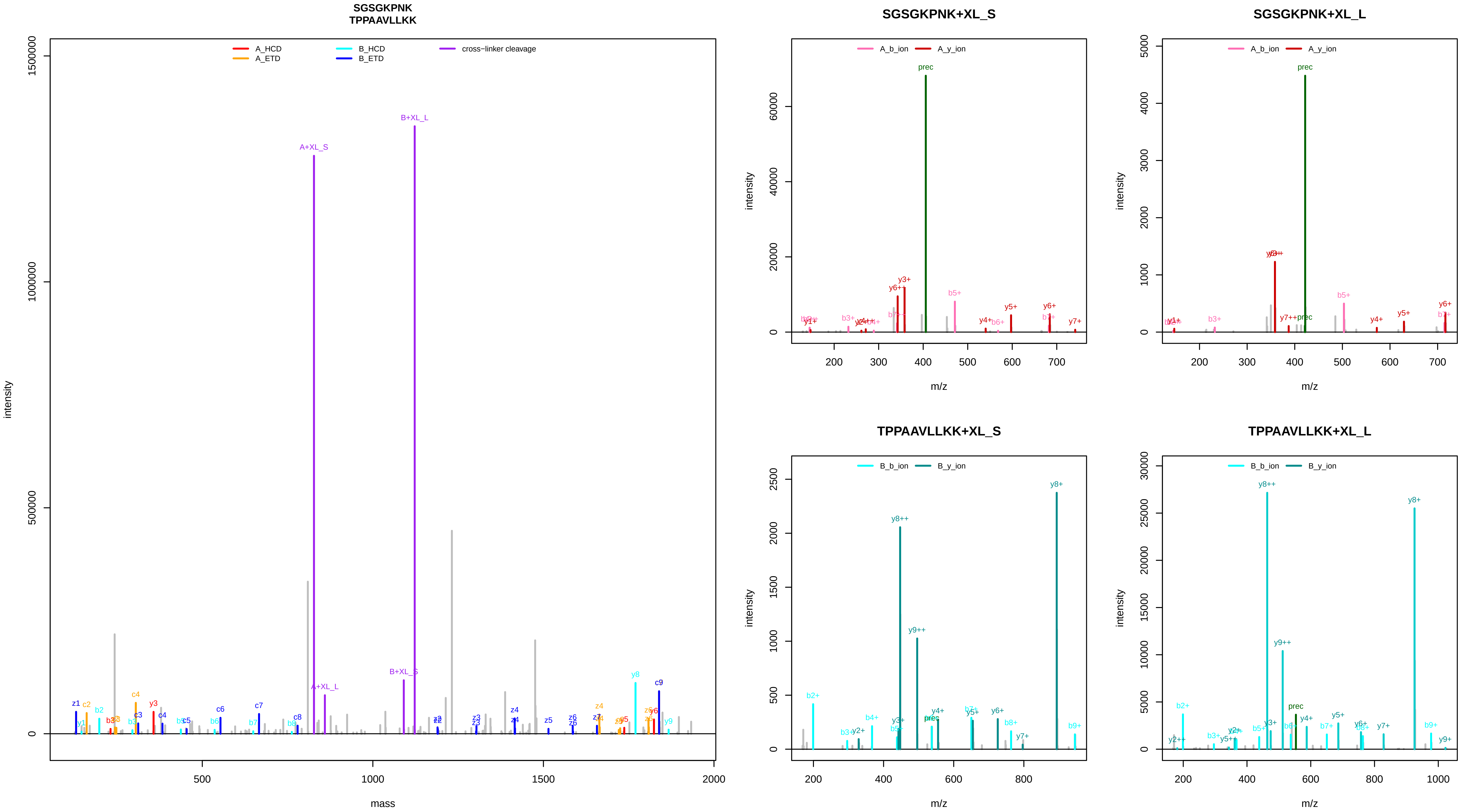
SGSGKPNK
TPPAAVLLKK
A_HCD
A_ETD
B_HCD
B_ETD
cross−linker cleavage
A+XL_S
B+XL_L
B+XL_S
A+XL_L
intensity
mass
SGSGKPNK+XL_S
SGSGKPNK+XL_L
TPPAAVLLKK+XL_S
TPPAAVLLKK+XL_L
A_b_ion
A_y_ion
B_b_ion
B_y_ion
prec
m/z

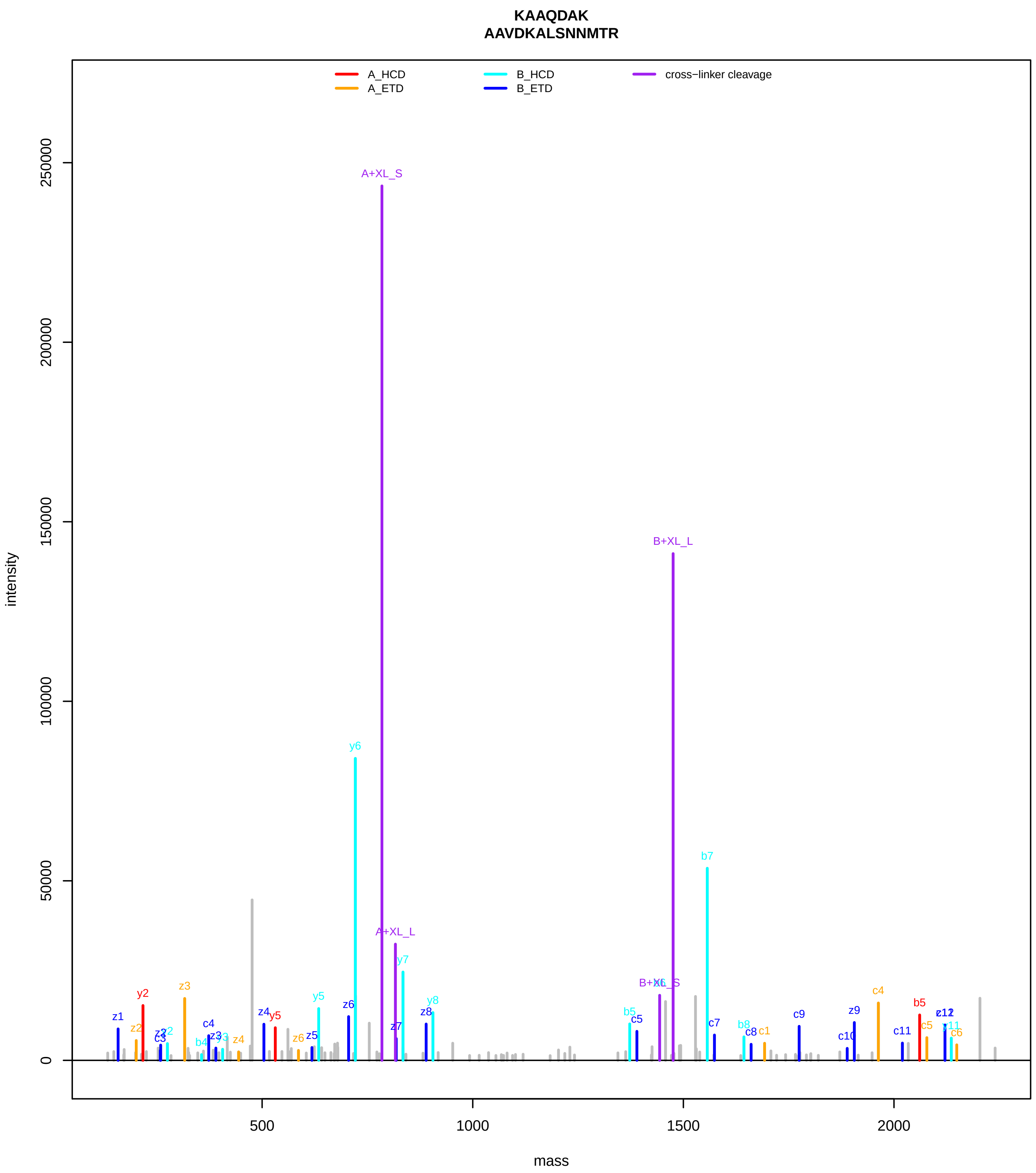

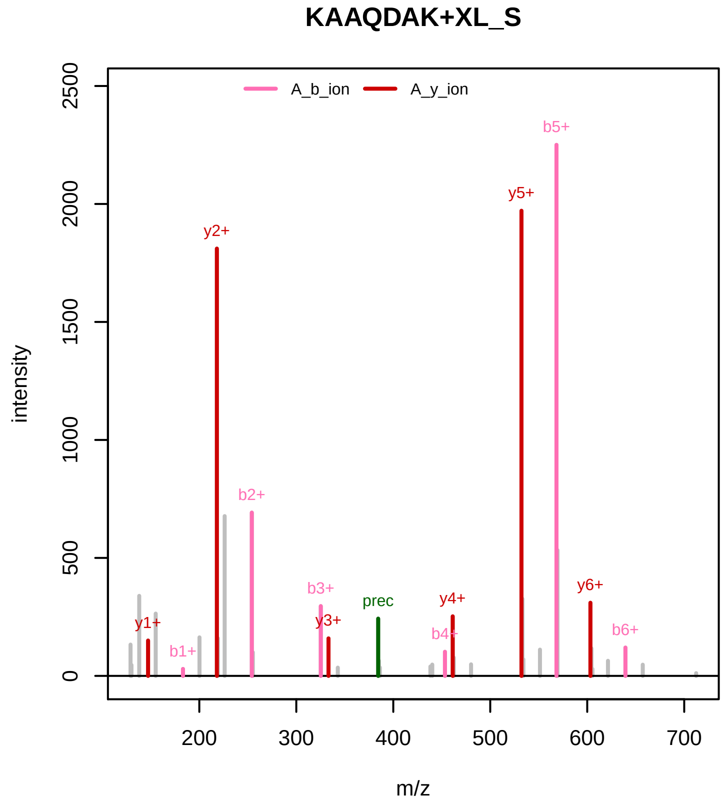

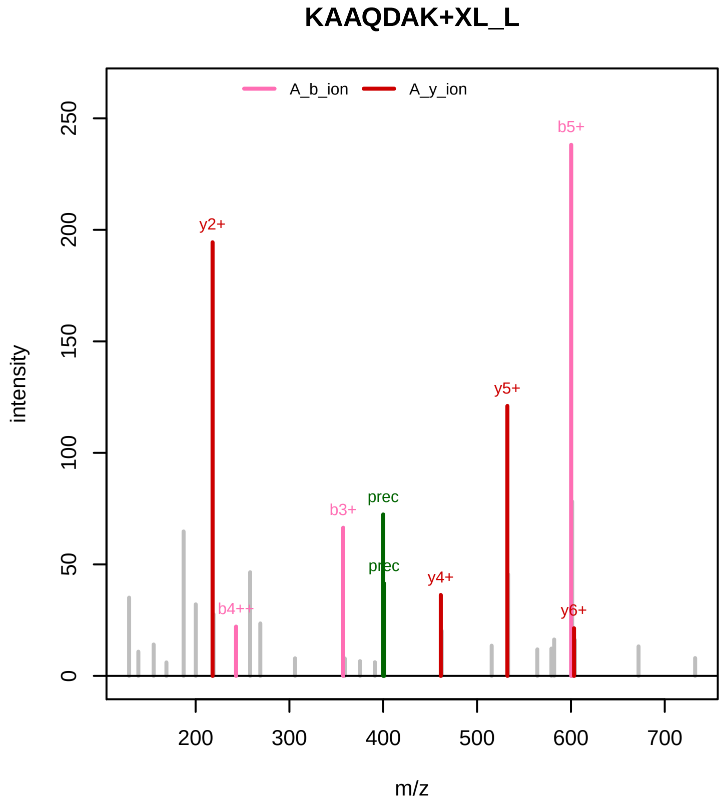

AAVDKALSNNMTR+XL_S

AAVDKALSNNMTR+XL_L

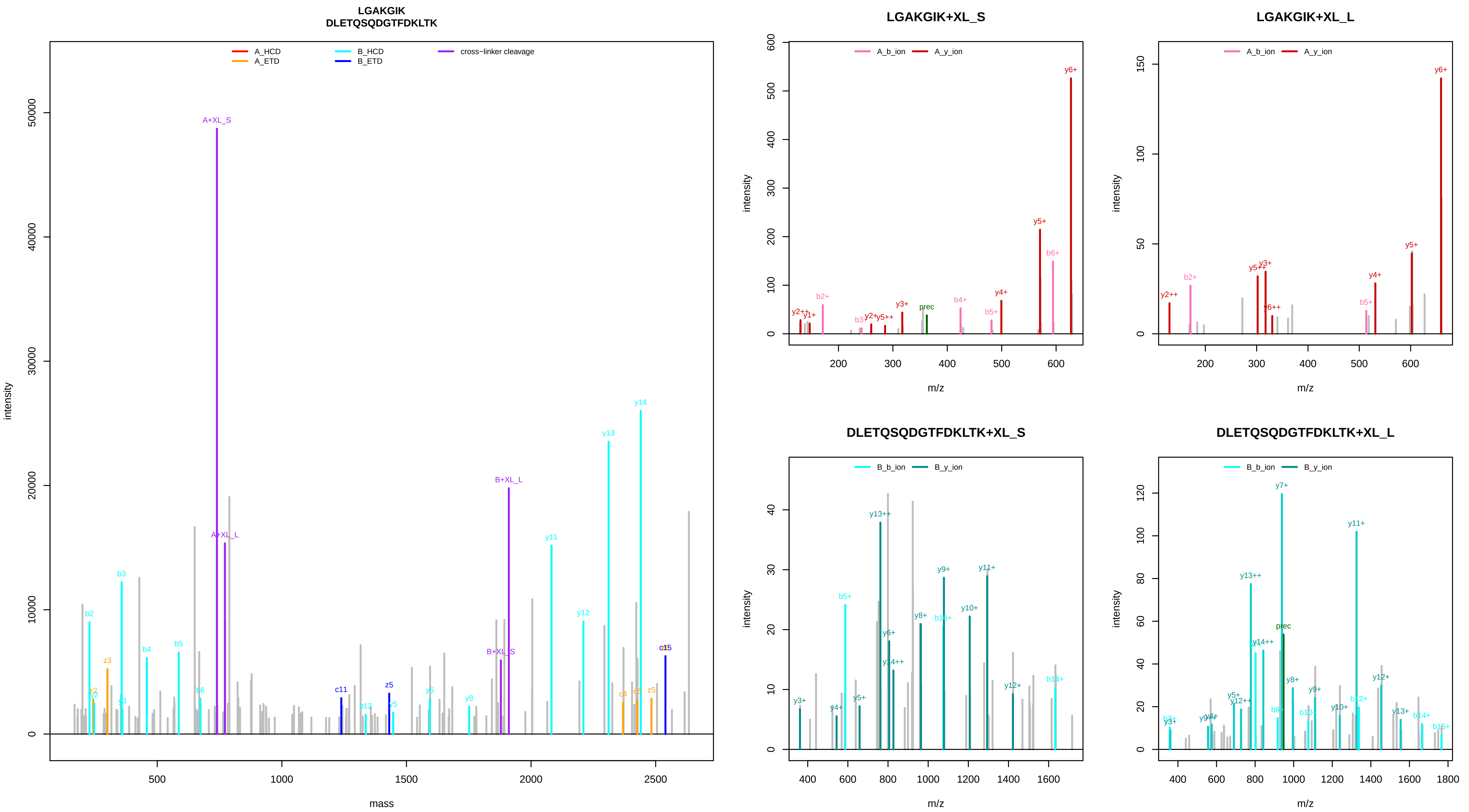

LGAKGIK
DLETQSQDGTFDKLTK
A_HCD
A_ETD
B_HCD
B_ETD
cross−linker cleavage
A+XL_S
A+XL_L
B+XL_L
B+XL_S
intensity
mass
LGAKGIK+XL_S
LGAKGIK+XL_L
A_b_ion
A_y_ion
DLETQSQDGTFDKLTK+XL_S
DLETQSQDGTFDKLTK+XL_L
B_b_ion
B_y_ion
prec
m/z

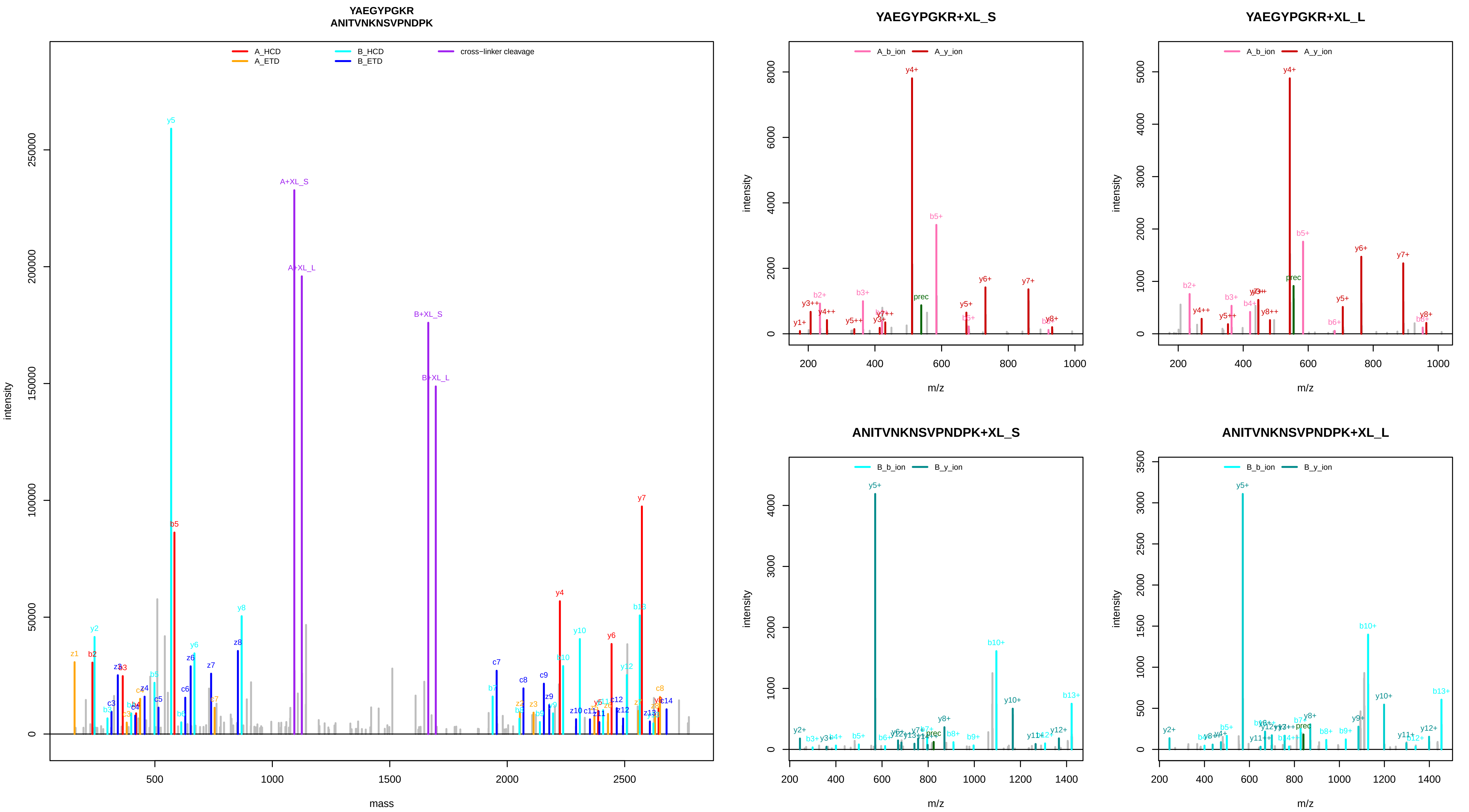
YAEGYPGKR
ANITVNKNSVPNDPK
A_HCD
A_ETD
B_HCD
B_ETD
cross−linker cleavage
A+XL_S
A+XL_L
B+XL_S
B+XL_L
intensity
mass
YAEGYPGKR+XL_S
YAEGYPGKR+XL_L
ANITVNKNSVPNDPK+XL_S
ANITVNKNSVPNDPK+XL_L
A_b_ion
A_y_ion
B_b_ion
B_y_ion
prec
m/z

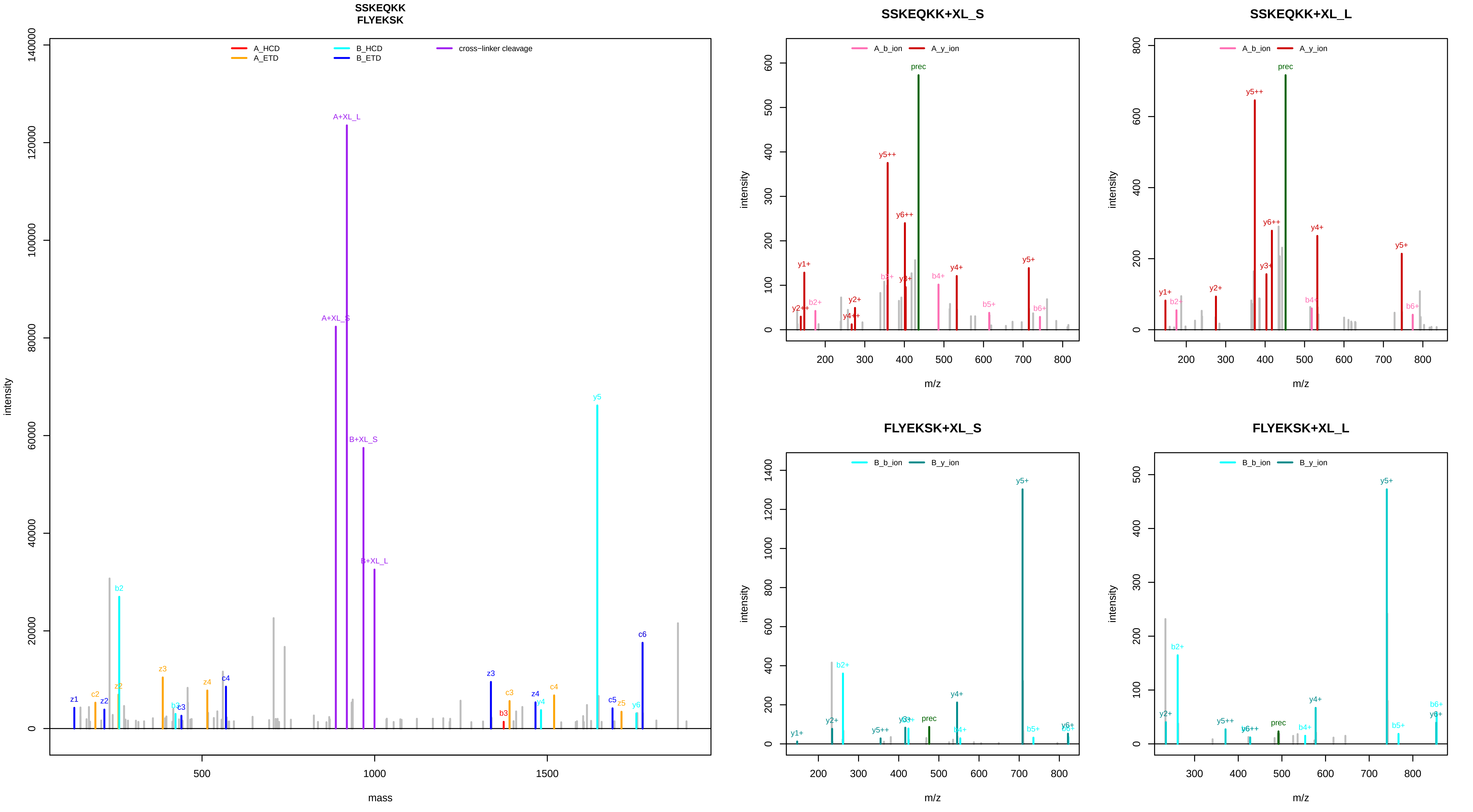

SSKEQKK
FLYEKSK
A_HCD
A_ETD
B_HCD
B_ETD
cross−linker cleavage
A+XL_L
A+XL_S
B+XL_S
B+XL_L
y5
c6
intensity
mass
SSKEQKK+XL_S
SSKEQKK+XL_L
FLYEKSK+XL_S
FLYEKSK+XL_L
A_b_ion
A_y_ion
B_b_ion
B_y_ion
prec
m/z

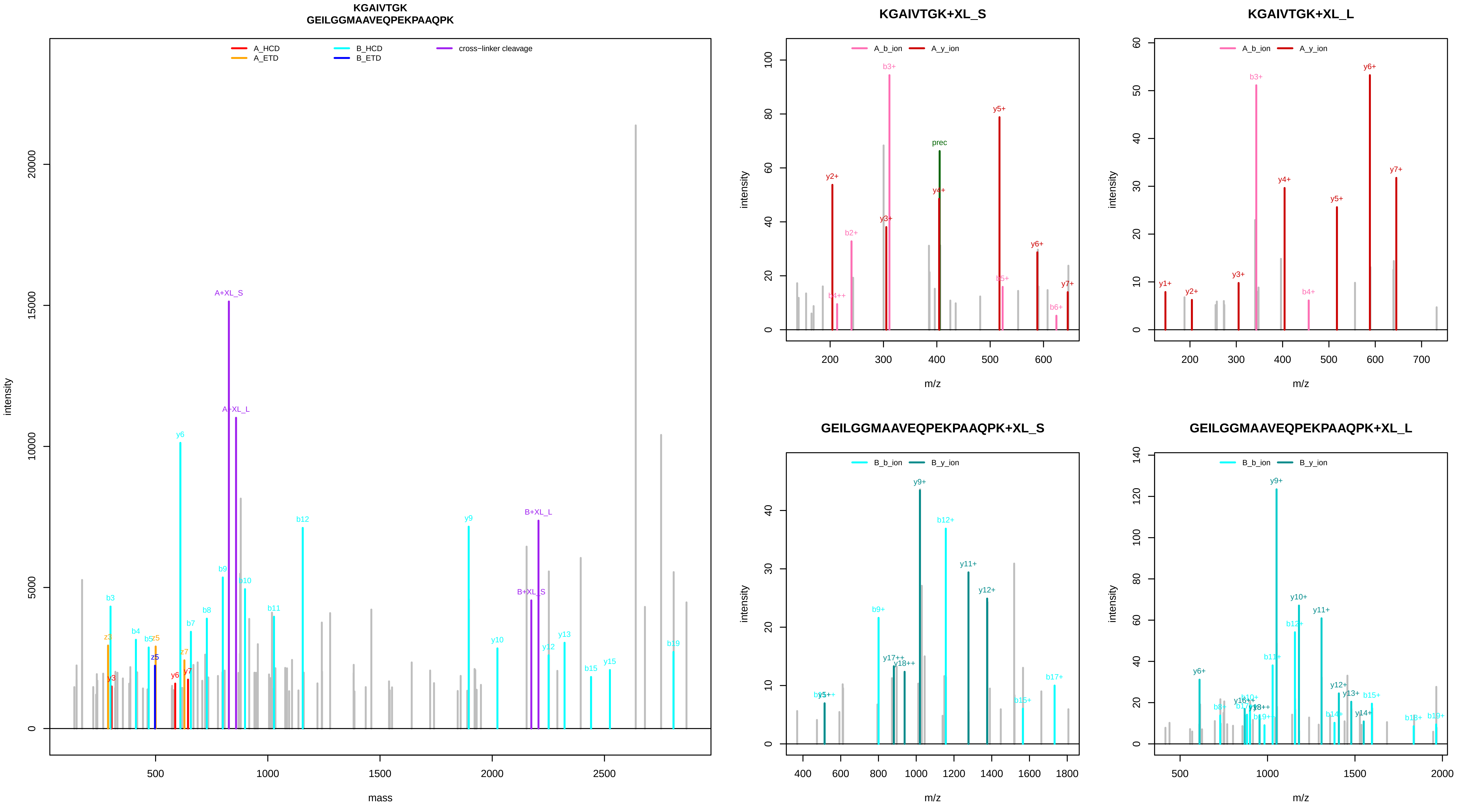
KGAIVTGK
GEILGGMAAVEQPEKPAAQPK
A_HCD
A_ETD
B_HCD
B_ETD
cross−linker cleavage
intensity
mass
A+XL_S
A+XL_L
B+XL_L
B+XL_S
KGAIVTGK+XL_S
KGAIVTGK+XL_L
GEILGGMAAVEQPEKPAAQPK+XL_S
GEILGGMAAVEQPEKPAAQPK+XL_L
A_b_ion
A_y_ion
B_b_ion
B_y_ion
prec
m/z

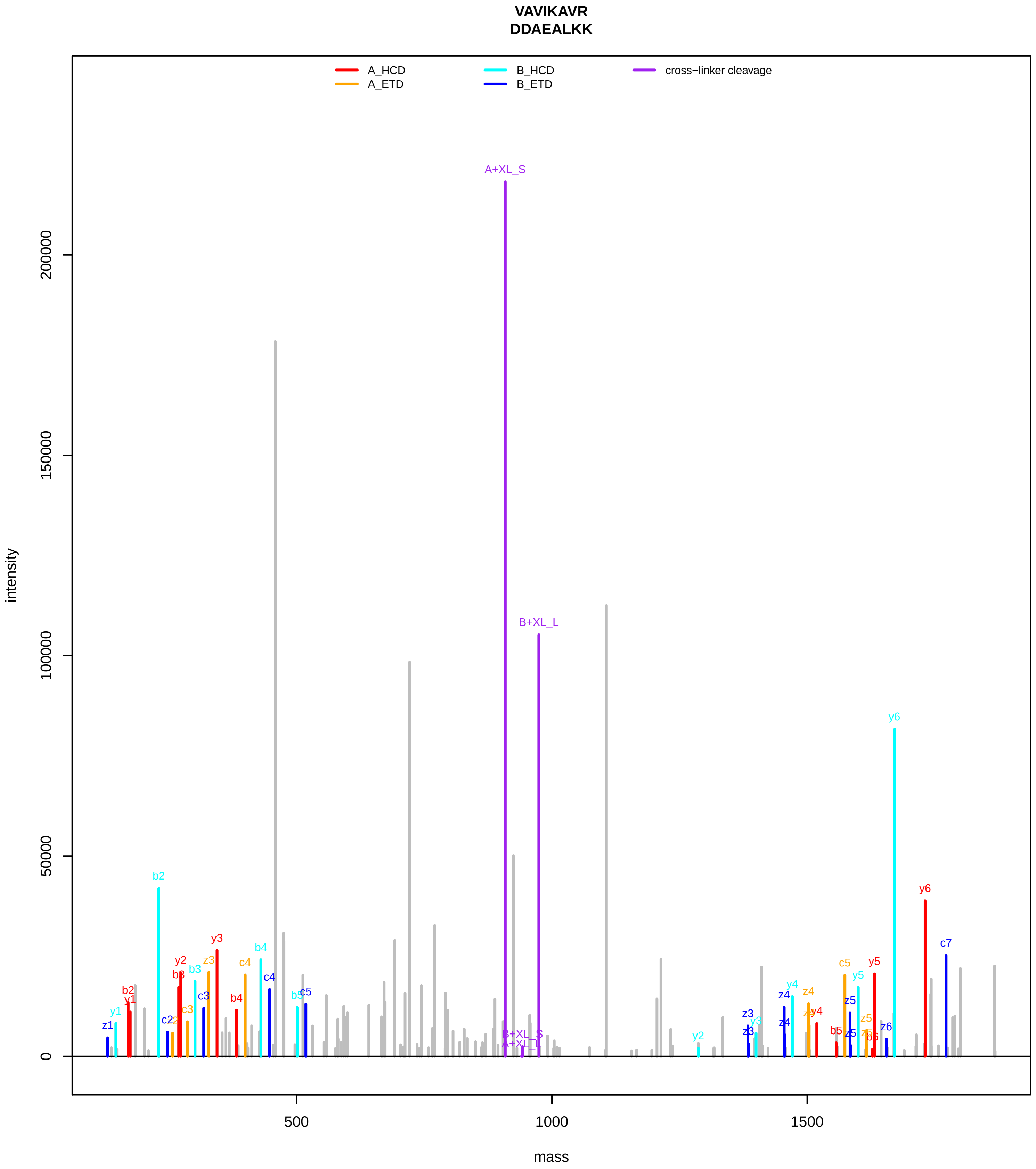

VAVIKAVR+XL_S

VAVIKAVR+XL_L

DDAEALKK+XL_S

DDAEALKK+XL_L

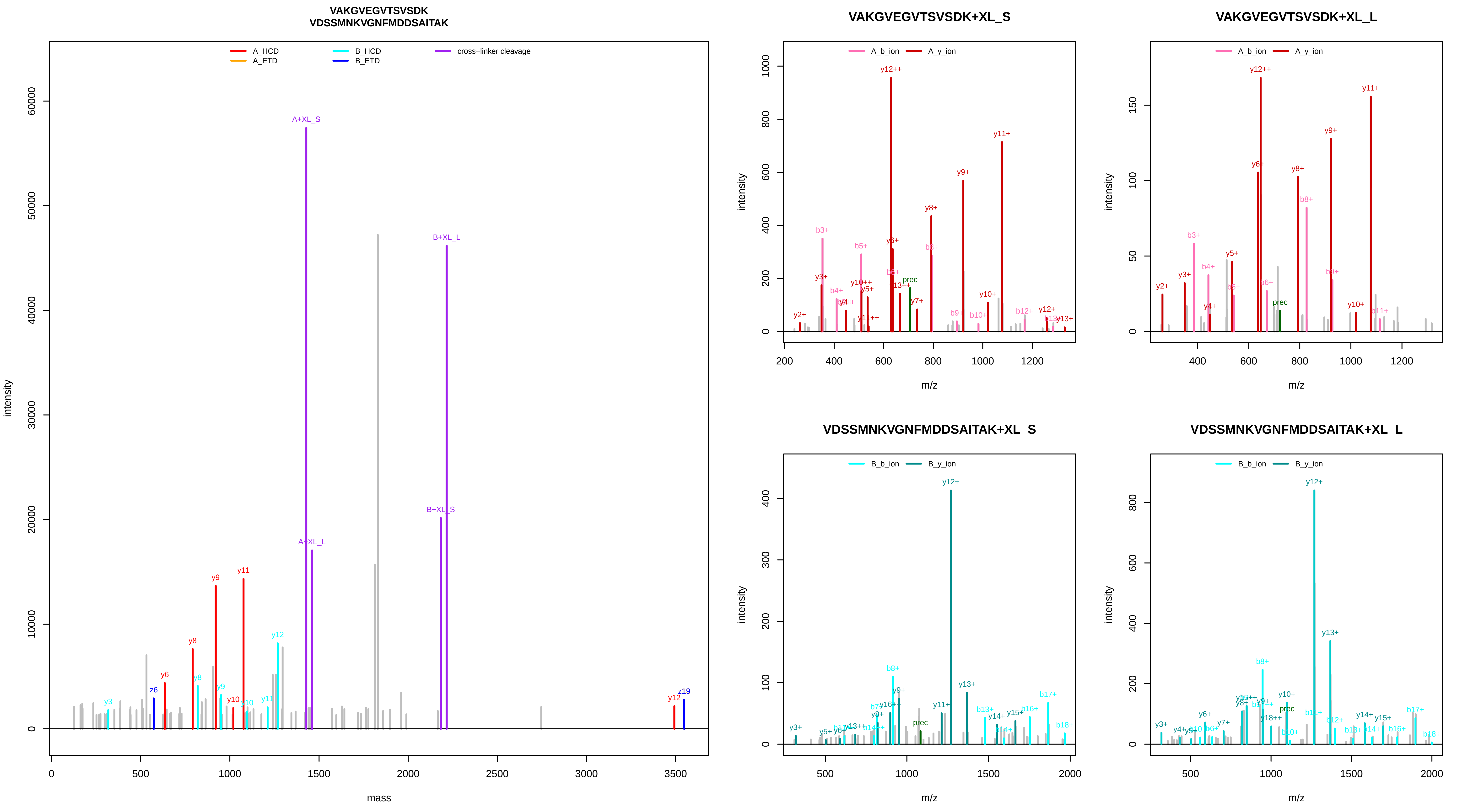
VAKGVEGVTSVSDK
VDSSMNKVGNFMDDSAITAK
A_HCD
A_ETD
B_HCD
B_ETD
cross−linker cleavage
A+XL_S
B+XL_L
A+XL_L
B+XL_S
intensity
mass
VAKGVEGVTSVSDK+XL_S
VAKGVEGVTSVSDK+XL_L
A_b_ion
A_y_ion
VDSSMNKVGNFMDDSAITAK+XL_S
VDSSMNKVGNFMDDSAITAK+XL_L
B_b_ion
B_y_ion
prec
m/z

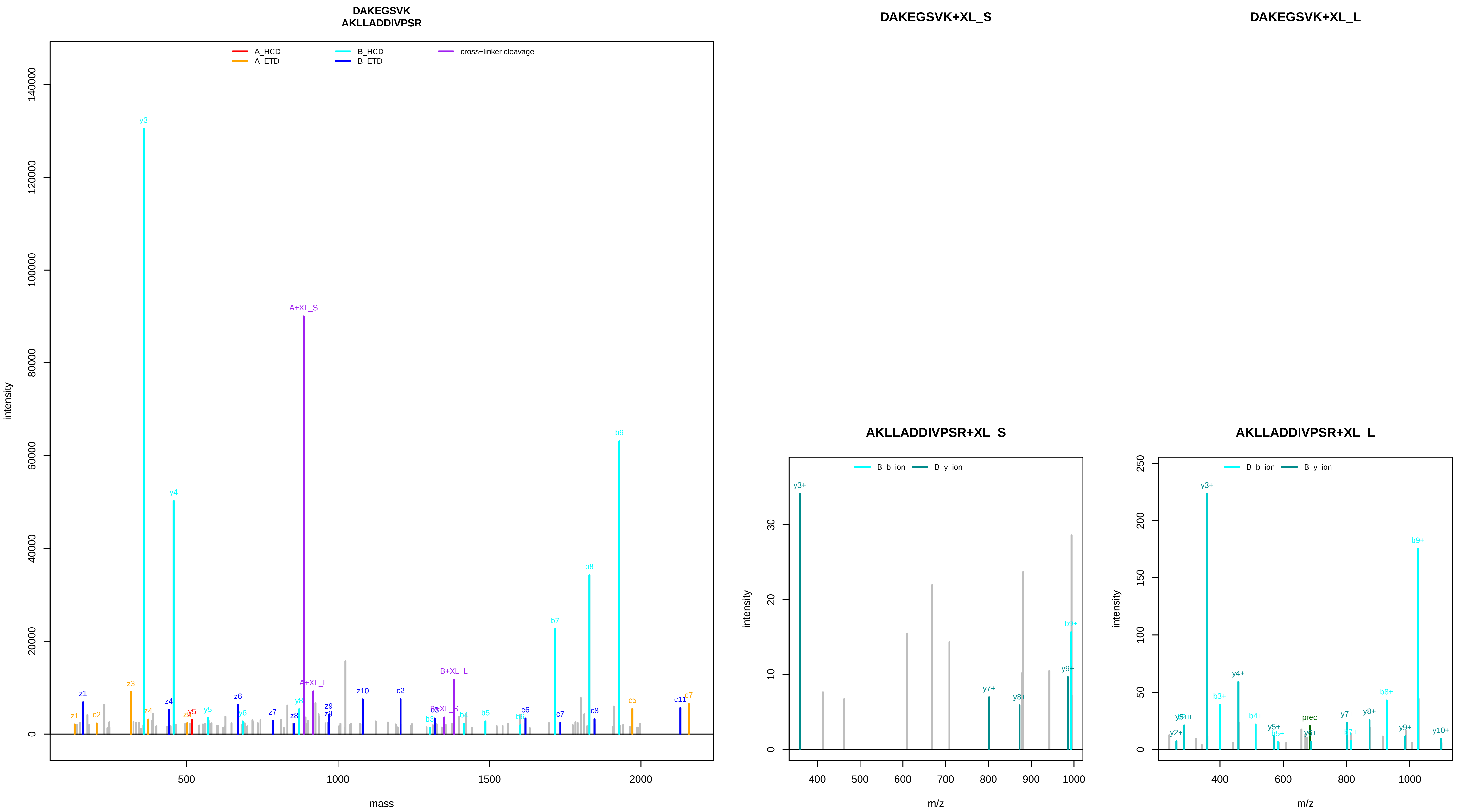
DAKEGSVK
AKLLADDIVPSR
A_HCD
A_ETD
B_HCD
B_ETD
cross−linker cleavage
intensity
mass
DAKEGSVK+XL_S
DAKEGSVK+XL_L
AKLLADDIVPSR+XL_S
AKLLADDIVPSR+XL_L
B_b_ion
B_y_ion
m/z

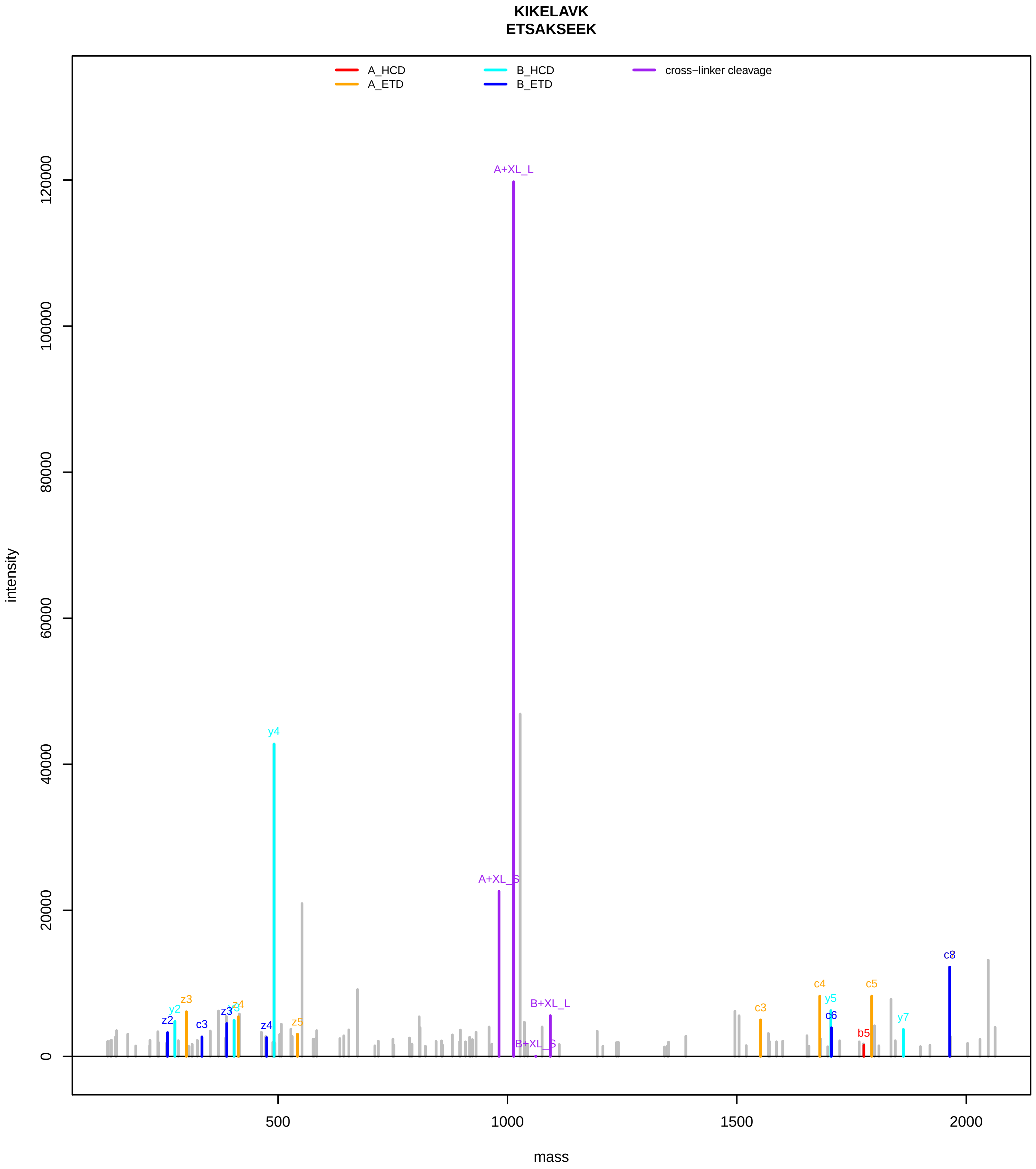

KIKELAVK+XL_S

KIKELAVK+XL_L

ETSAKSEEK+XL_S

ETSAKSEEK+XL_L

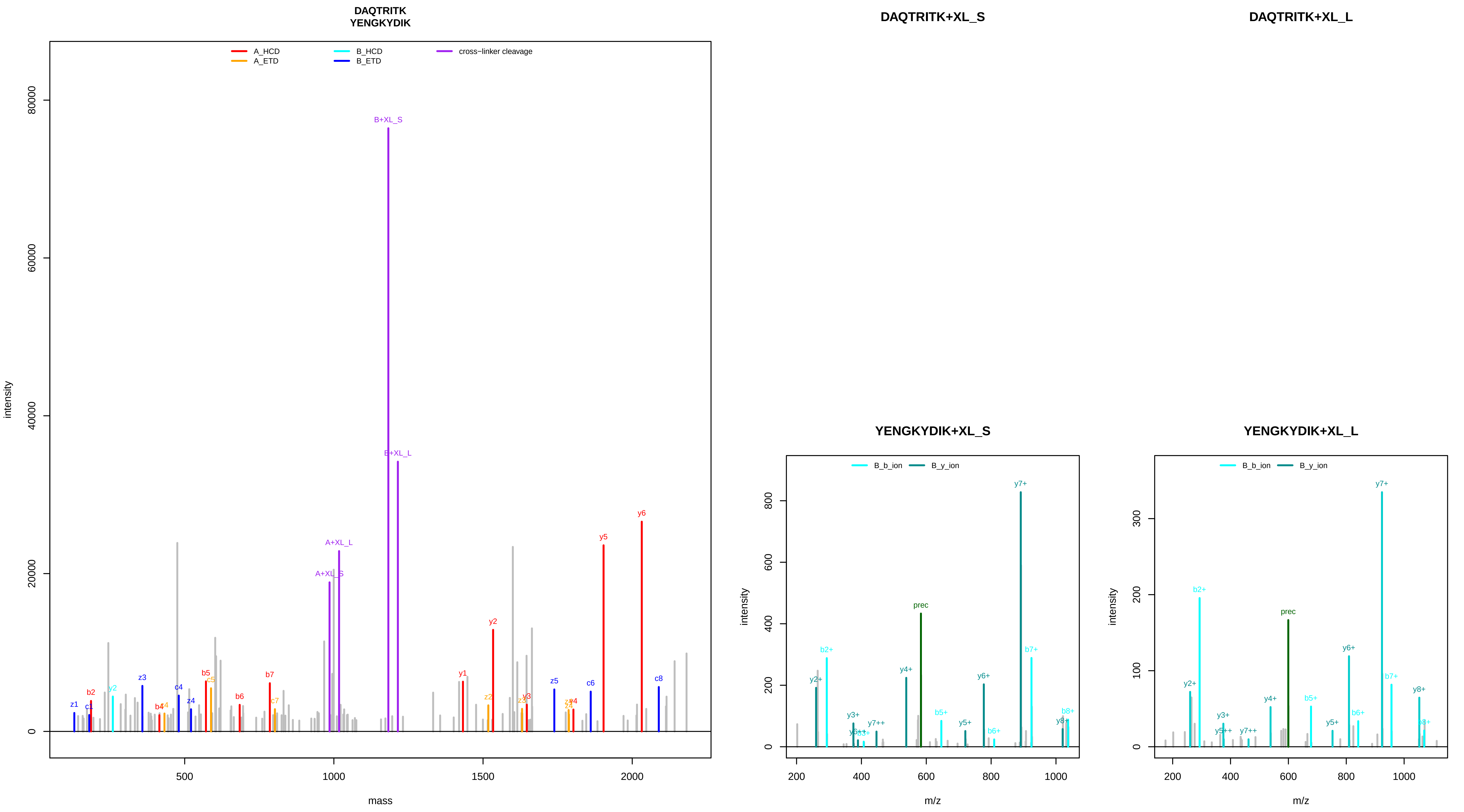
DAQTRITK
YENGKYDIK
A_HCD
A_ETD
B_HCD
B_ETD
cross−linker cleavage
B+XL_S
B+XL_L
A+XL_L
A+XL_S
intensity
mass
DAQTRITK+XL_S
DAQTRITK+XL_L
YENGKYDIK+XL_S
YENGKYDIK+XL_L
B_b_ion
B_y_ion
prec
m/z

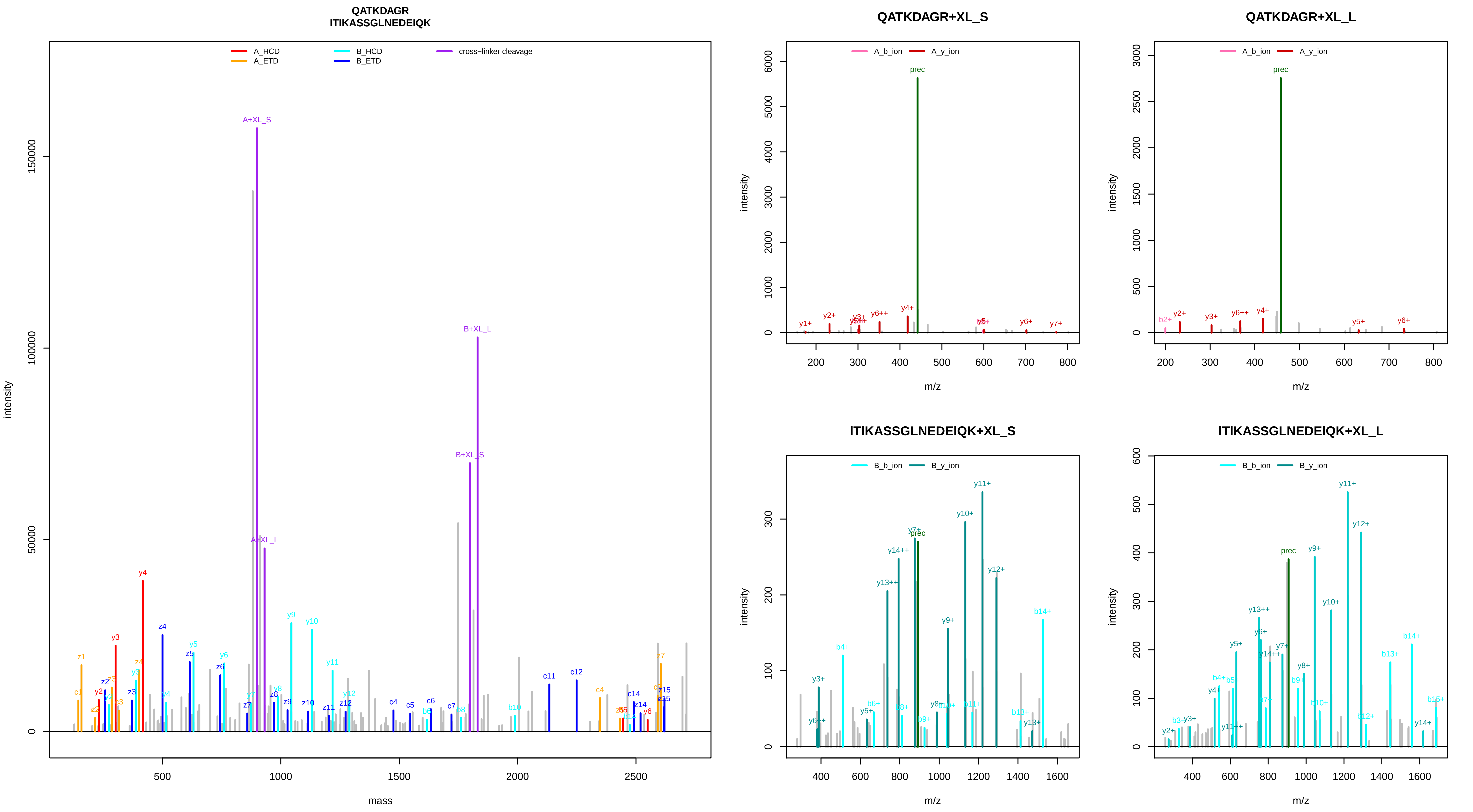
QATKDAGR
ITIKASSGLNEDEIQK
A_HCD
A_ETD
B_HCD
B_ETD
cross−linker cleavage
A+XL_S
A+XL_L
B+XL_S
B+XL_L
intensity
mass
QATKDAGR+XL_S
QATKDAGR+XL_L
A_b_ion
A_y_ion
prec
ITIKASSGLNEDEIQK+XL_S
ITIKASSGLNEDEIQK+XL_L
B_b_ion
B_y_ion
m/z

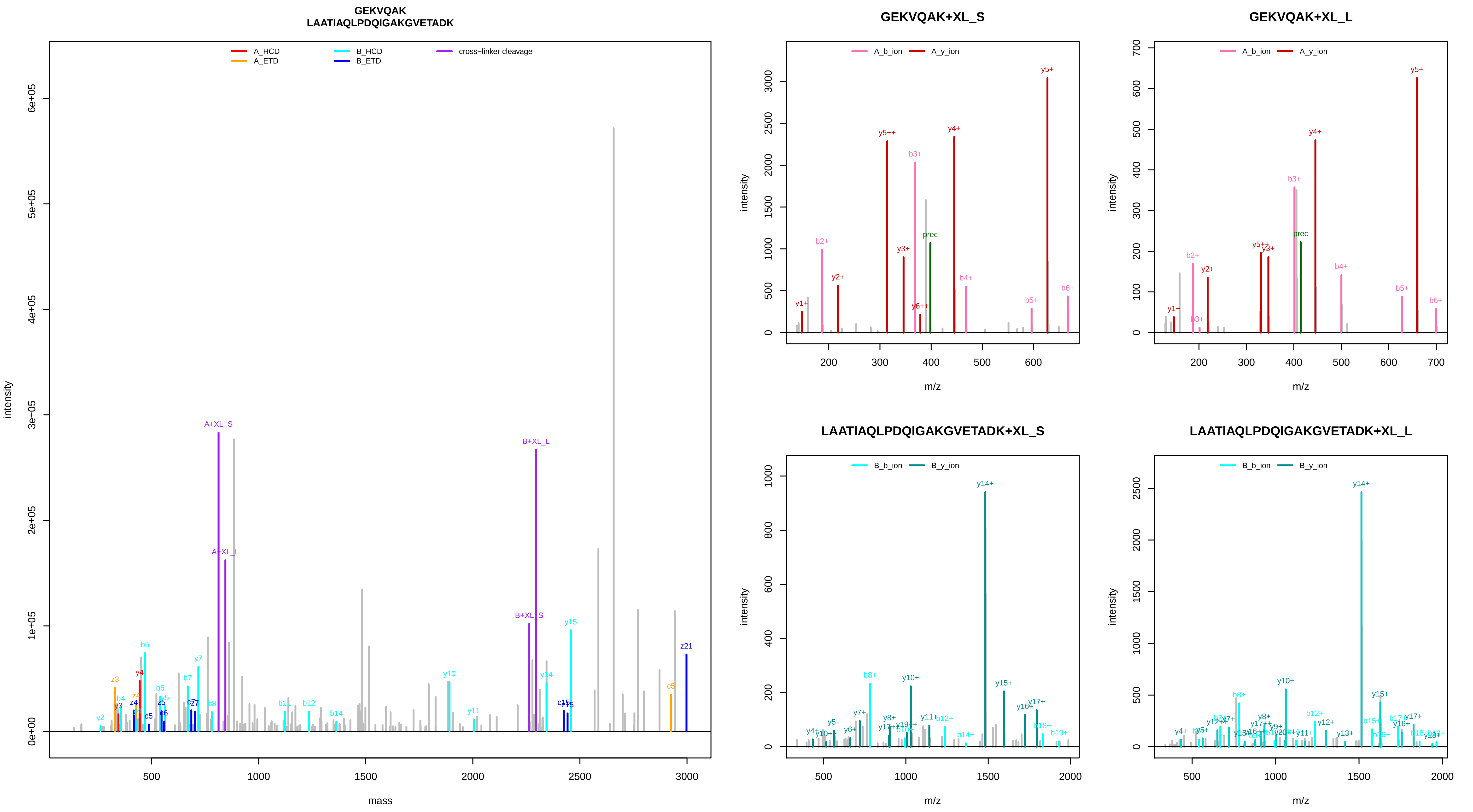
GEKVQAK
LAATIAQLPDQIGAKGVETADK
A_HCD
A_ETD
B_HCD
B_ETD
cross−linker cleavage
A+XL_S
A+XL_L
B+XL_L
B+XL_S
intensity
mass
GEKVQAK+XL_S
GEKVQAK+XL_L
LAATIAQLPDQIGAKGVETADK+XL_S
LAATIAQLPDQIGAKGVETADK+XL_L
A_b_ion
A_y_ion
B_b_ion
B_y_ion
prec
m/z

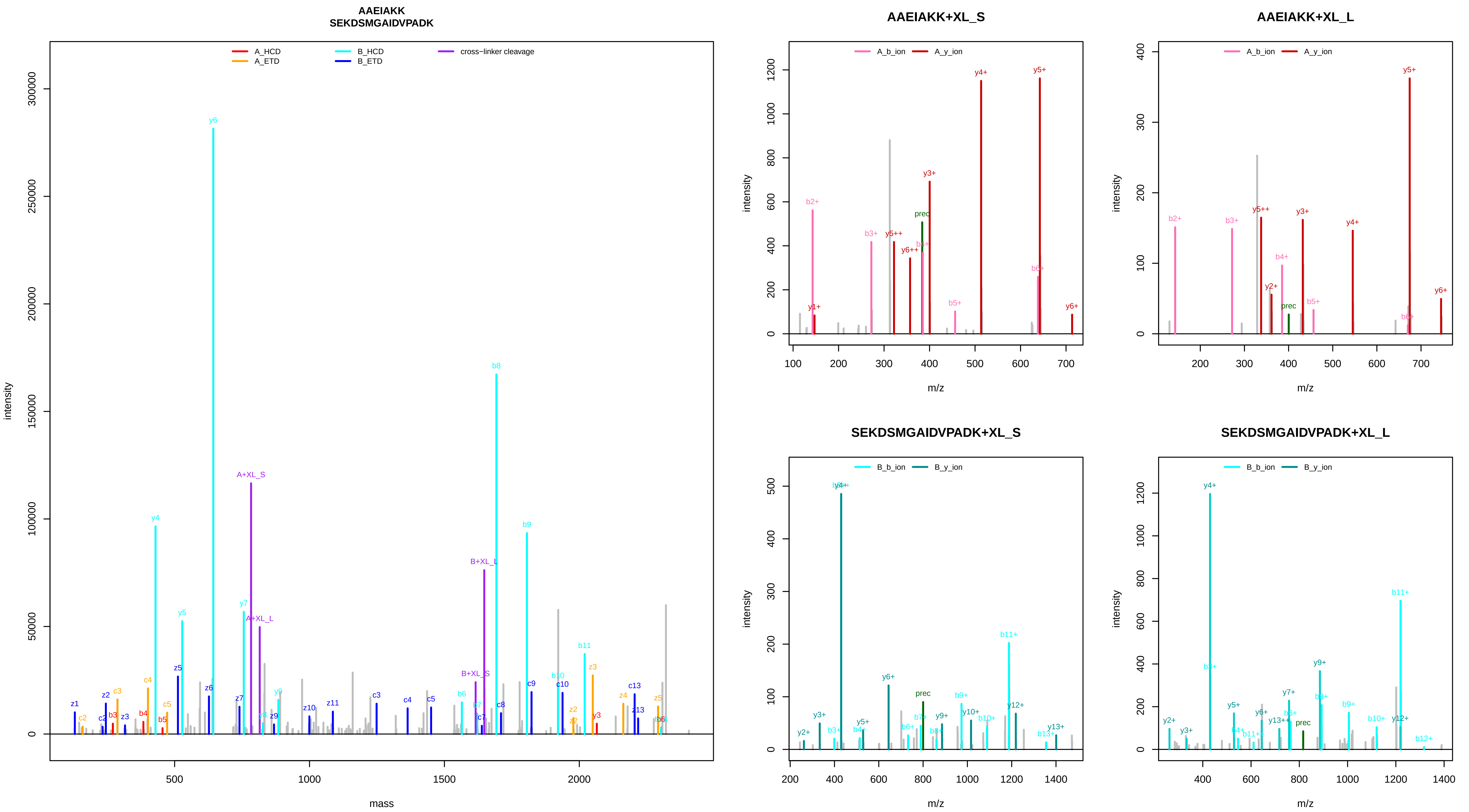

AAEIAKK
SEKDSMGAIDVPADK
A_HCD
A_ETD
B_HCD
B_ETD
cross−linker cleavage
intensity
mass
AAEIAKK+XL_S
AAEIAKK+XL_L
SEKDSMGAIDVPADK+XL_S
SEKDSMGAIDVPADK+XL_L
A_b_ion
A_y_ion
B_b_ion
B_y_ion
m/z

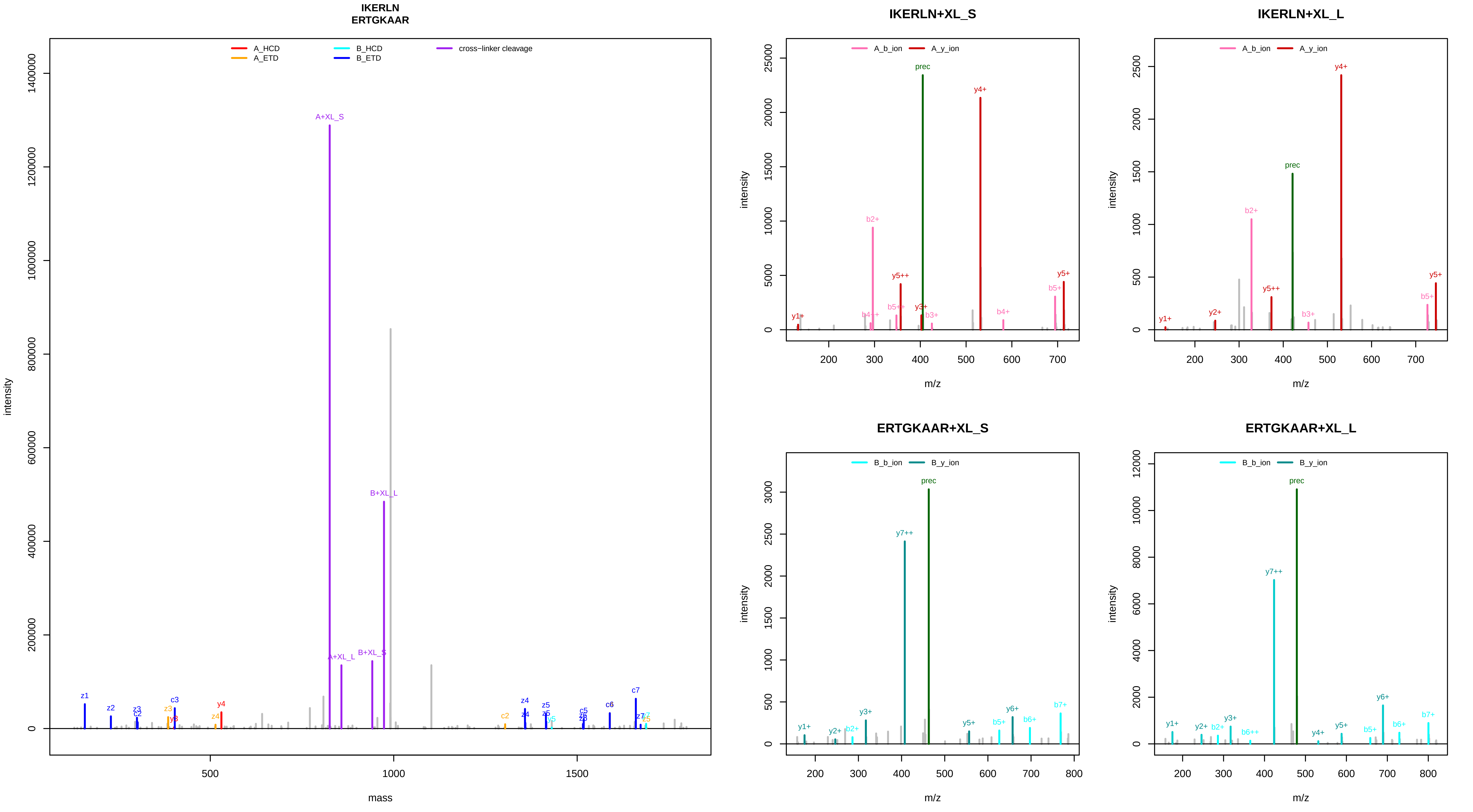
IKERLN
ERTGKAAR
A_HCD
A_ETD
B_HCD
B_ETD
cross−linker cleavage
A+XL_S
B+XL_L
A+XL_L
B+XL_S
intensity
mass
IKERLN+XL_S
IKERLN+XL_L
A_b_ion
A_y_ion
prec
ERTGKAAR+XL_S
ERTGKAAR+XL_L
B_b_ion
B_y_ion
m/z

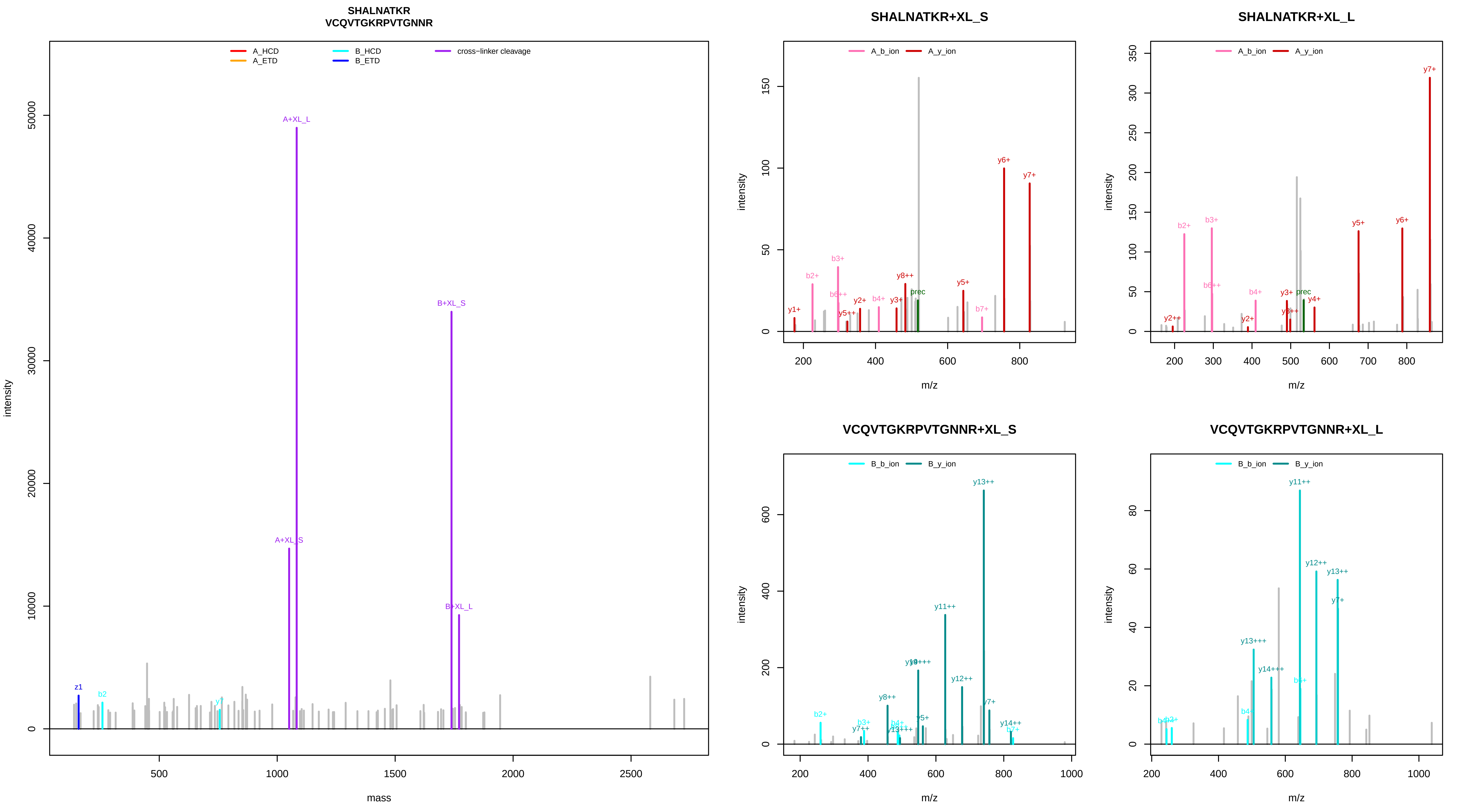

SHALNATKR
VCQVTGKRPVTGNNR
A_HCD
A_ETD
B_HCD
B_ETD
cross−linker cleavage
A+XL_L
B+XL_S
A+XL_S
B+XL_L
z1
b2
intensity
mass
SHALNATKR+XL_S
SHALNATKR+XL_L
VCQVTGKRPVTGNNR+XL_S
VCQVTGKRPVTGNNR+XL_L
A_b_ion
A_y_ion
B_b_ion
B_y_ion
m/z
prec
y13++
y11++
y12++
y13+++
y14+++
y14++
y8++
y7+
y6+
y5+
y3+
y2+
y1+
b2+
b3+
b4+
b7+

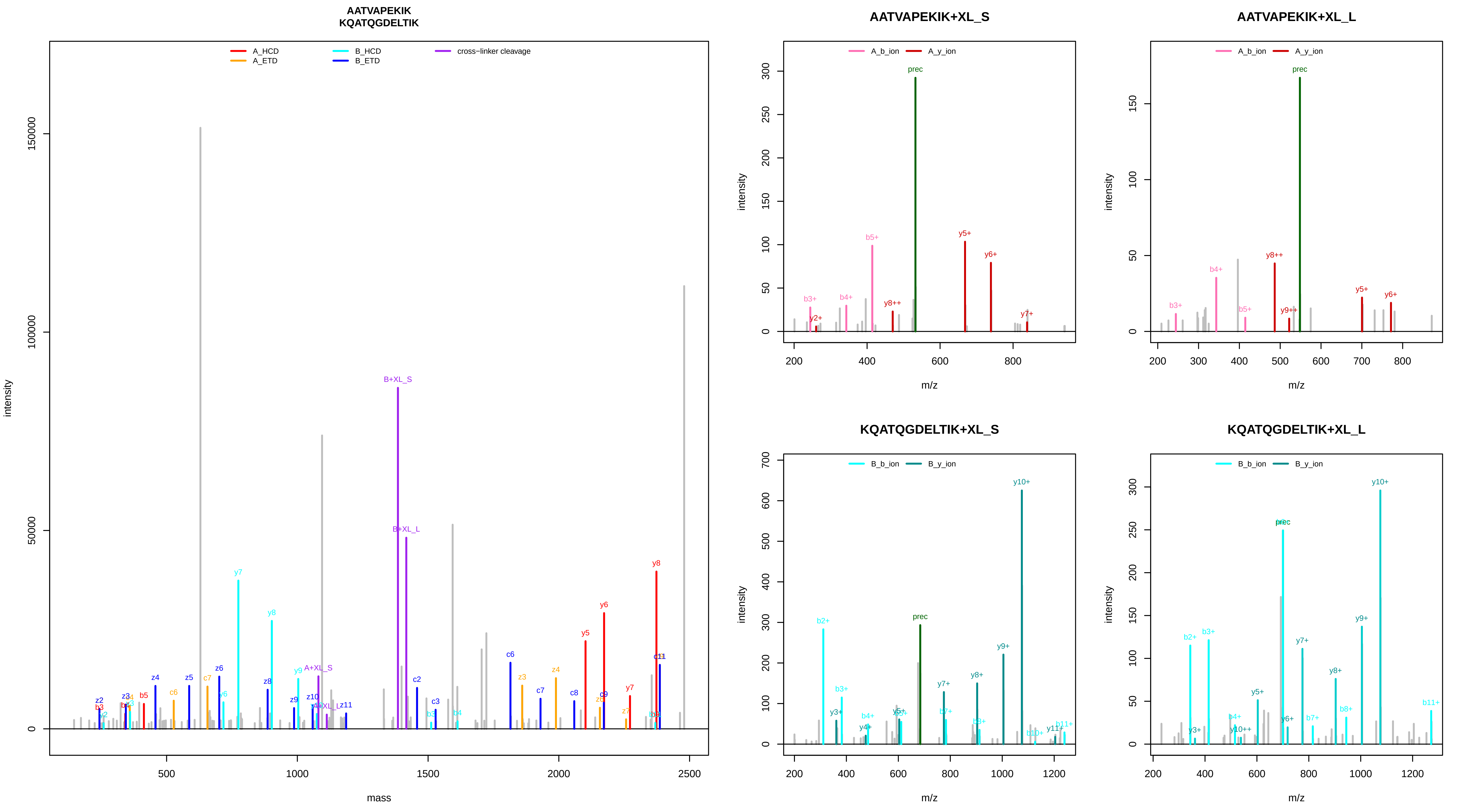
AATVAPEKIK
KQATQGDELTIK
A_HCD
A_ETD
B_HCD
B_ETD
cross−linker cleavage
intensity
mass
B+XL_S
B+XL_L
A+XL_S
AATVAPEKIK+XL_S
AATVAPEKIK+XL_L
KQATQGDELTIK+XL_S
KQATQGDELTIK+XL_L
A_b_ion
A_y_ion
B_b_ion
B_y_ion
prec
m/z

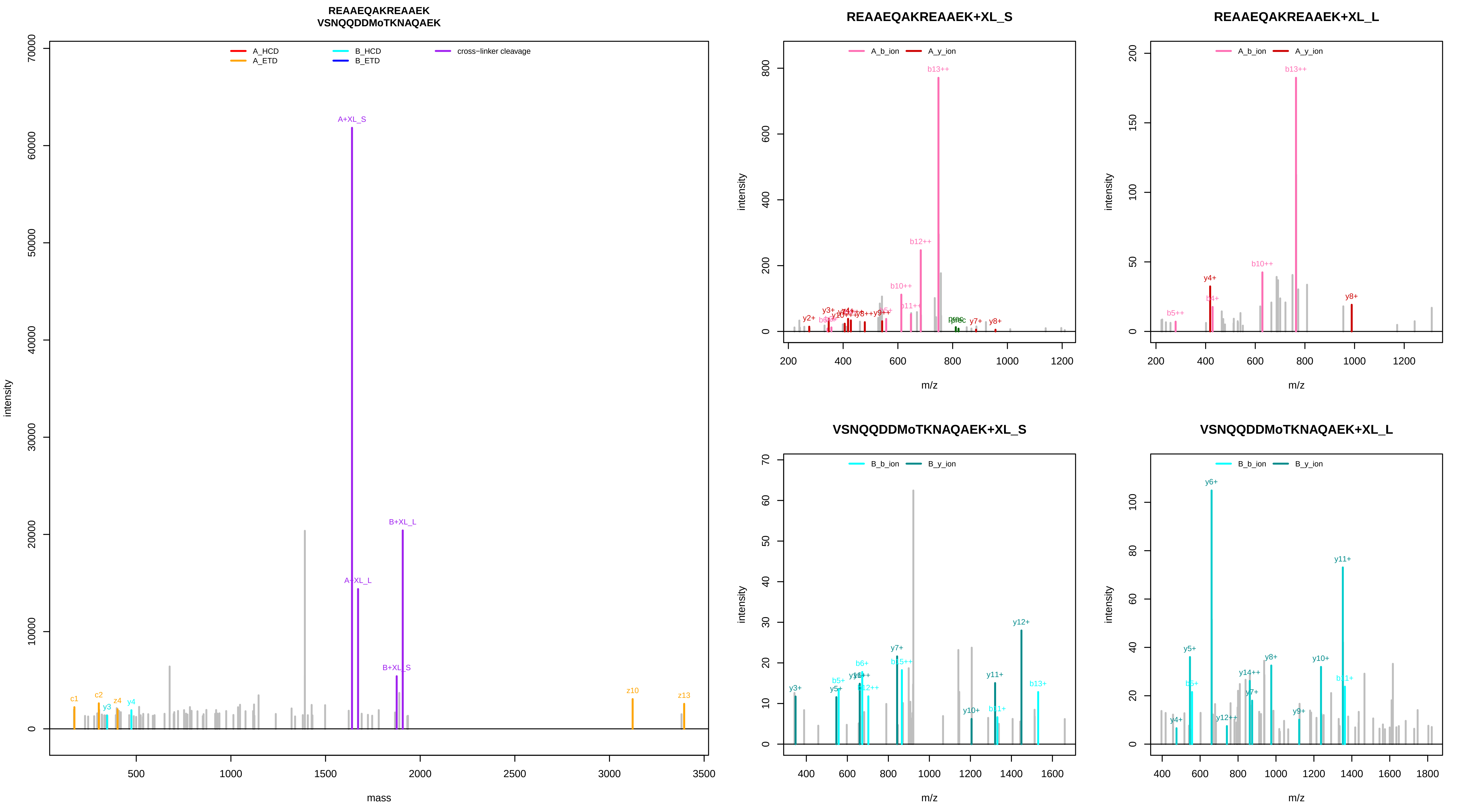
REAAEQAKREAAEK
VSNQQDDMoTKNAQAEK
A_HCD
A_ETD
B_HCD
B_ETD
cross−linker cleavage
A+XL_S
A+XL_L
B+XL_L
B+XL_S
c1
c2
y3
z4
y4
z10
z13
intensity
mass
REAAEQAKREAAEK+XL_S
A_b_ion
A_y_ion
b13++
b12++
b10++
b11++
y2+
y3+
y7+
y8+
m/z
REAAEQAKREAAEK+XL_L
b13++
b10++
y4+
b4+
b5++
y8+
VSNQQDDMoTKNAQAEK+XL_S
B_b_ion
B_y_ion
y3+
b5+
y5+
b6+
y7+
b15++
b12++
y10+
y11+
b11+
y12+
b13+
VSNQQDDMoTKNAQAEK+XL_L
y6+
y11+
y5+
b5+
y4+
y12++
y14++
y7+
y8+
y10+
b11+
y9+

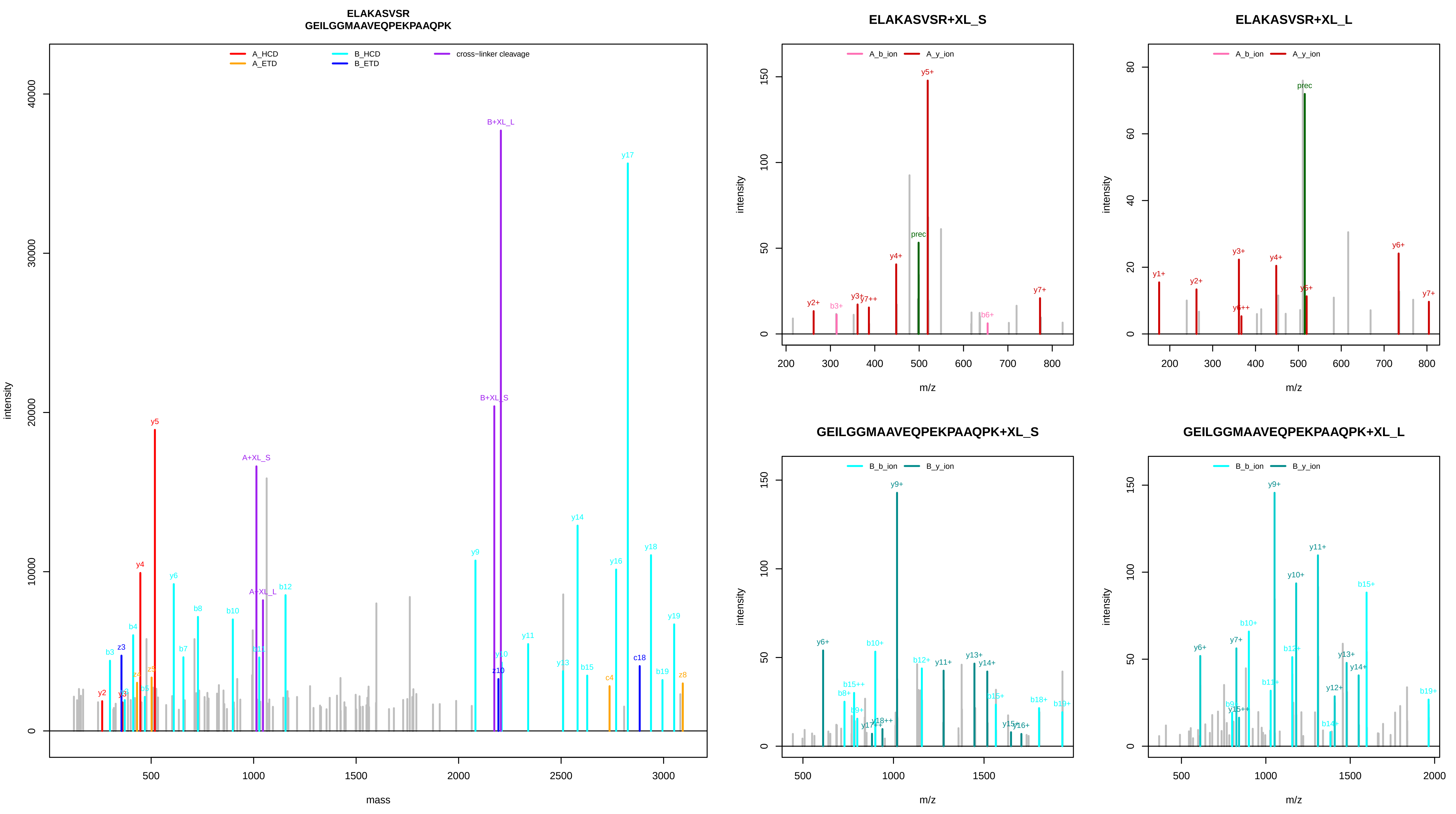
ELAKASVSR
GEILGGMAAVEQPEKPAAQPK
A_HCD
A_ETD
B_HCD
B_ETD
cross−linker cleavage
B+XL_L
B+XL_S
A+XL_S
A+XL_L
intensity
mass
ELAKASVSR+XL_S
ELAKASVSR+XL_L
GEILGGMAAVEQPEKPAAQPK+XL_S
GEILGGMAAVEQPEKPAAQPK+XL_L
A_b_ion
A_y_ion
B_b_ion
B_y_ion
prec
m/z

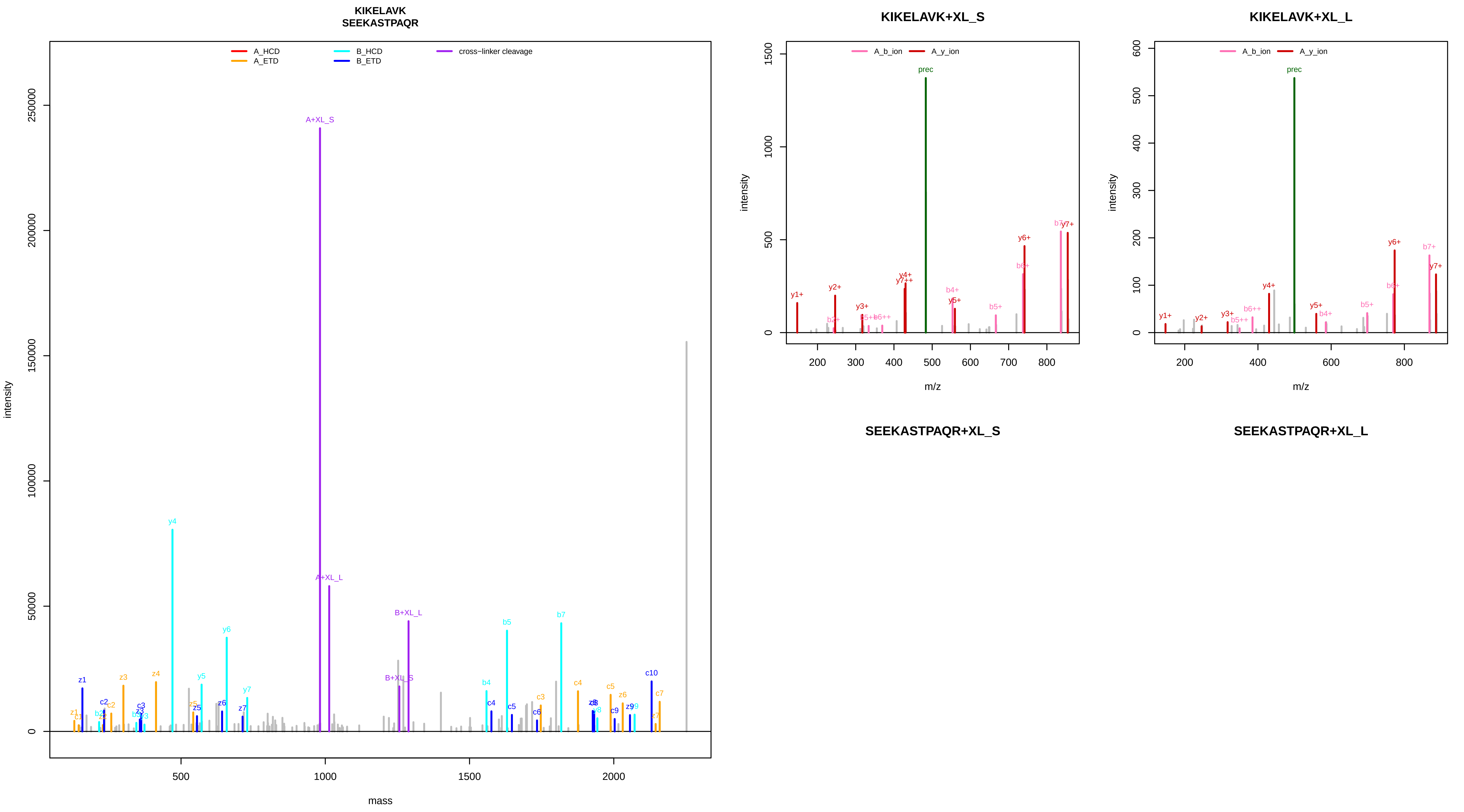
KIKELAVK
SEEKASTPAQR
A_HCD
A_ETD
B_HCD
B_ETD
cross−linker cleavage
A+XL_S
A+XL_L
B+XL_L
B+XL_S
intensity
mass
KIKELAVK+XL_S
KIKELAVK+XL_L
A_b_ion
A_y_ion
prec
m/z
SEEKASTPAQR+XL_S
SEEKASTPAQR+XL_L

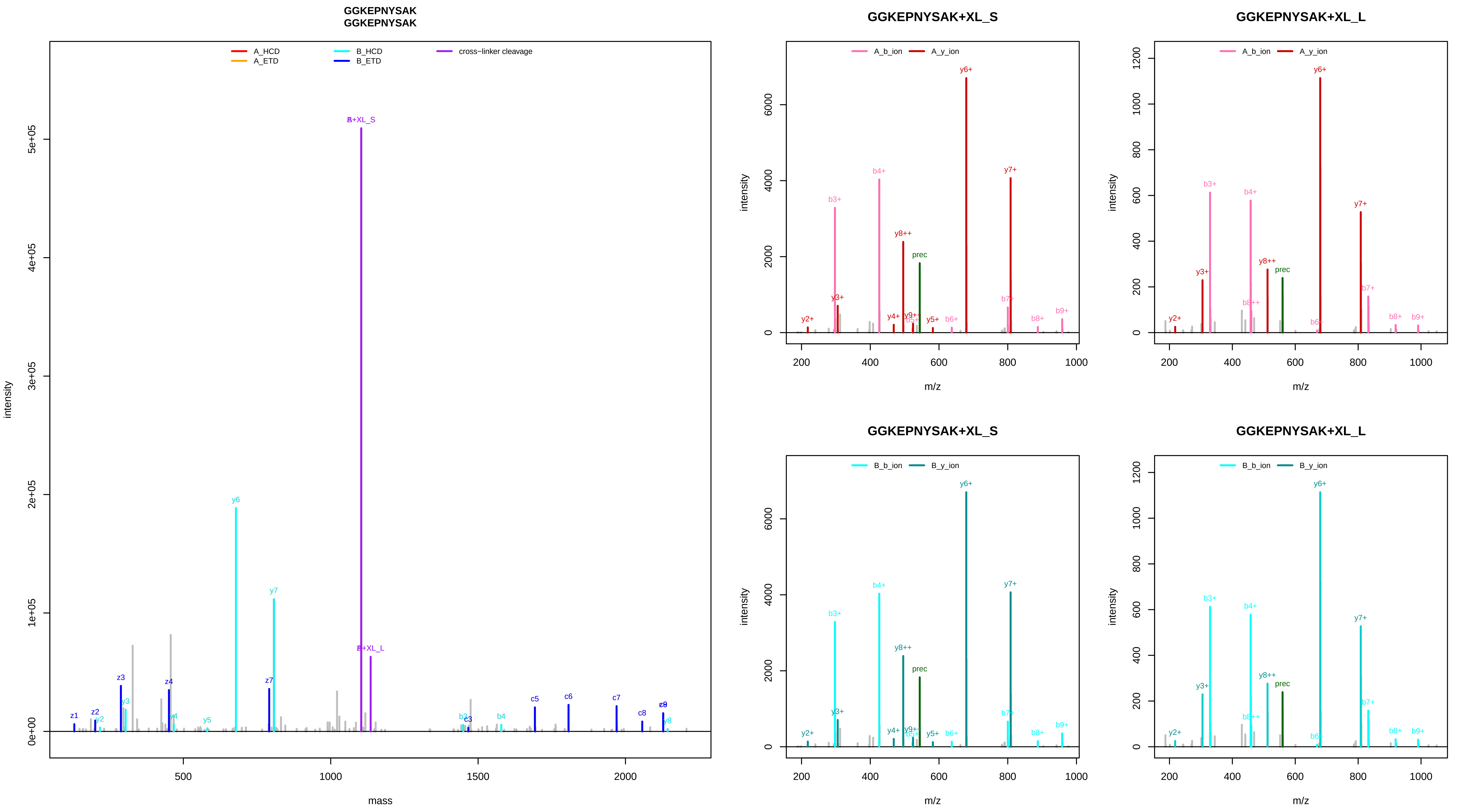
GGKEPNYSAK
GGKEPNYSAK
A_HCD
A_ETD
B_HCD
B_ETD
cross−linker cleavage
intensity
mass
GGKEPNYSAK+XL_S
GGKEPNYSAK+XL_L
A_b_ion
A_y_ion
B_b_ion
B_y_ion
m/z
intensity
prec

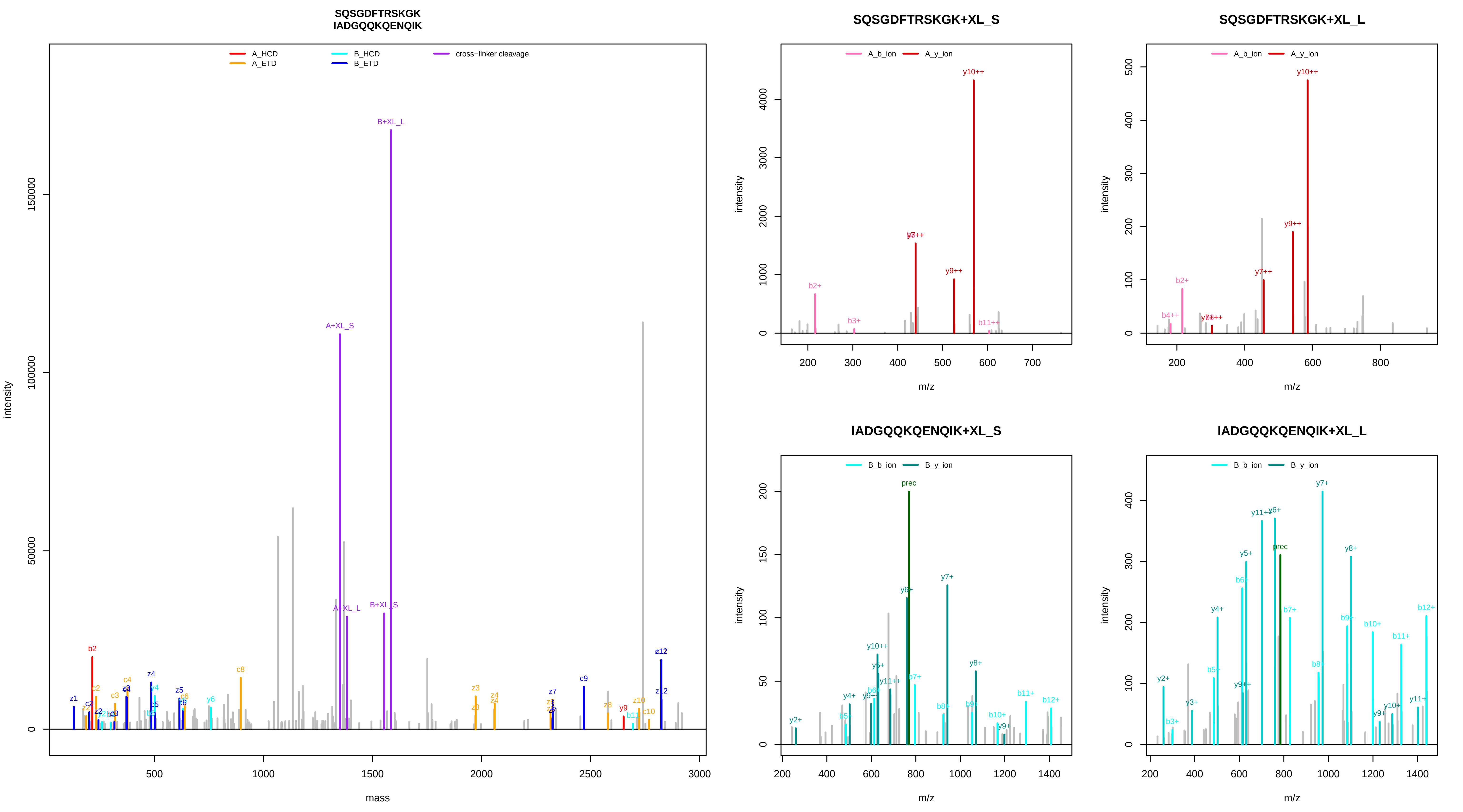
SQSGDFTRSKGK
IADGQQKQENQIK
A_HCD
A_ETD
B_HCD
B_ETD
cross−linker cleavage
B+XL_L
A+XL_S
A+XL_L
B+XL_S
intensity
mass
SQSGDFTRSKGK+XL_S
SQSGDFTRSKGK+XL_L
A_b_ion
A_y_ion
IADGQQKQENQIK+XL_S
IADGQQKQENQIK+XL_L
B_b_ion
B_y_ion
prec
m/z

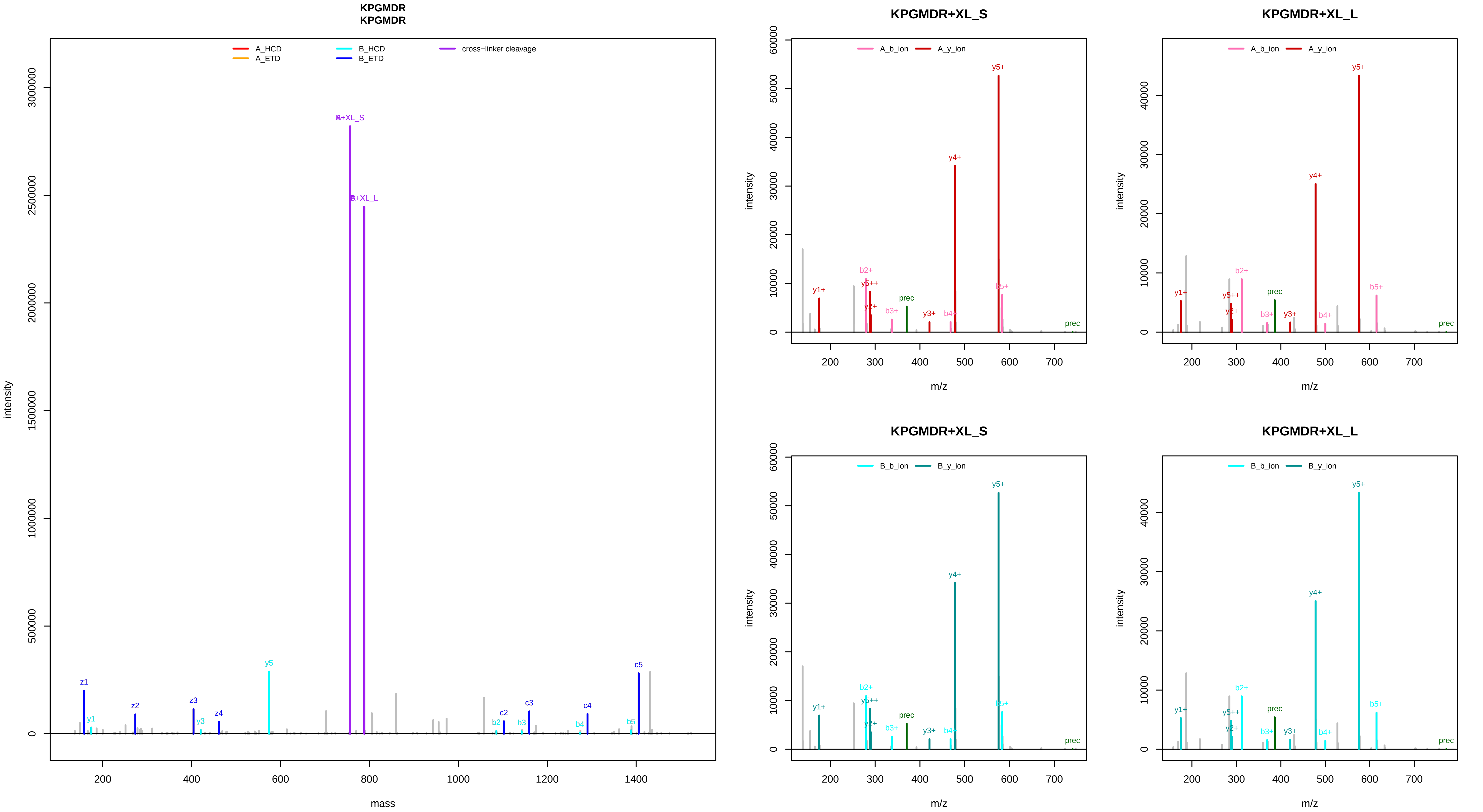
KPGMDR
KPGMDR
A_HCD
A_ETD
B_HCD
B_ETD
cross−linker cleavage
intensity
mass
KPGMDR+XL_S
KPGMDR+XL_L
A_b_ion
A_y_ion
B_b_ion
B_y_ion
m/z
intensity

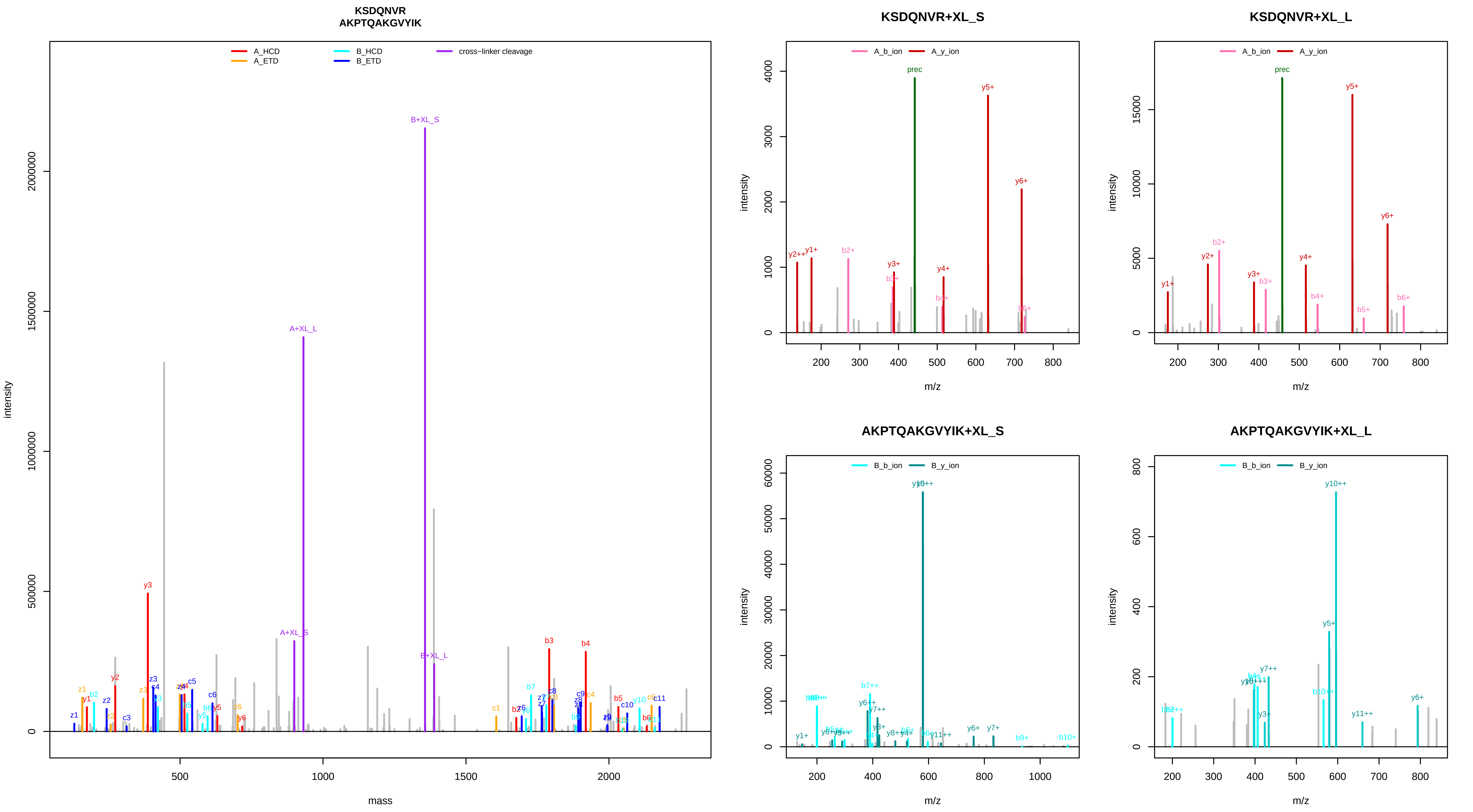
KSDQNVR
AKPTQAKGVYIK
A_HCD
A_ETD
B_HCD
B_ETD
cross−linker cleavage
B+XL_S
A+XL_L
A+XL_S
B+XL_L
intensity
mass
KSDQNVR+XL_S
KSDQNVR+XL_L
AKPTQAKGVYIK+XL_S
AKPTQAKGVYIK+XL_L
A_b_ion
A_y_ion
B_b_ion
B_y_ion
prec
m/z

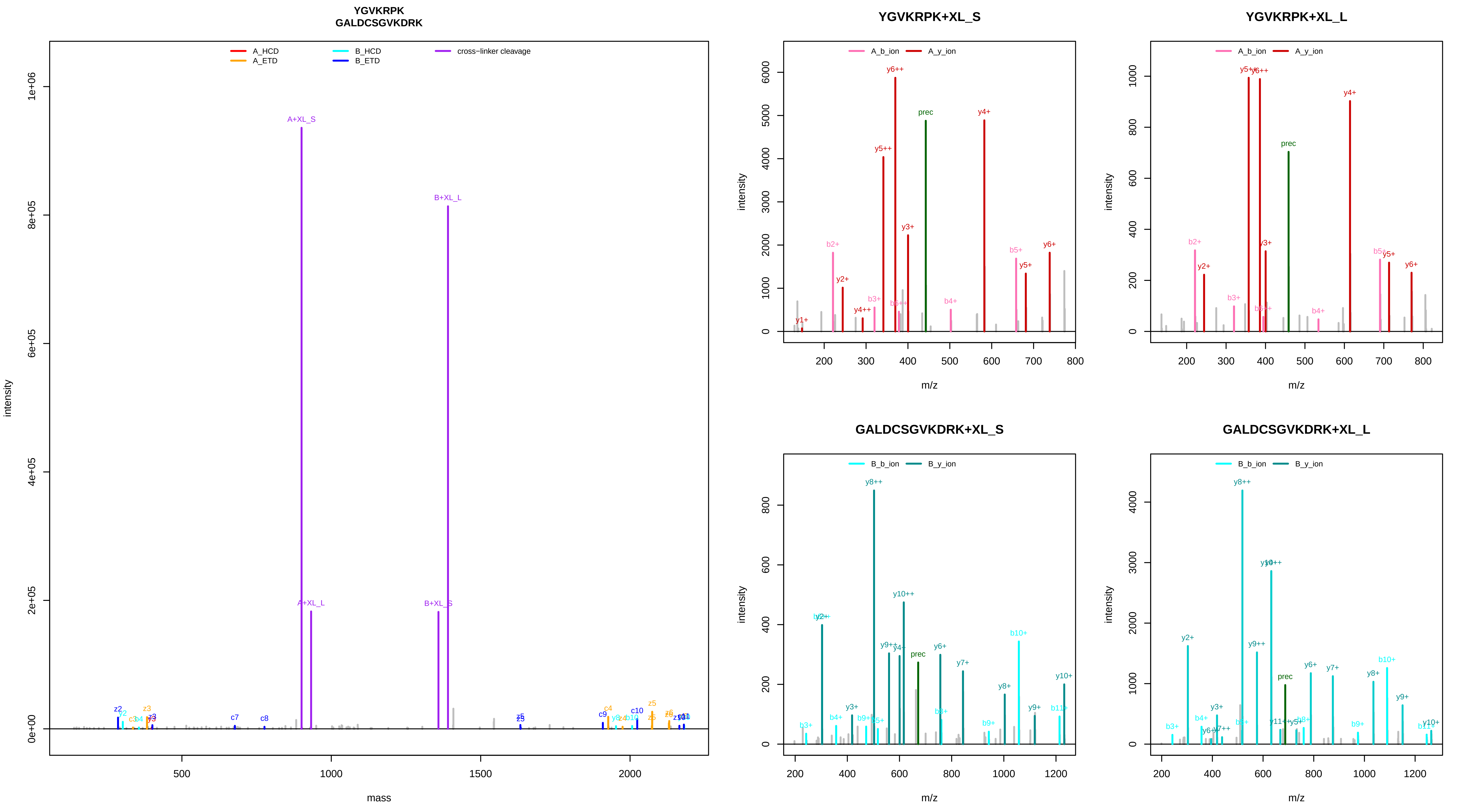

YGVKRPK
GALDCSGVKDRK
A_HCD
A_ETD
B_HCD
B_ETD
cross−linker cleavage
A+XL_S
B+XL_L
A+XL_L
B+XL_S
intensity
mass
YGVKRPK+XL_S
YGVKRPK+XL_L
A_b_ion
A_y_ion
prec
intensity
m/z
GALDCSGVKDRK+XL_S
GALDCSGVKDRK+XL_L
B_b_ion
B_y_ion

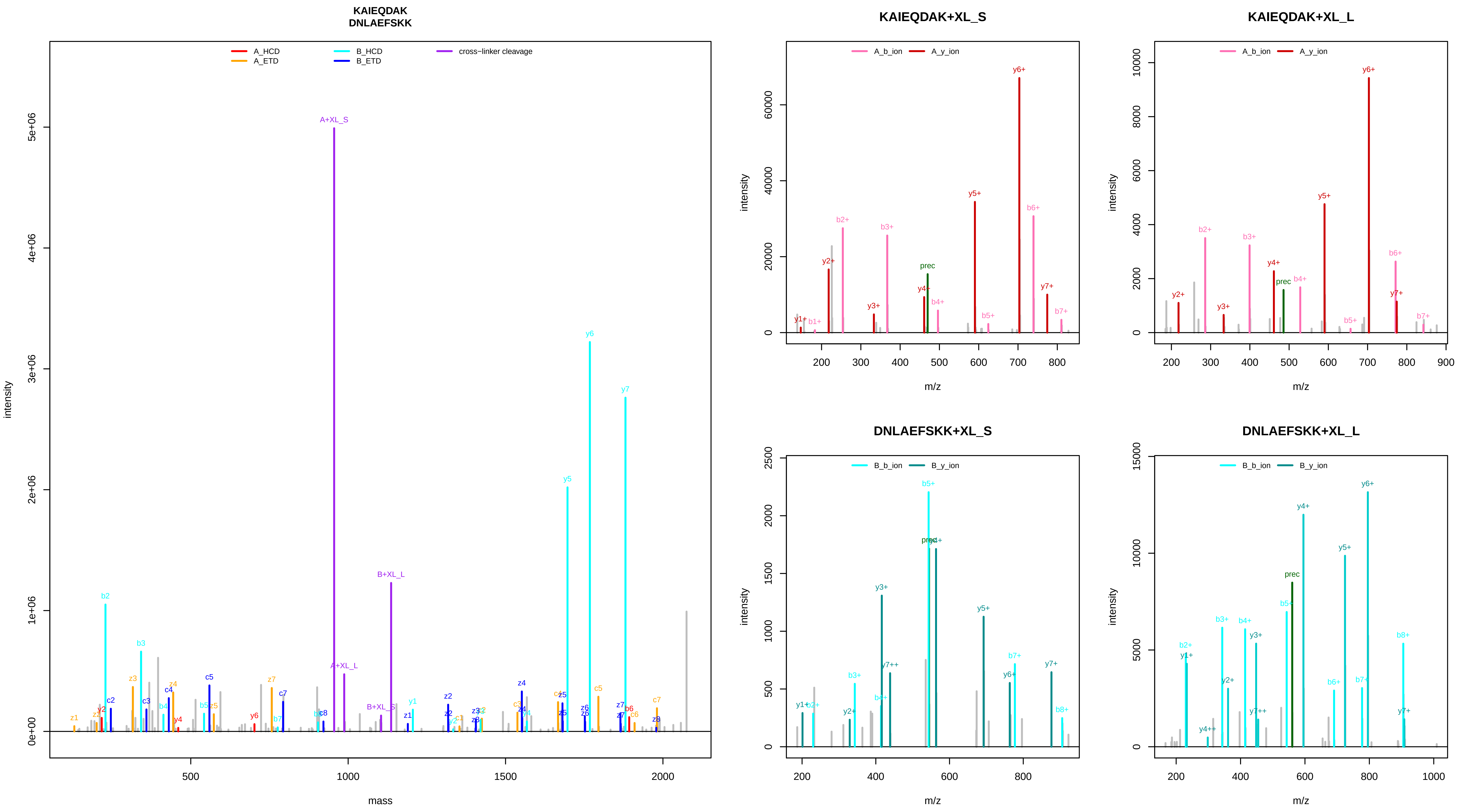
KAIEQDAK
DNLAEFSKK
A_HCD
A_ETD
B_HCD
B_ETD
cross−linker cleavage
A+XL_S
B+XL_L
A+XL_L
B+XL_S
intensity
mass
KAIEQDAK+XL_S
KAIEQDAK+XL_L
DNLAEFSKK+XL_S
DNLAEFSKK+XL_L
A_b_ion
A_y_ion
B_b_ion
B_y_ion
prec
m/z

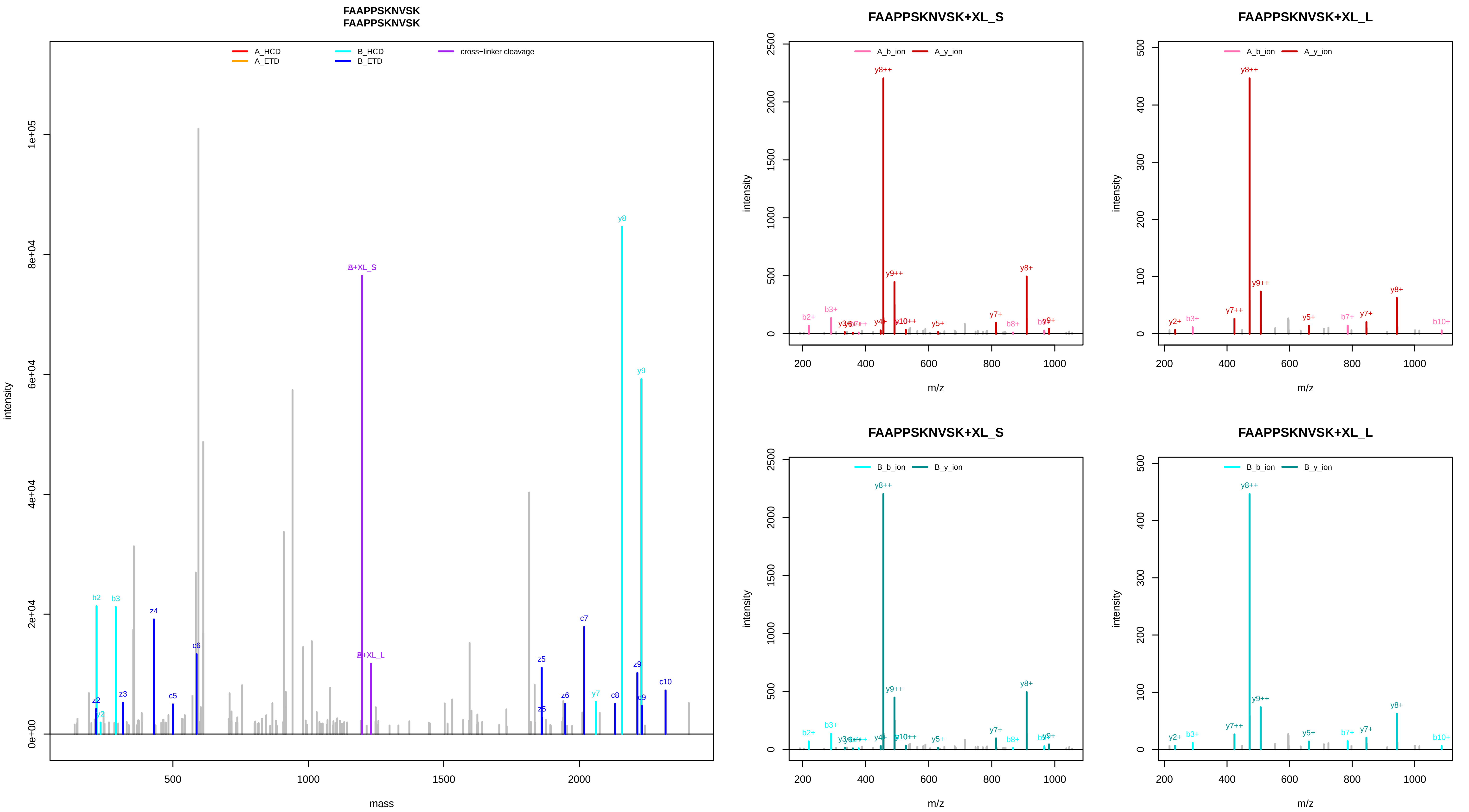
FAAPPSKNVSK
FAAPPSKNVSK
A_HCD
A_ETD
B_HCD
B_ETD
cross−linker cleavage
intensity
mass
B+XL_S
B+XL_L
y8
y9
y7
b2
b3
y2
z2
z3
z4
z5
z6
z9
c5
c6
c7
c8
c9
c10
FAAPPSKNVSK+XL_S
FAAPPSKNVSK+XL_L
A_b_ion
A_y_ion
B_b_ion
B_y_ion
m/z
y8++
y9++
y8+
y7+
y7++
y5+
y4+
y10++
y9+
y2+
b2+
b3+
b7+
b8+
b10+

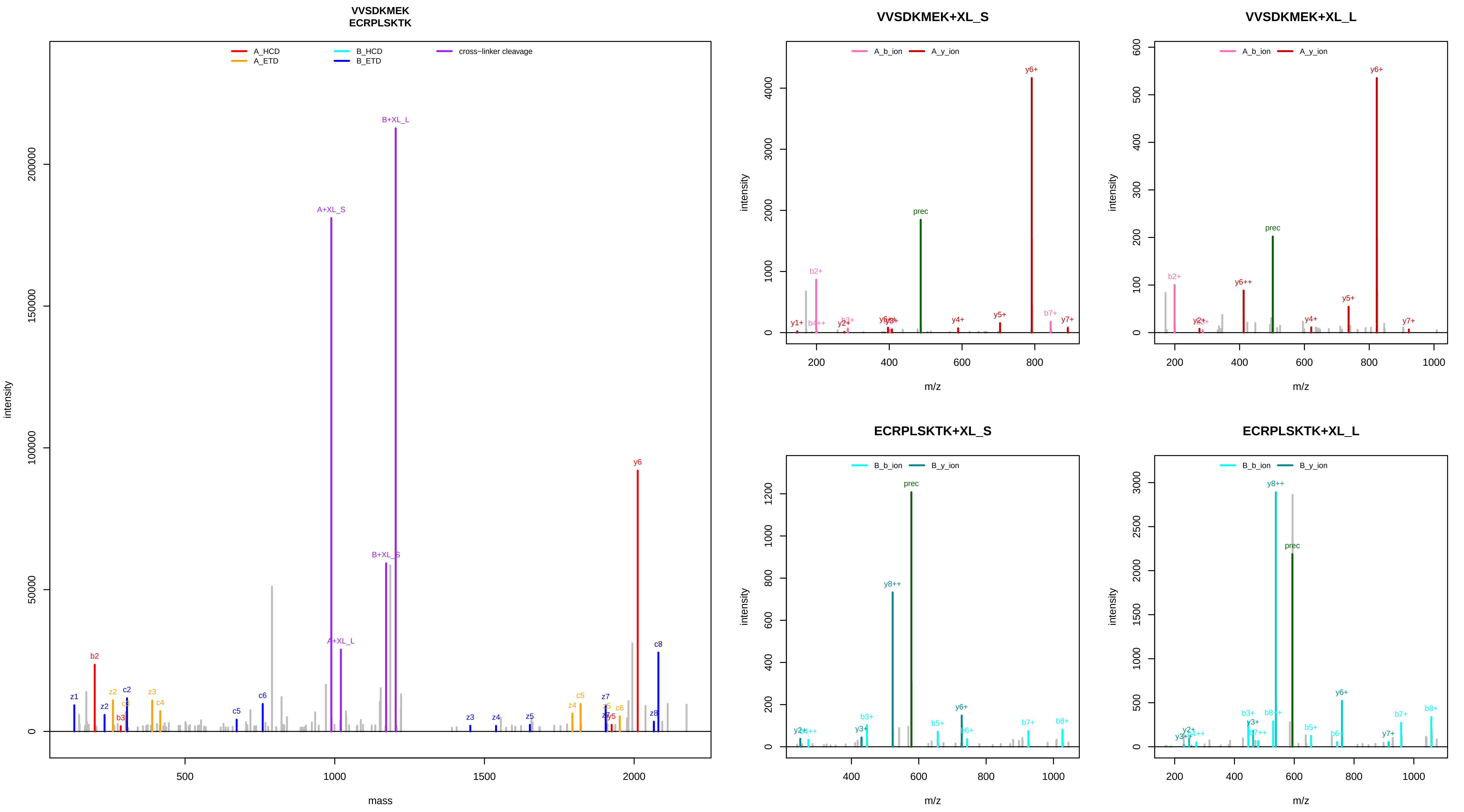

VVSDKMEK
ECRPLSKTK
A_HCD
A_ETD
B_HCD
B_ETD
cross−linker cleavage
B+XL_L
A+XL_S
B+XL_S
A+XL_L
y6
c8
b2
intensity
mass
VVSDKMEK+XL_S
VVSDKMEK+XL_L
ECRPLSKTK+XL_S
ECRPLSKTK+XL_L
A_b_ion
A_y_ion
B_b_ion
B_y_ion
prec
y6+
y8++
b8+
y6++
y5+
y4+
y7+
b2+
b7+
b3+
b5+
b6+
b7+
y3+
y2+
m/z

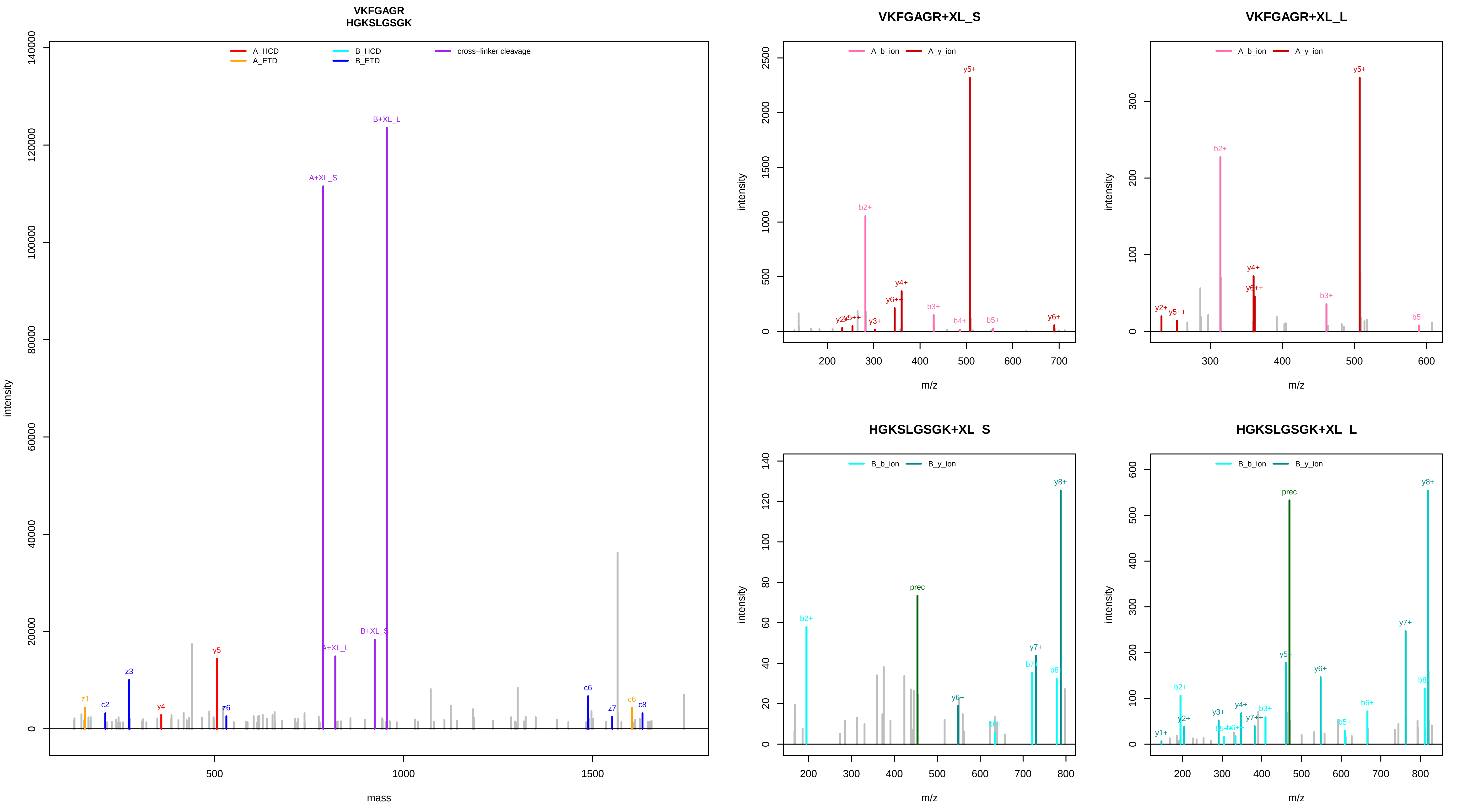

VKFGAGR
HGKSLGSGK
A_HCD
A_ETD
B_HCD
B_ETD
cross−linker cleavage
B+XL_L
A+XL_S
A+XL_L
B+XL_S
intensity
mass
VKFGAGR+XL_S
VKFGAGR+XL_L
HGKSLGSGK+XL_S
HGKSLGSGK+XL_L
A_b_ion
A_y_ion
B_b_ion
B_y_ion
prec
m/z

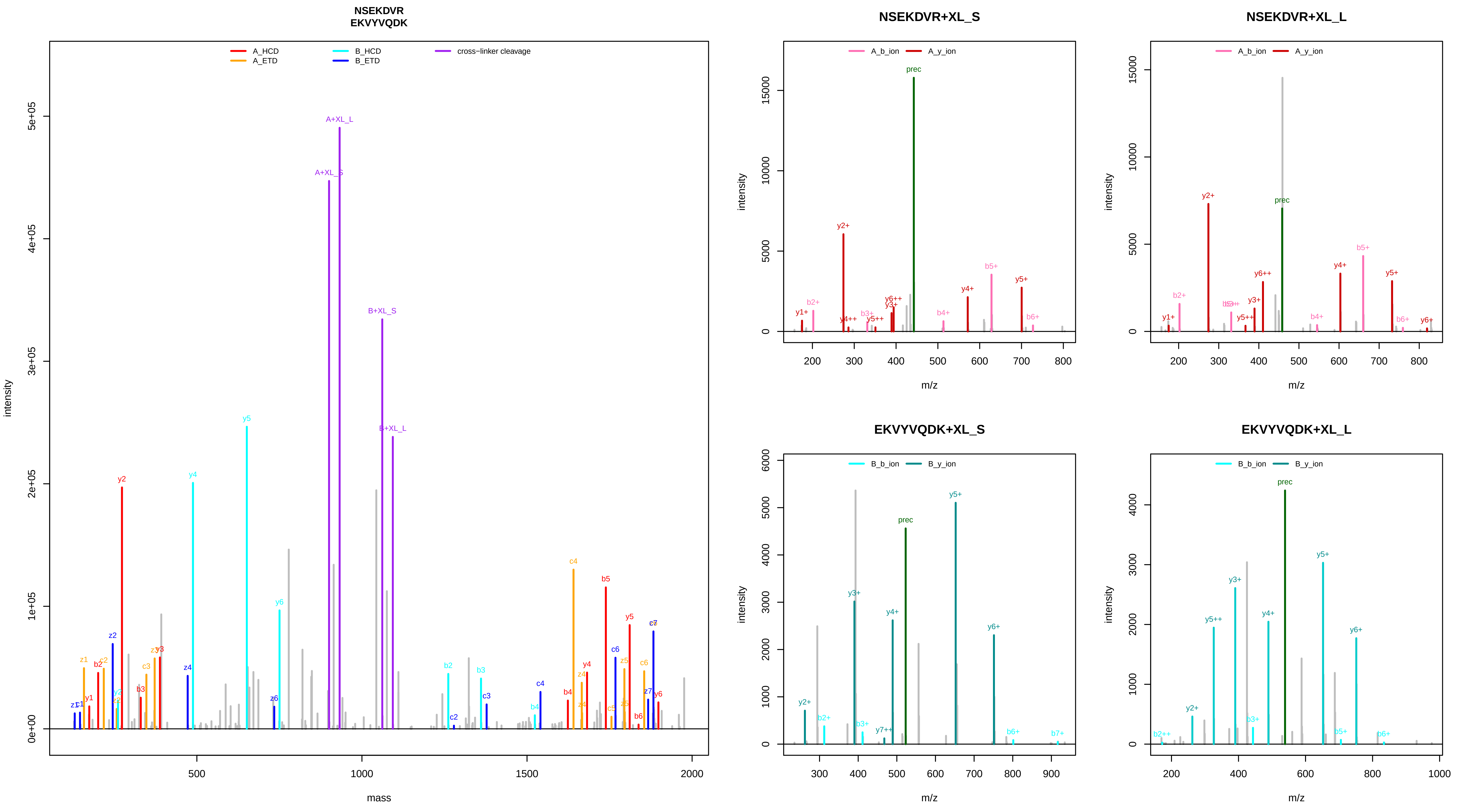
NSEKDVR
EKVYVQDK
A_HCD
A_ETD
B_HCD
B_ETD
cross−linker cleavage
A+XL_L
A+XL_S
B+XL_S
B+XL_L
intensity
mass
NSEKDVR+XL_S
NSEKDVR+XL_L
EKVYVQDK+XL_S
EKVYVQDK+XL_L
A_b_ion
A_y_ion
B_b_ion
B_y_ion
prec
m/z
intensity

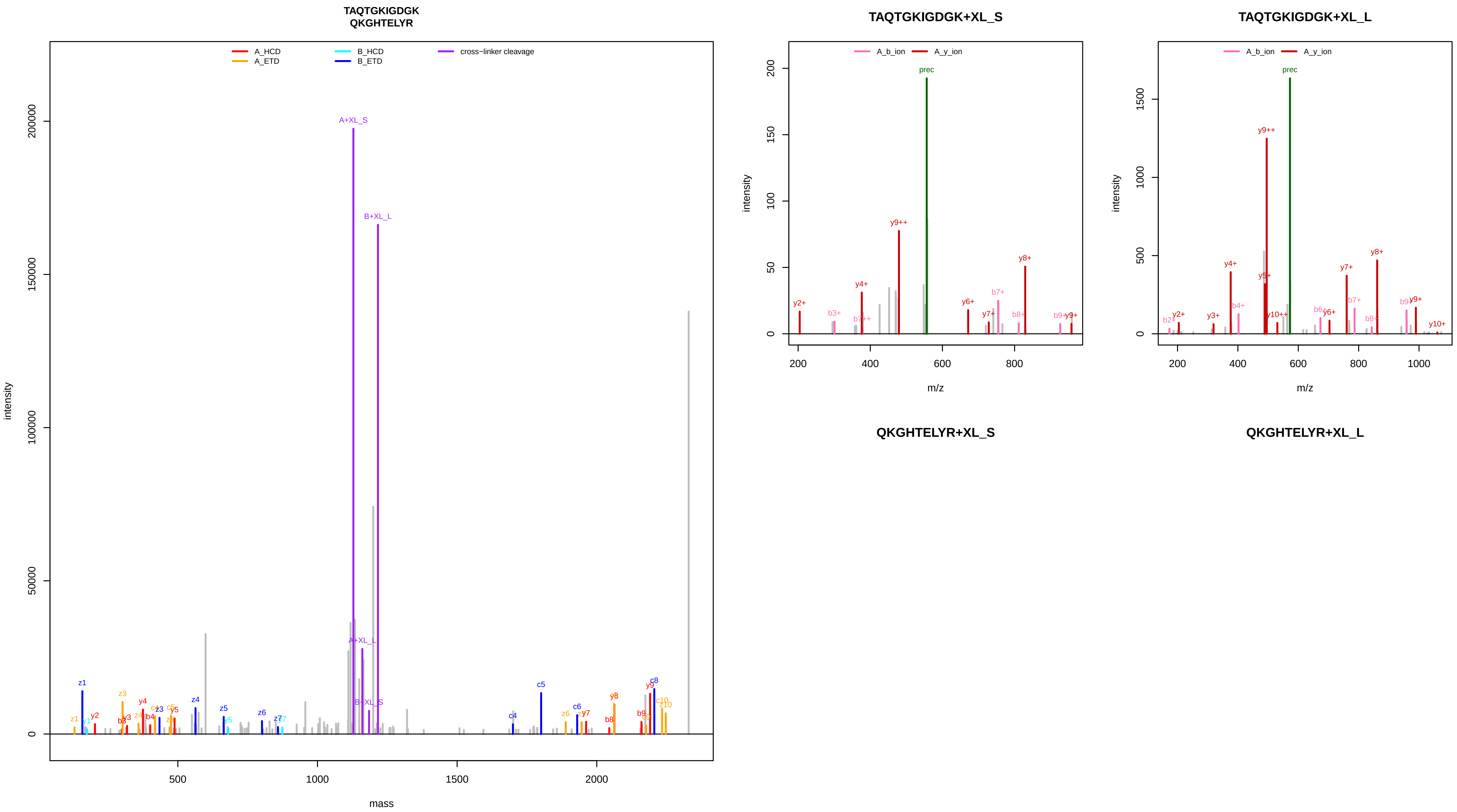
TAQTGKIGDGK
QKGHTELYR
A_HCD
A_ETD
B_HCD
B_ETD
cross−linker cleavage
A+XL_S
B+XL_L
A+XL_L
B+XL_S
intensity
mass
TAQTGKIGDGK+XL_S
TAQTGKIGDGK+XL_L
A_b_ion
A_y_ion
prec
intensity
m/z
QKGHTELYR+XL_S
QKGHTELYR+XL_L

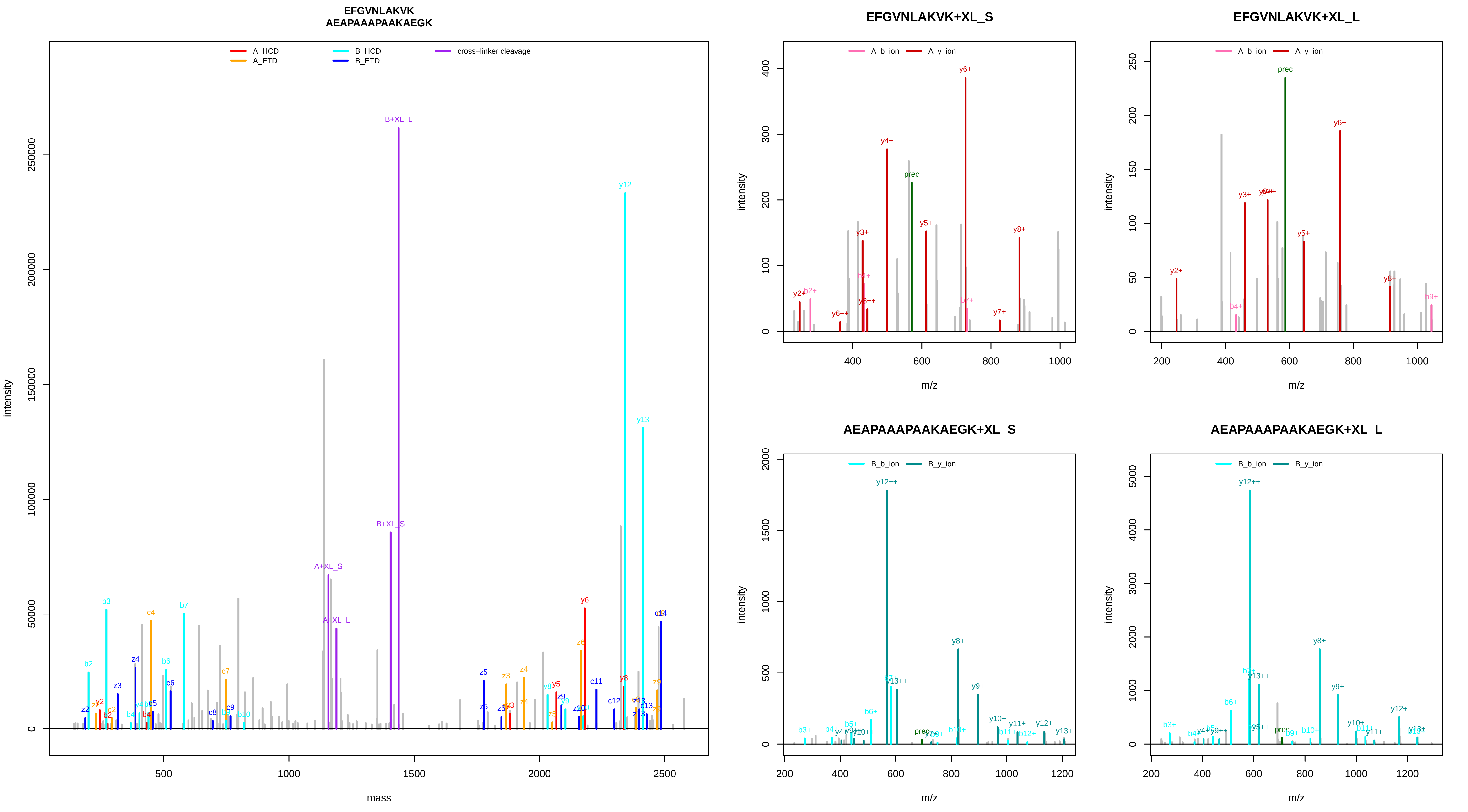

EFGVNLAKVK
AEAPAAAPAAKAEGK
A_HCD
A_ETD
B_HCD
B_ETD
cross−linker cleavage
B+XL_L
B+XL_S
A+XL_S
A+XL_L
y12
y13
intensity
mass
EFGVNLAKVK+XL_S
EFGVNLAKVK+XL_L
AEAPAAAPAAKAEGK+XL_S
AEAPAAAPAAKAEGK+XL_L
A_b_ion
A_y_ion
B_b_ion
B_y_ion
m/z
prec
y12++
y8+
y6+

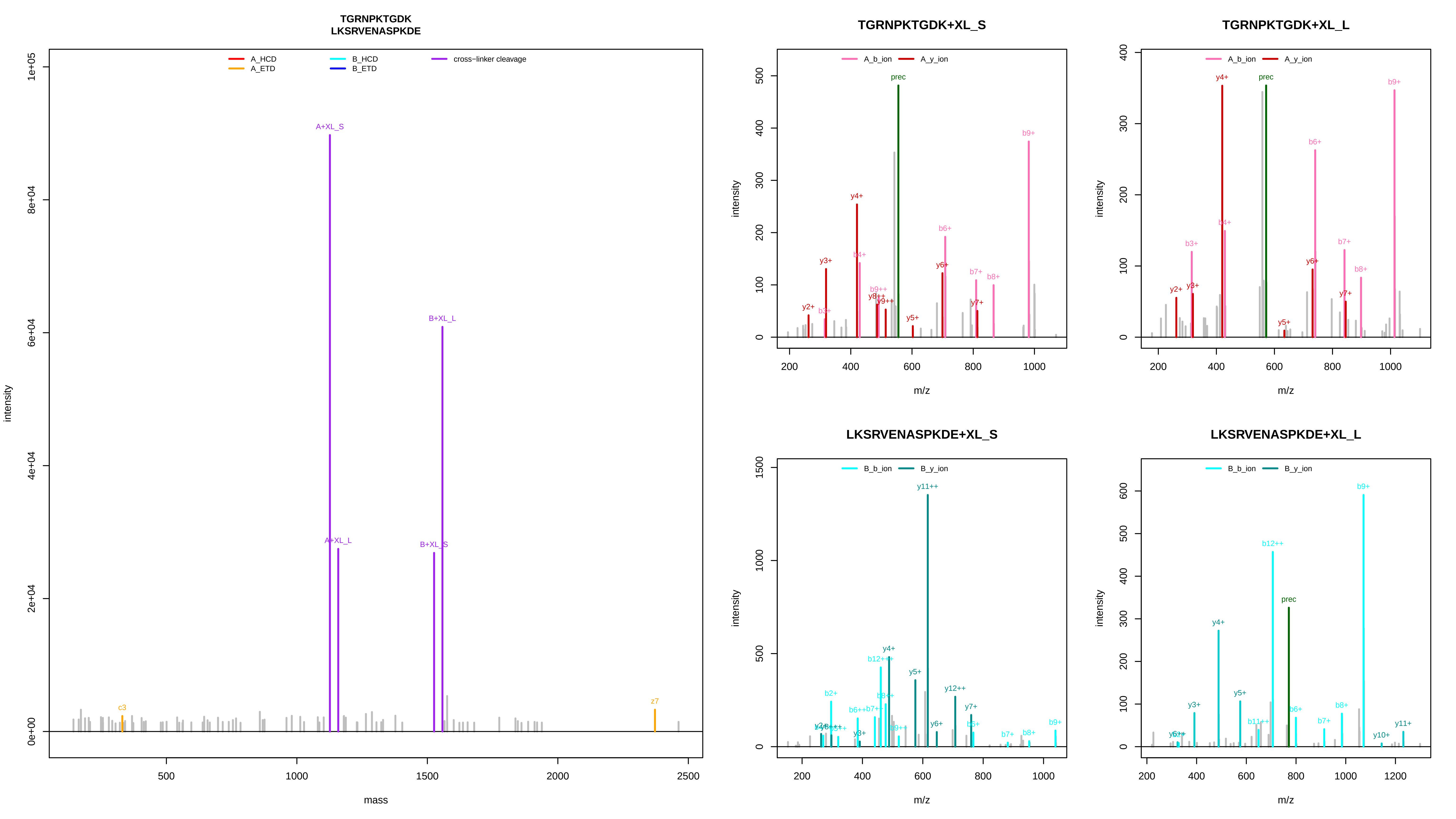
TGRNPKTGDK
LKSRVENASPKDE
A_HCD
A_ETD
B_HCD
B_ETD
cross−linker cleavage
A+XL_S
B+XL_L
A+XL_L
B+XL_S
c3
z7
intensity
mass
TGRNPKTGDK+XL_S
A_b_ion
A_y_ion
prec
y4+
y3+
y2+
b3+
b4+
b9++
y8++
y9++
y5+
y6+
b6+
b7+
y7+
b8+
b9+
m/z
TGRNPKTGDK+XL_L
LKSRVENASPKDE+XL_S
B_b_ion
B_y_ion
y11++
b12+++
b2+
b6++
b7++
y12++
LKSRVENASPKDE+XL_L
b12++
b11++
y10+
y11+

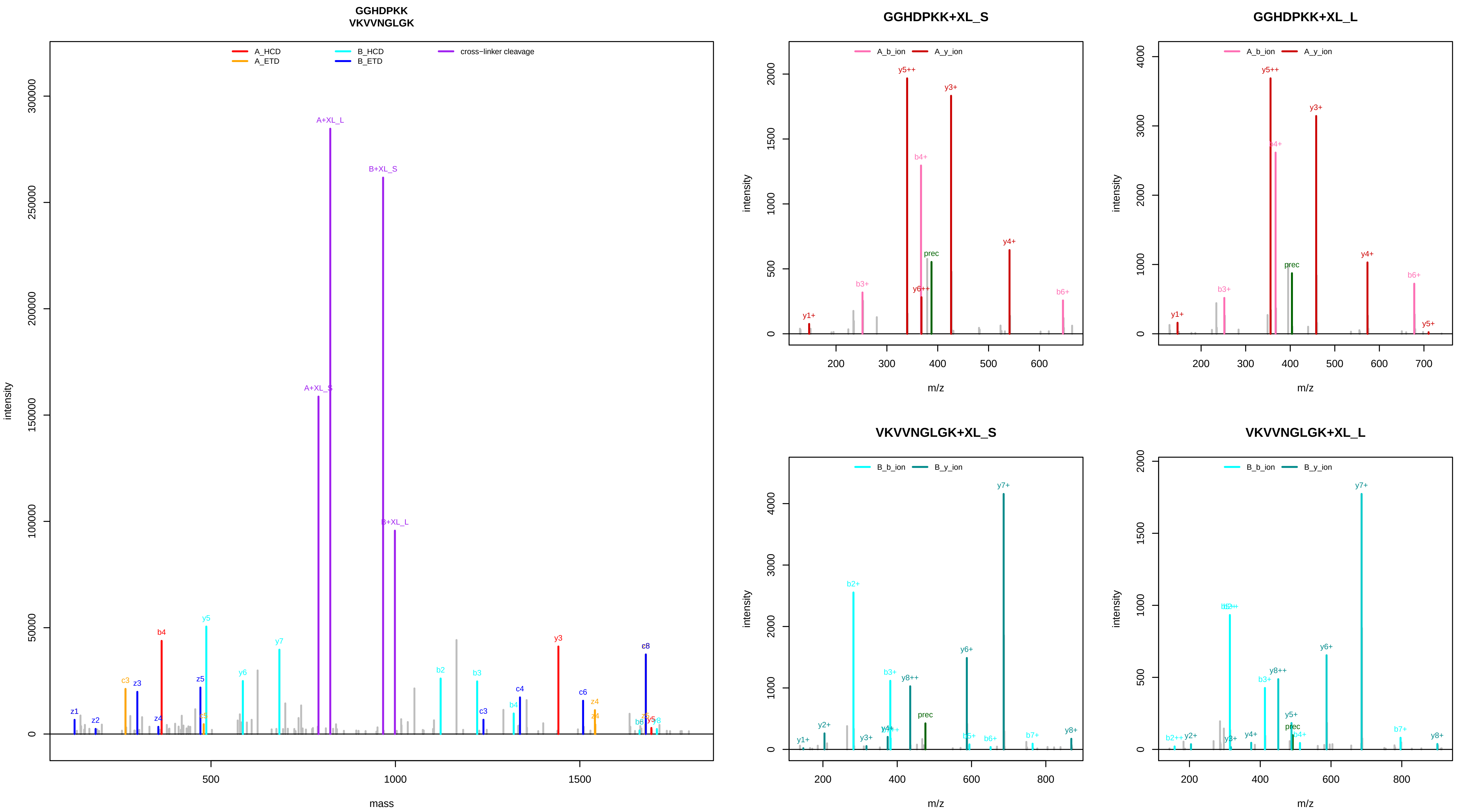
GGHDPKK
VKVVNGLGK
A_HCD
A_ETD
B_HCD
B_ETD
cross−linker cleavage
A+XL_L
B+XL_S
A+XL_S
B+XL_L
intensity
mass
GGHDPKK+XL_S
GGHDPKK+XL_L
VKVVNGLGK+XL_S
VKVVNGLGK+XL_L
A_b_ion
A_y_ion
B_b_ion
B_y_ion
m/z
prec

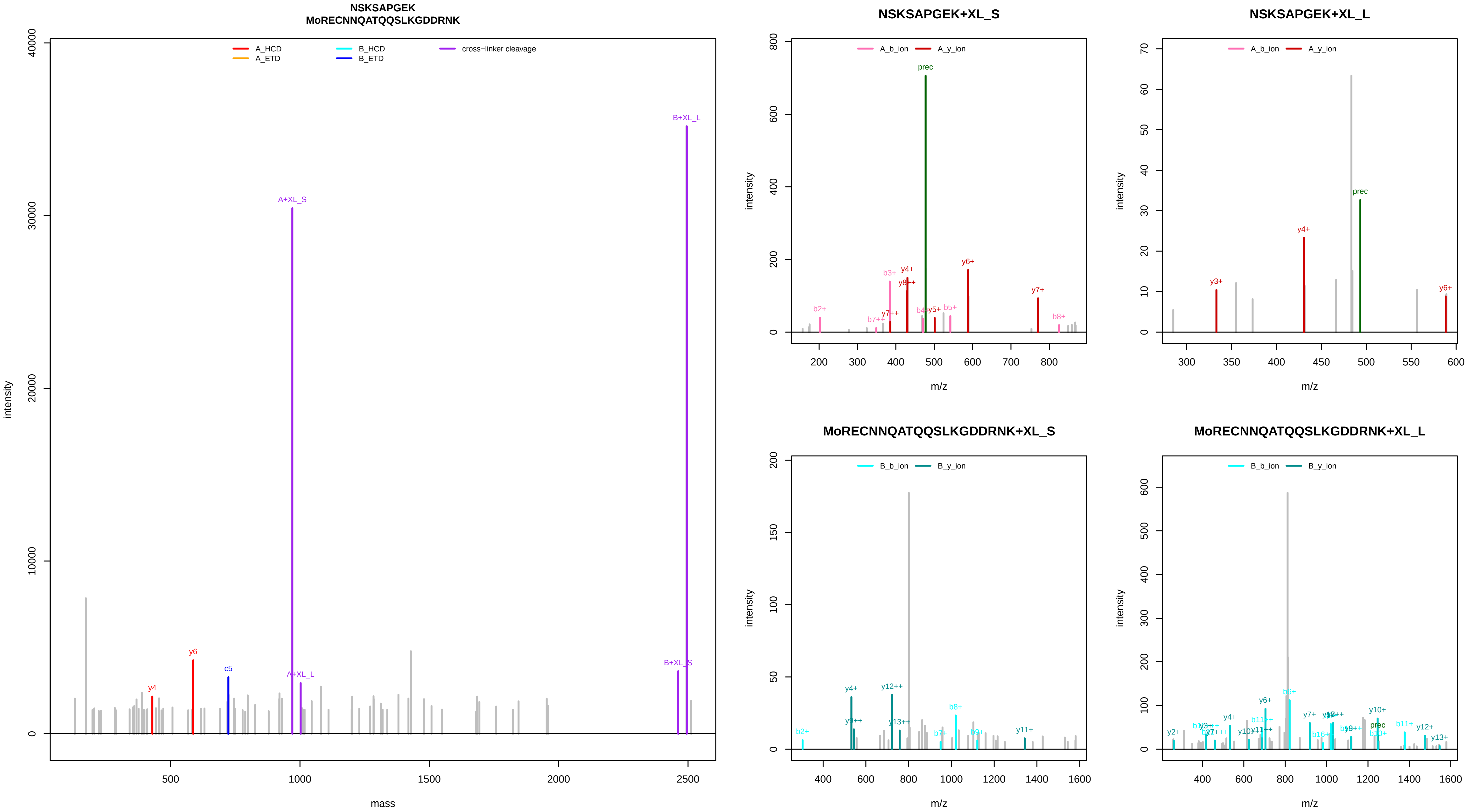
NSKSAPGEK
MoRECNNQATQQSLKGDDRNK
A_HCD
A_ETD
B_HCD
B_ETD
cross−linker cleavage
B+XL_L
A+XL_S
A+XL_L
B+XL_S
y4
y6
c5
intensity
mass
NSKSAPGEK+XL_S
A_b_ion
A_y_ion
prec
b2+
b3+
y4+
y6+
y7+
b8+
b5+
intensity
m/z
NSKSAPGEK+XL_L
A_b_ion
A_y_ion
prec
y3+
y4+
y6+
intensity
m/z
MoRECNNQATQQSLKGDDRNK+XL_S
B_b_ion
B_y_ion
y4+
y12++
y9++
y13++
b2+
b8+
b7+
y11+
intensity
m/z
MoRECNNQATQQSLKGDDRNK+XL_L
B_b_ion
B_y_ion
y2+
y4+
y6+
b6+
y7+
y10+
b11+
y12+
y13+
prec
intensity
m/z

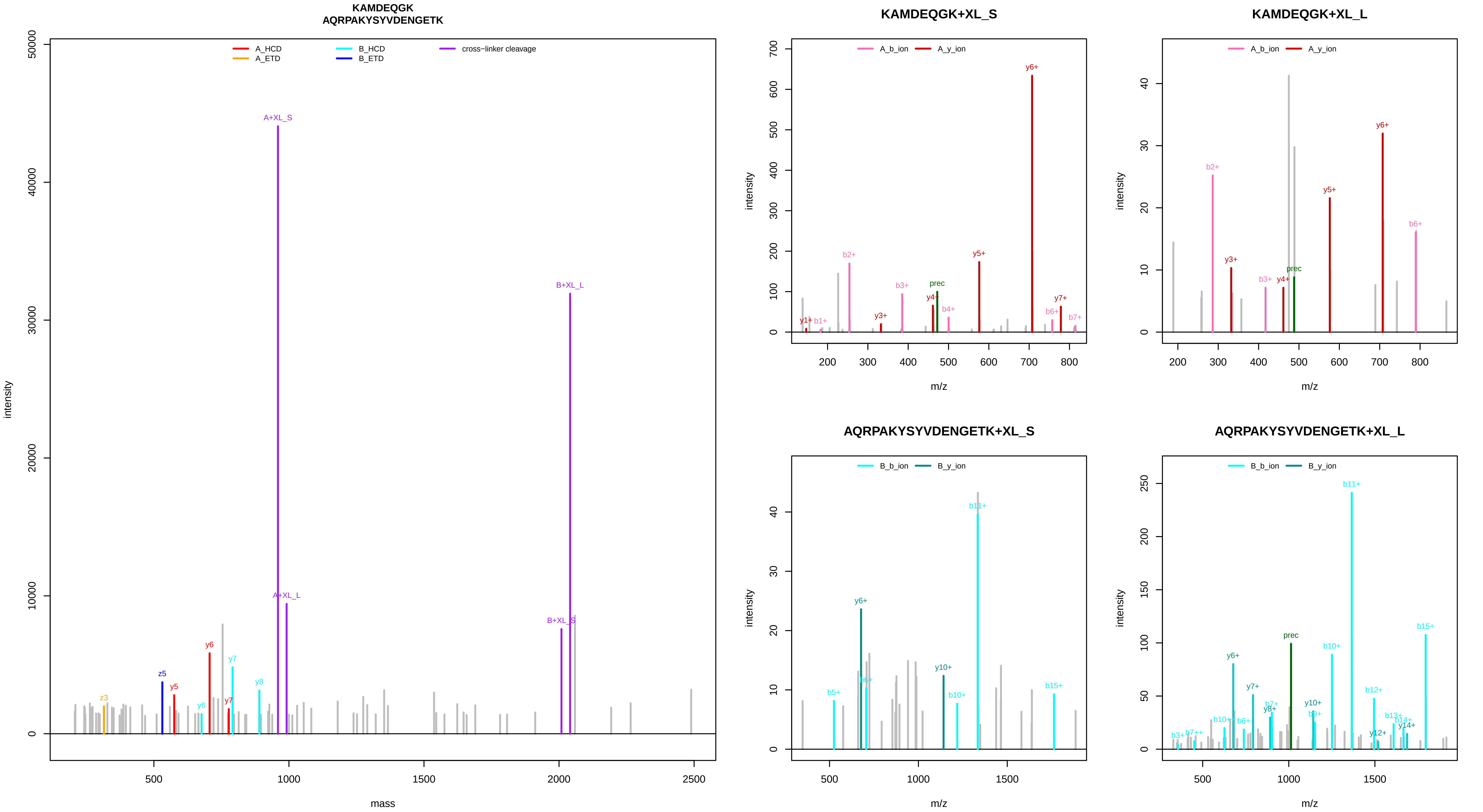
KAMDEQGK
AQRPAKYSYVDENGETK
A_HCD
A_ETD
B_HCD
B_ETD
cross−linker cleavage
A+XL_S
B+XL_L
A+XL_L
B+XL_S
intensity
mass
KAMDEQGK+XL_S
KAMDEQGK+XL_L
A_b_ion
A_y_ion
AQRPAKYSYVDENGETK+XL_S
AQRPAKYSYVDENGETK+XL_L
B_b_ion
B_y_ion
prec
m/z

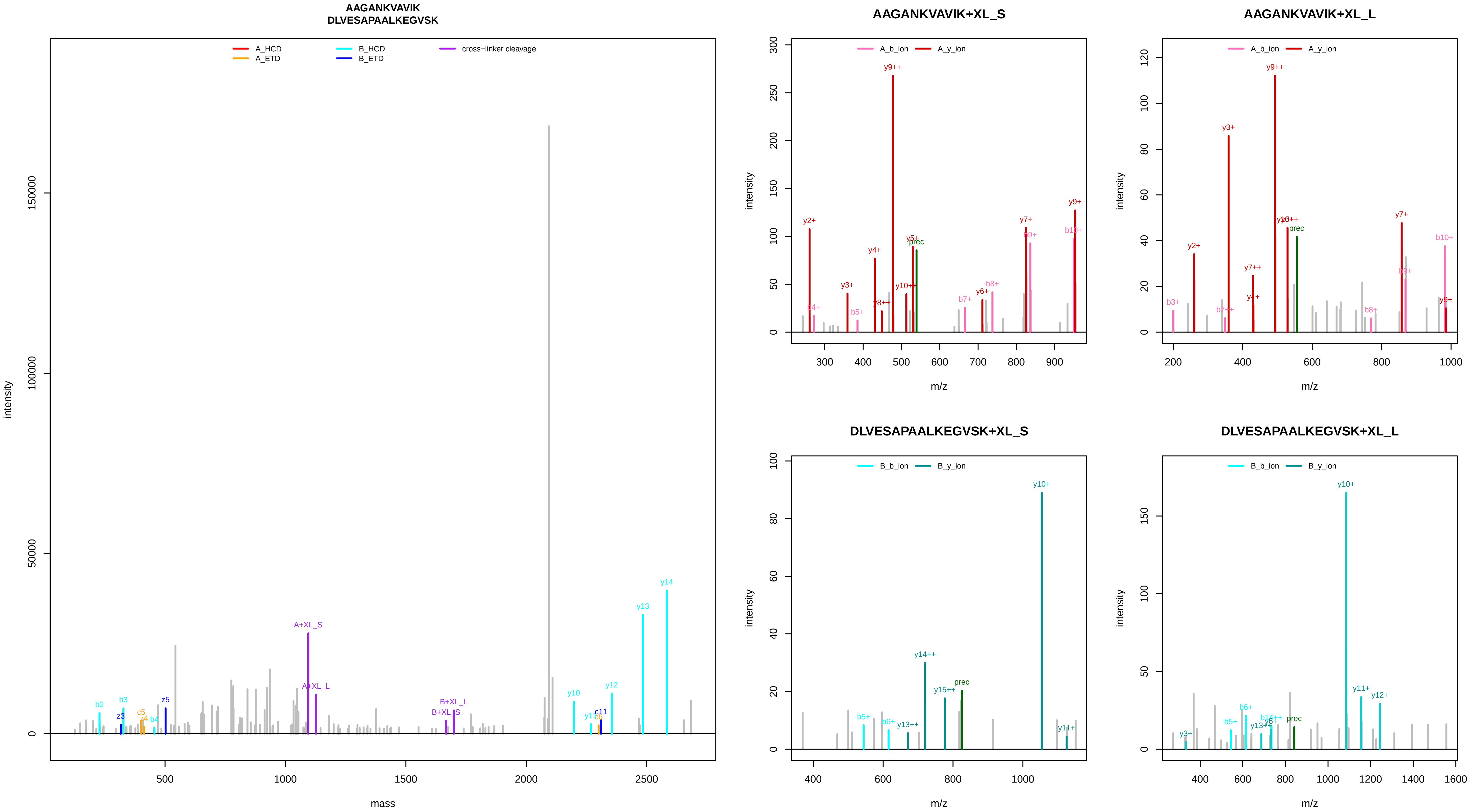
AAGANKVAVIK
DLVESAPAALKEGVSK
A_HCD
A_ETD
B_HCD
B_ETD
cross−linker cleavage
intensity
mass
A+XL_S
A+XL_L
B+XL_L
B+XL_S
AAGANKVAVIK+XL_S
AAGANKVAVIK+XL_L
DLVESAPAALKEGVSK+XL_S
DLVESAPAALKEGVSK+XL_L
A_b_ion
A_y_ion
B_b_ion
B_y_ion
m/z
prec

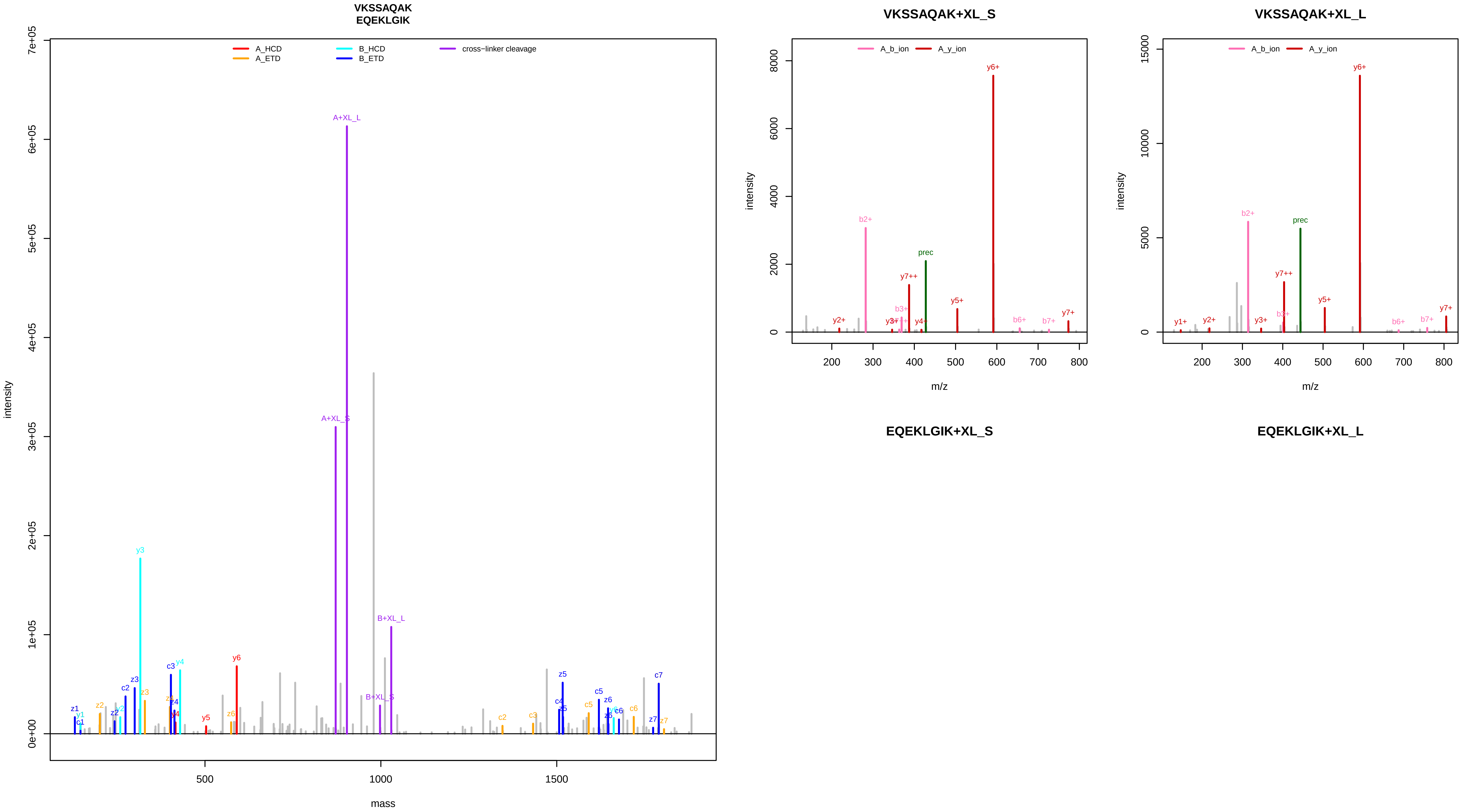
VKSSAQAK
EQEKLGIK
A_HCD
A_ETD
B_HCD
B_ETD
cross−linker cleavage
A+XL_L
A+XL_S
B+XL_L
B+XL_S
intensity
mass
VKSSAQAK+XL_S
VKSSAQAK+XL_L
A_b_ion
A_y_ion
prec
m/z
EQEKLGIK+XL_S
EQEKLGIK+XL_L

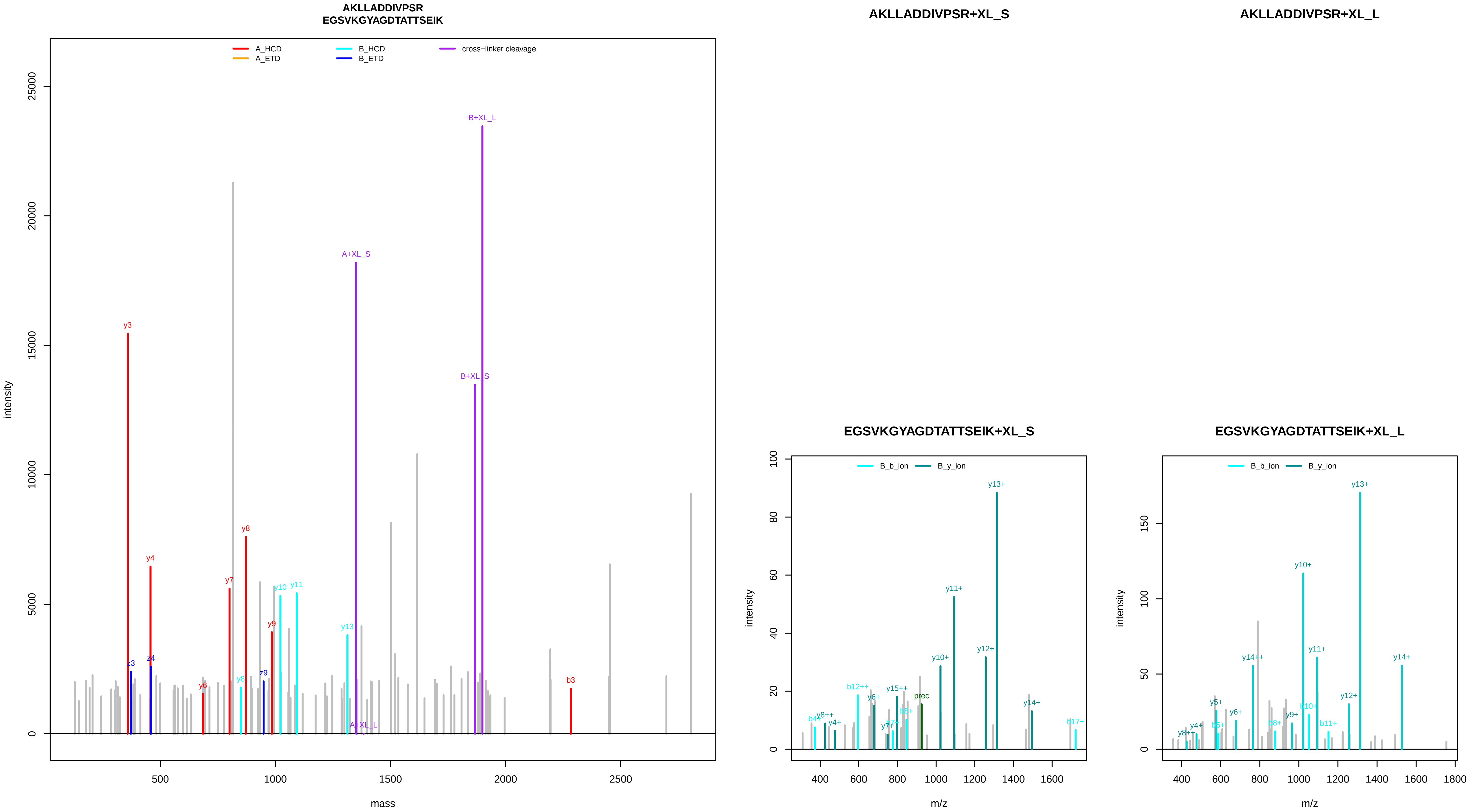

AKLLADDIVPSR
EGSVKGYAGDTATTSEIK
A_HCD
A_ETD
B_HCD
B_ETD
cross−linker cleavage
B+XL_L
A+XL_S
B+XL_S
A+XL_L
y3
y4
y6
y7
y8
y9
z3
z4
z9
y10
y11
y13
b3
intensity
mass
AKLLADDIVPSR+XL_S
AKLLADDIVPSR+XL_L
EGSVKGYAGDTATTSEIK+XL_S
EGSVKGYAGDTATTSEIK+XL_L
B_b_ion
B_y_ion
y13+
y11+
y12+
y10+
y14+
prec
y15++
b12++
y6+
y8++
y4+
y7+
b17+
y14++
y5+
y9+
b10+
b11+
y12+
m/z

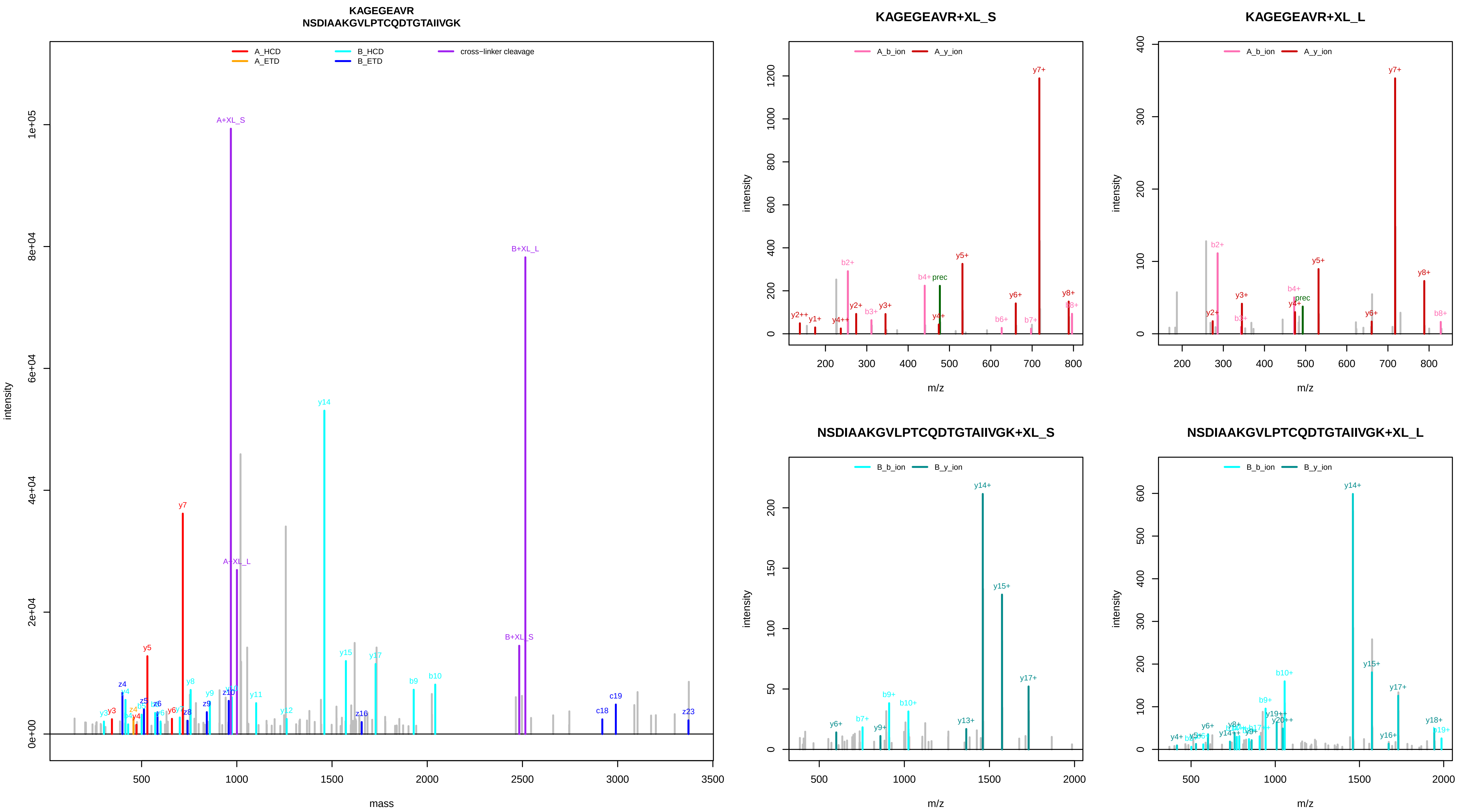
KAGEGEAVR
NSDIAAKGVLPTCQDTGTAIIVGK
A_HCD
A_ETD
B_HCD
B_ETD
cross−linker cleavage
A+XL_S
B+XL_L
A+XL_L
B+XL_S
y14
y7
y5
y15
y17
b9
b10
c19
c18
z23
z16
intensity
mass
KAGEGEAVR+XL_S
KAGEGEAVR+XL_L
NSDIAAKGVLPTCQDTGTAIIVGK+XL_S
NSDIAAKGVLPTCQDTGTAIIVGK+XL_L
A_b_ion
A_y_ion
B_b_ion
B_y_ion
prec
m/z
intensity

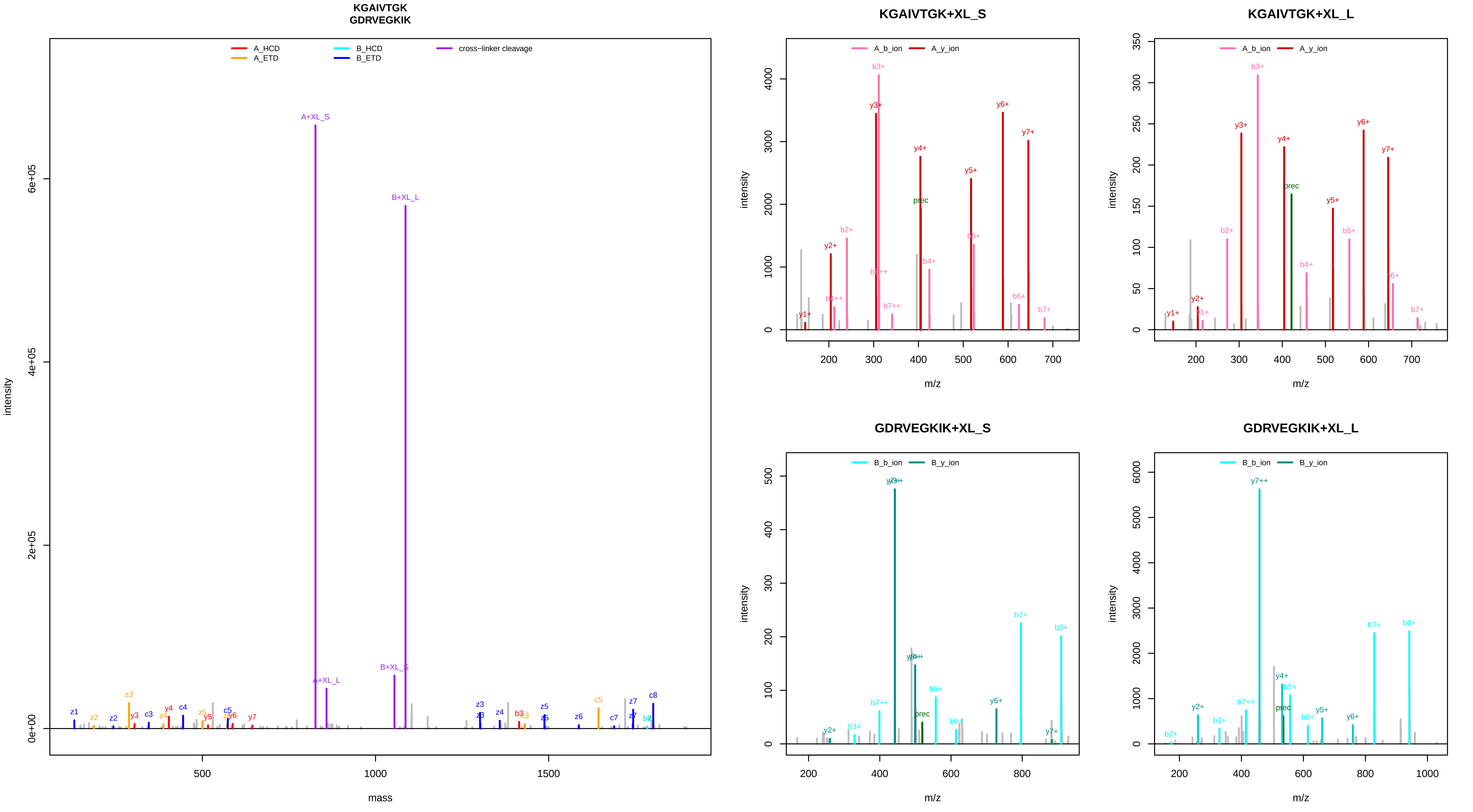
KGAIVTGK
GDRVEGKIK
A_HCD
A_ETD
B_HCD
B_ETD
cross−linker cleavage
A+XL_S
B+XL_L
A+XL_L
B+XL_S
intensity
mass
KGAIVTGK+XL_S
KGAIVTGK+XL_L
A_b_ion
A_y_ion
GDRVEGKIK+XL_S
GDRVEGKIK+XL_L
B_b_ion
B_y_ion
prec
m/z

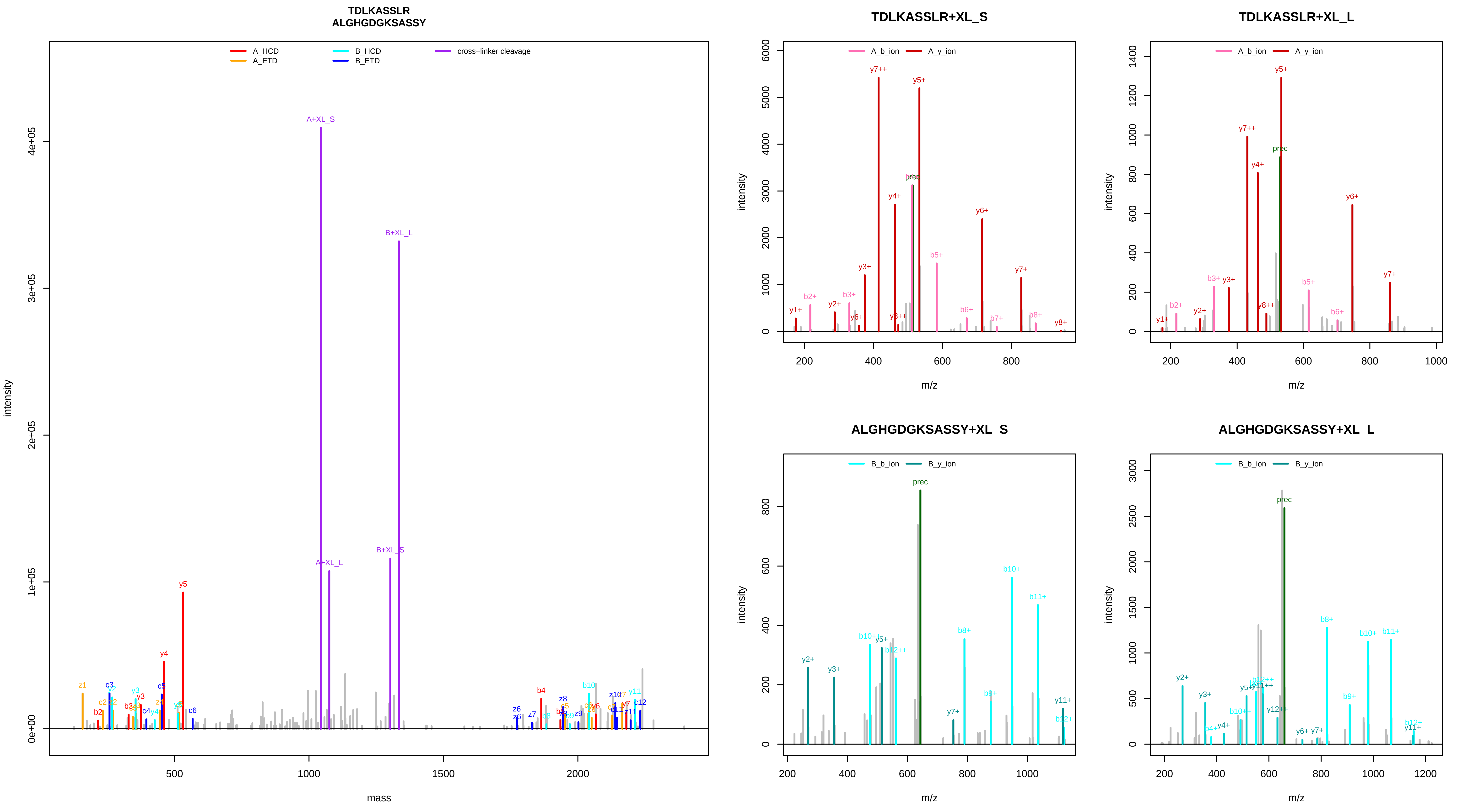
TDLKASSLR
ALGHGDGKSASSY
A_HCD
A_ETD
B_HCD
B_ETD
cross−linker cleavage
A+XL_S
B+XL_L
A+XL_L
B+XL_S
intensity
mass
TDLKASSLR+XL_S
TDLKASSLR+XL_L
A_b_ion
A_y_ion
ALGHGDGKSASSY+XL_S
ALGHGDGKSASSY+XL_L
B_b_ion
B_y_ion
prec
m/z

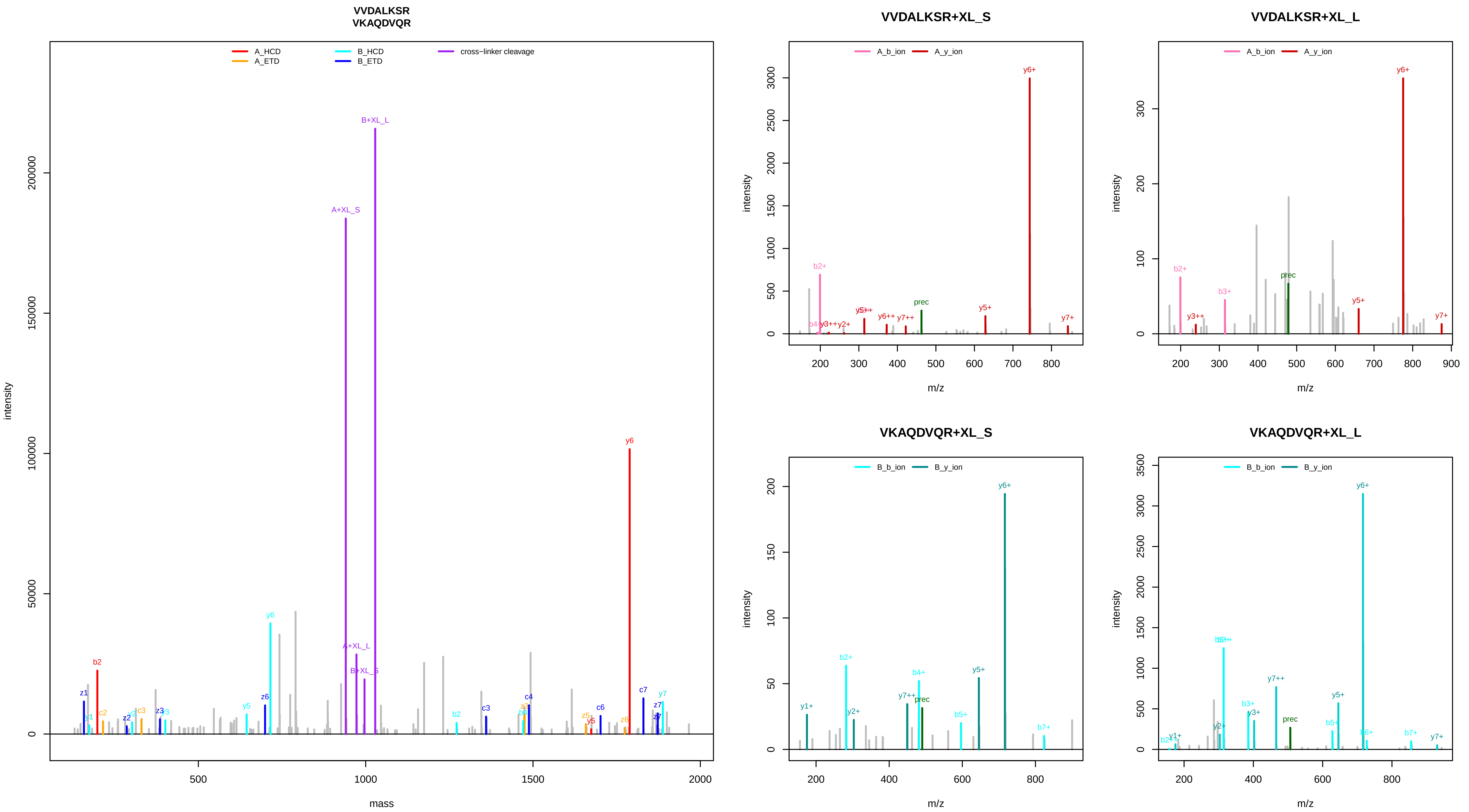
VVDALKSR
VKAQDVQR
A_HCD
A_ETD
B_HCD
B_ETD
cross−linker cleavage
B+XL_L
A+XL_S
A+XL_L
B+XL_S
y6
b2
c2
c3
z1
z2
z3
z6
y5
c3
c4
c6
c7
z7
y7
y5
z5
z6
intensity
mass
VVDALKSR+XL_S
VVDALKSR+XL_L
VKAQDVQR+XL_S
VKAQDVQR+XL_L
A_b_ion
A_y_ion
B_b_ion
B_y_ion
y6+
b2+
b3+
y5+
y7+
y3++
y5++
y6++
y7++
prec
y1+
y2+
y3+
b4+
b5+
b7+
m/z

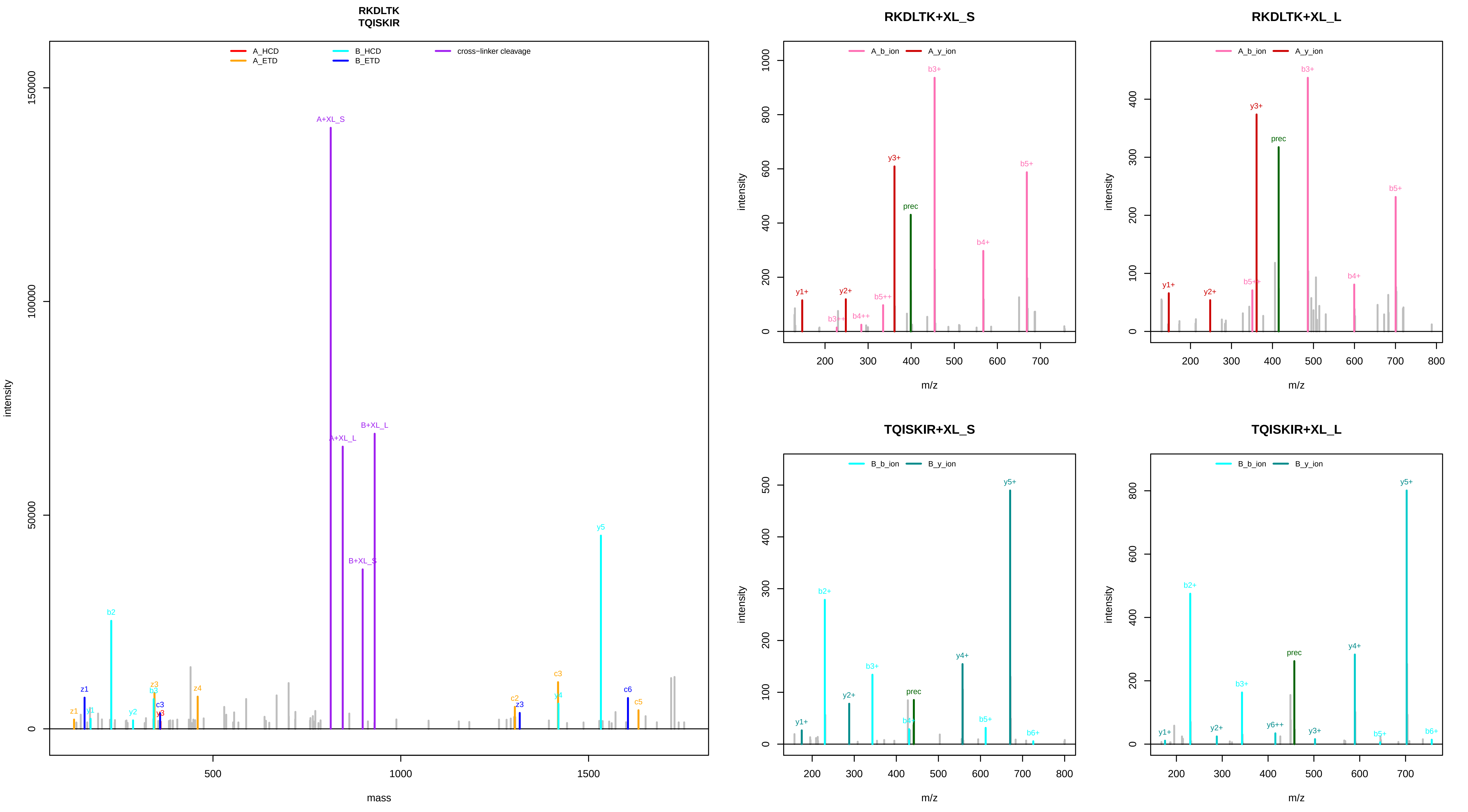

RKDLTK
TQISKIR
A_HCD
A_ETD
B_HCD
B_ETD
cross−linker cleavage
A+XL_S
A+XL_L
B+XL_L
B+XL_S
intensity
mass
RKDLTK+XL_S
RKDLTK+XL_L
TQISKIR+XL_S
TQISKIR+XL_L
A_b_ion
A_y_ion
B_b_ion
B_y_ion
prec
m/z

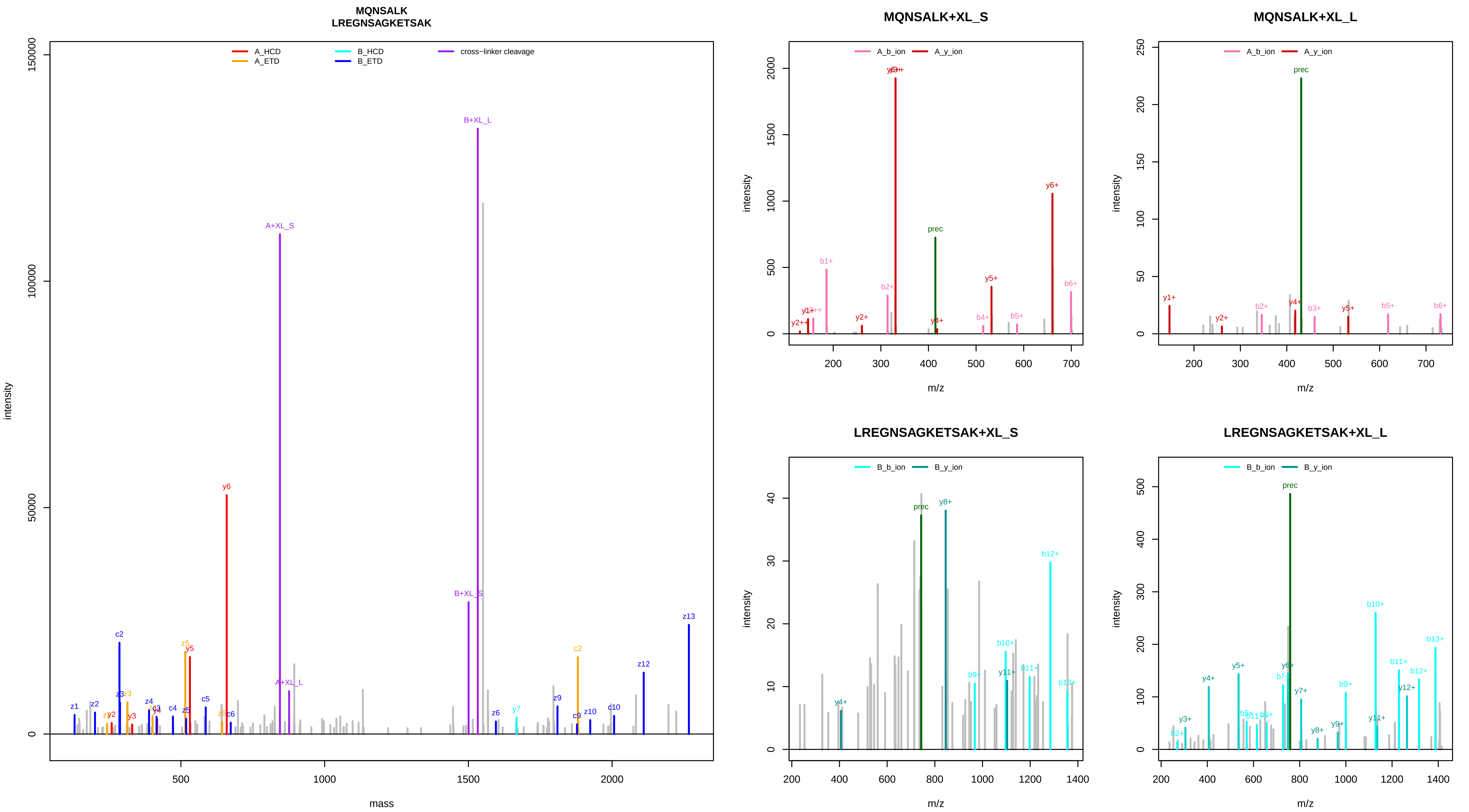
MQNSALK
LREGNSAGKETSAK
A_HCD
A_ETD
B_HCD
B_ETD
cross−linker cleavage
B+XL_L
A+XL_S
B+XL_S
A+XL_L
intensity
mass
MQNSALK+XL_S
MQNSALK+XL_L
LREGNSAGKETSAK+XL_S
LREGNSAGKETSAK+XL_L
A_b_ion
A_y_ion
B_b_ion
B_y_ion
prec
m/z

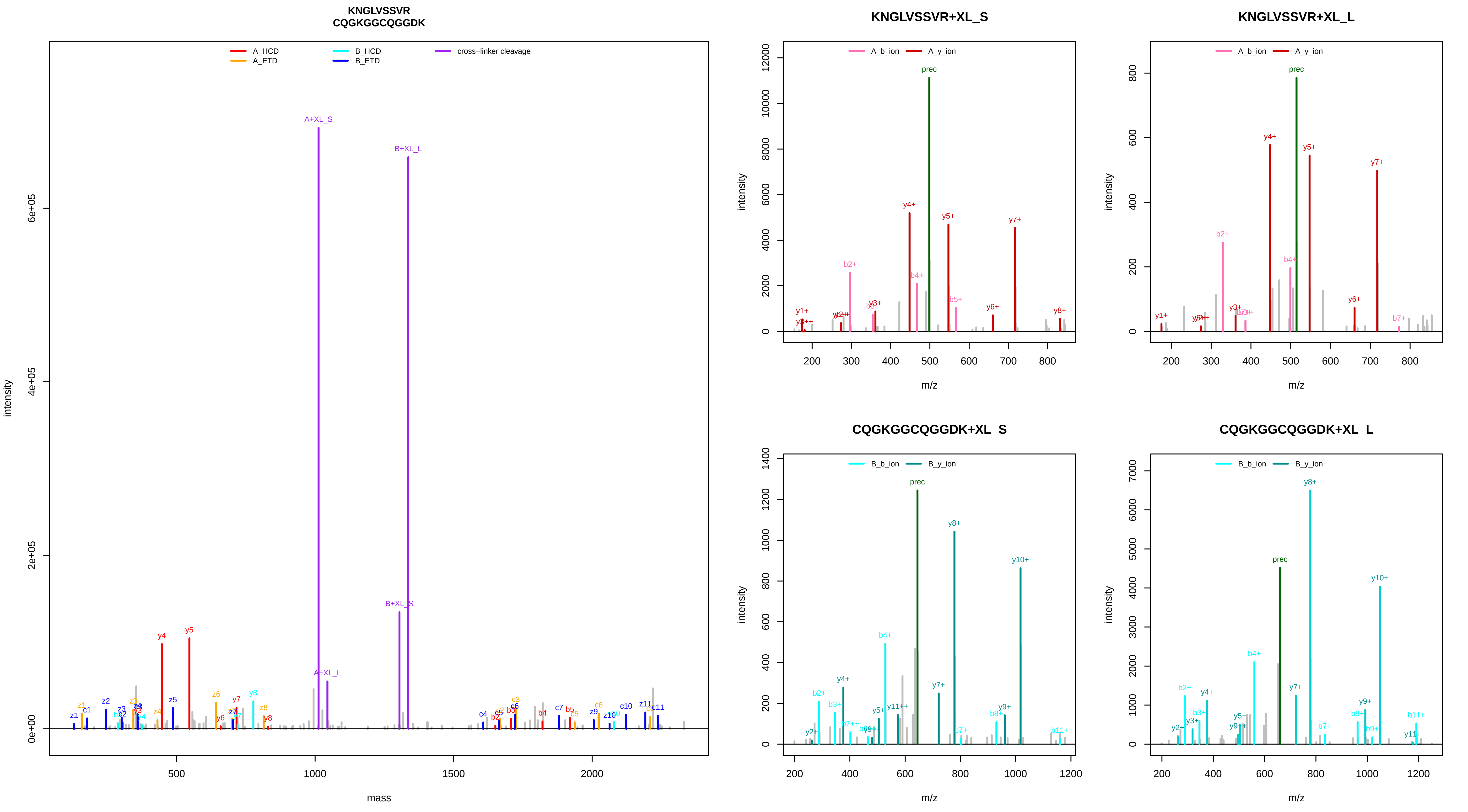
KNGLVSSVR
CQGKGGCQGGDK
A_HCD
A_ETD
B_HCD
B_ETD
cross−linker cleavage
A+XL_S
B+XL_L
B+XL_S
A+XL_L
intensity
mass
KNGLVSSVR+XL_S
KNGLVSSVR+XL_L
A_b_ion
A_y_ion
prec
CQGKGGCQGGDK+XL_S
CQGKGGCQGGDK+XL_L
B_b_ion
B_y_ion
m/z

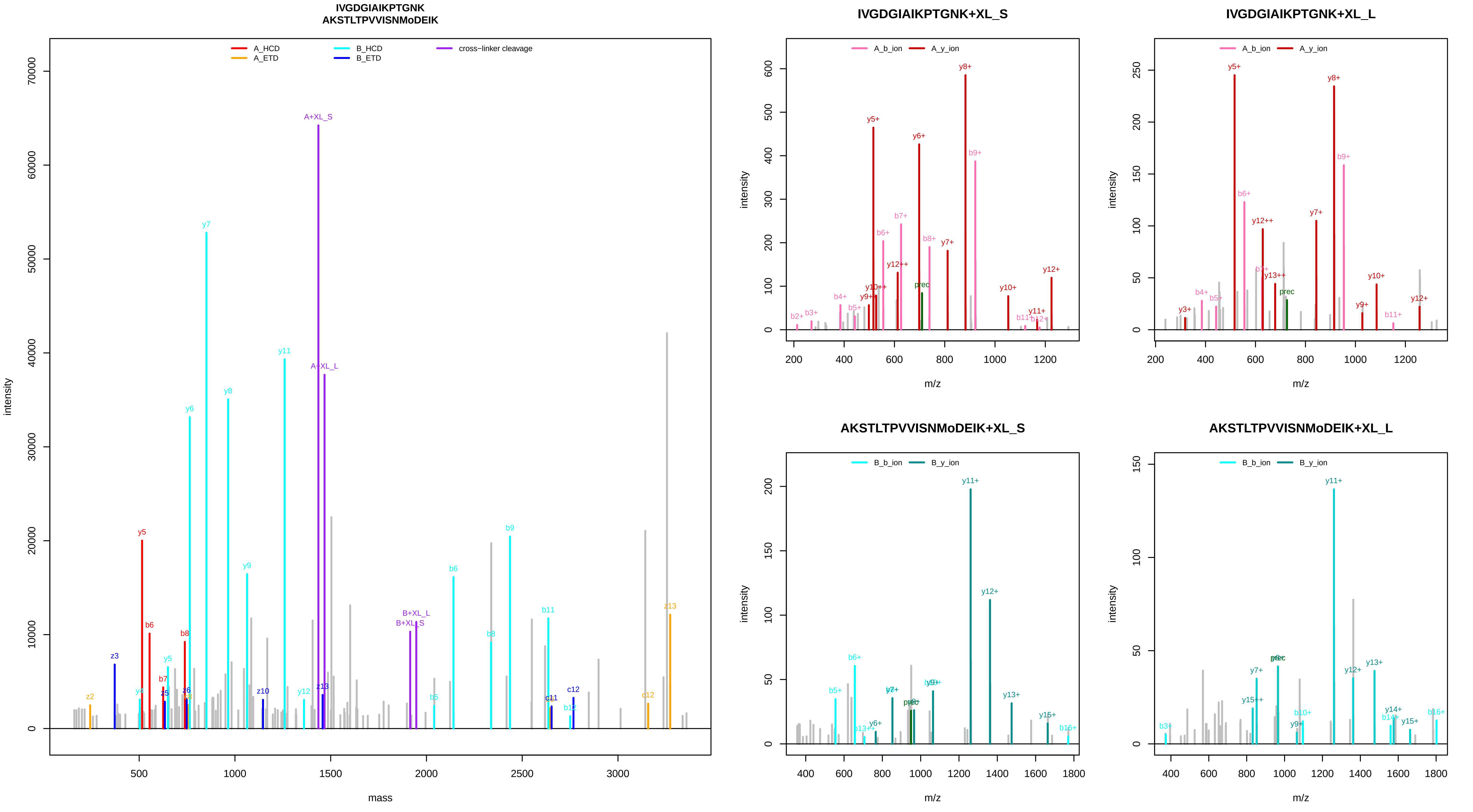
IVGDGIAIKPTGNK
AKSTLTPVVISNMoDEIK
A_HCD
A_ETD
B_HCD
B_ETD
cross−linker cleavage
A+XL_S
A+XL_L
B+XL_L
B+XL_S
intensity
mass
IVGDGIAIKPTGNK+XL_S
IVGDGIAIKPTGNK+XL_L
AKSTLTPVVISNMoDEIK+XL_S
AKSTLTPVVISNMoDEIK+XL_L
A_b_ion
A_y_ion
B_b_ion
B_y_ion
prec
m/z

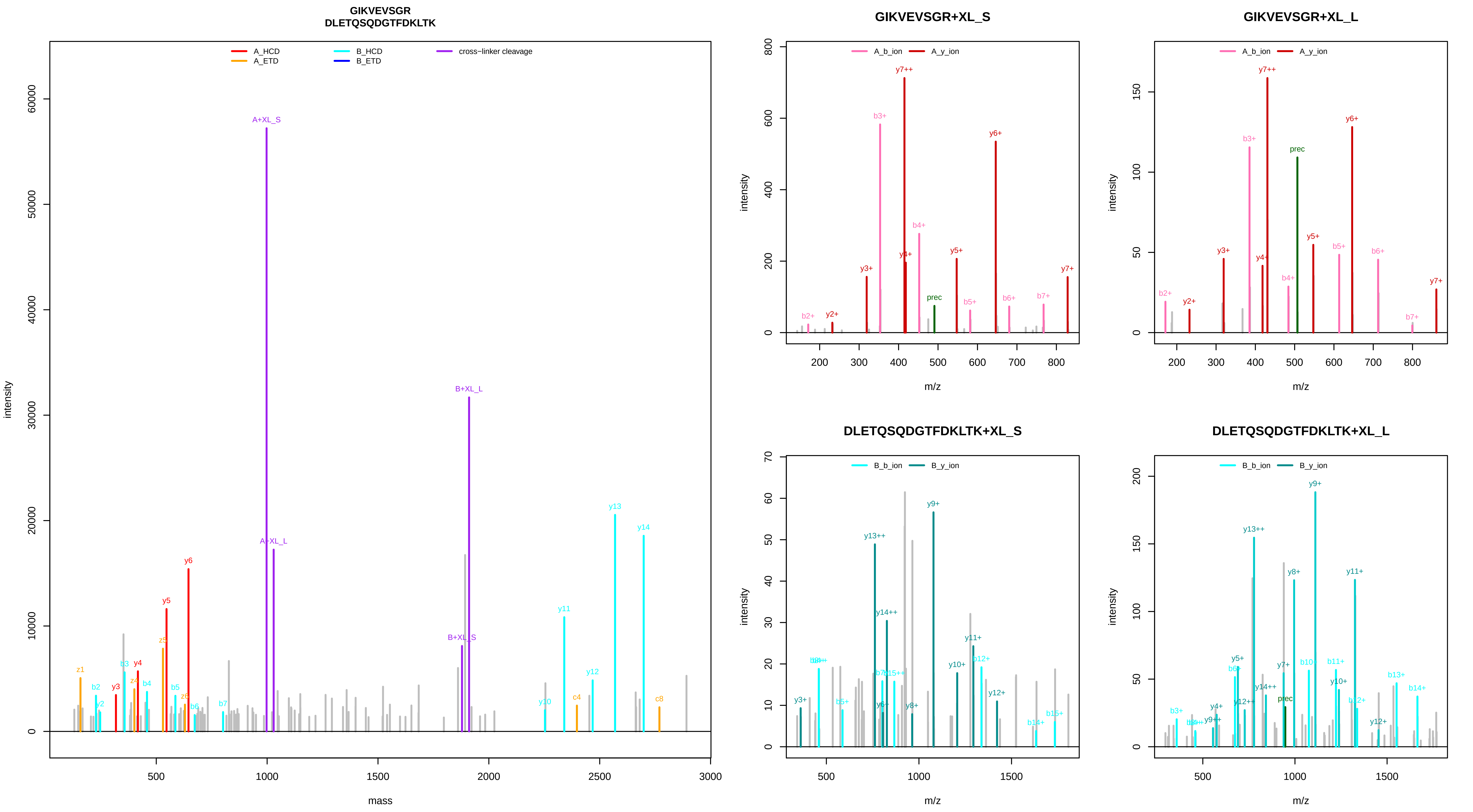
GIKVEVSGR
DLETQSQDGTFDKLTK
A_HCD
A_ETD
B_HCD
B_ETD
cross−linker cleavage
A+XL_S
A+XL_L
B+XL_L
B+XL_S
intensity
mass
GIKVEVSGR+XL_S
GIKVEVSGR+XL_L
DLETQSQDGTFDKLTK+XL_S
DLETQSQDGTFDKLTK+XL_L
A_b_ion
A_y_ion
B_b_ion
B_y_ion
m/z

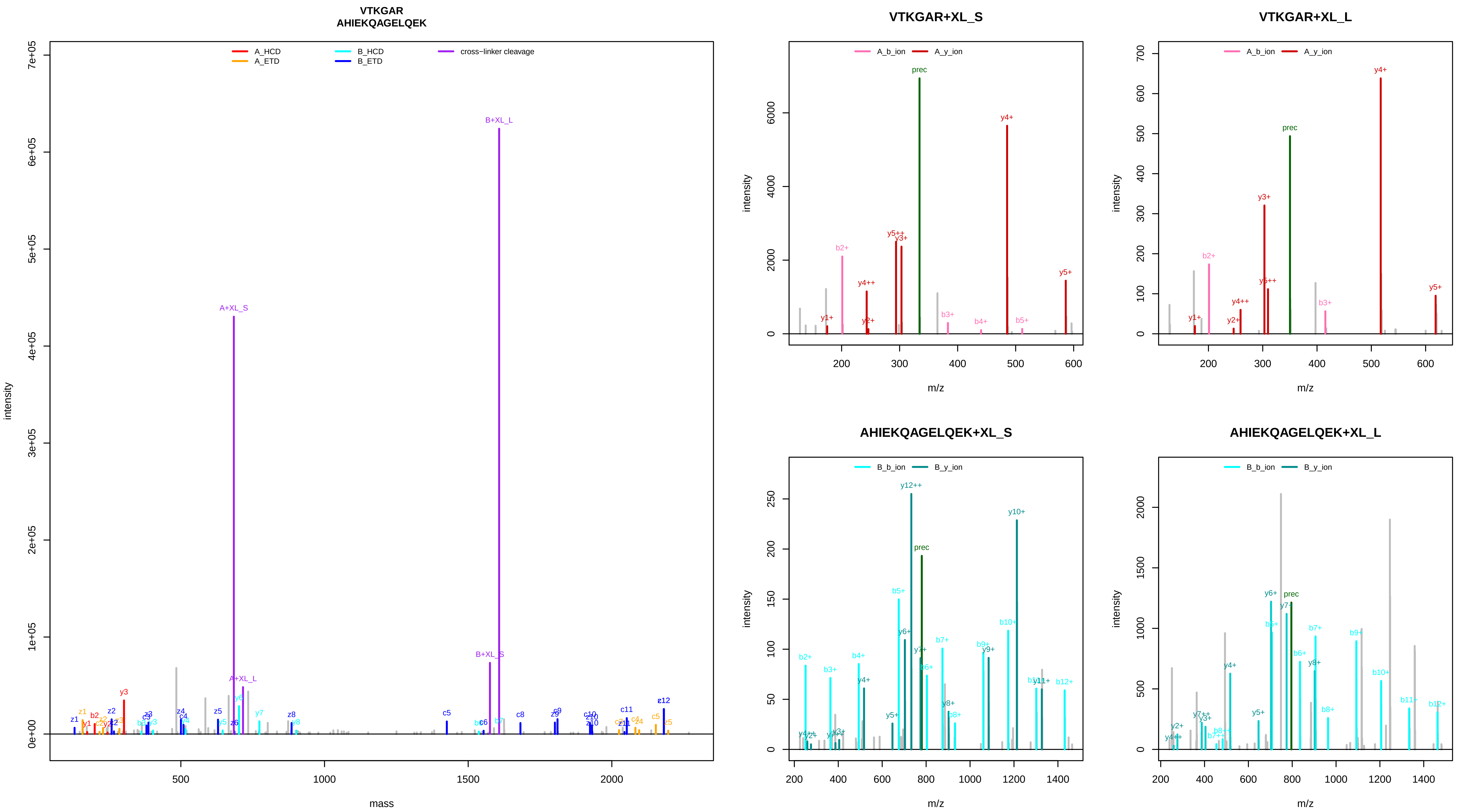

VTKGAR
AHIEKQAGELQEK
A_HCD
A_ETD
B_HCD
B_ETD
cross−linker cleavage
B+XL_L
A+XL_S
B+XL_S
A+XL_L
intensity
mass
VTKGAR+XL_S
VTKGAR+XL_L
A_b_ion
A_y_ion
prec
AHIEKQAGELQEK+XL_S
AHIEKQAGELQEK+XL_L
B_b_ion
B_y_ion
m/z

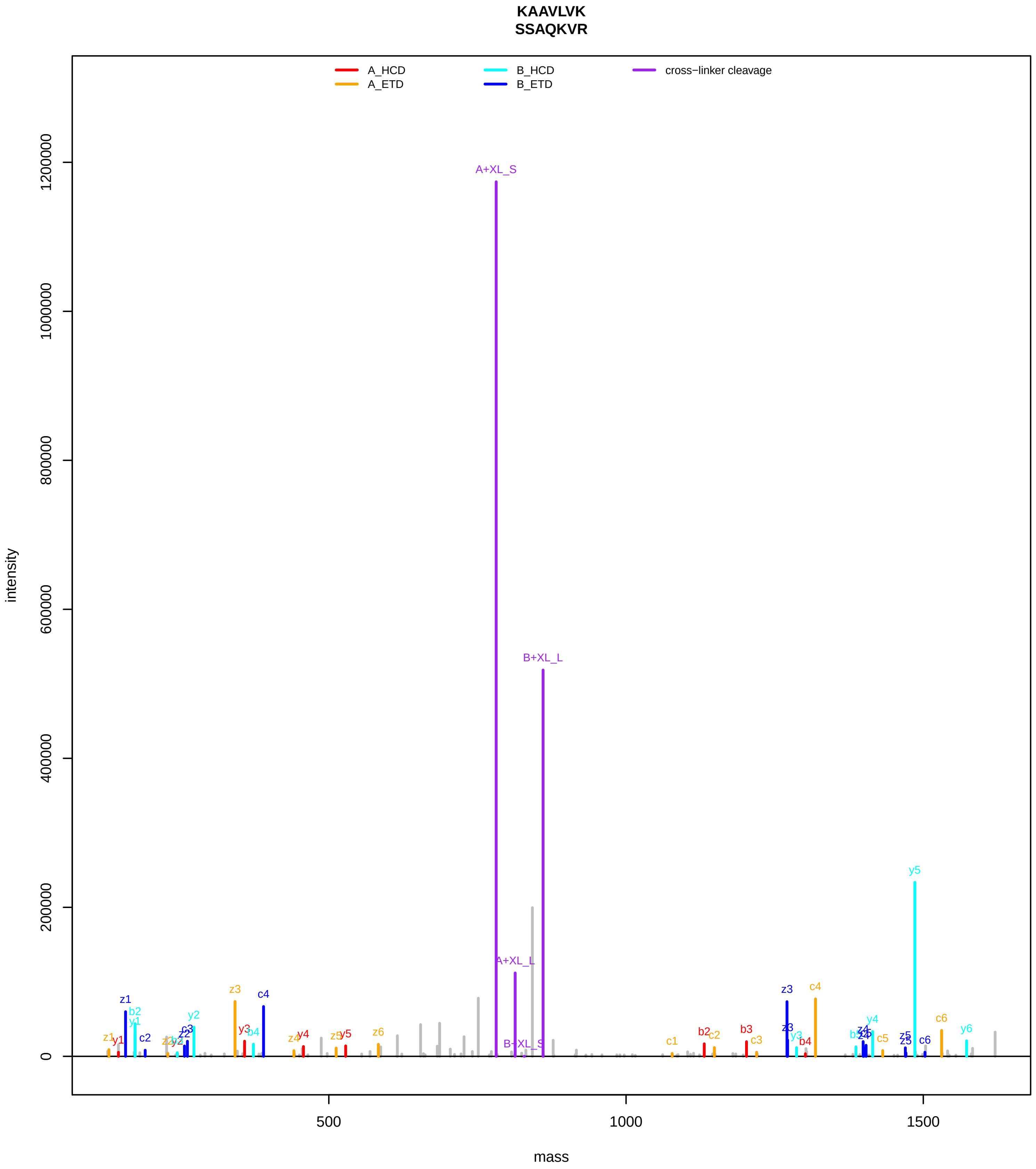

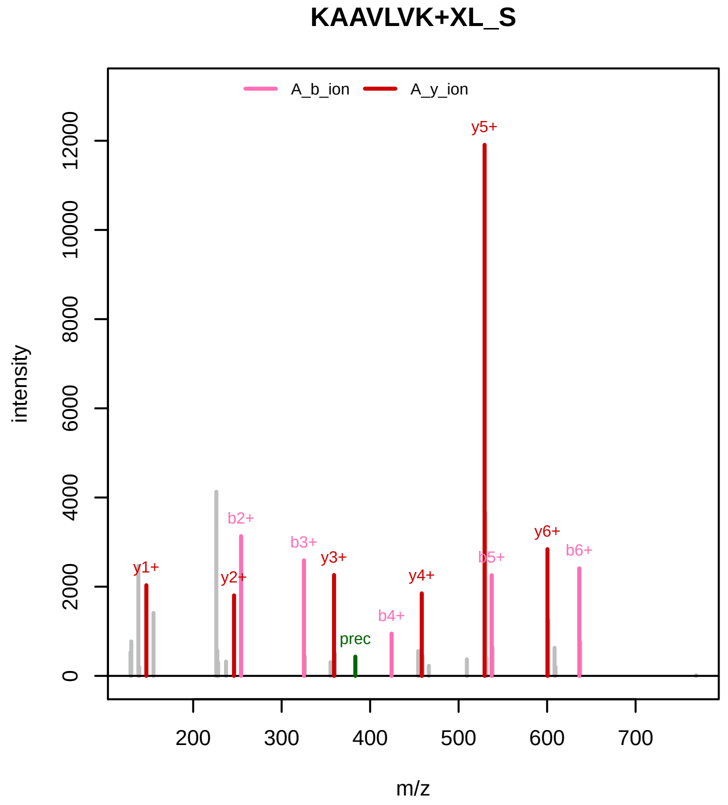

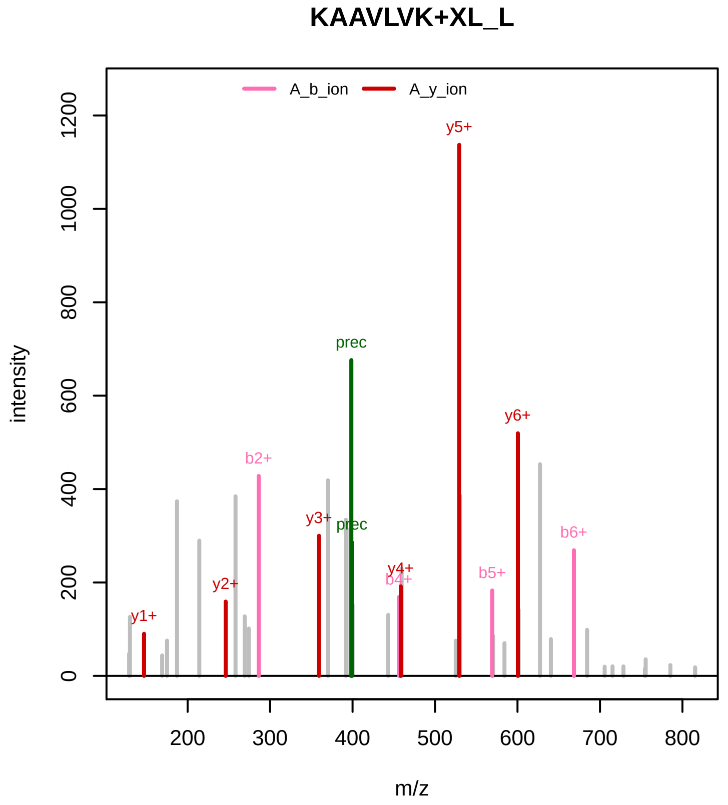

SSAQKVR+XL_S

SSAQKVR+XL_L

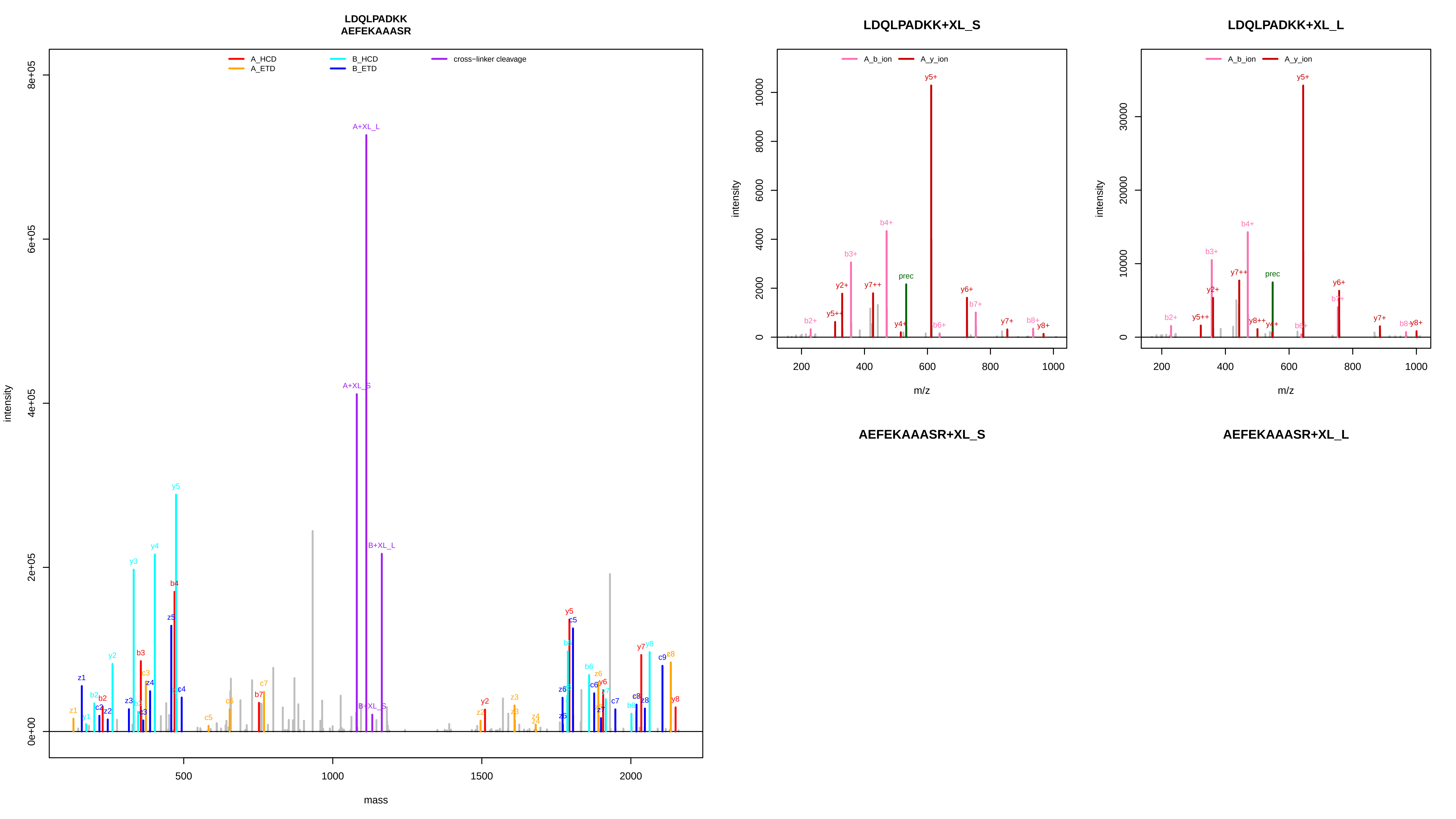
LDQLPADKK
AEFEKAAASR
A_HCD
A_ETD
B_HCD
B_ETD
cross−linker cleavage
A+XL_L
A+XL_S
B+XL_L
B+XL_S
intensity
mass
LDQLPADKK+XL_S
LDQLPADKK+XL_L
A_b_ion
A_y_ion
prec
m/z
AEFEKAAASR+XL_S
AEFEKAAASR+XL_L

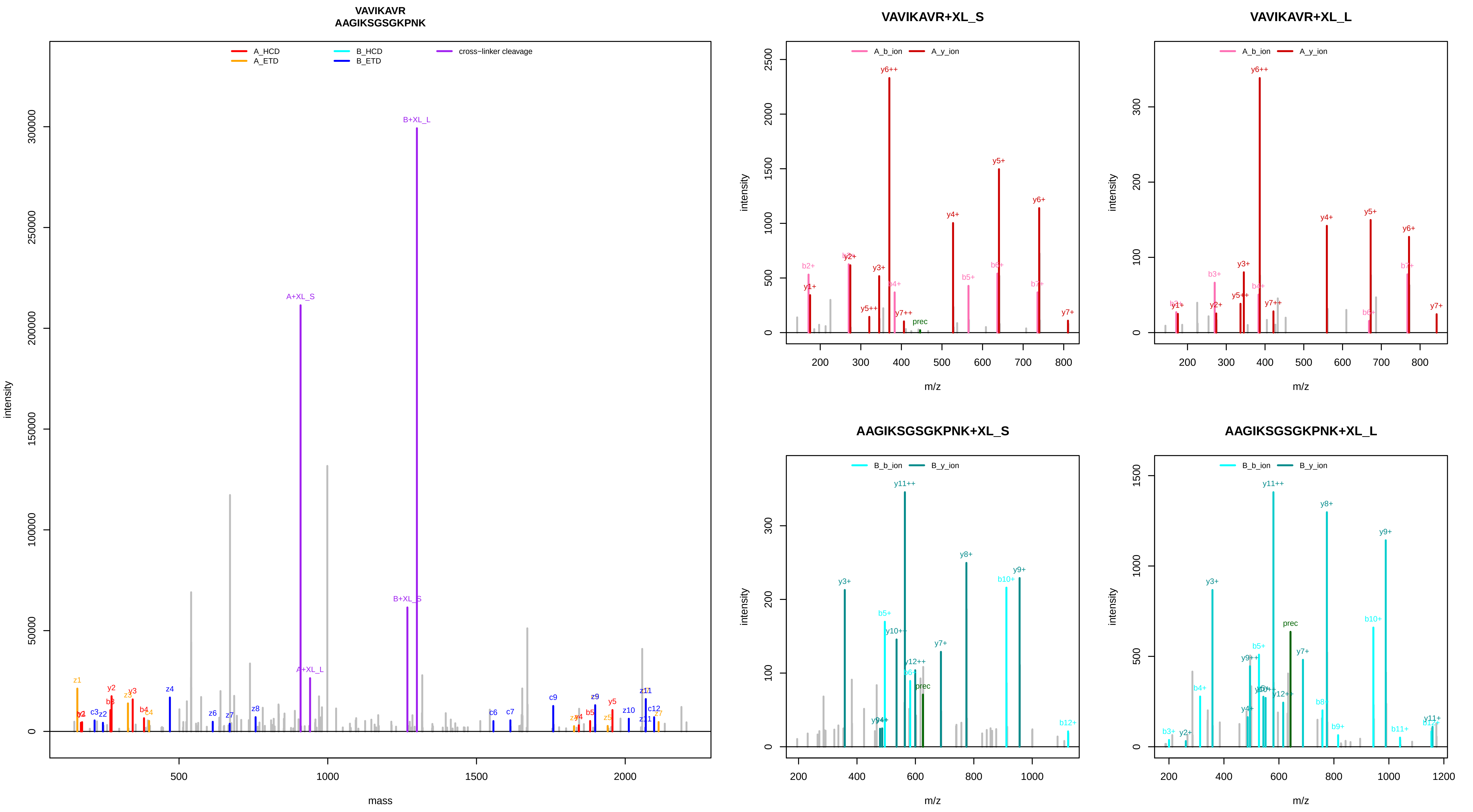
VAVIKAVR
AAGIKSGSGKPNK
A_HCD
A_ETD
B_HCD
B_ETD
cross−linker cleavage
B+XL_L
A+XL_S
B+XL_S
A+XL_L
intensity
mass
VAVIKAVR+XL_S
VAVIKAVR+XL_L
A_b_ion
A_y_ion
AAGIKSGSGKPNK+XL_S
AAGIKSGSGKPNK+XL_L
B_b_ion
B_y_ion
m/z
prec

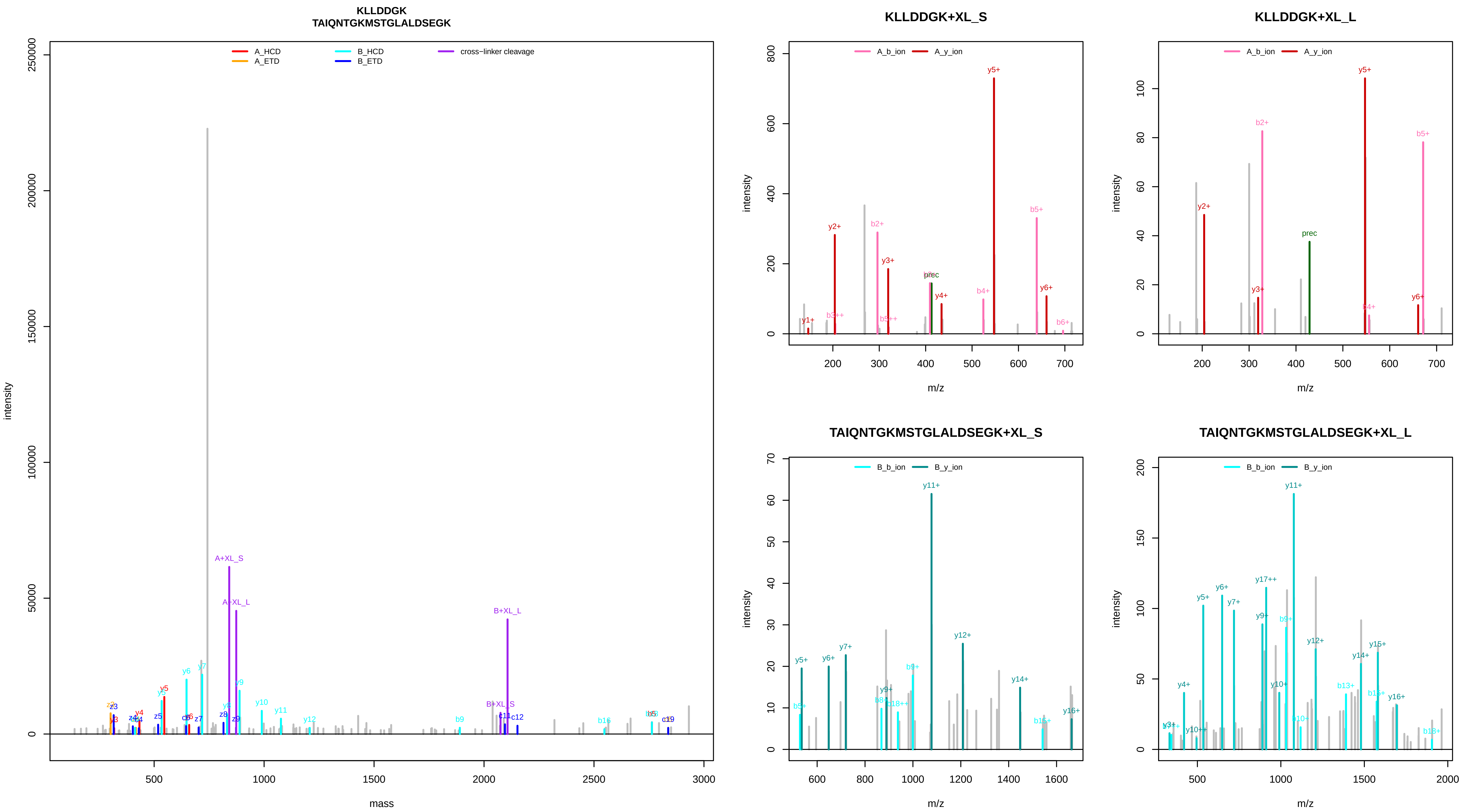
KLLDDGK
TAIQNTGKMSTGLALDSEGK
A_HCD
A_ETD
B_HCD
B_ETD
cross−linker cleavage
A+XL_S
A+XL_L
B+XL_L
B+XL_S
intensity
mass
KLLDDGK+XL_S
KLLDDGK+XL_L
A_b_ion
A_y_ion
prec
TAIQNTGKMSTGLALDSEGK+XL_S
TAIQNTGKMSTGLALDSEGK+XL_L
B_b_ion
B_y_ion
m/z

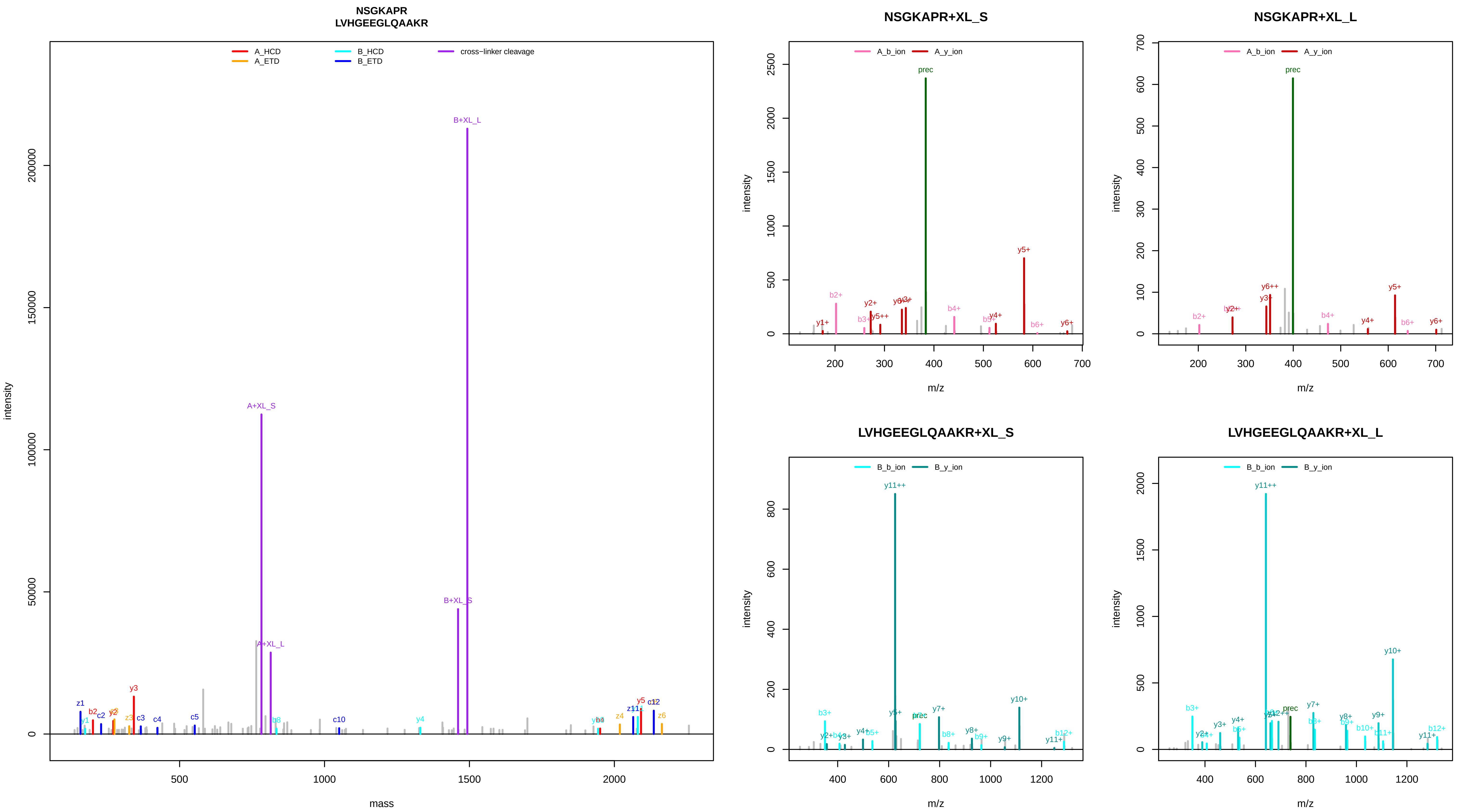
NSGKAPR
LVHGEEGLQAAKR
A_HCD
A_ETD
B_HCD
B_ETD
cross−linker cleavage
B+XL_L
A+XL_S
B+XL_S
A+XL_L
intensity
mass
NSGKAPR+XL_S
NSGKAPR+XL_L
LVHGEEGLQAAKR+XL_S
LVHGEEGLQAAKR+XL_L
A_b_ion
A_y_ion
B_b_ion
B_y_ion
prec
m/z

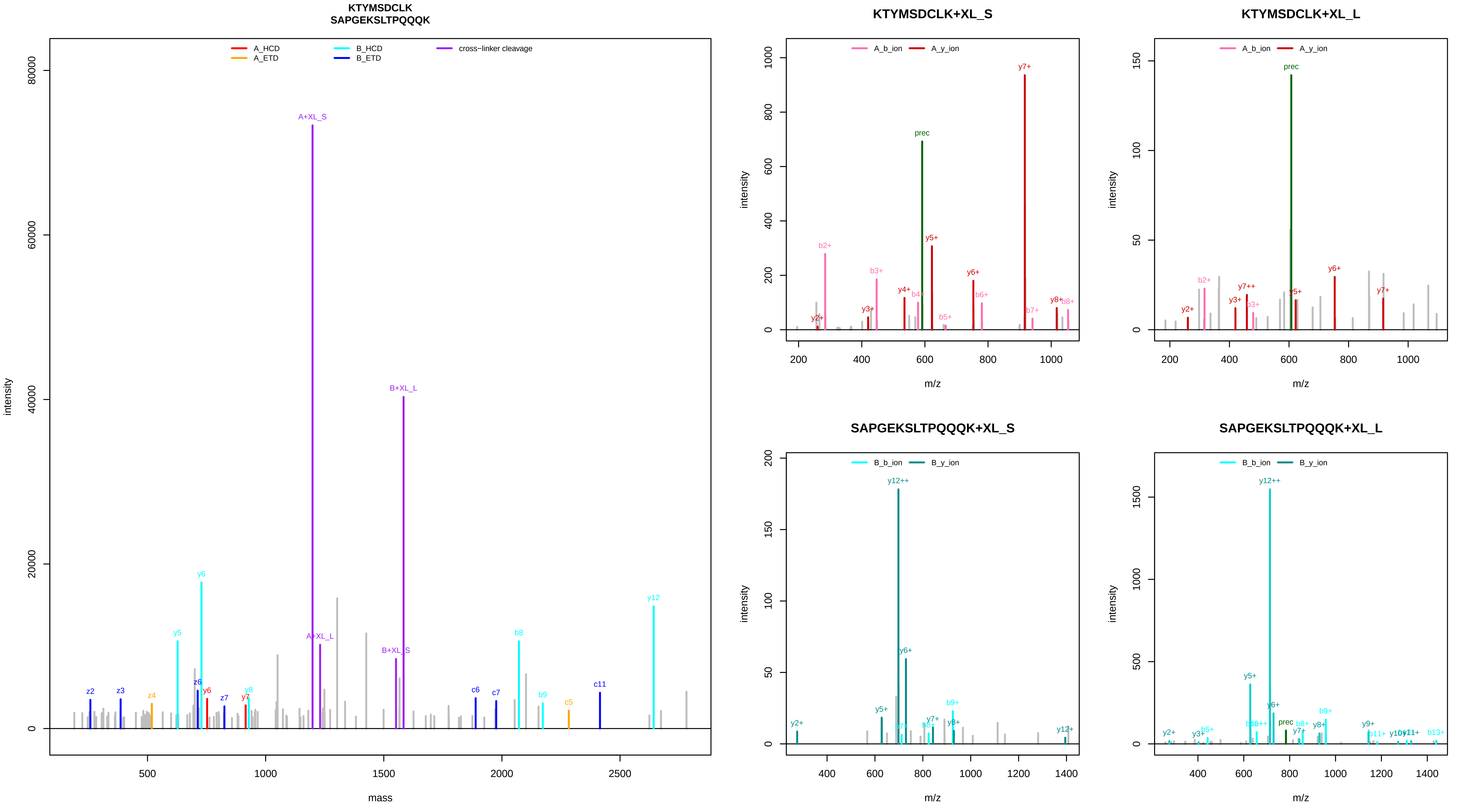
KTYMSDCLK
SAPGEKSLTPQQQK
A_HCD
A_ETD
B_HCD
B_ETD
cross−linker cleavage
intensity
mass
KTYMSDCLK+XL_S
KTYMSDCLK+XL_L
SAPGEKSLTPQQQK+XL_S
SAPGEKSLTPQQQK+XL_L
A_b_ion
A_y_ion
B_b_ion
B_y_ion
m/z

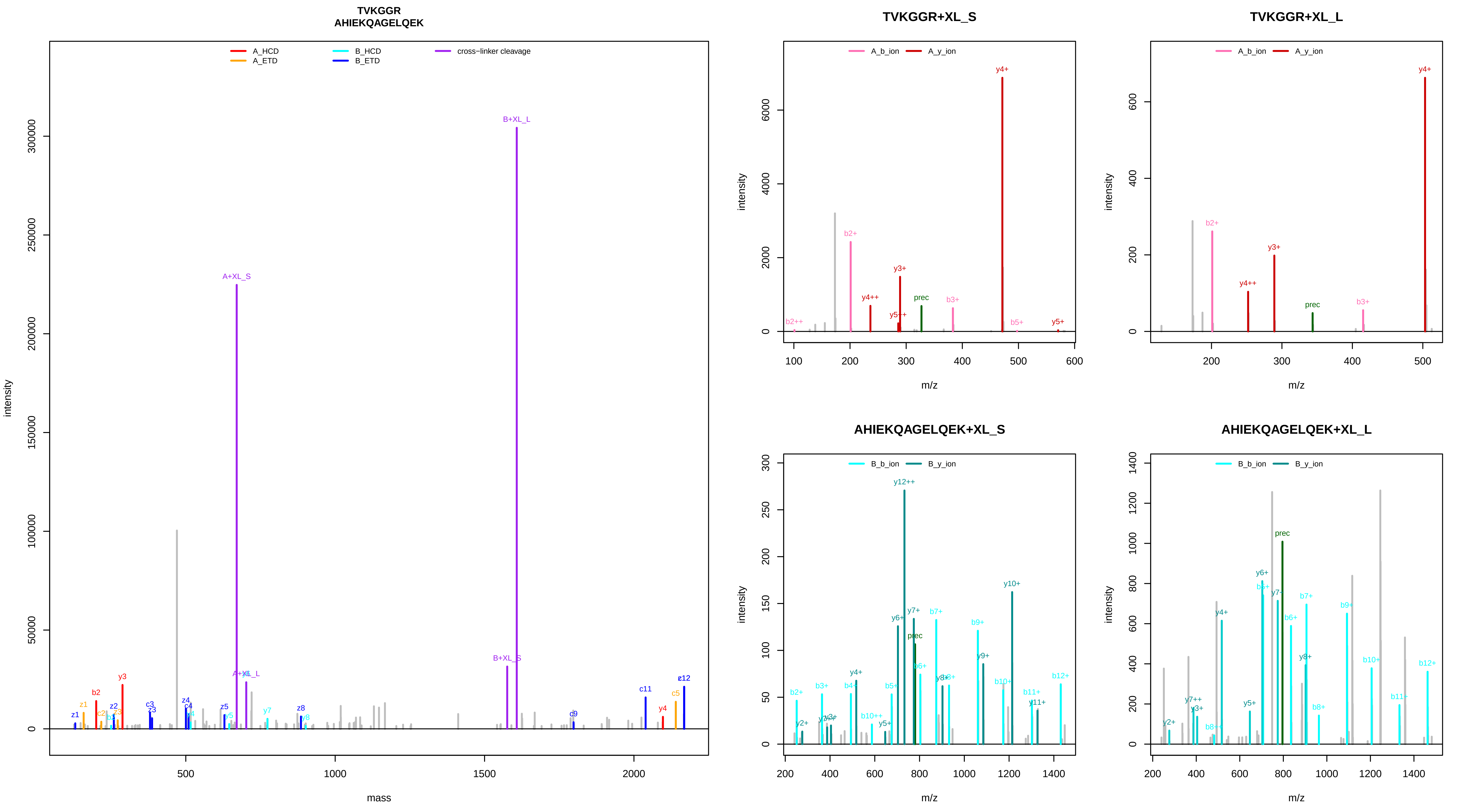
TVKGGR
AHIEKQAGELQEK
A_HCD
A_ETD
B_HCD
B_ETD
cross−linker cleavage
B+XL_L
A+XL_S
B+XL_S
A+XL_L
intensity
mass
TVKGGR+XL_S
TVKGGR+XL_L
AHIEKQAGELQEK+XL_S
AHIEKQAGELQEK+XL_L
A_b_ion
A_y_ion
B_b_ion
B_y_ion
prec
m/z

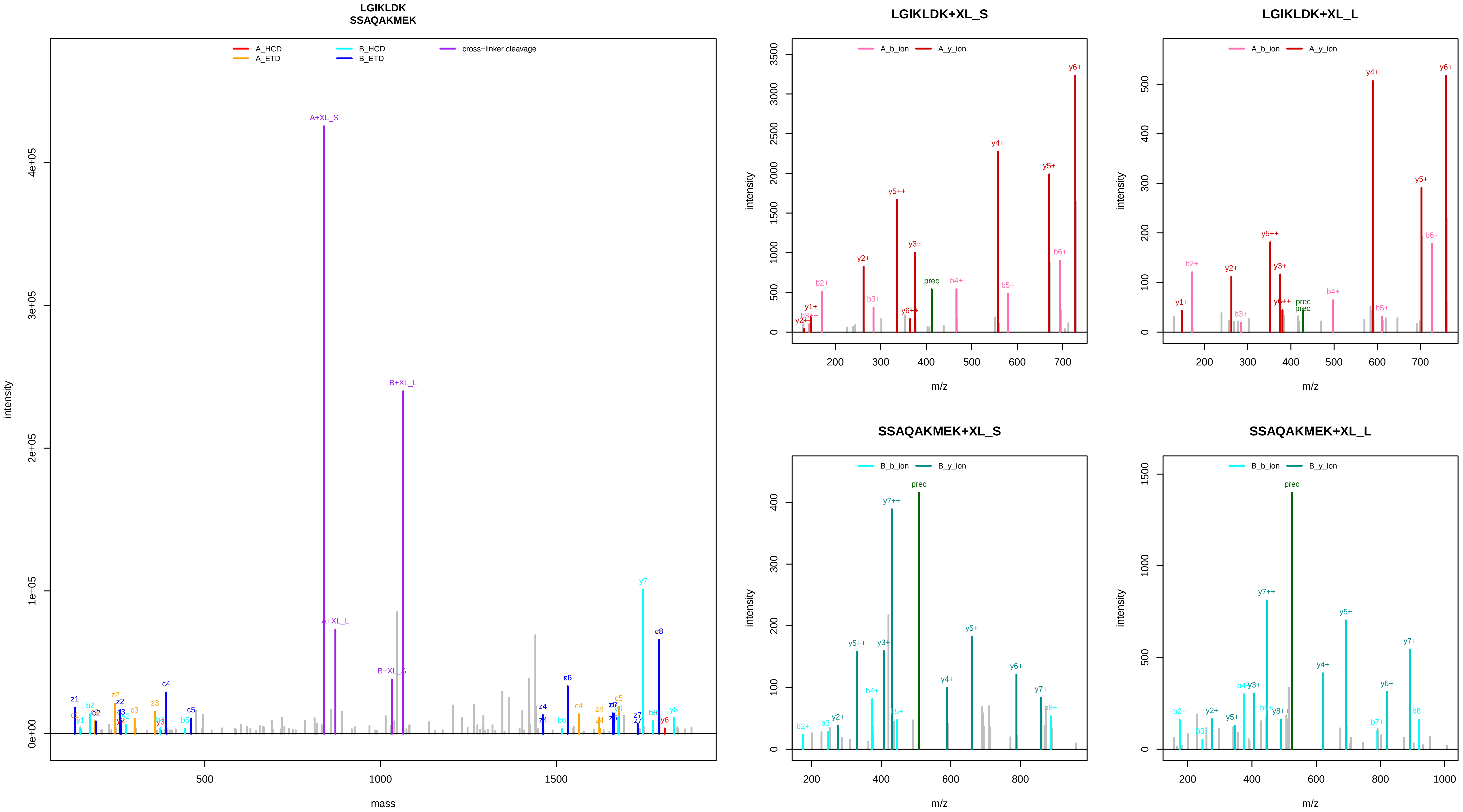
LGIKLDK
SSAQAKMEK
A_HCD
A_ETD
B_HCD
B_ETD
cross−linker cleavage
A+XL_S
B+XL_L
A+XL_L
B+XL_S
intensity
mass
LGIKLDK+XL_S
LGIKLDK+XL_L
A_b_ion
A_y_ion
SSAQAKMEK+XL_S
SSAQAKMEK+XL_L
B_b_ion
B_y_ion
prec
m/z

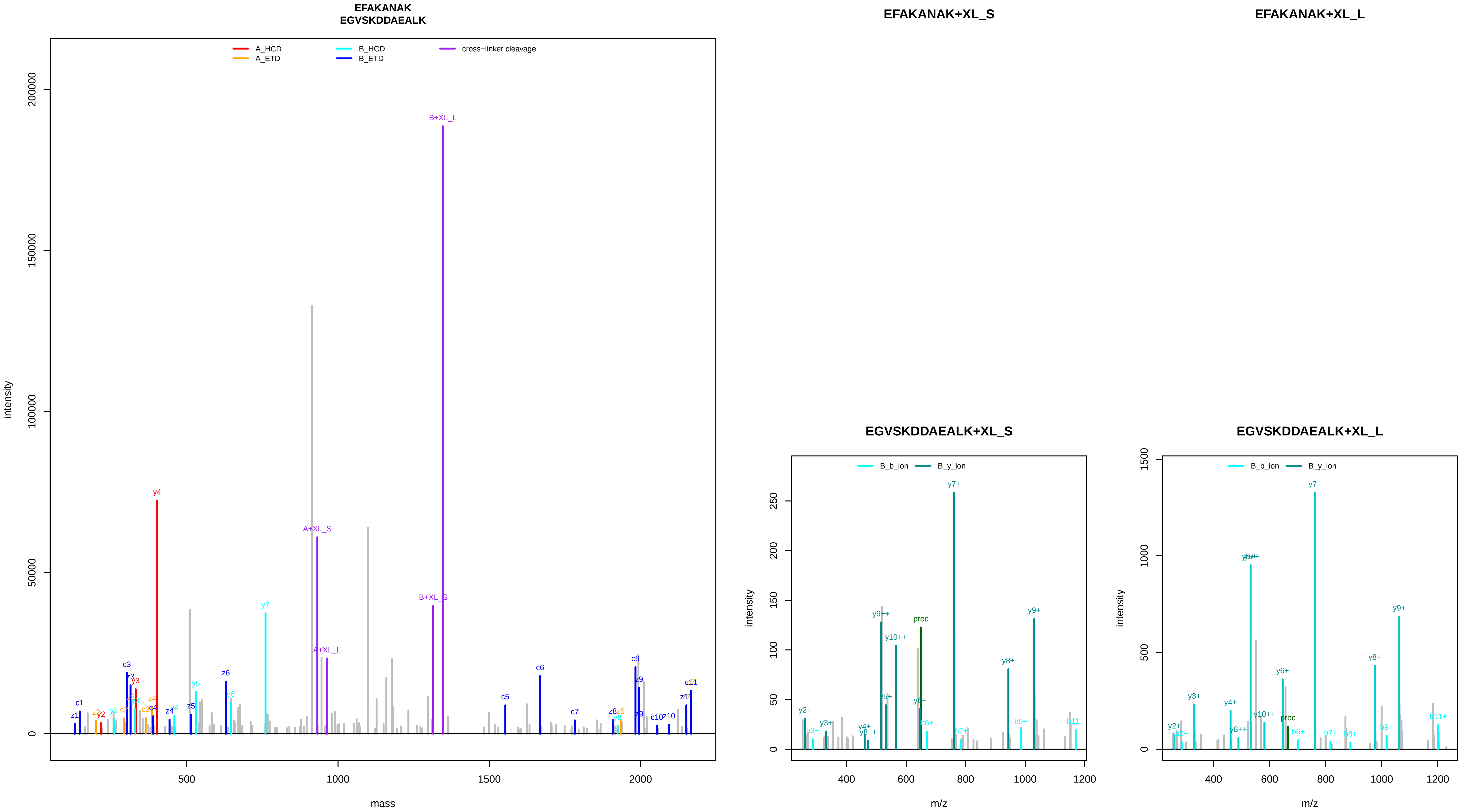
EFAKANAK
EGVSKDDAEALK
A_HCD
A_ETD
B_HCD
B_ETD
cross−linker cleavage
B+XL_L
A+XL_S
B+XL_S
A+XL_L
intensity
mass
EFAKANAK+XL_S
EFAKANAK+XL_L
EGVSKDDAEALK+XL_S
EGVSKDDAEALK+XL_L
B_b_ion
B_y_ion
prec
m/z

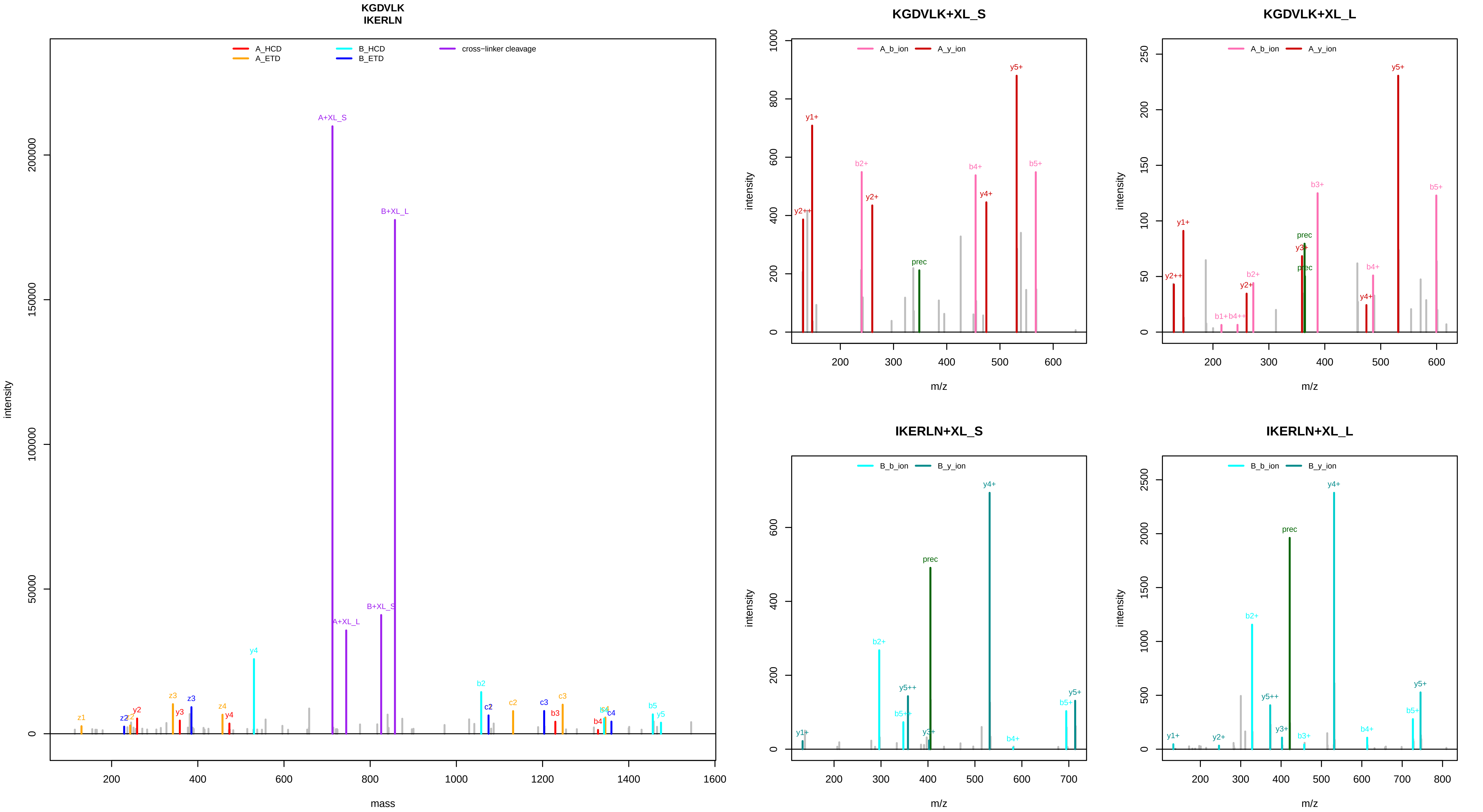

KGDVLK
IKERLN
A_HCD
A_ETD
B_HCD
B_ETD
cross−linker cleavage
A+XL_S
B+XL_L
A+XL_L
B+XL_S
intensity
mass
KGDVLK+XL_S
KGDVLK+XL_L
IKERLN+XL_S
IKERLN+XL_L
A_b_ion
A_y_ion
B_b_ion
B_y_ion
prec
m/z

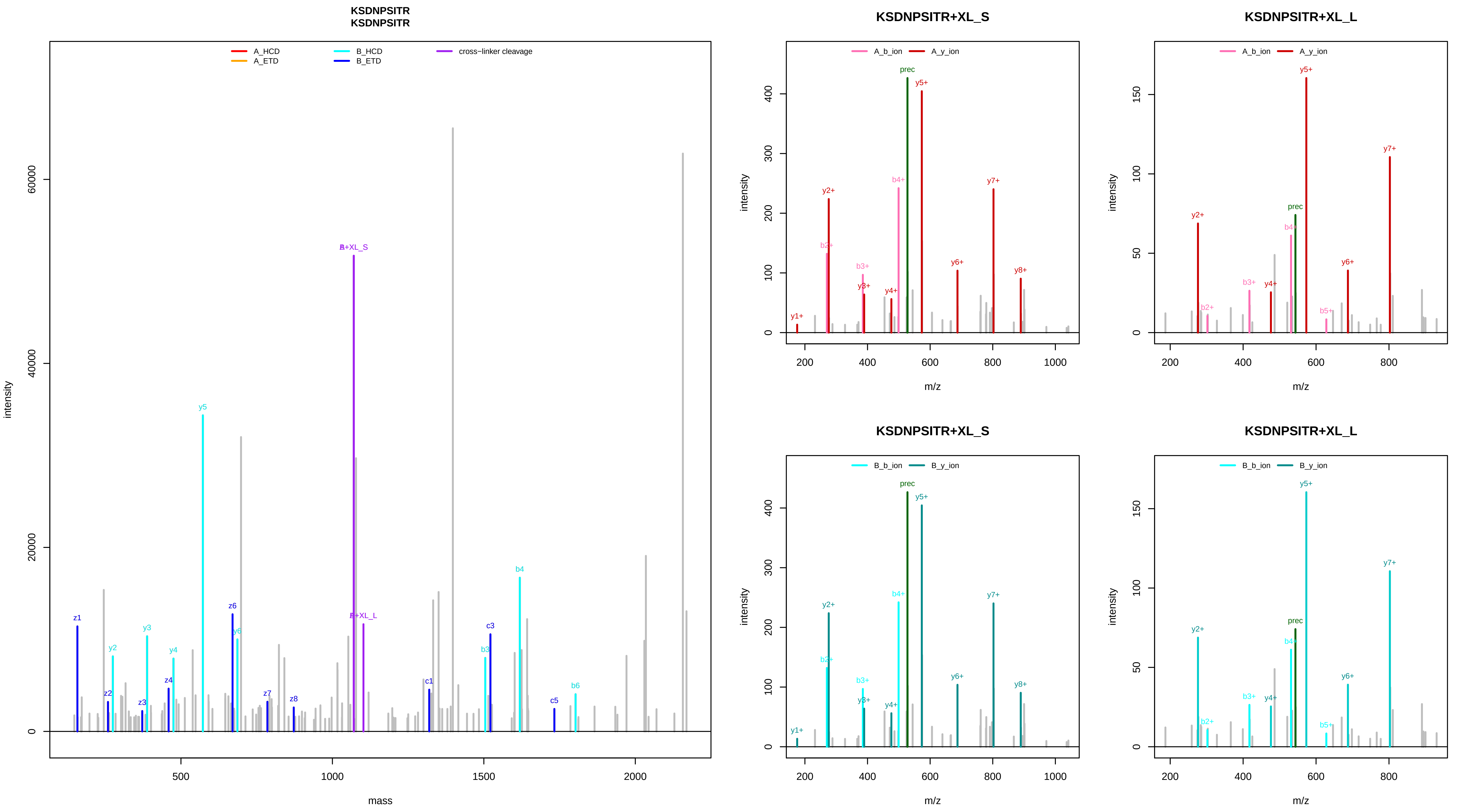
KSDNPSITR
KSDNPSITR
A_HCD
A_ETD
B_HCD
B_ETD
cross−linker cleavage
intensity
mass
KSDNPSITR+XL_S
KSDNPSITR+XL_L
A_b_ion
A_y_ion
B_b_ion
B_y_ion
prec
intensity
m/z

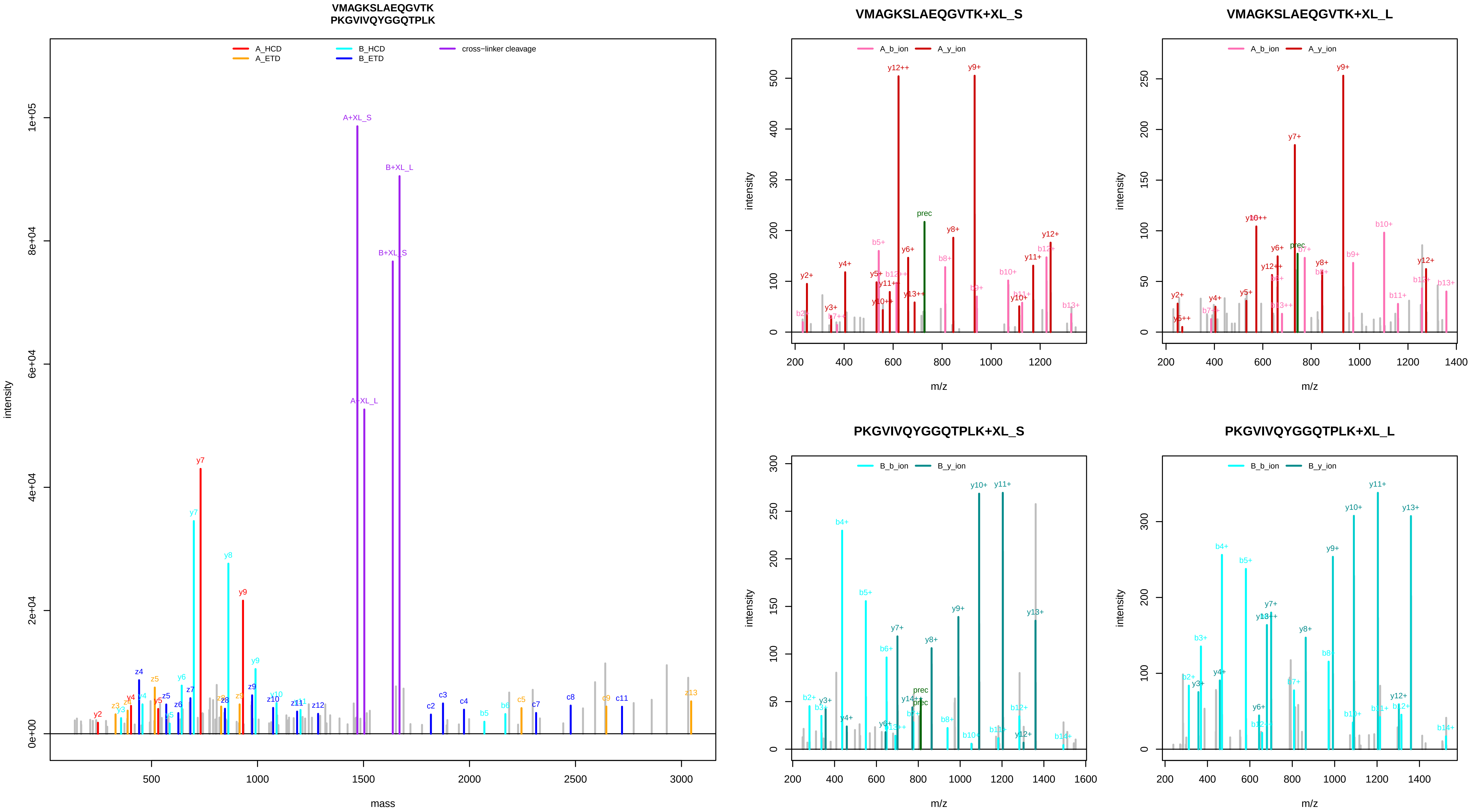
VMAGKSLAEQGVTK
PKGVIVQYGGQTPLK
A_HCD
A_ETD
B_HCD
B_ETD
cross−linker cleavage
intensity
mass
VMAGKSLAEQGVTK+XL_S
VMAGKSLAEQGVTK+XL_L
PKGVIVQYGGQTPLK+XL_S
PKGVIVQYGGQTPLK+XL_L
A_b_ion
A_y_ion
B_b_ion
B_y_ion
m/z

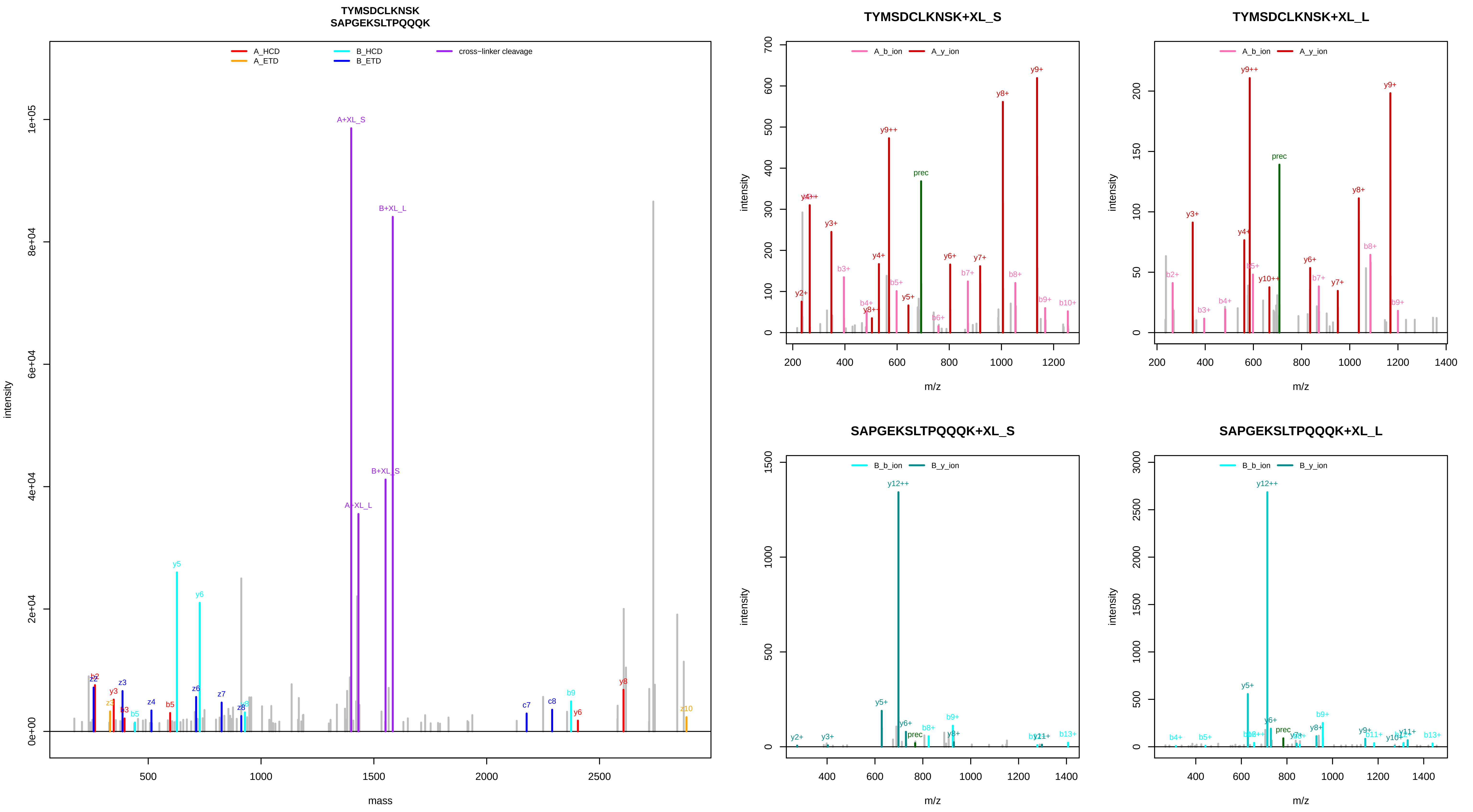
TYMSDCLKNSK
SAPGEKSLTPQQQK
A_HCD
A_ETD
B_HCD
B_ETD
cross−linker cleavage
A+XL_S
B+XL_L
B+XL_S
A+XL_L
intensity
mass
TYMSDCLKNSK+XL_S
TYMSDCLKNSK+XL_L
SAPGEKSLTPQQQK+XL_S
SAPGEKSLTPQQQK+XL_L
A_b_ion
A_y_ion
B_b_ion
B_y_ion
prec
m/z

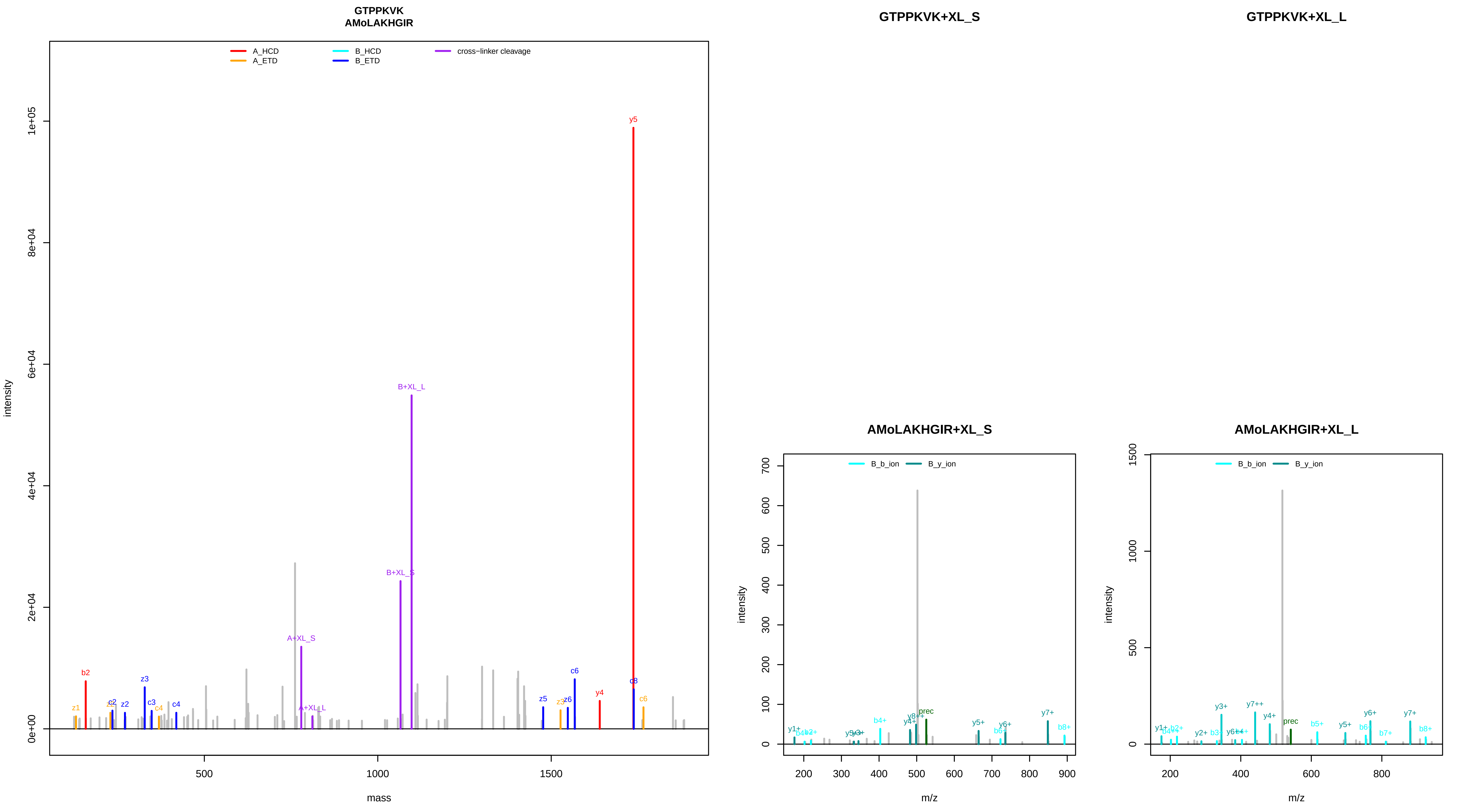
GTPPKVK
AMoLAKHGIR
A_HCD
A_ETD
B_HCD
B_ETD
cross−linker cleavage
intensity
mass
y5
B+XL_L
B+XL_S
A+XL_S
A+XL_L
b2
z3
c6
y4
c8
c6
z5
z6
GTPPKVK+XL_S
GTPPKVK+XL_L
AMoLAKHGIR+XL_S
AMoLAKHGIR+XL_L
B_b_ion
B_y_ion
prec
m/z
y7+
y6+
y5+
b8+
b4+

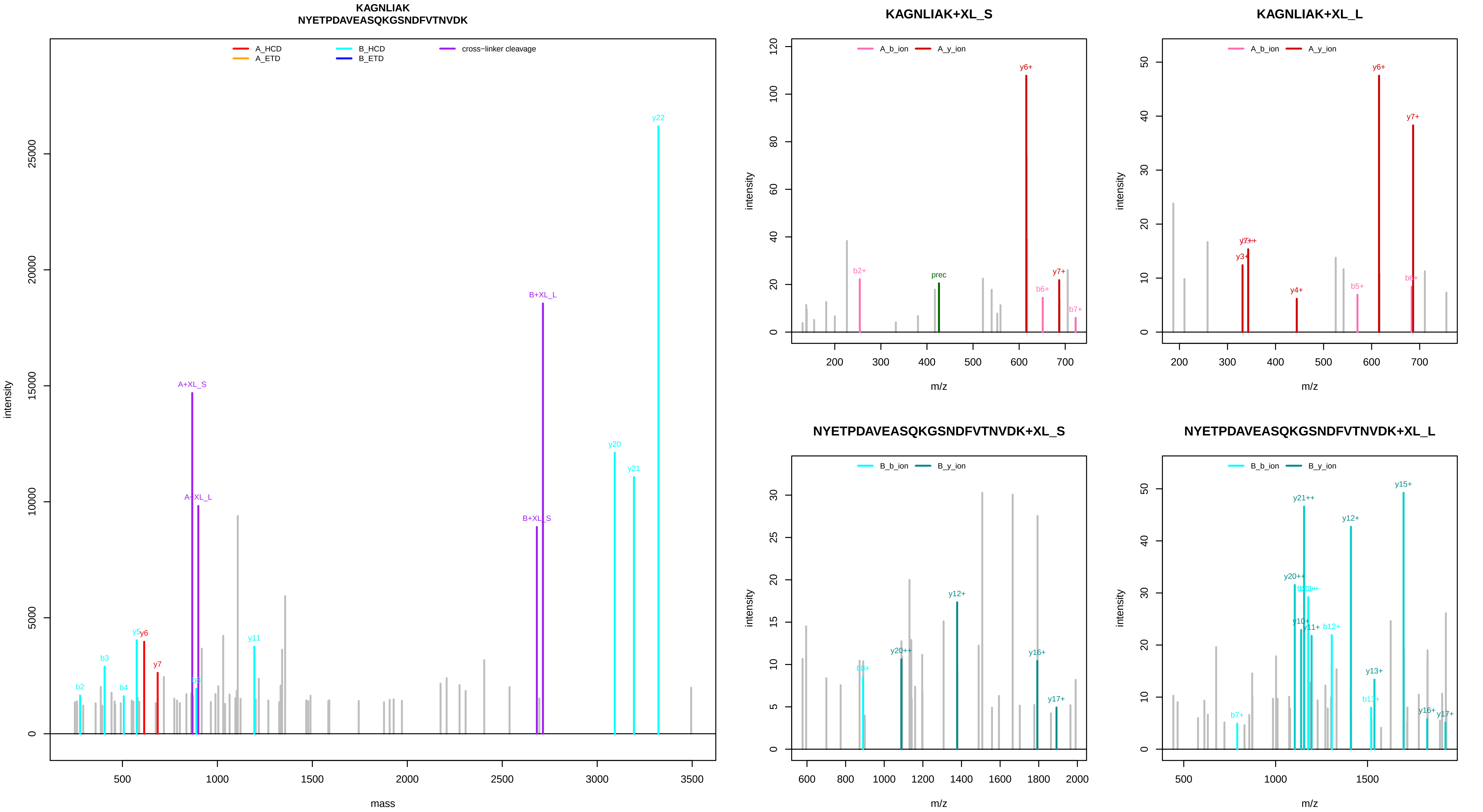
KAGNLIAK
NYETPDAVEASQKGSNDFVTNVDK
A_HCD
A_ETD
B_HCD
B_ETD
cross−linker cleavage
intensity
mass
A+XL_S
A+XL_L
B+XL_L
B+XL_S
y22
y20
y21
y11
y5
y6
y7
b2
b3
b4
KAGNLIAK+XL_S
KAGNLIAK+XL_L
NYETPDAVEASQKGSNDFVTNVDK+XL_S
NYETPDAVEASQKGSNDFVTNVDK+XL_L
A_b_ion
A_y_ion
B_b_ion
B_y_ion
m/z
y6+
y7+
b2+
prec
b6+
b7+
y3+
y4+
b5+
y12+
y20++
y16+
y17+
y15+
y21++
y10+
y11+
b12+
y13+
b7+
b13+

GKIFTK
AAQDAKR

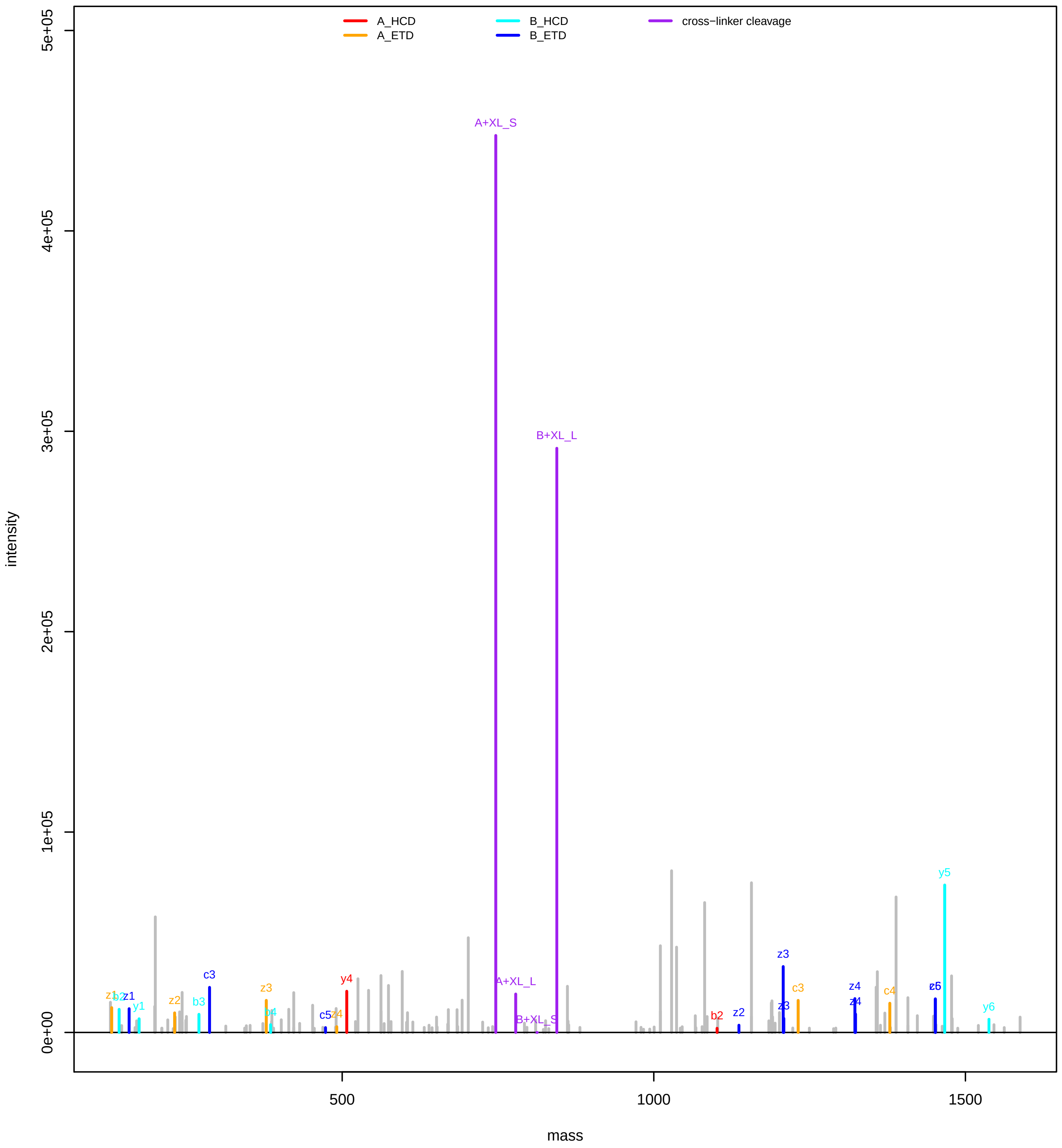

GKIFTK+XL_S

GKIFTK+XL_L

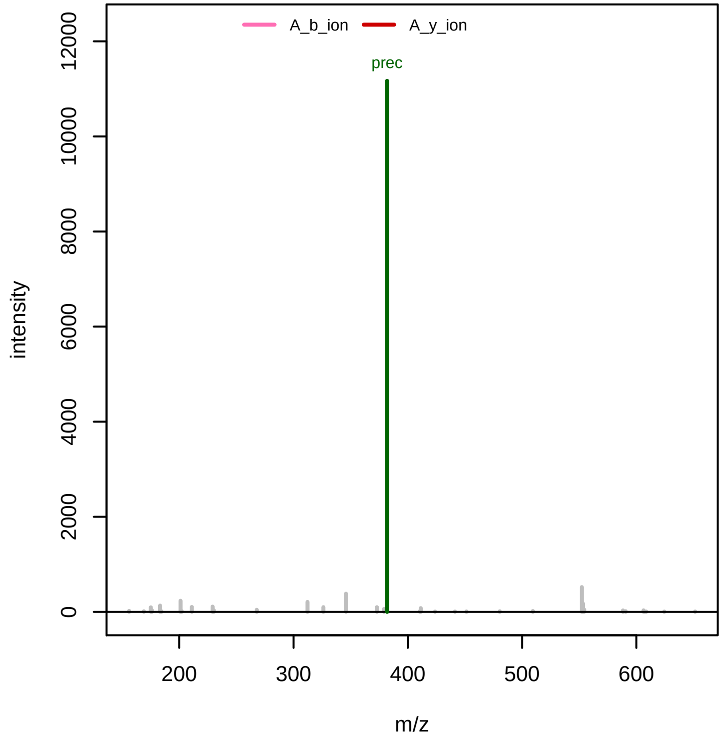

AAQDAKR+XL_S

AAQDAKR+XL_L

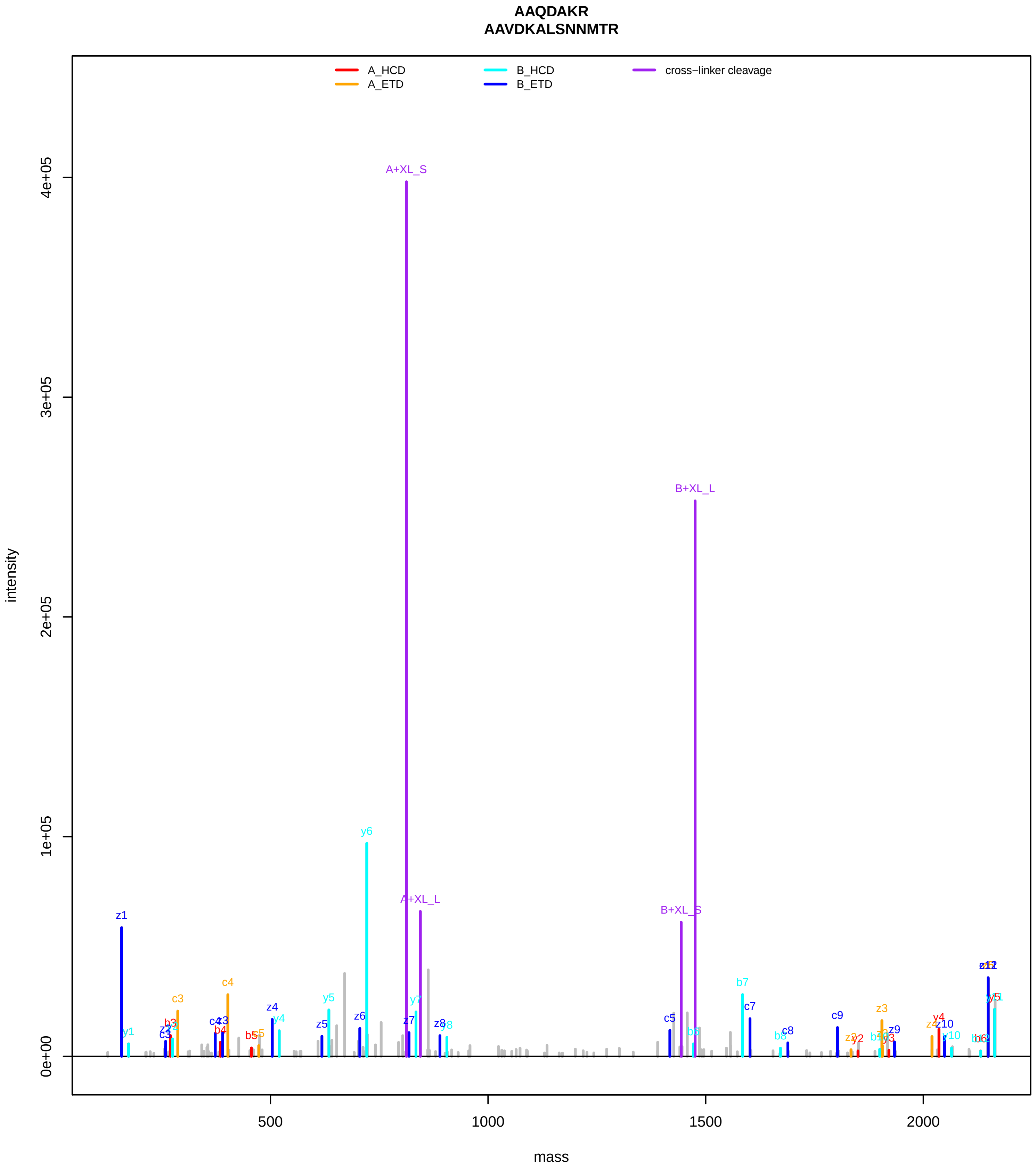

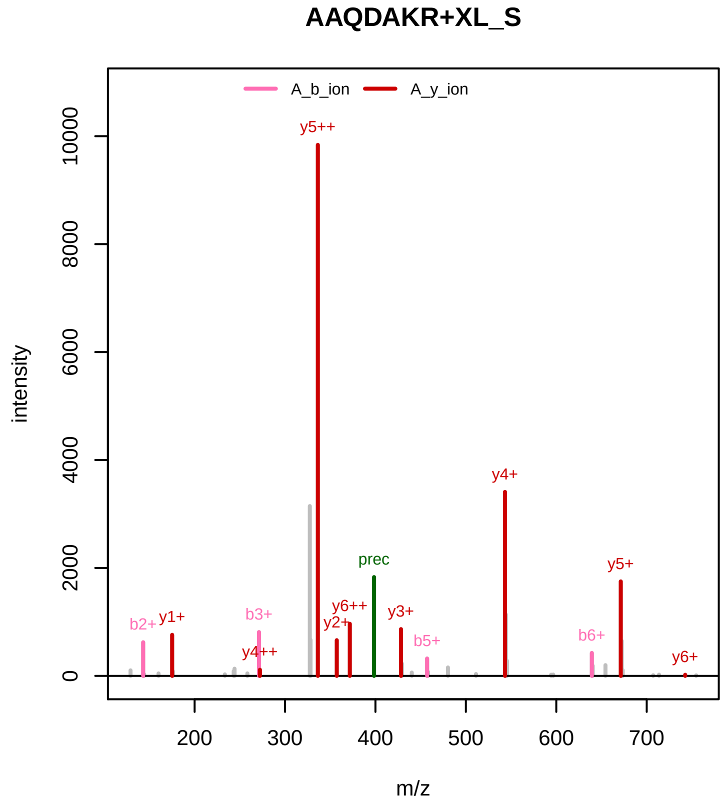

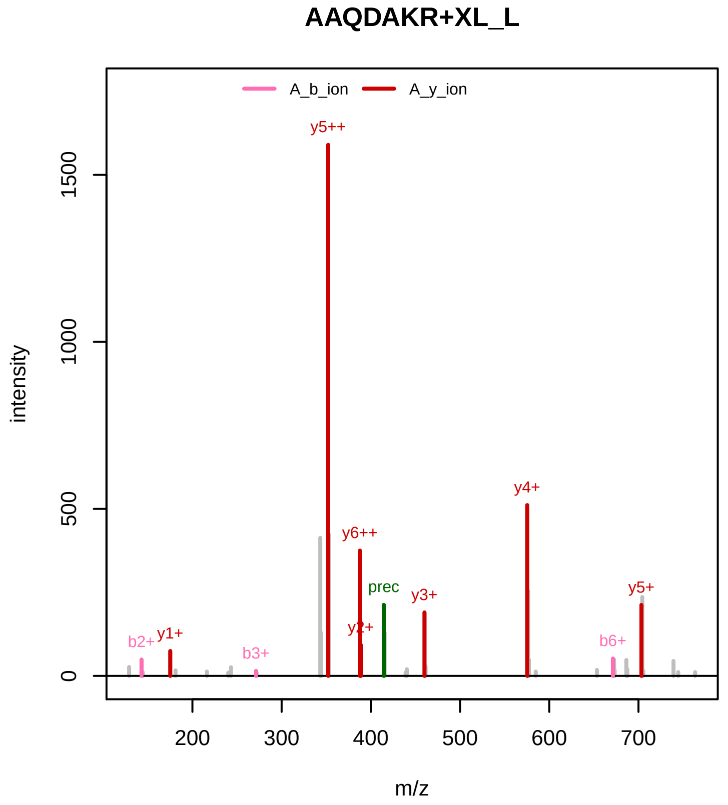

AAVDKALSNNMTR+XL_S

AAVDKALSNNMTR+XL_L

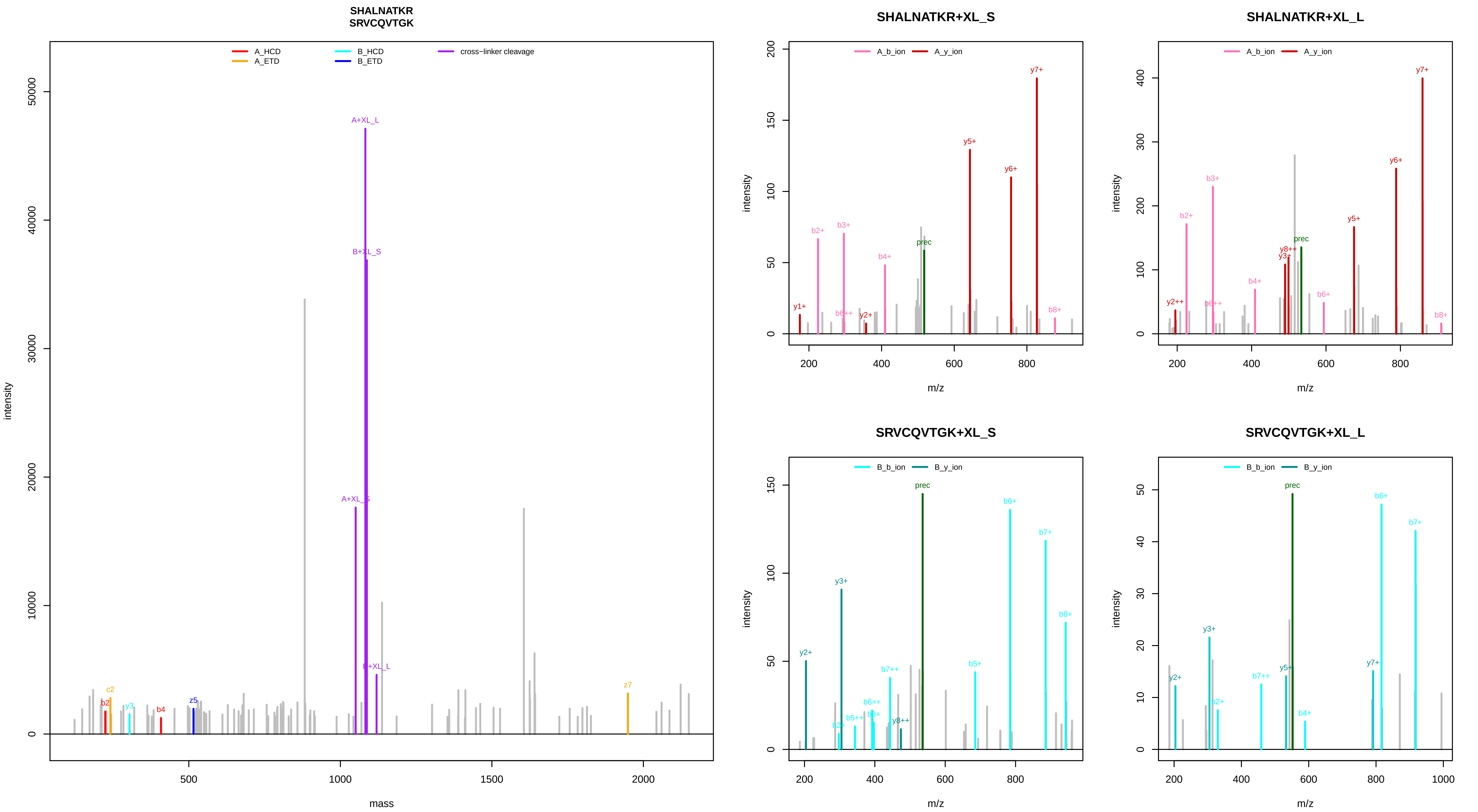
SHALNATKR
SRVCQVTGK
A_HCD
A_ETD
B_HCD
B_ETD
cross−linker cleavage
A+XL_L
B+XL_S
A+XL_S
B+XL_L
b2
c2
y3
b4
z5
z7
intensity
mass
SHALNATKR+XL_S
A_b_ion
A_y_ion
y1+
b2+
b3+
b6++
y2+
b4+
prec
y5+
y6+
y7+
b8+
m/z
SHALNATKR+XL_L
y2++
b2+
b3+
b6++
b4+
y8++
y3+
prec
b6+
y5+
y6+
y7+
b8+
SRVCQVTGK+XL_S
B_b_ion
B_y_ion
y2+
b2+
y3+
b5++
b6++
b3+
b7++
y8++
prec
b5+
b6+
b7+
b8+
SRVCQVTGK+XL_L
y2+
y3+
b2+
b7++
y5+
prec
b4+
y7+
b6+
b7+

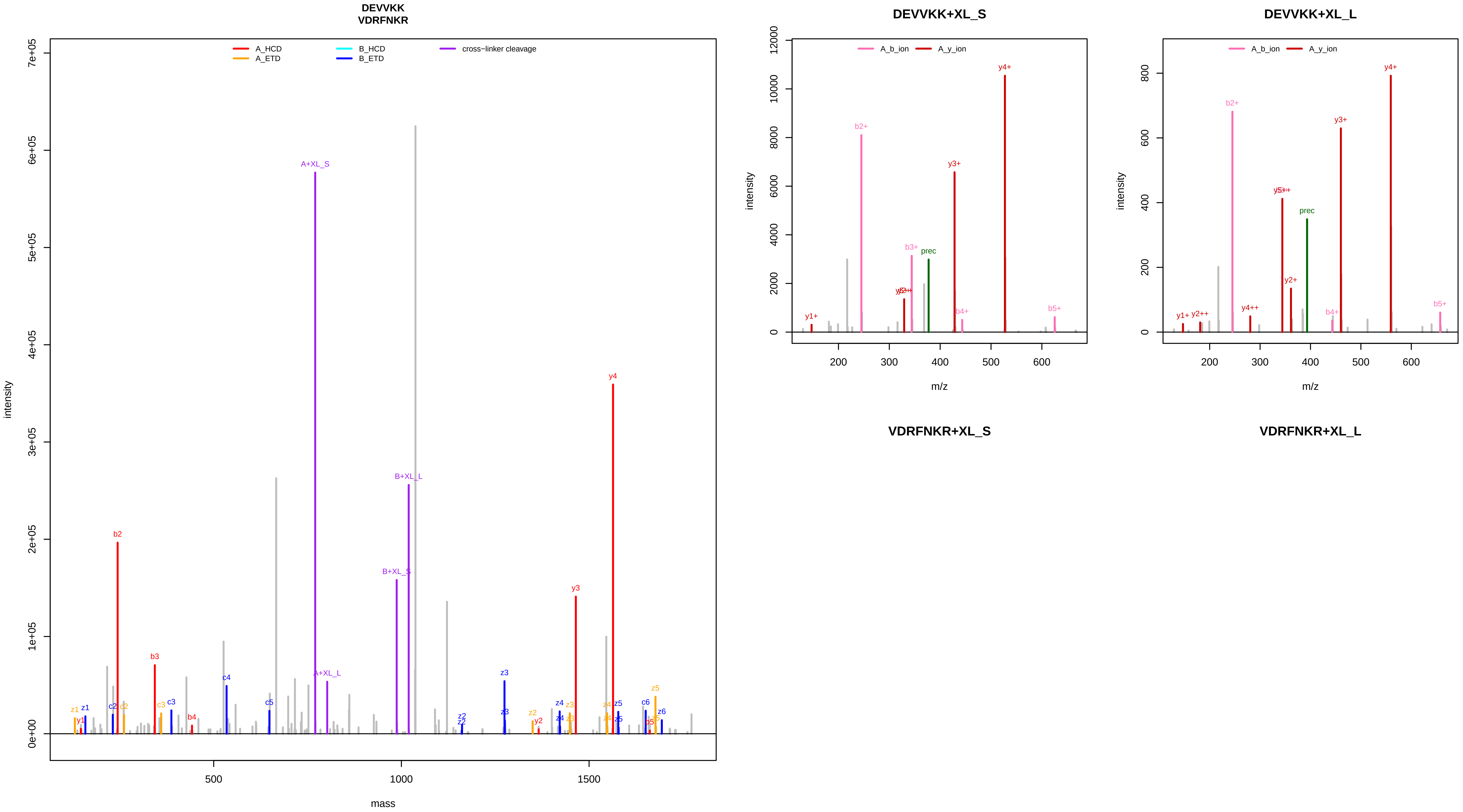
DEVVKK
VDRFNKR
A_HCD
A_ETD
B_HCD
B_ETD
cross−linker cleavage
A+XL_S
B+XL_L
B+XL_S
A+XL_L
b2
b3
b4
b5
y1
y2
y3
y4
c2
c3
c4
c5
c6
z1
z2
z3
z4
z5
z6
intensity
mass
DEVVKK+XL_S
DEVVKK+XL_L
A_b_ion
A_y_ion
b2+
b3+
b4+
b5+
y1+
y2++
y2+
y3+
y4+
y4++
y5++
prec
intensity
m/z
VDRFNKR+XL_S
VDRFNKR+XL_L

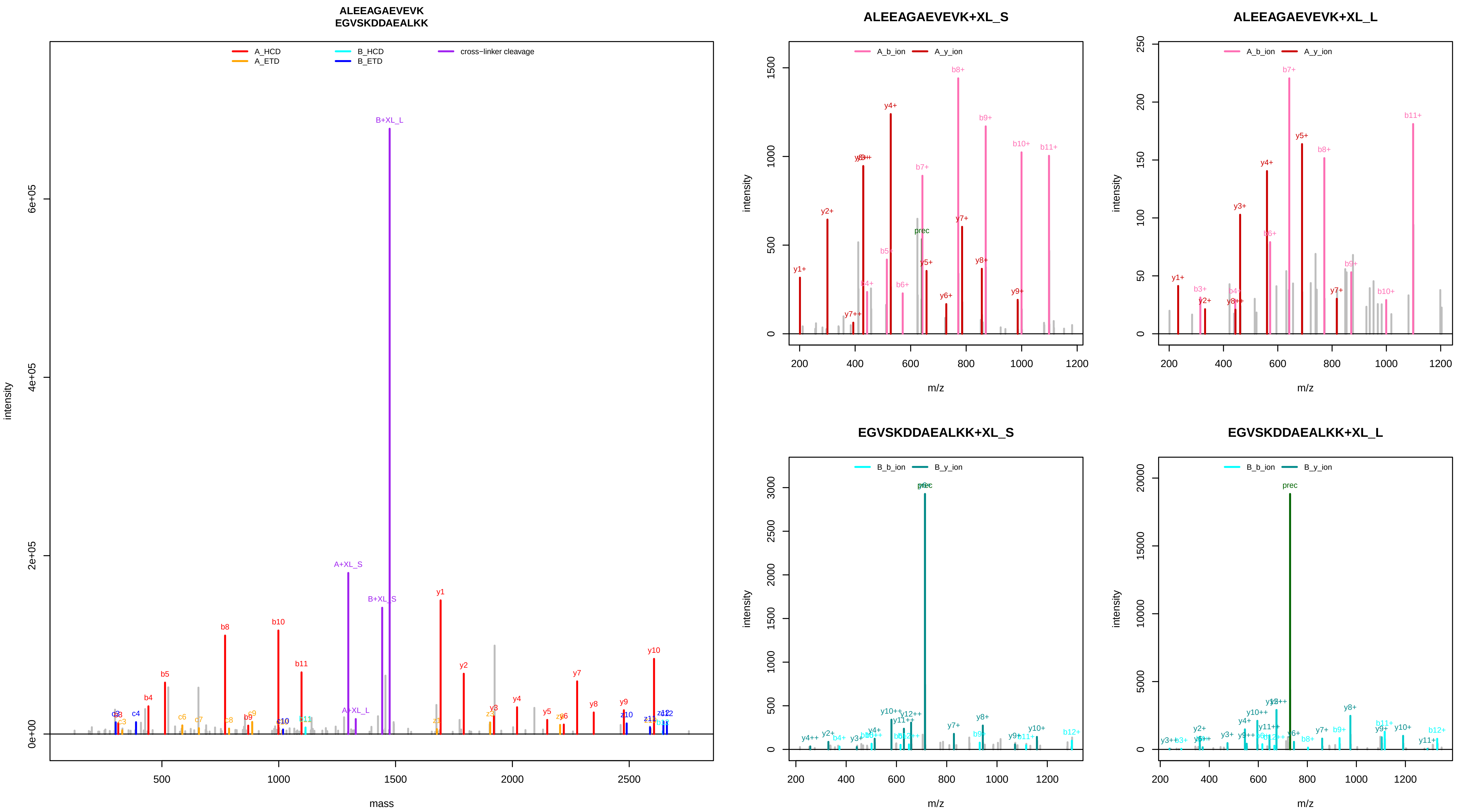

ALEEAGAEVEVK
EGVSKDDAEALKK
A_HCD
A_ETD
B_HCD
B_ETD
cross−linker cleavage
B+XL_L
A+XL_S
B+XL_S
A+XL_L
intensity
mass
ALEEAGAEVEVK+XL_S
ALEEAGAEVEVK+XL_L
EGVSKDDAEALKK+XL_S
EGVSKDDAEALKK+XL_L
A_b_ion
A_y_ion
B_b_ion
B_y_ion
prec
m/z

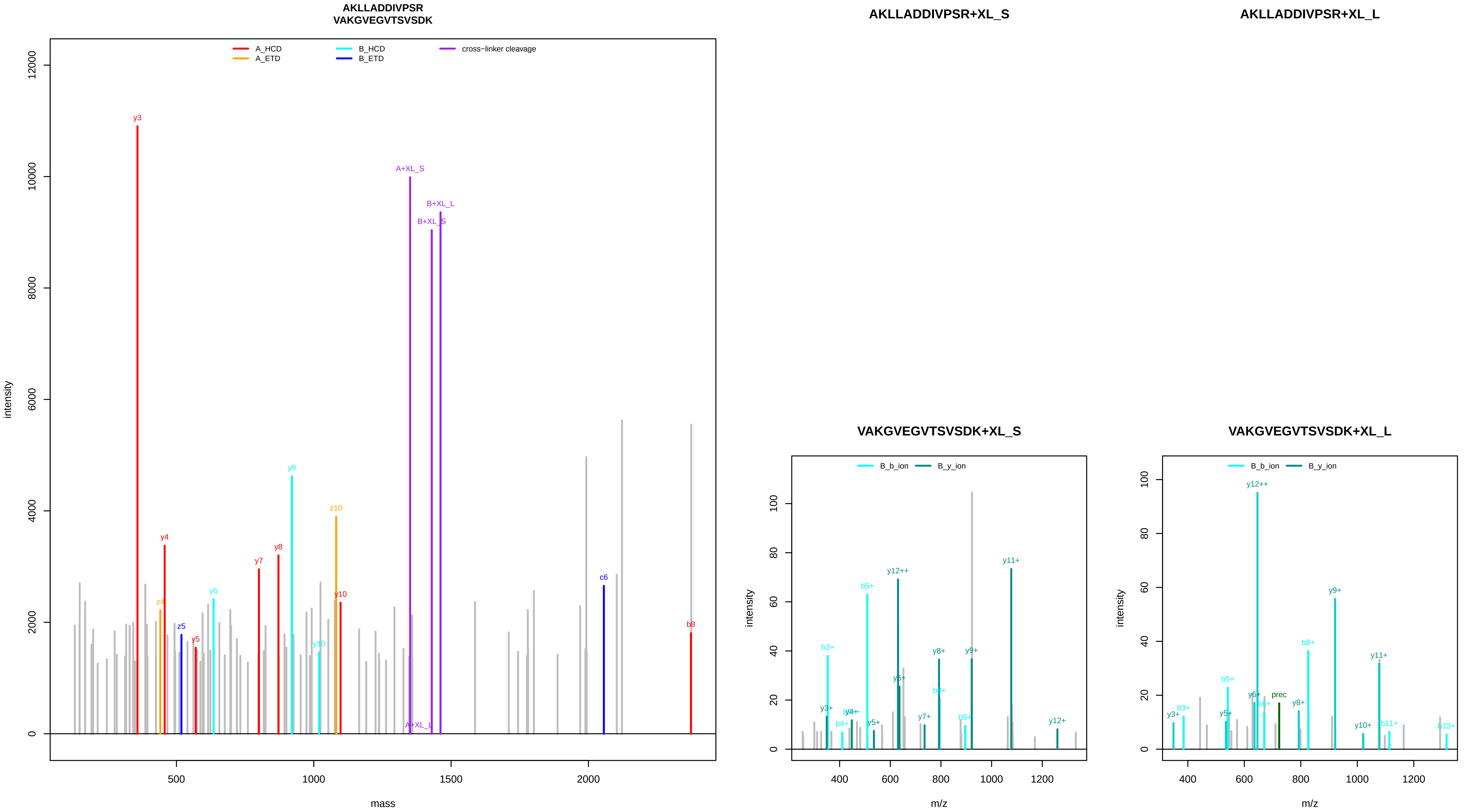
AKLLADDIVPSR
VAKGVEGVTSVSDK
A_HCD
A_ETD
B_HCD
B_ETD
cross−linker cleavage
intensity
mass
y3
y4
z4
z5
y5
y6
y7
y8
y9
y10
z10
y10
A+XL_S
B+XL_L
B+XL_S
A+XL_L
c6
b8
AKLLADDIVPSR+XL_S
AKLLADDIVPSR+XL_L
VAKGVEGVTSVSDK+XL_S
VAKGVEGVTSVSDK+XL_L
B_b_ion
B_y_ion
m/z
b3+
b5+
y12++
y11+
y8+
y9+
y12+
y3+
b4+
y5+
y7+
b9+
b8+
b11+
b13+
y10+
prec
y6+

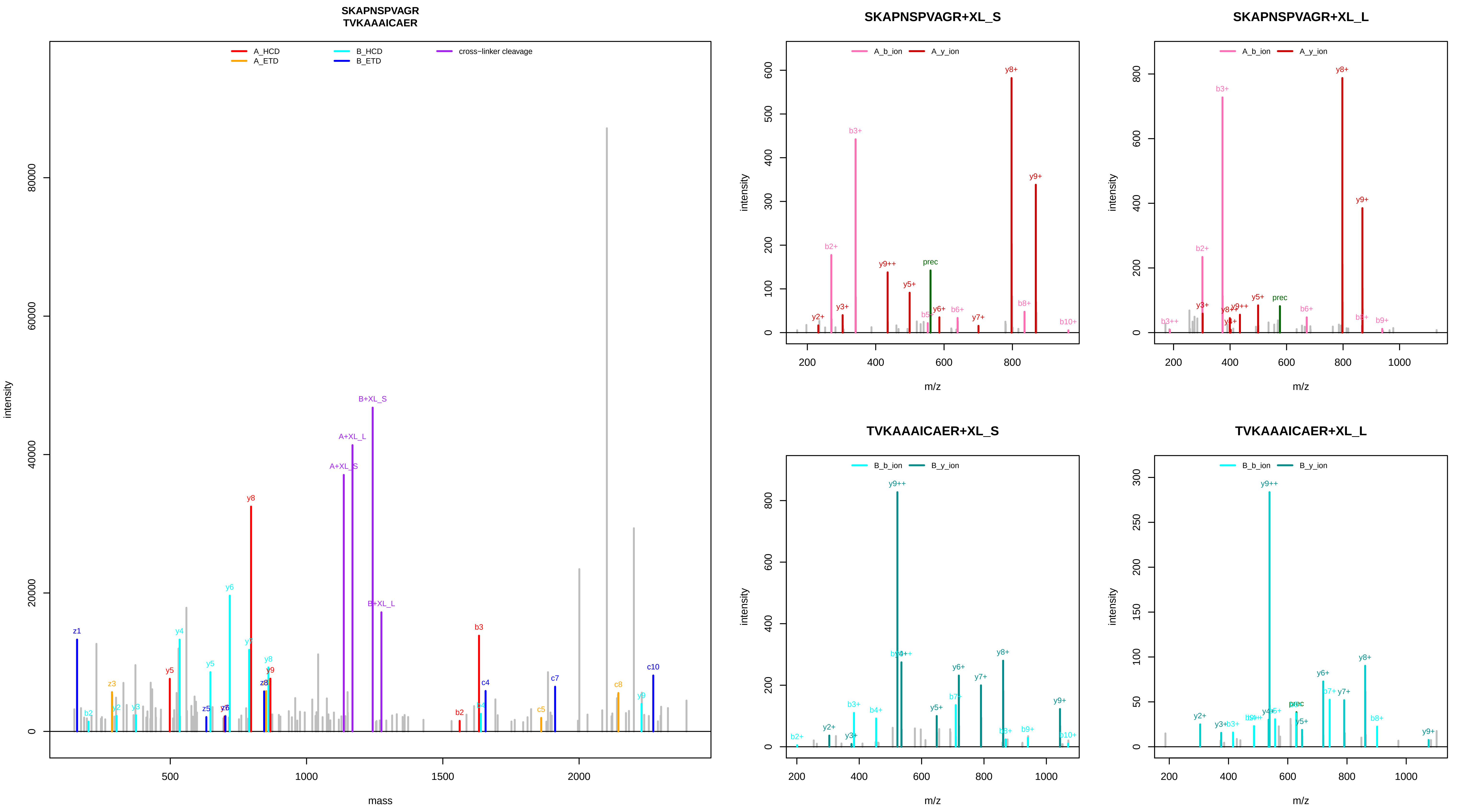

SKAPNSPVAGR
TVKAAAICAER
A_HCD
A_ETD
B_HCD
B_ETD
cross−linker cleavage
intensity
mass
B+XL_S
A+XL_L
A+XL_S
B+XL_L
SKAPNSPVAGR+XL_S
A_b_ion
A_y_ion
m/z
SKAPNSPVAGR+XL_L
TVKAAAICAER+XL_S
B_b_ion
B_y_ion
TVKAAAICAER+XL_L

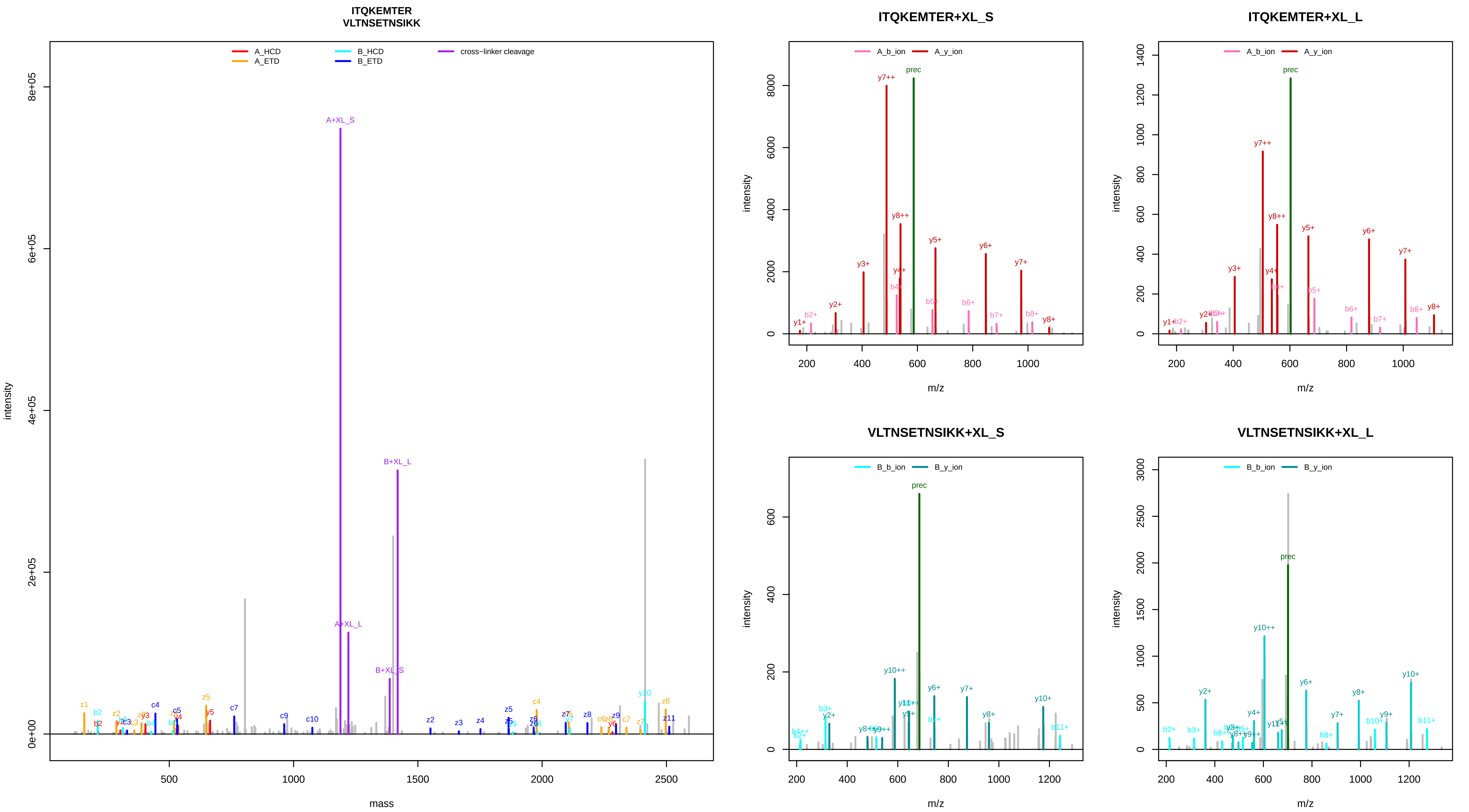

ITQKEMTER
VLTNSETNSIKK
A_HCD
A_ETD
B_HCD
B_ETD
cross−linker cleavage
A+XL_S
B+XL_L
A+XL_L
B+XL_S
intensity
mass
ITQKEMTER+XL_S
ITQKEMTER+XL_L
VLTNSETNSIKK+XL_S
VLTNSETNSIKK+XL_L
A_b_ion
A_y_ion
B_b_ion
B_y_ion
prec
m/z

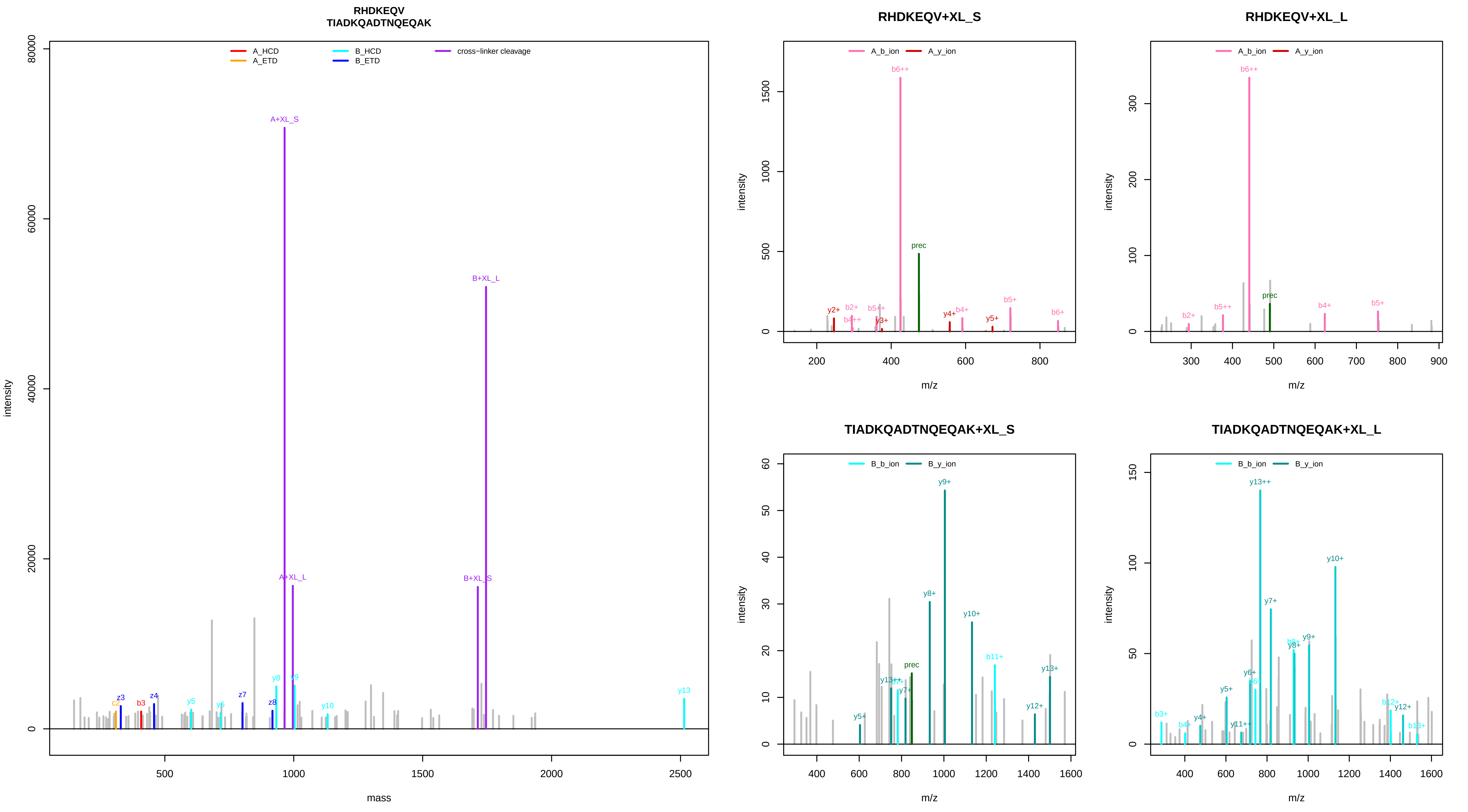
RHDKEQV
TIADKQADTNQEQAK
A_HCD
A_ETD
B_HCD
B_ETD
cross−linker cleavage
A+XL_S
B+XL_L
A+XL_L
B+XL_S
intensity
mass
RHDKEQV+XL_S
RHDKEQV+XL_L
TIADKQADTNQEQAK+XL_S
TIADKQADTNQEQAK+XL_L
A_b_ion
A_y_ion
B_b_ion
B_y_ion
prec
m/z

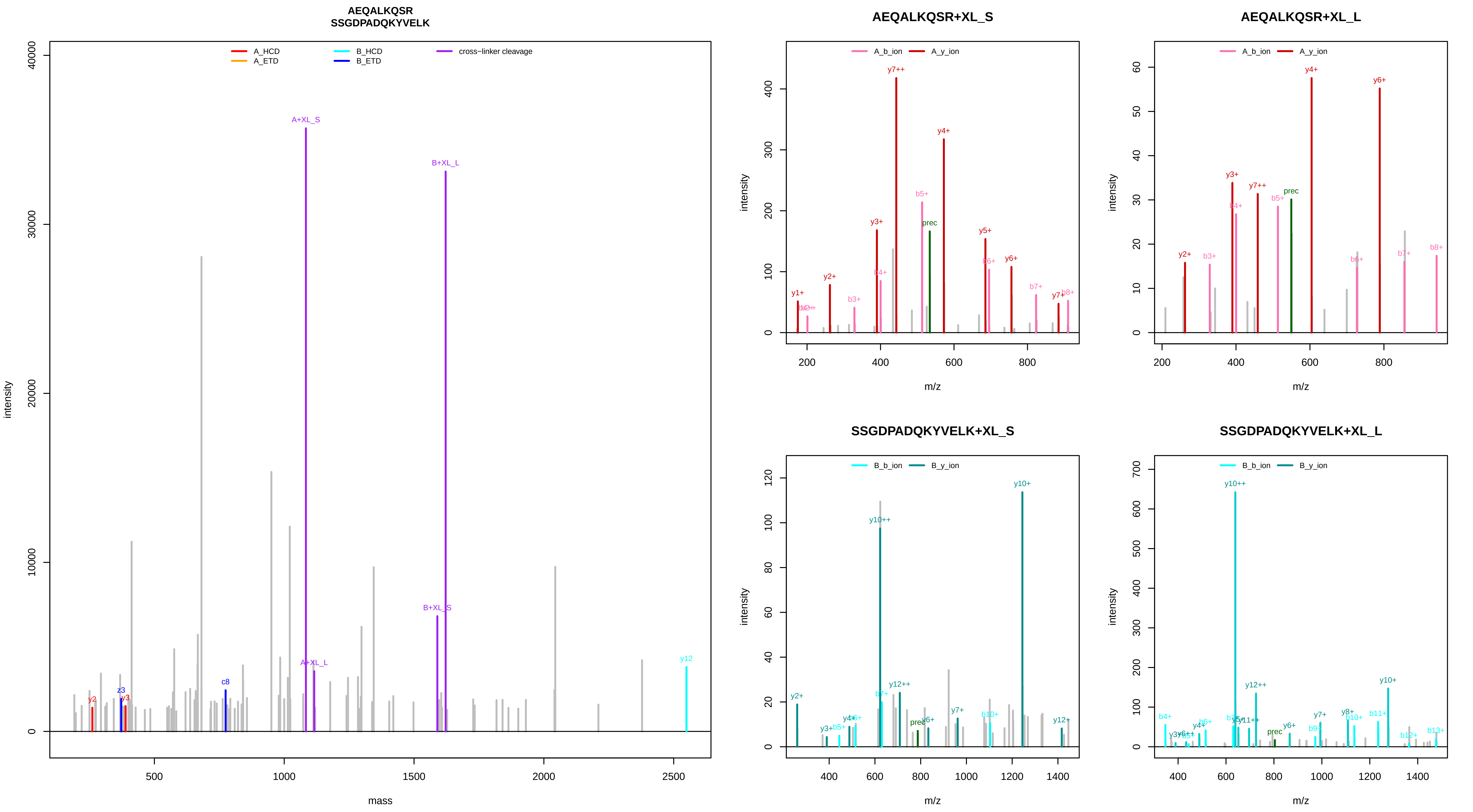

AEQALKQSR
SSGDPADQKYVELK
A_HCD
A_ETD
B_HCD
B_ETD
cross−linker cleavage
A+XL_S
B+XL_L
B+XL_S
A+XL_L
y12
c8
z3
y2
y3
intensity
mass
AEQALKQSR+XL_S
AEQALKQSR+XL_L
SSGDPADQKYVELK+XL_S
SSGDPADQKYVELK+XL_L
A_b_ion
A_y_ion
B_b_ion
B_y_ion
prec
m/z

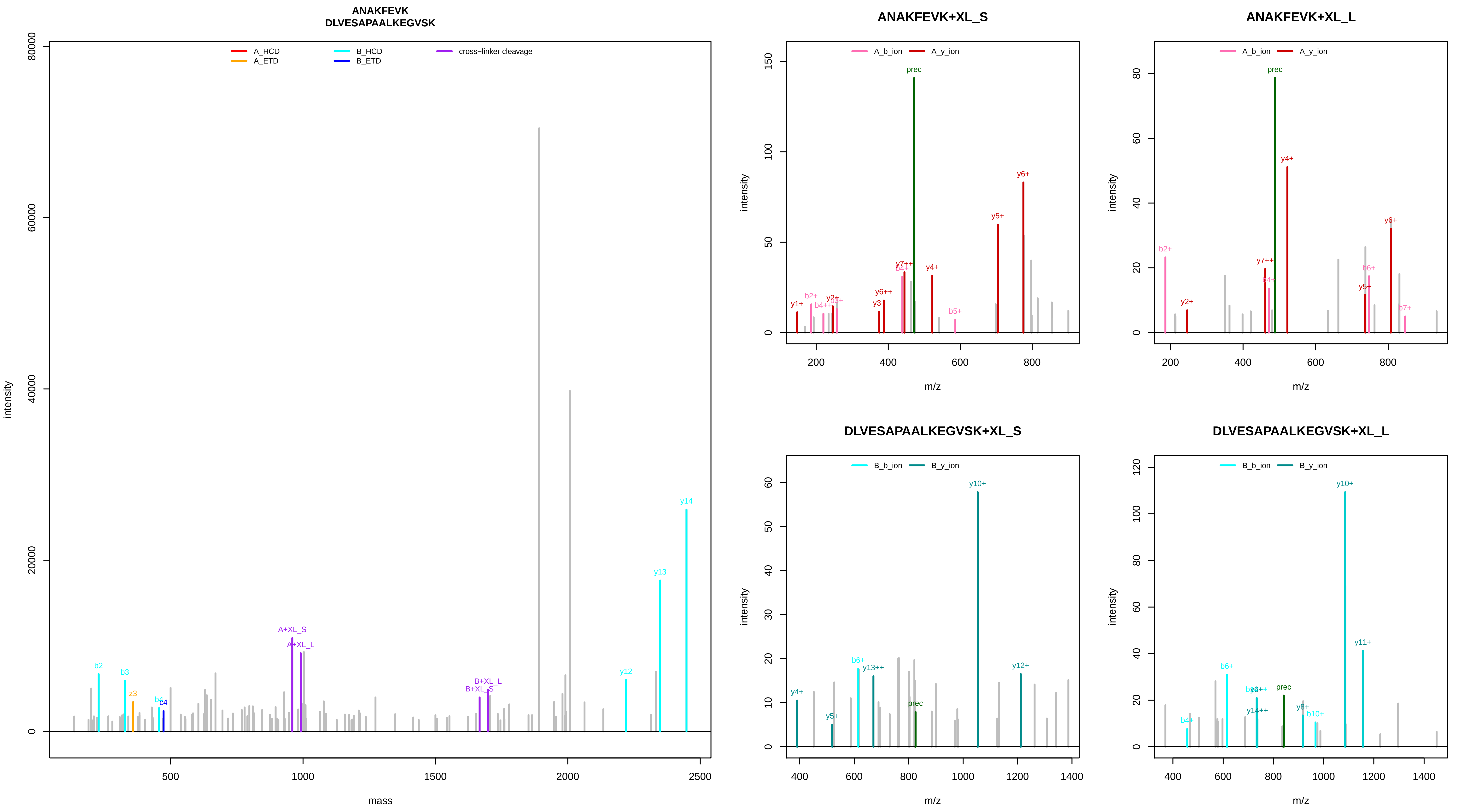
ANAKFEVK
DLVESAPAALKEGVSK
A_HCD
A_ETD
B_HCD
B_ETD
cross−linker cleavage
intensity
mass
ANAKFEVK+XL_S
ANAKFEVK+XL_L
DLVESAPAALKEGVSK+XL_S
DLVESAPAALKEGVSK+XL_L
A_b_ion
A_y_ion
B_b_ion
B_y_ion
prec
m/z

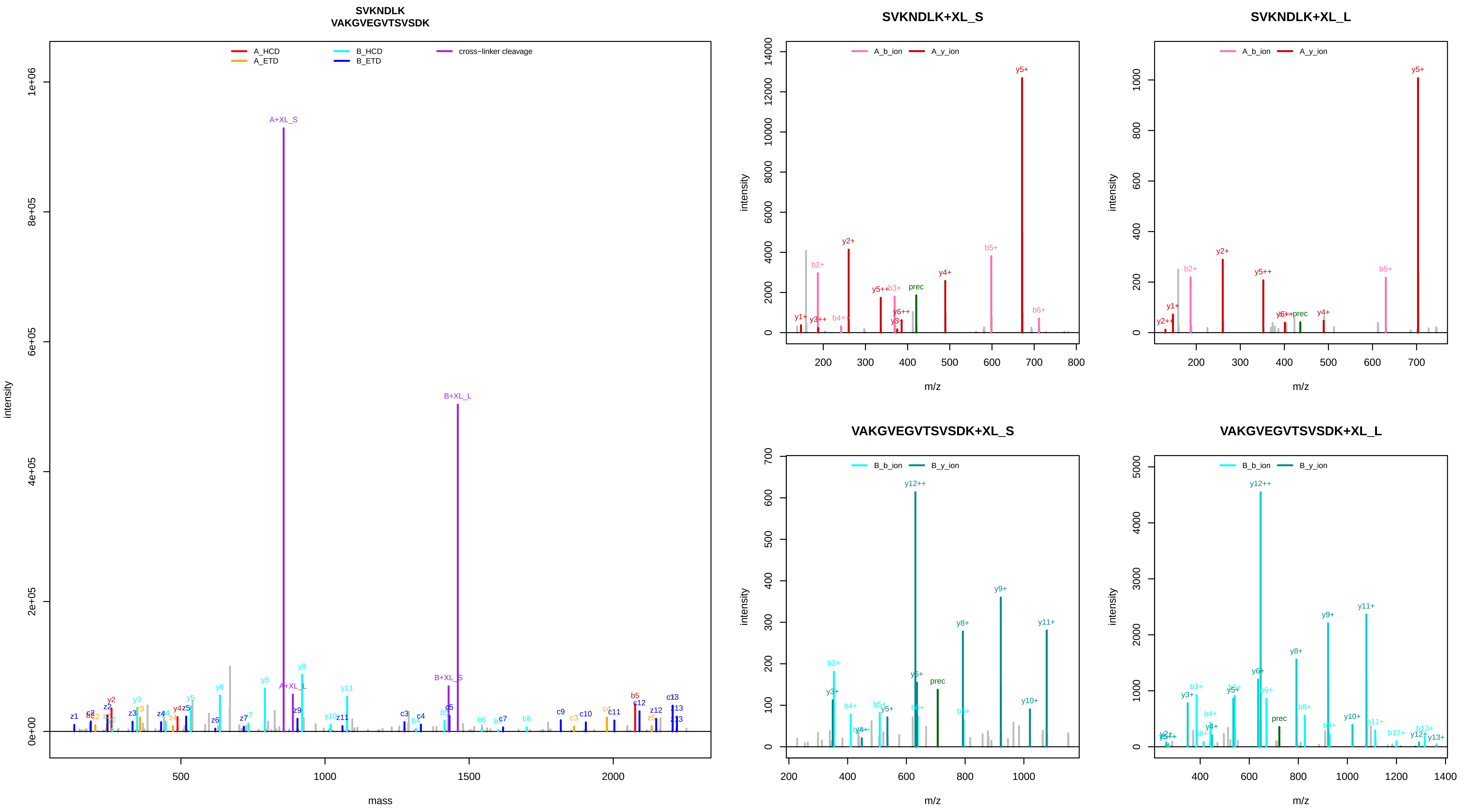
SVKNDLK
VAKGVEGVTSVSDK
A_HCD
A_ETD
B_HCD
B_ETD
cross−linker cleavage
A+XL_S
B+XL_L
A+XL_L
B+XL_S
intensity
mass
SVKNDLK+XL_S
SVKNDLK+XL_L
A_b_ion
A_y_ion
VAKGVEGVTSVSDK+XL_S
VAKGVEGVTSVSDK+XL_L
B_b_ion
B_y_ion
y12++
prec
m/z

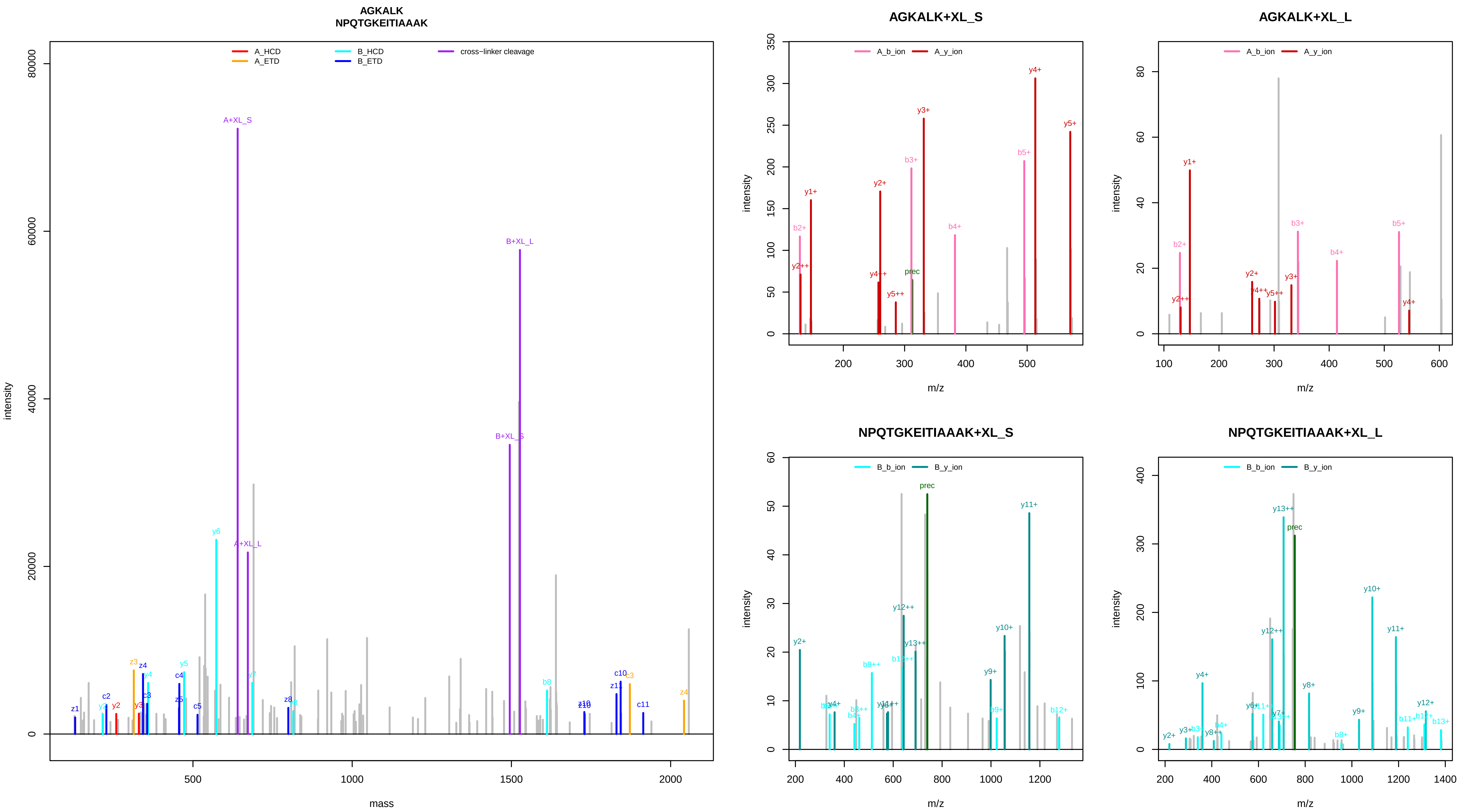

AGKALK
NPQTGKEITIAAAK
A_HCD
A_ETD
B_HCD
B_ETD
cross−linker cleavage
A+XL_S
B+XL_L
B+XL_S
A+XL_L
intensity
mass
AGKALK+XL_S
AGKALK+XL_L
NPQTGKEITIAAAK+XL_S
NPQTGKEITIAAAK+XL_L
A_b_ion
A_y_ion
B_b_ion
B_y_ion
prec
m/z

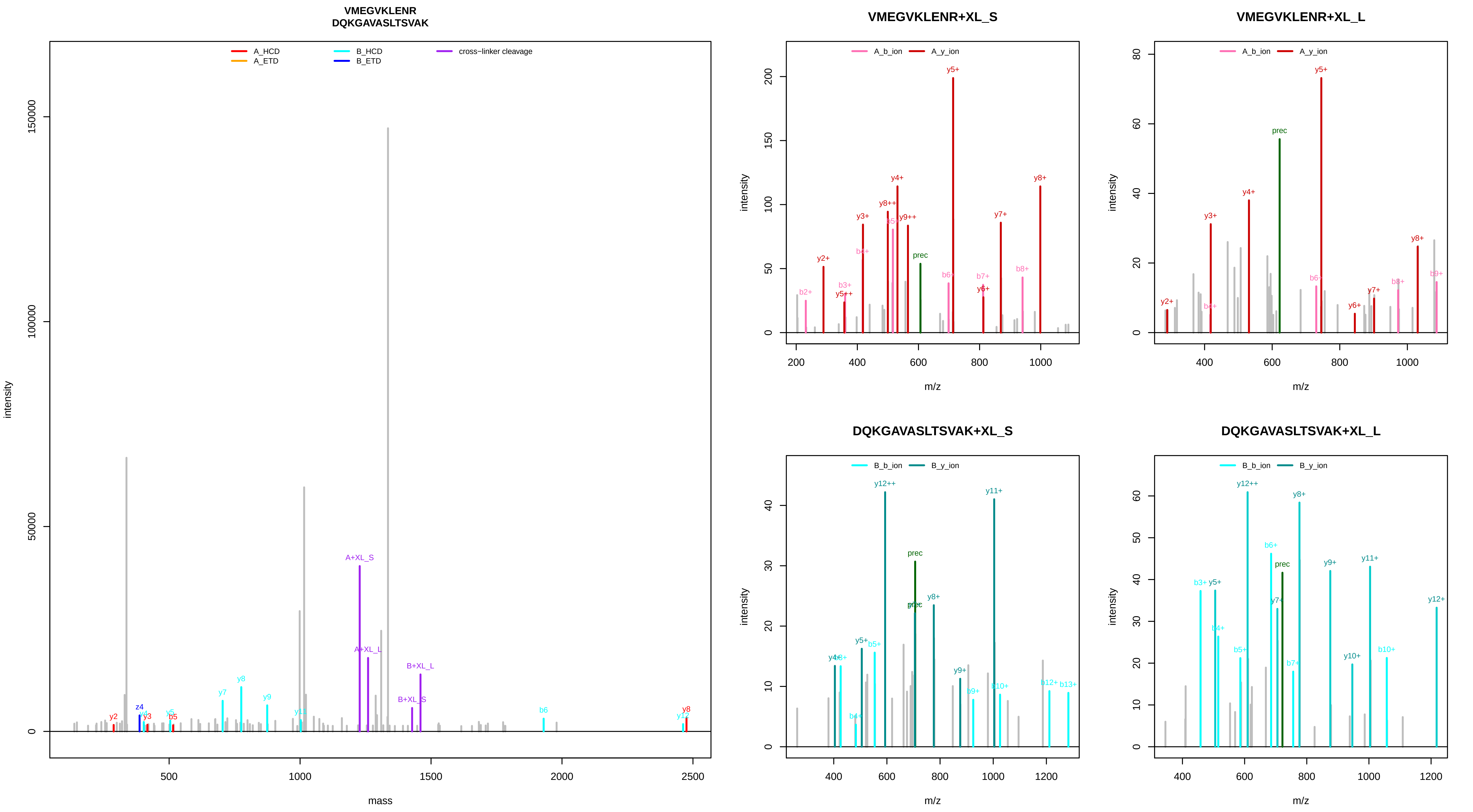
VMEGVKLENR
DQKGAVASLTSVAK
A_HCD
A_ETD
B_HCD
B_ETD
cross−linker cleavage
intensity
mass
A+XL_S
A+XL_L
B+XL_L
B+XL_S
VMEGVKLENR+XL_S
VMEGVKLENR+XL_L
A_b_ion
A_y_ion
DQKGAVASLTSVAK+XL_S
DQKGAVASLTSVAK+XL_L
B_b_ion
B_y_ion
prec
m/z

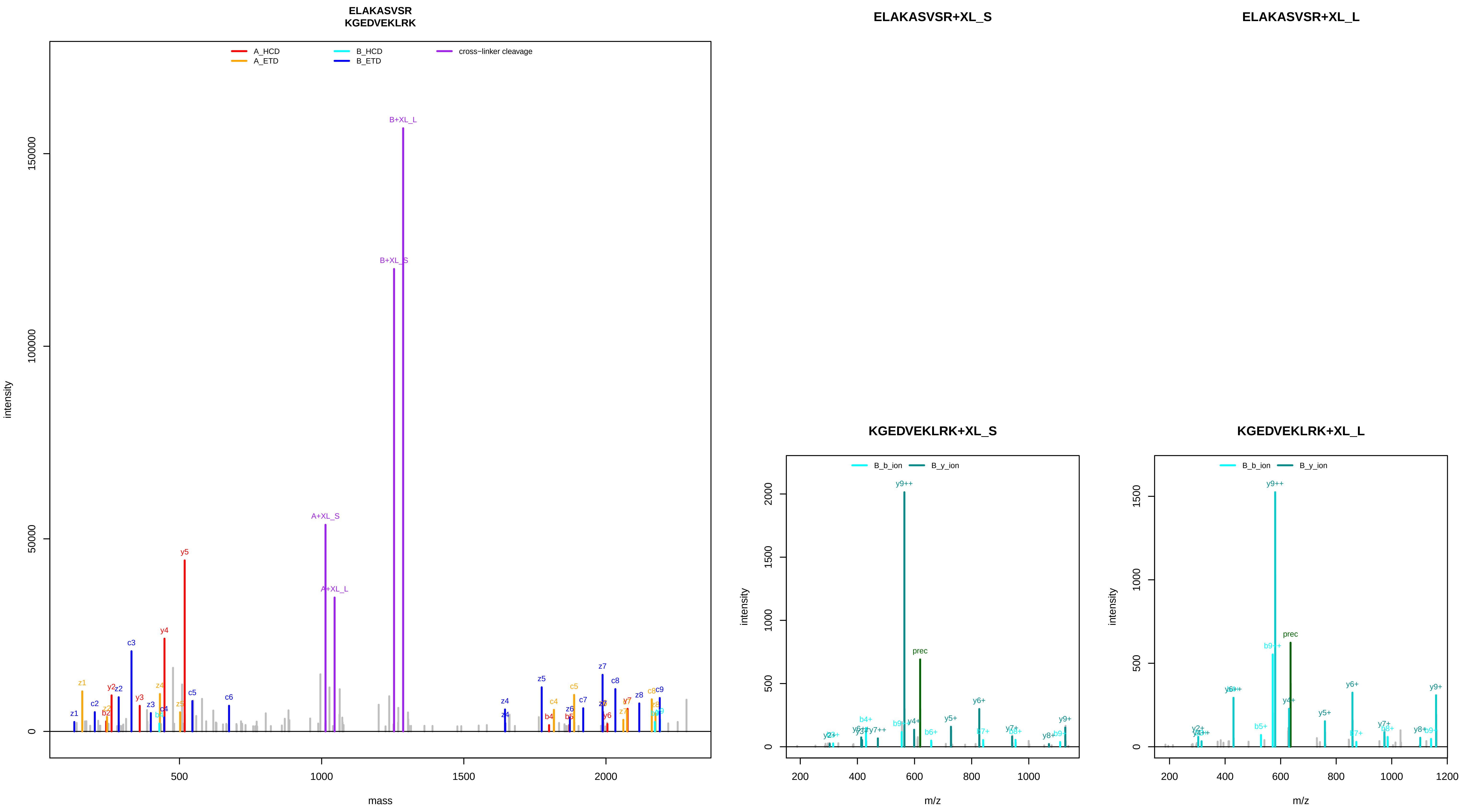
ELAKASVSR
KGEDVEKLRK
A_HCD
A_ETD
B_HCD
B_ETD
cross−linker cleavage
B+XL_L
B+XL_S
A+XL_S
A+XL_L
intensity
mass
ELAKASVSR+XL_S
ELAKASVSR+XL_L
KGEDVEKLRK+XL_S
KGEDVEKLRK+XL_L
B_b_ion
B_y_ion
y9++
prec
b9++
intensity
m/z

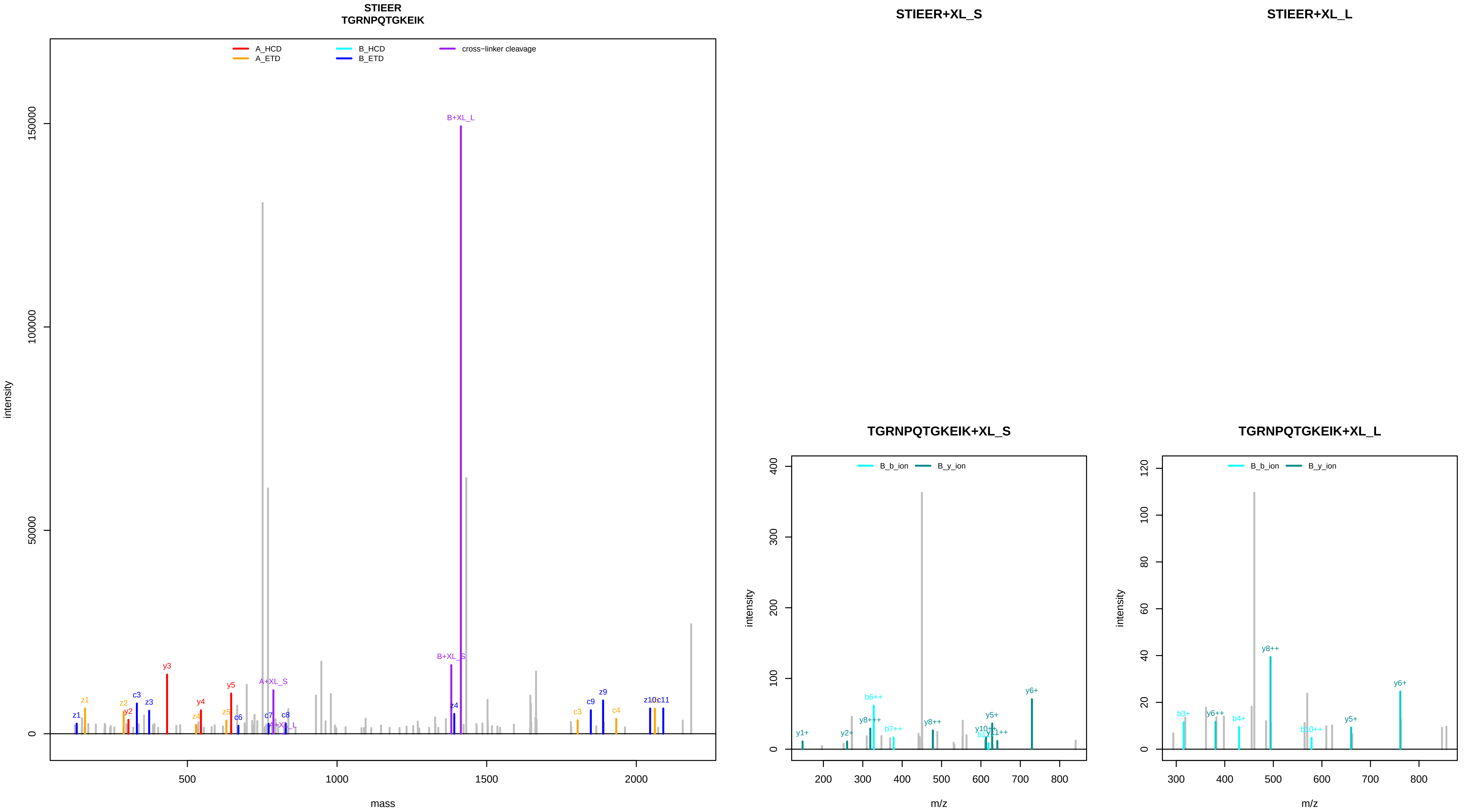
STIEER
TGRNPQTGKEIK
A_HCD
A_ETD
B_HCD
B_ETD
cross−linker cleavage
B+XL_L
B+XL_S
A+XL_S
A+XL_L
y3
y4
y5
y2
z1
z2
z3
z4
z5
z9
z10
c3
c6
c7
c8
c9
c11
c3
c4
150000
100000
50000
0
intensity
500
1000
1500
2000
mass
STIEER+XL_S
STIEER+XL_L
TGRNPQTGKEIK+XL_S
TGRNPQTGKEIK+XL_L
B_b_ion
B_y_ion
y1+
y2+
y8+++
b6++
b7++
y8++
y5+
y6+
y10++
y11++
intensity
m/z
b3+
y6++
b4+
y8++
b10++
y5+
y6+
m/z

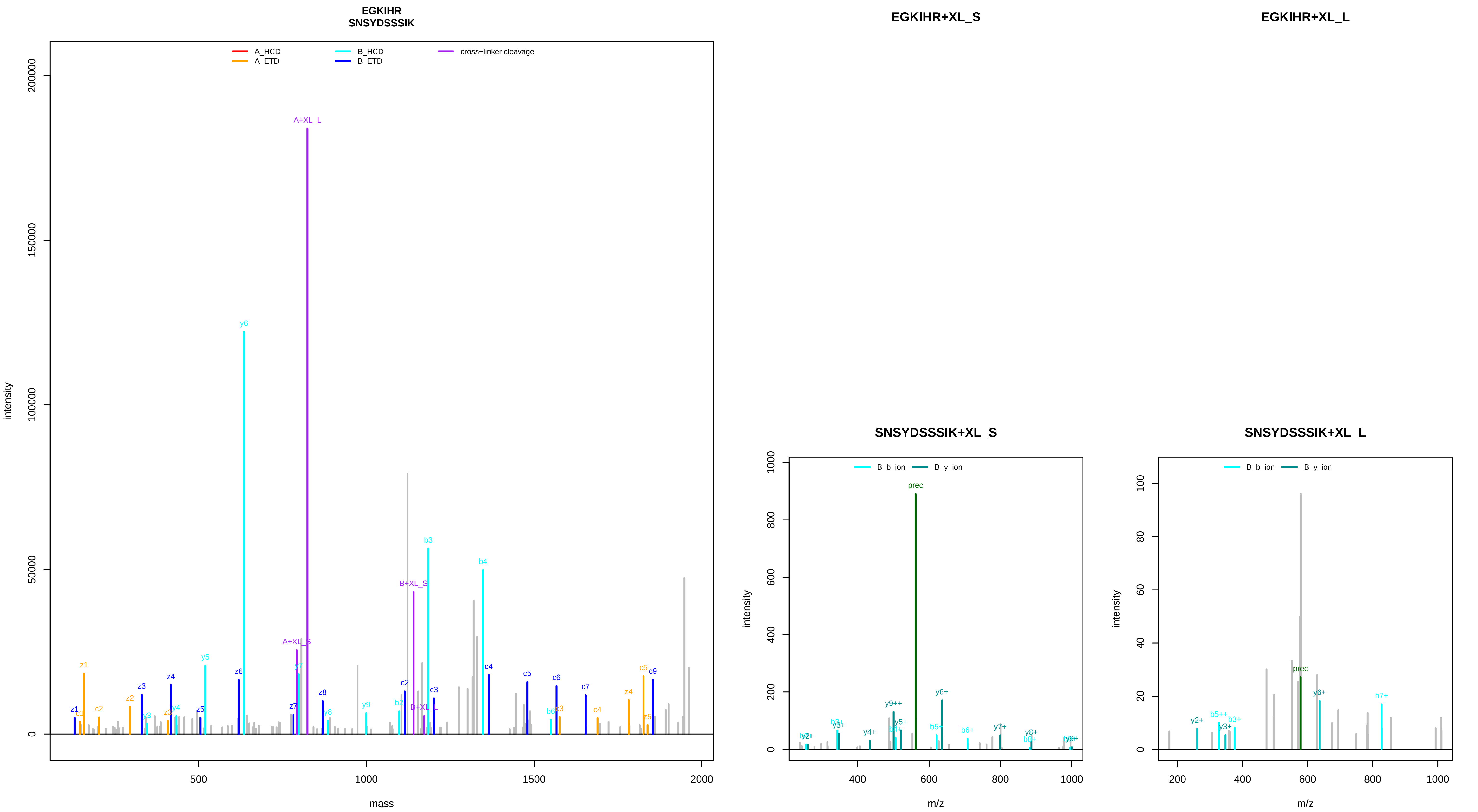
EGKIHR
SNSYDSSSIK
A_HCD
A_ETD
B_HCD
B_ETD
cross−linker cleavage
A+XL_L
A+XL_S
B+XL_S
intensity
mass
EGKIHR+XL_S
EGKIHR+XL_L
SNSYDSSSIK+XL_S
SNSYDSSSIK+XL_L
B_b_ion
B_y_ion
prec
m/z

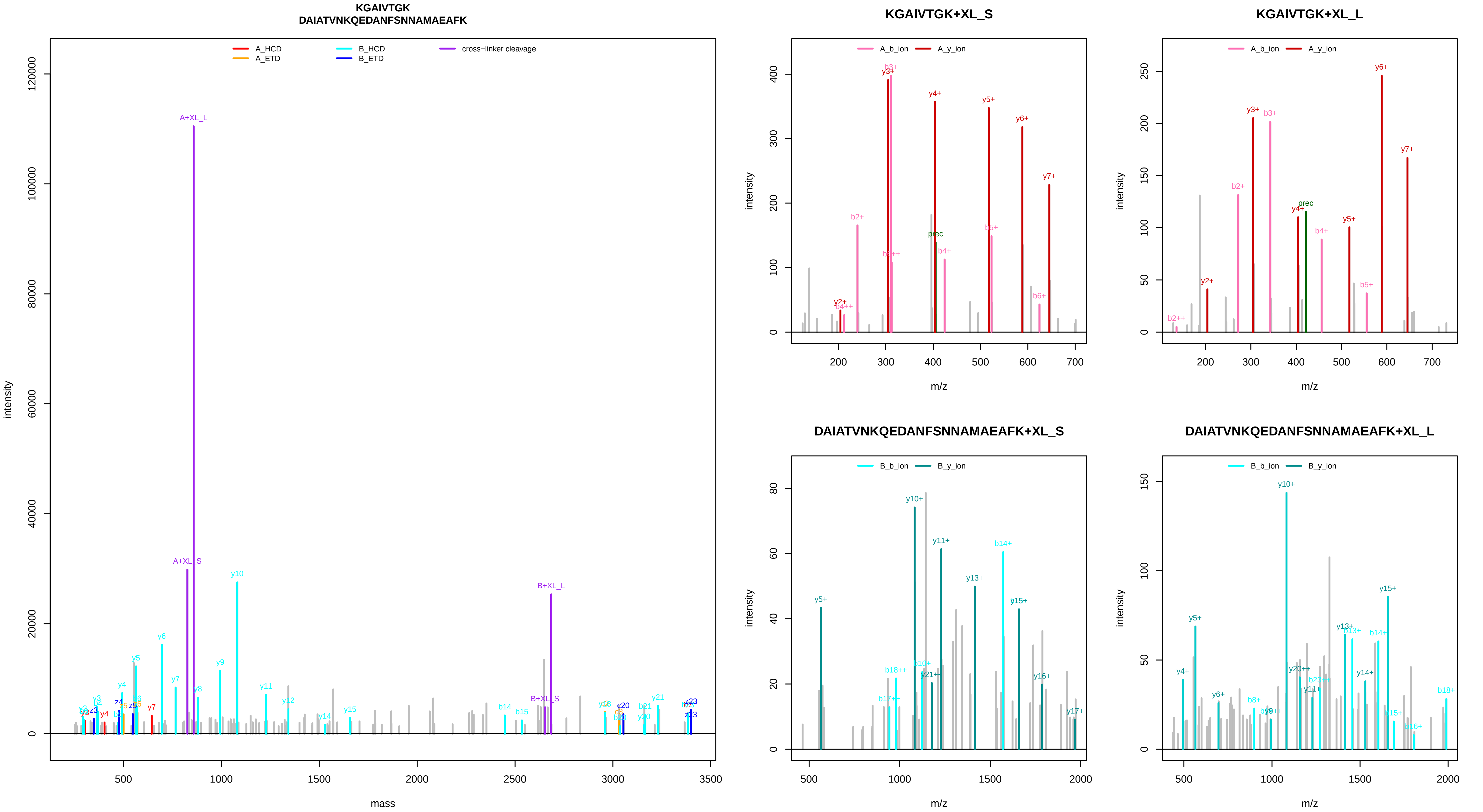
KGAIVTGK
DAIATVNKQEDANFSNNAMAEAFK
A_HCD
A_ETD
B_HCD
B_ETD
cross−linker cleavage
A+XL_L
A+XL_S
B+XL_L
B+XL_S
intensity
mass
KGAIVTGK+XL_S
KGAIVTGK+XL_L
A_b_ion
A_y_ion
DAIATVNKQEDANFSNNAMAEAFK+XL_S
DAIATVNKQEDANFSNNAMAEAFK+XL_L
B_b_ion
B_y_ion
m/z
prec

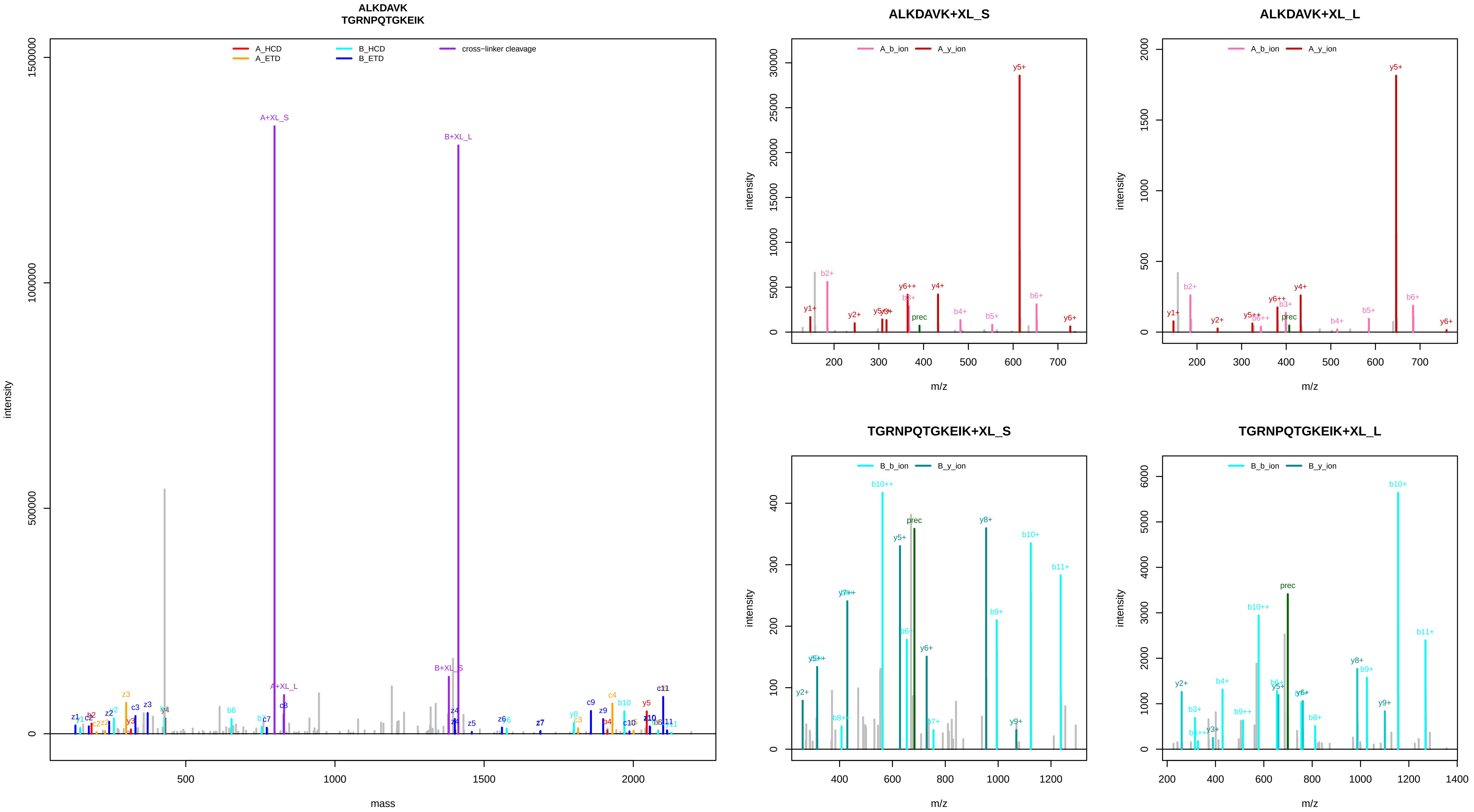
ALKDAVK
TGRNPQTGKEIK
A_HCD
A_ETD
B_HCD
B_ETD
cross−linker cleavage
A+XL_S
B+XL_L
B+XL_S
A+XL_L
intensity
mass
ALKDAVK+XL_S
ALKDAVK+XL_L
A_b_ion
A_y_ion
y5+
prec
m/z
intensity
TGRNPQTGKEIK+XL_S
TGRNPQTGKEIK+XL_L
B_b_ion
B_y_ion
b10++
b10+
b11+
y8+
m/z
intensity

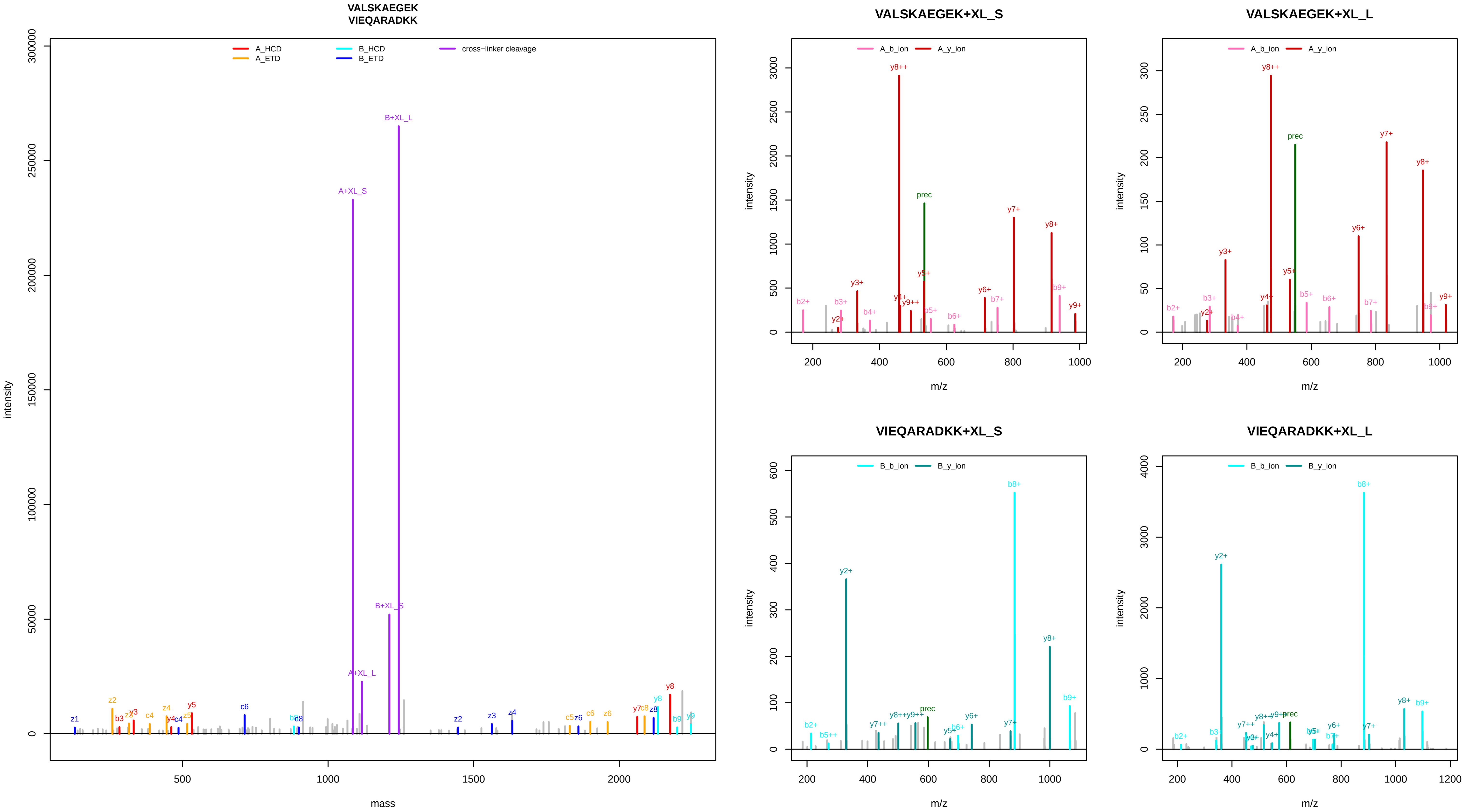

VALSKAEGEK
VIEQARADKK
A_HCD
A_ETD
B_HCD
B_ETD
cross−linker cleavage
intensity
mass
A+XL_S
B+XL_L
B+XL_S
A+XL_L
VALSKAEGEK+XL_S
VALSKAEGEK+XL_L
VIEQARADKK+XL_S
VIEQARADKK+XL_L
A_b_ion
A_y_ion
B_b_ion
B_y_ion
prec
m/z

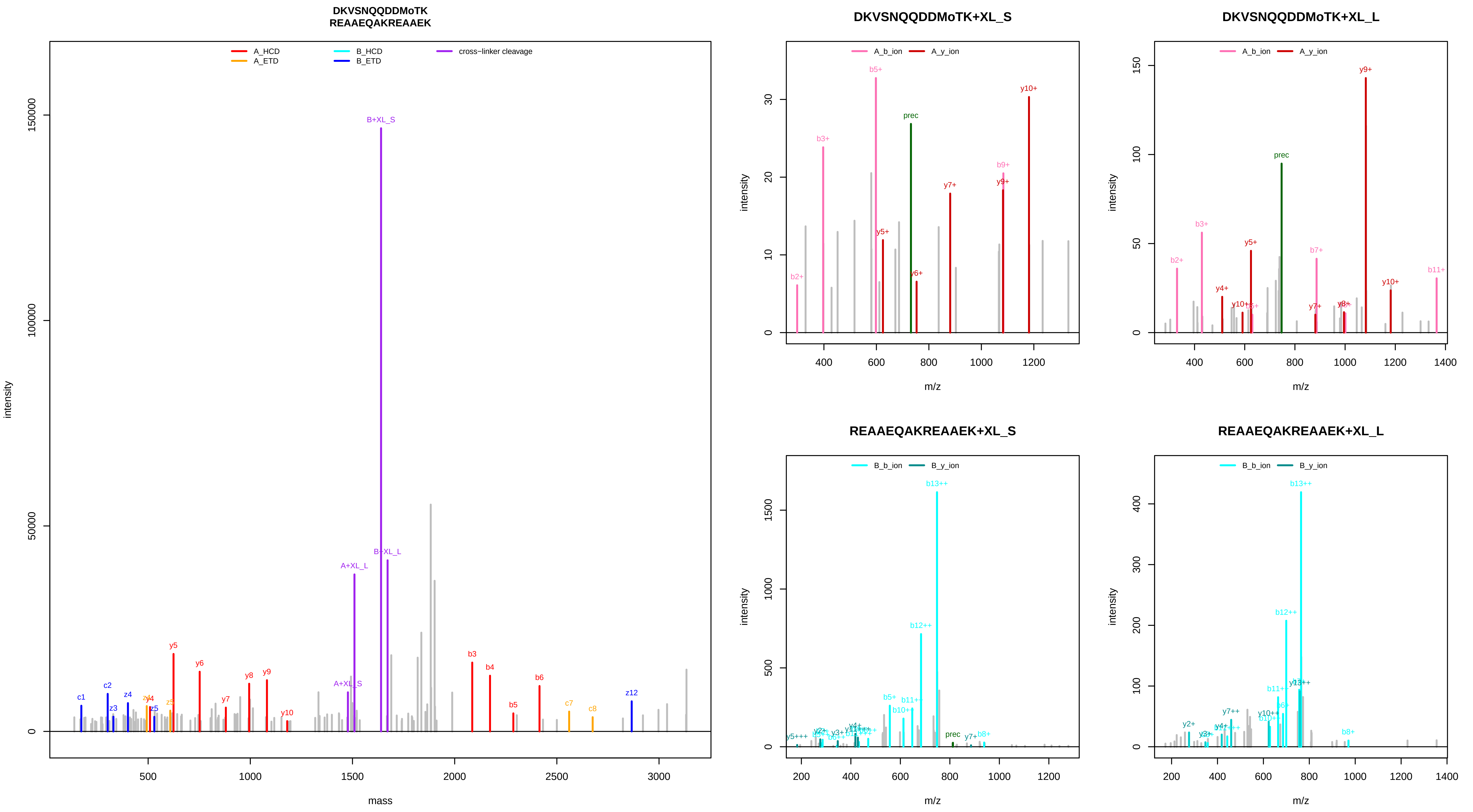
DKVSNQQDDMoTK
REAAEQAKREAAEK
A_HCD
A_ETD
B_HCD
B_ETD
cross−linker cleavage
B+XL_S
B+XL_L
A+XL_L
A+XL_S
intensity
mass
DKVSNQQDDMoTK+XL_S
DKVSNQQDDMoTK+XL_L
REAAEQAKREAAEK+XL_S
REAAEQAKREAAEK+XL_L
A_b_ion
A_y_ion
B_b_ion
B_y_ion
prec
m/z

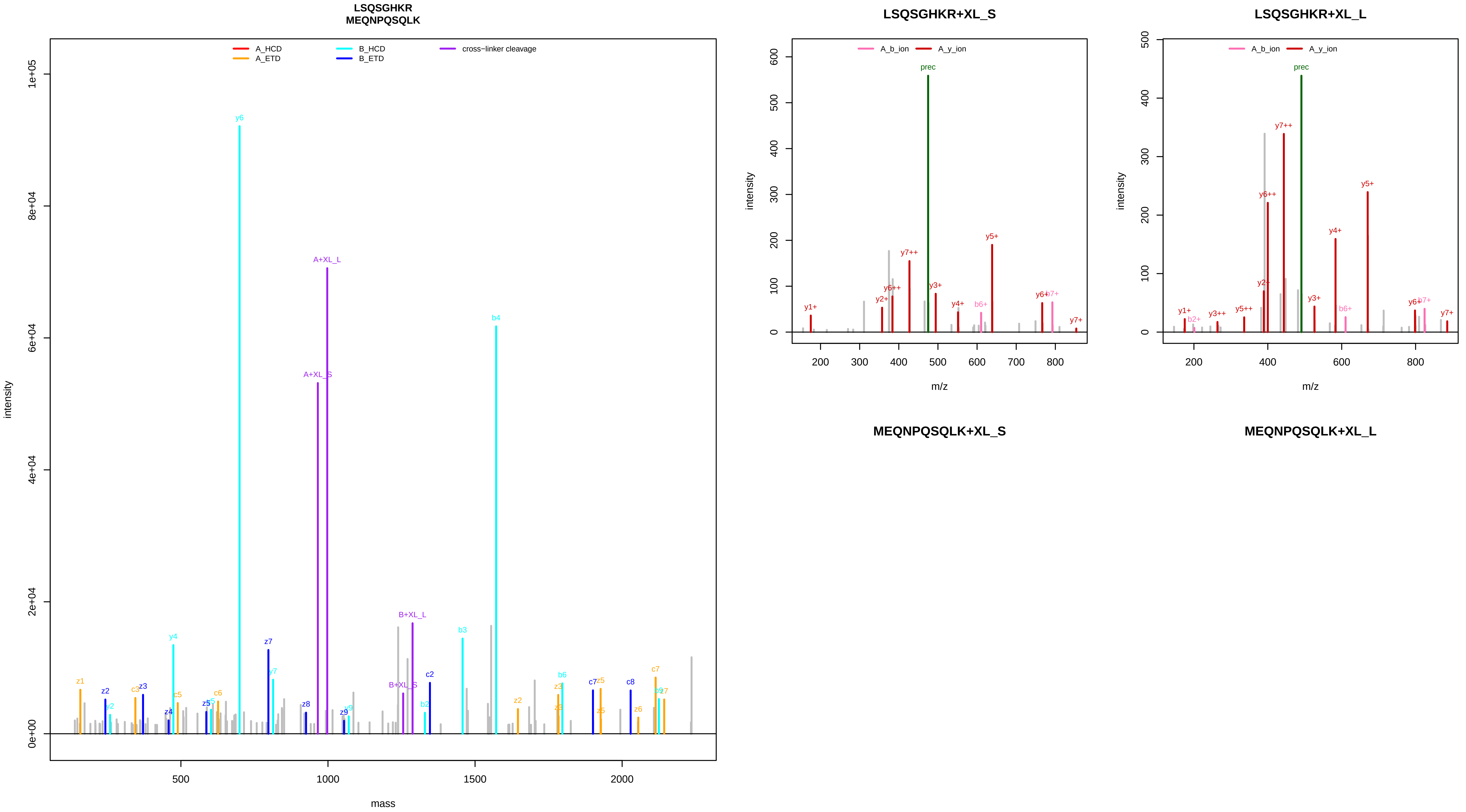
LSQSGHKR
MEQNPQSQLK
A_HCD
A_ETD
B_HCD
B_ETD
cross−linker cleavage
A+XL_L
A+XL_S
B+XL_L
B+XL_S
intensity
mass
LSQSGHKR+XL_S
LSQSGHKR+XL_L
A_b_ion
A_y_ion
prec
m/z
MEQNPQSQLK+XL_S
MEQNPQSQLK+XL_L

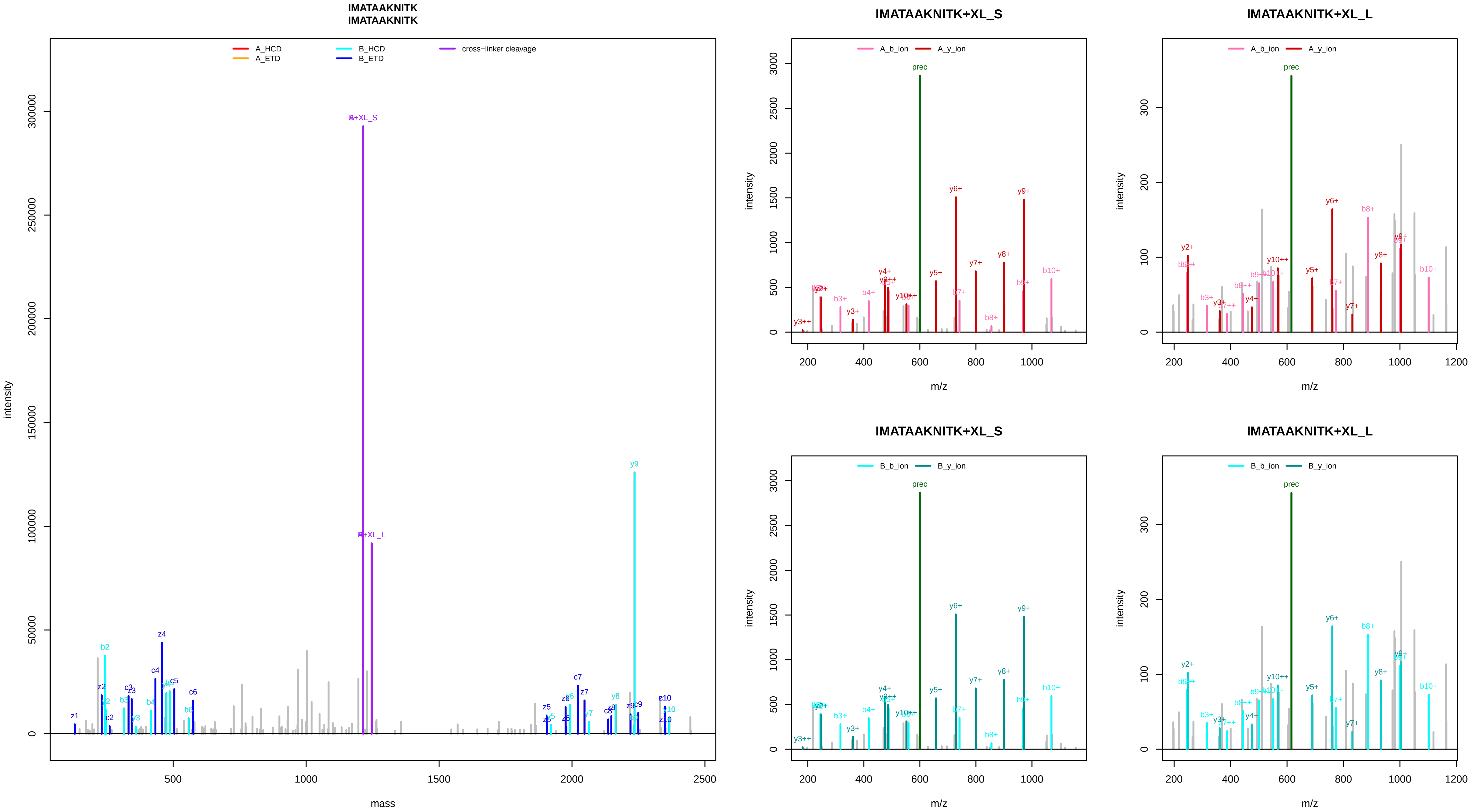
IMATAAKNITK
IMATAAKNITK
A_HCD
A_ETD
B_HCD
B_ETD
cross−linker cleavage
intensity
mass
IMATAAKNITK+XL_S
IMATAAKNITK+XL_L
A_b_ion
A_y_ion
B_b_ion
B_y_ion
prec
intensity
m/z

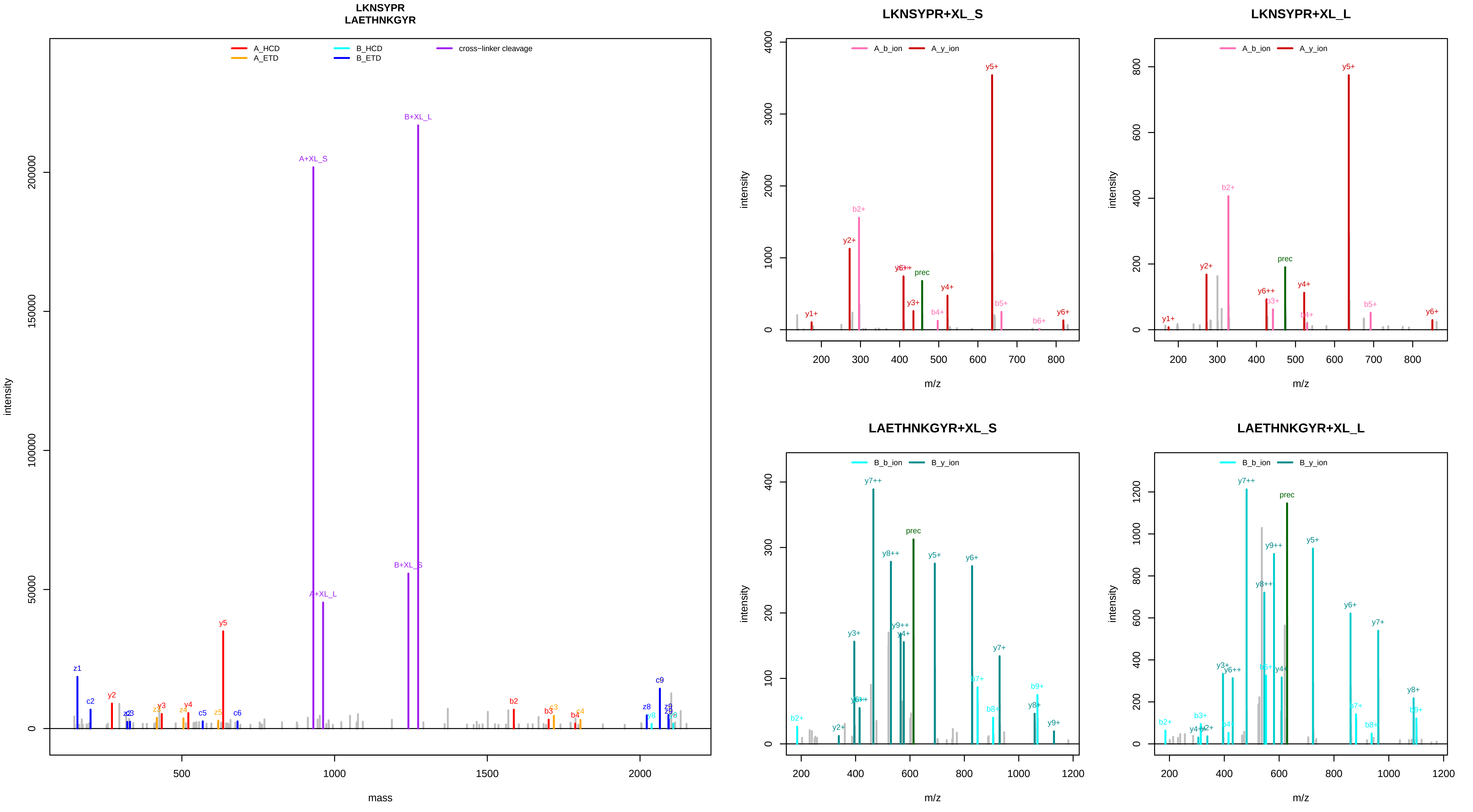

LKNSYPR
LAETHNKGYR
A_HCD
A_ETD
B_HCD
B_ETD
cross−linker cleavage
B+XL_L
A+XL_S
B+XL_S
A+XL_L
intensity
mass
LKNSYPR+XL_S
LKNSYPR+XL_L
LAETHNKGYR+XL_S
LAETHNKGYR+XL_L
A_b_ion
A_y_ion
B_b_ion
B_y_ion
prec
m/z

ASATDQLKK
AKAEAQVIIEQANK

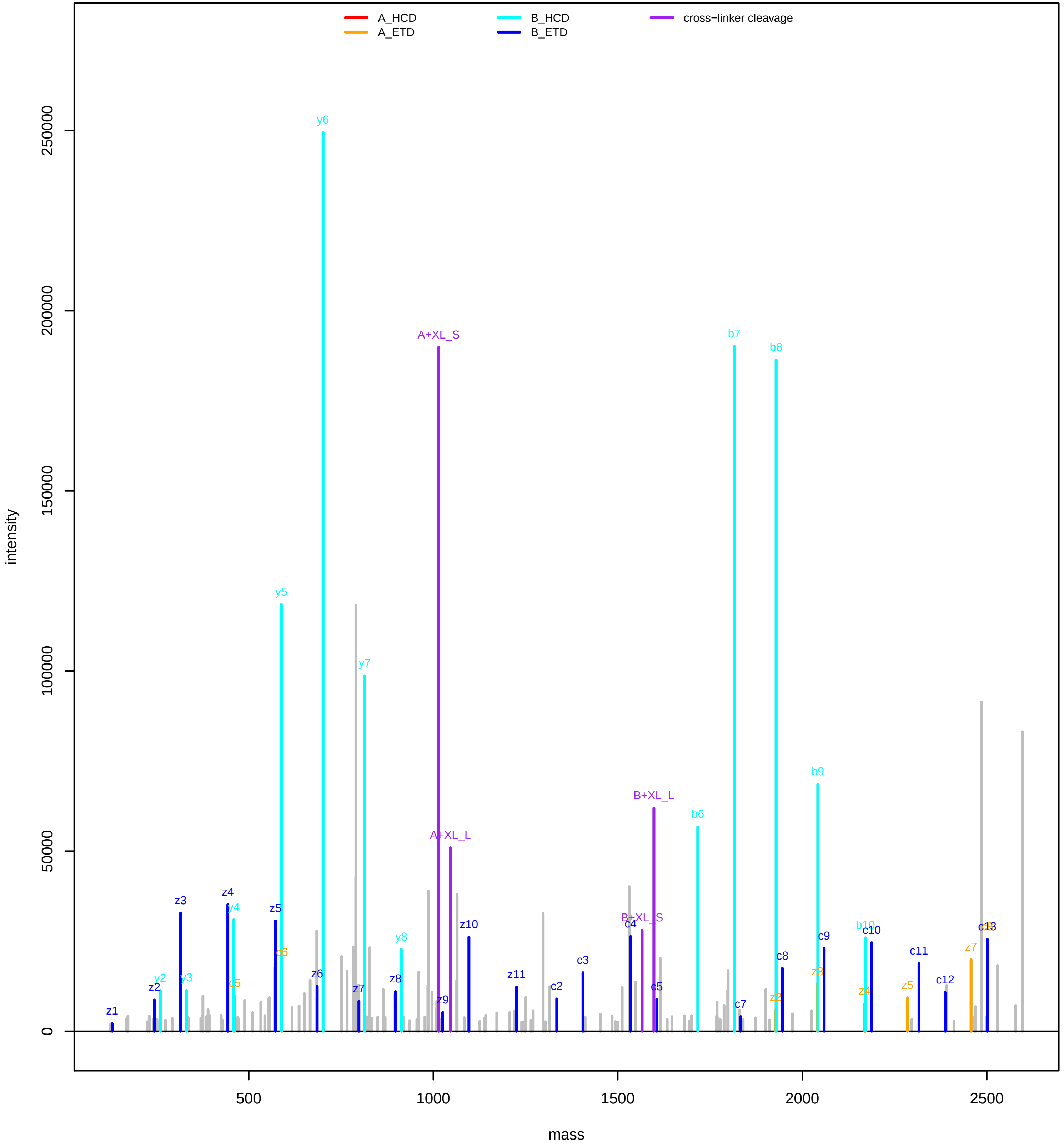

ASATDQLKK+XL_S

ASATDQLKK+XL_L

AKAEAQVIIEQANK+XL_S

AKAEAQVIIEQANK+XL_L

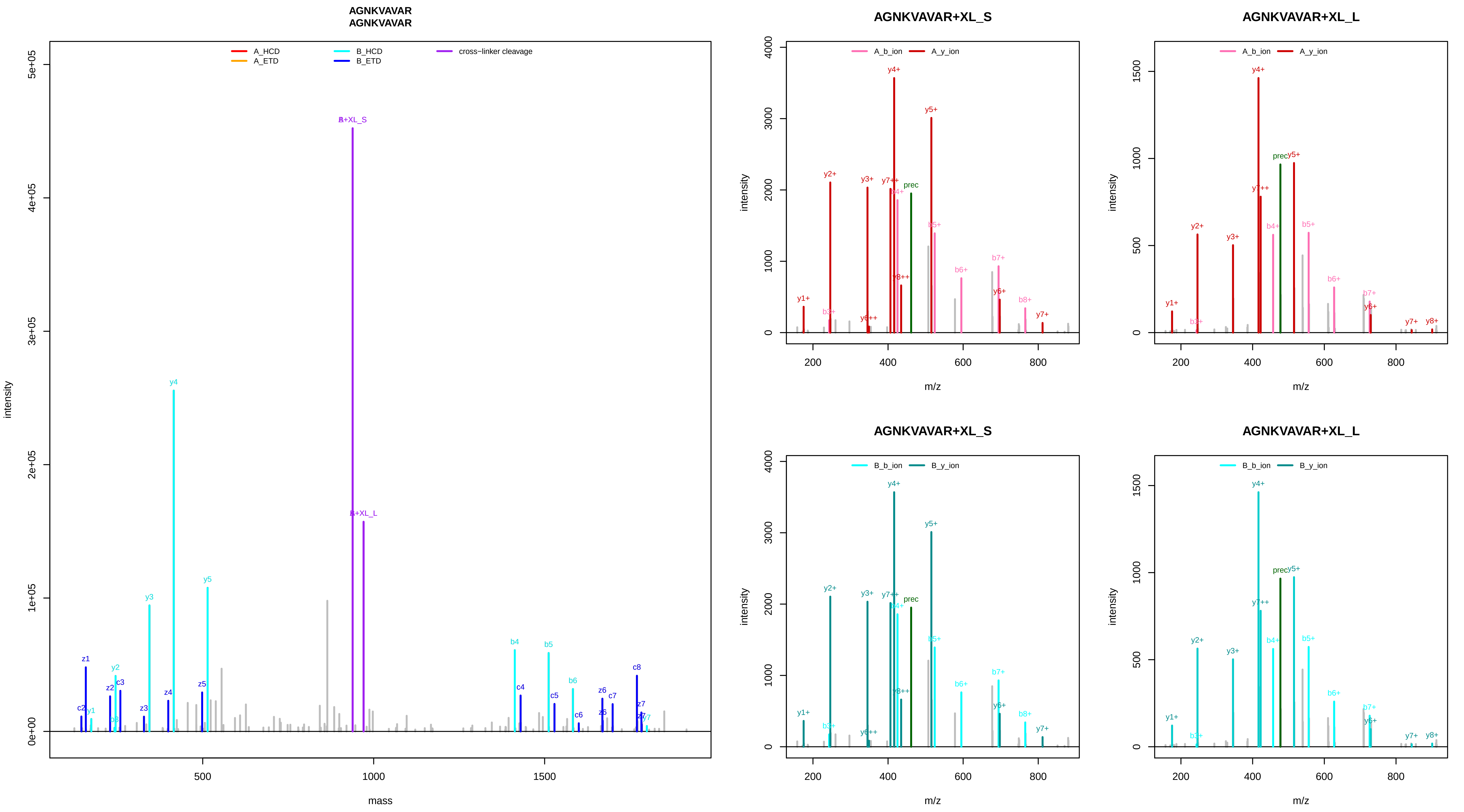
AGNKVAVAR
AGNKVAVAR
A_HCD
A_ETD
B_HCD
B_ETD
cross−linker cleavage
A+XL_S
A+XL_L
intensity
mass
0e+00
1e+05
2e+05
3e+05
4e+05
5e+05
500
1000
1500
y1
y2
y3
y4
y5
b3
b4
b5
b6
y7
z1
z2
z3
z4
z5
z6
z7
c2
c3
c4
c5
c6
c7
c8
AGNKVAVAR+XL_S
AGNKVAVAR+XL_L
A_b_ion
A_y_ion
B_b_ion
B_y_ion
prec
y1+
y2+
y3+
y4+
y5+
y6+
y7+
y8+
y6++
y7++
y8++
b3+
b4+
b5+
b6+
b7+
b8+
intensity
m/z
200
400
600
800
0
500
1000
1500
2000
3000
4000

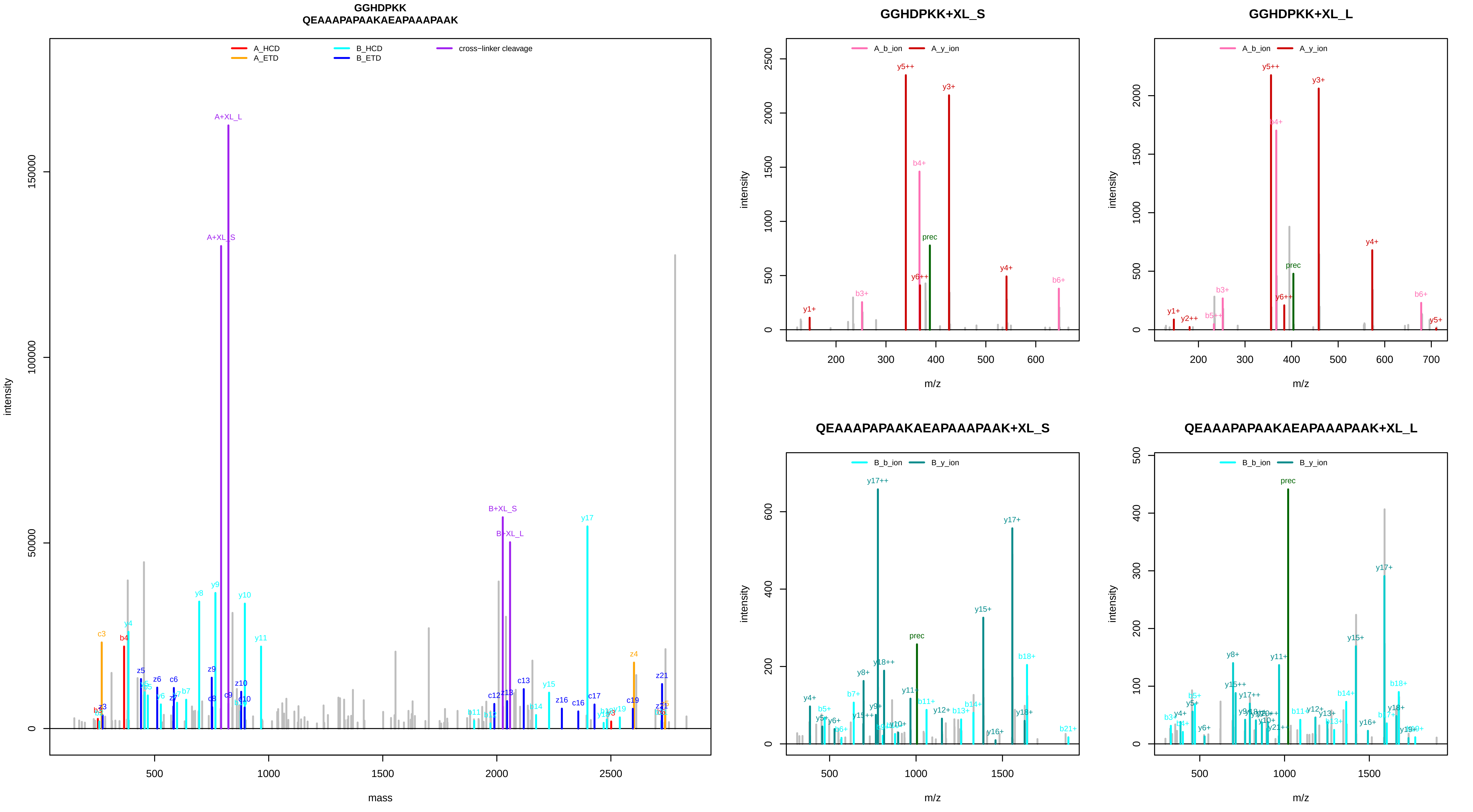

GGHDPKK
QEAAAPAPAAKAEAPAAAPAAK
A_HCD
A_ETD
B_HCD
B_ETD
cross−linker cleavage
A+XL_L
A+XL_S
B+XL_S
B+XL_L
intensity
mass
GGHDPKK+XL_S
GGHDPKK+XL_L
QEAAAPAPAAKAEAPAAAPAAK+XL_S
QEAAAPAPAAKAEAPAAAPAAK+XL_L
A_b_ion
A_y_ion
B_b_ion
B_y_ion
prec
m/z

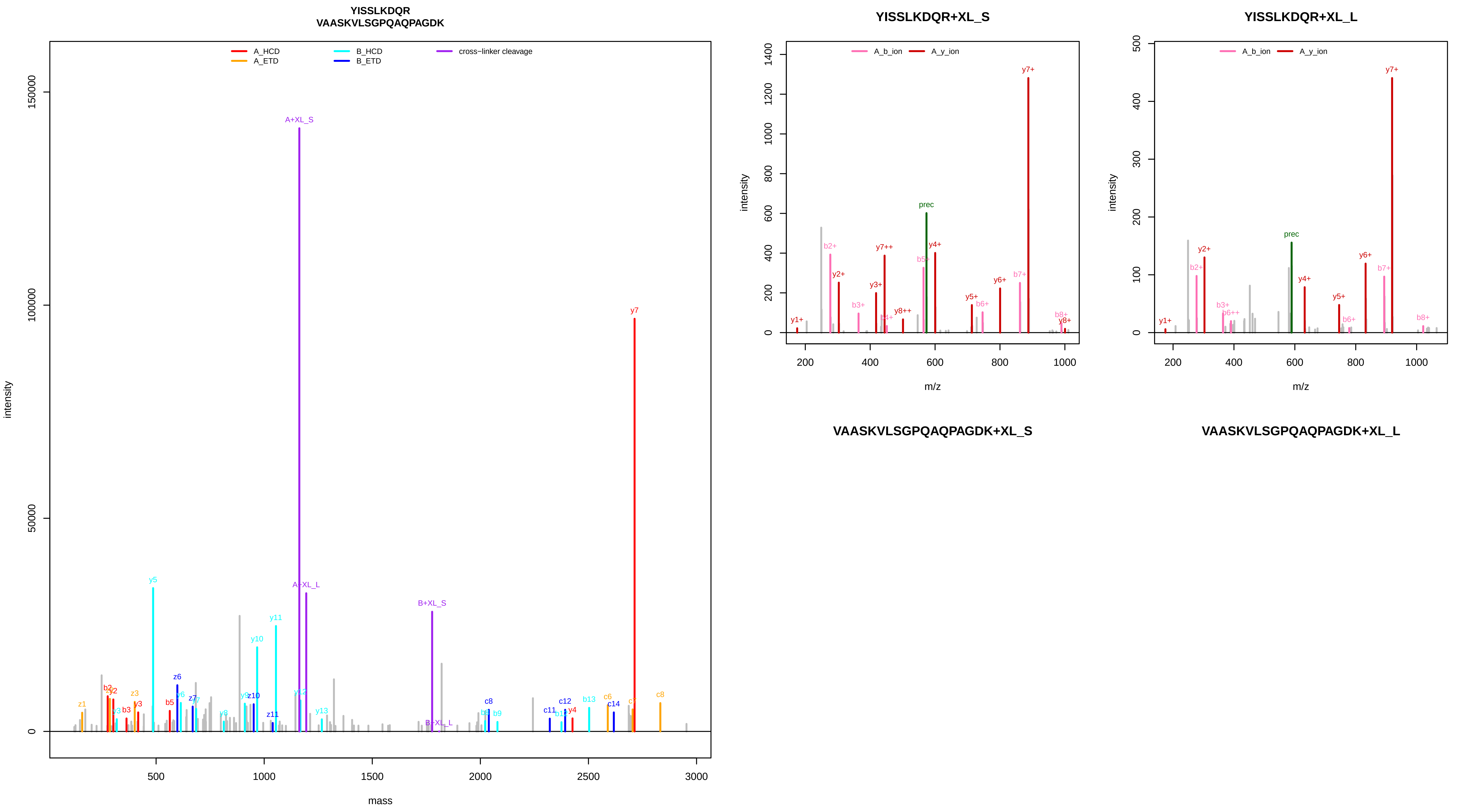
YISSLKDQR
VAASKVLSGPQAQPAGDK
A_HCD
A_ETD
B_HCD
B_ETD
cross−linker cleavage
A+XL_S
A+XL_L
B+XL_S
B+XL_L
intensity
mass
YISSLKDQR+XL_S
YISSLKDQR+XL_L
A_b_ion
A_y_ion
prec
m/z
VAASKVLSGPQAQPAGDK+XL_S
VAASKVLSGPQAQPAGDK+XL_L

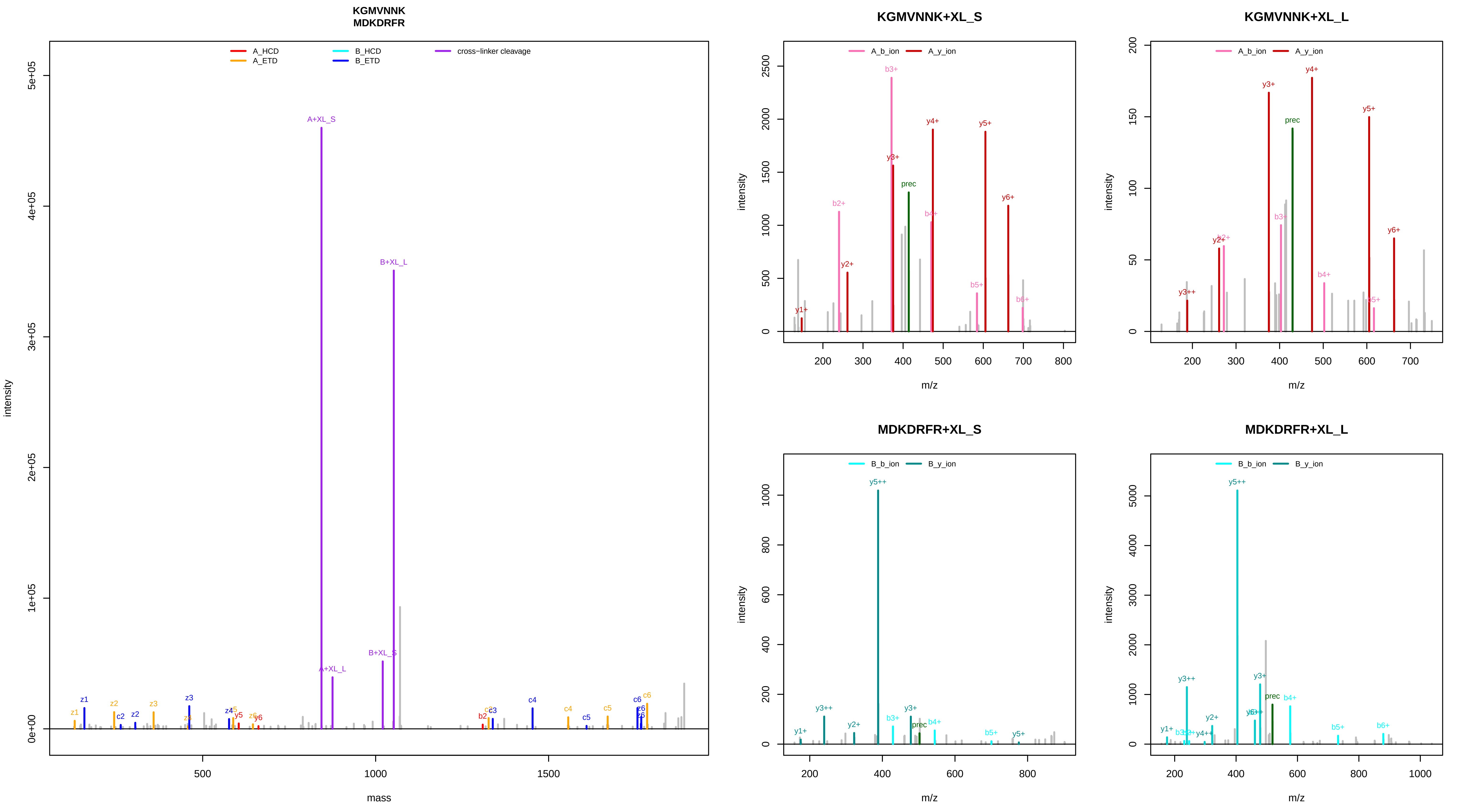
KGMVNNK
MDKDRFR
A_HCD
A_ETD
B_HCD
B_ETD
cross−linker cleavage
A+XL_S
B+XL_L
B+XL_S
A+XL_L
intensity
mass
KGMVNNK+XL_S
KGMVNNK+XL_L
A_b_ion
A_y_ion
MDKDRFR+XL_S
MDKDRFR+XL_L
B_b_ion
B_y_ion
m/z

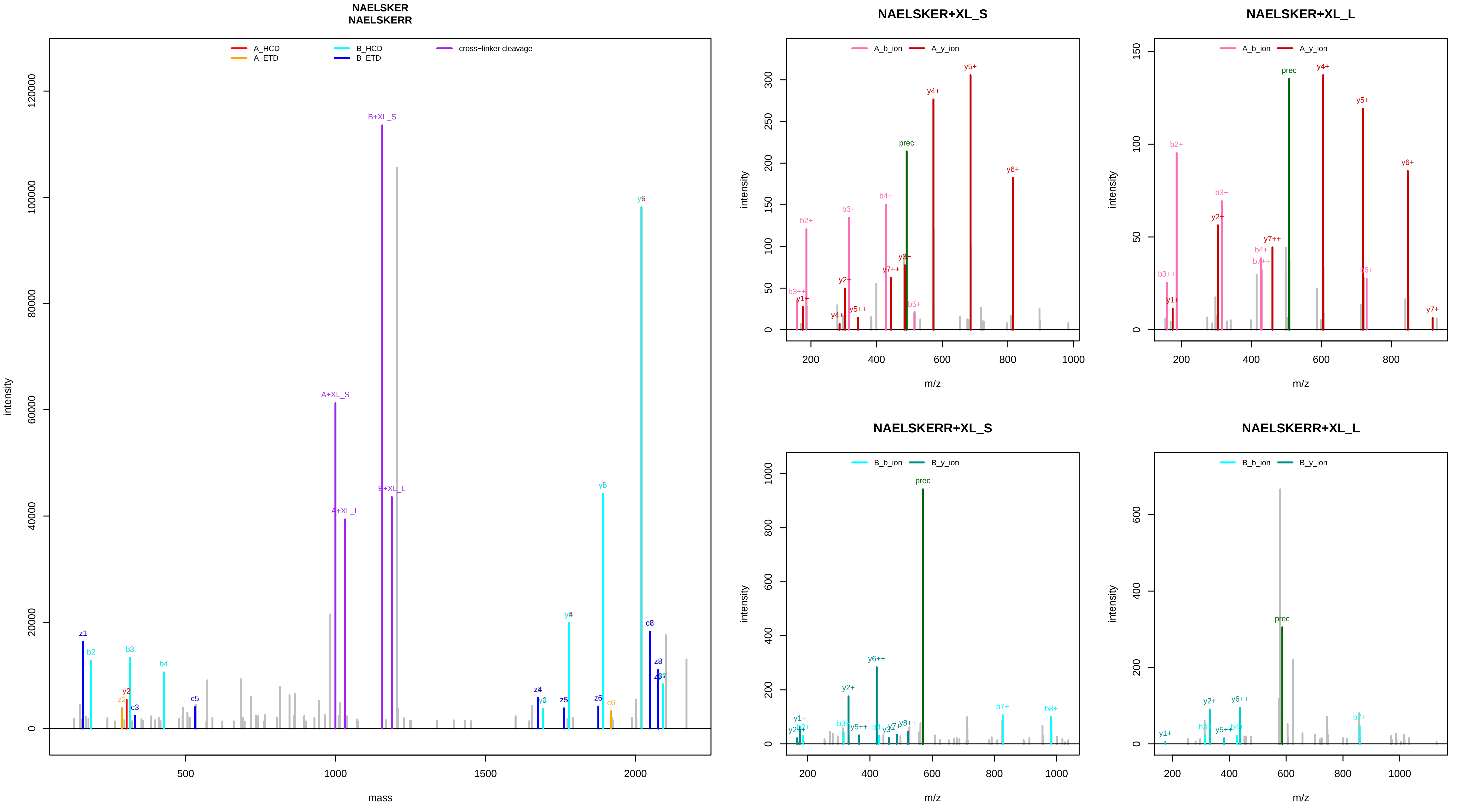
NAELSKER
NAELSKERR
A_HCD
A_ETD
B_HCD
B_ETD
cross−linker cleavage
B+XL_S
A+XL_S
B+XL_L
A+XL_L
intensity
mass
NAELSKER+XL_S
NAELSKER+XL_L
NAELSKERR+XL_S
NAELSKERR+XL_L
A_b_ion
A_y_ion
B_b_ion
B_y_ion
prec
m/z

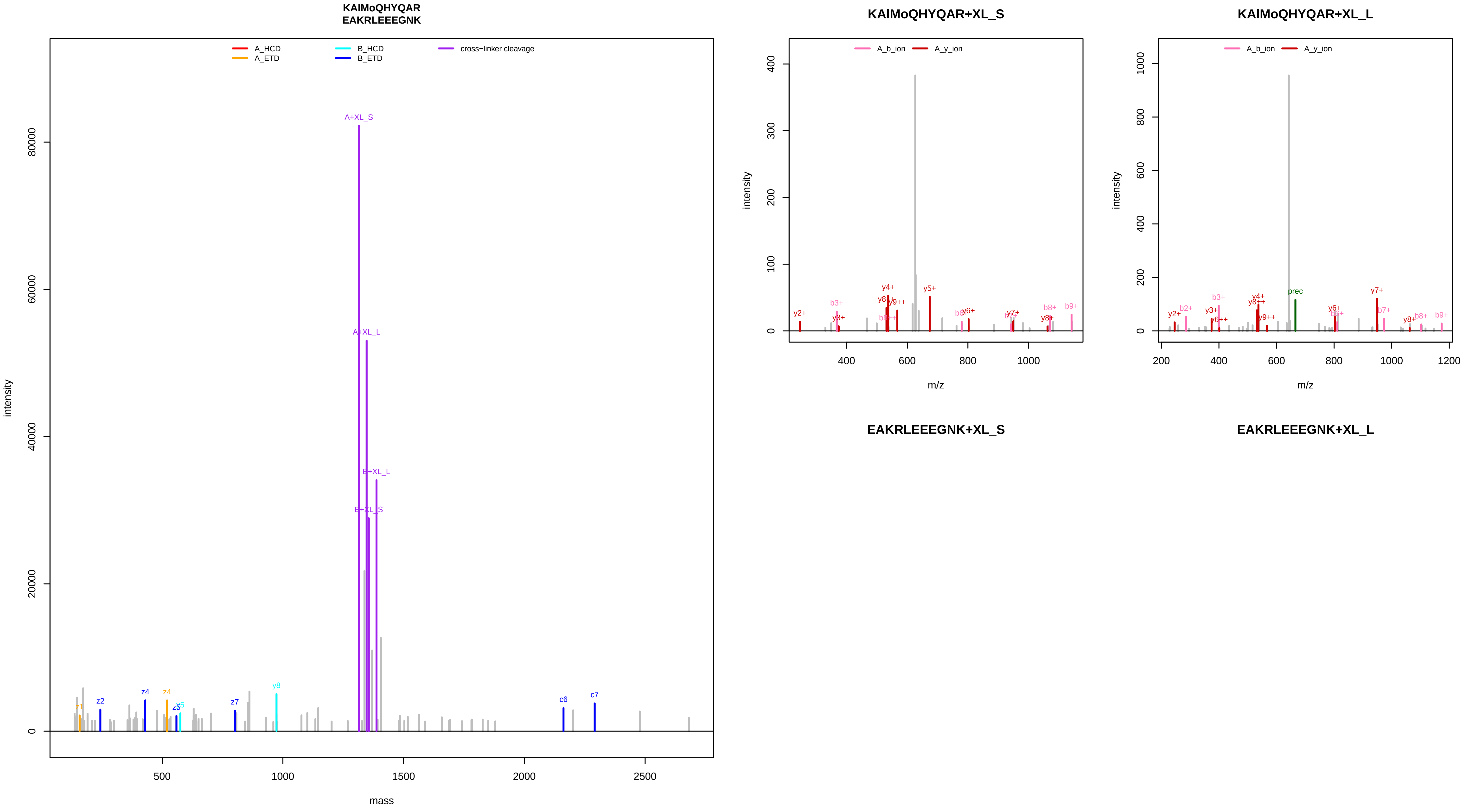

KAIMoQHYQAR
EAKRLEEEGNK
A_HCD
A_ETD
B_HCD
B_ETD
cross−linker cleavage
A+XL_S
A+XL_L
B+XL_L
B+XL_S
intensity
mass
KAIMoQHYQAR+XL_S
KAIMoQHYQAR+XL_L
A_b_ion
A_y_ion
prec
m/z
EAKRLEEEGNK+XL_S
EAKRLEEEGNK+XL_L

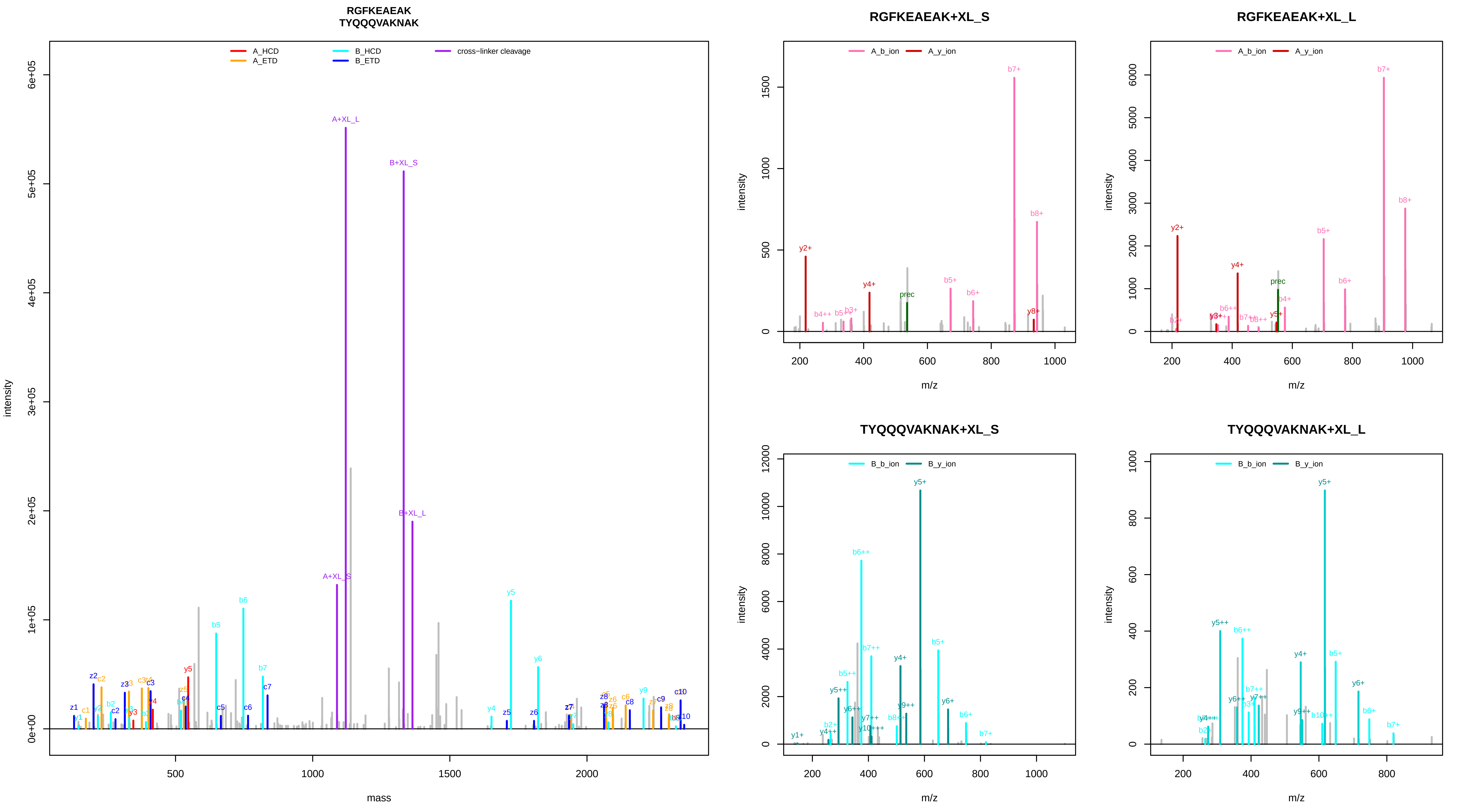

RGFKEAEAK
TYQQQVAKNAK
A_HCD
A_ETD
B_HCD
B_ETD
cross−linker cleavage
A+XL_L
B+XL_S
B+XL_L
A+XL_S
intensity
mass
RGFKEAEAK+XL_S
RGFKEAEAK+XL_L
A_b_ion
A_y_ion
TYQQQVAKNAK+XL_S
TYQQQVAKNAK+XL_L
B_b_ion
B_y_ion
m/z

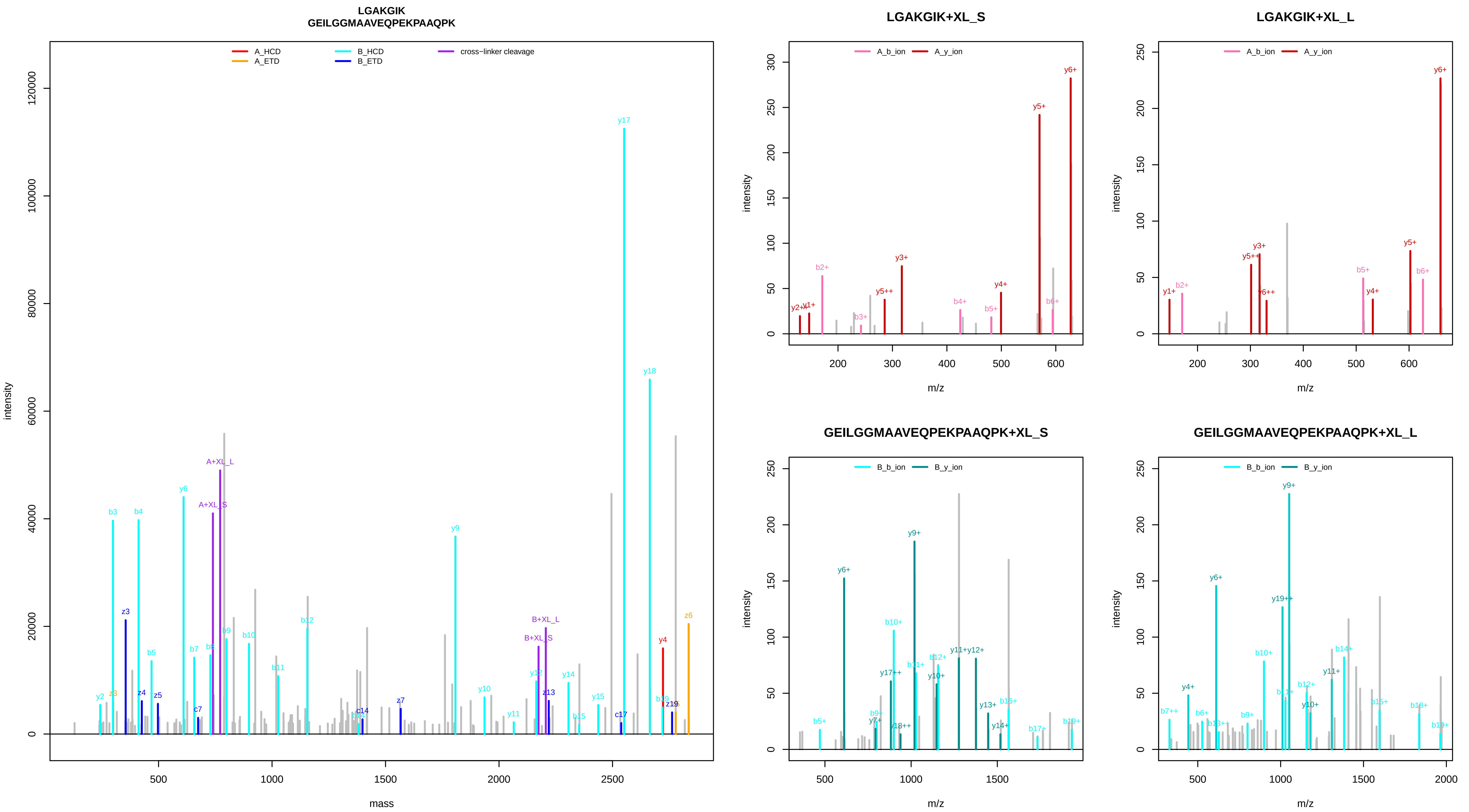

LGAKGIK
GEILGGMAAVEQPEKPAAQPK
A_HCD
A_ETD
B_HCD
B_ETD
cross−linker cleavage
intensity
mass
A+XL_L
A+XL_S
B+XL_L
B+XL_S
LGAKGIK+XL_S
LGAKGIK+XL_L
A_b_ion
A_y_ion
m/z
GEILGGMAAVEQPEKPAAQPK+XL_S
GEILGGMAAVEQPEKPAAQPK+XL_L
B_b_ion
B_y_ion

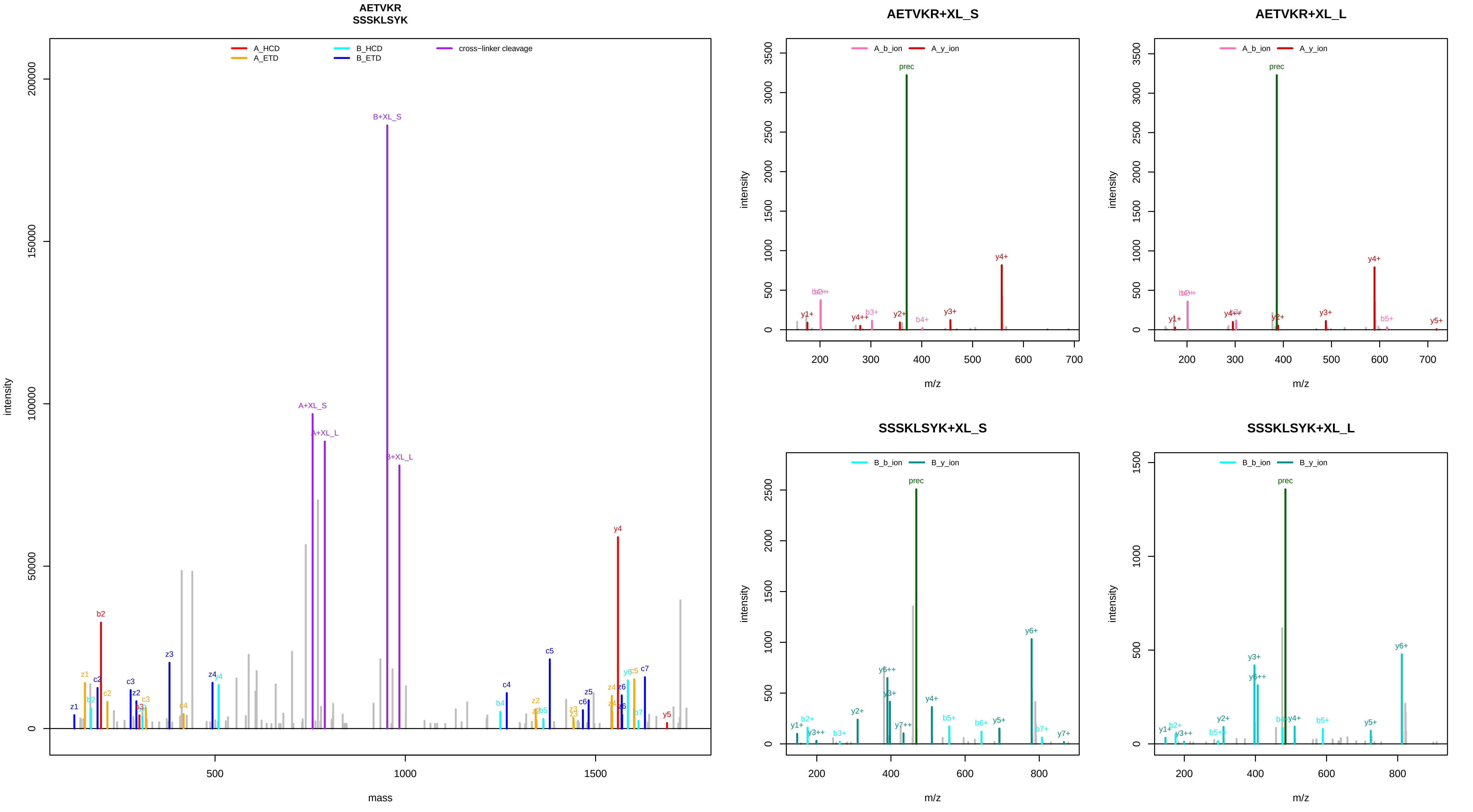

AETVKR
SSSKLSYK
A_HCD
A_ETD
B_HCD
B_ETD
cross−linker cleavage
B+XL_S
A+XL_S
A+XL_L
B+XL_L
intensity
mass
AETVKR+XL_S
AETVKR+XL_L
SSSKLSYK+XL_S
SSSKLSYK+XL_L
A_b_ion
A_y_ion
B_b_ion
B_y_ion
prec
m/z

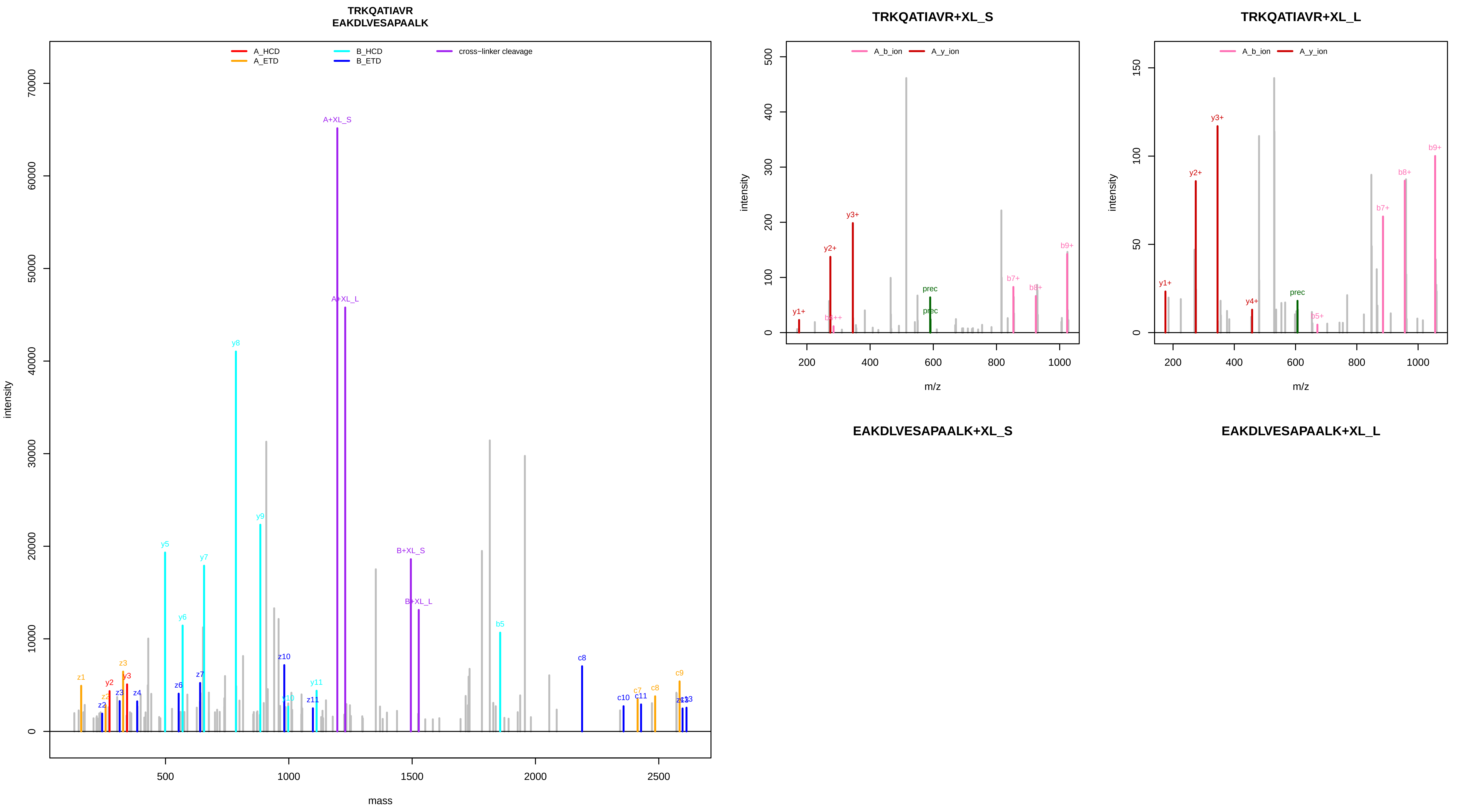
TRKQATIAVR
EAKDLVESAPAALK
A_HCD
A_ETD
B_HCD
B_ETD
cross−linker cleavage
A+XL_S
A+XL_L
B+XL_S
B+XL_L
intensity
mass
TRKQATIAVR+XL_S
TRKQATIAVR+XL_L
A_b_ion
A_y_ion
prec
m/z
EAKDLVESAPAALK+XL_S
EAKDLVESAPAALK+XL_L

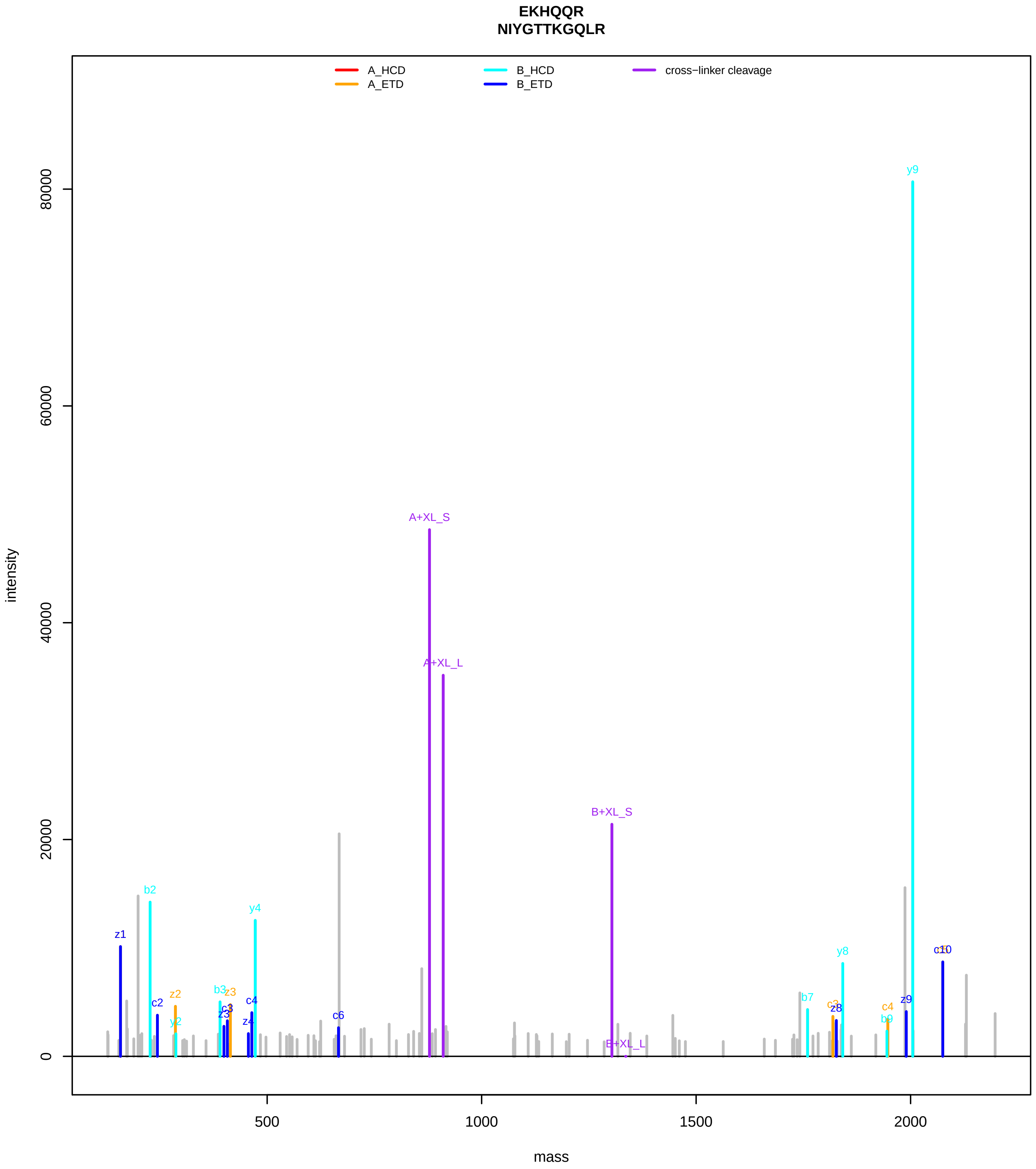

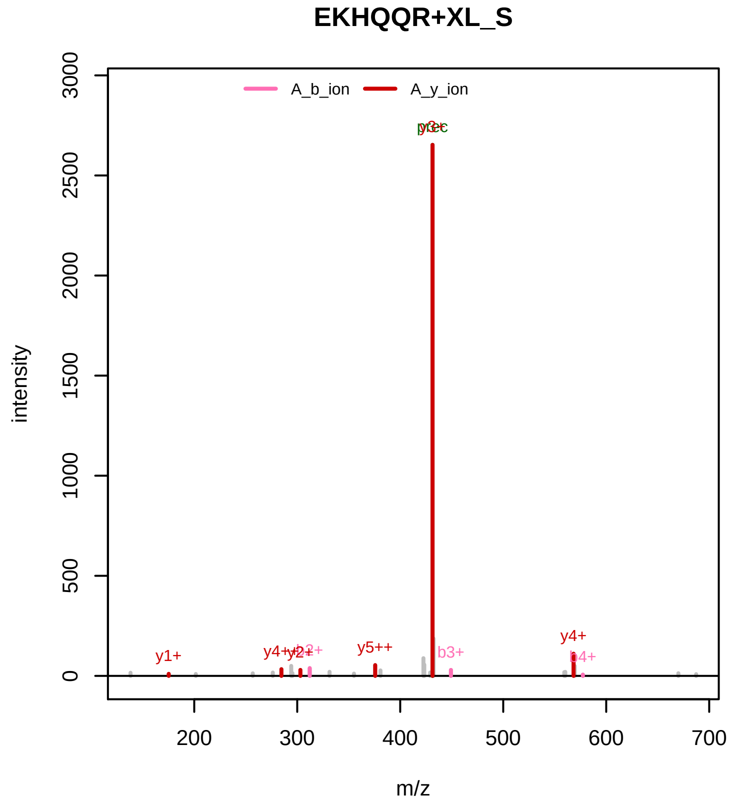

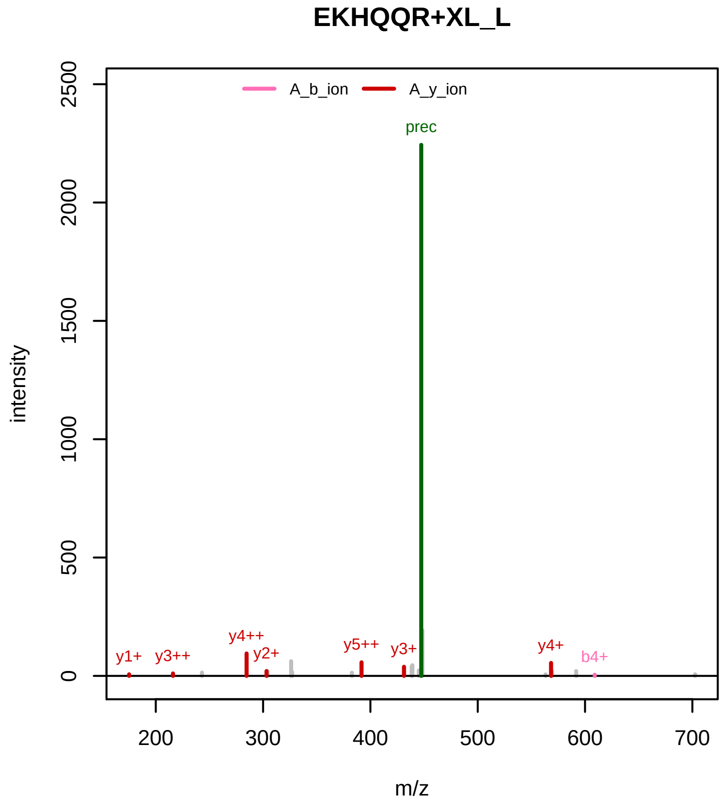

NIYGTTKGQLR+XL_S

NIYGTTKGQLR+XL_L

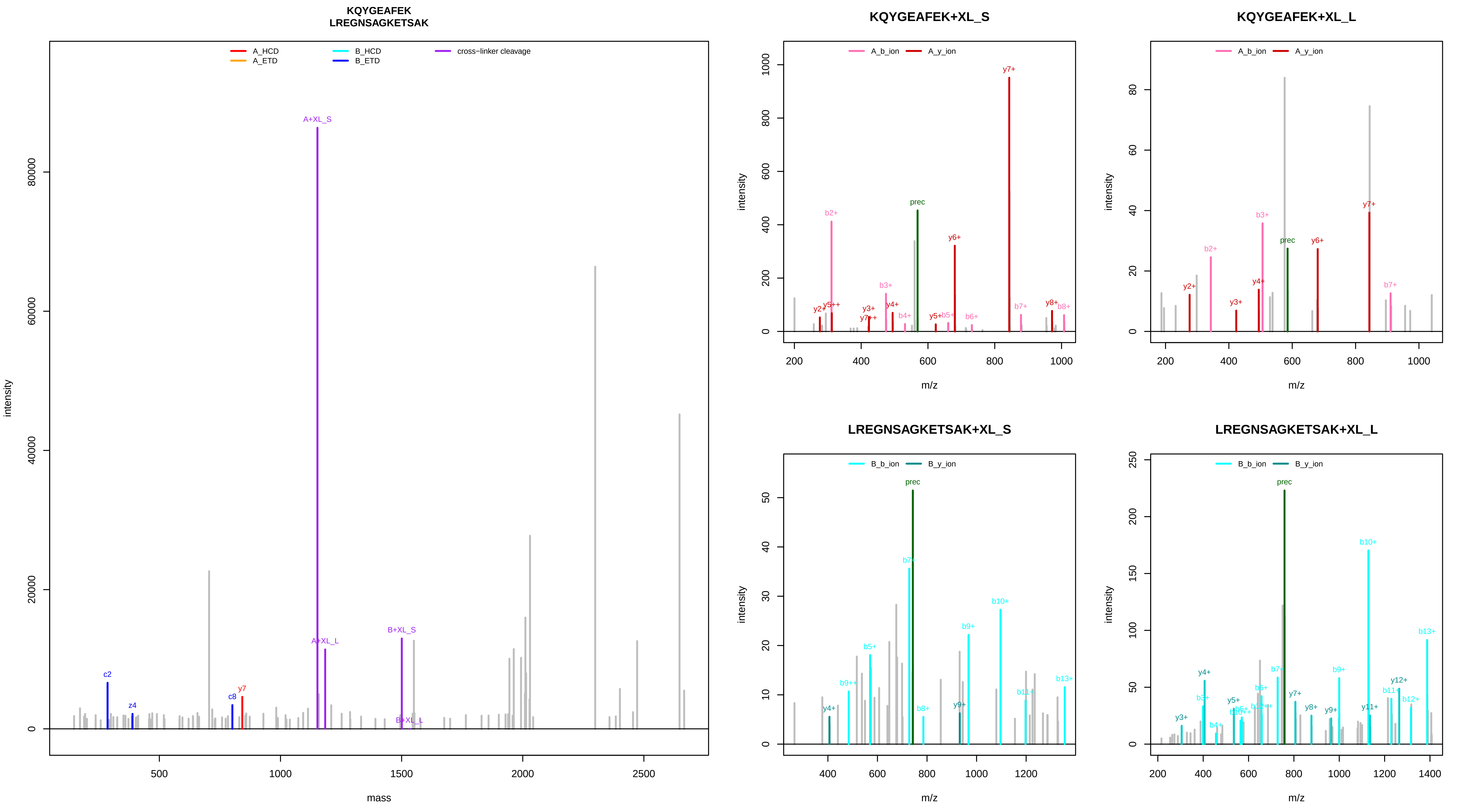
KQYGEAFEK
LREGNSAGKETSAK
A_HCD
A_ETD
B_HCD
B_ETD
cross−linker cleavage
A+XL_S
A+XL_L
B+XL_S
B+XL_L
c2
z4
c8
y7
intensity
mass
KQYGEAFEK+XL_S
KQYGEAFEK+XL_L
LREGNSAGKETSAK+XL_S
LREGNSAGKETSAK+XL_L
A_b_ion
A_y_ion
B_b_ion
B_y_ion
prec
m/z

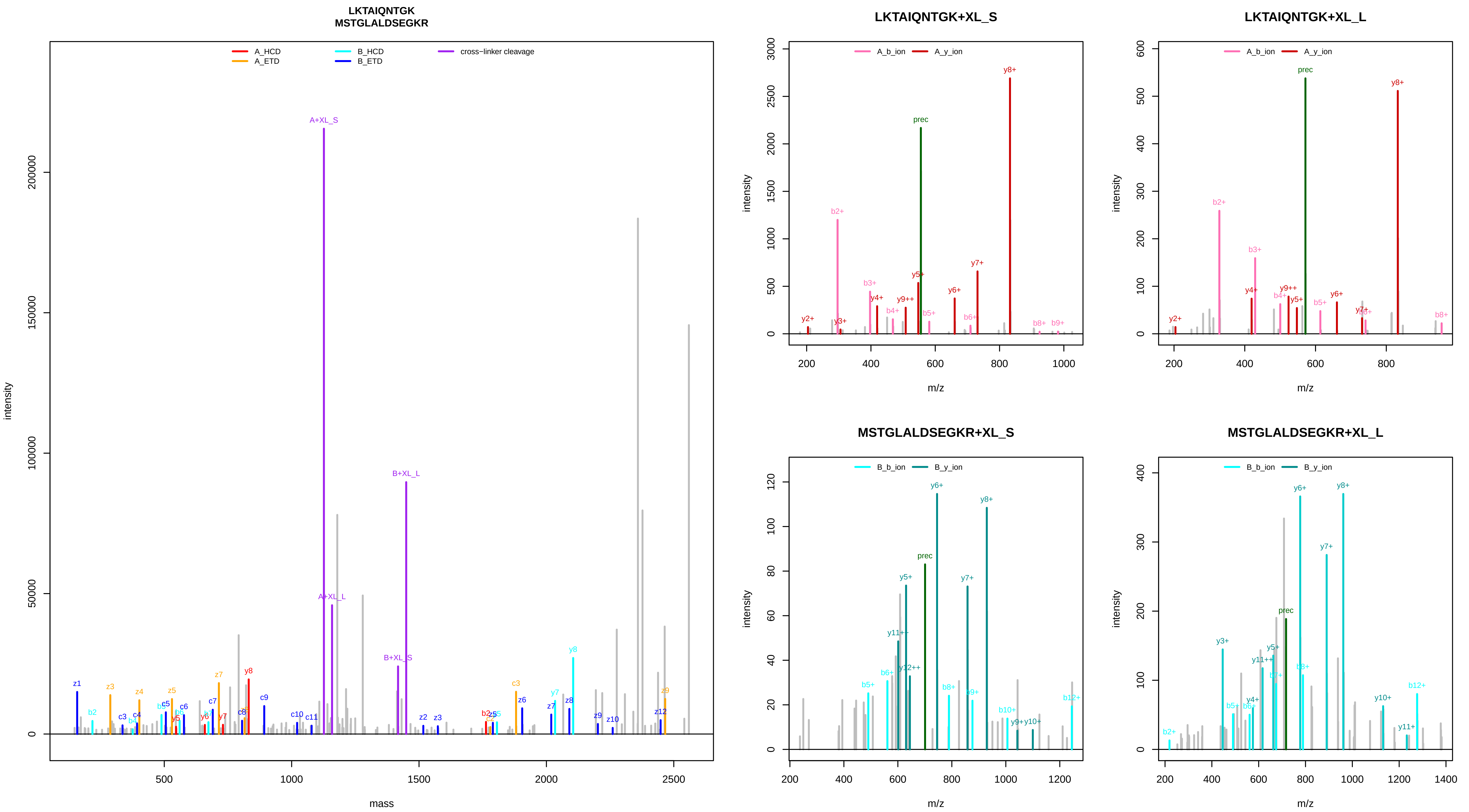
LKTAIQNTGK
MSTGLALDSEGKR
A_HCD
A_ETD
B_HCD
B_ETD
cross−linker cleavage
A+XL_S
A+XL_L
B+XL_L
B+XL_S
intensity
mass
LKTAIQNTGK+XL_S
LKTAIQNTGK+XL_L
A_b_ion
A_y_ion
prec
y8+
b2+
m/z
MSTGLALDSEGKR+XL_S
MSTGLALDSEGKR+XL_L
B_b_ion
B_y_ion
y6+
y8+

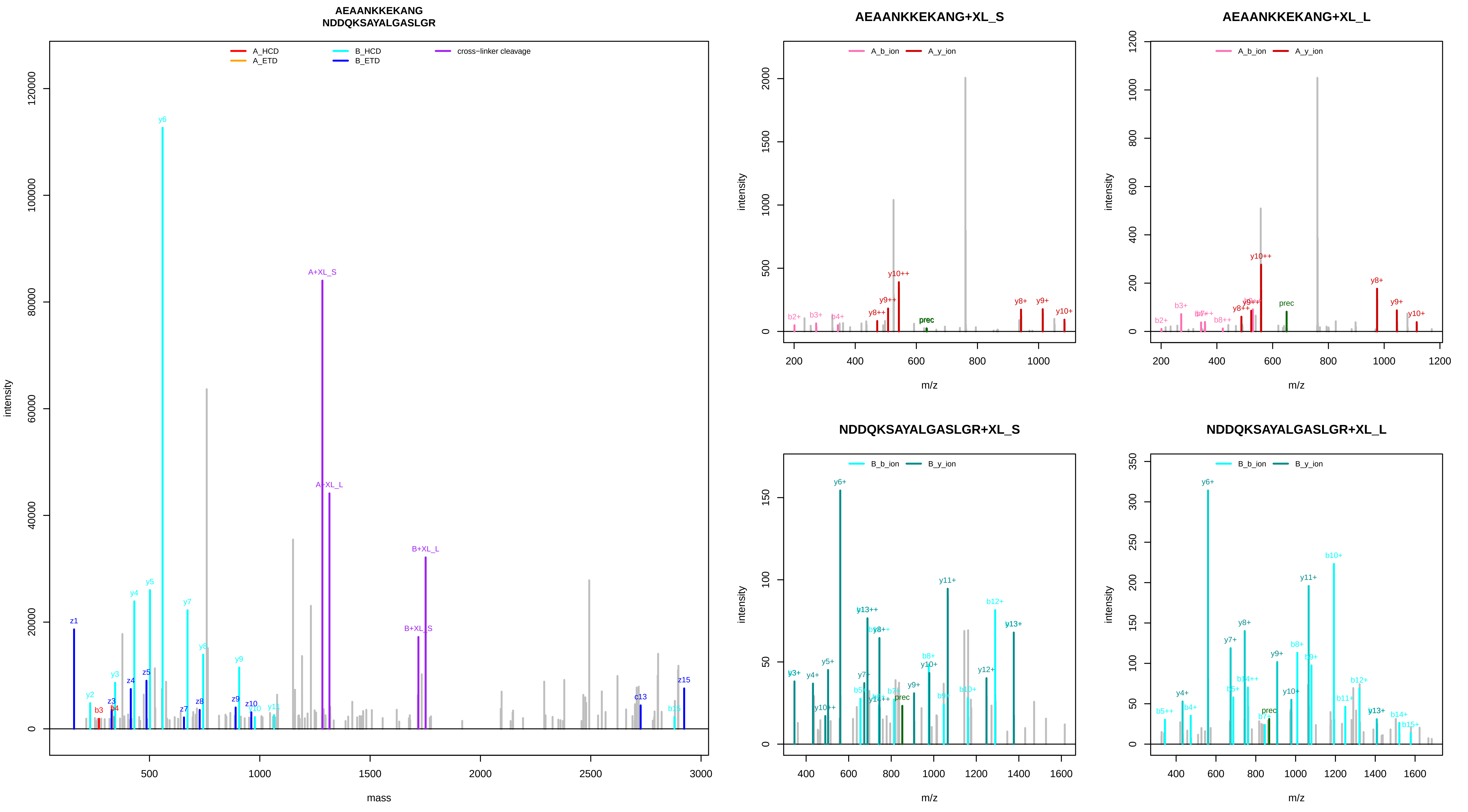
AEAANKKEKANG
NDDQKSAYALGASLGR
A_HCD
A_ETD
B_HCD
B_ETD
cross−linker cleavage
intensity
mass
AEAANKKEKANG+XL_S
AEAANKKEKANG+XL_L
A_b_ion
A_y_ion
NDDQKSAYALGASLGR+XL_S
NDDQKSAYALGASLGR+XL_L
B_b_ion
B_y_ion
m/z

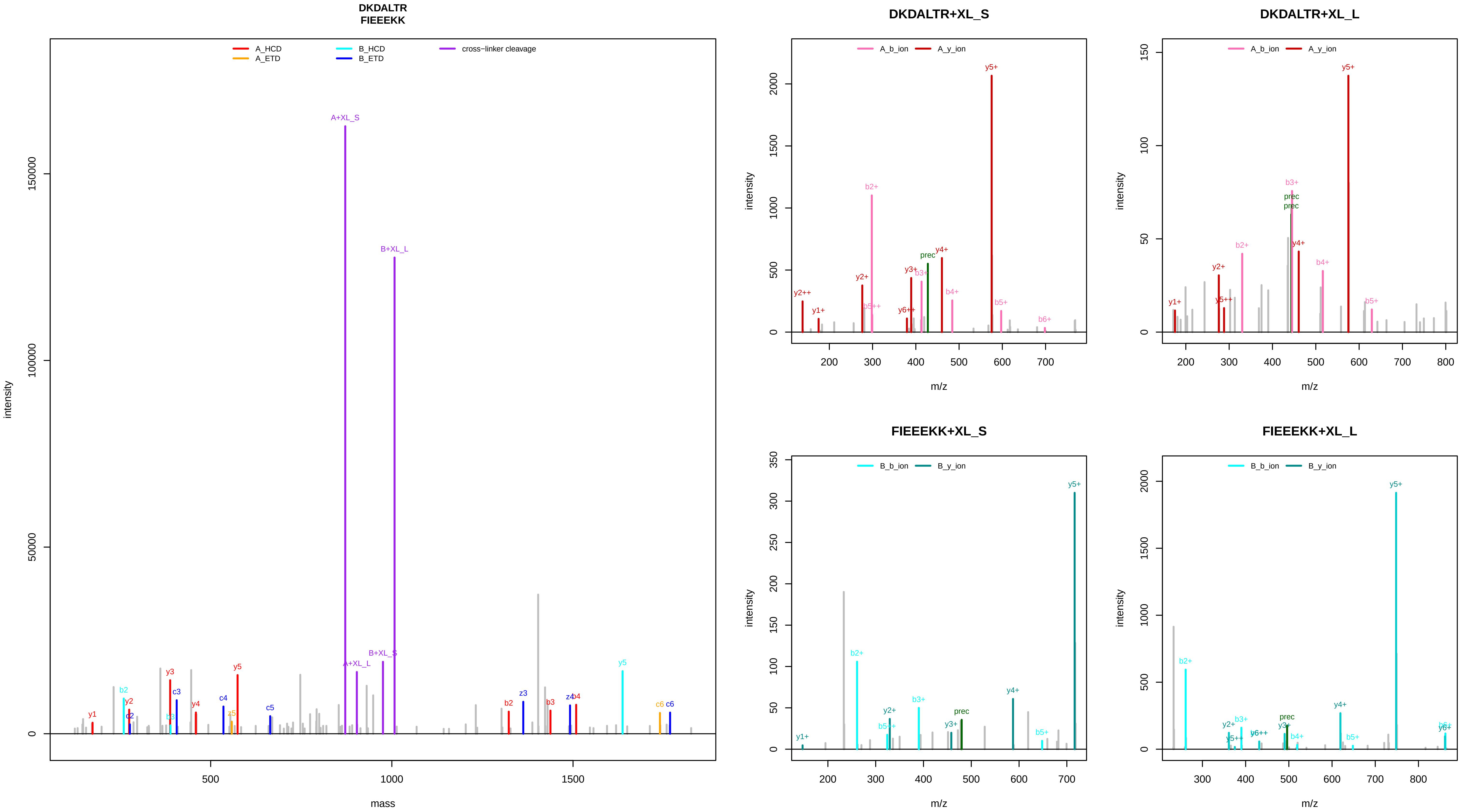
DKDALTR
FIEEEKK
A_HCD
A_ETD
B_HCD
B_ETD
cross−linker cleavage
A+XL_S
B+XL_L
A+XL_L
B+XL_S
intensity
mass
DKDALTR+XL_S
DKDALTR+XL_L
FIEEEKK+XL_S
FIEEEKK+XL_L
A_b_ion
A_y_ion
B_b_ion
B_y_ion
prec
m/z

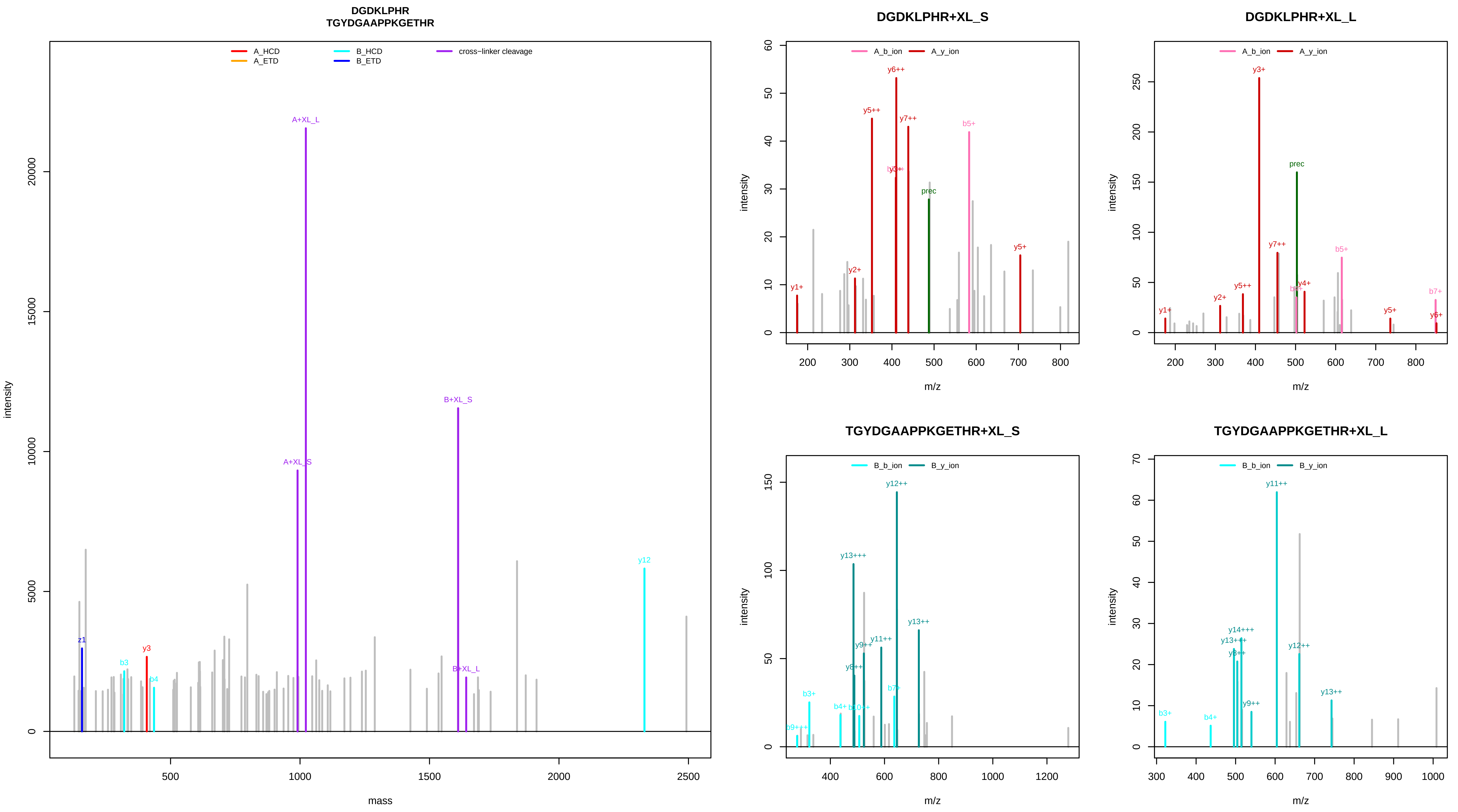
DGDKLPHR
TGYDGAAPPKGETHR
A_HCD
A_ETD
B_HCD
B_ETD
cross−linker cleavage
A+XL_L
A+XL_S
B+XL_S
B+XL_L
y12
z1
y3
b3
b4
intensity
mass
DGDKLPHR+XL_S
DGDKLPHR+XL_L
TGYDGAAPPKGETHR+XL_S
TGYDGAAPPKGETHR+XL_L
A_b_ion
A_y_ion
B_b_ion
B_y_ion
prec
m/z

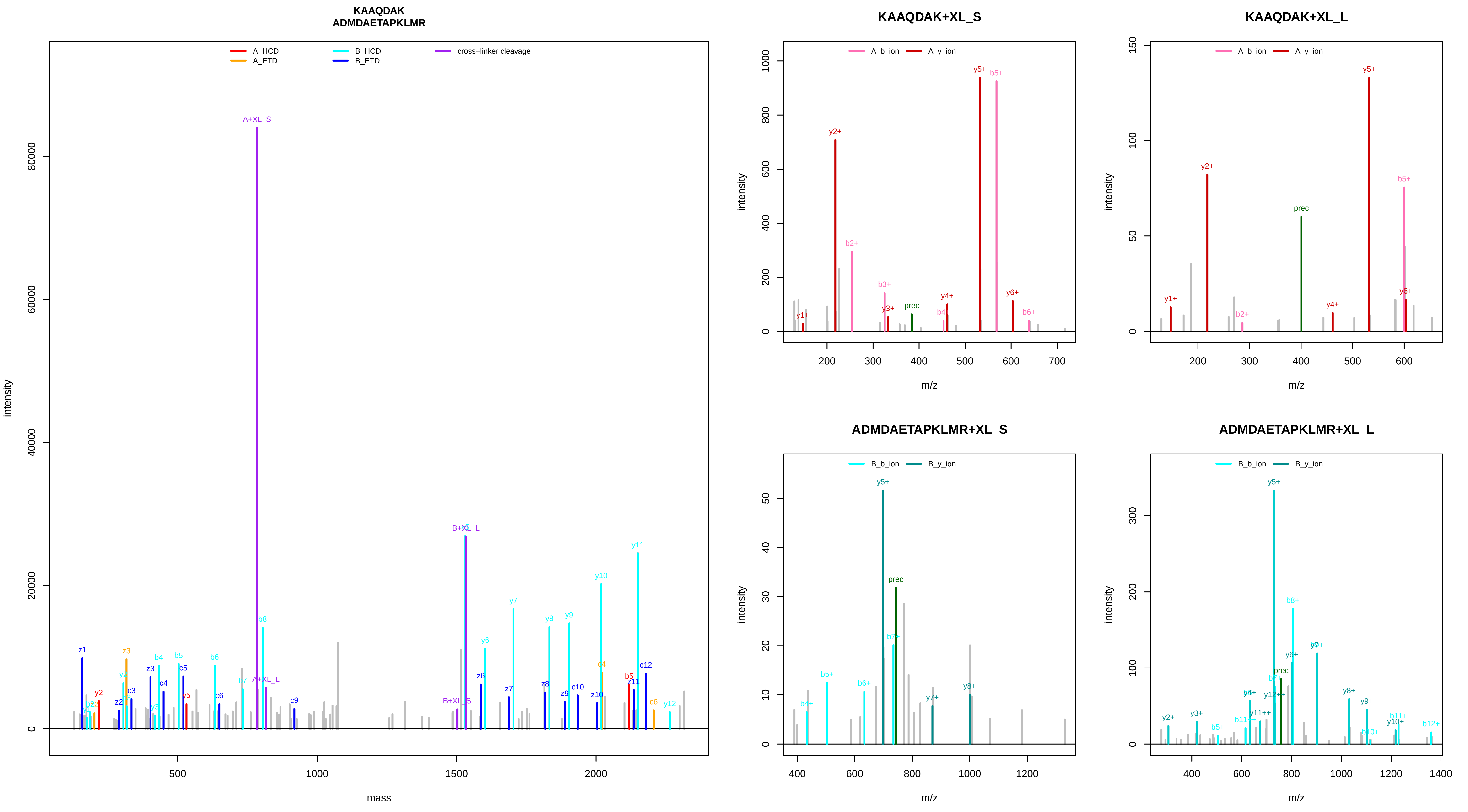

KAAQDAK
ADMDAETAPKLMR
A_HCD
A_ETD
B_HCD
B_ETD
cross−linker cleavage
A+XL_S
B+XL_L
A+XL_L
B+XL_S
intensity
mass
KAAQDAK+XL_S
KAAQDAK+XL_L
A_b_ion
A_y_ion
ADMDAETAPKLMR+XL_S
ADMDAETAPKLMR+XL_L
B_b_ion
B_y_ion
prec
m/z

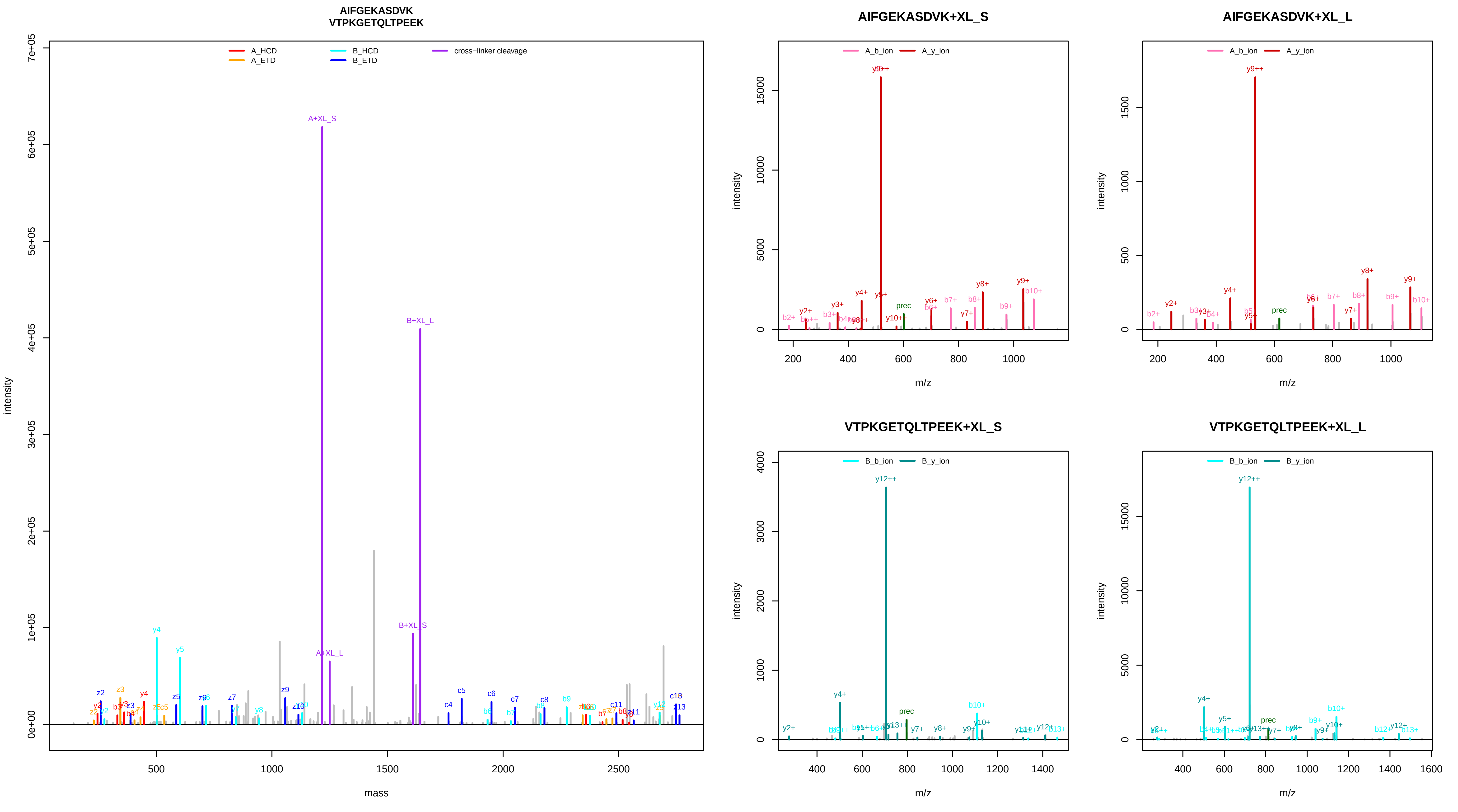
AIFGEKASDVK
VTPKGETQLTPEEK
A_HCD
A_ETD
B_HCD
B_ETD
cross−linker cleavage
A+XL_S
B+XL_L
B+XL_S
A+XL_L
intensity
mass
0e+00
1e+05
2e+05
3e+05
4e+05
5e+05
6e+05
7e+05
500
1000
1500
2000
2500
AIFGEKASDVK+XL_S
AIFGEKASDVK+XL_L
VTPKGETQLTPEEK+XL_S
VTPKGETQLTPEEK+XL_L
A_b_ion
A_y_ion
B_b_ion
B_y_ion
prec
y9++
y12++
m/z
intensity

TDVTIK
SADDSVSAQEKQTLIDHLNQK

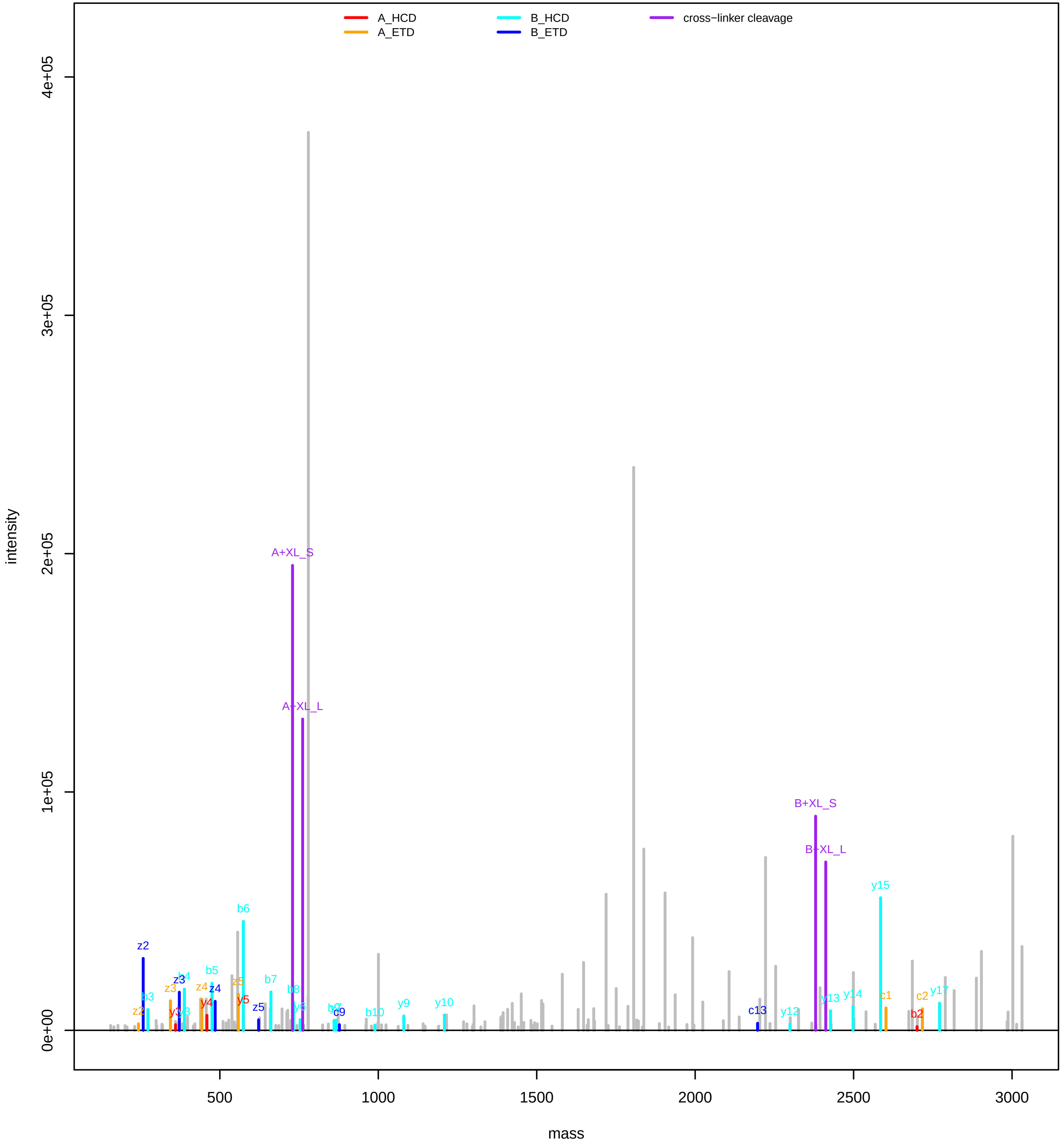

TDVTIK+XL_S

TDVTIK+XL_L

SADDSVSAQEKQTLIDHLNQK+XL_S

SADDSVSAQEKQTLIDHLNQK+XL_L

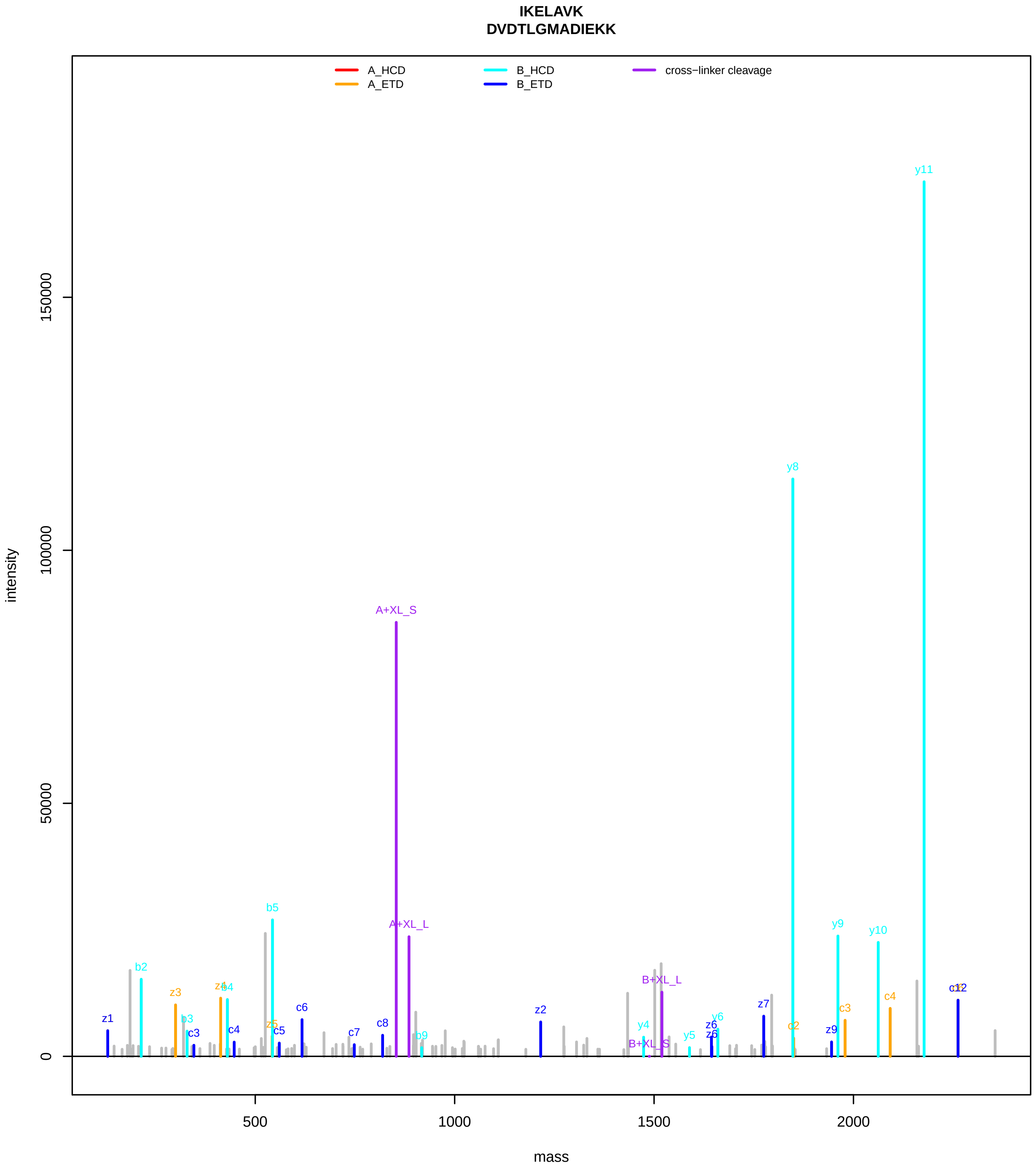

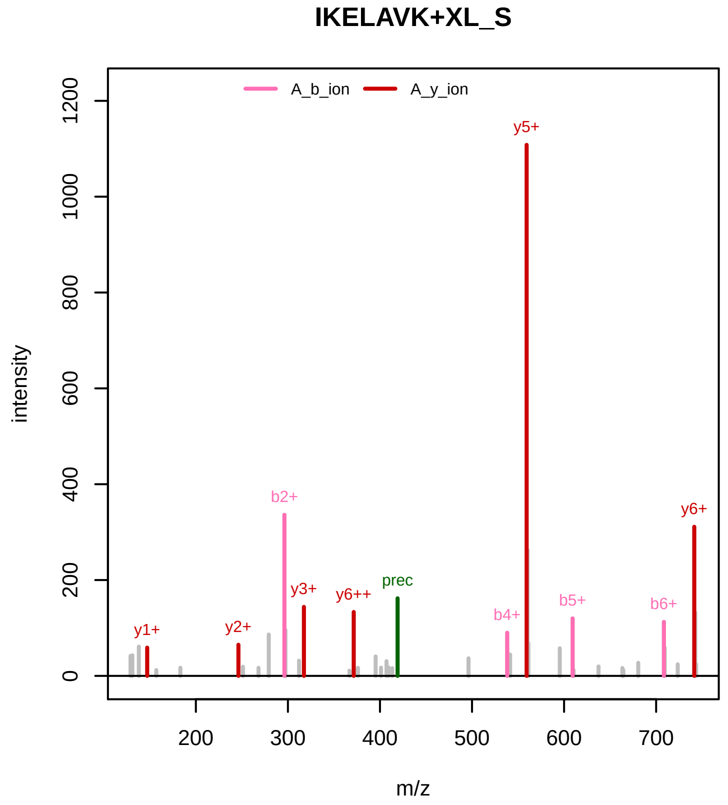

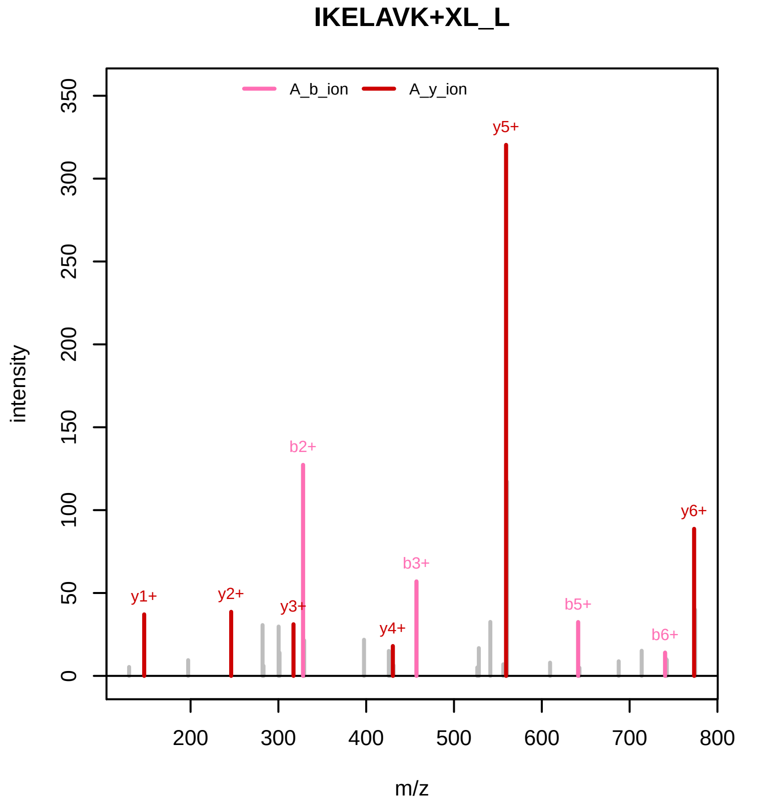

DVDTLGMADIEKK+XL_S

DVDTLGMADIEKK+XL_L

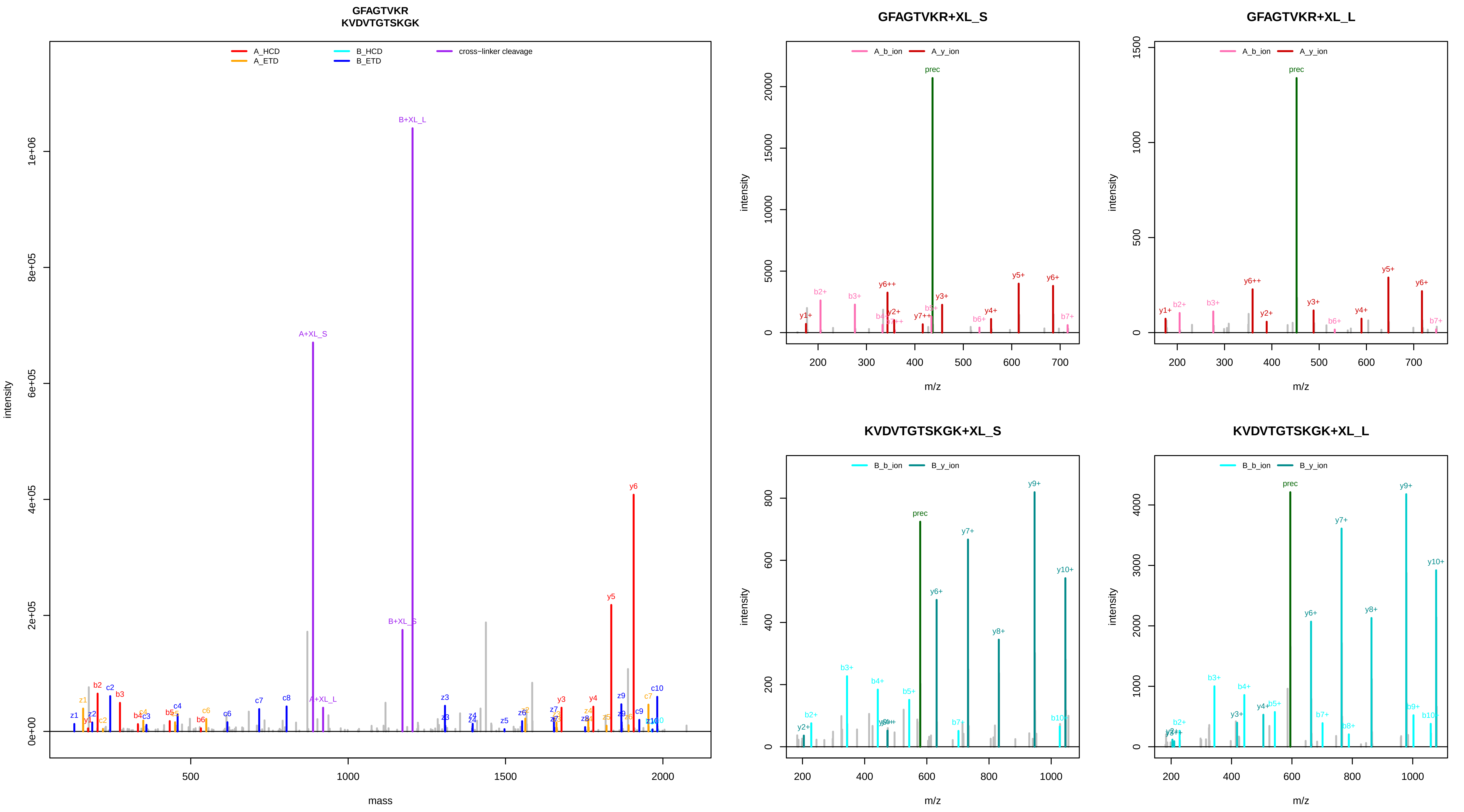
GFAGTVKR
KVDVTGTSKGK
A_HCD
A_ETD
B_HCD
B_ETD
cross−linker cleavage
intensity
mass
GFAGTVKR+XL_S
GFAGTVKR+XL_L
KVDVTGTSKGK+XL_S
KVDVTGTSKGK+XL_L
A_b_ion
A_y_ion
B_b_ion
B_y_ion
m/z

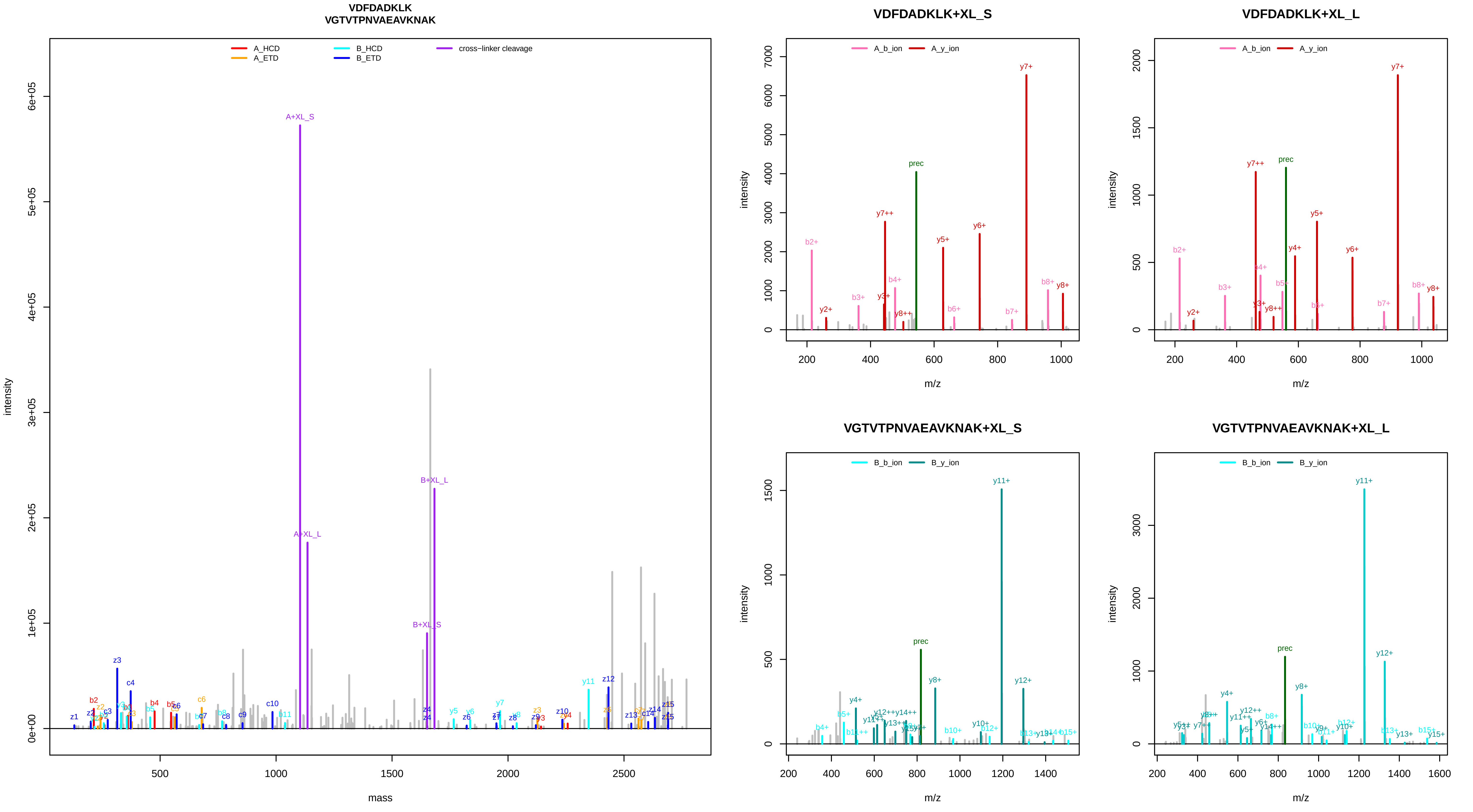
VDFDADKLK
VGTVTPNVAEAVKNAK
A_HCD
A_ETD
B_HCD
B_ETD
cross−linker cleavage
A+XL_S
A+XL_L
B+XL_L
B+XL_S
intensity
mass
VDFDADKLK+XL_S
VDFDADKLK+XL_L
VGTVTPNVAEAVKNAK+XL_S
VGTVTPNVAEAVKNAK+XL_L
A_b_ion
A_y_ion
B_b_ion
B_y_ion
prec
m/z

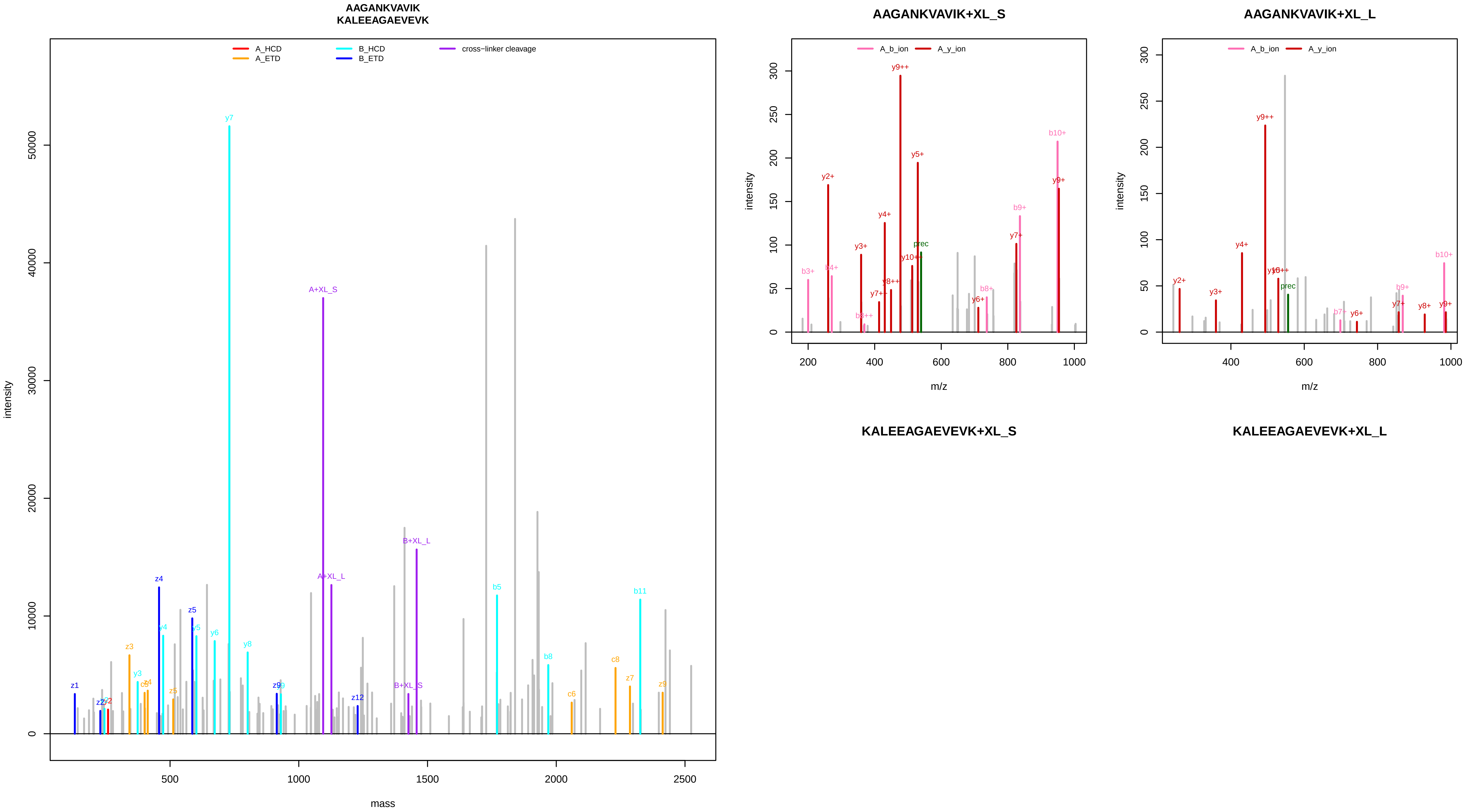
AAGANKVAVIK
KALEEAGAEVEVK
A_HCD
A_ETD
B_HCD
B_ETD
cross−linker cleavage
intensity
mass
A+XL_S
A+XL_L
B+XL_L
B+XL_S
AAGANKVAVIK+XL_S
AAGANKVAVIK+XL_L
A_b_ion
A_y_ion
intensity
m/z
prec
KALEEAGAEVEVK+XL_S
KALEEAGAEVEVK+XL_L

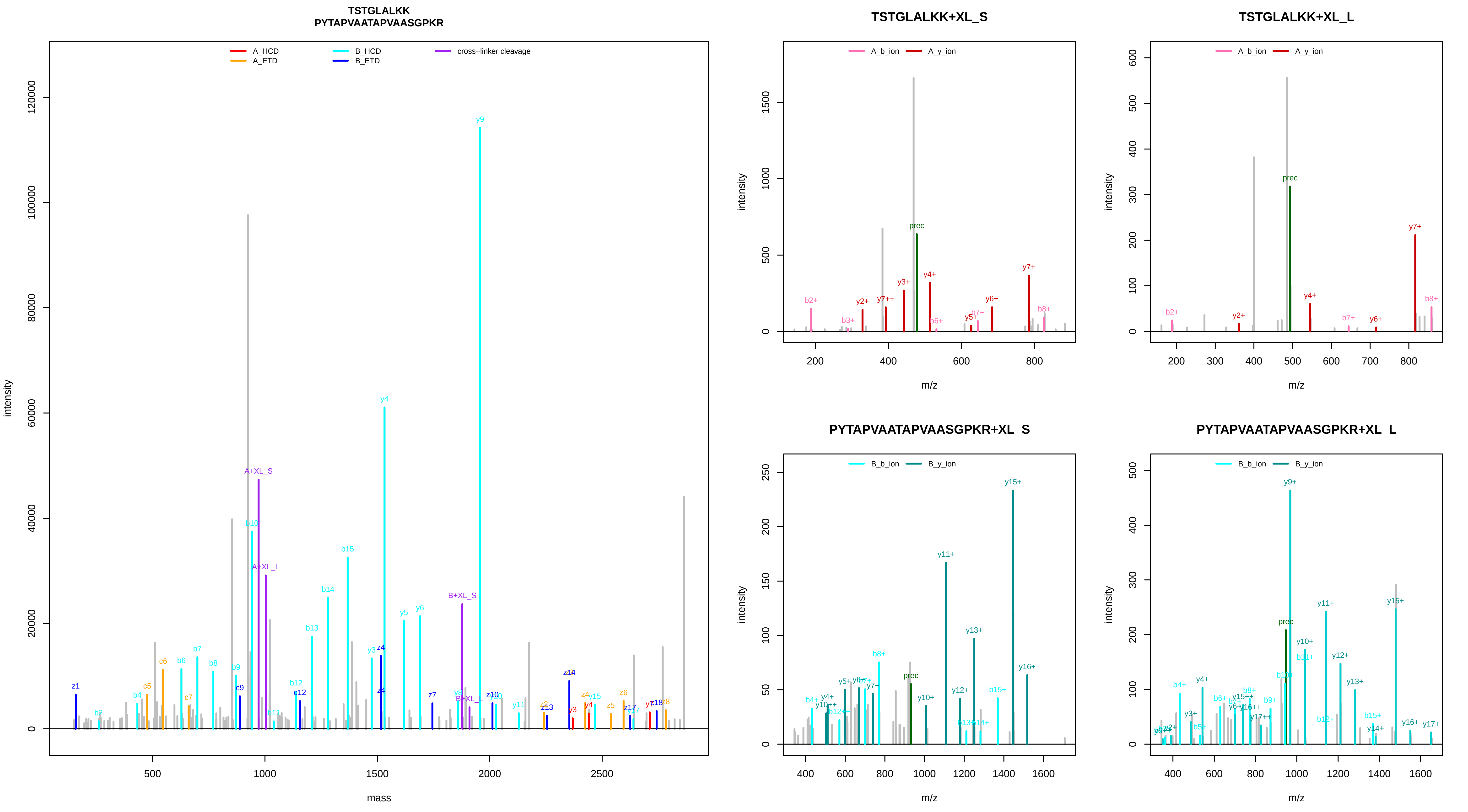

TSTGLALKK
PYTAPVAATAPVAASGPKR
A_HCD
A_ETD
B_HCD
B_ETD
cross−linker cleavage
A+XL_S
A+XL_L
B+XL_S
B+XL_L
intensity
mass
TSTGLALKK+XL_S
TSTGLALKK+XL_L
A_b_ion
A_y_ion
prec
m/z
PYTAPVAATAPVAASGPKR+XL_S
PYTAPVAATAPVAASGPKR+XL_L
B_b_ion
B_y_ion

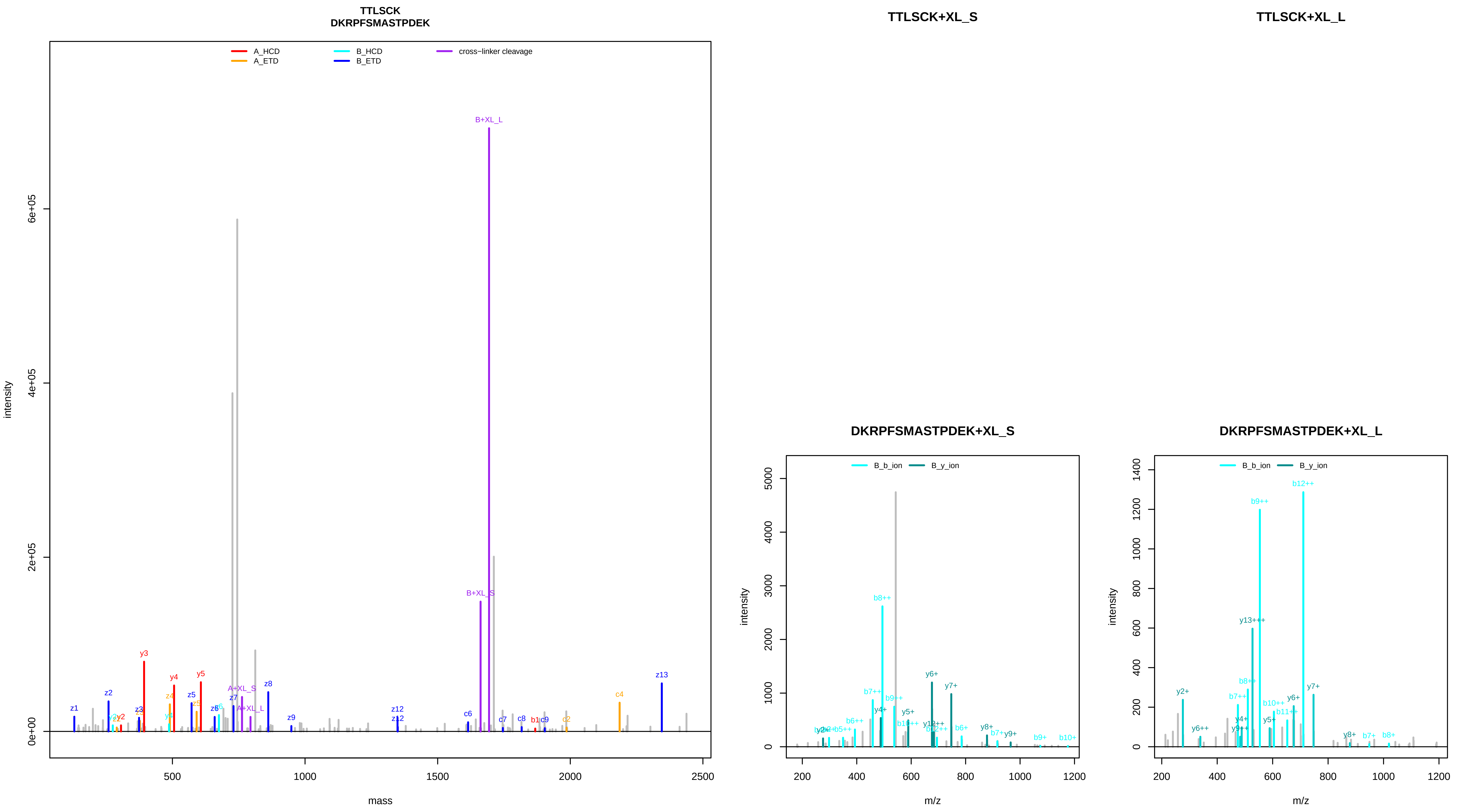

TTLSCK
DKRPFSMASTPDEK
A_HCD
A_ETD
B_HCD
B_ETD
cross−linker cleavage
B+XL_L
B+XL_S
A+XL_S
A+XL_L
intensity
mass
TTLSCK+XL_S
TTLSCK+XL_L
DKRPFSMASTPDEK+XL_S
DKRPFSMASTPDEK+XL_L
B_b_ion
B_y_ion
intensity
m/z

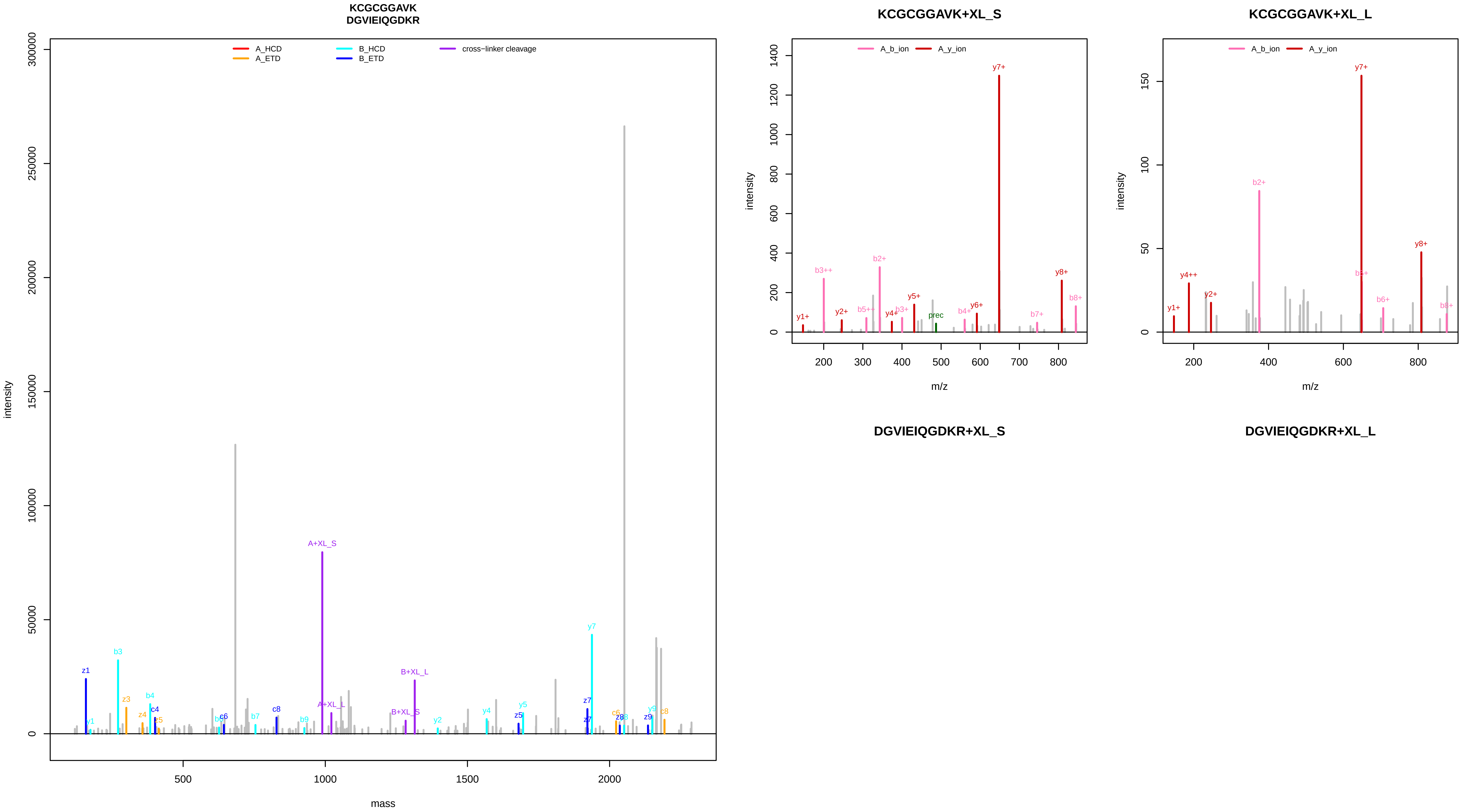
KCGCGGAVK
DGVIEIQGDKR
A_HCD
A_ETD
B_HCD
B_ETD
cross−linker cleavage
A+XL_S
B+XL_L
A+XL_L
B+XL_S
intensity
mass
KCGCGGAVK+XL_S
KCGCGGAVK+XL_L
A_b_ion
A_y_ion
prec
intensity
m/z
DGVIEIQGDKR+XL_S
DGVIEIQGDKR+XL_L

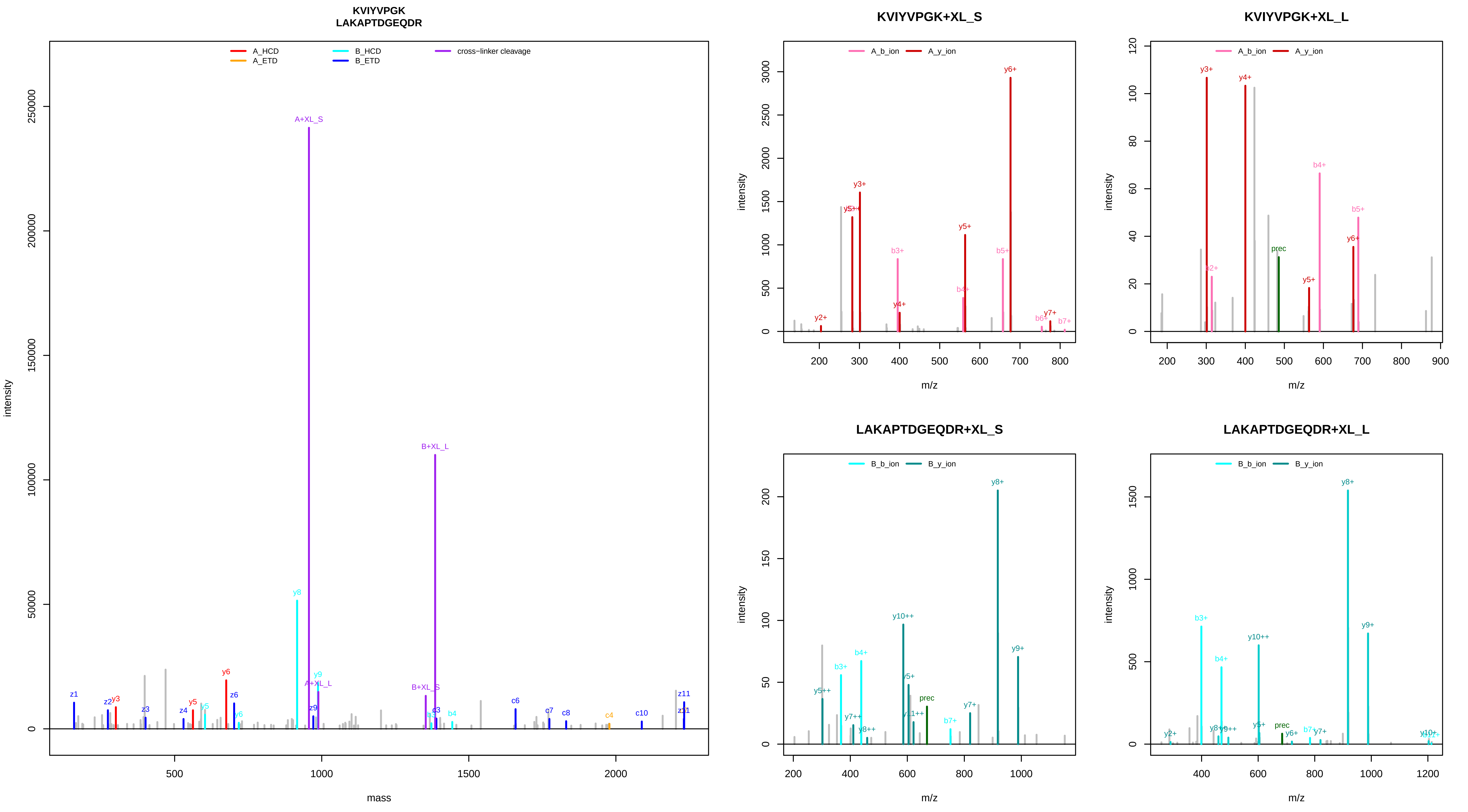
KVIYVPGK
LAKAPTDGEQDR
A_HCD
A_ETD
B_HCD
B_ETD
cross−linker cleavage
A+XL_S
B+XL_L
A+XL_L
B+XL_S
intensity
mass
KVIYVPGK+XL_S
KVIYVPGK+XL_L
LAKAPTDGEQDR+XL_S
LAKAPTDGEQDR+XL_L
A_b_ion
A_y_ion
B_b_ion
B_y_ion
prec
m/z

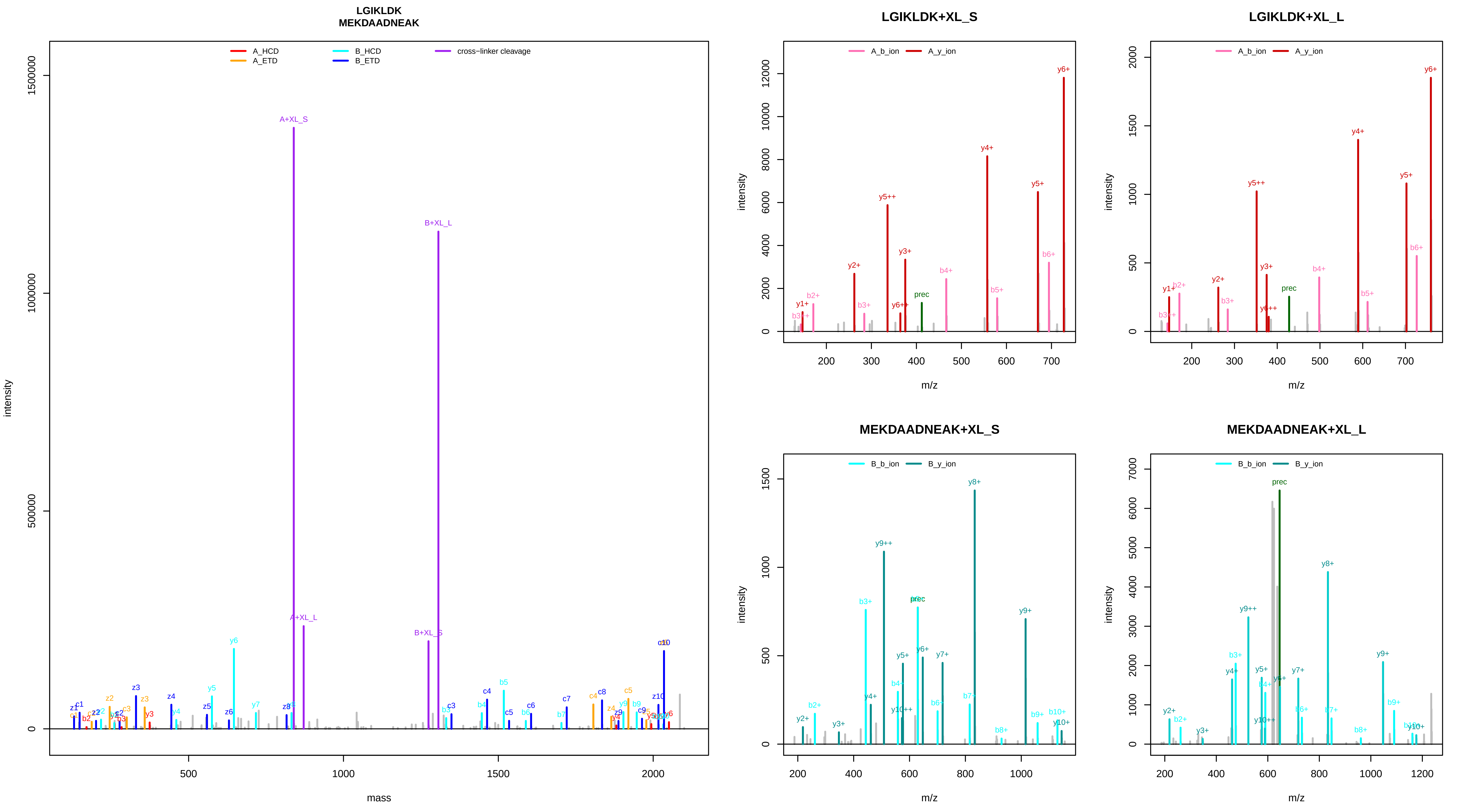

LGIKLDK
MEKDAADNEAK
A_HCD
A_ETD
B_HCD
B_ETD
cross−linker cleavage
A+XL_S
B+XL_L
A+XL_L
B+XL_S
intensity
mass
LGIKLDK+XL_S
LGIKLDK+XL_L
MEKDAADNEAK+XL_S
MEKDAADNEAK+XL_L
A_b_ion
A_y_ion
B_b_ion
B_y_ion
prec
m/z

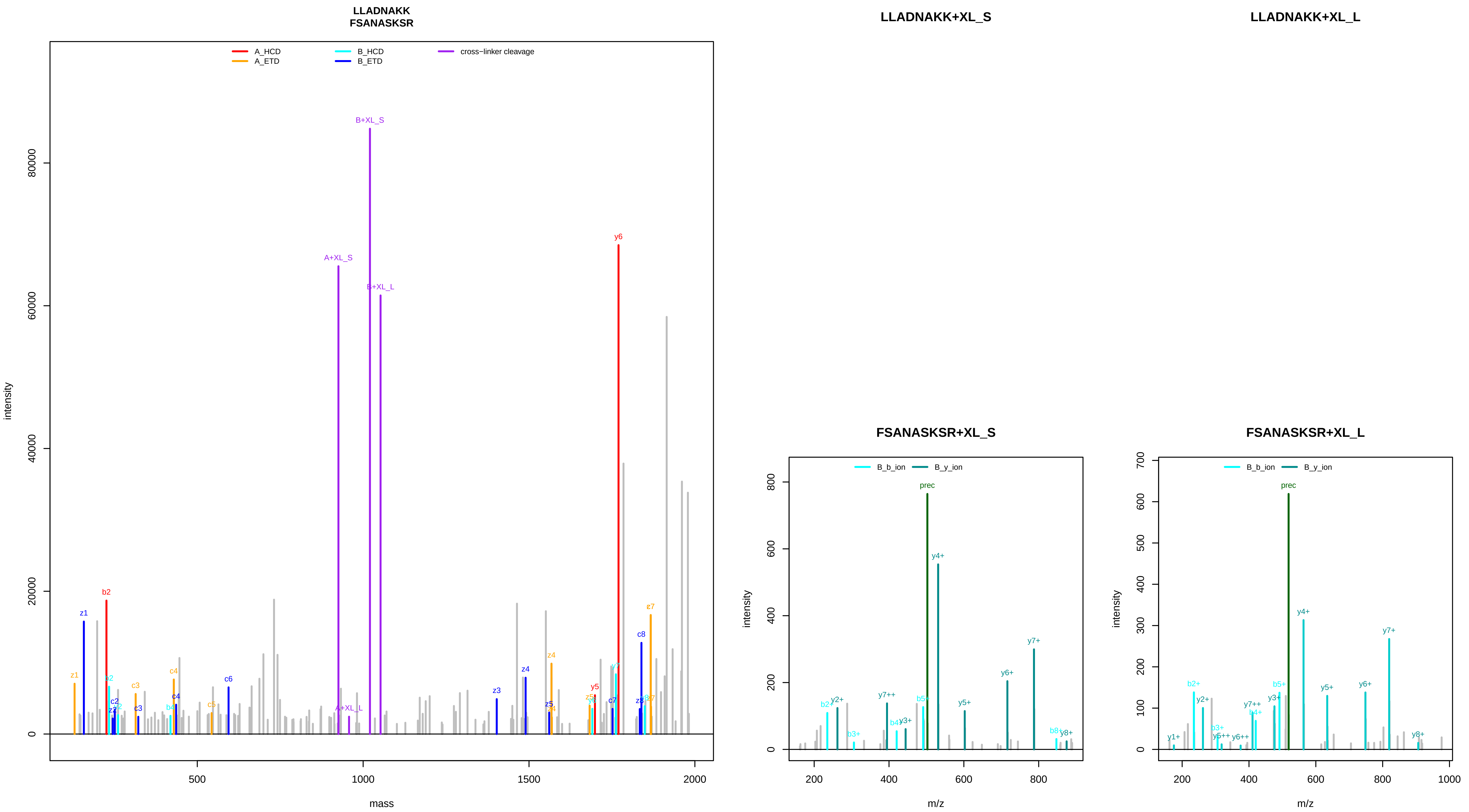
LLADNAKK
FSANASKSR
A_HCD
A_ETD
B_HCD
B_ETD
cross−linker cleavage
B+XL_S
A+XL_S
B+XL_L
A+XL_L
y6
y5
b2
z1
c8
z7
z4
c6
z3
z4
c4
c3
intensity
mass
LLADNAKK+XL_S
LLADNAKK+XL_L
FSANASKSR+XL_S
FSANASKSR+XL_L
B_b_ion
B_y_ion
prec
y4+
y7+
y6+
y5+
y2+
b2+
m/z

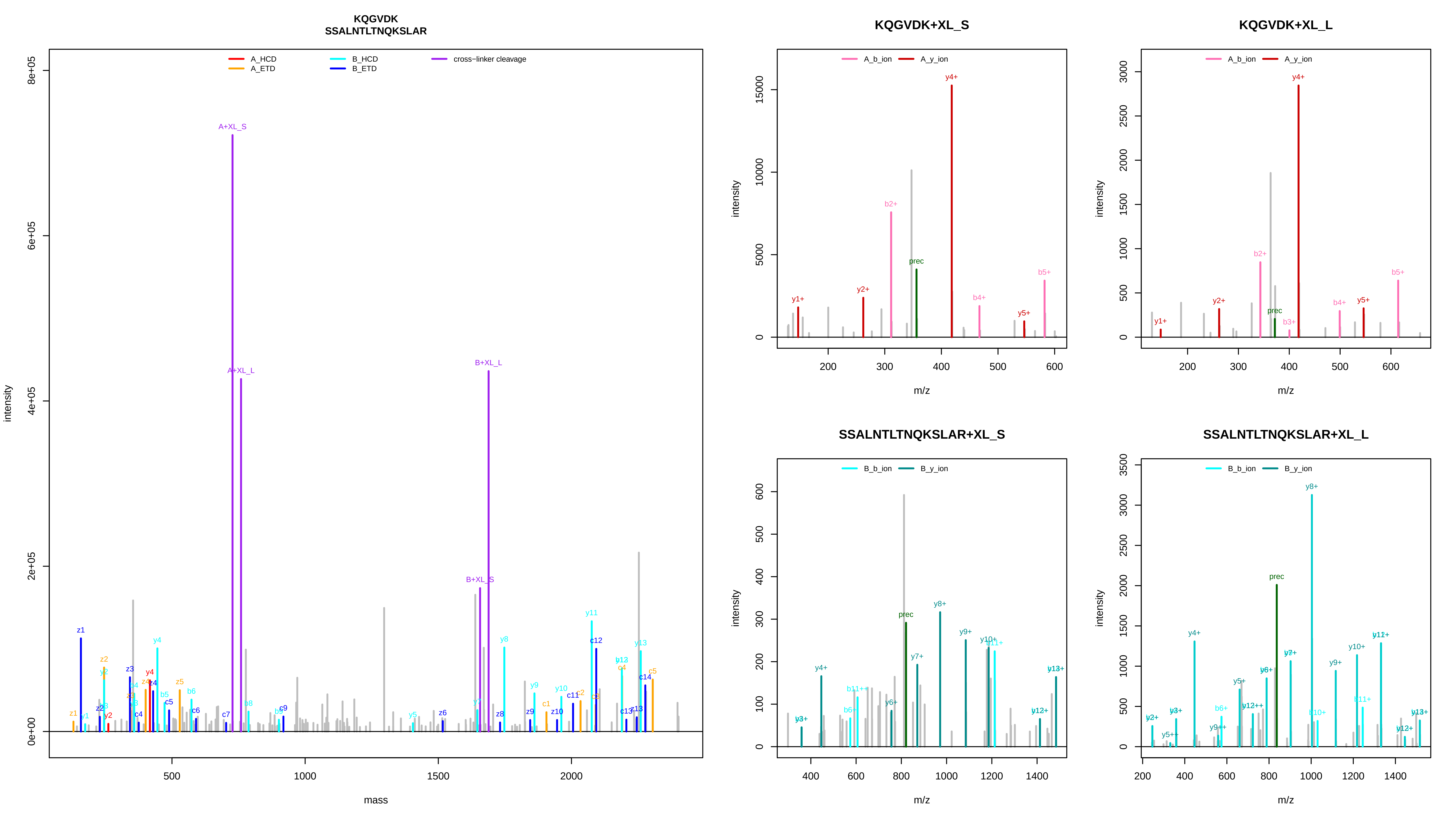
KQGVDK
SSALNTLTNQKSLAR
A_HCD
A_ETD
B_HCD
B_ETD
cross−linker cleavage
A+XL_S
A+XL_L
B+XL_L
B+XL_S
intensity
mass
0e+00
2e+05
4e+05
6e+05
8e+05
500
1000
1500
2000
KQGVDK+XL_S
KQGVDK+XL_L
A_b_ion
A_y_ion
SSALNTLTNQKSLAR+XL_S
SSALNTLTNQKSLAR+XL_L
B_b_ion
B_y_ion
prec
m/z

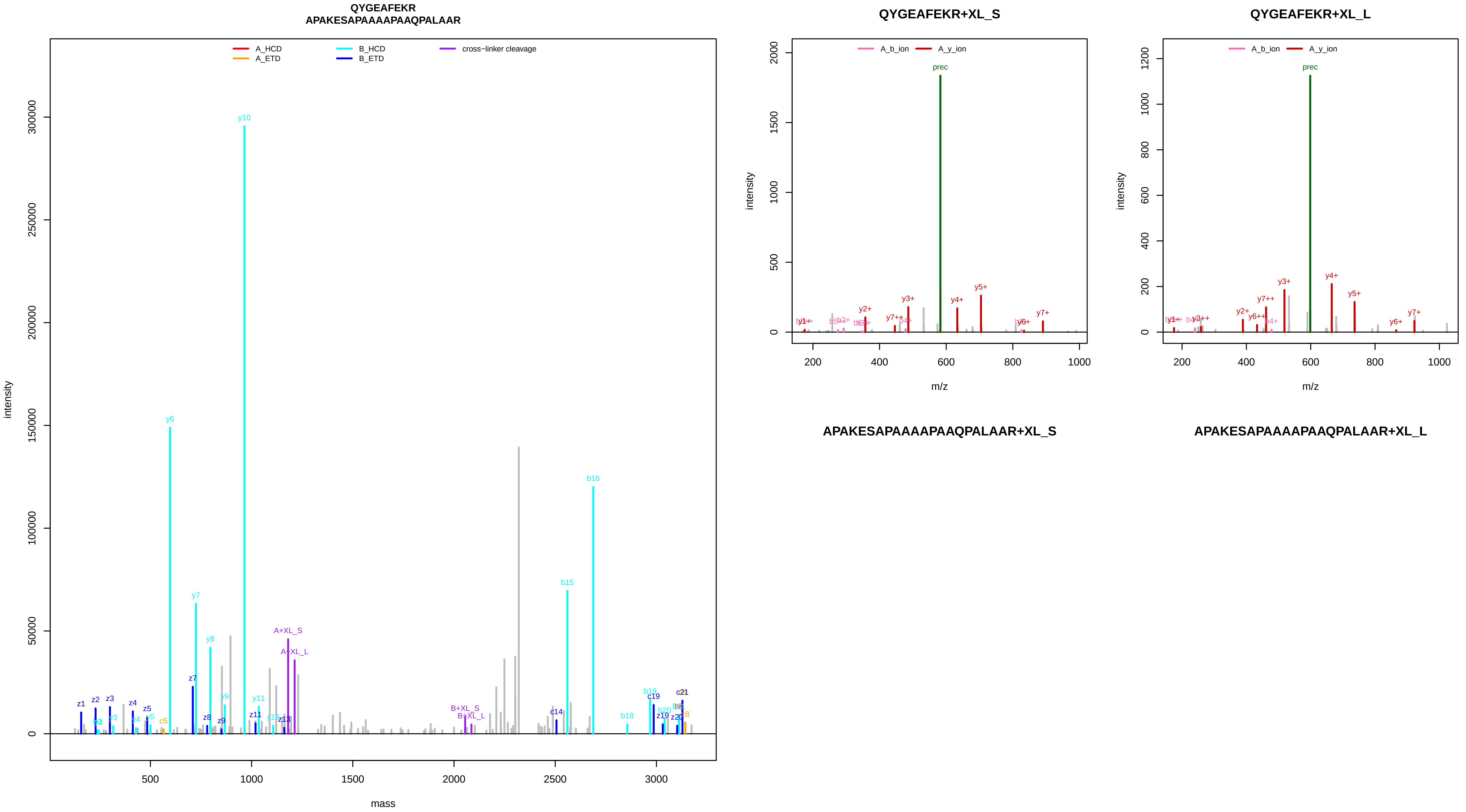
QYGEAFEKR
APAKESAPAAAAPAAQPALAAR
A_HCD
A_ETD
B_HCD
B_ETD
cross−linker cleavage
intensity
mass
A+XL_S
A+XL_L
B+XL_S
B+XL_L
QYGEAFEKR+XL_S
QYGEAFEKR+XL_L
A_b_ion
A_y_ion
prec
m/z
APAKESAPAAAAPAAQPALAAR+XL_S
APAKESAPAAAAPAAQPALAAR+XL_L

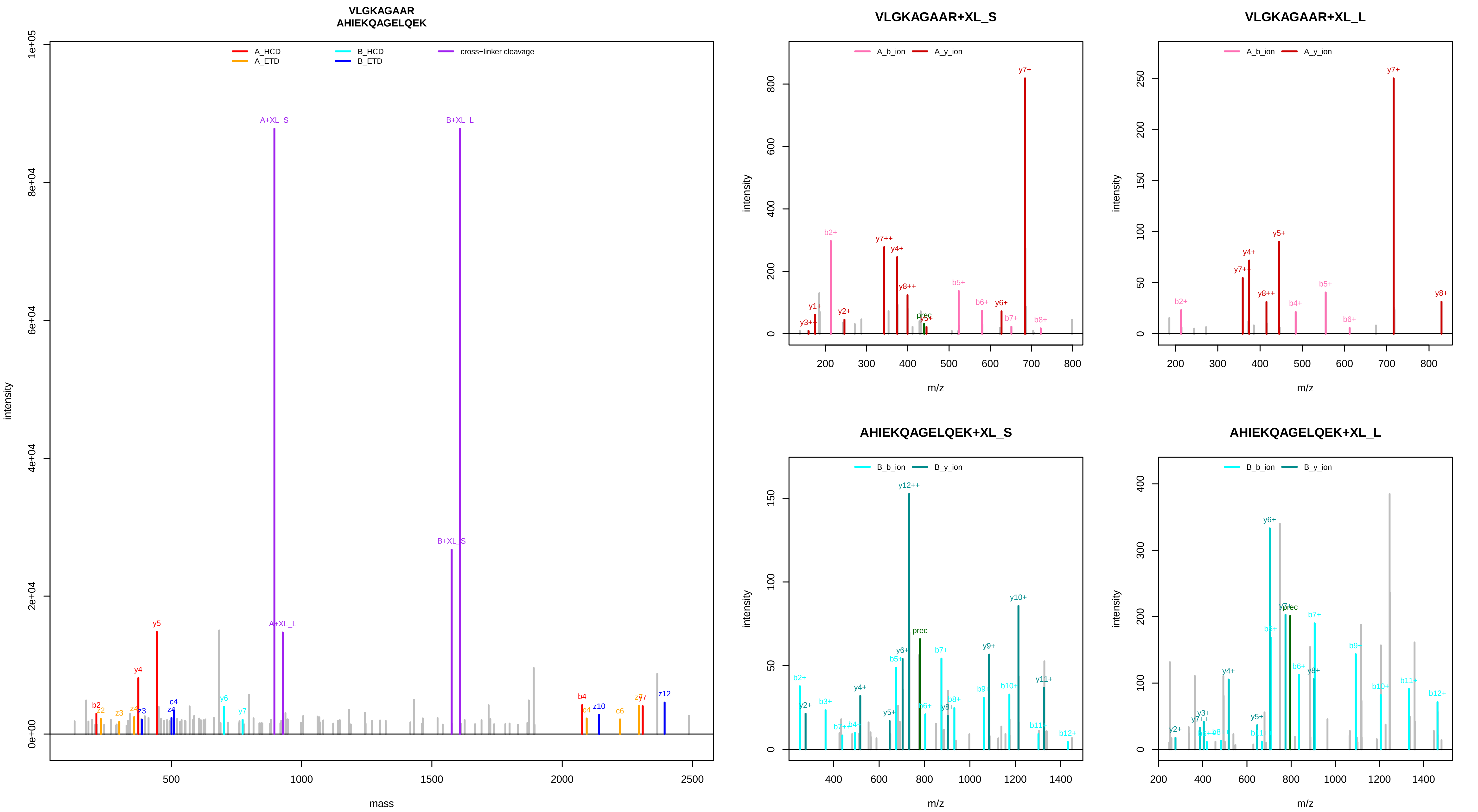
VLGKAGAAR
AHIEKQAGELQEK
A_HCD
A_ETD
B_HCD
B_ETD
cross−linker cleavage
A+XL_S
B+XL_L
B+XL_S
A+XL_L
intensity
mass
VLGKAGAAR+XL_S
VLGKAGAAR+XL_L
AHIEKQAGELQEK+XL_S
AHIEKQAGELQEK+XL_L
A_b_ion
A_y_ion
B_b_ion
B_y_ion
m/z
prec

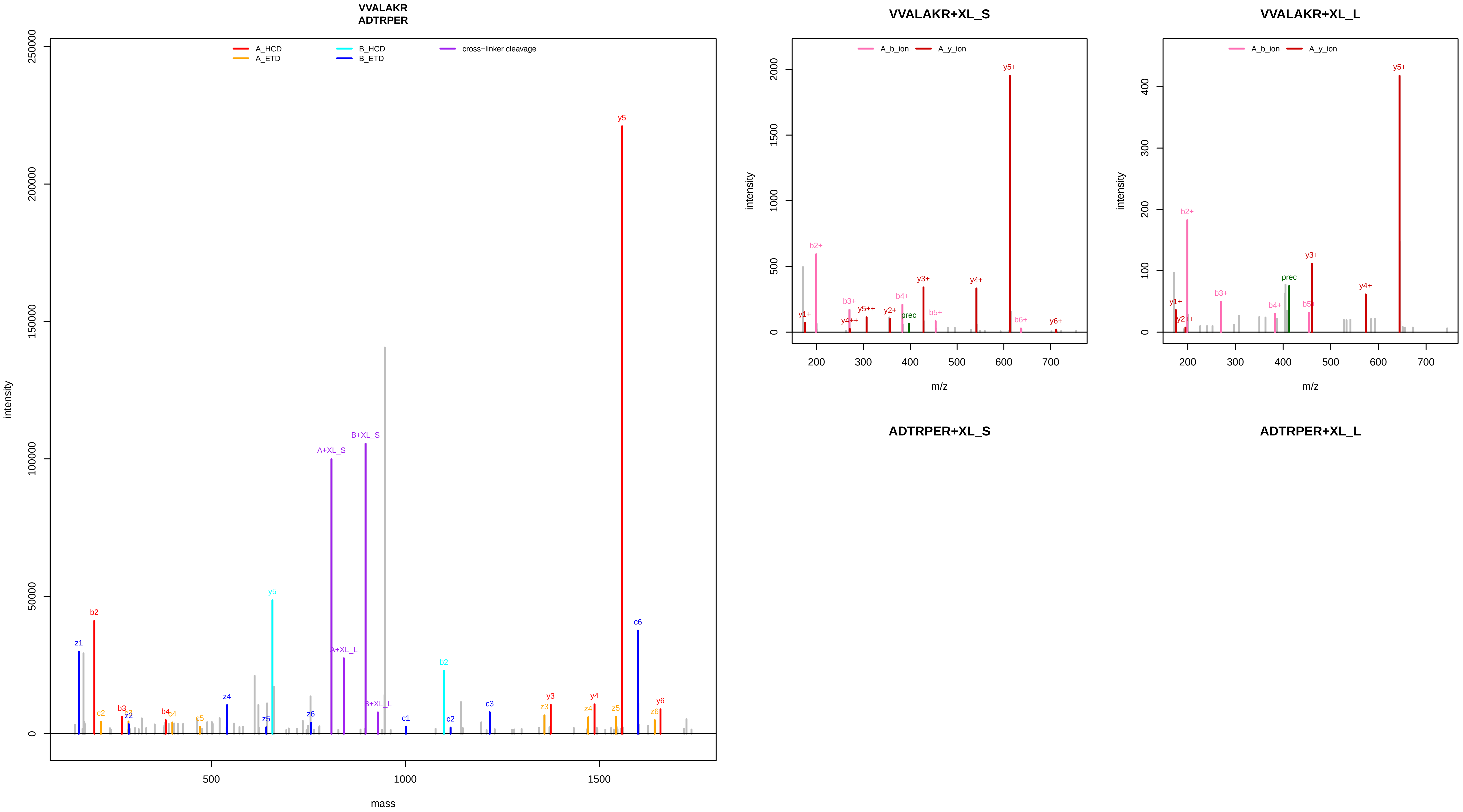
VVALAKR
ADTRPER
A_HCD
A_ETD
B_HCD
B_ETD
cross−linker cleavage
intensity
mass
y5
B+XL_S
A+XL_S
y5
b2
z1
c6
A+XL_L
b2
c3
B+XL_L
y3
y4
y6
VVALAKR+XL_S
A_b_ion
A_y_ion
y5+
b2+
y3+
y4+
b4+
b3+
y5++
y2+
prec
b5+
y1+
y4++
b6+
y6+
intensity
m/z
VVALAKR+XL_L
A_b_ion
A_y_ion
y5+
b2+
y3+
prec
b3+
y4+
b4+
b5+
y1+
y2++
intensity
m/z
ADTRPER+XL_S
ADTRPER+XL_L

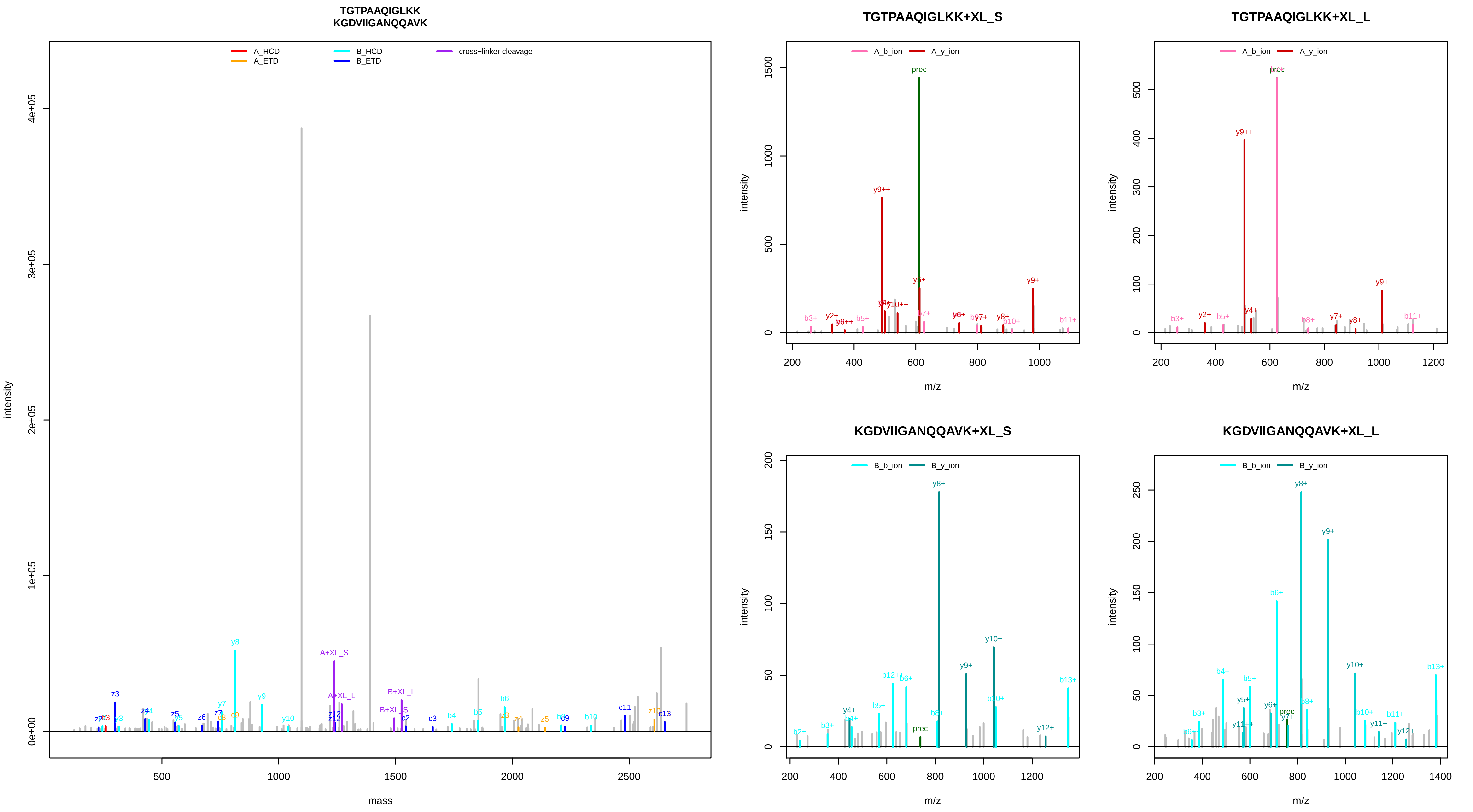
TGTPAAQIGLKK
KGDVIIGANQQAVK
A_HCD
A_ETD
B_HCD
B_ETD
cross−linker cleavage
A+XL_S
A+XL_L
B+XL_L
B+XL_S
intensity
mass
TGTPAAQIGLKK+XL_S
TGTPAAQIGLKK+XL_L
KGDVIIGANQQAVK+XL_S
KGDVIIGANQQAVK+XL_L
A_b_ion
A_y_ion
B_b_ion
B_y_ion
prec
m/z

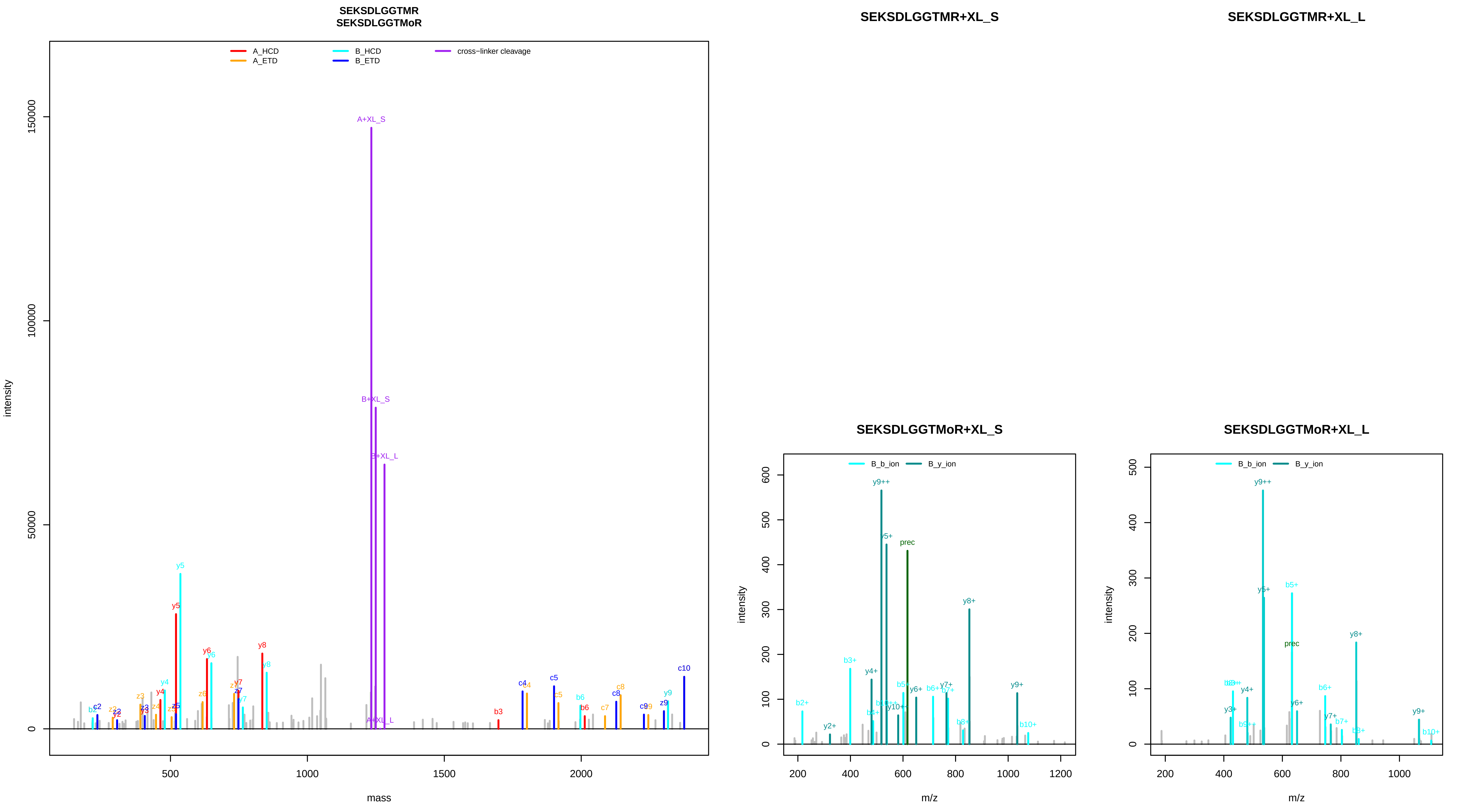
SEKSDLGGTMR
SEKSDLGGTMoR
A_HCD
A_ETD
B_HCD
B_ETD
cross−linker cleavage
A+XL_S
B+XL_S
B+XL_L
A+XL_L
intensity
mass
SEKSDLGGTMR+XL_S
SEKSDLGGTMR+XL_L
SEKSDLGGTMoR+XL_S
SEKSDLGGTMoR+XL_L
B_b_ion
B_y_ion
y9++
y5+
prec
y8+
b3+
y4+
b2+
y2+
y9+
b10+
b5+
b6+
y6+
y7+
b7+
b8+
intensity
m/z

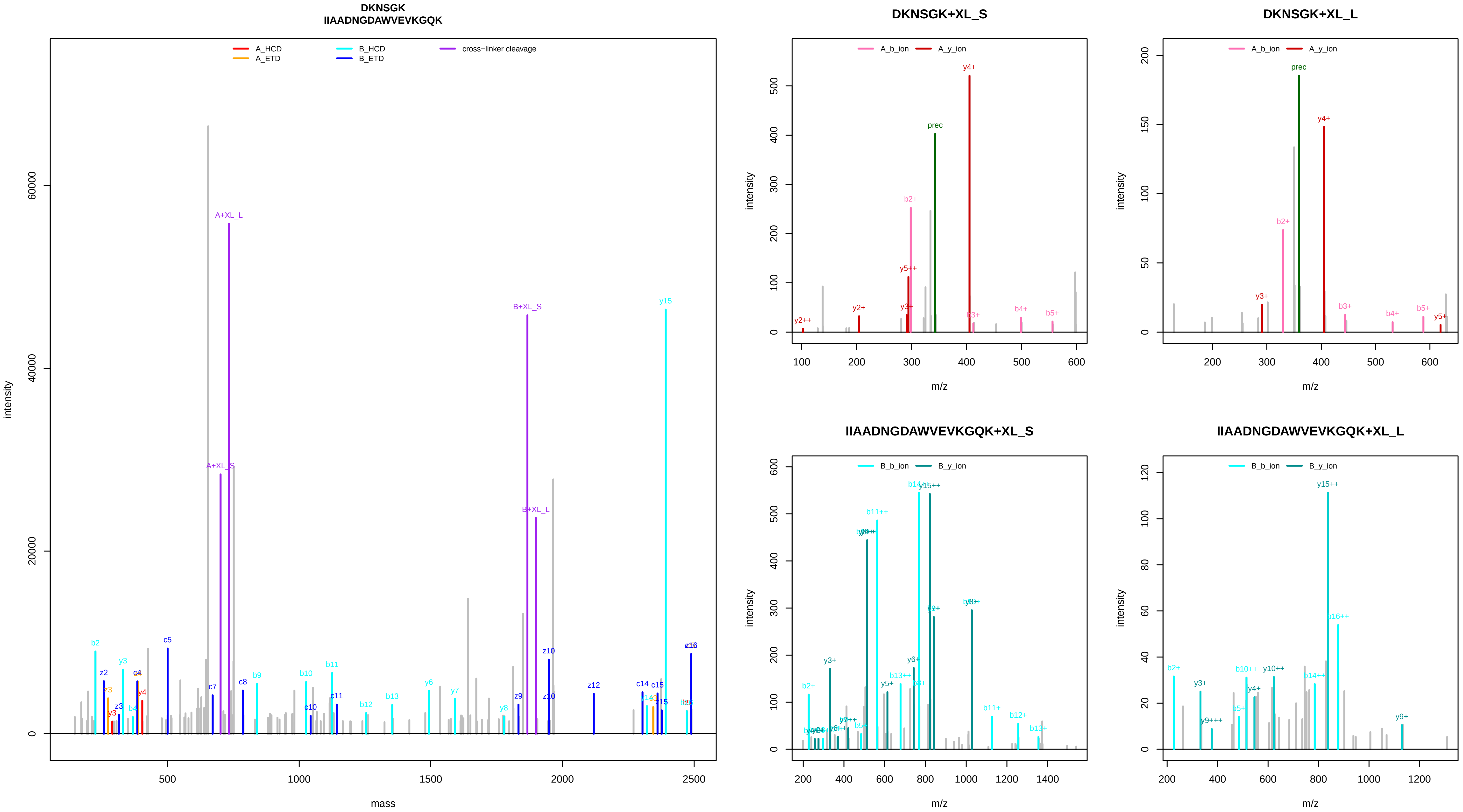

DKNSGK
IIAADNGDAWVEVKGQK
A_HCD
A_ETD
B_HCD
B_ETD
cross−linker cleavage
A+XL_L
A+XL_S
B+XL_S
B+XL_L
intensity
mass
DKNSGK+XL_S
DKNSGK+XL_L
IIAADNGDAWVEVKGQK+XL_S
IIAADNGDAWVEVKGQK+XL_L
A_b_ion
A_y_ion
B_b_ion
B_y_ion
prec
m/z

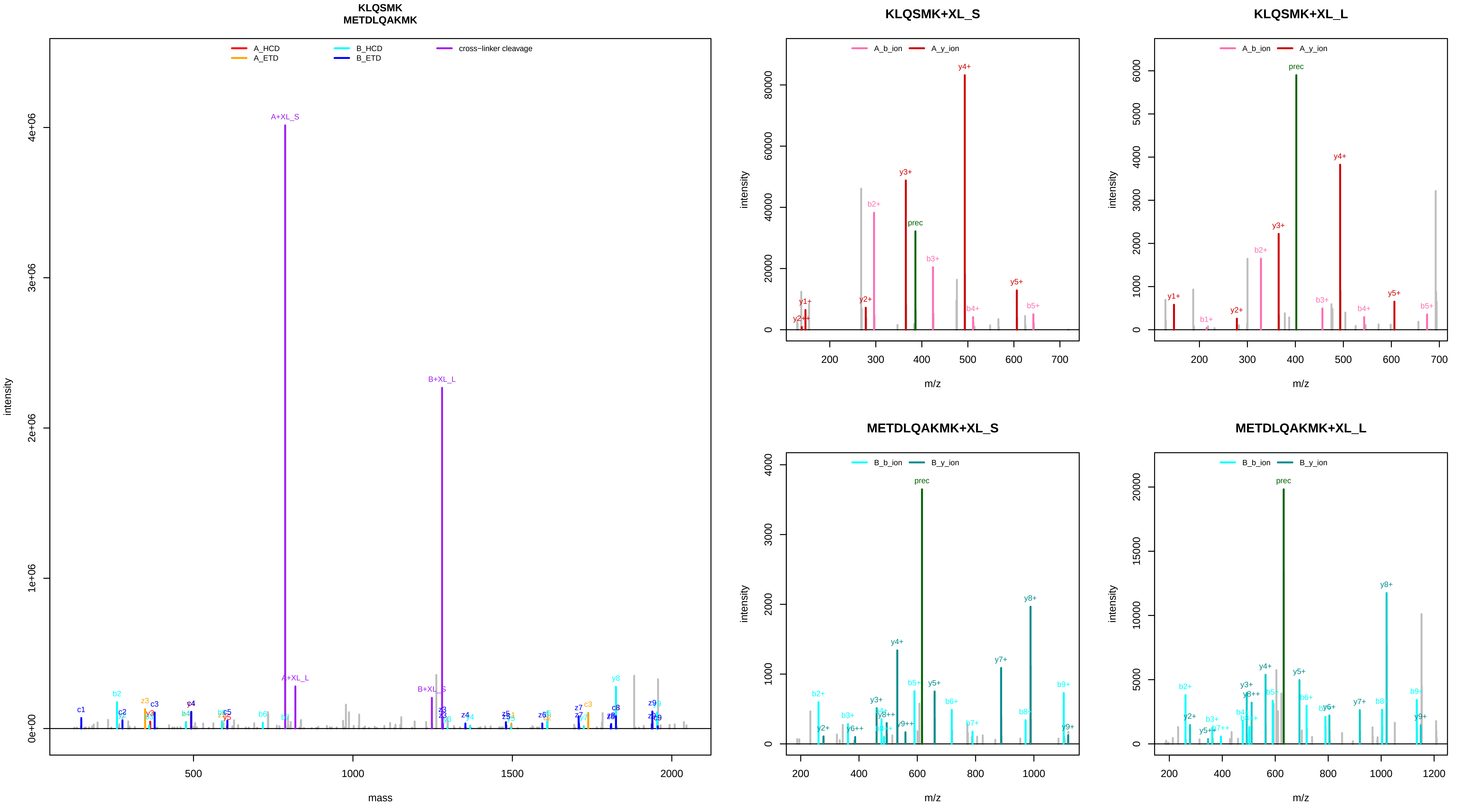
KLQSMK
METDLQAKMK
A_HCD
A_ETD
B_HCD
B_ETD
cross−linker cleavage
A+XL_S
B+XL_L
A+XL_L
B+XL_S
intensity
mass
KLQSMK+XL_S
KLQSMK+XL_L
A_b_ion
A_y_ion
METDLQAKMK+XL_S
METDLQAKMK+XL_L
B_b_ion
B_y_ion
prec
m/z

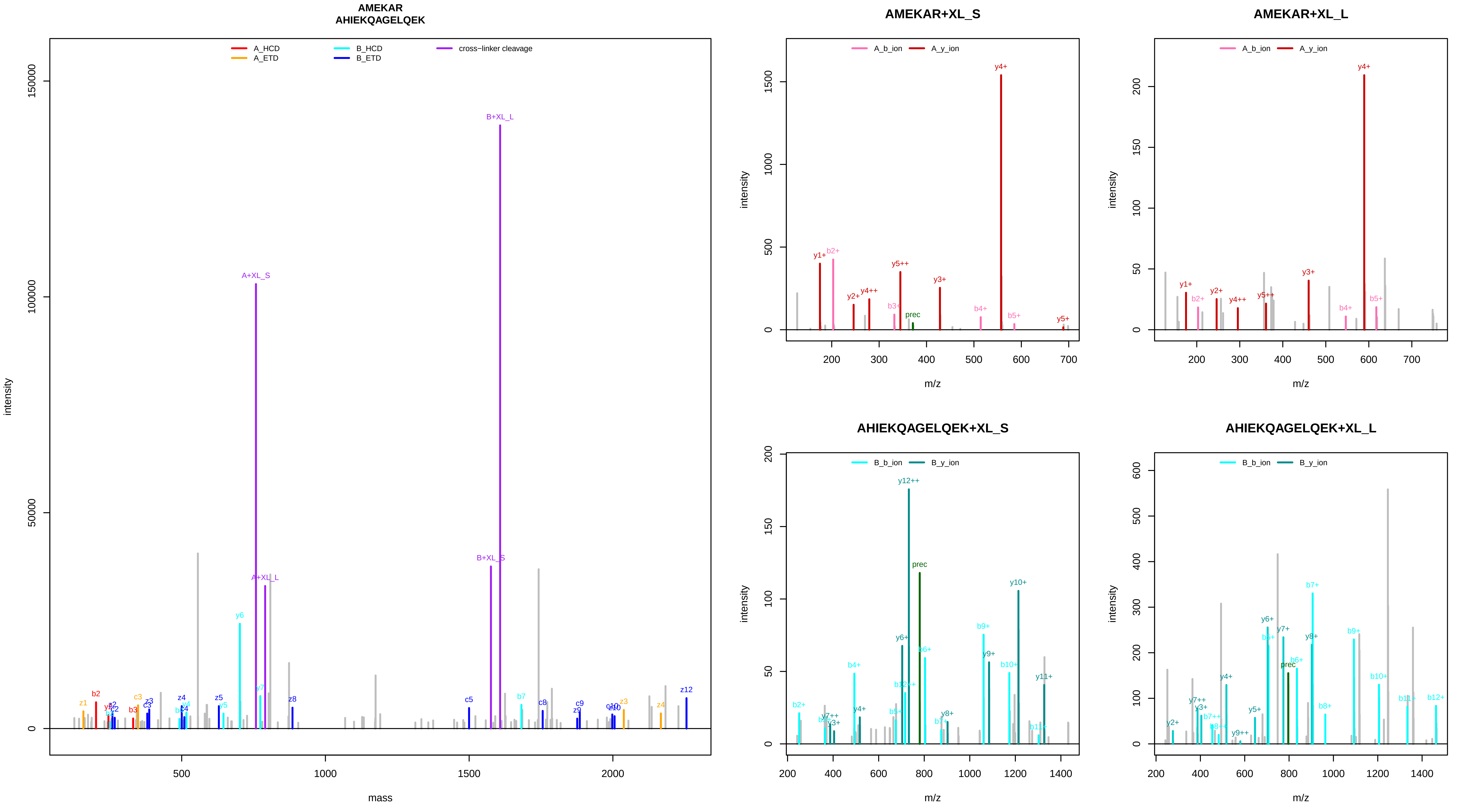
AMEKAR
AHIEKQAGELQEK
A_HCD
A_ETD
B_HCD
B_ETD
cross−linker cleavage
B+XL_L
A+XL_S
B+XL_S
A+XL_L
intensity
mass
AMEKAR+XL_S
AMEKAR+XL_L
A_b_ion
A_y_ion
AHIEKQAGELQEK+XL_S
AHIEKQAGELQEK+XL_L
B_b_ion
B_y_ion
prec
m/z

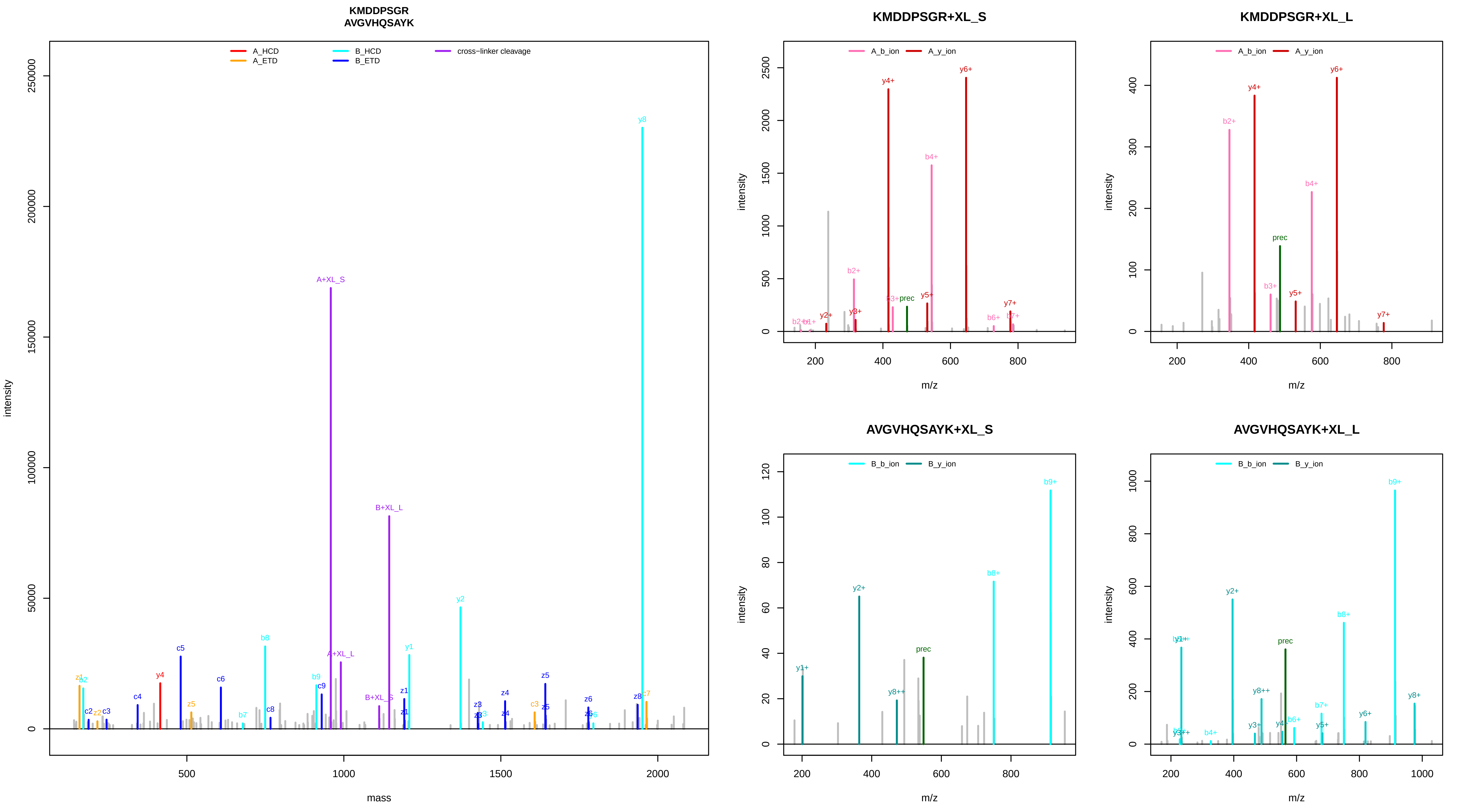
KMDDPSGR
AVGVHQSAYK
A_HCD
A_ETD
B_HCD
B_ETD
cross−linker cleavage
intensity
mass
KMDDPSGR+XL_S
KMDDPSGR+XL_L
AVGVHQSAYK+XL_S
AVGVHQSAYK+XL_L
A_b_ion
A_y_ion
B_b_ion
B_y_ion
m/z

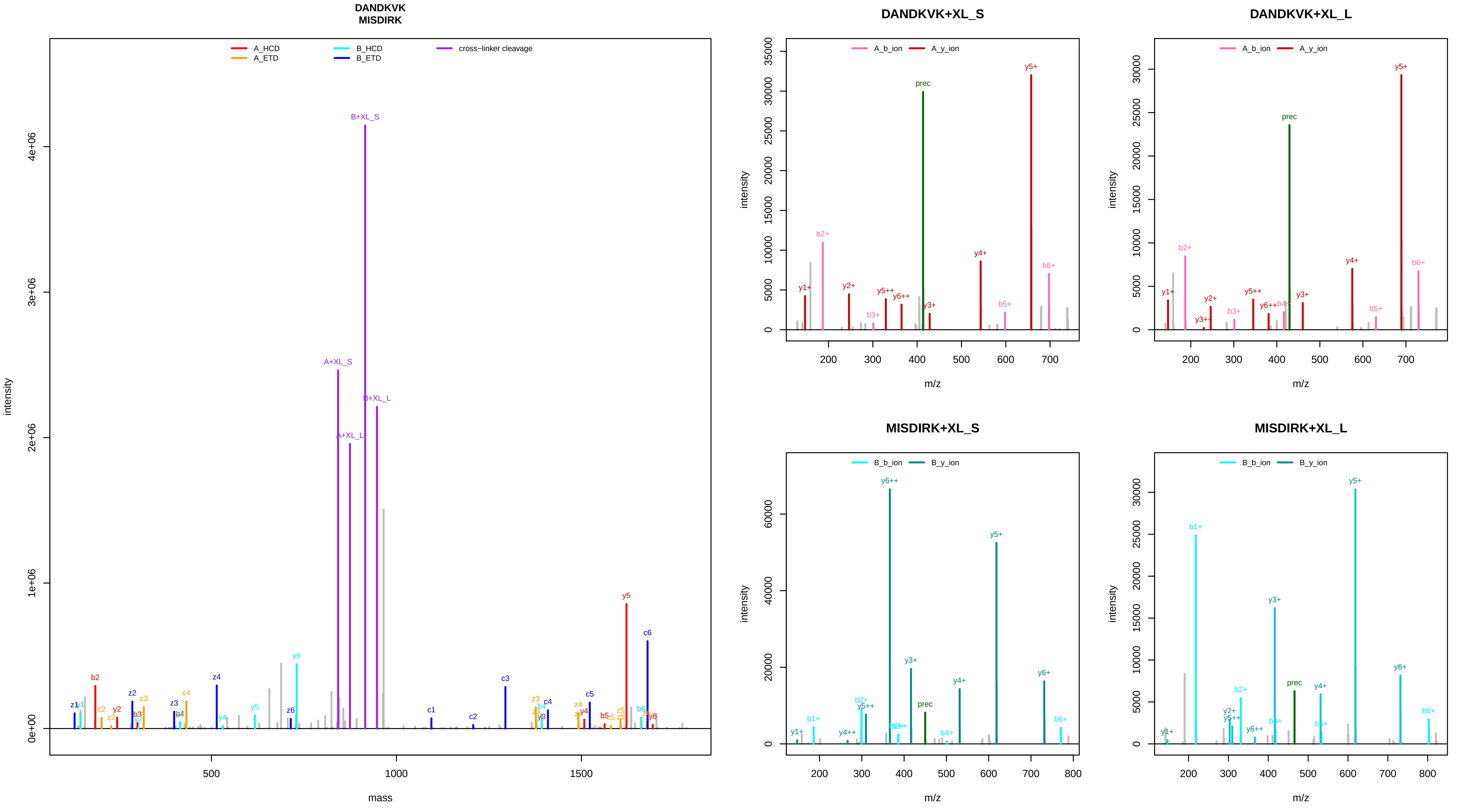
DANDKVK
MISDIRK
A_HCD
A_ETD
B_HCD
B_ETD
cross−linker cleavage
B+XL_S
A+XL_S
B+XL_L
A+XL_L
intensity
mass
DANDKVK+XL_S
DANDKVK+XL_L
MISDIRK+XL_S
MISDIRK+XL_L
A_b_ion
A_y_ion
B_b_ion
B_y_ion
prec
m/z

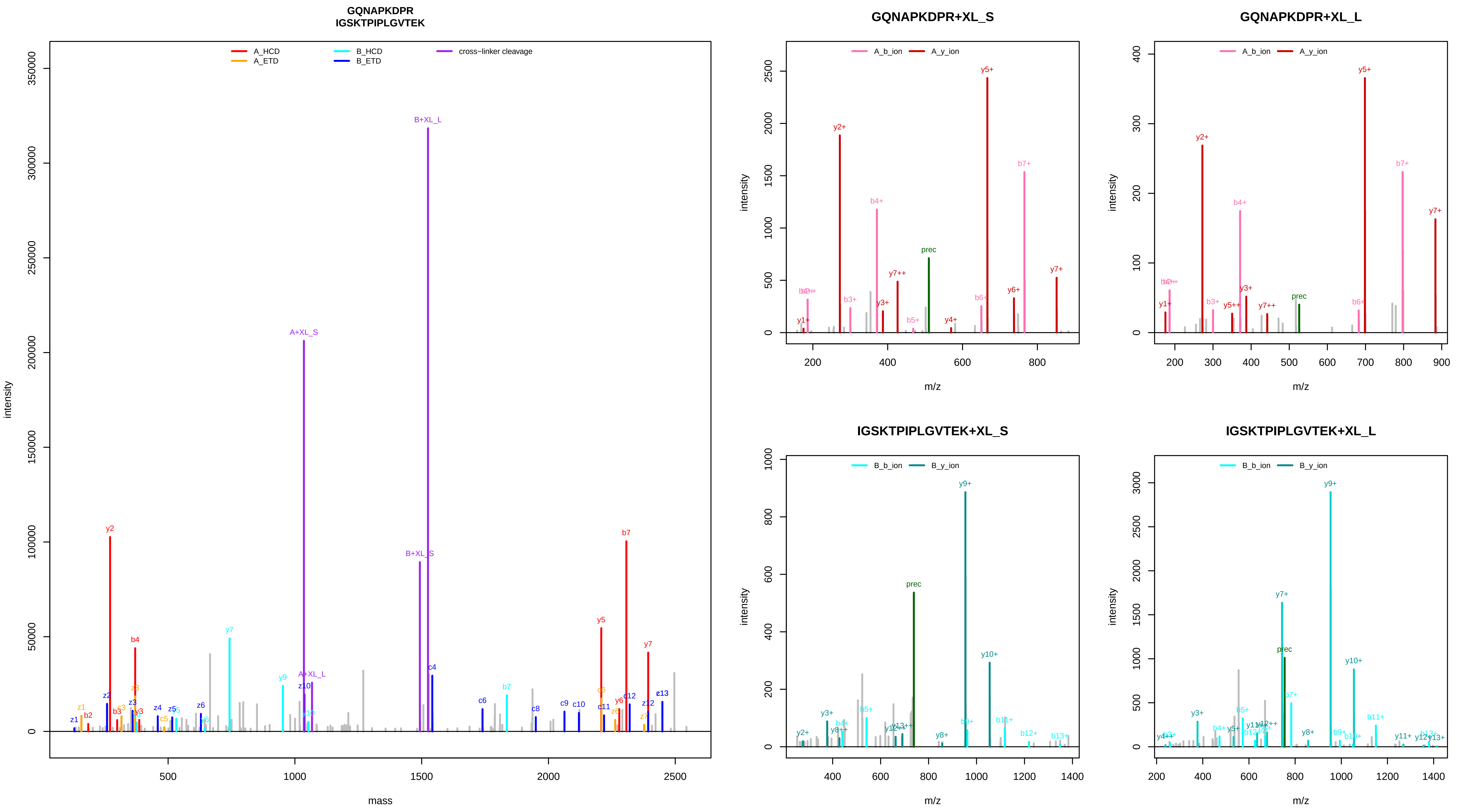

GQNAPKDPR
IGSKTPIPLGVTEK
A_HCD
A_ETD
B_HCD
B_ETD
cross−linker cleavage
B+XL_L
A+XL_S
B+XL_S
A+XL_L
intensity
mass
GQNAPKDPR+XL_S
GQNAPKDPR+XL_L
IGSKTPIPLGVTEK+XL_S
IGSKTPIPLGVTEK+XL_L
A_b_ion
A_y_ion
B_b_ion
B_y_ion
prec
m/z

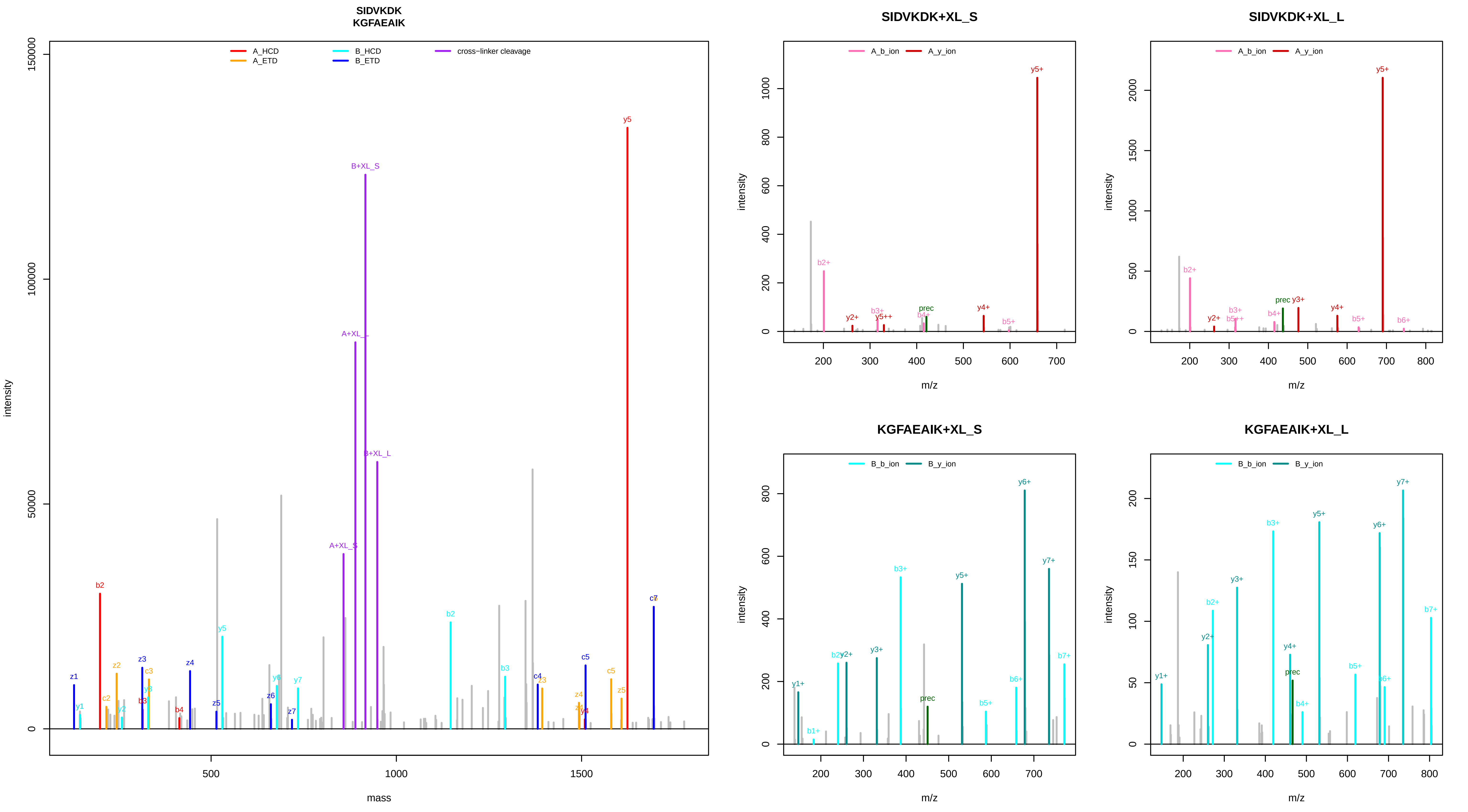

SIDVKDK
KGFAEAIK
A_HCD
A_ETD
B_HCD
B_ETD
cross−linker cleavage
intensity
mass
B+XL_S
A+XL_L
B+XL_L
A+XL_S
y5
b2
c7
c5
z5
z1
y1
c2
z2
z3
c3
b3
z4
b4
z6
z7
y7
b3
c4
z3
z4
SIDVKDK+XL_S
SIDVKDK+XL_L
A_b_ion
A_y_ion
y5+
b2+
y2+
b3+
y5++
b4+
prec
y4+
b5+
y3+
b6+
m/z
KGFAEAIK+XL_S
KGFAEAIK+XL_L
B_b_ion
B_y_ion
y6+
y7+
b3+
y5+
b7+
y3+
b2+
y2+
y1+
b1+
b5+
b6+
y4+
b4+

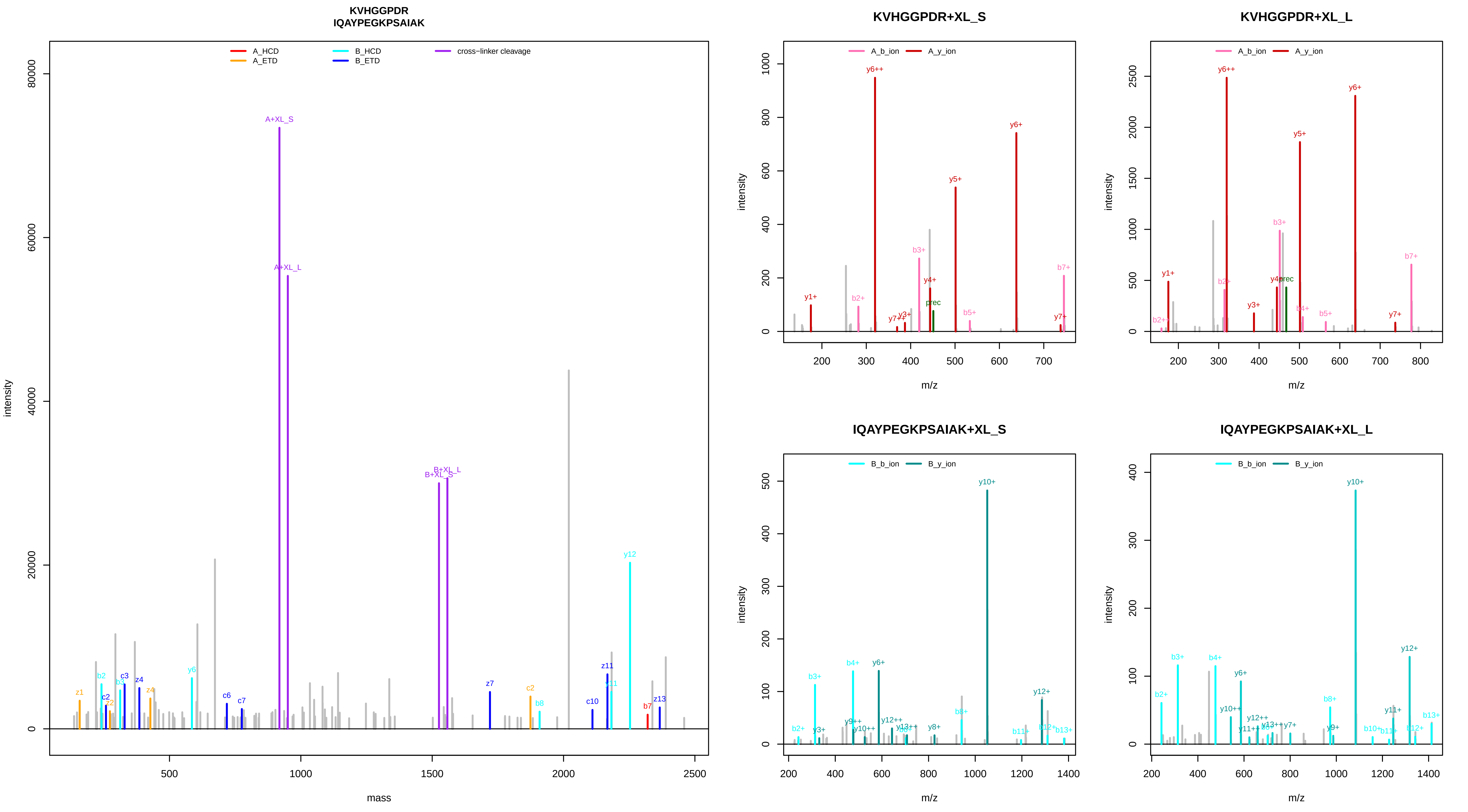
KVHGGPDR
IQAYPEGKPSAIAK
A_HCD
A_ETD
B_HCD
B_ETD
cross−linker cleavage
A+XL_S
A+XL_L
B+XL_S
B+XL_L
intensity
mass
KVHGGPDR+XL_S
KVHGGPDR+XL_L
A_b_ion
A_y_ion
IQAYPEGKPSAIAK+XL_S
IQAYPEGKPSAIAK+XL_L
B_b_ion
B_y_ion
m/z

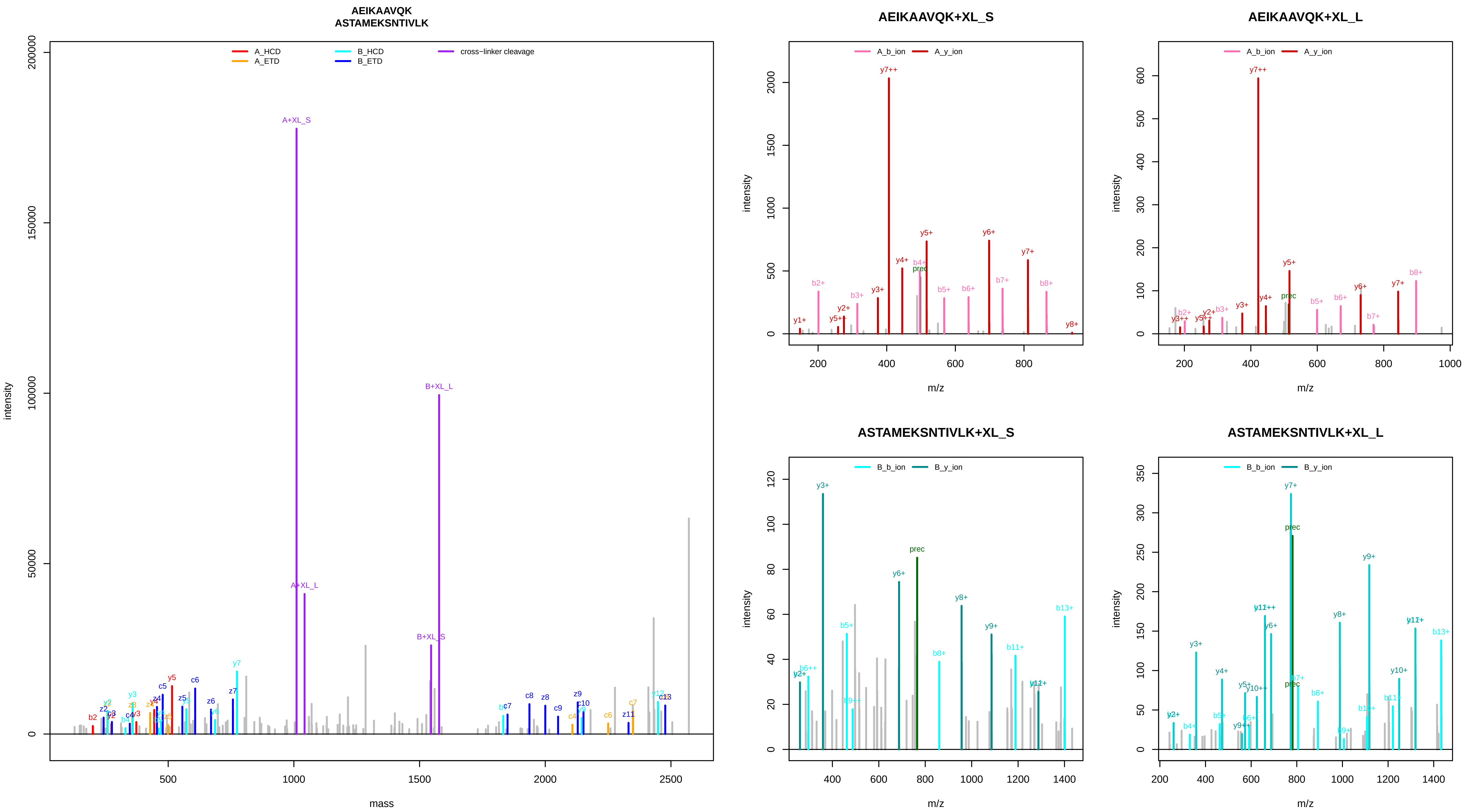

AEIKAAVQK
ASTAMEKSNTIVLK
A_HCD
A_ETD
B_HCD
B_ETD
cross−linker cleavage
A+XL_S
A+XL_L
B+XL_L
B+XL_S
intensity
mass
AEIKAAVQK+XL_S
AEIKAAVQK+XL_L
ASTAMEKSNTIVLK+XL_S
ASTAMEKSNTIVLK+XL_L
A_b_ion
A_y_ion
B_b_ion
B_y_ion
prec
m/z

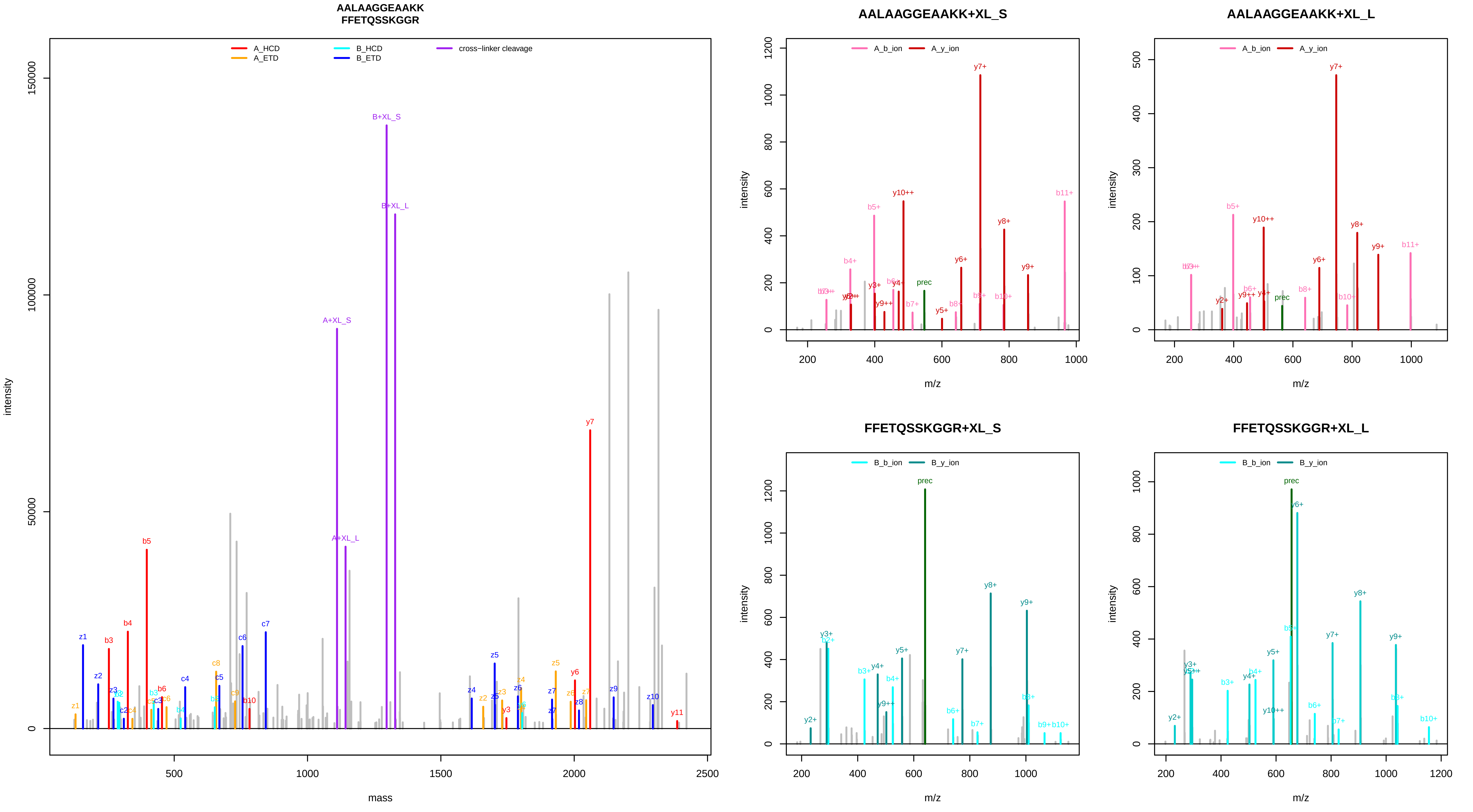
AALAAGGEAAKK
FFETQSSKGGR
A_HCD
A_ETD
B_HCD
B_ETD
cross−linker cleavage
B+XL_S
B+XL_L
A+XL_S
A+XL_L
intensity
mass
AALAAGGEAAKK+XL_S
AALAAGGEAAKK+XL_L
A_b_ion
A_y_ion
FFETQSSKGGR+XL_S
FFETQSSKGGR+XL_L
B_b_ion
B_y_ion
prec
m/z

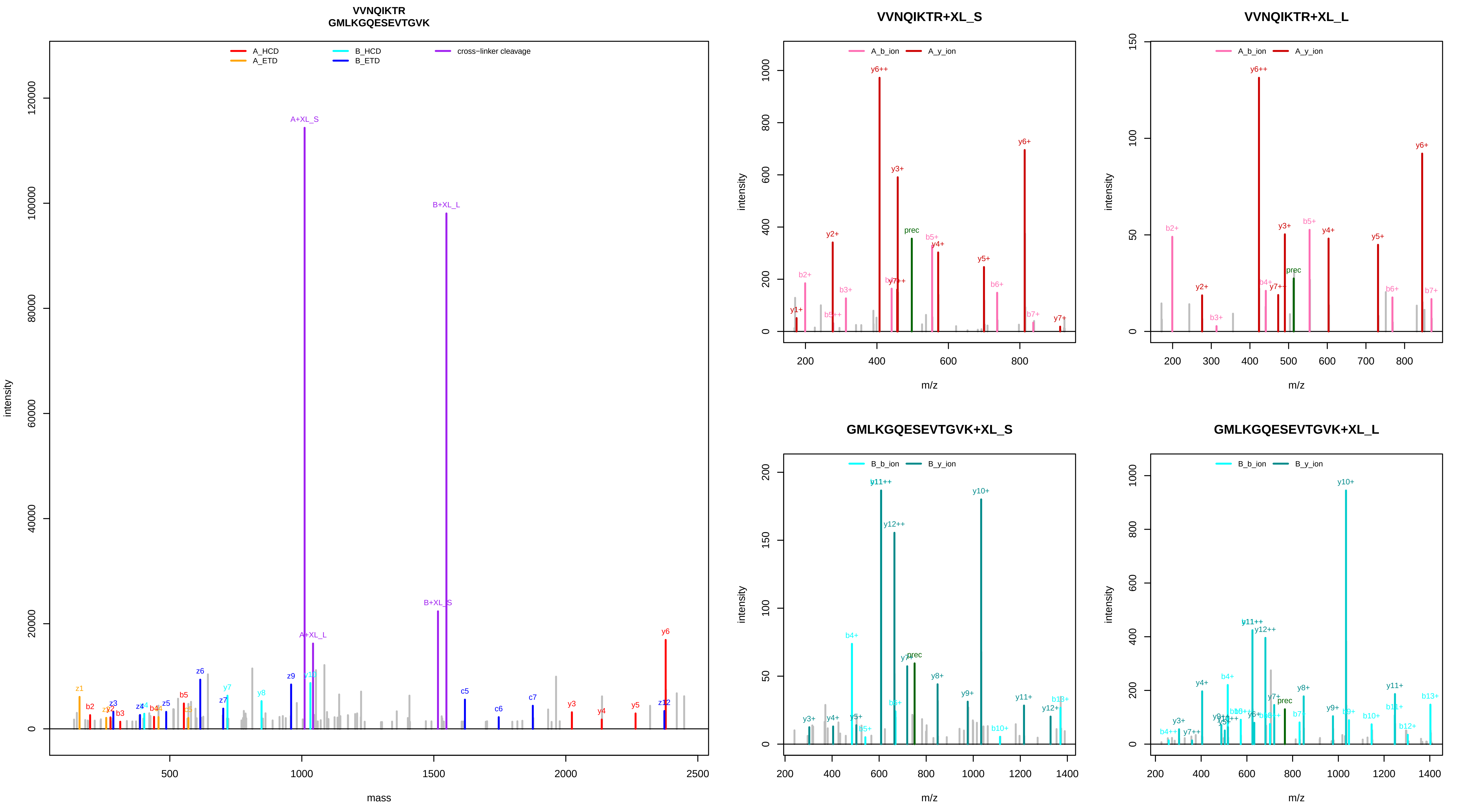
VVNQIKTR
GMLKGQESEVTGVK
A_HCD
A_ETD
B_HCD
B_ETD
cross−linker cleavage
A+XL_S
B+XL_L
B+XL_S
A+XL_L
intensity
mass
VVNQIKTR+XL_S
VVNQIKTR+XL_L
A_b_ion
A_y_ion
GMLKGQESEVTGVK+XL_S
GMLKGQESEVTGVK+XL_L
B_b_ion
B_y_ion
prec
m/z

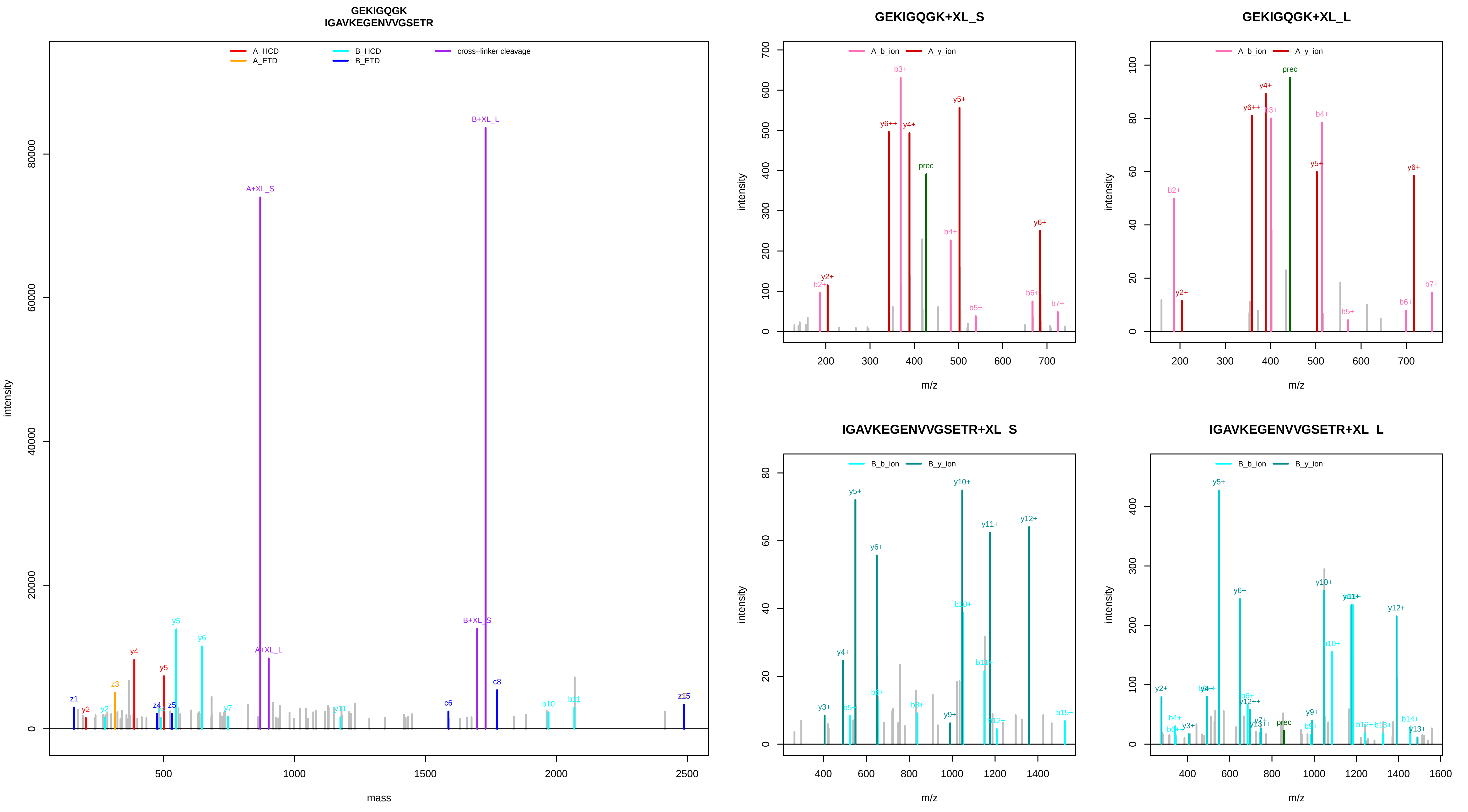
GEKIGQGK
IGAVKEGENVVGSETR
A_HCD
A_ETD
B_HCD
B_ETD
cross−linker cleavage
A+XL_S
B+XL_L
A+XL_L
B+XL_S
intensity
mass
GEKIGQGK+XL_S
GEKIGQGK+XL_L
A_b_ion
A_y_ion
prec
IGAVKEGENVVGSETR+XL_S
IGAVKEGENVVGSETR+XL_L
B_b_ion
B_y_ion
m/z

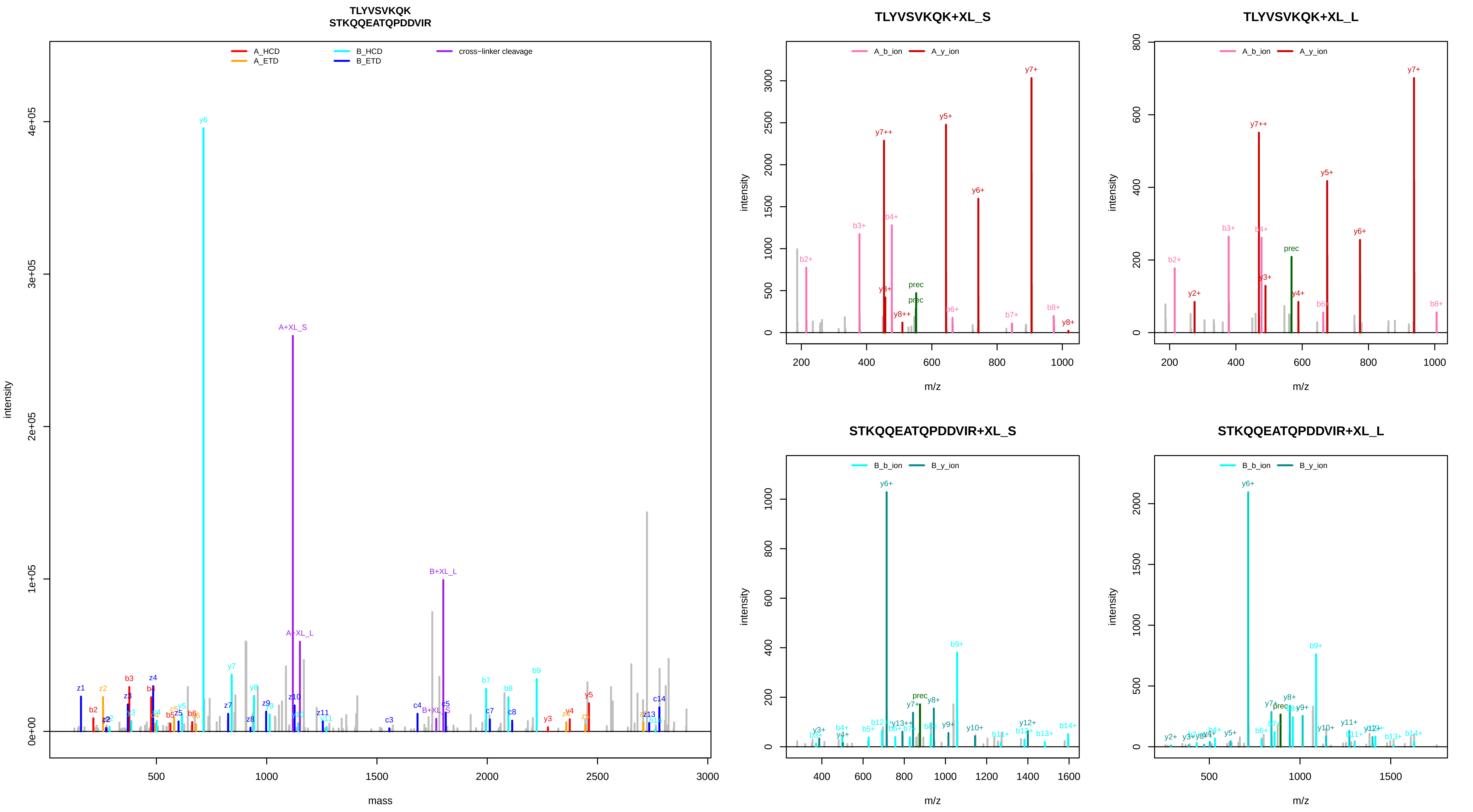
TLYVSVKQK
STKQQEATQPDDVIR
A_HCD
A_ETD
B_HCD
B_ETD
cross−linker cleavage
intensity
mass
y6
A+XL_S
A+XL_L
B+XL_L
B+XL_S
TLYVSVKQK+XL_S
TLYVSVKQK+XL_L
A_b_ion
A_y_ion
STKQQEATQPDDVIR+XL_S
STKQQEATQPDDVIR+XL_L
B_b_ion
B_y_ion
m/z
prec

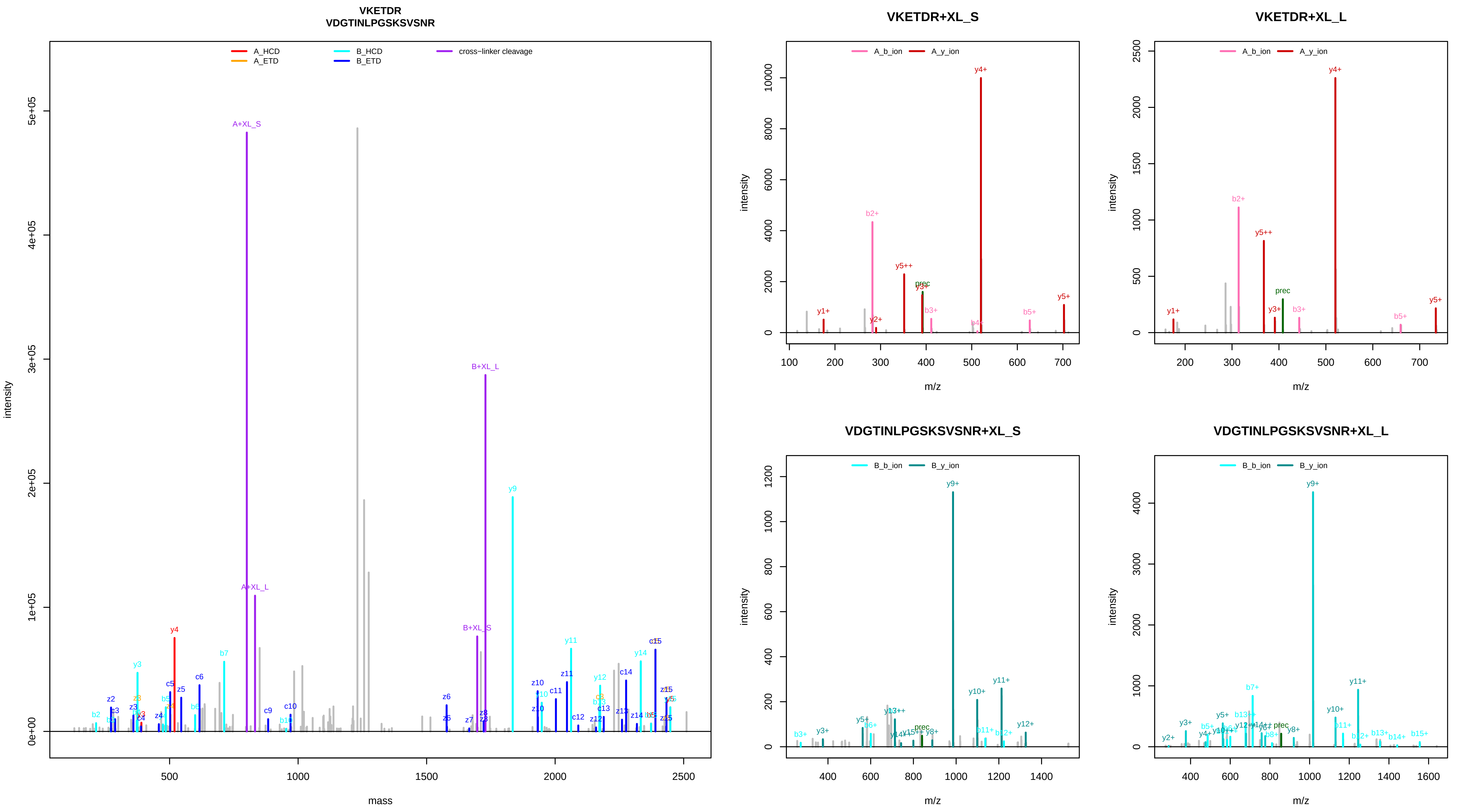

VKETDR
VDGTINLPGSKSVSNR
A_HCD
A_ETD
B_HCD
B_ETD
cross−linker cleavage
A+XL_S
A+XL_L
B+XL_L
B+XL_S
intensity
mass
VKETDR+XL_S
VKETDR+XL_L
A_b_ion
A_y_ion
VDGTINLPGSKSVSNR+XL_S
VDGTINLPGSKSVSNR+XL_L
B_b_ion
B_y_ion
prec
intensity
m/z

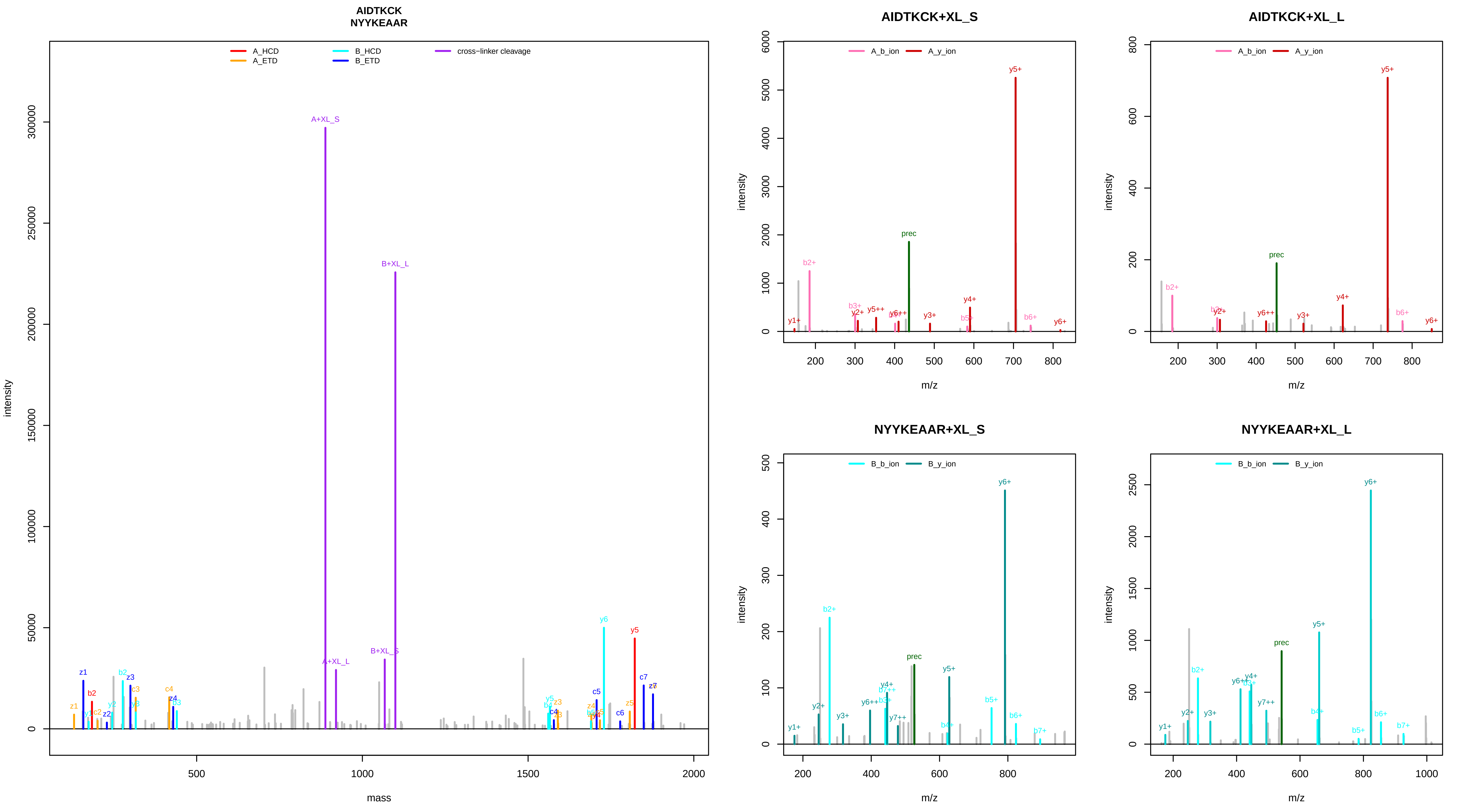

AIDTKCK
NYYKEAAR
A_HCD
A_ETD
B_HCD
B_ETD
cross−linker cleavage
A+XL_S
B+XL_L
A+XL_L
B+XL_S
intensity
mass
AIDTKCK+XL_S
AIDTKCK+XL_L
A_b_ion
A_y_ion
NYYKEAAR+XL_S
NYYKEAAR+XL_L
B_b_ion
B_y_ion
prec
intensity
m/z

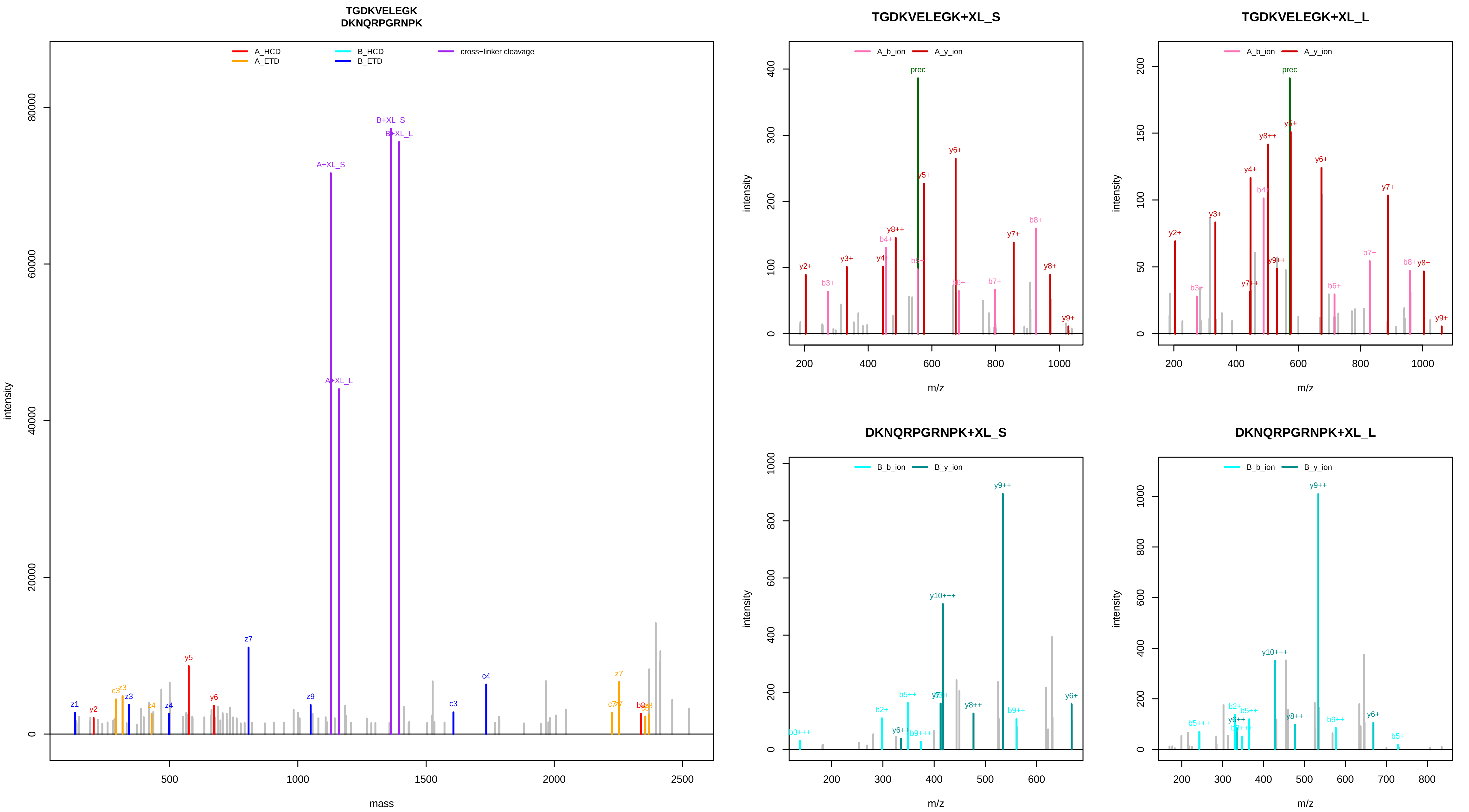

TGDKVELEGK
DKNQRPGRNPK
A_HCD
A_ETD
B_HCD
B_ETD
cross−linker cleavage
B+XL_S
B+XL_L
A+XL_S
A+XL_L
intensity
mass
TGDKVELEGK+XL_S
TGDKVELEGK+XL_L
A_b_ion
A_y_ion
prec
intensity
m/z
DKNQRPGRNPK+XL_S
DKNQRPGRNPK+XL_L
B_b_ion
B_y_ion
y9++
y10+++
intensity
m/z

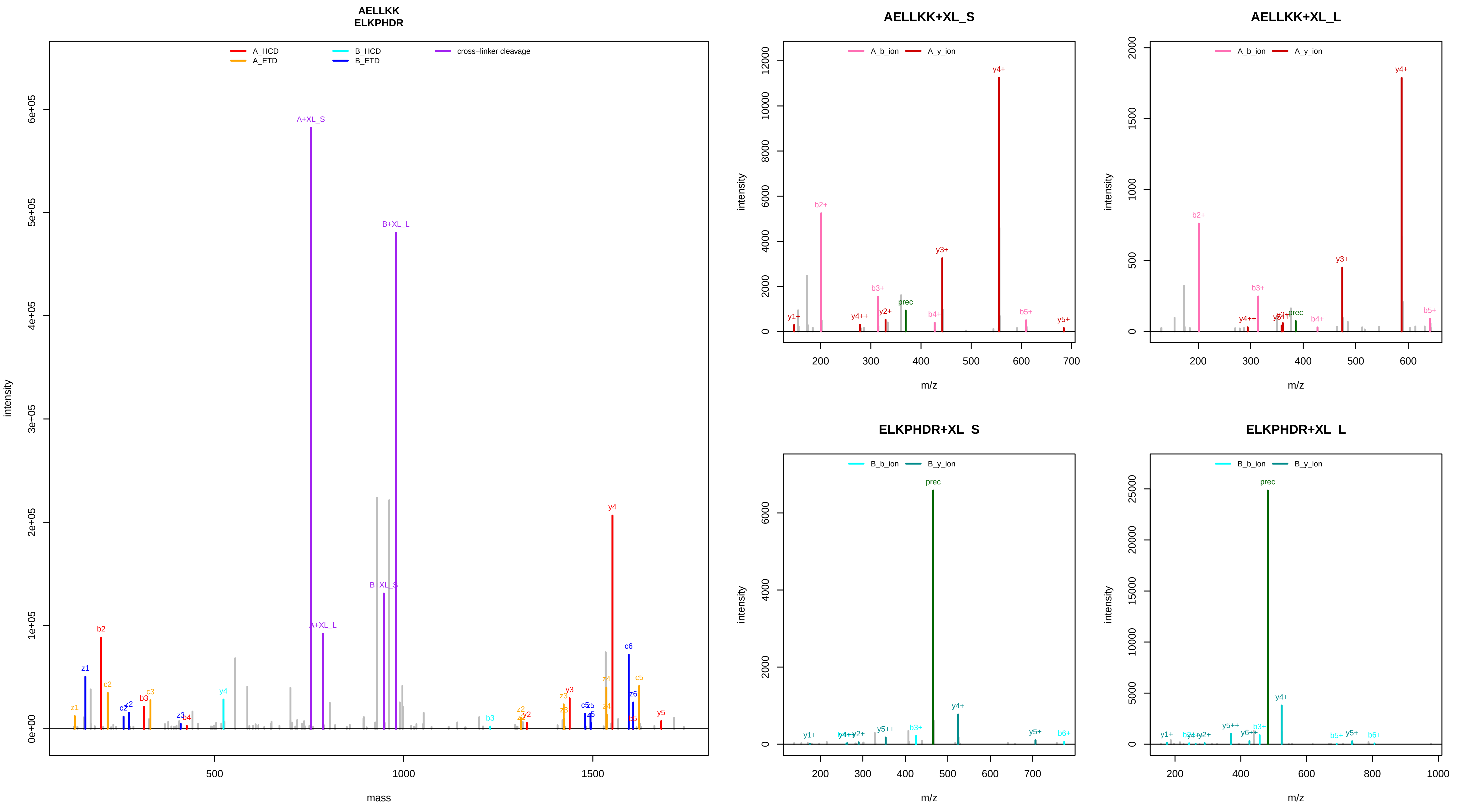
AELLKK
ELKPHDR
A_HCD
A_ETD
B_HCD
B_ETD
cross−linker cleavage
A+XL_S
B+XL_L
B+XL_S
A+XL_L
intensity
mass
AELLKK+XL_S
AELLKK+XL_L
ELKPHDR+XL_S
ELKPHDR+XL_L
A_b_ion
A_y_ion
B_b_ion
B_y_ion
prec
m/z
intensity

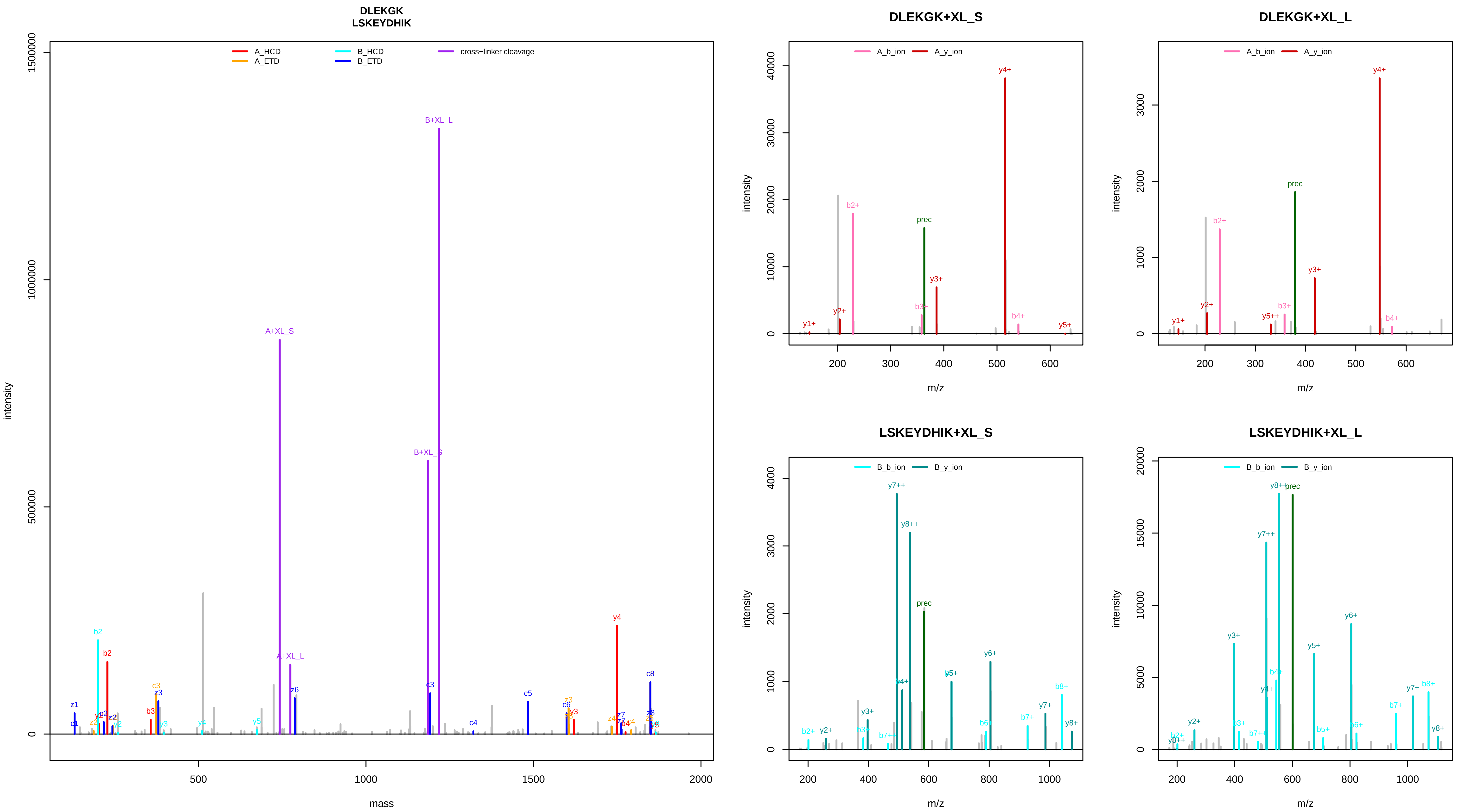

DLEKGK
LSKEYDHIK
A_HCD
A_ETD
B_HCD
B_ETD
cross−linker cleavage
B+XL_L
A+XL_S
B+XL_S
A+XL_L
intensity
mass
DLEKGK+XL_S
DLEKGK+XL_L
LSKEYDHIK+XL_S
LSKEYDHIK+XL_L
A_b_ion
A_y_ion
B_b_ion
B_y_ion
prec
m/z

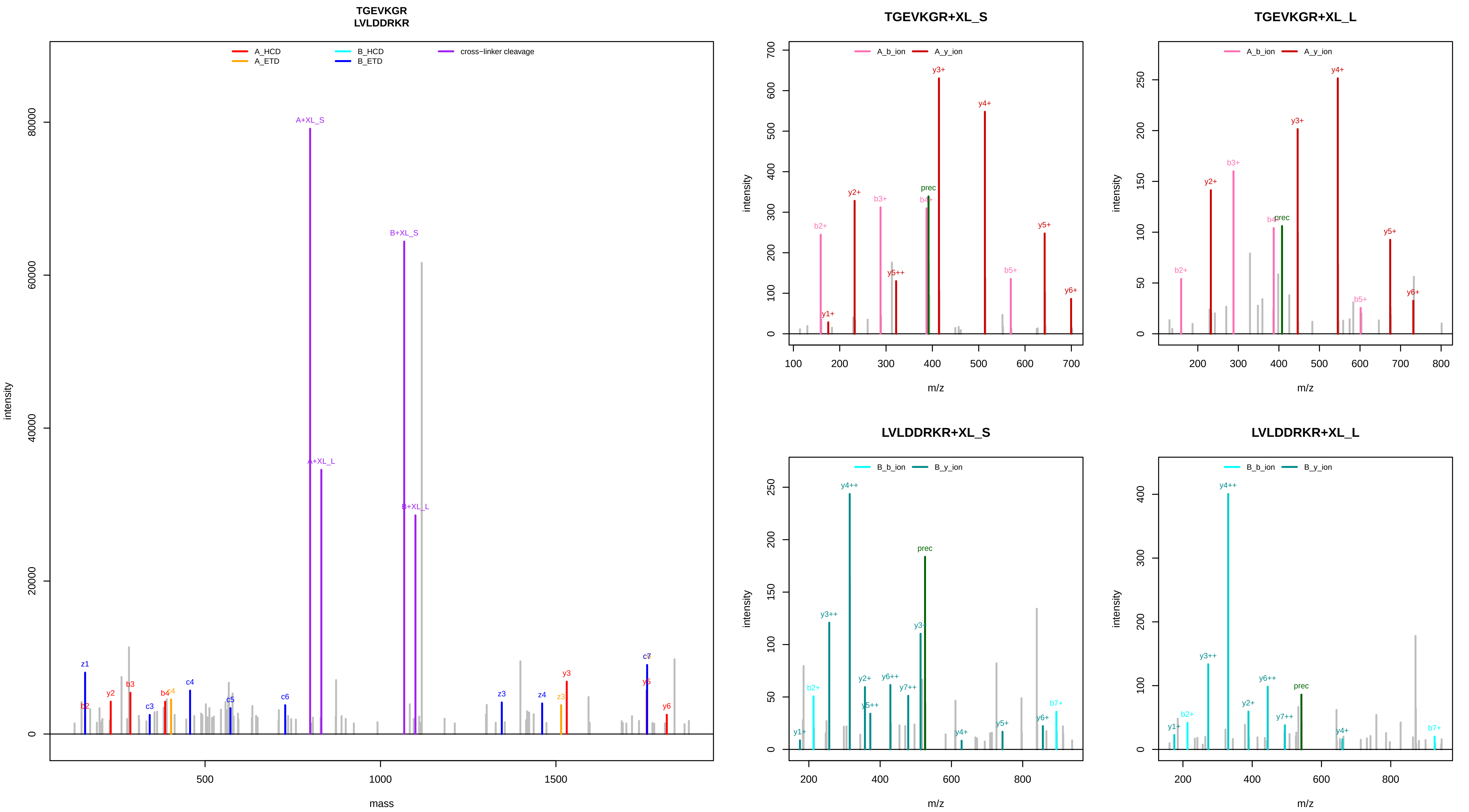

TGEVKGR
LVLDDRKR
A_HCD
A_ETD
B_HCD
B_ETD
cross−linker cleavage
A+XL_S
B+XL_S
A+XL_L
B+XL_L
intensity
mass
TGEVKGR+XL_S
TGEVKGR+XL_L
LVLDDRKR+XL_S
LVLDDRKR+XL_L
A_b_ion
A_y_ion
B_b_ion
B_y_ion
prec
m/z

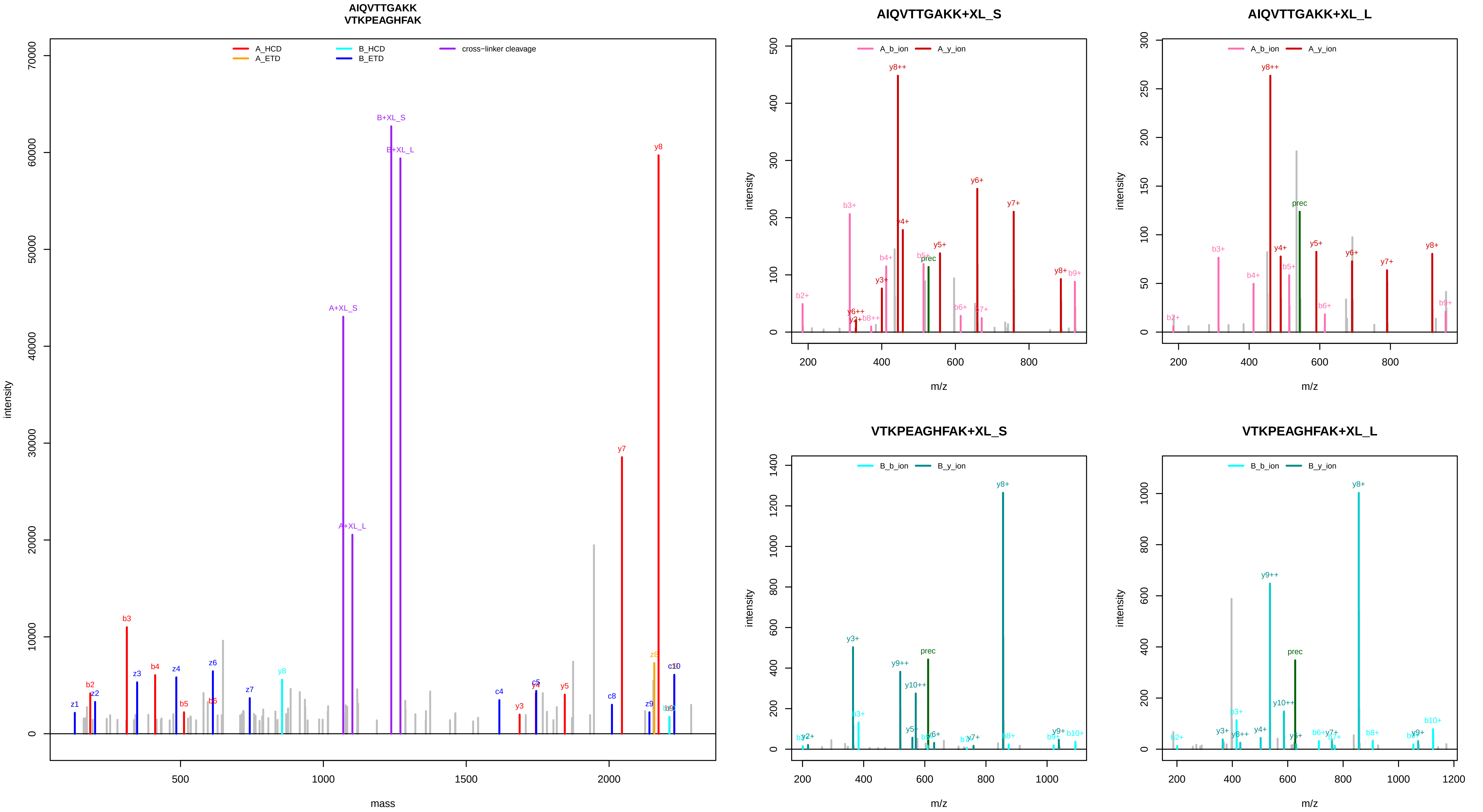

AIQVTTGAKK
VTKPEAGHFAK
A_HCD
A_ETD
B_HCD
B_ETD
cross−linker cleavage
intensity
mass
AIQVTTGAKK+XL_S
AIQVTTGAKK+XL_L
VTKPEAGHFAK+XL_S
VTKPEAGHFAK+XL_L
A_b_ion
A_y_ion
B_b_ion
B_y_ion
m/z

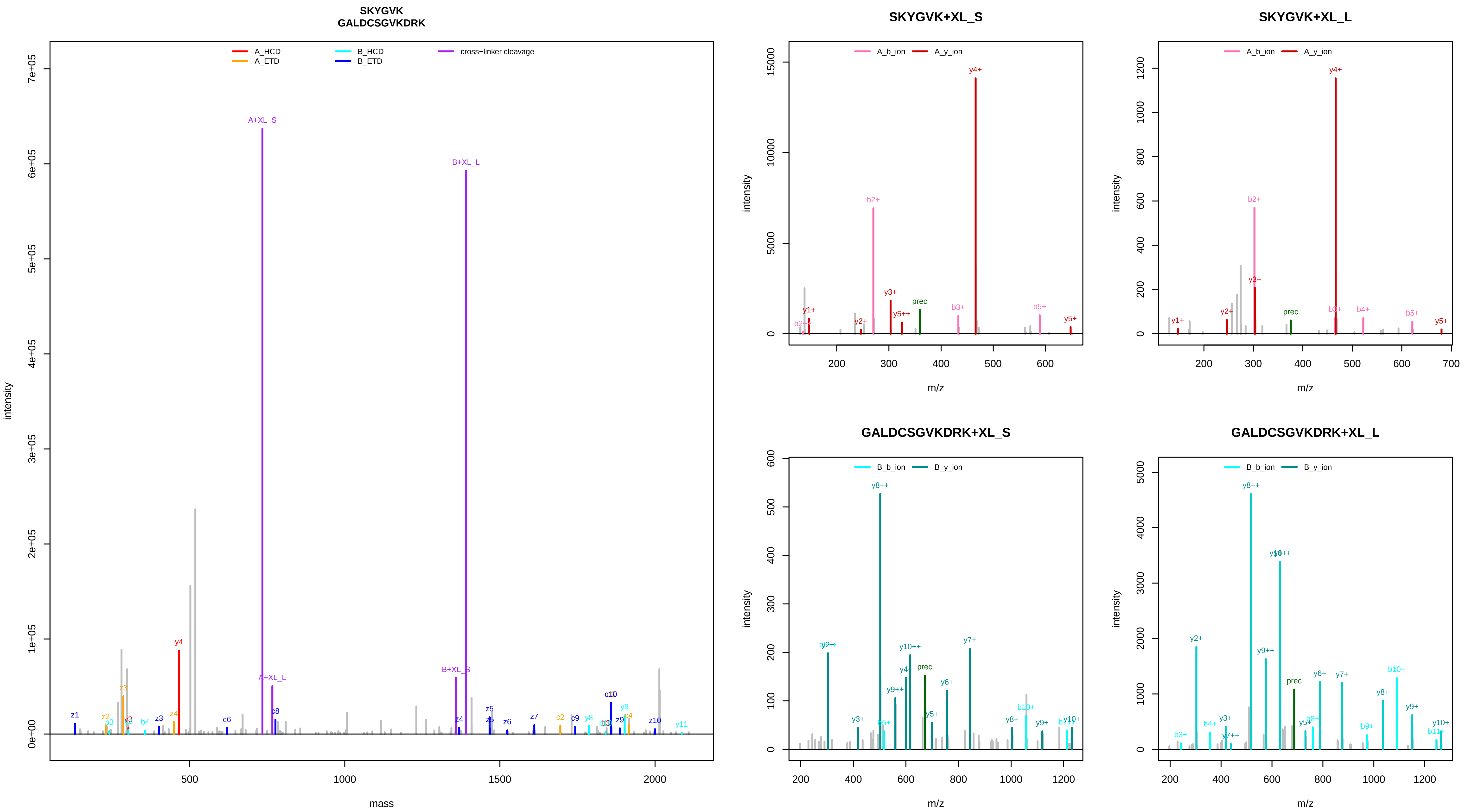

SKYGVK
GALDCSGVKDRK
A_HCD
A_ETD
B_HCD
B_ETD
cross−linker cleavage
A+XL_S
B+XL_L
A+XL_L
B+XL_S
intensity
mass
SKYGVK+XL_S
SKYGVK+XL_L
A_b_ion
A_y_ion
GALDCSGVKDRK+XL_S
GALDCSGVKDRK+XL_L
B_b_ion
B_y_ion
prec
m/z

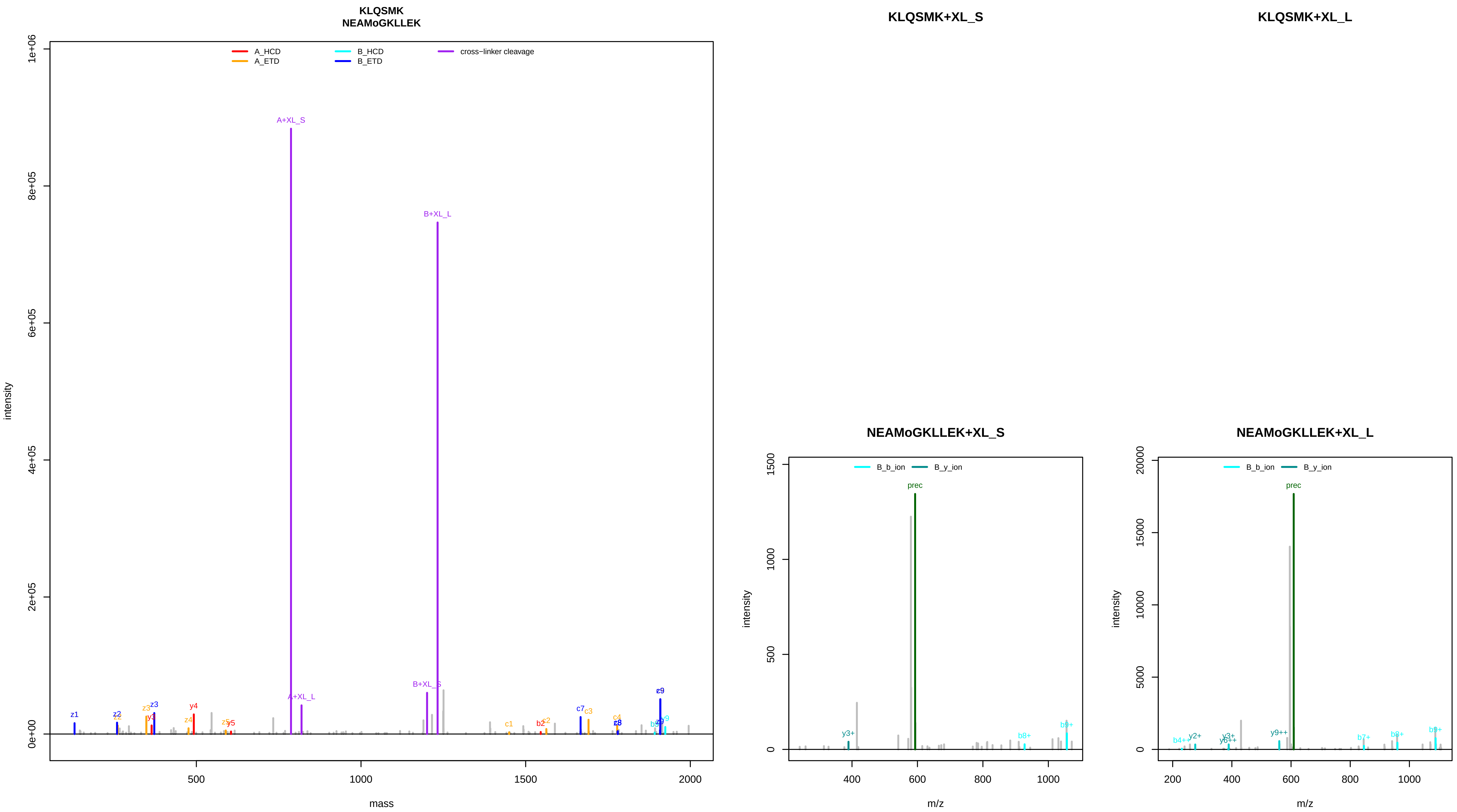
KLQSMK
NEAMoGKLLEK
A_HCD
A_ETD
B_HCD
B_ETD
cross−linker cleavage
A+XL_S
B+XL_L
B+XL_S
A+XL_L
intensity
mass
KLQSMK+XL_S
KLQSMK+XL_L
NEAMoGKLLEK+XL_S
NEAMoGKLLEK+XL_L
B_b_ion
B_y_ion
prec
m/z

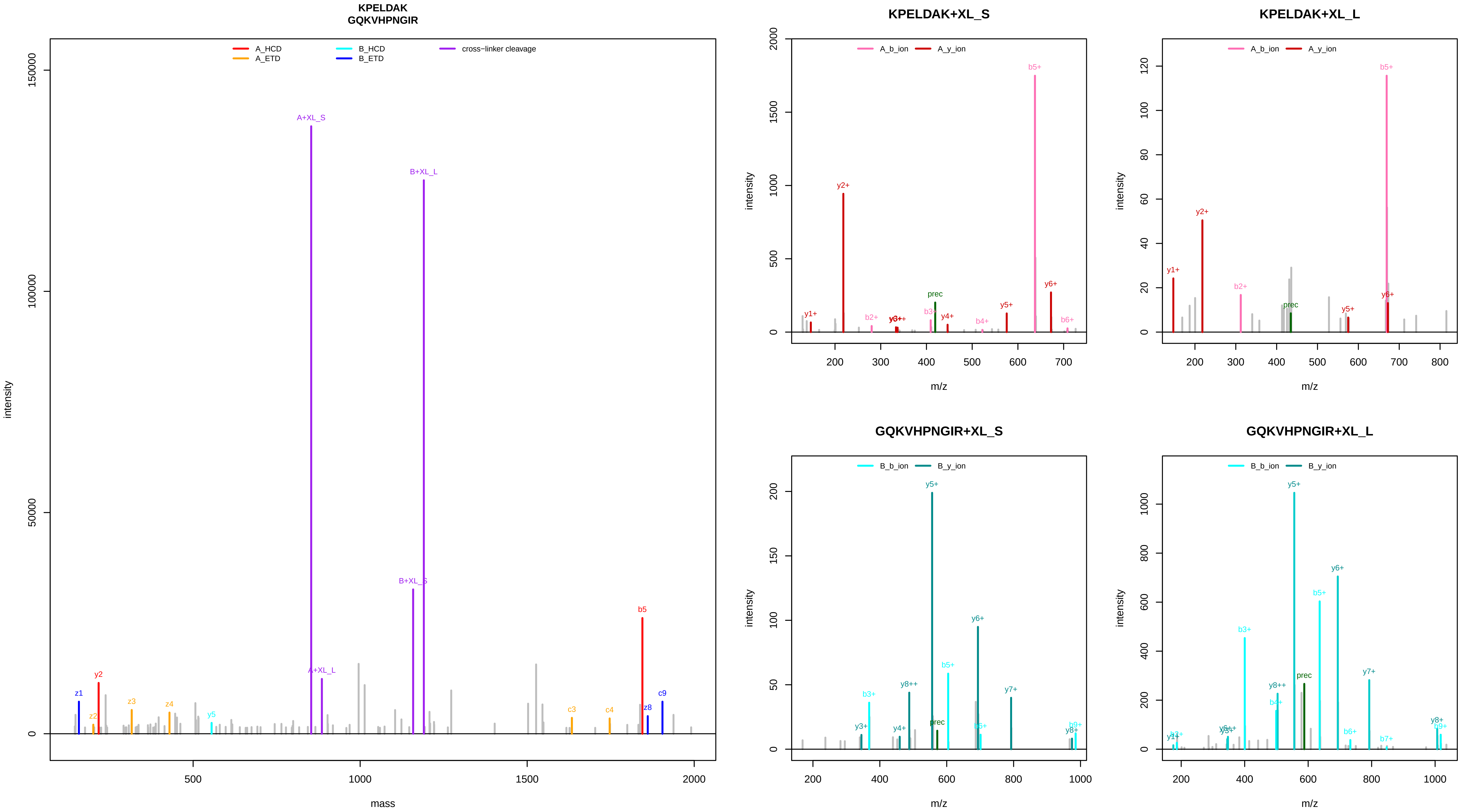
KPELDAK
GQKVHPNGIR
A_HCD
A_ETD
B_HCD
B_ETD
cross−linker cleavage
A+XL_S
B+XL_L
B+XL_S
A+XL_L
intensity
mass
KPELDAK+XL_S
KPELDAK+XL_L
GQKVHPNGIR+XL_S
GQKVHPNGIR+XL_L
A_b_ion
A_y_ion
B_b_ion
B_y_ion
m/z

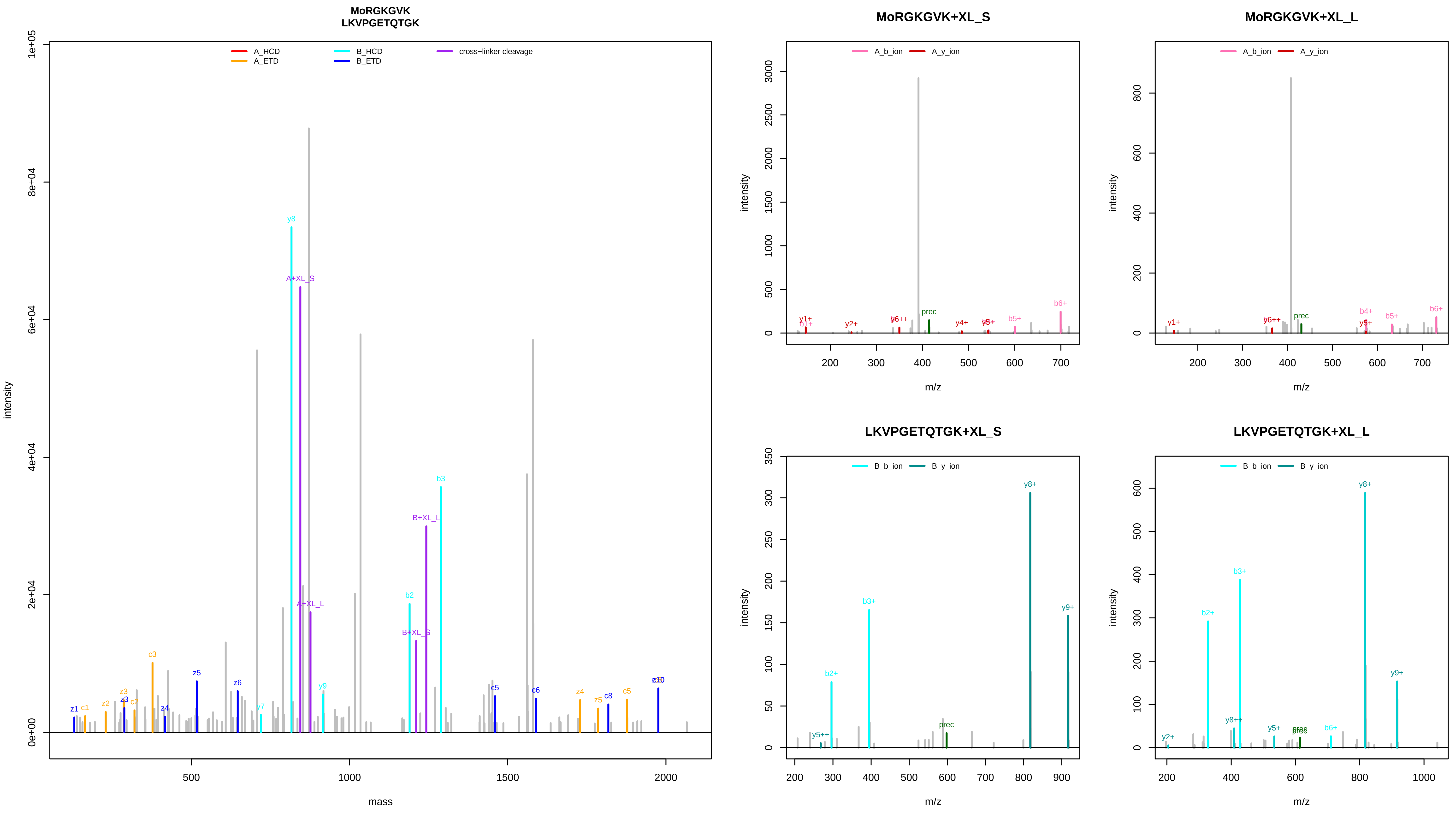

MoRGKGVK
LKVPGETQTGK
A_HCD
A_ETD
B_HCD
B_ETD
cross−linker cleavage
intensity
mass
A+XL_S
A+XL_L
B+XL_L
B+XL_S
MoRGKGVK+XL_S
MoRGKGVK+XL_L
A_b_ion
A_y_ion
prec
m/z
LKVPGETQTGK+XL_S
LKVPGETQTGK+XL_L
B_b_ion
B_y_ion

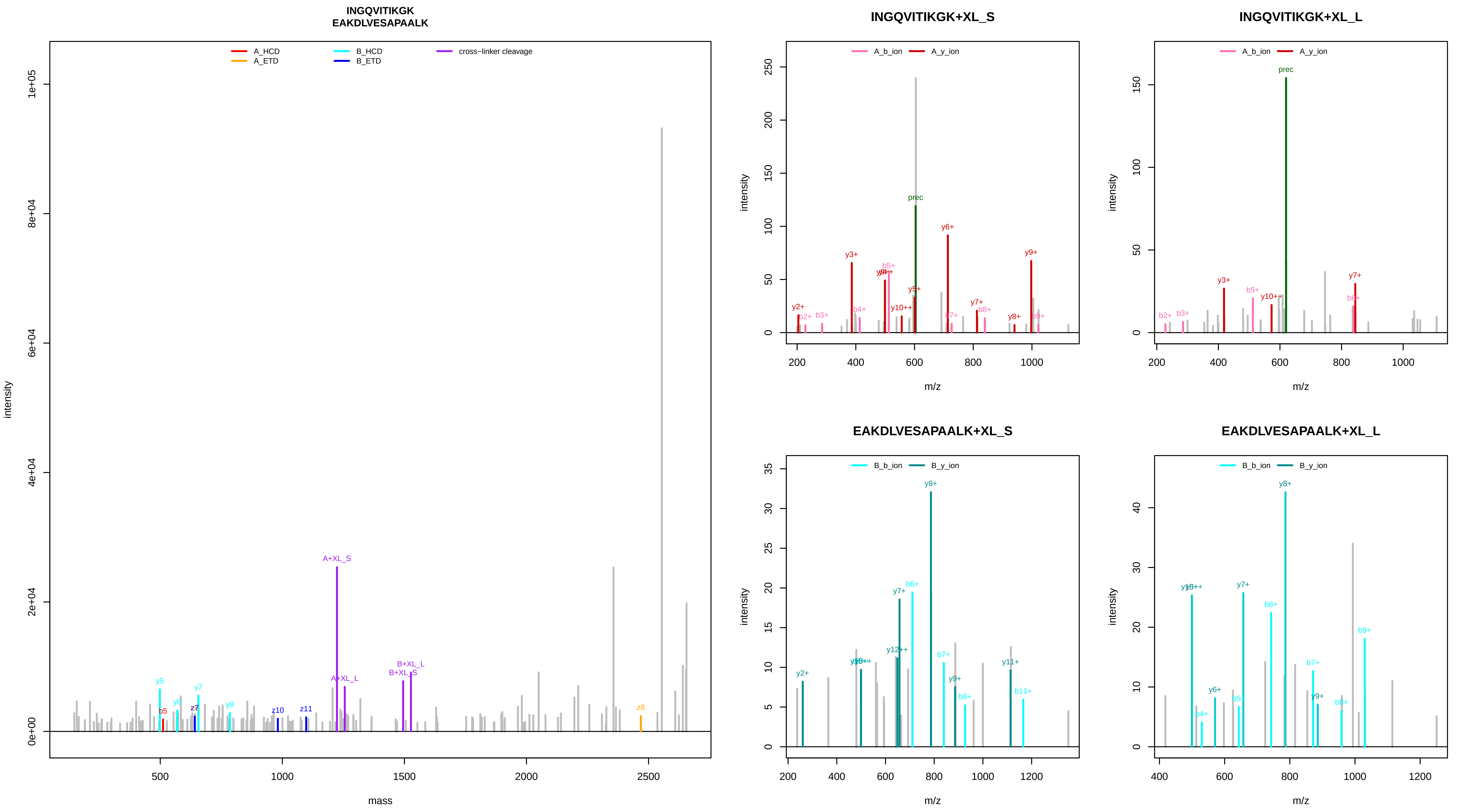
INGQVITIKGK
EAKDLVESAPAALK
A_HCD
A_ETD
B_HCD
B_ETD
cross−linker cleavage
A+XL_S
A+XL_L
B+XL_L
B+XL_S
intensity
mass
INGQVITIKGK+XL_S
INGQVITIKGK+XL_L
A_b_ion
A_y_ion
prec
EAKDLVESAPAALK+XL_S
EAKDLVESAPAALK+XL_L
B_b_ion
B_y_ion
m/z

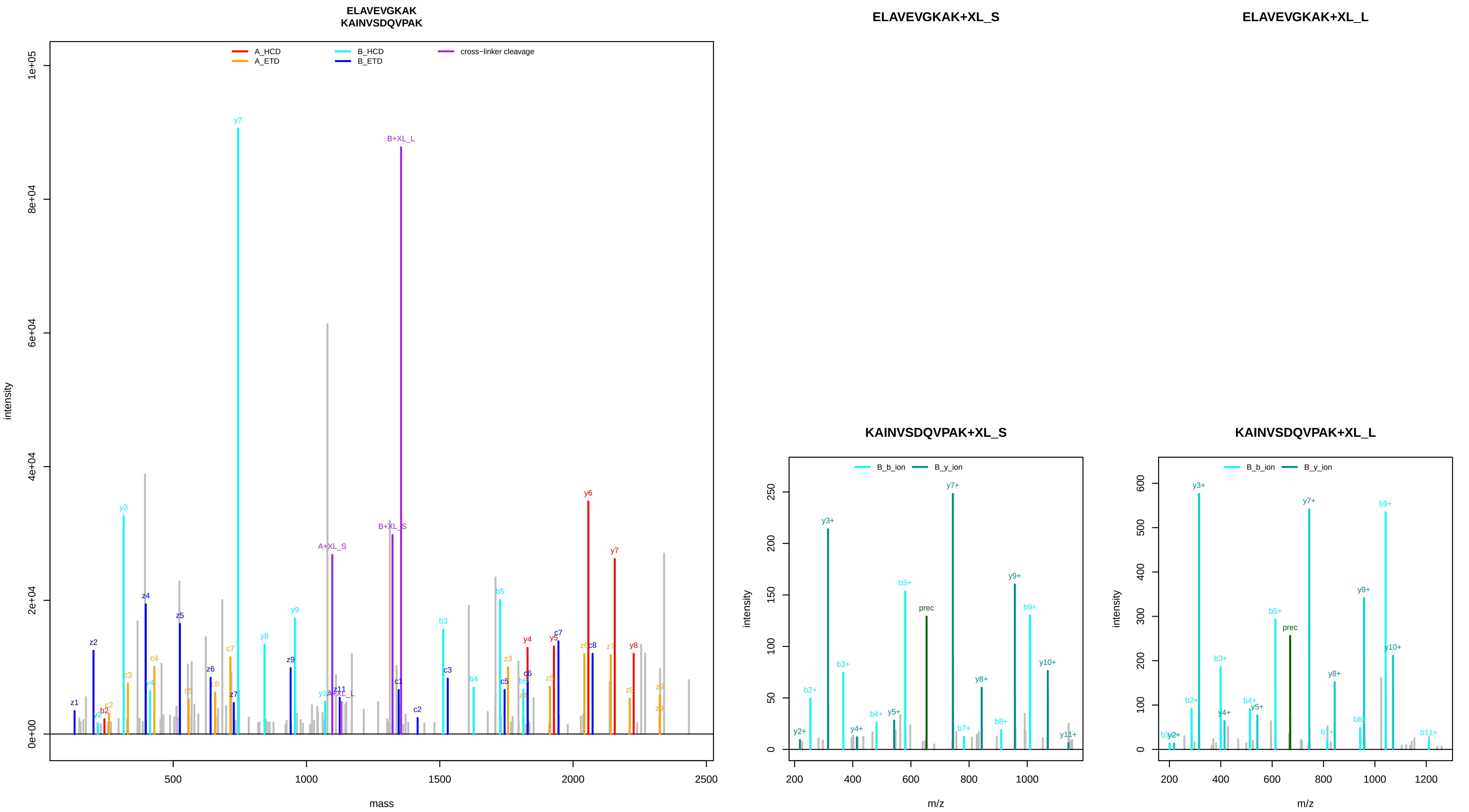
ELAVEVGKAK
KAINVSDQVPAK
A_HCD
A_ETD
B_HCD
B_ETD
cross−linker cleavage
intensity
mass
0e+00
2e+04
4e+04
6e+04
8e+04
1e+05
500
1000
1500
2000
2500
ELAVEVGKAK+XL_S
ELAVEVGKAK+XL_L
KAINVSDQVPAK+XL_S
KAINVSDQVPAK+XL_L
B_b_ion
B_y_ion
prec
m/z
intensity

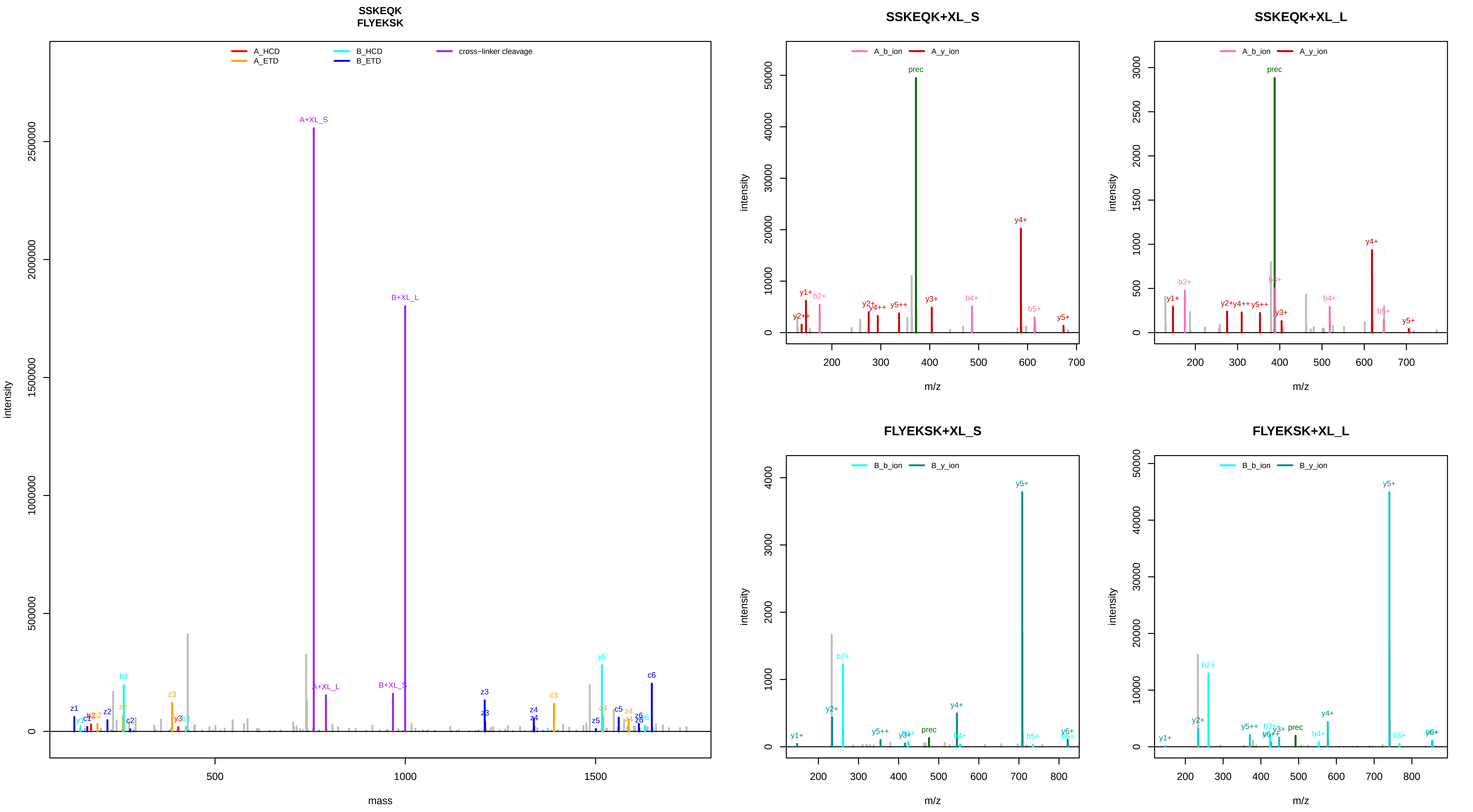
SSKEQK
FLYEKSK
A_HCD
A_ETD
B_HCD
B_ETD
cross−linker cleavage
A+XL_S
B+XL_L
A+XL_L
B+XL_S
intensity
mass
SSKEQK+XL_S
SSKEQK+XL_L
FLYEKSK+XL_S
FLYEKSK+XL_L
A_b_ion
A_y_ion
B_b_ion
B_y_ion
prec
m/z

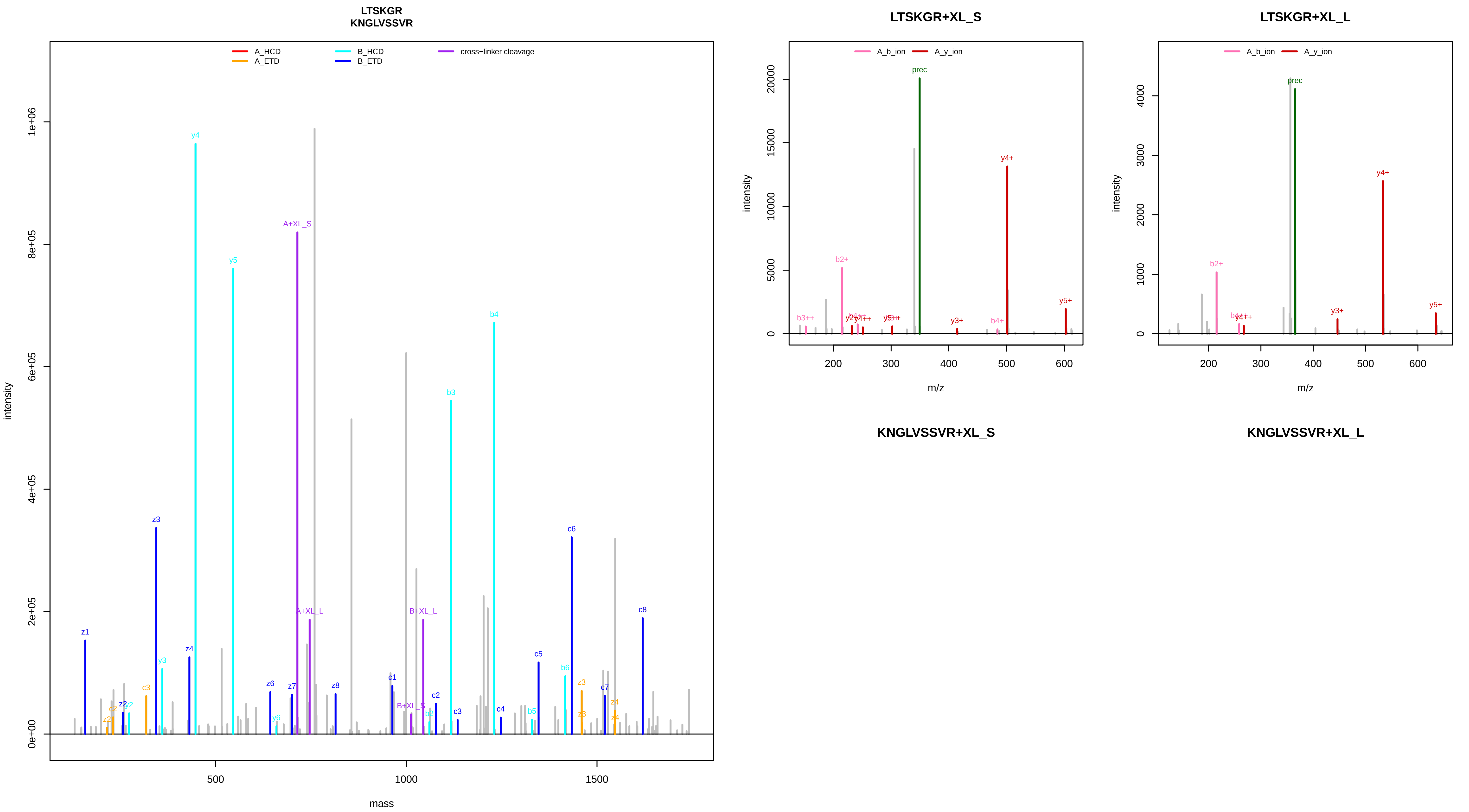
LTSKGR
KNGLVSSVR
A_HCD
A_ETD
B_HCD
B_ETD
cross−linker cleavage
intensity
mass
LTSKGR+XL_S
LTSKGR+XL_L
A_b_ion
A_y_ion
m/z
KNGLVSSVR+XL_S
KNGLVSSVR+XL_L

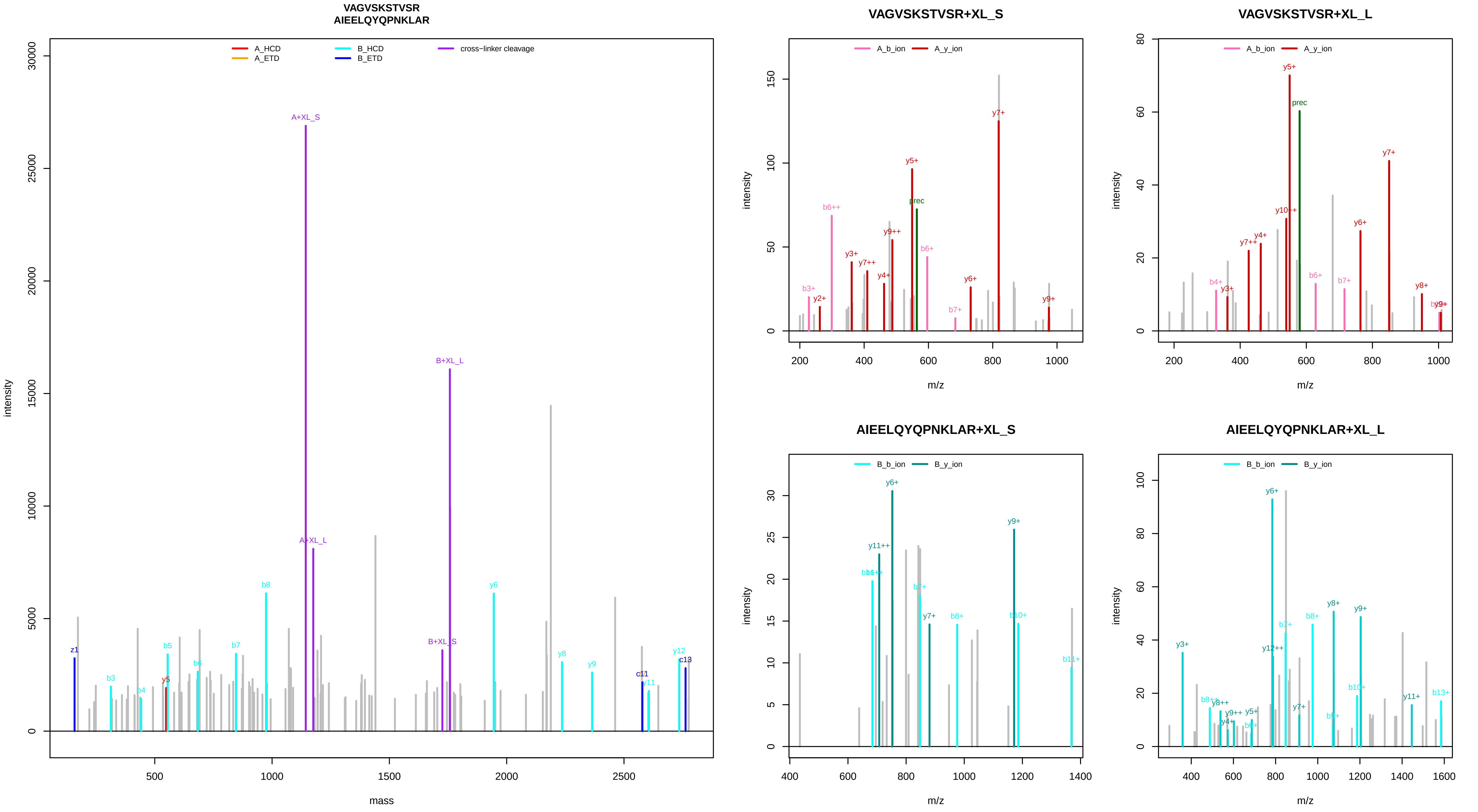
VAGVSKSTVSR
AIEELQYQPNKLAR
A_HCD
A_ETD
B_HCD
B_ETD
cross−linker cleavage
A+XL_S
B+XL_L
A+XL_L
B+XL_S
intensity
mass
VAGVSKSTVSR+XL_S
VAGVSKSTVSR+XL_L
AIEELQYQPNKLAR+XL_S
AIEELQYQPNKLAR+XL_L
A_b_ion
A_y_ion
B_b_ion
B_y_ion
prec
m/z

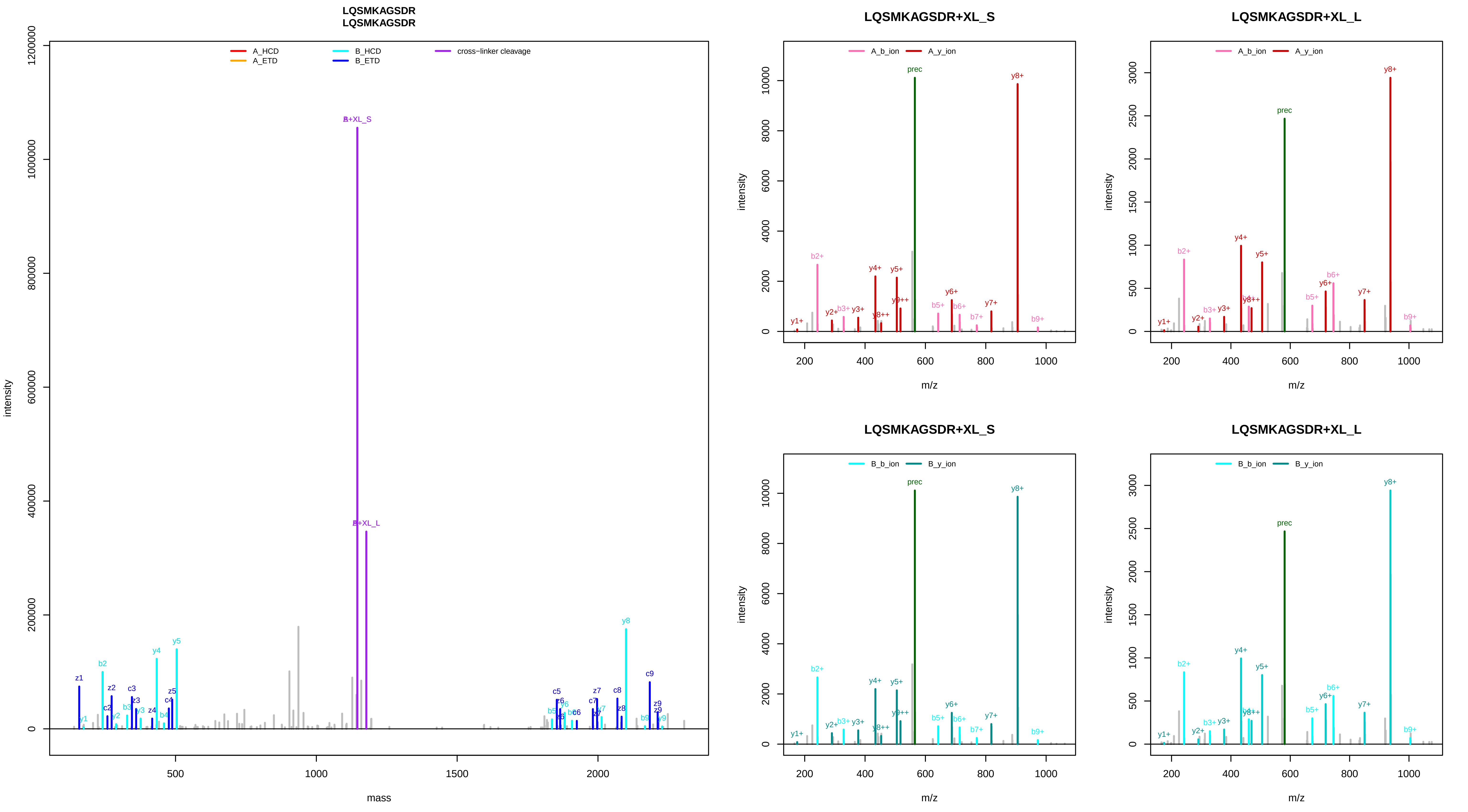

LQSMKAGSDR
LQSMKAGSDR
A_HCD
A_ETD
B_HCD
B_ETD
cross−linker cleavage
intensity
mass
LQSMKAGSDR+XL_S
LQSMKAGSDR+XL_L
A_b_ion
A_y_ion
B_b_ion
B_y_ion
prec
intensity
m/z

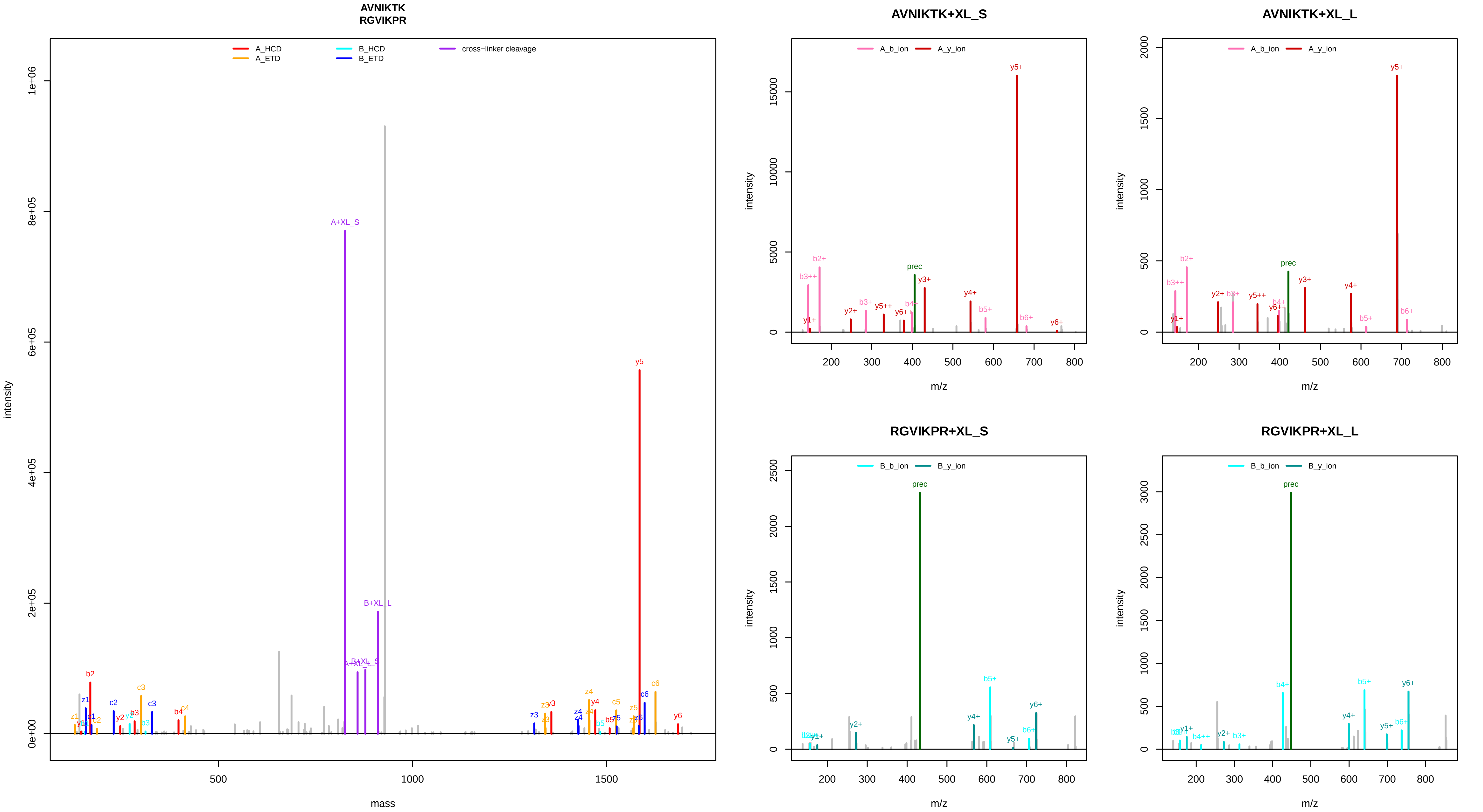
AVNIKTK
RGVIKPR
A_HCD
A_ETD
B_HCD
B_ETD
cross−linker cleavage
A+XL_S
B+XL_L
y5
intensity
mass
AVNIKTK+XL_S
AVNIKTK+XL_L
A_b_ion
A_y_ion
RGVIKPR+XL_S
RGVIKPR+XL_L
B_b_ion
B_y_ion
prec
m/z

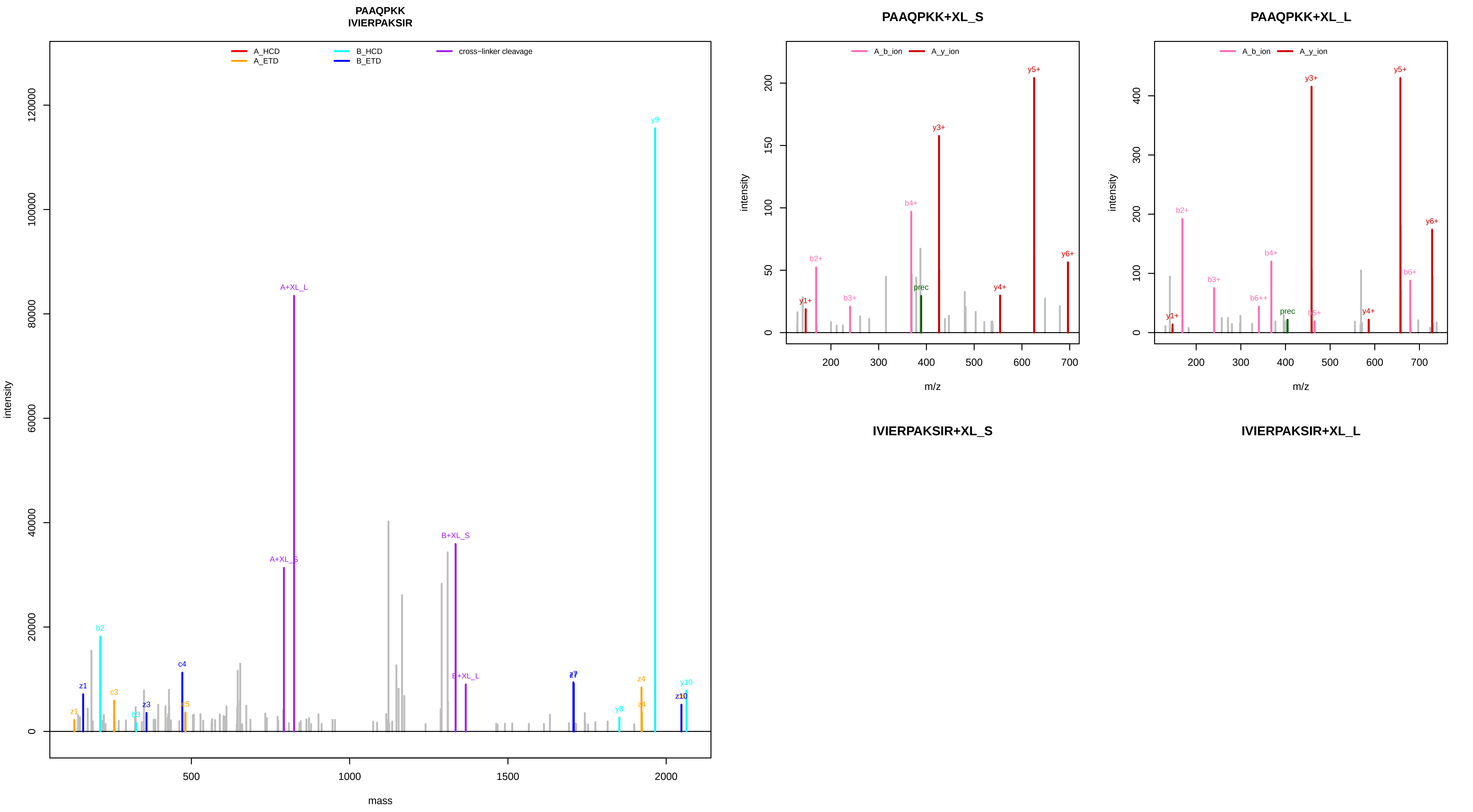
PAAQPKK
IVIERPAKSIR
A_HCD
A_ETD
B_HCD
B_ETD
cross−linker cleavage
intensity
mass
y9
A+XL_L
A+XL_S
B+XL_S
B+XL_L
b2
z1
c3
b3
z3
c4
c5
z7
y8
z4
z10
y10
PAAQPKK+XL_S
PAAQPKK+XL_L
A_b_ion
A_y_ion
m/z
y1+
b2+
b3+
b4+
prec
y3+
y4+
y5+
y6+
b6++
b5+
b6+
IVIERPAKSIR+XL_S
IVIERPAKSIR+XL_L

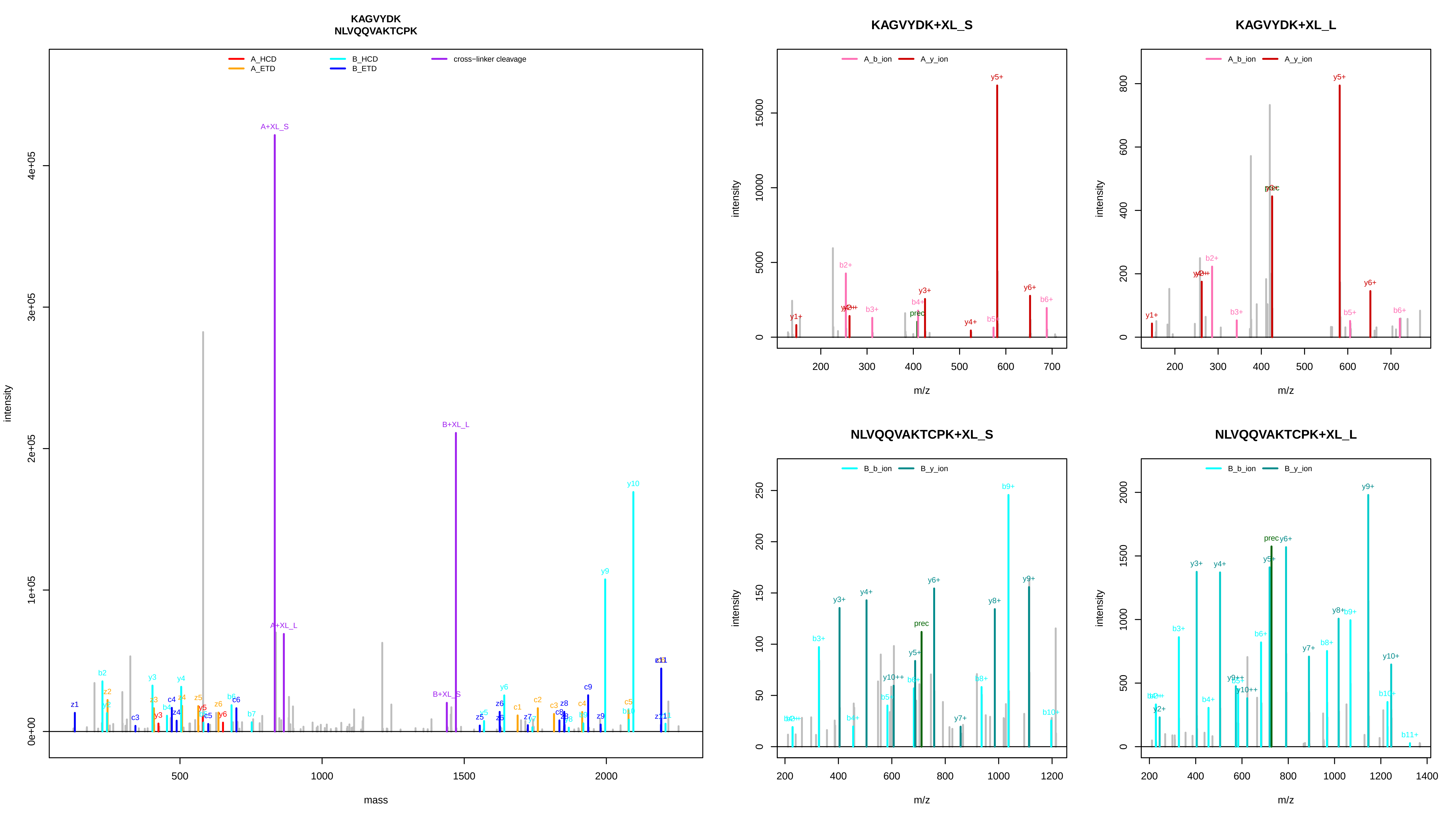
KAGVYDK
NLVQQVAKTCPK
A_HCD
A_ETD
B_HCD
B_ETD
cross−linker cleavage
A+XL_S
B+XL_L
A+XL_L
B+XL_S
y10
y9
intensity
mass
KAGVYDK+XL_S
KAGVYDK+XL_L
A_b_ion
A_y_ion
y5+
y6+
b6+
b2+
prec
m/z
NLVQQVAKTCPK+XL_S
NLVQQVAKTCPK+XL_L
B_b_ion
B_y_ion
b9+
y9+
y6+
y4+
y3+
y8+
b3+

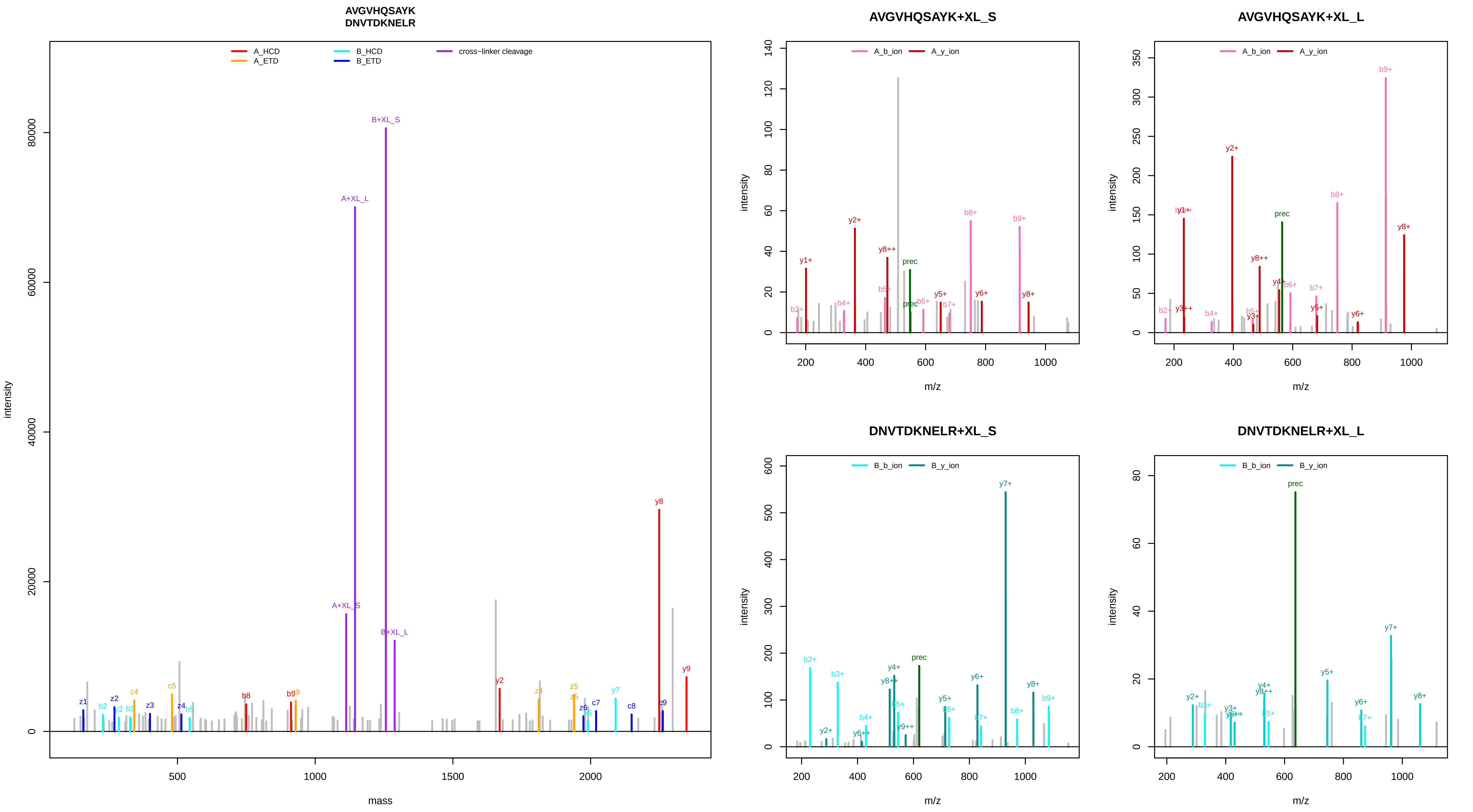

AVGVHQSAYK
DNVTDKNELR
A_HCD
A_ETD
B_HCD
B_ETD
cross−linker cleavage
B+XL_S
A+XL_L
A+XL_S
B+XL_L
intensity
mass
AVGVHQSAYK+XL_S
AVGVHQSAYK+XL_L
DNVTDKNELR+XL_S
DNVTDKNELR+XL_L
A_b_ion
A_y_ion
B_b_ion
B_y_ion
prec
m/z

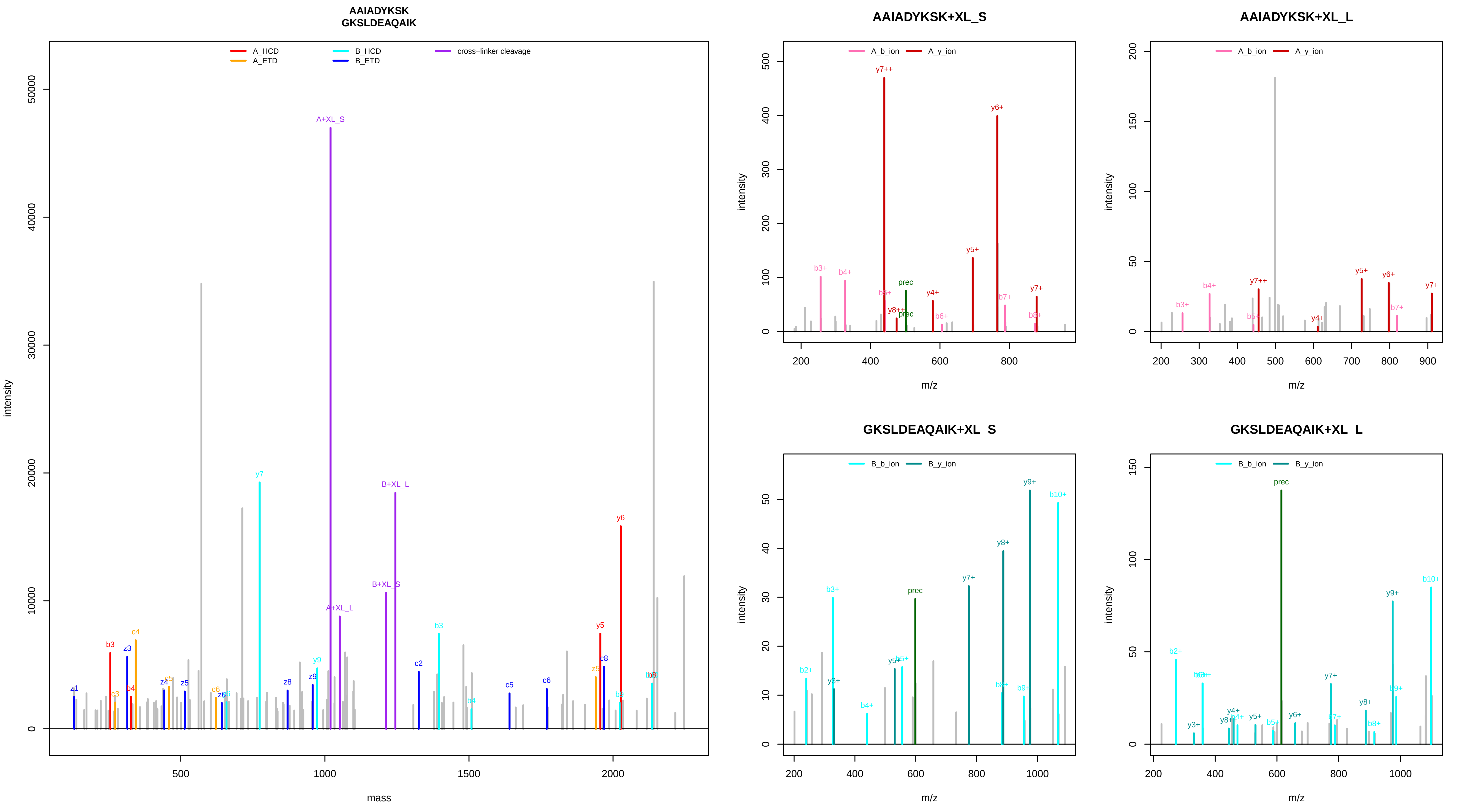

AAIADYKSK
GKSLDEAQAIK
A_HCD
A_ETD
B_HCD
B_ETD
cross−linker cleavage
A+XL_S
B+XL_L
B+XL_S
A+XL_L
intensity
mass
AAIADYKSK+XL_S
AAIADYKSK+XL_L
GKSLDEAQAIK+XL_S
GKSLDEAQAIK+XL_L
A_b_ion
A_y_ion
B_b_ion
B_y_ion
prec
m/z

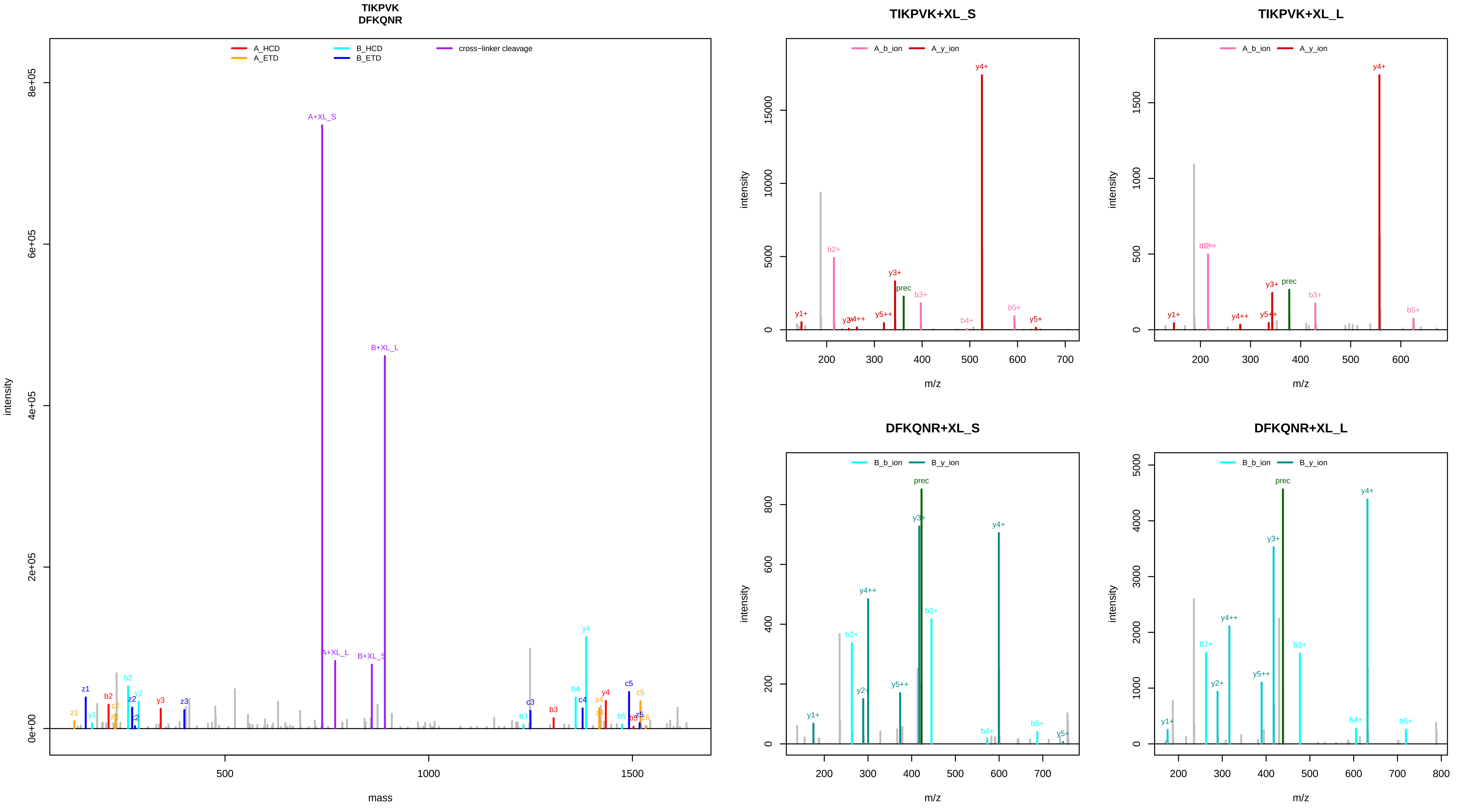
TIKPVK
DFKQNR
A_HCD
A_ETD
B_HCD
B_ETD
cross−linker cleavage
A+XL_S
B+XL_L
A+XL_L
B+XL_S
intensity
mass
TIKPVK+XL_S
TIKPVK+XL_L
DFKQNR+XL_S
DFKQNR+XL_L
A_b_ion
A_y_ion
B_b_ion
B_y_ion
prec
m/z

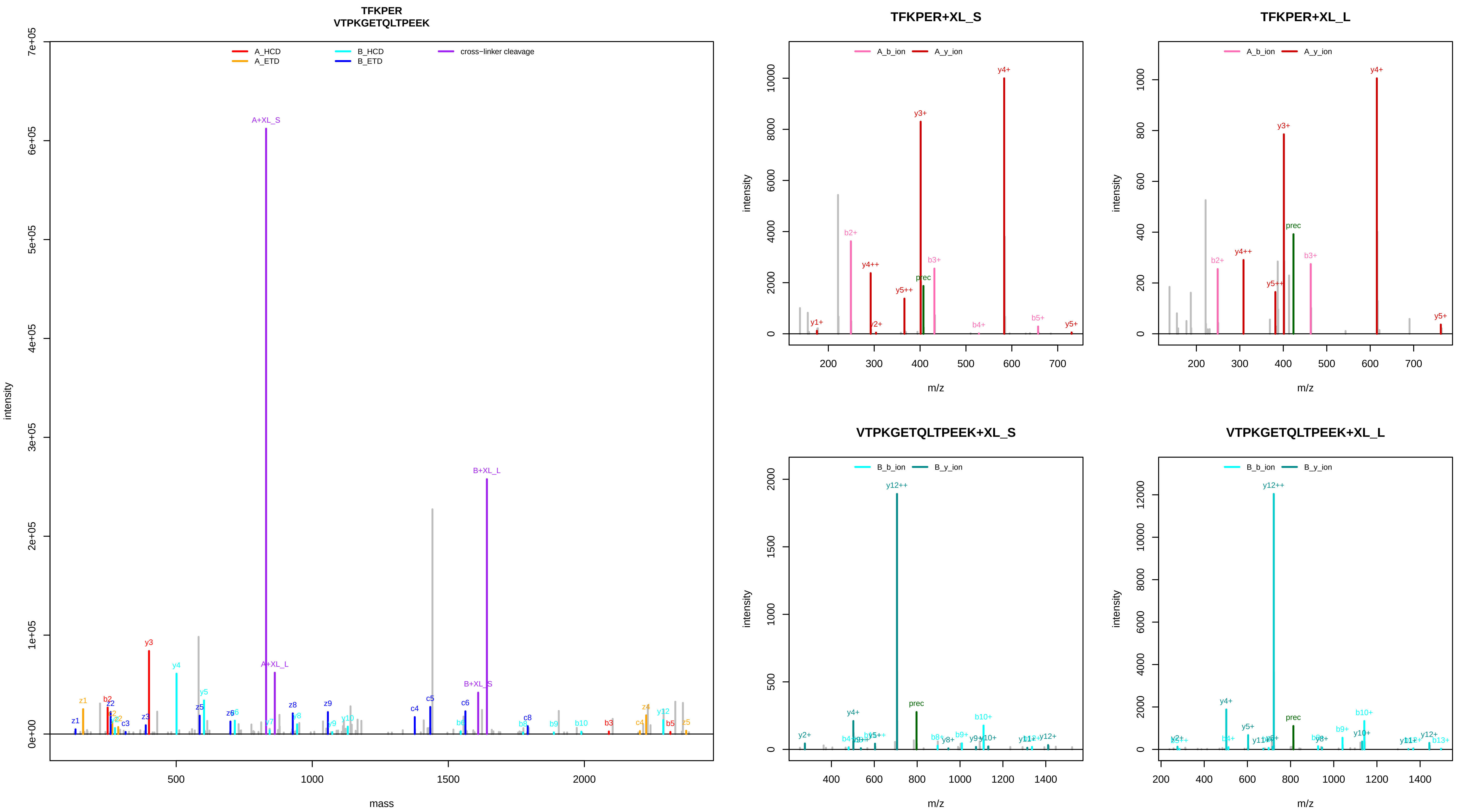

TFKPER
VTPKGETQLTPEEK
A_HCD
A_ETD
B_HCD
B_ETD
cross−linker cleavage
intensity
mass
A+XL_S
B+XL_L
A+XL_L
B+XL_S
TFKPER+XL_S
TFKPER+XL_L
VTPKGETQLTPEEK+XL_S
VTPKGETQLTPEEK+XL_L
A_b_ion
A_y_ion
B_b_ion
B_y_ion
m/z
prec

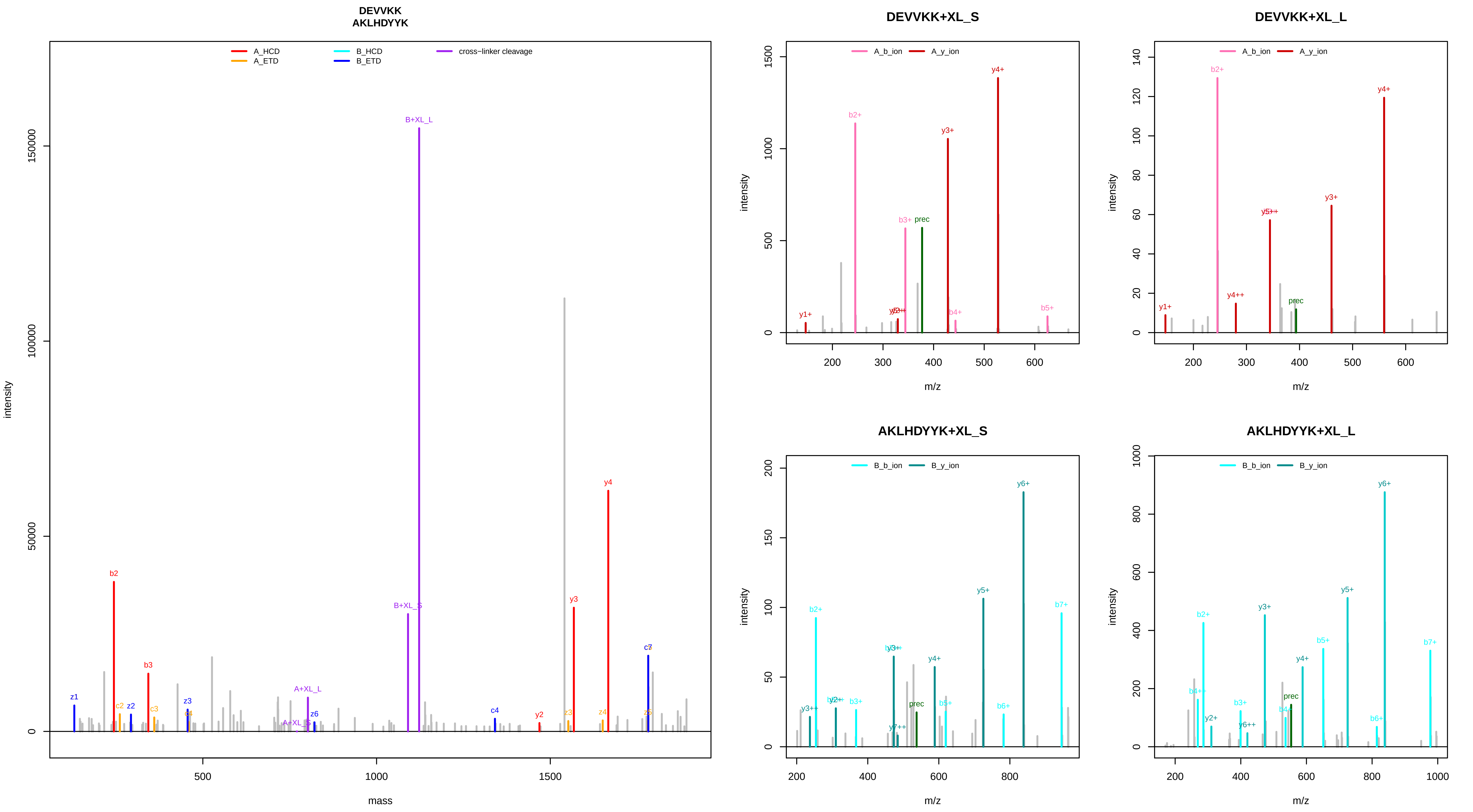

DEVVKK
AKLHDYYK
A_HCD
A_ETD
B_HCD
B_ETD
cross−linker cleavage
B+XL_L
B+XL_S
A+XL_L
A+XL_S
intensity
mass
DEVVKK+XL_S
DEVVKK+XL_L
AKLHDYYK+XL_S
AKLHDYYK+XL_L
A_b_ion
A_y_ion
B_b_ion
B_y_ion
prec
m/z

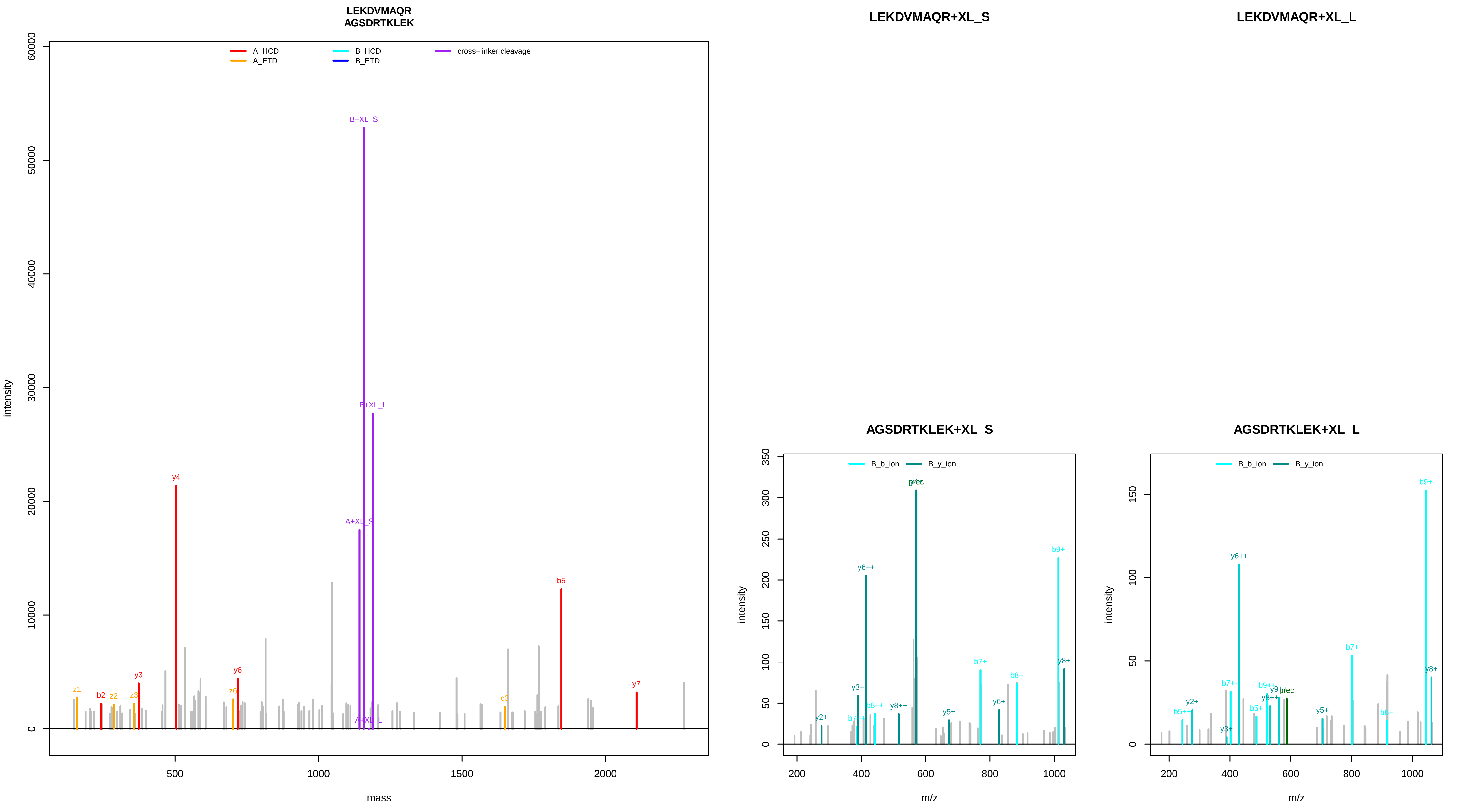

LEKDVMAQR
AGSDRTKLEK
A_HCD
A_ETD
B_HCD
B_ETD
cross−linker cleavage
B+XL_S
B+XL_L
A+XL_S
A+XL_L
y4
b5
y7
y3
y6
b2
z1
z2
z3
z6
c3
intensity
mass
LEKDVMAQR+XL_S
LEKDVMAQR+XL_L
AGSDRTKLEK+XL_S
AGSDRTKLEK+XL_L
B_b_ion
B_y_ion
prec
y6++
b9+
b7+
b8+
y8+
y3+
y6+
y5+
y2+
y8++
b8++
b7+
y2+
b5++
b7++
b9++
y8++
b5+
y3+
y5+
b8+
m/z

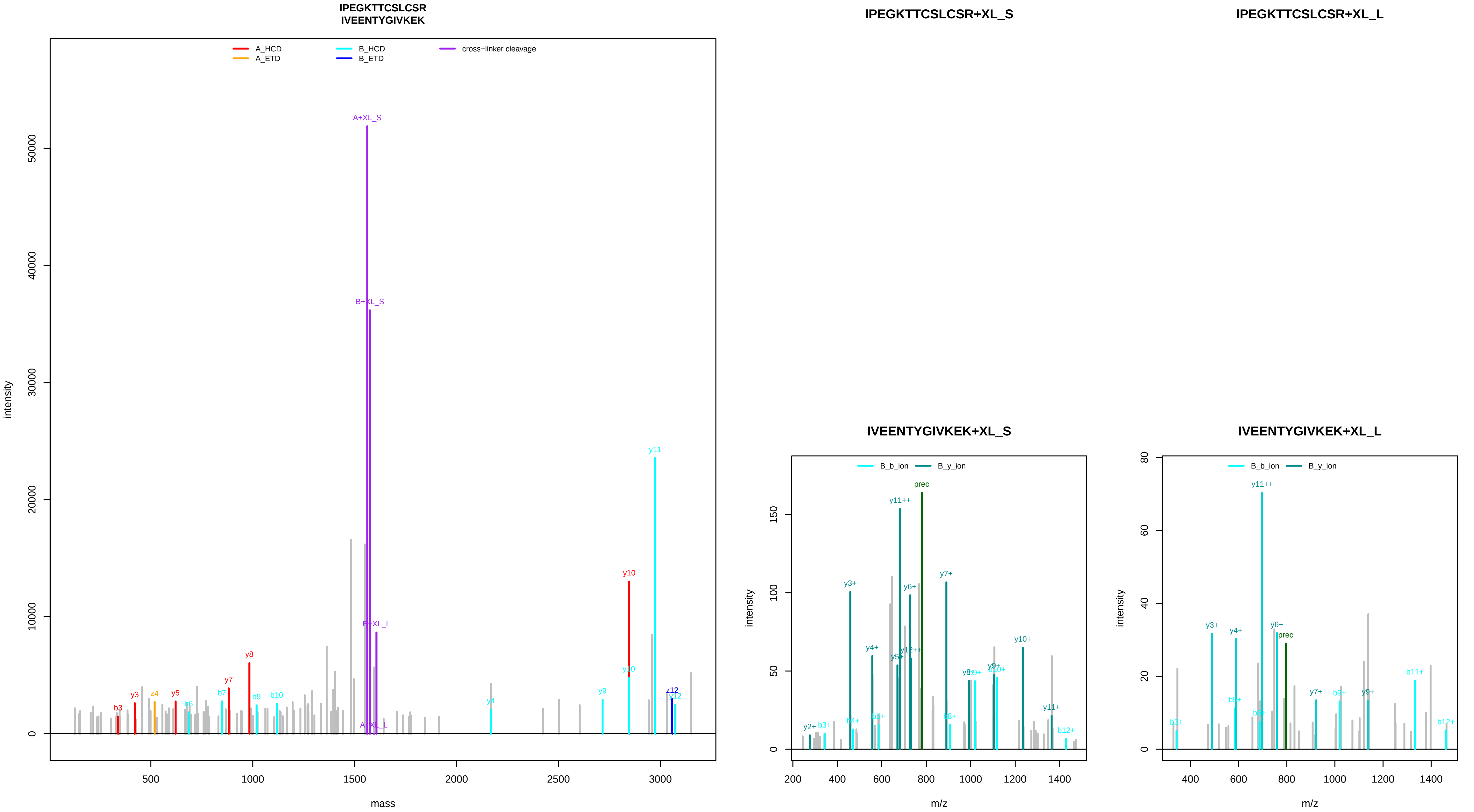

IPEGKTTCSLCSR
IVEENTYGIVKEK
A_HCD
A_ETD
B_HCD
B_ETD
cross−linker cleavage
A+XL_S
B+XL_S
B+XL_L
A+XL_L
intensity
mass
IPEGKTTCSLCSR+XL_S
IPEGKTTCSLCSR+XL_L
IVEENTYGIVKEK+XL_S
IVEENTYGIVKEK+XL_L
B_b_ion
B_y_ion
prec
m/z

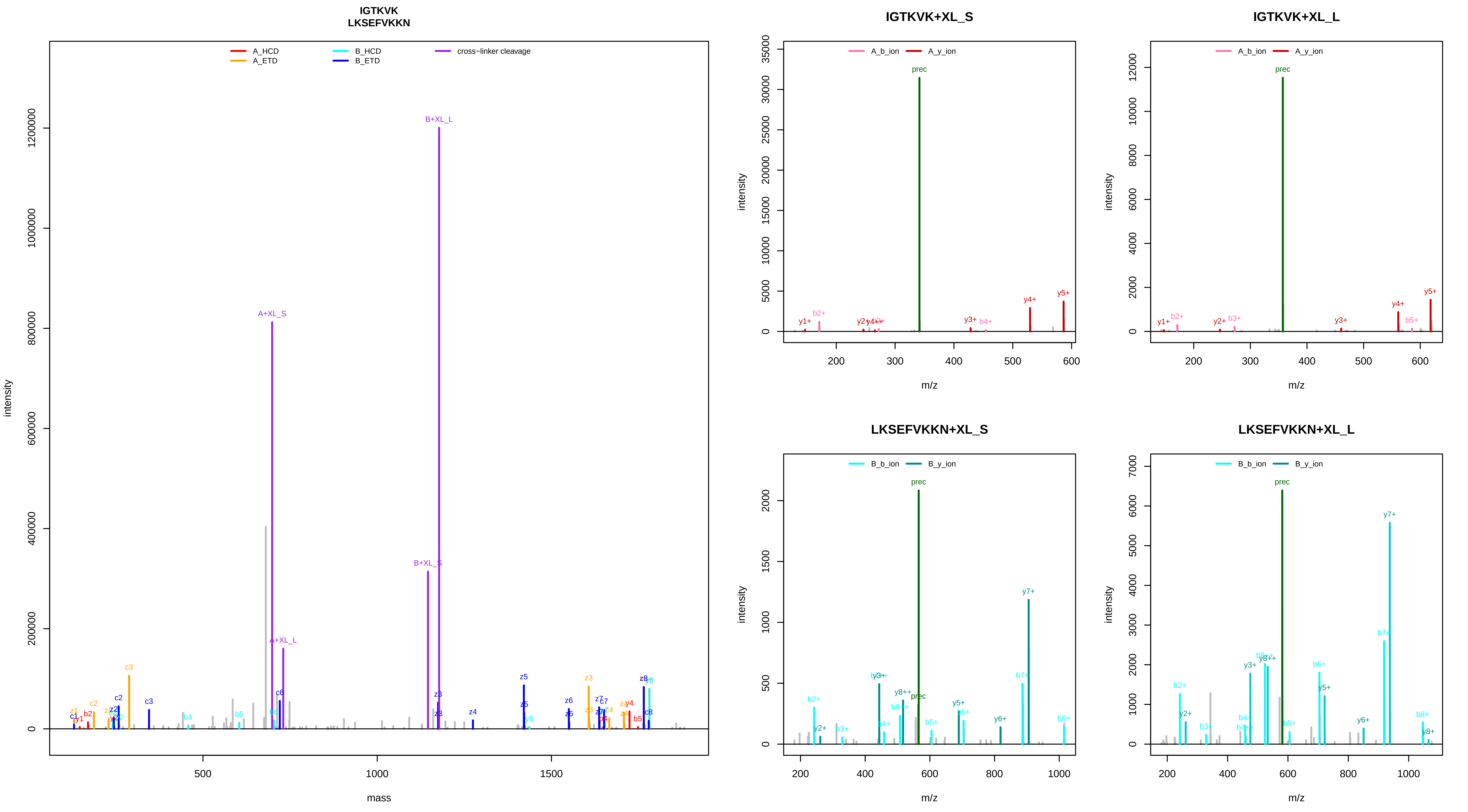
IGTKVK
LKSEFVKKN
A_HCD
A_ETD
B_HCD
B_ETD
cross−linker cleavage
intensity
mass
IGTKVK+XL_S
IGTKVK+XL_L
LKSEFVKKN+XL_S
LKSEFVKKN+XL_L
A_b_ion
A_y_ion
B_b_ion
B_y_ion
m/z
A+XL_S
A+XL_L
B+XL_S
B+XL_L
prec

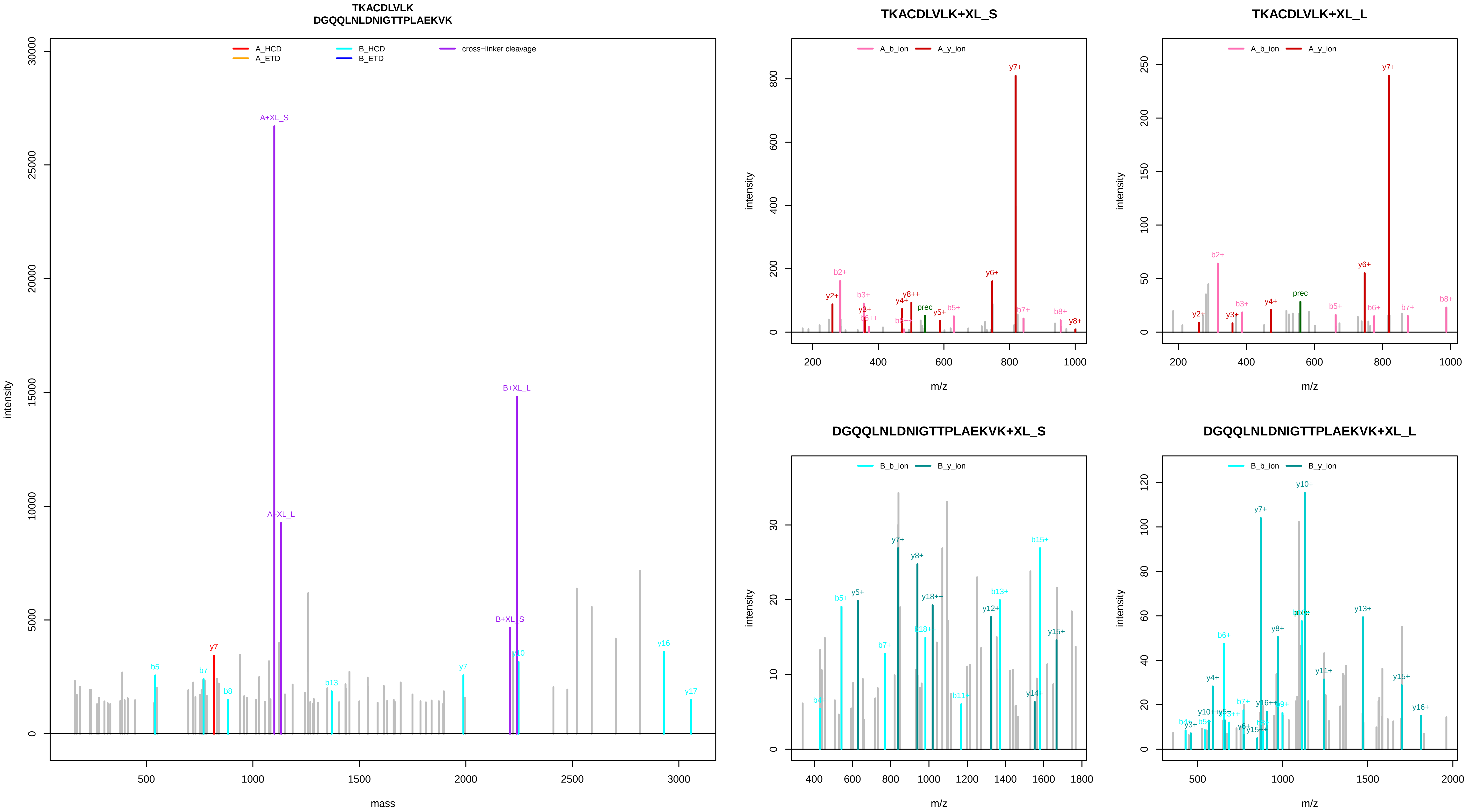

TKACDLVLK
DGQQLNLDNIGTTPLAEKVK
A_HCD
A_ETD
B_HCD
B_ETD
cross−linker cleavage
A+XL_S
B+XL_L
A+XL_L
B+XL_S
y7
b5
b7
b8
b13
y10
y16
y17
intensity
mass
TKACDLVLK+XL_S
TKACDLVLK+XL_L
A_b_ion
A_y_ion
prec
m/z
DGQQLNLDNIGTTPLAEKVK+XL_S
DGQQLNLDNIGTTPLAEKVK+XL_L
B_b_ion
B_y_ion

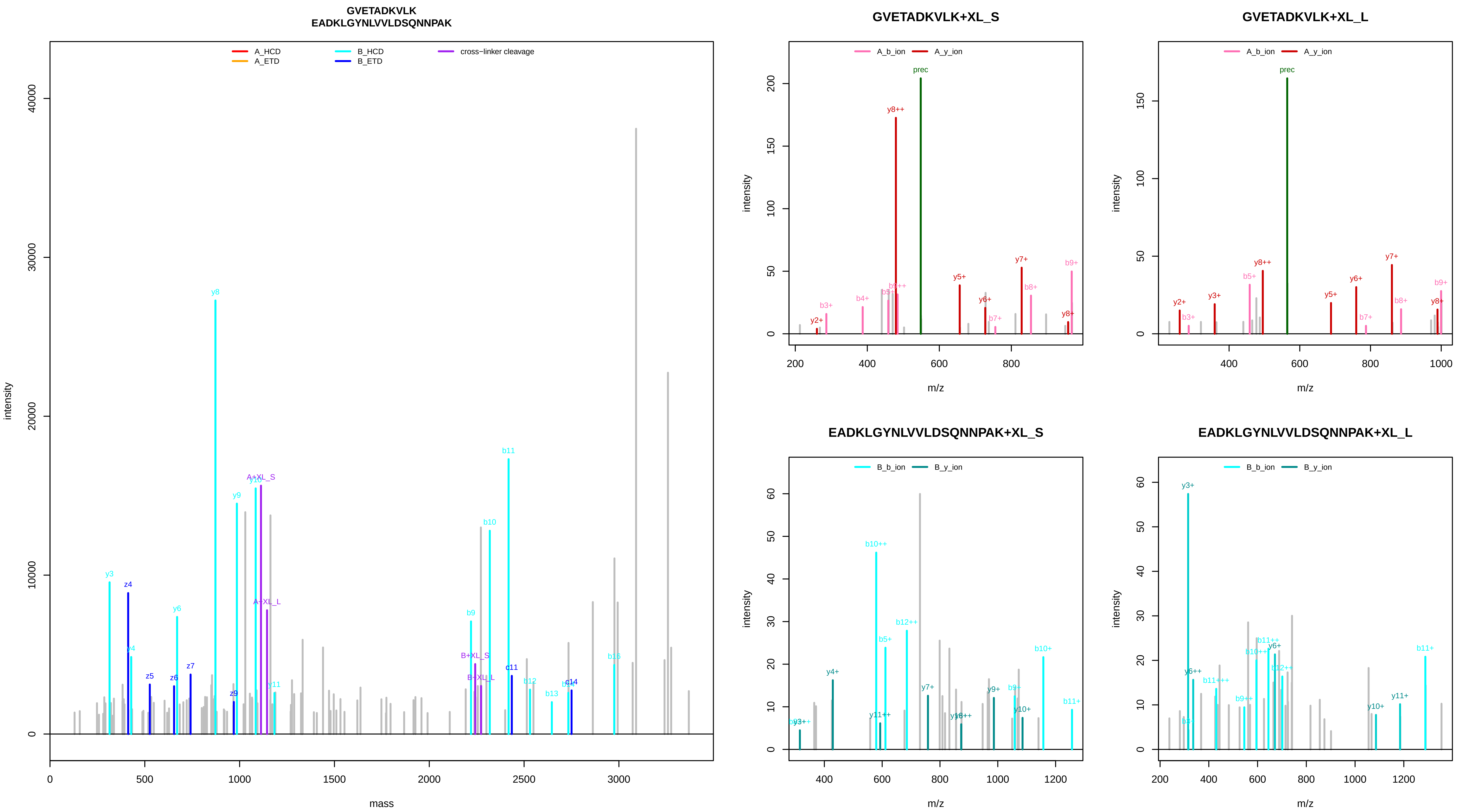
GVETADKVLK
EADKLGYNLVVLDSQNNPAK
A_HCD
A_ETD
B_HCD
B_ETD
cross−linker cleavage
intensity
mass
GVETADKVLK+XL_S
GVETADKVLK+XL_L
EADKLGYNLVVLDSQNNPAK+XL_S
EADKLGYNLVVLDSQNNPAK+XL_L
A_b_ion
A_y_ion
B_b_ion
B_y_ion
prec
m/z

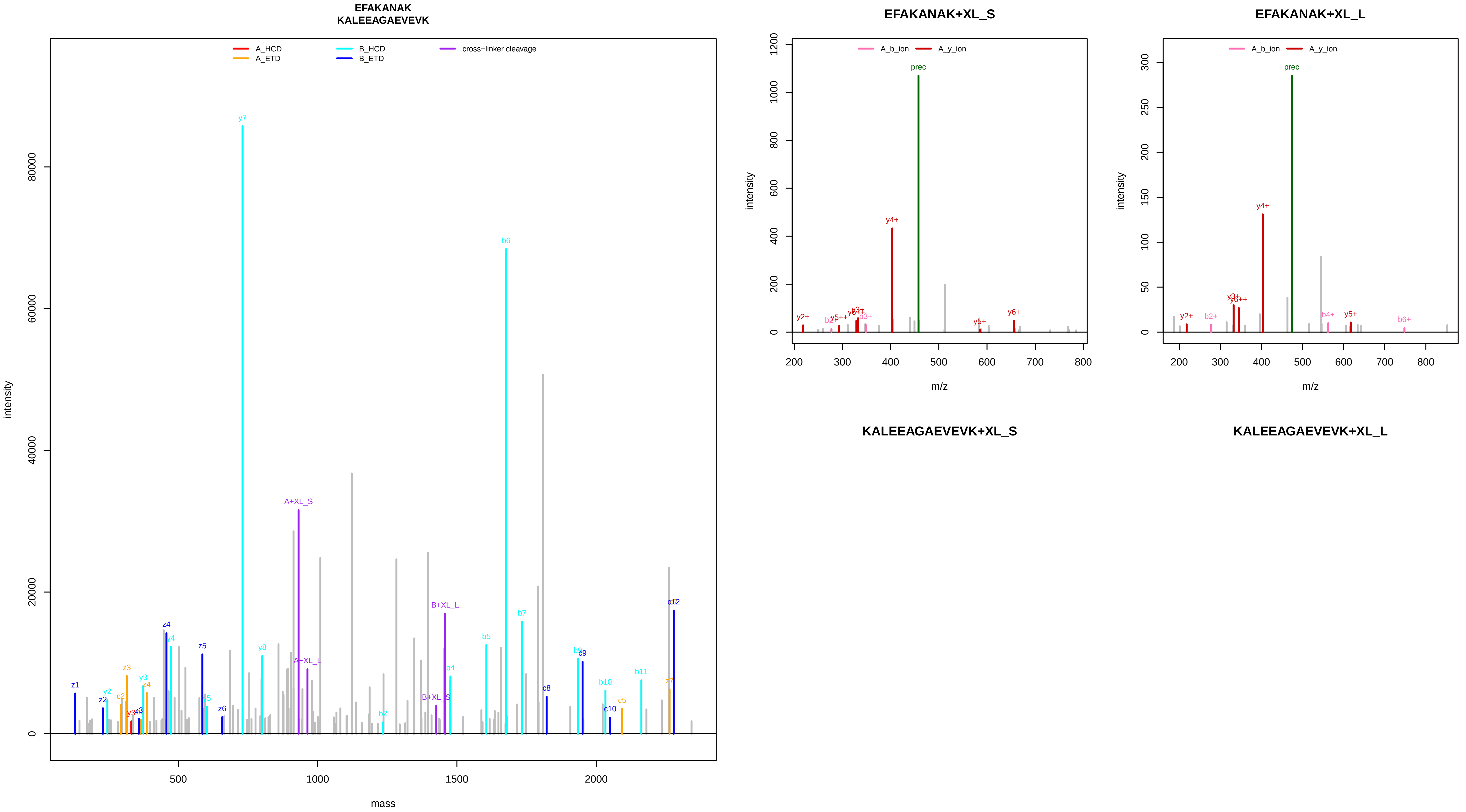
EFAKANAK
KALEEAGAEVEVK
A_HCD
A_ETD
B_HCD
B_ETD
cross−linker cleavage
y7
b6
A+XL_S
B+XL_L
A+XL_L
B+XL_S
intensity
mass
EFAKANAK+XL_S
EFAKANAK+XL_L
A_b_ion
A_y_ion
prec
y4+
m/z
KALEEAGAEVEVK+XL_S
KALEEAGAEVEVK+XL_L

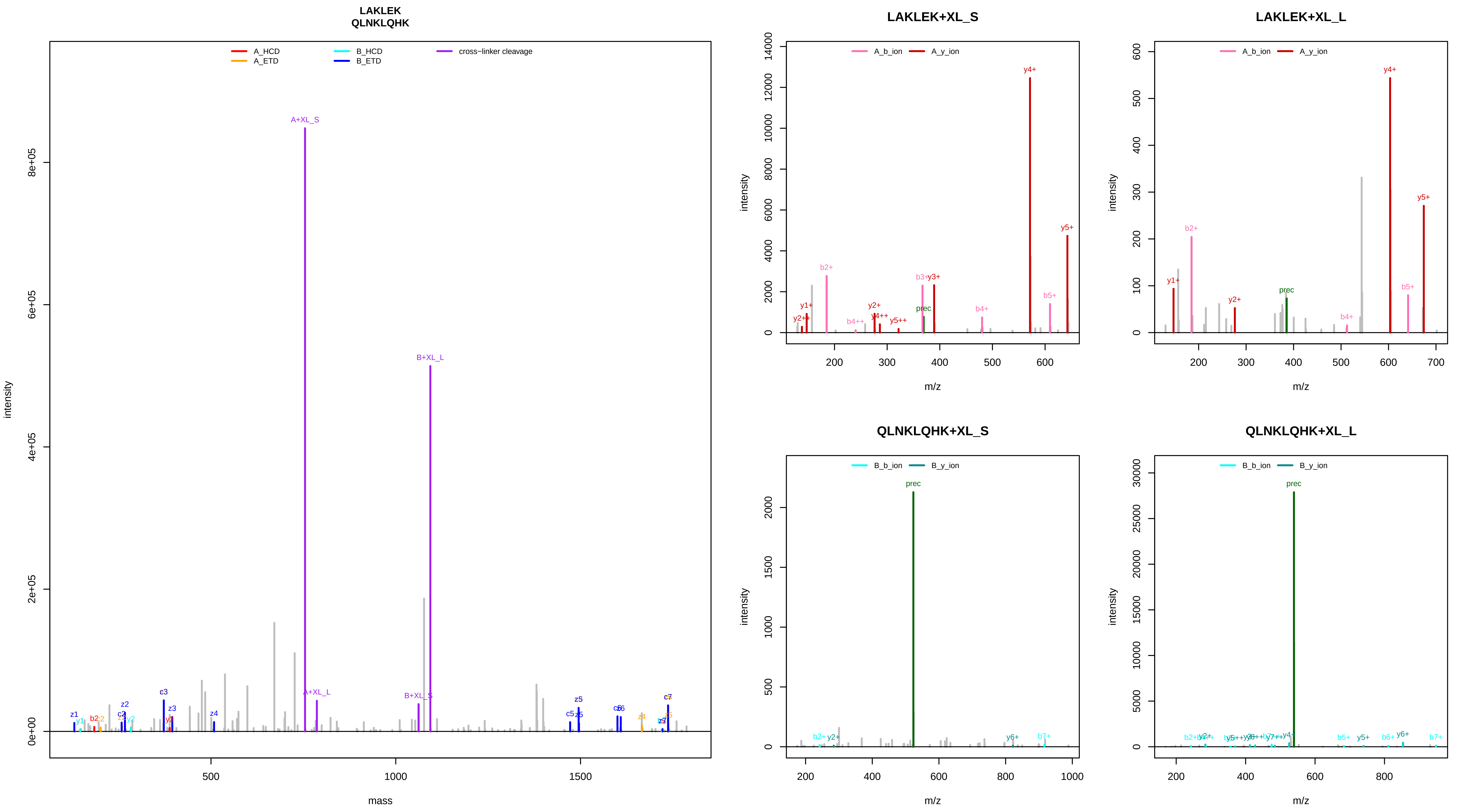
LAKLEK
QLNKLQHK
A_HCD
A_ETD
B_HCD
B_ETD
cross−linker cleavage
A+XL_S
B+XL_L
A+XL_L
B+XL_S
intensity
mass
LAKLEK+XL_S
LAKLEK+XL_L
QLNKLQHK+XL_S
QLNKLQHK+XL_L
A_b_ion
A_y_ion
B_b_ion
B_y_ion
prec
m/z

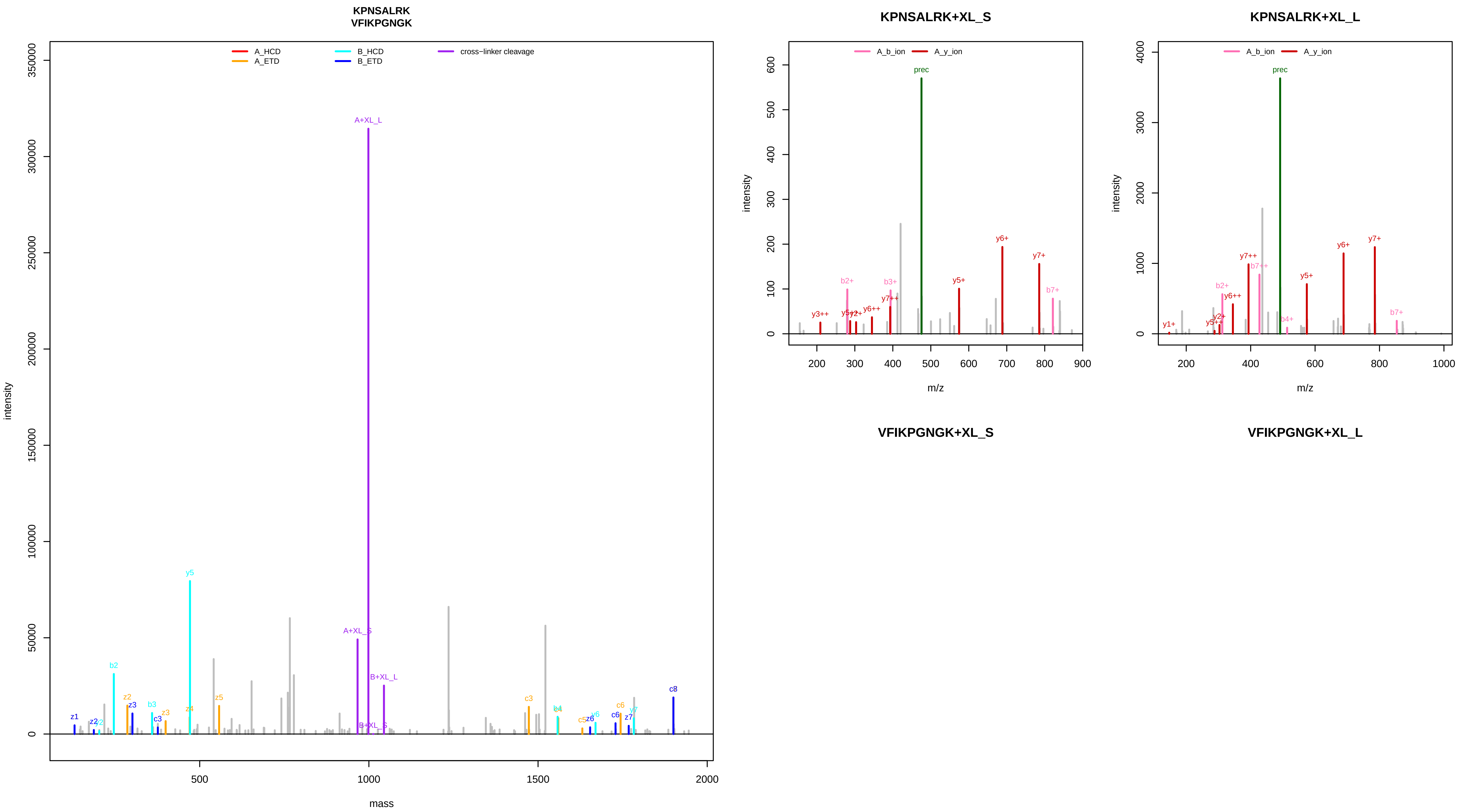
KPNSALRK
VFIKPGNGK
A_HCD
A_ETD
B_HCD
B_ETD
cross−linker cleavage
A+XL_L
A+XL_S
B+XL_L
B+XL_S
intensity
mass
KPNSALRK+XL_S
KPNSALRK+XL_L
A_b_ion
A_y_ion
prec
m/z
VFIKPGNGK+XL_S
VFIKPGNGK+XL_L

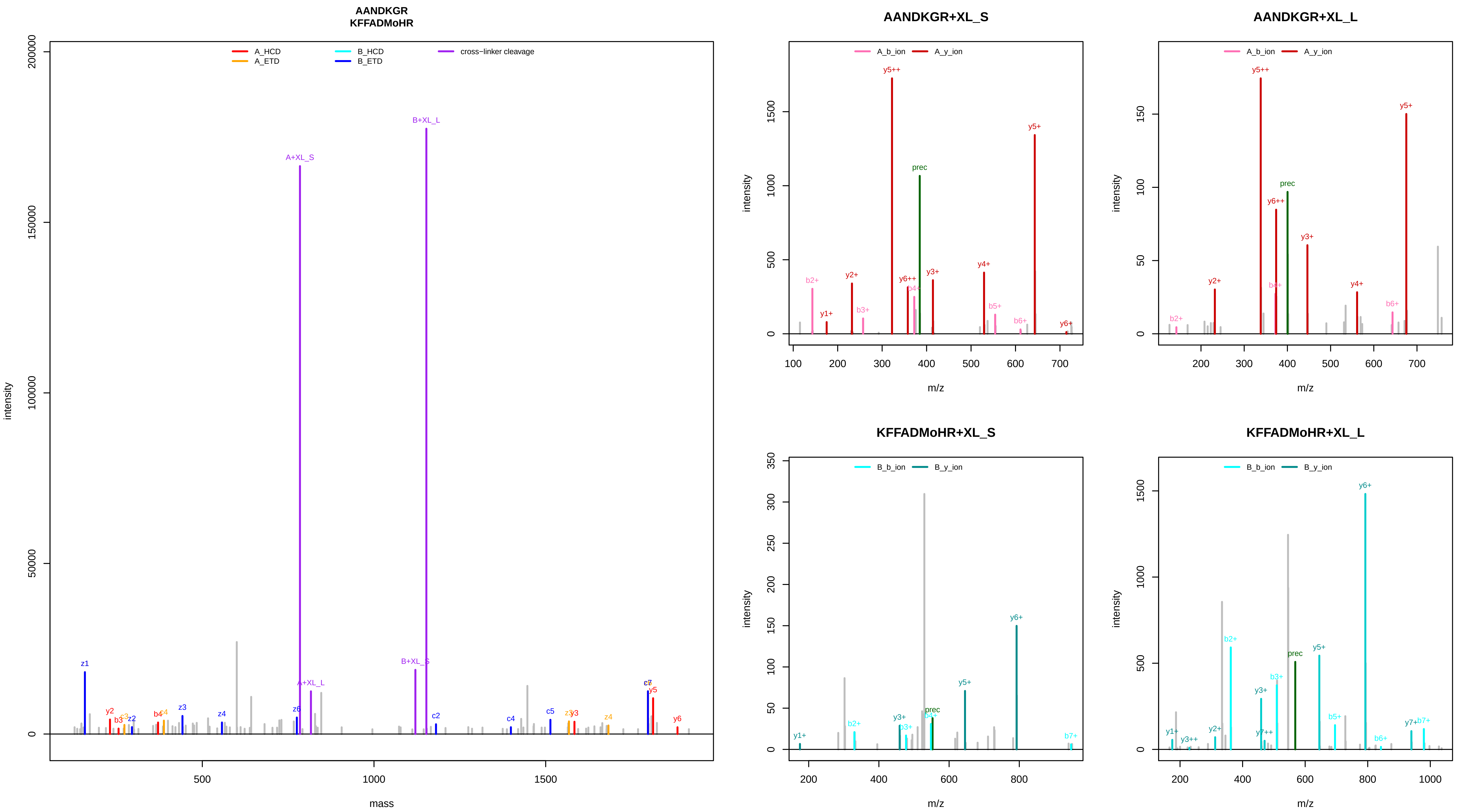
AANDKGR
KFFADMoHR
A_HCD
A_ETD
B_HCD
B_ETD
cross−linker cleavage
intensity
mass
B+XL_L
A+XL_S
B+XL_S
A+XL_L
AANDKGR+XL_S
AANDKGR+XL_L
KFFADMoHR+XL_S
KFFADMoHR+XL_L
A_b_ion
A_y_ion
B_b_ion
B_y_ion
prec
m/z
intensity

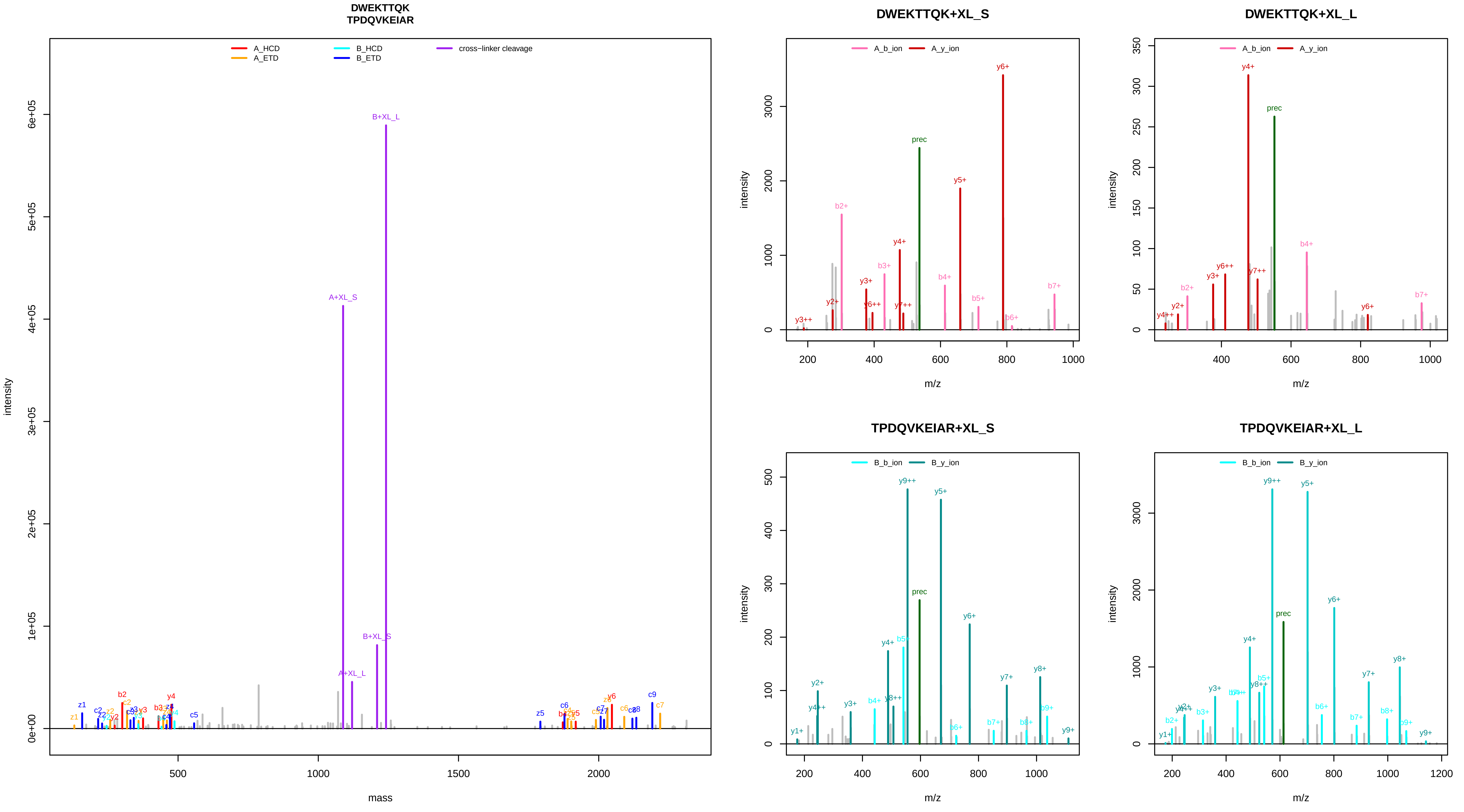

DWEKTTQK
TPDQVKEIAR
A_HCD
A_ETD
B_HCD
B_ETD
cross−linker cleavage
B+XL_L
A+XL_S
B+XL_S
A+XL_L
intensity
mass
DWEKTTQK+XL_S
DWEKTTQK+XL_L
TPDQVKEIAR+XL_S
TPDQVKEIAR+XL_L
A_b_ion
A_y_ion
B_b_ion
B_y_ion
prec
m/z

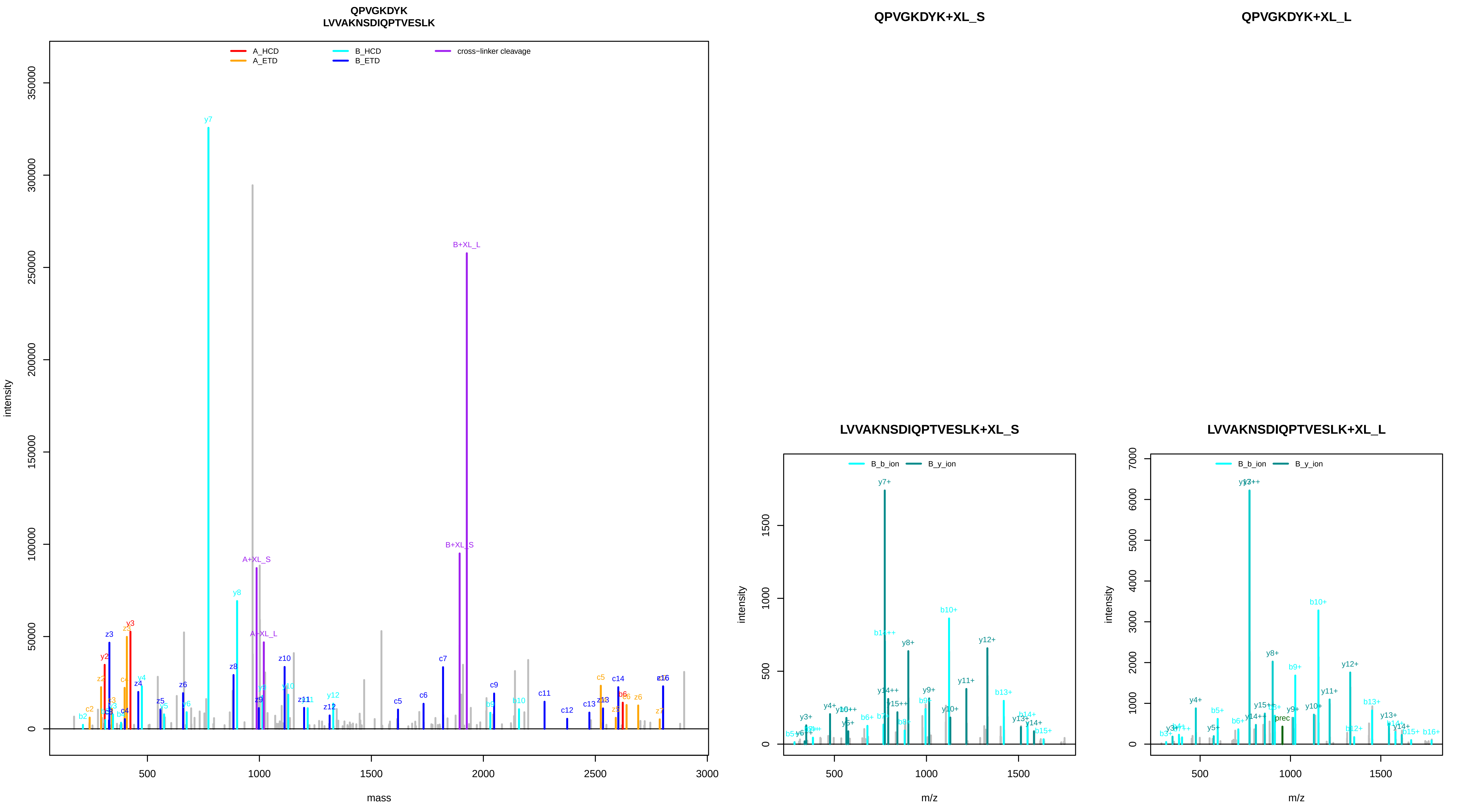
QPVGKDYK
LVVAKNSDIQPTVESLK
A_HCD
A_ETD
B_HCD
B_ETD
cross−linker cleavage
intensity
mass
QPVGKDYK+XL_S
QPVGKDYK+XL_L
LVVAKNSDIQPTVESLK+XL_S
LVVAKNSDIQPTVESLK+XL_L
B_b_ion
B_y_ion
intensity
m/z

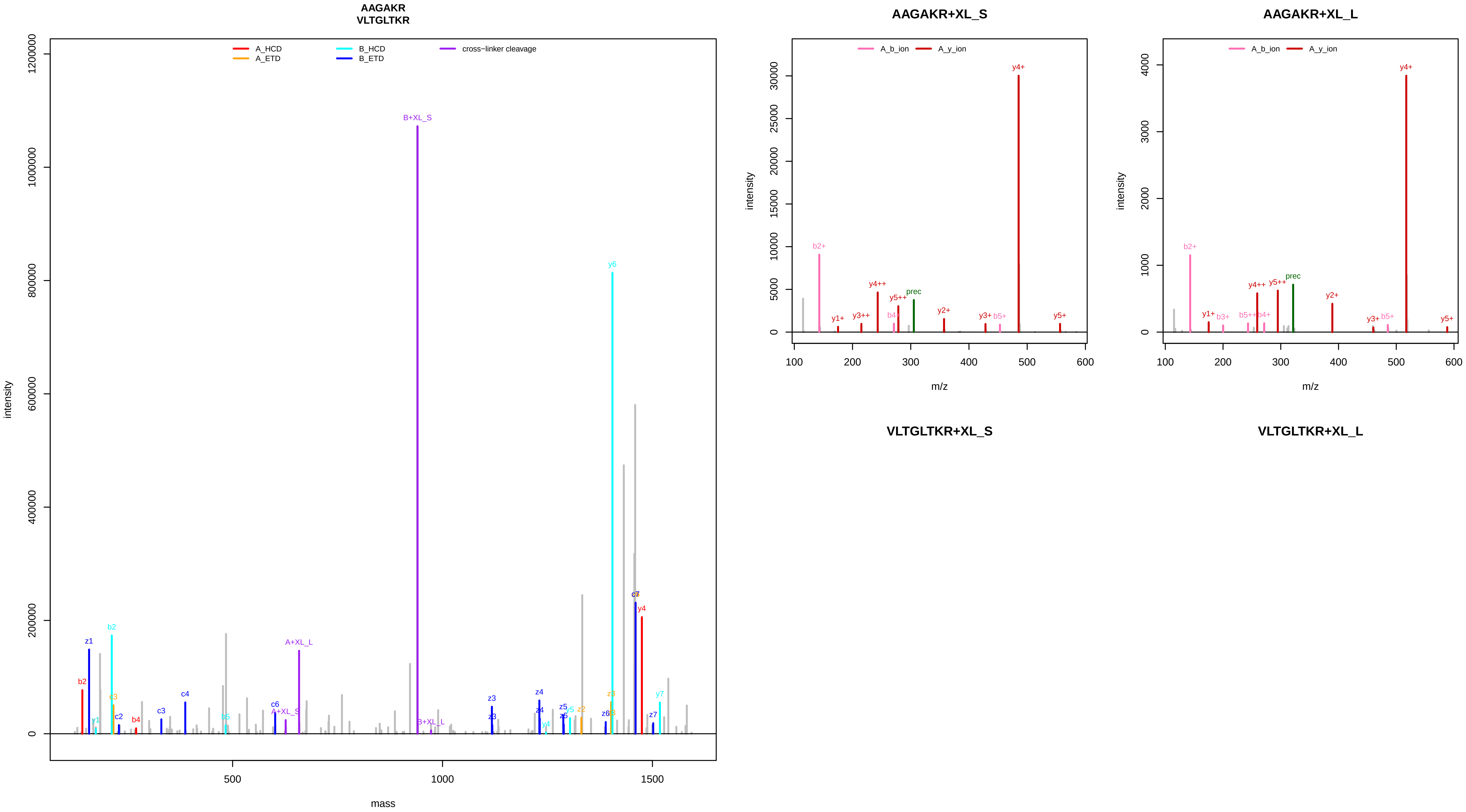
AAGAKR
VLTGLTKR
A_HCD
A_ETD
B_HCD
B_ETD
cross−linker cleavage
B+XL_S
A+XL_L
B+XL_L
A+XL_S
intensity
mass
AAGAKR+XL_S
AAGAKR+XL_L
A_b_ion
A_y_ion
prec
m/z
VLTGLTKR+XL_S
VLTGLTKR+XL_L

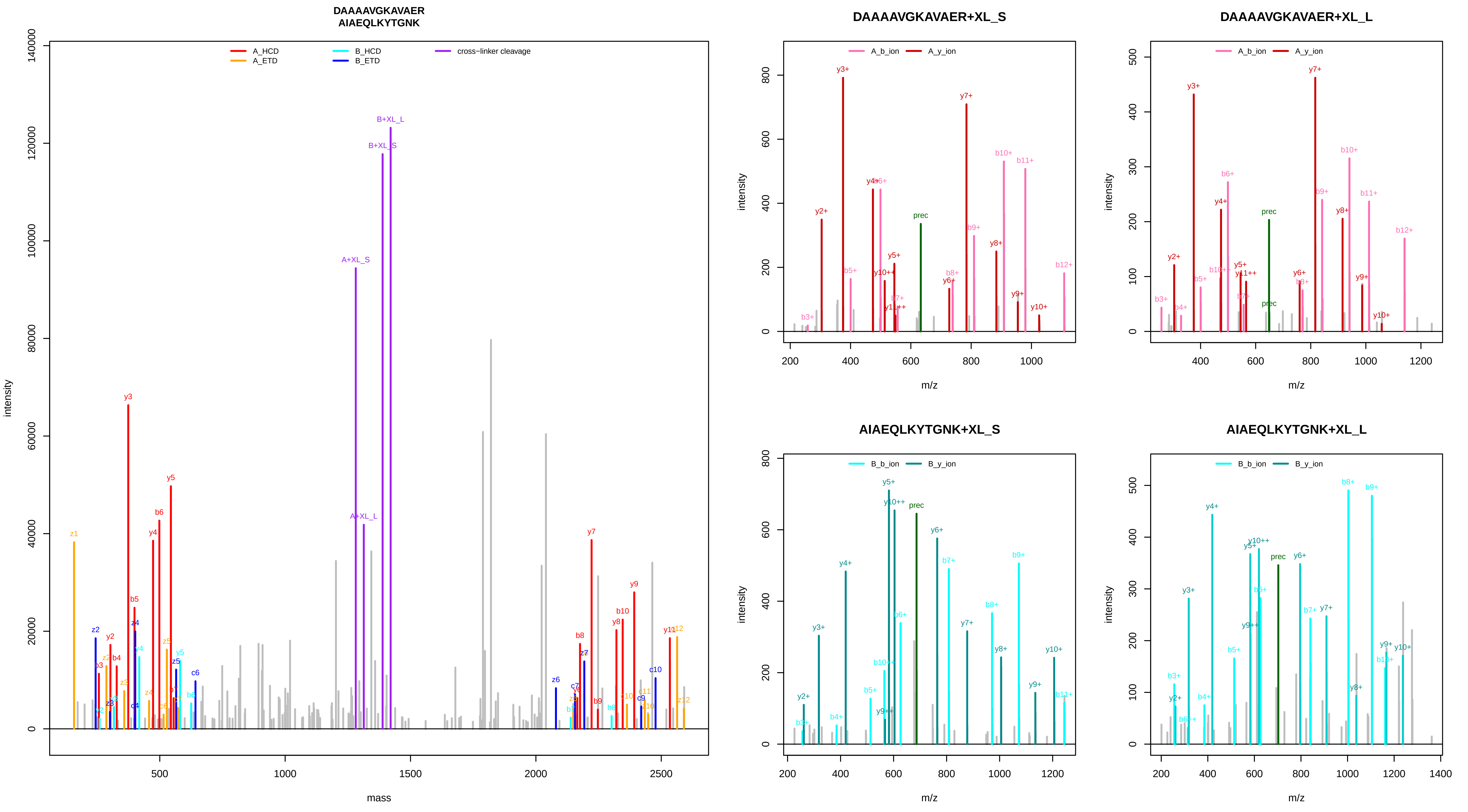
DAAAAVGKAVAER
AIAEQLKYTGNK
A_HCD
A_ETD
B_HCD
B_ETD
cross−linker cleavage
B+XL_L
B+XL_S
A+XL_S
A+XL_L
intensity
mass
DAAAAVGKAVAER+XL_S
DAAAAVGKAVAER+XL_L
AIAEQLKYTGNK+XL_S
AIAEQLKYTGNK+XL_L
A_b_ion
A_y_ion
B_b_ion
B_y_ion
prec
m/z

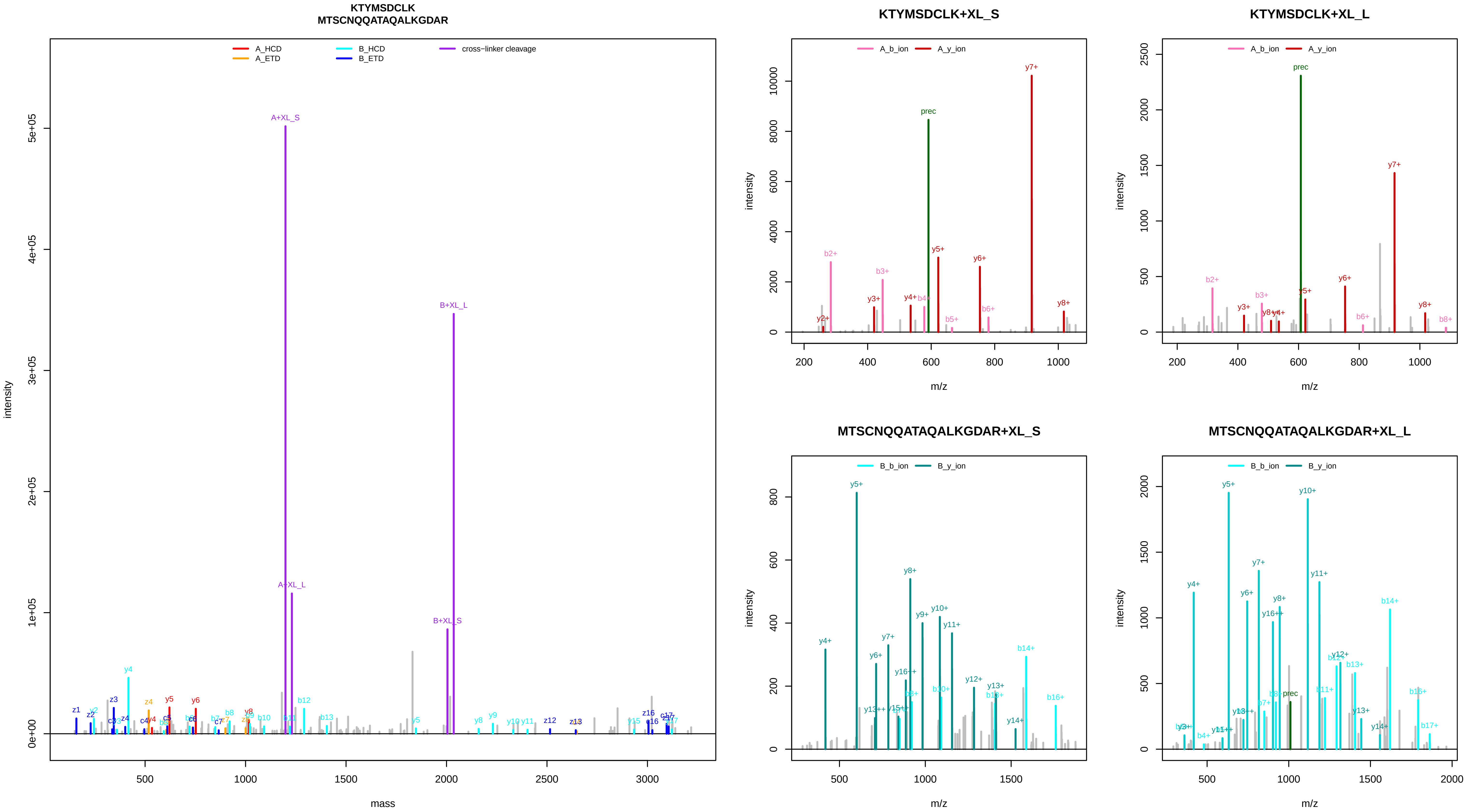

KTYMSDCLK
MTSCNQQATAQALKGDAR
A_HCD
A_ETD
B_HCD
B_ETD
cross−linker cleavage
A+XL_S
B+XL_L
A+XL_L
B+XL_S
intensity
mass
KTYMSDCLK+XL_S
KTYMSDCLK+XL_L
A_b_ion
A_y_ion
prec
MTSCNQQATAQALKGDAR+XL_S
MTSCNQQATAQALKGDAR+XL_L
B_b_ion
B_y_ion
m/z

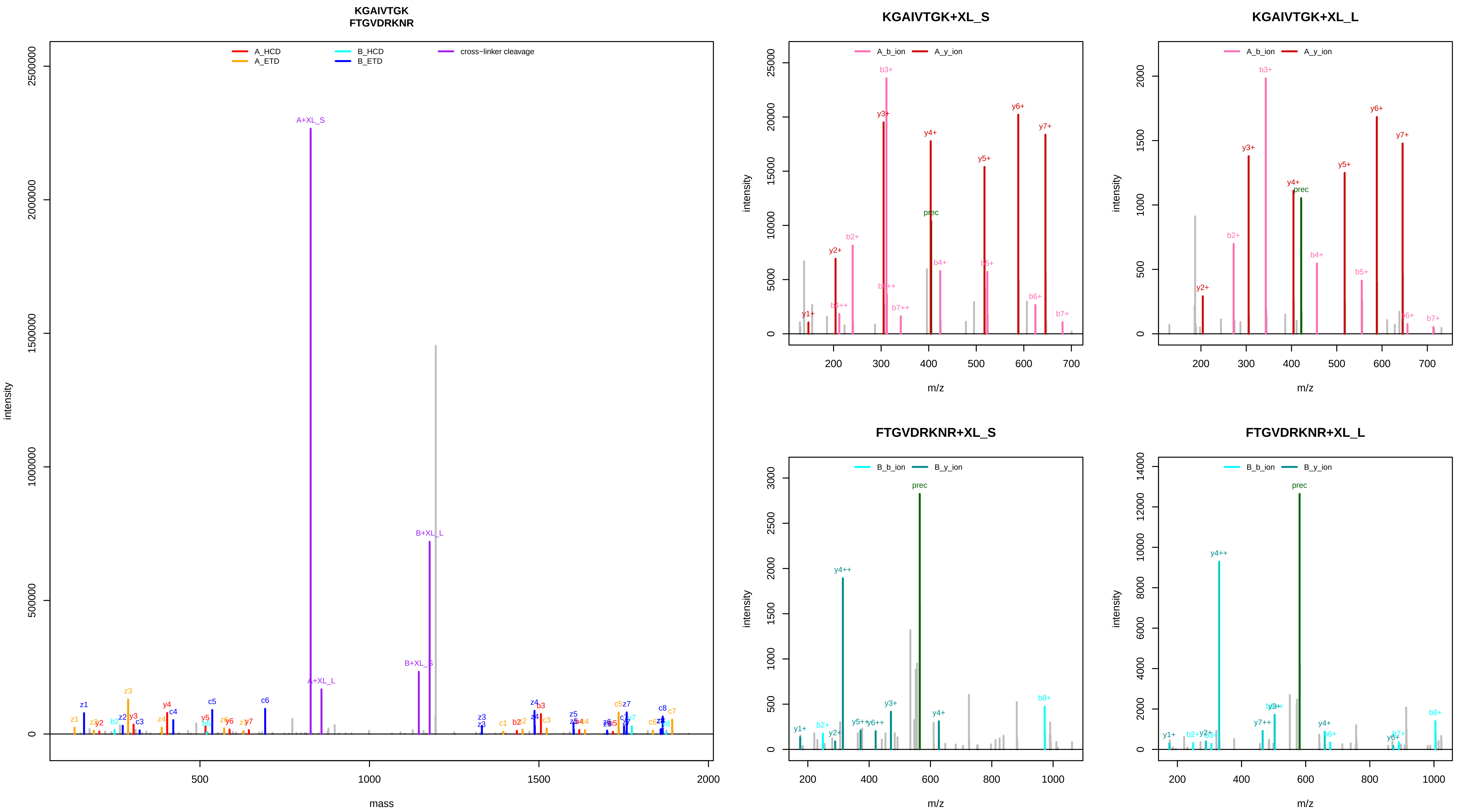
KGAIVTGK
FTGVDRKNR
A_HCD
A_ETD
B_HCD
B_ETD
cross−linker cleavage
A+XL_S
B+XL_L
B+XL_S
A+XL_L
intensity
mass
KGAIVTGK+XL_S
KGAIVTGK+XL_L
FTGVDRKNR+XL_S
FTGVDRKNR+XL_L
A_b_ion
A_y_ion
B_b_ion
B_y_ion
prec
m/z

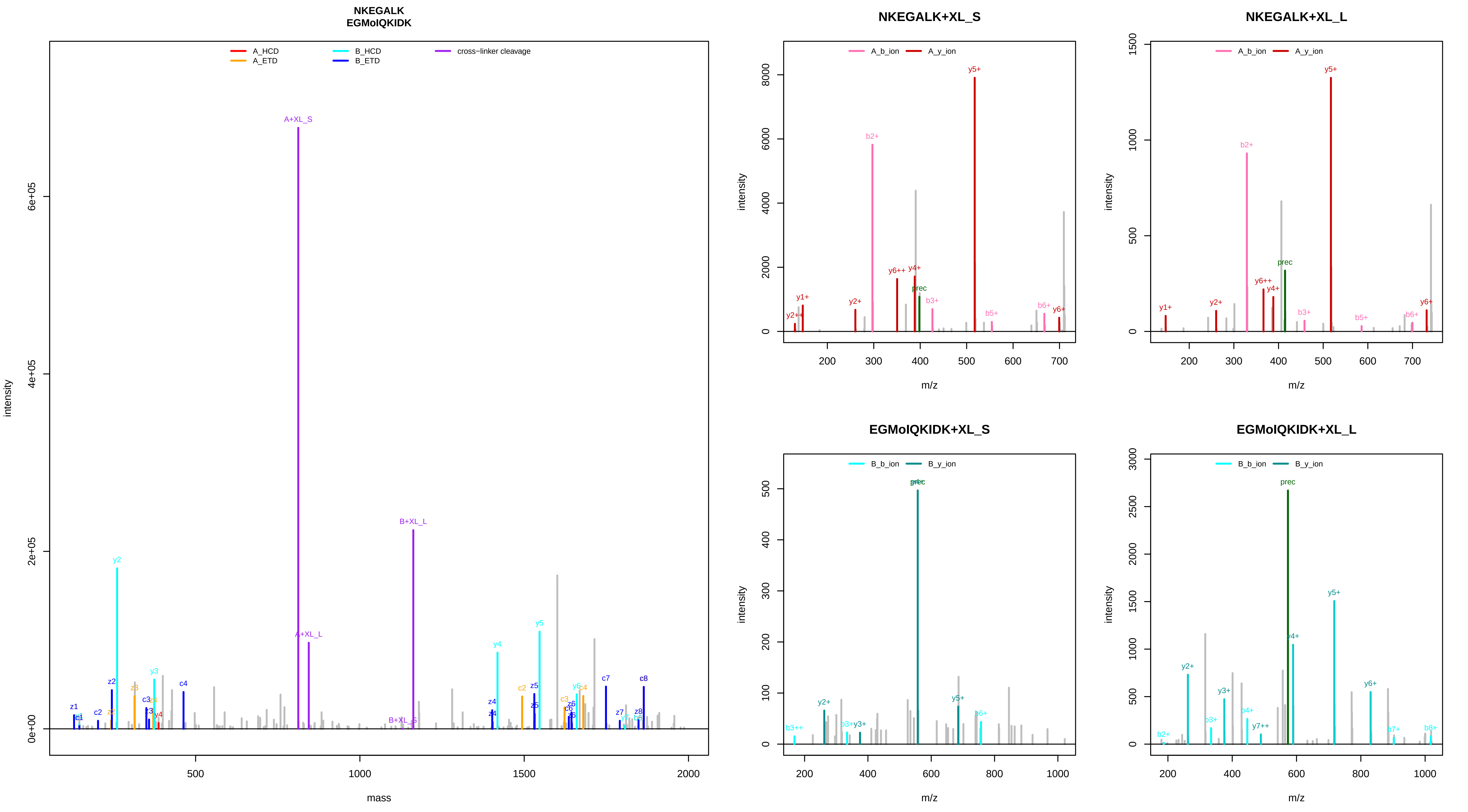
NKEGALK
EGMoIQKIDK
A_HCD
A_ETD
B_HCD
B_ETD
cross−linker cleavage
A+XL_S
B+XL_L
A+XL_L
B+XL_S
intensity
mass
NKEGALK+XL_S
NKEGALK+XL_L
EGMoIQKIDK+XL_S
EGMoIQKIDK+XL_L
A_b_ion
A_y_ion
B_b_ion
B_y_ion
prec
m/z
intensity

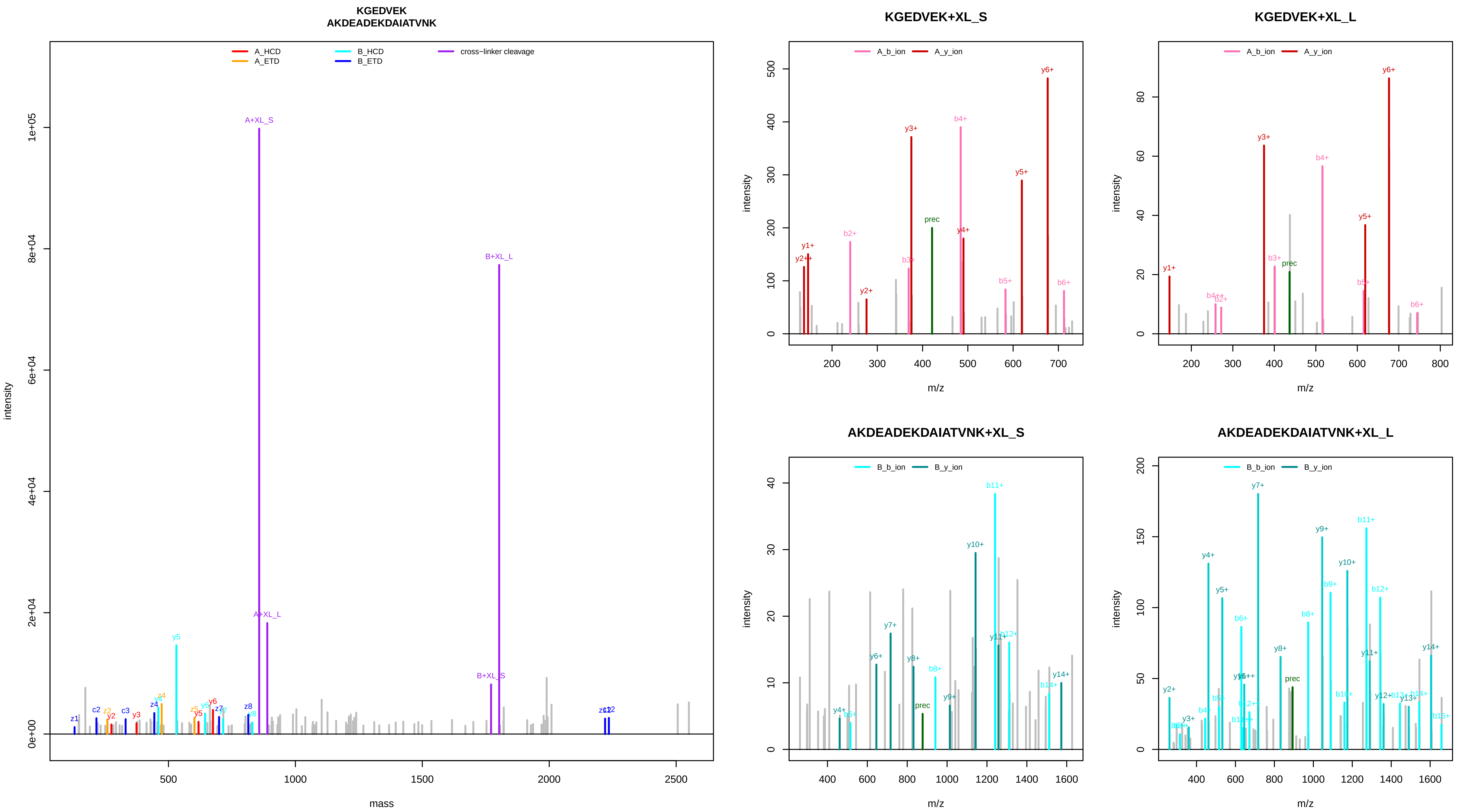
KGEDVEK
AKDEADEKDAIATVNK
A_HCD
A_ETD
B_HCD
B_ETD
cross−linker cleavage
A+XL_S
B+XL_L
A+XL_L
B+XL_S
intensity
mass
KGEDVEK+XL_S
KGEDVEK+XL_L
AKDEADEKDAIATVNK+XL_S
AKDEADEKDAIATVNK+XL_L
A_b_ion
A_y_ion
B_b_ion
B_y_ion
prec
m/z

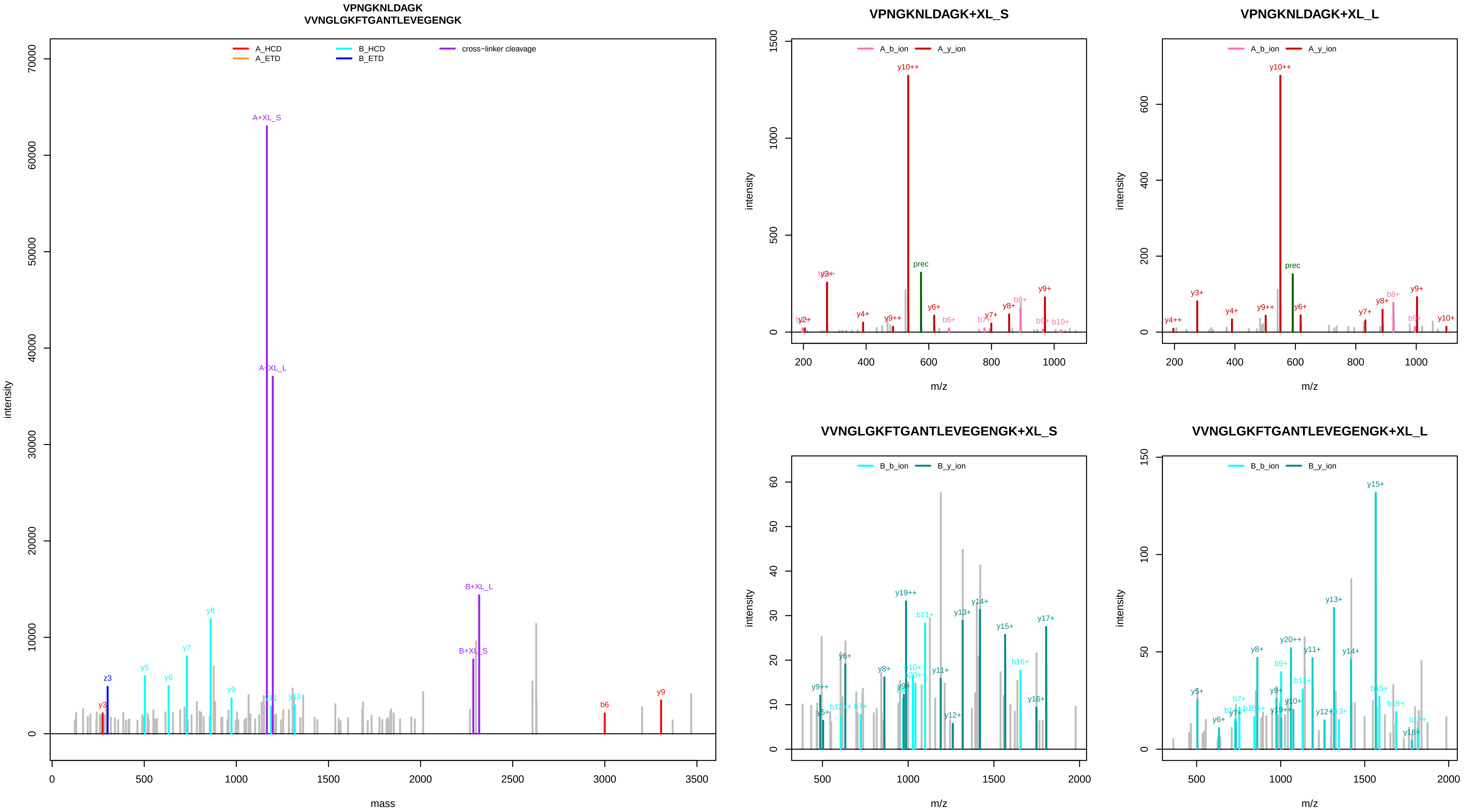
VPNGKNLDAGK
VVNGLGKFTGANTLEVEGENGK
A_HCD
A_ETD
B_HCD
B_ETD
cross−linker cleavage
A+XL_S
A+XL_L
B+XL_L
B+XL_S
intensity
mass
VPNGKNLDAGK+XL_S
VPNGKNLDAGK+XL_L
VVNGLGKFTGANTLEVEGENGK+XL_S
VVNGLGKFTGANTLEVEGENGK+XL_L
A_b_ion
A_y_ion
B_b_ion
B_y_ion
prec
m/z

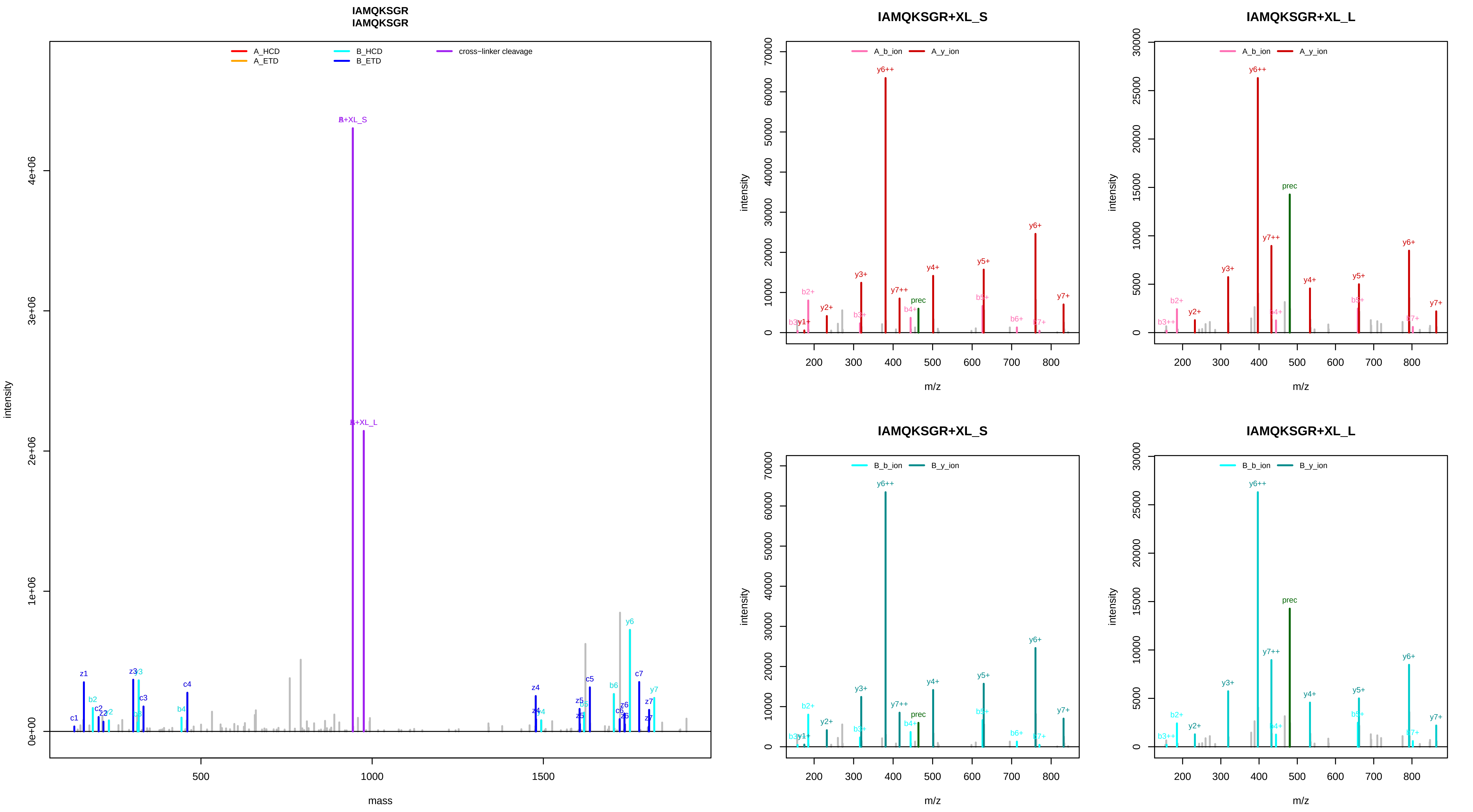
IAMQKSGR
IAMQKSGR
A_HCD
A_ETD
B_HCD
B_ETD
cross−linker cleavage
intensity
mass
IAMQKSGR+XL_S
IAMQKSGR+XL_L
A_b_ion
A_y_ion
B_b_ion
B_y_ion
m/z
prec

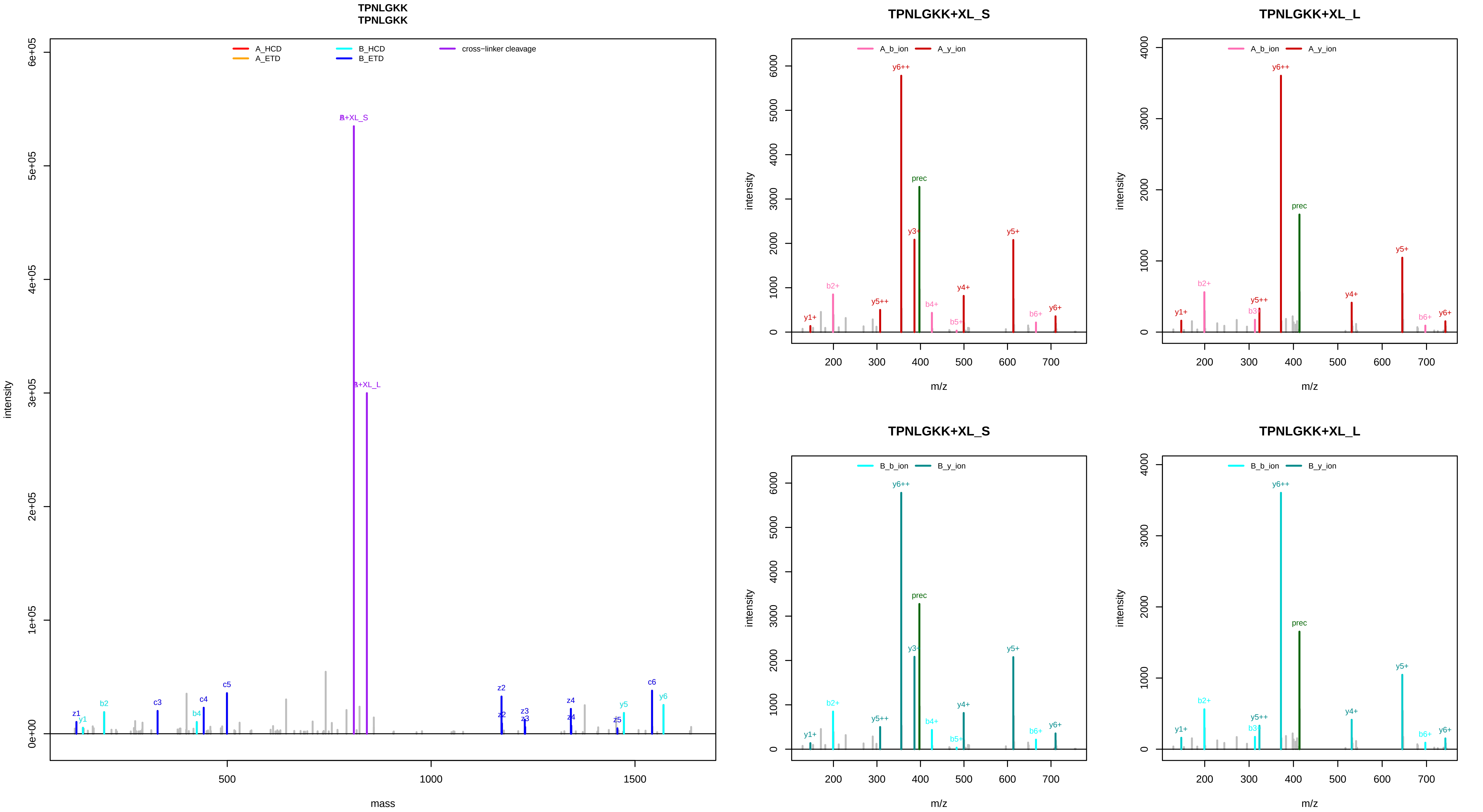
TPNLGKK
TPNLGKK
A_HCD
A_ETD
B_HCD
B_ETD
cross−linker cleavage
B+XL_S
B+XL_L
intensity
mass
TPNLGKK+XL_S
TPNLGKK+XL_L
A_b_ion
A_y_ion
B_b_ion
B_y_ion
prec
m/z

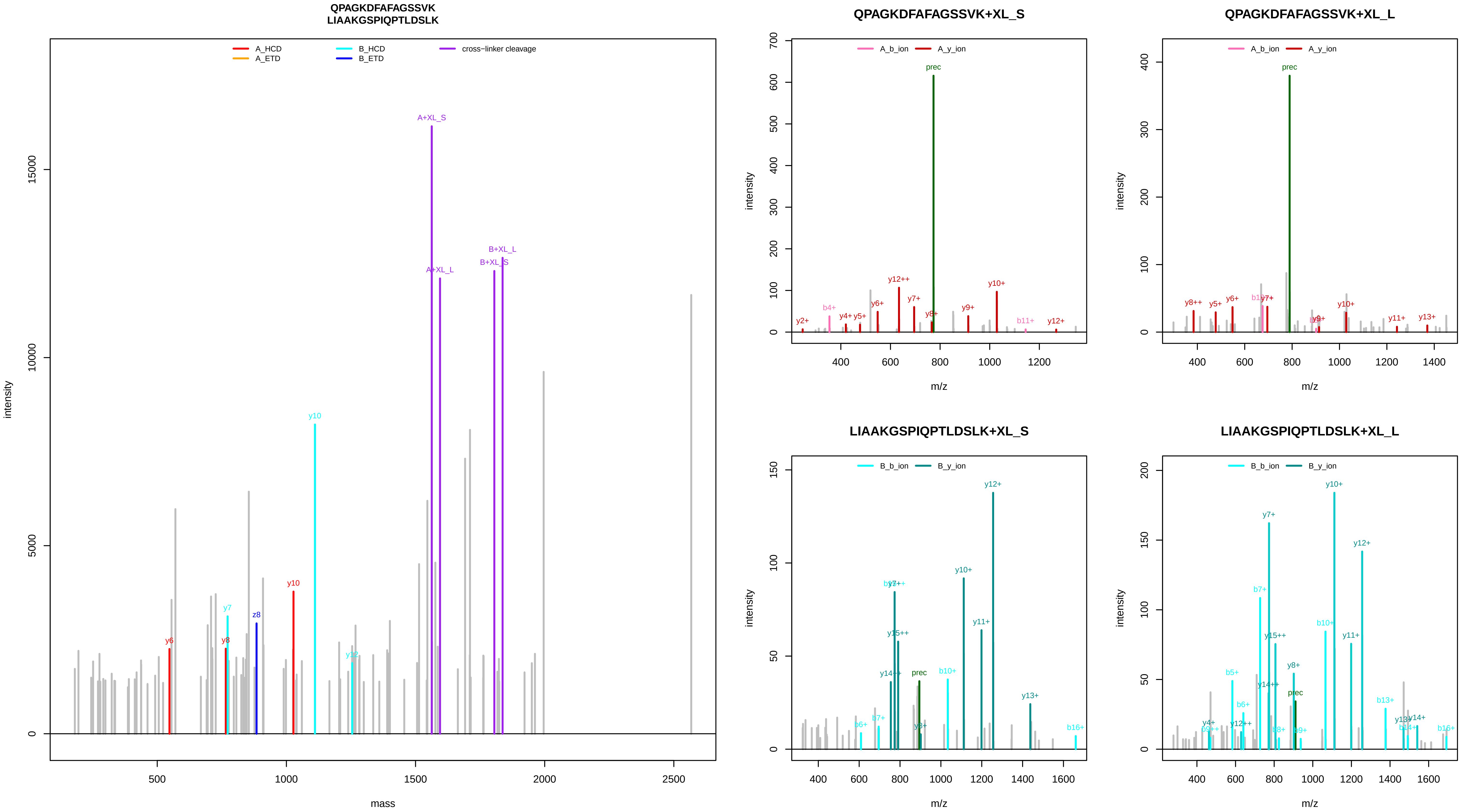
QPAGKDFAFAGSSVK
LIAAKGSPIQPTLDSLK
A_HCD
A_ETD
B_HCD
B_ETD
cross−linker cleavage
A+XL_S
A+XL_L
B+XL_L
B+XL_S
y10
y7
z8
y6
y8
y10
y12
intensity
mass
QPAGKDFAFAGSSVK+XL_S
QPAGKDFAFAGSSVK+XL_L
LIAAKGSPIQPTLDSLK+XL_S
LIAAKGSPIQPTLDSLK+XL_L
A_b_ion
A_y_ion
B_b_ion
B_y_ion
prec
m/z
intensity

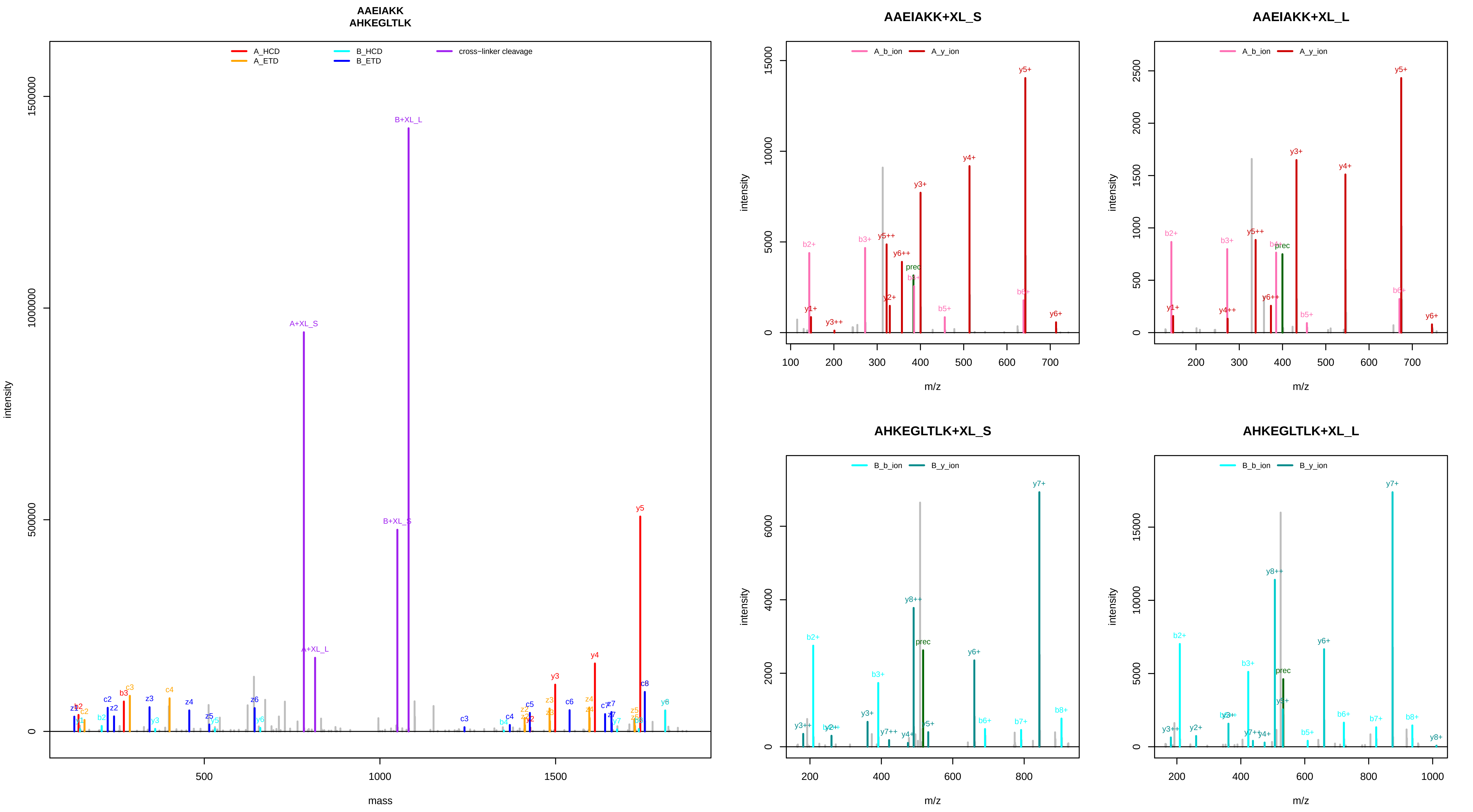
AAEIAKK
AHKEGLTLK
A_HCD
A_ETD
B_HCD
B_ETD
cross−linker cleavage
B+XL_L
A+XL_S
B+XL_S
A+XL_L
intensity
mass
AAEIAKK+XL_S
AAEIAKK+XL_L
AHKEGLTLK+XL_S
AHKEGLTLK+XL_L
A_b_ion
A_y_ion
B_b_ion
B_y_ion
m/z

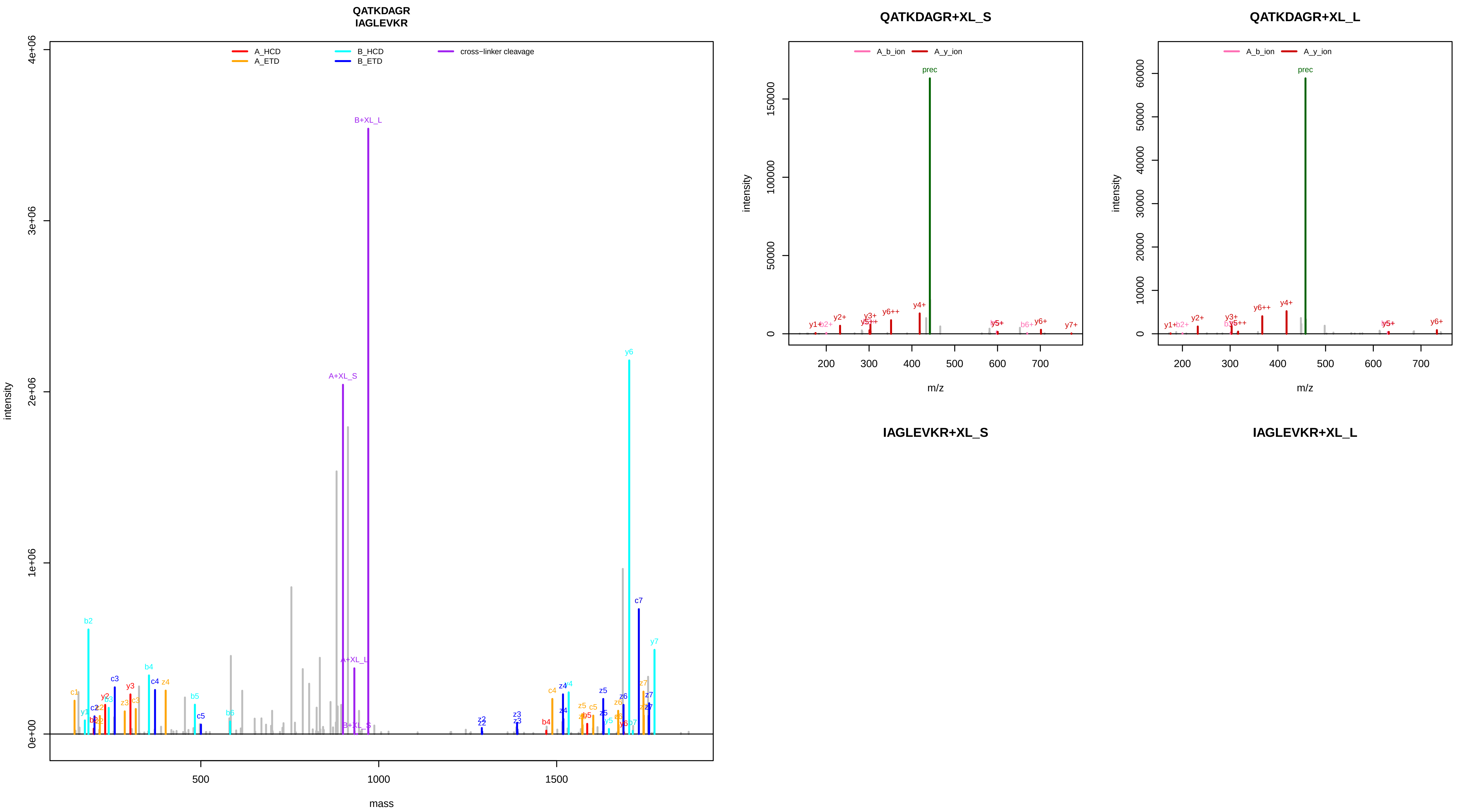
QATKDAGR
IAGLEVKR
A_HCD
A_ETD
B_HCD
B_ETD
cross−linker cleavage
B+XL_L
A+XL_S
A+XL_L
B+XL_S
intensity
mass
0e+00
1e+06
2e+06
3e+06
4e+06
500
1000
1500
QATKDAGR+XL_S
QATKDAGR+XL_L
A_b_ion
A_y_ion
prec
intensity
m/z
IAGLEVKR+XL_S
IAGLEVKR+XL_L

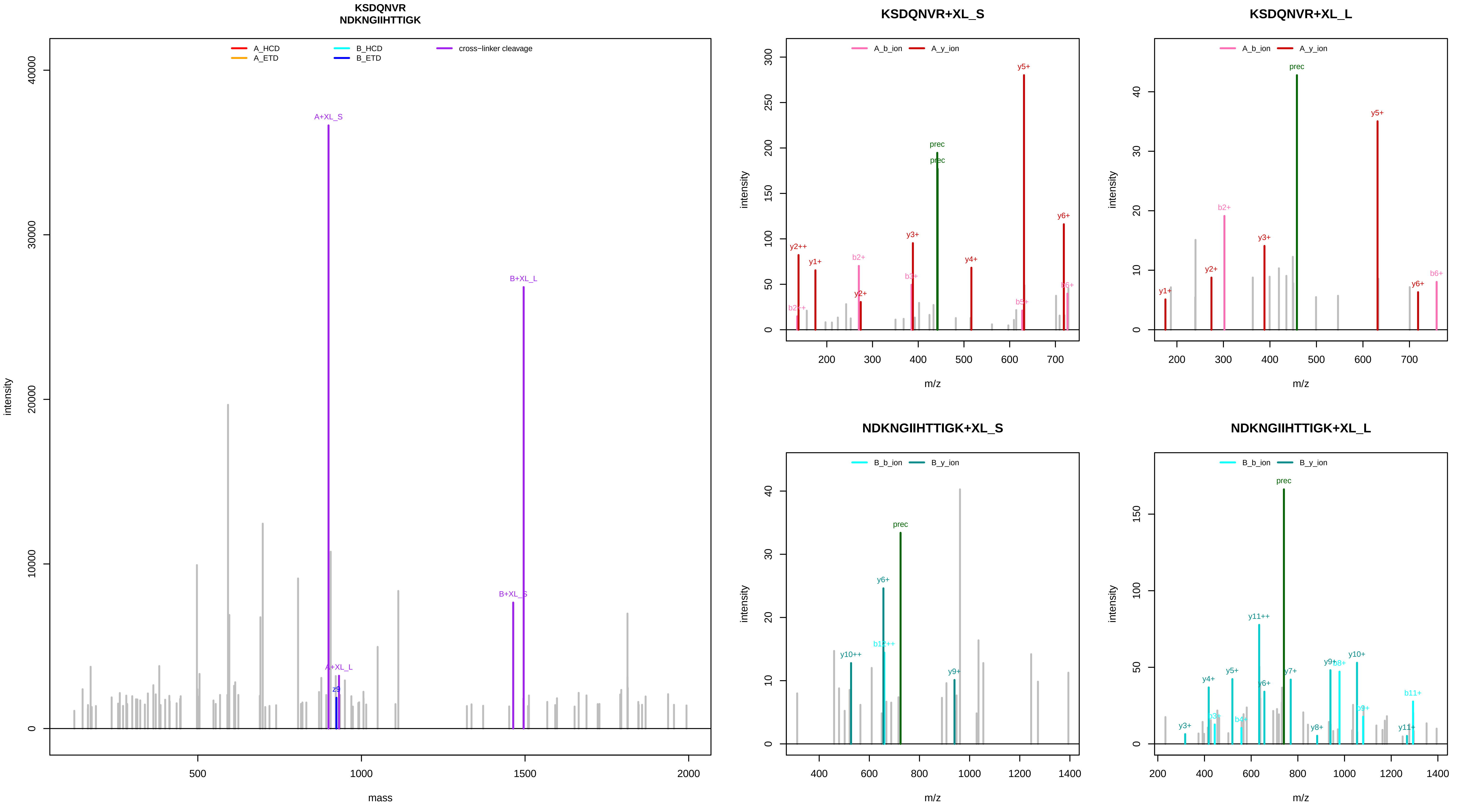
KSDQNVR
NDKNGIIHTTIGK
A_HCD
A_ETD
B_HCD
B_ETD
cross−linker cleavage
A+XL_S
B+XL_L
B+XL_S
A+XL_L
z9
intensity
mass
KSDQNVR+XL_S
KSDQNVR+XL_L
A_b_ion
A_y_ion
prec
y5+
y6+
y3+
y4+
y2++
y1+
b2+
y2+
b3+
b5+
b6+
b2++
m/z
NDKNGIIHTTIGK+XL_S
NDKNGIIHTTIGK+XL_L
B_b_ion
B_y_ion
y6+
y10++
b12++
y9+
y11++
y4+
y5+
y7+
y9+
y10+
b8+
y3+
y8+
y11+
b11+

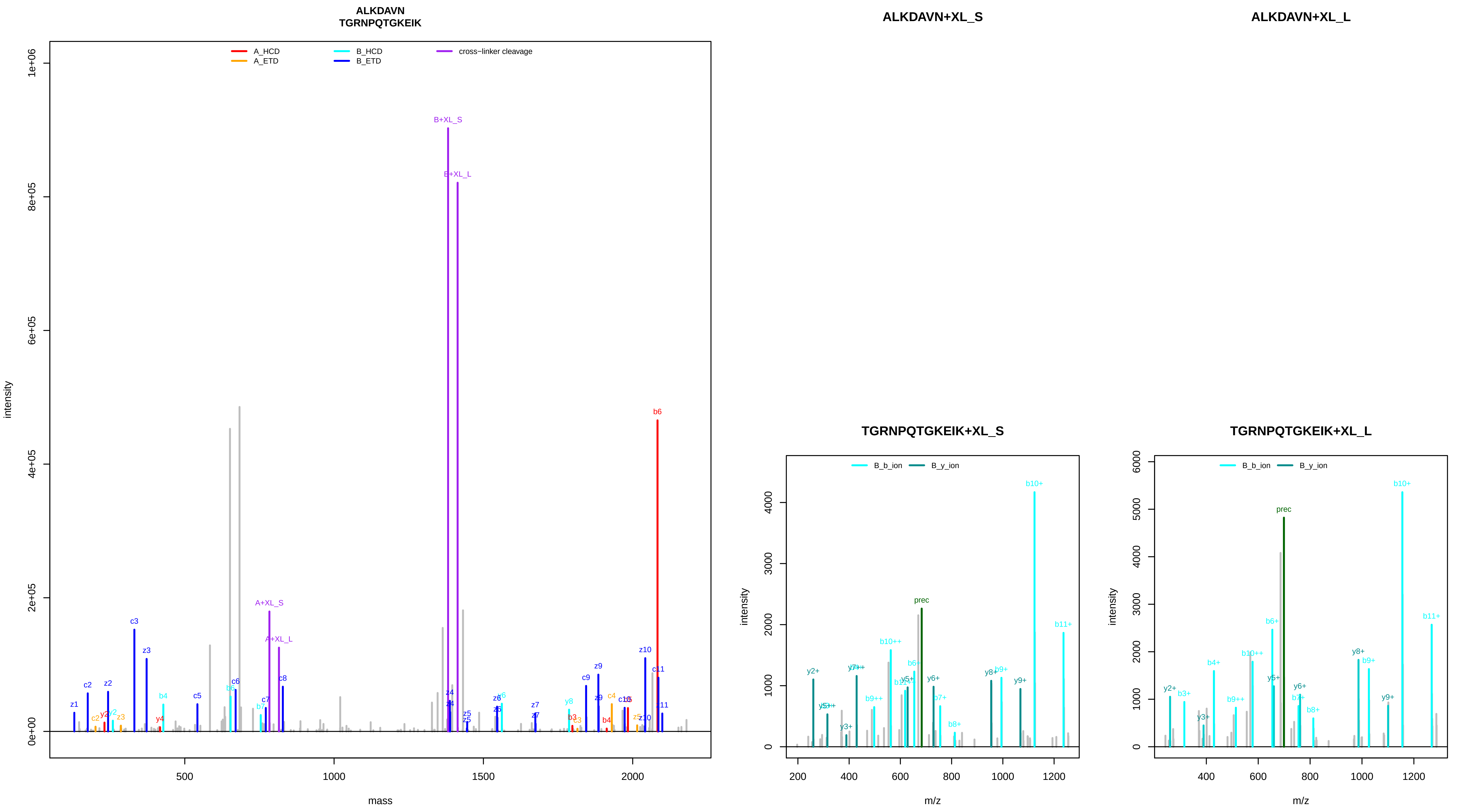

ALKDAVN
TGRNPQTGKEIK
A_HCD
A_ETD
B_HCD
B_ETD
cross−linker cleavage
B+XL_S
B+XL_L
A+XL_S
A+XL_L
intensity
mass
0e+00
2e+05
4e+05
6e+05
8e+05
1e+06
500
1000
1500
2000
ALKDAVN+XL_S
ALKDAVN+XL_L
TGRNPQTGKEIK+XL_S
TGRNPQTGKEIK+XL_L
B_b_ion
B_y_ion
prec
b10+
b11+
b10++
b9+
b9++
y2+
y3+
y5+
y6+
y8+
y9+
b6+
b7+
b8+
b4+
b3+
intensity
m/z

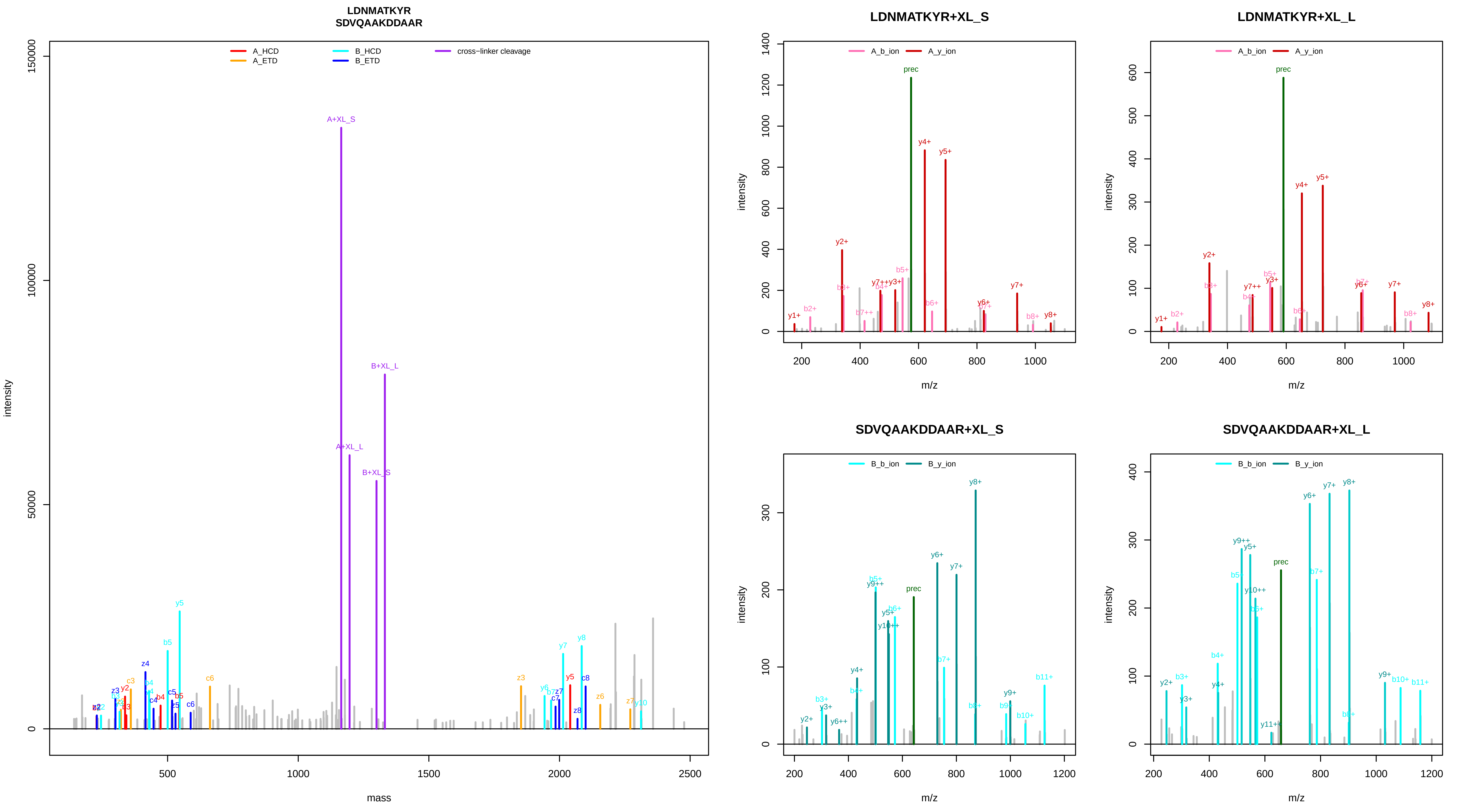
LDNMATKYR
SDVQAAKDDAAR
A_HCD
A_ETD
B_HCD
B_ETD
cross−linker cleavage
A+XL_S
B+XL_L
A+XL_L
B+XL_S
intensity
mass
LDNMATKYR+XL_S
LDNMATKYR+XL_L
SDVQAAKDDAAR+XL_S
SDVQAAKDDAAR+XL_L
A_b_ion
A_y_ion
B_b_ion
B_y_ion
prec
m/z

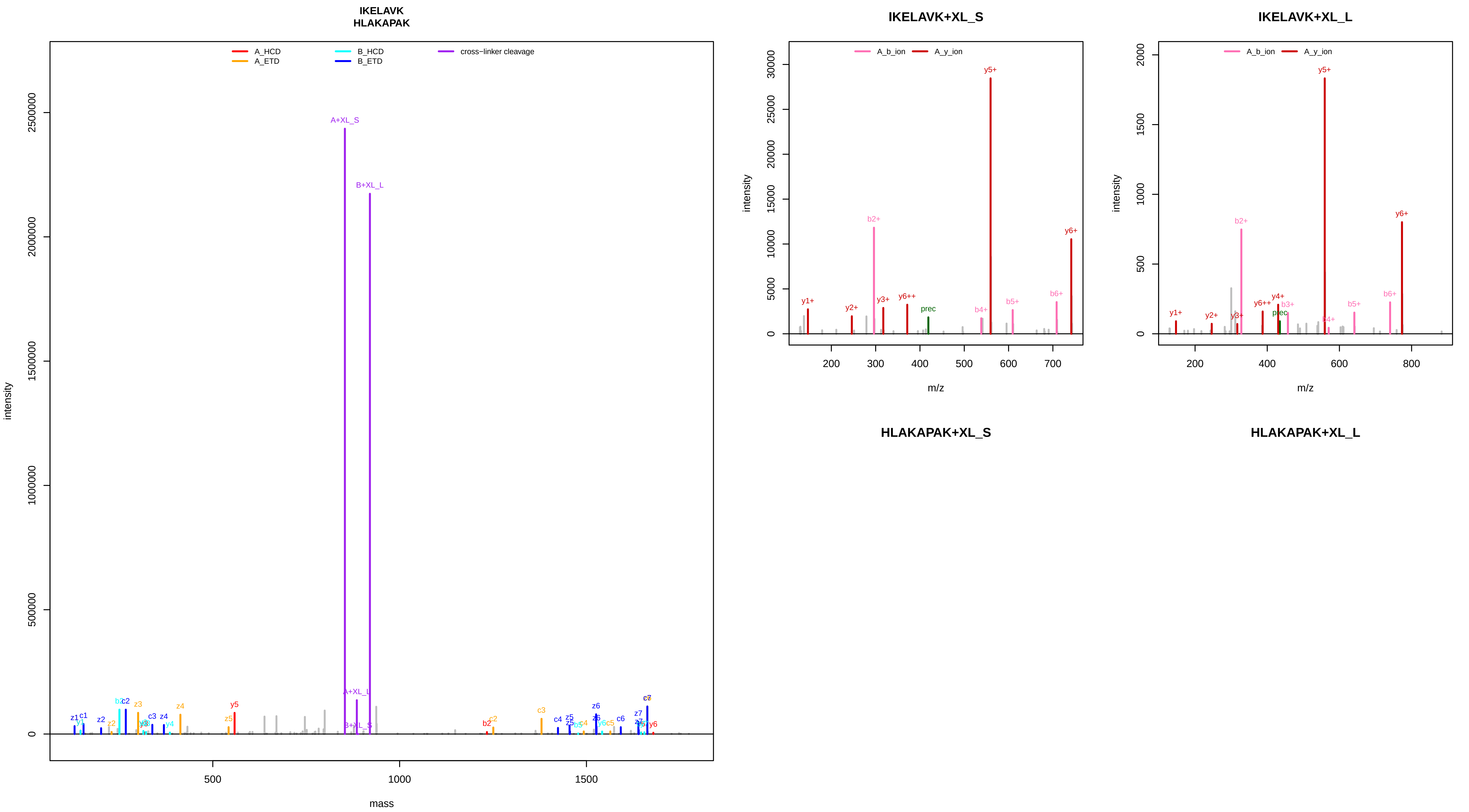

IKELAVK
HLAKAPAK
A_HCD
A_ETD
B_HCD
B_ETD
cross−linker cleavage
A+XL_S
B+XL_L
A+XL_L
intensity
mass
IKELAVK+XL_S
IKELAVK+XL_L
A_b_ion
A_y_ion
prec
m/z
HLAKAPAK+XL_S
HLAKAPAK+XL_L

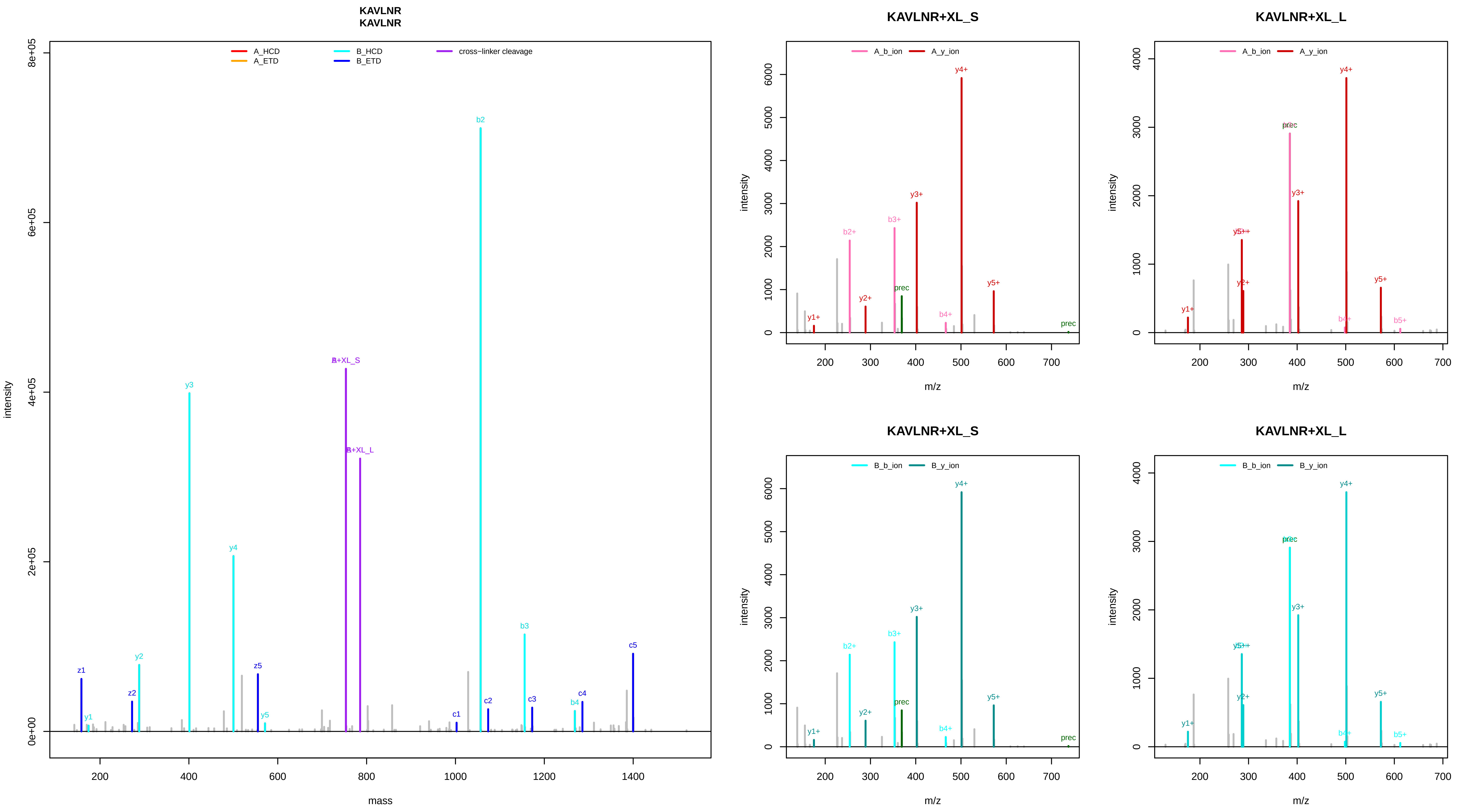

KAVLNR
KAVLNR
A_HCD
A_ETD
B_HCD
B_ETD
cross−linker cleavage
intensity
mass
KAVLNR+XL_S
KAVLNR+XL_L
A_b_ion
A_y_ion
B_b_ion
B_y_ion
intensity
m/z

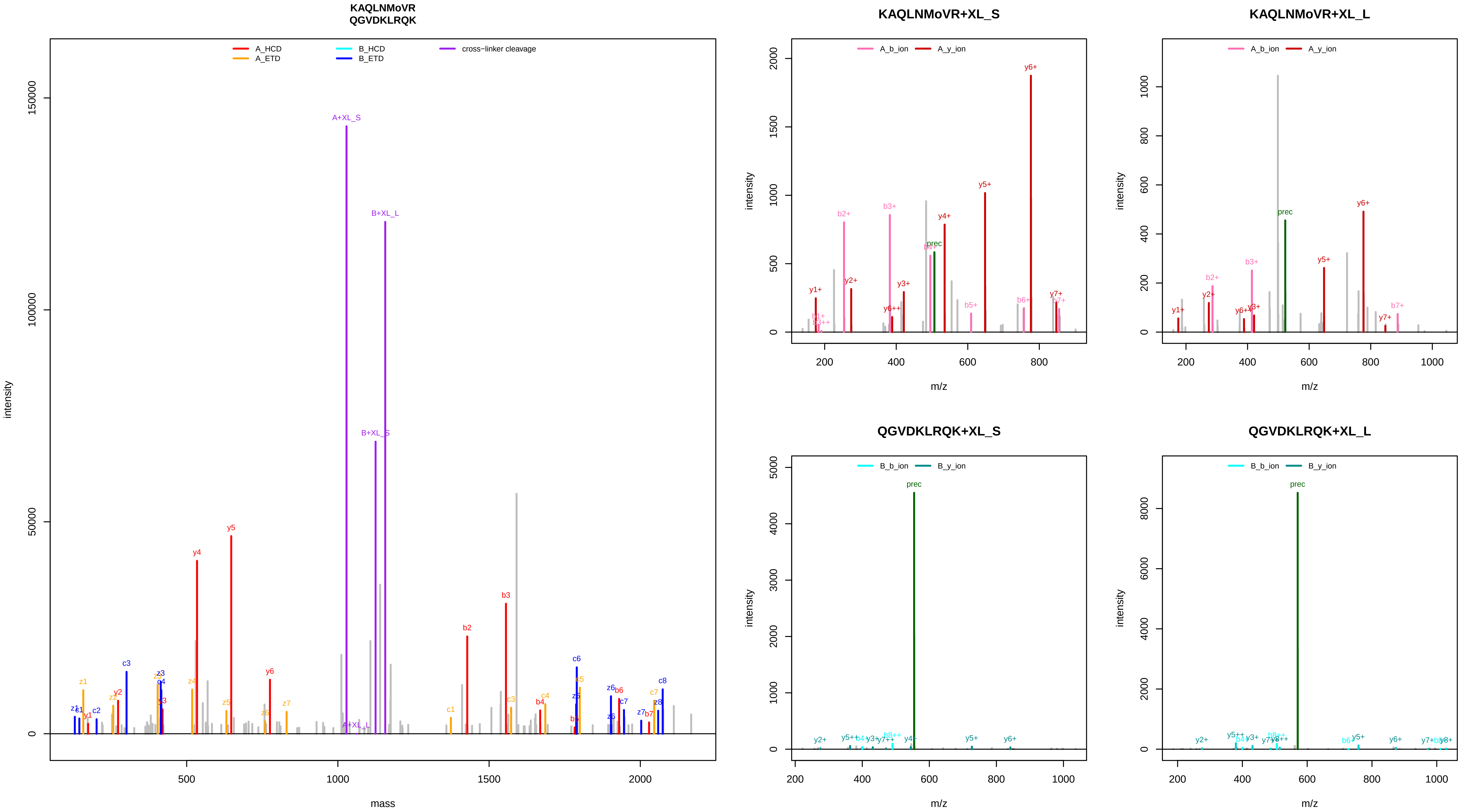
KAQLNMoVR
QGVDKLRQK
A_HCD
A_ETD
B_HCD
B_ETD
cross−linker cleavage
A+XL_S
B+XL_L
B+XL_S
A+XL_L
y4
y5
y6
b2
b3
c3
c6
c8
intensity
mass
0
50000
100000
150000
500
1000
1500
2000
KAQLNMoVR+XL_S
KAQLNMoVR+XL_L
QGVDKLRQK+XL_S
QGVDKLRQK+XL_L
A_b_ion
A_y_ion
B_b_ion
B_y_ion
prec
y6+
y5+
y4+
b2+
b3+
m/z
intensity

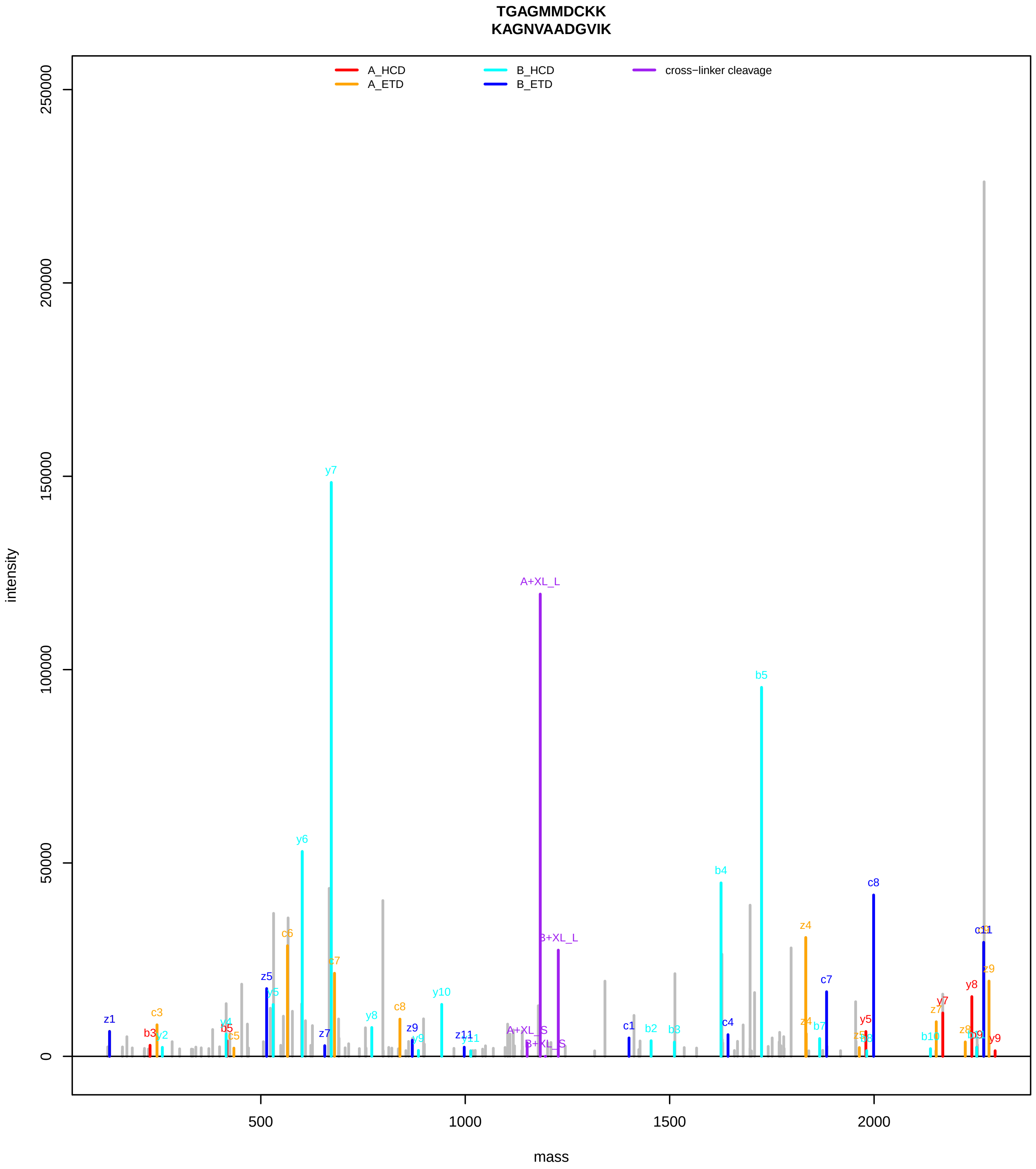

TGAGMMDCKK+XL_S

TGAGMMDCKK+XL_L

KAGNVAADGVIK+XL_S

KAGNVAADGVIK+XL_L

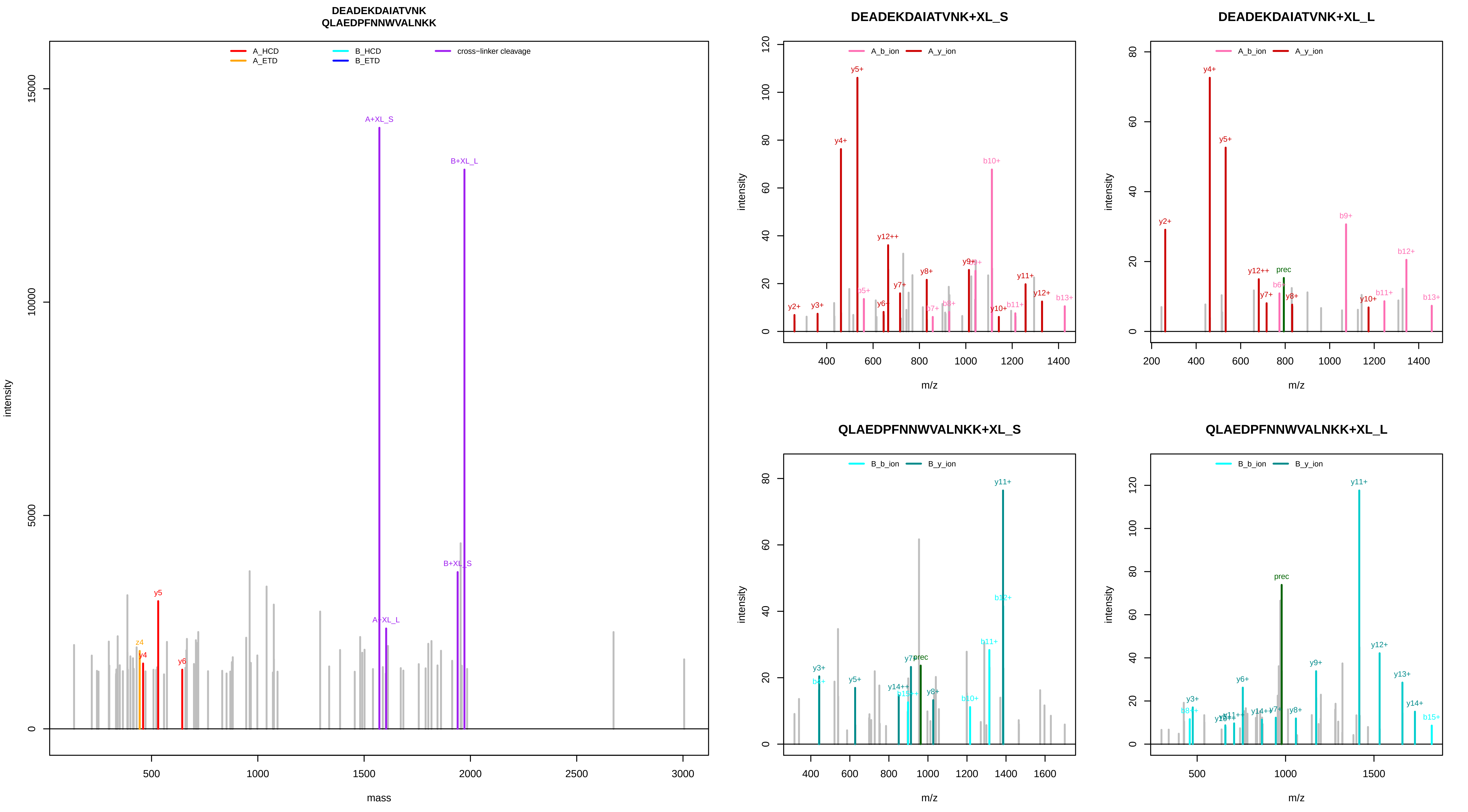
DEADEKDAIATVNK
QLAEDPFNNWVALNKK
A_HCD
A_ETD
B_HCD
B_ETD
cross−linker cleavage
A+XL_S
B+XL_L
B+XL_S
A+XL_L
intensity
mass
DEADEKDAIATVNK+XL_S
DEADEKDAIATVNK+XL_L
QLAEDPFNNWVALNKK+XL_S
QLAEDPFNNWVALNKK+XL_L
A_b_ion
A_y_ion
B_b_ion
B_y_ion
m/z
prec

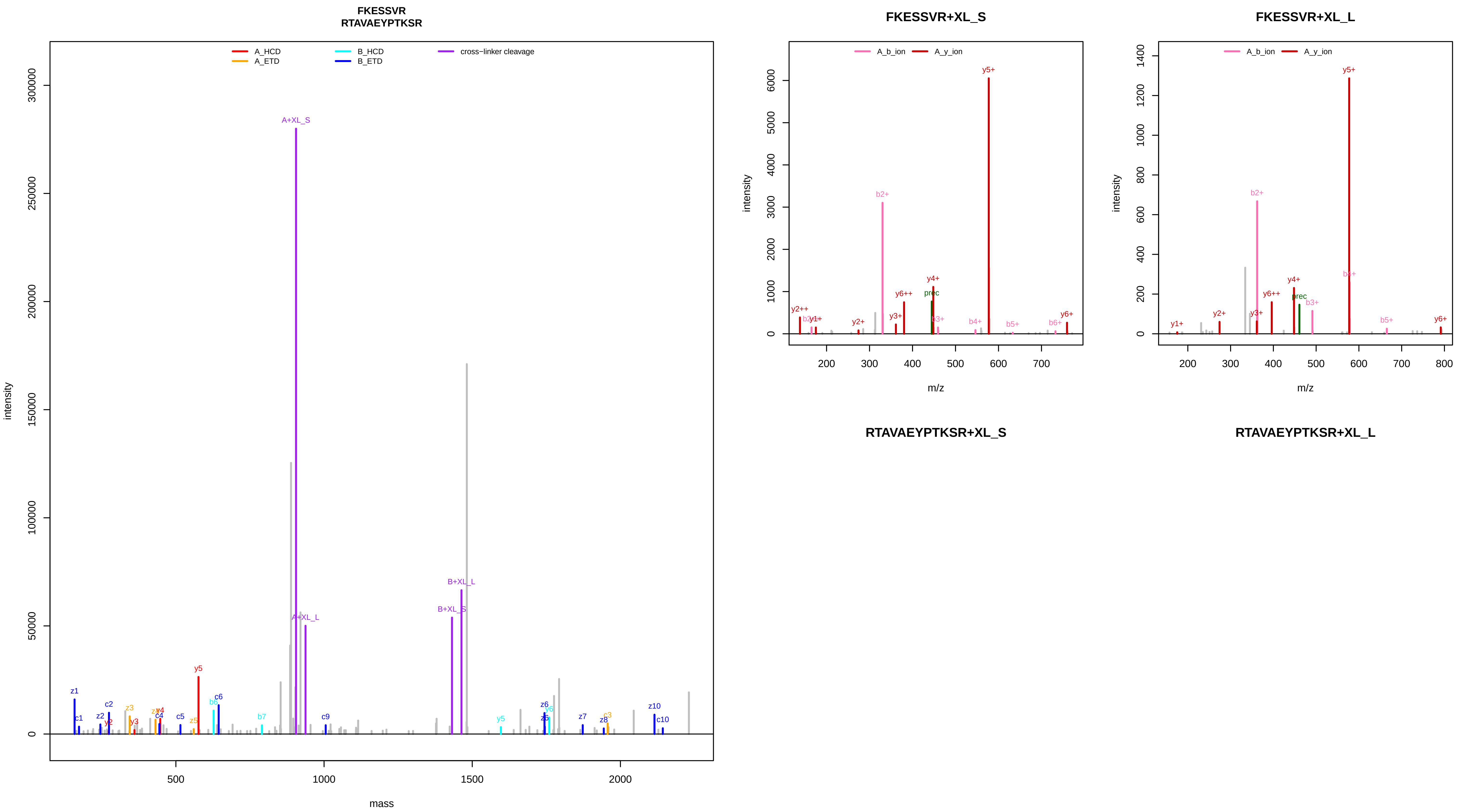
FKESSVR
RTAVAEYPTKSR
A_HCD
A_ETD
B_HCD
B_ETD
cross−linker cleavage
A+XL_S
A+XL_L
B+XL_S
B+XL_L
intensity
mass
FKESSVR+XL_S
A_b_ion
A_y_ion
prec
intensity
m/z
FKESSVR+XL_L
A_b_ion
A_y_ion
prec
intensity
m/z
RTAVAEYPTKSR+XL_S
RTAVAEYPTKSR+XL_L

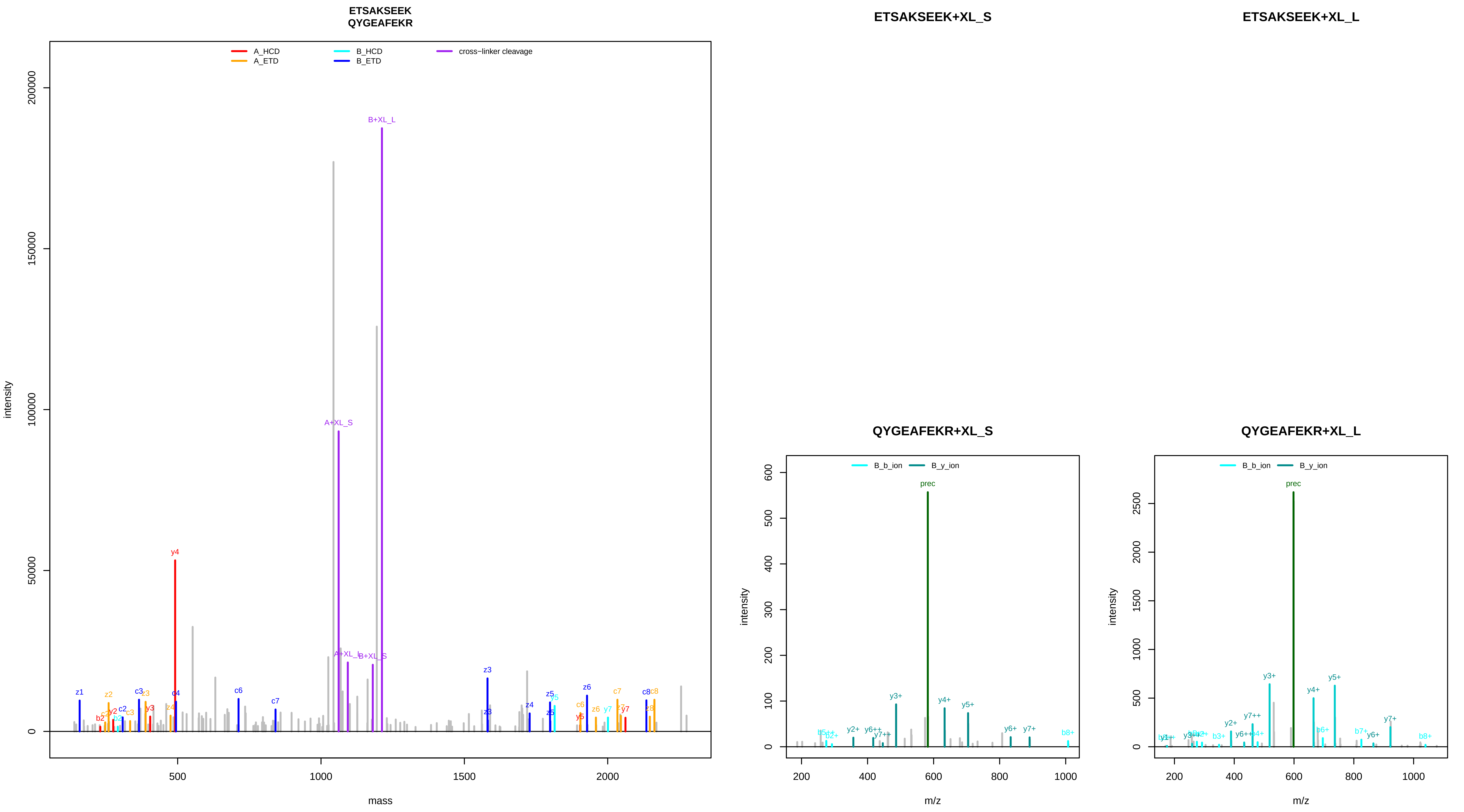

ETSAKSEEK
QYGEAFEKR
A_HCD
A_ETD
B_HCD
B_ETD
cross−linker cleavage
B+XL_L
A+XL_S
A+XL_L
B+XL_S
intensity
mass
ETSAKSEEK+XL_S
ETSAKSEEK+XL_L
QYGEAFEKR+XL_S
QYGEAFEKR+XL_L
B_b_ion
B_y_ion
prec
intensity
m/z

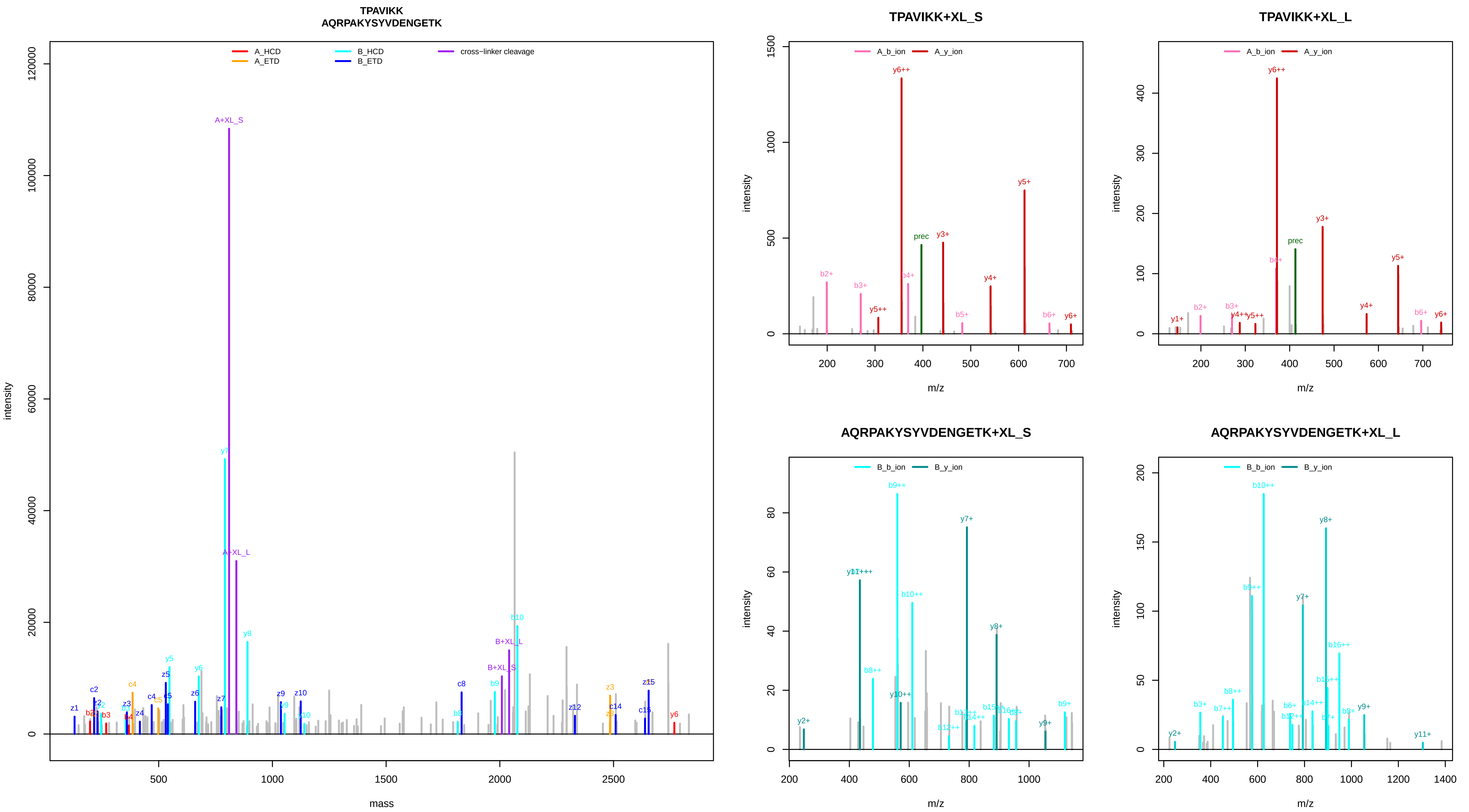
TPAVIKK
AQRPAKYSYVDENGETK
A_HCD
A_ETD
B_HCD
B_ETD
cross−linker cleavage
A+XL_S
A+XL_L
B+XL_L
B+XL_S
intensity
mass
TPAVIKK+XL_S
TPAVIKK+XL_L
AQRPAKYSYVDENGETK+XL_S
AQRPAKYSYVDENGETK+XL_L
A_b_ion
A_y_ion
B_b_ion
B_y_ion
prec
m/z

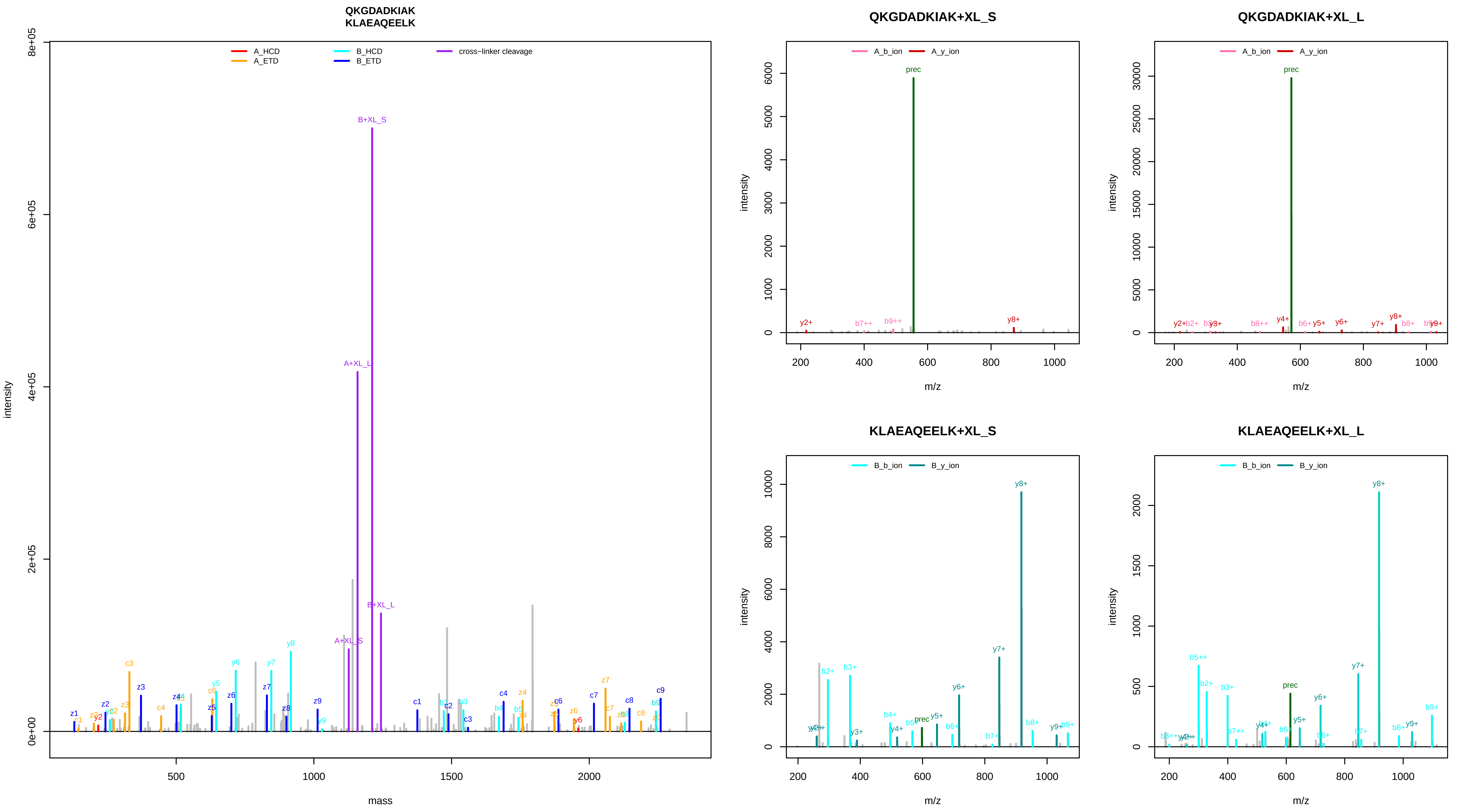
QKGDADKIAK
KLAEAQEELK
A_HCD
A_ETD
B_HCD
B_ETD
cross−linker cleavage
B+XL_S
A+XL_L
B+XL_L
A+XL_S
intensity
mass
QKGDADKIAK+XL_S
QKGDADKIAK+XL_L
KLAEAQEELK+XL_S
KLAEAQEELK+XL_L
A_b_ion
A_y_ion
B_b_ion
B_y_ion
prec
m/z

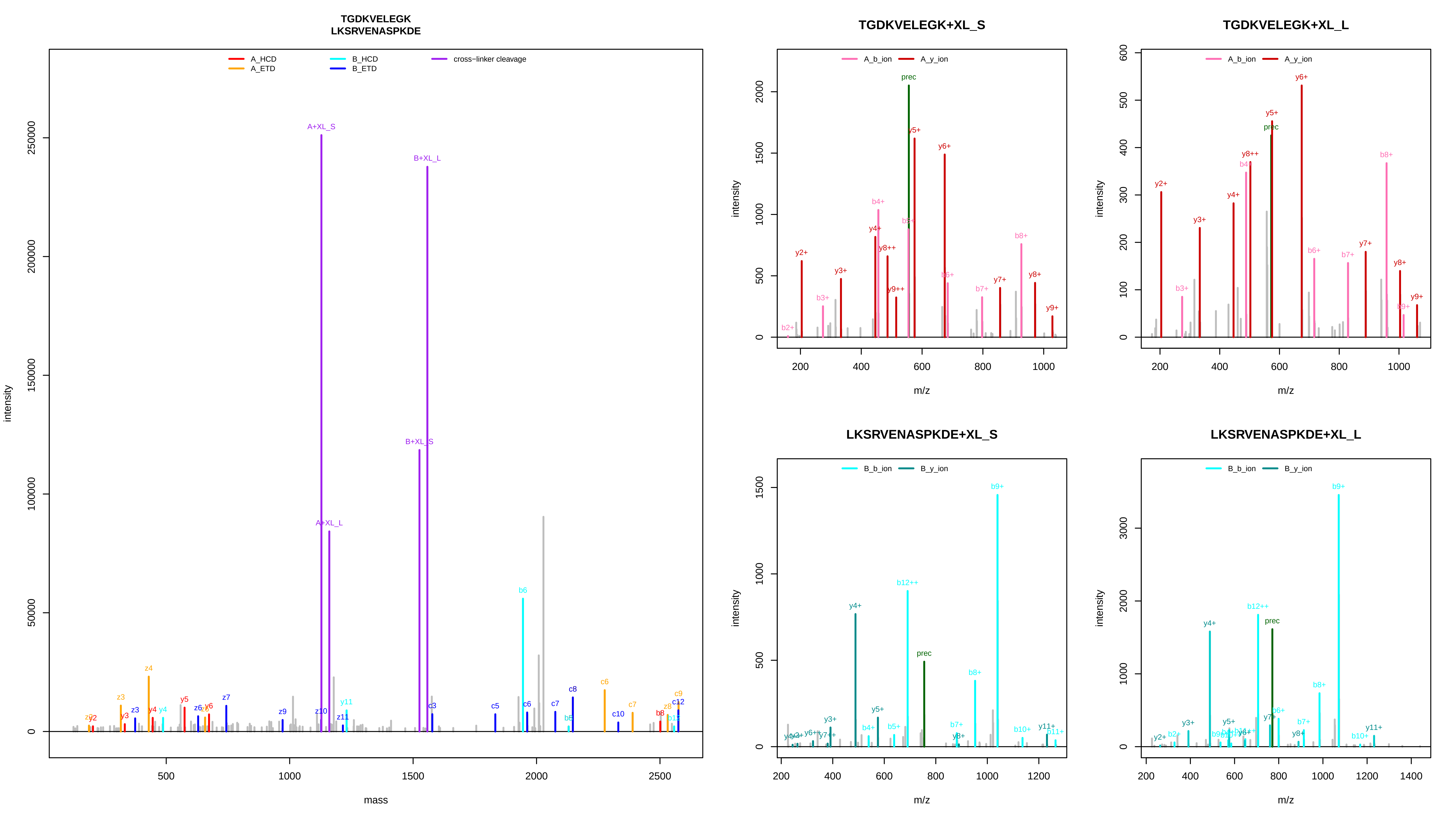
TGDKVELEGK
LKSRVENASPKDE
A_HCD
A_ETD
B_HCD
B_ETD
cross−linker cleavage
A+XL_S
B+XL_L
B+XL_S
A+XL_L
intensity
mass
TGDKVELEGK+XL_S
TGDKVELEGK+XL_L
LKSRVENASPKDE+XL_S
LKSRVENASPKDE+XL_L
A_b_ion
A_y_ion
B_b_ion
B_y_ion
prec
m/z

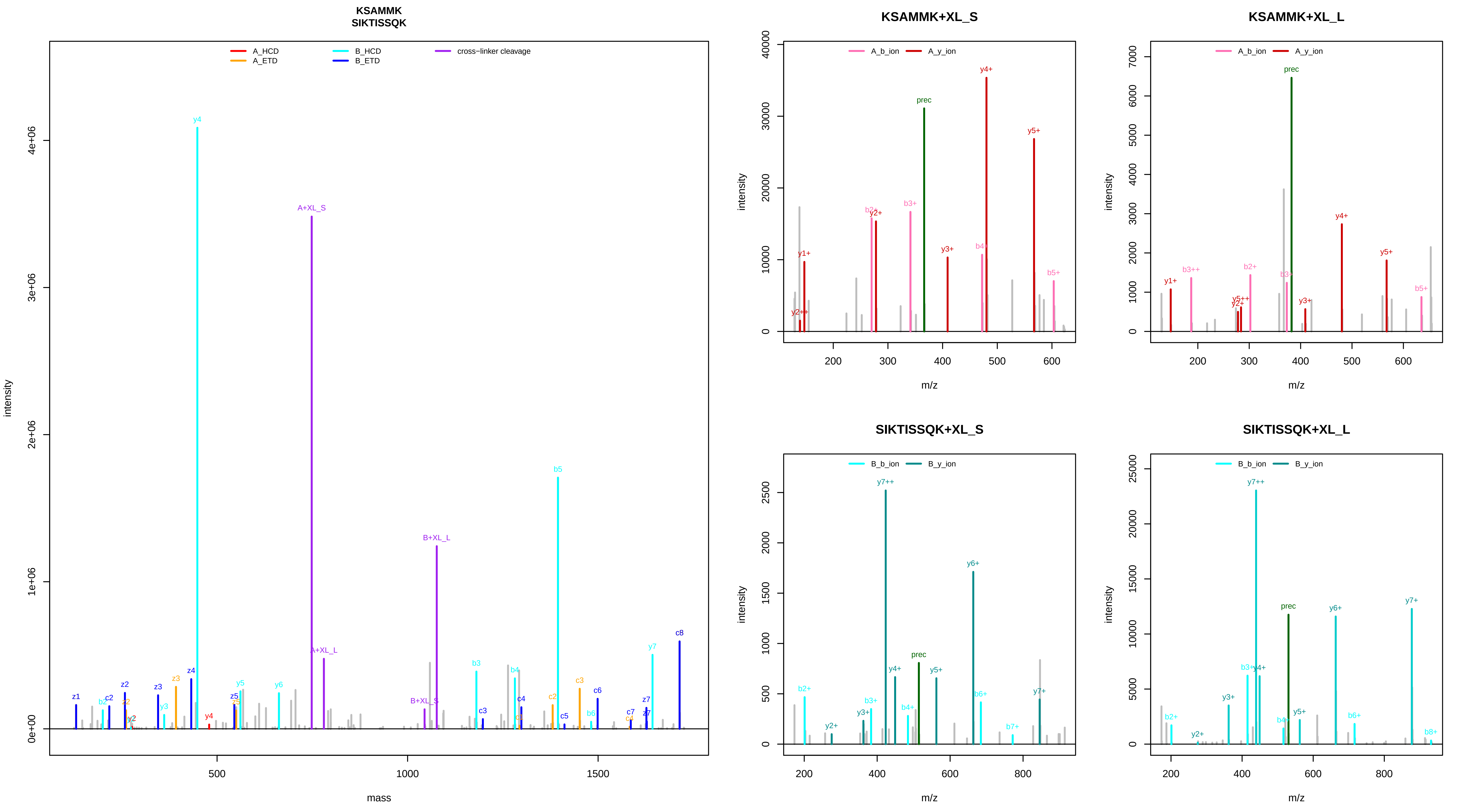
KSAMMK
SIKTISSQK
A_HCD
A_ETD
B_HCD
B_ETD
cross−linker cleavage
A+XL_S
A+XL_L
B+XL_L
B+XL_S
intensity
mass
KSAMMK+XL_S
KSAMMK+XL_L
SIKTISSQK+XL_S
SIKTISSQK+XL_L
A_b_ion
A_y_ion
B_b_ion
B_y_ion
prec
intensity
m/z

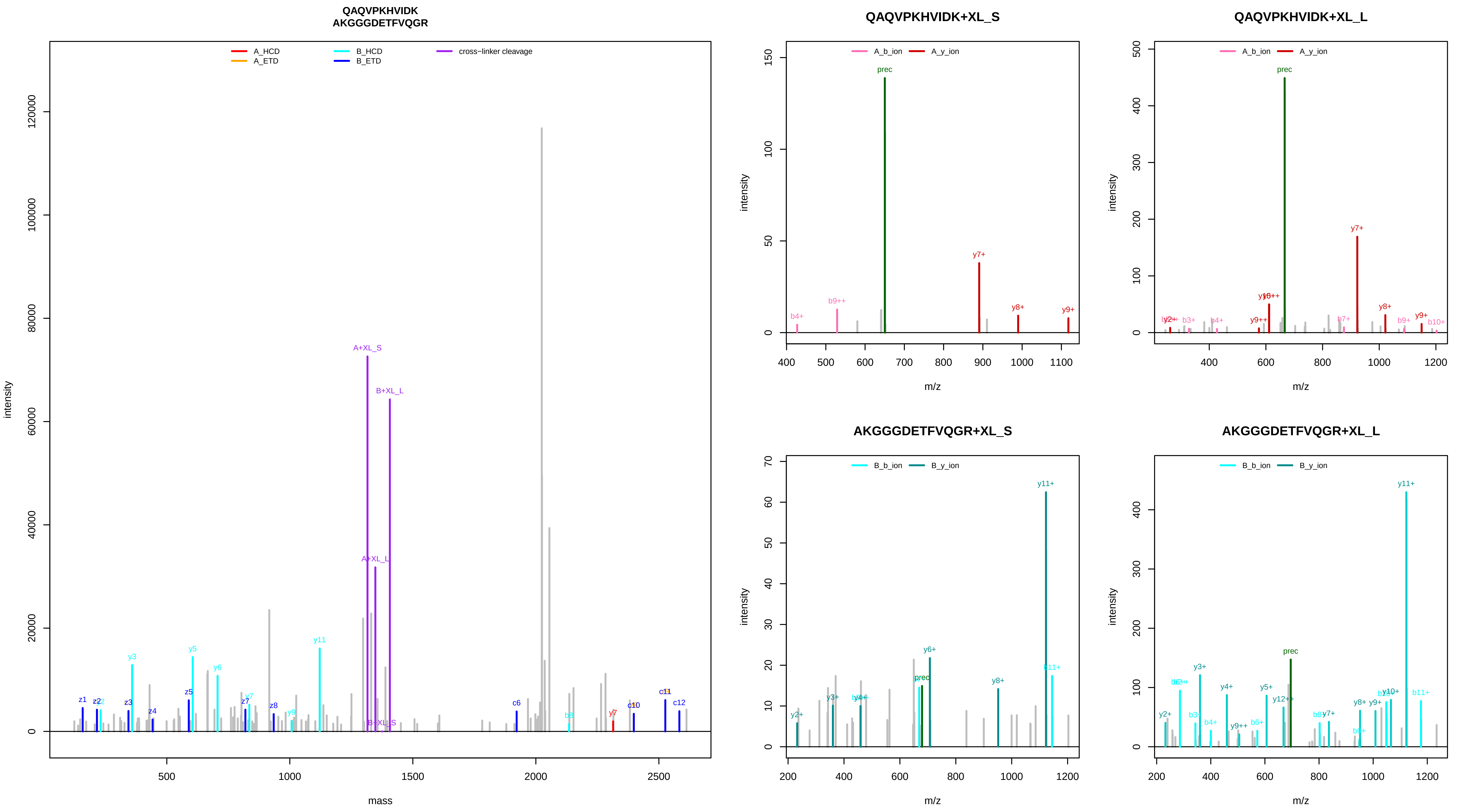

QAQVPKHVIDK
AKGGGDETFVQGR
A_HCD
A_ETD
B_HCD
B_ETD
cross−linker cleavage
A+XL_S
B+XL_L
A+XL_L
B+XL_S
intensity
mass
QAQVPKHVIDK+XL_S
QAQVPKHVIDK+XL_L
AKGGGDETFVQGR+XL_S
AKGGGDETFVQGR+XL_L
A_b_ion
A_y_ion
B_b_ion
B_y_ion
prec
m/z

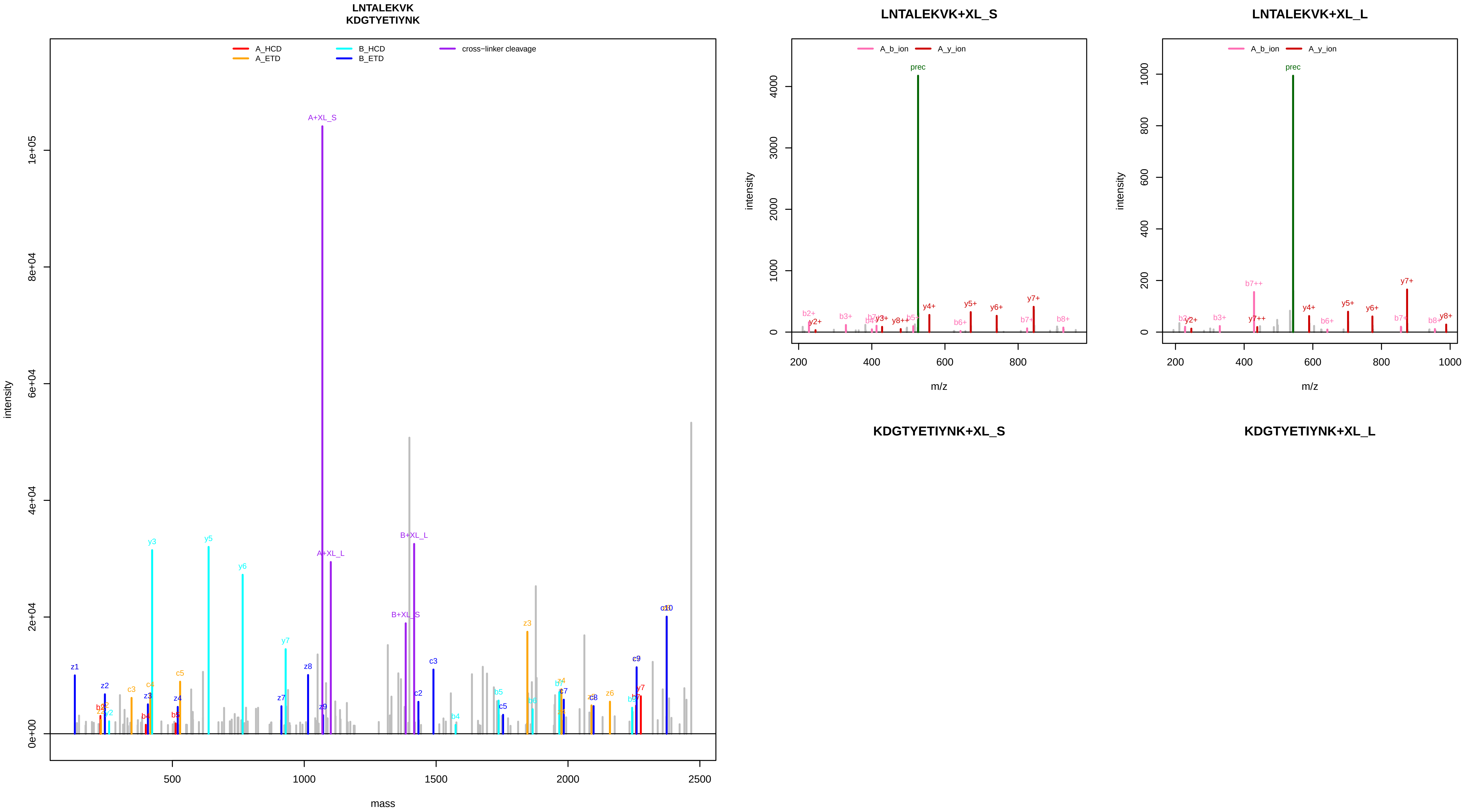

LNTALEKVK
KDGTYETIYNK
A_HCD
A_ETD
B_HCD
B_ETD
cross−linker cleavage
A+XL_S
A+XL_L
B+XL_L
B+XL_S
intensity
mass
LNTALEKVK+XL_S
LNTALEKVK+XL_L
A_b_ion
A_y_ion
prec
intensity
m/z
KDGTYETIYNK+XL_S
KDGTYETIYNK+XL_L

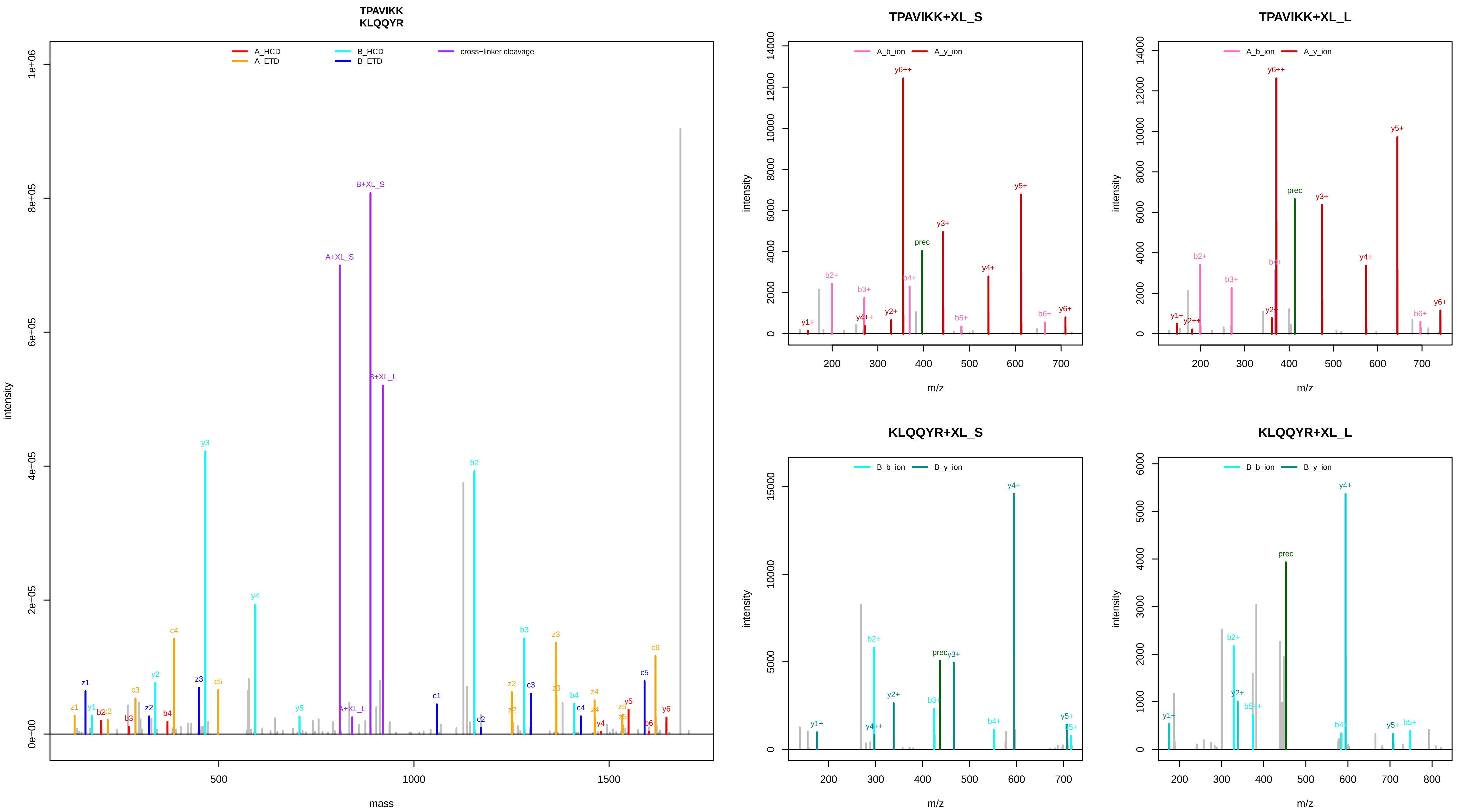
TPAVIKK
KLQQYR
A_HCD
A_ETD
B_HCD
B_ETD
cross−linker cleavage
B+XL_S
A+XL_S
B+XL_L
A+XL_L
intensity
mass
TPAVIKK+XL_S
TPAVIKK+XL_L
KLQQYR+XL_S
KLQQYR+XL_L
A_b_ion
A_y_ion
B_b_ion
B_y_ion
prec
m/z

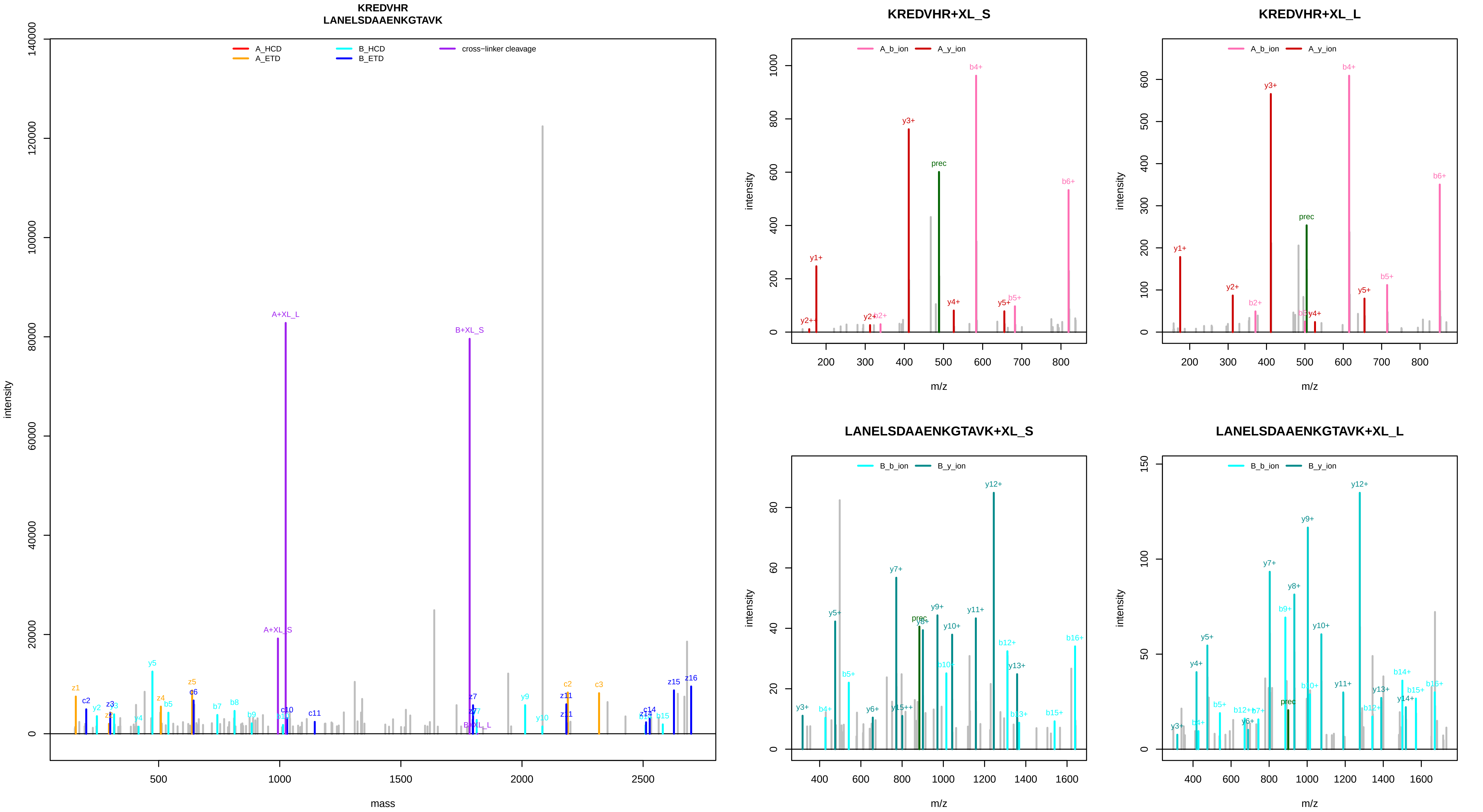

KREDVHR
LANELSDAAENKGTAVK
A_HCD
A_ETD
B_HCD
B_ETD
cross−linker cleavage
A+XL_L
B+XL_S
A+XL_S
B+XL_L
intensity
mass
KREDVHR+XL_S
KREDVHR+XL_L
A_b_ion
A_y_ion
prec
m/z
LANELSDAAENKGTAVK+XL_S
LANELSDAAENKGTAVK+XL_L
B_b_ion
B_y_ion

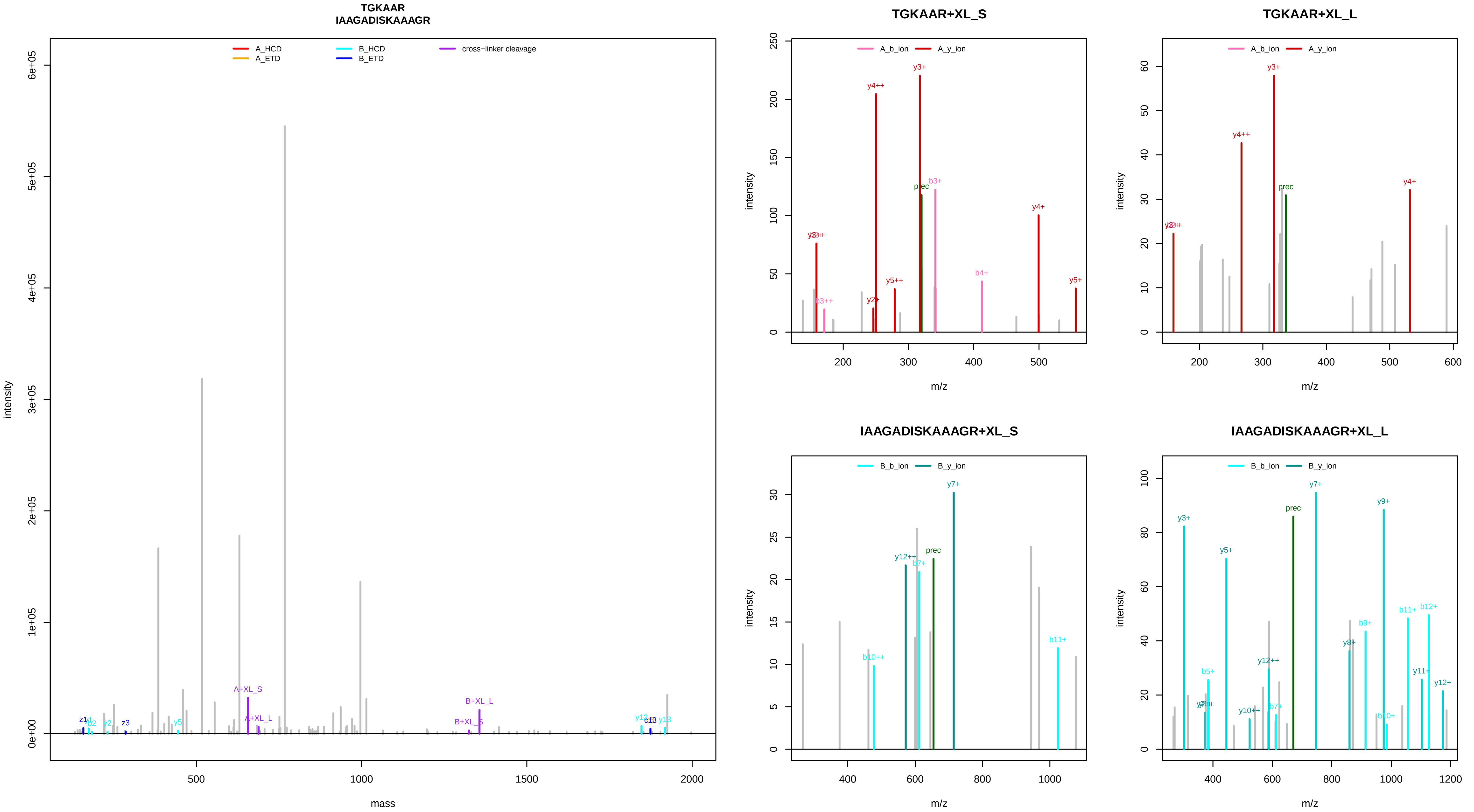
TGKAAR
IAAGADISKAAAGR
A_HCD
A_ETD
B_HCD
B_ETD
cross−linker cleavage
intensity
mass
A+XL_S
A+XL_L
B+XL_L
B+XL_S
TGKAAR+XL_S
TGKAAR+XL_L
IAAGADISKAAAGR+XL_S
IAAGADISKAAAGR+XL_L
A_b_ion
A_y_ion
B_b_ion
B_y_ion
m/z
prec

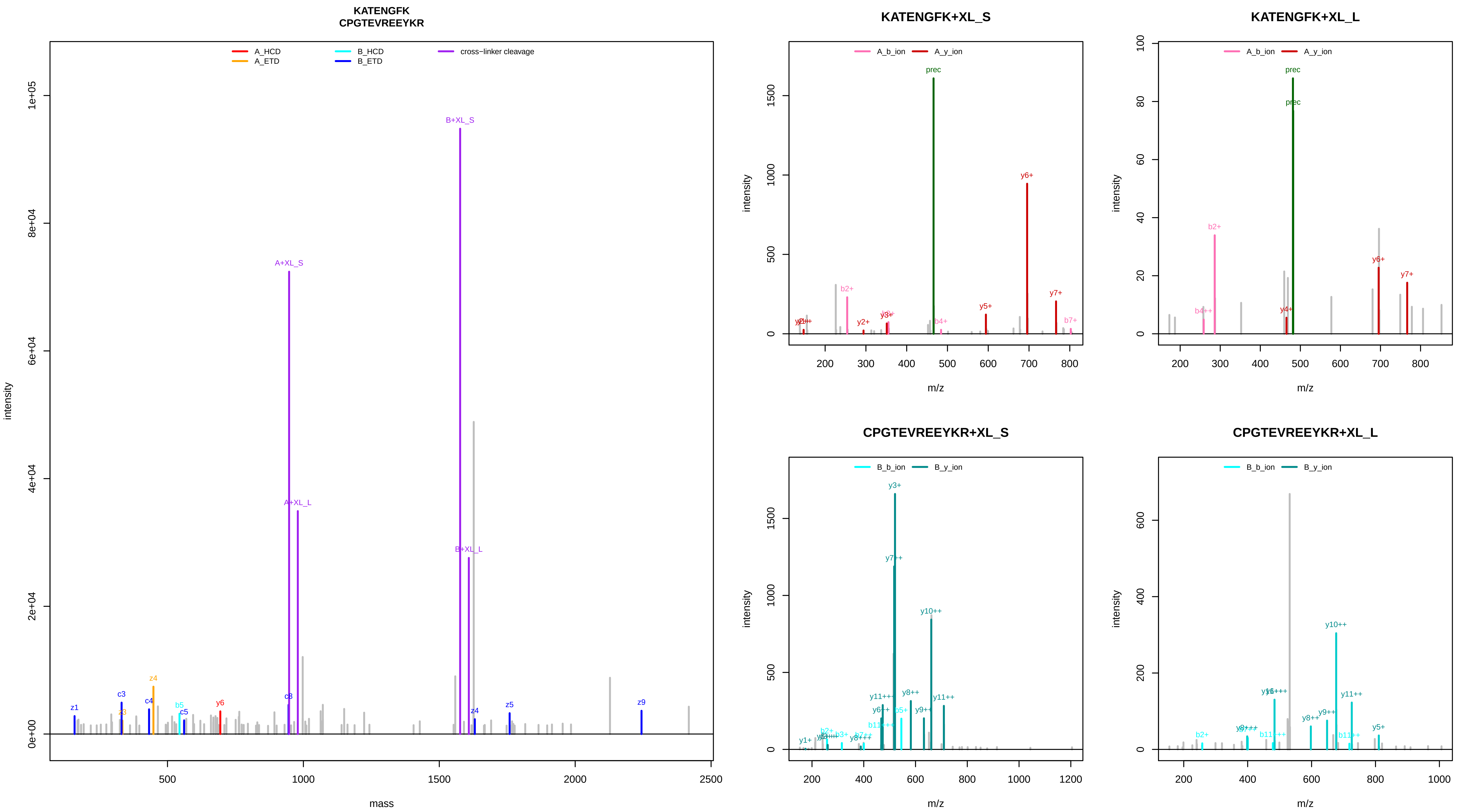
KATENGFK
CPGTEVREEYKR
A_HCD
A_ETD
B_HCD
B_ETD
cross−linker cleavage
intensity
mass
KATENGFK+XL_S
KATENGFK+XL_L
CPGTEVREEYKR+XL_S
CPGTEVREEYKR+XL_L
A_b_ion
A_y_ion
B_b_ion
B_y_ion
m/z
prec

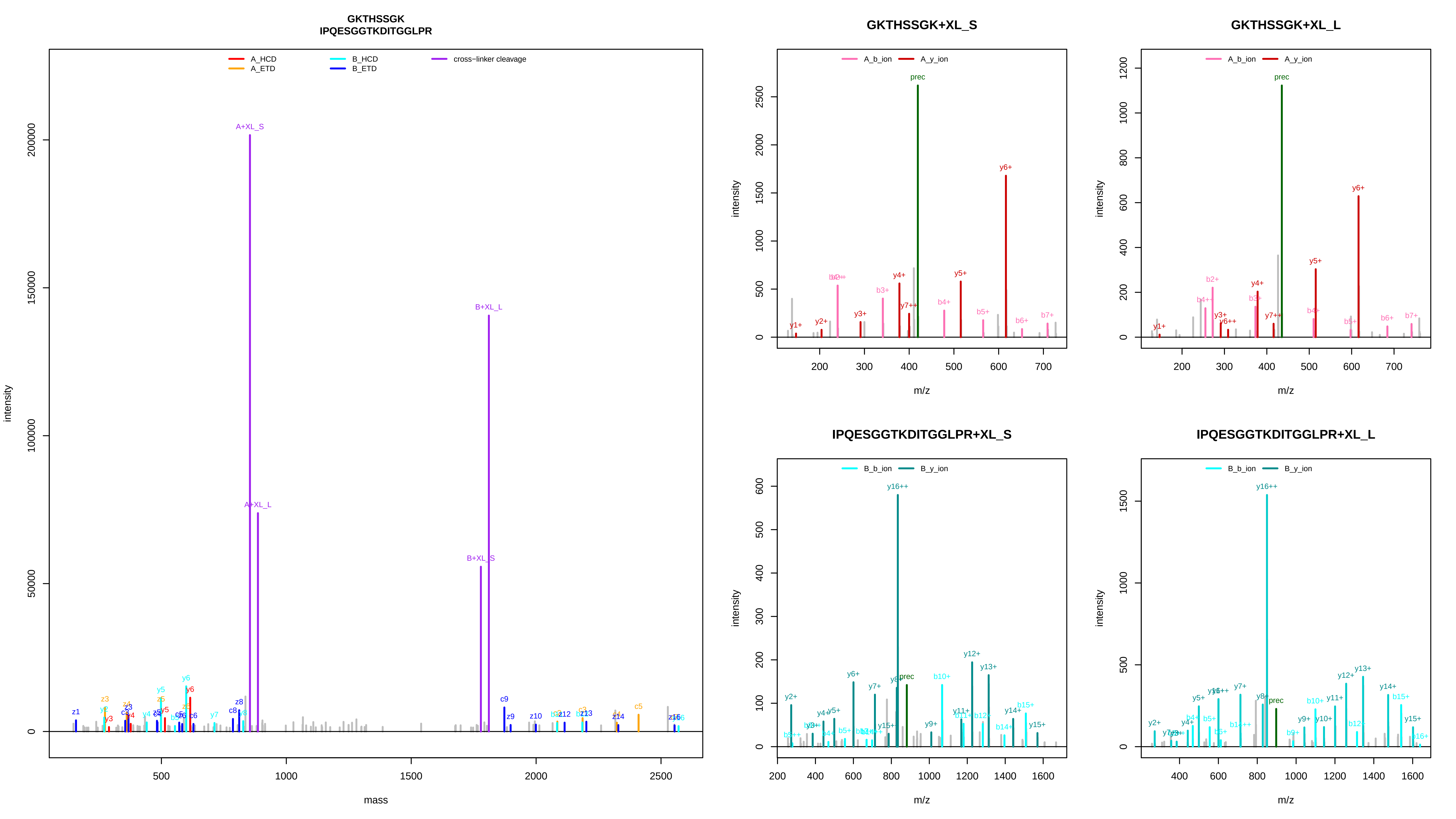
GKTHSSGK
IPQESGGTKDITGGLPR
A_HCD
A_ETD
B_HCD
B_ETD
cross−linker cleavage
A+XL_S
A+XL_L
B+XL_L
B+XL_S
intensity
mass
GKTHSSGK+XL_S
GKTHSSGK+XL_L
A_b_ion
A_y_ion
prec
y6+
y5+
y4+
m/z
IPQESGGTKDITGGLPR+XL_S
IPQESGGTKDITGGLPR+XL_L
B_b_ion
B_y_ion
y16++
y12+
y13+
b10+
b15+
y14+

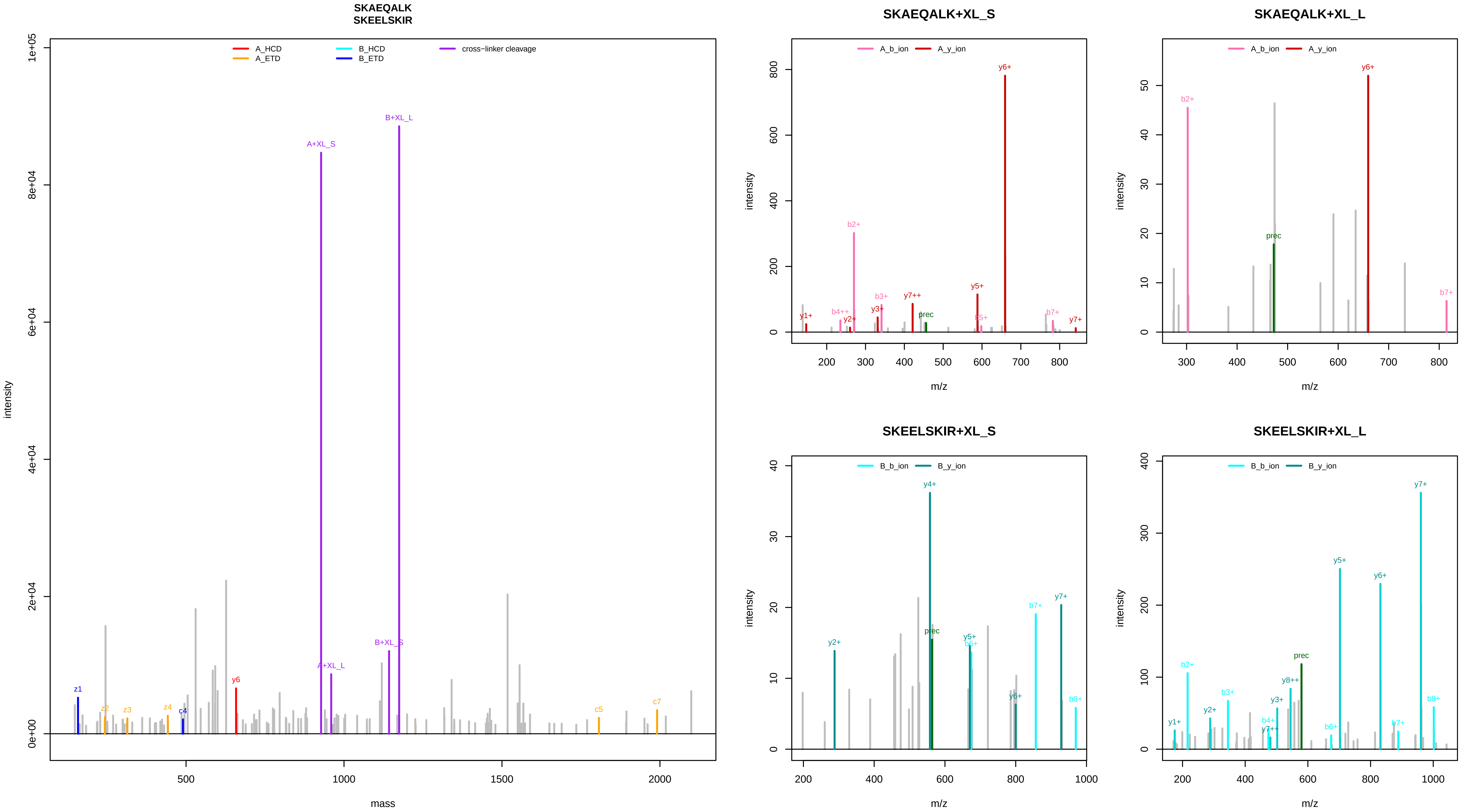
SKAEQALK
SKEELSKIR
A_HCD
A_ETD
B_HCD
B_ETD
cross−linker cleavage
A+XL_S
B+XL_L
B+XL_S
A+XL_L
intensity
mass
SKAEQALK+XL_S
SKAEQALK+XL_L
SKEELSKIR+XL_S
SKEELSKIR+XL_L
A_b_ion
A_y_ion
B_b_ion
B_y_ion
prec
m/z

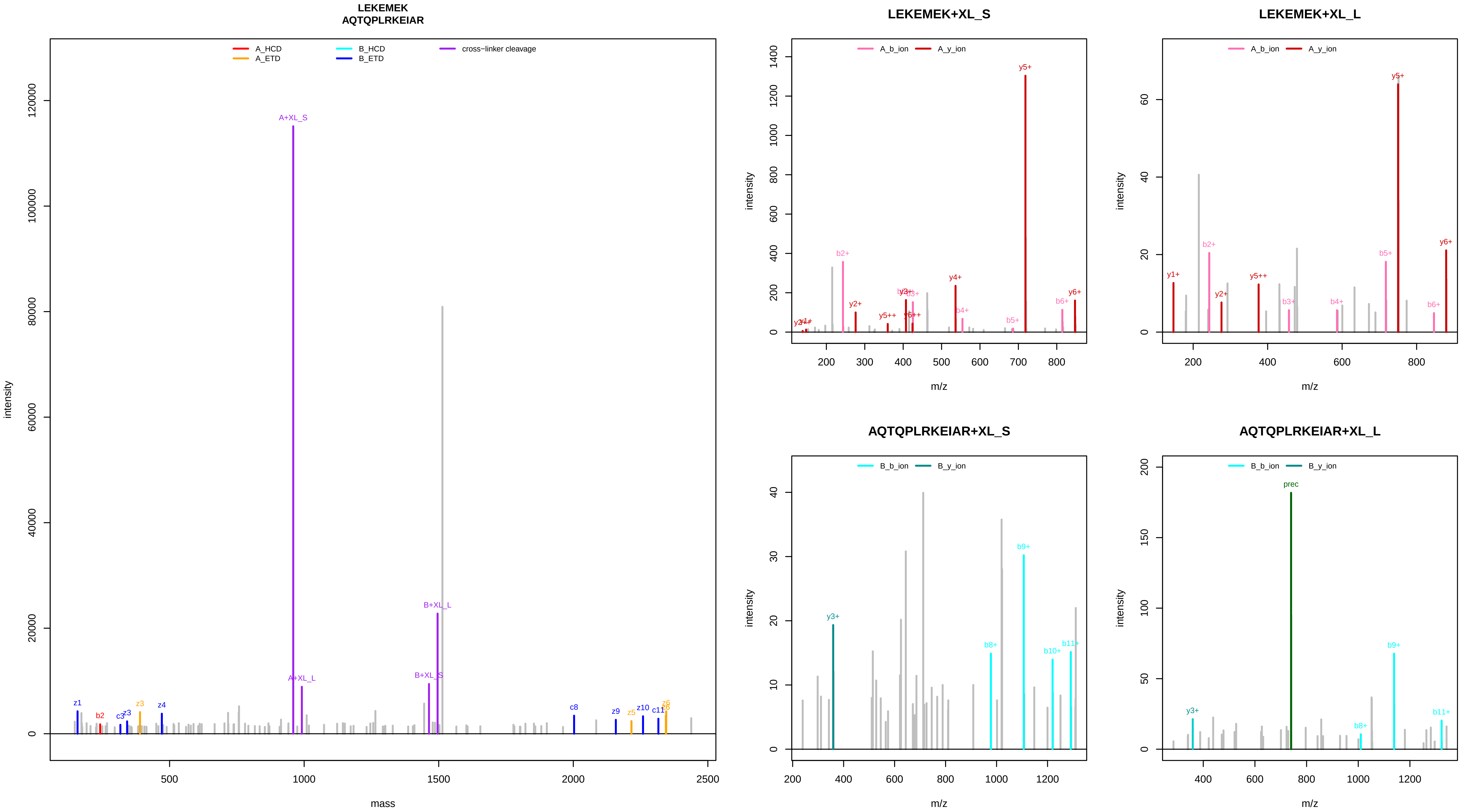
LEKEMEK
AQTQPLRKEIAR
A_HCD
A_ETD
B_HCD
B_ETD
cross−linker cleavage
A+XL_S
A+XL_L
B+XL_L
B+XL_S
z1
b2
c3
z3
z3
z4
c8
z9
z5
z10
c11
z6
intensity
mass
LEKEMEK+XL_S
A_b_ion
A_y_ion
y5+
b2+
y2+
y5++
y3+
b3+
y6++
y4+
b4+
b5+
b6+
y6+
intensity
m/z
LEKEMEK+XL_L
A_b_ion
A_y_ion
y5+
y1+
b2+
y2+
y5++
b3+
b4+
b5+
b6+
y6+
intensity
m/z
AQTQPLRKEIAR+XL_S
B_b_ion
B_y_ion
y3+
b8+
b9+
b10+
b11+
intensity
m/z
AQTQPLRKEIAR+XL_L
B_b_ion
B_y_ion
prec
y3+
b8+
b9+
b11+
intensity
m/z

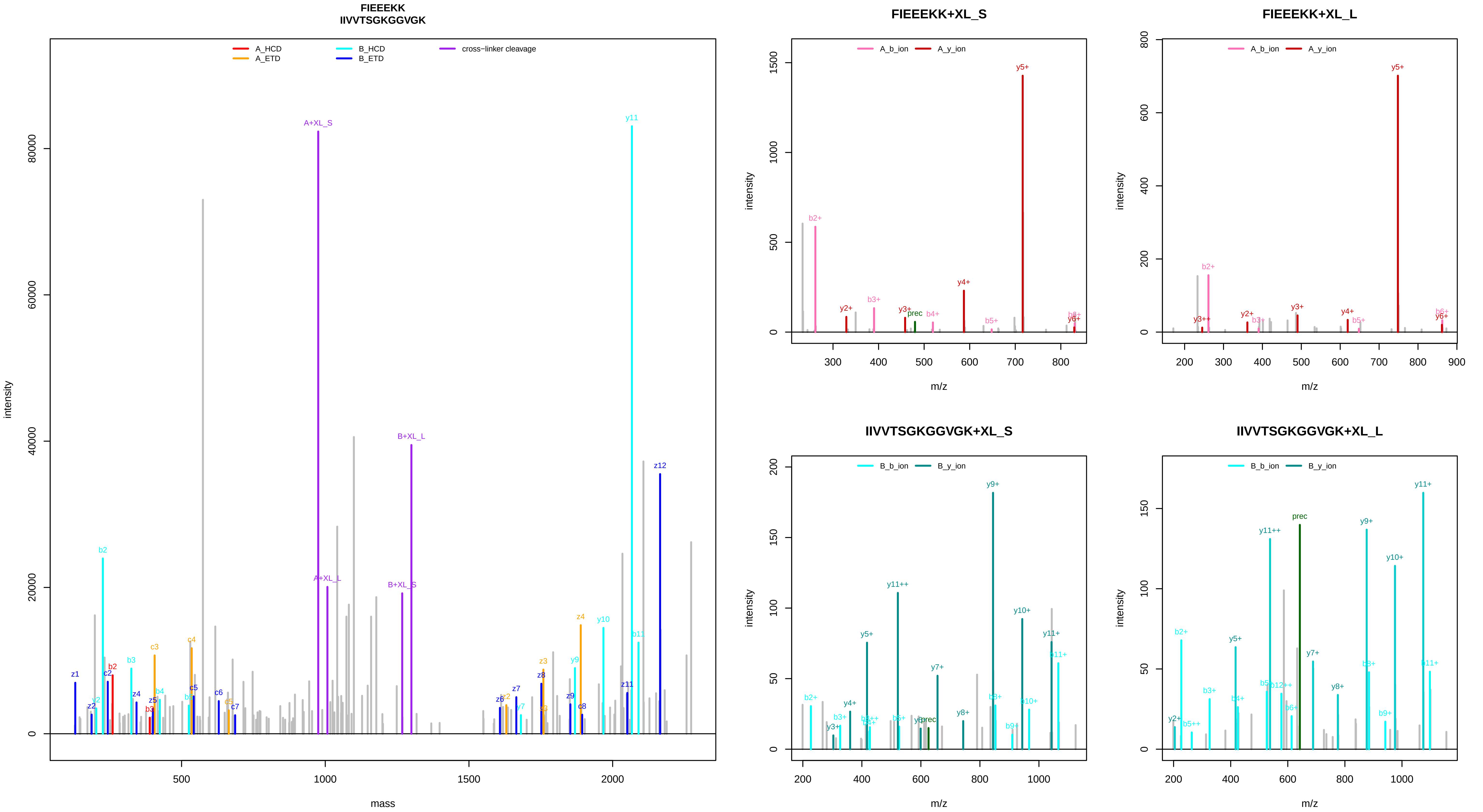
FIEEEKK
IIVVTSGKGGVGK
A_HCD
A_ETD
B_HCD
B_ETD
cross−linker cleavage
A+XL_S
A+XL_L
B+XL_S
B+XL_L
intensity
mass
FIEEEKK+XL_S
FIEEEKK+XL_L
A_b_ion
A_y_ion
IIVVTSGKGGVGK+XL_S
IIVVTSGKGGVGK+XL_L
B_b_ion
B_y_ion
prec
m/z

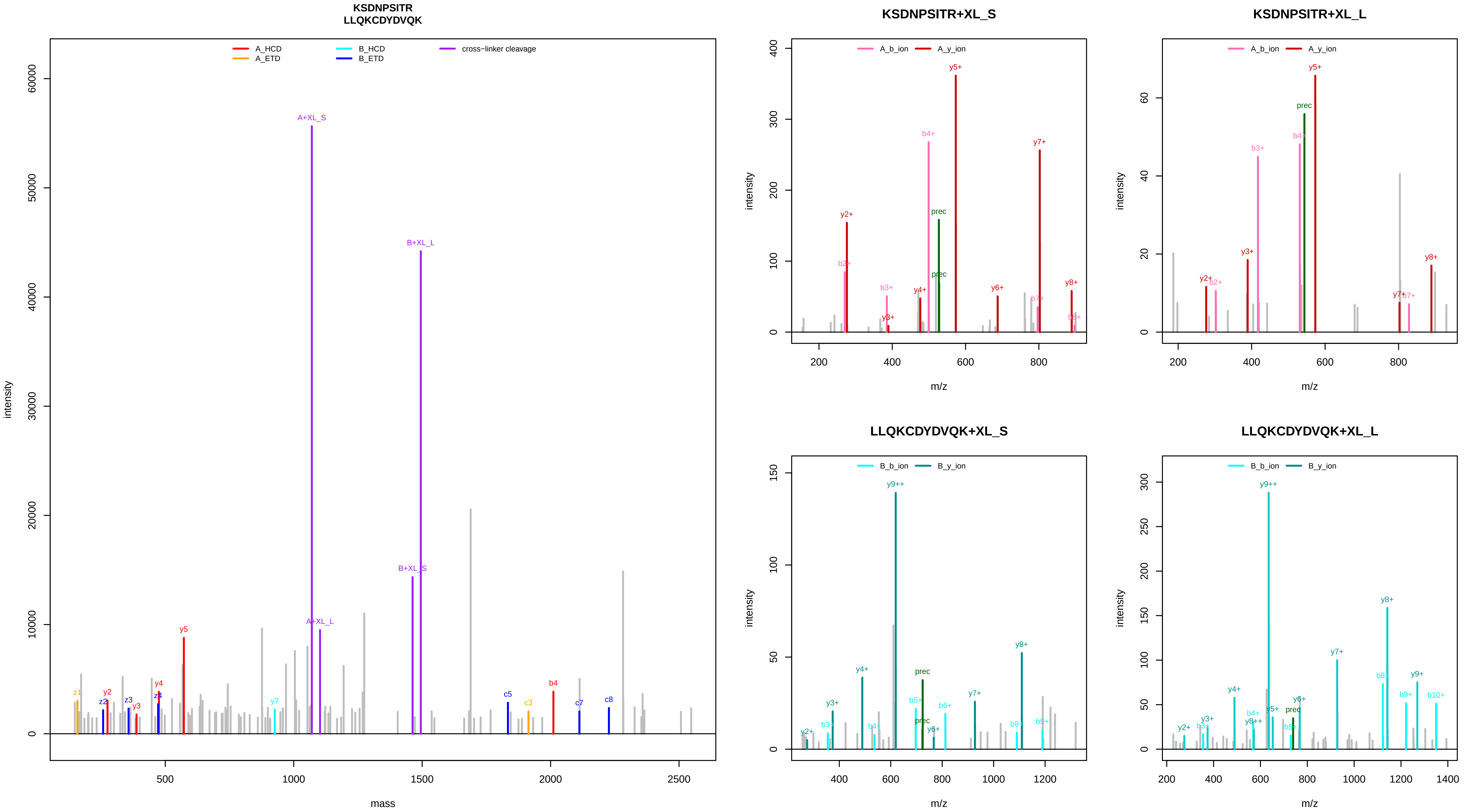
KSDNPSITR
LLQKCDYDVQK
A_HCD
A_ETD
B_HCD
B_ETD
cross−linker cleavage
A+XL_S
B+XL_L
B+XL_S
A+XL_L
intensity
mass
KSDNPSITR+XL_S
KSDNPSITR+XL_L
LLQKCDYDVQK+XL_S
LLQKCDYDVQK+XL_L
A_b_ion
A_y_ion
B_b_ion
B_y_ion
prec
m/z

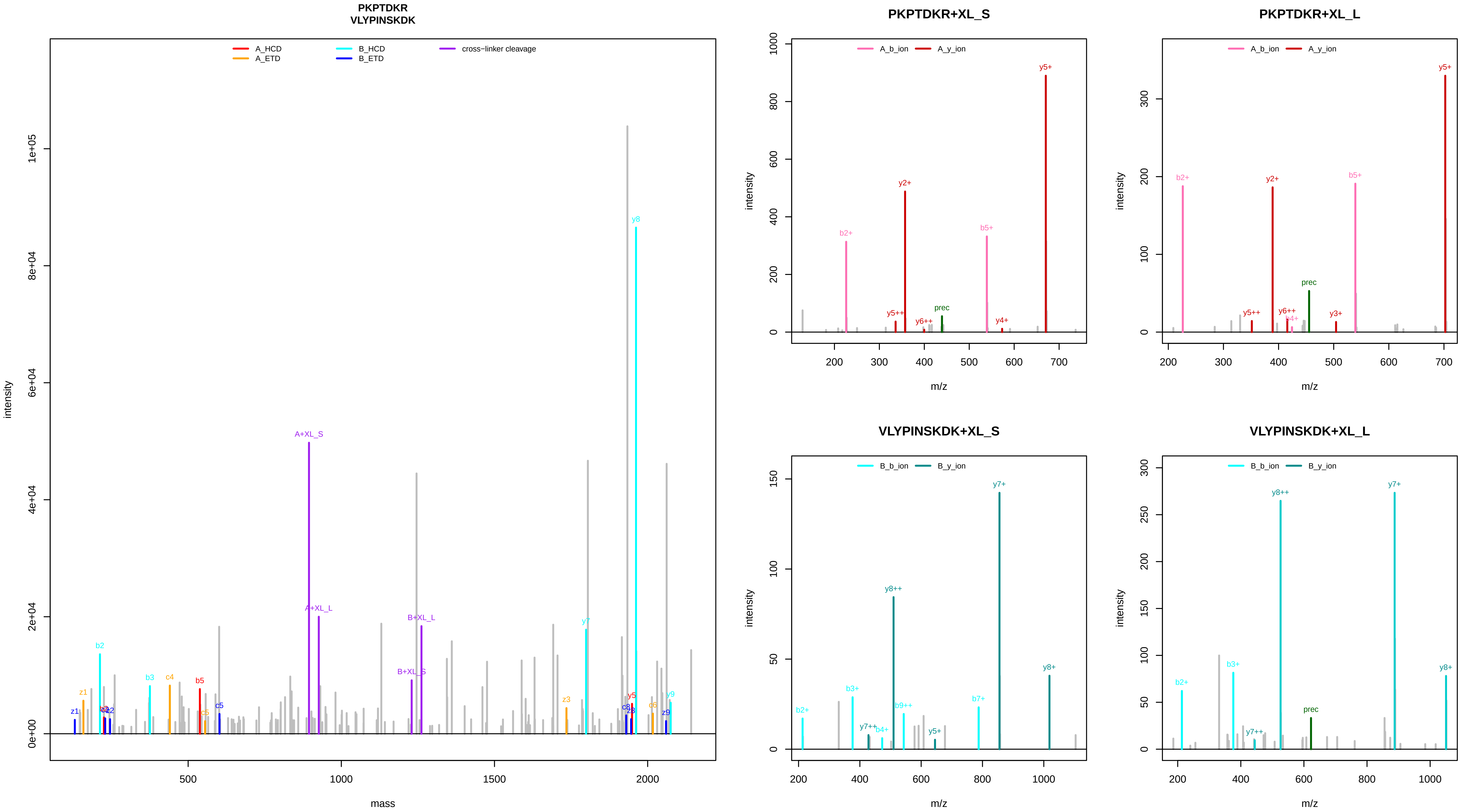
PKPTDKR
VLYPINSKDK
A_HCD
A_ETD
B_HCD
B_ETD
cross−linker cleavage
intensity
mass
A+XL_S
A+XL_L
B+XL_L
B+XL_S
y8
y7
y9
b2
b3
b5
c4
z1
z3
c5
c6
c8
z9
y5
PKPTDKR+XL_S
PKPTDKR+XL_L
A_b_ion
A_y_ion
prec
m/z
VLYPINSKDK+XL_S
VLYPINSKDK+XL_L
B_b_ion
B_y_ion

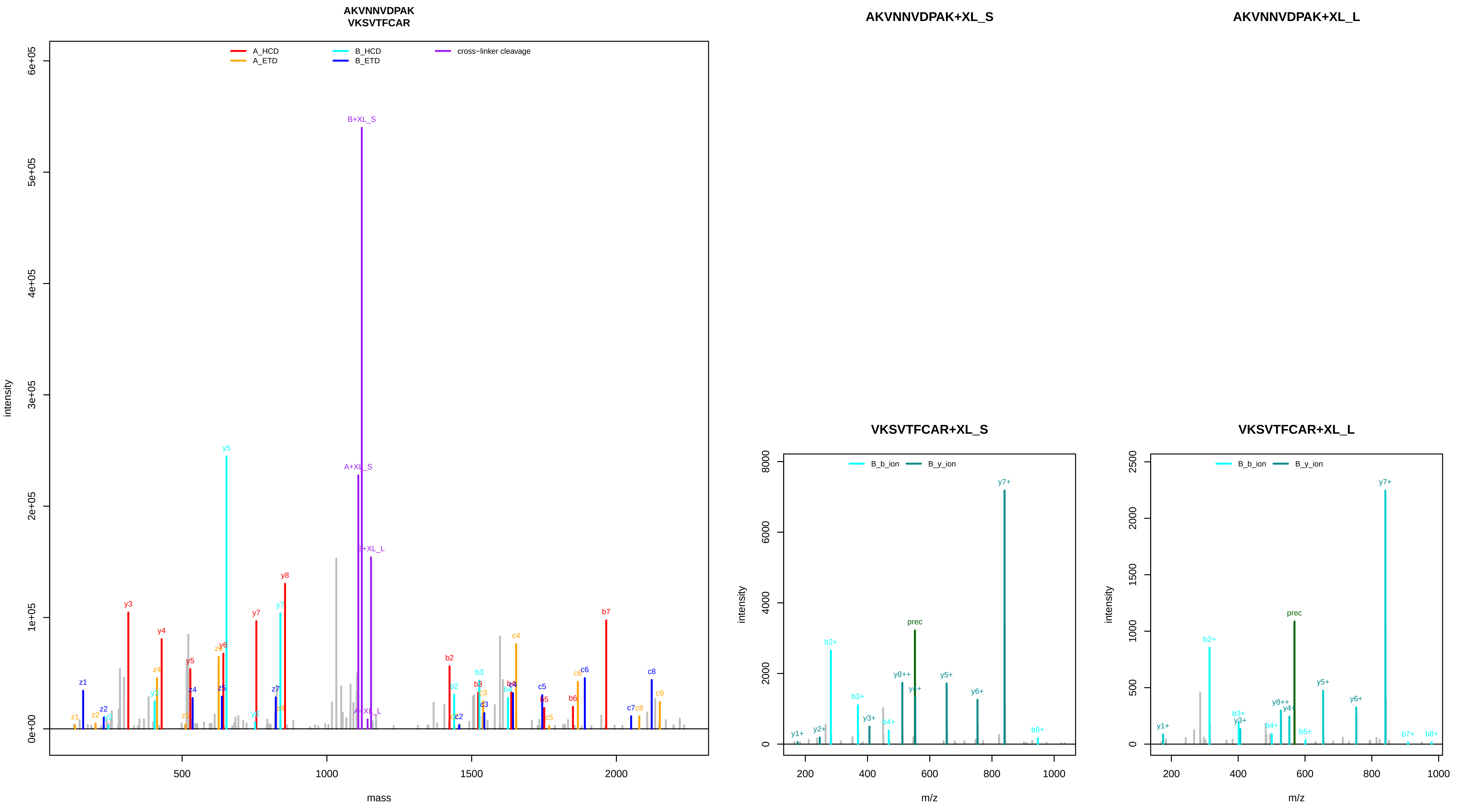

AKVNNVDPAK
VKSVTFCAR
A_HCD
A_ETD
B_HCD
B_ETD
cross−linker cleavage
B+XL_S
A+XL_S
B+XL_L
A+XL_L
intensity
mass
AKVNNVDPAK+XL_S
AKVNNVDPAK+XL_L
VKSVTFCAR+XL_S
VKSVTFCAR+XL_L
B_b_ion
B_y_ion
prec
m/z

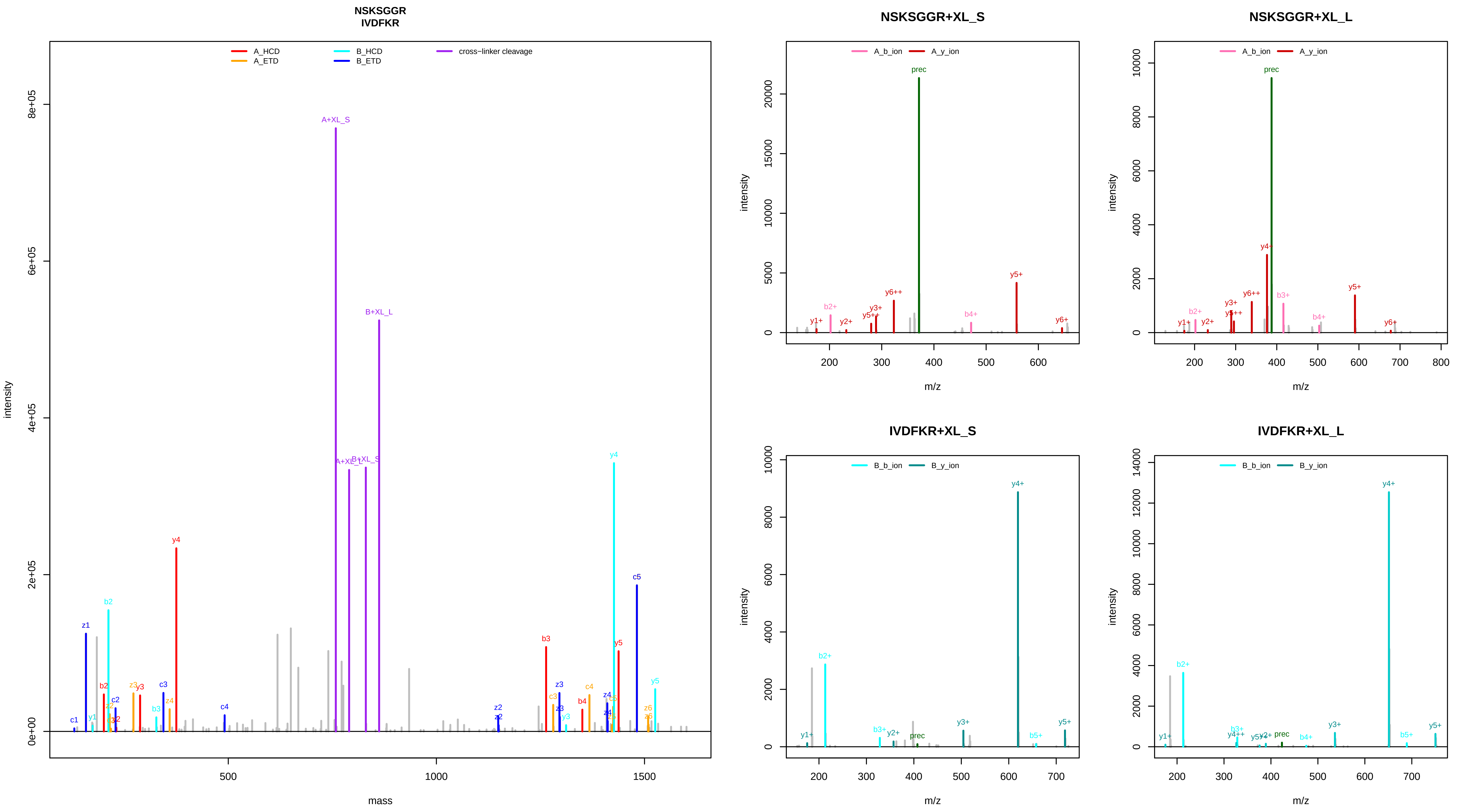

NSKSGGR
IVDFKR
A_HCD
A_ETD
B_HCD
B_ETD
cross−linker cleavage
A+XL_S
B+XL_L
intensity
mass
NSKSGGR+XL_S
NSKSGGR+XL_L
IVDFKR+XL_S
IVDFKR+XL_L
A_b_ion
A_y_ion
B_b_ion
B_y_ion
prec
m/z

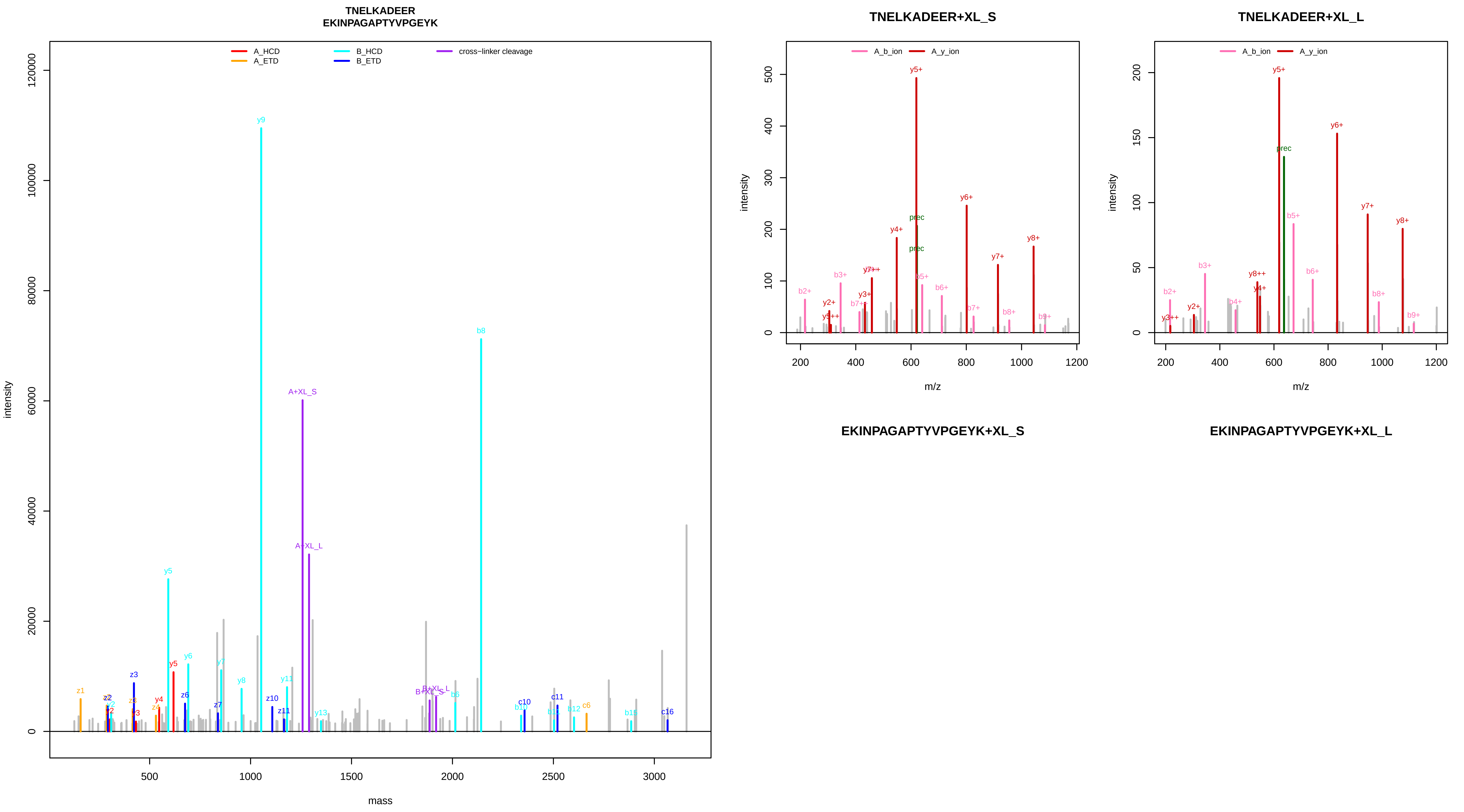

TNELKADEER
EKINPAGAPTYVPGEYK
A_HCD
A_ETD
B_HCD
B_ETD
cross−linker cleavage
intensity
mass
A+XL_S
A+XL_L
TNELKADEER+XL_S
TNELKADEER+XL_L
A_b_ion
A_y_ion
m/z
EKINPAGAPTYVPGEYK+XL_S
EKINPAGAPTYVPGEYK+XL_L

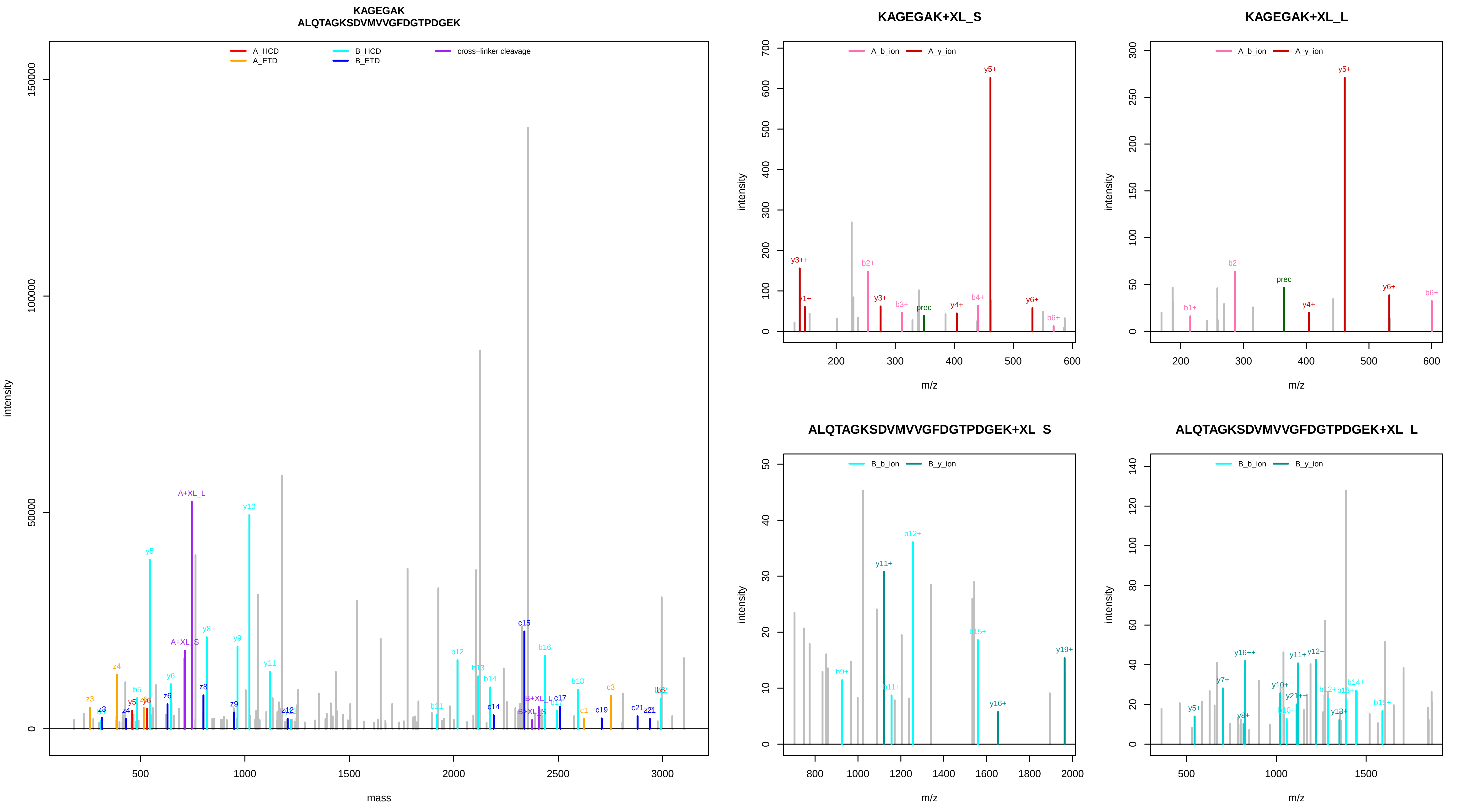

KAGEGAK
ALQTAGKSDVMVVGFDGTPDGEK
A_HCD
A_ETD
B_HCD
B_ETD
cross−linker cleavage
intensity
mass
A+XL_L
A+XL_S
y5
y6
y8
y9
y10
y11
b5
b12
b13
b14
b16
b18
z3
z4
z6
z8
z9
z12
c1
c3
c14
c15
c17
c19
c21
z21
KAGEGAK+XL_S
KAGEGAK+XL_L
A_b_ion
A_y_ion
y5+
y3++
y3+
y4+
y6+
b2+
b3+
b4+
b6+
b1+
prec
m/z
ALQTAGKSDVMVVGFDGTPDGEK+XL_S
ALQTAGKSDVMVVGFDGTPDGEK+XL_L
B_b_ion
B_y_ion
b9+
b11+
b12+
b15+
y11+
y16+
y19+
y5+
y7+
y8+
y10+
y11+
y12+
y13+
y16++
y21++
b10+
b12+
b13+
b14+
b15+

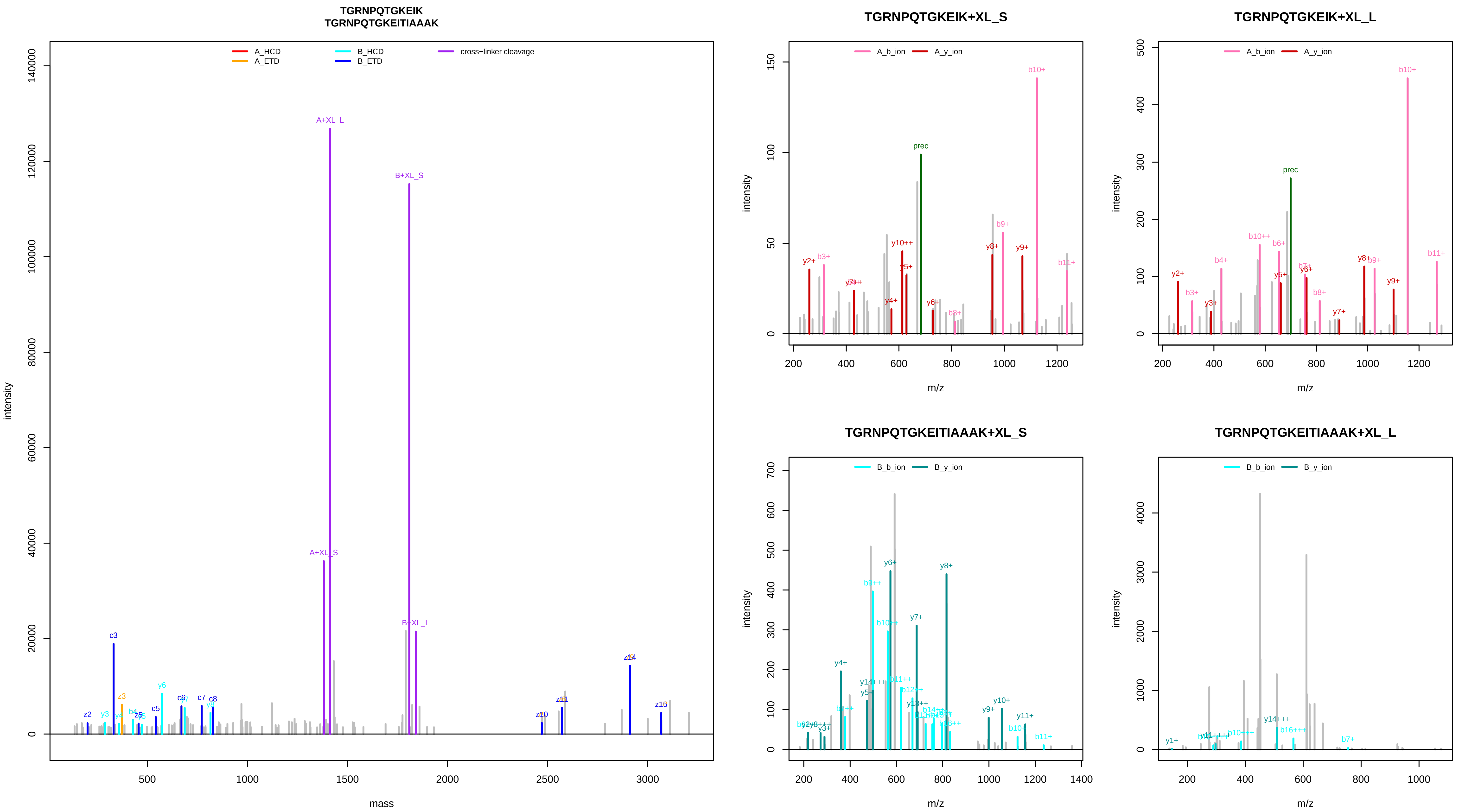
TGRNPQTGKEIK
TGRNPQTGKEITIAAAK
A_HCD
A_ETD
B_HCD
B_ETD
cross−linker cleavage
A+XL_L
B+XL_S
A+XL_S
B+XL_L
intensity
mass
TGRNPQTGKEIK+XL_S
TGRNPQTGKEIK+XL_L
TGRNPQTGKEITIAAAK+XL_S
TGRNPQTGKEITIAAAK+XL_L
A_b_ion
A_y_ion
B_b_ion
B_y_ion
prec
m/z

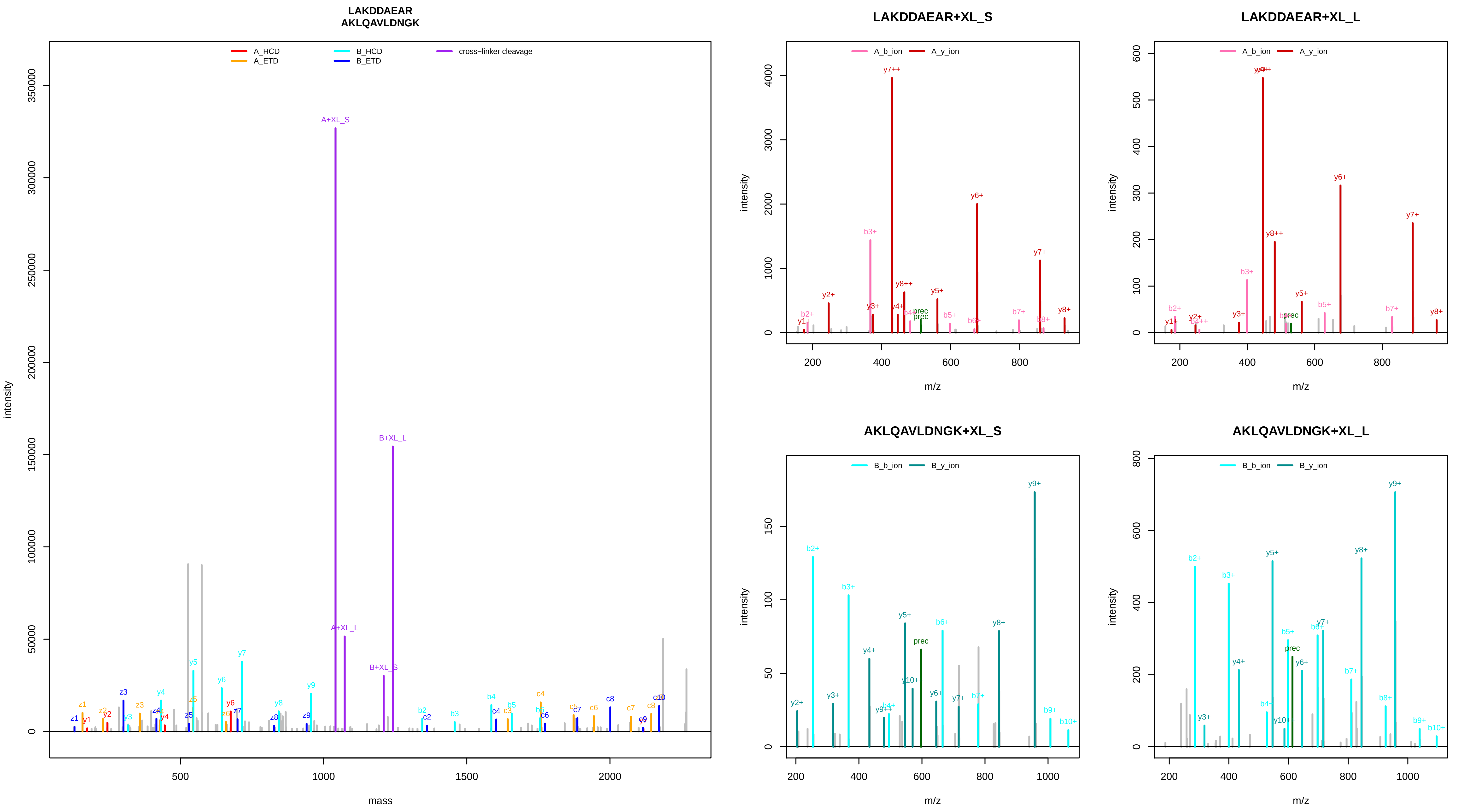
LAKDDAEAR
AKLQAVLDNGK
A_HCD
A_ETD
B_HCD
B_ETD
cross−linker cleavage
A+XL_S
B+XL_L
A+XL_L
B+XL_S
intensity
mass
LAKDDAEAR+XL_S
LAKDDAEAR+XL_L
AKLQAVLDNGK+XL_S
AKLQAVLDNGK+XL_L
A_b_ion
A_y_ion
B_b_ion
B_y_ion
prec
m/z

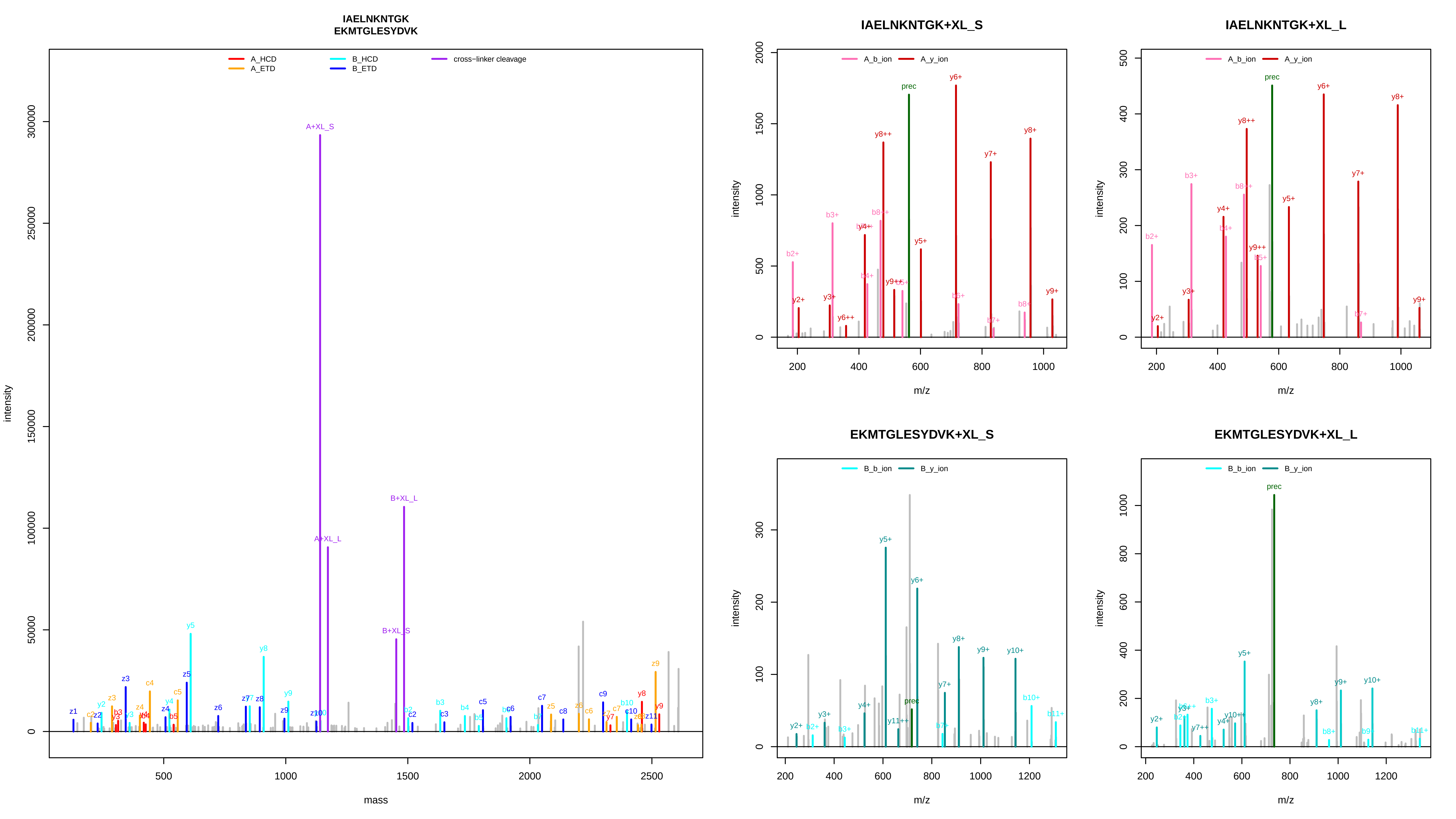
IAELNKNTGK
EKMTGLESYDVK
A_HCD
A_ETD
B_HCD
B_ETD
cross−linker cleavage
A+XL_S
A+XL_L
B+XL_L
B+XL_S
intensity
mass
IAELNKNTGK+XL_S
IAELNKNTGK+XL_L
EKMTGLESYDVK+XL_S
EKMTGLESYDVK+XL_L
A_b_ion
A_y_ion
B_b_ion
B_y_ion
prec
intensity
m/z

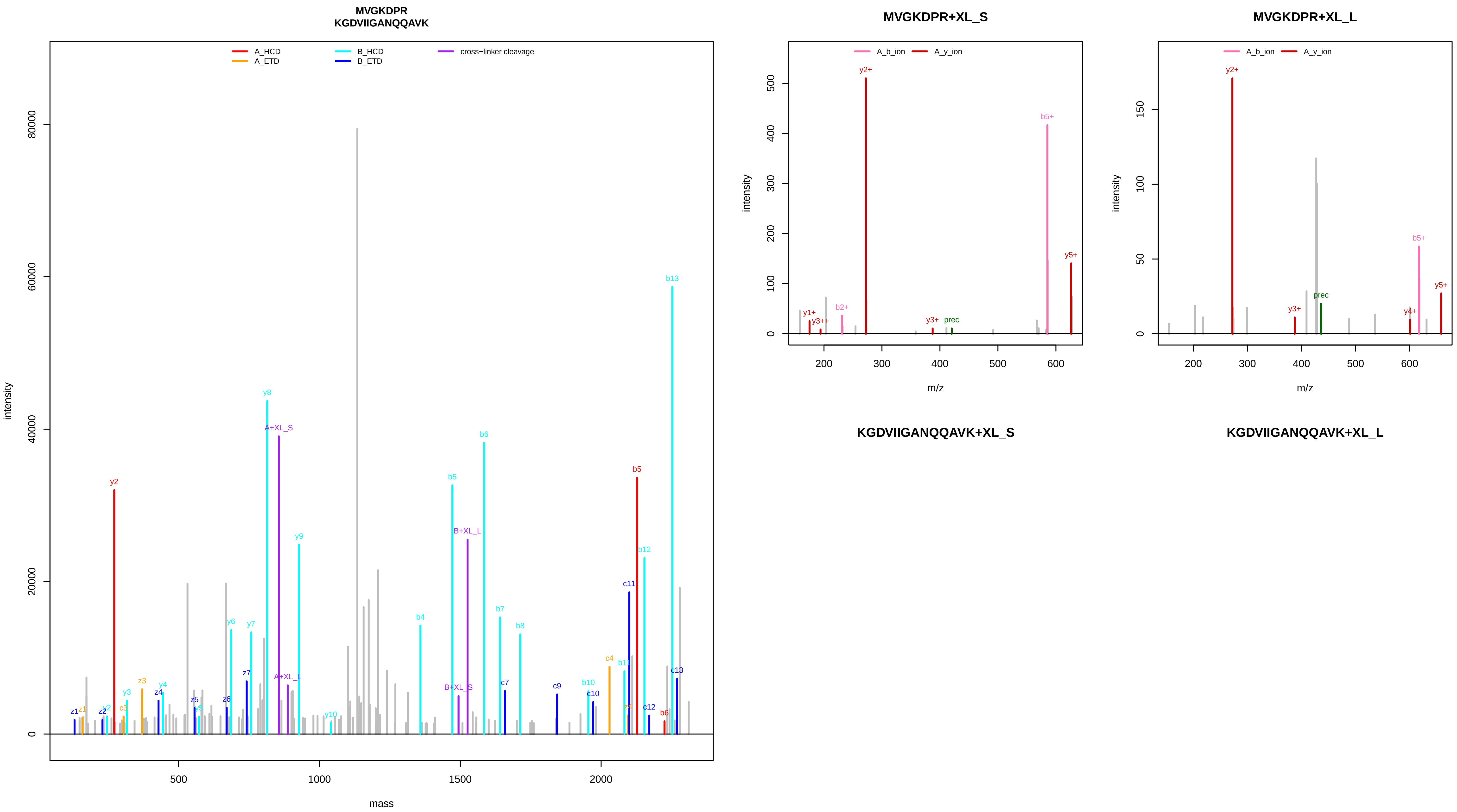
MVGKDPR
KGDVIIGANQQAVK
A_HCD
A_ETD
B_HCD
B_ETD
cross−linker cleavage
A+XL_S
A+XL_L
B+XL_L
B+XL_S
intensity
mass
MVGKDPR+XL_S
MVGKDPR+XL_L
A_b_ion
A_y_ion
m/z
prec
KGDVIIGANQQAVK+XL_S
KGDVIIGANQQAVK+XL_L

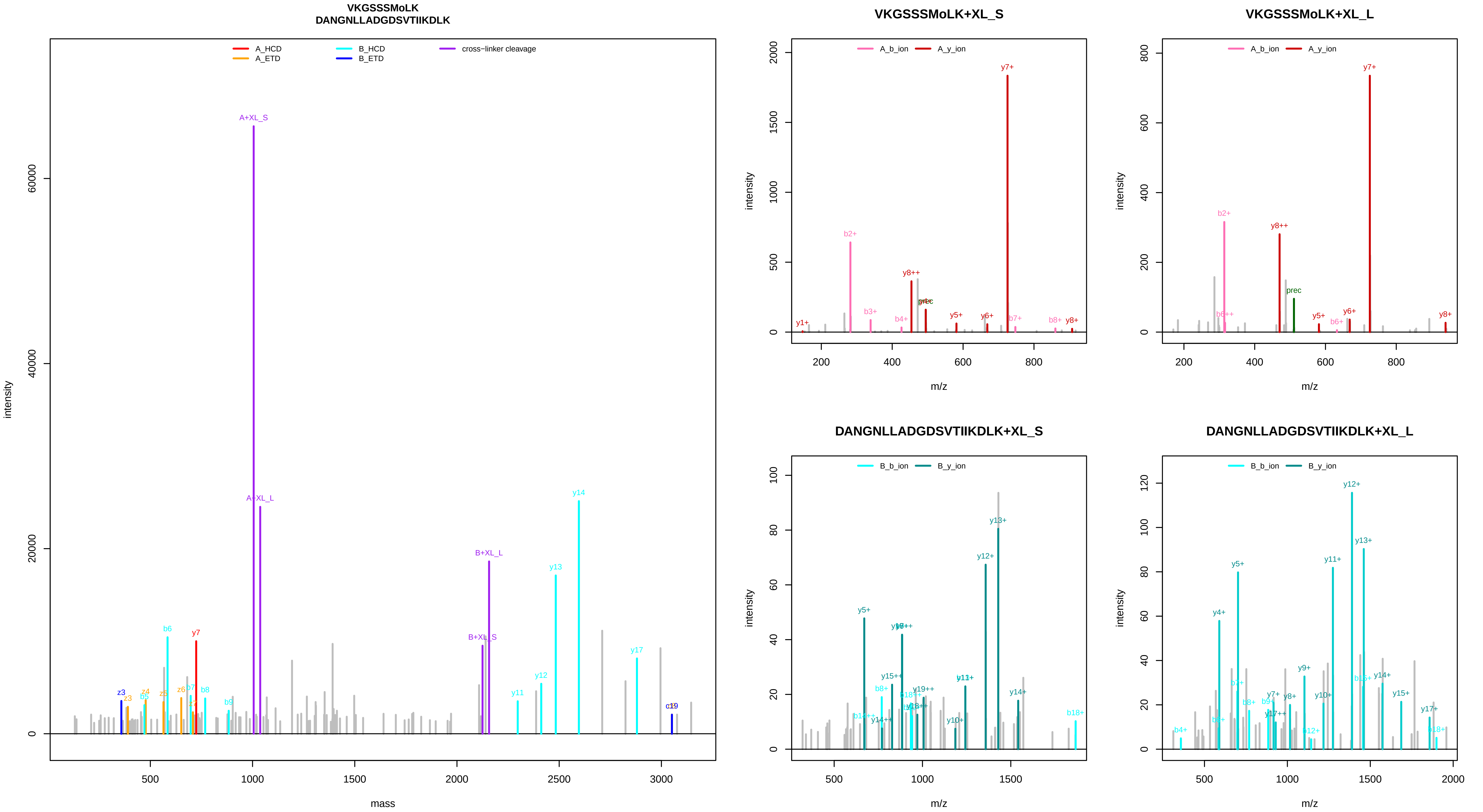

VKGSSSMoLK
DANGNLLADGDSVTIIKDLK
A_HCD
A_ETD
B_HCD
B_ETD
cross−linker cleavage
A+XL_S
A+XL_L
B+XL_L
B+XL_S
intensity
mass
VKGSSSMoLK+XL_S
VKGSSSMoLK+XL_L
A_b_ion
A_y_ion
DANGNLLADGDSVTIIKDLK+XL_S
DANGNLLADGDSVTIIKDLK+XL_L
B_b_ion
B_y_ion
m/z

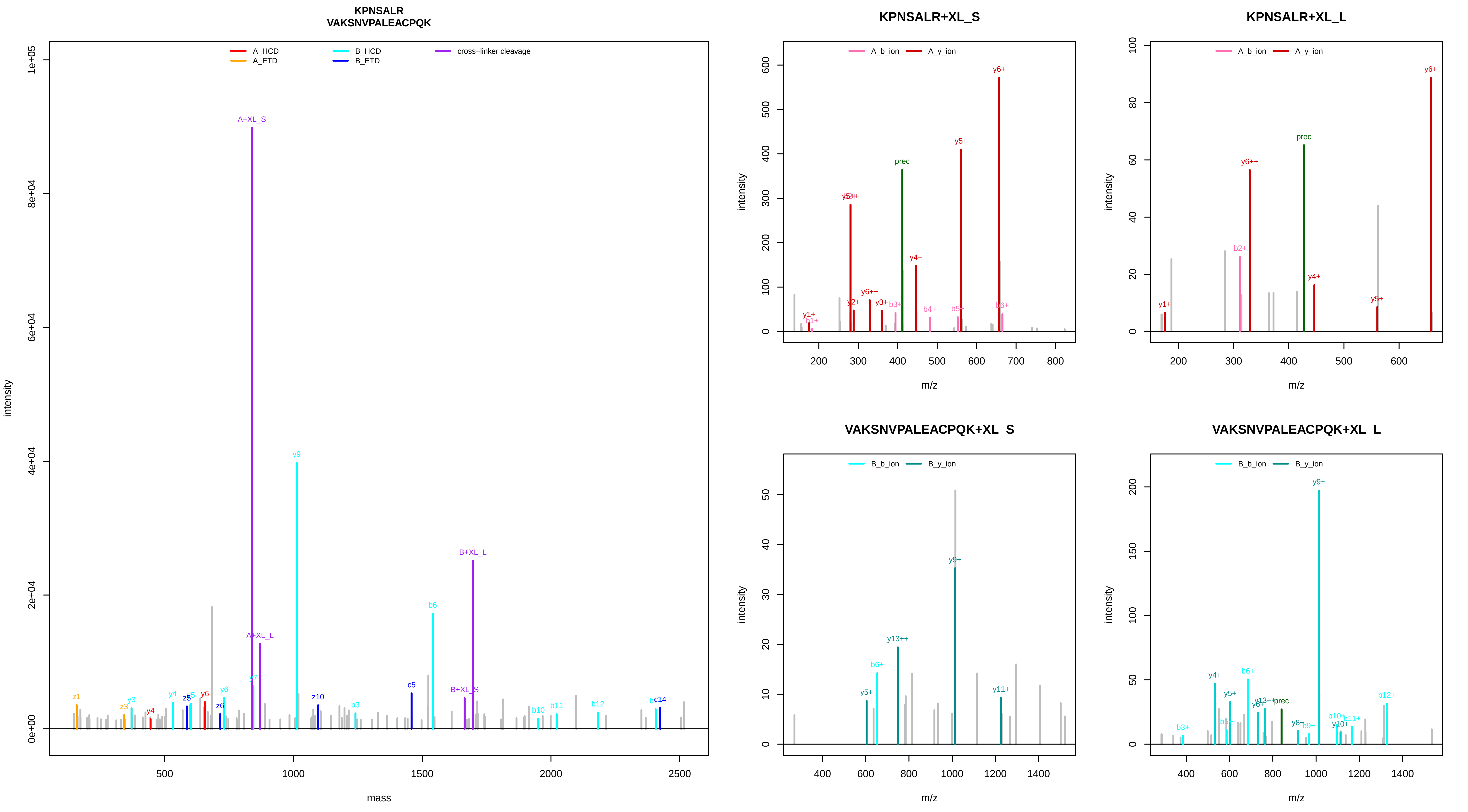

KPNSALR
VAKSNVPALEACPQK
A_HCD
A_ETD
B_HCD
B_ETD
cross−linker cleavage
mass
intensity
KPNSALR+XL_S
KPNSALR+XL_L
A_b_ion
A_y_ion
VAKSNVPALEACPQK+XL_S
VAKSNVPALEACPQK+XL_L
B_b_ion
B_y_ion
m/z

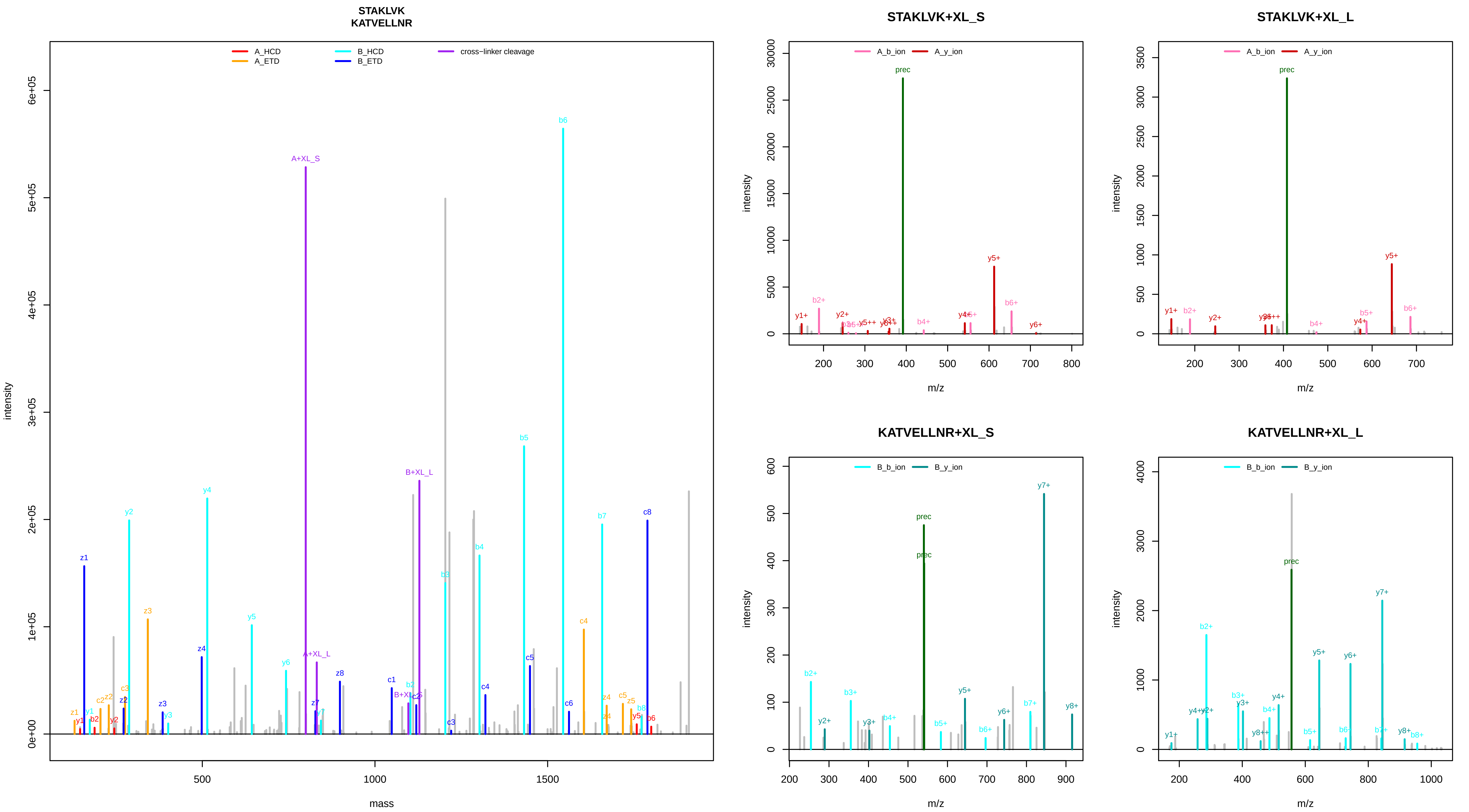
STAKLVK
KATVELLNR
A_HCD
A_ETD
B_HCD
B_ETD
cross−linker cleavage
intensity
mass
STAKLVK+XL_S
STAKLVK+XL_L
A_b_ion
A_y_ion
KATVELLNR+XL_S
KATVELLNR+XL_L
B_b_ion
B_y_ion
m/z
prec

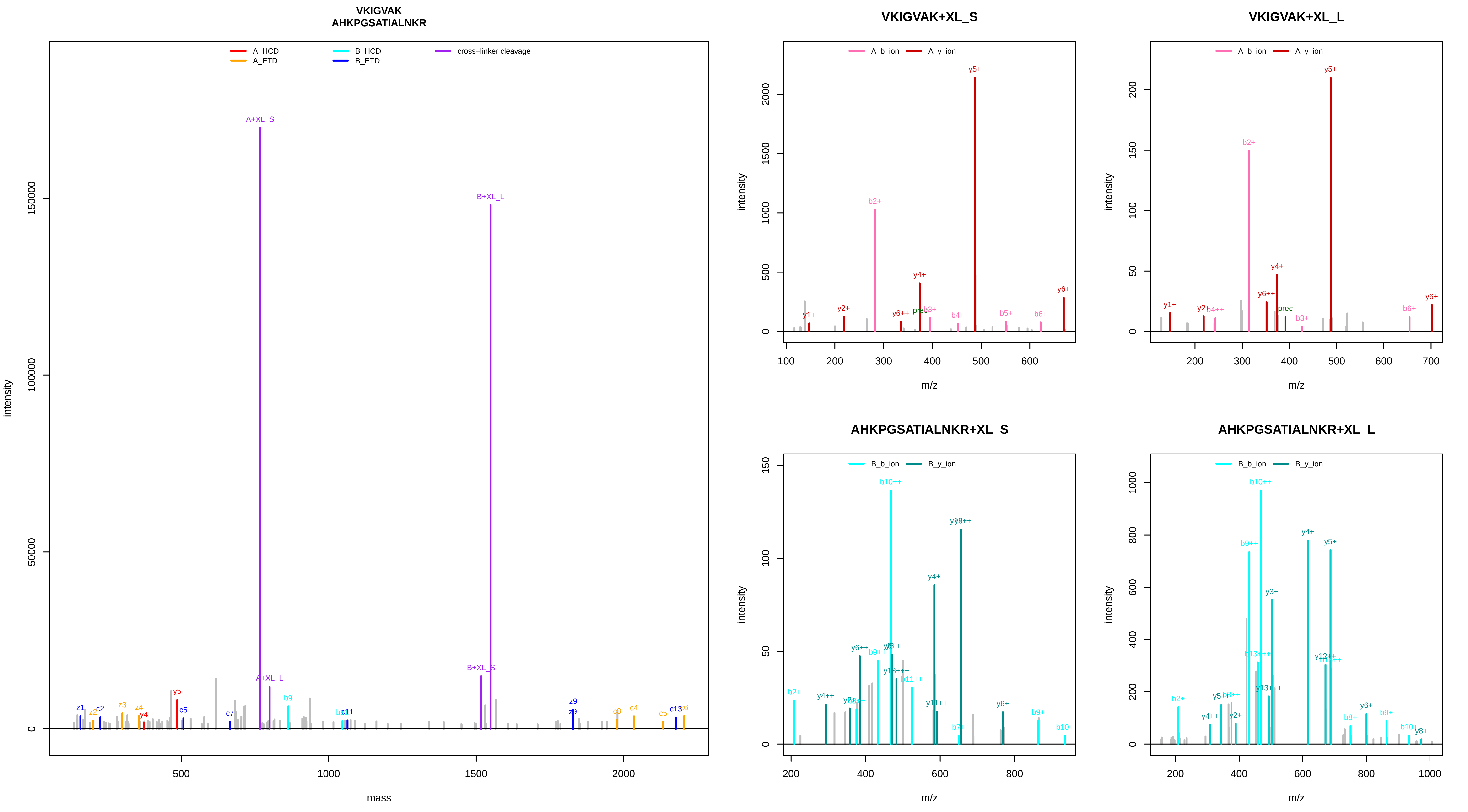
VKIGVAK
AHKPGSATIALNKR
A_HCD
A_ETD
B_HCD
B_ETD
cross−linker cleavage
A+XL_S
B+XL_L
B+XL_S
A+XL_L
intensity
mass
VKIGVAK+XL_S
VKIGVAK+XL_L
A_b_ion
A_y_ion
AHKPGSATIALNKR+XL_S
AHKPGSATIALNKR+XL_L
B_b_ion
B_y_ion
m/z

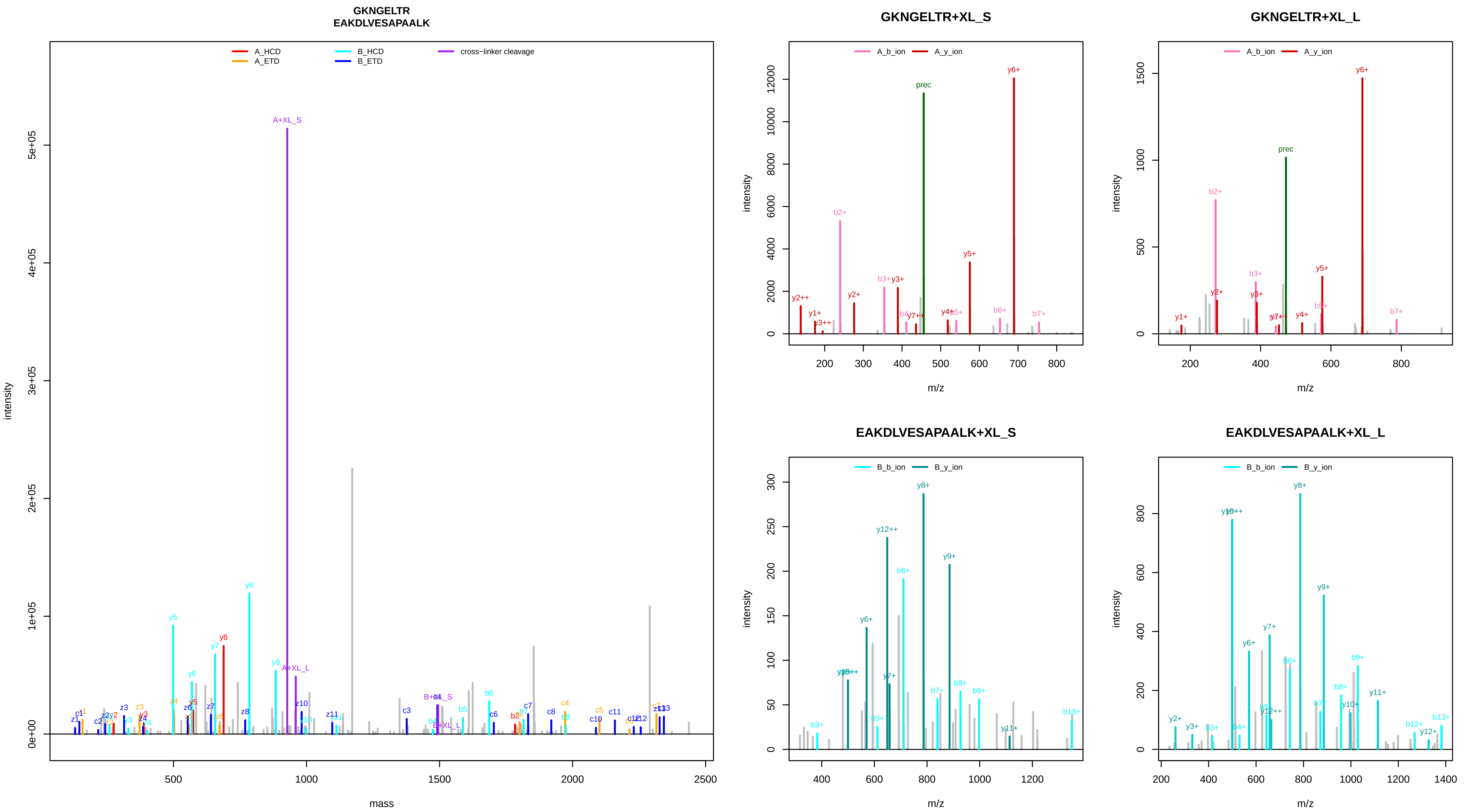

GKNGELTR
EAKDLVESAPAALK
A_HCD
A_ETD
B_HCD
B_ETD
cross−linker cleavage
A+XL_S
A+XL_L
intensity
mass
GKNGELTR+XL_S
GKNGELTR+XL_L
EAKDLVESAPAALK+XL_S
EAKDLVESAPAALK+XL_L
A_b_ion
A_y_ion
B_b_ion
B_y_ion
prec
m/z

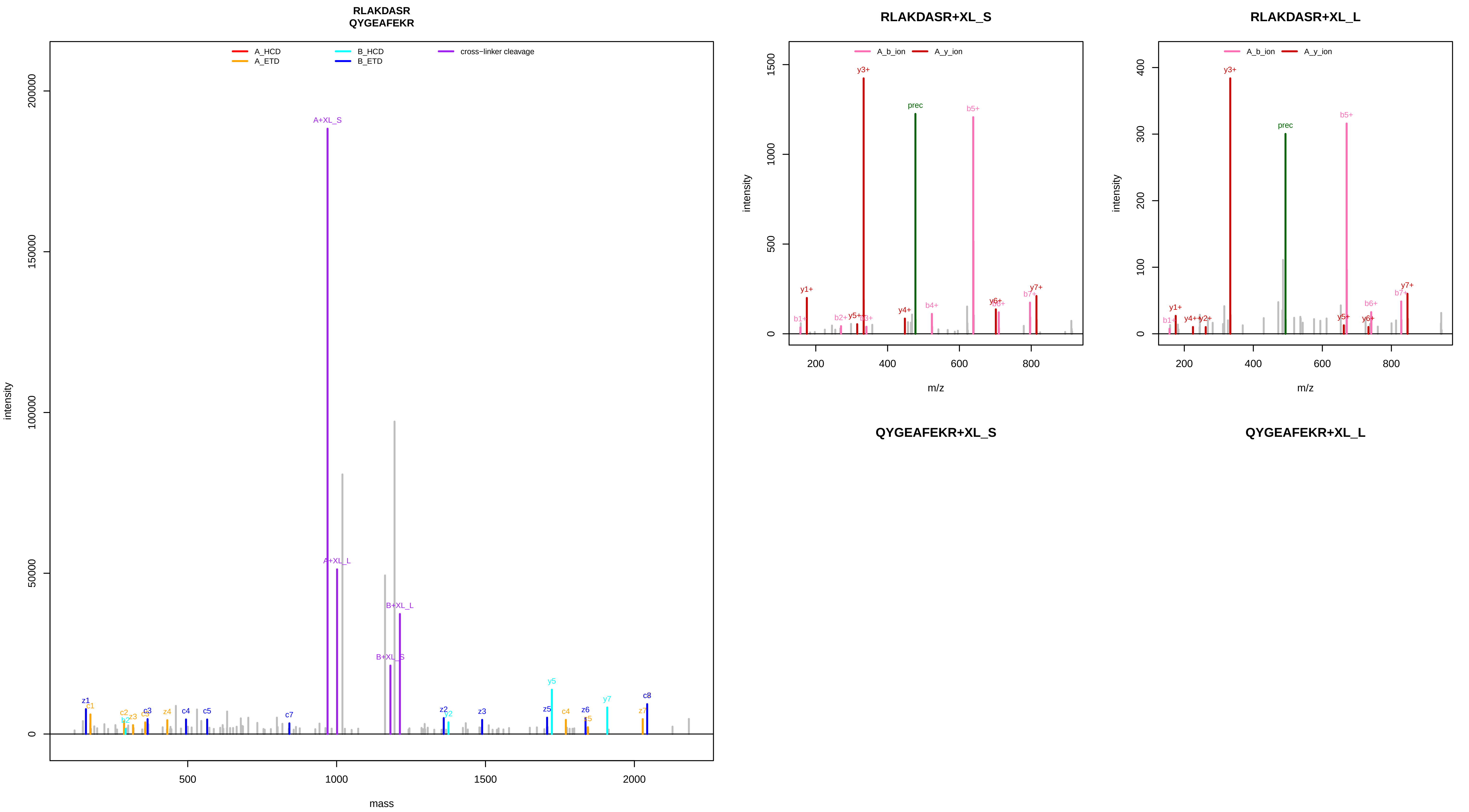
RLAKDASR
QYGEAFEKR
A_HCD
A_ETD
B_HCD
B_ETD
cross−linker cleavage
A+XL_S
A+XL_L
B+XL_L
B+XL_S
intensity
mass
RLAKDASR+XL_S
RLAKDASR+XL_L
A_b_ion
A_y_ion
prec
m/z
QYGEAFEKR+XL_S
QYGEAFEKR+XL_L

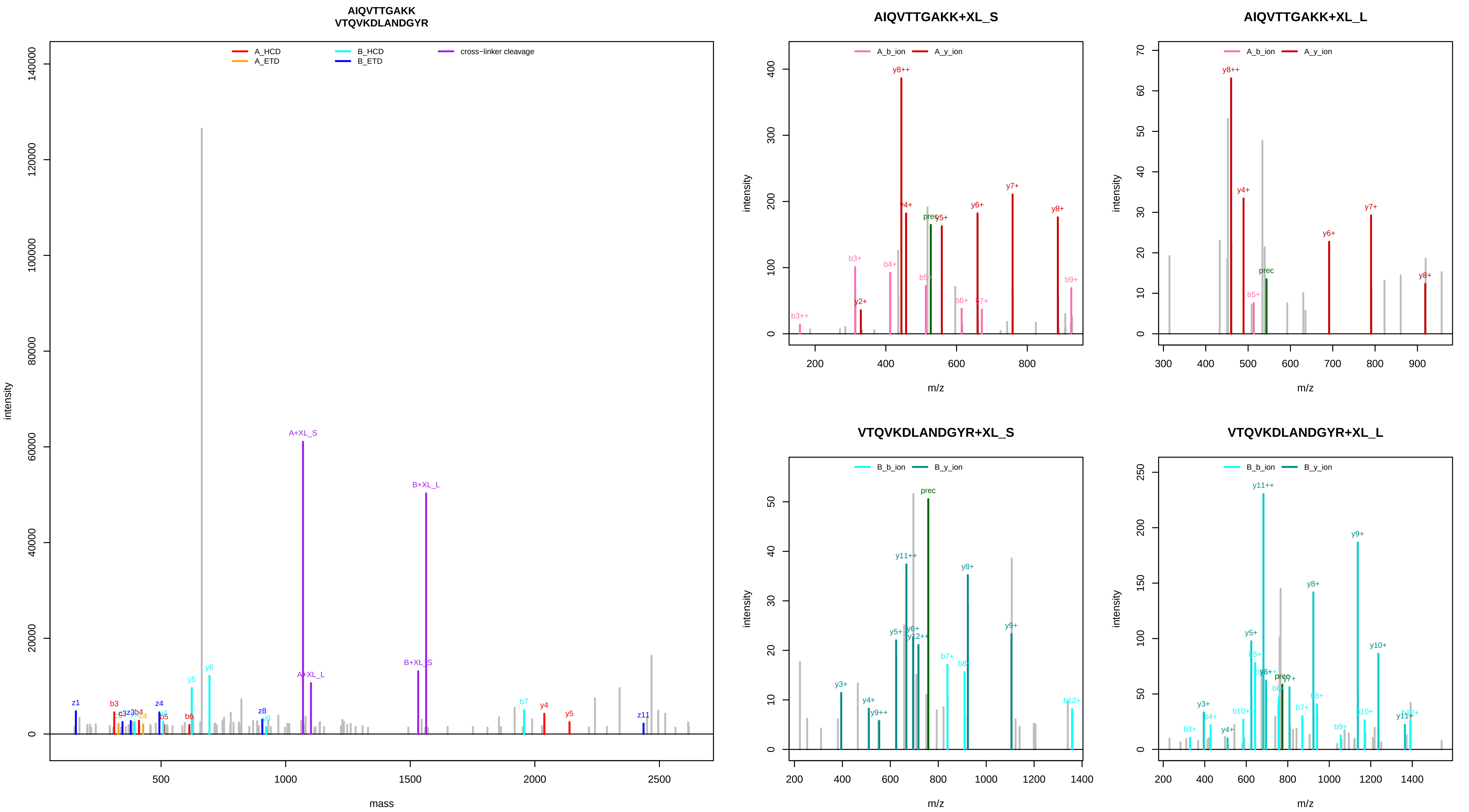
AIQVTTGAKK
VTQVKDLANDGYR
A_HCD
A_ETD
B_HCD
B_ETD
cross−linker cleavage
intensity
mass
A+XL_S
B+XL_L
A+XL_L
B+XL_S
AIQVTTGAKK+XL_S
AIQVTTGAKK+XL_L
VTQVKDLANDGYR+XL_S
VTQVKDLANDGYR+XL_L
A_b_ion
A_y_ion
B_b_ion
B_y_ion
prec
m/z
intensity

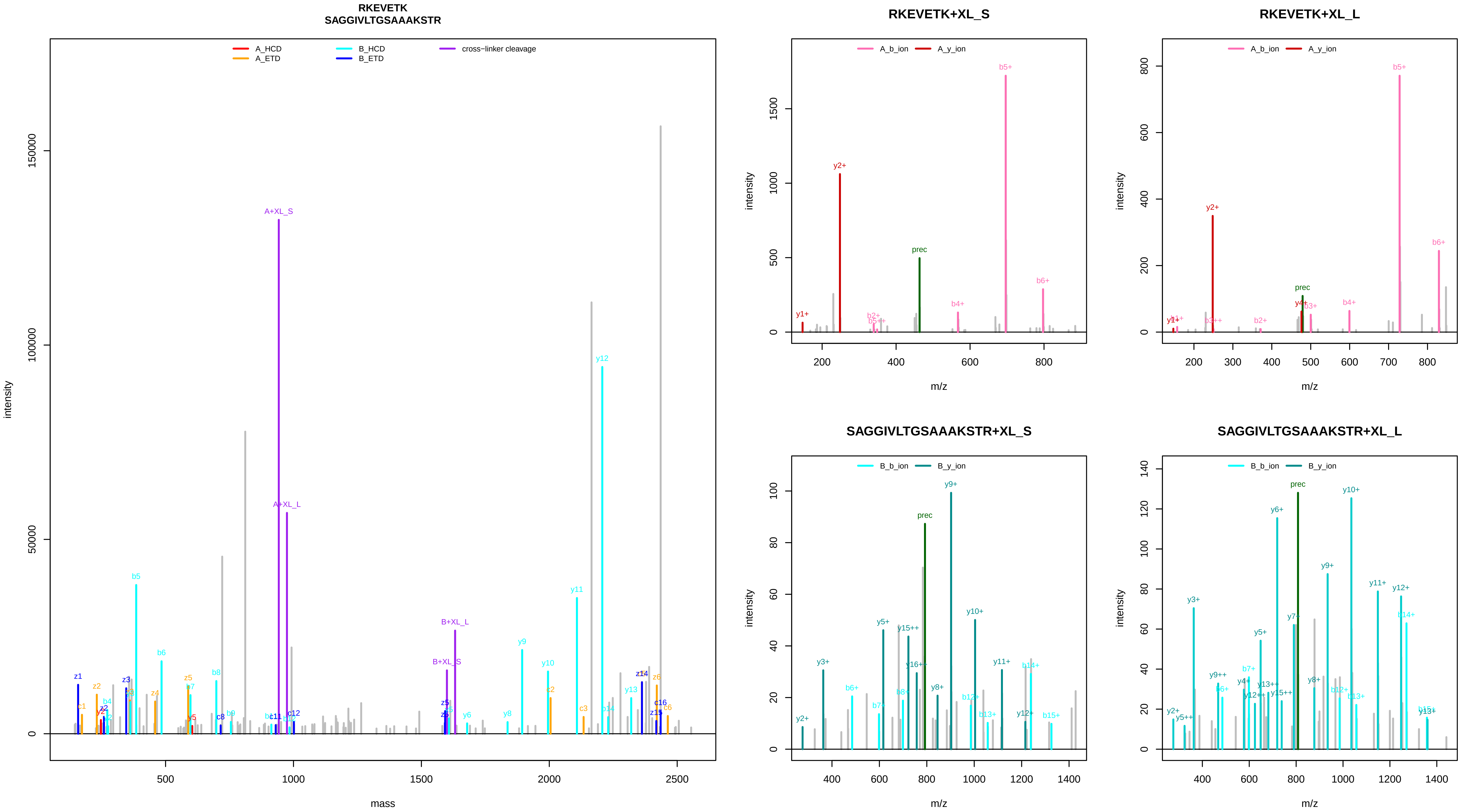

RKEVETK
SAGGIVLTGSAAAKSTR
A_HCD
A_ETD
B_HCD
B_ETD
cross−linker cleavage
A+XL_S
A+XL_L
B+XL_L
B+XL_S
intensity
mass
RKEVETK+XL_S
RKEVETK+XL_L
A_b_ion
A_y_ion
prec
intensity
m/z
SAGGIVLTGSAAAKSTR+XL_S
SAGGIVLTGSAAAKSTR+XL_L
B_b_ion
B_y_ion

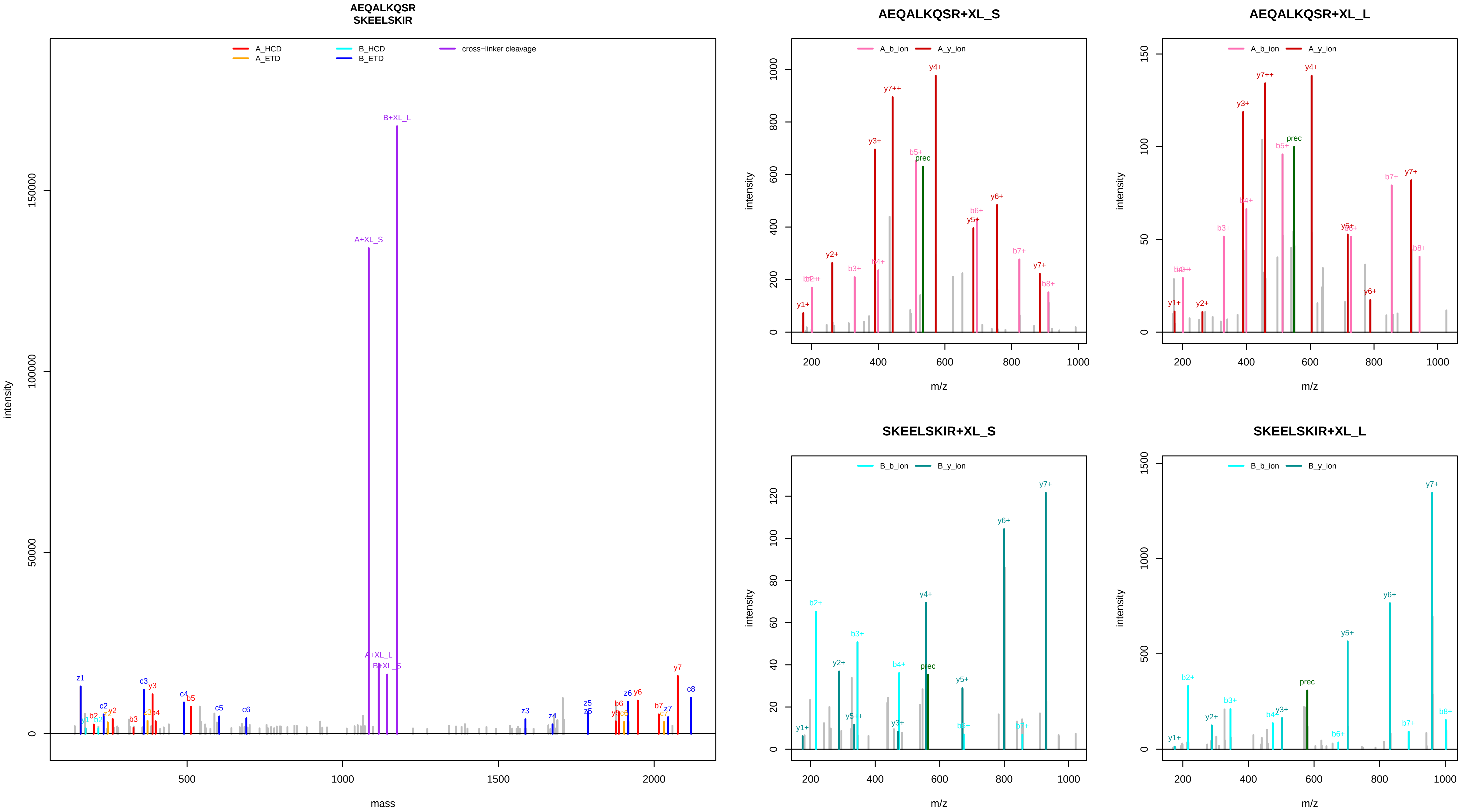
AEQALKQSR
SKEELSKIR
A_HCD
A_ETD
B_HCD
B_ETD
cross−linker cleavage
B+XL_L
A+XL_S
A+XL_L
B+XL_S
intensity
mass
0
50000
100000
150000
500
1000
1500
2000
AEQALKQSR+XL_S
AEQALKQSR+XL_L
A_b_ion
A_y_ion
SKEELSKIR+XL_S
SKEELSKIR+XL_L
B_b_ion
B_y_ion
prec
intensity
m/z

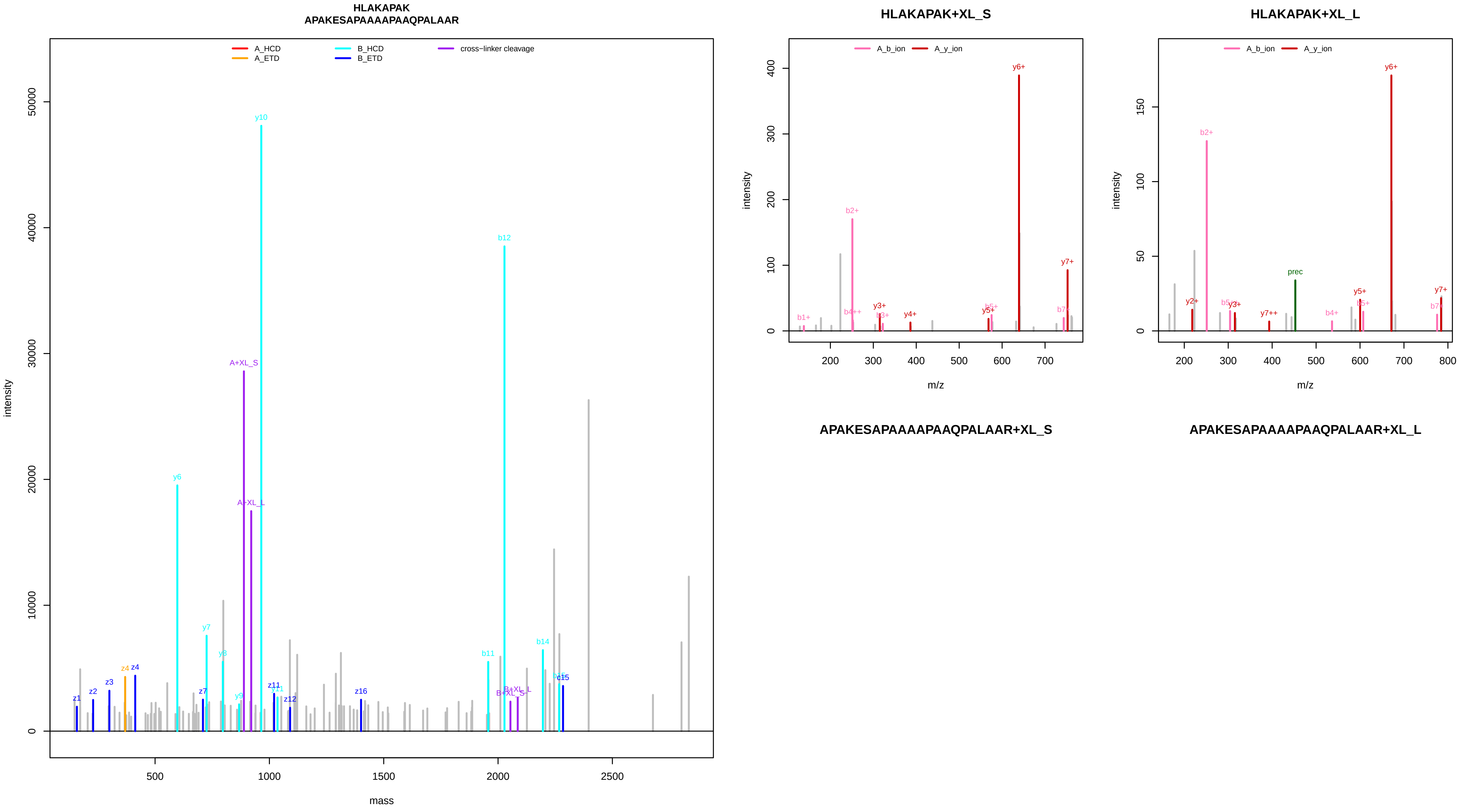
HLAKAPAK
APAKESAPAAAAPAAQPALAAR
A_HCD
A_ETD
B_HCD
B_ETD
cross−linker cleavage
intensity
mass
HLAKAPAK+XL_S
HLAKAPAK+XL_L
A_b_ion
A_y_ion
m/z
APAKESAPAAAAPAAQPALAAR+XL_S
APAKESAPAAAAPAAQPALAAR+XL_L

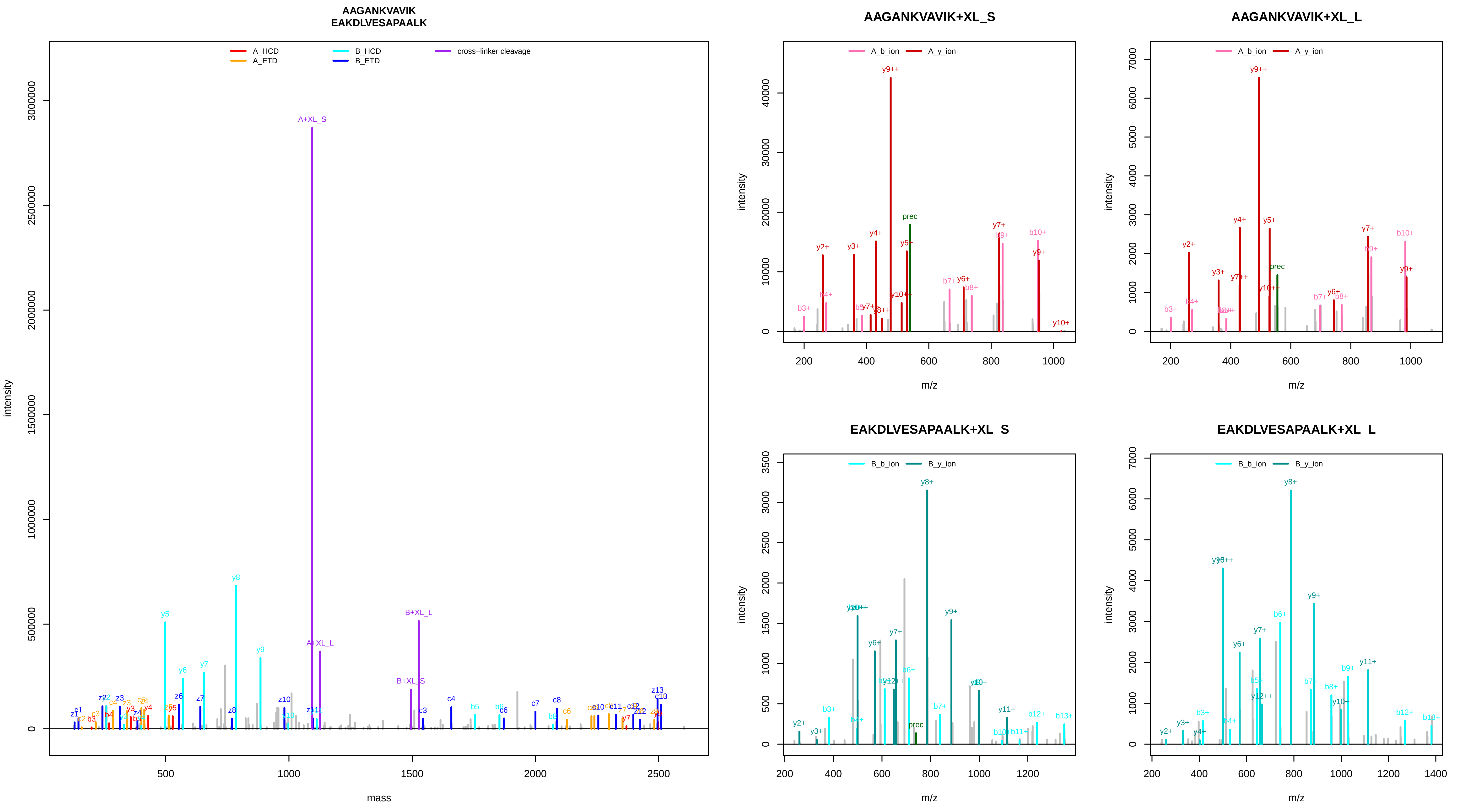
AAGANKVAVIK
EAKDLVESAPAALK
A_HCD
A_ETD
B_HCD
B_ETD
cross−linker cleavage
A+XL_S
A+XL_L
B+XL_L
B+XL_S
intensity
mass
AAGANKVAVIK+XL_S
AAGANKVAVIK+XL_L
EAKDLVESAPAALK+XL_S
EAKDLVESAPAALK+XL_L
A_b_ion
A_y_ion
B_b_ion
B_y_ion
prec
m/z

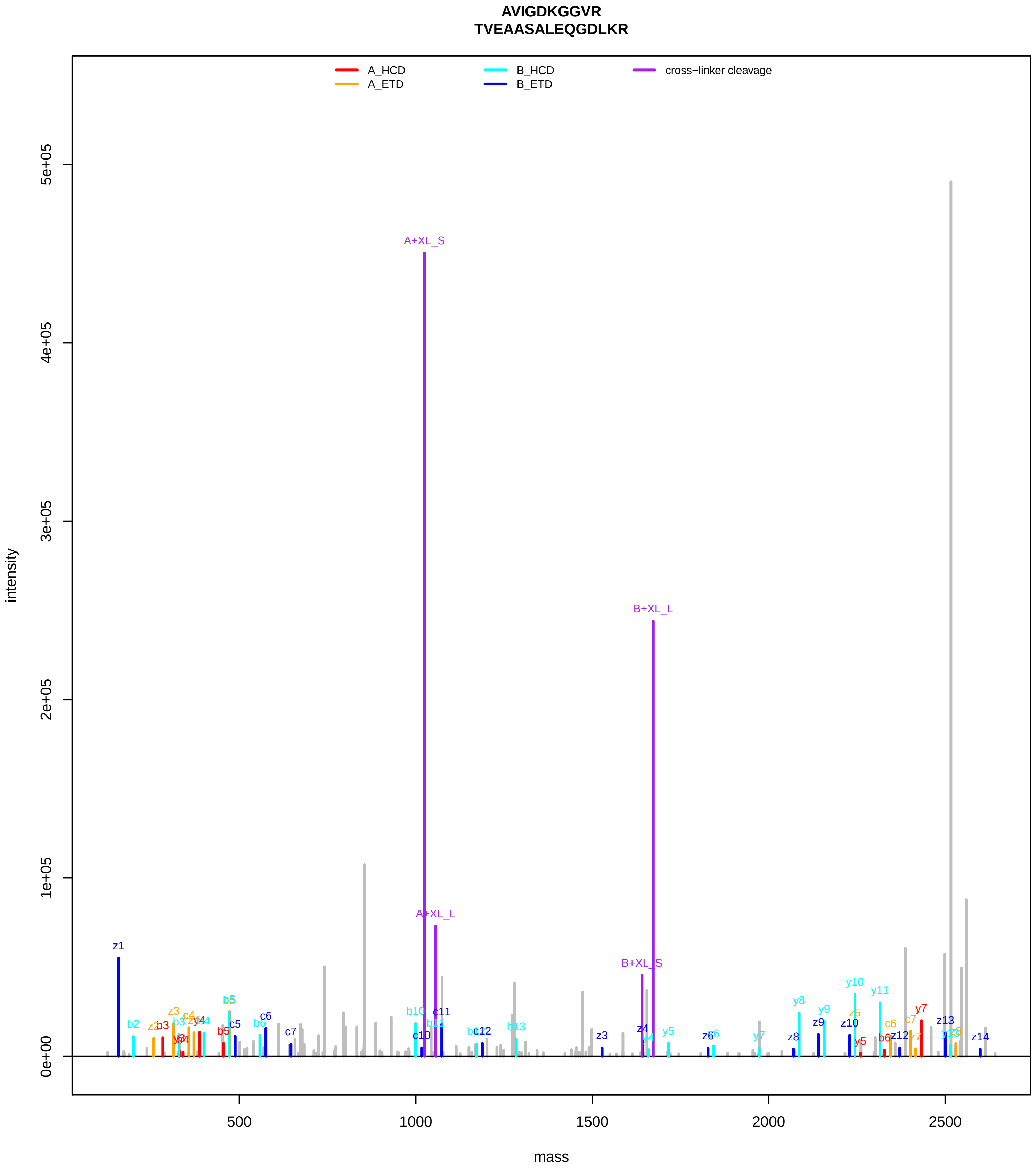

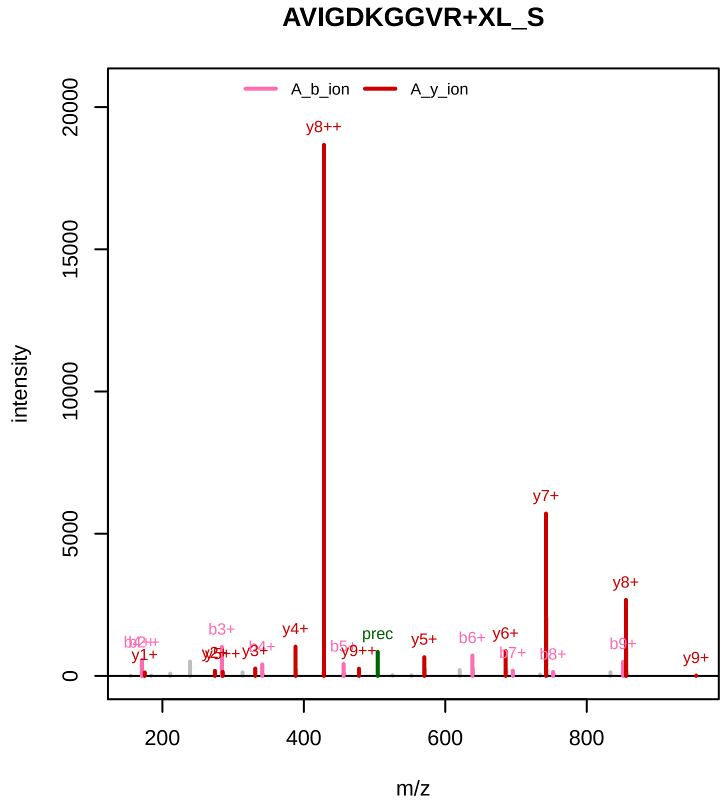

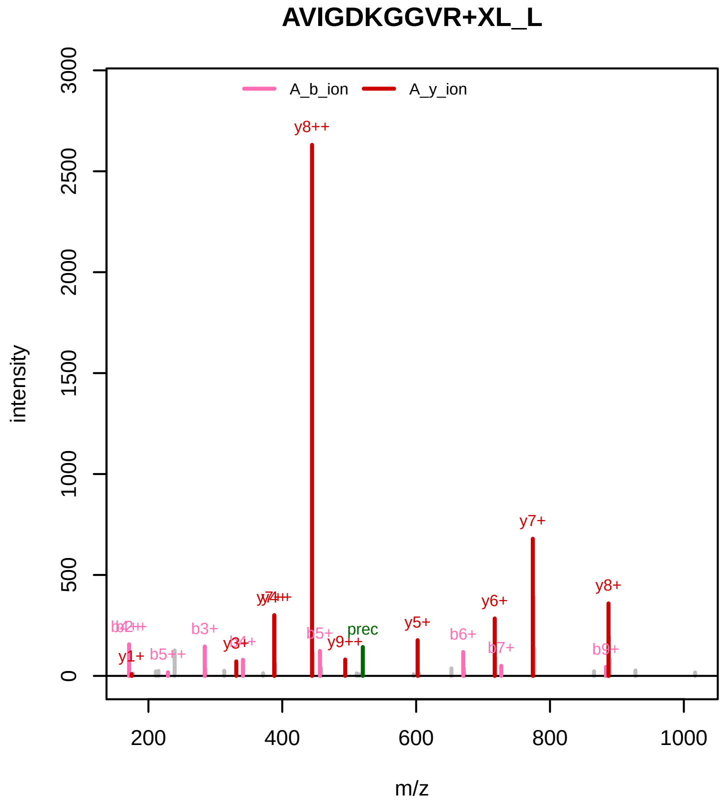

TVEAASALEQGDLKR+XL_S

TVEAASALEQGDLKR+XL_L

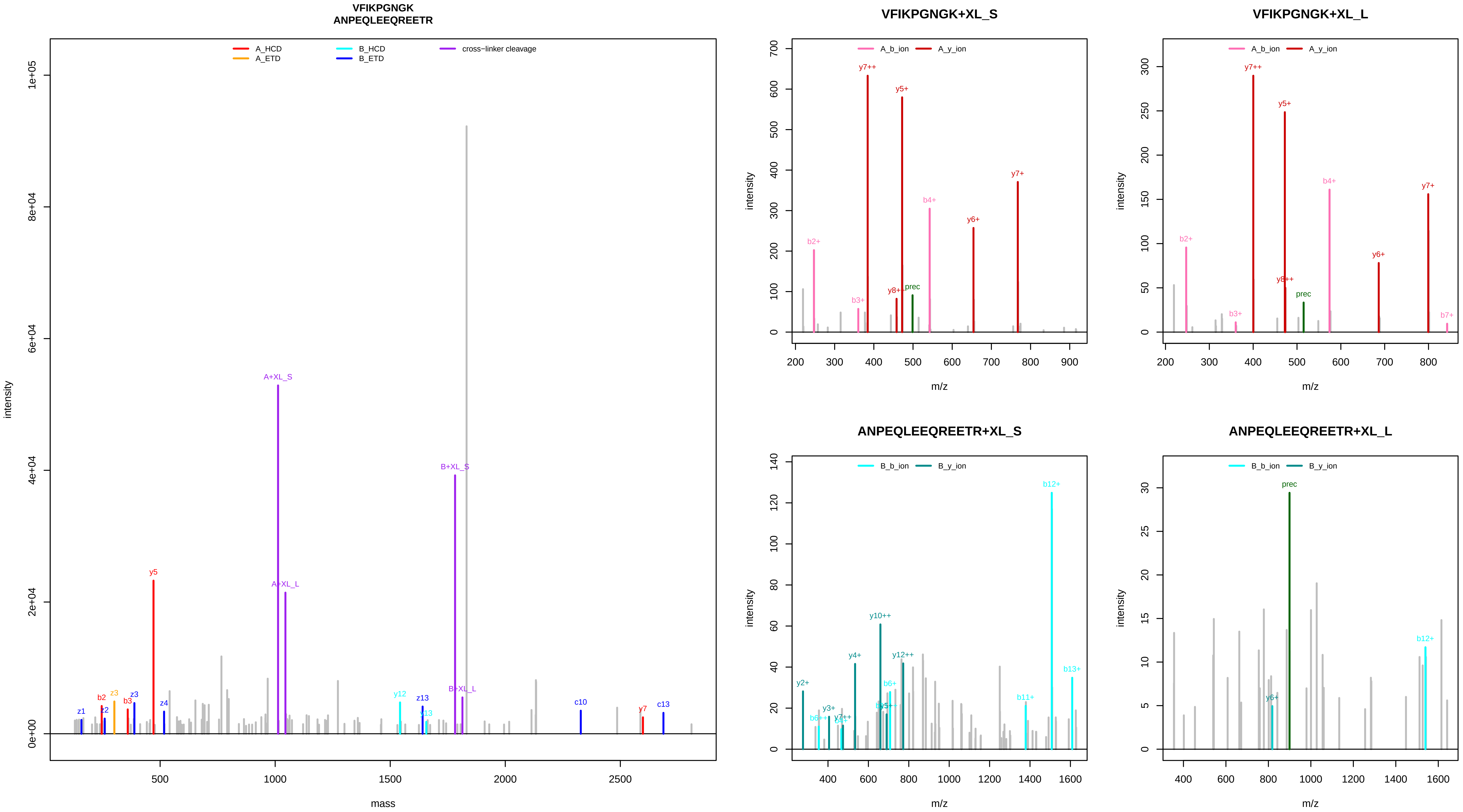
VFIKPGNGK
ANPEQLEEQREETR
A_HCD
A_ETD
B_HCD
B_ETD
cross−linker cleavage
intensity
mass
A+XL_S
A+XL_L
B+XL_S
B+XL_L
y5
b2
z3
b3
z1
z2
z4
y12
z13
c10
y7
c13
VFIKPGNGK+XL_S
VFIKPGNGK+XL_L
A_b_ion
A_y_ion
y7++
y5+
b4+
y6+
y7+
b2+
b3+
y8++
prec
b7+
m/z
ANPEQLEEQREETR+XL_S
ANPEQLEEQREETR+XL_L
B_b_ion
B_y_ion
b12+
y10++
y4+
y12++
y2+
y3+
b6+
b11+
b13+
y6+

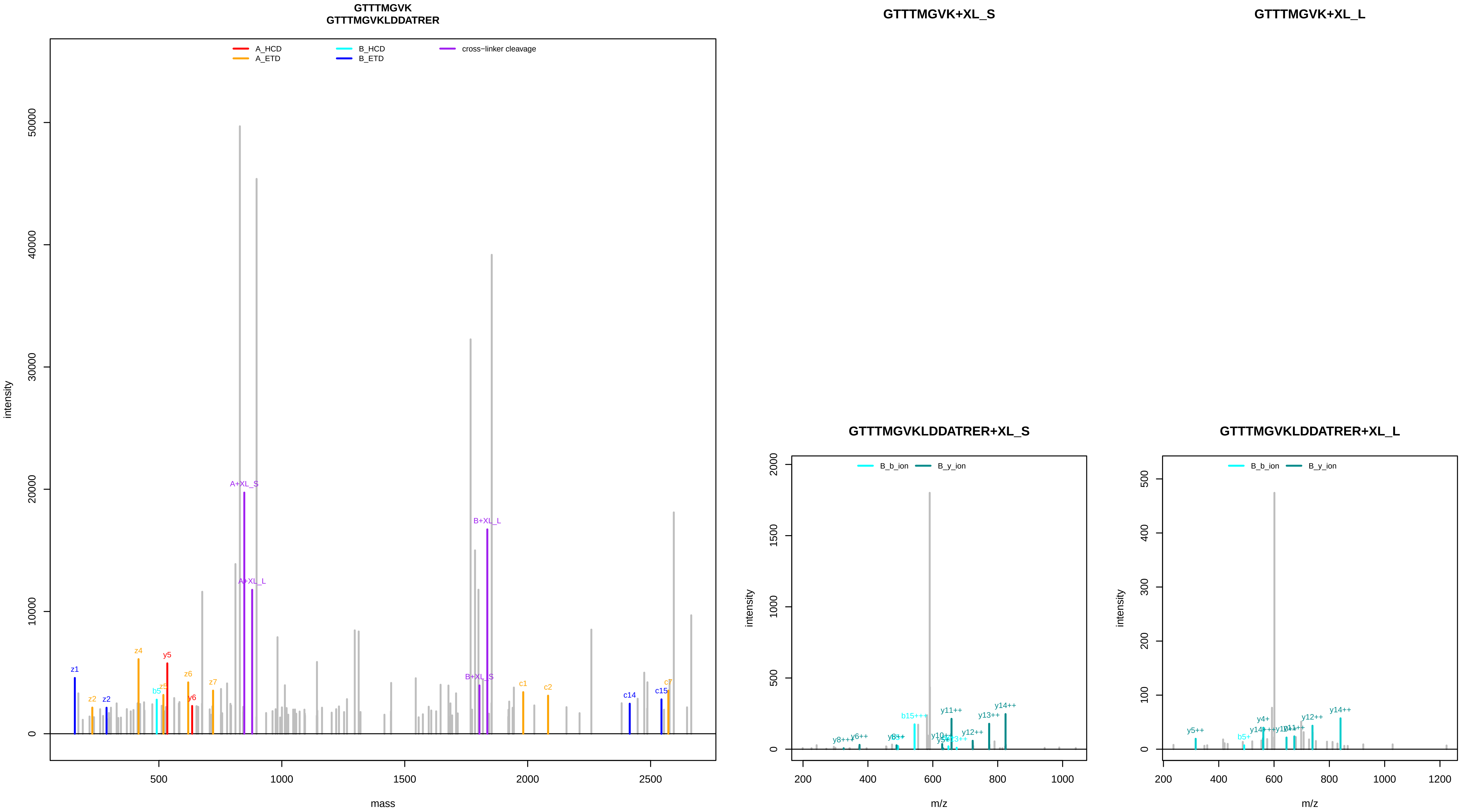
GTTTMGVK
GTTTMGVKLDDATRER
A_HCD
A_ETD
B_HCD
B_ETD
cross−linker cleavage
intensity
mass
A+XL_S
A+XL_L
B+XL_L
B+XL_S
z1
z2
z4
y5
b5
z5
z6
y6
z7
c1
c2
c14
c15
c7
GTTTMGVK+XL_S
GTTTMGVK+XL_L
GTTTMGVKLDDATRER+XL_S
GTTTMGVKLDDATRER+XL_L
B_b_ion
B_y_ion
m/z
b15+++
y11++
y14++
y13++
y12++
y8+++
y6++
y5++
y4+
b5+

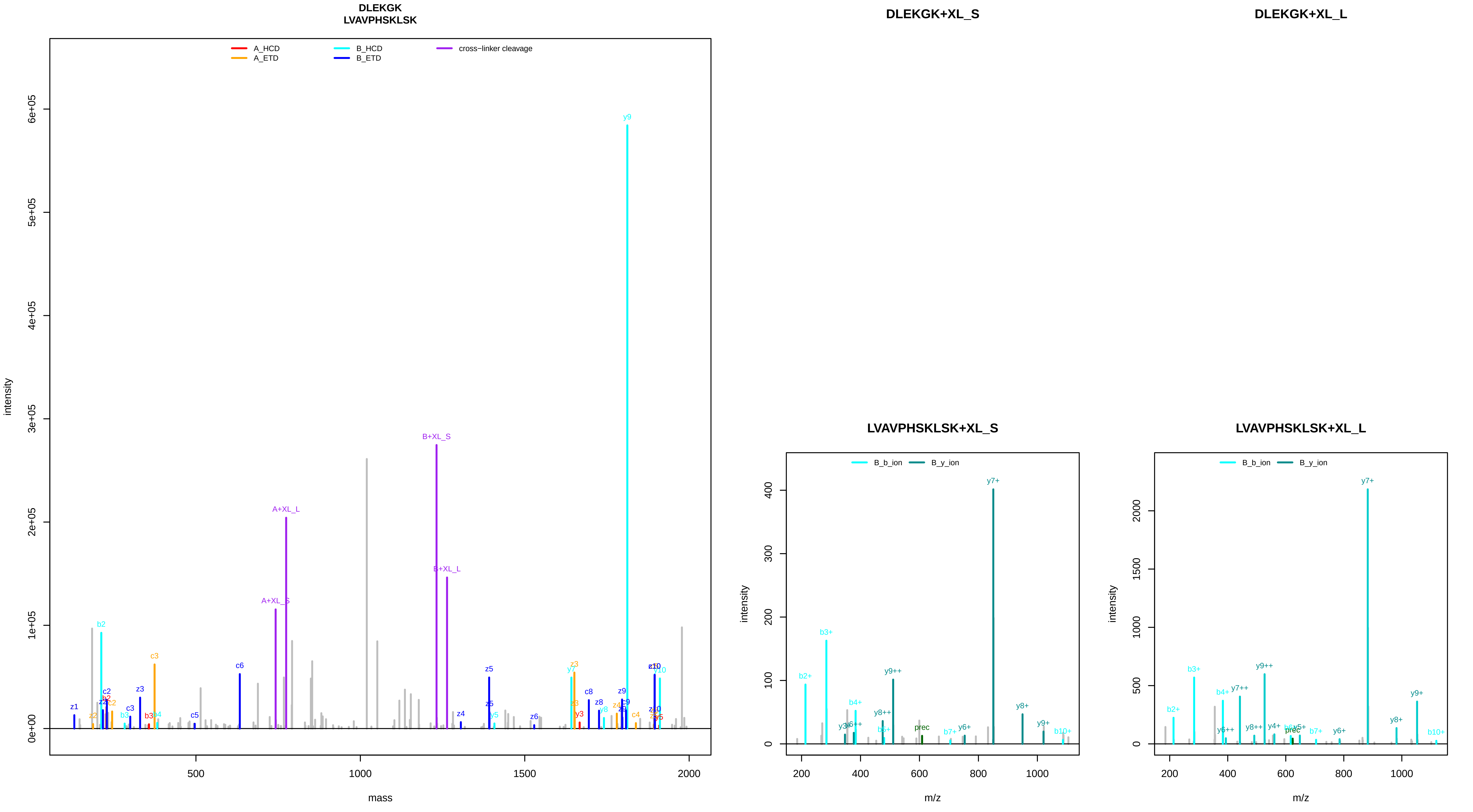
DLEKGK
LVAVPHSKLSK
A_HCD
A_ETD
B_HCD
B_ETD
cross−linker cleavage
intensity
mass
y9
B+XL_S
A+XL_L
B+XL_L
A+XL_S
b2
c3
c6
z5
DLEKGK+XL_S
DLEKGK+XL_L
LVAVPHSKLSK+XL_S
LVAVPHSKLSK+XL_L
B_b_ion
B_y_ion
y7+
b3+
b2+
y9++
y8+
prec
m/z
intensity

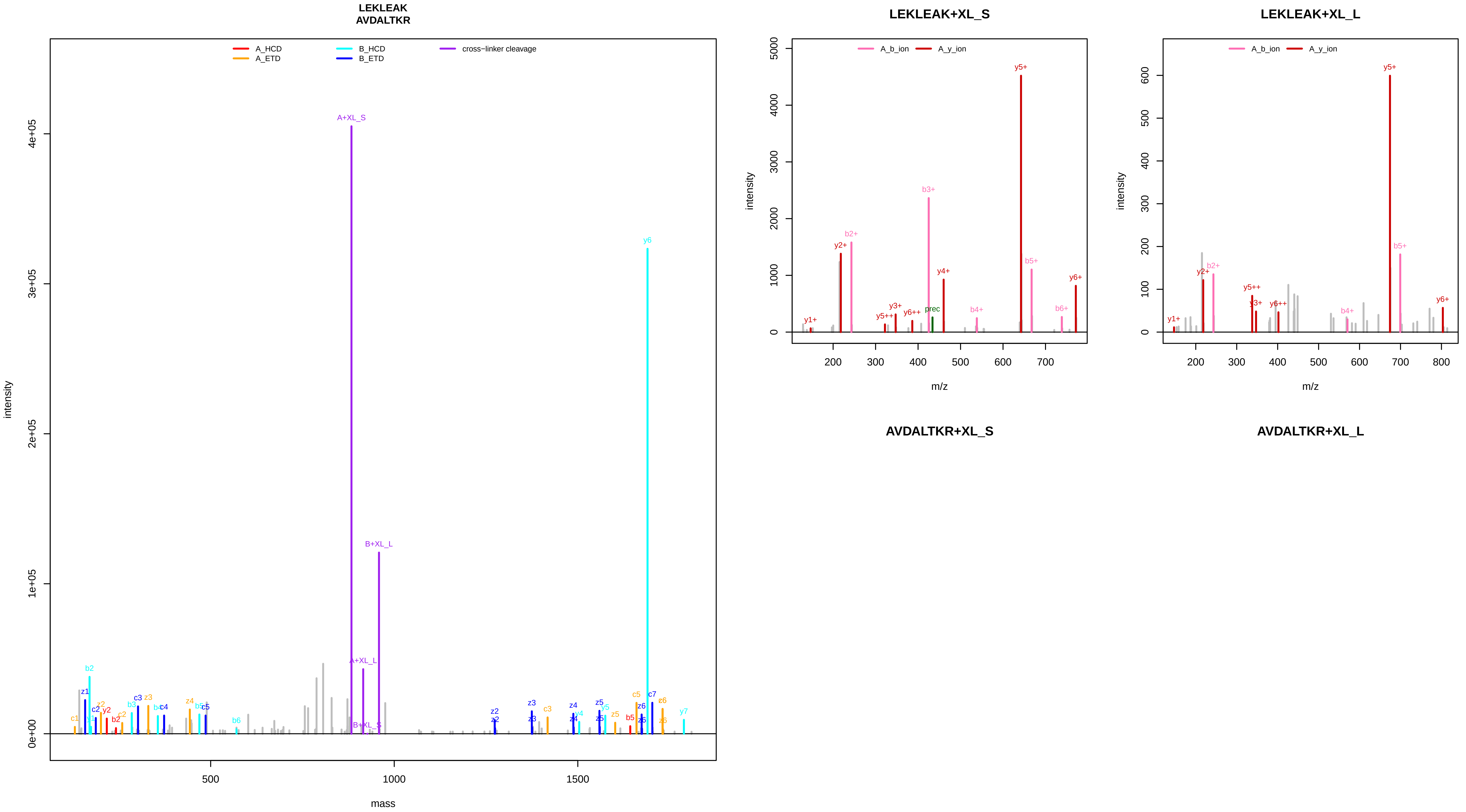
LEKLEAK
AVDALTKR
A_HCD
A_ETD
B_HCD
B_ETD
cross−linker cleavage
A+XL_S
B+XL_L
A+XL_L
B+XL_S
y6
b2
y7
intensity
mass
LEKLEAK+XL_S
LEKLEAK+XL_L
A_b_ion
A_y_ion
y5+
b3+
b2+
y2+
y4+
b5+
y6+
prec
b4+
b6+
y1+
y3+
y5++
y6++
m/z
AVDALTKR+XL_S
AVDALTKR+XL_L

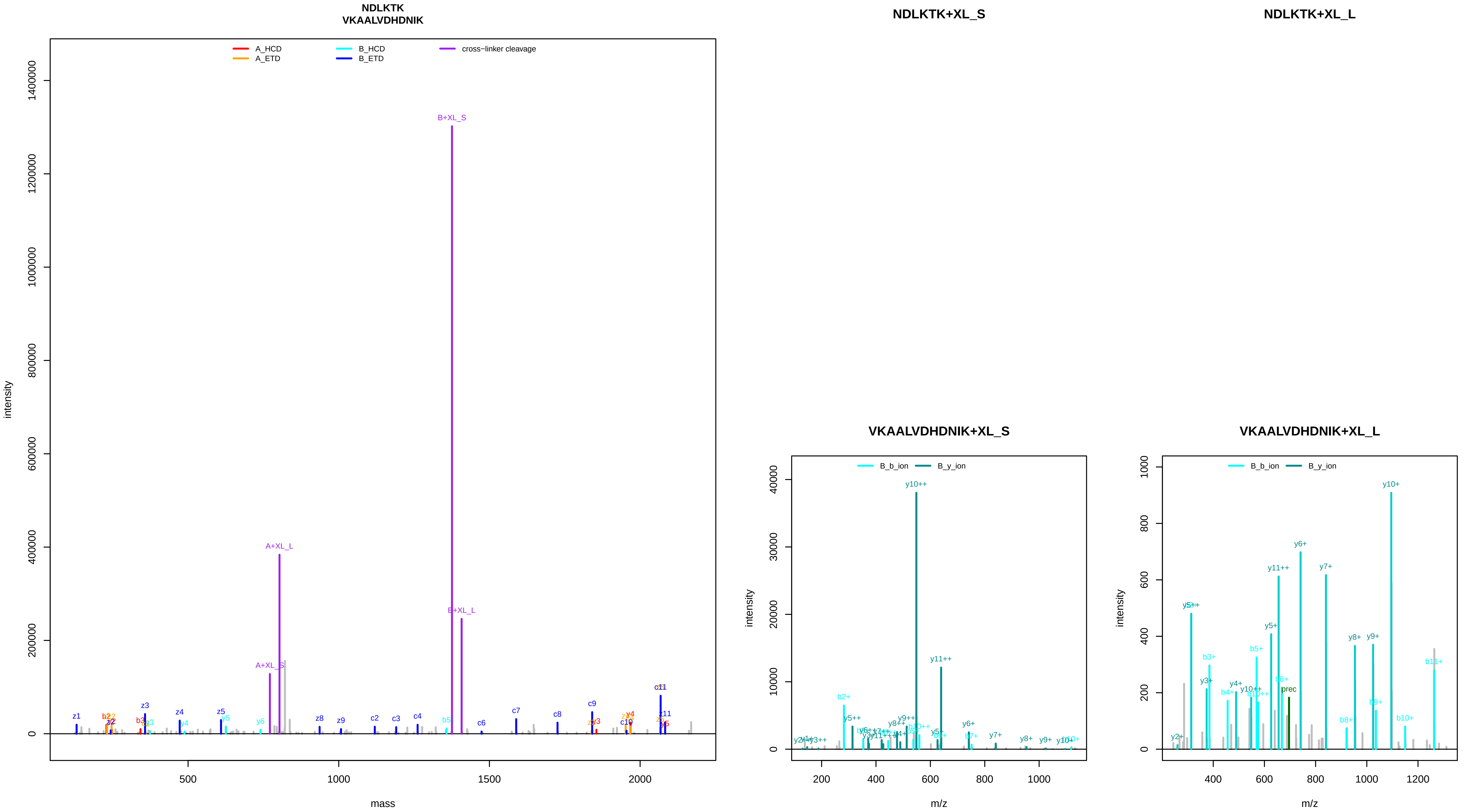
NDLKTK
VKAALVDHDNIK
A_HCD
A_ETD
B_HCD
B_ETD
cross−linker cleavage
B+XL_S
A+XL_L
B+XL_L
A+XL_S
intensity
mass
NDLKTK+XL_S
NDLKTK+XL_L
VKAALVDHDNIK+XL_S
VKAALVDHDNIK+XL_L
B_b_ion
B_y_ion
y10++
y11++
b2+
y10+
y6+
y7+
y11++
intensity
m/z

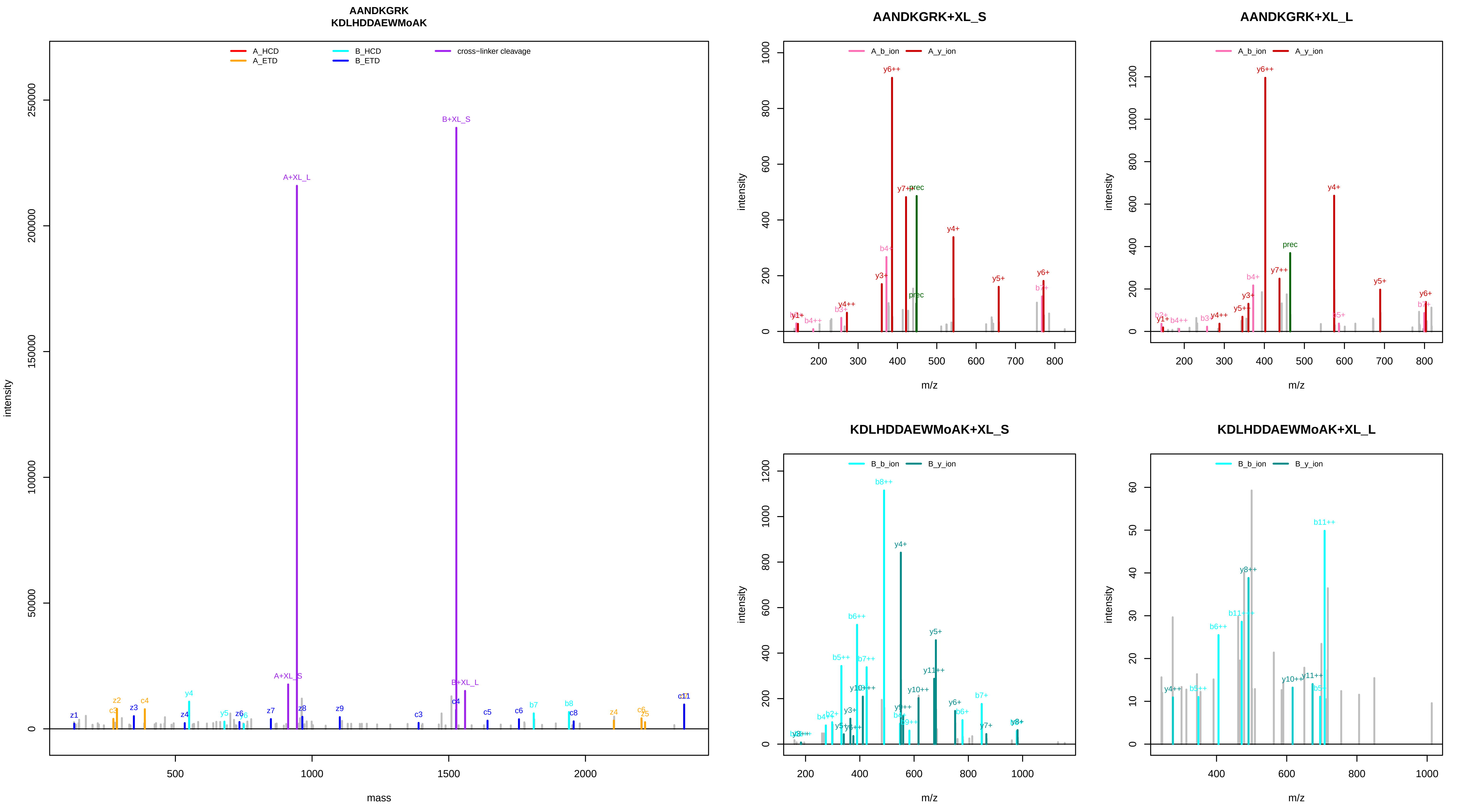
AANDKGRK
KDLHDDAEWMoAK
A_HCD
A_ETD
B_HCD
B_ETD
cross−linker cleavage
B+XL_S
A+XL_L
A+XL_S
B+XL_L
intensity
mass
AANDKGRK+XL_S
AANDKGRK+XL_L
A_b_ion
A_y_ion
prec
KDLHDDAEWMoAK+XL_S
KDLHDDAEWMoAK+XL_L
B_b_ion
B_y_ion
m/z

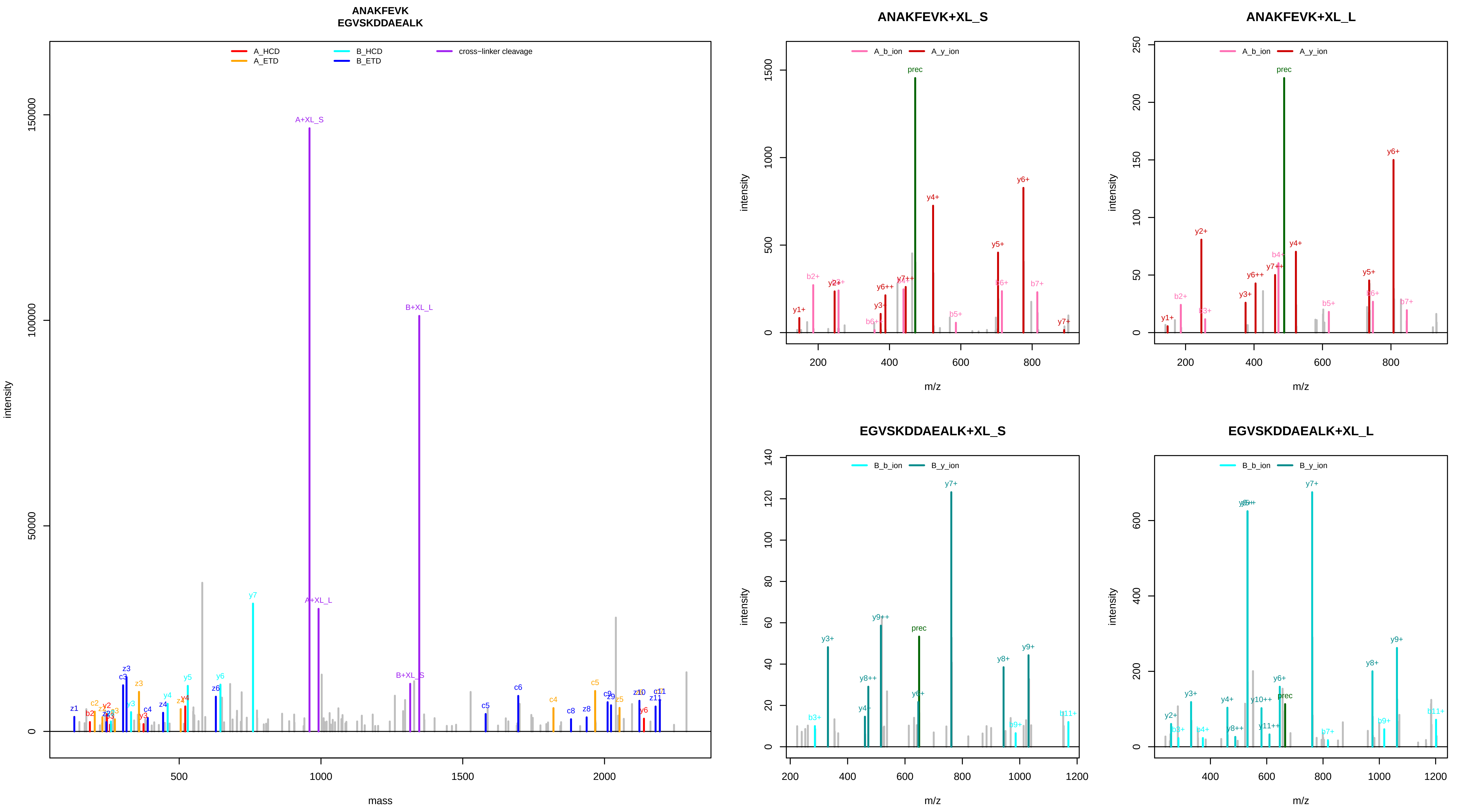
ANAKFEVK
EGVSKDDAEALK
A_HCD
A_ETD
B_HCD
B_ETD
cross−linker cleavage
A+XL_S
B+XL_L
A+XL_L
B+XL_S
y7
intensity
mass
0
50000
100000
150000
500
1000
1500
2000
ANAKFEVK+XL_S
A_b_ion
A_y_ion
prec
y1+
b2+
y2+
y3+
y6++
y7++
y4+
b5+
y5+
b6+
y6+
b7+
y7+
ANAKFEVK+XL_L
y2+
b2+
y3+
y6++
y7++
b4+
y4+
b5+
y5+
b6+
y6+
b7+
m/z
EGVSKDDAEALK+XL_S
B_b_ion
B_y_ion
y7+
y3+
y9++
prec
y8++
y4+
y6+
b3+
y8+
y9+
b9+
b11+
EGVSKDDAEALK+XL_L
y3+
y4+
y10++
y11++
y8++
y6+
y2+
b3+
b4+
b7+
y8+
y9+
b9+
b11+

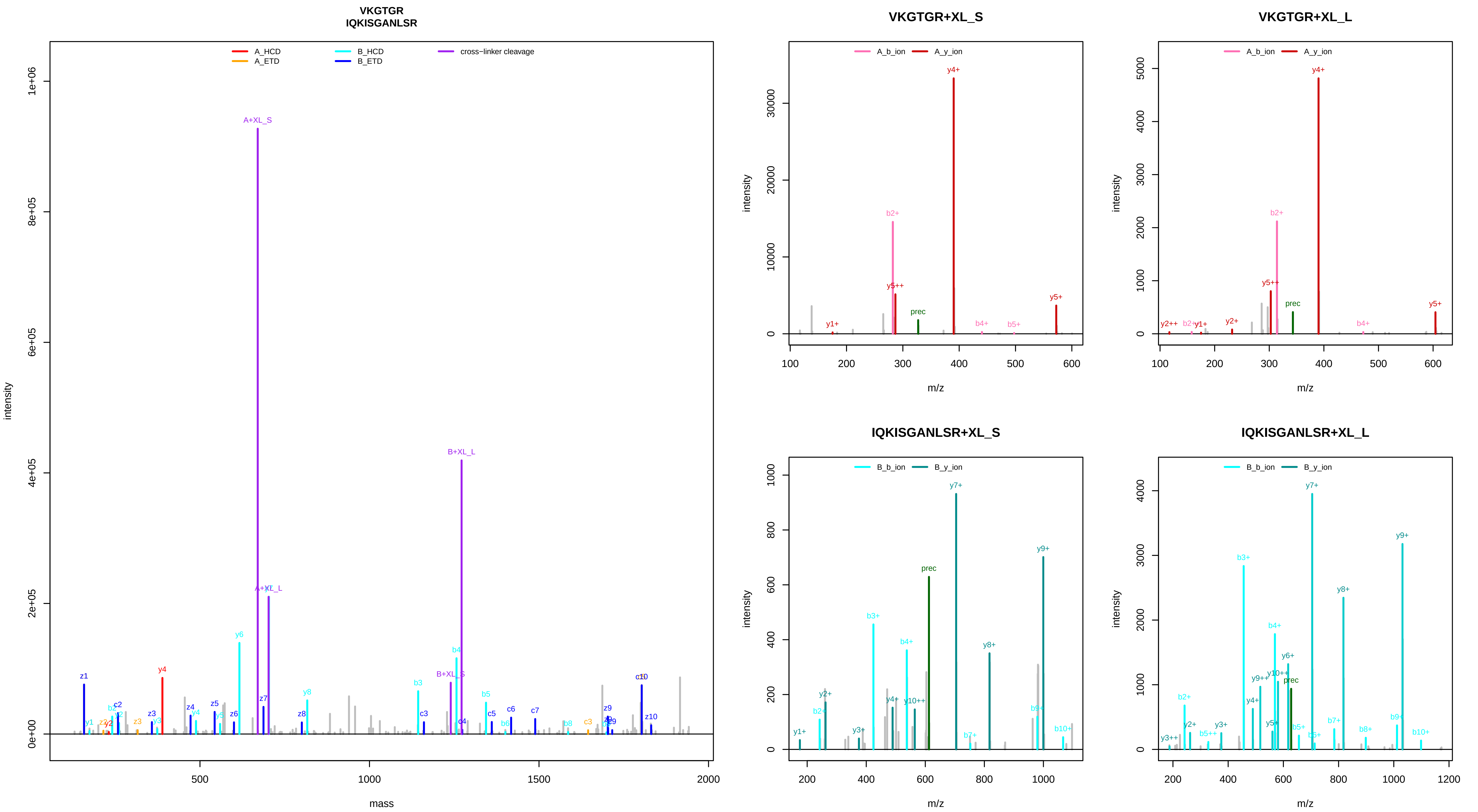
VKGTGR
IQKISGANLSR
A_HCD
A_ETD
B_HCD
B_ETD
cross−linker cleavage
A+XL_S
B+XL_L
A+XL_L
B+XL_S
intensity
mass
VKGTGR+XL_S
VKGTGR+XL_L
IQKISGANLSR+XL_S
IQKISGANLSR+XL_L
A_b_ion
A_y_ion
B_b_ion
B_y_ion
prec
m/z

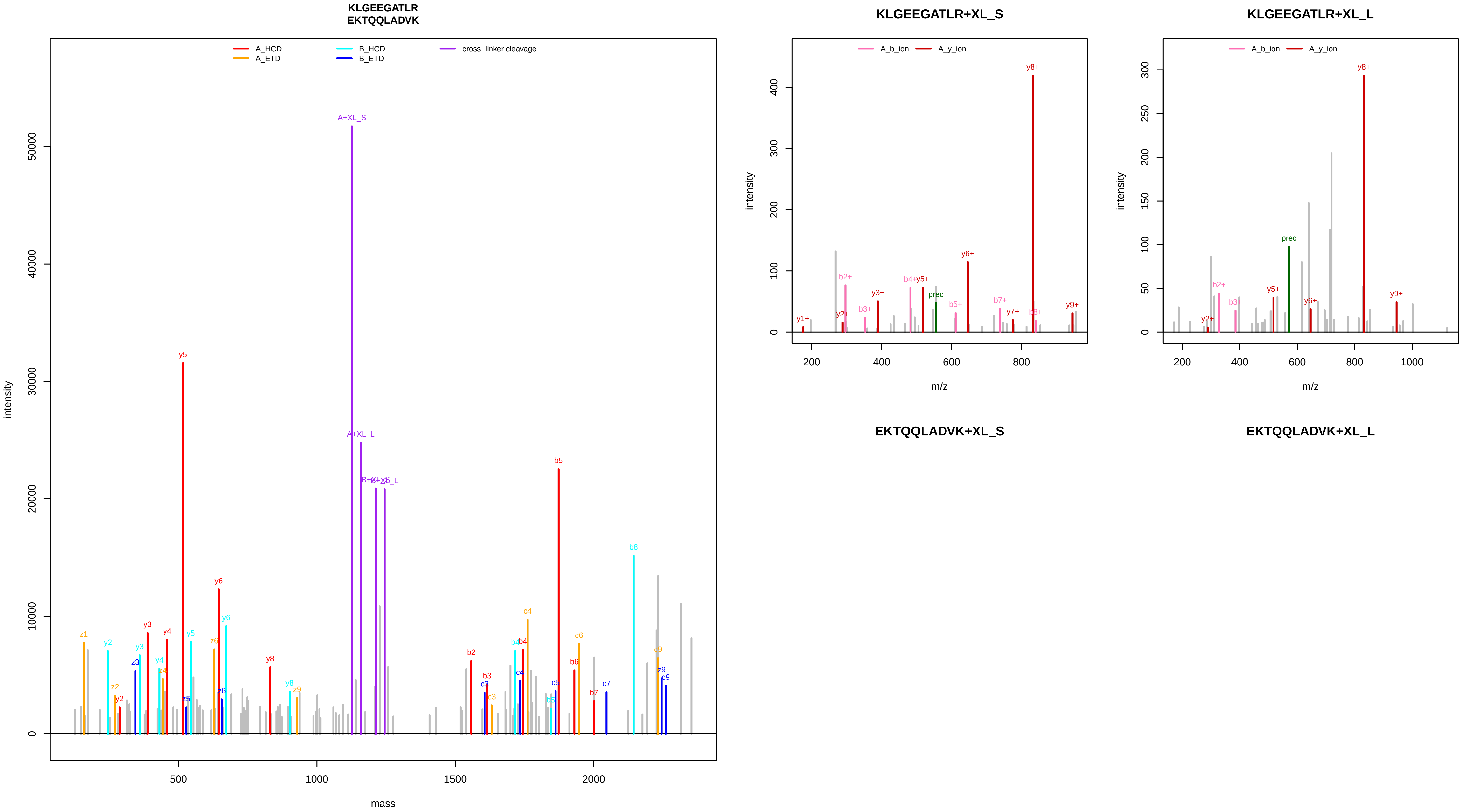
KLGEEGATLR
EKTQQLADVK
A_HCD
A_ETD
B_HCD
B_ETD
cross−linker cleavage
A+XL_S
A+XL_L
intensity
mass
KLGEEGATLR+XL_S
KLGEEGATLR+XL_L
A_b_ion
A_y_ion
prec
m/z
EKTQQLADVK+XL_S
EKTQQLADVK+XL_L

GFAGTVKR
KVDVTGTSK

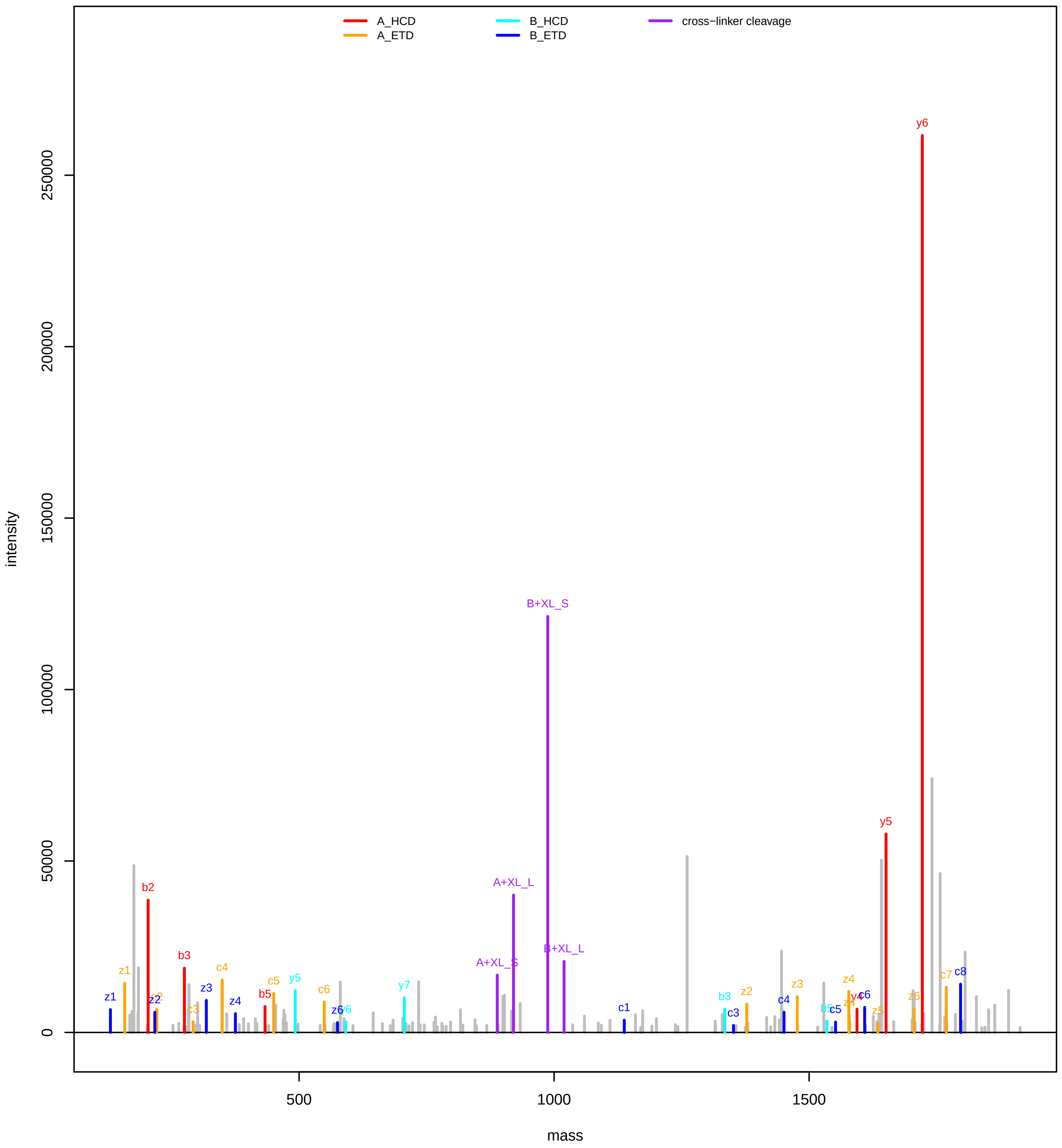

GFAGTVKR+XL_S

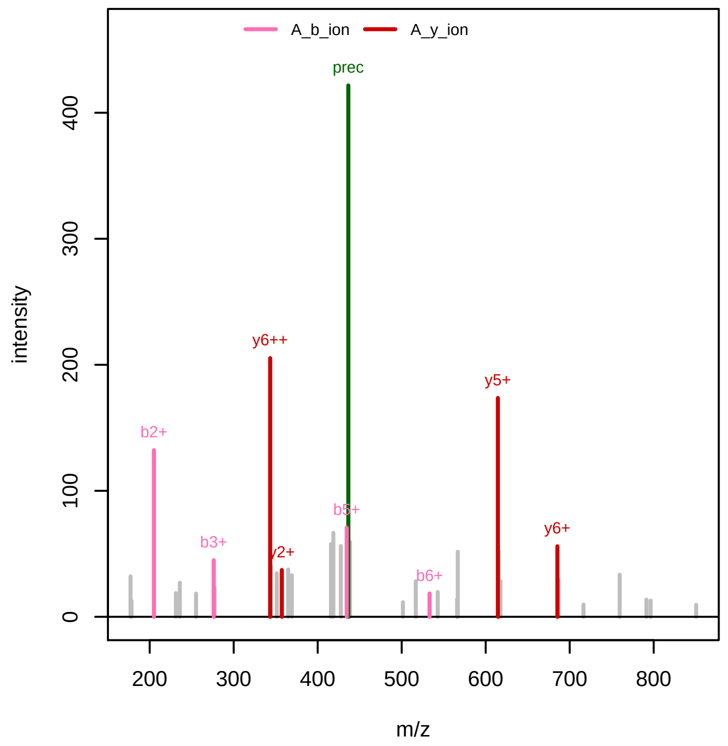

GFAGTVKR+XL_L

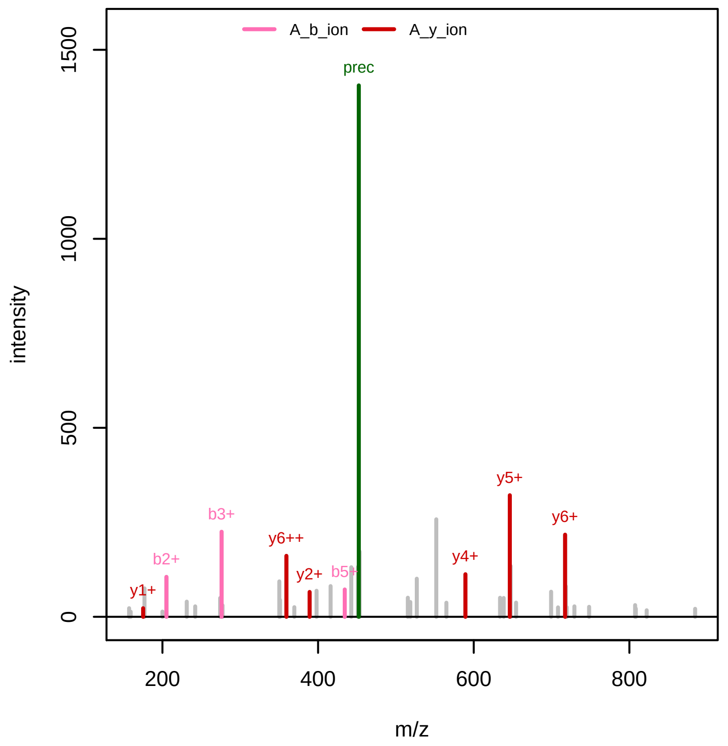

KVDVTGTSK+XL_S

KVDVTGTSK+XL_L

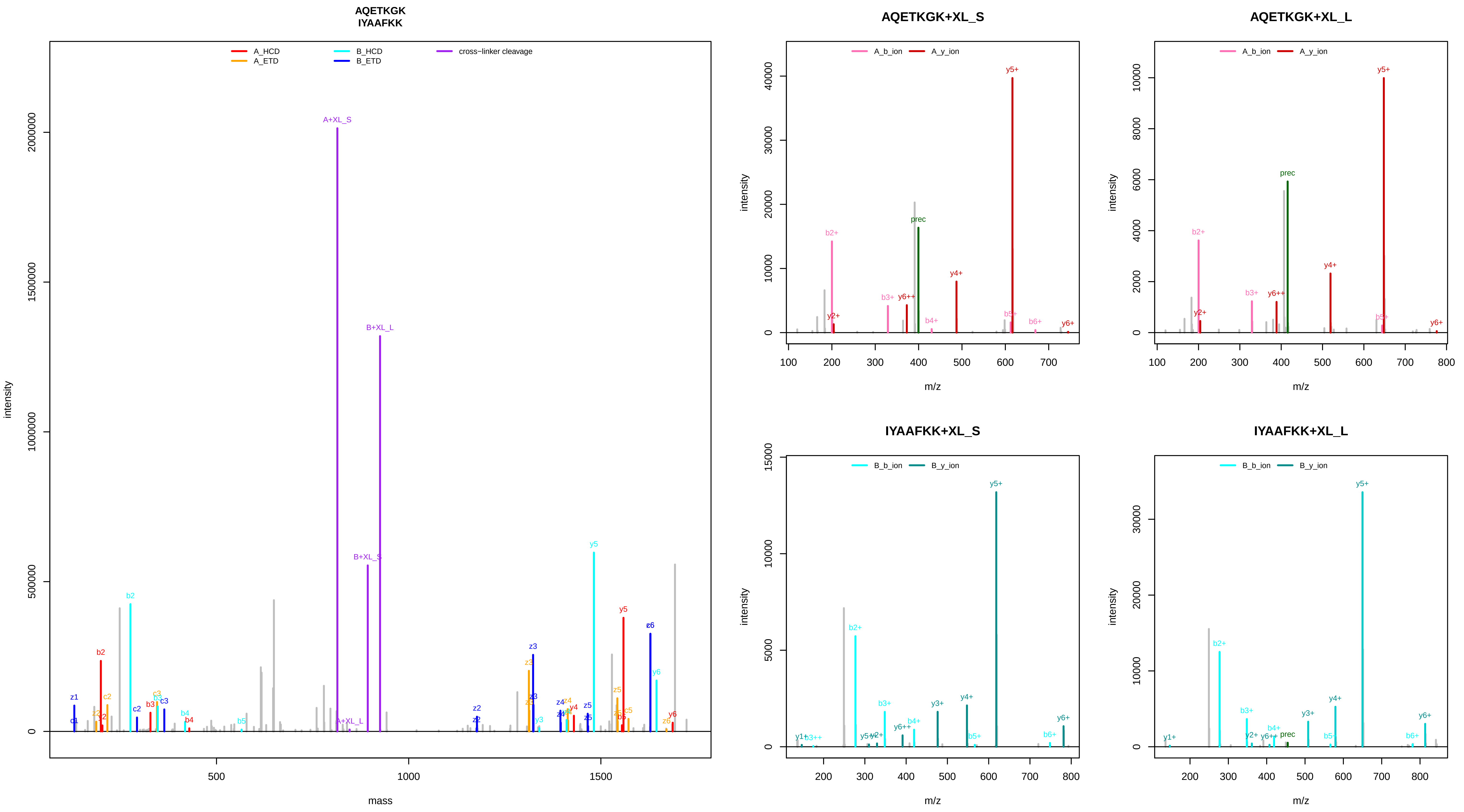

AQETKGK
IYAAFKK
A_HCD
A_ETD
B_HCD
B_ETD
cross−linker cleavage
A+XL_S
B+XL_L
B+XL_S
A+XL_L
intensity
mass
AQETKGK+XL_S
AQETKGK+XL_L
IYAAFKK+XL_S
IYAAFKK+XL_L
A_b_ion
A_y_ion
B_b_ion
B_y_ion
prec
intensity
m/z

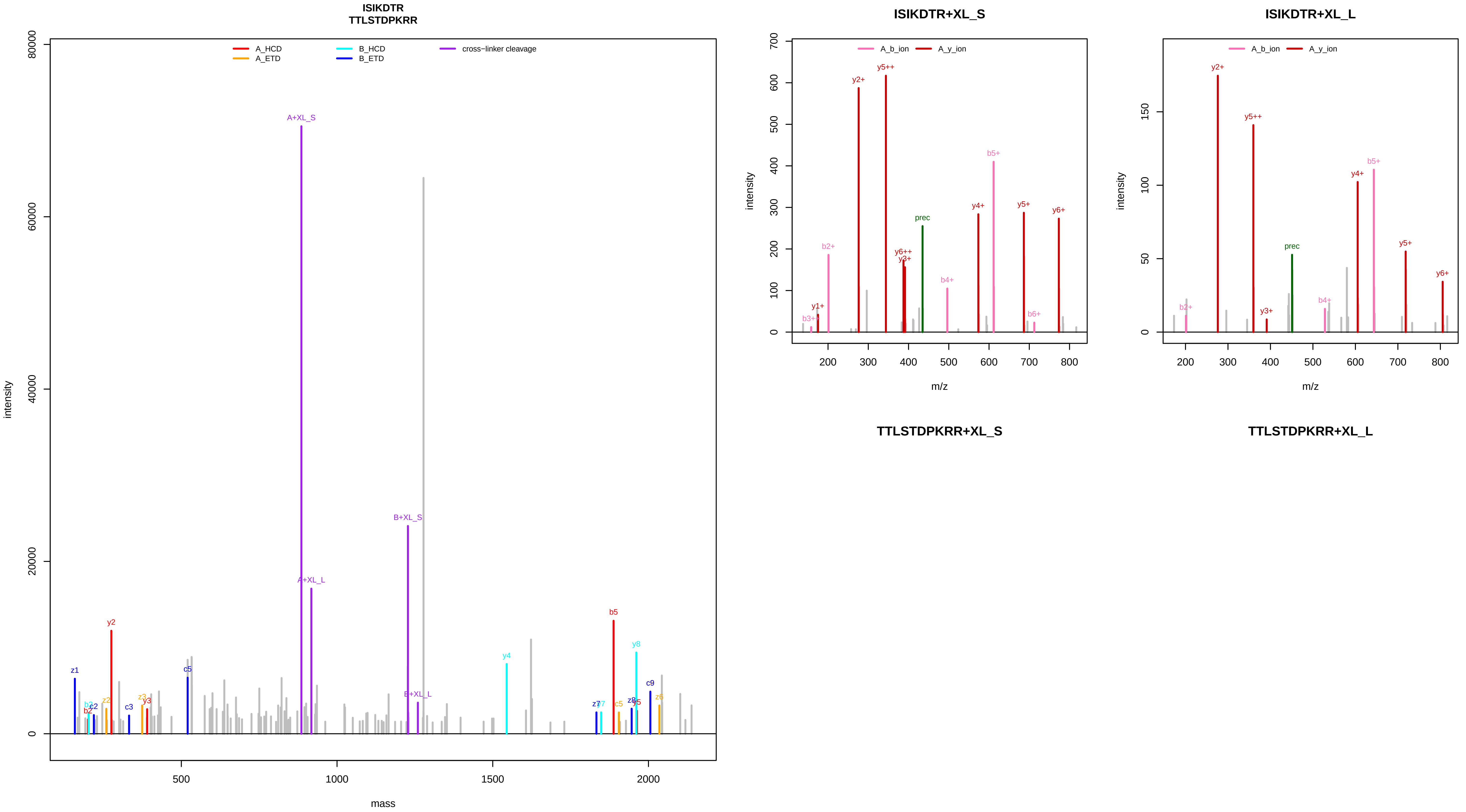

ISIKDTR
TTLSTDPKRR
A_HCD
A_ETD
B_HCD
B_ETD
cross−linker cleavage
A+XL_S
A+XL_L
B+XL_S
B+XL_L
intensity
mass
ISIKDTR+XL_S
ISIKDTR+XL_L
A_b_ion
A_y_ion
prec
m/z
TTLSTDPKRR+XL_S
TTLSTDPKRR+XL_L

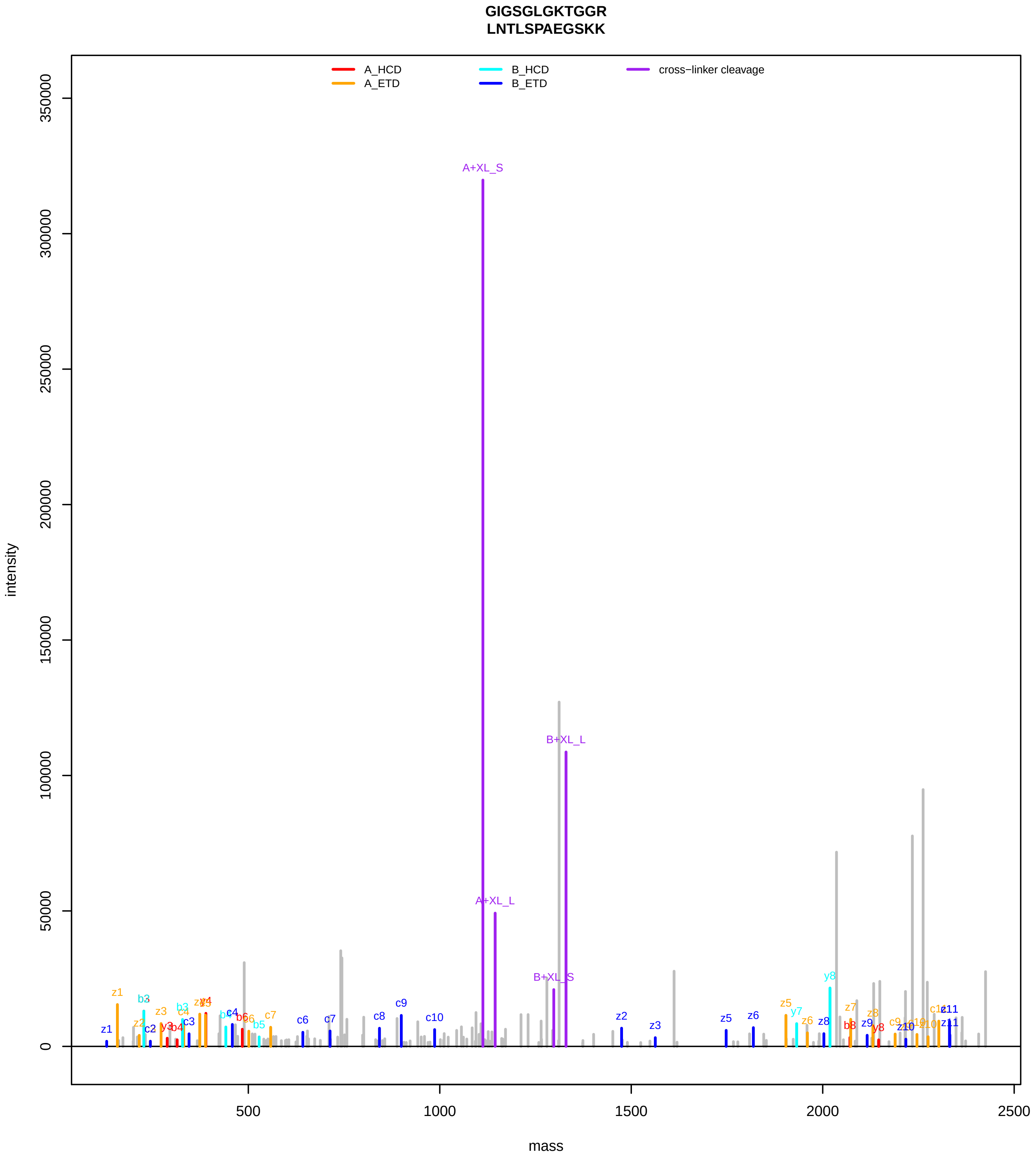

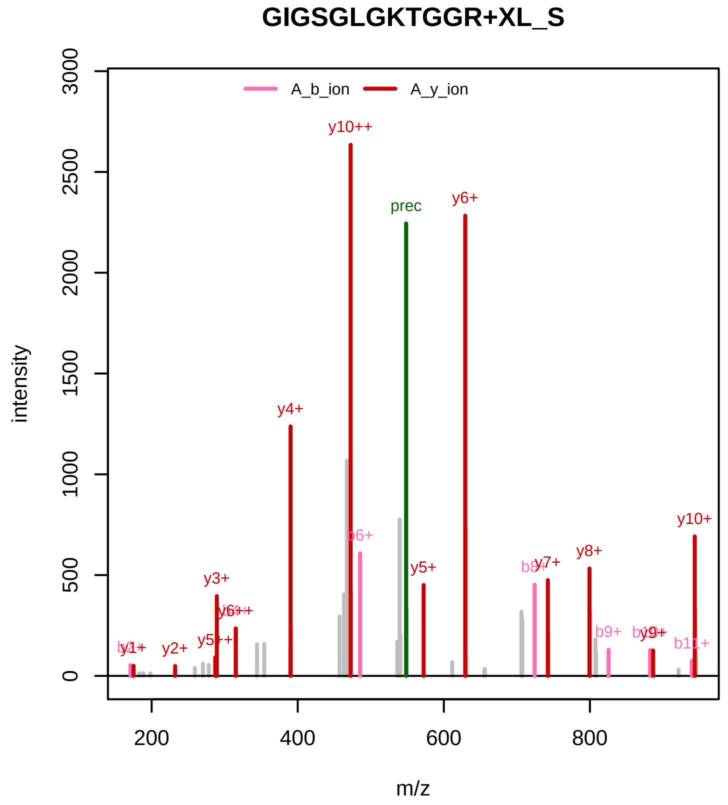

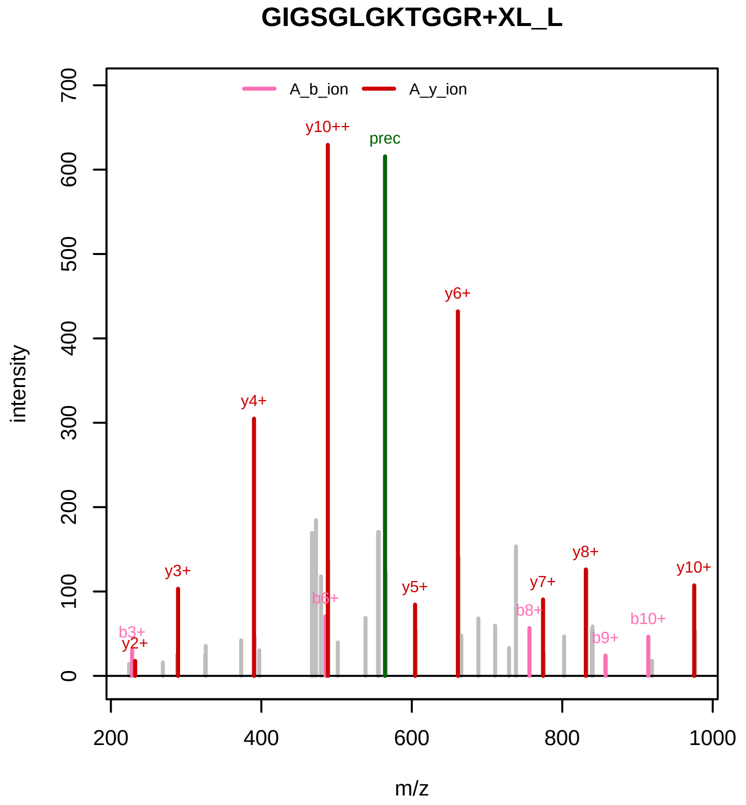

LNTLSPAEGSKK+XL_S

LNTLSPAEGSKK+XL_L

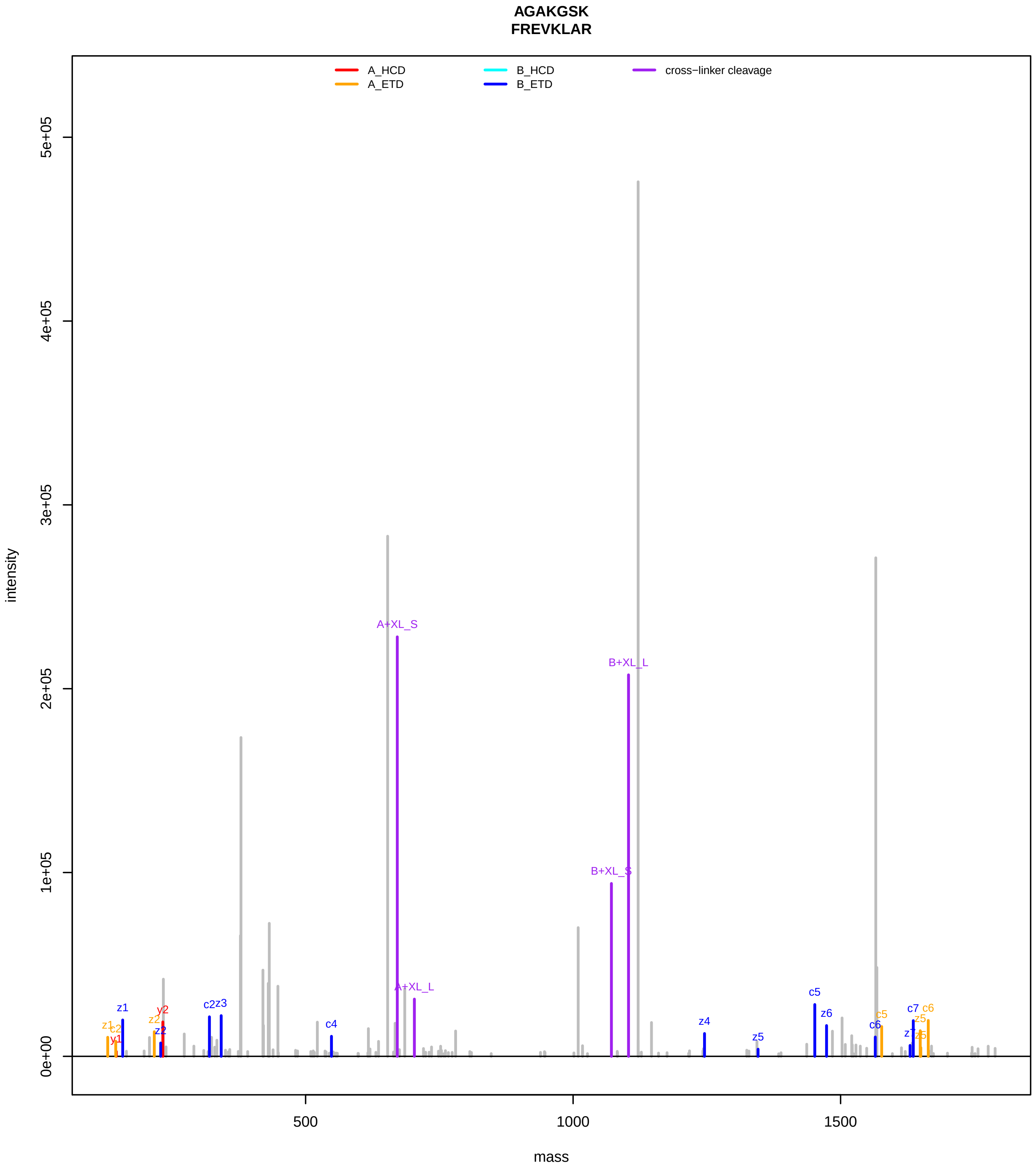

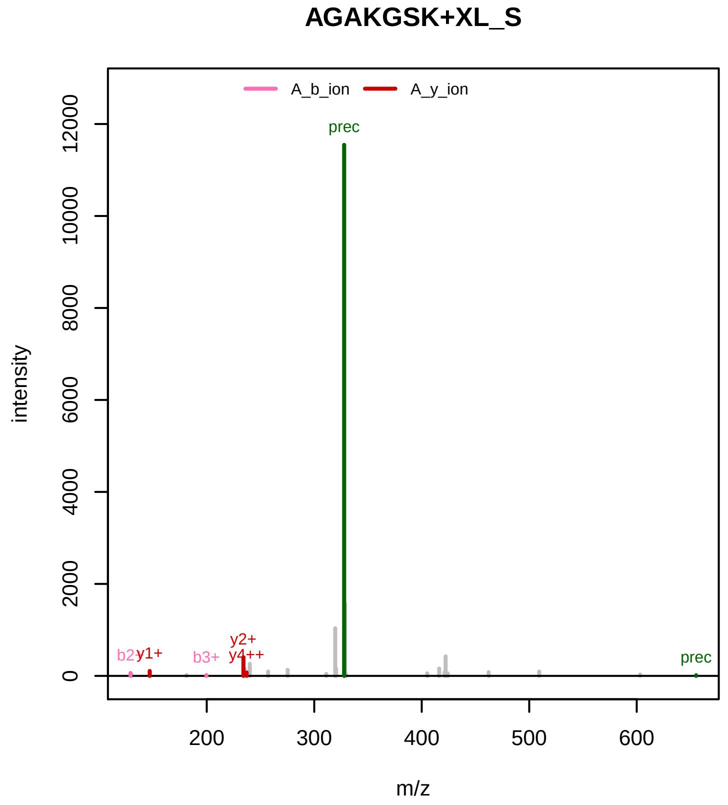

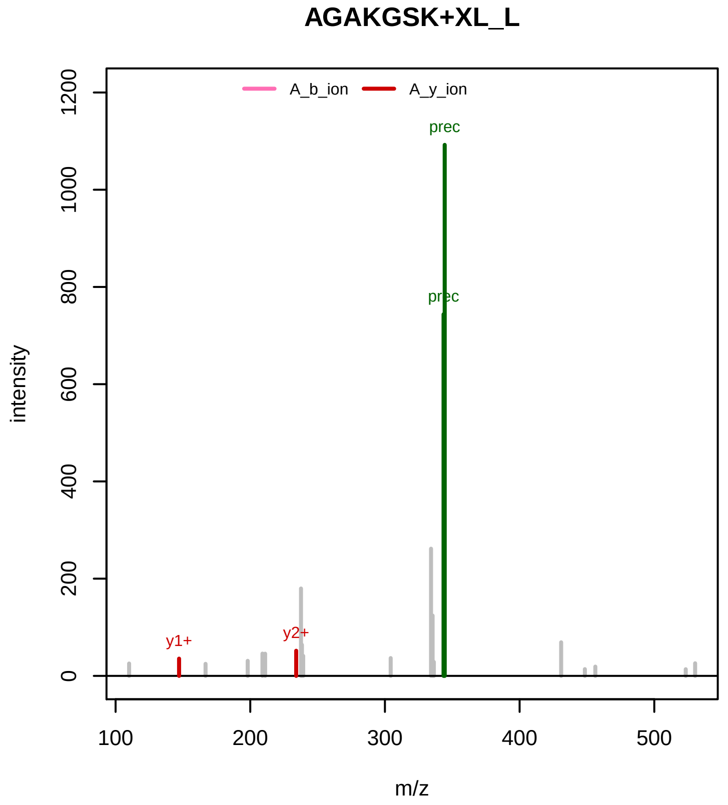

FREVKLAR+XL_S

FREVKLAR+XL_L

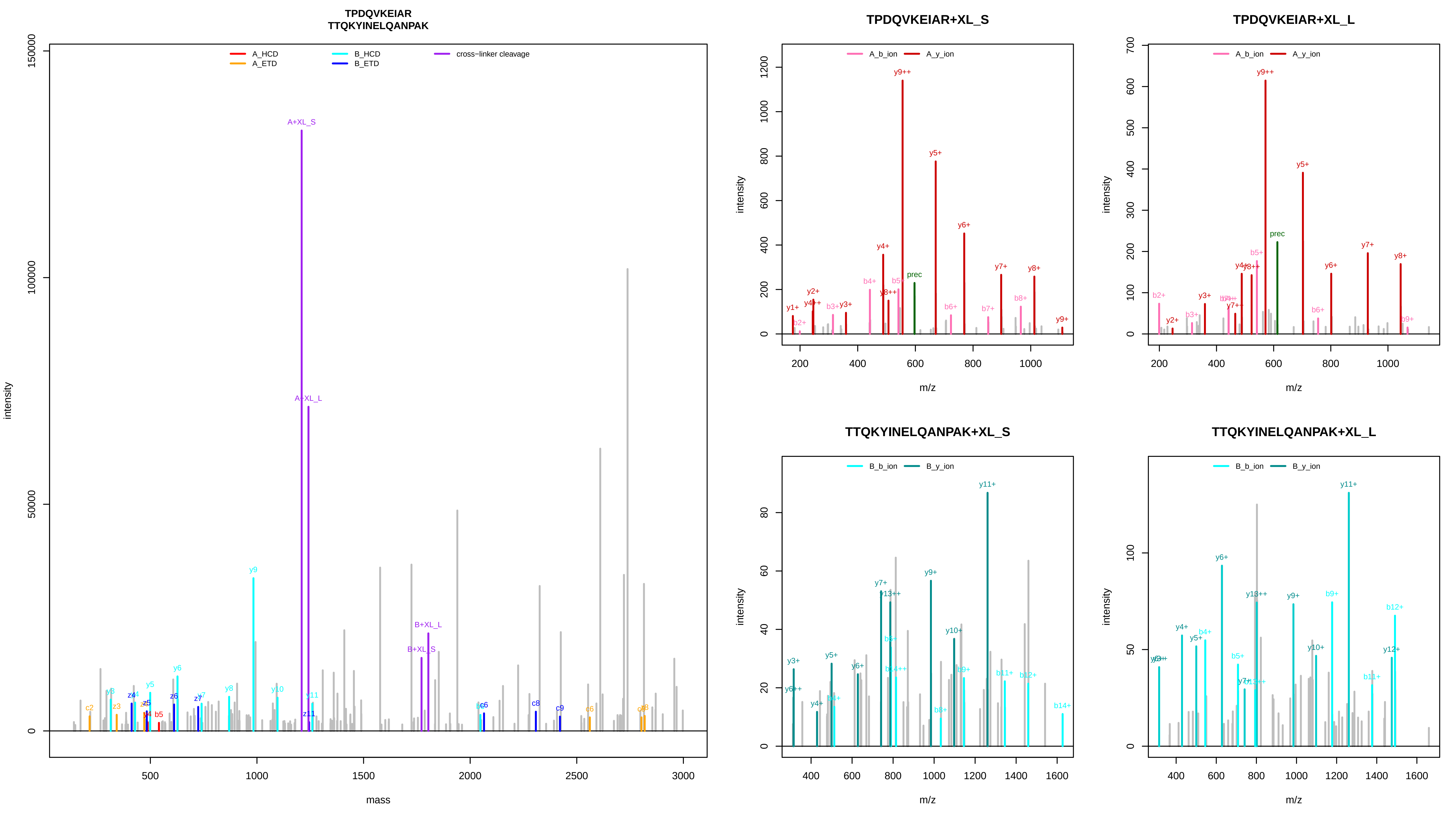

TPDQVKEIAR
TTQKYINELQANPAK
A_HCD
A_ETD
B_HCD
B_ETD
cross−linker cleavage
A+XL_S
A+XL_L
B+XL_L
B+XL_S
intensity
mass
TPDQVKEIAR+XL_S
TPDQVKEIAR+XL_L
TTQKYINELQANPAK+XL_S
TTQKYINELQANPAK+XL_L
A_b_ion
A_y_ion
B_b_ion
B_y_ion
prec
m/z

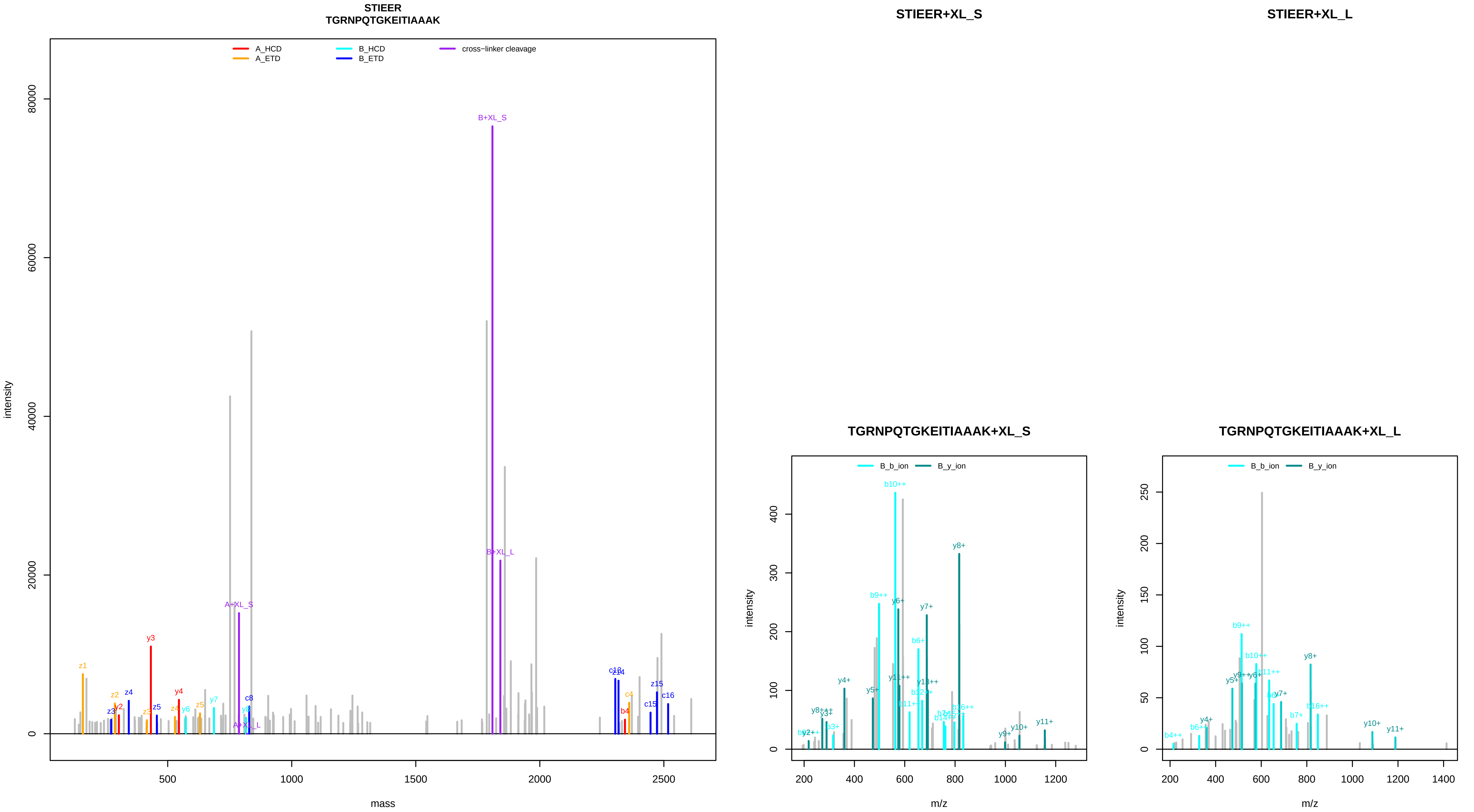
STIEER
TGRNPQTGKEITIAAAK
A_HCD
A_ETD
B_HCD
B_ETD
cross−linker cleavage
B+XL_S
B+XL_L
A+XL_S
A+XL_L
intensity
mass
STIEER+XL_S
STIEER+XL_L
TGRNPQTGKEITIAAAK+XL_S
TGRNPQTGKEITIAAAK+XL_L
B_b_ion
B_y_ion
m/z
intensity

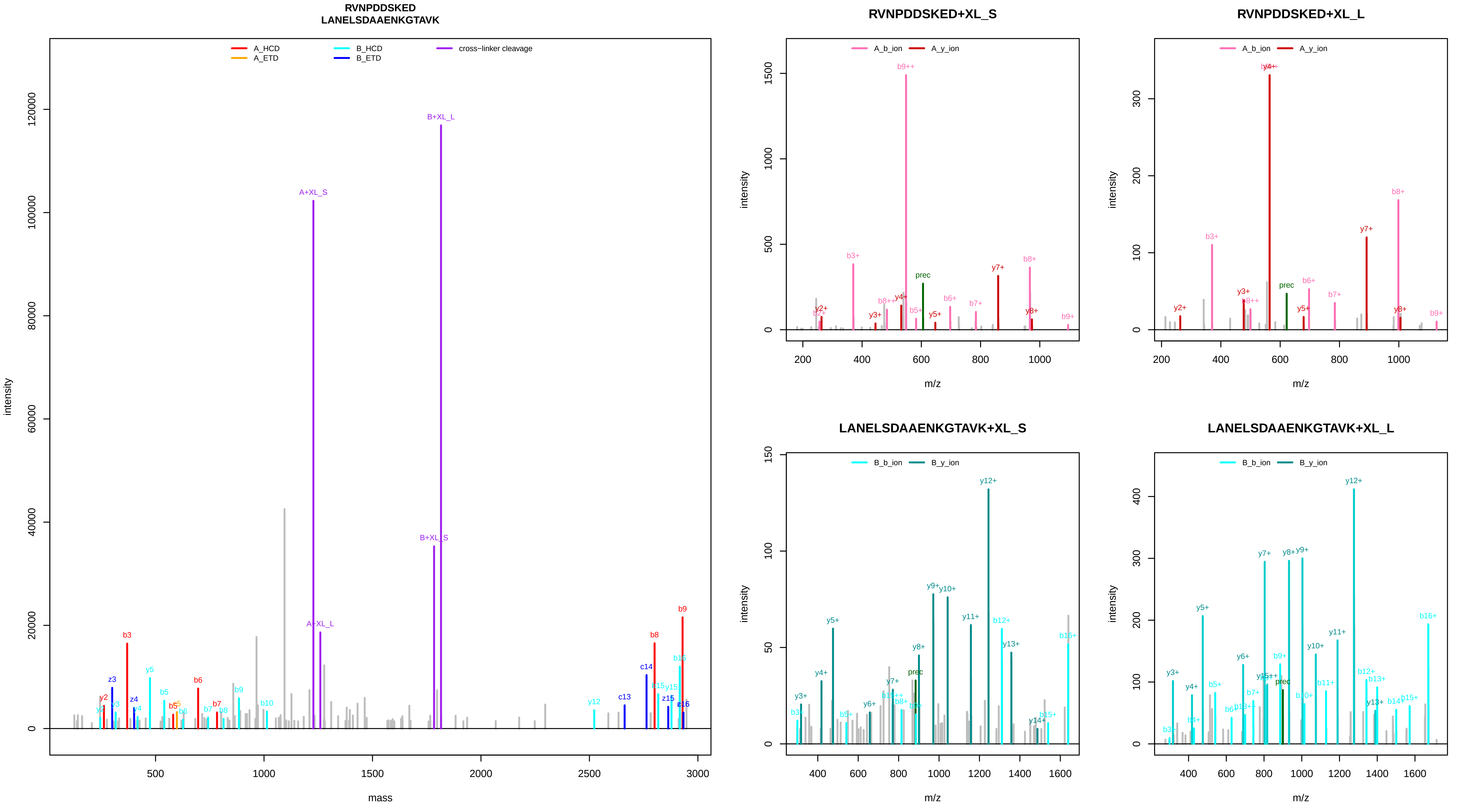

RVNPDDSKED
LANELSDAAENKGTAVK
A_HCD
A_ETD
B_HCD
B_ETD
cross−linker cleavage
A+XL_S
B+XL_L
B+XL_S
A+XL_L
intensity
mass
RVNPDDSKED+XL_S
RVNPDDSKED+XL_L
LANELSDAAENKGTAVK+XL_S
LANELSDAAENKGTAVK+XL_L
A_b_ion
A_y_ion
B_b_ion
B_y_ion
prec
m/z

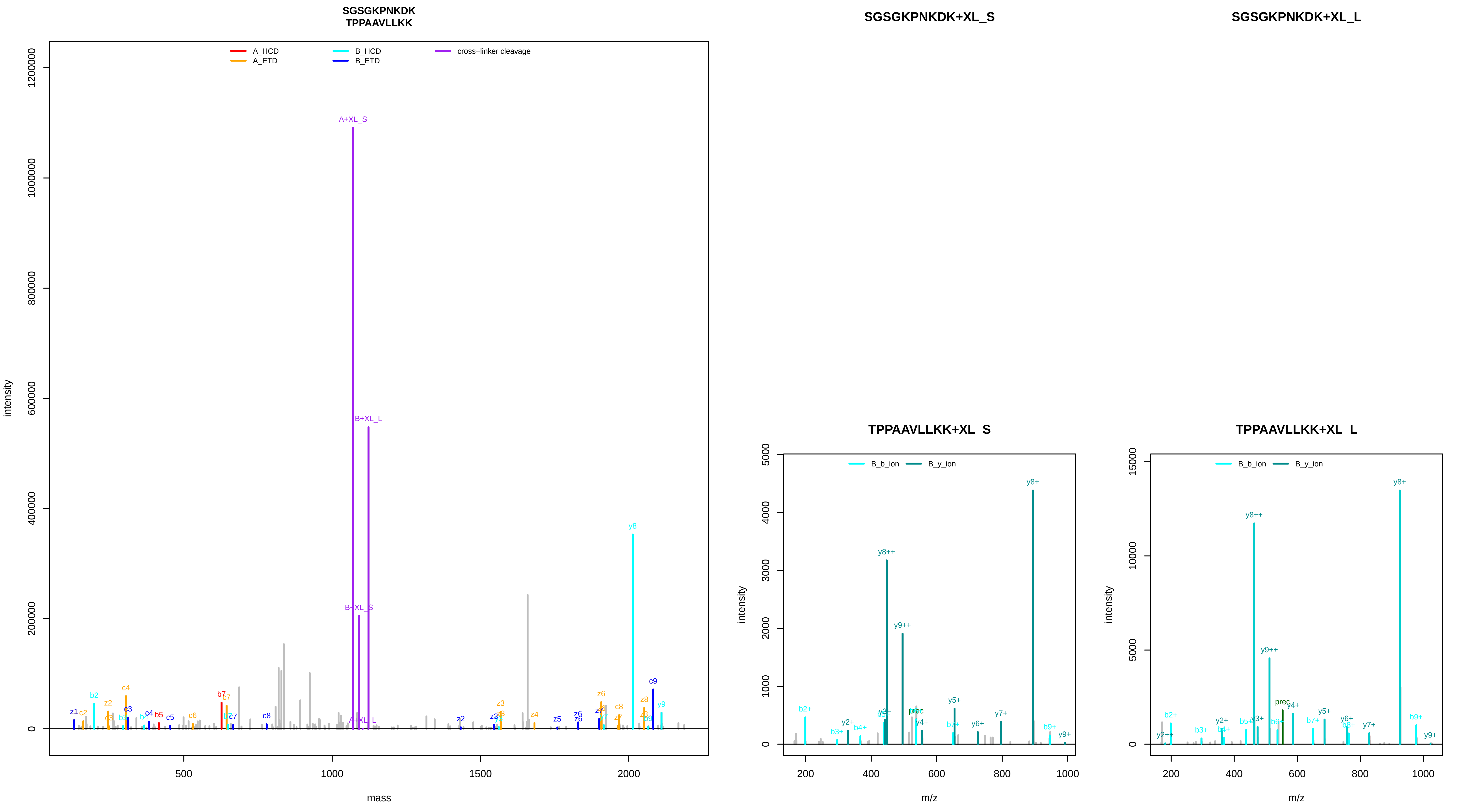

SGSGKPNKDK
TPPAAVLLKK
A_HCD
A_ETD
B_HCD
B_ETD
cross−linker cleavage
A+XL_S
B+XL_L
B+XL_S
A+XL_L
y8
y9
c9
intensity
mass
SGSGKPNKDK+XL_S
SGSGKPNKDK+XL_L
TPPAAVLLKK+XL_S
TPPAAVLLKK+XL_L
B_b_ion
B_y_ion
y8+
y8++
y9++
prec
m/z

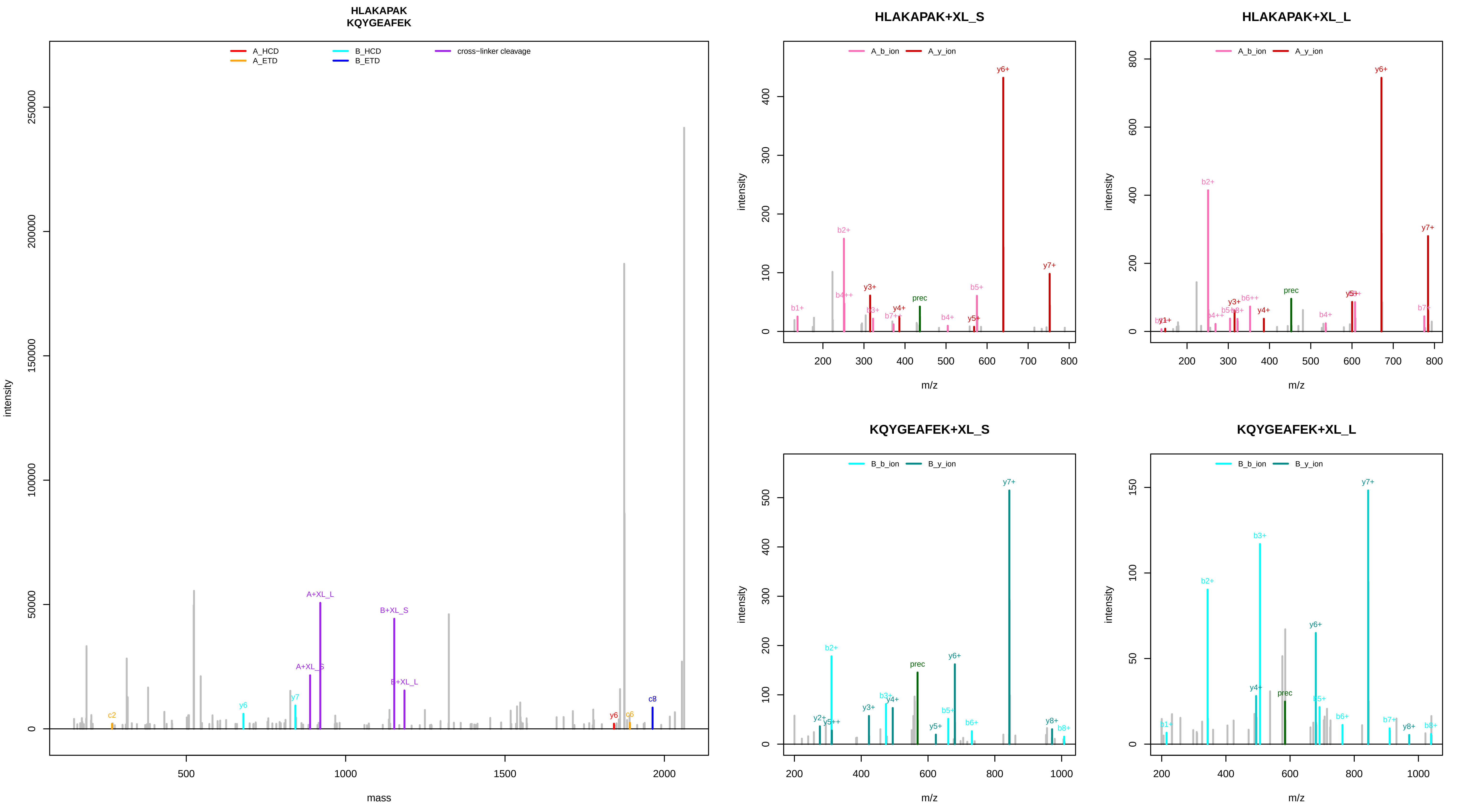
HLAKAPAK
KQYGEAFEK
A_HCD
A_ETD
B_HCD
B_ETD
cross−linker cleavage
intensity
mass
A+XL_L
A+XL_S
B+XL_S
B+XL_L
c2
y6
y7
c6
c8
HLAKAPAK+XL_S
HLAKAPAK+XL_L
A_b_ion
A_y_ion
KQYGEAFEK+XL_S
KQYGEAFEK+XL_L
B_b_ion
B_y_ion
m/z
prec

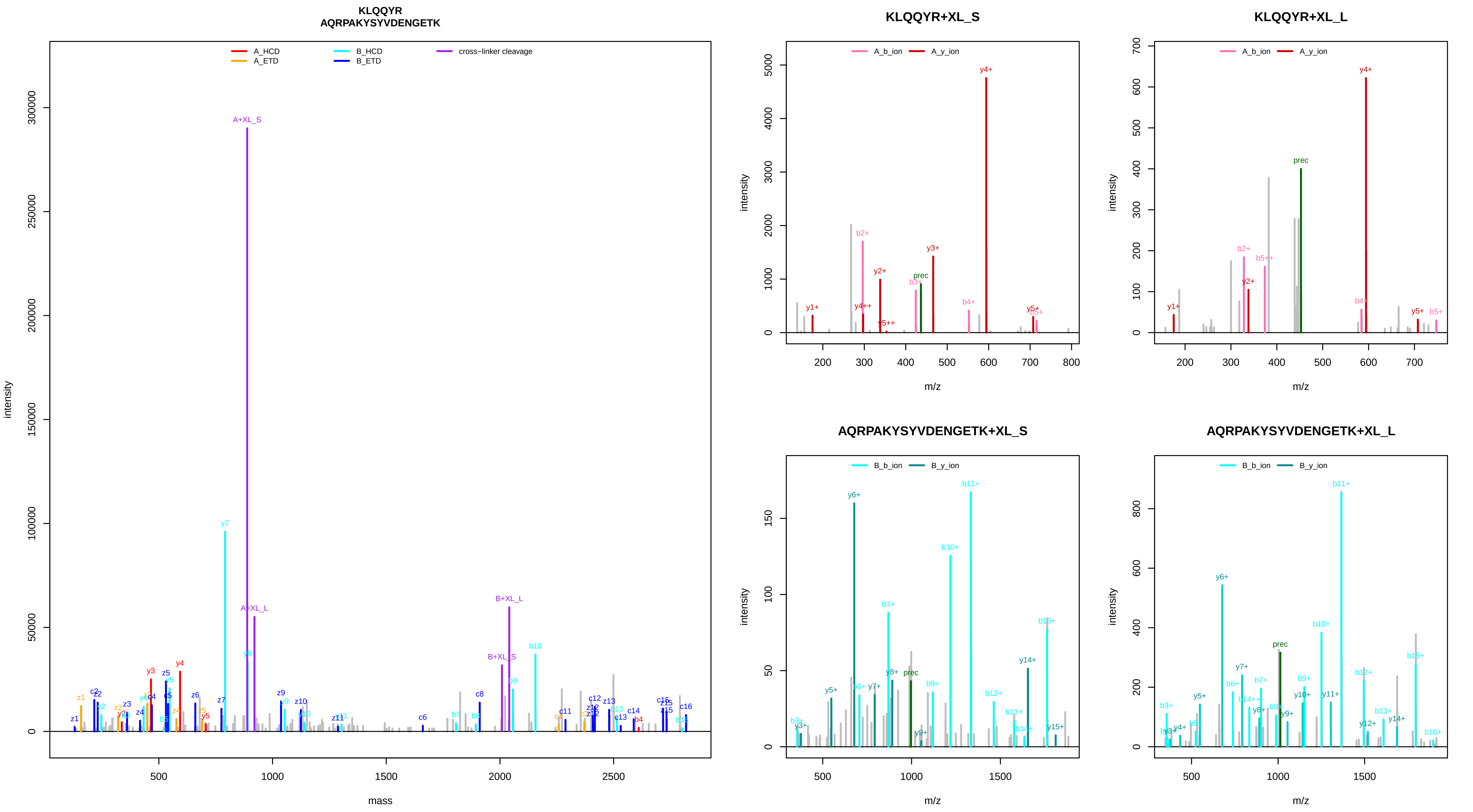
KLQQYR
AQRPAKYSYVDENGETK
A_HCD
A_ETD
B_HCD
B_ETD
cross−linker cleavage
A+XL_S
A+XL_L
B+XL_L
B+XL_S
intensity
mass
KLQQYR+XL_S
KLQQYR+XL_L
A_b_ion
A_y_ion
AQRPAKYSYVDENGETK+XL_S
AQRPAKYSYVDENGETK+XL_L
B_b_ion
B_y_ion
prec
m/z

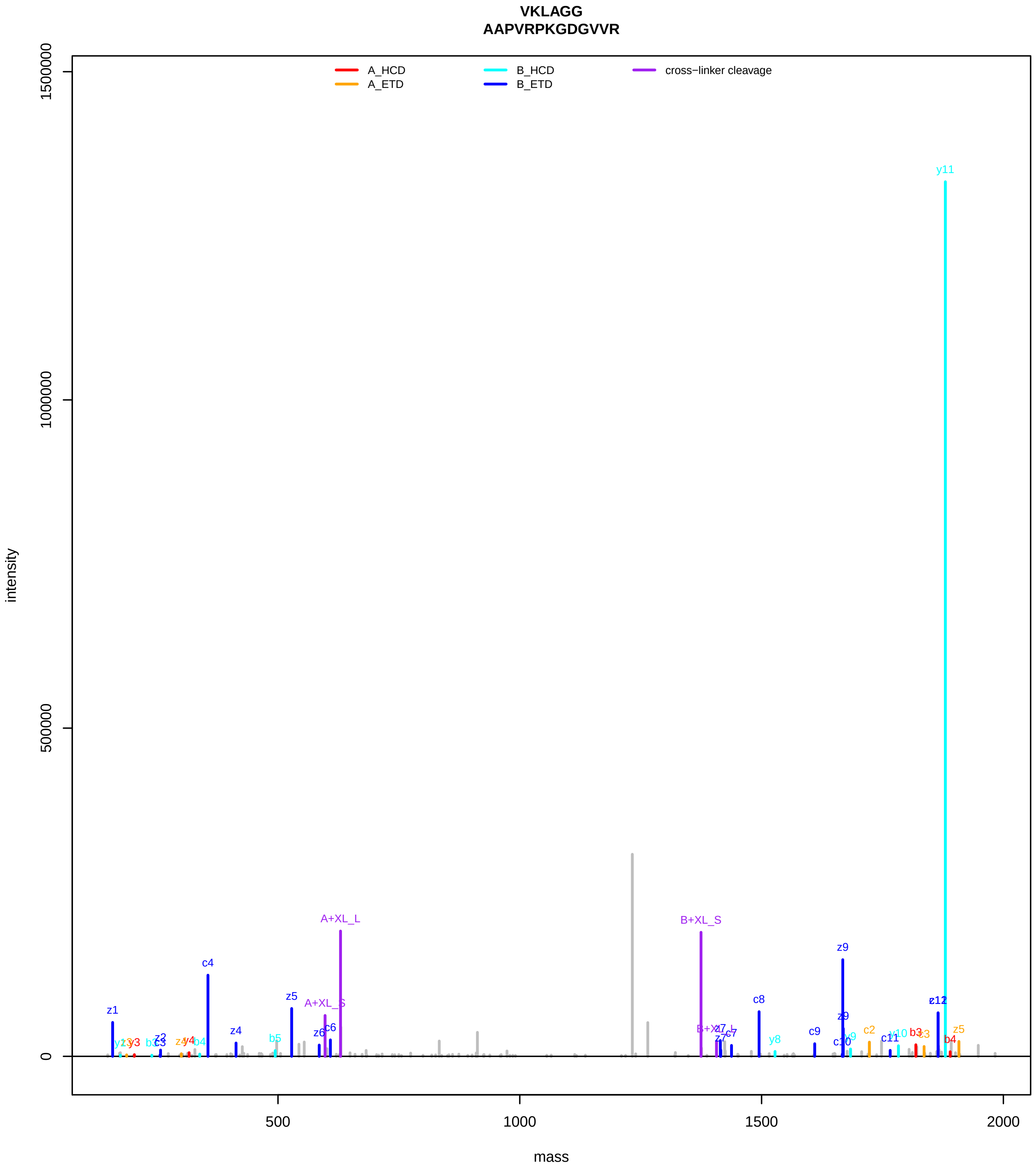

VKLAGG+XL_S

VKLAGG+XL_L

AAPVRPKGDGVVR+XL_S

AAPVRPKGDGVVR+XL_L

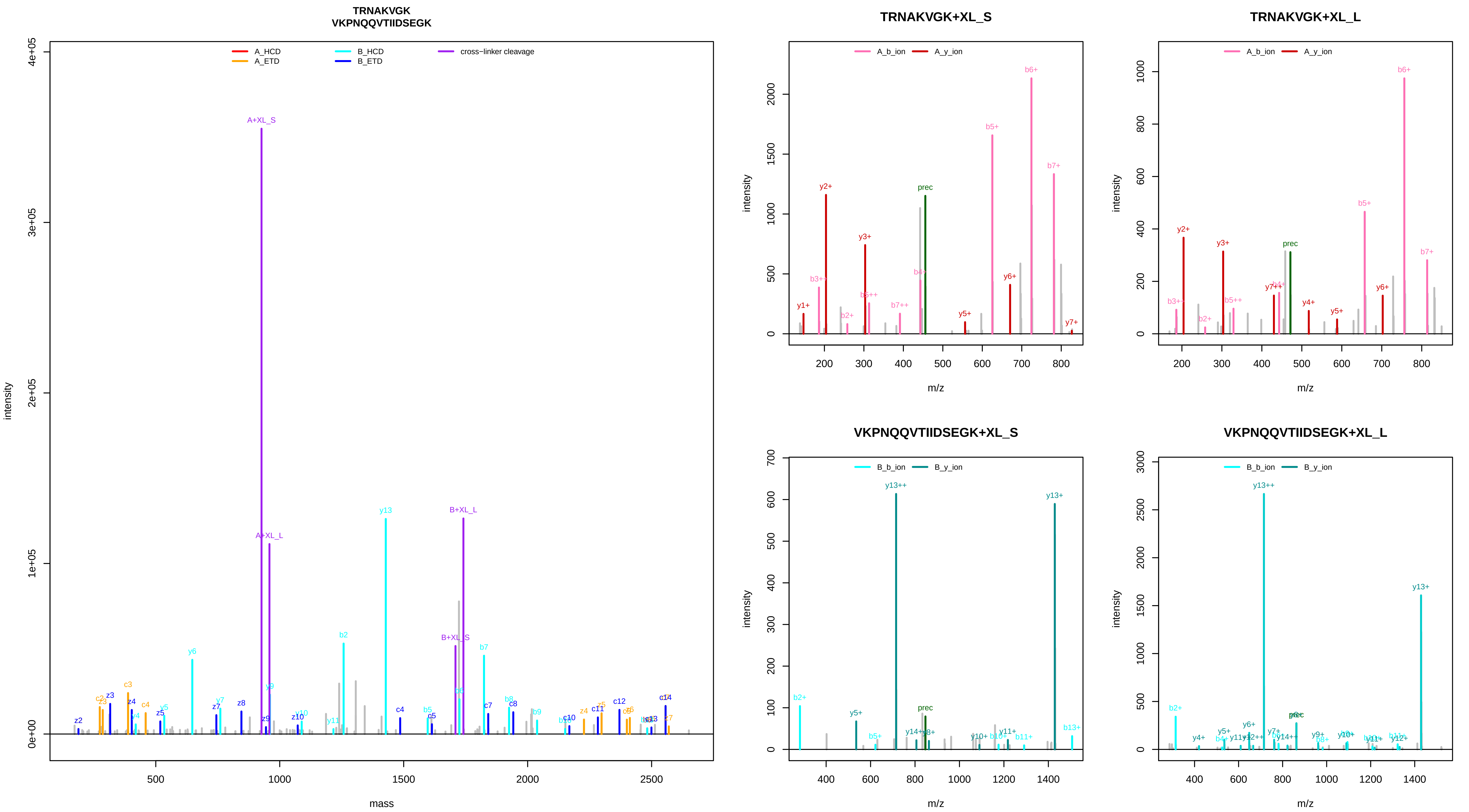

TRNAKVGK
VKPNQQVTIIDSEGK
A_HCD
A_ETD
B_HCD
B_ETD
cross−linker cleavage
A+XL_S
A+XL_L
B+XL_S
B+XL_L
intensity
mass
TRNAKVGK+XL_S
TRNAKVGK+XL_L
A_b_ion
A_y_ion
VKPNQQVTIIDSEGK+XL_S
VKPNQQVTIIDSEGK+XL_L
B_b_ion
B_y_ion
prec
m/z

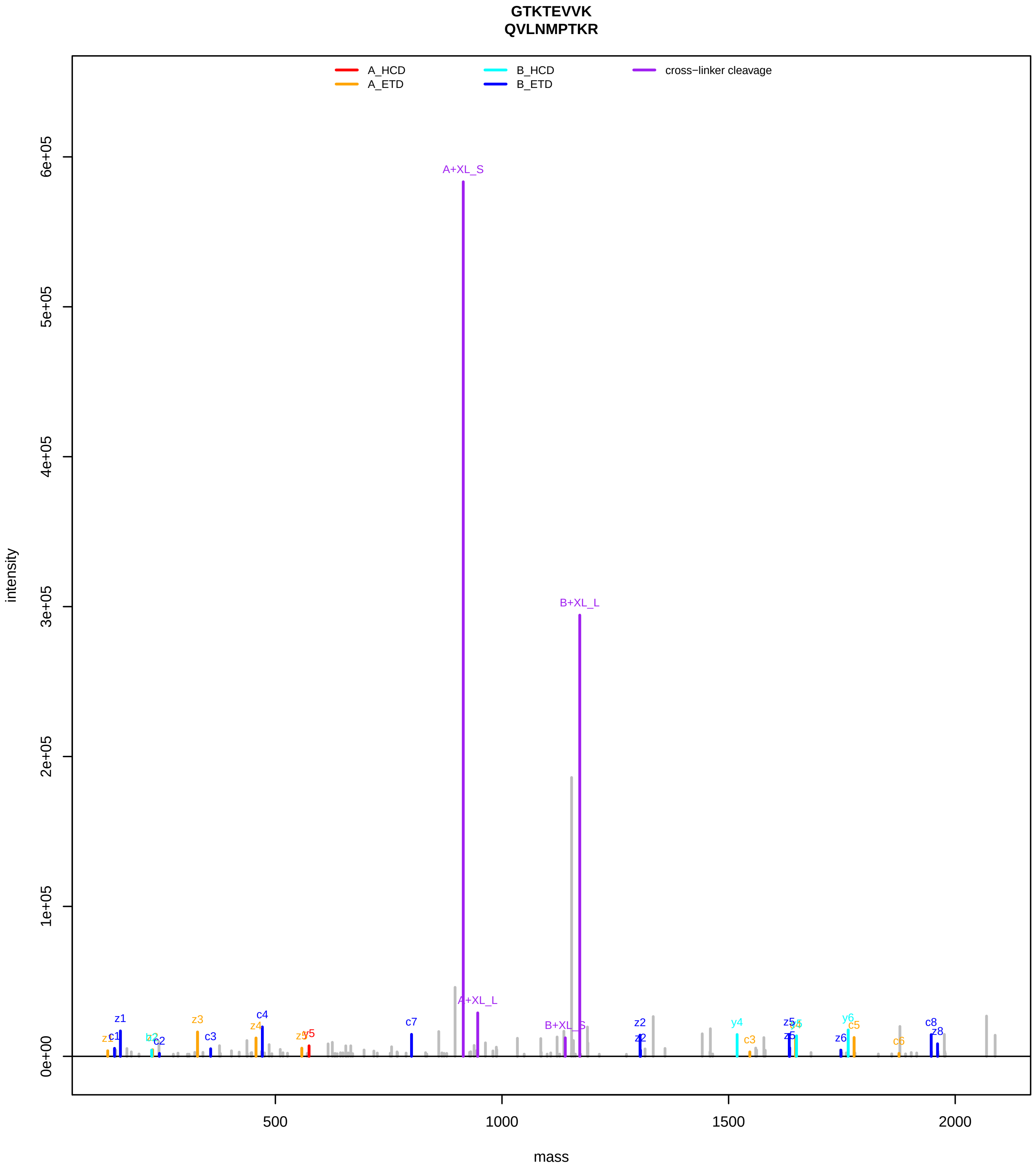

GTKTEVVK+XL_S

GTKTEVVK+XL_L

QVLNMPTKR+XL_S

QVLNMPTKR+XL_L

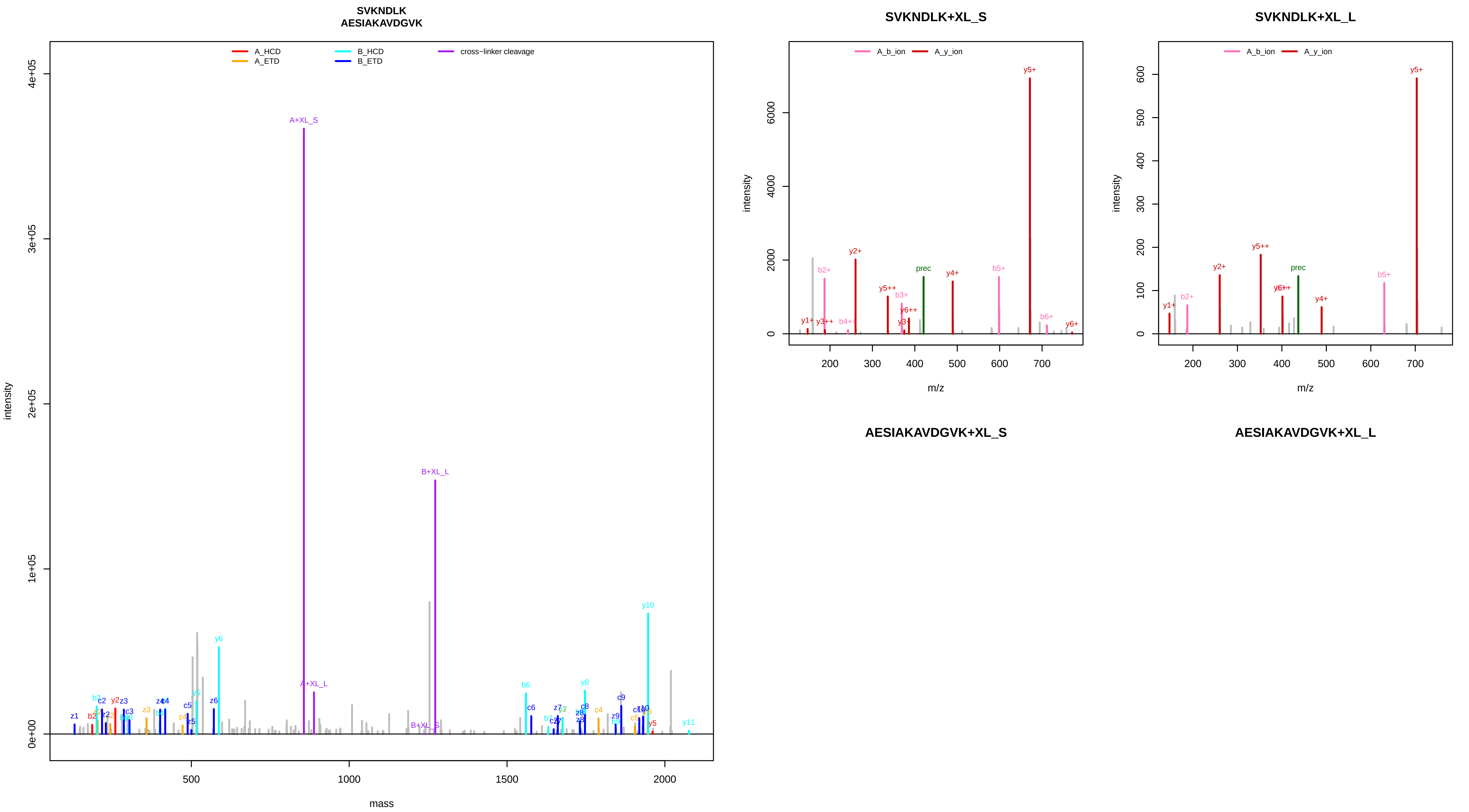
SVKNDLK
AESIAKAVDGVK
A_HCD
A_ETD
B_HCD
B_ETD
cross−linker cleavage
A+XL_S
B+XL_L
A+XL_L
B+XL_S
y6
y10
y8
b6
c6
c8
c9
y11
intensity
mass
SVKNDLK+XL_S
A_b_ion
A_y_ion
y5+
y2+
b5+
prec
y4+
b2+
y5++
b3+
y6++
b6+
y6+
y1+
y3++
b4++
intensity
m/z
SVKNDLK+XL_L
A_b_ion
A_y_ion
y5+
y5++
prec
y2+
b5+
b2+
y1+
y4+
intensity
m/z
AESIAKAVDGVK+XL_S
AESIAKAVDGVK+XL_L

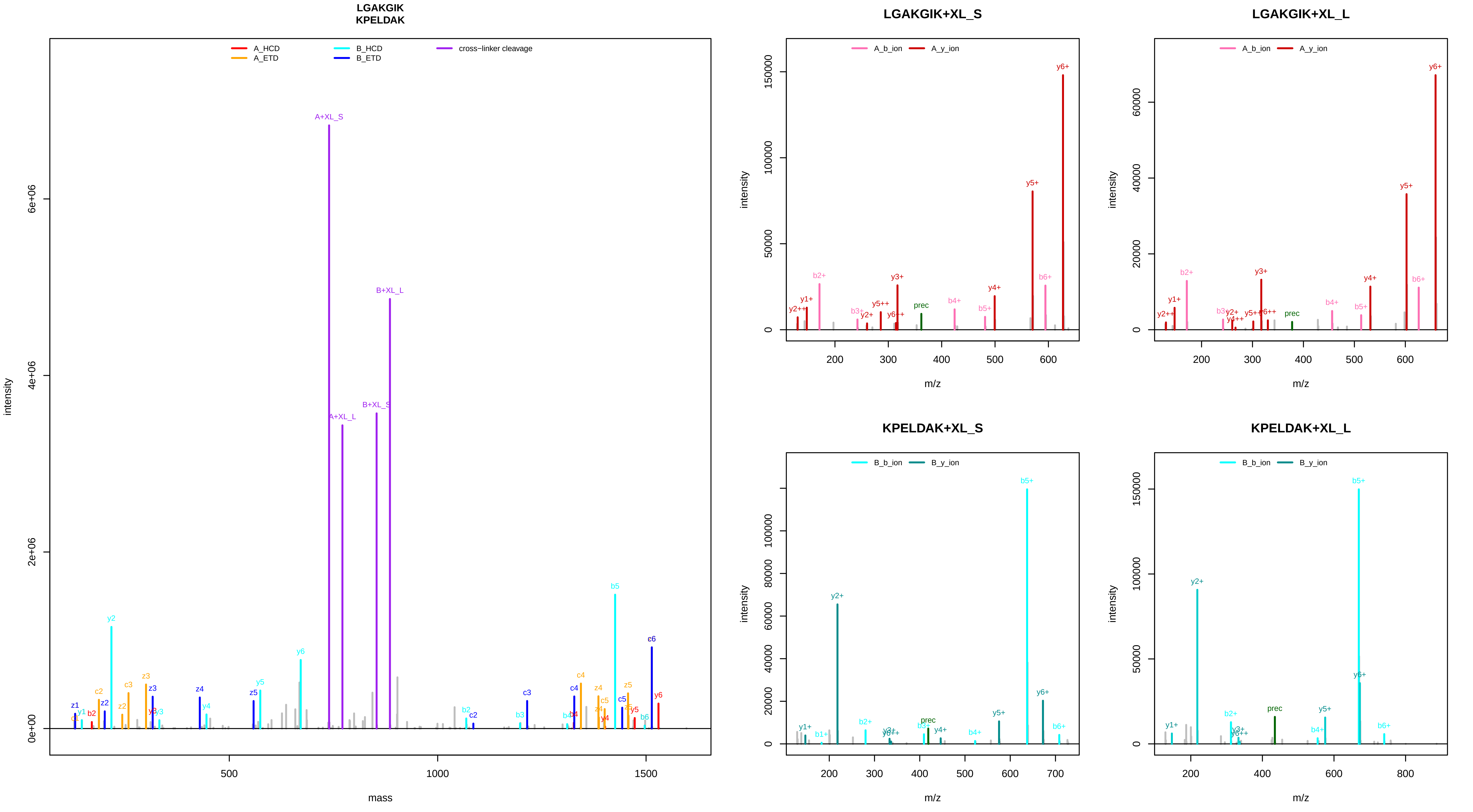

LGAKGIK
KPELDAK
A_HCD
A_ETD
B_HCD
B_ETD
cross−linker cleavage
A+XL_S
B+XL_L
A+XL_L
B+XL_S
intensity
mass
LGAKGIK+XL_S
LGAKGIK+XL_L
KPELDAK+XL_S
KPELDAK+XL_L
A_b_ion
A_y_ion
B_b_ion
B_y_ion
prec
m/z

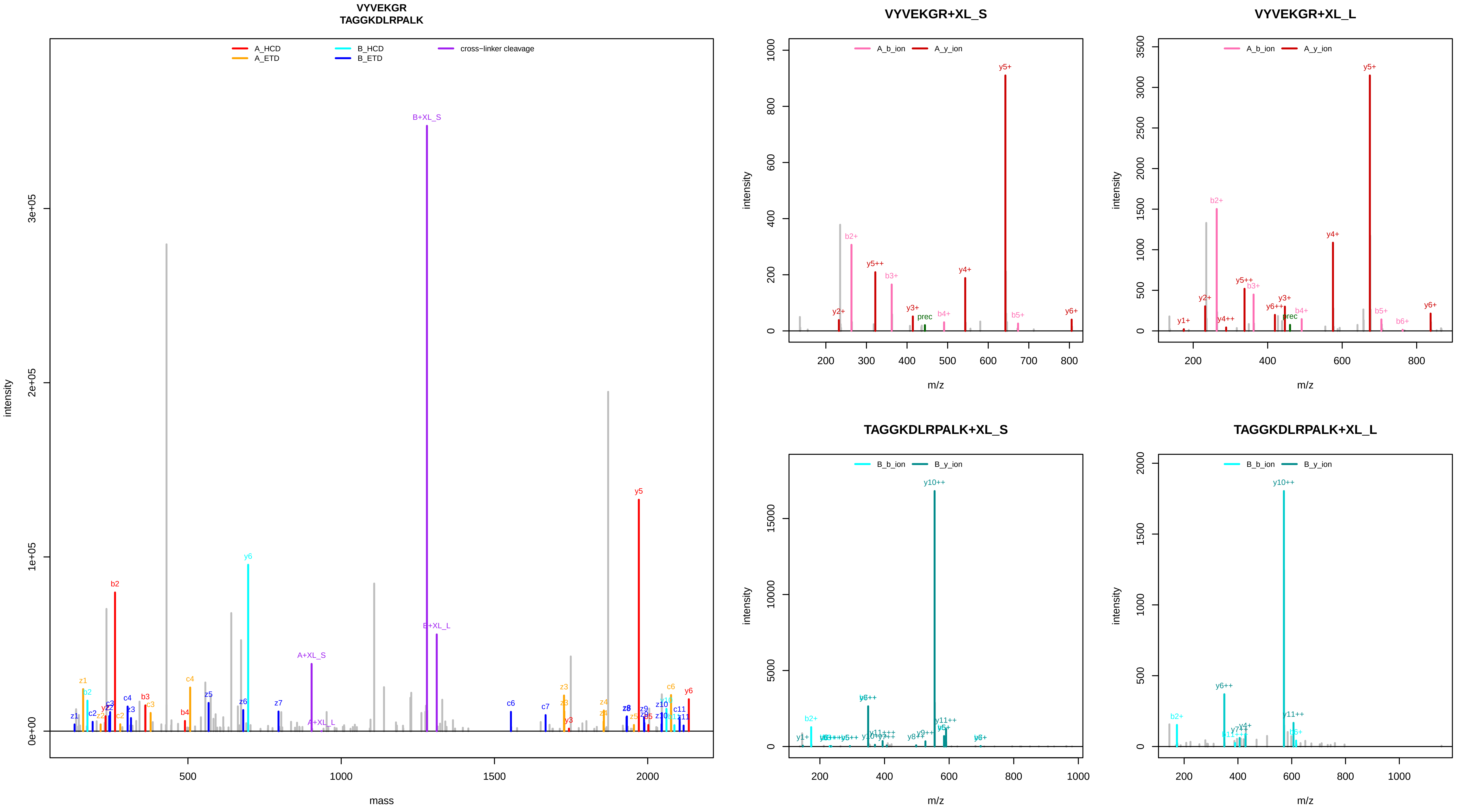

VYVEKGR
TAGGKDLRPALK
A_HCD
A_ETD
B_HCD
B_ETD
cross−linker cleavage
intensity
mass
B+XL_S
B+XL_L
A+XL_S
A+XL_L
VYVEKGR+XL_S
VYVEKGR+XL_L
TAGGKDLRPALK+XL_S
TAGGKDLRPALK+XL_L
A_b_ion
A_y_ion
B_b_ion
B_y_ion
m/z

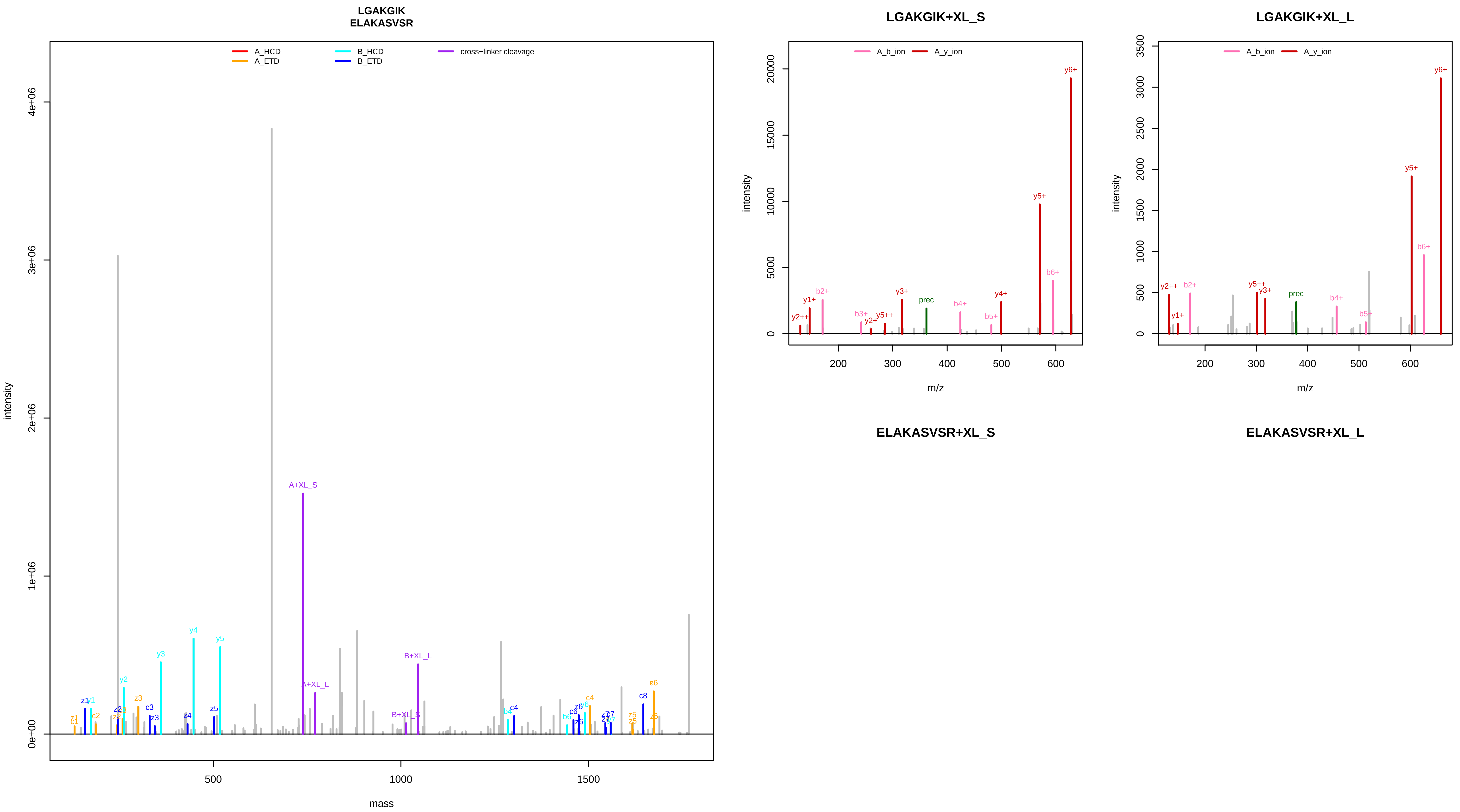
LGAKGIK
ELAKASVSR
A_HCD
A_ETD
B_HCD
B_ETD
cross−linker cleavage
intensity
mass
A+XL_S
A+XL_L
B+XL_S
B+XL_L
LGAKGIK+XL_S
LGAKGIK+XL_L
A_b_ion
A_y_ion
m/z
prec
ELAKASVSR+XL_S
ELAKASVSR+XL_L

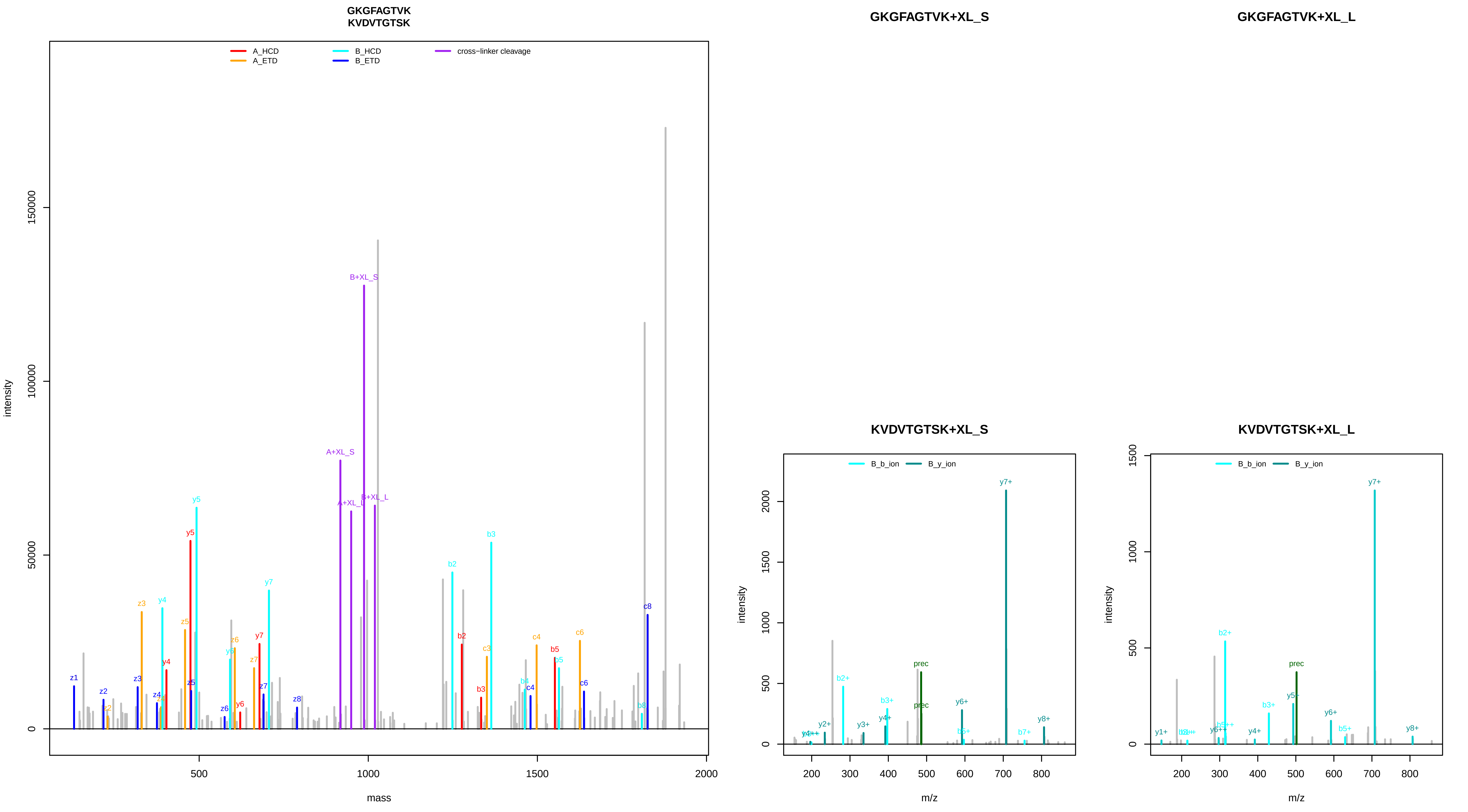
GKGFAGTVK
KVDVTGTSK
A_HCD
A_ETD
B_HCD
B_ETD
cross−linker cleavage
intensity
mass
GKGFAGTVK+XL_S
GKGFAGTVK+XL_L
KVDVTGTSK+XL_S
KVDVTGTSK+XL_L
B_b_ion
B_y_ion
m/z

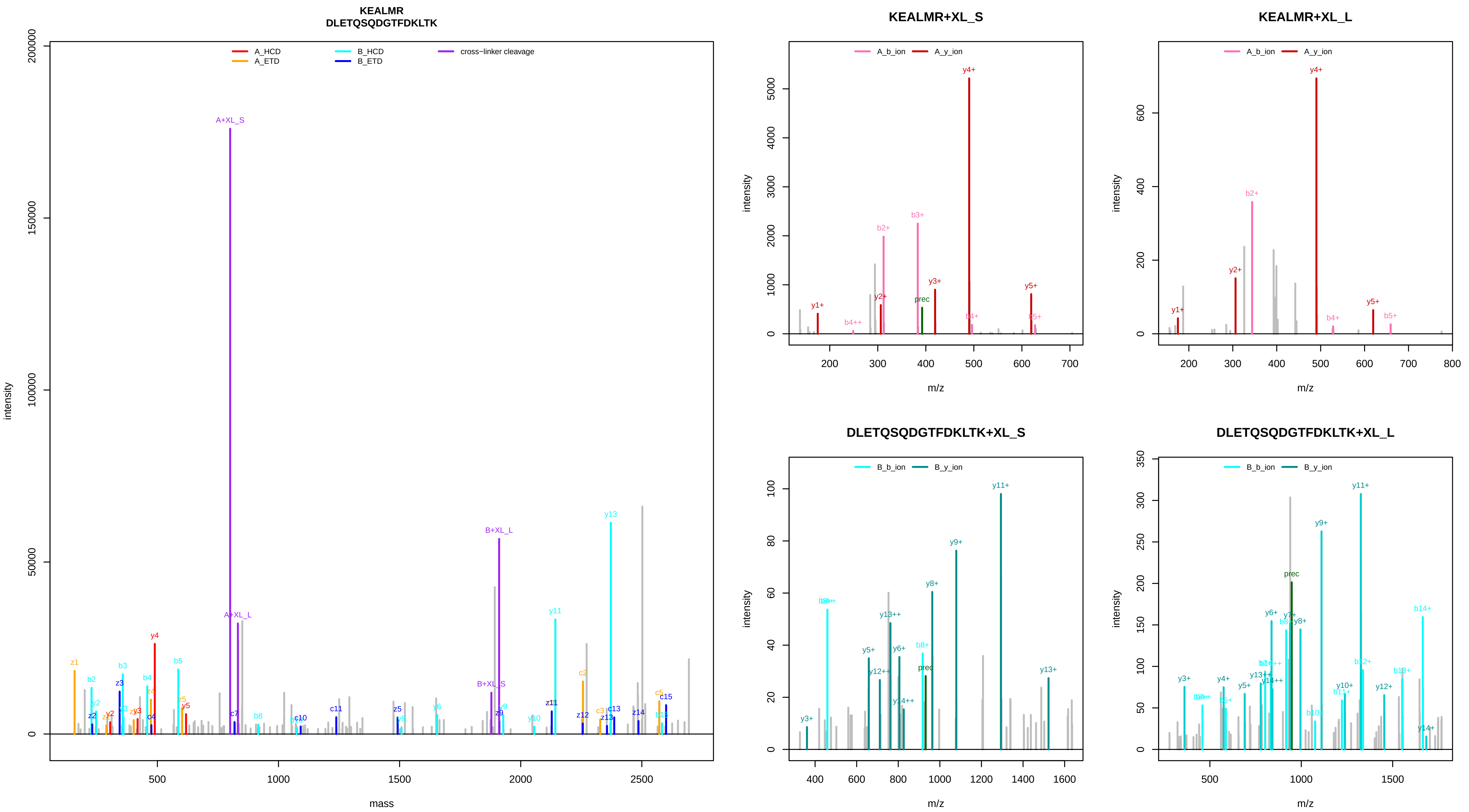

KEALMR
DLETQSQDGTFDKLTK
A_HCD
A_ETD
B_HCD
B_ETD
cross−linker cleavage
A+XL_S
A+XL_L
B+XL_L
B+XL_S
intensity
mass
KEALMR+XL_S
KEALMR+XL_L
DLETQSQDGTFDKLTK+XL_S
DLETQSQDGTFDKLTK+XL_L
A_b_ion
A_y_ion
B_b_ion
B_y_ion
m/z

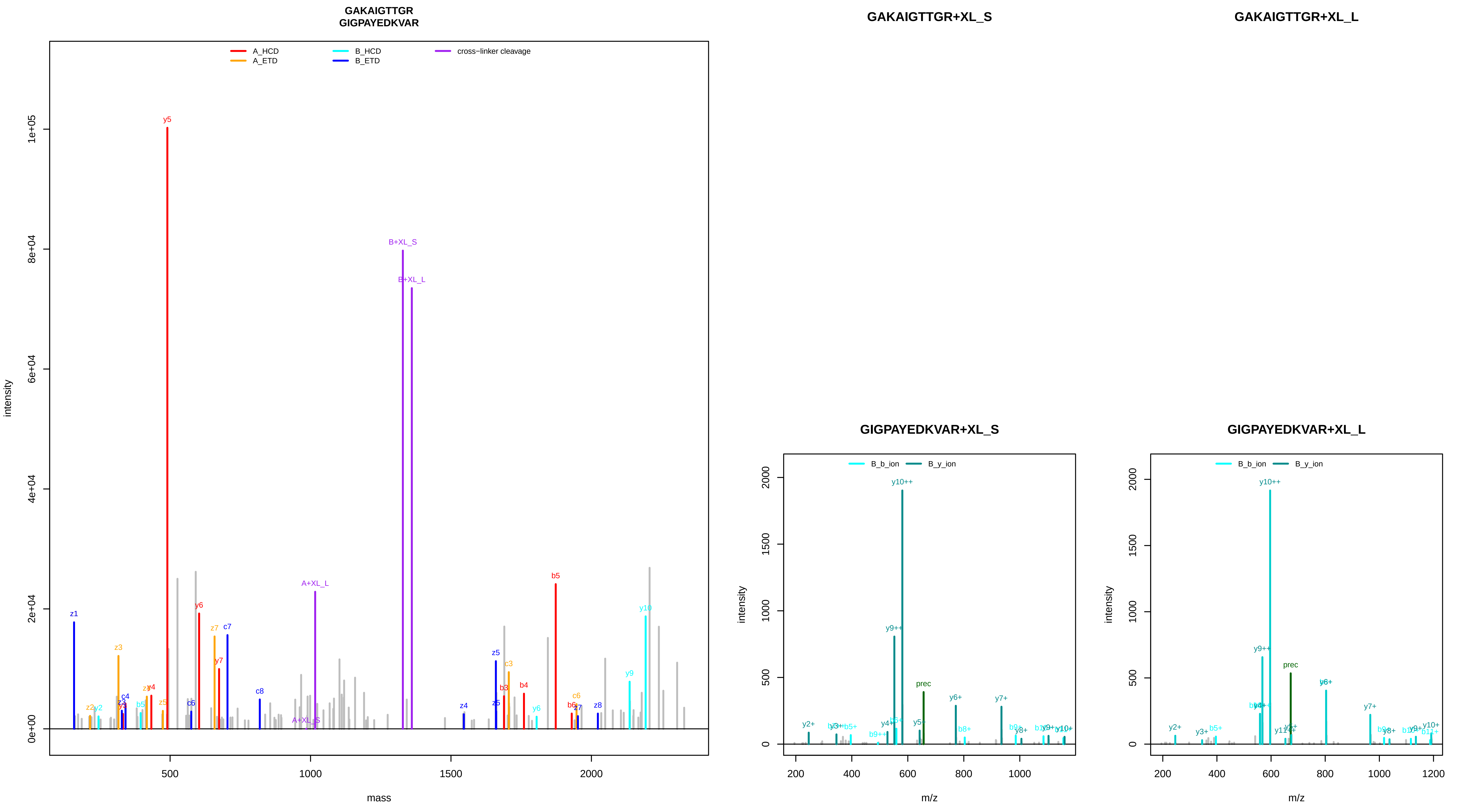
GAKAIGTTGR
GIGPAYEDKVAR
A_HCD
A_ETD
B_HCD
B_ETD
cross−linker cleavage
intensity
mass
B+XL_S
B+XL_L
A+XL_L
A+XL_S
GAKAIGTTGR+XL_S
GAKAIGTTGR+XL_L
GIGPAYEDKVAR+XL_S
GIGPAYEDKVAR+XL_L
B_b_ion
B_y_ion
y10++
y9++
prec
y6+
y7+
m/z

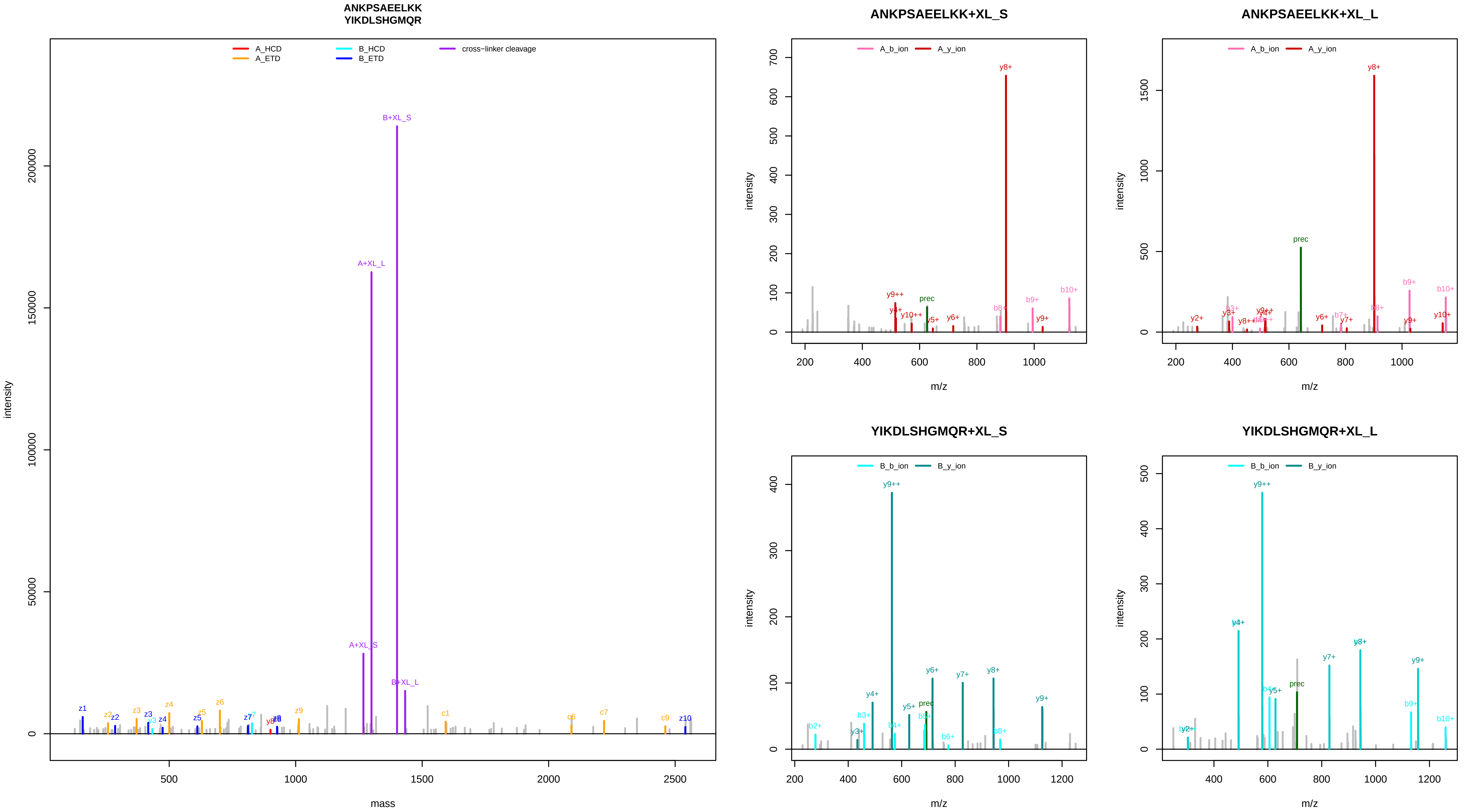

ANKPSAEELKK
YIKDLSHGMQR
A_HCD
A_ETD
B_HCD
B_ETD
cross−linker cleavage
B+XL_S
A+XL_L
A+XL_S
B+XL_L
intensity
mass
ANKPSAEELKK+XL_S
ANKPSAEELKK+XL_L
YIKDLSHGMQR+XL_S
YIKDLSHGMQR+XL_L
A_b_ion
A_y_ion
B_b_ion
B_y_ion
prec
m/z

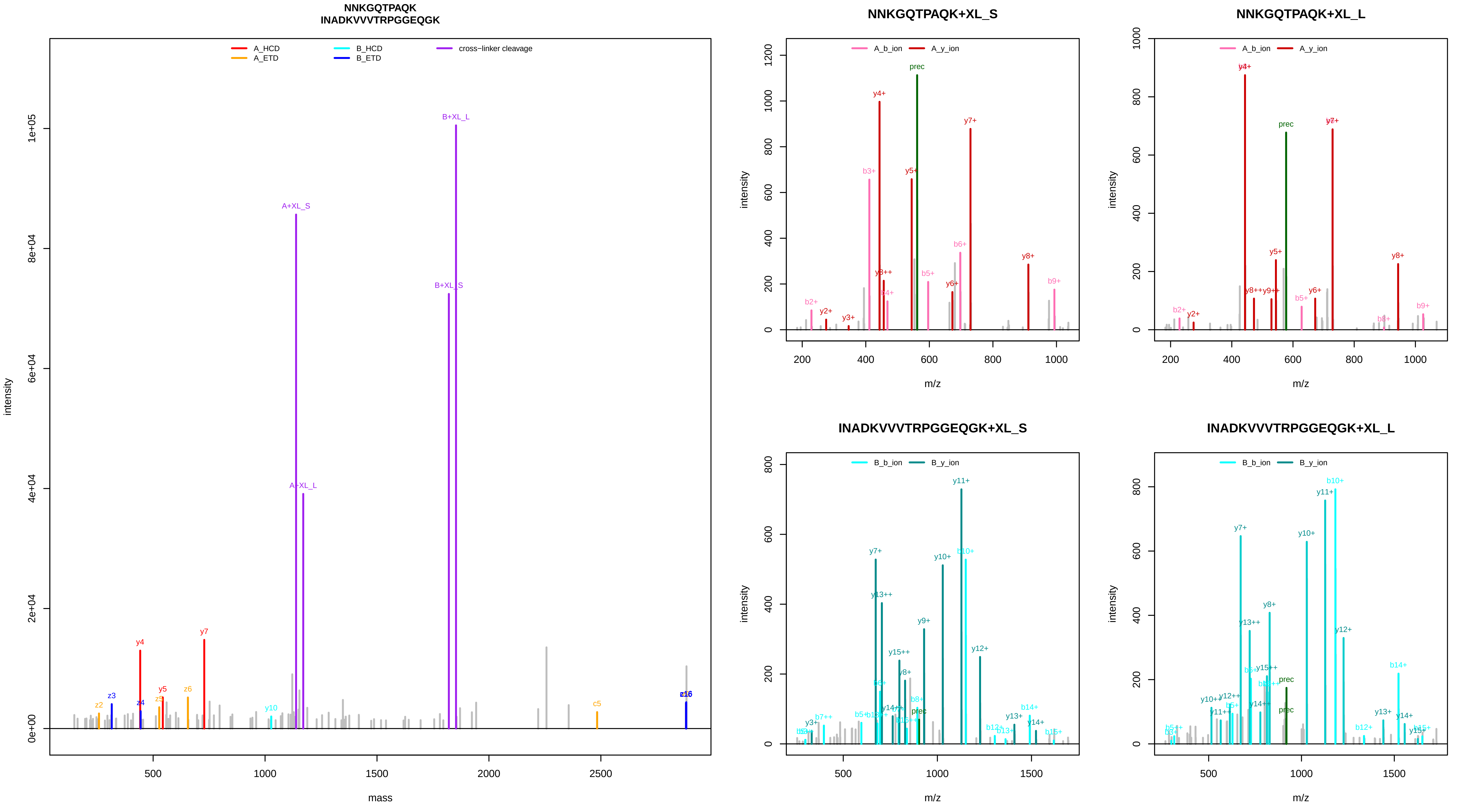
NNKGQTPAQK
INADKVVVTRPGGEQGK
A_HCD
A_ETD
B_HCD
B_ETD
cross−linker cleavage
intensity
mass
A+XL_S
A+XL_L
B+XL_L
B+XL_S
y4
y5
y7
z2
z3
z4
z5
z6
y10
c5
z16
NNKGQTPAQK+XL_S
NNKGQTPAQK+XL_L
A_b_ion
A_y_ion
prec
m/z
INADKVVVTRPGGEQGK+XL_S
INADKVVVTRPGGEQGK+XL_L
B_b_ion
B_y_ion

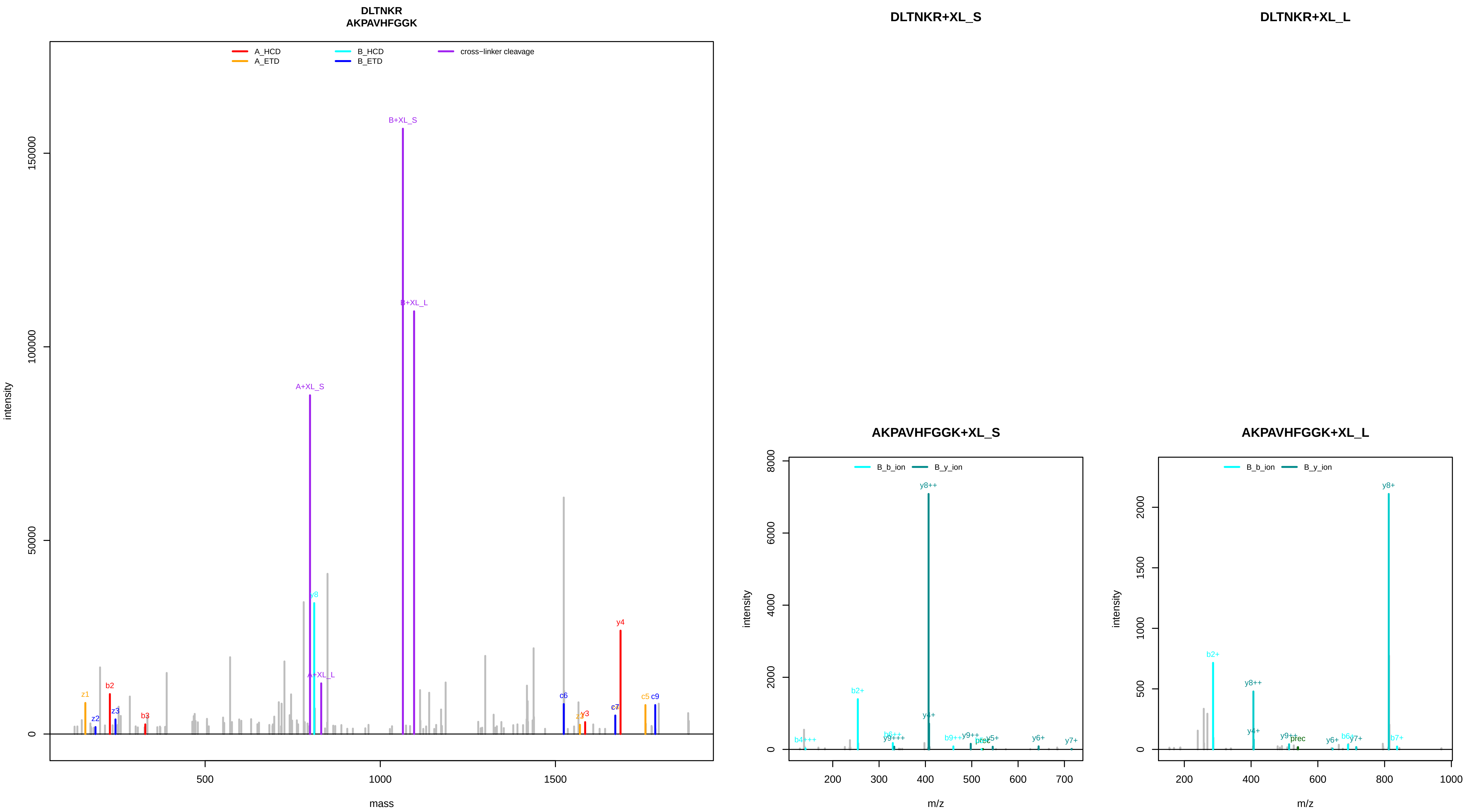
DLTNKR
AKPAVHFGGK
A_HCD
A_ETD
B_HCD
B_ETD
cross−linker cleavage
B+XL_S
B+XL_L
A+XL_S
A+XL_L
y8
y4
z1
b2
z2
z3
b3
c6
z3
y3
c7
c5
c9
intensity
mass
0
50000
100000
150000
500
1000
1500
DLTNKR+XL_S
DLTNKR+XL_L
AKPAVHFGGK+XL_S
AKPAVHFGGK+XL_L
B_b_ion
B_y_ion
y8++
b2+
y4+
b9++
y9++
prec
y5+
y6+
y7+
y9+++
b4+++
intensity
m/z
0
2000
4000
6000
8000
200
300
400
500
600
700
y8+
y8++
b2+
y4+
y9++
prec
y6+
b6+
y7+
b7+
0
500
1000
1500
2000
200
400
600
800
1000

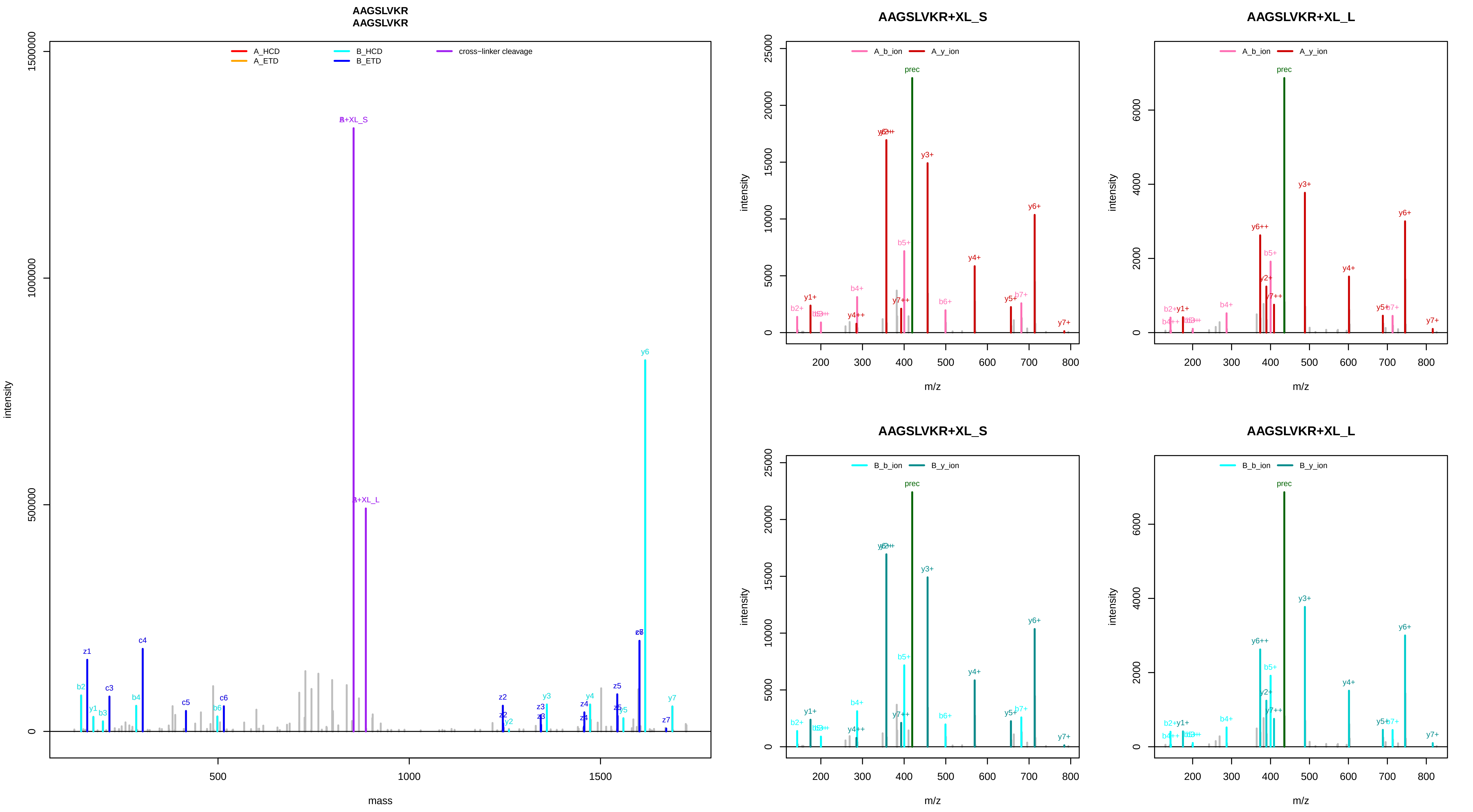
AAGSLVKR
AAGSLVKR
A_HCD
A_ETD
B_HCD
B_ETD
cross−linker cleavage
intensity
mass
AAGSLVKR+XL_S
AAGSLVKR+XL_L
A_b_ion
A_y_ion
B_b_ion
B_y_ion
prec
intensity
m/z

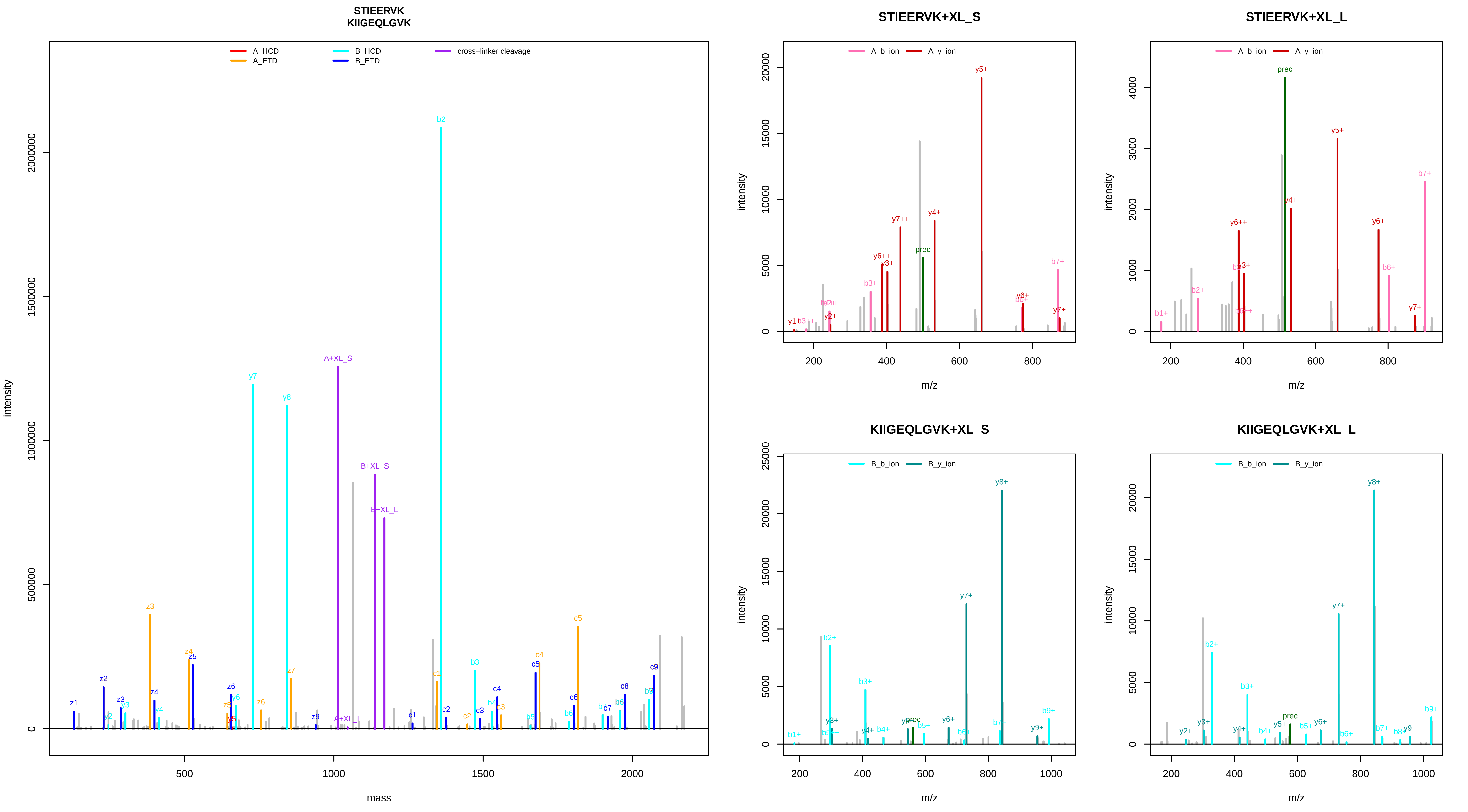
STIEERVK
KIIGEQLGVK
A_HCD
A_ETD
B_HCD
B_ETD
cross−linker cleavage
intensity
mass
STIEERVK+XL_S
STIEERVK+XL_L
KIIGEQLGVK+XL_S
KIIGEQLGVK+XL_L
A_b_ion
A_y_ion
B_b_ion
B_y_ion
m/z
A+XL_S
B+XL_S
B+XL_L
A+XL_L
prec

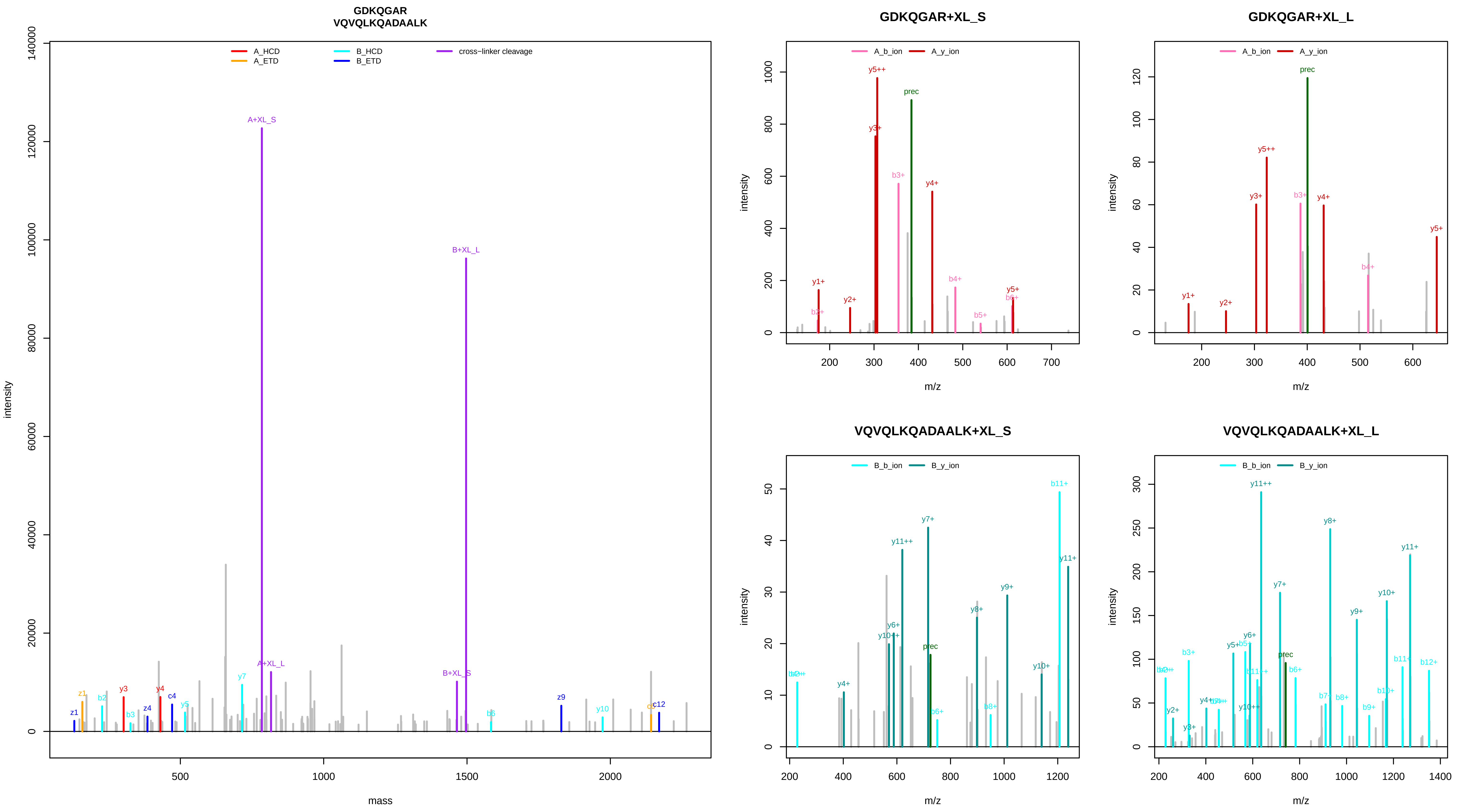
GDKQGAR
VQVQLKQADAALK
A_HCD
A_ETD
B_HCD
B_ETD
cross−linker cleavage
A+XL_S
B+XL_L
A+XL_L
B+XL_S
intensity
mass
GDKQGAR+XL_S
GDKQGAR+XL_L
VQVQLKQADAALK+XL_S
VQVQLKQADAALK+XL_L
A_b_ion
A_y_ion
B_b_ion
B_y_ion
prec
m/z

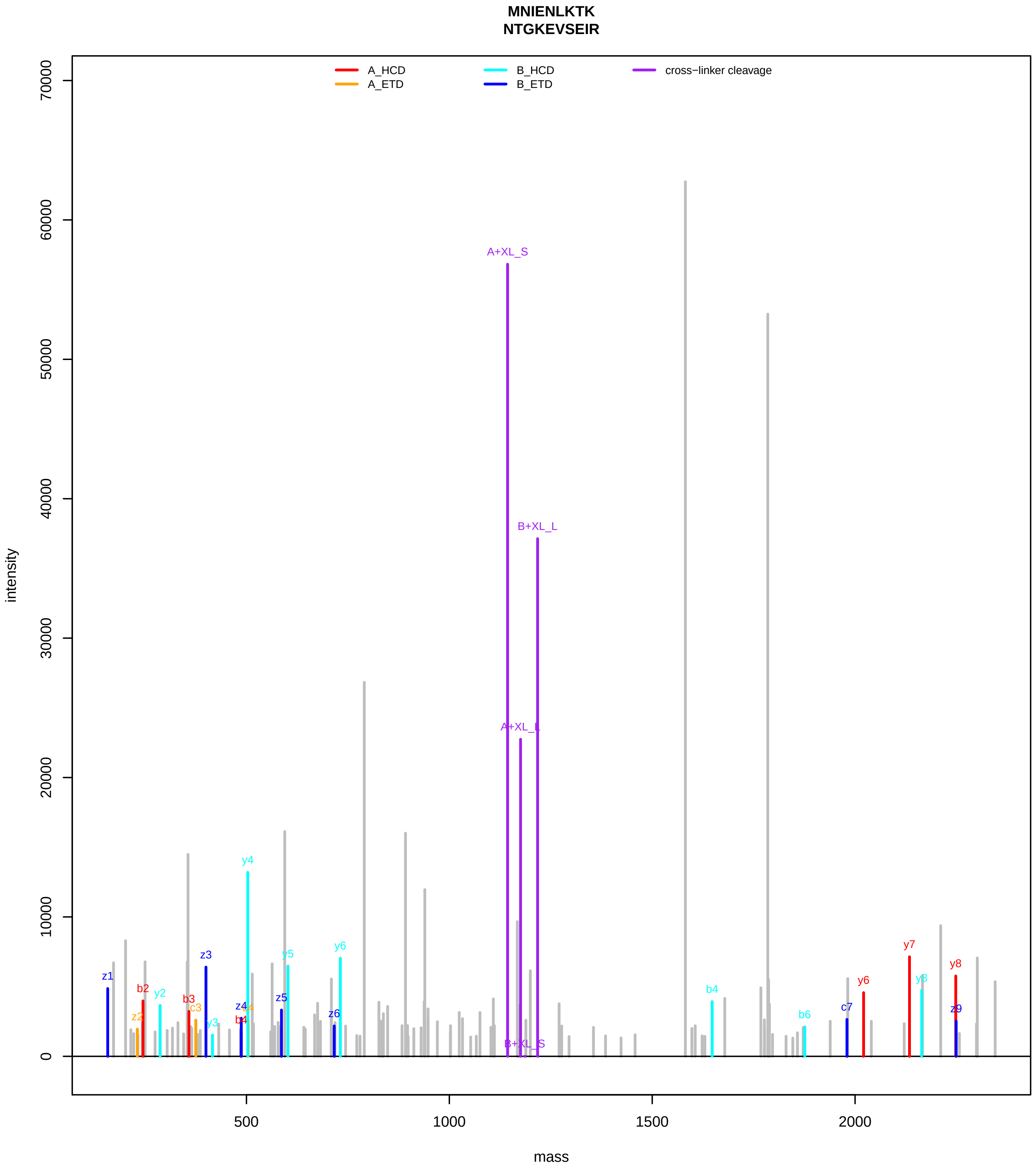

MNIENLKTK+XL_S

MNIENLKTK+XL_L

NTGKEVSEIR+XL_S

NTGKEVSEIR+XL_L

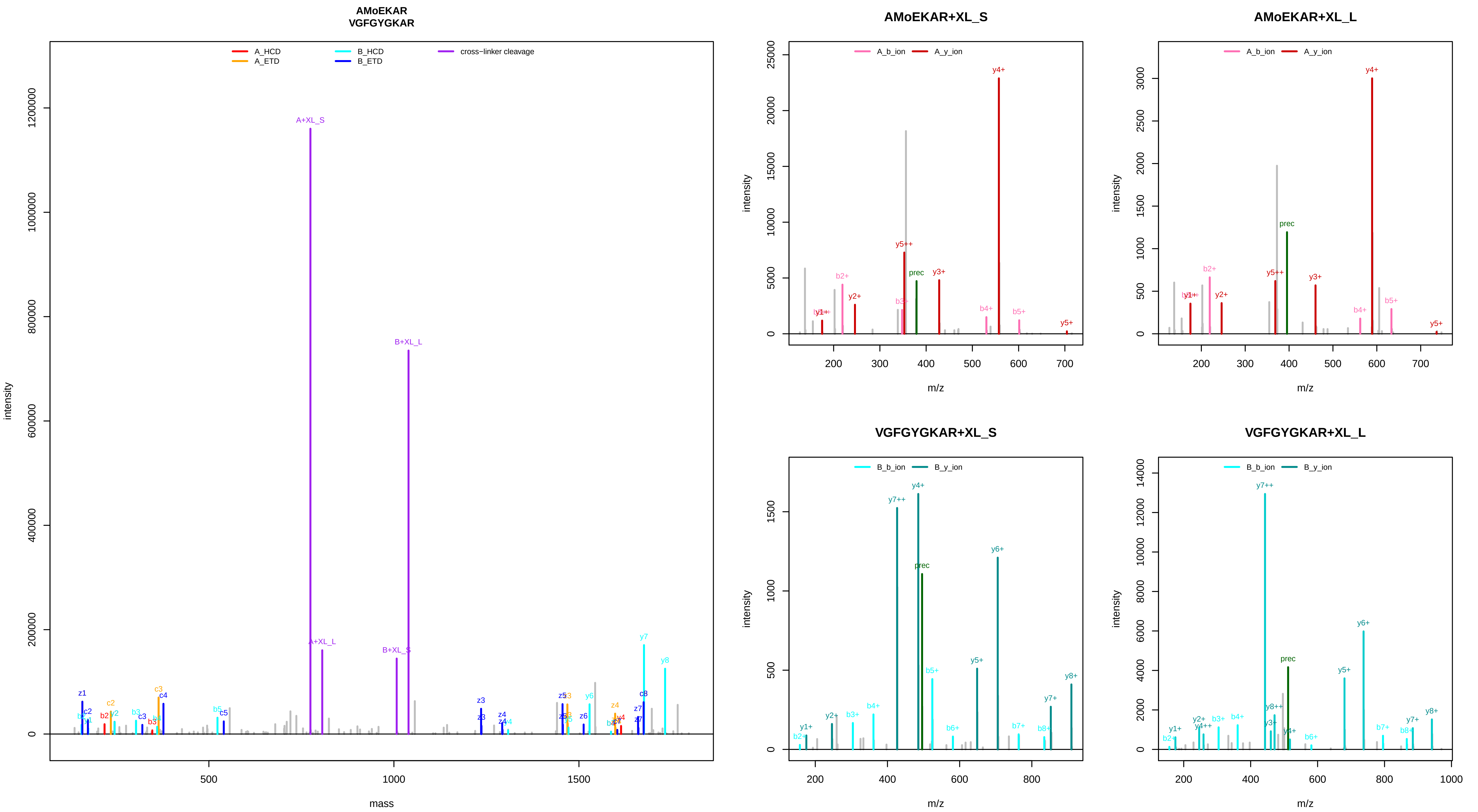
AMoEKAR
VGFGYGKAR
A_HCD
A_ETD
B_HCD
B_ETD
cross−linker cleavage
A+XL_S
B+XL_L
A+XL_L
B+XL_S
intensity
mass
AMoEKAR+XL_S
AMoEKAR+XL_L
VGFGYGKAR+XL_S
VGFGYGKAR+XL_L
A_b_ion
A_y_ion
B_b_ion
B_y_ion
prec
m/z
intensity

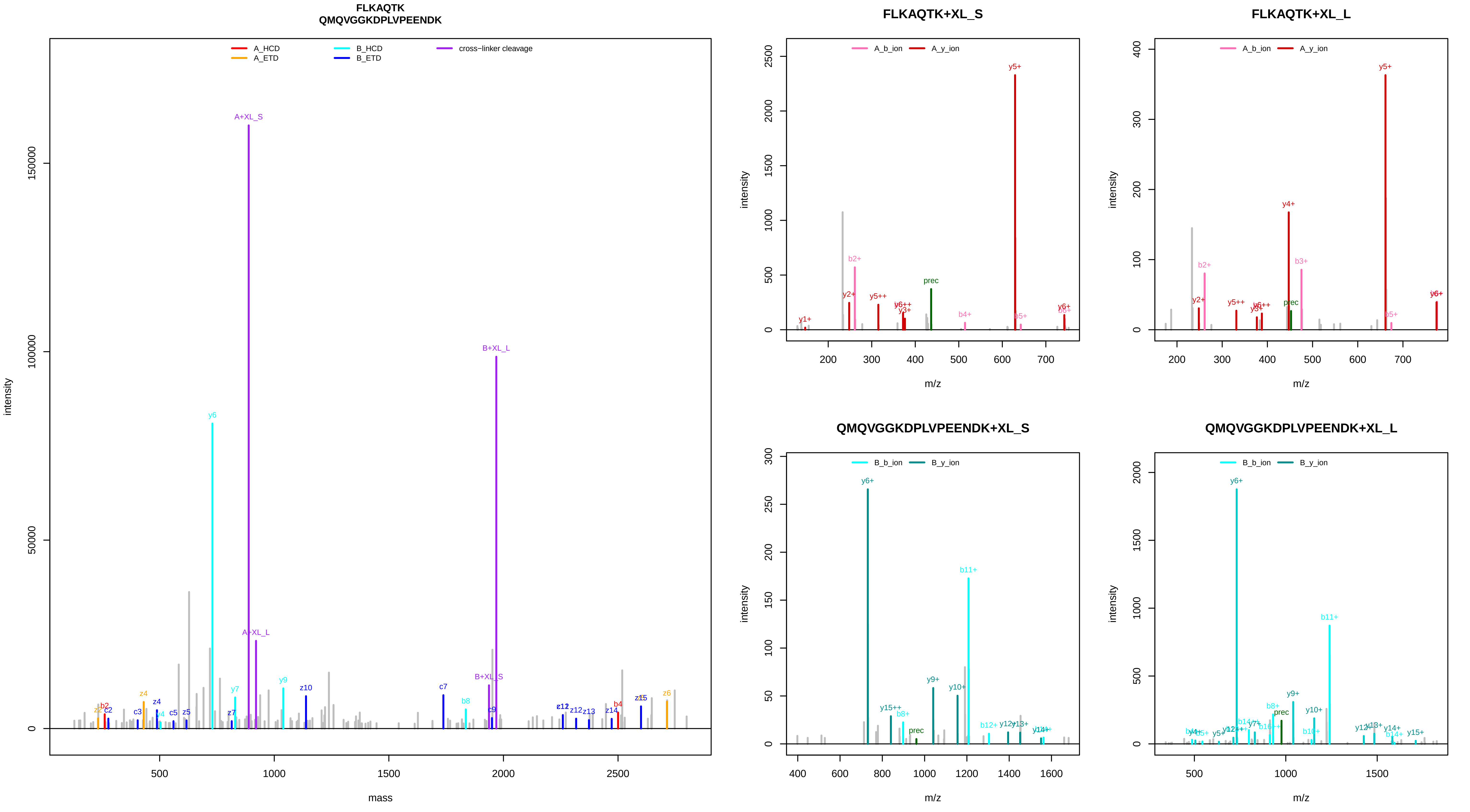
FLKAQTK
QMQVGGKDPLVPEENDK
A_HCD
A_ETD
B_HCD
B_ETD
cross−linker cleavage
A+XL_S
B+XL_L
A+XL_L
B+XL_S
intensity
mass
FLKAQTK+XL_S
FLKAQTK+XL_L
QMQVGGKDPLVPEENDK+XL_S
QMQVGGKDPLVPEENDK+XL_L
A_b_ion
A_y_ion
B_b_ion
B_y_ion
m/z
prec

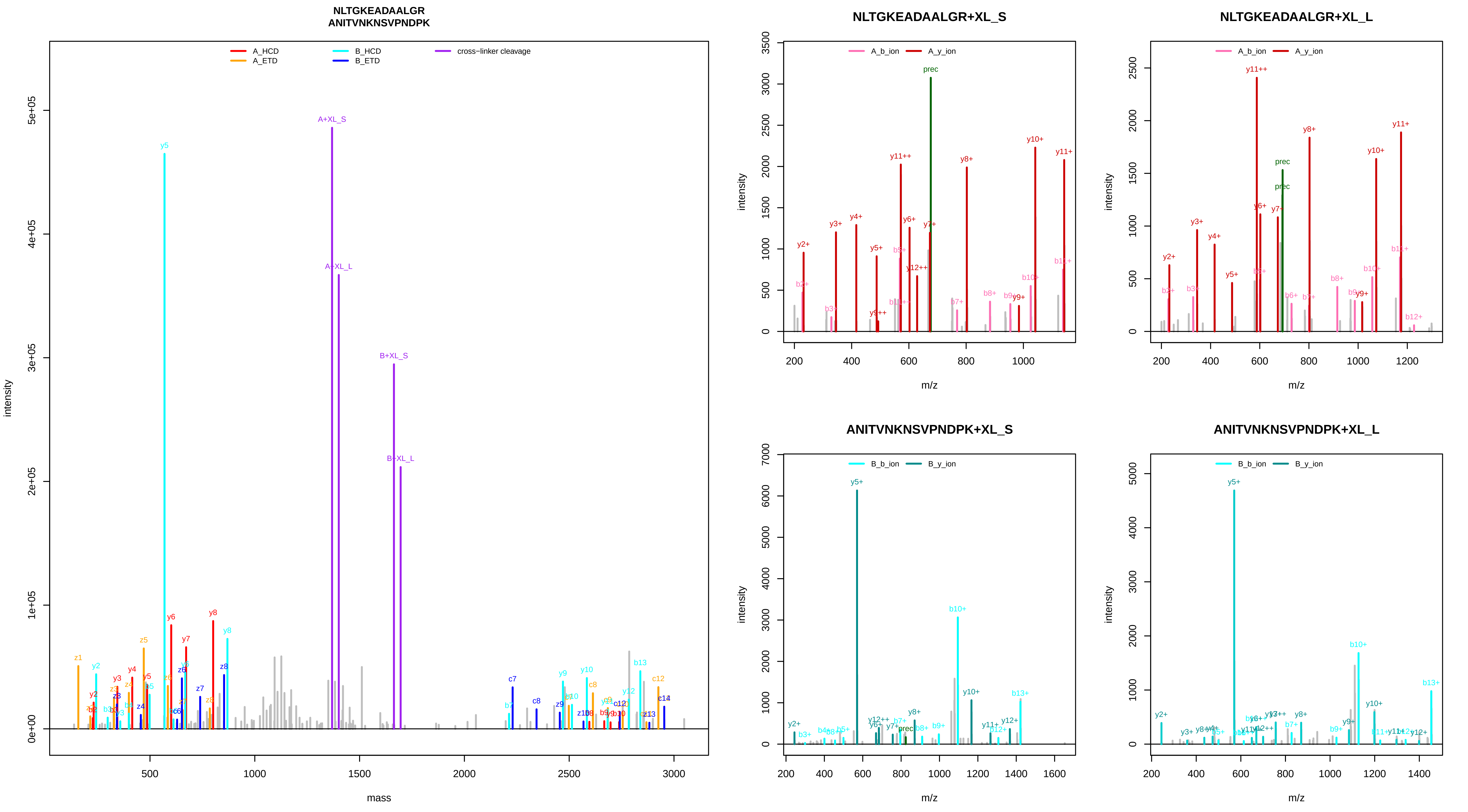
NLTGKEADAALGR
ANITVNKNSVPNDPK
A_HCD
A_ETD
B_HCD
B_ETD
cross−linker cleavage
A+XL_S
A+XL_L
B+XL_S
B+XL_L
intensity
mass
NLTGKEADAALGR+XL_S
NLTGKEADAALGR+XL_L
ANITVNKNSVPNDPK+XL_S
ANITVNKNSVPNDPK+XL_L
A_b_ion
A_y_ion
B_b_ion
B_y_ion
prec
m/z

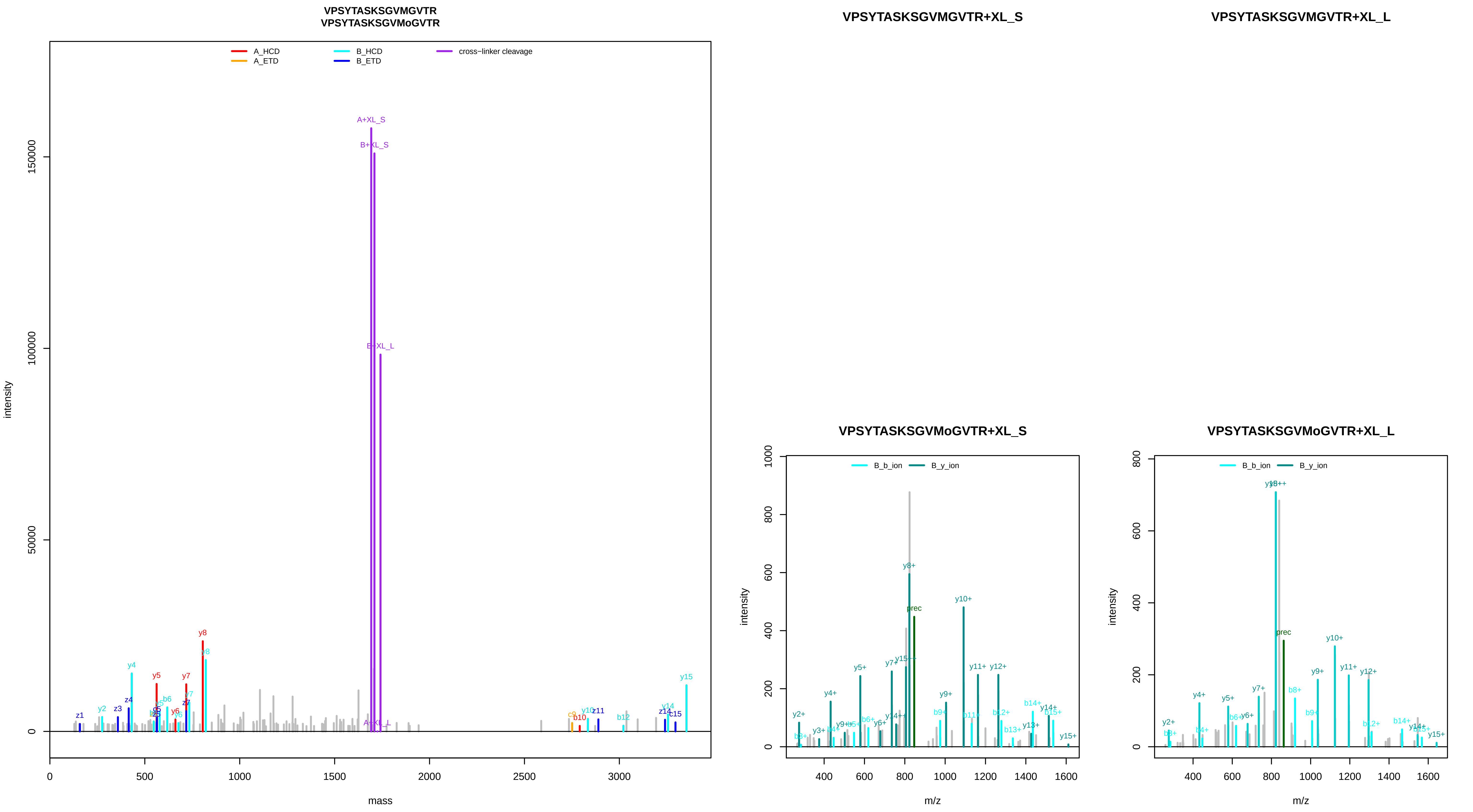
VPSYTASKSGVMGVTR
VPSYTASKSGVMoGVTR
A_HCD
A_ETD
B_HCD
B_ETD
cross−linker cleavage
A+XL_S
B+XL_S
B+XL_L
A+XL_L
intensity
mass
VPSYTASKSGVMGVTR+XL_S
VPSYTASKSGVMGVTR+XL_L
VPSYTASKSGVMoGVTR+XL_S
VPSYTASKSGVMoGVTR+XL_L
B_b_ion
B_y_ion
prec
intensity
m/z

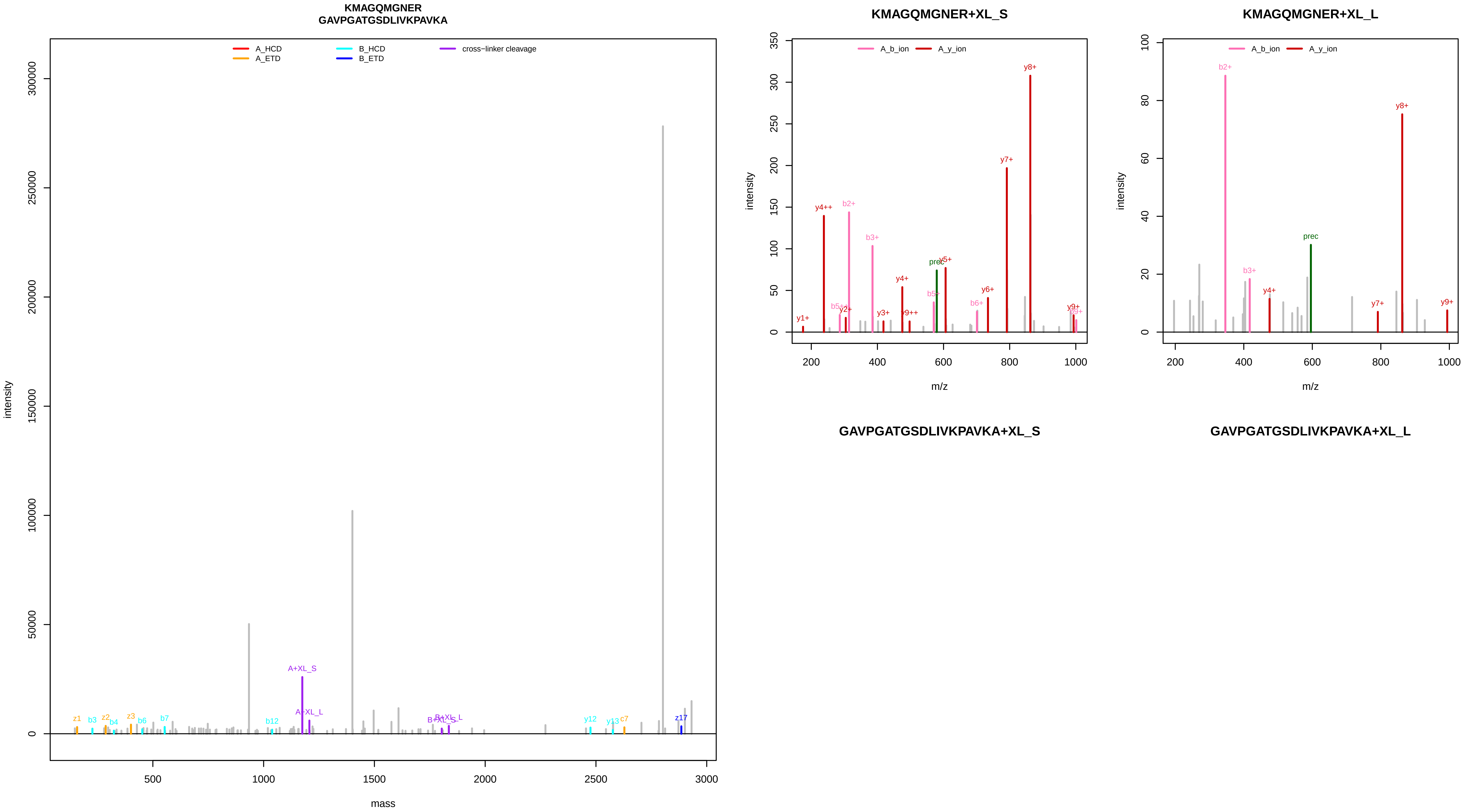
KMAGQMGNER
GAVPGATGSDLIVKPAVKA
A_HCD
A_ETD
B_HCD
B_ETD
cross−linker cleavage
intensity
mass
A+XL_S
A+XL_L
KMAGQMGNER+XL_S
KMAGQMGNER+XL_L
A_b_ion
A_y_ion
prec
m/z
GAVPGATGSDLIVKPAVKA+XL_S
GAVPGATGSDLIVKPAVKA+XL_L

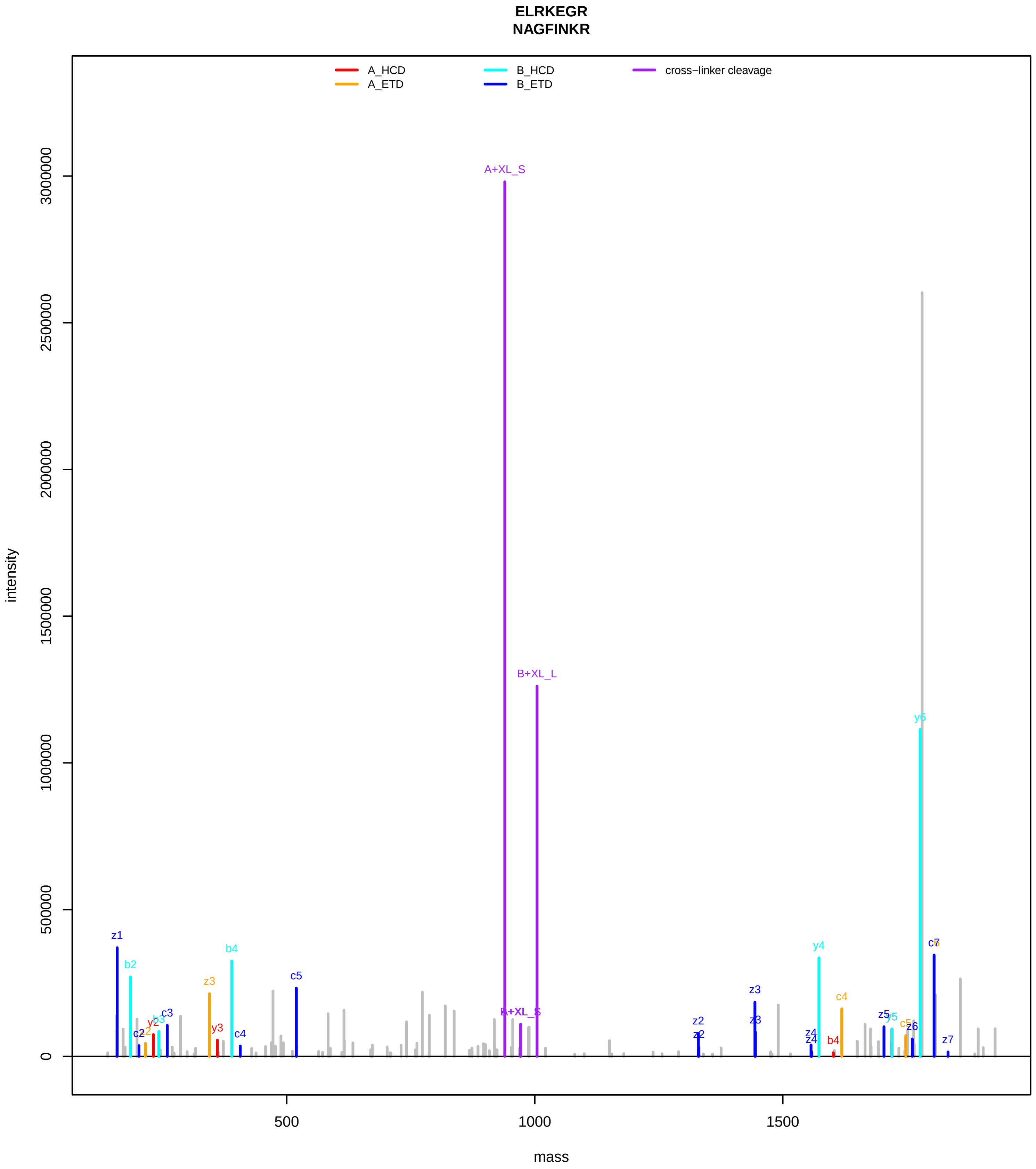

ELRKEGR+XL_S

ELRKEGR+XL_L

NAGFINKR+XL_S

NAGFINKR+XL_L

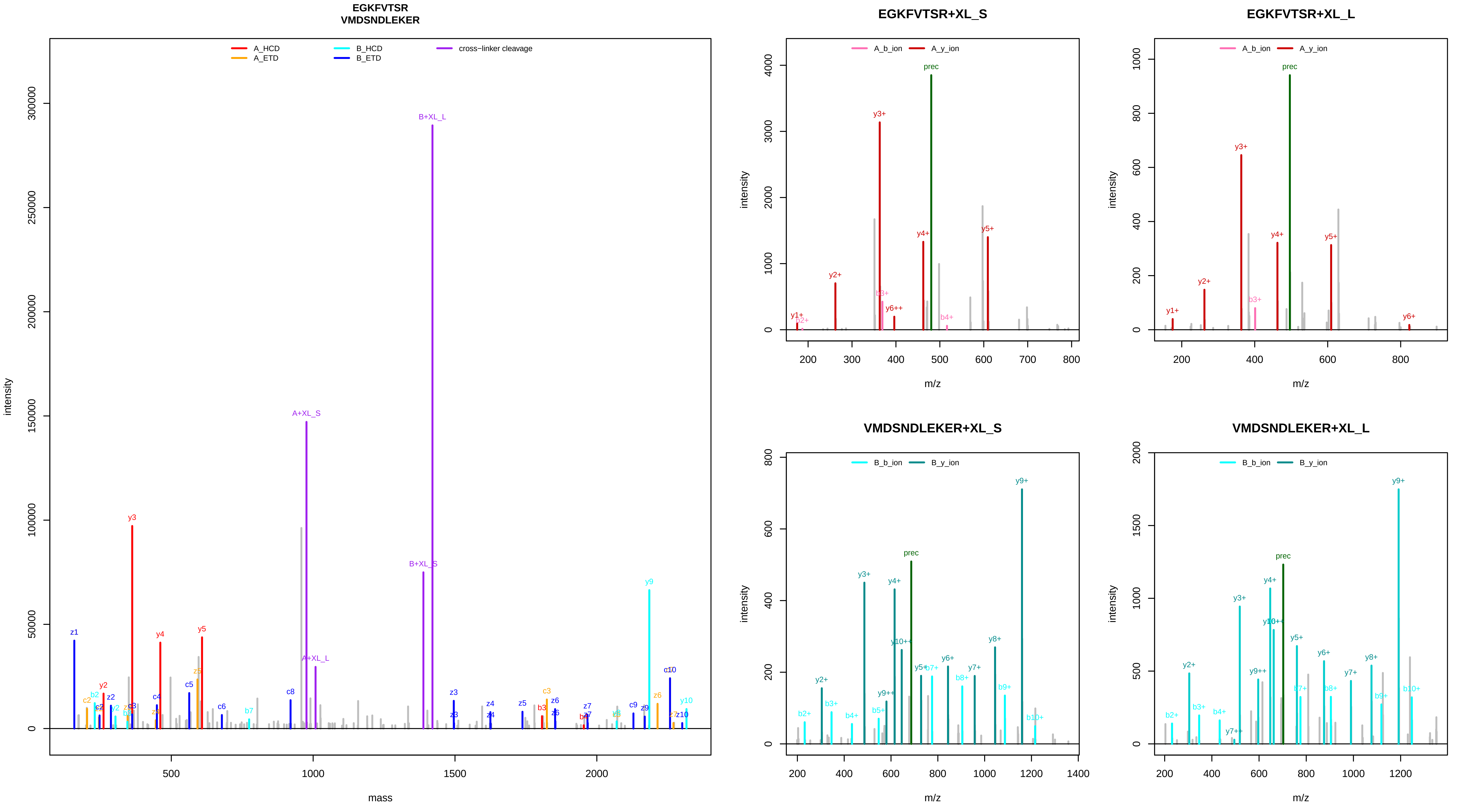
EGKFVTSR
VMDSNDLEKER
A_HCD
A_ETD
B_HCD
B_ETD
cross−linker cleavage
intensity
mass
EGKFVTSR+XL_S
EGKFVTSR+XL_L
VMDSNDLEKER+XL_S
VMDSNDLEKER+XL_L
A_b_ion
A_y_ion
B_b_ion
B_y_ion
prec
m/z

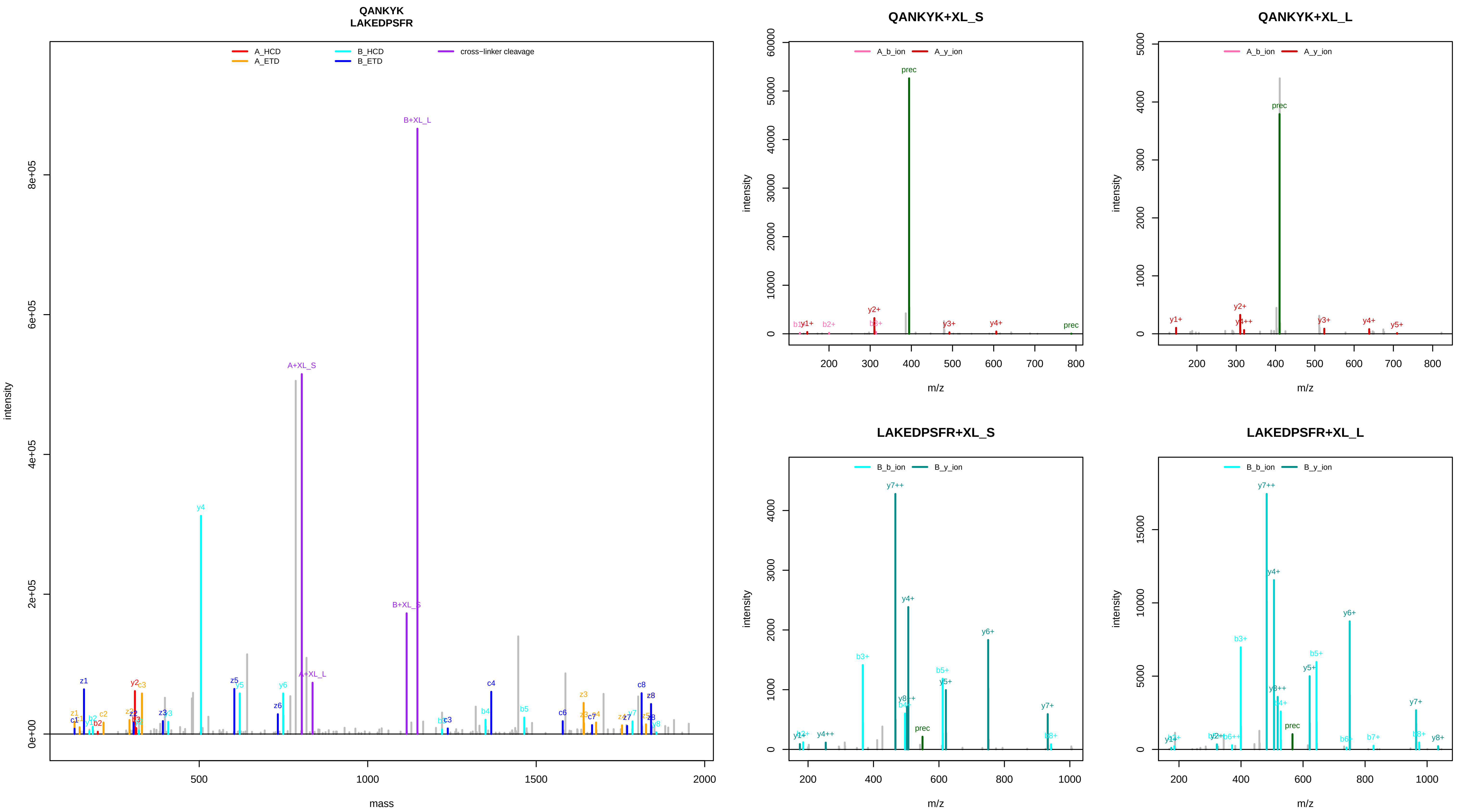

QANKYK
LAKEDPSFR
A_HCD
A_ETD
B_HCD
B_ETD
cross−linker cleavage
B+XL_L
A+XL_S
B+XL_S
A+XL_L
intensity
mass
QANKYK+XL_S
QANKYK+XL_L
LAKEDPSFR+XL_S
LAKEDPSFR+XL_L
A_b_ion
A_y_ion
B_b_ion
B_y_ion
prec
m/z

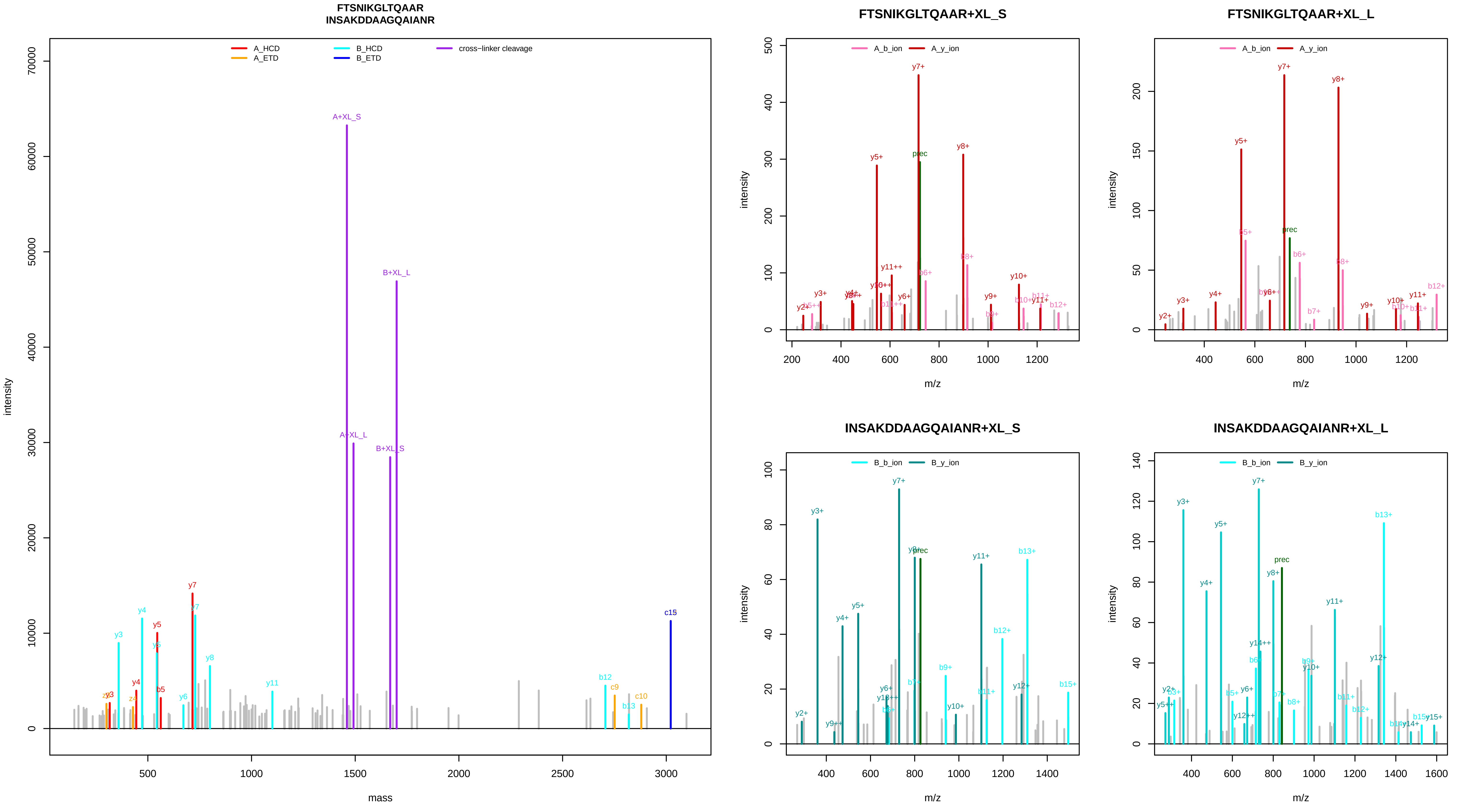

FTSNIKGLTQAAR
INSAKDDAAGQAIANR
A_HCD
A_ETD
B_HCD
B_ETD
cross−linker cleavage
A+XL_S
B+XL_L
A+XL_L
B+XL_S
c15
mass
intensity
FTSNIKGLTQAAR+XL_S
FTSNIKGLTQAAR+XL_L
INSAKDDAAGQAIANR+XL_S
INSAKDDAAGQAIANR+XL_L
A_b_ion
A_y_ion
B_b_ion
B_y_ion
prec
m/z

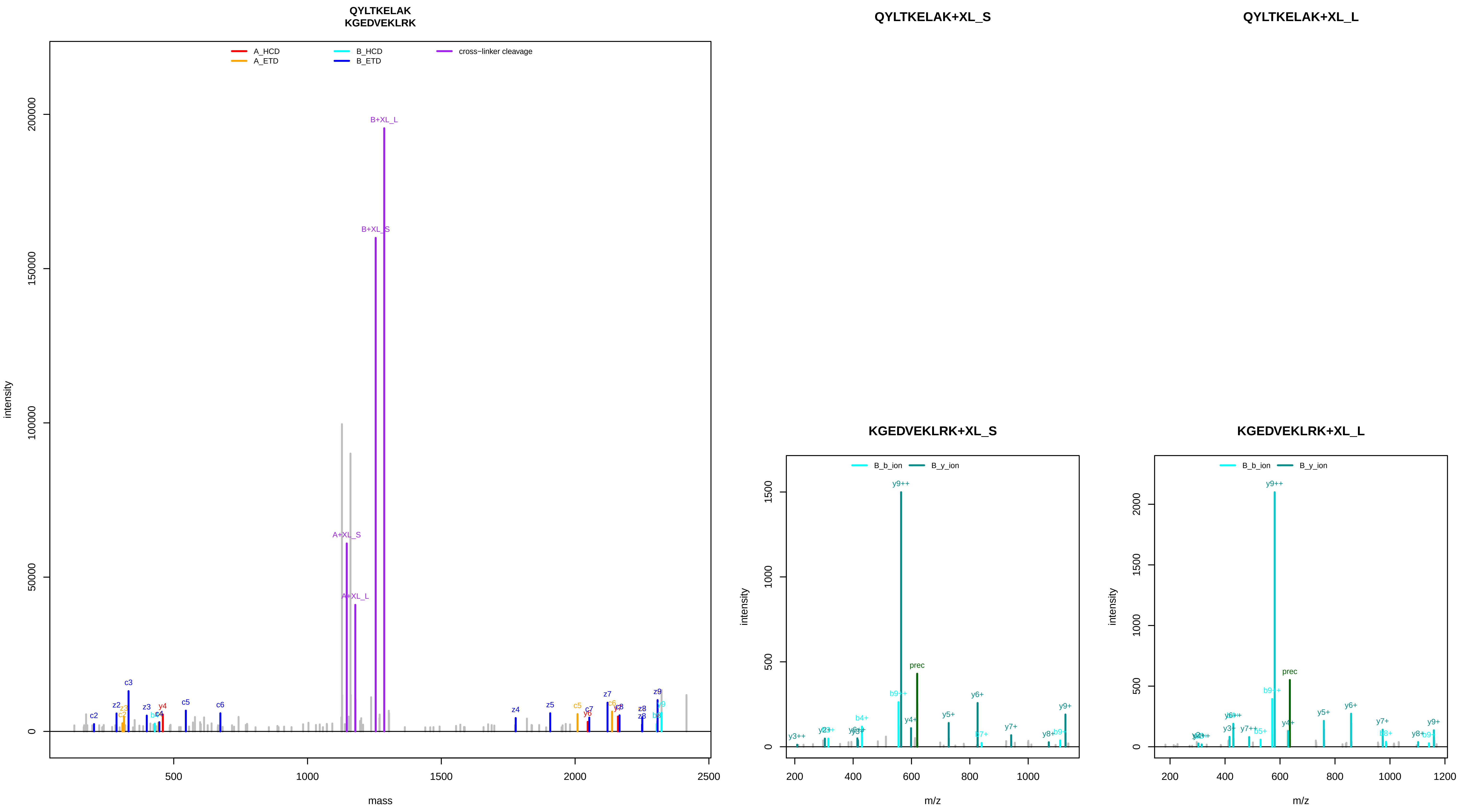

QYLTKELAK
KGEDVEKLRK
A_HCD
A_ETD
B_HCD
B_ETD
cross−linker cleavage
B+XL_L
B+XL_S
A+XL_S
A+XL_L
intensity
mass
QYLTKELAK+XL_S
QYLTKELAK+XL_L
KGEDVEKLRK+XL_S
KGEDVEKLRK+XL_L
B_b_ion
B_y_ion
y9++
prec
m/z

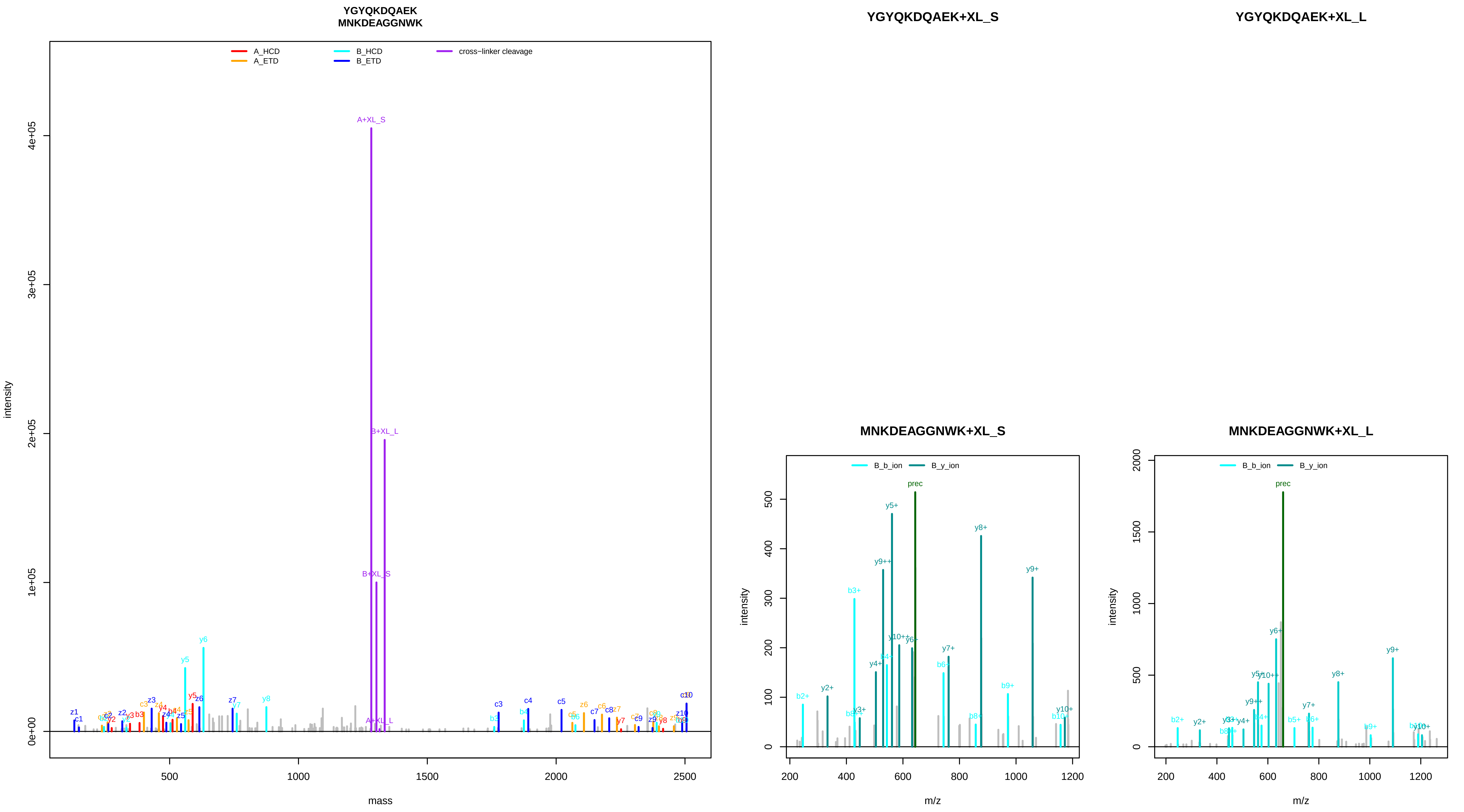
YGYQKDQAEK
MNKDEAGGNWK
A_HCD
A_ETD
B_HCD
B_ETD
cross−linker cleavage
A+XL_S
B+XL_L
B+XL_S
A+XL_L
intensity
mass
YGYQKDQAEK+XL_S
YGYQKDQAEK+XL_L
MNKDEAGGNWK+XL_S
MNKDEAGGNWK+XL_L
B_b_ion
B_y_ion
prec
m/z

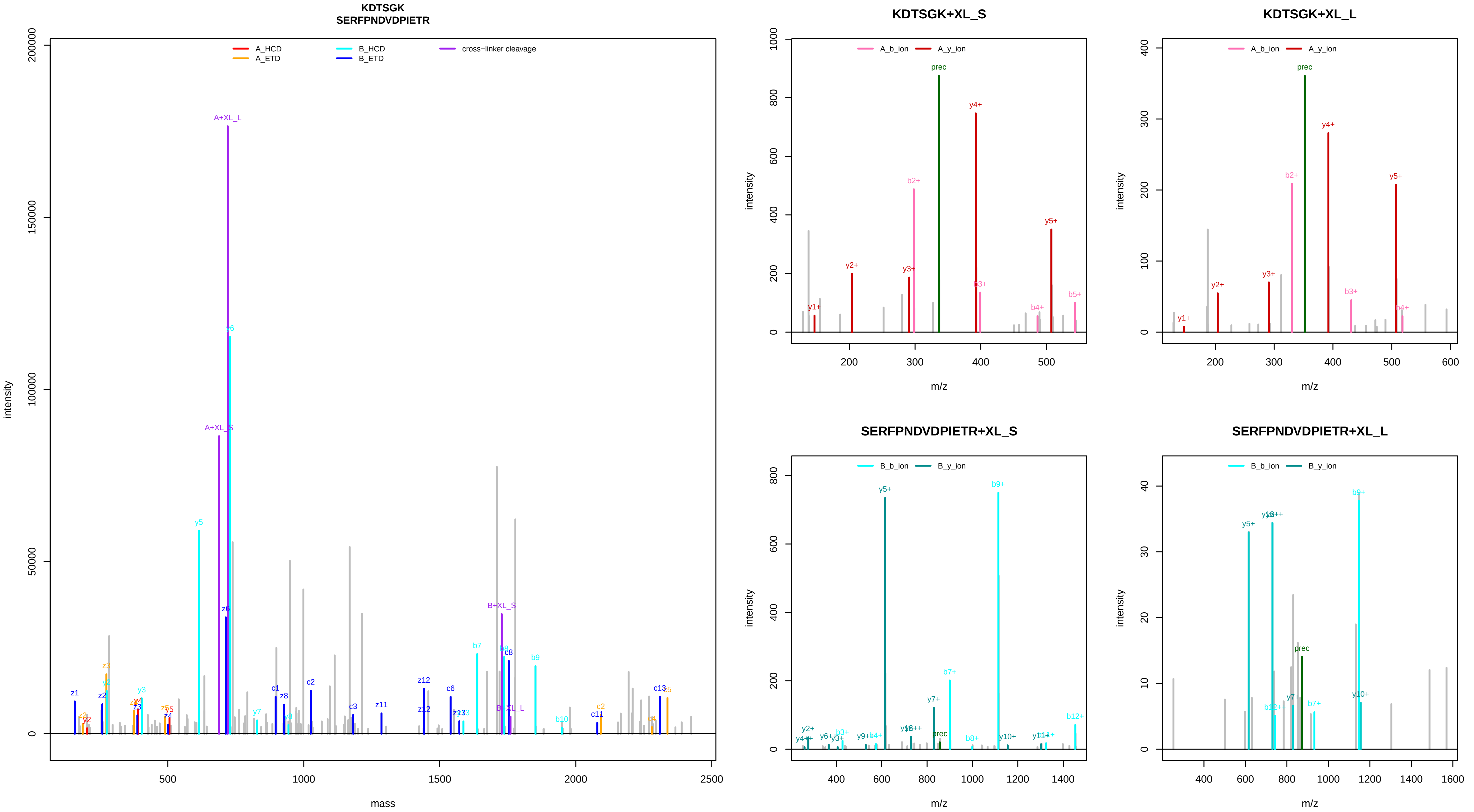
KDTSGK
SERFPNDVDPIETR
A_HCD
A_ETD
B_HCD
B_ETD
cross−linker cleavage
intensity
mass
A+XL_L
A+XL_S
B+XL_S
B+XL_L
KDTSGK+XL_S
KDTSGK+XL_L
SERFPNDVDPIETR+XL_S
SERFPNDVDPIETR+XL_L
A_b_ion
A_y_ion
B_b_ion
B_y_ion
prec
m/z

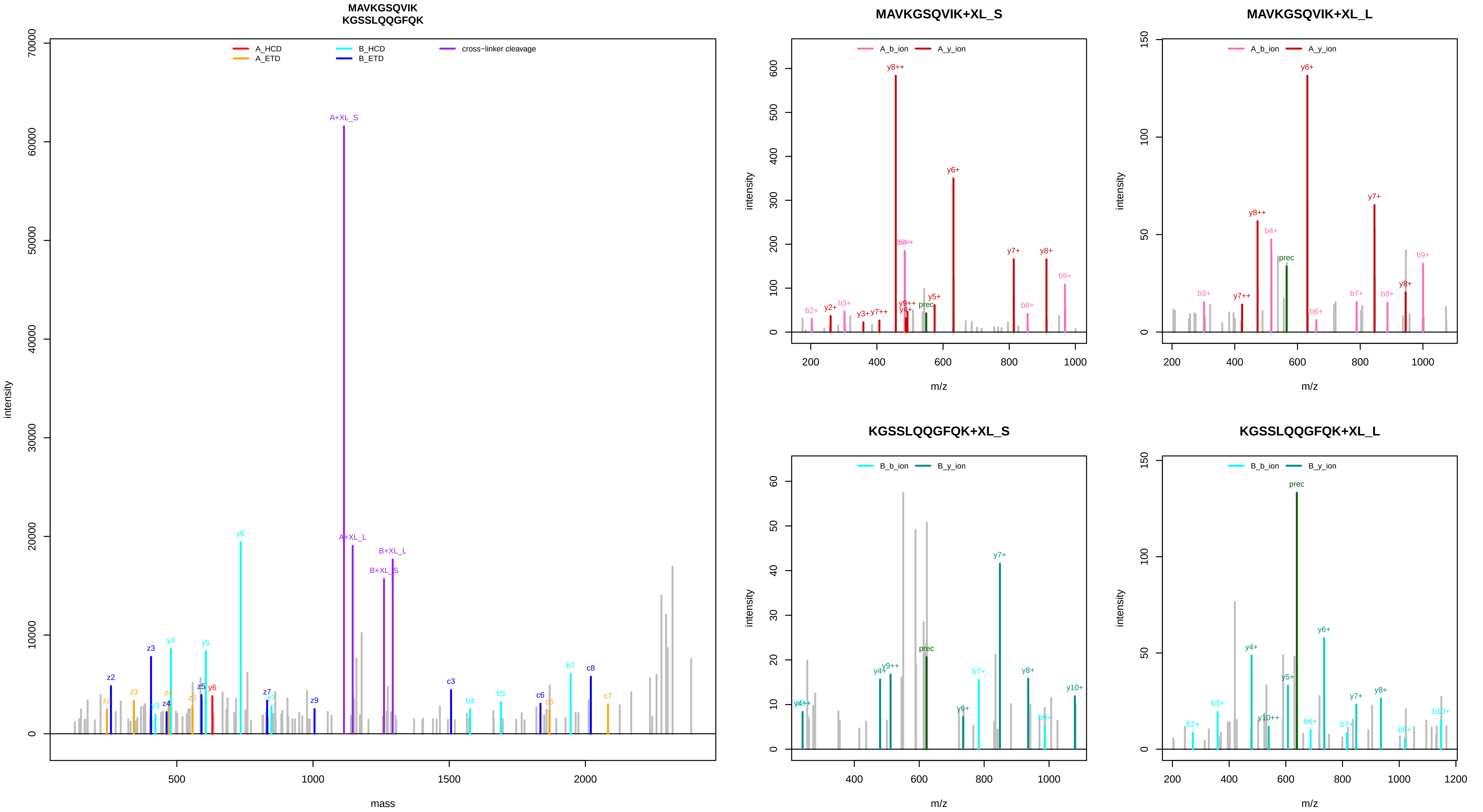
MAVKGSQVIK
KGSSLQQGFQK
A_HCD
A_ETD
B_HCD
B_ETD
cross−linker cleavage
A+XL_S
A+XL_L
B+XL_L
B+XL_S
intensity
mass
MAVKGSQVIK+XL_S
MAVKGSQVIK+XL_L
KGSSLQQGFQK+XL_S
KGSSLQQGFQK+XL_L
A_b_ion
A_y_ion
B_b_ion
B_y_ion
prec
m/z

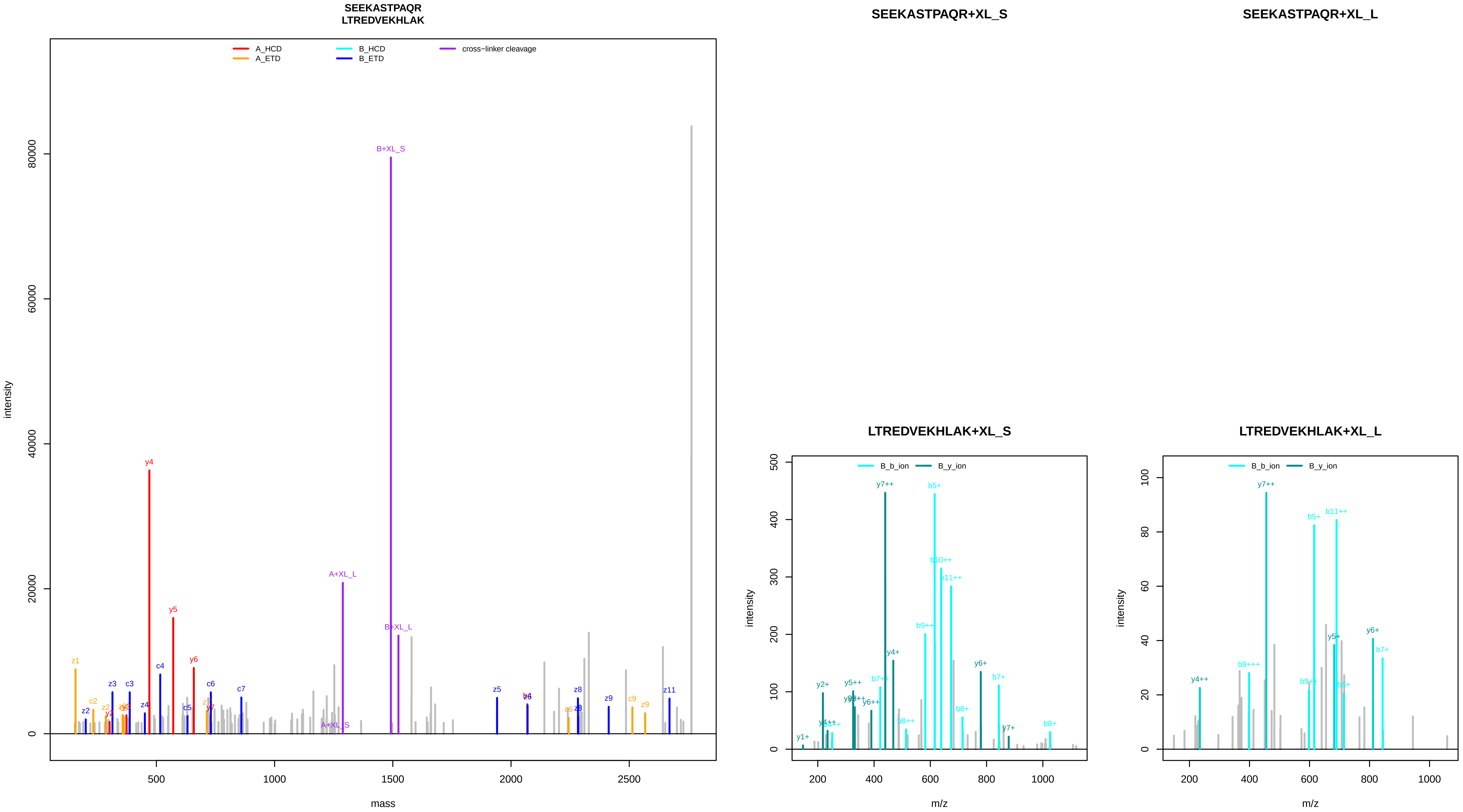
SEEKASTPAQR
LTREDVEKHLAK
A_HCD
A_ETD
B_HCD
B_ETD
cross−linker cleavage
B+XL_S
A+XL_L
B+XL_L
A+XL_S
y4
y5
y6
y7
z1
c2
z2
c3
z3
c4
z4
c5
c6
c7
z5
z8
z9
c9
z11
intensity
mass
0
20000
40000
60000
80000
500
1000
1500
2000
2500
SEEKASTPAQR+XL_S
SEEKASTPAQR+XL_L
LTREDVEKHLAK+XL_S
B_b_ion
B_y_ion
y7++
b5+
b10++
b11++
b9++
y4+
y6+
b7+
y2+
y5++
y6++
b6+
b8+
y1+
y7+
intensity
m/z
0
100
200
300
400
500
200
400
600
800
1000
LTREDVEKHLAK+XL_L
B_b_ion
B_y_ion
y7++
b5+
b11++
b9+++
y4++
y6+
y5+
b7+
intensity
m/z
0
20
40
60
80
100
200
400
600
800
1000

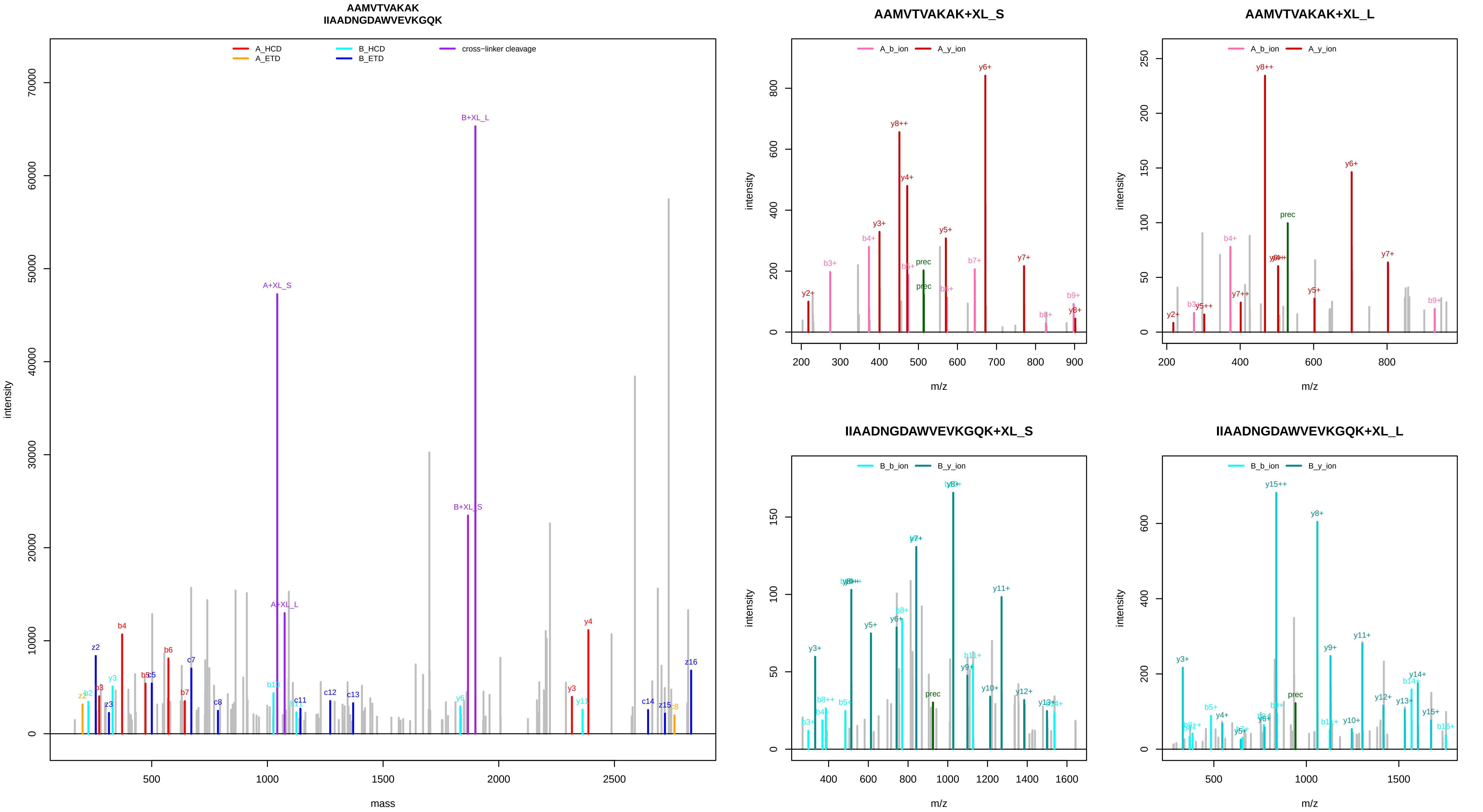
AAMVTVAKAK
IIAADNGDAWVEVKGQK
A_HCD
A_ETD
B_HCD
B_ETD
cross−linker cleavage
A+XL_S
A+XL_L
B+XL_L
B+XL_S
intensity
mass
AAMVTVAKAK+XL_S
AAMVTVAKAK+XL_L
IIAADNGDAWVEVKGQK+XL_S
IIAADNGDAWVEVKGQK+XL_L
A_b_ion
A_y_ion
B_b_ion
B_y_ion
prec
m/z

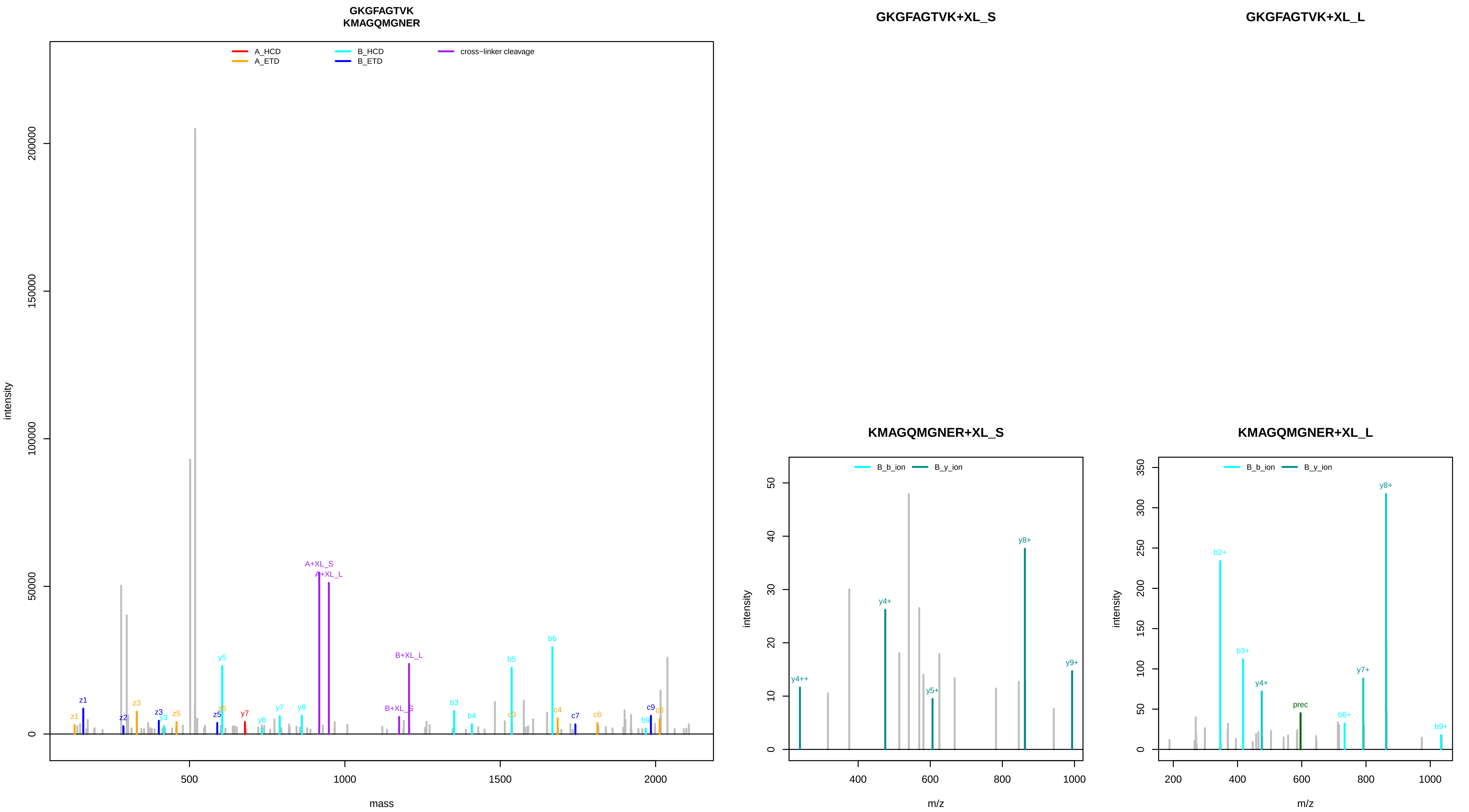
GKGFAGTVK
KMAGQMGNER
A_HCD
A_ETD
B_HCD
B_ETD
cross−linker cleavage
intensity
mass
A+XL_S
A+XL_L
B+XL_L
B+XL_S
GKGFAGTVK+XL_S
GKGFAGTVK+XL_L
KMAGQMGNER+XL_S
KMAGQMGNER+XL_L
B_b_ion
B_y_ion
m/z
prec

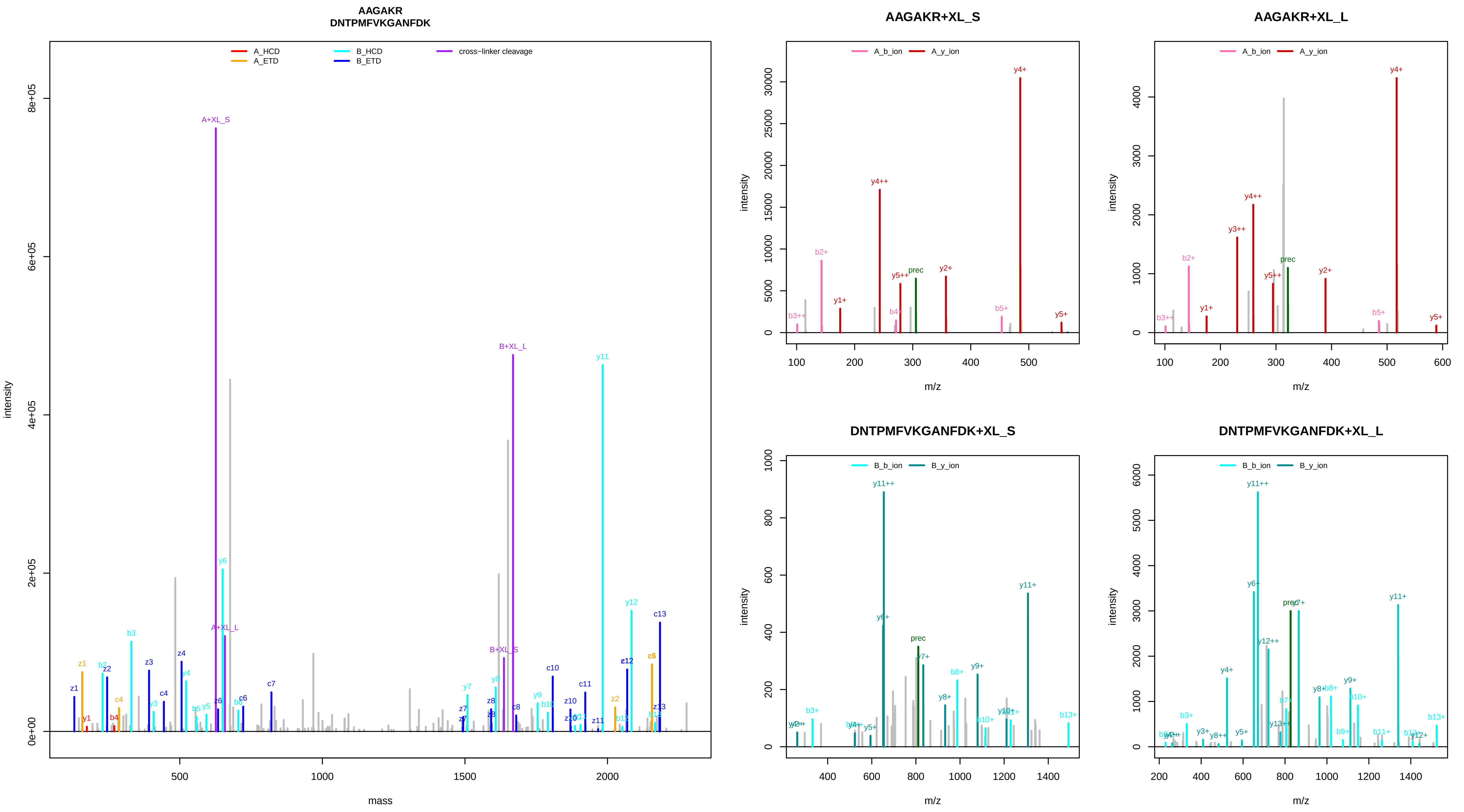
AAGAKR
DNTPMFVKGANFDK
A_HCD
A_ETD
B_HCD
B_ETD
cross−linker cleavage
intensity
mass
AAGAKR+XL_S
AAGAKR+XL_L
DNTPMFVKGANFDK+XL_S
DNTPMFVKGANFDK+XL_L
A_b_ion
A_y_ion
B_b_ion
B_y_ion
m/z

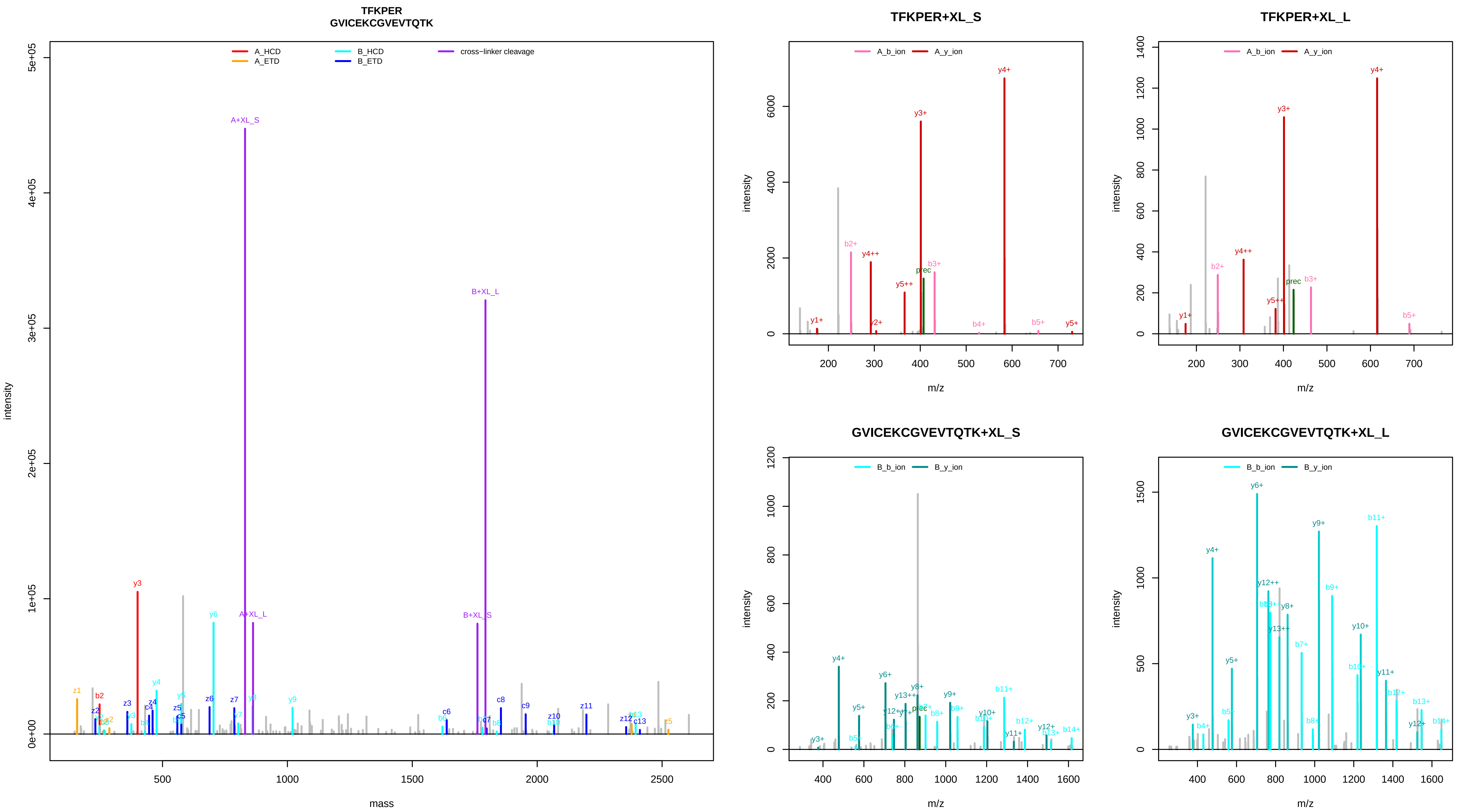

TFKPER
GVICEKCGVEVTQTK
A_HCD
A_ETD
B_HCD
B_ETD
cross−linker cleavage
A+XL_S
B+XL_L
A+XL_L
B+XL_S
intensity
mass
TFKPER+XL_S
TFKPER+XL_L
GVICEKCGVEVTQTK+XL_S
GVICEKCGVEVTQTK+XL_L
A_b_ion
A_y_ion
B_b_ion
B_y_ion
m/z
prec

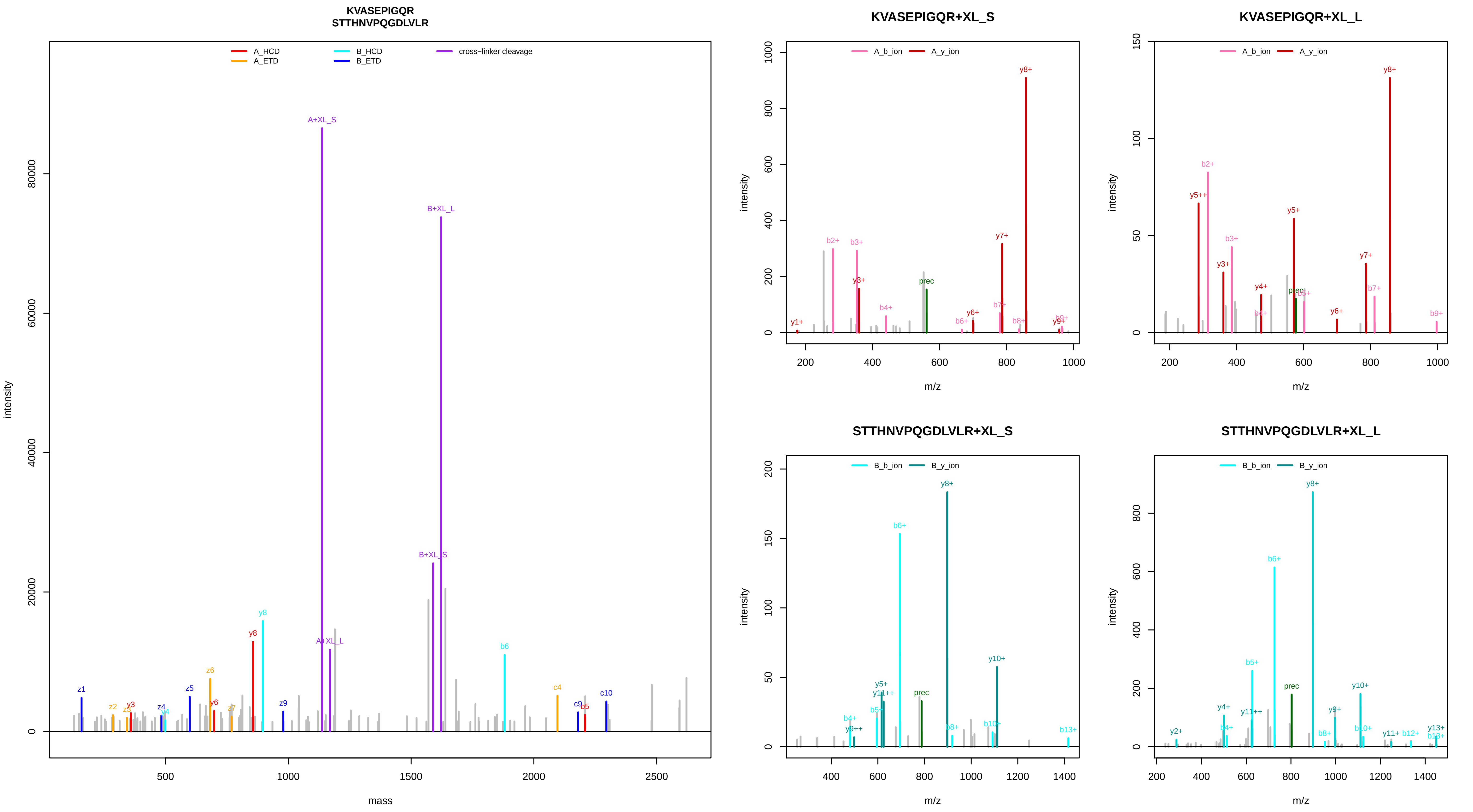
KVASEPIGQR
STTHNVPQGDLVLR
A_HCD
A_ETD
B_HCD
B_ETD
cross−linker cleavage
A+XL_S
B+XL_L
B+XL_S
A+XL_L
intensity
mass
KVASEPIGQR+XL_S
KVASEPIGQR+XL_L
STTHNVPQGDLVLR+XL_S
STTHNVPQGDLVLR+XL_L
A_b_ion
A_y_ion
B_b_ion
B_y_ion
prec
m/z

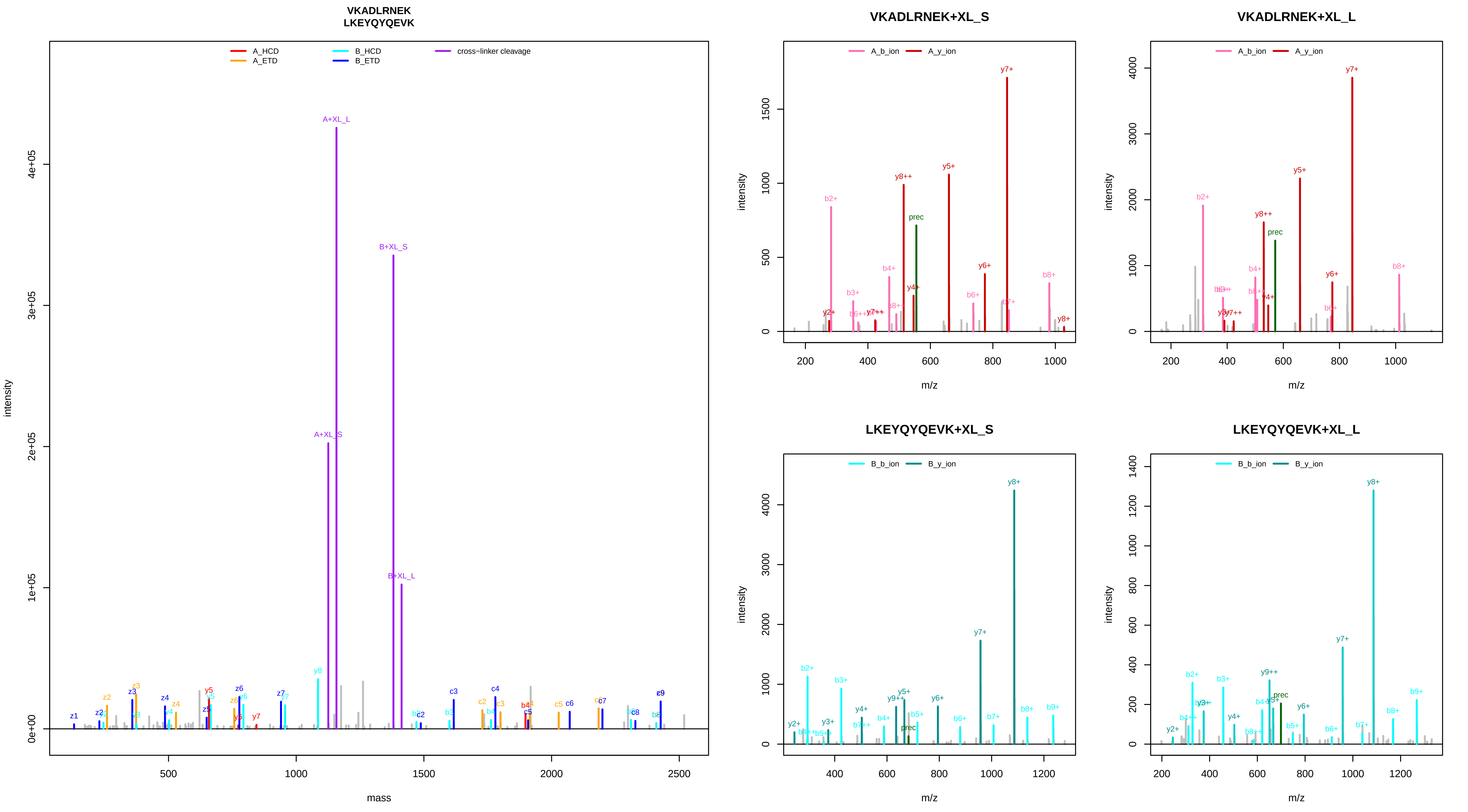
VKADLRNEK
LKEYQYQEVK
A_HCD
A_ETD
B_HCD
B_ETD
cross−linker cleavage
A+XL_L
B+XL_S
A+XL_S
B+XL_L
intensity
mass
VKADLRNEK+XL_S
VKADLRNEK+XL_L
LKEYQYQEVK+XL_S
LKEYQYQEVK+XL_L
A_b_ion
A_y_ion
B_b_ion
B_y_ion
prec
m/z

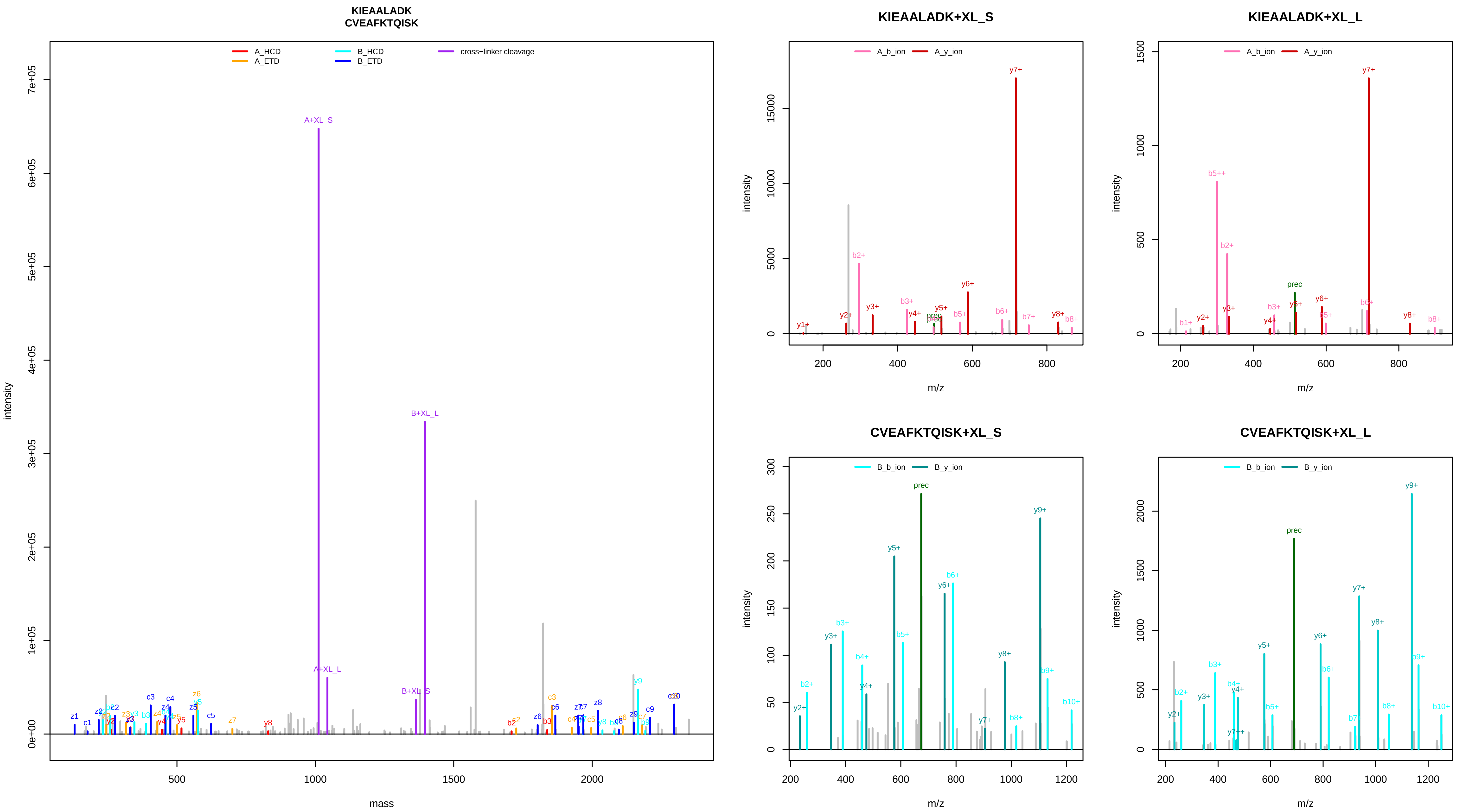
KIEAALADK
CVEAFKTQISK
A_HCD
A_ETD
B_HCD
B_ETD
cross−linker cleavage
A+XL_S
B+XL_L
A+XL_L
B+XL_S
intensity
mass
KIEAALADK+XL_S
KIEAALADK+XL_L
A_b_ion
A_y_ion
CVEAFKTQISK+XL_S
CVEAFKTQISK+XL_L
B_b_ion
B_y_ion
prec
m/z
intensity

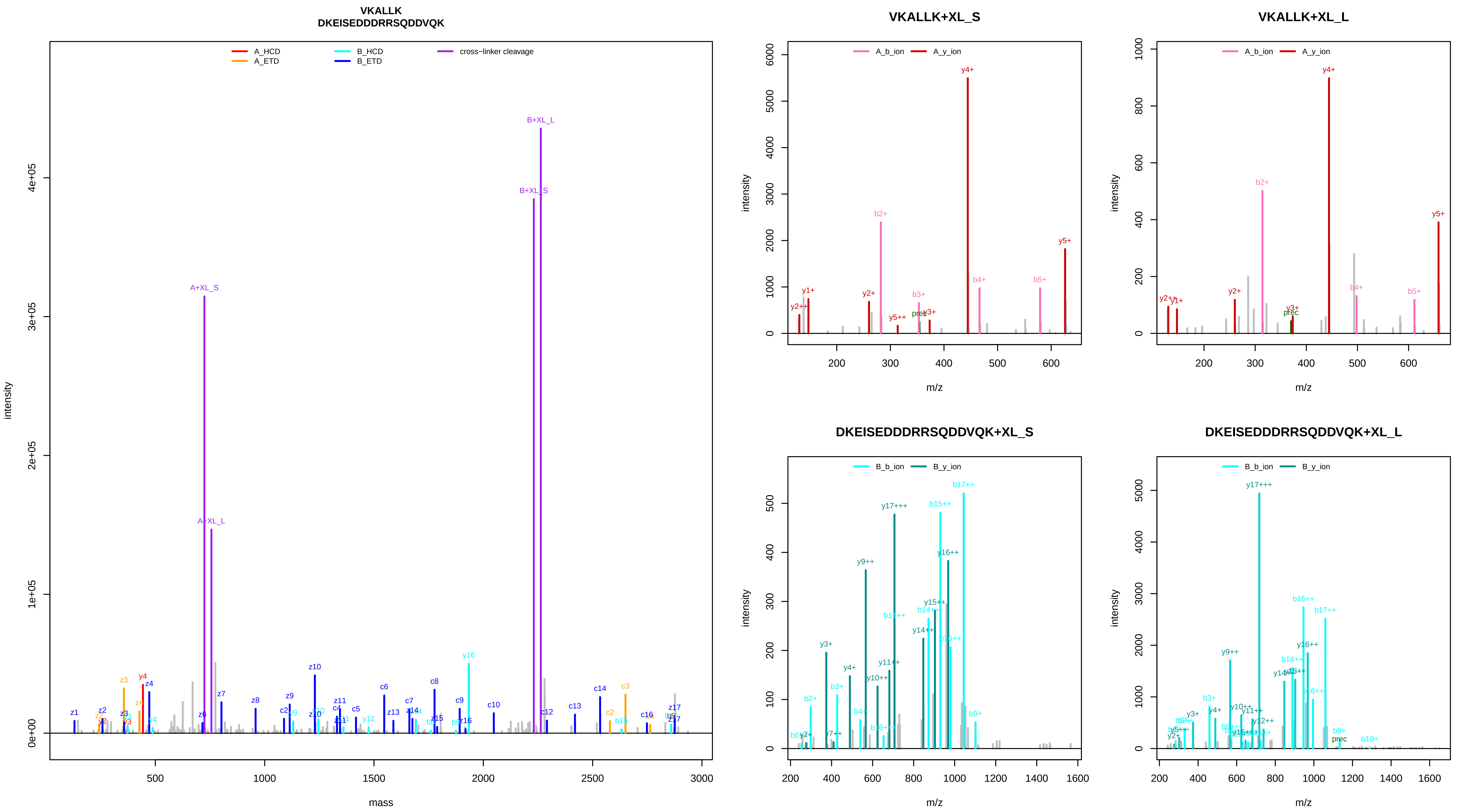
VKALLK
DKEISEDDDRRSQDDVQK
A_HCD
A_ETD
B_HCD
B_ETD
cross−linker cleavage
B+XL_L
B+XL_S
A+XL_S
A+XL_L
intensity
mass
VKALLK+XL_S
VKALLK+XL_L
A_b_ion
A_y_ion
DKEISEDDDRRSQDDVQK+XL_S
DKEISEDDDRRSQDDVQK+XL_L
B_b_ion
B_y_ion
m/z

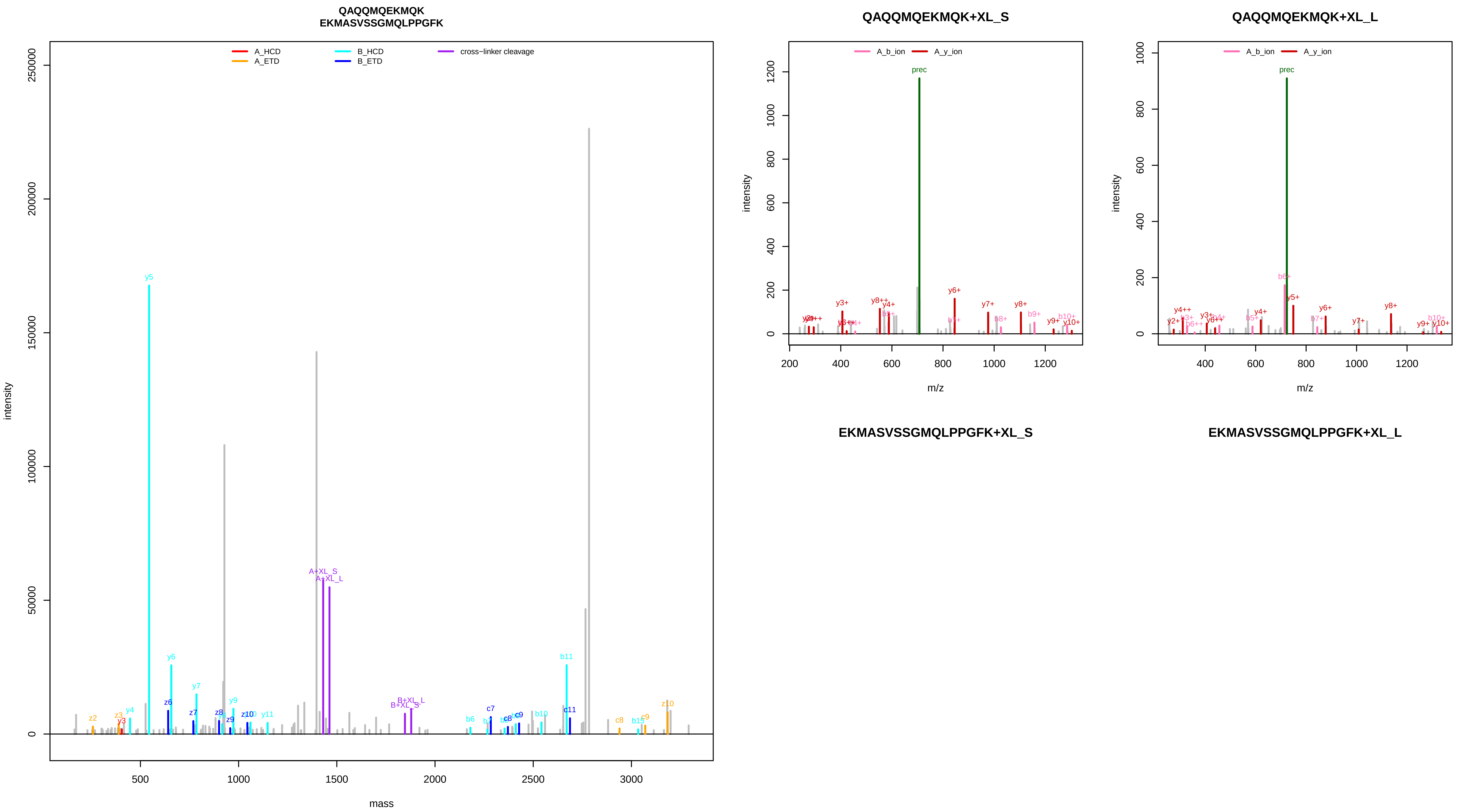
QAQQMQEKMQK
EKMASVSSGMQLPPGFK
A_HCD
A_ETD
B_HCD
B_ETD
cross−linker cleavage
A+XL_S
A+XL_L
B+XL_L
B+XL_S
intensity
mass
QAQQMQEKMQK+XL_S
A_b_ion
A_y_ion
prec
intensity
m/z
QAQQMQEKMQK+XL_L
A_b_ion
A_y_ion
prec
intensity
m/z
EKMASVSSGMQLPPGFK+XL_S
EKMASVSSGMQLPPGFK+XL_L

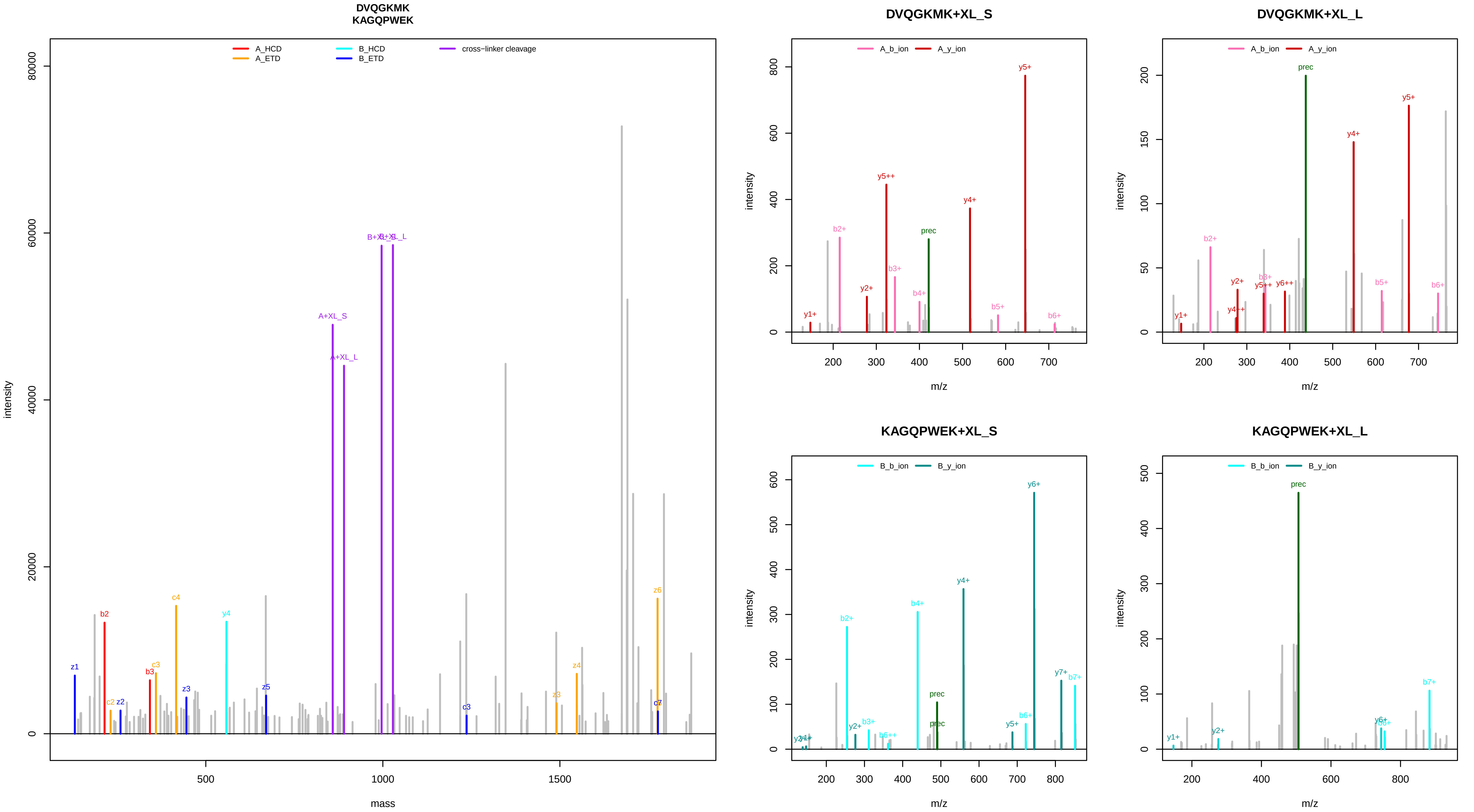

DVQGKMK
KAGQPWEK
A_HCD
A_ETD
B_HCD
B_ETD
cross−linker cleavage
intensity
mass
DVQGKMK+XL_S
DVQGKMK+XL_L
KAGQPWEK+XL_S
KAGQPWEK+XL_L
A_b_ion
A_y_ion
B_b_ion
B_y_ion
m/z

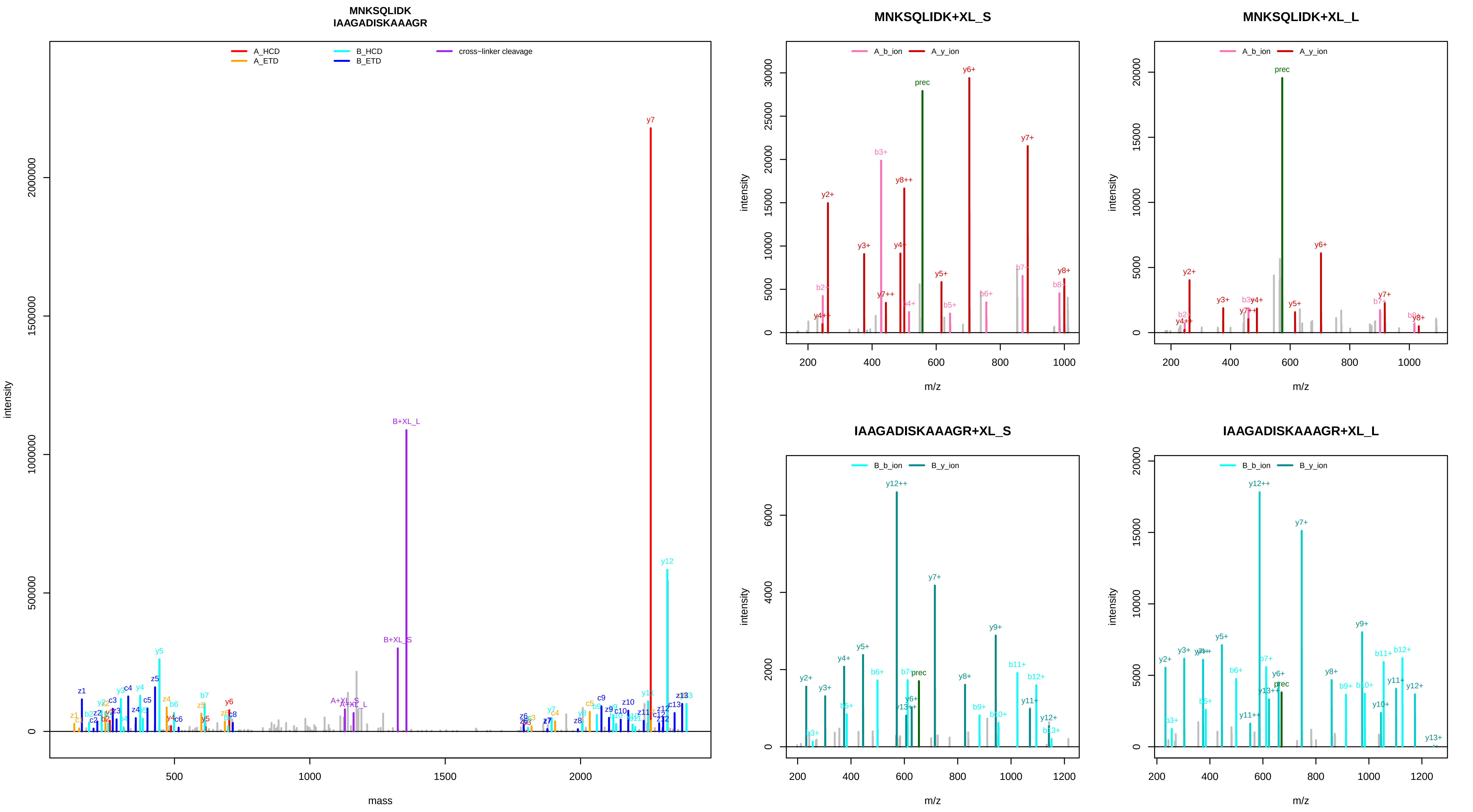
MNKSQLIDK
IAAGADISKAAAGR
A_HCD
A_ETD
B_HCD
B_ETD
cross−linker cleavage
B+XL_L
B+XL_S
A+XL_S
A+XL_L
intensity
mass
MNKSQLIDK+XL_S
MNKSQLIDK+XL_L
IAAGADISKAAAGR+XL_S
IAAGADISKAAAGR+XL_L
A_b_ion
A_y_ion
B_b_ion
B_y_ion
prec
m/z

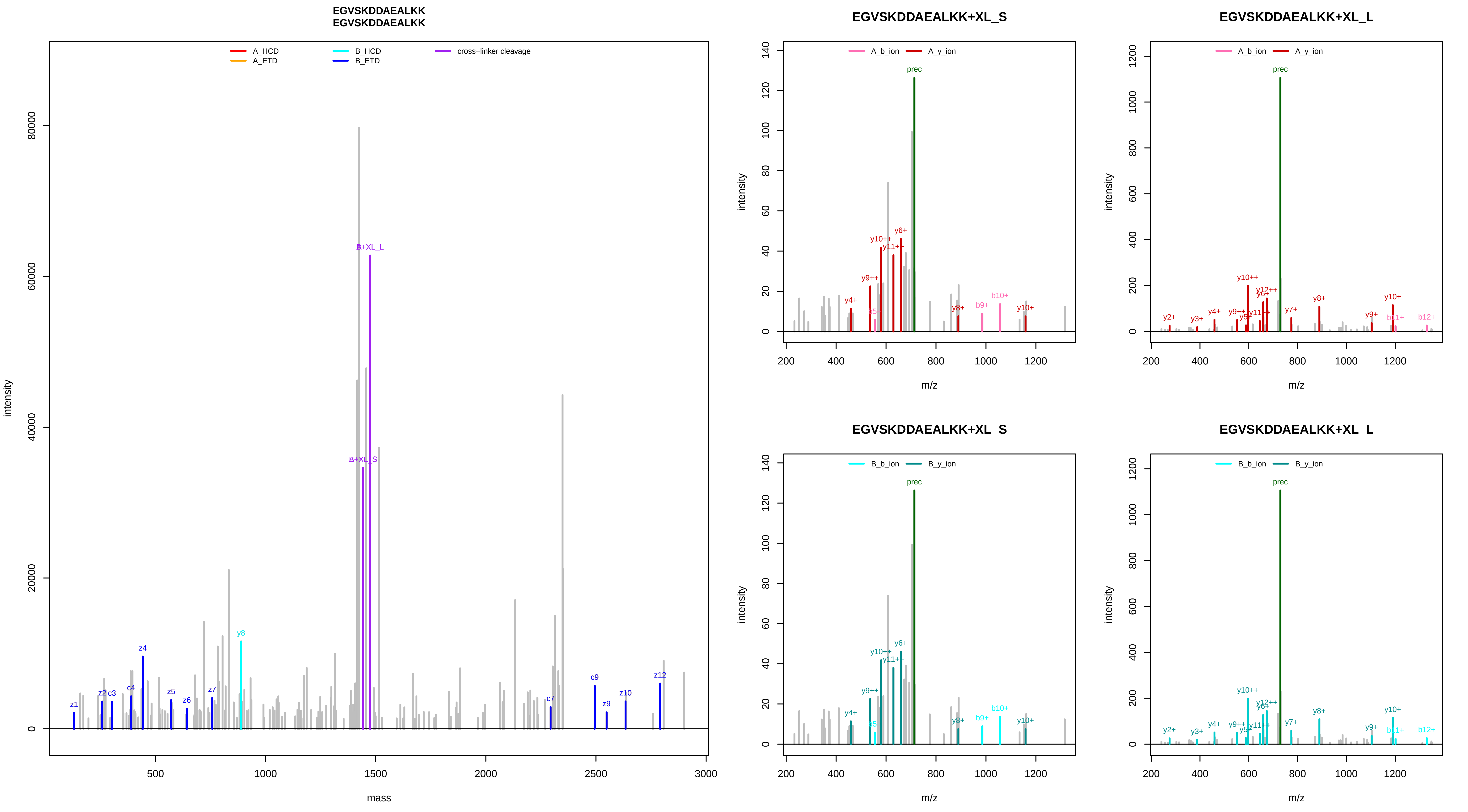
EGVSKDDAEALKK
EGVSKDDAEALKK
A_HCD
A_ETD
B_HCD
B_ETD
cross−linker cleavage
intensity
mass
EGVSKDDAEALKK+XL_S
EGVSKDDAEALKK+XL_L
A_b_ion
A_y_ion
B_b_ion
B_y_ion
prec
intensity
m/z

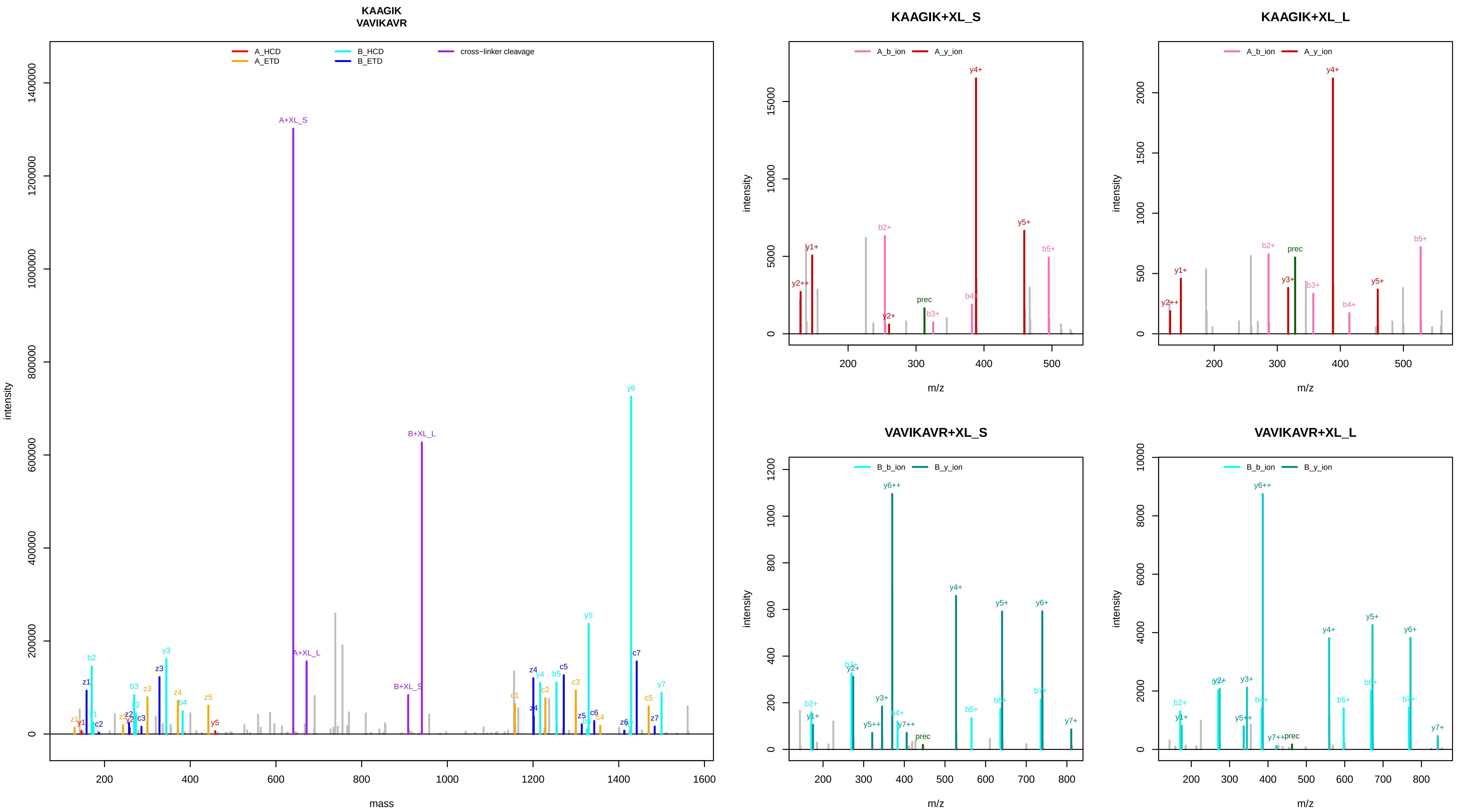
KAAGIK
VAVIKAVR
A_HCD
A_ETD
B_HCD
B_ETD
cross−linker cleavage
A+XL_S
B+XL_L
A+XL_L
B+XL_S
intensity
mass
KAAGIK+XL_S
KAAGIK+XL_L
VAVIKAVR+XL_S
VAVIKAVR+XL_L
A_b_ion
A_y_ion
B_b_ion
B_y_ion
prec
m/z

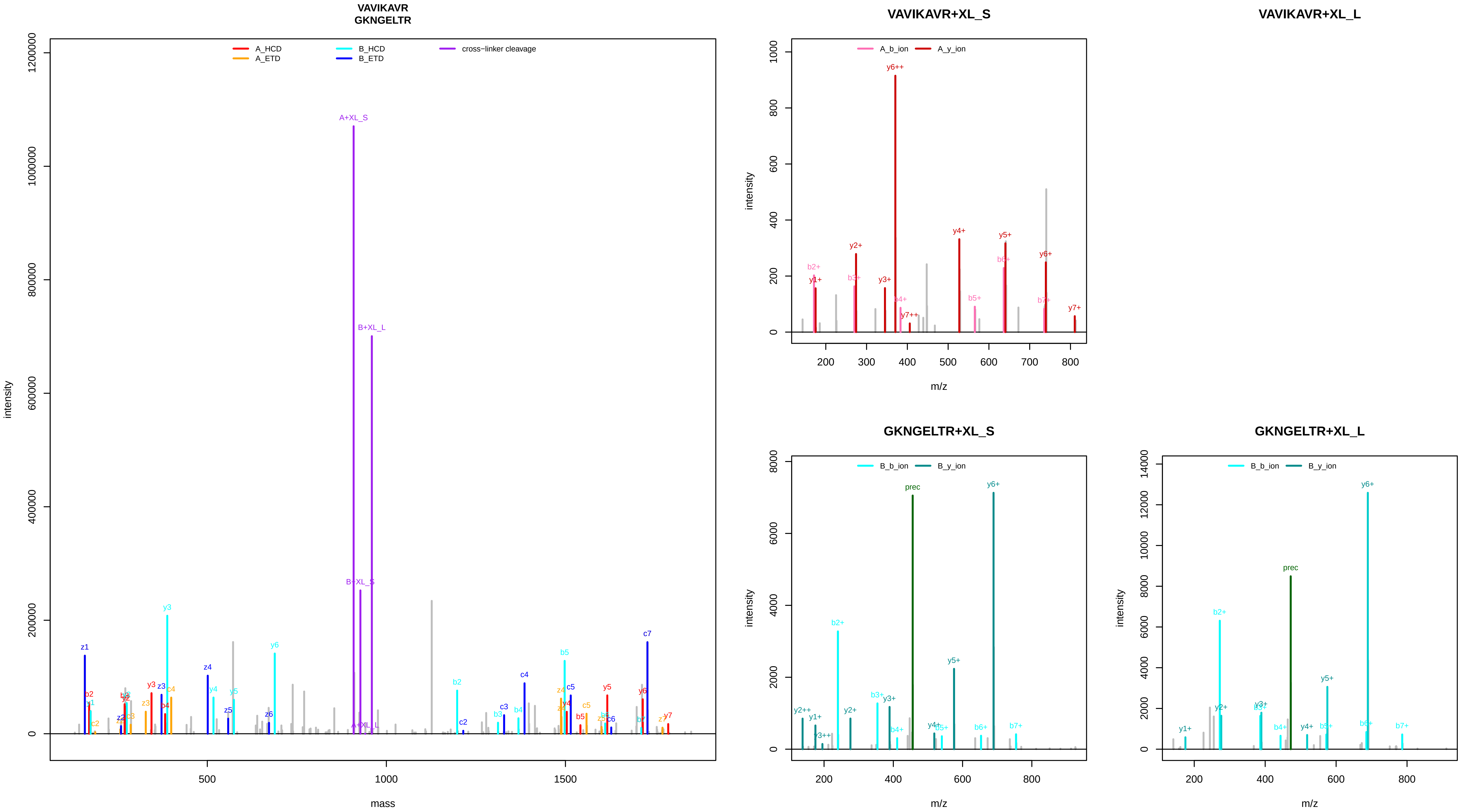

VAVIKAVR
GKNGELTR
A_HCD
A_ETD
B_HCD
B_ETD
cross−linker cleavage
A+XL_S
B+XL_L
B+XL_S
A+XL_L
intensity
mass
VAVIKAVR+XL_S
VAVIKAVR+XL_L
A_b_ion
A_y_ion
m/z
GKNGELTR+XL_S
GKNGELTR+XL_L
B_b_ion
B_y_ion
prec

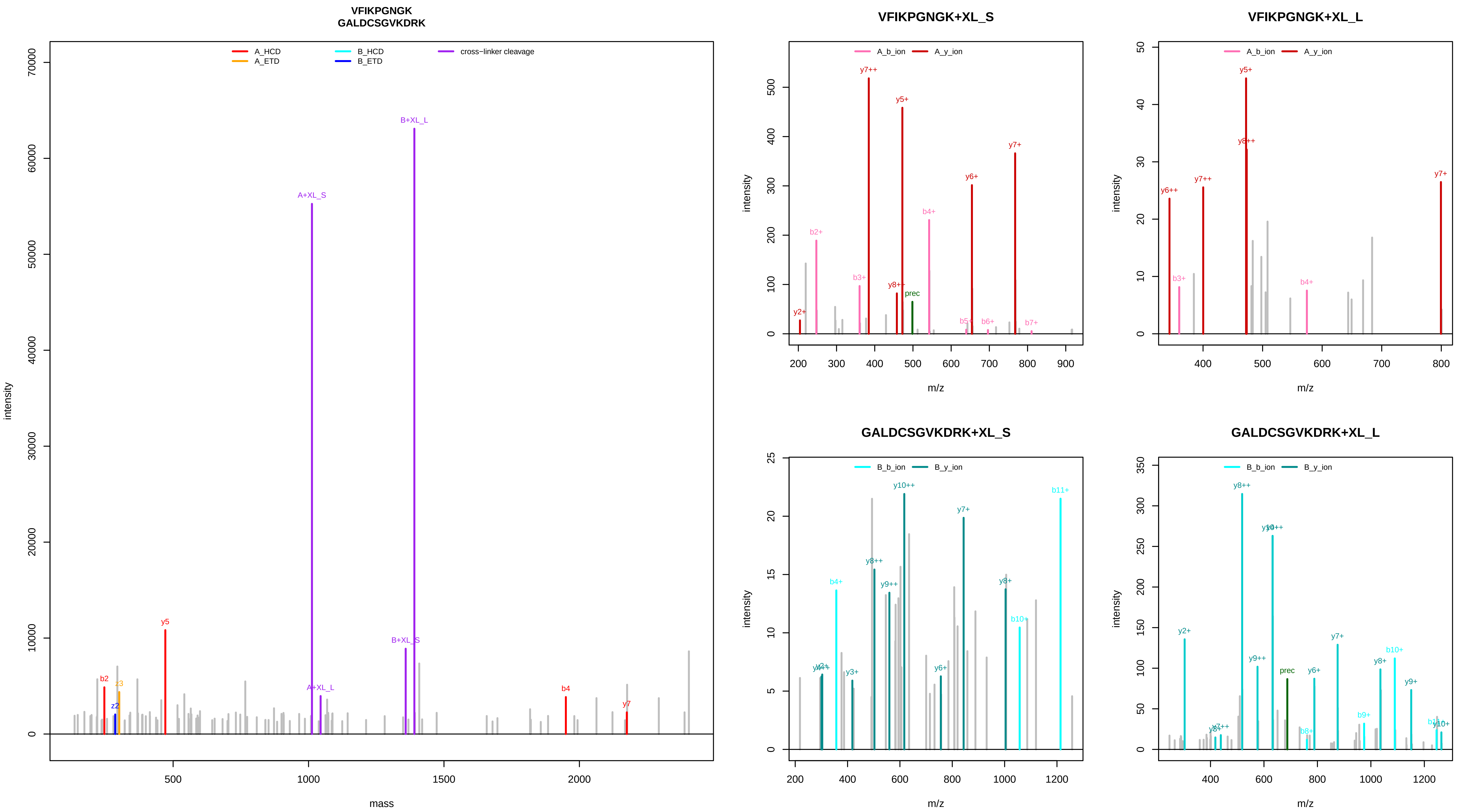

VFIKPGNGK
GALDCSGVKDRK
A_HCD
A_ETD
B_HCD
B_ETD
cross−linker cleavage
B+XL_L
A+XL_S
B+XL_S
A+XL_L
y5
b2
z3
z2
b4
y7
intensity
mass
VFIKPGNGK+XL_S
VFIKPGNGK+XL_L
GALDCSGVKDRK+XL_S
GALDCSGVKDRK+XL_L
A_b_ion
A_y_ion
B_b_ion
B_y_ion
prec
m/z

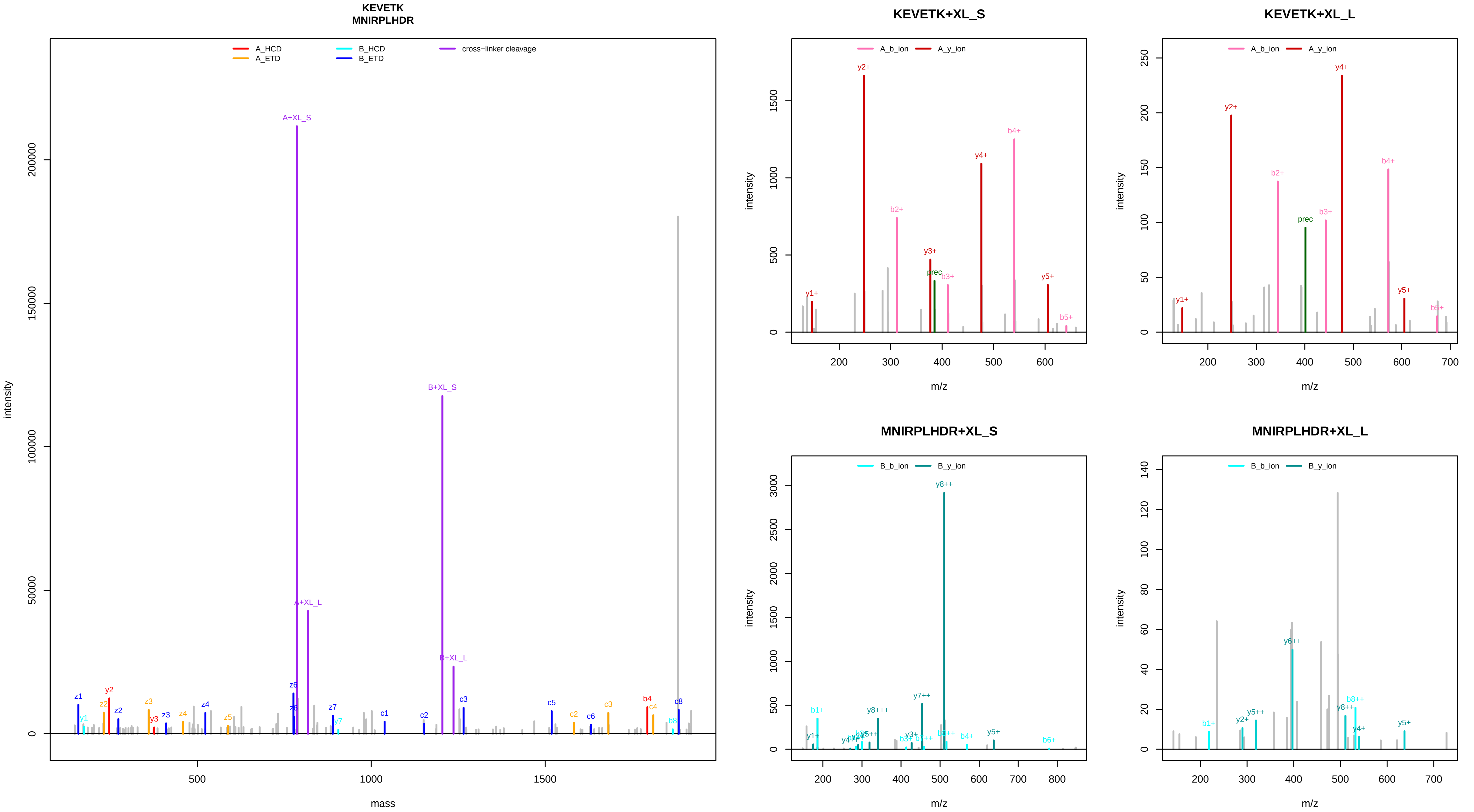

KEVETK
MNIRPLHDR
A_HCD
A_ETD
B_HCD
B_ETD
cross−linker cleavage
A+XL_S
B+XL_S
A+XL_L
B+XL_L
intensity
mass
KEVETK+XL_S
KEVETK+XL_L
MNIRPLHDR+XL_S
MNIRPLHDR+XL_L
A_b_ion
A_y_ion
B_b_ion
B_y_ion
prec
m/z

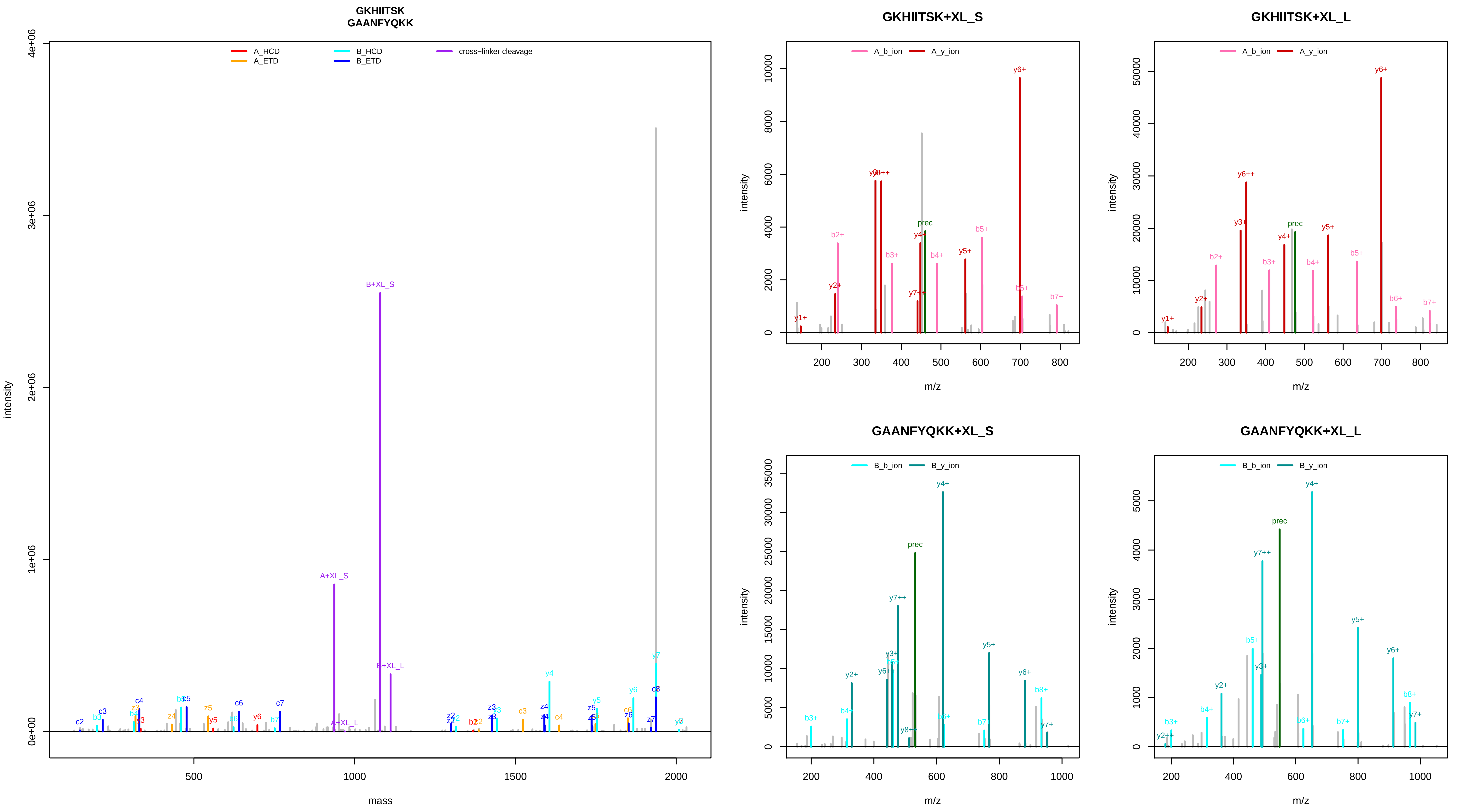
GKHIITSK
GAANFYQKK
A_HCD
A_ETD
B_HCD
B_ETD
cross−linker cleavage
B+XL_S
A+XL_S
B+XL_L
A+XL_L
intensity
mass
GKHIITSK+XL_S
GKHIITSK+XL_L
GAANFYQKK+XL_S
GAANFYQKK+XL_L
A_b_ion
A_y_ion
B_b_ion
B_y_ion
prec
m/z

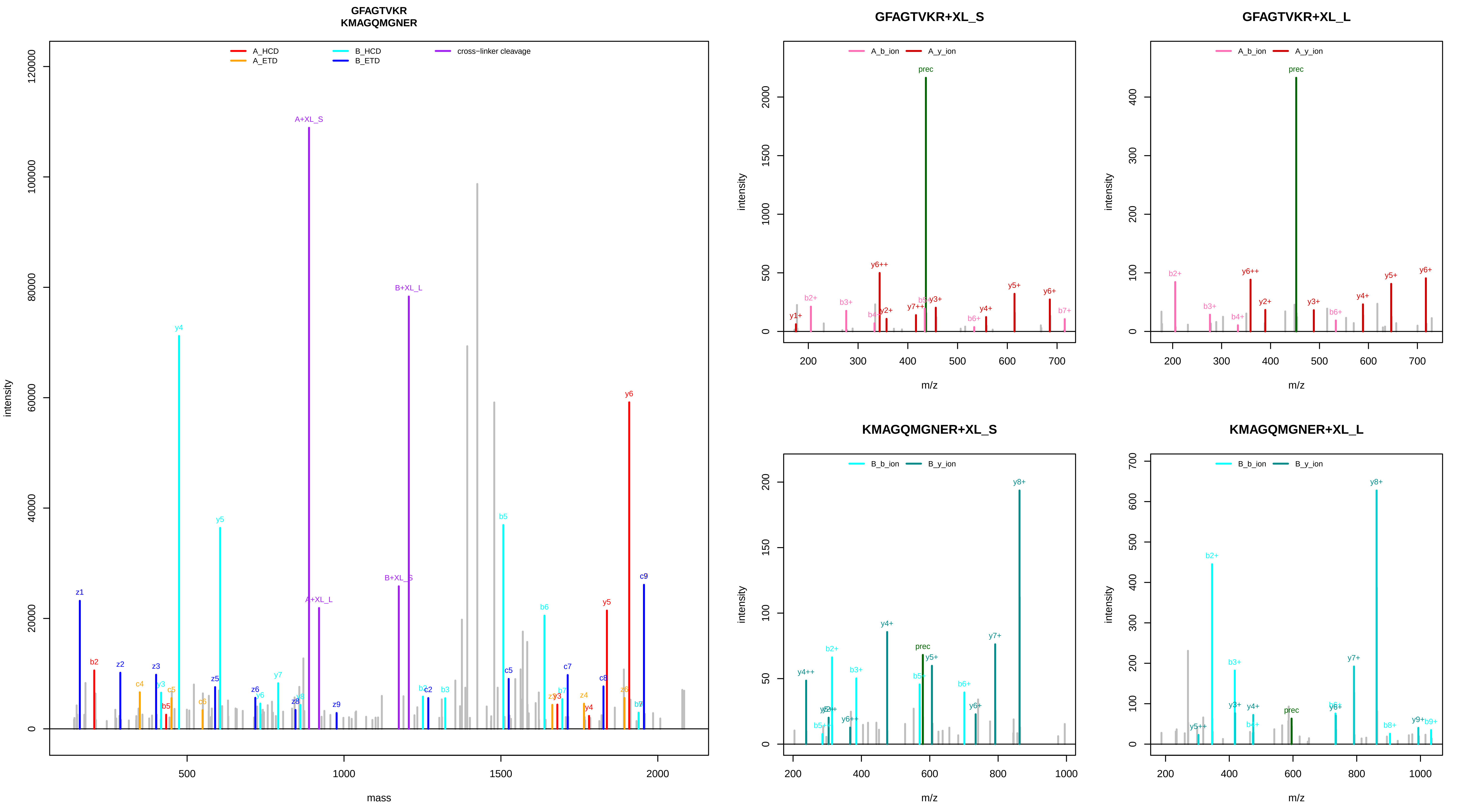

GFAGTVKR
KMAGQMGNER
A_HCD
A_ETD
B_HCD
B_ETD
cross−linker cleavage
intensity
mass
GFAGTVKR+XL_S
GFAGTVKR+XL_L
KMAGQMGNER+XL_S
KMAGQMGNER+XL_L
A_b_ion
A_y_ion
B_b_ion
B_y_ion
prec
m/z

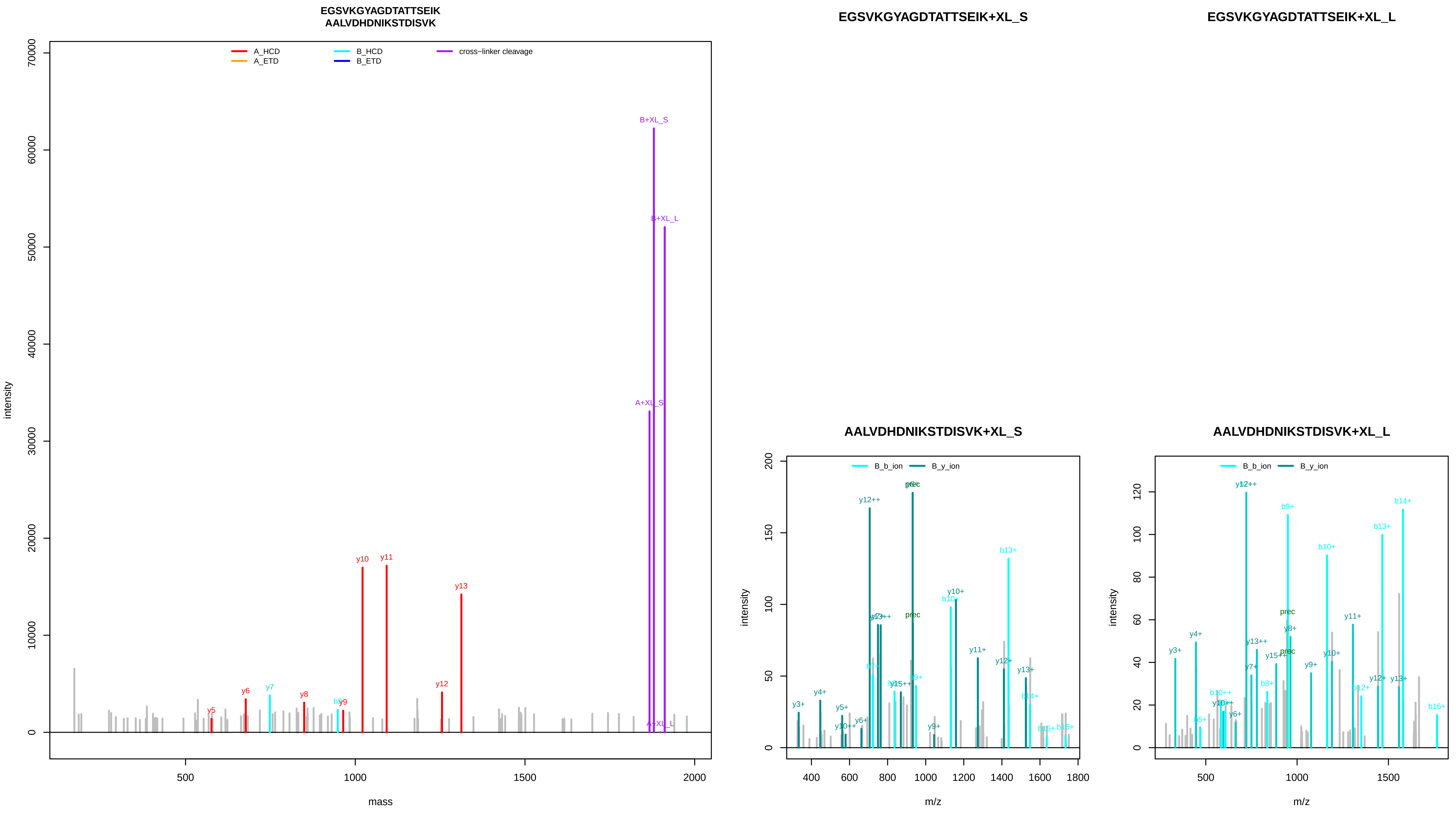
EGSVKGYAGDTATTSEIK
AALVDHDNIKSTDISVK
A_HCD
A_ETD
B_HCD
B_ETD
cross−linker cleavage
B+XL_S
B+XL_L
A+XL_S
A+XL_L
y5
y6
y7
y8
y9
y10
y11
y12
y13
intensity
mass
EGSVKGYAGDTATTSEIK+XL_S
EGSVKGYAGDTATTSEIK+XL_L
AALVDHDNIKSTDISVK+XL_S
AALVDHDNIKSTDISVK+XL_L
B_b_ion
B_y_ion
prec
m/z

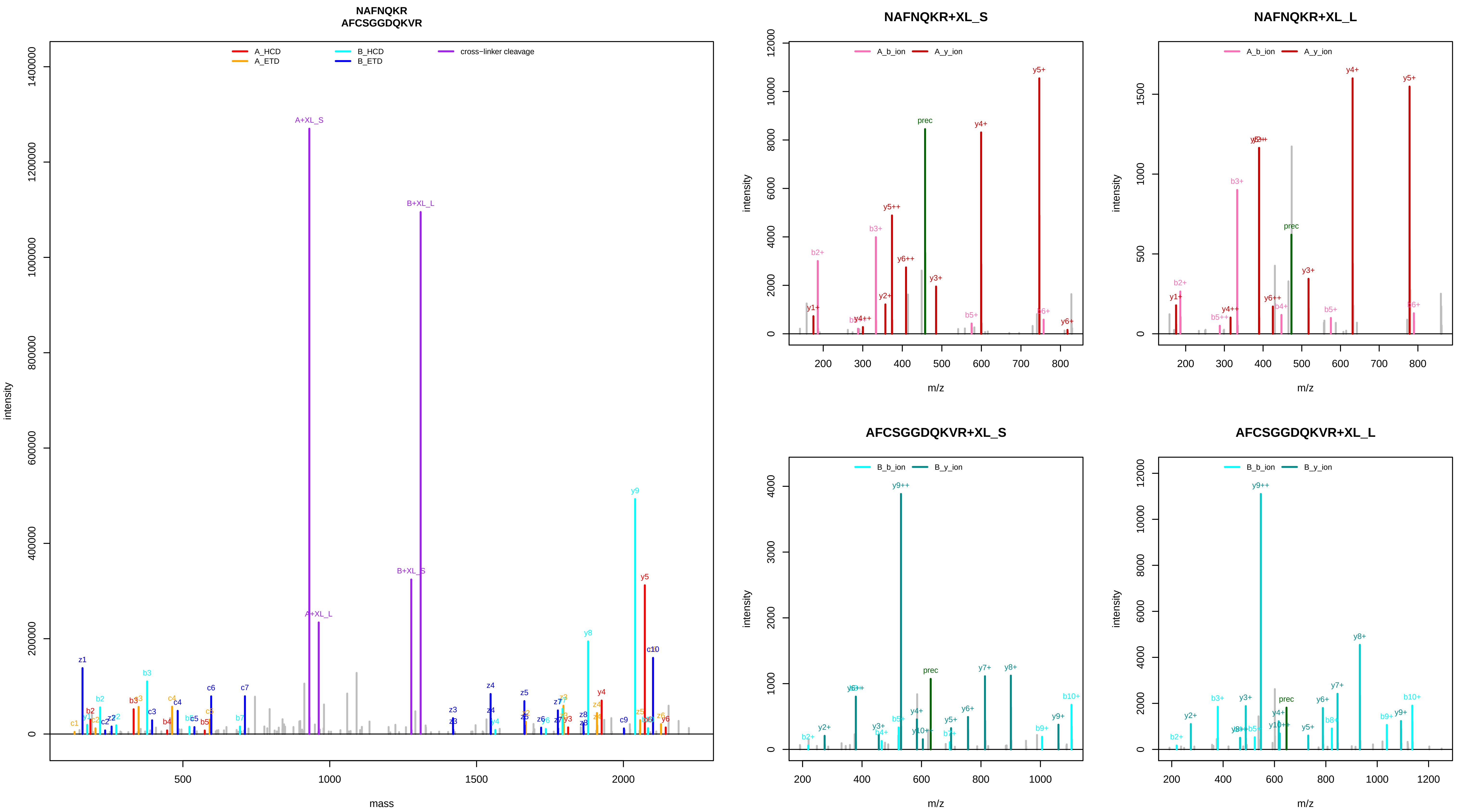
NAFNQKR
AFCSGGDQKVR
A_HCD
A_ETD
B_HCD
B_ETD
cross−linker cleavage
A+XL_S
B+XL_L
B+XL_S
A+XL_L
intensity
mass
NAFNQKR+XL_S
NAFNQKR+XL_L
AFCSGGDQKVR+XL_S
AFCSGGDQKVR+XL_L
A_b_ion
A_y_ion
B_b_ion
B_y_ion
prec
m/z

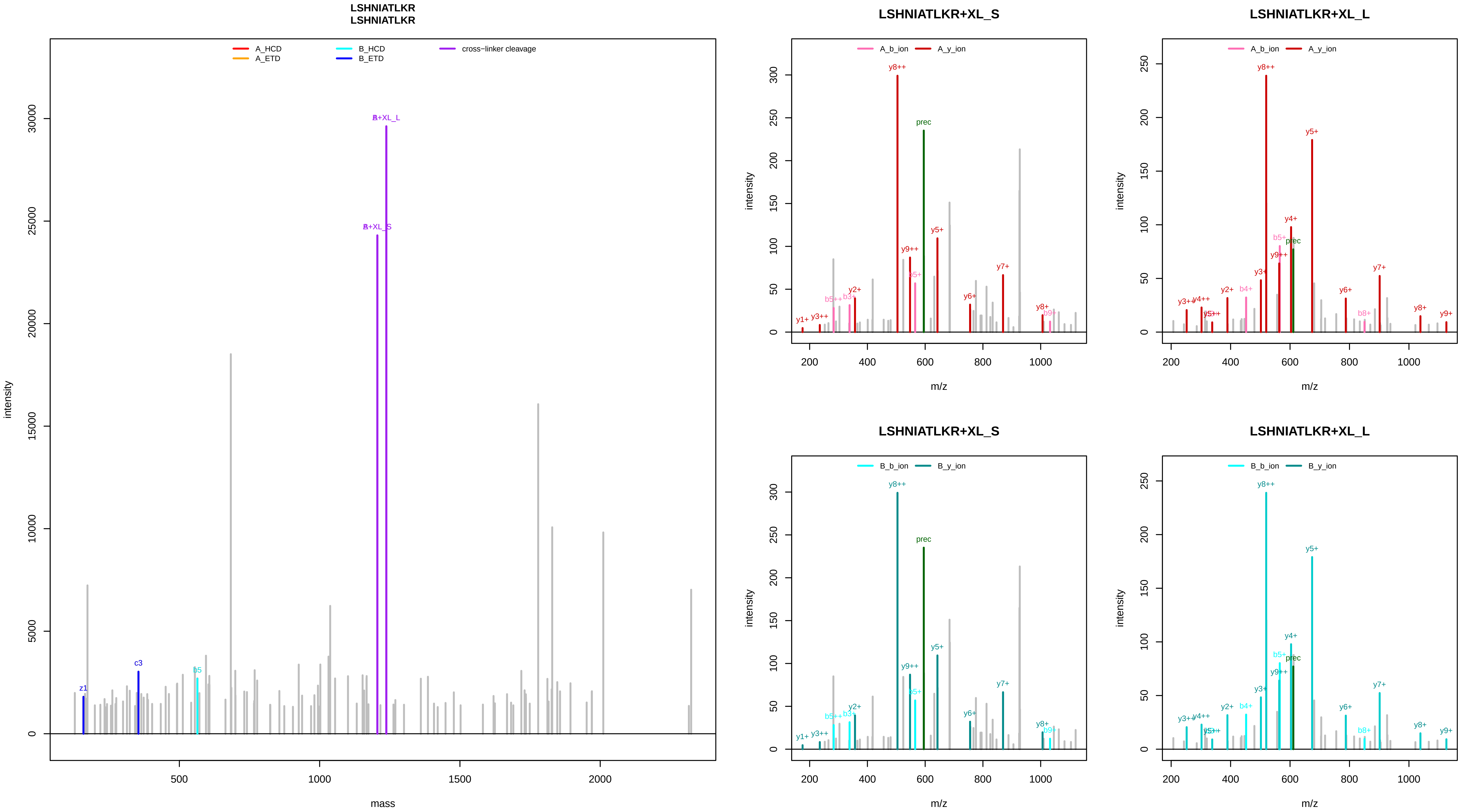

LSHNIATLKR
LSHNIATLKR
A_HCD
A_ETD
B_HCD
B_ETD
cross−linker cleavage
intensity
mass
LSHNIATLKR+XL_S
LSHNIATLKR+XL_L
A_b_ion
A_y_ion
B_b_ion
B_y_ion
prec
m/z

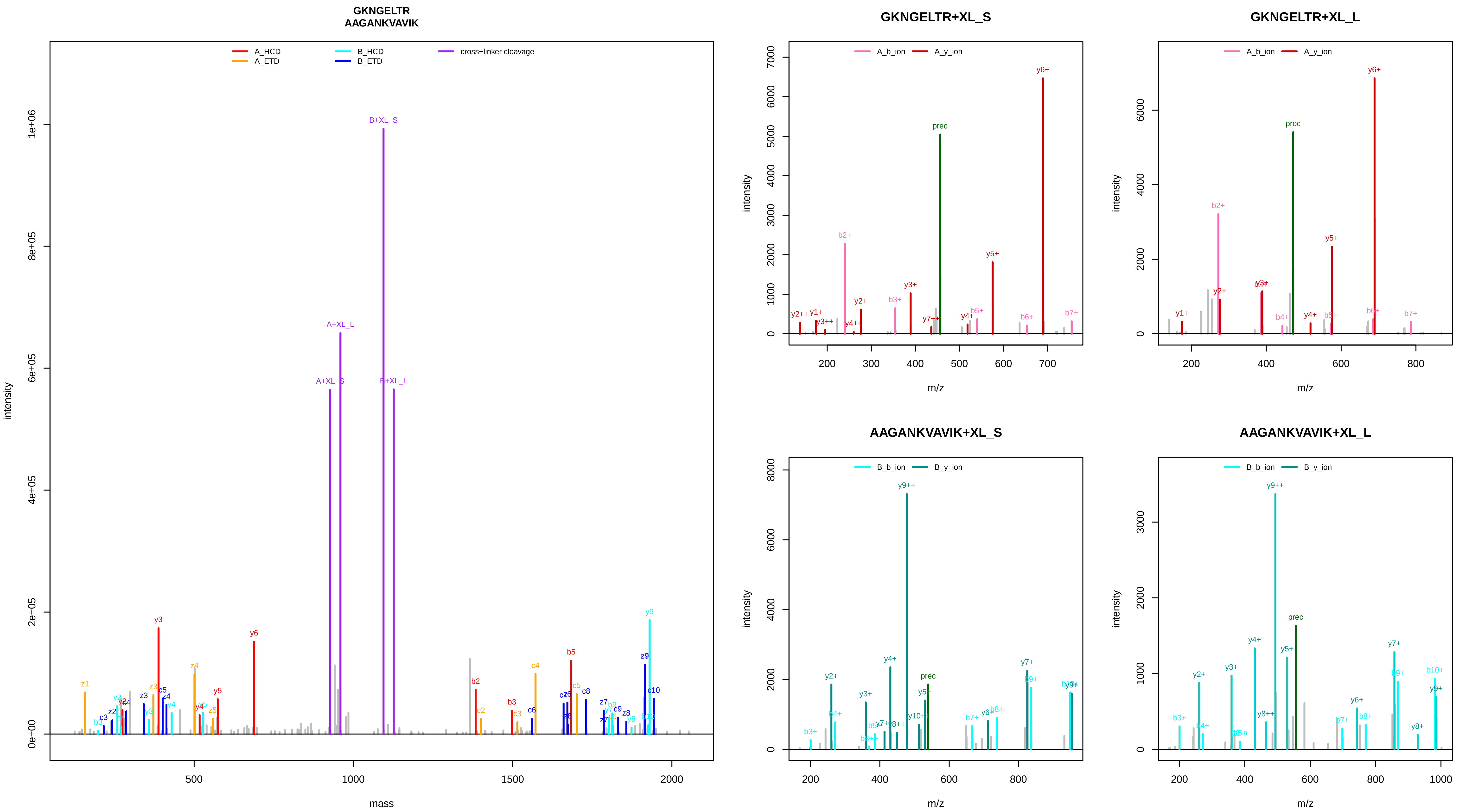
GKNGELTR
AAGANKVAVIK
A_HCD
A_ETD
B_HCD
B_ETD
cross−linker cleavage
B+XL_S
A+XL_L
A+XL_S
B+XL_L
intensity
mass
GKNGELTR+XL_S
GKNGELTR+XL_L
AAGANKVAVIK+XL_S
AAGANKVAVIK+XL_L
A_b_ion
A_y_ion
B_b_ion
B_y_ion
prec
m/z

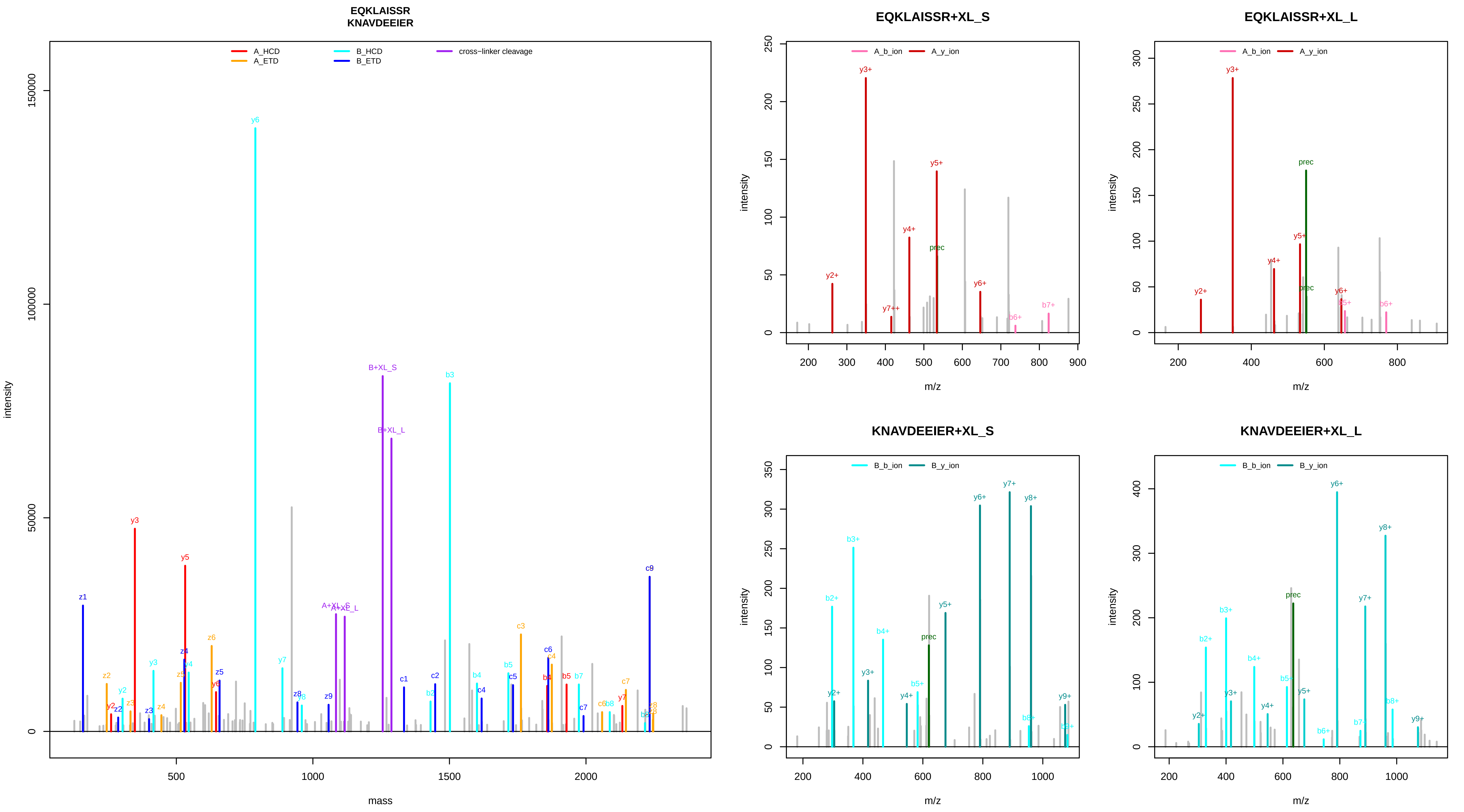
EQKLAISSR
KNAVDEEIER
A_HCD
A_ETD
B_HCD
B_ETD
cross−linker cleavage
intensity
mass
B+XL_S
B+XL_L
A+XL_S
A+XL_L
EQKLAISSR+XL_S
EQKLAISSR+XL_L
KNAVDEEIER+XL_S
KNAVDEEIER+XL_L
A_b_ion
A_y_ion
B_b_ion
B_y_ion
prec
m/z

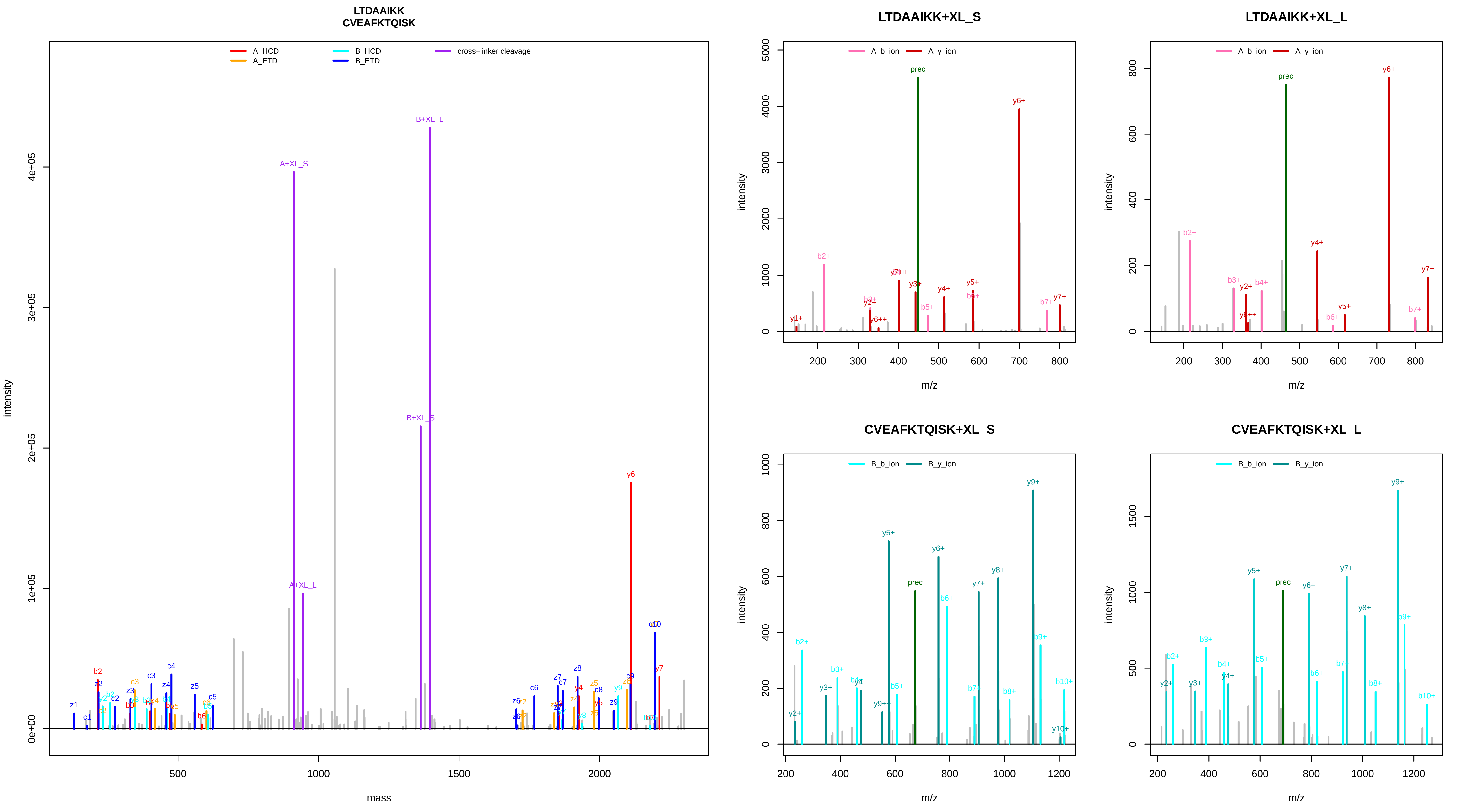
LTDAAIKK
CVEAFKTQISK
A_HCD
A_ETD
B_HCD
B_ETD
cross−linker cleavage
A+XL_S
B+XL_L
B+XL_S
A+XL_L
y6
y7
c10
intensity
mass
LTDAAIKK+XL_S
LTDAAIKK+XL_L
A_b_ion
A_y_ion
prec
y6+
y7+
b2+
y4+
y5+
m/z
CVEAFKTQISK+XL_S
CVEAFKTQISK+XL_L
B_b_ion
B_y_ion
y9+
y5+
y6+
y7+
y8+
b10+

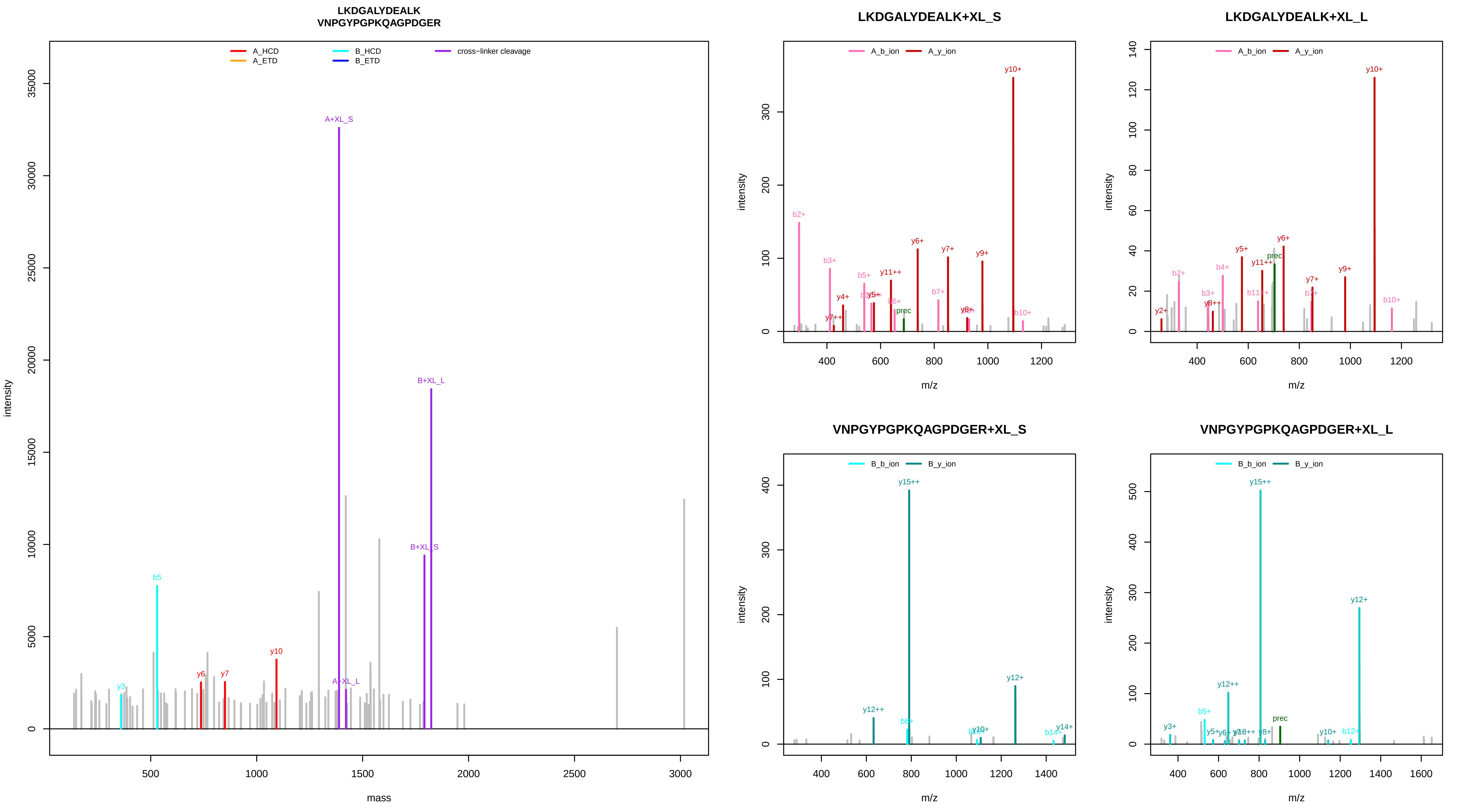
LKDGALYDEALK
VNPGYPGPKQAGPDGER
A_HCD
A_ETD
B_HCD
B_ETD
cross−linker cleavage
A+XL_S
B+XL_L
B+XL_S
A+XL_L
b5
y3
y6
y7
y10
intensity
mass
LKDGALYDEALK+XL_S
LKDGALYDEALK+XL_L
VNPGYPGPKQAGPDGER+XL_S
VNPGYPGPKQAGPDGER+XL_L
A_b_ion
A_y_ion
B_b_ion
B_y_ion
y10+
y6+
y7+
y9+
b2+
b3+
b5+
y11++
y4+
y7++
y5+
b6+
b7+
y8+
b10+
prec
y2+
y8++
b4+
b11++
y12+
y12++
y15++
y14+
b14+
b8+
y10+
y3+
y5+
y8+
b12+
m/z
intensity

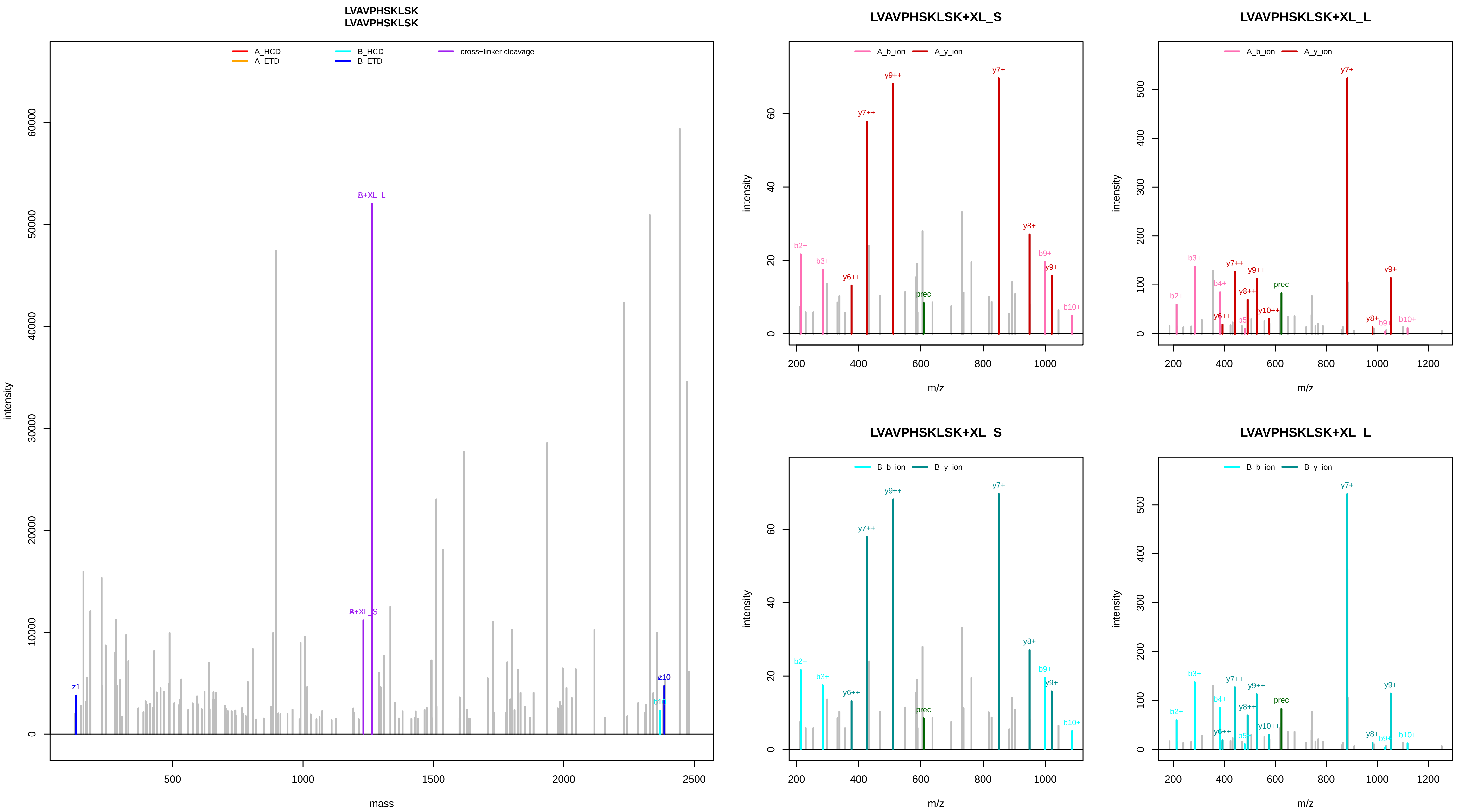
LVAVPHSKLSK
LVAVPHSKLSK
A_HCD
A_ETD
B_HCD
B_ETD
cross−linker cleavage
intensity
mass
LVAVPHSKLSK+XL_S
LVAVPHSKLSK+XL_L
A_b_ion
A_y_ion
B_b_ion
B_y_ion
prec
intensity
m/z

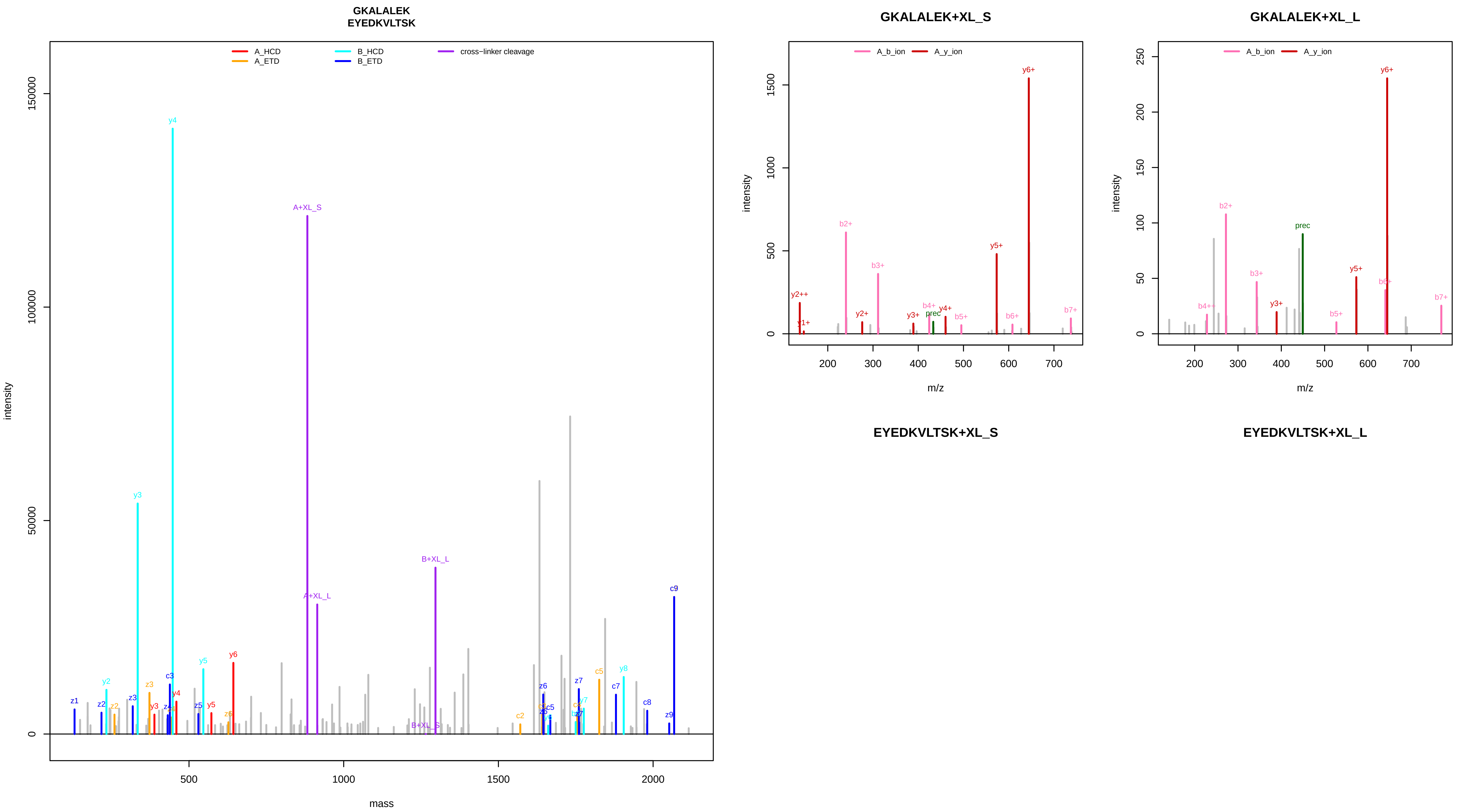
GKALALEK
EYEDKVLTSK
A_HCD
A_ETD
B_HCD
B_ETD
cross−linker cleavage
A+XL_S
A+XL_L
B+XL_L
B+XL_S
intensity
mass
GKALALEK+XL_S
GKALALEK+XL_L
A_b_ion
A_y_ion
prec
m/z
EYEDKVLTSK+XL_S
EYEDKVLTSK+XL_L

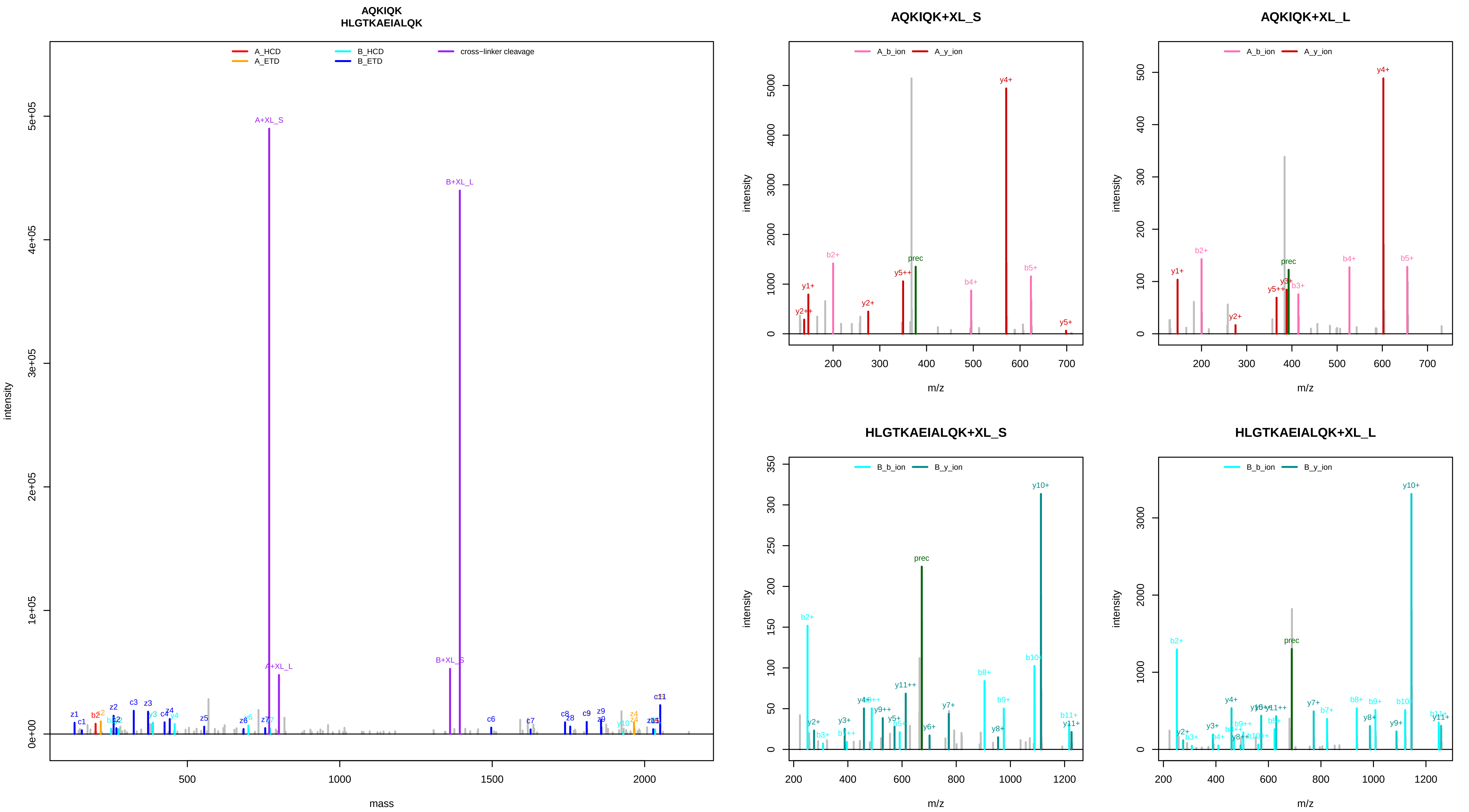
AQKIQK
HLGTKAEIALQK
A_HCD
A_ETD
B_HCD
B_ETD
cross−linker cleavage
A+XL_S
B+XL_L
A+XL_L
B+XL_S
intensity
mass
AQKIQK+XL_S
AQKIQK+XL_L
A_b_ion
A_y_ion
HLGTKAEIALQK+XL_S
HLGTKAEIALQK+XL_L
B_b_ion
B_y_ion
prec
m/z

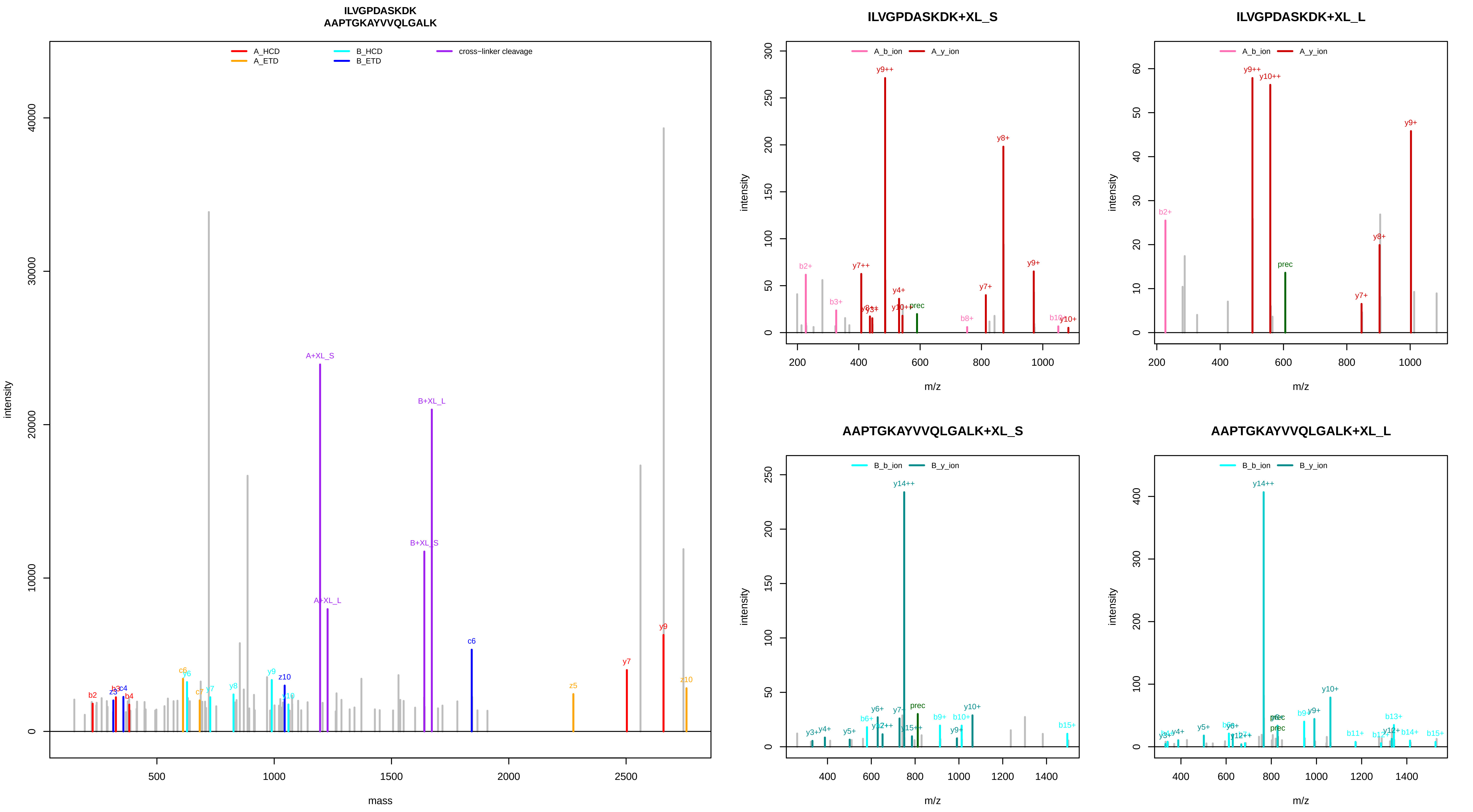
ILVGPDASKDK
AAPTGKAYVVQLGALK
A_HCD
A_ETD
B_HCD
B_ETD
cross−linker cleavage
A+XL_S
A+XL_L
B+XL_L
B+XL_S
intensity
mass
ILVGPDASKDK+XL_S
ILVGPDASKDK+XL_L
AAPTGKAYVVQLGALK+XL_S
AAPTGKAYVVQLGALK+XL_L
A_b_ion
A_y_ion
B_b_ion
B_y_ion
prec
m/z

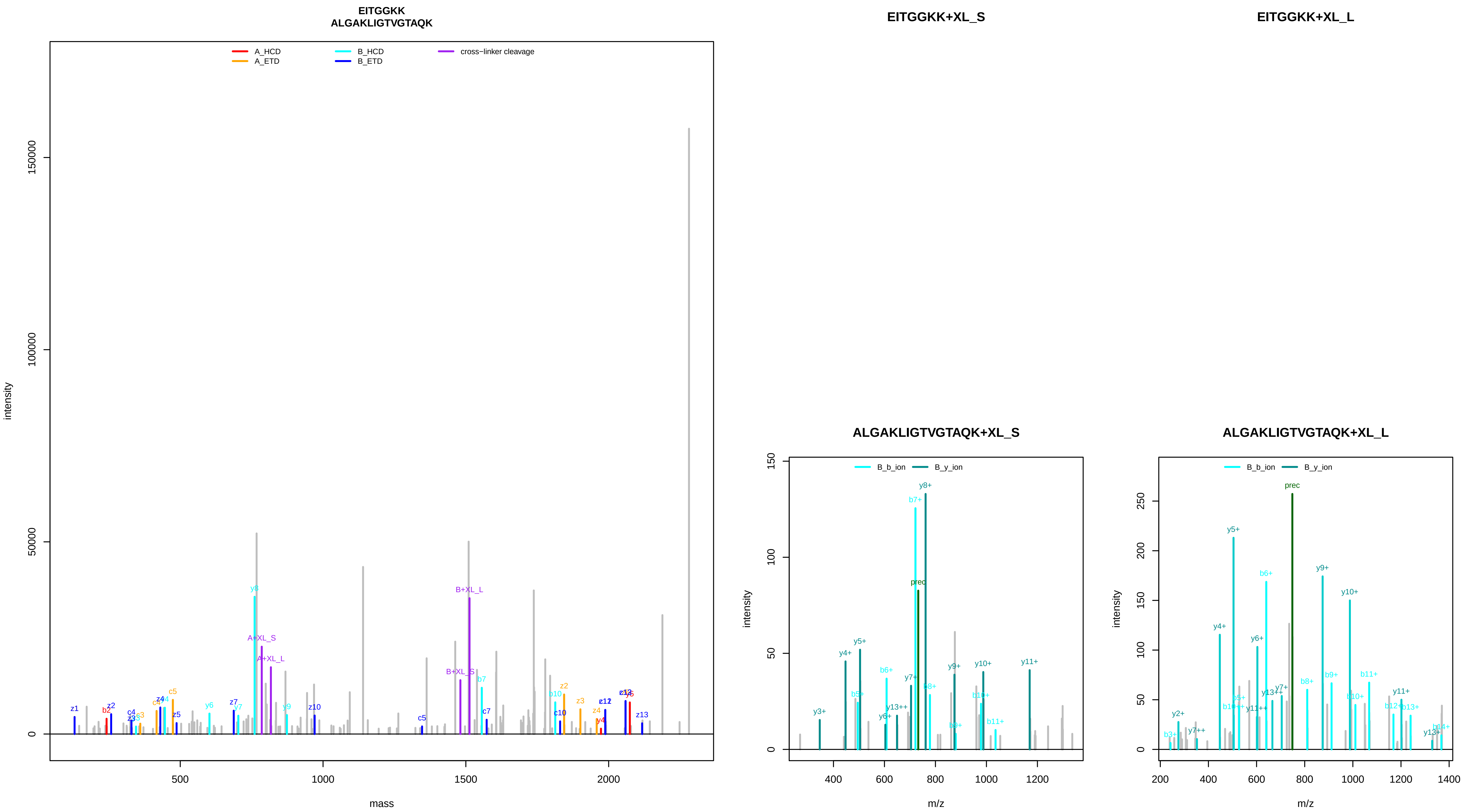
EITGGKK
ALGAKLIGTVGTAQK
A_HCD
A_ETD
B_HCD
B_ETD
cross−linker cleavage
intensity
mass
A+XL_S
A+XL_L
B+XL_S
B+XL_L
EITGGKK+XL_S
EITGGKK+XL_L
ALGAKLIGTVGTAQK+XL_S
ALGAKLIGTVGTAQK+XL_L
B_b_ion
B_y_ion
prec
m/z
intensity

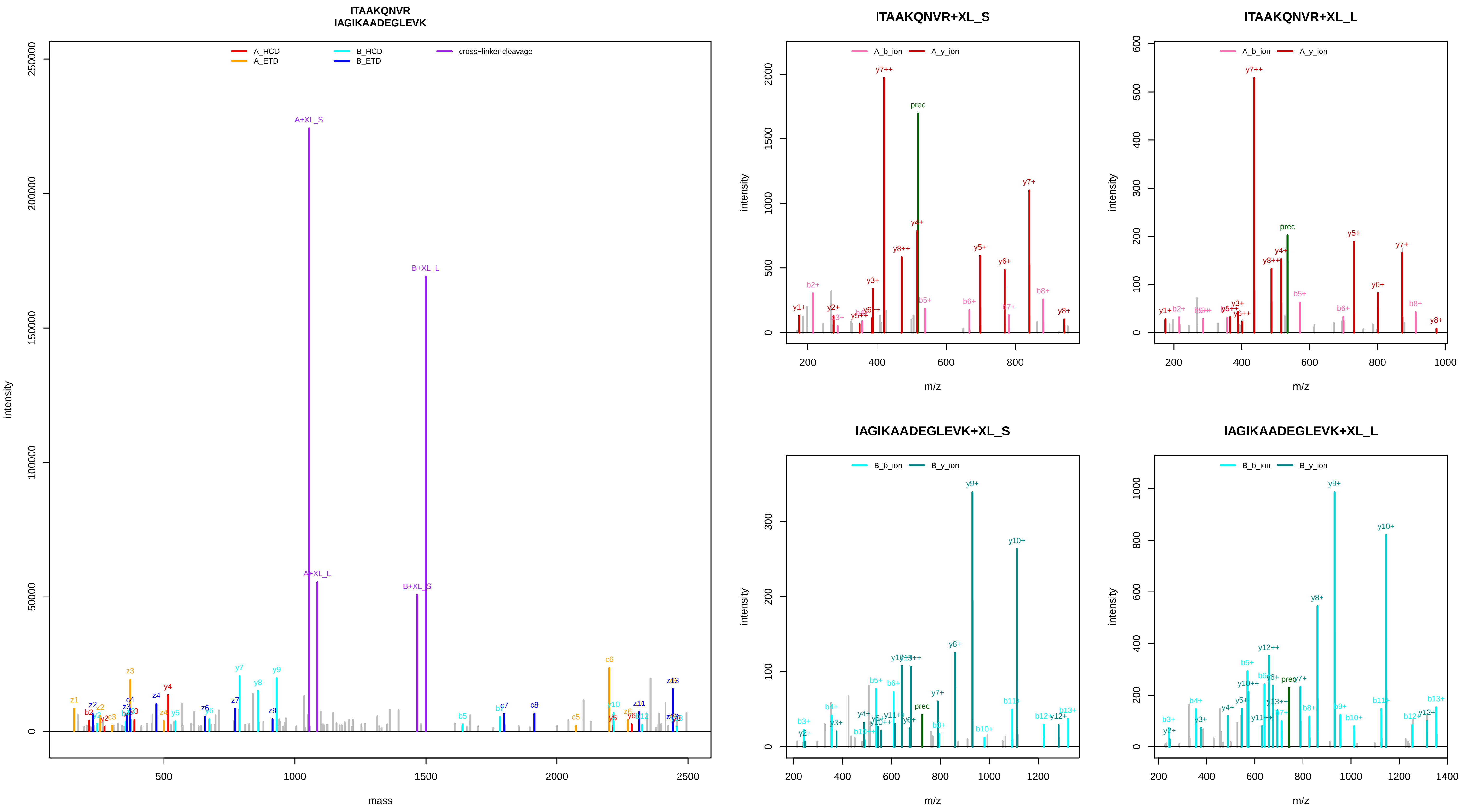
ITAAKQNVR
IAGIKAADEGLEVK
A_HCD
A_ETD
B_HCD
B_ETD
cross−linker cleavage
A+XL_S
B+XL_L
A+XL_L
B+XL_S
intensity
mass
ITAAKQNVR+XL_S
ITAAKQNVR+XL_L
IAGIKAADEGLEVK+XL_S
IAGIKAADEGLEVK+XL_L
A_b_ion
A_y_ion
B_b_ion
B_y_ion
prec
m/z

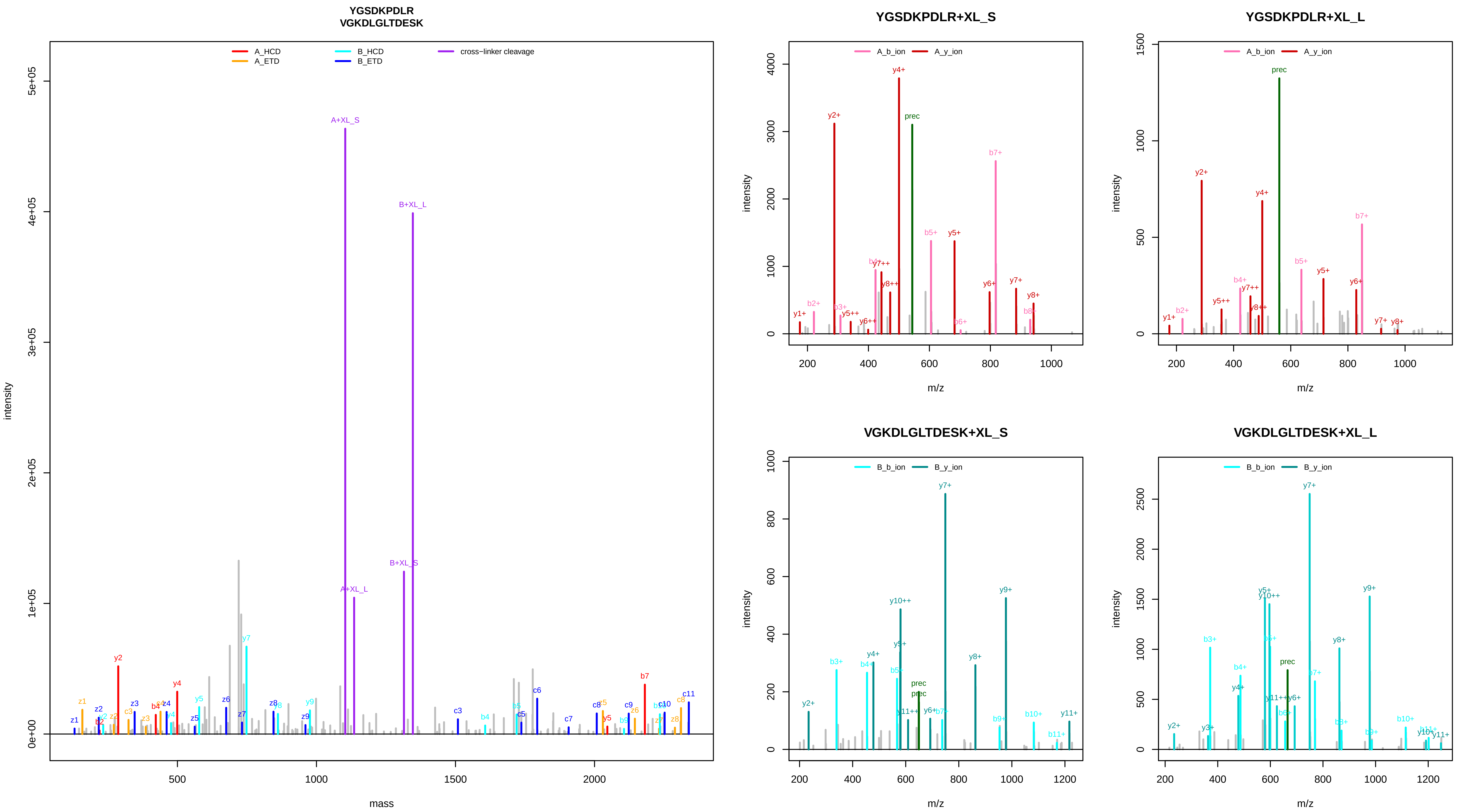
YGSDKPDLR
VGKDLGLTDESK
A_HCD
A_ETD
B_HCD
B_ETD
cross−linker cleavage
A+XL_S
B+XL_L
B+XL_S
A+XL_L
intensity
mass
YGSDKPDLR+XL_S
YGSDKPDLR+XL_L
A_b_ion
A_y_ion
VGKDLGLTDESK+XL_S
VGKDLGLTDESK+XL_L
B_b_ion
B_y_ion
prec
m/z

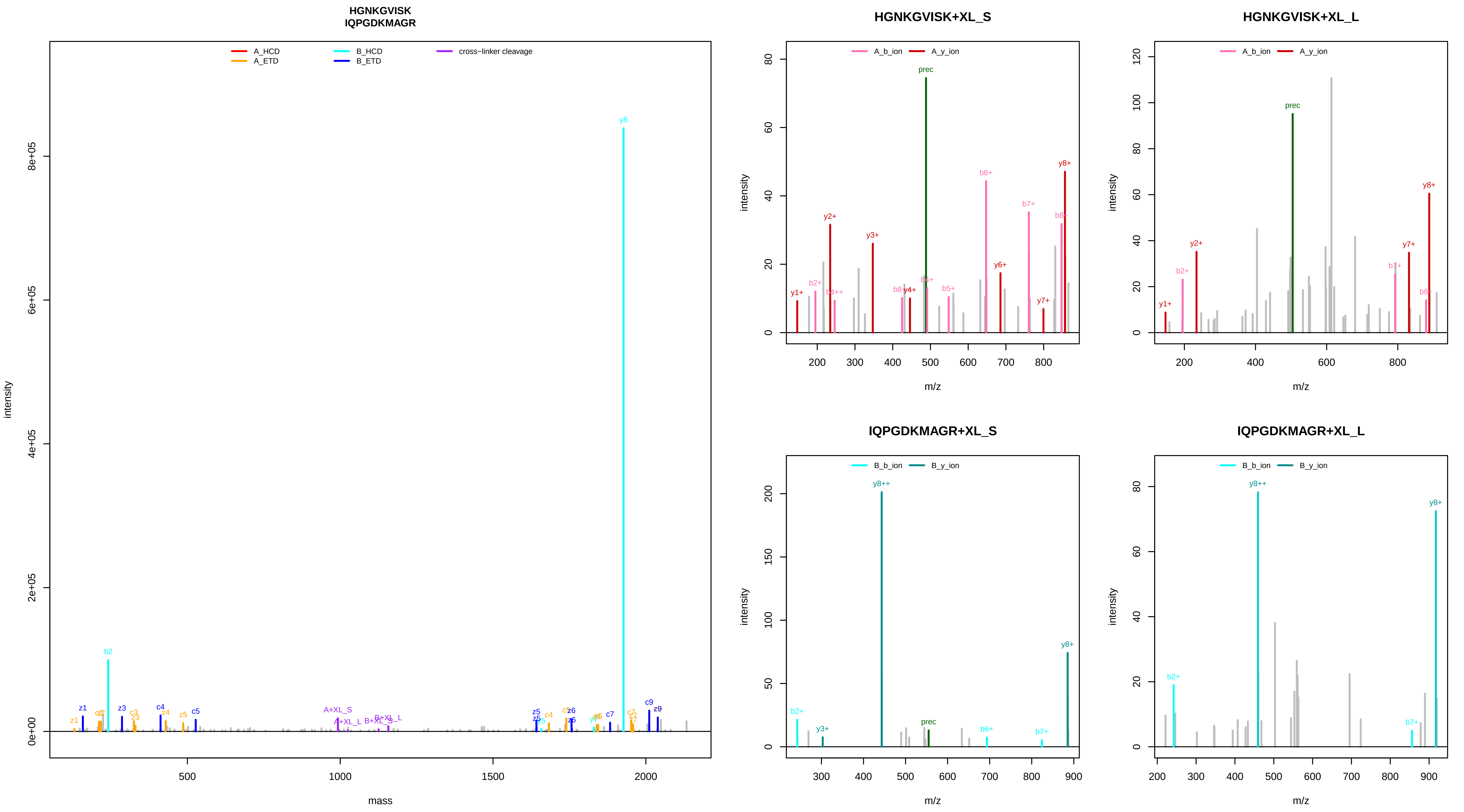
HGNKGVISK
IQPGDKMAGR
A_HCD
A_ETD
B_HCD
B_ETD
cross−linker cleavage
intensity
mass
0e+00
2e+05
4e+05
6e+05
8e+05
500
1000
1500
2000
y8
b2
z1
z3
c4
z4
c5
z5
A+XL_S
A+XL_L
B+XL_S
B+XL_L
z6
c7
c9
z9
HGNKGVISK+XL_S
HGNKGVISK+XL_L
IQPGDKMAGR+XL_S
IQPGDKMAGR+XL_L
A_b_ion
A_y_ion
B_b_ion
B_y_ion
prec
y1+
y2+
y3+
y4+
y6+
y7+
y8+
b2+
b5+
b6+
b7+
y8++
b7+
m/z
intensity

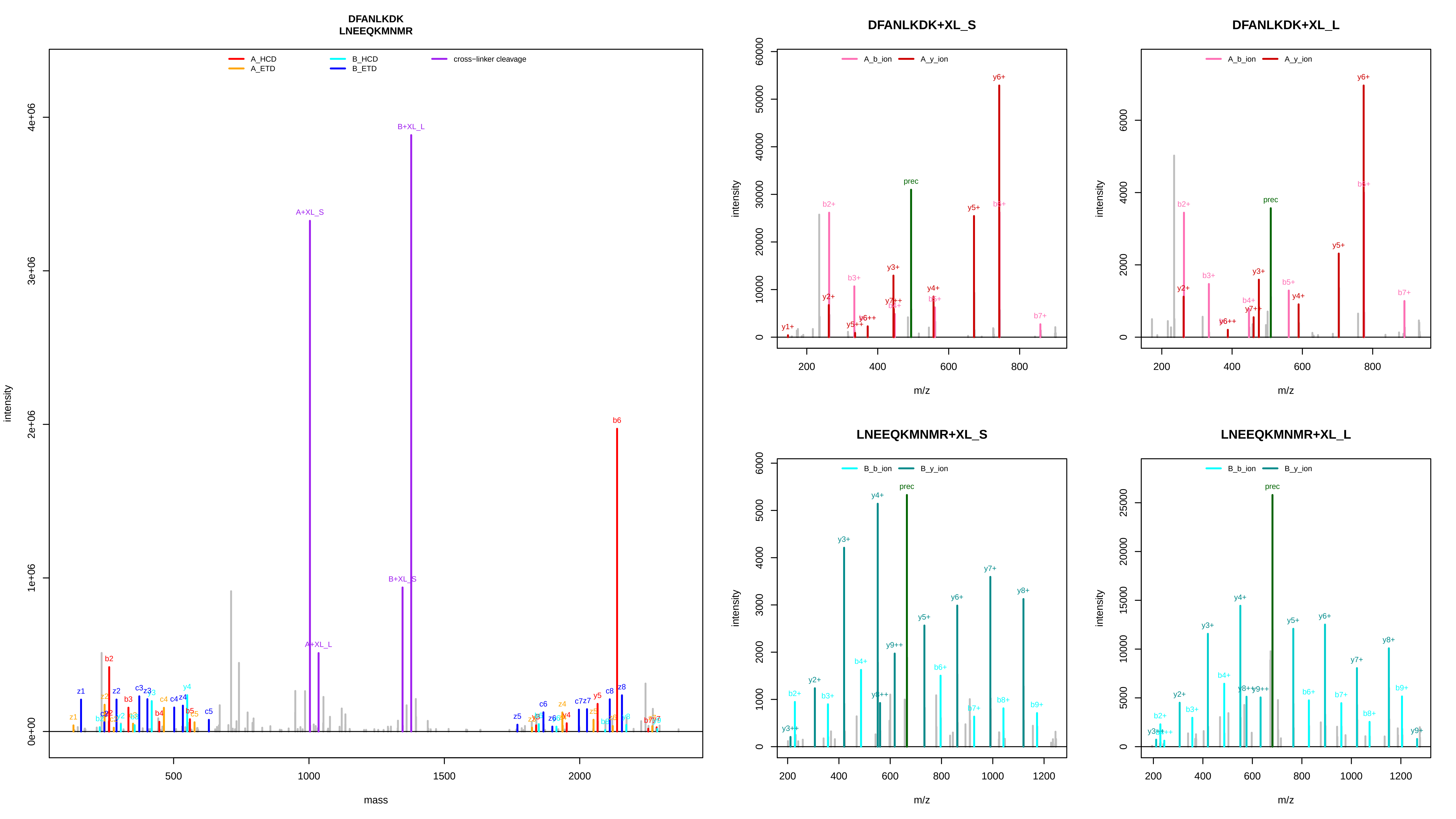
DFANLKDK
LNEEQKMNMR
A_HCD
A_ETD
B_HCD
B_ETD
cross−linker cleavage
A+XL_S
A+XL_L
B+XL_S
B+XL_L
intensity
mass
DFANLKDK+XL_S
DFANLKDK+XL_L
LNEEQKMNMR+XL_S
LNEEQKMNMR+XL_L
A_b_ion
A_y_ion
B_b_ion
B_y_ion
prec
m/z

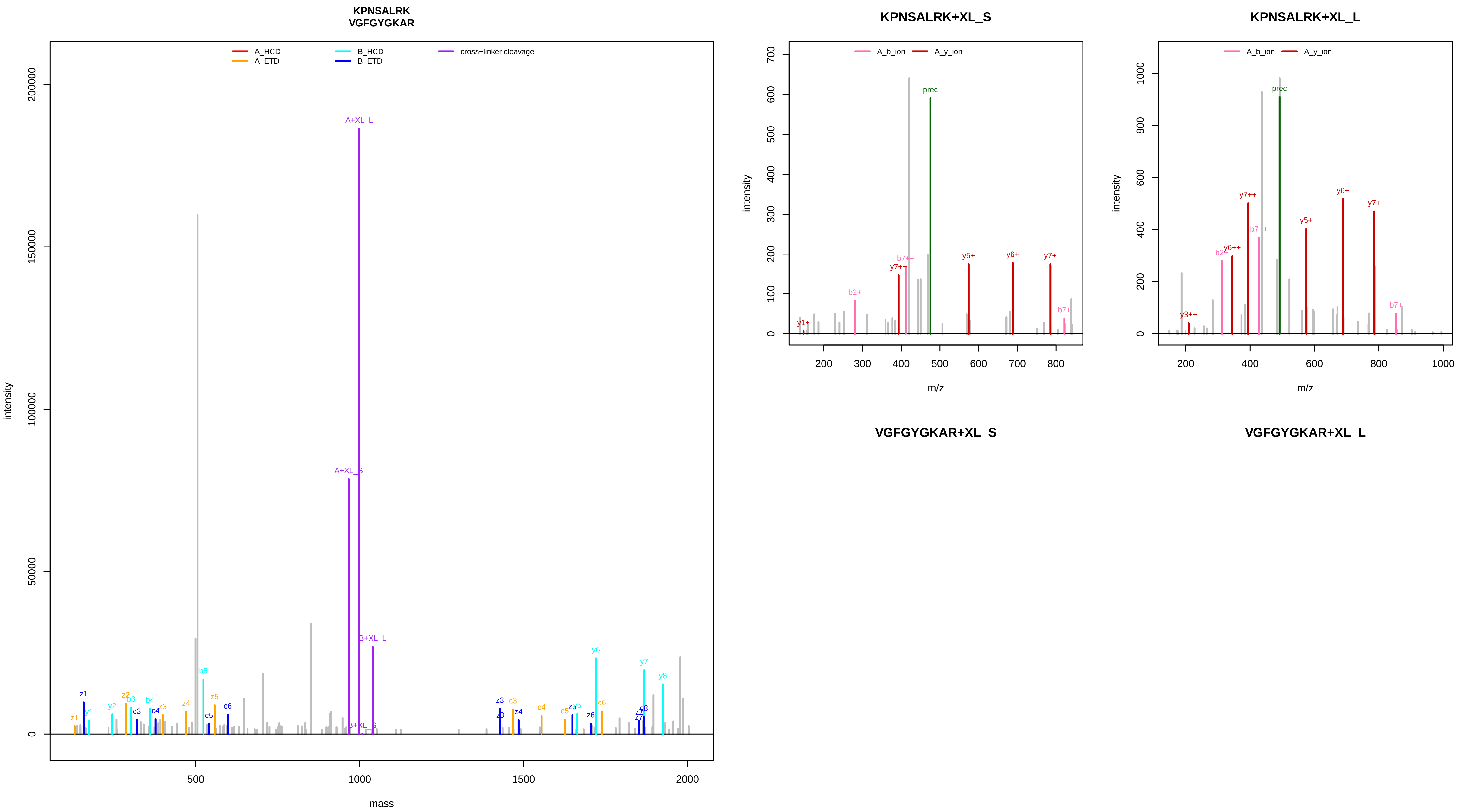

KPNSALRK
VGFGYGKAR
A_HCD
A_ETD
B_HCD
B_ETD
cross−linker cleavage
intensity
mass
A+XL_L
A+XL_S
B+XL_L
B+XL_S
KPNSALRK+XL_S
KPNSALRK+XL_L
A_b_ion
A_y_ion
prec
m/z
VGFGYGKAR+XL_S
VGFGYGKAR+XL_L

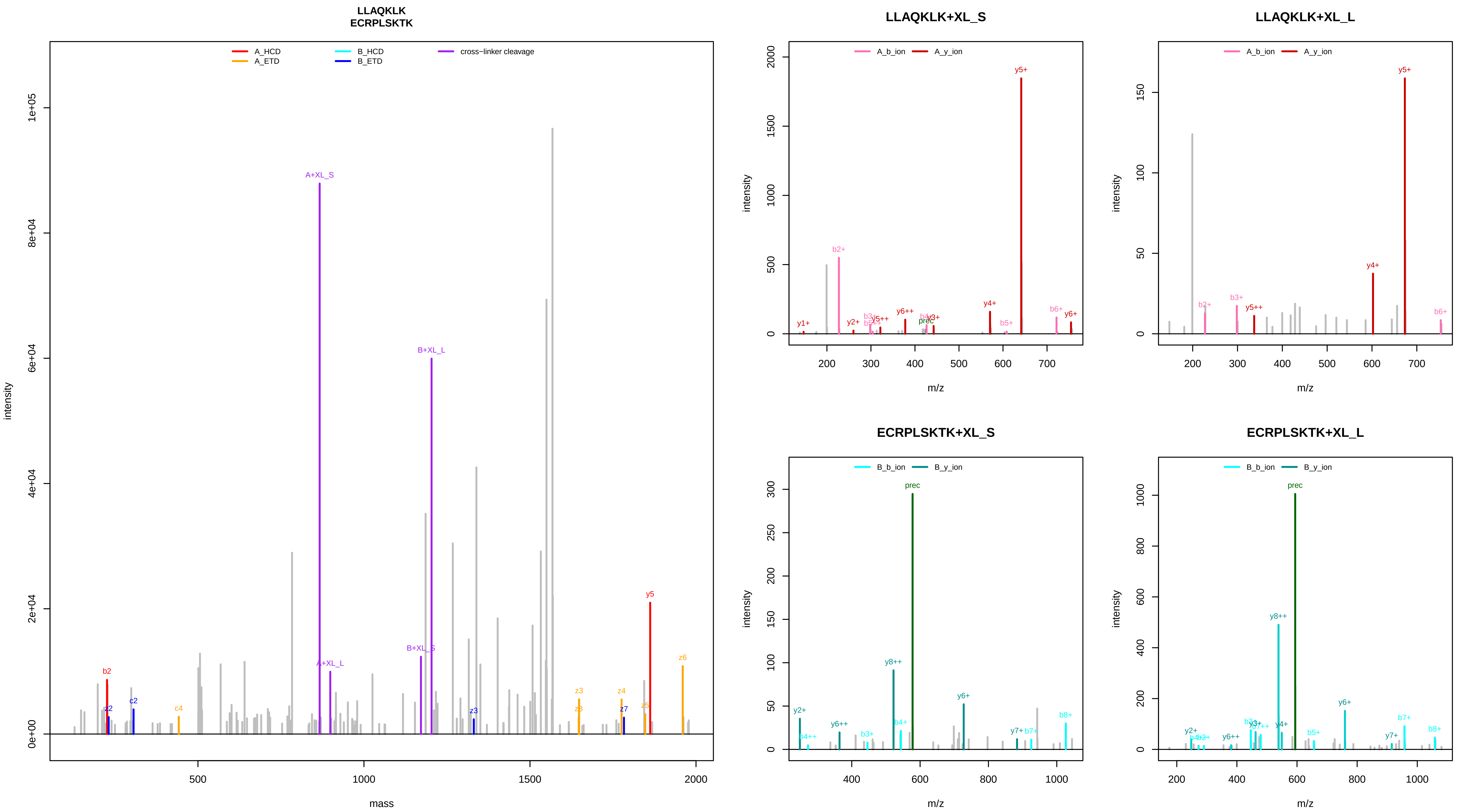

LLAQKLK
ECRPLSKTK
A_HCD
A_ETD
B_HCD
B_ETD
cross−linker cleavage
A+XL_S
B+XL_L
B+XL_S
A+XL_L
b2
c2
z2
c4
z3
y5
z6
z4
z7
z5
intensity
mass
LLAQKLK+XL_S
LLAQKLK+XL_L
ECRPLSKTK+XL_S
ECRPLSKTK+XL_L
A_b_ion
A_y_ion
B_b_ion
B_y_ion
y5+
y4+
b2+
b6+
y6+
y6++
prec
y8++
y6+
b8+
b7+
m/z

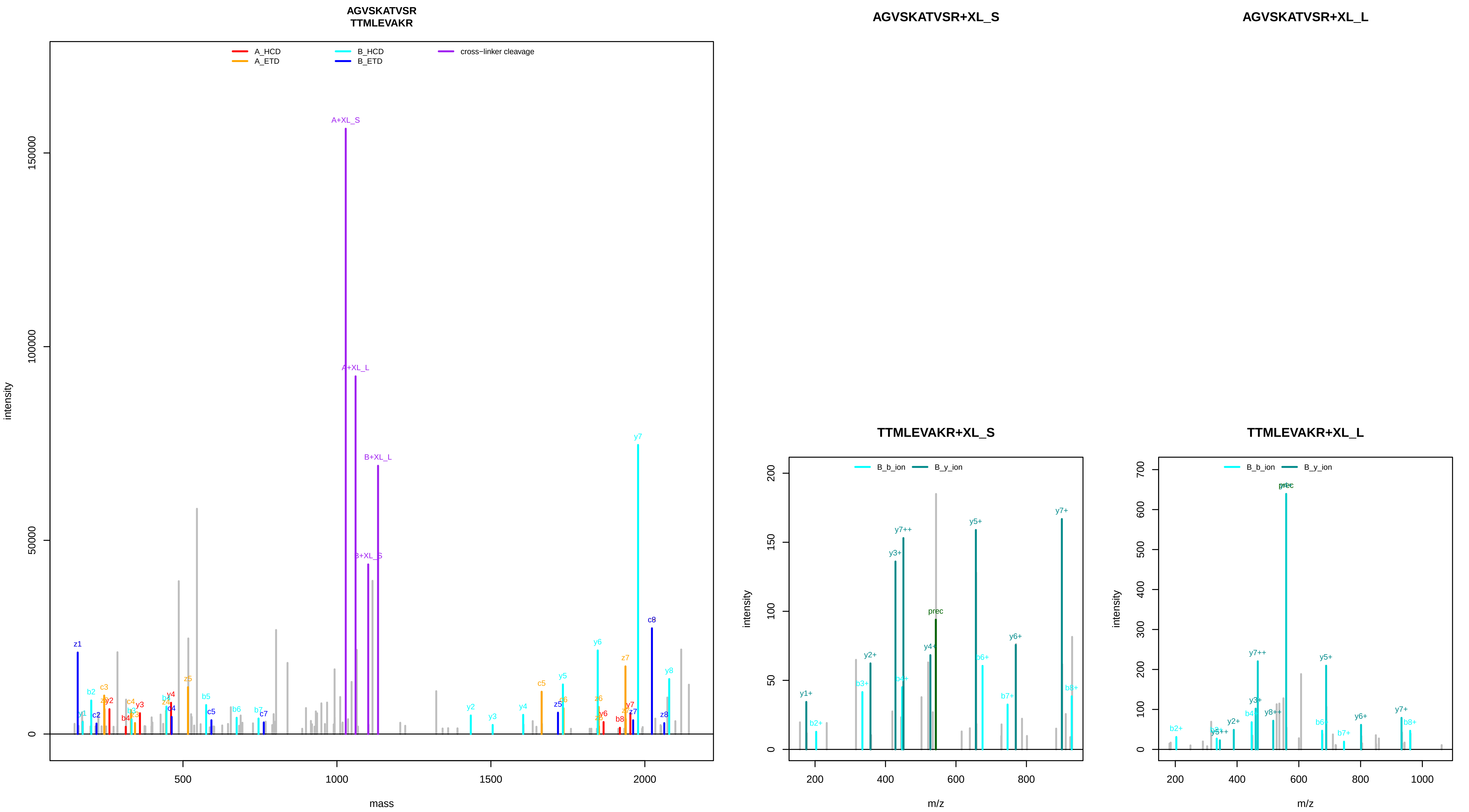

AGVSKATVSR
TTMLEVAKR
A_HCD
A_ETD
B_HCD
B_ETD
cross−linker cleavage
A+XL_S
A+XL_L
B+XL_L
B+XL_S
intensity
mass
AGVSKATVSR+XL_S
AGVSKATVSR+XL_L
TTMLEVAKR+XL_S
TTMLEVAKR+XL_L
B_b_ion
B_y_ion
prec
intensity
m/z

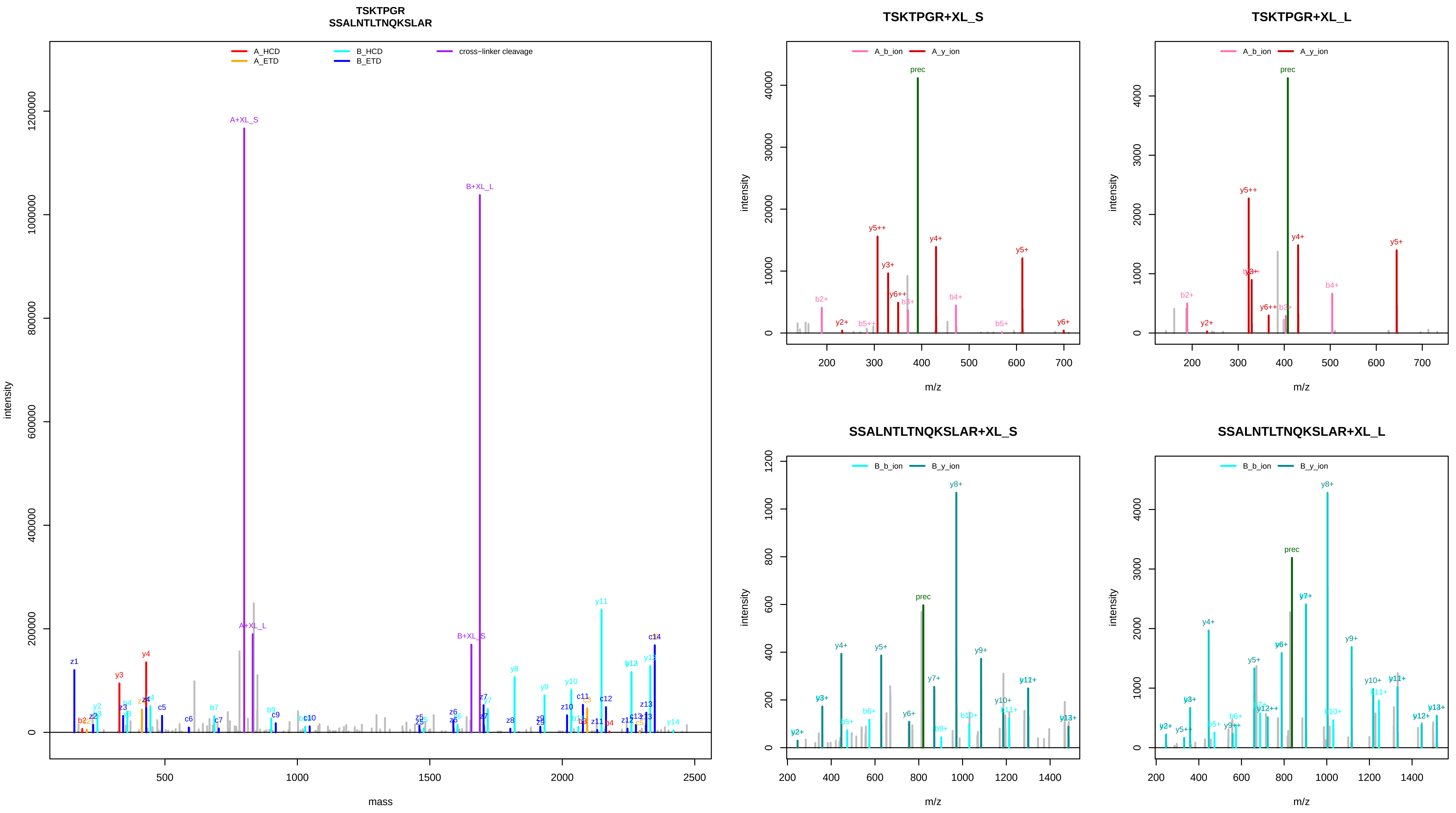
TSKTPGR
SSALNTLTNQKSLAR
A_HCD
A_ETD
B_HCD
B_ETD
cross−linker cleavage
A+XL_S
B+XL_L
A+XL_L
B+XL_S
intensity
mass
TSKTPGR+XL_S
TSKTPGR+XL_L
A_b_ion
A_y_ion
prec
m/z
SSALNTLTNQKSLAR+XL_S
SSALNTLTNQKSLAR+XL_L
B_b_ion
B_y_ion

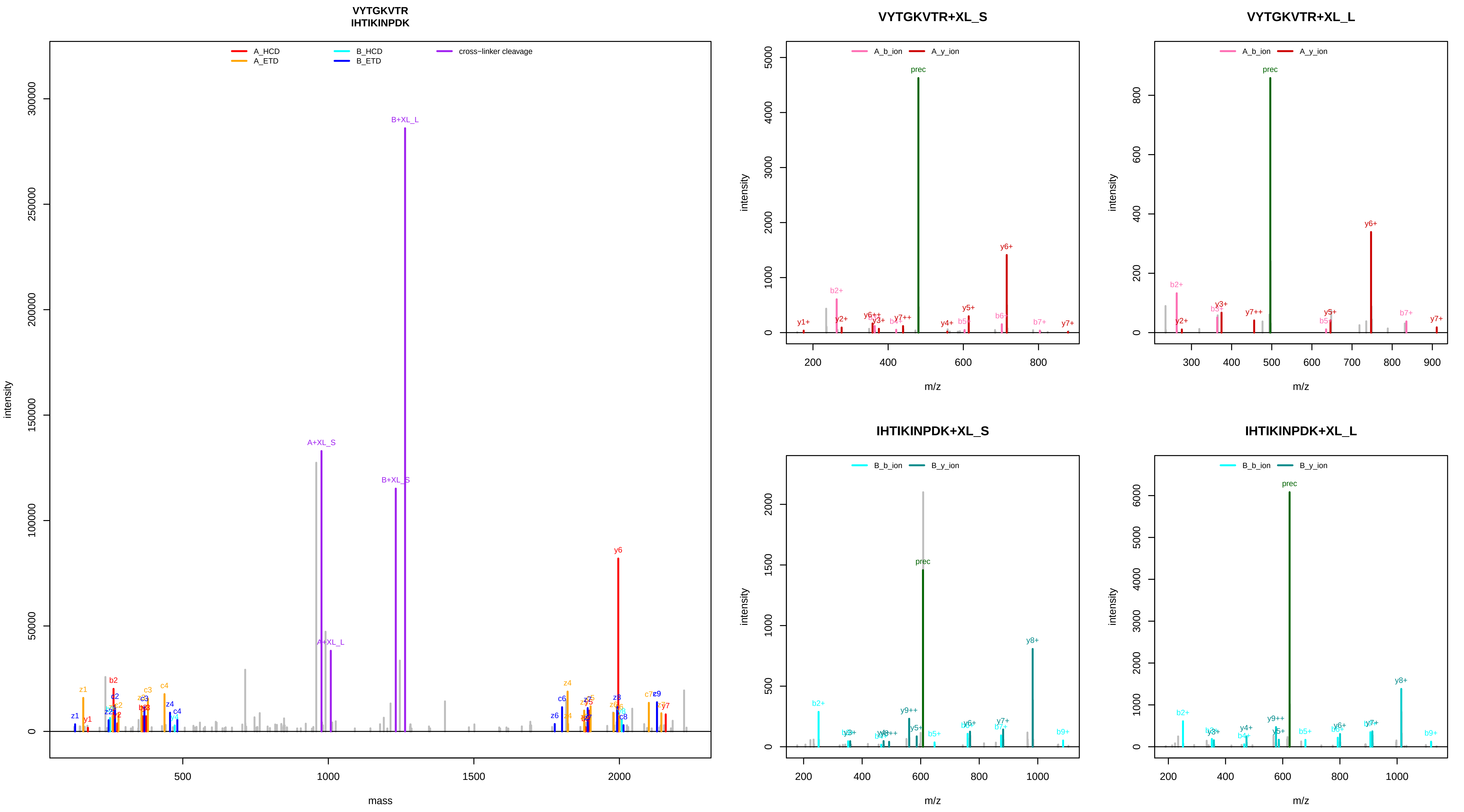

VYTGKVTR
IHTIKINPDK
A_HCD
A_ETD
B_HCD
B_ETD
cross−linker cleavage
B+XL_L
A+XL_S
B+XL_S
A+XL_L
y6
intensity
mass
VYTGKVTR+XL_S
VYTGKVTR+XL_L
IHTIKINPDK+XL_S
IHTIKINPDK+XL_L
A_b_ion
A_y_ion
B_b_ion
B_y_ion
prec
m/z

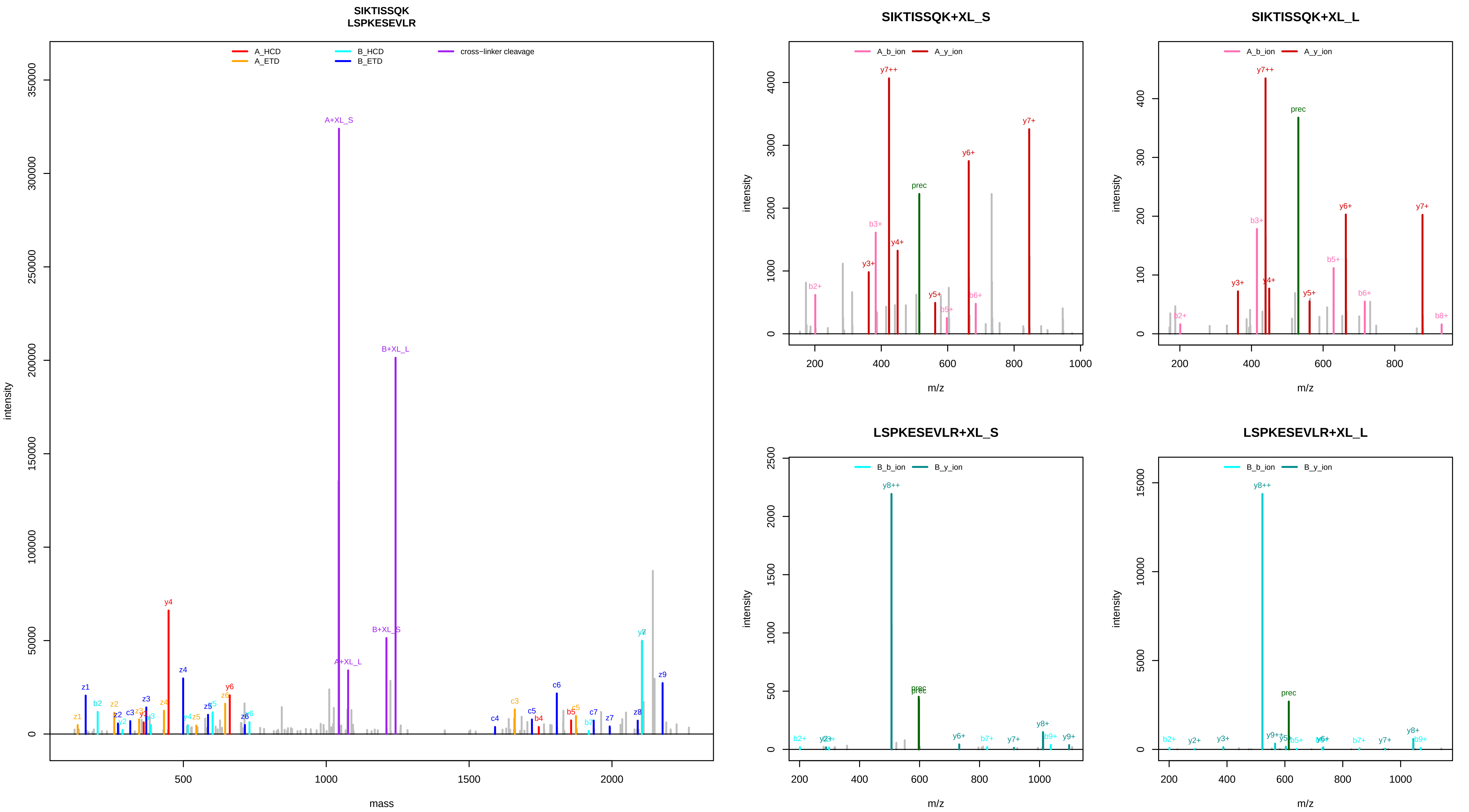

SIKTISSQK
LSPKESEVLR
A_HCD
A_ETD
B_HCD
B_ETD
cross−linker cleavage
A+XL_S
B+XL_L
B+XL_S
A+XL_L
intensity
mass
SIKTISSQK+XL_S
SIKTISSQK+XL_L
A_b_ion
A_y_ion
LSPKESEVLR+XL_S
LSPKESEVLR+XL_L
B_b_ion
B_y_ion
m/z
prec
y7++
y7+
y6+
y8++

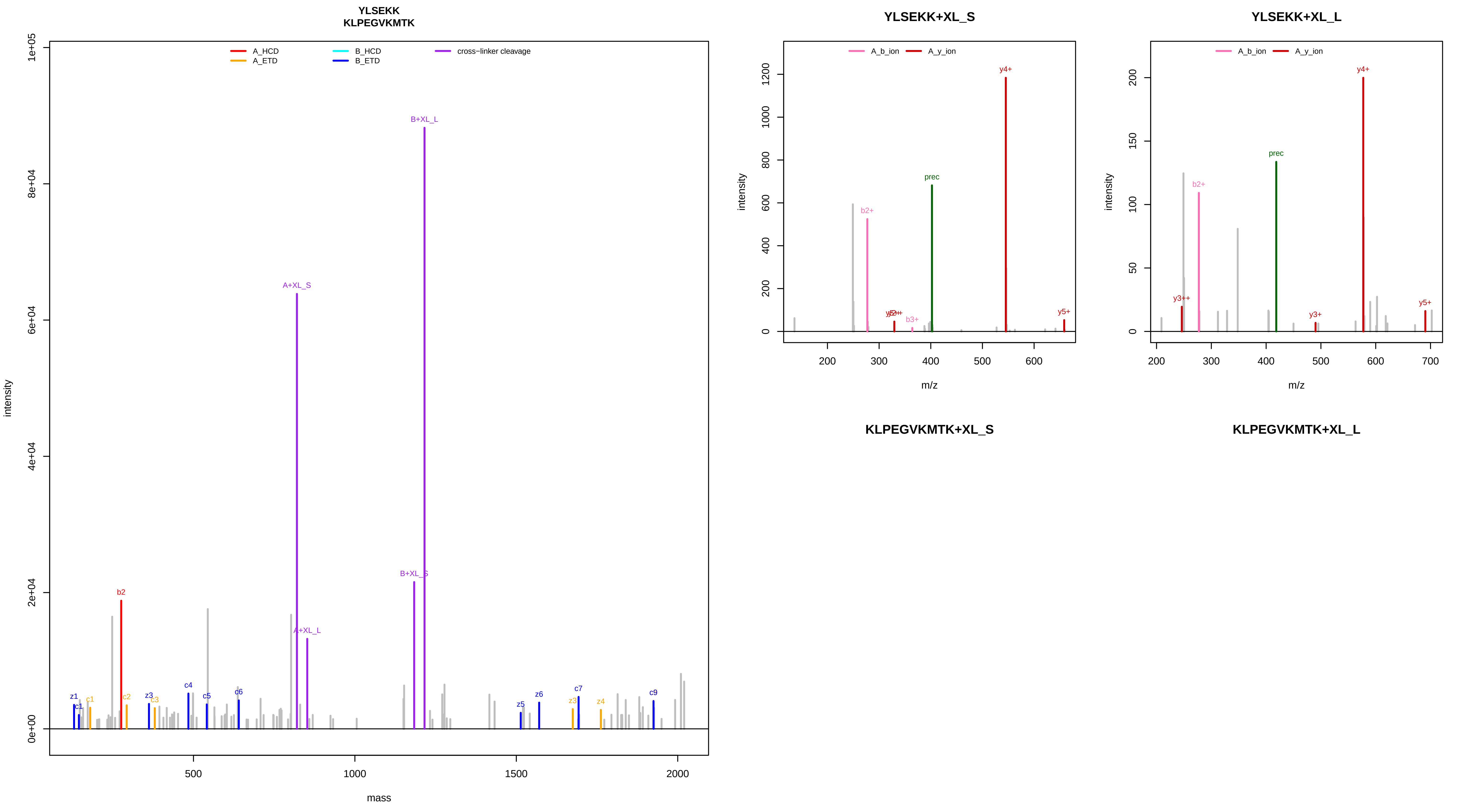
YLSEKK
KLPEGVKMTK
A_HCD
A_ETD
B_HCD
B_ETD
cross−linker cleavage
B+XL_L
A+XL_S
B+XL_S
A+XL_L
b2
z1
c1
c2
z3
c3
c4
c5
c6
z5
z6
c7
z3
z4
c9
intensity
mass
YLSEKK+XL_S
A_b_ion
A_y_ion
y4+
prec
b2+
b3+
y5+
m/z
YLSEKK+XL_L
y3++
y3+
KLPEGVKMTK+XL_S
KLPEGVKMTK+XL_L

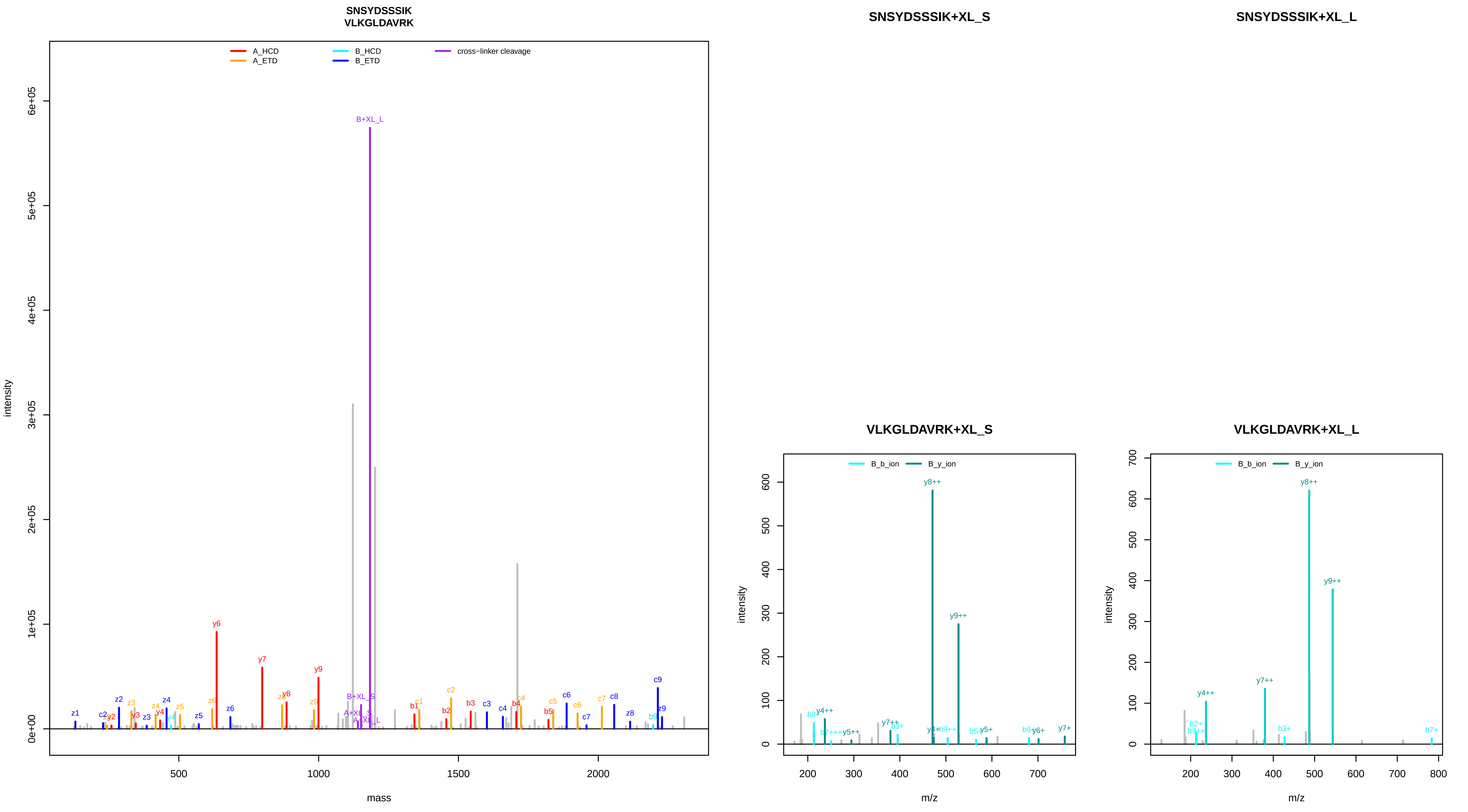
SNSYDSSSIK
VLKGLDAVRK
A_HCD
A_ETD
B_HCD
B_ETD
cross−linker cleavage
B+XL_L
B+XL_S
A+XL_S
A+XL_L
intensity
mass
SNSYDSSSIK+XL_S
SNSYDSSSIK+XL_L
VLKGLDAVRK+XL_S
VLKGLDAVRK+XL_L
B_b_ion
B_y_ion
y8++
y9++
y4++
y7++
m/z

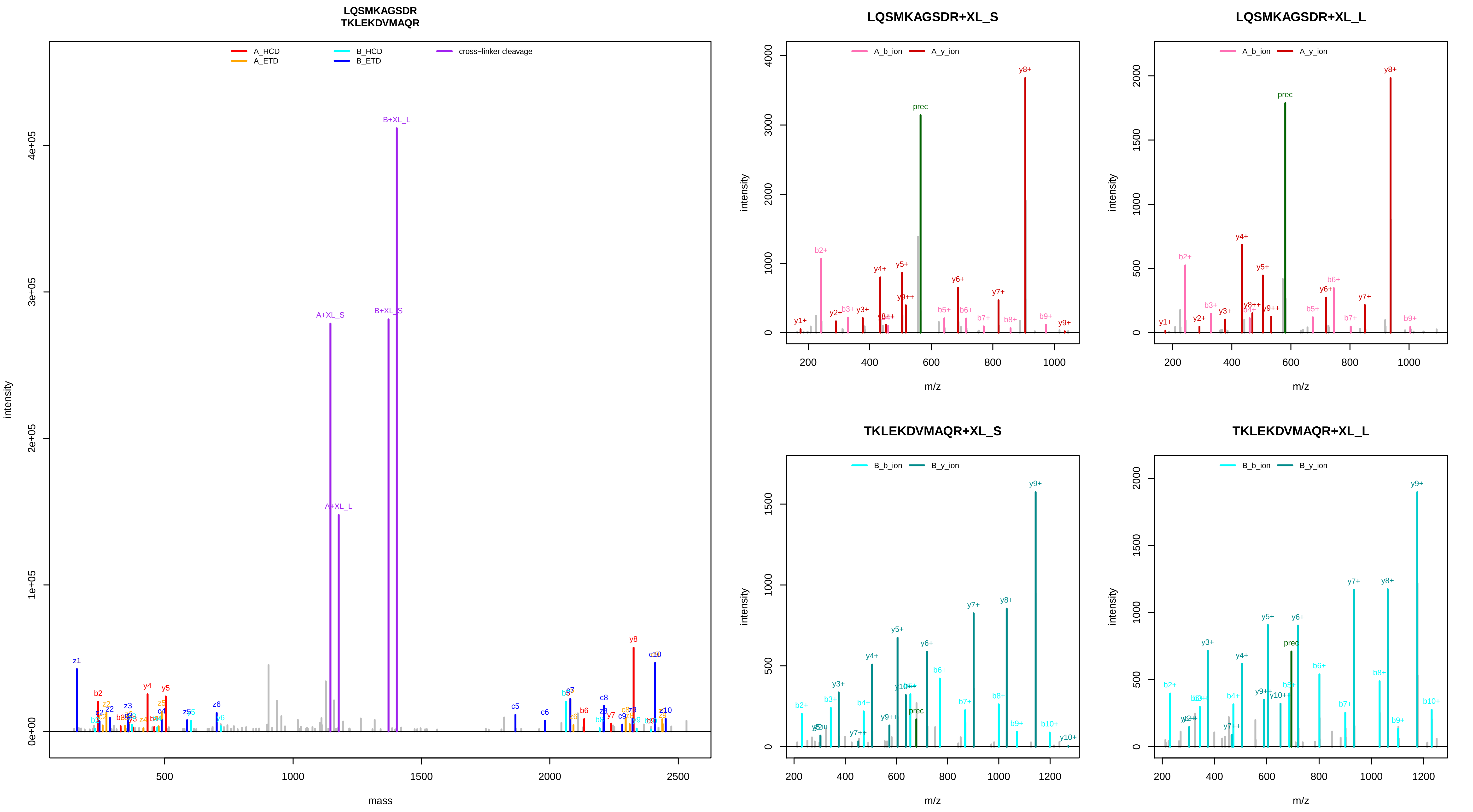

LQSMKAGSDR
TKLEKDVMAQR
A_HCD
A_ETD
B_HCD
B_ETD
cross−linker cleavage
B+XL_L
A+XL_S
B+XL_S
A+XL_L
intensity
mass
LQSMKAGSDR+XL_S
LQSMKAGSDR+XL_L
TKLEKDVMAQR+XL_S
TKLEKDVMAQR+XL_L
A_b_ion
A_y_ion
B_b_ion
B_y_ion
prec
m/z

GGSEELYKK
YAEGYPGKR

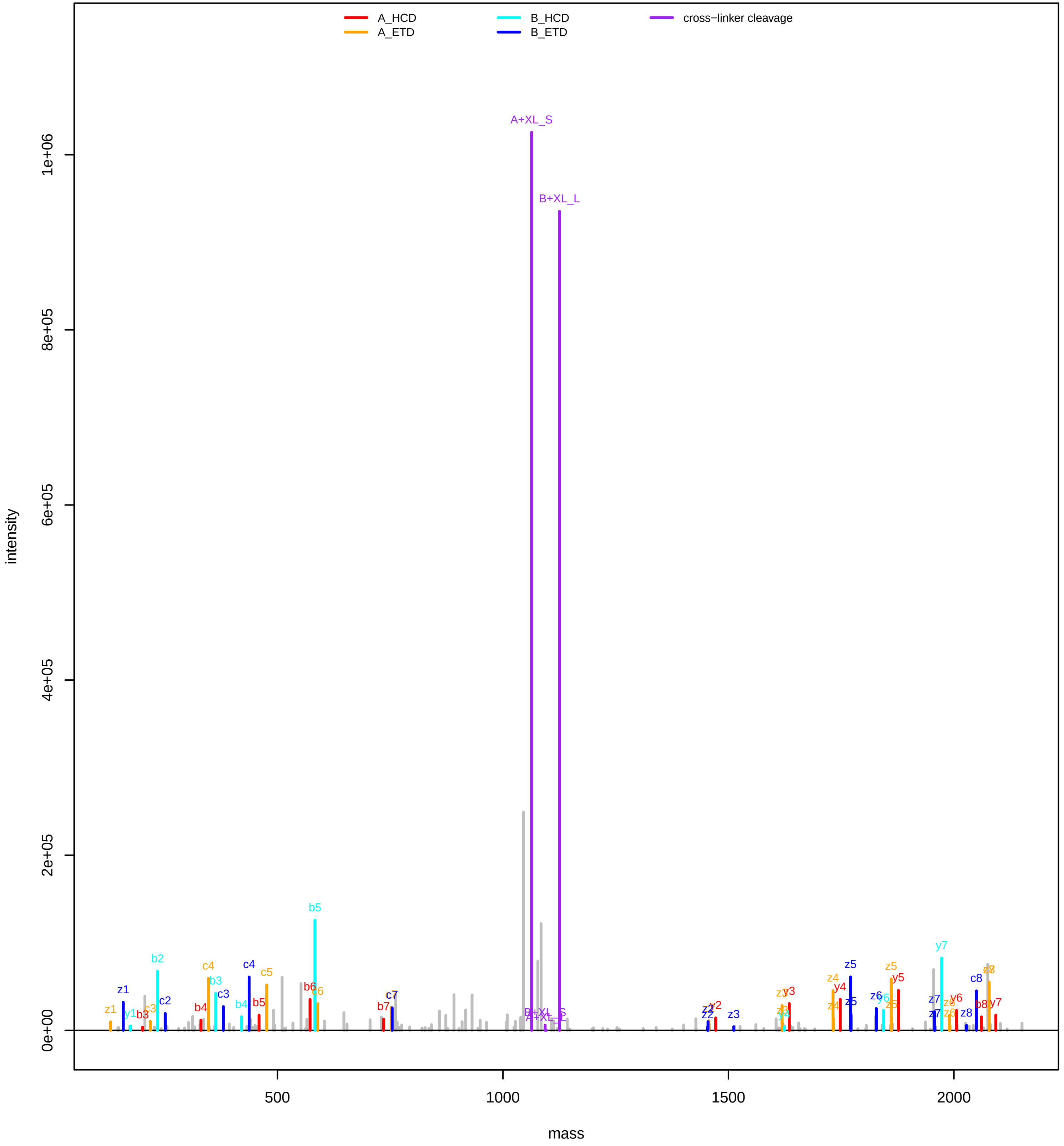

GGSEELYKK+XL_S

GGSEELYKK+XL_L

YAEGYPGKR+XL_S

YAEGYPGKR+XL_L

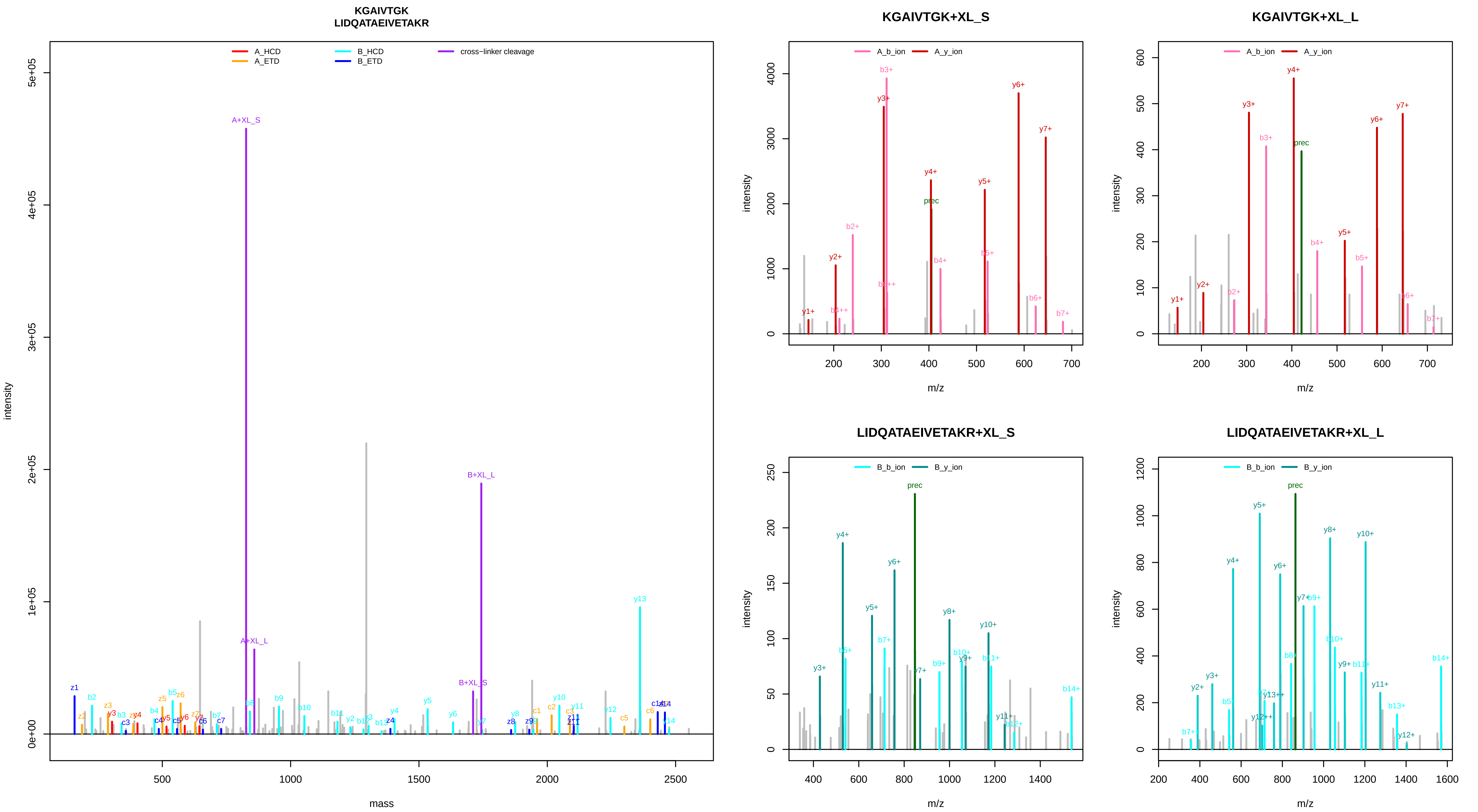

KGAIVTGK
LIDQATAEIVETAKR
A_HCD
A_ETD
B_HCD
B_ETD
cross−linker cleavage
A+XL_S
A+XL_L
B+XL_L
B+XL_S
intensity
mass
KGAIVTGK+XL_S
KGAIVTGK+XL_L
LIDQATAEIVETAKR+XL_S
LIDQATAEIVETAKR+XL_L
A_b_ion
A_y_ion
B_b_ion
B_y_ion
prec
m/z

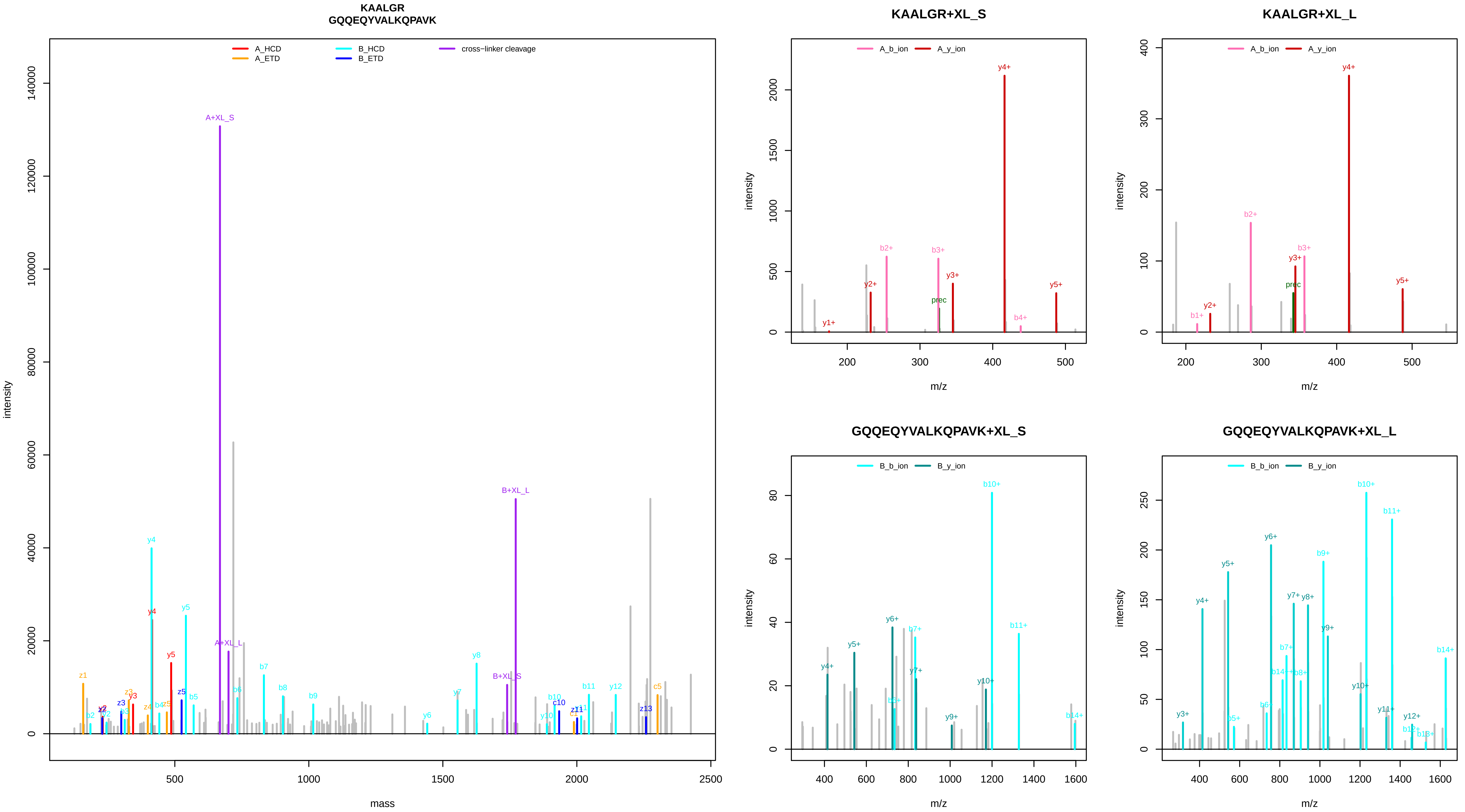

KAALGR
GQQEQYVALKQPAVK
A_HCD
A_ETD
B_HCD
B_ETD
cross−linker cleavage
A+XL_S
A+XL_L
B+XL_L
B+XL_S
intensity
mass
KAALGR+XL_S
KAALGR+XL_L
A_b_ion
A_y_ion
prec
m/z
GQQEQYVALKQPAVK+XL_S
GQQEQYVALKQPAVK+XL_L
B_b_ion
B_y_ion

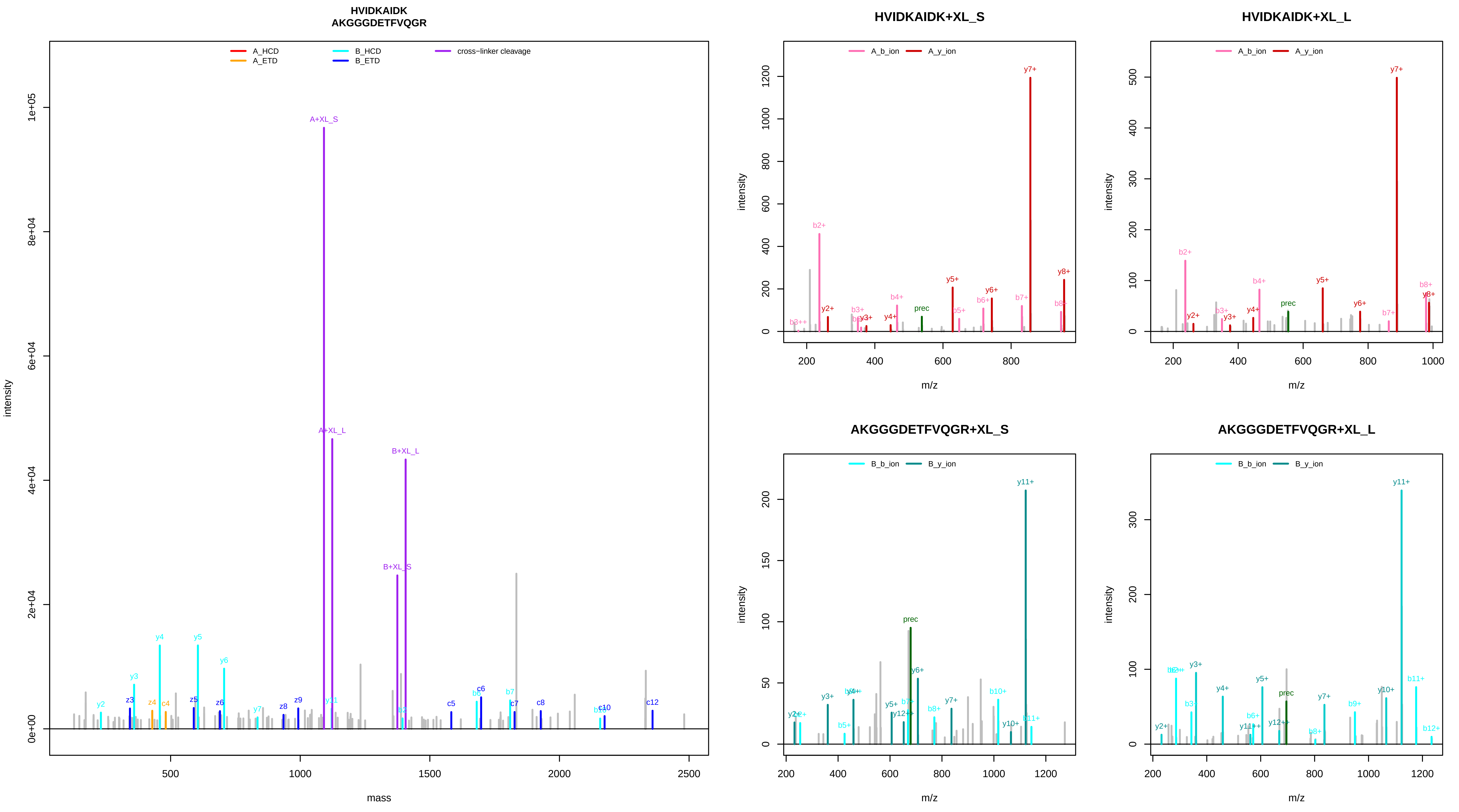

HVIDKAIDK
AKGGGDETFVQGR
A_HCD
A_ETD
B_HCD
B_ETD
cross−linker cleavage
A+XL_S
A+XL_L
B+XL_L
B+XL_S
intensity
mass
HVIDKAIDK+XL_S
HVIDKAIDK+XL_L
A_b_ion
A_y_ion
AKGGGDETFVQGR+XL_S
AKGGGDETFVQGR+XL_L
B_b_ion
B_y_ion
prec
m/z

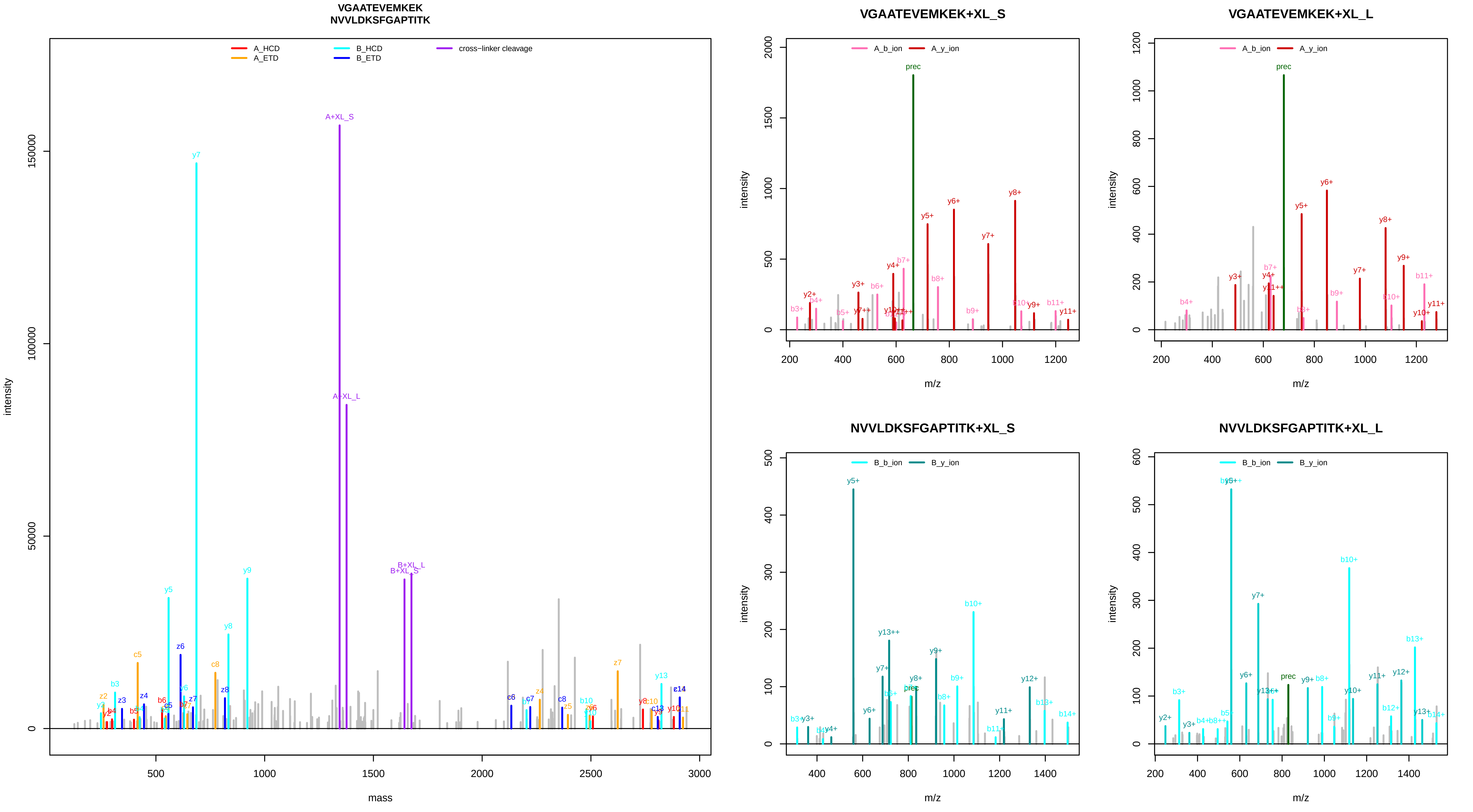
VGAATEVEMKEK
NVVLDKSFGAPTITK
A_HCD
A_ETD
B_HCD
B_ETD
cross−linker cleavage
A+XL_S
A+XL_L
B+XL_L
B+XL_S
intensity
mass
VGAATEVEMKEK+XL_S
VGAATEVEMKEK+XL_L
NVVLDKSFGAPTITK+XL_S
NVVLDKSFGAPTITK+XL_L
A_b_ion
A_y_ion
B_b_ion
B_y_ion
prec
m/z

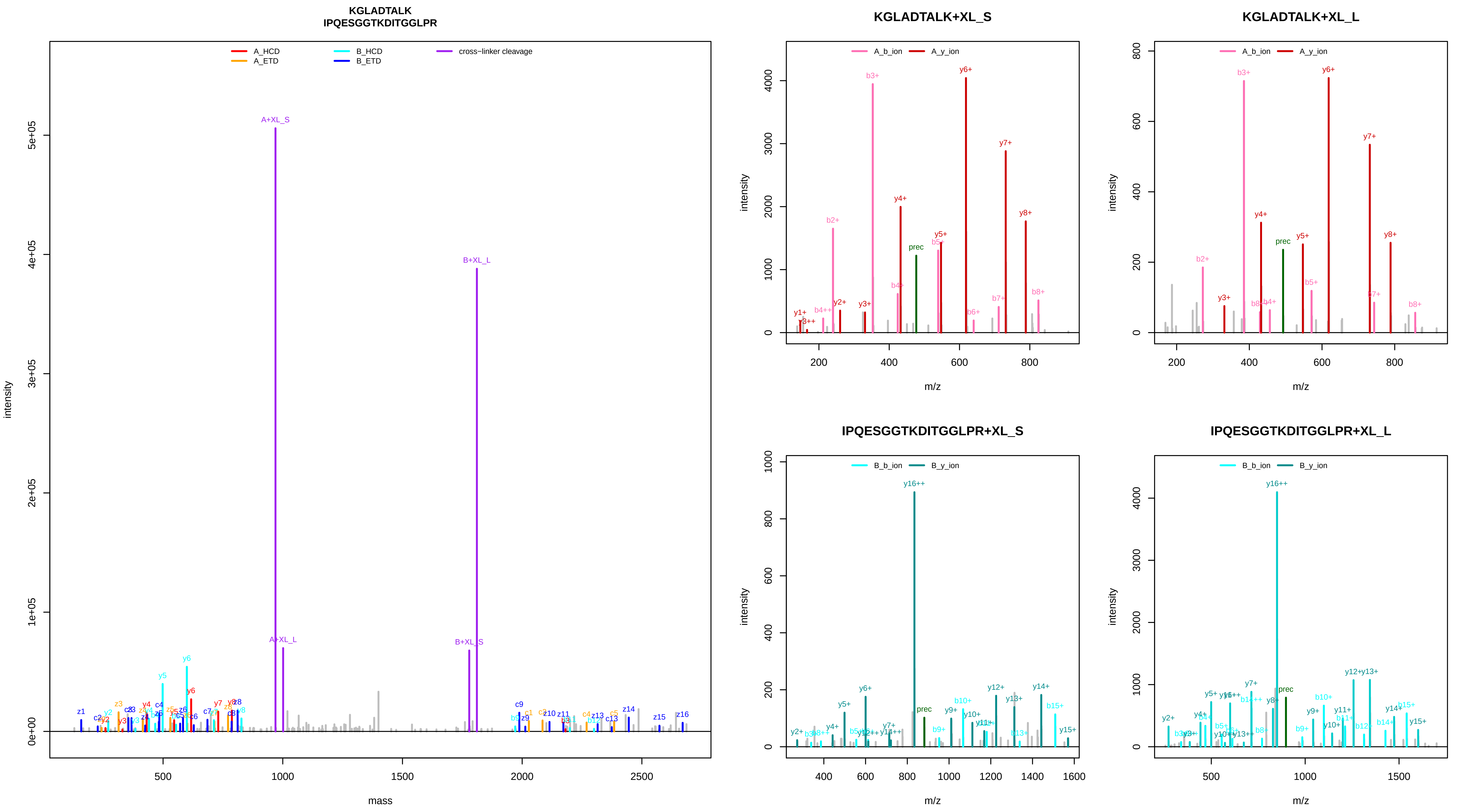
KGLADTALK
IPQESGGTKDITGGLPR
A_HCD
A_ETD
B_HCD
B_ETD
cross−linker cleavage
A+XL_S
B+XL_L
A+XL_L
B+XL_S
intensity
mass
KGLADTALK+XL_S
KGLADTALK+XL_L
A_b_ion
A_y_ion
IPQESGGTKDITGGLPR+XL_S
IPQESGGTKDITGGLPR+XL_L
B_b_ion
B_y_ion
prec
m/z

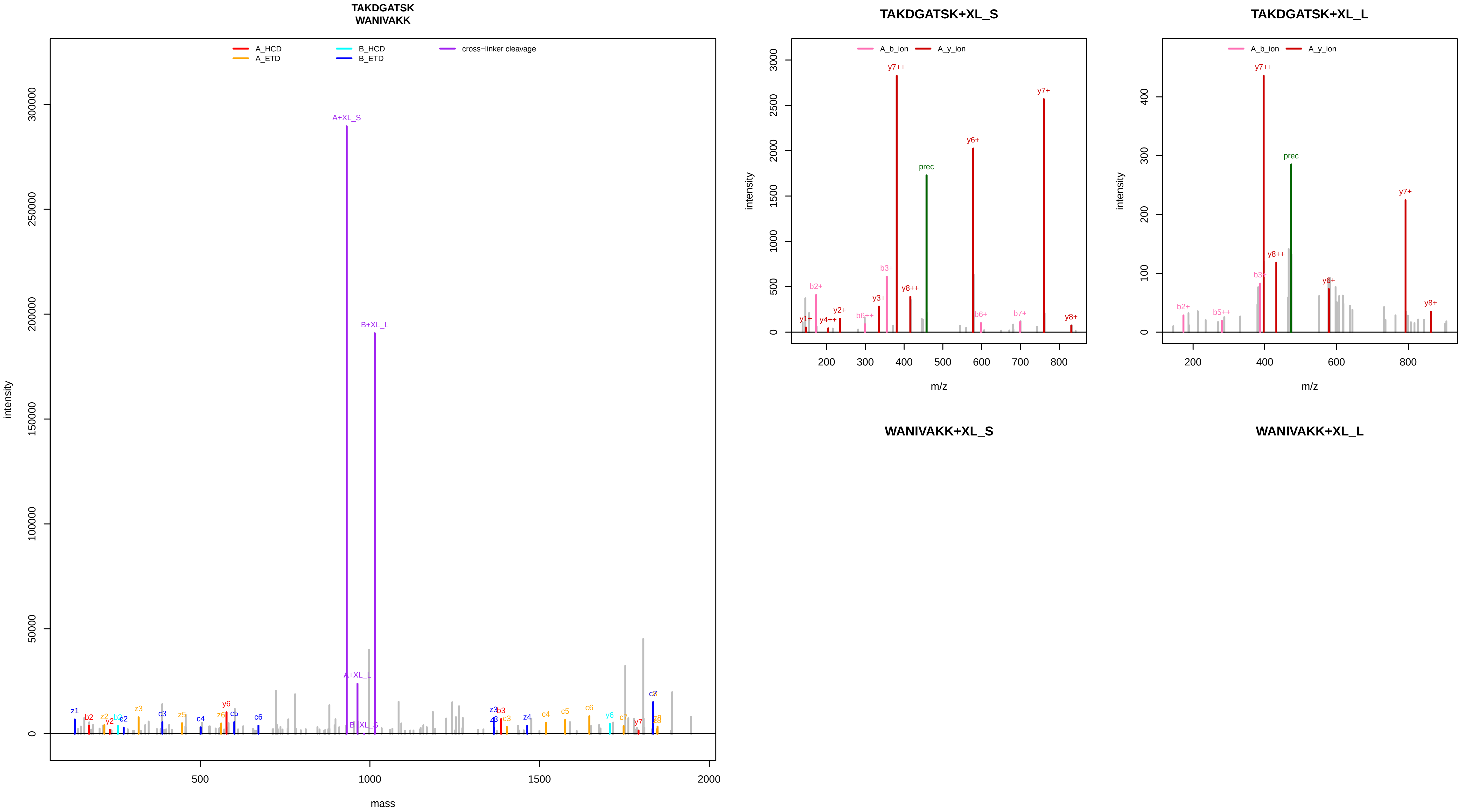
TAKDGATSK
WANIVAKK
A_HCD
A_ETD
B_HCD
B_ETD
cross−linker cleavage
A+XL_S
B+XL_L
A+XL_L
B+XL_S
intensity
mass
TAKDGATSK+XL_S
A_b_ion
A_y_ion
y7++
y7+
y6+
prec
b3+
y8++
y3+
b2+
y2+
y1+
y4++
b6++
b6+
b7+
y8+
m/z
TAKDGATSK+XL_L
b5++
y6+
WANIVAKK+XL_S
WANIVAKK+XL_L

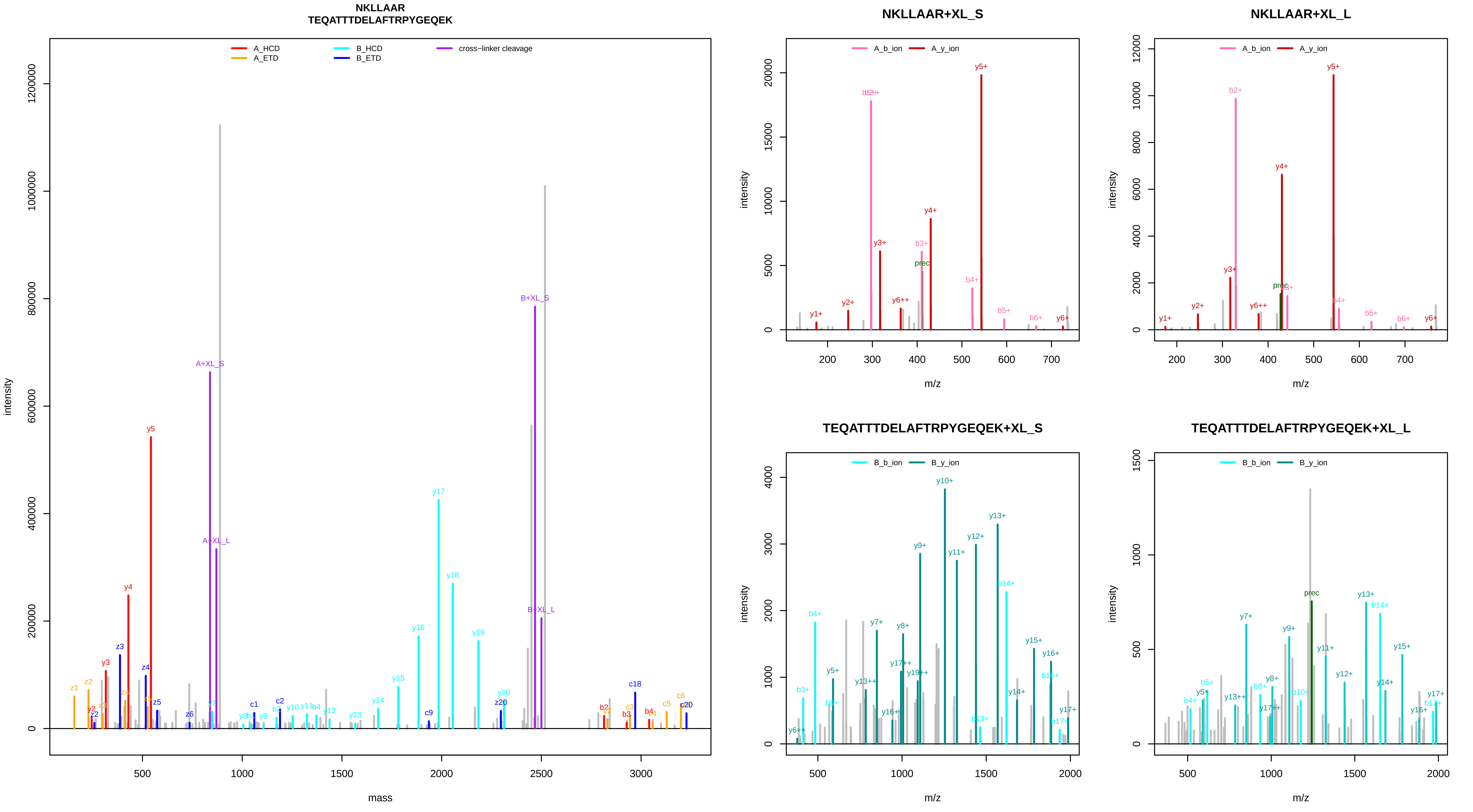
NKLLAAR
TEQATTTDELAFTRPYGEQEK
A_HCD
A_ETD
B_HCD
B_ETD
cross−linker cleavage
A+XL_S
A+XL_L
B+XL_S
B+XL_L
intensity
mass
NKLLAAR+XL_S
NKLLAAR+XL_L
A_b_ion
A_y_ion
TEQATTTDELAFTRPYGEQEK+XL_S
TEQATTTDELAFTRPYGEQEK+XL_L
B_b_ion
B_y_ion
prec
m/z

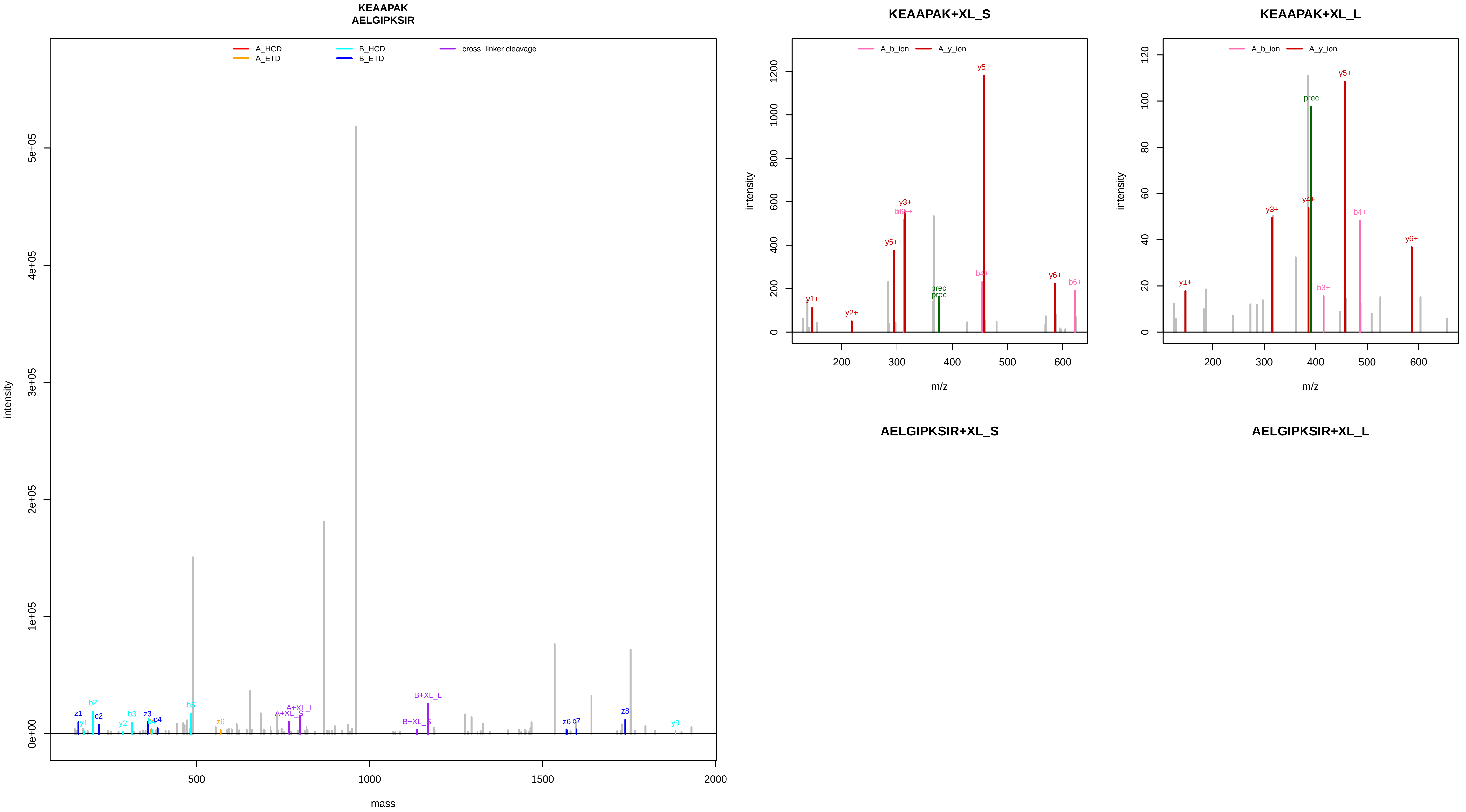
KEAAPAK
AELGIPKSIR
A_HCD
A_ETD
B_HCD
B_ETD
cross−linker cleavage
intensity
mass
KEAAPAK+XL_S
KEAAPAK+XL_L
A_b_ion
A_y_ion
m/z
AELGIPKSIR+XL_S
AELGIPKSIR+XL_L

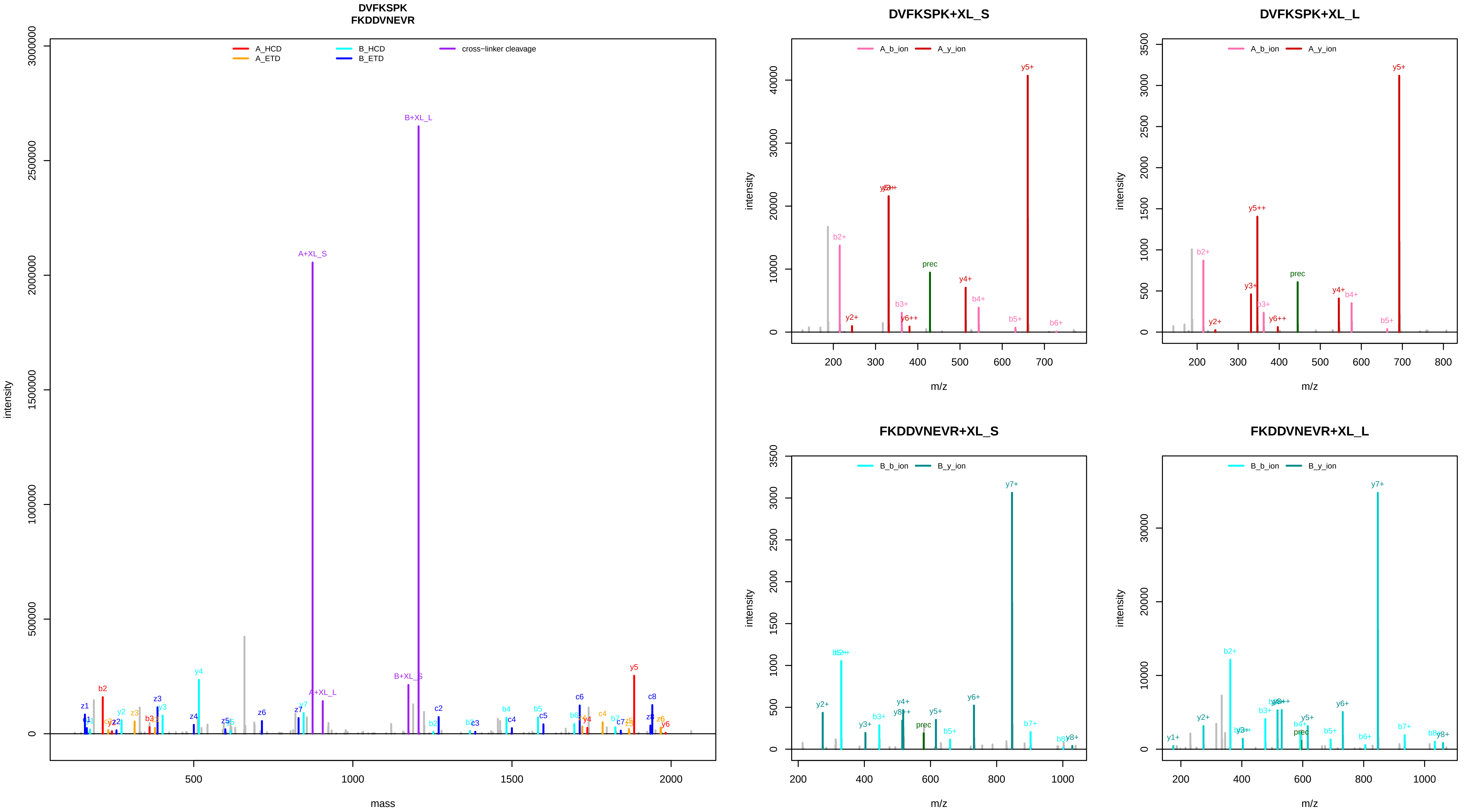
DVFKSPK
FKDDVNEVR
A_HCD
A_ETD
B_HCD
B_ETD
cross−linker cleavage
B+XL_L
A+XL_S
B+XL_S
A+XL_L
intensity
mass
DVFKSPK+XL_S
DVFKSPK+XL_L
A_b_ion
A_y_ion
FKDDVNEVR+XL_S
FKDDVNEVR+XL_L
B_b_ion
B_y_ion
intensity
m/z

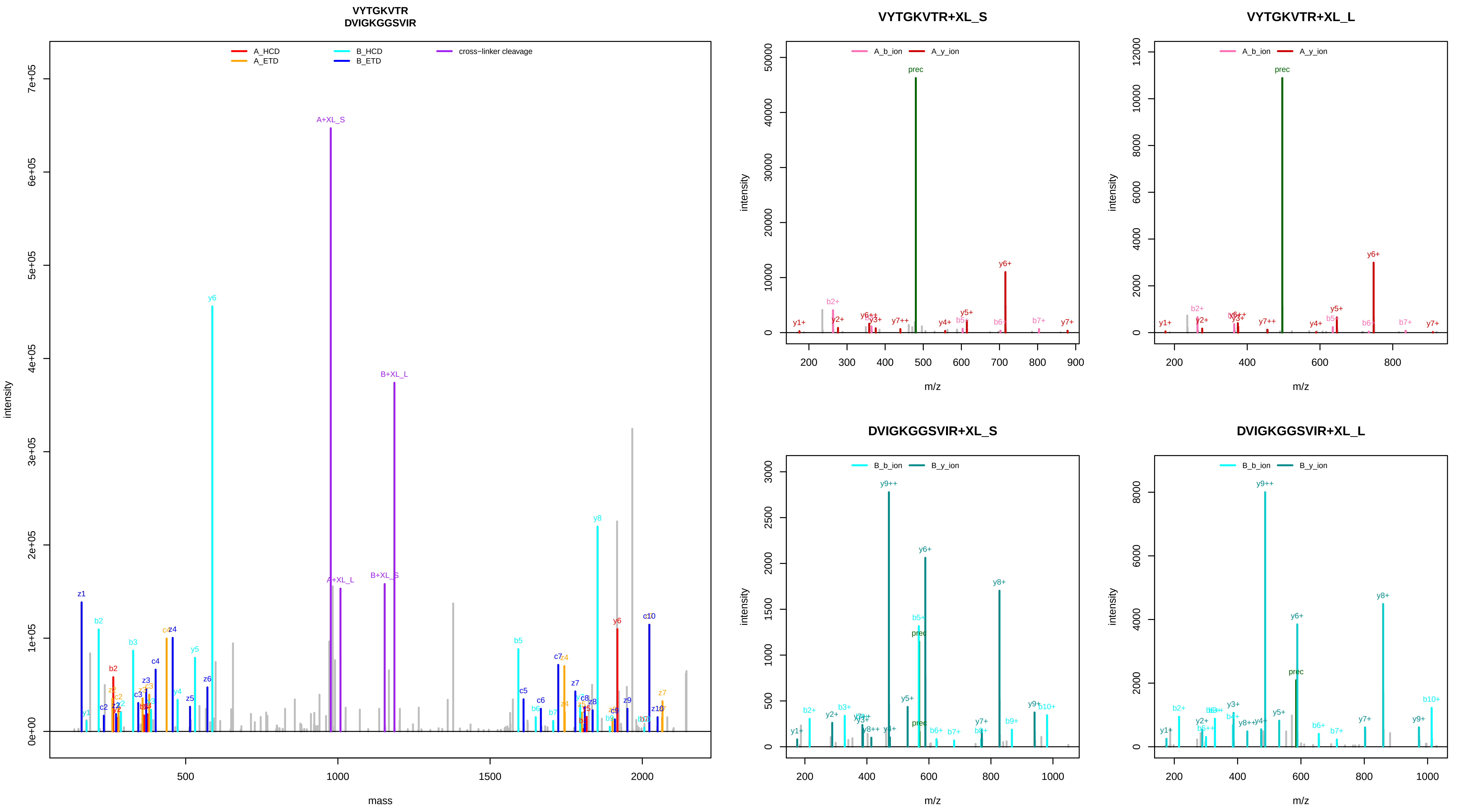
VYTGKVTR
DVIGKGGSVIR
A_HCD
A_ETD
B_HCD
B_ETD
cross−linker cleavage
A+XL_S
B+XL_L
A+XL_L
B+XL_S
intensity
mass
VYTGKVTR+XL_S
VYTGKVTR+XL_L
DVIGKGGSVIR+XL_S
DVIGKGGSVIR+XL_L
A_b_ion
A_y_ion
B_b_ion
B_y_ion
prec
m/z

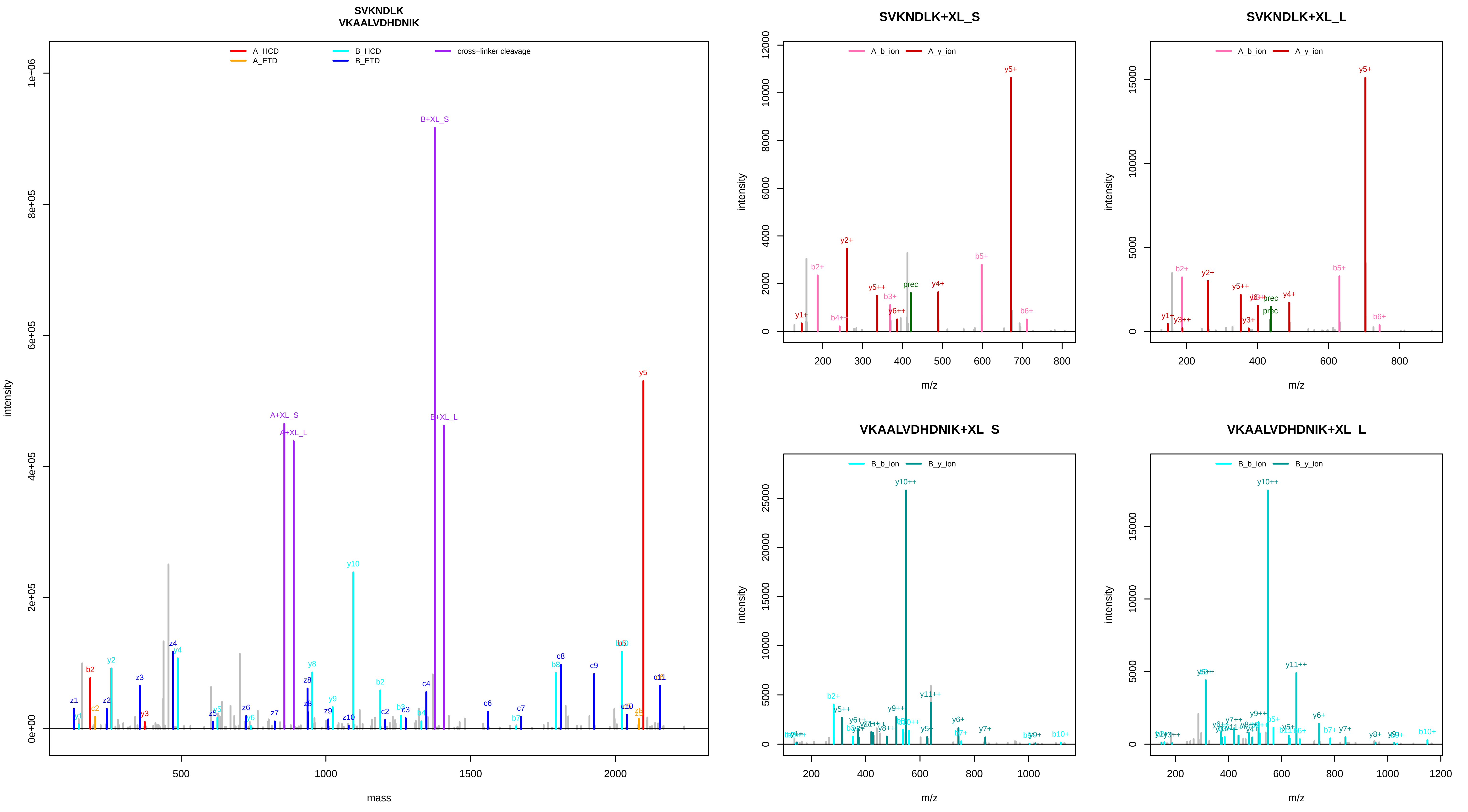
SVKNDLK
VKAALVDHDNIK
A_HCD
A_ETD
B_HCD
B_ETD
cross−linker cleavage
B+XL_S
A+XL_S
A+XL_L
B+XL_L
intensity
mass
SVKNDLK+XL_S
SVKNDLK+XL_L
VKAALVDHDNIK+XL_S
VKAALVDHDNIK+XL_L
A_b_ion
A_y_ion
B_b_ion
B_y_ion
prec
m/z

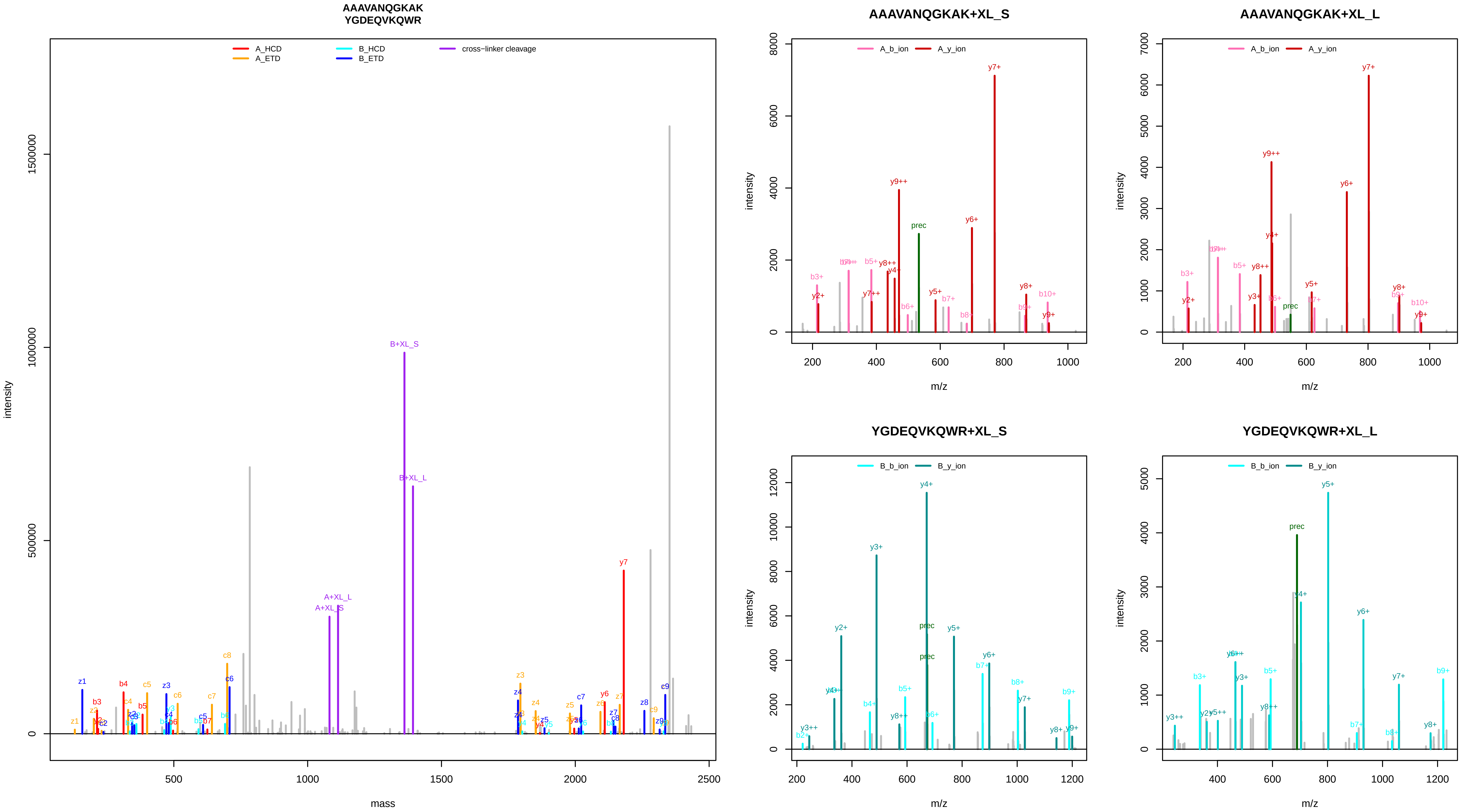
AAAVANQGKAK
YGDEQVKQWR
A_HCD
A_ETD
B_HCD
B_ETD
cross−linker cleavage
B+XL_S
B+XL_L
A+XL_L
A+XL_S
intensity
mass
AAAVANQGKAK+XL_S
AAAVANQGKAK+XL_L
YGDEQVKQWR+XL_S
YGDEQVKQWR+XL_L
A_b_ion
A_y_ion
B_b_ion
B_y_ion
prec
m/z

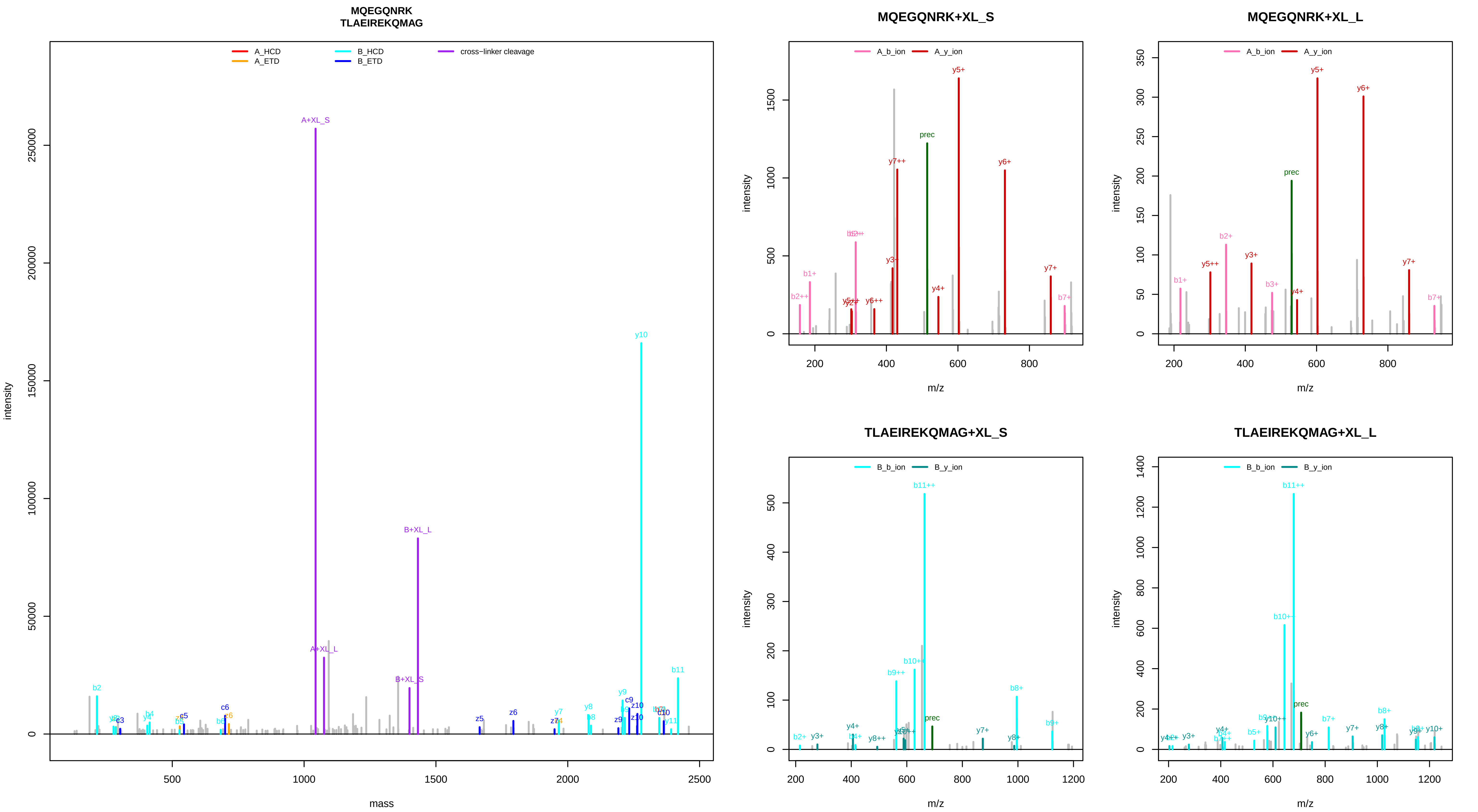

MQEGQNRK
TLAEIREKQMAG
A_HCD
A_ETD
B_HCD
B_ETD
cross−linker cleavage
A+XL_S
A+XL_L
B+XL_L
B+XL_S
intensity
mass
MQEGQNRK+XL_S
MQEGQNRK+XL_L
TLAEIREKQMAG+XL_S
TLAEIREKQMAG+XL_L
A_b_ion
A_y_ion
B_b_ion
B_y_ion
prec
m/z

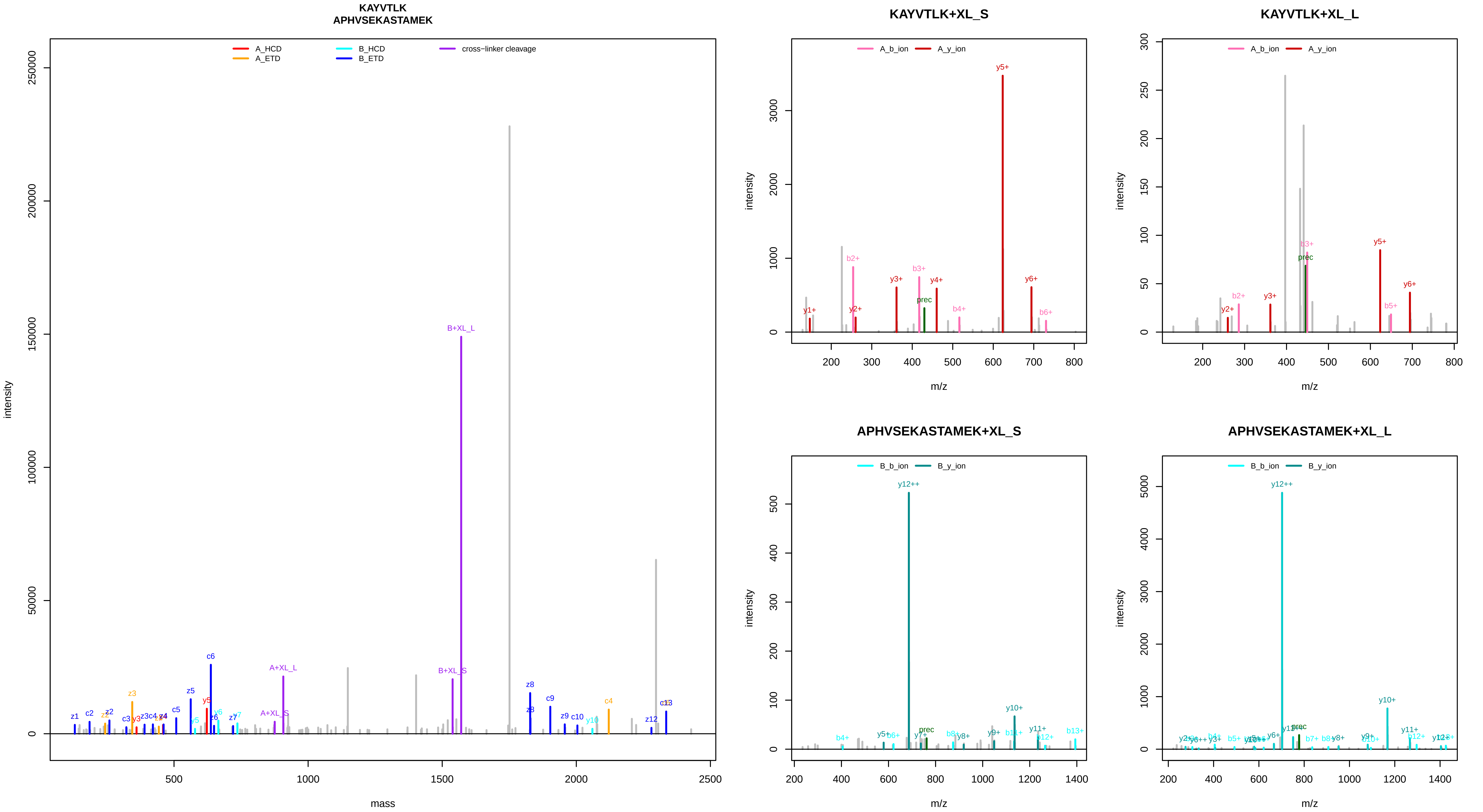

KAYVTLK
APHVSEKASTAMEK
A_HCD
A_ETD
B_HCD
B_ETD
cross−linker cleavage
B+XL_L
B+XL_S
A+XL_L
A+XL_S
intensity
mass
KAYVTLK+XL_S
KAYVTLK+XL_L
A_b_ion
A_y_ion
APHVSEKASTAMEK+XL_S
APHVSEKASTAMEK+XL_L
B_b_ion
B_y_ion
prec
y12++
y10+
y5+
m/z

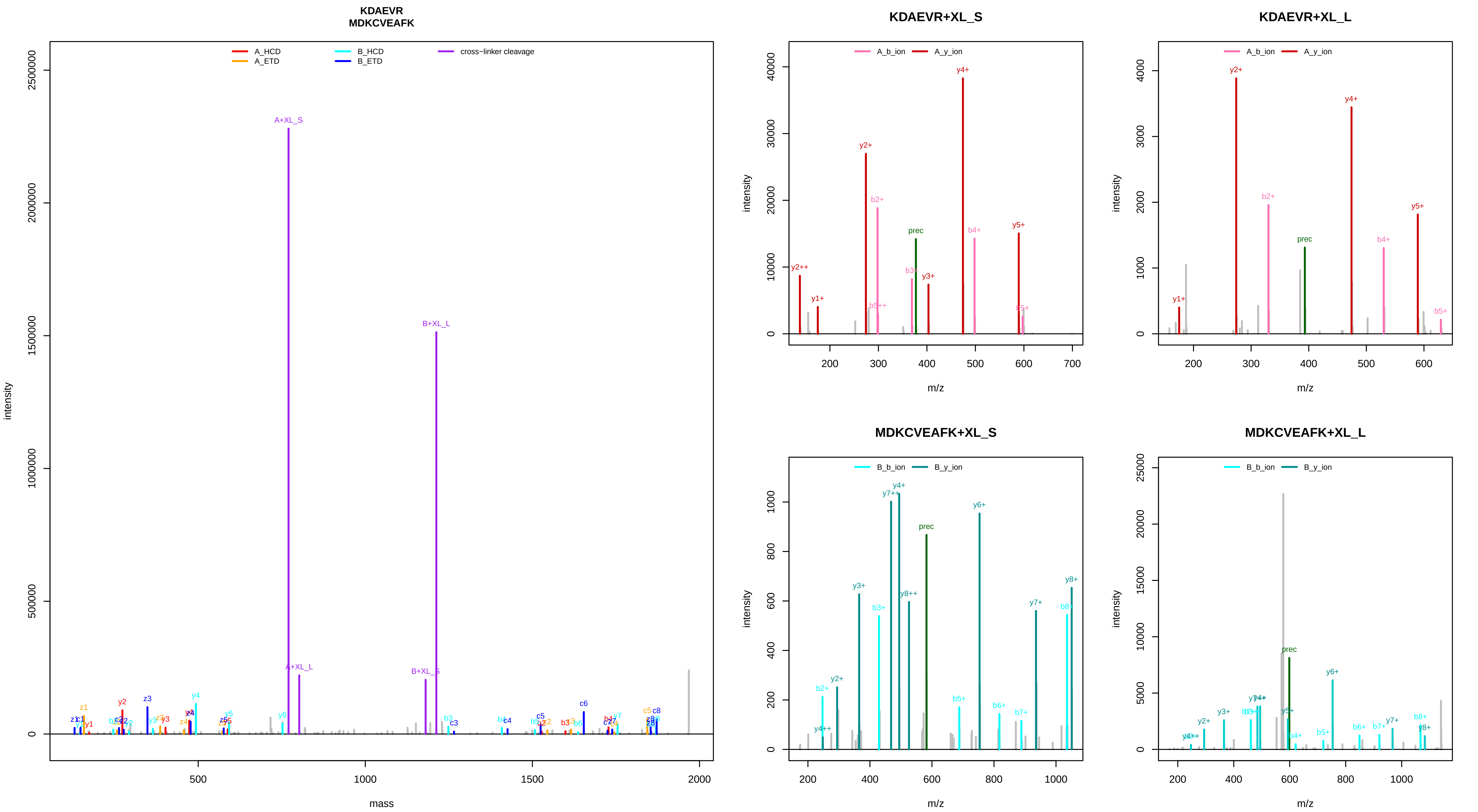
KDAEVR
MDKCVEAFK
A_HCD
A_ETD
B_HCD
B_ETD
cross−linker cleavage
A+XL_S
B+XL_L
A+XL_L
B+XL_S
intensity
mass
KDAEVR+XL_S
KDAEVR+XL_L
MDKCVEAFK+XL_S
MDKCVEAFK+XL_L
A_b_ion
A_y_ion
B_b_ion
B_y_ion
prec
m/z
intensity

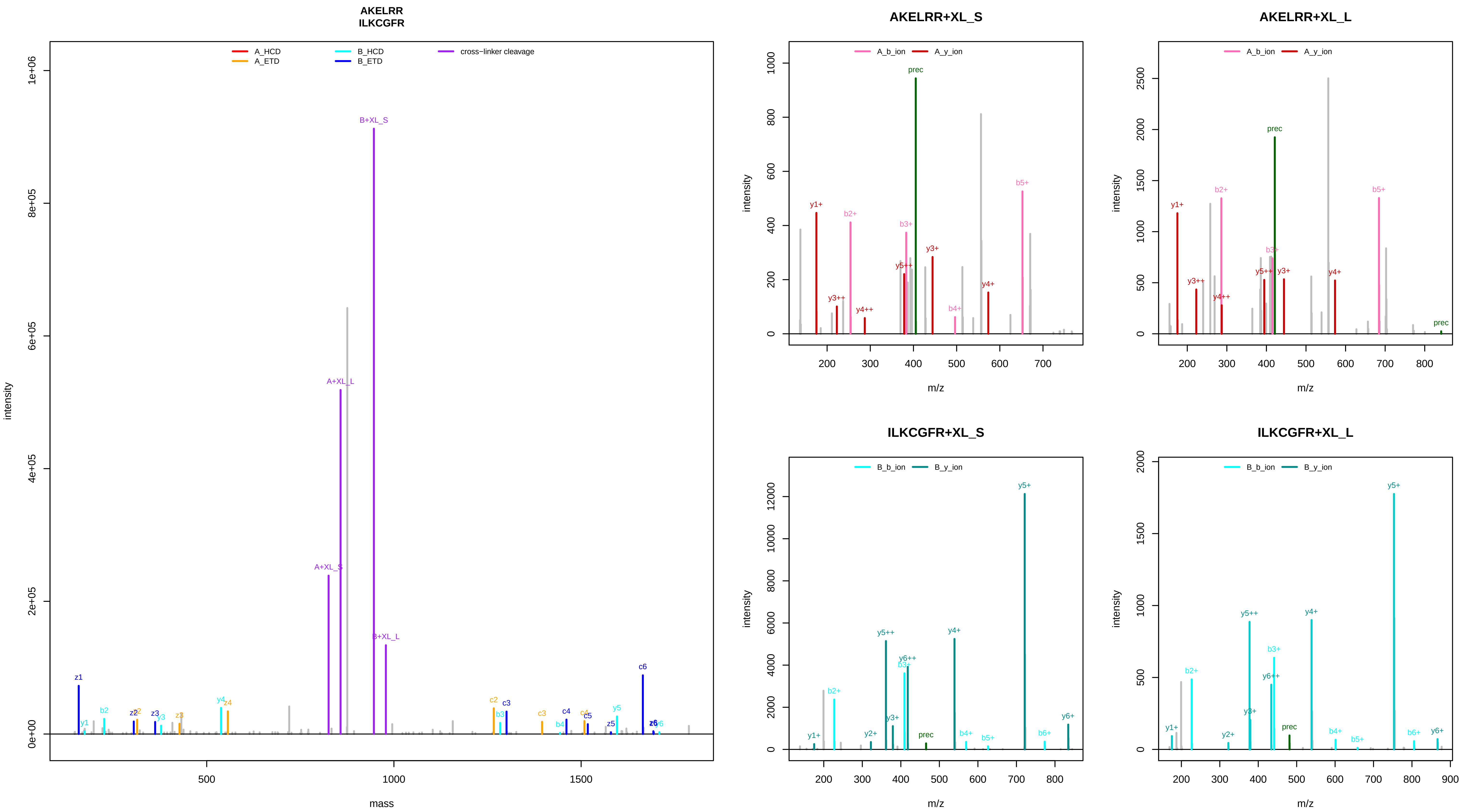

AKELRR
ILKCGFR
A_HCD
A_ETD
B_HCD
B_ETD
cross−linker cleavage
B+XL_S
A+XL_L
A+XL_S
B+XL_L
intensity
mass
AKELRR+XL_S
AKELRR+XL_L
ILKCGFR+XL_S
ILKCGFR+XL_L
A_b_ion
A_y_ion
B_b_ion
B_y_ion
prec
m/z

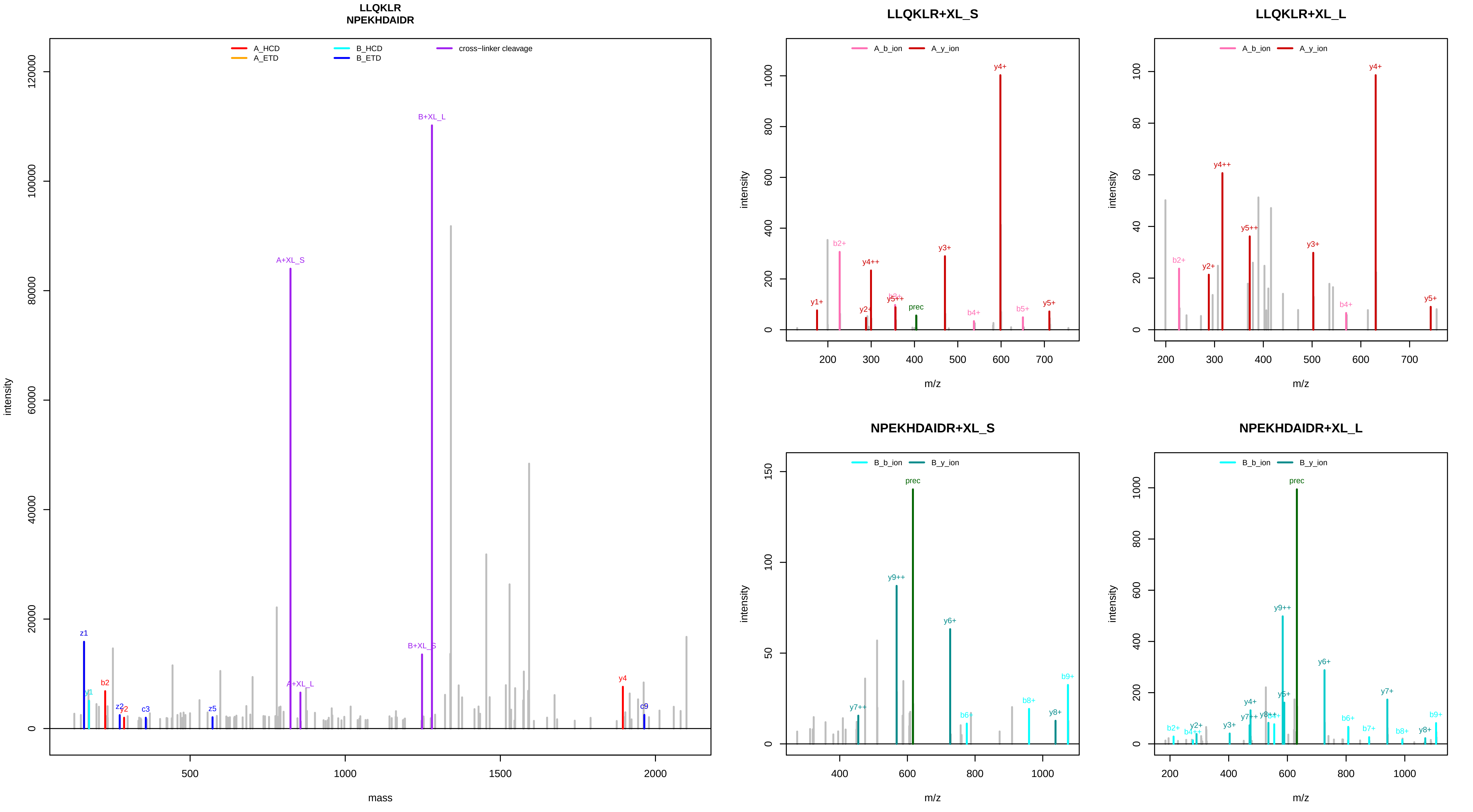

LLQKLR
NPEKHDAIDR
A_HCD
A_ETD
B_HCD
B_ETD
cross−linker cleavage
B+XL_L
A+XL_S
B+XL_S
A+XL_L
z1
y1
b2
z2
y2
c3
z5
y4
c9
intensity
mass
LLQKLR+XL_S
A_b_ion
A_y_ion
y4+
b2+
y4++
y3+
y1+
y2+
y5++
prec
b4+
b5+
y5+
m/z
LLQKLR+XL_L
y4++
y5++
y2+
y3+
b4+
NPEKHDAIDR+XL_S
B_b_ion
B_y_ion
y9++
y6+
y7++
b6+
b8+
y8+
b9+
NPEKHDAIDR+XL_L
y6+
y7+
y4+
y3+
b6+
b7+
b8+
y8+

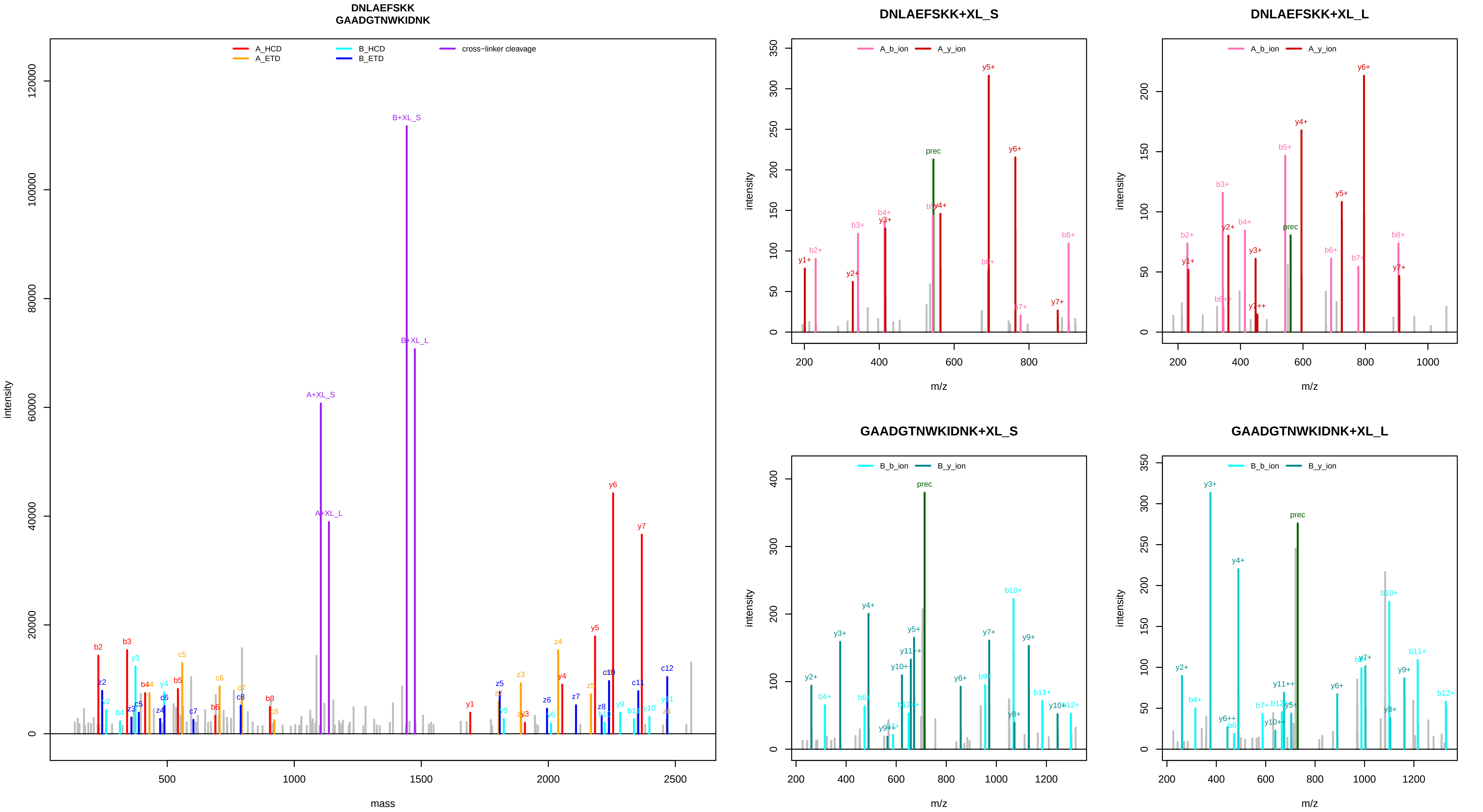

DNLAEFSKK
GAADGTNWKIDNK
A_HCD
A_ETD
B_HCD
B_ETD
cross−linker cleavage
B+XL_S
B+XL_L
A+XL_S
A+XL_L
intensity
mass
DNLAEFSKK+XL_S
DNLAEFSKK+XL_L
GAADGTNWKIDNK+XL_S
GAADGTNWKIDNK+XL_L
A_b_ion
A_y_ion
B_b_ion
B_y_ion
prec
m/z

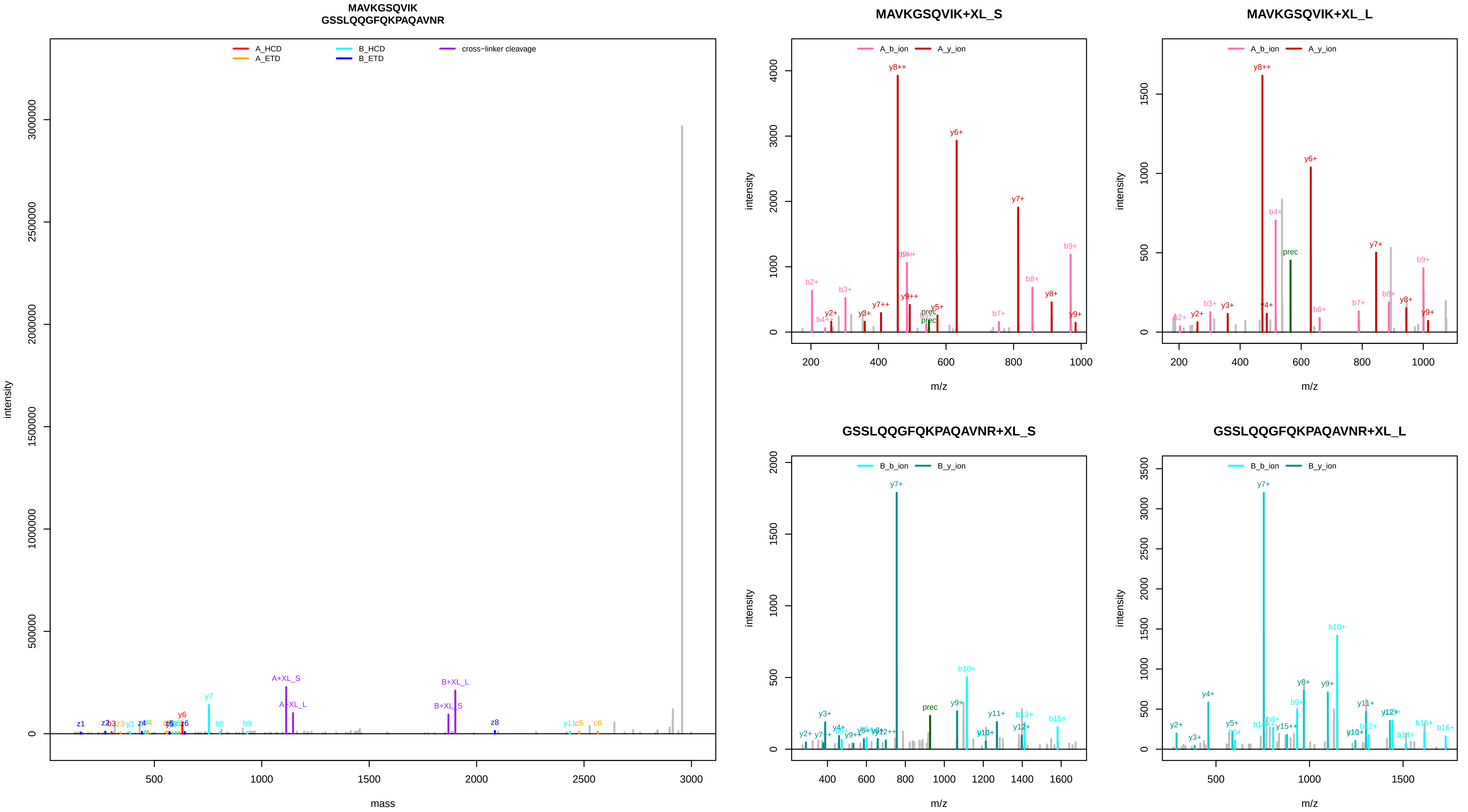

MAVKGSQVIK
GSSLQQGFQKPAQAVNR
A_HCD
A_ETD
B_HCD
B_ETD
cross−linker cleavage
A+XL_S
A+XL_L
B+XL_L
B+XL_S
intensity
mass
MAVKGSQVIK+XL_S
MAVKGSQVIK+XL_L
GSSLQQGFQKPAQAVNR+XL_S
GSSLQQGFQKPAQAVNR+XL_L
A_b_ion
A_y_ion
B_b_ion
B_y_ion
prec
m/z

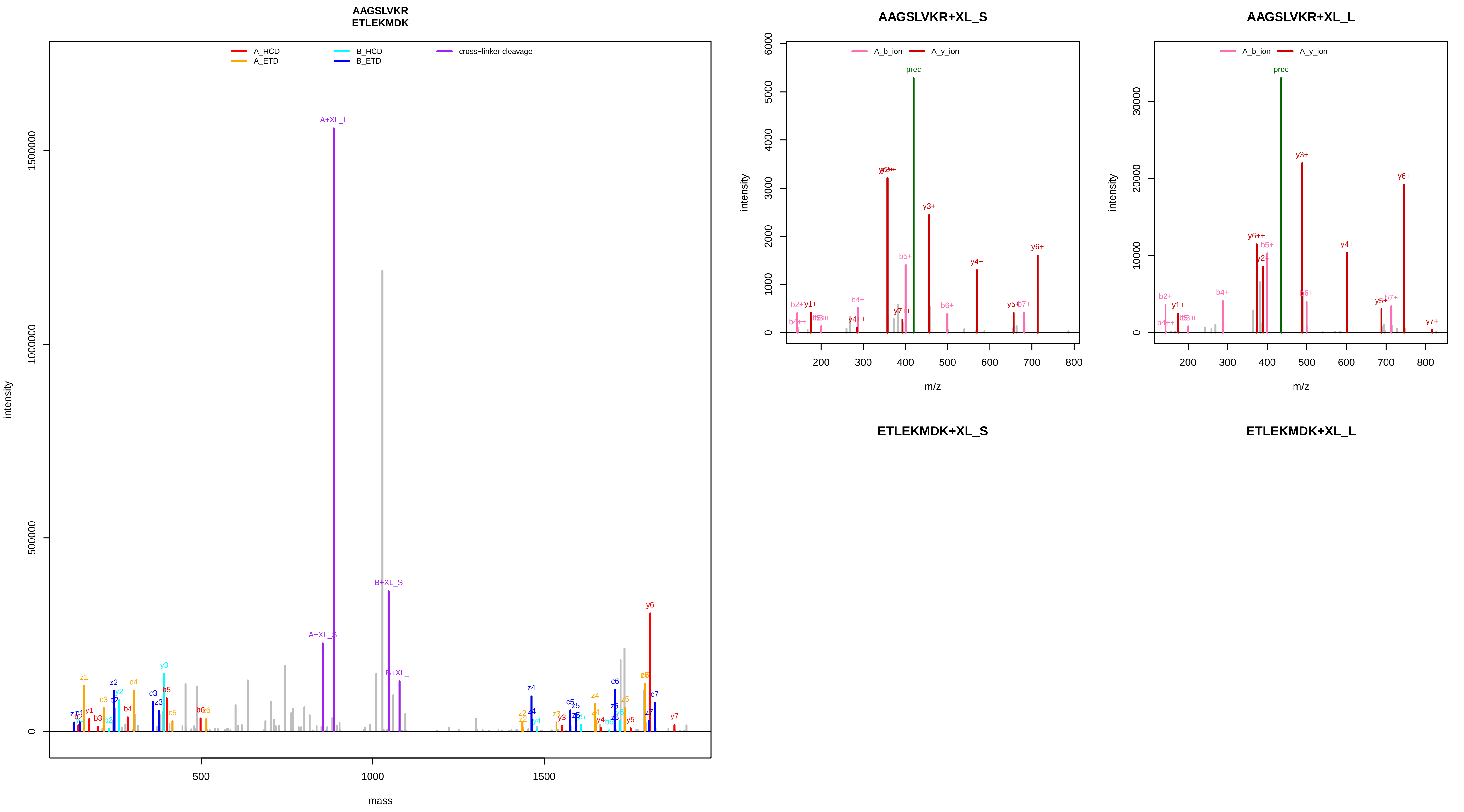
AAGSLVKR
ETLEKMDK
A_HCD
A_ETD
B_HCD
B_ETD
cross−linker cleavage
A+XL_L
A+XL_S
B+XL_S
B+XL_L
intensity
mass
AAGSLVKR+XL_S
AAGSLVKR+XL_L
A_b_ion
A_y_ion
prec
intensity
m/z
ETLEKMDK+XL_S
ETLEKMDK+XL_L

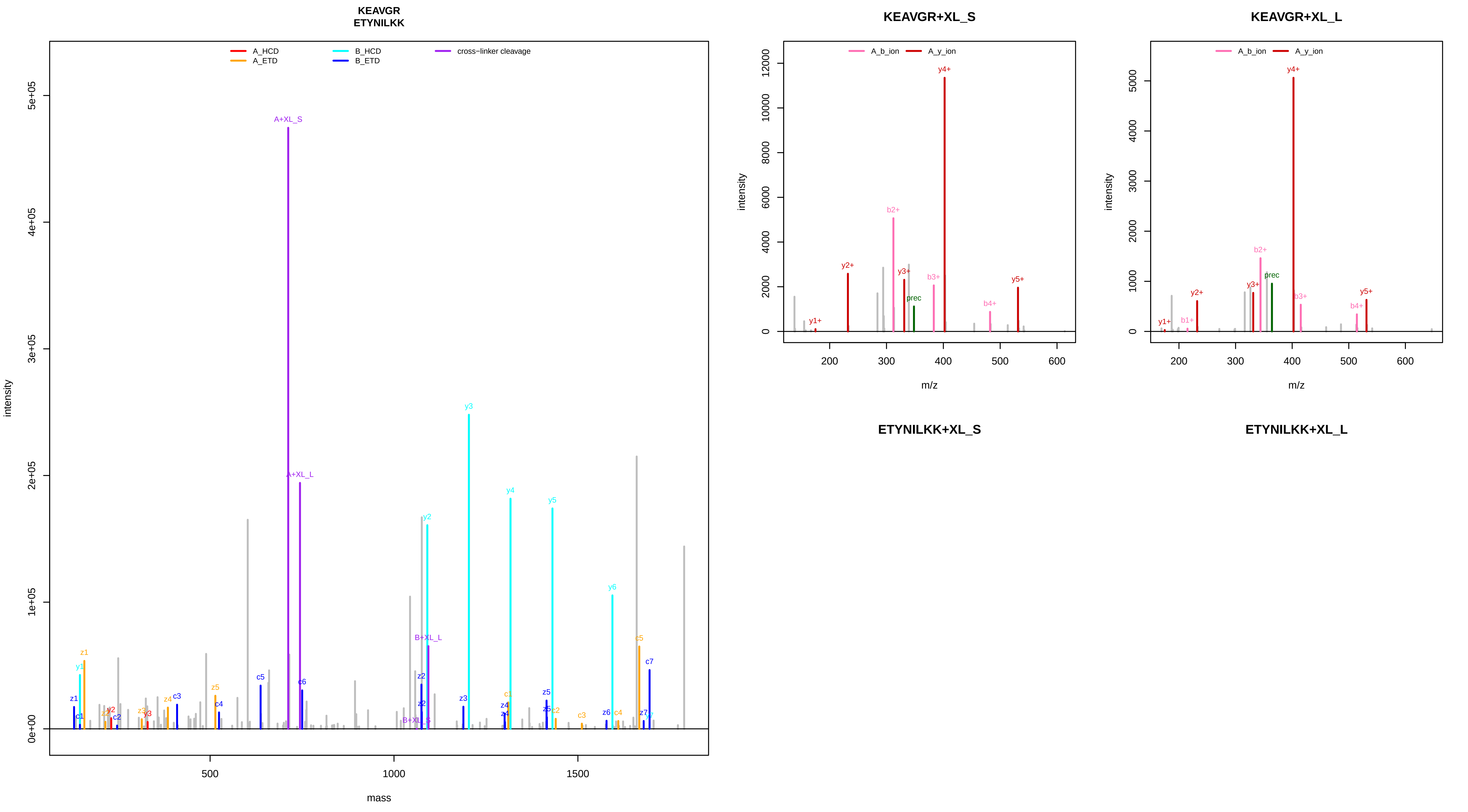
KEAVGR
ETYNILKK
A_HCD
A_ETD
B_HCD
B_ETD
cross−linker cleavage
A+XL_S
A+XL_L
B+XL_L
B+XL_S
intensity
mass
KEAVGR+XL_S
KEAVGR+XL_L
A_b_ion
A_y_ion
prec
m/z
ETYNILKK+XL_S
ETYNILKK+XL_L

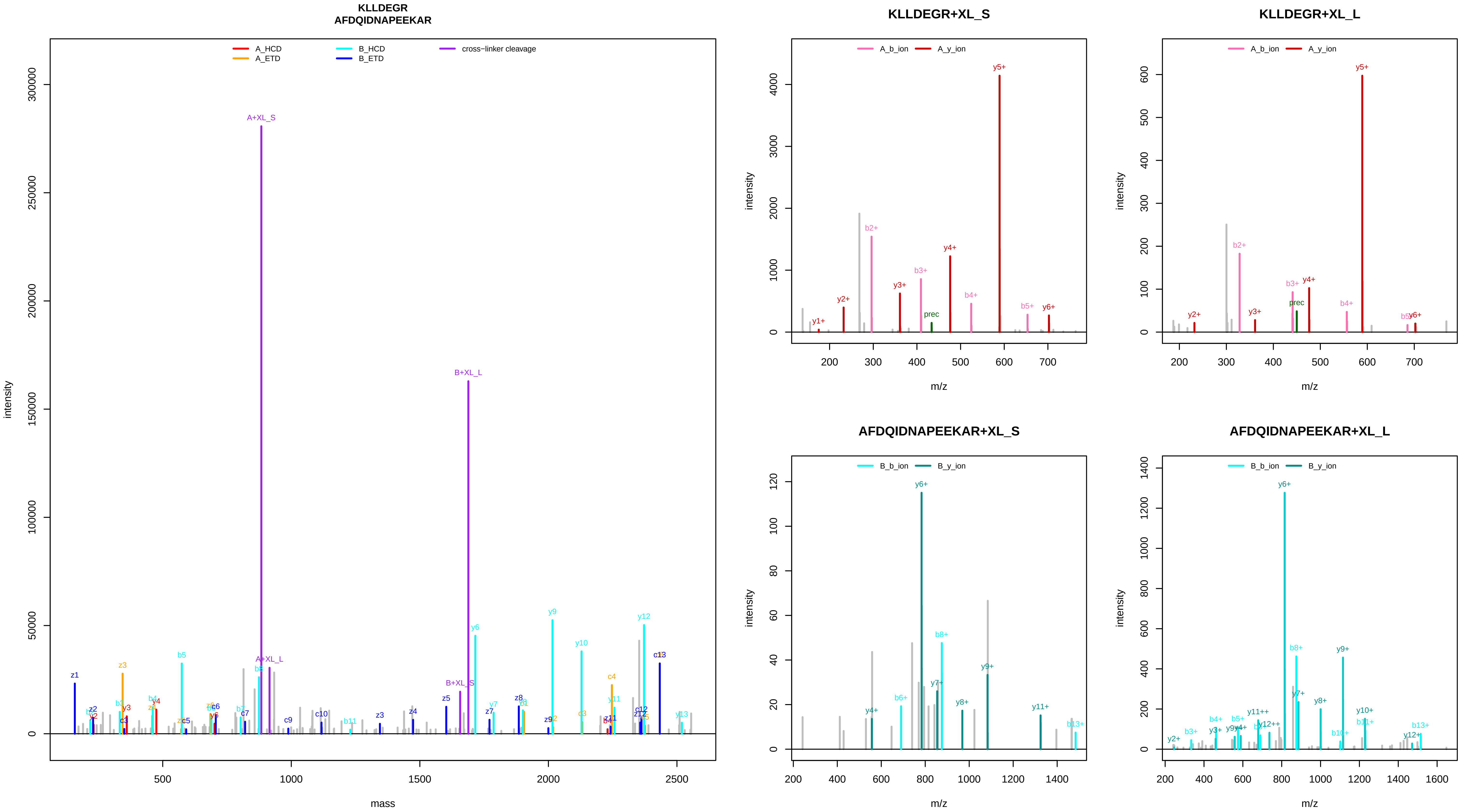
KLLDEGR
AFDQIDNAPEEKAR
A_HCD
A_ETD
B_HCD
B_ETD
cross−linker cleavage
A+XL_S
B+XL_L
intensity
mass
KLLDEGR+XL_S
KLLDEGR+XL_L
AFDQIDNAPEEKAR+XL_S
AFDQIDNAPEEKAR+XL_L
A_b_ion
A_y_ion
B_b_ion
B_y_ion
prec
m/z

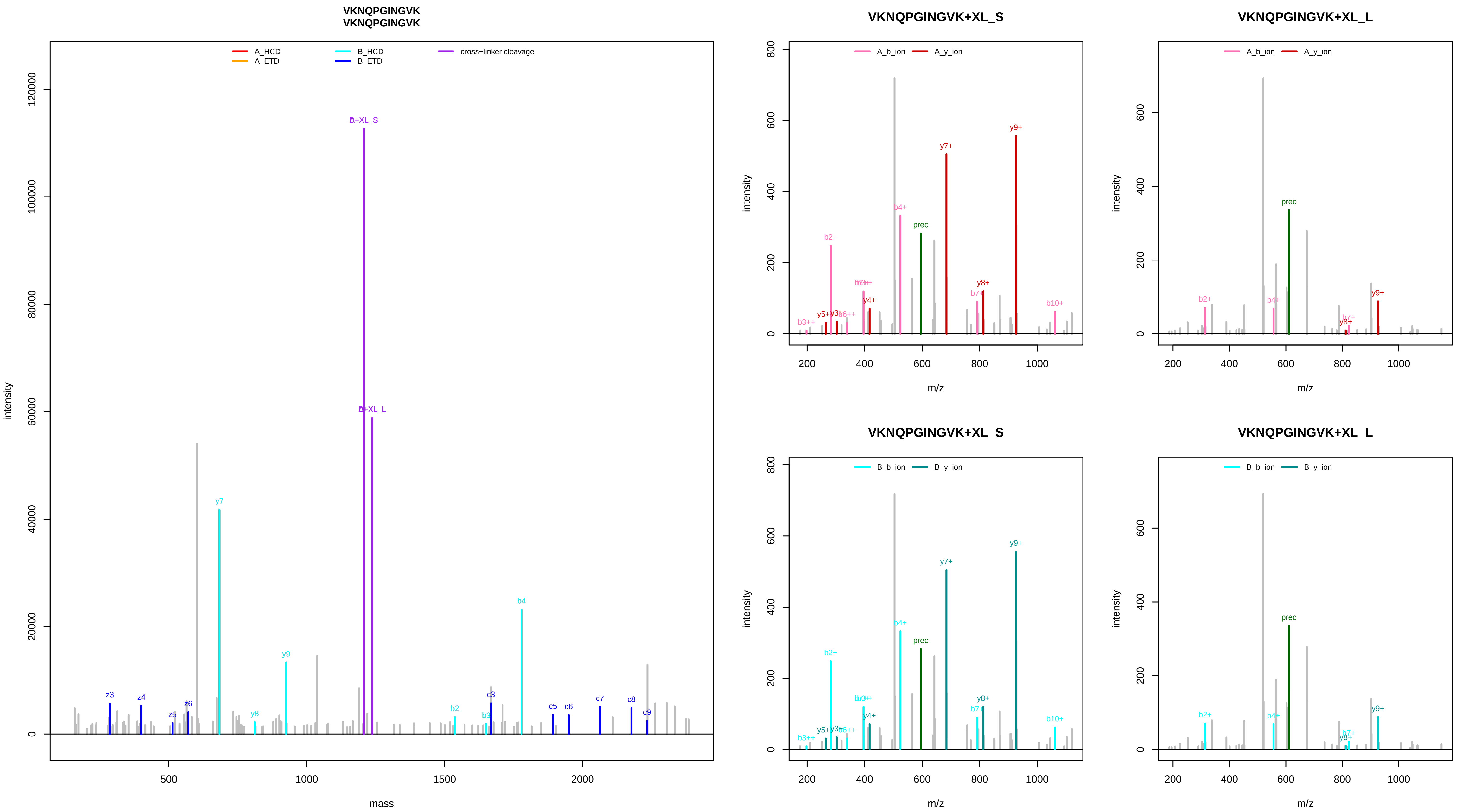
VKNQPGINGVK
VKNQPGINGVK
A_HCD
A_ETD
B_HCD
B_ETD
cross−linker cleavage
intensity
mass
VKNQPGINGVK+XL_S
VKNQPGINGVK+XL_L
A_b_ion
A_y_ion
B_b_ion
B_y_ion
prec
m/z
intensity

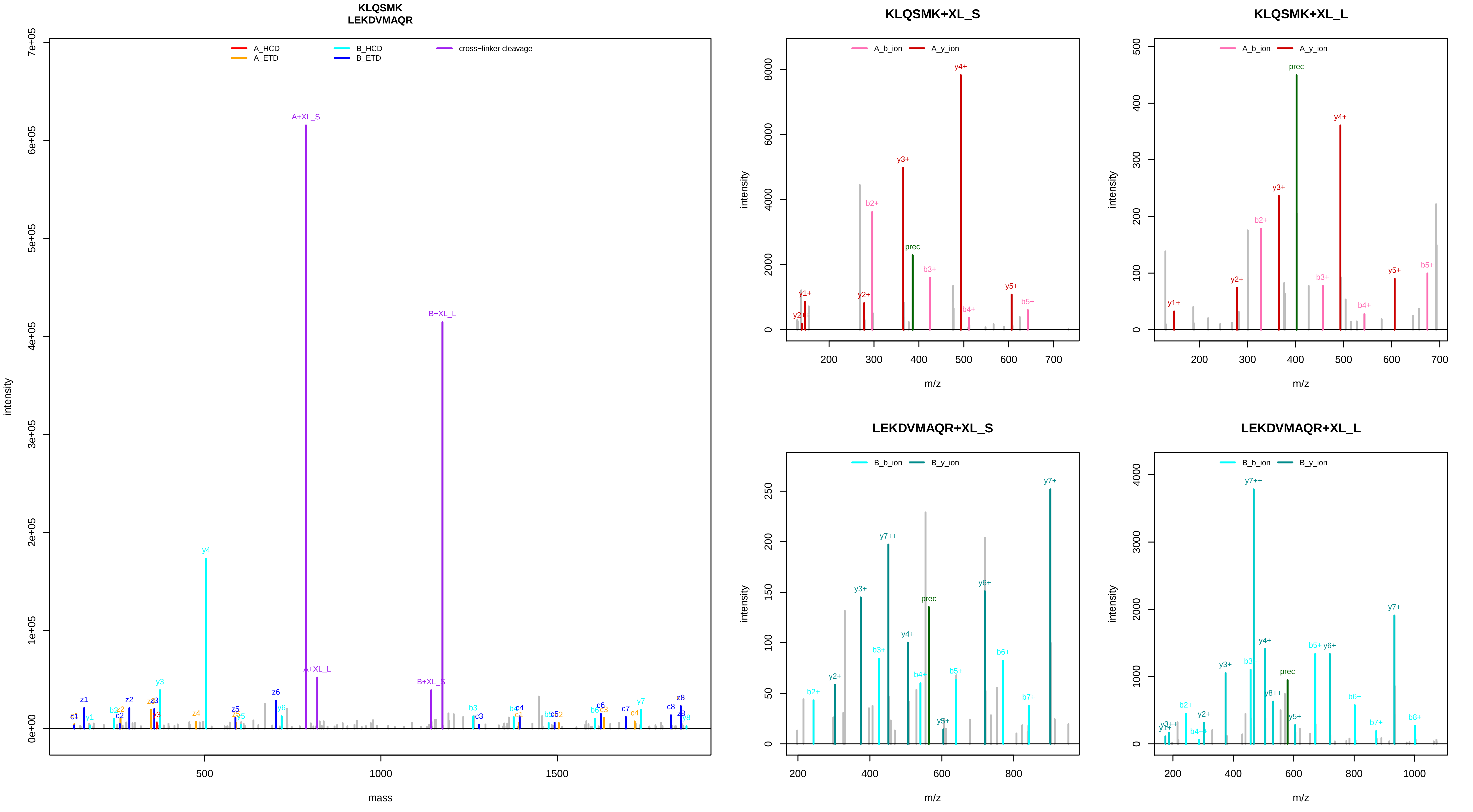
KLQSMK
LEKDVMAQR
A_HCD
A_ETD
B_HCD
B_ETD
cross−linker cleavage
A+XL_S
B+XL_L
A+XL_L
B+XL_S
intensity
mass
KLQSMK+XL_S
KLQSMK+XL_L
LEKDVMAQR+XL_S
LEKDVMAQR+XL_L
A_b_ion
A_y_ion
B_b_ion
B_y_ion
prec
intensity
m/z

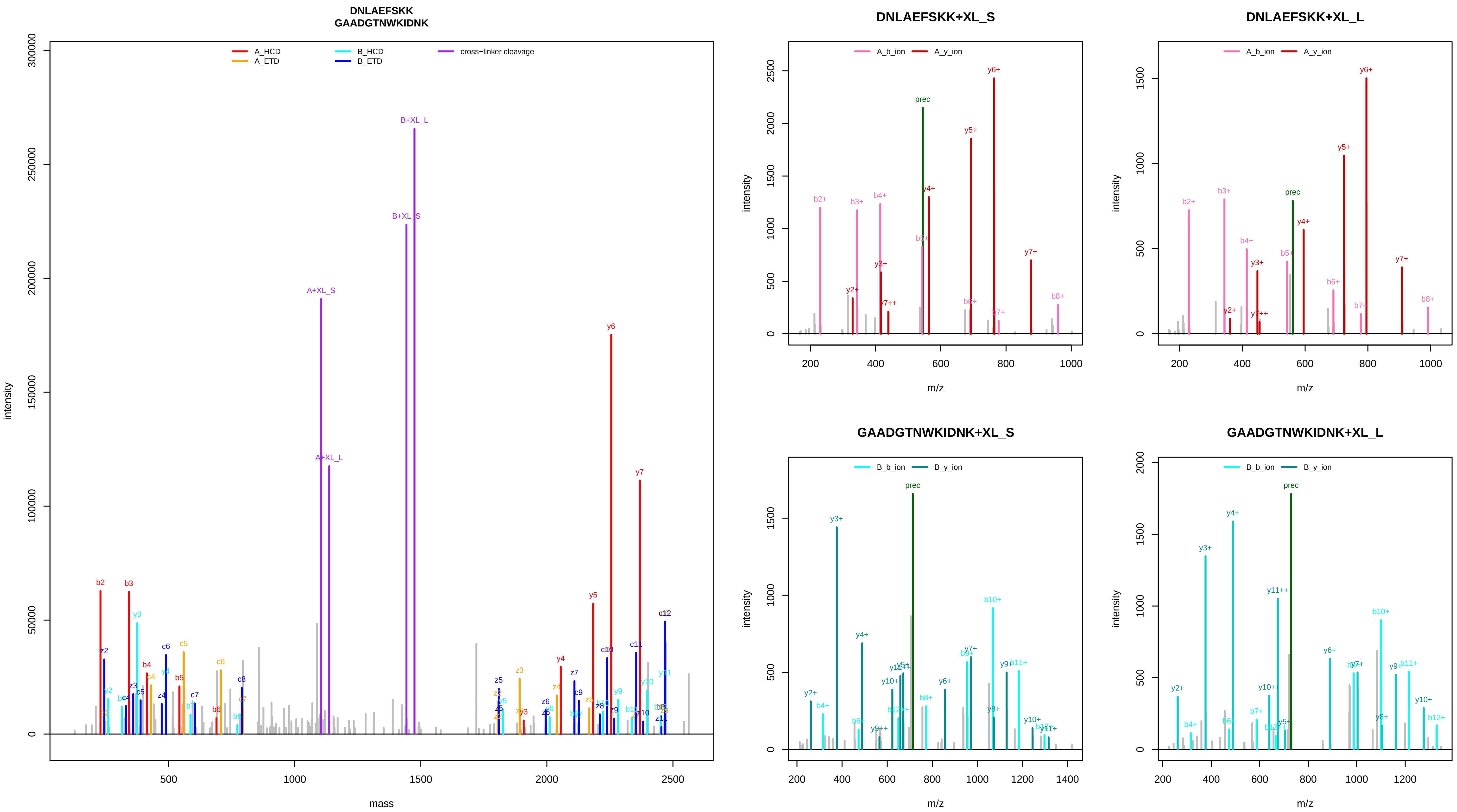
DNLAEFSKK
GAADGTNWKIDNK
A_HCD
A_ETD
B_HCD
B_ETD
cross−linker cleavage
B+XL_L
B+XL_S
A+XL_S
A+XL_L
y6
y7
y5
b2
b3
y3
intensity
mass
DNLAEFSKK+XL_S
DNLAEFSKK+XL_L
GAADGTNWKIDNK+XL_S
GAADGTNWKIDNK+XL_L
A_b_ion
A_y_ion
B_b_ion
B_y_ion
prec
m/z

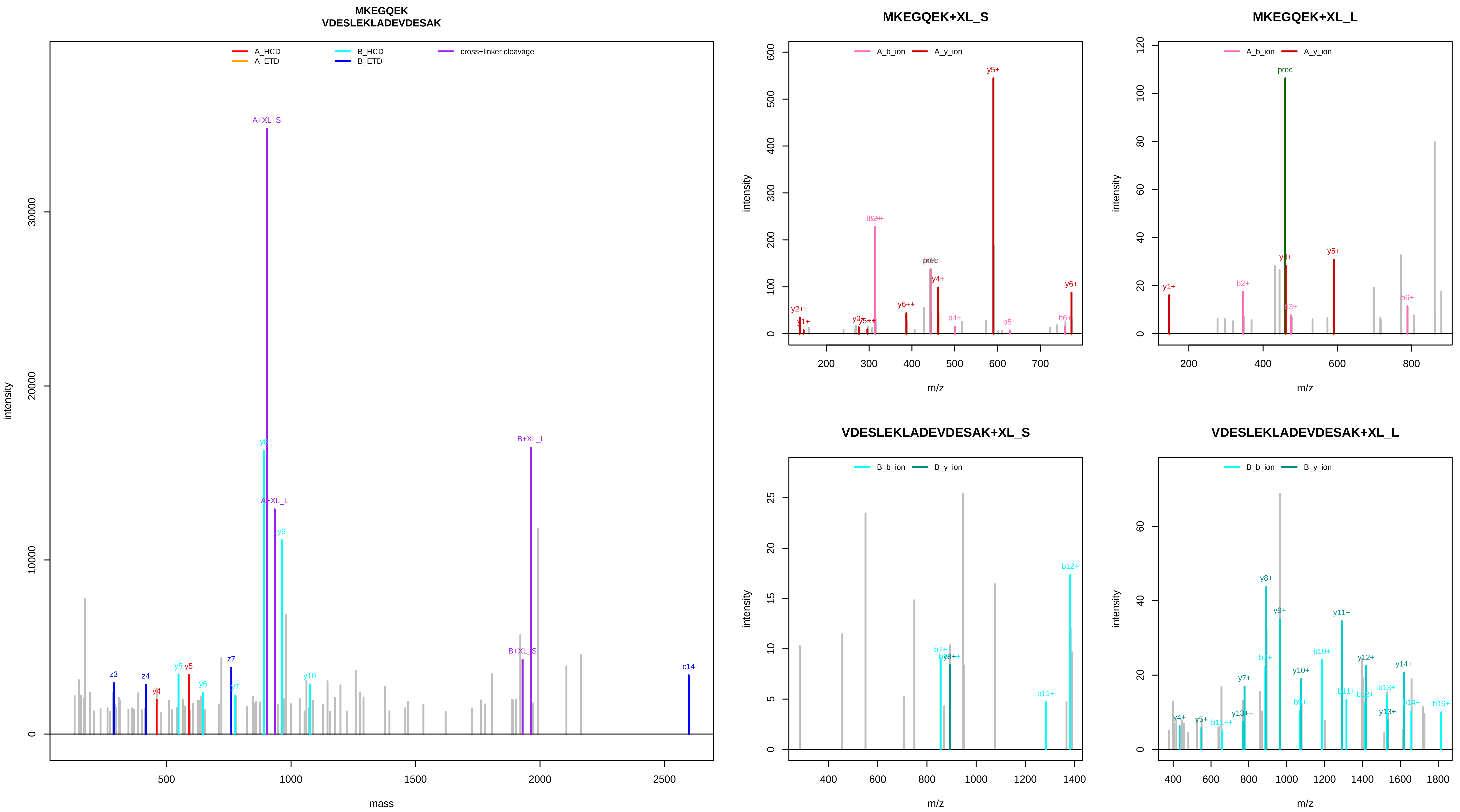
MKEGQEK
VDESLEKLADEVDESAK
A_HCD
A_ETD
B_HCD
B_ETD
cross−linker cleavage
A+XL_S
B+XL_L
A+XL_L
B+XL_S
intensity
mass
MKEGQEK+XL_S
MKEGQEK+XL_L
VDESLEKLADEVDESAK+XL_S
VDESLEKLADEVDESAK+XL_L
A_b_ion
A_y_ion
B_b_ion
B_y_ion
prec
m/z

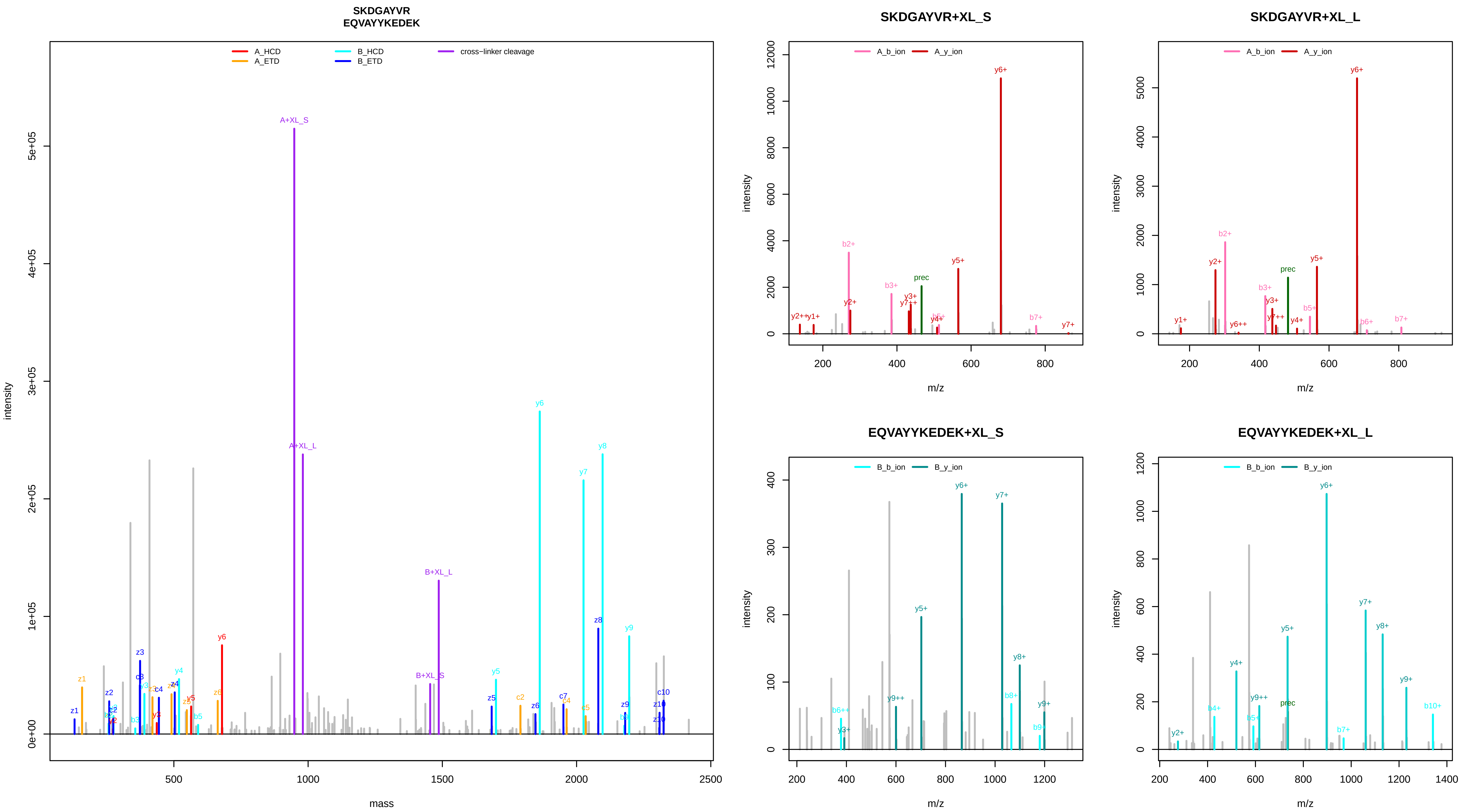
SKDGAYVR
EQVAYYKEDEK
A_HCD
A_ETD
B_HCD
B_ETD
cross−linker cleavage
A+XL_S
A+XL_L
B+XL_L
B+XL_S
intensity
mass
SKDGAYVR+XL_S
SKDGAYVR+XL_L
EQVAYYKEDEK+XL_S
EQVAYYKEDEK+XL_L
A_b_ion
A_y_ion
B_b_ion
B_y_ion
prec
m/z

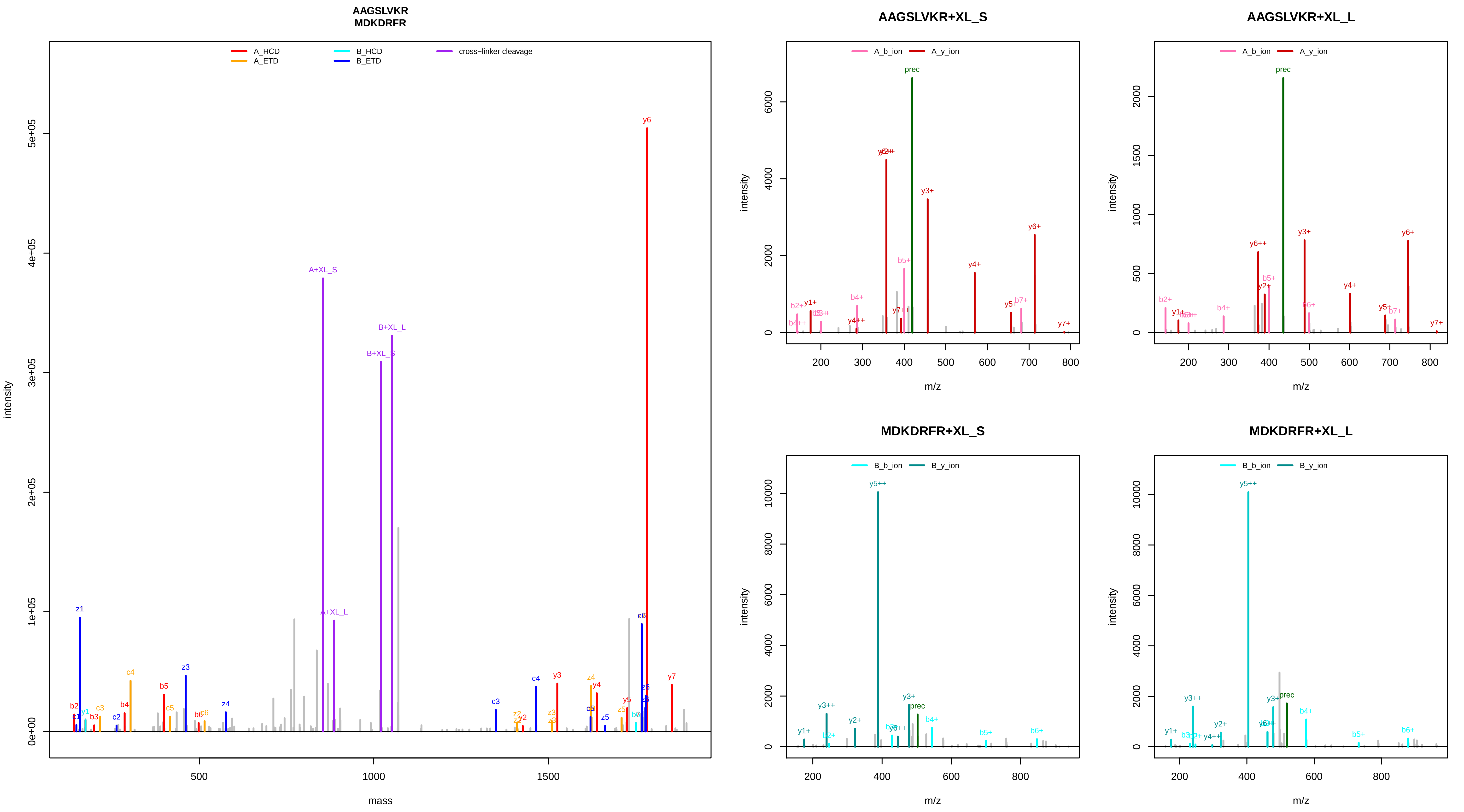
AAGSLVKR
MDKDRFR
A_HCD
A_ETD
B_HCD
B_ETD
cross−linker cleavage
intensity
mass
AAGSLVKR+XL_S
AAGSLVKR+XL_L
MDKDRFR+XL_S
MDKDRFR+XL_L
A_b_ion
A_y_ion
B_b_ion
B_y_ion
prec
m/z
A+XL_S
B+XL_L
B+XL_S
A+XL_L

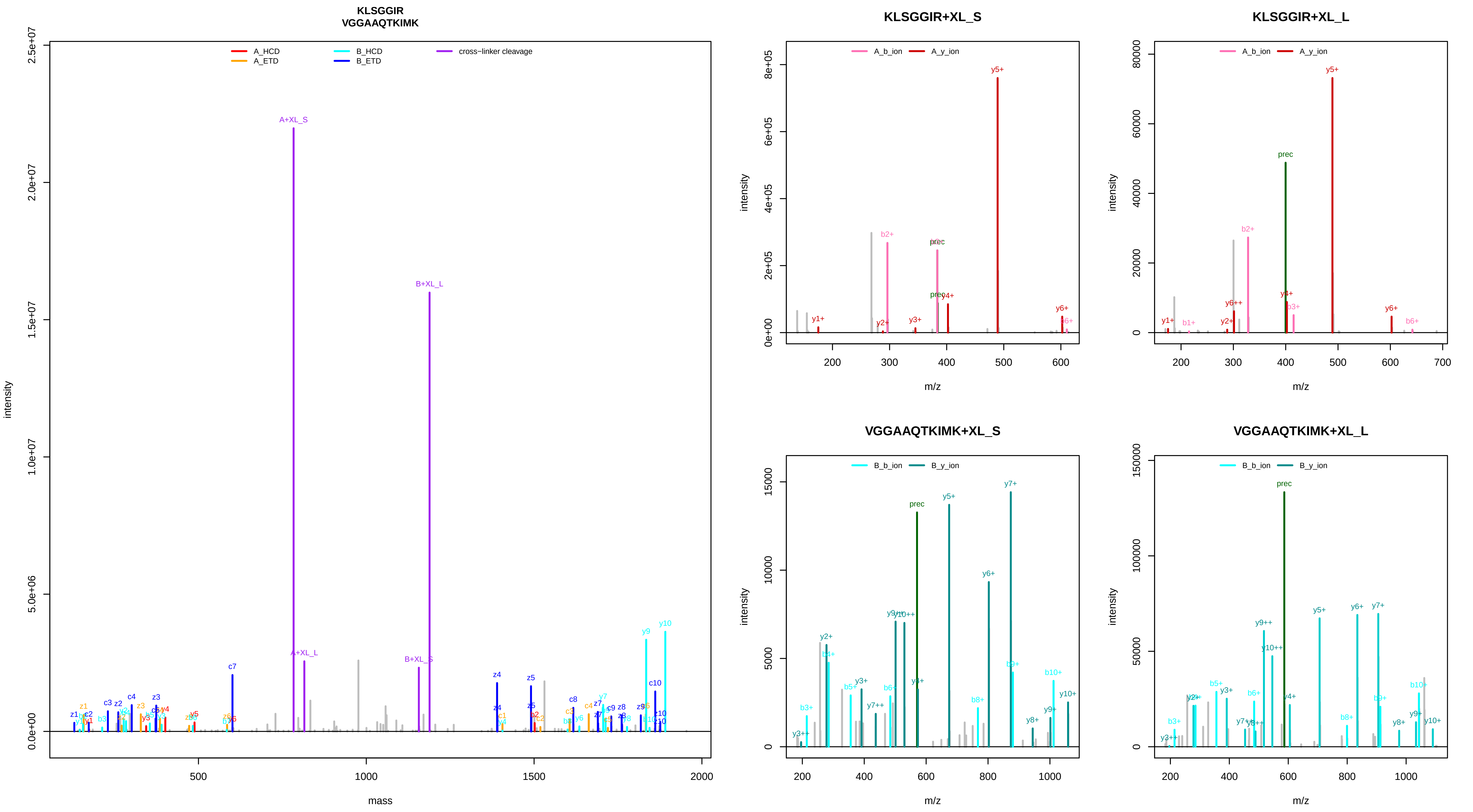

KLSGGIR
VGGAAQTKIMK
A_HCD
A_ETD
B_HCD
B_ETD
cross−linker cleavage
A+XL_S
B+XL_L
A+XL_L
B+XL_S
intensity
mass
KLSGGIR+XL_S
KLSGGIR+XL_L
VGGAAQTKIMK+XL_S
VGGAAQTKIMK+XL_L
A_b_ion
A_y_ion
B_b_ion
B_y_ion
prec
m/z

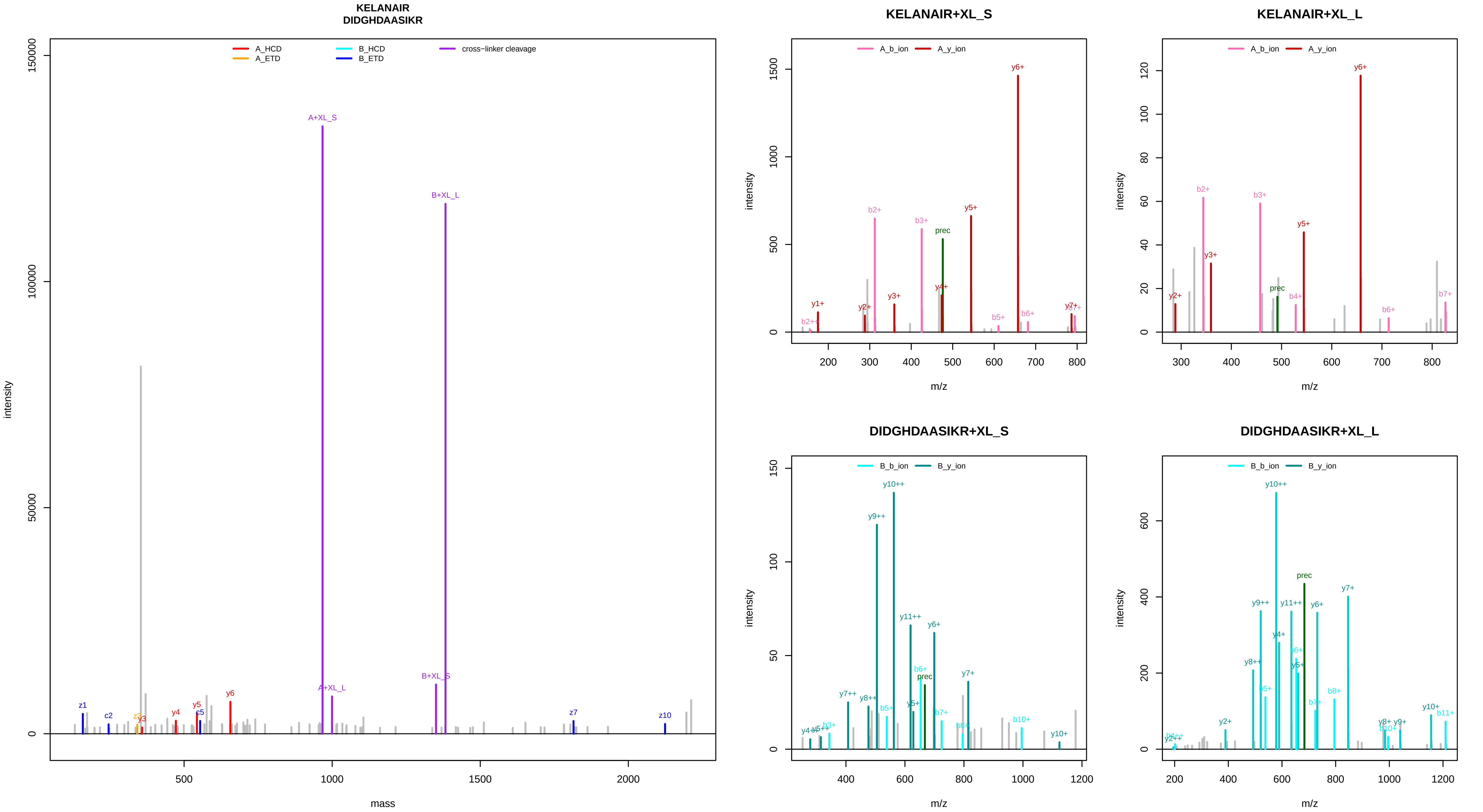
KELANAIR
DIDGHDAASIKR
A_HCD
A_ETD
B_HCD
B_ETD
cross−linker cleavage
A+XL_S
B+XL_L
A+XL_L
B+XL_S
intensity
mass
KELANAIR+XL_S
KELANAIR+XL_L
DIDGHDAASIKR+XL_S
DIDGHDAASIKR+XL_L
A_b_ion
A_y_ion
B_b_ion
B_y_ion
prec
m/z

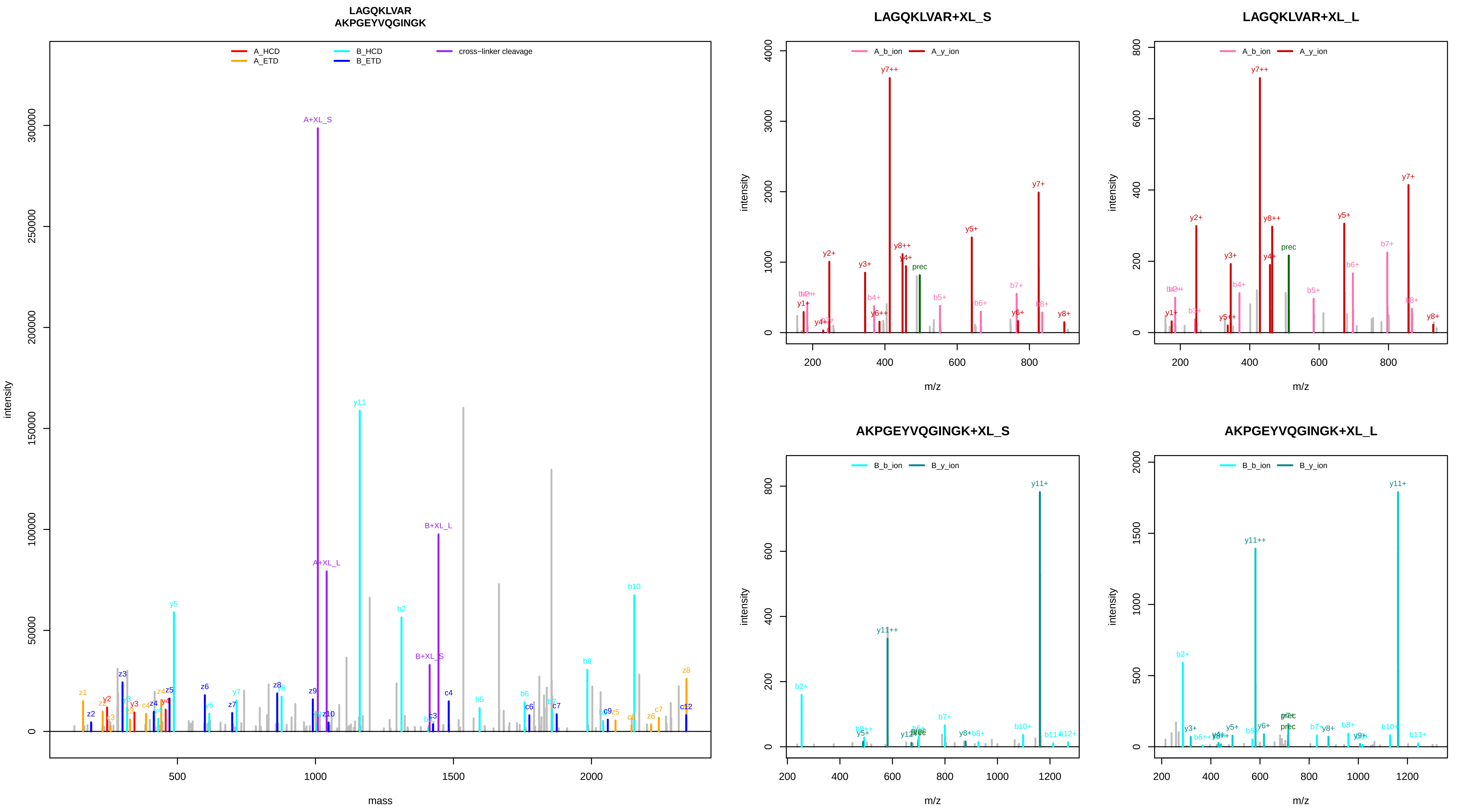

LAGQKLVAR
AKPGEYVQGINGK
A_HCD
A_ETD
B_HCD
B_ETD
cross−linker cleavage
A+XL_S
A+XL_L
B+XL_L
B+XL_S
intensity
mass
LAGQKLVAR+XL_S
LAGQKLVAR+XL_L
A_b_ion
A_y_ion
prec
intensity
m/z
AKPGEYVQGINGK+XL_S
AKPGEYVQGINGK+XL_L
B_b_ion
B_y_ion
intensity
m/z

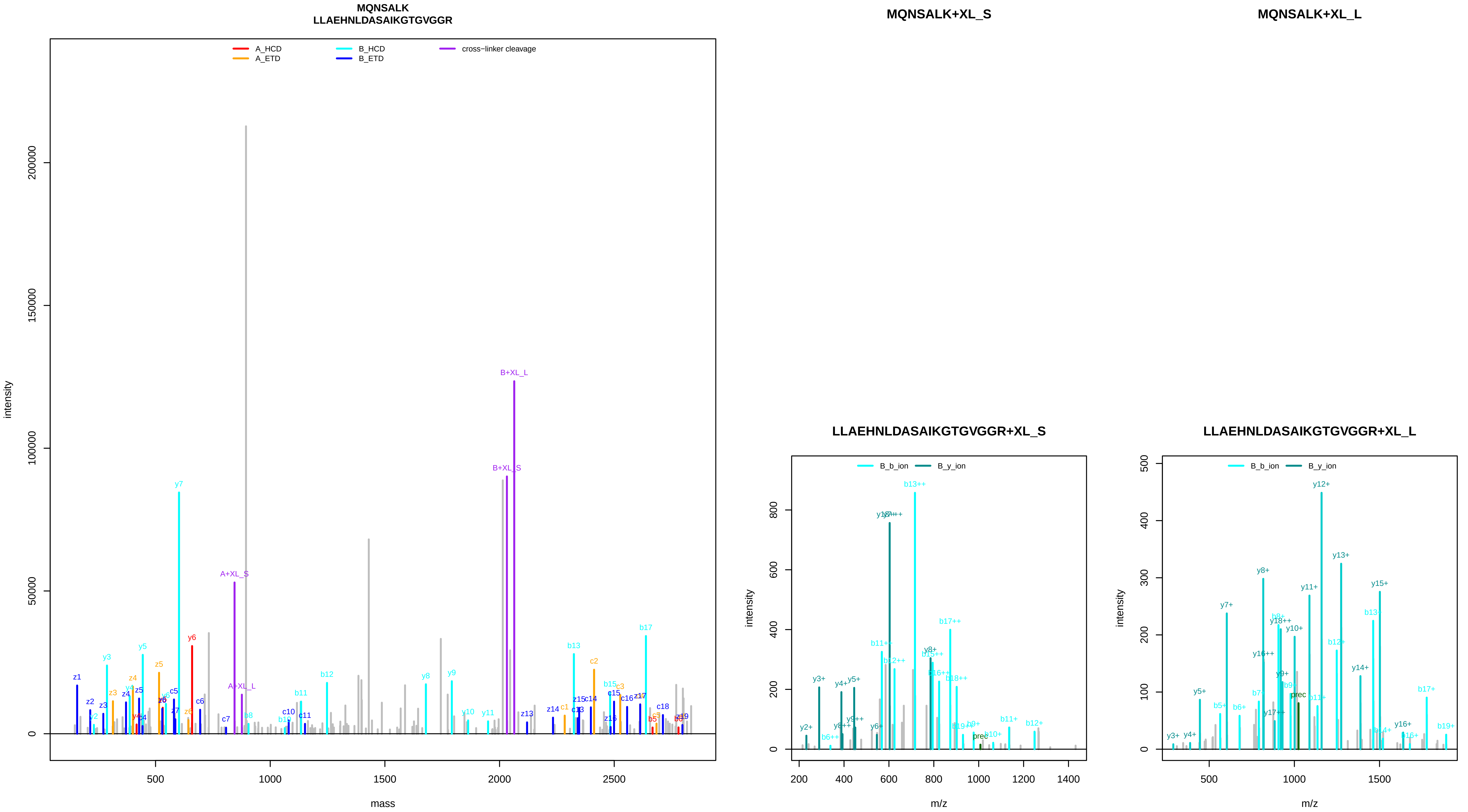
MQNSALK
LLAEHNLDASAIKGTGVGGR
A_HCD
A_ETD
B_HCD
B_ETD
cross−linker cleavage
intensity
mass
B+XL_L
B+XL_S
A+XL_S
A+XL_L
MQNSALK+XL_S
MQNSALK+XL_L
LLAEHNLDASAIKGTGVGGR+XL_S
LLAEHNLDASAIKGTGVGGR+XL_L
B_b_ion
B_y_ion
m/z
prec

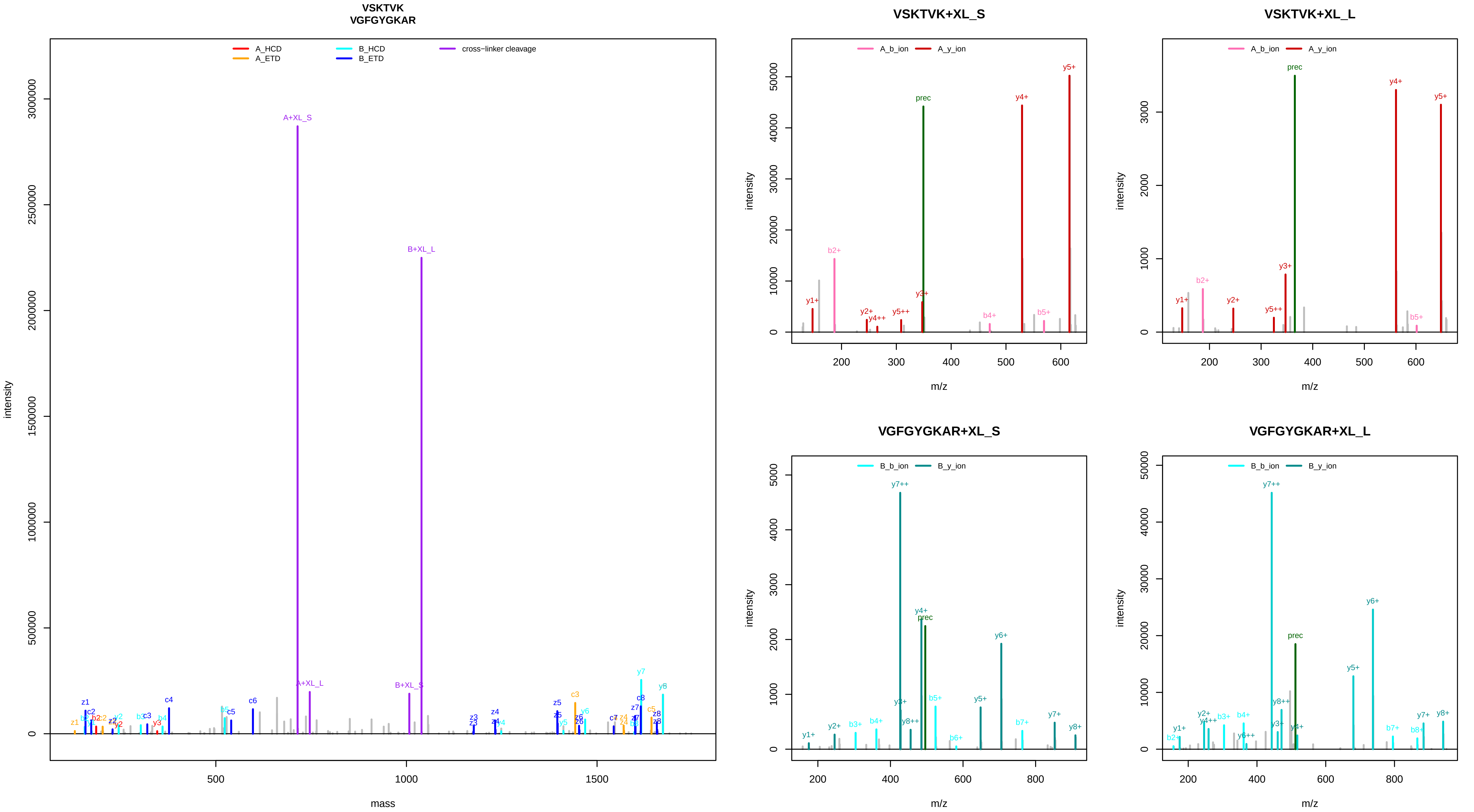

VSKTVK
VGFGYGKAR
A_HCD
A_ETD
B_HCD
B_ETD
cross−linker cleavage
A+XL_S
B+XL_L
A+XL_L
B+XL_S
intensity
mass
VSKTVK+XL_S
VSKTVK+XL_L
A_b_ion
A_y_ion
prec
m/z
VGFGYGKAR+XL_S
VGFGYGKAR+XL_L
B_b_ion
B_y_ion

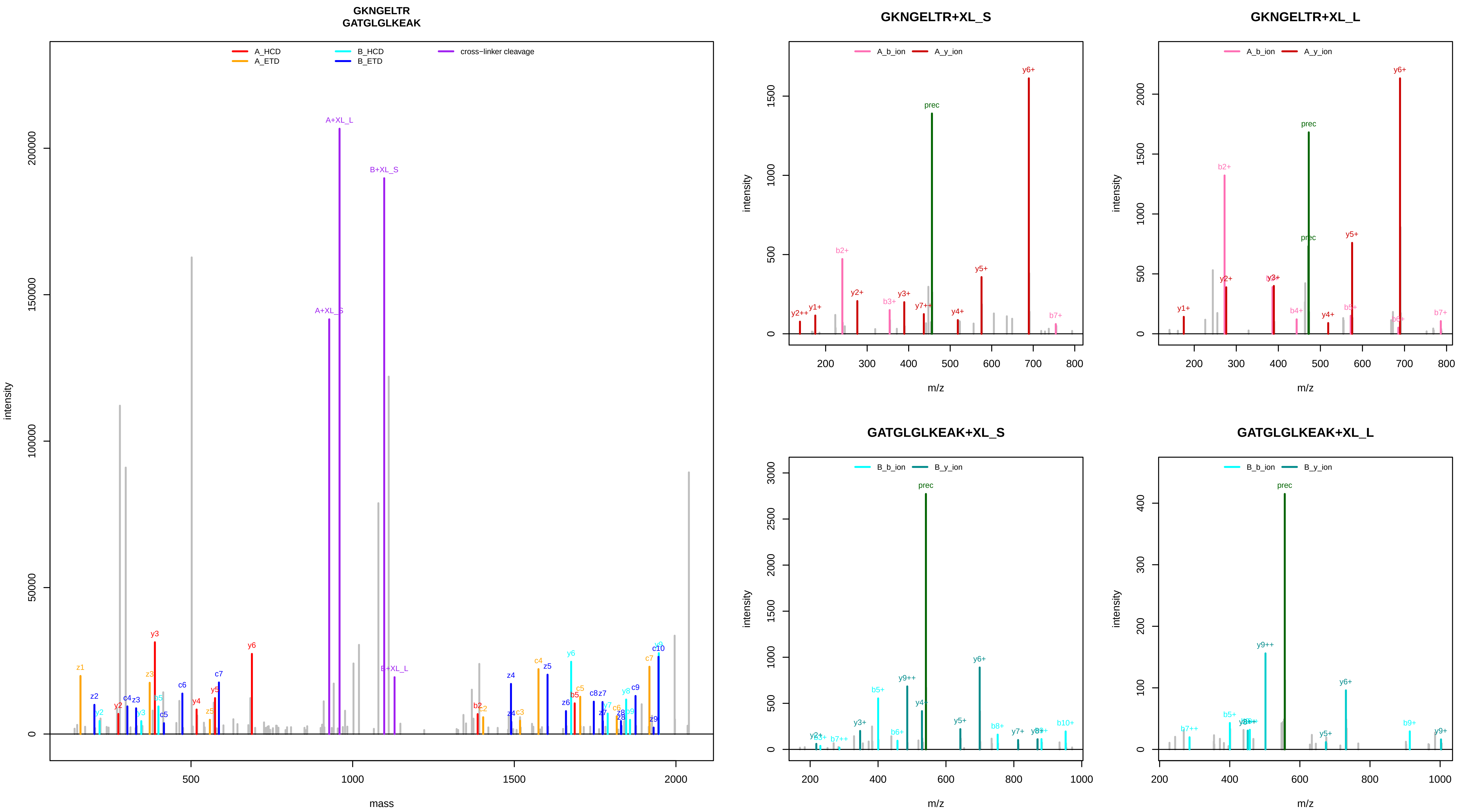
GKNGELTR
GATGLGLKEAK
A_HCD
A_ETD
B_HCD
B_ETD
cross−linker cleavage
A+XL_L
B+XL_S
A+XL_S
B+XL_L
intensity
mass
GKNGELTR+XL_S
GKNGELTR+XL_L
GATGLGLKEAK+XL_S
GATGLGLKEAK+XL_L
A_b_ion
A_y_ion
B_b_ion
B_y_ion
prec
m/z

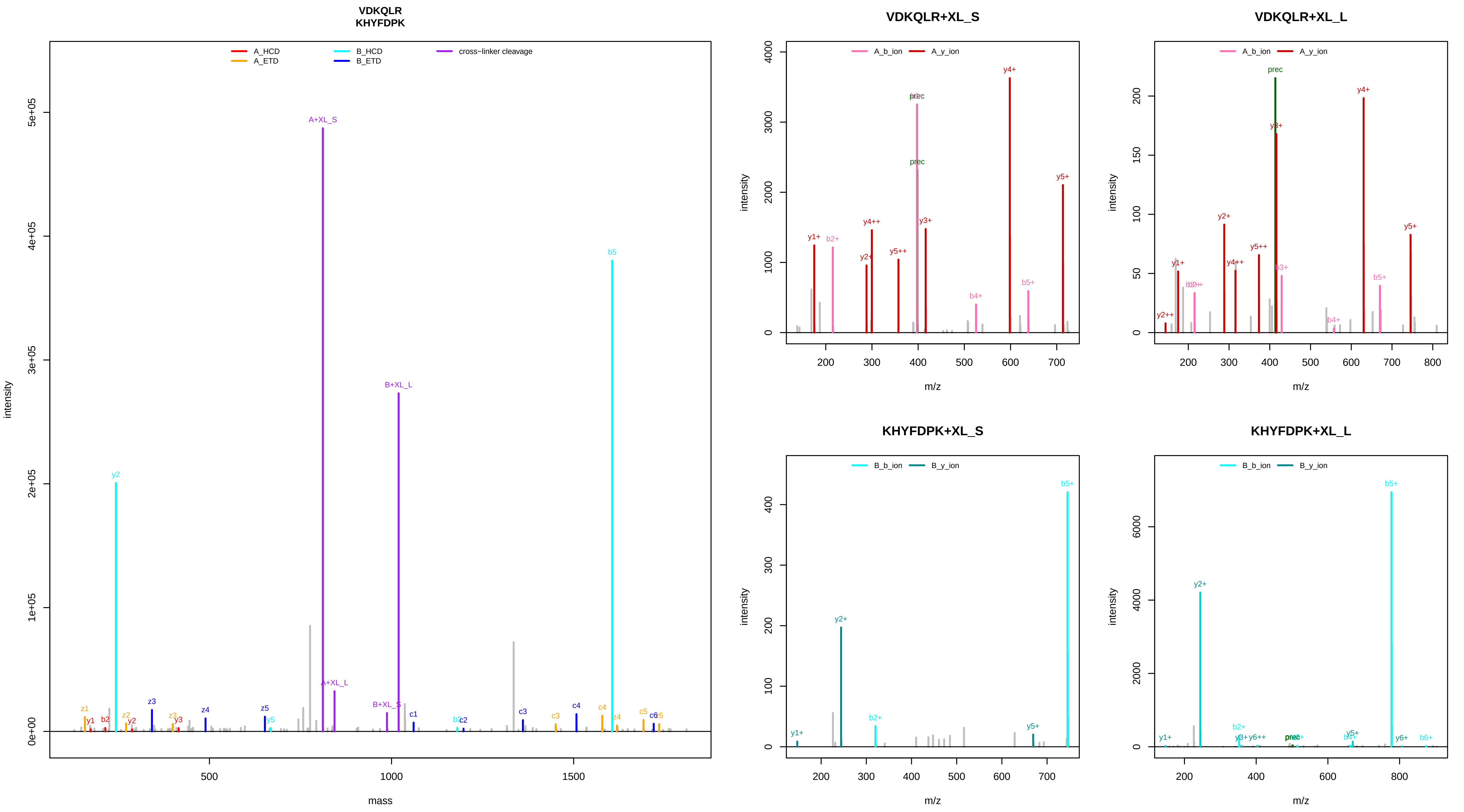

VDKQLR
KHYFDPK
A_HCD
A_ETD
B_HCD
B_ETD
cross−linker cleavage
A+XL_S
B+XL_L
A+XL_L
B+XL_S
intensity
mass
VDKQLR+XL_S
VDKQLR+XL_L
KHYFDPK+XL_S
KHYFDPK+XL_L
A_b_ion
A_y_ion
B_b_ion
B_y_ion
prec
m/z

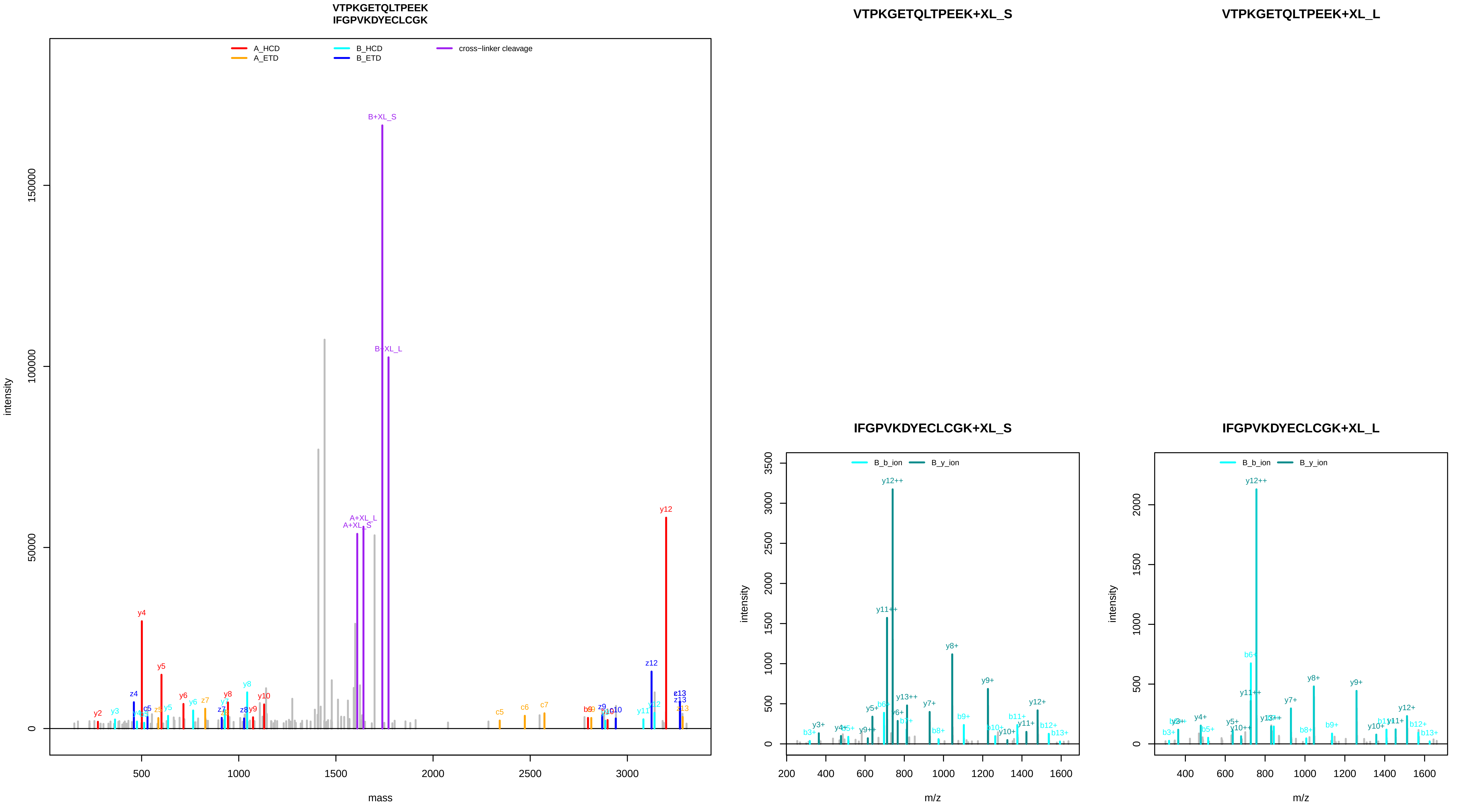

VTPKGETQLTPEEK
IFGPVKDYECLCGK
A_HCD
A_ETD
B_HCD
B_ETD
cross−linker cleavage
B+XL_S
B+XL_L
A+XL_L
A+XL_S
y2
y3
z4
y4
c5
y5
z5
y6
z7
y7
y8
z8
y9
y10
c5
c6
c7
b9
z9
y11
z12
y12
z13
c13
intensity
mass
VTPKGETQLTPEEK+XL_S
VTPKGETQLTPEEK+XL_L
IFGPVKDYECLCGK+XL_S
IFGPVKDYECLCGK+XL_L
B_b_ion
B_y_ion
y12++
y11++
y13++
b6+
y5+
y6+
y7+
y8+
y9+
y10+
y11+
y12+
b3+
y3+
y4+
b5+
b8+
b9+
b11+
b12+
b13+
m/z

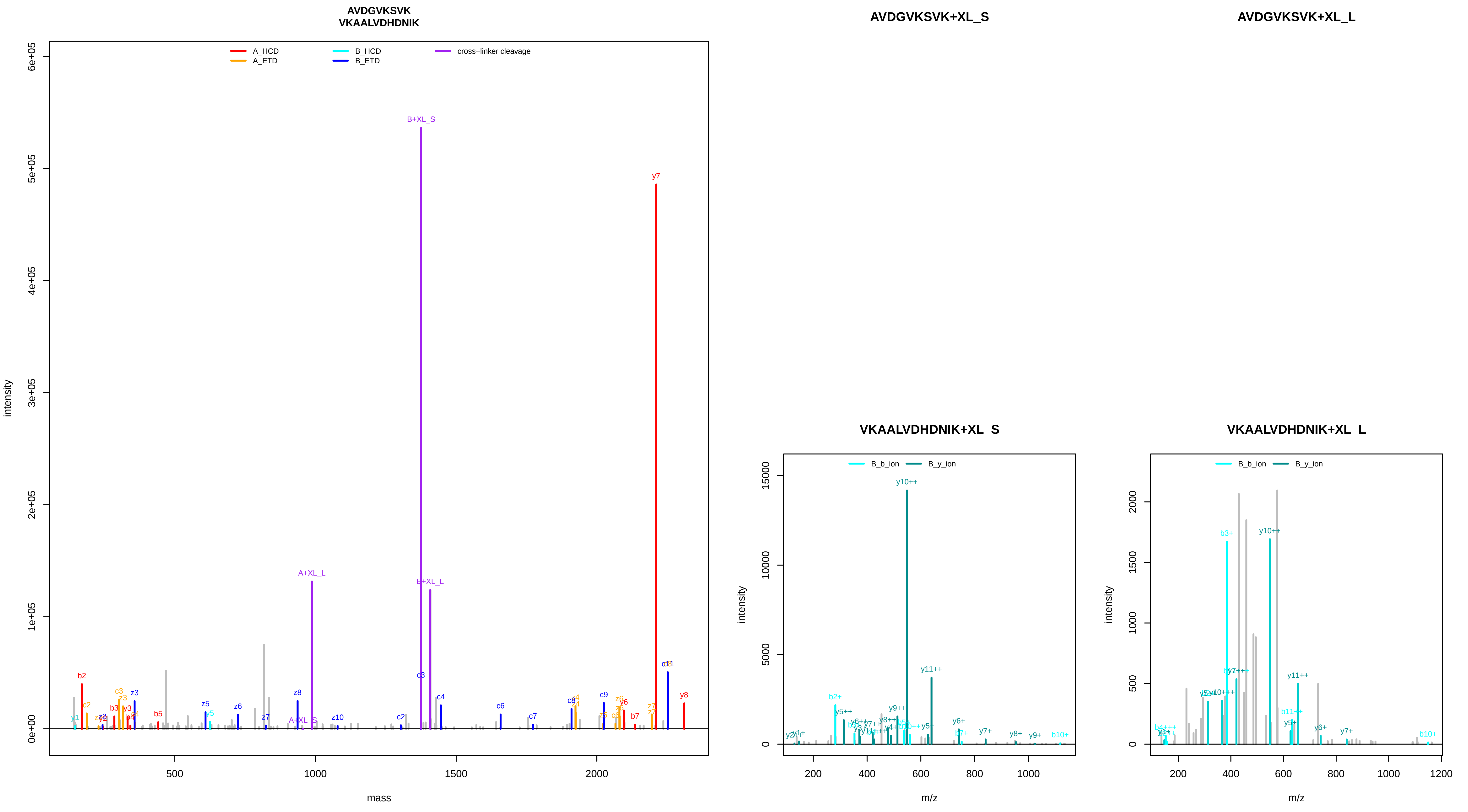

AVDGVKSVK
VKAALVDHDNIK
A_HCD
A_ETD
B_HCD
B_ETD
cross−linker cleavage
B+XL_S
y7
A+XL_L
B+XL_L
intensity
mass
AVDGVKSVK+XL_S
AVDGVKSVK+XL_L
VKAALVDHDNIK+XL_S
VKAALVDHDNIK+XL_L
B_b_ion
B_y_ion
y10++
y11++
b3+
m/z

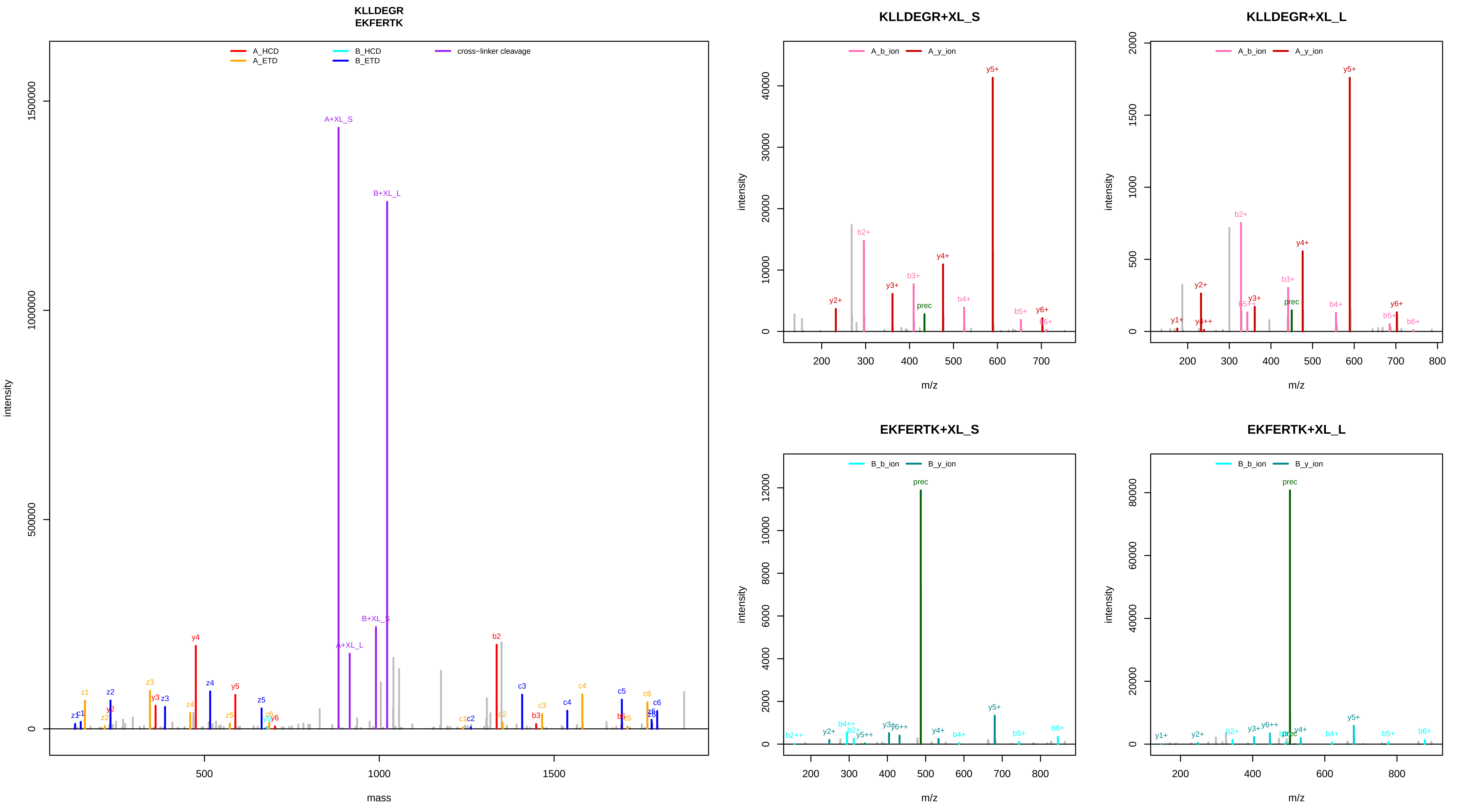
KLLDEGR
EKFERTK
A_HCD
A_ETD
B_HCD
B_ETD
cross−linker cleavage
A+XL_S
B+XL_L
B+XL_S
A+XL_L
intensity
mass
KLLDEGR+XL_S
KLLDEGR+XL_L
EKFERTK+XL_S
EKFERTK+XL_L
A_b_ion
A_y_ion
B_b_ion
B_y_ion
prec
intensity
m/z

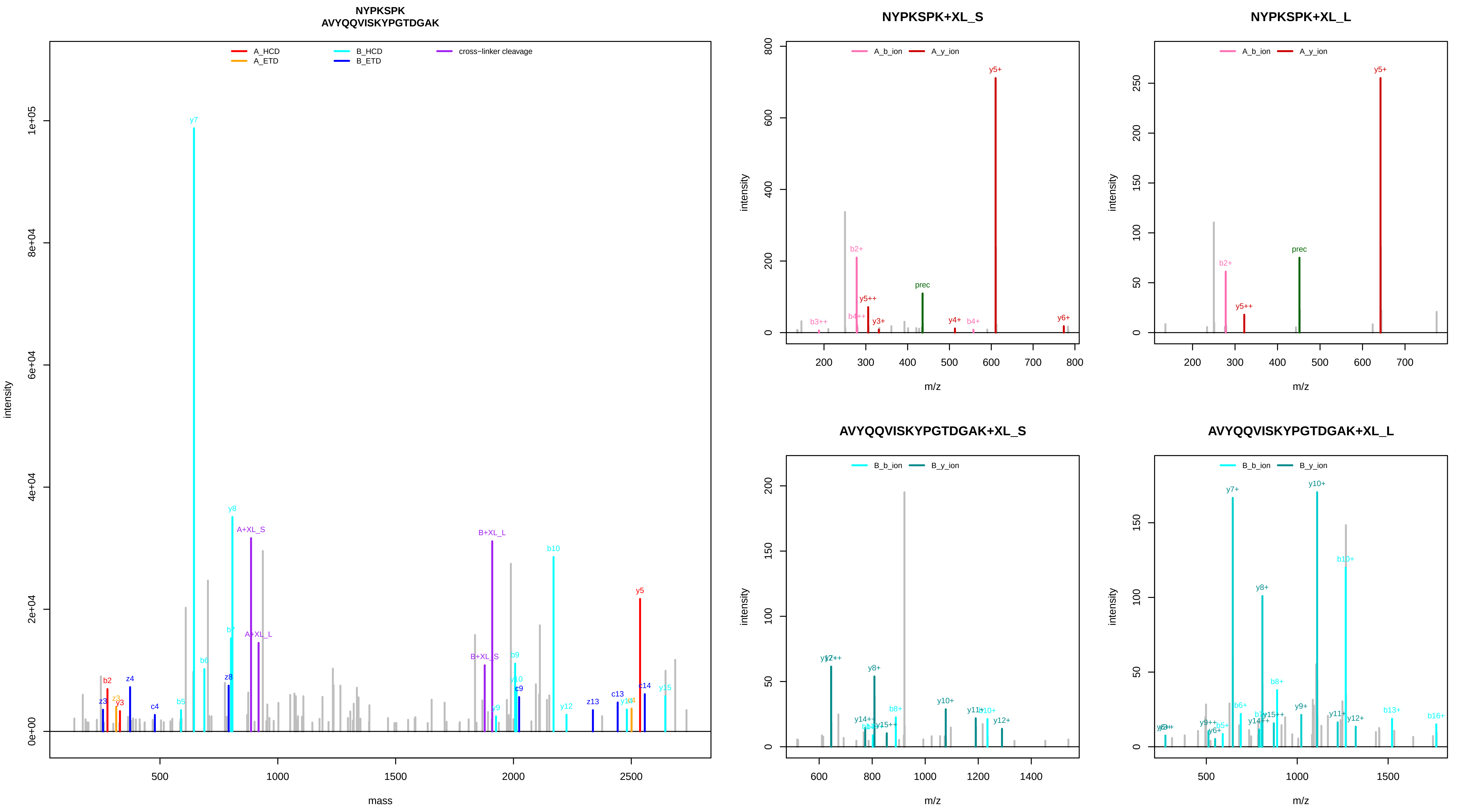
NYPKSPK
AVYQQVISKYPGTDGAK
A_HCD
A_ETD
B_HCD
B_ETD
cross−linker cleavage
intensity
mass
y7
y8
A+XL_S
A+XL_L
B+XL_L
B+XL_S
b10
y5
b9
y10
c9
y9
b7
b6
b5
z8
z4
c4
b2
z3
y3
z13
c13
c14
y15
y12
NYPKSPK+XL_S
NYPKSPK+XL_L
A_b_ion
A_y_ion
y5+
b2+
y5++
prec
y3+
y4+
b4+
y6+
b3++
b4++
m/z
AVYQQVISKYPGTDGAK+XL_S
AVYQQVISKYPGTDGAK+XL_L
B_b_ion
B_y_ion
y8+
b8+
y10+
y11+
y12+
y14++
y15++
y7+
b10+
y9+
b6+
b13+
b16+
y9++
y6+
y15++
y14++

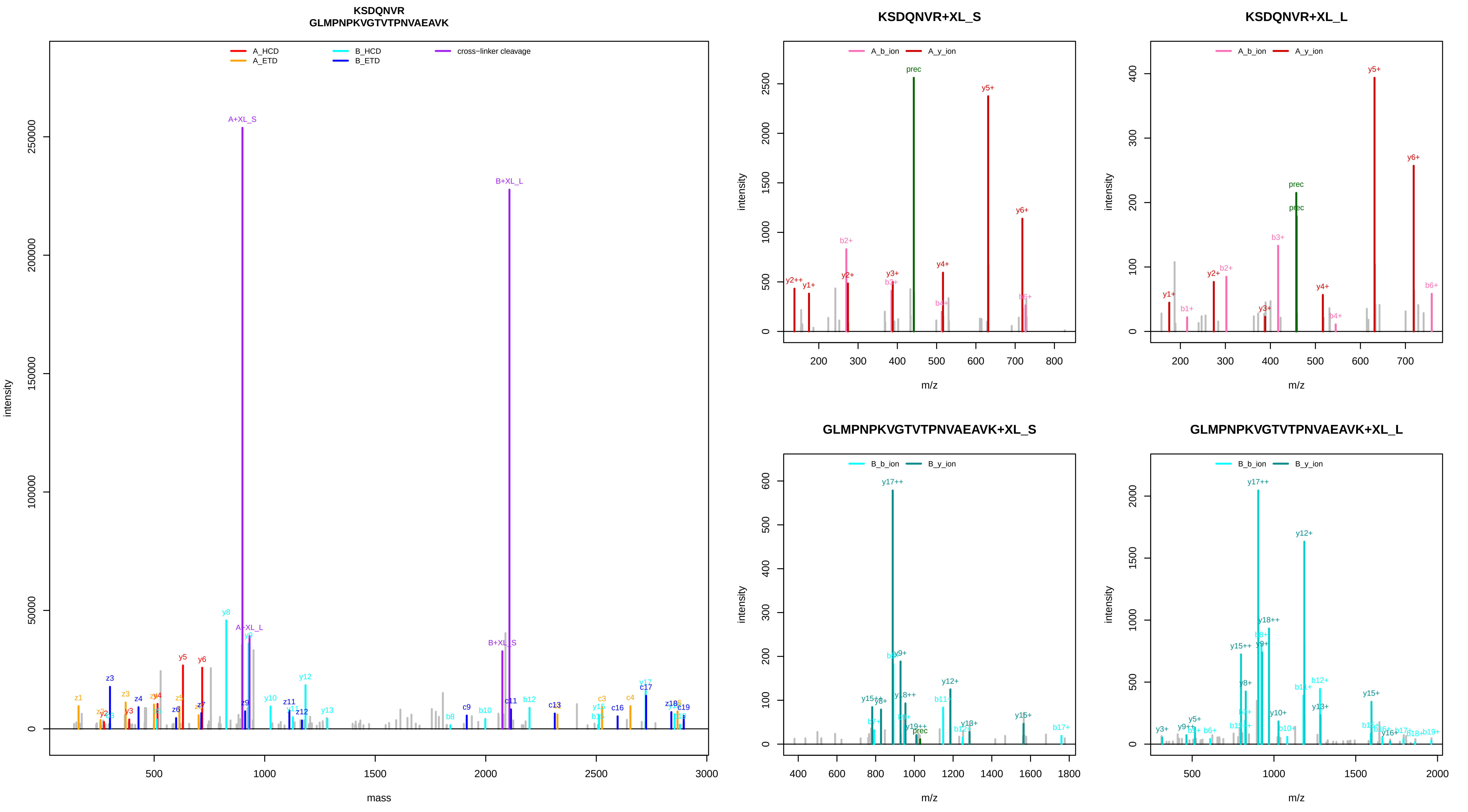
KSDQNVR
GLMPNPKVGTVTPNVAEAVK
A_HCD
A_ETD
B_HCD
B_ETD
cross−linker cleavage
A+XL_S
B+XL_L
A+XL_L
B+XL_S
y8
y5
y6
y12
y17
c17
z3
intensity
mass
KSDQNVR+XL_S
KSDQNVR+XL_L
A_b_ion
A_y_ion
prec
y5+
y6+
y4+
y3+
b2+
b3+
y2+
y1+
y2++
m/z
GLMPNPKVGTVTPNVAEAVK+XL_S
GLMPNPKVGTVTPNVAEAVK+XL_L
B_b_ion
B_y_ion
y17++
y12+
y18++
y15++
y9+
y13+
y15+
b17+

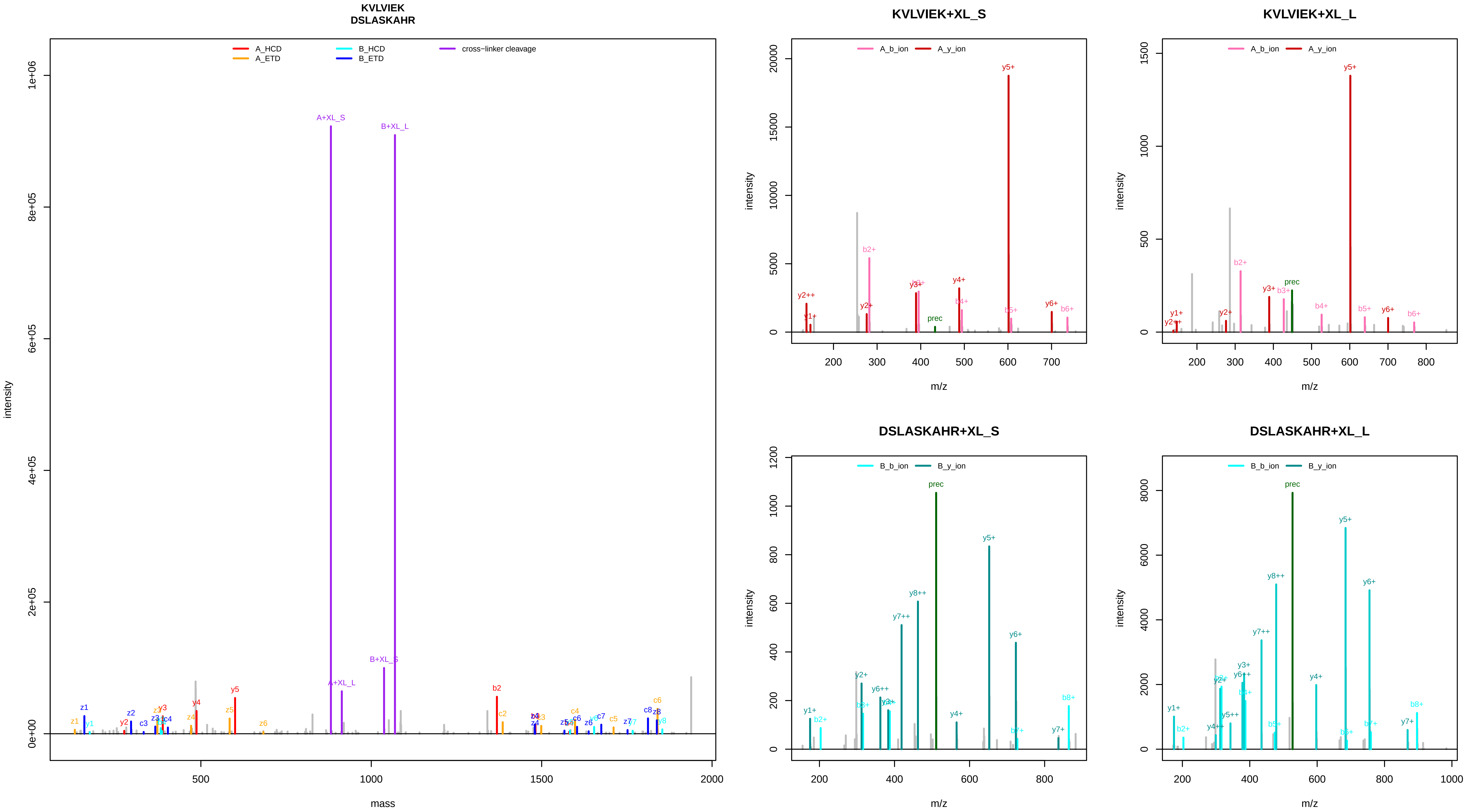
KVLVIEK
DSLASKAHR
A_HCD
A_ETD
B_HCD
B_ETD
cross−linker cleavage
A+XL_S
B+XL_L
B+XL_S
A+XL_L
intensity
mass
KVLVIEK+XL_S
KVLVIEK+XL_L
DSLASKAHR+XL_S
DSLASKAHR+XL_L
A_b_ion
A_y_ion
B_b_ion
B_y_ion
prec
m/z

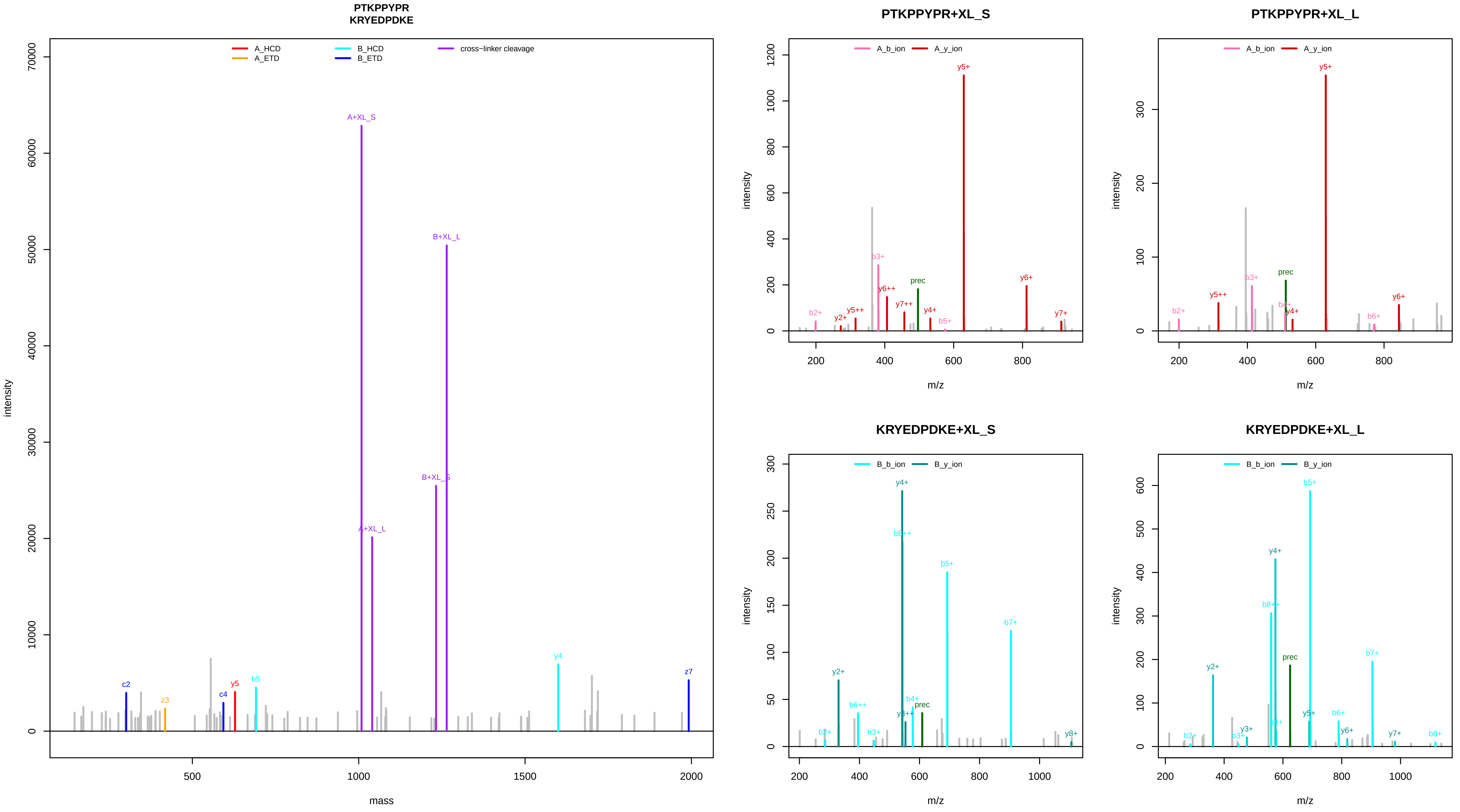
PTKPPYPR
KRYEDPDKE
A_HCD
A_ETD
B_HCD
B_ETD
cross−linker cleavage
A+XL_S
B+XL_L
B+XL_S
A+XL_L
c2
z3
y5
c4
b5
y4
z7
intensity
mass
PTKPPYPR+XL_S
A_b_ion
A_y_ion
y5+
b3+
y6++
prec
y6+
b2+
y2+
y5++
y7++
y4+
b5+
y7+
m/z
PTKPPYPR+XL_L
y5++
b4+
b6+
KRYEDPDKE+XL_S
B_b_ion
B_y_ion
y4+
b8++
b5+
b7+
y2+
b6++
b3+
y3++
y8+
KRYEDPDKE+XL_L
y3+
b6+
y6+
b8+

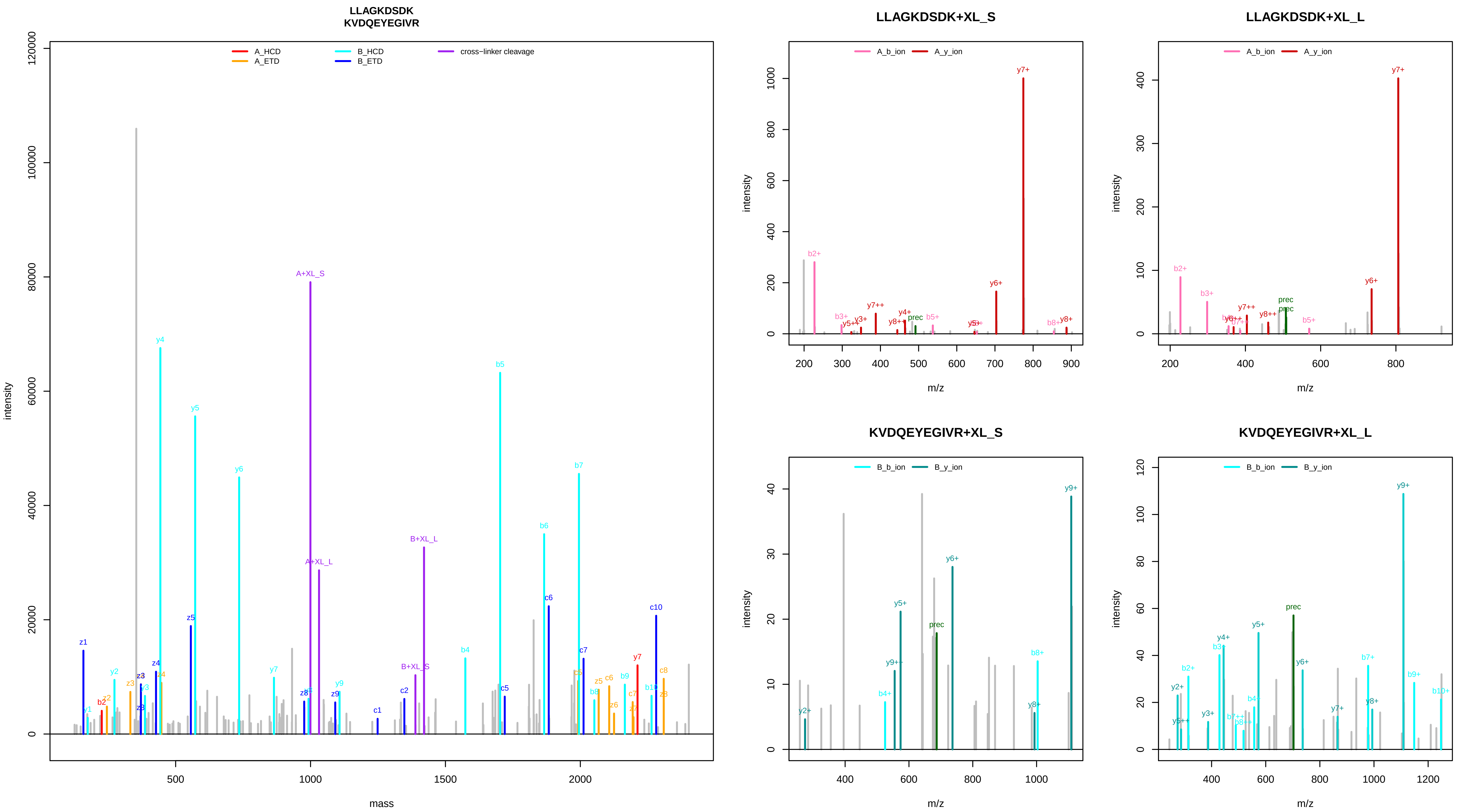

LLAGKDSDK
KVDQEYEGIVR
A_HCD
A_ETD
B_HCD
B_ETD
cross−linker cleavage
intensity
mass
A+XL_S
A+XL_L
B+XL_L
B+XL_S
LLAGKDSDK+XL_S
LLAGKDSDK+XL_L
KVDQEYEGIVR+XL_S
KVDQEYEGIVR+XL_L
A_b_ion
A_y_ion
B_b_ion
B_y_ion
m/z
prec

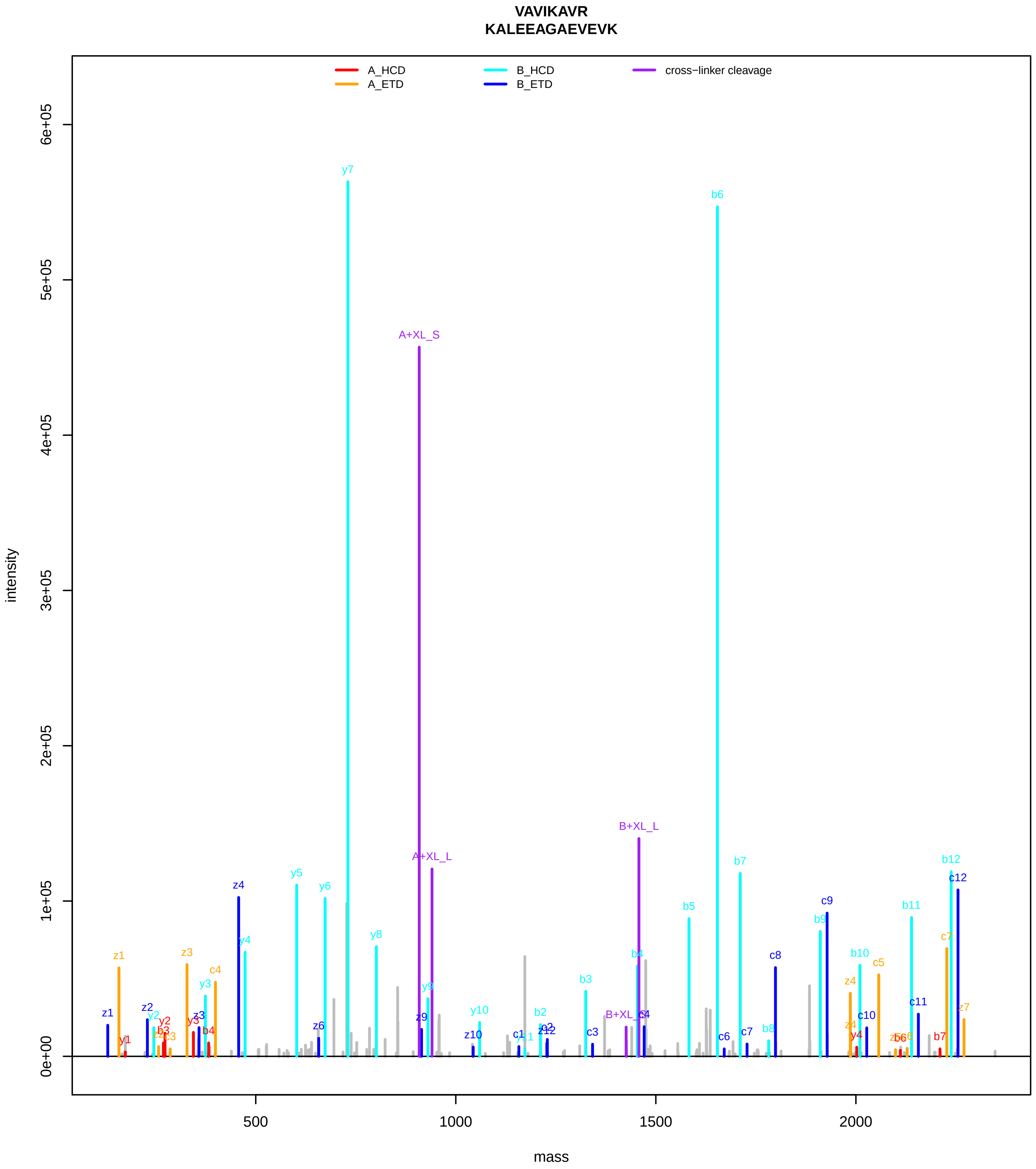

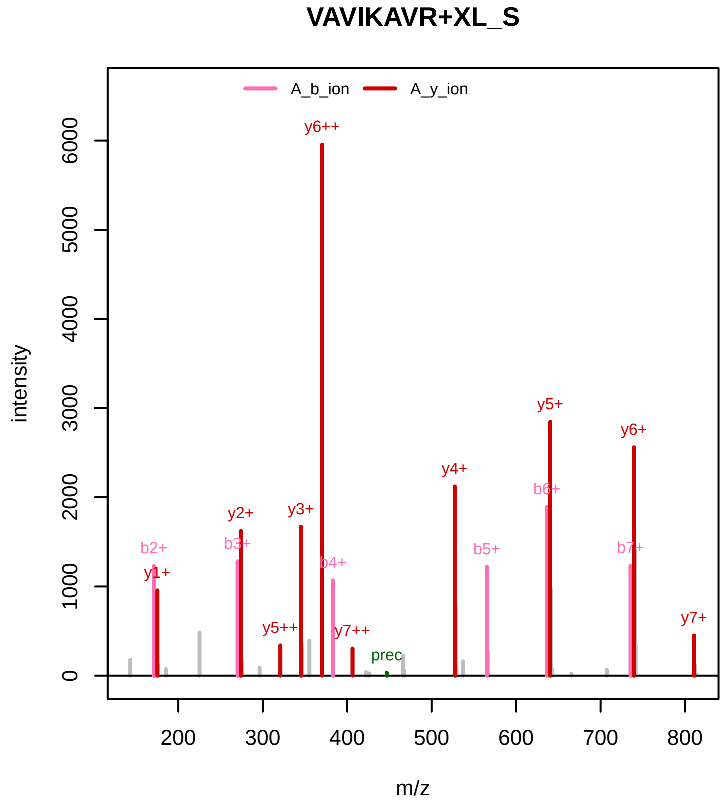

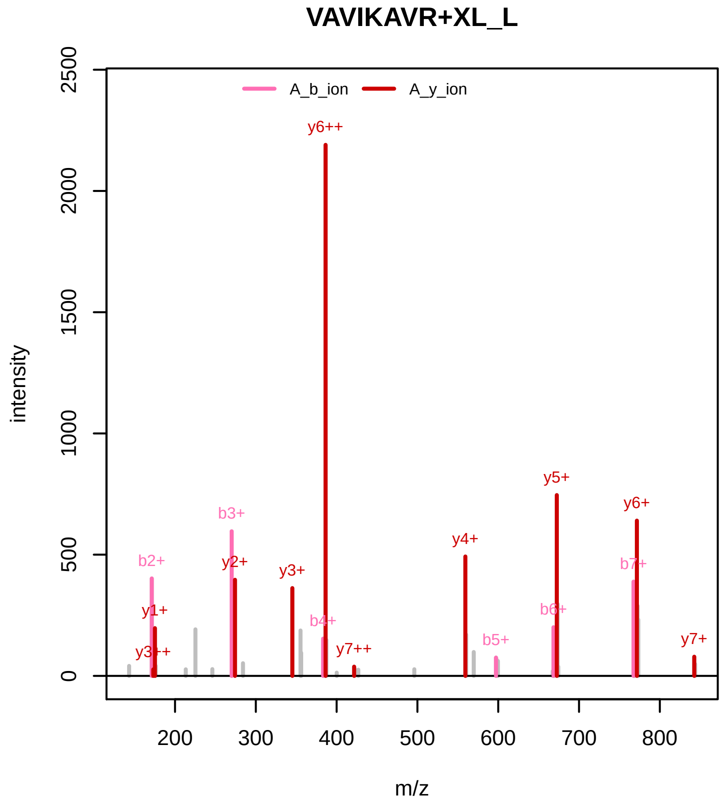

KALEEAGAEVEVK+XL_S

KALEEAGAEVEVK+XL_L

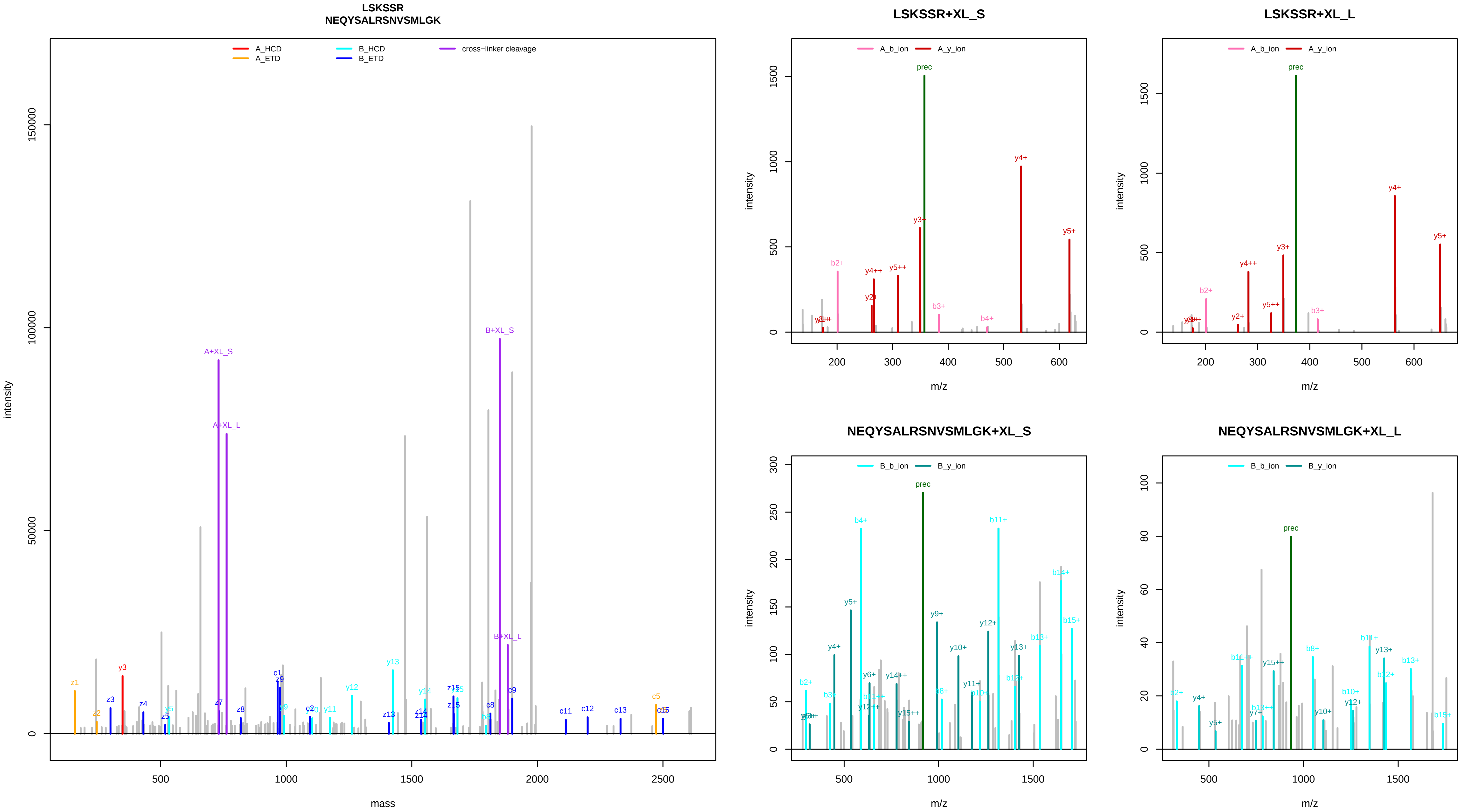
LSKSSR
NEQYSALRSNVSMLGK
A_HCD
A_ETD
B_HCD
B_ETD
cross−linker cleavage
A+XL_S
A+XL_L
B+XL_S
B+XL_L
intensity
mass
LSKSSR+XL_S
LSKSSR+XL_L
NEQYSALRSNVSMLGK+XL_S
NEQYSALRSNVSMLGK+XL_L
A_b_ion
A_y_ion
B_b_ion
B_y_ion
prec
m/z

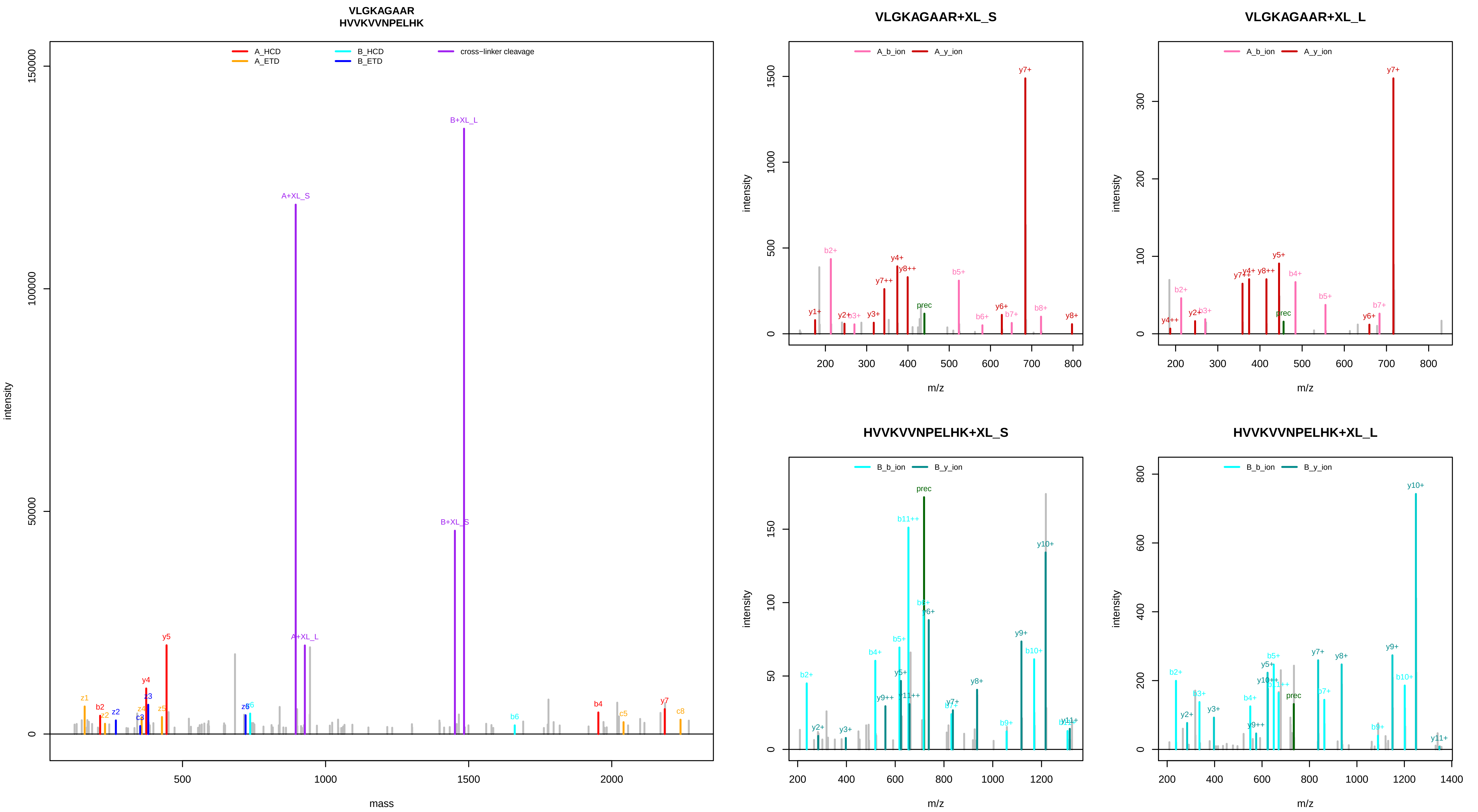

VLGKAGAAR
HVVKVVNPELHK
A_HCD
A_ETD
B_HCD
B_ETD
cross−linker cleavage
B+XL_L
A+XL_S
B+XL_S
A+XL_L
intensity
mass
VLGKAGAAR+XL_S
VLGKAGAAR+XL_L
HVVKVVNPELHK+XL_S
HVVKVVNPELHK+XL_L
A_b_ion
A_y_ion
B_b_ion
B_y_ion
prec
m/z

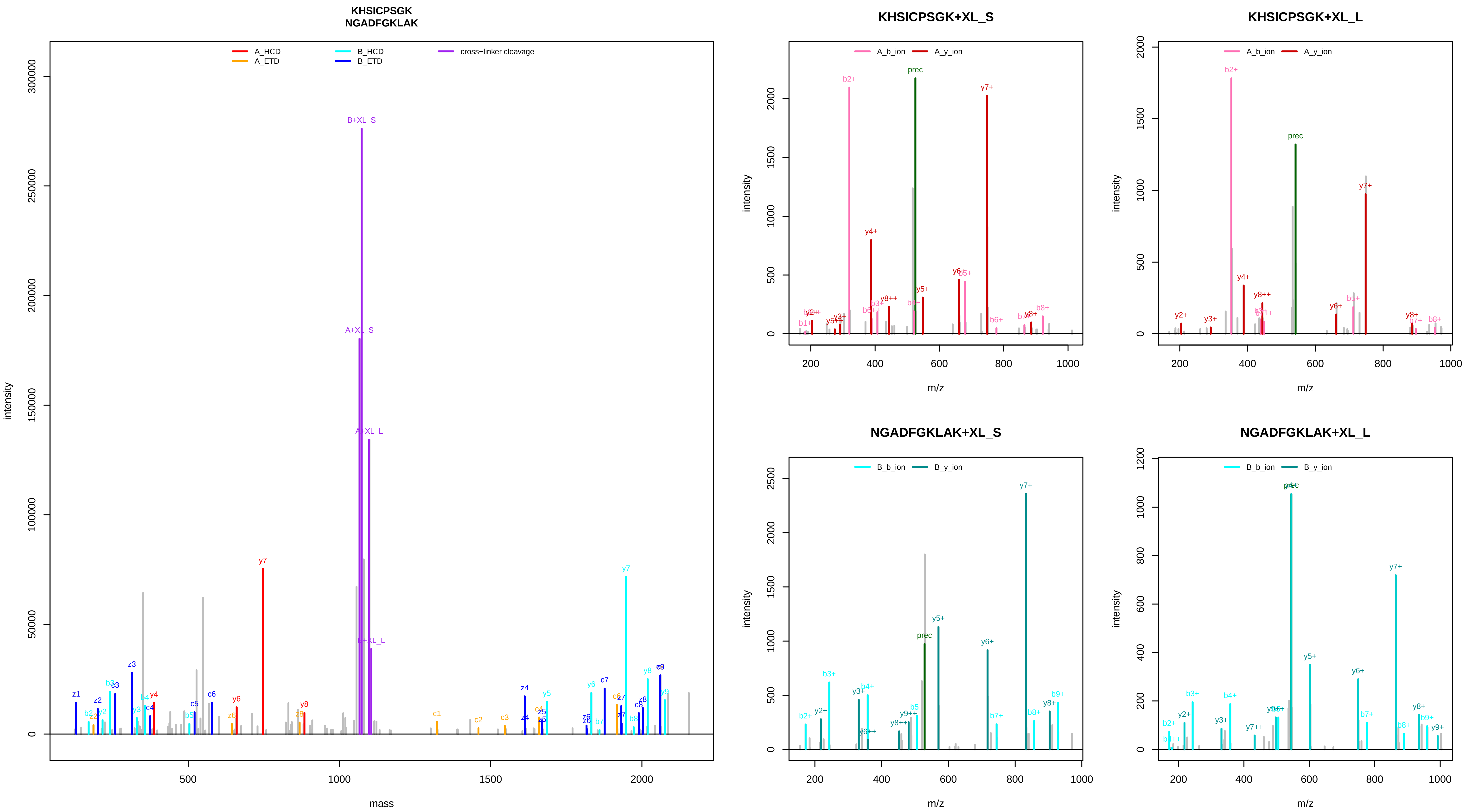
KHSICPSGK
NGADFGKLAK
A_HCD
A_ETD
B_HCD
B_ETD
cross−linker cleavage
B+XL_S
A+XL_S
A+XL_L
B+XL_L
intensity
mass
KHSICPSGK+XL_S
KHSICPSGK+XL_L
A_b_ion
A_y_ion
NGADFGKLAK+XL_S
NGADFGKLAK+XL_L
B_b_ion
B_y_ion
prec
m/z

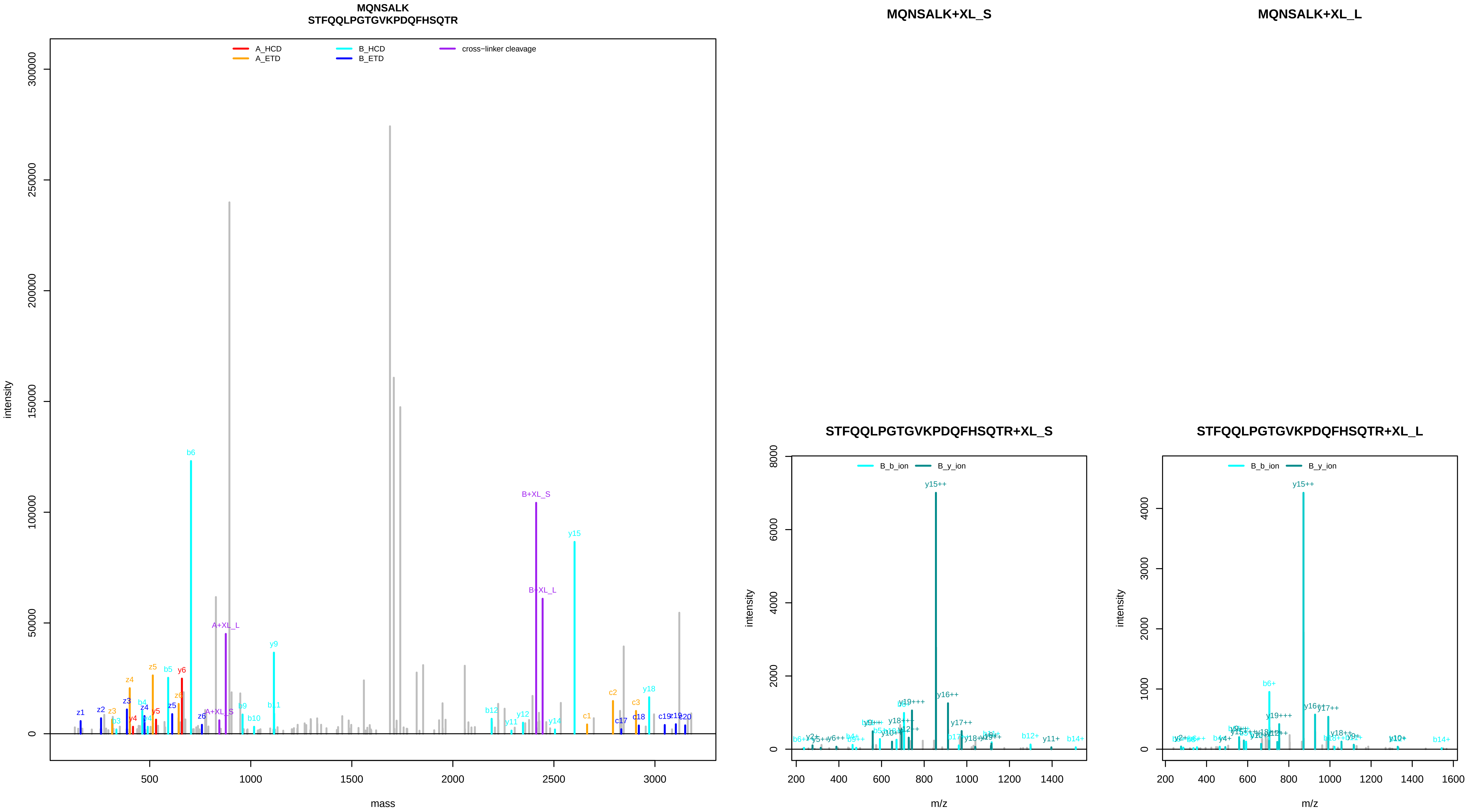

MQNSALK
STFQQLPGTGVKPDQFHSQTR
A_HCD
A_ETD
B_HCD
B_ETD
cross−linker cleavage
intensity
mass
b6
B+XL_S
B+XL_L
y15
A+XL_L
A+XL_S
y9
y18
MQNSALK+XL_S
MQNSALK+XL_L
STFQQLPGTGVKPDQFHSQTR+XL_S
STFQQLPGTGVKPDQFHSQTR+XL_L
B_b_ion
B_y_ion
y15++
y16++
b6+
m/z

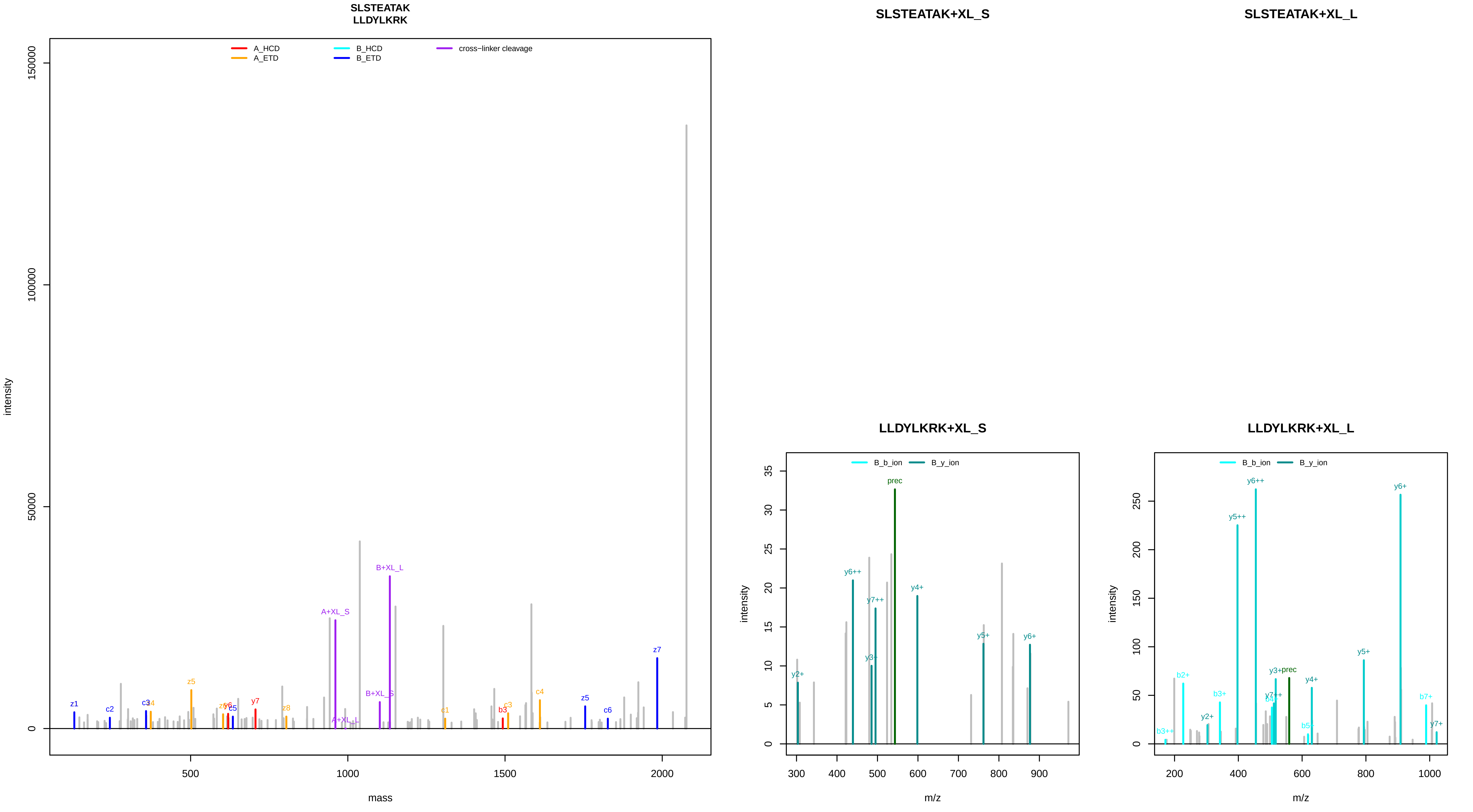
SLSTEATAK
LLDYLKRK
A_HCD
A_ETD
B_HCD
B_ETD
cross−linker cleavage
intensity
mass
A+XL_S
B+XL_L
B+XL_S
A+XL_L
z1
c2
c3
z5
y7
z8
c1
b3
c3
c4
z5
c6
z7
c5
SLSTEATAK+XL_S
SLSTEATAK+XL_L
LLDYLKRK+XL_S
LLDYLKRK+XL_L
B_b_ion
B_y_ion
prec
y6++
y7++
y4+
y3+
y2+
y5+
y6+
m/z
y6++
y5++
y6+
y5+
b2+
b3+
y2+
b3++
y3+prec
y4+
b7+
y7+
b5+

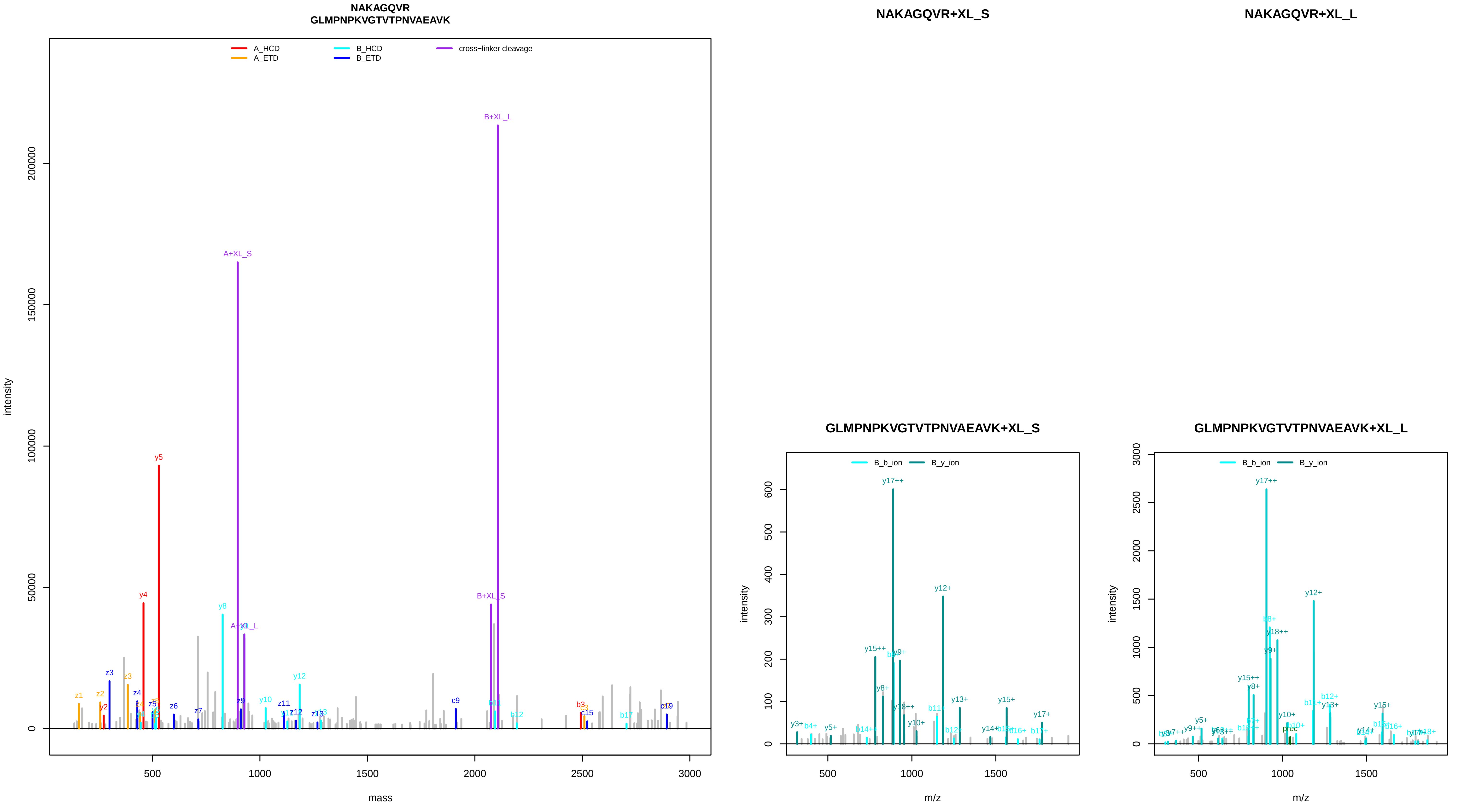
NAKAGQVR
GLMPNPKVGTVTPNVAEAVK
A_HCD
A_ETD
B_HCD
B_ETD
cross−linker cleavage
B+XL_L
A+XL_S
y5
y4
y8
A+XL_L
B+XL_S
intensity
mass
NAKAGQVR+XL_S
NAKAGQVR+XL_L
GLMPNPKVGTVTPNVAEAVK+XL_S
GLMPNPKVGTVTPNVAEAVK+XL_L
B_b_ion
B_y_ion
y17++
y12+
m/z

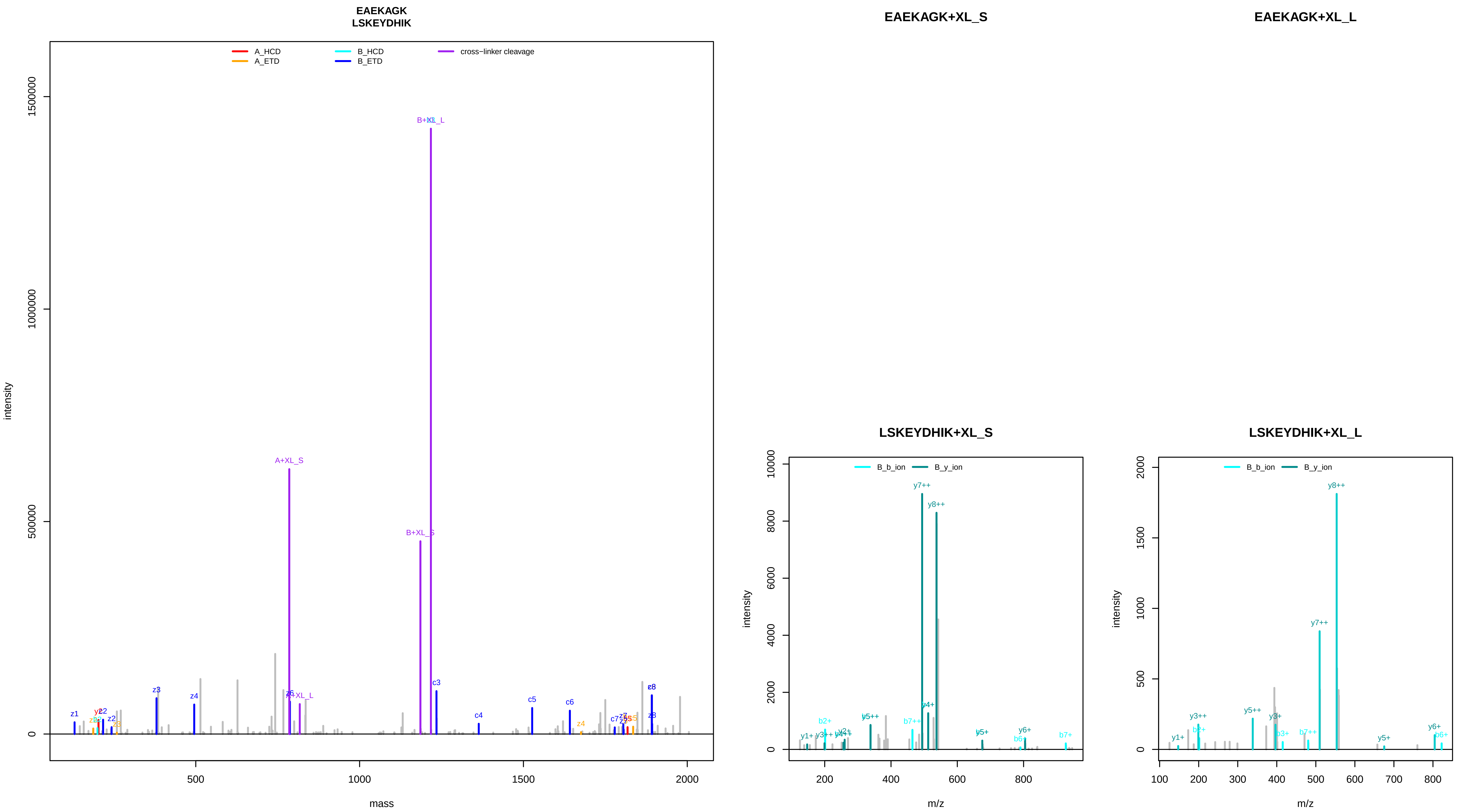

EAEKAGK
LSKEYDHIK
A_HCD
A_ETD
B_HCD
B_ETD
cross−linker cleavage
B+XL_L
A+XL_S
B+XL_S
A+XL_L
intensity
mass
EAEKAGK+XL_S
EAEKAGK+XL_L
LSKEYDHIK+XL_S
LSKEYDHIK+XL_L
B_b_ion
B_y_ion
y7++
y8++
m/z

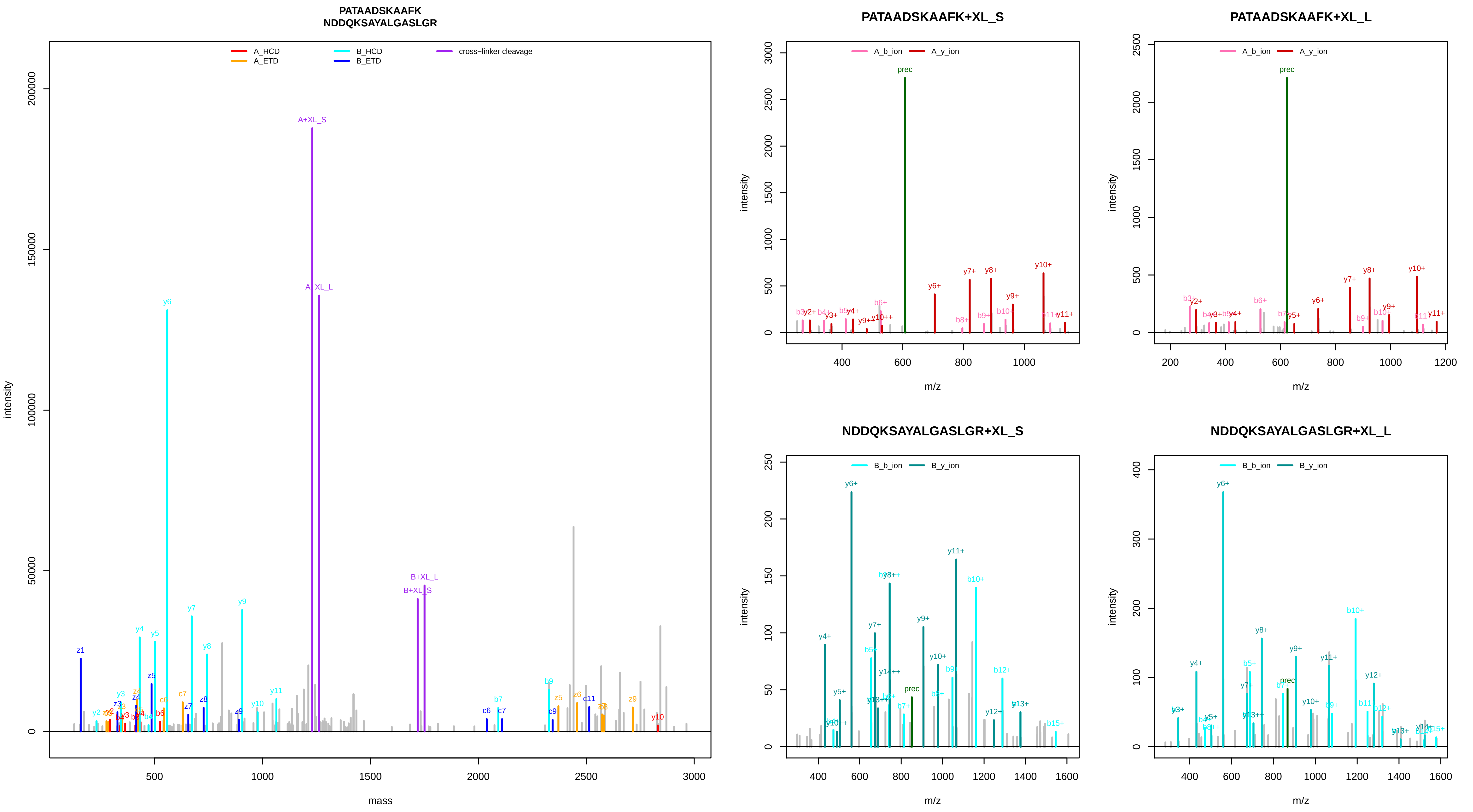

PATAADSKAAFK
NDDQKSAYALGASLGR
A_HCD
A_ETD
B_HCD
B_ETD
cross−linker cleavage
A+XL_S
A+XL_L
B+XL_L
B+XL_S
intensity
mass
PATAADSKAAFK+XL_S
PATAADSKAAFK+XL_L
A_b_ion
A_y_ion
prec
intensity
m/z
NDDQKSAYALGASLGR+XL_S
NDDQKSAYALGASLGR+XL_L
B_b_ion
B_y_ion
intensity
m/z

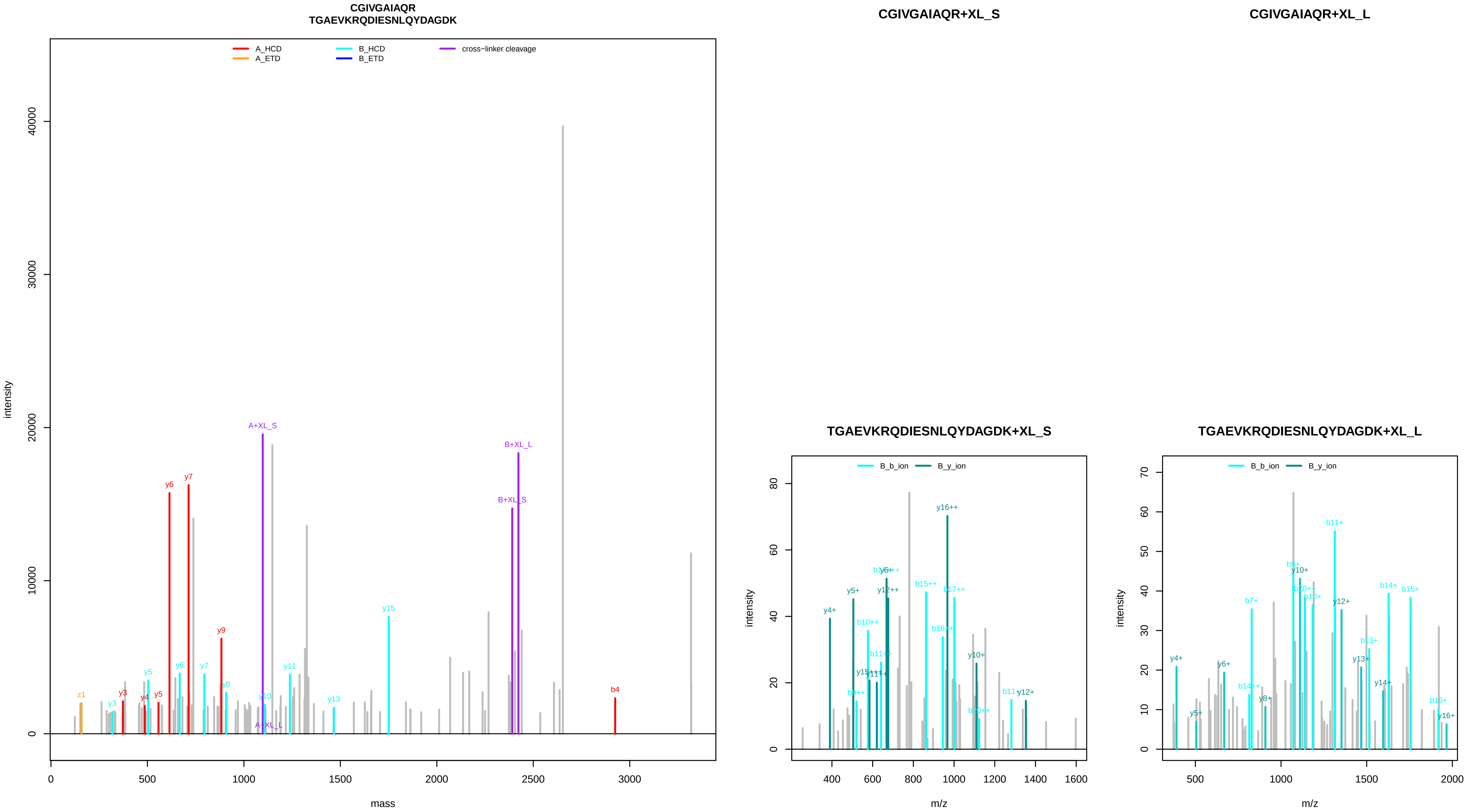
CGIVGAIAQR
TGAEVKRQDIESNLQYDAGDK
A_HCD
A_ETD
B_HCD
B_ETD
cross−linker cleavage
intensity
mass
A+XL_S
A+XL_L
B+XL_L
B+XL_S
y6
y7
y9
y3
y4
y5
z1
b4
y8
y10
y11
y13
y15
CGIVGAIAQR+XL_S
CGIVGAIAQR+XL_L
TGAEVKRQDIESNLQYDAGDK+XL_S
TGAEVKRQDIESNLQYDAGDK+XL_L
B_b_ion
B_y_ion
m/z
y16++
b15++
b17++
b16++
y10+
y12+
y4+
y5+
y6+
y12++
b10++
b11+
b7+
b14+
b15+
y13+
y14+
y16+
y8+

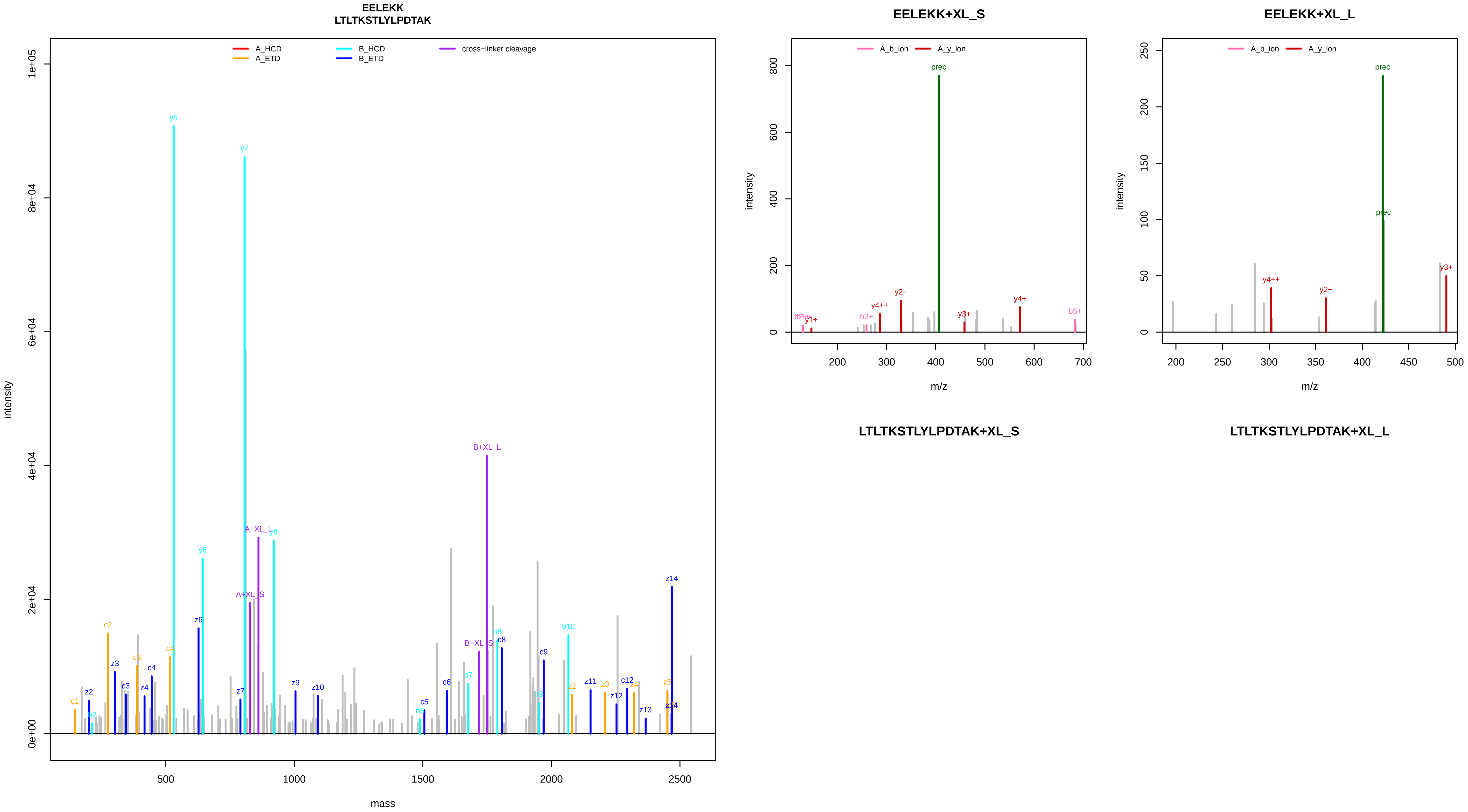
EELEKK
LTLTKSTLYLPDTAK
A_HCD
A_ETD
B_HCD
B_ETD
cross−linker cleavage
intensity
mass
y5
y7
y6
y8
A+XL_L
A+XL_S
B+XL_L
B+XL_S
z14
b10
b8
c8
c9
z6
c2
c4
c3
z3
c4
z2
c1
z4
z7
z9
z10
c5
c6
b7
z11
z3
z12
c12
z4
z13
z5
z2
EELEKK+XL_S
A_b_ion
A_y_ion
prec
y2+
y4++
y4+
y3+
b2+
b5+
y1+
m/z
EELEKK+XL_L
A_b_ion
A_y_ion
prec
y4++
y2+
y3+
m/z
LTLTKSTLYLPDTAK+XL_S
LTLTKSTLYLPDTAK+XL_L

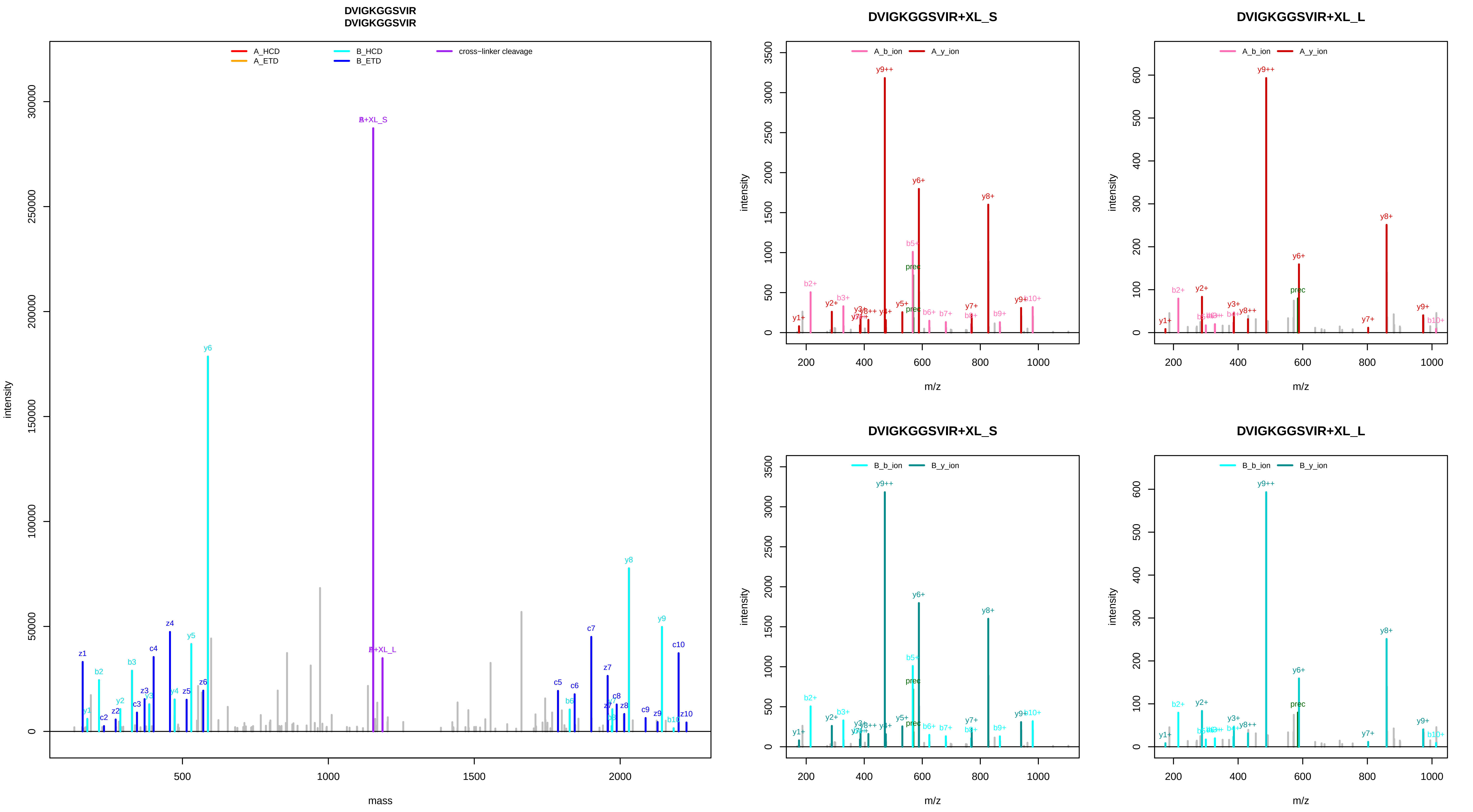
DVIGKGGSVIR
DVIGKGGSVIR
A_HCD
A_ETD
B_HCD
B_ETD
cross−linker cleavage
intensity
mass
0
50000
100000
150000
200000
250000
300000
500
1000
1500
2000
B+XL_S
B+XL_L
y6
y8
y9
y5
z4
c4
z1
b2
b3
c7
c10
c5
c6
z7
c8
z8
c9
z9
z10
b10
b6
y2
y1
c2
z2
c3
z3
y3
y4
z5
z6
DVIGKGGSVIR+XL_S
DVIGKGGSVIR+XL_L
A_b_ion
A_y_ion
B_b_ion
B_y_ion
intensity
m/z
0
500
1000
1500
2000
2500
3000
3500
100
200
300
400
600
800
1000
y9++
y6+
y8+
b5+
prec
b2+
b3+
y2+
y1+
y5+
b6+
b7+
y7+
b8+
b9+
y9+
b10+
y3+
y8++
b4+

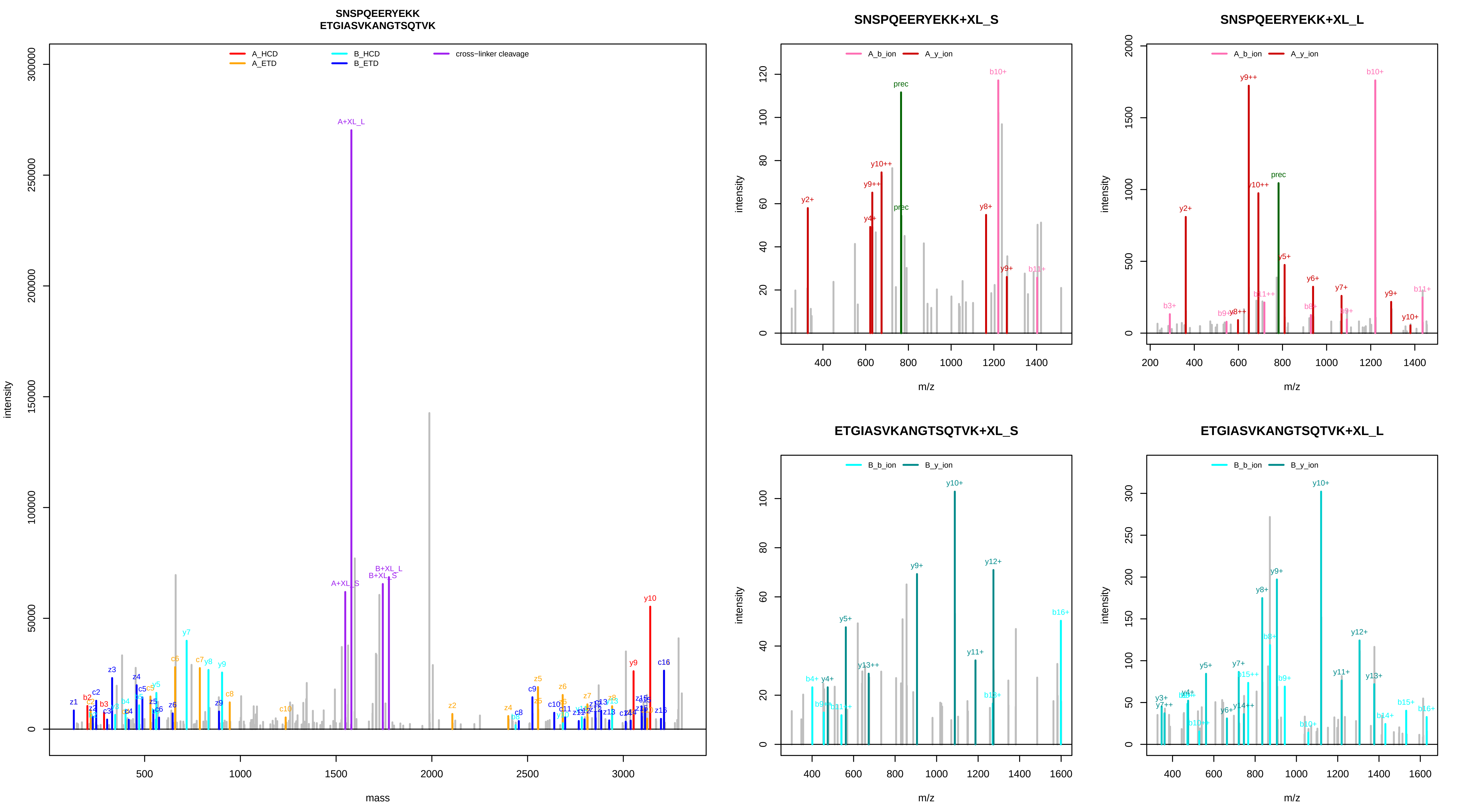

SNSPQEERYEKK
ETGIASVKANGTSQTVK
A_HCD
A_ETD
B_HCD
B_ETD
cross−linker cleavage
A+XL_L
A+XL_S
B+XL_L
B+XL_S
intensity
mass
SNSPQEERYEKK+XL_S
SNSPQEERYEKK+XL_L
A_b_ion
A_y_ion
ETGIASVKANGTSQTVK+XL_S
ETGIASVKANGTSQTVK+XL_L
B_b_ion
B_y_ion
m/z

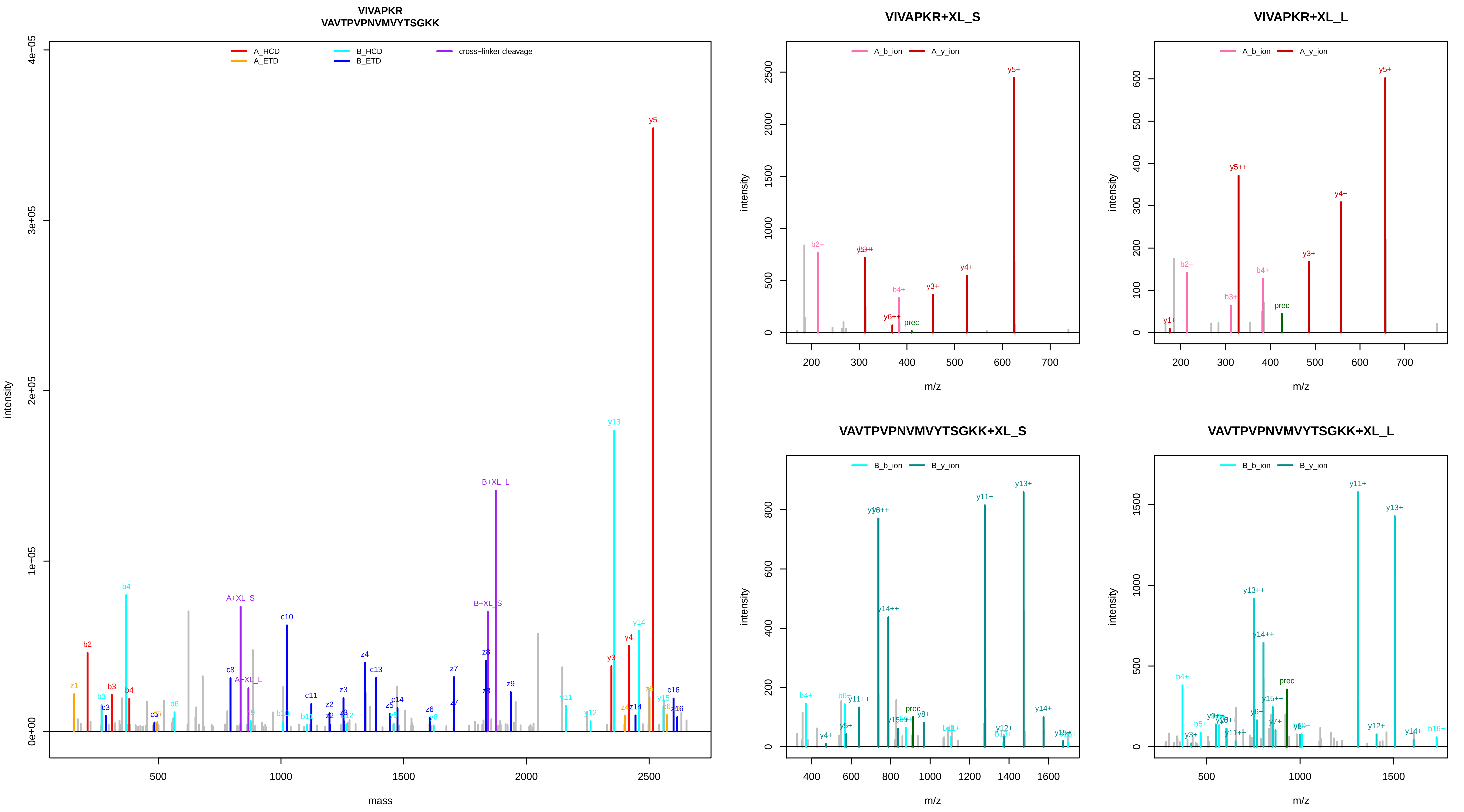
VIVAPKR
VAVTPVPNVMVYTSGKK
A_HCD
A_ETD
B_HCD
B_ETD
cross−linker cleavage
intensity
mass
A+XL_S
A+XL_L
B+XL_S
B+XL_L
VIVAPKR+XL_S
VIVAPKR+XL_L
VAVTPVPNVMVYTSGKK+XL_S
VAVTPVPNVMVYTSGKK+XL_L
A_b_ion
A_y_ion
B_b_ion
B_y_ion
m/z
prec

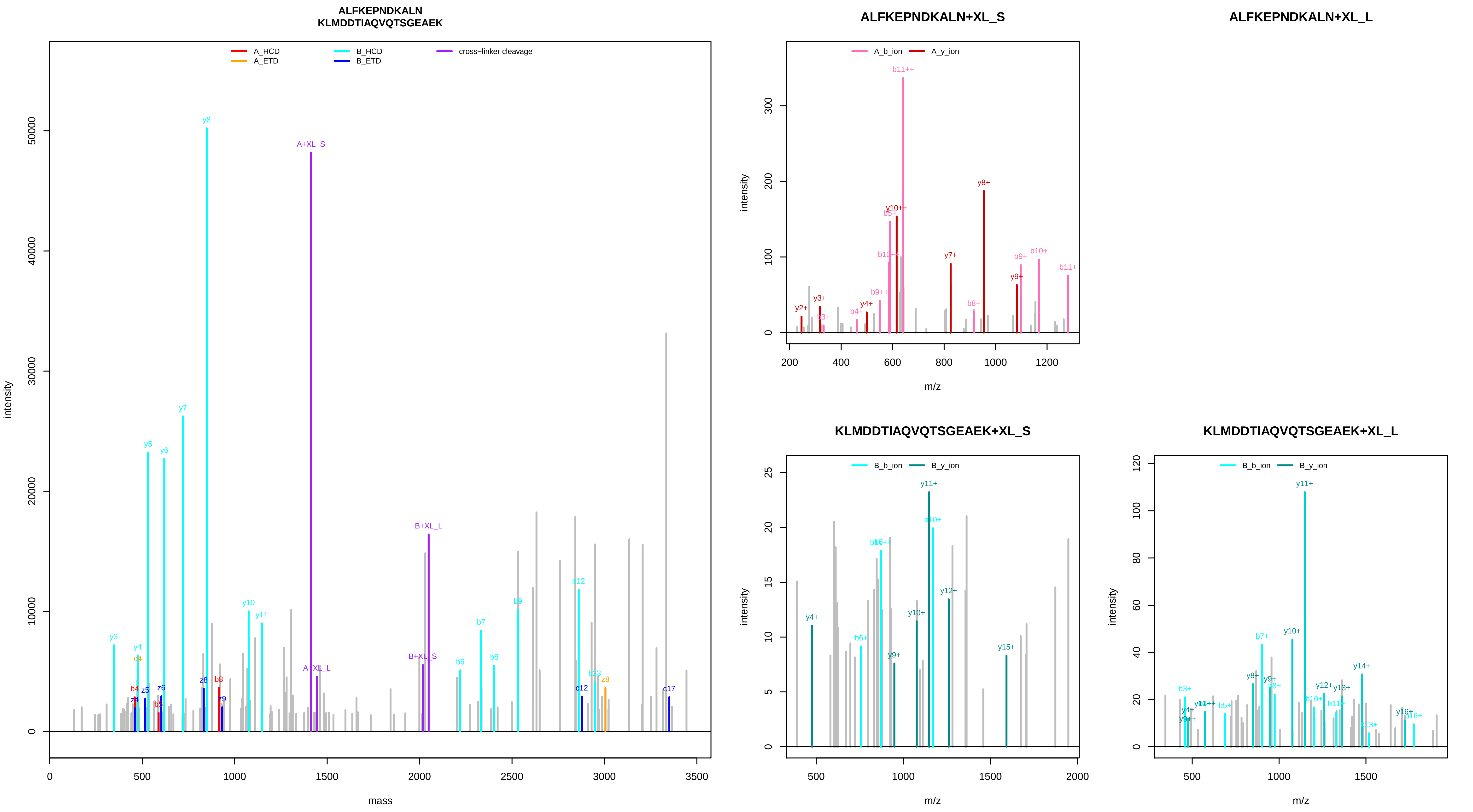

ALFKEPNDKALN
KLMDDTIAQVQTSGEAEK
A_HCD
A_ETD
B_HCD
B_ETD
cross−linker cleavage
intensity
mass
y8
A+XL_S
y7
y5
y6
B+XL_L
y10
y11
y3
y4
B+XL_S
A+XL_L
b6
b7
b8
b9
b12
b13
z8
c12
c17
ALFKEPNDKALN+XL_S
A_b_ion
A_y_ion
m/z
ALFKEPNDKALN+XL_L
KLMDDTIAQVQTSGEAEK+XL_S
B_b_ion
B_y_ion
KLMDDTIAQVQTSGEAEK+XL_L

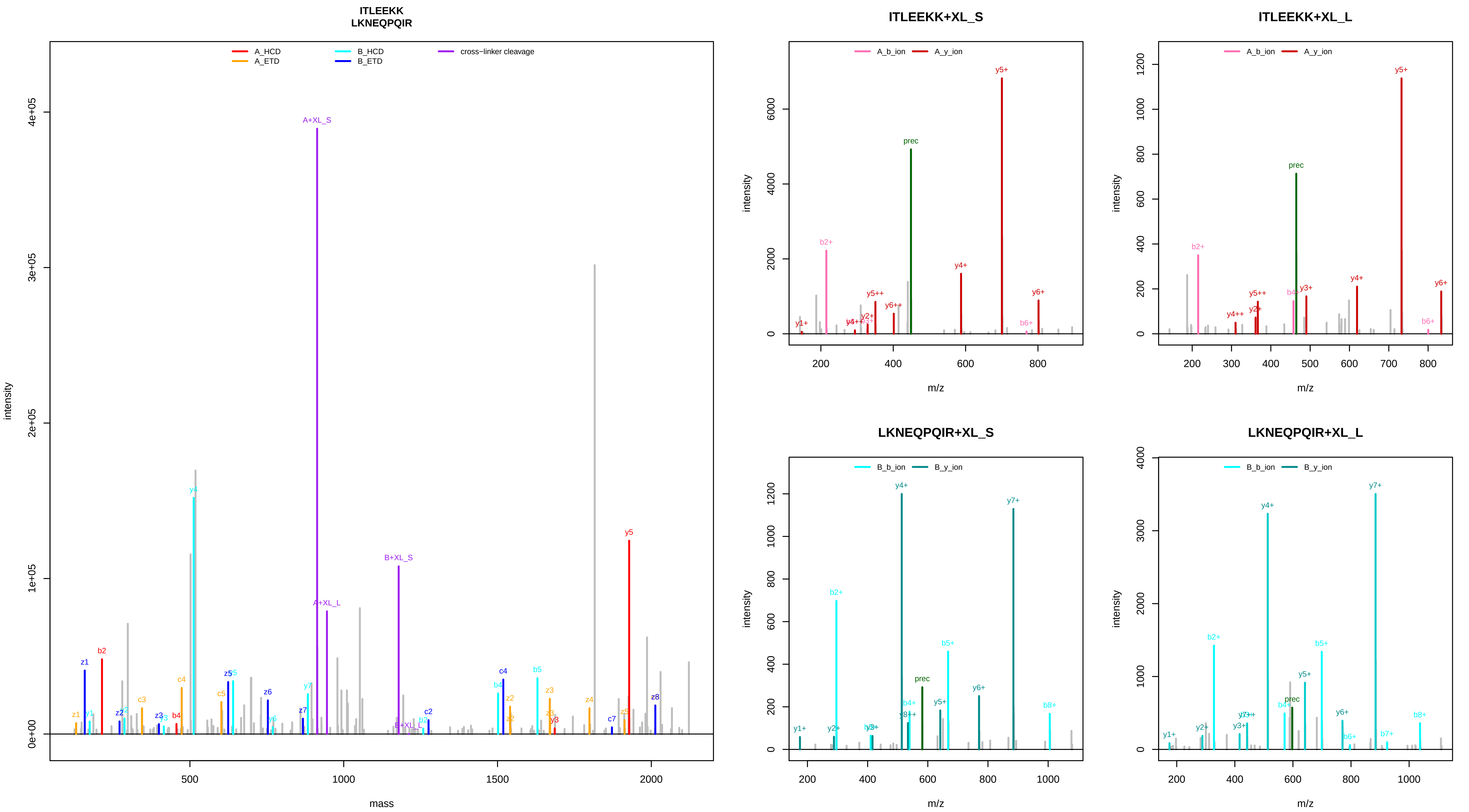
ITLEEKK
LKNEQPQIR
A_HCD
A_ETD
B_HCD
B_ETD
cross−linker cleavage
A+XL_S
A+XL_L
B+XL_S
B+XL_L
intensity
mass
ITLEEKK+XL_S
ITLEEKK+XL_L
LKNEQPQIR+XL_S
LKNEQPQIR+XL_L
A_b_ion
A_y_ion
B_b_ion
B_y_ion
prec
m/z

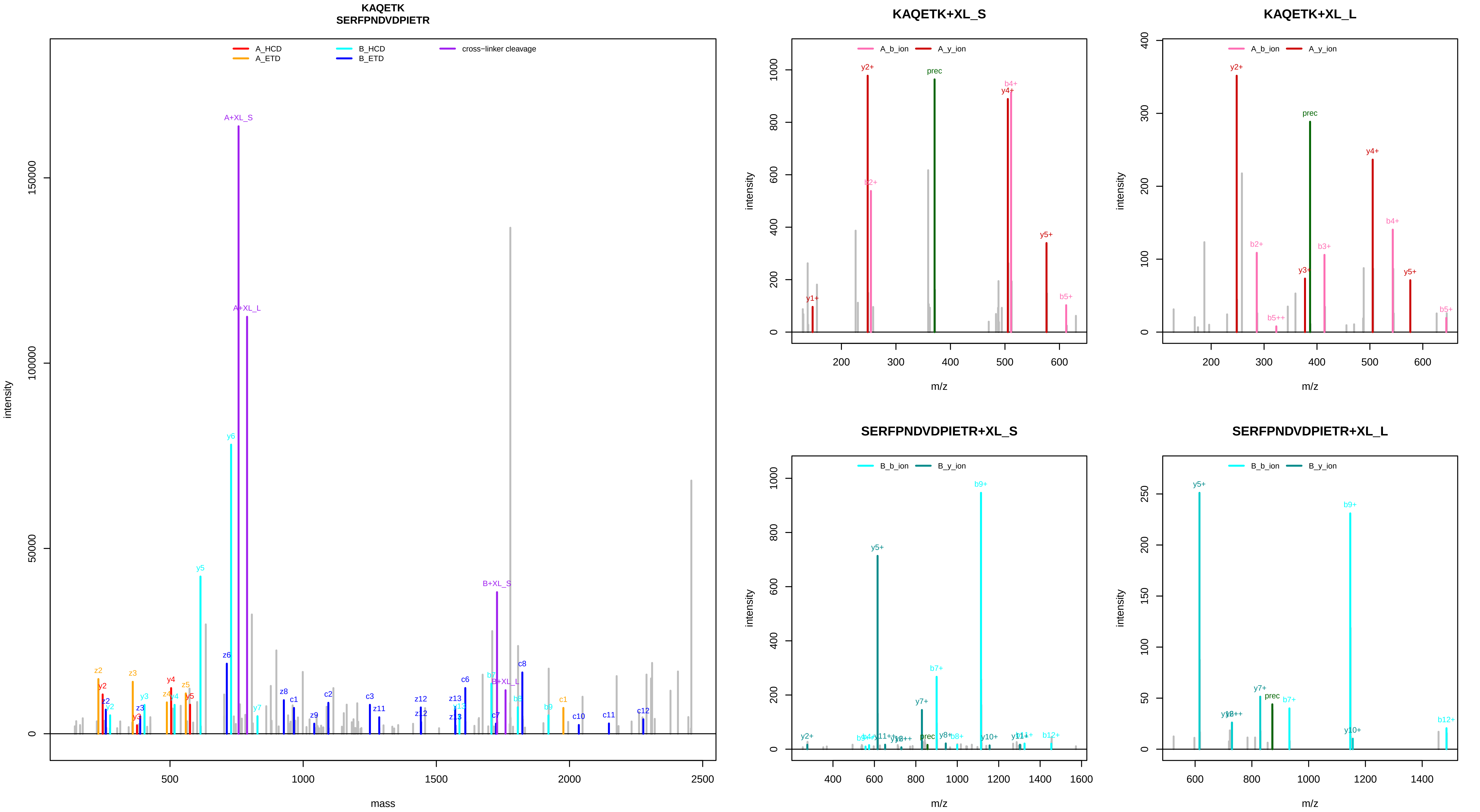

KAQETK
SERFPNDVDPIETR
A_HCD
A_ETD
B_HCD
B_ETD
cross−linker cleavage
intensity
mass
KAQETK+XL_S
KAQETK+XL_L
SERFPNDVDPIETR+XL_S
SERFPNDVDPIETR+XL_L
A_b_ion
A_y_ion
B_b_ion
B_y_ion
m/z

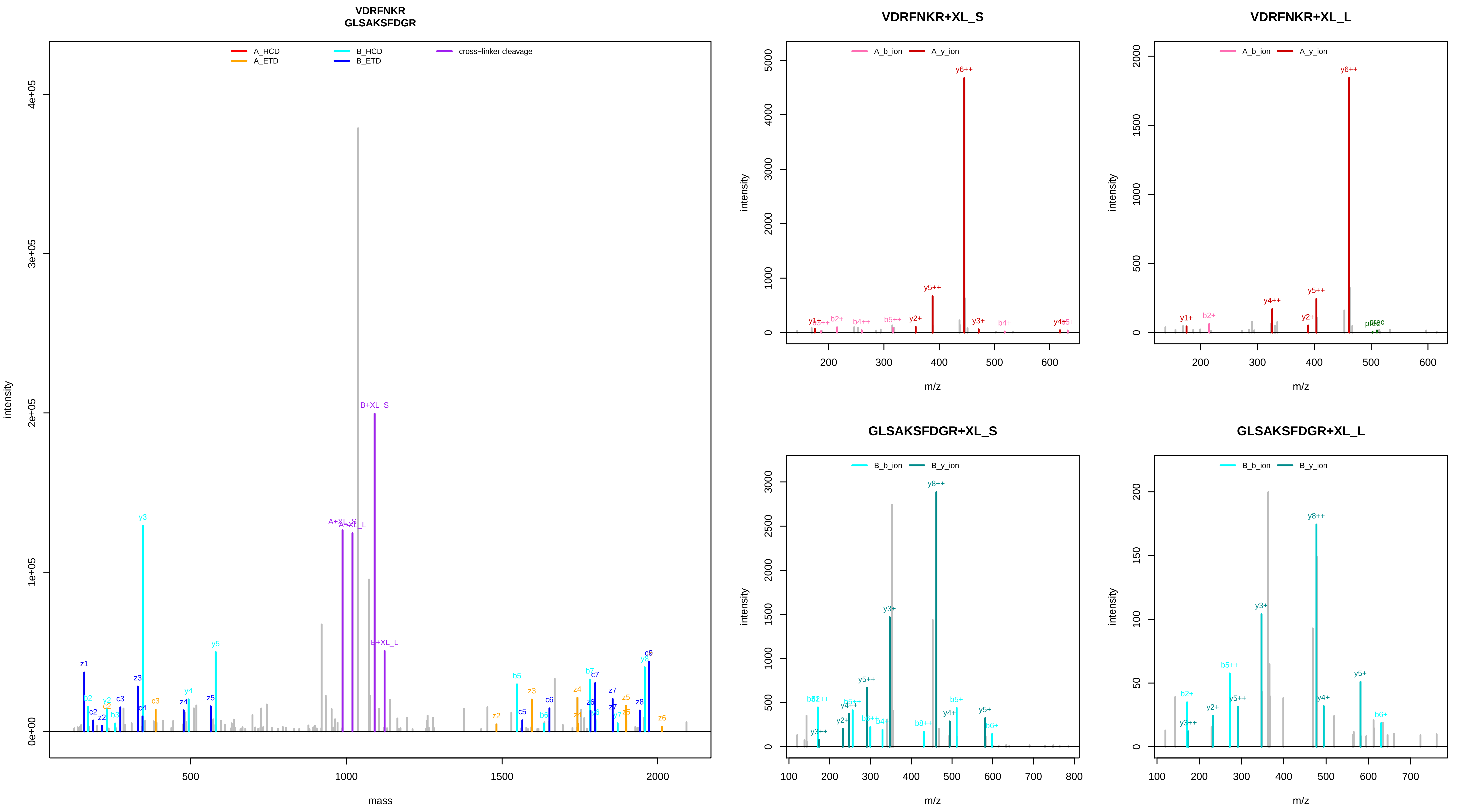
VDRFNKR
GLSAKSFDGR
A_HCD
A_ETD
B_HCD
B_ETD
cross−linker cleavage
intensity
mass
B+XL_S
A+XL_S
A+XL_L
B+XL_L
VDRFNKR+XL_S
VDRFNKR+XL_L
GLSAKSFDGR+XL_S
GLSAKSFDGR+XL_L
A_b_ion
A_y_ion
B_b_ion
B_y_ion
m/z

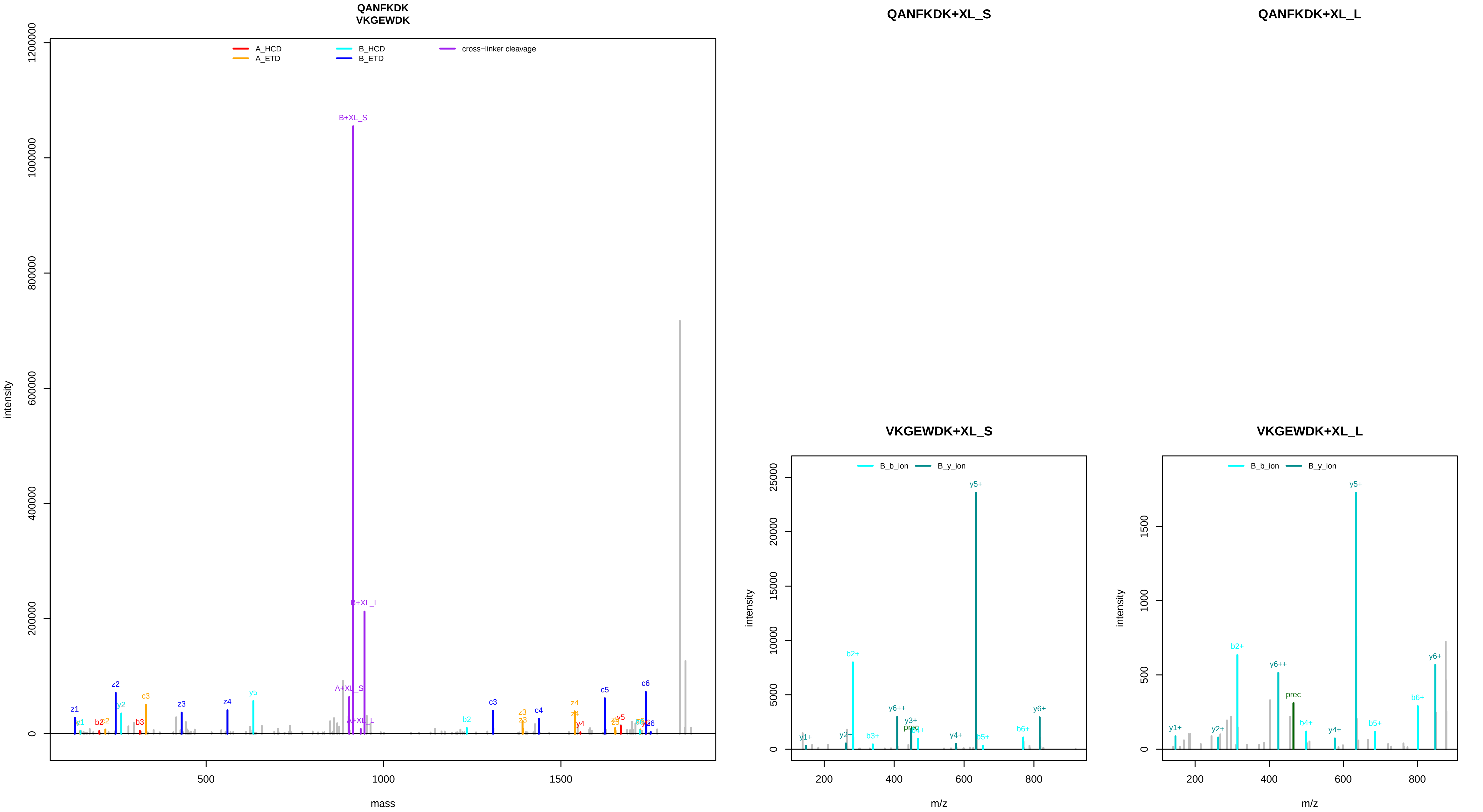
QANFKDK
VKGEWDK
A_HCD
A_ETD
B_HCD
B_ETD
cross−linker cleavage
B+XL_S
B+XL_L
A+XL_S
A+XL_L
intensity
mass
QANFKDK+XL_S
QANFKDK+XL_L
VKGEWDK+XL_S
VKGEWDK+XL_L
B_b_ion
B_y_ion
prec
intensity
m/z

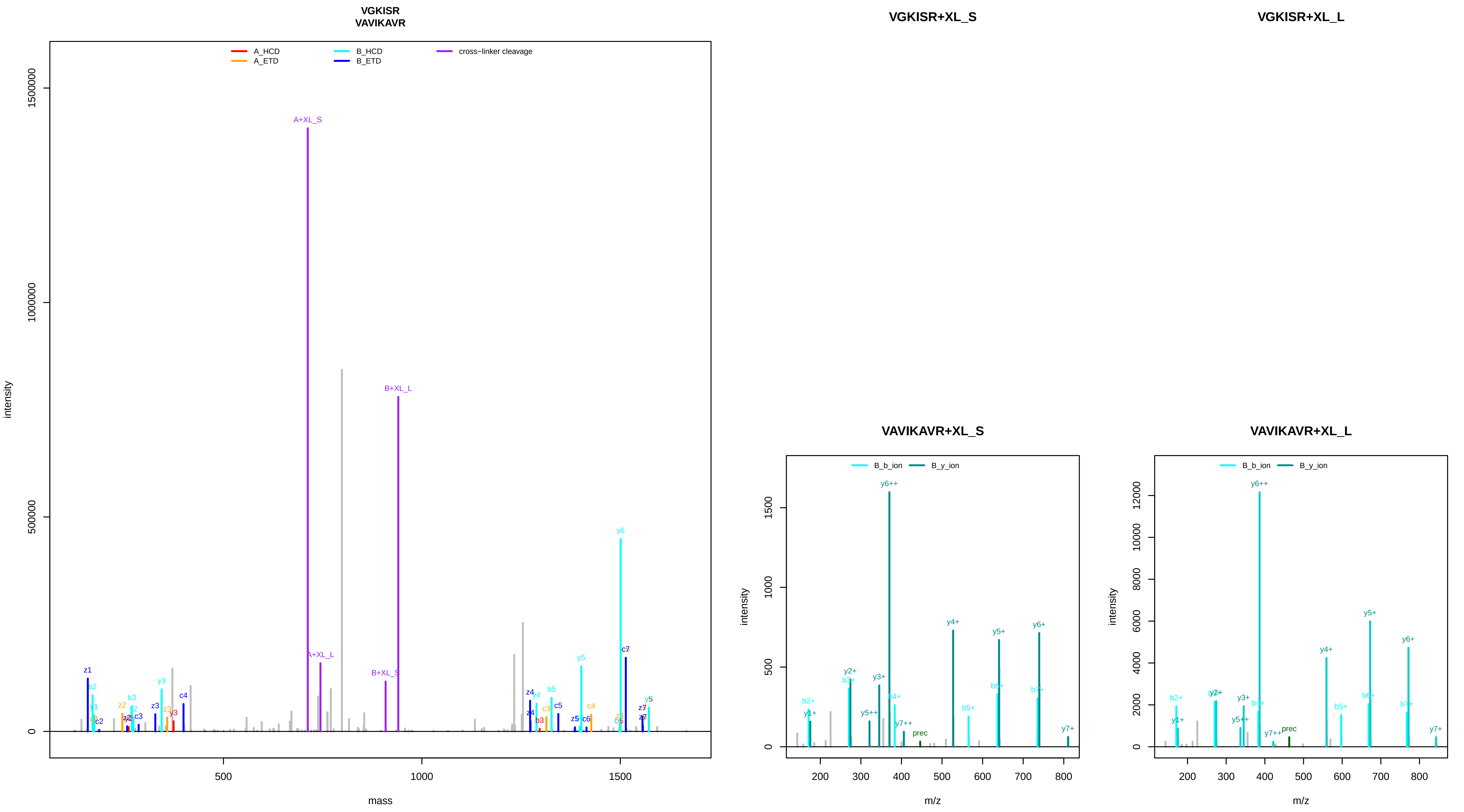
VGKISR
VAVIKAVR
A_HCD
A_ETD
B_HCD
B_ETD
cross−linker cleavage
A+XL_S
B+XL_L
A+XL_L
B+XL_S
intensity
mass
VGKISR+XL_S
VGKISR+XL_L
VAVIKAVR+XL_S
VAVIKAVR+XL_L
B_b_ion
B_y_ion
y6++
y4+
y5+
y6+
y7+
prec
intensity
m/z

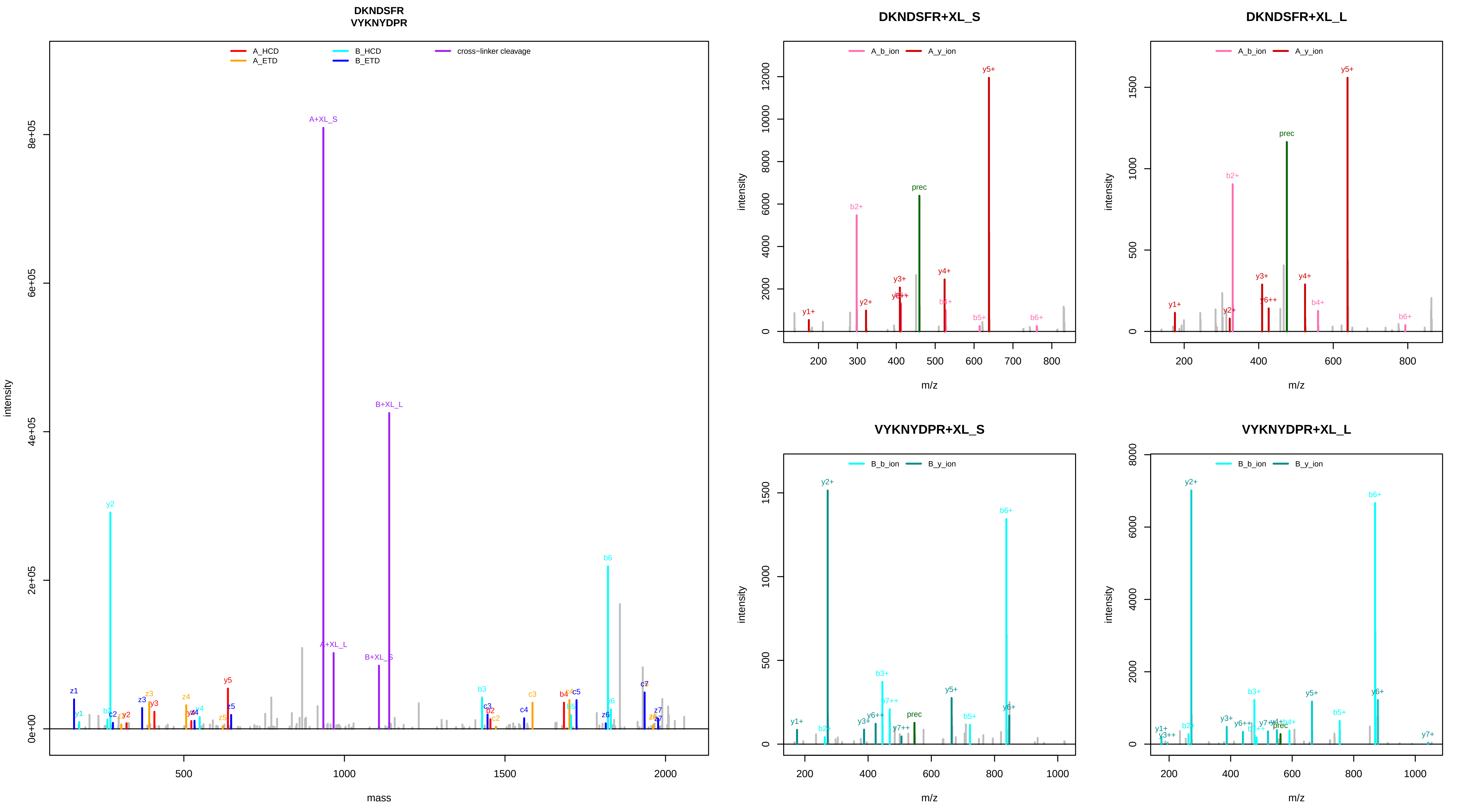
DKNDSFR
VYKNYDPR
A_HCD
A_ETD
B_HCD
B_ETD
cross−linker cleavage
A+XL_S
B+XL_L
A+XL_L
B+XL_S
intensity
mass
DKNDSFR+XL_S
DKNDSFR+XL_L
A_b_ion
A_y_ion
VYKNYDPR+XL_S
VYKNYDPR+XL_L
B_b_ion
B_y_ion
prec
intensity
m/z

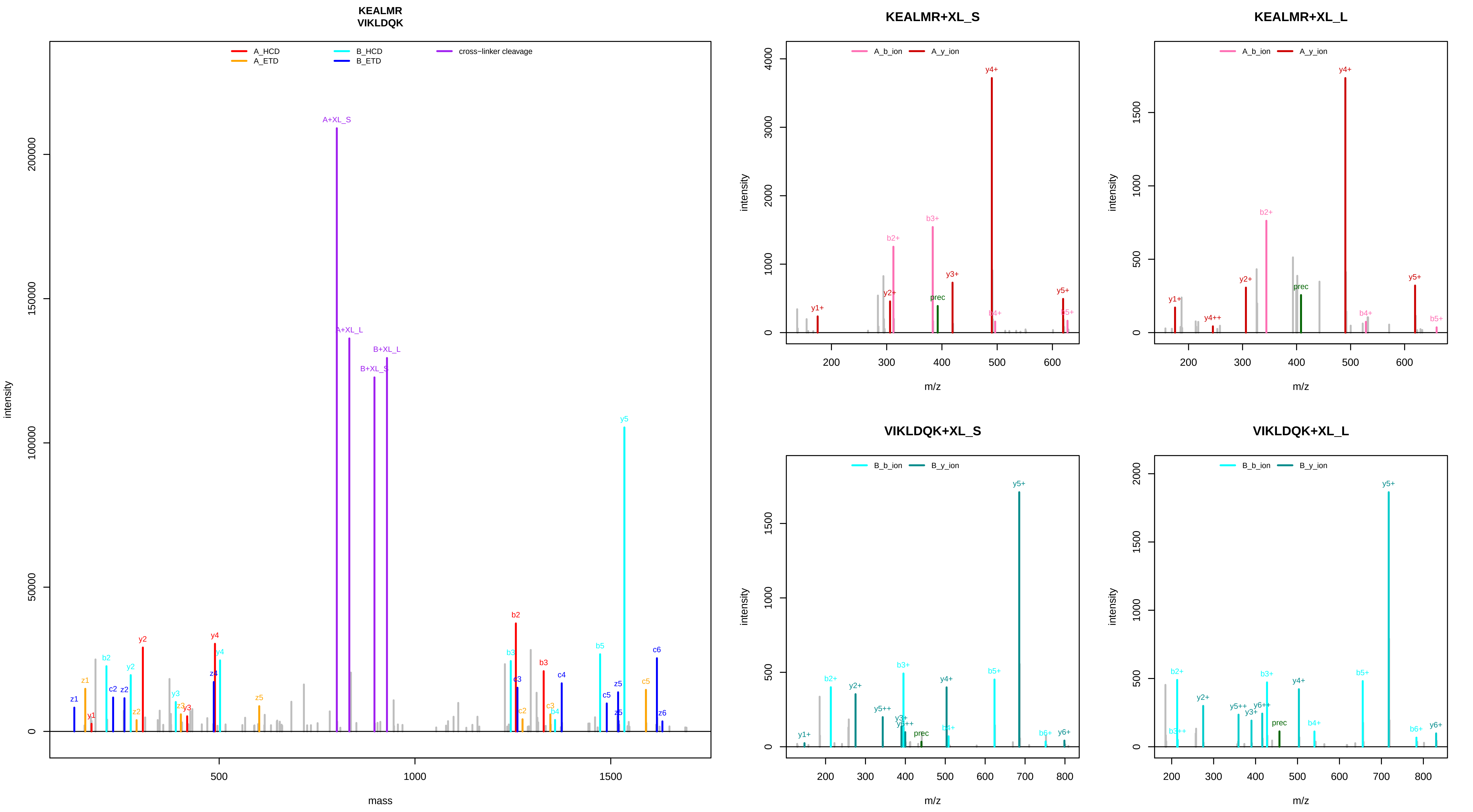
KEALMR
VIKLDQK
A_HCD
A_ETD
B_HCD
B_ETD
cross−linker cleavage
A+XL_S
A+XL_L
B+XL_L
B+XL_S
intensity
mass
KEALMR+XL_S
KEALMR+XL_L
VIKLDQK+XL_S
VIKLDQK+XL_L
A_b_ion
A_y_ion
B_b_ion
B_y_ion
prec
m/z

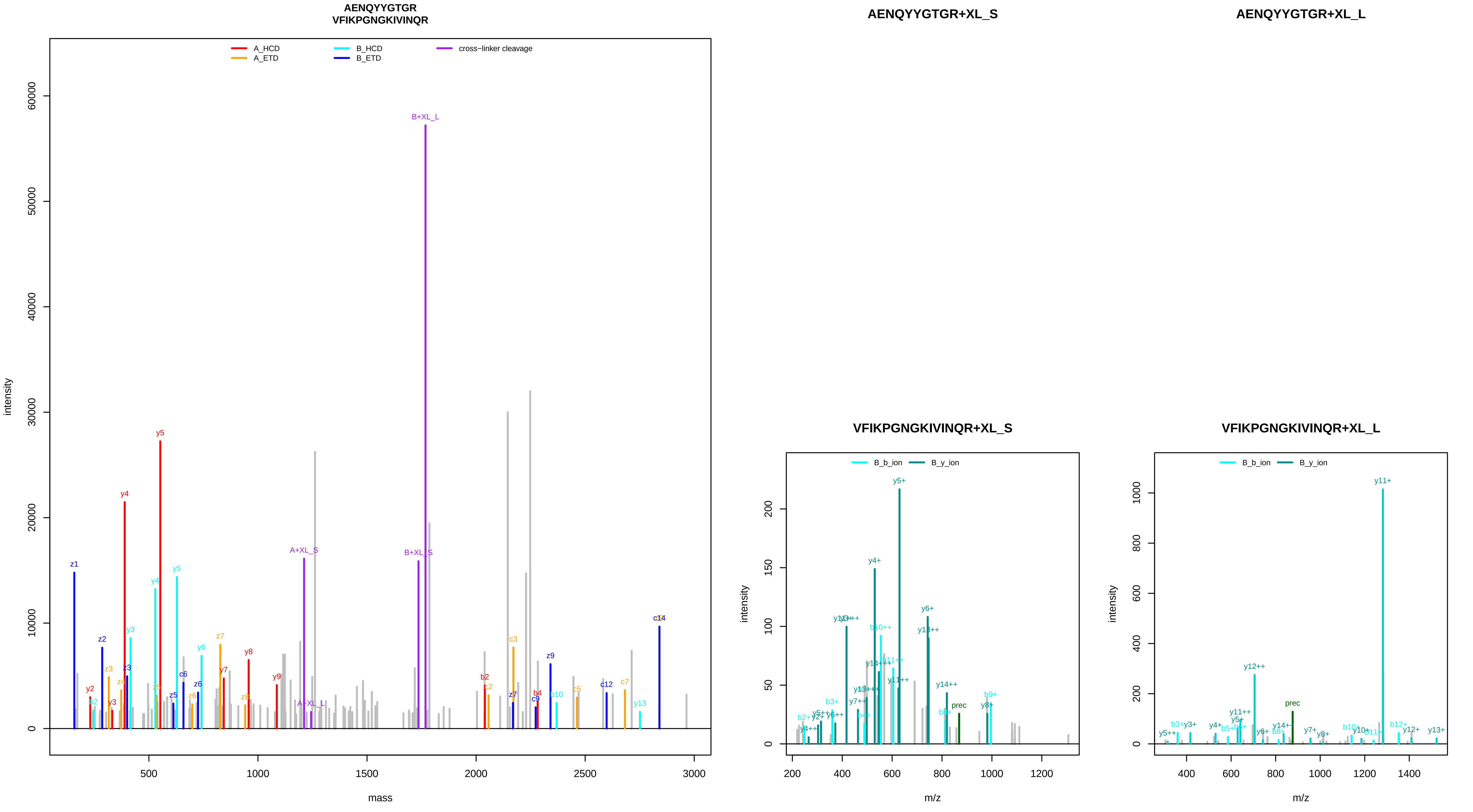
AENQYYGTGR
VFIKPGNGKIVINQR
A_HCD
A_ETD
B_HCD
B_ETD
cross−linker cleavage
B+XL_L
A+XL_S
B+XL_S
intensity
mass
AENQYYGTGR+XL_S
AENQYYGTGR+XL_L
VFIKPGNGKIVINQR+XL_S
VFIKPGNGKIVINQR+XL_L
B_b_ion
B_y_ion
m/z
prec

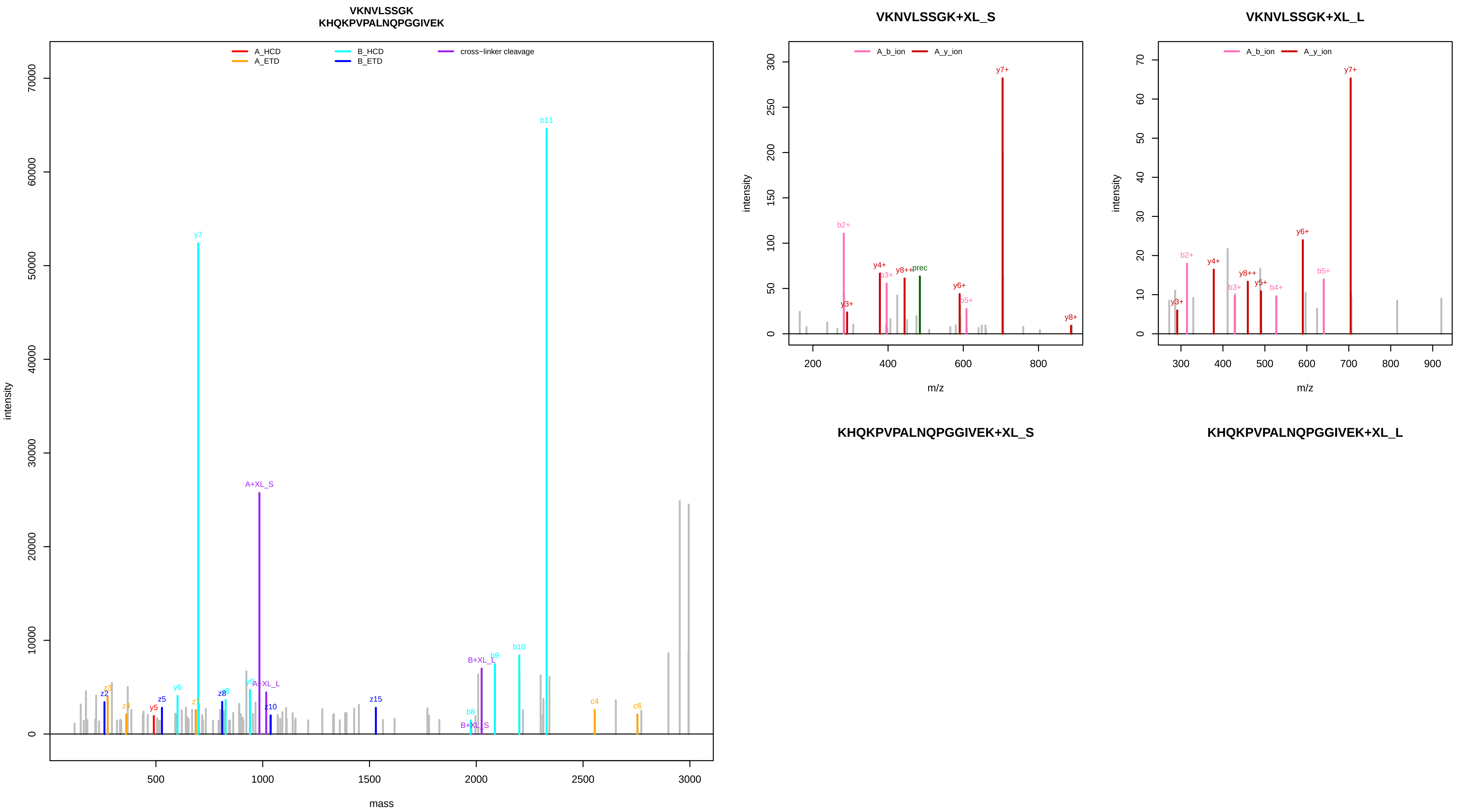

VKNVLSSGK
KHQKPVPALNQPGGIVEK
A_HCD
A_ETD
B_HCD
B_ETD
cross−linker cleavage
intensity
mass
b11
y7
A+XL_S
A+XL_L
B+XL_L
B+XL_S
b10
b9
b8
y6
y9
y8
y5
z2
z3
z4
z5
z7
z8
z10
z15
c4
c6
VKNVLSSGK+XL_S
A_b_ion
A_y_ion
y7+
b2+
y4+
b3+
y8++
prec
y3+
y6+
b5+
y8+
m/z
VKNVLSSGK+XL_L
A_b_ion
A_y_ion
y7+
y6+
b2+
y4+
y8++
b3+
y5+
b4+
b5+
y3+
m/z
KHQKPVPALNQPGGIVEK+XL_S
KHQKPVPALNQPGGIVEK+XL_L

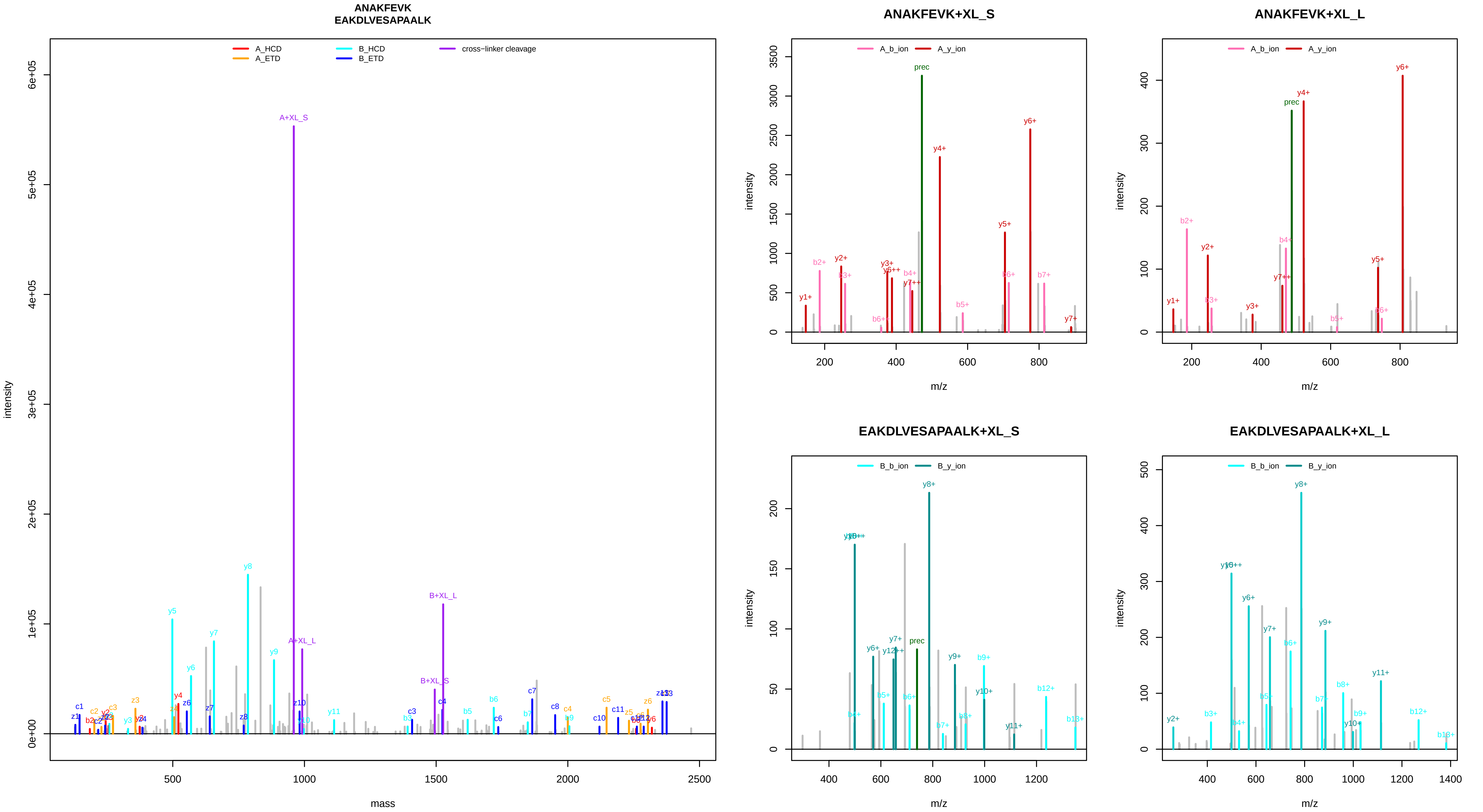
ANAKFEVK
EAKDLVESAPAALK
A_HCD
A_ETD
B_HCD
B_ETD
cross−linker cleavage
A+XL_S
A+XL_L
B+XL_L
B+XL_S
intensity
mass
ANAKFEVK+XL_S
ANAKFEVK+XL_L
EAKDLVESAPAALK+XL_S
EAKDLVESAPAALK+XL_L
A_b_ion
A_y_ion
B_b_ion
B_y_ion
prec
m/z

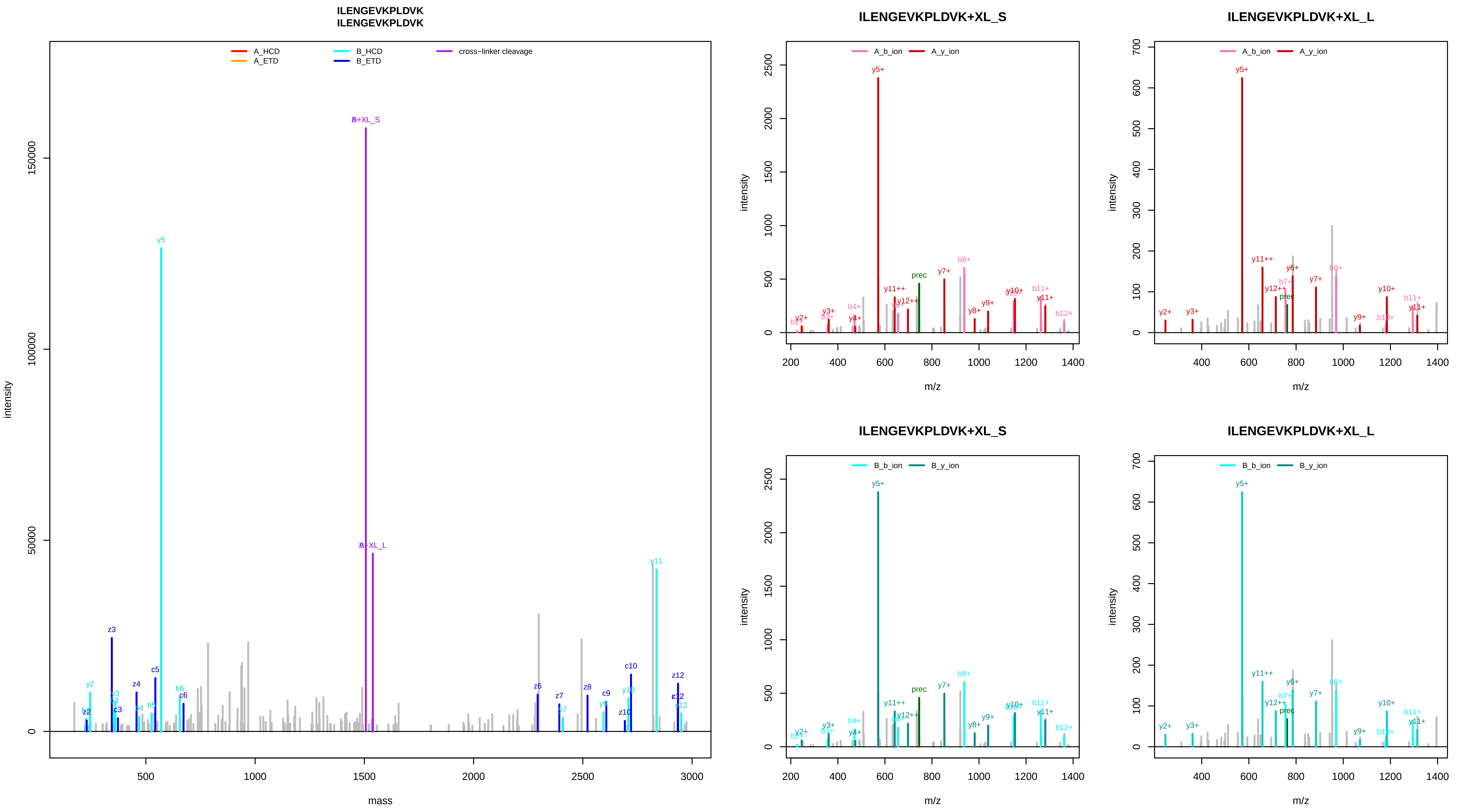

ILENGEVKPLDVK
ILENGEVKPLDVK
A_HCD
A_ETD
B_HCD
B_ETD
cross−linker cleavage
intensity
mass
ILENGEVKPLDVK+XL_S
ILENGEVKPLDVK+XL_L
A_b_ion
A_y_ion
B_b_ion
B_y_ion
prec
m/z
intensity

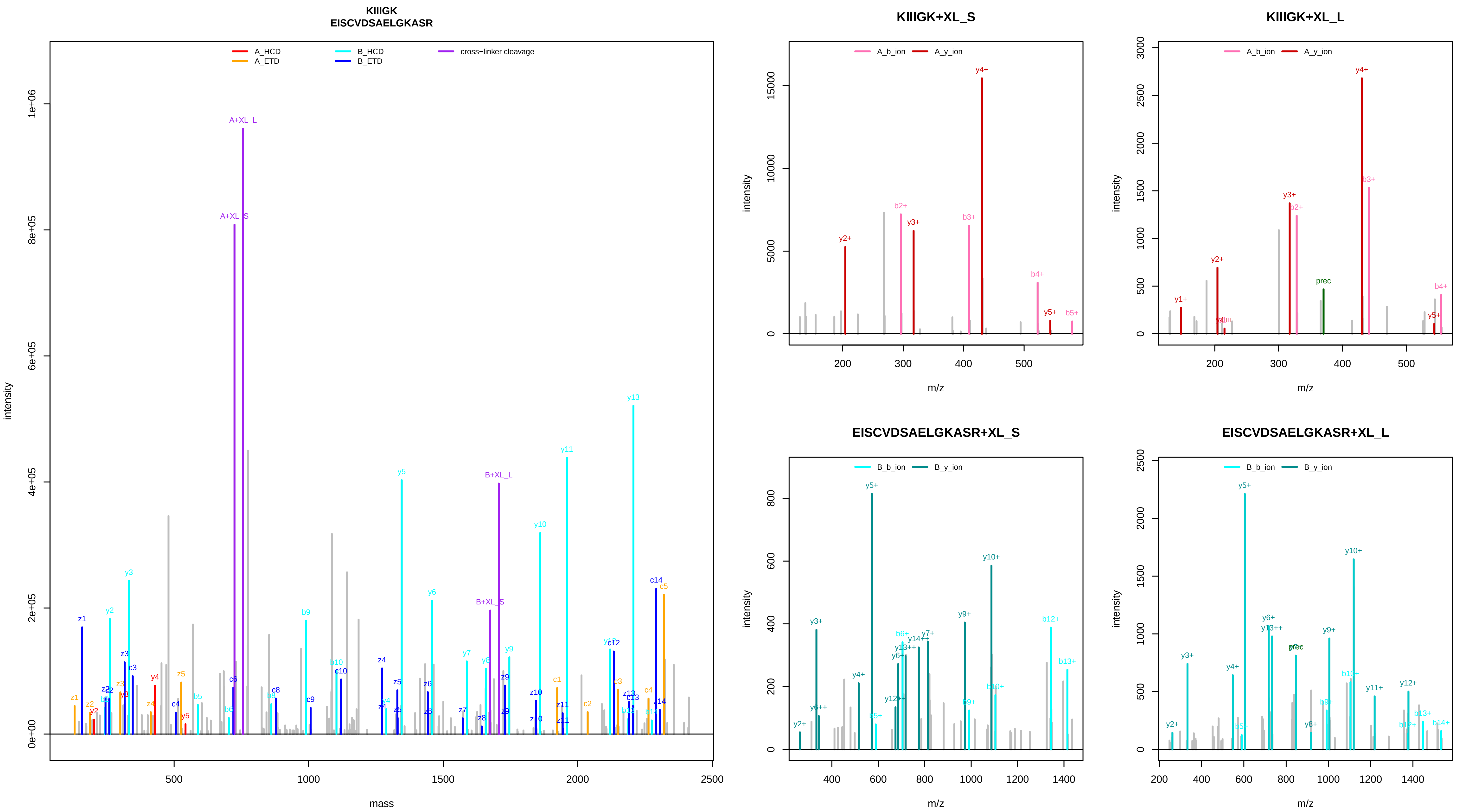
KIIIGK
EISCVDSAELGKASR
A_HCD
A_ETD
B_HCD
B_ETD
cross−linker cleavage
A+XL_L
A+XL_S
B+XL_L
B+XL_S
intensity
mass
KIIIGK+XL_S
KIIIGK+XL_L
EISCVDSAELGKASR+XL_S
EISCVDSAELGKASR+XL_L
A_b_ion
A_y_ion
B_b_ion
B_y_ion
m/z

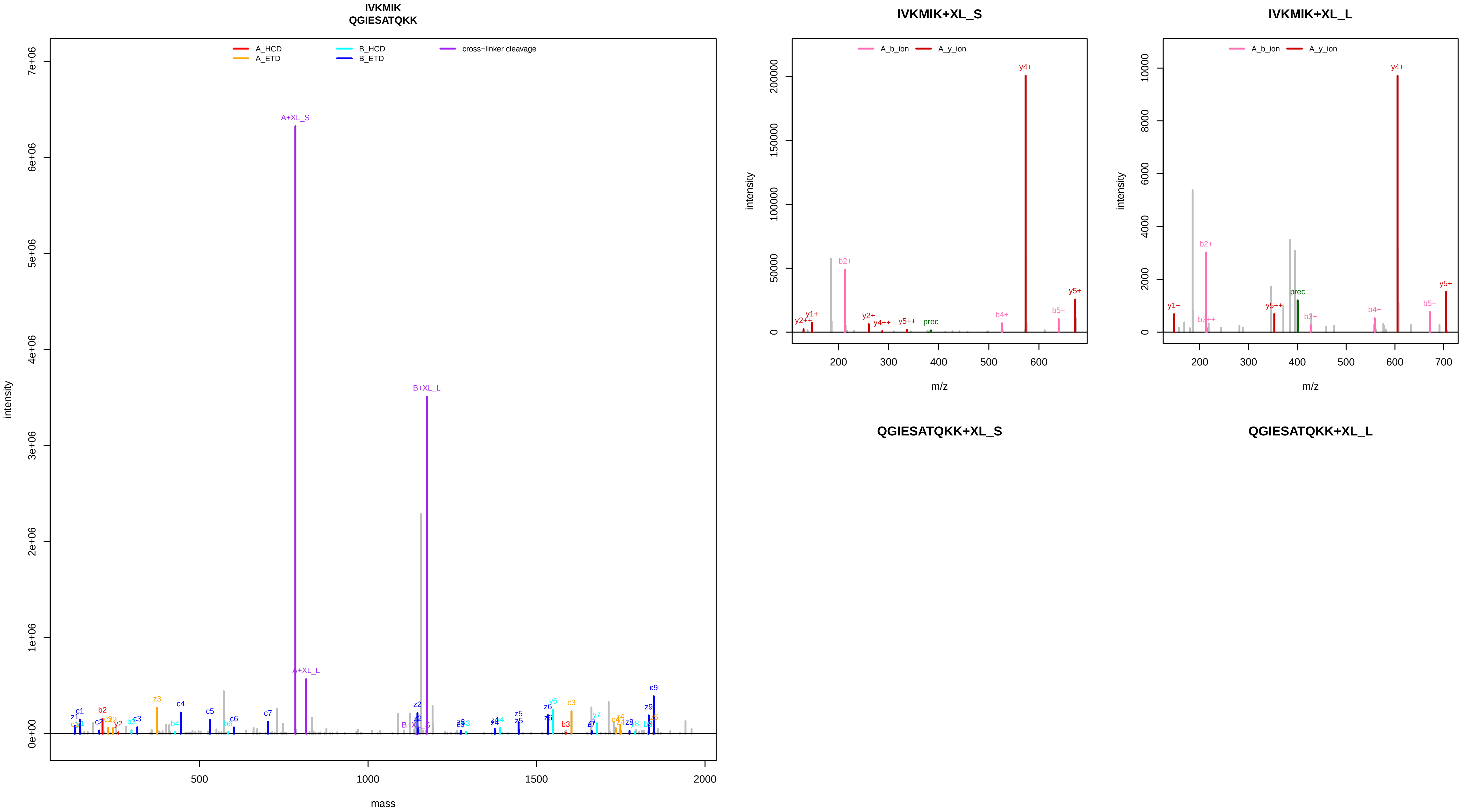
IVKMIK
QGIESATQKK
A_HCD
A_ETD
B_HCD
B_ETD
cross−linker cleavage
A+XL_S
B+XL_L
A+XL_L
intensity
mass
IVKMIK+XL_S
IVKMIK+XL_L
A_b_ion
A_y_ion
m/z
QGIESATQKK+XL_S
QGIESATQKK+XL_L

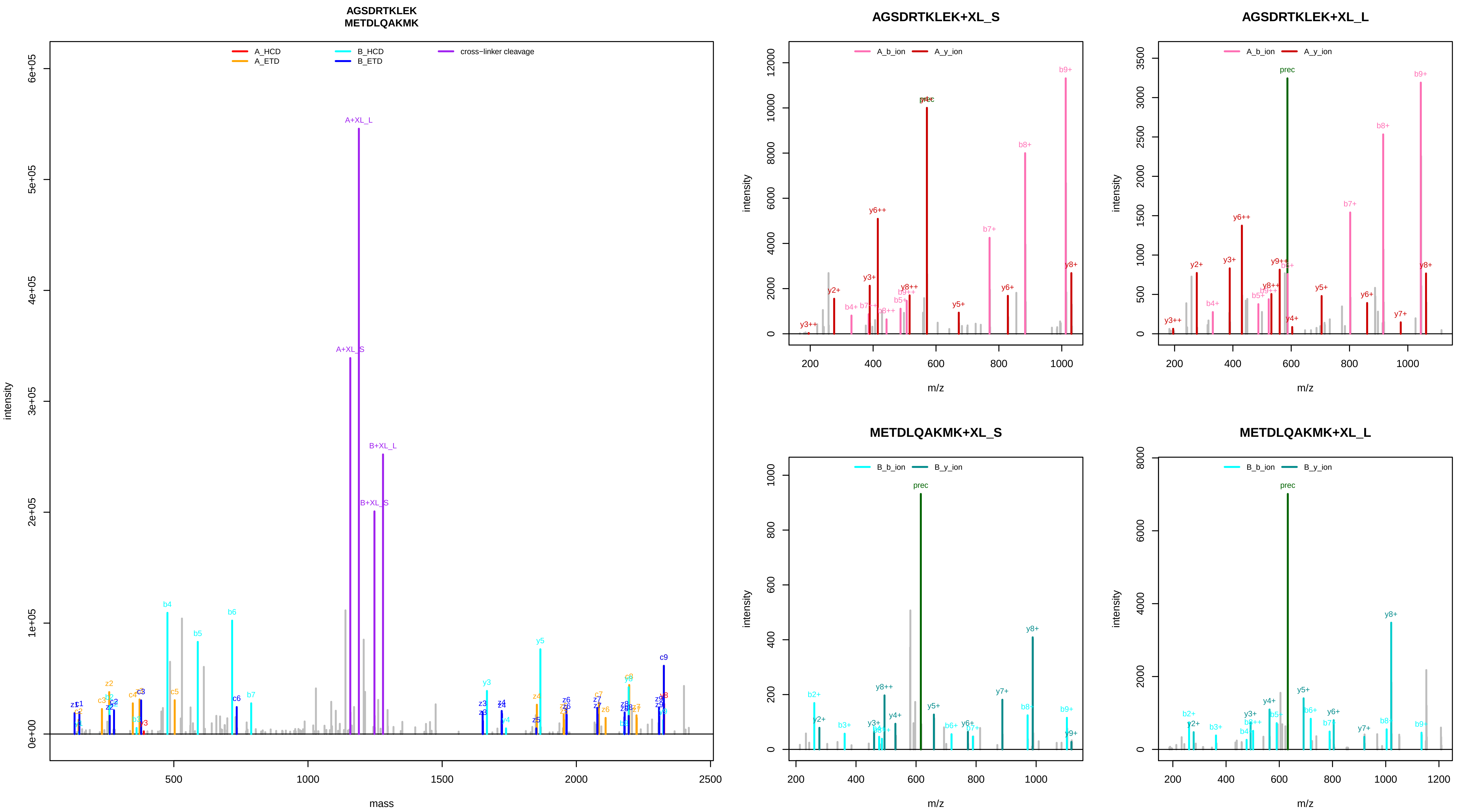
AGSDRTKLEK
METDLQAKMK
A_HCD
A_ETD
B_HCD
B_ETD
cross−linker cleavage
A+XL_L
A+XL_S
B+XL_L
B+XL_S
intensity
mass
AGSDRTKLEK+XL_S
AGSDRTKLEK+XL_L
METDLQAKMK+XL_S
METDLQAKMK+XL_L
A_b_ion
A_y_ion
B_b_ion
B_y_ion
prec
m/z

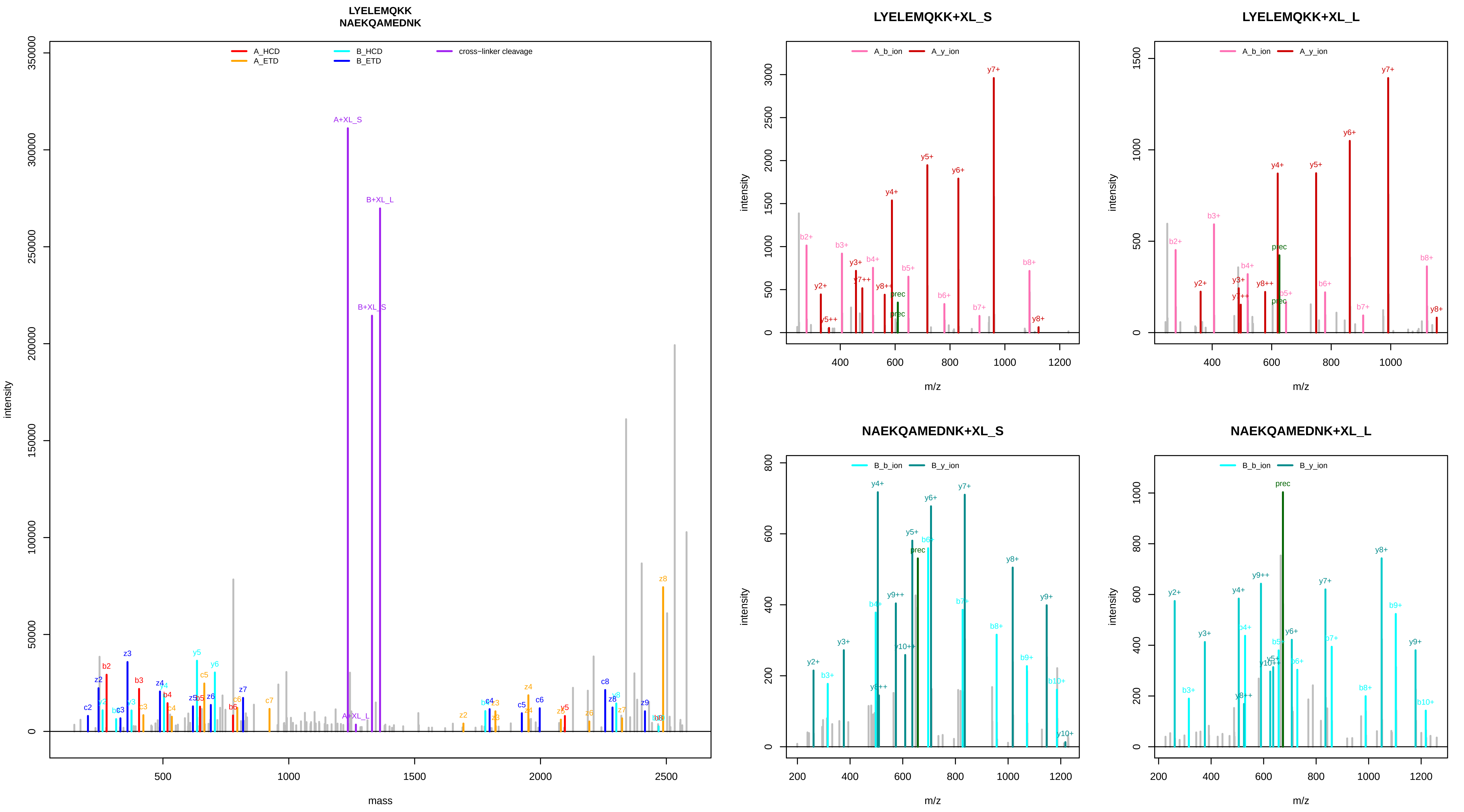
LYELEMQKK
NAEKQAMEDNK
A_HCD
A_ETD
B_HCD
B_ETD
cross−linker cleavage
A+XL_S
B+XL_L
B+XL_S
intensity
mass
LYELEMQKK+XL_S
LYELEMQKK+XL_L
A_b_ion
A_y_ion
NAEKQAMEDNK+XL_S
NAEKQAMEDNK+XL_L
B_b_ion
B_y_ion
prec
m/z

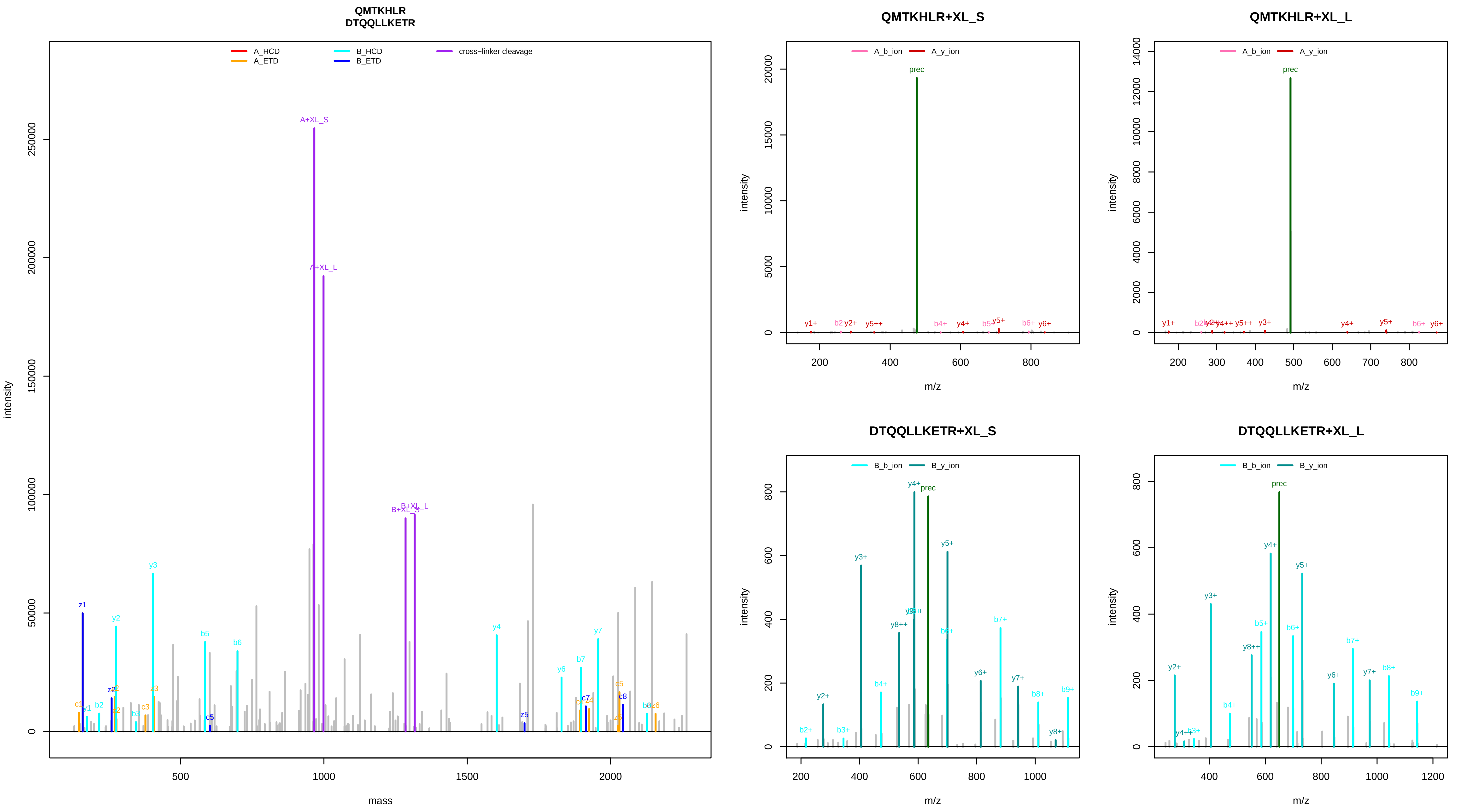
QMTKHLR
DTQQLLKETR
A_HCD
A_ETD
B_HCD
B_ETD
cross−linker cleavage
A+XL_S
A+XL_L
B+XL_S
B+XL_L
intensity
mass
QMTKHLR+XL_S
QMTKHLR+XL_L
A_b_ion
A_y_ion
prec
DTQQLLKETR+XL_S
DTQQLLKETR+XL_L
B_b_ion
B_y_ion
m/z

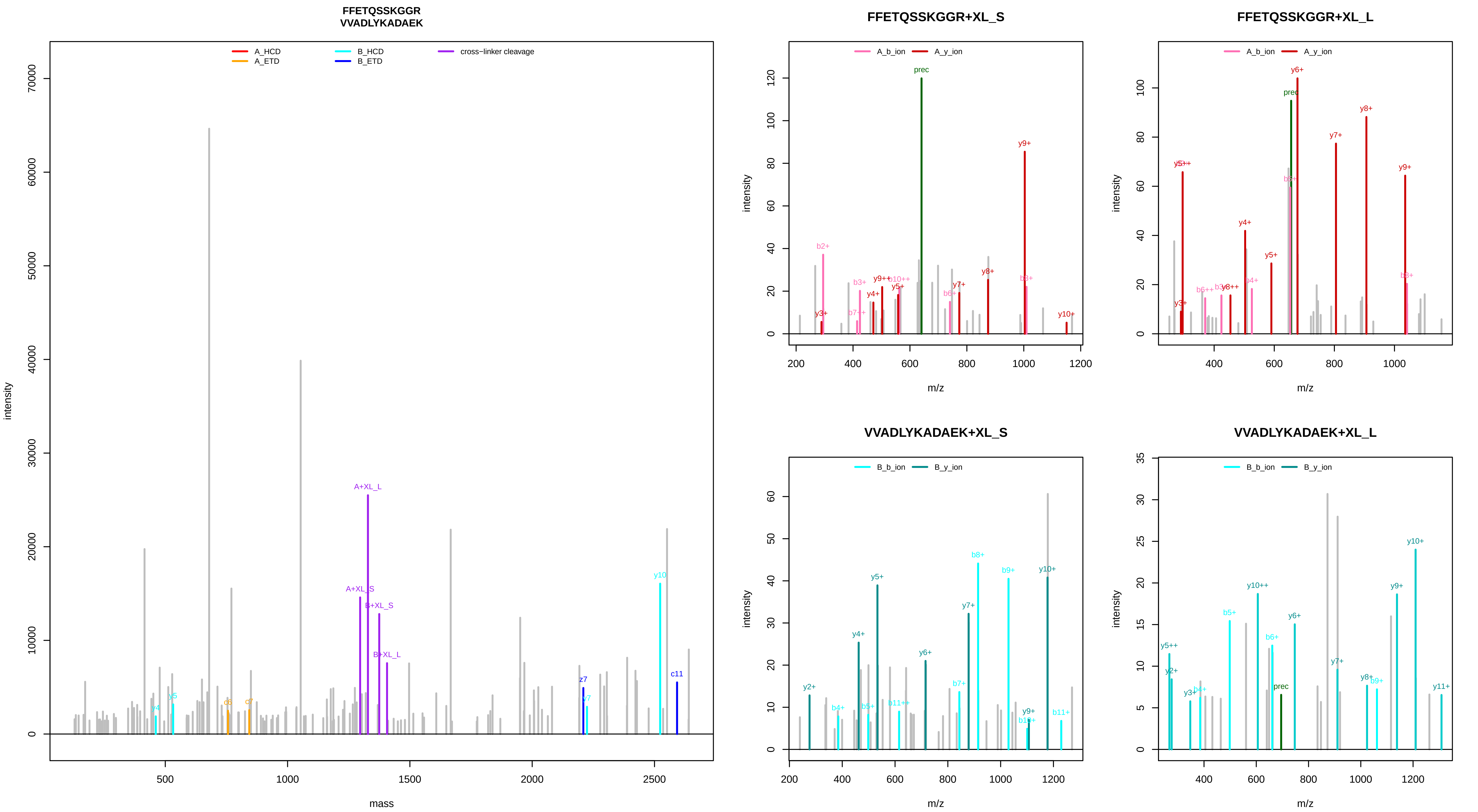

FFETQSSKGGR
VVADLYKADAEK
A_HCD
A_ETD
B_HCD
B_ETD
cross−linker cleavage
A+XL_L
A+XL_S
B+XL_S
B+XL_L
y10
c11
z7
intensity
mass
FFETQSSKGGR+XL_S
FFETQSSKGGR+XL_L
VVADLYKADAEK+XL_S
VVADLYKADAEK+XL_L
A_b_ion
A_y_ion
B_b_ion
B_y_ion
prec
m/z

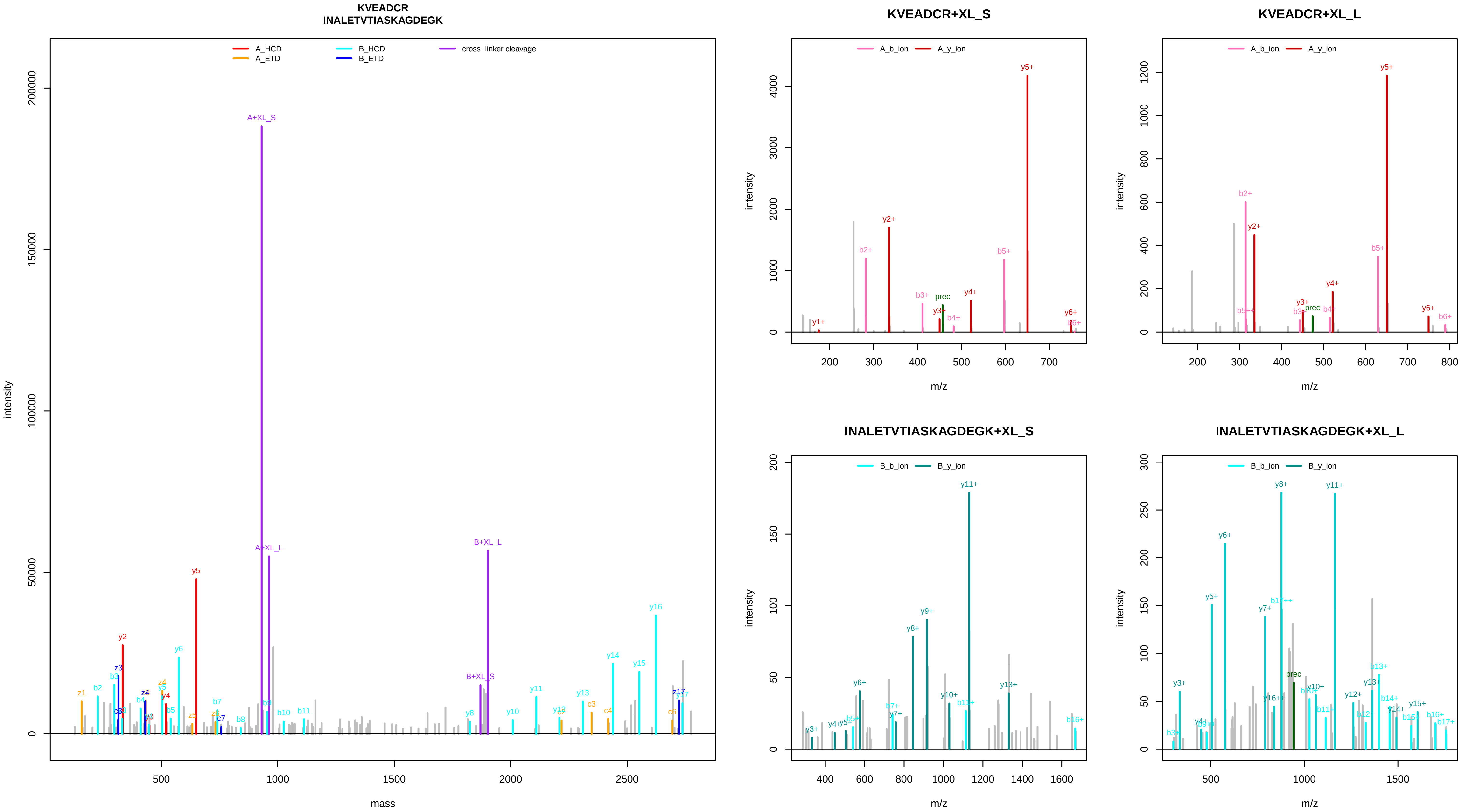

KVEADCR
INALETVTIASKAGDEGK
A_HCD
A_ETD
B_HCD
B_ETD
cross−linker cleavage
A+XL_S
A+XL_L
B+XL_L
B+XL_S
intensity
mass
KVEADCR+XL_S
KVEADCR+XL_L
INALETVTIASKAGDEGK+XL_S
INALETVTIASKAGDEGK+XL_L
A_b_ion
A_y_ion
B_b_ion
B_y_ion
prec
m/z

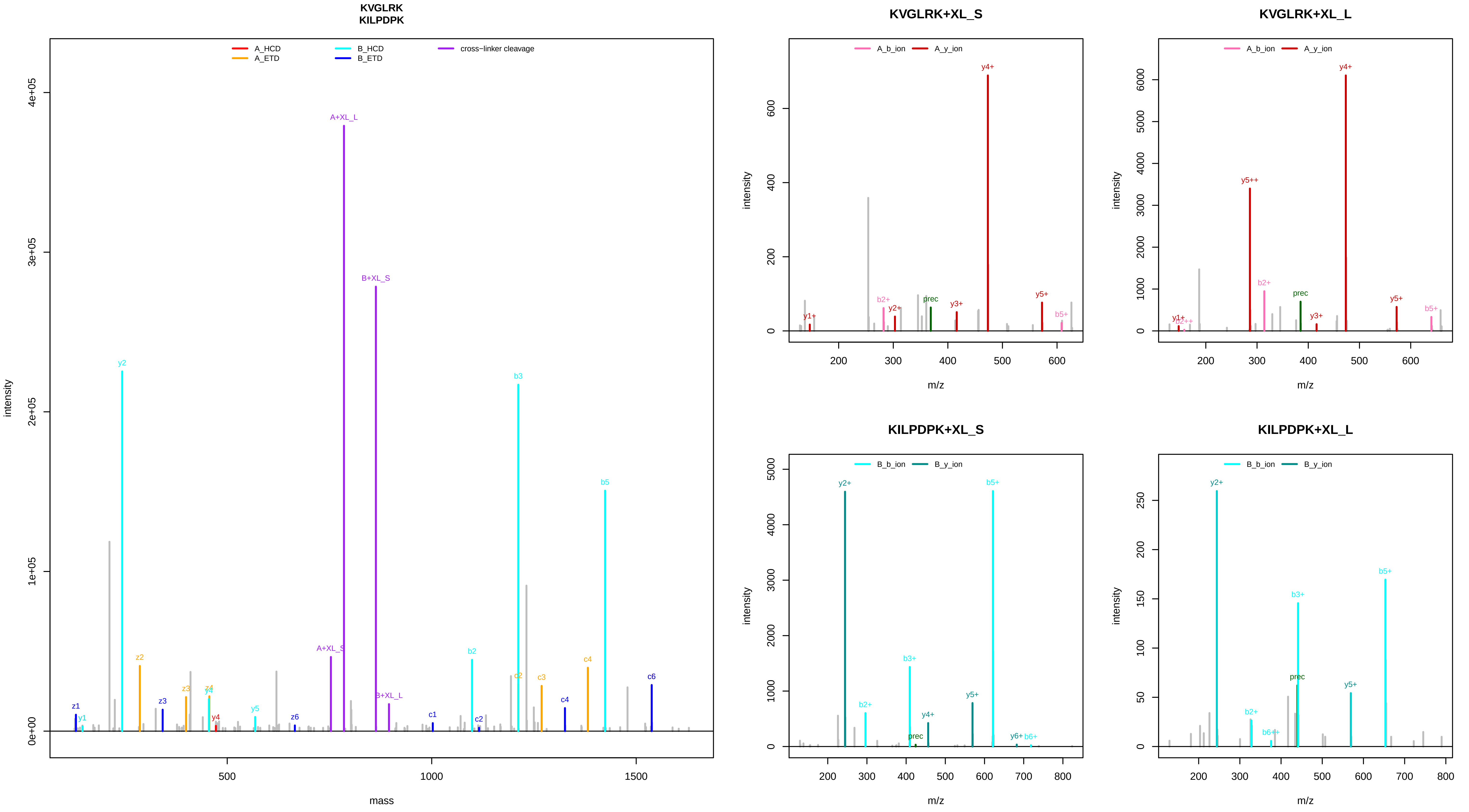
KVGLRK
KILPDPK
A_HCD
A_ETD
B_HCD
B_ETD
cross−linker cleavage
intensity
mass
A+XL_L
B+XL_S
A+XL_S
B+XL_L
y2
b3
b5
b2
z1
y1
z2
z3
y4
y5
z6
c1
c2
c3
c4
c6
KVGLRK+XL_S
KVGLRK+XL_L
KILPDPK+XL_S
KILPDPK+XL_L
A_b_ion
A_y_ion
B_b_ion
B_y_ion
prec
m/z

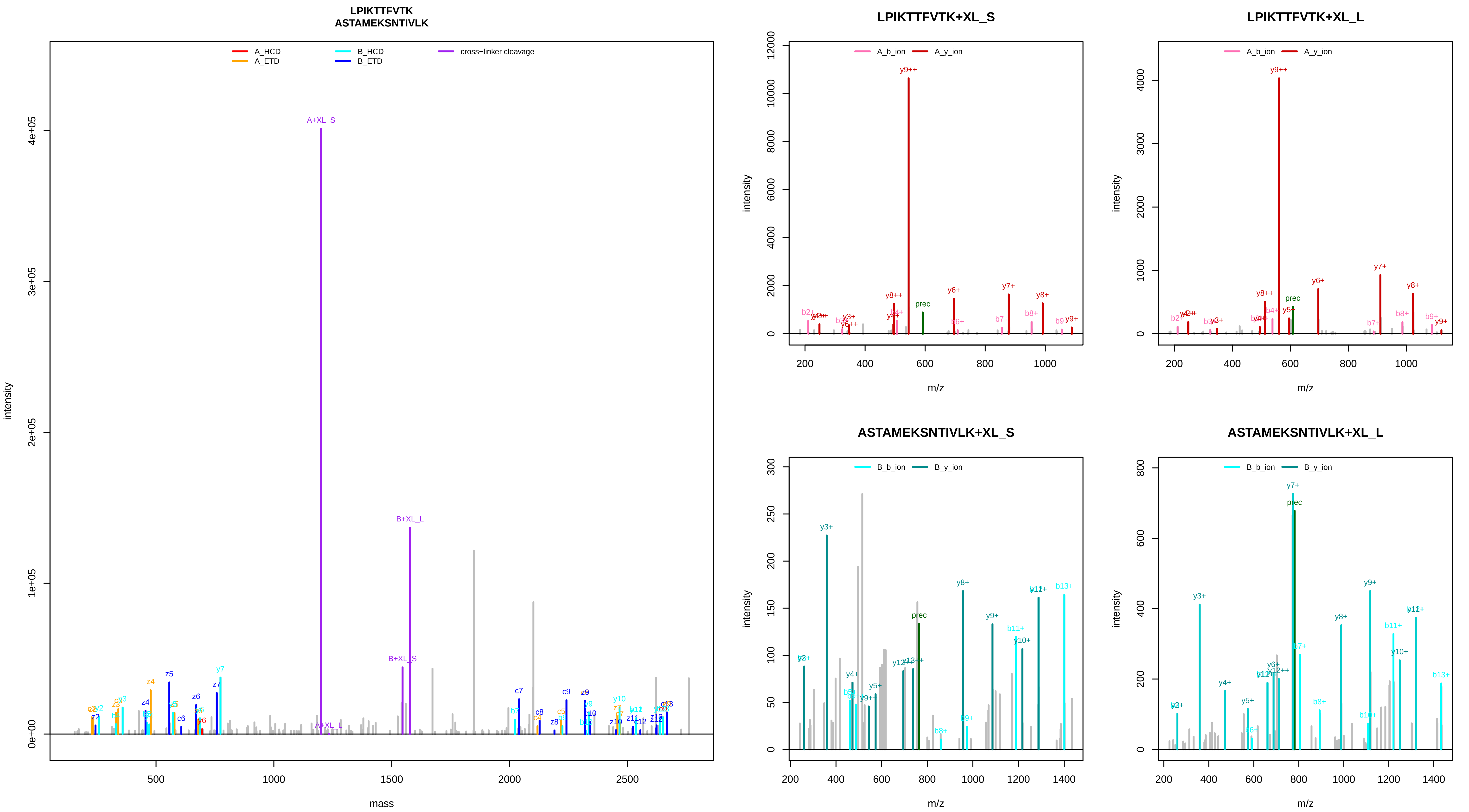
LPIKTTFVTK
ASTAMEKSNTIVLK
A_HCD
A_ETD
B_HCD
B_ETD
cross−linker cleavage
A+XL_S
B+XL_L
B+XL_S
A+XL_L
intensity
mass
LPIKTTFVTK+XL_S
LPIKTTFVTK+XL_L
ASTAMEKSNTIVLK+XL_S
ASTAMEKSNTIVLK+XL_L
A_b_ion
A_y_ion
B_b_ion
B_y_ion
prec
m/z

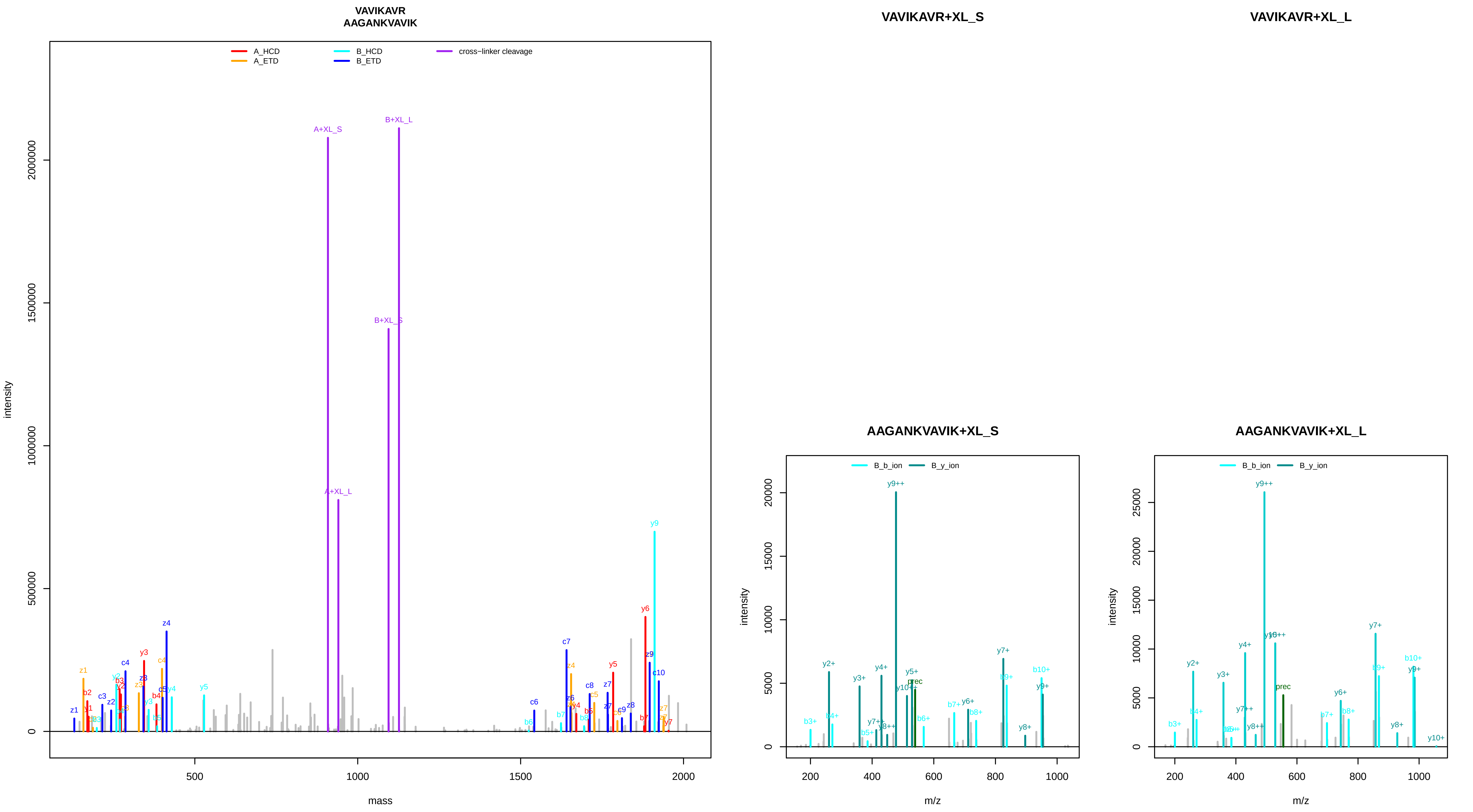
VAVIKAVR
AAGANKVAVIK
A_HCD
A_ETD
B_HCD
B_ETD
cross−linker cleavage
A+XL_S
B+XL_L
B+XL_S
A+XL_L
intensity
mass
VAVIKAVR+XL_S
VAVIKAVR+XL_L
AAGANKVAVIK+XL_S
AAGANKVAVIK+XL_L
B_b_ion
B_y_ion
y9++
prec
m/z

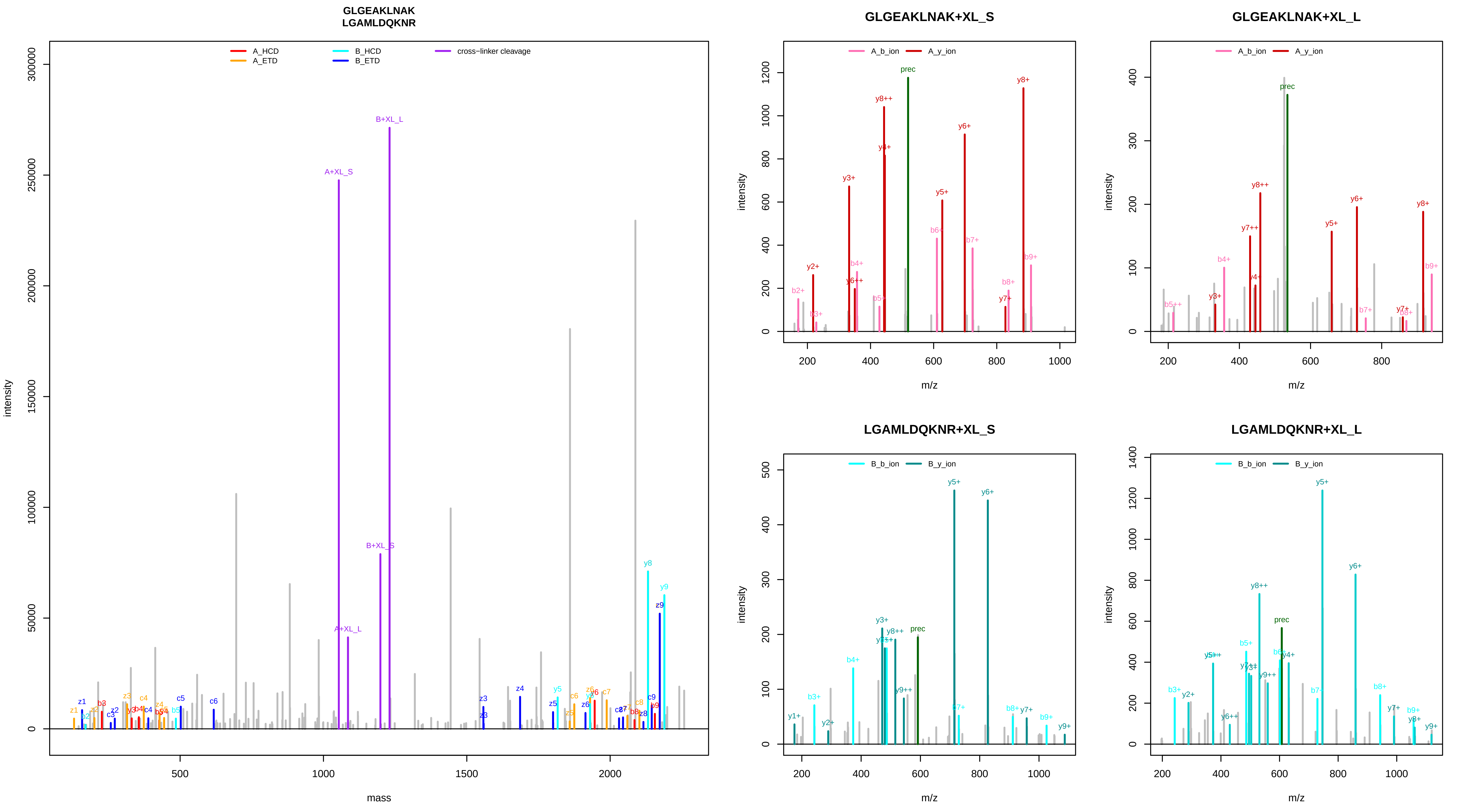
GLGEAKLNAK
LGAMLDQKNR
A_HCD
A_ETD
B_HCD
B_ETD
cross−linker cleavage
A+XL_S
B+XL_L
B+XL_S
A+XL_L
intensity
mass
0
50000
100000
150000
200000
250000
300000
500
1000
1500
2000
GLGEAKLNAK+XL_S
A_b_ion
A_y_ion
prec
intensity
m/z
0
200
400
600
800
1000
1200
200
400
600
800
1000
GLGEAKLNAK+XL_L
A_b_ion
A_y_ion
prec
intensity
m/z
0
100
200
300
400
200
400
600
800
LGAMLDQKNR+XL_S
B_b_ion
B_y_ion
prec
intensity
m/z
0
100
200
300
400
500
200
400
600
800
1000
LGAMLDQKNR+XL_L
B_b_ion
B_y_ion
prec
intensity
m/z
0
200
400
600
800
1000
1200
1400
200
400
600
800
1000

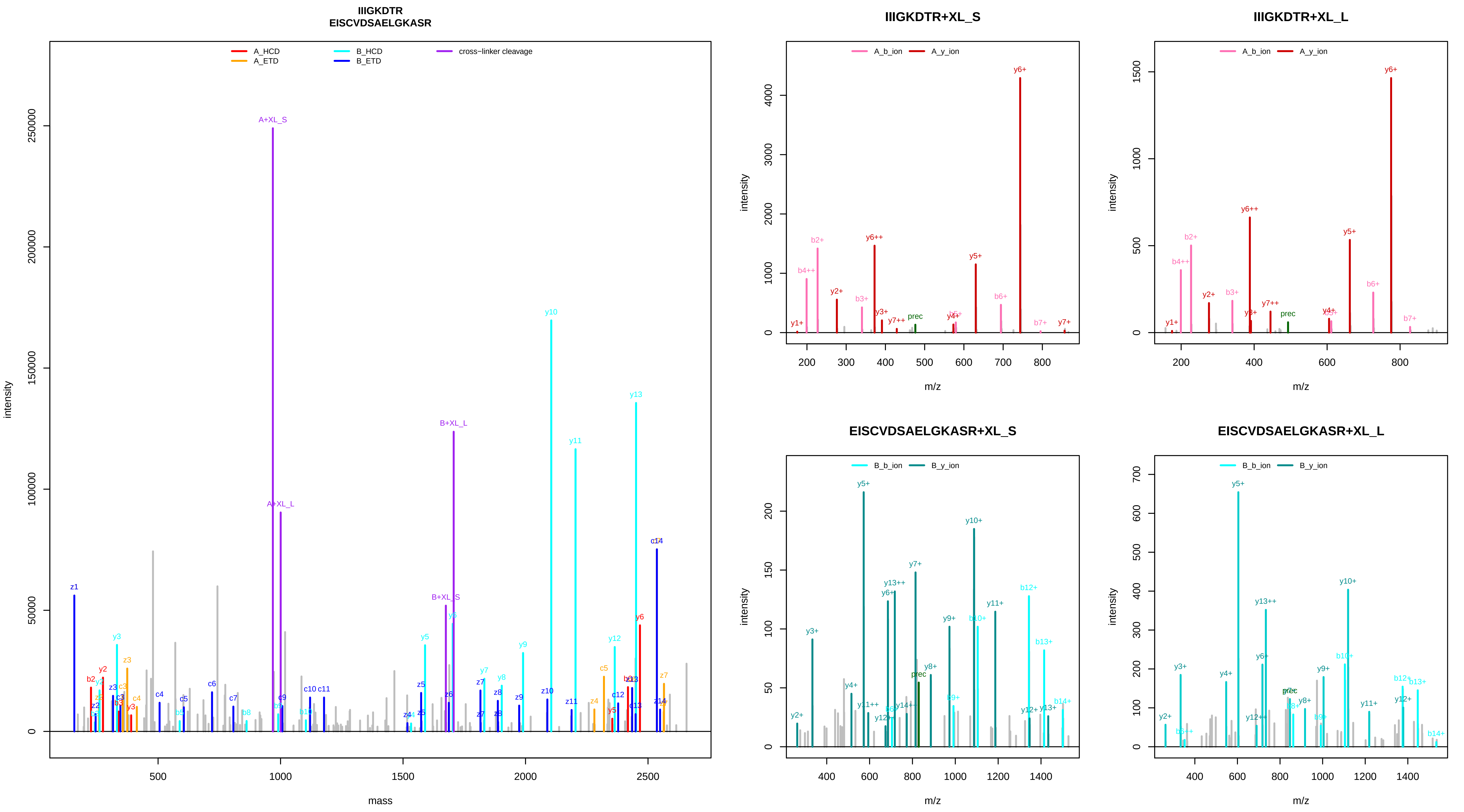
IIIGKDTR
EISCVDSAELGKASR
A_HCD
A_ETD
B_HCD
B_ETD
cross−linker cleavage
A+XL_S
A+XL_L
B+XL_L
B+XL_S
intensity
mass
IIIGKDTR+XL_S
IIIGKDTR+XL_L
A_b_ion
A_y_ion
EISCVDSAELGKASR+XL_S
EISCVDSAELGKASR+XL_L
B_b_ion
B_y_ion
prec
m/z

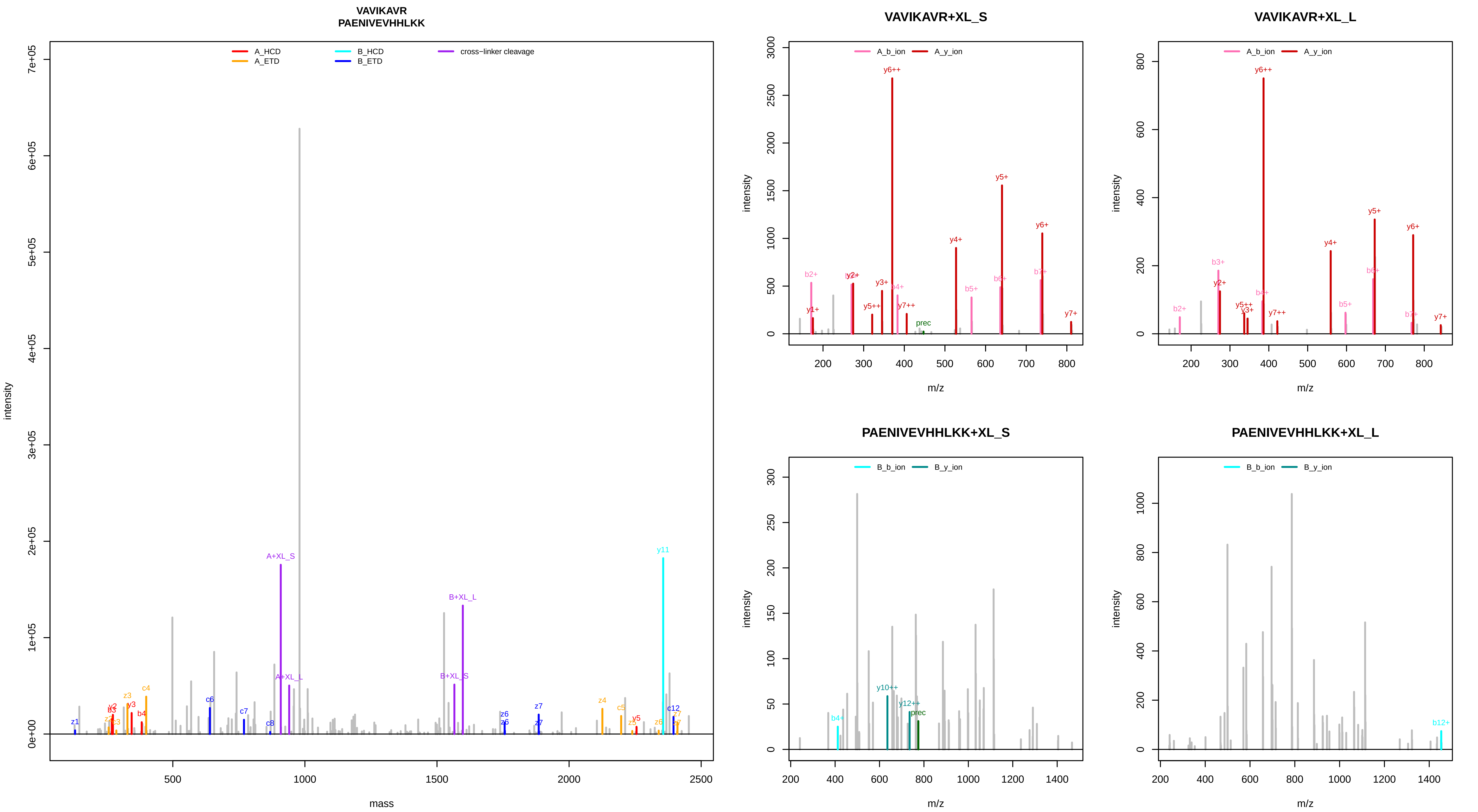

VAVIKAVR
PAENIVEVHHLKK
A_HCD
A_ETD
B_HCD
B_ETD
cross−linker cleavage
A+XL_S
A+XL_L
B+XL_L
B+XL_S
y11
intensity
mass
VAVIKAVR+XL_S
VAVIKAVR+XL_L
A_b_ion
A_y_ion
PAENIVEVHHLKK+XL_S
PAENIVEVHHLKK+XL_L
B_b_ion
B_y_ion
m/z

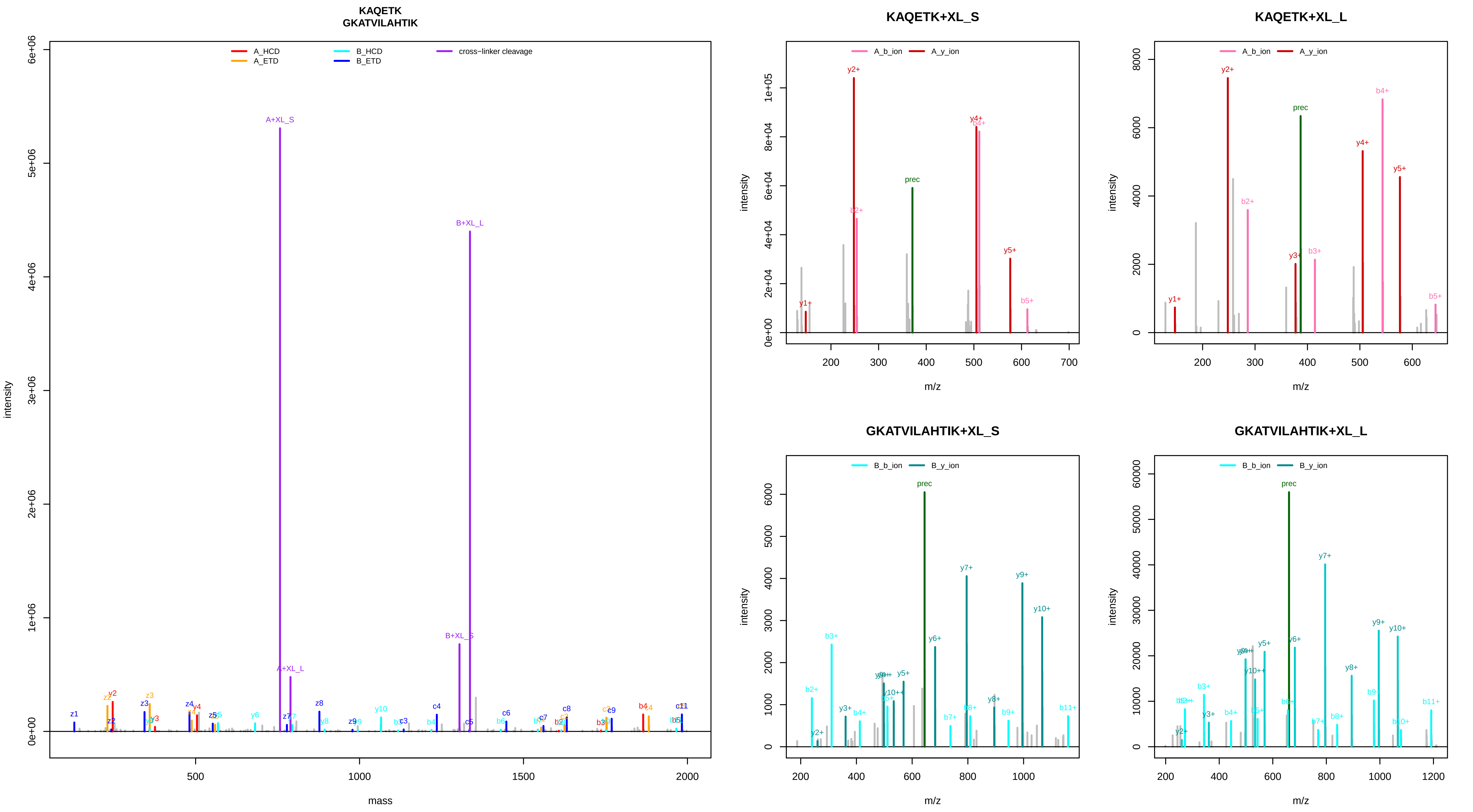

KAQETK
GKATVILAHTIK
A_HCD
A_ETD
B_HCD
B_ETD
cross−linker cleavage
A+XL_S
B+XL_L
B+XL_S
A+XL_L
intensity
mass
KAQETK+XL_S
KAQETK+XL_L
GKATVILAHTIK+XL_S
GKATVILAHTIK+XL_L
A_b_ion
A_y_ion
B_b_ion
B_y_ion
prec
m/z

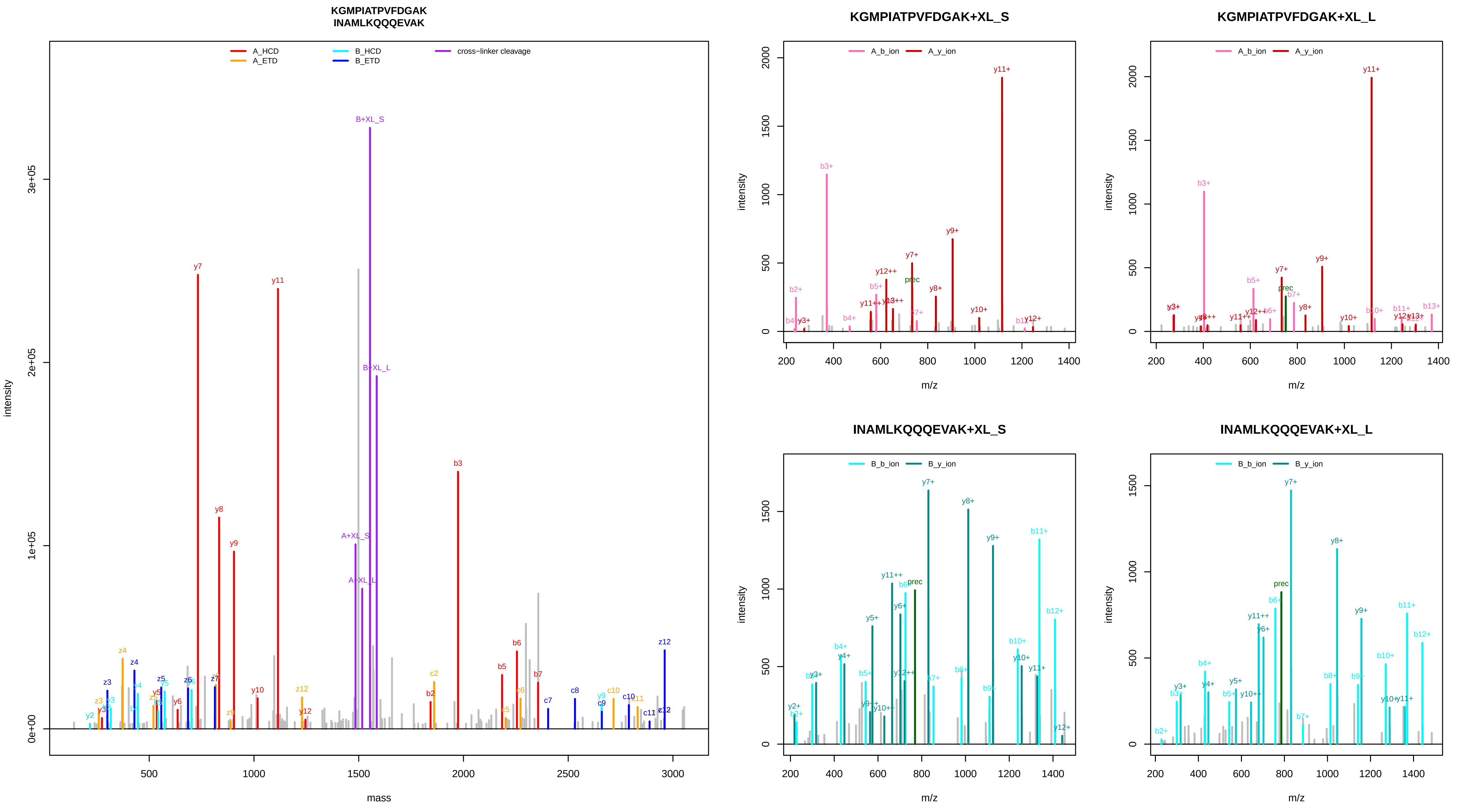
KGMPIATPVFDGAK
INAMLKQQQEVAK
A_HCD
A_ETD
B_HCD
B_ETD
cross−linker cleavage
B+XL_S
B+XL_L
A+XL_S
A+XL_L
intensity
mass
KGMPIATPVFDGAK+XL_S
KGMPIATPVFDGAK+XL_L
A_b_ion
A_y_ion
INAMLKQQQEVAK+XL_S
INAMLKQQQEVAK+XL_L
B_b_ion
B_y_ion
prec
m/z

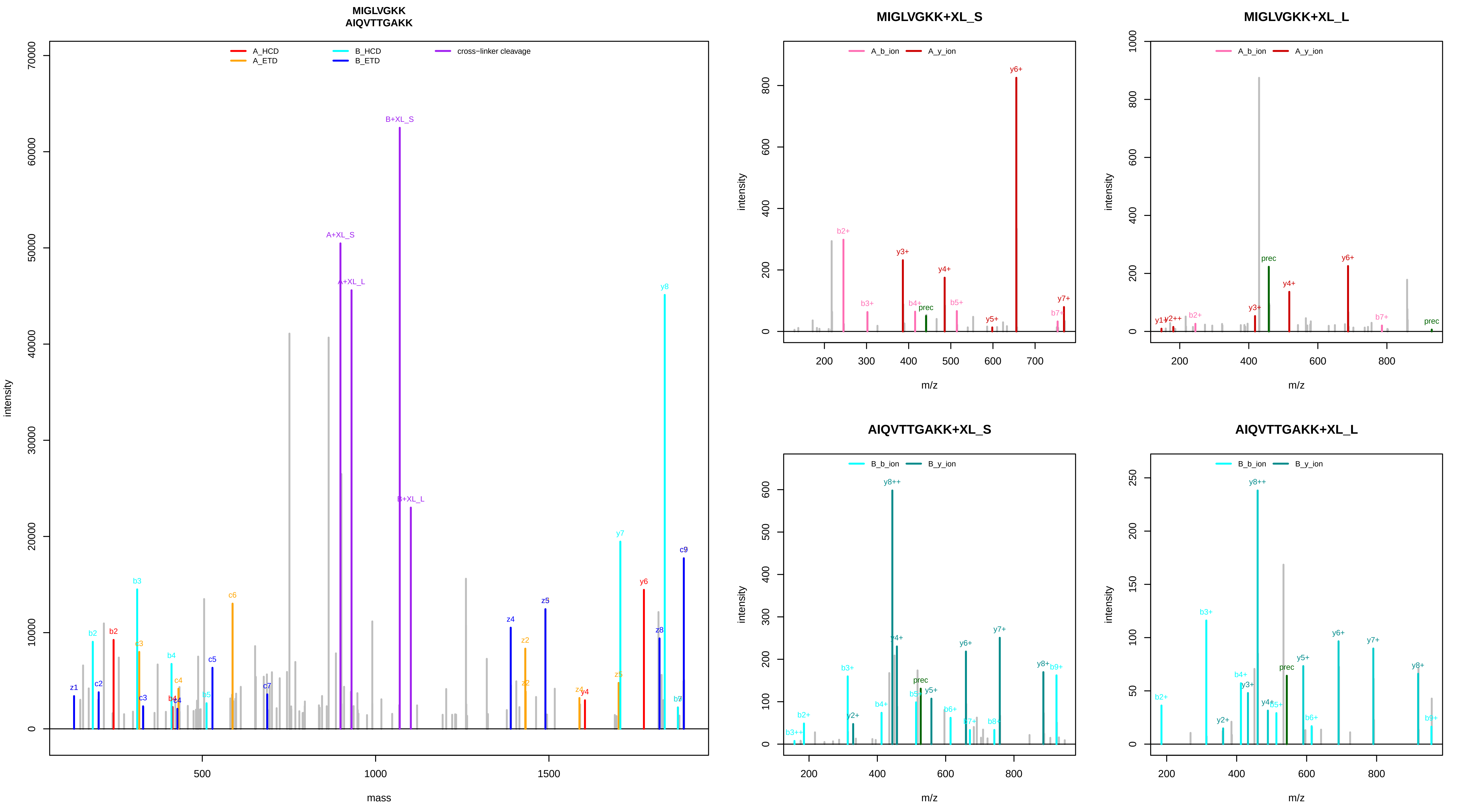

MIGLVGKK
AIQVTTGAKK
A_HCD
A_ETD
B_HCD
B_ETD
cross−linker cleavage
intensity
mass
MIGLVGKK+XL_S
MIGLVGKK+XL_L
AIQVTTGAKK+XL_S
AIQVTTGAKK+XL_L
A_b_ion
A_y_ion
B_b_ion
B_y_ion
m/z

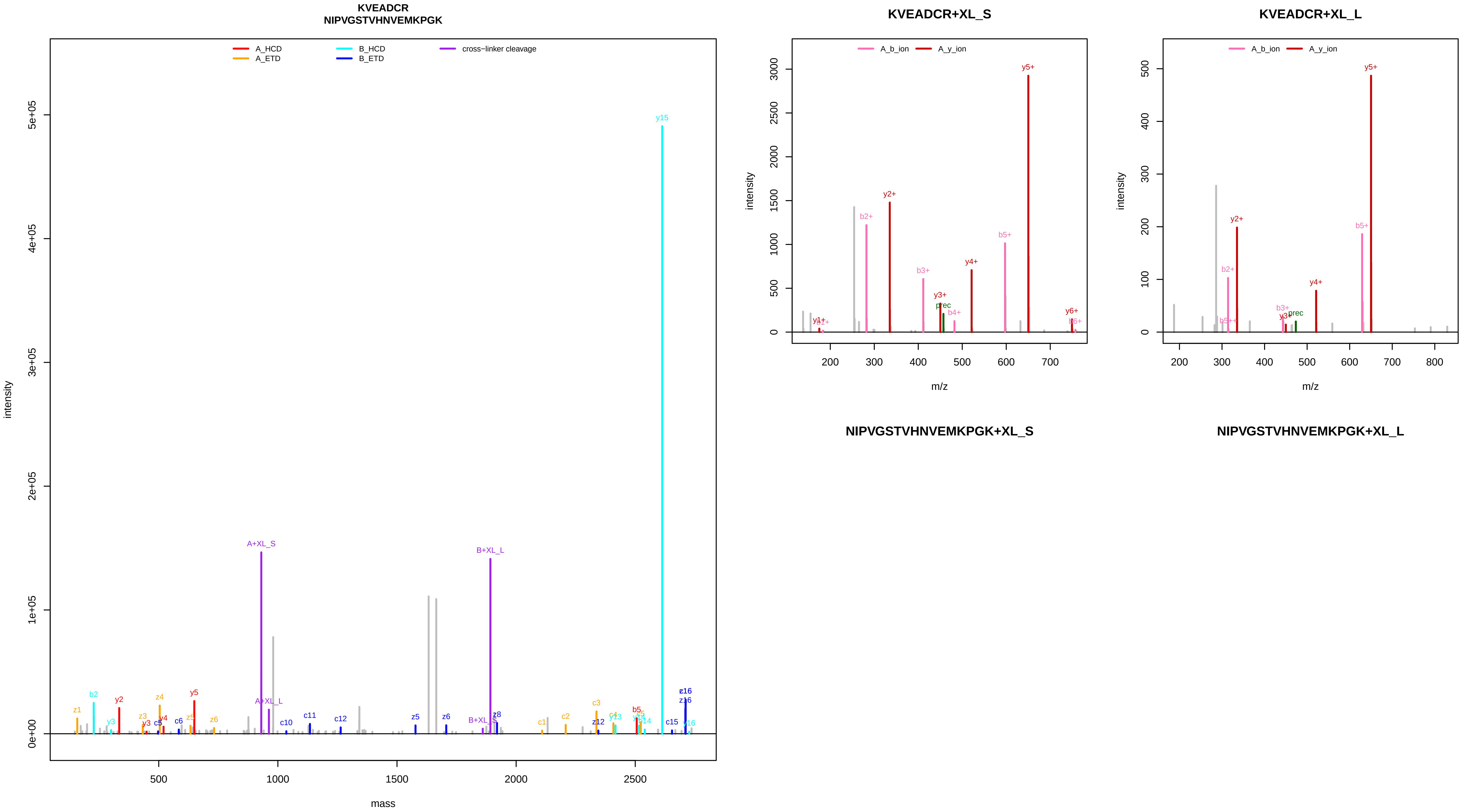
KVEADCR
NIPVGSTVHNVEMKPGK
A_HCD
A_ETD
B_HCD
B_ETD
cross−linker cleavage
intensity
mass
y15
A+XL_S
B+XL_L
A+XL_L
KVEADCR+XL_S
KVEADCR+XL_L
A_b_ion
A_y_ion
m/z
y5+
y2+
b2+
b3+
b4+
b5+
y3+
y4+
y6+
prec
NIPVGSTVHNVEMKPGK+XL_S
NIPVGSTVHNVEMKPGK+XL_L

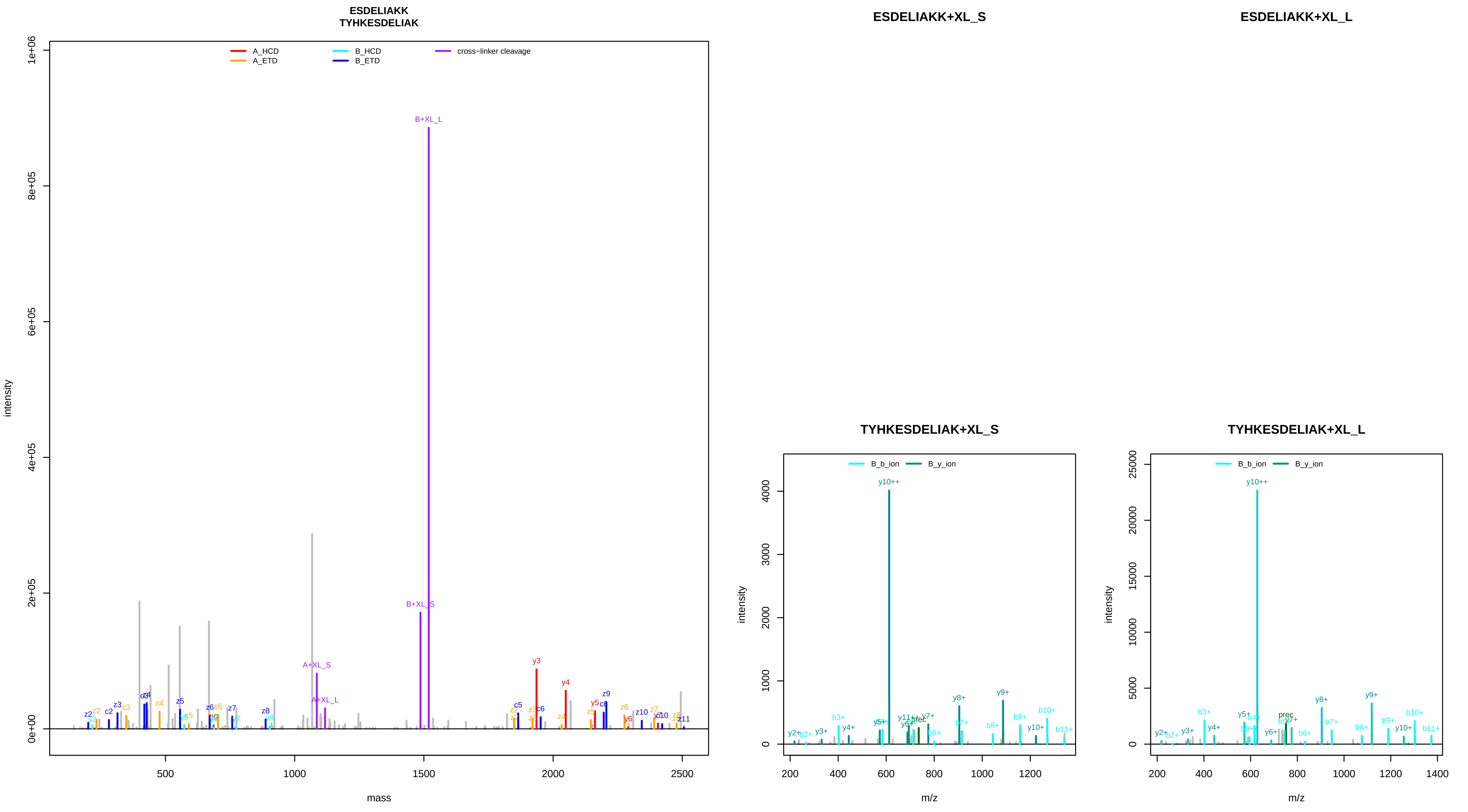
ESDELIAKK
TYHKESDELIAK
A_HCD
A_ETD
B_HCD
B_ETD
cross−linker cleavage
B+XL_L
B+XL_S
A+XL_S
A+XL_L
intensity
mass
ESDELIAKK+XL_S
ESDELIAKK+XL_L
TYHKESDELIAK+XL_S
TYHKESDELIAK+XL_L
B_b_ion
B_y_ion
y10++
intensity
m/z

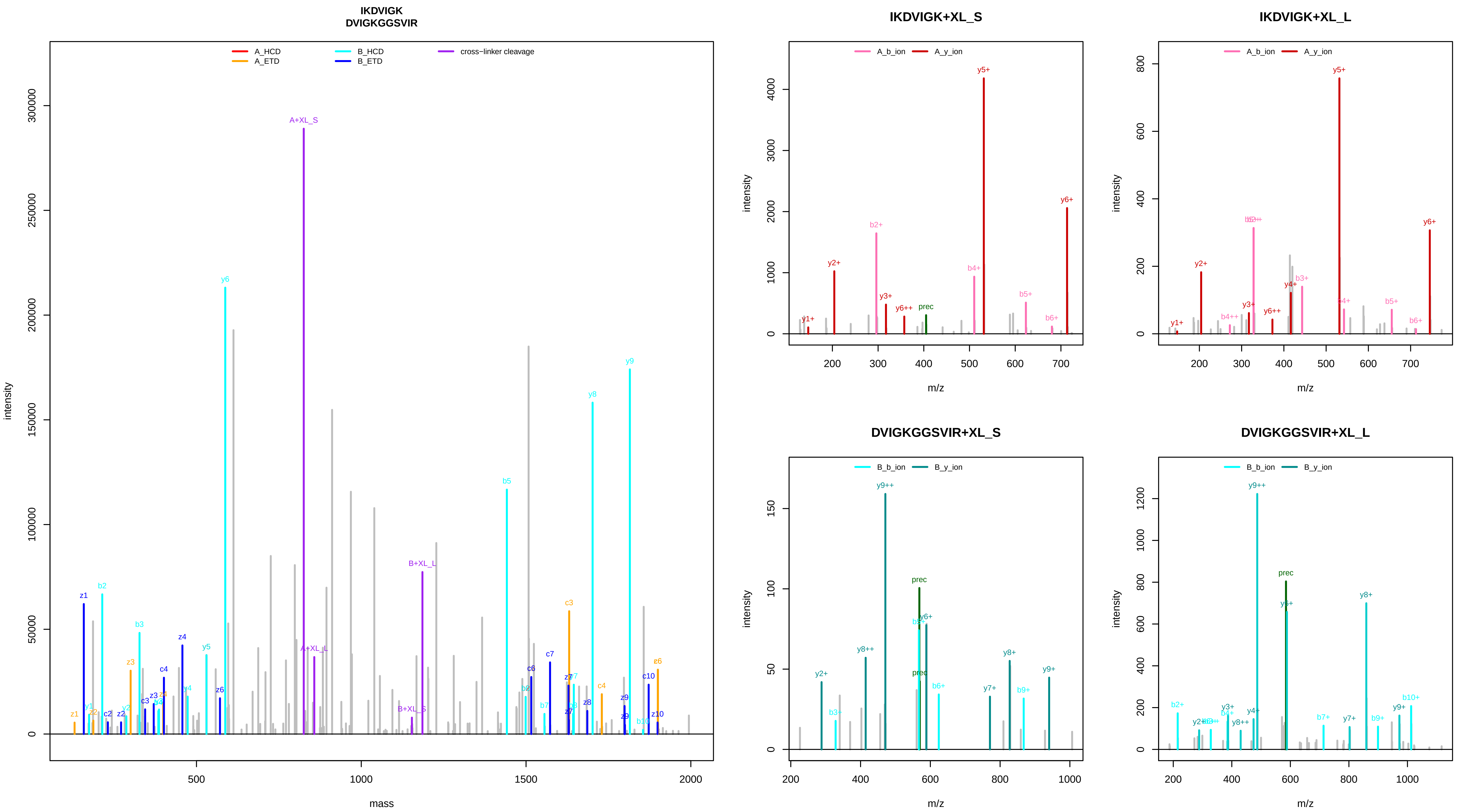

IKDVIGK
DVIGKGGSVIR
A_HCD
A_ETD
B_HCD
B_ETD
cross−linker cleavage
intensity
mass
A+XL_S
A+XL_L
B+XL_L
B+XL_S
y6
y8
y9
b5
b2
b3
z1
z4
y5
c3
c4
c6
c7
c10
z6
z8
z9
z10
IKDVIGK+XL_S
IKDVIGK+XL_L
A_b_ion
A_y_ion
y5+
y6+
y2+
b2+
b4+
b5+
b6+
y3+
y6++
prec
m/z
DVIGKGGSVIR+XL_S
DVIGKGGSVIR+XL_L
B_b_ion
B_y_ion
y9++
y8++
y2+
y6+
y7+
y8+
y9+
b3+
b6+
b9+
b2+
b7+
b10+

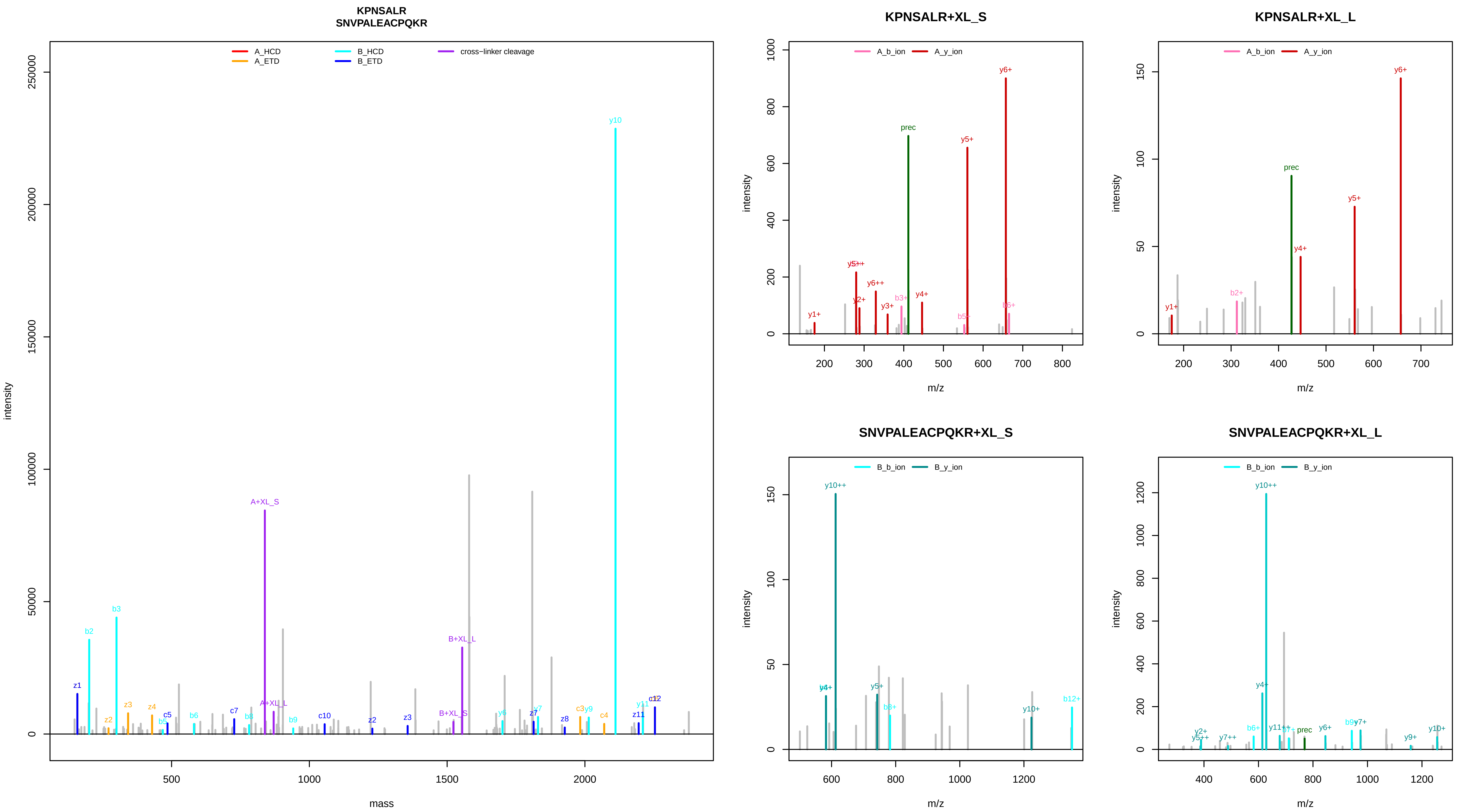
KPNSALR
SNVPALEACPQKR
A_HCD
A_ETD
B_HCD
B_ETD
cross−linker cleavage
intensity
mass
y10
A+XL_S
B+XL_L
b2
b3
z1
z2
z3
z4
c5
c7
c10
c12
z7
z8
z11
y11
y9
c3
c4
KPNSALR+XL_S
KPNSALR+XL_L
A_b_ion
A_y_ion
prec
y6+
y5+
y4+
y1+
b2+
m/z
SNVPALEACPQKR+XL_S
SNVPALEACPQKR+XL_L
B_b_ion
B_y_ion
y10++
y10+
b12+
b9
y7+
y6+

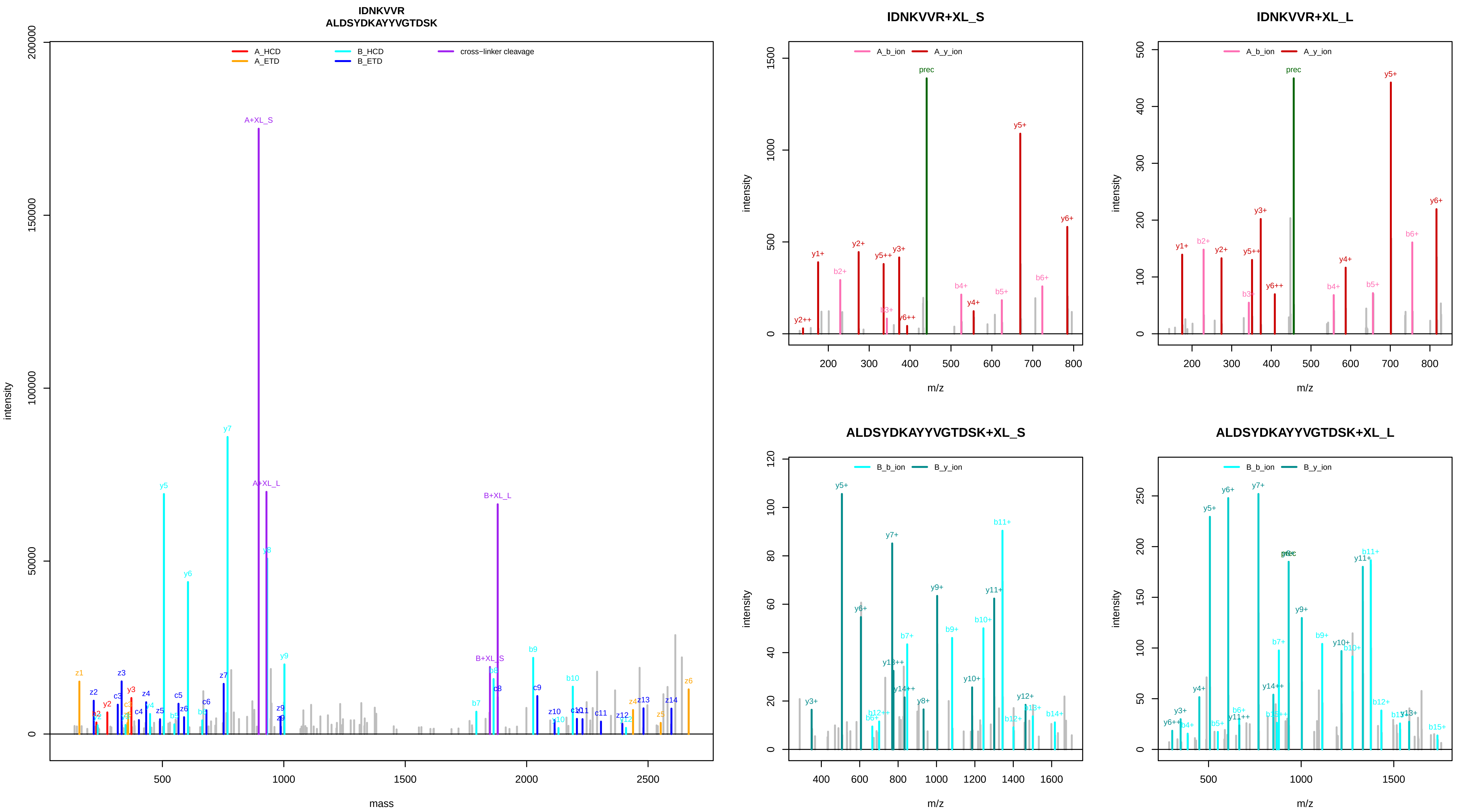

IDNKVVR
ALDSYDKAYYVGTDSK
A_HCD
A_ETD
B_HCD
B_ETD
cross−linker cleavage
A+XL_S
A+XL_L
B+XL_L
B+XL_S
intensity
mass
IDNKVVR+XL_S
IDNKVVR+XL_L
ALDSYDKAYYVGTDSK+XL_S
ALDSYDKAYYVGTDSK+XL_L
A_b_ion
A_y_ion
B_b_ion
B_y_ion
prec
m/z

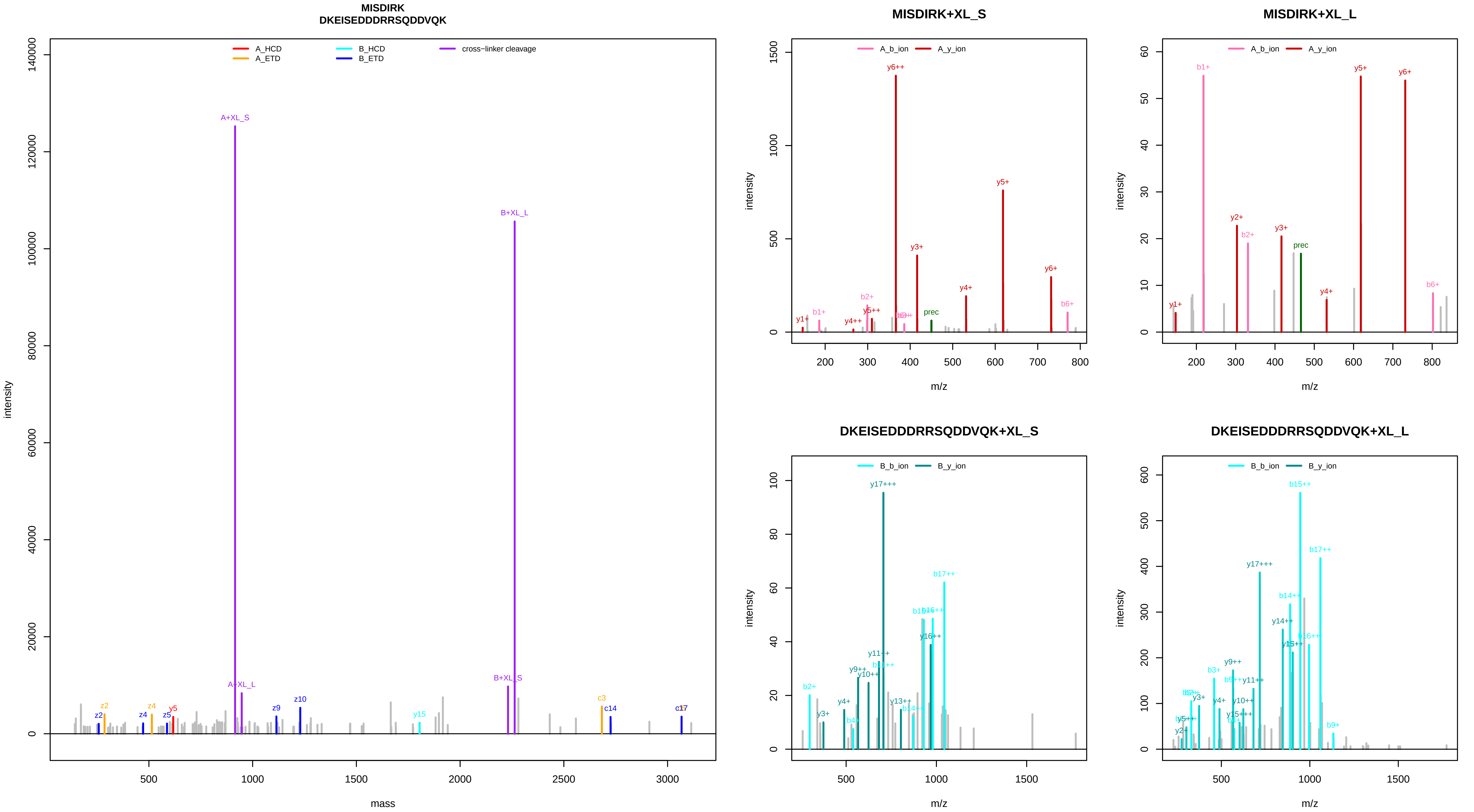
MISDIRK
DKEISEDDDRRSQDDVQK
A_HCD
A_ETD
B_HCD
B_ETD
cross−linker cleavage
A+XL_S
B+XL_L
A+XL_L
B+XL_S
z2
z4
z5
y5
z9
z10
y15
c3
c14
c17
intensity
mass
MISDIRK+XL_S
MISDIRK+XL_L
A_b_ion
A_y_ion
y6++
y5+
y3+
y6+
y4+
b2+
b1+
y1+
y4++
y5++
b6+
prec
y2+
intensity
m/z
DKEISEDDDRRSQDDVQK+XL_S
DKEISEDDDRRSQDDVQK+XL_L
B_b_ion
B_y_ion
y17+++
b17++
b15++
b16++
y16++
y11++
b11++
y9++
y10++
b2+
y4+
y3+
y13++
b3+
b4+
y14++
y15++
y11++
y9++
y10++
y15+++
b14++
b9+
b9++
b5+
y5++
y2+
intensity
m/z

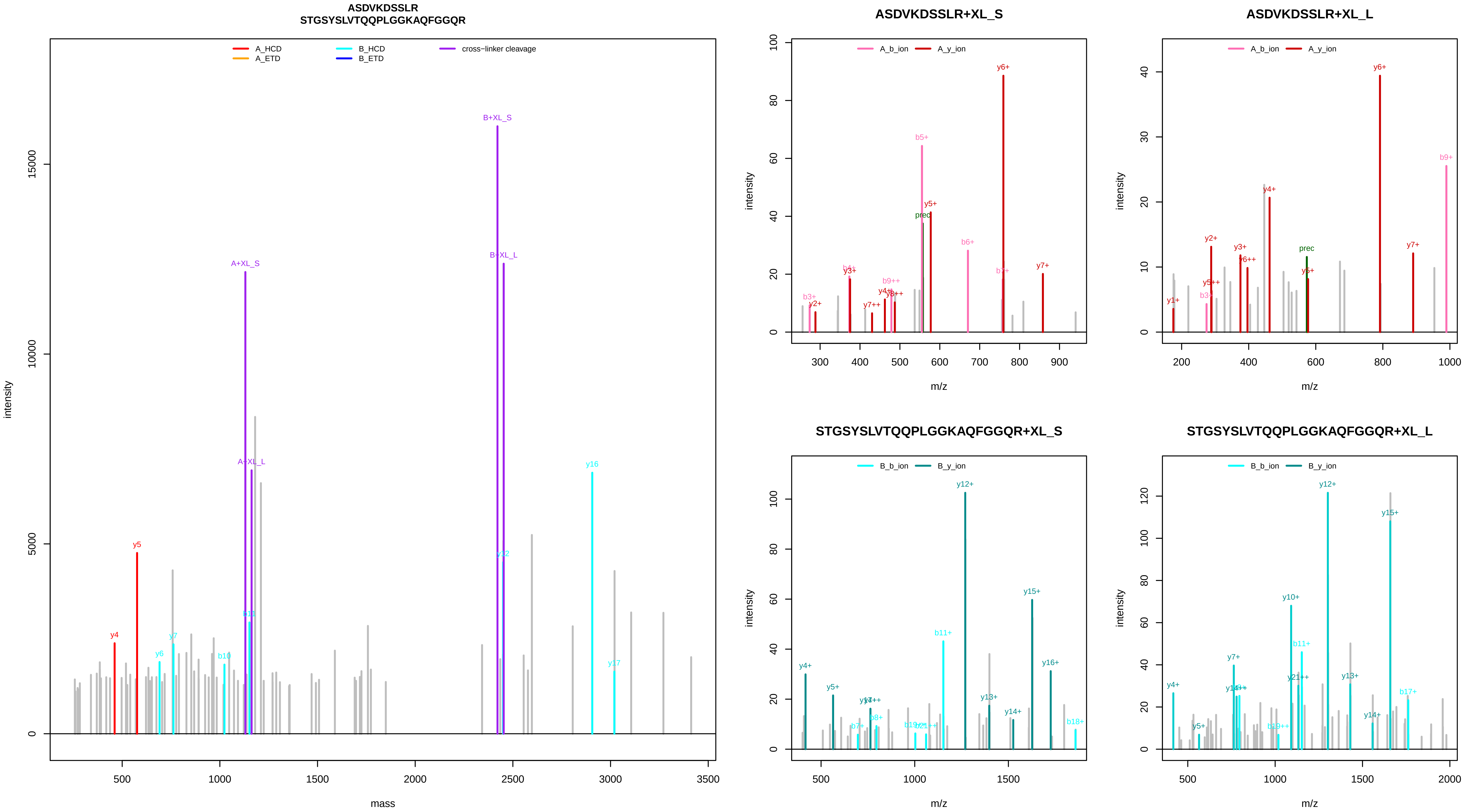

ASDVKDSSLR
STGSYSLVTQQPLGGKAQFGGQR
A_HCD
A_ETD
B_HCD
B_ETD
cross−linker cleavage
B+XL_S
B+XL_L
A+XL_S
A+XL_L
y16
y17
y12
y5
y4
y6
y7
b10
b11
intensity
mass
ASDVKDSSLR+XL_S
ASDVKDSSLR+XL_L
A_b_ion
A_y_ion
prec
m/z
STGSYSLVTQQPLGGKAQFGGQR+XL_S
STGSYSLVTQQPLGGKAQFGGQR+XL_L
B_b_ion
B_y_ion
y12+
y15+
y16+
y10+
y13+
y14+
b11+
b18+
b17+
y4+
y5+
y7+
y6+
y3+
y2+
y1+
b5+
b6+
b9+
y7+

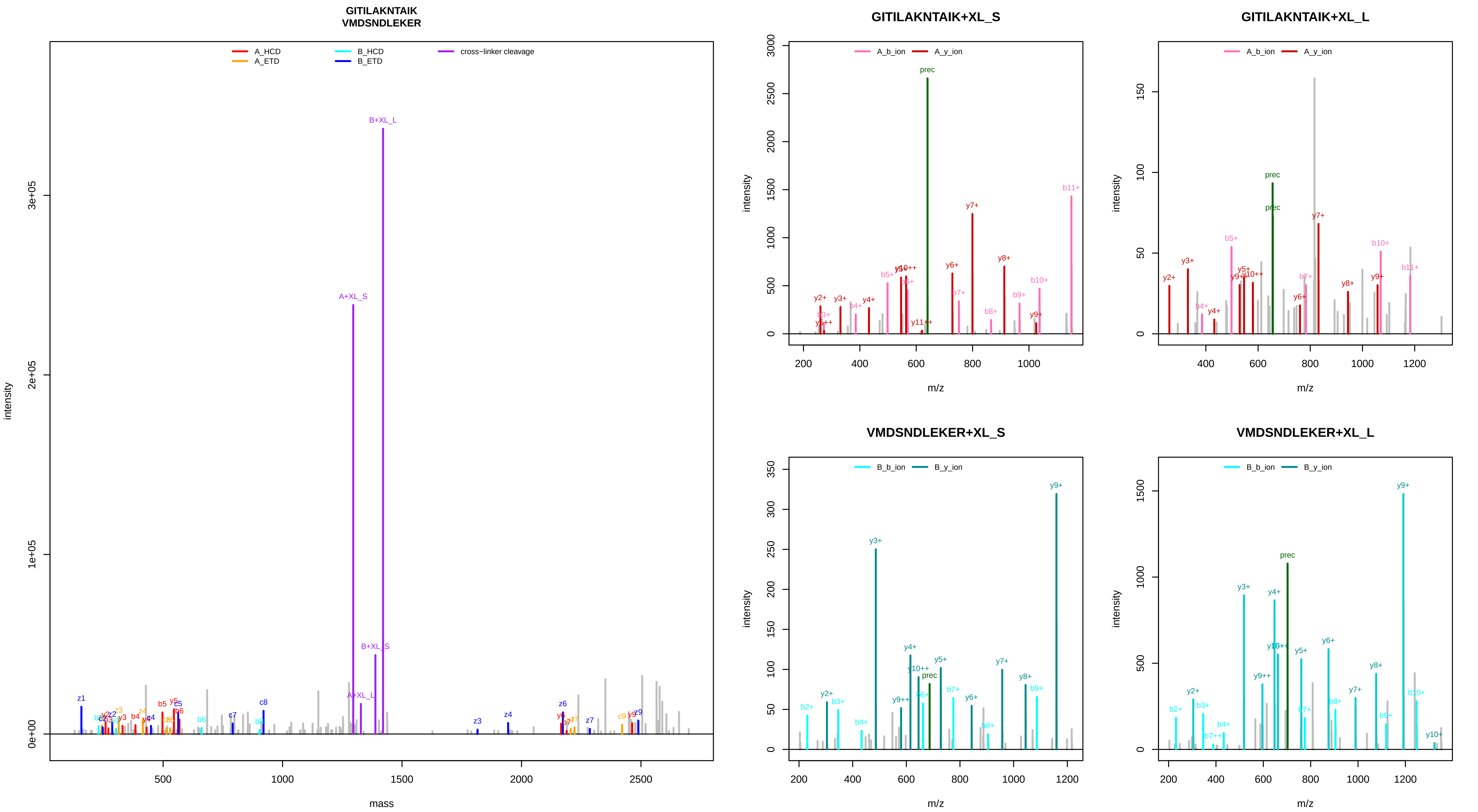
GITILAKNTAIK
VMDSNDLEKER
A_HCD
A_ETD
B_HCD
B_ETD
cross−linker cleavage
B+XL_L
A+XL_S
B+XL_S
A+XL_L
intensity
mass
GITILAKNTAIK+XL_S
GITILAKNTAIK+XL_L
VMDSNDLEKER+XL_S
VMDSNDLEKER+XL_L
A_b_ion
A_y_ion
B_b_ion
B_y_ion
prec
m/z

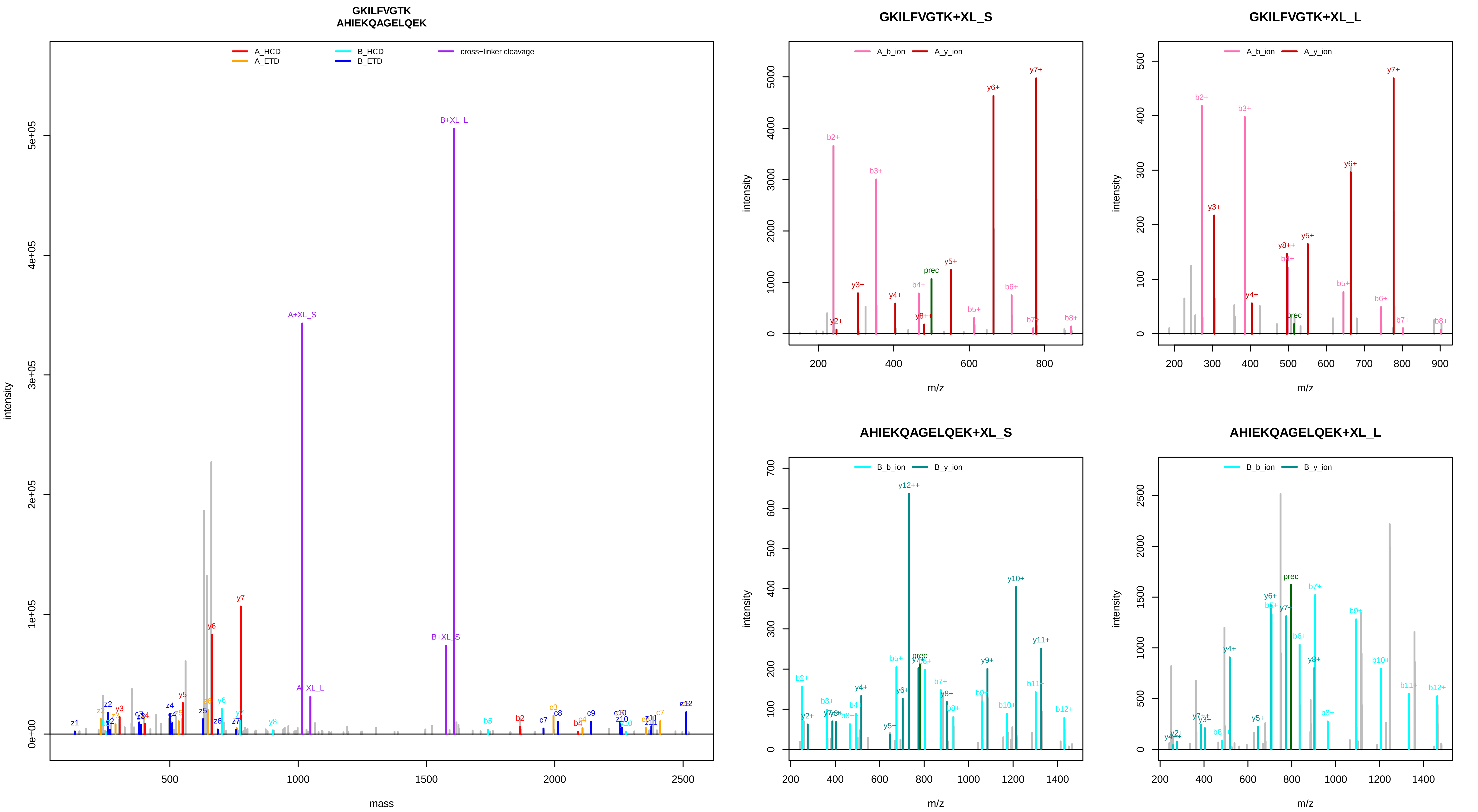

GKILFVGTK
AHIEKQAGELQEK
A_HCD
A_ETD
B_HCD
B_ETD
cross−linker cleavage
B+XL_L
A+XL_S
B+XL_S
A+XL_L
intensity
mass
GKILFVGTK+XL_S
GKILFVGTK+XL_L
A_b_ion
A_y_ion
AHIEKQAGELQEK+XL_S
AHIEKQAGELQEK+XL_L
B_b_ion
B_y_ion
prec
m/z

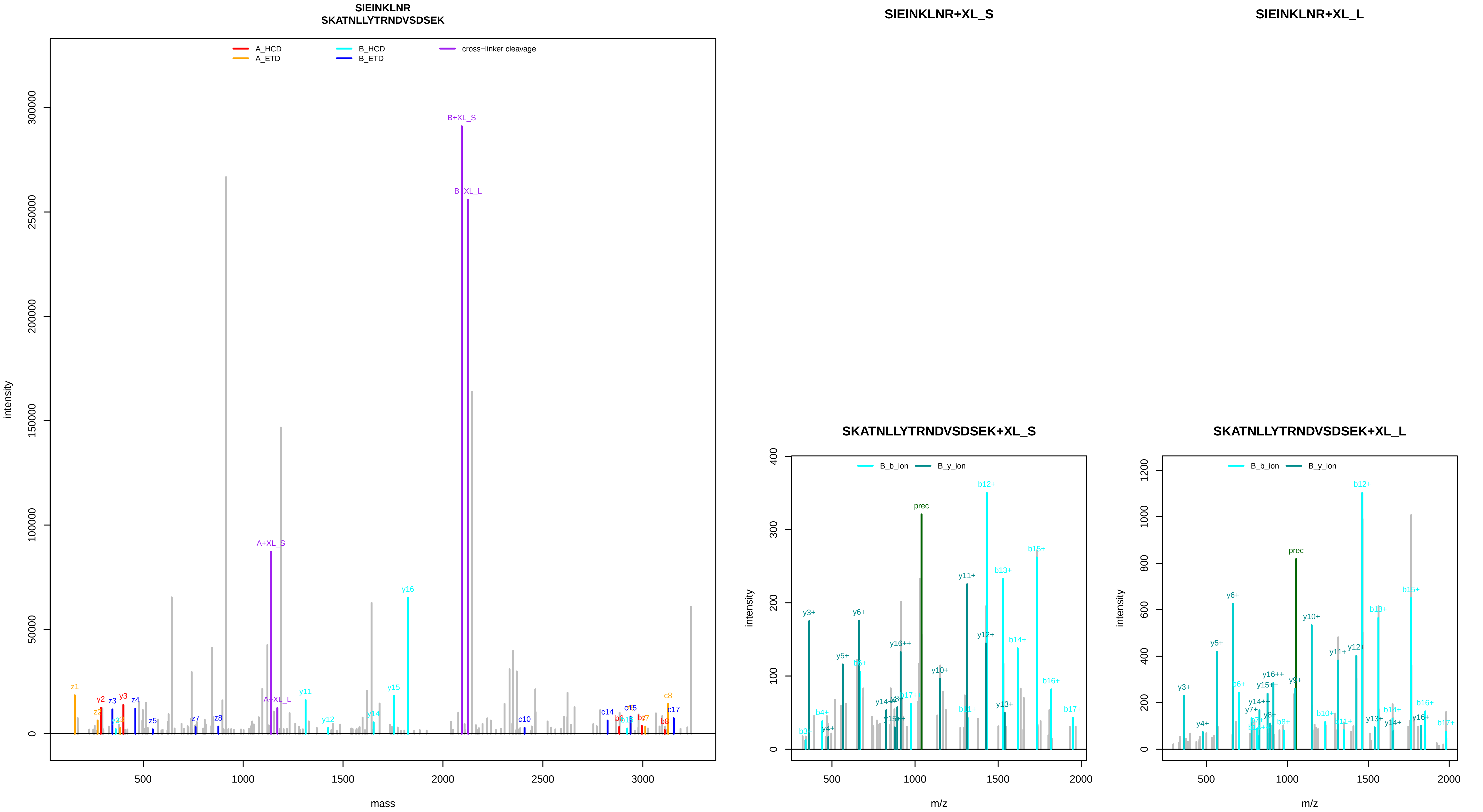

SIEINKLNR
SKATNLLYTRNDVSDSEK
A_HCD
A_ETD
B_HCD
B_ETD
cross−linker cleavage
B+XL_S
B+XL_L
A+XL_S
A+XL_L
intensity
mass
SIEINKLNR+XL_S
SIEINKLNR+XL_L
SKATNLLYTRNDVSDSEK+XL_S
SKATNLLYTRNDVSDSEK+XL_L
B_b_ion
B_y_ion
prec
intensity
m/z

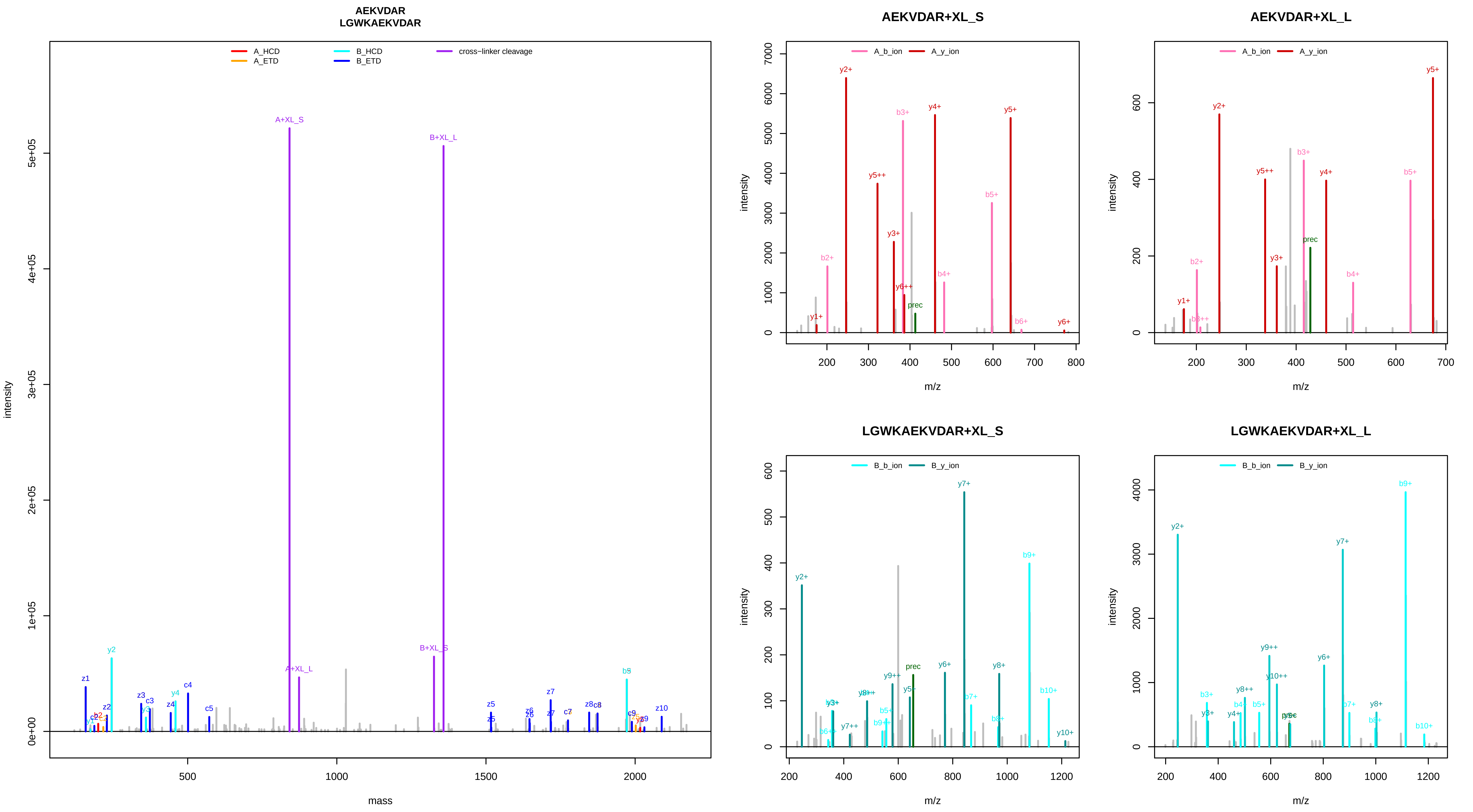

AEKVDAR
LGWKAEKVDAR
A_HCD
A_ETD
B_HCD
B_ETD
cross−linker cleavage
A+XL_S
B+XL_L
B+XL_S
A+XL_L
intensity
mass
0e+00
1e+05
2e+05
3e+05
4e+05
5e+05
500
1000
1500
2000
z1
y2
z2
z3
c3
y4
z4
c4
c5
z5
z6
z7
c7
z8
c8
b9
c9
z9
z10
AEKVDAR+XL_S
A_b_ion
A_y_ion
y1+
y2+
b2+
y5++
y3+
b3+
y6++
prec
y4+
b4+
b5+
y5+
b6+
y6+
m/z
AEKVDAR+XL_L
b3++
LGWKAEKVDAR+XL_S
B_b_ion
B_y_ion
y7++
y8++
y9++
y5+
y6+
y7+
b7+
y8+
b8+
b9+
b10+
y10+
LGWKAEKVDAR+XL_L
y10++

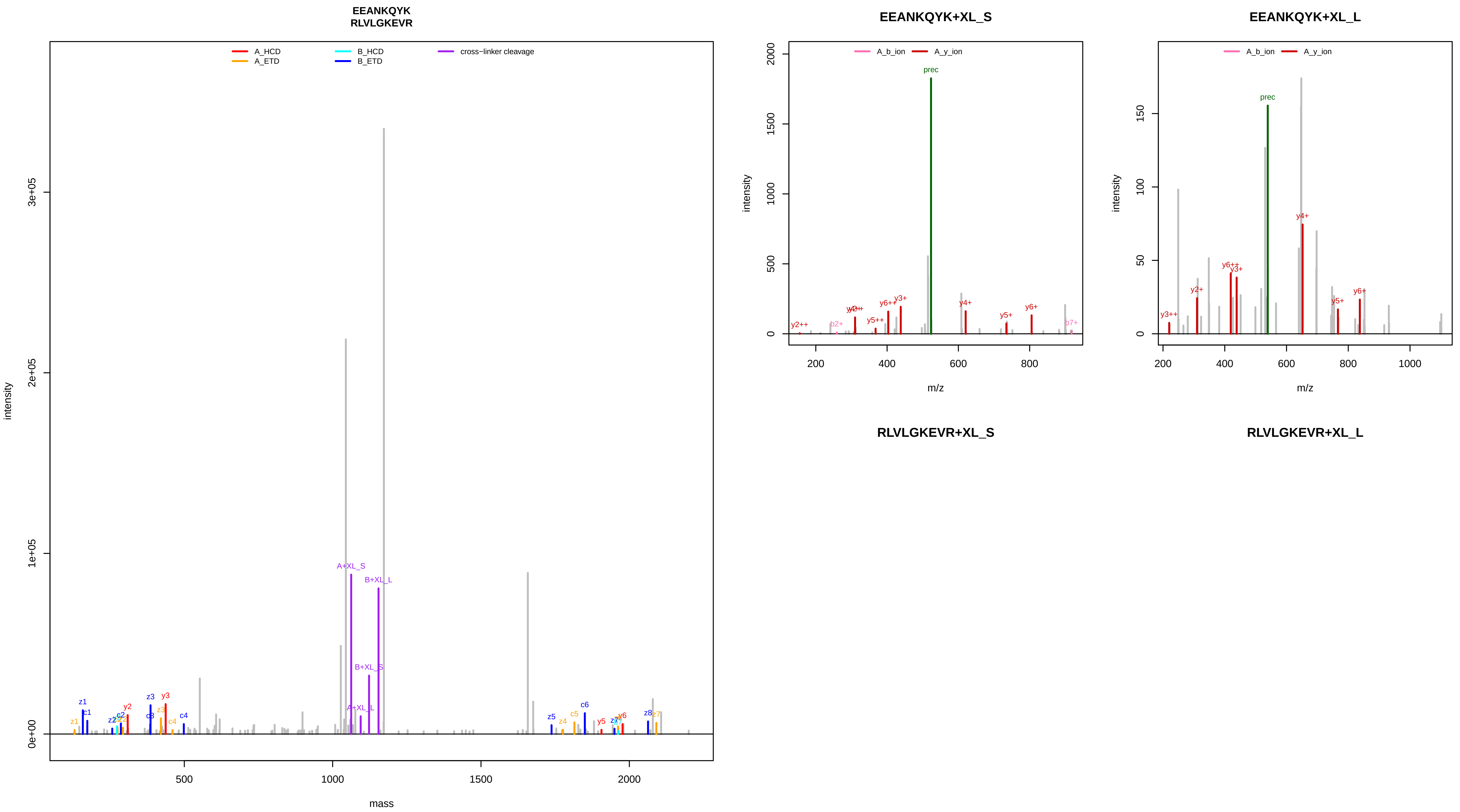
EEANKQYK
RLVLGKEVR
A_HCD
A_ETD
B_HCD
B_ETD
cross−linker cleavage
A+XL_S
B+XL_L
B+XL_S
A+XL_L
intensity
mass
0e+00
1e+05
2e+05
3e+05
500
1000
1500
2000
EEANKQYK+XL_S
EEANKQYK+XL_L
A_b_ion
A_y_ion
prec
intensity
m/z
RLVLGKEVR+XL_S
RLVLGKEVR+XL_L

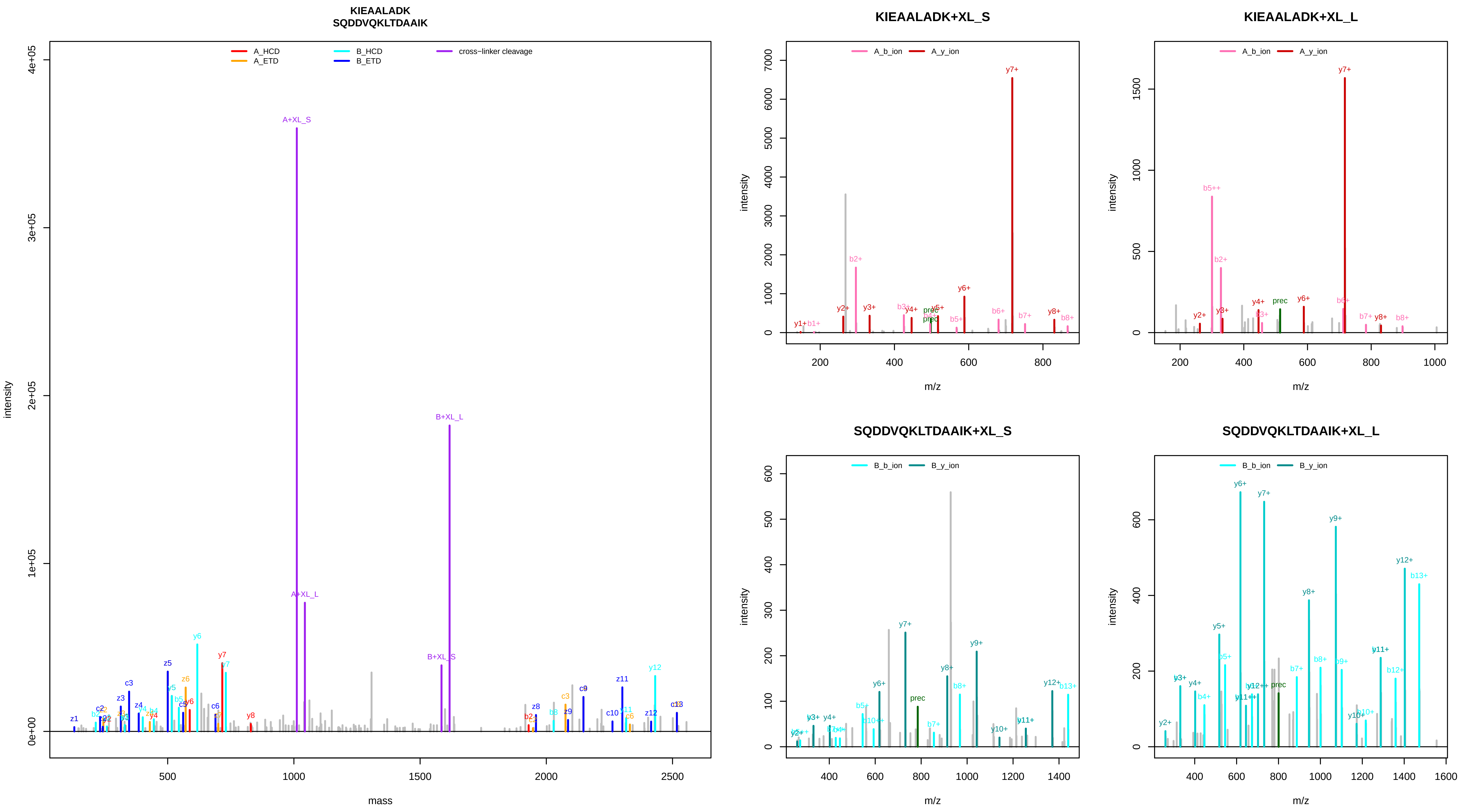
KIEAALADK
SQDDVQKLTDAAIK
A_HCD
A_ETD
B_HCD
B_ETD
cross−linker cleavage
A+XL_S
B+XL_L
A+XL_L
B+XL_S
intensity
mass
KIEAALADK+XL_S
KIEAALADK+XL_L
A_b_ion
A_y_ion
SQDDVQKLTDAAIK+XL_S
SQDDVQKLTDAAIK+XL_L
B_b_ion
B_y_ion
prec
m/z

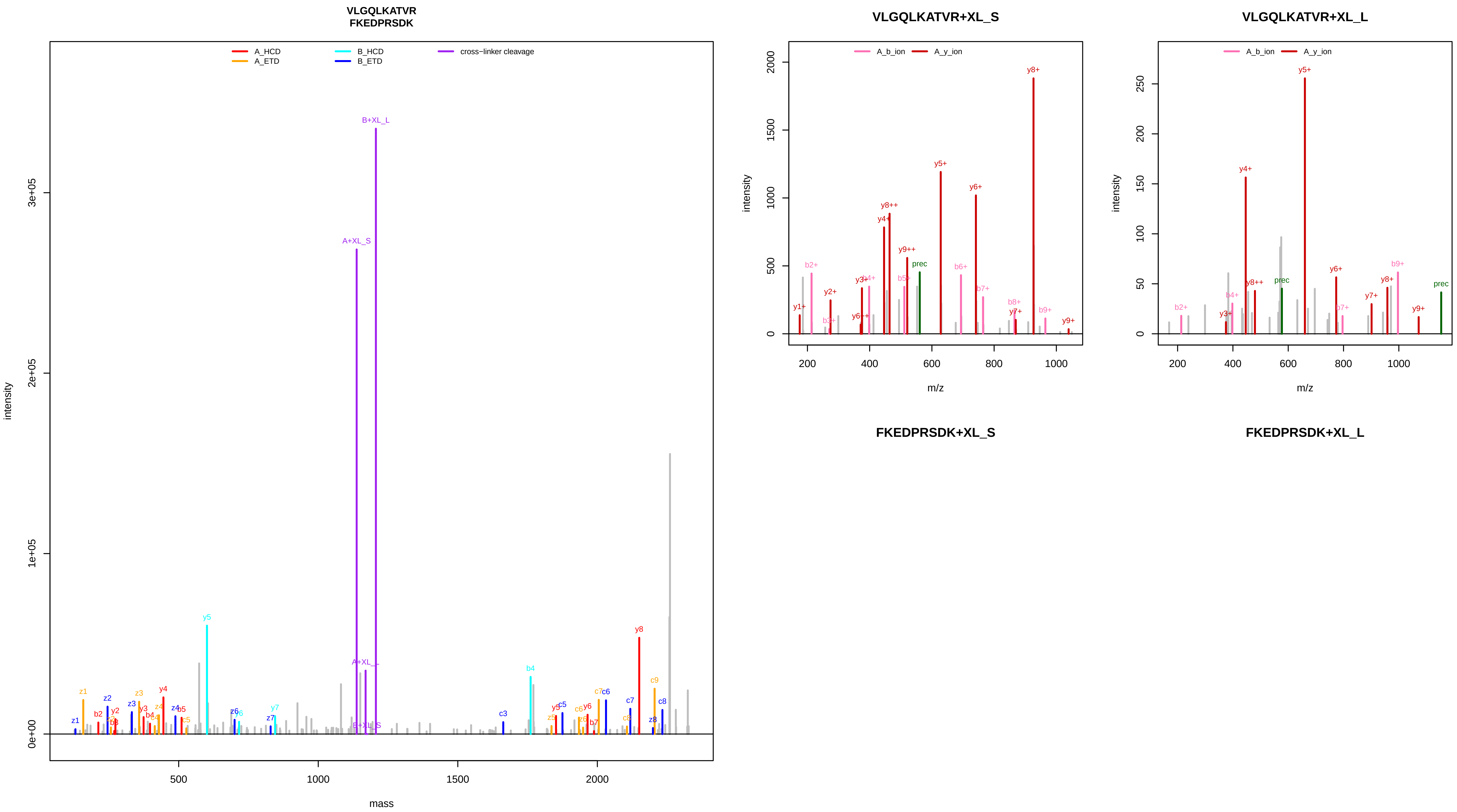
VLGQLKATVR
FKEDPRSDK
A_HCD
A_ETD
B_HCD
B_ETD
cross−linker cleavage
B+XL_L
A+XL_S
A+XL_L
B+XL_S
intensity
mass
VLGQLKATVR+XL_S
VLGQLKATVR+XL_L
A_b_ion
A_y_ion
prec
m/z
FKEDPRSDK+XL_S
FKEDPRSDK+XL_L

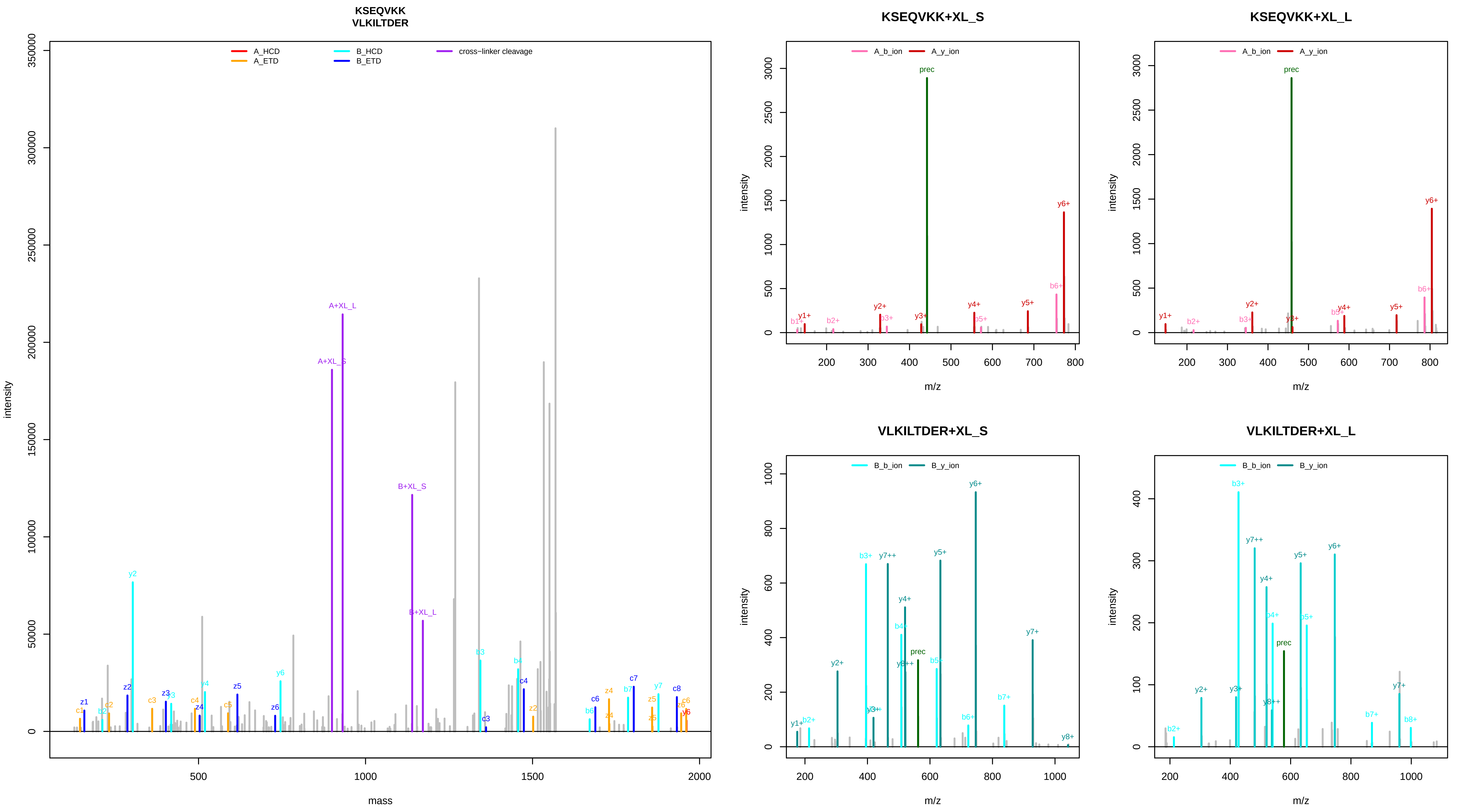
KSEQVKK
VLKILTDER
A_HCD
A_ETD
B_HCD
B_ETD
cross−linker cleavage
A+XL_L
A+XL_S
B+XL_S
B+XL_L
intensity
mass
KSEQVKK+XL_S
KSEQVKK+XL_L
A_b_ion
A_y_ion
prec
VLKILTDER+XL_S
VLKILTDER+XL_L
B_b_ion
B_y_ion
m/z

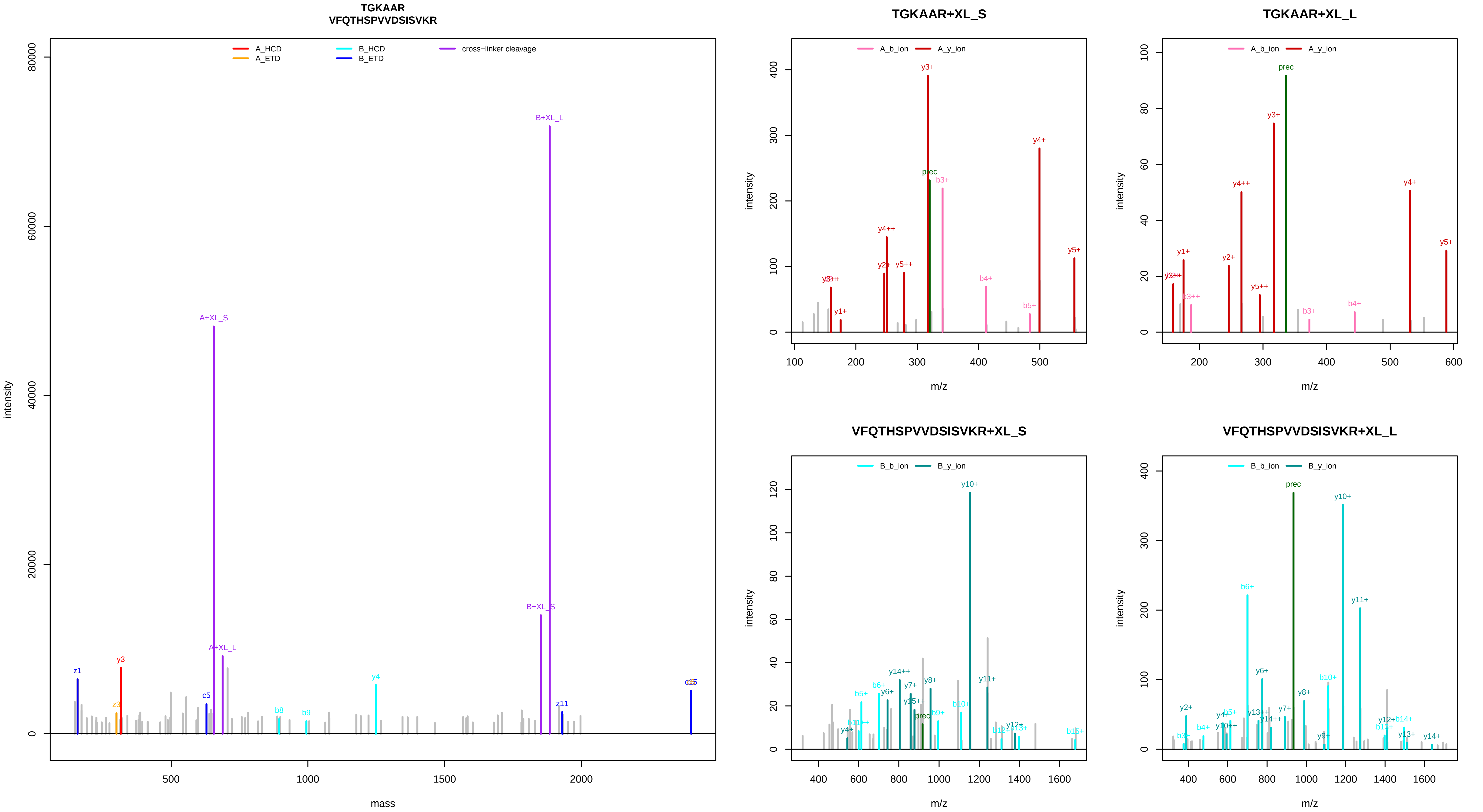
TGKAAR
VFQTHSPVVDSISVKR
A_HCD
A_ETD
B_HCD
B_ETD
cross−linker cleavage
B+XL_L
A+XL_S
B+XL_S
A+XL_L
z1
y3
z3
c5
b8
b9
y4
z11
c15
intensity
mass
TGKAAR+XL_S
A_b_ion
A_y_ion
y3+
y4+
prec
b3+
y4++
y5+
y2+
y5++
b4+
b5+
y1+
intensity
m/z
TGKAAR+XL_L
A_b_ion
A_y_ion
prec
y3+
y4++
y4+
y5+
y1+
y2+
b3++
y5++
b4+
b3+
intensity
m/z
VFQTHSPVVDSISVKR+XL_S
B_b_ion
B_y_ion
y10+
y14++
y11+
y8+
y7+
b6+
b5+
y6+
y15++
prec
b10+
b9+
y4+
y12+
b15+
intensity
m/z
VFQTHSPVVDSISVKR+XL_L
B_b_ion
B_y_ion
prec
y10+
y11+
b6+
y6+
b10+
y8+
y2+
y7+
y13++
y14++
b5+
y4+
y10++
b4+
b3+
y12+
b14+
b13+
y13+
y9+
y14+
intensity
m/z

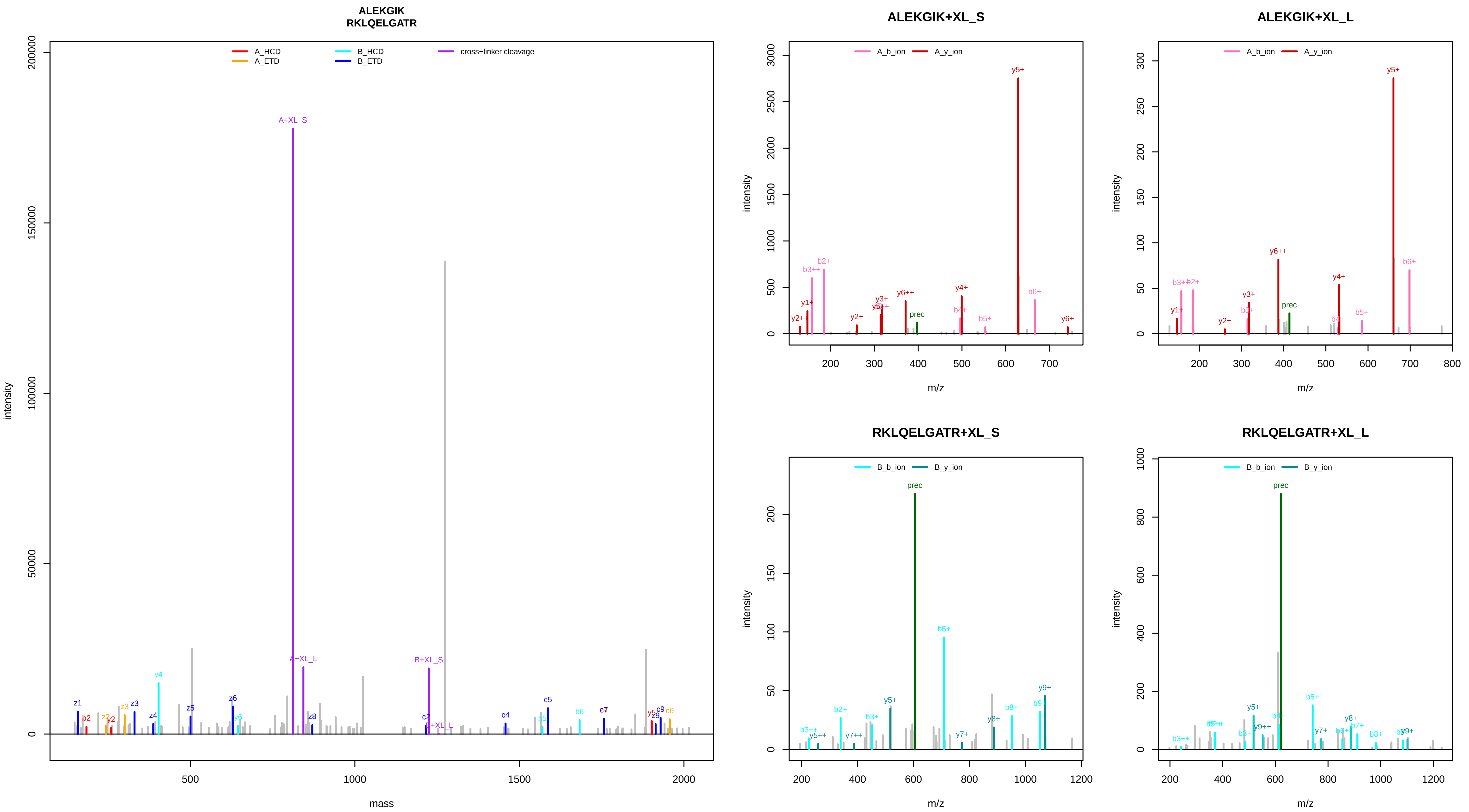
ALEKGIK
RKLQELGATR
A_HCD
A_ETD
B_HCD
B_ETD
cross−linker cleavage
A+XL_S
A+XL_L
B+XL_S
intensity
mass
ALEKGIK+XL_S
ALEKGIK+XL_L
RKLQELGATR+XL_S
RKLQELGATR+XL_L
A_b_ion
A_y_ion
B_b_ion
B_y_ion
y5+
b2+
b6+
y4+
y6++
prec
m/z
b5+
y9+

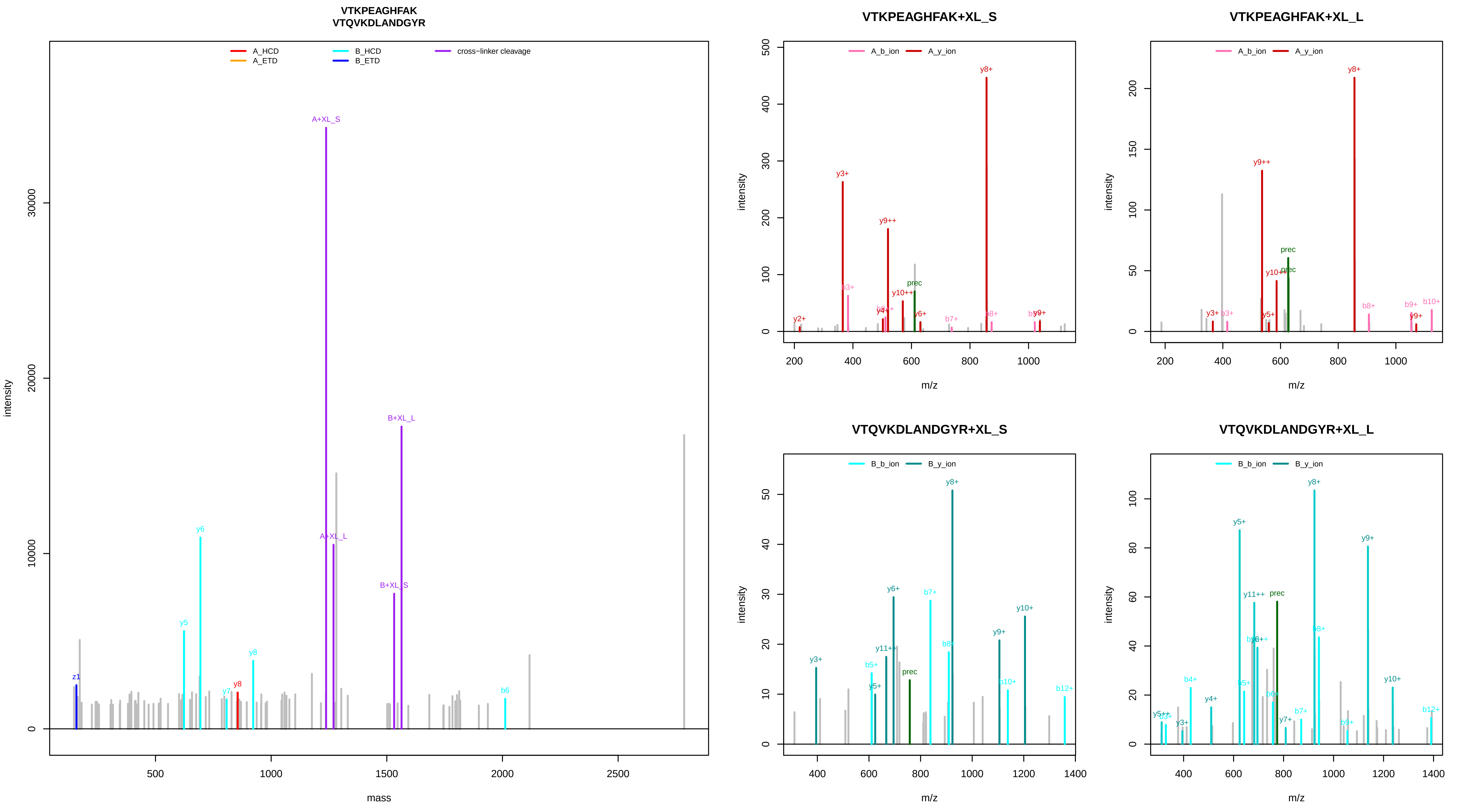

VTKPEAGHFAK
VTQVKDLANDGYR
A_HCD
A_ETD
B_HCD
B_ETD
cross−linker cleavage
A+XL_S
B+XL_L
A+XL_L
B+XL_S
intensity
mass
VTKPEAGHFAK+XL_S
VTKPEAGHFAK+XL_L
A_b_ion
A_y_ion
VTQVKDLANDGYR+XL_S
VTQVKDLANDGYR+XL_L
B_b_ion
B_y_ion
m/z

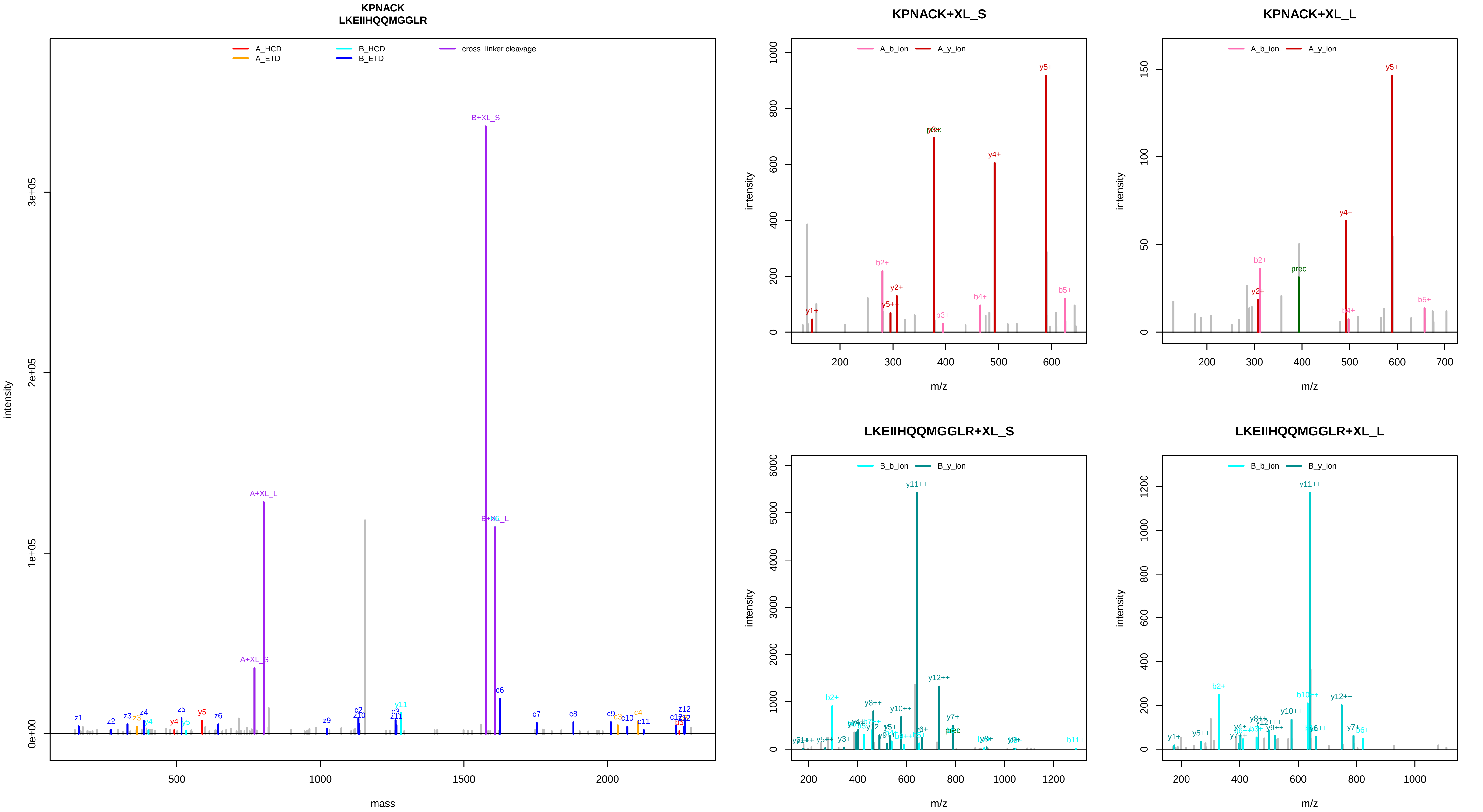

KPNACK
LKEIIHQQMGGLR
A_HCD
A_ETD
B_HCD
B_ETD
cross−linker cleavage
B+XL_S
A+XL_L
B+XL_L
A+XL_S
intensity
mass
0e+00
1e+05
2e+05
3e+05
500
1000
1500
2000
KPNACK+XL_S
KPNACK+XL_L
LKEIIHQQMGGLR+XL_S
LKEIIHQQMGGLR+XL_L
A_b_ion
A_y_ion
B_b_ion
B_y_ion
m/z
y5+
y4+
y2+
b2+
b4+
b5+
b3+
y1+
prec
y11++
y12++
y10++
y8++
y7+
b11+
b10++

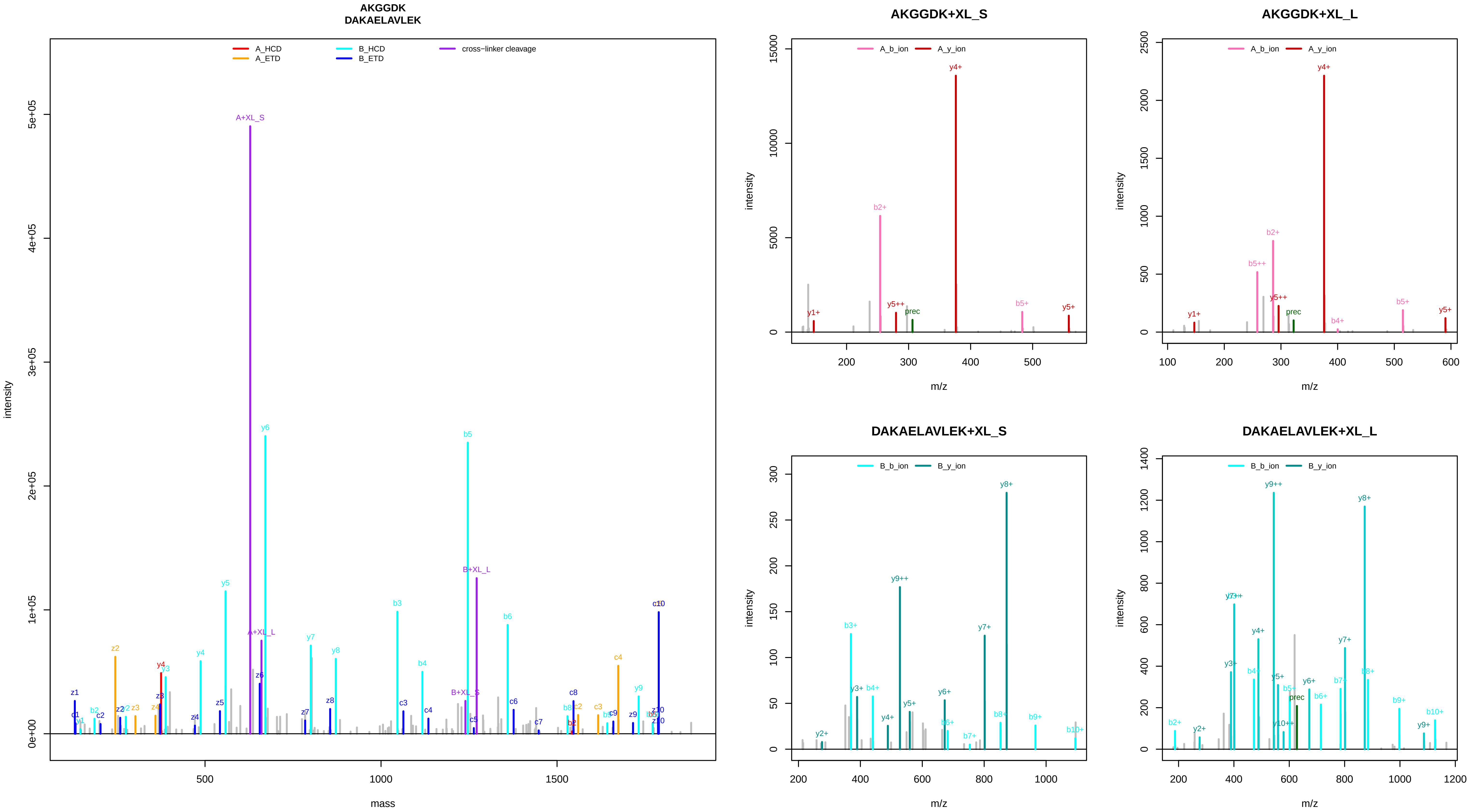

AKGGDK
DAKAELAVLEK
A_HCD
A_ETD
B_HCD
B_ETD
cross−linker cleavage
A+XL_S
B+XL_L
A+XL_L
B+XL_S
intensity
mass
AKGGDK+XL_S
AKGGDK+XL_L
A_b_ion
A_y_ion
DAKAELAVLEK+XL_S
DAKAELAVLEK+XL_L
B_b_ion
B_y_ion
m/z
prec

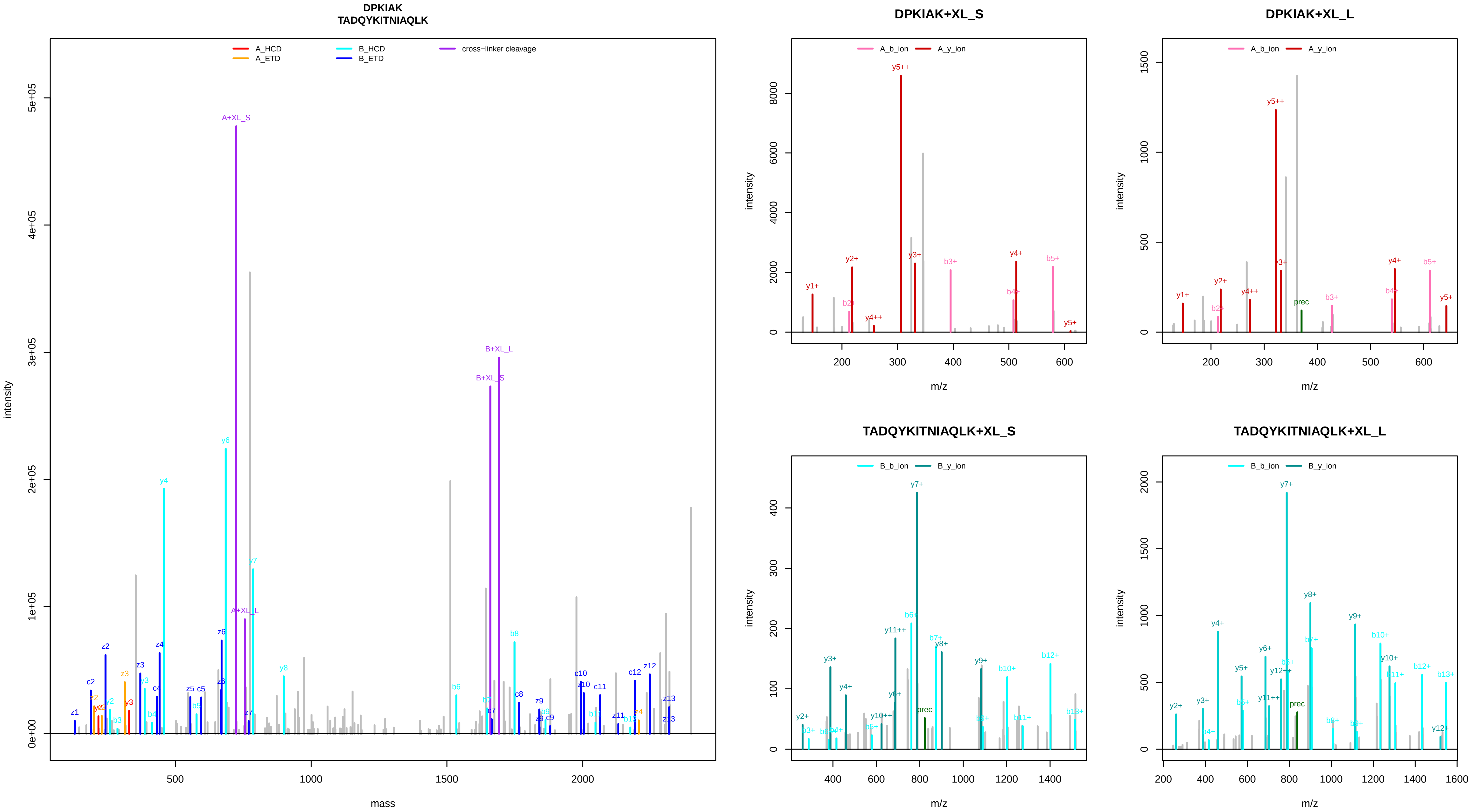
DPKIAK
TADQYKITNIAQLK
A_HCD
A_ETD
B_HCD
B_ETD
cross−linker cleavage
A+XL_S
B+XL_L
B+XL_S
A+XL_L
intensity
mass
DPKIAK+XL_S
DPKIAK+XL_L
TADQYKITNIAQLK+XL_S
TADQYKITNIAQLK+XL_L
A_b_ion
A_y_ion
B_b_ion
B_y_ion
m/z
prec

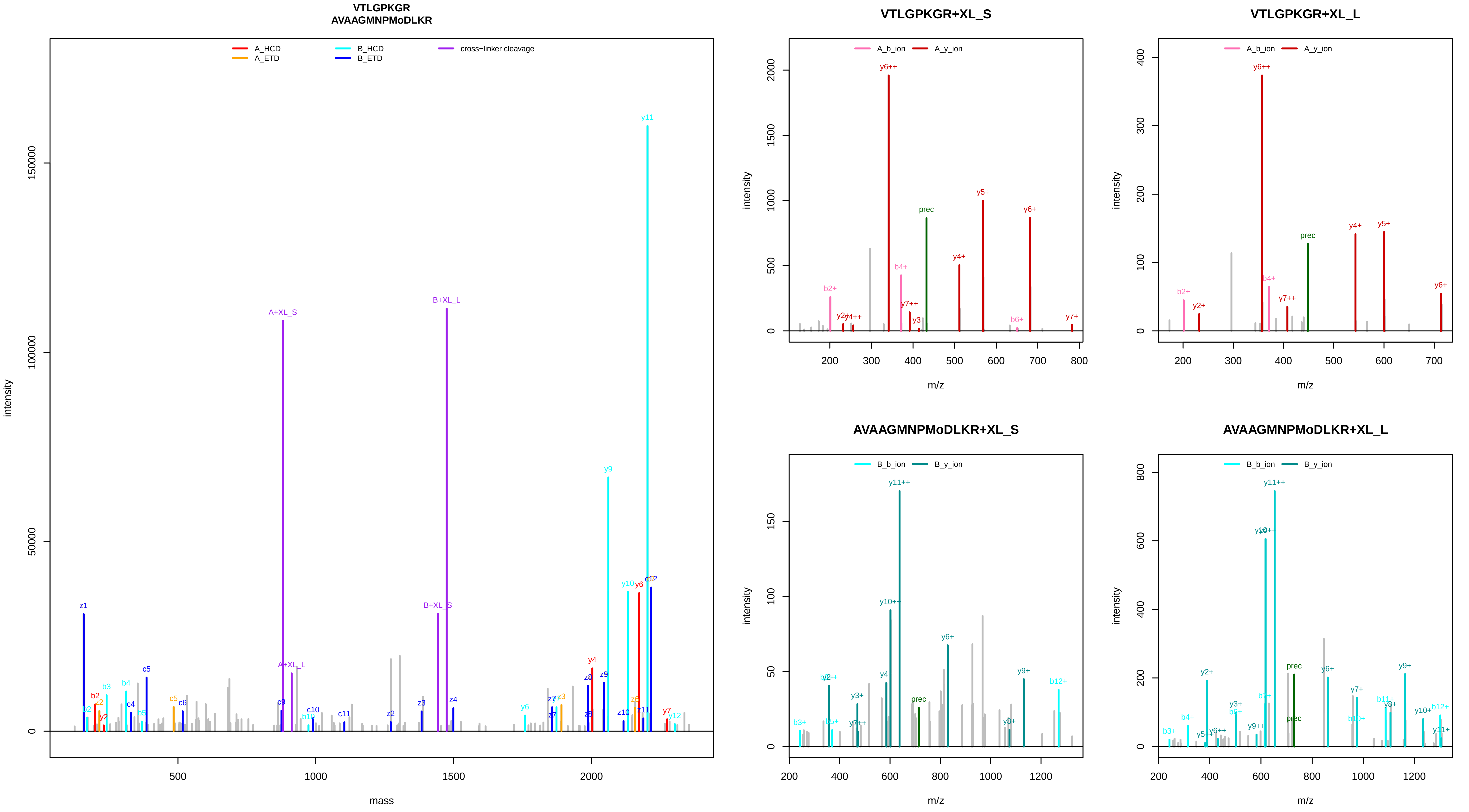

VTLGPKGR
AVAAGMNPMoDLKR
A_HCD
A_ETD
B_HCD
B_ETD
cross−linker cleavage
intensity
mass
0
50000
100000
150000
500
1000
1500
2000
y11
A+XL_S
B+XL_L
B+XL_S
A+XL_L
y9
y10
y6
c12
z1
c5
b3
b4
c4
b2
z2
y2
c5
c6
c9
c10
b10
c11
z2
z3
z4
y6
z7
z3
z8
z9
y4
z10
z11
y7
y12
VTLGPKGR+XL_S
VTLGPKGR+XL_L
A_b_ion
A_y_ion
intensity
m/z
0
500
1000
1500
2000
200
300
400
500
600
700
800
y6++
prec
y5+
y6+
y4+
b4+
b2+
y7++
y3+
b6+
y7+
0
100
200
300
400
y4+
y5+
y2+
AVAAGMNPMoDLKR+XL_S
AVAAGMNPMoDLKR+XL_L
B_b_ion
B_y_ion
0
50
100
150
200
400
600
800
1000
1200
y11++
y10++
y6+
y9+
b12+
y4+
y3+
b3+
b5+
y7++
y8+
0
200
400
600
800
y2+
y6+
y9+
y7+
b4+
b3+
y3+
y10+
b12+
y11+
b11+
b10+

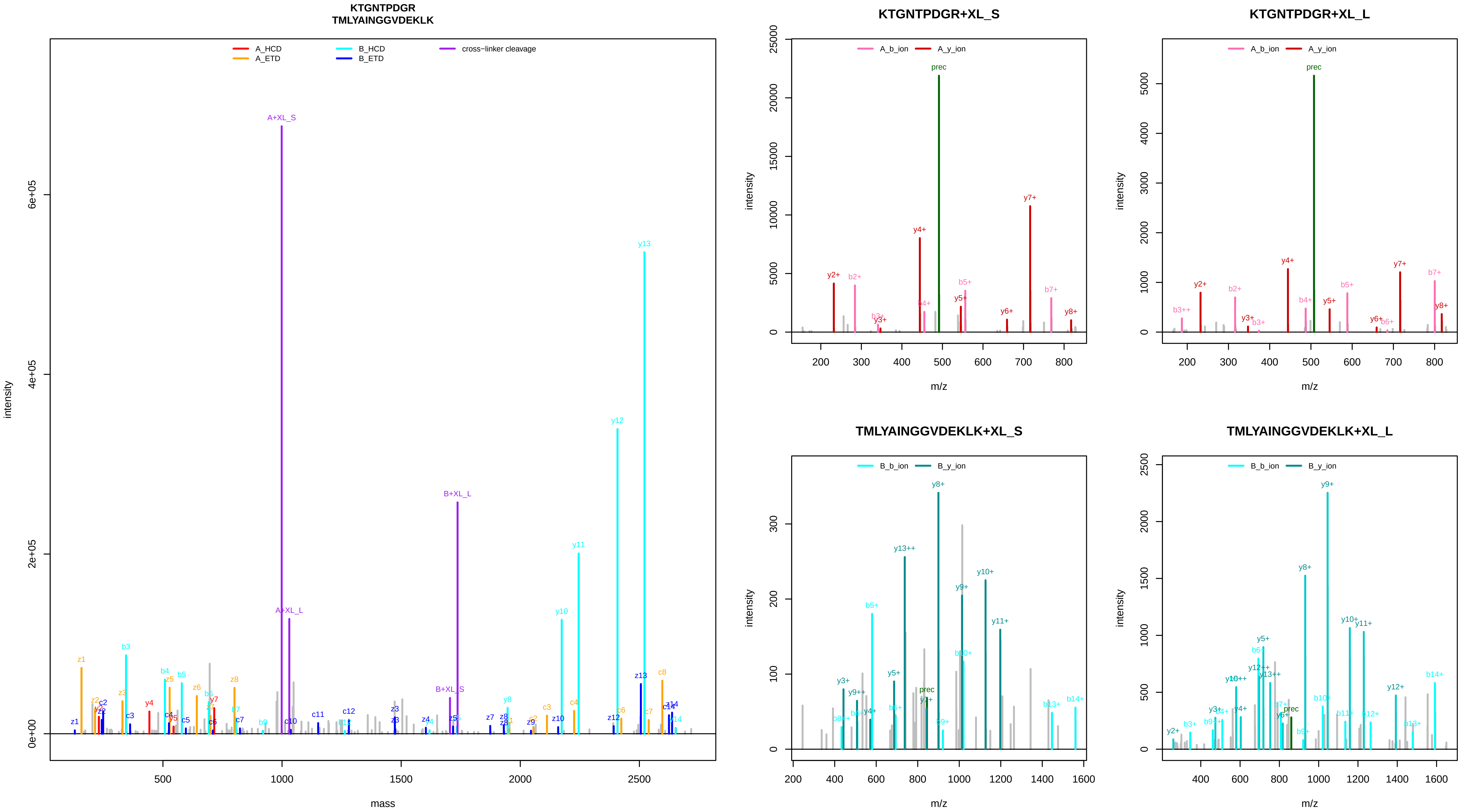
KTGNTPDGR
TMLYAINGGVDEKLK
A_HCD
A_ETD
B_HCD
B_ETD
cross−linker cleavage
A+XL_S
A+XL_L
B+XL_L
B+XL_S
intensity
mass
KTGNTPDGR+XL_S
KTGNTPDGR+XL_L
TMLYAINGGVDEKLK+XL_S
TMLYAINGGVDEKLK+XL_L
A_b_ion
A_y_ion
B_b_ion
B_y_ion
prec
m/z

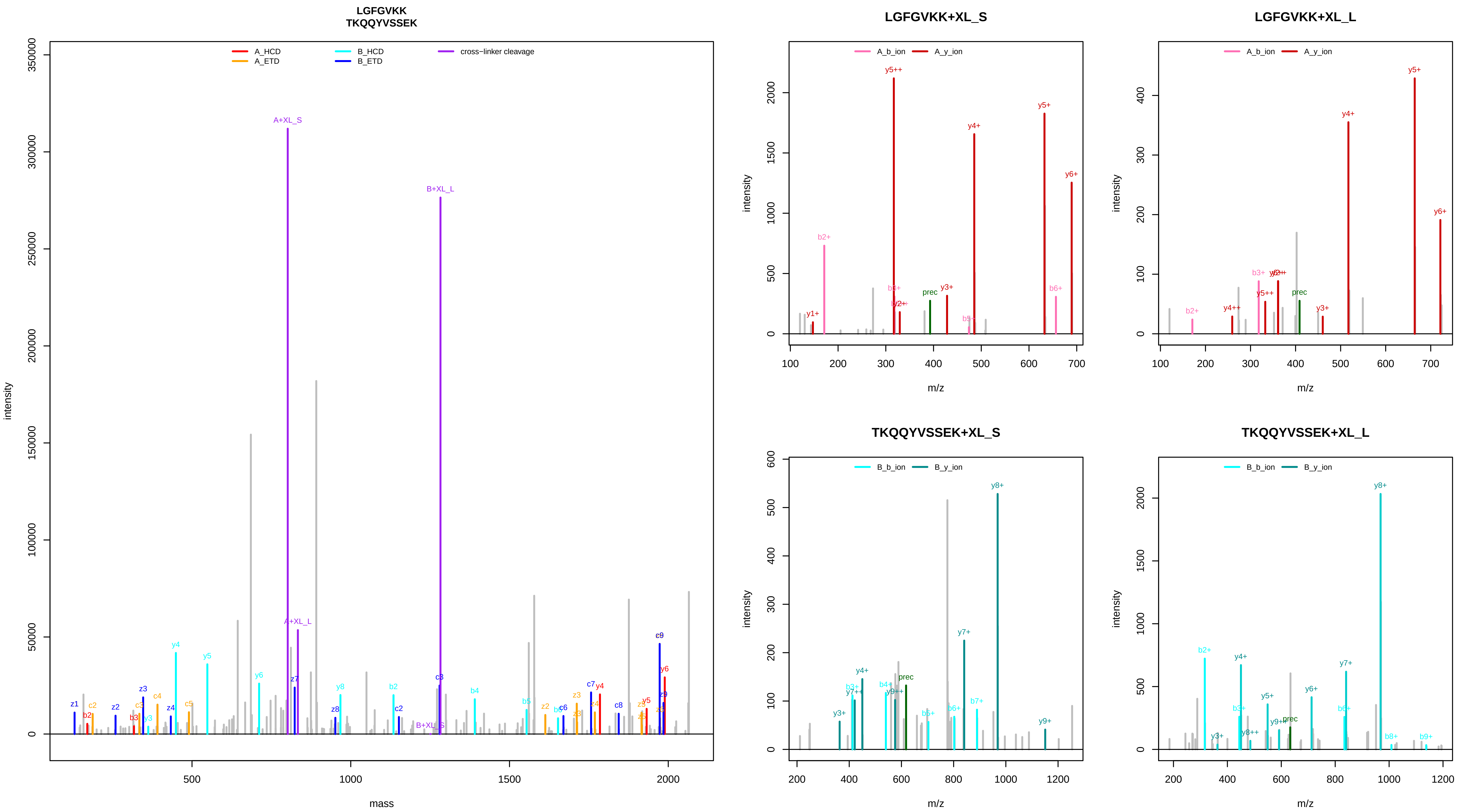

LGFGVKK
TKQQYVSSEK
A_HCD
A_ETD
B_HCD
B_ETD
cross−linker cleavage
A+XL_S
B+XL_L
A+XL_L
B+XL_S
intensity
mass
LGFGVKK+XL_S
LGFGVKK+XL_L
TKQQYVSSEK+XL_S
TKQQYVSSEK+XL_L
A_b_ion
A_y_ion
B_b_ion
B_y_ion
prec
m/z

MQAFSDKGK
GDKITYLVK

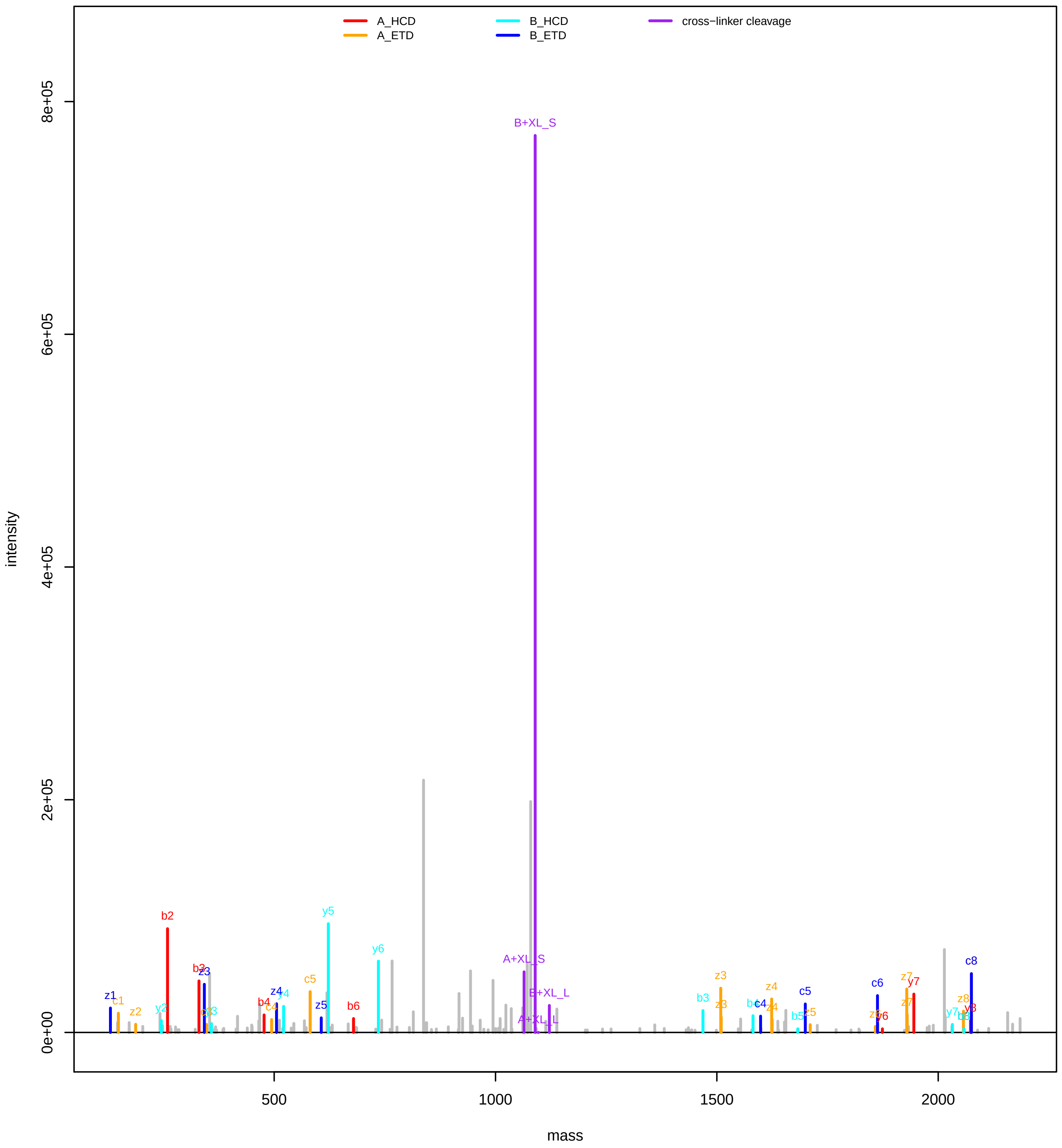

MQAFSDKGK+XL_S

MQAFSDKGK+XL_L

GDKITYLVK+XL_S

GDKITYLVK+XL_L

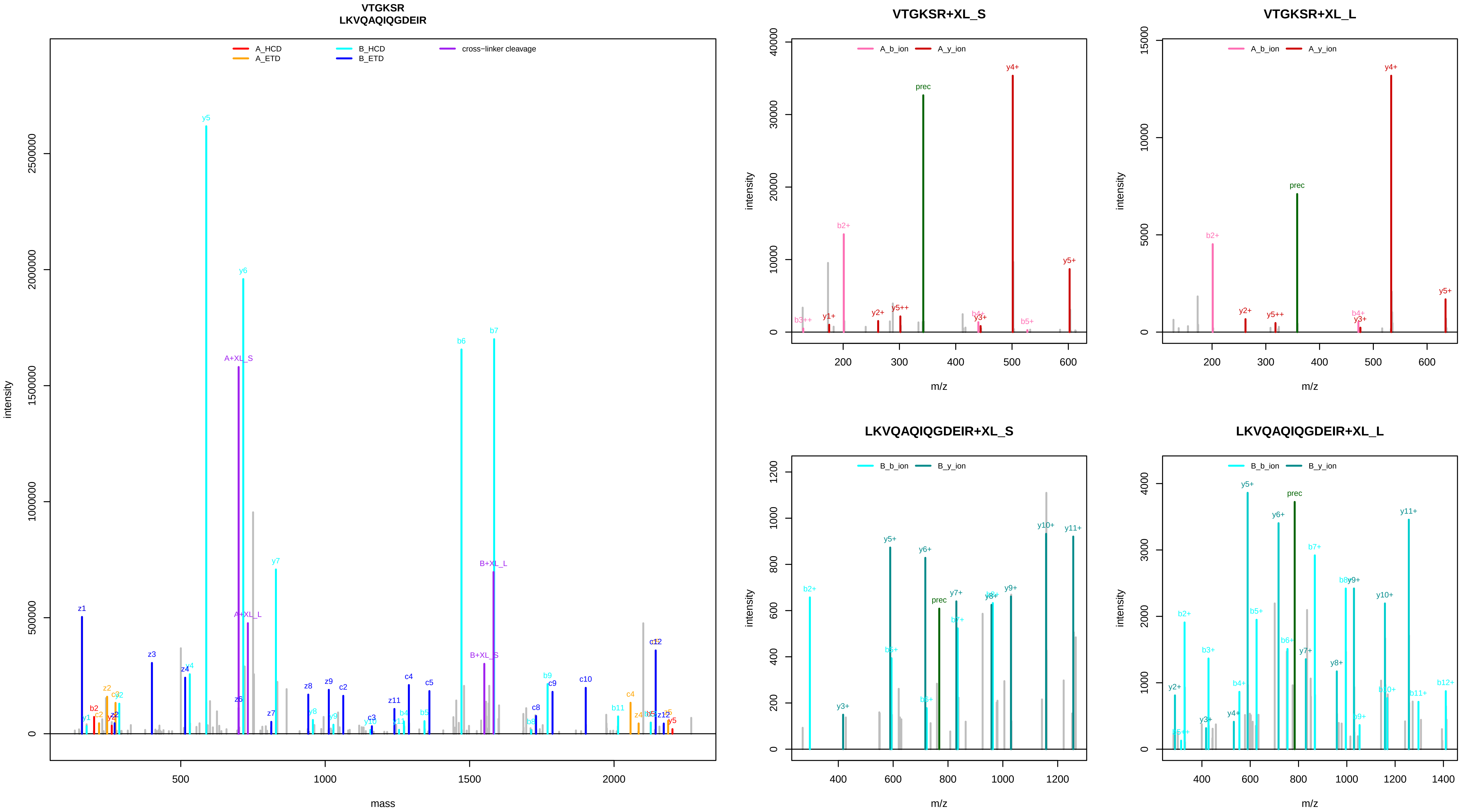

VTGKSR
LKVQAQIQGDEIR
A_HCD
A_ETD
B_HCD
B_ETD
cross−linker cleavage
intensity
mass
VTGKSR+XL_S
VTGKSR+XL_L
LKVQAQIQGDEIR+XL_S
LKVQAQIQGDEIR+XL_L
A_b_ion
A_y_ion
B_b_ion
B_y_ion
intensity
m/z

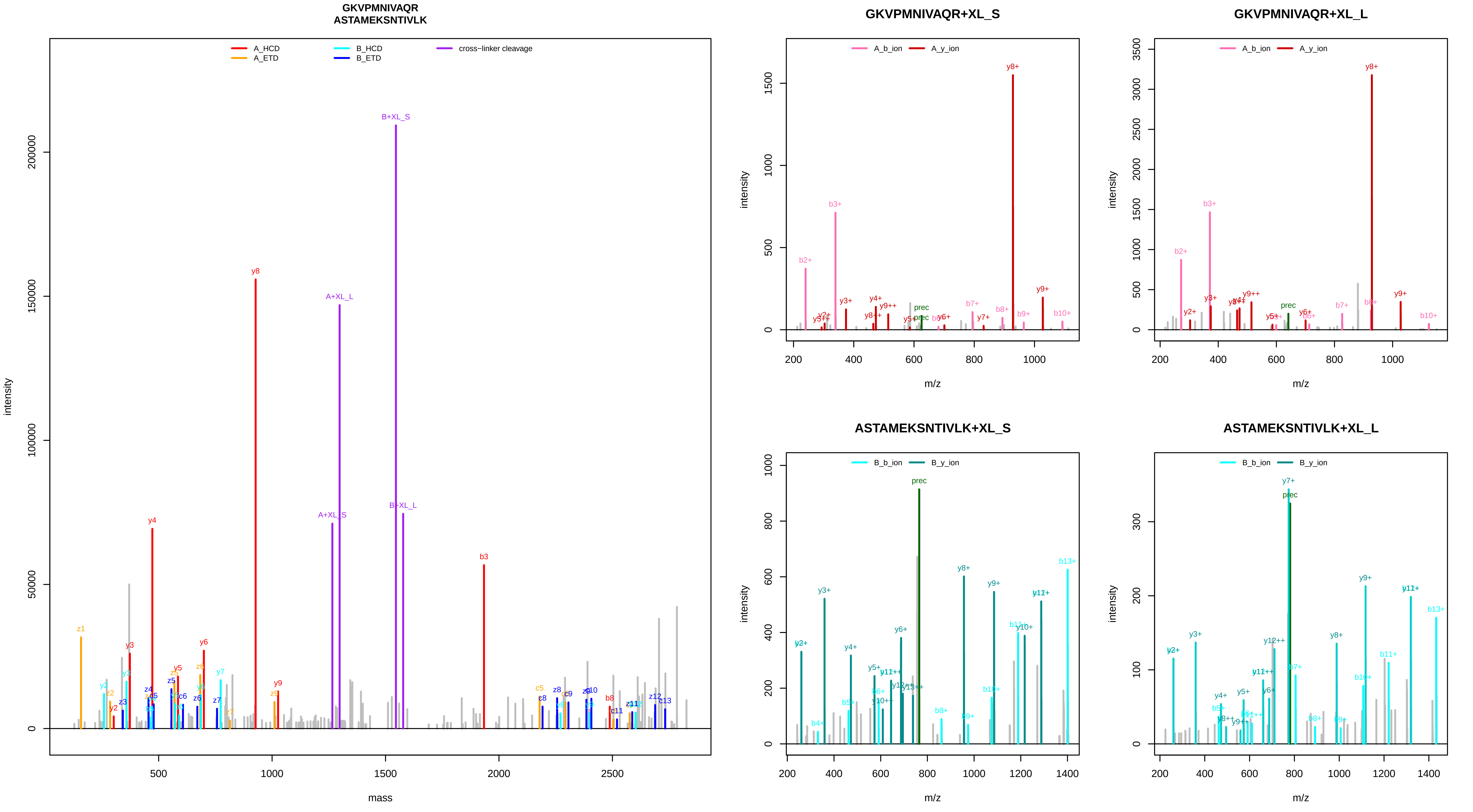
GKVPMNIVAQR
ASTAMEKSNTIVLK
A_HCD
A_ETD
B_HCD
B_ETD
cross−linker cleavage
B+XL_S
A+XL_L
A+XL_S
B+XL_L
y8
y4
b3
y3
y6
y5
y9
z1
intensity
mass
GKVPMNIVAQR+XL_S
GKVPMNIVAQR+XL_L
A_b_ion
A_y_ion
y8+
b3+
b2+
y9+
b10+
prec
m/z
ASTAMEKSNTIVLK+XL_S
ASTAMEKSNTIVLK+XL_L
B_b_ion
B_y_ion
y7+
y8+
y9+
y3+
b13+
y11+

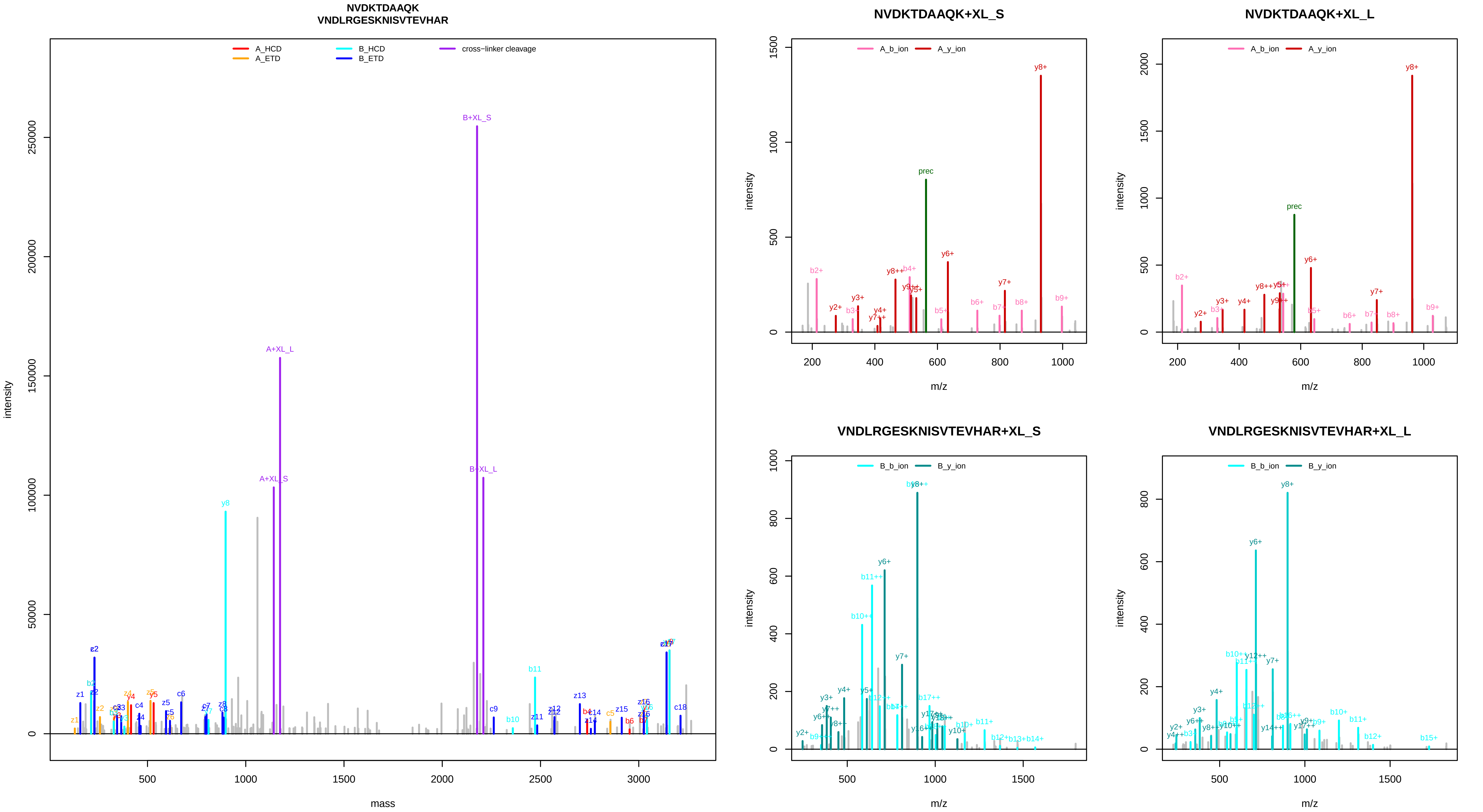
NVDKTDAAQK
VNDLRGESKNISVTEVHAR
A_HCD
A_ETD
B_HCD
B_ETD
cross−linker cleavage
B+XL_S
A+XL_L
B+XL_L
A+XL_S
intensity
mass
NVDKTDAAQK+XL_S
NVDKTDAAQK+XL_L
A_b_ion
A_y_ion
prec
VNDLRGESKNISVTEVHAR+XL_S
VNDLRGESKNISVTEVHAR+XL_L
B_b_ion
B_y_ion
m/z

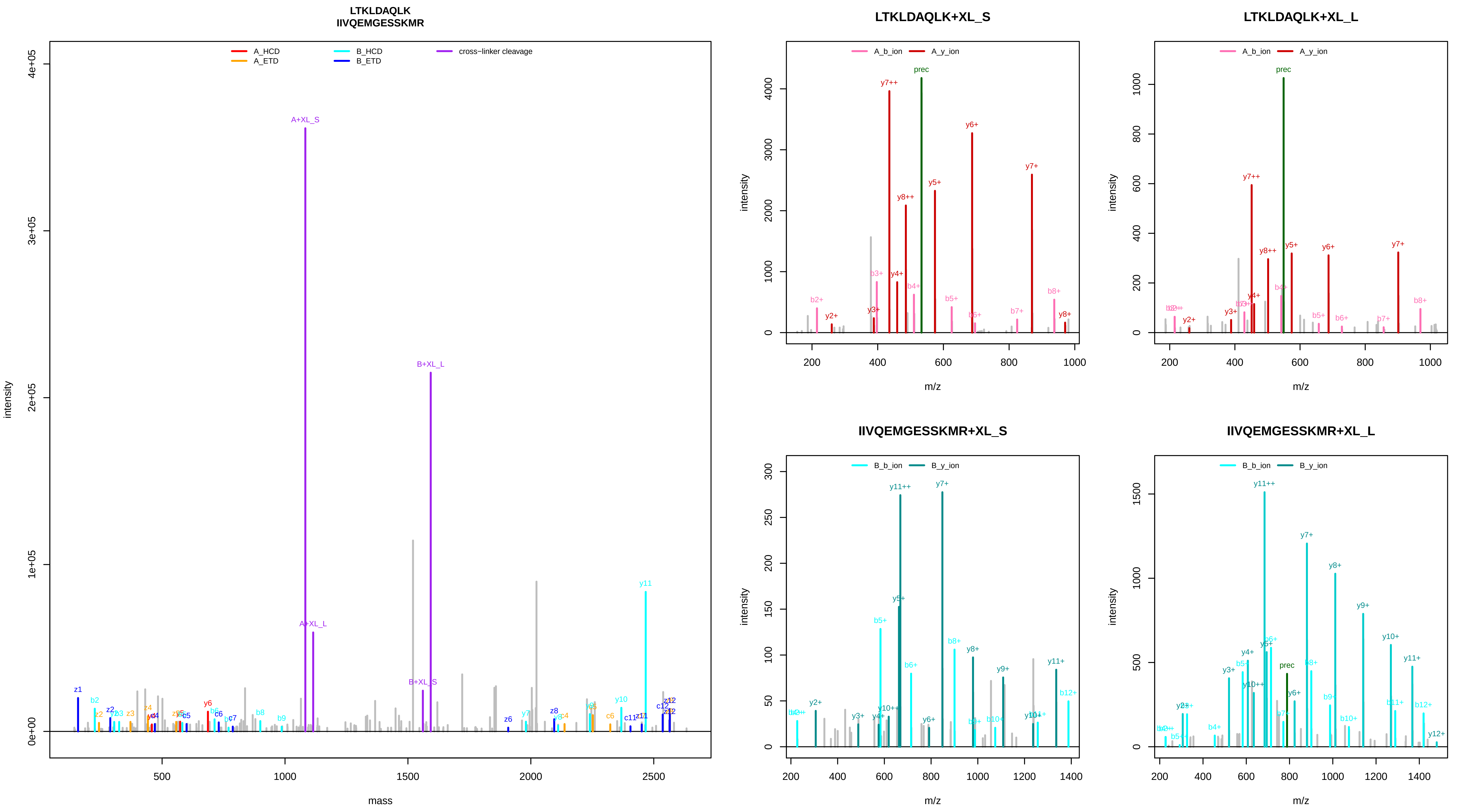

LTKLDAQLK
IIVQEMGESSKMR
A_HCD
A_ETD
B_HCD
B_ETD
cross−linker cleavage
A+XL_S
B+XL_L
A+XL_L
B+XL_S
intensity
mass
LTKLDAQLK+XL_S
LTKLDAQLK+XL_L
A_b_ion
A_y_ion
prec
IIVQEMGESSKMR+XL_S
IIVQEMGESSKMR+XL_L
B_b_ion
B_y_ion
m/z

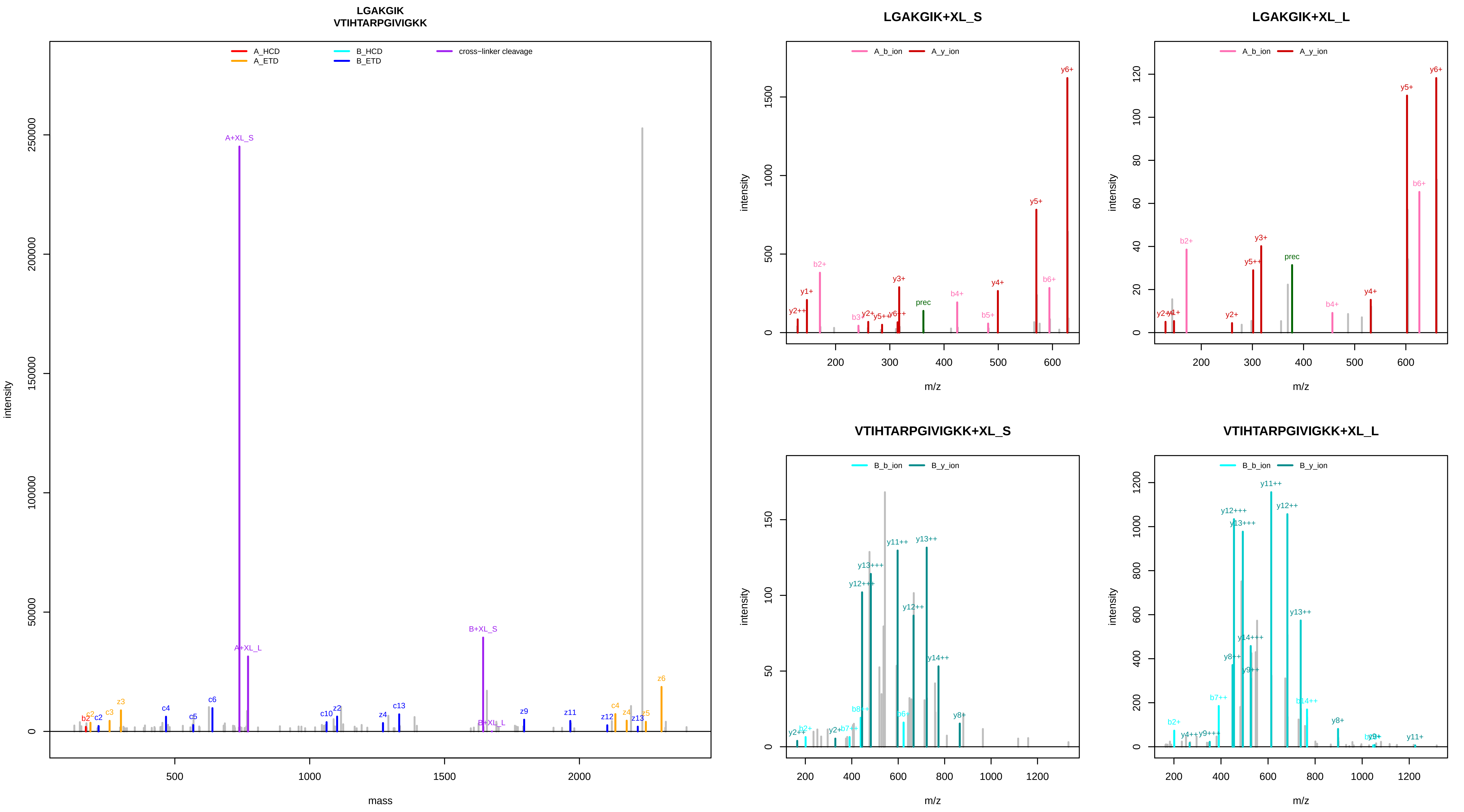
LGAKGIK
VTIHTARPGIVIGKK
A_HCD
A_ETD
B_HCD
B_ETD
cross−linker cleavage
A+XL_S
A+XL_L
B+XL_S
B+XL_L
intensity
mass
LGAKGIK+XL_S
LGAKGIK+XL_L
A_b_ion
A_y_ion
prec
VTIHTARPGIVIGKK+XL_S
VTIHTARPGIVIGKK+XL_L
B_b_ion
B_y_ion
m/z

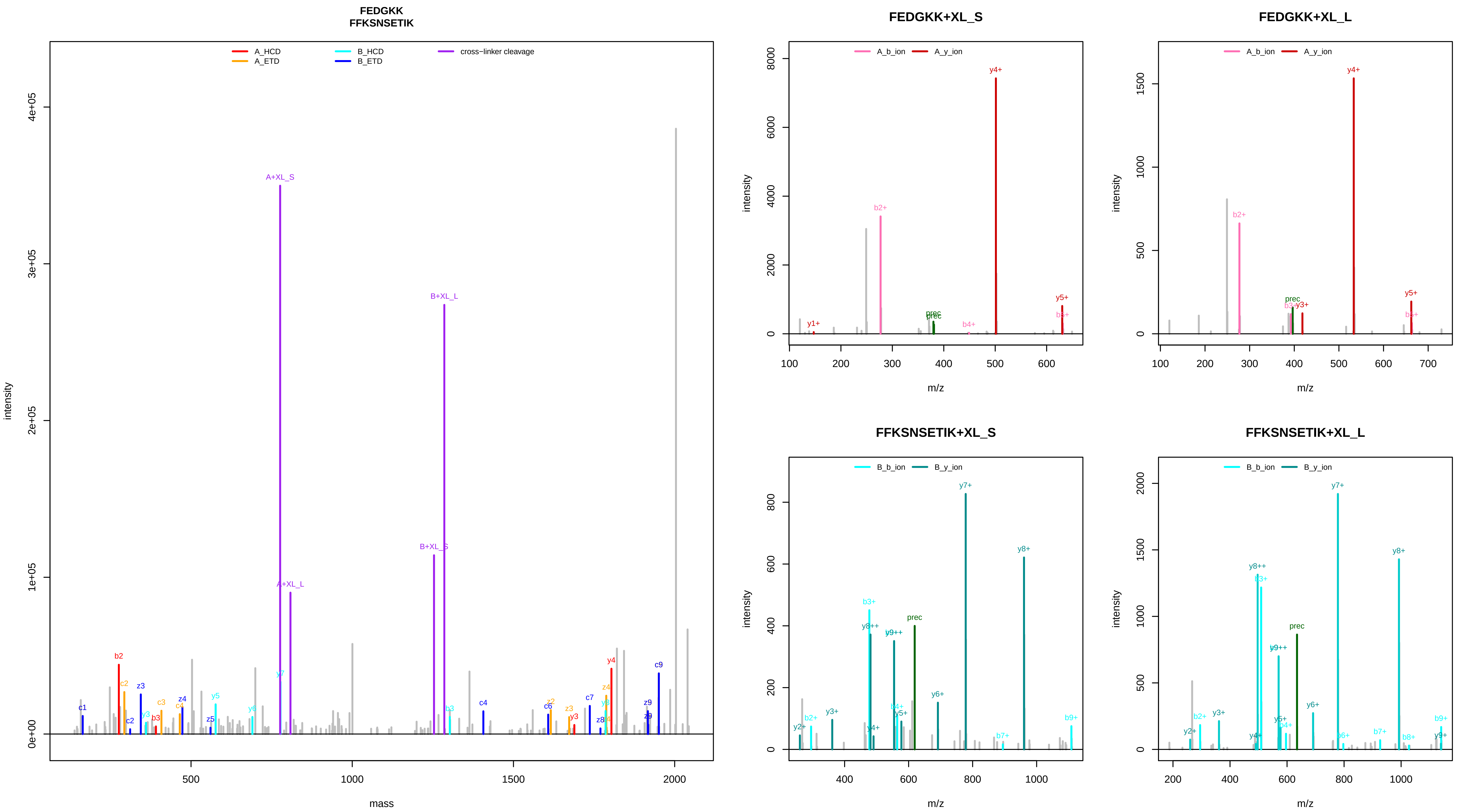

FEDGKK
FFKSNSETIK
A_HCD
A_ETD
B_HCD
B_ETD
cross−linker cleavage
A+XL_S
B+XL_L
B+XL_S
A+XL_L
intensity
mass
FEDGKK+XL_S
FEDGKK+XL_L
FFKSNSETIK+XL_S
FFKSNSETIK+XL_L
A_b_ion
A_y_ion
B_b_ion
B_y_ion
m/z
prec
y4+
y5+
b2+
y7+
y8+

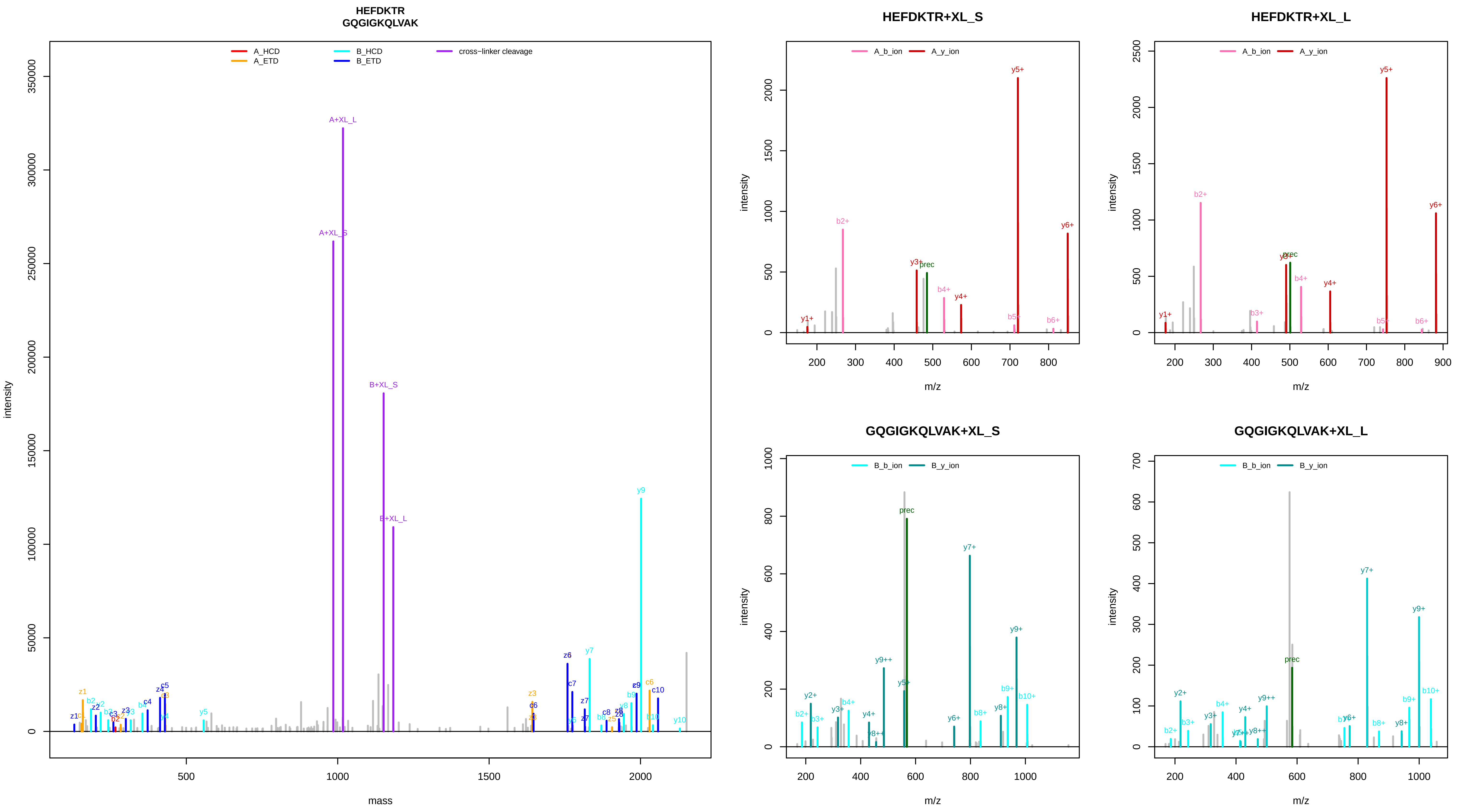

HEFDKTR
GQGIGKQLVAK
A_HCD
A_ETD
B_HCD
B_ETD
cross−linker cleavage
A+XL_L
A+XL_S
B+XL_S
B+XL_L
intensity
mass
HEFDKTR+XL_S
HEFDKTR+XL_L
A_b_ion
A_y_ion
GQGIGKQLVAK+XL_S
GQGIGKQLVAK+XL_L
B_b_ion
B_y_ion
prec
m/z

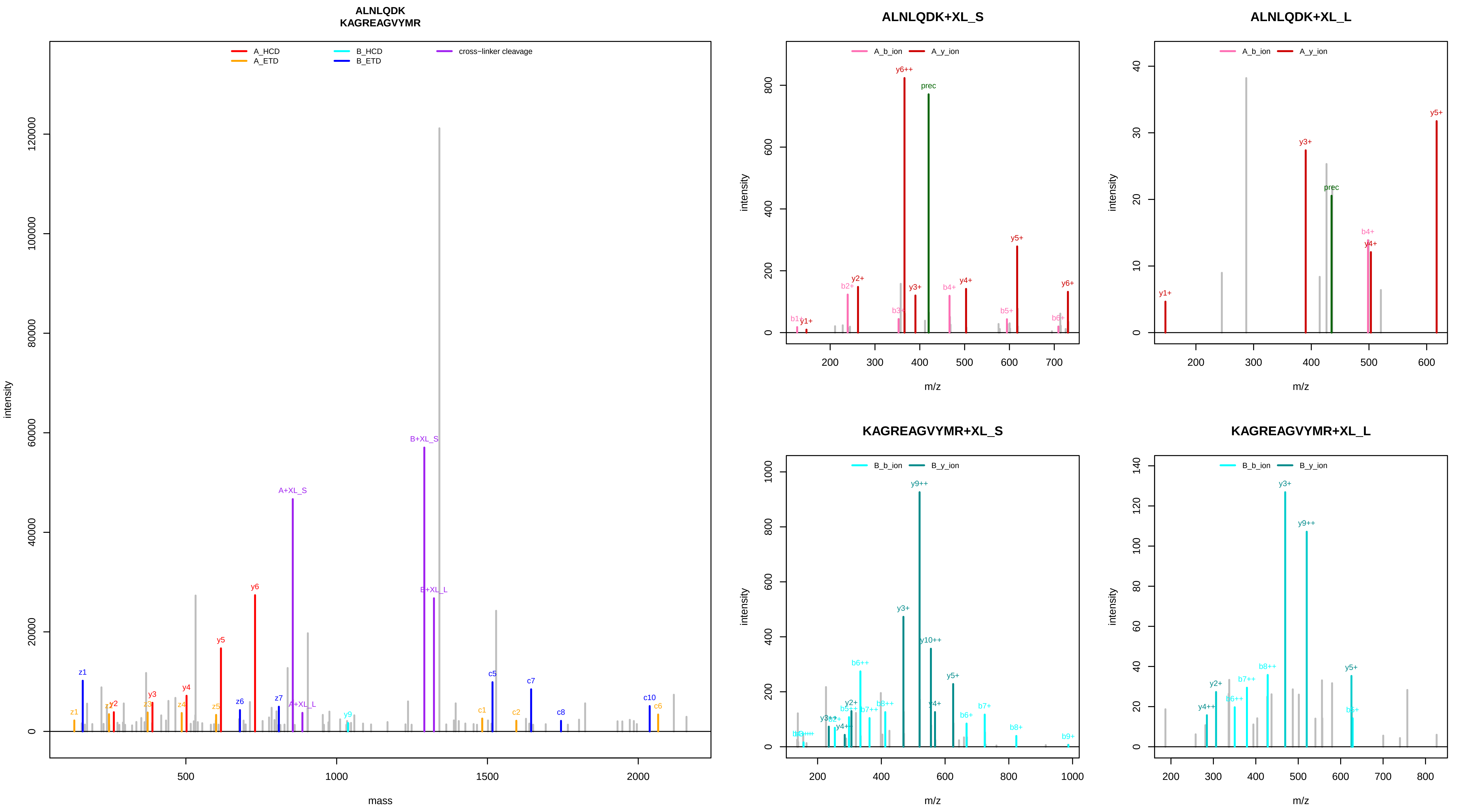
ALNLQDK
KAGREAGVYMR
A_HCD
A_ETD
B_HCD
B_ETD
cross−linker cleavage
B+XL_S
A+XL_S
B+XL_L
A+XL_L
intensity
mass
ALNLQDK+XL_S
A_b_ion
A_y_ion
intensity
m/z
ALNLQDK+XL_L
A_b_ion
A_y_ion
intensity
m/z
KAGREAGVYMR+XL_S
B_b_ion
B_y_ion
intensity
m/z
KAGREAGVYMR+XL_L
B_b_ion
B_y_ion
intensity
m/z

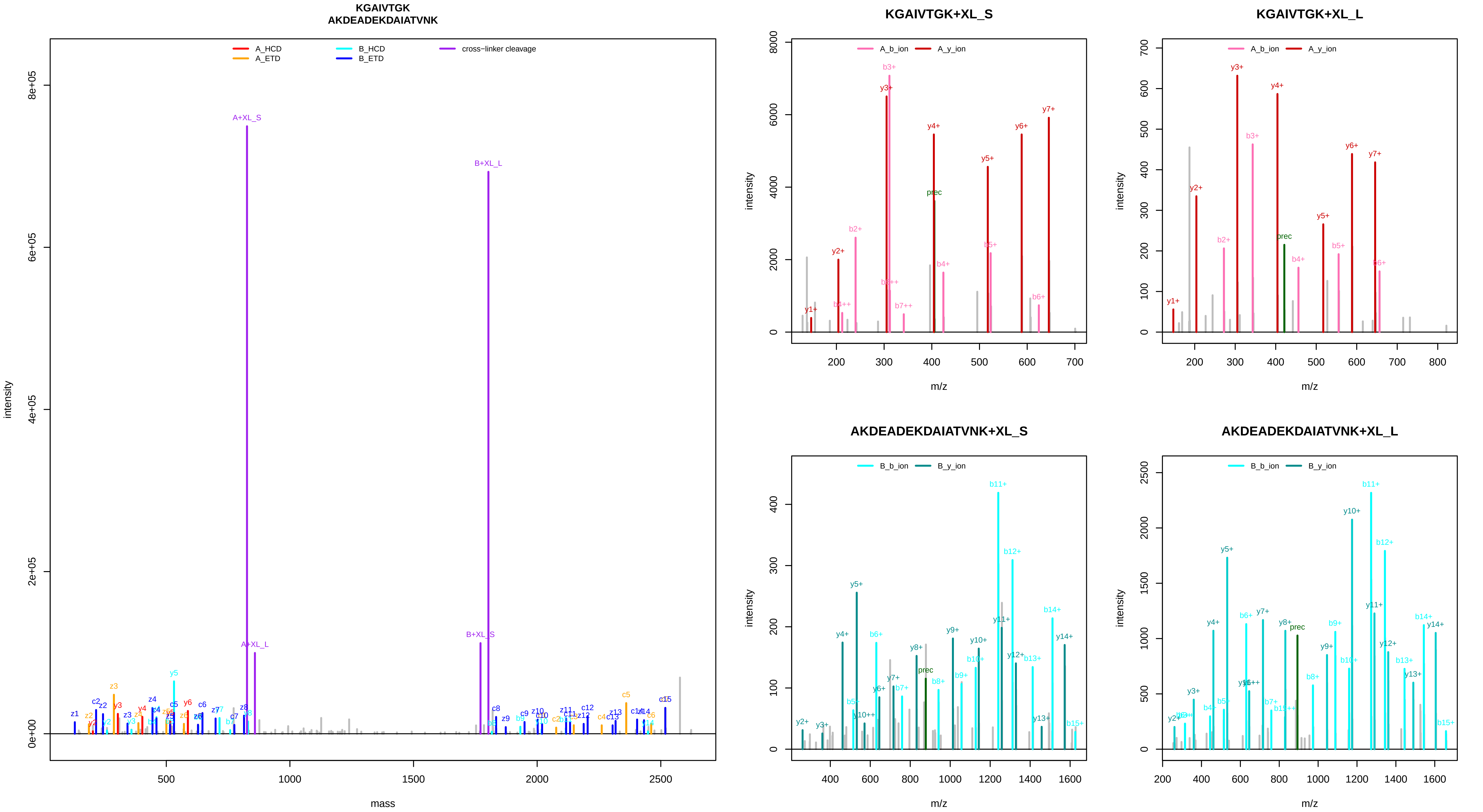
KGAIVTGK
AKDEADEKDAIATVNK
A_HCD
A_ETD
B_HCD
B_ETD
cross−linker cleavage
A+XL_S
B+XL_L
A+XL_L
B+XL_S
intensity
mass
KGAIVTGK+XL_S
KGAIVTGK+XL_L
AKDEADEKDAIATVNK+XL_S
AKDEADEKDAIATVNK+XL_L
A_b_ion
A_y_ion
B_b_ion
B_y_ion
prec
m/z

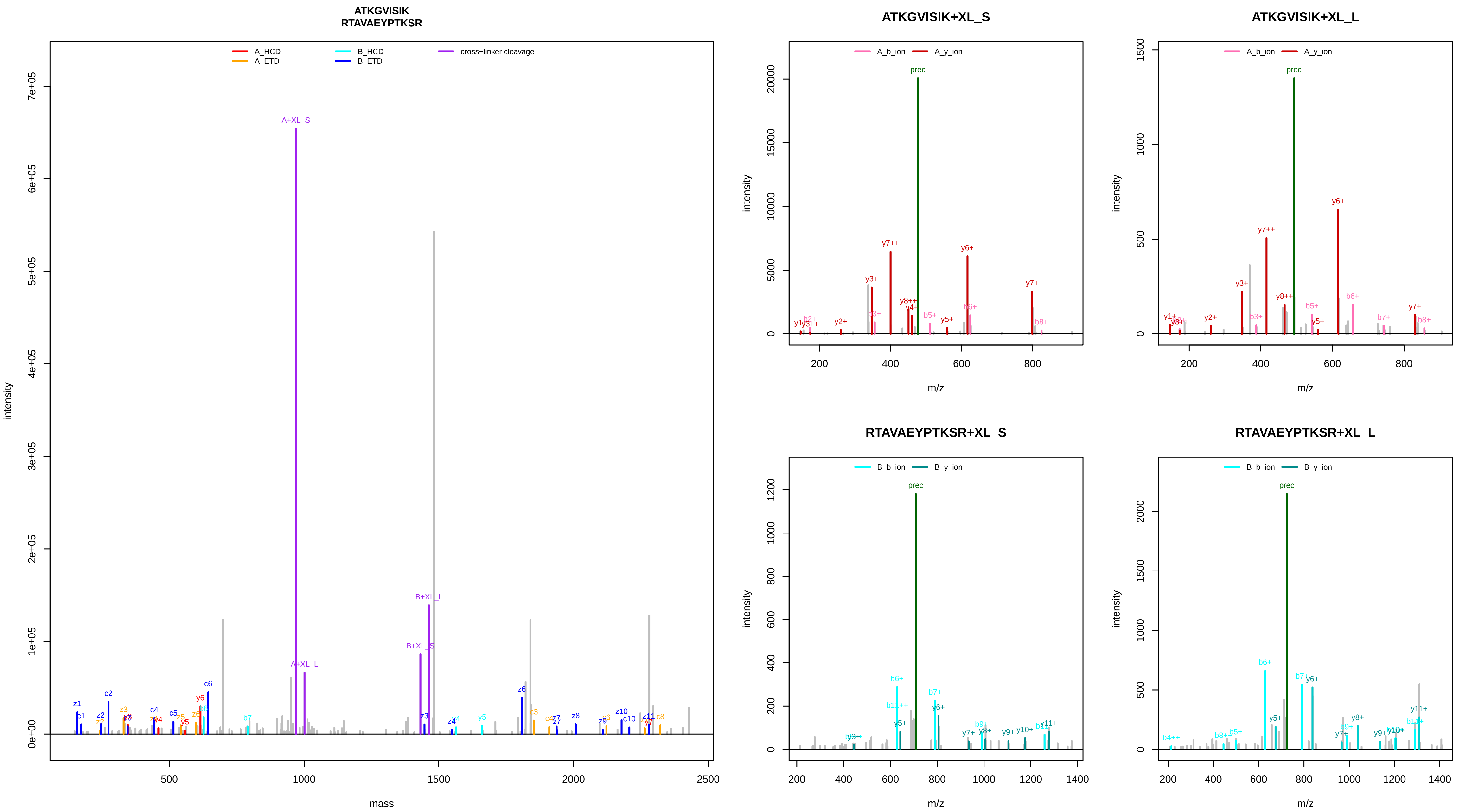
ATKGVISIK
RTAVAEYPTKSR
A_HCD
A_ETD
B_HCD
B_ETD
cross−linker cleavage
A+XL_S
A+XL_L
B+XL_L
B+XL_S
intensity
mass
ATKGVISIK+XL_S
ATKGVISIK+XL_L
A_b_ion
A_y_ion
prec
RTAVAEYPTKSR+XL_S
RTAVAEYPTKSR+XL_L
B_b_ion
B_y_ion
m/z

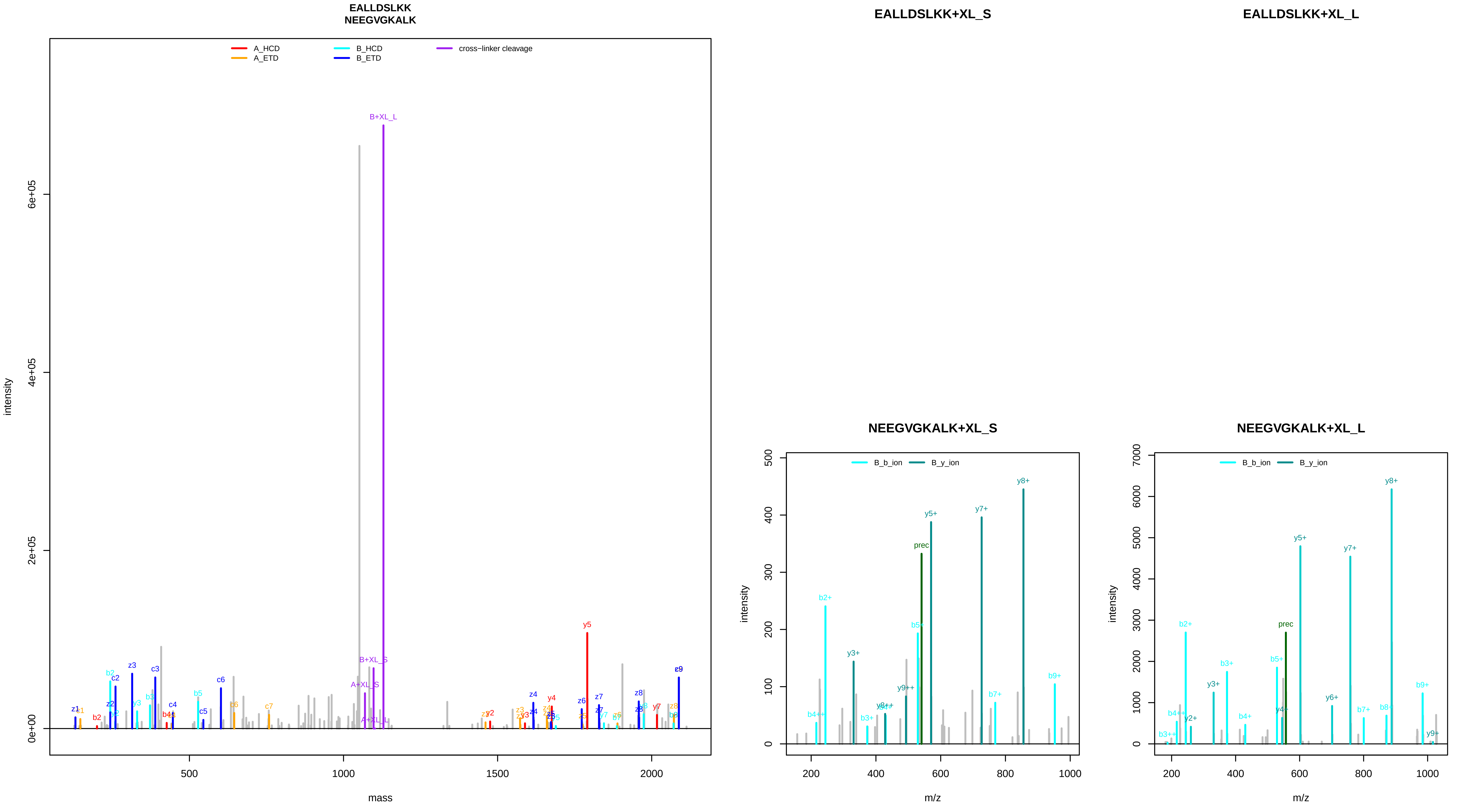
EALLDSLKK
NEEGVGKALK
A_HCD
A_ETD
B_HCD
B_ETD
cross−linker cleavage
B+XL_L
B+XL_S
A+XL_S
A+XL_L
y5
intensity
mass
EALLDSLKK+XL_S
EALLDSLKK+XL_L
NEEGVGKALK+XL_S
B_b_ion
B_y_ion
y8+
y7+
y5+
prec
b2+
b9+
intensity
m/z
NEEGVGKALK+XL_L
B_b_ion
B_y_ion
y8+
y7+
y5+
prec
b2+
intensity
m/z

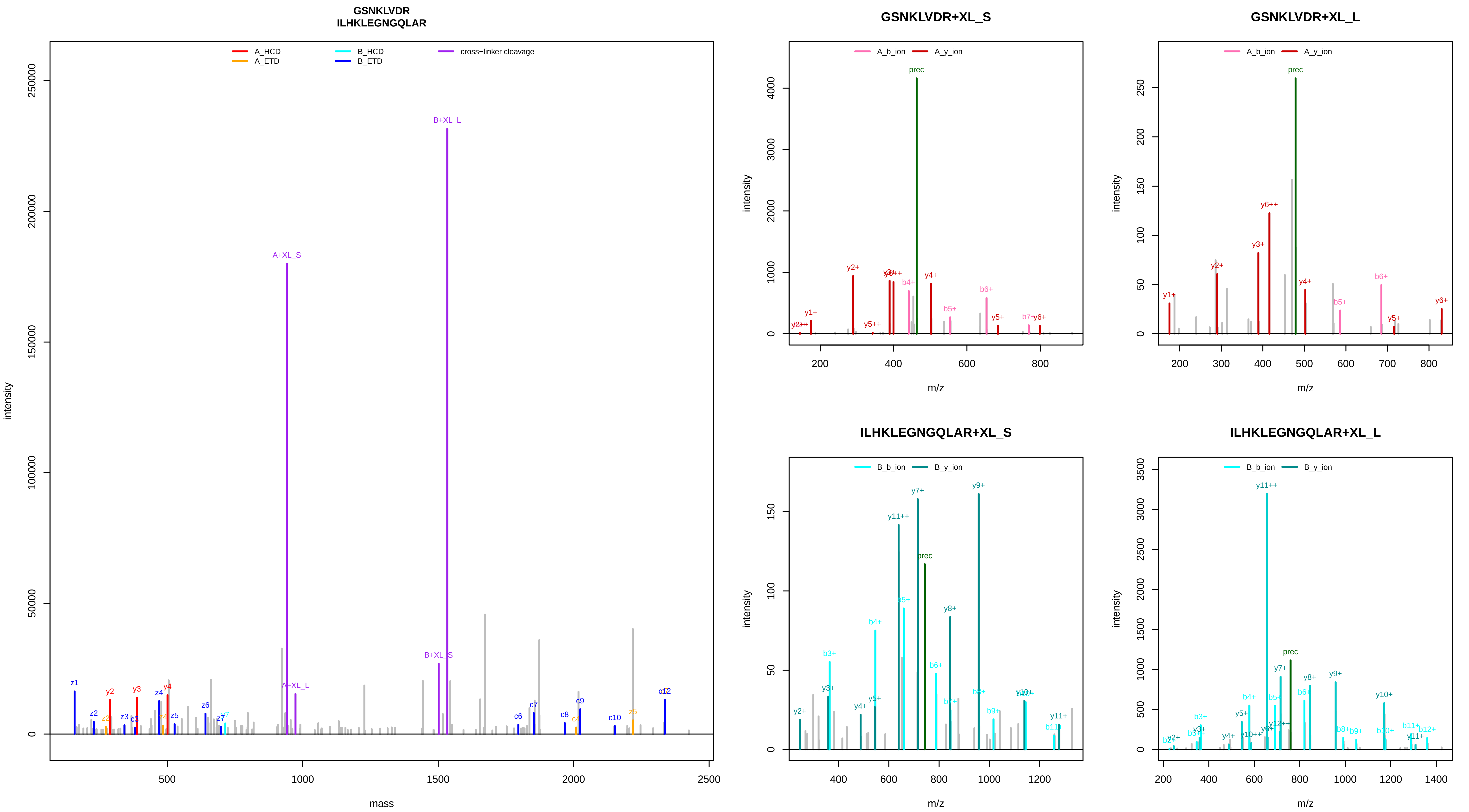
GSNKLVDR
ILHKLEGNGQLAR
A_HCD
A_ETD
B_HCD
B_ETD
cross−linker cleavage
B+XL_L
A+XL_S
B+XL_S
A+XL_L
intensity
mass
GSNKLVDR+XL_S
GSNKLVDR+XL_L
A_b_ion
A_y_ion
prec
ILHKLEGNGQLAR+XL_S
ILHKLEGNGQLAR+XL_L
B_b_ion
B_y_ion
m/z

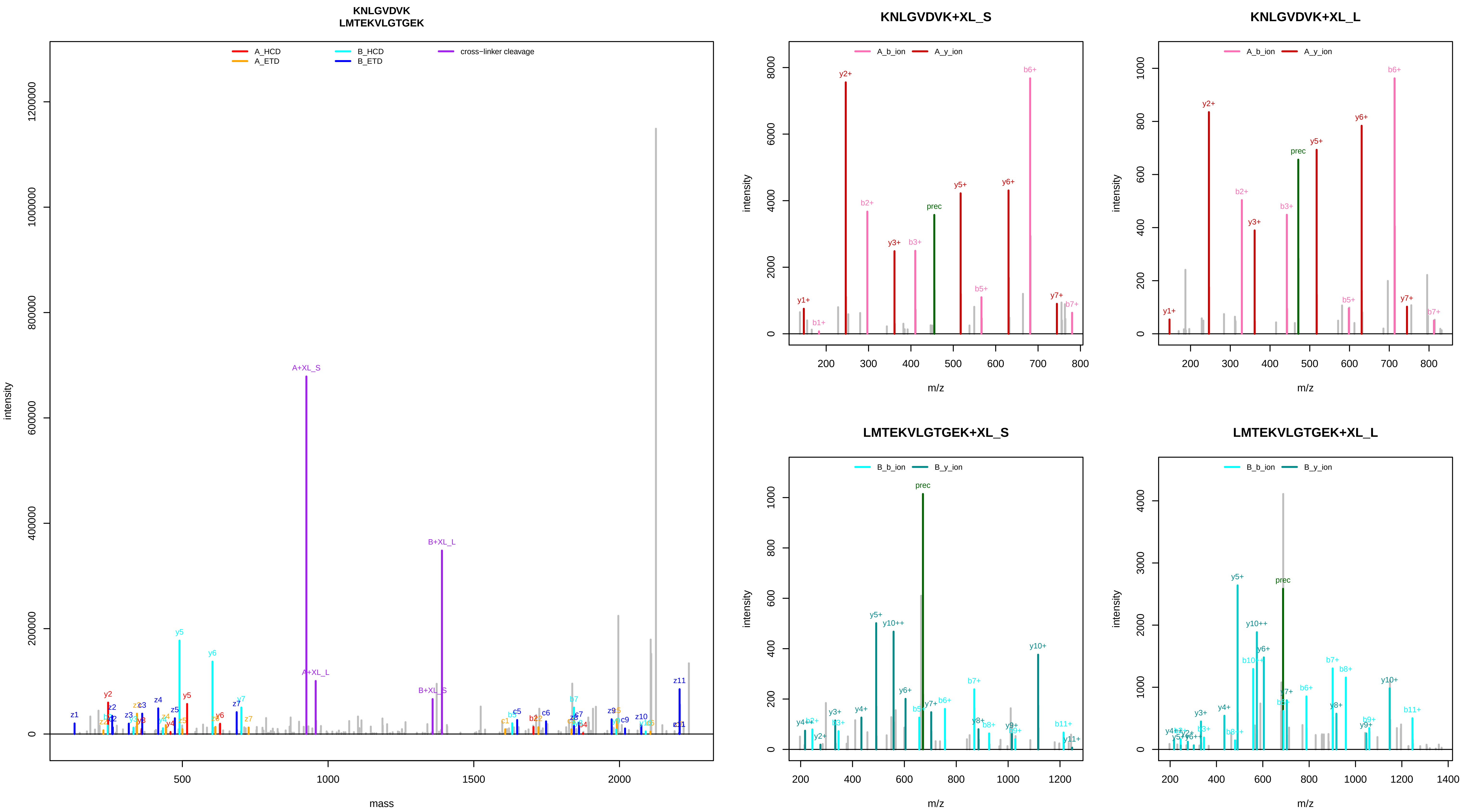
KNLGVDVK
LMTEKVLGTGEK
A_HCD
A_ETD
B_HCD
B_ETD
cross−linker cleavage
A+XL_S
B+XL_L
A+XL_L
B+XL_S
intensity
mass
KNLGVDVK+XL_S
KNLGVDVK+XL_L
LMTEKVLGTGEK+XL_S
LMTEKVLGTGEK+XL_L
A_b_ion
A_y_ion
B_b_ion
B_y_ion
prec
m/z

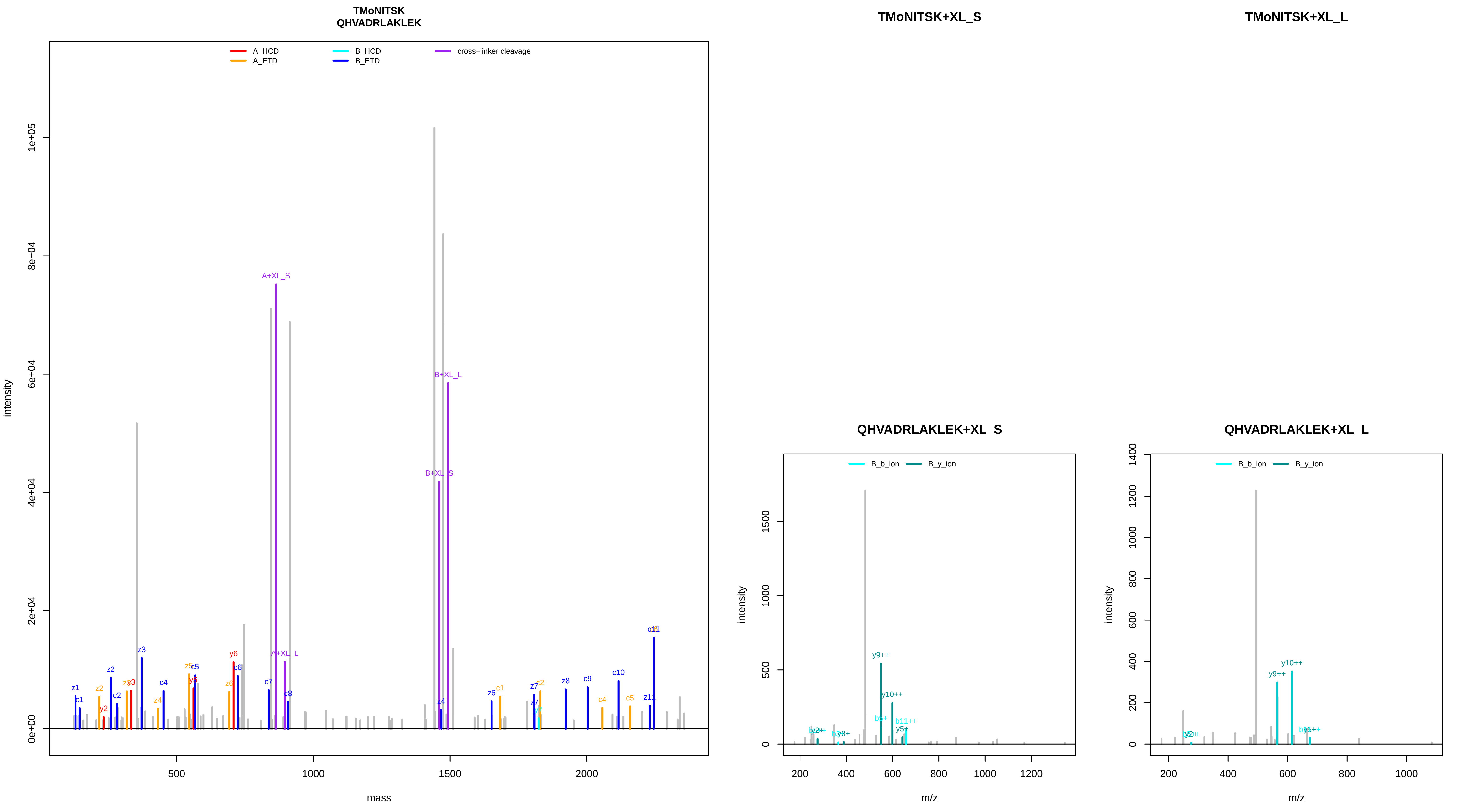

TMoNITSK
QHVADRLAKLEK
A_HCD
A_ETD
B_HCD
B_ETD
cross−linker cleavage
A+XL_S
B+XL_L
B+XL_S
A+XL_L
intensity
mass
TMoNITSK+XL_S
TMoNITSK+XL_L
QHVADRLAKLEK+XL_S
QHVADRLAKLEK+XL_L
B_b_ion
B_y_ion
y9++
y10++
intensity
m/z

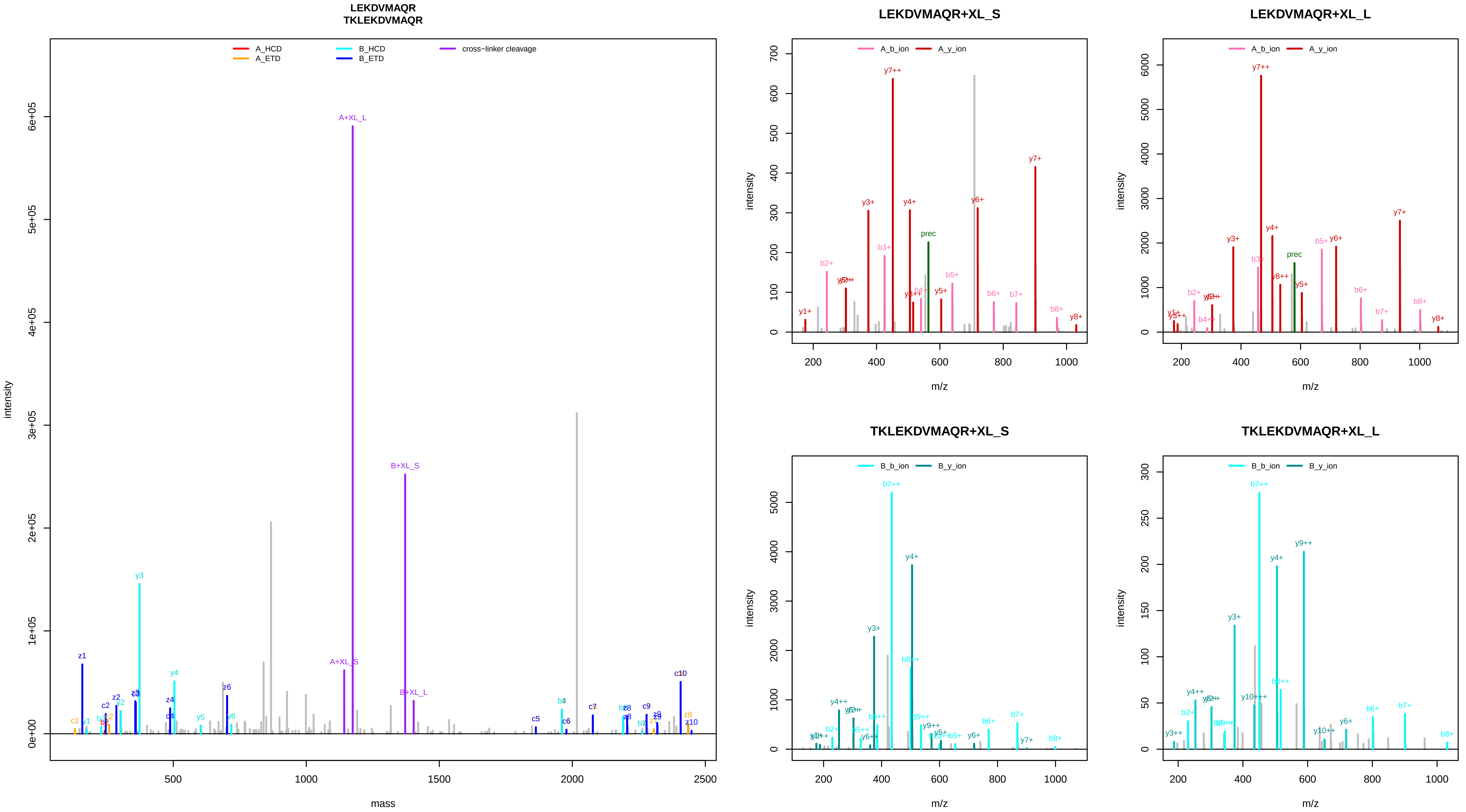
LEKDVMAQR
TKLEKDVMAQR
A_HCD
A_ETD
B_HCD
B_ETD
cross−linker cleavage
A+XL_L
B+XL_S
A+XL_S
B+XL_L
intensity
mass
LEKDVMAQR+XL_S
LEKDVMAQR+XL_L
TKLEKDVMAQR+XL_S
TKLEKDVMAQR+XL_L
A_b_ion
A_y_ion
B_b_ion
B_y_ion
prec
m/z

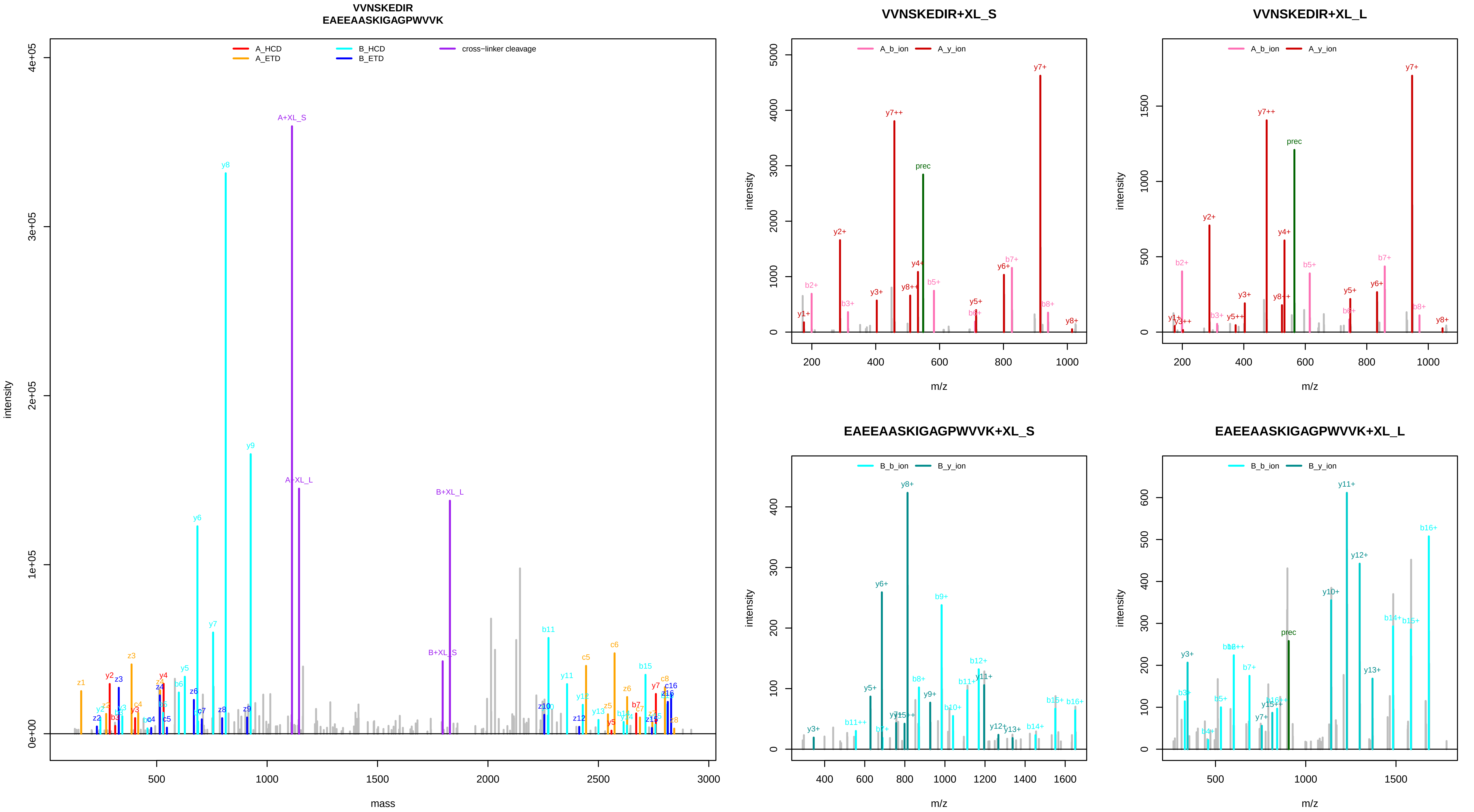
VVNSKEDIR
EAEEAASKIGAGPWVVK
A_HCD
A_ETD
B_HCD
B_ETD
cross−linker cleavage
A+XL_S
A+XL_L
B+XL_L
B+XL_S
intensity
mass
VVNSKEDIR+XL_S
VVNSKEDIR+XL_L
A_b_ion
A_y_ion
prec
EAEEAASKIGAGPWVVK+XL_S
EAEEAASKIGAGPWVVK+XL_L
B_b_ion
B_y_ion
m/z

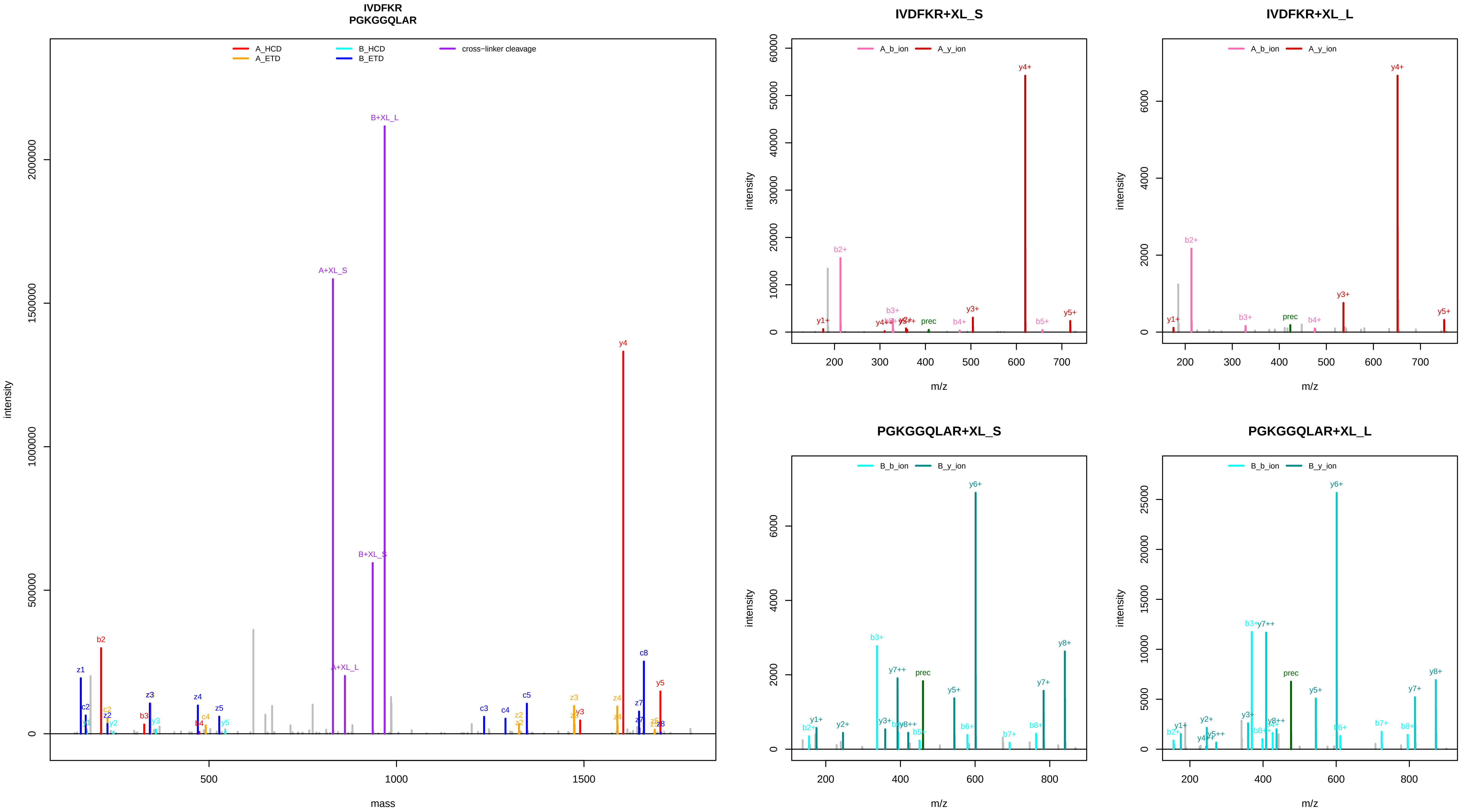
IVDFKR
PGKGGQLAR
A_HCD
A_ETD
B_HCD
B_ETD
cross−linker cleavage
B+XL_L
A+XL_S
B+XL_S
A+XL_L
intensity
mass
IVDFKR+XL_S
IVDFKR+XL_L
A_b_ion
A_y_ion
PGKGGQLAR+XL_S
PGKGGQLAR+XL_L
B_b_ion
B_y_ion
prec
m/z
intensity

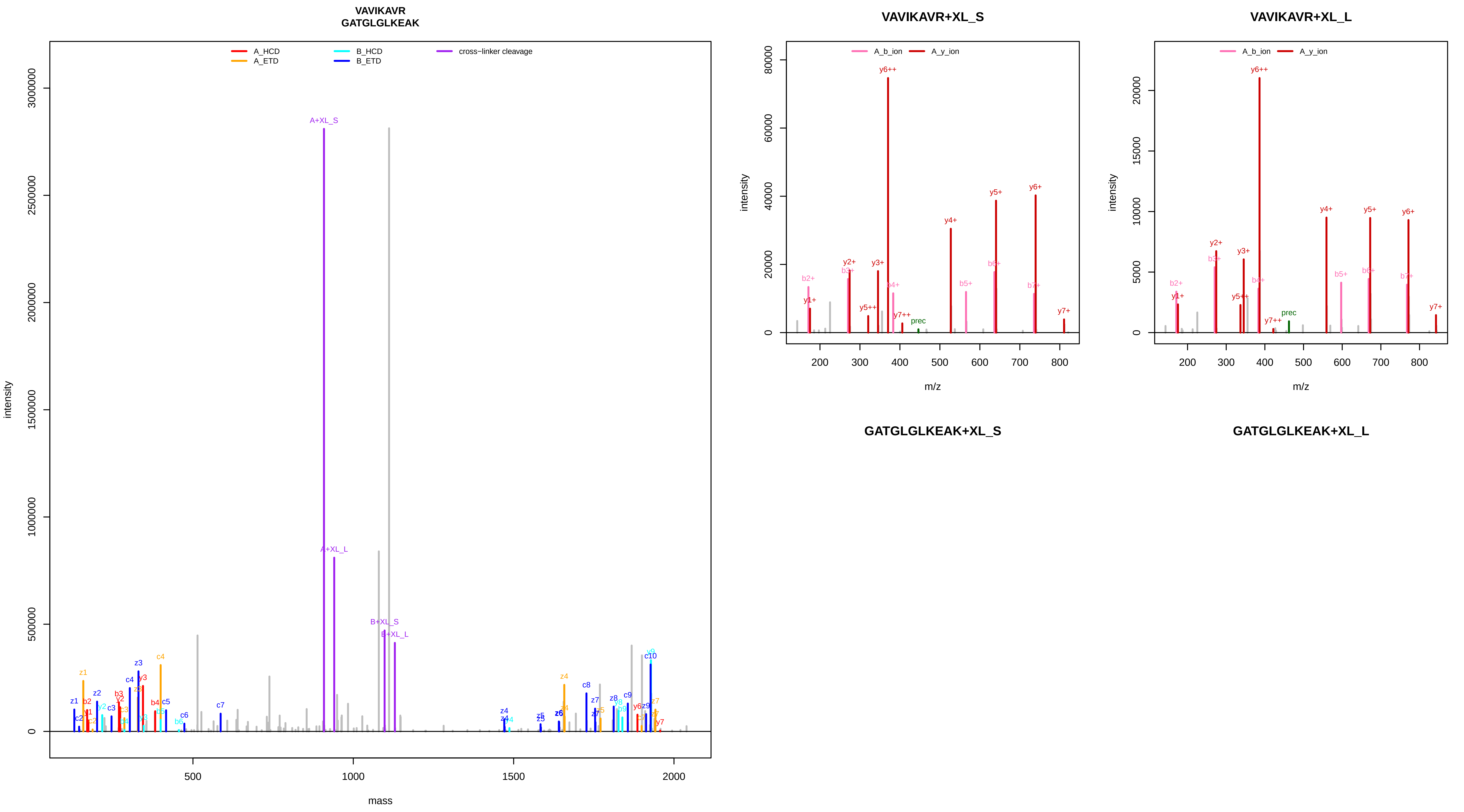
VAVIKAVR
GATGLGLKEAK
A_HCD
A_ETD
B_HCD
B_ETD
cross−linker cleavage
A+XL_S
A+XL_L
B+XL_S
B+XL_L
intensity
mass
VAVIKAVR+XL_S
VAVIKAVR+XL_L
A_b_ion
A_y_ion
y6++
y5+
y6+
y4+
prec
m/z
GATGLGLKEAK+XL_S
GATGLGLKEAK+XL_L

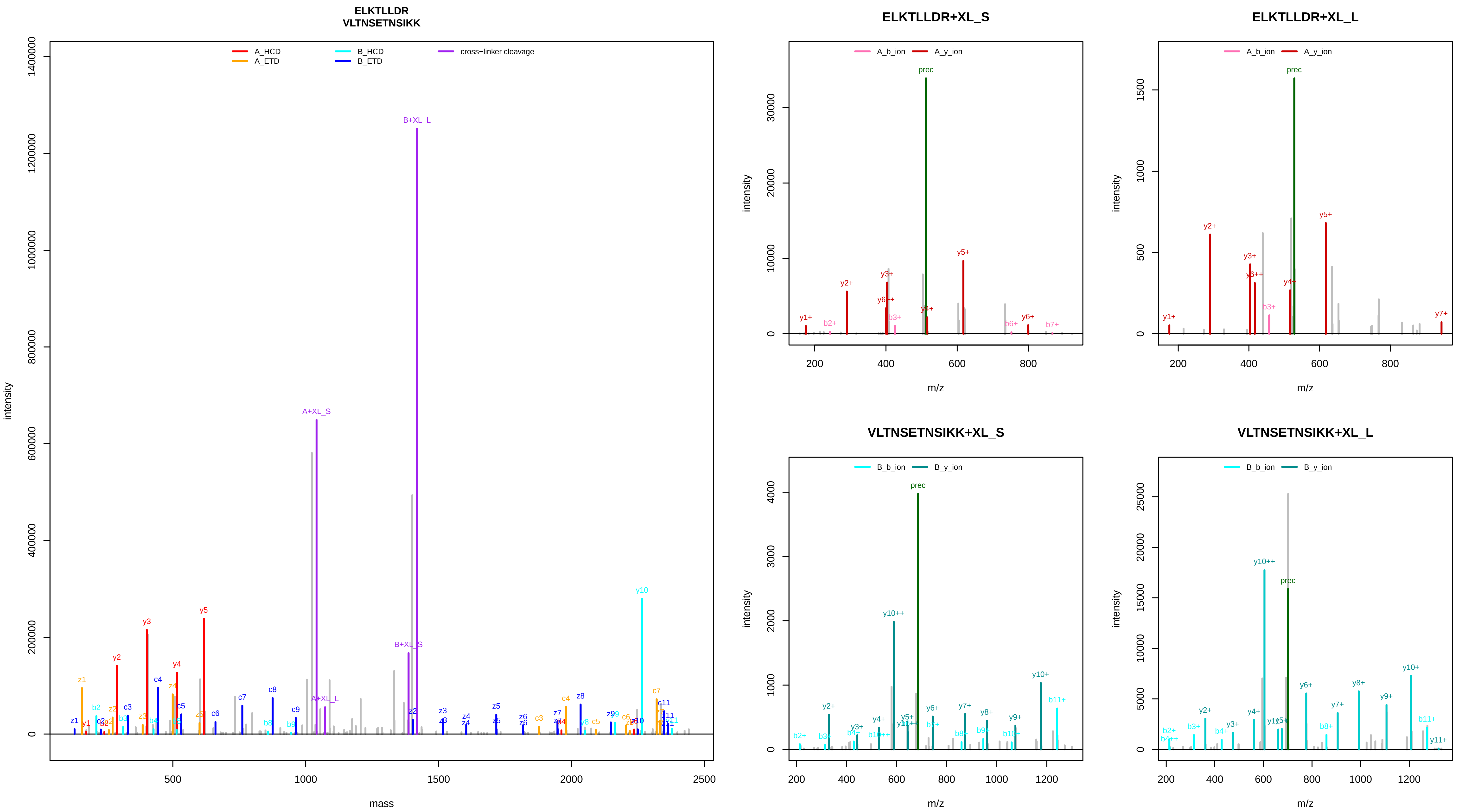
ELKTLLDR
VLTNSETNSIKK
A_HCD
A_ETD
B_HCD
B_ETD
cross−linker cleavage
B+XL_L
A+XL_S
B+XL_S
A+XL_L
intensity
mass
ELKTLLDR+XL_S
ELKTLLDR+XL_L
VLTNSETNSIKK+XL_S
VLTNSETNSIKK+XL_L
A_b_ion
A_y_ion
B_b_ion
B_y_ion
prec
m/z

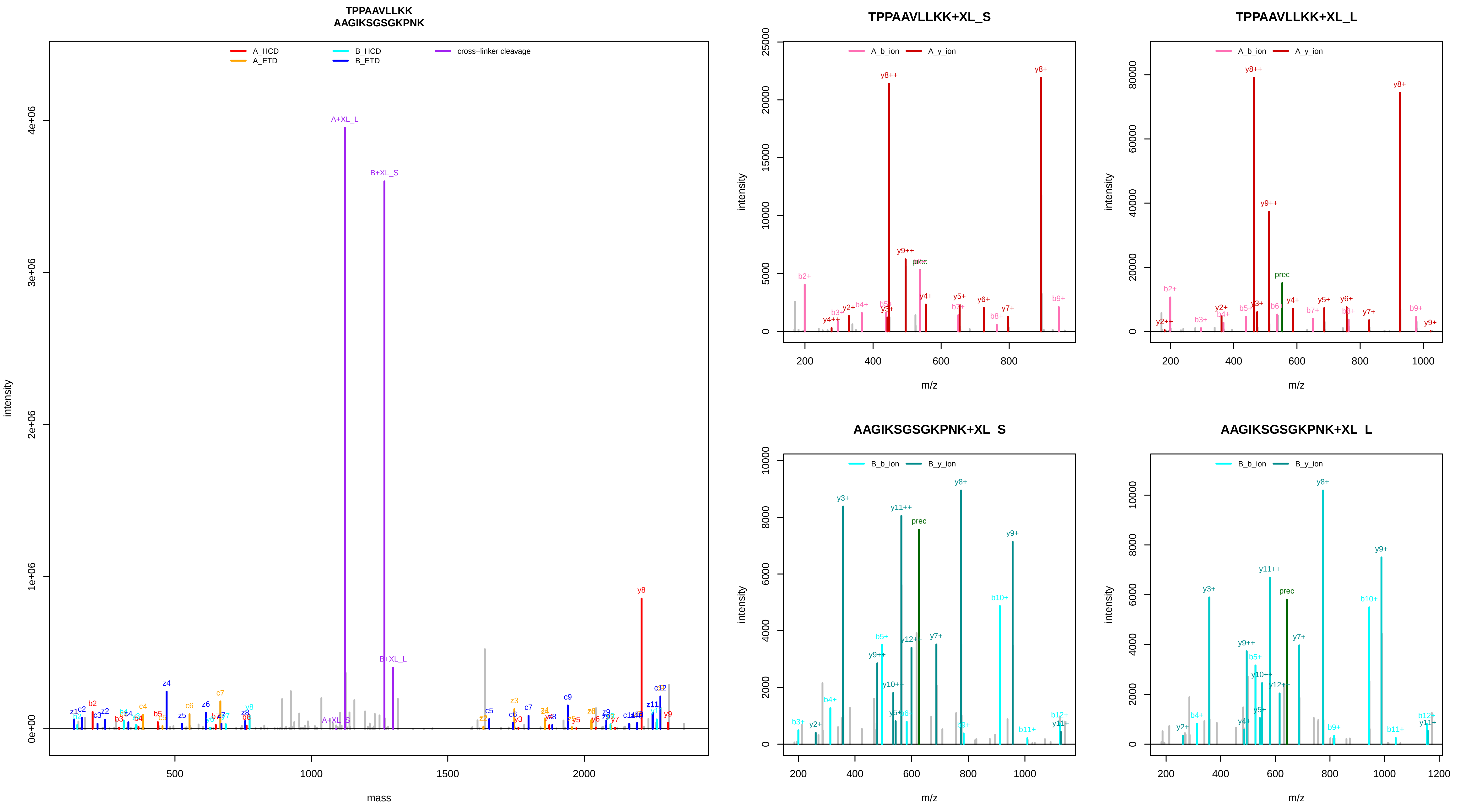

TPPAAVLLKK
AAGIKSGSGKPNK
A_HCD
A_ETD
B_HCD
B_ETD
cross−linker cleavage
A+XL_L
B+XL_S
B+XL_L
A+XL_S
intensity
mass
0e+00
1e+06
2e+06
3e+06
4e+06
500
1000
1500
2000
TPPAAVLLKK+XL_S
TPPAAVLLKK+XL_L
AAGIKSGSGKPNK+XL_S
AAGIKSGSGKPNK+XL_L
A_b_ion
A_y_ion
B_b_ion
B_y_ion
prec
m/z
intensity

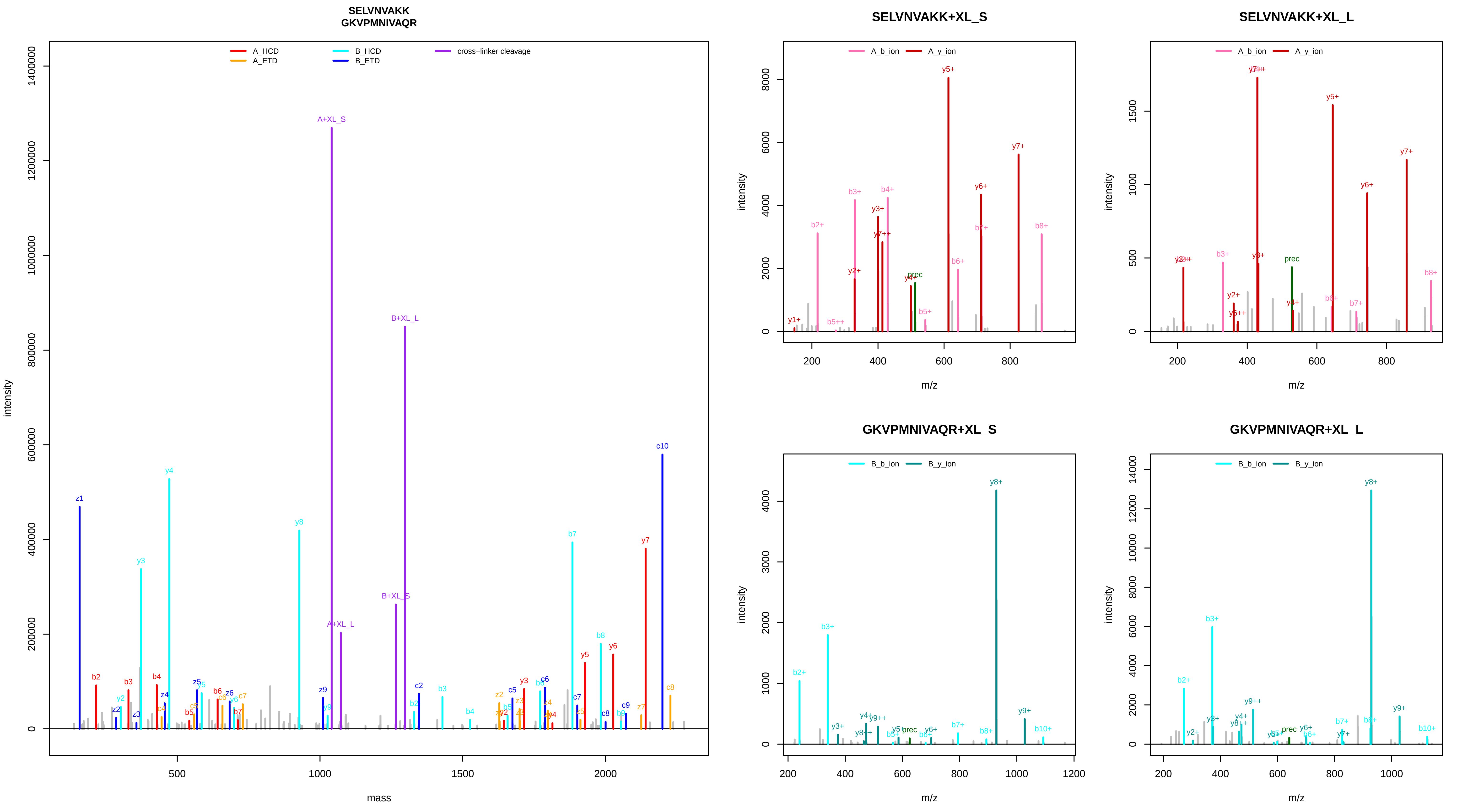
SELVNVAKK
GKVPMNIVAQR
A_HCD
A_ETD
B_HCD
B_ETD
cross−linker cleavage
A+XL_S
B+XL_L
B+XL_S
A+XL_L
intensity
mass
0
200000
400000
600000
800000
1000000
1200000
1400000
500
1000
1500
2000
SELVNVAKK+XL_S
SELVNVAKK+XL_L
GKVPMNIVAQR+XL_S
GKVPMNIVAQR+XL_L
A_b_ion
A_y_ion
B_b_ion
B_y_ion
prec
m/z
intensity

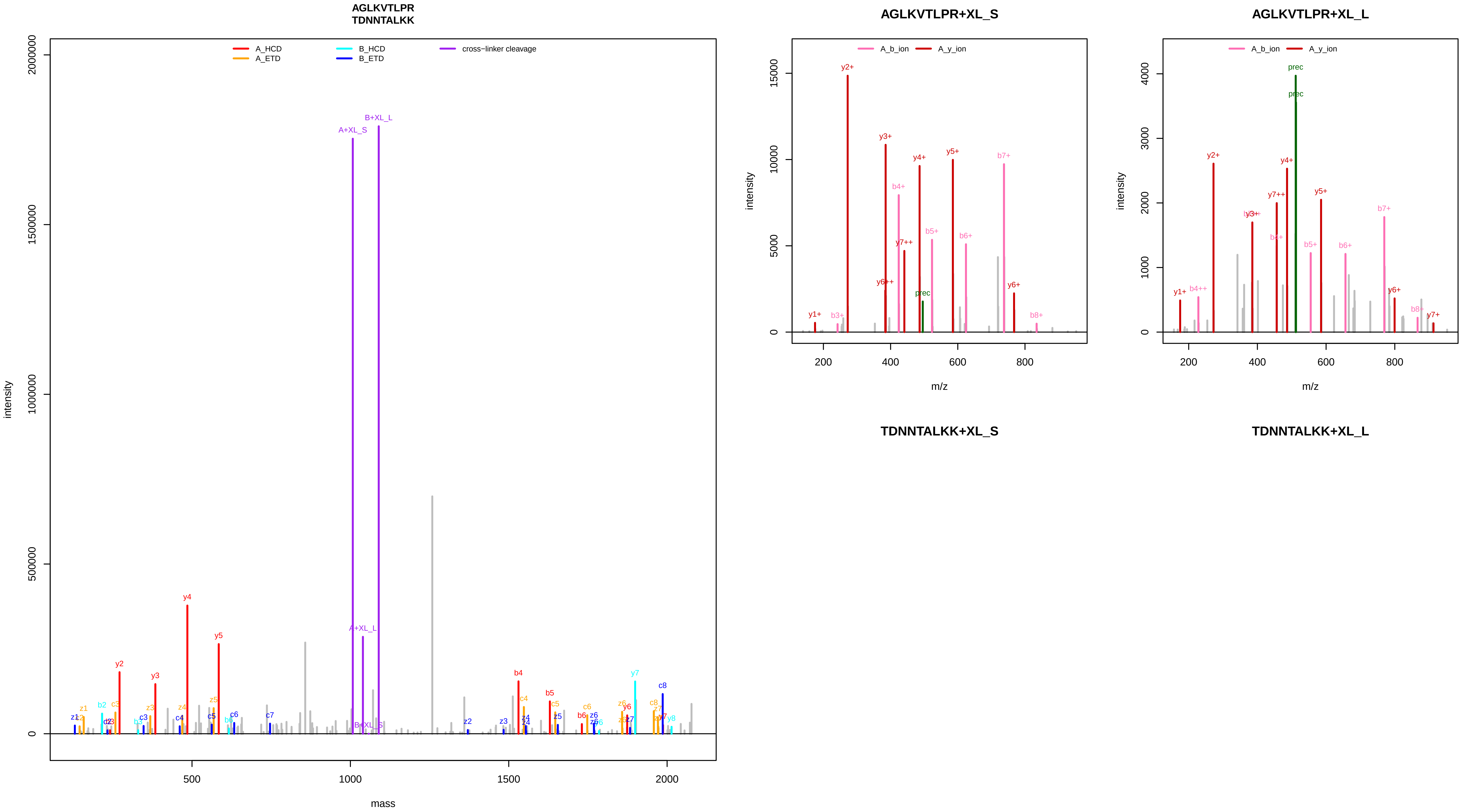

TDNNTALKK+XL_S

TDNNTALKK+XL_L

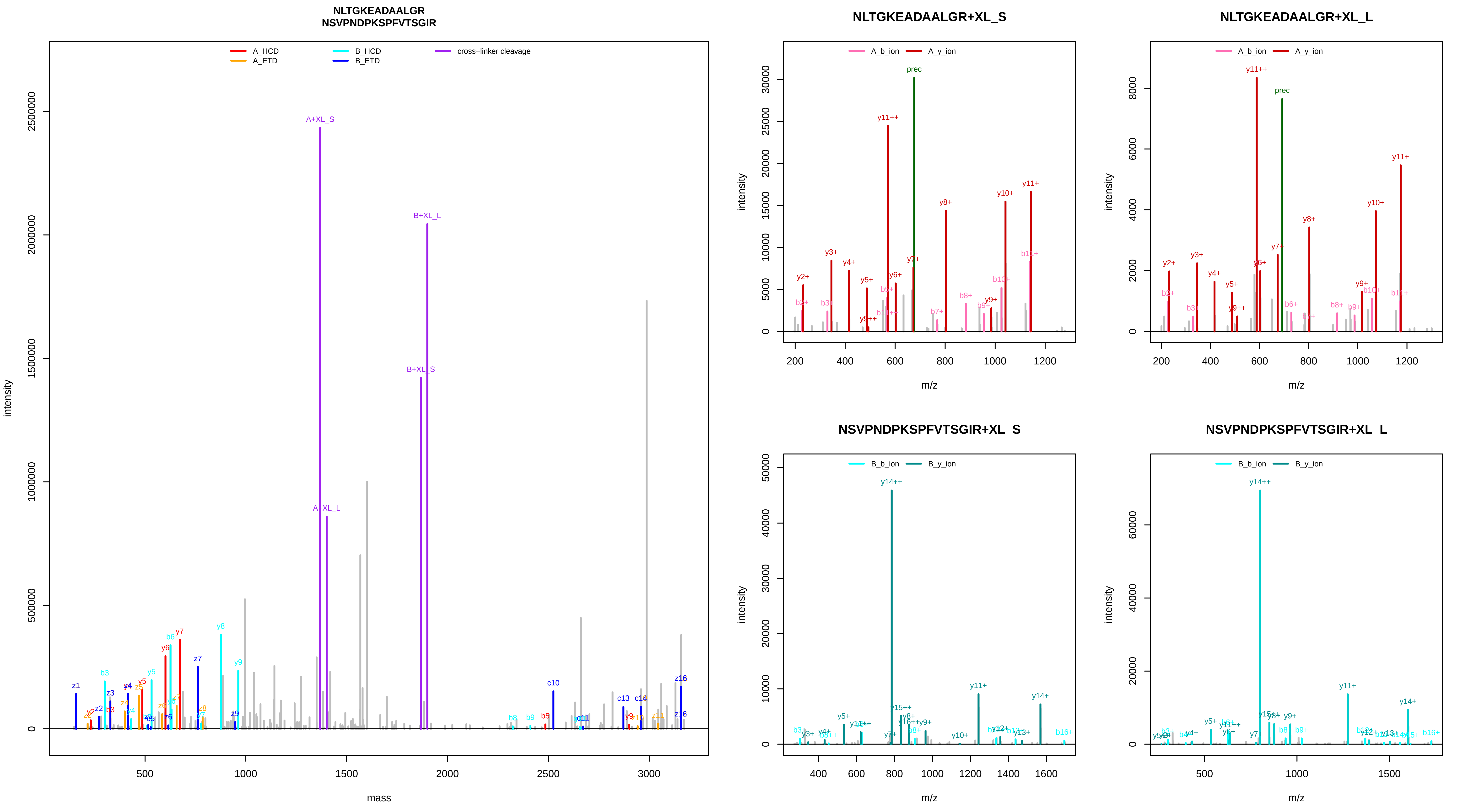
NLTGKEADAALGR
NSVPNDPKSPFVTSGIR
A_HCD
A_ETD
B_HCD
B_ETD
cross−linker cleavage
A+XL_S
B+XL_L
B+XL_S
A+XL_L
intensity
mass
NLTGKEADAALGR+XL_S
NLTGKEADAALGR+XL_L
A_b_ion
A_y_ion
prec
y11++
y11+
y10+
y8+
y7+
y6+
y5+
y4+
y3+
y2+
y9+
intensity
m/z
NSVPNDPKSPFVTSGIR+XL_S
NSVPNDPKSPFVTSGIR+XL_L
B_b_ion
B_y_ion
y14++
y14+
y11+
y5+
y9+
intensity
m/z

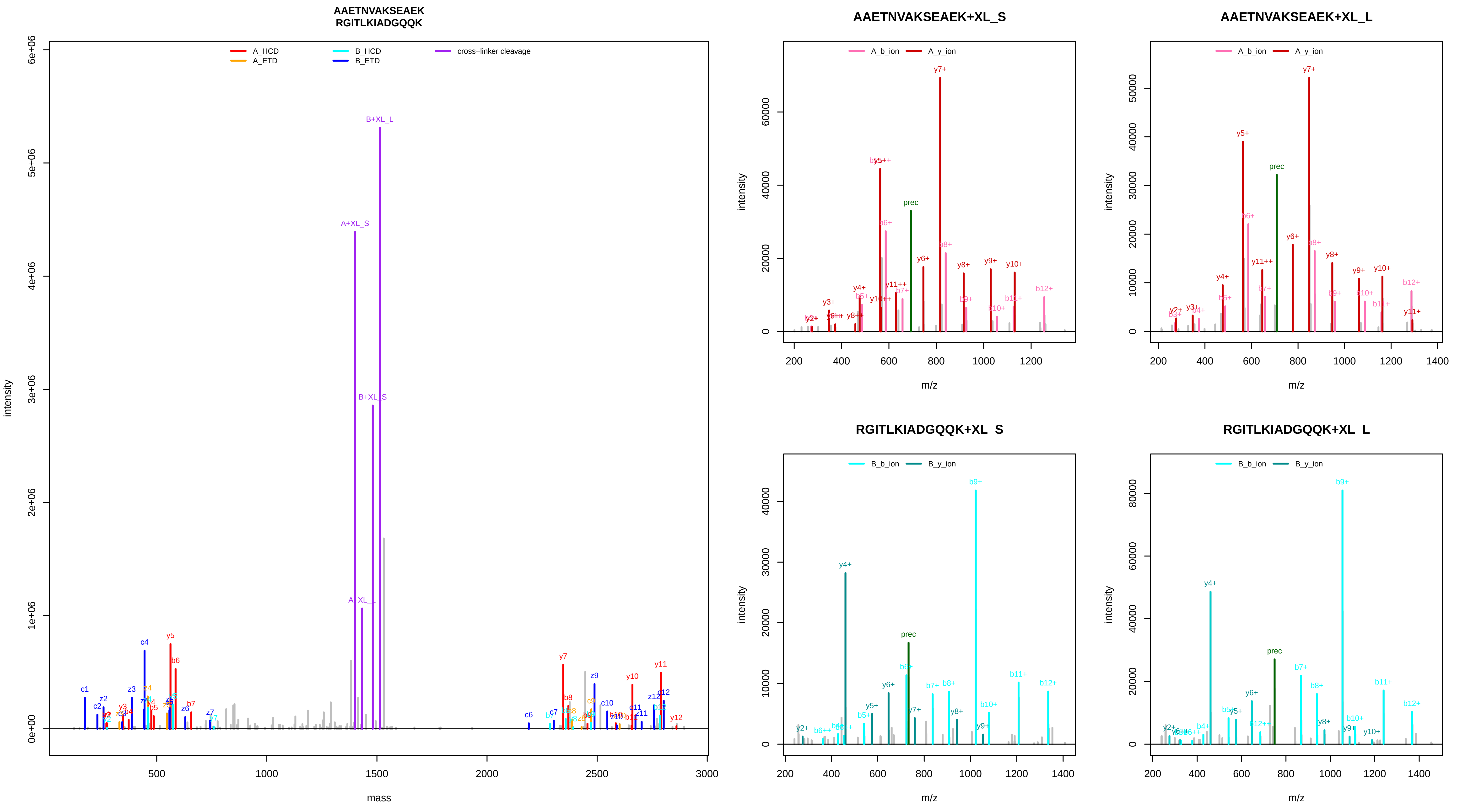
AAETNVAKSEAEK
RGITLKIADGQQK
A_HCD
A_ETD
B_HCD
B_ETD
cross−linker cleavage
B+XL_L
A+XL_S
B+XL_S
intensity
mass
AAETNVAKSEAEK+XL_S
AAETNVAKSEAEK+XL_L
RGITLKIADGQQK+XL_S
RGITLKIADGQQK+XL_L
A_b_ion
A_y_ion
B_b_ion
B_y_ion
prec
m/z

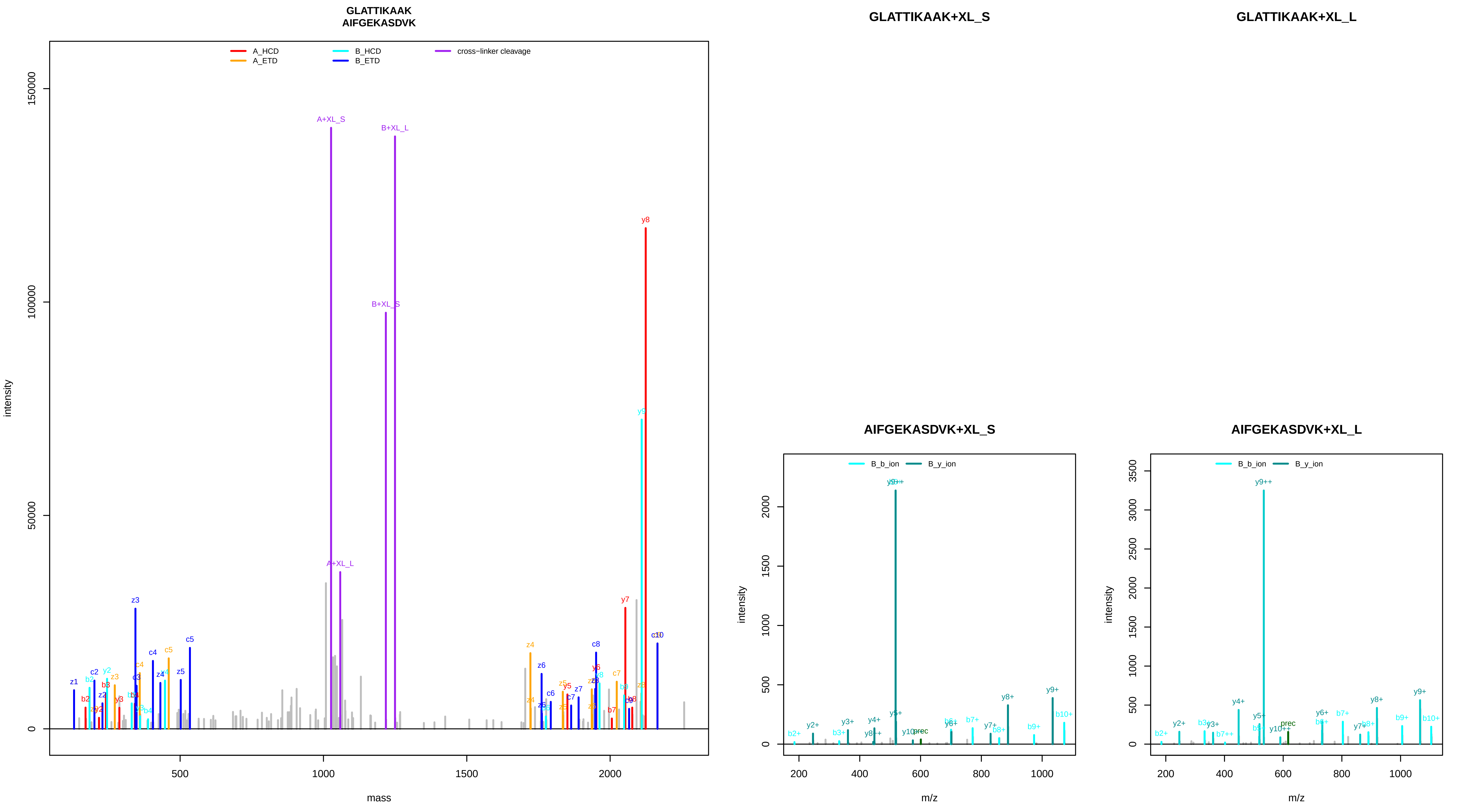
GLATTIKAAK
AIFGEKASDVK
A_HCD
A_ETD
B_HCD
B_ETD
cross−linker cleavage
A+XL_S
B+XL_L
B+XL_S
A+XL_L
y8
y9
y7
c10
c8
z4
z3
c5
c4
intensity
mass
150000
100000
50000
0
500
1000
1500
2000
GLATTIKAAK+XL_S
GLATTIKAAK+XL_L
AIFGEKASDVK+XL_S
AIFGEKASDVK+XL_L
B_b_ion
B_y_ion
y9++
prec
intensity
m/z

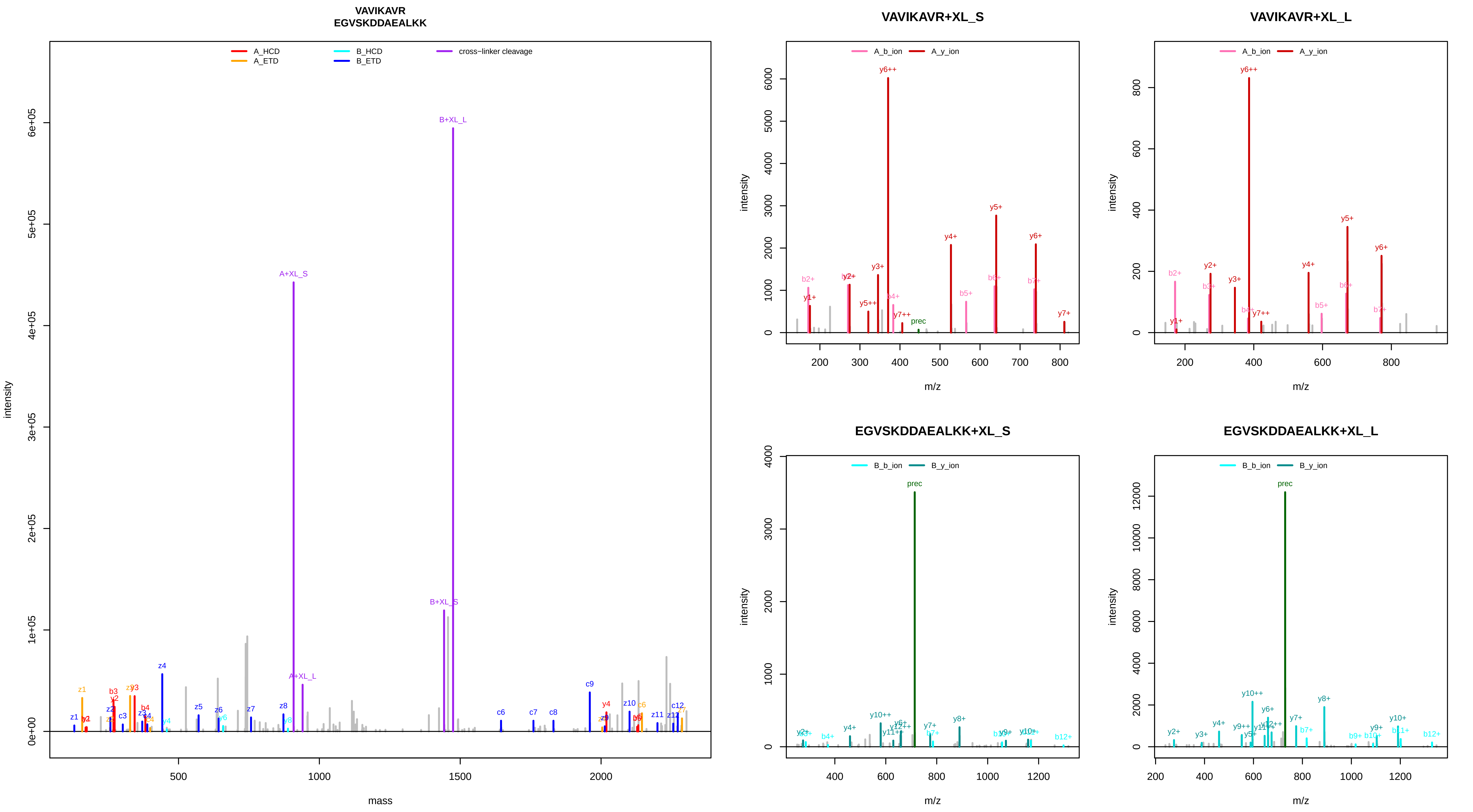

VAVIKAVR
EGVSKDDAEALKK
A_HCD
A_ETD
B_HCD
B_ETD
cross−linker cleavage
A+XL_S
B+XL_L
B+XL_S
A+XL_L
intensity
mass
VAVIKAVR+XL_S
VAVIKAVR+XL_L
A_b_ion
A_y_ion
EGVSKDDAEALKK+XL_S
EGVSKDDAEALKK+XL_L
B_b_ion
B_y_ion
prec
m/z

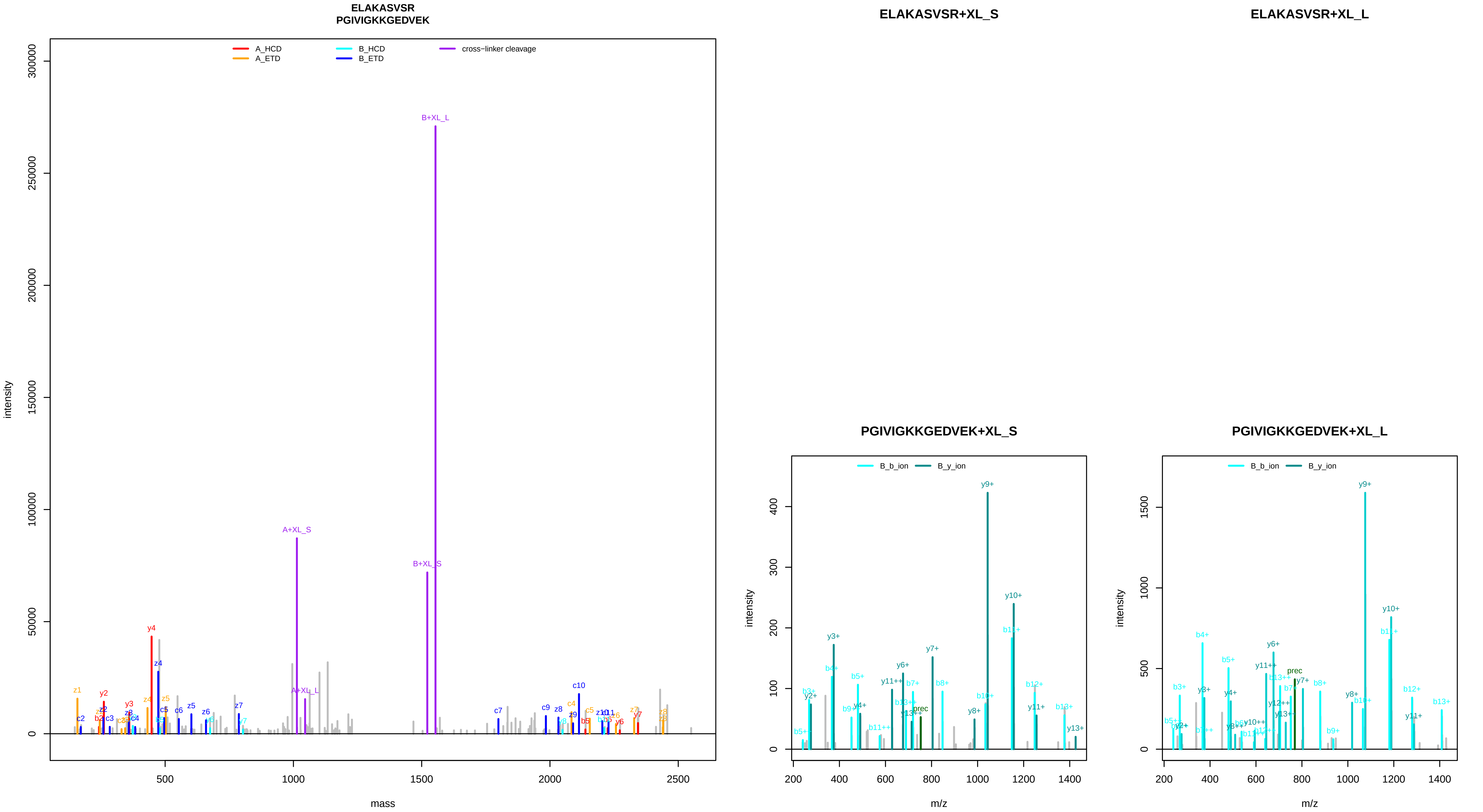
ELAKASVSR
PGIVIGKKGEDVEK
A_HCD
A_ETD
B_HCD
B_ETD
cross−linker cleavage
B+XL_L
A+XL_S
B+XL_S
A+XL_L
intensity
mass
0
50000
100000
150000
200000
250000
300000
500
1000
1500
2000
2500
ELAKASVSR+XL_S
ELAKASVSR+XL_L
PGIVIGKKGEDVEK+XL_S
PGIVIGKKGEDVEK+XL_L
B_b_ion
B_y_ion
m/z
intensity
y9+
y10+
y3+
y7+
y6+
prec

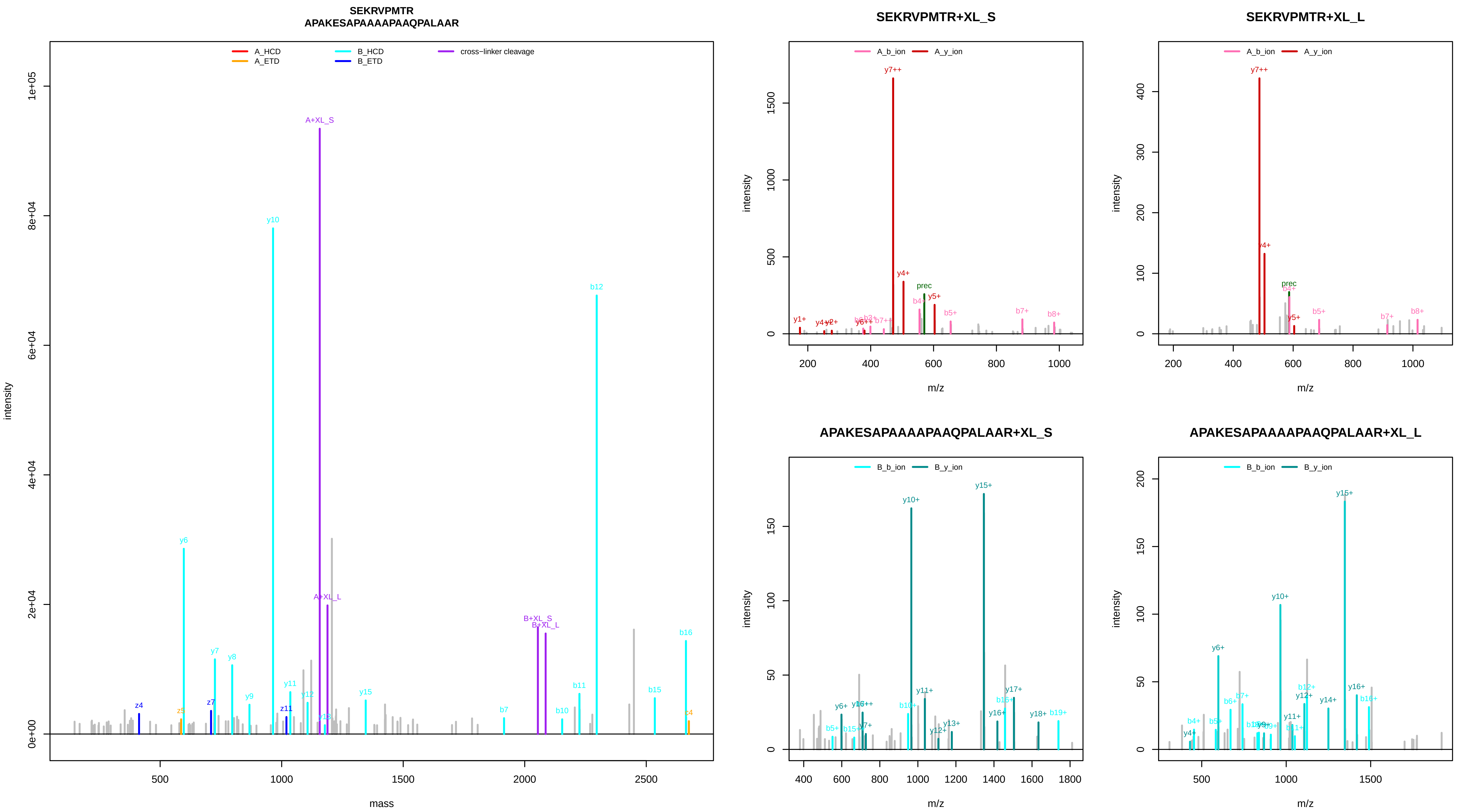
SEKRVPMTR
APAKESAPAAAAPAAQPALAAR
A_HCD
A_ETD
B_HCD
B_ETD
cross−linker cleavage
A+XL_S
A+XL_L
B+XL_S
B+XL_L
intensity
mass
SEKRVPMTR+XL_S
SEKRVPMTR+XL_L
APAKESAPAAAAPAAQPALAAR+XL_S
APAKESAPAAAAPAAQPALAAR+XL_L
A_b_ion
A_y_ion
B_b_ion
B_y_ion
prec
m/z

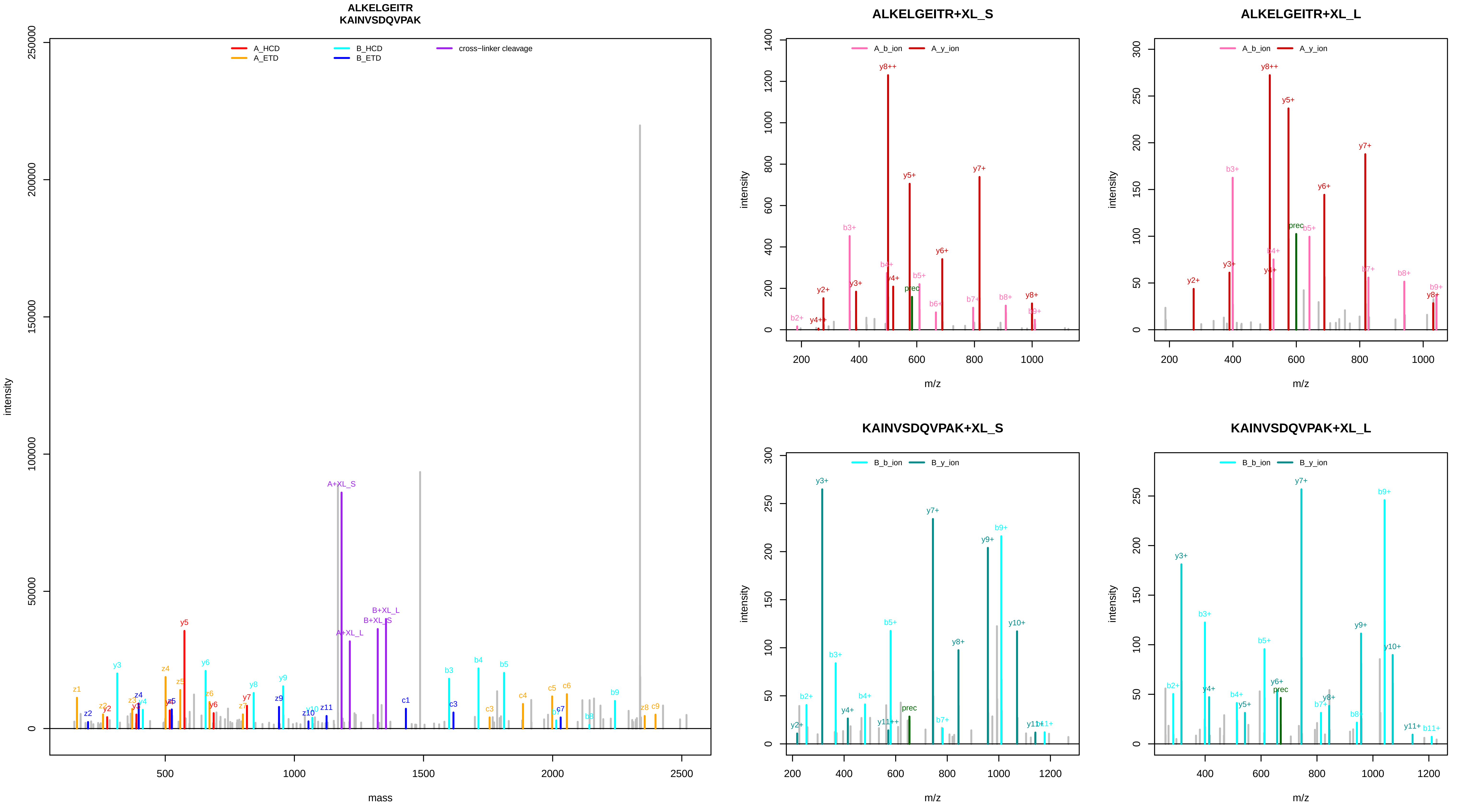
ALKELGEITR
KAINVSDQVPAK
A_HCD
A_ETD
B_HCD
B_ETD
cross−linker cleavage
intensity
mass
ALKELGEITR+XL_S
ALKELGEITR+XL_L
KAINVSDQVPAK+XL_S
KAINVSDQVPAK+XL_L
A_b_ion
A_y_ion
B_b_ion
B_y_ion
m/z
A+XL_S
A+XL_L
B+XL_L
B+XL_S
prec

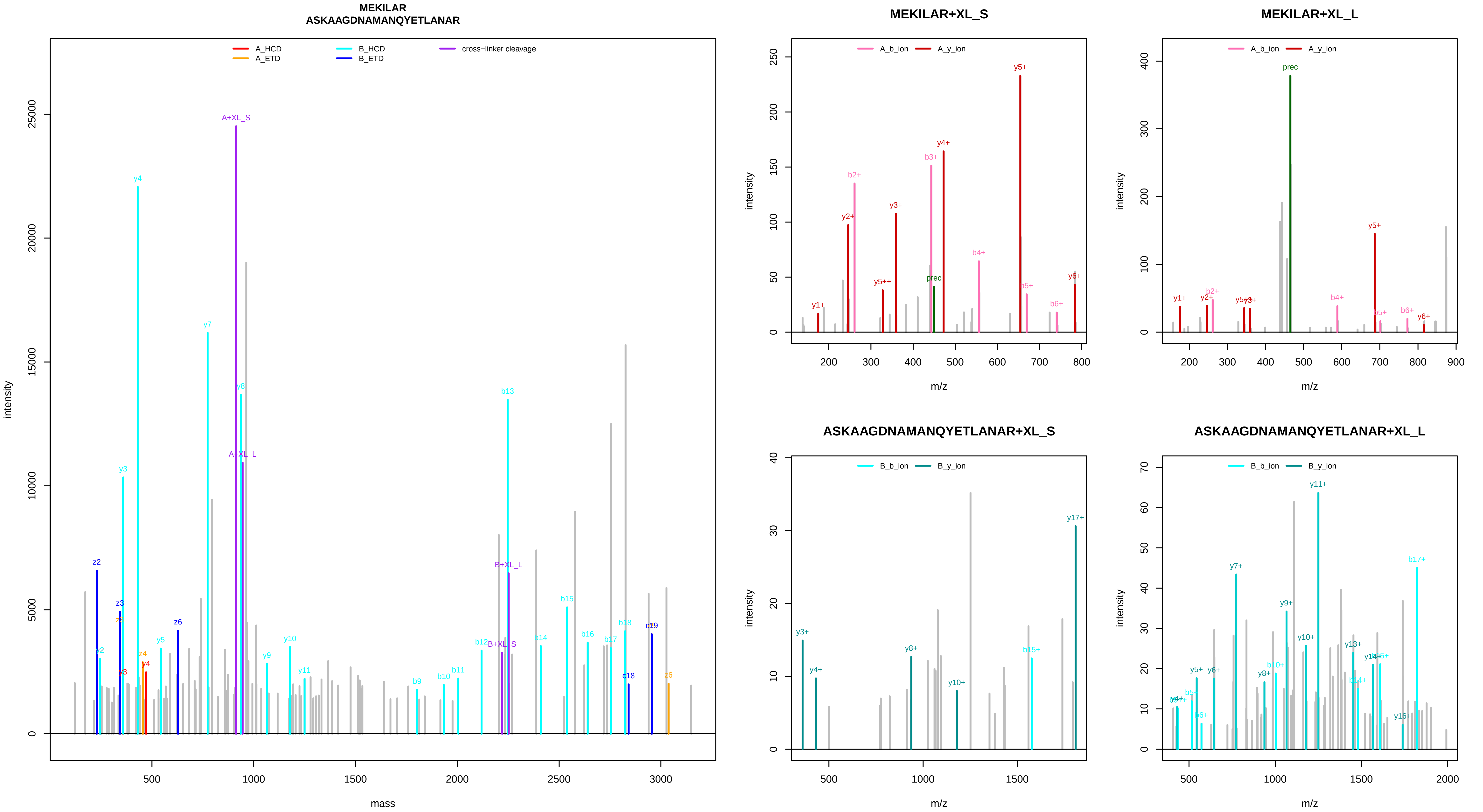

MEKILAR
ASKAAGDNAMANQYETLANAR
A_HCD
A_ETD
B_HCD
B_ETD
cross−linker cleavage
A+XL_S
A+XL_L
B+XL_L
B+XL_S
intensity
mass
MEKILAR+XL_S
MEKILAR+XL_L
ASKAAGDNAMANQYETLANAR+XL_S
ASKAAGDNAMANQYETLANAR+XL_L
A_b_ion
A_y_ion
B_b_ion
B_y_ion
prec
m/z

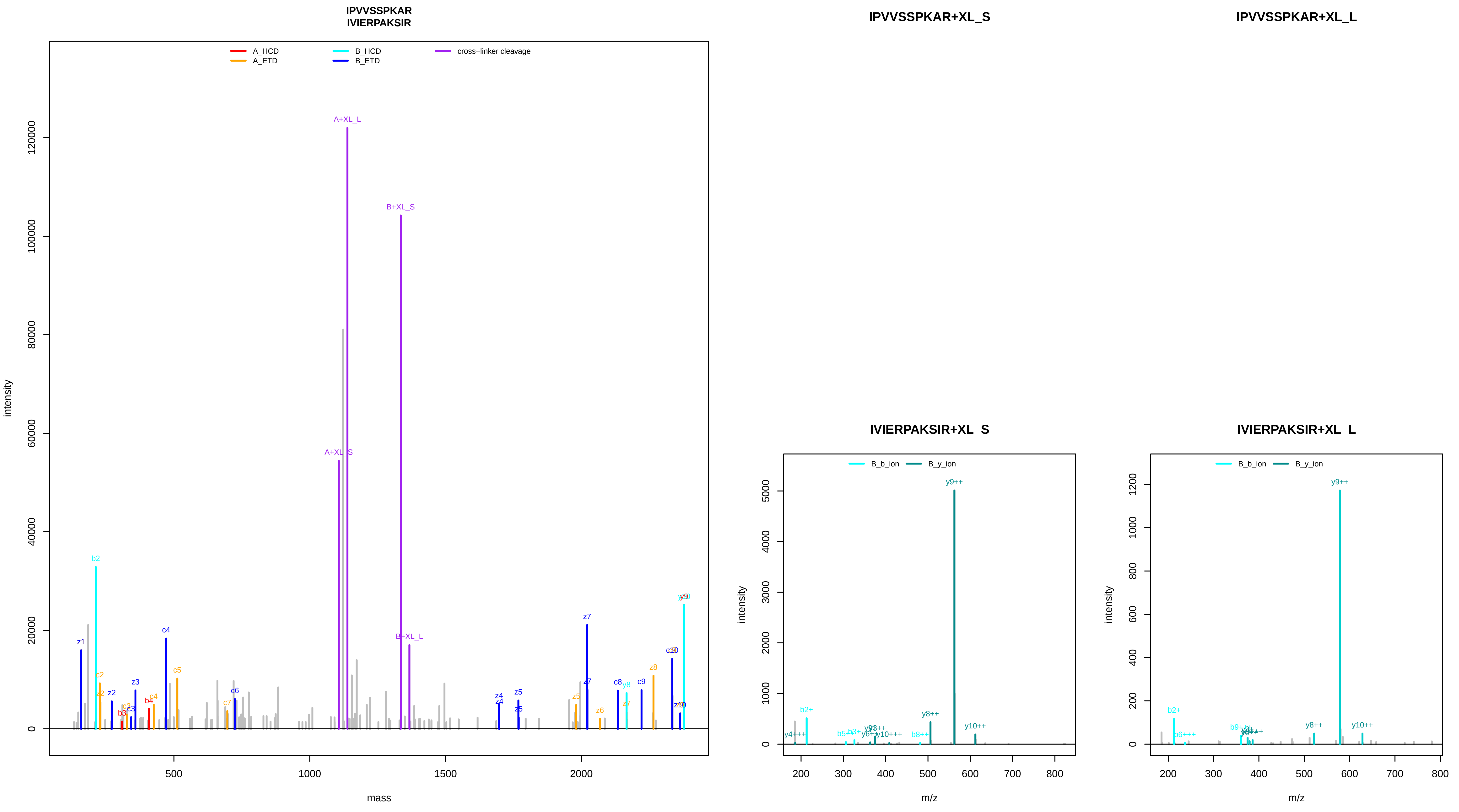

IPVVSSPKAR
IVIERPAKSIR
A_HCD
A_ETD
B_HCD
B_ETD
cross−linker cleavage
A+XL_L
B+XL_S
A+XL_S
B+XL_L
intensity
mass
IPVVSSPKAR+XL_S
IPVVSSPKAR+XL_L
IVIERPAKSIR+XL_S
IVIERPAKSIR+XL_L
B_b_ion
B_y_ion
y9++
y8++
y10++
b2+
m/z

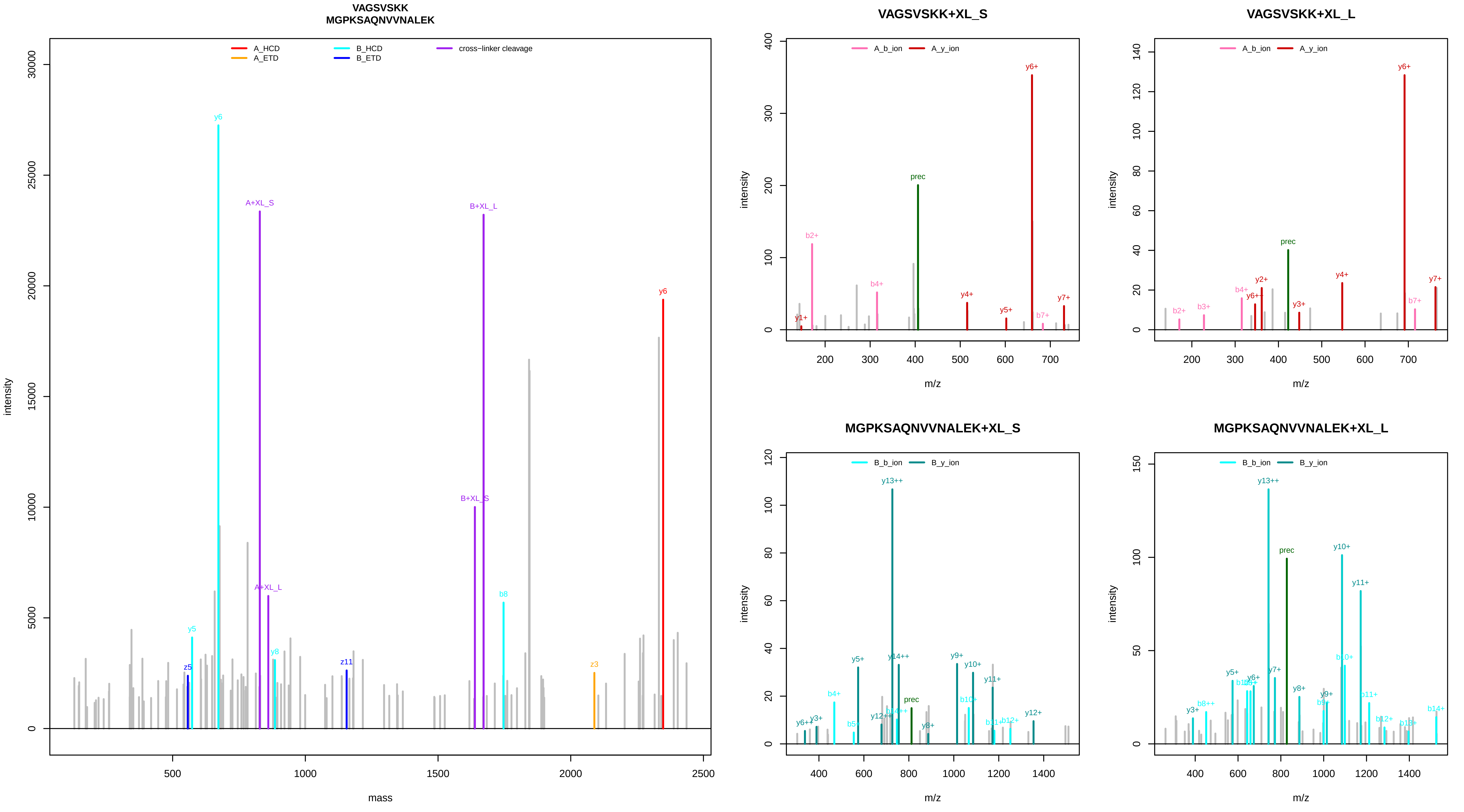
VAGSVSKK
MGPKSAQNVVNALEK
A_HCD
A_ETD
B_HCD
B_ETD
cross−linker cleavage
intensity
mass
y6
A+XL_S
B+XL_L
B+XL_S
A+XL_L
b8
y5
z5
y8
z11
z3
VAGSVSKK+XL_S
VAGSVSKK+XL_L
A_b_ion
A_y_ion
m/z
prec
b2+
b3+
b4+
b7+
y1+
y2+
y3+
y4+
y5+
y6+
y6++
y7+
MGPKSAQNVVNALEK+XL_S
MGPKSAQNVVNALEK+XL_L
B_b_ion
B_y_ion
y13++
y14++
y12++
y3+
y5+
y6++
y6+
y7+
y8+
y9+
y10+
y11+
y12+
b4+
b5+
b8++
b10+
b11+
b12+
b14+

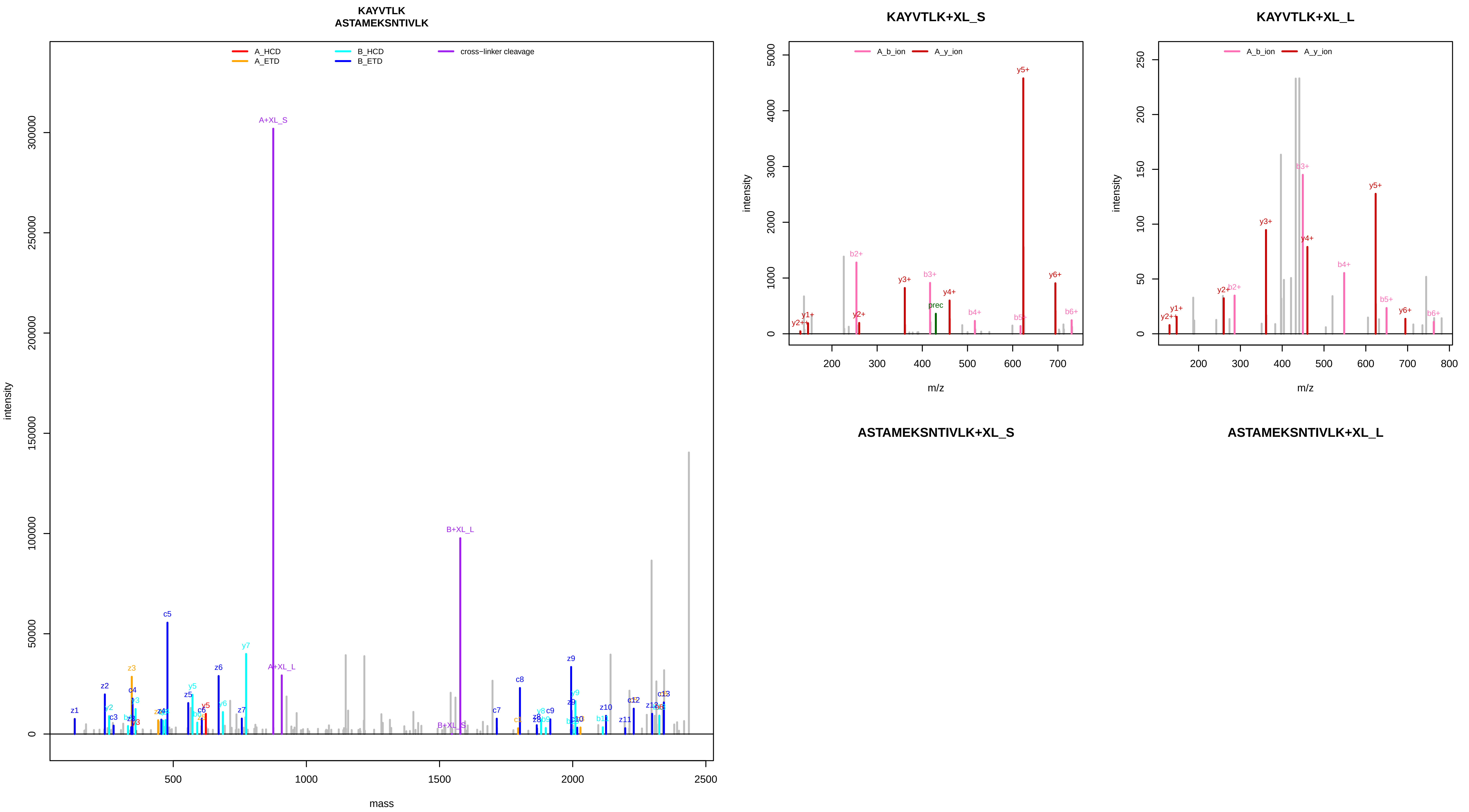

KAYVTLK
ASTAMEKSNTIVLK
A_HCD
A_ETD
B_HCD
B_ETD
cross−linker cleavage
A+XL_S
B+XL_L
A+XL_L
B+XL_S
intensity
mass
KAYVTLK+XL_S
KAYVTLK+XL_L
A_b_ion
A_y_ion
prec
m/z
ASTAMEKSNTIVLK+XL_S
ASTAMEKSNTIVLK+XL_L

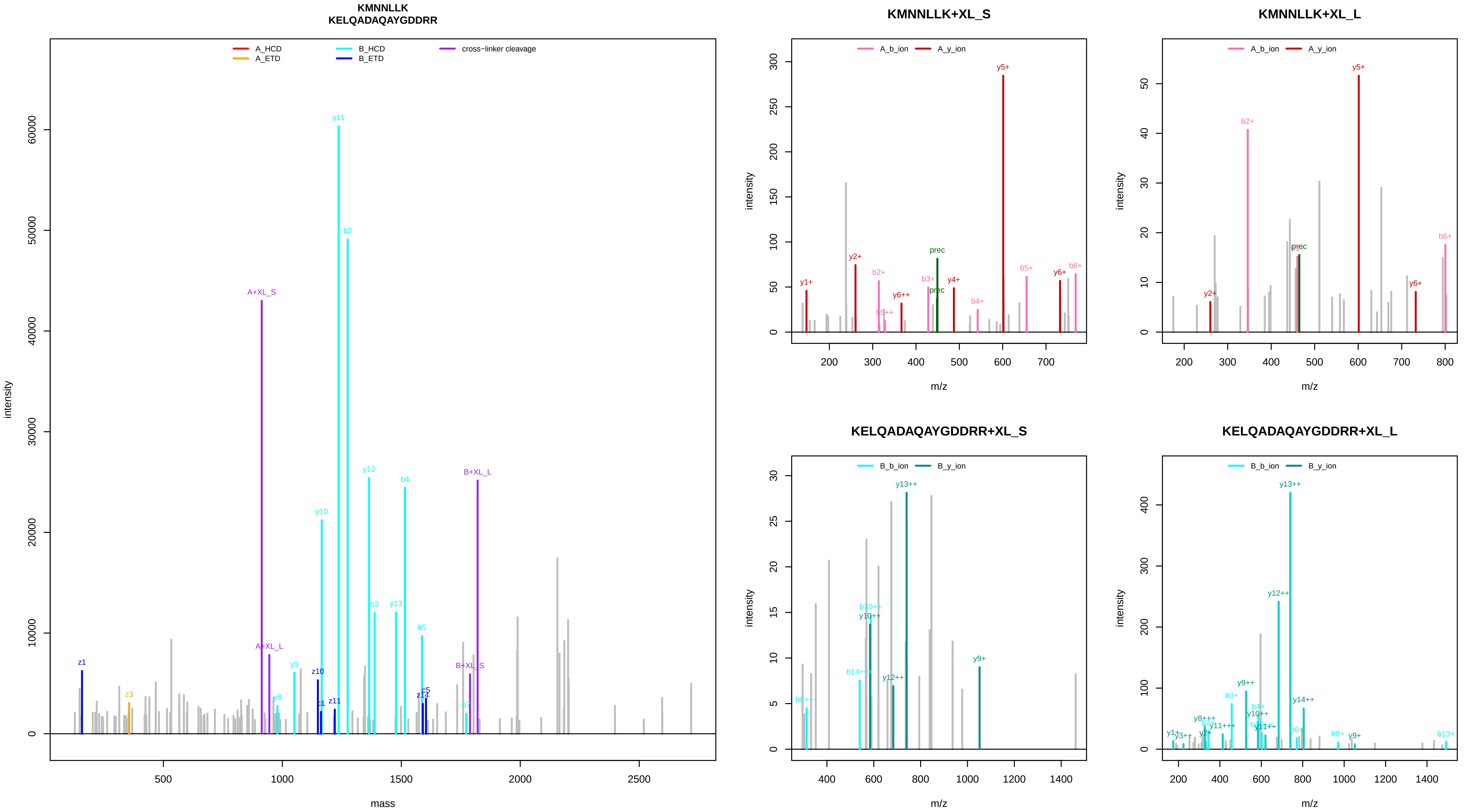

KMNNLLK
KELQADAQAYGDDRR
A_HCD
A_ETD
B_HCD
B_ETD
cross−linker cleavage
intensity
mass
KMNNLLK+XL_S
KMNNLLK+XL_L
KELQADAQAYGDDRR+XL_S
KELQADAQAYGDDRR+XL_L
A_b_ion
A_y_ion
B_b_ion
B_y_ion
m/z

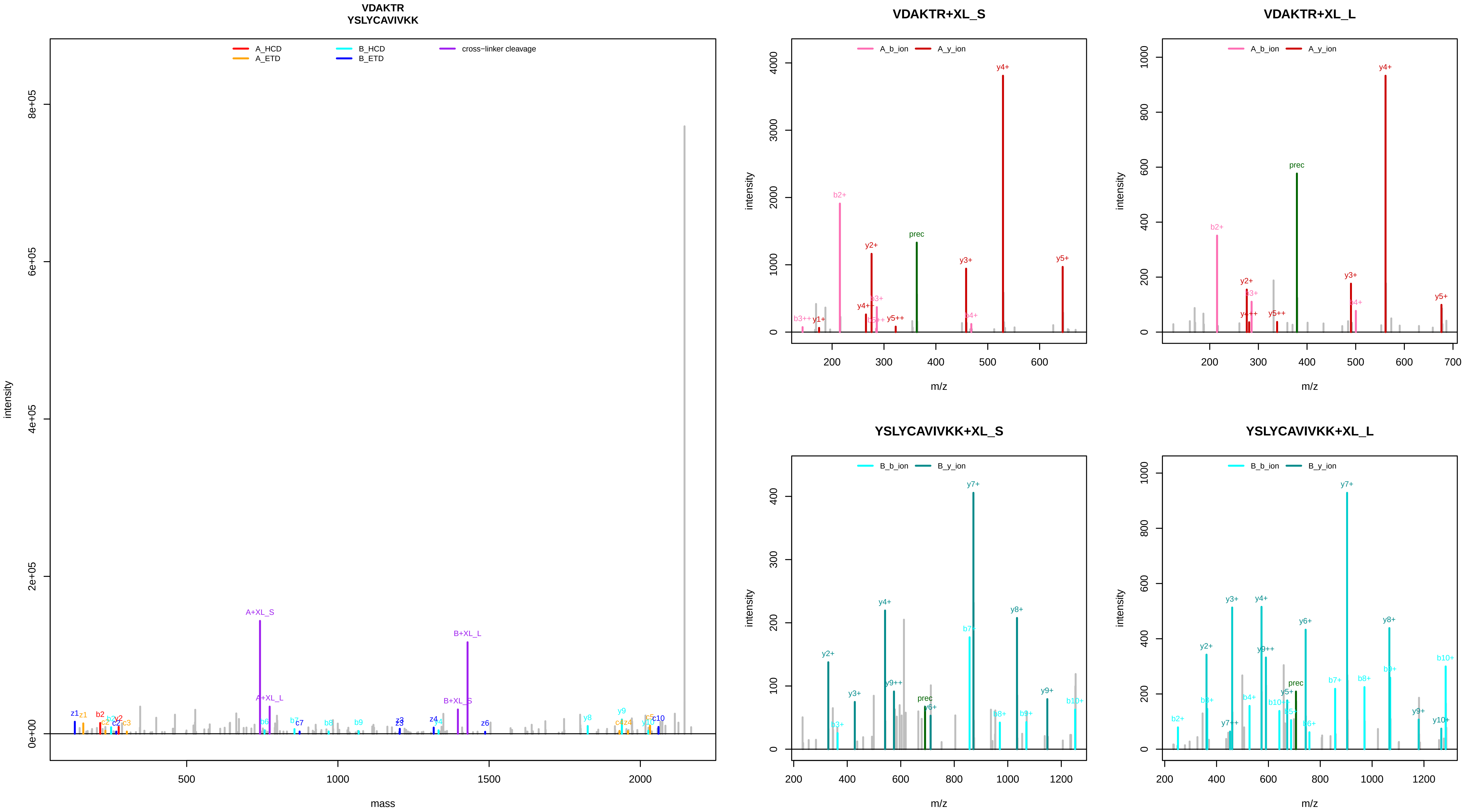

VDAKTR
YSLYCAVIVKK
A_HCD
A_ETD
B_HCD
B_ETD
cross−linker cleavage
A+XL_S
A+XL_L
B+XL_L
B+XL_S
intensity
mass
VDAKTR+XL_S
VDAKTR+XL_L
YSLYCAVIVKK+XL_S
YSLYCAVIVKK+XL_L
A_b_ion
A_y_ion
B_b_ion
B_y_ion
prec
m/z

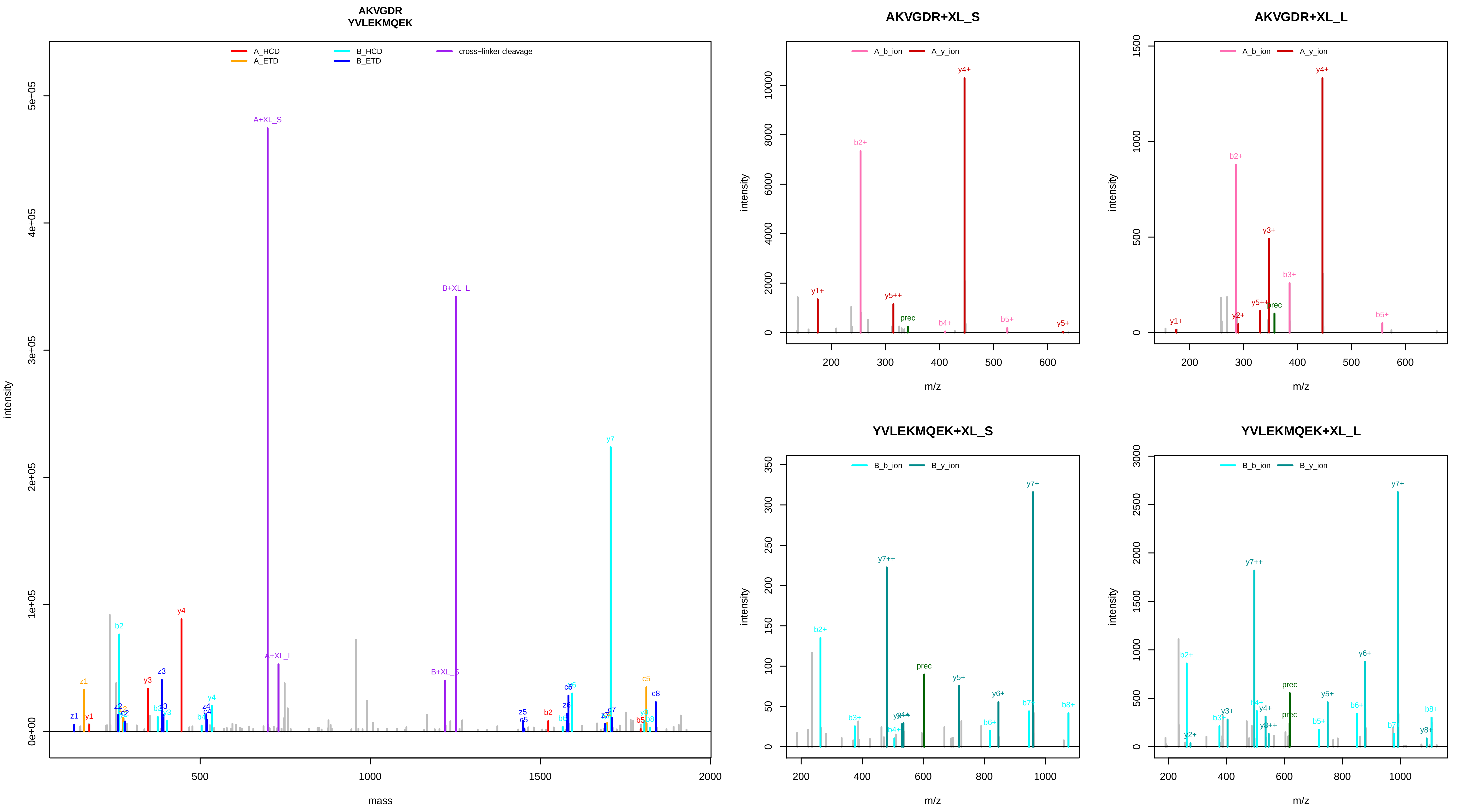

AKVGDR
YVLEKMQEK
A_HCD
A_ETD
B_HCD
B_ETD
cross−linker cleavage
A+XL_S
B+XL_L
A+XL_L
B+XL_S
intensity
mass
AKVGDR+XL_S
AKVGDR+XL_L
YVLEKMQEK+XL_S
YVLEKMQEK+XL_L
A_b_ion
A_y_ion
B_b_ion
B_y_ion
prec
m/z

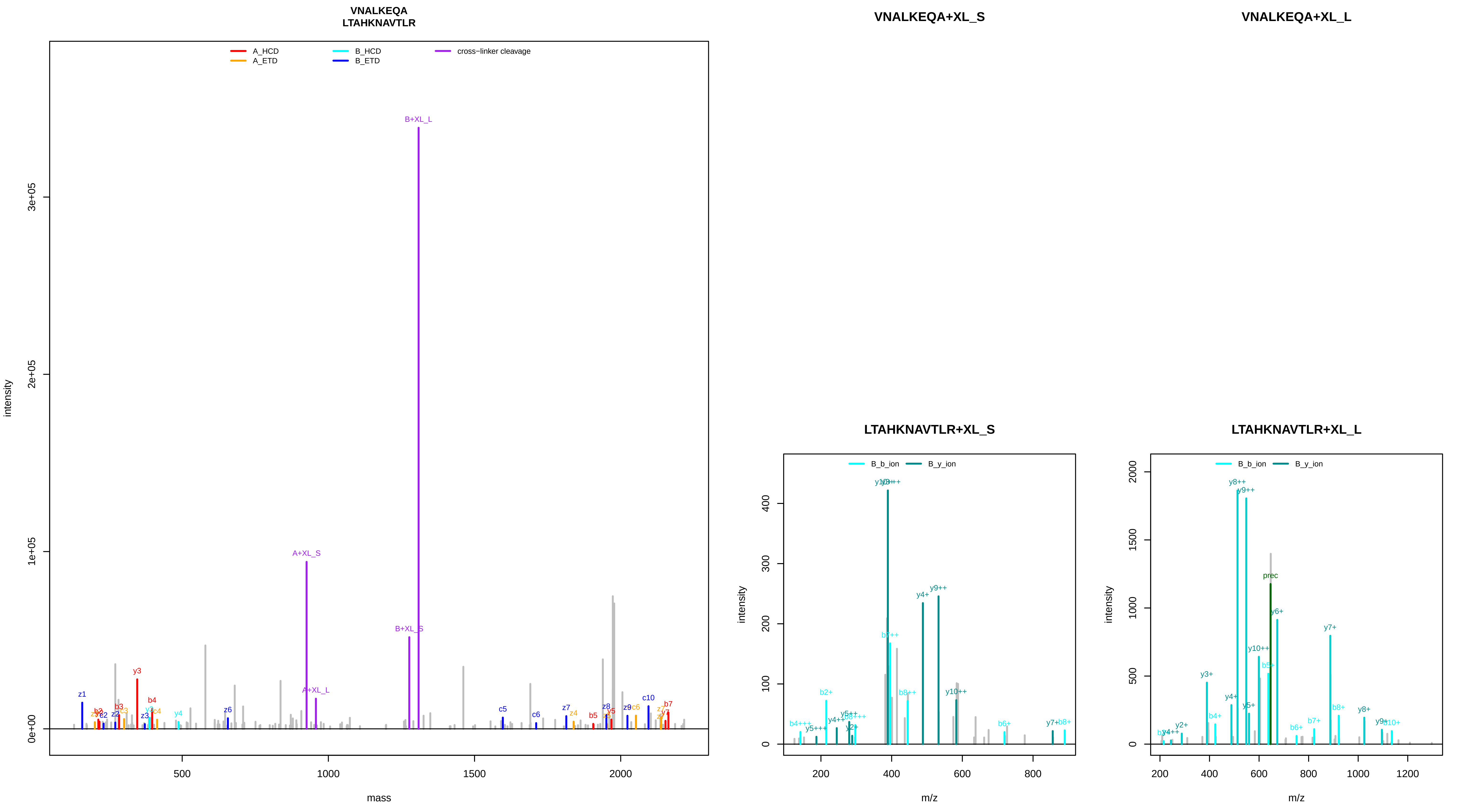
VNALKEQA
LTAHKNAVTLR
A_HCD
A_ETD
B_HCD
B_ETD
cross−linker cleavage
B+XL_L
A+XL_S
B+XL_S
A+XL_L
intensity
mass
VNALKEQA+XL_S
VNALKEQA+XL_L
LTAHKNAVTLR+XL_S
LTAHKNAVTLR+XL_L
B_b_ion
B_y_ion
prec
m/z

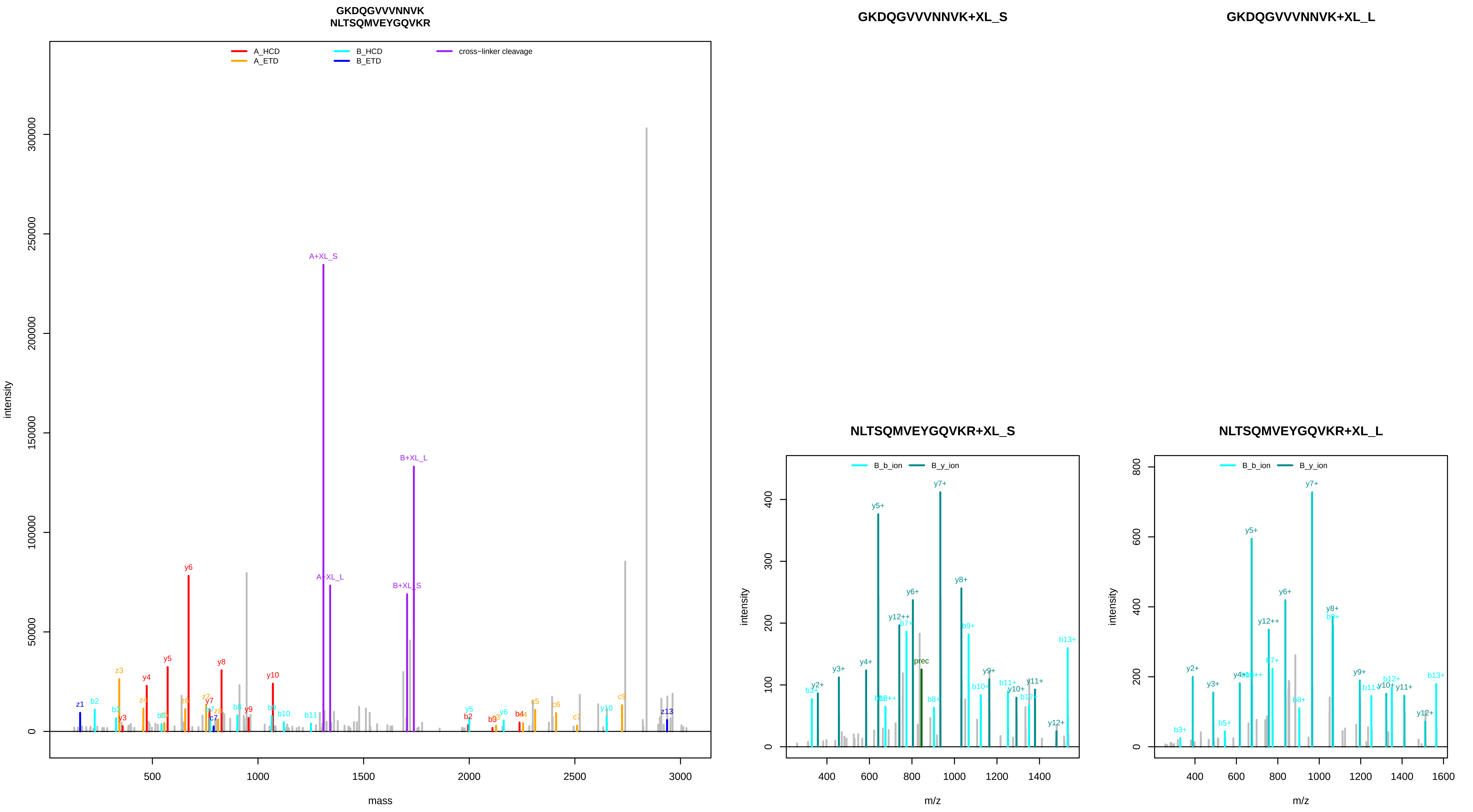
GKDQGVVVNNVK
NLTSQMVEYGQVKR
A_HCD
A_ETD
B_HCD
B_ETD
cross−linker cleavage
A+XL_S
B+XL_L
A+XL_L
B+XL_S
intensity
mass
GKDQGVVVNNVK+XL_S
GKDQGVVVNNVK+XL_L
NLTSQMVEYGQVKR+XL_S
NLTSQMVEYGQVKR+XL_L
B_b_ion
B_y_ion
intensity
m/z

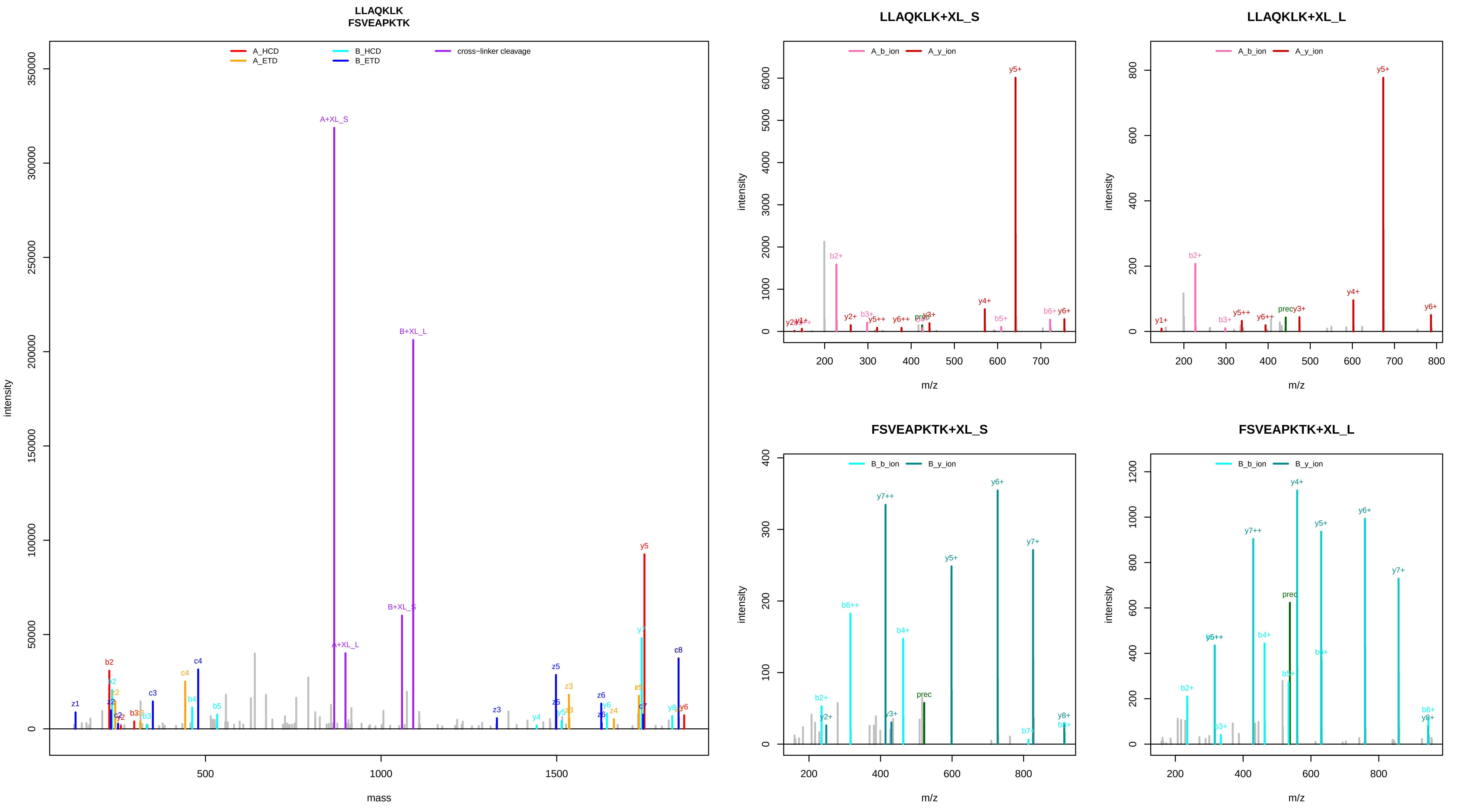
LLAQKLK
FSVEAPKTK
A_HCD
A_ETD
B_HCD
B_ETD
cross−linker cleavage
A+XL_S
B+XL_L
B+XL_S
A+XL_L
intensity
mass
LLAQKLK+XL_S
LLAQKLK+XL_L
A_b_ion
A_y_ion
FSVEAPKTK+XL_S
FSVEAPKTK+XL_L
B_b_ion
B_y_ion
intensity
m/z

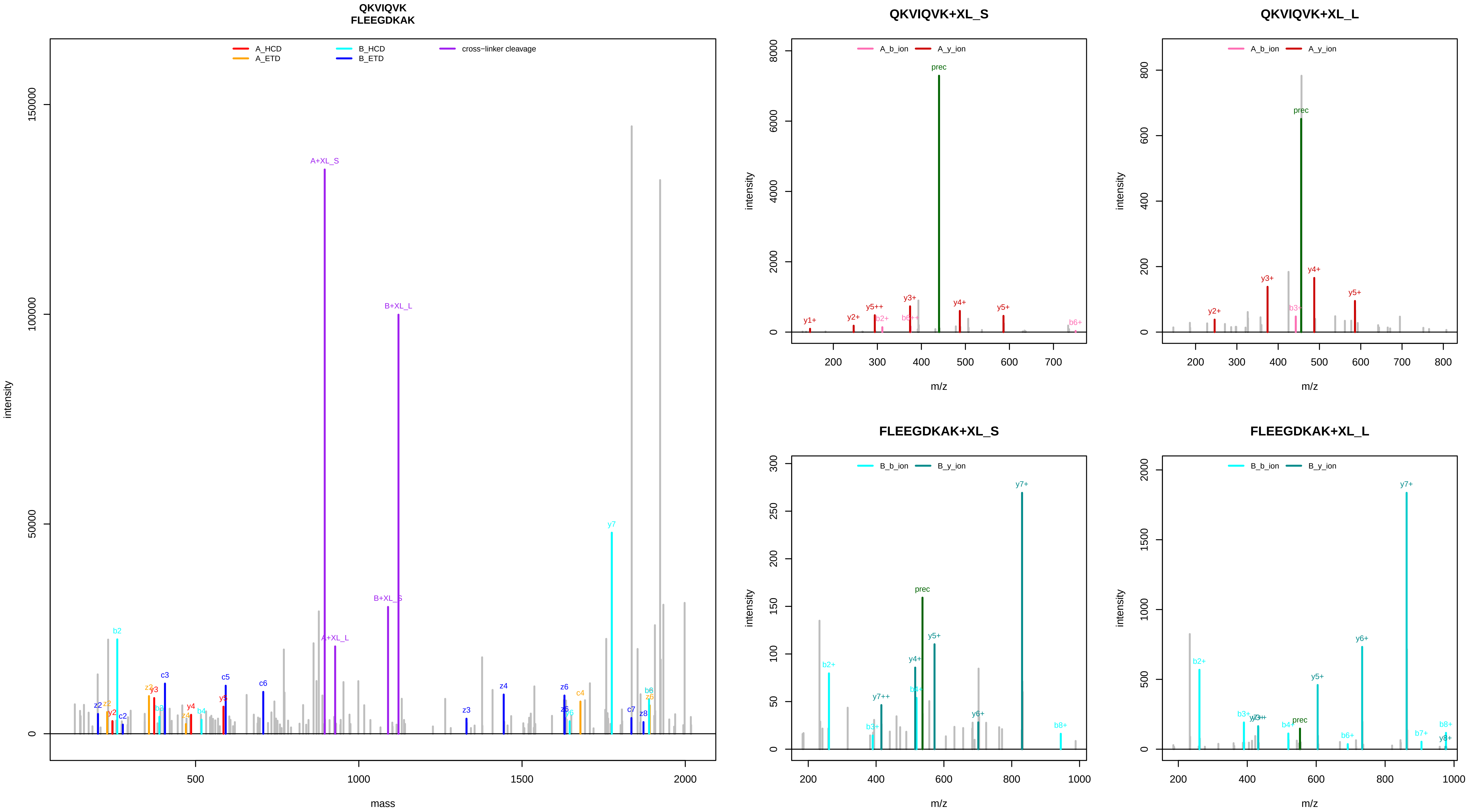

QKVIQVK
FLEEGDKAK
A_HCD
A_ETD
B_HCD
B_ETD
cross−linker cleavage
A+XL_S
B+XL_L
B+XL_S
A+XL_L
intensity
mass
QKVIQVK+XL_S
QKVIQVK+XL_L
A_b_ion
A_y_ion
prec
FLEEGDKAK+XL_S
FLEEGDKAK+XL_L
B_b_ion
B_y_ion
m/z

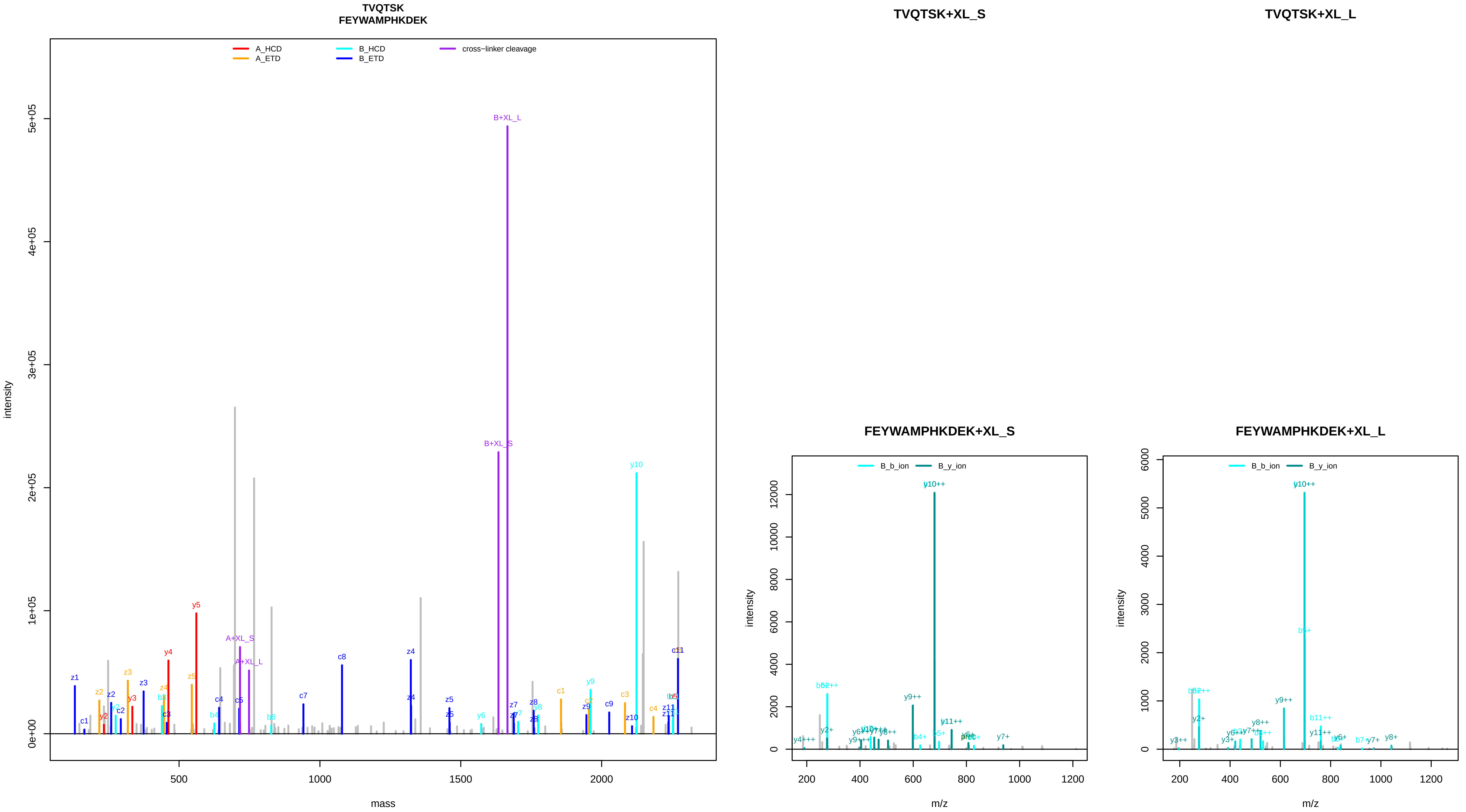
TVQTSK
FEYWAMPHKDEK
A_HCD
A_ETD
B_HCD
B_ETD
cross−linker cleavage
B+XL_L
B+XL_S
A+XL_S
A+XL_L
intensity
mass
0e+00
1e+05
2e+05
3e+05
4e+05
5e+05
500
1000
1500
2000
TVQTSK+XL_S
TVQTSK+XL_L
FEYWAMPHKDEK+XL_S
FEYWAMPHKDEK+XL_L
B_b_ion
B_y_ion
y10++
y9++
intensity
m/z
200
400
600
800
1000
1200

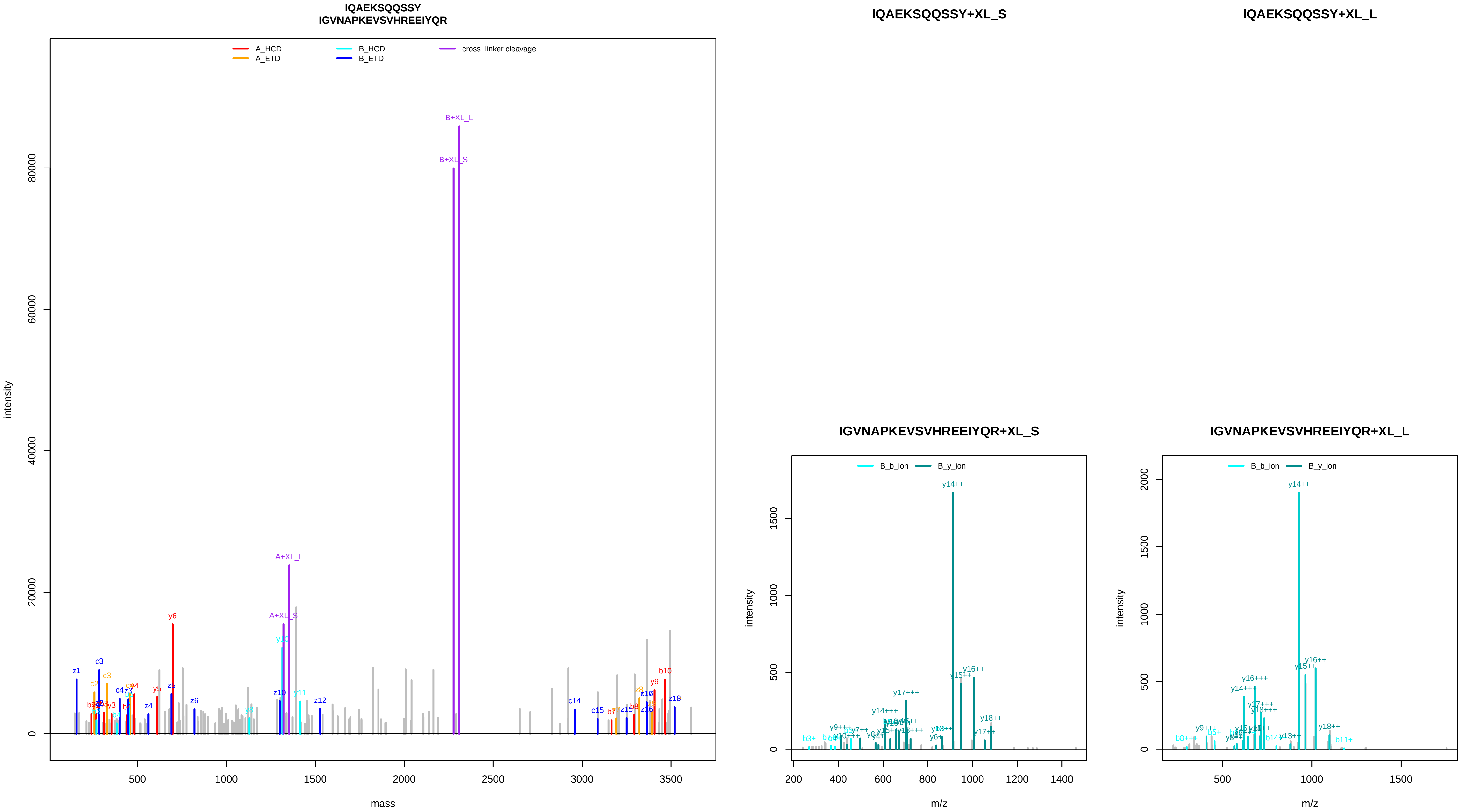
IQAEKSQQSSY
IGVNAPKEVSVHREEIYQR
A_HCD
A_ETD
B_HCD
B_ETD
cross−linker cleavage
B+XL_L
B+XL_S
A+XL_L
A+XL_S
intensity
mass
IQAEKSQQSSY+XL_S
IQAEKSQQSSY+XL_L
IGVNAPKEVSVHREEIYQR+XL_S
IGVNAPKEVSVHREEIYQR+XL_L
B_b_ion
B_y_ion
y14++
y15++
y16++
y17+++
y18++
y16+++
y14+++
intensity
m/z

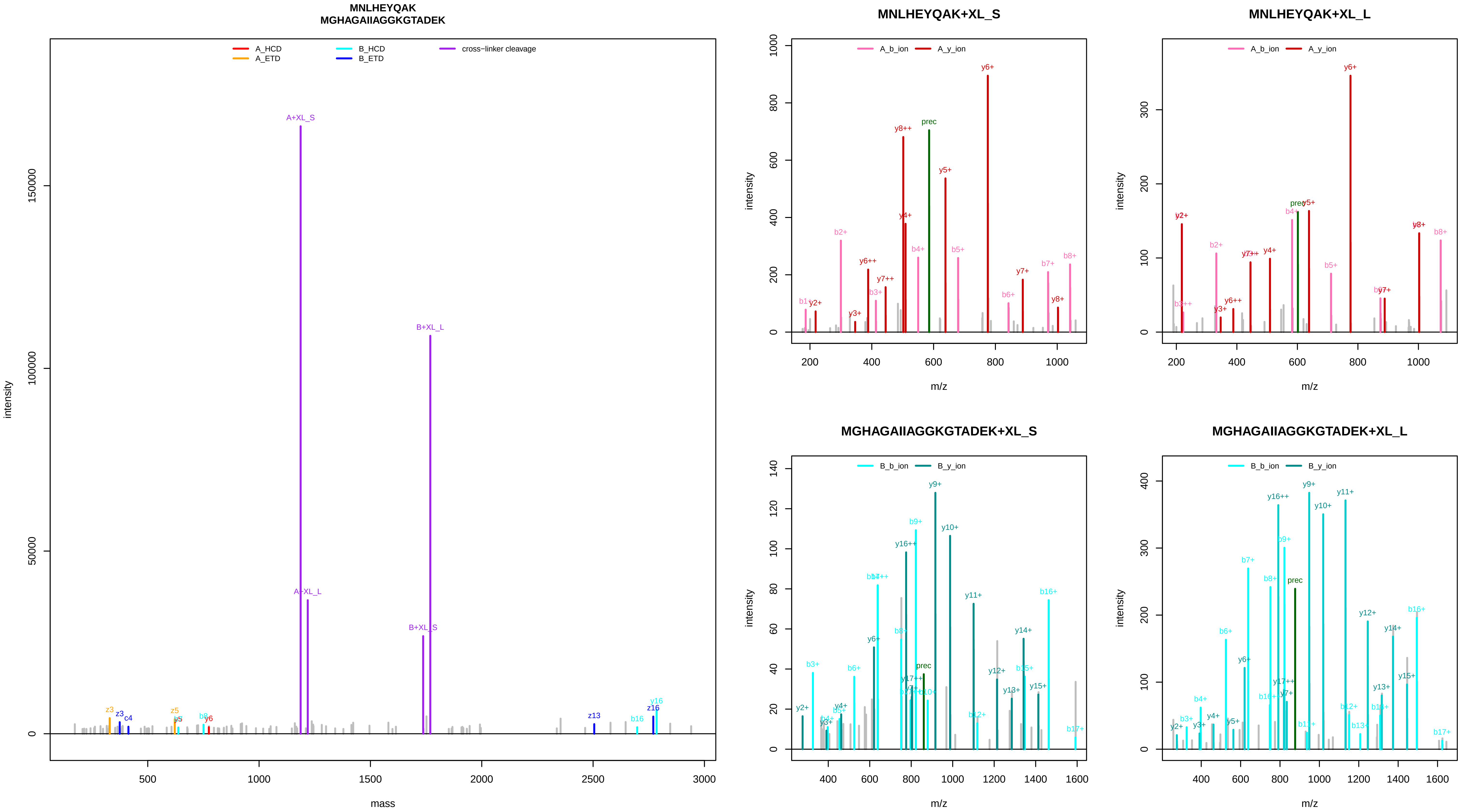

MNLHEYQAK
MGHAGAIIAGGKGTADEK
A_HCD
A_ETD
B_HCD
B_ETD
cross−linker cleavage
A+XL_S
B+XL_L
A+XL_L
B+XL_S
intensity
mass
MNLHEYQAK+XL_S
MNLHEYQAK+XL_L
A_b_ion
A_y_ion
MGHAGAIIAGGKGTADEK+XL_S
MGHAGAIIAGGKGTADEK+XL_L
B_b_ion
B_y_ion
prec
m/z

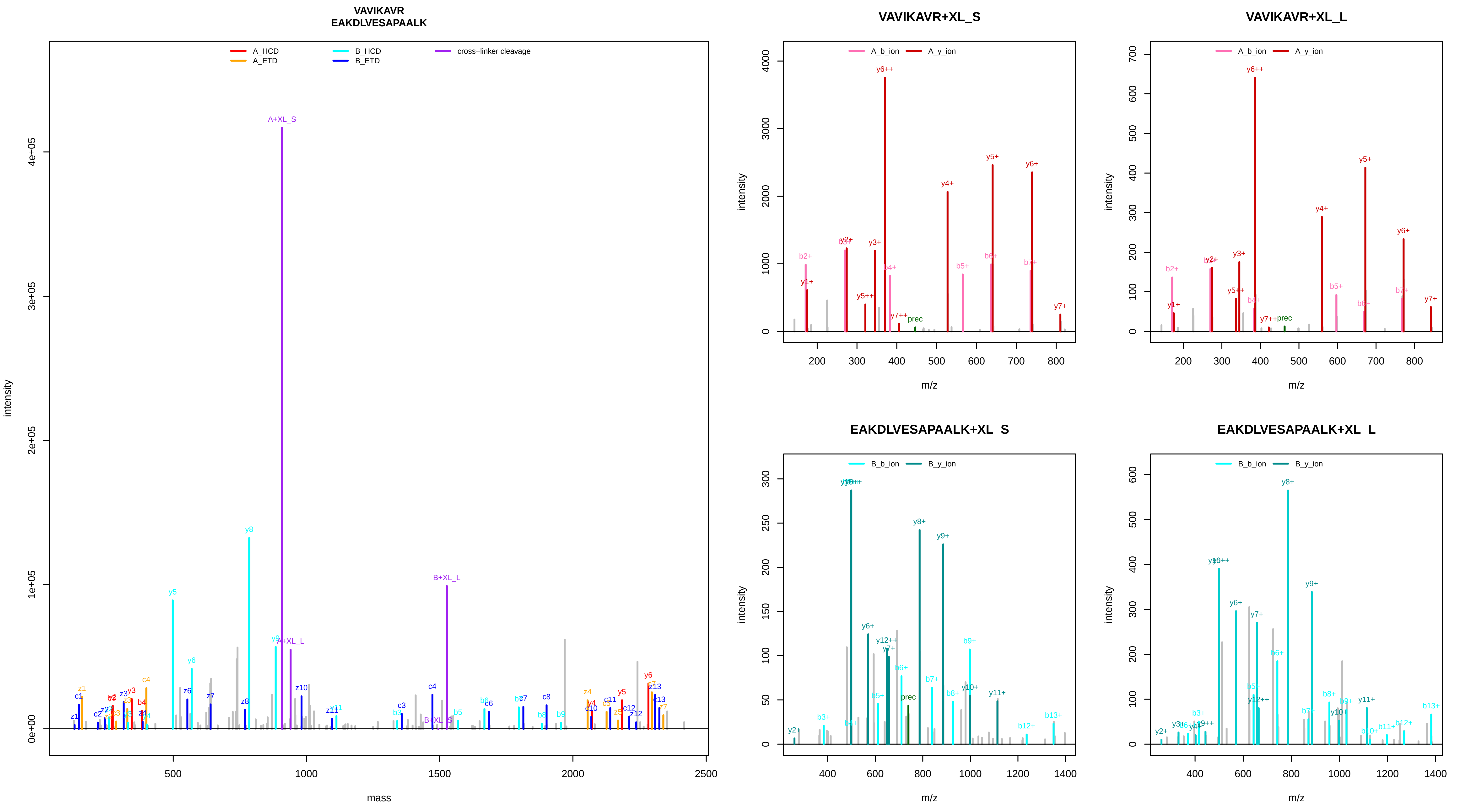
VAVIKAVR
EAKDLVESAPAALK
A_HCD
A_ETD
B_HCD
B_ETD
cross−linker cleavage
A+XL_S
A+XL_L
B+XL_L
B+XL_S
intensity
mass
VAVIKAVR+XL_S
VAVIKAVR+XL_L
A_b_ion
A_y_ion
EAKDLVESAPAALK+XL_S
EAKDLVESAPAALK+XL_L
B_b_ion
B_y_ion
prec
m/z

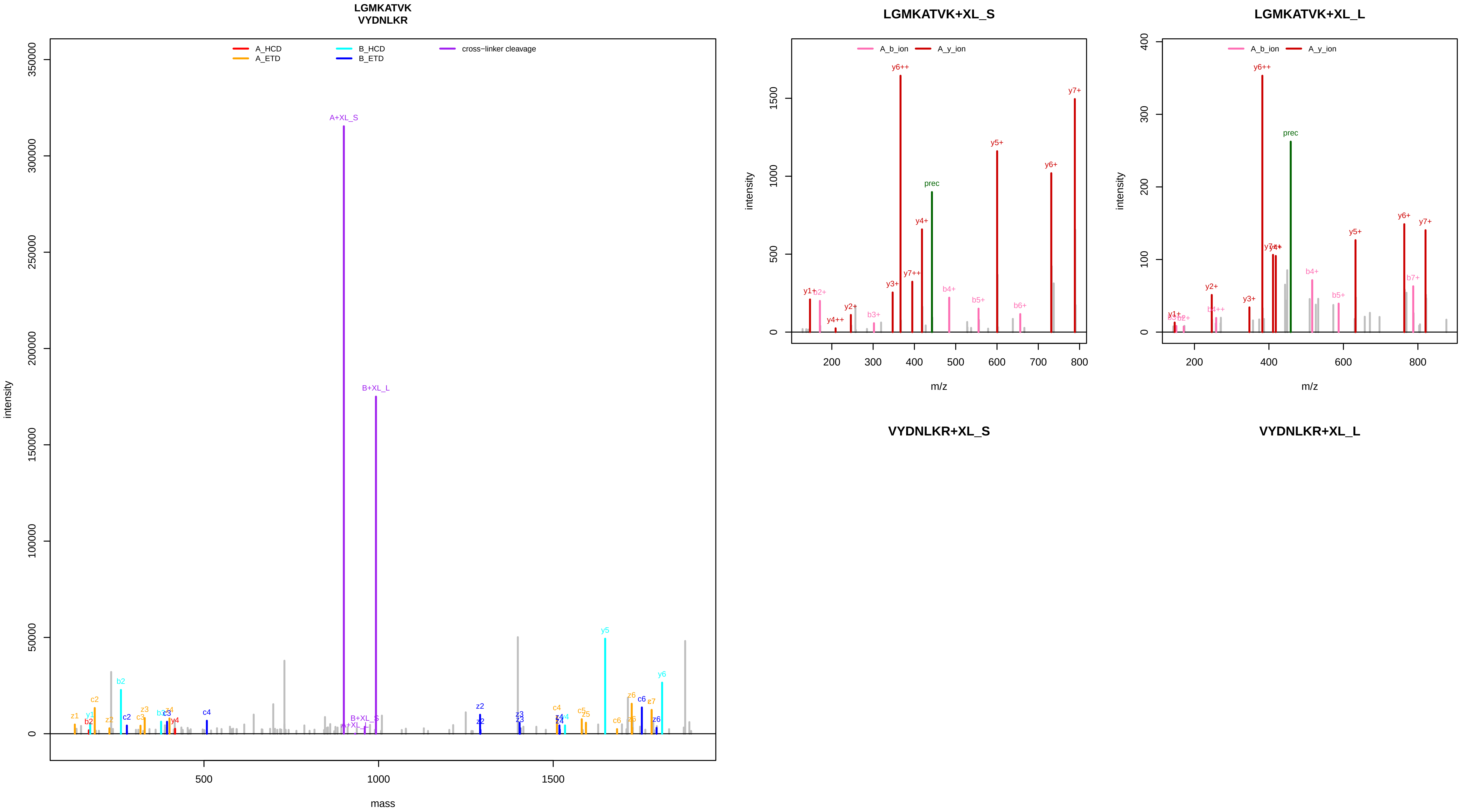

LGMKATVK
VYDNLKR
A_HCD
A_ETD
B_HCD
B_ETD
cross−linker cleavage
A+XL_S
B+XL_L
intensity
mass
LGMKATVK+XL_S
LGMKATVK+XL_L
A_b_ion
A_y_ion
prec
m/z
VYDNLKR+XL_S
VYDNLKR+XL_L

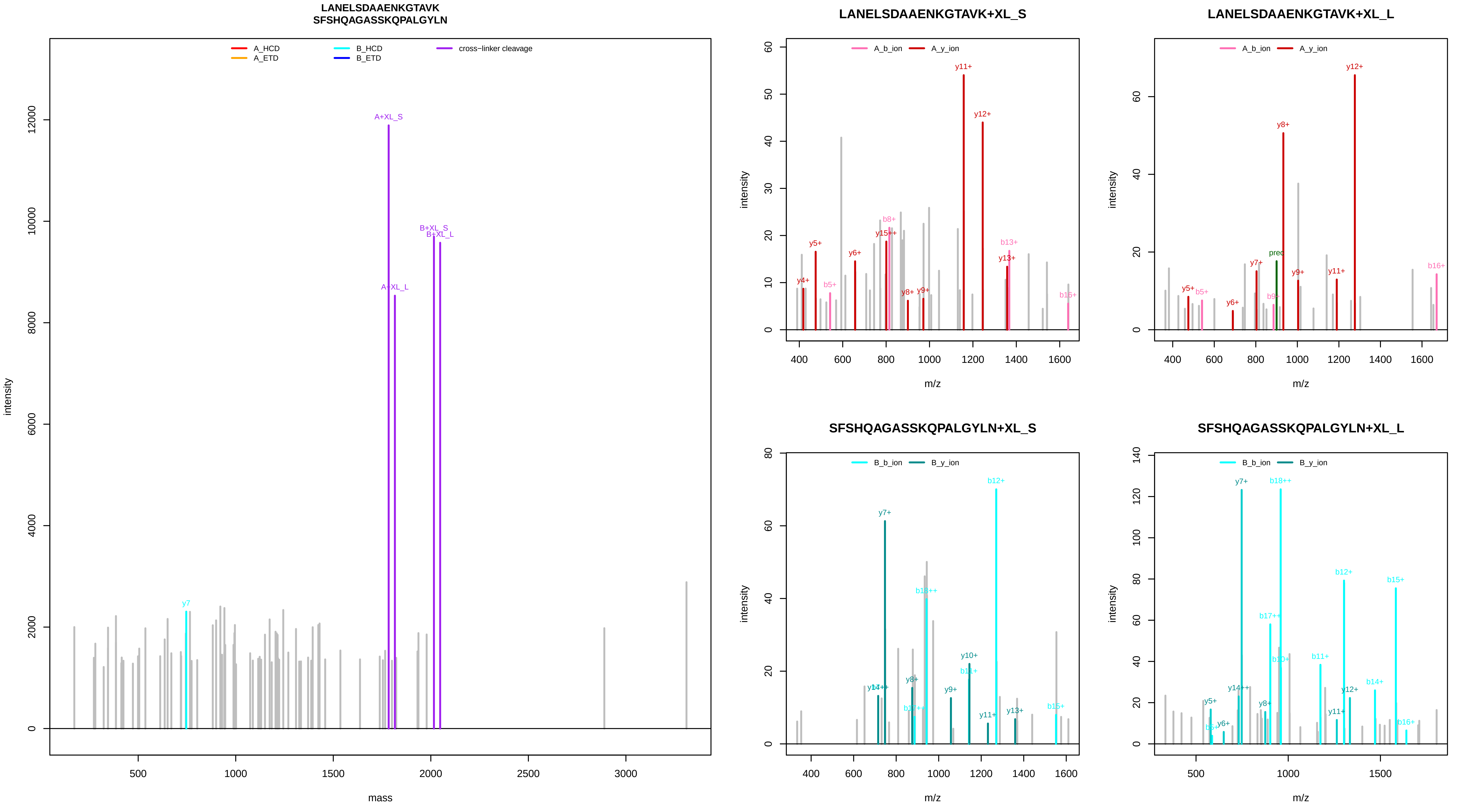
LANELSDAAENKGTAVK
SFSHQAGASSKQPALGYLN
A_HCD
A_ETD
B_HCD
B_ETD
cross−linker cleavage
A+XL_S
A+XL_L
B+XL_S
B+XL_L
y7
intensity
mass
LANELSDAAENKGTAVK+XL_S
LANELSDAAENKGTAVK+XL_L
SFSHQAGASSKQPALGYLN+XL_S
SFSHQAGASSKQPALGYLN+XL_L
A_b_ion
A_y_ion
B_b_ion
B_y_ion
m/z

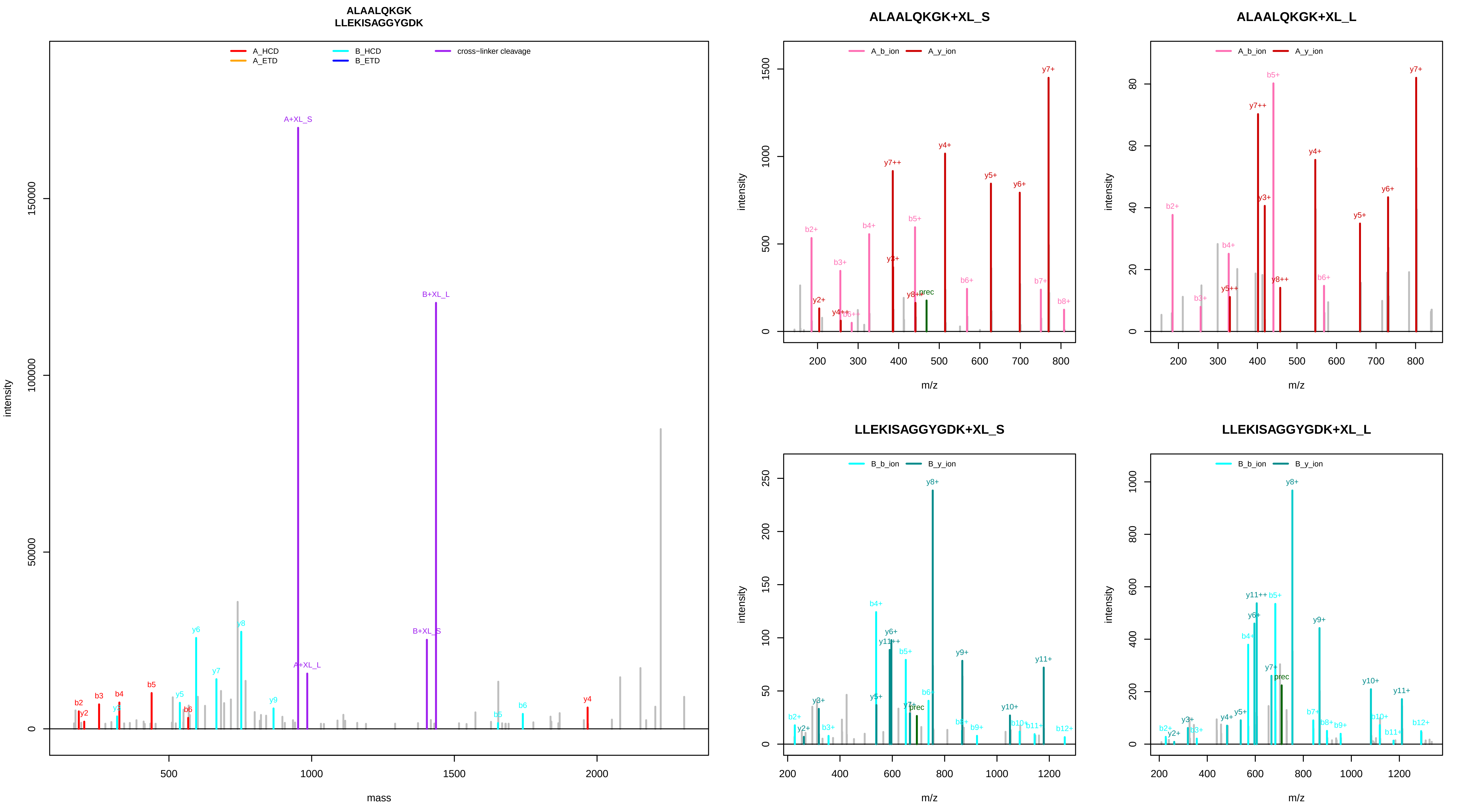
ALAALQKGK
LLEKISAGGYGDK
A_HCD
A_ETD
B_HCD
B_ETD
cross−linker cleavage
A+XL_S
B+XL_L
B+XL_S
A+XL_L
intensity
mass
ALAALQKGK+XL_S
ALAALQKGK+XL_L
LLEKISAGGYGDK+XL_S
LLEKISAGGYGDK+XL_L
A_b_ion
A_y_ion
B_b_ion
B_y_ion
m/z

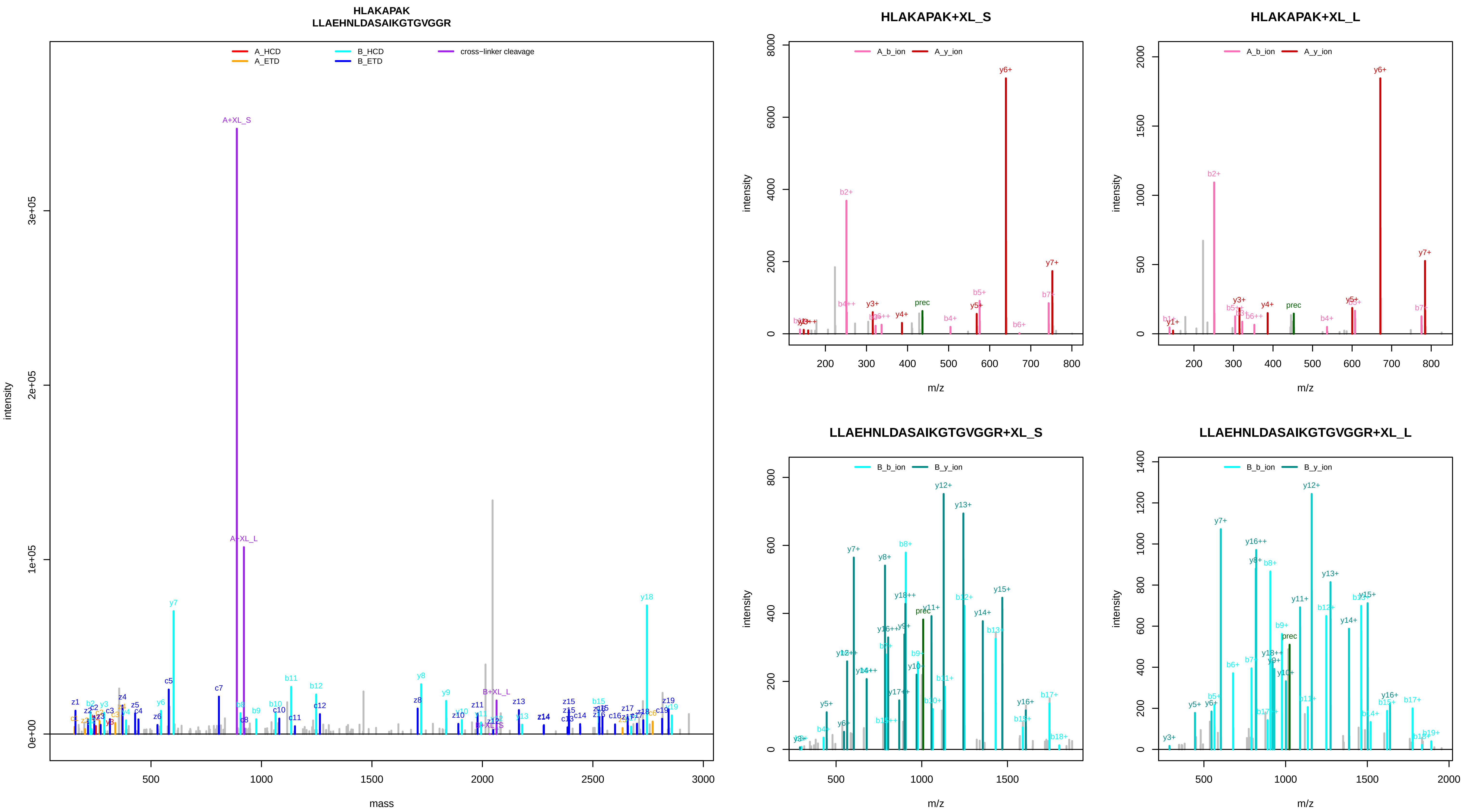

HLAKAPAK
LLAEHNLDASAIKGTGVGGR
A_HCD
A_ETD
B_HCD
B_ETD
cross−linker cleavage
A+XL_S
A+XL_L
B+XL_L
intensity
mass
HLAKAPAK+XL_S
HLAKAPAK+XL_L
A_b_ion
A_y_ion
prec
m/z
LLAEHNLDASAIKGTGVGGR+XL_S
LLAEHNLDASAIKGTGVGGR+XL_L
B_b_ion
B_y_ion

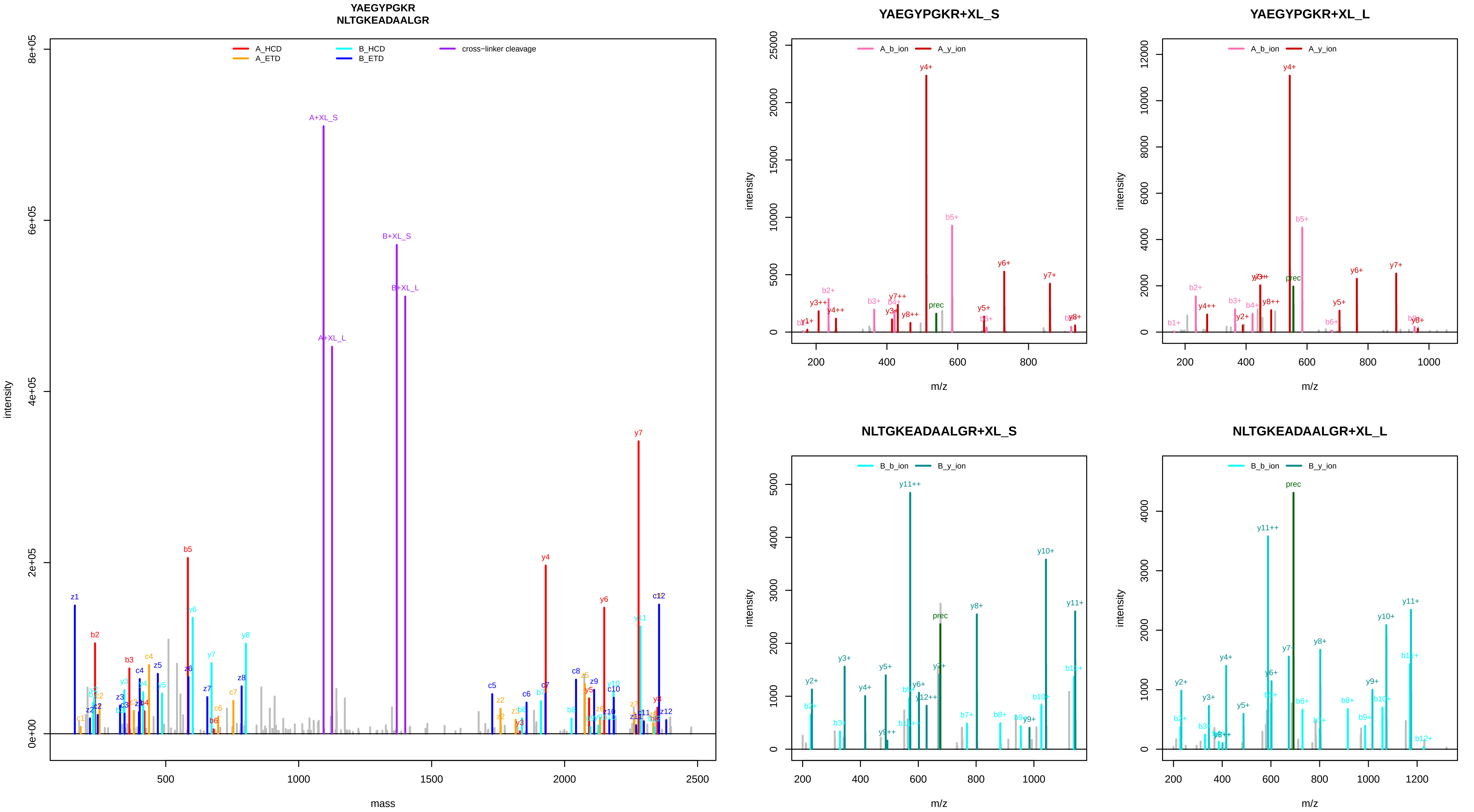

YAEGYPGKR
NLTGKEADAALGR
A_HCD
A_ETD
B_HCD
B_ETD
cross−linker cleavage
intensity
mass
YAEGYPGKR+XL_S
YAEGYPGKR+XL_L
NLTGKEADAALGR+XL_S
NLTGKEADAALGR+XL_L
A_b_ion
A_y_ion
B_b_ion
B_y_ion
m/z

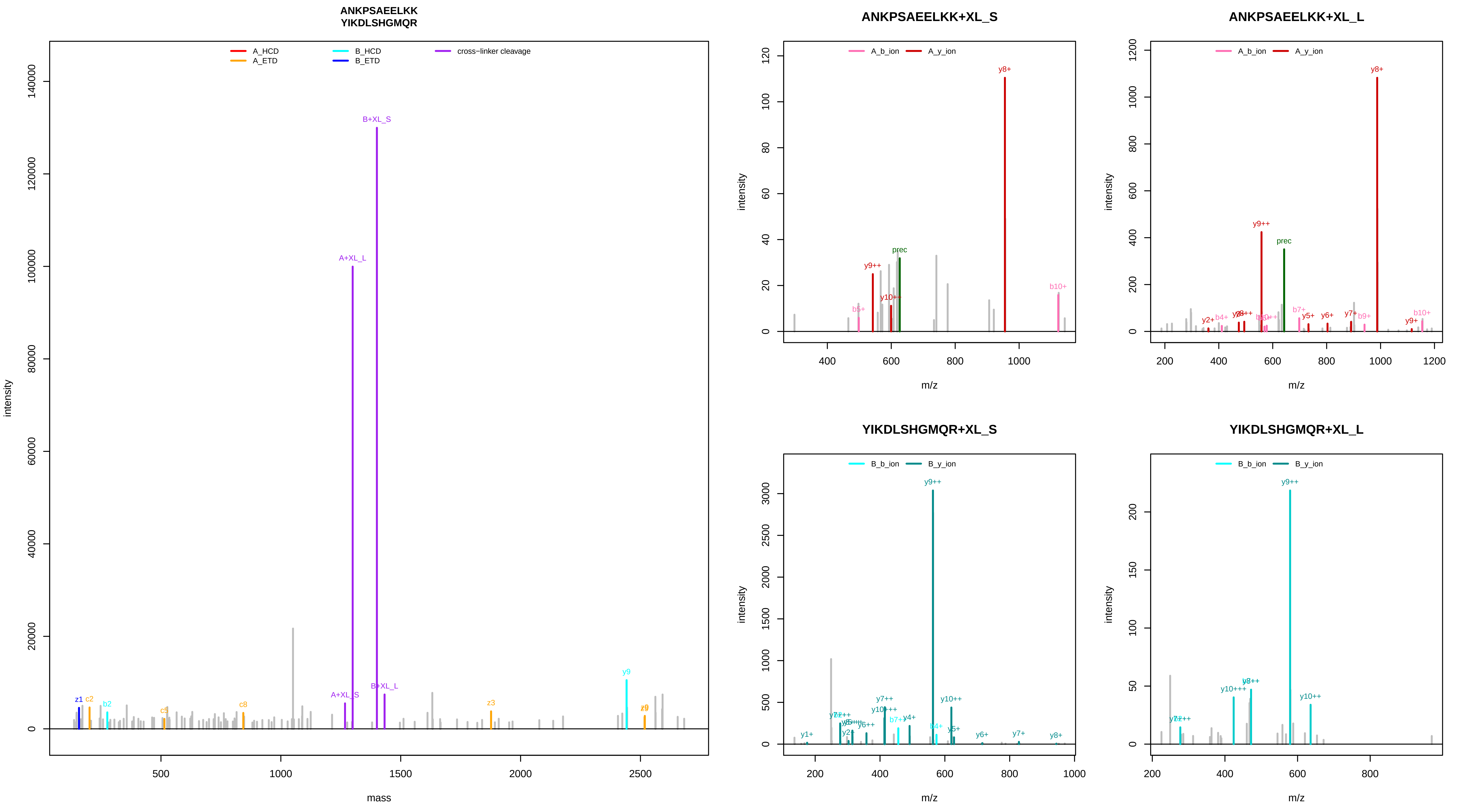
ANKPSAEELKK
YIKDLSHGMQR
A_HCD
A_ETD
B_HCD
B_ETD
cross−linker cleavage
B+XL_S
A+XL_L
A+XL_S
B+XL_L
z1
c2
b2
c5
c8
z3
y9
z9
intensity
mass
ANKPSAEELKK+XL_S
ANKPSAEELKK+XL_L
A_b_ion
A_y_ion
y8+
y9++
y10++
prec
b5+
b10+
y2+
b4+
b7+
y5+
y6+
y7+
b9+
y9+
m/z
YIKDLSHGMQR+XL_S
YIKDLSHGMQR+XL_L
B_b_ion
B_y_ion
y9++
y10++
y7++
y10+++
y8++
y4+
b7+
b4+
y5+
y6+
y7+
y8+
y1+
y2+

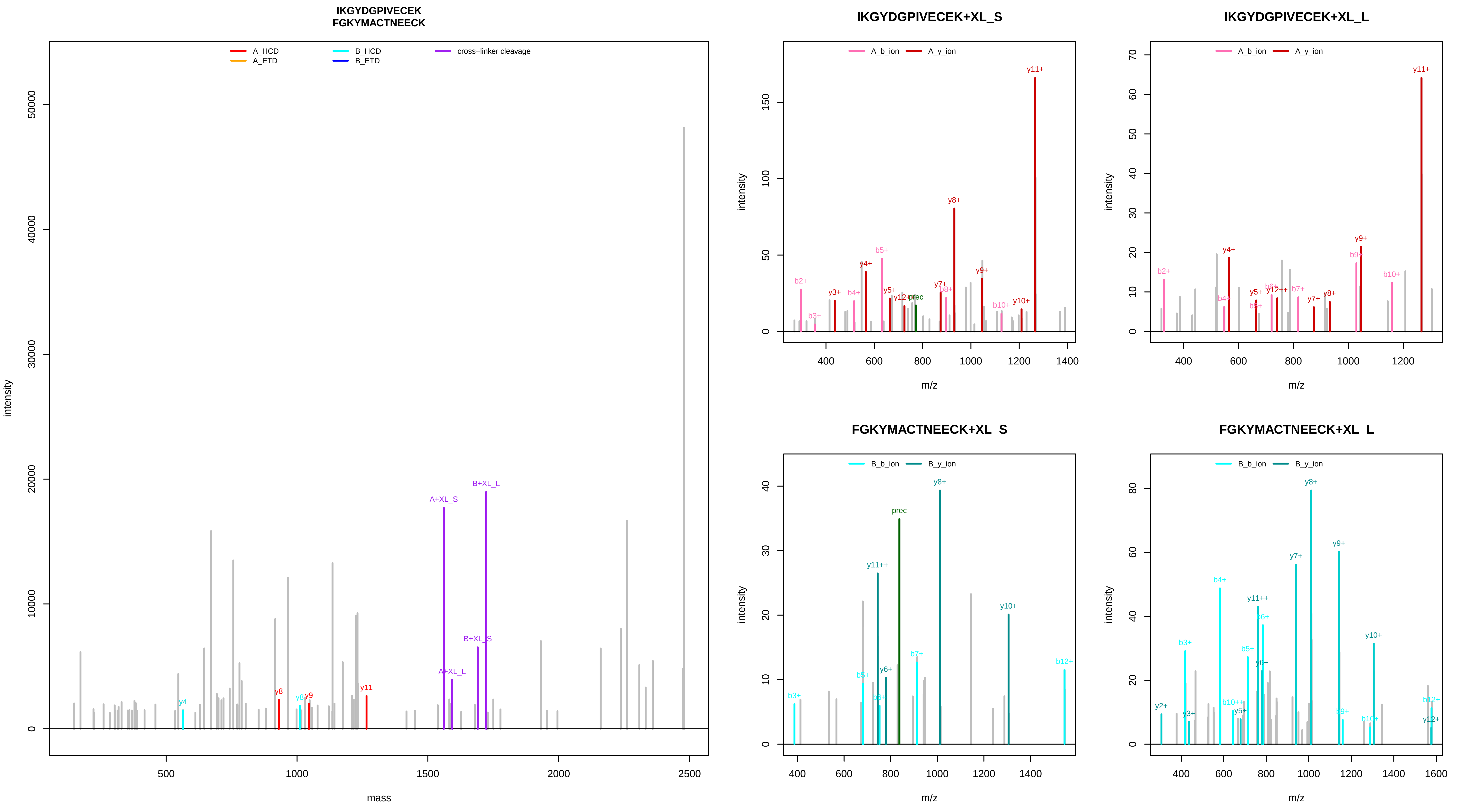
IKGYDGPIVECEK
FGKYMACTNEECK
A_HCD
A_ETD
B_HCD
B_ETD
cross−linker cleavage
A+XL_S
B+XL_L
B+XL_S
A+XL_L
intensity
mass
IKGYDGPIVECEK+XL_S
IKGYDGPIVECEK+XL_L
FGKYMACTNEECK+XL_S
FGKYMACTNEECK+XL_L
A_b_ion
A_y_ion
B_b_ion
B_y_ion
m/z

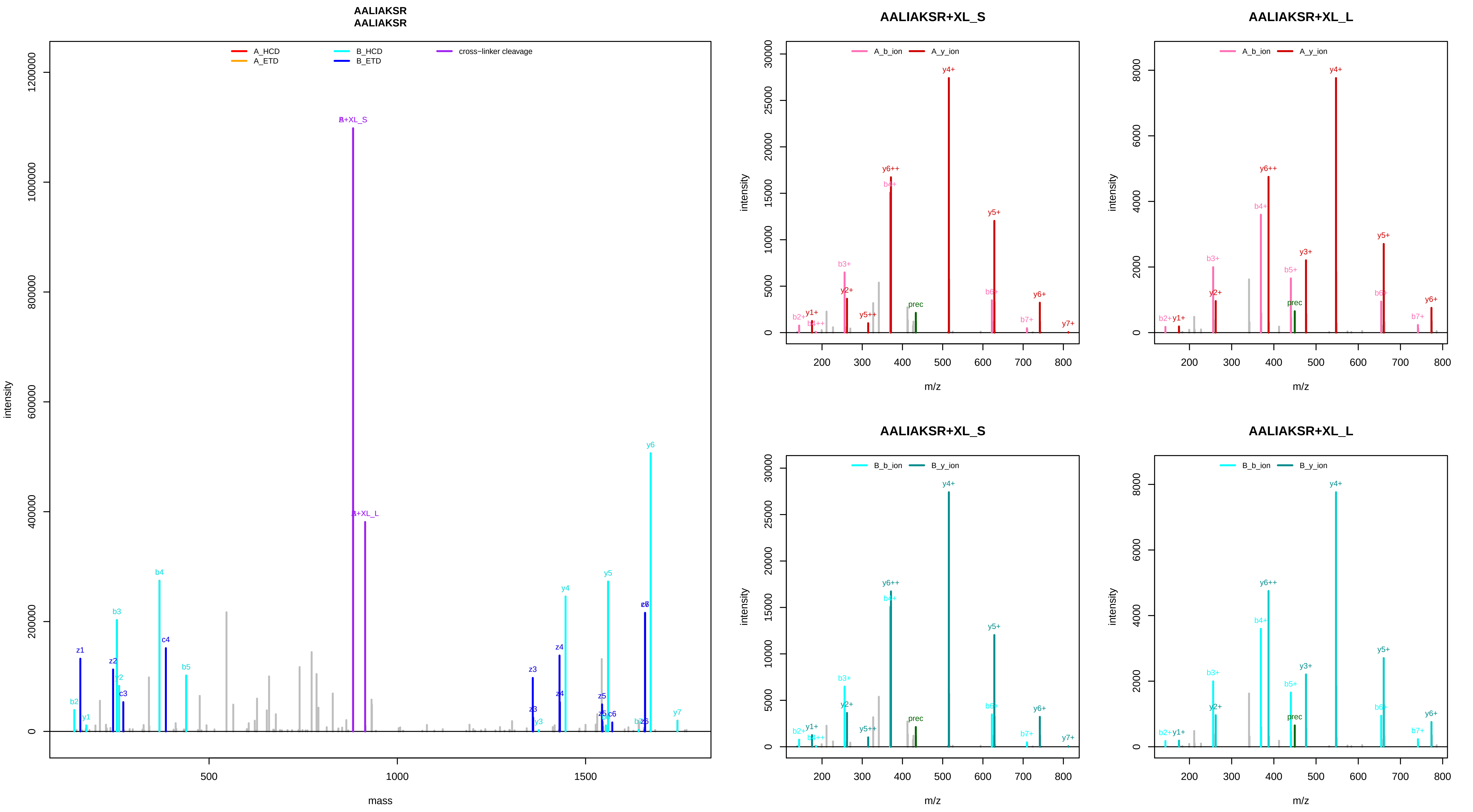
AALIAKSR
AALIAKSR
A_HCD
A_ETD
B_HCD
B_ETD
cross−linker cleavage
intensity
mass
0
200000
400000
600000
800000
1000000
1200000
500
1000
1500
B+XL_S
B+XL_L
b2
y1
z1
z2
b3
y2
c3
b4
c4
b5
z3
y3
z4
y4
z5
c5
c6
y5
b7
z6
c7
y6
y7
AALIAKSR+XL_S
A_b_ion
A_y_ion
AALIAKSR+XL_L
A_b_ion
A_y_ion
AALIAKSR+XL_S
B_b_ion
B_y_ion
AALIAKSR+XL_L
B_b_ion
B_y_ion
intensity
m/z
b2+
y1+
b4++
b3+
y2+
y5++
y6++
b4+
prec
y4+
b5+
y3+
b6+
y5+
b7+
y6+
y7+
0
5000
10000
15000
20000
25000
30000
2000
4000
6000
8000
200
300
400
500
600
700
800

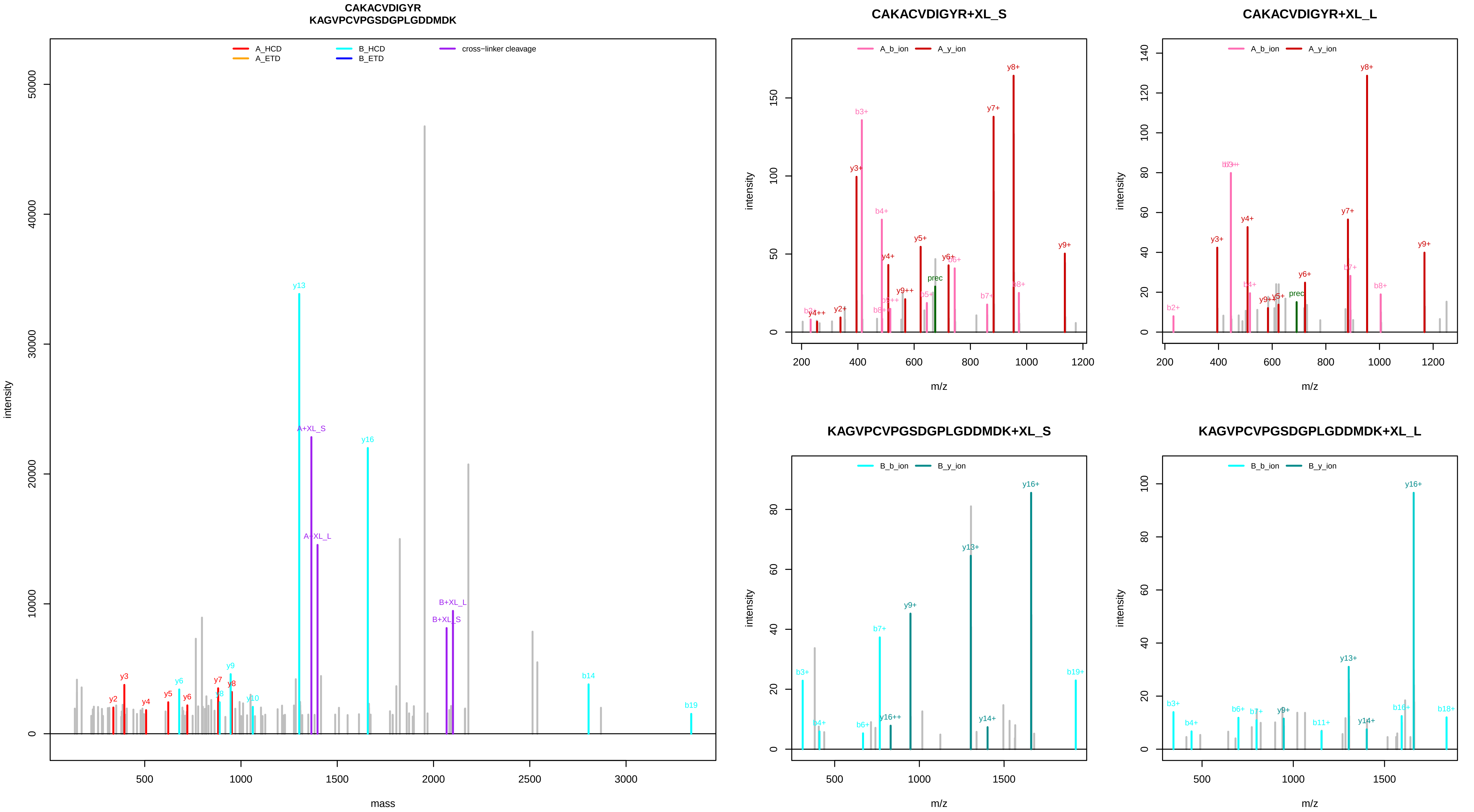

CAKACVDIGYR
KAGVPCVPGSDGPLGDDMDK
A_HCD
A_ETD
B_HCD
B_ETD
cross−linker cleavage
intensity
mass
CAKACVDIGYR+XL_S
CAKACVDIGYR+XL_L
A_b_ion
A_y_ion
m/z
KAGVPCVPGSDGPLGDDMDK+XL_S
KAGVPCVPGSDGPLGDDMDK+XL_L
B_b_ion
B_y_ion

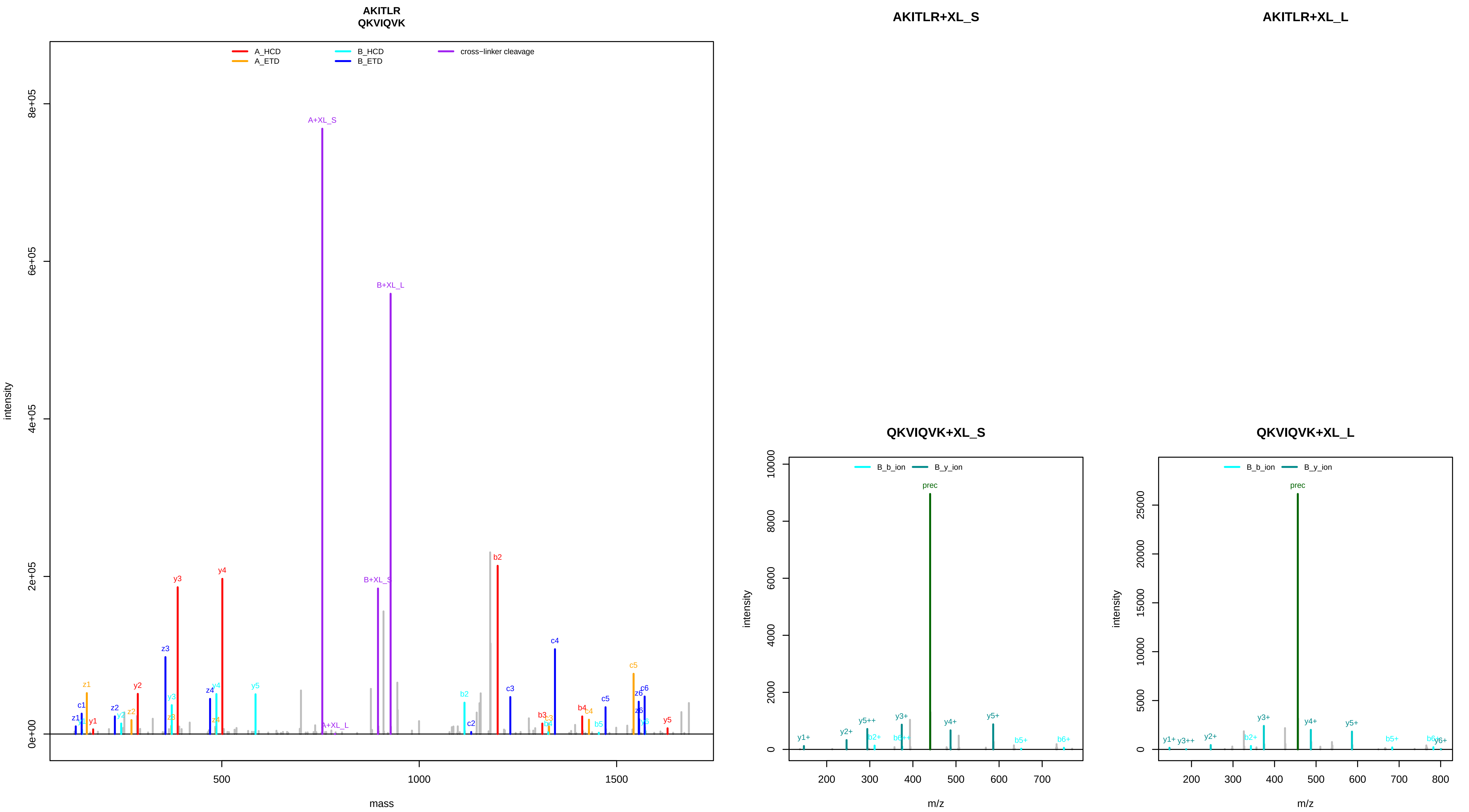

AKITLR
QKVIQVK
A_HCD
A_ETD
B_HCD
B_ETD
cross−linker cleavage
A+XL_S
B+XL_L
B+XL_S
A+XL_L
intensity
mass
AKITLR+XL_S
AKITLR+XL_L
QKVIQVK+XL_S
QKVIQVK+XL_L
B_b_ion
B_y_ion
prec
m/z

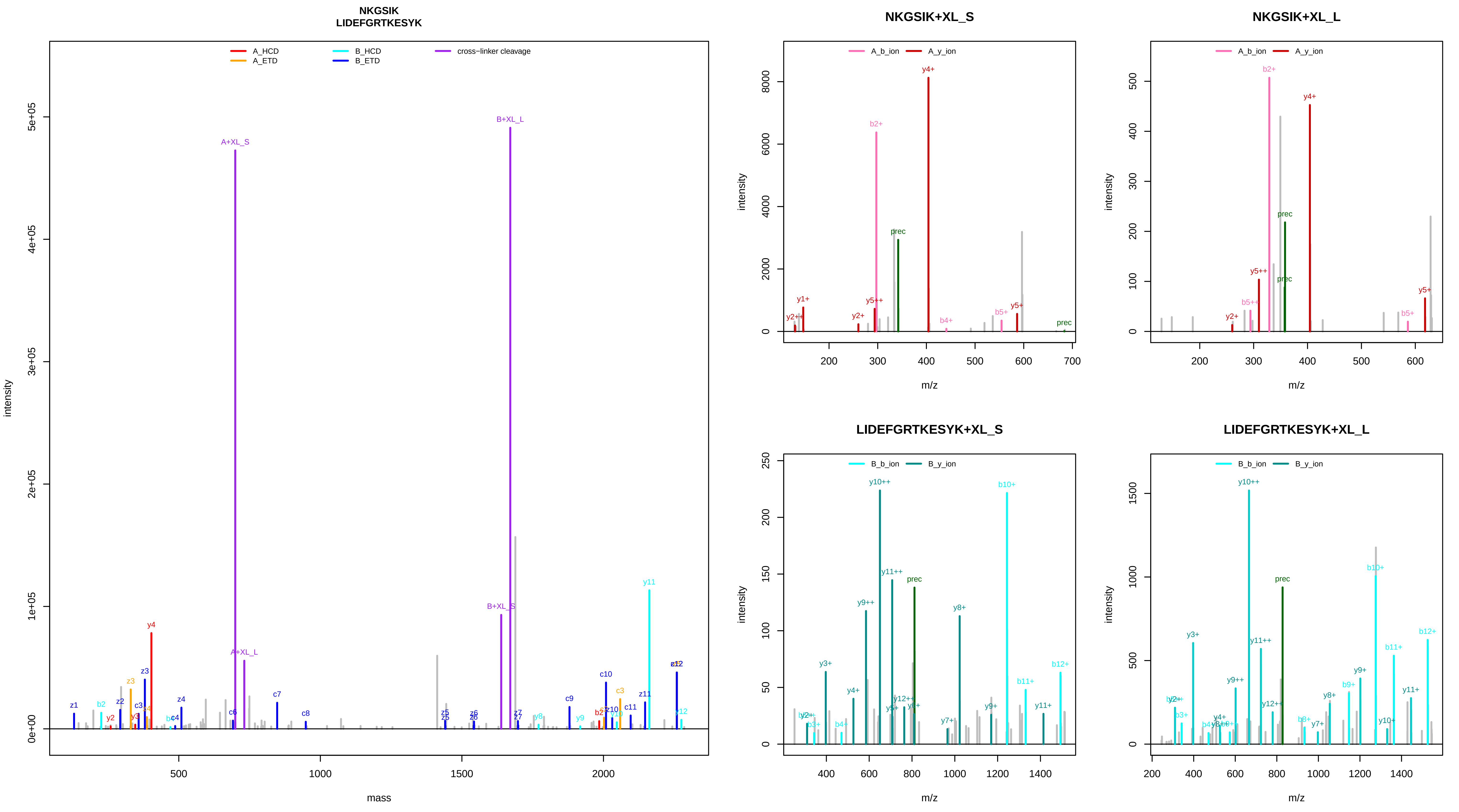
NKGSIK
LIDEFGRTKESYK
A_HCD
A_ETD
B_HCD
B_ETD
cross−linker cleavage
A+XL_S
B+XL_L
B+XL_S
A+XL_L
intensity
mass
NKGSIK+XL_S
NKGSIK+XL_L
LIDEFGRTKESYK+XL_S
LIDEFGRTKESYK+XL_L
A_b_ion
A_y_ion
B_b_ion
B_y_ion
prec
m/z

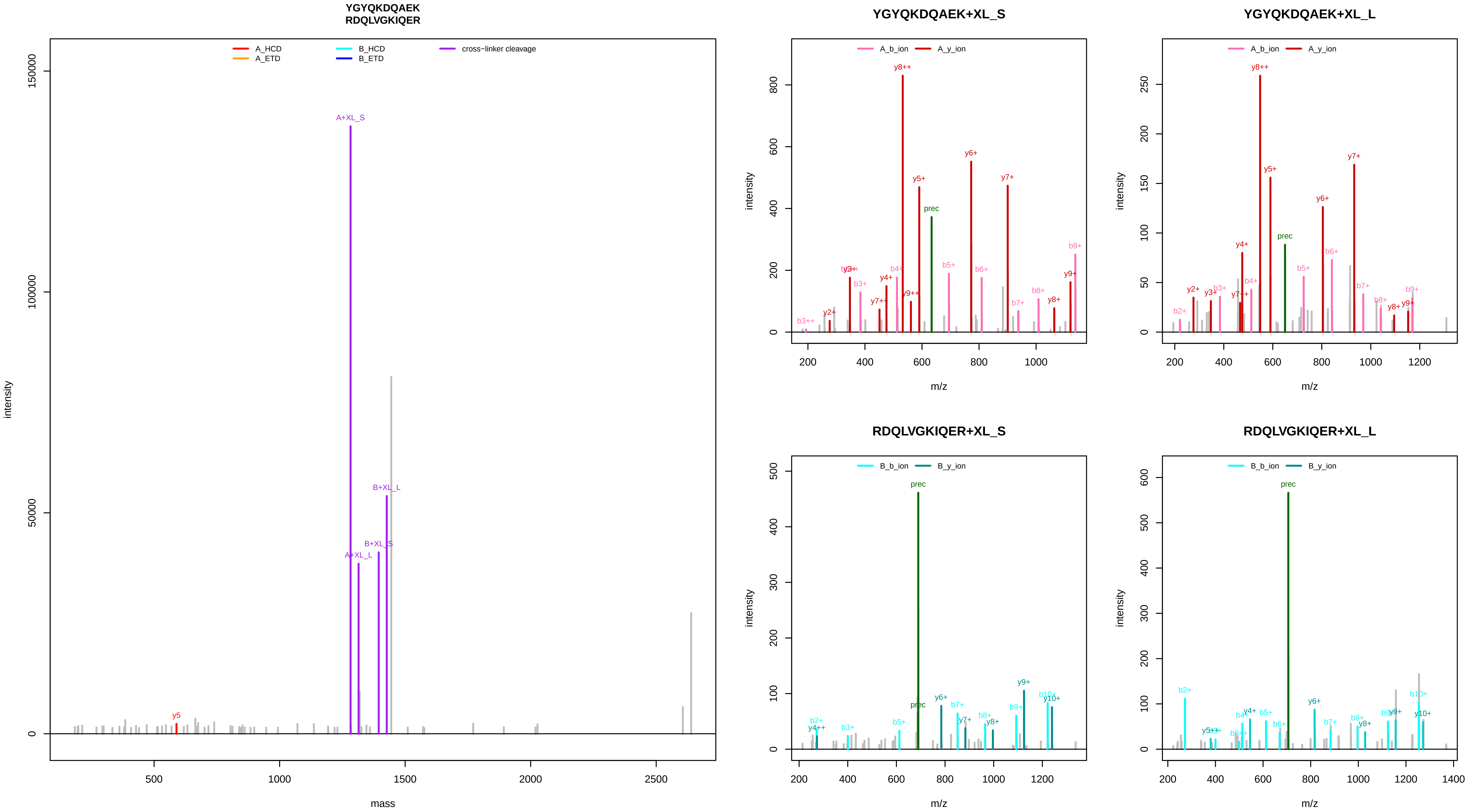

YGYQKDQAEK
RDQLVGKIQER
A_HCD
A_ETD
B_HCD
B_ETD
cross−linker cleavage
A+XL_S
A+XL_L
B+XL_S
B+XL_L
y5
intensity
mass
YGYQKDQAEK+XL_S
YGYQKDQAEK+XL_L
RDQLVGKIQER+XL_S
RDQLVGKIQER+XL_L
A_b_ion
A_y_ion
B_b_ion
B_y_ion
prec
m/z

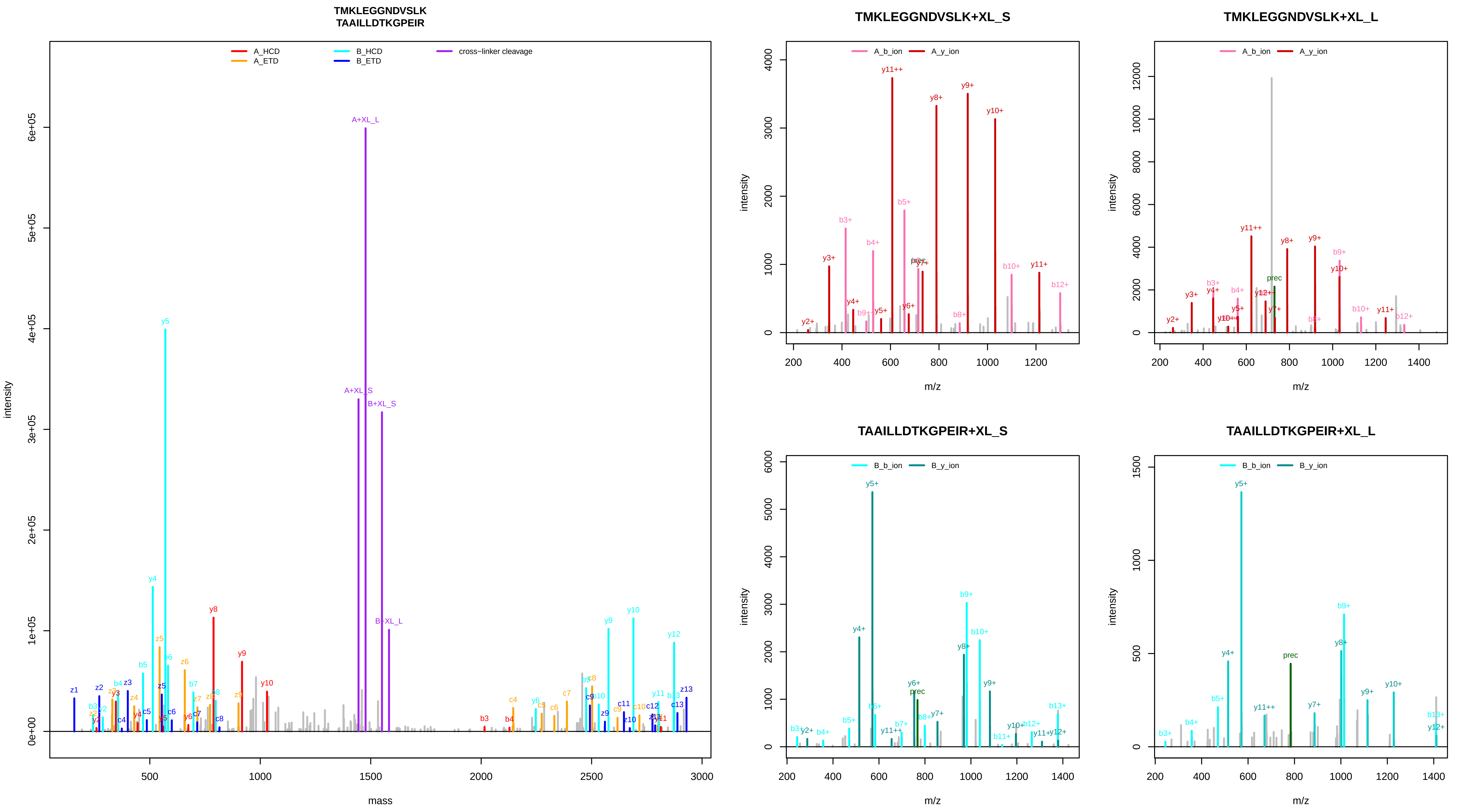
TMKLEGGNDVSLK
TAAILLDTKGPEIR
A_HCD
A_ETD
B_HCD
B_ETD
cross−linker cleavage
A+XL_L
A+XL_S
B+XL_S
B+XL_L
intensity
mass
TMKLEGGNDVSLK+XL_S
TMKLEGGNDVSLK+XL_L
A_b_ion
A_y_ion
TAAILLDTKGPEIR+XL_S
TAAILLDTKGPEIR+XL_L
B_b_ion
B_y_ion
prec
m/z

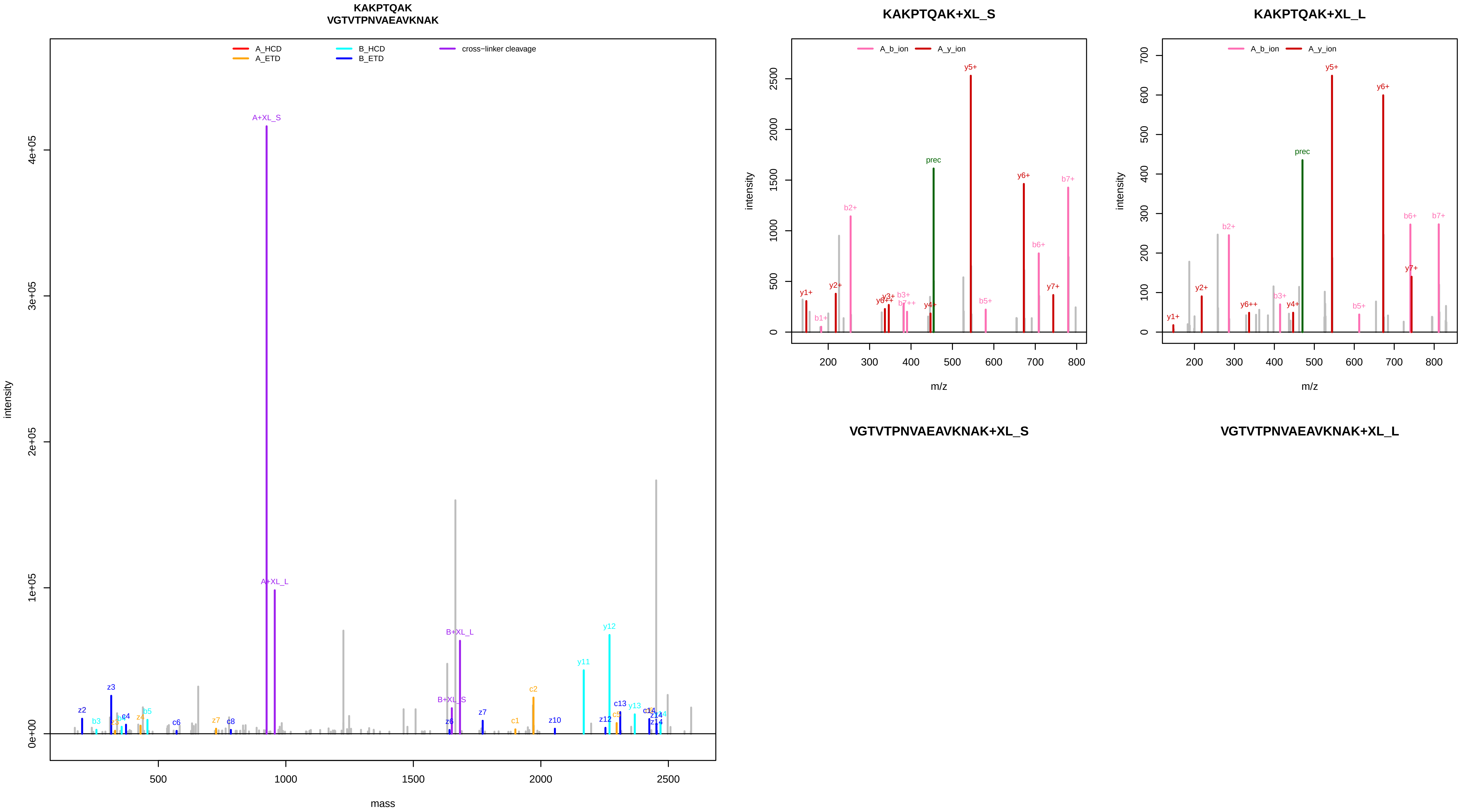
KAKPTQAK
VGTVTPNVAEAVKNAK
A_HCD
A_ETD
B_HCD
B_ETD
cross−linker cleavage
A+XL_S
A+XL_L
B+XL_L
B+XL_S
y12
y11
y13
c2
c13
z3
z2
z7
z10
z12
intensity
mass
KAKPTQAK+XL_S
KAKPTQAK+XL_L
A_b_ion
A_y_ion
prec
y5+
y6+
y7+
b2+
b6+
b7+
y1+
y2+
b5+
m/z
VGTVTPNVAEAVKNAK+XL_S
VGTVTPNVAEAVKNAK+XL_L

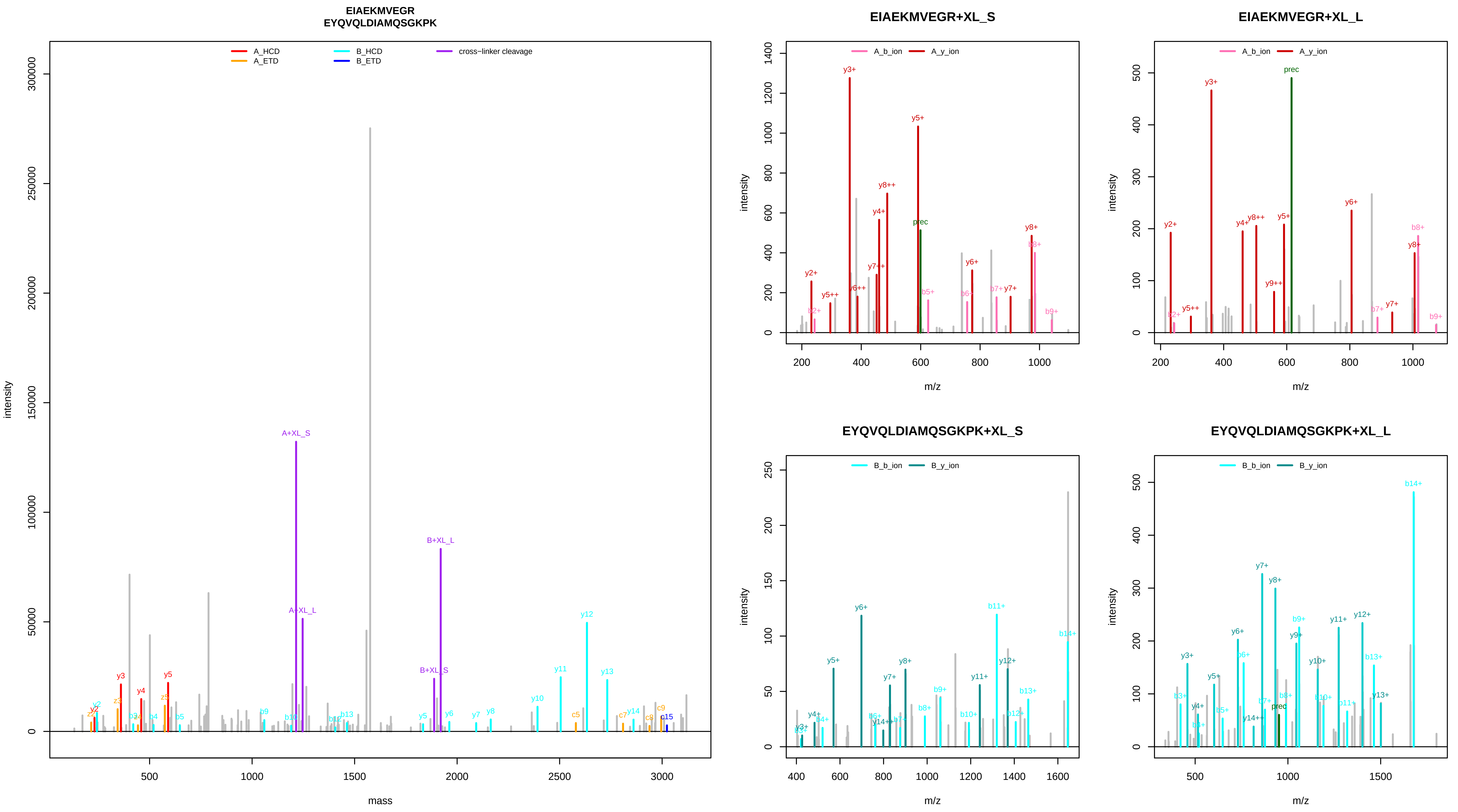
EIAEKMVEGR
EYQVQLDIAMQSGKPK
A_HCD
A_ETD
B_HCD
B_ETD
cross−linker cleavage
A+XL_S
A+XL_L
B+XL_L
B+XL_S
intensity
mass
EIAEKMVEGR+XL_S
EIAEKMVEGR+XL_L
EYQVQLDIAMQSGKPK+XL_S
EYQVQLDIAMQSGKPK+XL_L
A_b_ion
A_y_ion
B_b_ion
B_y_ion
prec
m/z

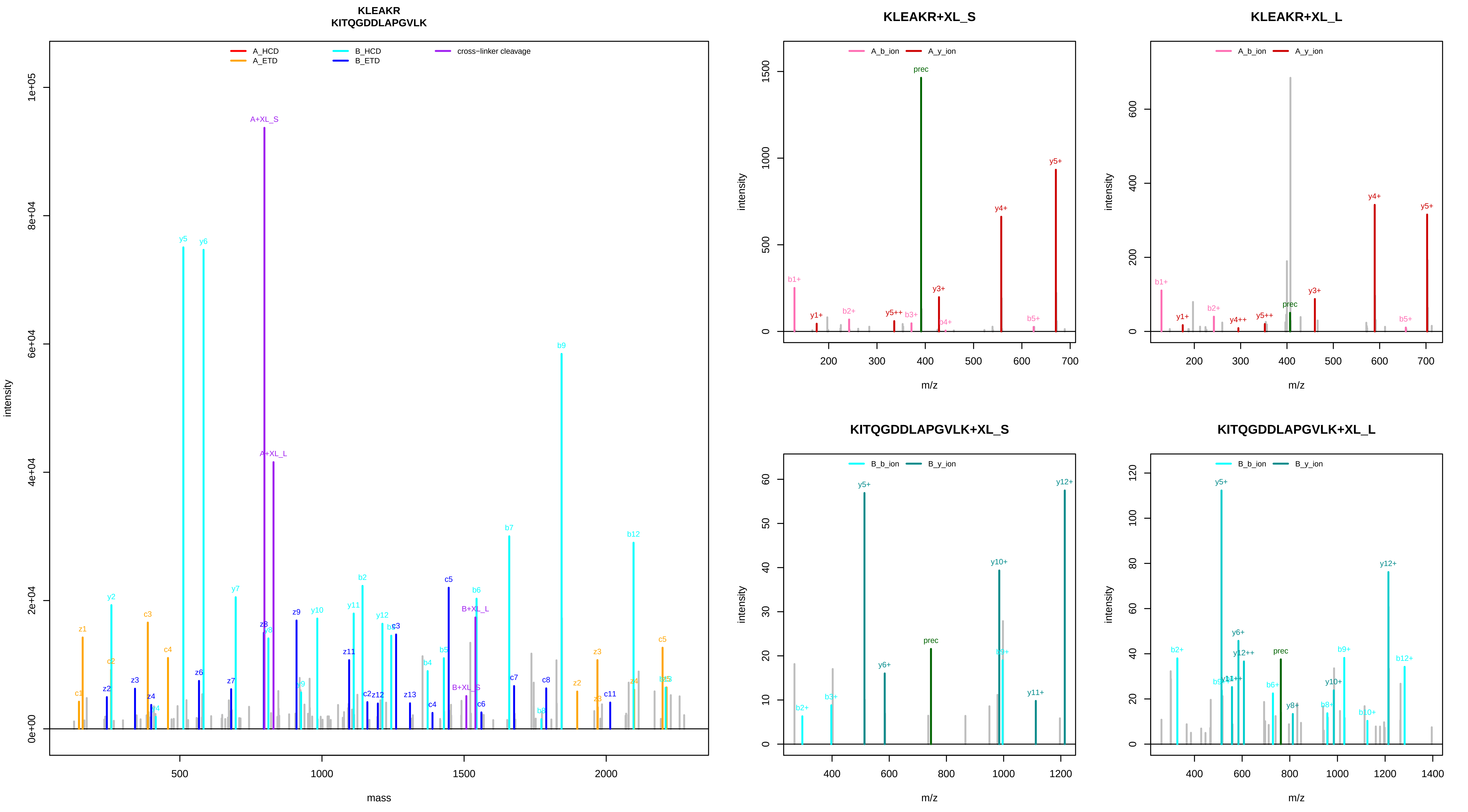
KLEAKR
KITQGDDLAPGVLK
A_HCD
A_ETD
B_HCD
B_ETD
cross−linker cleavage
intensity
mass
A+XL_S
A+XL_L
B+XL_L
B+XL_S
KLEAKR+XL_S
KLEAKR+XL_L
KITQGDDLAPGVLK+XL_S
KITQGDDLAPGVLK+XL_L
A_b_ion
A_y_ion
B_b_ion
B_y_ion
prec
m/z

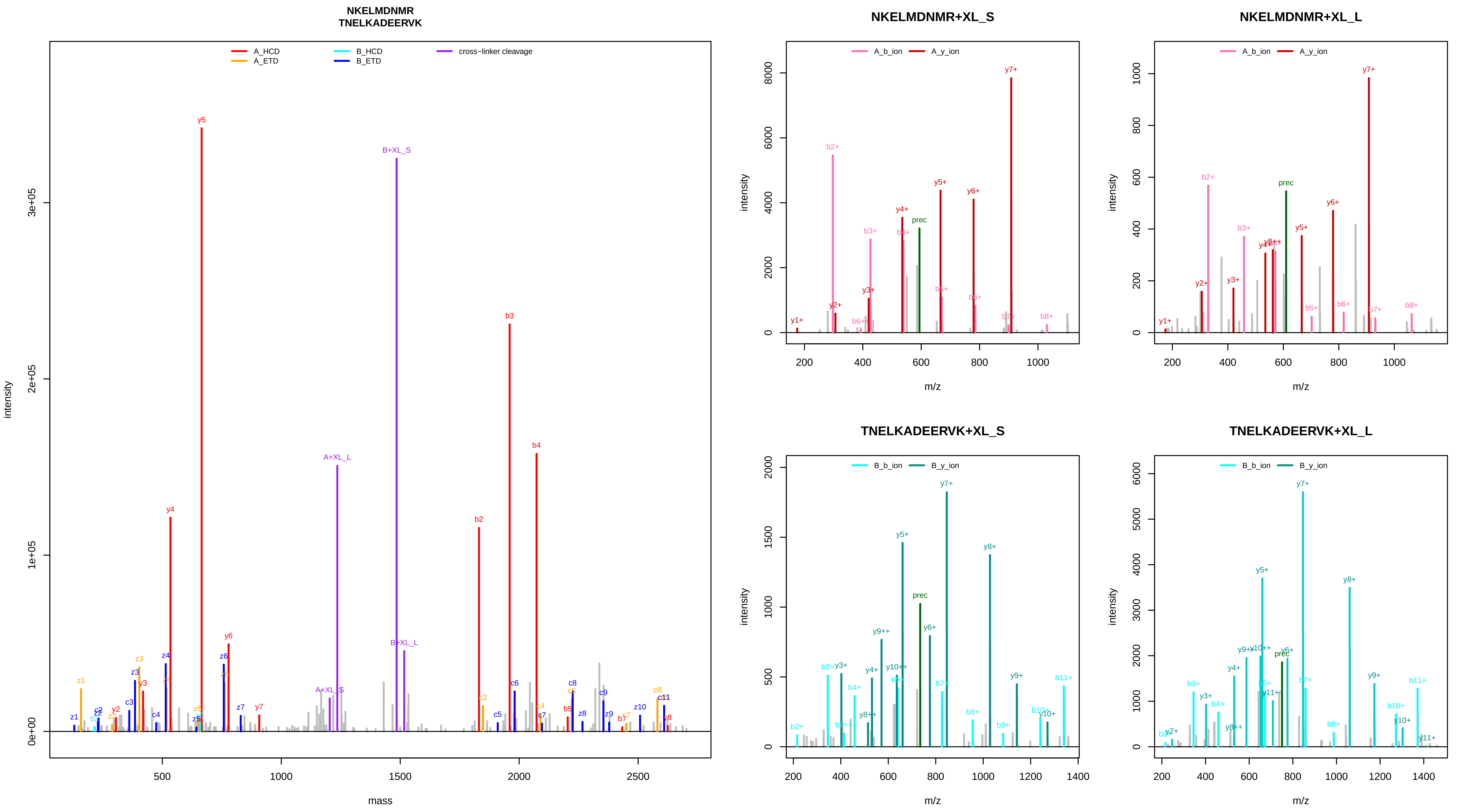
NKELMDNMR
TNELKADEERVK
A_HCD
A_ETD
B_HCD
B_ETD
cross−linker cleavage
intensity
mass
NKELMDNMR+XL_S
NKELMDNMR+XL_L
TNELKADEERVK+XL_S
TNELKADEERVK+XL_L
A_b_ion
A_y_ion
B_b_ion
B_y_ion
m/z
B+XL_S
A+XL_L
B+XL_L
A+XL_S
prec

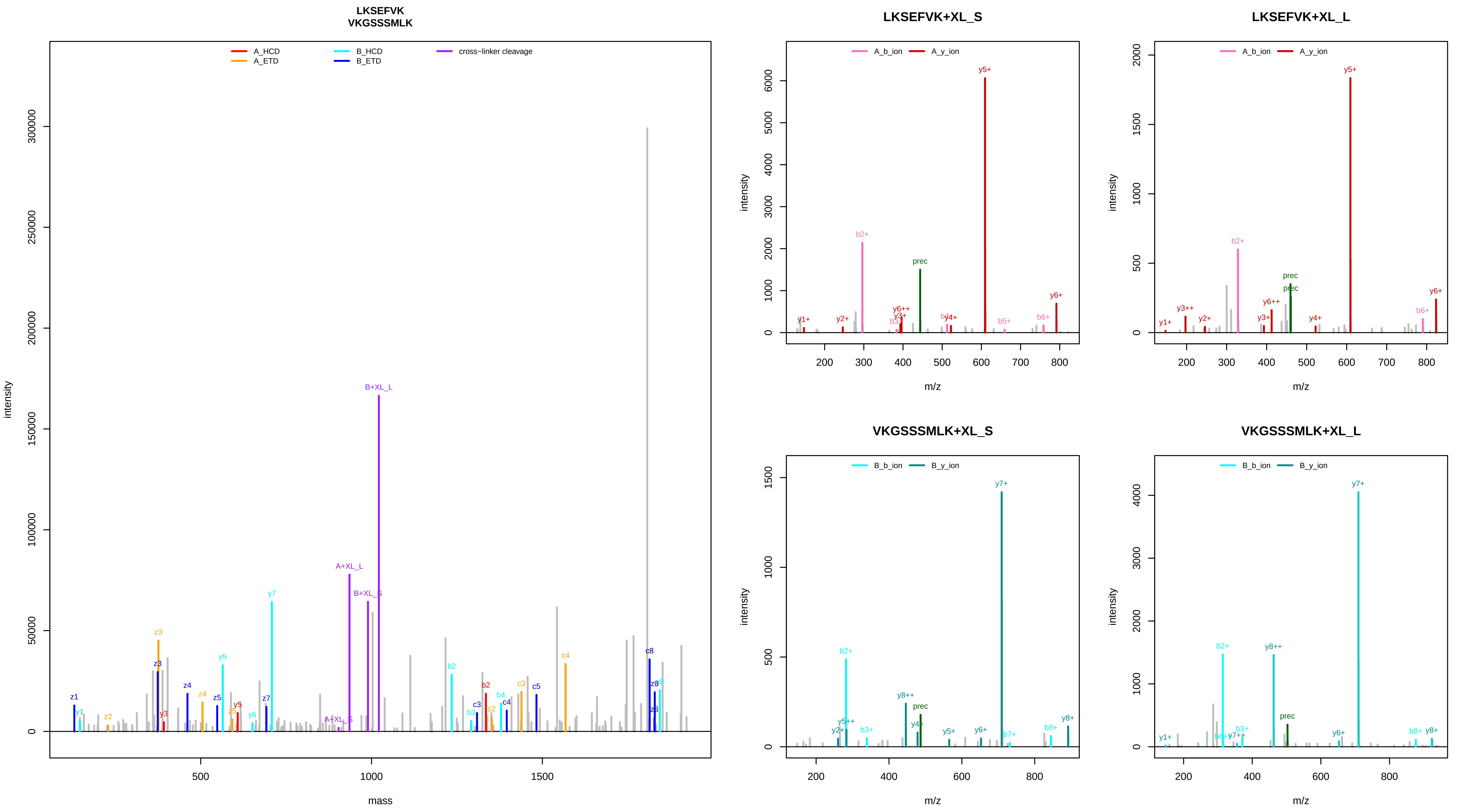
LKSEFVK
VKGSSSMLK
A_HCD
A_ETD
B_HCD
B_ETD
cross−linker cleavage
intensity
mass
LKSEFVK+XL_S
LKSEFVK+XL_L
VKGSSSMLK+XL_S
VKGSSSMLK+XL_L
A_b_ion
A_y_ion
B_b_ion
B_y_ion
m/z
B+XL_L
A+XL_L
B+XL_S
A+XL_S
prec

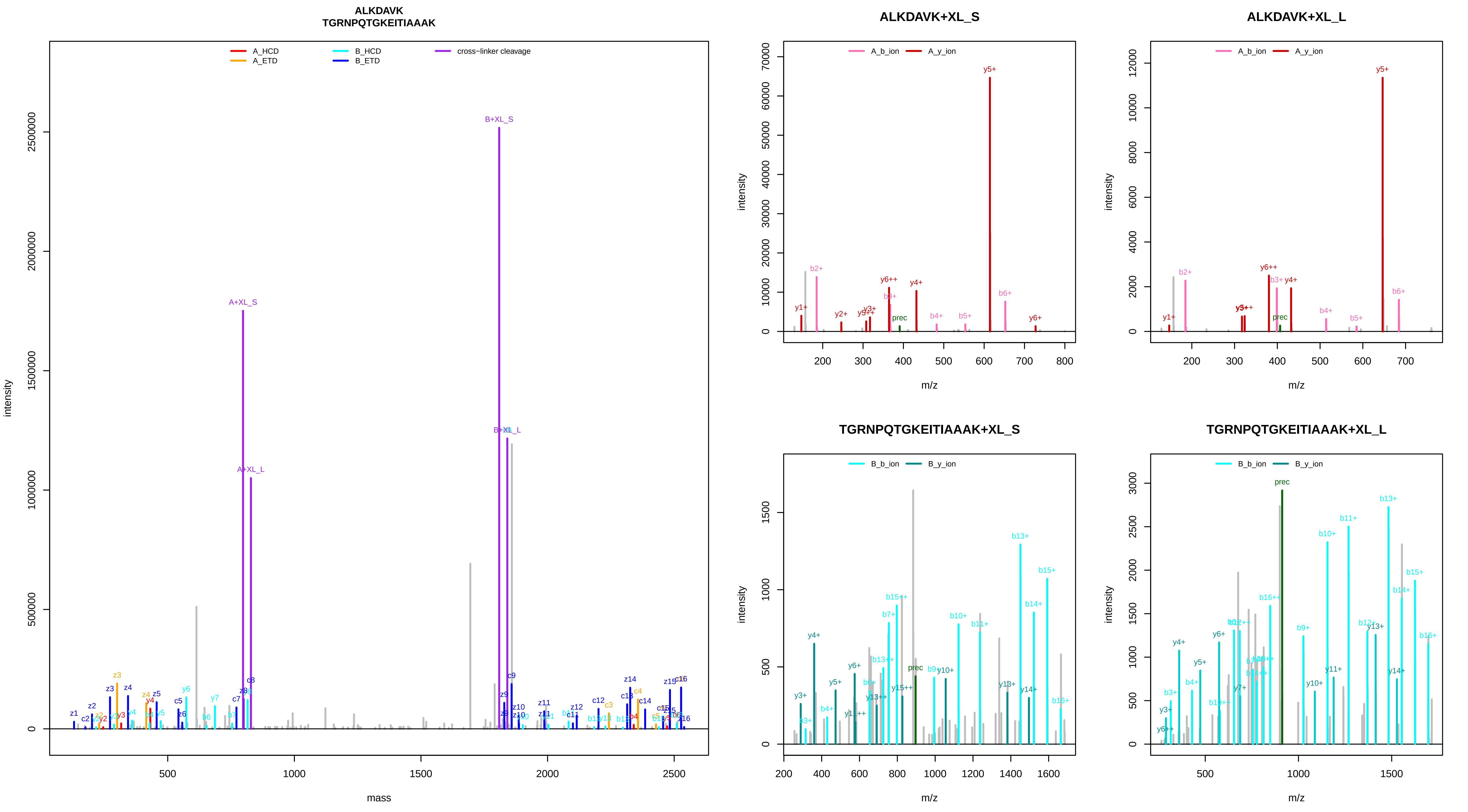
ALKDAVK
TGRNPQTGKEITIAAAK
A_HCD
A_ETD
B_HCD
B_ETD
cross−linker cleavage
B+XL_S
A+XL_S
B+XL_L
A+XL_L
intensity
mass
ALKDAVK+XL_S
ALKDAVK+XL_L
A_b_ion
A_y_ion
TGRNPQTGKEITIAAAK+XL_S
TGRNPQTGKEITIAAAK+XL_L
B_b_ion
B_y_ion
prec
m/z

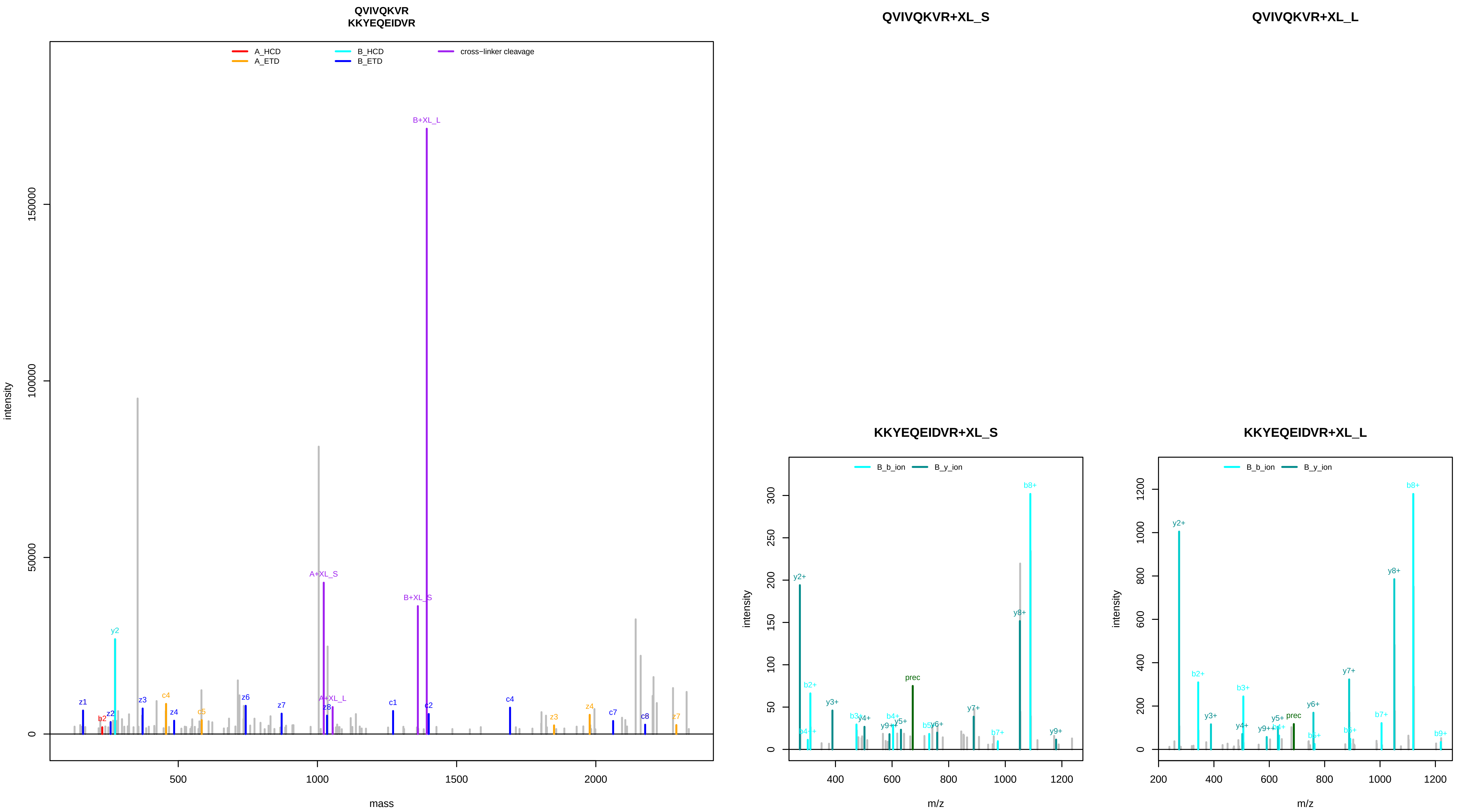
QVIVQKVR
KKYEQEIDVR
A_HCD
A_ETD
B_HCD
B_ETD
cross−linker cleavage
B+XL_L
A+XL_S
B+XL_S
A+XL_L
intensity
mass
QVIVQKVR+XL_S
QVIVQKVR+XL_L
KKYEQEIDVR+XL_S
KKYEQEIDVR+XL_L
B_b_ion
B_y_ion
prec
m/z

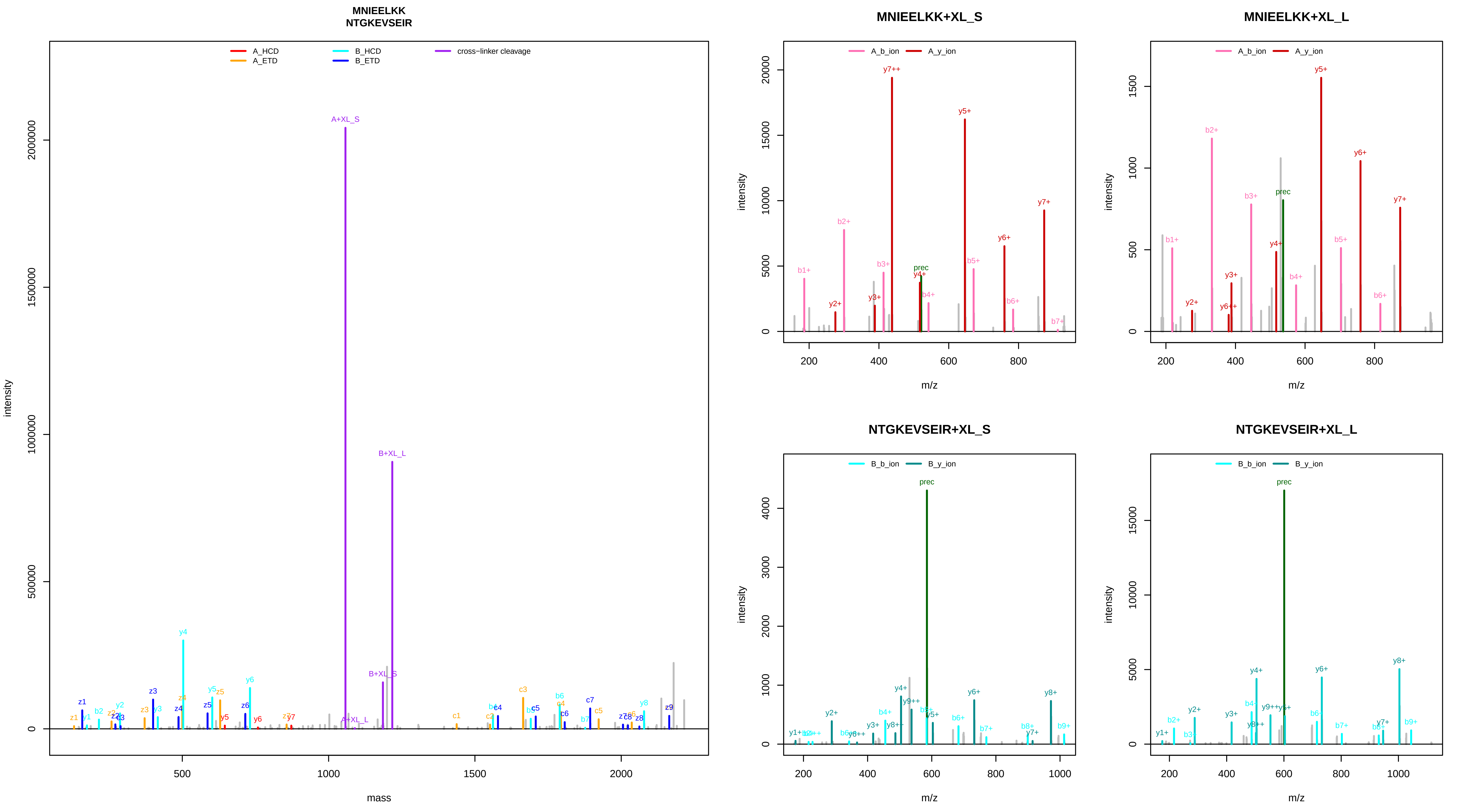
MNIEELKK
NTGKEVSEIR
A_HCD
A_ETD
B_HCD
B_ETD
cross−linker cleavage
A+XL_S
B+XL_L
B+XL_S
A+XL_L
intensity
mass
MNIEELKK+XL_S
MNIEELKK+XL_L
A_b_ion
A_y_ion
prec
m/z
NTGKEVSEIR+XL_S
NTGKEVSEIR+XL_L
B_b_ion
B_y_ion

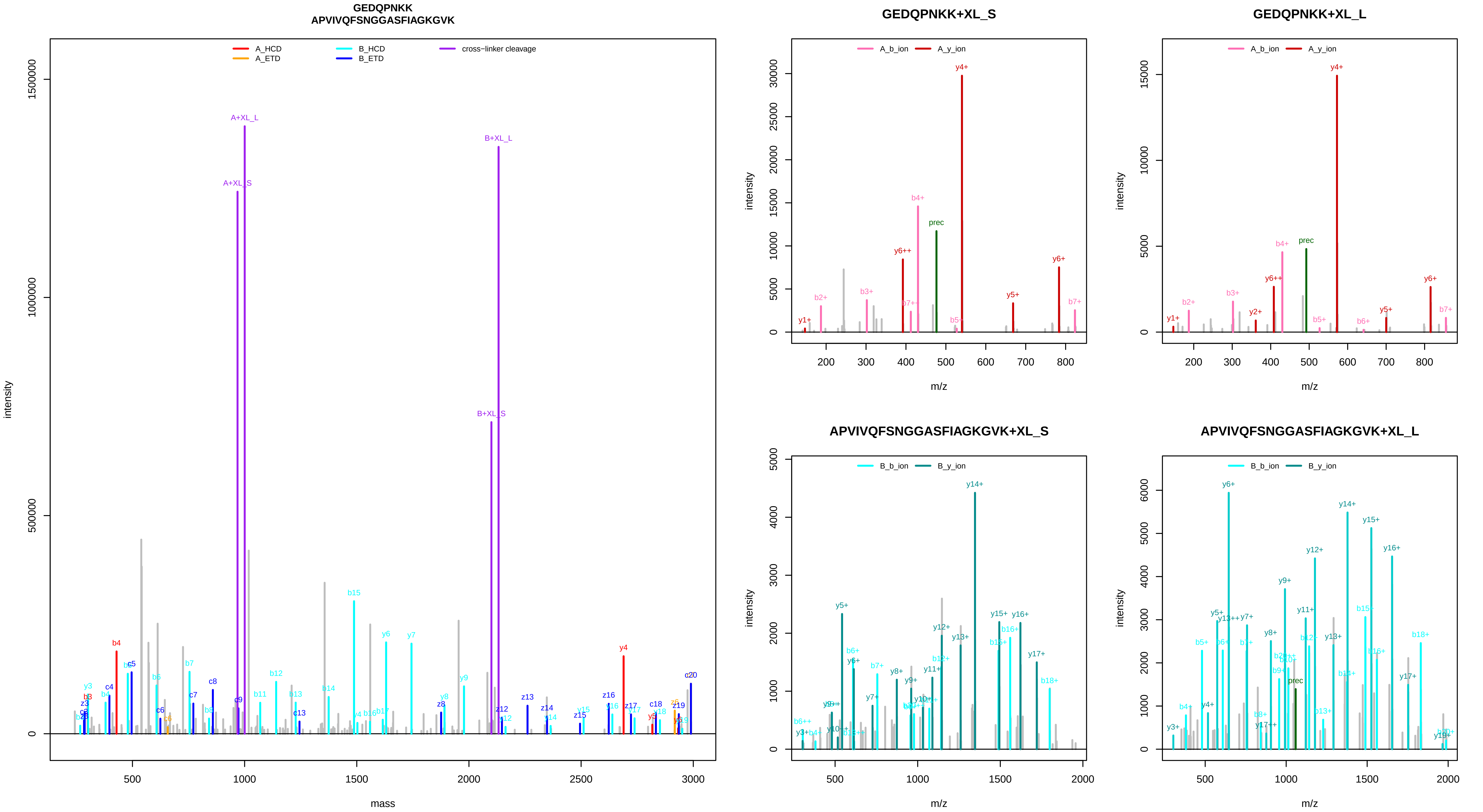
GEDQPNKK
APVIVQFSNGGASFIAGKGVK
A_HCD
A_ETD
B_HCD
B_ETD
cross−linker cleavage
A+XL_L
A+XL_S
B+XL_L
B+XL_S
intensity
mass
GEDQPNKK+XL_S
GEDQPNKK+XL_L
A_b_ion
A_y_ion
prec
APVIVQFSNGGASFIAGKGVK+XL_S
APVIVQFSNGGASFIAGKGVK+XL_L
B_b_ion
B_y_ion
m/z

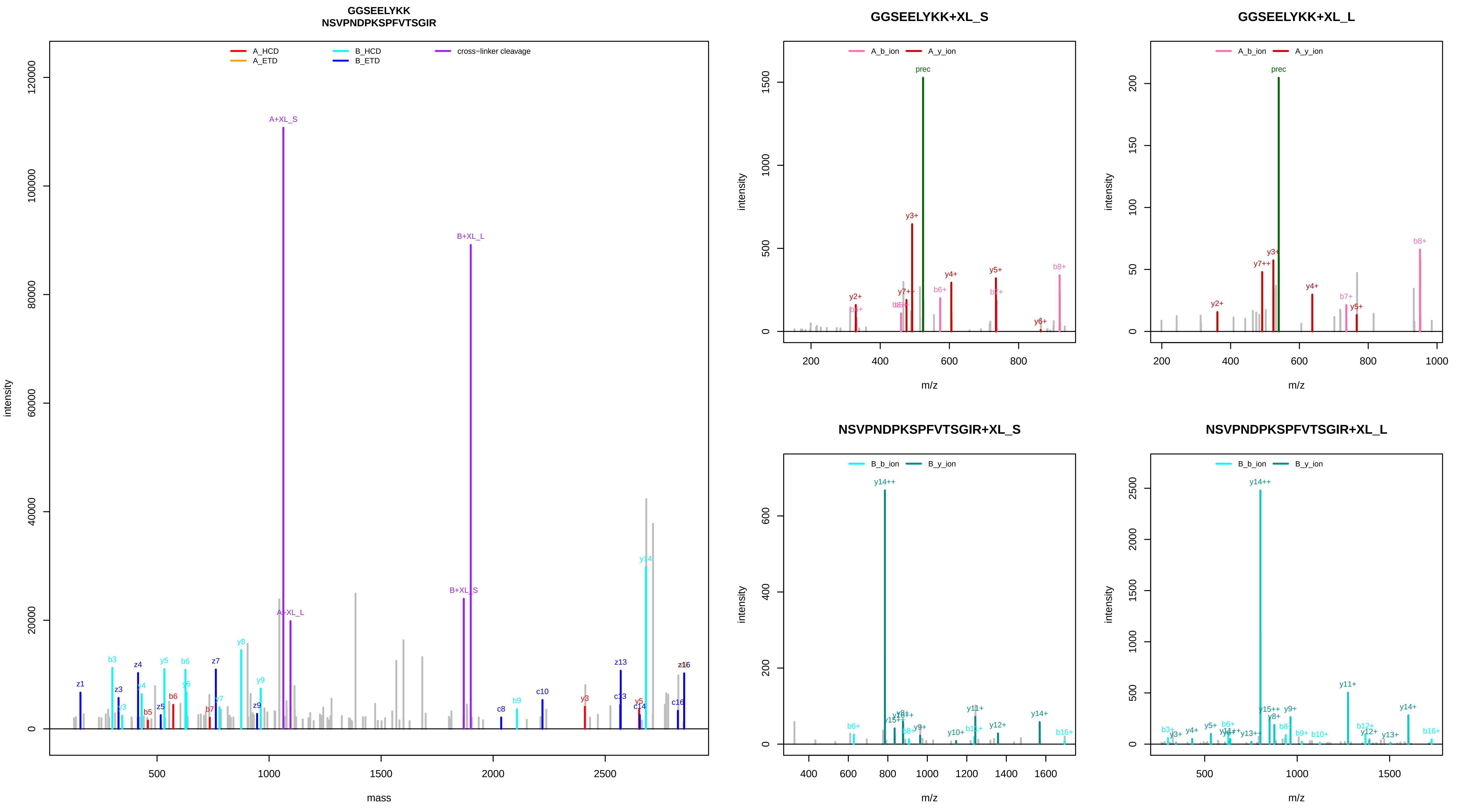
GGSEELYKK
NSVPNDPKSPFVTSGIR
A_HCD
A_ETD
B_HCD
B_ETD
cross−linker cleavage
A+XL_S
B+XL_L
B+XL_S
A+XL_L
intensity
mass
GGSEELYKK+XL_S
GGSEELYKK+XL_L
NSVPNDPKSPFVTSGIR+XL_S
NSVPNDPKSPFVTSGIR+XL_L
A_b_ion
A_y_ion
B_b_ion
B_y_ion
prec
m/z

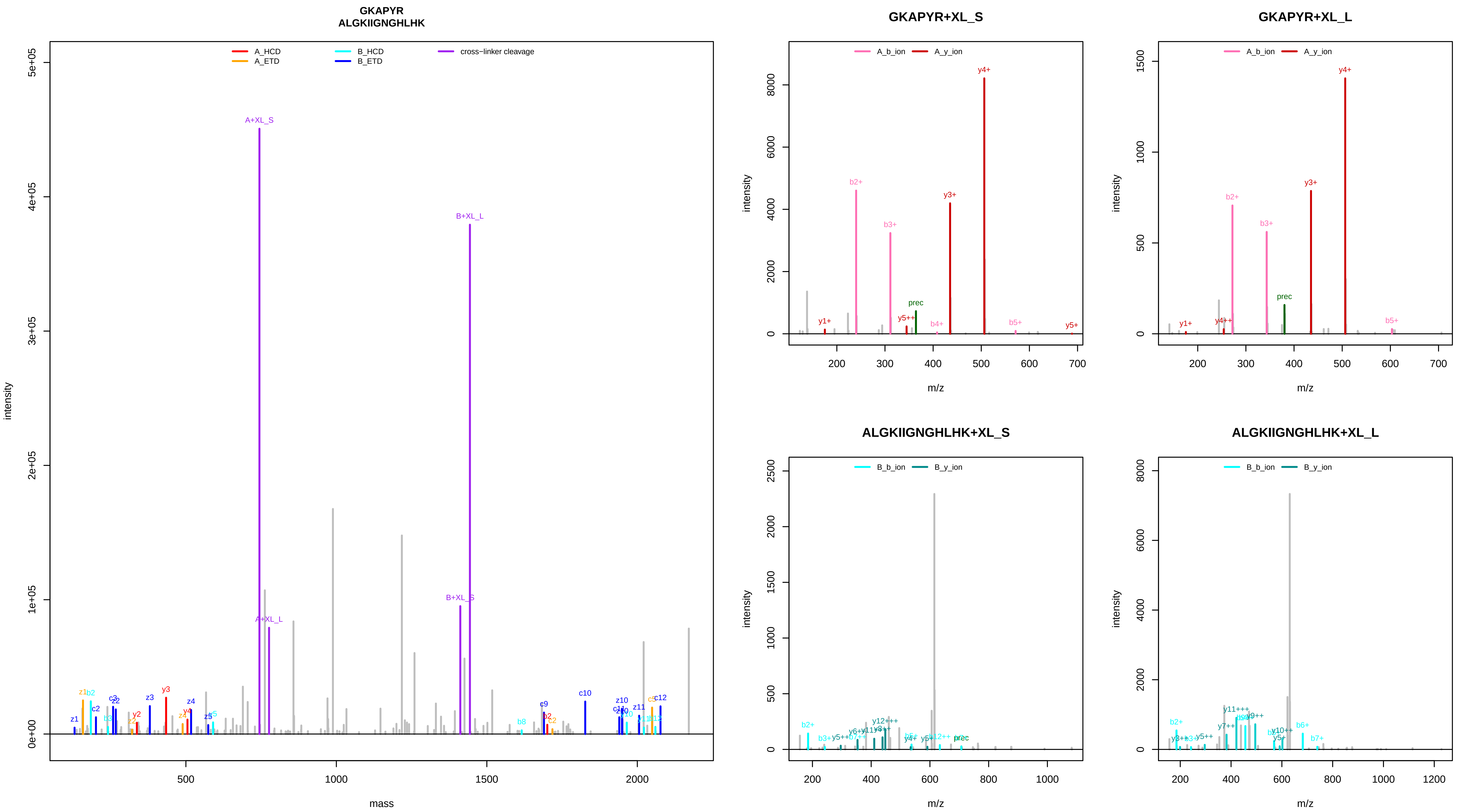
GKAPYR
ALGKIIGNGHLHK
A_HCD
A_ETD
B_HCD
B_ETD
cross−linker cleavage
A+XL_S
B+XL_L
B+XL_S
A+XL_L
intensity
mass
GKAPYR+XL_S
GKAPYR+XL_L
A_b_ion
A_y_ion
ALGKIIGNGHLHK+XL_S
ALGKIIGNGHLHK+XL_L
B_b_ion
B_y_ion
m/z
prec

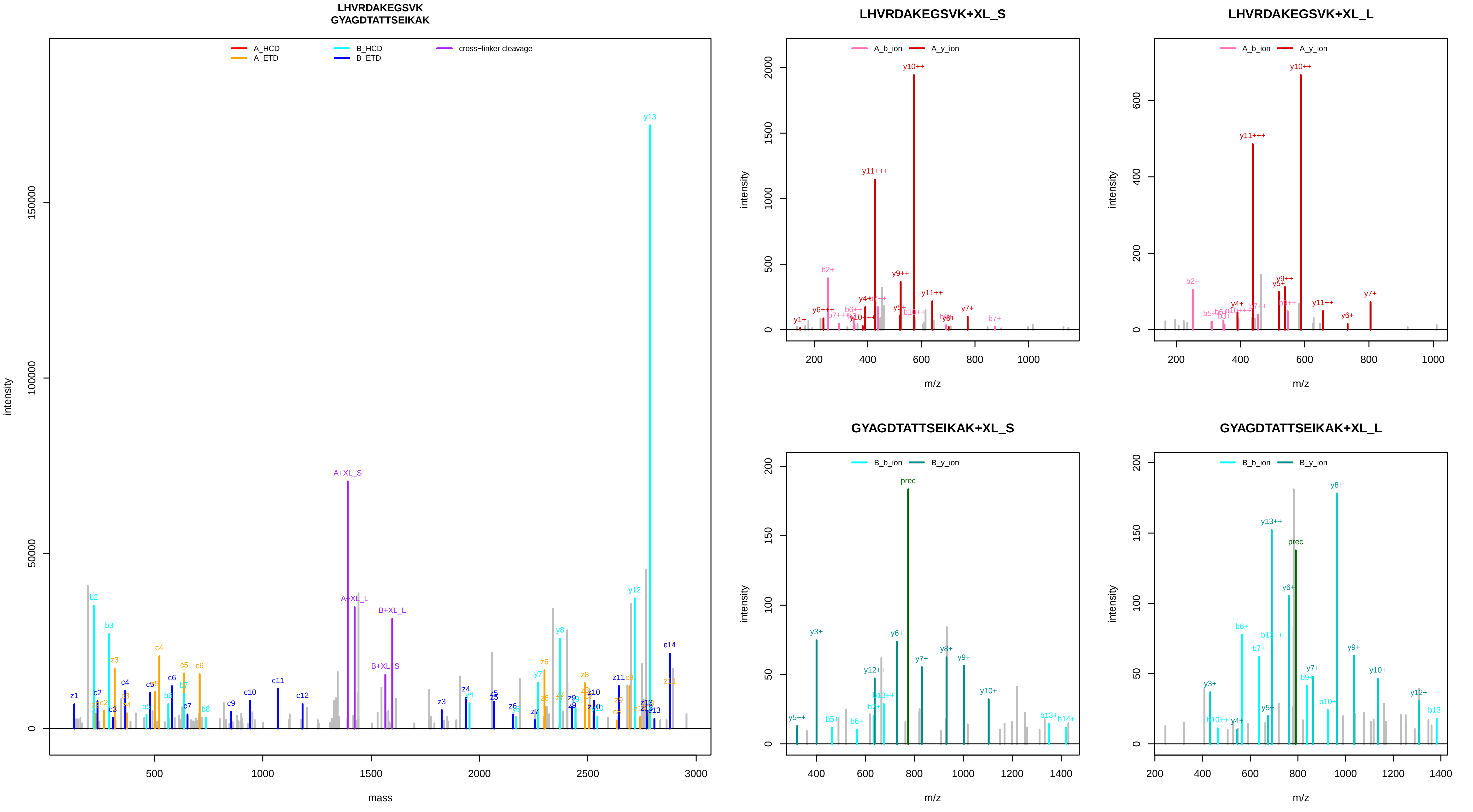
LHVRDAKEGSVK
GYAGDTATTSEIKAK
A_HCD
A_ETD
B_HCD
B_ETD
cross−linker cleavage
A+XL_S
A+XL_L
B+XL_L
B+XL_S
y13
y12
intensity
mass
LHVRDAKEGSVK+XL_S
LHVRDAKEGSVK+XL_L
GYAGDTATTSEIKAK+XL_S
GYAGDTATTSEIKAK+XL_L
A_b_ion
A_y_ion
B_b_ion
B_y_ion
prec
m/z

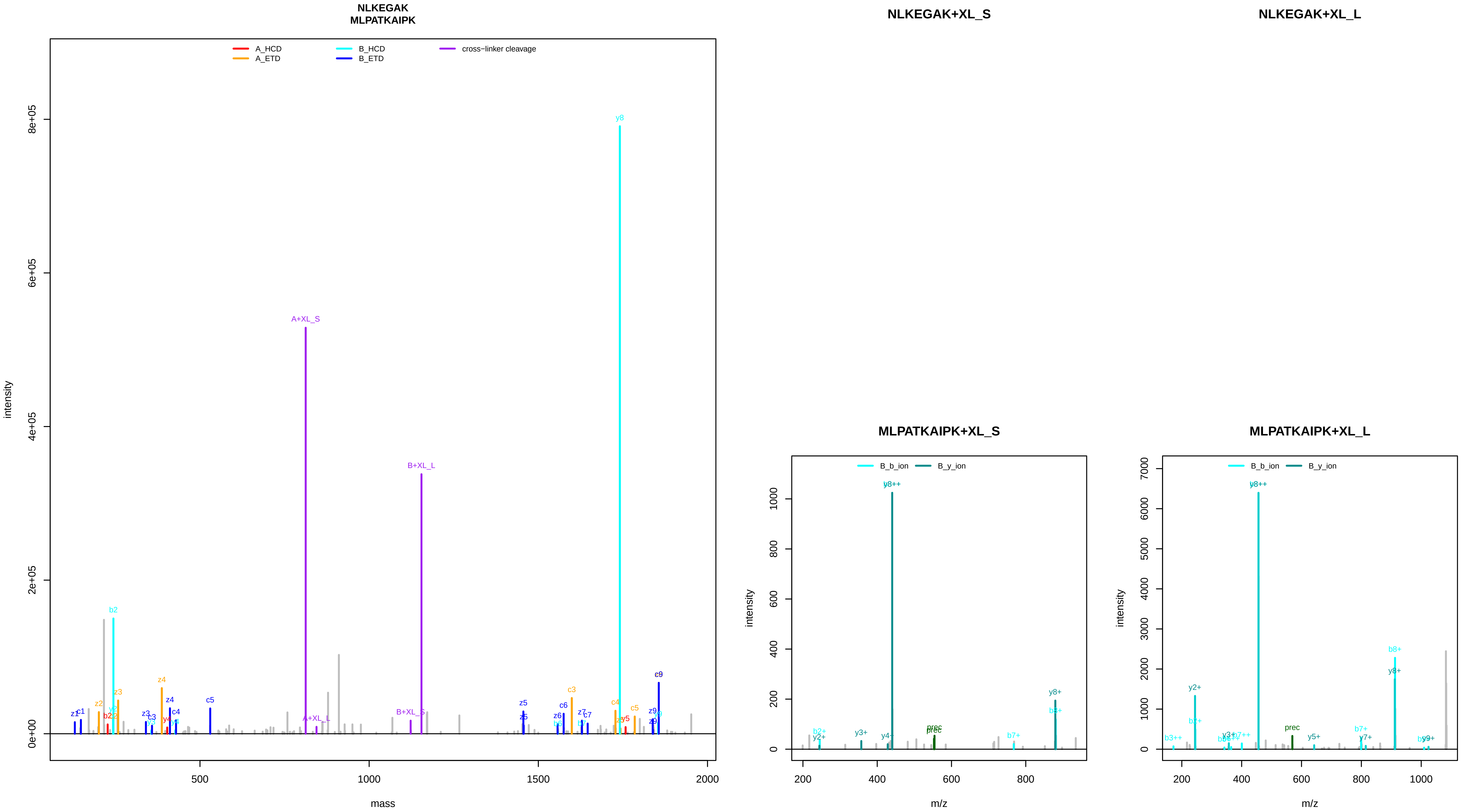
NLKEGAK
MLPATKAIPK
A_HCD
A_ETD
B_HCD
B_ETD
cross−linker cleavage
intensity
mass
A+XL_S
B+XL_L
y8
b2
NLKEGAK+XL_S
NLKEGAK+XL_L
MLPATKAIPK+XL_S
MLPATKAIPK+XL_L
B_b_ion
B_y_ion
m/z
prec
y8++
y8+
b8+
y2+

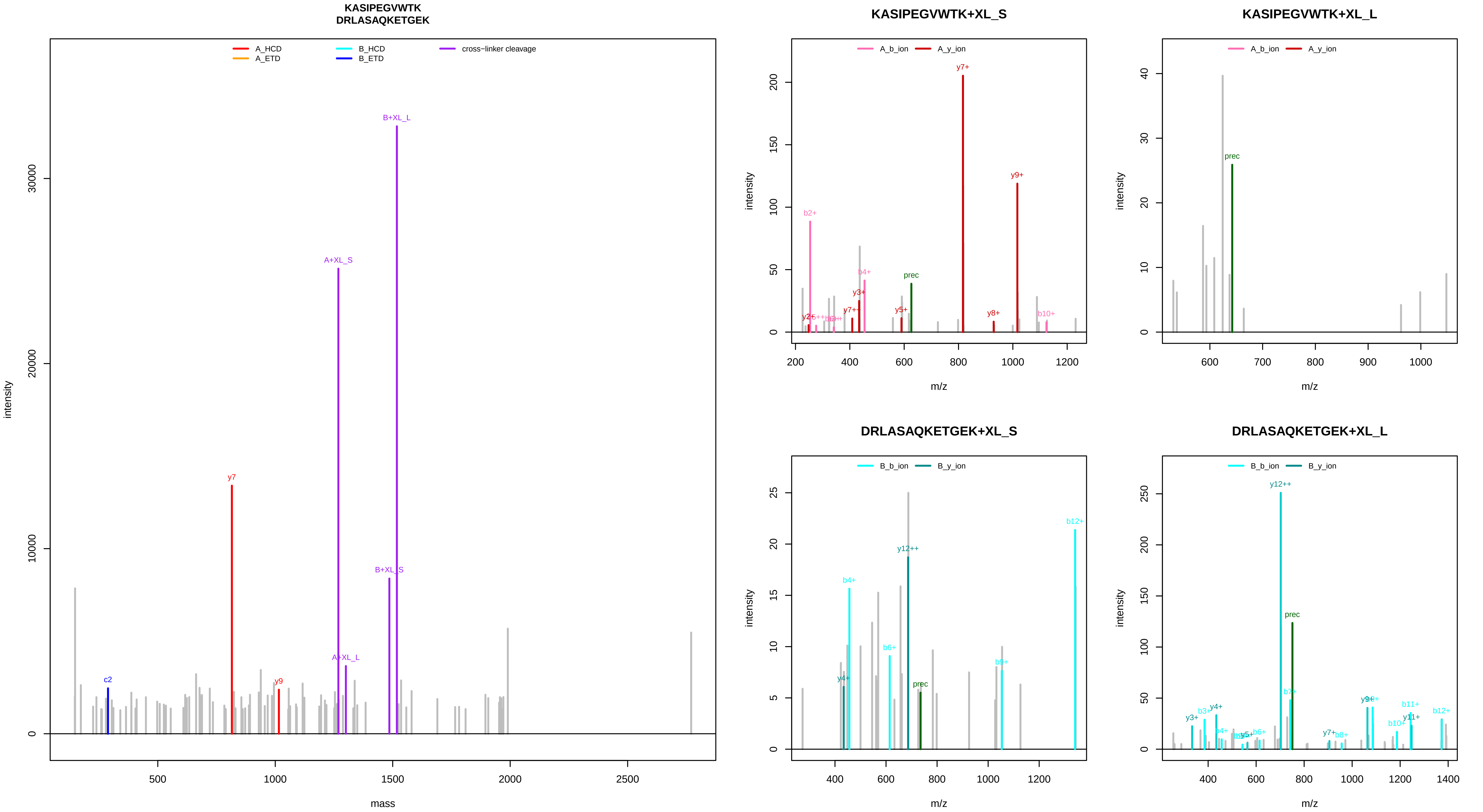
KASIPEGVWTK
DRLASAQKETGEK
A_HCD
A_ETD
B_HCD
B_ETD
cross−linker cleavage
B+XL_L
A+XL_S
y7
B+XL_S
A+XL_L
c2
y9
intensity
mass
KASIPEGVWTK+XL_S
KASIPEGVWTK+XL_L
DRLASAQKETGEK+XL_S
DRLASAQKETGEK+XL_L
A_b_ion
A_y_ion
B_b_ion
B_y_ion
y7+
y9+
b2+
b4+
prec
y3+
y7++
y5+
y2+
y8+
b10+
b4+
y12++
b6+
b9+
b12+
y4+
b3+
y4+
b7+
y7+
b8+
b10+
b11+
y11+
b12+
m/z

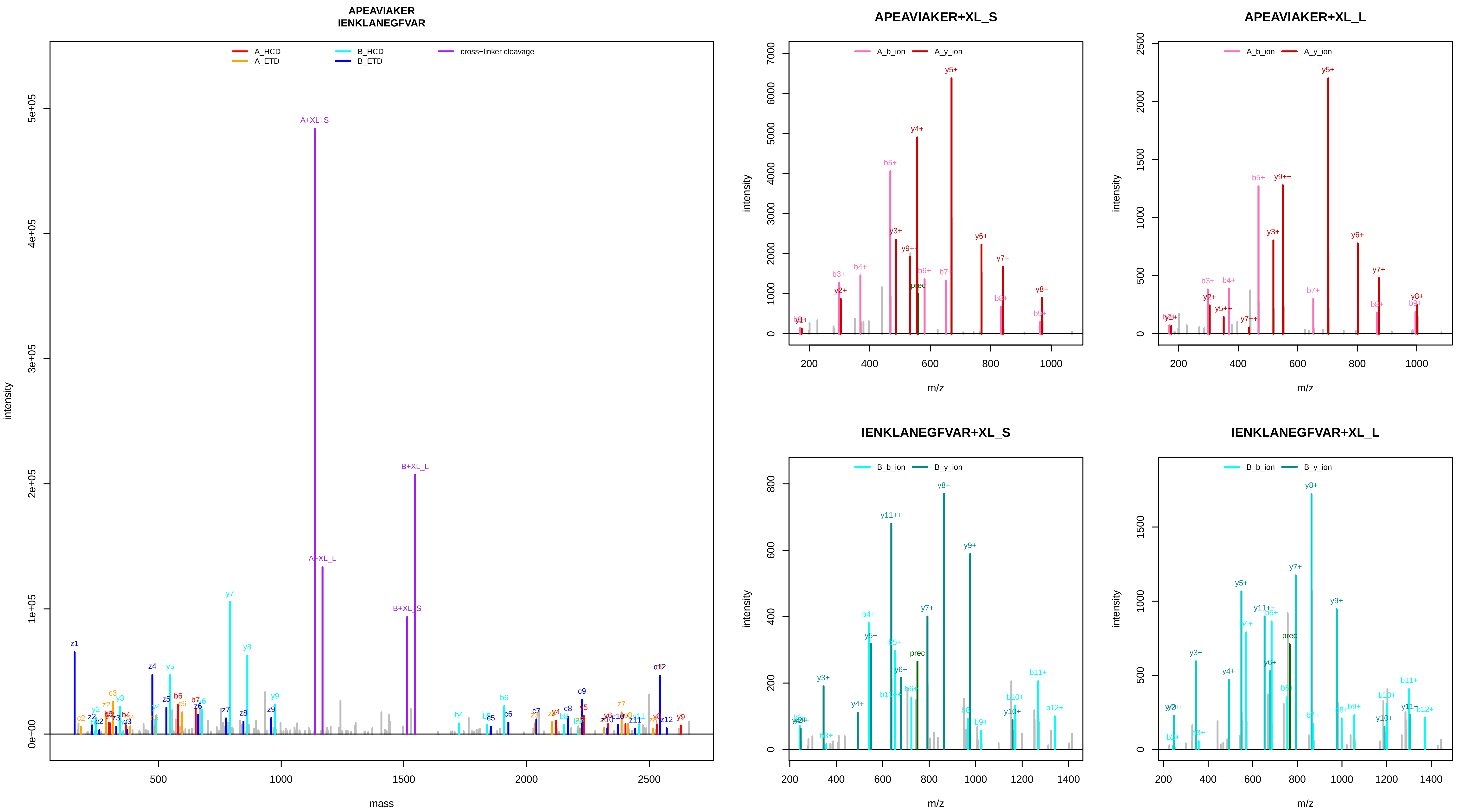
APEAVIAKER
IENKLANEGFVAR
A_HCD
A_ETD
B_HCD
B_ETD
cross−linker cleavage
A+XL_S
A+XL_L
B+XL_L
B+XL_S
intensity
mass
APEAVIAKER+XL_S
APEAVIAKER+XL_L
IENKLANEGFVAR+XL_S
IENKLANEGFVAR+XL_L
A_b_ion
A_y_ion
B_b_ion
B_y_ion
prec
m/z

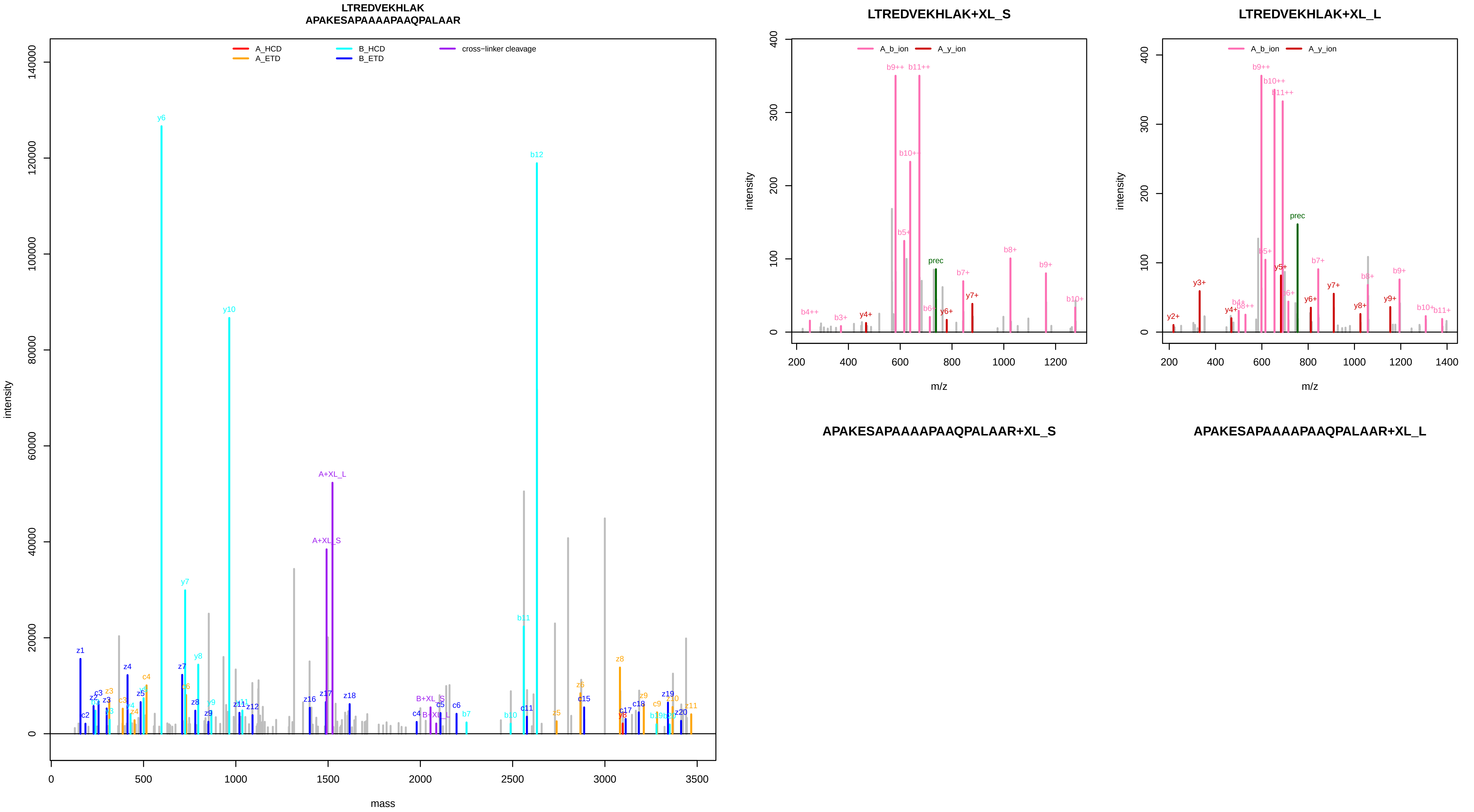
LTREDVEKHLAK
APAKESAPAAAAPAAQPALAAR
A_HCD
A_ETD
B_HCD
B_ETD
cross−linker cleavage
intensity
mass
A+XL_L
A+XL_S
B+XL_S
B+XL_L
LTREDVEKHLAK+XL_S
LTREDVEKHLAK+XL_L
A_b_ion
A_y_ion
prec
m/z
APAKESAPAAAAPAAQPALAAR+XL_S
APAKESAPAAAAPAAQPALAAR+XL_L

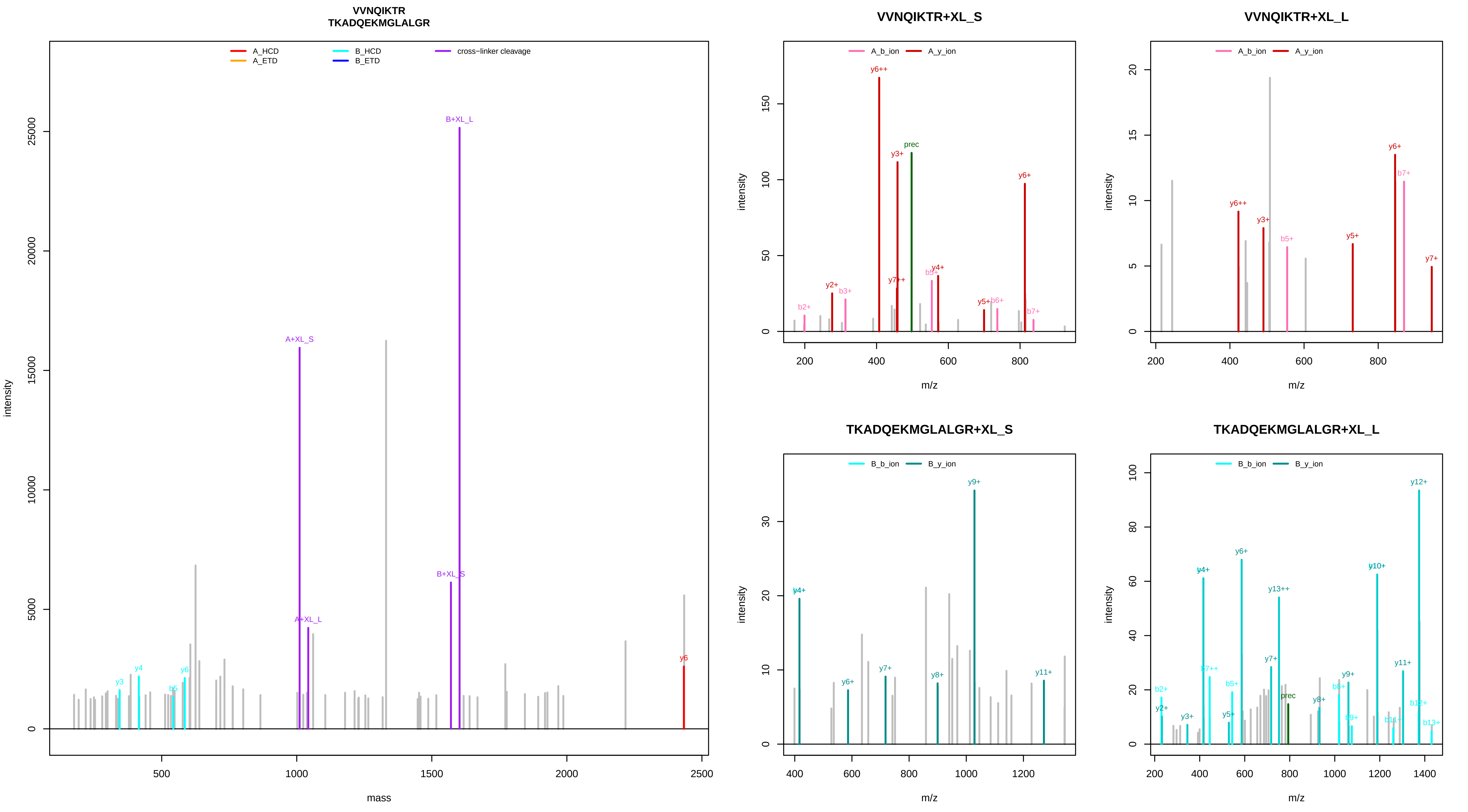

VVNQIKTR
TKADQEKMGLALGR
A_HCD
A_ETD
B_HCD
B_ETD
cross−linker cleavage
B+XL_L
A+XL_S
B+XL_S
A+XL_L
y3
y4
y6
b5
y6
intensity
mass
VVNQIKTR+XL_S
A_b_ion
A_y_ion
y6++
prec
y3+
y6+
y2+
b3+
b2+
y4+
y5+
b6+
b7+
intensity
m/z
VVNQIKTR+XL_L
A_b_ion
A_y_ion
y6+
b7+
y6++
y3+
b5+
y5+
y7+
intensity
m/z
TKADQEKMGLALGR+XL_S
B_b_ion
B_y_ion
y9+
y4+
y6+
y7+
y8+
y11+
intensity
m/z
TKADQEKMGLALGR+XL_L
B_b_ion
B_y_ion
y12+
y10+
y6+
y4+
y13++
y7+
y11+
y9+
b5+
y8+
prec
b2+
y2+
y3+
y5+
b12+
b13+
intensity
m/z

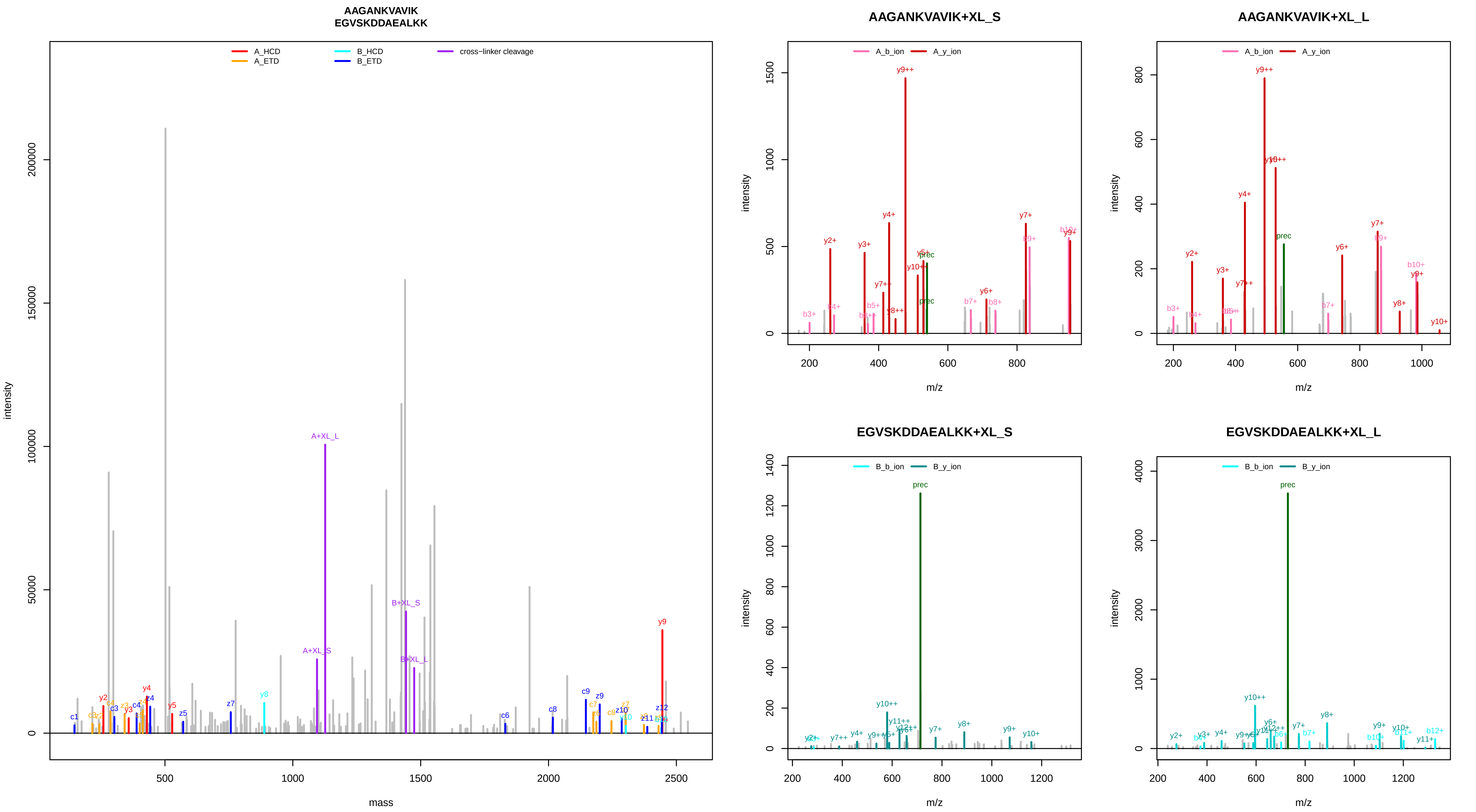

AAGANKVAVIK
EGVSKDDAEALKK
A_HCD
A_ETD
B_HCD
B_ETD
cross−linker cleavage
A+XL_L
A+XL_S
B+XL_S
B+XL_L
y9
intensity
mass
AAGANKVAVIK+XL_S
AAGANKVAVIK+XL_L
A_b_ion
A_y_ion
y9++
prec
intensity
m/z
EGVSKDDAEALKK+XL_S
EGVSKDDAEALKK+XL_L
B_b_ion
B_y_ion
prec
y10++
y8+
intensity
m/z

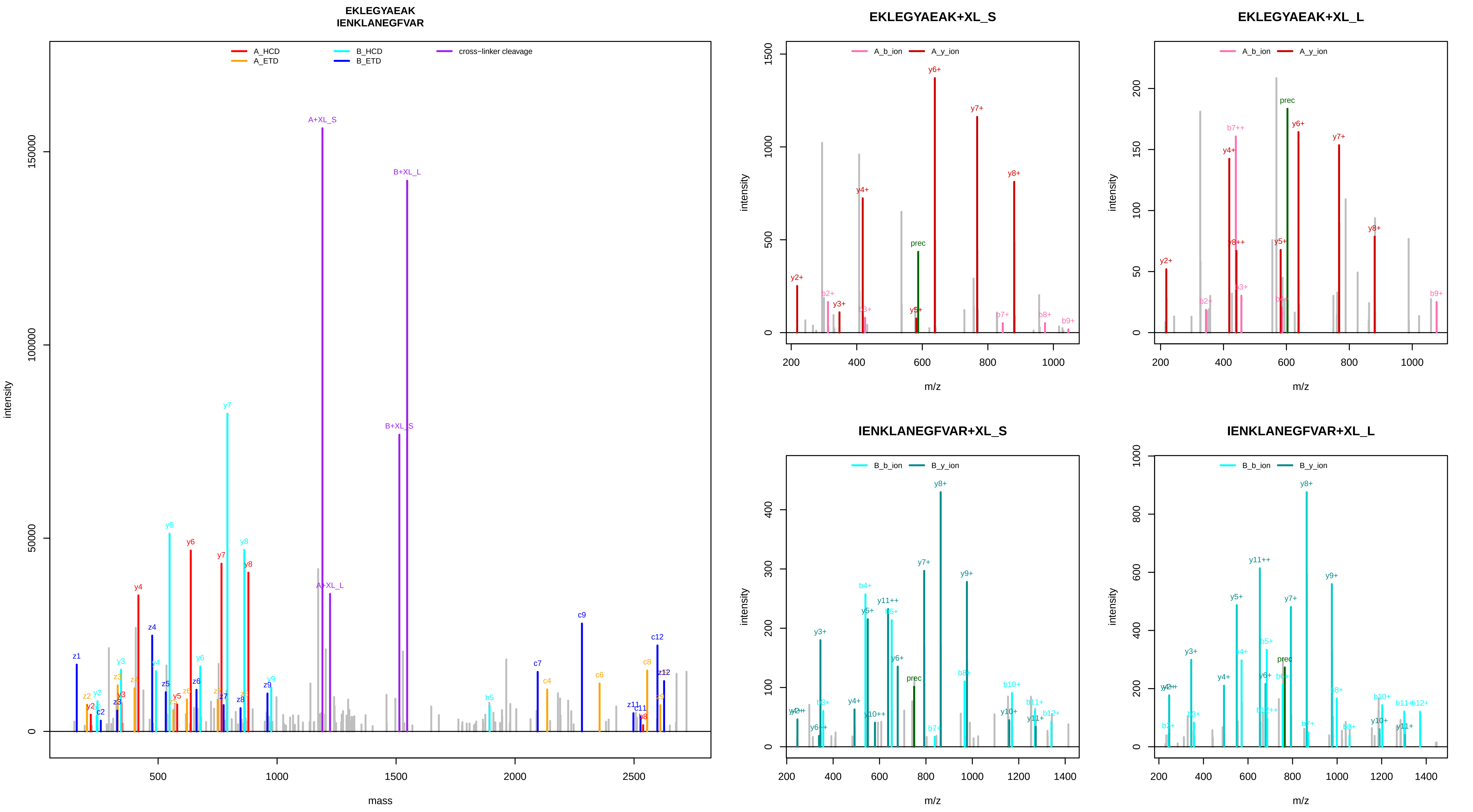
EKLEGYAEAK
IENKLANEGFVAR
A_HCD
A_ETD
B_HCD
B_ETD
cross−linker cleavage
A+XL_S
B+XL_L
B+XL_S
A+XL_L
intensity
mass
EKLEGYAEAK+XL_S
EKLEGYAEAK+XL_L
A_b_ion
A_y_ion
prec
m/z
IENKLANEGFVAR+XL_S
IENKLANEGFVAR+XL_L
B_b_ion
B_y_ion

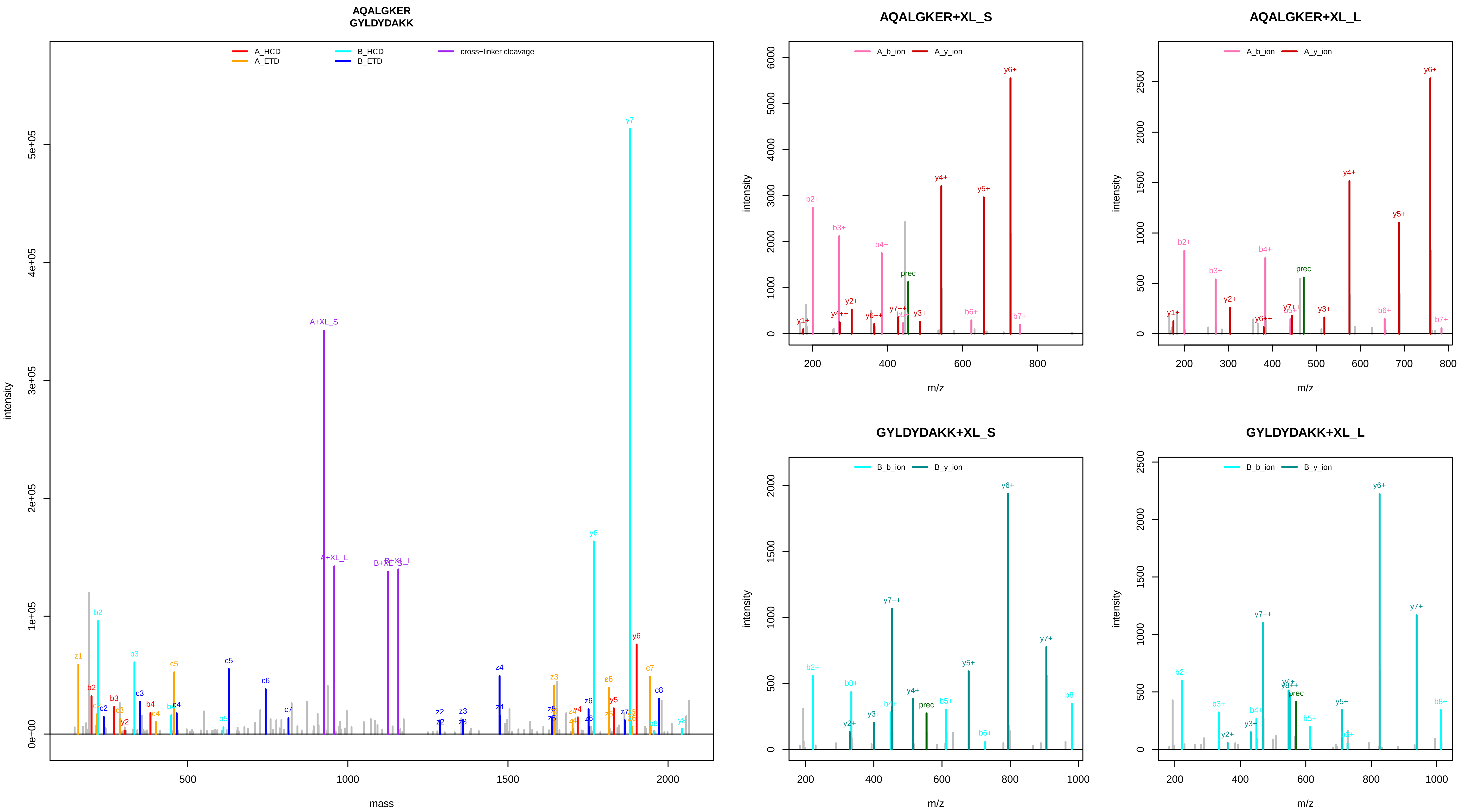
AQALGKER
GYLDYDAKK
A_HCD
A_ETD
B_HCD
B_ETD
cross−linker cleavage
intensity
mass
A+XL_S
A+XL_L
B+XL_S
B+XL_L
AQALGKER+XL_S
AQALGKER+XL_L
GYLDYDAKK+XL_S
GYLDYDAKK+XL_L
A_b_ion
A_y_ion
B_b_ion
B_y_ion
prec
m/z

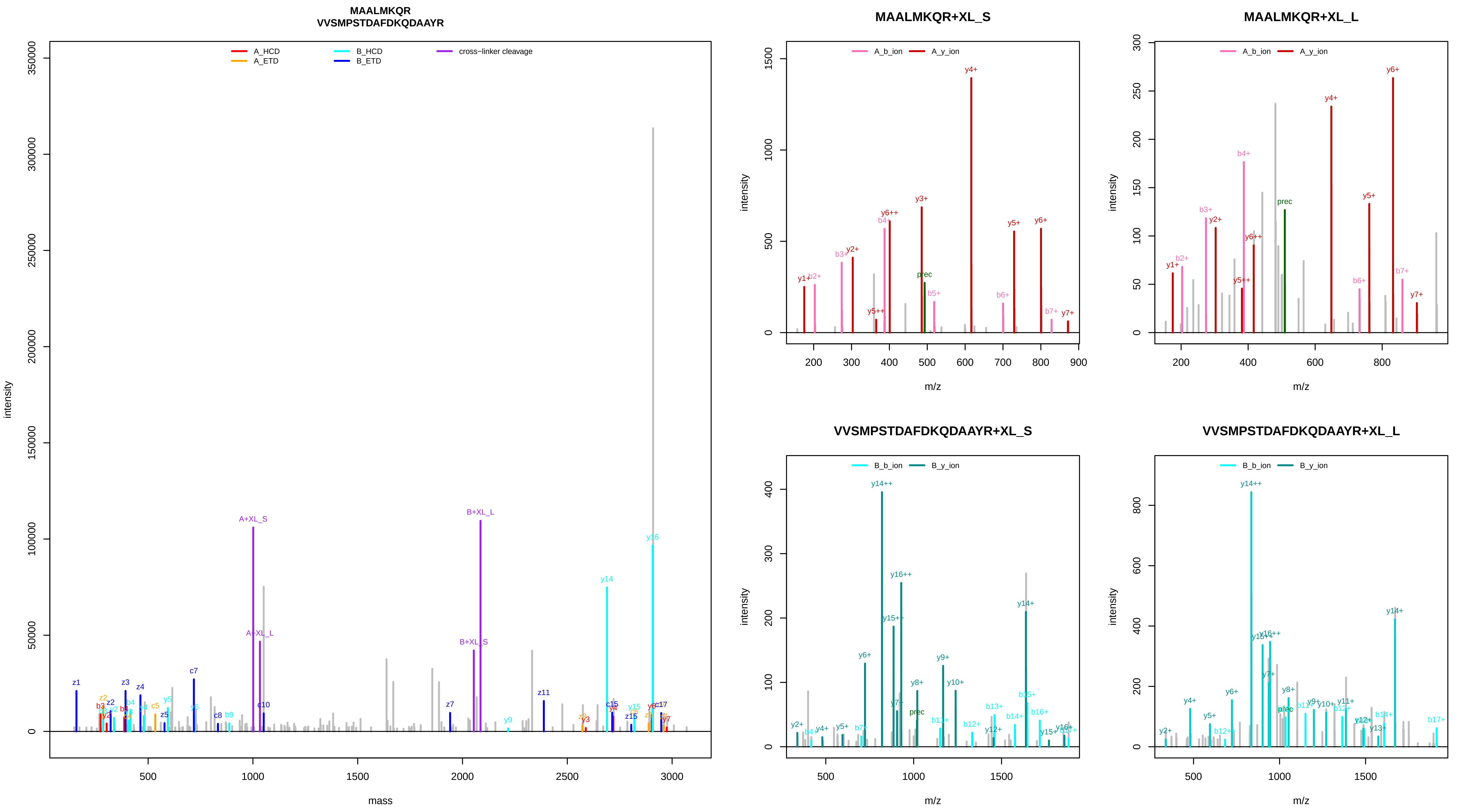

MAALMKQR
VVSMPSTDAFDKQDAAYR
A_HCD
A_ETD
B_HCD
B_ETD
cross−linker cleavage
A+XL_S
A+XL_L
B+XL_L
B+XL_S
intensity
mass
MAALMKQR+XL_S
MAALMKQR+XL_L
VVSMPSTDAFDKQDAAYR+XL_S
VVSMPSTDAFDKQDAAYR+XL_L
A_b_ion
A_y_ion
B_b_ion
B_y_ion
m/z
prec

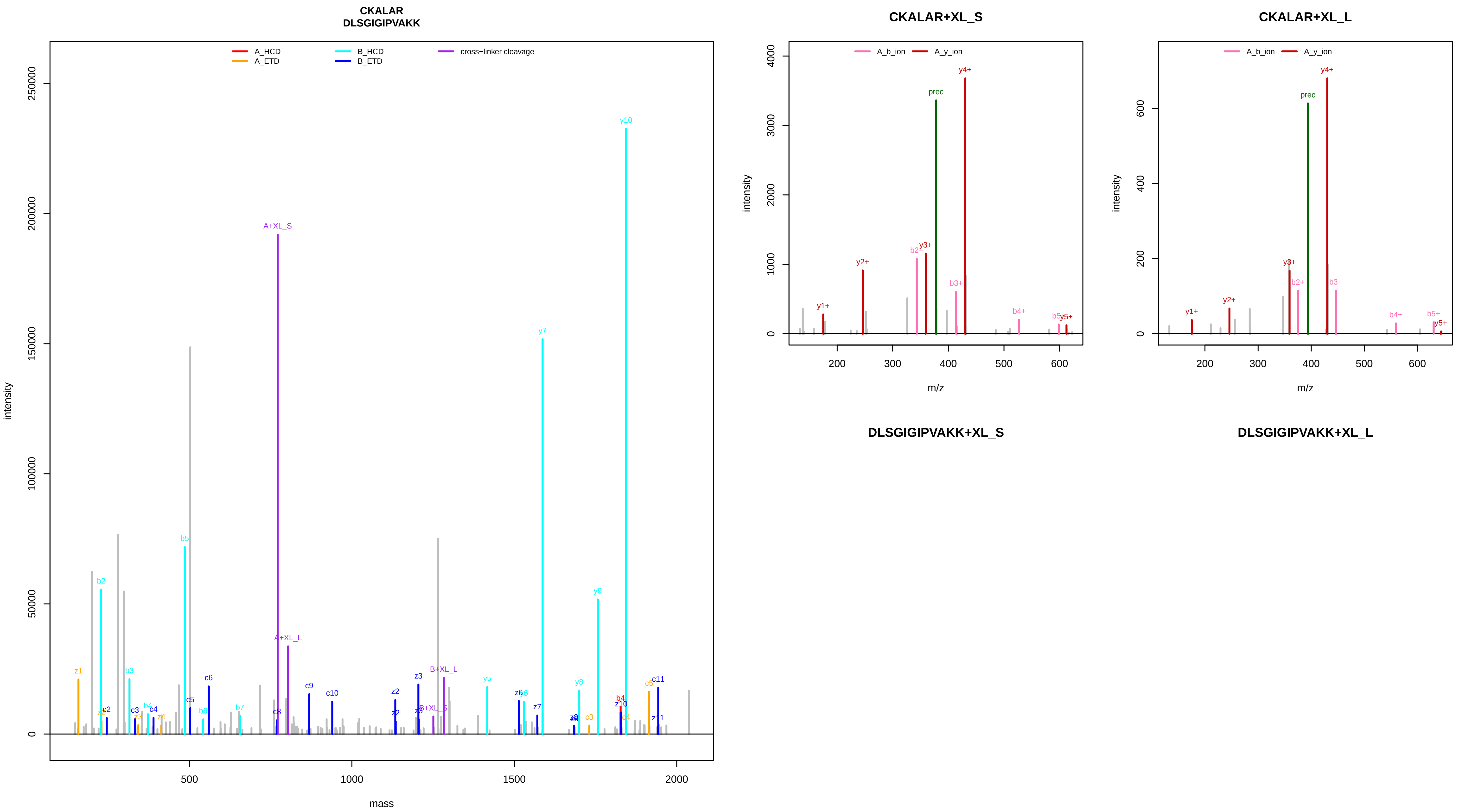
CKALAR
DLSGIGIPVAKK
A_HCD
A_ETD
B_HCD
B_ETD
cross−linker cleavage
A+XL_S
A+XL_L
B+XL_S
B+XL_L
intensity
mass
CKALAR+XL_S
CKALAR+XL_L
A_b_ion
A_y_ion
prec
m/z
DLSGIGIPVAKK+XL_S
DLSGIGIPVAKK+XL_L

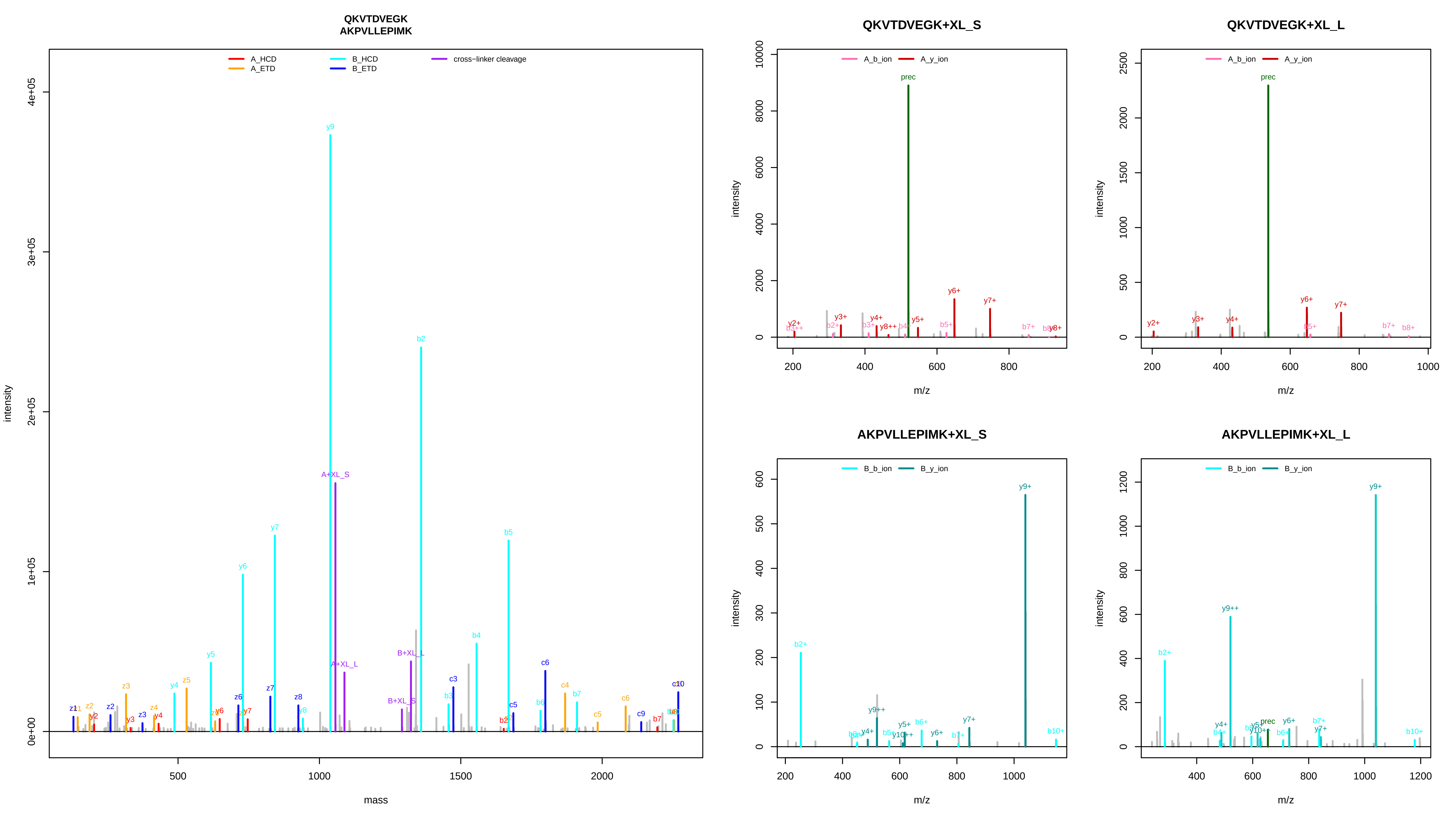
QKVTDVEGK
AKPVLLEPIMK
A_HCD
A_ETD
B_HCD
B_ETD
cross−linker cleavage
intensity
mass
QKVTDVEGK+XL_S
QKVTDVEGK+XL_L
AKPVLLEPIMK+XL_S
AKPVLLEPIMK+XL_L
A_b_ion
A_y_ion
B_b_ion
B_y_ion
prec
m/z

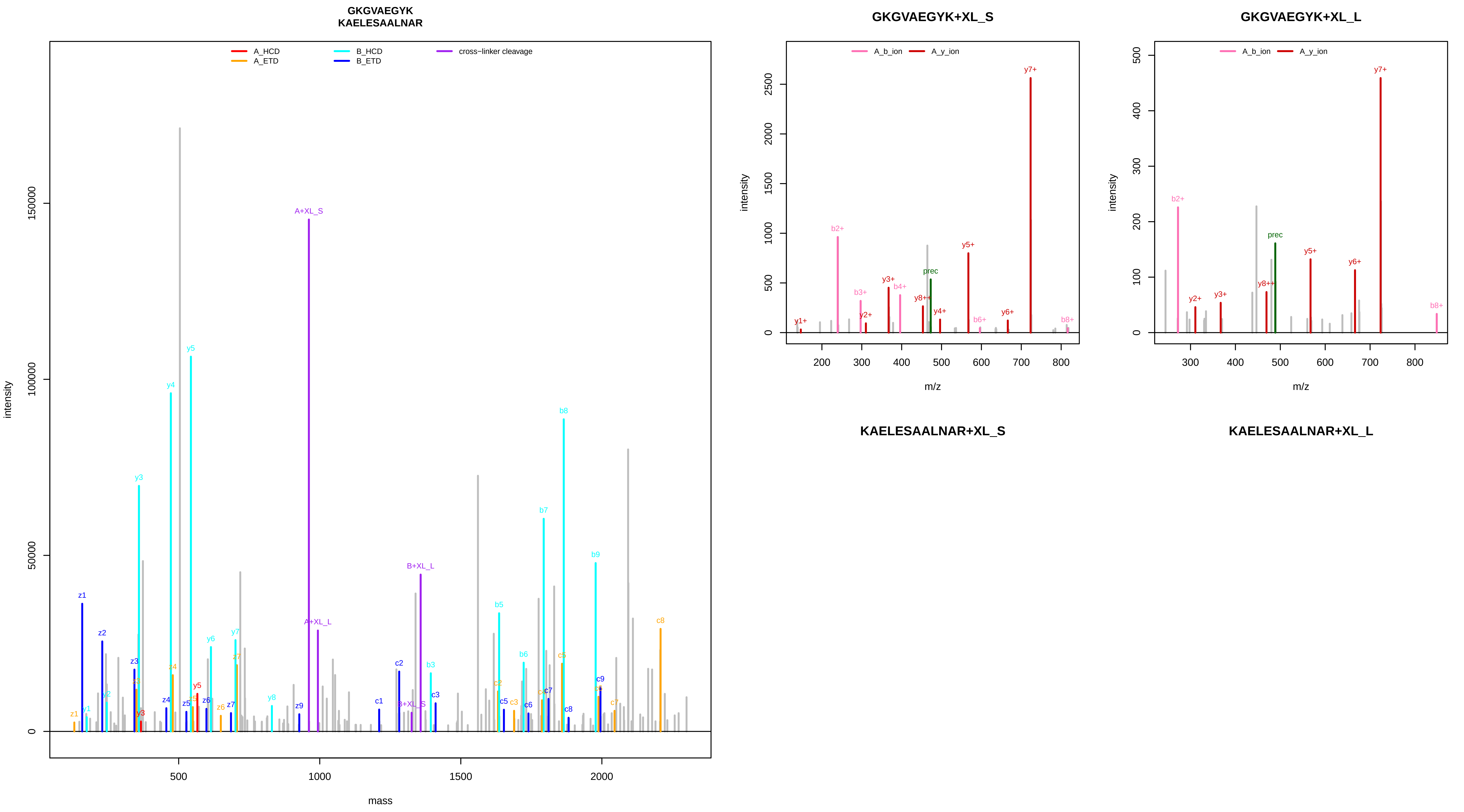
GKGVAEGYK
KAELESAALNAR
A_HCD
A_ETD
B_HCD
B_ETD
cross−linker cleavage
A+XL_S
A+XL_L
B+XL_L
B+XL_S
intensity
mass
GKGVAEGYK+XL_S
GKGVAEGYK+XL_L
A_b_ion
A_y_ion
prec
m/z
KAELESAALNAR+XL_S
KAELESAALNAR+XL_L

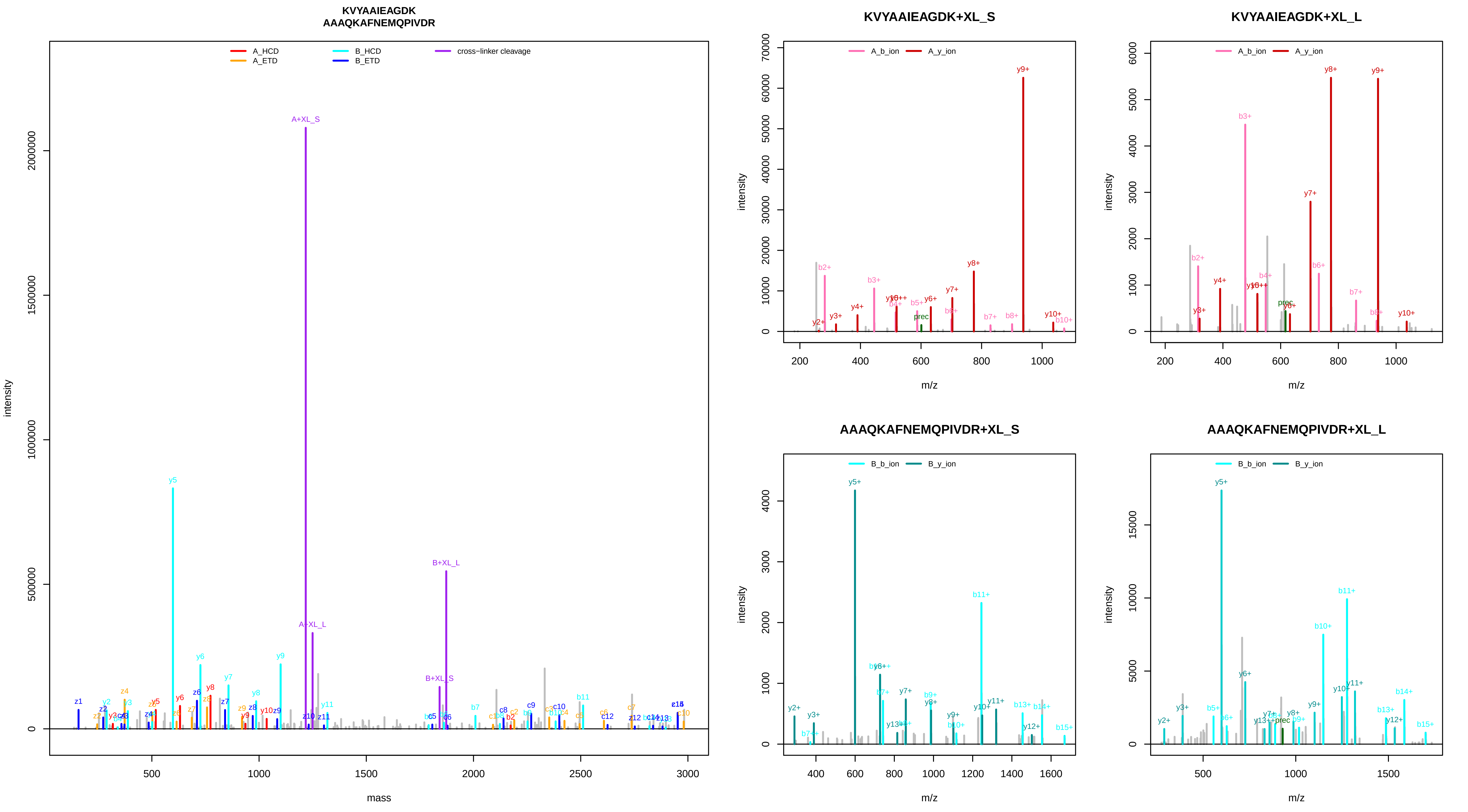
KVYAAIEAGDK
AAAQKAFNEMQPIVDR
A_HCD
A_ETD
B_HCD
B_ETD
cross−linker cleavage
A+XL_S
A+XL_L
B+XL_L
B+XL_S
intensity
mass
KVYAAIEAGDK+XL_S
KVYAAIEAGDK+XL_L
AAAQKAFNEMQPIVDR+XL_S
AAAQKAFNEMQPIVDR+XL_L
A_b_ion
A_y_ion
B_b_ion
B_y_ion
prec
m/z

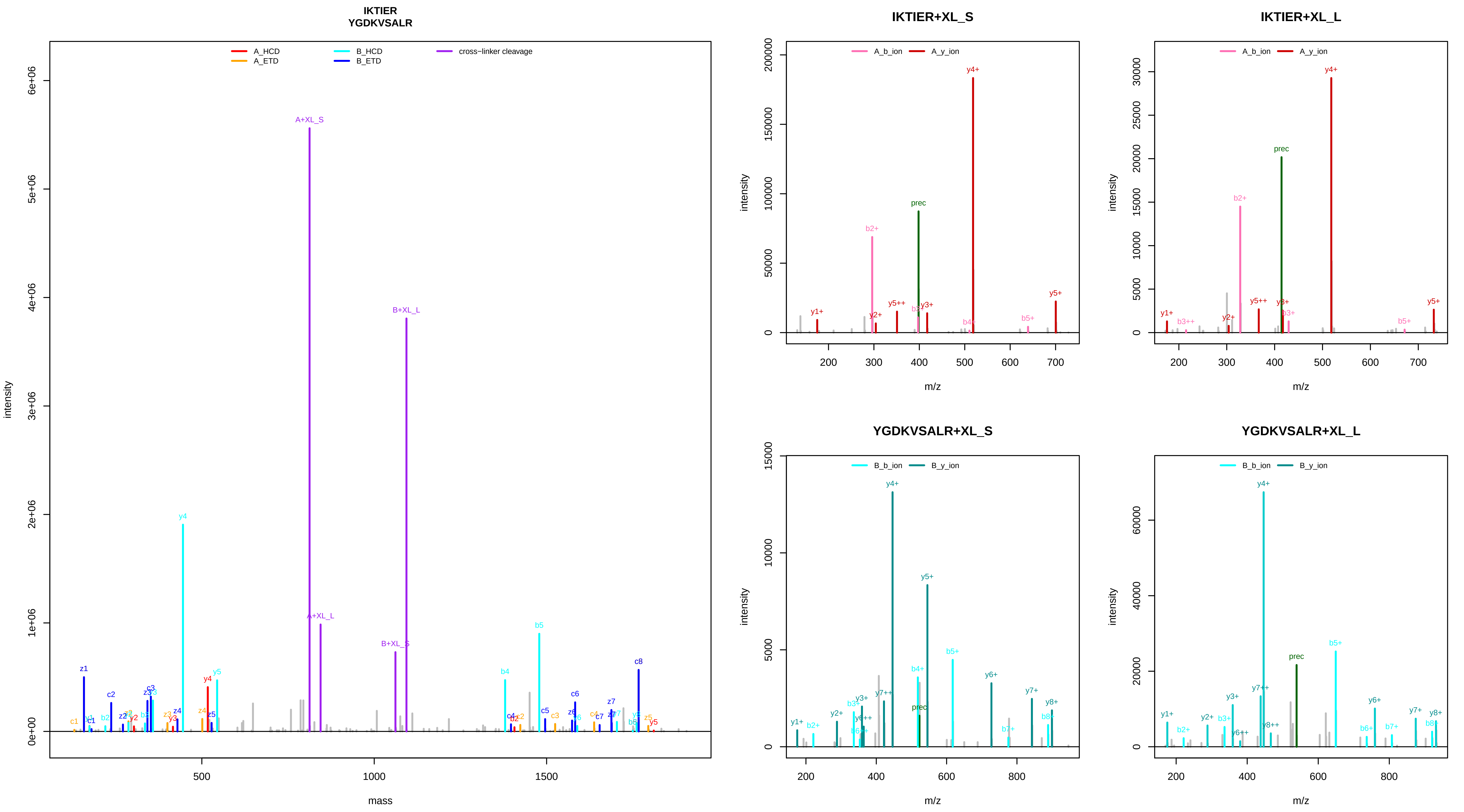
IKTIER
YGDKVSALR
A_HCD
A_ETD
B_HCD
B_ETD
cross−linker cleavage
A+XL_S
B+XL_L
A+XL_L
B+XL_S
y4
y5
b4
b5
c8
z1
c2
c3
z3
z4
z5
c6
c5
z7
c7
y2
y3
c1
y1
b2
z2
c4
c3
z5
y5
intensity
mass
0e+00
1e+06
2e+06
3e+06
4e+06
5e+06
6e+06
500
1000
1500
IKTIER+XL_S
A_b_ion
A_y_ion
y4+
prec
b2+
y1+
y2+
y5++
y3+
b4+
b5+
y5+
intensity
m/z
0
50000
100000
150000
200000
200
300
400
500
600
700
IKTIER+XL_L
A_b_ion
A_y_ion
y4+
prec
b2+
y1+
b3++
y2+
y5++
y3+
b3+
b5+
y5+
intensity
m/z
0
5000
10000
15000
20000
25000
30000
200
300
400
500
600
700
YGDKVSALR+XL_S
B_b_ion
B_y_ion
y4+
y5+
b5+
b4+
y6+
y7+
y8+
y3+
b3+
y7++
y6++
y2+
y1+
b2+
prec
b7+
intensity
m/z
0
5000
10000
15000
200
400
600
800
YGDKVSALR+XL_L
B_b_ion
B_y_ion
y4+
b5+
prec
y7++
y3+
y6+
y7+
y8+
y1+
y2+
b2+
b3+
y8++
y6++
b6+
b7+
intensity
m/z
0
20000
40000
60000
200
400
600
800

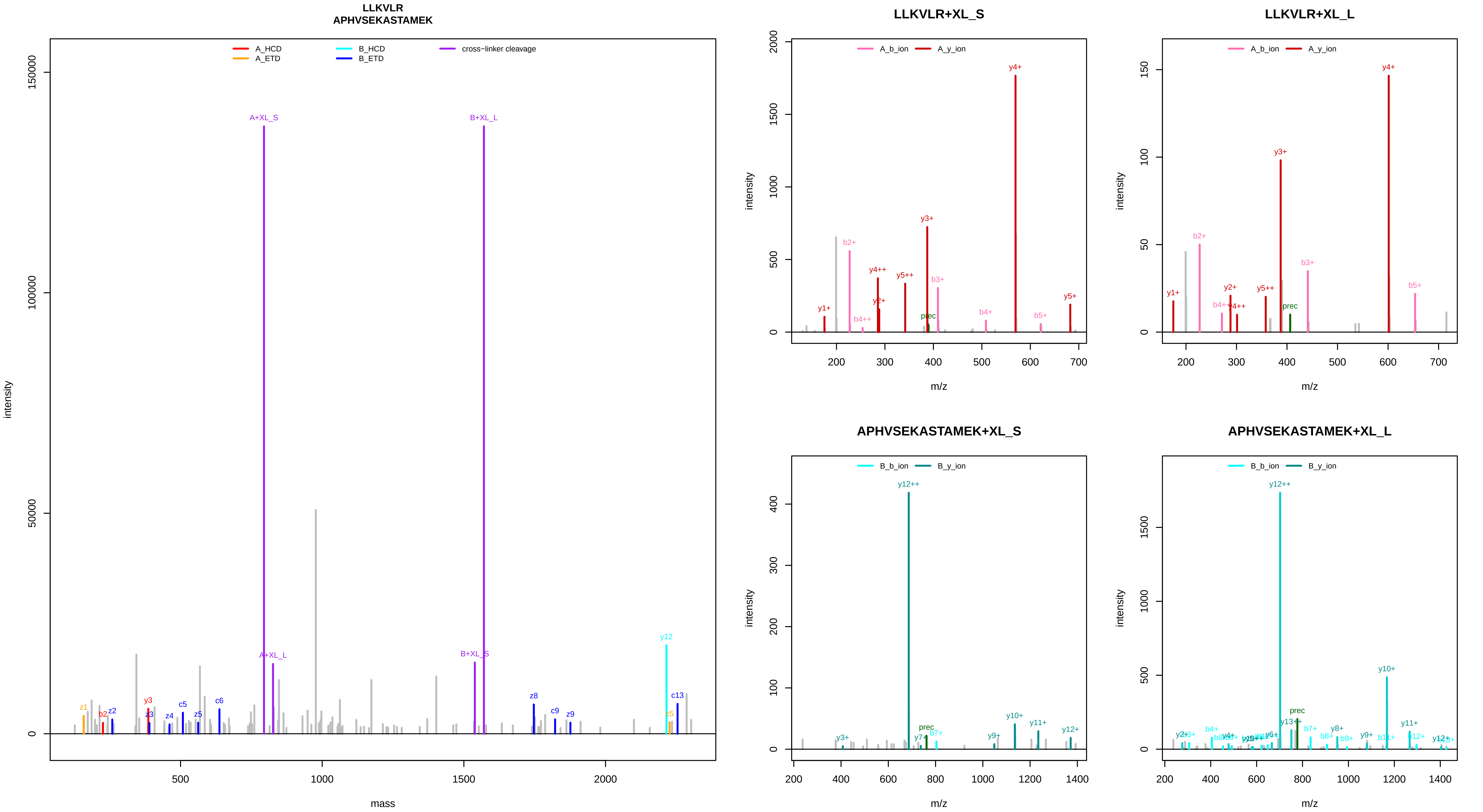

LLKVLR
APHVSEKASTAMEK
A_HCD
A_ETD
B_HCD
B_ETD
cross−linker cleavage
A+XL_S
B+XL_L
A+XL_L
B+XL_S
intensity
mass
LLKVLR+XL_S
LLKVLR+XL_L
APHVSEKASTAMEK+XL_S
APHVSEKASTAMEK+XL_L
A_b_ion
A_y_ion
B_b_ion
B_y_ion
m/z

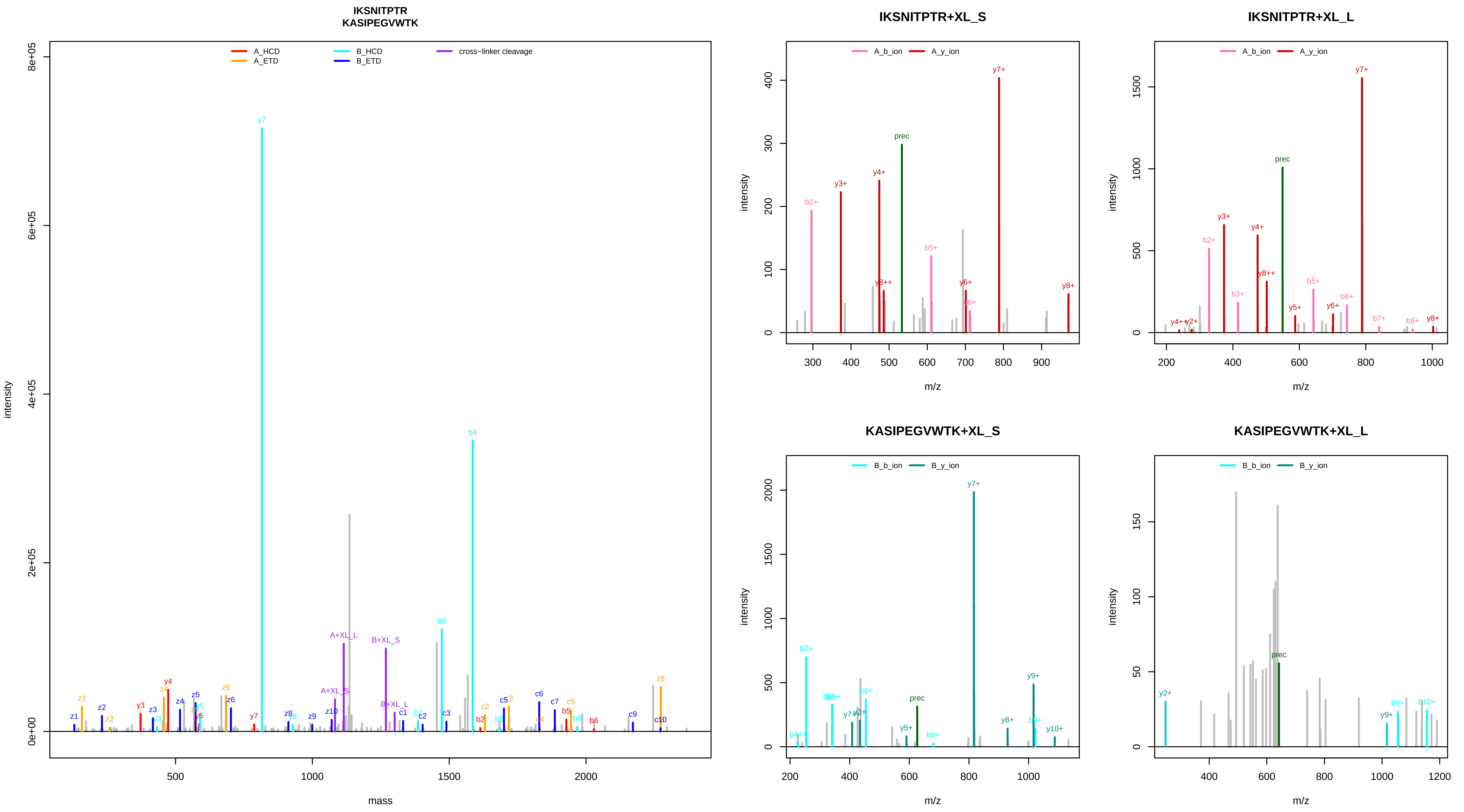
IKSNITPTR
KASIPEGVWTK
A_HCD
A_ETD
B_HCD
B_ETD
cross−linker cleavage
intensity
mass
IKSNITPTR+XL_S
IKSNITPTR+XL_L
KASIPEGVWTK+XL_S
KASIPEGVWTK+XL_L
A_b_ion
A_y_ion
B_b_ion
B_y_ion
prec
m/z
intensity

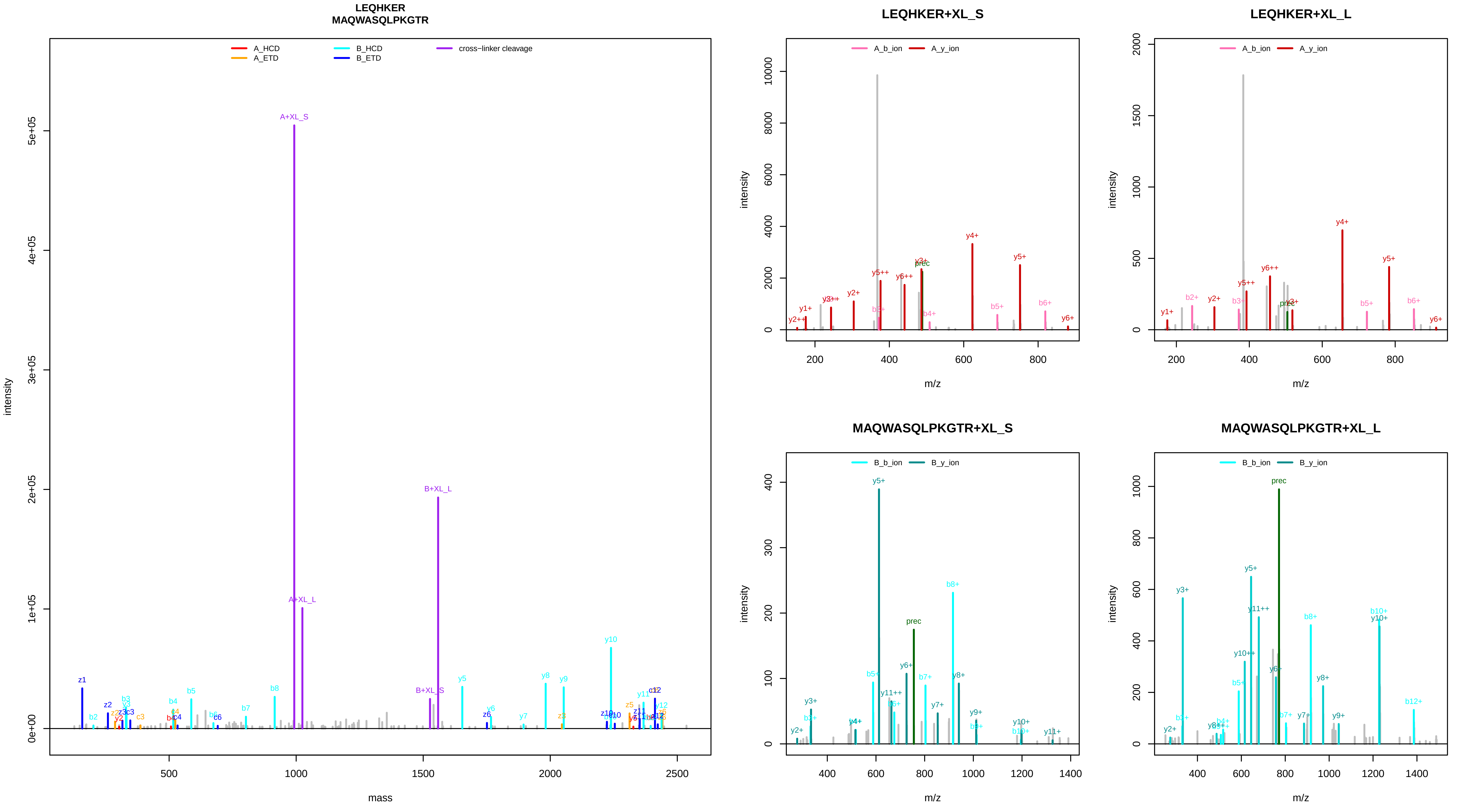
LEQHKER
MAQWASQLPKGTR
A_HCD
A_ETD
B_HCD
B_ETD
cross−linker cleavage
A+XL_S
A+XL_L
B+XL_L
B+XL_S
intensity
mass
0e+00
1e+05
2e+05
3e+05
4e+05
5e+05
500
1000
1500
2000
2500
LEQHKER+XL_S
LEQHKER+XL_L
MAQWASQLPKGTR+XL_S
MAQWASQLPKGTR+XL_L
A_b_ion
A_y_ion
B_b_ion
B_y_ion
prec
m/z
intensity

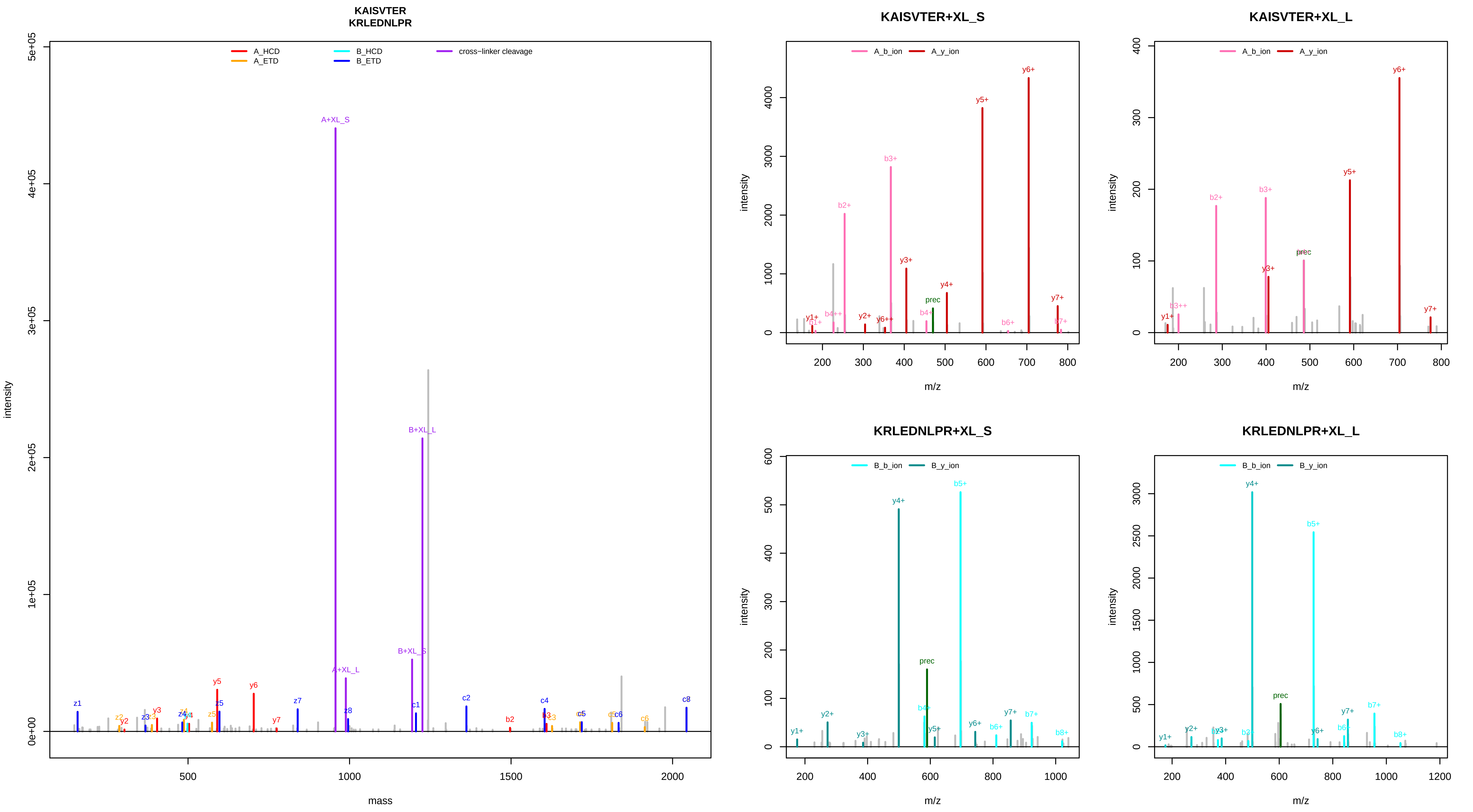
KAISVTER
KRLEDNLPR
A_HCD
A_ETD
B_HCD
B_ETD
cross−linker cleavage
A+XL_S
B+XL_L
B+XL_S
A+XL_L
intensity
mass
KAISVTER+XL_S
KAISVTER+XL_L
A_b_ion
A_y_ion
KRLEDNLPR+XL_S
KRLEDNLPR+XL_L
B_b_ion
B_y_ion
prec
m/z

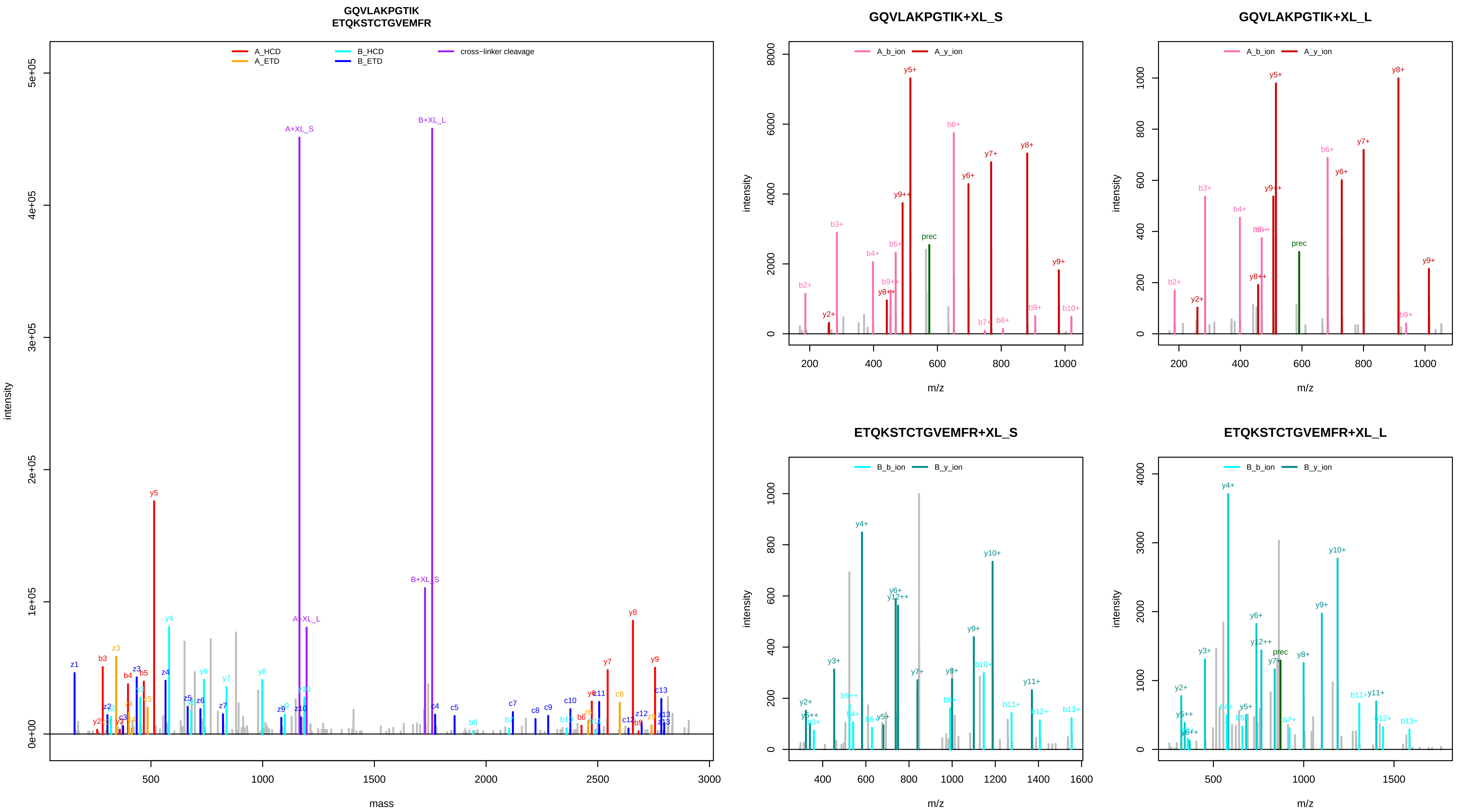
GQVLAKPGTIK
ETQKSTCTGVEMFR
A_HCD
A_ETD
B_HCD
B_ETD
cross−linker cleavage
A+XL_S
B+XL_L
B+XL_S
A+XL_L
intensity
mass
GQVLAKPGTIK+XL_S
GQVLAKPGTIK+XL_L
A_b_ion
A_y_ion
prec
m/z
ETQKSTCTGVEMFR+XL_S
ETQKSTCTGVEMFR+XL_L
B_b_ion
B_y_ion

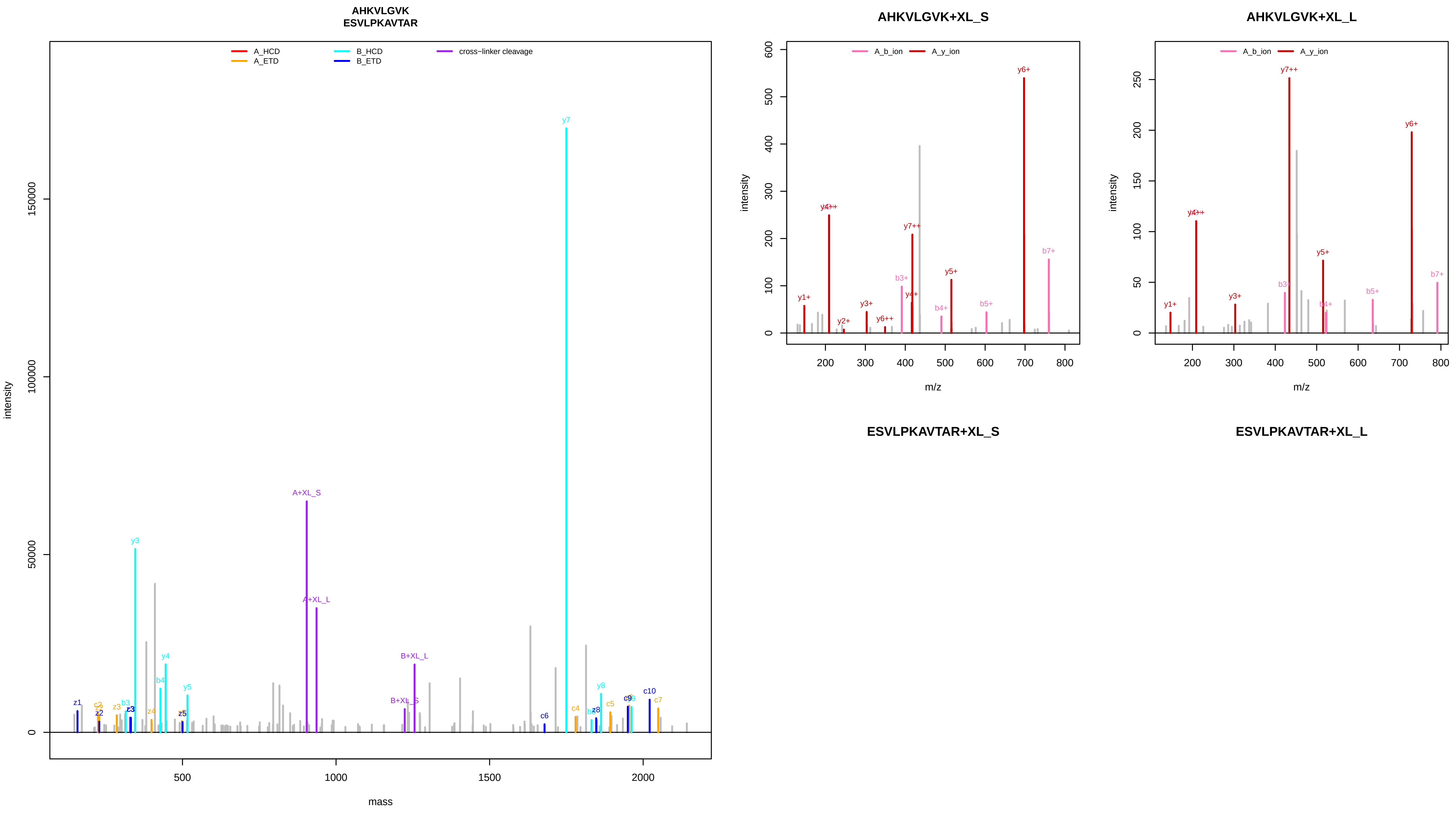
AHKVLGVK
ESVLPKAVTAR
A_HCD
A_ETD
B_HCD
B_ETD
cross−linker cleavage
intensity
mass
A+XL_S
A+XL_L
B+XL_L
B+XL_S
AHKVLGVK+XL_S
AHKVLGVK+XL_L
A_b_ion
A_y_ion
m/z
ESVLPKAVTAR+XL_S
ESVLPKAVTAR+XL_L

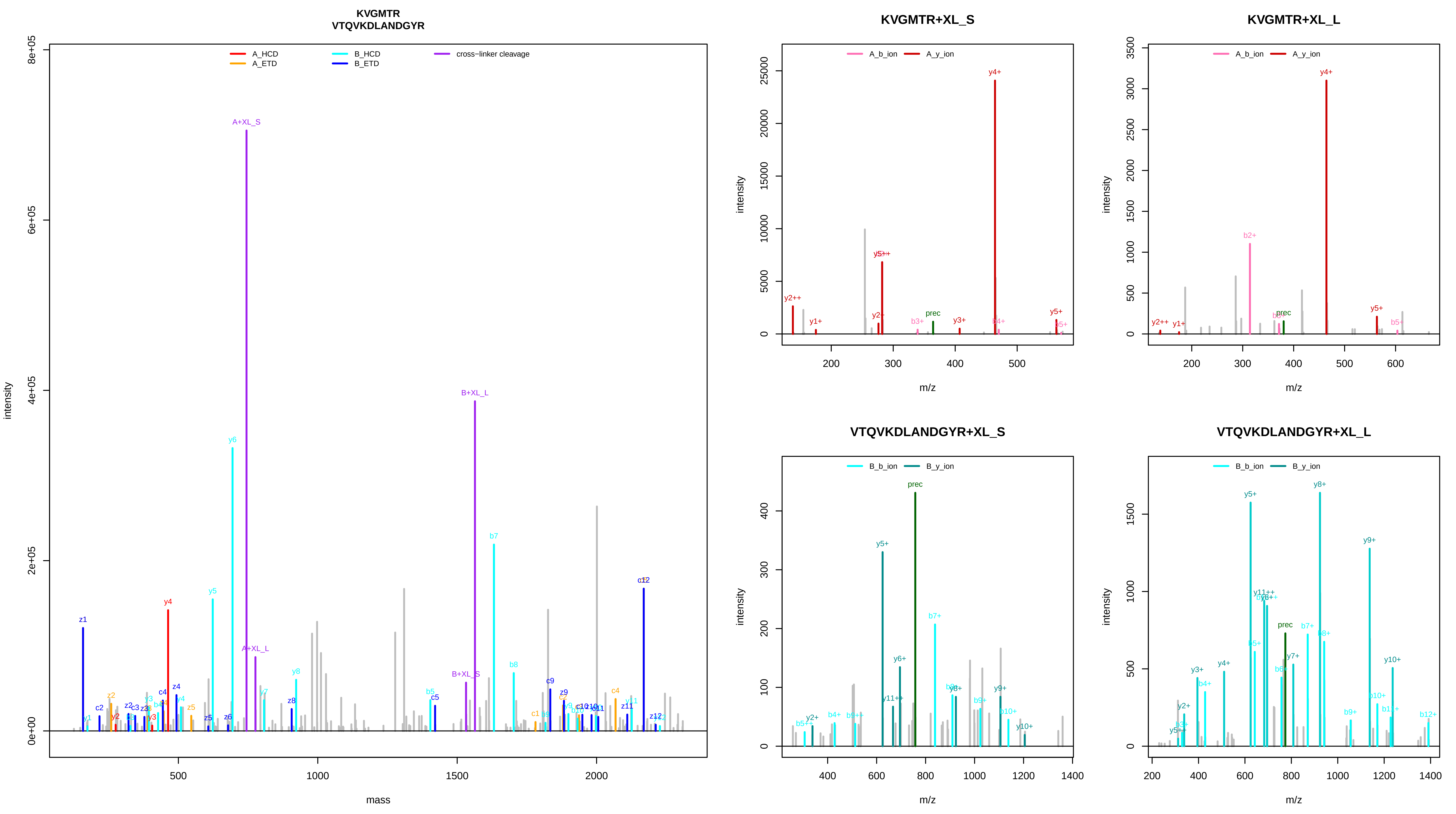
KVGMTR
VTQVKDLANDGYR
A_HCD
A_ETD
B_HCD
B_ETD
cross−linker cleavage
A+XL_S
B+XL_L
A+XL_L
B+XL_S
intensity
mass
KVGMTR+XL_S
KVGMTR+XL_L
VTQVKDLANDGYR+XL_S
VTQVKDLANDGYR+XL_L
A_b_ion
A_y_ion
B_b_ion
B_y_ion
prec
m/z

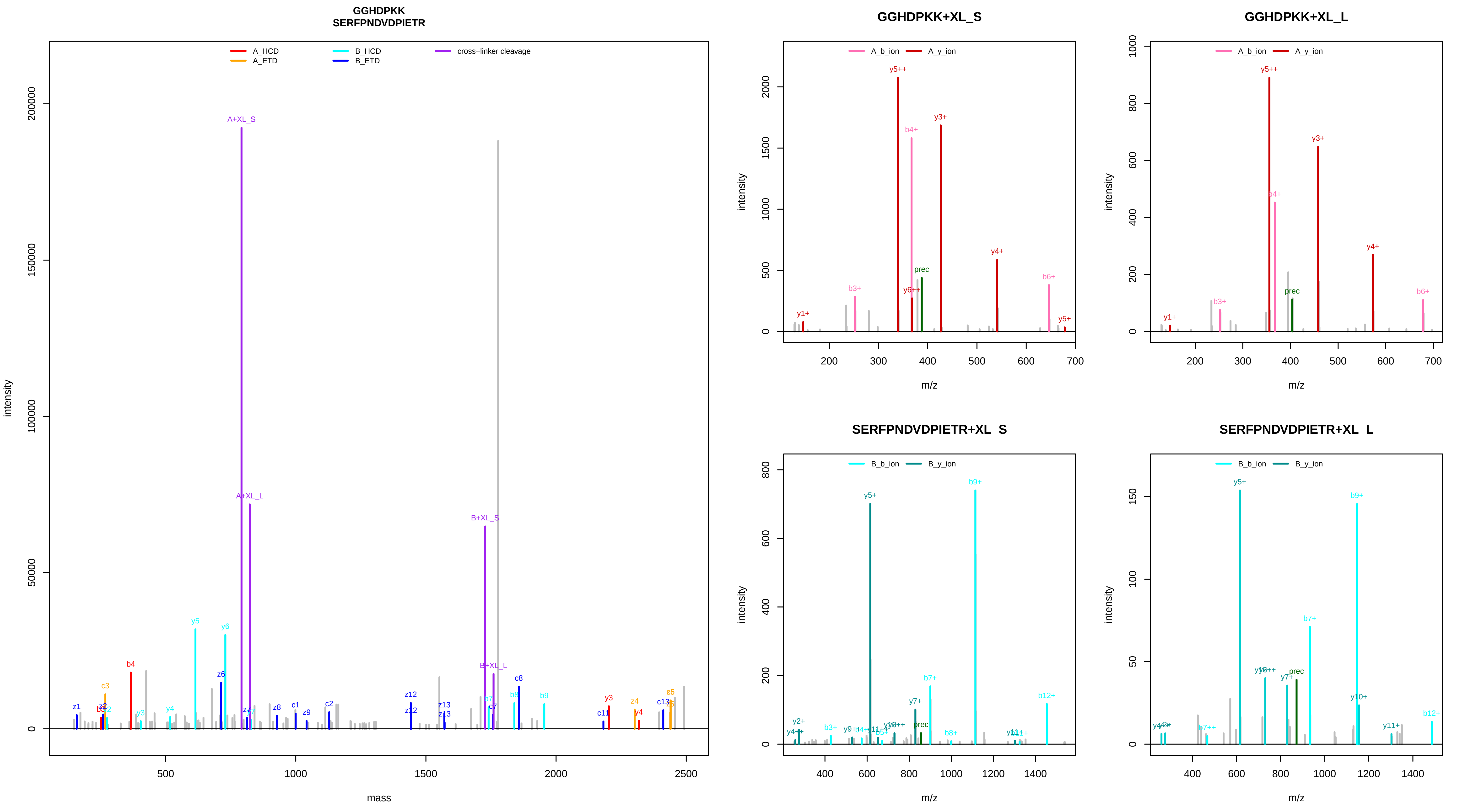

GGHDPKK
SERFPNDVDPIETR
A_HCD
A_ETD
B_HCD
B_ETD
cross−linker cleavage
A+XL_S
A+XL_L
B+XL_S
B+XL_L
intensity
mass
GGHDPKK+XL_S
GGHDPKK+XL_L
SERFPNDVDPIETR+XL_S
SERFPNDVDPIETR+XL_L
A_b_ion
A_y_ion
B_b_ion
B_y_ion
prec
m/z

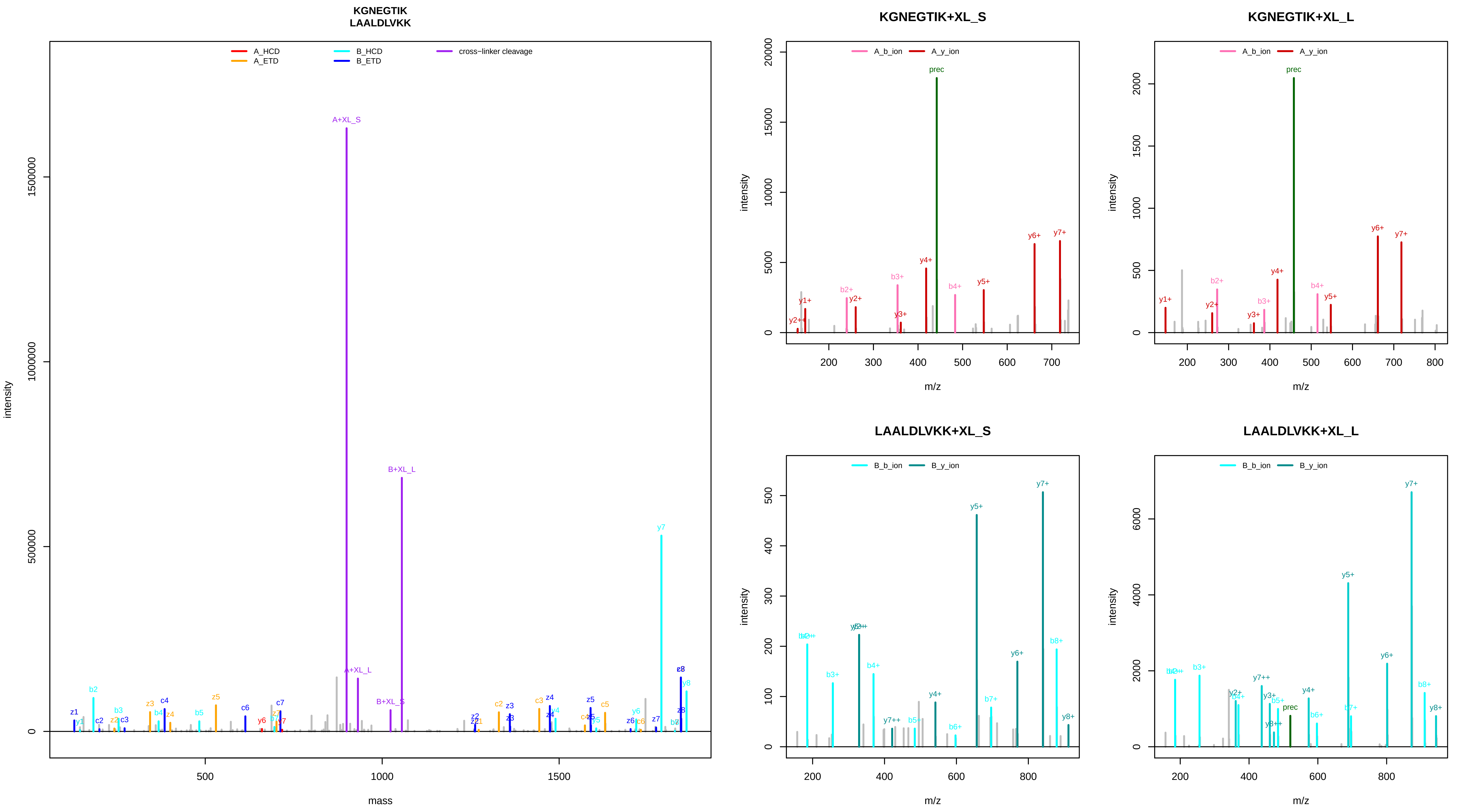

KGNEGTIK
LAALDLVKK
A_HCD
A_ETD
B_HCD
B_ETD
cross−linker cleavage
A+XL_S
B+XL_L
A+XL_L
B+XL_S
intensity
mass
KGNEGTIK+XL_S
KGNEGTIK+XL_L
LAALDLVKK+XL_S
LAALDLVKK+XL_L
A_b_ion
A_y_ion
B_b_ion
B_y_ion
prec
m/z

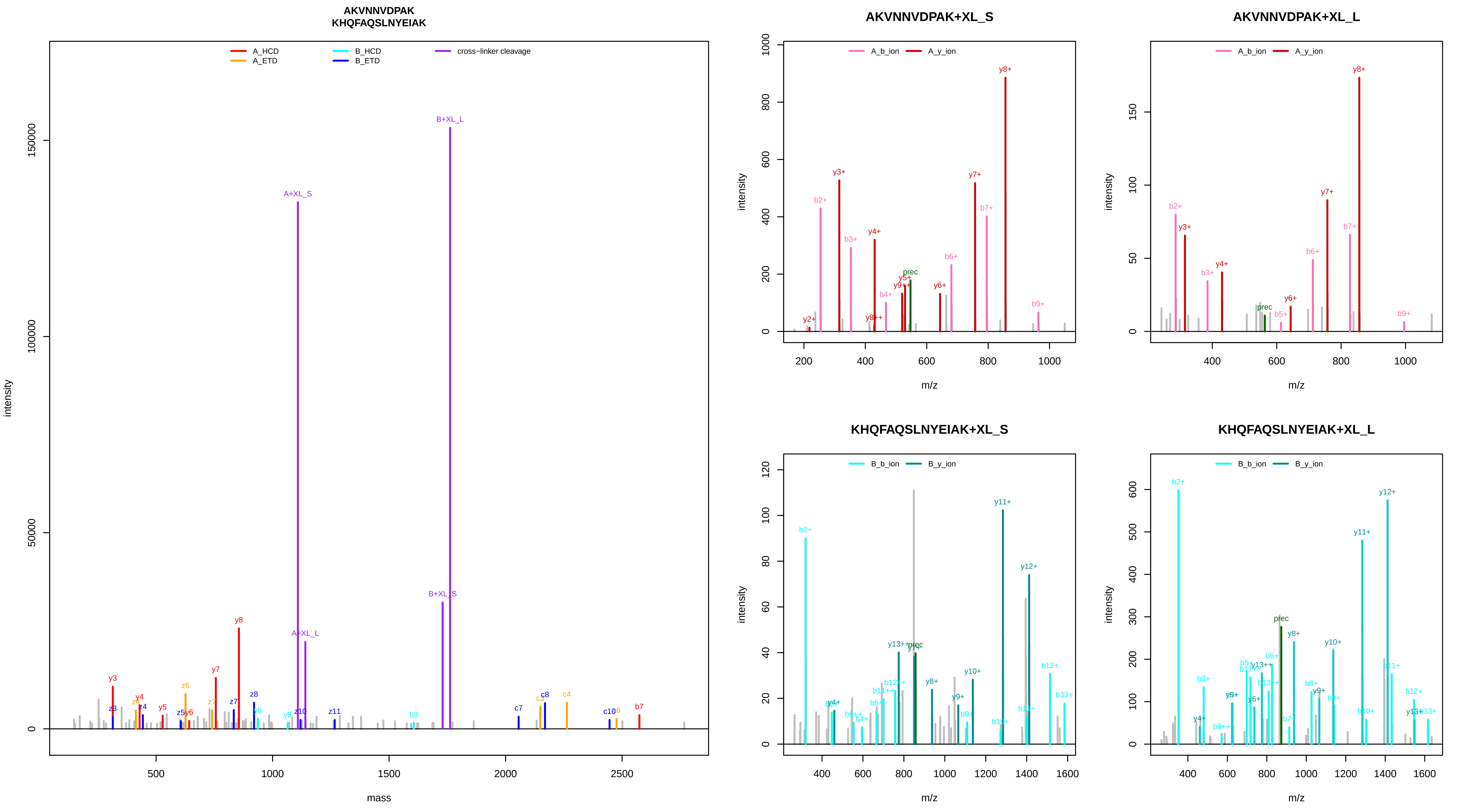
AKVNNVDPAK
KHQFAQSLNYEIAK
A_HCD
A_ETD
B_HCD
B_ETD
cross−linker cleavage
B+XL_L
A+XL_S
B+XL_S
A+XL_L
intensity
mass
AKVNNVDPAK+XL_S
AKVNNVDPAK+XL_L
KHQFAQSLNYEIAK+XL_S
KHQFAQSLNYEIAK+XL_L
A_b_ion
A_y_ion
B_b_ion
B_y_ion
prec
m/z

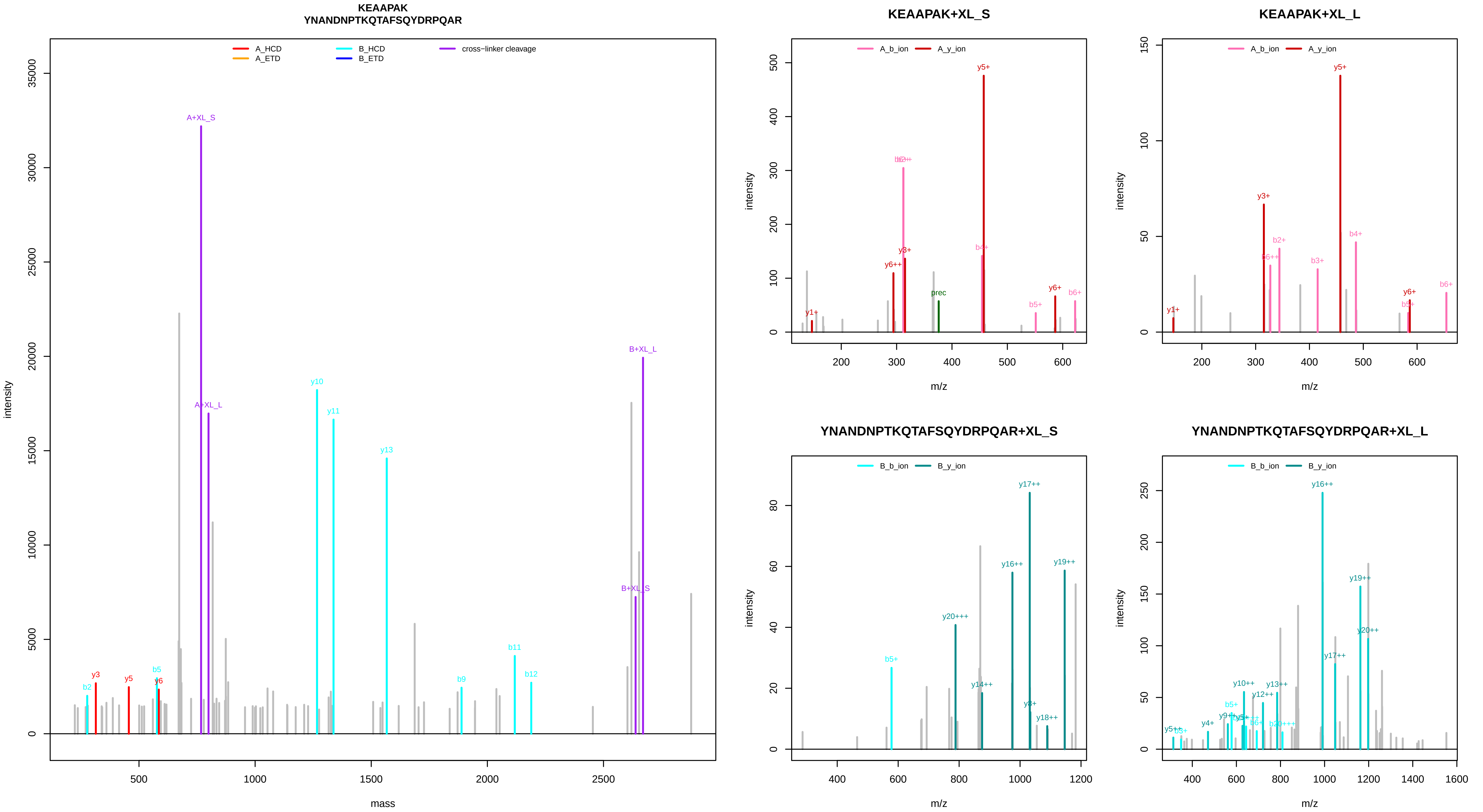
KEAAPAK
YNANDNPTKQTAFSQYDRPQAR
A_HCD
A_ETD
B_HCD
B_ETD
cross−linker cleavage
A+XL_S
A+XL_L
B+XL_L
B+XL_S
y10
y11
y13
b11
b12
b9
y3
y5
y6
b2
b5
intensity
mass
KEAAPAK+XL_S
KEAAPAK+XL_L
YNANDNPTKQTAFSQYDRPQAR+XL_S
YNANDNPTKQTAFSQYDRPQAR+XL_L
A_b_ion
A_y_ion
B_b_ion
B_y_ion
prec
m/z

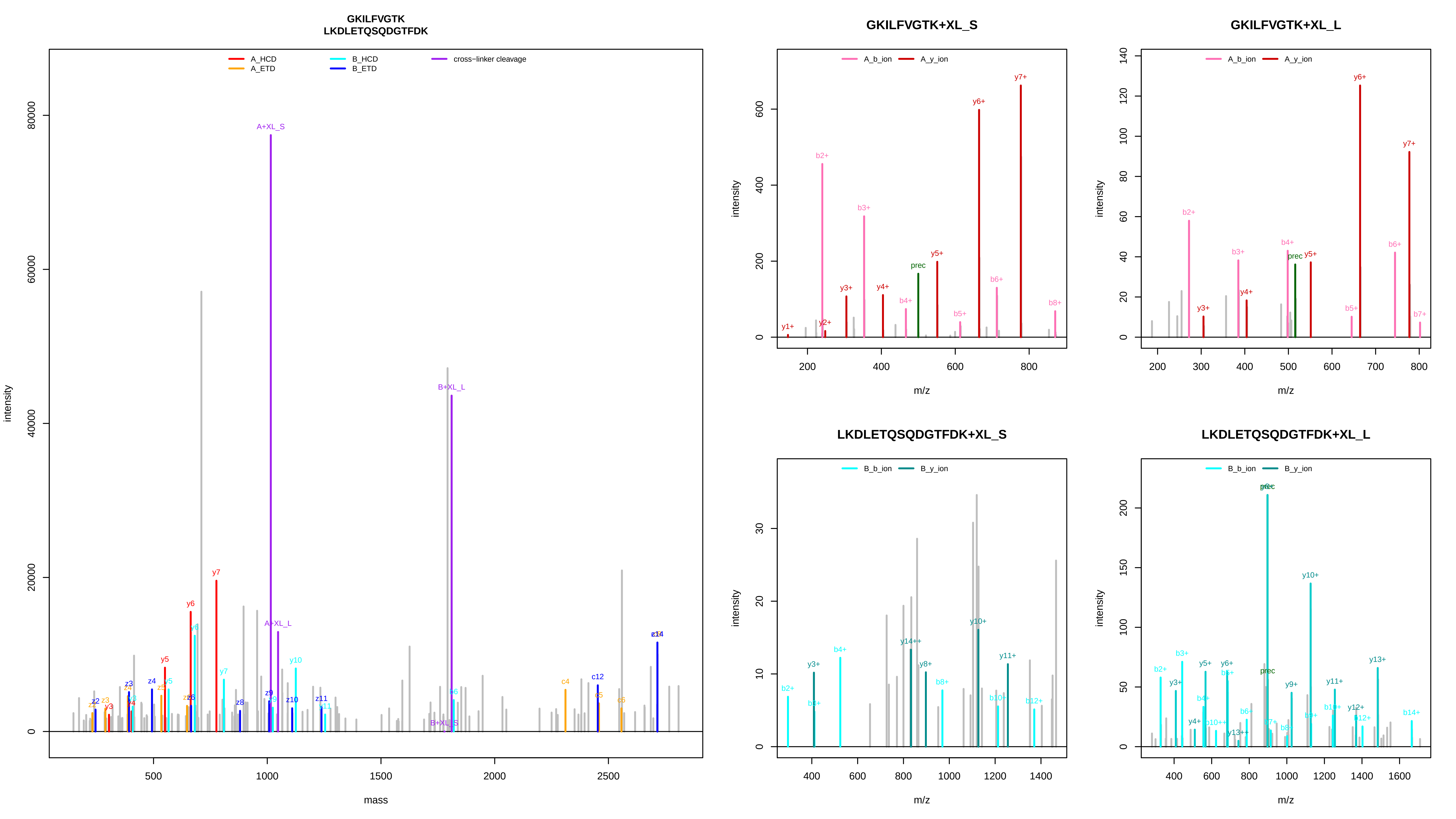
GKILFVGTK
LKDLETQSQDGTFDK
A_HCD
A_ETD
B_HCD
B_ETD
cross−linker cleavage
A+XL_S
B+XL_L
A+XL_L
B+XL_S
intensity
mass
GKILFVGTK+XL_S
GKILFVGTK+XL_L
LKDLETQSQDGTFDK+XL_S
LKDLETQSQDGTFDK+XL_L
A_b_ion
A_y_ion
B_b_ion
B_y_ion
prec
m/z

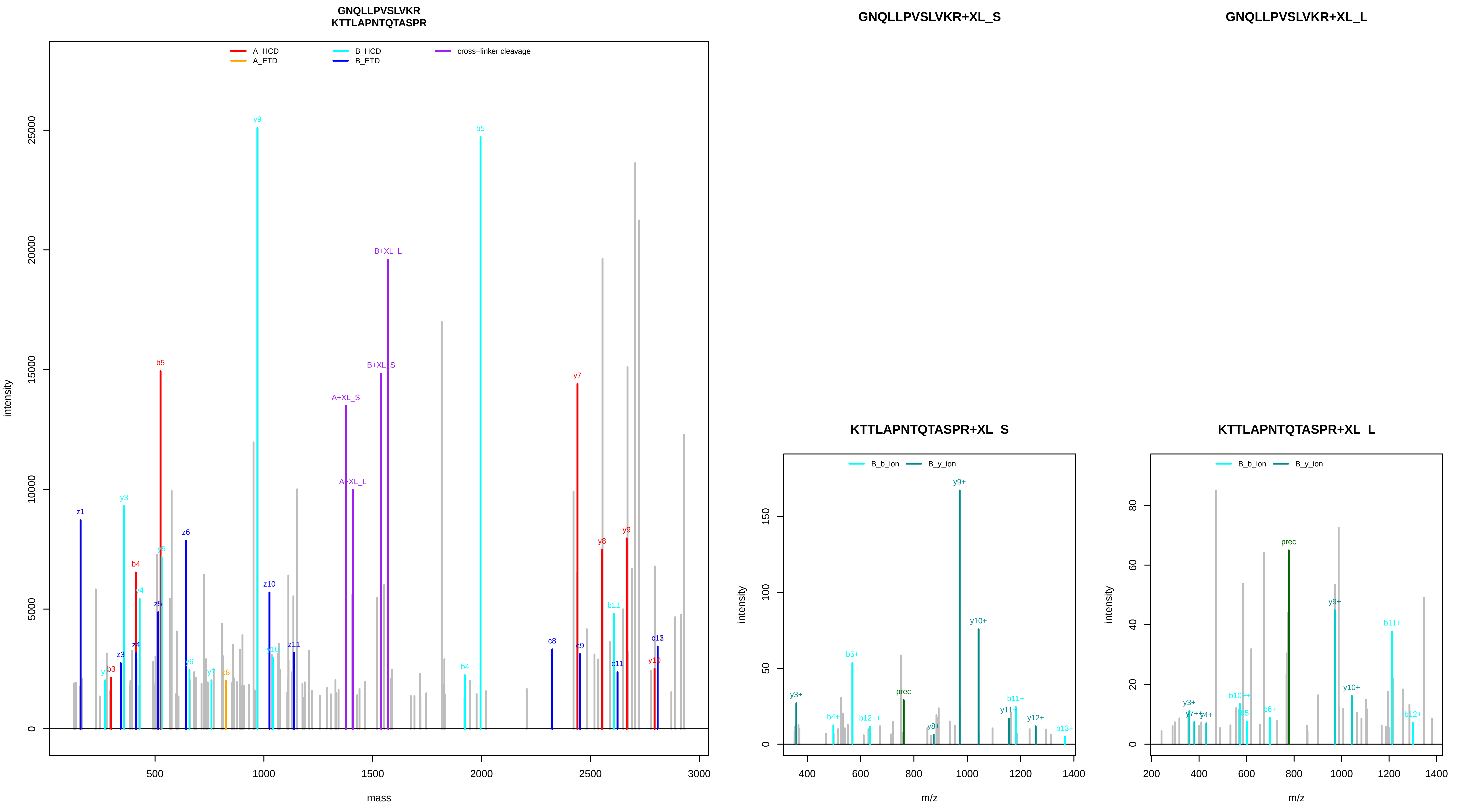
GNQLLPVSLVKR
KTTLAPNTQTASPR
A_HCD
A_ETD
B_HCD
B_ETD
cross−linker cleavage
intensity
mass
y9
b5
B+XL_L
B+XL_S
A+XL_S
A+XL_L
y7
y8
y9
y10
b11
c11
c8
c9
c13
z1
z3
z4
z5
z6
z10
z11
y2
y3
y4
y5
y6
y7
y10
b3
b4
c8
b4
GNQLLPVSLVKR+XL_S
GNQLLPVSLVKR+XL_L
KTTLAPNTQTASPR+XL_S
KTTLAPNTQTASPR+XL_L
B_b_ion
B_y_ion
m/z
y9+
y10+
b5+
prec
y3+
b4+
b12++
y8+
y11+
b11+
y12+
b13+
y9+
b11+
y10+
b10++
b5+
b6+
y3+
y7++
y4+
b12+

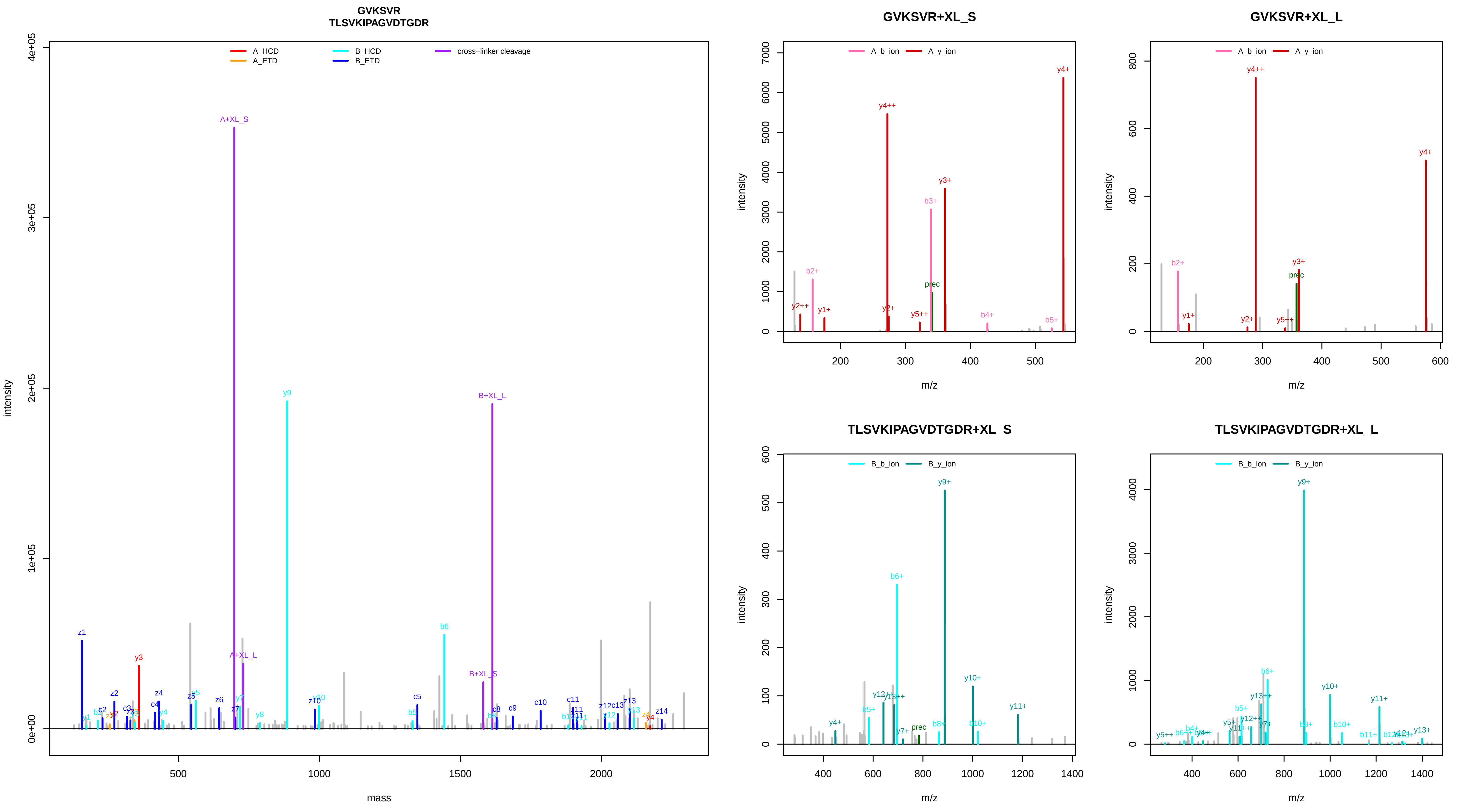
GVKSVR
TLSVKIPAGVDTGDR
A_HCD
A_ETD
B_HCD
B_ETD
cross−linker cleavage
A+XL_S
B+XL_L
A+XL_L
B+XL_S
intensity
mass
GVKSVR+XL_S
GVKSVR+XL_L
TLSVKIPAGVDTGDR+XL_S
TLSVKIPAGVDTGDR+XL_L
A_b_ion
A_y_ion
B_b_ion
B_y_ion
m/z
prec

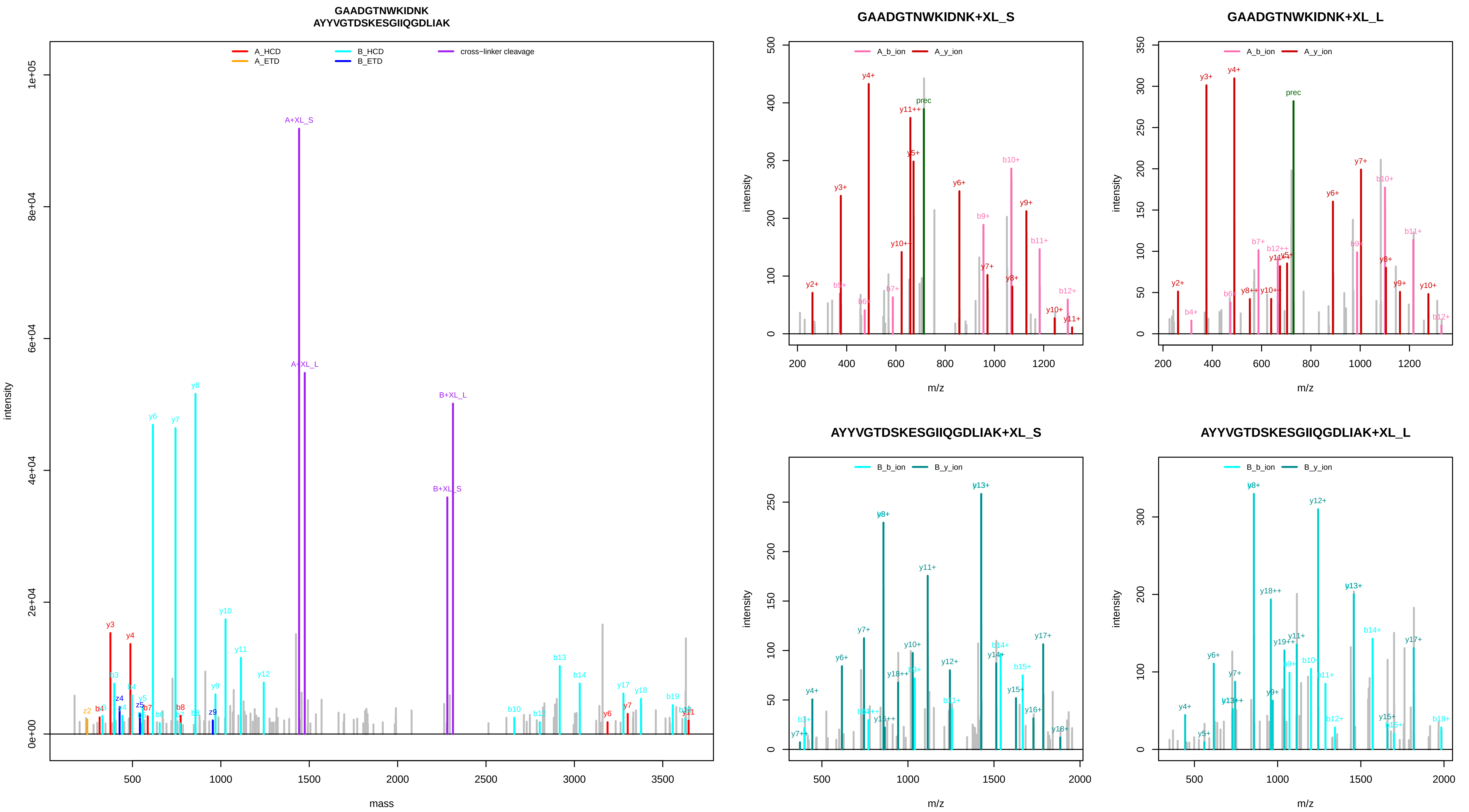
GAADGTNWKIDNK
AYYVGTDSKESGIIQGDLIAK
A_HCD
A_ETD
B_HCD
B_ETD
cross−linker cleavage
A+XL_S
A+XL_L
B+XL_L
B+XL_S
intensity
mass
GAADGTNWKIDNK+XL_S
GAADGTNWKIDNK+XL_L
A_b_ion
A_y_ion
prec
AYYVGTDSKESGIIQGDLIAK+XL_S
AYYVGTDSKESGIIQGDLIAK+XL_L
B_b_ion
B_y_ion
m/z

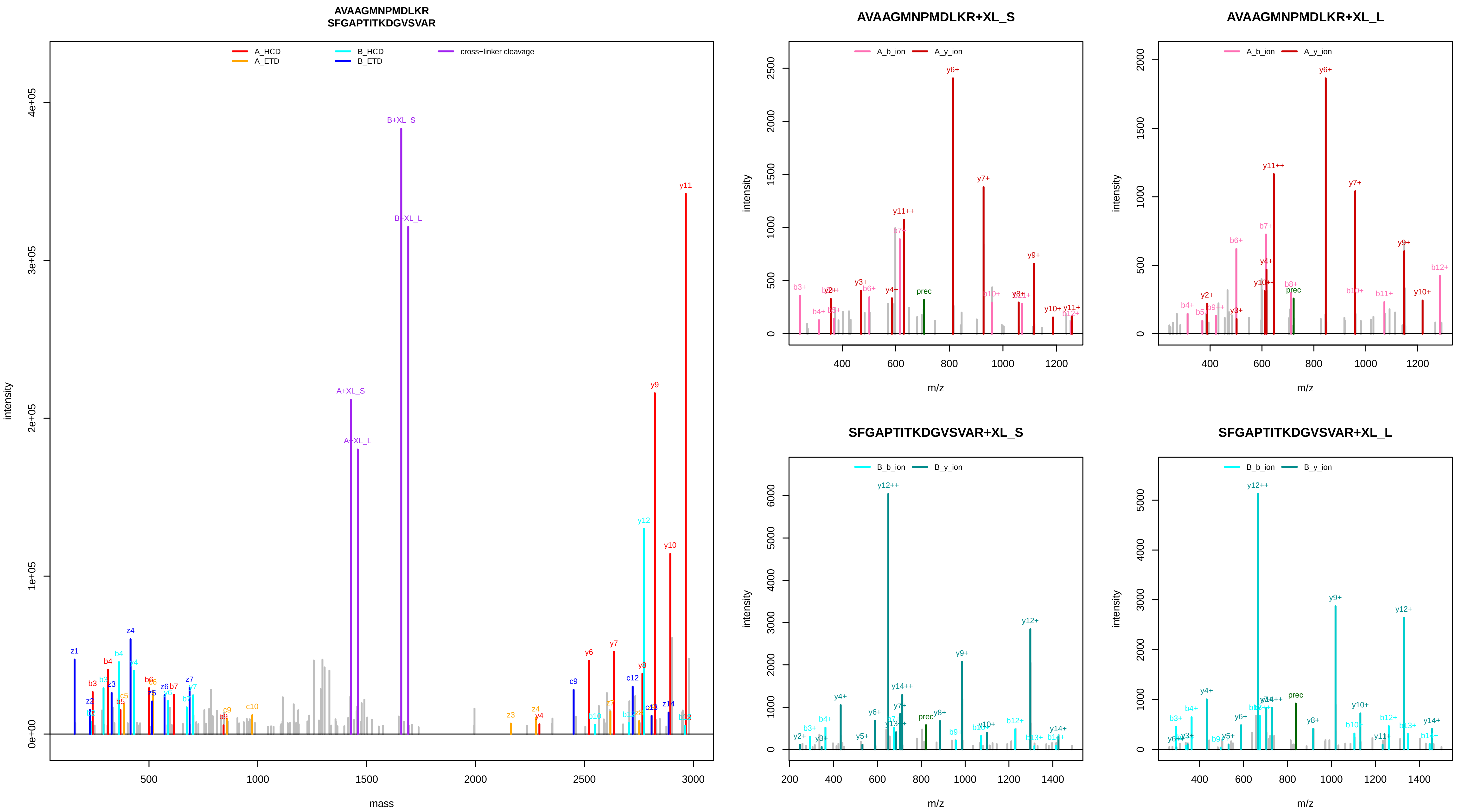

AVAAGMNPMDLKR
SFGAPTITKDGVSVAR
A_HCD
A_ETD
B_HCD
B_ETD
cross−linker cleavage
B+XL_S
B+XL_L
A+XL_S
A+XL_L
intensity
mass
AVAAGMNPMDLKR+XL_S
AVAAGMNPMDLKR+XL_L
SFGAPTITKDGVSVAR+XL_S
SFGAPTITKDGVSVAR+XL_L
A_b_ion
A_y_ion
B_b_ion
B_y_ion
prec
m/z

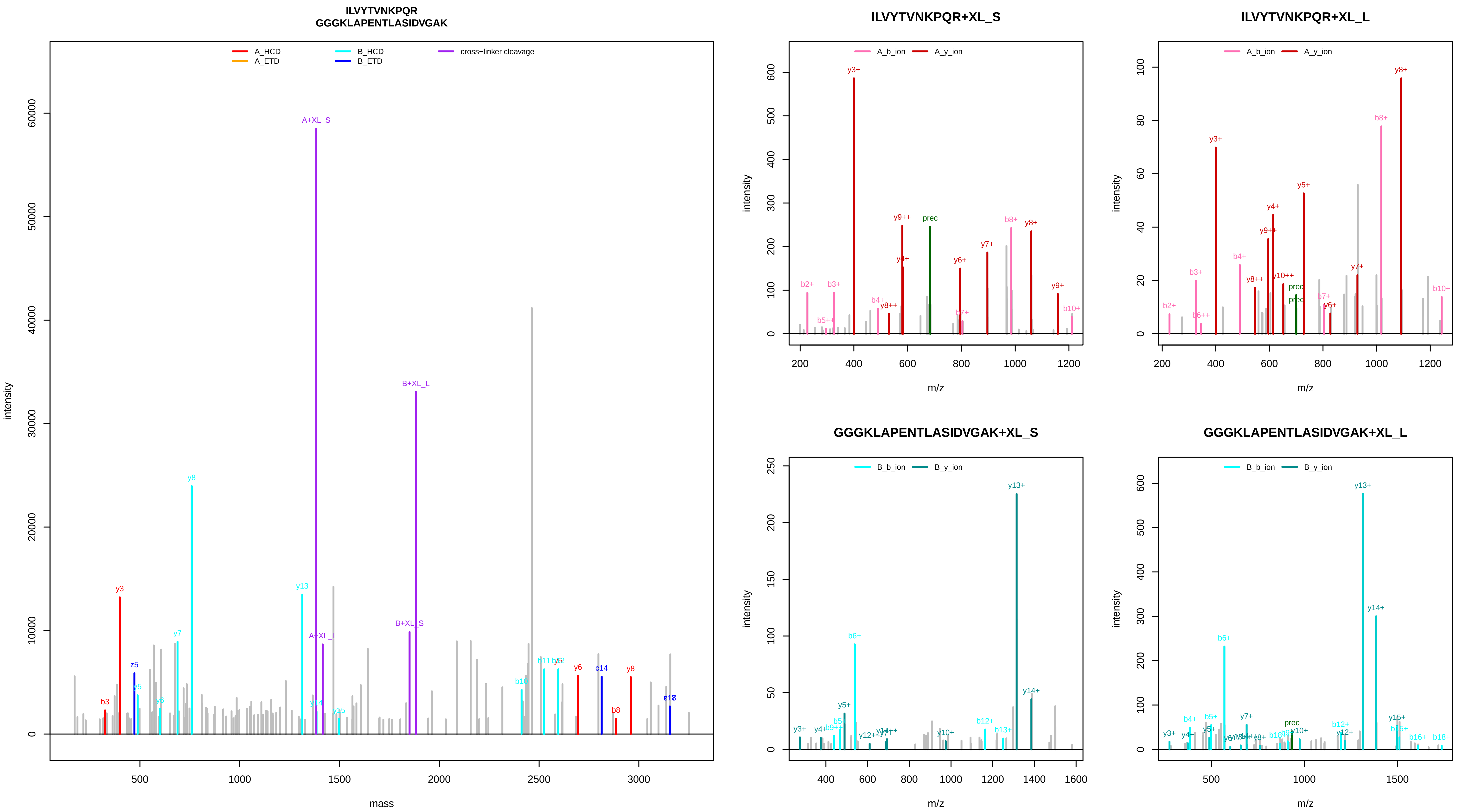
ILVYTVNKPQR
GGGKLAPENTLASIDVGAK
A_HCD
A_ETD
B_HCD
B_ETD
cross−linker cleavage
A+XL_S
B+XL_L
B+XL_S
A+XL_L
intensity
mass
ILVYTVNKPQR+XL_S
ILVYTVNKPQR+XL_L
A_b_ion
A_y_ion
GGGKLAPENTLASIDVGAK+XL_S
GGGKLAPENTLASIDVGAK+XL_L
B_b_ion
B_y_ion
prec
m/z

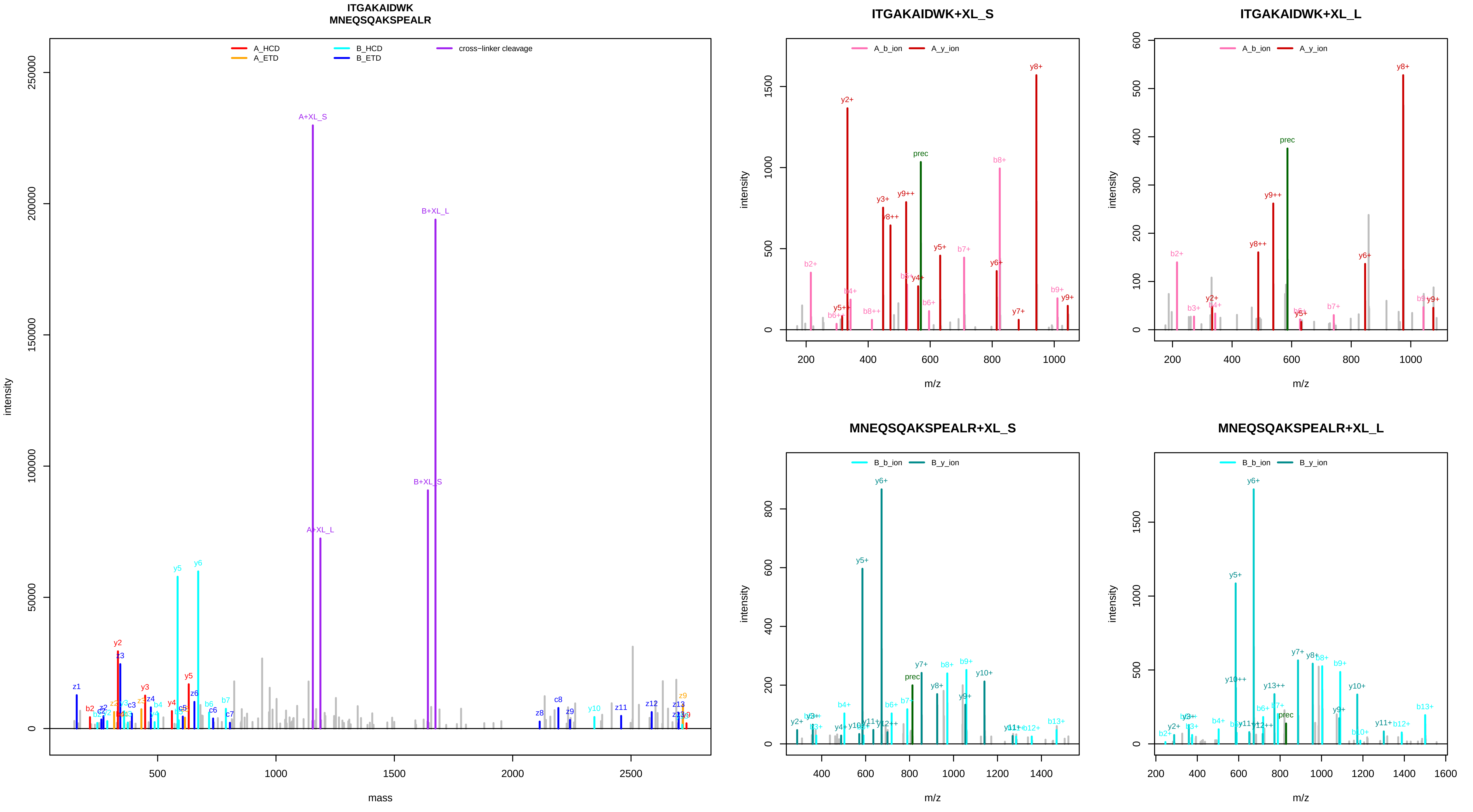
ITGAKAIDWK
MNEQSQAKSPEALR
A_HCD
A_ETD
B_HCD
B_ETD
cross−linker cleavage
A+XL_S
B+XL_L
B+XL_S
A+XL_L
intensity
mass
ITGAKAIDWK+XL_S
ITGAKAIDWK+XL_L
MNEQSQAKSPEALR+XL_S
MNEQSQAKSPEALR+XL_L
A_b_ion
A_y_ion
B_b_ion
B_y_ion
prec
m/z

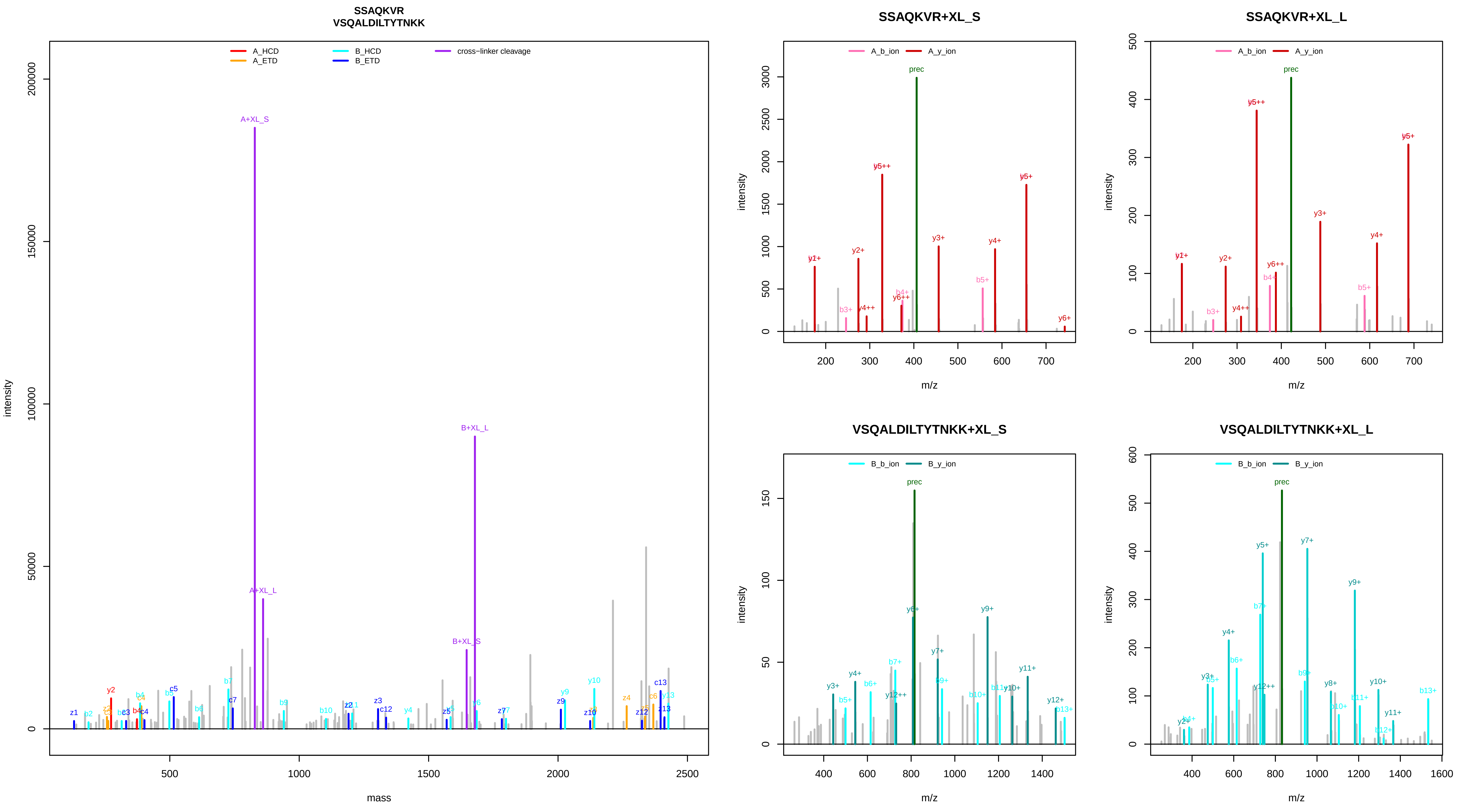

SSAQKVR
VSQALDILTYTNKK
A_HCD
A_ETD
B_HCD
B_ETD
cross−linker cleavage
A+XL_S
A+XL_L
B+XL_L
B+XL_S
intensity
mass
SSAQKVR+XL_S
SSAQKVR+XL_L
A_b_ion
A_y_ion
prec
m/z
VSQALDILTYTNKK+XL_S
VSQALDILTYTNKK+XL_L
B_b_ion
B_y_ion

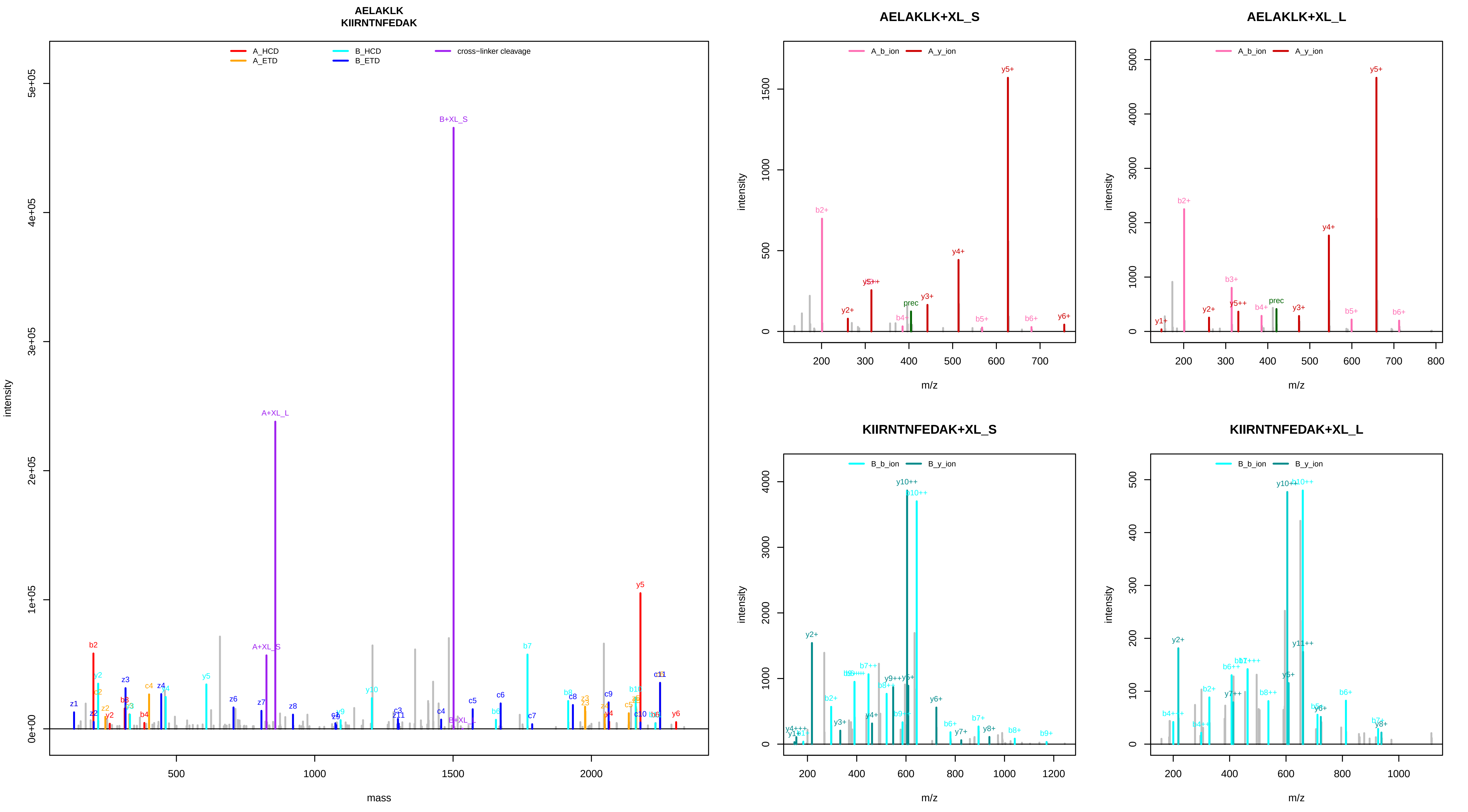
AELAKLK
KIIRNTNFEDAK
A_HCD
A_ETD
B_HCD
B_ETD
cross−linker cleavage
B+XL_S
A+XL_L
A+XL_S
intensity
mass
AELAKLK+XL_S
AELAKLK+XL_L
A_b_ion
A_y_ion
KIIRNTNFEDAK+XL_S
KIIRNTNFEDAK+XL_L
B_b_ion
B_y_ion
prec
m/z

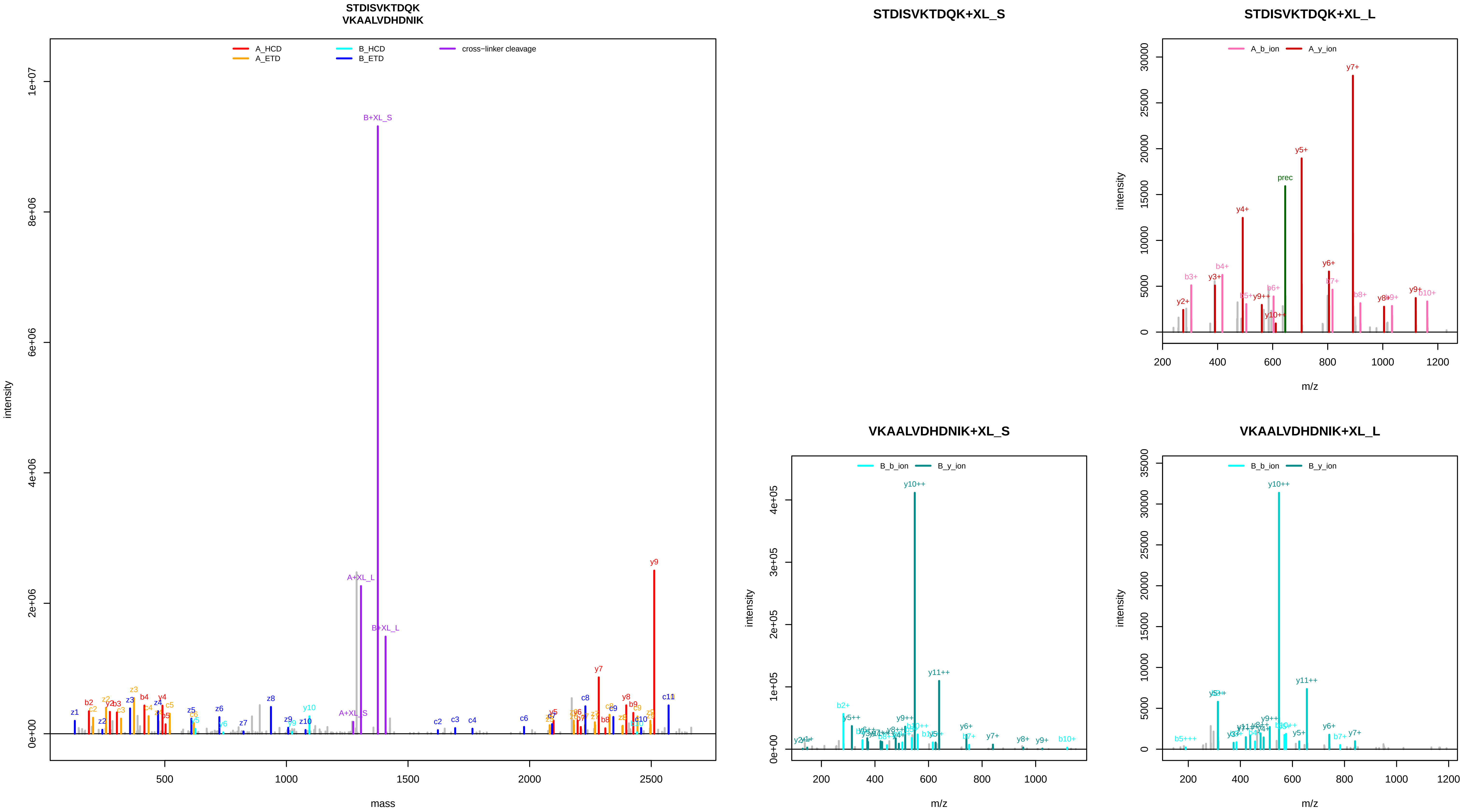

STDISVKTDQK
VKAALVDHDNIK
A_HCD
A_ETD
B_HCD
B_ETD
cross−linker cleavage
B+XL_S
A+XL_L
B+XL_L
A+XL_S
y9
y7
intensity
mass
STDISVKTDQK+XL_S
STDISVKTDQK+XL_L
A_b_ion
A_y_ion
y7+
y5+
prec
y4+
y6+
intensity
m/z
VKAALVDHDNIK+XL_S
B_b_ion
B_y_ion
y10++
y11++
intensity
m/z
VKAALVDHDNIK+XL_L
B_b_ion
B_y_ion
y10++
y11++
intensity
m/z

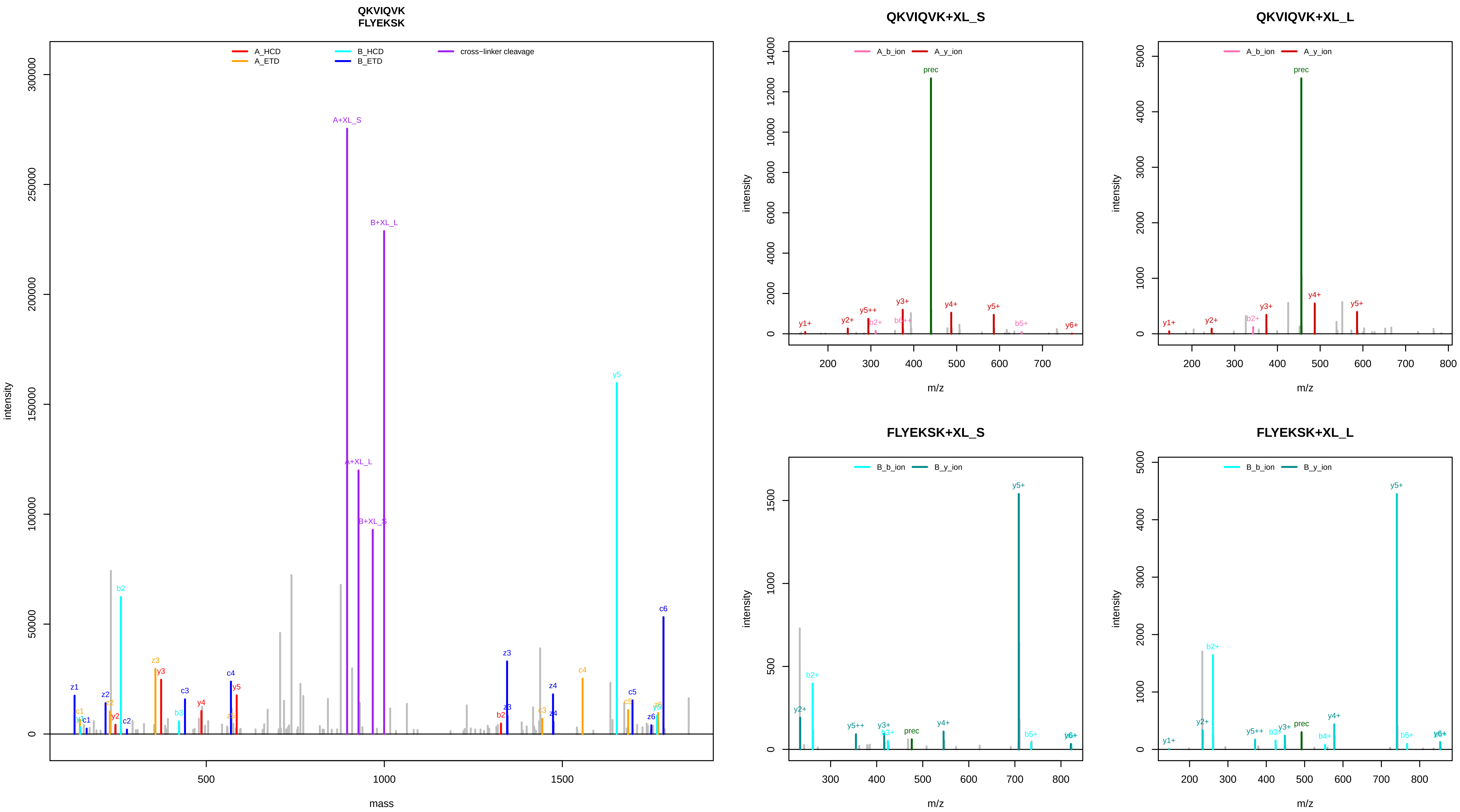
QKVIQVK
FLYEKSK
A_HCD
A_ETD
B_HCD
B_ETD
cross−linker cleavage
A+XL_S
B+XL_L
A+XL_L
B+XL_S
intensity
mass
QKVIQVK+XL_S
QKVIQVK+XL_L
FLYEKSK+XL_S
FLYEKSK+XL_L
A_b_ion
A_y_ion
B_b_ion
B_y_ion
prec
m/z
intensity

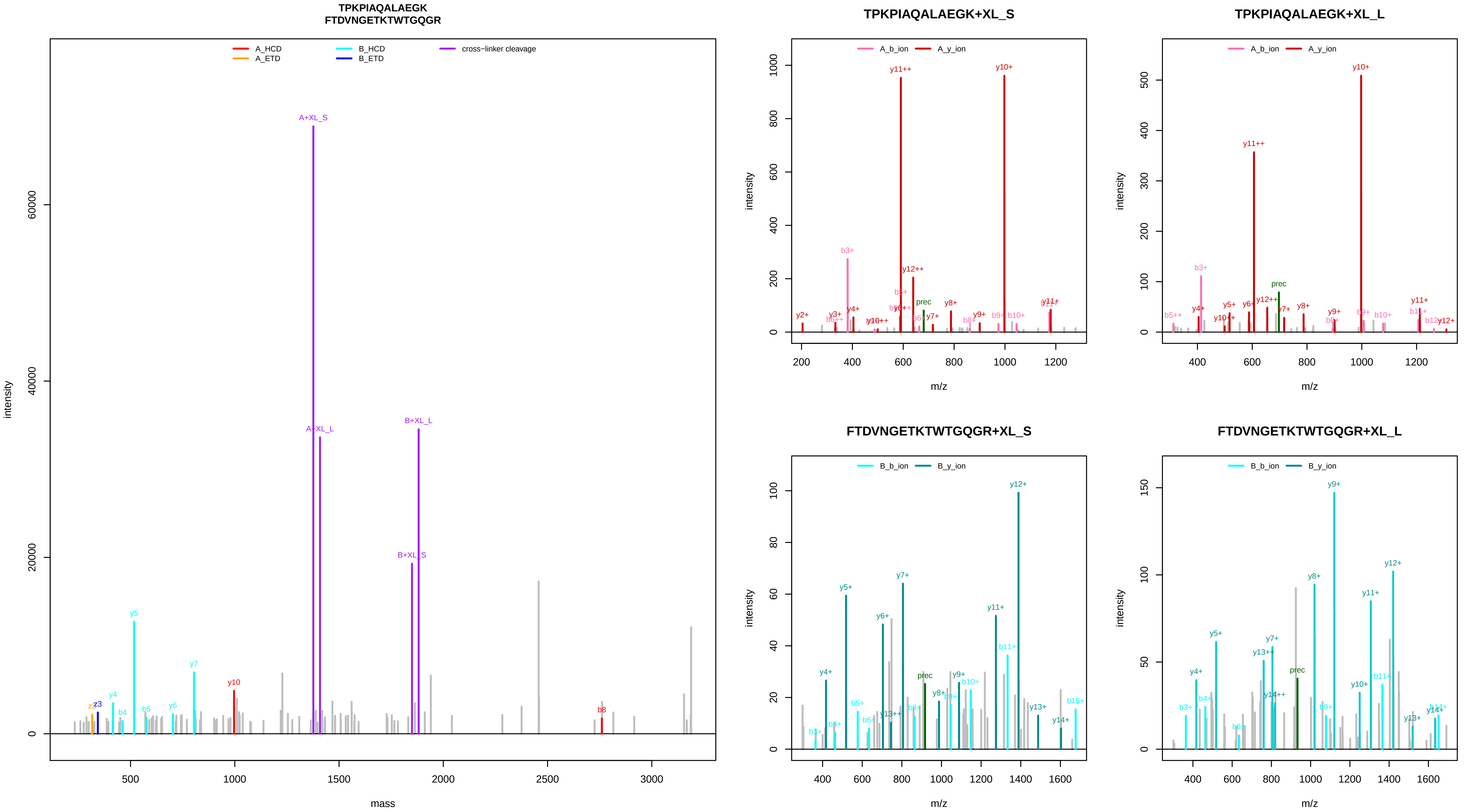

TPKPIAQALAEGK
FTDVNGETKTWTGQGR
A_HCD
A_ETD
B_HCD
B_ETD
cross−linker cleavage
A+XL_S
A+XL_L
B+XL_L
B+XL_S
y5
y7
y10
b8
y4
b4
b5
y6
z3
intensity
mass
TPKPIAQALAEGK+XL_S
TPKPIAQALAEGK+XL_L
A_b_ion
A_y_ion
FTDVNGETKTWTGQGR+XL_S
FTDVNGETKTWTGQGR+XL_L
B_b_ion
B_y_ion
prec
m/z

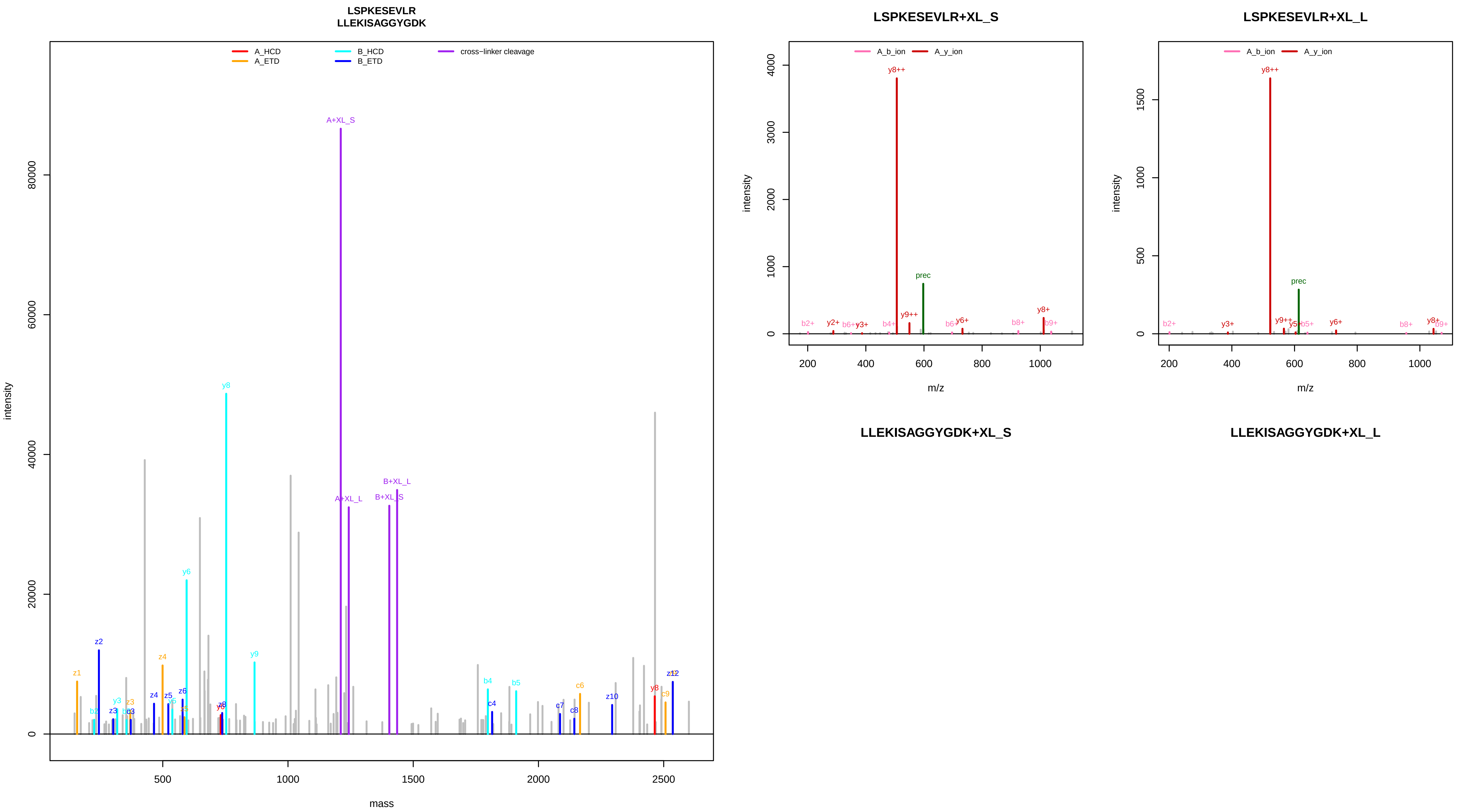

LSPKESEVLR
LLEKISAGGYGDK
A_HCD
A_ETD
B_HCD
B_ETD
cross−linker cleavage
A+XL_S
A+XL_L
B+XL_S
B+XL_L
intensity
mass
LSPKESEVLR+XL_S
LSPKESEVLR+XL_L
A_b_ion
A_y_ion
y8++
prec
m/z
LLEKISAGGYGDK+XL_S
LLEKISAGGYGDK+XL_L

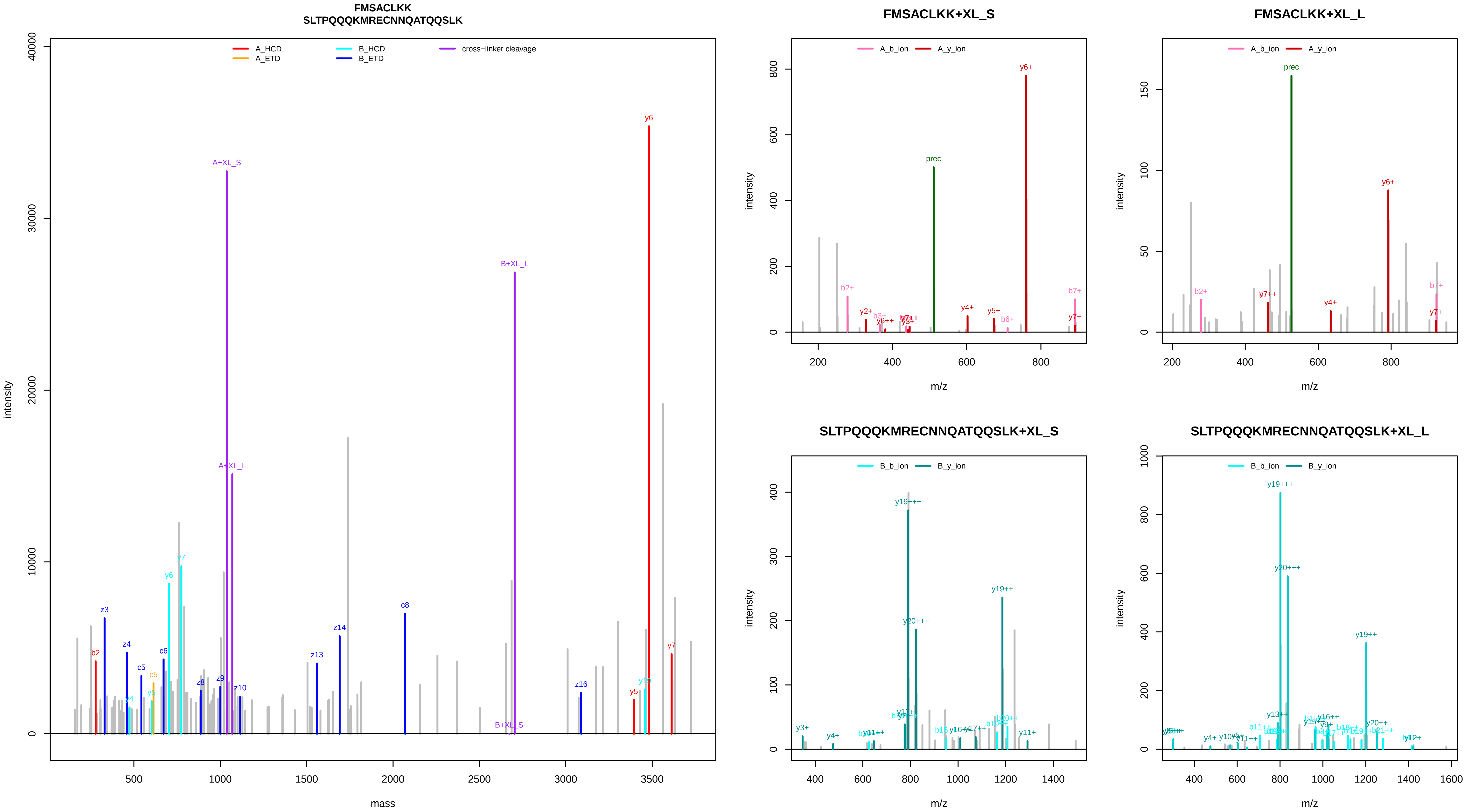
FMSACLKK
SLTPQQQKMRECNNQATQQSLK
A_HCD
A_ETD
B_HCD
B_ETD
cross−linker cleavage
y6
A+XL_S
B+XL_L
A+XL_L
B+XL_S
b2
z3
z4
c5
c6
y6
y7
z8
z9
z10
z13
z14
c8
z16
y5
y7
intensity
mass
FMSACLKK+XL_S
A_b_ion
A_y_ion
prec
y6+
b2+
y2+
y4+
y5+
b6+
b7+
y7+
m/z
FMSACLKK+XL_L
prec
y6+
b2+
y4+
b7+
y7+
SLTPQQQKMRECNNQATQQSLK+XL_S
B_b_ion
B_y_ion
y19+++
y20+++
y19++
y3+
y4+
y11+
SLTPQQQKMRECNNQATQQSLK+XL_L
y19+++
y20+++
y19++
y13++
y20++
y4+
intensity
m/z

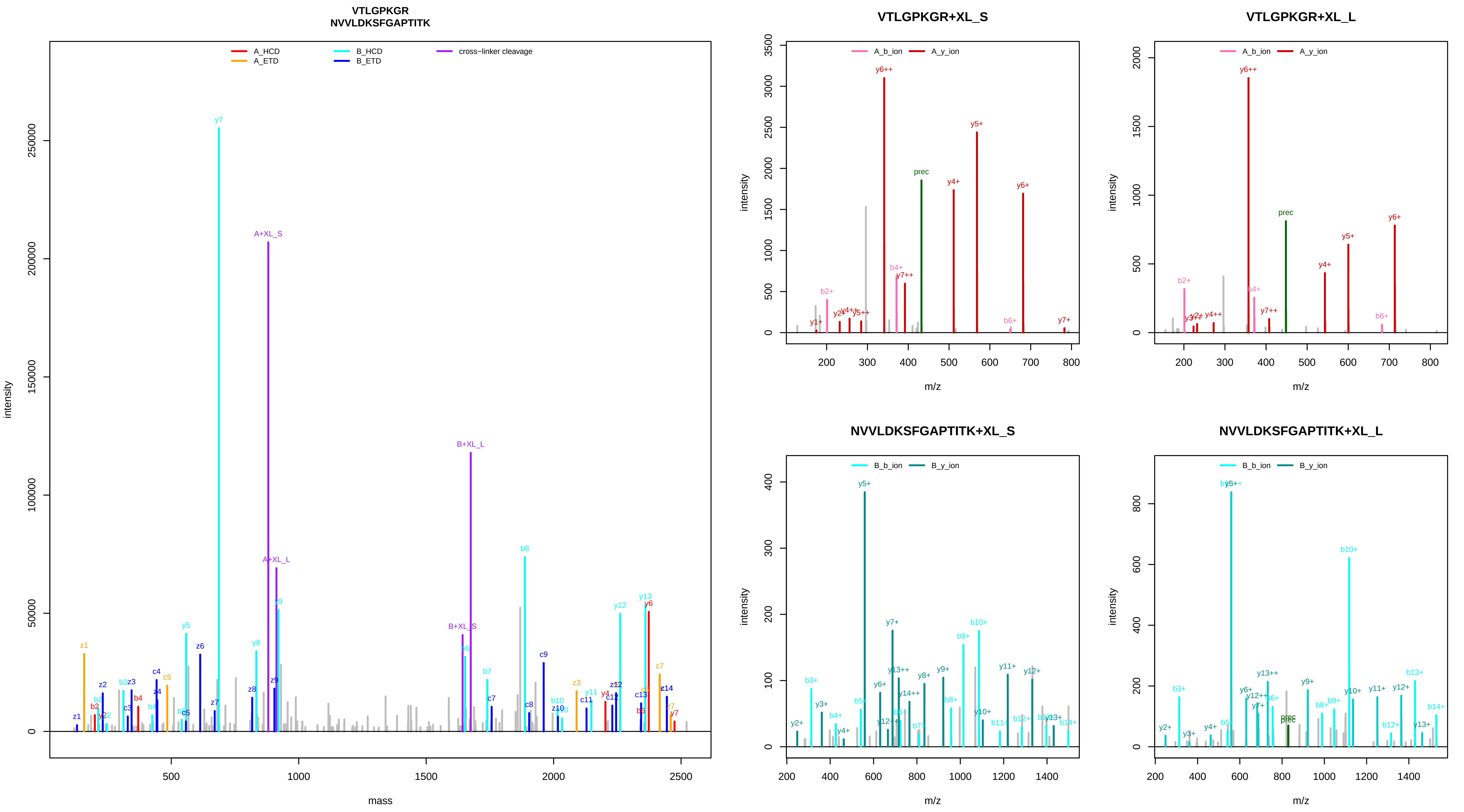
VTLGPKGR
NVVLDKSFGAPTITK
A_HCD
A_ETD
B_HCD
B_ETD
cross−linker cleavage
intensity
mass
VTLGPKGR+XL_S
VTLGPKGR+XL_L
NVVLDKSFGAPTITK+XL_S
NVVLDKSFGAPTITK+XL_L
A_b_ion
A_y_ion
B_b_ion
B_y_ion
m/z

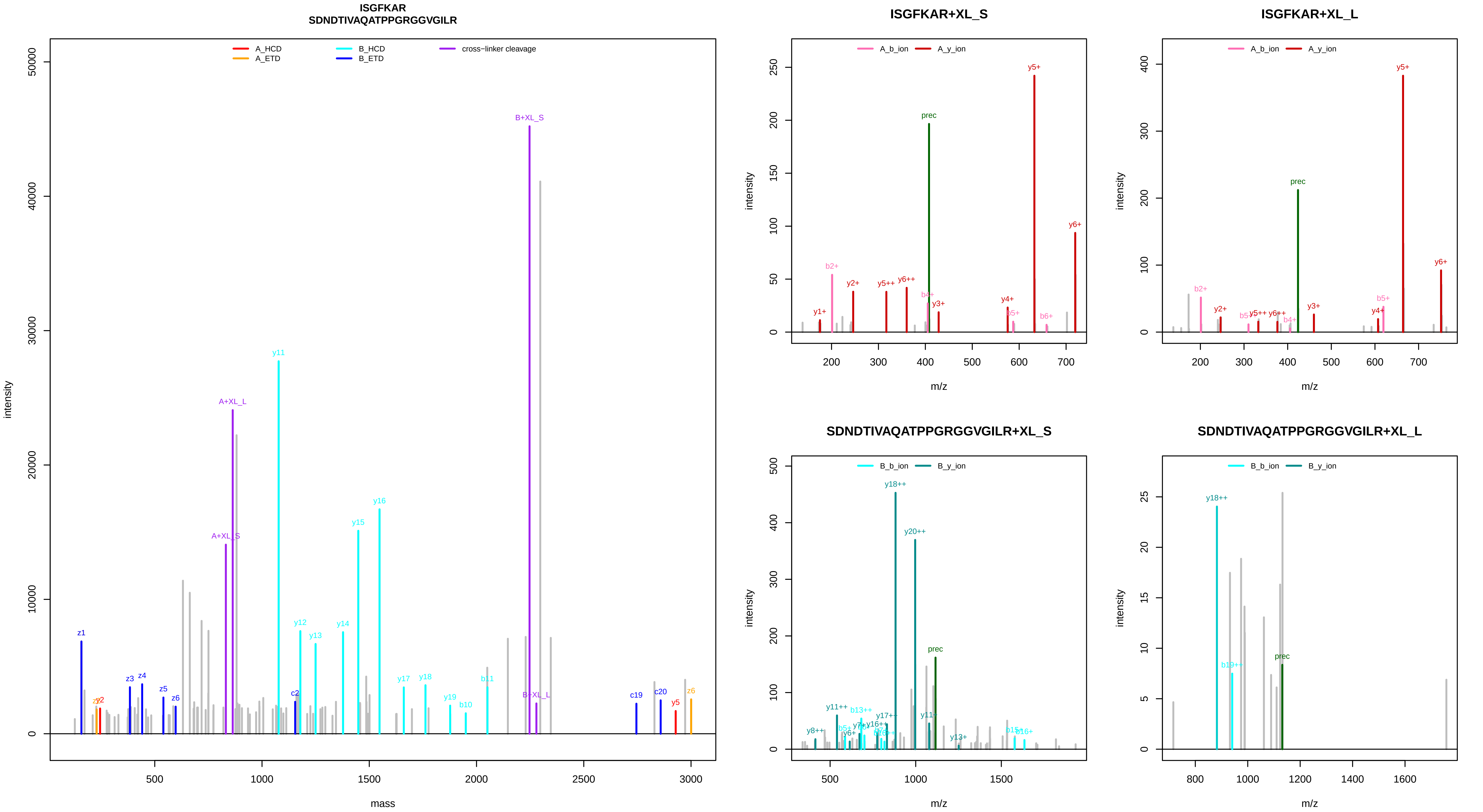
ISGFKAR
SDNDTIVAQATPPGRGGVGILR
A_HCD
A_ETD
B_HCD
B_ETD
cross−linker cleavage
B+XL_S
A+XL_L
A+XL_S
B+XL_L
y11
y12
y13
y14
y15
y16
y17
y18
y19
b10
b11
z1
z2
z3
z4
z5
z6
y2
y5
c2
c19
c20
intensity
mass
ISGFKAR+XL_S
ISGFKAR+XL_L
A_b_ion
A_y_ion
prec
b2+
b4+
b5+
b6+
y1+
y2+
y3+
y4+
y5+
y6+
y5++
y6++
m/z
SDNDTIVAQATPPGRGGVGILR+XL_S
SDNDTIVAQATPPGRGGVGILR+XL_L
B_b_ion
B_y_ion
y18++
y20++
y11++
y13++
y8++
y17++
y16++
y6+
y7+
b5+
b13++
b15+
b16+
y11+
y13+
b19++

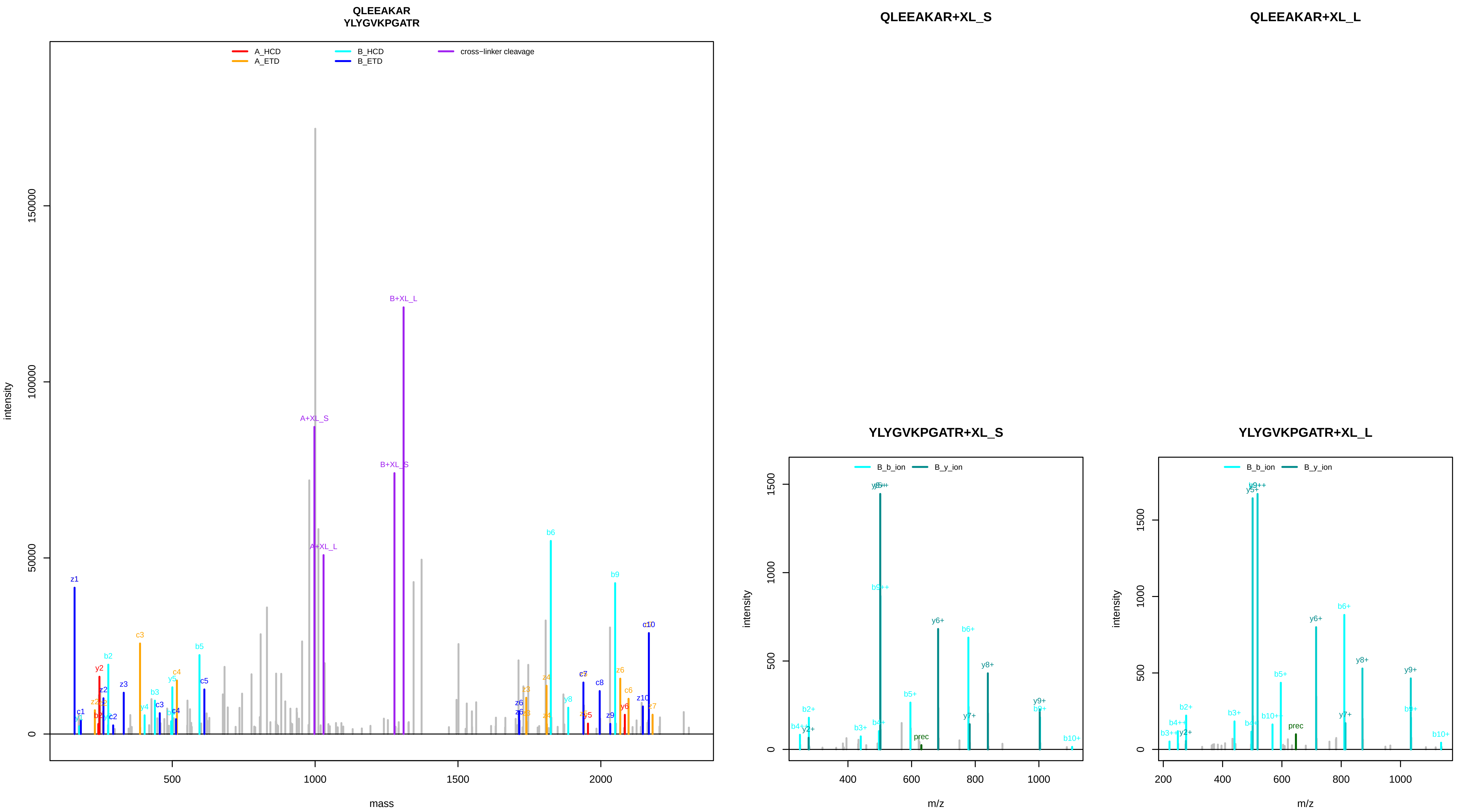
QLEEAKAR
YLYGVKPGATR
A_HCD
A_ETD
B_HCD
B_ETD
cross−linker cleavage
intensity
mass
A+XL_S
A+XL_L
B+XL_S
B+XL_L
QLEEAKAR+XL_S
QLEEAKAR+XL_L
YLYGVKPGATR+XL_S
YLYGVKPGATR+XL_L
B_b_ion
B_y_ion
m/z
prec

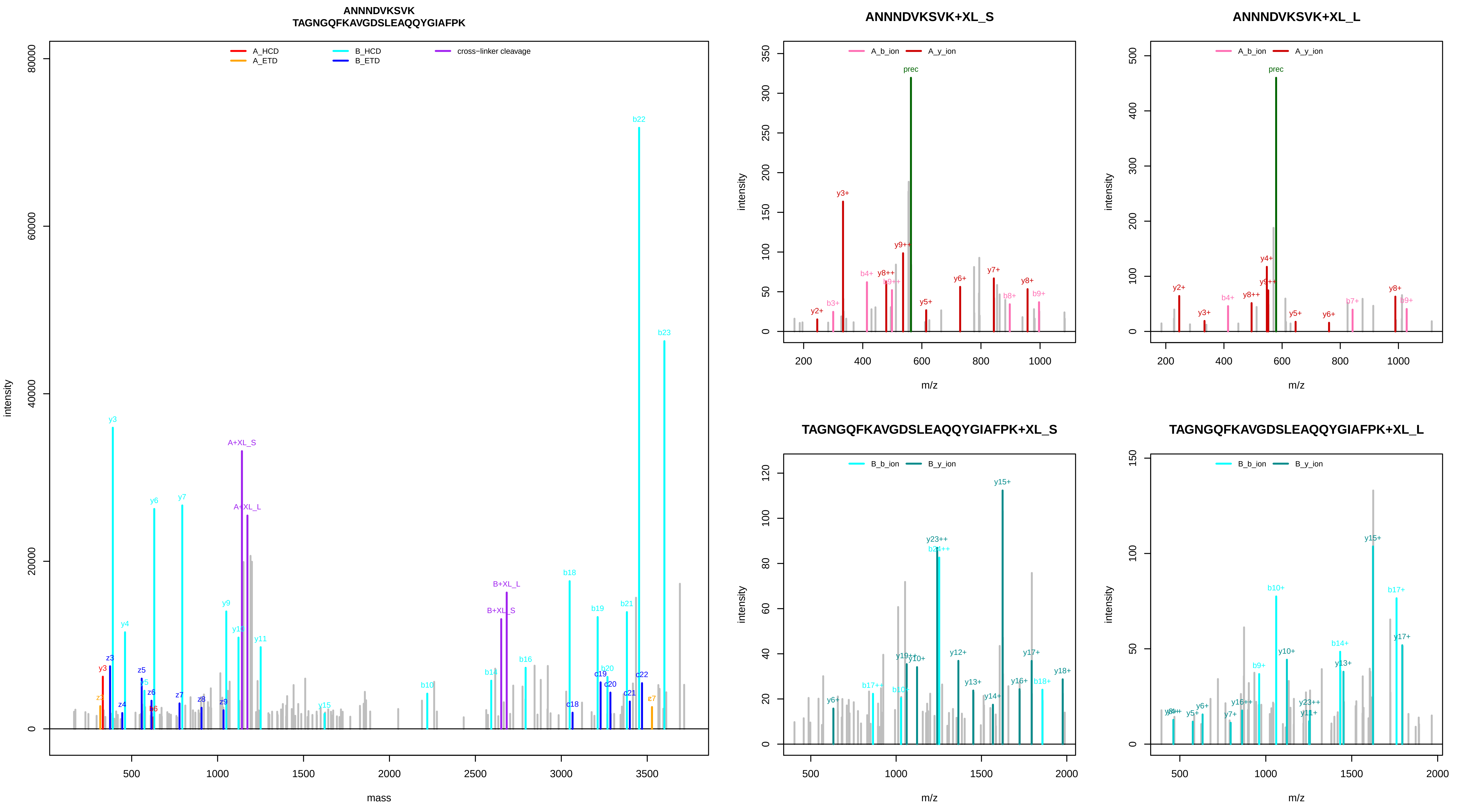
ANNNDVKSVK
TAGNGQFKAVGDSLEAQQYGIAFPK
A_HCD
A_ETD
B_HCD
B_ETD
cross−linker cleavage
A+XL_S
A+XL_L
B+XL_L
B+XL_S
intensity
mass
ANNNDVKSVK+XL_S
ANNNDVKSVK+XL_L
TAGNGQFKAVGDSLEAQQYGIAFPK+XL_S
TAGNGQFKAVGDSLEAQQYGIAFPK+XL_L
A_b_ion
A_y_ion
B_b_ion
B_y_ion
prec
m/z

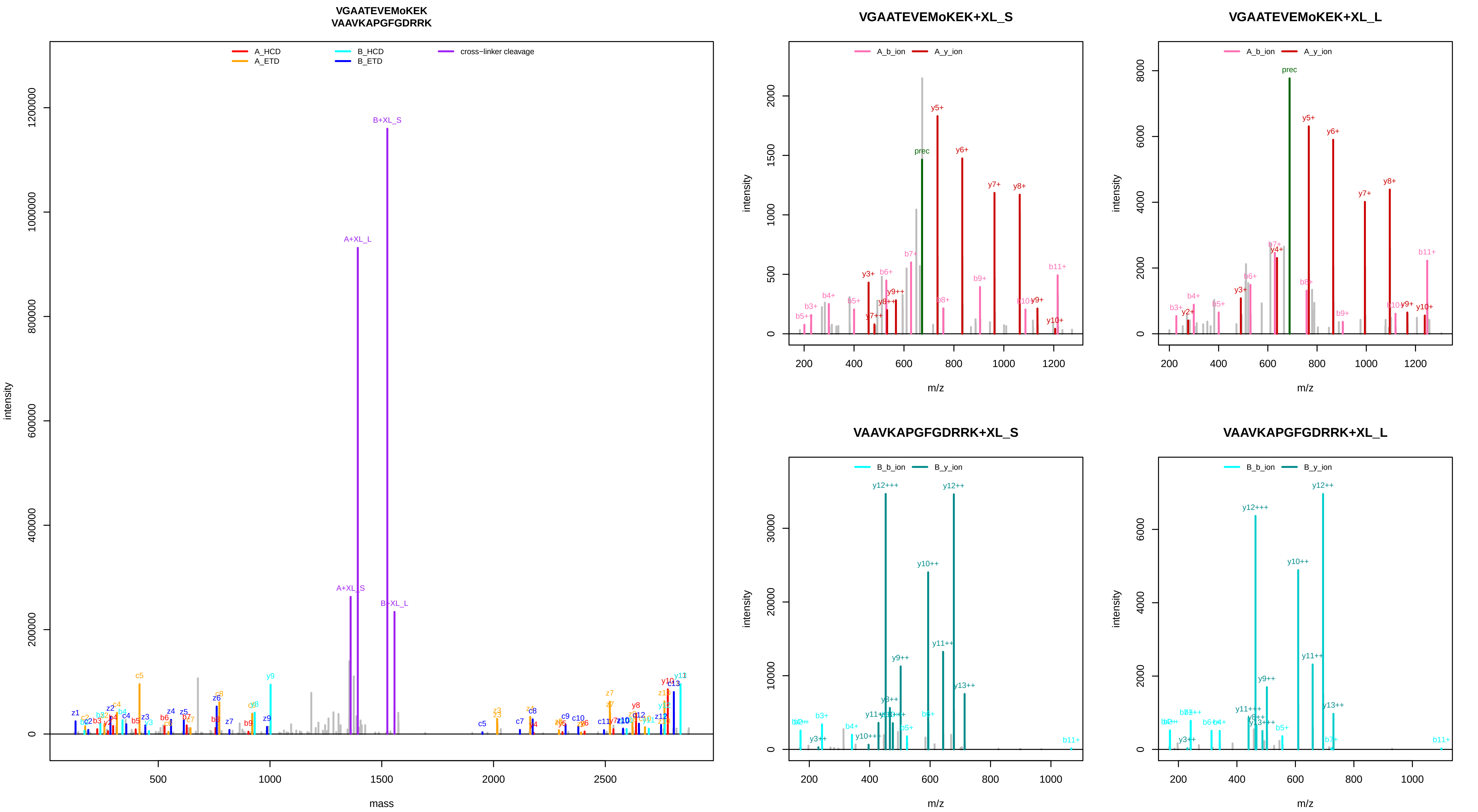
VGAATEVEMoKEK
VAAVKAPGFGDRRK
A_HCD
A_ETD
B_HCD
B_ETD
cross−linker cleavage
B+XL_S
A+XL_L
A+XL_S
B+XL_L
intensity
mass
VGAATEVEMoKEK+XL_S
VGAATEVEMoKEK+XL_L
VAAVKAPGFGDRRK+XL_S
VAAVKAPGFGDRRK+XL_L
A_b_ion
A_y_ion
B_b_ion
B_y_ion
prec
m/z

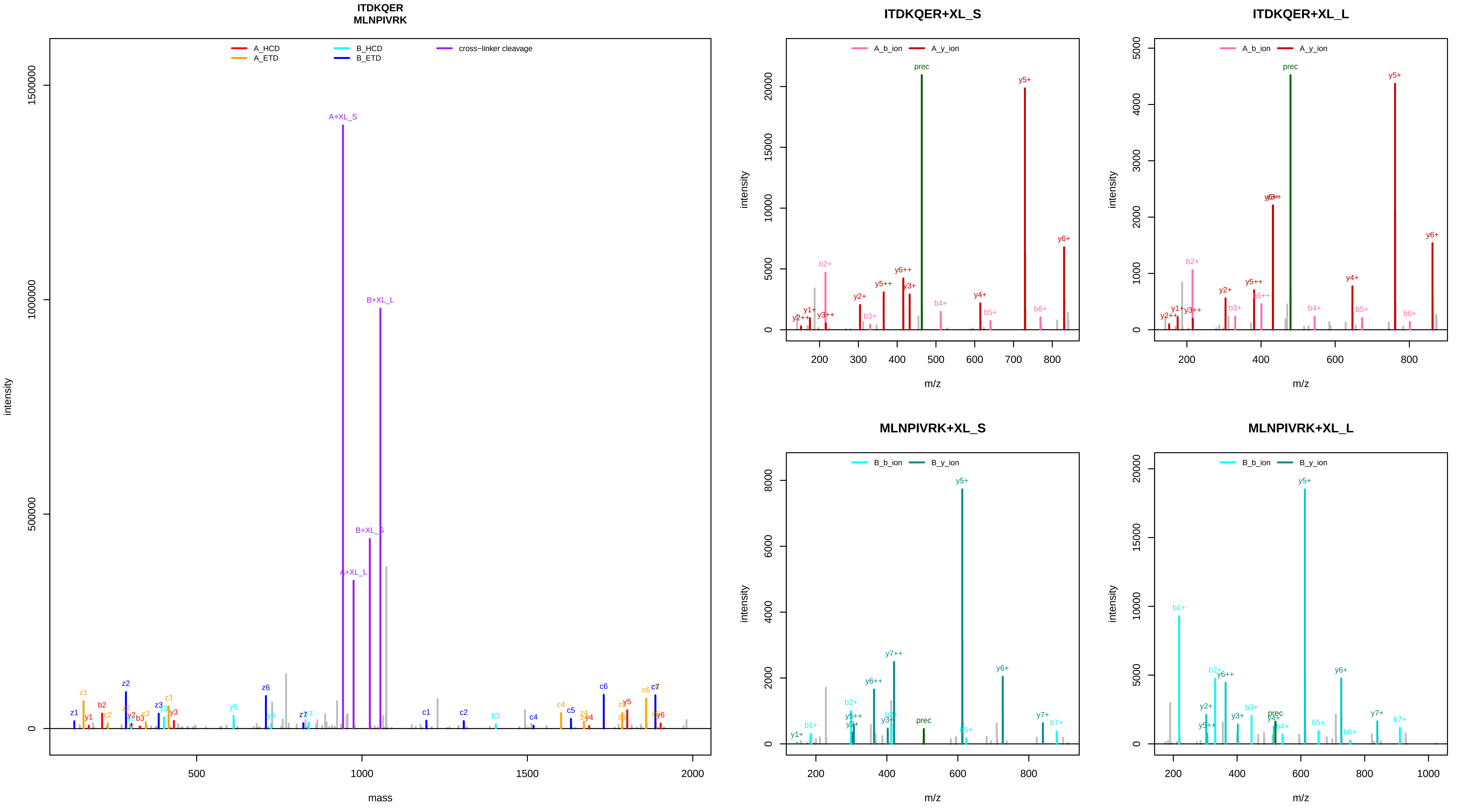
ITDKQER
MLNPIVRK
A_HCD
A_ETD
B_HCD
B_ETD
cross−linker cleavage
A+XL_S
B+XL_L
B+XL_S
A+XL_L
intensity
mass
ITDKQER+XL_S
ITDKQER+XL_L
A_b_ion
A_y_ion
MLNPIVRK+XL_S
MLNPIVRK+XL_L
B_b_ion
B_y_ion
prec
intensity
m/z

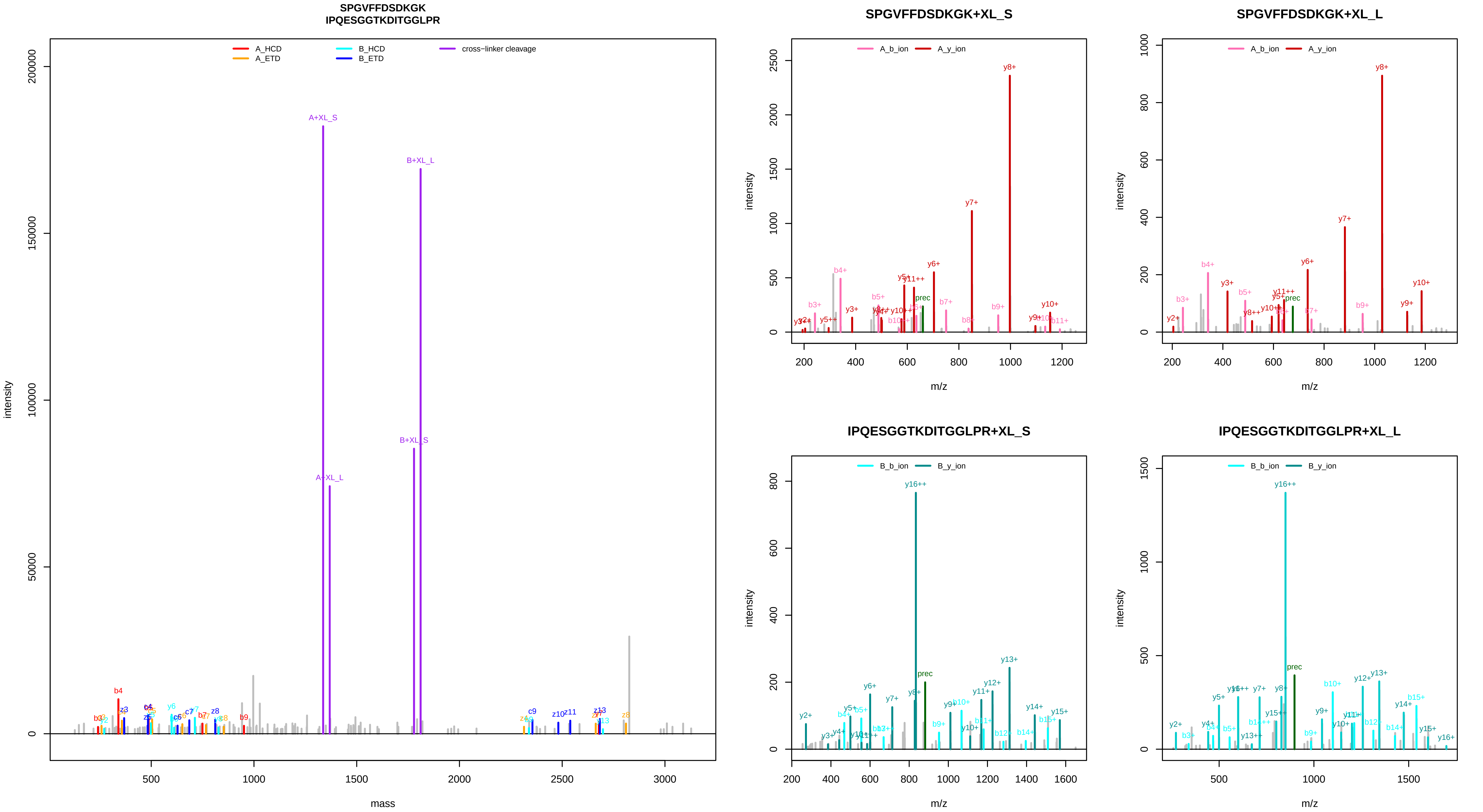

SPGVFFDSDKGK
IPQESGGTKDITGGLPR
A_HCD
A_ETD
B_HCD
B_ETD
cross−linker cleavage
A+XL_S
B+XL_L
B+XL_S
A+XL_L
intensity
mass
SPGVFFDSDKGK+XL_S
SPGVFFDSDKGK+XL_L
A_b_ion
A_y_ion
m/z
IPQESGGTKDITGGLPR+XL_S
IPQESGGTKDITGGLPR+XL_L
B_b_ion
B_y_ion

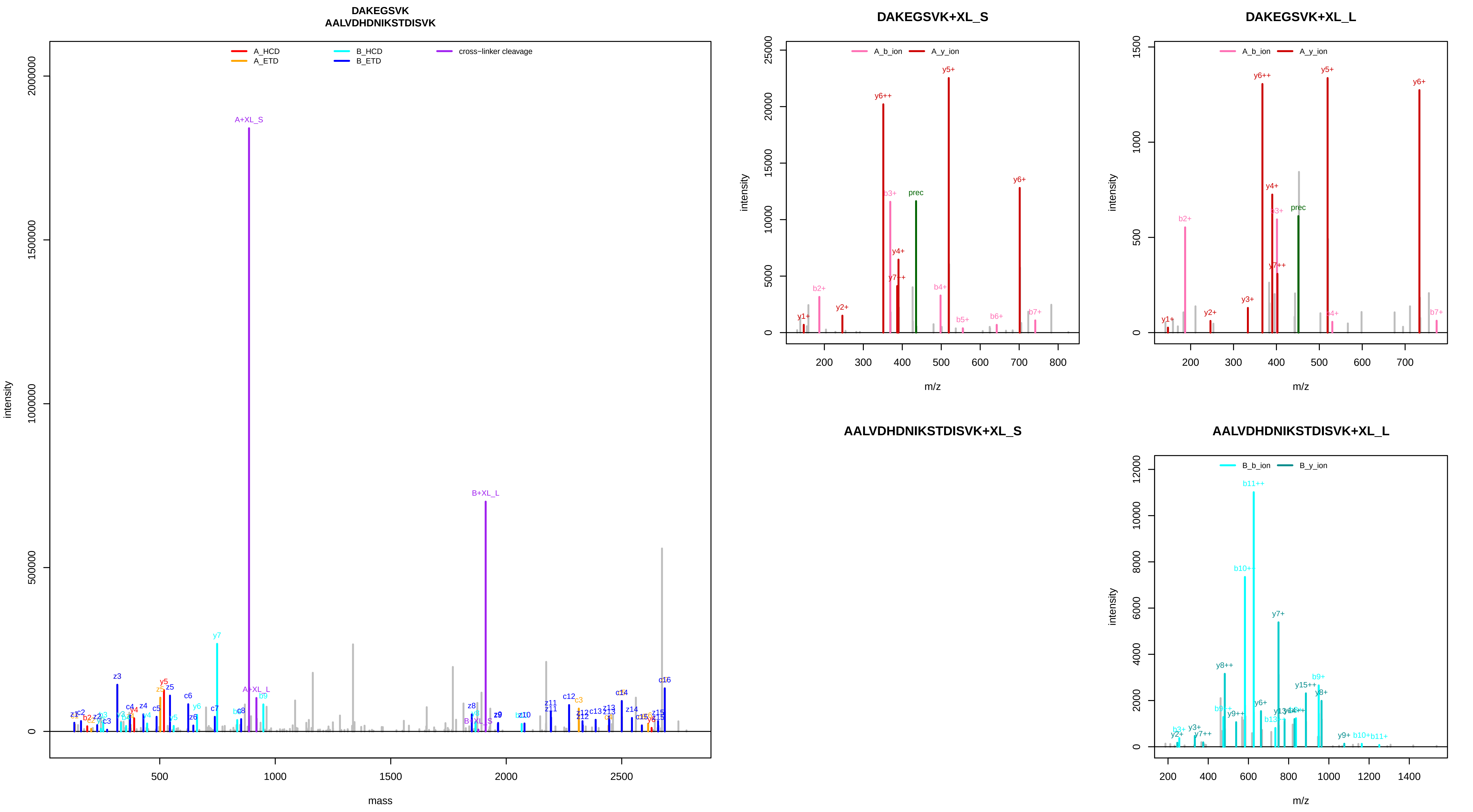
DAKEGSVK
AALVDHDNIKSTDISVK
A_HCD
A_ETD
B_HCD
B_ETD
cross−linker cleavage
A+XL_S
B+XL_L
intensity
mass
DAKEGSVK+XL_S
DAKEGSVK+XL_L
A_b_ion
A_y_ion
prec
m/z
AALVDHDNIKSTDISVK+XL_S
AALVDHDNIKSTDISVK+XL_L
B_b_ion
B_y_ion

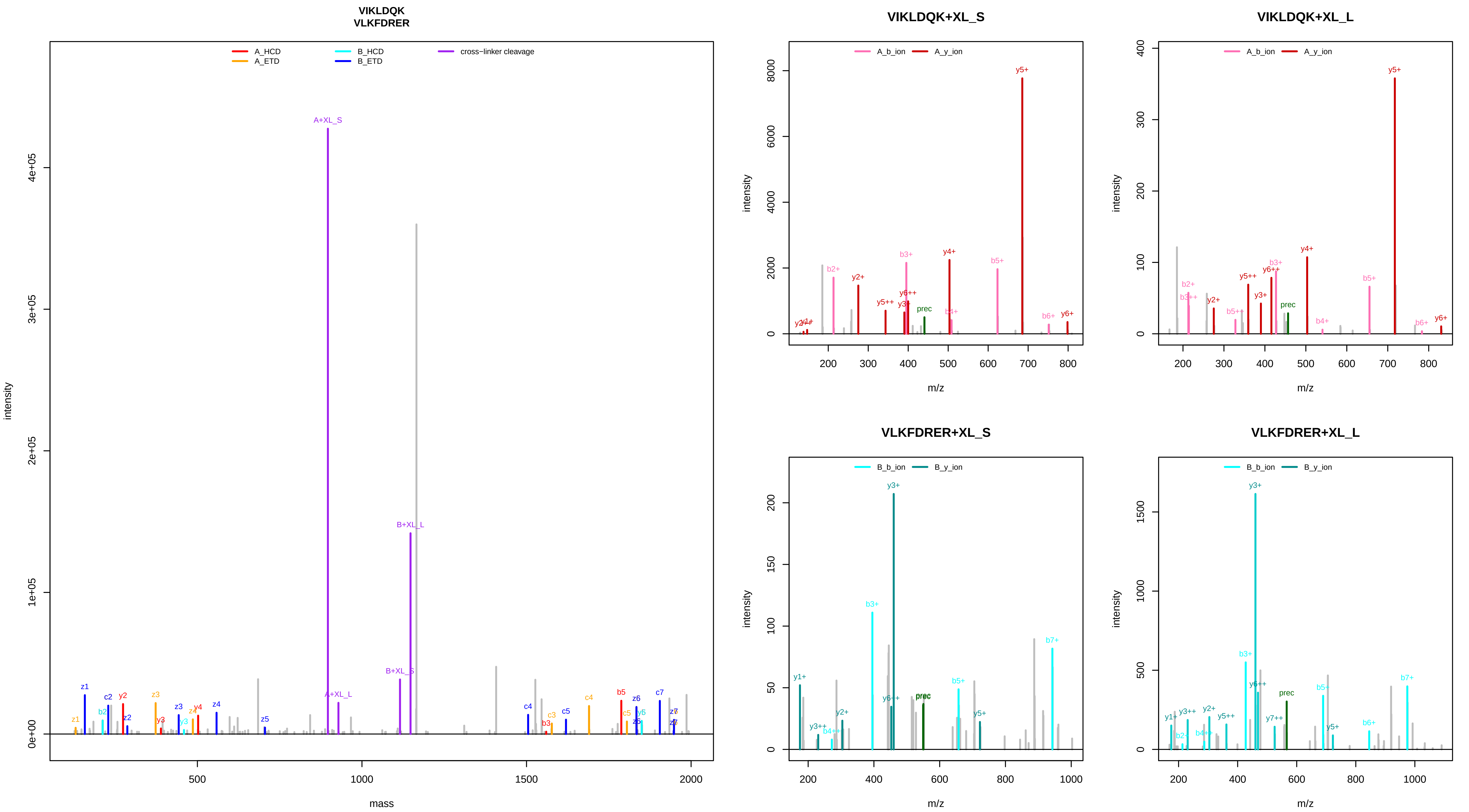
VIKLDQK
VLKFDRER
A_HCD
A_ETD
B_HCD
B_ETD
cross−linker cleavage
A+XL_S
B+XL_L
B+XL_S
A+XL_L
intensity
mass
VIKLDQK+XL_S
VIKLDQK+XL_L
VLKFDRER+XL_S
VLKFDRER+XL_L
A_b_ion
A_y_ion
B_b_ion
B_y_ion
prec
m/z

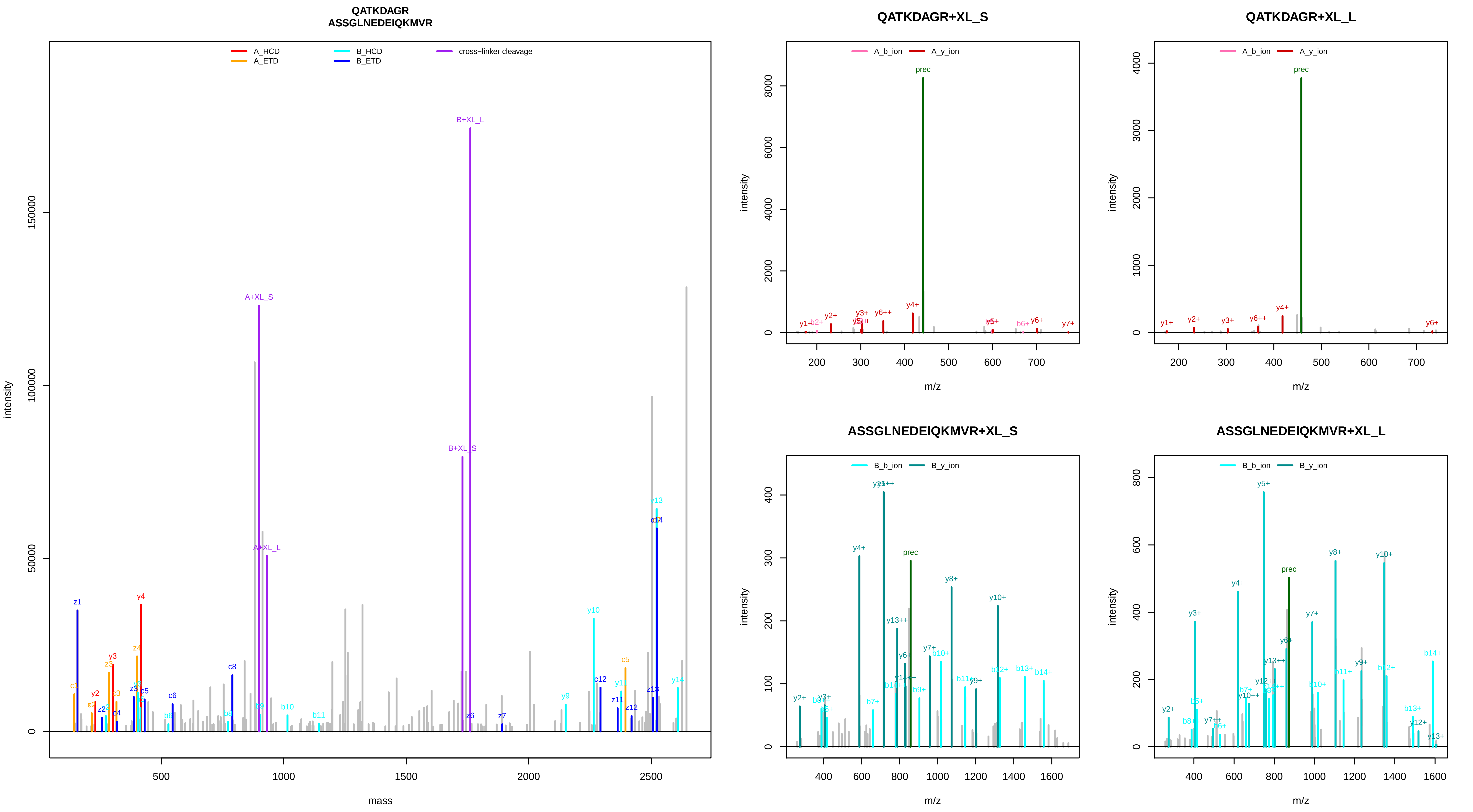

QATKDAGR
ASSGLNEDEIQKMVR
A_HCD
A_ETD
B_HCD
B_ETD
cross−linker cleavage
intensity
mass
A+XL_S
A+XL_L
B+XL_L
B+XL_S
QATKDAGR+XL_S
QATKDAGR+XL_L
ASSGLNEDEIQKMVR+XL_S
ASSGLNEDEIQKMVR+XL_L
A_b_ion
A_y_ion
B_b_ion
B_y_ion
prec
m/z

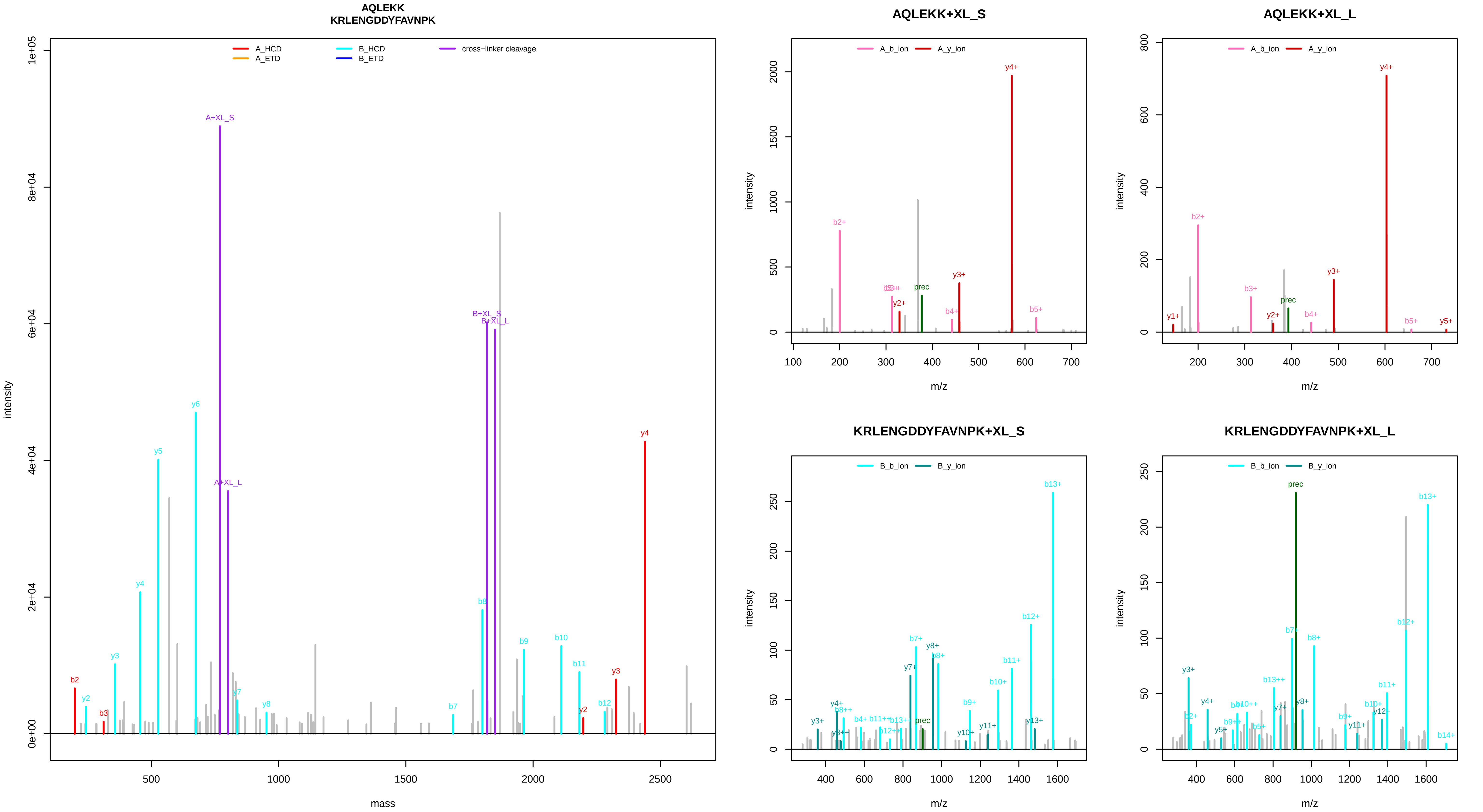

AQLEKK
KRLENGDDYFAVNPK
A_HCD
A_ETD
B_HCD
B_ETD
cross−linker cleavage
A+XL_S
A+XL_L
B+XL_S
B+XL_L
intensity
mass
AQLEKK+XL_S
AQLEKK+XL_L
KRLENGDDYFAVNPK+XL_S
KRLENGDDYFAVNPK+XL_L
A_b_ion
A_y_ion
B_b_ion
B_y_ion
prec
m/z

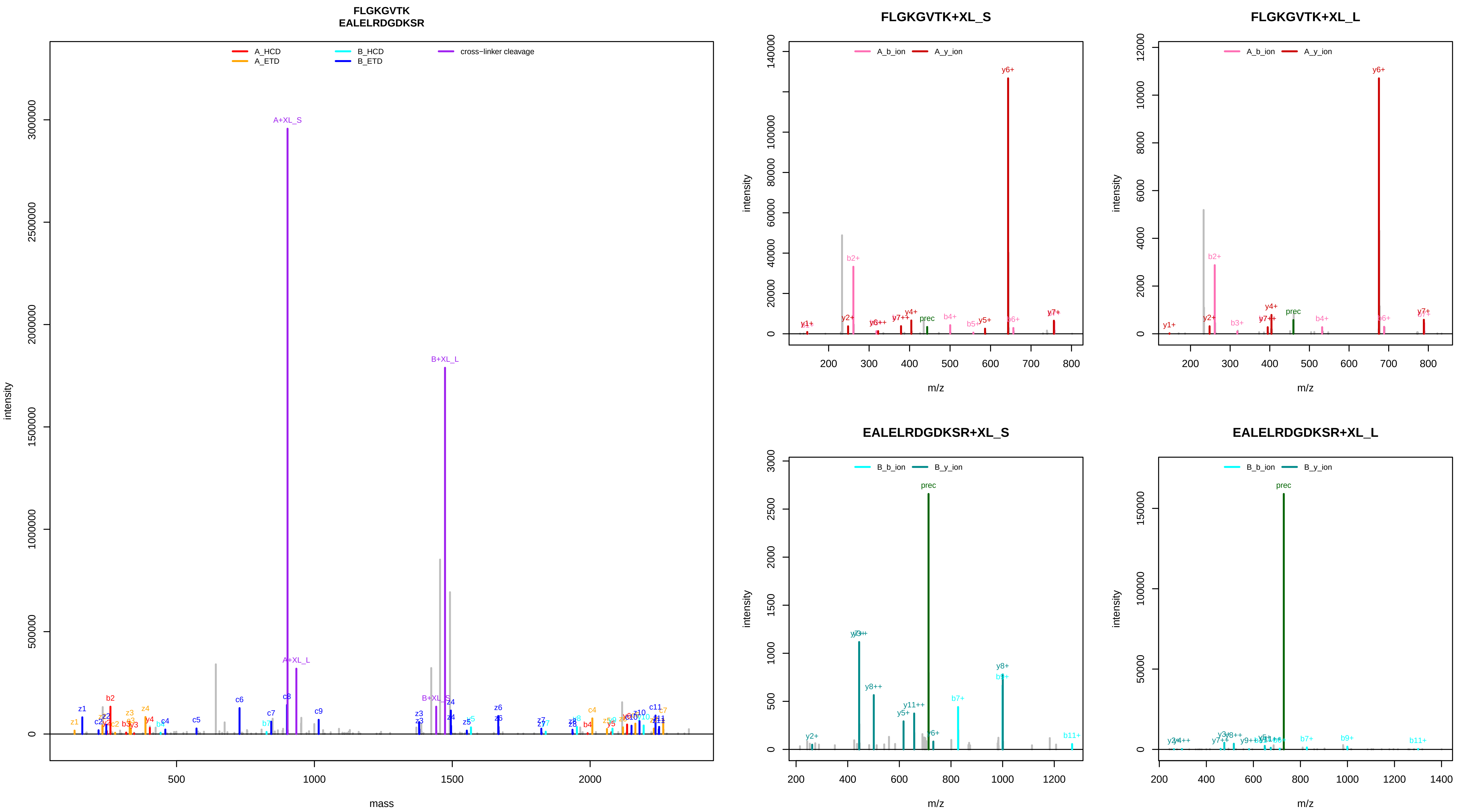
FLGKGVTK
EALELRDGDKSR
A_HCD
A_ETD
B_HCD
B_ETD
cross−linker cleavage
A+XL_S
B+XL_L
A+XL_L
B+XL_S
intensity
mass
FLGKGVTK+XL_S
FLGKGVTK+XL_L
A_b_ion
A_y_ion
y6+
b2+
y4+
prec
y7+
EALELRDGDKSR+XL_S
EALELRDGDKSR+XL_L
B_b_ion
B_y_ion
prec
y8+
b9+
b7+
b11+
m/z

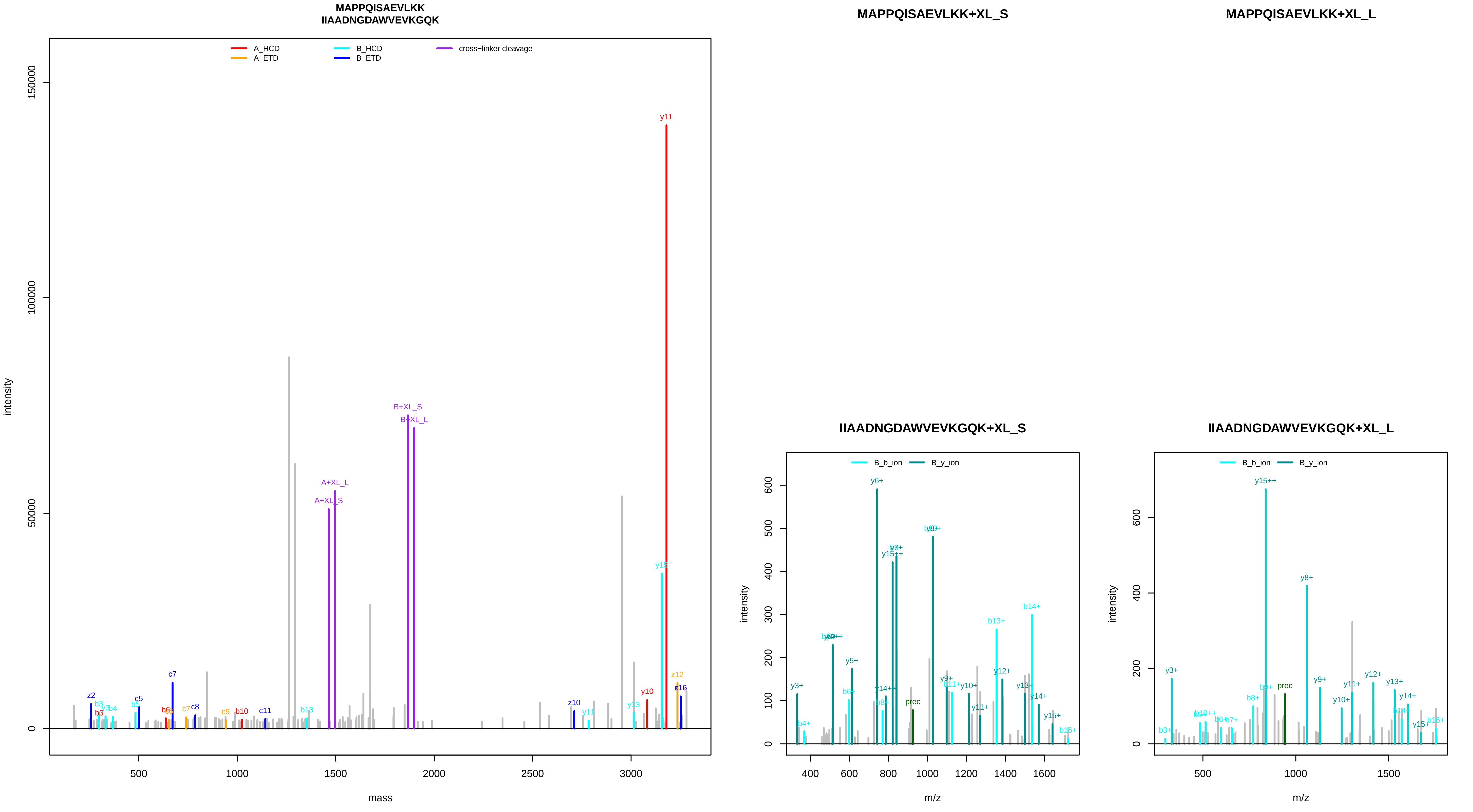
MAPPQISAEVLKK
IIAADNGDAWVEVKGQK
A_HCD
A_ETD
B_HCD
B_ETD
cross−linker cleavage
intensity
mass
y11
B+XL_S
B+XL_L
A+XL_L
A+XL_S
MAPPQISAEVLKK+XL_S
MAPPQISAEVLKK+XL_L
IIAADNGDAWVEVKGQK+XL_S
IIAADNGDAWVEVKGQK+XL_L
B_b_ion
B_y_ion
m/z
prec

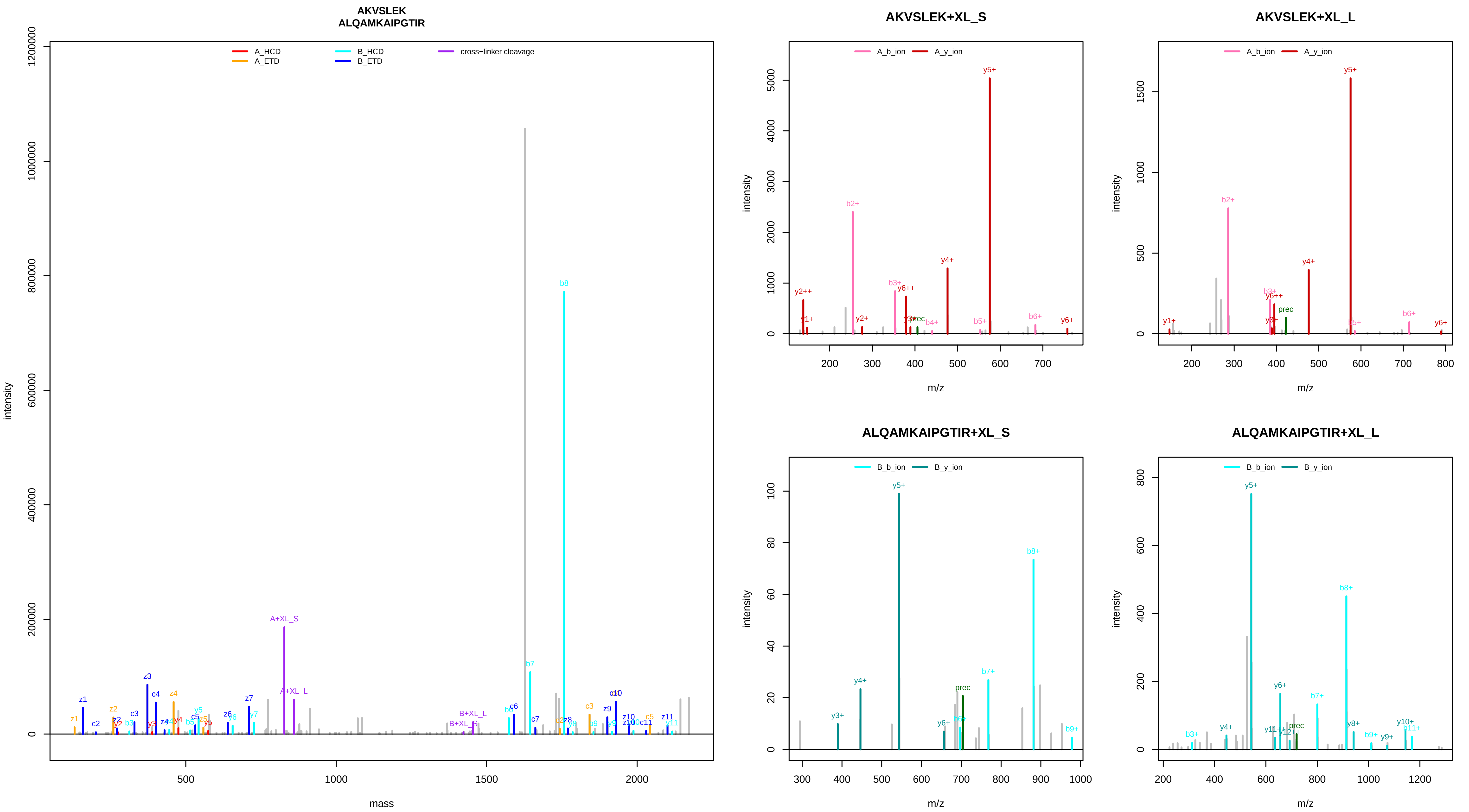

AKVSLEK
ALQAMKAIPGTIR
A_HCD
A_ETD
B_HCD
B_ETD
cross−linker cleavage
A+XL_S
A+XL_L
B+XL_L
B+XL_S
intensity
mass
AKVSLEK+XL_S
AKVSLEK+XL_L
A_b_ion
A_y_ion
ALQAMKAIPGTIR+XL_S
ALQAMKAIPGTIR+XL_L
B_b_ion
B_y_ion
m/z

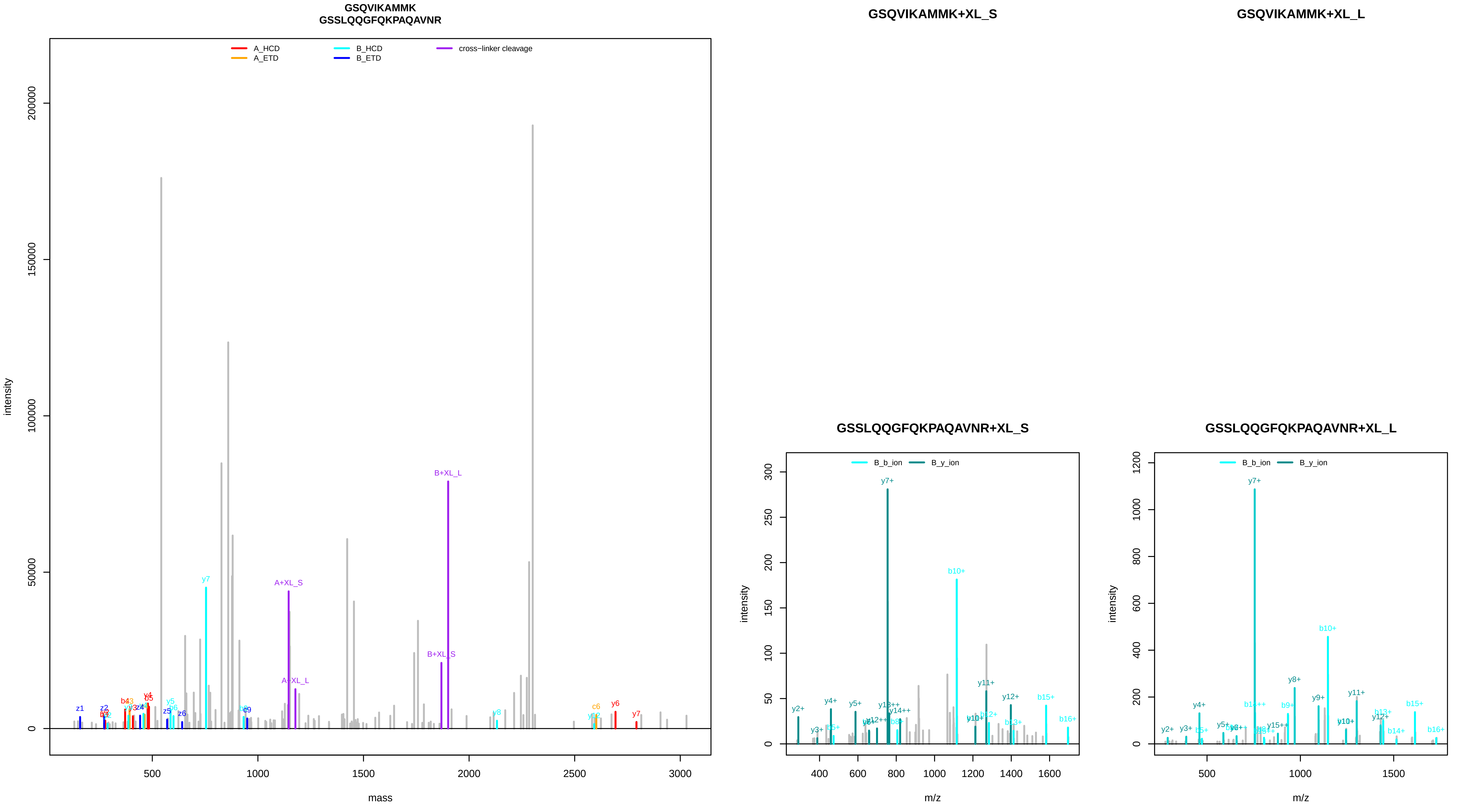

GSQVIKAMMK
GSSLQQGFQKPAQAVNR
A_HCD
A_ETD
B_HCD
B_ETD
cross−linker cleavage
intensity
mass
A+XL_S
A+XL_L
B+XL_L
B+XL_S
GSQVIKAMMK+XL_S
GSQVIKAMMK+XL_L
GSSLQQGFQKPAQAVNR+XL_S
GSSLQQGFQKPAQAVNR+XL_L
B_b_ion
B_y_ion
intensity
m/z

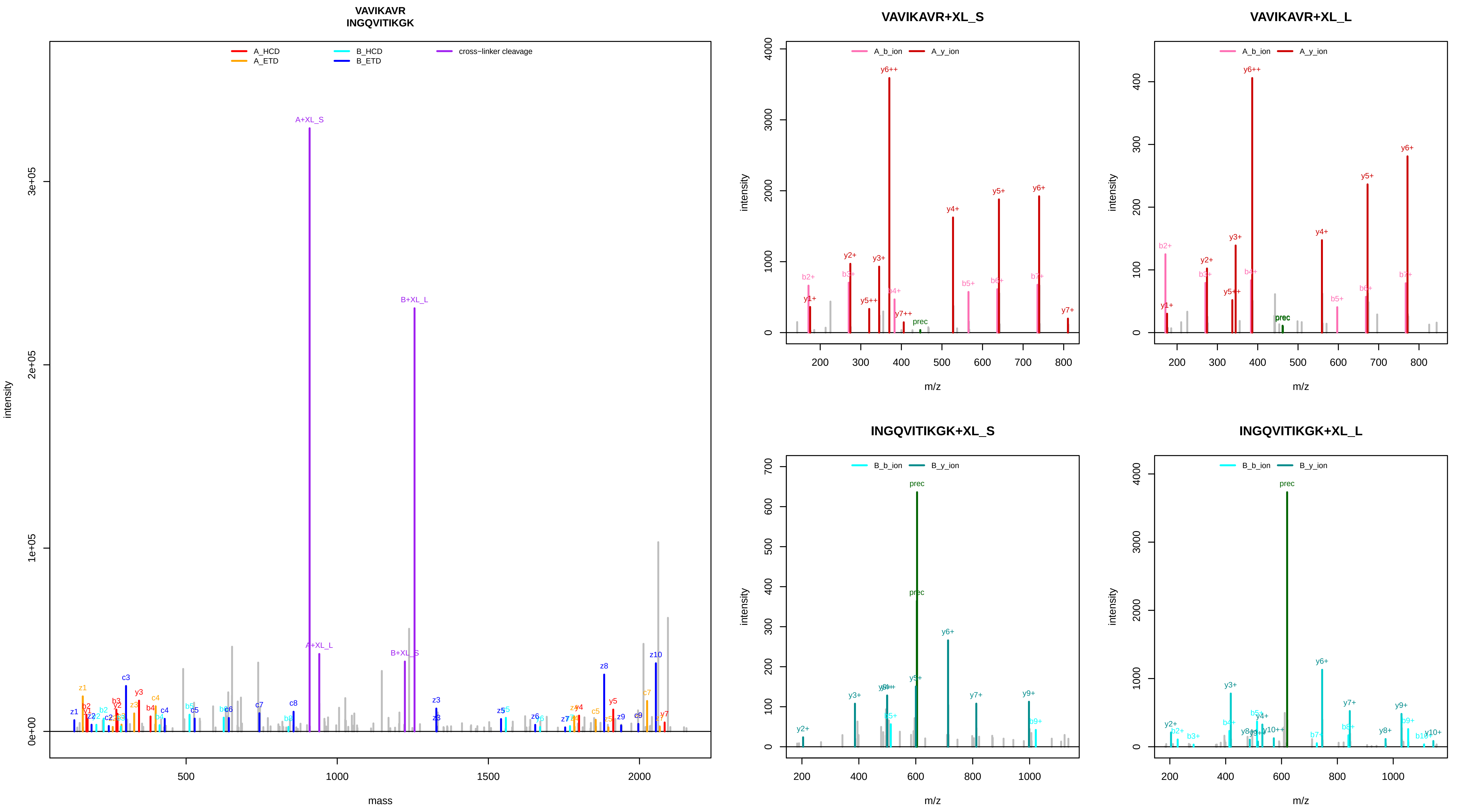

VAVIKAVR
INGQVITIKGK
A_HCD
A_ETD
B_HCD
B_ETD
cross−linker cleavage
A+XL_S
B+XL_L
A+XL_L
B+XL_S
intensity
mass
VAVIKAVR+XL_S
VAVIKAVR+XL_L
A_b_ion
A_y_ion
INGQVITIKGK+XL_S
INGQVITIKGK+XL_L
B_b_ion
B_y_ion
prec
m/z

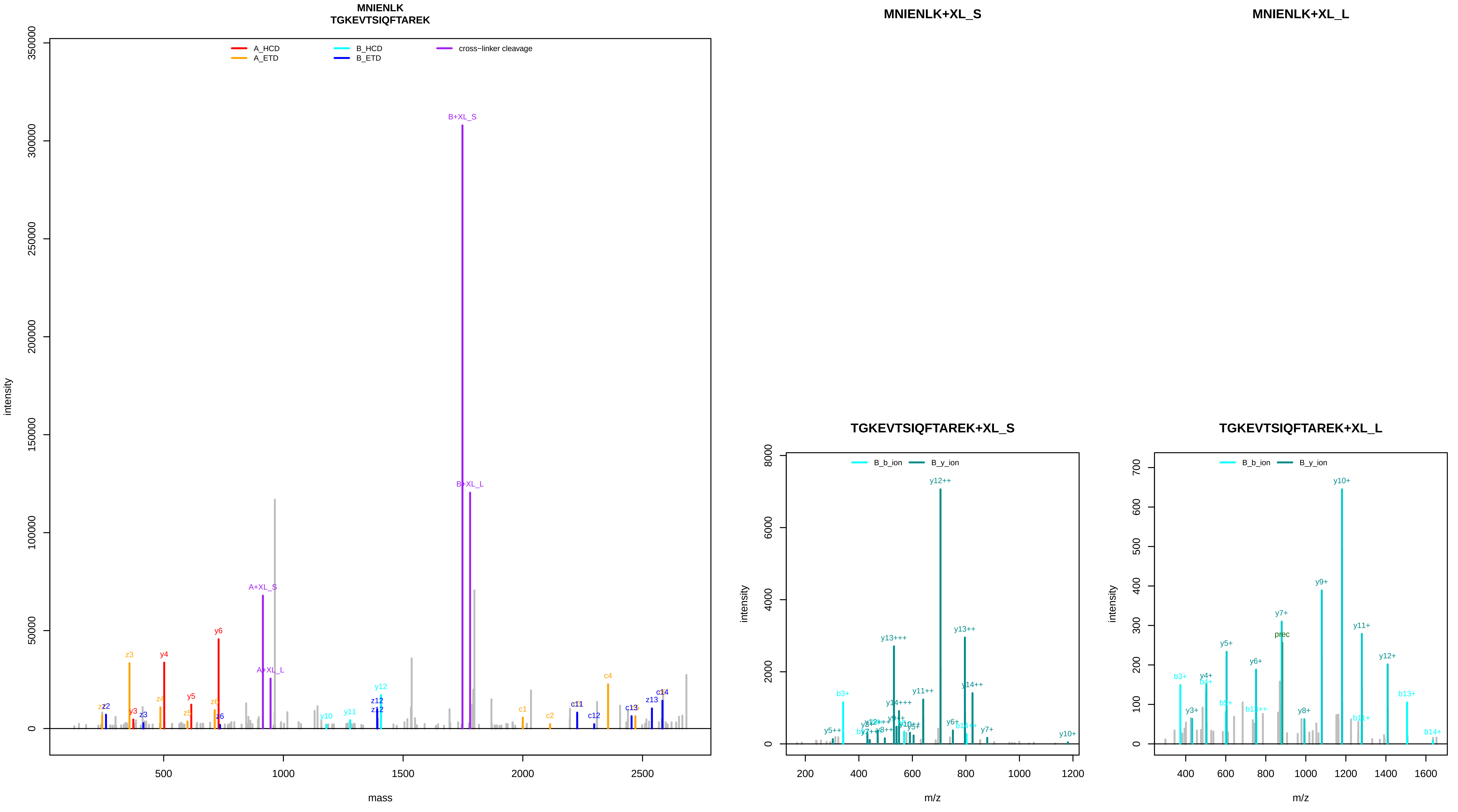
MNIENLK
TGKEVTSIQFTAREK
A_HCD
A_ETD
B_HCD
B_ETD
cross−linker cleavage
B+XL_S
B+XL_L
A+XL_S
A+XL_L
intensity
mass
MNIENLK+XL_S
MNIENLK+XL_L
TGKEVTSIQFTAREK+XL_S
TGKEVTSIQFTAREK+XL_L
B_b_ion
B_y_ion
y12++
y13++
y13+++
y11++
y14++
y14+++
b3+
y5++
y6+
y7+
y10+
y9+
y7+
prec
y11+
y12+
y5+
y6+
b3+
y4+
y3+
y8+
b13+
b14+
m/z

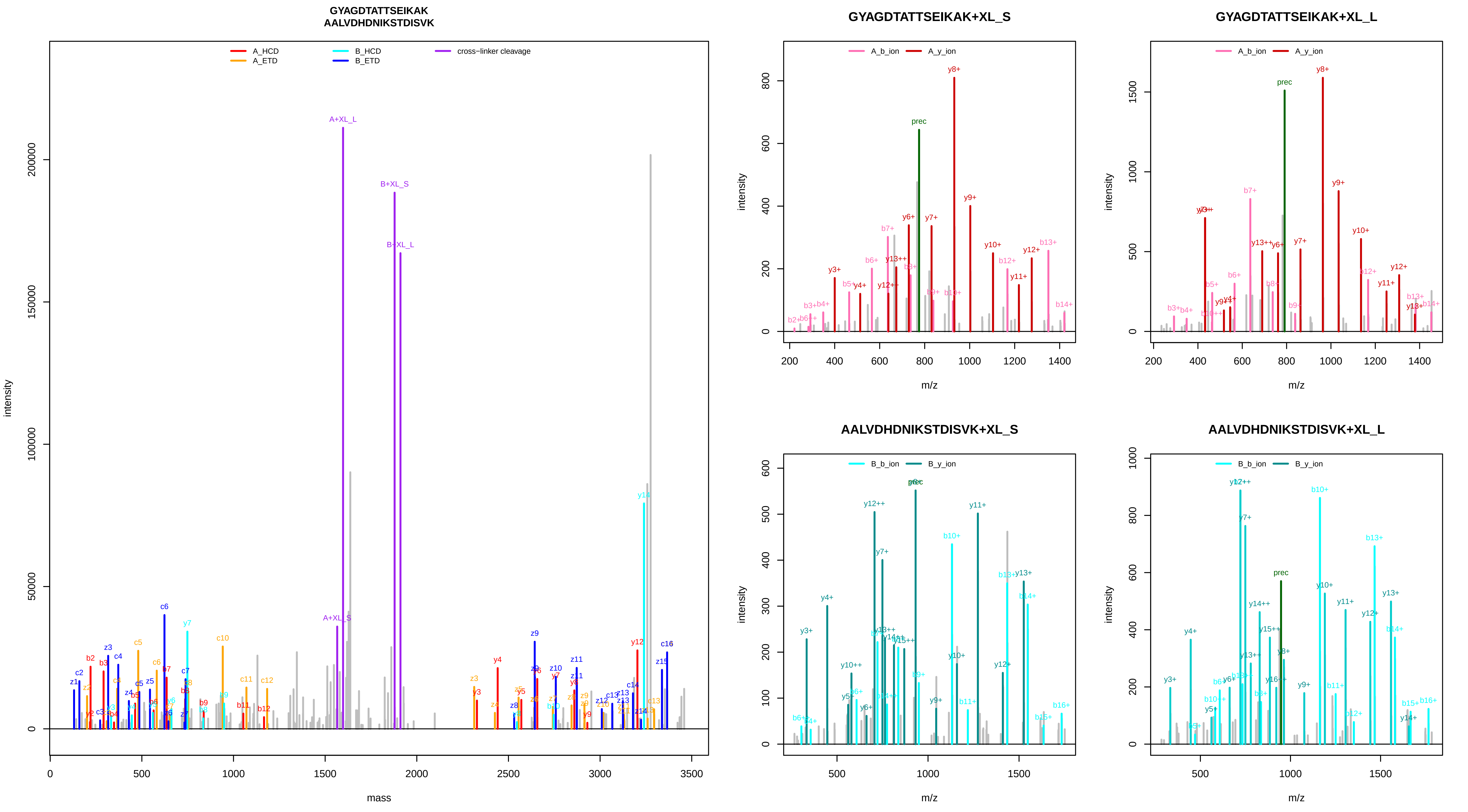
GYAGDTATTSEIKAK
AALVDHDNIKSTDISVK
A_HCD
A_ETD
B_HCD
B_ETD
cross−linker cleavage
A+XL_L
B+XL_S
B+XL_L
A+XL_S
intensity
mass
GYAGDTATTSEIKAK+XL_S
GYAGDTATTSEIKAK+XL_L
A_b_ion
A_y_ion
prec
AALVDHDNIKSTDISVK+XL_S
AALVDHDNIKSTDISVK+XL_L
B_b_ion
B_y_ion
m/z

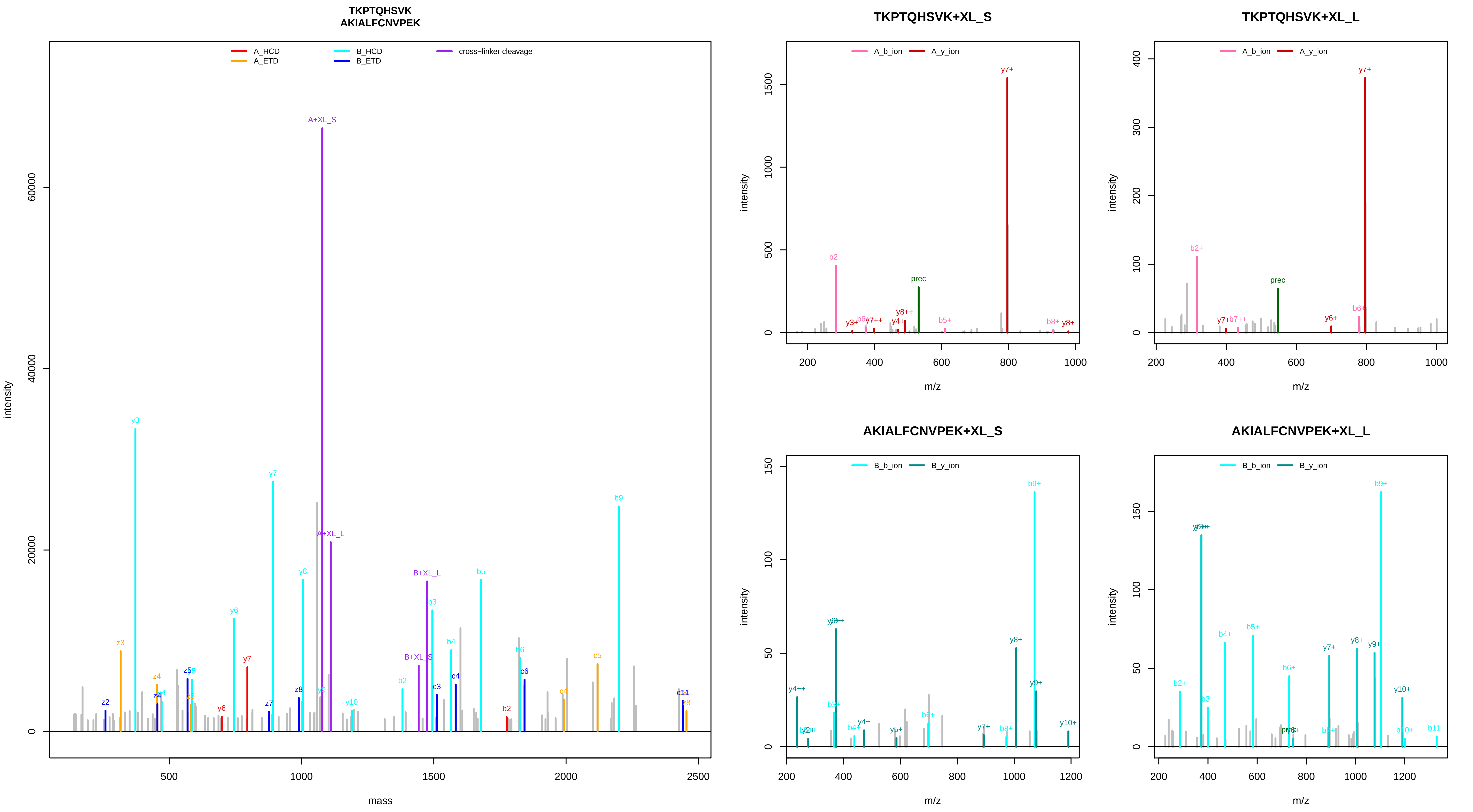
TKPTQHSVK
AKIALFCNVPEK
A_HCD
A_ETD
B_HCD
B_ETD
cross−linker cleavage
A+XL_S
A+XL_L
B+XL_L
B+XL_S
intensity
mass
TKPTQHSVK+XL_S
TKPTQHSVK+XL_L
AKIALFCNVPEK+XL_S
AKIALFCNVPEK+XL_L
A_b_ion
A_y_ion
B_b_ion
B_y_ion
prec
m/z

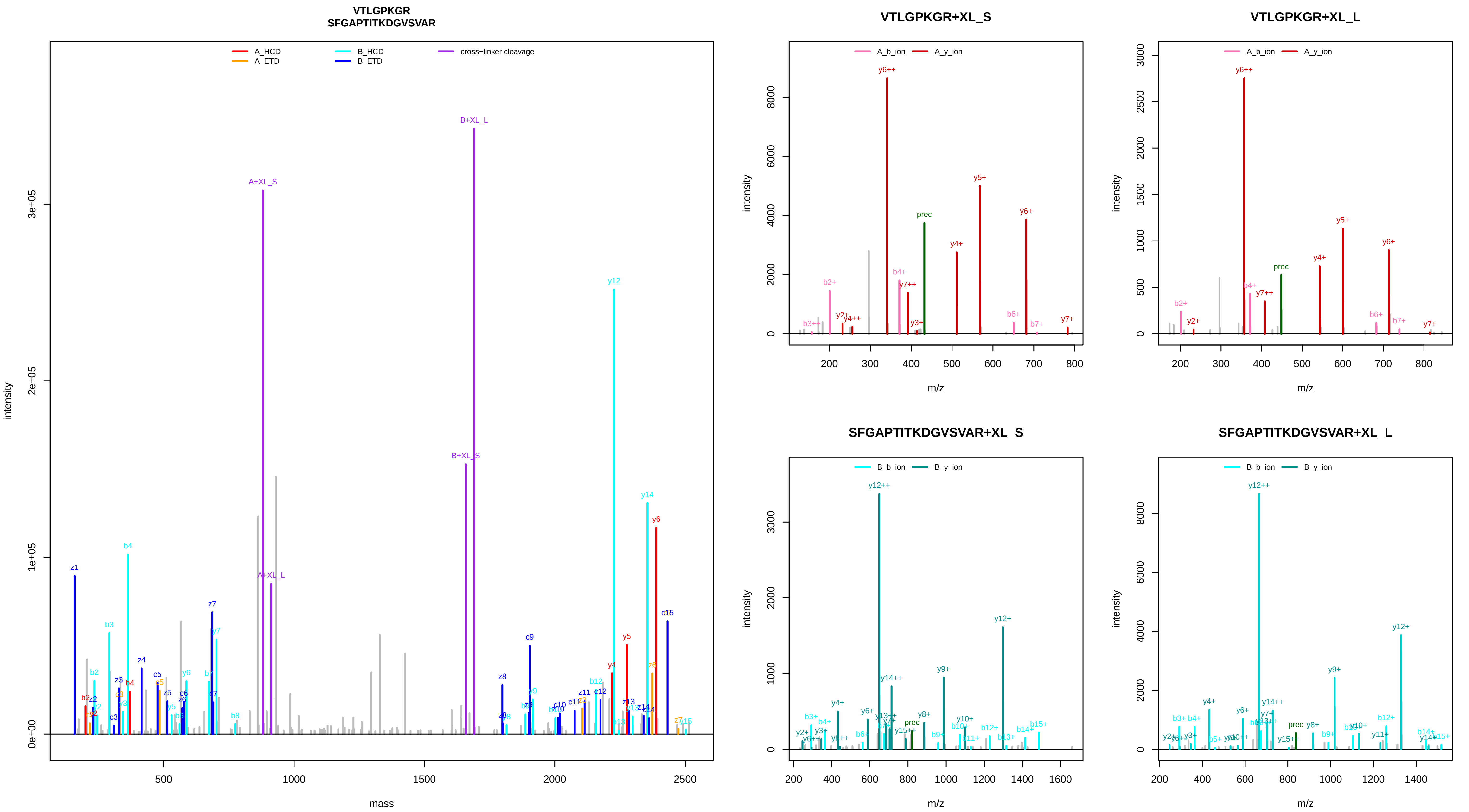
VTLGPKGR
SFGAPTITKDGVSVAR
A_HCD
A_ETD
B_HCD
B_ETD
cross−linker cleavage
B+XL_L
A+XL_S
B+XL_S
A+XL_L
intensity
mass
VTLGPKGR+XL_S
VTLGPKGR+XL_L
SFGAPTITKDGVSVAR+XL_S
SFGAPTITKDGVSVAR+XL_L
A_b_ion
A_y_ion
B_b_ion
B_y_ion
prec
m/z

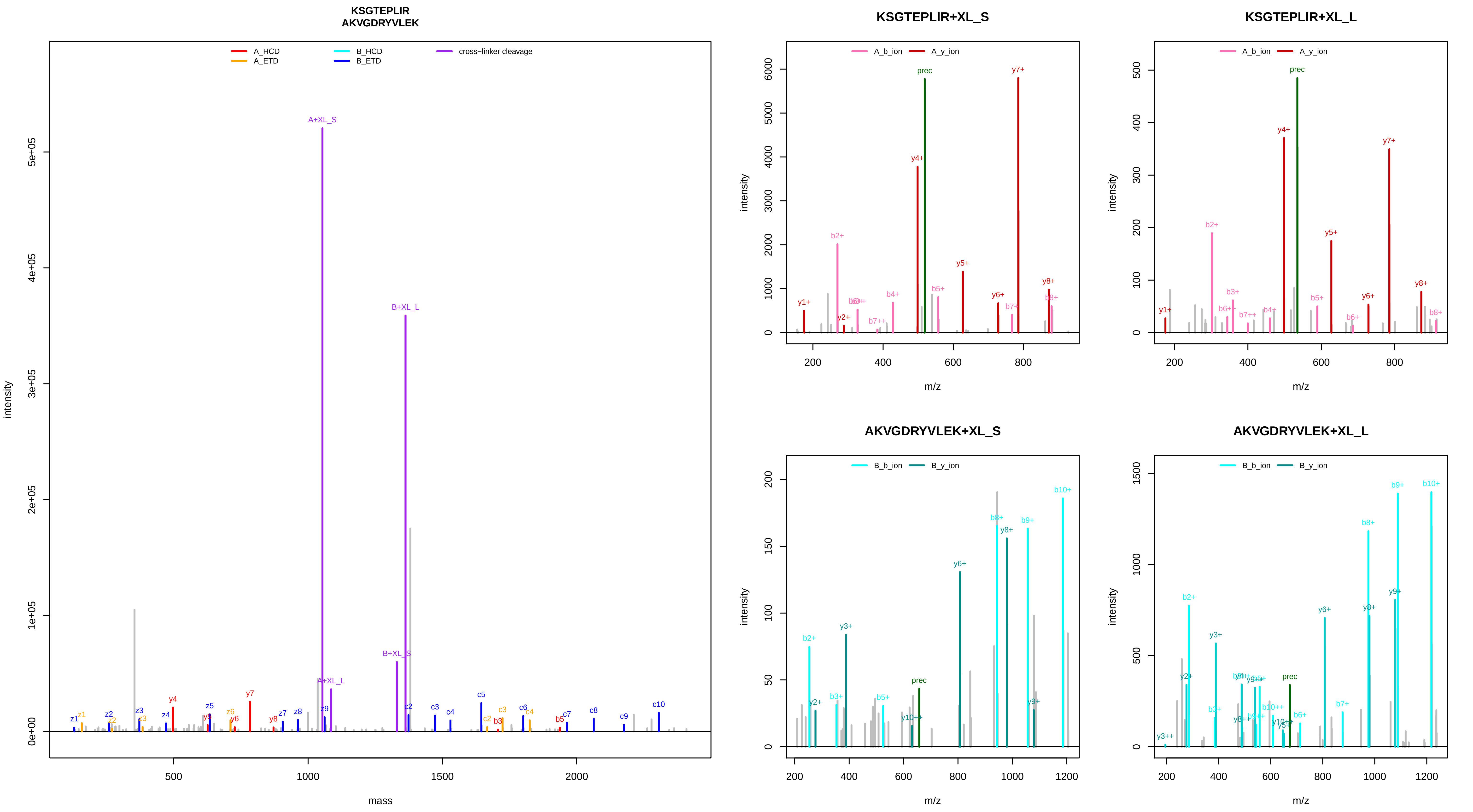
KSGTEPLIR
AKVGDRYVLEK
A_HCD
A_ETD
B_HCD
B_ETD
cross−linker cleavage
A+XL_S
B+XL_L
B+XL_S
A+XL_L
intensity
mass
0e+00
1e+05
2e+05
3e+05
4e+05
5e+05
500
1000
1500
2000
KSGTEPLIR+XL_S
KSGTEPLIR+XL_L
A_b_ion
A_y_ion
prec
AKVGDRYVLEK+XL_S
AKVGDRYVLEK+XL_L
B_b_ion
B_y_ion
m/z

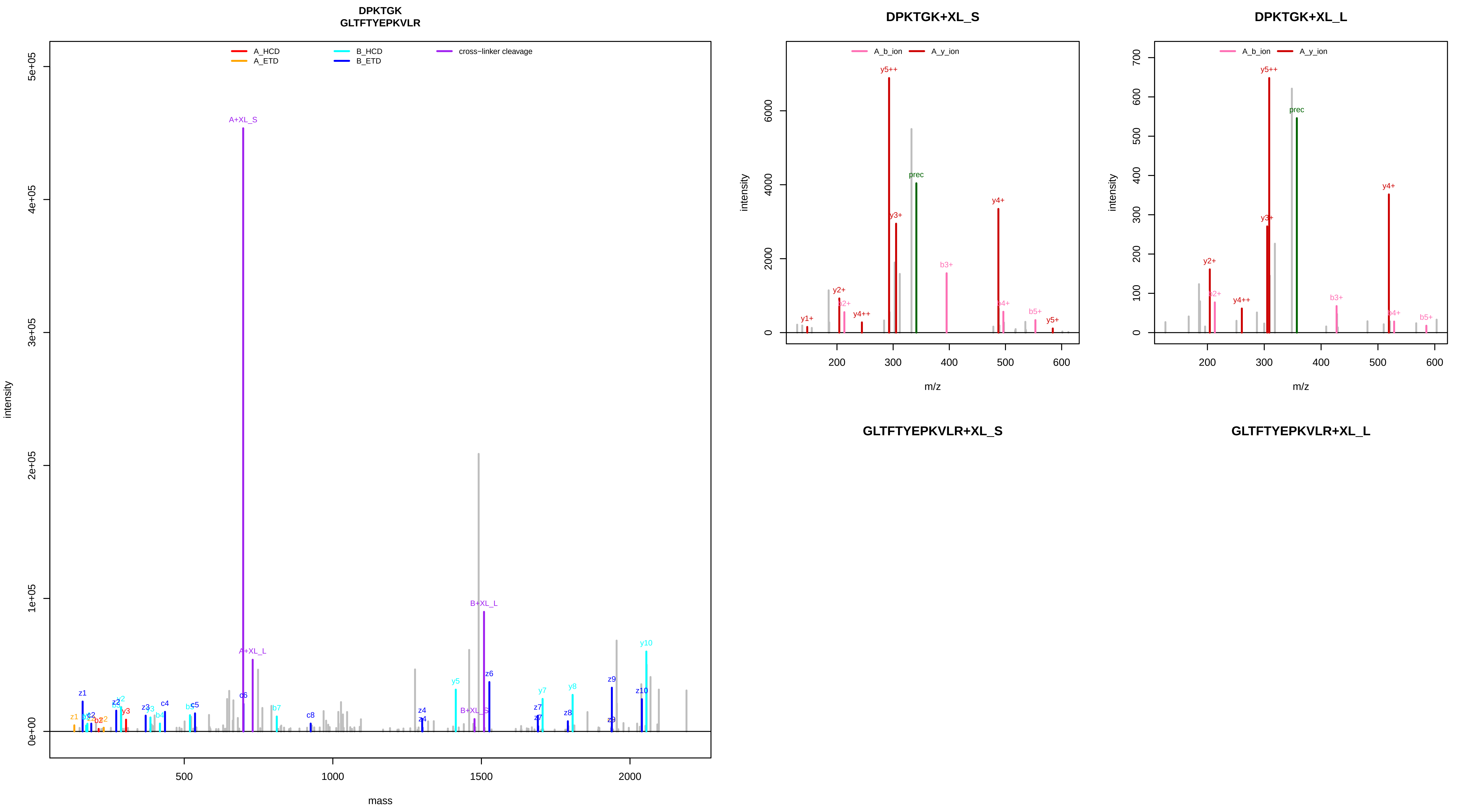
DPKTGK
GLTFTYEPKVLR
A_HCD
A_ETD
B_HCD
B_ETD
cross−linker cleavage
A+XL_S
A+XL_L
B+XL_L
B+XL_S
intensity
mass
DPKTGK+XL_S
DPKTGK+XL_L
A_b_ion
A_y_ion
prec
intensity
m/z
GLTFTYEPKVLR+XL_S
GLTFTYEPKVLR+XL_L

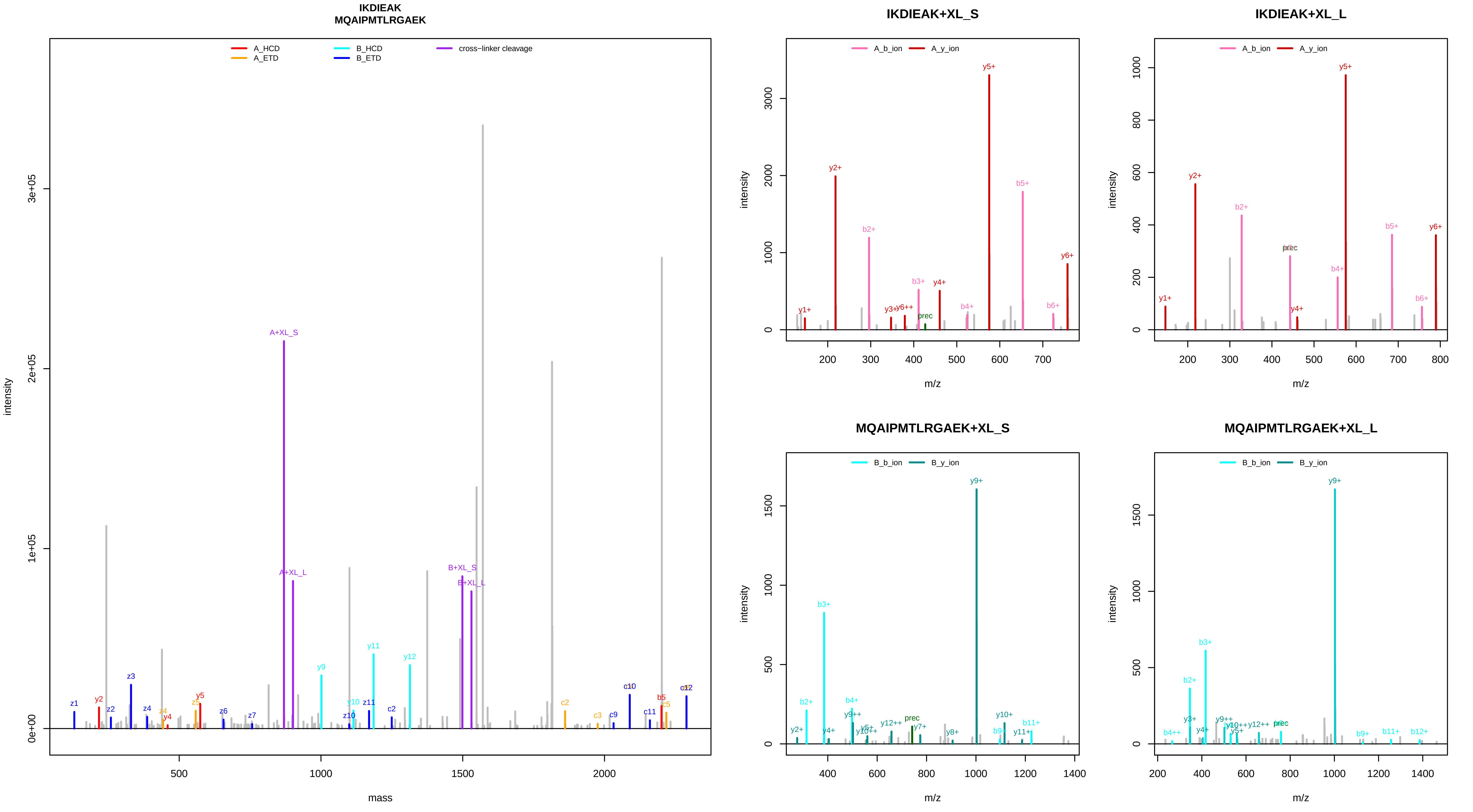

IKDIEAK
MQAIPMTLRGAEK
A_HCD
A_ETD
B_HCD
B_ETD
cross−linker cleavage
A+XL_S
A+XL_L
B+XL_S
B+XL_L
intensity
mass
IKDIEAK+XL_S
IKDIEAK+XL_L
MQAIPMTLRGAEK+XL_S
MQAIPMTLRGAEK+XL_L
A_b_ion
A_y_ion
B_b_ion
B_y_ion
m/z

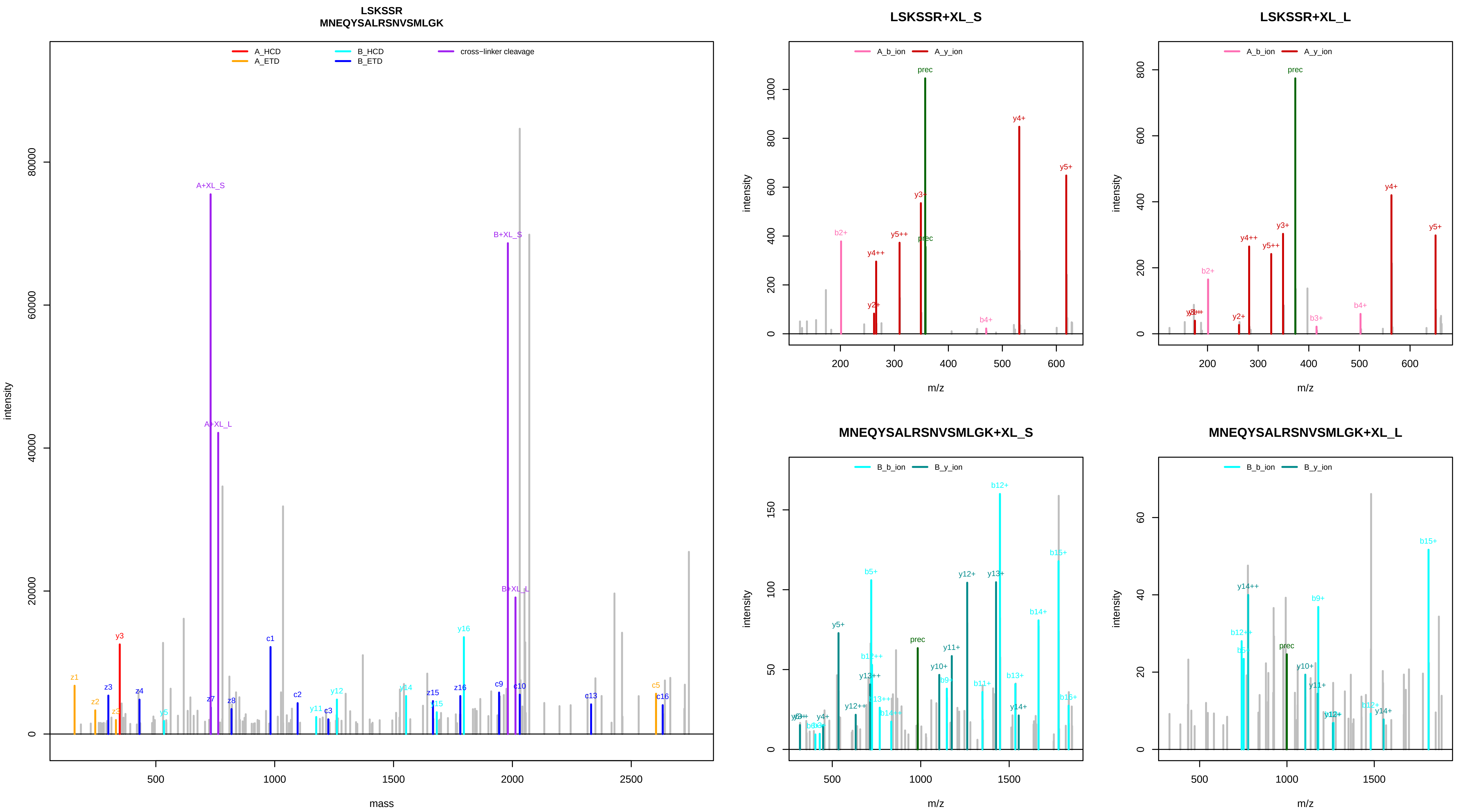

LSKSSR
MNEQYSALRSNVSMLGK
A_HCD
A_ETD
B_HCD
B_ETD
cross−linker cleavage
A+XL_S
A+XL_L
B+XL_S
B+XL_L
intensity
mass
LSKSSR+XL_S
LSKSSR+XL_L
A_b_ion
A_y_ion
prec
m/z
MNEQYSALRSNVSMLGK+XL_S
MNEQYSALRSNVSMLGK+XL_L
B_b_ion
B_y_ion

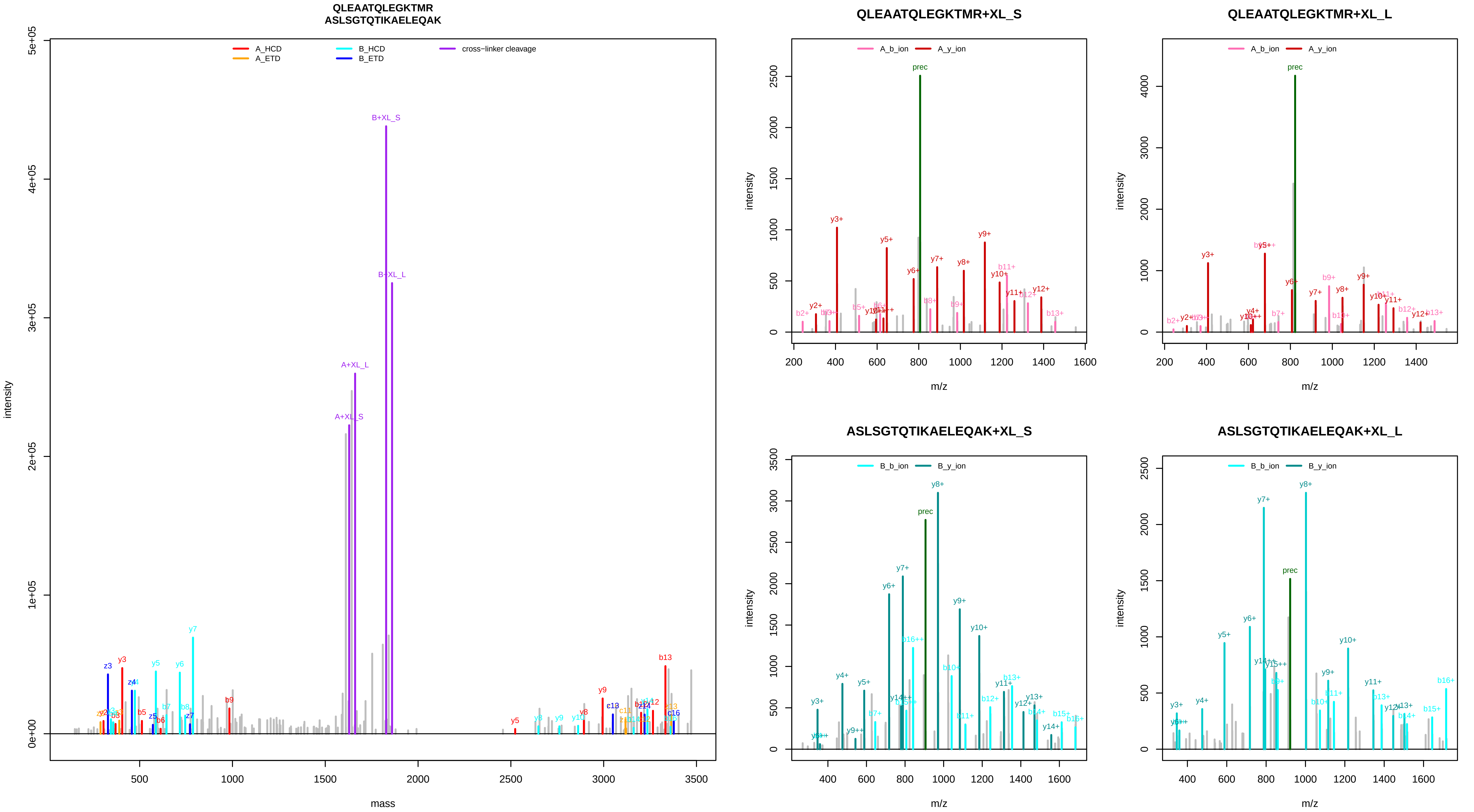

QLEAATQLEGKTMR
ASLSGTQTIKAELEQAK
A_HCD
A_ETD
B_HCD
B_ETD
cross−linker cleavage
B+XL_S
B+XL_L
A+XL_L
A+XL_S
intensity
mass
QLEAATQLEGKTMR+XL_S
QLEAATQLEGKTMR+XL_L
A_b_ion
A_y_ion
prec
m/z
ASLSGTQTIKAELEQAK+XL_S
ASLSGTQTIKAELEQAK+XL_L
B_b_ion
B_y_ion

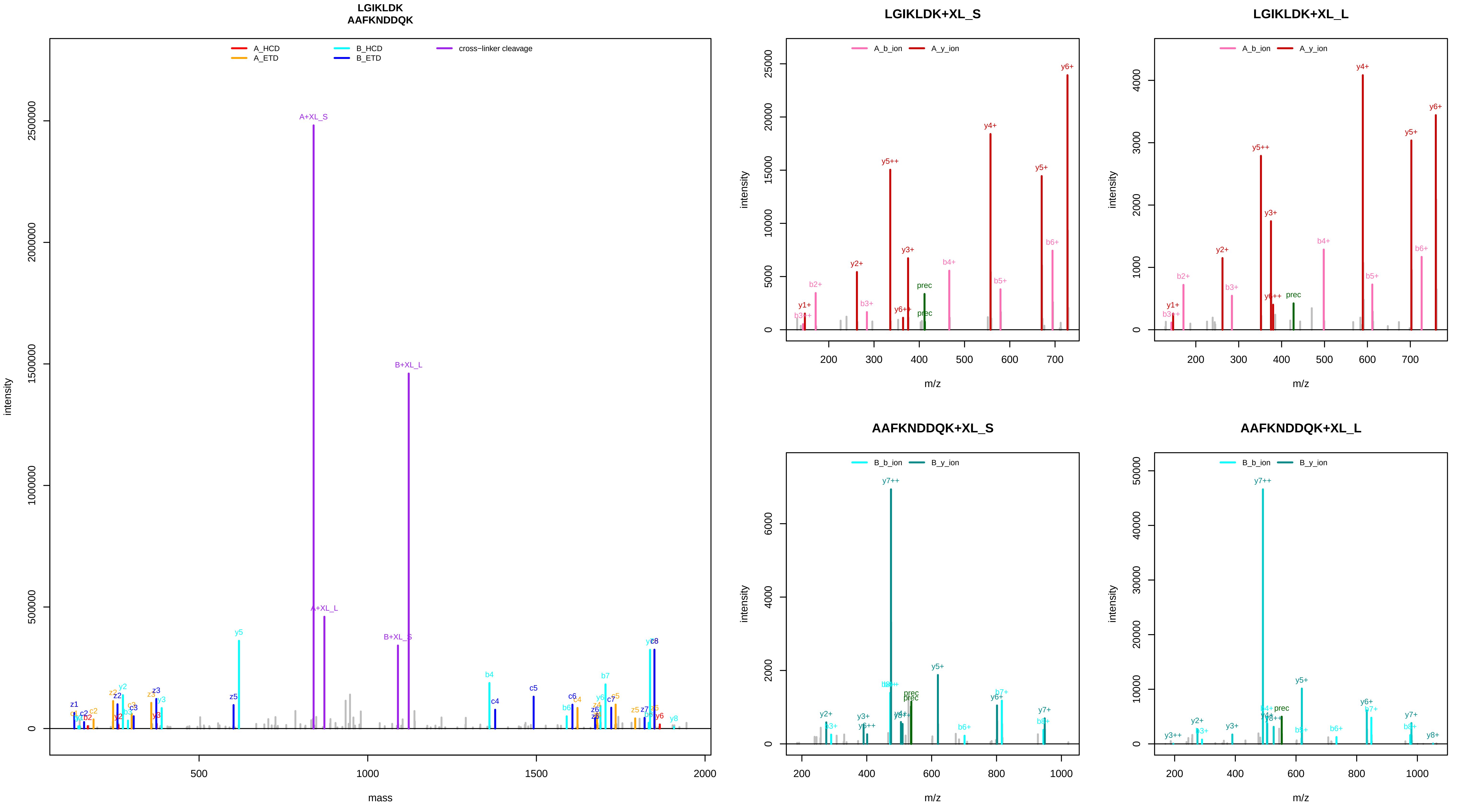

LGIKLDK
AAFKNDDQK
A_HCD
A_ETD
B_HCD
B_ETD
cross−linker cleavage
A+XL_S
B+XL_L
A+XL_L
B+XL_S
intensity
mass
LGIKLDK+XL_S
LGIKLDK+XL_L
AAFKNDDQK+XL_S
AAFKNDDQK+XL_L
A_b_ion
A_y_ion
B_b_ion
B_y_ion
prec
m/z

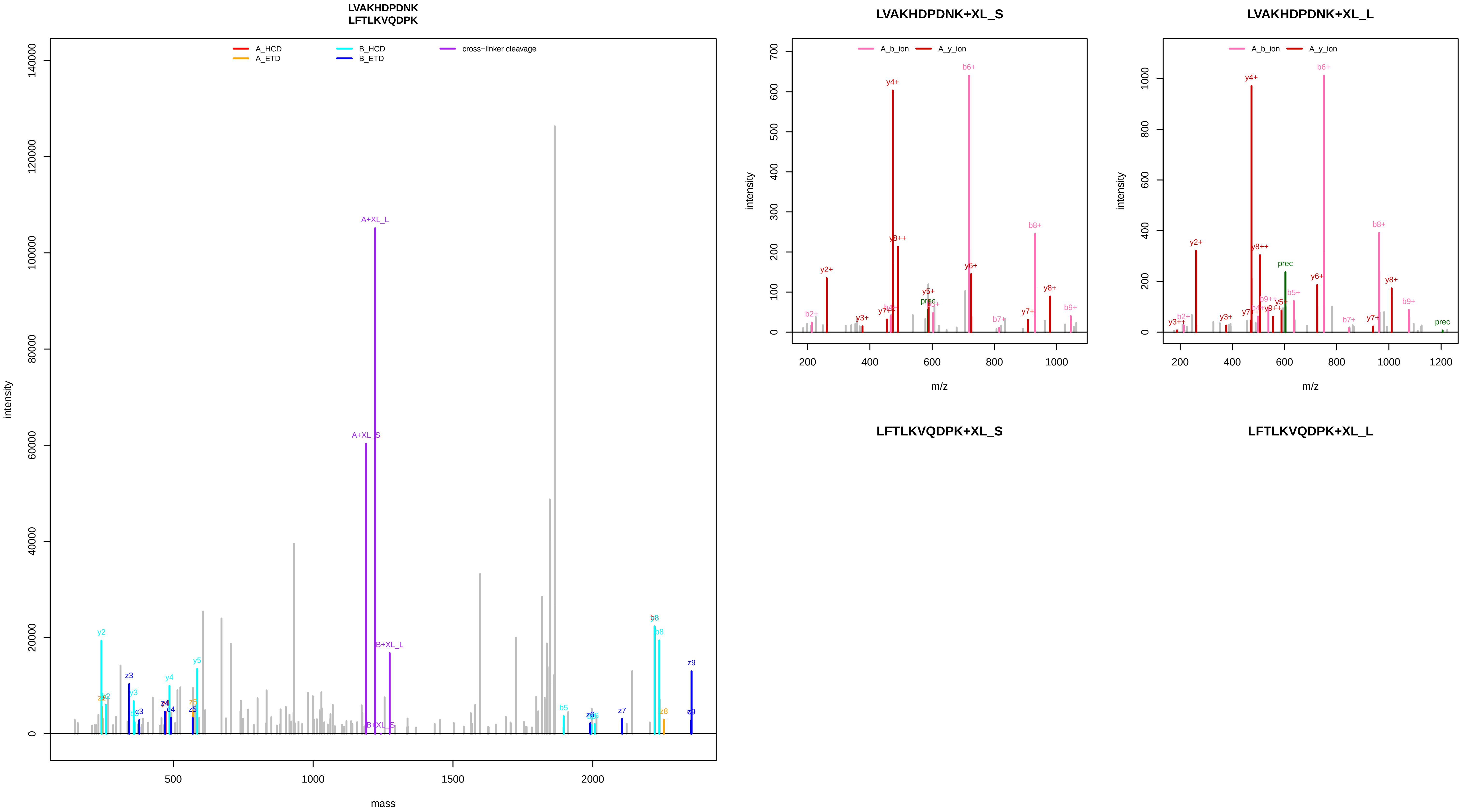

LVAKHDPDNK
LFTLKVQDPK
A_HCD
A_ETD
B_HCD
B_ETD
cross−linker cleavage
A+XL_L
A+XL_S
B+XL_L
B+XL_S
intensity
mass
LVAKHDPDNK+XL_S
LVAKHDPDNK+XL_L
A_b_ion
A_y_ion
m/z
LFTLKVQDPK+XL_S
LFTLKVQDPK+XL_L

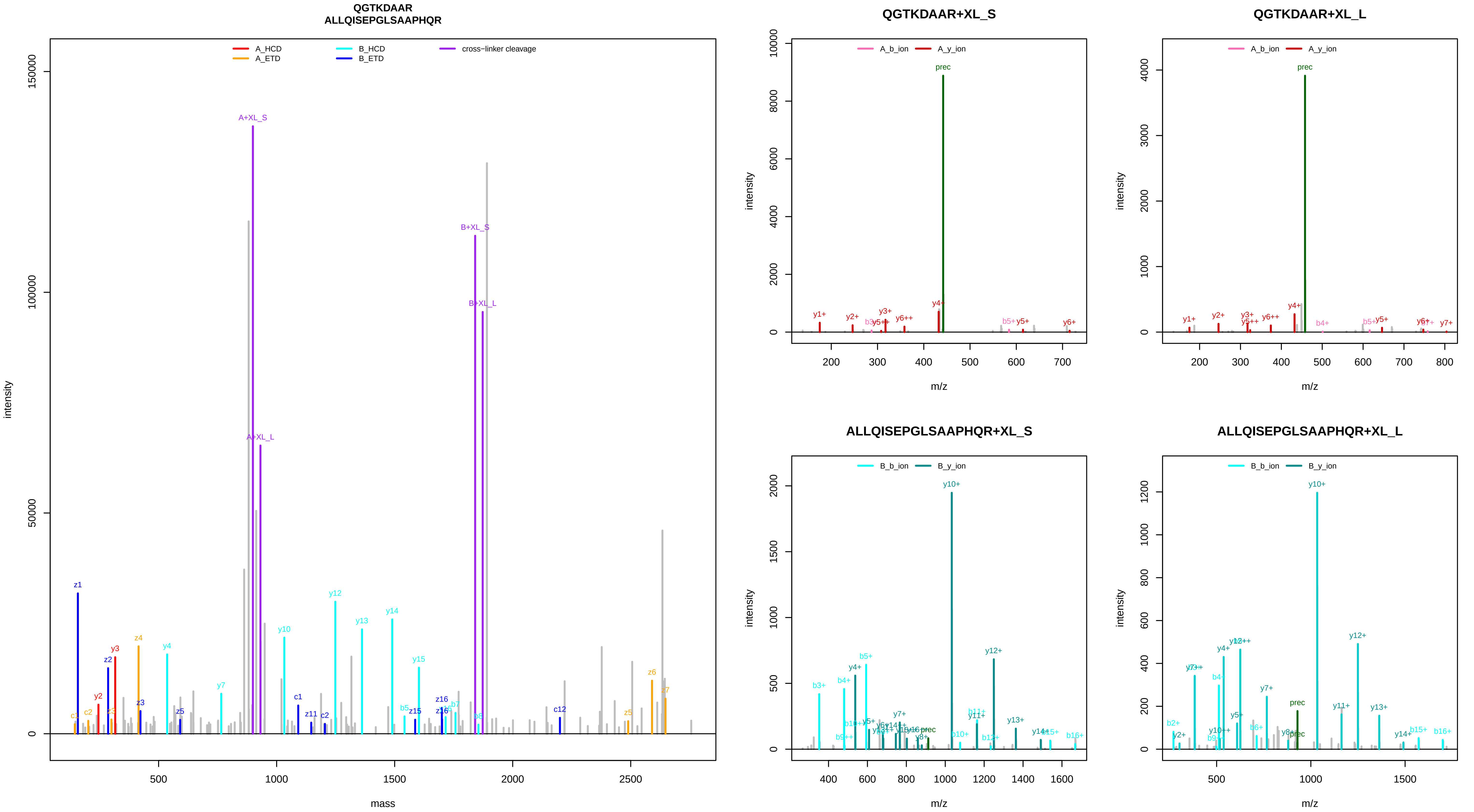
QGTKDAAR
ALLQISEPGLSAAPHQR
A_HCD
A_ETD
B_HCD
B_ETD
cross−linker cleavage
A+XL_S
A+XL_L
B+XL_S
B+XL_L
intensity
mass
QGTKDAAR+XL_S
QGTKDAAR+XL_L
A_b_ion
A_y_ion
prec
m/z
ALLQISEPGLSAAPHQR+XL_S
ALLQISEPGLSAAPHQR+XL_L
B_b_ion
B_y_ion
y10+
y12+

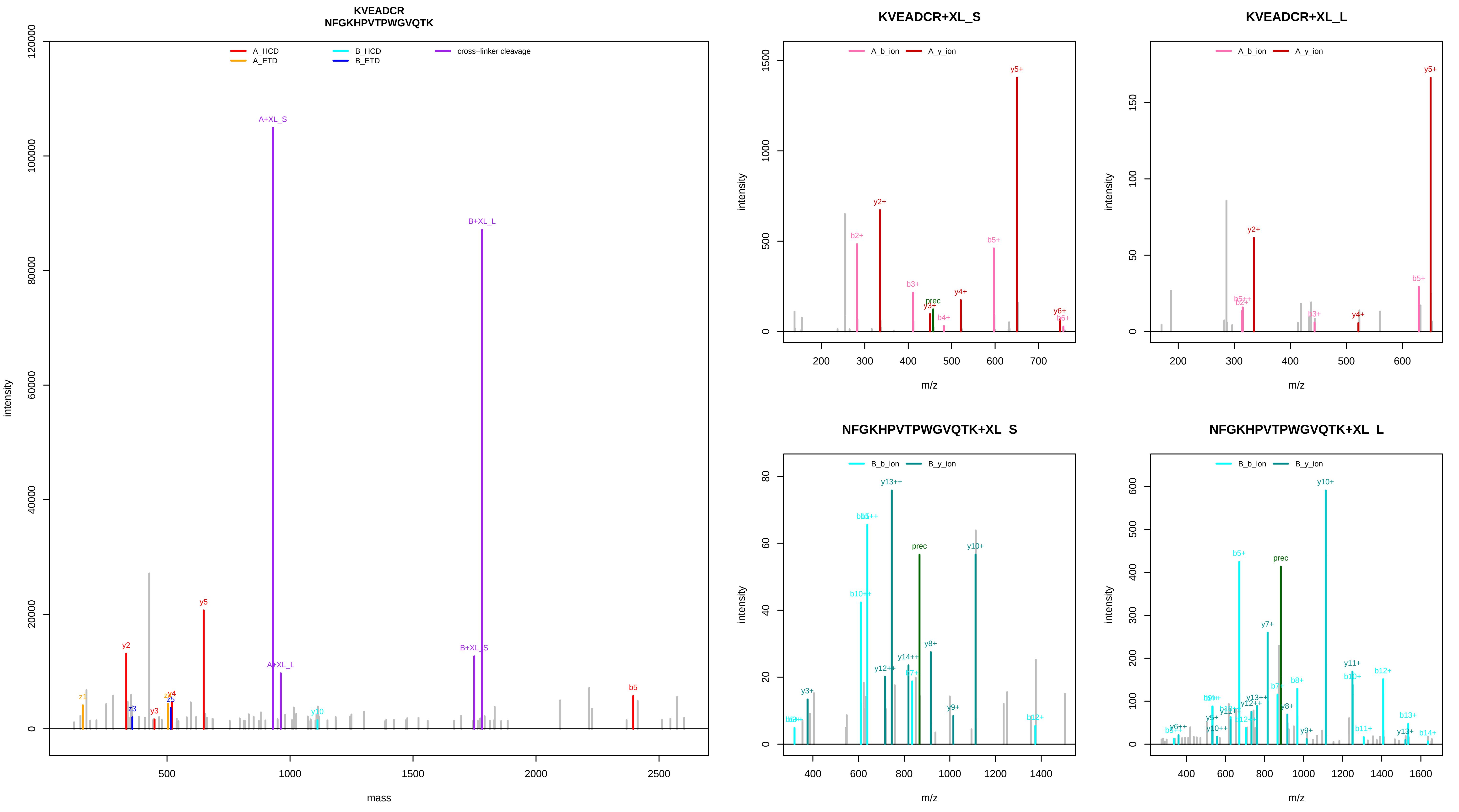
KVEADCR
NFGKHPVTPWGVQTK
A_HCD
A_ETD
B_HCD
B_ETD
cross−linker cleavage
A+XL_S
B+XL_L
B+XL_S
A+XL_L
y2
y3
y4
y5
b5
z1
z3
z5
y10
intensity
mass
KVEADCR+XL_S
A_b_ion
A_y_ion
y5+
y2+
b2+
b3+
b4+
b5+
y3+
y4+
y6+
b6+
prec
intensity
m/z
KVEADCR+XL_L
A_b_ion
A_y_ion
y5+
y2+
b5++
b2+
b3+
y4+
b5+
intensity
m/z
NFGKHPVTPWGVQTK+XL_S
B_b_ion
B_y_ion
y13++
prec
y10+
b10++
y3+
y12++
y14++
y8+
y9+
b7+
b12+
intensity
m/z
NFGKHPVTPWGVQTK+XL_L
B_b_ion
B_y_ion
y10+
b5+
prec
y7+
y11+
b10+
b12+
b8+
b7+
y8+
y13++
y12++
y11++
y6++
y5+
y10++
y9+
b11+
b13+
y13+
b14+
intensity
m/z

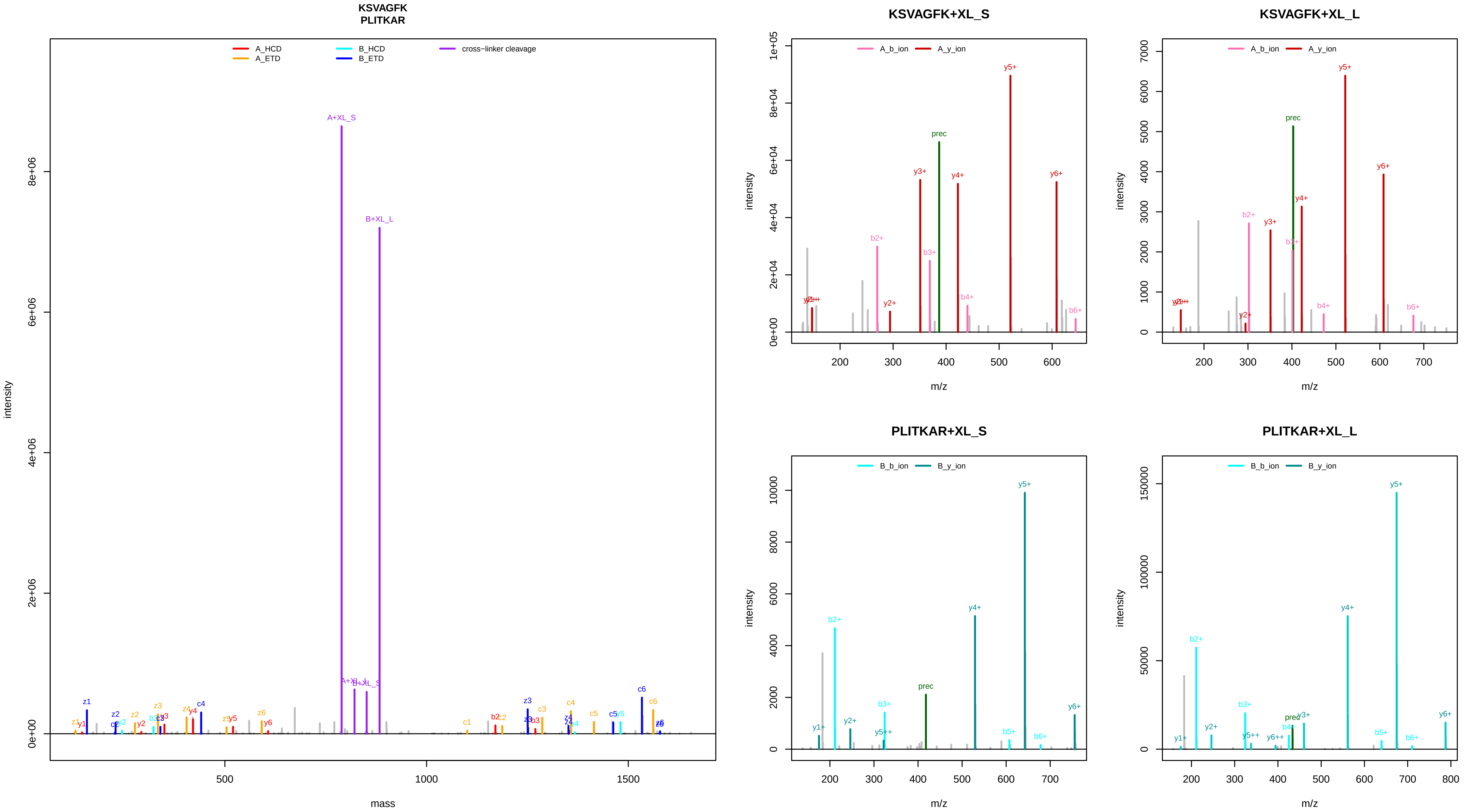
KSVAGFK
PLITKAR
A_HCD
A_ETD
B_HCD
B_ETD
cross−linker cleavage
A+XL_S
B+XL_L
intensity
mass
KSVAGFK+XL_S
KSVAGFK+XL_L
PLITKAR+XL_S
PLITKAR+XL_L
A_b_ion
A_y_ion
B_b_ion
B_y_ion
prec
m/z
intensity

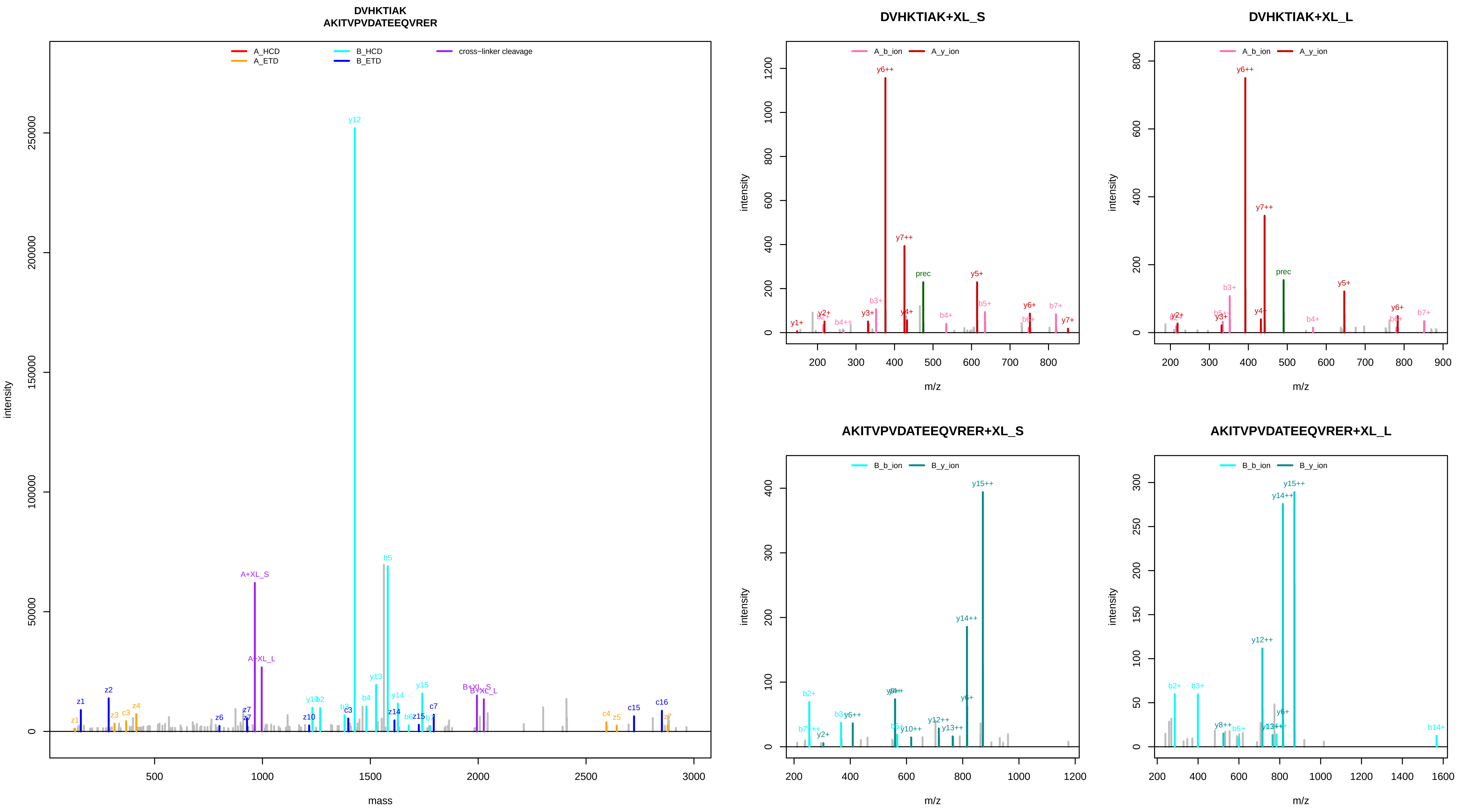
DVHKTIAK
AKITVPVDATEEQVRER
A_HCD
A_ETD
B_HCD
B_ETD
cross−linker cleavage
intensity
mass
DVHKTIAK+XL_S
DVHKTIAK+XL_L
AKITVPVDATEEQVRER+XL_S
AKITVPVDATEEQVRER+XL_L
A_b_ion
A_y_ion
B_b_ion
B_y_ion
m/z

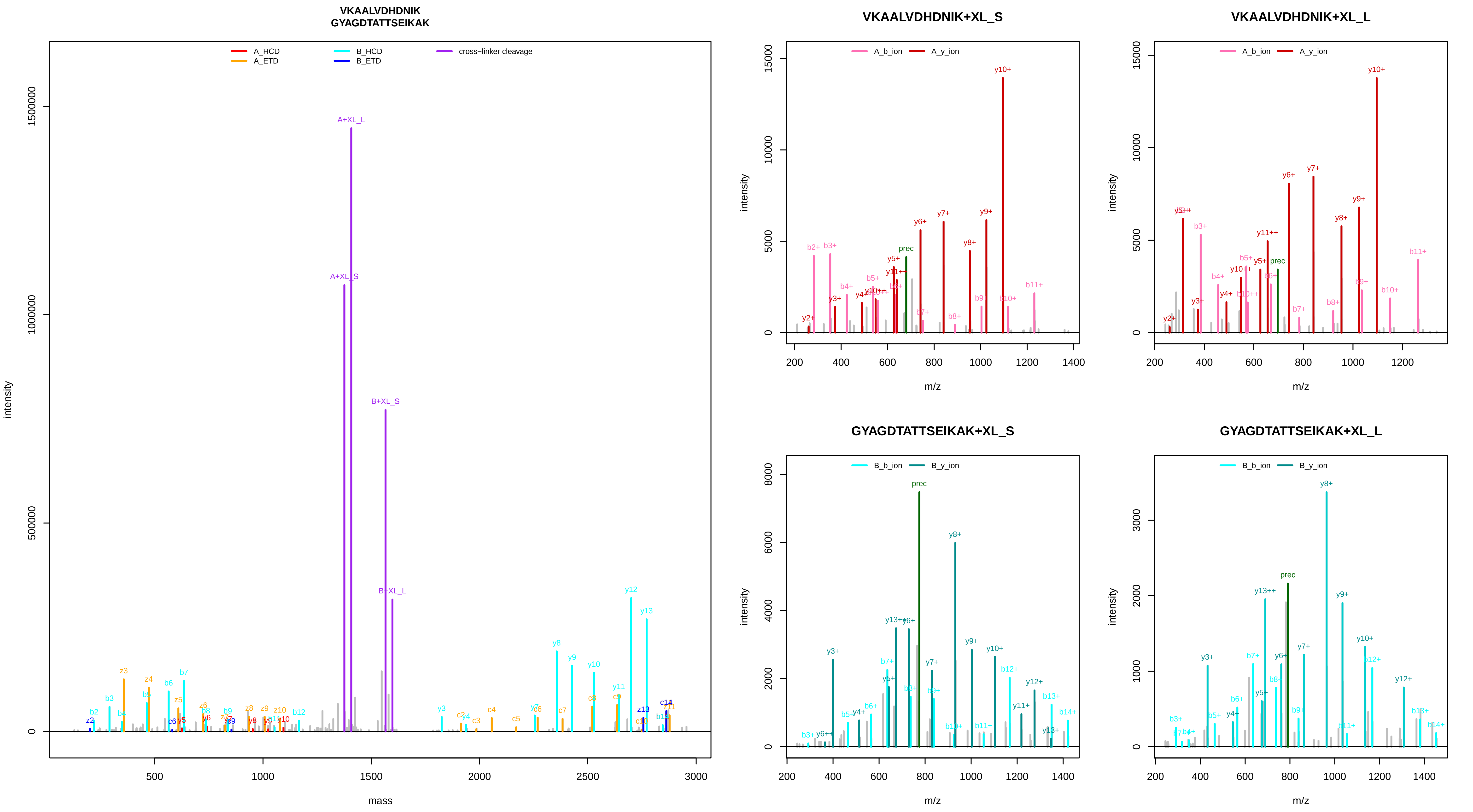
VKAALVDHDNIK
GYAGDTATTSEIKAK
A_HCD
A_ETD
B_HCD
B_ETD
cross−linker cleavage
A+XL_L
A+XL_S
B+XL_S
B+XL_L
intensity
mass
VKAALVDHDNIK+XL_S
VKAALVDHDNIK+XL_L
A_b_ion
A_y_ion
GYAGDTATTSEIKAK+XL_S
GYAGDTATTSEIKAK+XL_L
B_b_ion
B_y_ion
prec
m/z

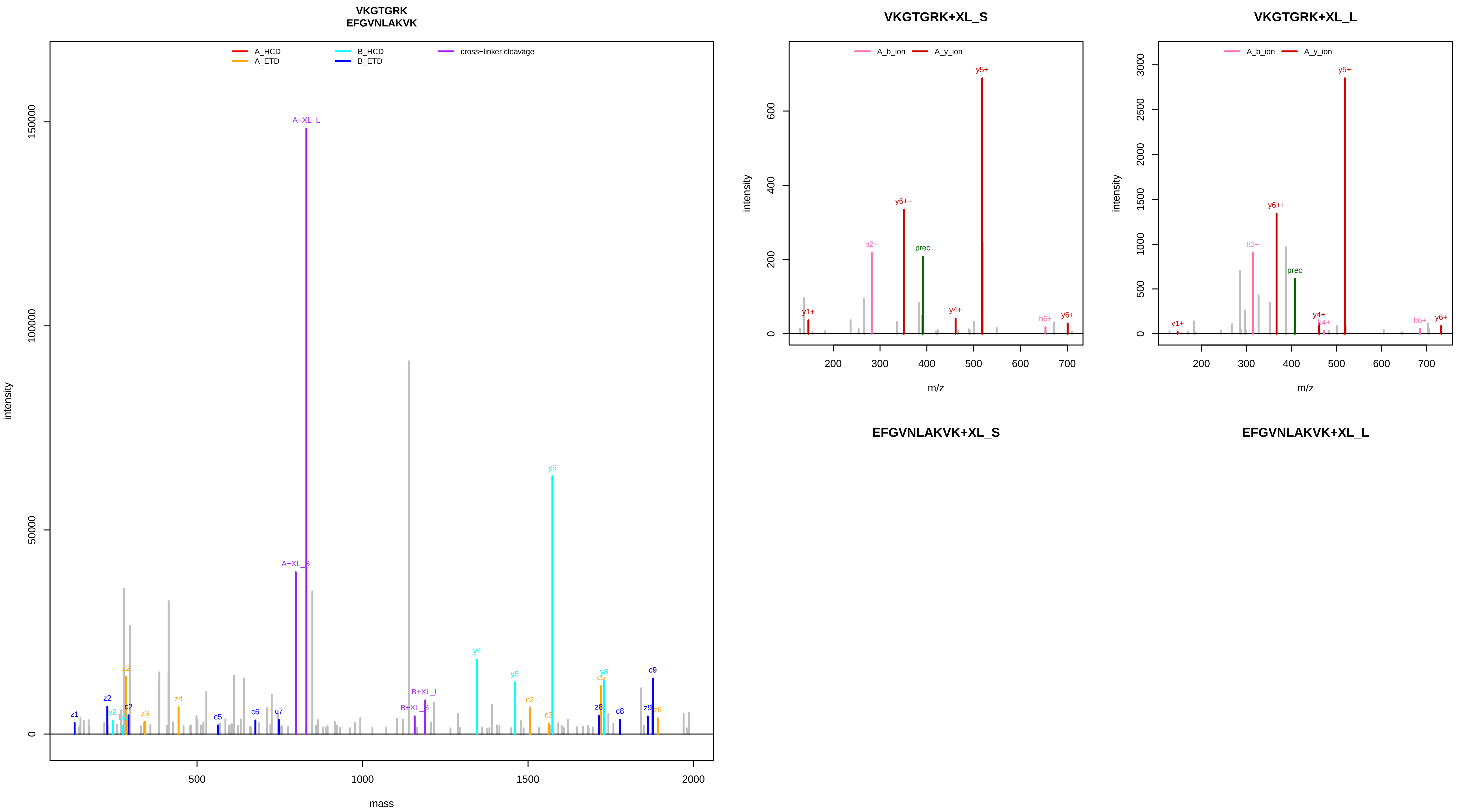
VKGTGRK
EFGVNLAKVK
A_HCD
A_ETD
B_HCD
B_ETD
cross−linker cleavage
A+XL_L
A+XL_S
B+XL_L
B+XL_S
intensity
mass
VKGTGRK+XL_S
VKGTGRK+XL_L
A_b_ion
A_y_ion
prec
m/z
EFGVNLAKVK+XL_S
EFGVNLAKVK+XL_L

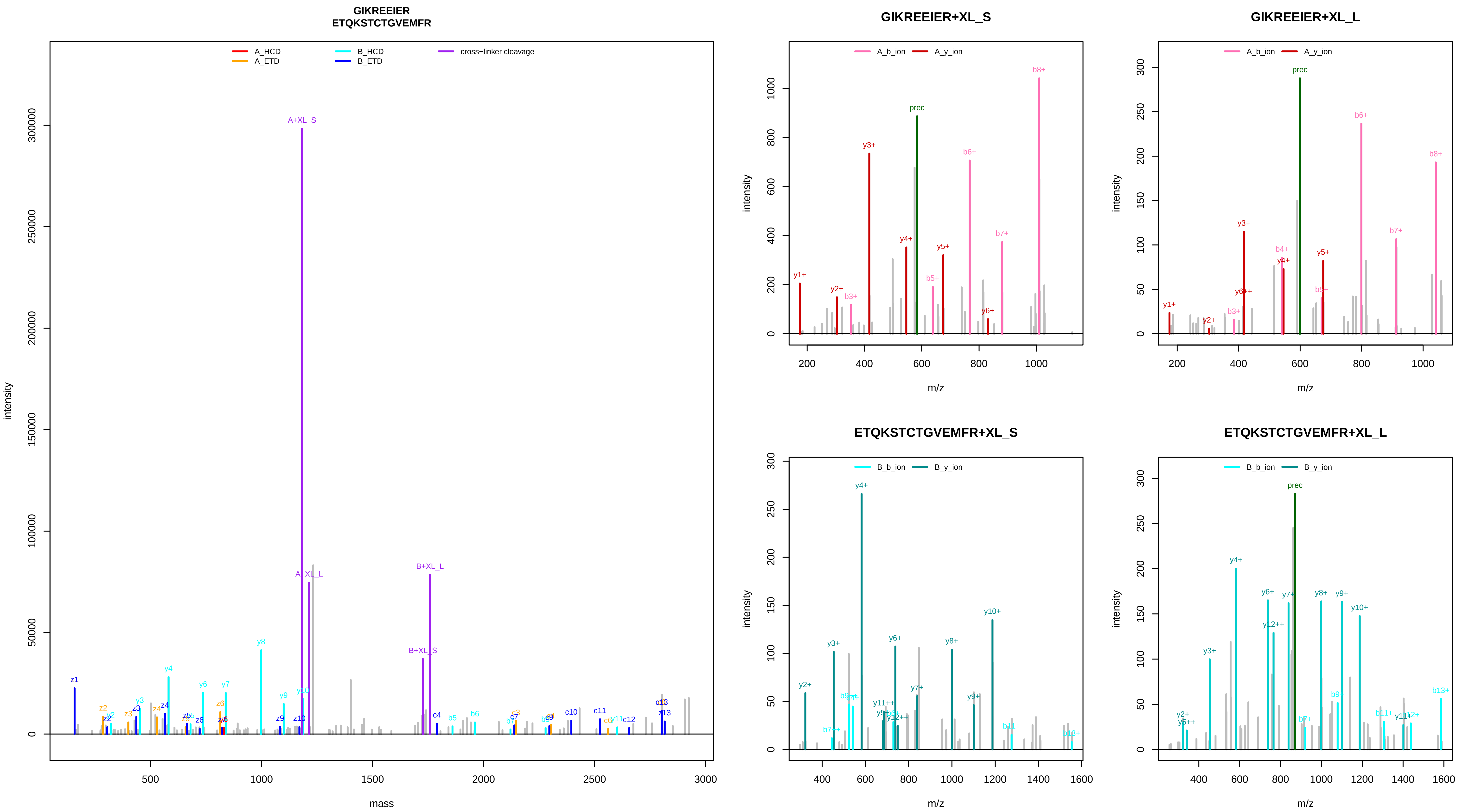
GIKREEIER
ETQKSTCTGVEMFR
A_HCD
A_ETD
B_HCD
B_ETD
cross−linker cleavage
intensity
mass
GIKREEIER+XL_S
GIKREEIER+XL_L
ETQKSTCTGVEMFR+XL_S
ETQKSTCTGVEMFR+XL_L
A_b_ion
A_y_ion
B_b_ion
B_y_ion
m/z
prec

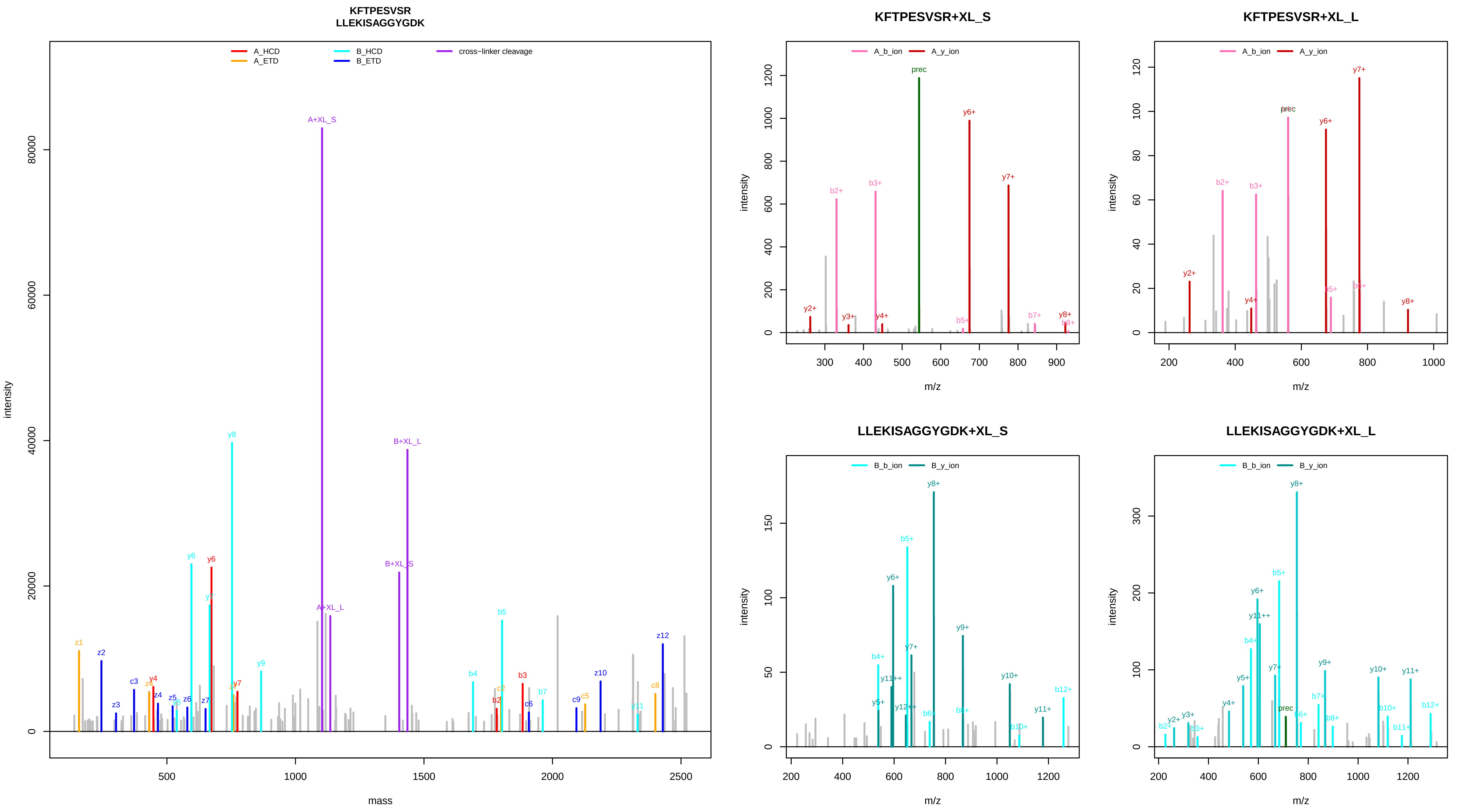

KFTPESVSR
LLEKISAGGYGDK
A_HCD
A_ETD
B_HCD
B_ETD
cross−linker cleavage
A+XL_S
B+XL_L
B+XL_S
A+XL_L
intensity
mass
KFTPESVSR+XL_S
KFTPESVSR+XL_L
LLEKISAGGYGDK+XL_S
LLEKISAGGYGDK+XL_L
A_b_ion
A_y_ion
B_b_ion
B_y_ion
prec
m/z

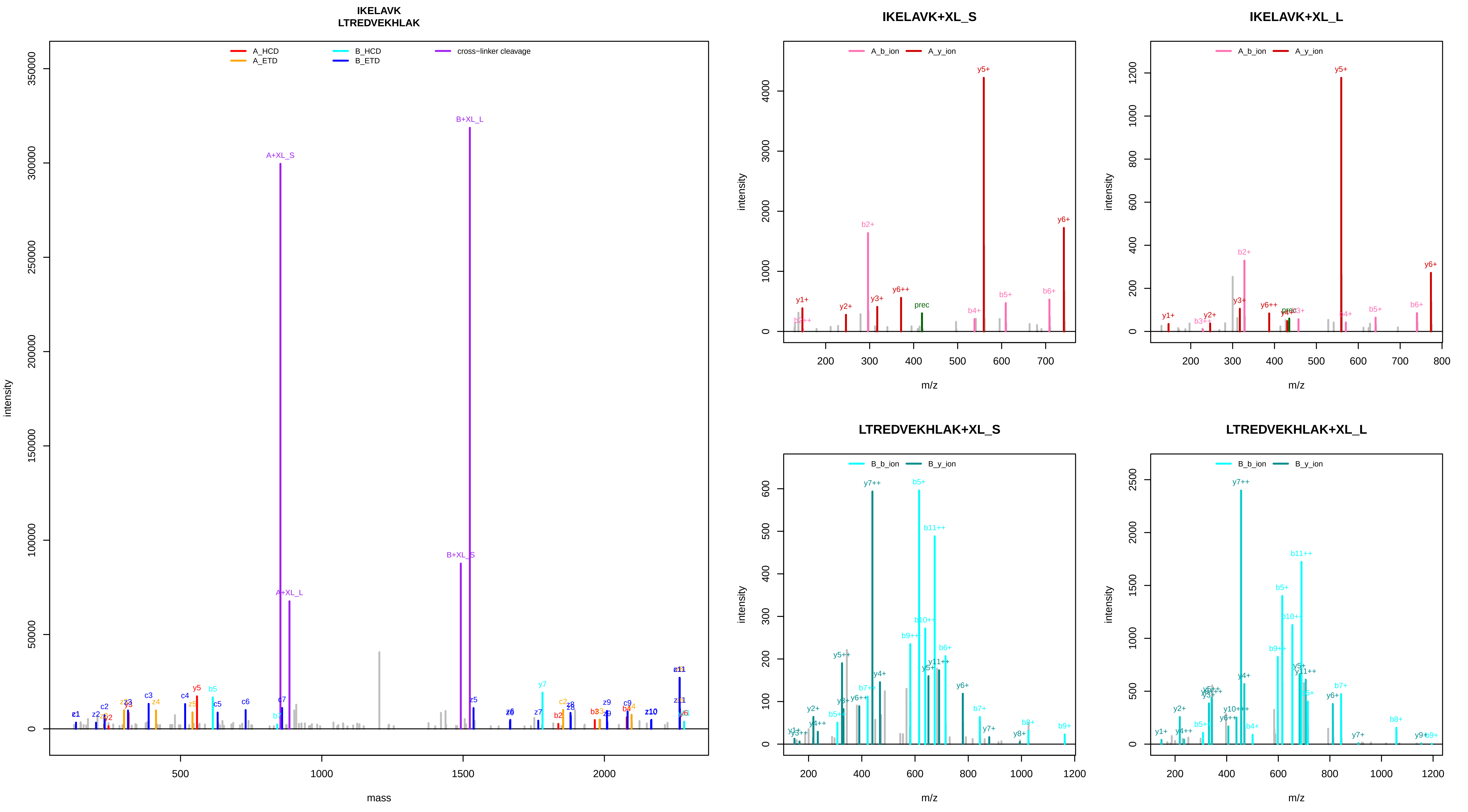

IKELAVK
LTREDVEKHLAK
A_HCD
A_ETD
B_HCD
B_ETD
cross−linker cleavage
A+XL_S
B+XL_L
B+XL_S
A+XL_L
intensity
mass
IKELAVK+XL_S
IKELAVK+XL_L
A_b_ion
A_y_ion
LTREDVEKHLAK+XL_S
LTREDVEKHLAK+XL_L
B_b_ion
B_y_ion
m/z

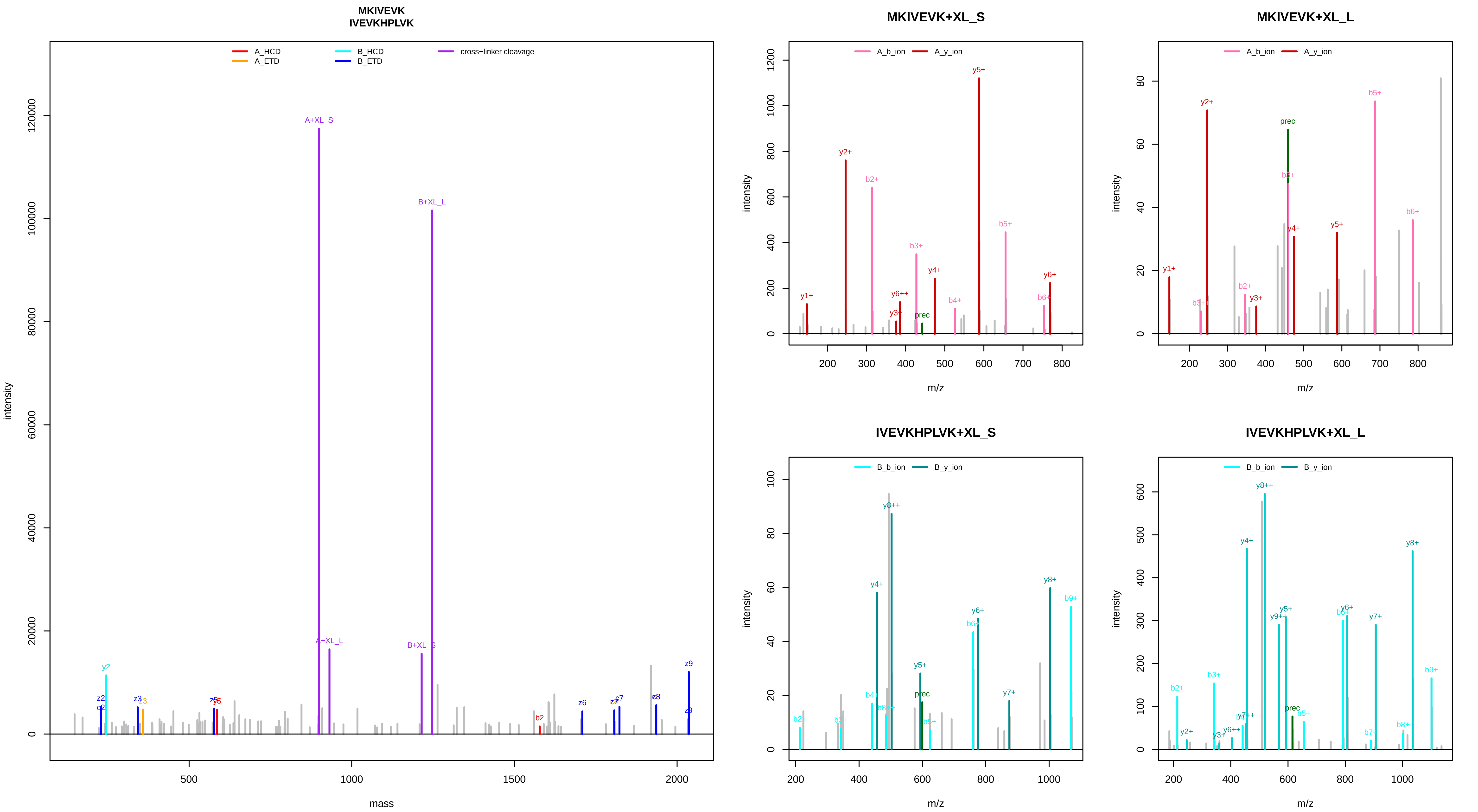
MKIVEVK
IVEVKHPLVK
A_HCD
A_ETD
B_HCD
B_ETD
cross−linker cleavage
A+XL_S
B+XL_L
A+XL_L
B+XL_S
intensity
mass
MKIVEVK+XL_S
MKIVEVK+XL_L
IVEVKHPLVK+XL_S
IVEVKHPLVK+XL_L
A_b_ion
A_y_ion
B_b_ion
B_y_ion
prec
m/z

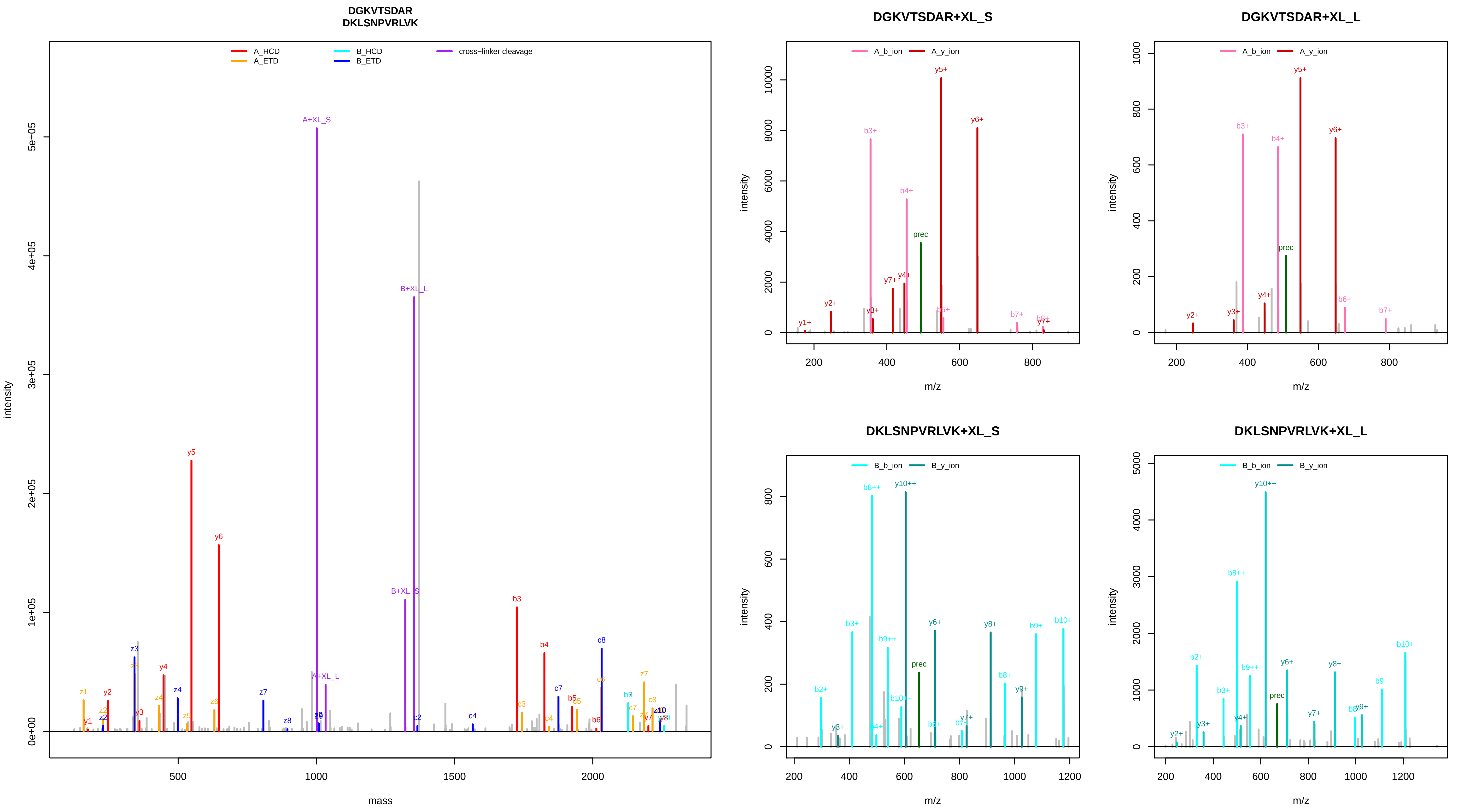
DGKVTSDAR
DKLSNPVRLVK
A_HCD
A_ETD
B_HCD
B_ETD
cross−linker cleavage
A+XL_S
B+XL_L
B+XL_S
A+XL_L
intensity
mass
DGKVTSDAR+XL_S
DGKVTSDAR+XL_L
A_b_ion
A_y_ion
prec
m/z
DKLSNPVRLVK+XL_S
DKLSNPVRLVK+XL_L
B_b_ion
B_y_ion

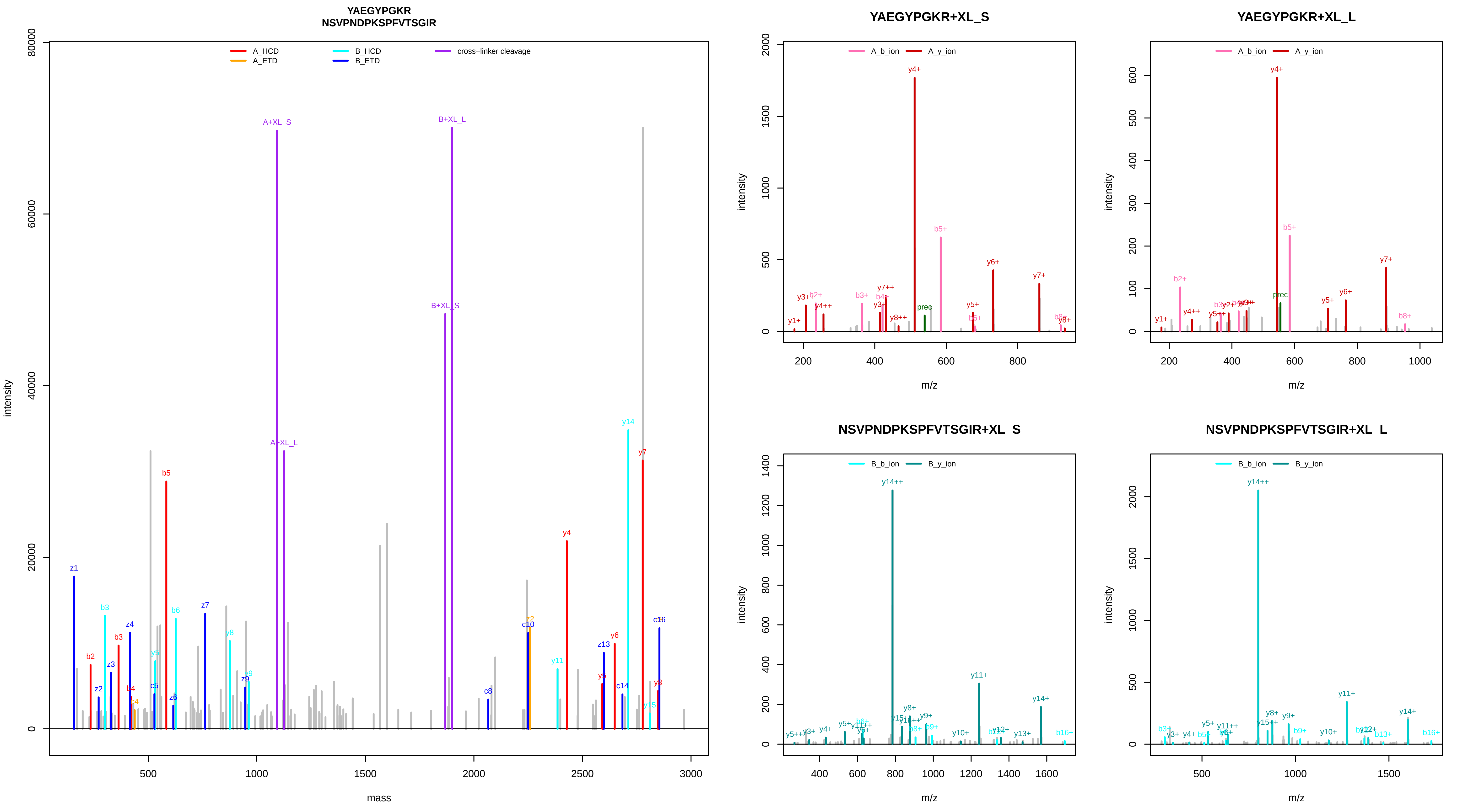
YAEGYPGKR
NSVPNDPKSPFVTSGIR
A_HCD
A_ETD
B_HCD
B_ETD
cross−linker cleavage
A+XL_S
B+XL_L
B+XL_S
A+XL_L
intensity
mass
YAEGYPGKR+XL_S
YAEGYPGKR+XL_L
NSVPNDPKSPFVTSGIR+XL_S
NSVPNDPKSPFVTSGIR+XL_L
A_b_ion
A_y_ion
B_b_ion
B_y_ion
prec
m/z

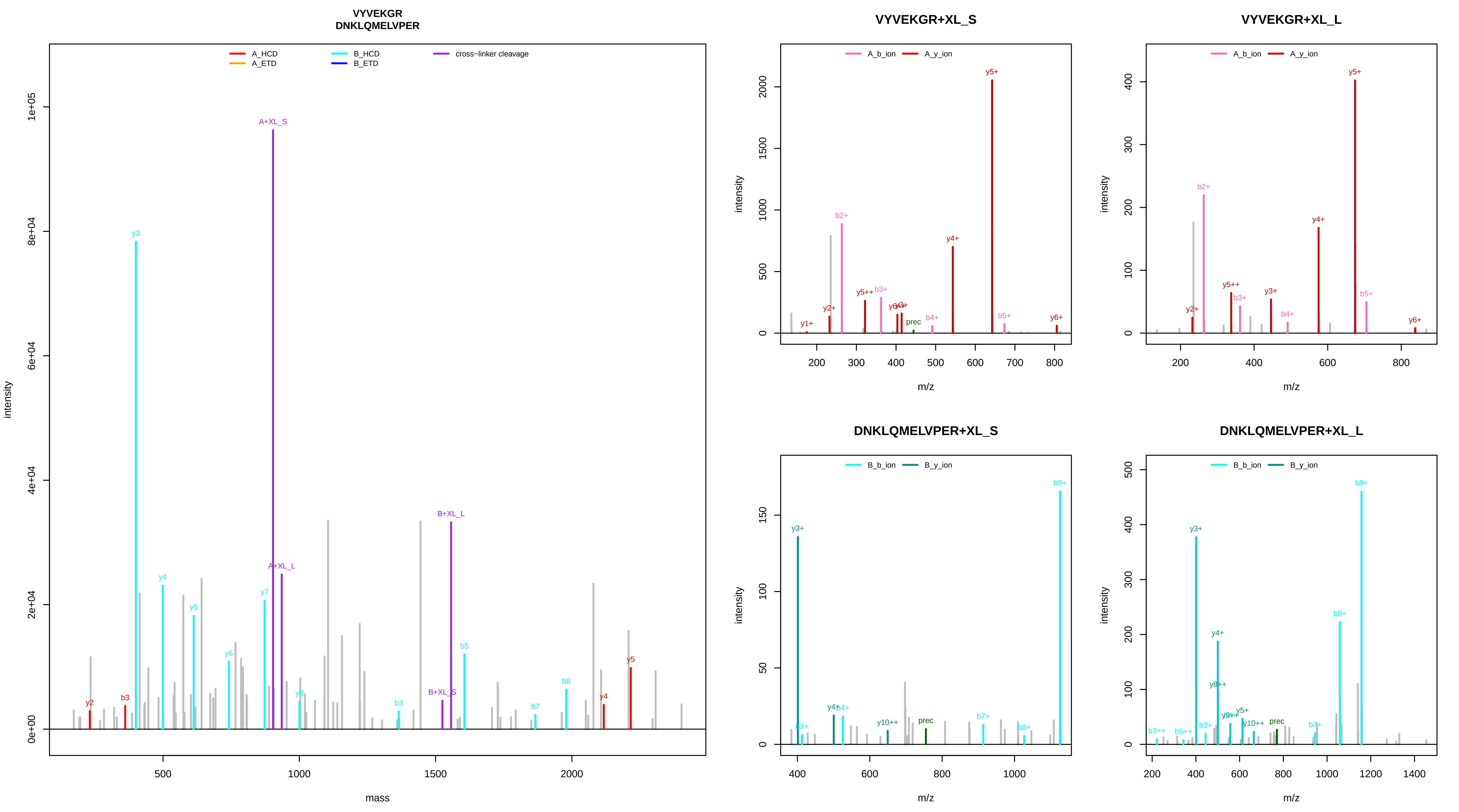

VYVEKGR
DNKLQMELVPER
A_HCD
A_ETD
B_HCD
B_ETD
cross−linker cleavage
A+XL_S
A+XL_L
B+XL_L
B+XL_S
intensity
mass
VYVEKGR+XL_S
VYVEKGR+XL_L
DNKLQMELVPER+XL_S
DNKLQMELVPER+XL_L
A_b_ion
A_y_ion
B_b_ion
B_y_ion
prec
m/z

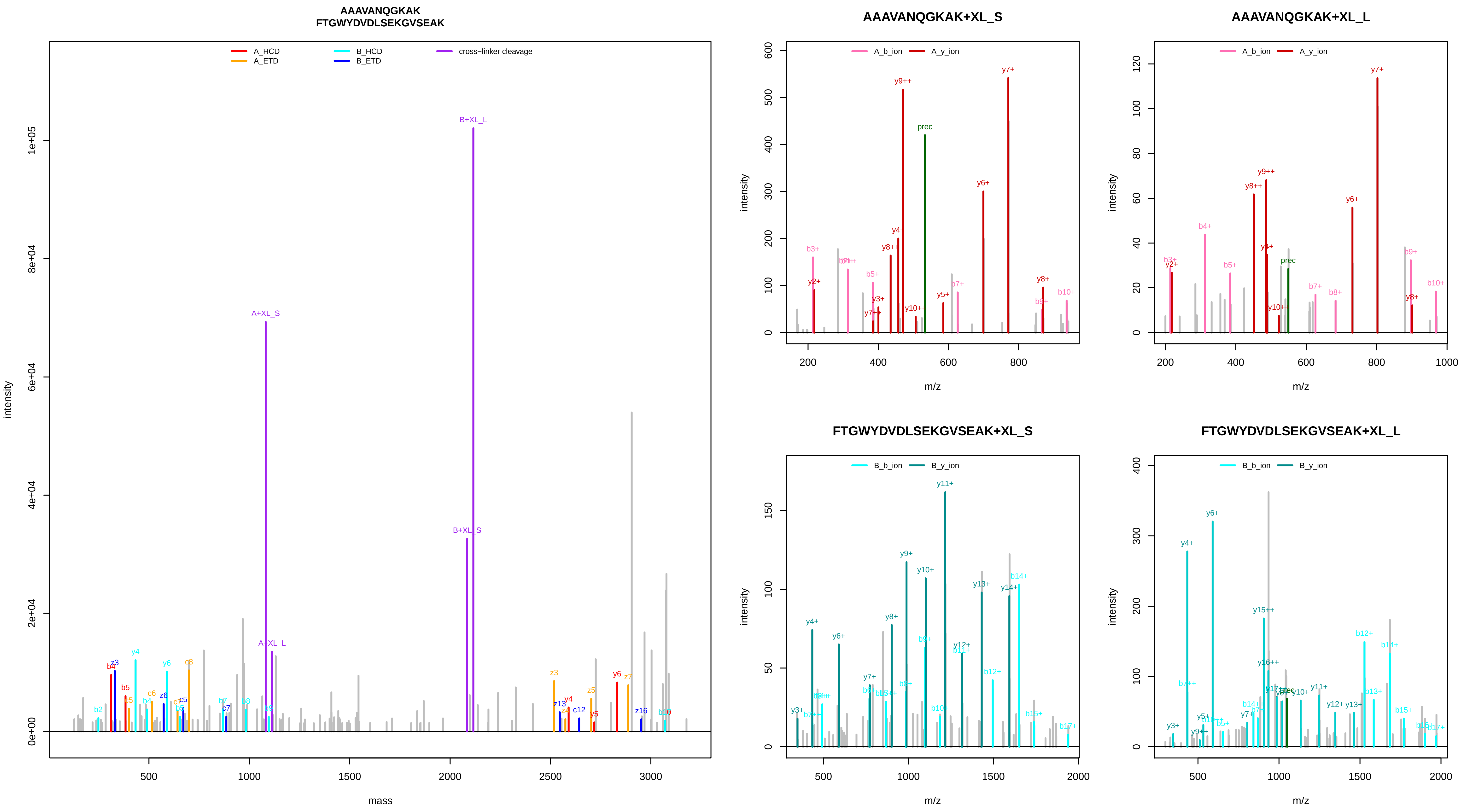

AAAVANQGKAK
FTGWYDVDLSEKGVSEAK
A_HCD
A_ETD
B_HCD
B_ETD
cross−linker cleavage
B+XL_L
A+XL_S
B+XL_S
A+XL_L
intensity
mass
AAAVANQGKAK+XL_S
AAAVANQGKAK+XL_L
A_b_ion
A_y_ion
prec
FTGWYDVDLSEKGVSEAK+XL_S
FTGWYDVDLSEKGVSEAK+XL_L
B_b_ion
B_y_ion
intensity
m/z

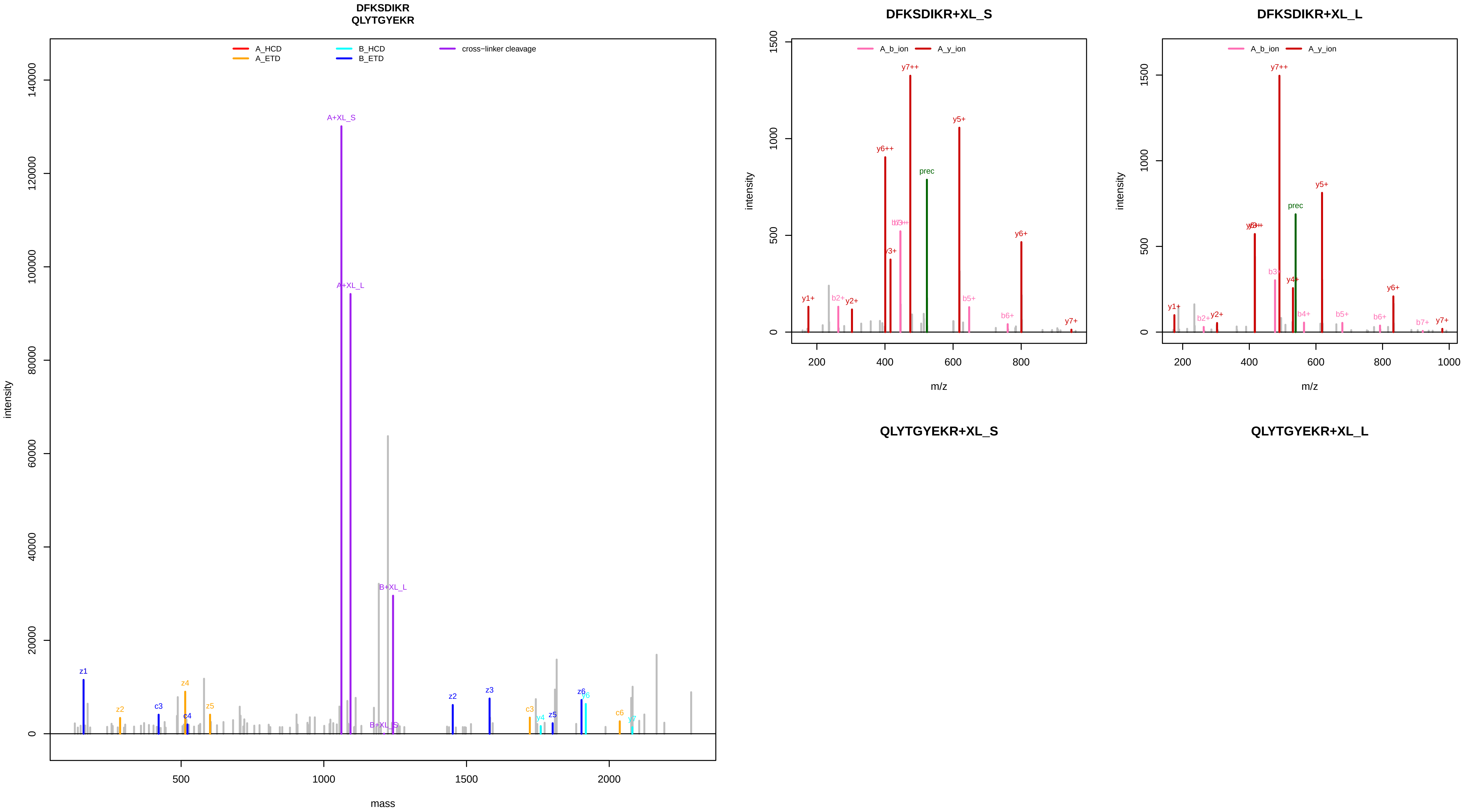
DFKSDIKR
QLYTGYEKR
A_HCD
A_ETD
B_HCD
B_ETD
cross−linker cleavage
A+XL_S
A+XL_L
B+XL_L
B+XL_S
intensity
mass
DFKSDIKR+XL_S
DFKSDIKR+XL_L
A_b_ion
A_y_ion
prec
m/z
QLYTGYEKR+XL_S
QLYTGYEKR+XL_L

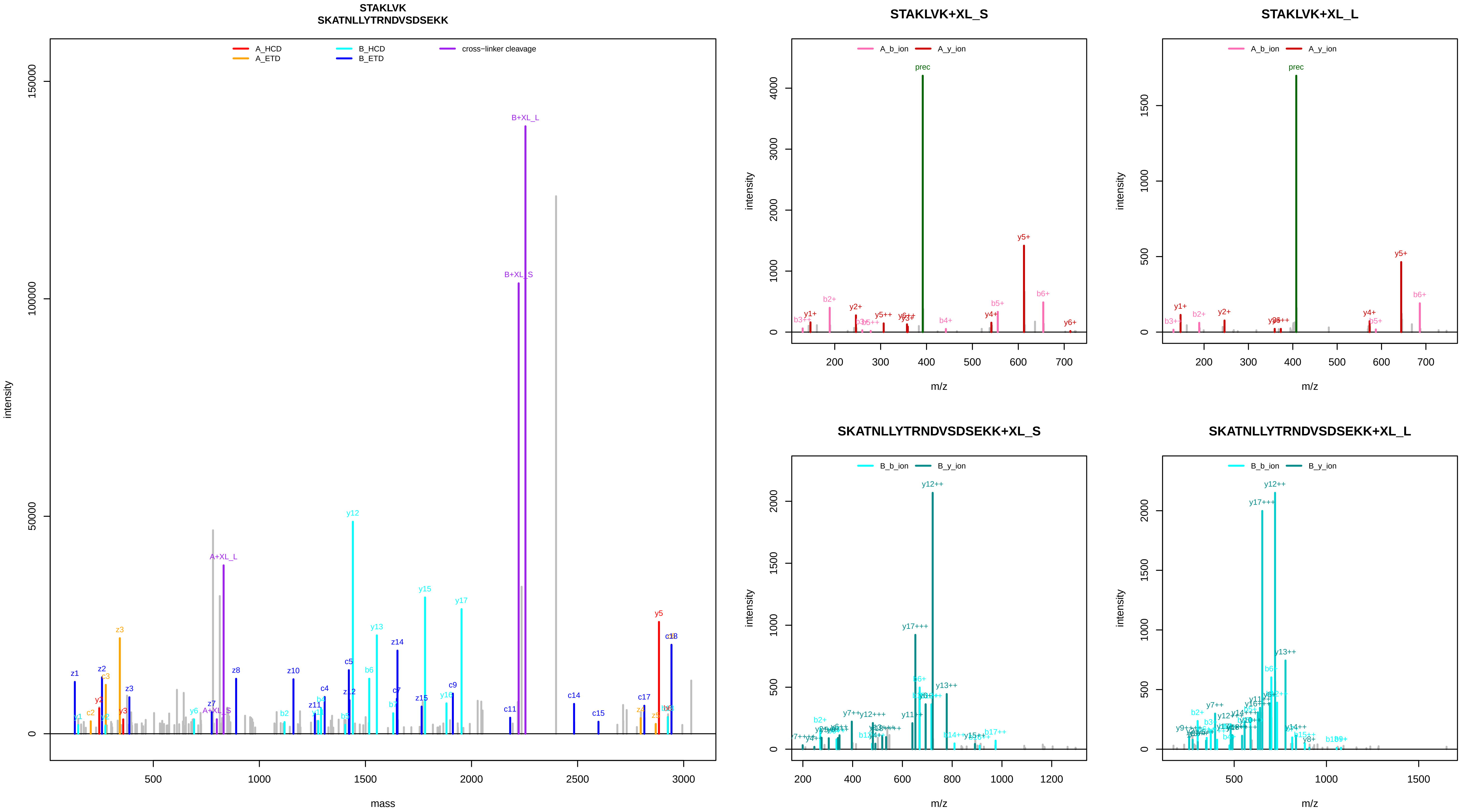
STAKLVK
SKATNLLYTRNDVSDSEKK
A_HCD
A_ETD
B_HCD
B_ETD
cross−linker cleavage
B+XL_L
B+XL_S
A+XL_L
A+XL_S
intensity
mass
STAKLVK+XL_S
STAKLVK+XL_L
A_b_ion
A_y_ion
prec
m/z
SKATNLLYTRNDVSDSEKK+XL_S
SKATNLLYTRNDVSDSEKK+XL_L
B_b_ion
B_y_ion

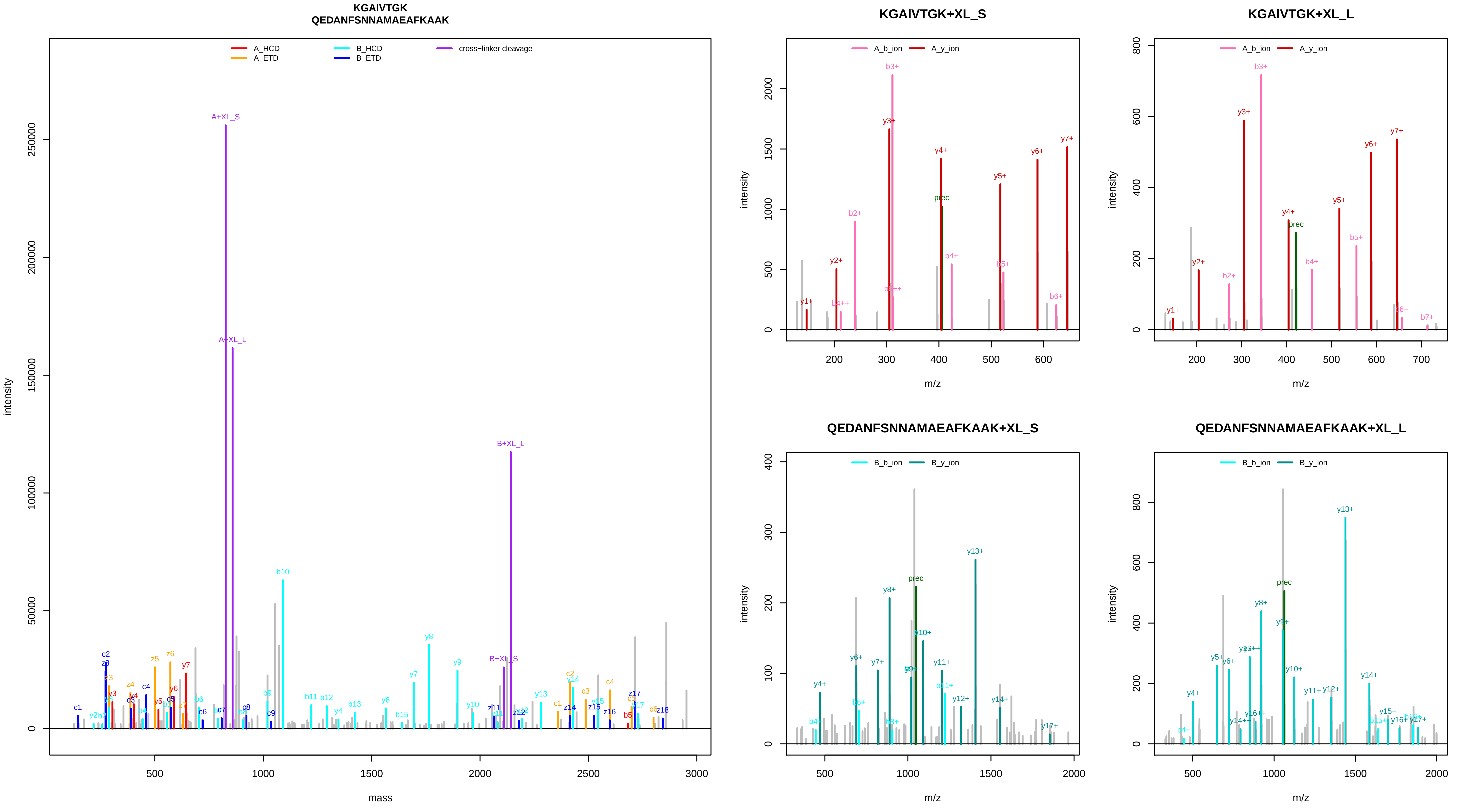
KGAIVTGK
QEDANFSNNAMAEAFKAAK
A_HCD
A_ETD
B_HCD
B_ETD
cross−linker cleavage
A+XL_S
A+XL_L
B+XL_L
B+XL_S
intensity
mass
KGAIVTGK+XL_S
KGAIVTGK+XL_L
QEDANFSNNAMAEAFKAAK+XL_S
QEDANFSNNAMAEAFKAAK+XL_L
A_b_ion
A_y_ion
B_b_ion
B_y_ion
prec
m/z

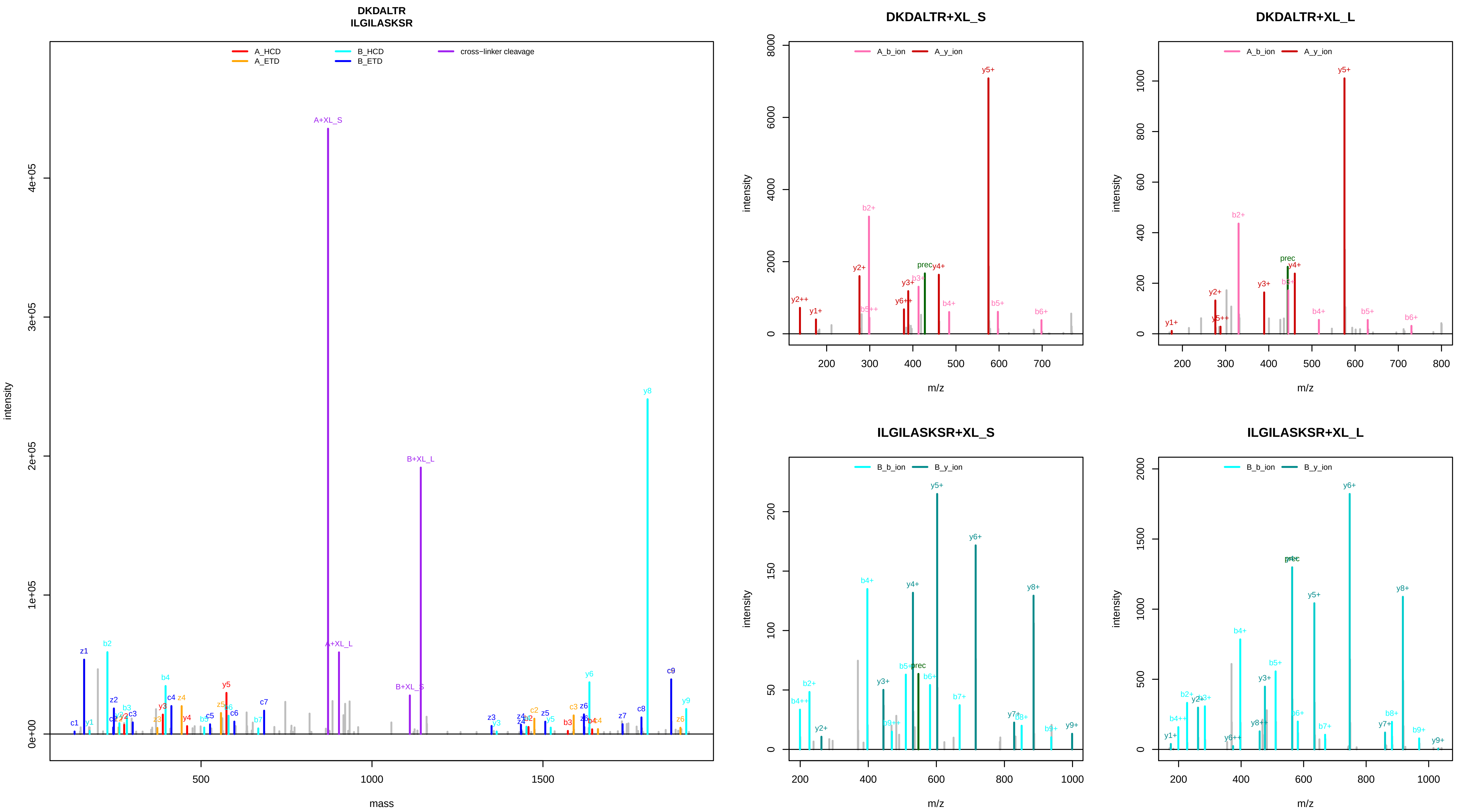
DKDALTR
ILGILASKSR
A_HCD
A_ETD
B_HCD
B_ETD
cross−linker cleavage
A+XL_S
B+XL_L
A+XL_L
B+XL_S
y8
c9
y9
z6
intensity
mass
0e+00
1e+05
2e+05
3e+05
4e+05
500
1000
1500
DKDALTR+XL_S
DKDALTR+XL_L
A_b_ion
A_y_ion
y5+
b2+
y4+
y3+
y2+
y1+
prec
b4+
b5+
b6+
m/z
ILGILASKSR+XL_S
ILGILASKSR+XL_L
B_b_ion
B_y_ion
y5+
y6+
y8+
y4+
b4+
y3+
b7+
y9+
prec

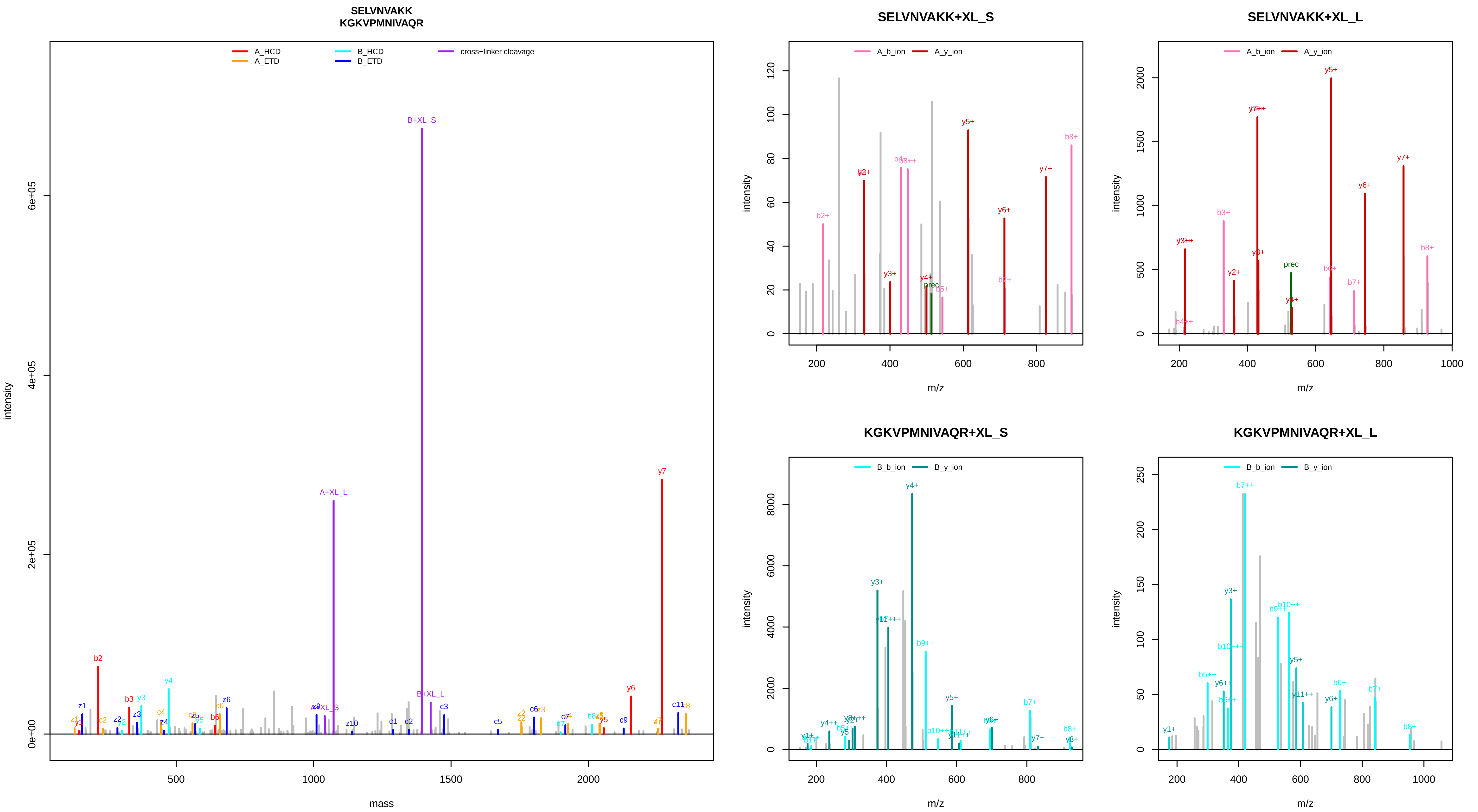
SELVNVAKK
KGKVPMNIVAQR
A_HCD
A_ETD
B_HCD
B_ETD
cross−linker cleavage
B+XL_S
A+XL_L
B+XL_L
A+XL_S
y7
y6
b2
b3
y4
intensity
mass
SELVNVAKK+XL_S
SELVNVAKK+XL_L
KGKVPMNIVAQR+XL_S
KGKVPMNIVAQR+XL_L
A_b_ion
A_y_ion
B_b_ion
B_y_ion
prec
m/z

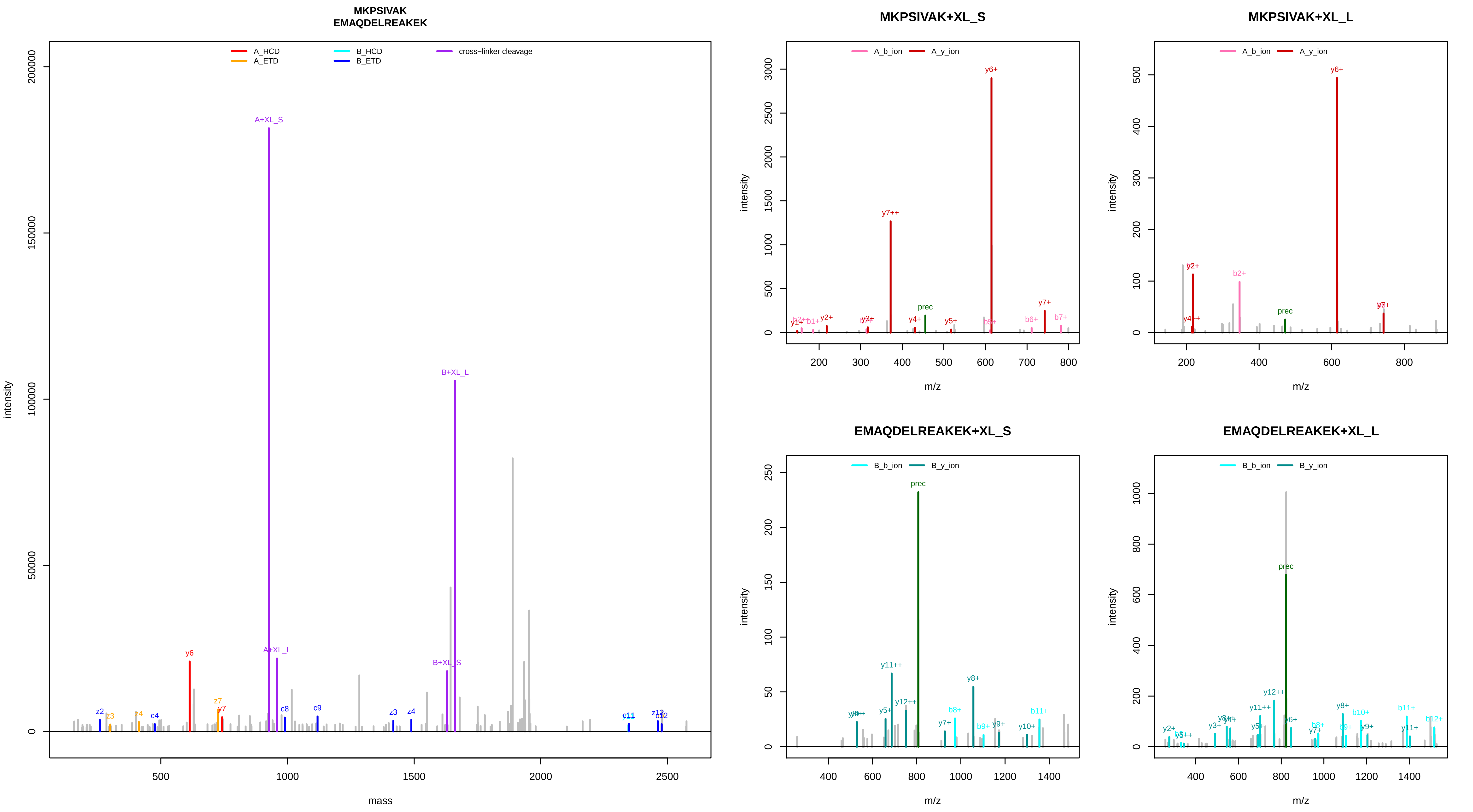
MKPSIVAK
EMAQDELREAKEK
A_HCD
A_ETD
B_HCD
B_ETD
cross−linker cleavage
A+XL_S
B+XL_L
A+XL_L
B+XL_S
intensity
mass
MKPSIVAK+XL_S
MKPSIVAK+XL_L
A_b_ion
A_y_ion
EMAQDELREAKEK+XL_S
EMAQDELREAKEK+XL_L
B_b_ion
B_y_ion
prec
m/z

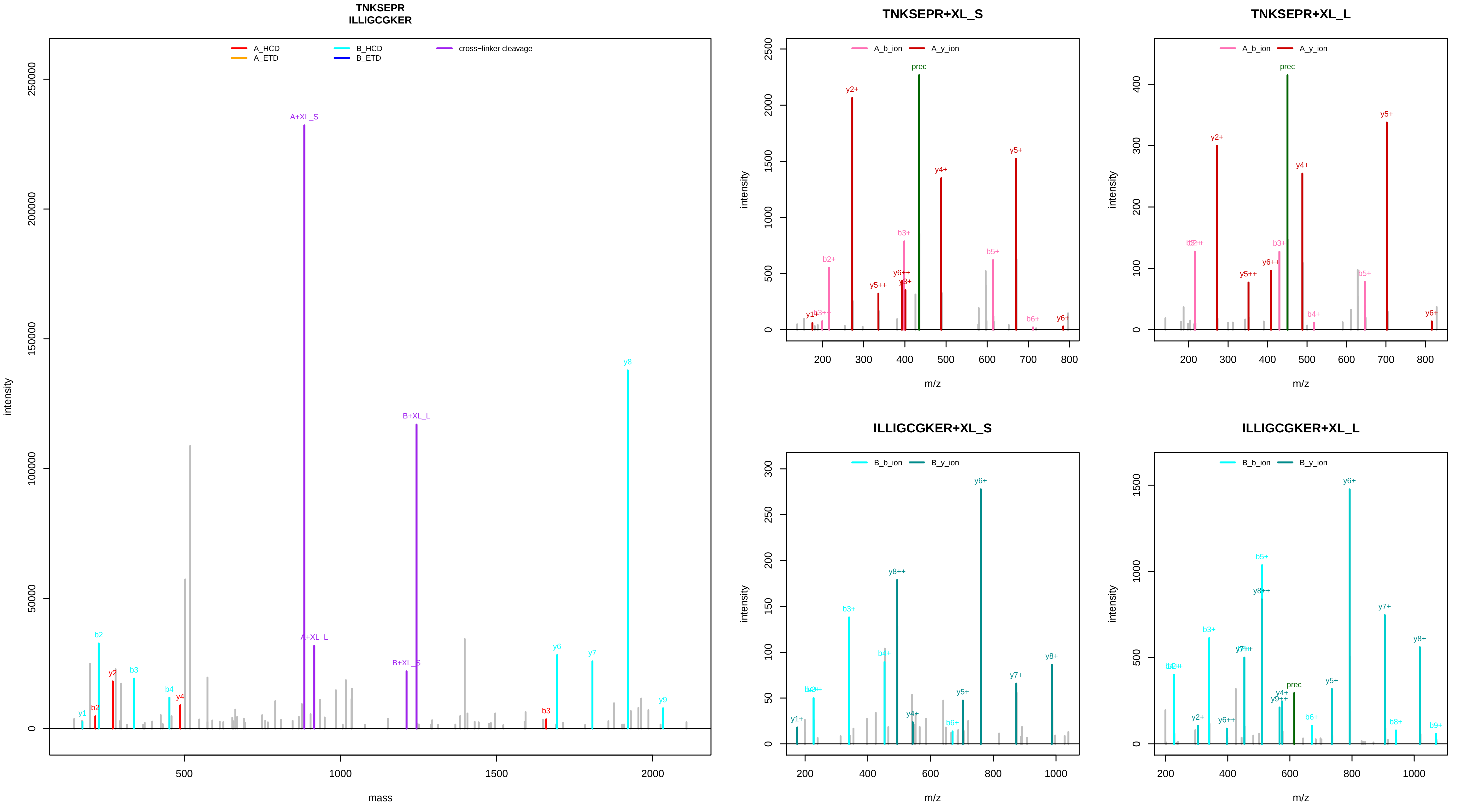

TNKSEPR
ILLIGCGKER
A_HCD
A_ETD
B_HCD
B_ETD
cross−linker cleavage
A+XL_S
B+XL_L
A+XL_L
B+XL_S
intensity
mass
TNKSEPR+XL_S
TNKSEPR+XL_L
ILLIGCGKER+XL_S
ILLIGCGKER+XL_L
A_b_ion
A_y_ion
B_b_ion
B_y_ion
prec
m/z

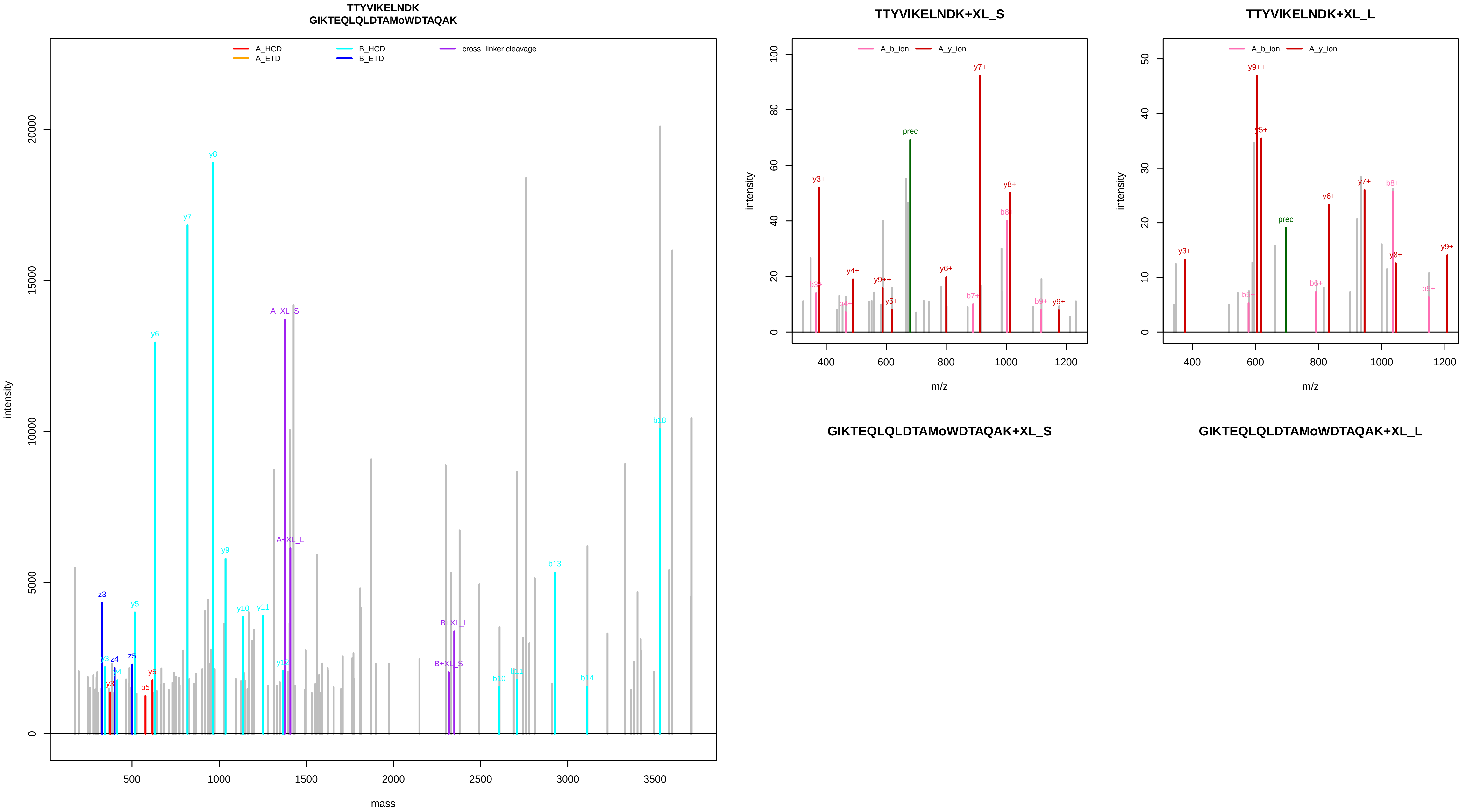
TTYVIKELNDK
GIKTEQLQLDTAMoWDTAQAK
A_HCD
A_ETD
B_HCD
B_ETD
cross−linker cleavage
intensity
mass
A+XL_S
A+XL_L
B+XL_L
B+XL_S
TTYVIKELNDK+XL_S
TTYVIKELNDK+XL_L
A_b_ion
A_y_ion
prec
m/z
GIKTEQLQLDTAMoWDTAQAK+XL_S
GIKTEQLQLDTAMoWDTAQAK+XL_L

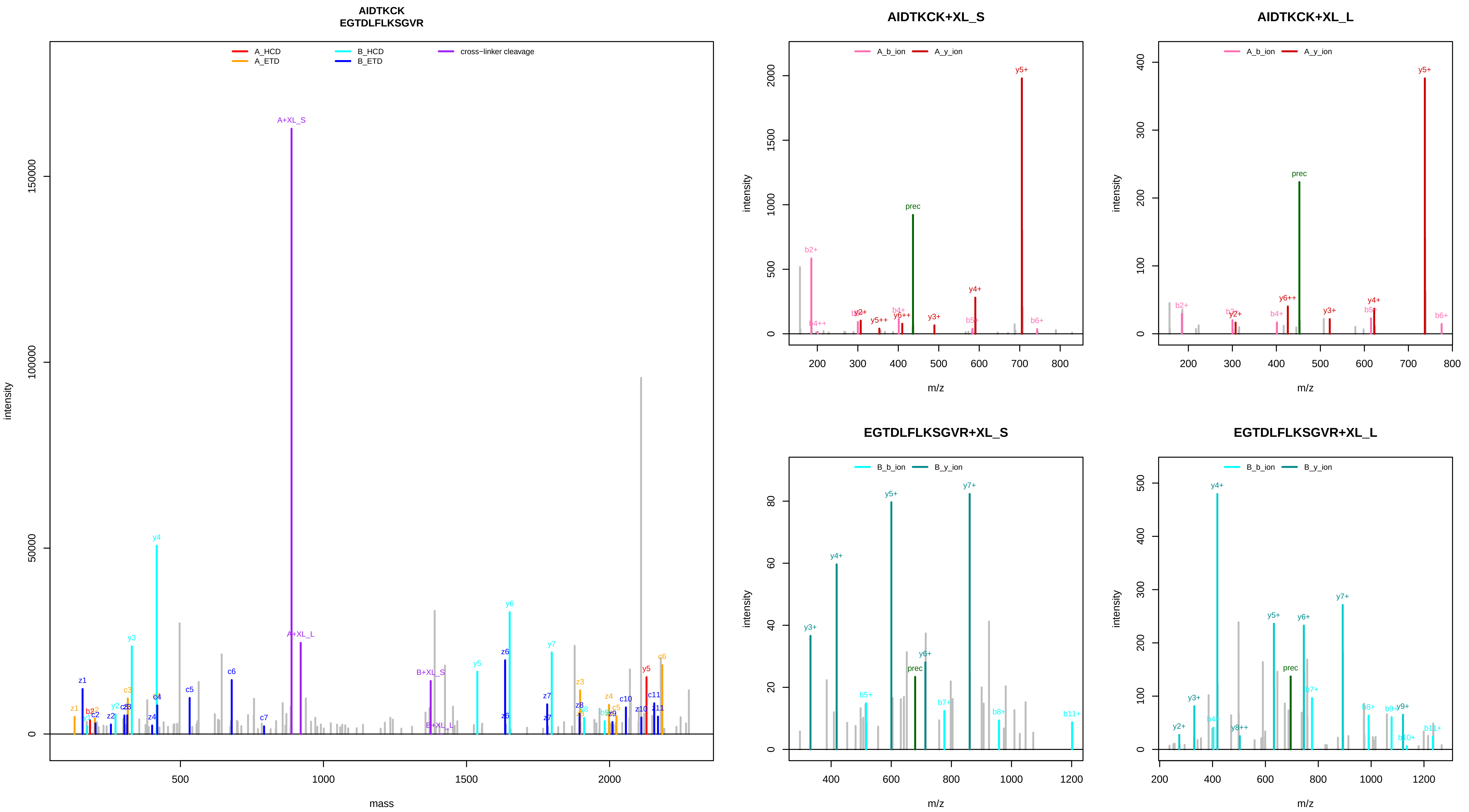

AIDTKCK
EGTDLFLKSGVR
A_HCD
A_ETD
B_HCD
B_ETD
cross−linker cleavage
A+XL_S
A+XL_L
B+XL_S
B+XL_L
intensity
mass
AIDTKCK+XL_S
AIDTKCK+XL_L
EGTDLFLKSGVR+XL_S
EGTDLFLKSGVR+XL_L
A_b_ion
A_y_ion
B_b_ion
B_y_ion
prec
m/z

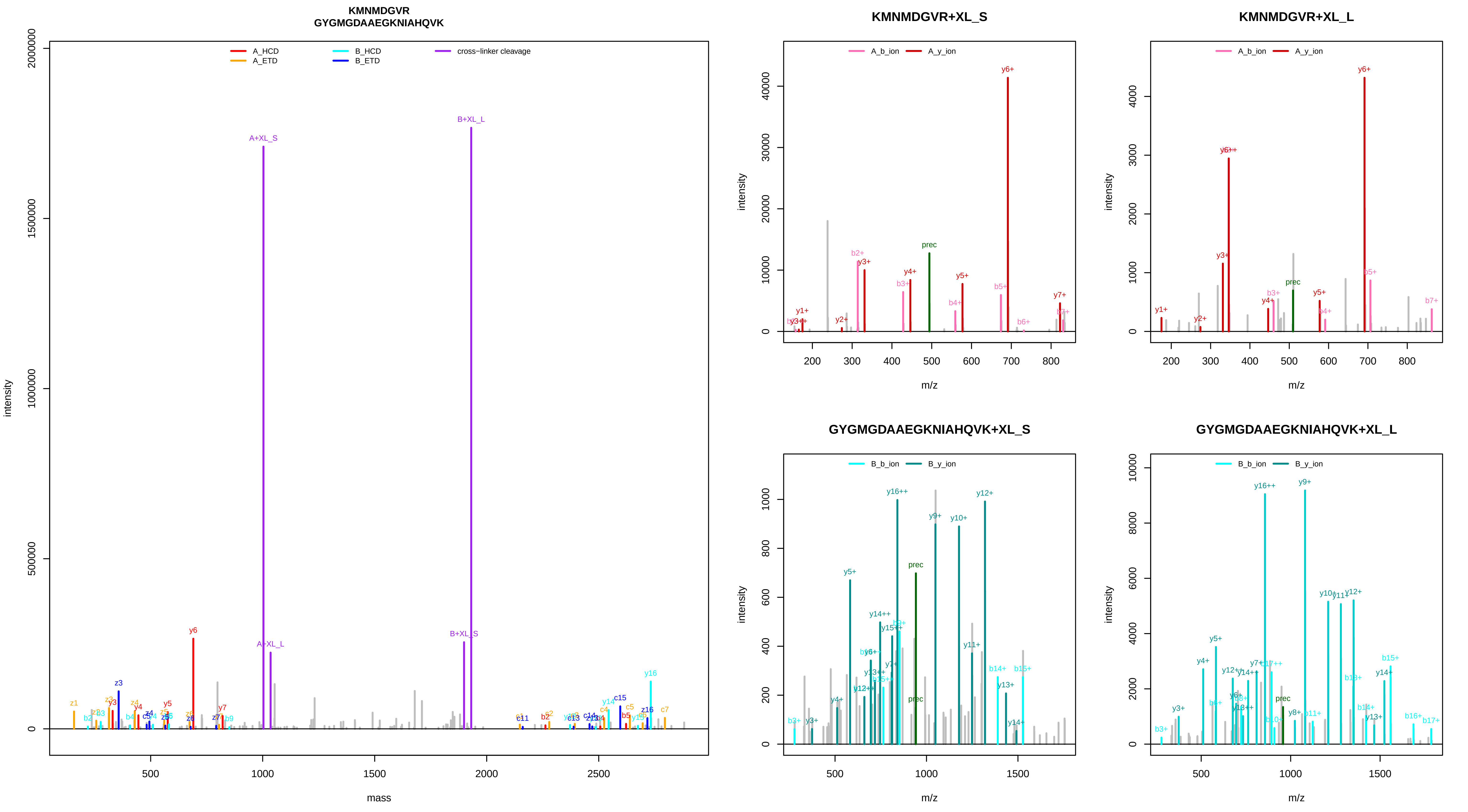
KMNMDGVR
GYGMGDAAEGKNIAHQVK
A_HCD
A_ETD
B_HCD
B_ETD
cross−linker cleavage
A+XL_S
B+XL_L
A+XL_L
B+XL_S
y6
y16
c15
z16
z3
intensity
mass
KMNMDGVR+XL_S
KMNMDGVR+XL_L
A_b_ion
A_y_ion
y6+
prec
y3+
y4+
y5+
y7+
b2+
b3+
b5+
intensity
m/z
GYGMGDAAEGKNIAHQVK+XL_S
GYGMGDAAEGKNIAHQVK+XL_L
B_b_ion
B_y_ion
y16++
y12+
y9+
y10+
y5+
prec
y11+
b14+
b15+
y14++
intensity
m/z

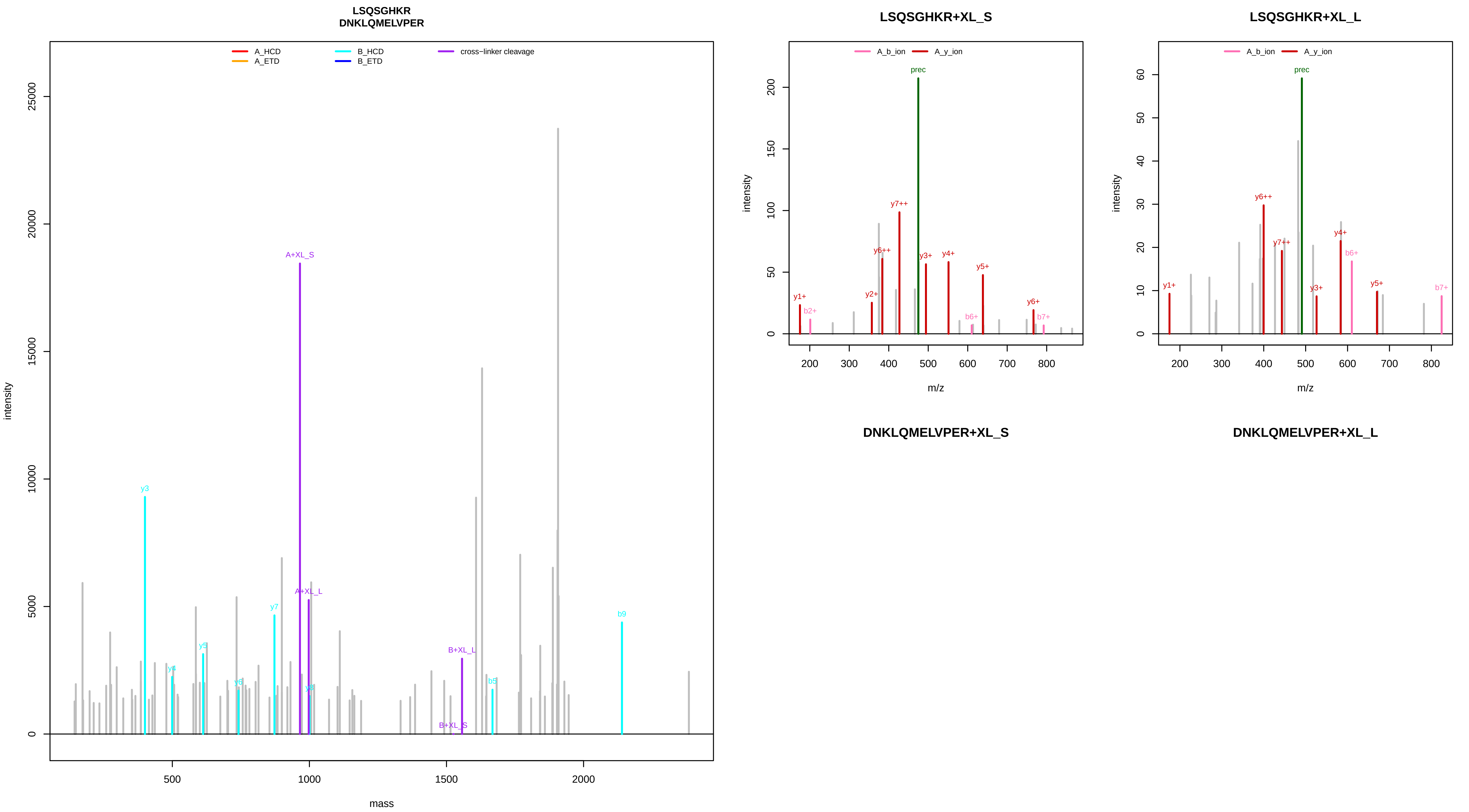
LSQSGHKR
DNKLQMELVPER
A_HCD
A_ETD
B_HCD
B_ETD
cross−linker cleavage
A+XL_S
A+XL_L
B+XL_L
B+XL_S
y3
y4
y5
y6
y7
y8
b5
b9
intensity
mass
LSQSGHKR+XL_S
LSQSGHKR+XL_L
A_b_ion
A_y_ion
prec
y1+
b2+
y2+
y6++
y7++
y3+
y4+
b6+
y5+
y6+
b7+
m/z
DNKLQMELVPER+XL_S
DNKLQMELVPER+XL_L

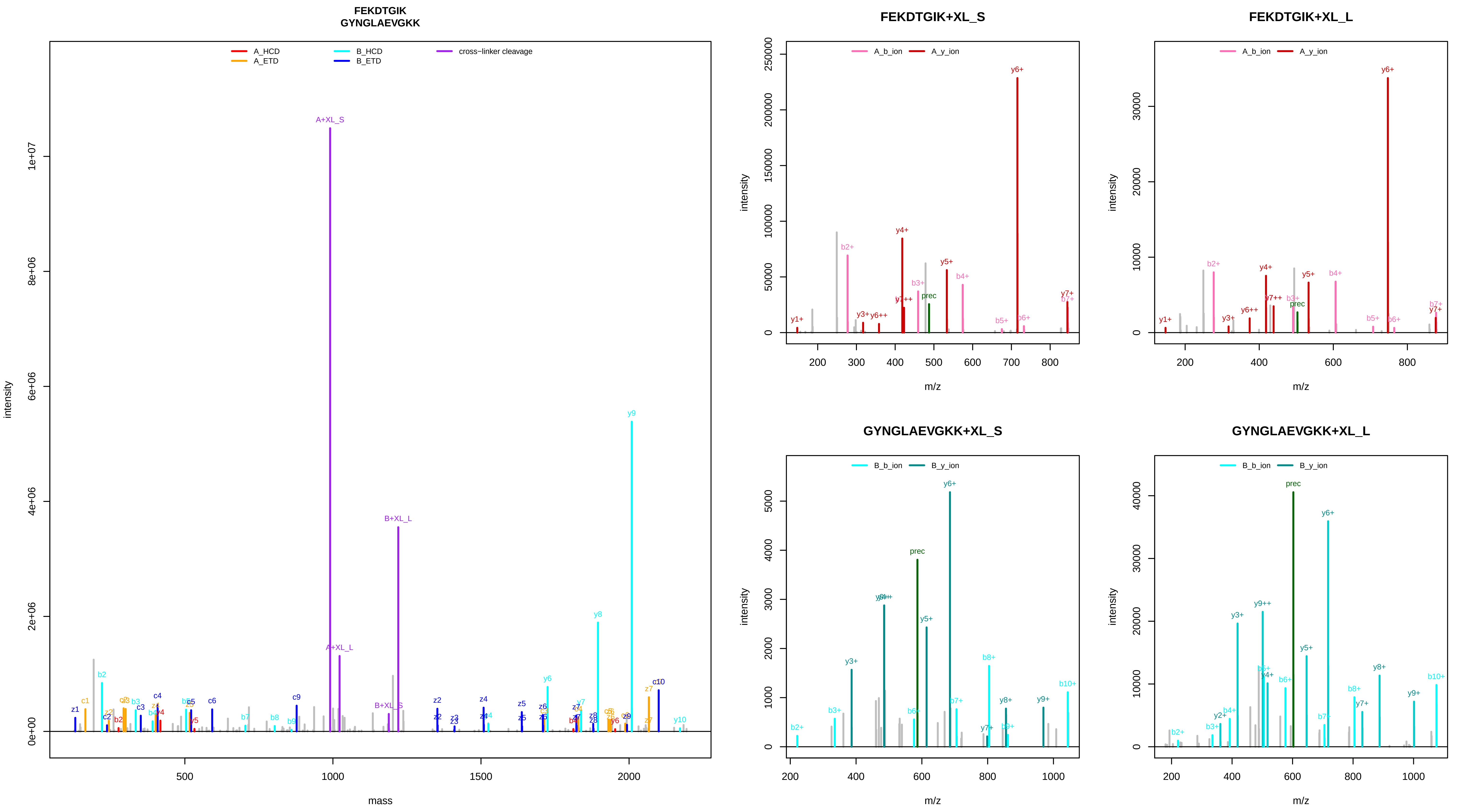
FEKDTGIK
GYNGLAEVGKK
A_HCD
A_ETD
B_HCD
B_ETD
cross−linker cleavage
A+XL_S
B+XL_L
A+XL_L
B+XL_S
intensity
mass
FEKDTGIK+XL_S
FEKDTGIK+XL_L
GYNGLAEVGKK+XL_S
GYNGLAEVGKK+XL_L
A_b_ion
A_y_ion
B_b_ion
B_y_ion
prec
m/z

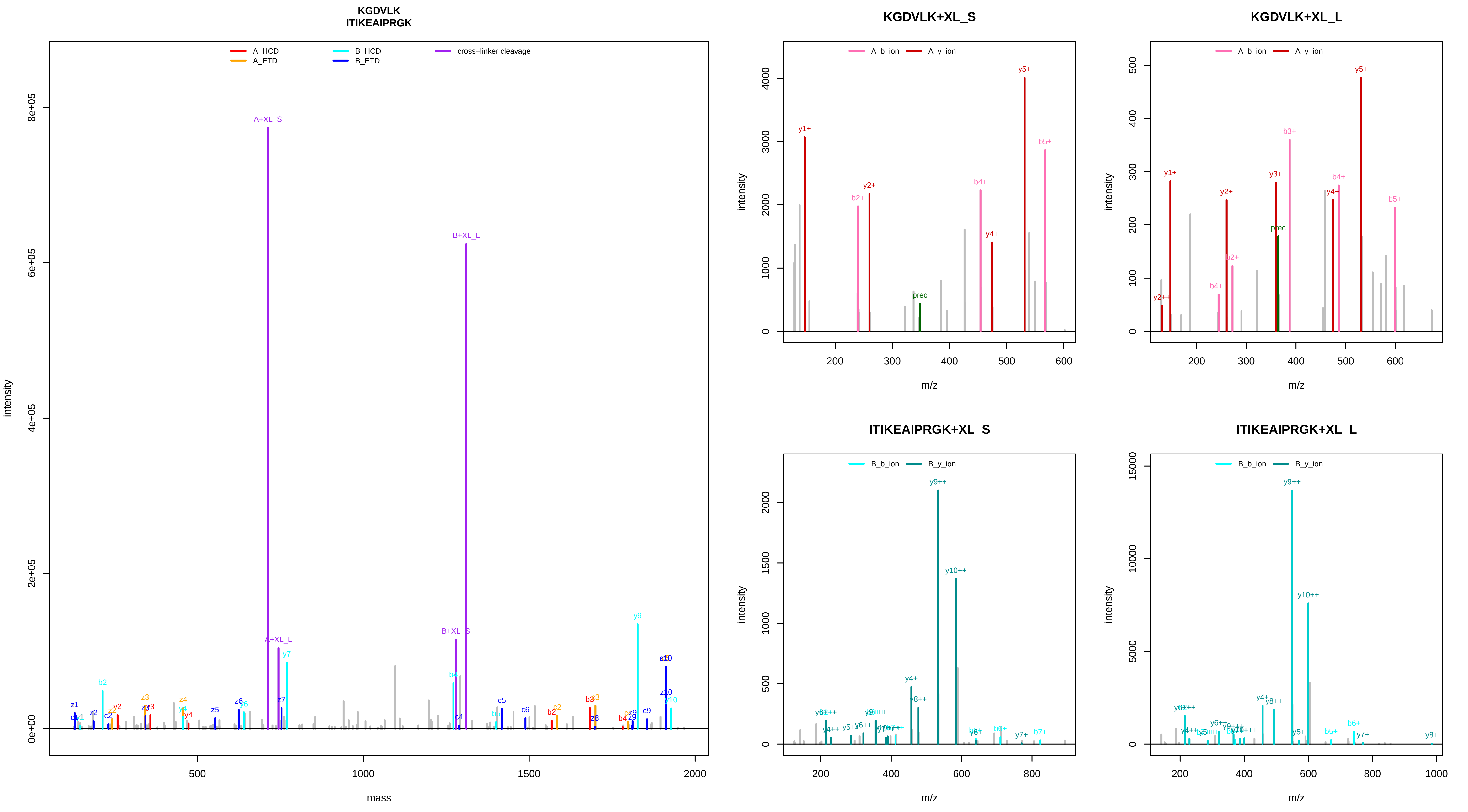

KGDVLK
ITIKEAIPRGK
A_HCD
A_ETD
B_HCD
B_ETD
cross−linker cleavage
A+XL_S
B+XL_L
A+XL_L
B+XL_S
intensity
mass
KGDVLK+XL_S
KGDVLK+XL_L
ITIKEAIPRGK+XL_S
ITIKEAIPRGK+XL_L
A_b_ion
A_y_ion
B_b_ion
B_y_ion
prec
m/z

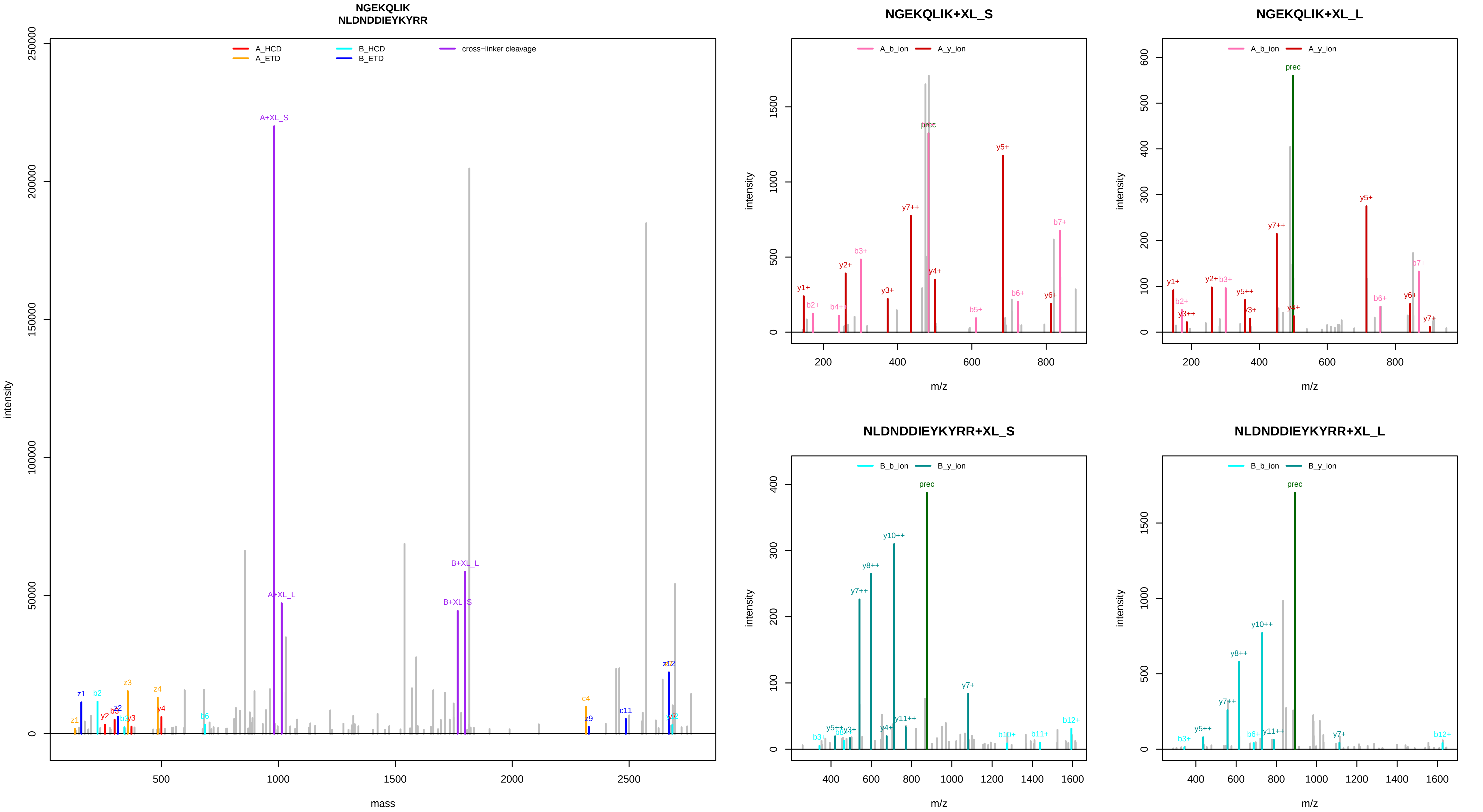
NGEKQLIK
NLDNDDIEYKYRR
A_HCD
A_ETD
B_HCD
B_ETD
cross−linker cleavage
A+XL_S
A+XL_L
B+XL_L
B+XL_S
intensity
mass
NGEKQLIK+XL_S
NGEKQLIK+XL_L
A_b_ion
A_y_ion
prec
m/z
NLDNDDIEYKYRR+XL_S
NLDNDDIEYKYRR+XL_L
B_b_ion
B_y_ion

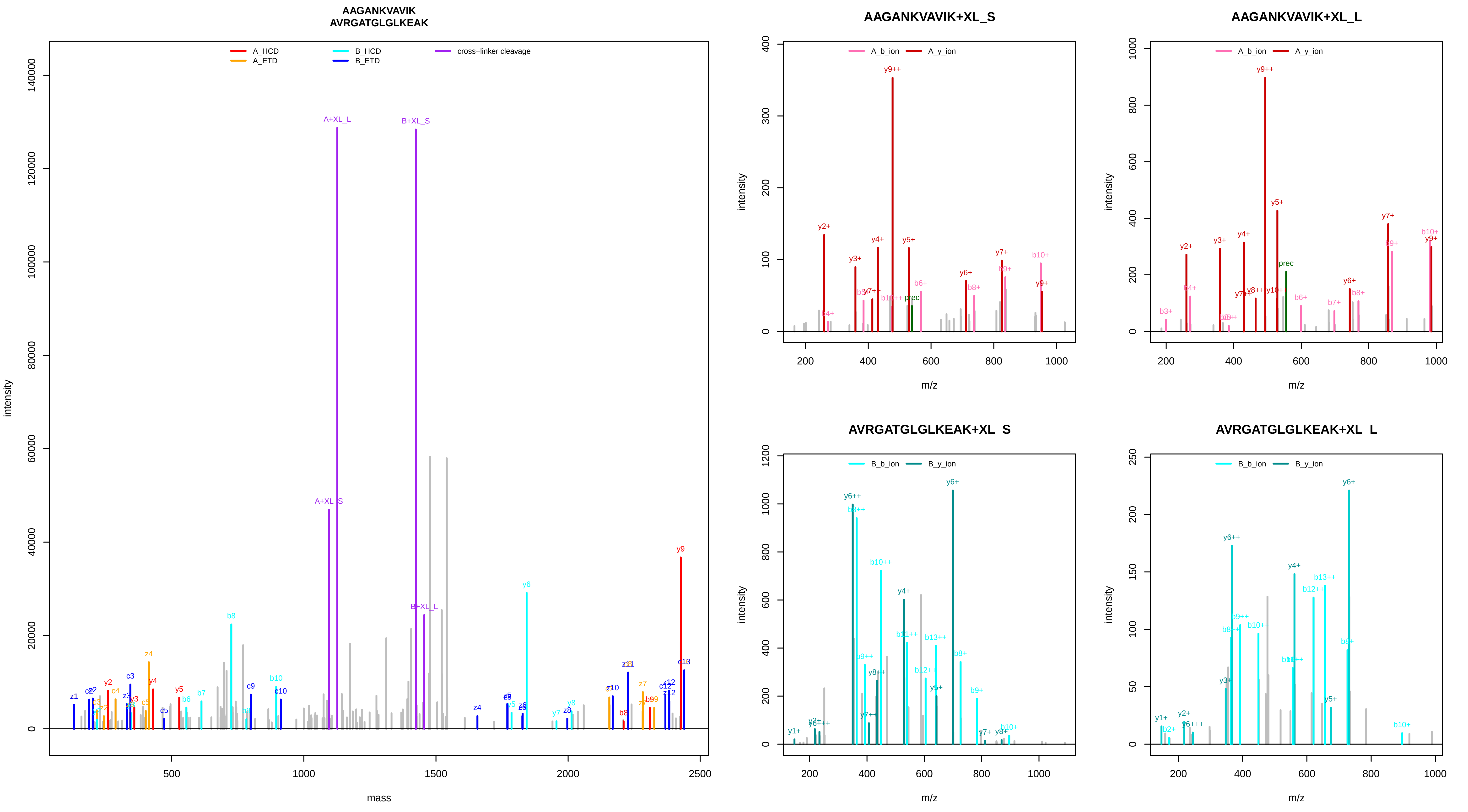
AAGANKVAVIK
AVRGATGLGLKEAK
A_HCD
A_ETD
B_HCD
B_ETD
cross−linker cleavage
A+XL_L
B+XL_S
A+XL_S
B+XL_L
intensity
mass
AAGANKVAVIK+XL_S
AAGANKVAVIK+XL_L
A_b_ion
A_y_ion
AVRGATGLGLKEAK+XL_S
AVRGATGLGLKEAK+XL_L
B_b_ion
B_y_ion
m/z

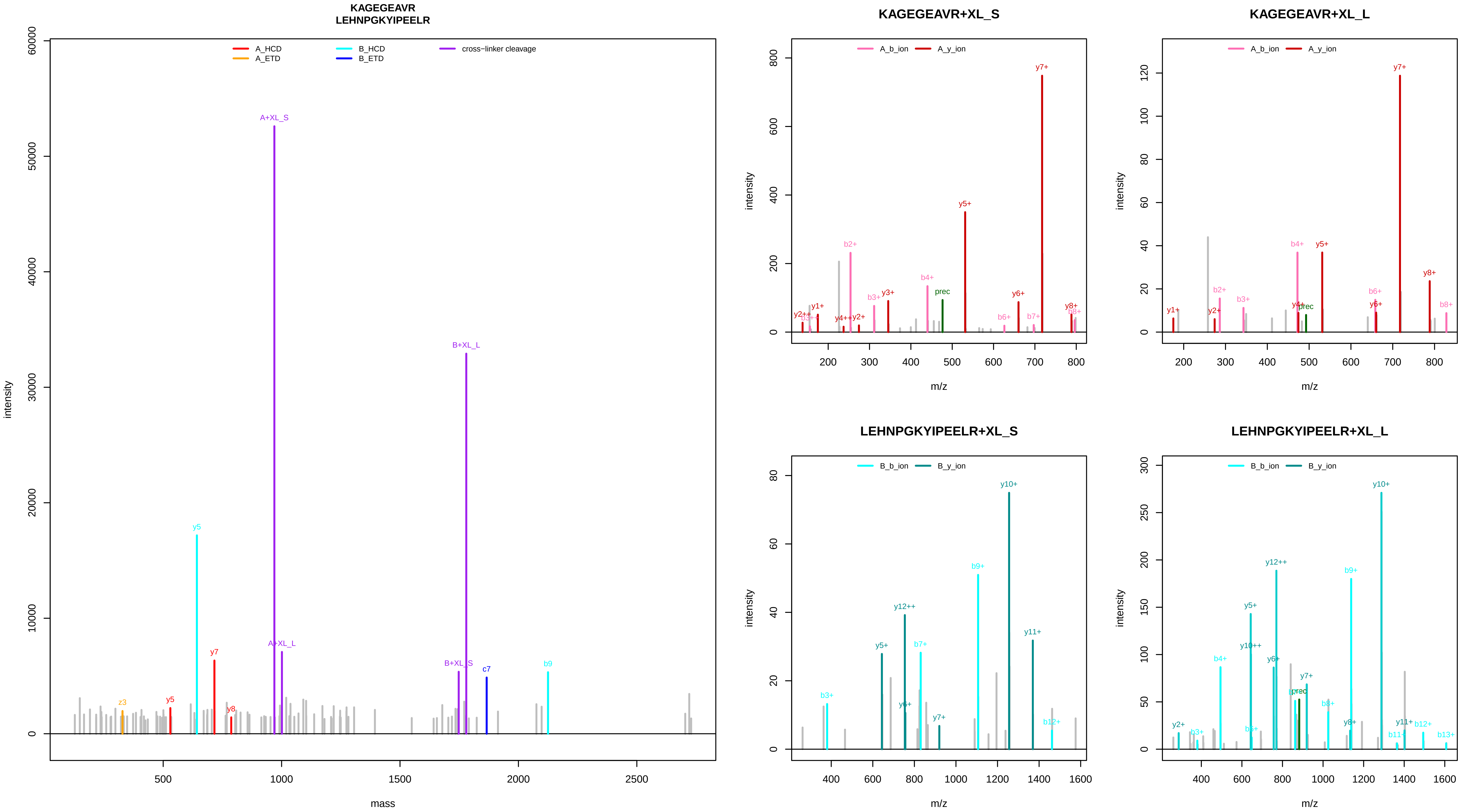

KAGEGEAVR
LEHNPGKYIPEELR
A_HCD
A_ETD
B_HCD
B_ETD
cross−linker cleavage
A+XL_S
B+XL_L
A+XL_L
B+XL_S
intensity
mass
KAGEGEAVR+XL_S
KAGEGEAVR+XL_L
LEHNPGKYIPEELR+XL_S
LEHNPGKYIPEELR+XL_L
A_b_ion
A_y_ion
B_b_ion
B_y_ion
m/z
prec

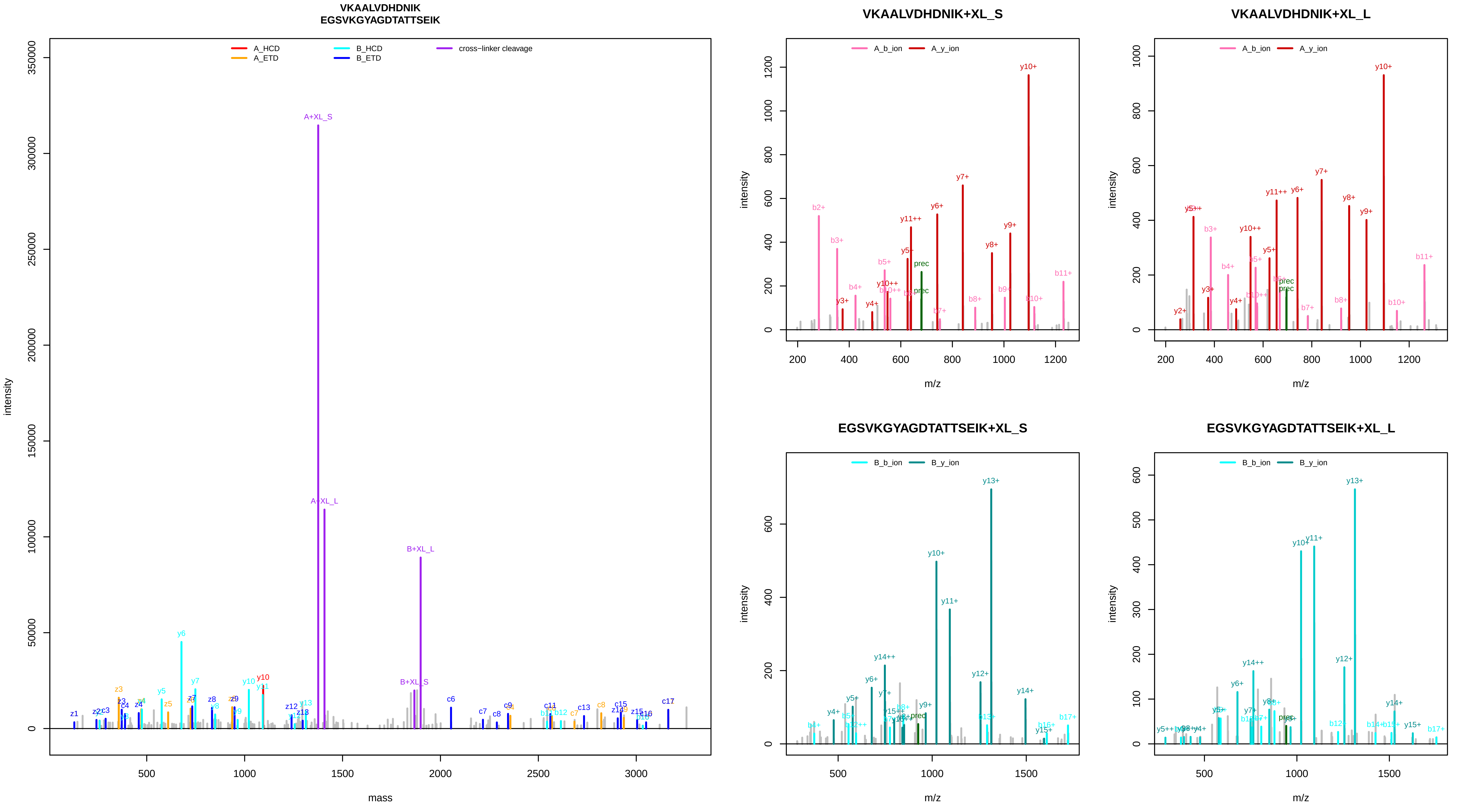

VKAALVDHDNIK
EGSVKGYAGDTATTSEIK
A_HCD
A_ETD
B_HCD
B_ETD
cross−linker cleavage
A+XL_S
A+XL_L
B+XL_L
B+XL_S
intensity
mass
VKAALVDHDNIK+XL_S
VKAALVDHDNIK+XL_L
A_b_ion
A_y_ion
EGSVKGYAGDTATTSEIK+XL_S
EGSVKGYAGDTATTSEIK+XL_L
B_b_ion
B_y_ion
m/z

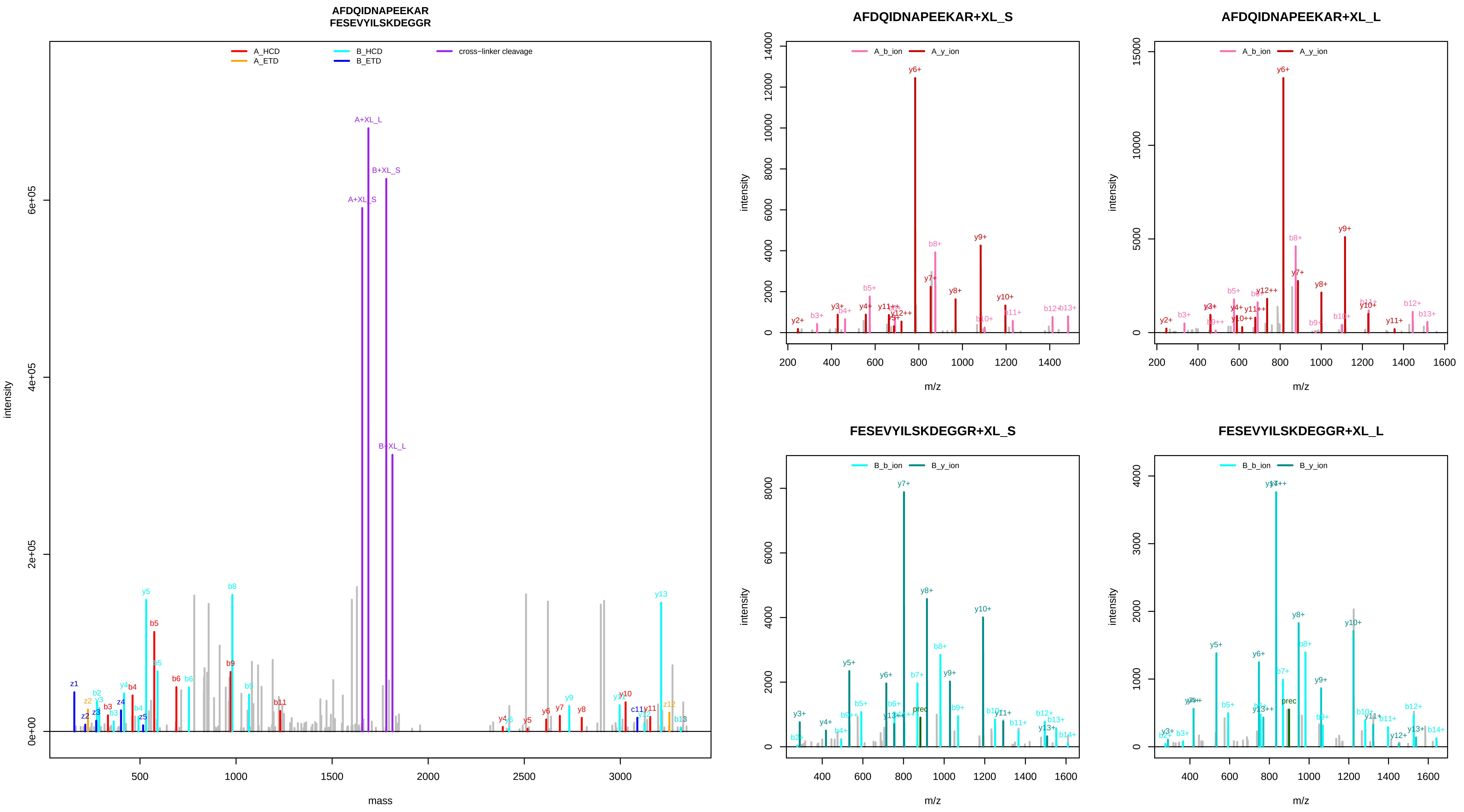
AFDQIDNAPEEKAR
FESEVYILSKDEGGR
A_HCD
A_ETD
B_HCD
B_ETD
cross−linker cleavage
A+XL_L
B+XL_S
A+XL_S
B+XL_L
intensity
mass
AFDQIDNAPEEKAR+XL_S
AFDQIDNAPEEKAR+XL_L
FESEVYILSKDEGGR+XL_S
FESEVYILSKDEGGR+XL_L
A_b_ion
A_y_ion
B_b_ion
B_y_ion
m/z

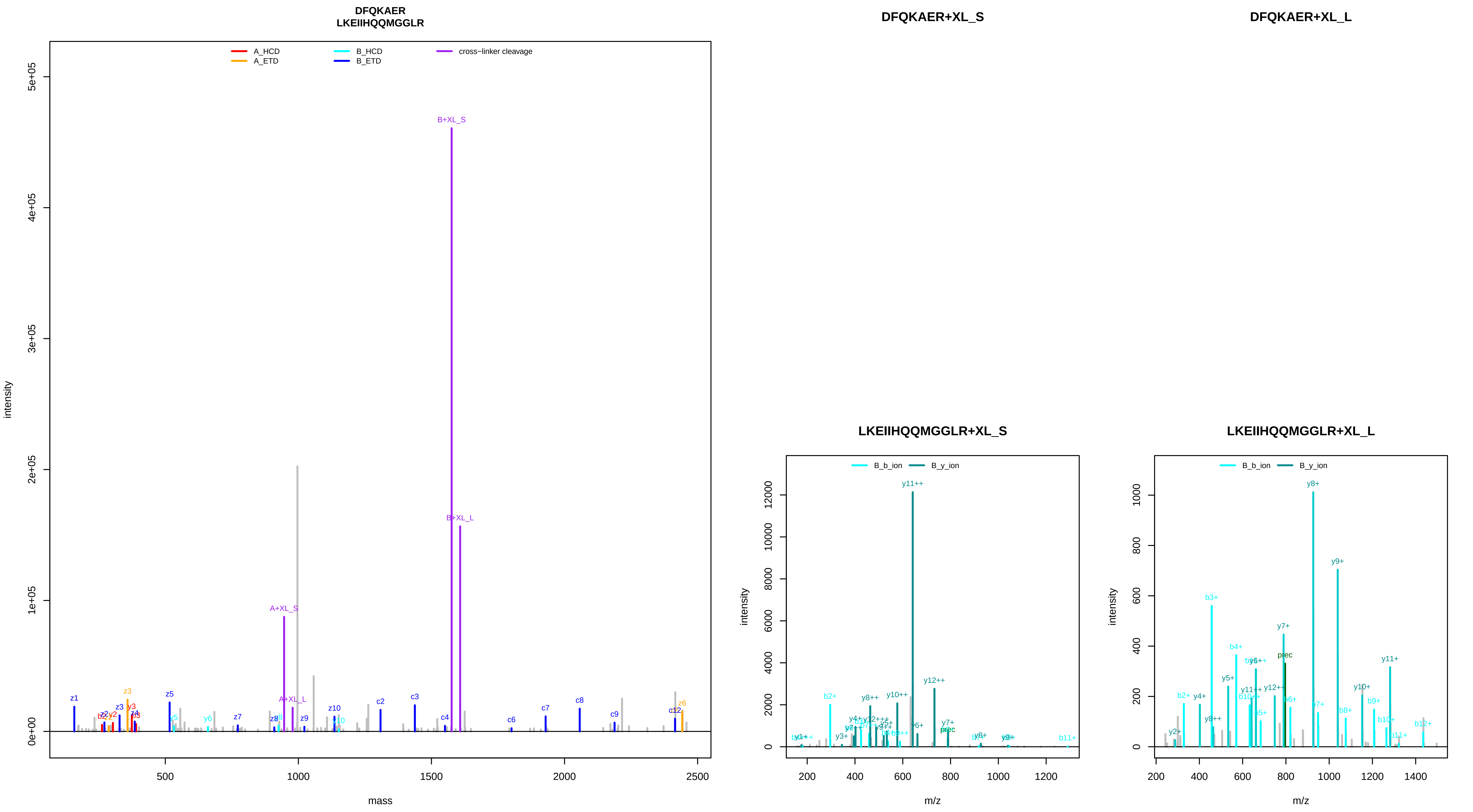
DFQKAER
LKEIIHQQMGGLR
A_HCD
A_ETD
B_HCD
B_ETD
cross−linker cleavage
B+XL_S
B+XL_L
A+XL_S
A+XL_L
intensity
mass
DFQKAER+XL_S
DFQKAER+XL_L
LKEIIHQQMGGLR+XL_S
LKEIIHQQMGGLR+XL_L
B_b_ion
B_y_ion
y11++
y12++
y10++
y8++
b2+
y8+
y9+
b3+
y7+
prec
y11+
intensity
m/z
m/z

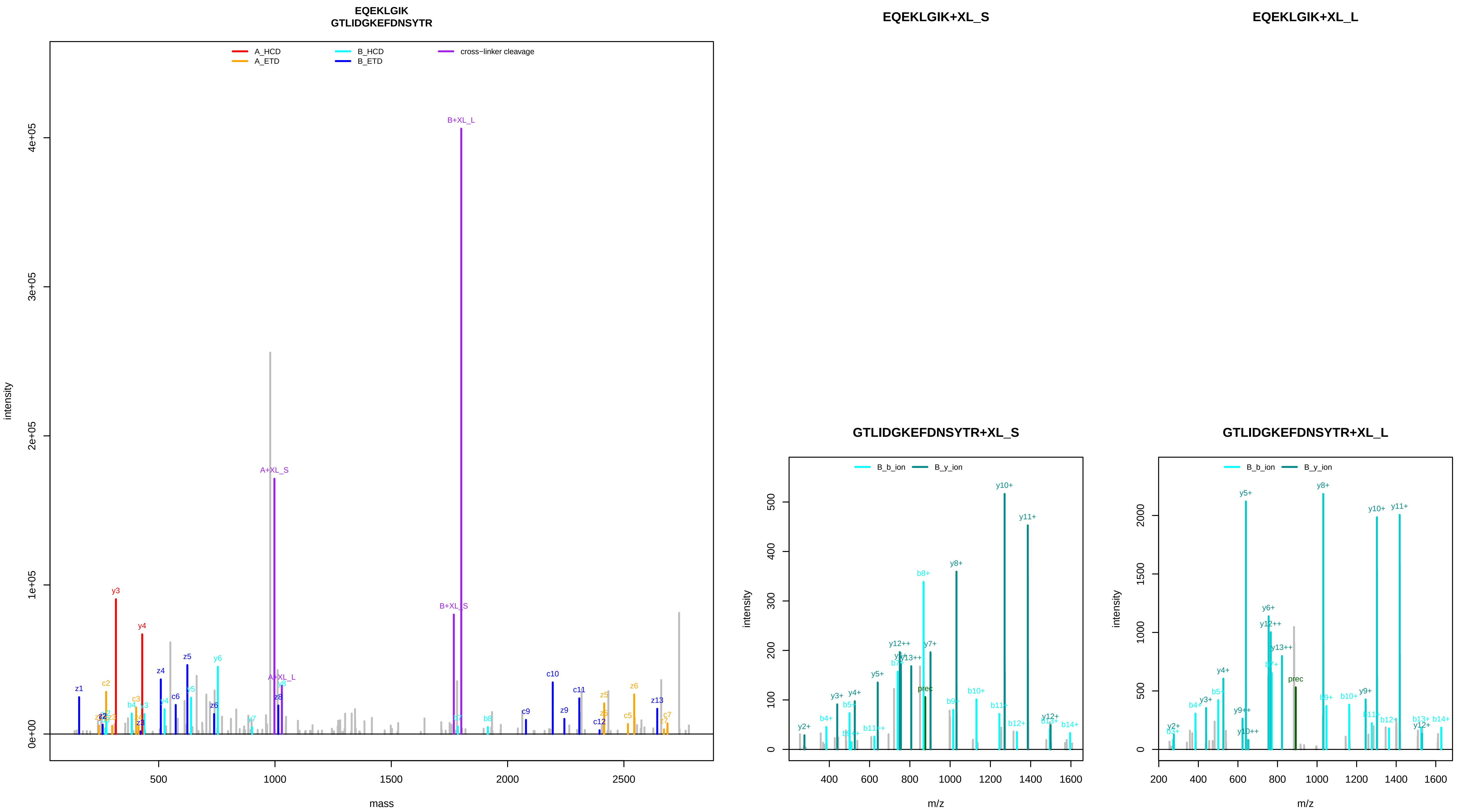
EQEKLGIK
GTLIDGKEFDNSYTR
A_HCD
A_ETD
B_HCD
B_ETD
cross−linker cleavage
B+XL_L
A+XL_S
B+XL_S
A+XL_L
intensity
mass
EQEKLGIK+XL_S
EQEKLGIK+XL_L
GTLIDGKEFDNSYTR+XL_S
GTLIDGKEFDNSYTR+XL_L
B_b_ion
B_y_ion
prec
m/z

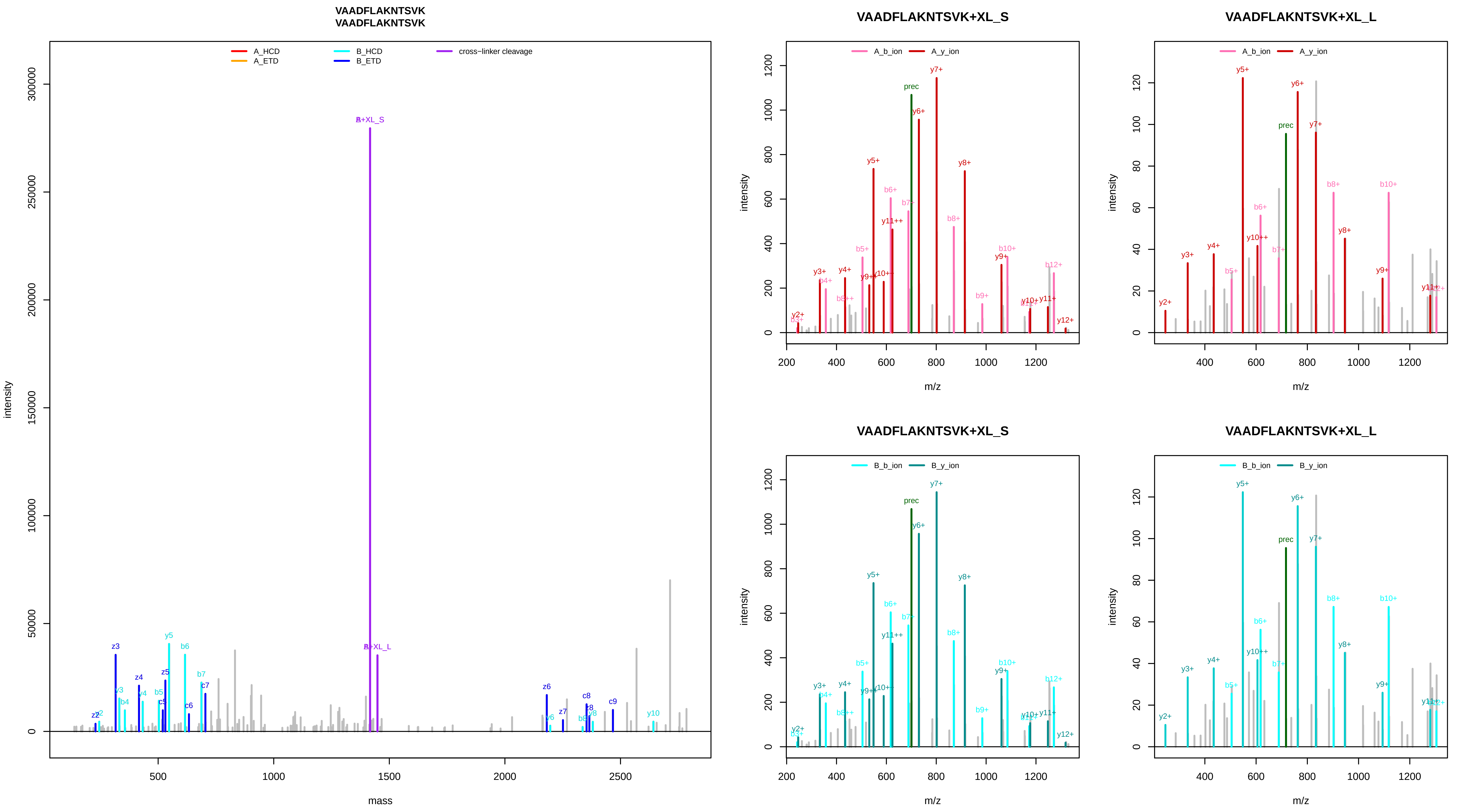
VAADFLAKNTSVK
VAADFLAKNTSVK
A_HCD
A_ETD
B_HCD
B_ETD
cross−linker cleavage
intensity
mass
VAADFLAKNTSVK+XL_S
VAADFLAKNTSVK+XL_L
A_b_ion
A_y_ion
B_b_ion
B_y_ion
prec
intensity
m/z

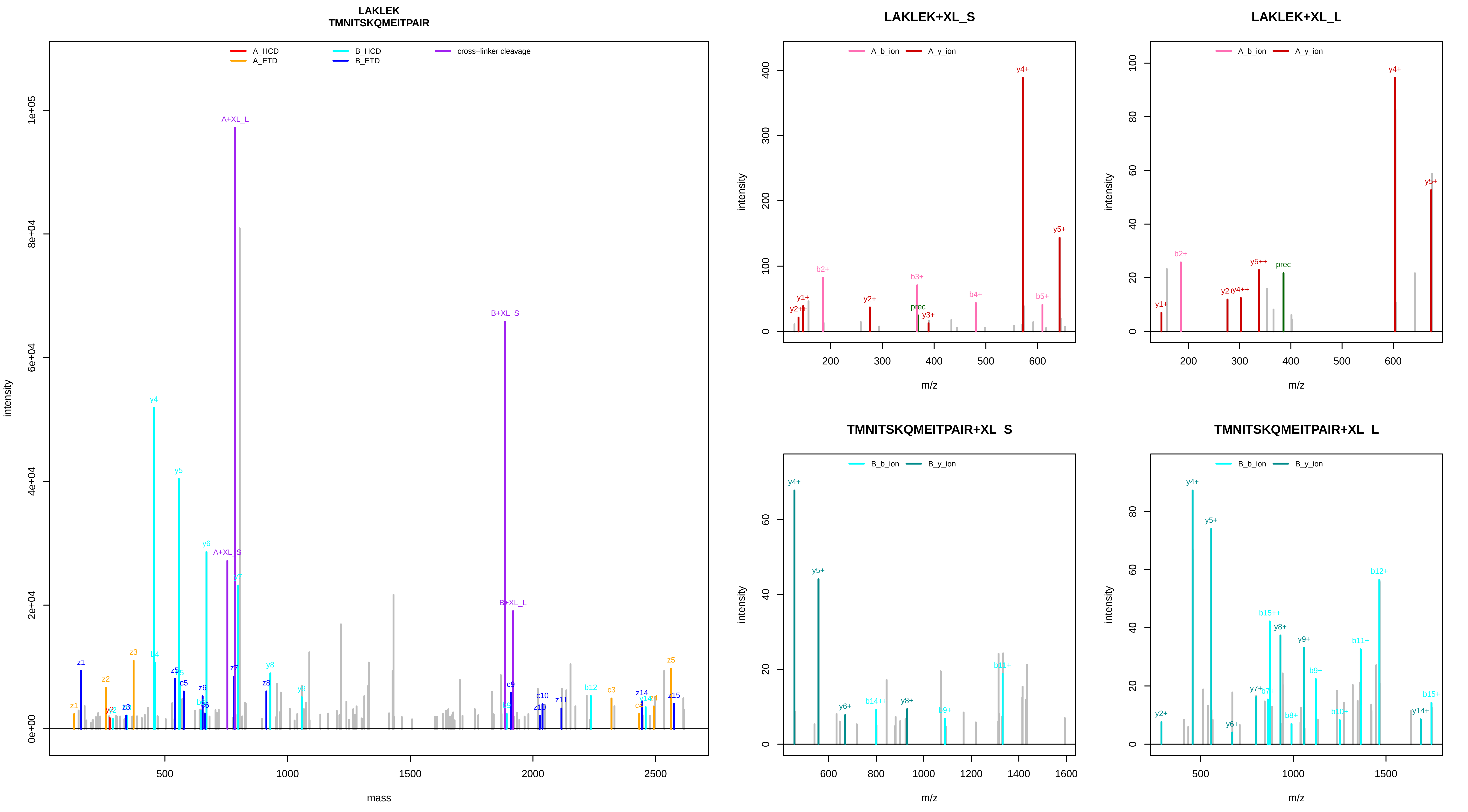

LAKLEK
TMNITSKQMEITPAIR
A_HCD
A_ETD
B_HCD
B_ETD
cross−linker cleavage
A+XL_L
B+XL_S
A+XL_S
B+XL_L
intensity
mass
LAKLEK+XL_S
LAKLEK+XL_L
A_b_ion
A_y_ion
TMNITSKQMEITPAIR+XL_S
TMNITSKQMEITPAIR+XL_L
B_b_ion
B_y_ion
m/z

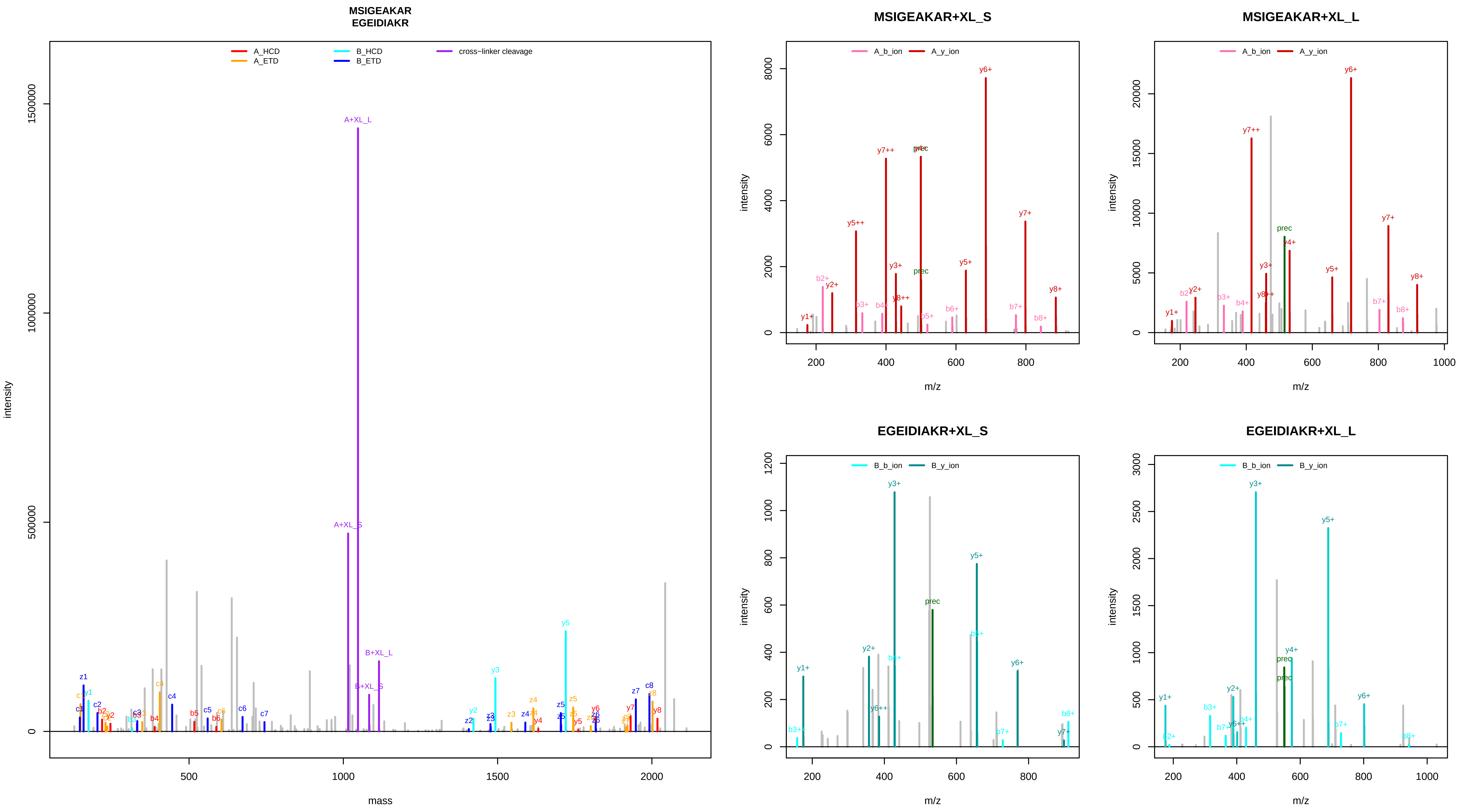

MSIGEAKAR
EGEIDIAKR
A_HCD
A_ETD
B_HCD
B_ETD
cross−linker cleavage
A+XL_L
A+XL_S
B+XL_L
B+XL_S
y5
y3
intensity
mass
0
500000
1000000
1500000
500
1000
1500
2000
MSIGEAKAR+XL_S
MSIGEAKAR+XL_L
EGEIDIAKR+XL_S
EGEIDIAKR+XL_L
A_b_ion
A_y_ion
B_b_ion
B_y_ion
prec
y6+
y7+
y7++
y3+
y5+
y4+
intensity
m/z

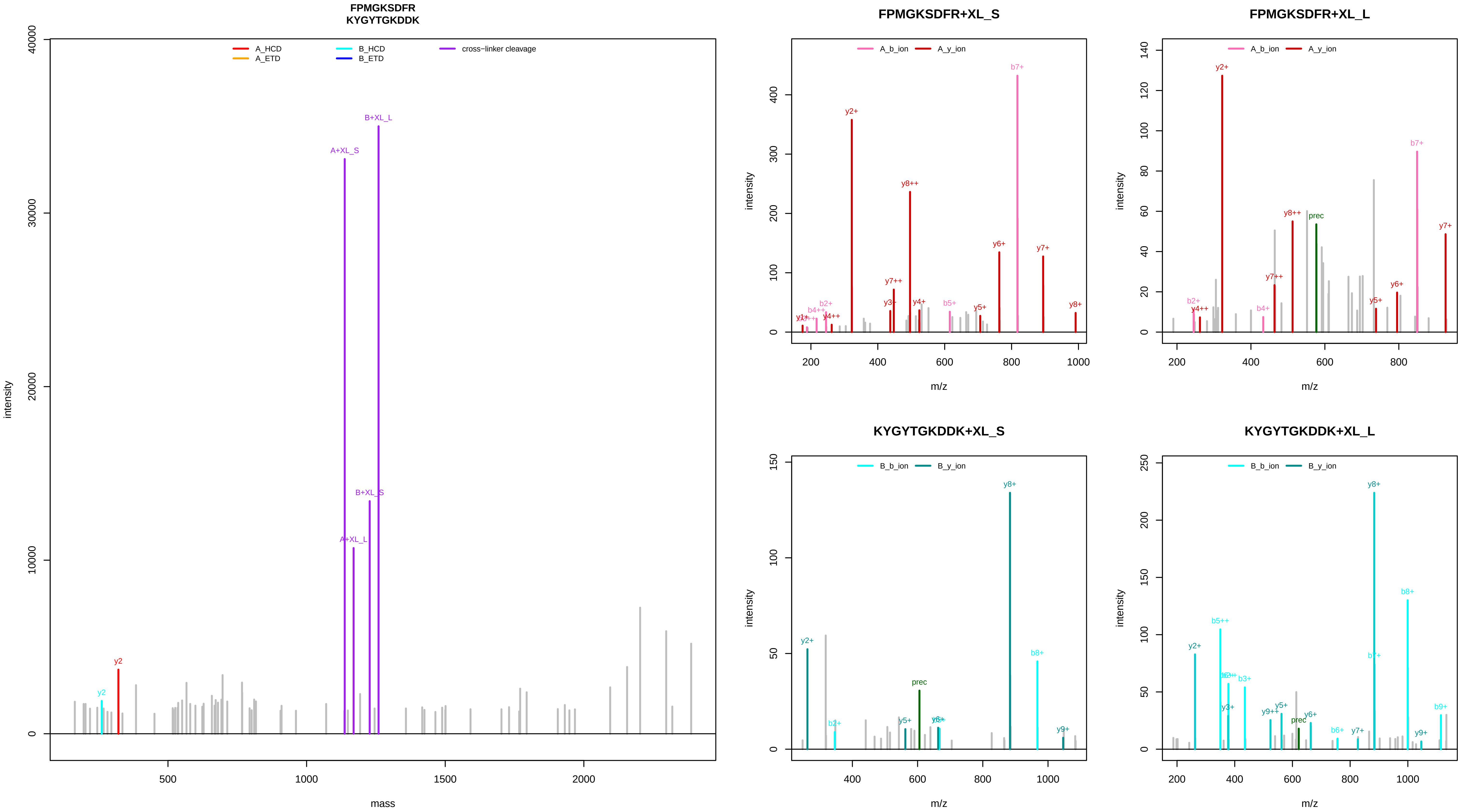
FPMGKSDFR
KYGYTGKDDK
A_HCD
A_ETD
B_HCD
B_ETD
cross−linker cleavage
B+XL_L
A+XL_S
B+XL_S
A+XL_L
y2
intensity
mass
FPMGKSDFR+XL_S
A_b_ion
A_y_ion
FPMGKSDFR+XL_L
KYGYTGKDDK+XL_S
B_b_ion
B_y_ion
KYGYTGKDDK+XL_L
prec
m/z

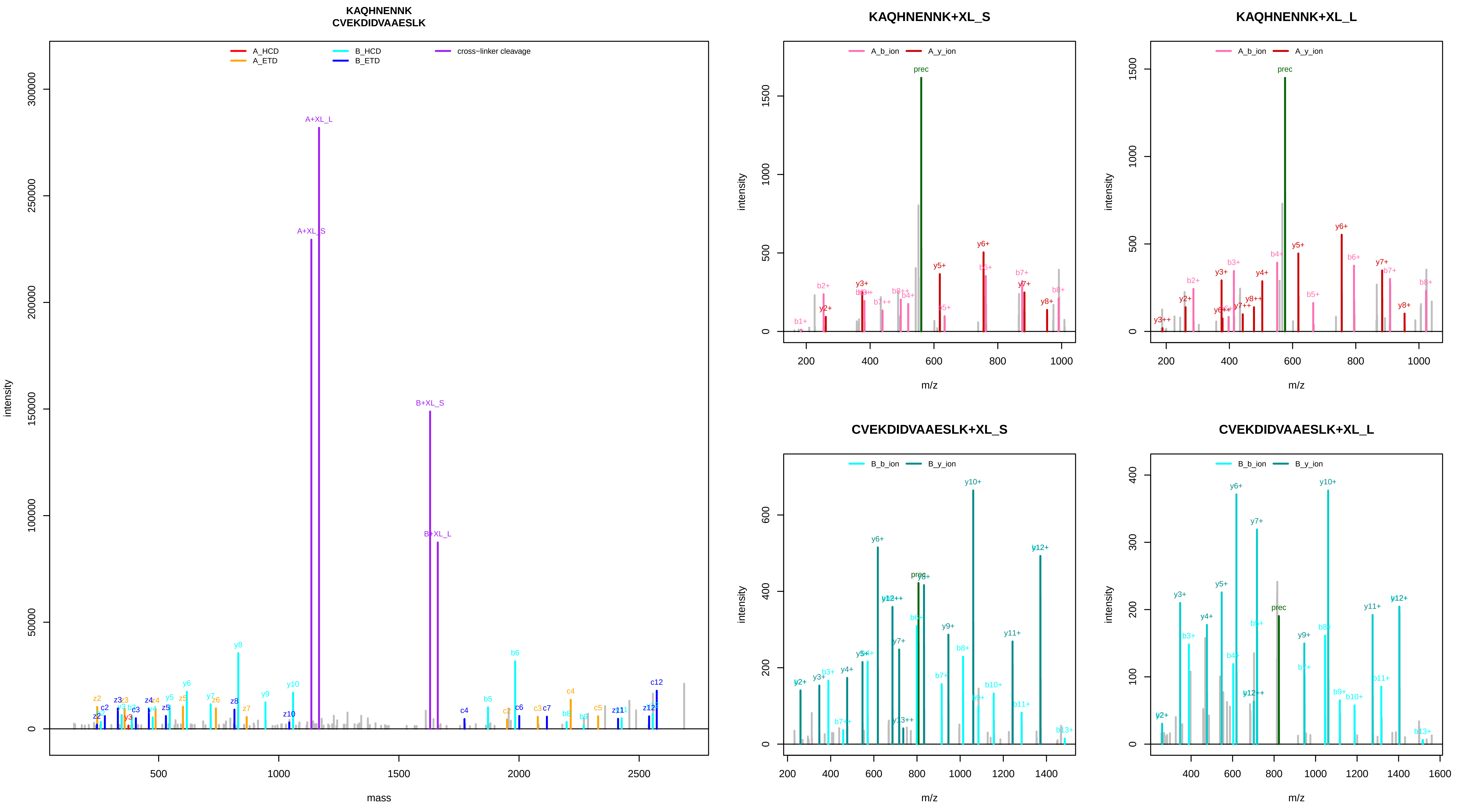
KAQHNENNK
CVEKDIDVAAESLK
A_HCD
A_ETD
B_HCD
B_ETD
cross−linker cleavage
A+XL_L
A+XL_S
B+XL_S
B+XL_L
intensity
mass
KAQHNENNK+XL_S
KAQHNENNK+XL_L
CVEKDIDVAAESLK+XL_S
CVEKDIDVAAESLK+XL_L
A_b_ion
A_y_ion
B_b_ion
B_y_ion
prec
m/z

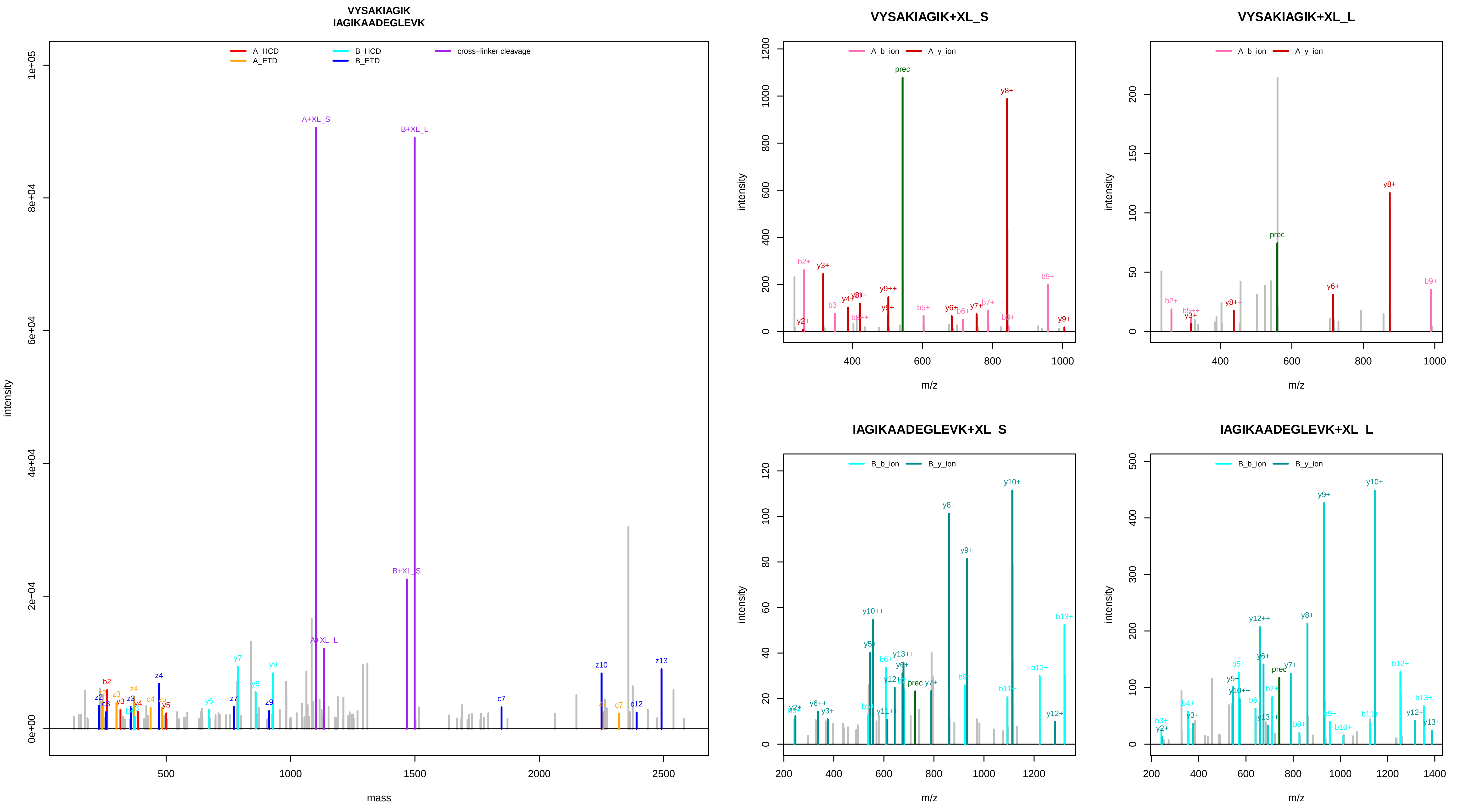

VYSAKIAGIK
IAGIKAADEGLEVK
A_HCD
A_ETD
B_HCD
B_ETD
cross−linker cleavage
A+XL_S
B+XL_L
B+XL_S
A+XL_L
intensity
mass
VYSAKIAGIK+XL_S
VYSAKIAGIK+XL_L
IAGIKAADEGLEVK+XL_S
IAGIKAADEGLEVK+XL_L
A_b_ion
A_y_ion
B_b_ion
B_y_ion
prec
m/z

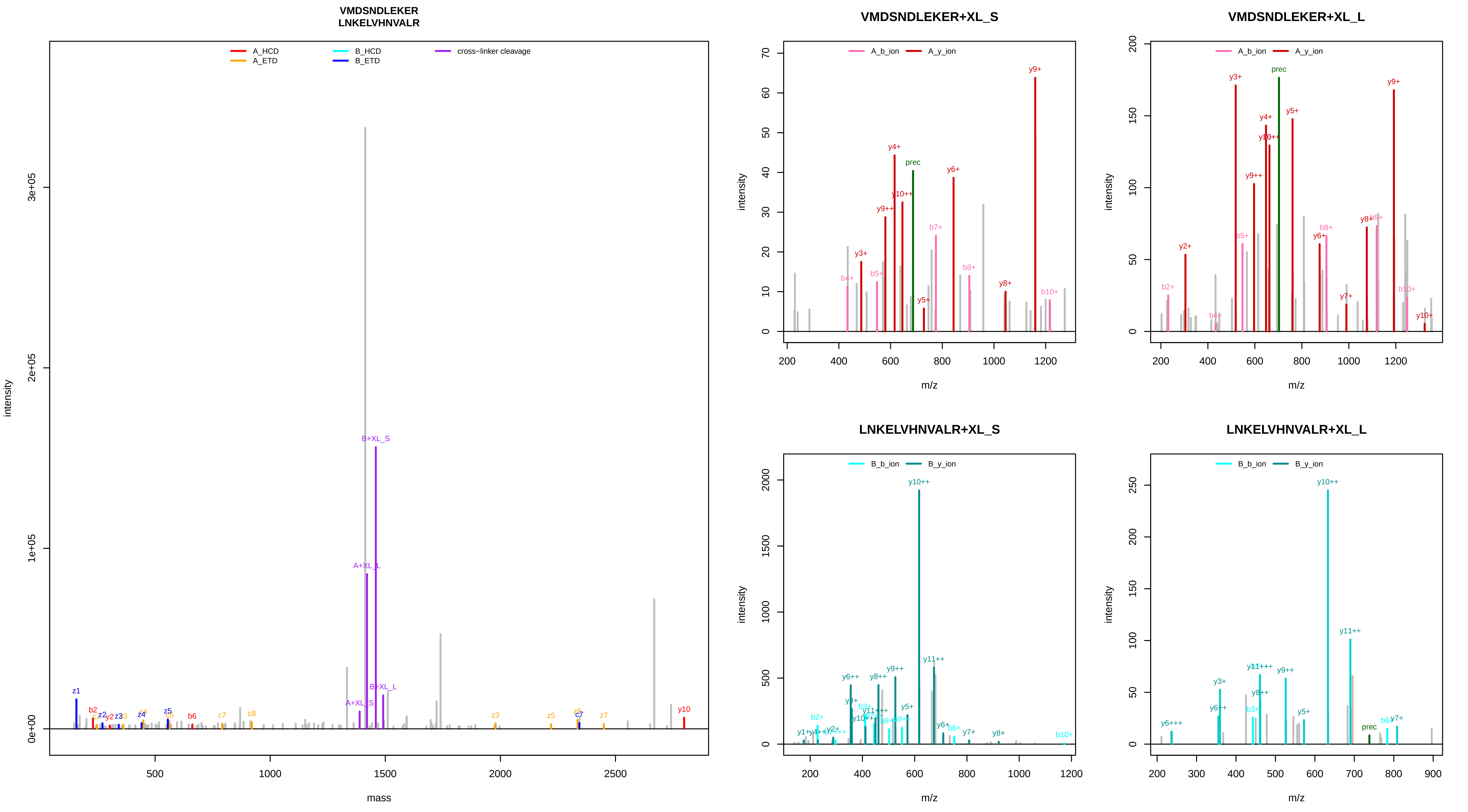
VMDSNDLEKER
LNKELVHNVALR
A_HCD
A_ETD
B_HCD
B_ETD
cross−linker cleavage
intensity
mass
B+XL_S
A+XL_L
B+XL_L
A+XL_S
VMDSNDLEKER+XL_S
VMDSNDLEKER+XL_L
LNKELVHNVALR+XL_S
LNKELVHNVALR+XL_L
A_b_ion
A_y_ion
B_b_ion
B_y_ion
prec
m/z

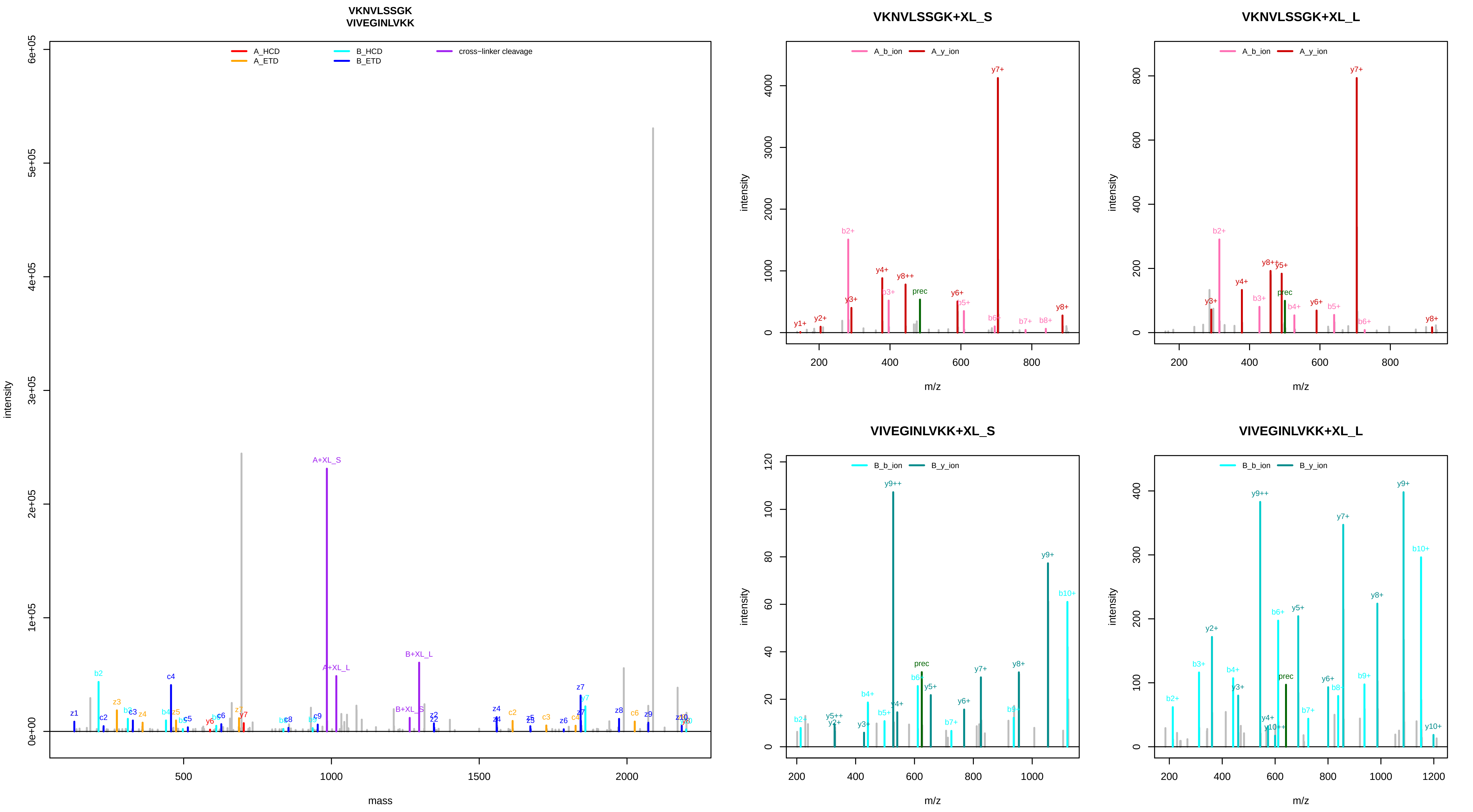

VKNVLSSGK
VIVEGINLVKK
A_HCD
A_ETD
B_HCD
B_ETD
cross−linker cleavage
A+XL_S
A+XL_L
B+XL_L
B+XL_S
intensity
mass
VKNVLSSGK+XL_S
VKNVLSSGK+XL_L
A_b_ion
A_y_ion
VIVEGINLVKK+XL_S
VIVEGINLVKK+XL_L
B_b_ion
B_y_ion
prec
m/z

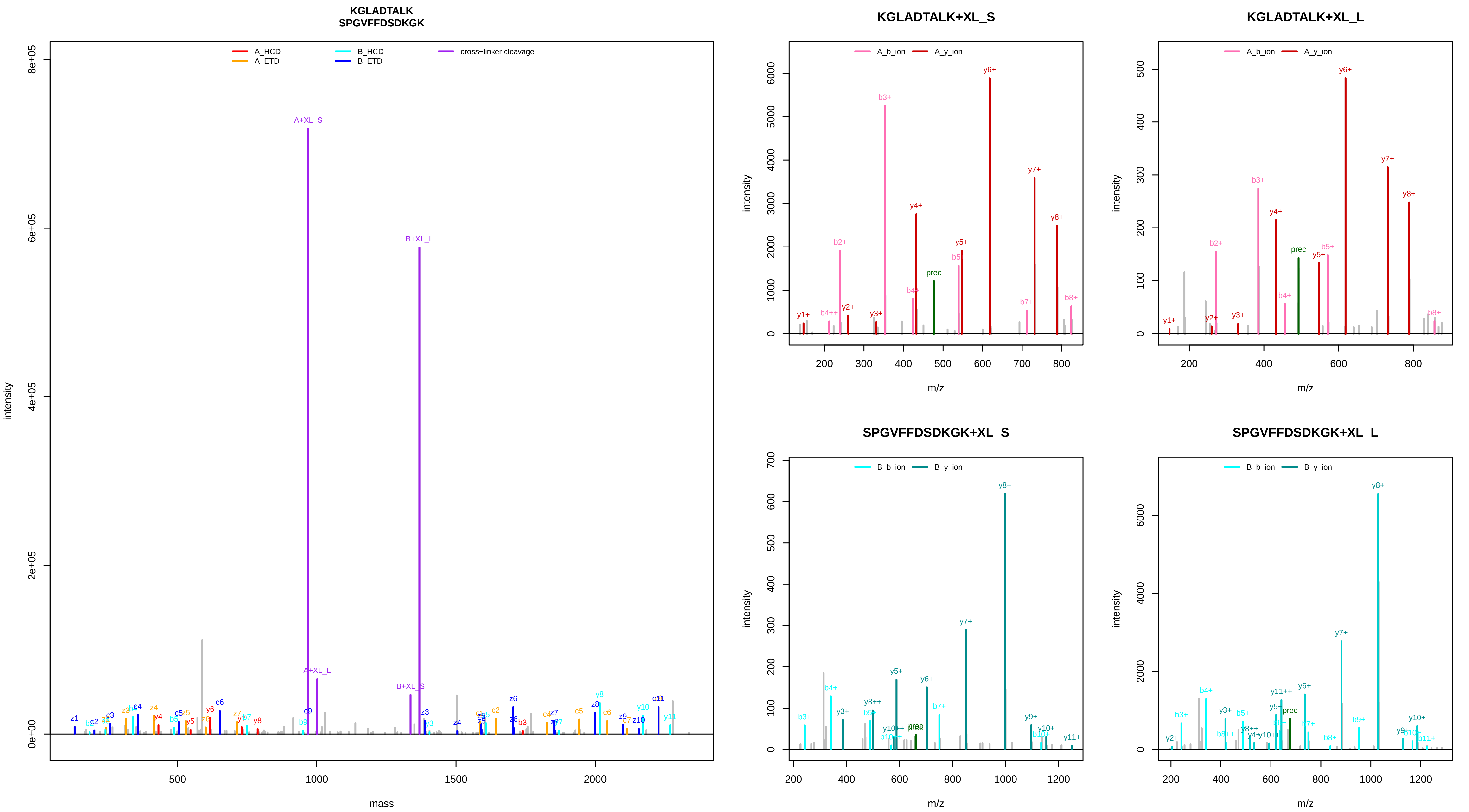
KGLADTALK
SPGVFFDSDKGK
A_HCD
A_ETD
B_HCD
B_ETD
cross−linker cleavage
A+XL_S
B+XL_L
A+XL_L
B+XL_S
intensity
mass
KGLADTALK+XL_S
KGLADTALK+XL_L
A_b_ion
A_y_ion
prec
SPGVFFDSDKGK+XL_S
SPGVFFDSDKGK+XL_L
B_b_ion
B_y_ion
m/z

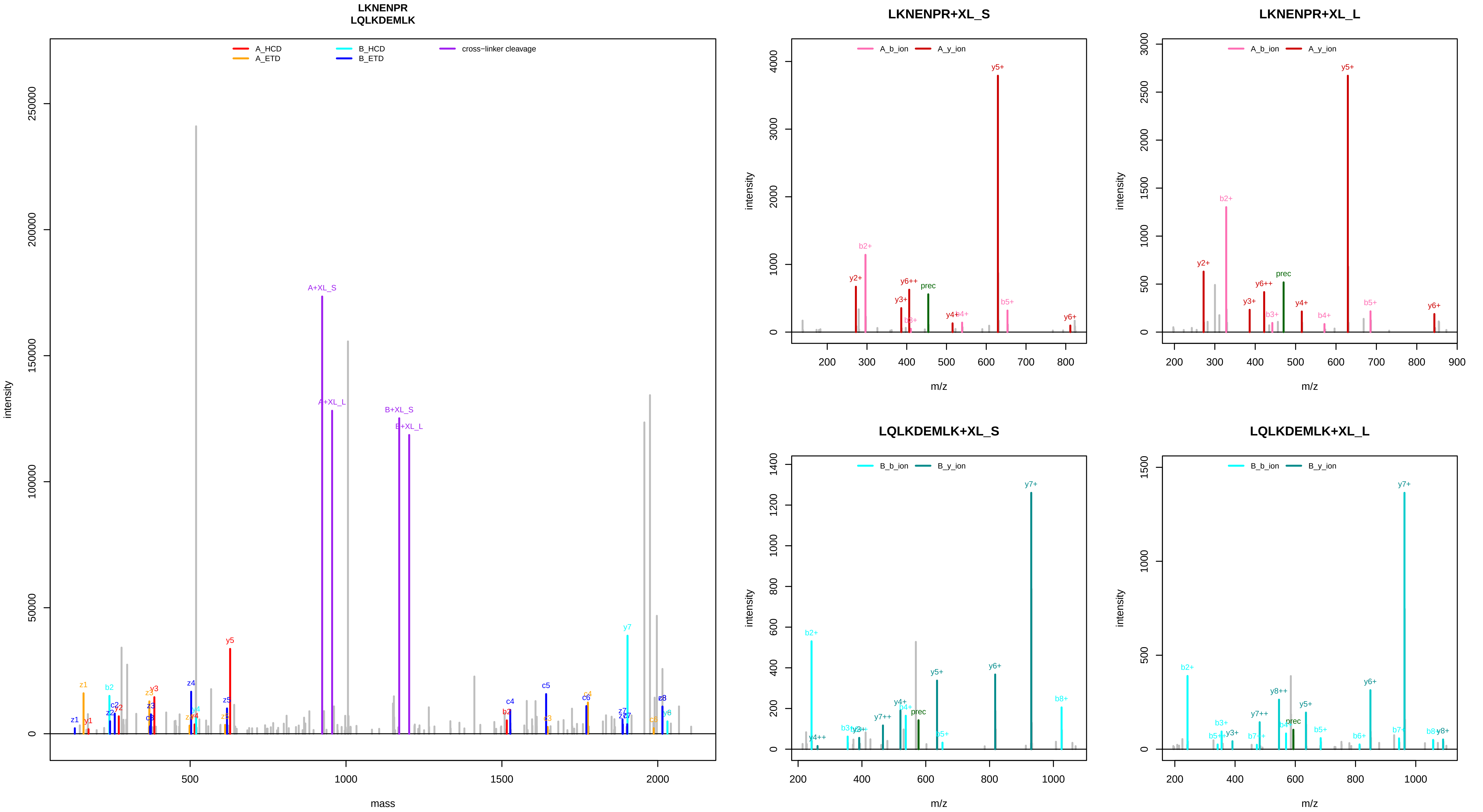

LKNENPR
LQLKDEMLK
A_HCD
A_ETD
B_HCD
B_ETD
cross−linker cleavage
intensity
mass
LKNENPR+XL_S
LKNENPR+XL_L
LQLKDEMLK+XL_S
LQLKDEMLK+XL_L
A_b_ion
A_y_ion
B_b_ion
B_y_ion
m/z

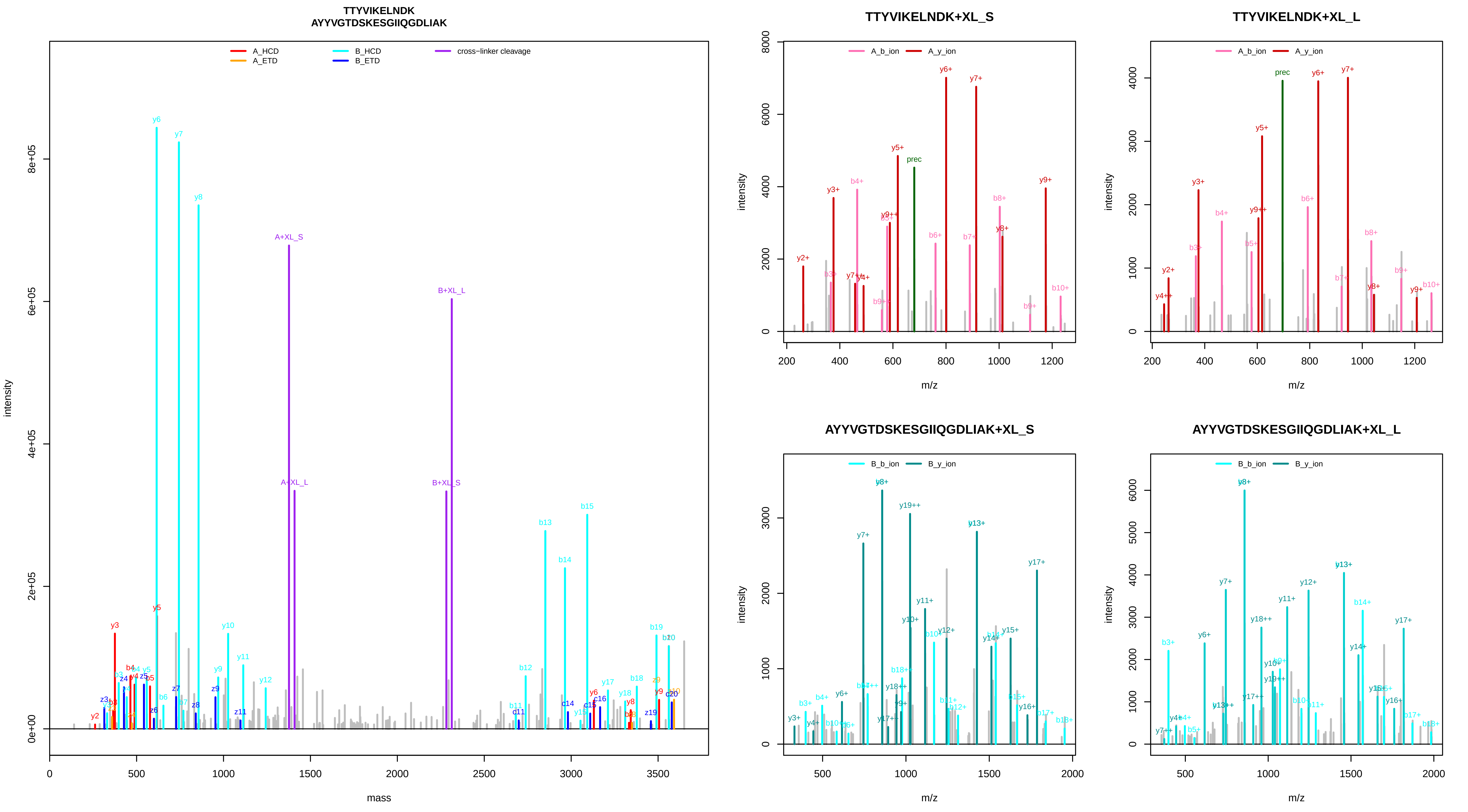
TTYVIKELNDK
AYYVGTDSKESGIIQGDLIAK
A_HCD
A_ETD
B_HCD
B_ETD
cross−linker cleavage
A+XL_S
A+XL_L
B+XL_L
B+XL_S
intensity
mass
TTYVIKELNDK+XL_S
TTYVIKELNDK+XL_L
AYYVGTDSKESGIIQGDLIAK+XL_S
AYYVGTDSKESGIIQGDLIAK+XL_L
A_b_ion
A_y_ion
B_b_ion
B_y_ion
prec
m/z

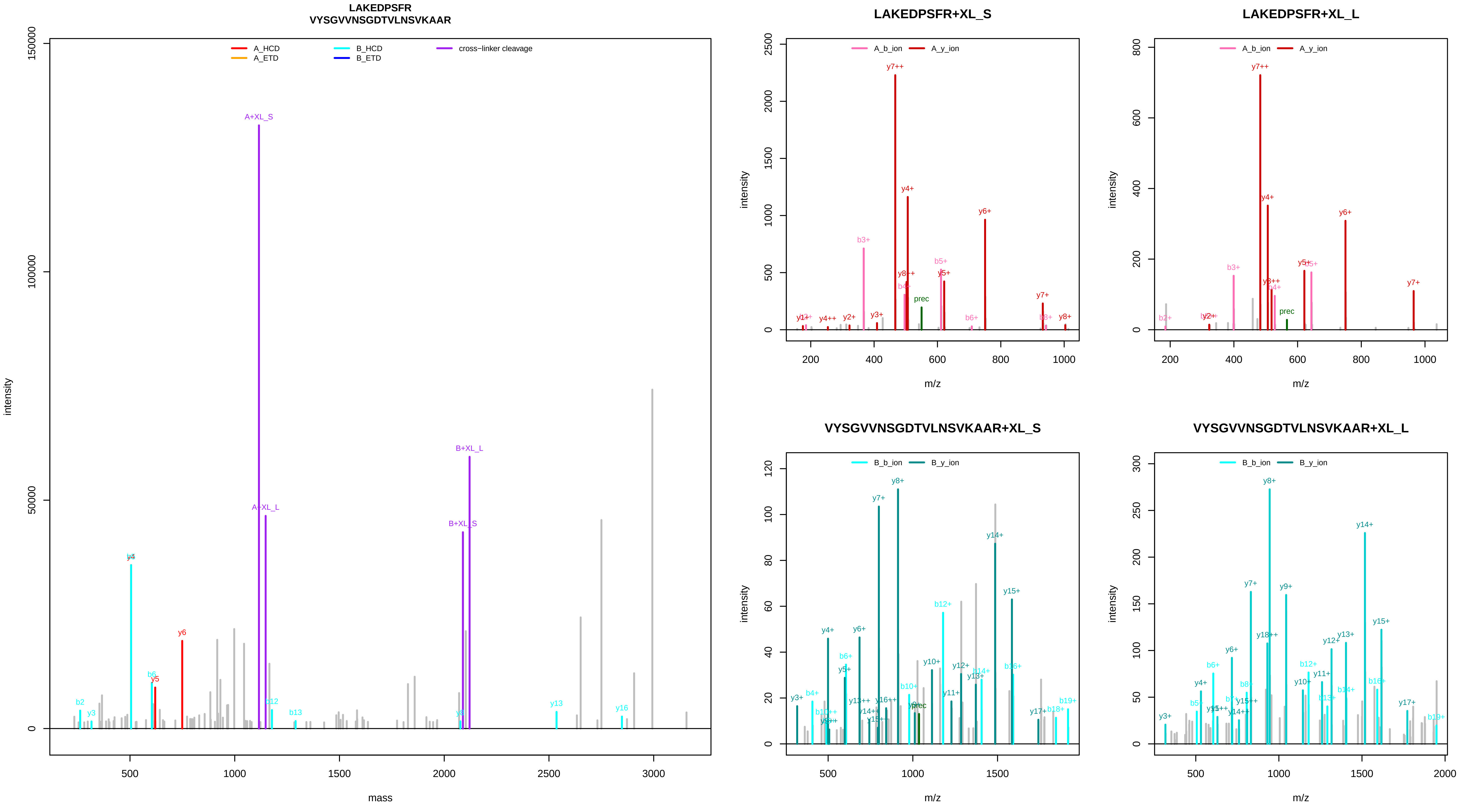

LAKEDPSFR
VYSGVVNSGDTVLNSVKAAR
A_HCD
A_ETD
B_HCD
B_ETD
cross−linker cleavage
A+XL_S
A+XL_L
B+XL_L
B+XL_S
intensity
mass
LAKEDPSFR+XL_S
LAKEDPSFR+XL_L
VYSGVVNSGDTVLNSVKAAR+XL_S
VYSGVVNSGDTVLNSVKAAR+XL_L
A_b_ion
A_y_ion
B_b_ion
B_y_ion
prec
m/z

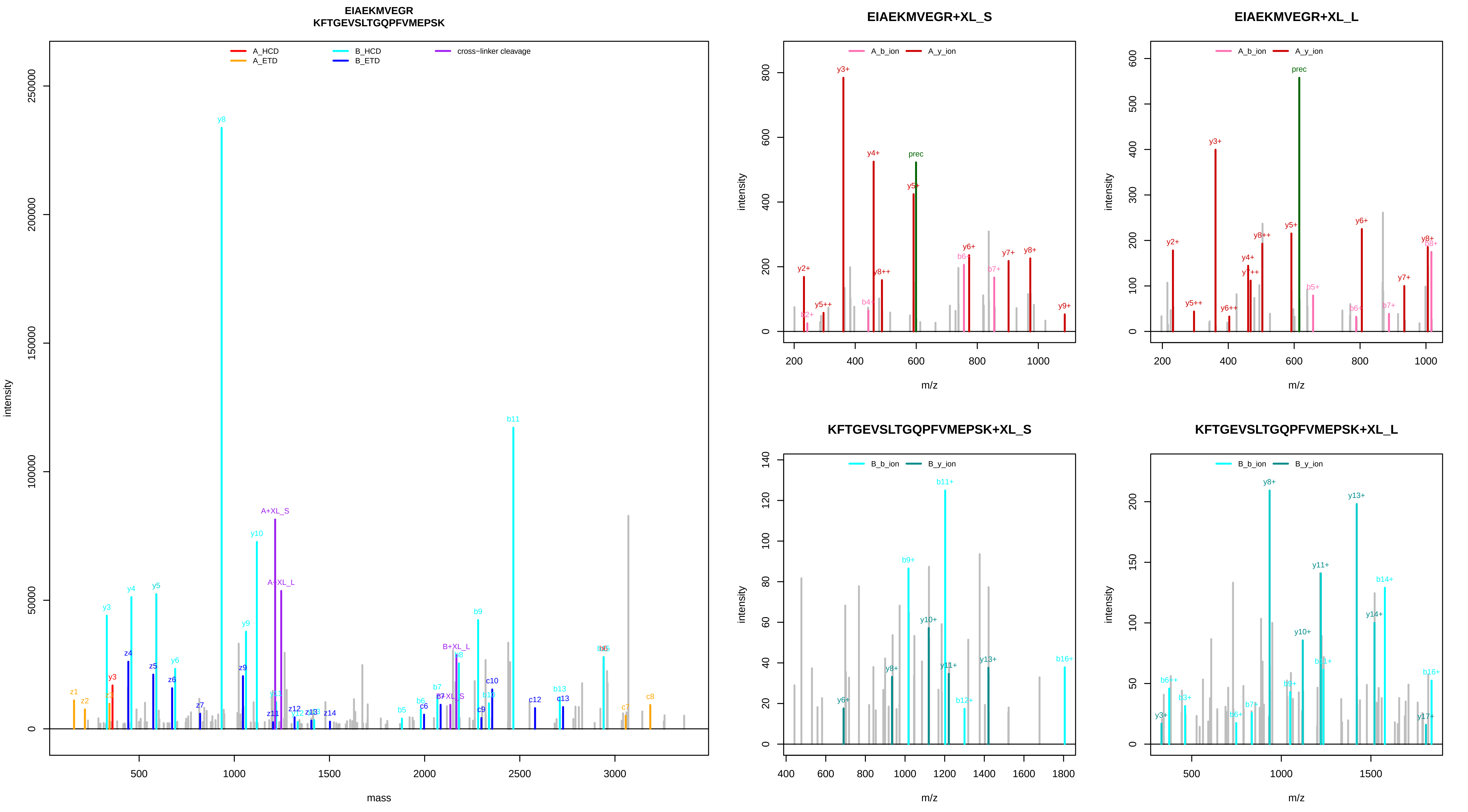

EIAEKMVEGR
KFTGEVSLTGQPFVMEPSK
A_HCD
A_ETD
B_HCD
B_ETD
cross−linker cleavage
intensity
mass
EIAEKMVEGR+XL_S
EIAEKMVEGR+XL_L
KFTGEVSLTGQPFVMEPSK+XL_S
KFTGEVSLTGQPFVMEPSK+XL_L
A_b_ion
A_y_ion
B_b_ion
B_y_ion
m/z

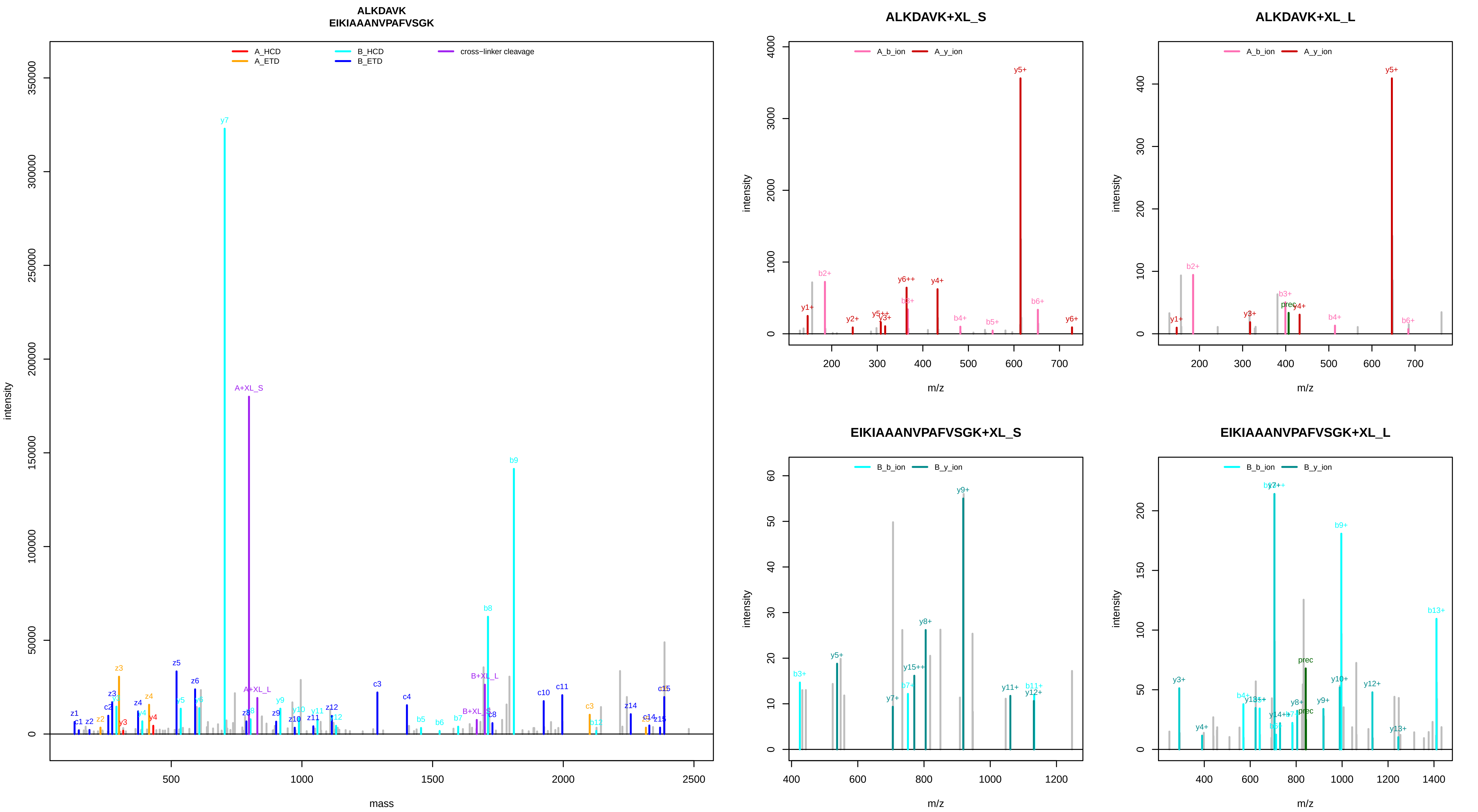
ALKDAVK
EIKIAAANVPAFVSGK
A_HCD
A_ETD
B_HCD
B_ETD
cross−linker cleavage
intensity
mass
ALKDAVK+XL_S
ALKDAVK+XL_L
EIKIAAANVPAFVSGK+XL_S
EIKIAAANVPAFVSGK+XL_L
A_b_ion
A_y_ion
B_b_ion
B_y_ion
m/z

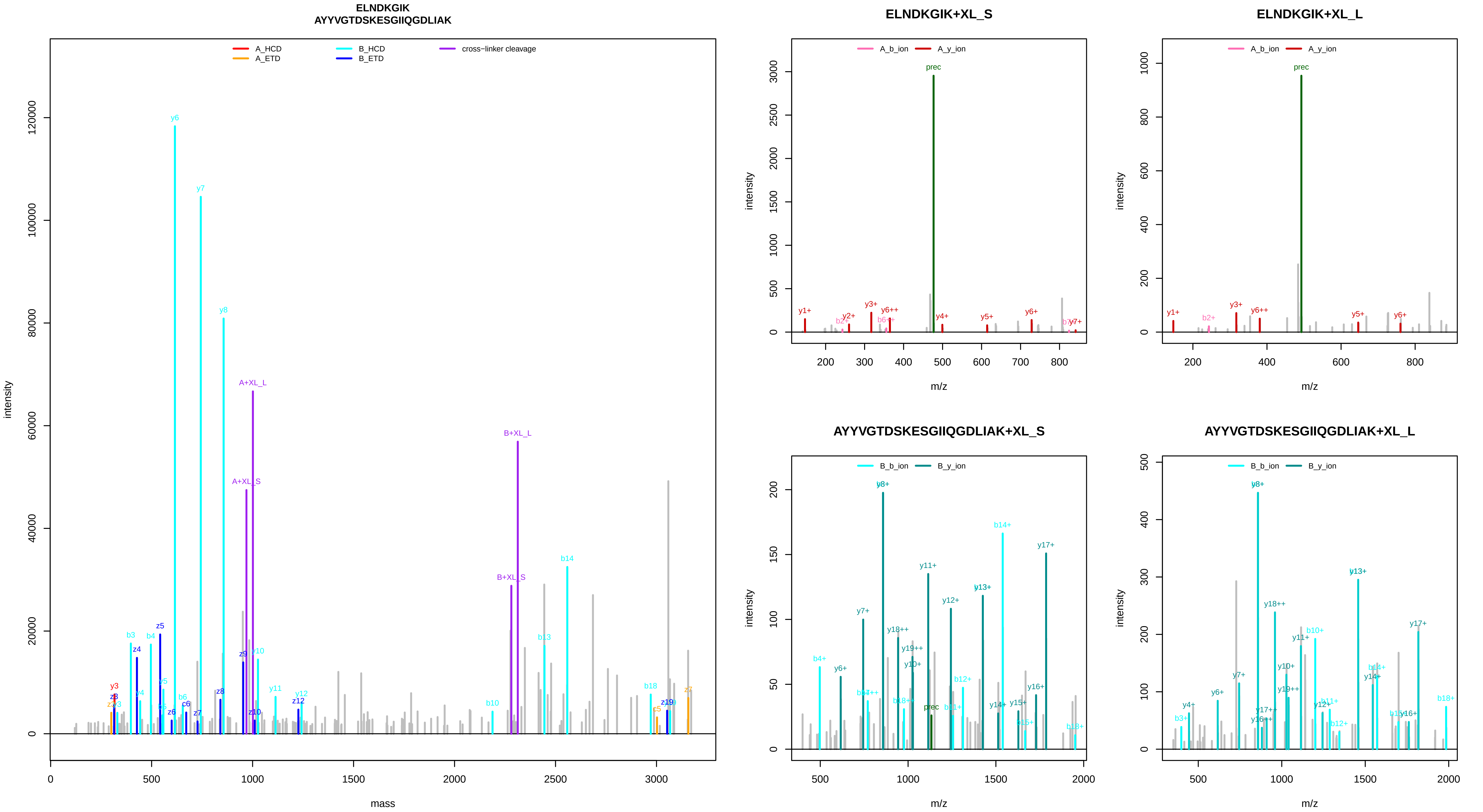
ELNDKGIK
AYYVGTDSKESGIIQGDLIAK
A_HCD
A_ETD
B_HCD
B_ETD
cross−linker cleavage
intensity
mass
A+XL_L
A+XL_S
B+XL_L
B+XL_S
ELNDKGIK+XL_S
ELNDKGIK+XL_L
A_b_ion
A_y_ion
prec
m/z
AYYVGTDSKESGIIQGDLIAK+XL_S
AYYVGTDSKESGIIQGDLIAK+XL_L
B_b_ion
B_y_ion

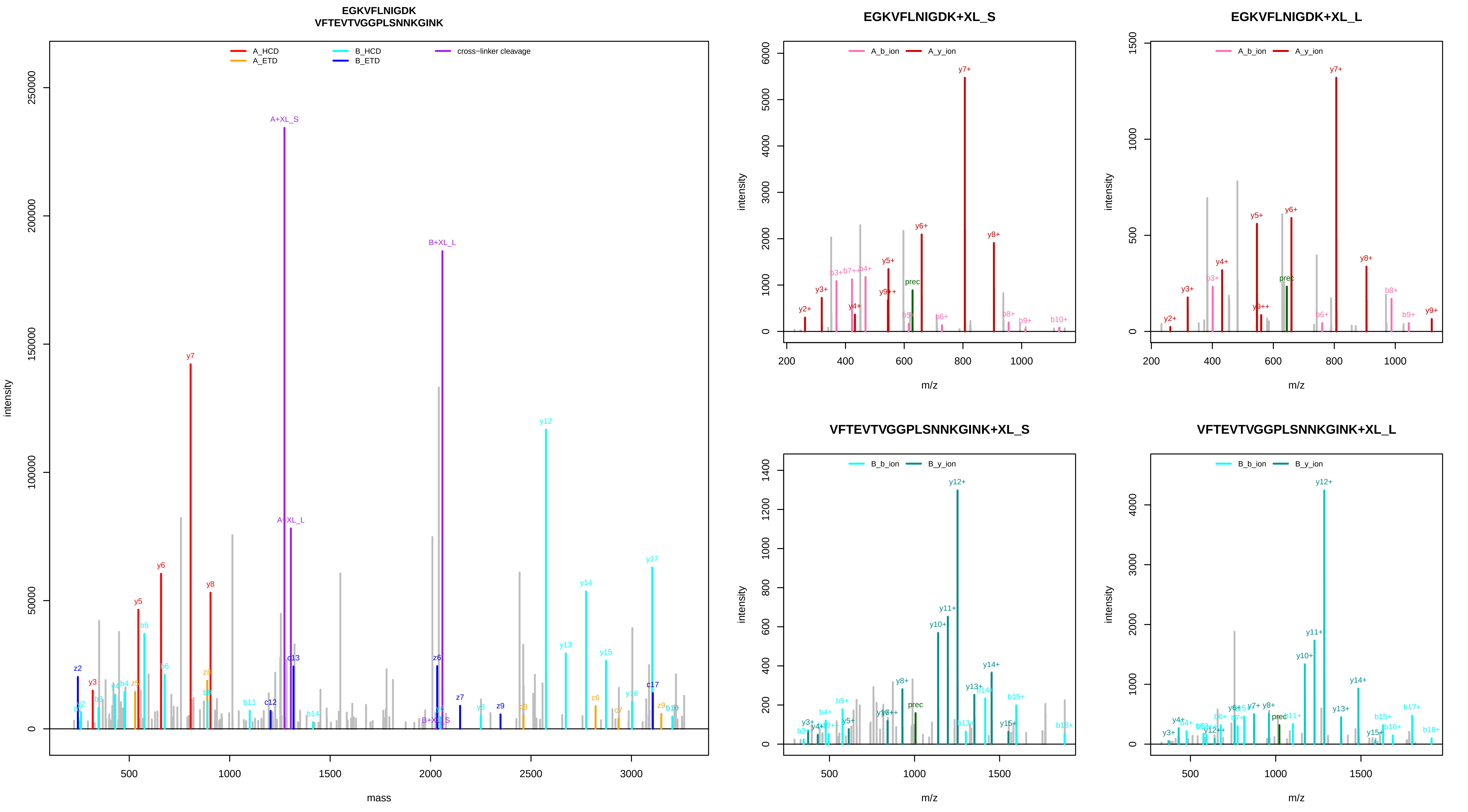
EGKVFLNIGDK
VFTEVTVGGPLSNNKGINK
A_HCD
A_ETD
B_HCD
B_ETD
cross−linker cleavage
A+XL_S
B+XL_L
A+XL_L
B+XL_S
intensity
mass
EGKVFLNIGDK+XL_S
EGKVFLNIGDK+XL_L
VFTEVTVGGPLSNNKGINK+XL_S
VFTEVTVGGPLSNNKGINK+XL_L
A_b_ion
A_y_ion
B_b_ion
B_y_ion
prec
m/z

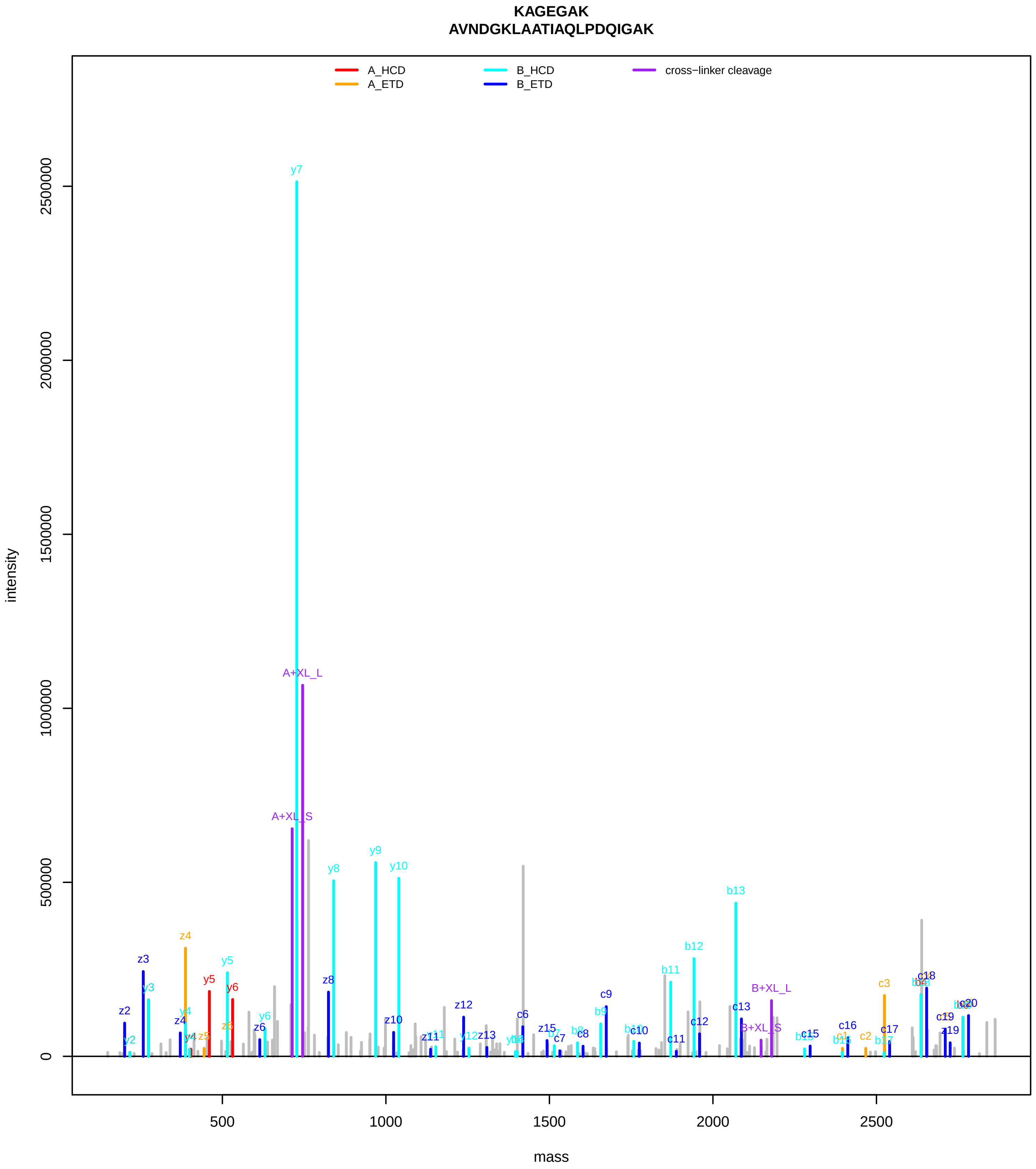

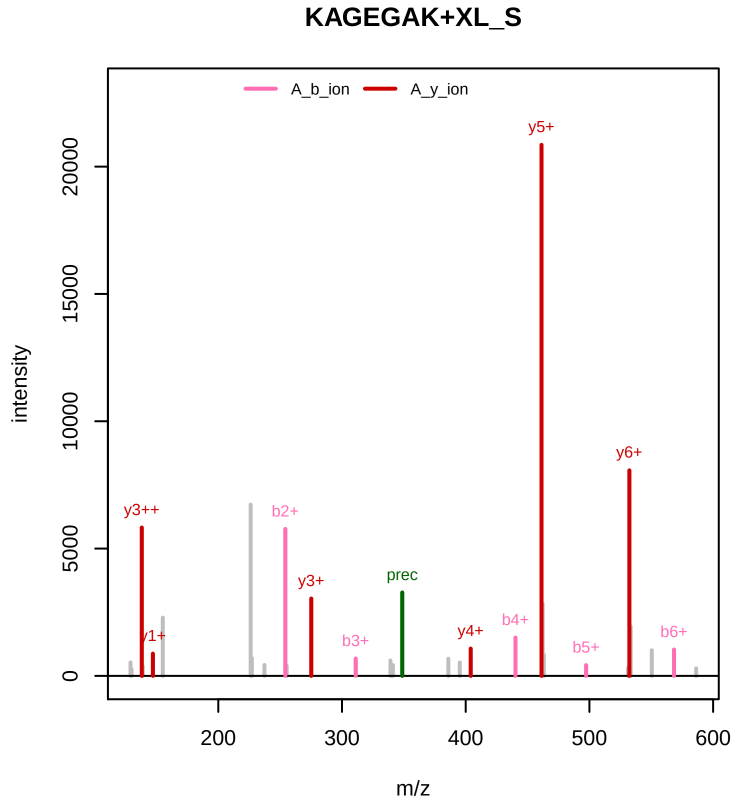

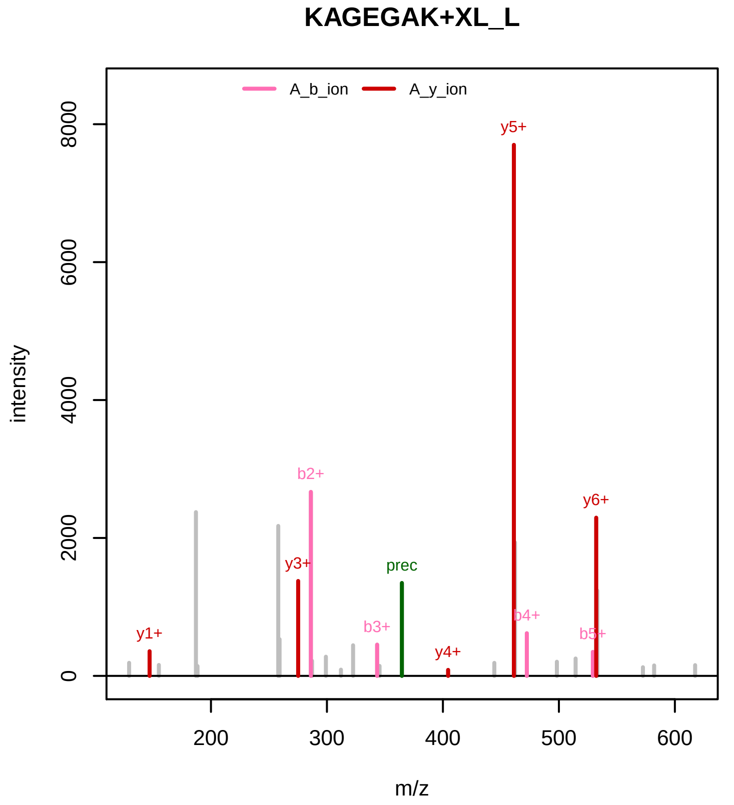

AVNDGKLAATIAQLPDQIGAK+XL_S

AVNDGKLAATIAQLPDQIGAK+XL_L

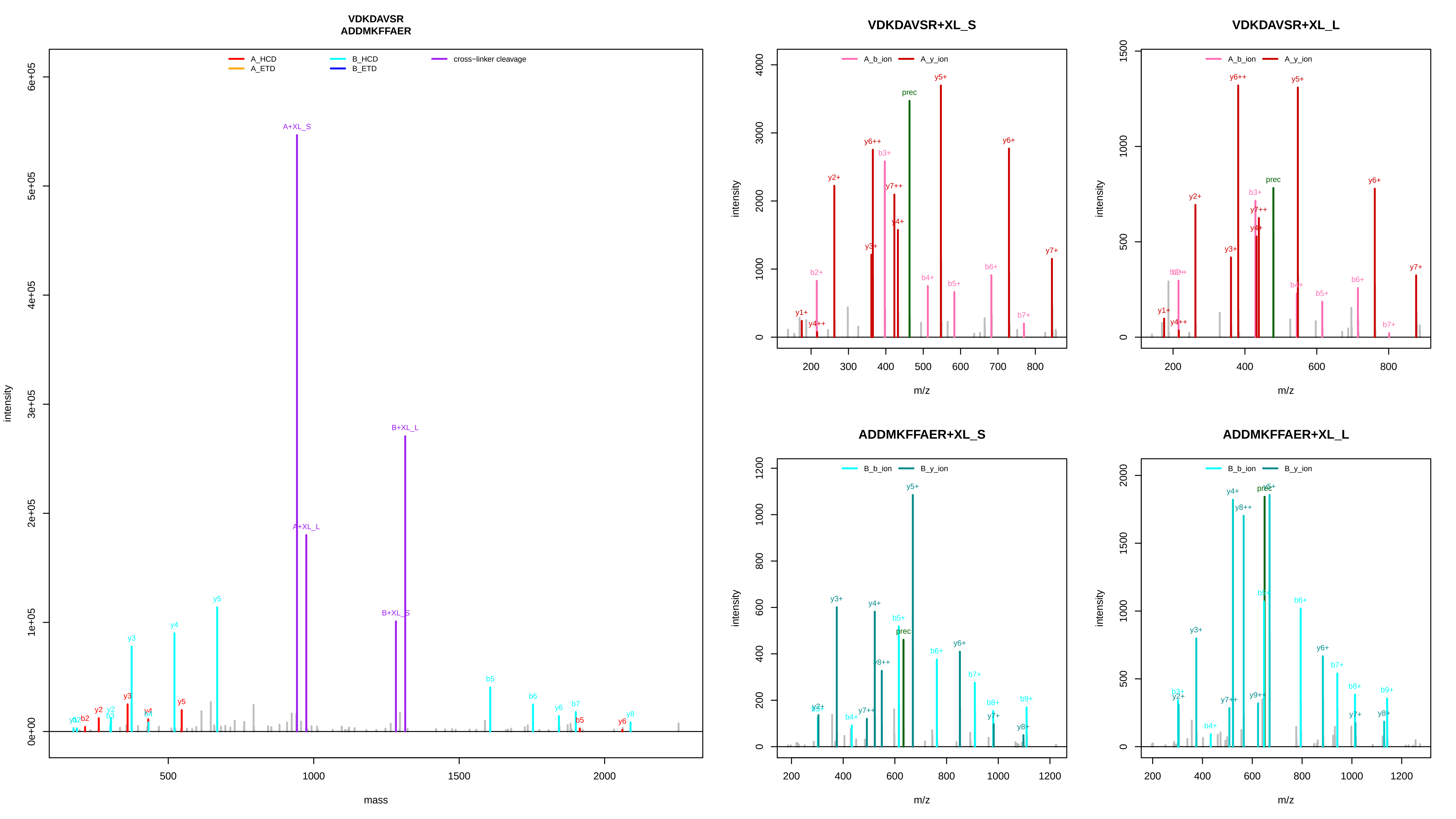

VDKDAVSR
ADDMKFFAER
A_HCD
A_ETD
B_HCD
B_ETD
cross−linker cleavage
A+XL_S
A+XL_L
B+XL_L
B+XL_S
y1 b2 y2 b3 y3 y4 y5 b4 b5 b6 b7 y6 y7 y8
intensity
mass
VDKDAVSR+XL_S
A_b_ion
A_y_ion
y1+ y4++ b2+ y2+ y3+ y6++ b3+ y7++ y4+ prec y5+ b4+ b5+ b6+ y6+ b7+ y7+
intensity
m/z
VDKDAVSR+XL_L
A_b_ion
A_y_ion
y1+ y4++ b2+ y2+ y3+ y6++ b3+ y7++ y4+ prec y5+ b4+ b5+ b6+ y6+ b7+ y7+
intensity
m/z
ADDMKFFAER+XL_S
B_b_ion
B_y_ion
y2+ y3+ b4+ y7++ y4+ y8++ b5+ prec y5+ b6+ y6+ b7+ b8+ y7+ y8+ b9+
intensity
m/z
ADDMKFFAER+XL_L
B_b_ion
B_y_ion
y2+ y3+ b4+ y7++ y4+ y8++ y9++ b5+ prec y5+ b6+ y6+ b7+ b8+ y7+ y8+ b9+
intensity
m/z

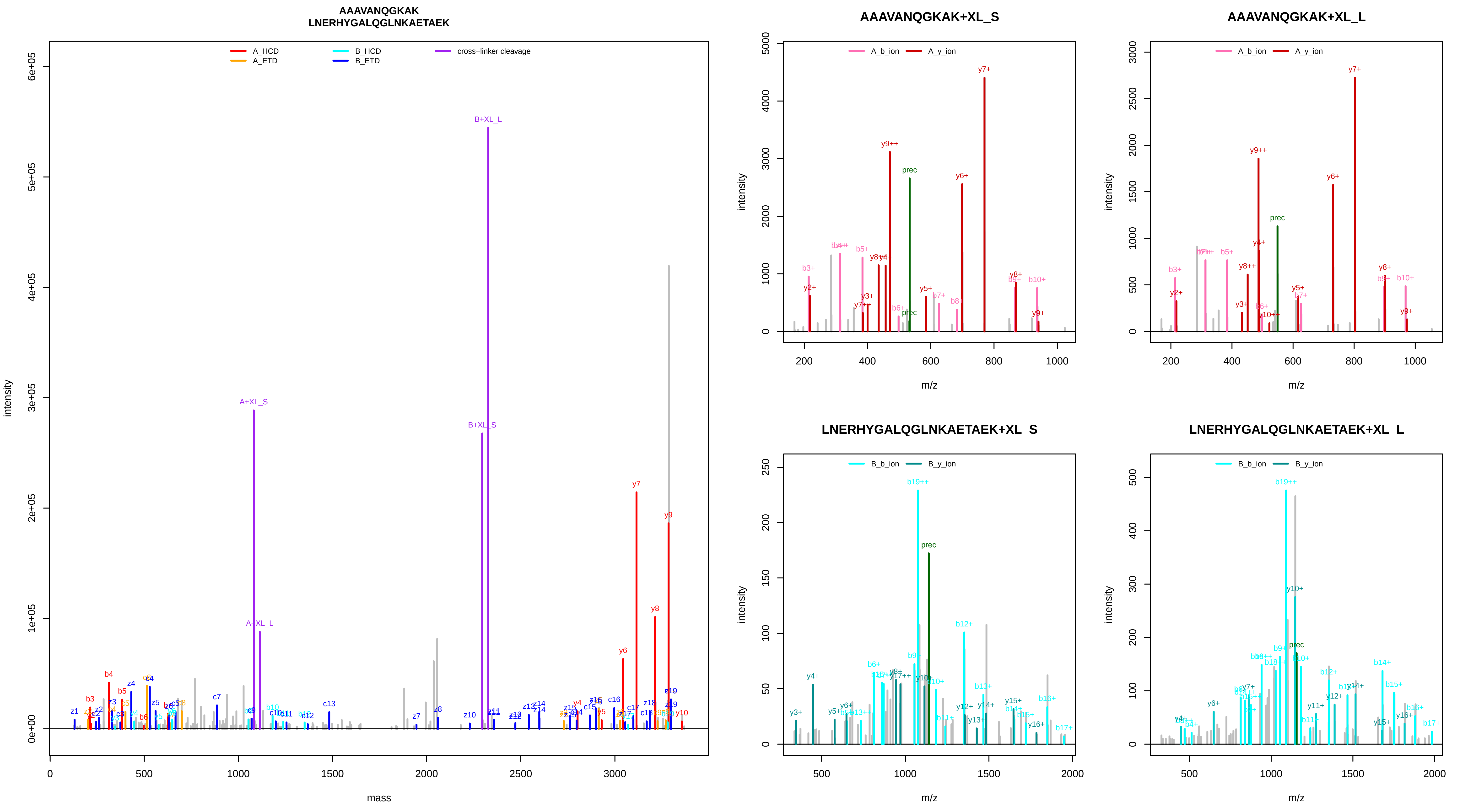
AAAVANQGKAK
LNERHYGALQGLNKAETAEK
A_HCD
A_ETD
B_HCD
B_ETD
cross−linker cleavage
B+XL_L
A+XL_S
B+XL_S
A+XL_L
intensity
mass
AAAVANQGKAK+XL_S
AAAVANQGKAK+XL_L
A_b_ion
A_y_ion
LNERHYGALQGLNKAETAEK+XL_S
LNERHYGALQGLNKAETAEK+XL_L
B_b_ion
B_y_ion
prec
m/z
intensity

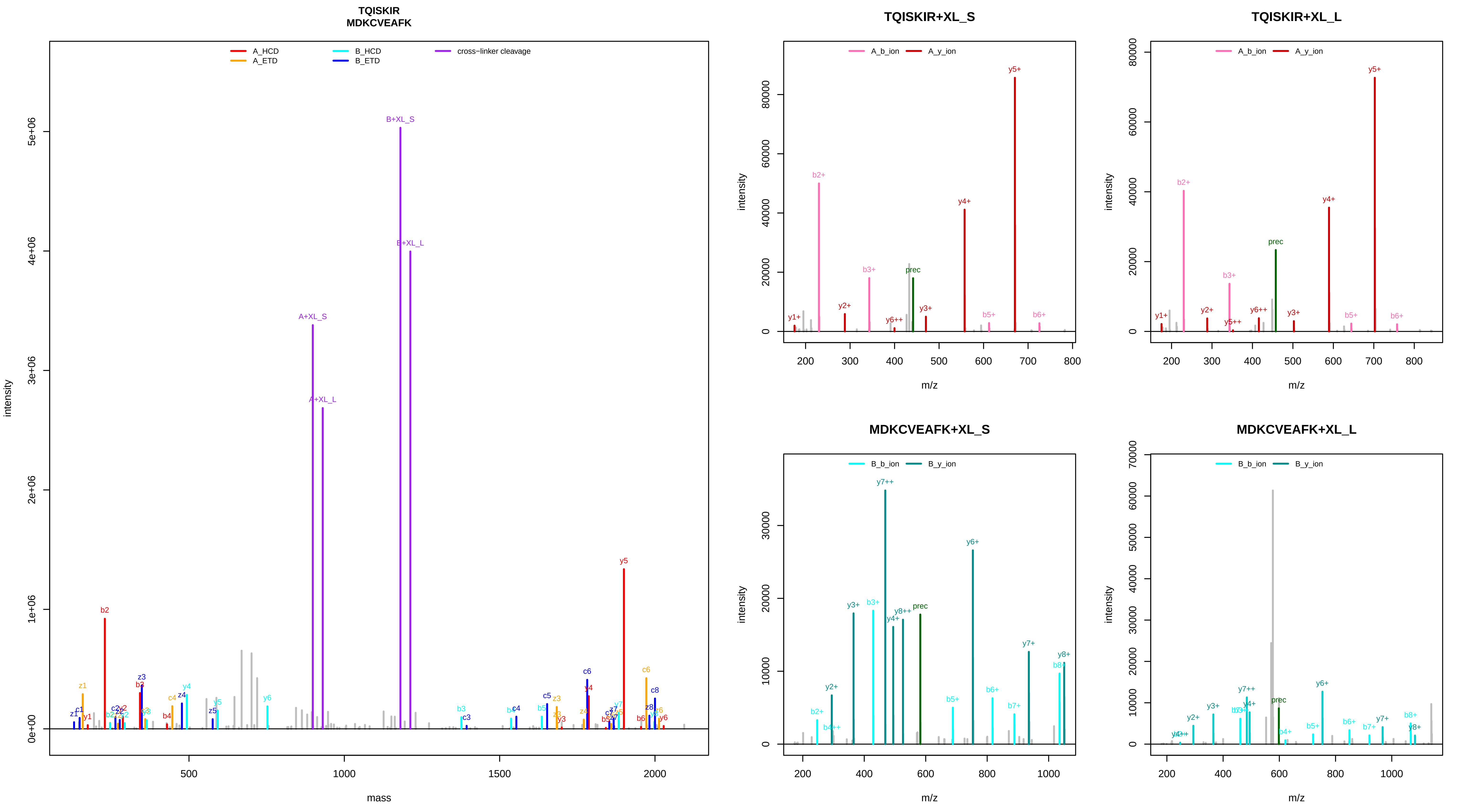

TQISKIR
MDKCVEAFK
A_HCD
A_ETD
B_HCD
B_ETD
cross−linker cleavage
B+XL_S
B+XL_L
A+XL_S
A+XL_L
intensity
mass
TQISKIR+XL_S
TQISKIR+XL_L
A_b_ion
A_y_ion
MDKCVEAFK+XL_S
MDKCVEAFK+XL_L
B_b_ion
B_y_ion
prec
m/z

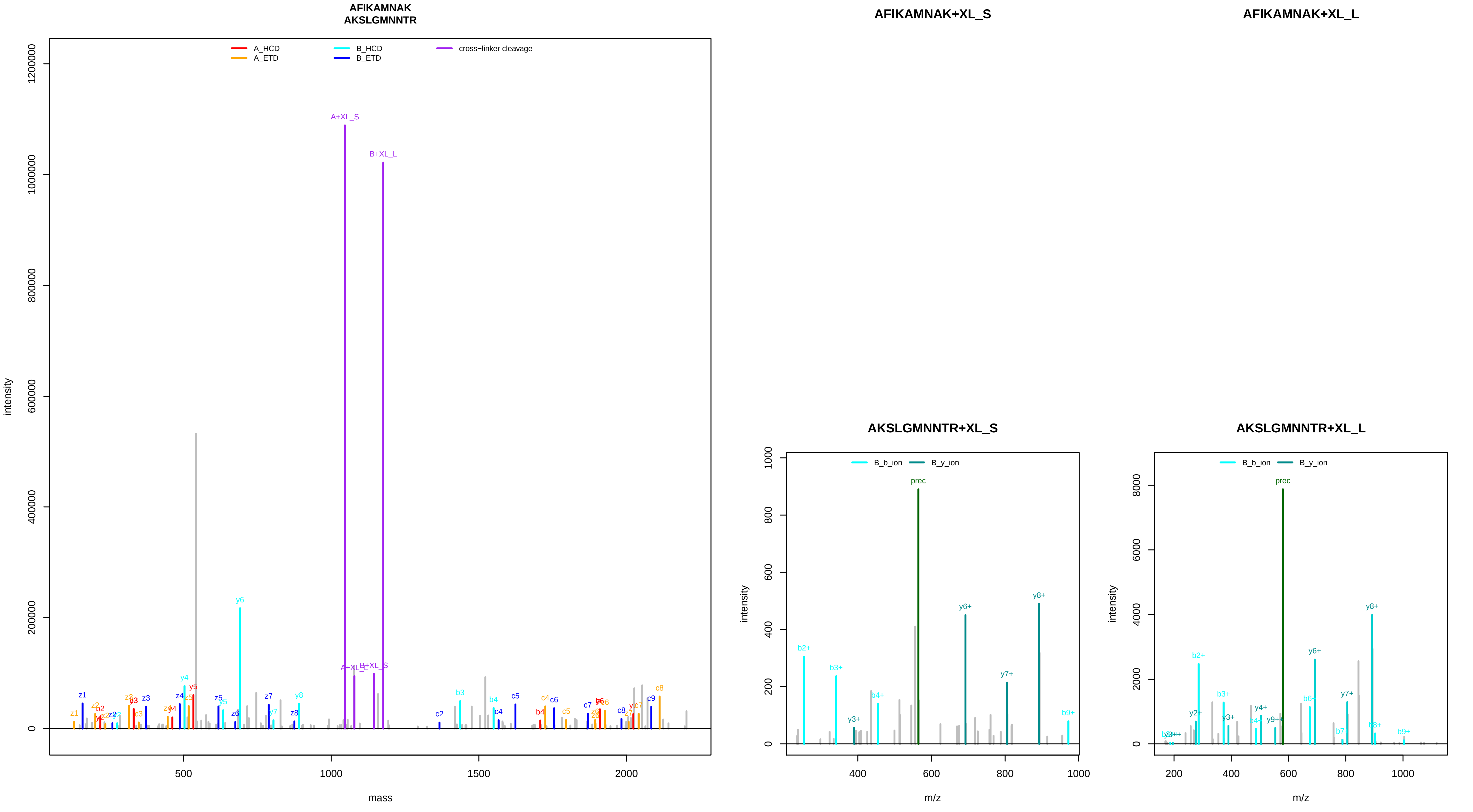
AFIKAMNAK
AKSLGMNNTR
A_HCD
A_ETD
B_HCD
B_ETD
cross−linker cleavage
A+XL_S
B+XL_L
intensity
mass
AFIKAMNAK+XL_S
AFIKAMNAK+XL_L
AKSLGMNNTR+XL_S
AKSLGMNNTR+XL_L
B_b_ion
B_y_ion
prec
m/z

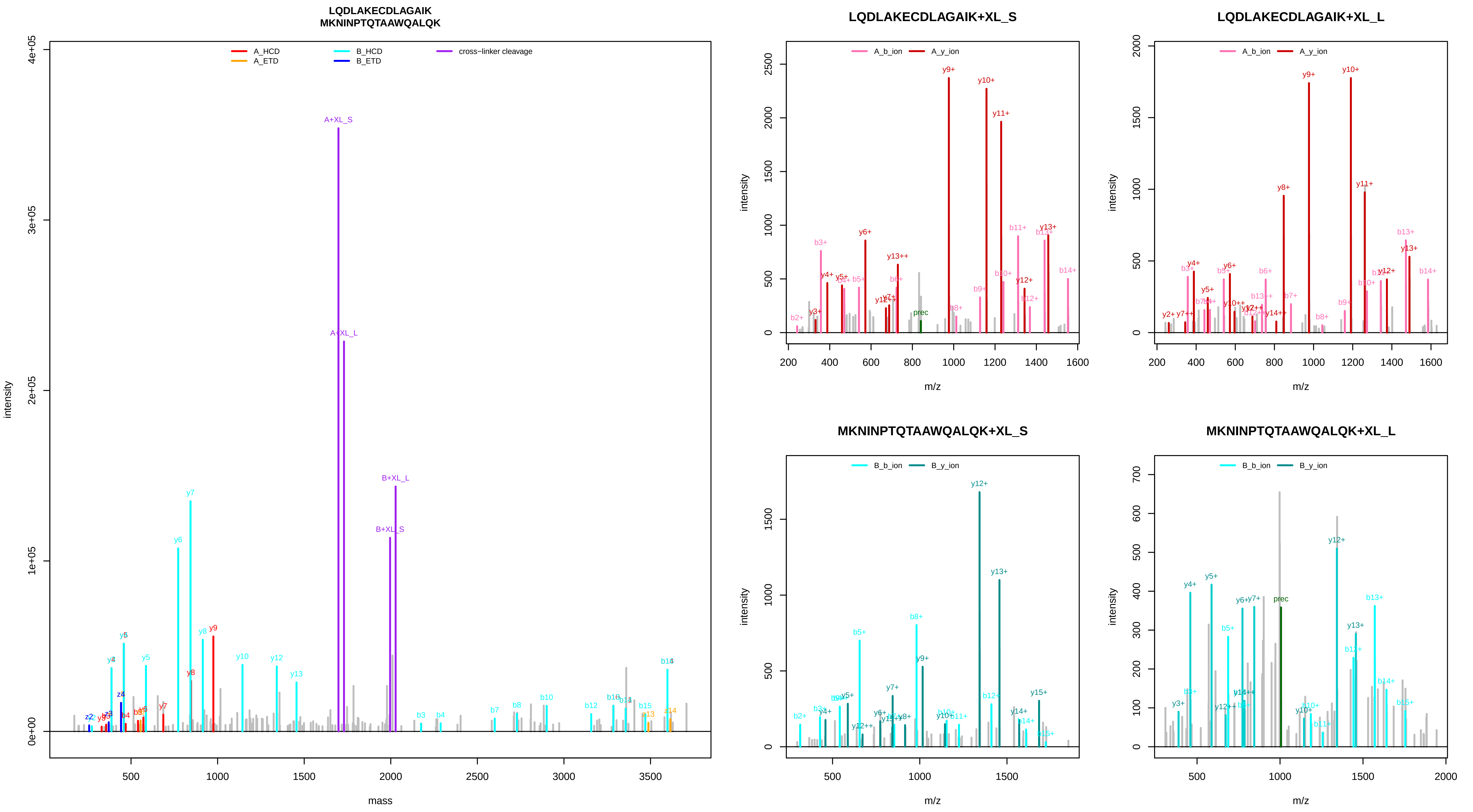
LQDLAKECDLAGAIK
MKNINPTQTAAWQALQK
A_HCD
A_ETD
B_HCD
B_ETD
cross−linker cleavage
A+XL_S
A+XL_L
B+XL_L
B+XL_S
intensity
mass
LQDLAKECDLAGAIK+XL_S
LQDLAKECDLAGAIK+XL_L
MKNINPTQTAAWQALQK+XL_S
MKNINPTQTAAWQALQK+XL_L
A_b_ion
A_y_ion
B_b_ion
B_y_ion
prec
m/z

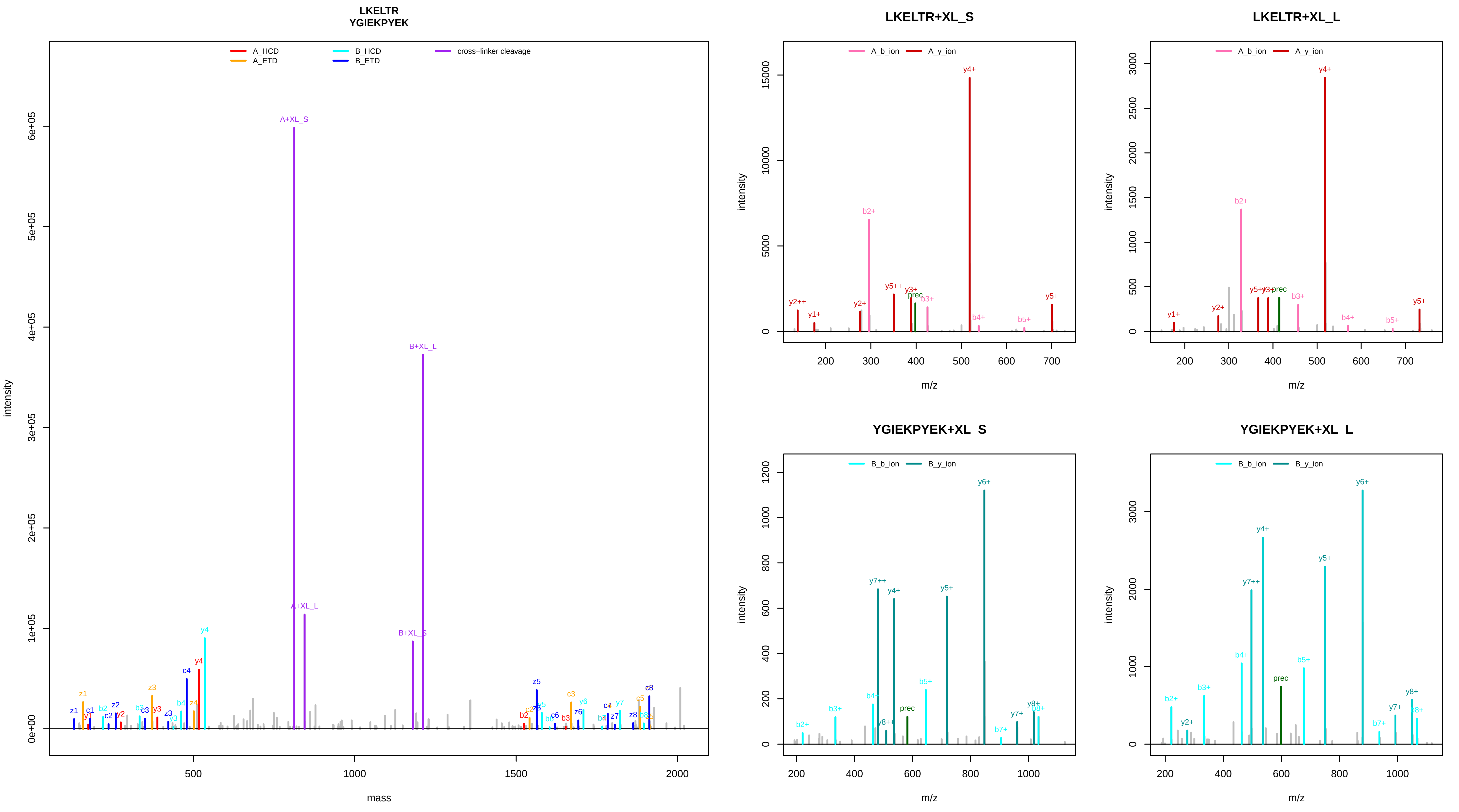

LKELTR
YGIEKPYEK
A_HCD
A_ETD
B_HCD
B_ETD
cross−linker cleavage
A+XL_S
B+XL_L
A+XL_L
B+XL_S
intensity
mass
LKELTR+XL_S
LKELTR+XL_L
YGIEKPYEK+XL_S
YGIEKPYEK+XL_L
A_b_ion
A_y_ion
B_b_ion
B_y_ion
m/z

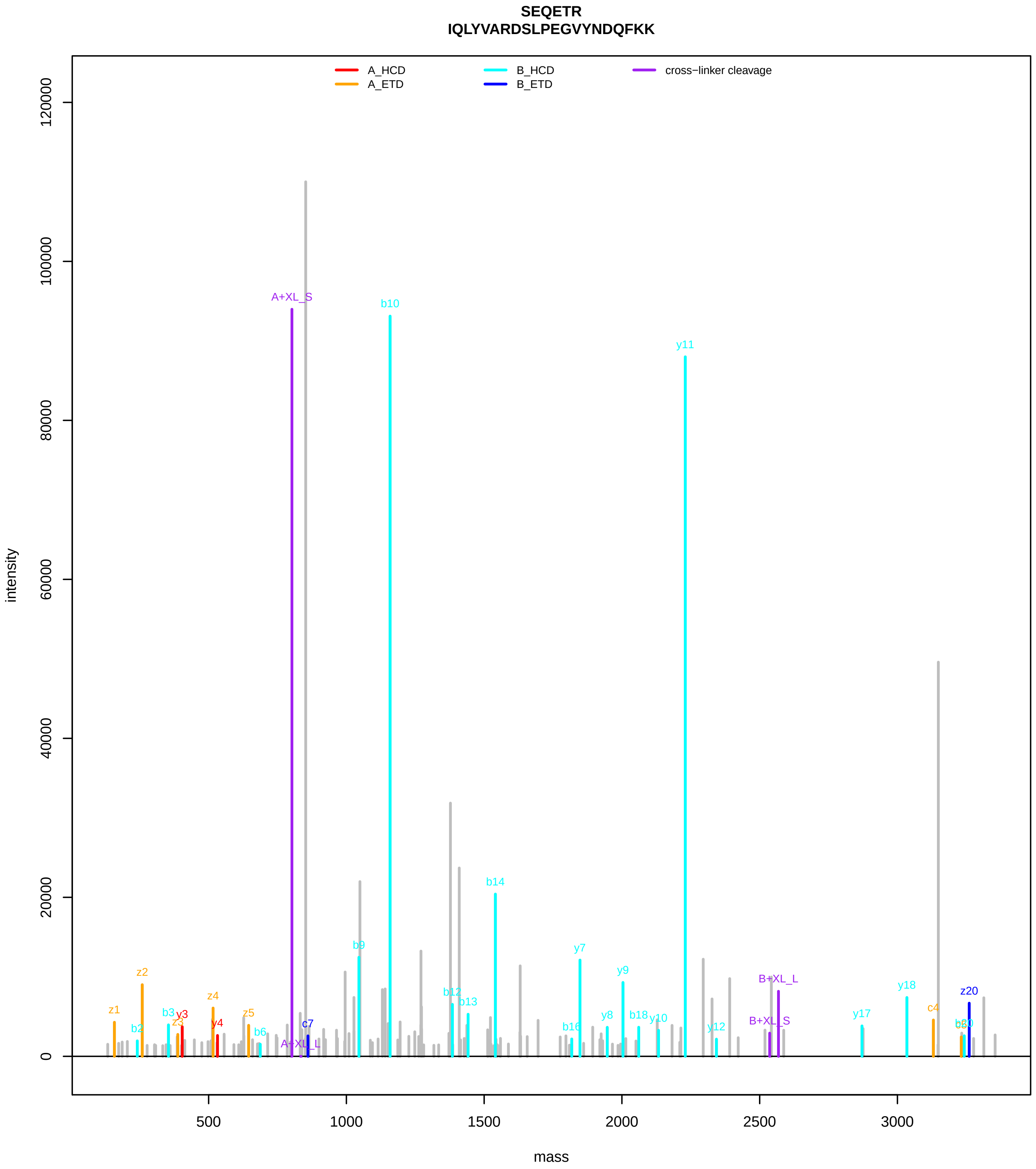

SEQETR+XL_S

SEQETR+XL_L

IQLYVARDSLPEGVYNDQFKK+XL_S

IQLYVARDSLPEGVYNDQFKK+XL_L

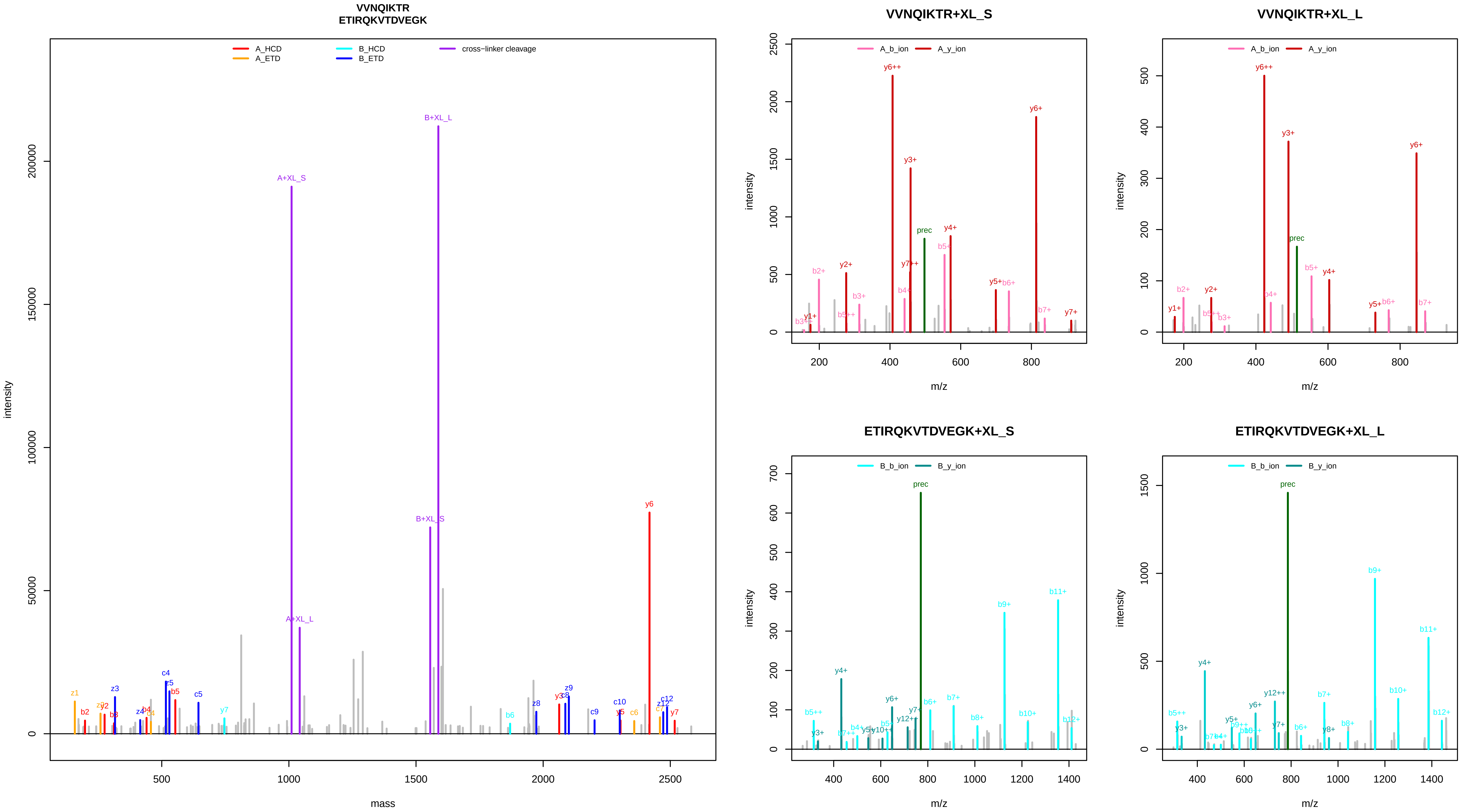
VVNQIKTR
ETIRQKVTDVEGK
A_HCD
A_ETD
B_HCD
B_ETD
cross−linker cleavage
intensity
mass
VVNQIKTR+XL_S
VVNQIKTR+XL_L
A_b_ion
A_y_ion
ETIRQKVTDVEGK+XL_S
ETIRQKVTDVEGK+XL_L
B_b_ion
B_y_ion
m/z

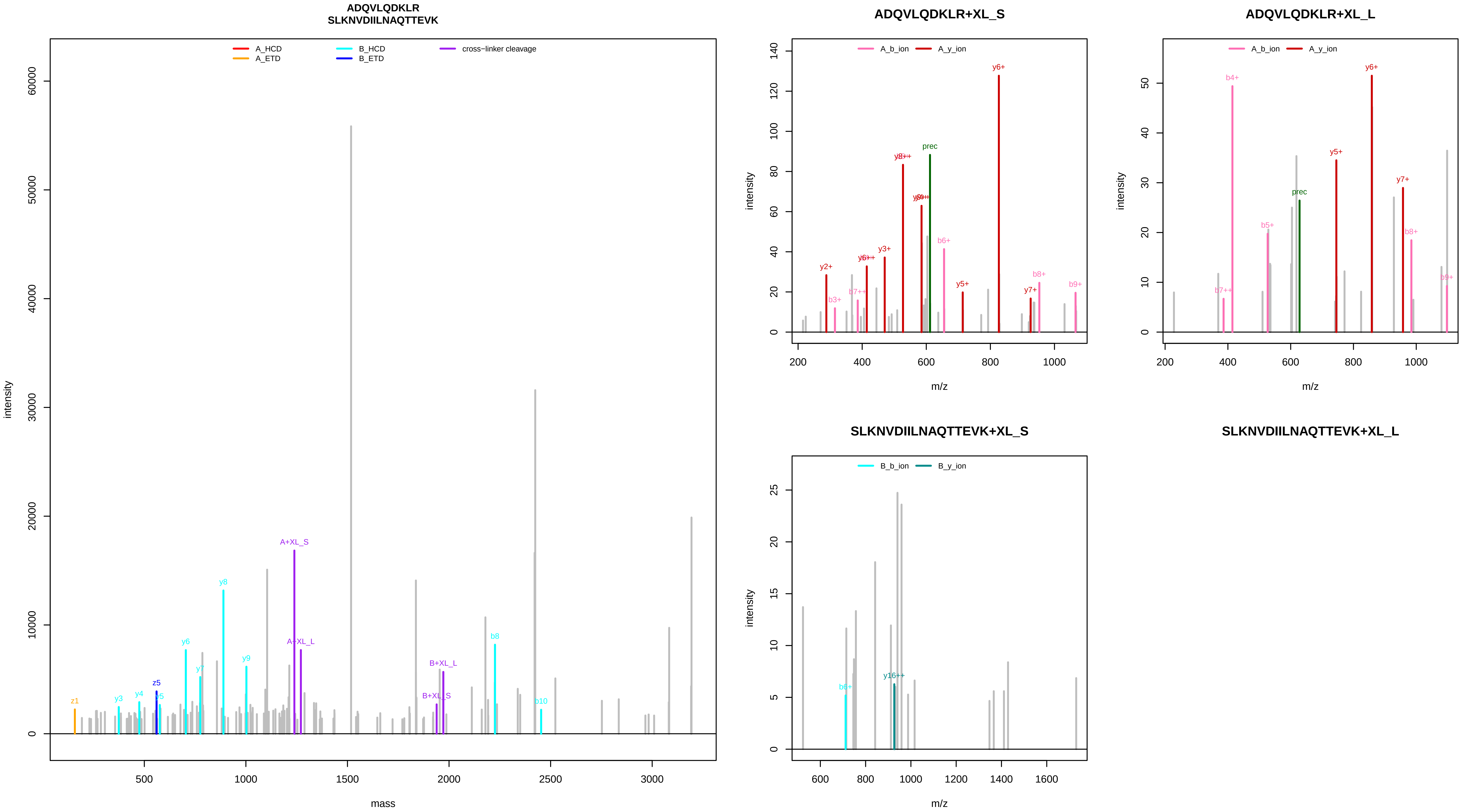

ADQVLQDKLR
SLKNVDIILNAQTTEVK
A_HCD
A_ETD
B_HCD
B_ETD
cross−linker cleavage
intensity
mass
A+XL_S
A+XL_L
B+XL_L
B+XL_S
ADQVLQDKLR+XL_S
ADQVLQDKLR+XL_L
A_b_ion
A_y_ion
prec
m/z
SLKNVDIILNAQTTEVK+XL_S
SLKNVDIILNAQTTEVK+XL_L
B_b_ion
B_y_ion
y16++

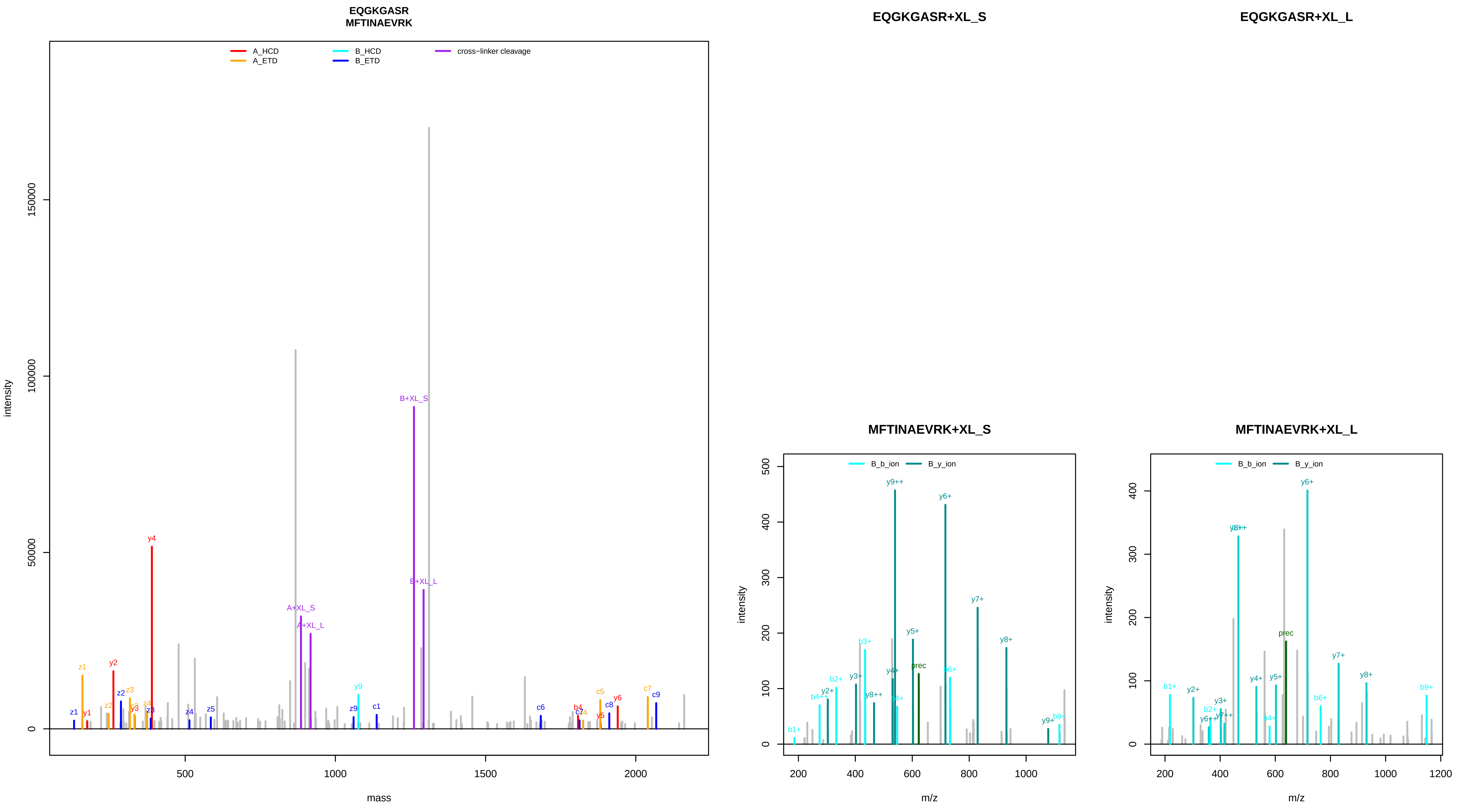

EQGKGASR
MFTINAEVRK
A_HCD
A_ETD
B_HCD
B_ETD
cross−linker cleavage
B+XL_S
B+XL_L
A+XL_S
A+XL_L
intensity
mass
EQGKGASR+XL_S
EQGKGASR+XL_L
MFTINAEVRK+XL_S
MFTINAEVRK+XL_L
B_b_ion
B_y_ion
prec
m/z

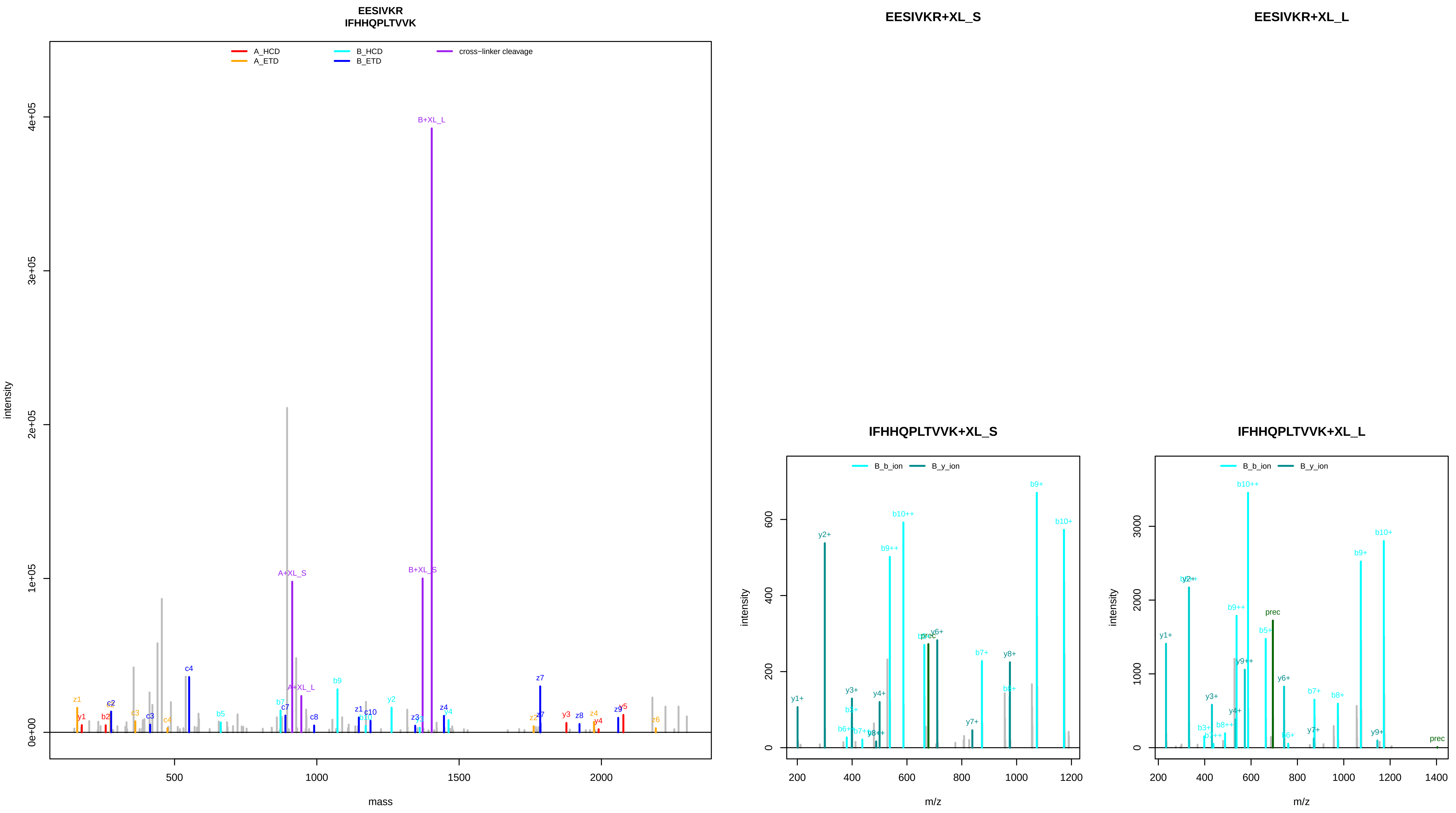
EESIVKR
IFHHQPLTVVK
A_HCD
A_ETD
B_HCD
B_ETD
cross−linker cleavage
intensity
mass
0e+00
1e+05
2e+05
3e+05
4e+05
500
1000
1500
2000
B+XL_L
A+XL_S
B+XL_S
A+XL_L
z1
y1
b2
c2
c3
c3
c4
c4
b5
b7
c7
c8
b9
z1
c10
b10
y2
z3
z4
y4
z7
z7
z2
y3
z8
z4
y4
z9
y5
z6
EESIVKR+XL_S
EESIVKR+XL_L
IFHHQPLTVVK+XL_S
IFHHQPLTVVK+XL_L
B_b_ion
B_y_ion
m/z
0
200
400
600
800
1000
1200
1400
3000
2000
1000
y1+
y2+
y3+
y4+
y6+
y7+
y8+
y9+
b5+
b6+
b7+
b8+
b9+
b10+
b9++
b10++
prec

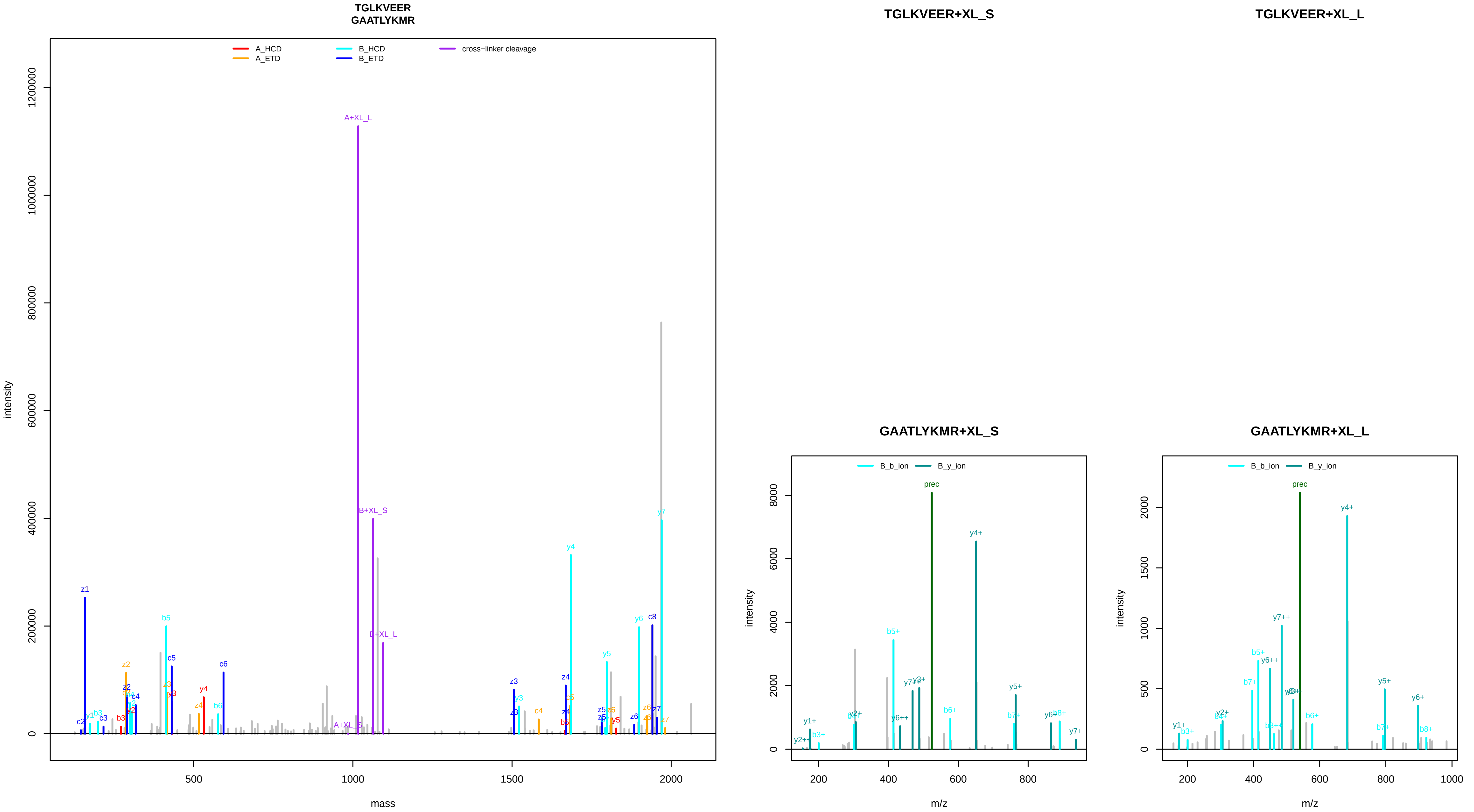
TGLKVEER
GAATLYKMR
A_HCD
A_ETD
B_HCD
B_ETD
cross−linker cleavage
A+XL_L
B+XL_S
B+XL_L
A+XL_S
intensity
mass
TGLKVEER+XL_S
TGLKVEER+XL_L
GAATLYKMR+XL_S
GAATLYKMR+XL_L
B_b_ion
B_y_ion
prec
intensity
m/z

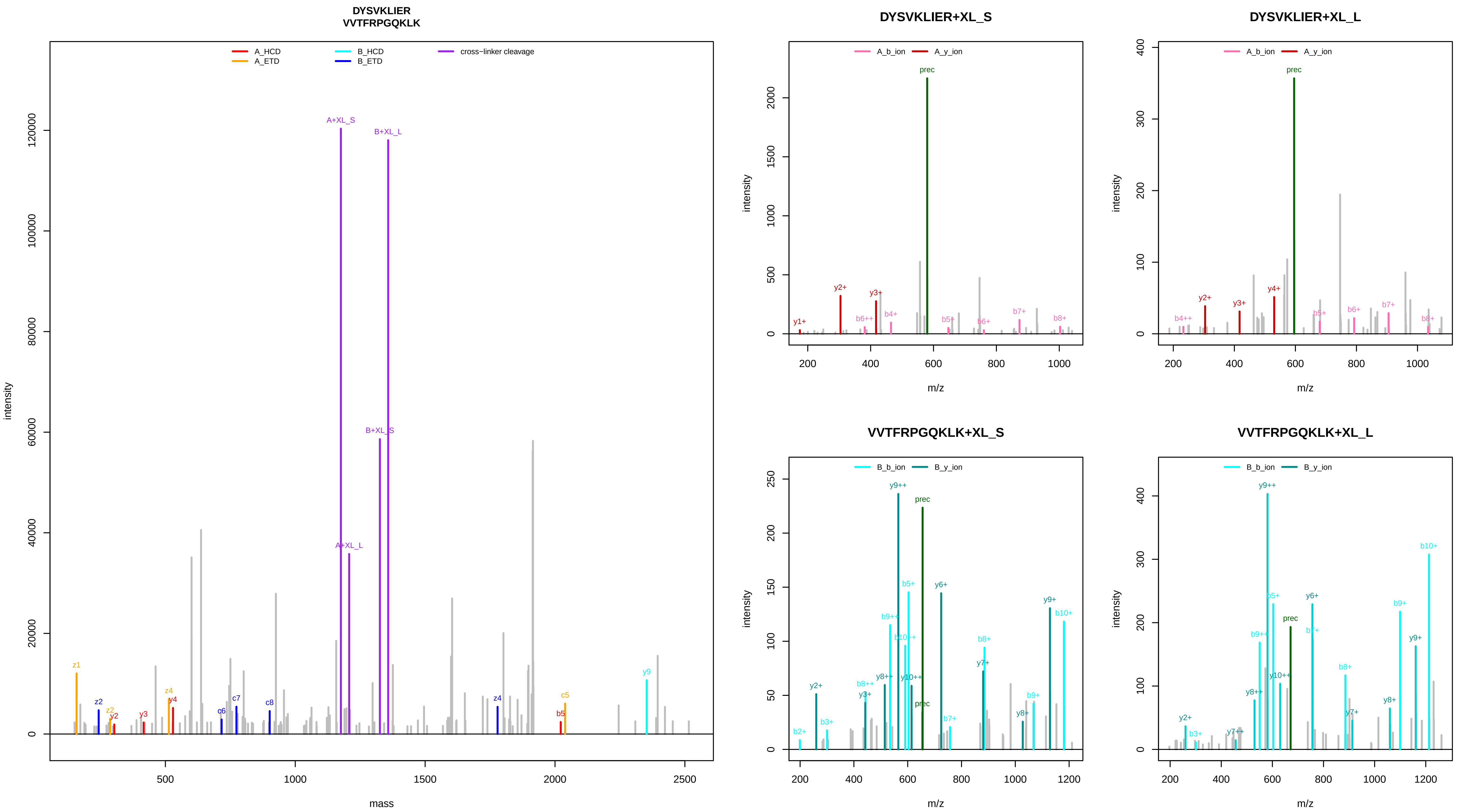
DYSVKLIER
VVTFRPGQKLK
A_HCD
A_ETD
B_HCD
B_ETD
cross−linker cleavage
A+XL_S
B+XL_L
B+XL_S
A+XL_L
intensity
mass
DYSVKLIER+XL_S
DYSVKLIER+XL_L
A_b_ion
A_y_ion
prec
m/z
VVTFRPGQKLK+XL_S
VVTFRPGQKLK+XL_L
B_b_ion
B_y_ion

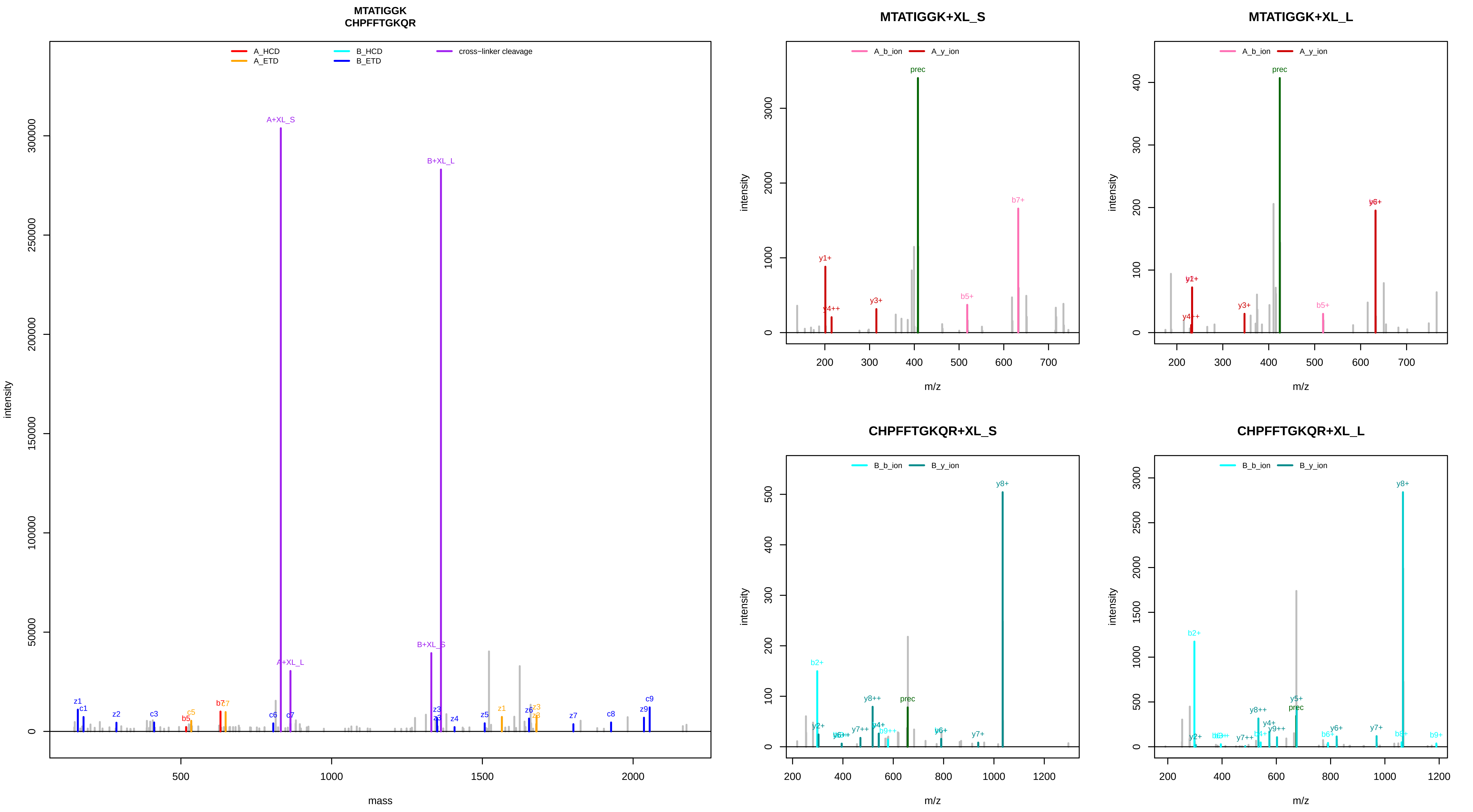

MTATIGGK
CHPFFTGKQR
A_HCD
A_ETD
B_HCD
B_ETD
cross−linker cleavage
A+XL_S
B+XL_L
B+XL_S
A+XL_L
intensity
mass
MTATIGGK+XL_S
MTATIGGK+XL_L
A_b_ion
A_y_ion
prec
CHPFFTGKQR+XL_S
CHPFFTGKQR+XL_L
B_b_ion
B_y_ion
y8+
b2+
m/z

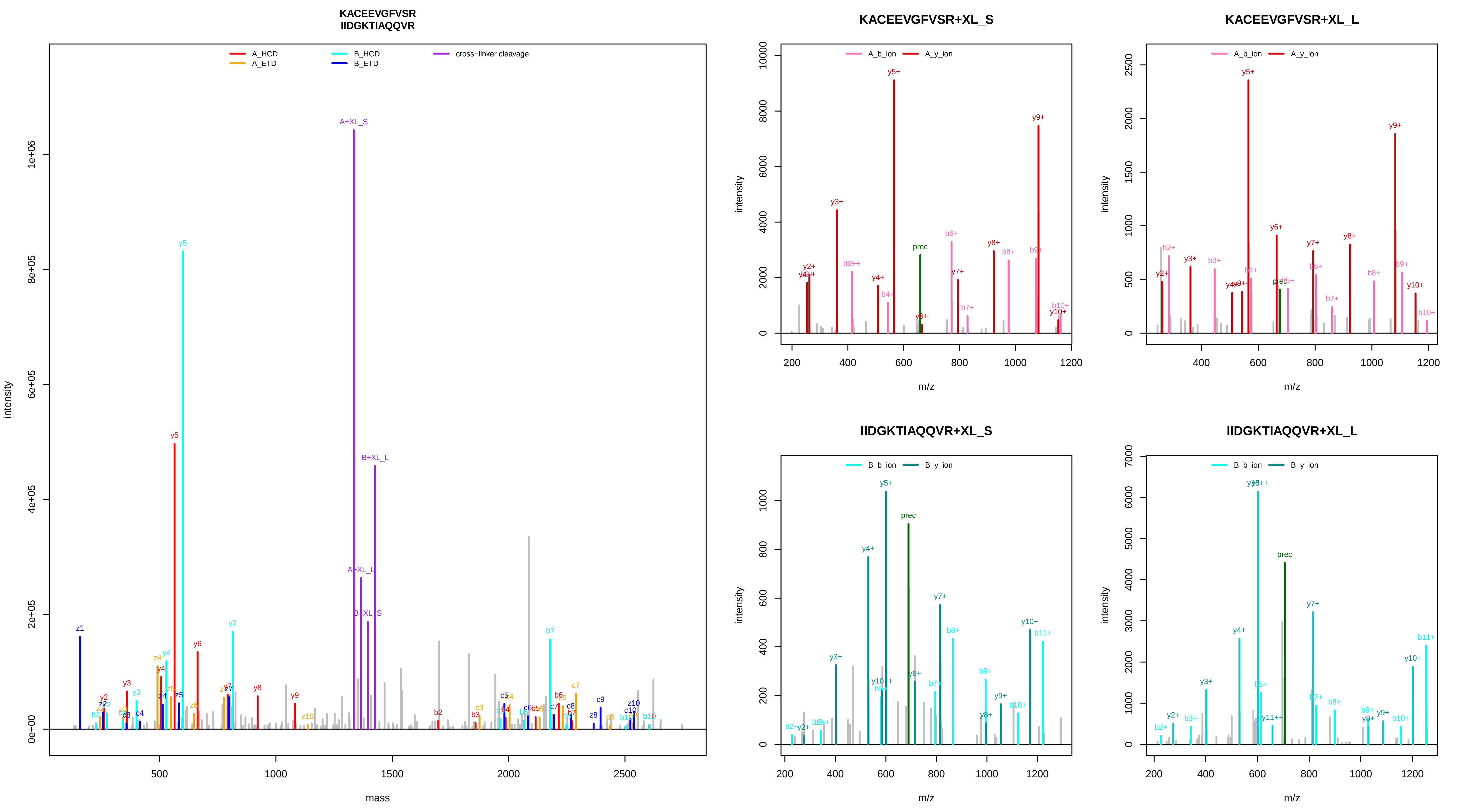
KACEEVGFVSR
IIDGKTIAQQVR
A_HCD
A_ETD
B_HCD
B_ETD
cross−linker cleavage
A+XL_S
B+XL_L
A+XL_L
B+XL_S
intensity
mass
KACEEVGFVSR+XL_S
KACEEVGFVSR+XL_L
IIDGKTIAQQVR+XL_S
IIDGKTIAQQVR+XL_L
A_b_ion
A_y_ion
B_b_ion
B_y_ion
prec
m/z

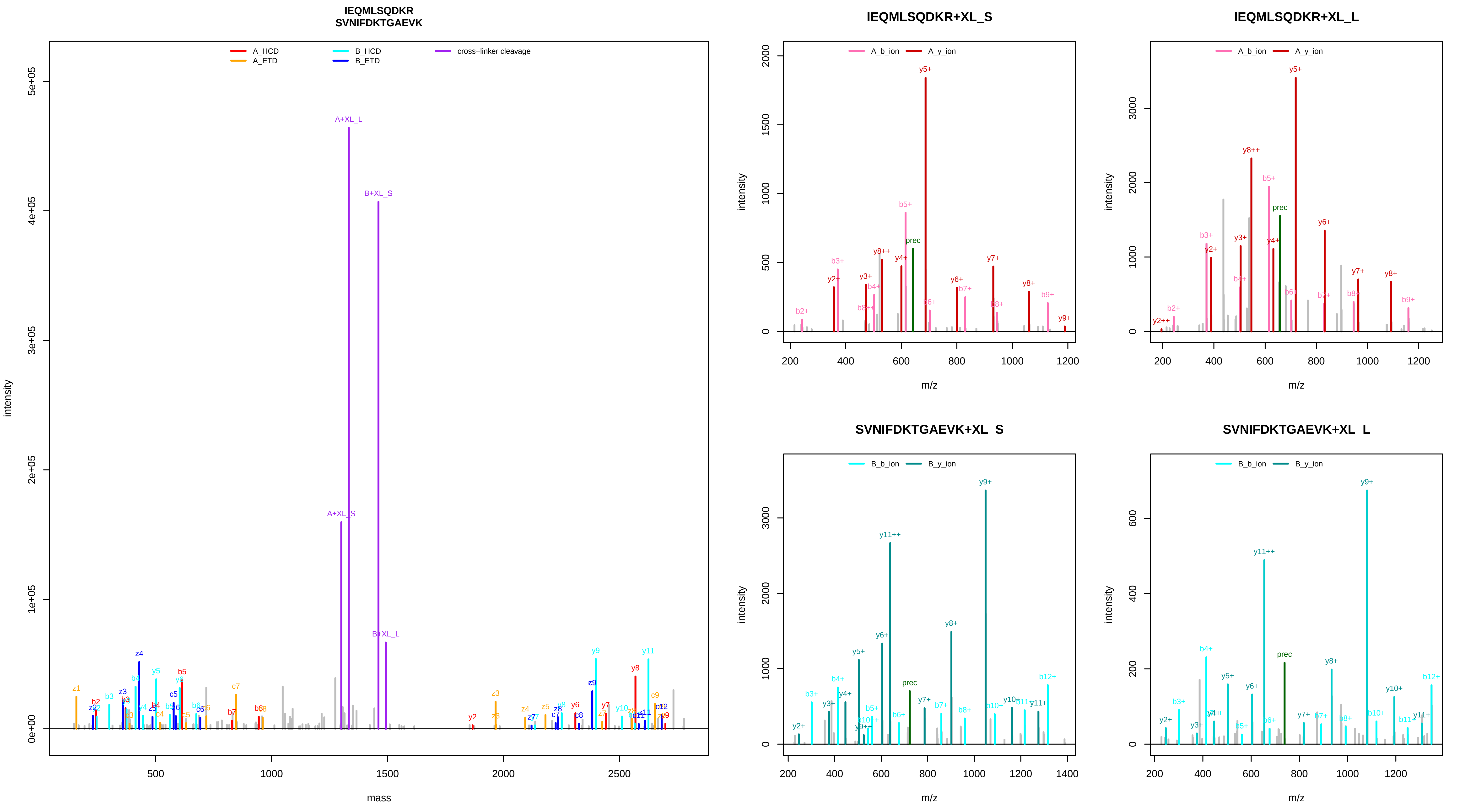

IEQMLSQDKR
SVNIFDKTGAEVK
A_HCD
A_ETD
B_HCD
B_ETD
cross−linker cleavage
A+XL_L
B+XL_S
A+XL_S
B+XL_L
intensity
mass
IEQMLSQDKR+XL_S
IEQMLSQDKR+XL_L
A_b_ion
A_y_ion
SVNIFDKTGAEVK+XL_S
SVNIFDKTGAEVK+XL_L
B_b_ion
B_y_ion
prec
m/z

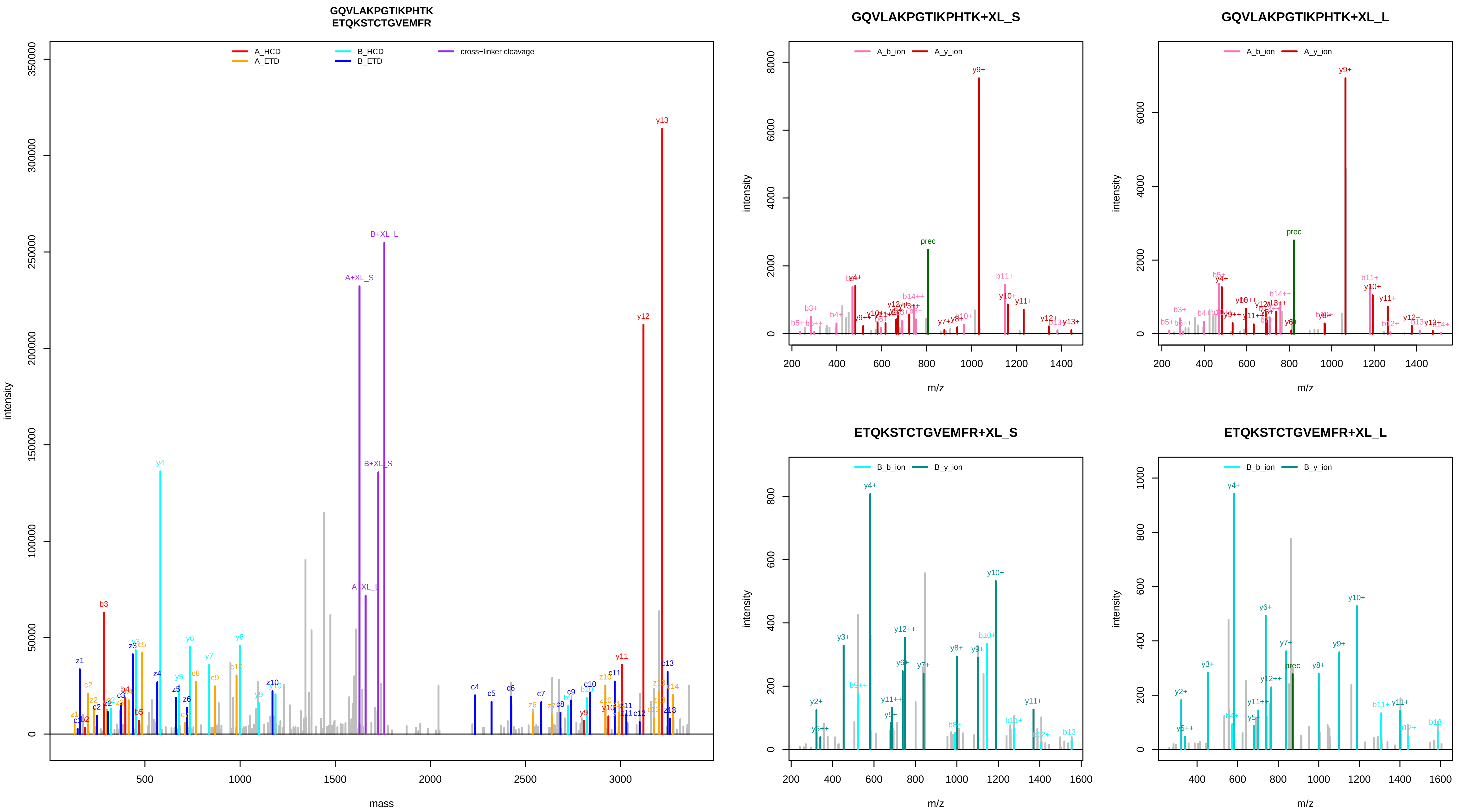

GQVLAKPGTIKPHTK
ETQKSTCTGVEMFR
A_HCD
A_ETD
B_HCD
B_ETD
cross−linker cleavage
intensity
mass
A+XL_S
B+XL_L
B+XL_S
A+XL_L
y13
y12
y11
y4
b3
GQVLAKPGTIKPHTK+XL_S
GQVLAKPGTIKPHTK+XL_L
A_b_ion
A_y_ion
y9+
prec
m/z
ETQKSTCTGVEMFR+XL_S
ETQKSTCTGVEMFR+XL_L
B_b_ion
B_y_ion
y4+
y10+

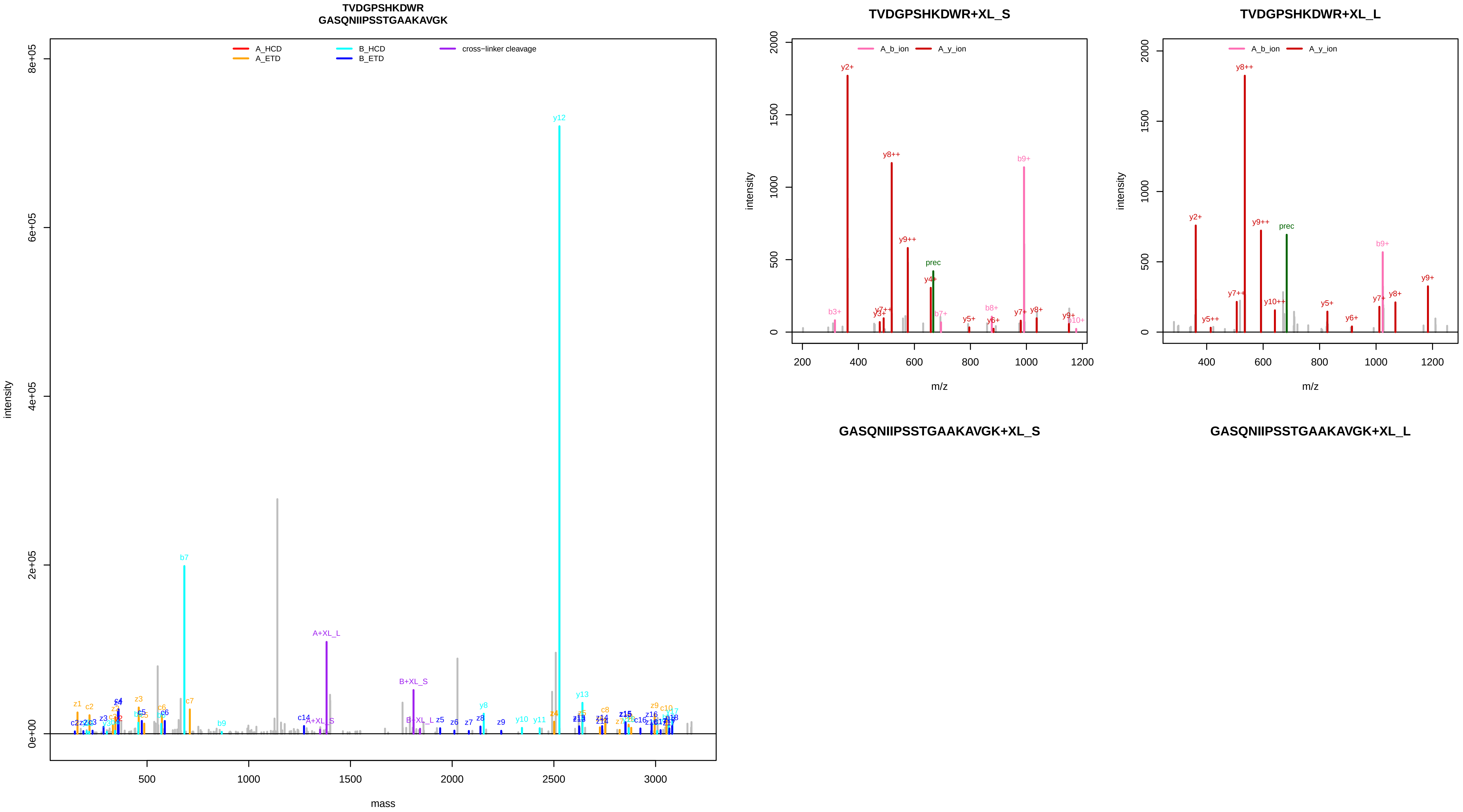

TVDGPSHKDWR
GASQNIIPSSTGAAKAVGK
A_HCD
A_ETD
B_HCD
B_ETD
cross−linker cleavage
intensity
mass
y12
b7
A+XL_L
B+XL_S
TVDGPSHKDWR+XL_S
TVDGPSHKDWR+XL_L
A_b_ion
A_y_ion
m/z
prec
GASQNIIPSSTGAAKAVGK+XL_S
GASQNIIPSSTGAAKAVGK+XL_L

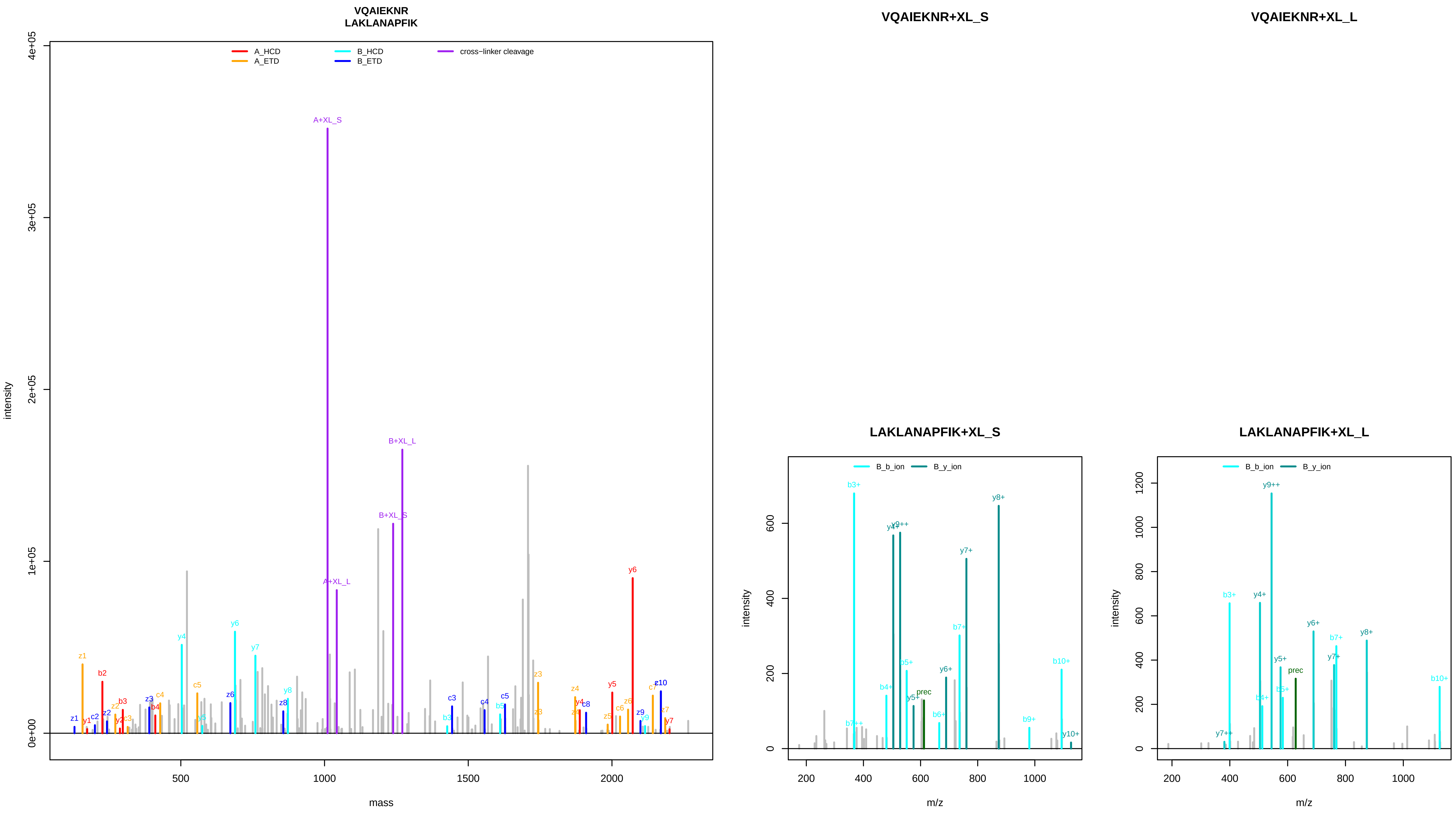
VQAIEKNR
LAKLANAPFIK
A_HCD
A_ETD
B_HCD
B_ETD
cross−linker cleavage
A+XL_S
B+XL_L
B+XL_S
A+XL_L
intensity
mass
VQAIEKNR+XL_S
VQAIEKNR+XL_L
LAKLANAPFIK+XL_S
LAKLANAPFIK+XL_L
B_b_ion
B_y_ion
prec
m/z

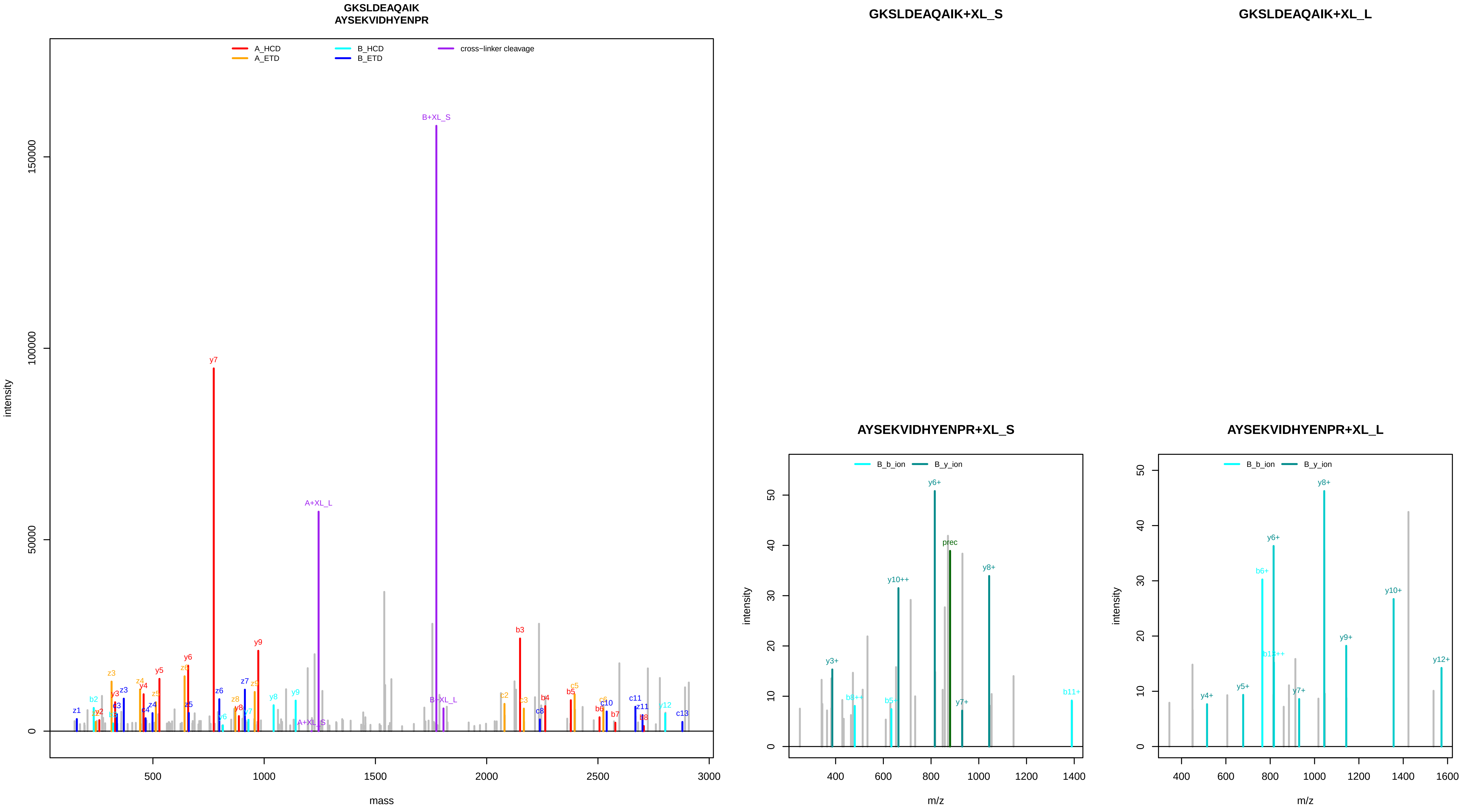

GKSLDEAQAIK
AYSEKVIDHYENPR
A_HCD
A_ETD
B_HCD
B_ETD
cross−linker cleavage
B+XL_S
A+XL_L
B+XL_L
A+XL_S
intensity
mass
GKSLDEAQAIK+XL_S
GKSLDEAQAIK+XL_L
AYSEKVIDHYENPR+XL_S
AYSEKVIDHYENPR+XL_L
B_b_ion
B_y_ion
prec
intensity
m/z

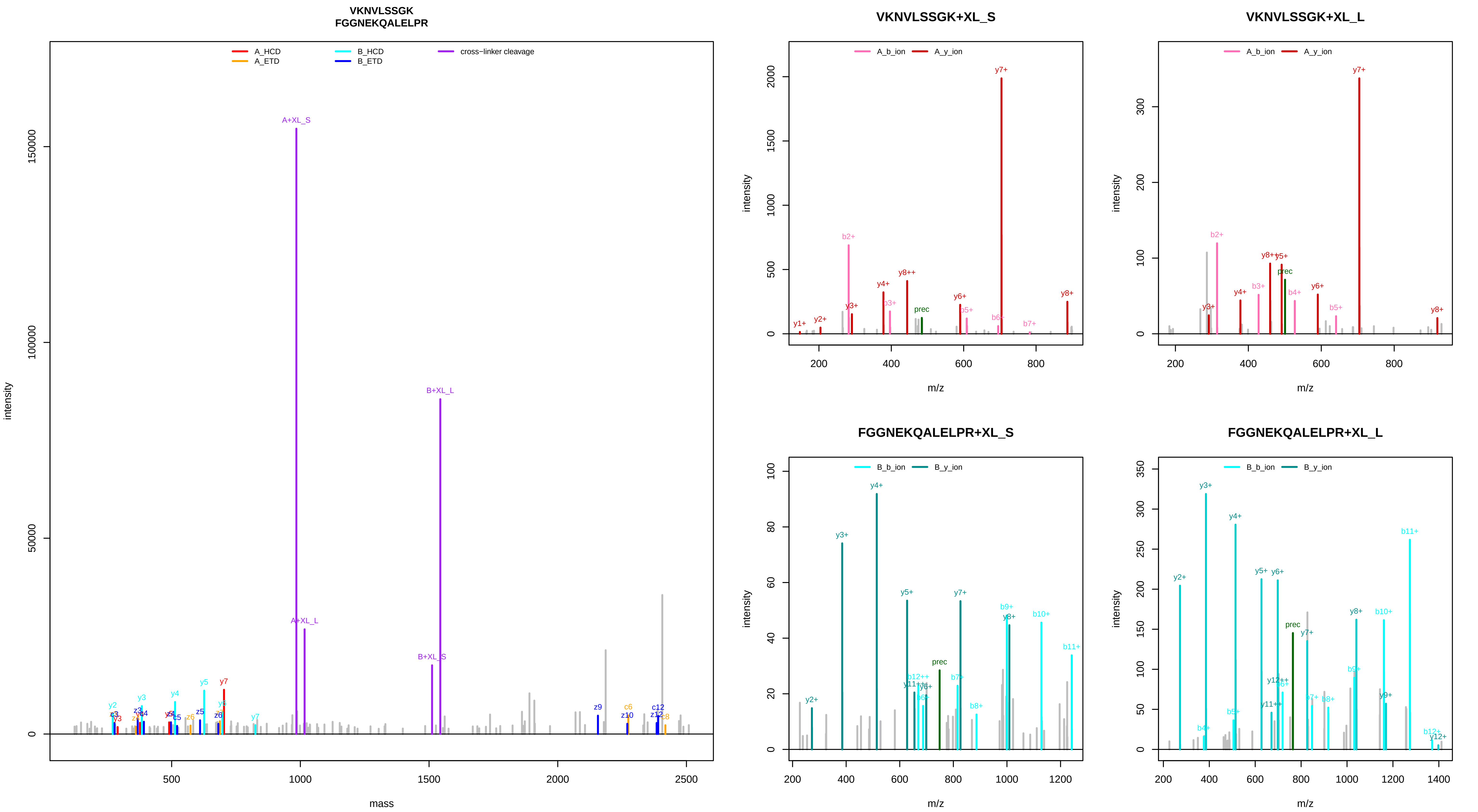
VKNVLSSGK
FGGNEKQALELPR
A_HCD
A_ETD
B_HCD
B_ETD
cross−linker cleavage
A+XL_S
B+XL_L
A+XL_L
B+XL_S
intensity
mass
VKNVLSSGK+XL_S
VKNVLSSGK+XL_L
FGGNEKQALELPR+XL_S
FGGNEKQALELPR+XL_L
A_b_ion
A_y_ion
B_b_ion
B_y_ion
prec
m/z

KGIVMFEK
NIIAVSSGKGGVGK

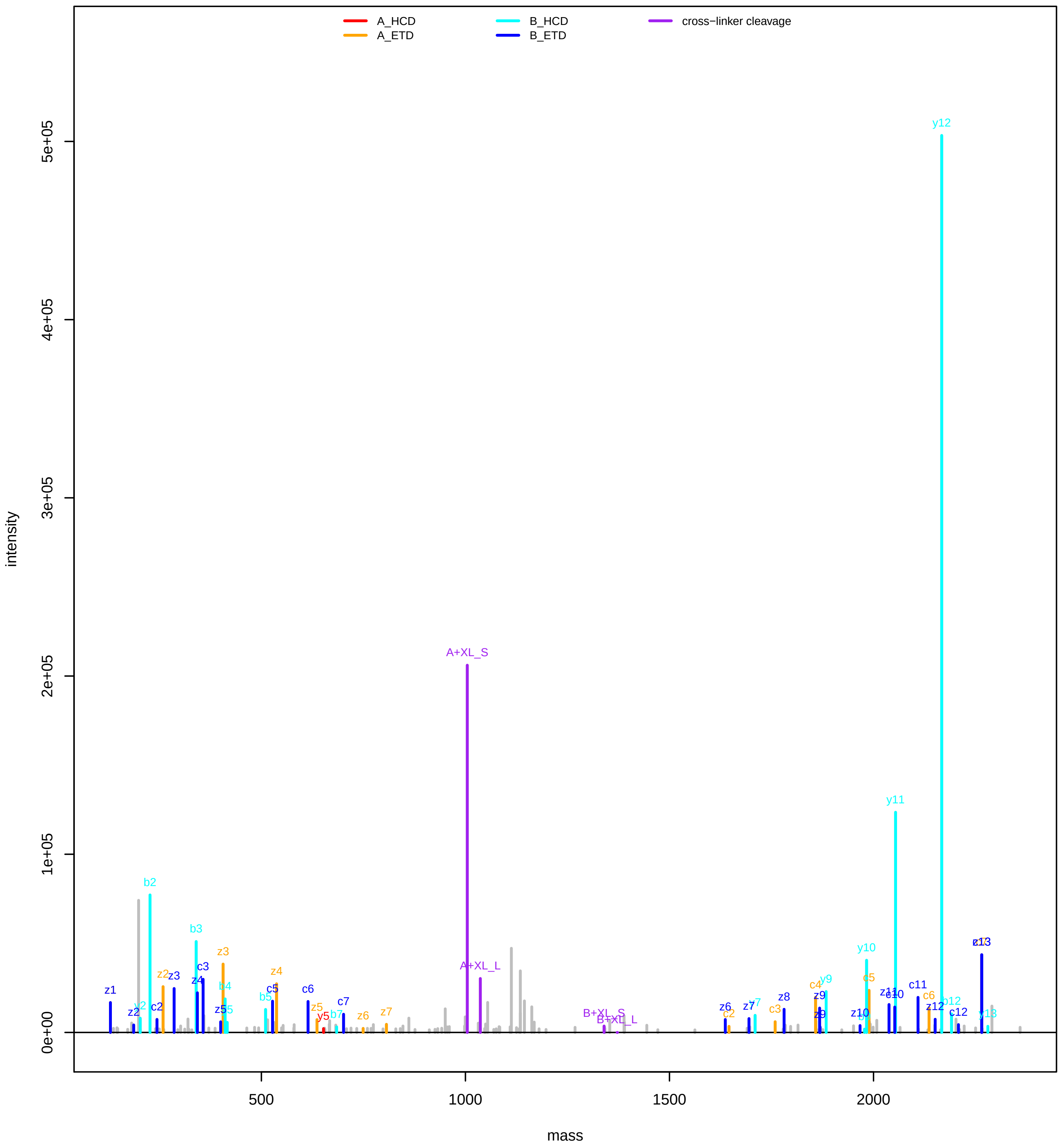

KGIVMFEK+XL_S

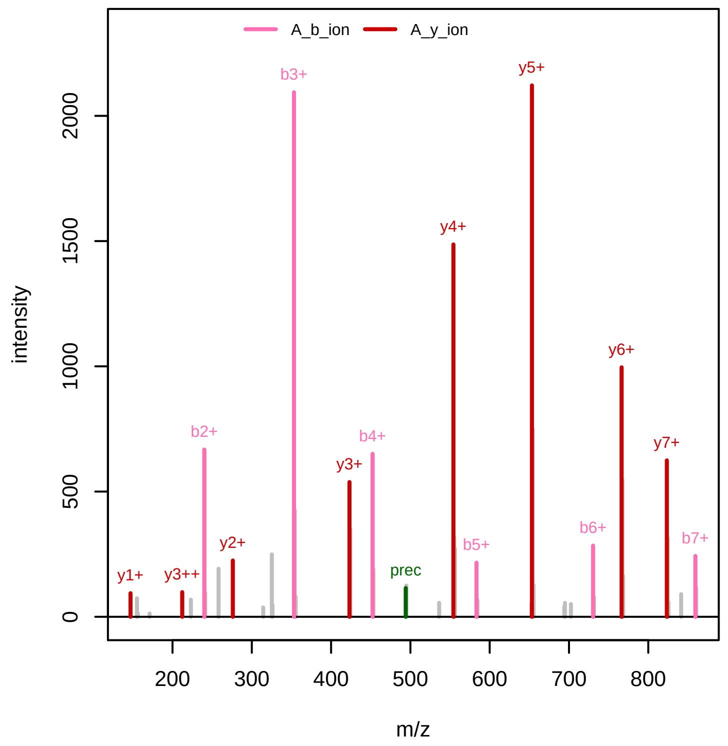

KGIVMFEK+XL_L

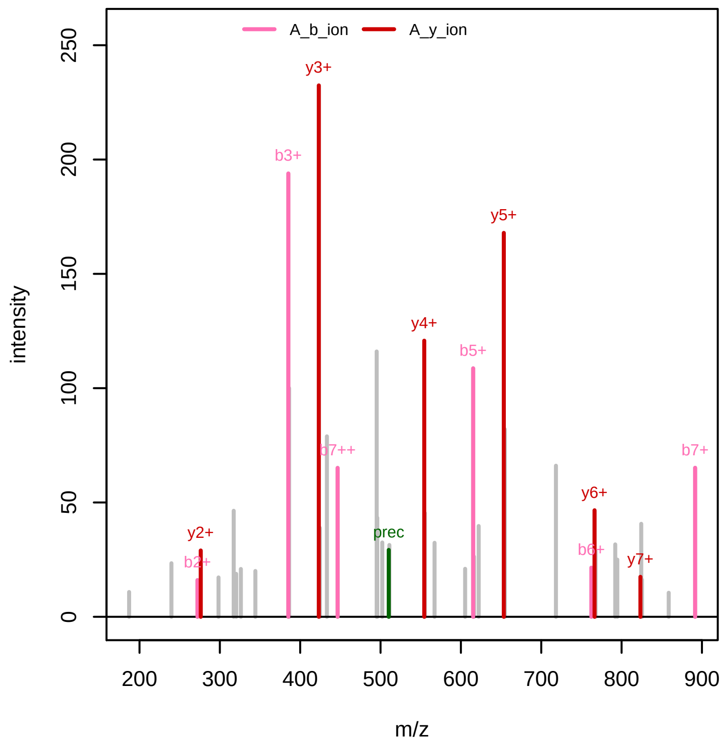

NIIAVSSGKGGVGK+XL_S

NIIAVSSGKGGVGK+XL_L

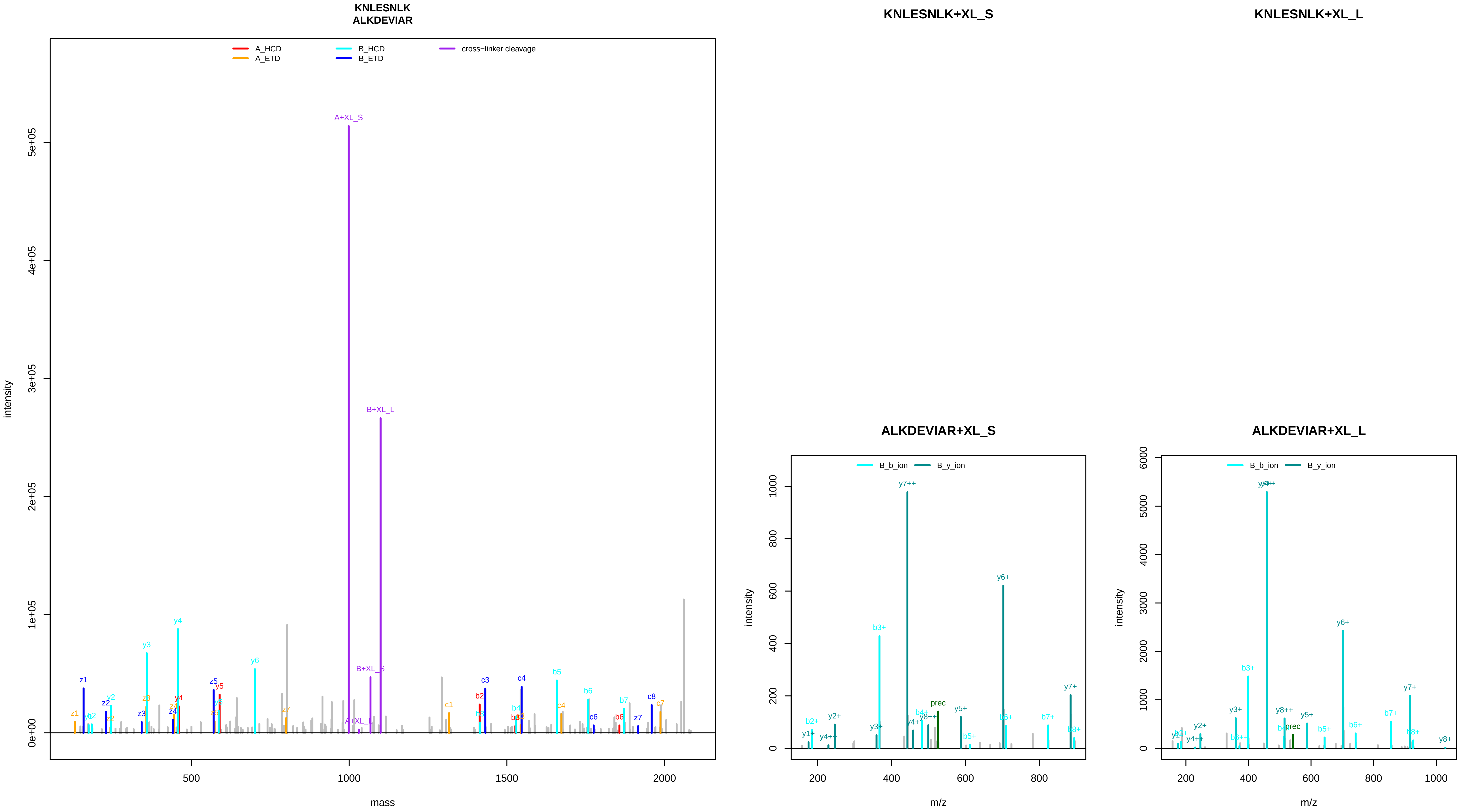
KNLESNLK
ALKDEVIAR
A_HCD
A_ETD
B_HCD
B_ETD
cross−linker cleavage
A+XL_S
B+XL_L
B+XL_S
A+XL_L
intensity
mass
0e+00
1e+05
2e+05
3e+05
4e+05
5e+05
500
1000
1500
2000
KNLESNLK+XL_S
KNLESNLK+XL_L
ALKDEVIAR+XL_S
ALKDEVIAR+XL_L
B_b_ion
B_y_ion
y7++
y6+
b3+
y7+
prec
m/z
intensity
0
200
400
600
800
1000
200
400
600
800
1000
2000
3000
4000
5000
6000

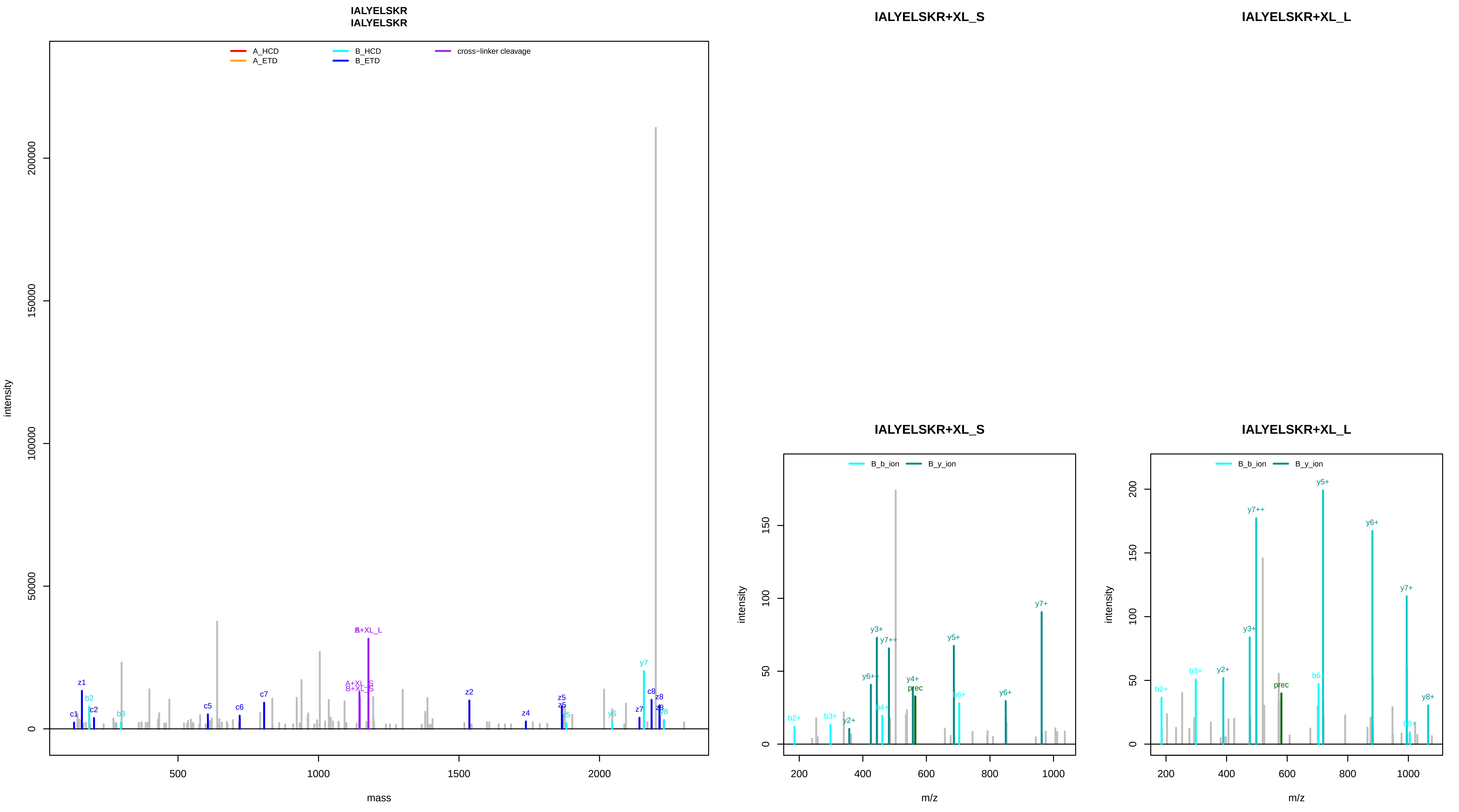
IALYELSKR
IALYELSKR
A_HCD
A_ETD
B_HCD
B_ETD
cross−linker cleavage
intensity
mass
IALYELSKR+XL_S
IALYELSKR+XL_L
IALYELSKR+XL_S
B_b_ion
B_y_ion
intensity
m/z
IALYELSKR+XL_L
B_b_ion
B_y_ion
intensity
m/z

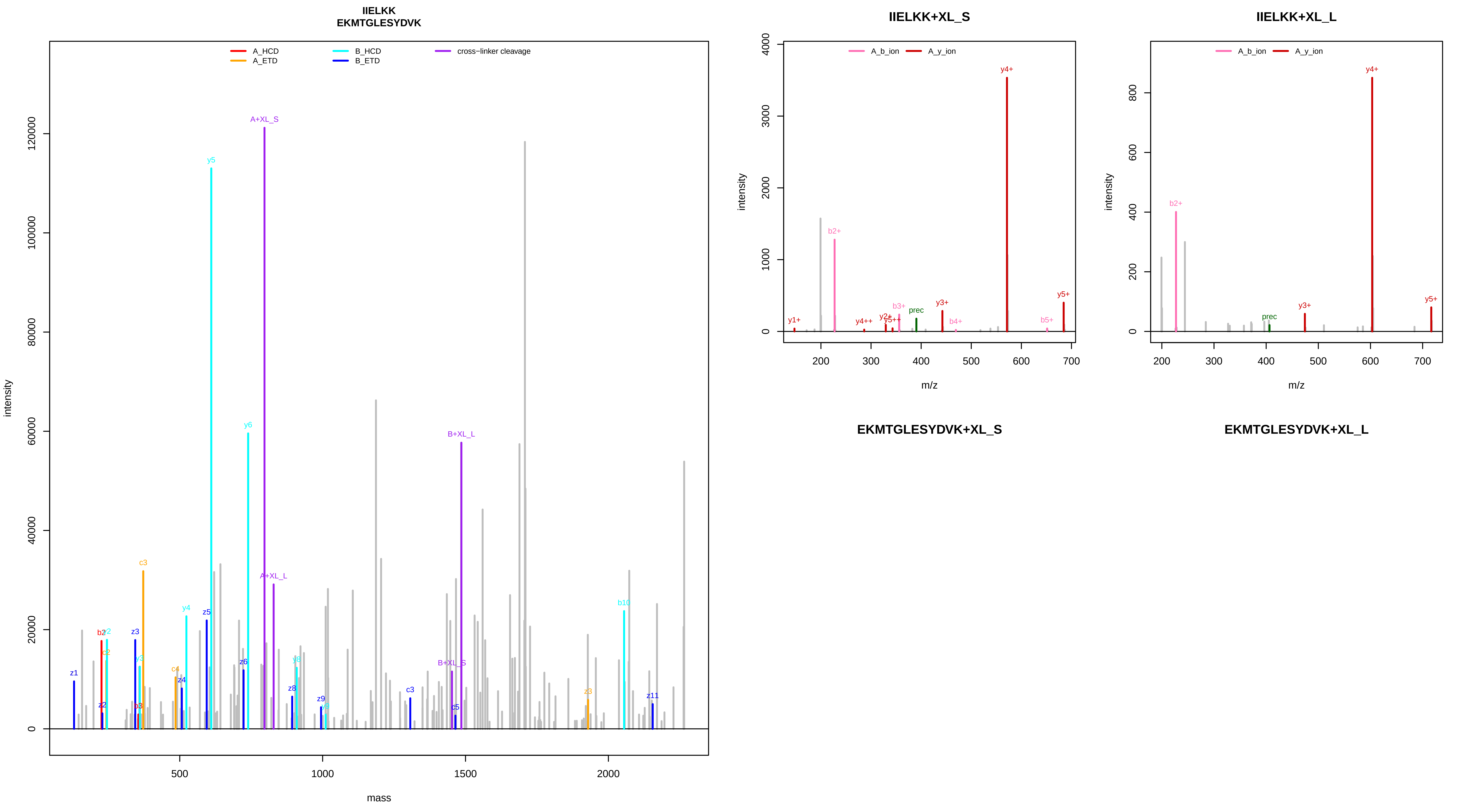
IIELKK
EKMTGLESYDVK
A_HCD
A_ETD
B_HCD
B_ETD
cross−linker cleavage
A+XL_S
A+XL_L
B+XL_L
B+XL_S
intensity
mass
IIELKK+XL_S
IIELKK+XL_L
A_b_ion
A_y_ion
prec
m/z
EKMTGLESYDVK+XL_S
EKMTGLESYDVK+XL_L

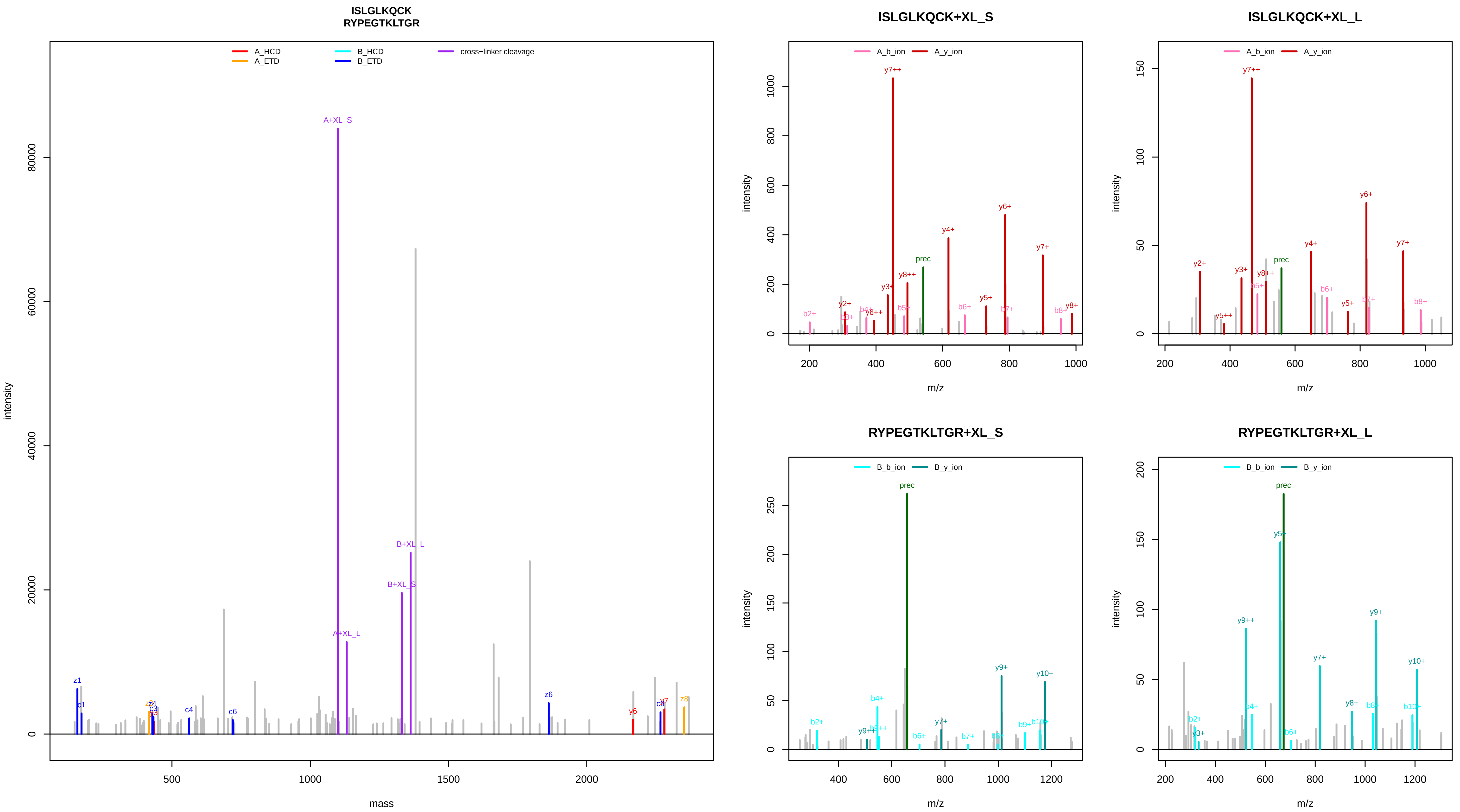
ISLGLKQCK
RYPEGTKLTGR
A_HCD
A_ETD
B_HCD
B_ETD
cross−linker cleavage
A+XL_S
A+XL_L
B+XL_S
B+XL_L
intensity
mass
ISLGLKQCK+XL_S
ISLGLKQCK+XL_L
RYPEGTKLTGR+XL_S
RYPEGTKLTGR+XL_L
A_b_ion
A_y_ion
B_b_ion
B_y_ion
prec
m/z

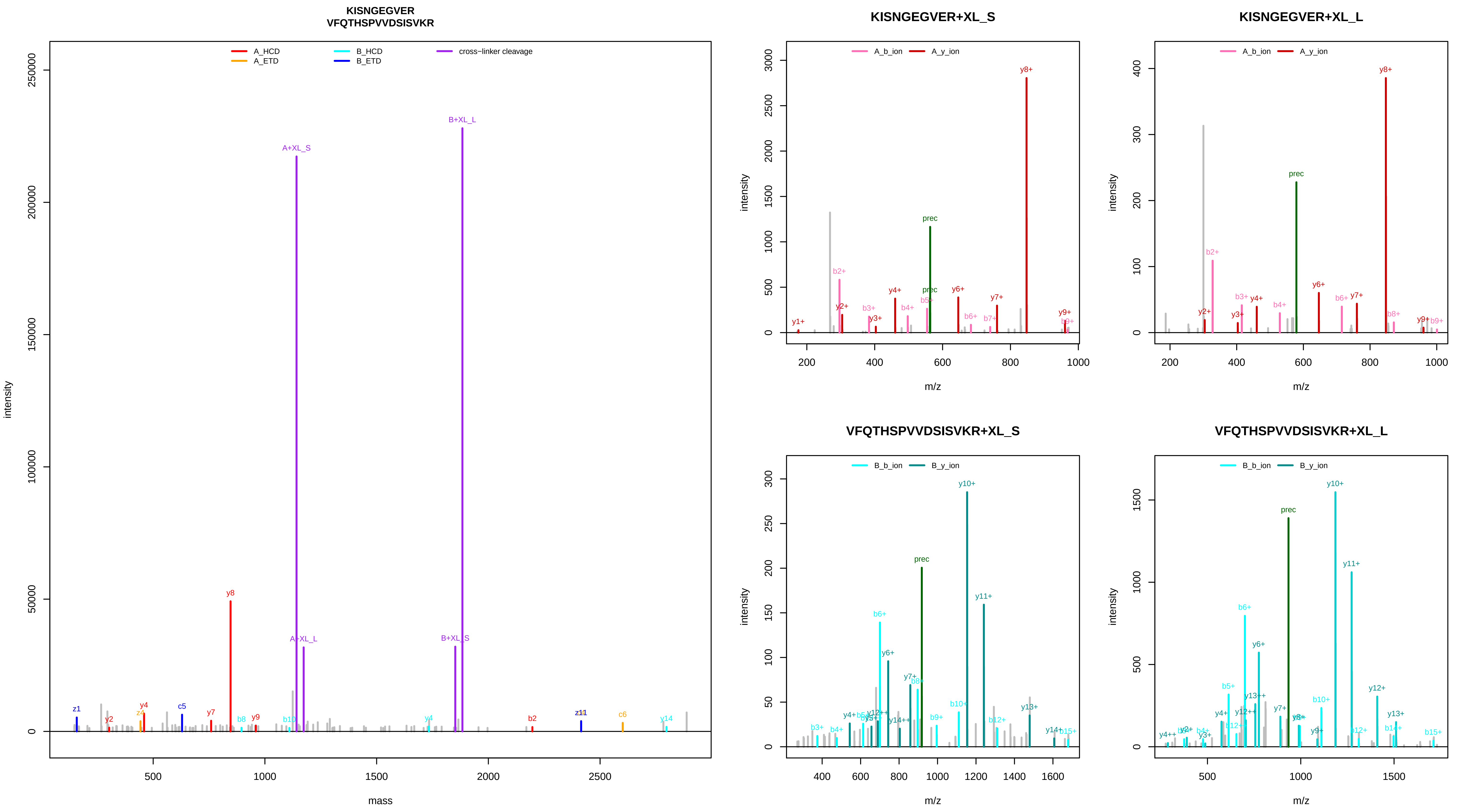

KISNGEGVER
VFQTHSPVVDSISVKR
A_HCD
A_ETD
B_HCD
B_ETD
cross−linker cleavage
A+XL_S
B+XL_L
A+XL_L
B+XL_S
intensity
mass
KISNGEGVER+XL_S
KISNGEGVER+XL_L
VFQTHSPVVDSISVKR+XL_S
VFQTHSPVVDSISVKR+XL_L
A_b_ion
A_y_ion
B_b_ion
B_y_ion
prec
m/z

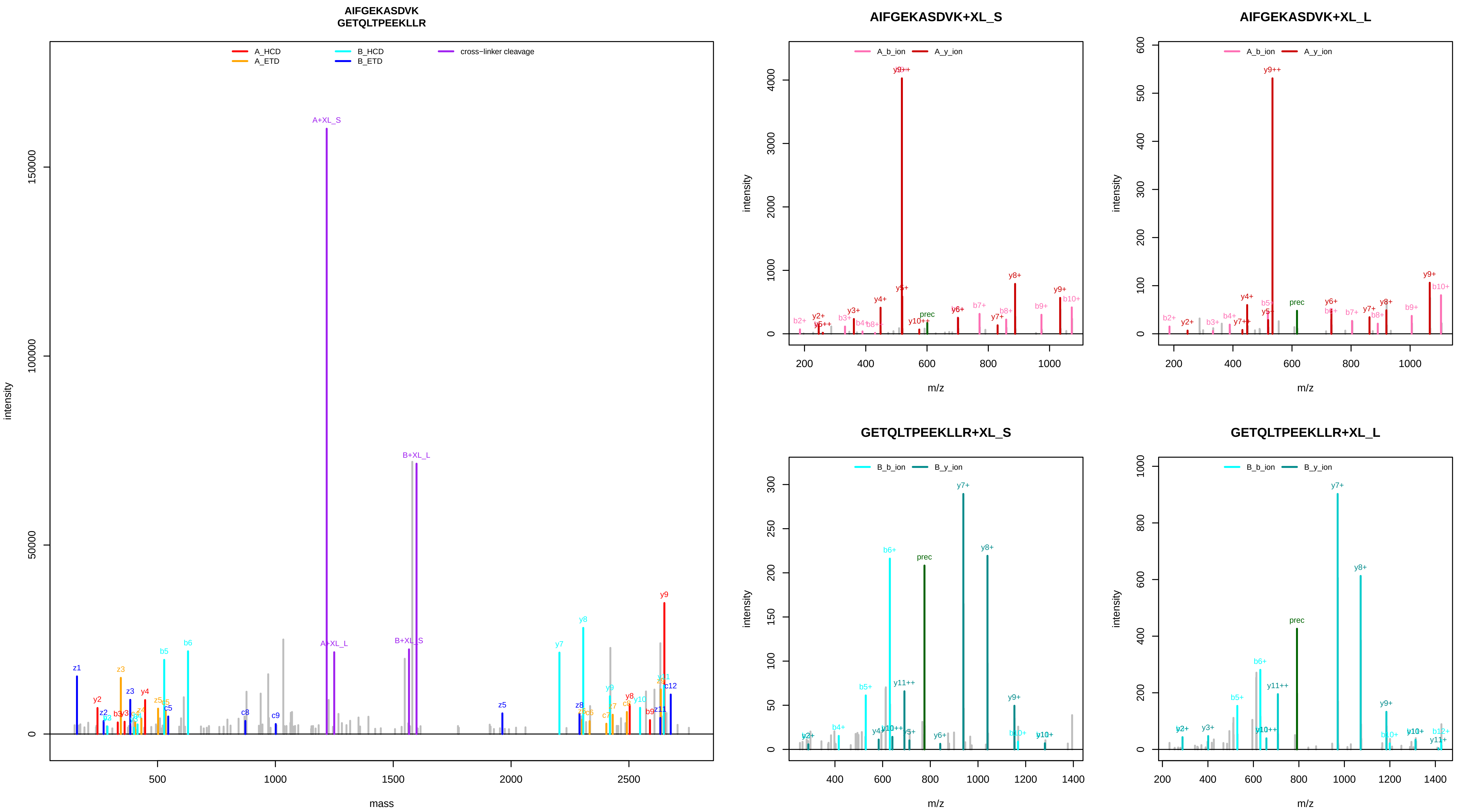
AIFGEKASDVK
GETQLTPEEKLLR
A_HCD
A_ETD
B_HCD
B_ETD
cross−linker cleavage
A+XL_S
B+XL_L
A+XL_L
B+XL_S
intensity
mass
AIFGEKASDVK+XL_S
AIFGEKASDVK+XL_L
GETQLTPEEKLLR+XL_S
GETQLTPEEKLLR+XL_L
A_b_ion
A_y_ion
B_b_ion
B_y_ion
prec
m/z

DSLPEGVYNDQFKK
IMGKASFVTLQDVGGR

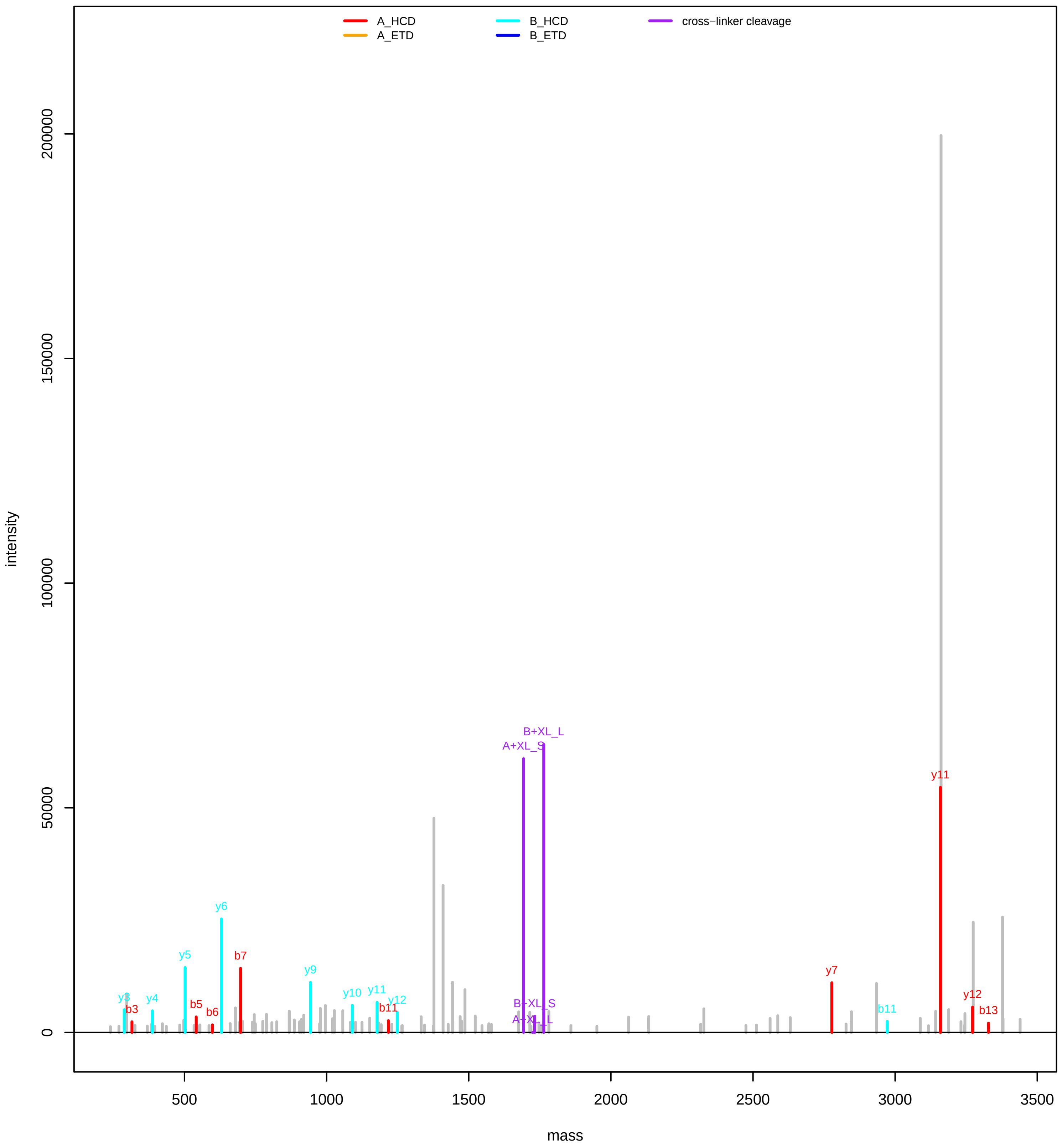

DSLPEGVYNDQFKK+XL_S

DSLPEGVYNDQFKK+XL_L

IMGKASFVTLQDVGGR+XL_S

IMGKASFVTLQDVGGR+XL_L

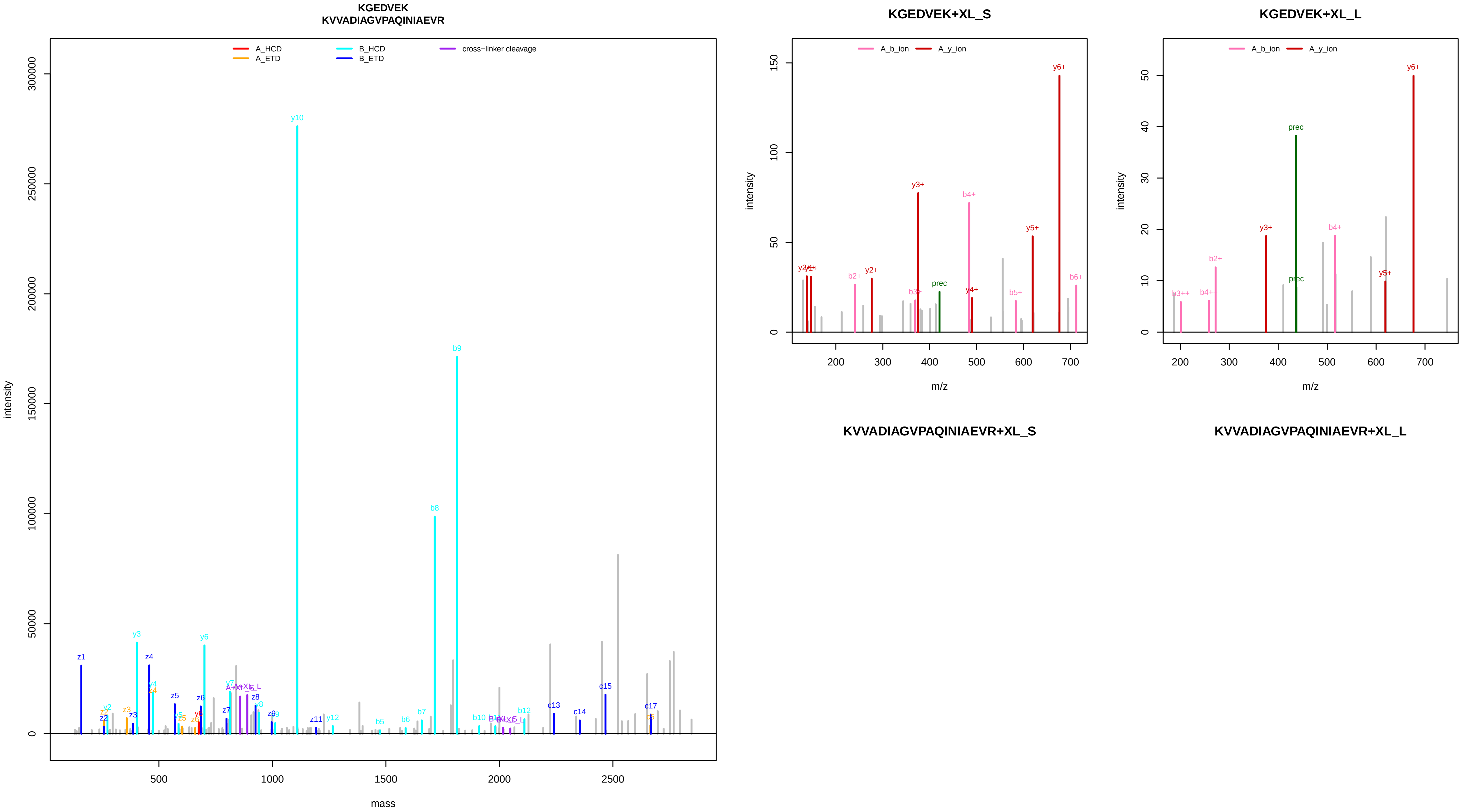
KGEDVEK
KVVADIAGVPAQINIAEVR
A_HCD
A_ETD
B_HCD
B_ETD
cross−linker cleavage
intensity
mass
KGEDVEK+XL_S
KGEDVEK+XL_L
A_b_ion
A_y_ion
m/z
KVVADIAGVPAQINIAEVR+XL_S
KVVADIAGVPAQINIAEVR+XL_L

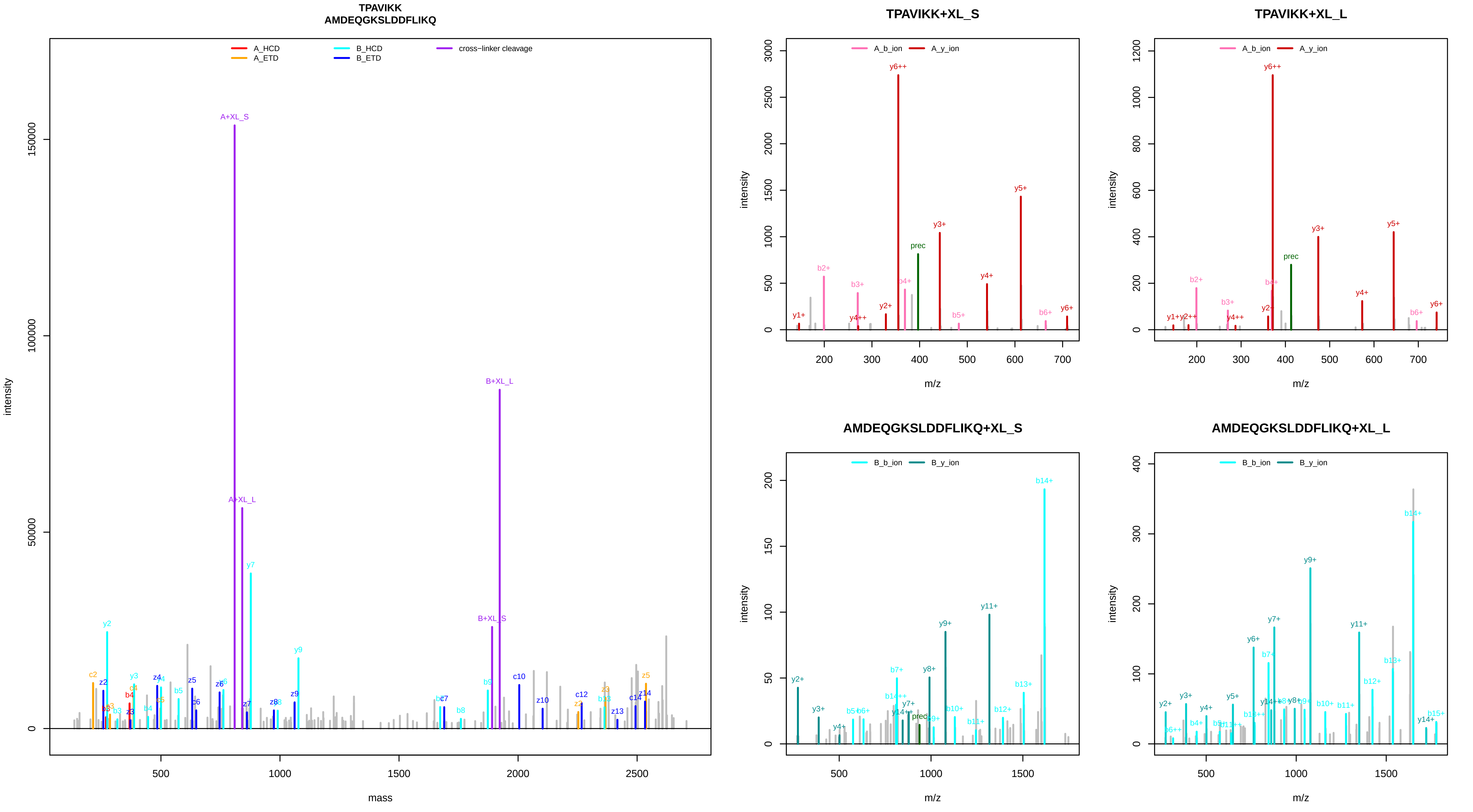

TPAVIKK
AMDEQGKSLDDFLIKQ
A_HCD
A_ETD
B_HCD
B_ETD
cross−linker cleavage
A+XL_S
A+XL_L
B+XL_L
B+XL_S
intensity
mass
TPAVIKK+XL_S
TPAVIKK+XL_L
A_b_ion
A_y_ion
AMDEQGKSLDDFLIKQ+XL_S
AMDEQGKSLDDFLIKQ+XL_L
B_b_ion
B_y_ion
m/z

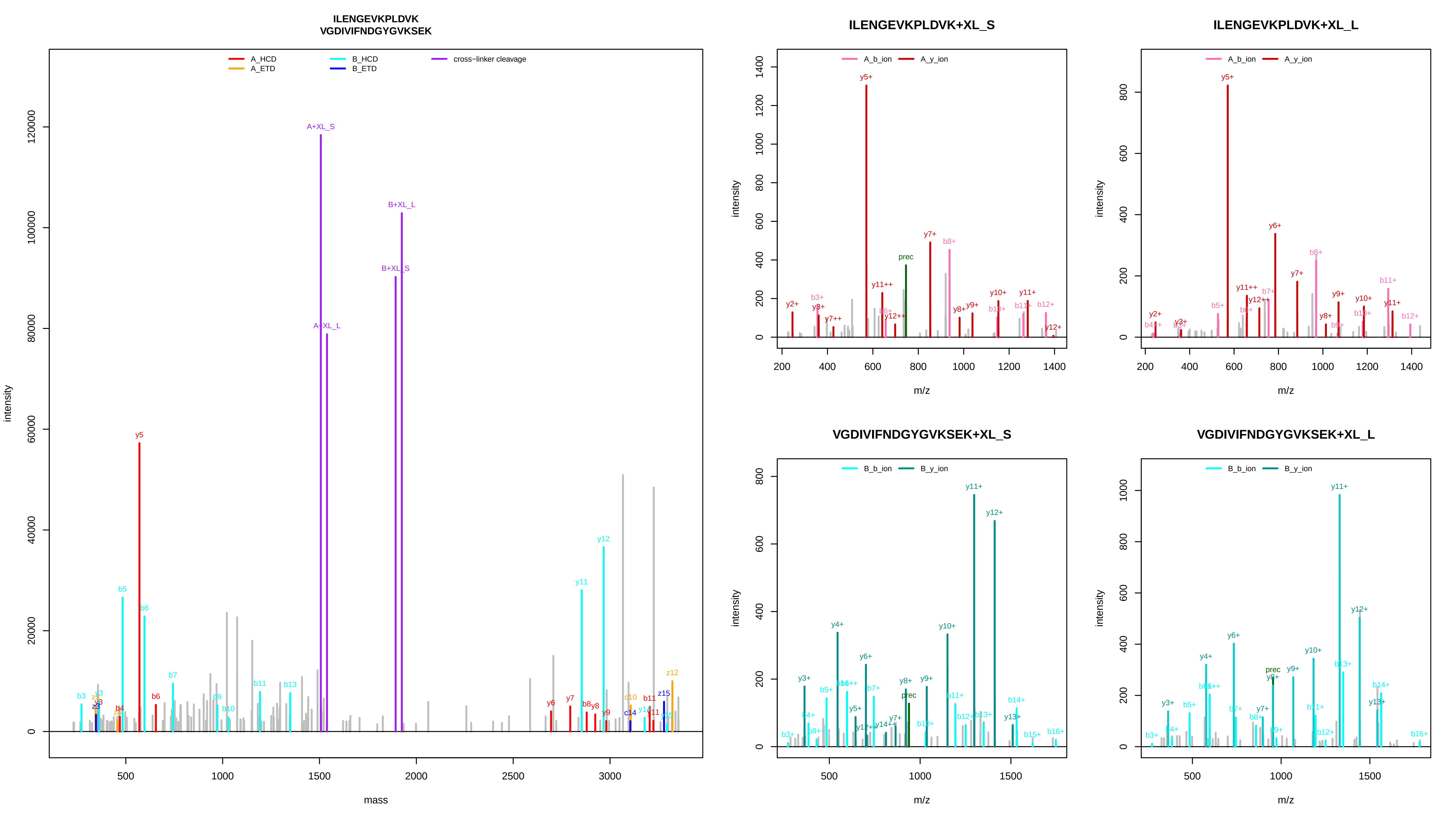
ILENGEVKPLDVK
VGDIVIFNDGYGVKSEK
A_HCD
A_ETD
B_HCD
B_ETD
cross−linker cleavage
A+XL_S
B+XL_L
B+XL_S
A+XL_L
intensity
mass
ILENGEVKPLDVK+XL_S
ILENGEVKPLDVK+XL_L
A_b_ion
A_y_ion
VGDIVIFNDGYGVKSEK+XL_S
VGDIVIFNDGYGVKSEK+XL_L
B_b_ion
B_y_ion
prec
m/z

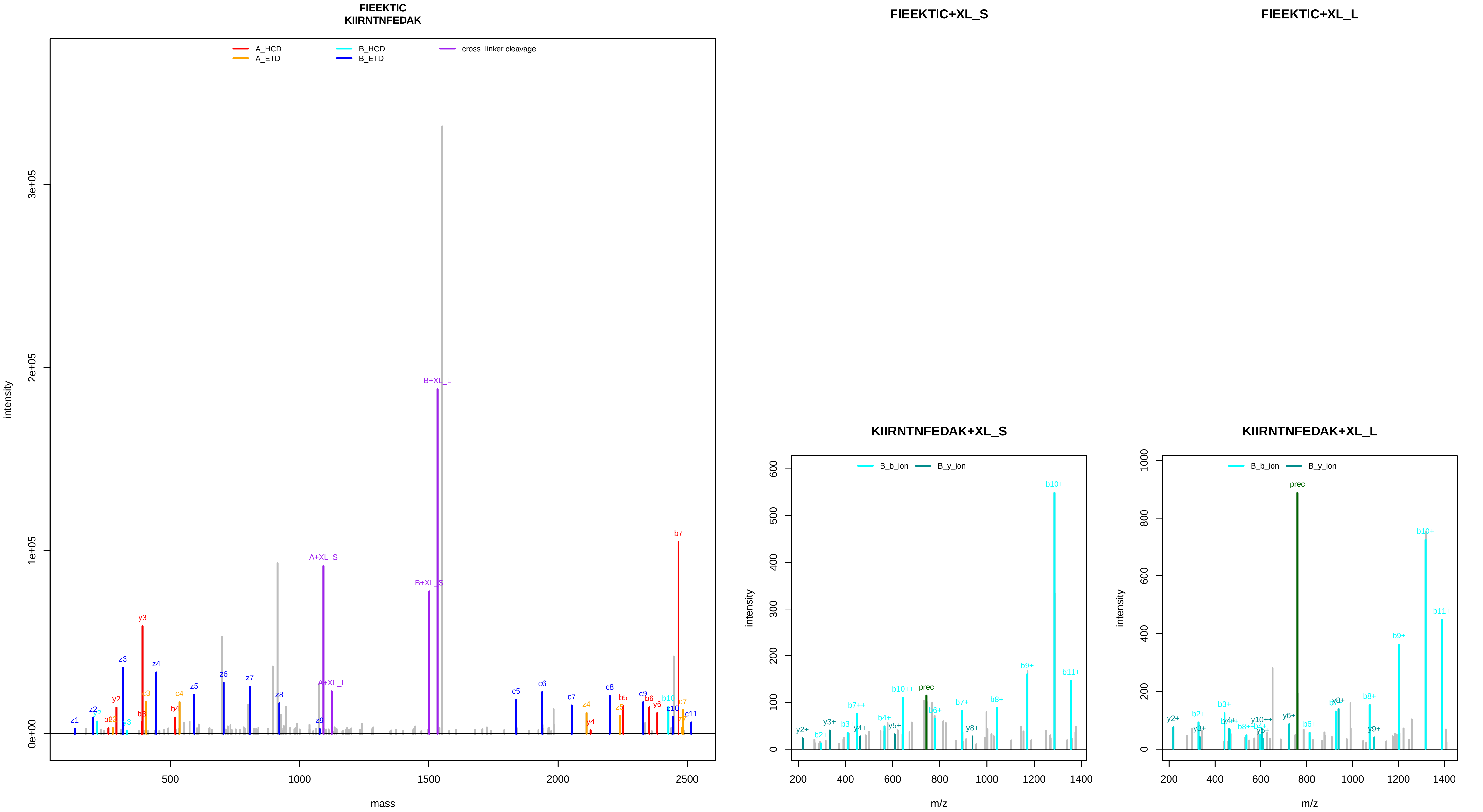
FIEEKTIC
KIIRNTNFEDAK
A_HCD
A_ETD
B_HCD
B_ETD
cross−linker cleavage
intensity
mass
B+XL_L
B+XL_S
A+XL_S
A+XL_L
FIEEKTIC+XL_S
FIEEKTIC+XL_L
KIIRNTNFEDAK+XL_S
KIIRNTNFEDAK+XL_L
B_b_ion
B_y_ion
prec
m/z

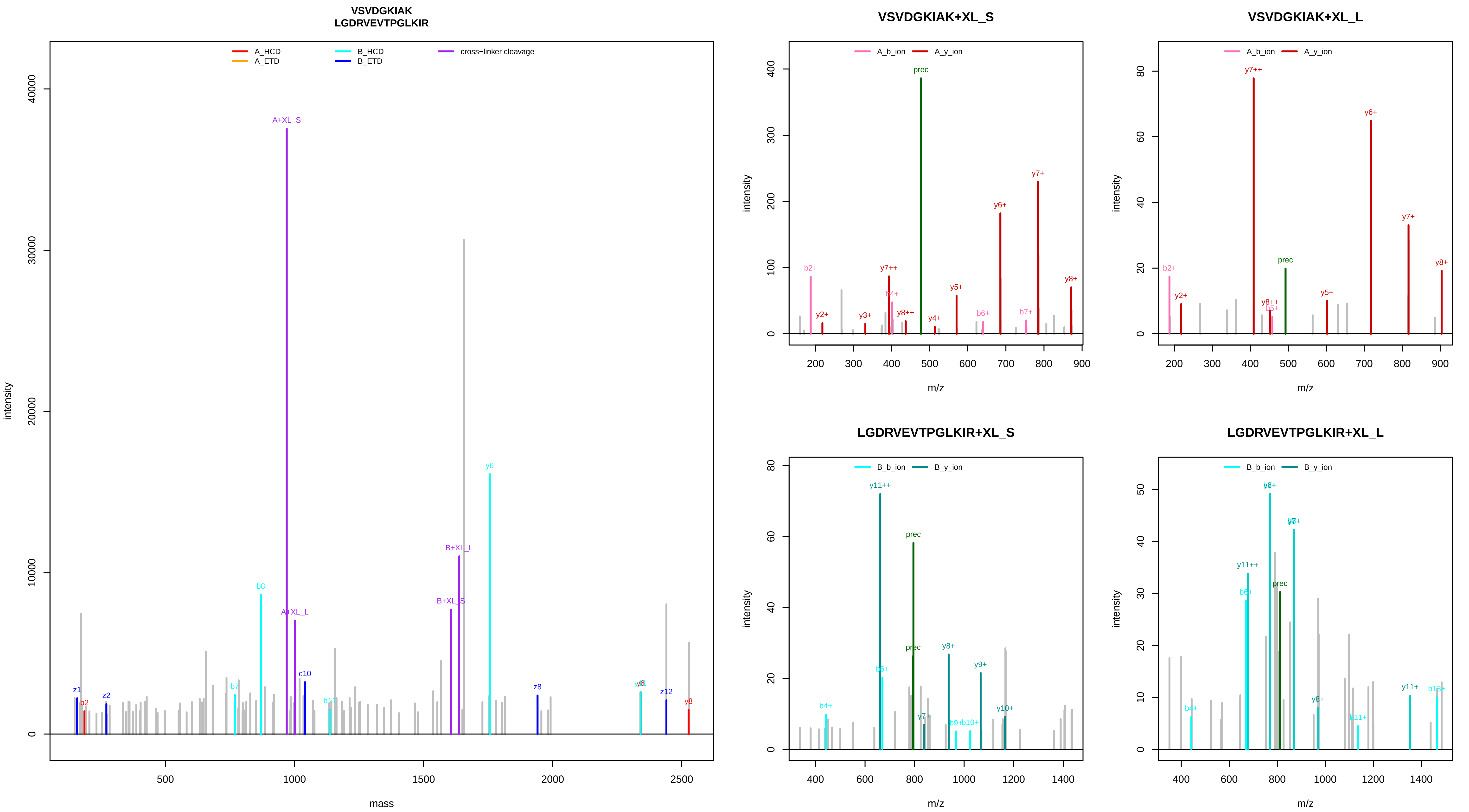
VSVDGKIAK
LGDRVEVTPGLKIR
A_HCD
A_ETD
B_HCD
B_ETD
cross−linker cleavage
A+XL_S
A+XL_L
B+XL_L
B+XL_S
y6
b8
c10
z1
b2
z2
b7
b11
z8
z12
y8
intensity
mass
VSVDGKIAK+XL_S
VSVDGKIAK+XL_L
LGDRVEVTPGLKIR+XL_S
LGDRVEVTPGLKIR+XL_L
A_b_ion
A_y_ion
B_b_ion
B_y_ion
prec
m/z

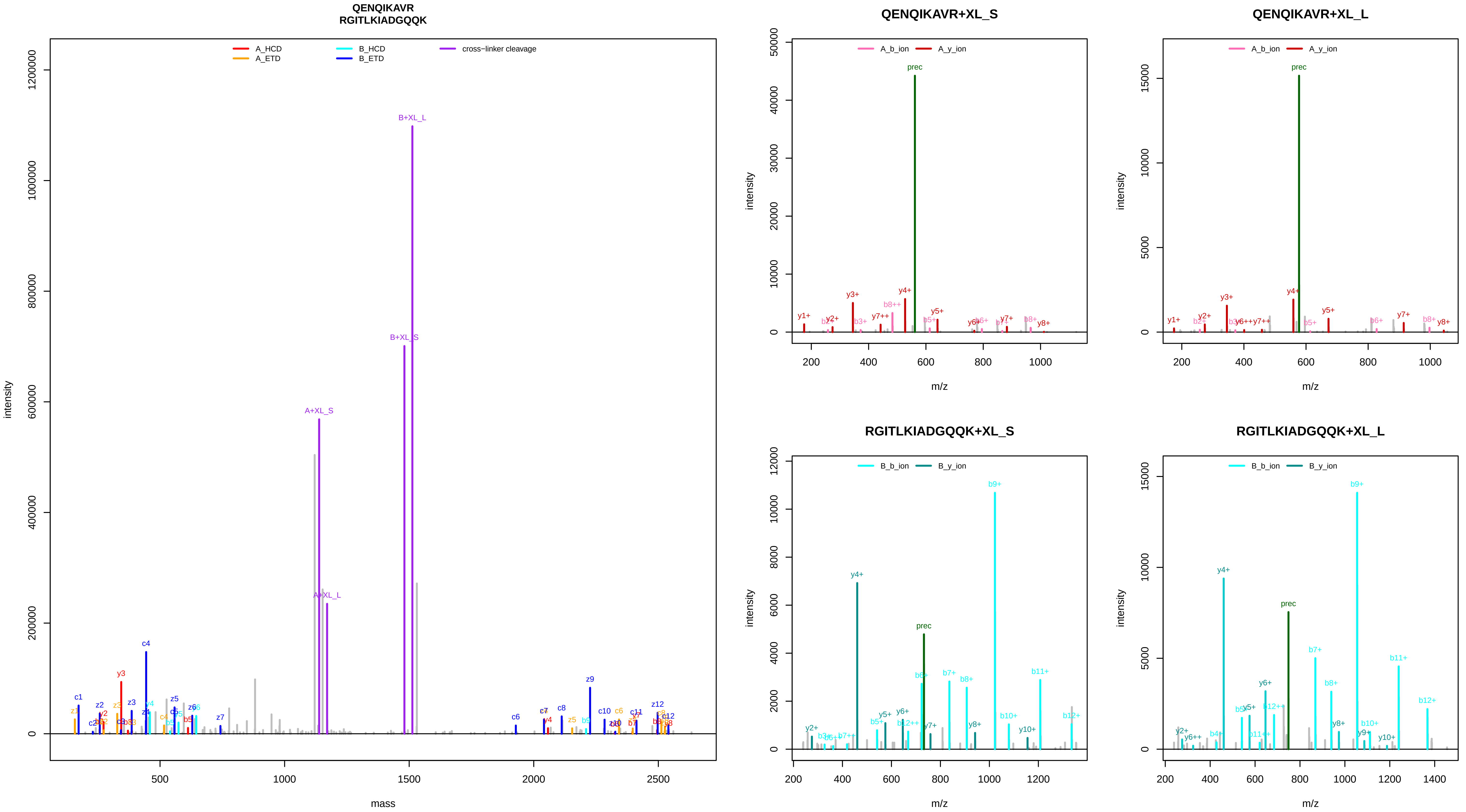
QENQIKAVR
RGITLKIADGQQK
A_HCD
A_ETD
B_HCD
B_ETD
cross−linker cleavage
B+XL_L
B+XL_S
A+XL_S
A+XL_L
intensity
mass
QENQIKAVR+XL_S
QENQIKAVR+XL_L
A_b_ion
A_y_ion
prec
m/z
RGITLKIADGQQK+XL_S
RGITLKIADGQQK+XL_L
B_b_ion
B_y_ion

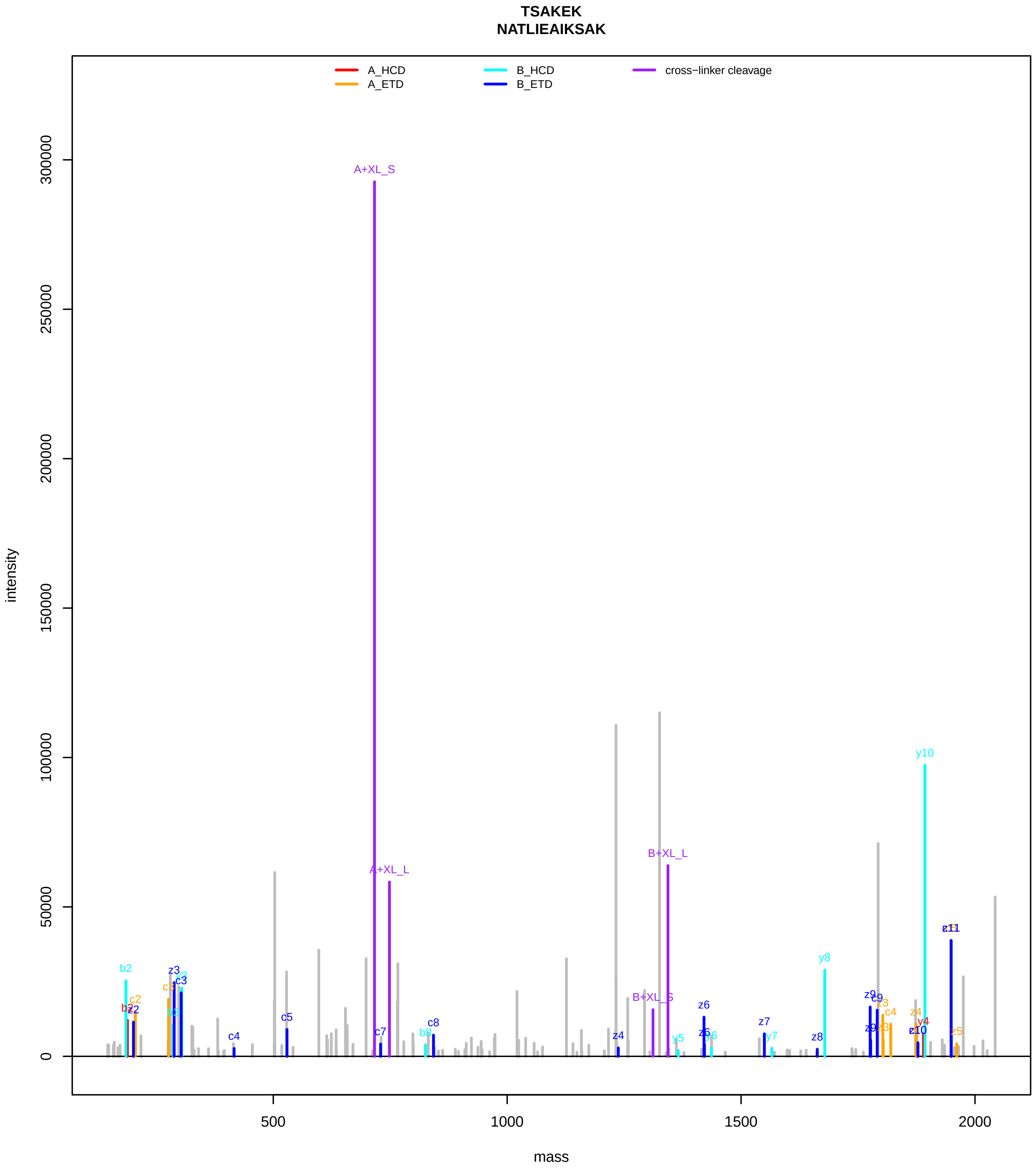

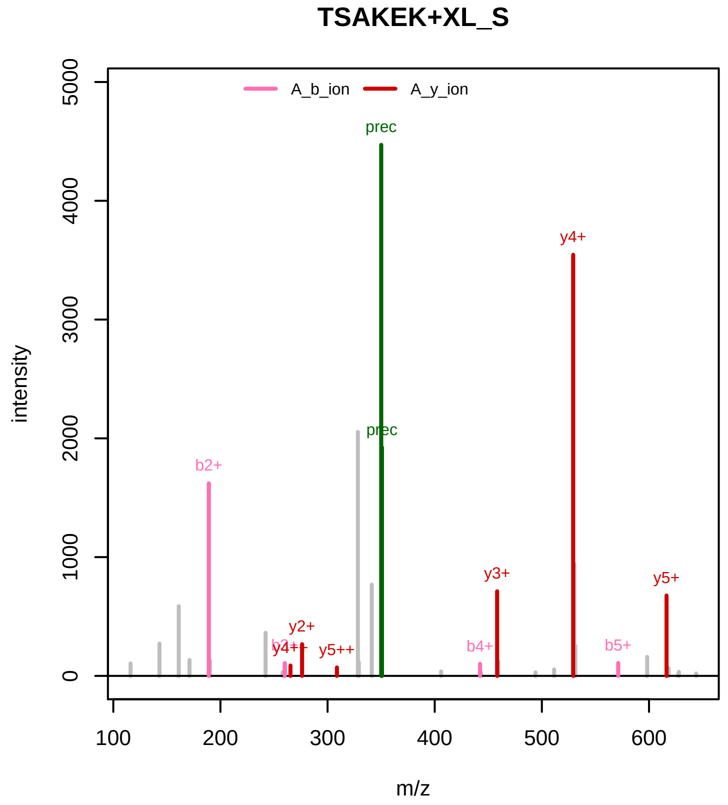

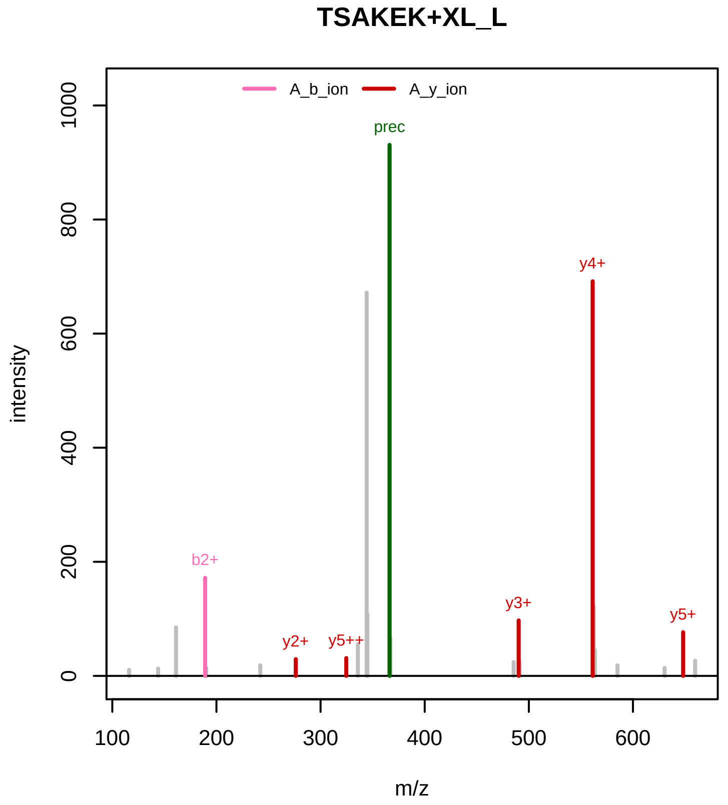

NATLIEAIKSAK+XL_S

NATLIEAIKSAK+XL_L

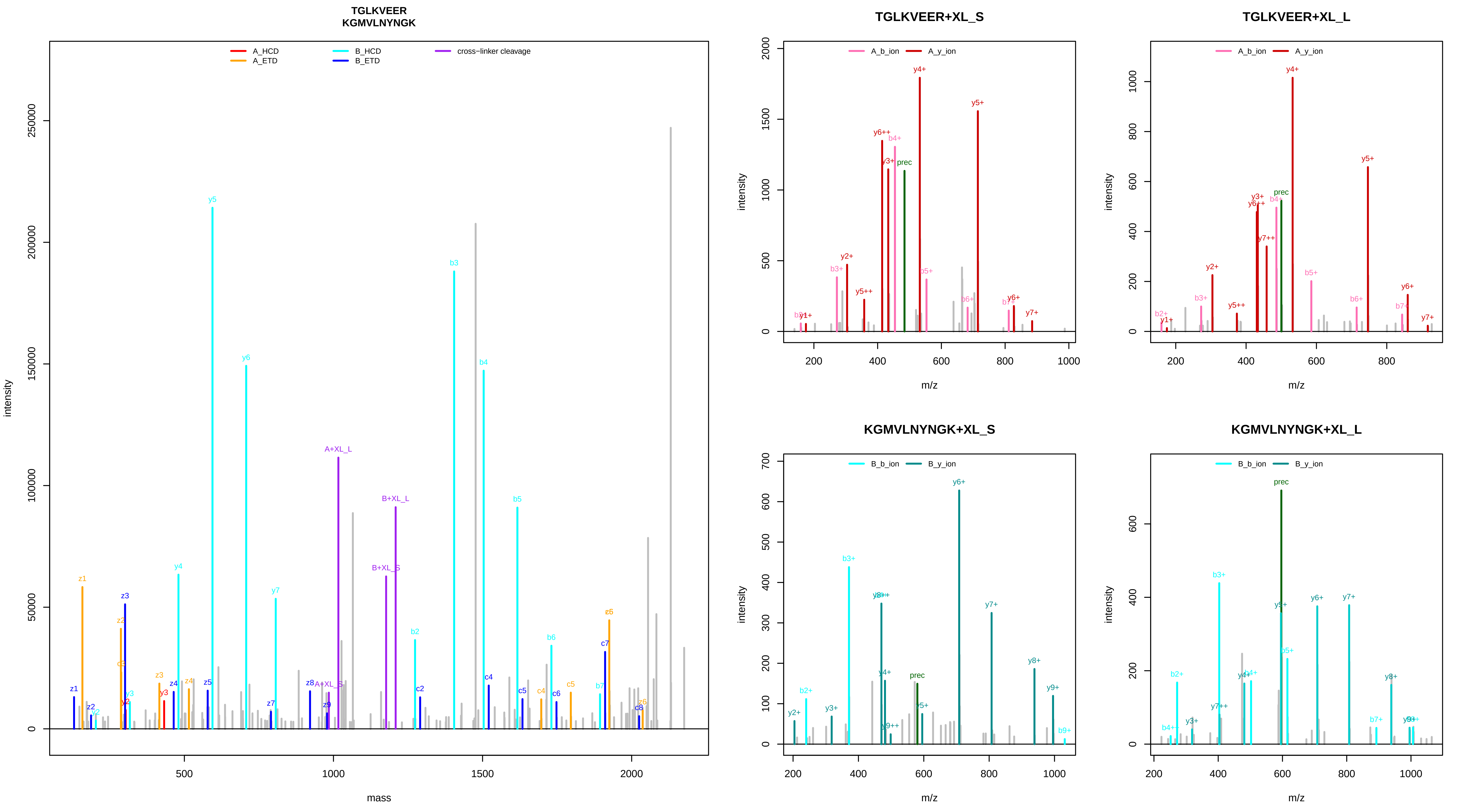
TGLKVEER
KGMVLNYNGK
A_HCD
A_ETD
B_HCD
B_ETD
cross−linker cleavage
intensity
mass
A+XL_L
B+XL_L
B+XL_S
A+XL_S
TGLKVEER+XL_S
TGLKVEER+XL_L
KGMVLNYNGK+XL_S
KGMVLNYNGK+XL_L
A_b_ion
A_y_ion
B_b_ion
B_y_ion
prec
m/z

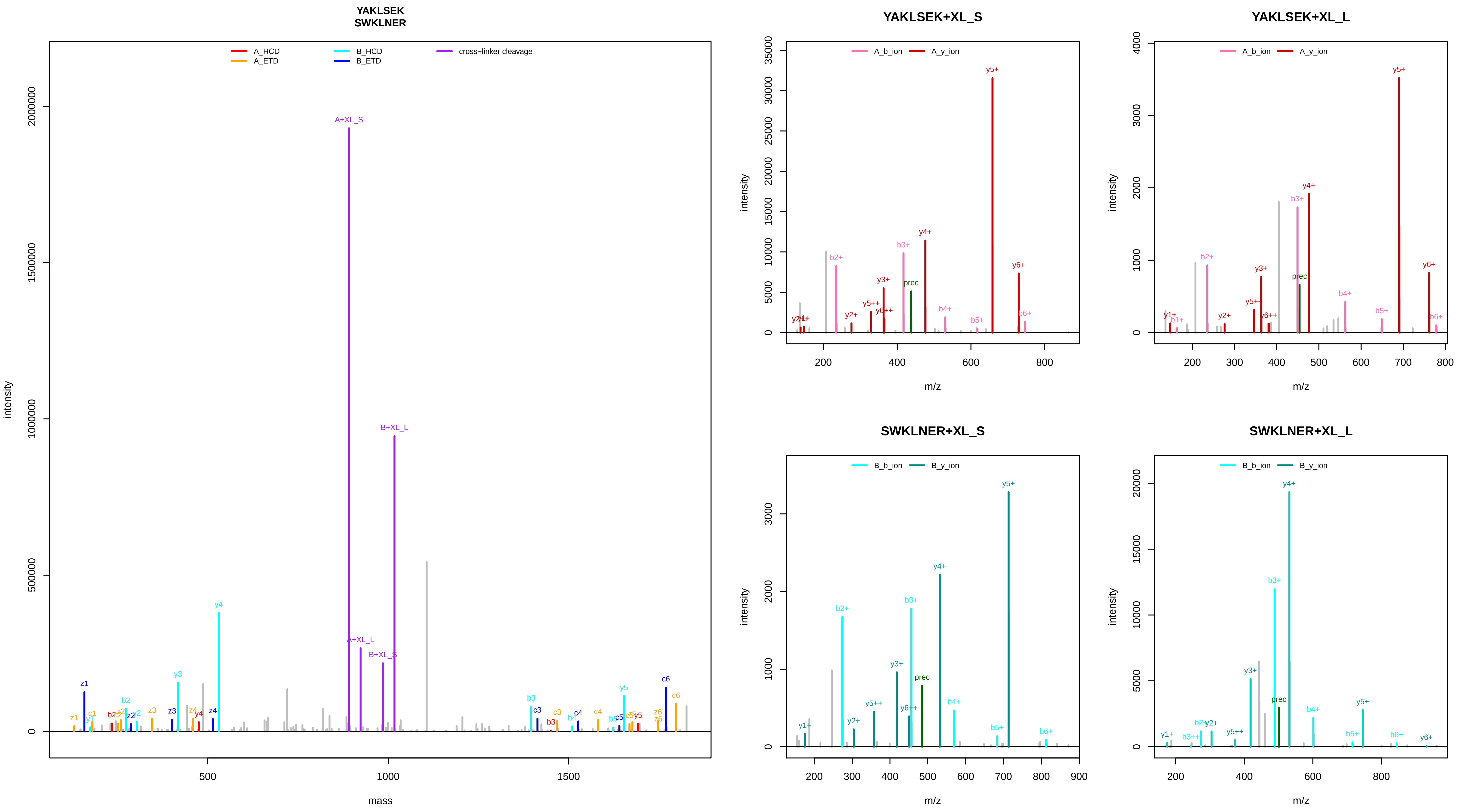

YAKLSEK
SWKLNER
A_HCD
A_ETD
B_HCD
B_ETD
cross−linker cleavage
A+XL_S
B+XL_L
A+XL_L
B+XL_S
intensity
mass
YAKLSEK+XL_S
YAKLSEK+XL_L
SWKLNER+XL_S
SWKLNER+XL_L
A_b_ion
A_y_ion
B_b_ion
B_y_ion
prec
m/z
intensity

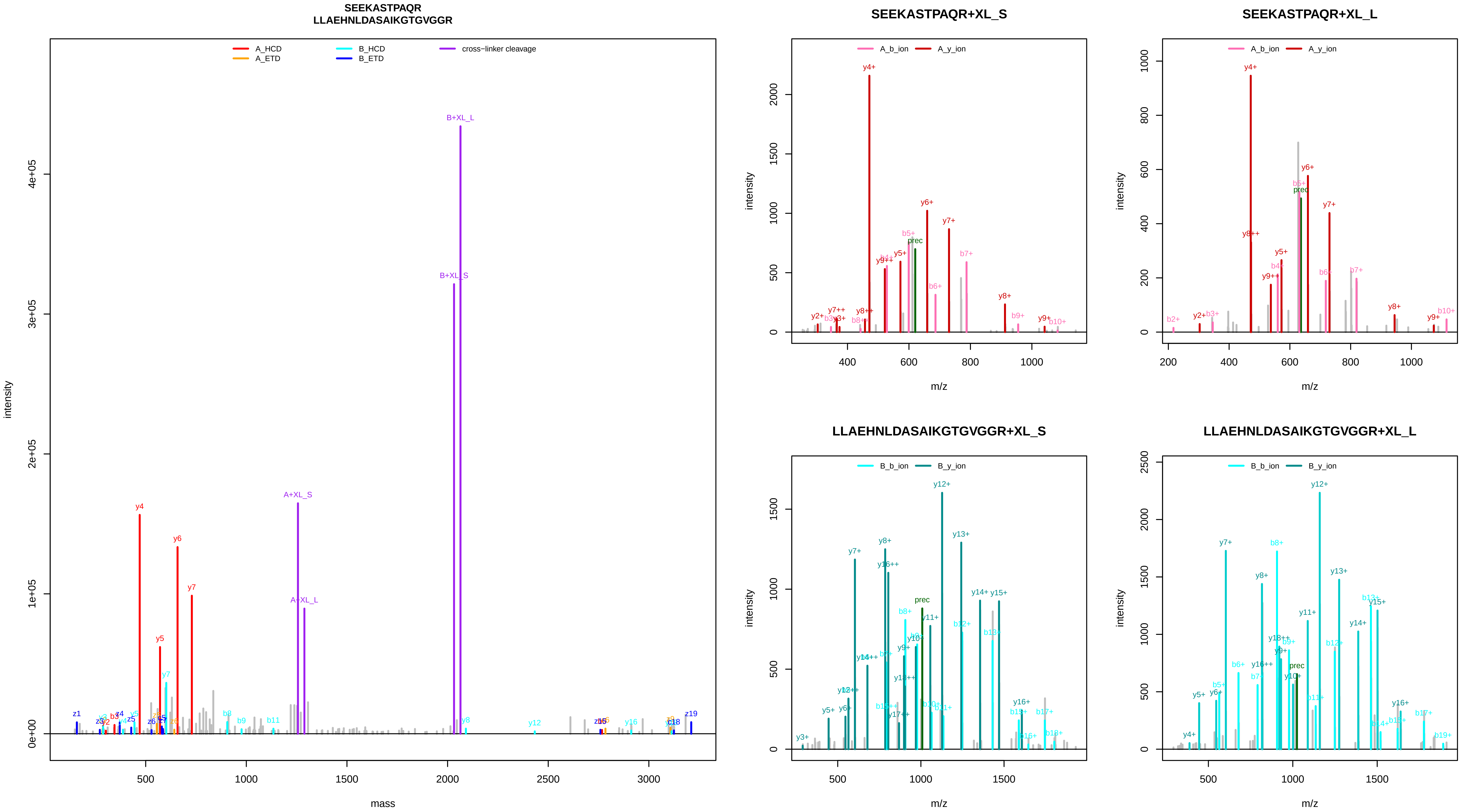
SEEKASTPAQR
LLAEHNLDASAIKGTGVGGR
A_HCD
A_ETD
B_HCD
B_ETD
cross−linker cleavage
B+XL_L
B+XL_S
A+XL_S
A+XL_L
intensity
mass
SEEKASTPAQR+XL_S
SEEKASTPAQR+XL_L
A_b_ion
A_y_ion
LLAEHNLDASAIKGTGVGGR+XL_S
LLAEHNLDASAIKGTGVGGR+XL_L
B_b_ion
B_y_ion
prec
m/z

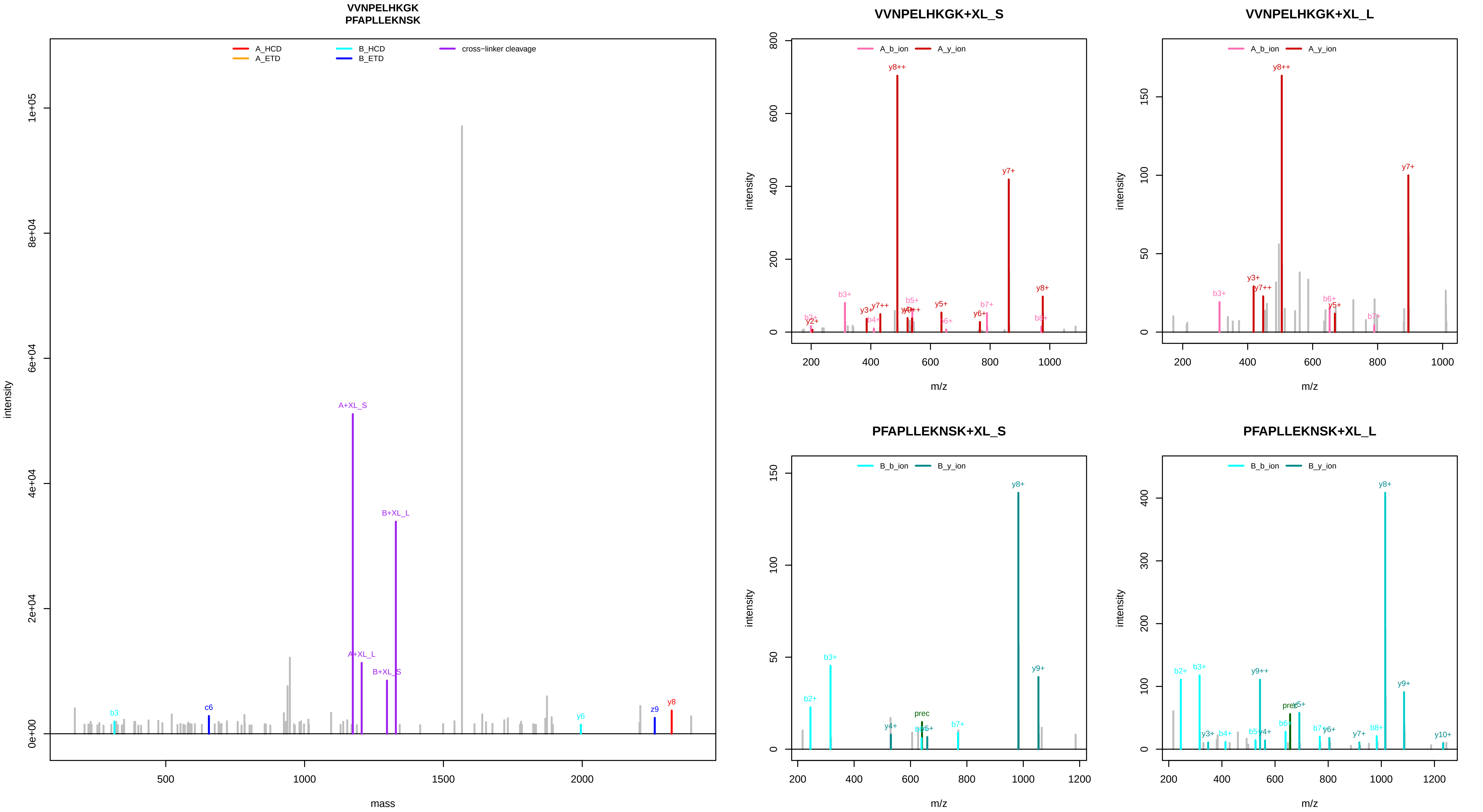
VVNPELHKGK
PFAPLLEKNSK
A_HCD
A_ETD
B_HCD
B_ETD
cross−linker cleavage
A+XL_S
B+XL_L
A+XL_L
B+XL_S
b3
c6
y6
z9
y8
intensity
mass
VVNPELHKGK+XL_S
VVNPELHKGK+XL_L
A_b_ion
A_y_ion
PFAPLLEKNSK+XL_S
PFAPLLEKNSK+XL_L
B_b_ion
B_y_ion
prec
m/z

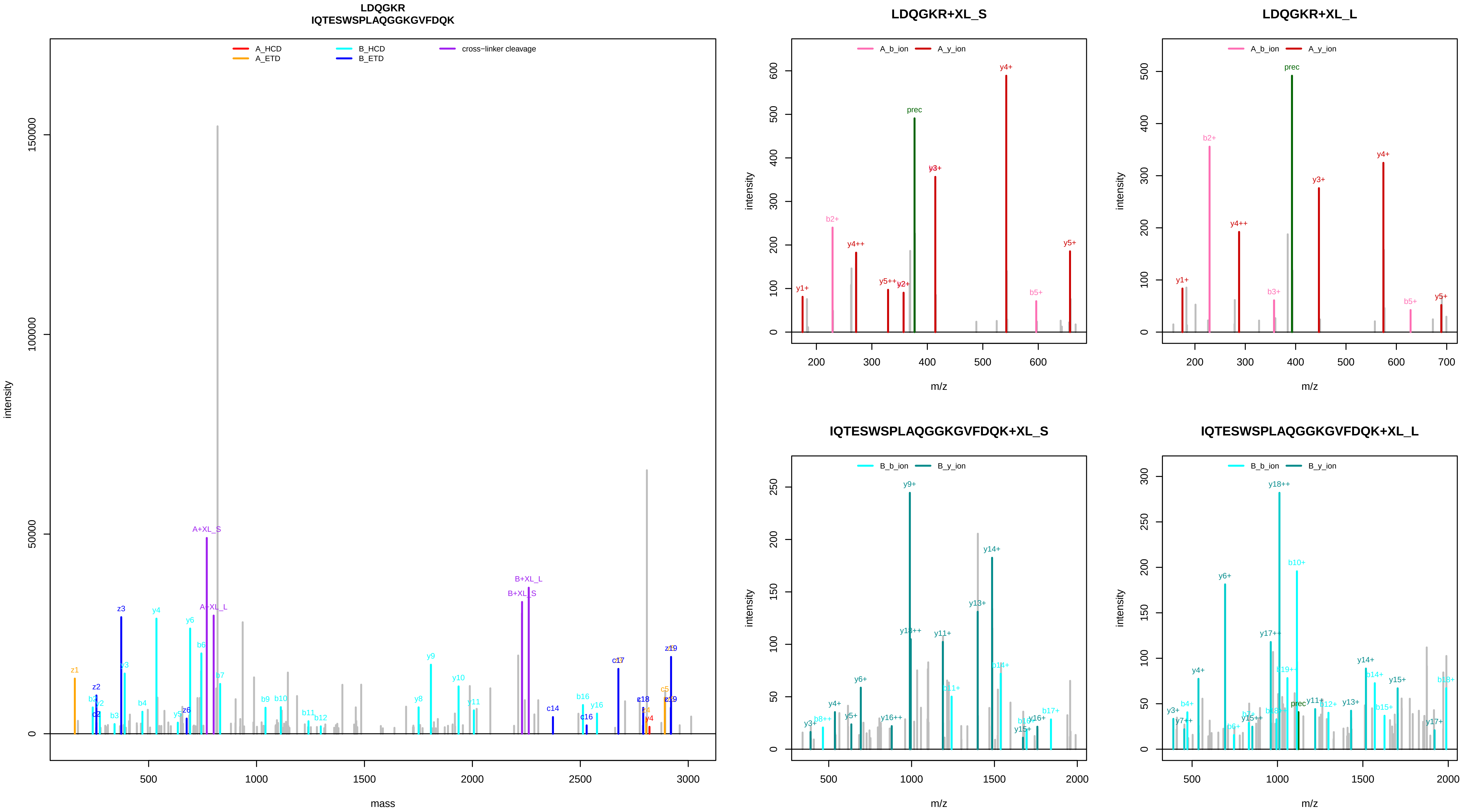
LDQGKR
IQTESWSPLAQGGKGVFDQK
A_HCD
A_ETD
B_HCD
B_ETD
cross−linker cleavage
intensity
mass
A+XL_S
A+XL_L
B+XL_L
B+XL_S
LDQGKR+XL_S
LDQGKR+XL_L
A_b_ion
A_y_ion
prec
m/z
IQTESWSPLAQGGKGVFDQK+XL_S
IQTESWSPLAQGGKGVFDQK+XL_L
B_b_ion
B_y_ion

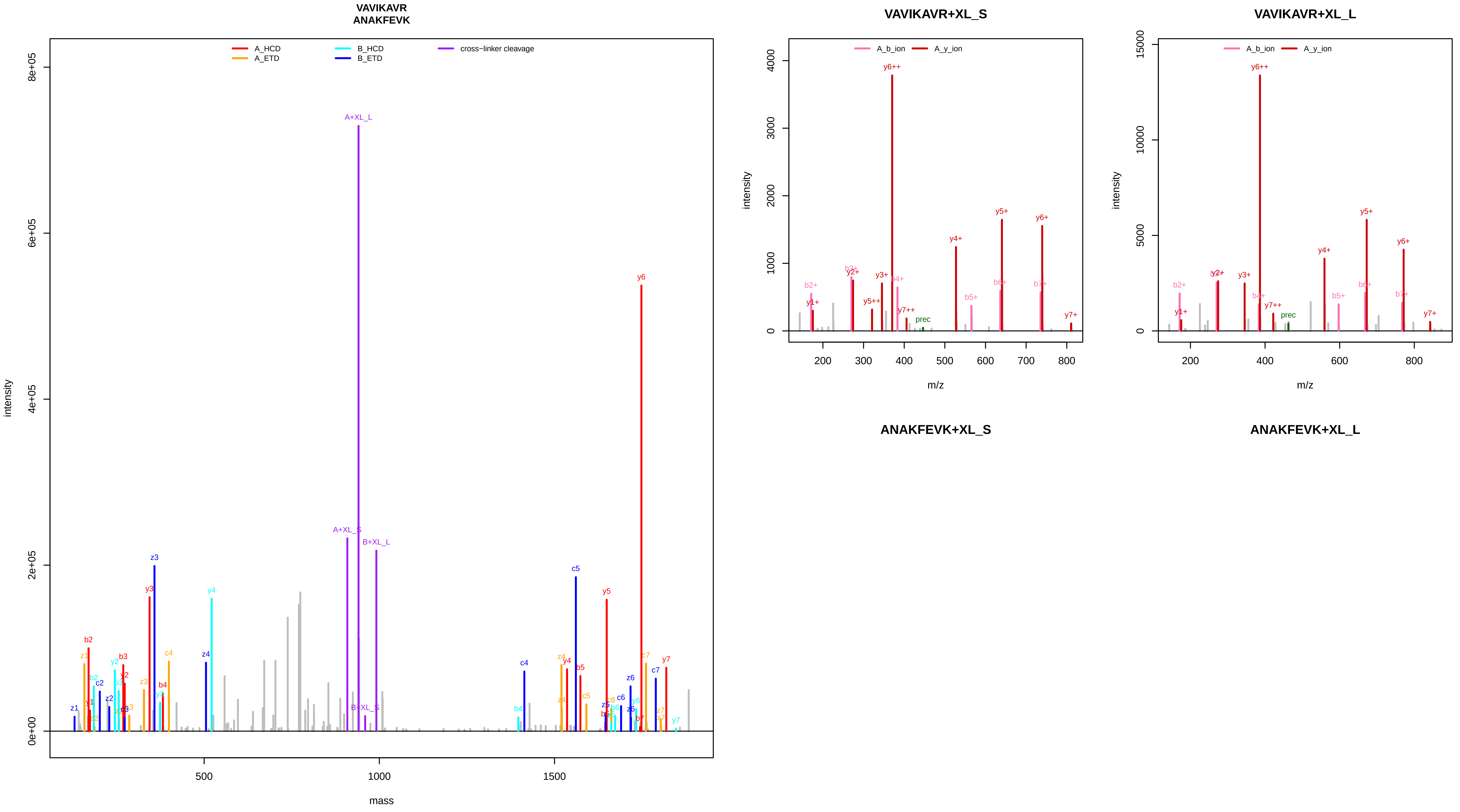
VAVIKAVR
ANAKFEVK
A_HCD
A_ETD
B_HCD
B_ETD
cross−linker cleavage
A+XL_L
A+XL_S
B+XL_L
B+XL_S
intensity
mass
VAVIKAVR+XL_S
VAVIKAVR+XL_L
A_b_ion
A_y_ion
prec
m/z
ANAKFEVK+XL_S
ANAKFEVK+XL_L

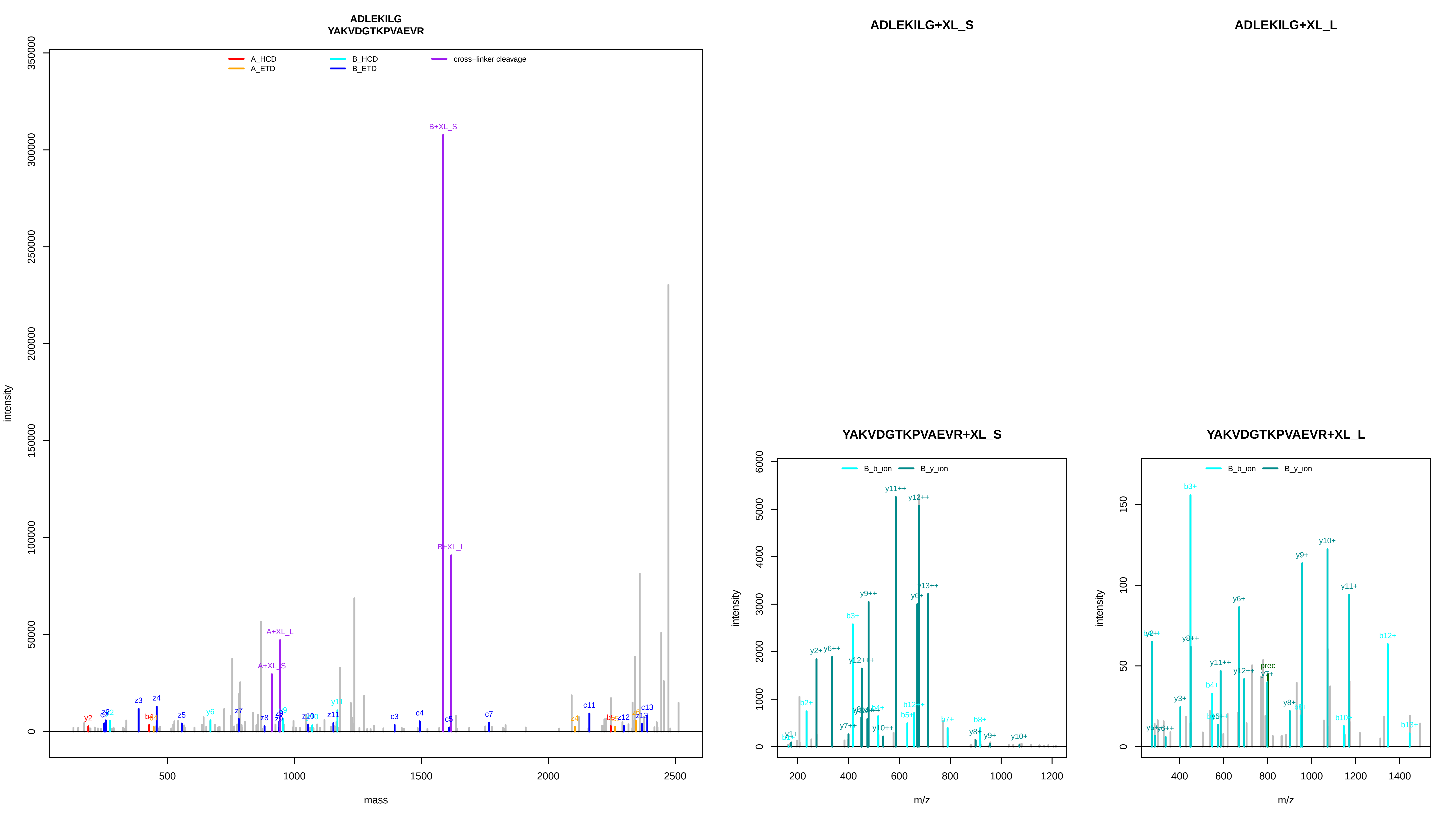
ADLEKILG
YAKVDGTKPVAEVR
A_HCD
A_ETD
B_HCD
B_ETD
cross−linker cleavage
B+XL_S
B+XL_L
A+XL_L
A+XL_S
intensity
mass
ADLEKILG+XL_S
ADLEKILG+XL_L
YAKVDGTKPVAEVR+XL_S
YAKVDGTKPVAEVR+XL_L
B_b_ion
B_y_ion
intensity
m/z

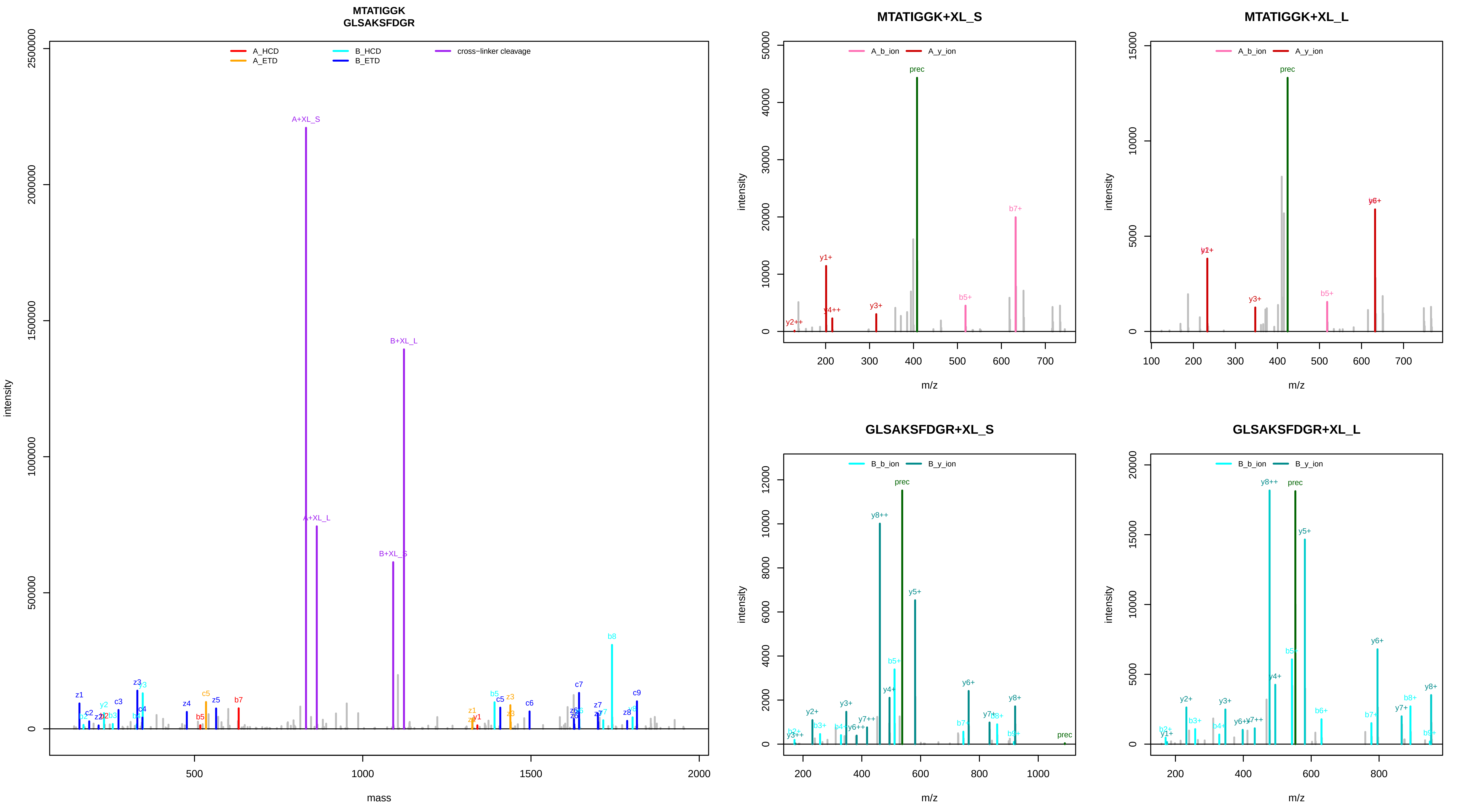
MTATIGGK
GLSAKSFDGR
A_HCD
A_ETD
B_HCD
B_ETD
cross−linker cleavage
A+XL_S
B+XL_L
A+XL_L
B+XL_S
intensity
mass
MTATIGGK+XL_S
MTATIGGK+XL_L
GLSAKSFDGR+XL_S
GLSAKSFDGR+XL_L
A_b_ion
A_y_ion
B_b_ion
B_y_ion
prec
m/z

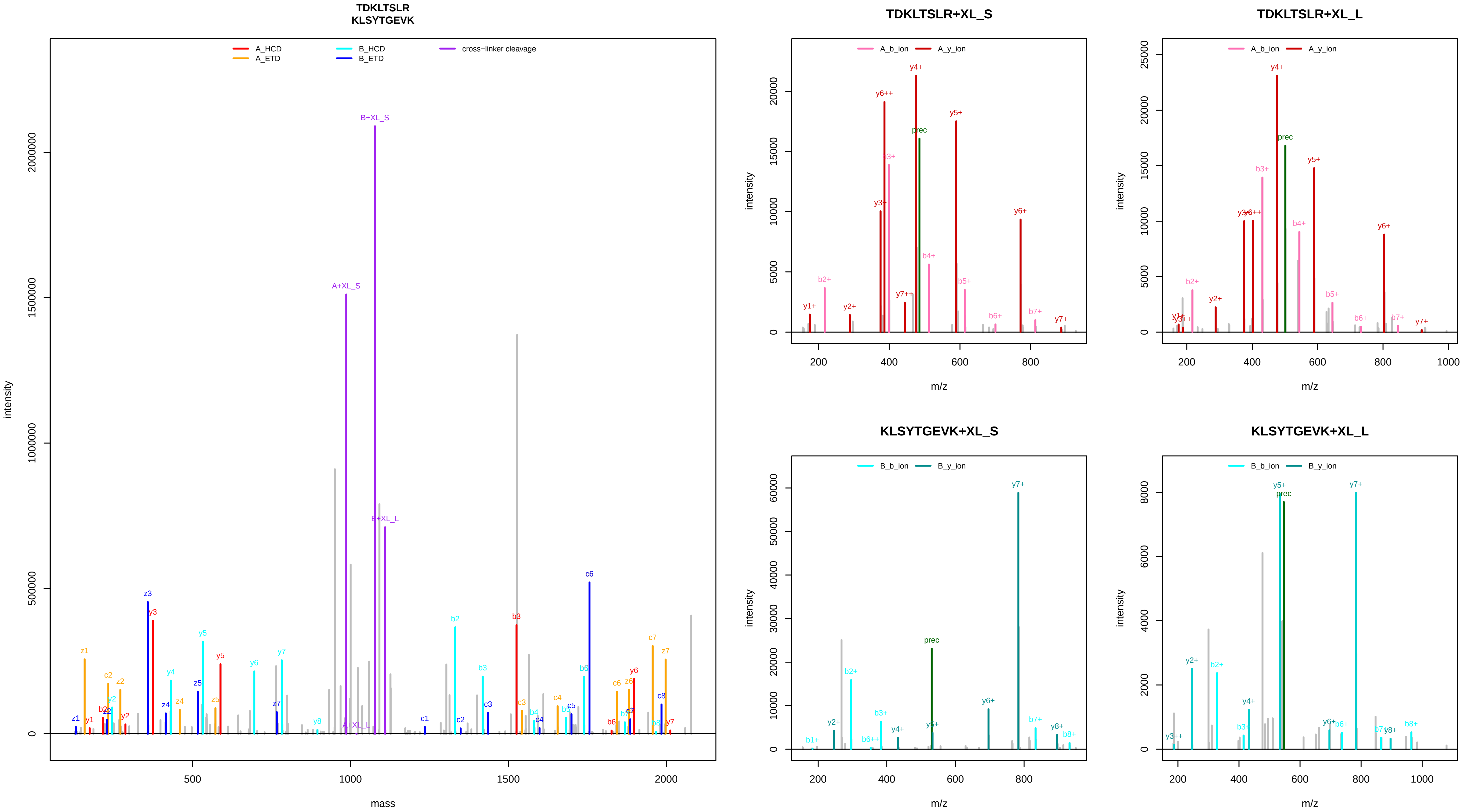
TDKLTSLR
KLSYTGEVK
A_HCD
A_ETD
B_HCD
B_ETD
cross−linker cleavage
B+XL_S
A+XL_S
B+XL_L
A+XL_L
intensity
mass
TDKLTSLR+XL_S
TDKLTSLR+XL_L
KLSYTGEVK+XL_S
KLSYTGEVK+XL_L
A_b_ion
A_y_ion
B_b_ion
B_y_ion
prec
m/z

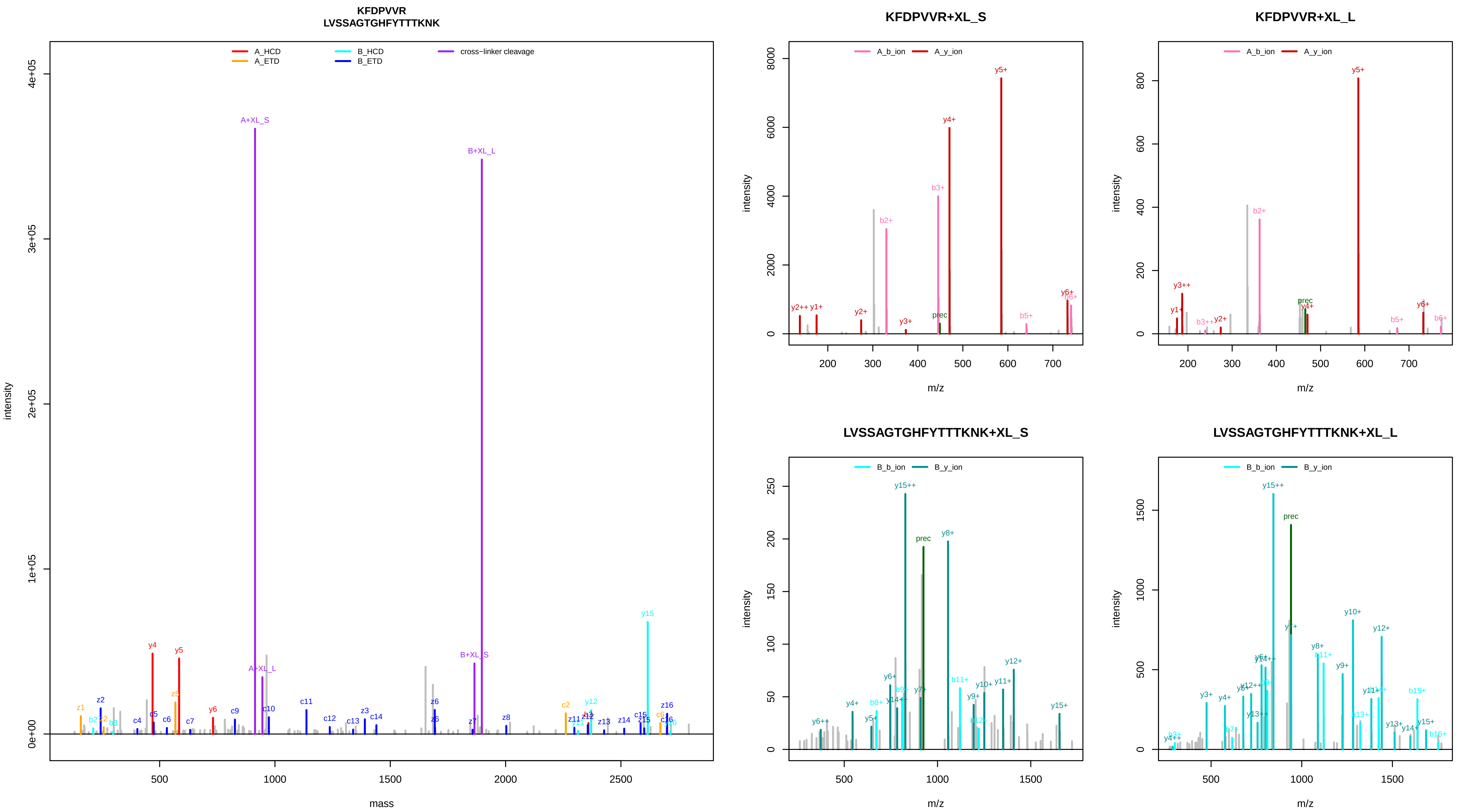
KFDPVVR
LVSSAGTGHFYTTTKNK
A_HCD
A_ETD
B_HCD
B_ETD
cross−linker cleavage
A+XL_S
B+XL_L
A+XL_L
B+XL_S
intensity
mass
KFDPVVR+XL_S
KFDPVVR+XL_L
LVSSAGTGHFYTTTKNK+XL_S
LVSSAGTGHFYTTTKNK+XL_L
A_b_ion
A_y_ion
B_b_ion
B_y_ion
prec
m/z

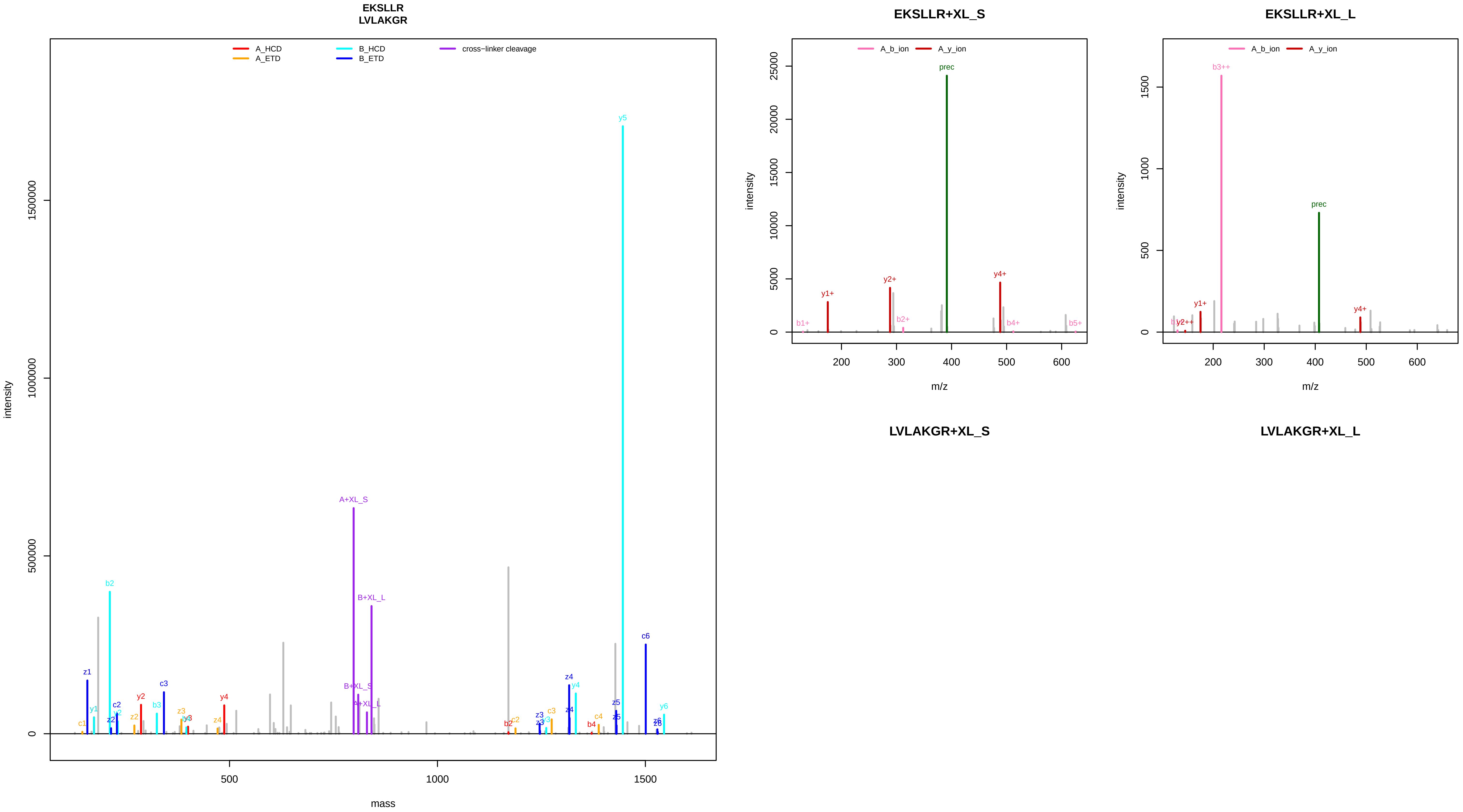
EKSLLR
LVLAKGR
A_HCD
A_ETD
B_HCD
B_ETD
cross−linker cleavage
intensity
mass
0
500000
1000000
1500000
500
1000
1500
y5
A+XL_S
B+XL_L
B+XL_S
A+XL_L
b2
c6
z1
c3
y2
y4
z4
y4
y6
EKSLLR+XL_S
A_b_ion
A_y_ion
prec
y1+
y2+
y4+
b1+
b2+
b4+
b5+
m/z
0
5000
10000
15000
20000
25000
200
300
400
500
600
EKSLLR+XL_L
A_b_ion
A_y_ion
b3++
prec
y1+
y2++
y4+
m/z
0
500
1000
1500
200
300
400
500
600
LVLAKGR+XL_S
LVLAKGR+XL_L

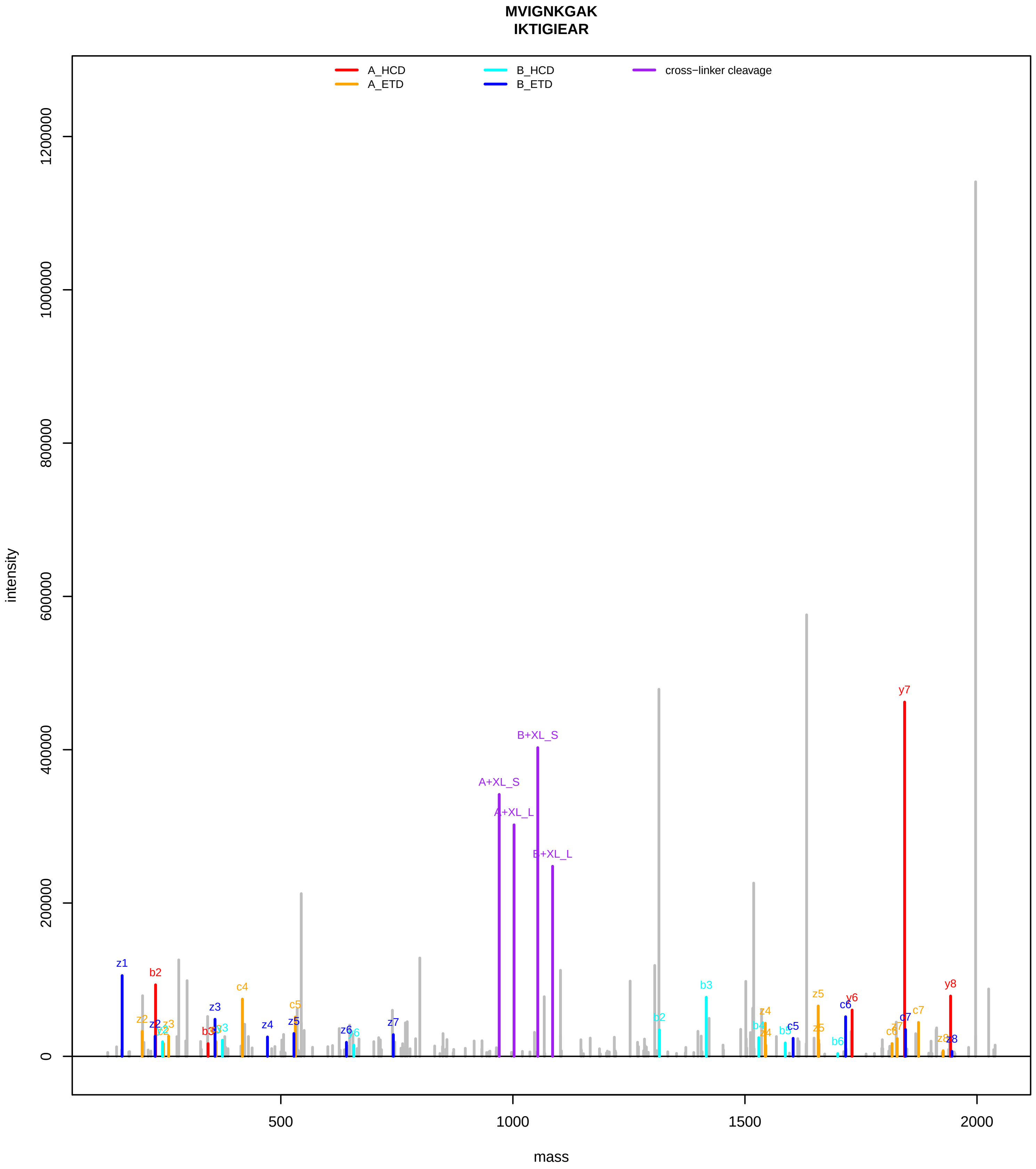

MVIGNKGAK+XL_S

MVIGNKGAK+XL_L

IKTIGIEAR+XL_S

IKTIGIEAR+XL_L

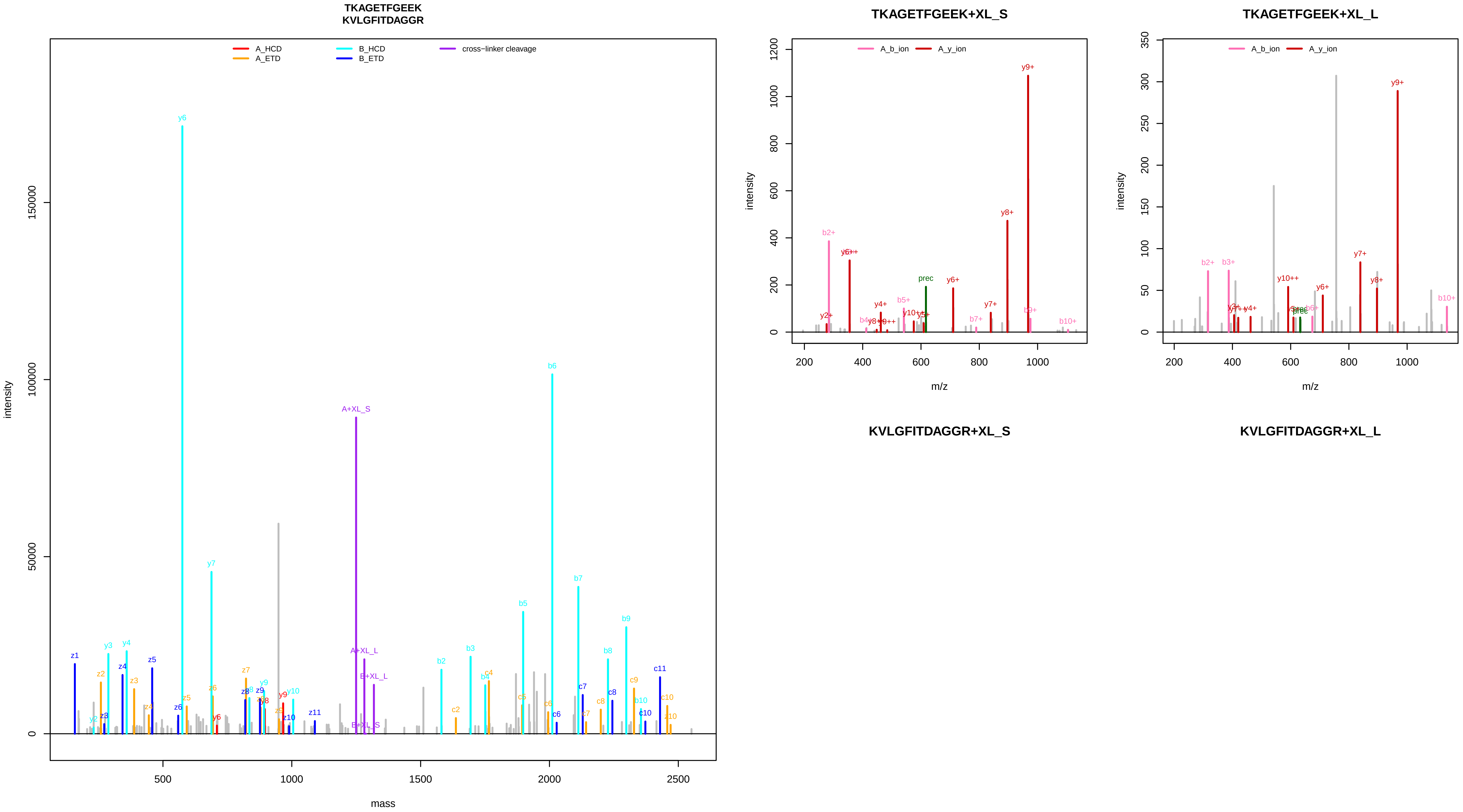

TKAGETFGEEK
KVLGFITDAGGR
A_HCD
A_ETD
B_HCD
B_ETD
cross−linker cleavage
intensity
mass
0
50000
100000
150000
500
1000
1500
2000
2500
y6
b6
A+XL_S
A+XL_L
B+XL_L
B+XL_S
y7
b7
b5
b9
b8
b3
b2
b4
c4
c5
c6
c7
c8
c9
c10
c11
y3
y4
y9
y10
b10
z1
z2
z3
z4
z5
z6
z7
z8
z9
z10
z11
TKAGETFGEEK+XL_S
TKAGETFGEEK+XL_L
A_b_ion
A_y_ion
intensity
m/z
0
200
400
600
800
1000
1200
y9+
y8+
y7+
y6+
y4+
y2+
b2+
b3+
b5+
b7+
b10+
prec
y10++
0
50
100
150
200
250
300
350
KVLGFITDAGGR+XL_S
KVLGFITDAGGR+XL_L

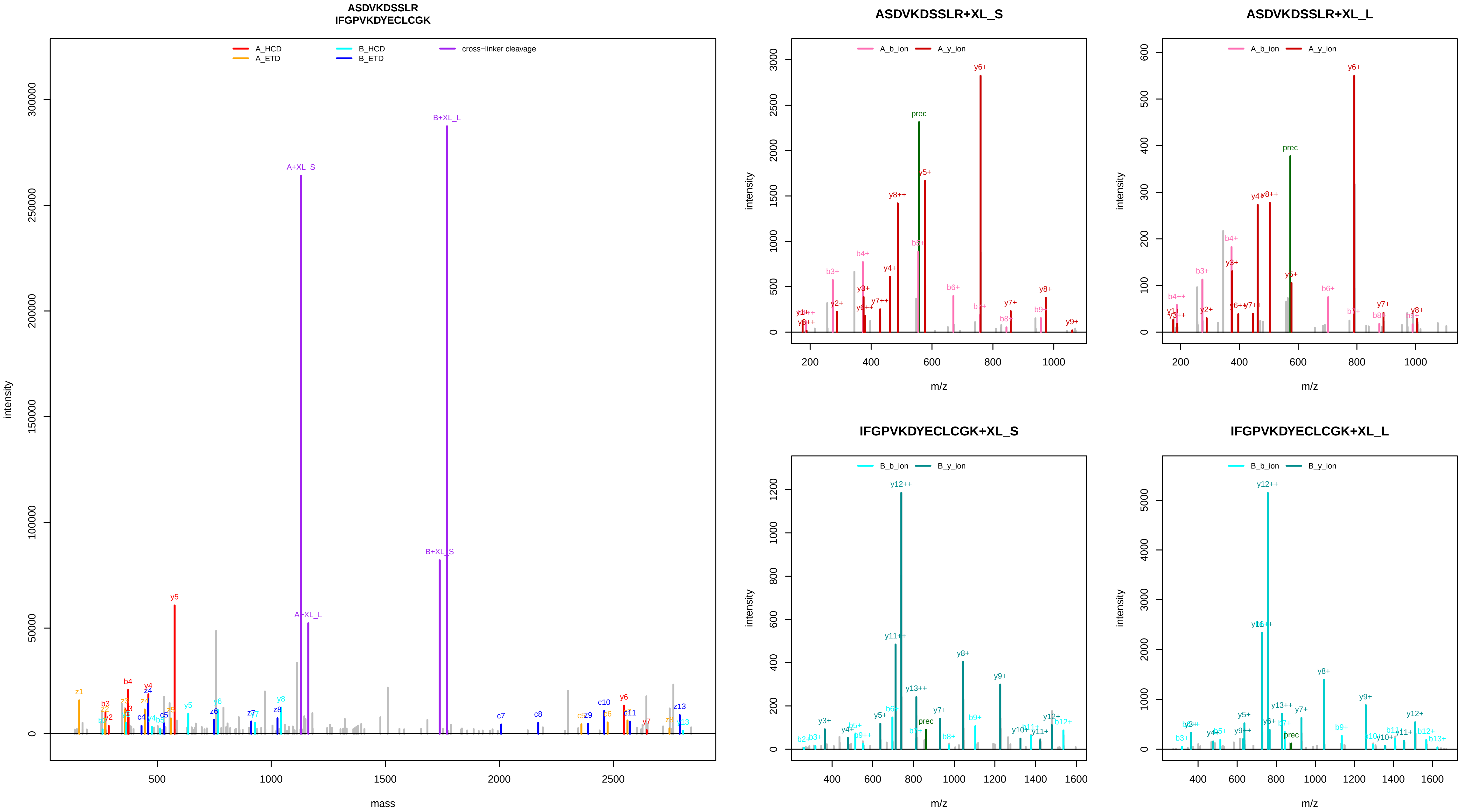
ASDVKDSSLR
IFGPVKDYECLCGK
A_HCD
A_ETD
B_HCD
B_ETD
cross−linker cleavage
A+XL_S
B+XL_L
B+XL_S
A+XL_L
intensity
mass
ASDVKDSSLR+XL_S
ASDVKDSSLR+XL_L
A_b_ion
A_y_ion
IFGPVKDYECLCGK+XL_S
IFGPVKDYECLCGK+XL_L
B_b_ion
B_y_ion
prec
m/z

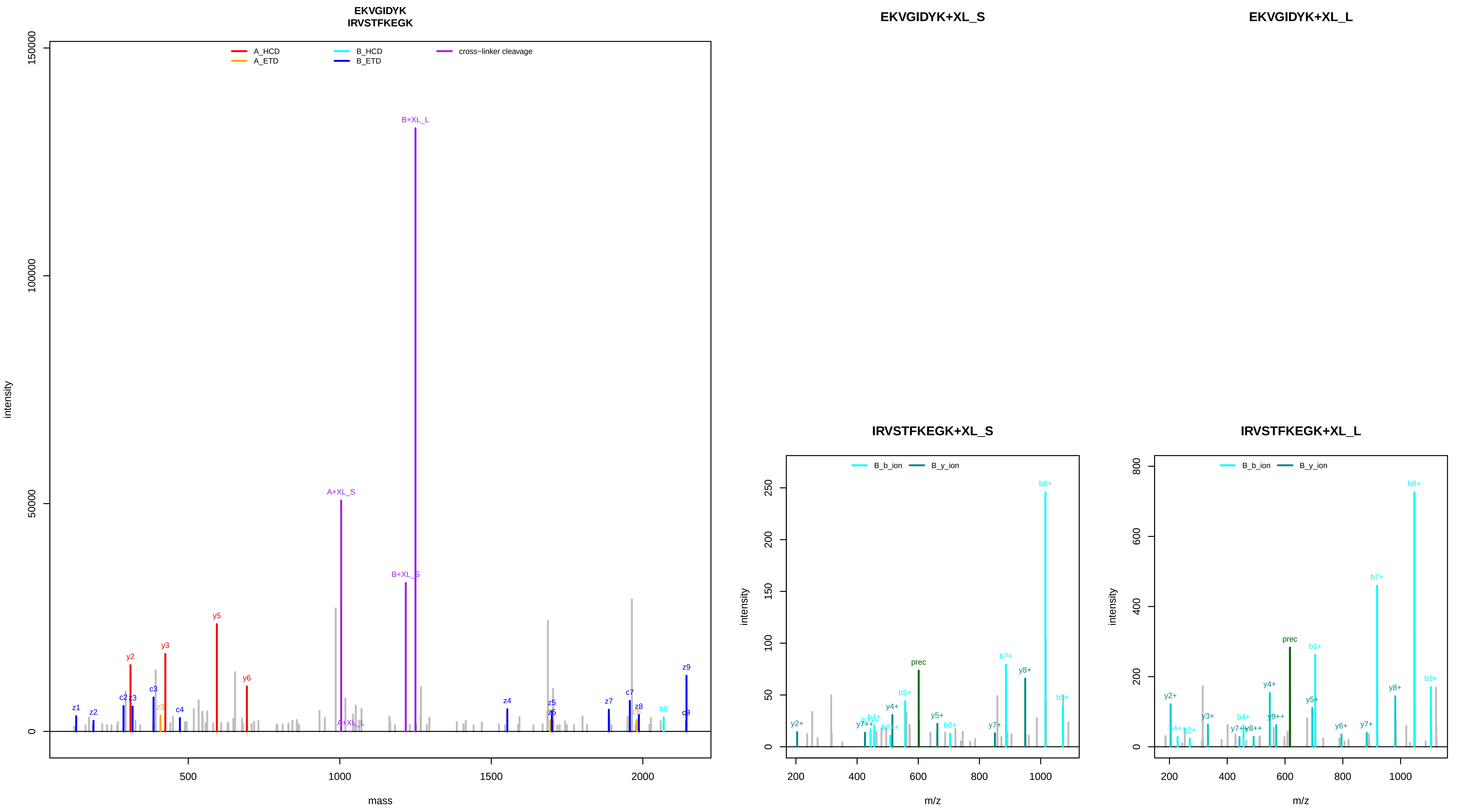
EKVGIDYK
IRVSTFKEGK
A_HCD
A_ETD
B_HCD
B_ETD
cross−linker cleavage
B+XL_L
A+XL_S
B+XL_S
A+XL_L
intensity
mass
EKVGIDYK+XL_S
EKVGIDYK+XL_L
IRVSTFKEGK+XL_S
IRVSTFKEGK+XL_L
B_b_ion
B_y_ion
prec
m/z

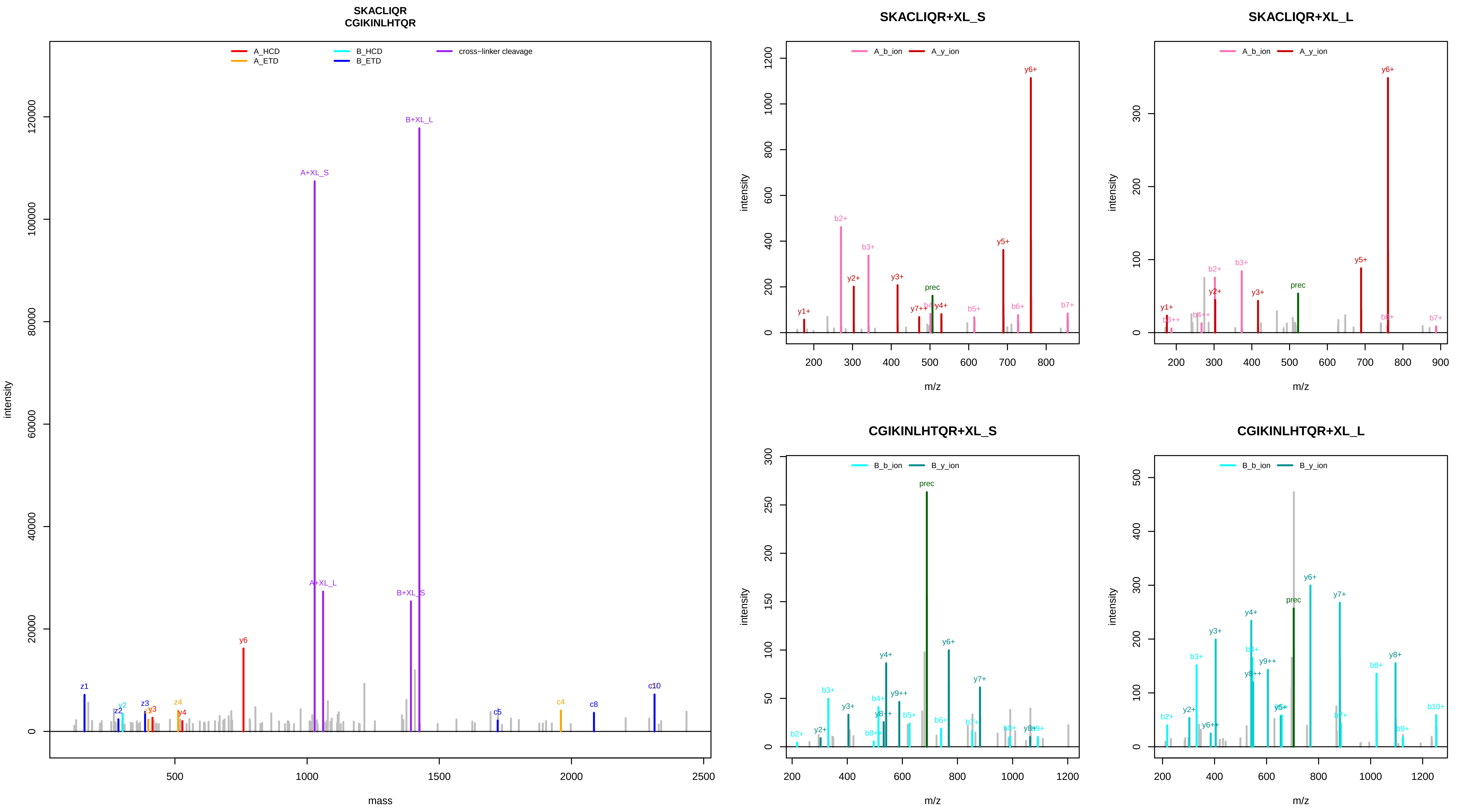
SKACLIQR
CGIKINLHTQR
A_HCD
A_ETD
B_HCD
B_ETD
cross−linker cleavage
B+XL_L
A+XL_S
A+XL_L
B+XL_S
intensity
mass
SKACLIQR+XL_S
SKACLIQR+XL_L
CGIKINLHTQR+XL_S
CGIKINLHTQR+XL_L
A_b_ion
A_y_ion
B_b_ion
B_y_ion
prec
m/z

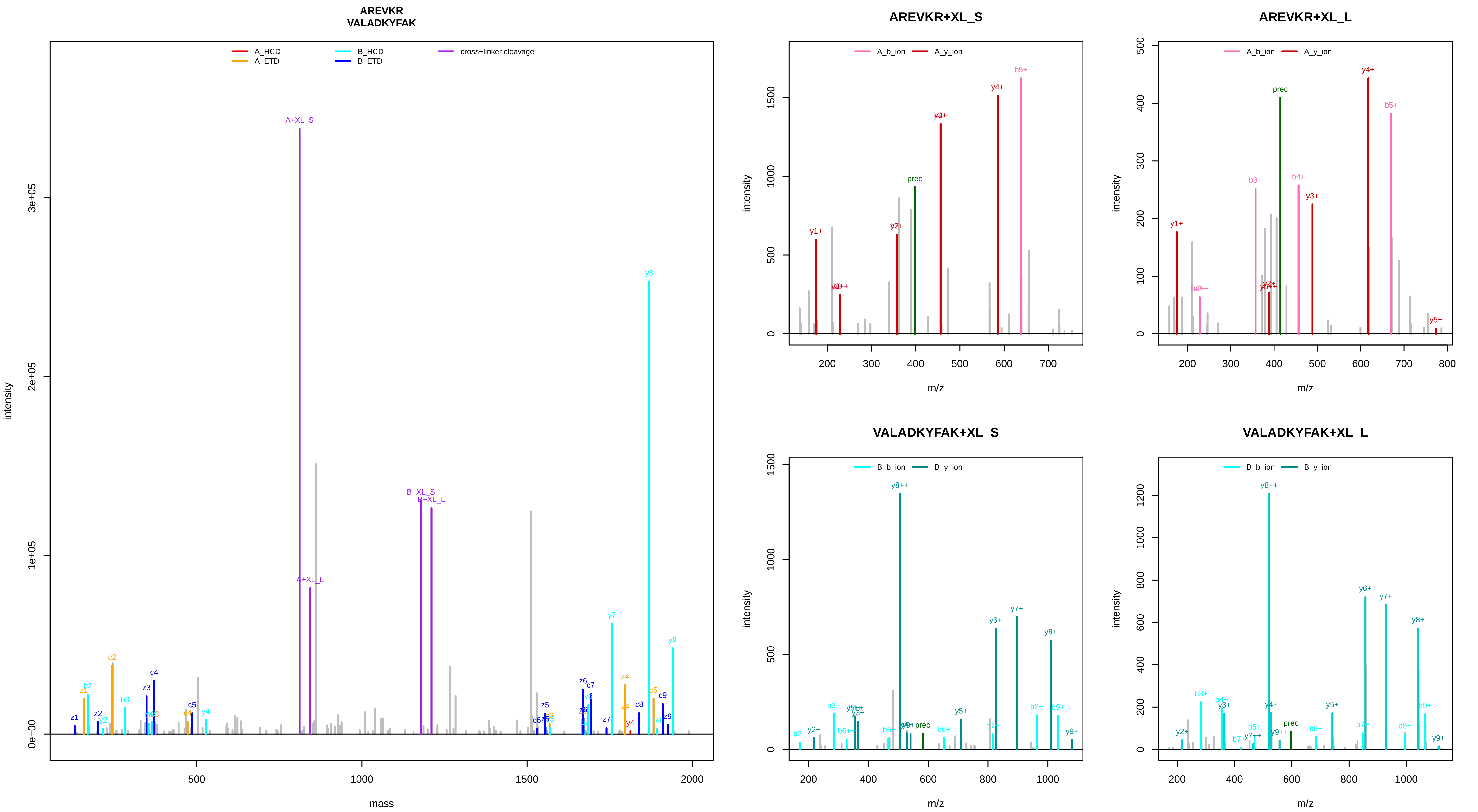

AREVKR
VALADKYFAK
A_HCD
A_ETD
B_HCD
B_ETD
cross−linker cleavage
A+XL_S
A+XL_L
B+XL_S
B+XL_L
intensity
mass
AREVKR+XL_S
AREVKR+XL_L
VALADKYFAK+XL_S
VALADKYFAK+XL_L
A_b_ion
A_y_ion
B_b_ion
B_y_ion
prec
m/z

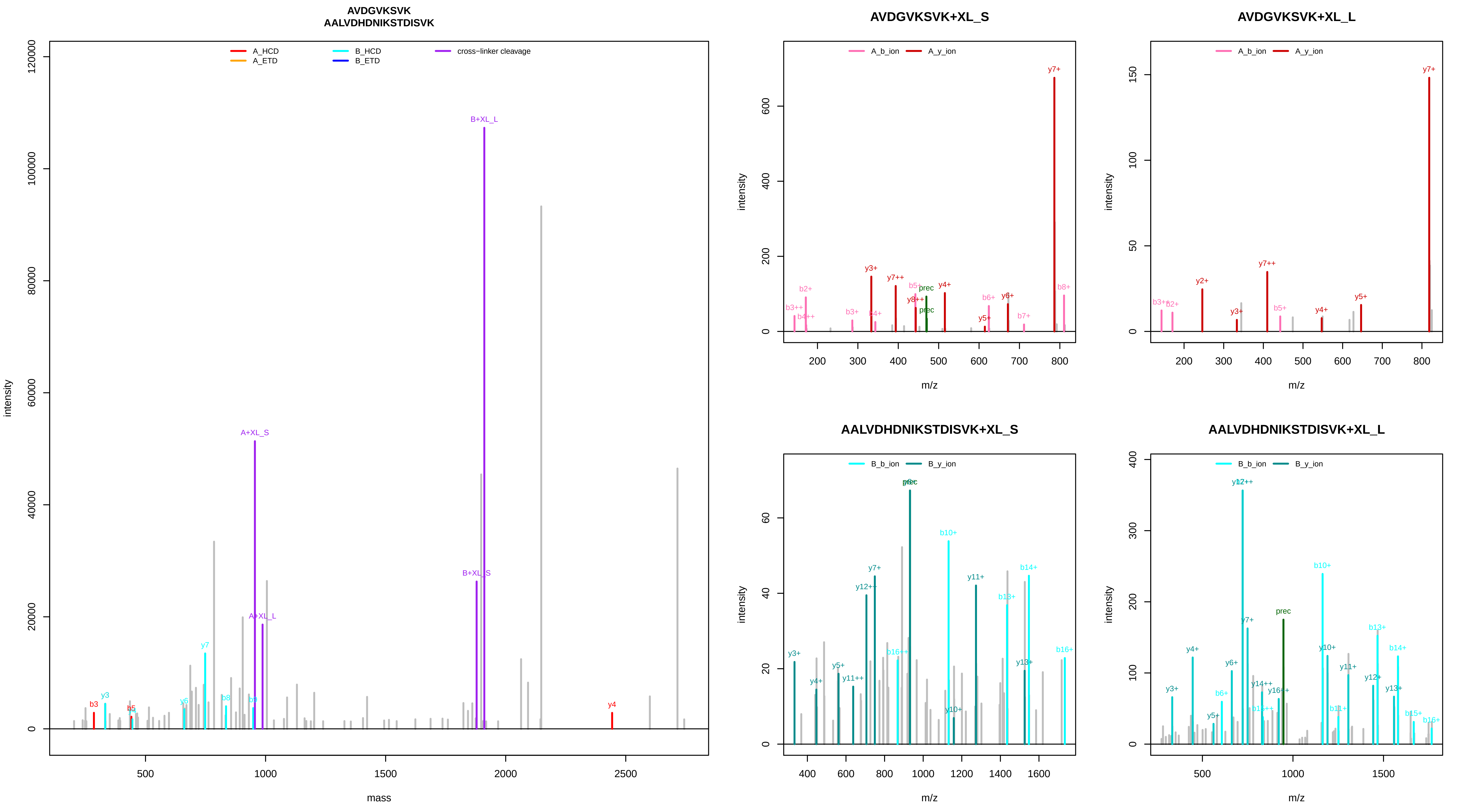
AVDGVKSVK
AALVDHDNIKSTDISVK
A_HCD
A_ETD
B_HCD
B_ETD
cross−linker cleavage
B+XL_L
A+XL_S
B+XL_S
A+XL_L
intensity
mass
AVDGVKSVK+XL_S
AVDGVKSVK+XL_L
A_b_ion
A_y_ion
AALVDHDNIKSTDISVK+XL_S
AALVDHDNIKSTDISVK+XL_L
B_b_ion
B_y_ion
m/z

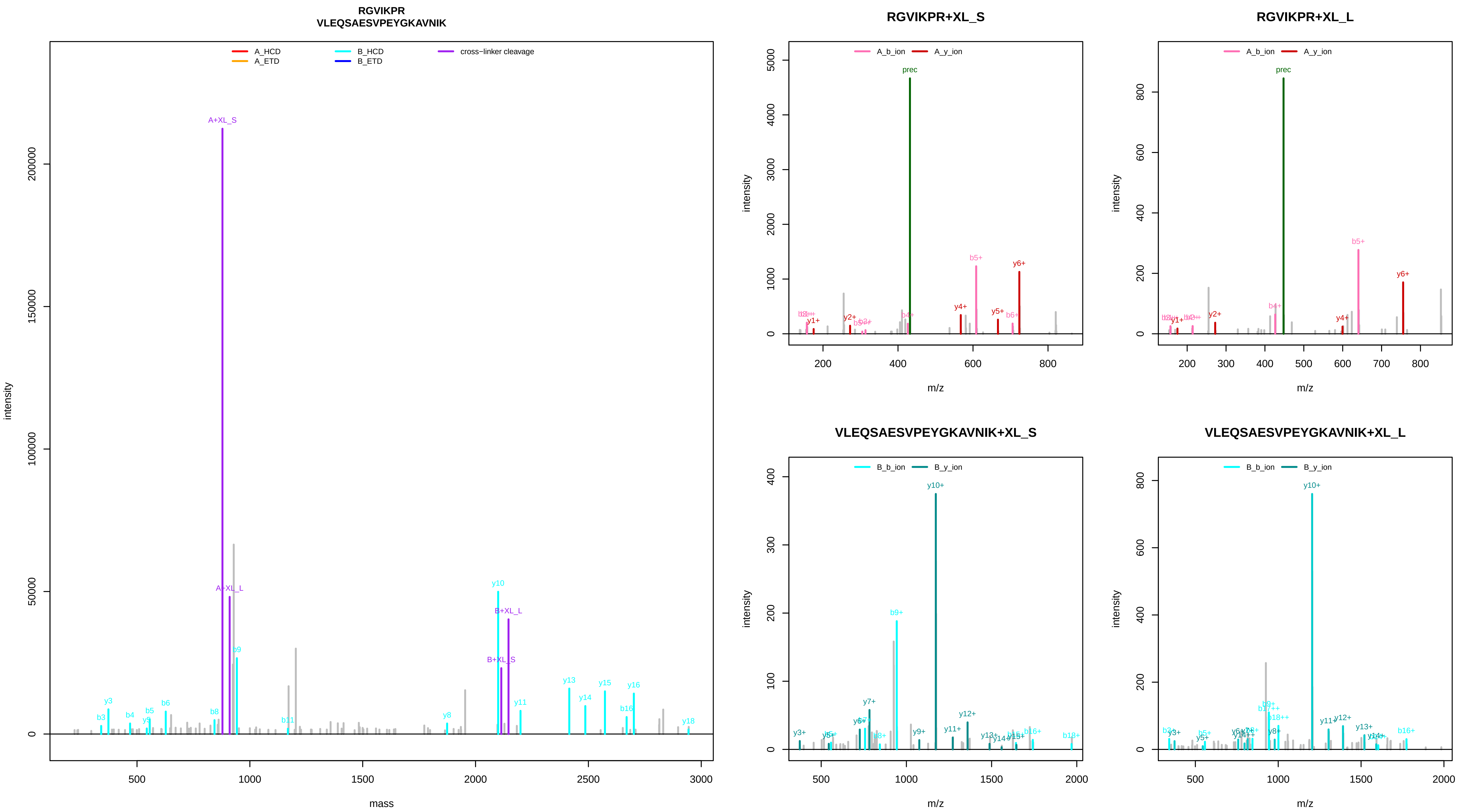

RGVIKPR
VLEQSAESVPEYGKAVNIK
A_HCD
A_ETD
B_HCD
B_ETD
cross−linker cleavage
intensity
mass
RGVIKPR+XL_S
RGVIKPR+XL_L
VLEQSAESVPEYGKAVNIK+XL_S
VLEQSAESVPEYGKAVNIK+XL_L
A_b_ion
A_y_ion
B_b_ion
B_y_ion
m/z

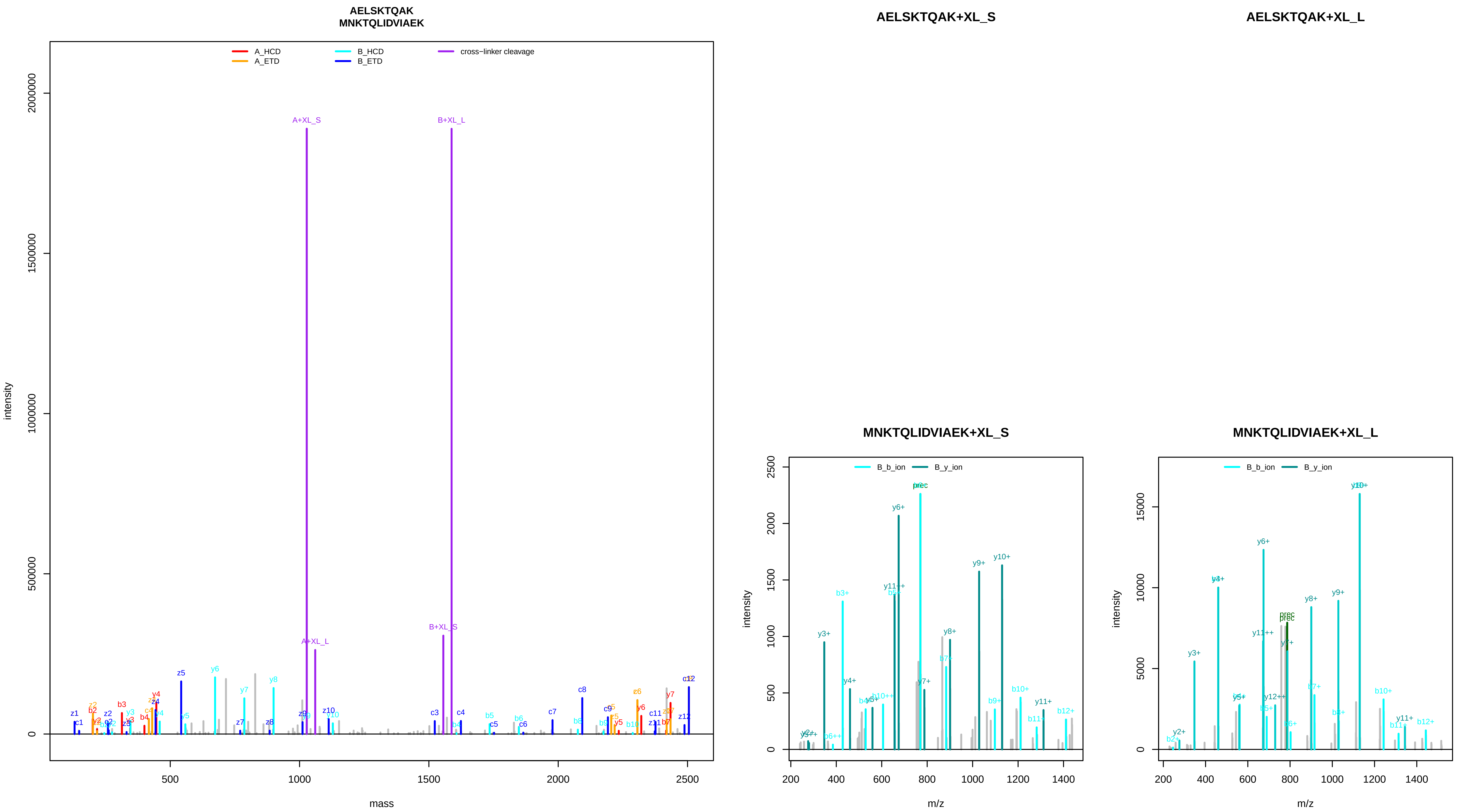

AELSKTQAK
MNKTQLIDVIAEK
A_HCD
A_ETD
B_HCD
B_ETD
cross−linker cleavage
A+XL_S
B+XL_L
B+XL_S
A+XL_L
intensity
mass
AELSKTQAK+XL_S
AELSKTQAK+XL_L
MNKTQLIDVIAEK+XL_S
MNKTQLIDVIAEK+XL_L
B_b_ion
B_y_ion
intensity
m/z

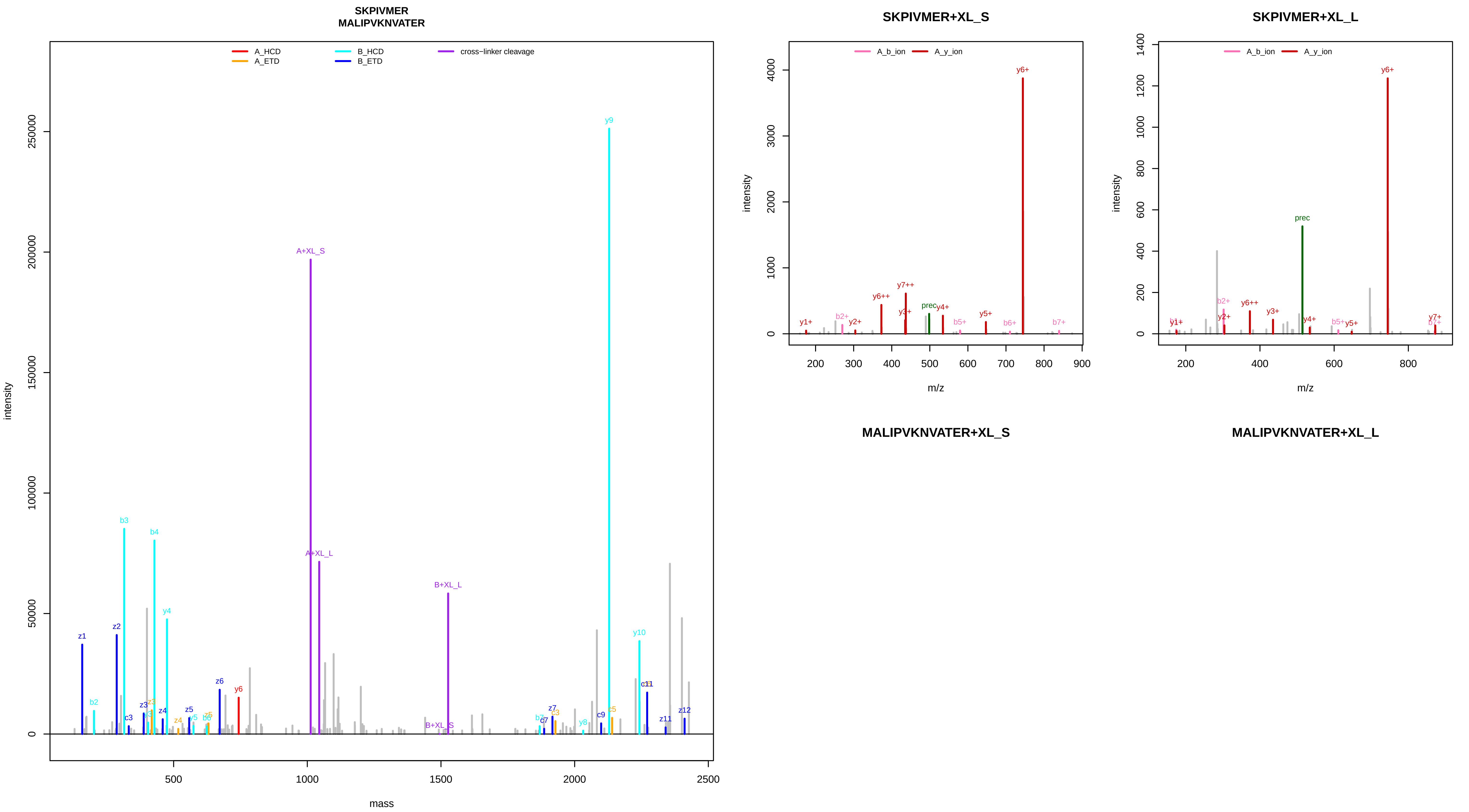
SKPIVMER
MALIPVKNVATER
A_HCD
A_ETD
B_HCD
B_ETD
cross−linker cleavage
intensity
mass
A+XL_S
A+XL_L
B+XL_L
B+XL_S
y9
y10
b3
b4
y4
SKPIVMER+XL_S
SKPIVMER+XL_L
A_b_ion
A_y_ion
m/z
prec
MALIPVKNVATER+XL_S
MALIPVKNVATER+XL_L

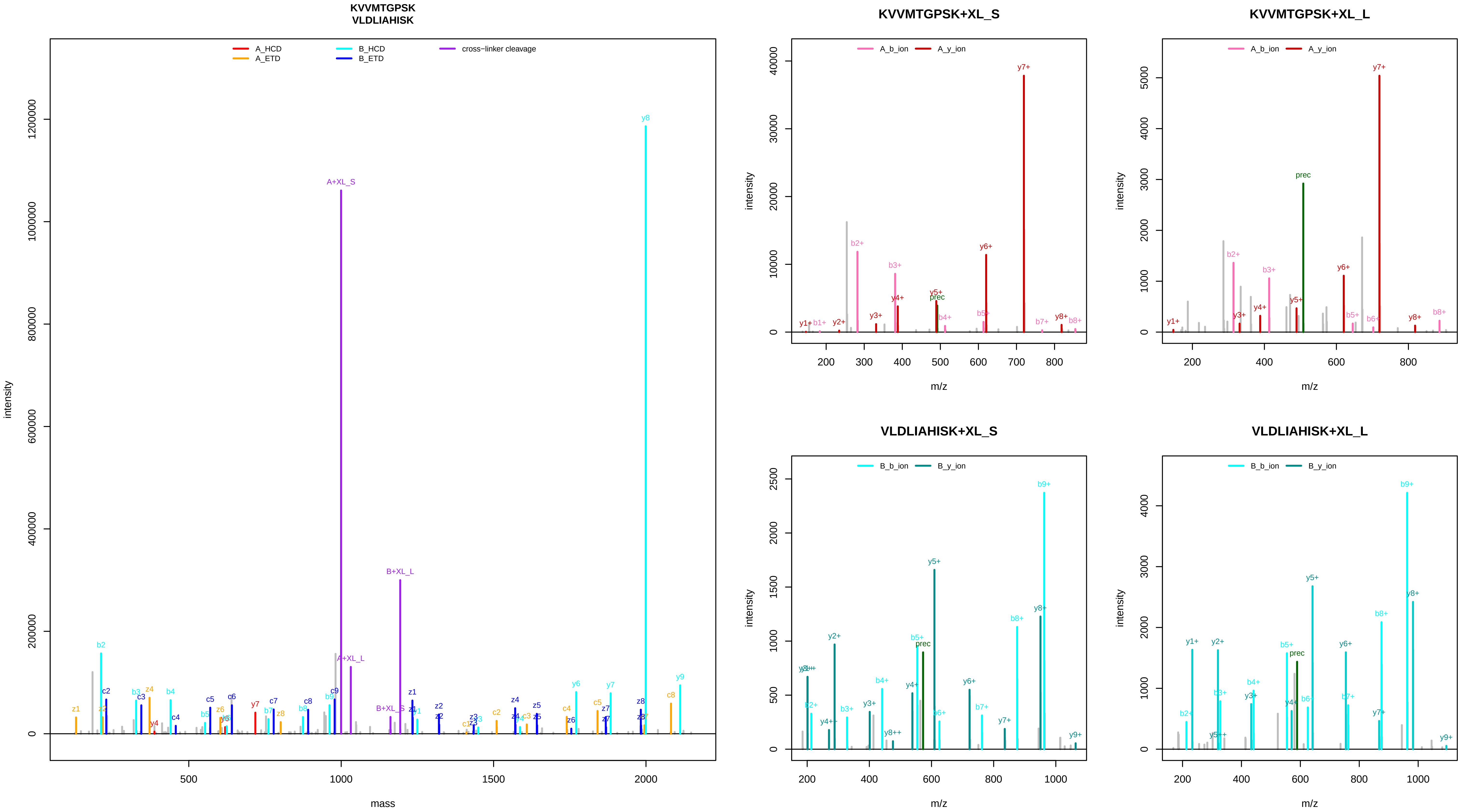
KVVMTGPSK
VLDLIAHISK
A_HCD
A_ETD
B_HCD
B_ETD
cross−linker cleavage
A+XL_S
B+XL_L
A+XL_L
B+XL_S
intensity
mass
KVVMTGPSK+XL_S
KVVMTGPSK+XL_L
VLDLIAHISK+XL_S
VLDLIAHISK+XL_L
A_b_ion
A_y_ion
B_b_ion
B_y_ion
prec
m/z

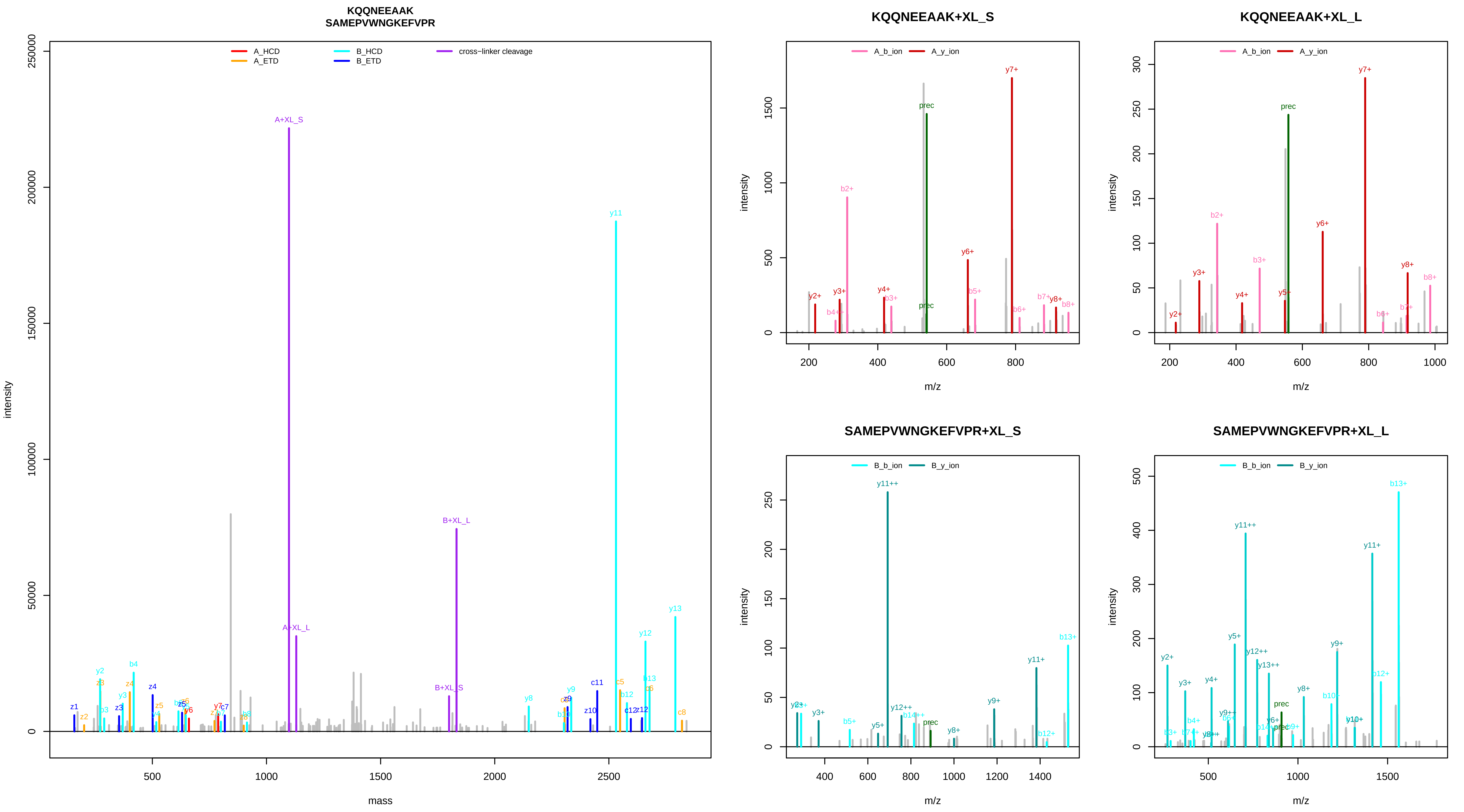
KQQNEEAAK
SAMEPVWNGKEFVPR
A_HCD
A_ETD
B_HCD
B_ETD
cross−linker cleavage
A+XL_S
A+XL_L
B+XL_L
B+XL_S
intensity
mass
KQQNEEAAK+XL_S
KQQNEEAAK+XL_L
A_b_ion
A_y_ion
SAMEPVWNGKEFVPR+XL_S
SAMEPVWNGKEFVPR+XL_L
B_b_ion
B_y_ion
prec
m/z

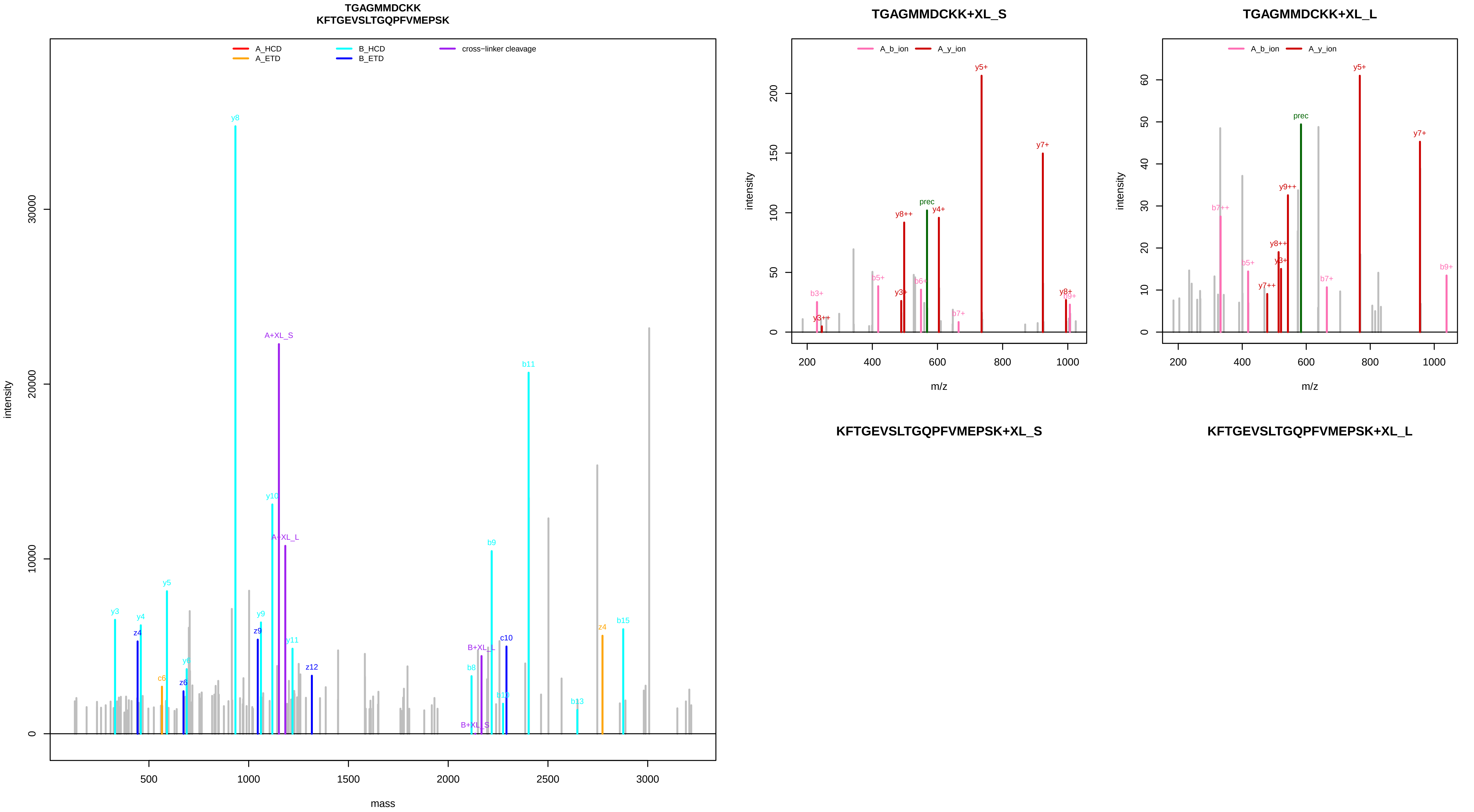

TGAGMMDCKK
KFTGEVSLTGQPFVMEPSK
A_HCD
A_ETD
B_HCD
B_ETD
cross−linker cleavage
intensity
mass
TGAGMMDCKK+XL_S
TGAGMMDCKK+XL_L
A_b_ion
A_y_ion
m/z
KFTGEVSLTGQPFVMEPSK+XL_S
KFTGEVSLTGQPFVMEPSK+XL_L

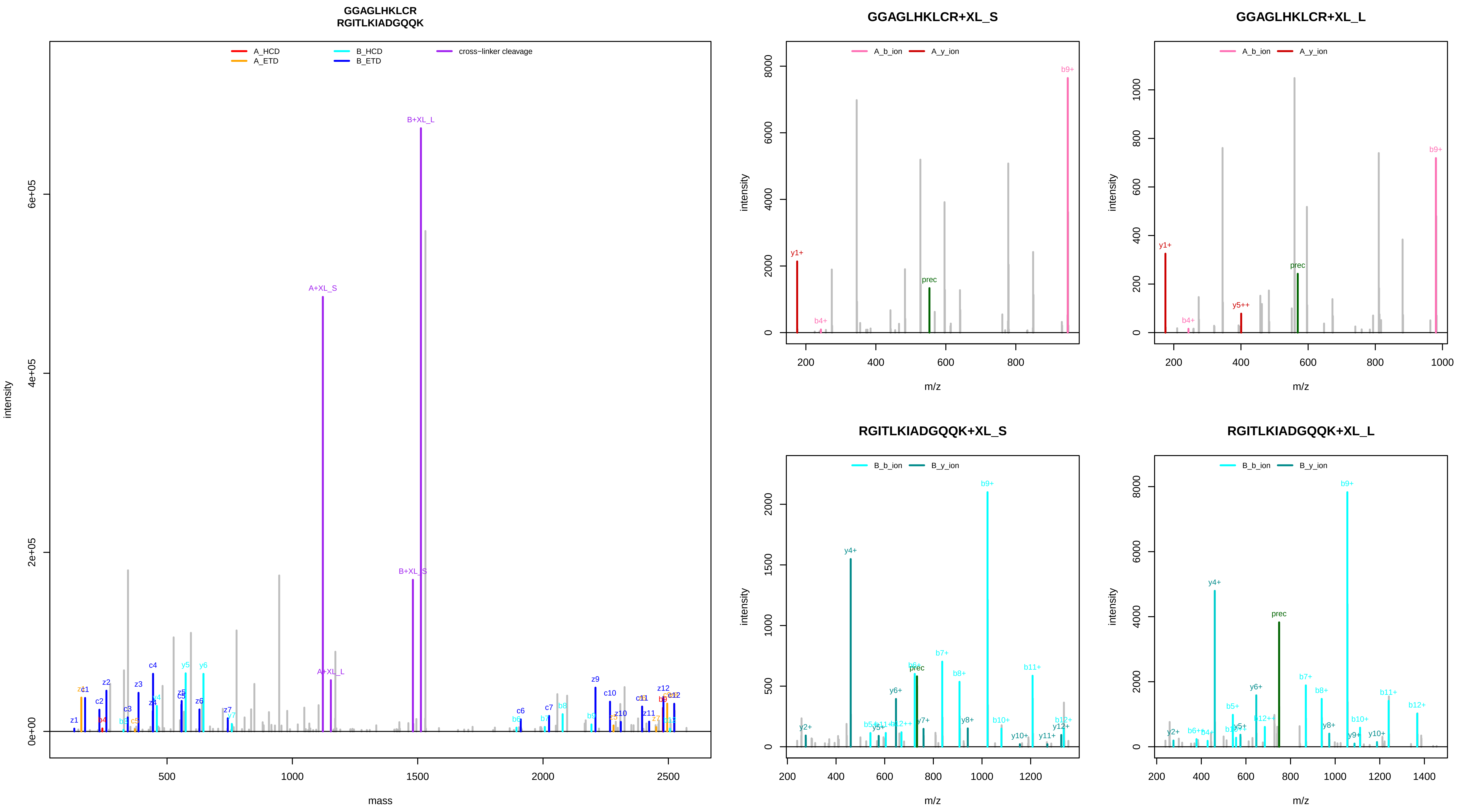
GGAGLHKLCR
RGITLKIADGQQK
A_HCD
A_ETD
B_HCD
B_ETD
cross−linker cleavage
B+XL_L
A+XL_S
B+XL_S
A+XL_L
intensity
mass
GGAGLHKLCR+XL_S
GGAGLHKLCR+XL_L
A_b_ion
A_y_ion
RGITLKIADGQQK+XL_S
RGITLKIADGQQK+XL_L
B_b_ion
B_y_ion
prec
m/z

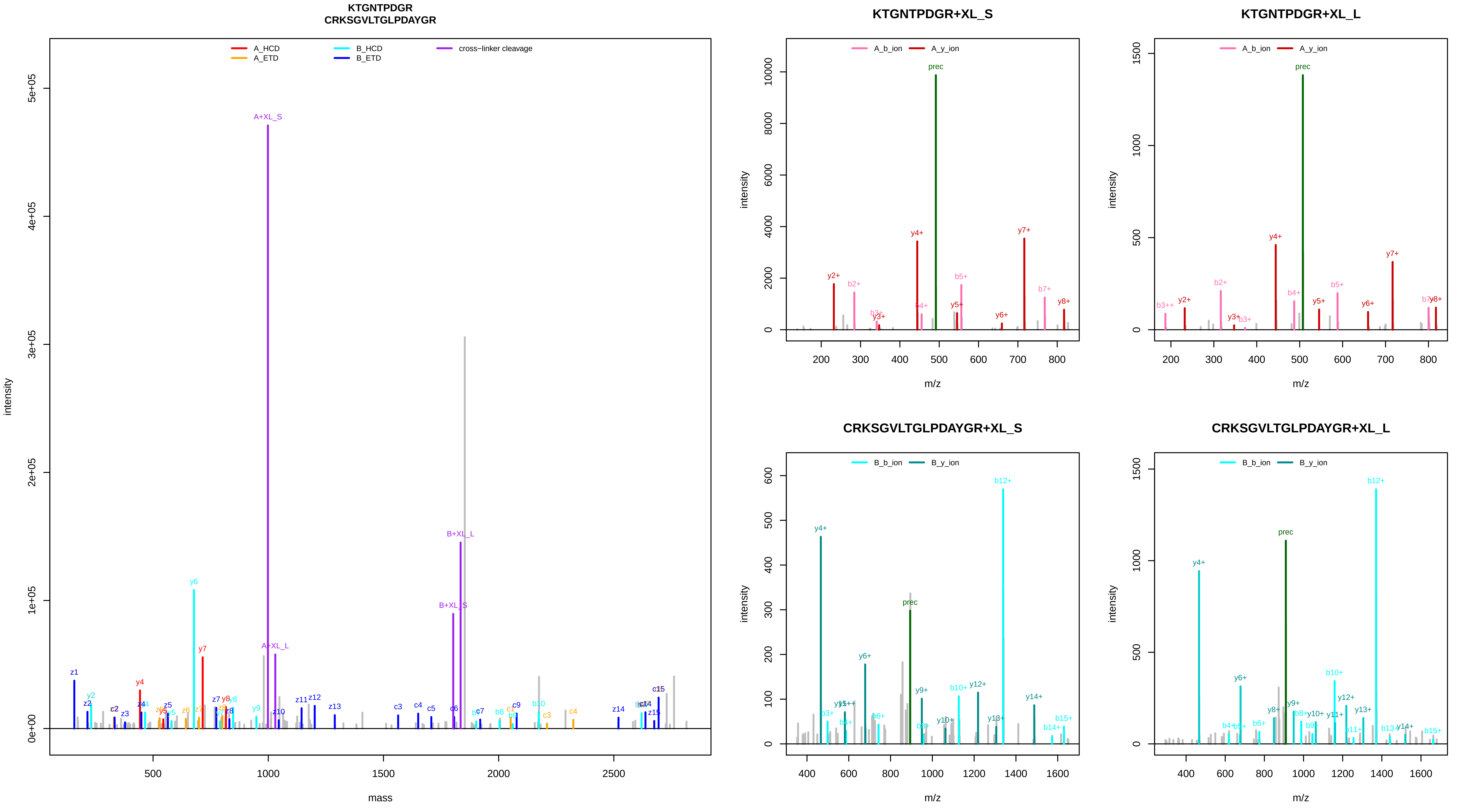

KTGNTPDGR
CRKSGVLTGLPDAYGR
A_HCD
A_ETD
B_HCD
B_ETD
cross−linker cleavage
A+XL_S
A+XL_L
B+XL_L
B+XL_S
intensity
mass
KTGNTPDGR+XL_S
KTGNTPDGR+XL_L
CRKSGVLTGLPDAYGR+XL_S
CRKSGVLTGLPDAYGR+XL_L
A_b_ion
A_y_ion
B_b_ion
B_y_ion
prec
m/z

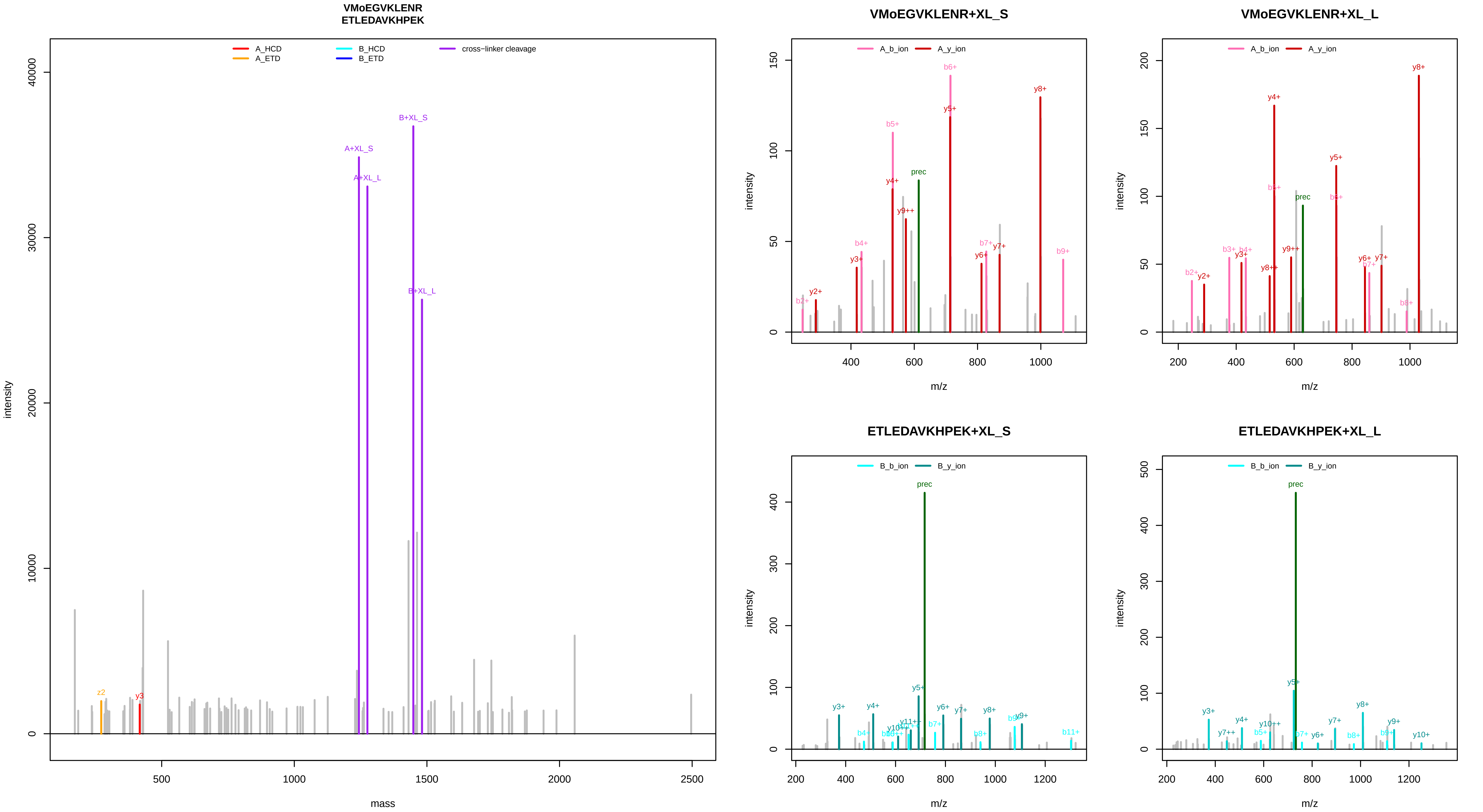
VMoEGVKLENR
ETLEDAVKHPEK
A_HCD
A_ETD
B_HCD
B_ETD
cross−linker cleavage
A+XL_S
A+XL_L
B+XL_S
B+XL_L
intensity
mass
VMoEGVKLENR+XL_S
VMoEGVKLENR+XL_L
ETLEDAVKHPEK+XL_S
ETLEDAVKHPEK+XL_L
A_b_ion
A_y_ion
B_b_ion
B_y_ion
prec
m/z

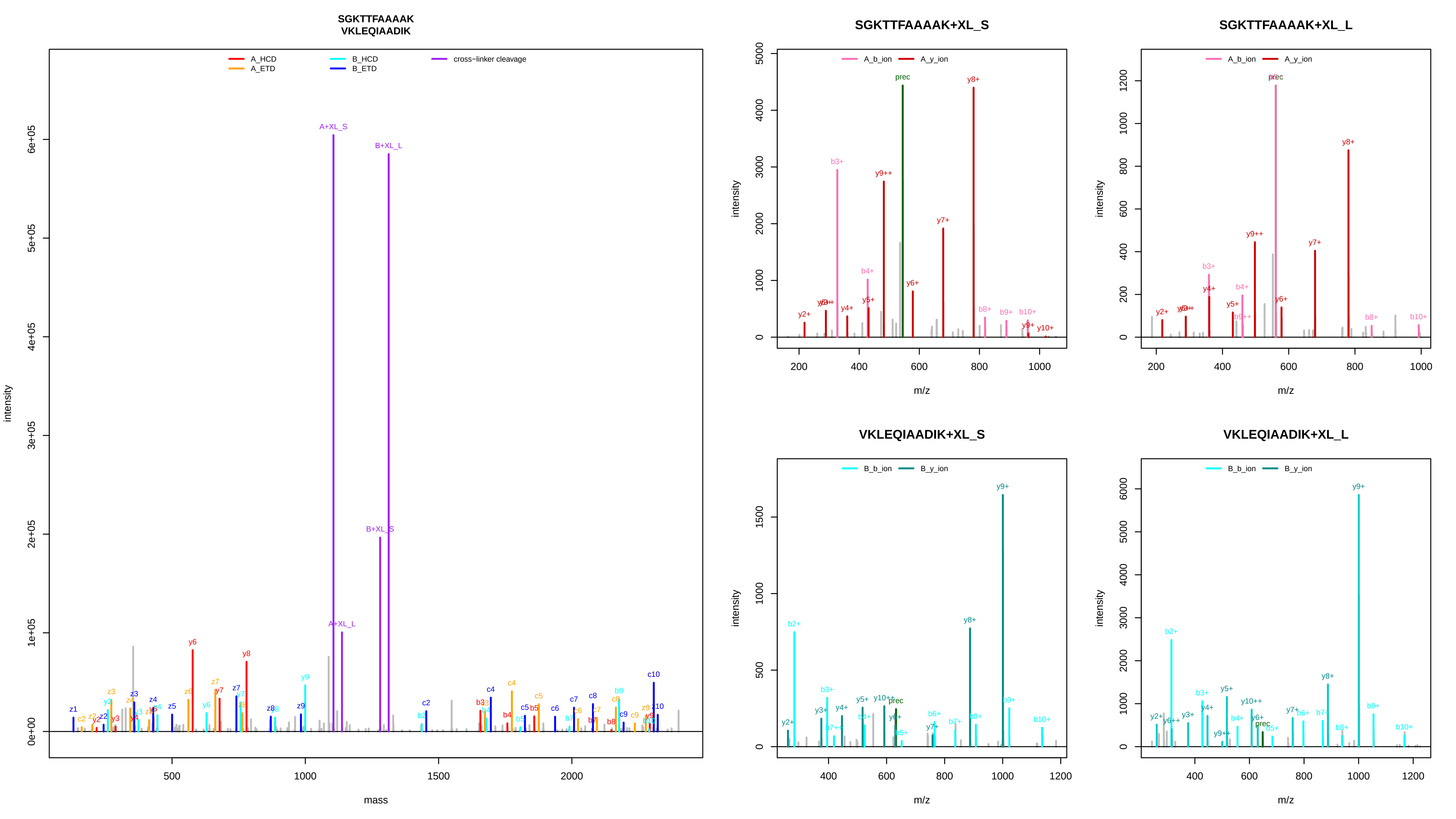
SGKTTFAAAAK
VKLEQIAADIK
A_HCD
A_ETD
B_HCD
B_ETD
cross−linker cleavage
A+XL_S
B+XL_L
B+XL_S
A+XL_L
intensity
mass
0e+00
1e+05
2e+05
3e+05
4e+05
5e+05
6e+05
500
1000
1500
2000
SGKTTFAAAAK+XL_S
SGKTTFAAAAK+XL_L
VKLEQIAADIK+XL_S
VKLEQIAADIK+XL_L
A_b_ion
A_y_ion
B_b_ion
B_y_ion
prec
intensity
m/z

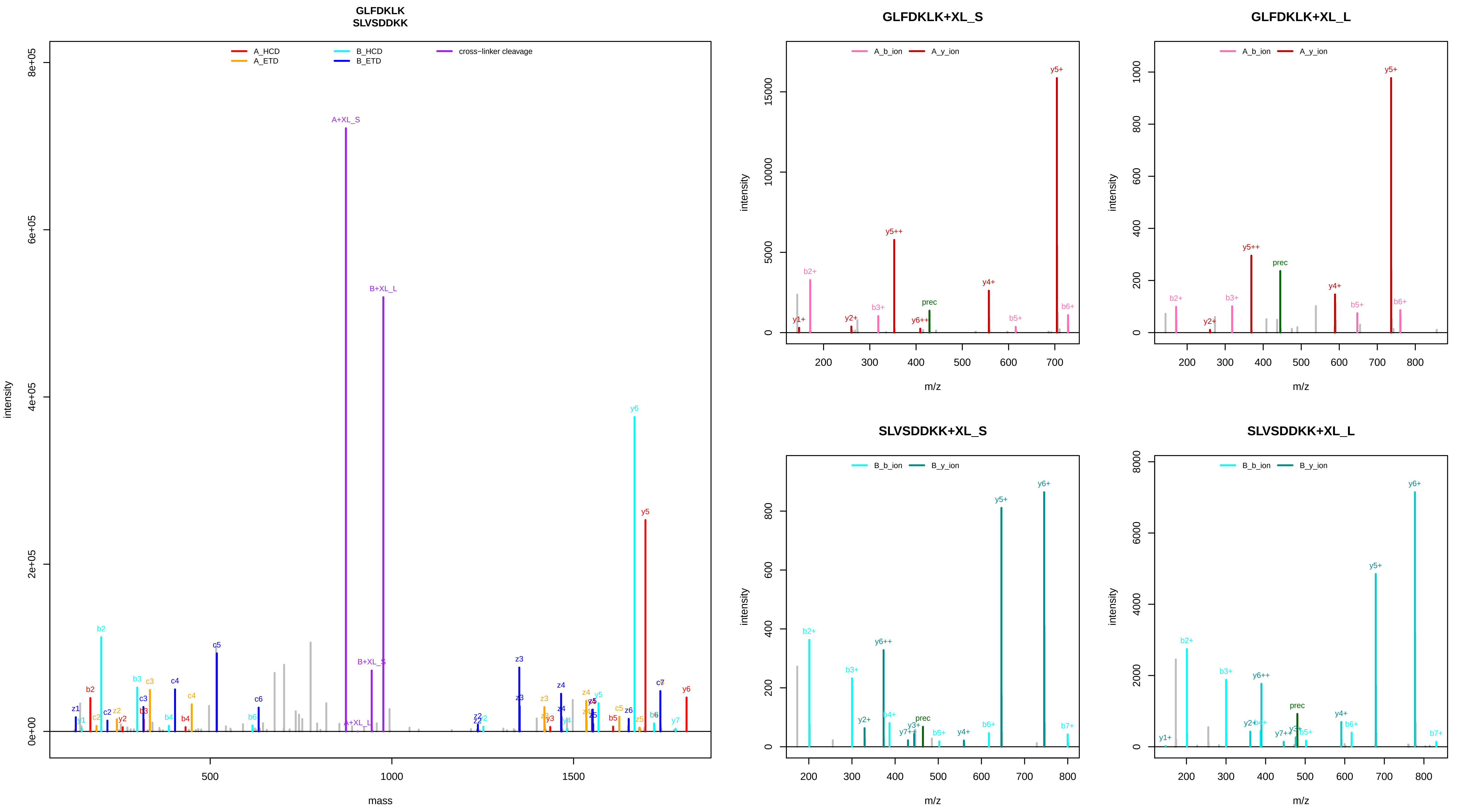
GLFDKLK
SLVSDDKK
A_HCD
A_ETD
B_HCD
B_ETD
cross−linker cleavage
A+XL_S
B+XL_L
B+XL_S
A+XL_L
intensity
mass
GLFDKLK+XL_S
GLFDKLK+XL_L
SLVSDDKK+XL_S
SLVSDDKK+XL_L
A_b_ion
A_y_ion
B_b_ion
B_y_ion
prec
m/z

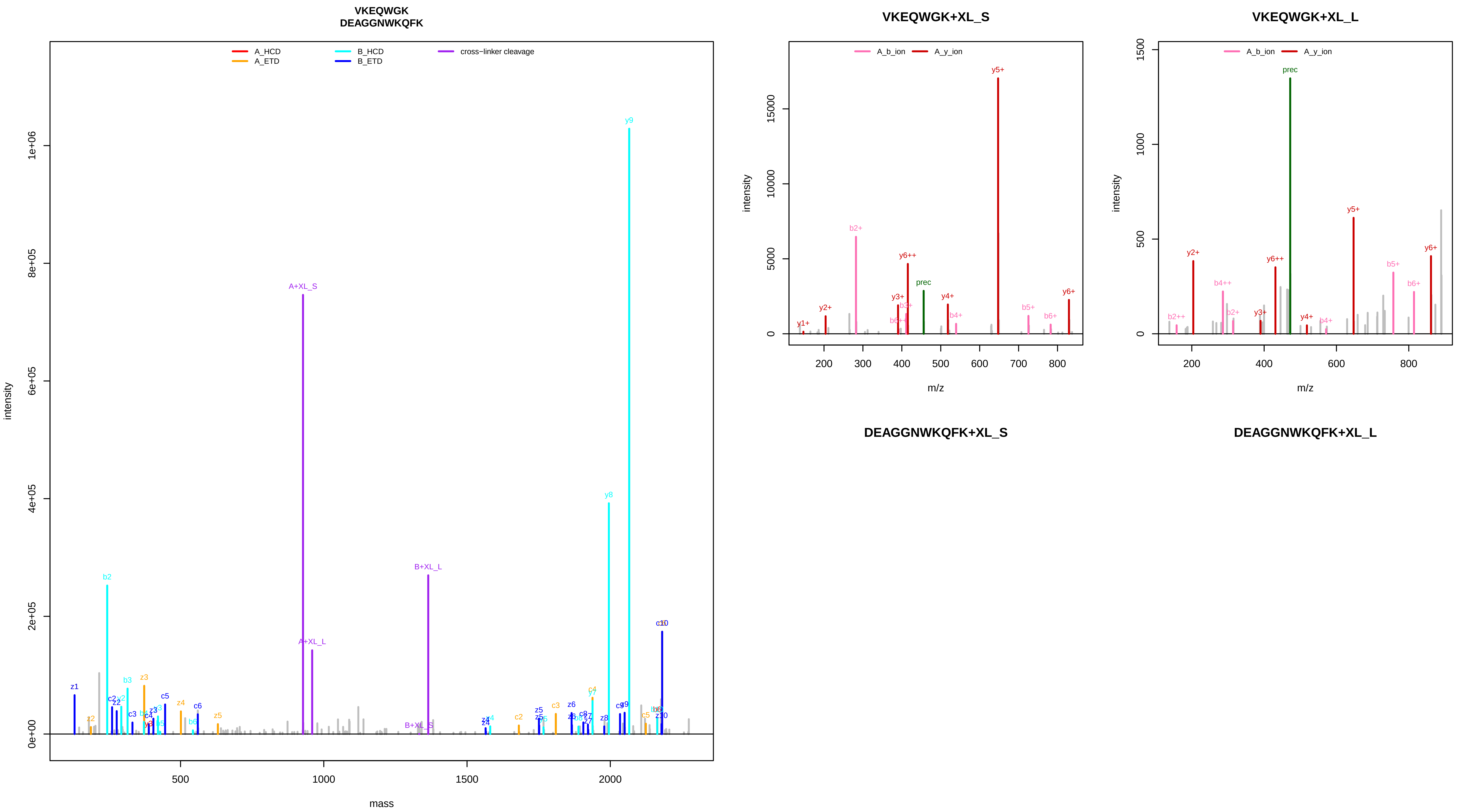

VKEQWGK
DEAGGNWKQFK
A_HCD
A_ETD
B_HCD
B_ETD
cross−linker cleavage
A+XL_S
A+XL_L
B+XL_L
B+XL_S
intensity
mass
VKEQWGK+XL_S
VKEQWGK+XL_L
A_b_ion
A_y_ion
prec
m/z
DEAGGNWKQFK+XL_S
DEAGGNWKQFK+XL_L

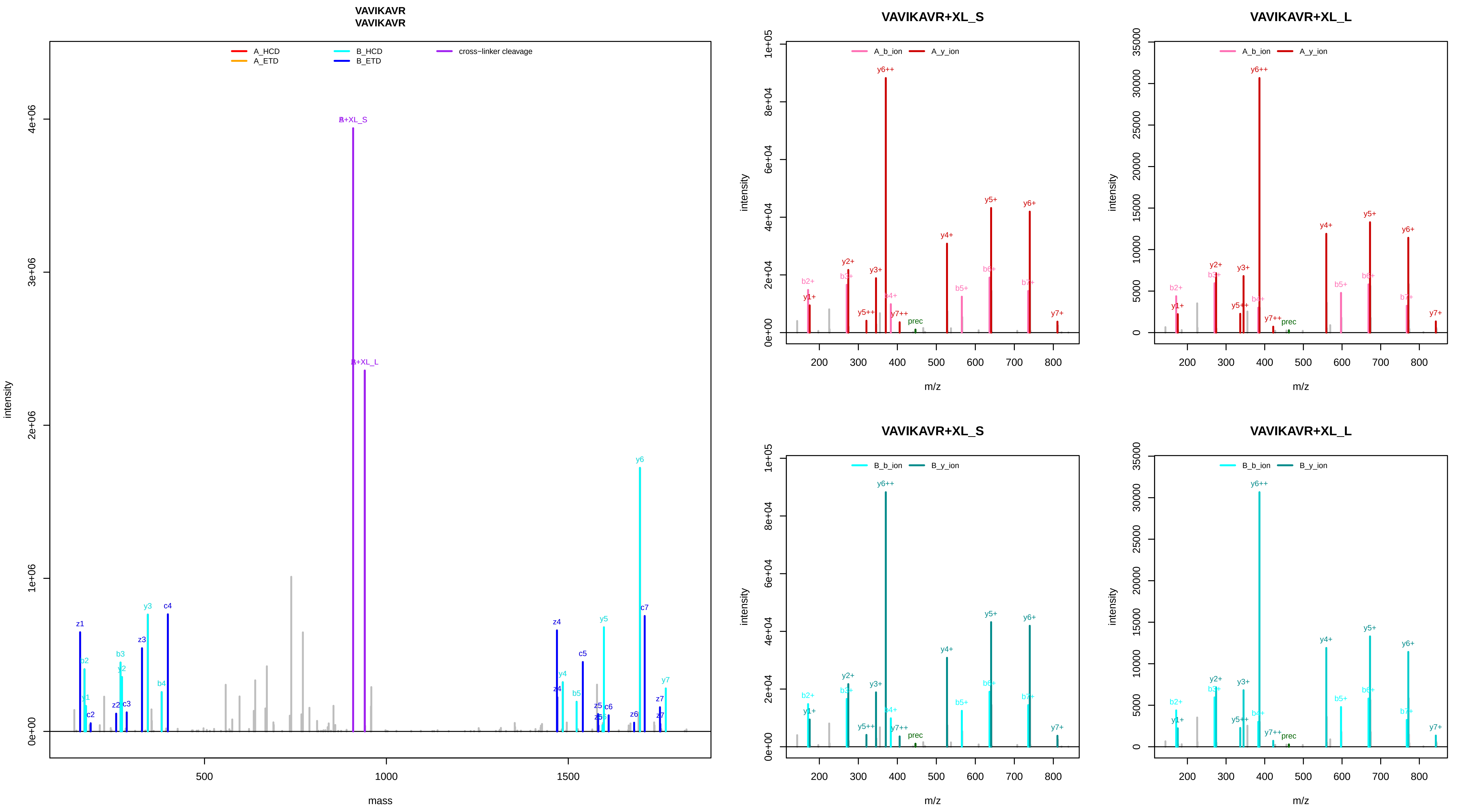

VAVIKAVR
VAVIKAVR
A_HCD
A_ETD
B_HCD
B_ETD
cross−linker cleavage
intensity
mass
VAVIKAVR+XL_S
VAVIKAVR+XL_L
A_b_ion
A_y_ion
B_b_ion
B_y_ion
m/z
intensity

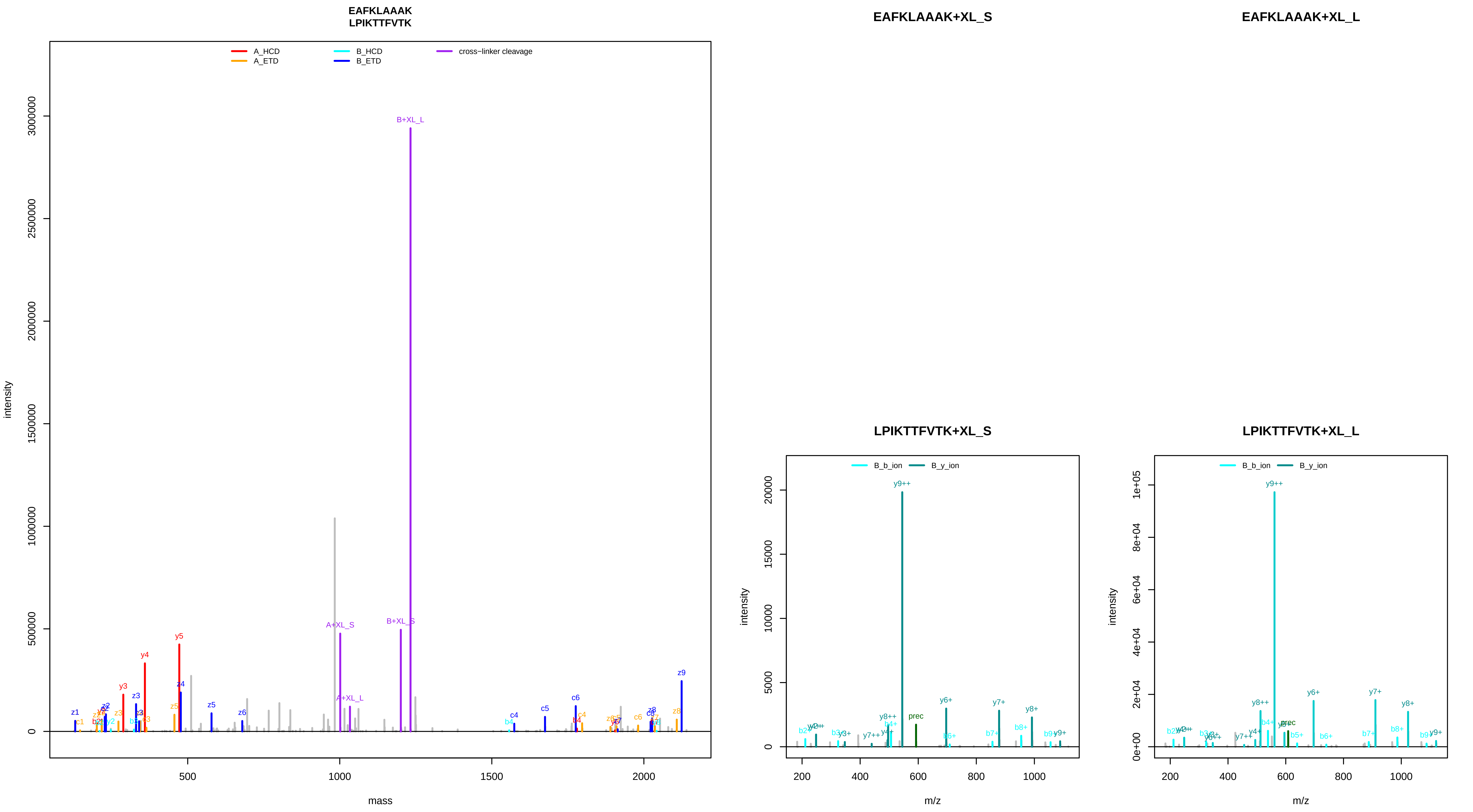
EAFKLAAAK
LPIKTTFVTK
A_HCD
A_ETD
B_HCD
B_ETD
cross−linker cleavage
B+XL_L
A+XL_S
B+XL_S
A+XL_L
intensity
mass
EAFKLAAAK+XL_S
EAFKLAAAK+XL_L
LPIKTTFVTK+XL_S
LPIKTTFVTK+XL_L
B_b_ion
B_y_ion
y9++
prec
m/z
intensity

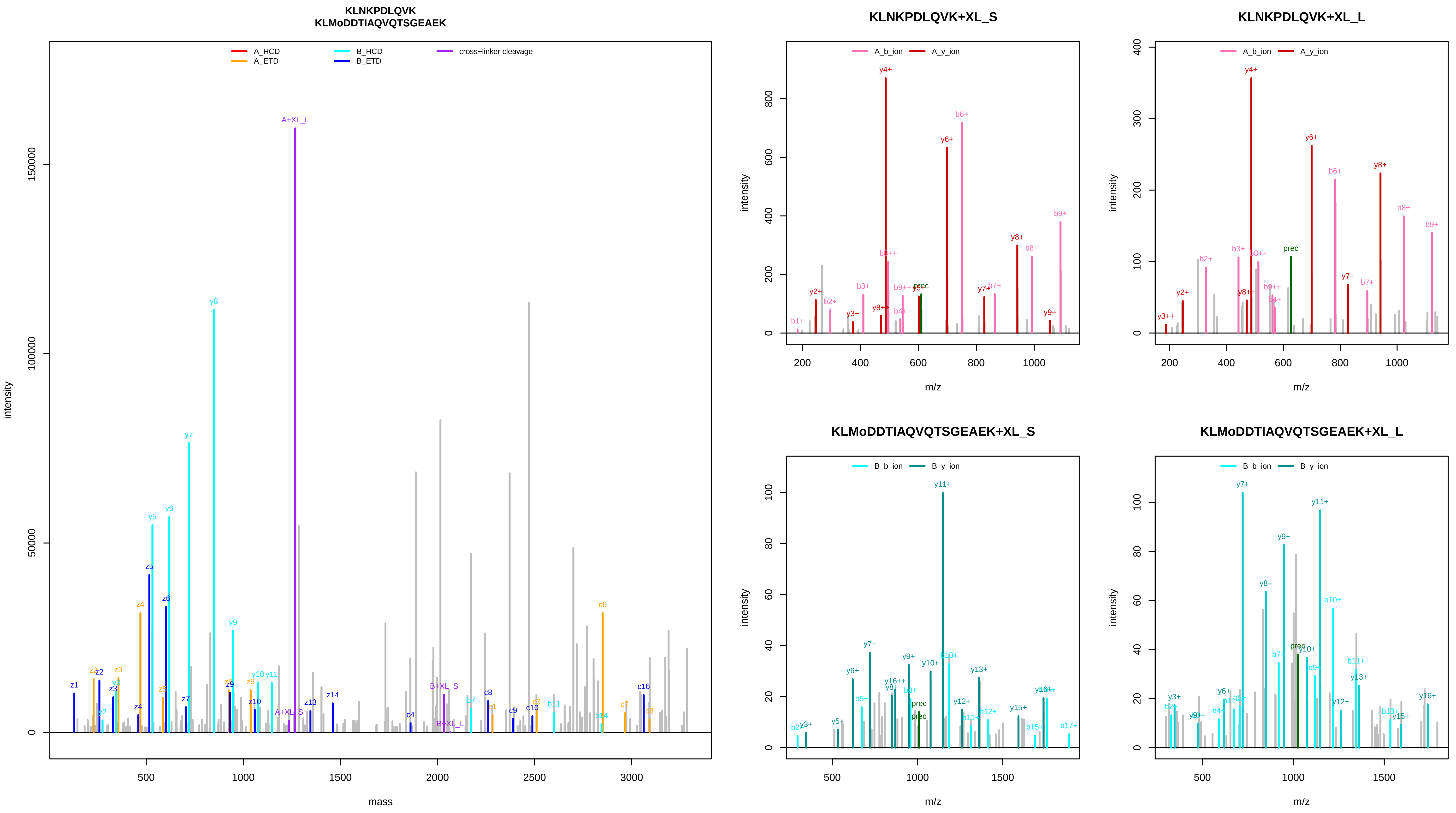
KLNKPDLQVK
KLMoDDTIAQVQTSGEAEK
A_HCD
A_ETD
B_HCD
B_ETD
cross−linker cleavage
A+XL_L
A+XL_S
B+XL_S
B+XL_L
intensity
mass
KLNKPDLQVK+XL_S
KLNKPDLQVK+XL_L
A_b_ion
A_y_ion
KLMoDDTIAQVQTSGEAEK+XL_S
KLMoDDTIAQVQTSGEAEK+XL_L
B_b_ion
B_y_ion
prec
m/z

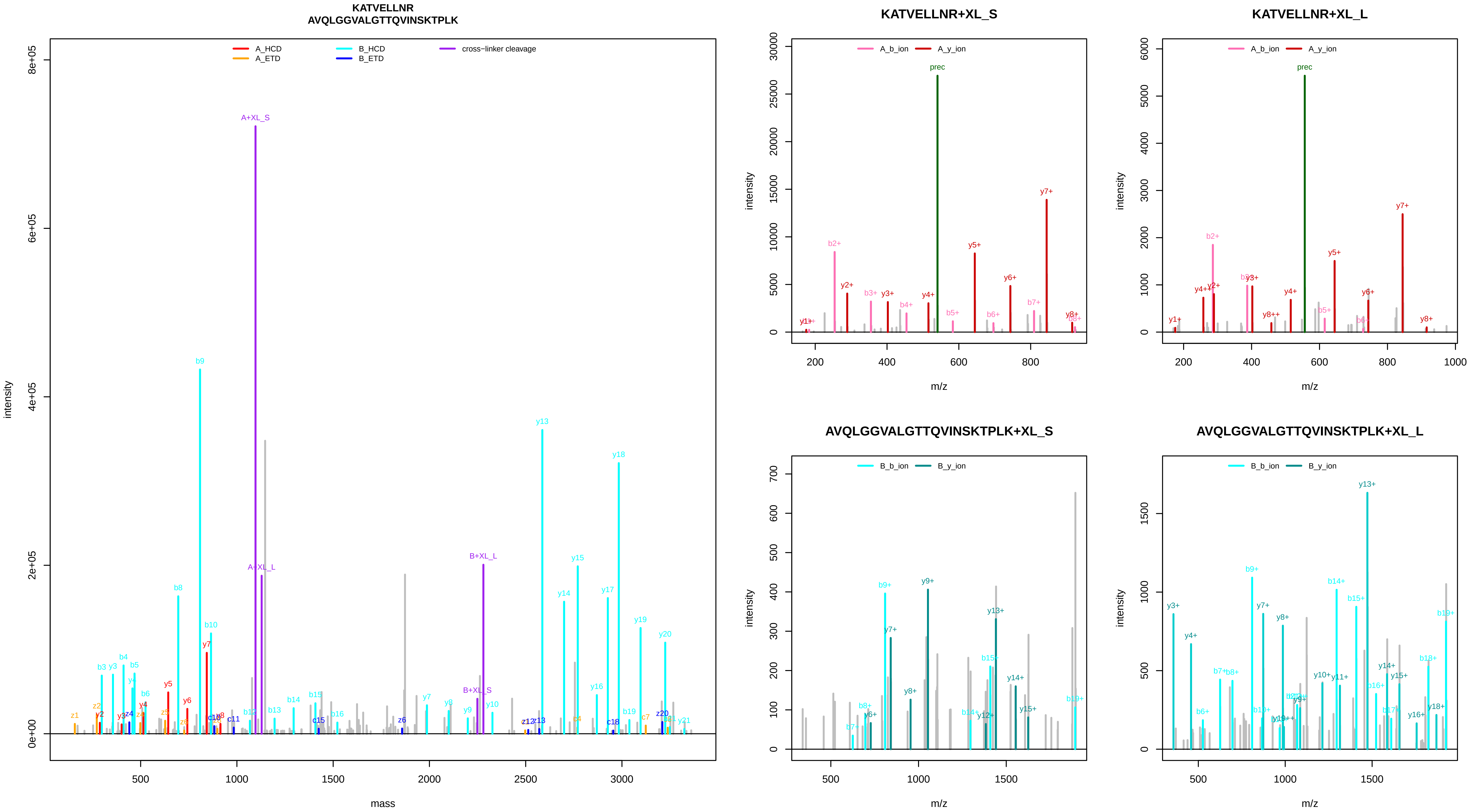
KATVELLNR
AVQLGGVALGTTQVINSKTPLK
A_HCD
A_ETD
B_HCD
B_ETD
cross−linker cleavage
A+XL_S
A+XL_L
B+XL_L
B+XL_S
intensity
mass
KATVELLNR+XL_S
KATVELLNR+XL_L
A_b_ion
A_y_ion
prec
m/z
AVQLGGVALGTTQVINSKTPLK+XL_S
AVQLGGVALGTTQVINSKTPLK+XL_L
B_b_ion
B_y_ion

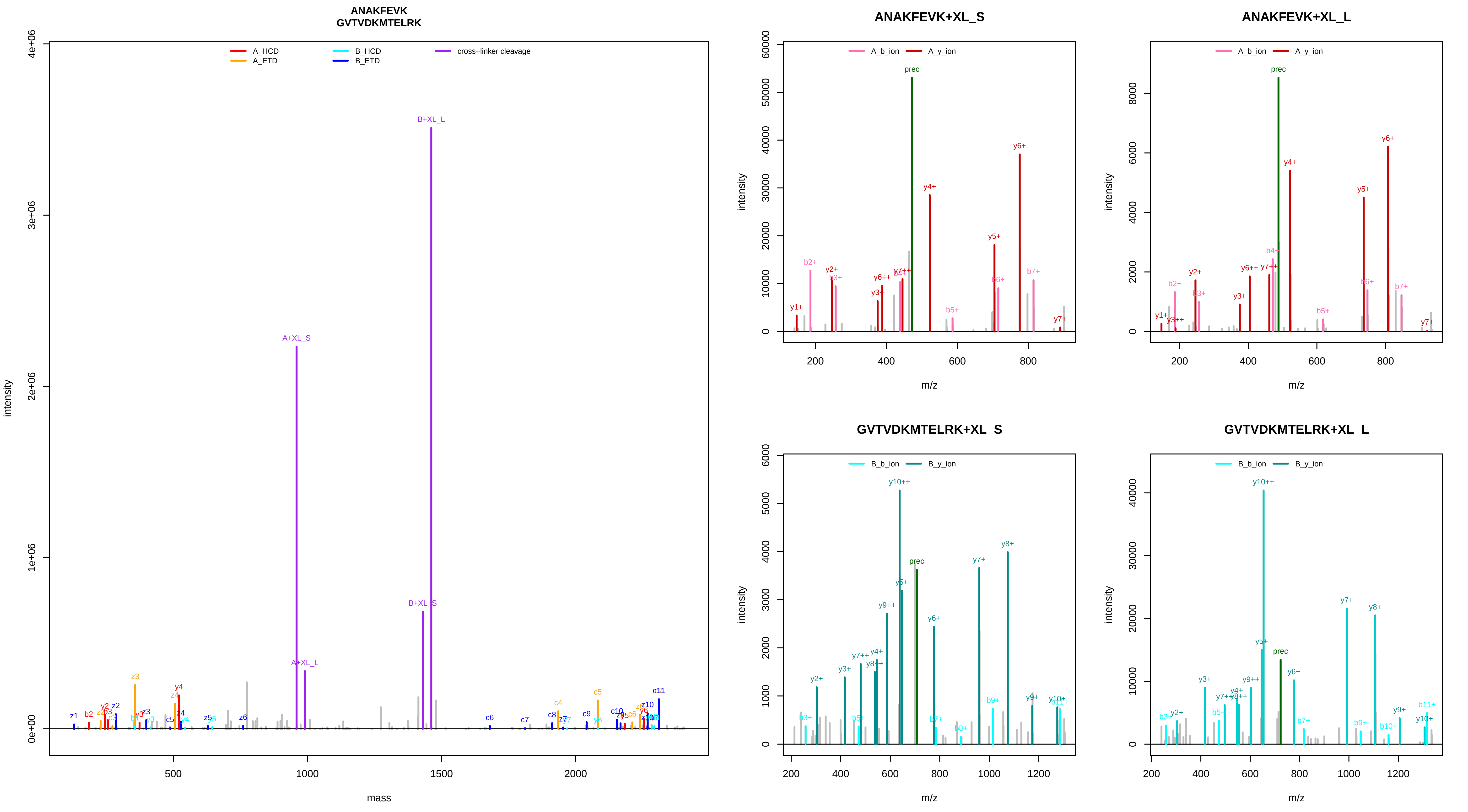

ANAKFEVK
GVTVDKMTELRK
A_HCD
A_ETD
B_HCD
B_ETD
cross−linker cleavage
B+XL_L
A+XL_S
B+XL_S
A+XL_L
intensity
mass
ANAKFEVK+XL_S
ANAKFEVK+XL_L
A_b_ion
A_y_ion
prec
GVTVDKMTELRK+XL_S
GVTVDKMTELRK+XL_L
B_b_ion
B_y_ion
m/z

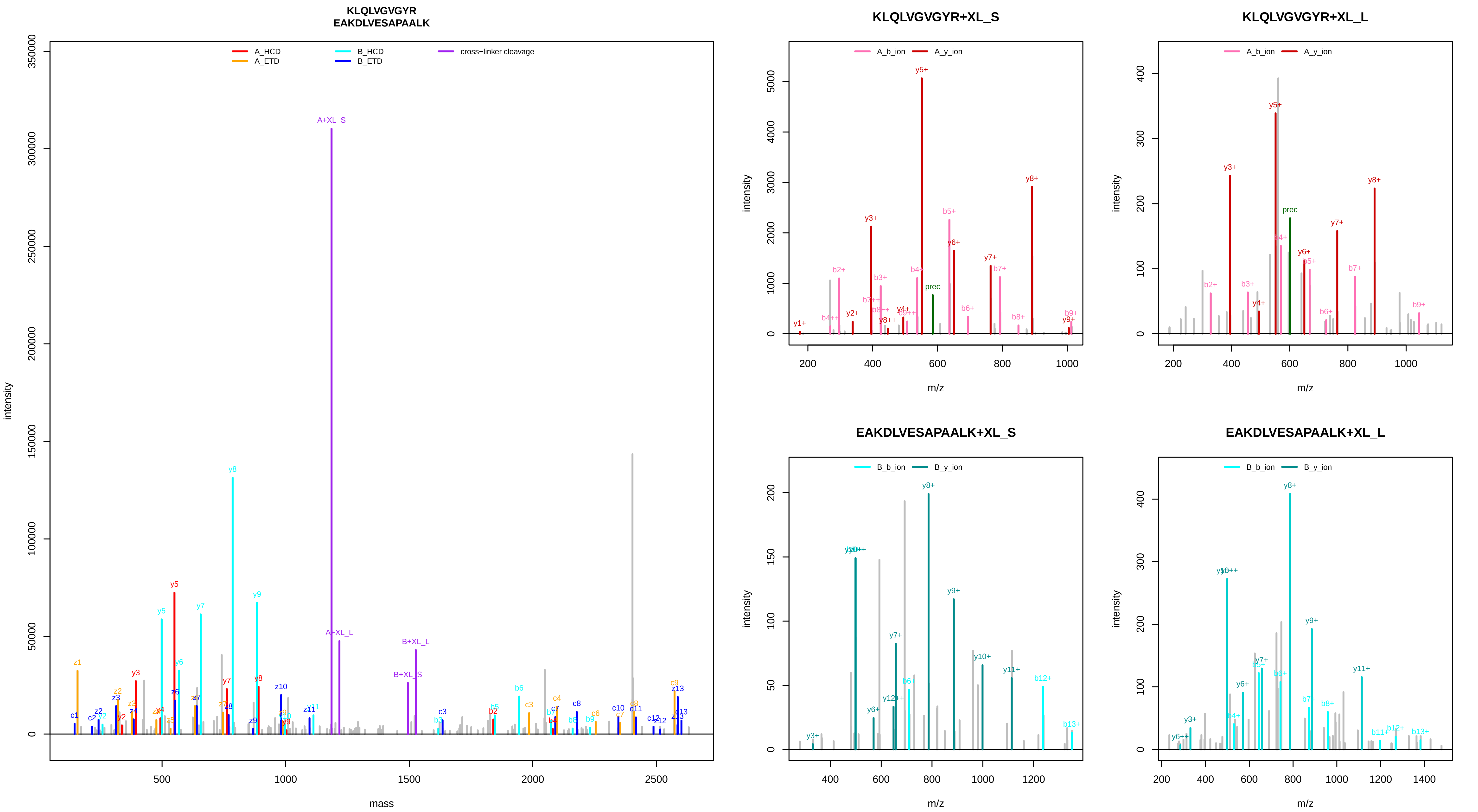

KLQLVGVGYR
EAKDLVESAPAALK
A_HCD
A_ETD
B_HCD
B_ETD
cross−linker cleavage
A+XL_S
A+XL_L
B+XL_L
B+XL_S
intensity
mass
KLQLVGVGYR+XL_S
KLQLVGVGYR+XL_L
A_b_ion
A_y_ion
EAKDLVESAPAALK+XL_S
EAKDLVESAPAALK+XL_L
B_b_ion
B_y_ion
prec
m/z

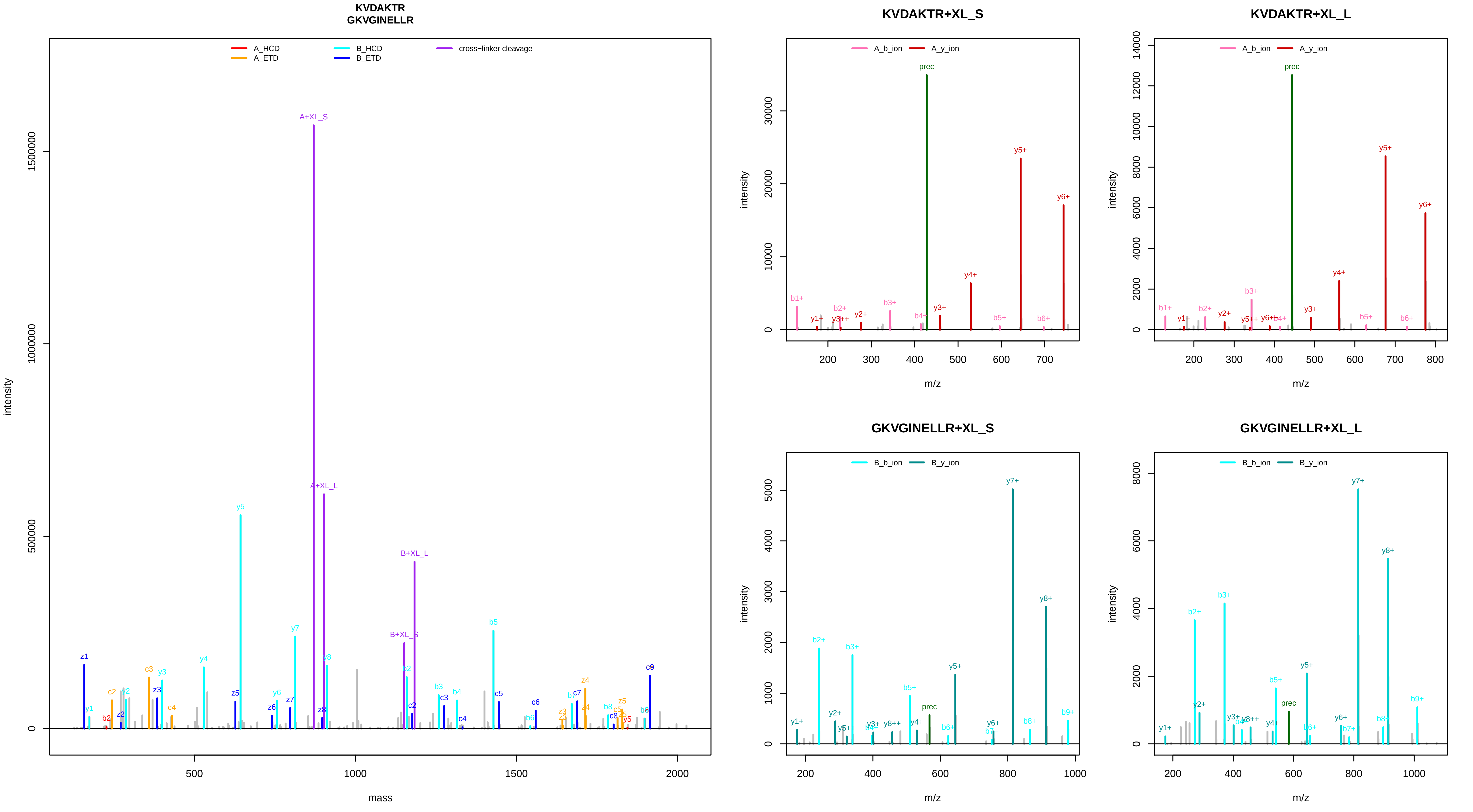

KVDAKTR
GKVGINELLR
A_HCD
A_ETD
B_HCD
B_ETD
cross−linker cleavage
A+XL_S
A+XL_L
B+XL_L
B+XL_S
intensity
mass
KVDAKTR+XL_S
KVDAKTR+XL_L
GKVGINELLR+XL_S
GKVGINELLR+XL_L
A_b_ion
A_y_ion
B_b_ion
B_y_ion
prec
m/z

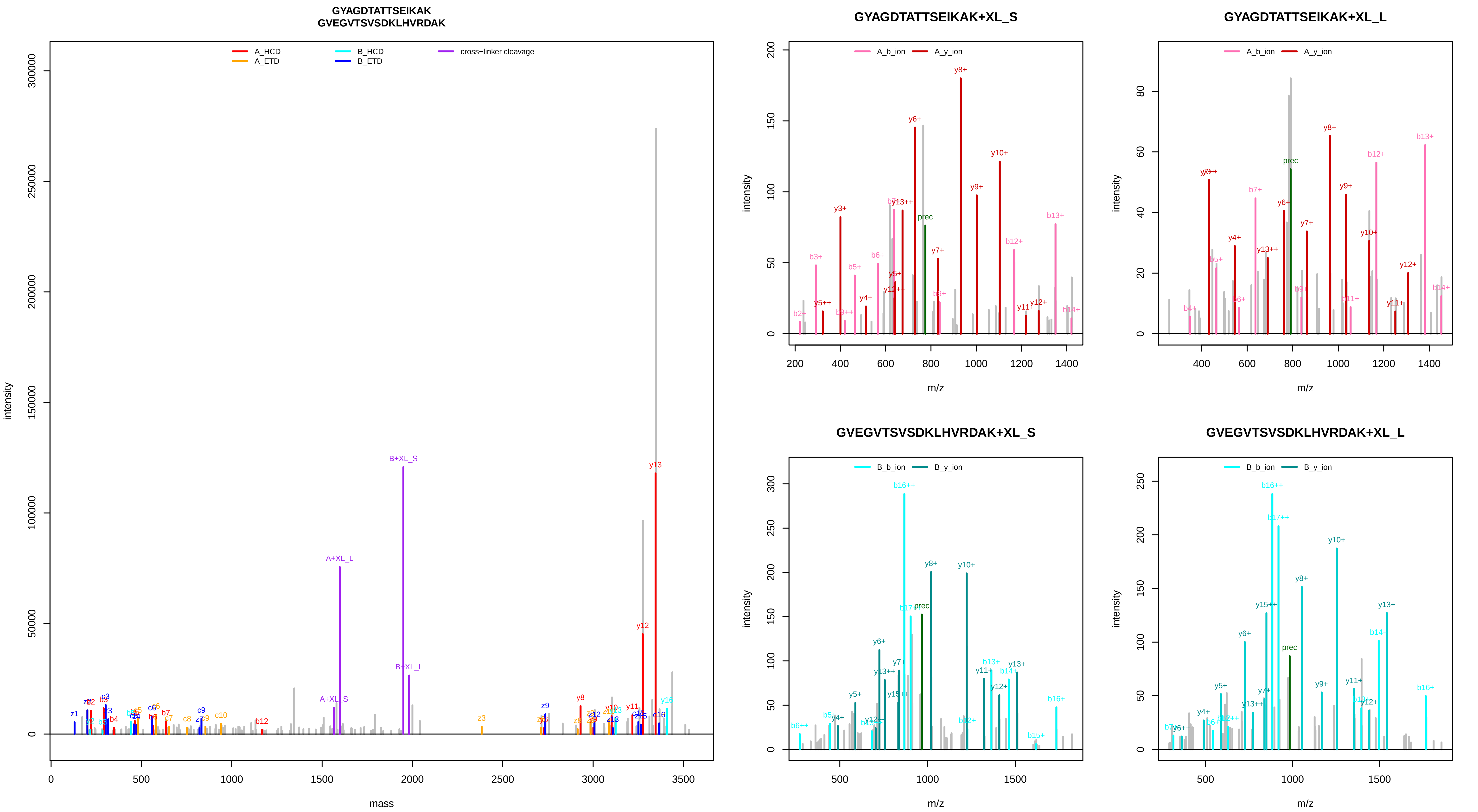
GYAGDTATTSEIKAK
GVEGVTSVSDKLHVRDAK
A_HCD
A_ETD
B_HCD
B_ETD
cross−linker cleavage
intensity
mass
GYAGDTATTSEIKAK+XL_S
GYAGDTATTSEIKAK+XL_L
GVEGVTSVSDKLHVRDAK+XL_S
GVEGVTSVSDKLHVRDAK+XL_L
A_b_ion
A_y_ion
B_b_ion
B_y_ion
m/z

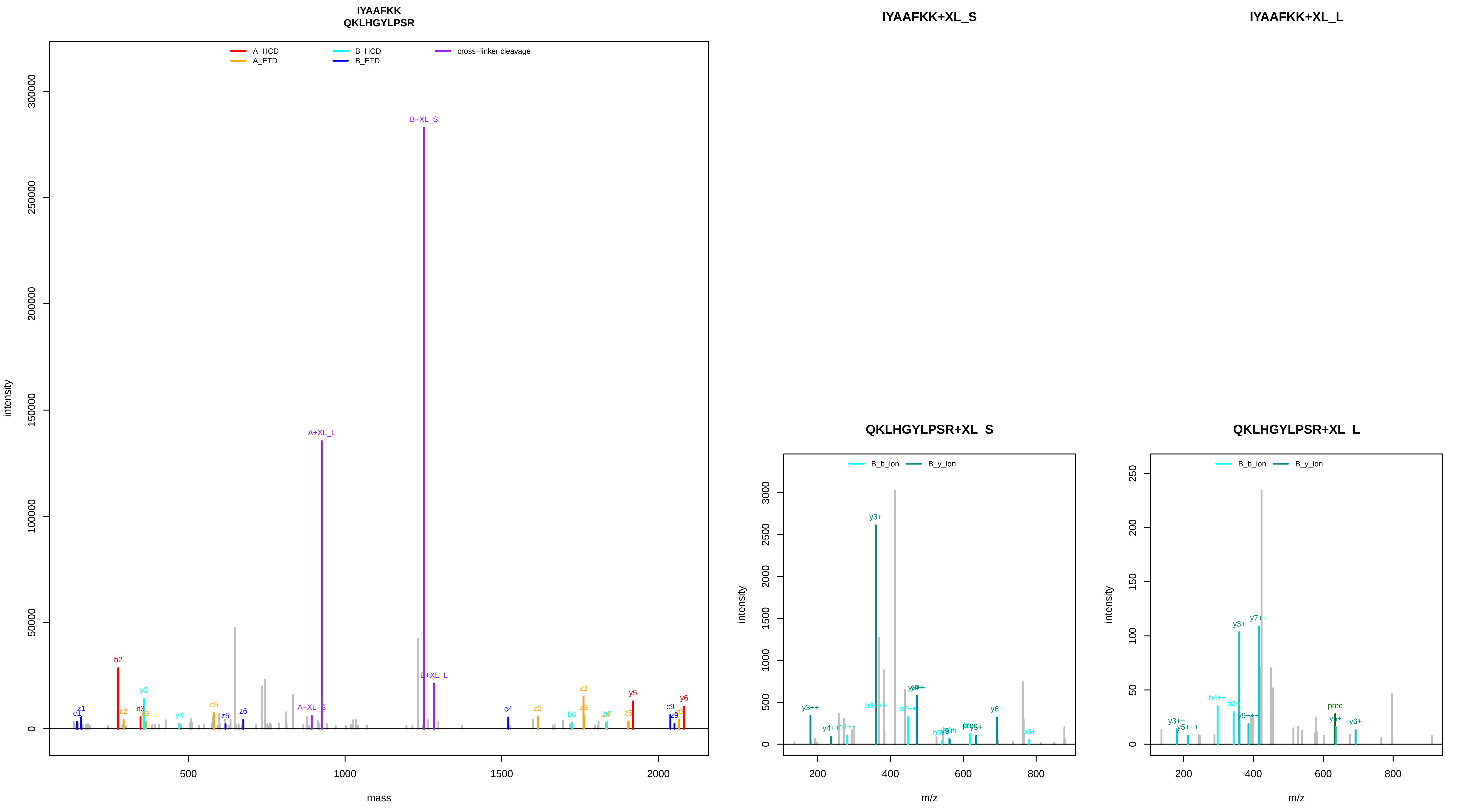
IYAAFKK
QKLHGYLPSR
A_HCD
A_ETD
B_HCD
B_ETD
cross−linker cleavage
B+XL_S
A+XL_L
B+XL_L
A+XL_S
intensity
mass
IYAAFKK+XL_S
IYAAFKK+XL_L
QKLHGYLPSR+XL_S
QKLHGYLPSR+XL_L
B_b_ion
B_y_ion
m/z

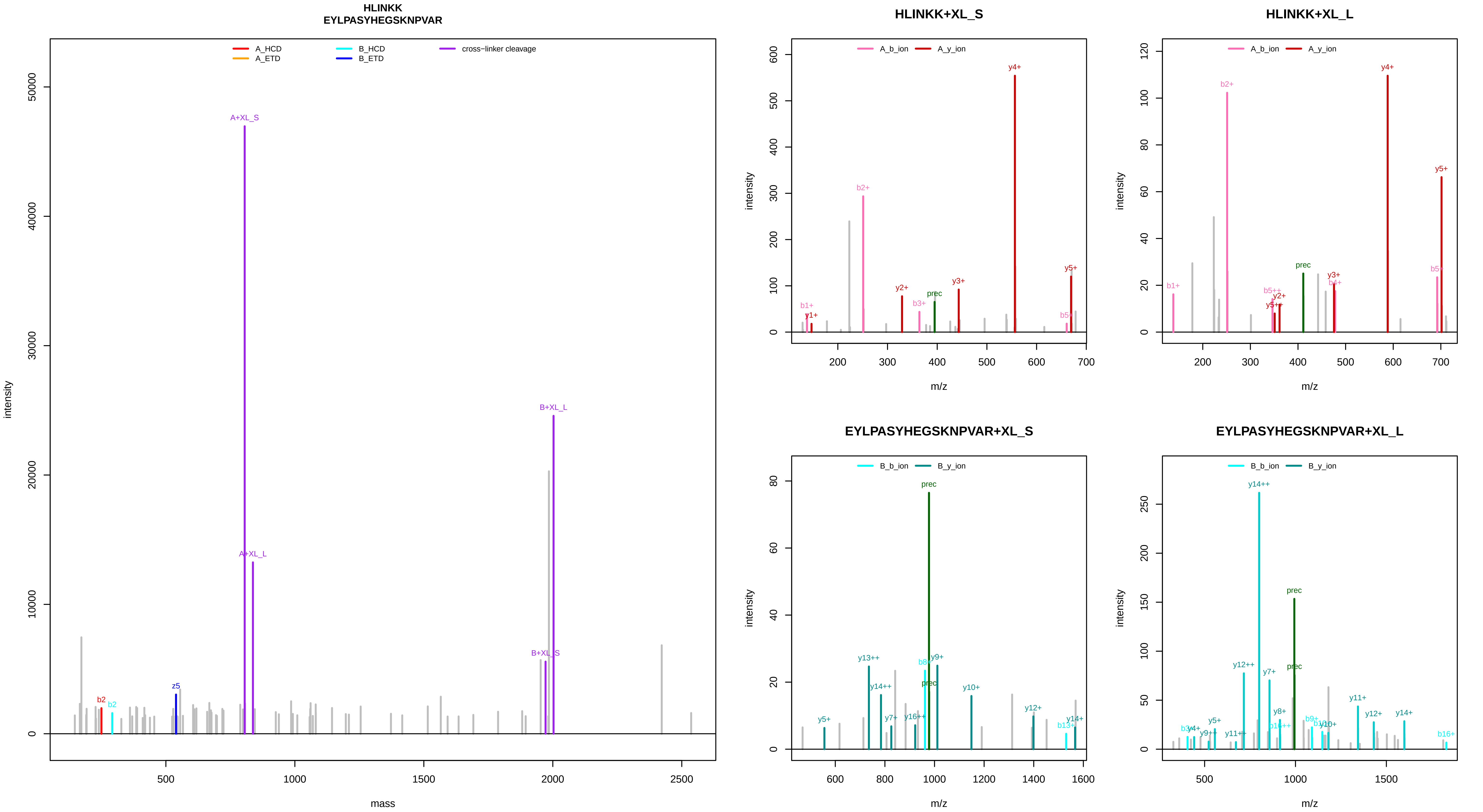
HLINKK
EYLPASYHEGSKNPVAR
A_HCD
A_ETD
B_HCD
B_ETD
cross−linker cleavage
A+XL_S
A+XL_L
B+XL_L
B+XL_S
b2
z5
intensity
mass
HLINKK+XL_S
HLINKK+XL_L
A_b_ion
A_y_ion
EYLPASYHEGSKNPVAR+XL_S
EYLPASYHEGSKNPVAR+XL_L
B_b_ion
B_y_ion
prec
m/z

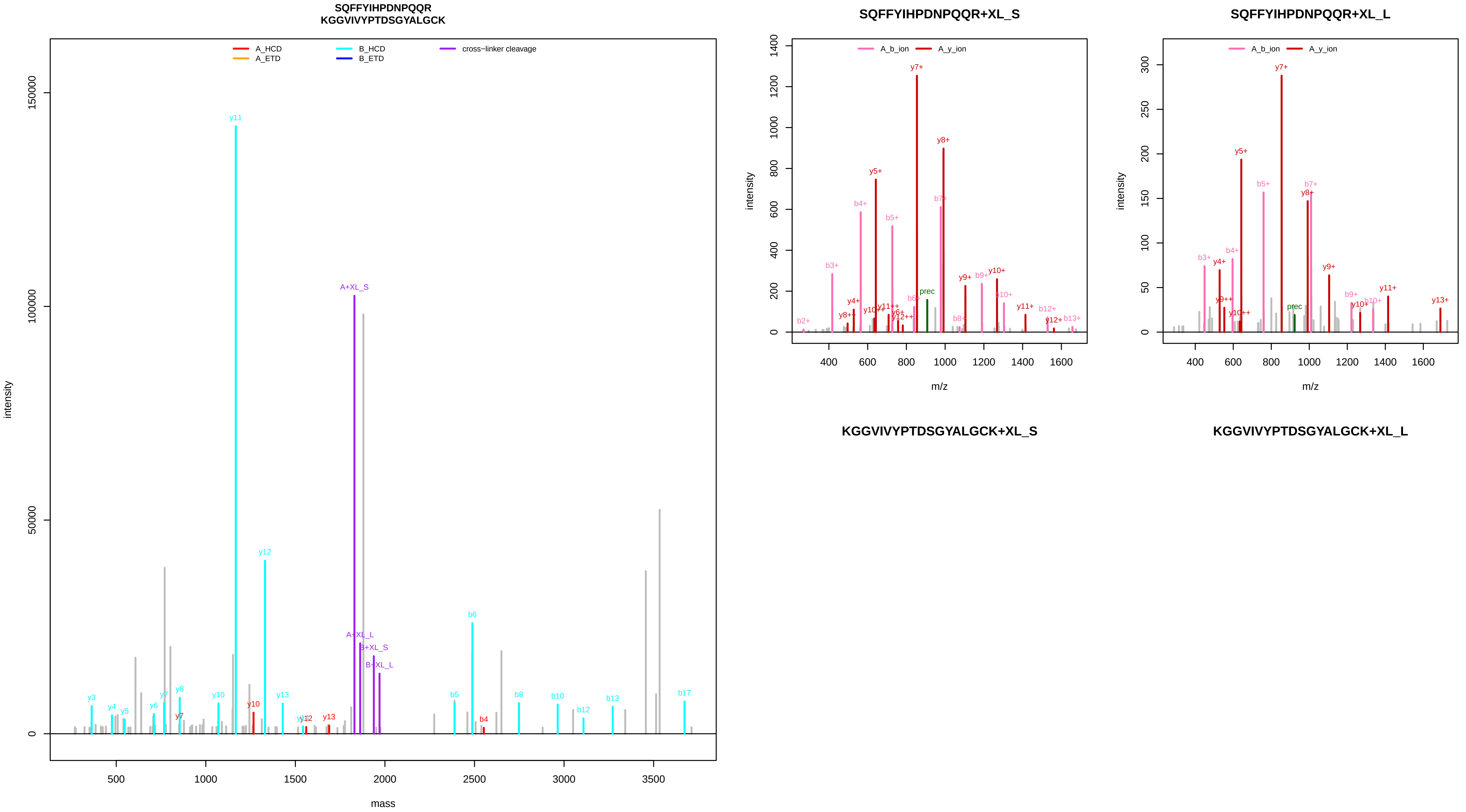

SQFFYIHPDNPQQR
KGGVIVYPTDSGYALGCK
A_HCD
A_ETD
B_HCD
B_ETD
cross−linker cleavage
intensity
mass
y11
A+XL_S
A+XL_L
B+XL_S
B+XL_L
y12
b6
y3
y4
y5
y6
y7
y8
y10
y13
b5
b8
b10
b12
b13
b17
b4
SQFFYIHPDNPQQR+XL_S
SQFFYIHPDNPQQR+XL_L
A_b_ion
A_y_ion
m/z
prec
KGGVIVYPTDSGYALGCK+XL_S
KGGVIVYPTDSGYALGCK+XL_L

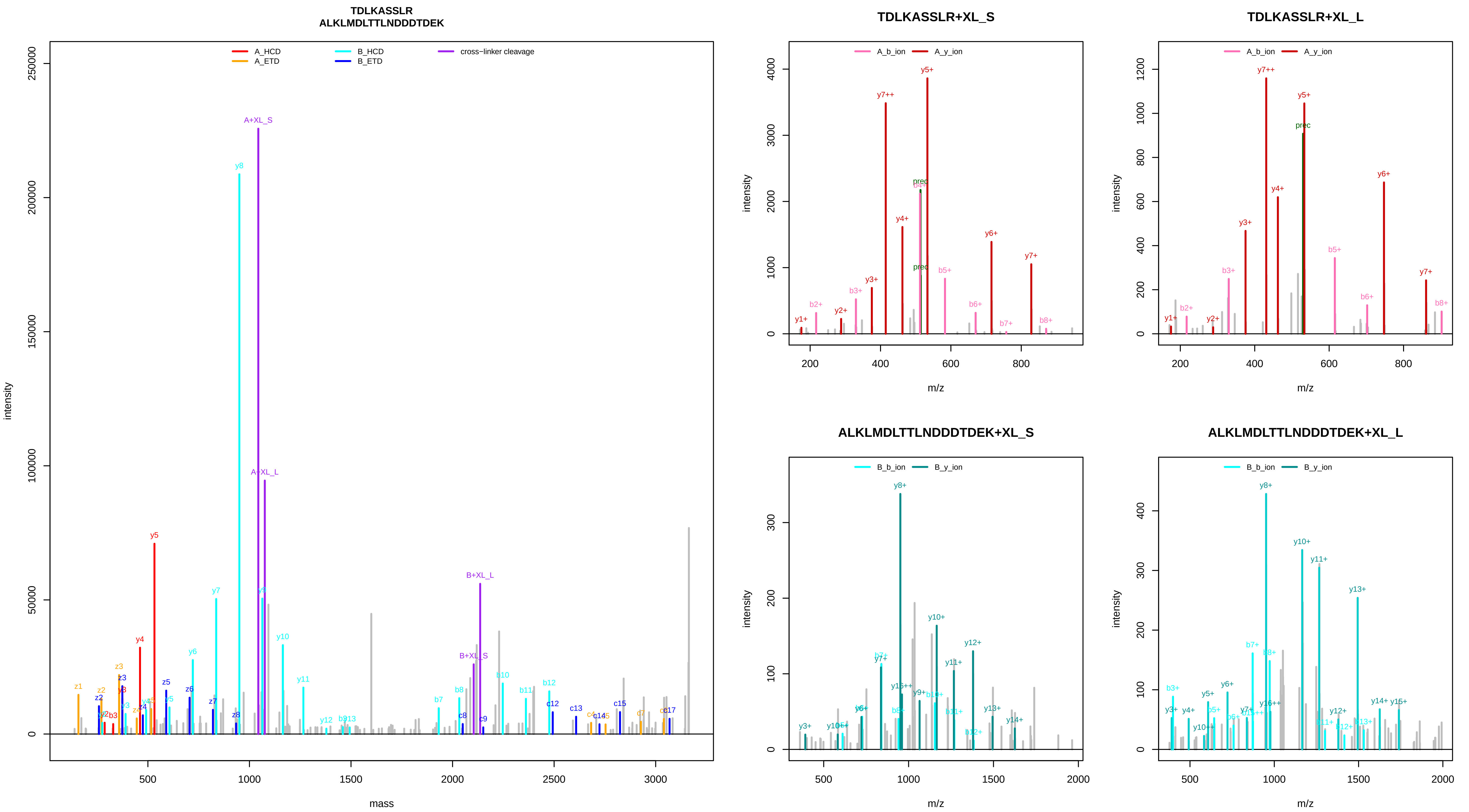
TDLKASSLR
ALKLMDLTTLNDDDTDEK
A_HCD
A_ETD
B_HCD
B_ETD
cross−linker cleavage
A+XL_S
A+XL_L
B+XL_L
B+XL_S
intensity
mass
TDLKASSLR+XL_S
TDLKASSLR+XL_L
ALKLMDLTTLNDDDTDEK+XL_S
ALKLMDLTTLNDDDTDEK+XL_L
A_b_ion
A_y_ion
B_b_ion
B_y_ion
m/z

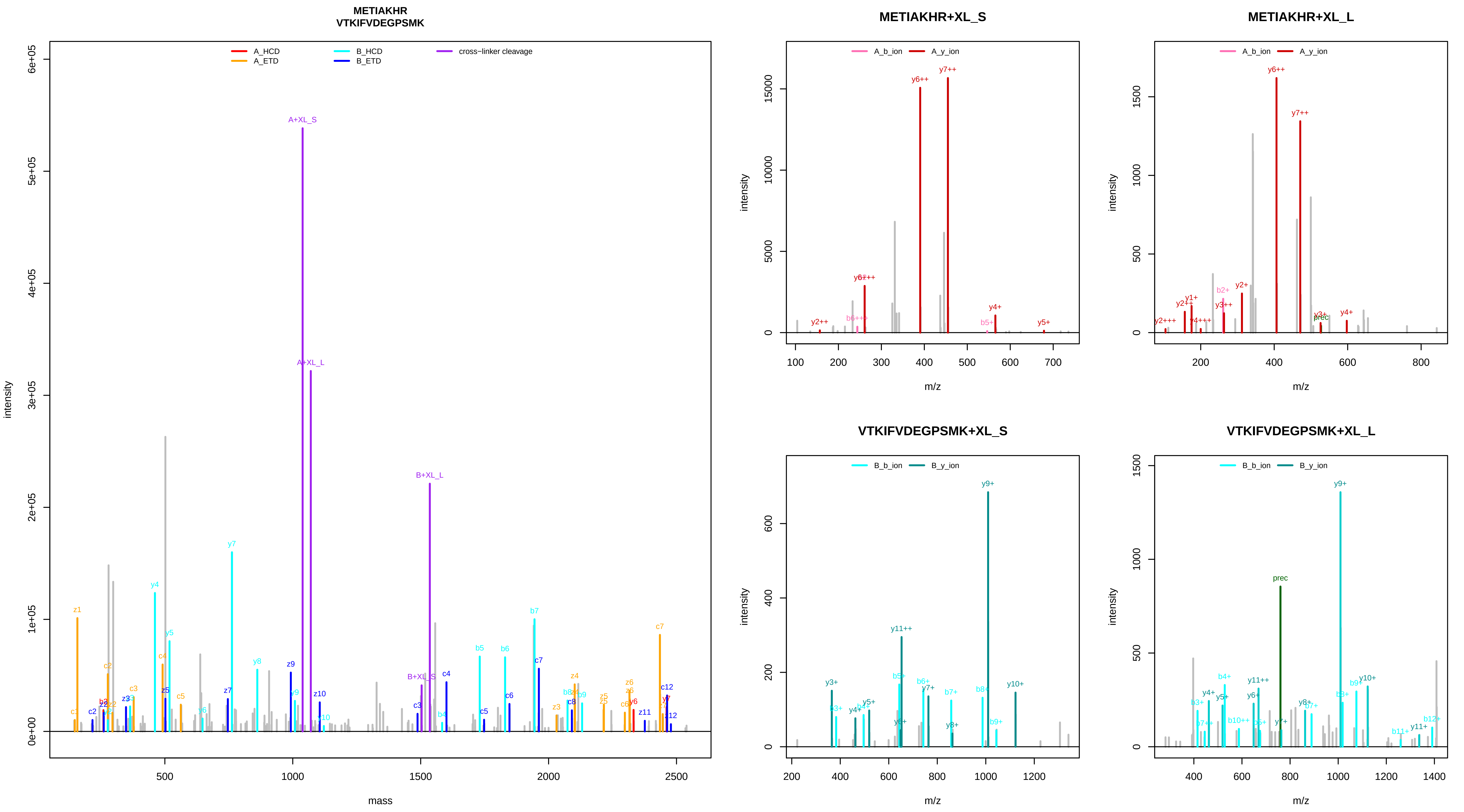

METIAKHR
VTKIFVDEGPSMK
A_HCD
A_ETD
B_HCD
B_ETD
cross−linker cleavage
A+XL_S
A+XL_L
B+XL_L
B+XL_S
intensity
mass
METIAKHR+XL_S
METIAKHR+XL_L
VTKIFVDEGPSMK+XL_S
VTKIFVDEGPSMK+XL_L
A_b_ion
A_y_ion
B_b_ion
B_y_ion
m/z

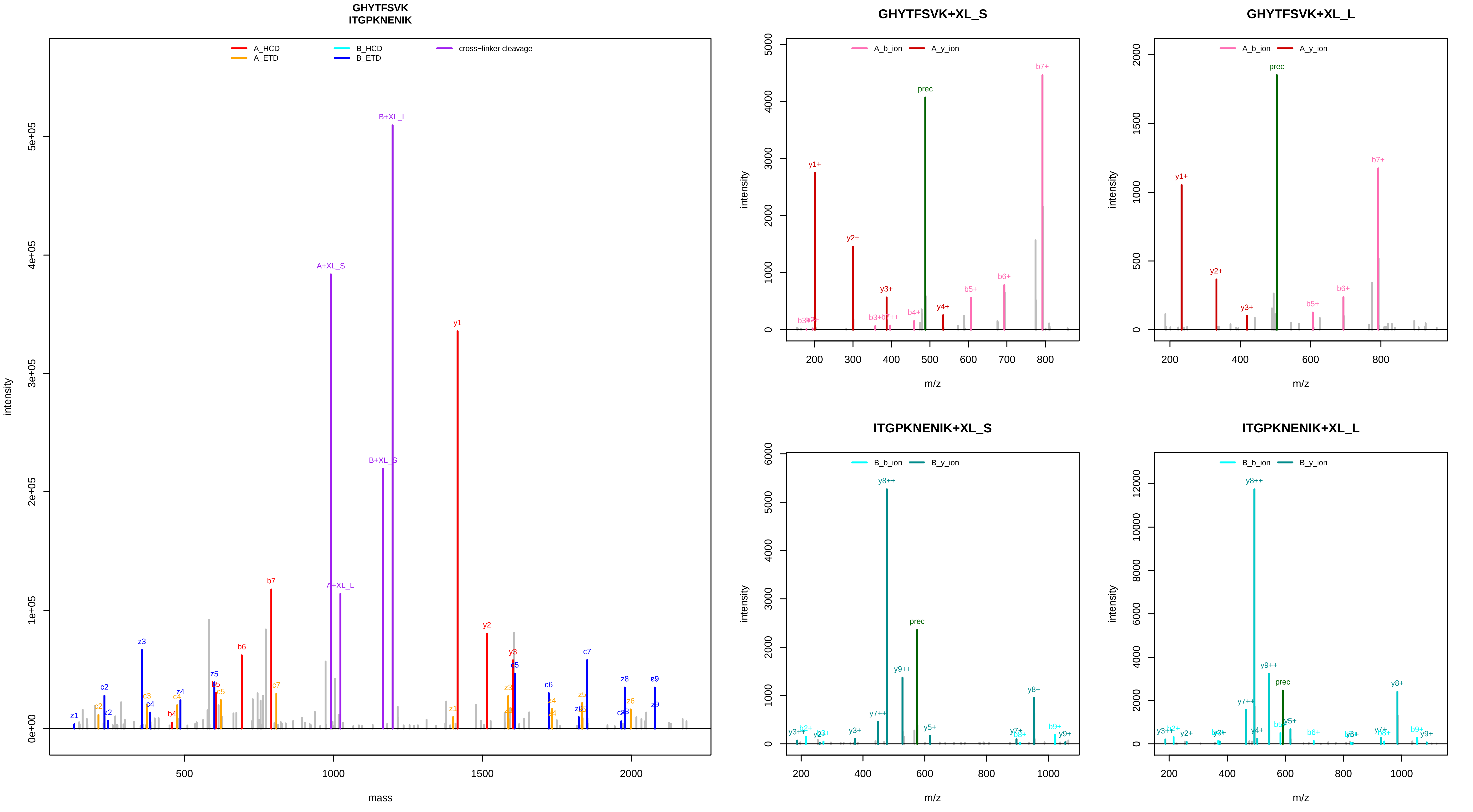
GHYTFSVK
ITGPKNENIK
A_HCD
A_ETD
B_HCD
B_ETD
cross−linker cleavage
intensity
mass
GHYTFSVK+XL_S
GHYTFSVK+XL_L
ITGPKNENIK+XL_S
ITGPKNENIK+XL_L
A_b_ion
A_y_ion
B_b_ion
B_y_ion
m/z
prec

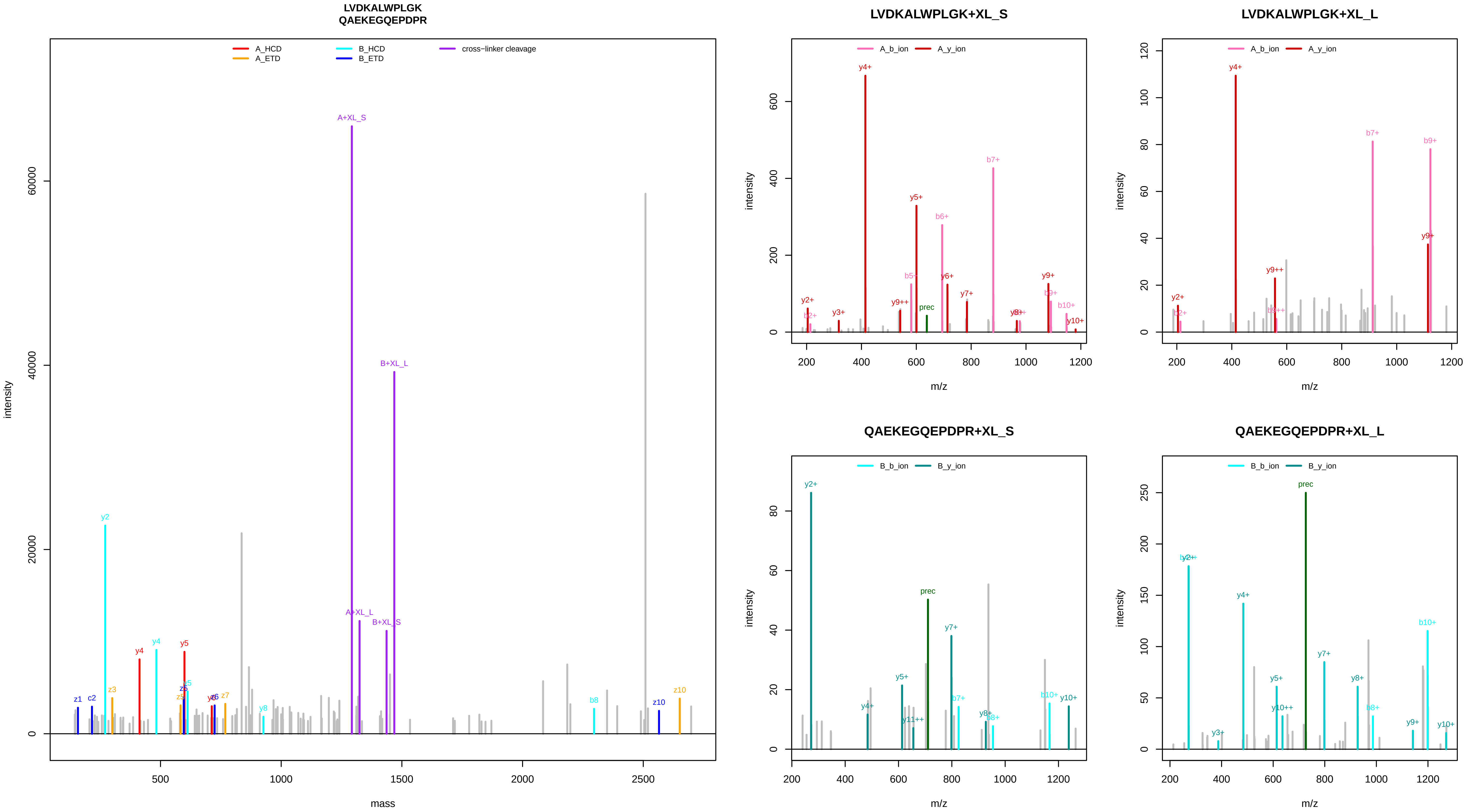

LVDKALWPLGK
QAEKEGQEPDPR
A_HCD
A_ETD
B_HCD
B_ETD
cross−linker cleavage
A+XL_S
B+XL_L
A+XL_L
B+XL_S
intensity
mass
LVDKALWPLGK+XL_S
LVDKALWPLGK+XL_L
QAEKEGQEPDPR+XL_S
QAEKEGQEPDPR+XL_L
A_b_ion
A_y_ion
B_b_ion
B_y_ion
prec
m/z

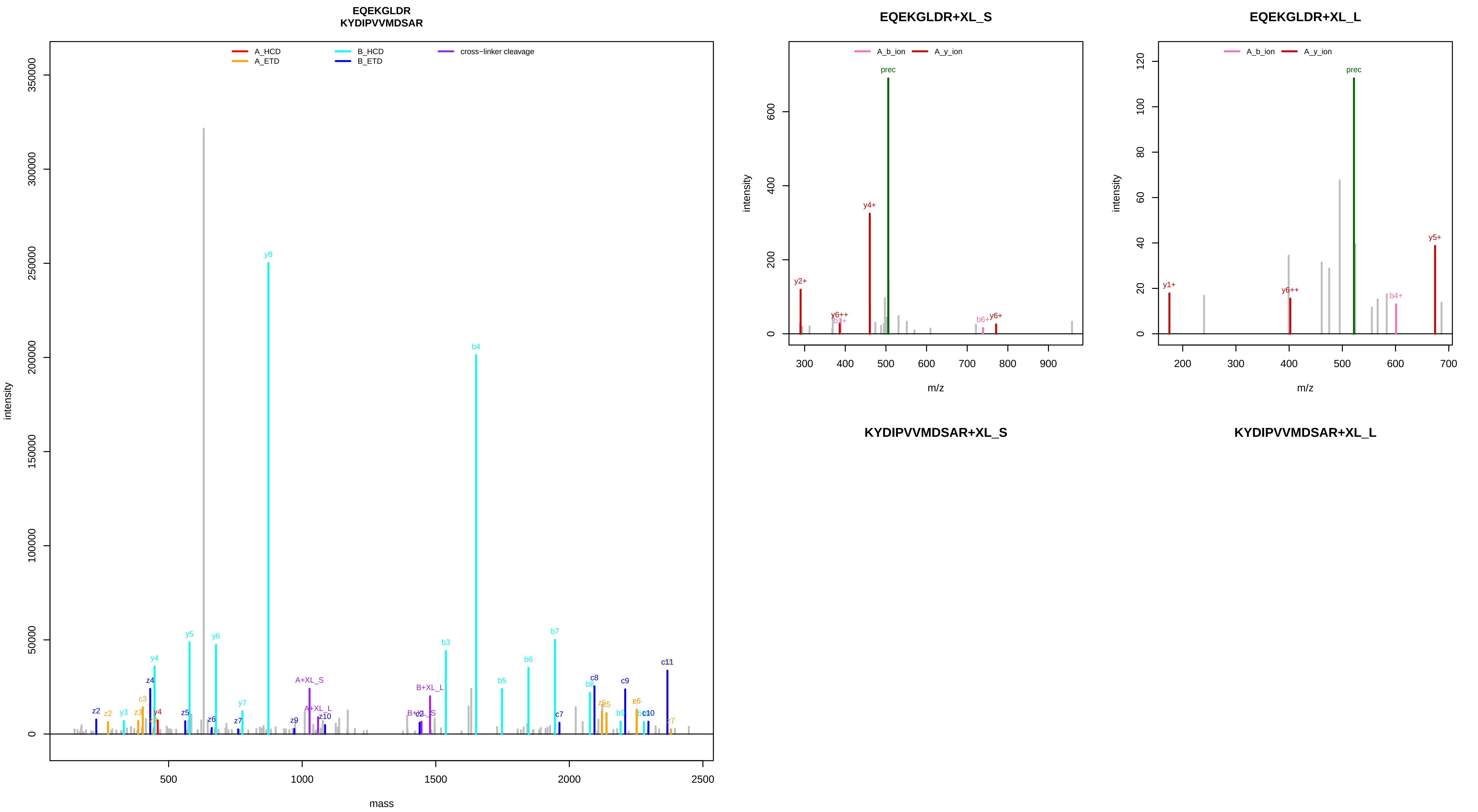

EQEKGLDR
KYDIPVVMDSAR
A_HCD
A_ETD
B_HCD
B_ETD
cross−linker cleavage
intensity
mass
EQEKGLDR+XL_S
A_b_ion
A_y_ion
prec
m/z
EQEKGLDR+XL_L
KYDIPVVMDSAR+XL_S
KYDIPVVMDSAR+XL_L

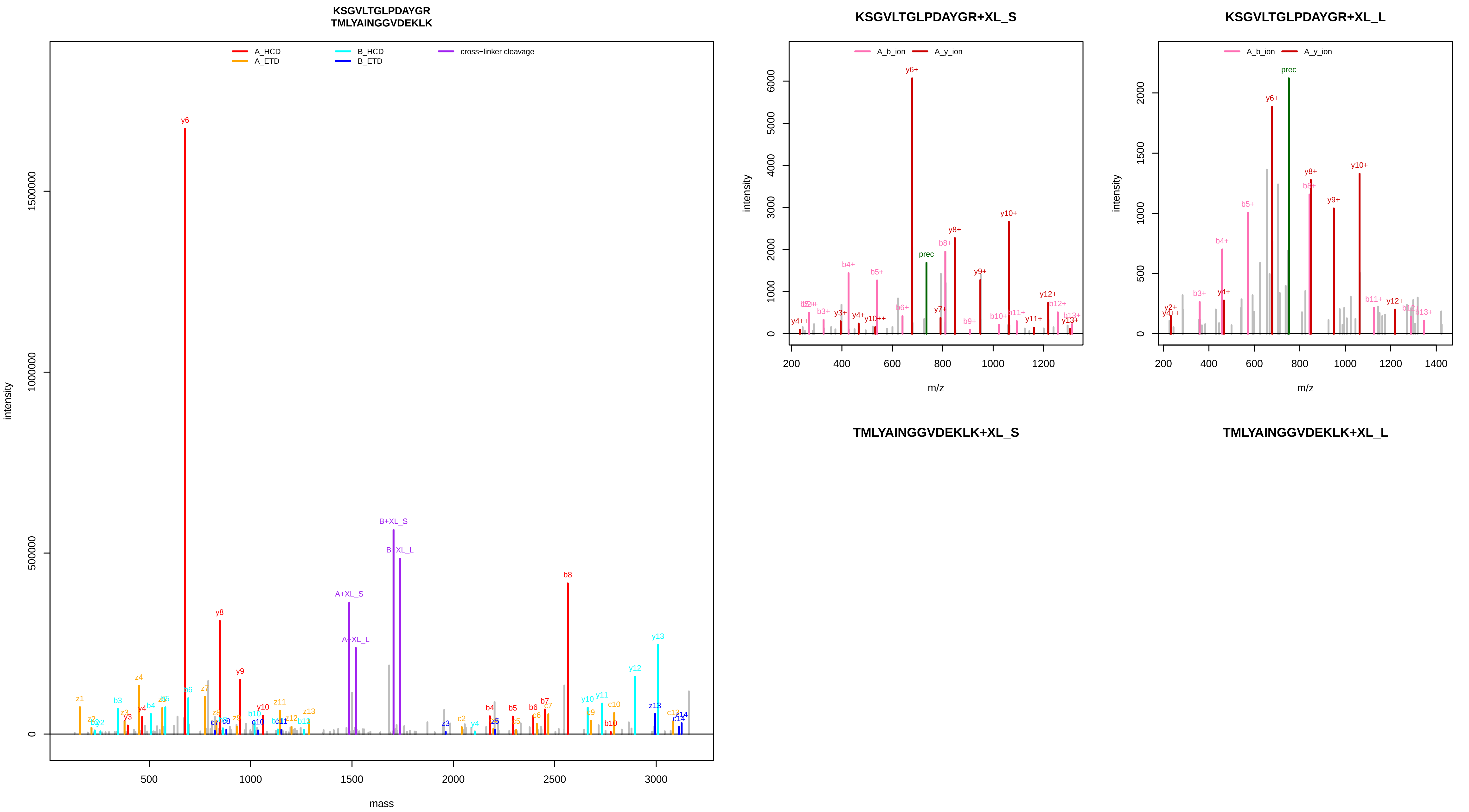
KSGVLTGLPDAYGR
TMLYAINGGVDEKLK
A_HCD
A_ETD
B_HCD
B_ETD
cross−linker cleavage
intensity
mass
y6
y8
y9
y10
b8
A+XL_S
A+XL_L
B+XL_S
B+XL_L
y12
y13
KSGVLTGLPDAYGR+XL_S
KSGVLTGLPDAYGR+XL_L
A_b_ion
A_y_ion
prec
m/z
TMLYAINGGVDEKLK+XL_S
TMLYAINGGVDEKLK+XL_L

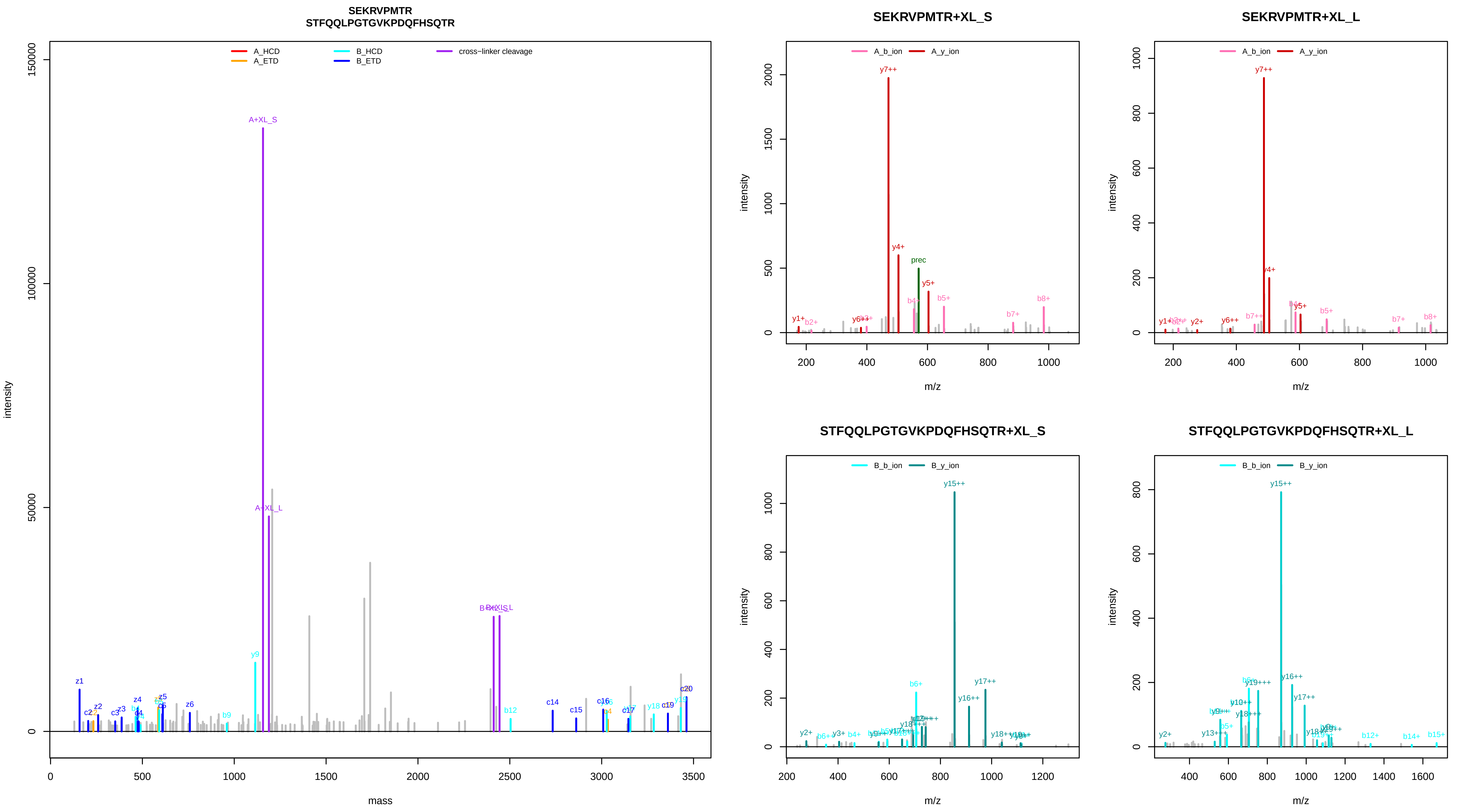
SEKRVPMTR
STFQQLPGTGVKPDQFHSQTR
A_HCD
A_ETD
B_HCD
B_ETD
cross−linker cleavage
A+XL_S
A+XL_L
intensity
mass
SEKRVPMTR+XL_S
SEKRVPMTR+XL_L
A_b_ion
A_y_ion
prec
m/z
STFQQLPGTGVKPDQFHSQTR+XL_S
STFQQLPGTGVKPDQFHSQTR+XL_L
B_b_ion
B_y_ion

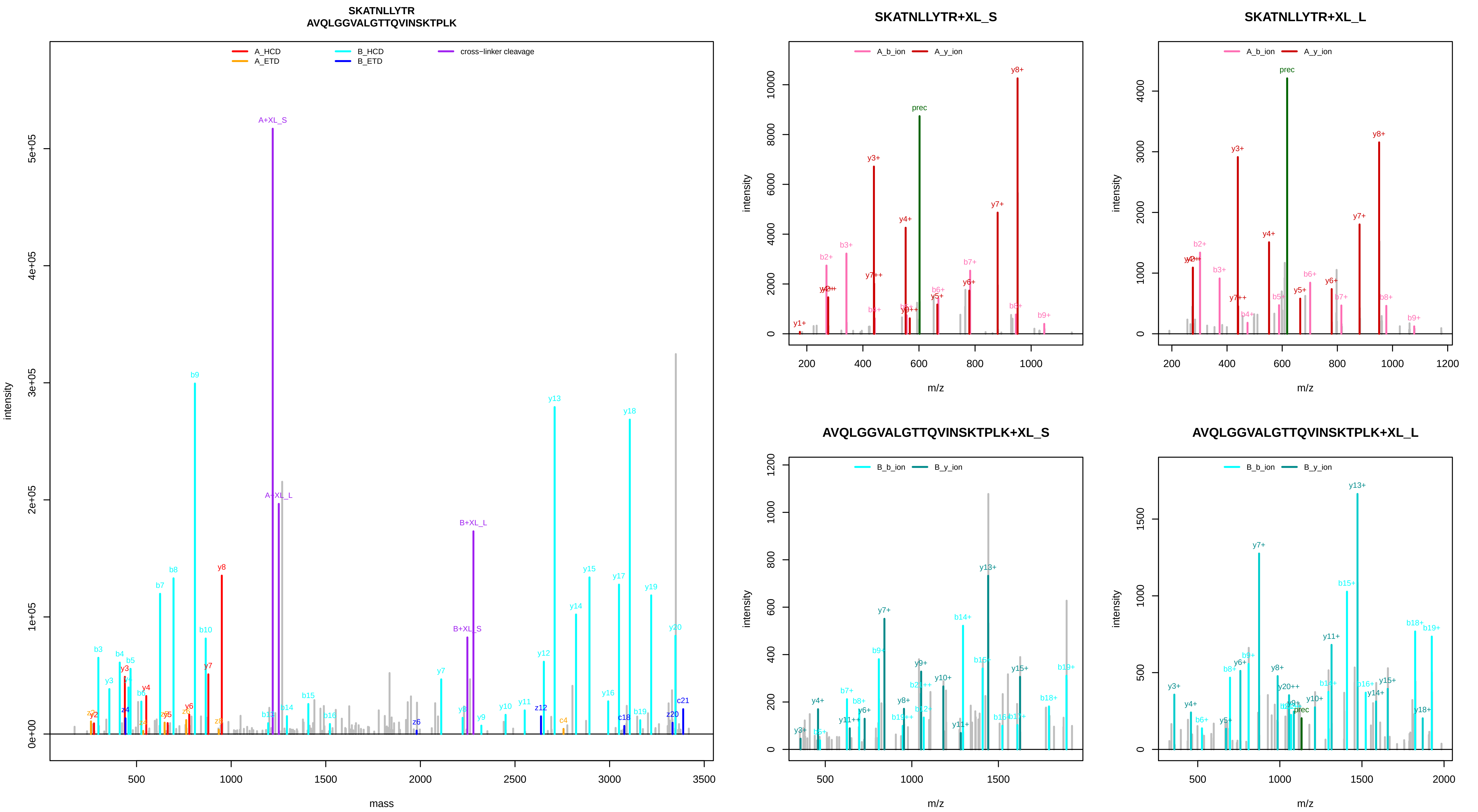
SKATNLLYTR
AVQLGGVALGTTQVINSKTPLK
A_HCD
A_ETD
B_HCD
B_ETD
cross−linker cleavage
A+XL_S
A+XL_L
B+XL_L
B+XL_S
intensity
mass
SKATNLLYTR+XL_S
SKATNLLYTR+XL_L
AVQLGGVALGTTQVINSKTPLK+XL_S
AVQLGGVALGTTQVINSKTPLK+XL_L
A_b_ion
A_y_ion
B_b_ion
B_y_ion
prec
m/z

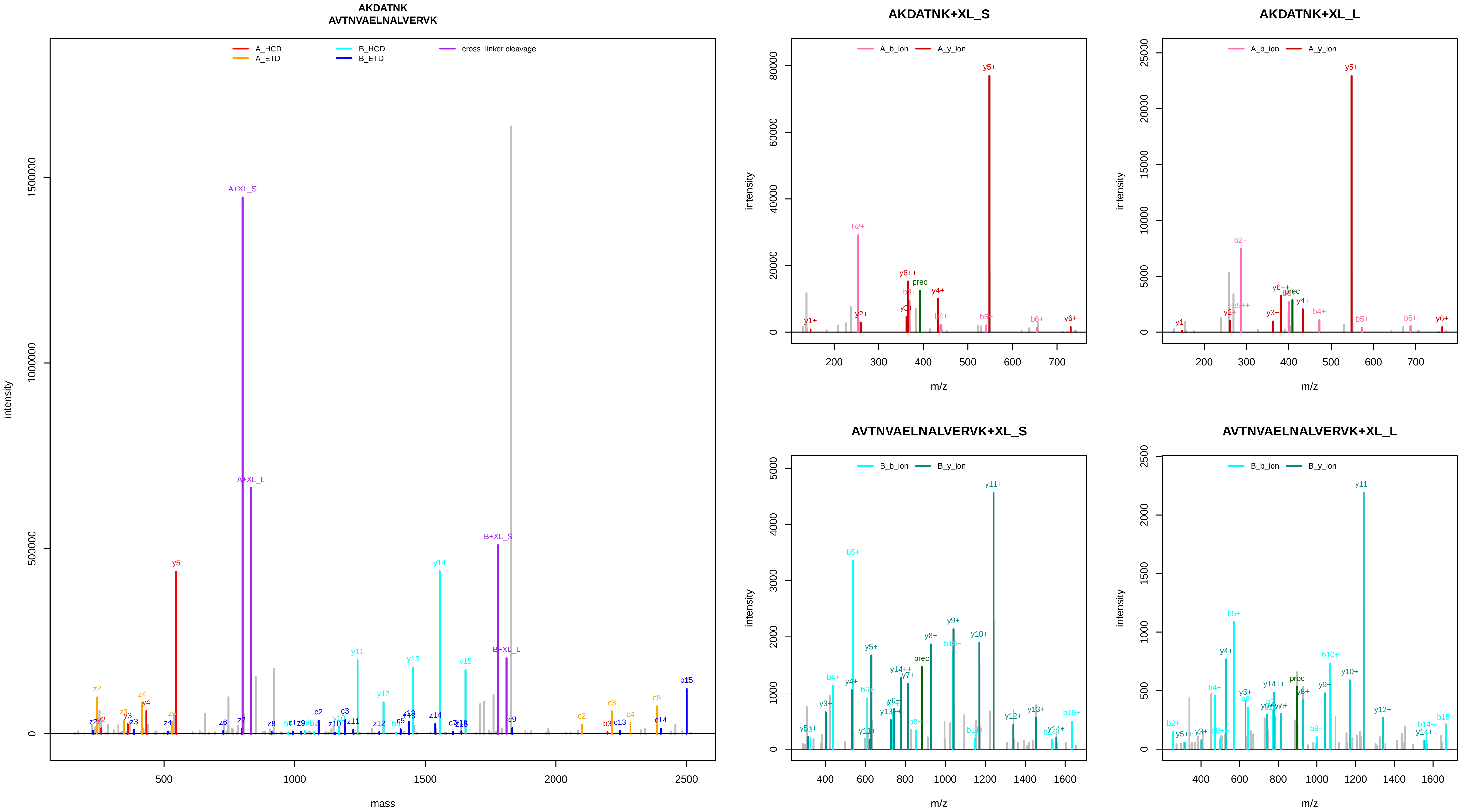

AKDATNK
AVTNVAELNALVERVK
A_HCD
A_ETD
B_HCD
B_ETD
cross−linker cleavage
A+XL_S
A+XL_L
B+XL_S
B+XL_L
intensity
mass
AKDATNK+XL_S
AKDATNK+XL_L
A_b_ion
A_y_ion
AVTNVAELNALVERVK+XL_S
AVTNVAELNALVERVK+XL_L
B_b_ion
B_y_ion
prec
m/z

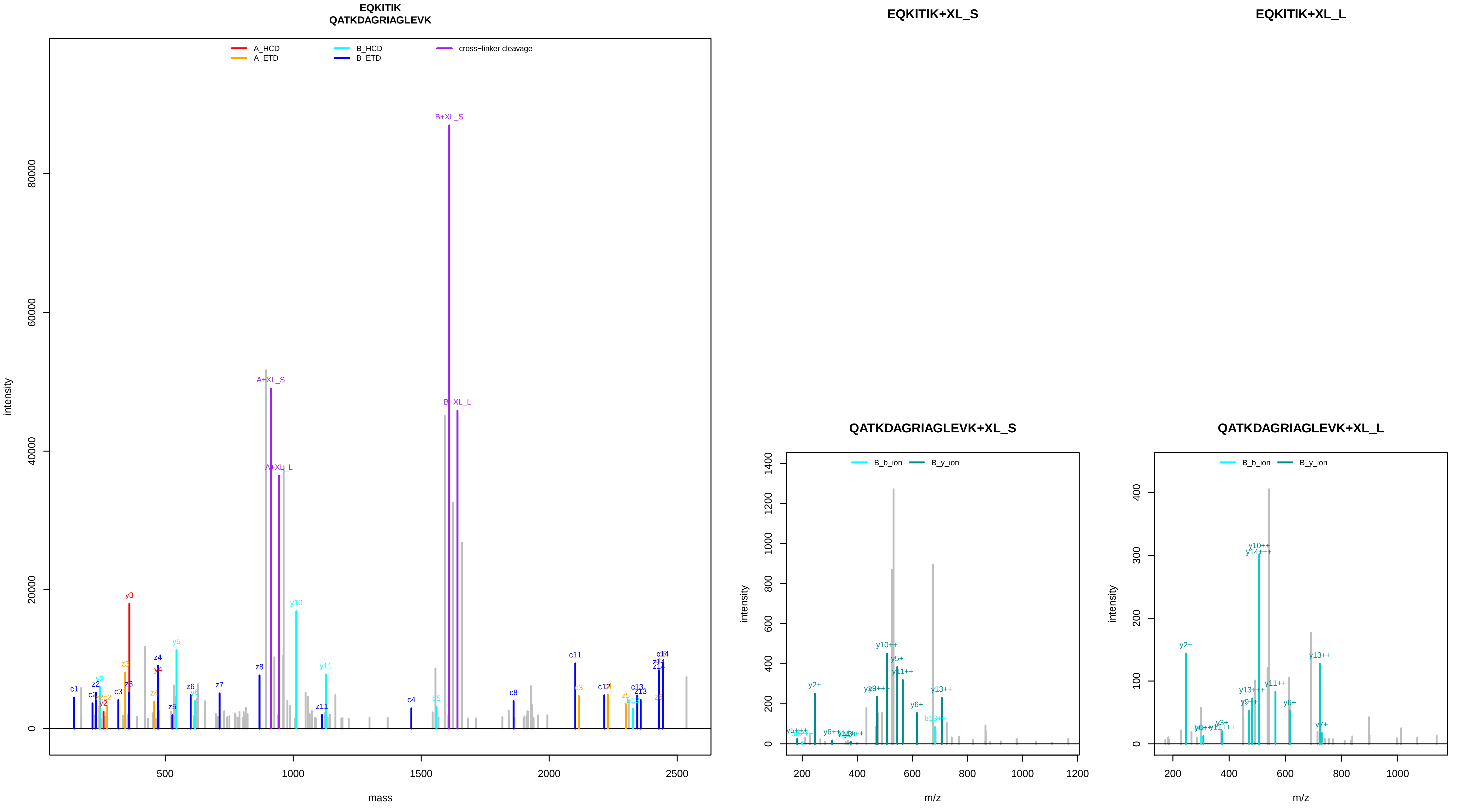
EQKITIK
QATKDAGRIAGLEVK
A_HCD
A_ETD
B_HCD
B_ETD
cross−linker cleavage
B+XL_S
B+XL_L
A+XL_S
A+XL_L
intensity
mass
EQKITIK+XL_S
EQKITIK+XL_L
QATKDAGRIAGLEVK+XL_S
QATKDAGRIAGLEVK+XL_L
B_b_ion
B_y_ion
m/z

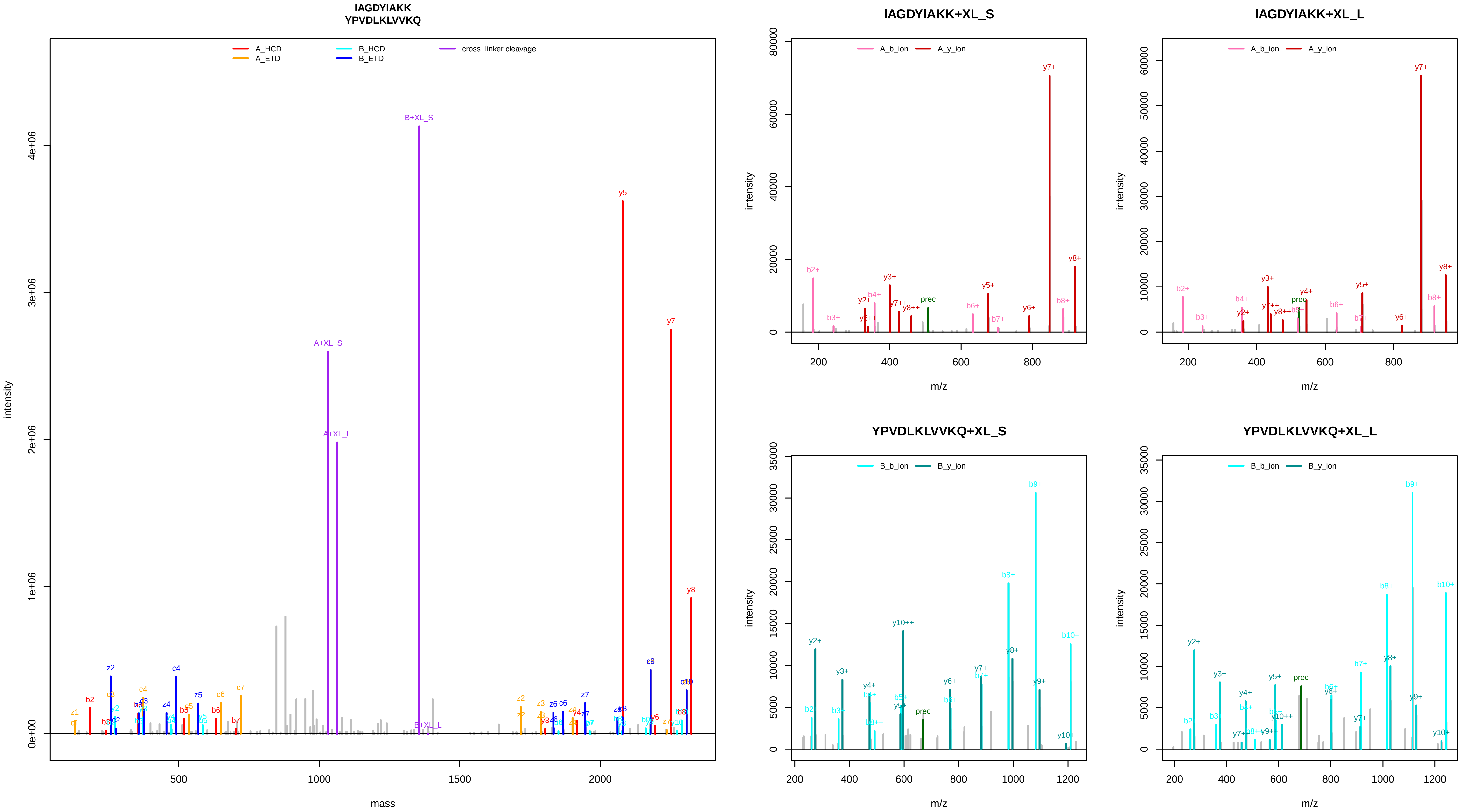
IAGDYIAKK
YPVDLKLVVKQ
A_HCD
A_ETD
B_HCD
B_ETD
cross−linker cleavage
B+XL_S
A+XL_S
A+XL_L
B+XL_L
intensity
mass
IAGDYIAKK+XL_S
IAGDYIAKK+XL_L
A_b_ion
A_y_ion
YPVDLKLVVKQ+XL_S
YPVDLKLVVKQ+XL_L
B_b_ion
B_y_ion
prec
m/z

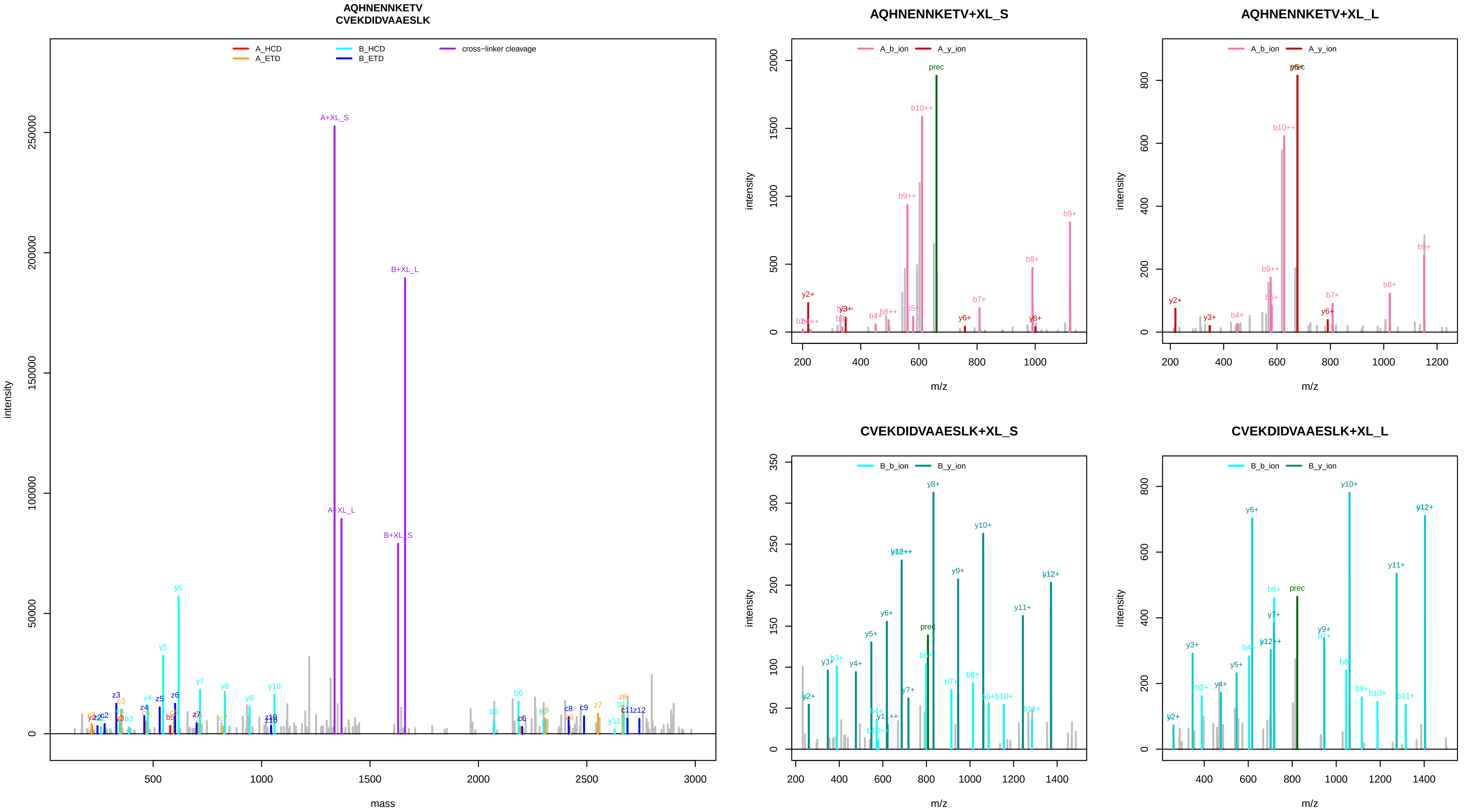
AQHNENNKETV
CVEKDIDVAAESLK
A_HCD
A_ETD
B_HCD
B_ETD
cross−linker cleavage
A+XL_S
B+XL_L
A+XL_L
B+XL_S
intensity
mass
AQHNENNKETV+XL_S
AQHNENNKETV+XL_L
A_b_ion
A_y_ion
prec
CVEKDIDVAAESLK+XL_S
CVEKDIDVAAESLK+XL_L
B_b_ion
B_y_ion
m/z

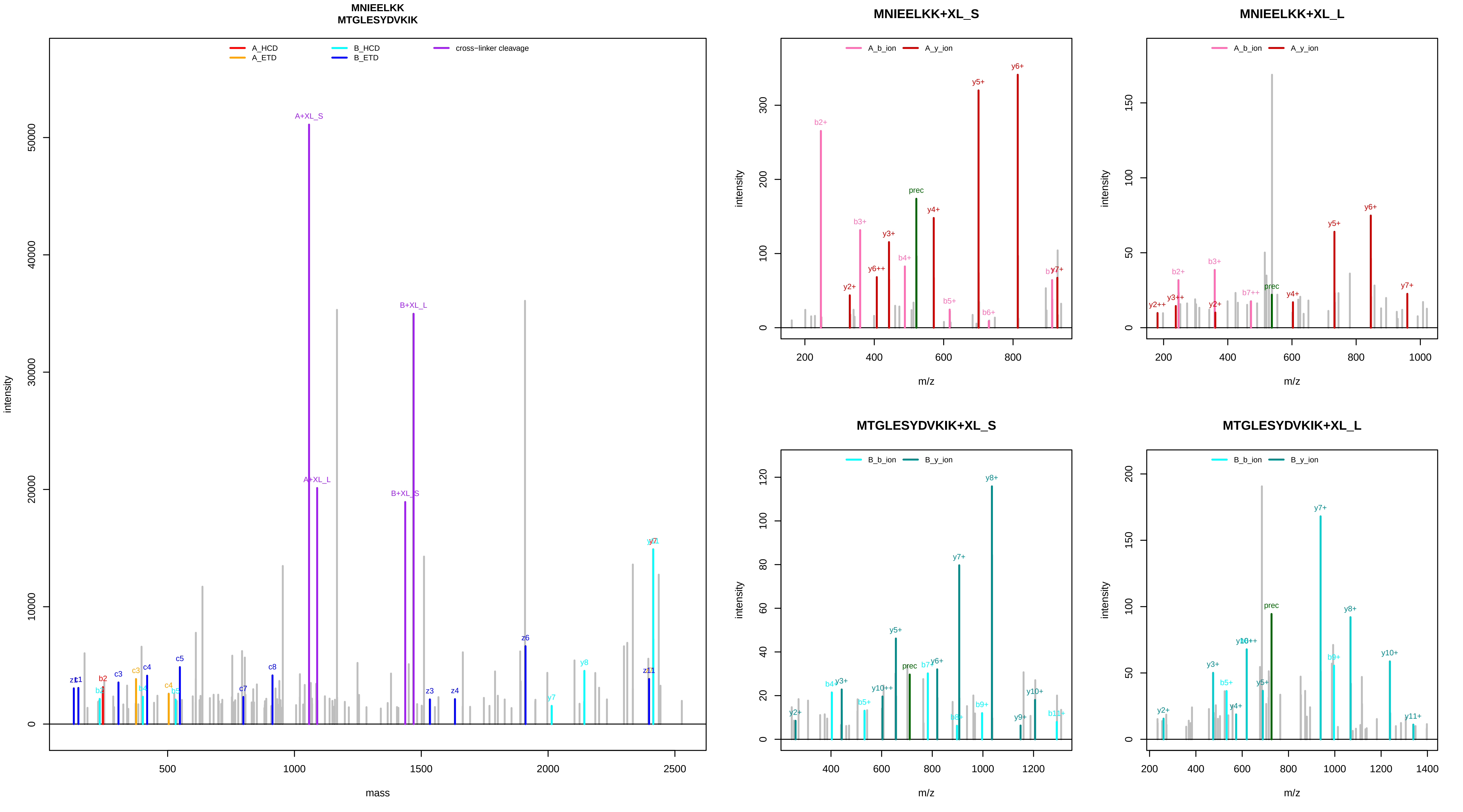

MNIEELKK
MTGLESYDVKIK
A_HCD
A_ETD
B_HCD
B_ETD
cross−linker cleavage
A+XL_S
A+XL_L
B+XL_L
B+XL_S
intensity
mass
MNIEELKK+XL_S
MNIEELKK+XL_L
MTGLESYDVKIK+XL_S
MTGLESYDVKIK+XL_L
A_b_ion
A_y_ion
B_b_ion
B_y_ion
prec
m/z

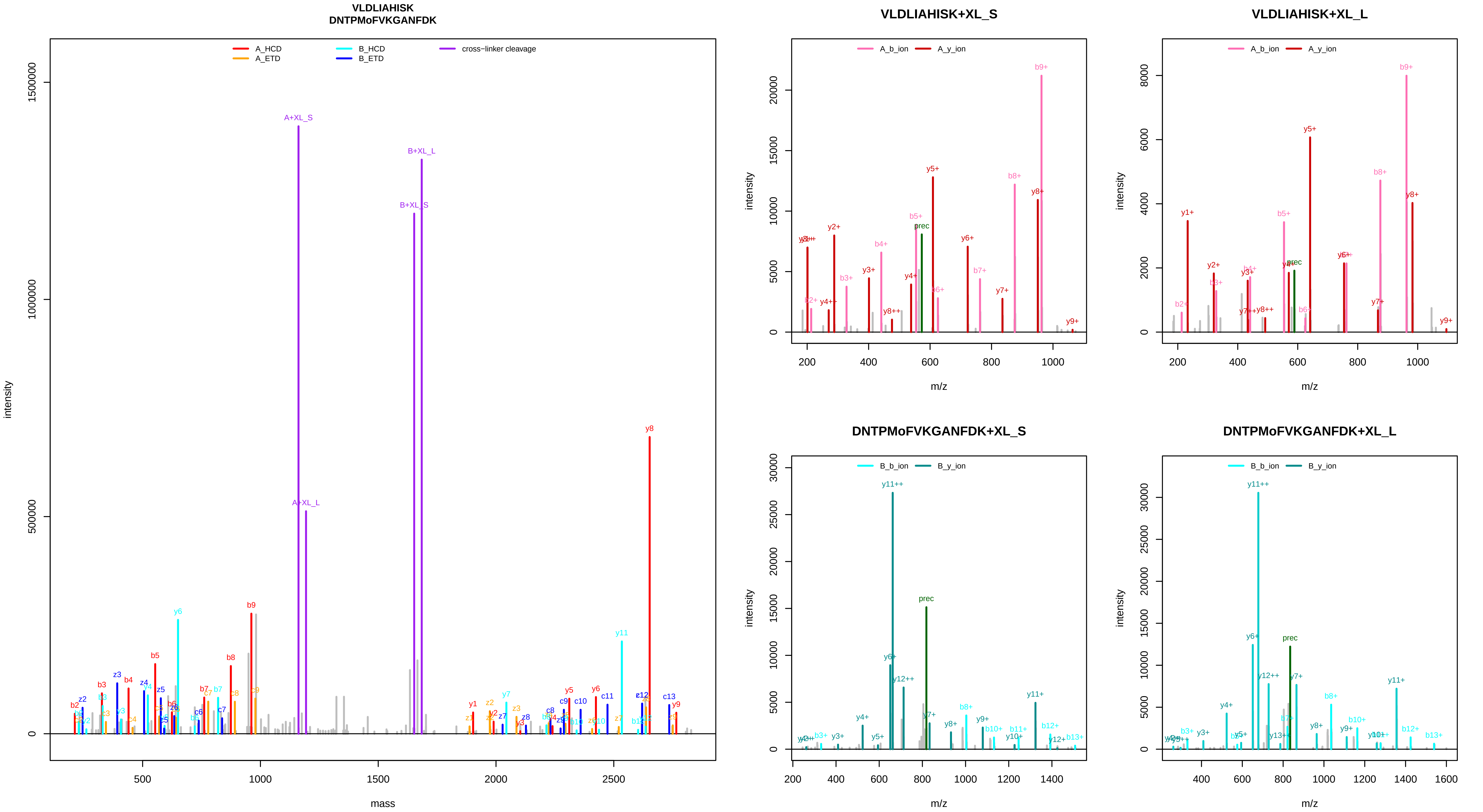

VLDLIAHISK
DNTPMoFVKGANFDK
A_HCD
A_ETD
B_HCD
B_ETD
cross−linker cleavage
A+XL_S
B+XL_L
B+XL_S
A+XL_L
intensity
mass
VLDLIAHISK+XL_S
VLDLIAHISK+XL_L
DNTPMoFVKGANFDK+XL_S
DNTPMoFVKGANFDK+XL_L
A_b_ion
A_y_ion
B_b_ion
B_y_ion
prec
m/z

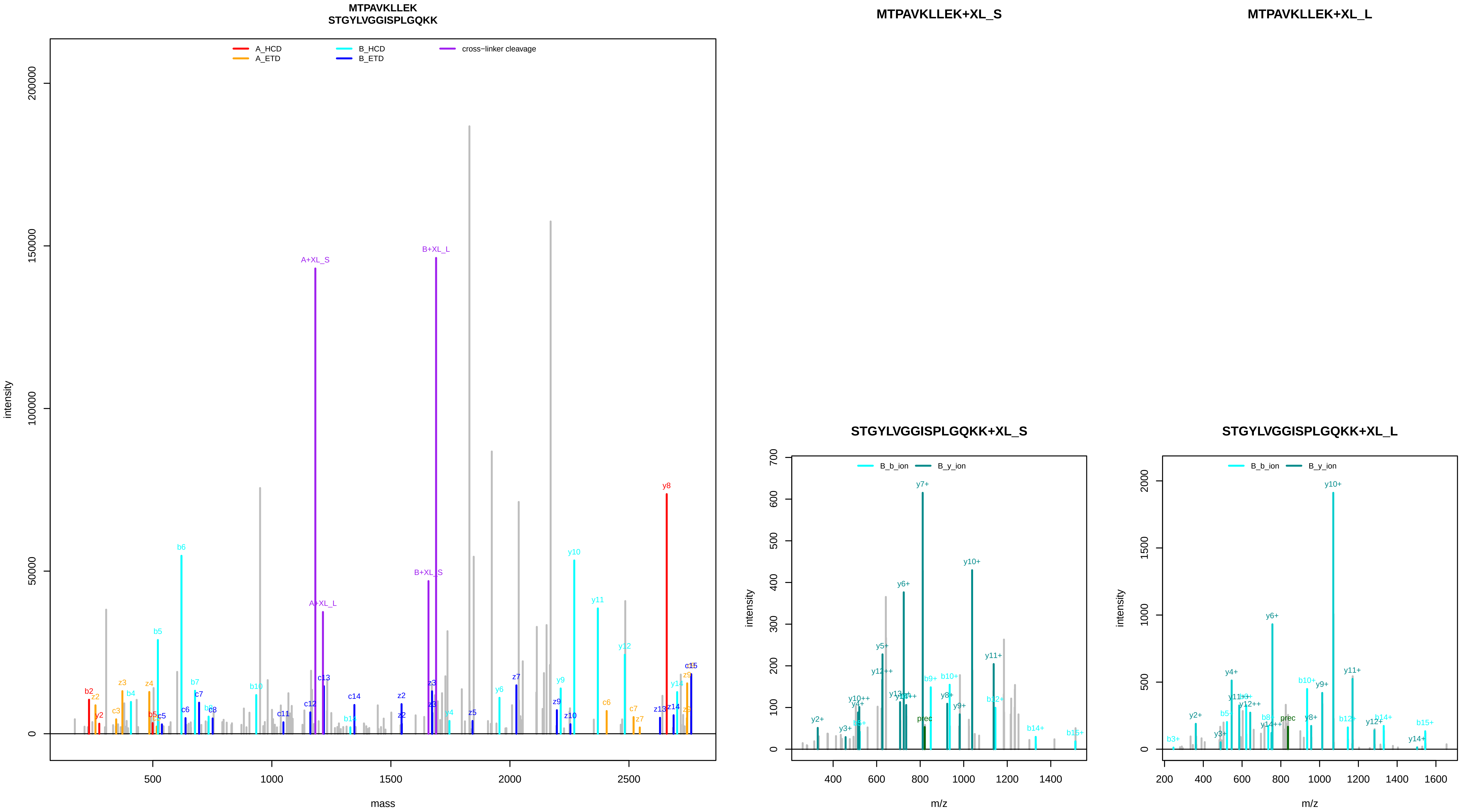

MTPAVKLLEK
STGYLVGGISPLGQKK
A_HCD
A_ETD
B_HCD
B_ETD
cross−linker cleavage
A+XL_S
B+XL_L
B+XL_S
A+XL_L
intensity
mass
MTPAVKLLEK+XL_S
MTPAVKLLEK+XL_L
STGYLVGGISPLGQKK+XL_S
STGYLVGGISPLGQKK+XL_L
B_b_ion
B_y_ion
prec
m/z

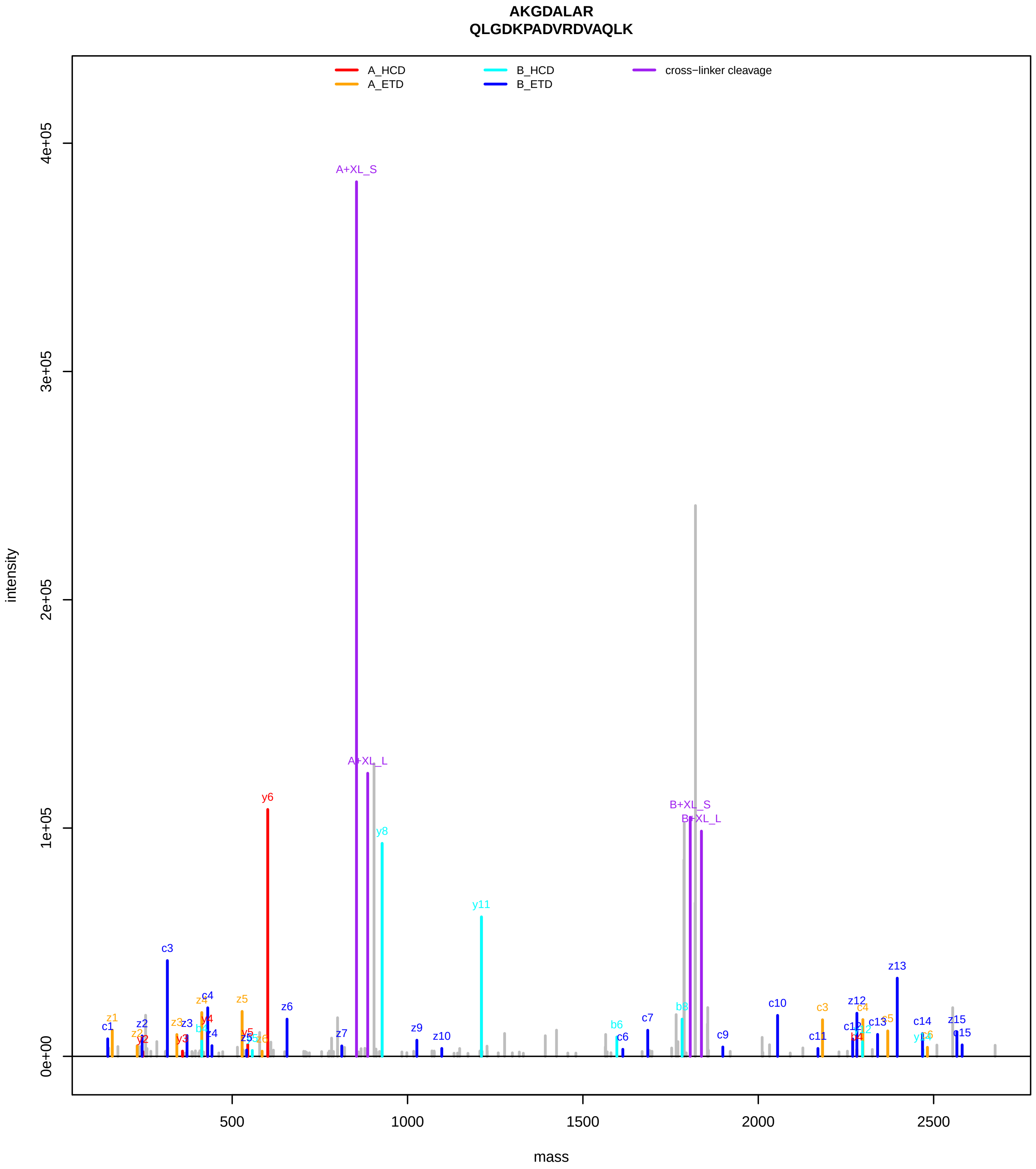

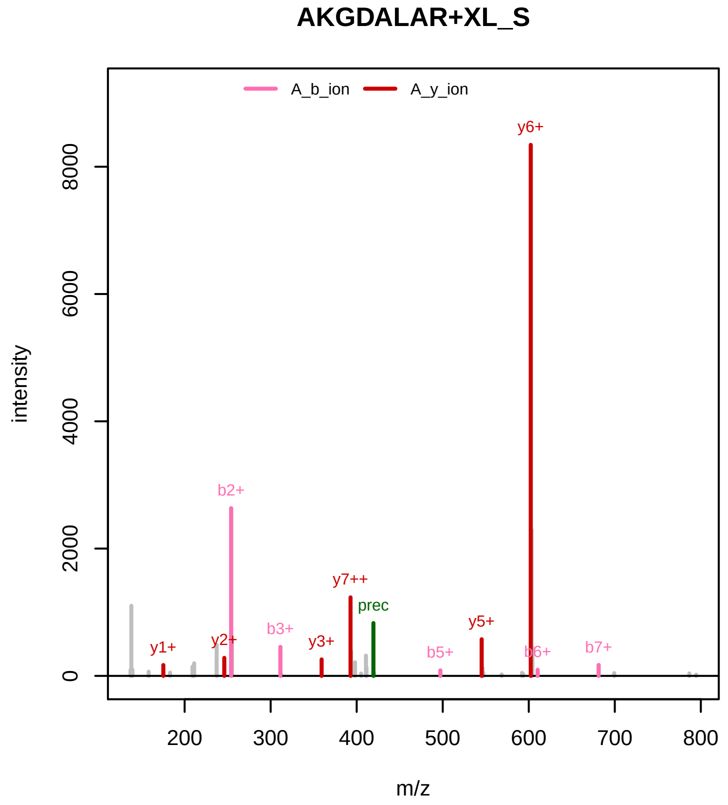

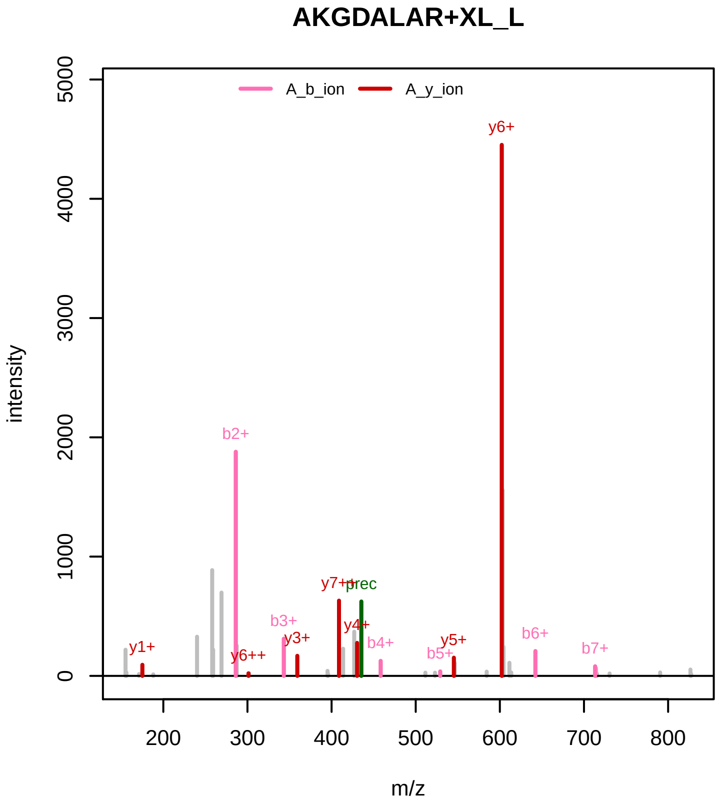

QLGDKPADVRDVAQLK+XL_S

QLGDKPADVRDVAQLK+XL_L

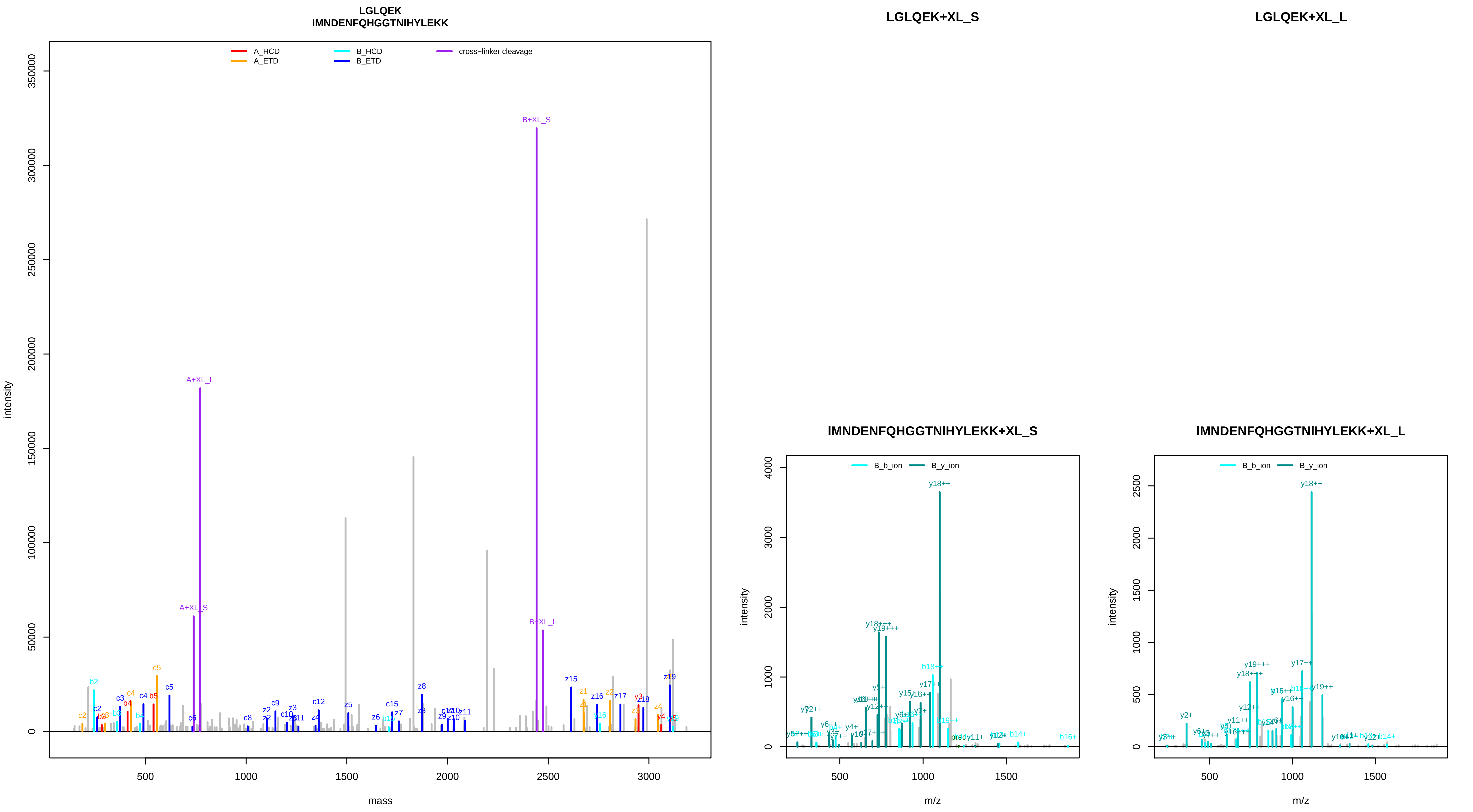
LGLQEK
IMNDENFQHGGTNIHYLEKK
A_HCD
A_ETD
B_HCD
B_ETD
cross−linker cleavage
A+XL_L
A+XL_S
B+XL_S
B+XL_L
intensity
mass
LGLQEK+XL_S
LGLQEK+XL_L
IMNDENFQHGGTNIHYLEKK+XL_S
IMNDENFQHGGTNIHYLEKK+XL_L
B_b_ion
B_y_ion
y18++
y18+++
y19+++
y17++
y19++
intensity
m/z

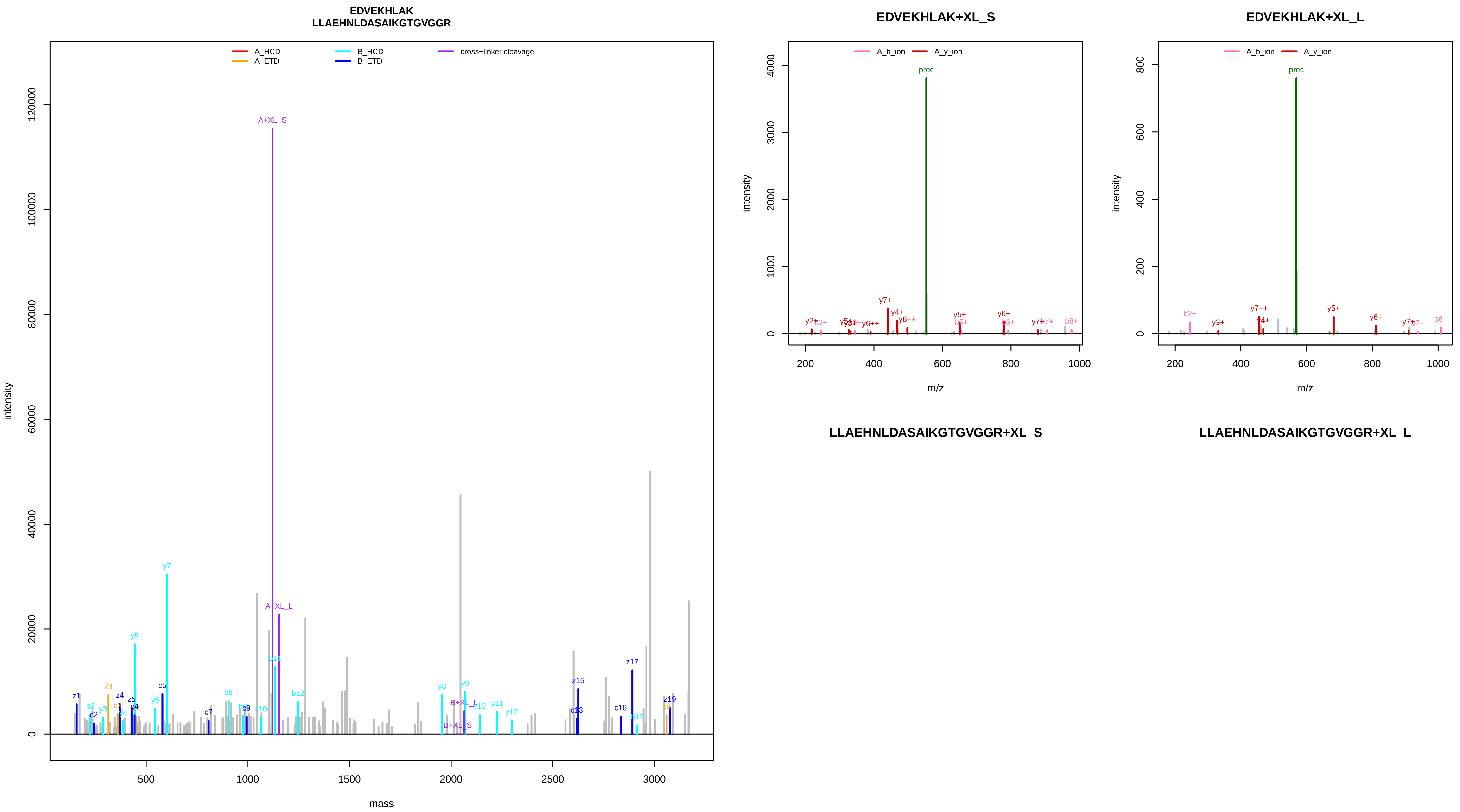
EDVEKHLAK
LLAEHNLDASAIKGTGVGGR
A_HCD
A_ETD
B_HCD
B_ETD
cross−linker cleavage
A+XL_S
A+XL_L
B+XL_L
B+XL_S
intensity
mass
EDVEKHLAK+XL_S
EDVEKHLAK+XL_L
A_b_ion
A_y_ion
prec
m/z
LLAEHNLDASAIKGTGVGGR+XL_S
LLAEHNLDASAIKGTGVGGR+XL_L

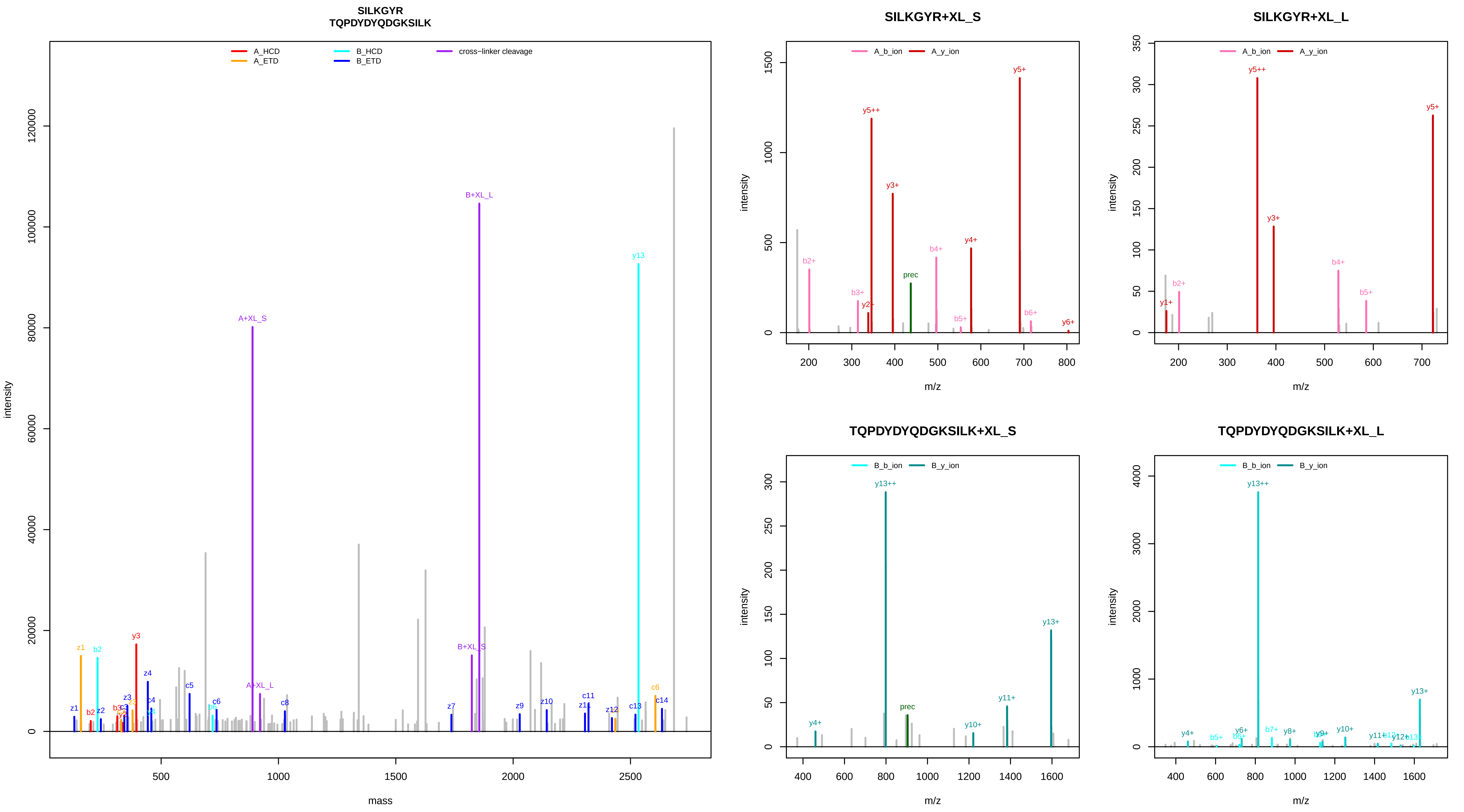
SILKGYR
TQPDYDYQDGKSILK
A_HCD
A_ETD
B_HCD
B_ETD
cross−linker cleavage
intensity
mass
SILKGYR+XL_S
SILKGYR+XL_L
TQPDYDYQDGKSILK+XL_S
TQPDYDYQDGKSILK+XL_L
A_b_ion
A_y_ion
B_b_ion
B_y_ion
m/z
B+XL_L
A+XL_S
B+XL_S
A+XL_L
y13
y5+
y5++
y3+
y4+
b4+
b2+
b3+
prec
b5+
b6+
y6+
y1+
y13++
y13+
y11+
y10+
y4+

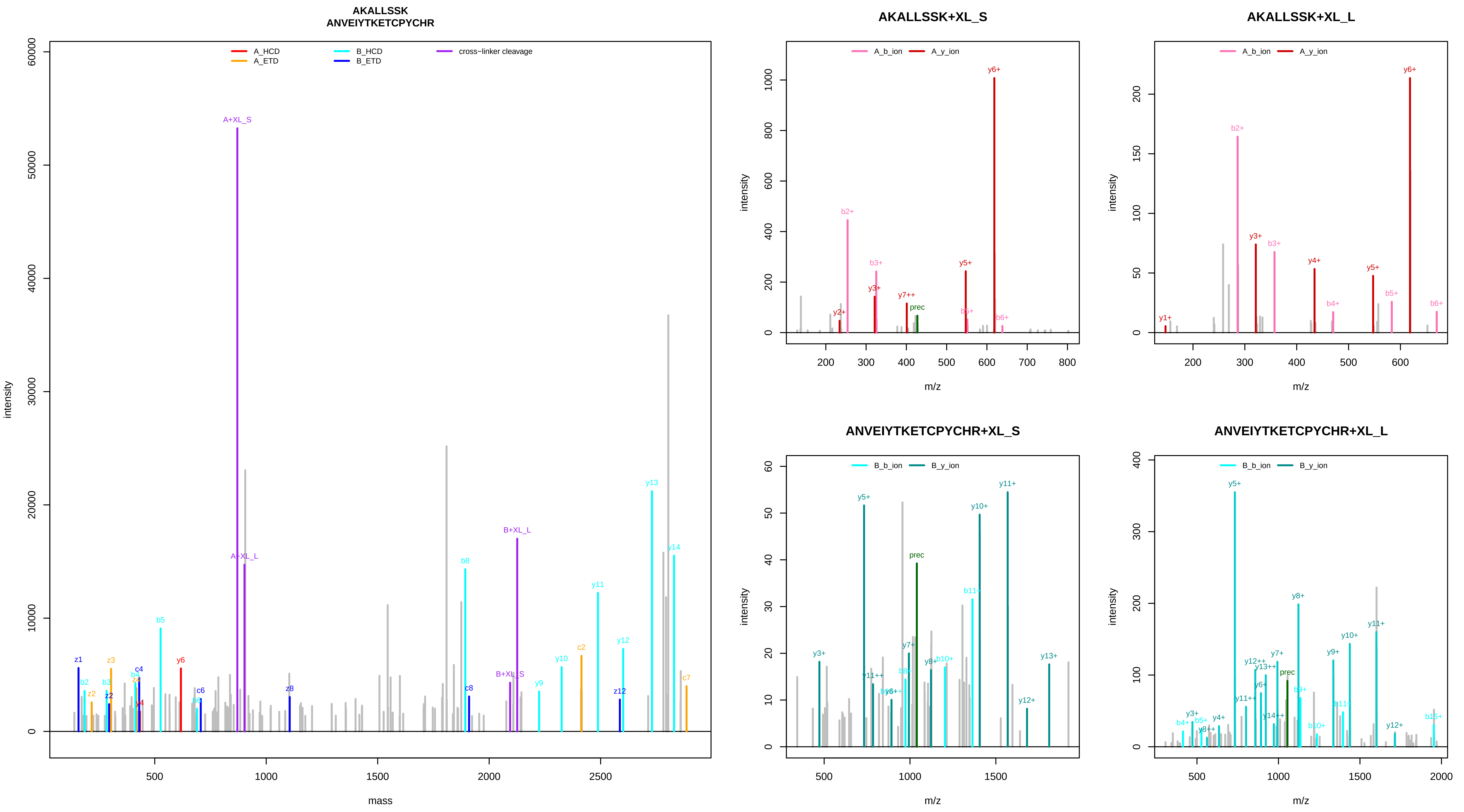
AKALLSSK
ANVEIYTKETCPYCHR
A_HCD
A_ETD
B_HCD
B_ETD
cross−linker cleavage
A+XL_S
A+XL_L
B+XL_L
B+XL_S
intensity
mass
AKALLSSK+XL_S
AKALLSSK+XL_L
ANVEIYTKETCPYCHR+XL_S
ANVEIYTKETCPYCHR+XL_L
A_b_ion
A_y_ion
B_b_ion
B_y_ion
prec
m/z

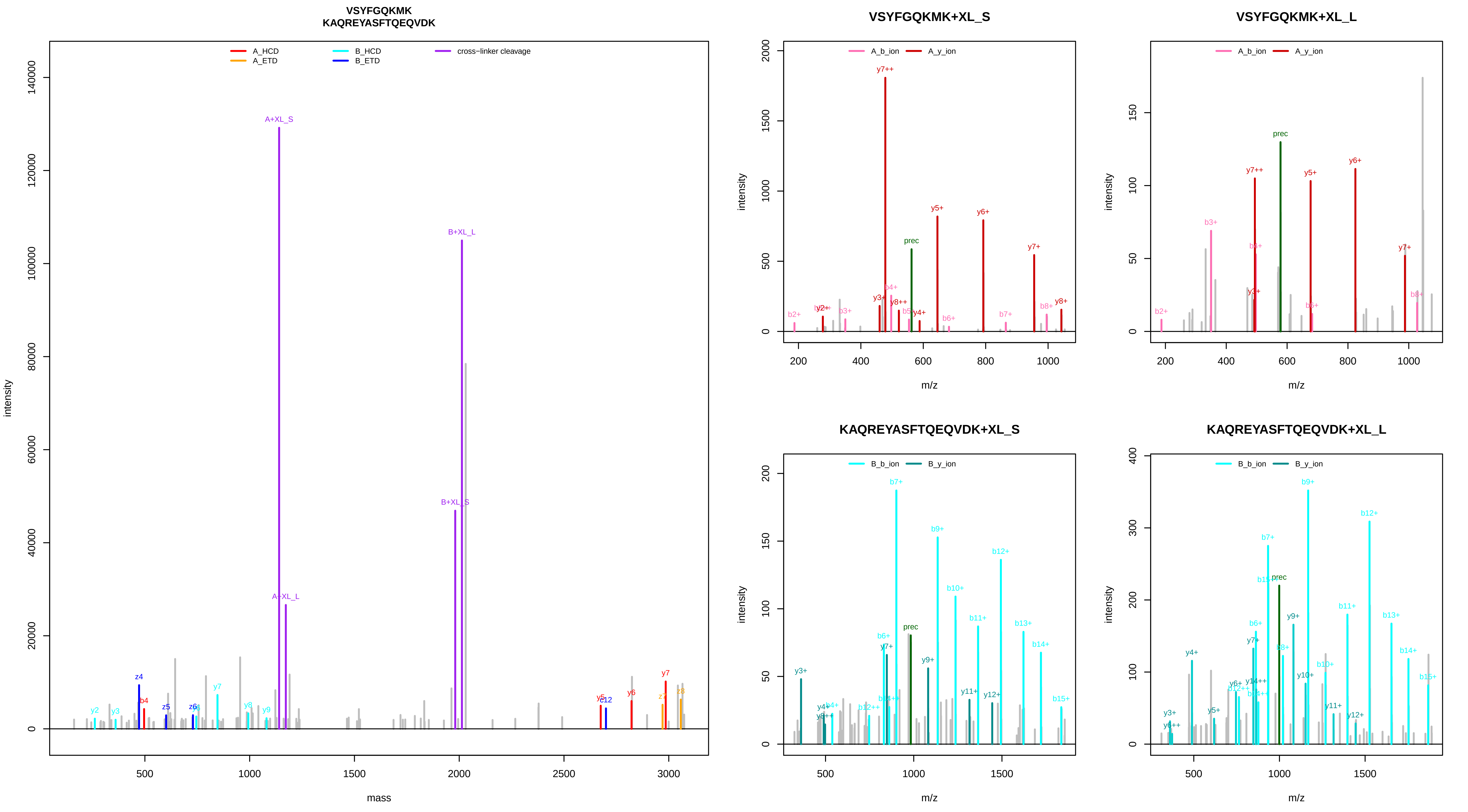
VSYFGQKMK
KAQREYASFTQEQVDK
A_HCD
A_ETD
B_HCD
B_ETD
cross−linker cleavage
A+XL_S
B+XL_L
B+XL_S
A+XL_L
intensity
mass
VSYFGQKMK+XL_S
VSYFGQKMK+XL_L
A_b_ion
A_y_ion
prec
m/z
KAQREYASFTQEQVDK+XL_S
KAQREYASFTQEQVDK+XL_L
B_b_ion
B_y_ion

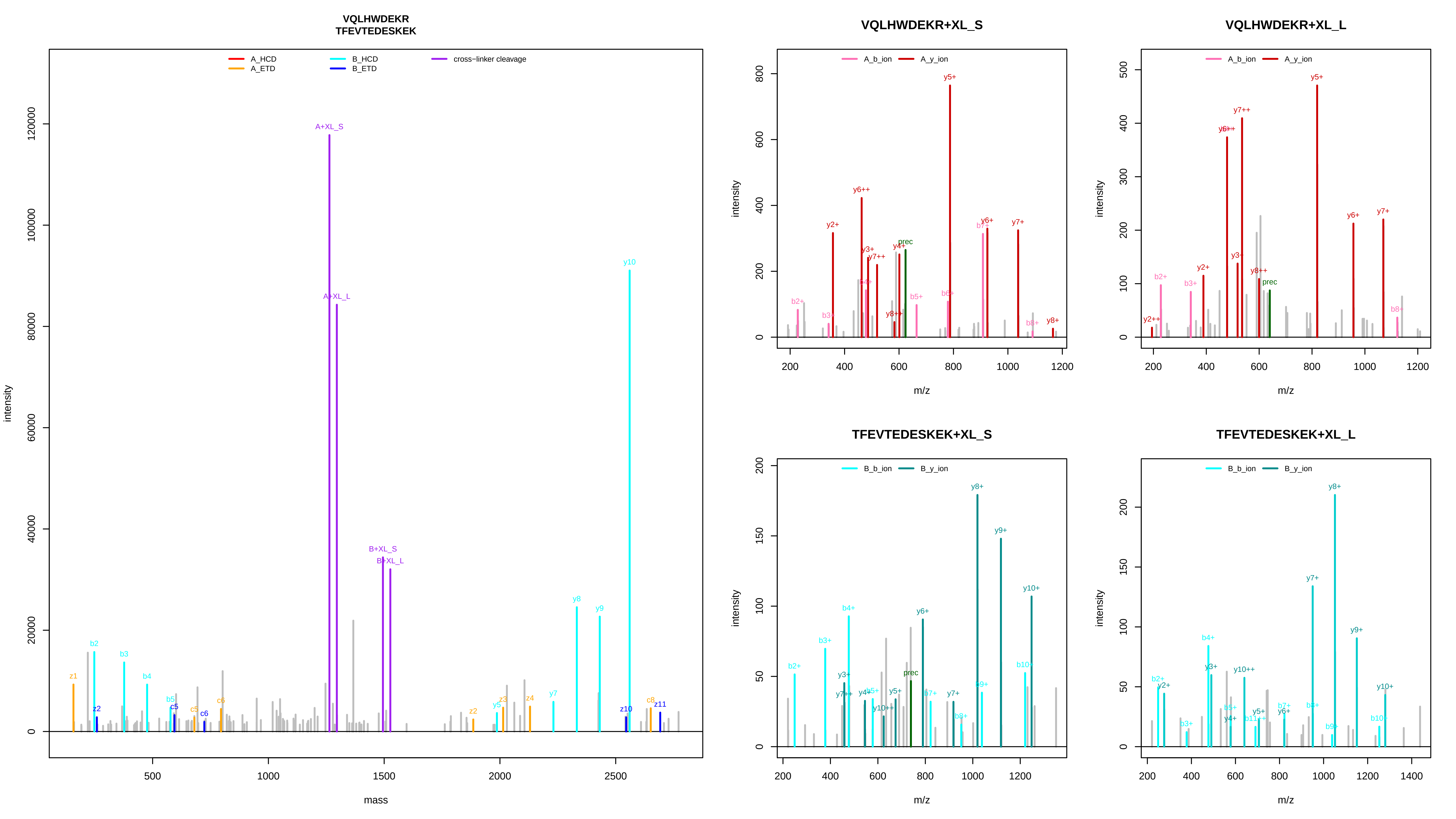
VQLHWDEKR
TFEVTEDESKEK
A_HCD
A_ETD
B_HCD
B_ETD
cross−linker cleavage
A+XL_S
A+XL_L
B+XL_S
B+XL_L
y10
y8
y9
y7
y5
z1
b2
z2
b3
b4
b5
c5
c5
c6
c6
z2
z3
z4
z10
c8
z11
intensity
mass
VQLHWDEKR+XL_S
A_b_ion
A_y_ion
y5+
y6++
y2+
y3+
y7++
y4+
prec
y6+
b7+
y7+
b2+
b3+
b5+
b6+
y8++
b8+
y8+
intensity
m/z
VQLHWDEKR+XL_L
A_b_ion
A_y_ion
y5+
y7++
y6++
y2+
y3+
y8++
prec
b2+
b3+
y2++
y6+
y7+
b8+
intensity
m/z
TFEVTEDESKEK+XL_S
B_b_ion
B_y_ion
y8+
y9+
y10+
b4+
y6+
b3+
b2+
y3+
y7++
y4+
b5+
y5+
y10++
prec
b7+
y7+
b8+
b9+
b10+
intensity
m/z
TFEVTEDESKEK+XL_L
B_b_ion
B_y_ion
y8+
y7+
y9+
b4+
y3+
y10++
b2+
y2+
b5+
y4+
y5+
b11++
b7+
y6+
b8+
b3+
b9+
y10+
b10+
intensity
m/z

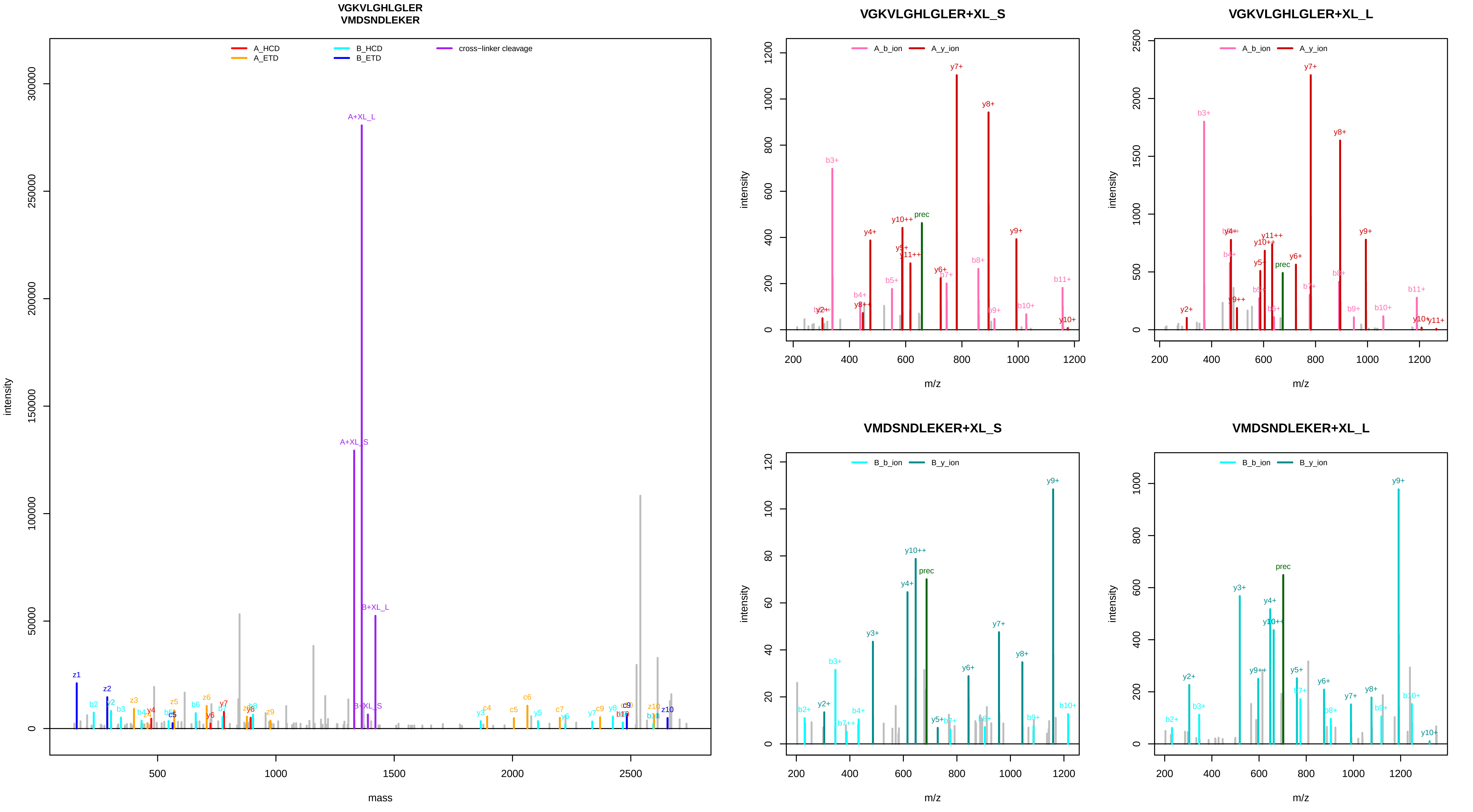
VGKVLGHLGLER
VMDSNDLEKER
A_HCD
A_ETD
B_HCD
B_ETD
cross−linker cleavage
A+XL_L
A+XL_S
B+XL_L
B+XL_S
intensity
mass
VGKVLGHLGLER+XL_S
VGKVLGHLGLER+XL_L
VMDSNDLEKER+XL_S
VMDSNDLEKER+XL_L
A_b_ion
A_y_ion
B_b_ion
B_y_ion
prec
m/z

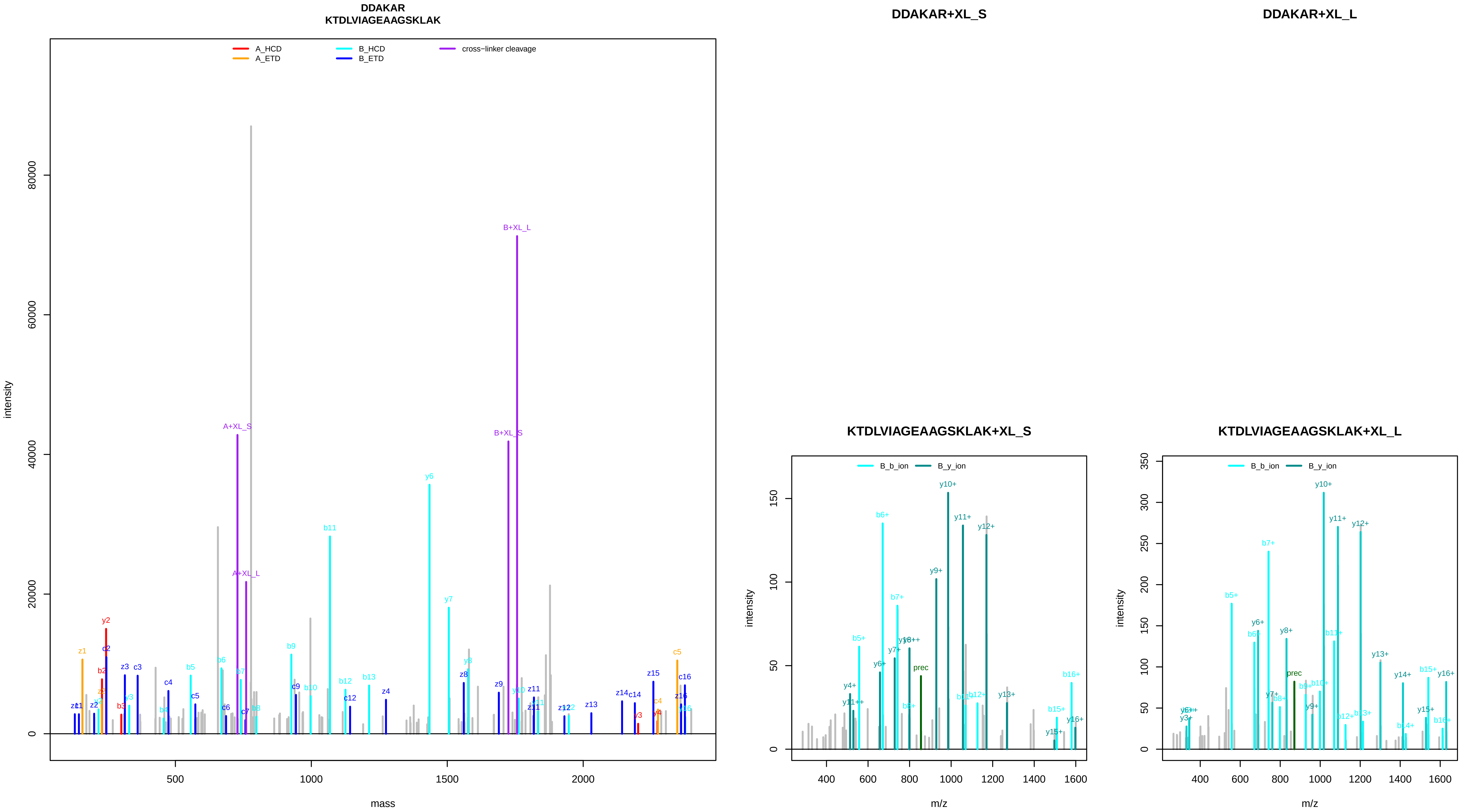

DDAKAR
KTDLVIAGEAAGSKLAK
A_HCD
A_ETD
B_HCD
B_ETD
cross−linker cleavage
intensity
mass
DDAKAR+XL_S
DDAKAR+XL_L
KTDLVIAGEAAGSKLAK+XL_S
KTDLVIAGEAAGSKLAK+XL_L
B_b_ion
B_y_ion
m/z

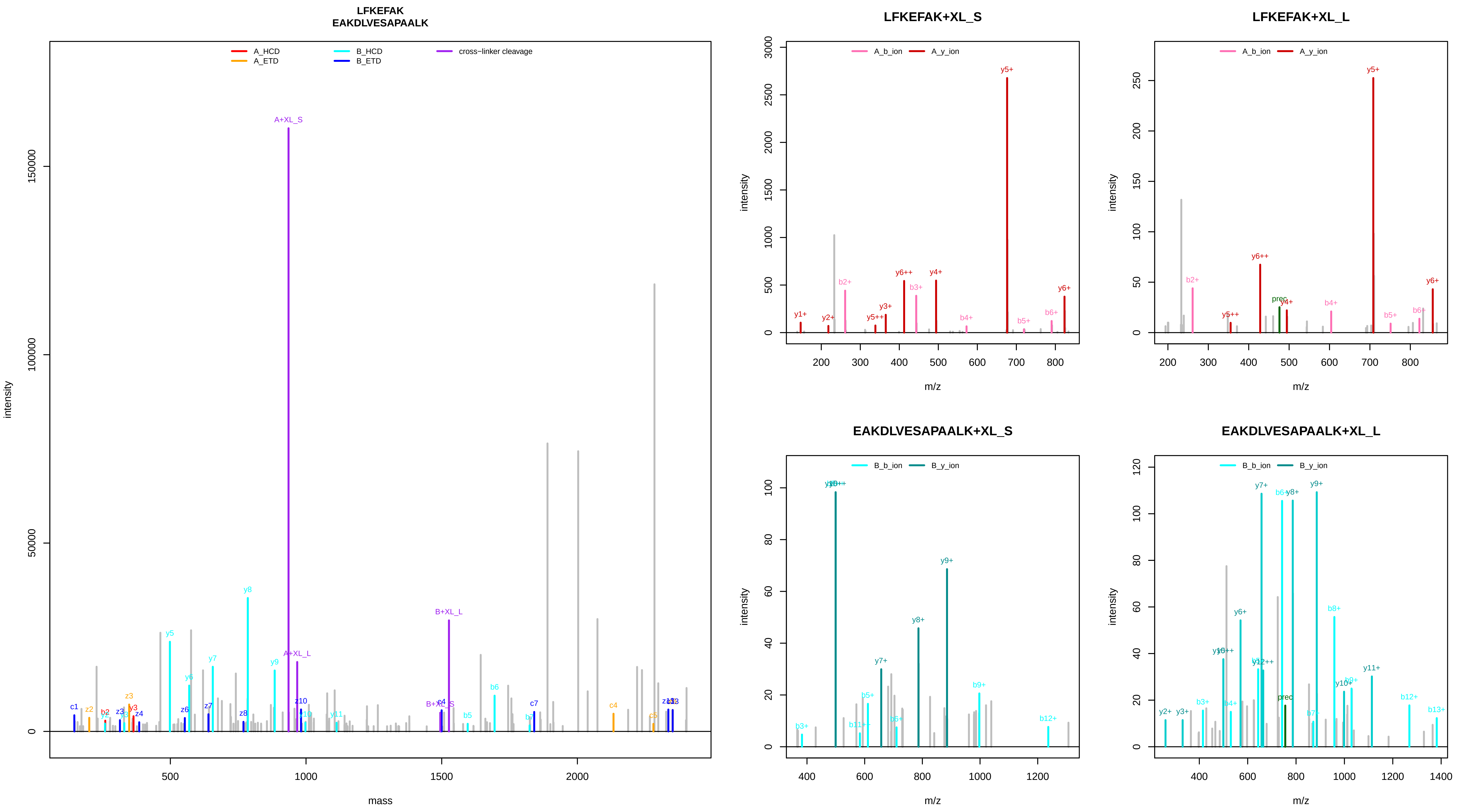

LFKEFAK
EAKDLVESAPAALK
A_HCD
A_ETD
B_HCD
B_ETD
cross−linker cleavage
A+XL_S
A+XL_L
B+XL_L
B+XL_S
mass
intensity
LFKEFAK+XL_S
LFKEFAK+XL_L
A_b_ion
A_y_ion
EAKDLVESAPAALK+XL_S
EAKDLVESAPAALK+XL_L
B_b_ion
B_y_ion
m/z

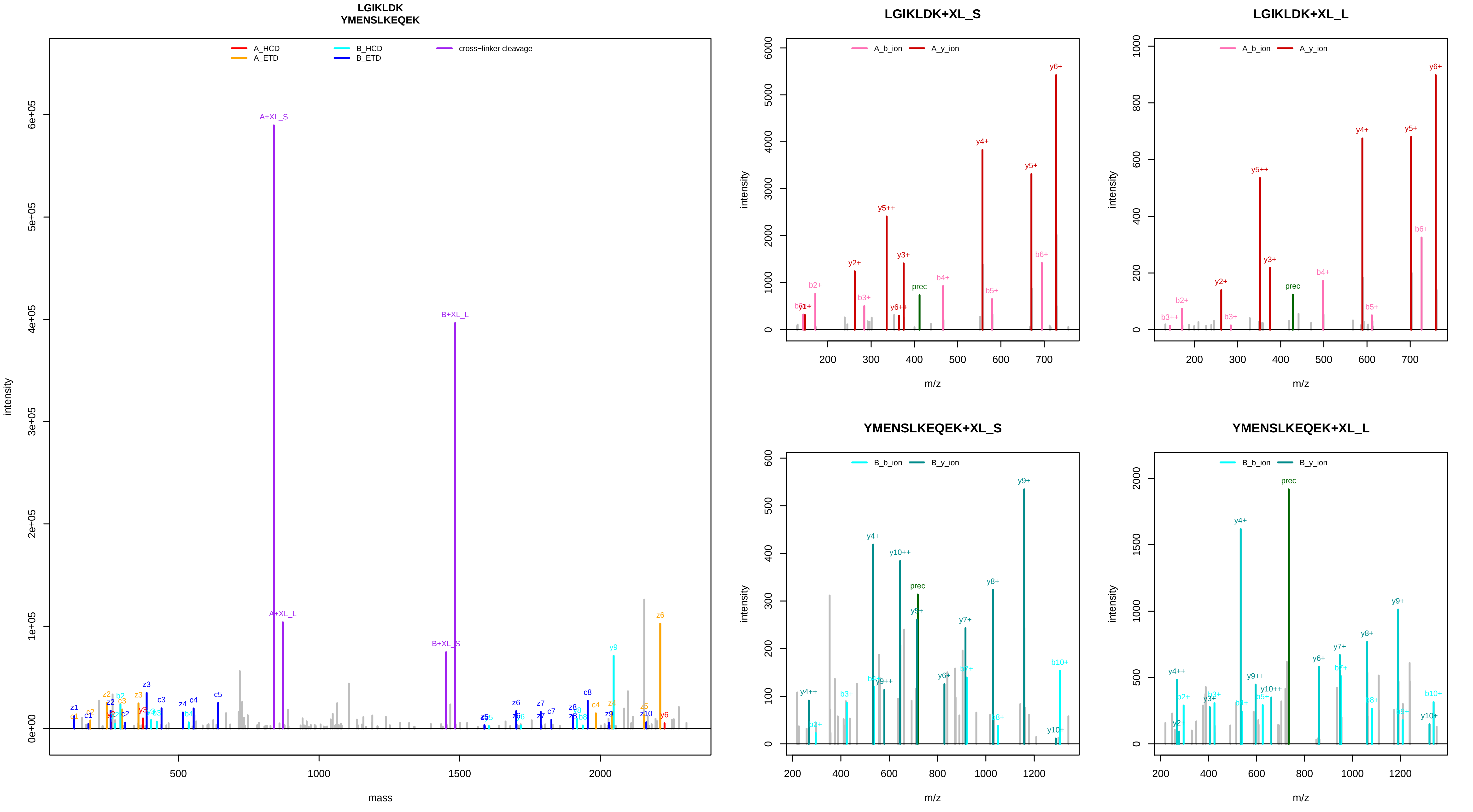

LGIKLDK
YMENSLKEQEK
A_HCD
A_ETD
B_HCD
B_ETD
cross−linker cleavage
A+XL_S
B+XL_L
A+XL_L
B+XL_S
intensity
mass
LGIKLDK+XL_S
LGIKLDK+XL_L
YMENSLKEQEK+XL_S
YMENSLKEQEK+XL_L
A_b_ion
A_y_ion
B_b_ion
B_y_ion
prec
m/z

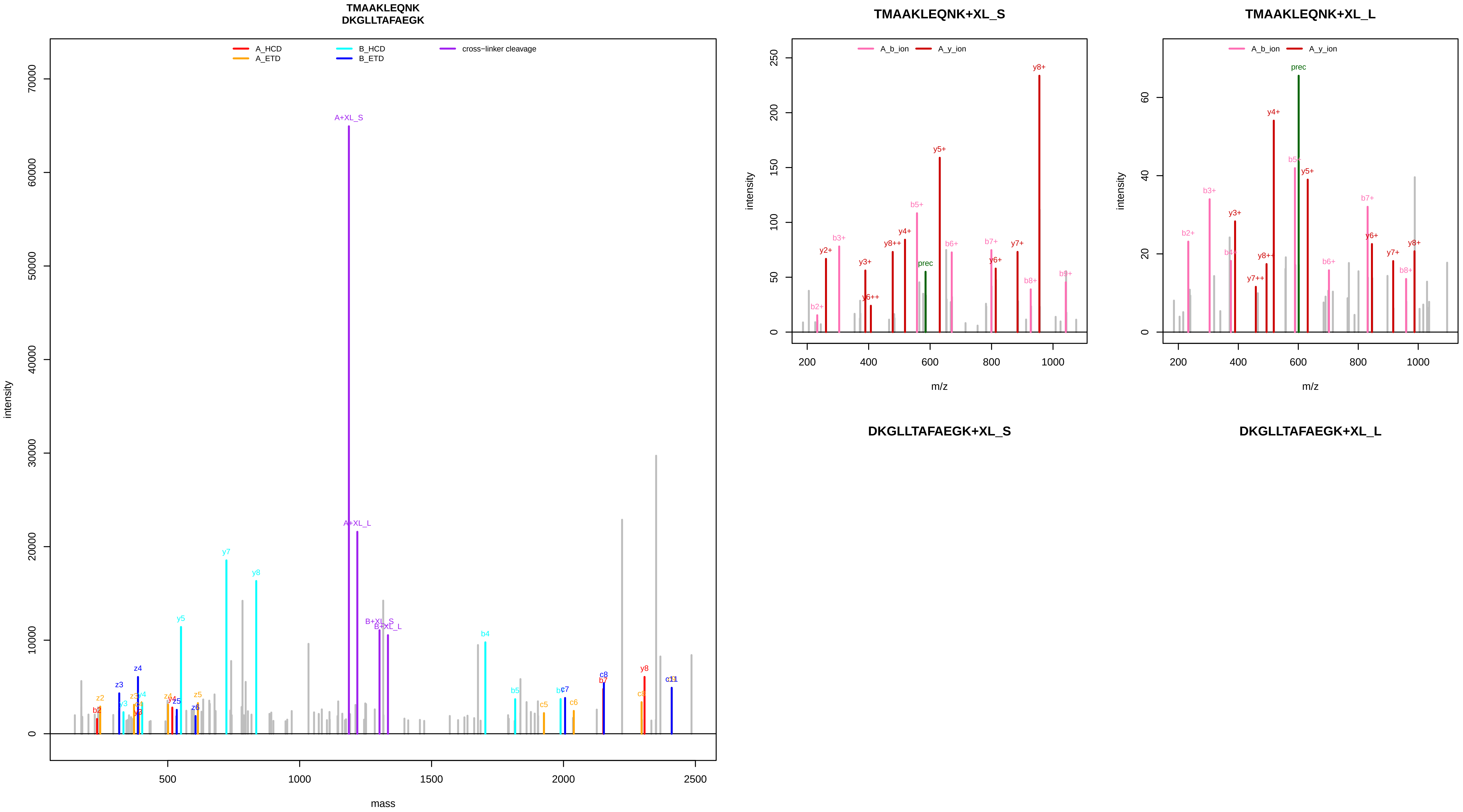

TMAAKLEQNK
DKGLLTAFAEGK
A_HCD
A_ETD
B_HCD
B_ETD
cross−linker cleavage
A+XL_S
A+XL_L
B+XL_S
B+XL_L
intensity
mass
TMAAKLEQNK+XL_S
TMAAKLEQNK+XL_L
A_b_ion
A_y_ion
prec
m/z
DKGLLTAFAEGK+XL_S
DKGLLTAFAEGK+XL_L

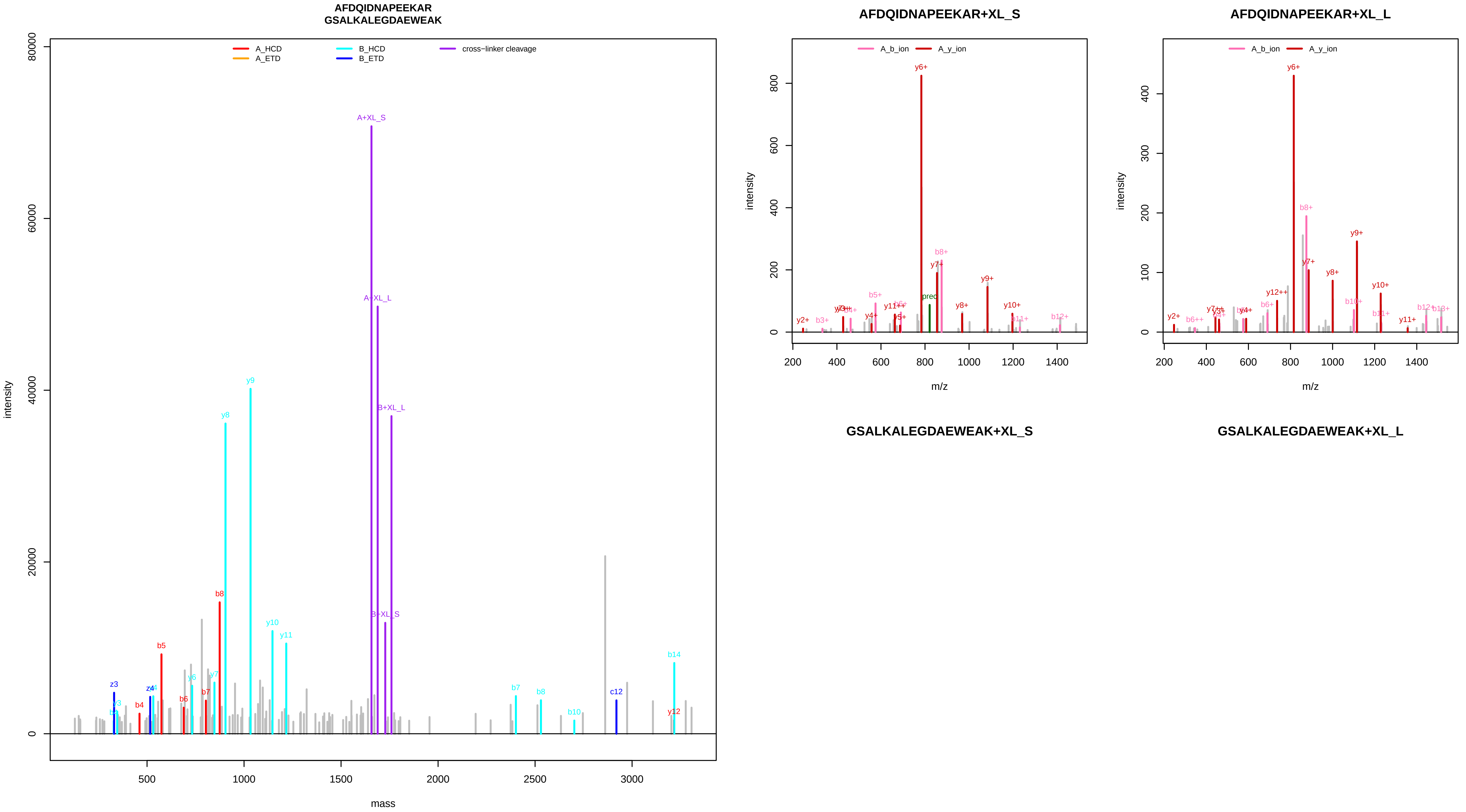
AFDQIDNAPEEKAR
GSALKALEGDAEWEAK
A_HCD
A_ETD
B_HCD
B_ETD
cross−linker cleavage
A+XL_S
A+XL_L
B+XL_L
B+XL_S
intensity
mass
0
20000
40000
60000
80000
500
1000
1500
2000
2500
3000
AFDQIDNAPEEKAR+XL_S
AFDQIDNAPEEKAR+XL_L
A_b_ion
A_y_ion
m/z
200
400
600
800
1000
1200
1400
GSALKALEGDAEWEAK+XL_S
GSALKALEGDAEWEAK+XL_L

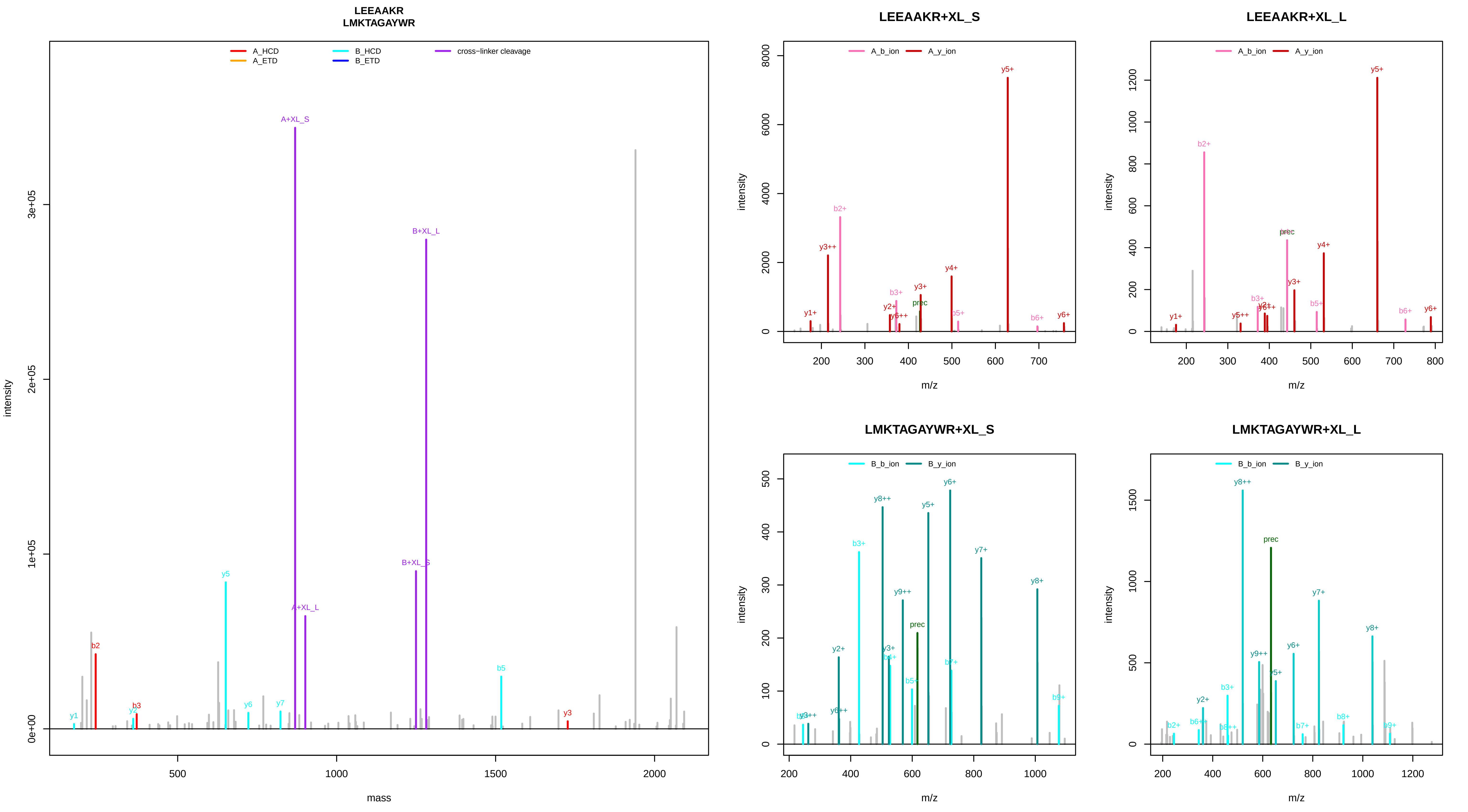

LEEAAKR
LMKTAGAYWR
A_HCD
A_ETD
B_HCD
B_ETD
cross−linker cleavage
A+XL_S
B+XL_L
B+XL_S
A+XL_L
intensity
mass
LEEAAKR+XL_S
LEEAAKR+XL_L
LMKTAGAYWR+XL_S
LMKTAGAYWR+XL_L
A_b_ion
A_y_ion
B_b_ion
B_y_ion
m/z
prec

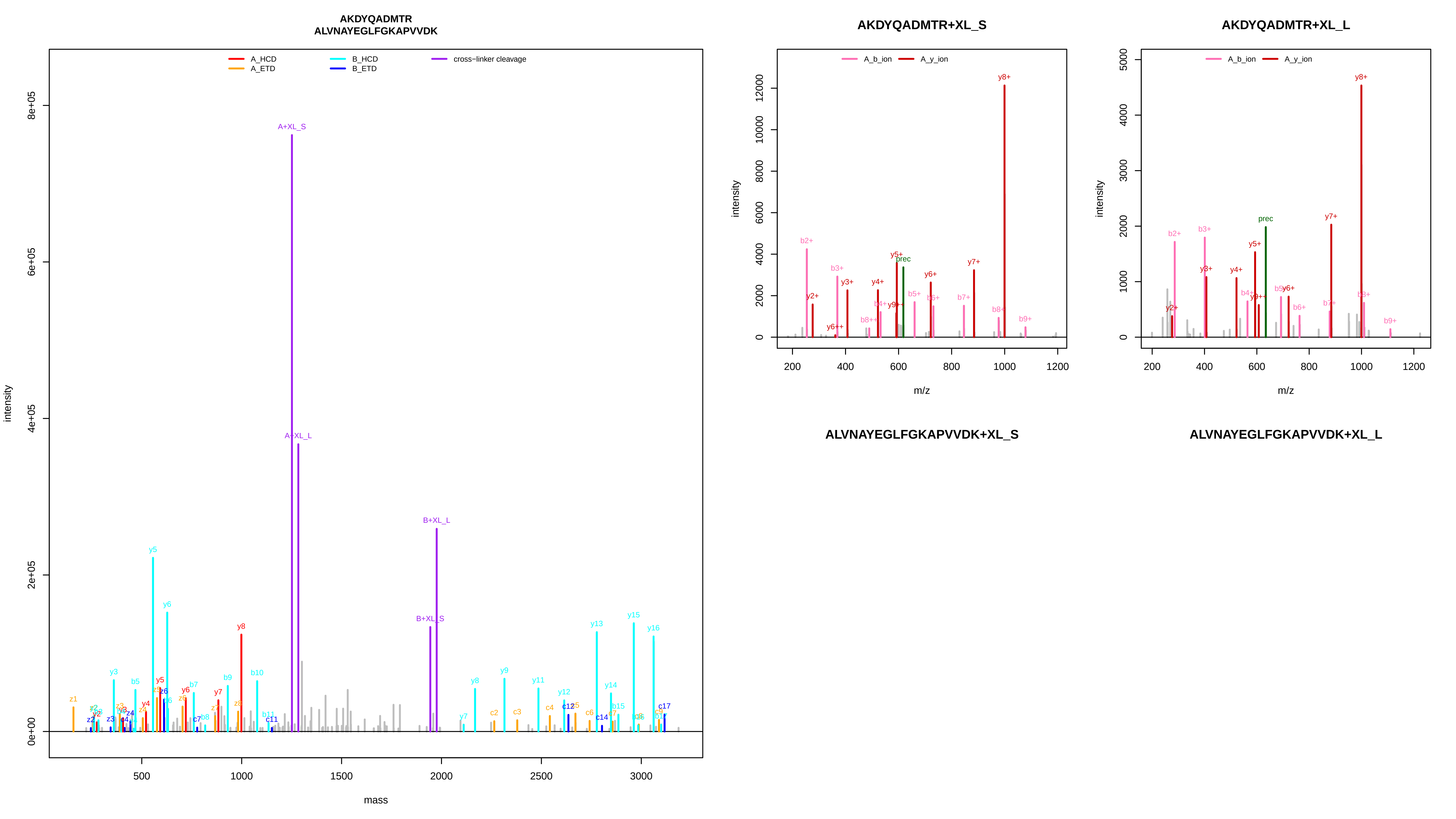
AKDYQADMTR
ALVNAYEGLFGKAPVVDK
A_HCD
A_ETD
B_HCD
B_ETD
cross−linker cleavage
A+XL_S
A+XL_L
B+XL_L
B+XL_S
intensity
mass
AKDYQADMTR+XL_S
AKDYQADMTR+XL_L
A_b_ion
A_y_ion
prec
m/z
ALVNAYEGLFGKAPVVDK+XL_S
ALVNAYEGLFGKAPVVDK+XL_L

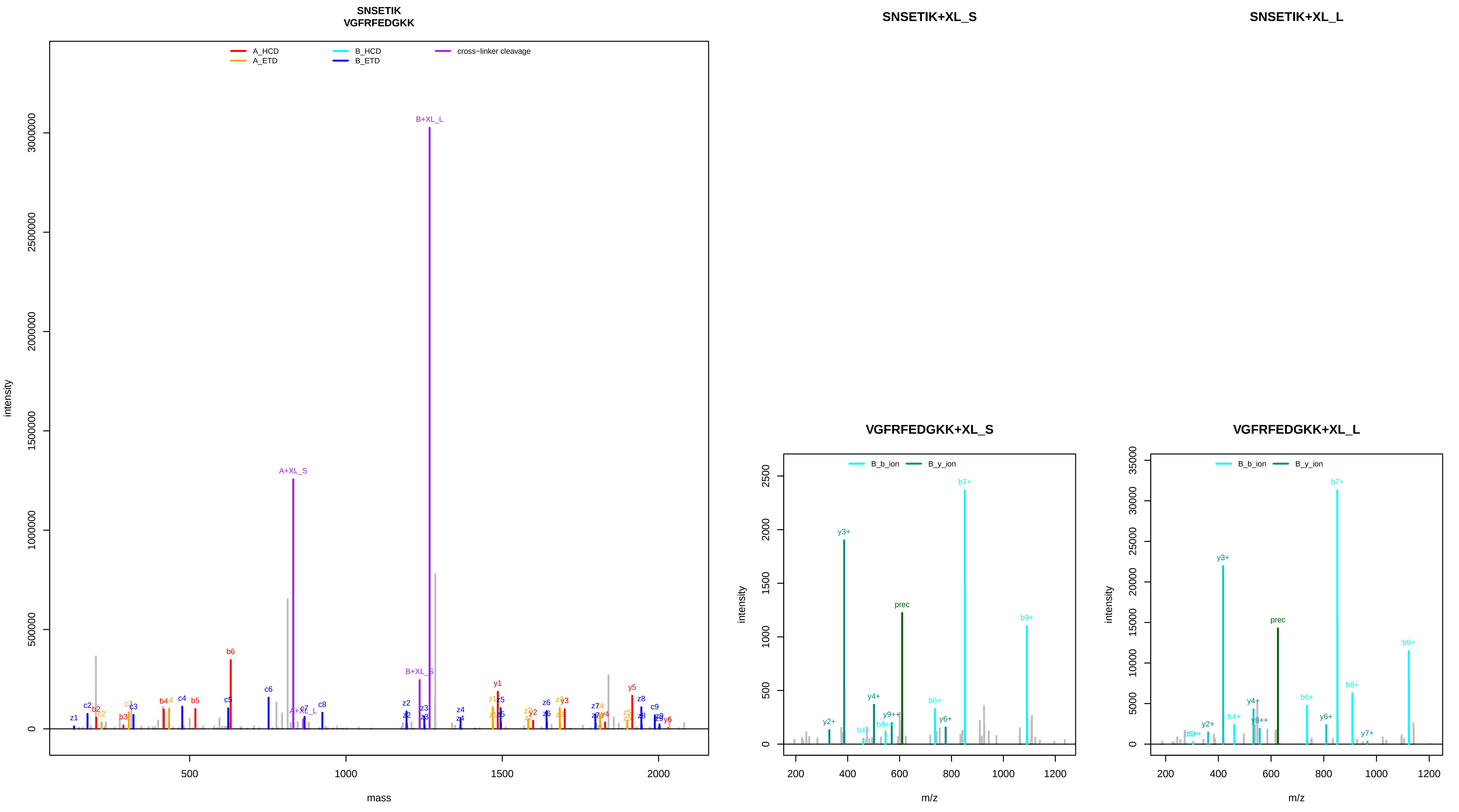
SNSETIK
VGFRFEDGKK
A_HCD
A_ETD
B_HCD
B_ETD
cross−linker cleavage
B+XL_L
A+XL_S
B+XL_S
A+XL_L
intensity
mass
SNSETIK+XL_S
SNSETIK+XL_L
VGFRFEDGKK+XL_S
VGFRFEDGKK+XL_L
B_b_ion
B_y_ion
prec
m/z

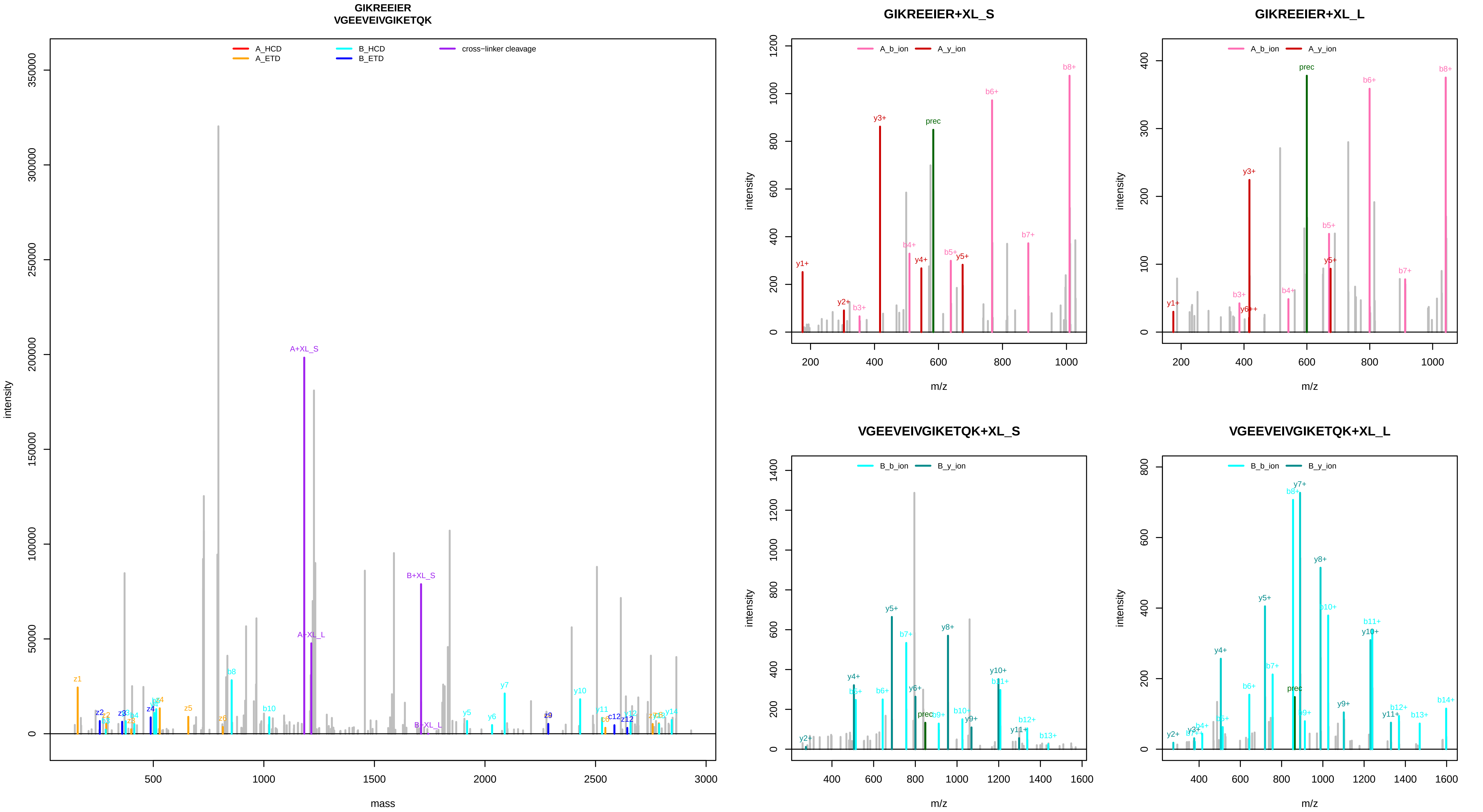
GIKREEIER
VGEEVEIVGIKETQK
A_HCD
A_ETD
B_HCD
B_ETD
cross−linker cleavage
A+XL_S
A+XL_L
B+XL_S
B+XL_L
intensity
mass
GIKREEIER+XL_S
GIKREEIER+XL_L
A_b_ion
A_y_ion
prec
VGEEVEIVGIKETQK+XL_S
VGEEVEIVGIKETQK+XL_L
B_b_ion
B_y_ion
m/z

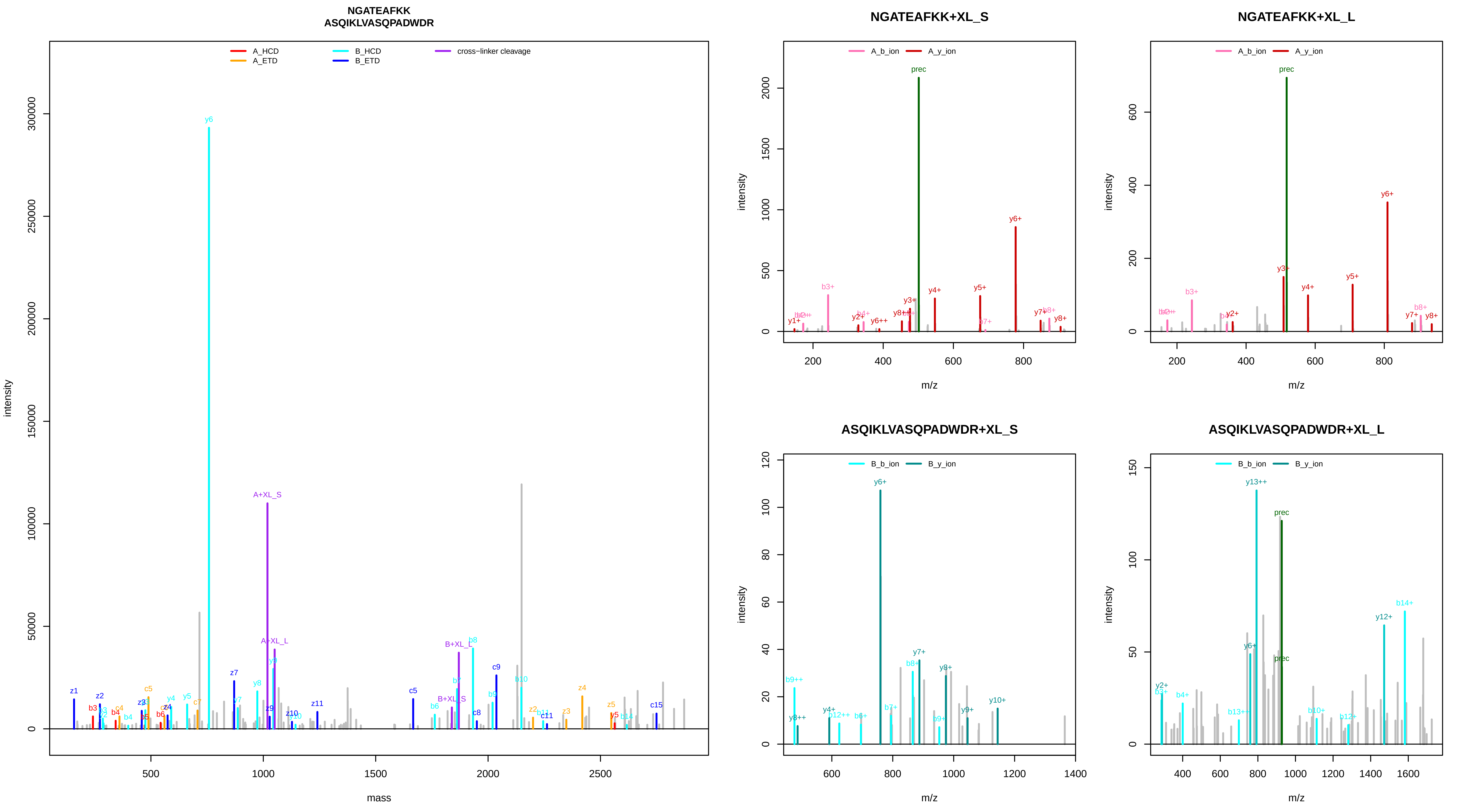
NGATEAFKK
ASQIKLVASQPADWDR
A_HCD
A_ETD
B_HCD
B_ETD
cross−linker cleavage
intensity
mass
A+XL_S
A+XL_L
B+XL_L
B+XL_S
NGATEAFKK+XL_S
NGATEAFKK+XL_L
A_b_ion
A_y_ion
prec
m/z
ASQIKLVASQPADWDR+XL_S
ASQIKLVASQPADWDR+XL_L
B_b_ion
B_y_ion

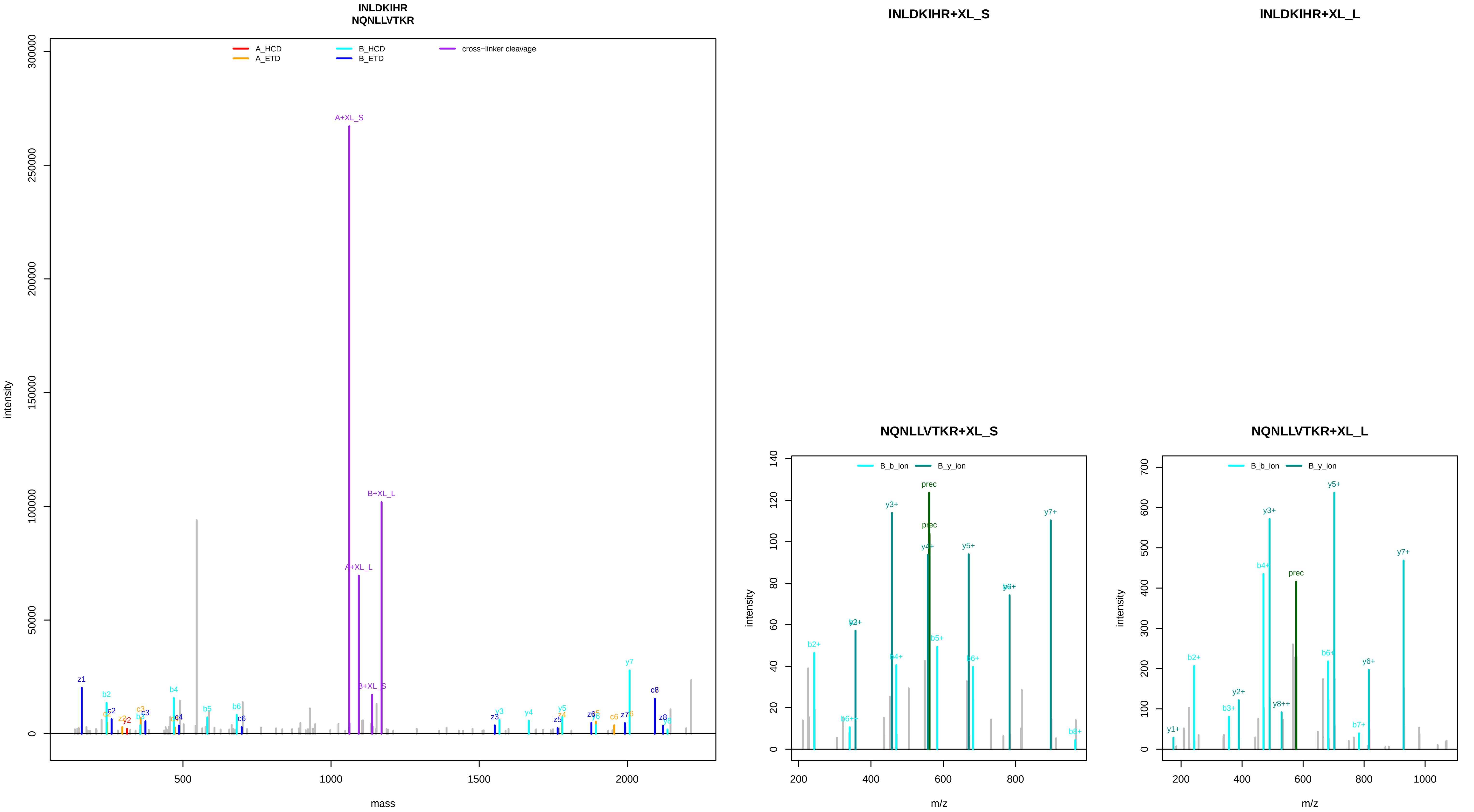

INLDKIHR
NQNLLVTKR
A_HCD
A_ETD
B_HCD
B_ETD
cross−linker cleavage
A+XL_S
B+XL_L
A+XL_L
B+XL_S
intensity
mass
0
50000
100000
150000
200000
250000
300000
500
1000
1500
2000
z1
b2
c2
c3
b4
b5
b6
c6
c4
y2
z3
y3
y4
z5
y5
z6
z7
y7
c6
c8
z8
INLDKIHR+XL_S
INLDKIHR+XL_L
NQNLLVTKR+XL_S
NQNLLVTKR+XL_L
B_b_ion
B_y_ion
prec
y3+
y4+
y5+
y6+
y7+
y2+
b2+
b5+
b6+
b4+
b8+
b3+
b7+
y1+
y8++
intensity
m/z
0
20
40
60
80
100
120
140
200
400
600
800
1000
300
500
700

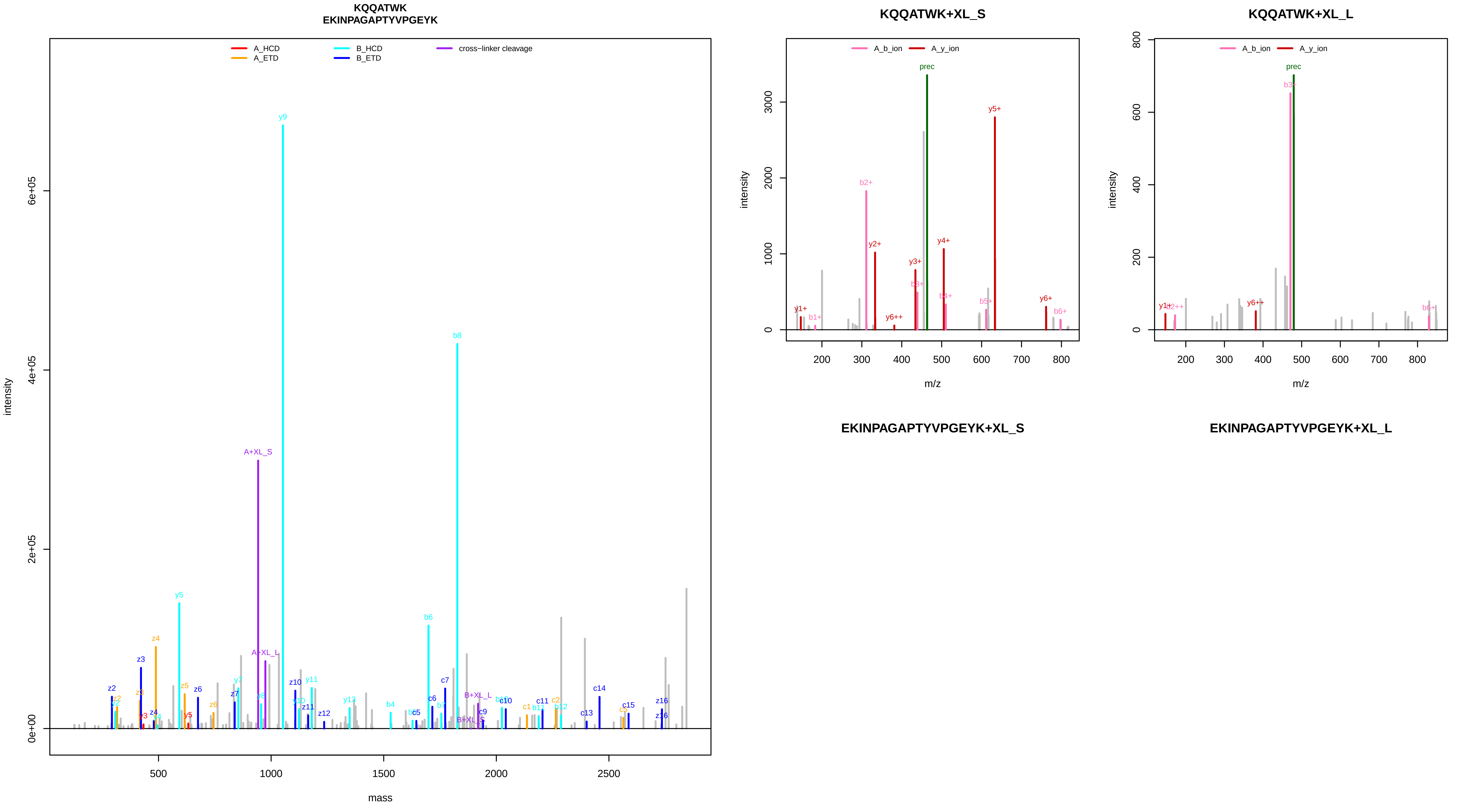
KQQATWK
EKINPAGAPTYVPGEYK
A_HCD
A_ETD
B_HCD
B_ETD
cross−linker cleavage
intensity
mass
KQQATWK+XL_S
A_b_ion
A_y_ion
prec
m/z
KQQATWK+XL_L
EKINPAGAPTYVPGEYK+XL_S
EKINPAGAPTYVPGEYK+XL_L

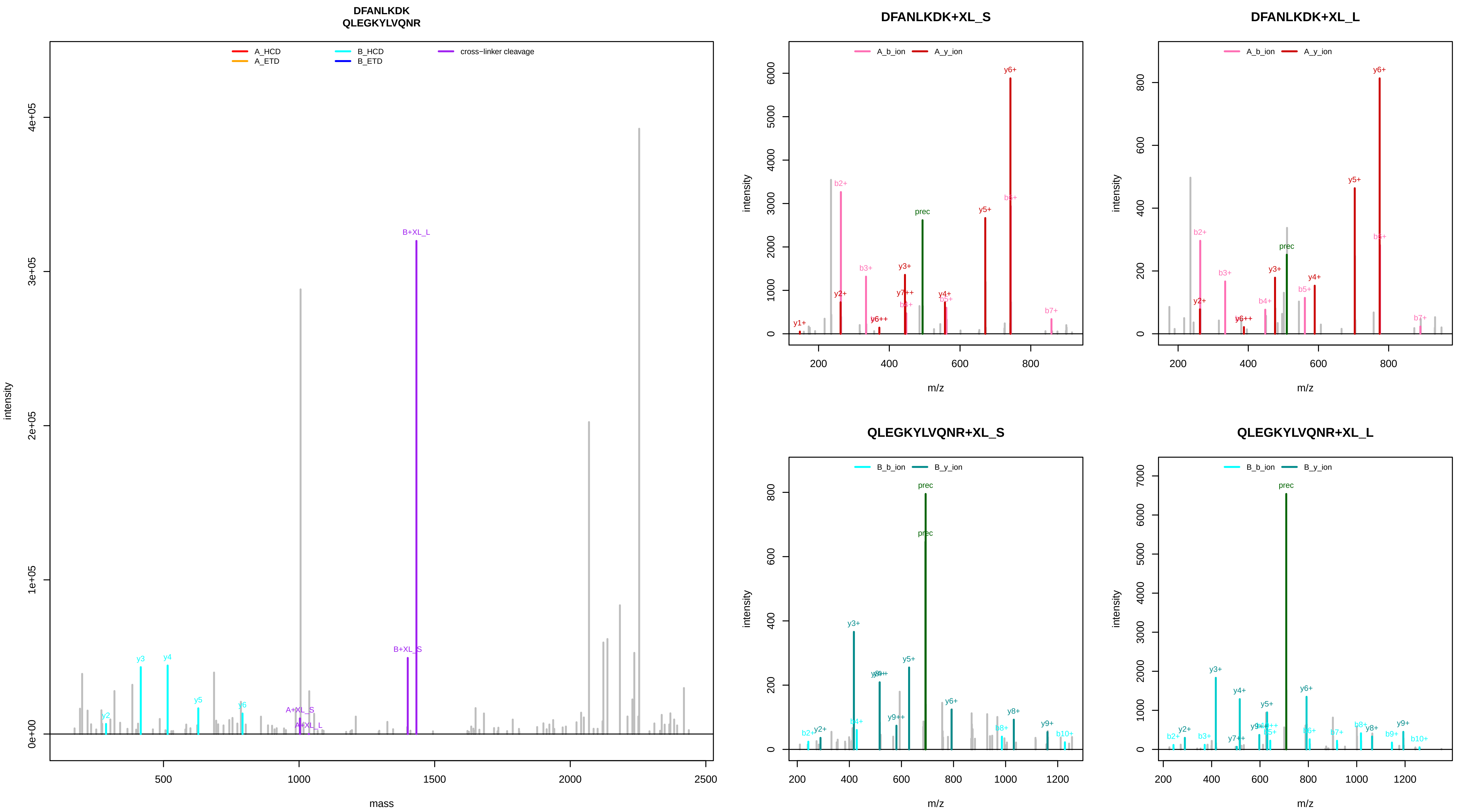

DFANLKDK
QLEGKYLVQNR
A_HCD
A_ETD
B_HCD
B_ETD
cross−linker cleavage
B+XL_L
B+XL_S
A+XL_S
A+XL_L
intensity
mass
DFANLKDK+XL_S
DFANLKDK+XL_L
QLEGKYLVQNR+XL_S
QLEGKYLVQNR+XL_L
A_b_ion
A_y_ion
B_b_ion
B_y_ion
prec
m/z

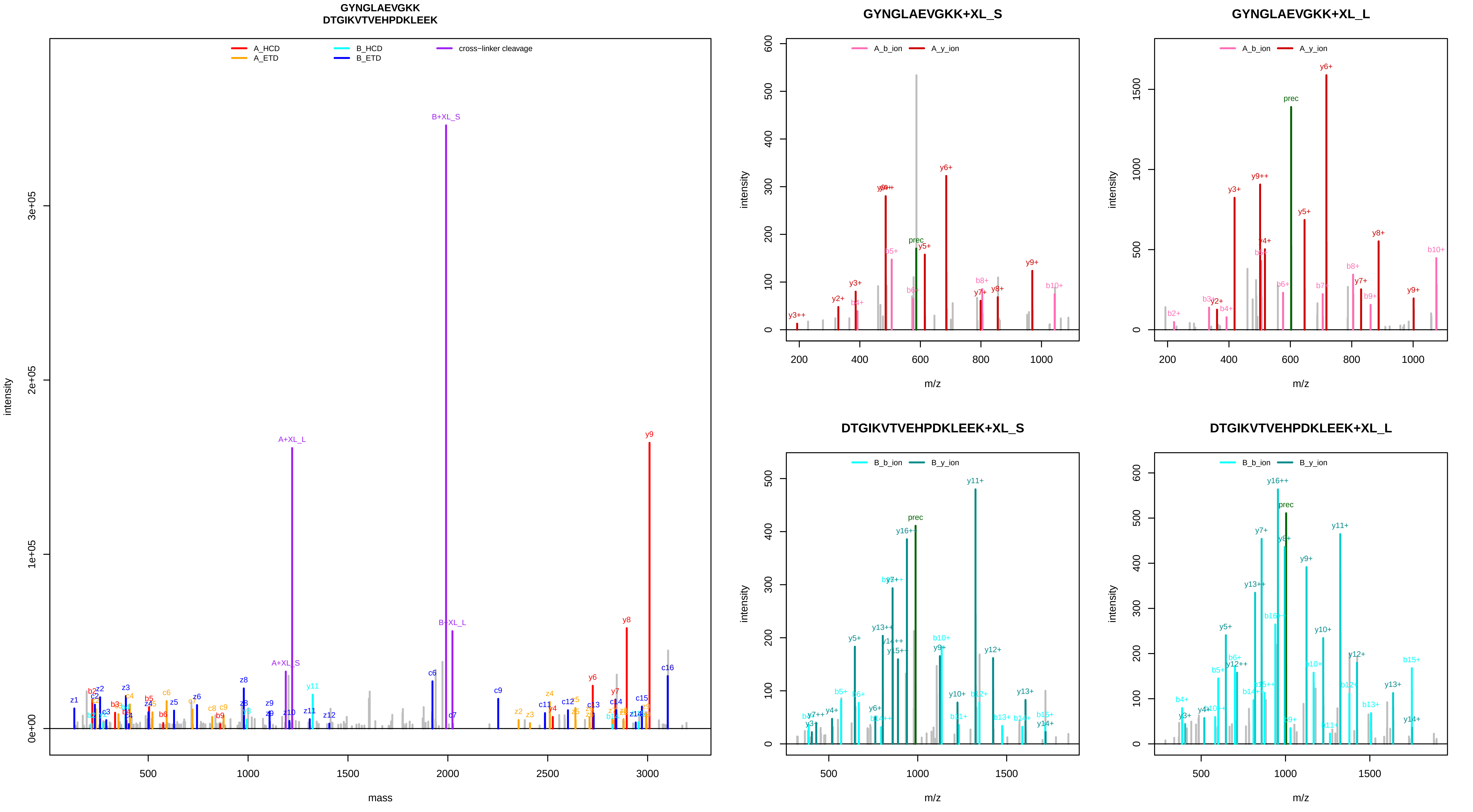
GYNGLAEVGKK
DTGIKVTVEHPDKLEEK
A_HCD
A_ETD
B_HCD
B_ETD
cross−linker cleavage
B+XL_S
A+XL_L
B+XL_L
A+XL_S
y9
y8
intensity
mass
GYNGLAEVGKK+XL_S
GYNGLAEVGKK+XL_L
A_b_ion
A_y_ion
prec
DTGIKVTVEHPDKLEEK+XL_S
DTGIKVTVEHPDKLEEK+XL_L
B_b_ion
B_y_ion
m/z

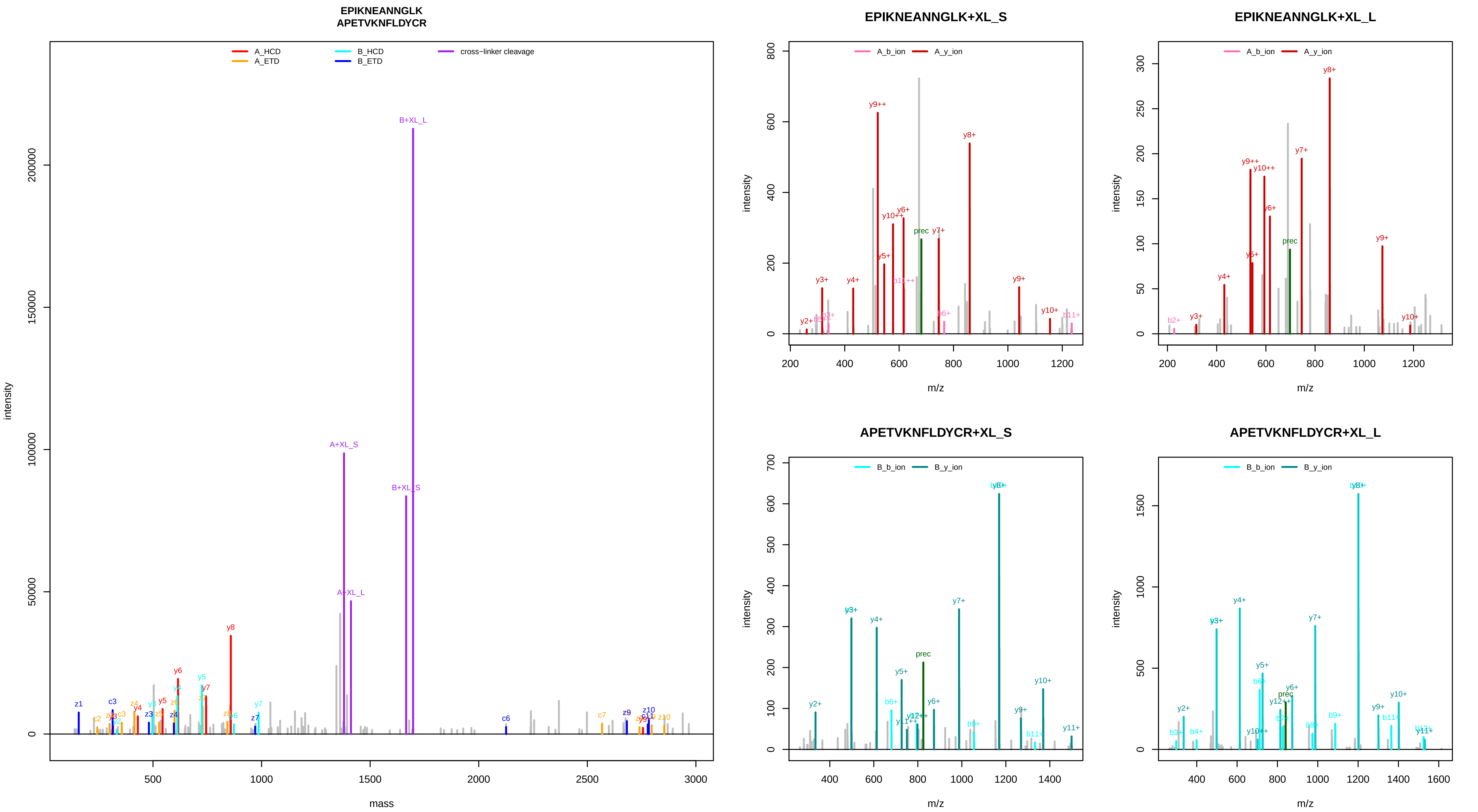

EPIKNEANNGLK
APETVKNFLDYCR
A_HCD
A_ETD
B_HCD
B_ETD
cross−linker cleavage
B+XL_L
A+XL_S
B+XL_S
A+XL_L
intensity
mass
EPIKNEANNGLK+XL_S
EPIKNEANNGLK+XL_L
APETVKNFLDYCR+XL_S
APETVKNFLDYCR+XL_L
A_b_ion
A_y_ion
B_b_ion
B_y_ion
prec
m/z

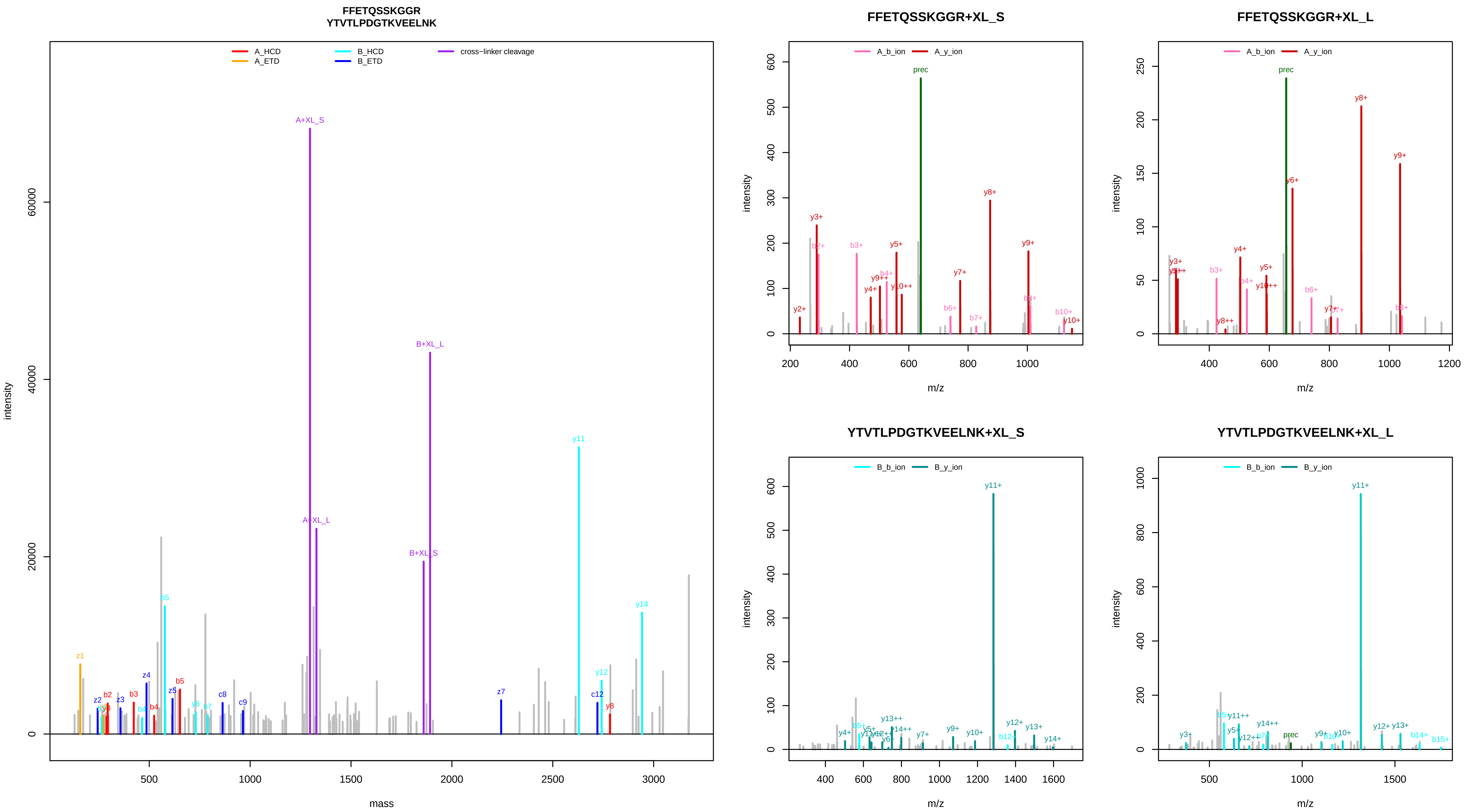
FFETQSSKGGR
YTVTLPDGTKVEELNK
A_HCD
A_ETD
B_HCD
B_ETD
cross−linker cleavage
A+XL_S
B+XL_L
A+XL_L
B+XL_S
y11
y14
y12
c12
y8
z7
b5
z4
z5
c8
c9
intensity
mass
FFETQSSKGGR+XL_S
FFETQSSKGGR+XL_L
A_b_ion
A_y_ion
prec
m/z
YTVTLPDGTKVEELNK+XL_S
YTVTLPDGTKVEELNK+XL_L
B_b_ion
B_y_ion
y11+

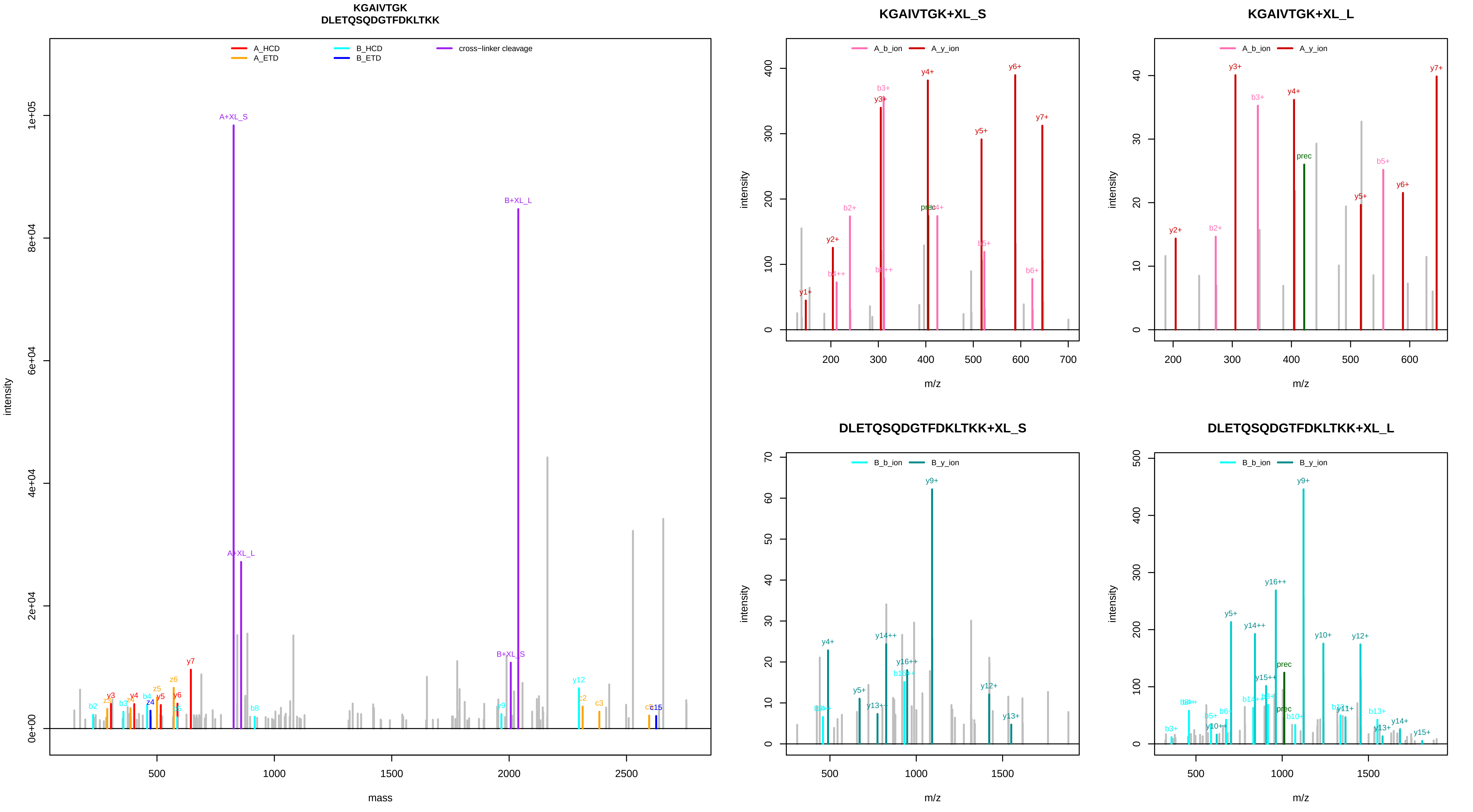

KGAIVTGK
DLETQSQDGTFDKLTKK
A_HCD
A_ETD
B_HCD
B_ETD
cross−linker cleavage
A+XL_S
B+XL_L
A+XL_L
B+XL_S
intensity
mass
KGAIVTGK+XL_S
KGAIVTGK+XL_L
DLETQSQDGTFDKLTKK+XL_S
DLETQSQDGTFDKLTKK+XL_L
A_b_ion
A_y_ion
B_b_ion
B_y_ion
prec
m/z

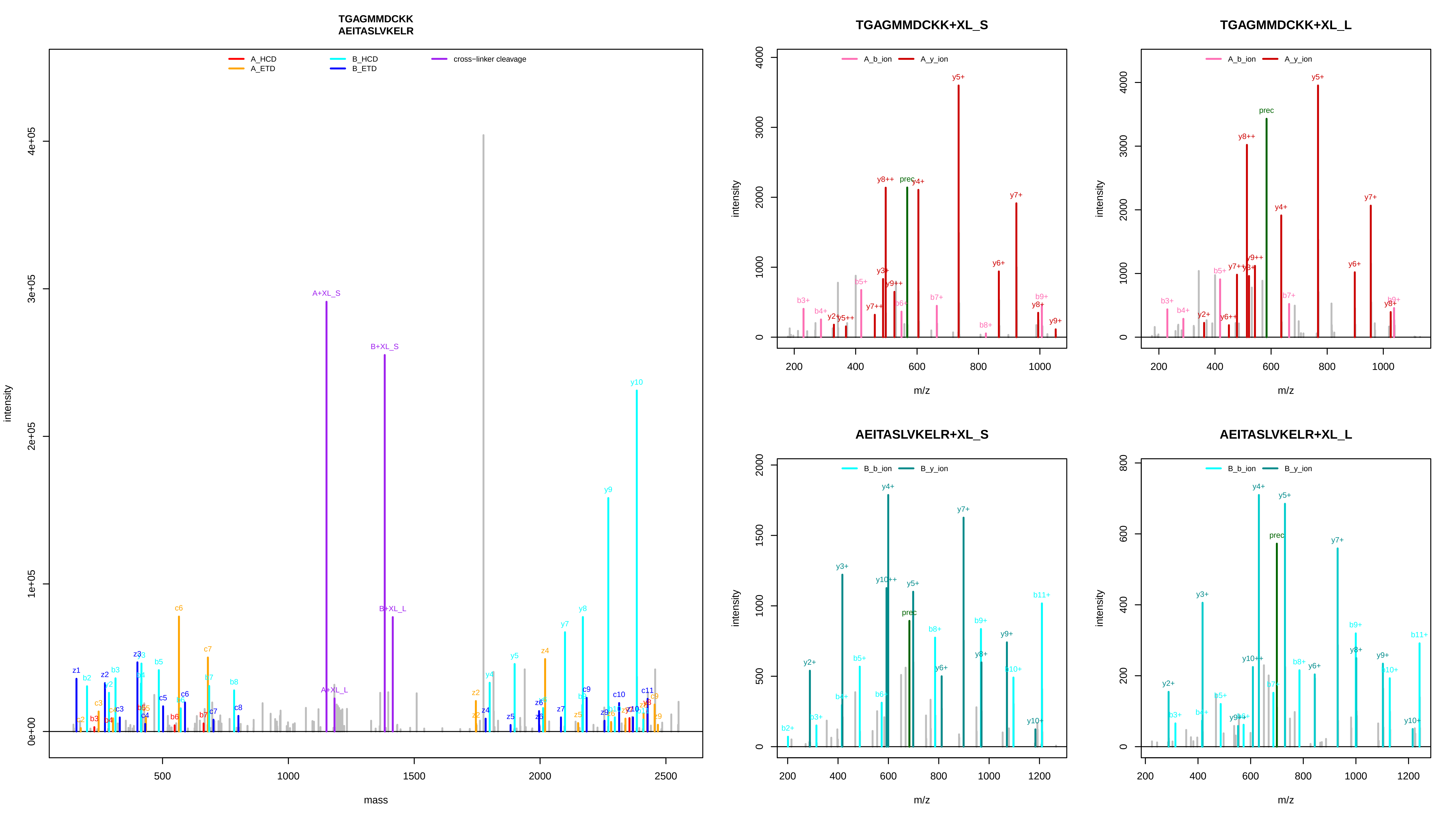

TGAGMMDCKK
AEITASLVKELR
A_HCD
A_ETD
B_HCD
B_ETD
cross−linker cleavage
A+XL_S
B+XL_S
A+XL_L
B+XL_L
intensity
mass
TGAGMMDCKK+XL_S
TGAGMMDCKK+XL_L
AEITASLVKELR+XL_S
AEITASLVKELR+XL_L
A_b_ion
A_y_ion
B_b_ion
B_y_ion
prec
m/z

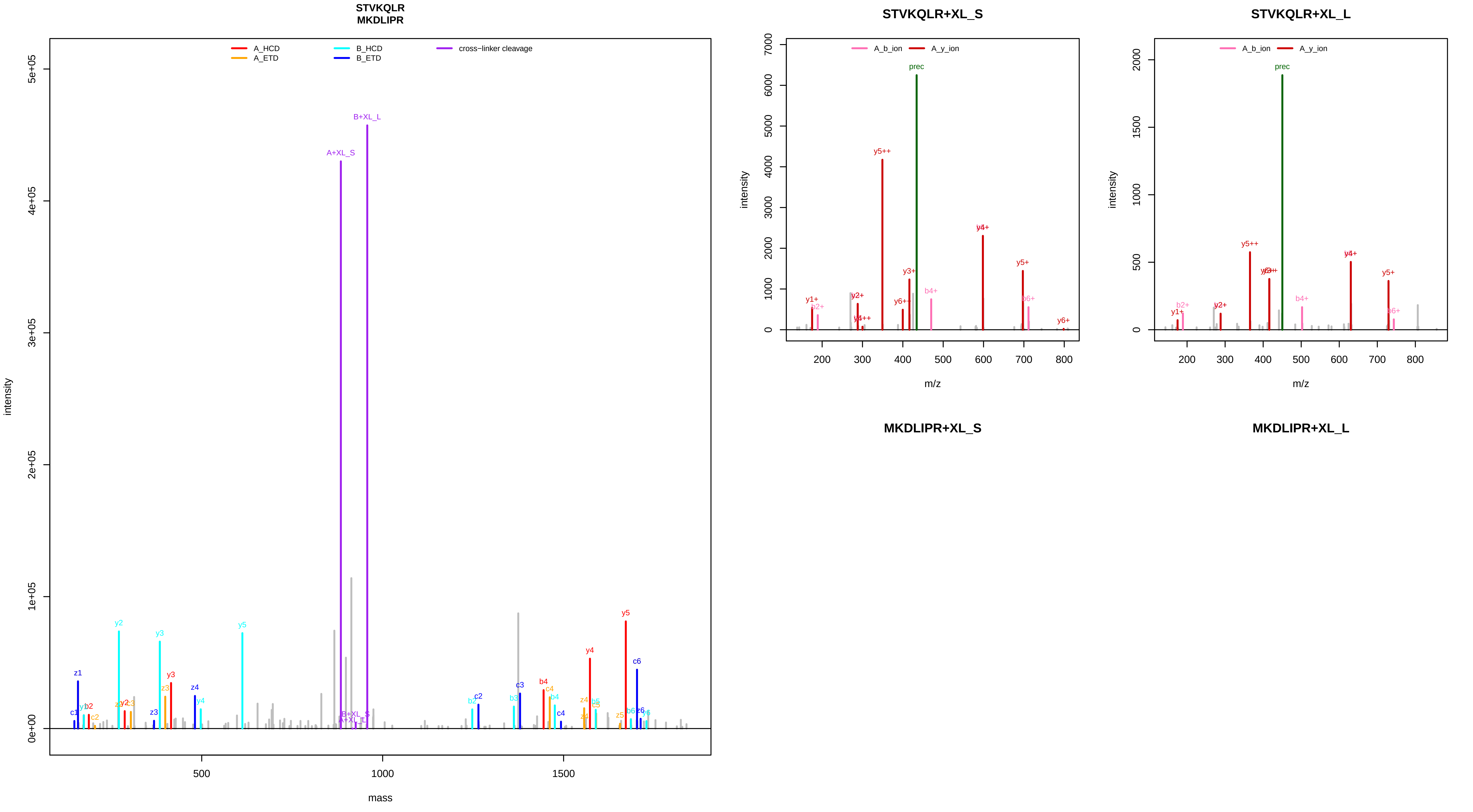
STVKQLR
MKDLIPR
A_HCD
A_ETD
B_HCD
B_ETD
cross−linker cleavage
B+XL_L
A+XL_S
intensity
mass
STVKQLR+XL_S
STVKQLR+XL_L
A_b_ion
A_y_ion
prec
m/z
MKDLIPR+XL_S
MKDLIPR+XL_L

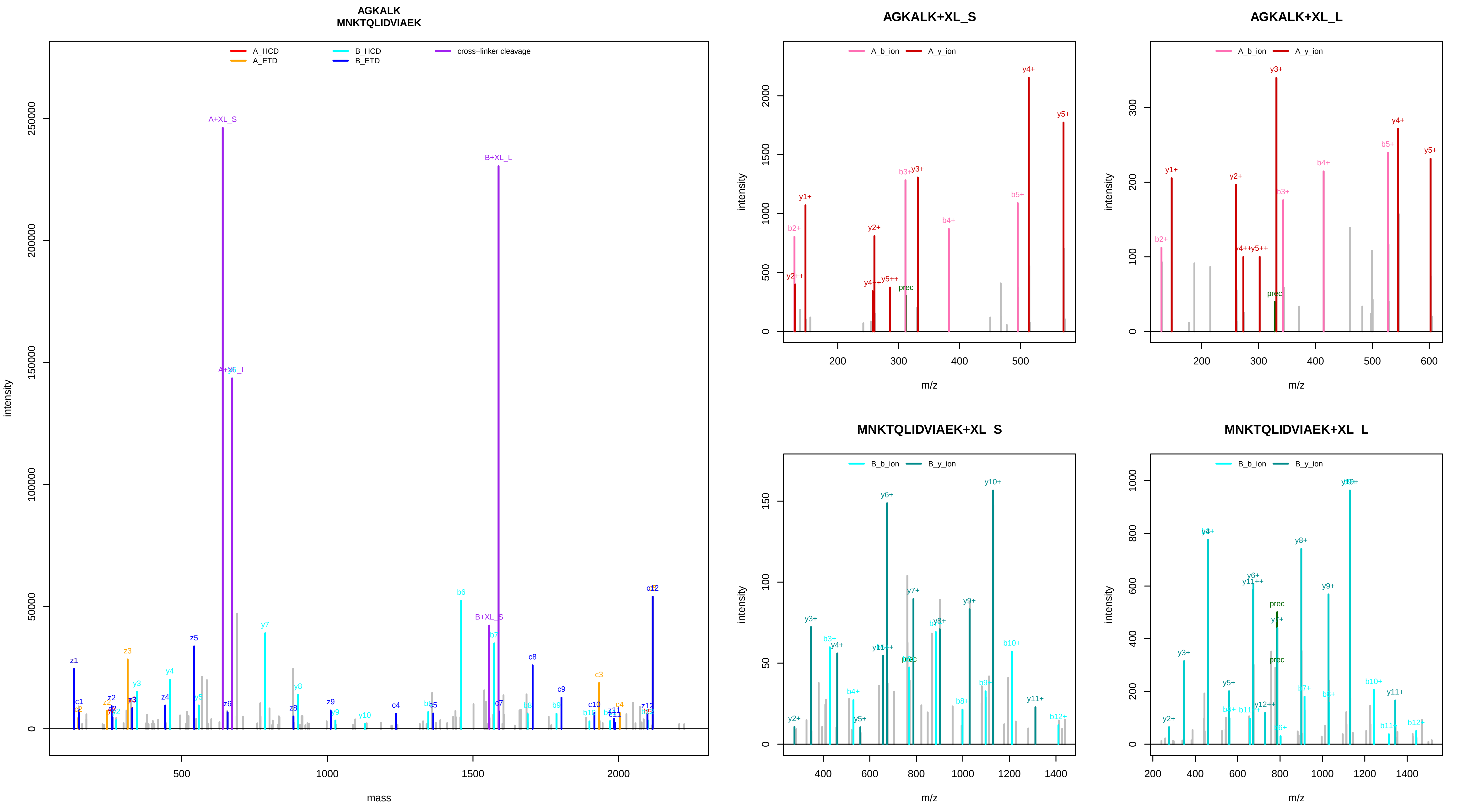
AGKALK
MNKTQLIDVIAEK
A_HCD
A_ETD
B_HCD
B_ETD
cross−linker cleavage
A+XL_S
B+XL_L
A+XL_L
B+XL_S
intensity
mass
AGKALK+XL_S
AGKALK+XL_L
A_b_ion
A_y_ion
MNKTQLIDVIAEK+XL_S
MNKTQLIDVIAEK+XL_L
B_b_ion
B_y_ion
prec
m/z

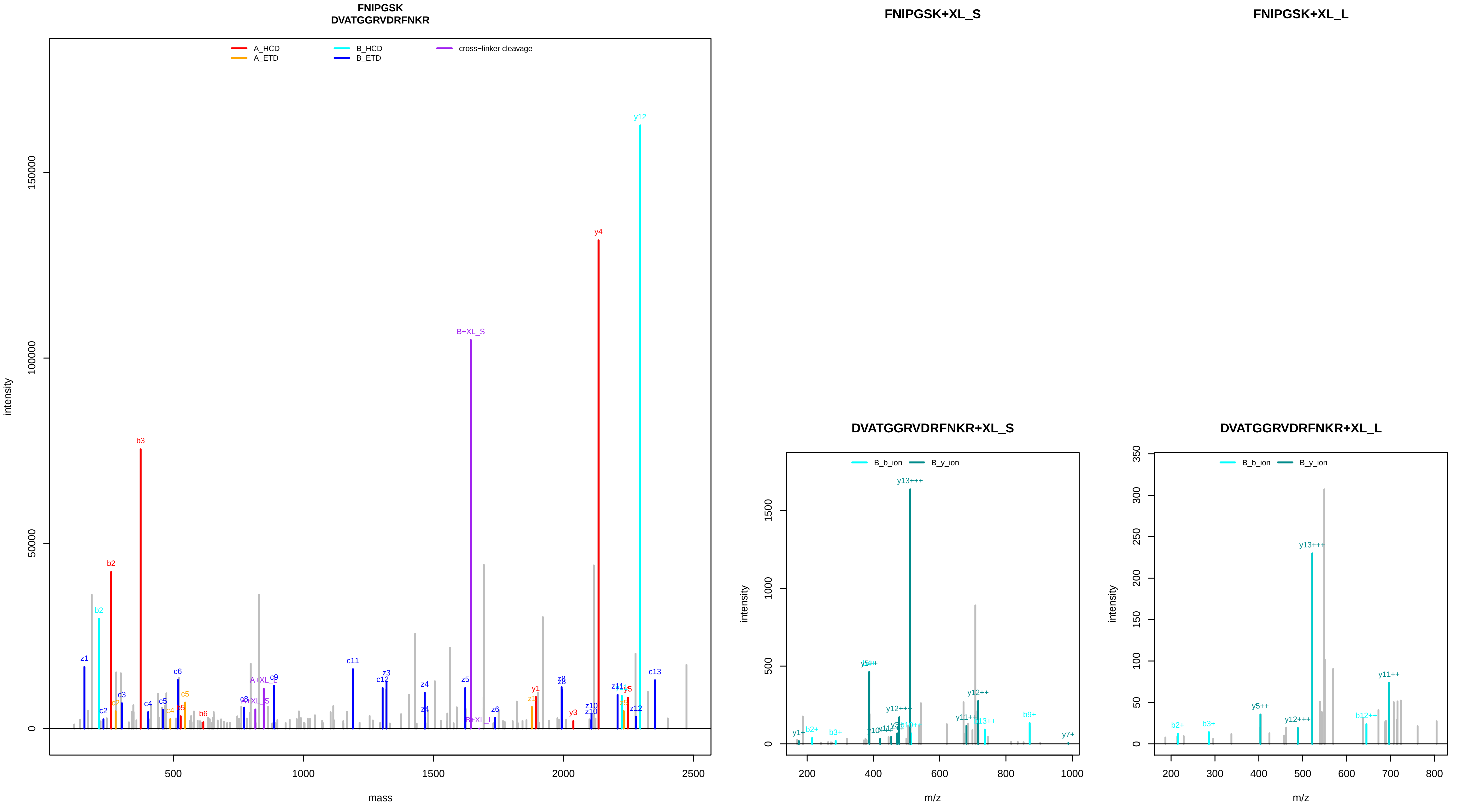

FNIPGSK
DVATGGRVDRFNKR
A_HCD
A_ETD
B_HCD
B_ETD
cross−linker cleavage
intensity
mass
FNIPGSK+XL_S
FNIPGSK+XL_L
DVATGGRVDRFNKR+XL_S
DVATGGRVDRFNKR+XL_L
B_b_ion
B_y_ion
intensity
m/z

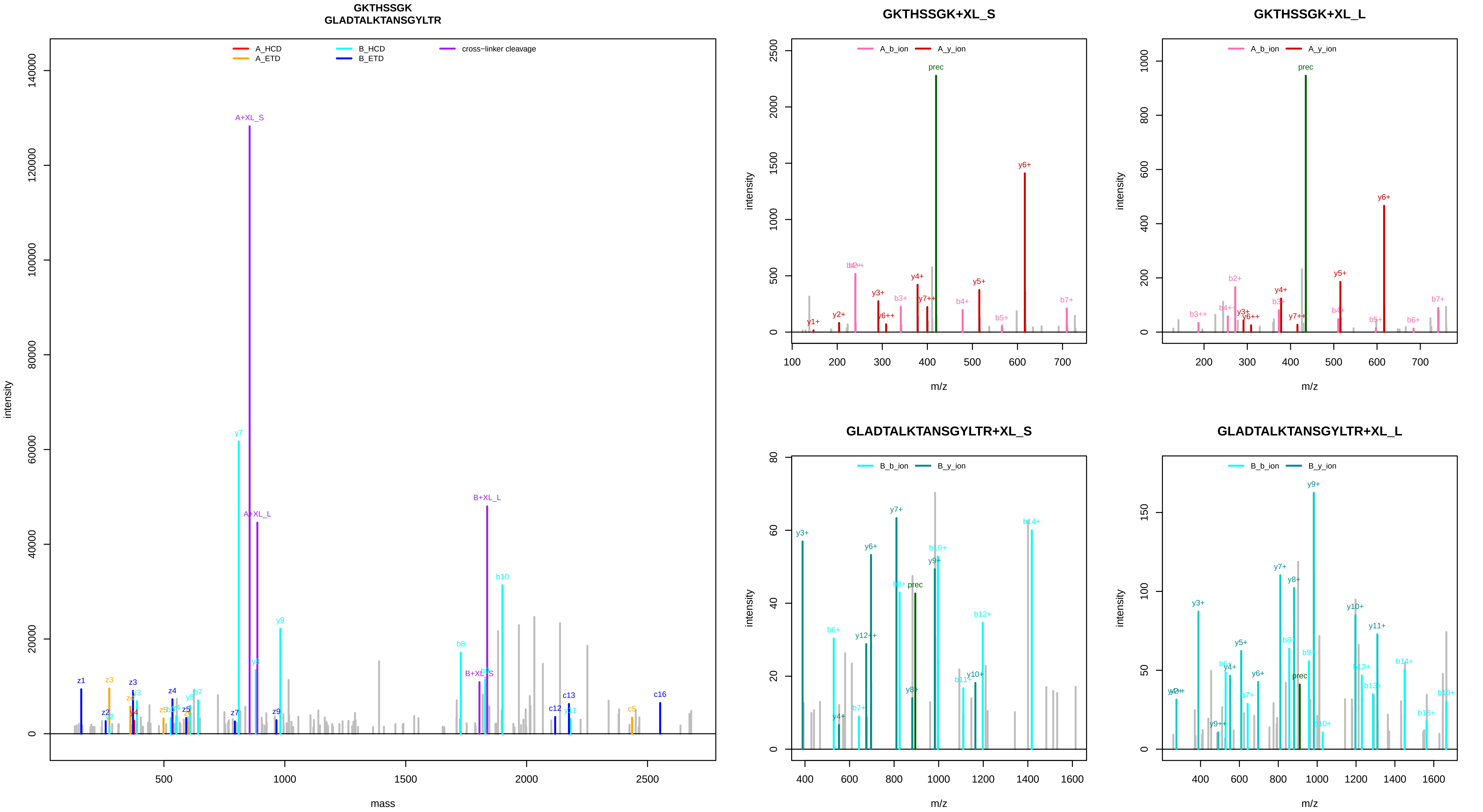

GKTHSSGK
GLADTALKTANSGYLTR
A_HCD
A_ETD
B_HCD
B_ETD
cross−linker cleavage
A+XL_S
A+XL_L
B+XL_L
B+XL_S
intensity
mass
GKTHSSGK+XL_S
GKTHSSGK+XL_L
GLADTALKTANSGYLTR+XL_S
GLADTALKTANSGYLTR+XL_L
A_b_ion
A_y_ion
B_b_ion
B_y_ion
prec
m/z

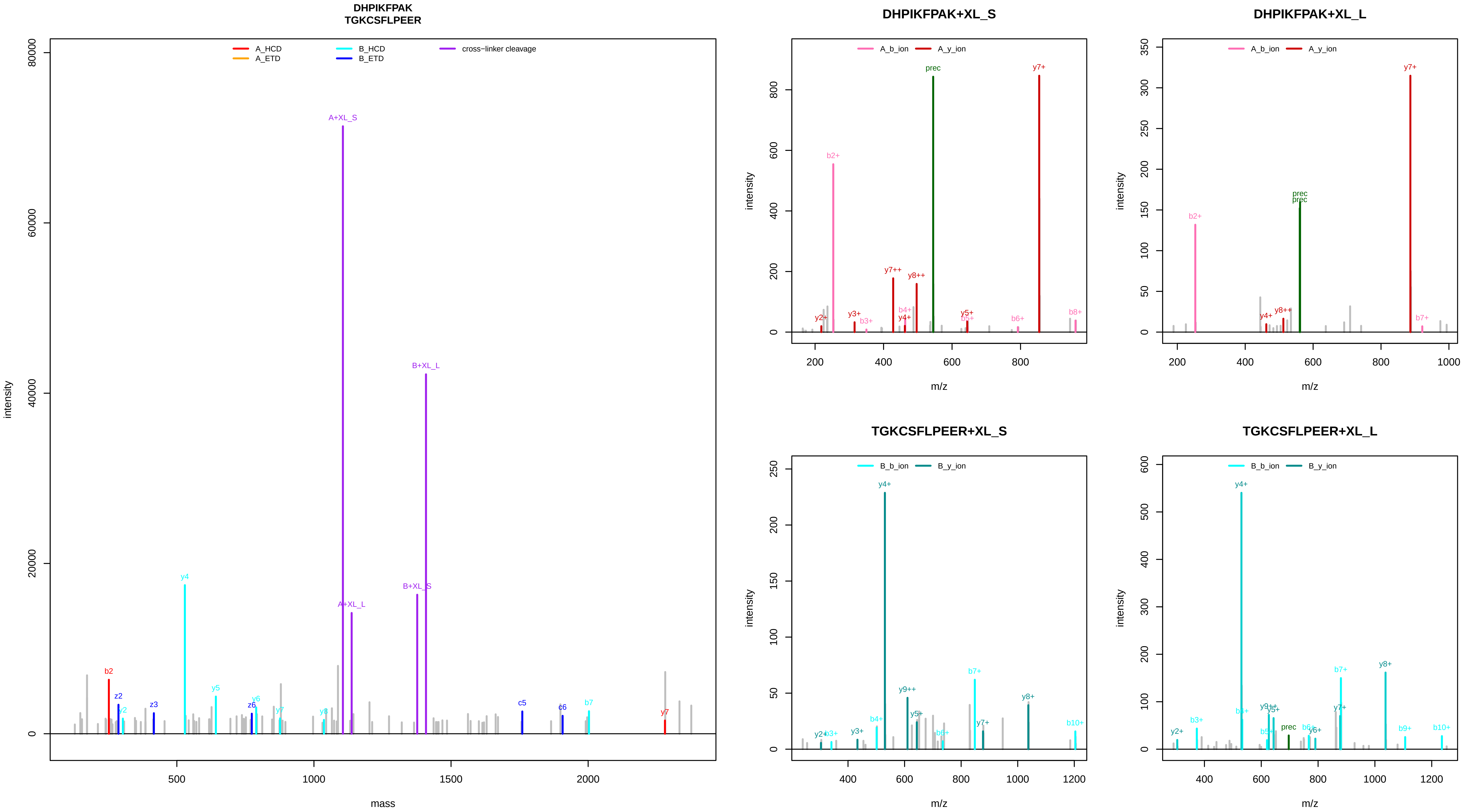
DHPIKFPAK
TGKCSFLPEER
A_HCD
A_ETD
B_HCD
B_ETD
cross−linker cleavage
A+XL_S
B+XL_L
B+XL_S
A+XL_L
intensity
mass
DHPIKFPAK+XL_S
DHPIKFPAK+XL_L
TGKCSFLPEER+XL_S
TGKCSFLPEER+XL_L
A_b_ion
A_y_ion
B_b_ion
B_y_ion
prec
m/z

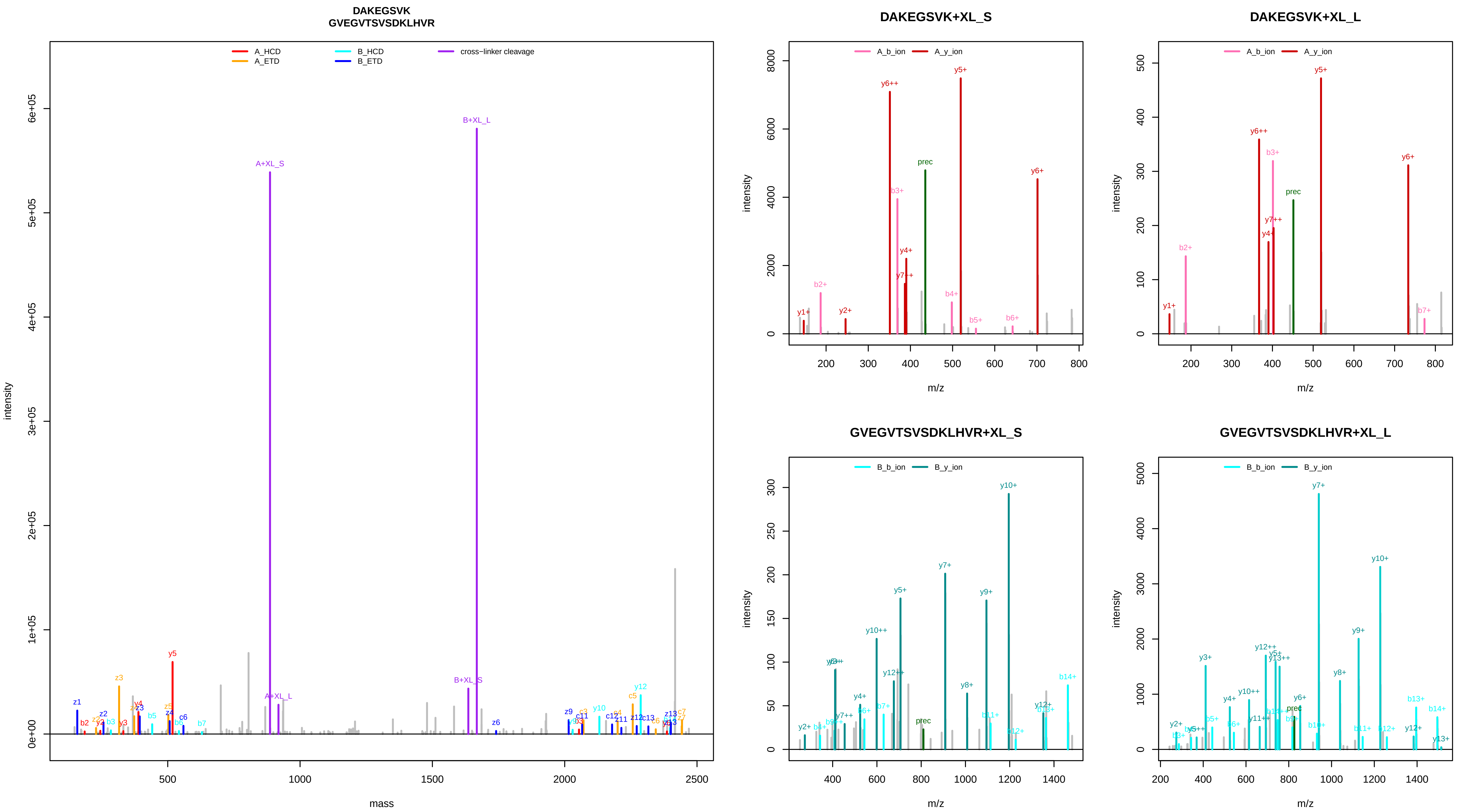
DAKEGSVK
GVEGVTSVSDKLHVR
A_HCD
A_ETD
B_HCD
B_ETD
cross−linker cleavage
A+XL_S
B+XL_L
A+XL_L
B+XL_S
intensity
mass
DAKEGSVK+XL_S
DAKEGSVK+XL_L
A_b_ion
A_y_ion
GVEGVTSVSDKLHVR+XL_S
GVEGVTSVSDKLHVR+XL_L
B_b_ion
B_y_ion
prec
m/z

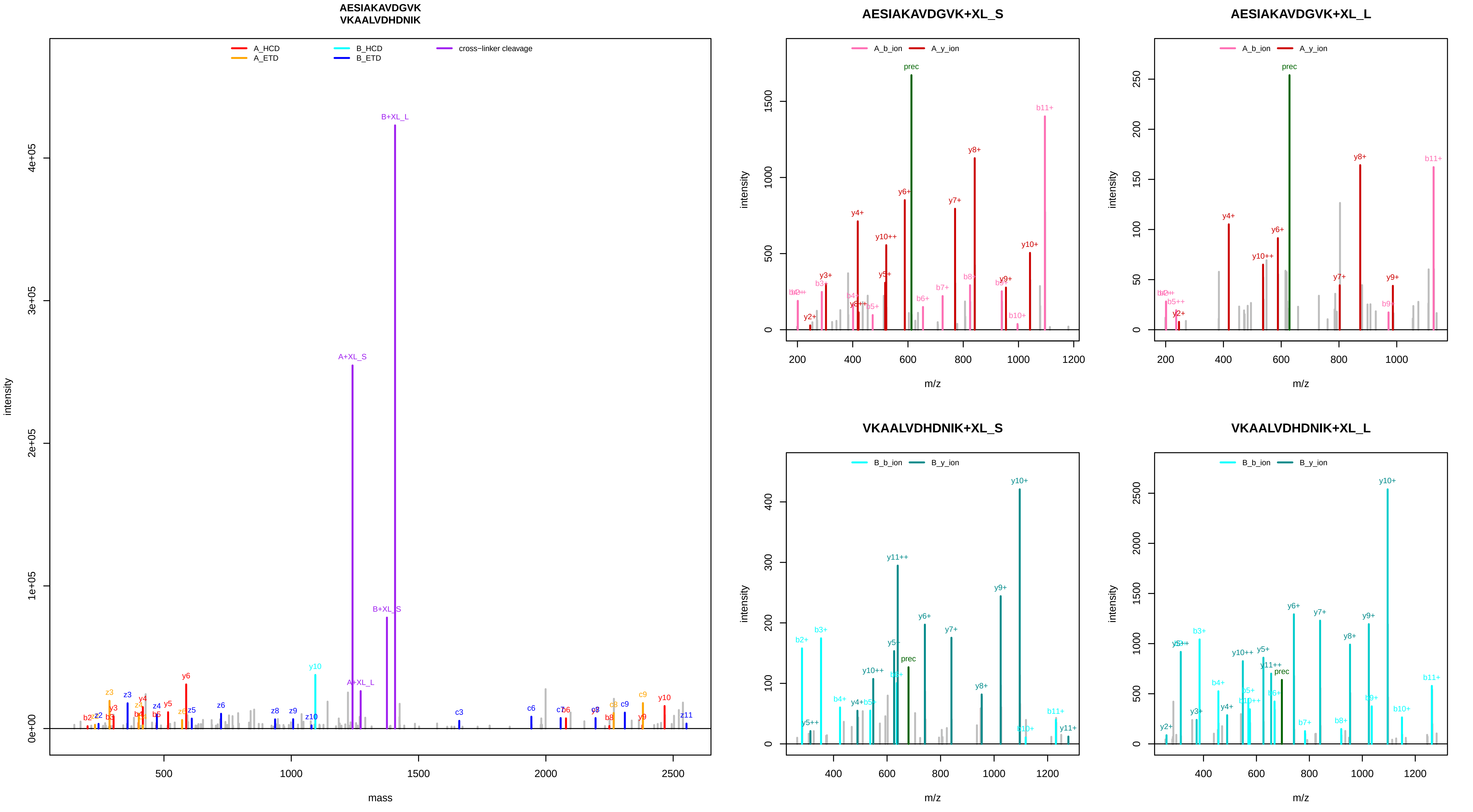
AESIAKAVDGVK
VKAALVDHDNIK
A_HCD
A_ETD
B_HCD
B_ETD
cross−linker cleavage
B+XL_L
A+XL_S
B+XL_S
A+XL_L
intensity
mass
AESIAKAVDGVK+XL_S
AESIAKAVDGVK+XL_L
VKAALVDHDNIK+XL_S
VKAALVDHDNIK+XL_L
A_b_ion
A_y_ion
B_b_ion
B_y_ion
prec
m/z
