# Supplementary material for: Optimized fragmentation schemes and data analysis strategies for proteome-wide cross-link identification
Source: Nat Commun. 2017 May 19;8:15473. doi: 10.1038/ncomms15473 (PMC5454533; doi:10.1038/ncomms15473)
Supplement: Supplementary Information — Supplementary Figures, Supplementary Discussion and Supplementary References [file ncomms15473-s1.pdf]

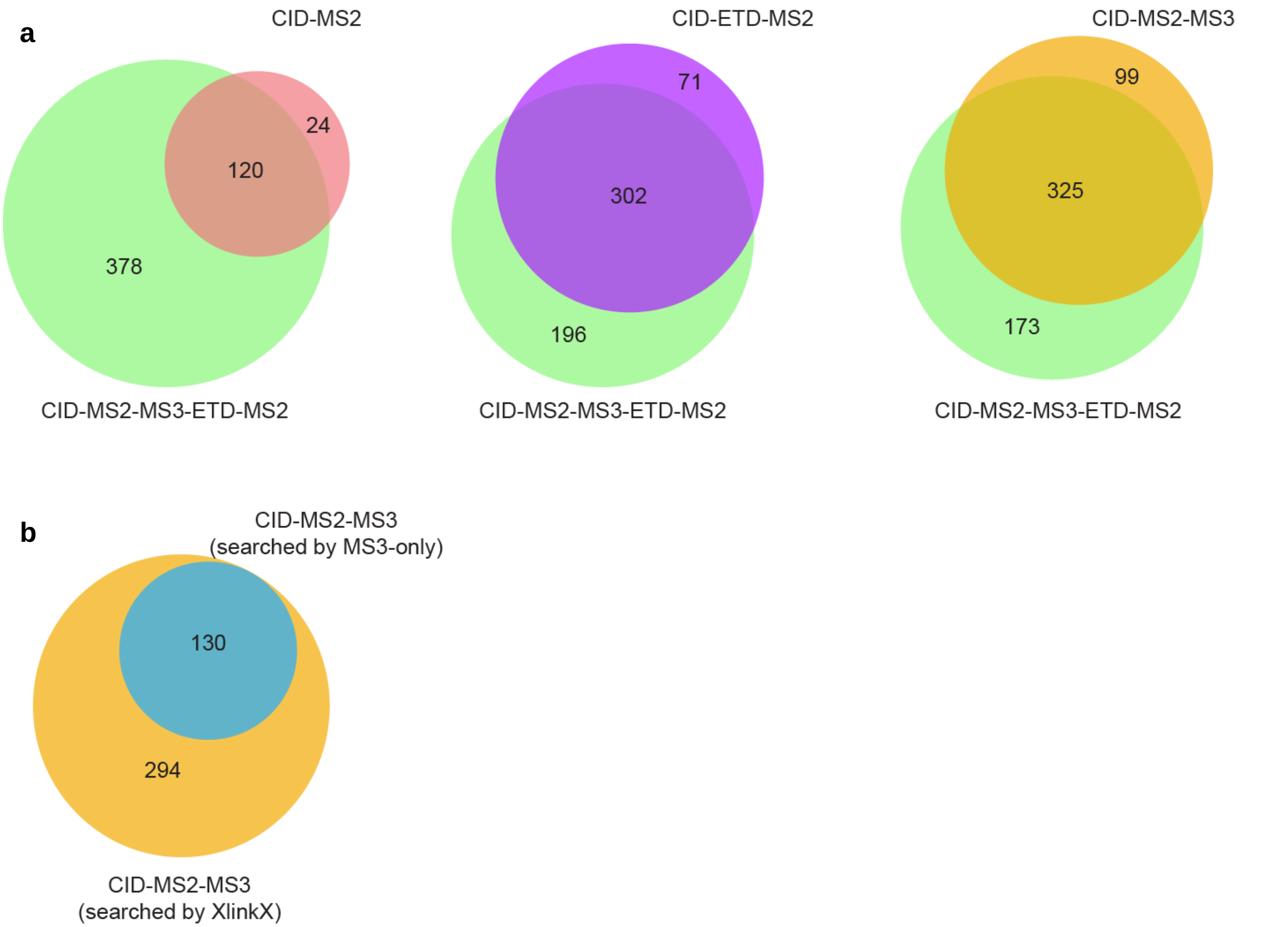

Supplementary Figure 1. Venn diagram of the identified cross-links in different MS acquisition and data analysis strategies presented in Figure 2a of the main text. (a) Comparison of different XlinkX v2.0-compatible MS acquisition strategies. (b) Comparative analysis of CID-MS2-MS3 data using either XlinkX v2.0 or an MS3-only approach.

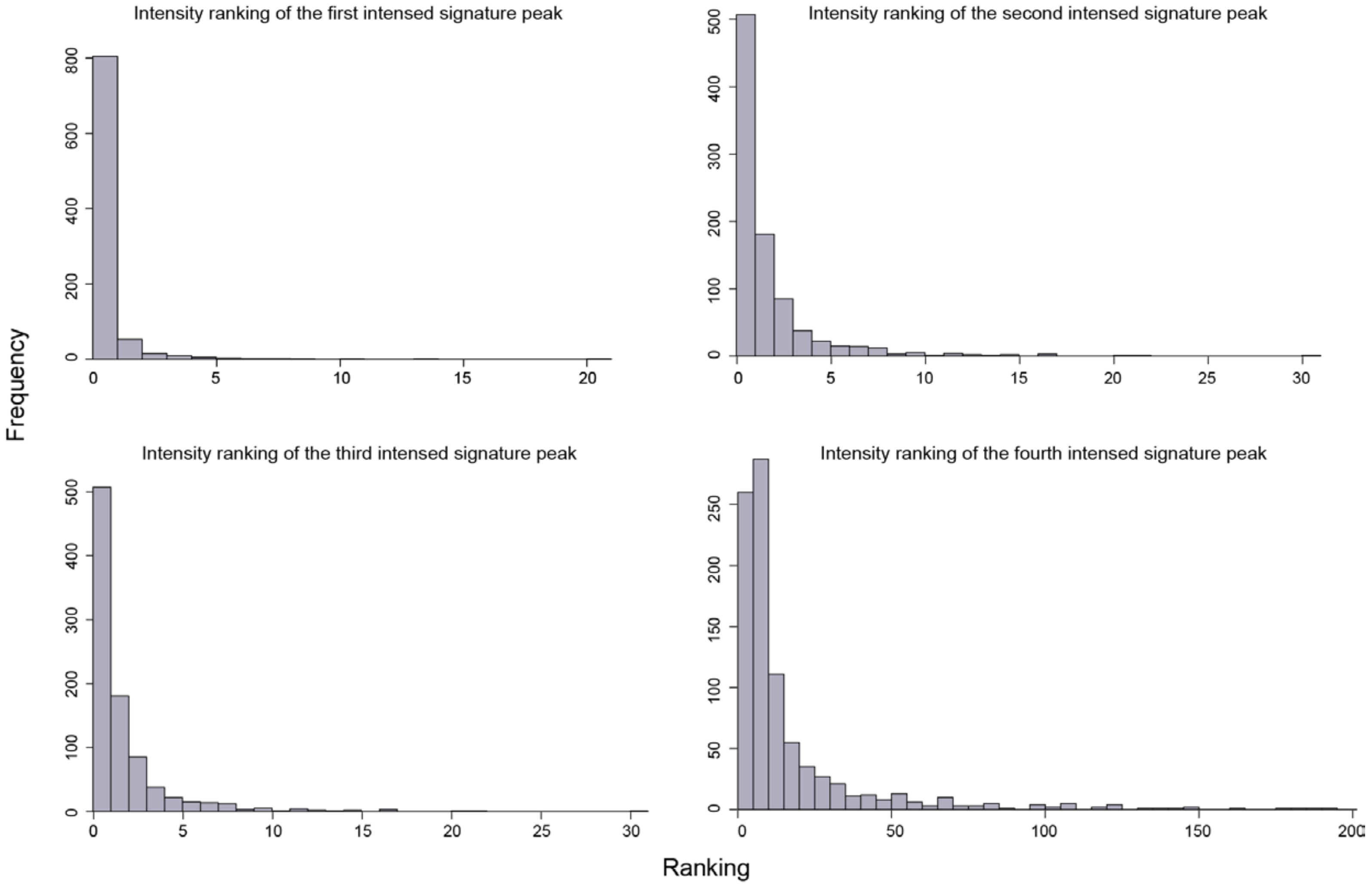

Supplementary Figure 2. Histograms of intensity rankings of each of the four signature peaks among all fragment ions in the CID MS2 spectrum.

.

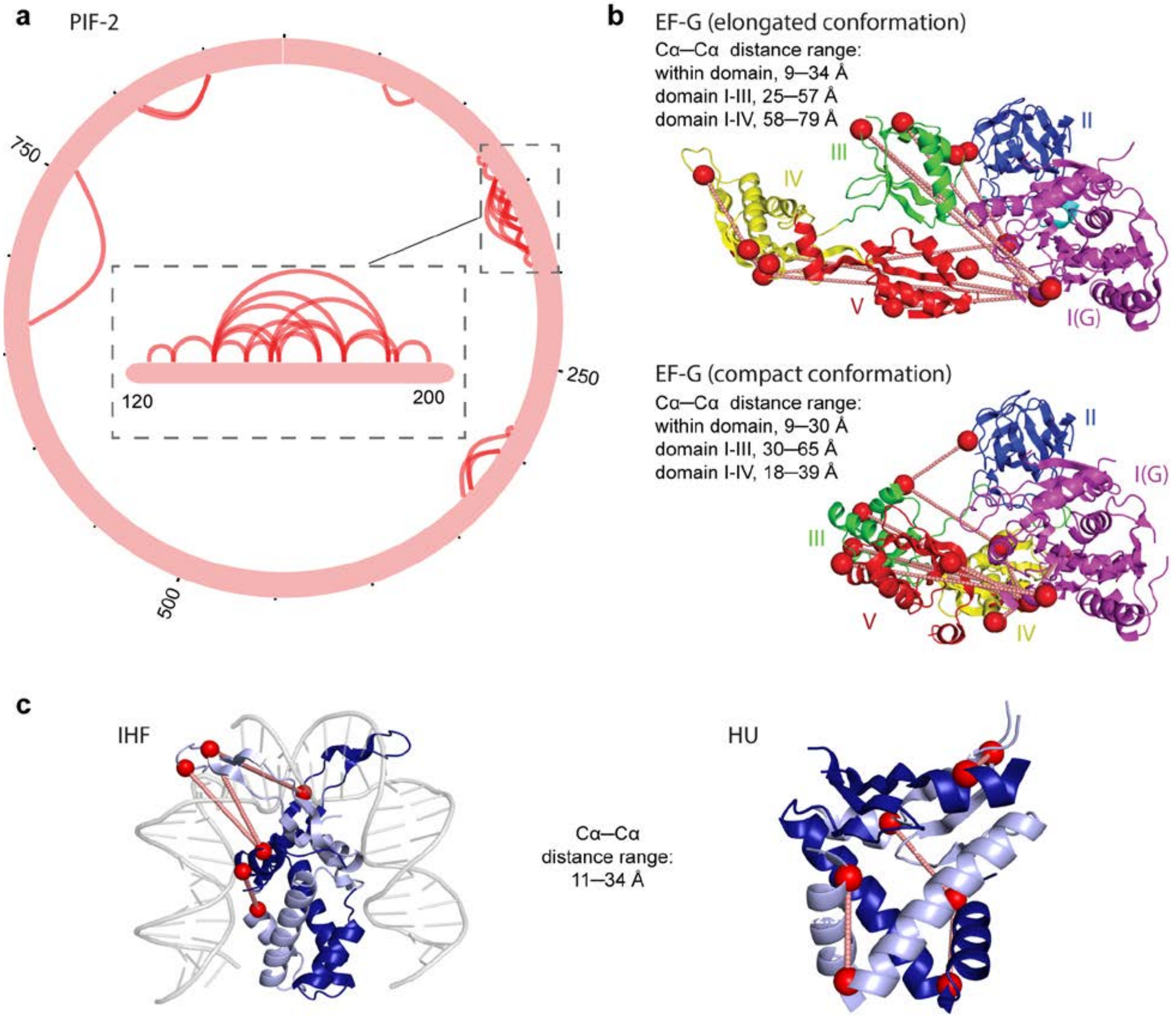

Supplementary Figure 3. Mapping of cross-links onto the structural models of translation and transciption regulators. (a) Cross-links of translation initiation factor 2 shown in a circular plot. The inset shows a zoom-in into a region of the N domain with a very high cross-link density. (b) Cross-link mapping of the elongated and compact conformation of elongation factor G (PDB entries 3JCD and 3JCE). (c) A crystal structure of the DNA-bound IHF α/β-heterodimer (left, PDB entry 1IHF) and a crystal structure of the HU α/β-heterodimer (right, PDB entry 2O97). Cross-links are shown as red lines connecting two red spheres that represent the Lys Cα atoms.

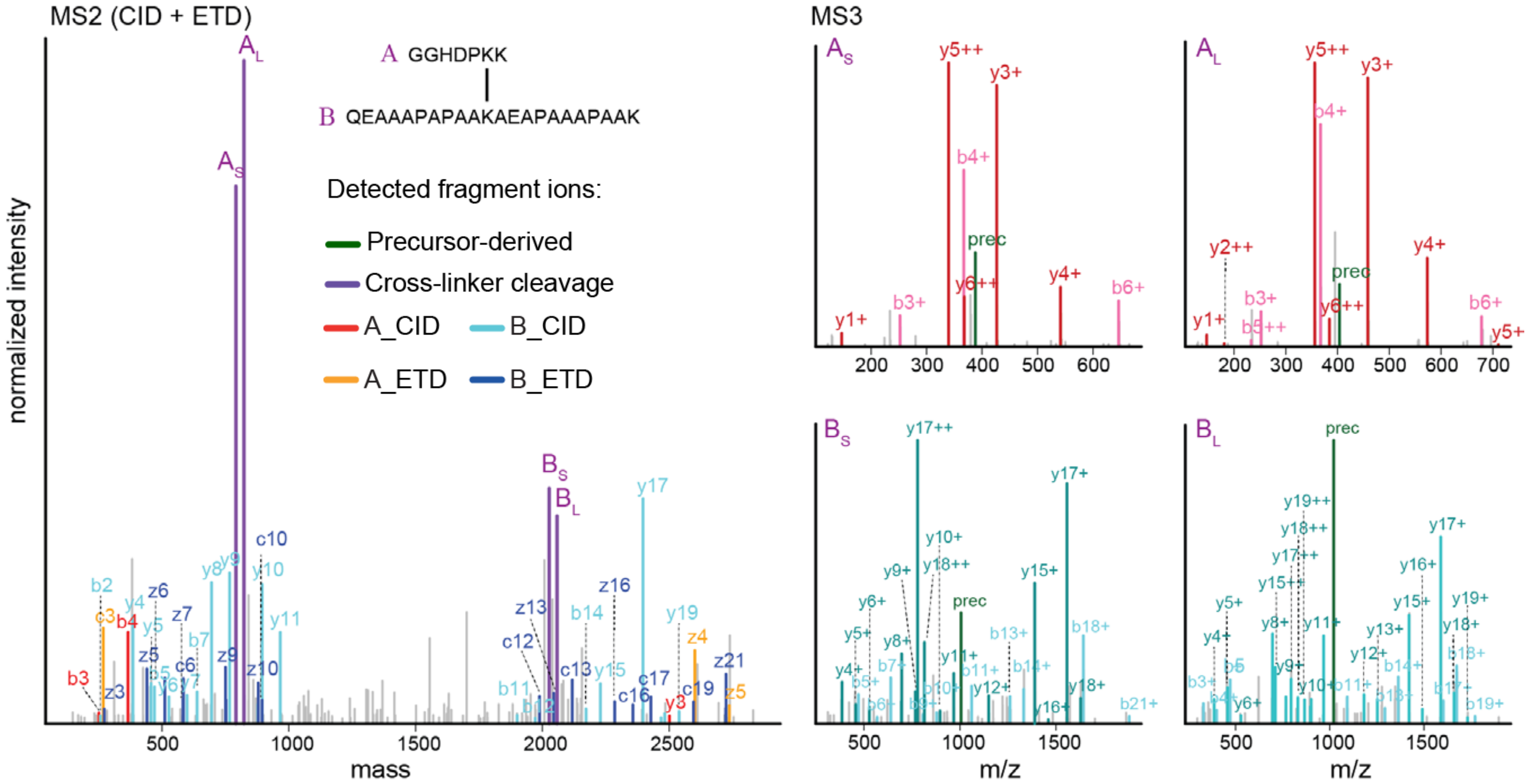

Supplementary Figure 4. Example spectra of a cross-link identification (pyruvate dehydrogenase ODP2 K302 cross-linked to ODP1 K368) from the hybrid CID-MS2-MS3-ETD-MS2 strategy. In this cross-link, both CID and ETD MS2 fragmentation is largely tailored towards peptide B thus resulting inefficient fragmentation of peptide A. The sequence information of peptide A is significantly improved by the MS3 acquisitions targeting both signature peaks derived from peptide A (A_s and A_L), highlighting the unique feature of MS3 in solving the fragmentation bias during cross-link identification.

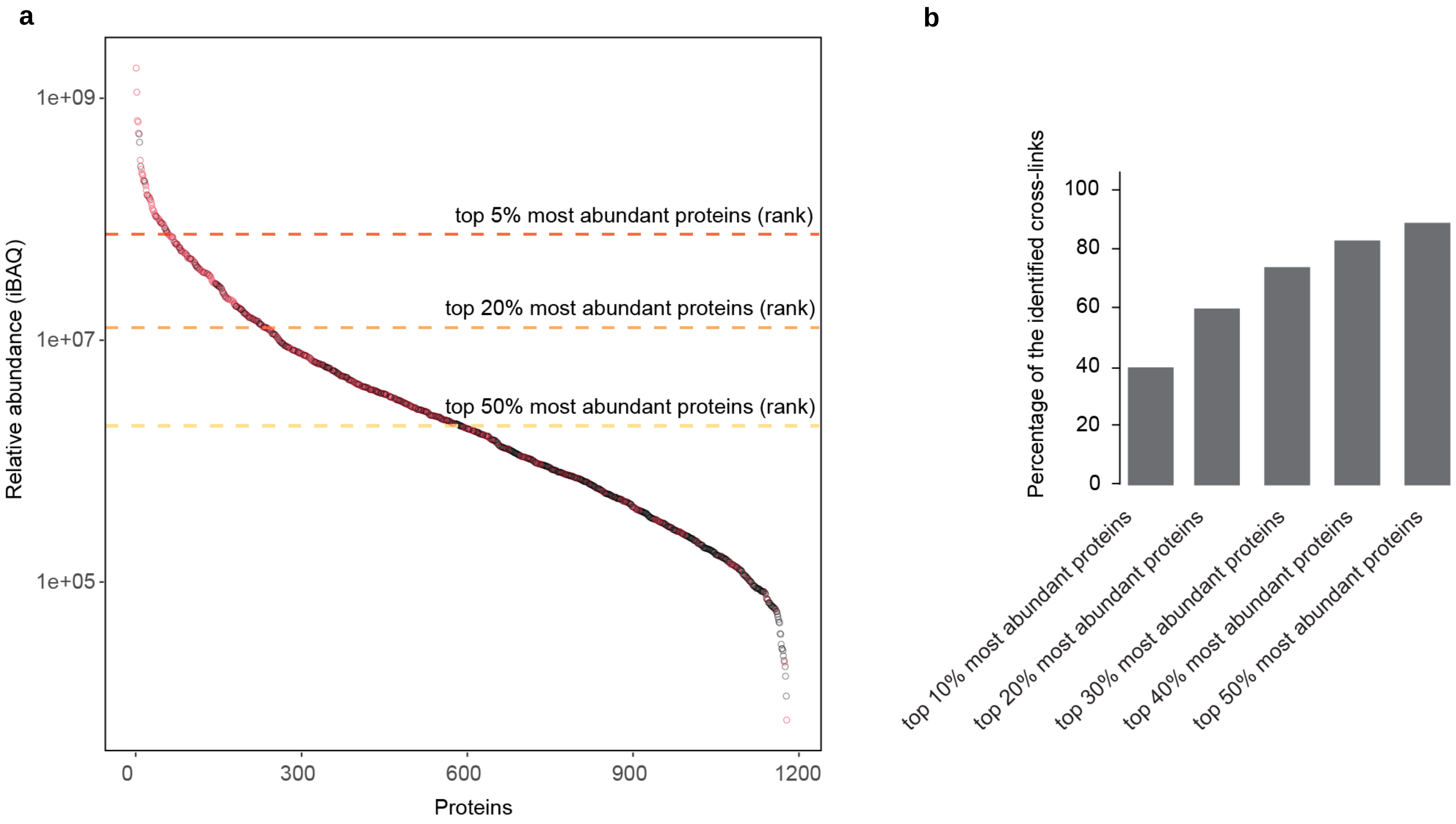

Supplementary Figure 5. Characterization of the proteome coverage of our *E. coli* cross-linking dataset. (a) Protein relative abundances are plotted based on their iBAQ values. Proteins labeled in red are the ones included in our XL-MS dataset. (b). A bar diagram of the number of cross-links in different categories of protein abundances.

a

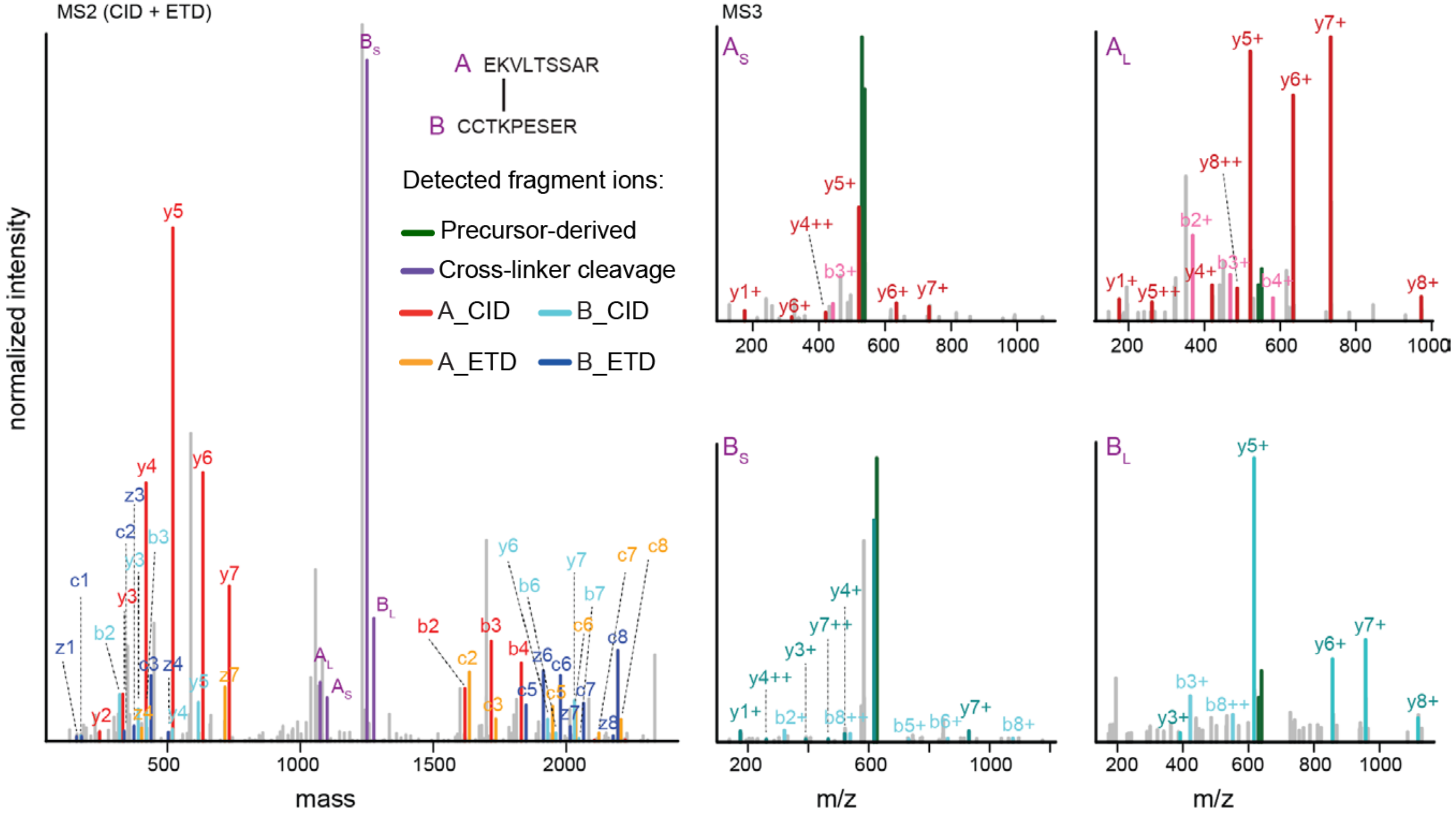

MS2 (CID + ETD)
normalized intensity
mass
A EKVLTSSAR
B CCTKPESER
Detected fragment ions:
Precursor-derived
Cross-linker cleavage
A_CID
B_CID
A_ETD
B_ETD
MS3
m/z

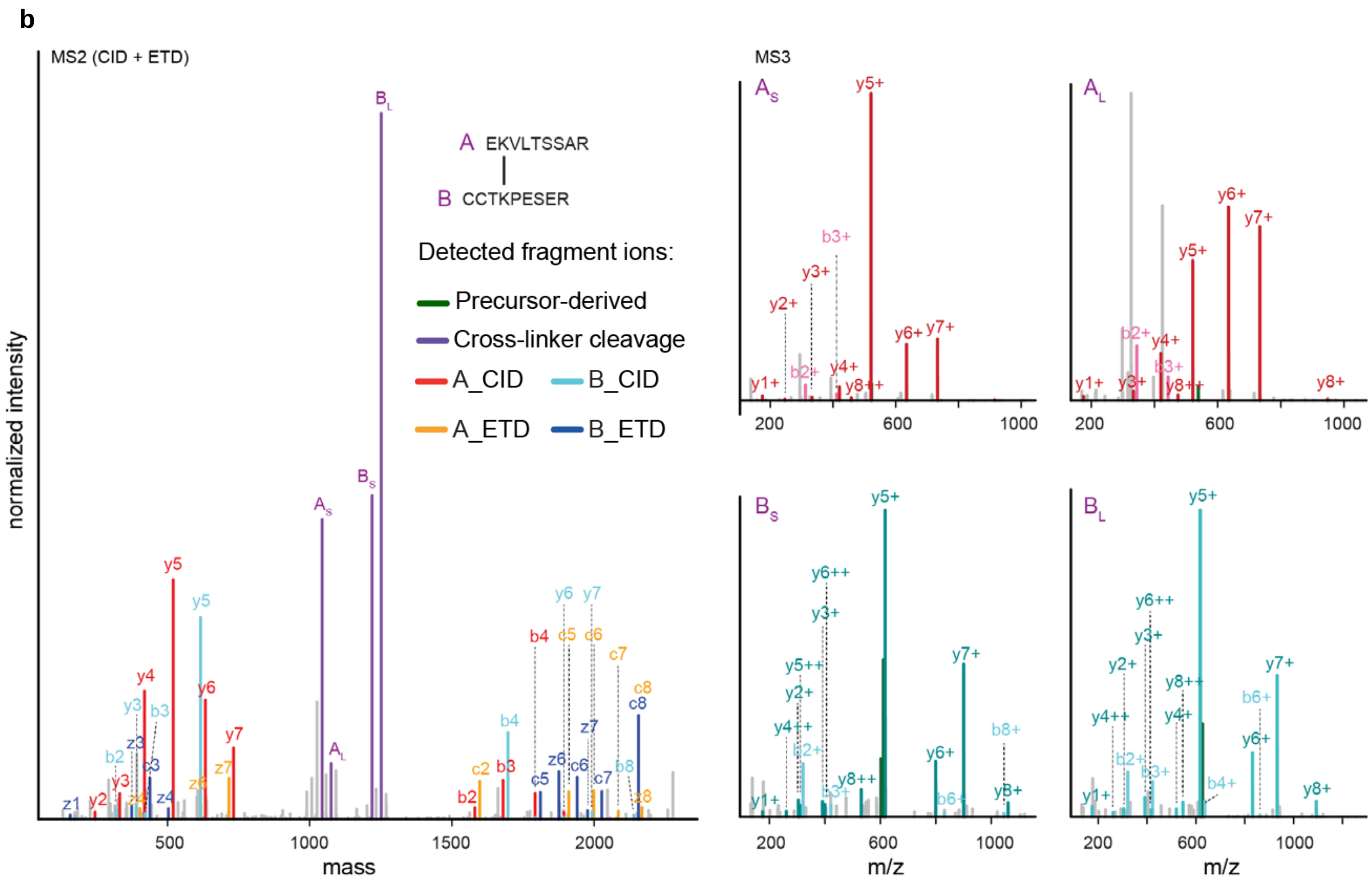

Supplementary Figure 6. Example spectra of the same cross-link identification using BuUrBu (a) and DSSO (b) cross-linkers. Both cross-linkers provide signature fragmentation patterns (shown in purple) and are thus applicable to the data analysis workflow of XlinkX v2.0.

Supplementary Discussion

Optimizing the number of most intense ions (top n) in the intensity-based precursor determination strategy

In the intensity-based strategy, the precursor mass of each linked peptide is calculated from the top *n* (*n* is a user defined parameter) most intense ions in the CID MS2 spectrum, provided that they contain at least 1 of the 4 signature peaks. To optimize the number of *n* to be used, we plotted intensity rankings of each of the signature peaks among all ions in the corresponding CID-MS2 spectrum. From a total of 898 spectra being examined, 95% of them contain at least one signature peak within top three most intense fragment ions (Supplementary Figure 2). Therefore, we chose top_n=3 for the intensity-based approach in our study. However, this parameter needs to be adjusted depending on the type of the applied cross-linker (some cross-linkers have more prominent signature peaks than others), the MS instrumentation, and the acquisition parameters (e.g. MS2 collision energy). To consider the differences during data acquisition, we suggest the users to perform a first search using Δm-based approach. Next, an intensity ranking plot can be generated based on results from the first search to optimize *n* for a subsequent intensity-based search.

Structural characterization of various endogenous proteins complexes in E. coli

To confirm that our XL-MS approach captured the native architecture of endogenous proteins and biomolecular complexes, we mapped our cross-linking data on several publicly available high-resolution structures or highly confident homology models of *E. coli* protein assemblies.

70S ribosome and translational regulators

The first example we focus on is the *E. coli* 70S ribosome. We identified 236 cross-links involving subunits of the 70S core ribosome, and were able to map 149 of these cross-links onto structurally characterized regions. Out of the 149 cross-links, 136 show a Cα–Cα distance of less than 38 Å, which approximates the maximum distance restraint imposed by DSSO or BS3 cross-linkers[1]. 8 cross-links are in a range of 38 Å to 56 Å, which can be partially explained by the known high mobility of the ribosome during transcription[2]. In addition, we confidently identified 5 ribosomal cross-links, ranging from 102 Å to 182 Å, which would traverse through the center of

the ribonucleoprotein assembly if mapped on a single ribosome. We carefully examined these cross-links and noticed that all connected Lys residues are positioned in close proximity to the ribosome surface (Figure 3a). Since surface exposed Lys are highly prone to cross-linking, the observed connections are likely to be real, possibly originating from polysomes (ensembles of several ribosomes translating a single mRNA simultaneously and forming flexible, higher-order structures). The existence of *E. coli* polysomes was also proposed in another recent XL-MS study that likewise identified high-confidence cross-links violating the expected distance restraints[3]. Of note, cryo-electron tomography studies on polysome structures have shown that the neighboring ribosomes have various configurations, which is in agreement with the multiple contact sites observed in our cross-linking study[4]. We conclude thus that the vast majority (91.2%) of the identified ribosome cross-links agree with available ribosomal structures, 5.4% are likely to reflect ribosomal mobility during translation, and 3.4% hint at the presence of polysomes.

Next, we identified many cross-links between the ribosome and translation-associated factors, including two translation initiation factors (IF-2 and IF-3) and three translation elongation factors (EF-Tu, EF-Ts, and EF-G). IF-2 and IF-3 are the first factors to bind to the ribosome during the assembly of the 30S pre-initiation complex[5]. IF-2 comprises three isoforms due to the presence of three alternative start codons[6]. IF-2 alpha, the longest isoform, was shown to be twofold more abundant than the N-terminally truncated beta1 and beta2 isoforms[6]. Correspondingly, IF-2 alpha is represented by 16 of the 27 unique IF-2 cross-links as they involve Lys residues that are absent in the beta isoforms. It is of special interest that the IF-2 N domain (residues 1–391) is involved in all but three IF-2 cross-links (Supplementary Figure 3a). This domain engages in the assembly of ribosomal subunits to form the 70S initiation complex[7]. However, as the N domains of different species show great sequence and length variability[7], the *E. coli* homolog is still awaiting structural characterization. In this regard, our cross-linking data represent a valuable repository that, in combination with complementary structural biology studies, may eventually unravel the N domain structure and, thereby, the full architecture of *E. coli* IF-2. IF-3 is substantially smaller than IF-2 (20 versus 97 kDa), yet, the full-length structure of *E. coli* IF-3 also has not been determined. Therefore, we built an IF-3 homology model using the Phyre2 web server[8], which entirely complies with the XL-MS derived distance restraints (Figure 3b).

Prokaryotic translation is aided by the elongation factors EF-P, EF-G, EF-Tu and EF-Ts. The EF-Tu and EF-Ts structures were captured by 14 unique cross-links. We mapped 6 EF-Tu

cross-links and 4 EF-Ts cross-links onto the crystal structure of the EF Tu-Ts complex (PDB entry 4PC1), showing full concurrence between XL-MS and X-ray crystallography (Figure 3c). EF-G, a five-domain protein containing ~700 amino acids[9,10], was found to be involved in 13 cross-links. It has been suggested that EF-G undergoes numerous extensive conformational rearrangements during translocation but high-resolution structures primarily captured the so-called elongated conformation[11,12]. Interestingly, this structure explains only 7 out of 13 EF-G cross-links, whereas the remaining 6 cross-links have anomalous distances ranging from 52 Å to 79 Å (Supplementary Figure 3b). All these 6 cross-links connect different domains of EF-G (3 cross-links between domain I and IV; 3 cross-links between domain I and III), indicating extensive inter-domain rearrangement. In support of this notion, Lin *et al.* recently reported the crystal structure of a previously unseen conformation of EF-G, providing high-resolution structural evidence for a much more compact structure than previously expected[12]. In this compact conformation, domain IV is in close proximity to domains I and II, complying with the 3 cross-links we observe between domain I and IV (Supplementary Figure 3b). The remaining 3 cross-links between domain I and III fit in none of the crystallized conformations, which suggests that EF-G can adopt additional conformations or transition states that have so far been refractory to X-ray crystallography.

Chaperone complexes and their interaction with the 70S ribosome

In *E. coli*, three major chaperone systems, the trigger factor (TF), DnaK and the GroES-GroEL complex, are known to be involved in the folding of translated proteins. The prevailing view suggests that TF, which associates with the ribosome, assists the initial folding of nascent polypeptide chains. Subsequently, the ATP-dependent DnaK and GroES-GroEL chaperone systems continue to fold the protein *de novo*[13,14]. Next to unveiling many connections within all three chaperone systems, including 7 TF cross-links, 10 DnaK-GrpE cross-links and 14 GroES-GroEL cross-links, our XL-MS data give direct evidence for ribosome-chaperone interactions (Figure 3a). We identified three cross-links between 50S ribosomal proteins and TF (TF-RPL23 and TF-RPL24) as well as DnaK (DnaK-RPL25). We mapped the two ribosome-TF cross-links onto the available cryo-electron microscopy structure of the full-length TF associated with ribosome-nascent chain complexes (PDB entry 2VRH), finding them to be in full agreement with the TF-ribosome binding interface (Figure 3a). These results show the transient engagement of the ribosome with chaperone complexes and provide structural insights into co-translational chaperone-assisted folding *in vivo*.

Carbohydrate-degrading enzymes

Our study revealed 122 unique cross-links among proteins involved in the bacterial carbohydrate catabolism. According to the Uniprot Knowledgebase, *E. coli* glycolysis and Krebs cycle comprise 44 proteins, 26 of which were found in our cross-linking study. The structurally best characterized proteins, i.e., those involved in five or more unique cross-links, were subjected to a more detailed analysis.

Most cross-links were identified within the pyruvate dehydrogenase complex and the α-ketoglutarate dehydrogenase complex that catalyze the oxidative decarboxylation of pyruvate or α-ketoglutarate[15]. Both protein assemblies comprise multiple copies of three enzyme components E1, E2 and E3. The α-ketoglutarate dehydrogenase complex can form 24-mers. In the pyruvate dehydrogenase complex, the E1 component (OPD1) forms a dodecamer of homo-dimers, the E2 component (ODP2) forms an octamer of homo-trimers and the E3 component (DLDH) assembles as a dodecamer of homo-dimers[16]. The subunits of both enzyme complexes also contain several flexible linker regions since their catalytic action is characterized by large domain movements[15]. Consequently, their complete architecture is yet to be deciphered. In Figure 4a and 4b, we assembled publicly available high-resolution structures of the individual enzyme components and compared them with our cross-linking data (PDB entries listed in the Figure legend). The high-resolution structures and the XL-MS results are fully reconcilable. Since cross-linking is able to capture transient interactions and contacts within flexible protein regions, we also obtained insights into the quaternary organization of both multi-enzyme complexes. This is evidenced by cross-links between the pyruvate dehydrogenase E1 (ODP1) and E3 (DLDH) crystal structures and between the α-ketoglutarate dehydrogenase E1 (ODO1) and E2 (ODO2) crystal structures. Moreover, several cross-links between structurally uncharacterized regions of the three enzymes may serve as valuable input for future cryo-electron microscopy or computational modeling studies.

Furthermore, we obtained structural information on several other catabolic enzymes and enzyme complexes (Figure 4c-g). In all cases, the cross-links accurately reflect the existing crystal structures, evidencing that our data give insights into naturally occurring protein folds and non-covalent contacts. Besides confirming well-characterized binding events, we also identified so far unknown interactions. For example, we verified two cross-links between glyceraldehyde 3-phopshate dehydrogenase, a catabolic enzyme acting during glycolysis, and malonyl-coenzyme A

acyl carrier protein transacylase, which is involved in fatty acid anabolism. This hints at a structural linkage between these pathways, possibly through a transiently formed metabolon.

Histone-like proteins

We identified 30 unique cross-links – 12 inter-species and 18 intra-species connections – that give insights into the structural organization of the nucleoid-associated proteins H-NS, StpA, IHF and HU. This class of proteins fulfill histone-like functions in *E. coli*, contributing to an efficient packing of the bacterial chromosome[17,18].

H-NS and its less abundant paralogue StpA preferentially associate with bent DNA but also have RNA binding capacity[17]. In our study, H-NS is characterized by 10 intra-molecular cross-links. Since H-NS has the potential to form homo-oligomers, we analyzed whether the identified cross-links suggest H-NS oligomerization. Full-length H-NS has not been structurally characterized so far, therefore, we used the Phyre2 web server[8] to generate an H-NS homology model based on high-resolution structures of H-NS subdomains and homologous proteins. Comparing the H-NS model and our XL-MS-derived restraints shows that all cross-links can be explained by one H-NS monomer (Figure 3d) which, however, does not rule out the possible presence of H-NS homo-oligomers. The less prevalent H-NS homolog StpA is represented by 1 unique cross-link, which complies with the NMR structure of StpA (PDB entry 2LRX, Figure 3d).

IHF and HU are closely related, both belonging to the DNABII protein family. Both proteins have similar cellular functions including preservation of DNA supercoiling and transcription regulation through DNA bending, however, their mechanism of DNA recognition are partially different[18]. IHF and HU form structurally very similar α/β-heterodimers, consisting of an α-helical body and two 'β-ribbon arms' that wrap around the DNA[19]. In case of IHF, 4 unique cross-links were verified and mapped onto a crystal structure (PDB entry 1IHF). All links are in full agreement with the Cα–Cα distance restraint imposed by DSSO, however the length of the only link connecting the 'β-ribbon arms' (34 Å), indicates backbone flexibility in this region (Supplementary Figure 3c). Interestingly, this cross-link traverses through the DNA binding region of IHF, indicating that our experiment also captured DNA-free IHF complexes. This would directly explain the increased flexibility of the 'β-ribbon arms', which are known to be the most mobile part of IHF and HU, especially in absence of DNA[19,20]. A high mobility of this protein region is also evident from the *E. coli* HU crystal structure (PDB entry 2O97) since electron density for the 'β-ribbon arms' is entirely missing. Therefore, only four out of 15 identified HU cross-links

could be compared to high-resolution structural data. Reassuringly, all four cross-links were in full agreement with the crystallographic data (Supplementary Figure 3c).

In conclusion, all cross-links that could be mapped onto atomic models are structurally sound, confirming that our XL-MS approach provides insights into the natively existing structural proteome of *E. coli*.

Statistical analysis on the proteome coverage of our cross-linking dataset

To assess the proteome coverage of our *E. coli* cross-linking dataset, we compared relative protein abundances (obtained by the iBAQ approach[21] implemented in the MaxQuant software [22]) to the proteins involved in cross-links (Supplementary Figure 5a) and number of cross-links (Supplementary Figure 5b). These analyses show 10%, 35% and 64% of proteins involved in cross-links are among the top 5%, 10% and 30% most abundant proteins, respectively, based on the rankings from iBAQ values. Furthermore, 39%, 60%, 74%, 83% and 88% of cross-links are detected within the top 10%, 20%, 30%, 40% and 50% most abundant proteins, respectively. These results indicate that cross-links are enriched in proteins with higher abundances. Increasing the sensitivity of the XL-MS approach will require further methodological improvements, such as advancements of the MS instrumentation (enabling faster scanning rates and a higher dynamic range) and the development of highly specific cross-link enrichment strategies (e.g., affinity-tagged cross-linkers).

Supplementary References

1. Merkley, E. D. *et al.* Distance restraints from crosslinking mass spectrometry: Mining a molecular dynamics simulation database to evaluate lysine-lysine distances. *Protein Sci.* **23,** 747–759 (2014).

2. Bock, L. V *et al.* Energy barriers and driving forces in tRNA translocation through the ribosome. *Nat. Struct. Mol. Biol.* **20,** 1390–6 (2013).

3. Tan, D. *et al.* Trifunctional cross-linker for mapping protein-protein interaction networks and comparing protein conformational states. eLife **5**, e12509 (2016).

4. Brandt, F. *et al.* The Native 3D Organization of Bacterial Polysomes. *Cell* **136,** 261–271 (2009).

5. Milón, P., Maracci, C., Filonava, L., Gualerzi, C. O. & Rodnina, M. V. Real-time assembly landscape of bacterial 30S translation initiation complex. *Nat. Struct. Mol. Biol.* **19,** 609–615 (2012).

6. Sacerdot, C. *et al.* Both forms of translational initiation factor IF2 (alpha and beta) are required for maximal growth of Escherichia coli. Evidence for two translational initiation codons for IF2 beta. *J. Mol. Biol.* **225,** 67–80 (1992).

7. Simonetti, A. *et al.* Involvement of protein IF2 N domain in ribosomal subunit joining revealed from architecture and function of the full-length initiation factor. *Proc. Natl. Acad. Sci. U. S. A.* **110,** 15656–61 (2013).

8. Kelley, L. A., Mezulis, S., Yates, C. M., Wass, M. N. & Sternberg, M. J. E. The Phyre2 web portal for protein modeling, prediction and analysis. *Nat. Protoc.* **10,** 845–858 (2015).

9. AEvarsson, A. *et al.* Three-dimensional structure of the ribosomal translocase: elongation factor G from Thermus thermophilus. *EMBO J.* **13,** 3669–3677 (1994).

10. Czworkowski, J., Wang, J., Steitz, T. A. & Moore, P. B. The crystal structure of elongation factor G complexed with GDP, at 2.7 A resolution. *EMBO J.* **13,** 3661–3668 (1994).

11. Salsi, E., Farah, E., Netter, Z., Dann, J. & Ermolenko, D. N. Movement of elongation factor G between compact and extended conformations. *J. Mol. Biol.* **427,** 454–467 (2015).

12. Lin, J., Gagnon, M. G., Bulkley, D. & Steitz, T. A. Conformational changes of elongation factor g on the ribosome during tRNA translocation. *Cell* **160,** 219–227 (2015).

13. Bukau, B., Deuerling, E., Pfund, C. & Craig, E. A. Getting newly synthesized proteins into shape. *Cell* **101,** 119–122 (2000).

14. Hartl, F. U. & Hayer-Hartl, M. Molecular chaperones in the cytosol: from nascent chain to folded protein. *Science* **295,** 1852–8 (2002).

15. Perham, R. N. Swinging arms and swinging domains in multifunctional enzymes: catalytic machines for multistep reactions. *Annu. Rev. Biochem.* **69,** 961–1004 (2000).

16. Wang, J. *et al.* Structure and function of the catalytic domain of the dihydrolipoyl acetyltransferase component in Escherichia coli pyruvate dehydrogenase complex. *J. Biol. Chem.* **289,** 15215–15230 (2014).

17. Dorman, C. J. H-NS: a universal regulator for a dynamic genome. *Nat. Rev. Microbiol.* **2,** 391–400 (2004).

18. Swinger, K. K. & Rice, P. A. IHF and HU: Flexible architects of bent DNA. *Curr. Opin. Struct. Biol.* **14,** 28–35 (2004).

19. Swinger, K. K., Lemberg, K. M., Zhang, Y. & Rice, P. A. Flexible DNA bending in HU-DNA cocrystal structures. *EMBO J.* **22,** 3749–3760 (2003).

20. Vis, H. *et al.* Solution structure of the HU protein from Bacillus stearothermophilus. *J. Mol. Biol.* **254,** 692–703 (1995).

21. Schwanhäusser, B. *et al.* Global quantification of mammalian gene expression control. *Nature* **473,** 337–342 (2011).

22. Cox, J., Mann, M. MaxQuant enables high peptide identification rates, individualized p.p.b.-range mass accuracies and proteome-wide protein quantification. *Nat Biotechnol* **26**, 1367 - 1372 (2008).
